# Proteomic Analysis of Chicken Chorioallantoic Membrane (CAM) during Embryonic Development Provides Functional Insight

**DOI:** 10.1155/2022/7813921

**Published:** 2022-06-19

**Authors:** Tamer A. E. Ahmed, Cristianne M. M. Cordeiro, Oluwadara Elebute, Maxwell T. Hincke

**Affiliations:** ^1^Department of Cellular and Molecular Medicine, Faculty of Medicine, University of Ottawa, Ottawa, Ontario, Canada; ^2^Department of Innovation in Medical Education, Faculty of Medicine, University of Ottawa, Ottawa, Ontario, Canada

## Abstract

In oviparous animals, the egg contains all resources required for embryonic development. The chorioallantoic membrane (CAM) is a placenta-like structure produced by the embryo for acid-base balance, respiration, and calcium solubilization from the eggshell for bone mineralization. The CAM is a valuable *in vivo* model in cancer research for development of drug delivery systems and has been used to study tissue grafts, tumor metastasis, toxicology, angiogenesis, and assessment of bacterial invasion. However, the protein constituents involved in different CAM functions are poorly understood. Therefore, we have characterized the CAM proteome at two stages of development (ED12 and ED19) and assessed the contribution of the embryonic blood serum (EBS) proteome to identify CAM-unique proteins. LC/MS/MS-based proteomics allowed the identification of 1470, 1445, and 791 proteins in CAM (ED12), CAM (ED19), and EBS, respectively. In total, 1796 unique proteins were identified. Of these, 175 (ED12), 177 (ED19), and 105 (EBS) were specific to these stages/compartments. This study attributed specific CAM protein constituents to functions such as calcium ion transport, gas exchange, vasculature development, and chemical protection against invading pathogens. Defining the complex nature of the CAM proteome provides a crucial basis to expand its biomedical applications for pharmaceutical and cancer research.

## 1. Introduction

Development of the avian embryo is a highly sophisticated and integrated process that starts in the proximal hen oviduct immediately after fertilization and before egg laying [1–3]. The developing chicken embryo is a valuable research model for understanding vertebrate embryonic development [4, 5]. Critical events that take place during embryonic development [1, 2, 6–13] are summarized in table S1.

The formation of essential extraembryonic membranes (amnion, chorion, allantois, chorioallantoic membrane (CAM), and yolk sac) occurs during the establishment of the germ layer stage (ED1-7) [6, 7, 14]. The full development of the embryo's main respiratory organ, the CAM, occurs during the embryo completion stage (ED8-18) [6, 7]. The CAM promotes gaseous exchange (O_2_-CO_2_), protects against pathogen invasion, and enables calcium mobilization and transport that support essential metabolic needs [5, 6, 14–24]. Finally, the initiation of pulmonary respiration and degeneration of the CAM are the hallmarks of the emergence stage (ED19-21) [6, 7].

**Figure 1:**
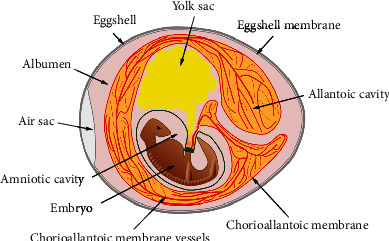
Stylized image of the fertilized egg showing embryo and the supporting extraembryonic membranes (CAM, yolk sac, and amnion), at approximately ED9-12. The CAM is a highly vascularized membrane, which is connected to the embryo through a continuous circulatory system.

The CAM is a thin translucent extraembryonic membrane that is connected to the embryo through a continuous circulatory system (Figure 1) [25, 26]. The CAM vasculature is accessible and can be easily imaged, making it a useful model for evaluation of angiogenesis and for investigating tumorigenesis [27, 28]. In addition, the CAM system enables the injection of pharmacological agents into the vasculature and direct assessment of regional responses. Furthermore, its *ex ovo* format has been utilized to study vascular permeability and vascular leakage [27]. Moreover, fertilized chicken eggs are readily available, and the embryo grows rapidly, making the CAM model ideal for the growth of human and murine xenografts and large-scale studies that do not trigger animal welfare concerns [28]. Its biomedical applications include the assessment of the angiogenic response of tumor tissue and malignant cells after implantation onto the CAM [5, 20] or evaluating tumor metastasis capacity, for instance, of carcinosarcoma, melanoma, and sarcomas [5]. The impact of compounds (for example, biologics, anticancer agents, gases, and organometallic compounds) on CAM development can be assessed [11]. In addition, the CAM model has been used to evaluate various drug delivery systems (DDS) [13, 27]. Furthermore, CAM has been utilized as a model tissue to evaluate two-photon excitation photodynamic therapy of age-related macular degeneration [26] and for surgical retina [22] and neurosurgery research [24].

The primary goal of the current study was to identify the protein constituents of the active CAM of fertilized eggs at two different time points of incubation (embryonic days 12 and 19) using proteomic analysis, in order to identify the molecular actors in CAM function and gain insight into further biomedical applications of this model system. The functionalities of CAM-specific proteins were identified using a bioinformatics approach.

## 2. Material and Methods

Unless otherwise specified, all reagents were purchased from Millipore-Sigma (Oakville, ON, Canada). The BCA protein assay reagent (bicinchoninic acid), NuPAGE® 4–12% Bis-Tris gels, NuPAGE® MES SDS running buffer, NuPAGE® sample reducing agent, NuPAGE® antioxidant, NuPAGE® LDS sample buffer, prestained protein standard, methanol, sodium chloride, and Corning clear polystyrene 96-well microplates were purchased from ThermoFisher Scientific (Nepean, ON). Glacial acetic acid, glycerol, sodium phosphate dibasic, and trizma base were from Bioshop Canada (Burlington, ON, Canada). Bromophenol blue, Coomassie blue R250, potassium chloride, potassium phosphate monobasic, and sodium dodecyl sulfate were from VWR (Mississauga, ON, Canada). Petri dishes (150 mm) were purchased from Ultident Scientific (St. Laurent, QC, Canada).

### 2.1. CAM and Embryonic Blood Serum Sampling from Fertilized Eggs

Collection of CAM and embryonic blood serum (EBS) samples from fertilized eggs was approved by the University of Ottawa Animal Care Ethics Committee (CMM-129) [6]. Fertilized eggs from White Leghorn hens, laid within a single 24 h period, were obtained from the Animal Diseases Research Institute (ADRI, Ottawa, Canada). Twelve eggs were incubated broad end up at 37°C in a humidified, rocking Petersime Model 1 incubator for up to 19 days. Viable fertilized eggs were collected at days 12 (*n* = 6) and 19 (*n* = 3) and cracked open at the equatorial region. The CAM samples were dissected free of adhering embryo and egg material and then gently washed with 30 mL phosphate-buffered saline (PBS, 10 mM sodium phosphate buffer, 0.15 M NaCl, and pH 7.4), followed by centrifugation at 5000 rpm for 20 min at 4°C, three times, with resuspension. The washed CAM pellets were sonicated in 5 mL of PBS on ice with five bursts of 30 s (Sonic Dismembrator, Fisher Scientific, Nepean, ON). Blood was sampled from the beating chick embryo heart at day 19 using a syringe (28G 1/2-0.36 × 13 mm) and cleared by centrifugation at 13,000 rpm for 10 min at 4°C to collect EBS. CAM and EBS samples were stored at −20°C until further processing.

### 2.2. Characterization of CAM and EBS by Proteomics

#### 2.2.1. Electrophoretic Fractionation

The protein concentrations in CAM and EBS-derived extracts were determined using the bicinchoninic acid (BCA) protein assay, with bovine serum albumin as a standard. Replicate CAM samples at each stage were combined for electrophoresis and then mixed (13 *μ*L, load of 50 *μ*g protein equivalent) with 5 *μ*L of 4X NuPAGE LDS sample buffer (2% lithium dodecyl sulfate, pH 8.4) and 2 *μ*L of 10× NuPAGE reducing agent (500 mM) dithiothreitol (DTT) followed by heating at 70°C for 10 min. The embryonic CAM and blood serum samples were fractionated on a 4–12% Bis-Tris gel for 35 minutes (200 V), and gel lanes were stained with Coomassie blue before sectioning into 10 equal gel slices (Figure S1). The variability between individual samples was reduced by tissue pooling to produce CAM samples (days 12 and 19) that were then subjected to proteomic analysis, as shown in figure S2.

#### 2.2.2. LC/MS/MS Analysis

The excised gel samples were sent to the Proteomics Platform of the Eastern Quebec Genomics Centre (Laval, QC, Canada) for LC/MS/MS analysis (in-gel digestion, mass spectrometry analysis, and Mascot database searching). The procedures for these analyses were performed as previously described [29, 30]. Briefly, protein in-gel was digested with trypsin and peptides were separated by reversed-phase (RP) nanoscale capillary liquid chromatography (nanoLC) performed using an Agilent 1200 nanopump connected to a 5600 mass spectrometer (AB Sciex, Framingham, Massachusetts, USA) with a nanoelectrospray ion source. Mass spectra were detected using the Analyst software (Version 1.6, AB Sciex, Framingham, Massachusetts, USA) (ES-MS/MS). MS/MS peak lists were generated using Protein Pilot (Version 4.5, AB Sciex, Framingham, Massachusetts, USA) and analyzed using Mascot (Version 2.4.0, Matrix Science, London, UK) and X! Tandem (CYCLONE version, 2010.12.01.1), both programmed to search the TAX_GallusGallus_9031_20141114 database (unknown version, 222,250 entries) with carbamidomethyl (C) as a fixed modification and deamidation (NQ), Gln-> pyro-Glu (N-term Q), and oxidation (MP) as variable modifications.

For detection of posttranscriptional modifications (PTMs), all MS/MS samples were analyzed using Mascot (Matrix Science, London, UK; version 2.5.1). Mascot was set up to process the data using Uniprot Gallus gallus reference proteome (https://www.uniprot.org/proteomes/UP000000539, 2020.06.09 version) (Unknown version, 27863 entries) assuming the digestion enzyme trypsin. Mascot was searched with a fragment ion mass tolerance of 0.100 Da and a parent ion tolerance of 0.100 Da. Assessed PTMs included carboxylation of glutamic acid (E) and phosphorylation of serine and threonine (S and T) amino acids. PTM abundance was expressed as total spectral count and exponentially modified protein abundance index (emPAI) values [31].

#### 2.2.3. Criteria for Protein Identification

Validation of MS/MS-based peptide and protein identification was performed using Scaffold (version Scaffold_4.8.7, Proteome Software Inc., Portland, OR, USA). MS/MS spectra were searched against the Uniprot (http://www.uniprot.org) and NCBI (http://www.ncbi.nlm.nih.gov/protein) chicken databases. Peptide Decoy was selected to detect false-positive results (see below). Peptide identifications were accepted if they could be established at >95% probability by the Peptide Prophet algorithm [32]. Protein identification was accepted at a false discovery rate (FDR) of 1% at protein and peptide level, with at least one unique peptide. Protein probabilities were assigned by the Protein Prophet algorithm [32]. To validate the false discovery rate % of the identified peptides and proteins, a random sequence (Decoy) was generated to serve as a false positive control. In total, 19 decoys were added during the spectrometric procedures. Several keratins and trypsin protein identifications were deleted from our dataset as they result from external contaminants (human skin keratin) and the digestion procedure (porcine trypsin). The porcine trypsin used for the sample protein digestion served as an internal positive control and confirmed the specificity of the mass spectrometric technique. In addition, cytochrome C and Hela cell digests served as intrasample and intracycle positive controls, respectively, and helped optimize/validate instrumental features such as retention time, peak intensity, and sensitivity. Finally, the embryonic blood serum sample served as a tissue control to facilitate the identification of CAM-specific proteins. The relative abundance of identified proteins was calculated using the emPAI values [31].

### 2.3. Bioinformatics Analysis

Various protein families were identified via submitting the entire entrez gene ID list identified in this study to the Uniprot database (http://www.uniprot.org, retrieve/ID mapping). Gene ontology (GO) terms for proteins identified in the CAM (days 12 and 19; specific or common to both time points) and EBS of fertilized eggs were obtained from the Database for Annotation, Visualization, and Integrated Discovery (DAVID) Bioinformatics Functional Annotation Tool (DAVID Bioinformatics Resources 6.7, NIAID/NIH) [6, 29, 30]. Each GO term corresponded to an EASE score (a modified Fisher Exact *P* value and high enrichment value), using GOTERM_BP_FAT and GOTERM_MF_FAT. Only GO terms with an EASE score ≤ 0.05 were considered to be significantly enriched [33]. DAVID Bioinformatics Resources was also used for pathway mapping of the entire proteins list via the Kyoto Encyclopedia of Genes and Genomes (KEGG) enrichment analyses [34] with a threshold of <0.05 for the *P* values.

Proteins identified in CAM samples were compared to Vesiclepedia to determine CAM proteins on this list related to extracellular vesicles (EVs). Vesiclepedia is a web-based compendium of RNA, proteins, lipids, and metabolites identified in EVs (http://www.microvesicles.org) and lists the top 100 proteins that have been identified in different EV studies [35]. The identified CAM proteins on the EV list were used as input for STRING (http://string-db.org/) to depict their possible protein-protein interactions, according to computational and experimental predications [36]. Various proteins involved in the different CAM functions were also used as input for STRING to determine the possible protein-protein interaction networks based on computational or experimental predictions along from published data.

All the figures were designed using Microsoft 365® Apps for enterprise (Excel and Power Point) along with Adobe photoshop CS (version 8).

## 3. Results

**Figure 2:**
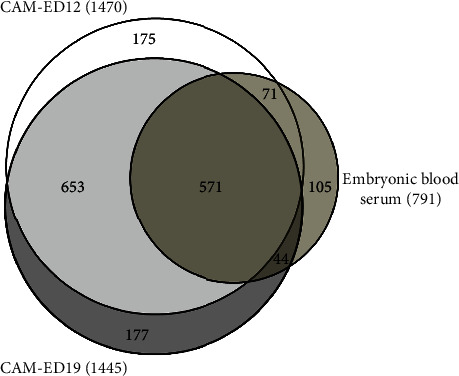
Venn chart showing the differential distribution of identified CAM proteins (ED12 and ED19) as compared to those of the embryonic blood serum.

LC/MS/MS-based proteomics enabled the identification of an extensive inventory of proteins in the CAM and EBS samples. A list of all proteins and their abundances along with peptides identified in the CAM and EBS samples is provided in table S2. In addition, a list of PTM abundance in CAM and EBS samples, in terms of total spectral count and emPAI values, is provided in table S3. The EBS proteome was determined for comparison to CAM tissue samples collected at two different time points (ED12 and ED19) and served as a tissue control, in order to identify proteins that are specific to the CAM. In total, 1796 different proteins were identified in CAM (1691 proteins in ED12+ED19 samples) and EBS (791 proteins) (Table S4). Similar numbers of proteins were identified at both CAM time points (ED12: 1470 and ED19: 1445). Of these, 571 proteins were common to the CAM at both time points and to EBS, while 1005 proteins were CAM-specific as compared to EBS. On the other hand, 653 of these 1005 proteins were common to CAM samples only at both stages of embryonic development, while 175 and 177 of these 1005 proteins were specific to CAM at ED12 and ED19, respectively. Finally, 105 proteins of the complete inventory (1796 proteins) were only detected in the EBS (Figure 2 and table S4).

A vast range of protein families was identified in the CAM and EBS samples, including actin, aldehyde dehydrogenase, aminoacyl-tRNA synthetase (classes I and II), annexins, various ATPases, collagens, DEAD-box helicase, heat shock protein 70, globin, heterogeneous nuclear ribonucleoproteins, histones (H1/5, H2A, H2B, H3, and H4), integrins (*α* and *β* chains), intermediate filament, myosin, serpin, peptidases, solute carrier family, TCP-1 chaperonin family, tropomyosin, tubulin, and ubiquitin-conjugating enzyme (Table S5).

Bioinformatics analysis was carried out for CAM proteins common to both ages and those specific to CAM at each age, along with proteins identified in the EBS, using the Database for Annotation, Visualization, and Integrated Discovery (DAVID, https://david.ncifcrf.gov/) that provides a comprehensive set of functional annotation tools. Functional annotation of proteins that are common to CAM at ED12 and ED19 was performed using Gene ontology (GO) term enrichment analysis and allowed the identification of 33 functionalities reflecting the CAM roles during embryonic development. These include, but are not limited to, proteins involved in ATP metabolic process (10 proteins), cell-substrate adhesion (8 proteins), cell junction organization (4 proteins), cytoskeleton organization (15 proteins), enzyme inhibitor activity (14 proteins), vesicle-mediated transport (26 proteins), structural molecule activity (41 proteins), and vasculature development (13 proteins). The enriched functionality with the highest number of proteins (102 proteins) is nucleotide binding (GO:0,000,166) (Table S6).

GO term enrichment analysis of proteins that are specific to CAM (D12) showed 5 different functionalities including oxidation-reduction (GO:0,055,114) (11 proteins), cellular macromolecular complex subunit organization (GO:0,034,621) (7 proteins), carboxylic acid binding (GO:0,031,406) (5 proteins), vitamin-binding (GO:0,019,842) (5 proteins), and actin filament organization (GO:0,007,015) (3 proteins) (Table S7). GO term enrichment analysis of proteins that are specific to CAM (D19) showed 3 different functionalities, including phosphorylation (GO:0,016,310) (12 proteins), enzyme inhibitor activity (GO:0,004,857) (5 proteins), and small GTPase-mediated signal transduction (GO:0,007,264) (5 proteins) (Table S7). GO term enrichment analysis of proteins that were identified in the EBS showed 59 different functionalities reflecting the diverse roles of blood serum in embryonic development. These include, but are not limited to, proteins involved in antioxidant activity, ATPase activity, cellular response to stress, enzyme inhibitor activity, gas transport, homeostatic process, in utero embryonic development, oxygen binding, proteolysis, regulation of apoptosis, response to inorganic substance, response to oxidative stress, response to wounding, and sterol metabolic process (Table S8).

**Figure 3:**
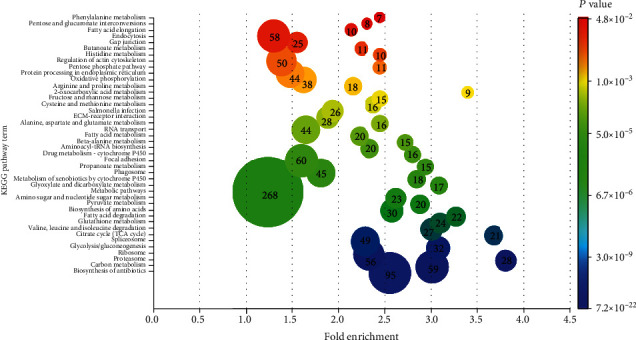
Pathway enrichment of the study proteome. Kyoto Encyclopedia of Genes and Genomes (KEGG) analyses showed 42 enriched terms with a *P* value of <0.05. The number enclosed in the circle and circle size reflects the number of proteins involved in each pathway. The color gradient indicates the significant KEGG pathway with the highest *P* value (Blue highlighted) towards the pathway with the lowest *P* value (red highlighted). The *Y*-axis represents the identified KEGG pathways, while the *X*-axis denotes the fold enrichment score.

**Figure 4:**
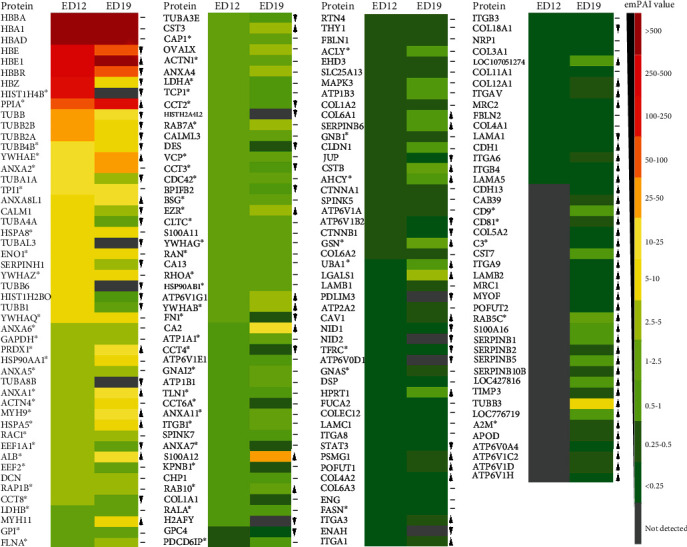
Heat map showing the abundance of proteins (emPAI values) detected in CAM samples, with indication of those present on the Vesiclepedia top 100 EV protein list (^∗^). Up arrow (▲) indicates proteins that were at least 2-fold greater in abundance at ED19 as compared to ED12, while the down arrow (▼) indicates proteins that were at least 2-fold more abundant at ED12 as compared to ED19. The dash (─) indicates proteins that showed similar levels (1.00 to 1.99-fold change) at both time points. The color gradient indicates protein abundance from red (highest) to green (lowest). Proteins not detected in a specific CAM sample are shown as grey.

**Figure 5:**
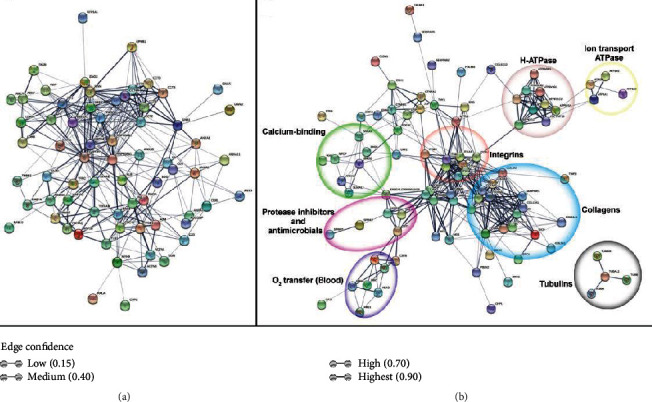
STRING-based protein-protein network analysis. (a) CAM proteins identified in the EV list (71); (b) CAM-specific proteins that are identified at both time points (ED12 and ED19) and attributed to different CAM functions. The thickness of the connection line reflects the edge confidence value, where thin lines indicate low edge confidence while thick lines indicate high edge confidence.

KEGG enrichment analyses of the entire proteomic list revealed 42 significantly enriched pathway terms, including and not limited to metabolic pathways (268 proteins), biosynthesis of antibiotics (95 proteins), focal adhesion (60 proteins), carbon metabolism (59 proteins), endocytosis (58 proteins), ribosomes (56 proteins), regulation of actin cytoskeleton (50 proteins), spliceosomes (49 proteins), protein processing in endoplasmic reticulum (44 proteins), oxidative phosphorylation (38 proteins), ECM-receptor interaction (28 proteins), proteasome (28 proteins), Salmonella infection (26 proteins), gap junction (25 proteins), and fructose and mannose metabolism (15 proteins) (Figure 3). Compared to the list of top 100 protein constituents of extracellular vesicles, we identified 71 CAM proteins (Figure 4), which were used as input for STRING to show all their possible protein-protein interactions (Figure 5(a)). The input of various proteins involved in the different CAM functions for STRING allowed the determination of protein-protein interaction networks, including H-ATPases, ion transport ATPases, calcium-binding, O_2_ transfer, collagens, integrins, tubulins, proteinase inhibitors, and antimicrobials (Figure 5(b)).

**Figure 6:**
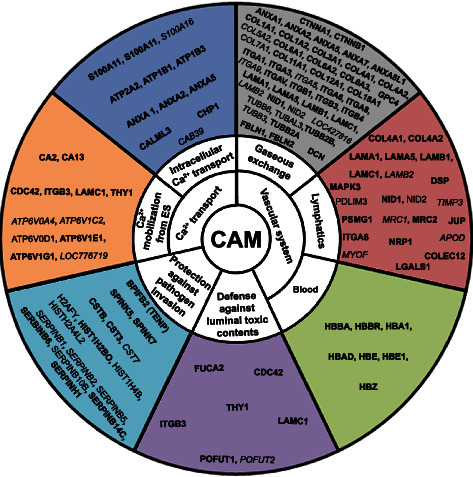
Summary of CAM functions. Ca^+2^ transport: Ca^+2^ mobilization from ES and intracellular Ca^+2^ transport, vascular system (gaseous exchange, lymphatics, and blood), protection against pathogen invasion, and defense against luminal toxic contents, with assignment of proteins (as official gene symbol) identified in this study. Proteins shown in regular font were identified only in CAM ED12, while proteins displayed in italics were solely in CAM ED19. Proteins in bold were identified in CAM tissue samples at both time points (ED12 and ED19).

The association between the protein identified in CAM tissue samples and the underlying functions, including Ca^2+^ transport, protection against pathogen invasion, vascular system, allantoic epithelium, and allantoic fluid, will be discussed in the following section. The emPAI values of CAM proteins involved in these functions and their relative change between ED12 and 19 are presented in Figure 4. The association between proteins identified in CAM samples and various functions is summarized in Figure 6 and table S9.

## 4. Discussion

The CAM of the developing chick embryo CAM has traditionally been used as an angiogenic assay, since it provides a noninnervated rapidly growing vascular bed, which can serve as a surrogate blood supply for organ culture and hence a platform for biomaterial testing [37]. The CAM is also an *in vitro* alternative to the Draize rabbit eye test to assess the irritancy potential of chemicals, since the CAM responds to injury with an inflammatory process similar to that in the rabbit eye's conjunctival tissue [38]. Moreover, the CAM is a suitable model to study drug delivery systems for chemotherapy applications to target cancer cells [39]. However, the protein constituents of this complex organ have not yet been fully characterized; such information is necessary to fully exploit the biomedical potential of this useful *in vitro* assay.

The CAM is the avian homologue of the mammalian placenta [40] and is the main respiratory organ of the chick embryo for about 2 weeks [6, 41, 42]. The growth of the CAM is a dynamic process that lasts for about 12 days of embryo life, starting at ED3 and becoming fully differentiated by ED12 [11, 13, 20]. The mature CAM begins to degrade after ED19 following the initiation of lung respiration [8, 40, 43].

Two time points were selected in the current study to evaluate the protein constituents of the active CAM at different stages of development: ED12, when CAM becomes fully differentiated, and ED19, when it is highly functional as indicated by the maximal daily Ca accumulation by the embryo [44]. The EBS proteome collected at ED19 was determined for comparison to CAM tissue samples, in order to distinguish proteins that are relatively or highly specific to the CAM. In contrast, the collection of blood samples at ED12 was technically challenging, as the embryo size is tiny, and the blood volume until ED12 is less than 100 *μ*L [45, 46]. In addition, it has been shown that the plasma protein composition does not change during embryonic development; rather, the cellular composition and morphology vary at different stages of embryonic development [13, 45, 47, 48]. The CAM main functions include eggshell solubilization, Ca^2+^ and nutrient transport, gaseous exchange, and protection against pathogen invasion [14].

### 4.1. Ca^2+^ Transport

The villus cavity (VC) cells of the chorionic epithelium actively secrete protons to dissolve the inner eggshell [49], while the CC cells are thought to transport the calcium ions (Ca^2+^) necessary for embryo tissue growth and bone mineralization [16, 20, 22]. Over 100 mg of calcium is mobilized from the eggshell (ES) to support skeletal calcification over the course of embryonic development [23]. Calcium dissolution from the ES occurs at the calcium reserve body at the base of the mammillary cones [6, 17]. The Ca^2+^-transport function of the CAM starts at ED10 to 12 and reaches a maximum by ED17. The CAM exhibits biochemical activities associated with calcium transport, including calcium-binding proteins (CaBP), Ca^2+^-activated ATPase, and carbonic anhydrase (CA) [14, 16]. Four different strategies have been proposed to explain the transport of calcium through the chorionic epithelium [50]. The most widely accepted scheme is that calcium transport across the CAM is a three-stage process including the following: (1) mobilization of calcium from the ES, (2) calcium transport through the cytoplasm to the basolateral (blood) side, and (3) expulsion into the circulation [14, 21].

#### 4.1.1. Calcium Mobilization from ES

Vacuolar-type H+-ATPase (V-ATPase) and carbonic anhydrase (CA) present in the VC cells of the chorionic epithelium mediate the mobilization of calcium from ES [16, 49]. In this study, we identified two cytoplasmic isozymes of the CA family with high confidence, CA2 and CA13, in the proteomes of CAM tissues (ED12 and ED19) and the EBS. CA2 was 14-fold more abundant at ED19 as compared to ED12, while CA13 was equally abundant at both time points (Figure 4). CA catalyzes the interconversion of carbon dioxide (CO_2_) and bicarbonate (H^−^CO_3_) [19] and is involved in acid/base regulation, bone resorption, and calcification, gas transport, and ion transport [18, 19]. Cytoplasmic CA generates protons that are pumped via the H^+^-ATPase towards the ES [51]. This results in regional acidification and dissolves eggshell mineral at the bases of the mammillary cones (calcium reserve body). The liberated Ca^2+^ is transferred to the growing embryo [51, 52]. Globally, at least 16 different CA isozymes have been identified [53], which may be cytoplasmic (1, 2, 3, 7, and 13), membrane-associated (4, 9, 12, 14, 15, and 17), mitochondrial (5), secreted (6), or have no known physiological function (8, 10, and 11) [52, 54]. While CA2 and CA4 were previously detected immunohistochemically in the VC cells of the chorionic epithelium [18, 19, 55], we did not identify CA4 in our study.

In this study, functional annotation clustering of proteins relatively specific to CAM tissue samples (common proteins at ED12 and ED19), using DAVID bioinformatics resources, demonstrated the presence of ATP metabolic process functionality (GO:0,046,034) containing the V-ATPase family member, ATPase H+ transporting V1 subunit E1 (*ATP6V1E1*). In addition, we identified another V-ATPase family member, ATPase H^+^ transporting V1 subunit G1 (*ATP6V1G1*). *ATP6V1G1* was 3-fold more abundant in ED19 as compared to ED12, while *ATP6V1E1* was equally abundant at both time points (Figure 4). Furthermore, four V-ATPases were identified at ED19, including ATPase H+ transporting V0 subunit a4 (*ATP6V0A4*), ATPase H^+^ transporting V1 subunit C2 (*ATP6V1C2*), D (*ATP6V1D*), and A-like (LOC776719). Moreover, an additional V-ATPase family member was identified at ED12, the ATPase H+ transporting V0 subunit d1 (*ATP6V0D1*) (Figures 4 and 6). Overall, we identified 10 members of the ATPase H+ transporting complex, of which seven of them were specifically identified in the CAM. The eukaryotic V-ATPases are multisubunit proteins of up to 14 different polypeptides, composed of a peripheral V1 complex (A to H subunits) that hydrolyzes ATP and an integral membrane V0 complex (a to e subunits) that mediates the transport of H^+^ or Na+ [56].

#### 4.1.2. Intracellular Ca^2+^ Transport

The second stage in Ca^2+^ transport is intracellular translocation involving the endocytic internalization of immobilized Ca^2+^ at the apical surface and vesicle-mediated delivery to the basolateral side of the chorion epithelium [57]. In this study, functional annotation clustering of proteins relatively specific to CAM tissue samples (proteins common to ED12 and ED19) demonstrated the presence of ATP metabolic process functionality (GO:0,046,034) containing ATPase sarcoplasmic/endoplasmic reticulum Ca^2+^ transporting 2 (*ATP2A2*) along with ATPase Na+/K+ transporting subunits beta 1 and 3 (*ATP1B1* and *ATP1B3*). *ATP1B3*, *ATP2A2*, and *ATP1B1* showed similar abundance at both time points (Figure 4). Avian ATPase sarcoplasmic/endoplasmic reticulum Ca^2+^ transporting 2 has been shown to pump Ca^2+^ into membrane-bound compartments [58]. Both sodium/potassium and calcium ATPases are required for calcium entry into the CAM vascular system [21]. The term “CaBP” is applied generically to all Ca^2+^- binding proteins [59]. CaBP at the apical surface binds Ca^2+^ and induces adsorptive endocytosis and creation of pinocytic vesicles that contain the CaBP and the membrane-associated Ca^2+^-ATPase [16]. KEGG pathway analysis showed that endocytosis is an enriched term (58 proteins). Calcium isotope studies have revealed that CAM ectodermal cells sequester calcium into endosome-like vesicles during the initial phase of uptake and transport [50]. Alternatively, Ca^2+^ translocation involves calcium-uptake-competent microsomal vesicles containing CaBP at the internal space of the vesicles and integral membrane protein Ca^2+^-ATPase associated with inward translocation of calcium via ATP hydrolysis [57].

In CAM explants, vitamin K elicited a seven to eightfold increase in CaBP activity, which was dose-dependent, inhibited by vitamin K antagonists, and maximal at the developmental stage (13-15 days of incubation) that corresponds to the onset of calcium transport by the CAM *in vivo* [60]. A posttranslational, vitamin K-dependent y-glutamyl carboxylase activity has been reported in the CAM [61, 62]. Although the *γ*-glutamyl carboxylase enzyme was not detected in our proteomic study, we did detect y-glutamyl carboxylation (posttranslational modification) in G protein subunit alpha i1 (GNAI1) and myocardial zonula adherens protein (MYZAP) (ED12), along with heat shock 70 kDa protein 8 (HSPA8) and Splicing factor 3b subunit 3 (SF3B3) (ED19) (Table S3).

#### 4.1.3. Calcium Binding Proteins (CaBPs)

CaBPs control cytoplasmic Ca^2+^ concentration using pumps, channels, sequestering agents, and buffers. In addition, CaBPs include Ca^2+^ transporters and calcium-sensors [63]. Ca^2+^ transporters include calbindins, calretinin (CR), and parvalbumin, while Ca^2+^ sensors include calmodulin [64]. In this study, we identified various calcium-binding proteins (CaPBs) at ED12 and ED19, including annexins 1, 2, and 5 (*ANXA 1*, *ANXA2*, and *ANXA5*), actinin alpha 1 (*ACTN1*), cadherin 1 (*CDH1*), signal transducer and activator of transcription 3 (*STAT3*), integrin subunit alpha 6, 8, and V (*ITGA6*, *ITGA8*, and *ITGAV*), fibulins 1 and 2 (*FBLN1* and *FBLN2*), calmodulin-like 3 (*CALML3*), S100 calcium binding proteins A11 and A12 (*S100A11* and *S100A12*), calcineurin-like EF-hand protein 1 (*CHP1*), collectin subfamily member 12 (*COLEC12*), solute carrier family 25 member 13 (*SLC25A13*), EH domain containing 3 (*EHD3*), and nidogen 1 (*NID1*). In addition, the calcium binding protein 39 (CAB39) was identified in the CAM tissue sample at ED19 (Figure 6). A number of CaBPs were more abundant at ED19 as compared to ED12: *S100 A12* (43-fold), *ITGA6* (14-fold), *CDH1* (5-fold), *ANXA1* (4-fold), and *ITGAV* (4-fold). Others were more abundant at ED12: *NID1* and *STAT3* (3-fold). The remaining proteins were equally abundant at both time points (Figure 4). Ca^2+^ availability in the chorionic epithelium may be correlated with changes in the expression patterns and functions of CaBPs [65]. Intracellular Ca^2+^ mobilization triggers the recruitment of different annexins to membranes in several cell models [66]. Annexins can function as calcium channels, for example, to promote the uptake of the Ca^2+^ required for the formation of intravesicular amorphous calcium carbonate required for ES calcification [67]. Annexins 1, 2, 5, and 6 were previously identified in the chorionic epithelium at ED8 and 12 [65]. CaBPs containing the EF-hand domain are involved mainly in Ca^2+^ sensing functions, including calmodulin, S100, and calcineurin superfamilies [59].

Finally, Ca^2+^-mediated signaling is involved in different cellular machinery ranging from muscle contraction to neurotransmission. An intracellular calcium increase has been shown to trigger plasma membrane EV biogenesis [68]. Most proteins constituting the EV list (71 proteins) were identified in the CAM tissue samples (Figure 4).

### 4.2. Protection against Pathogen Invasion

During embryonic development, the CAM is the second physical barrier after the ES, as its chorionic epithelium is directly applied to the ESM. In addition, the CAM provides chemical protection to the developing embryo due to its inherent antimicrobial protein constituents [14]. In this study, various antimicrobial proteins were detected at both ED12 and 19, including histone H2B (*HIST1H2BO*), cystatins (B and C), SERPINs (*SERPINB6*, *SERPINB14C*, and *SERPINH1*), SPINKs (ovomucoid and ovoinhibitor), and TENP (BPI fold containing family B member 2). *HIST1H2BO* (6-fold), *SERPINH1* (2-fold), and TENP (3-olds) were more abundant at ED12 as compared to ED19, while cystatin B (5-fold), cystatin C (2-fold), and *SERPINB6* (2-fold) were more abundant at ED19 as compared to ED12. *SERPINB14C and* SPINKs showed similar abundance at both time points (Figure 4). In addition, histones H2A (*H2AFY* and *HISTH2A4L2*) and H4 (*HIST1H4B*) were identified at ED12, and cystatin F and SERBINs (*SERPINB1*, *SERPINB2*, *SERPINB5*, and *SERPINB10B*) were detected at ED19 (Figure 6).

#### 4.2.1. Histones

In addition to their nuclear role in chromatin folding [29, 69], histones can also function as cationic antimicrobial peptides (CAMPs), since they are hydrophobic, and cationic and can form amphipathic *α*-helical structures [70, 71]. Histones have been shown to possess potent antimicrobial properties. Chicken histones H1, H2A, and H2B possess broad-spectrum antimicrobial activity suggesting their potential roles in immune defense mechanisms [29]. The histone mixture (H1, H2A, H2B, H3, H4, and H5) isolated from chicken erythrocytes exhibits antimicrobial activity against various Gram-negative and Gram-positive bacteria [29, 70]. Similarly, a mixture of chicken histones (H1, H2A, and H2B) isolated from hen ovary and oviduct displays broad-spectrum antimicrobial activities [72].

#### 4.2.2. Cystatins

The cystatin superfamily exhibits diverse biological activities, including protease inhibition, along with antimicrobial, antiviral, and immunomodulatory properties [73, 74]. Cystatins superfamily can be subdivided into 3 major families; family I (stefins including cystatins A and B), family II (cystatins including cystatins C, D, E, F, G, S, and chicken cystatin), and family III (kininogens) [73, 75]. Cystatin extracted from horse-shoe crab hemocytes showed antimicrobial activity against *Salmonella typhimrium* (*S. typhimrium*), *Escherichia coli* (*E. coli*), and *Klebsiella penumoniae* (*K. penumoniae*) [76]. In addition, chicken and human cystatins (family II) showed bactericidal activity against *Porphyromonas gingivalis* [74]. Furthermore, cystatins A, C, D, and S and chicken cystatin inhibit the replication of certain viruses and bacteria [75]. Moreover, cystatins C and D displayed antiviral activity against coronavirus [77].

#### 4.2.3. Serpins

Serpins are a superfamily of protease inhibitors that can induce the expression of host antimicrobial peptides and cytokines. Serpins also have been shown to exert antimicrobial activity via a membrane disruption mechanism. In addition, noninhibitory serpins such as ovalbumin-related protein X (OVALX) (*SERPINB14C*) possess antibacterial activity [78]. OVALX, unlike ovalbumin (*SERPINB14*), exhibits antibacterial activities against both *Listeria monocytogenes* and *Salmonella enterica* [79]. KEGG pathway analysis showed Salmonella infection as an enriched term (26 proteins), which identified protein constituents known to interact with Salmonella during infection, and underscores the utility of the CAM as a model to evaluate the invasiveness of various bacterial strains including *S*. *typhimurium* [80].

Finally, TENP (*BPIFB2*) [81] and SPINKs, including ovoinhibitor (*SPINK5*) and ovomucoid (*SPINK7*), possess antimicrobial activities [82].

### 4.3. Vascular System

In addition to its respiratory functions, the CAM vasculature contains fully developed lymphatics. The CAM tissue promotes gas exchange via the area vasculosa until ED6 that is gradually replaced by the CAM vessels. The blood vasculature and lymphatics reside within the CAM stroma [11].

#### 4.3.1. Gaseous Exchange

A crucial function of CAM is O_2_-CO_2_ gaseous exchange [5, 15, 24, 34]. The CAM capillary plexus develops into two stages. From ED6 to 10, the capillary network density increases by sprouting from preexisting capillaries. After ED10, the dense capillary network expands as the CAM grows [83]. Our proteomics of CAM tissues identified proteins involved in CAM vascularization, which were functionally categorized as vasculature development (annexin A2, caveolin 1, collagen I *α*1, catenin *β*1, integrin *α*V, neuropilin 1, and reticulon 4), cellular macromolecular complex subunit organization (tubulin (*β*6 class V and *α* like 3) and ENAH actin regulator), structural molecule activity (annexin A1, claudin 1, collagen (I (*α*1 and *α*2), III (*α*1), IV (*α*1 and *α*2), XI (*α*1), XII (*α*1), and XVIII (*α*1)), desmin, laminin (*α*1 and *γ*1), myosin (heavy chain 11, smooth muscle), and tubulin *β* (2A class IIa and 2B class IIb)), and cell substrate adhesion (catenin *β*1, integrin (*α*6, *β*1, and *β*3), laminin *γ*1, and nidogen 1) (Figure 6).

In the chick embryo CAM, we detected various annexins (A1, A2, A5, A7, and A8-like), catenins (*α*1 and *β*1), collagen subunits (1*α*1, 1*α*2, 3*α*1, 4*α*1, 4*α*2, 6*α*1, 6*α*2, 6*α*3,11*α*1,12*α*1, and 18*α*1), decorin, fibulin (1 and 2), glypican 4, integrin subunits (*α*1, *α*3, *α*6, *α*8, *α*V, *β*1, *β*3, and *β*4), laminin subunits (*α*1, *α*5, *β*1, and *γ*1), nidogen 1, and tubulins (*β*2A and *β*2B) in the CAM tissue proteomes at ED12 and ED19. A number of these vasculature-relevant proteins were more abundant at ED19 as compared to ED12: annexin A1 (4-fold), annexin A8-like (2-fold), collagen IV *α*2 chain (2-fold), collagen VI *α*1 chain (2-fold), collagen XII *α*1 chain (5-fold), integrin subunit *α*1 (4-fold), integrin subunit *α*3 (3-fold), integrin subunit *α*V (4-fold), integrin subunit *α*6 (14-fold), integrin subunit *β*4 (4-folds), and laminin subunit *α*5 (2-fold). Others were more abundant at ED12 as compared to ED19: catenin subunit *β*1 (2-fold), collagen XVIII subunit *α*1 (2-fold), glypican 4 (8-fold), laminin subunit *α*1 (2-fold), nidogen 1 (3-fold), tubulin *β*2A (3-fold), and tubulin *β*2B (4-fold). The remaining proteins showed similar abundance at both time points (Figure 4). In addition, collagen subunit 7*α*1, nidogen 2, tubulins (*β* 6 class V, *α* like 3) were present at ED12. Furthermore, collagen subunit 5*α*2, integrin subunits (*α*5 and *α*9), laminin subunit *β*2, tenascin XB, and tubulin *β*3 class III were detected at ED19. Mammalian angiogenesis is regulated by a vast array of structural proteins, including annexins, catenins, collagens, decorin, fibulins, fibronectin, glypican, integrins, laminins, nidogens, tenascins, tubulins, and vitronectin [84–90].

It has been shown that the expression of collagen IV, fibronectin, and laminin in the extracellular matrix (ECM) between the chorionic epithelium and the mesodermal blood promotes the angiogenic process [5, 91]. As discussed earlier, annexins 1, 2, 5, and 6 have been identified in the CAM tissue at ED8 and 12 using immunohistochemistry [65]. Laminin subunits (*α*5, *β*1, and *γ*1) and nidogen (1 and 2) are associated with cellular adhesion at ED15 [6]. Deposition of laminin and collagen I is detected in the CAM tissues as indicated by immunoassays [91, 92]. Nidogen might be associated with the directional migration of cells during chick embryogenesis and may be critical for the formation of most embryonic tissues. Nidogen is detected simultaneously and colocalizes with laminin during embryonic development [93]. The CAM capillary plexus lines the entire inner surface of the ES, which contains approximately 12,000 respiratory pores with an effective pore size of 7.7 *μ*m [41]. At ED16, when the oxygen permeability of the ESM is maximal, the gas exchange area of the CAM capillaries is around 81 cm^2^ [83, 94, 95]. The O_2_-CO_2_ gases are exchanged between the external air and the capillary blood by diffusion through the porous ES and eggshell membrane (ESM) [11, 83]. KEGG pathway analysis revealed that focal adhesion (60 proteins) and gap junction (25 proteins) are enriched terms. Focal adhesions promote endothelial cell adhesion and migration and subsequently angiogenesis [96], while gap junctions (GJ) regulate the vessel diameter [97]. The CAM is a valuable model to assess the vascular-disrupting action of anticancer drugs (combretastatin analogues) via reducing the activity of focal adhesion kinase (FAK) [96]. Moreover, the pharmacological effect of GJ blockers on arterial and venous vessel diameters can be assessed using the CAM model [97].

#### 4.3.2. Lymphatics

The CAM contains a fully developed lymphatic system with high similarities to the mammalian lymphatic system [11]. The CAM is characterized by having a high density of blood and lymphatic vessels, which are localized in the CAM stroma [20]. Various proteins that are involved in the lymphatics structure and functions were identified in the current study including collagen IV, laminins (*LAMA1*, *LAMA5*, *LAMB1*, and *LAMC1*), nidogen 1 (*NID1*), endoglin (*ENG*), galectin 1 (*LGALS1*), mannose receptor C type 2 (*MRC2*), mitogen-activated protein kinases-3 (*MAPK3*), collectin subfamily member 12 (*COLEC12*), desmoplakin (*DSP*), proteosome assembly chaperone 1 (*PSMG1*), junction plakoglobin (*JUP*), integrin subunit *α*6 (*ITGA6*), and neuropilin 1 (*NRP1*) have been identified in the CAM tissue (D12 and D19). A variety of lymphatic system proteins were more abundant at ED19 as compared to ED12: collagen IV *α*2 chain (2-fold), integrin subunit *α*6 (14-fold), laminin subunit *α*5 (2-fold), galectin 1 (13-fold), mannose receptor C type 2 (3-fold), and proteosome assembly chaperone 1 (2-fold). Others were more abundant at ED12 as compared to ED19: laminin subunit *α*1 (2-fold), nidogen 1 (3-fold), and junction plakoglobin (4-fold). The remaining proteins showed similar abundance at both time points (Figure 4). Nidogen 2 (*NID2*) and PDZ and LIM domain 3 (*PDLIM3*) have been detected in CAM (ED12), while laminin (*LAMB2*), apolipoprotein D (*APOD*), mannose receptor C type 1 (*MRC1*), metalloproteinase inhibitor 3 (*TIMP3*), and myoferlin (*MYOF*) have been identified in CAM (ED19) (Figure 6).

Lymphangiogenesis in the CAM takes place between ED5 and 9 [11]. The CAM lymphatics are characterized by a thin endothelial lining, pores, and the absence of a basal lamina [98]. The CAM lymphatic endothelial cells express vascular endothelial growth factor receptor-2 and 3 (VEGFR2 and VEGFR3) [99] along with prospero homeobox protein 1 (PROX1) [11]. Vascular endothelial growth factor-C (VEGF-C) has been shown to induce the development of lymphatics in the CAM [42]. The lymphatics basement membrane is comprised mainly of collagen IV, laminins, perlecan, fibronectin, and nidogen [100]. The structure-function relationship of the lymphatic system is dependent on a vast array of proteins including endoglin [101, 102], galectin 1 [103, 104], mannose receptors [105], mitogen-activated protein kinases-3 [106], collectin subfamily member 12, desmoplakin, nidogen 1, apolipoprotein D, PDZ and LIM domain 3, metalloproteinase inhibitor 3, proteosome assembly chaperone 1, junction plakoglobin, and integrin subunit *α*6 [107], myoferlin [43], and neuropilin 1 [108].

It has been shown that Erk mitogen-activated protein kinases (MAPK3 and MAPK1) maintain lymphatic cell identity [106], while the endoglin-mediated signaling promotes the development of blood and lymphatic vessels [101]. Endoglin is capable of modulating the angiogenic process of the CAM in the developing chicken embryos [101]. Similarly, galectin 1 promotes angiogenesis in the CAM tissue [103]. Galectin-1 is highly expressed by human lymphatic endothelial cells [104]. Mannose receptors have been detected in the lymphatic endothelium and are involved in the fluid-collection function of the lymphatic vasculature [105]. Collectin subfamily member 12, mannose receptor 1, desmoplakin, nidogen 1, apolipoprotein D, PDZ and LIM domain 3, metalloproteinase inhibitor 3, proteosome assembly chaperone 1, junction plakoglobin, and integrin subunit *α*6 are recognized as lymphatic endothelial cell markers [107]. Neuropilin 1 acts as a coreceptor with VEGFR2 for signaling by the VEGF family [108, 109]. Finally, myoferlin was shown to be also expressed in endothelial cells to modulate VEGFR-2 and EGFR signaling by enhancing their stability [43].

#### 4.3.3. Embryonic Blood

In the chicken embryo, blood begins to circulate at ED2 [15]. The serum contains a vast array of distinct proteins actively secreted or leaked from blood cells and tissues. Identifying the protein constituents of serum facilitates the development of innovative protein biomarkers for specific economic traits in livestock species [110].

In previous studies, proteomic analysis of blood serum samples collected from single-comb white Leghorn hens using MALDI-TOF MS (matrix assisted laser desorption ionization (MALDI)-time-of-flight (TOF) mass spectrometry) enabled the identification of 30 proteins [110]. Similarly, 84 proteins were identified in chicken blood serum using 2D LC ESI MS/MS (two-dimensional liquid chromatography electrospray ionization tandem mass spectrometry) [111]. In addition, proteomics profiling of chicken serum using LC-MS/MS after depletion of highly abundant proteins allowed the identification of 146 different proteins [112]. Recently, quantitative proteomics of chicken plasma using LC/MS/MS after lipopolysaccharide challenge identified 418 proteins [113]. In the current study, we identified 791 proteins in the EBS (ED19) using LC/MS/MS (Table S4) which is a greater number than previous proteomics analyses [110–112, 114]. On the other hand, this can be compared to the human serum protein constituents (>4000) identified using proteomic analysis based on the highly sensitive TMT–LC/LC-MS/MS (tandem-mass-tag (TMT) labeling, exhaustive two-dimensional liquid chromatography (LC/LC) fractionation coupled with high-resolution tandem mass spectrometry (MS/MS)) proteomics [115].

In the current study, we identified all *α* and *β* hemoglobin subunits in embryonic serum at ED19 including *π* (*HBZ*), *α*A (*HBA1*), *α*D (*HBAD*), *β*A (*HBBA*), *β*H (*HBE1*), *ε* (HBE), and *ρ* (*HBBR*). Whole blood is composed of 17-18% protein, where hemoglobin (RBCs O_2_-carrier protein) makes up approximately 70% of the total blood proteins. In the plasma fraction, the major proteins are albumin, globulins, and fibrinogen, representing about 56%, 40.2%, and 0.6%, respectively [116]. Vertebrate hemoglobins are generally referred to as *α* or *β* globins [117]. Chicken has three *α* and four *β* globins [118]. All seven hemoglobin subunits, including *α* (*α*A, *α*D, and *π*) and *β* (*β*A, *β*H, *ε*, and *ρ*), have been detected in chicken embryonic blood between ED3 and 17 using ESI-MS/MS [117, 118].

### 4.4. Allantoic Epithelium and Allantoic Fluid

In this study, CA2, CA13, Vacuolar H^+^-ATPase subunits, and annexin 2 were identified in the CAM proteome. CA has been identified histochemically and immunohistochemically at ED13, not only in the MR cells of the endodermal epithelium, but also in the basolateral membrane of adjacent granule cells [19]. As discussed earlier, CA has been identified in the chorionic epithelium. Therefore, the CA 2 and 13 isoforms that we identified in the CAM might be present in either of these layers or both. Vacuolar H^+^-ATPases were previously identified in the MR cells by ED17 [16, 49]. Furthermore, annexin 2 was detected using immunohistochemistry not only in the chorionic epithelium at ED12 but also in the basal cells of the allantoic epithelium facing the allantoic cavity that contains urine [65].

#### 4.4.1. Barrier Function

Enzymes involved in the metabolism of *α*-fucose were detected only in CAM tissue samples, at both ED12 and ED19, including protein O-fucosyltransferase (*POFUT1* and *POFUT1*) and *α*-L-fucosidase 2 (*FUCA2*). Protein O-fucosyltransferase was more abundant at ED19 (2-fold), while *α*-L-fucosidase 2 *showed* similar abundance at both time points (Figure 4). L-fucose (*α*-L-fucose) monosaccharide is a common component of glycoconjugates. Fucosyltransferases and fucosidases are the main enzymes involved in the incorporation and cleavage of L-fucose residues [119]. *POFUT1* and *POFUT2* encode an O-fucosyltransferase that adds fucose directly to polypeptide chains, while *α*-L-fucosidase recycles catabolized fucose-containing glycoconjugates [120]. The allantoic epithelium consists of a thin cell layer that serves as a selectively permeable barrier against the allantoic fluid. It permits the absorption of electrolytes and water while maintaining the toxic contents in the intraluminal space [121]. The presence of *α*-fucose in the CAM epithelium lining the allantoic cavity has been shown to prevent diffusion of toxic contents from the allantoic fluid to the embryo [16]. Moreover, the numerous cell-cell junctions between allantoic cells facing the lumen might explain this barrier function [16]. The allantoic epithelium is composed of three different cell types, including granule-rich cells (glycogen granules), mitochondria-rich (MR) cells, and basal cells [16, 65]. The MR cells are responsible for the progressive acidification of the allantoic fluid occurring during incubation. This apical extrusion of H^+^, analogous to a kidney mechanism, probably involves H^+^-ATPase pump and exchange with Na^+^ [19, 49]. Active transport of Na^+^ is thought to account for the gradual reabsorption of water from the allantoic fluid that is made available by concomitant urate precipitation. CA of the MR cells might also mediate bicarbonate reabsorption from the allantoic cavity that contributes to the ability of the embryo to buffer acid generated by metabolic processes [19].

Regarding cell-cell junction, functional annotation clustering of proteins at ED12 and ED19 indicated the presence of cell junction organization functionalities including cell division cycle 42 (*CDC42*), integrin *β*3 subunit (*ITGB3*), laminin *γ*1 subunit (*LAMC1*), and thy-1 cell surface antigen (*THY1*). All these proteins showed similar abundance at both time points (Figure 4). Cell-cell junction proteins might be involved in the establishment of apical tight junctions concomitant to the initiation of calcium transport across the chorionic epithelium [16] (Figure 6). STRING analysis of proteins involved in the different CAM functions identified interaction networks including H^+^-ATPases, ion transport ATPases, calcium-binding, O_2_ transfer, collagens, integrins, tubulins, proteinase inhibitors, and antimicrobials (Figure 4(b)).

#### 4.4.2. Waste Reservoir Function

The allantois serves as a reservoir for the waste products excreted by the embryo, mainly urea first and then uric acid later [24, 99, 122, 123]. In birds, uric acid is generated via the uric acid cycle. It is the excretory product of purine and amino acid metabolism. Hypoxanthinephosphoribosyl transferase (*HPRT1*) catalyzes the conversion of inosine monophosphate to hypoxanthine, which is then oxidized to xanthine and subsequently uric acid under the action of the enzyme xanthine oxidase (*XO*) [124]. The accumulation of uric acid in the allantoic fluid after ED13 leads to a decrease in its pH [52]. Although *XO* was not identified in the current study, *HPRT1* was detected in CAM at both time points (ED12 and 19), but not EBS, and was 4-fold more abundant at ED19 (Figure 4). Uric acid conversion to urates is combined with selective transport of sodium and chloride across the allantoic inner membrane from the allantoic fluid to the plasma on ED13 [16, 125]. This conversion is associated with a decrease in allantoic fluid osmolality and the creation of osmotic gradients to promote water flow to the blood stream and subsequently to the embryo [126]. In addition, the allantois is involved in the absorption of albumen [127]. Fluid accumulation expands the allantois such that its terminal portion resembles a balloon in embryos [99]. The endodermal (allantoic) epithelium lines the allantoic cavity and regulates water and electrolyte transport from its lumen [19].

## 5. Conclusion

The CAM is a transitional model between *in vitro* and *in vivo* studies and is increasingly utilized for cancer research, drug delivery, and toxicologic analysis, along with assessment of bacterial invasion. Two time points were selected in the current study to evaluate the protein constituents of the active CAM at different stages of development: ED12, when CAM becomes fully differentiated, and ED19, function when it is fully functional. This approach allowed the identification of 1470, 1445, and 791 proteins in CAM (D12), CAM (D19), and EBS, respectively. Of these, 175, 177, and 105 were highly specific to CAM (ED12), CAM (ED19), and EBS, respectively, while 653 proteins were detected in the CAM at both time points. The identified proteins include a large array of protein superfamilies with different functionalities that are related to CAM physiology. This proteomic approach, followed by functional annotation clustering, facilitated the identification of proteins directly responsible for CAM functionalities. This applied research strategy has helped to identify crucial CAM constituents deployed for calcium ion transport (ATPases, CaBPs, and carbonic anhydrases), gas exchange (annexins, hemoglobins, and tubulins), vasculature development (collagens, integrins, and laminins), and chemical protection against invading pathogens (cystatins, histones, and serpins). KEGG analysis highlighted the enriched terms of endocytosis, Salmonella infection, focal adhesion, and gap junctions. This study highlights the complex nature of the CAM proteome and identifies specific proteins responsible for its functionalities, in order to extend its biomedical applications for pharmaceutical and cancer research.

## Data Availability

The mass spectrometry proteomics data have been deposited to the ProteomeXchange Consortium via the PRIDE [128, 129] partner repository with the dataset identifier PXD027129, project name: Chorioallantoic membrane (CAM) and embryonic blood serum proteomics, project accession: PXD027129, username: reviewer_pxd027129@ebi.ac.uk, and password: gYFlommi. All processed data is available within the article. This article contains supplemental material: Supplementary tables 1-9 and two supplementary figures.

## Additional Points

*Significance Statement.* The chick embryo chorioallantoic membrane (CAM) is a valuable *in vivo* model in cancer research and to study tissue grafts, tumor growth and metastasis, drug delivery and toxicologic analysis, and angiogenic and antiangiogenic molecules. Comparative proteomic analyses of the CAM at two developmental stages (ED12 and ED19) revealed specific CAM functions that include gaseous exchange, Ca^2+^ transport, vasculature development, and protection against pathogen invasion. KEGG analysis highlighted the enriched terms of endocytosis, Salmonella infection, focal adhesion, and gap junctions. Overall, our results highlight the structure-function relationship of the CAM protein constituents that potentially could expand its biomedical applications.

## Supplementary Materials

Supplementary 1 Timing of chick embryo development phases and associated critical events.

Supplementary 2 Sample description and sample protein/peptide reports.

Supplementary 3 Posttranscriptional modifications (PTMs) abundance in CAM and EBS samples expressed as total spectral count and emPAI values. Detected PTMs included carboxylation of glutamic acid (E) and phosphorylation of serine and threonine (S and T) amino acids.

Supplementary 4 List of proteins identified in this study, for CAM proteomes (ED12 and ED19) and embryonic blood serum proteome (EBS).

Supplementary 5 Protein families using Uniprot.

Supplementary 6 Functional annotation analysis of the identified CAM protein constituents common to ED12 and ED19 and absent from EBS.

Supplementary 7 Functional annotation analysis of the identified CAM protein constituents specific to either ED12 or ED19 and absent from EBS.

Supplementary 8 Functional annotation analysis of the identified EBS protein constituents.

Supplementary 9 Various CAM functions and the associated proteins identified in this study.

Supplementary 10 Electrophoretic pattern of CAM and EBS samples, showing position of bands excised from NuPAGE 4–12% Bis-Tris gels and sent for proteomic analysis. All samples were run for 35 min, followed by Coomassie blue staining.

Supplementary 11 Electrophoretic pattern showing variability of samples pooled to produce the final CAM samples (D12 and D19) that were processed for proteomics.
