# Supplementary material for: Proteomic Analysis of Chicken Chorioallantoic Membrane (CAM) during Embryonic Development Provides Functional Insight
Source: Biomed Res Int. 2022 Jun 19;2022:7813921. doi: 10.1155/2022/7813921 (PMC9237712; doi:10.1155/2022/7813921)
Supplement: Supplementary 1 — Timing of chick embryo development phases and associated critical events. [file 7813921.f1.pdf]

Table S1. Timing of chick embryo development phases and associated critical events.

Embryonic development stage	Embryonic Event	Embryonic days (ED)																				
		1	2	3	4	5	6	7	8	9	10	11	12	13	14	15	16	17	18	19	20	21
Establishment of germ layers	Configuration of body plans	■																				
	Development of the vascular system and immunity	■	■	■	■	■	■	■	■	■	■	■	■	■	■	■	■	■	■			
	Development of yolk sac vasculature		■	■	■	■	■	■	■													
	Development of cardiac structures and functions		■	■	■	■	■	■	■													
	Development of heart, gonads, and limb buds			■	■	■	■	■														
	Development of liver			■	■	■	■	■	■	■												
	Evolution of chorioallantoic membrane (CAM)			■	■	■	■	■	■	■	■	■	■	■	■							
	Expansion of the CAM vasculature				■	■	■	■	■	■	■	■	■	■	■	■	■	■	■			
	Development of spleen				■	■	■															
	Mesonephric kidney starts discharging				■	■																
	Attachment of CAM to eggshell membrane				■	■																
	Start of the eye pigmentation				■																	
	Development of thymus					■	■	■														
	The appearance of chicken combs						■	■														
	Initiation of sex determination						■															
	The emergence of early feather buds							■														
Embryo completion	Progressive development of feathers and toes								■	■	■	■	■									
	The gradual development of eyelids and peaks								■	■	■	■	■									
	The emergence of T and B immune cells											■	■									
	Acceleration of heart, liver, and brain growth												■	■	■	■	■	■				
	Chick embryo is structurally complete														■	■						
	Feathers become visible															■						
	The embryo head faces the air sac															■	■	■				
Emergence	CAM vasculature reaches maximum volume																		■			
	Internal pipping																			■	■	
	Initiation of lung respiration																			■	■	
	The disappearance of the albumen and yolk sac																			■	■	
	Initiation of CAM degeneration																			■	■	
	Accumulation of glycogen																			■	■	■
	Oral consumption of amnion																			■	■	■
	External pipping and hatching																					■
