# Supplementary material for: Proteomic Analysis of Chicken Chorioallantoic Membrane (CAM) during Embryonic Development Provides Functional Insight
Source: Biomed Res Int. 2022 Jun 19;2022:7813921. doi: 10.1155/2022/7813921 (PMC9237712; doi:10.1155/2022/7813921)
Supplement: Supplementary 3 — Posttranscriptional modifications (PTMs) abundance in CAM and EBS samples expressed as total spectral count and emPAI values. Detected PTMs included carboxylation of glutamic acid (E) and phosphorylation of serine and threonine (S and T) amino acids. [file 7813921.f3.pdf]

Table S3. Posttranscriptional modifications (PTMs) abundance in CAM and EBS samples expressed as total spectral count and emPAI values. Detected PTMs included carboxylation of glutamic acid (E) and phosphorylation of serine and threonine (S, T) amino acids.

PTM / Protein Name	Official gene symbol	Gene ID	PTM total spectral count (emPAI)			General total spectral count (emPAI)		
			CAM (ED12)	CAM (ED19)	EBS	CAM (ED12)	CAM (ED19)	EBS
**γ-carboxylation**								
Heat shock 70kDa protein 8<br>RMVNHFIAEFKRK	HSPA8	395853	0(0)	4(0.117)	0(0)	105(6.1)	89(6.5)	61(3.0)
Splicing factor 3b subunit 3<br>KQKNVAEELDRT	SF3B3	415680	0(0)	1(0.019)	ND	15(0.2)	20(0.3)	ND
Centriolar coiled-coil protein 110<br>RVQEILENIQNRK	CCP110	416613	ND	ND	1(0.136)	ND	ND	1(0.1)
G protein subunit alpha i1<br>KIIHEAGYSEEECKQYKA	GNAI1	396368	1(0.069)	0(0)	ND	10(0.5)	10(0.5)	ND
Myocardial zonula adherens protein<br>KLAAQKLAERL	MYZAP	415404	1(0.067)	ND	ND	1(0.1)	ND	ND
**Phospho-serine / phospho-threonine**								
Ovalbumin (SERPINB14)<br>RFDKLPGFGDSIEAQCGTSVNVHSSLRD<br>REVVGSAEAGVDAASVSEEFRA	OVAL	396058	2(0.197)	12(0.209)	0(0)	400(54.2)	266(32.6)	34(2.0)
Heat shock protein 90 alpha family class A member 1<br>KTEDKPEIEDVGSDEEEEKKDGDKK	HSP90AA1	423463	0(0)	3(0.032)	0(0)	106(2.7)	94(3.3)	22(1.1)
Hepatoma-derived growth factor<br>RKAEDVLEDSPKRT	HDGF	776936	2(0.157)	0(0)	0(0)	8(0.6)	10(1.1)	48(10.2)
Heterogeneous nuclear ribonucleoprotein A3<br>RRSSQGHEPKEPEQLRK	HNRNPA3	100859627	0(0)	0(0)	3(0.132)	27(0.5)	16(0.4)	14(2.0)
Fibrinogen beta chain<br>-ASVEYDNEEDSPQIDARA	FGB	373926	0(0)	0(0)	1(0.098)	2(0.1)	10 (0.4)	36(3.4)
Calcium/calmodulin dependent protein kinase II delta<br>KESTESSNTTIEDEDVKARK	CAMK2D	422688	0(0)	1(0.049)	ND	11(0.2)	5(0.2)	ND
COP9 signalosome subunit 4<br>KLETYLKIARL	COPS4	422594	0(0)	1(0.059)	0(0)	2(0.1)	2(0.1)	9(0.6)
HYDIN, axonemal central pair apparatus protein<br>KAFVTQEEEDQQKLRL	HYDIN	427538	ND	1(0.005)	ND	ND	1(0.0)	ND
Growth differentiation factor 11<br>RLESIKSQILSKL	GDF11	107049246	ND	ND	1(0.124)	ND	ND	1(0.1)

ND, protein/peptide not detected in this sample.
0, PTM peptide not detected in this sample, which however contains non-PTM peptides.
Grey highlighted residues represent the modified amino acids.
