# Supplementary material for: Proteomic Analysis of Chicken Chorioallantoic Membrane (CAM) during Embryonic Development Provides Functional Insight
Source: Biomed Res Int. 2022 Jun 19;2022:7813921. doi: 10.1155/2022/7813921 (PMC9237712; doi:10.1155/2022/7813921)
Supplement: Supplementary 4 — List of proteins identified in this study, for CAM proteomes (ED12 and ED19) and embryonic blood serum proteome (EBS). [file 7813921.f4.pdf]

Table S4. List of proteins identified in this study, for CAM proteomes (ED12 and ED19) and embryonic blood serum proteome (EBS).

No.	Protein Name	Official gene symbol	Gene ID	CAM (ED12)	CAM (ED19)	Embryonic blood serum
1	1,4-alpha-glucan branching enzyme 1	GBE1	427964	NO	YES	NO
2	1-acylglycerol-3-phosphate O-acyltransferase 2	AGPAT2	772114	YES	YES	NO
3	2',3'-cyclic nucleotide 3' phosphodiesterase	CNP	395921	YES	YES	NO
4	2,4-dienoyl-CoA reductase 1, mitochondrial	DECR1	420218	YES	YES	NO
5	2,4-dienoyl-CoA reductase 2, peroxisomal	DECR2	100858408	YES	NO	NO
6	3-hydroxyacyl-CoA dehydratase 3	HACD3	415539	YES	YES	NO
7	3-hydroxybutyrate dehydrogenase, type 1 A	BDH1A	424891	YES	NO	NO
8	3-hydroxybutyrate dehydrogenase, type 2	BDH2	422715	YES	YES	NO
9	3-hydroxyisobutyrate dehydrogenase	HIBADH	420632	YES	YES	NO
10	3-hydroxyisobutyryl-CoA hydrolase	HIBCH	423979	NO	YES	NO
11	3-oxoacid CoA-transferase 1	OXCT1	427190	YES	YES	YES
12	3-phosphoinositide dependent protein kinase 1	PDPK1	416588	NO	YES	NO
13	4-aminobutyrate aminotransferase	ABAT	416642	NO	YES	NO
14	5-aminoimidazole-4-carboxamide ribonucleotide formyltransferase/IMP cyclohydrolase	ATIC	396091	YES	YES	YES
15	5'-nucleotidase domain containing 2	NT5DC2	415895	YES	YES	YES
16	5'-nucleotidase, cytosolic IB	NT5C1B	421954	YES	YES	NO
17	5'-nucleotidase, cytosolic II	NT5C2	423871	YES	YES	NO
18	6-phosphogluconolactonase	PGLS	430442	YES	YES	YES
19	Acetyl-CoA acetyltransferase 1	ACAT1	418968	YES	YES	YES
20	Acetyl-CoA acetyltransferase 2	ACAT2	421587	YES	YES	YES
21	Acetyl-CoA acyltransferase 1	ACAA1	770094	YES	YES	NO
22	Acetyl-CoA acyltransferase 2	ACAA2	426847	YES	YES	YES
23	Acid phosphatase 1, soluble	ACP1	421909	YES	YES	YES
24	Acidic (leucine-rich) nuclear phosphoprotein 32 family, member B	ANP32B	420087	YES	YES	NO
25	Acidic nuclear phosphoprotein 32 family member A	ANP32A	415562	YES	YES	YES
26	Acidic nuclear phosphoprotein 32 family member E	ANP32E	426109	YES	YES	YES
27	Acireductone dioxygenase 1	ADI1	421918	YES	YES	NO
28	Aconitase 1, soluble	ACO1	373916	YES	YES	YES
29	Aconitase 2	ACO2	374009	YES	YES	NO
30	Actin like 6A	ACTL6A	424975	YES	NO	YES
31	Actin related protein 2/3 complex subunit 1A	ARPC1A	770269	YES	YES	YES
32	Actin related protein 2/3 complex subunit 1B	ARPC1B	416490	YES	YES	NO
33	Actin related protein 2/3 complex subunit 2	ARPC2	429041	YES	YES	NO
34	Actin related protein 2/3 complex subunit 3	ARPC3	416837	YES	YES	YES
35	Actin related protein 2/3 complex subunit 4	ARPC4	416051	YES	YES	YES
36	Actin related protein 2/3 complex subunit 5	ARPC5	429075	YES	YES	YES
37	Actin related protein 2/3 complex subunit 5 like	ARPC5L	101752218	YES	YES	YES
38	Actin, alpha 2, smooth muscle, aorta	ACTA2	423787	YES	YES	NO
39	Actin, alpha, cardiac muscle 1	ACTC1	423298	NO	YES	YES
40	Actin, beta-like 2	ACTBL2	426652	YES	NO	YES
41	Actin, gamma 1	ACTG1	415296	YES	YES	YES
42	Actinin, alpha 1	ACTN1	373918	YES	YES	NO
43	Actinin, alpha 4	ACTN4	396024	YES	YES	YES

44	Activated leukocyte cell adhesion molecule	ALCAM	396092	YES	NO	NO
45	Activated RNA polymerase II transcriptional coactivator p15-like	LOC107055444	107055444	YES	YES	NO
46	Acylaminoacyl-peptide hydrolase	APEH	415926	YES	NO	YES
47	Acyl-CoA binding domain containing 3	ACBD3	421317	YES	YES	NO
48	Acyl-CoA dehydrogenase family member 9	ACAD9	415974	YES	YES	NO
49	Acyl-CoA dehydrogenase, C-2 to C-3 short chain	ACADS	416969	NO	YES	NO
50	Acyl-CoA dehydrogenase, long chain	ACADL	424005	YES	YES	NO
51	Acyl-CoA dehydrogenase, short/branched chain	ACADSB	423947	YES	NO	NO
52	Acyl-CoA synthetase family member 2	ACSF2	422102	NO	YES	NO
53	Acyl-CoA synthetase long-chain family member 1	ACSL1	422547	YES	YES	NO
54	Acyl-CoA synthetase short-chain family member 1A	ACSS1A	416714	YES	YES	YES
55	Acyl-CoA synthetase long chain family member 3	ACSL3	424810	YES	YES	NO
56	Acyl-CoA thioesterase 7	ACOT7	419371	YES	NO	NO
57	Acyl-CoA thioesterase 9	ACOT9	418600	YES	YES	NO
58	Acyl-CoA thioesterase 13	ACOT13	421006	NO	YES	NO
59	Adaptor protein, phosphotyrosine interacting with PH domain and leucine zipper 2	APPL2	418075	NO	YES	NO
60	Adaptor related protein complex 1 gamma 1 subunit	AP1G1	415880	NO	YES	NO
61	Adaptor related protein complex 1 mu 1 subunit	AP1M1	420149	YES	YES	NO
62	Adaptor related protein complex 2 alpha 2 subunit	AP2A2	423102	YES	YES	NO
63	Adaptor related protein complex 2 beta 1	AP2B1	417525	YES	YES	NO
64	Adaptor related protein complex 2 mu 1 subunit	AP2M1	770246	YES	YES	NO
65	Adducin 1	ADD1	422882	YES	NO	YES
66	Adenine phosphoribosyltransferase	APRT	100857155	YES	YES	YES
67	Adenosine deaminase	ADA	419194	YES	YES	YES
68	Adenosine deaminase like	ADAL	415391	NO	NO	YES
69	Adenosine kinase	ADK	423735	YES	YES	YES
70	Adenosine monophosphate deaminase 3	AMPD3	423041	NO	NO	YES
71	Adenosylhomocysteinase	AHCY	419146	YES	YES	YES
72	Adenosylhomocysteinase like 1	AHCYL1	419803	YES	YES	NO
73	Adenylate cyclase associated protein 1	CAP1	419680	YES	YES	YES
74	Adenylate kinase 1	AK1	396002	YES	YES	NO
75	Adenylate kinase 2	AK2	428227	YES	YES	YES
76	Adenylosuccinate lyase	ADSL	396540	YES	NO	YES
77	Adenylosuccinate synthase 1	ADSS1	776127	YES	NO	YES
78	Adenylosuccinate synthase 2	ADSS2	428579	YES	NO	NO
79	Adiponectin, C1Q and collagen domain containing	ADIPOQ	404536	NO	YES	NO
80	ADP dependent glucokinase	ADPGK	415317	YES	YES	NO
81	ADP ribosylation factor 3	ARF3	107049507	YES	YES	NO
82	ADP ribosylation factor 4	ARF4	769725	YES	YES	YES
83	ADP ribosylation factor GTPase activating protein 3	ARFGAP3	418224	YES	NO	NO
84	ADP ribosylation factor like GTPase 1	ARL1	417934	YES	YES	NO
85	ADP ribosylation factor like GTPase 8B	ARL8B	416109	YES	YES	NO
86	ADP-ribosylation factor 6	ARF6	428927	YES	YES	NO
87	AE binding protein 1	AEBP1	100859431	NO	YES	NO
88	Agrin	AGRN	396538	NO	YES	NO
89	AHNAK nucleoprotein 2	AHNAK2	100859120	YES	YES	NO
90	A-kinase anchoring protein 12	AKAP12	421634	YES	YES	NO
91	AKT serine/threonine kinase 1	AKT1	395928	YES	YES	NO

92	Alanyl aminopeptidase, membrane	ANPEP	395667	NO	YES	NO
93	Alanyl-tRNA synthetase	AARS	415668	NO	NO	YES
94	Alanyl-tRNA synthetase 1	AARS1	415668	YES	NO	NO
95	Albumin	ALB	396197	YES	YES	YES
96	Alcohol dehydrogenase 1C (class I), gamma polypeptide	ADH1C	771920	YES	YES	NO
97	Alcohol dehydrogenase 4 (class II), pi polypeptide	ADH4	422705	YES	YES	YES
98	Aldehyde dehydrogenase 1 family member A3	ALDH1A3	395389	YES	YES	NO
99	Aldehyde dehydrogenase 2 family (mitochondrial)	ALDH2	416880	YES	YES	YES
100	Aldehyde dehydrogenase 3 family member A2	ALDH3A2	417615	YES	YES	NO
101	Aldehyde dehydrogenase 3 family, member B2	ALDH3B2	428813	YES	YES	NO
102	Aldehyde dehydrogenase 4 family member A1	ALDH4A1	419467	YES	YES	NO
103	Aldehyde dehydrogenase 5 family member A1	ALDH5A1	420818	YES	YES	NO
104	Aldehyde dehydrogenase 6 family member A1	ALDH6A1	423345	YES	YES	NO
105	Aldehyde dehydrogenase 7 family member A1	ALDH7A1	426812	YES	YES	NO
106	Aldehyde dehydrogenase 9 family member A1	ALDH9A1	424405	YES	YES	YES
107	Aldehyde dehydrogenase 18 family member A1	ALDH18A1	423976	YES	NO	NO
108	Aldo-keto reductase family 1 member A1	AKR1A1	424599	YES	YES	YES
109	Aldo-keto reductase family 1, member B10	AKR1B1	418169	YES	YES	YES
110	Aldo-keto reductase family 1 member B10	AKR1B10	395338	YES	YES	NO
111	Aldo-keto reductase family 1, member B1-like	LOC425137	425137	YES	YES	NO
112	Aldo-keto reductase family 1 member B10-like	LOC418170	418170	YES	YES	YES
113	Aldo-keto reductase family 7 member A2	AKR7A2	419471	YES	YES	NO
114	Aldolase, fructose-bisphosphate B	ALDOB	427308	YES	YES	YES
115	Aldolase, fructose-bisphosphate C	ALDOC	395492	YES	YES	YES
116	ALG12, alpha-1,6-mannosyltransferase	ALG12	417733	YES	YES	NO
117	ALG14, UDP-N-acetylglucosaminyltransferase subunit	ALG14	424484	YES	YES	NO
118	Alpha 2-HS glycoprotein	AHSG	424956	NO	YES	YES
119	Alpha fetoprotein	AFP	422652	YES	YES	YES
120	Alpha-1-microglobulin/bikunin precursor	AMBP	770795	YES	YES	YES
121	Alpha-2-macroglobulin	A2M	418251	NO	YES	YES
122	Alpha-2-macroglobulin-like 1	A2ML1	418254	YES	YES	NO
123	Alpha-2-macroglobulin-like 2	A2ML2	427942	NO	YES	YES
124	Alpha-2-macroglobulin-like 4	A2ML4	100858010	NO	NO	YES
125	Alpha-L-fucosidase 2	FUCA2	421668	YES	YES	NO
126	Alpha-N-acetylgalactosaminidase	NAGA	396547	YES	YES	NO
127	Aly/REF export factor	ALYREF	769169	YES	NO	YES
128	Amine oxidase, copper containing 3	AOC3	420015	YES	YES	NO
129	Aminoacyl tRNA synthetase complex interacting multifunctional protein 1	AIMP1	422533	YES	YES	YES
130	Aminoacylase 1	ACY1	100858836	NO	YES	NO
131	Aminoadipate-semialdehyde synthase	AASS	417757	YES	YES	NO
132	Aminolevulinate dehydratase	ALAD	417273	YES	YES	YES
133	Aminomethyltransferase	AMT	395566	NO	YES	NO
134	Aminopeptidase puromycin sensitive	NPEPPS	426231	YES	YES	YES
135	Aminopeptidase-like 1	NPEPL1	419314	YES	YES	YES
136	Amylo-alpha-1, 6-glucosidase, 4-alpha-glucanotransferase	AGL	424474	NO	YES	NO
137	Angiotensinogen	AGT	421543	YES	YES	YES
138	Ankyrin 1	ANK1	396311	YES	YES	YES
139	Ankyrin repeat and FYVE domain containing 1	ANKFY1	417494	NO	NO	YES

140	Annexin A1	ANXA1	404271	YES	YES	NO
141	Annexin A2	ANXA2	396297	YES	YES	NO
142	Annexin A4	ANXA4	772393	YES	YES	YES
143	Annexin A5	ANXA5	428767	YES	YES	NO
144	Annexin A6	ANXA6	395481	YES	YES	YES
145	Annexin A7	ANXA7	423747	YES	YES	NO
146	Annexin A8-like	ANXA8L1	423774	YES	YES	NO
147	Annexin A11	ANXA11	423637	YES	YES	YES
148	Anterior gradient 2, protein disulphide isomerase family member	AGR2	420596	YES	YES	NO
149	Apolipoprotein A1	APOA1	396536	YES	YES	YES
150	Apolipoprotein A4	APOA4	395780	YES	YES	YES
151	Apolipoprotein A-I binding protein	APOA1BP	426569	YES	YES	NO
152	Apolipoprotein A-II	APOA2	100858842	NO	YES	YES
153	Apolipoprotein B	APOB1	396535	YES	YES	YES
154	Apolipoprotein D	APOD	424893	NO	YES	NO
155	Apolipoprotein H	APOH	417431	NO	YES	YES
156	Apolipoprotein O	APOO	418599	YES	NO	NO
157	Apoptosis inhibitor 5	API5	423168	YES	YES	YES
158	Apoptotic peptidase activating factor 1	APAF1	417926	NO	NO	YES
159	Apurinic/apyrimidinic endodeoxyribonuclease 1	APEX1	100431102	YES	YES	YES
160	Aquaporin 3	AQP3	426894	YES	YES	NO
161	Archain 1	ARCN1	770561	YES	YES	YES
162	ArfGAP with coiled-coil, ankyrin repeat and PH domains 2	ACAP2	424895	YES	NO	NO
163	Argininosuccinate lyase 2	ASL2	417545	YES	YES	NO
164	Arginyl aminopeptidase	RNPEP	421165	YES	YES	NO
165	Arginyl-tRNA synthetase	RARS	416168	YES	YES	YES
166	Argonaute 1, RISC catalytic component	AGO1	428222	YES	NO	YES
167	Argonaute 3, RISC catalytic component	AGO3	419628	NO	YES	NO
168	ARP1 actin-related protein 1 homolog A, centractin alpha	ACTR1A	423862	YES	YES	YES
169	ARP2 actin related protein 2 homolog	ACTR2	396147	YES	YES	YES
170	ARP3 actin related protein 3 homolog	ACTR3	374197	YES	YES	YES
171	ArsA arsenite transporter, ATP-binding, homolog 1 (bacterial)	ASNA1	100859771	YES	YES	YES
172	Arsenite methyltransferase	AS3MT	423870	YES	YES	NO
173	Asparagine synthetase [glutamine-hydrolyzing]	ASNS	420574	YES	YES	NO
174	Asparaginyl-tRNA synthetase	NARS	426856	YES	YES	NO
175	Aspartyl aminopeptidase	DNPEP	424200	YES	YES	YES
176	Aspartyl-tRNA synthetase	DARS	424296	YES	YES	YES
177	Astacin like metalloendopeptidase	ASTL	423176	YES	NO	NO
178	Atlastin GTPase 1	ATL1	423577	YES	YES	NO
179	ATP binding cassette subfamily B member 10	ABCB10	421537	YES	NO	YES
180	ATP binding cassette subfamily E member 1	ABCE1	422462	YES	YES	YES
181	ATP citrate lyase	ACLY	395373	YES	YES	YES
182	ATP synthase mitochondrial F1 complex assembly factor 2	ATPAF2	416511	YES	YES	NO
183	ATP synthase peripheral stalk-membrane subunit b	ATP5PB	419866	YES	YES	NO
184	ATP synthase, H+ transporting, mitochondrial F1 complex, alpha subunit 1, cardiac muscle	ATP5A1W	431564	YES	YES	YES
185	ATP synthase, H+ transporting, mitochondrial F1 complex, beta polypeptide	ATP5B	426673	YES	YES	YES
186	ATP synthase, H+ transporting, mitochondrial F1 complex, gamma polypeptide 1	ATP5C1	419108	YES	YES	NO

187	ATP synthase, H+ transporting, mitochondrial F1 complex, O subunit	ATP5O	418508	YES	YES	NO
188	ATP synthase, H+ transporting, mitochondrial Fo complex subunit D	ATP5H	422115	YES	YES	NO
189	ATPase H+ transporting V0 subunit a4	ATP6V0A4	418104	NO	YES	NO
190	ATPase H+ transporting V0 subunit d1	ATP6V0D1	415674	YES	NO	NO
191	ATPase H+ transporting V1 subunit A	ATP6V1A	395821	YES	YES	YES
192	ATPase H+ transporting V1 subunit B2	ATP6V1B2	395497	YES	YES	YES
193	ATPase H+ transporting V1 subunit C2	ATP6V1C2	421939	NO	YES	NO
194	ATPase H+ transporting V1 subunit D	ATP6V1D	423280	NO	YES	NO
195	ATPase H+ transporting V1 subunit E1	ATP6V1E1	418162	YES	YES	NO
196	ATPase H+ transporting V1 subunit G1	ATP6V1G1	417249	YES	YES	NO
197	ATPase H+ transporting V1 subunit H	ATP6V1H	426199	NO	YES	YES
198	ATPase Na+/K+ transporting subunit alpha 1	ATP1A1	396530	YES	YES	YES
199	ATPase Na+/K+ transporting subunit beta 1	ATP1B1	396529	YES	YES	NO
200	ATPase Na+/K+ transporting subunit beta-3	ATP1B3	396549	YES	YES	NO
201	ATPase plasma membrane Ca2+ transporting 4	ATP2B4	419934	YES	YES	YES
202	ATPase sarcoplasmic/endoplasmic reticulum Ca2+ transporting 2	ATP2A2	396446	YES	YES	NO
203	Attachment region binding protein	ARBP	395523	YES	NO	NO
204	AU RNA binding methylglutaconyl-CoA hydratase	AUH	427269	YES	YES	NO
205	Autophagy related 3	ATG3	418369	NO	NO	YES
206	Basic leucine zipper and W2 domains 1	BZW1	424073	YES	YES	NO
207	Basic leucine zipper and W2 domains 2	BZW2	420594	YES	YES	NO
208	Basigin (Ok blood group)	BSG	770363	YES	YES	YES
209	B-cell receptor associated protein 29	BCAP29	417702	YES	YES	NO
210	BCL2 associated athanogene 2	BAG2	421880	YES	NO	NO
211	BCL2 interacting protein 1	BNIP1	416207	YES	NO	NO
212	BCL2 like 1	BCL2L1	373954	NO	NO	YES
213	BCL2 like 15	BCL2L15	772088	YES	YES	NO
214	Beta-arrestin 1	LOC107051449	107051449	YES	YES	NO
215	BH3 interacting domain death agonist	BID	395236	YES	YES	YES
216	Bile acid-CoA: amino acid N-acyltransferase	BAAT	769879	YES	YES	NO
217	Biliverdin reductase A	BLVRA	420776	YES	NO	YES
218	Bisphosphoglycerate mutase	BPGM	418172	YES	YES	YES
219	Bleomycin hydrolase	BLMH	395996	YES	YES	YES
220	BPI fold containing family B member 2	BPIFB2	395882	YES	YES	NO
221	Brain abundant membrane attached signal protein 1	BASP1	373905	YES	YES	NO
222	Brain and reproductive organ-expressed (TNFRSF1A modulator)	BRE	421417	YES	YES	YES
223	BRCA1/BRCA2-containing complex subunit 3	BRCC3	422201	YES	NO	NO
224	Breast carcinoma amplified sequence 2	BCAS2	419884	YES	YES	NO
225	Bridging integrator 1	BIN1	424288	YES	NO	NO
226	BRO1 domain and CAAX motif containing	BROX	421334	YES	YES	YES
227	BUB3, mitotic checkpoint protein	BUB3	423949	YES	YES	YES
228	BUD31 homolog	BUD31	416492	YES	YES	YES
229	Cadherin 1	CDH1	415860	YES	YES	NO
230	Cadherin 13	CDH13	414849	NO	YES	NO
231	Calcineurin like EF-hand protein 1	CHP1	423211	YES	YES	NO
232	Calcium binding protein 39	CAB39	424830	NO	YES	NO
233	Calcium regulated heat stable protein 1	CARHSP1	416640	YES	YES	NO
234	Calcium voltage-gated channel auxiliary subunit alpha2 delta 1	CACNA2D1	768444	NO	YES	NO

235	Calcium/calmodulin dependent protein kinase II delta	CAMK2D	422688	YES	YES	NO
236	Calcium/calmodulin dependent protein kinase II gamma	CAMK2G	423737	YES	YES	NO
237	Calcium/calmodulin dependent serine protein kinase	CASK	418565	NO	YES	NO
238	Calcium-binding protein	P22	396352	YES	YES	YES
239	Caldesmon 1	CALD1	373965	YES	YES	NO
240	Calmodulin 1 (phosphorylase kinase, delta)	CALM1	396523	YES	YES	YES
241	Calmodulin like 3	CALML3	416692	YES	YES	NO
242	Calpain 1	CAPN1	693249	YES	YES	YES
243	Calpain 11	CAPN11	396240	YES	YES	NO
244	Calpain small subunit	LOC395965	395956	YES	YES	YES
245	Calpain small subunit 2	CAPNS2	770982	NO	YES	NO
246	Calpastatin	CAST	427121	YES	YES	YES
247	Calponin 1	CNN1	396522	YES	YES	NO
248	Calponin 2	CNN2	100216000	YES	YES	NO
249	Calreticulin	CALR	100859104	YES	YES	YES
250	Canopy FGF signaling regulator 2	CNPY2	100858933	YES	YES	NO
251	CAP-Gly domain containing linker protein 1	CLIP1	395784	NO	YES	NO
252	Capping actin protein of muscle Z-line alpha subunit 1	CAPZA1	396521	YES	YES	YES
253	Capping actin protein of muscle Z-line alpha subunit 2	CAPZA2	417771	YES	YES	NO
254	Capping actin protein of muscle Z-line beta subunit	CAPZB	396418	YES	YES	YES
255	Capping actin protein, gelsolin like	CAPG	431244	YES	YES	YES
256	Caprin family member 2	CAPRIN2	418141	NO	NO	YES
257	Carbamoyl-phosphate synthetase 2, aspartate transcarbamylase, and dihydroorotase	CAD	428659	YES	YES	YES
258	Carbonic anhydrase 2	CA2	396257	YES	YES	YES
259	Carbonyl reductase 3	CBR3	418512	YES	YES	YES
260	Carbonic anhydrase 13	CA13	100858989	YES	YES	YES
261	Carboxylesterase 1 like 2	CES1L2	769339	NO	YES	NO
262	Carboxymethylenebutenolidase homolog	CMBL	420928	YES	YES	NO
263	Carboxypeptidase D	CPD	417590	YES	YES	NO
264	Carboxypeptidase M	CPM	417843	YES	YES	NO
265	Carnitine palmitoyltransferase 1A	CPT1A	423118	YES	YES	NO
266	Carnitine palmitoyltransferase 2	CPT2	424649	YES	NO	NO
267	Carnosine synthase 1	CARNS1	100359387	NO	NO	YES
268	Cartilage associated protein	CRTAP	395992	YES	NO	NO
269	Casein kinase2 alpha 1	CSNK2A1	432370	YES	YES	NO
270	Caspase 3	CASP3	395476	YES	NO	YES
271	Catalase	CAT	423600	YES	YES	YES
272	Catenin alpha 1	CTNNA1	416184	YES	YES	NO
273	Catenin beta 1	CTNNB1	395964	YES	YES	NO
274	Cathelicidin antimicrobial peptide	CAMP	420407	YES	YES	YES
275	Cathelicidin-1	CATH1	414337	YES	YES	YES
276	Cathepsin A	CTSA	428163	YES	YES	NO
277	Cathepsin B	CTSB	396329	YES	YES	NO
278	Cathepsin C	CTSC	419014	YES	YES	NO
279	Cathepsin D	CTSD	396090	YES	YES	NO
280	Cathepsin E-A-like	CTSEAL	417848	YES	NO	NO
281	Cathepsin Z	CTSZ	419311	NO	YES	NO
282	Caveolin 1	CAV1	373996	YES	YES	NO

283	CCHC-type zinc finger nucleic acid binding protein	CNBP	395731	YES	YES	NO
284	CD9 molecule	CD9	395527	NO	YES	NO
285	CD55 molecule (Cromer blood group)	CD55	419852	NO	YES	YES
286	CD81 molecule	CD81	374256	NO	YES	NO
287	CD109 molecule	CD109	421862	NO	YES	NO
288	CD151 molecule (Raph blood group)	CD151	423105	NO	YES	NO
289	CD276 molecule	CD276	415315	YES	NO	NO
290	Cell cycle associated protein 1	CAPRIN1	426151	YES	NO	NO
291	Cell division cycle 37	CDC37	395430	YES	NO	NO
292	Cell division cycle 42	CDC42	395917	YES	YES	NO
293	Cell division cycle and apoptosis regulator 1	CCAR1	423692	NO	NO	YES
294	Cell wall biogenesis 43 C-terminal homolog	CWH43	422763	NO	YES	NO
295	Centrosomal protein 250	CEP250	419138	YES	NO	NO
296	C-factor-like	LOC515662	415662	NO	YES	NO
297	Chaperonin containing TCP1 subunit 2	CCT2	417846	YES	YES	YES
298	Chaperonin containing TCP1 subunit 3	CCT3	425644	YES	YES	YES
299	Chaperonin containing TCP1 subunit 4	CCT4	395414	YES	YES	YES
300	Chaperonin containing TCP1 subunit 5	CCT5	420930	YES	YES	YES
301	Chaperonin containing TCP1 subunit 6A	CCT6A	417541	YES	YES	YES
302	Chaperonin containing TCP1 subunit 7	CCT7	428806	YES	YES	YES
303	Chaperonin containing TCP1 subunit 8	CCT8	418486	YES	YES	YES
304	Charged multivesicular body protein 6	CHMP6	428673	YES	YES	NO
305	Chloride intracellular channel 2	CLIC2	422178	YES	YES	YES
306	Chloride intracellular channel 3	CLIC3	417293	YES	YES	NO
307	Chloride intracellular channel 4	CLIC4	419595	YES	YES	NO
308	Chondroitin sulfate proteoglycan 4	CSPG4	425524	YES	YES	NO
309	Chromobox 3	CBX3	395355	YES	YES	NO
310	chromosome 1 open reading frame, human C11orf54	C1H11ORF54	419001	YES	YES	YES
311	Chromosome 5 C14orf166 homolog	C5H14ORF166	396326	YES	YES	NO
312	Chromosome 8 C1orf123 homolog	C8H1orf123	424650	NO	YES	NO
313	Chromosome 33 C12orf10 homolog	C33H12orf10	426187	YES	YES	YES
314	Chromosome segregation 1 like	CSE1L	419212	YES	YES	YES
315	Chromosome transmission fidelity factor 18	CHTF18	416531	YES	YES	NO
316	Citrate synthase	CS	100858903	YES	YES	YES
317	Clanexin	CANX	416288	YES	YES	YES
318	Clathrin heavy chain	CLTC	395272	YES	YES	YES
319	Clathrin interactor 1	CLINT1	416240	YES	YES	NO
320	Clathrin light chain A	CLTA	427284	YES	NO	NO
321	Clathrin light chain B	CLTB	416226	YES	YES	NO
322	Clathrin, heavy chain-like 1	CLTCL1	416765	YES	YES	NO
323	Claudin 1	CLDN1	424910	YES	YES	NO
324	Cleavage and polyadenylation specific factor 6	CPSF6	417844	NO	NO	YES
325	Cleavage stimulation factor subunit 3	CSTF3	421595	YES	NO	YES
326	Clustered mitochondria homolog	CLUH	417675	NO	YES	NO
327	Clusterin	CLU	395722	YES	YES	NO
328	CNDP dipeptidase 2 (metallopeptidase M20 family)	CNDP2	421013	YES	YES	YES
329	Coagulation factor XIII A chain	F13A1	395420	YES	YES	YES
330	Coatomer protein complex subunit alpha	COPA	426906	YES	YES	YES

331	Coatomer protein complex subunit beta 1	COPB1	423063	YES	YES	YES
332	Coatomer protein complex subunit beta 2	COPB2	424823	YES	YES	YES
333	Coatomer protein complex subunit gamma 1	COPG1	416014	YES	YES	NO
334	Coenzyme Q4	COQ4	417219	YES	YES	NO
335	Coenzyme Q8A	COQ8A	416745	YES	NO	NO
336	Cofilin-2	CFL2	423320	YES	YES	YES
337	Coiled-coil domain containing 25	CCDC25	422005	YES	YES	NO
338	Coiled-coil domain containing 93	CCDC93	424277	YES	YES	NO
339	Coiled-coil-helix-coiled-coil-helix domain containing 3	CHCHD3	418435	YES	YES	NO
340	Coiled-coil-helix-coiled-coil-helix domain containing 4	CHCHD4	416037	YES	YES	NO
341	Cold shock domain containing E1	CSDE1	419886	YES	YES	NO
342	Collagen alpha-2(IV) chain-like	LOC107051274	107051274	YES	YES	YES
343	Collagen type I alpha 1 chain	COL1A1	395532	YES	YES	NO
344	Collagen type I alpha 2 chain	COL1A2	396243	YES	YES	NO
345	Collagen type III alpha 1 chain	COL3A1	396340	YES	YES	NO
346	Collagen type IV alpha 1 chain	COL4A1	395530	YES	YES	NO
347	Collagen type IV alpha 2 chain	COL4A2	418752	YES	YES	NO
348	collagen type V alpha 2 chain	COL5A2	423986	NO	YES	NO
349	Collagen type VI alpha 1 chain	COL6A1	396000	YES	YES	NO
350	Collagen type VI alpha 2 chain	COL6A2	396292	YES	YES	NO
351	Collagen type VI alpha 3 chain	COL6A3	396548	YES	YES	NO
352	Collagen, type VII, alpha 1	COL7A1	427584	YES	NO	NO
353	Collagen type XI alpha 1 chain	COL11A1	374046	YES	YES	NO
354	Collagen type XII alpha 1 chain	COL12A1	395875	YES	YES	NO
355	Collagen, type XVIII, alpha 1	COL18A1	373978	YES	YES	NO
356	Collectin subfamily member 12	COLEC12	421061	YES	YES	NO
357	COMM domain containing 4	COMMD4	770048	NO	YES	NO
358	COMM domain containing 5	COMMD5	422376	YES	YES	NO
359	COMM domain containing 6	COMMD6	769778	YES	YES	NO
360	COMM domain containing 7	COMMD7	419286	YES	YES	NO
361	Complement C1q binding protein	C1QBP	395538	YES	YES	NO
362	Complement component 3	C3	396370	NO	YES	YES
363	Complement component 4 binding protein, alpha	C4BPA	395384	YES	YES	YES
364	Complement factor D	CFD	100857593	YES	YES	NO
365	Complement factor H	CFH	429057	NO	YES	YES
366	COP9 signalosome subunit 2	COPS2	430917	YES	NO	YES
367	COP9 signalosome subunit 4	COPS4	422594	YES	YES	YES
368	COP9 signalosome subunit 5	COPS5	426579	NO	NO	YES
369	COP9 signalosome subunit 7A	COPS7A	418283	YES	YES	YES
370	Copine 1	CPNE1	419134	YES	YES	YES
371	Copine 3	CPNE3	420214	YES	YES	YES
372	Copper metabolism domain containing 1	COMMD1	769006	NO	YES	NO
373	Coproporphyrinogen oxidase	CPOX	418377	YES	YES	YES
374	Core-binding factor beta subunit	CBFB	395257	NO	YES	NO
375	Coronin 1C	CORO1C	416893	YES	YES	YES
376	Coronin-1B-like	LOC107056441	107056441	NO	YES	NO
377	Cortactin	CTTN	396455	YES	NO	NO
378	COX20, cytochrome c oxidase assembly factor	COX20	421493	YES	NO	NO

379	C-reactive protein, pentraxin-related	LOC776376	776376	NO	NO	YES
380	Creatine kinase B	CKB	396248	YES	YES	YES
381	CRK proto-oncogene, adaptor protein	CRK	417553	YES	YES	YES
382	Crystallin lambda 1	CRYL1	418953	NO	YES	NO
383	Crystallin zeta	CRYZ	772289	NO	YES	YES
384	C-terminal binding protein 1	CTBP1	422904	YES	NO	YES
385	C-terminal Src kinase	CSK	396396	YES	YES	NO
386	CTP synthase 1	CTPS1	419561	NO	NO	YES
387	Cullin 3	CUL3	424804	NO	YES	NO
388	Cullin 4A	CUL4A	418744	YES	YES	NO
389	Cullin associated and neddylation dissociated 1	CAND1	417837	YES	YES	YES
390	CWF19-like 1, cell cycle control (S. pombe)	CWF19L1	101749626	YES	NO	NO
391	Cyclin dependent kinase 1	CDK1	396252	YES	NO	NO
392	Cyclin dependent kinase 5	CDK5	100190948	YES	YES	NO
393	Cystathionine gamma-lyase	CTH	424716	YES	YES	YES
394	Cystathionine-beta-synthase	CBS	418545	YES	NO	YES
395	Cystatin B	CSTB	418267	YES	YES	NO
396	Cystatin C	CST3	396497	YES	YES	NO
397	Cystatin F	CST7	416716	NO	YES	NO
398	Cysteine and glycine rich protein 1	CSRP1	396176	YES	YES	NO
399	Cysteine and glycine rich protein 2	CSRP2	396128	YES	YES	NO
400	Cysteinyl-tRNA synthetase	CARS	423086	YES	YES	NO
401	Cytidine/uridine monophosphate kinase 1	CMPK1	429100	YES	YES	YES
402	Cytochrome b5 reductase 1	CYB5R1	418220	YES	YES	NO
403	Cytochrome b5 type A	CYB5A	414798	YES	YES	YES
404	Cytochrome b5 type B	CYB5B	415865	YES	YES	NO
405	Cytochrome c oxidase subunit 4I1	COX4I1	415826	YES	YES	YES
406	Cytochrome c oxidase subunit II	COX2	39116936	YES	YES	NO
407	Cytochrome c, somatic	CYCS	420624	YES	YES	NO
408	Cytochrome p450 oxidoreductase	POR	417520	YES	YES	NO
409	Cytoglobin	CYGB	427802	YES	YES	NO
410	Cytokine induced apoptosis inhibitor 1	CIAPIN1	415637	YES	YES	YES
411	Cytoplasmic FMR1 interacting protein 1	CYFIP1	418677	YES	YES	NO
412	Cytoskeleton associated protein 4	CKAP4	418073	YES	YES	YES
413	Cytosolic iron-sulfur assembly component 1	CIAO1	426332	NO	NO	YES
414	Damage specific DNA binding protein 1	DDB1	374050	YES	YES	YES
415	DAZ associated protein 1	DAZAP1	427266	YES	NO	YES
416	DEAD (Asp-Glu-Ala-Asp) box polypeptide 39B	DDX39B	100529080	YES	YES	YES
417	DEAD-box helicase 1	DDX1	395249	YES	YES	YES
418	DEAD-box helicase 3, X-linked	DDX3X	418567	YES	NO	YES
419	DEAD-box helicase 5	DDX5	395629	YES	NO	NO
420	DEAD-box helicase 6	DDX6	419783	YES	YES	YES
421	DEAH-box helicase 15	DHX15	422813	YES	NO	YES
422	DEAD-box helicase 17	DDX17	418024	YES	YES	YES
423	DEAD-box helicase 19	DDX19	426504	YES	YES	YES
424	DEAD-box helicase 23	DDX23	100858001	YES	NO	NO
425	Decorin	DCN	417892	YES	YES	NO
426	Dedicator of cytokinesis 1	DOCK1	423960	YES	YES	NO

427	Dedicator of cytokinesis 10	DOCK10	424803	NO	YES	NO
428	Dehydrogenase/reductase 4	DHRS4	426247	YES	YES	NO
429	Dehydrogenase/reductase 7	DHRS7	423527	YES	YES	YES
430	DEK proto-oncogene	DEK	420828	NO	NO	YES
431	Dematin actin binding protein	DMTN	776542	NO	NO	YES
432	Dephospho-CoA kinase domain containing	DCAKD	419967	NO	YES	NO
433	Desmin	DES	395906	YES	YES	NO
434	Desmoglein 2	DSG2	428529	YES	NO	NO
435	Desmoplakin	DSP	420869	YES	YES	NO
436	Destrin, actin depolymerizing factor	DSTN	396539	YES	YES	YES
437	Developmentally regulated GTP binding protein 1	DRG1	416962	YES	NO	NO
438	Diablo IAP-binding mitochondrial protein	DIABLO	416860	YES	YES	NO
439	Dicarbonyl and L-xylulose reductase	DCXR	374066	YES	YES	YES
440	Dihydrofolate reductase	DHFR	427317	YES	NO	NO
441	Dihydrolipoamide branched chain transacylase E2	DBT	395374	YES	YES	NO
442	Dihydrolipoamide dehydrogenase	DLD	417699	YES	YES	NO
443	Dihydrolipoamide S-acetyltransferase	DLAT	419796	YES	YES	NO
444	Dihydrolipoamide S-succinyltransferase	DLST	423357	YES	YES	NO
445	Dihydropyrimidinase like 2	DPYSL2	395155	YES	YES	NO
446	Dihydropyrimidinase like 3	DPYSL3	395154	YES	YES	NO
447	Dipeptidyl peptidase 4	DPP4	424187	NO	YES	NO
448	Dipeptidyl peptidase 7	DPP7	417297	YES	YES	NO
449	DNA damage inducible 1 homolog 2	DDI2	425541	NO	NO	YES
450	DNA polymerase theta	POLQ	418326	YES	YES	NO
451	DNA replication regulator and spliceosomal factor	SMU1	427377	YES	NO	NO
452	DNA topoisomerase I	TOP1	396004	YES	NO	YES
453	DnaJ heat shock protein family (Hsp40) member B4	DNAJB4	424550	NO	YES	NO
454	DnaJ heat shock protein family (Hsp40) member C3	DNAJC3	418787	YES	YES	NO
455	DnaJ heat shock protein family (Hsp40) member C7	DNAJC7	428312	YES	NO	NO
456	DnaJ heat shock protein family (Hsp40) member C9	DNAJC9	423640	YES	NO	YES
457	DnaJ heat shock protein family (Hsp40) member C13	DNAJC13	420688	NO	YES	NO
458	Dolichyl-diphosphooligosaccharide--protein glycosyltransferase non-catalytic subunit	DDOST	425542	YES	YES	YES
459	Drebrin like	DBNL	425994	NO	NO	YES
460	Dynactin subunit 1	DCTN1	426238	NO	YES	YES
461	Dynactin subunit 2	DCTN2	395587	NO	YES	YES
462	Dynactin subunit 3	DCTN3	427404	YES	YES	NO
463	Dynactin subunit 4	DCTN4	416269	YES	NO	NO
464	Dynamin 1	DNM1	417217	YES	NO	NO
465	Dynamin 1 like	DNM1L	418132	YES	YES	NO
466	Dynamin 2-like	LOC430067	430067	YES	NO	NO
467	Dynamin 3	DNM3	424389	NO	YES	NO
468	Dynein cytoplasmic 1 heavy chain 1	DYNC1H1	423461	YES	YES	NO
469	Dynein cytoplasmic 1 intermediate chain 2	DYNC1I2	424151	YES	YES	NO
470	Dynein cytoplasmic 1 light intermediate chain 1	DYNC1LI1	420668	YES	YES	NO
471	Dynein cytoplasmic 1 light intermediate chain 2	DYNC1LI2	415793	YES	YES	NO
472	Dyskerin pseudouridine synthase 1	DKC1	422196	YES	YES	NO
473	Early endosome antigen 1	EEA1	417896	NO	YES	NO
474	Echinoderm microtubule associated protein like 1	EML1	423452	YES	YES	YES

475	Ectonucleoside triphosphate diphosphohydrolase 1	ENTPD1	423826	YES	YES	YES
476	Ectonucleoside triphosphate diphosphohydrolase 5	ENTPD5	423343	NO	NO	YES
477	EGF like, fibronectin type III and laminin G domains	EGFLAM	427445	NO	YES	NO
478	EH domain containing 3	EHD3	421306	YES	YES	NO
479	EH domain containing 4	EHD4	100859860	YES	NO	YES
480	ELAV like RNA binding protein 1	ELAVL1	395637	YES	YES	YES
481	Electron transfer flavoprotein dehydrogenase	ETFDH	428724	YES	YES	NO
482	Electron transfer flavoprotein, alpha subunit	ETFA	415353	YES	YES	NO
483	Elongation factor like GTPase 1	EFL1	415467	NO	NO	YES
484	Elongation factor Tu GTP binding domain containing 2	EFTUD2	428281	YES	YES	NO
485	ELOVL fatty acid elongase 1	ELOVL1	424564	YES	YES	NO
486	EMAP like 4	EML4	771345	YES	YES	NO
487	EMG1, N1-specific pseudouridine methyltransferase	EMG1	418292	YES	NO	NO
488	ENAH actin regulator	ENAH	374180	YES	NO	NO
489	Endoglin	ENG	771557	YES	YES	NO
490	Endonuclease domain containing 1	ENDOD1	428090	YES	YES	NO
491	Endoplasmic reticulum aminopeptidase 1	ERAP1	427122	YES	YES	NO
492	Endoplasmic reticulum protein 29	ERP29	416882	YES	YES	YES
493	Endoplasmic reticulum protein 44	ERP44	420994	YES	YES	YES
494	Endoplasmic reticulum-golgi intermediate compartment 1	ERGIC1	416205	YES	YES	NO
495	Enolase 1, (alpha)	ENO1	396017	YES	YES	YES
496	Enoyl-CoA delta isomerase 1	ECI1	416657	YES	YES	NO
497	Enoyl-CoA hydratase 1	ECH1	100858352	YES	YES	NO
498	Enoyl-CoA hydratase domain containing 2	ECHDC2	424646	YES	YES	NO
499	Enoyl-CoA hydratase, short chain 1	ECHS1	770828	YES	YES	NO
500	Envoplakin	EVPL	427805	YES	YES	NO
501	Epithelial cell adhesion molecule	EPCAM	421292	YES	YES	NO
502	Epoxide hydrolase 1-like	EPHX1L	421447	YES	YES	NO
503	Epoxide hydrolase 2	EPHX2	421999	NO	YES	NO
504	ER lipid raft associated 1	ERLIN1	423670	YES	NO	NO
505	ER lipid raft associated 2	ERLIN2	426769	YES	YES	YES
506	ER membrane protein complex subunit 1	EMC1	419470	YES	YES	NO
507	ER membrane protein complex subunit 2	EMC2	420273	YES	YES	NO
508	ER membrane protein complex subunit 3	EMC3	415936	YES	YES	NO
509	ERI1 exoribonuclease family member 3	ERI3	424579	YES	YES	NO
510	Erythrocyte membrane protein band 4.1	EPB41	396493	NO	YES	YES
511	Erythrocyte membrane protein band 4.1 like 2	EPB41L2	421703	YES	YES	NO
512	Erythrocyte membrane protein band 4.2	EPB42	419216	YES	YES	YES
513	Esterase D	ESD	418856	YES	YES	YES
514	ETHE1, persulfide dioxygenase	ETHE1	426450	YES	YES	YES
515	Eukaryotic translation elongation factor 1 alpha 1	EEF1A1	373963	YES	YES	YES
516	Eukaryotic translation elongation factor 1 beta 2	EEF1B2	395723	YES	YES	YES
517	Eukaryotic translation elongation factor 1 delta	EEF1D	107049004	YES	YES	YES
518	Eukaryotic translation elongation factor 1 gamma	EEF1G	100526660	YES	YES	YES
519	Eukaryotic translation elongation factor 2	EEF2	396325	YES	YES	YES
520	Eukaryotic translation initiation factor 1A domain containing	EIF1AD	414740	YES	YES	NO
521	Eukaryotic translation initiation factor 1A, X-linked	EIF1AX	418606	YES	YES	YES
522	Eukaryotic translation initiation factor 2 subunit alpha	EIF2S1	423279	YES	YES	YES

523	Eukaryotic translation initiation factor 2 subunit beta	EIF2S2	395295	YES	YES	YES
524	Eukaryotic translation initiation factor 2 subunit gamma	EIF2S3	418597	YES	YES	YES
525	Eukaryotic translation initiation factor 2B subunit delta	EIF2B4	425800	YES	YES	YES
526	Eukaryotic translation initiation factor 2D	EIF2D	419845	YES	YES	NO
527	Eukaryotic translation initiation factor 3 subunit A	EIF3A	423927	YES	YES	YES
528	Eukaryotic translation initiation factor 3 subunit B	EIF3B	769783	YES	NO	NO
529	Eukaryotic translation initiation factor 3 subunit D	EIF3D	771356	YES	YES	YES
530	Eukaryotic translation initiation factor 3 subunit E	EIF3E	420272	YES	YES	YES
531	Eukaryotic translation initiation factor 3 subunit F	EIF3F	423748	YES	YES	YES
532	Eukaryotic translation initiation factor 3 subunit J	EIF3J	415573	YES	NO	YES
533	Eukaryotic translation initiation factor 3 subunit L	EIF3L	418033	YES	NO	NO
534	Eukaryotic translation initiation factor 3 subunit M	EIF3M	421602	YES	YES	YES
535	Eukaryotic translation initiation factor 4 gamma 1	EIF4G1	424953	YES	YES	NO
536	Eukaryotic translation initiation factor 4 gamma 2	EIF4G2	395905	YES	NO	NO
537	Eukaryotic translation initiation factor 4A2	EIF4A2	395232	YES	YES	YES
538	Eukaryotic translation initiation factor 4A3	EIF4A3	416704	YES	YES	YES
539	Eukaryotic translation initiation factor 4B	EIF4B	100858349	YES	NO	NO
540	Eukaryotic translation initiation factor 4E	EIF4E	422703	YES	YES	YES
541	Eukaryotic translation initiation factor 4H	EIF4H	417490	YES	YES	YES
542	Eukaryotic translation initiation factor 5	EIF5	423479	YES	YES	YES
543	Eukaryotic translation initiation factor 5A-1-like	LOC107050352	107050352	YES	YES	YES
544	eukaryotic translation initiation factor 5B	EIF5B	395514	NO	YES	YES
545	Eukaryotic translation initiation factor 6	EIF6	100550619	YES	YES	YES
546	Eukaryotic translation termination factor 1	ETF1	416182	YES	YES	YES
547	Exportin 1	XPO1	421192	YES	YES	NO
548	Exportin 7	XPO7	426925	YES	YES	YES
549	Extracellular fatty acid-binding protein	EXFABP	396393	YES	YES	NO
550	Ezrin	EZR	395701	YES	YES	NO
551	Family with sequence similarity 120C	FAM120C	415983	YES	YES	NO
552	Family with sequence similarity 129 member B	FAM129B	771307	YES	YES	NO
553	Family with sequence similarity 175 member B	FAM175B	423954	YES	NO	NO
554	Family with sequence similarity 188 member A	FAM188A	420526	YES	YES	YES
555	Family with sequence similarity 213 member A	FAM213A	423625	YES	YES	NO
556	Family with sequence similarity 49 member A	FAM49A	421949	YES	NO	NO
557	Family with sequence similarity 49 member B	FAM49B	420330	YES	YES	YES
558	Family with sequence similarity 96 member A	FAM96A	415571	NO	NO	YES
559	Far upstream element binding protein 1	FUBP1	424551	YES	YES	YES
560	Far upstream element binding protein 3	FUBP3	417184	YES	YES	NO
561	Farnesyl diphosphate synthase	FDPS	425061	YES	YES	YES
562	Fas associated factor family member 2	FAF2	416225	YES	YES	NO
563	Fas associated via death domain	FADD	423146	YES	YES	YES
564	Fascin actin-bundling protein 1	FSCN1	416485	YES	YES	NO
565	Fatty acid binding protein 3	FABP3	419557	YES	YES	NO
566	Fatty acid binding protein 5	FABP5	420197	YES	YES	NO
567	Fatty acid synthase	FASN	396061	YES	YES	NO
568	Fatty acid-binding protein 7	FABP7	396246	YES	YES	NO
569	F-box protein 3	FBXO3	429495	YES	NO	YES
570	F-box protein 44	FBXO44	419494	NO	YES	NO

571	FERM, ARH/RhoGEF and pleckstrin domain protein 1	FARP1	418781	YES	YES	NO
572	Fermitin family member 2	FERMT2	423589	YES	YES	NO
573	Ferritin heavy chain 1	FTH1	395970	YES	YES	YES
574	Ferrochelatase	FECH	374020	YES	NO	YES
575	Fetuin B	FETUB	395404	NO	YES	YES
576	Fibrinogen alpha chain	FGA	396307	NO	YES	YES
577	Fibrinogen beta chain	FGB	373926	YES	YES	YES
578	Fibrinogen gamma chain	FGG	395837	NO	YES	YES
579	Fibrinogen-like protein 1-like	LOC430700	430700	NO	YES	NO
580	Fibronectin 1	FN1	396133	YES	YES	YES
581	Fibulin 1	FBLN1	373979	YES	YES	NO
582	Fibulin 2	FBLN2	427583	YES	YES	NO
583	Filamin A	FLNA	395261	YES	YES	YES
584	Filamin B	FLNB	378913	YES	YES	YES
585	Filamin C	FLNC	395260	YES	YES	NO
586	FK506 binding protein 3	FKBP3	395353	YES	YES	YES
587	FK506 binding protein 4	FKBP4	418261	YES	YES	YES
588	FK506 binding protein 5	FKBP5	421186	NO	YES	NO
589	FK506 binding protein 9	FKBP9	395652	YES	YES	NO
590	FK506 binding protein 10	FKBP10	427013	YES	YES	NO
591	Flap structure-specific endonuclease 1	FEN1	769677	YES	YES	YES
592	Flavin containing monooxygenase 3	FMO3	395267	YES	YES	NO
593	Flotillin 2	FLOT2	417579	YES	YES	YES
594	Focadhesin	FOCAD	427233	NO	YES	NO
595	Fructose-bisphosphatase 1	FBP1	395218	YES	YES	YES
596	Fumarate hydratase	FH	420969	YES	YES	NO
597	Fumarylacetoacetate hydrolase domain containing 2A	FAHD2A	426684	YES	NO	NO
598	FUS RNA binding protein	FUS	414144	NO	NO	YES
599	G protein pathway suppressor 1	GPS1	417382	YES	NO	YES
600	G protein subunit alpha 11	GNA11	374077	YES	YES	NO
601	G protein subunit alpha 14	GNA14	431611	NO	NO	YES
602	G protein subunit alpha i2	GNAI2	396367	YES	YES	NO
603	G protein subunit alpha i3	GNAI3	374097	YES	YES	NO
604	G protein subunit alpha L	GNAL	474379	NO	NO	YES
605	G protein subunit beta 1	GNB1	419402	YES	YES	NO
606	G protein subunit beta 4	GNB4	424974	YES	YES	NO
607	G3BP stress granule assembly factor 1	G3BP1	416265	YES	NO	YES
608	GA binding protein transcription factor alpha subunit	GABPA	418478	YES	NO	YES
609	Galactokinase 2	GALK2	415596	YES	YES	NO
610	Galactosidase alpha	GLA	422188	NO	YES	NO
611	Galactosidase beta 1	GLB1	420720	YES	NO	NO
612	Galectin 1	LGALS1	396491	YES	YES	NO
613	Galectin like	LGALSL	421278	NO	YES	NO
614	GAR1 ribonucleoprotein	GAR1	422697	YES	NO	NO
615	GC, vitamin D binding protein	GC	395696	YES	YES	YES
616	GCN1, eIF2 alpha kinase activator homolog	GCN1	416982	YES	YES	NO
617	GDP dissociation inhibitor 1	GDI1	395854	YES	YES	YES
618	GDP-mannose pyrophosphorylase A	GMPPA	769292	YES	YES	NO

619	GDP-mannose pyrophosphorylase B	GMPPB	415924	YES	YES	YES
620	Gelsolin	GSN	395774	YES	YES	YES
621	Geranylgeranyl diphosphate synthase 1	GGPS1	427092	YES	NO	NO
622	Glactosidase beta 1	GLB1	420720	NO	YES	NO
623	GLG1 golgi glycoprotein 1	GLG1	396492	YES	NO	NO
624	GLI pathogenesis related 2	GLIPR2	420999	YES	YES	YES
625	Glucosamine-6-phosphate deaminase 1	GNPDA1	416341	YES	YES	NO
626	Glucosamine-6-phosphate deaminase 2	GNPDA2	422772	NO	YES	NO
627	Glucosamine-phosphate N-acetyltransferase 1	GNPNAT1	423588	NO	YES	NO
628	Glucose-6-phosphate dehydrogenase	G6PD	112530318	YES	YES	YES
629	Glucose-6-phosphate isomerase	GPI	415783	YES	YES	YES
630	Glutamate dehydrogenase 2	GLUD2	423612	YES	YES	NO
631	Glutamate-cysteine ligase catalytic subunit	GCLC	421894	NO	YES	YES
632	Glutamate-cysteine ligase modifier subunit	GCLM	424492	YES	YES	YES
633	Glutamic-oxaloacetic transaminase 1	GOT1	396261	YES	YES	YES
634	Glutamic-oxaloacetic transaminase 2	GOT2	396533	YES	YES	YES
635	Glutamine amidotransferase like class 1 domain containing 3A	GATD3A	418552	YES	YES	NO
636	Glutamine synthetase-like	GSL	417253	NO	NO	YES
637	Glutamine-fructose-6-phosphate transaminase 1	GFPT1	419514	YES	YES	NO
638	Glutamine-fructose-6-phosphate transaminase 2	GFPT2	426962	YES	NO	NO
639	Glutaminyl-tRNA synthetase	QARS	416057	YES	YES	YES
640	Glutamyl-prolyl-tRNA synthetase	EPRS	421348	YES	YES	YES
641	Glutamyl-tRNA amidotransferase subunit B	GATB	422480	YES	NO	NO
642	Glutaredoxin	GLRX	396069	NO	NO	YES
643	Glutaredoxin 3	GLRX3	423968	YES	YES	YES
644	Glutathione peroxidase 1	GPX1	100857115	YES	YES	YES
645	Glutathione peroxidase 3	GPX3	427638	NO	YES	YES
646	Glutathione peroxidase 4	GPX4	374056	YES	YES	YES
647	Glutathione peroxidase 7	GPX7	424643	YES	YES	NO
648	Glutathione S-transferase alpha 2	GSTA2	414895	YES	YES	NO
649	Glutathione S-transferase alpha 3	GSTA3	414896	YES	YES	YES
650	Glutathione S-transferase alpha 4	GSTA4	395612	YES	YES	YES
651	Glutathione S-transferase class-alpha	LOC395611	395611	YES	YES	NO
652	Glutathione S-transferase kappa 1	GSTK1	418302	YES	YES	NO
653	Glutathione S-transferase mu 2 (muscle)	GSTM2	395976	YES	YES	YES
654	Glutathione S-transferase omega 2	GSTO2	423881	YES	YES	YES
655	Glutathione S-transferase theta 1-like	GSTT1L	769847	YES	YES	NO
656	Glutathione synthetase	GSS	428135	YES	YES	YES
657	Glutathione transferase	LOC396380	396380	YES	YES	NO
658	Glutathione-disulfide reductase	GSR	771783	YES	YES	NO
659	Glyceraldehyde-3-phosphate dehydrogenase	GAPDH	374193	YES	YES	YES
660	Glycerol-3-phosphate dehydrogenase 1-like	GPDL1	420664	YES	YES	YES
661	Glycerophosphocholine phosphodiesterase 1	GPCPD1	421311	NO	NO	YES
662	Glycerophosphodiester phosphodiesterase domain containing 2	GDPD2	422217	NO	YES	NO
663	Glycine C-acetyltransferase	GCAT	101750333	YES	YES	NO
664	Glycogen synthase kinase 3 alpha	GSK3A	418335	YES	YES	NO
665	Glycogenin 1	GYG1	425050	NO	NO	YES
666	Glycosylphosphatidylinositol anchored high density lipoprotein binding protein 1	GPIHBP1	420302	NO	YES	NO

667	Glycyl-tRNA synthetase	GARS	428403	YES	YES	NO
668	Glyoxalase domain containing 4	GLOD4	101750166	YES	YES	YES
669	Glyoxalase I	GLO1	421428	YES	YES	YES
670	Glypican 4	GPC4	422234	YES	YES	NO
671	GNAS complex locus	GNAS	419313	YES	YES	NO
672	Golgi glycoprotein 1	GLG1	396492	NO	YES	NO
673	Golgi membrane protein 1	GOLM1	427462	YES	YES	NO
674	Golgin A2	GOLGA2	427766	YES	NO	NO
675	Golgin B1	GOLGB1	426868	NO	YES	NO
676	GRIP and coiled-coil domain containing 2	GCC2	428014	YES	NO	NO
677	Growth factor receptor bound protein 2	GRB2	386572	YES	NO	NO
678	Growth hormone inducible transmembrane protein	GHITM	423621	YES	YES	NO
679	GrpE like 1, mitochondrial	GRPEL1	422865	YES	YES	NO
680	GTPase IMAP family member 7-like	LOC112529948	112529948	YES	YES	YES
681	GTPase IMAP family member 8-like	LOC420368	420368	NO	YES	YES
682	Guanine monophosphate synthase	GMPS	425028	YES	YES	NO
683	Guanosine monophosphate reductase 2	GMPR2	420842	NO	NO	YES
684	GULP PTB domain containing engulfment adaptor 1	GULP1	423988	NO	YES	NO
685	H1 histone family member X	H1FX	107054440	YES	NO	YES
686	H1 histone family, member 0	H1F0	693250	YES	YES	YES
687	H2A histone family, member Y	H2AFY	395858	YES	NO	NO
688	H2A.Z variant histone 2	H2AZ2	426617	YES	NO	YES
689	Haloacid dehalogenase like hydrolase domain containing 2	HDHD2	416385	YES	YES	NO
690	Heat shock 70kDa protein 2	HSPA2	423504	YES	YES	YES
691	Heat shock 70kDa protein 5 (glucose-regulated protein, 78kDa)	HSPA5	396487	YES	YES	YES
692	Heat shock 70kDa protein 8	HSPA8	395853	YES	YES	YES
693	Heat shock protein 90 alpha family class A member 1	HSP90AA1	423463	YES	YES	YES
694	Heat shock protein 90 alpha family class B member 1	HSP90AB1	396188	YES	YES	NO
695	Heat shock protein 90 beta family member 1	HSP90B1	374163	YES	YES	NO
696	Heat shock protein family A (Hsp70) member 4	HSPA4	416339	YES	YES	YES
697	heat shock protein family A (Hsp70) member 4 like	HSP4AL	422496	YES	YES	YES
698	Heat shock protein family A (Hsp70) member 9	HSPA9	416183	YES	YES	YES
699	Heat shock protein family B (small) member 1	HSPB1	396227	YES	YES	NO
700	Heat shock protein family D (Hsp60) member 1	HSPD1	424059	YES	YES	YES
701	Heat shock protein family E (Hsp10) member 1	HSPE1	395948	NO	NO	YES
702	Hematological and neurological expressed 1 like	HN1L	416763	NO	YES	NO
703	Hematopoietic prostaglandin D synthase	HPGDS	395863	YES	YES	YES
704	Heme binding protein 1	HEBP1	417961	YES	YES	YES
705	Heme oxygenase 2	HMOX2	416663	YES	YES	NO
706	Hemoglobin beta, subunit A	HBBA	396485	YES	YES	YES
707	Hemoglobin beta, subunit rho	HBBR	419079	YES	YES	YES
708	Hemoglobin subunit alpha 1	HBA1	416652	YES	YES	YES
709	Hemoglobin subunit D	HBAD	416651	YES	YES	YES
710	Hemoglobin subunit epsilon	HBE	107049060	YES	YES	YES
711	Hemoglobin subunit epsilon 1	HBE1	428114	YES	YES	YES
712	Hemoglobin subunit mu	HBM	416651	YES	NO	YES
713	Hemoglobin subunit zeta	HBZ	416650	YES	YES	YES
714	Heparan sulfate proteoglycan 2	HSPG2	429806	YES	YES	NO

715	Hepatoma-derived growth factor	HDGF	776936	YES	YES	YES
716	Heterochromatin protein 1 binding protein 3	HP1BP3	425543	YES	NO	YES
717	Heterogeneous nuclear ribonucleoprotein A1	HNRNPA1	424136	YES	YES	YES
718	Heterogeneous nuclear ribonucleoprotein A/B	HNRNPAB	396268	YES	YES	YES
719	Heterogeneous nuclear ribonucleoprotein A2/B1	HNRNPA2B1	420627	YES	YES	YES
720	Heterogeneous nuclear ribonucleoprotein A3	HNRNPA3	100859627	YES	YES	YES
721	Heterogeneous nuclear ribonucleoprotein D	HNRNPD	422602	YES	YES	YES
722	Heterogeneous nuclear ribonucleoprotein D like	HNRNPDL	422601	NO	NO	YES
723	Heterogeneous nuclear ribonucleoprotein H1	HNRNPH2	395157	YES	YES	YES
724	Heterogeneous nuclear ribonucleoprotein H3	HNRNPH3	423686	YES	YES	YES
725	Heterogeneous nuclear ribonucleoprotein K	HNRNPK	427458	YES	NO	NO
726	Heterogeneous nuclear ribonucleoprotein K like	HNRNPKL	426516	NO	YES	YES
727	Heterogeneous nuclear ribonucleoprotein L	HNRNPL	101749377	YES	NO	NO
728	Heterogeneous nuclear ribonucleoprotein M	HNRNPM	420054	YES	YES	YES
729	Heterogeneous nuclear ribonucleoprotein R	HNRNPR	419570	YES	YES	YES
730	Heterogeneous nuclear ribonucleoprotein U	HNRNPU	421492	YES	YES	YES
731	Heterogeneous nuclear ribonucleoprotein U like 2	HNRNPUL2	395763	YES	YES	YES
732	Hexokinase 1	HK1	373889	YES	YES	NO
733	Hexokinase 2	HK2	374044	YES	YES	YES
734	Hexosaminidase subunit beta	HEXB	427204	YES	YES	NO
735	High density lipoprotein binding protein	HDLBP	395984	YES	YES	NO
736	High mobility group box 1	HMGB1	395724	YES	YES	YES
737	High mobility group box 2	HMGB2	396482	YES	YES	YES
738	High mobility group box 3	HMGB3	396232	NO	YES	YES
739	Hikeshi, heat shock protein nuclear import factor	HIKESHI	427034	YES	YES	NO
740	Histamine N-methyltransferase	HNMT	424298	NO	YES	NO
741	Histidine triad nucleotide binding protein 1	HINT1	100859112	NO	YES	NO
742	Histidine triad nucleotide binding protein 2	HINT2	395424	YES	YES	NO
743	Histidyl-tRNA synthetase	HARS	416132	YES	YES	NO
744	Histocompatibility minor 13	HM13	100859573	YES	NO	NO
745	Histone cluster 1, H1.03	HIST1H103	100858354	NO	NO	YES
746	Histone cluster 1, H1.11L (similar to human histone cluster 1, class H1 genes)	HIST1H111L	427892	YES	NO	YES
747	Histone cluster 1, H1.11R	HIST1H111R	427896	YES	YES	YES
748	Histone cluster 1, H2A, IV	HIST1H2A4	404299	NO	YES	YES
749	Histone cluster 1, H2bo	HIST1H2BO	100858607	YES	YES	NO
750	Histone cluster 1, H2B-VII (similar to human histone cluster 1, class H2B, member N)	HIST1H2B7	417956	NO	NO	YES
751	Histone cluster 1, H2B-VIII	HIST1H2B8	427886	YES	YES	YES
752	Histone cluster 1, H4b	HIST1H4B	770005	YES	NO	NO
753	Histone cluster 2, H4b	HIST2H4B	417950	YES	YES	YES
754	Histone deacetylase 1	HDAC1	373961	YES	YES	NO
755	Histone H2A-IV-like 2	HISTH2A4L2	101750582	YES	NO	NO
756	Histone H3.2-like	LOC769852	769852	YES	YES	YES
757	Histone PARylation factor 1	HPF1	422431	NO	NO	YES
758	Homer scaffolding protein 3	HOMER3	420115	NO	NO	YES
759	Hook microtubule tethering protein 3	HOOK3	427297	YES	YES	NO
760	HRas proto-oncogene, GTPase	HRAS	396229	YES	YES	NO
761	HSPA (Hsp70) binding protein 1	HSPBP1	100858988	YES	YES	YES
762	Hydroxyacid oxidase 2	HAO2	418311	NO	NO	YES

763	Hydroxyacyl-CoA dehydrogenase	HADH	420290	YES	YES	NO
764	Hydroxyacyl-CoA dehydrogenase/3-ketoacyl-CoA thiolase/enoyl-CoA hydratase (trifunctional protein), alpha subunit	HADHA	395929	YES	YES	NO
765	Hydroxyacylglutathione hydrolase	HAGH	416537	YES	YES	YES
766	Hydroxymethylbilane synthase	HMBS	419701	YES	YES	YES
767	Hydroxyprostaglandin dehydrogenase 15-(NAD)	HPGD	422567	NO	YES	NO
768	Hydroxypyruvate isomerase (putative)	HYI	424567	YES	YES	NO
769	Hydroxysteroid (11-beta) dehydrogenase 1a	HSD11B1A	771930	YES	YES	NO
770	Hydroxysteroid 17-beta dehydrogenase 4	HSD17B4	395785	YES	YES	NO
771	Hydroxysteroid (17-beta) dehydrogenase 10	HSD17B10	425758	YES	YES	NO
772	Hydroxysteroid (17-beta) dehydrogenase 12	HSD17B12	769787	YES	YES	NO
773	Hydroxysteroid dehydrogenase like 2	HSDL2	100858057	YES	YES	NO
774	Hypoxanthine phosphoribosyltransferase 1	HPRT1	395653	YES	YES	NO
775	Hypoxia up-regulated 1	HYOU1	428251	YES	YES	NO
776	IgGFc-binding protein-like	LOC429249	429249	NO	YES	YES
777	Immunoglobulin alpha heavy chain	N/A	N/A	YES	YES	NO
778	Immunoglobulin lambda-like polypeptide 1	IGLL1	416928	NO	NO	YES
779	Immunoglobulin superfamily member 9	IGSF9	425454	YES	YES	YES
780	Importin 5	IPO5	418783	YES	YES	YES
781	Importin 7	IPO7	423046	YES	YES	NO
782	Importin 9	IPO9	421167	NO	YES	NO
783	Inner membrane mitochondrial protein	IMMT	422912	YES	YES	NO
784	Inosine monophosphate dehydrogenase 2	IMPDH2	416058	YES	YES	NO
785	Inosine triphosphatase	ITPA	424390	NO	YES	NO
786	Inositol 1,4,5-trisphosphate receptor type 2	ITPR2	418212	NO	YES	NO
787	Inositol 1,4,5-trisphosphate receptor type 3	ITPR3	419910	NO	YES	NO
788	Inositol monophosphatase 1	IMPA1	420199	NO	YES	YES
789	Insulin degrading enzyme	IDE	423814	YES	NO	YES
790	Insulin like growth factor 2 mRNA binding protein 1	IGF2BP1	395953	YES	YES	NO
791	Insulin like growth factor 2 mRNA binding protein 3	IGF2BP3	420617	YES	YES	NO
792	Insulin like growth factor 2 receptor	IGF2R	395817	NO	YES	NO
793	Insulin like growth factor binding protein 7	IGFBP7	422620	YES	YES	NO
794	Integrin alpha 5	N/A	N/A	NO	YES	NO
795	Integrin linked kinase	ILK	374018	YES	YES	NO
796	Integrin subunit alpha 1	ITGA1	395951	YES	YES	NO
797	Integrin subunit alpha 3	ITGA3	373946	YES	YES	NO
798	Integrin subunit alpha 6	ITGA6	396226	YES	YES	NO
799	Integrin subunit alpha 8	ITGA8	396225	YES	YES	NO
800	Integrin subunit alpha 9	ITGA9	420757	NO	YES	NO
801	Integrin subunit alpha-V	ITGAV	396420	YES	YES	NO
802	Integrin subunit beta 1	ITGB1	374058	YES	YES	NO
803	Integrin subunit beta 3	ITGB3	374209	YES	YES	NO
804	Integrin subunit beta 4	ITGB4	417374	YES	YES	NO
805	Inter-alpha-trypsin inhibitor heavy chain 2	ITIH2	419110	NO	NO	YES
806	Interferon regulatory factor 2 binding protein-like	C5H14orf4	429422	YES	NO	NO
807	Interleukin enhancer binding factor 2	ILF2	425709	YES	YES	NO
808	Interleukin enhancer binding factor 3	ILF3	100858655	YES	YES	NO
809	IQ motif containing GTPase activating protein 2	IQGAP2	427211	YES	YES	NO

810	IQ motif containing GTPase activating protein 3	IQGAP3	415591	YES	YES	YES
811	Isoamyl acetate-hydrolyzing esterase 1 homolog	IAH1	421930	YES	YES	NO
812	Isochorismatase domain containing 1	ISOC1	415601	YES	YES	YES
813	Isocitrate dehydrogenase (NADP(+)) 1, cytosolic	IDH1	424112	YES	YES	YES
814	Isocitrate dehydrogenase (NADP(+)) 2, mitochondrial	IDH2	431056	YES	YES	YES
815	Isocitrate dehydrogenase 3 (NAD(+)) alpha	IDH3A	415362	YES	YES	NO
816	Isoleucyl-tRNA synthetase	IARS	415957	YES	YES	YES
817	Isoleucyl-tRNA synthetase 2, mitochondrial	IARS2	421346	YES	YES	NO
818	Junction plakoglobin	JUP	429710	YES	YES	NO
819	Karyopherin subunit alpha 3	KPNA3	418870	YES	NO	NO
820	Karyopherin subunit alpha 4	KPNA4	425012	YES	YES	NO
821	Karyopherin subunit beta 1	KPNB1	426499	YES	YES	YES
822	KDEL motif containing 1	KDELC1	418766	YES	NO	NO
823	Keratin 5	KRT5	407779	NO	NO	YES
824	Keratin 6A	KRT6A	408041	NO	YES	YES
825	Keratin 7	KRT7	395772	YES	YES	NO
826	Keratin 8	KRT8	426896	YES	YES	YES
827	Keratin 13	KRT13	408040	YES	YES	YES
828	Keratin 14	KRT14	408039	YES	YES	NO
829	Keratin 17	KRT17	100858439	YES	YES	NO
830	Keratin 18	KRT18	101749333	YES	YES	NO
831	Keratin 24	KRT24	395861	YES	YES	YES
832	Keratin 40	KRT40	420045	NO	YES	YES
833	Keratin, type II cytoskeletal 4-like	LOC112529929	112529929	NO	YES	NO
834	KH-type splicing regulatory protein	KHSRP	374140	YES	YES	YES
835	KIAA1671	KIAA1671	768701	NO	NO	YES
836	Kinectin 1	KTN1	396335	YES	YES	NO
837	Kinesin family member 5B	KIF5B	420472	YES	YES	NO
838	kinesin light chain 1	KLC1	423484	YES	NO	NO
839	Kinesin light chain 2	KLC2	769965	YES	NO	NO
840	Kininogen 1	KNG1	424957	NO	NO	YES
841	Lactate dehydrogenase A	LDHA	396221	YES	YES	YES
842	Lactate dehydrogenase B	LDHB	373997	YES	YES	YES
843	Lamin A/C	LMNA	396224	YES	YES	YES
844	Lamin B receptor	LBR	396285	YES	NO	YES
845	Lamin B1	LMNB1	396223	YES	YES	YES
846	Lamin B2	LMNB2	396222	YES	YES	YES
847	Laminin subunit alpha 1	LAMA1	374016	YES	YES	NO
848	Laminin subunit alpha 5	LAMA5	428148	YES	YES	NO
849	Laminin subunit beta 1	LAMB1	396478	YES	YES	NO
850	Laminin subunit gamma 1	LAMC1	424442	YES	YES	NO
851	Laminin, beta 2 (laminin S)	LAMB2	373980	NO	YES	NO
852	LanC like 1	LANCL1	424006	YES	YES	YES
853	Lanosterol synthase	LSS	424037	YES	NO	NO
854	Lecithin-cholesterol acyltransferase	LCAT	396136	NO	NO	YES
855	Lectin, galactoside-binding, soluble, 3	LGALS3	373917	NO	YES	NO
856	Lectin, mannose binding 1 like	LMAN1L	426849	YES	YES	NO
857	Lectin, mannose binding 2	LMAN2	100859676	YES	YES	YES

858	Legumain	LGMN	423418	YES	NO	NO
859	Leucine aminopeptidase 3	LAP3	425306	YES	YES	NO
860	Leucine rich pentatricopeptide repeat containing	LRPPRC	421403	YES	YES	NO
861	Leucine rich repeat containing 1	LRRC1	421891	YES	NO	NO
862	Leucine rich repeat containing 15	LRRC15	768899	YES	YES	YES
863	Leucine rich repeat containing 40	LRRC40	424713	YES	NO	YES
864	Leucine rich repeat containing 57	LRRC57	423236	YES	NO	NO
865	Leucine zipper and CTNNBIP1 domain containing	LZIC	419445	YES	YES	NO
866	Leucyl-tRNA synthetase	LARS	416347	YES	YES	YES
867	Leukocyte cell derived chemotaxin 2	LECT2	396471	YES	YES	YES
868	Leukocyte ribonuclease A-2	RSFR	423668	NO	YES	NO
869	Leukotriene A4 hydrolase	LTA4H	417918	YES	YES	YES
870	LIM and calponin homology domains 1	LIMCH1	422780	YES	NO	NO
871	LIM and SH3 protein 1	LASP1	420002	YES	YES	NO
872	LIM domain and actin binding 1	LIMA1	426878	YES	YES	NO
873	LIM domain containing preferred translocation partner in lipoma	LPP	429148	YES	YES	NO
874	LIM zinc finger domain containing 1	LIMS1	414880	NO	YES	NO
875	Lin-7 homolog C, crumbs cell polarity complex component	LIN7C	421608	YES	YES	YES
876	Lipase maturation factor 2	LMF2	429380	YES	NO	NO
877	lon peptidase 1, mitochondrial	LONP1	768942	YES	YES	NO
878	LSM3 homolog, U6 small nuclear RNA and mRNA degradation associated	LSM3	416040	YES	YES	NO
879	LSM6 homolog, U6 small nuclear RNA and mRNA degradation associated	LSM6	422466	NO	NO	YES
880	Lumican	LUM	417891	YES	YES	YES
881	Lymphocyte antigen 86	LY86	420872	NO	YES	NO
882	Lymphocyte cytosolic protein 1	LCP1	418852	YES	YES	NO
883	Lymphocyte-specific protein 1 pseudogene 1	LSP1P1	374254	YES	NO	NO
884	Lysine demethylase 1A	KDM1A	419571	YES	NO	NO
885	Lysophospholipase 2	LYPLA2	419685	YES	YES	NO
886	Lysozyme (renal amyloidosis)	LYZ	396218	YES	YES	YES
887	Lysozyme g2	LYG2	395708	YES	YES	YES
888	Lysyl-tRNA synthetase	KARS	415885	YES	YES	YES
889	Macrophage migration inhibitory factor (glycosylation-inhibiting factor)	MIF	100857237	NO	YES	NO
890	Macrophage stimulating 1 (hepatocyte growth factor-like)	MST1	396135	YES	YES	NO
891	Mago homolog, exon junction complex core component	MAGOH	374226	YES	YES	YES
892	Major vault protein	MVP	420049	YES	YES	NO
893	Malate dehydrogenase 1	MDH1	421281	YES	YES	YES
894	Malate dehydrogenase 2	MDH2	417517	YES	YES	YES
895	Mannose phosphate isomerase	MPI	769765	YES	YES	YES
896	Mannose receptor C type 1	MRC1	420516	NO	YES	NO
897	Mannose receptor C type 2	MRC2	419950	YES	YES	NO
898	MARCKS like 1	MARCKSL1	770764	YES	YES	NO
899	Matrin 3	MATR3	373948	YES	YES	NO
900	Matrix metallopeptidase 2	MMP2	386583	YES	YES	NO
901	Matrix remodeling associated 7	MXRA7	769894	YES	YES	NO
902	Mediator of cell motility 1	MEMO1	421483	YES	NO	NO
903	Melanoma cell adhesion molecule	MCAM	448832	YES	YES	NO
904	Membrane palmitoylated protein 6	MPP6	420622	NO	NO	YES
905	Mercaptopyruvate sulfurtransferase	MPST	418048	YES	YES	YES

906	Mesoderm development candidate 2	MESDC2	415475	YES	YES	NO
907	Mesothelin	MSLN	416534	NO	YES	NO
908	Metastasis associated 1 family member 2	MTA2	101747888	YES	NO	NO
909	Metastasis associated 1 family member 3	MTA3	421395	YES	NO	NO
910	Methionine adenosyltransferase 1A	MAT1A	423628	YES	NO	NO
911	Methionyl aminopeptidase 2	METAP2	417912	YES	YES	NO
912	Methylcrotonoyl-CoA carboxylase 2	MCCC2	427395	YES	YES	NO
913	Methylenetetrahydrofolate dehydrogenase, cyclohydrolase and formyltetrahydrofolate synthetase 1	MTHFD1	423508	YES	YES	YES
914	Methylmalonyl-CoA mutase	MUT	422049	YES	YES	NO
915	Methylthioadenosine phosphorylase	MTAP	431261	YES	YES	NO
916	MHC B-G antigen	BG8	417035	NO	NO	YES
917	Microsomal glutathione S-transferase 1	MGST1	418178	YES	YES	NO
918	Microsomal glutathione S-transferase 3	MGST3	424404	YES	YES	NO
919	Microtubule associated protein 4	MAP4	396097	YES	YES	NO
920	Microtubule associated protein RP/EB family member 1	MAPRE1	419288	YES	YES	YES
921	Microtubule associated protein tau	MAPT	426737	NO	NO	YES
922	Microtubule associated serine/threonine kinase 2	MAST2	424606	NO	NO	YES
923	Microtubule-actin crosslinking factor 1	MACF1	419671	NO	YES	NO
924	MIF4G domain containing	MIF4GD	100858848	YES	NO	NO
925	Minichromosome maintenance complex component 4	MCM4	426764	YES	NO	NO
926	Minichromosome maintenance complex component 6	MCM6	424295	YES	YES	NO
927	Mitochondria localized glutamic acid rich protein	MGARP	770869	NO	YES	NO
928	Mitochondrial calcium uniporter dominant negative beta subunit	MCUB	422698	YES	NO	NO
929	Mitochondrial carrier 2	MTCH2	395597	YES	YES	NO
930	Mitochondrial cytochrome c1, heme protein	N/A	N/A	YES	YES	NO
931	Mitogen-activated protein kinase 3	MAPK3	373953	YES	YES	NO
932	Mitogen-activated protein kinase 14	MAPK14	421183	YES	YES	NO
933	Mitochondrial ribosomal protein L12	MRPL12	769031	YES	YES	NO
934	Mitochondrial ribosomal protein L18	MRPL18	421585	NO	YES	NO
935	Mitochondrial ribosomal protein L39	MRPL39	418475	YES	NO	NO
936	Mitochondrial ribosomal protein L58	MRPL58	422114	YES	NO	NO
937	Mitogen-activated protein kinase kinase 2	MAP2K2	396349	NO	YES	NO
938	Mitotic spindle positioning	MISP	420097	NO	YES	YES
939	MMS19 homolog, cytosolic iron-sulfur assembly component	MMS19	423842	NO	YES	NO
940	MOB family member 4, phocein	MOB4	429007	YES	YES	NO
941	MOB kinase activator 2	MOB2	423097	NO	YES	NO
942	Monoamine oxidase A	MAOA	418563	YES	YES	NO
943	Monoglyceride lipase	MGLL	416024	YES	YES	NO
944	Mothers against decapentaplegic homolog 2-like	LOC769000	769000	YES	NO	NO
945	Mov10 RISC complex RNA helicase	MOV10	419872	YES	NO	YES
946	Mucin-5AC-like	MUC5ACL	107053416<br>100859916	YES	YES	NO
947	Mucin-5B-like	LOC112532570	112532570	YES	YES	NO
948	Muscleblind like splicing regulator 2	MBNL2	418784	NO	NO	YES
949	Myoferlin	MYOF	423805	NO	YES	NO
950	Myosin IB	MYO1B	424045	YES	YES	NO
951	Myosin IC	MYO1C	417555	YES	YES	NO

952	Myosin ID	MYO1D	419963	NO	YES	NO
953	Myosin light chain 12B	MYL12B	770011	YES	YES	YES
954	Myosin light chain kinase	MYLK	396445	YES	YES	NO
955	Myosin light chain kinase 2	MYLK2	396356	NO	NO	YES
956	Myosin light polypeptide 6	LOC654833	654833	YES	YES	NO
957	Myosin VI	MYO6	395487	YES	YES	NO
958	Myosin XVIIIA	MYO18A	417581	YES	YES	YES
959	Myosin, heavy chain 9, non-muscle	MYH9	396469	YES	YES	YES
960	Myosin, heavy chain 10, non-muscle	MYH10	396465	YES	YES	YES
961	Myosin, heavy chain 11, smooth muscle	MYH11	396211	YES	YES	NO
962	Myosin, heavy chain 1D, skeletal muscle (similar to human myosin, heavy chain 1, skeletal muscle, adult)	MYH1D	417306	YES	YES	NO
963	Myosin, heavy chain 1F, skeletal muscle (similar to human myosin, heavy chain 1, skeletal muscle, adult)	MYH1F	768566	NO	NO	YES
964	Myosin, light chain 4, alkali; atrial, embryonic	MYL4	396472	YES	YES	NO
965	Myosin, light chain 9, regulatory	MYL9	396215	YES	YES	NO
966	Myosin-XV-like	MYO15L	417377	YES	NO	YES
967	Myristoylated alanine rich protein kinase C substrate	MARCKS	396473	YES	YES	NO
968	N(alpha)-acetyltransferase 15, NatA auxiliary subunit	NAA15	422440	YES	YES	NO
969	N(alpha)-acetyltransferase 50, NatE catalytic subunit	NAA50	418319	YES	YES	NO
970	N-acetylneuraminate synthase	NANS	427283	YES	YES	YES
971	N-acetyltransferase 8B (GCN5-related, putative, gene/pseudogene)	NAT8B	776932	YES	YES	NO
972	N-acetyltransferase, pineal gland isozyme NAT-3	PNAT3	415809	YES	YES	NO
973	NACHT and WD repeat domain containing 1	NWD1	419556	YES	NO	NO
974	NAD(P)H quinone dehydrogenase 2	NQO2	420886	NO	YES	NO
975	NAD(P)HX dehydratase	NAXD	418755	NO	YES	NO
976	NADH:ubiquinone oxidoreductase core subunit S1	NDUFS1	424102	YES	YES	NO
977	NADH:ubiquinone oxidoreductase core subunit S3	NDUFS3	423179	YES	YES	NO
978	NADH:ubiquinone oxidoreductase core subunit S8	NDUFS8	769492	YES	YES	NO
979	NADH:ubiquinone oxidoreductase core subunit V1	NDUFV1	430210	YES	YES	NO
980	NADH:ubiquinone oxidoreductase core subunit V2	NDUFV2	426488	YES	YES	NO
981	NADH:ubiquinone oxidoreductase subunit A5	NDUFA5	417753	YES	YES	NO
982	NADH:ubiquinone oxidoreductase subunit A7	NDUFA7	420065	YES	YES	NO
983	NADH:ubiquinone oxidoreductase subunit A8	NDUFA8	417112	NO	YES	NO
984	NADH:ubiquinone oxidoreductase subunit A9	NDUFA9	419039	YES	YES	NO
985	NADH:ubiquinone oxidoreductase subunit A10	NDUFA10	424032	YES	YES	NO
986	NADH:ubiquinone oxidoreductase subunit A12	NDUFA12	417907	YES	YES	NO
987	NADH:ubiquinone oxidoreductase subunit B6	NDUFB6	416391	YES	YES	NO
988	NADH:ubiquinone oxidoreductase subunit B8	NDUFB8	423763	YES	YES	NO
989	NADH:ubiquinone oxidoreductase subunit B9	NDUFB9	420337	YES	YES	NO
990	NADH:ubiquinone oxidoreductase subunit B10	NDUFB10	416543	YES	YES	NO
991	NADH:ubiquinone oxidoreductase subunit S4	NDUFS4	374122	YES	YES	NO
992	Nascent polypeptide-associated complex alpha subunit	NACA	396544	YES	YES	YES
993	NCK adaptor protein 1	NCK1	418727	YES	NO	NO
994	NCK associated protein 1	NCKAP1	424000	NO	YES	NO
995	NDRG family member 4	NDRG4	768618	NO	YES	NO
996	NEDD8-activating enzyme E1 regulatory subunit	NAERS	107054305	NO	YES	YES
997	Nestin	NES	395890	YES	NO	NO

998	N-ethylmaleimide sensitive factor, vesicle fusing ATPase	NSF	419972	YES	YES	YES
999	Neuraminidase 2	NEU2	107049056	NO	YES	NO
1000	Neuroblast differentiation-associated protein AHNAK-like	LOC101750635	101750635	YES	YES	NO
1001	Neurofibromin 1	NF1	396085	NO	YES	NO
1002	Neurofilament, light polypeptide	NEFL	419528	NO	NO	YES
1003	Neurolysin	NLN	427167	YES	YES	NO
1004	Neuropilin 1	NRP1	395560	YES	YES	NO
1005	Neuroplastin	NPTN	415316	YES	NO	NO
1006	NFS1, cysteine desulfurase	NFS1	419133	YES	NO	YES
1007	NHP2 ribonucleoprotein	NHP2	416218	YES	YES	YES
1008	Nicalin	NCLN	426048	NO	YES	NO
1009	Nicotinamide nucleotide transhydrogenase	NNT	427196	YES	YES	NO
1010	Nicotinamide phosphoribosyltransferase pseudogene 1	NAMPTP1	417707	NO	YES	NO
1011	Nidogen 1	NID1	395531	YES	YES	NO
1012	Nidogen 2	NID2	423583	YES	NO	NO
1013	Nipsnap homolog 2	NIPSNAP2	417539	YES	YES	NO
1014	Nitrilase family member 2	NIT2	418386	YES	YES	NO
1015	NME/NM23 nucleoside diphosphate kinase 2	NME2	395916	YES	YES	YES
1016	NmrA like redox sensor 1	NMRAL1	416672	YES	YES	NO
1017	N-myristoyltransferase 1	NMT1	419966	YES	NO	NO
1018	Non-POU domain containing, octamer-binding	NONO	428701	YES	YES	YES
1019	Non-SMC condensin I complex subunit D2	NCAPD2	418275	NO	NO	YES
1020	NOP56 ribonucleoprotein	NOP56	426574	YES	NO	NO
1021	NOP58 ribonucleoprotein	NOP58	424087	YES	NO	NO
1022	NPL4 homolog, ubiquitin recognition factor	NPLOC4	417456	YES	NO	YES
1023	NSF attachment protein alpha	NAPA	100858304	YES	YES	YES
1024	NSF attachment protein gamma	NAPG	421051	NO	NO	YES
1025	NSFL1 cofactor	NSFL1C	419268	YES	YES	YES
1026	Nuclear autoantigenic sperm protein	NASP	424600	YES	YES	NO
1027	Nuclear casein kinase and cyclin dependent kinase substrate 1	NUCKS1	404775	NO	NO	YES
1028	Nuclear distribution C, dynein complex regulator	NUDC	419578	YES	NO	NO
1029	Nuclear mitotic apparatus protein 1	NUMA1	768717	YES	YES	YES
1030	Nucleobindin 2	NUCB2	423071	YES	YES	YES
1031	Nucleolin	NCL	396201	YES	YES	YES
1032	Nucleophosmin	NPM1	396203	YES	YES	YES
1033	Nucleophosmin/nucleoplasmin 3	NPM3	770430	NO	YES	YES
1034	Nucleoporin 85	NUP85	422120	YES	NO	NO
1035	Nucleoporin 93	NUP93	415693	YES	NO	NO
1036	Nucleoporin 133	NUP133	421535	YES	NO	YES
1037	Nucleoporin 155	NUP155	427443	YES	NO	YES
1038	Nucleoporin 205	NUP205	417935	YES	NO	YES
1039	Nucleoporin 210	NUP210	415977	YES	YES	YES
1040	Nucleoporin 214	NUP214	427760	NO	NO	YES
1041	Nucleosome assembly protein 1 like 1	NAP1L1	417864	YES	YES	YES
1042	Nucleosome assembly protein 1 like 4	NAP1L4	423087	YES	YES	YES
1043	NudC domain containing 1	NUDCD1	428379	YES	NO	NO
1044	NudC domain containing 2	NUDCD2	416162	NO	NO	YES
1045	Nudix hydrolase 1	NUDT1	416467	YES	YES	NO

1046	Nudix hydrolase 2	NUDT2	427399	YES	YES	NO
1047	Nudix hydrolase 3	NUDT3	419905	NO	YES	NO
1048	Nudix hydrolase 16 like 1	NUDT16L1	395557	YES	YES	YES
1049	Nudix hydrolase 21	NUDT21	100858636	YES	YES	YES
1050	Obg like ATPase 1	OLA1	424144	YES	YES	YES
1051	Ornithine aminotransferase	OAT	426430	YES	YES	NO
1052	Orosomucoid 1 (ovoglycoprotein)	ORM1	395220	YES	YES	YES
1053	Osteoclast stimulating factor 1	OSTF1	427258	YES	YES	NO
1054	Osteoglycin	OGN	374039	YES	YES	NO
1055	OTU deubiquitinase, ubiquitin aldehyde binding 1	OTUB1	777320	YES	YES	YES
1056	Ovalbumin (SERPINB14)	OVAL	396058	YES	YES	YES
1057	Ovalbumin-related protein X (SERPINB14C)	OVALX	420898	YES	YES	NO
1058	Ovalbumin-related protein Y (SERPINB14B)	OVALY	420897	YES	YES	NO
1059	Ovomucin, alpha subunit	LOC395381	395381	YES	YES	NO
1060	Ovostatin	OVST	396151	YES	YES	NO
1061	Oxoglutarate dehydrogenase	OGDH	426429	YES	YES	YES
1062	Oxysterol binding protein like 8	OSBPL8	417866	YES	NO	NO
1063	Oxysterol binding protein like 10	OSBPL10	420663	YES	NO	NO
1064	Palladin, cytoskeletal associated protein	PALLD	422428	YES	YES	NO
1065	Palmitoyl-protein thioesterase 1	PPT1	419681	YES	NO	NO
1066	Paraoxonase 1	PON1	395830	YES	YES	YES
1067	Paraspeckle component 1	PSPC1	418955	YES	NO	NO
1068	Parkinsonism associated deglycase	PARK7	395277	YES	YES	YES
1069	PBX homeobox interacting protein 1	PBXIP1	100857958	YES	YES	NO
1070	PC4 and SFRS1 interacting protein 1	PSIP1	431605	YES	NO	NO
1071	PDGFA associated protein 1	PDAP1	416491	NO	YES	YES
1072	PDS5 cohesin associated factor B	PDS5B	418910	YES	NO	YES
1073	PDZ and LIM domain 1	PDLIM1	428948	YES	YES	YES
1074	PDZ and LIM domain 3	PDLIM3	414873	YES	NO	NO
1075	PDZ and LIM domain 5	PDLIM5	422699	YES	NO	NO
1076	Penta-EF-hand domain containing 1	PEF1	419644	YES	YES	YES
1077	Peptidase D	PEPD	415776	YES	YES	NO
1078	Peptidase M20 domain containing 1	PM20D1	419838	NO	NO	YES
1079	Peptidase, mitochondrial processing alpha subunit	PMPCA	417134	YES	YES	NO
1080	Peptidylprolyl cis/trans isomerase, NIMA-interacting 4	PIN4	422134	NO	YES	NO
1081	Peptidylprolyl isomerase A	PPIA	776282	YES	YES	YES
1082	Peptidylprolyl isomerase B (cyclophilin B)	PPIB	396447	YES	YES	YES
1083	Peptidylprolyl isomerase D	PPID	428725	YES	YES	YES
1084	Peptidylprolyl isomerase H	PPIH	419507	YES	YES	NO
1085	Peptidylprolyl isomerase like 1	PPIL1	100859332	YES	YES	YES
1086	Perilipin-4	PLIN4	100857433	YES	YES	NO
1087	Periplakin	PPL	408044	YES	YES	NO
1088	Peroxiredoxin 1	PRDX1	424598	YES	YES	YES
1089	Peroxiredoxin 3	PRDX3	428986	YES	YES	YES
1090	Peroxiredoxin 4	PRDX4	418601	YES	YES	NO
1091	Peroxiredoxin 6	PRDX6	429062	YES	YES	YES
1092	Peroxisomal trans-2-enoyl-CoA reductase	PECR	424224	YES	YES	NO
1093	PEST proteolytic signal containing nuclear protein	PCNP	418398	YES	YES	YES

1094	Phenylalanyl-tRNA synthetase alpha subunit	FARSA	100859604	YES	YES	YES
1095	Phenylalanyl-tRNA synthetase beta subunit	FARSB	424812	YES	YES	YES
1096	Phosphatase domain containing, paladin 1	PALD1	373898	NO	NO	YES
1097	Phosphate cytidylyltransferase 1, choline, alpha	PCYT1A	424915	NO	NO	YES
1098	Phosphate regulating endopeptidase homolog, X-linked	PHEX	395777	YES	NO	NO
1099	Phosphatidylethanolamine binding protein 1	PEBP1	416990	YES	YES	YES
1100	Phosphatidylinositol transfer protein beta	PITPNB	416912	YES	NO	YES
1101	Phosphodiesterase 6D	PDE6D	424932	NO	YES	NO
1102	Phosphofructokinase, platelet	PFKP	428411	YES	YES	YES
1103	Phosphoglucomutase 1	PGM1	424691	YES	YES	NO
1104	Phosphoglucomutase 2	PGM2	426435	YES	YES	YES
1105	Phosphoglucomutase 2 like 1	PGM2L1	419052	NO	NO	YES
1106	Phosphoglucomutase 3	PGM3	421841	YES	YES	NO
1107	Phosphoglucomutase 5	PGM5	427215	YES	YES	NO
1108	Phosphogluconate dehydrogenase	PGD	419450	YES	YES	YES
1109	Phosphoglycerate dehydrogenase	PHGDH	424381	YES	YES	NO
1110	Phosphoglycerate kinase 2	PGK2	395833	YES	YES	YES
1111	Phosphoglycerate mutase 1	PGAM1	428969	YES	YES	NO
1112	Phosphoglycolate phosphatase	PGP	416559	YES	YES	YES
1113	phospholipase A2 receptor 1	PLA2R1	404304	YES	YES	NO
1114	Phospholipase C delta 1	PLCD1	420416	NO	YES	NO
1115	Phospholipase C gamma 1	PLCG1	419175	NO	NO	YES
1116	Phosphomannomutase 1	PMM1	417989	NO	YES	NO
1117	Phosphomannomutase 2	PMM2	427679	YES	YES	NO
1118	Phosphopantothenoylcysteine synthetase	PPCS	100859789	YES	YES	YES
1119	Phosphoribosyl pyrophosphate synthetase 1-like 1	PRPS1L1	422185	YES	YES	YES
1120	Phosphoribosyl pyrophosphate synthetase associated protein 1	PRPSAP1	417360	NO	YES	YES
1121	Phosphoribosyl pyrophosphate synthetase associated protein 2	PRPSAP2	416521	YES	NO	YES
1122	Phosphoribosylaminoimidazole carboxylase and phosphoribosylaminoimidazolesuccinocarboxamide synthase	PAICS	396534	YES	YES	YES
1123	Phosphoribosylglycinamide formyltransferase, phosphoribosylglycinamide synthetase, phosphoribosylaminoimidazole synthetase	GART	395315	YES	YES	YES
1124	Phosphorylase, glycogen, liver	PYGL	378909	YES	YES	NO
1125	phosphorylase, glycogen; brain	PYGB	421248	YES	YES	NO
1126	Phosphotriesterase related	PTER	420525	YES	YES	YES
1127	Piccolo presynaptic cytomatrix protein	PCLO	395319	NO	YES	NO
1128	Piezo type mechanosensitive ion channel component 1	PIEZO1	415849	NO	YES	NO
1129	PIT54 protein	PIT54	395364	NO	YES	YES
1130	Pitrilysin metallopeptidase 1	PITRM1	420462	YES	YES	NO
1131	Plasminogen	PLG	421580	YES	YES	YES
1132	Plastin 3	PLS3	422222	YES	YES	YES
1133	Platelet activating factor acetylhydrolase 1b catalytic subunit 2	PAFAH1B2	419765	YES	YES	NO
1134	Platelet activating factor acetylhydrolase 1b regulatory subunit 1	PAFAH1B1	374224	YES	YES	YES
1135	Platelet and endothelial cell adhesion molecule 1	PECAM1	771243	YES	YES	NO
1136	Pleckstrin	PLEK	395669	NO	NO	YES
1137	Pleckstrin homology and RhoGEF domain containing G1	PLEKHG1	421632	YES	YES	NO
1138	Plectin-like	LOC112531804, LOC107050437	112531804, 107050437	YES	YES	NO

1139	Plexin A1	PLXNA1	416030	NO	YES	NO
1140	Plexin B2	PLXNB2	425938	YES	YES	NO
1141	pM5 protein	PM5	416602	YES	YES	YES
1142	Podocalyxin like	PODXL	395755	NO	YES	NO
1143	Poly(A) binding protein cytoplasmic 1	PABPC1	430997	YES	YES	YES
1144	Poly(rC)-binding protein 2-like	LOC426023	426023	YES	YES	YES
1145	Poly(U) binding splicing factor 60	PUF60	426402	YES	NO	YES
1146	Polymerase (RNA) I polypeptide B, 128kDa	POLR1B	101749883	YES	YES	NO
1147	Polymerase I and transcript release factor	PTRF	396006	YES	YES	NO
1148	Polypeptide N-acetylgalactosaminyltransferase 7	GALNT7	422561	YES	YES	NO
1149	Polypyrimidine tract binding protein 1	PTBP1	420098	YES	YES	YES
1150	Potassium channel tetramerization domain containing 12	KCTD12	107051871	YES	YES	NO
1151	Potassium channel tetramerization domain containing 12B	KCTD12B	425504	NO	YES	NO
1152	Potassium channel tetramerization domain containing 16	KCTD16	427644	YES	YES	NO
1153	PPFIA binding protein 1	PPFIBP1	418219	YES	NO	NO
1154	Prefoldin subunit 2	PFDN2	100858383	YES	YES	NO
1155	Prefoldin subunit 4	PFDN4	419337	NO	YES	NO
1156	Prefoldin subunit 5	PFDN5	100859376	YES	YES	NO
1157	Pre-mRNA processing factor 4	PRPF4	417270	YES	NO	NO
1158	Pre-mRNA processing factor 6	PRPF6	419254	YES	NO	NO
1159	Pre-mRNA processing factor 8	PRPF8	417559	YES	YES	YES
1160	Pre-mRNA-processing factor 19	PRPF19	430767	YES	YES	YES
1161	pre-mRNA-processing factor 19-like	PRPF19L	107053341	YES	YES	YES
1162	pre-mRNA-splicing factor SYF1-like	LOC107051321	107051321	YES	NO	NO
1163	Prenylcysteine oxidase 1	PCYOX1	426297	YES	YES	NO
1164	Procollagen-lysine, 2-oxoglutarate 5-dioxygenase 1	PLOD1	419485	YES	YES	NO
1165	Procollagen-lysine,2-oxoglutarate 5-dioxygenase 2	PLOD2	424882	YES	NO	NO
1166	Profilin 2	PFN2	771904	YES	YES	NO
1167	Progesterone receptor membrane component 1	PGRMC1	772196	YES	YES	NO
1168	Progesterone receptor membrane component 2	PGRMC2	422501	YES	YES	NO
1169	programmed cell death 4	PDCD4	374191	YES	NO	NO
1170	Programmed cell death 6	PDCD6	420988	YES	YES	YES
1171	Programmed cell death 6 interacting protein	PDCD6IP	420725	YES	YES	NO
1172	Prohibitin	PHB	419980	YES	YES	YES
1173	Prohibitin-2	PHB2	771124	YES	YES	YES
1174	Proliferating cell nuclear antigen	PCNA	373984	YES	YES	NO
1175	Proliferation-associated 2G4, 38kDa	PA2G4	425279	YES	YES	YES
1176	Proline and arginine rich end leucine rich repeat protein	PRELP	419933	YES	YES	NO
1177	Proline rich coiled-coil 1	PRRC1	427127	YES	YES	NO
1178	Proline synthetase cotranscribed homolog (bacterial)	PROSC	426770	YES	YES	YES
1179	Prolyl 3-hydroxylase 1	P3H1	414142	YES	NO	NO
1180	Prolyl 3-hydroxylase 3	P3H3	418289	YES	NO	NO
1181	Prolyl 4-hydroxylase subunit alpha 1	P4HA1	423704	YES	NO	NO
1182	Prolyl 4-hydroxylase subunit beta	P4HB	374091	YES	YES	YES
1183	Prolyl endopeptidase	PREP	421785	YES	YES	YES
1184	Prolylcarboxypeptidase	PRCP	428096	YES	NO	NO
1185	Propionyl-CoA carboxylase alpha subunit	PCCA	418774	NO	YES	NO
1186	Propionyl-CoA carboxylase beta subunit	PCCB	768706	YES	YES	NO

1187	Prostaglandin D2 synthase 21kDa (brain)	PTGDS	374110	YES	YES	NO
1188	Prostaglandin E synthase 3	PTGES3	100859133	YES	YES	YES
1189	Prostaglandin F2 receptor inhibitor	PTGFRN	418449	YES	YES	NO
1190	Prostaglandin reductase 1	PTGR1	427337	YES	YES	YES
1191	Prostaglandin-endoperoxide synthase 1 (prostaglandin G/H synthase and cyclooxygenase)	PTGS1	427752	NO	YES	NO
1192	Proteasome (prosome, macropain) 26S subunit, non-ATPase, 13	LOC422989	422989	YES	YES	YES
1193	Proteasome 26S subunit, ATPase 1	PSMC1	395804	YES	YES	YES
1194	Proteasome 26S subunit, ATPase 2	PSMC2	417716	YES	YES	YES
1195	Proteasome 26S subunit, ATPase 3	PSMC3	423182	YES	YES	YES
1196	Proteasome 26S subunit, ATPase 5	PSMC5	428274	YES	YES	NO
1197	Proteasome 26S subunit, ATPase 6	PSMC6	423586	YES	YES	YES
1198	Proteasome 26S subunit, non-ATPase 1	PSMD1	424926	YES	YES	YES
1199	Proteasome 26S subunit, non-ATPase 2	PSMD2	425294	YES	YES	YES
1200	Proteasome 26S subunit, non-ATPase 3	PSMD3	426133	YES	YES	YES
1201	Proteasome 26S subunit, non-ATPase 4	PSMD4	100216364	YES	YES	YES
1202	Proteasome 26S subunit, non-ATPase 5	PSMD5	417125	YES	YES	YES
1203	Proteasome 26S subunit, non-ATPase 6	PSMD6	416079	YES	YES	YES
1204	Proteasome 26S subunit, non-ATPase 7	PSMD7	415869	YES	YES	YES
1205	Proteasome 26S subunit, non-ATPase 9	PSMD9	416853	YES	YES	YES
1206	Proteasome 26S subunit, non-ATPase 10	PSMD10	422351	YES	YES	YES
1207	Proteasome 26S subunit, non-ATPase 11	PSMD11	430878	YES	YES	YES
1208	Proteasome 26S subunit, non-ATPase 12	PSMD12	417425	YES	YES	YES
1209	Proteasome 26S subunit, non-ATPase 14	PSMD14	424189	YES	YES	YES
1210	Proteasome assembly chaperone 1	PSMG1	418520	YES	YES	NO
1211	Proteasome assembly chaperone 2	PSMG2	421040	YES	YES	NO
1212	Proteasome inhibitor subunit 1	PSMF1	430115	YES	YES	YES
1213	Proteasome subunit alpha 1	PSMA1	395874	YES	YES	YES
1214	Proteasome subunit alpha 2	PSMA2	420772	YES	YES	YES
1215	Proteasome subunit alpha 3	PSMA3	423542	YES	YES	YES
1216	Proteasome subunit alpha 4	PSMA4	415357	YES	YES	YES
1217	Proteasome subunit alpha 5	PSMA5	426937	YES	YES	YES
1218	Proteasome subunit alpha 6	PSMA6	423326	YES	YES	YES
1219	Proteasome subunit alpha 7	PSMA7	395318	YES	YES	YES
1220	Proteasome subunit beta 1	PSMB1	421551	YES	YES	YES
1221	Proteasome subunit beta 2	PSMB2	419630	YES	YES	YES
1222	Proteasome subunit beta 3	PSMB3	419997	YES	YES	YES
1223	Proteasome subunit beta 4	PSMB4	429986	YES	YES	YES
1224	Proteasome subunit beta 5	PSMB5	396003	YES	YES	YES
1225	Proteasome subunit beta 7	PSMB7	378915	YES	YES	YES
1226	Protein arginine methyltransferase 1 variant 1	N/A	N/A	YES	YES	YES
1227	Protein arginine methyltransferase 5	PRMT5	101750306	YES	YES	YES
1228	Protein disulfide isomerase family A member 3	PDIA3	373899	YES	YES	YES
1229	Protein disulfide isomerase family A member 4	PDIA4	420785	YES	YES	YES
1230	Protein disulfide isomerase family A member 5	PDIA5	424249	YES	YES	NO
1231	Protein disulfide isomerase family A member 6	PDIA6	421940	YES	YES	YES
1232	Protein kinase C and casein kinase substrate in neurons 2	PACSIN2	395975	NO	YES	NO
1233	Protein kinase C delta	PRKCD	415905	NO	YES	NO

1234	Protein kinase cAMP-activated catalytic subunit beta	PRKACB	424542	YES	NO	NO
1235	Protein kinase cAMP-dependent type I regulatory subunit alpha	PRKAR1A	417438	YES	YES	NO
1236	Protein O-fucosyltransferase 1	POFUT1	395070	YES	YES	NO
1237	Protein O-fucosyltransferase 2	POFUT2	395112	NO	YES	NO
1238	Protein phosphatase 1 catalytic subunit beta	PPP1CB	396019	YES	YES	YES
1239	Protein phosphatase 1 regulatory subunit 7	PPP1R7	424844	YES	NO	YES
1240	Protein phosphatase 1 regulatory subunit 21	PPP1R21	421287	YES	YES	YES
1241	Protein phosphatase 1, regulatory (inhibitor) subunit 9B	PPP1R9B	395762	YES	NO	NO
1242	Protein phosphatase 2 catalytic subunit beta	PPP2CB	396021	YES	YES	NO
1243	Protein phosphatase 2 phosphatase activator	PTPA	426329	YES	YES	YES
1244	Protein phosphatase 2 regulatory subunit B alpha	PPP2R2A	419523	YES	YES	YES
1245	Protein phosphatase 2 regulatory subunit B delta	PPP2R5D	421250	YES	NO	NO
1246	Protein phosphatase 3, catalytic subunit, alpha isozyme	PPP3CA	395113	YES	YES	NO
1247	Protein phosphatase 6 catalytic subunit	PPP6C	772354	YES	NO	YES
1248	Protein phosphatase, Mg2+/Mn2+ dependent 1B	PPM1B	421404	YES	YES	YES
1249	Protein S100-A9-like	LOC101747386	101747386	NO	YES	NO
1250	Protein tyrosine phosphatase non-receptor type 11	PTPN11	395815	NO	NO	YES
1251	Protein tyrosine phosphatase type 4A2	PTP4A2	100543852	YES	YES	NO
1252	Protein tyrosine phosphatase, receptor type D	PTPRD	431602	YES	NO	NO
1253	Protein-L-isoaspartate (D-aspartate) O-methyltransferase	PCMT1	428607	YES	YES	YES
1254	Putative glutamine amidotransferase like class 1 domain containing 3A-like 1	GATD3AL1	418811	YES	YES	YES
1255	Putative glutamine amidotransferase like class 1 domain containing 3A-like 2	GATD3AL2	422305	YES	YES	NO
1256	Putative methyltransferase DDB_G0268948	LOC107048987	107048987	YES	YES	YES
1257	Pyridoxal (pyridoxine, vitamin B6) kinase	PDXK	418549	NO	YES	NO
1258	Pyridoxal phosphatase	PDXP	771207	NO	YES	NO
1259	Pyrroline-5-carboxylate reductase 1	PYCR1	769203	YES	NO	NO
1260	Pyrroline-5-carboxylate reductase-like	PYCRL	420293	YES	YES	NO
1261	Pyruvate carboxylase	PC	374263	YES	YES	NO
1262	Pyruvate dehydrogenase (lipoamide) alpha 1	PDHA1	418610	YES	YES	YES
1263	Pyruvate dehydrogenase (lipoamide) beta	PDHB	416066	YES	YES	NO
1264	Pyruvate kinase, liver and RBC	PKLR	396456	YES	YES	YES
1265	QKI, KH domain containing RNA binding	QKI	374204	YES	YES	NO
1266	Quinoid dihydropteridine reductase	QDPR	426335	YES	YES	YES
1267	RAB1B, member RAS oncogene family	RAB1B	421273	YES	YES	YES
1268	RAB2A, member RAS oncogene family	RAB2A	396153	YES	YES	YES
1269	RAB2B, member RAS oncogene family	RAB2B	100858328	YES	YES	NO
1270	RAB3B, member RAS oncogene family	RAB3B	424636	YES	NO	NO
1271	RAB4A, member RAS oncogene family	RAB4A	421532	NO	YES	NO
1272	RAB5A, member RAS oncogene family	RAB5A	420649	YES	YES	NO
1273	RAB5B, member RAS oncogene family	RAB5B	100529061	YES	YES	YES
1274	RAB5C, member RAS oncogene family	RAB5C	395197	NO	YES	YES
1275	RAB6A, member RAS oncogene family	RAB6A	419063	YES	YES	NO
1276	RAB7A, member RAS oncogene family	RAB7A	416016	YES	YES	YES
1277	RAB8A, member RAS oncogene family	RAB8A	428352	NO	YES	NO
1278	RAB8B, member RAS oncogene family	RAB8B	415371	YES	YES	YES
1279	RAB9A, member RAS oncogene family	RAB9A	418635	YES	YES	YES
1280	RAB10, member RAS oncogene family	RAB10	421994	YES	YES	NO
1281	RAB11B, member RAS oncogene family	RAB11B	420063	YES	YES	YES

1282	RAB12, member RAS oncogene family	RAB12	421048	NO	NO	YES
1283	RAB14, member RAS oncogene family	RAB14	417119	YES	YES	YES
1284	RAB18, member RAS oncogene family	RAB18	420483	YES	YES	NO
1285	RAB21, member RAS oncogene family	RAB21	771383	YES	YES	YES
1286	RAB22A, member RAS oncogene family	RAB22A	419318	YES	YES	NO
1287	RAB27A, member RAS oncogene family	RAB27A	415410	YES	YES	NO
1288	RAB32, member RAS oncogene family	RAB32	421616	YES	YES	NO
1289	RAB33B, member RAS oncogene family	RAB33B	422441	YES	NO	NO
1290	RAB34, member RAS oncogene family	RAB34	100858849	YES	YES	NO
1291	RAD23 homolog B, nucleotide excision repair protein	RAD23B	431623	YES	YES	YES
1292	Radixin	RDX	395511	YES	YES	YES
1293	Raftlin, lipid raft linker 1	RFTN1	395109	YES	YES	NO
1294	RALY heterogeneous nuclear ribonucleoprotein	RALY	419148	YES	NO	YES
1295	RAN binding protein 1	RANBP1	416787	YES	NO	YES
1296	RAN binding protein 2	RANBP2	418730	YES	NO	NO
1297	RAN, member RAS oncogene family	RAN	396193	YES	YES	YES
1298	Rap1 GTPase-GDP dissociation stimulator 1	RAP1GDS1	422701	YES	YES	NO
1299	RAP1A, member of RAS oncogene family	RAP1A	419867	NO	YES	YES
1300	RAP1B, member of RAS oncogene family	RAP1B	417840	YES	YES	YES
1301	RAP2A, member of RAS oncogene family	RAP2A	769797	NO	YES	NO
1302	Ras homolog enriched in brain	RHEB	420434	NO	YES	NO
1303	Ras homolog family member A	RHOA	395442	YES	YES	NO
1304	RAS like proto-oncogene A	RALA	420765	YES	YES	YES
1305	Ras related GTP binding A	RRAGA	422311	YES	YES	NO
1306	Ras suppressor protein 1	RSU1	420524	YES	YES	NO
1307	Ras-related C3 botulinum toxin substrate 1 (rho family, small GTP binding protein Rac1)	RAC1	395871	YES	YES	YES
1308	RB binding protein 4, chromatin remodeling factor	RBBP4	395658	YES	YES	YES
1309	RB binding protein 7, chromatin remodeling factor	RBBP7	395390	YES	YES	NO
1310	Receptor accessory protein 5	REEP5	770021	YES	YES	NO
1311	Receptor for activated C kinase 1	RACK1	417044	YES	YES	YES
1312	Regulation of nuclear pre-mRNA domain containing 1B	RPRD1B	419295	YES	NO	YES
1313	Regulator of chromosome condensation 1	RCC1	429810	YES	NO	YES
1314	Regulator of chromosome condensation 2	RCC2	419361	YES	YES	YES
1315	Regulator of microtubule dynamics 1	RMDN1	420213	YES	YES	NO
1316	Related RAS viral (r-ras) oncogene homolog	RRAS	423062	YES	YES	NO
1317	Replication factor C subunit 4	RFC4	424958	YES	NO	NO
1318	Replication protein A1	RPA1	417563	YES	NO	YES
1319	Retention in endoplasmic reticulum sorting receptor 1	RER1	419397	YES	YES	NO
1320	Reticulocalbin 1	RCN1	428602	NO	YES	NO
1321	Reticulon 4	RTN4	378790	YES	YES	NO
1322	Retinoid X receptor alpha	RXRA	417143	YES	NO	NO
1323	Retinol binding protein 4 A, plasma	RBP4A	396166	YES	YES	YES
1324	Retinol dehydrogenase 16 (all-trans)	RDH16	776432	YES	YES	NO
1325	Rh family C glycoprotein	RHCG	415490	NO	YES	NO
1326	Rh-associated glycoprotein	RHAG	395118	NO	NO	YES
1327	Rho associated coiled-coil containing protein kinase 2	ROCK2	374182	NO	YES	NO
1328	Rho GDP dissociation inhibitor beta	ARHGDIB	417941	NO	YES	YES

1329	Rho GTPase activating protein 1	ARHGAP1	428865	YES	YES	YES
1330	Rho-associated, coiled-coil containing protein kinase 1	ROCK1	373970	NO	YES	NO
1331	Rho-related GTP-binding protein RhoG-like	RHOGL	422207	YES	YES	NO
1332	Riboflavin binding protein	RBP	396449	YES	NO	NO
1333	Ribonuclease/angiogenin inhibitor 1	RNH1	423111	YES	YES	NO
1334	Ribophorin I	RPN1	416017	YES	YES	NO
1335	Ribophorin II	RPN2	419177	YES	YES	NO
1336	Ribosomal protein L3	RPL3	418016	YES	YES	NO
1337	Ribosomal protein L4	RPL4	415551	YES	YES	NO
1338	Ribosomal protein L5	RPL5	395296	YES	YES	YES
1339	Ribosomal protein L6	RPL6	373957	YES	YES	NO
1340	Ribosomal protein L7	RPL7	420182	YES	YES	YES
1341	Ribosomal protein L7a	RPL7A	417158	YES	YES	NO
1342	Ribosomal protein L8	RPL8	418568	YES	YES	YES
1343	Ribosomal protein L9	RPL9	425468	YES	YES	YES
1344	Ribosomal protein L10a	RPL10A	419895	YES	YES	YES
1345	Ribosomal protein L10-like	RPL10L	396411	YES	NO	NO
1346	Ribosomal protein L11	RPL11	419682	YES	YES	YES
1347	Ribosomal protein L12	RPL12	417264	YES	YES	YES
1348	Ribosomal protein L13	RPL13	395849	YES	YES	NO
1349	Ribosomal protein L13a	RPL13a	560828	YES	YES	NO
1350	Ribosomal protein L14	RPL14	374134	YES	YES	NO
1351	Ribosomal protein L15	RPL15	428442	YES	YES	NO
1352	Ribosomal protein L17	RPL17	426845	YES	YES	NO
1353	Ribosomal protein L18 (Fragment)	N/A	N/A	YES	YES	NO
1354	Ribosomal protein L18a	RPL18A	417823	YES	YES	NO
1355	Ribosomal protein L19	RPL19	420003	YES	NO	NO
1356	Ribosomal protein L21	RPL21	418933	YES	YES	NO
1357	Ribosomal protein L22	RPL22	373937	YES	YES	YES
1358	Ribosomal protein L23	RPL23	420001	YES	YES	YES
1359	Ribosomal protein L23a	RPL23A	417574	YES	YES	NO
1360	Ribosomal protein L24	RPL24	418401	YES	YES	YES
1361	Ribosomal protein L26 like 1	RPL26L1	396400	YES	YES	NO
1362	Ribosomal protein L27	RPL27	396280	YES	YES	YES
1363	Ribosomal protein L27a	RPL27A	770018	YES	YES	YES
1364	Ribosomal protein L28	RPL28	107051012	YES	YES	NO
1365	Ribosomal protein L30	RPL30	425416	YES	YES	YES
1366	Ribosomal protein L31	RPL31	418710	YES	YES	NO
1367	Ribosomal protein L35a	RPL35A	424924	NO	YES	NO
1368	Ribosomal protein L36	RPL36	373936	YES	YES	NO
1369	Ribosomal protein lateral stalk subunit P0	RPLP0	395835	YES	YES	YES
1370	Ribosomal protein lateral stalk subunit P1	RPLP1	396262	YES	NO	NO
1371	Ribosomal protein lateral stalk subunit P2	RPLP2	426492	YES	YES	NO
1372	Ribosomal protein S2	RPS2	416544	YES	YES	NO
1373	Ribosomal protein S3	RPS3	419069	YES	YES	YES
1374	Ribosomal protein S3A	RPS3A	422477	YES	YES	YES
1375	Ribosomal protein S4,Y-linked 1	RPS4Y1	396001	YES	YES	YES
1376	Ribosomal protein S6	RPS6	396148	YES	YES	NO

1377	Ribosomal protein S6 kinase A3	RPS6KA3	418605	NO	YES	NO
1378	Ribosomal protein S7	RPS7	421919	YES	YES	YES
1379	Ribosomal protein S8	RPS8	424584	YES	YES	YES
1380	Ribosomal protein S10	RPS10	419904	YES	YES	YES
1381	Ribosomal protein S11	RPS11	419049	YES	YES	NO
1382	Ribosomal protein S12	RPS12	421698	YES	YES	NO
1383	Ribosomal protein S13	RPS13	414782	YES	YES	YES
1384	Ribosomal protein S14	RPS14	416275	YES	YES	NO
1385	Ribosomal protein S15a	RPS15A	427675	YES	YES	NO
1386	Ribosomal protein S16	RPS16	417871	YES	YES	YES
1387	Ribosomal protein S17	RPS17	374053	YES	YES	NO
1388	Ribosomal protein S19	RPS19	107050719	YES	YES	NO
1389	Ribosomal protein S20	RPS20	430990	YES	YES	YES
1390	Ribosomal protein S23	RPS23	427323	YES	YES	YES
1391	Ribosomal protein S24	RPS24	423726	YES	NO	NO
1392	Ribosomal protein S25	RPS25	770722	YES	YES	YES
1393	Ribosomal protein S26	RPS26	100857770	YES	YES	YES
1394	Ribosomal protein S27a	RPS27A	395796	YES	YES	YES
1395	Ribosomal protein SA pseudogene 58	RPSAP58	395181	YES	YES	YES
1396	Ribosomal RNA processing 12 homolog	RRP12	423845	YES	NO	NO
1397	Ribosome binding protein 1	RRBP1	396414	YES	YES	NO
1398	RIMS-binding protein 3A-like	RIMSBP3AL	101748341	NO	NO	YES
1399	RNA 2',3'-cyclic phosphate and 5'-OH ligase	RTCB	418064	YES	YES	YES
1400	RNA 3'-terminal phosphate cyclase	RTCA	424468	YES	YES	YES
1401	RNA binding motif (RNP1, RRM) protein 3	RBM3	425789	YES	YES	YES
1402	RNA binding motif protein 12	RBM12	404531	YES	YES	YES
1403	RNA binding motif protein 15B	RBM15B	770664	YES	YES	NO
1404	RNA binding motif protein 24	RBM24	420846	NO	NO	YES
1405	RNA binding motif protein 25	RBM25	423250	YES	YES	NO
1406	RNA binding motif protein, X-linked like 1	RBMXL1	422248	YES	YES	YES
1407	RNA guanine-7 methyltransferase	RNMT	421046	YES	YES	NO
1408	RNA polymerase II subunit B	POLR2B	422621	NO	NO	YES
1409	RNA polymerase II subunit H	POLR2H	424954	YES	YES	NO
1410	RNA-binding protein 4B-like	LOC100859276	100859276	YES	NO	YES
1411	RTN3w	RTN3	431037	YES	YES	NO
1412	RuvB like AAA ATPase 1	RUVBL1	416022	YES	YES	YES
1413	RuvB-like 2	LOC107051177	107051177	YES	YES	YES
1414	S100 calcium binding protein A11	S100A11	396075	YES	YES	NO
1415	S100 calcium binding protein A12	S100A12	426356	YES	YES	NO
1416	S100 calcium binding protein A16	S100A16	100859094	NO	YES	NO
1417	SAC1 suppressor of actin mutations 1-like (yeast)	SACM1L	420700	YES	YES	YES
1418	Saccharopine dehydrogenase (putative)	SCCPDH	421485	YES	YES	NO
1419	SAMM50 sorting and assembly machinery component	SAMM50	418234	YES	YES	NO
1420	SAP domain containing ribonucleoprotein	SARNP	425058	YES	NO	YES
1421	Sarcosine dehydrogenase	SARDH	417146	YES	NO	NO
1422	Scinderin	SCIN	420588	YES	YES	NO
1423	SCO2, cytochrome c oxidase assembly protein	SCO2	429671	NO	YES	NO
1424	Sec1 family domain containing 1	SCFD1	423307	YES	YES	NO

1425	SEC11 homolog C, signal peptidase complex subunit	SEC11C	426850	NO	YES	NO
1426	SEC22 homolog B, vesicle trafficking protein	SEC22B	424377	YES	YES	YES
1427	SEC23 homolog A, coat complex II component	SEC23A	423335	YES	YES	NO
1428	SEC24 homolog C, COPII coat complex component	SEC24C	423742	YES	NO	NO
1429	SEC31 homolog B, COPII coat complex component	SEC31B	423764	NO	NO	YES
1430	SEC61 translocon alpha 1 subunit	SEC61A1	416023	YES	YES	NO
1431	Secreted protein acidic and cysteine rich	SPARC	386571	YES	NO	NO
1432	Secretion associated Ras related GTPase 1A	SAR1A	423711	YES	YES	NO
1433	Secretory carrier membrane protein 2	SCAMP2	769853	YES	YES	YES
1434	SEH1 like nucleoporin	SEH1L	100859886	YES	YES	NO
1435	Selenium binding protein 1	SELENBP1	425664	YES	YES	YES
1436	Selenocysteine lyase	SCLY	424739	YES	NO	NO
1437	Selenophosphate synthetase 1	SEPHS1	426612	YES	YES	NO
1438	Sepiapterin reductase (7,8-dihydrobiopterin:NADP+ oxidoreductase)	SPR	425255	YES	YES	NO
1439	Septin 2	SEPT2	424843	YES	YES	YES
1440	Septin 2-like	SEPT2L	416777	YES	YES	NO
1441	Septin 6	SEPT6	422369	YES	YES	NO
1442	Septin 7	SEPT7	420741	YES	YES	NO
1443	Septin 8	SEPTIN8	416333	YES	YES	NO
1444	Septin 9	SEPTIN9	417347	YES	YES	NO
1445	Septin 11	SEPTIN11	422635	YES	YES	NO
1446	Serine and arginine rich splicing factor 1	SRSF1	772264	YES	YES	YES
1447	Serine and arginine rich splicing factor 2	SRSF2	396195	YES	YES	YES
1448	Serine and arginine rich splicing factor 3	SRSF3	419815	YES	YES	YES
1449	Serine and arginine rich splicing factor 6	SRSF6	419116	YES	YES	YES
1450	Serine and arginine rich splicing factor 7	SRSF7	100859609	YES	YES	YES
1451	Serine carboxypeptidase 1	SCPEP1	417403	YES	YES	NO
1452	Serine peptidase inhibitor, Kazal type 5	SPINK5	416235	YES	YES	NO
1453	Serine peptidase inhibitor, Kazal type 7 (putative)	SPINK7	416236	YES	YES	NO
1454	Serine peptidase inhibitor, Kunitz type 4	SPINT4	101751715	NO	YES	NO
1455	Serine/threonine kinase 25	STK25	378801	NO	YES	NO
1456	Serine/threonine kinase receptor associated protein	STRAP	418175	YES	YES	YES
1457	Serine/threonine protein kinase 26	STK26	422231	NO	NO	YES
1458	Serine/threonine-protein phosphatase 2A 65 kDa regulatory subunit A alpha isoform	LOC107050599	107050599	YES	NO	NO
1459	Serologically defined colon cancer antigen 8	SDCCAG8	421498	YES	NO	NO
1460	Serpin family B member 1	SERPINB1	420894	NO	YES	NO
1461	Serpin family B member 2	SERPINB2	420896	NO	YES	NO
1462	Serpin family B member 5	SERPINB5	420900	NO	YES	NO
1463	Serpin family F member 2	SERPINF2	100857105	NO	YES	YES
1464	Serpin family H member 1	SERPINH1	396228	YES	YES	NO
1465	Serpin peptidase inhibitor, clade A (alpha-1 antiproteinase, antitrypsin), member 1	SPIA1	423434	NO	NO	YES
1466	Serpin peptidase inhibitor, clade A (alpha-1 antiproteinase, antitrypsin), member 4	SPIA4	423433	YES	YES	YES
1467	Serpin peptidase inhibitor, clade B (ovalbumin), member 10 B	SERPINB10B	395715	NO	YES	NO
1468	Serpin peptidase inhibitor, clade B (ovalbumin), member 6	SERPINB6	420895	YES	YES	NO
1469	Serpin peptidase inhibitor, clade C (antithrombin), member 1	SERPINC1	424440	NO	NO	YES
1470	Serpin peptidase inhibitor, clade D (heparin cofactor), member 1	SERPIND1	395877	NO	NO	YES
1471	SERPINE1 mRNA binding protein 1	SERBP1	424706	YES	NO	NO
1472	Seryl-tRNA synthetase 1	SARS1	426697	YES	YES	YES

1473	SET nuclear proto-oncogene	SET	417210	YES	YES	YES
1474	SH3 domain binding glutamate rich protein like	SH3BGRL	422277	YES	YES	NO
1475	SH3 domain containing GRB2 like 1, endophilin A2	SH3GL1	395202	YES	YES	NO
1476	SH3 domain containing GRB2 like endophilin B1	SH3GLB1	424522	YES	NO	NO
1477	Shootin 1	SHTN1	423919	YES	YES	NO
1478	Sideroflexin 2	SFXN2	423866	YES	YES	NO
1479	Sideroflexin 3	SFXN3	428972	YES	YES	NO
1480	Signal peptidase complex subunit 3	SPCS3	396234	YES	YES	NO
1481	Signal sequence receptor subunit 1	SSR1	420871	YES	YES	NO
1482	Signal transducer and activator of transcription 3	STAT3	420027	YES	YES	NO
1483	Similar to Ciliogenesis and planar polarity effector 1 [Parambassis ranga (Indian glassy fish)]	CPLANE1	N/A	NO	YES	YES
1484	Similar to General transcription factor II-I repeat domain-containing protein 2-like [Xiphophorus hellerii (green swordtail)]	N/A	N/A	NO	NO	YES
1485	Similar to Eukaryotic translation initiation factor 3 subunit K [*Numida meleagris* (helmeted guineafowl)]	EIF3K	N/A	YES	YES	NO
1486	Similar to HEAT repeat protein [Ancylostoma ceylanicum]	N/A	N/A	YES	YES	NO
1487	Similar to Host cell factor 1-like [Apteryx mantelli mantelli]	N/A	N/A	NO	NO	YES
1488	Similar to Hydroxyacyl-CoA dehydrogenase trifunctional multienzyme complex subunit beta [Meleagris gallopavo (turkey)]	HADHB	N/A	YES	YES	YES
1489	Similar to KRAS proto-oncogene, GTPase [Pelodiscus sinensis (Chinese soft-shelled turtle)]	KRAS	N/A	NO	YES	NO
1490	Similar to Myeloid-associated differentiation marker homolog [*Phasianus colchicus* (Ring-necked pheasant)]	N/A	N/A	YES	YES	NO
1491	Similar to Plectin a [*Danio rerio* (zebrafish)]	PLECA	N/A	YES	YES	NO
1492	Similar to Protein phosphatase 4, catalytic subunit b [*Danio rerio* (zebrafish)]	PPP4CB	N/A	YES	YES	NO
1493	Similar to Proteasome 26S subunit, ATPase 4 [Danio rerio (zebrafish)]	PSMC4	N/A	YES	NO	NO
1494	Similar to protein S100-A12-like [Coturnix japonica (Japanese quail)]	N/A	N/A	YES	YES	NO
1495	Similar to RAB29, member RAS oncogene family [Anas platyrhynchos (mallard)]	RAB29	N/A	NO	YES	NO
1496	Similar to Rho GDP dissociation inhibitor alpha [Meleagris gallopavo (turkey)]	ARHGDIA	N/A	YES	YES	YES
1497	Similar to Ribosomal protein S9 of Zebrafish	RPS9	N/A	YES	YES	NO
1498	Similar to Ribosomal protein S18 of Zebrafish	RBS18	N/A	YES	YES	YES
1499	Similar to TRAF2 and NCK interacting kinase b of zebrafish	TNIKB	N/A	NO	NO	YES
1500	Similar to Ubiquitin like modifier activating enzyme 1 [Corvus moneduloides (New Caledonian crow)]	UBA1	N/A	YES	YES	YES
1501	Similar to Ubiquitin-like modifier activating enzyme 1 [*Danio rerio* (zebrafish)]	UBA1	N/A	YES	YES	NO
1502	Similar to Ubiquitin protein ligase E3 component n-recognin 4 of human	UBR4	N/A	NO	NO	YES
1503	Similar to Zinc finger MYM-type protein 1-like [Rhopalosiphum maidis (corn leaf aphid)]	N/A	N/A	NO	YES	NO
1504	Similar to YKT6 v-SNARE homolog (S. cerevisiae)	YKT6	N/A	YES	YES	YES
1505	Similar to Zgc:153322 [*Danio rerio* (zebrafish)]	zgc:153322	N/A	YES	YES	YES
1506	Sin3A associated protein 18	SAP18	374206	YES	YES	YES
1507	Single stranded DNA binding protein 1	SSBP1	418122	NO	YES	NO
1508	Sirtuin 2	SIRT2	548628	YES	YES	NO
1509	Sirtuin 5	SIRT5	420834	YES	YES	NO
1510	Sjogren syndrome antigen B	SSB	395268	YES	YES	YES
1511	SLC9A3 regulator 1	SLC9A3R1	422108	YES	YES	NO
1512	SLC9A3 regulator 2	SLC9A3R2	416550	NO	YES	NO

1513	Small glutamine rich tetratricopeptide repeat containing alpha	SGTA	426396	YES	NO	NO
1514	Small nuclear ribonucleoprotein D2 polypeptide	SNRPD2	107049207	NO	YES	YES
1515	Small nuclear ribonucleoprotein D3 polypeptide	SNRPD3	416947	YES	YES	YES
1516	Small nuclear ribonucleoprotein polypeptide A	SNRPA1	415523	YES	YES	YES
1517	Small nuclear ribonucleoprotein polypeptide E	SNRPE	396180	NO	NO	YES
1518	Small nuclear ribonucleoprotein polypeptide N	SNRPN	395298	YES	NO	YES
1519	Small nuclear ribonucleoprotein U5 200kDa subunit	SNRNP200	503574	YES	YES	YES
1520	Small ubiquitin-related modifier 1 precursor	SUMO1	373930	NO	YES	NO
1521	SNU13 homolog, small nuclear ribonucleoprotein (U4/U6.U5)	SNU13	417986	YES	NO	NO
1522	Solute carrier family 16 member 1	SLC16A1	419875	YES	YES	YES
1523	Solute carrier family 16 member 7	SLC16A7	417815	NO	YES	NO
1524	Solute carrier family 25 member 1	SLC25A1	416764	YES	YES	NO
1525	Solute carrier family 25 member 3	SLC25A3	417924	YES	YES	YES
1526	Solute carrier family 25 member 4	SLC25A4	422546	YES	YES	NO
1527	Solute carrier family 25 member 5	SLC25A5	772225	YES	YES	YES
1528	Solute carrier family 25 member 6	SLC25A6	374072	YES	YES	NO
1529	Solute carrier family 25 member 10	SLC25A10	769048	NO	YES	NO
1530	Solute carrier family 25 member 11	SLC25A11	107049718	YES	YES	NO
1531	Solute carrier family 25 member 13	SLC25A13	428427	YES	YES	NO
1532	Solute carrier family 25 member 20	SLC25A20	416062	YES	YES	NO
1533	Solute carrier family 25 member 24	SLC25A24	424337	YES	YES	YES
1534	Solute carrier family 27 member 4	SLC27A4	417220	YES	YES	NO
1535	Solute carrier family 29 member 1 (Augustine blood group)	SLC29A1	421439	NO	NO	YES
1536	Solute carrier family 4 member 1 (Diego blood group)	SLC4A1	396532	YES	YES	YES
1537	Solute carrier family 43 member 3	SLC43A3	423133	YES	YES	YES
1538	Solute carrier family 44 (choline transporter), member 1	SLC44A1	427301	YES	NO	NO
1539	Sorcin	SRI	420538	YES	YES	YES
1540	Sorting nexin 2	SNX2	426797	YES	YES	YES
1541	Sorting nexin 3	SNX3	421772	YES	YES	NO
1542	Sorting nexin 4	SNX4	424260	YES	YES	NO
1543	Sorting nexin 12	SNX12	428704	YES	YES	YES
1544	Sorting nexin 32	SNX32	771038	YES	YES	NO
1545	Spectrin alpha, non-erythrocytic 1	SPTAN1	374234	YES	YES	YES
1546	Spectrin beta, erythrocytic	SPTB	769410	YES	YES	YES
1547	Spectrin beta, non-erythrocytic 1	SPTBN1	421216	YES	YES	YES
1548	Sperm associated antigen 9	SPAG9	422095	NO	NO	YES
1549	Spermidine synthase	SRM	771447	YES	YES	NO
1550	S-phase kinase-associated protein 1	SKP1	416319	YES	YES	NO
1551	Splicing factor 3a subunit 1	SF3A1	427717	YES	NO	YES
1552	Splicing factor 3a subunit 3	SF3A3	419608	YES	NO	NO
1553	Splicing factor 3b subunit 1	SF3B1	424056	YES	NO	YES
1554	Splicing factor 3b subunit 3	SF3B3	415680	YES	YES	NO
1555	Splicing factor 3b subunit 6	SF3B6	421976	NO	YES	NO
1556	Splicing factor proline and glutamine rich	SFPQ	395803	YES	YES	YES
1557	SPRY domain containing 7	SPRYD7	418871	YES	YES	NO
1558	SPT5 homolog, DSIF elongation factor subunit	SUPT5H	426493	NO	NO	YES
1559	SRC proto-oncogene, non-receptor tyrosine kinase	SRC	396442	YES	YES	NO
1560	Staphylococcal nuclease and tudor domain containing	SND1	107050735	YES	YES	NO

1561	Stathmin 1	STMN1	396057	YES	YES	NO
1562	STE20 like kinase	SLK	423878	NO	YES	NO
1563	STEAP4 metalloreductase	STEAP4	772063	NO	YES	NO
1564	Sterol carrier protein 2	SCP2	396550	NO	YES	NO
1565	Stomatin	STOM	417118	YES	YES	YES
1566	Stratifin	SFN	408037	YES	YES	NO
1567	Stress induced phosphoprotein 1	STIP1	101748085	YES	YES	YES
1568	Stromal antigen 2	STAG2	422360	NO	NO	YES
1569	Structural maintenance of chromosomes 1A	SMC1A	395187	YES	YES	YES
1570	Structural maintenance of chromosomes 2	SMC2	396156	NO	NO	YES
1571	Structural maintenance of chromosomes 3	SMC3	395188	YES	YES	YES
1572	Structural maintenance of chromosomes 4	SMC4	395189	YES	NO	YES
1573	STT3A, catalytic subunit of the oligosaccharyltransferase complex	STT3A	100857165	YES	YES	NO
1574	Succinate dehydrogenase complex flavoprotein subunit A	SDHA	395758	YES	YES	NO
1575	Succinate dehydrogenase complex iron sulfur subunit B	SDHB	100859720	YES	YES	NO
1576	Succinate-CoA ligase ADP-forming beta subunit	SUCLA2	418857	YES	YES	NO
1577	Succinate-CoA ligase GDP-forming beta subunit	SUCLG2	416087	NO	YES	NO
1578	Sulfite oxidase	SUOX	107055404	NO	NO	YES
1579	Sulfotransferase family 1C member 3	SULT1C3	395300	YES	YES	NO
1580	Sulfotransferase family 1E member 1	SULT1E1	422662	YES	NO	NO
1581	Sulfotransferase family cytosolic 1B member 1	SULT1B1	395227	YES	YES	NO
1582	Sulfotransferase family cytosolic 2B member 1-like	LOC415852	415852	YES	YES	NO
1583	SUMO1 activating enzyme subunit 1	SAE1	101750090	YES	YES	NO
1584	SUN domain containing ossification factor	SUCO	424385	NO	YES	NO
1585	Superoxide dismutase 1, soluble	SOD1	395938	YES	YES	YES
1586	Superoxide dismutase 2, mitochondrial	SOD2	374042	YES	YES	YES
1587	Supervillin	SVIL	420475	YES	NO	NO
1588	Suppression of tumorigenicity 13 (colon carcinoma) (Hsp70 interacting protein) pseudogene 5	ST13P5	418003	YES	YES	YES
1589	Surfeit 4	SURF4	374273	YES	YES	NO
1590	Survival motor neuron domain containing 1	SMNDC1	423889	NO	NO	YES
1591	SWI/SNF related, matrix associated, actin dependent regulator of chromatin, subfamily a, member 5	SMARCA5	422457	YES	NO	NO
1592	SWI/SNF related, matrix associated, actin dependent regulator of chromatin, subfamily e, member 1	SMARCE1	420047	YES	NO	NO
1593	Switching B cell complex subunit SWAP70	SWAP70	423044	YES	NO	NO
1594	Synaptogyrin 1	SYNGR1	418015	NO	YES	NO
1595	Synaptogyrin 2	SYNGR2	100859110	YES	YES	NO
1596	Synaptosome associated protein 23	SNAP23	423235	YES	YES	YES
1597	Synaptosome associated protein 91	SNAP91	428635	YES	NO	NO
1598	Synaptotagmin binding cytoplasmic RNA interacting protein	SYNCRIP	421831	YES	YES	NO
1599	Synemin	SYNM	395599	NO	NO	YES
1600	Syntaxin 7	STX7	426930	YES	NO	NO
1601	Syntaxin binding protein 1	STXBP1	404293	YES	YES	NO
1602	Talin 1	TLN1	395194	YES	YES	YES
1603	Talin 2	TLN2	415374	NO	YES	YES
1604	Tandem PRG2/PRG3 gene pair	PGR2/3	423134	YES	YES	NO
1605	TBC1 domain family member 2B	TBC1D2B	415364	YES	NO	NO

1606	t-complex 1	TCP1	421586	YES	YES	YES
1607	Tenascin XB	LOC427816	427816	NO	YES	NO
1608	Tensin 1	TNS1	396439	NO	YES	NO
1609	Testin LIM domain protein	TES	395332	YES	YES	NO
1610	Tetraspanin 8	TSPAN8	417854	NO	YES	NO
1611	Tetratricopeptide repeat domain 17	TTC17	423167	NO	NO	YES
1612	Tetratricopeptide repeat domain 37	TTC37	427114	NO	YES	YES
1613	Tetratricopeptide repeat domain 38	TTC38	426193	YES	YES	YES
1614	Thimet oligopeptidase 1	THOP1	426398	YES	YES	NO
1615	Thiopurine S-methyltransferase	TPMT	420830	YES	NO	YES
1616	Thioredoxin	TXN	396437	NO	NO	YES
1617	Thioredoxin domain containing 12	TXNDC12	772208	YES	NO	NO
1618	Thioredoxin domain containing 17	TXNDC17	417680	YES	YES	NO
1619	Thioredoxin domain containing 5	TXNDC5	420867	YES	YES	NO
1620	Thioredoxin like 1	TXNL1	426854	YES	YES	YES
1621	Thioredoxin related transmembrane protein 3	TMX3	421027	YES	YES	NO
1622	Thioredoxin related transmembrane protein 4	TMX4	416729	YES	YES	YES
1623	Thiosulfate sulfurtransferase	TST	418049	YES	YES	NO
1624	THO complex 7	THOC7	416077	YES	NO	NO
1625	Threonyl-tRNA synthetase	TARS	427427	YES	YES	YES
1626	Thy-1 cell surface antigen	THY1	378897	YES	YES	NO
1627	Thymocyte nuclear protein 1	THYN1	374161	YES	YES	NO
1628	Thymopoietin	TMPO	417923	NO	NO	YES
1629	Thyroid hormone receptor associated protein 3	THRAP3	419623	NO	NO	YES
1630	TIMP metallopeptidase inhibitor 3	TIMP3	396483	NO	YES	NO
1631	Tissue specific transplantation antigen P35B	TSTA3	420292	YES	NO	NO
1632	Titin	TTN	424126	YES	YES	YES
1633	Topoisomerase (DNA) II beta	TOP2B	395966	YES	NO	YES
1634	Trafficking protein particle complex 3	TRAPPC3	419625	YES	YES	NO
1635	Trafficking protein particle complex 4	TRAPPC4	419788	NO	YES	NO
1636	Trafficking protein particle complex 5	TRAPPC5	100858483	YES	YES	NO
1637	Trafficking protein particle complex 11	TRAPPC11	428748	NO	YES	NO
1638	Transaldolase 1	TALDO1	423019	YES	YES	YES
1639	Transcription elongation factor A1	TCEA1	421119	NO	NO	YES
1640	Transcription elongation regulator 1	TCERG1	416349	YES	YES	YES
1641	Transcription factor A, mitochondrial	TFAM	373888	YES	YES	NO
1642	Transcriptional activator protein Pur-beta-like	LOC107049543	107049543	NO	NO	YES
1643	Transferrin (ovotransferrin)	TF	396241	YES	YES	YES
1644	Transferrin receptor	TFRC	396191	YES	YES	YES
1645	Transformer 2 alpha homolog	TRA2A	420618	YES	YES	YES
1646	Transformer 2 beta homolog	TRA2B	395403	NO	NO	YES
1647	Transforming growth factor beta induced	TGFBI	395897	YES	NO	NO
1648	Transgelin	TAGLN	396490	YES	YES	NO
1649	Transglutaminase 2	TGM2	396432	YES	YES	YES
1650	Transglutaminase 4	TGM4	420706	NO	YES	NO
1651	Transketolase like 1	TKTL1	415991	YES	YES	YES
1652	Translin	TSN	395955	YES	YES	YES
1653	Translocated promoter region, nuclear basket protein	TPR	424457	YES	NO	YES

1654	Translocation associated membrane protein 1-like 1	TRAM1L1	378918	YES	NO	NO
1655	Translocator protein	TSPO	418226	NO	YES	NO
1656	Translocator protein 2	TSPO2	419914	NO	YES	NO
1657	Transmembrane 9 superfamily member 2-like	TM9SF2L	422249	YES	NO	NO
1658	Transmembrane 9 superfamily member 3	TM9SF3	395460	YES	YES	NO
1659	Transmembrane and coiled-coil domains 1	TMCO1	427100	YES	YES	NO
1660	Transmembrane emp24 domain-containing protein 4-like	LOC107049088	107049088	YES	YES	NO
1661	Transmembrane p24 trafficking protein 5	TMED5	424499	YES	YES	NO
1662	Transmembrane p24 trafficking protein 7	TMED7	769360	YES	YES	NO
1663	Transmembrane p24 trafficking protein 10	TMED10	423362	YES	YES	NO
1664	Transmembrane protein 11	TMEM11	427663	YES	YES	NO
1665	Transmembrane protein 33	TMEM33	422779	YES	YES	NO
1666	Transmembrane protein 65	TMEM65	428387	NO	YES	NO
1667	Transmembrane protein 245	TMEM245	420957	NO	YES	NO
1668	Transportin 1	TNPO1	427218	YES	NO	NO
1669	Transthyretin	TTR	396277	YES	YES	YES
1670	Trimethyllysine hydroxylase, epsilon	TMLHE	422296	YES	YES	YES
1671	Triosephosphate isomerase 1	TPI1	396435	YES	YES	YES
1672	Tripeptidyl peptidase 2	TPP2	428020	YES	YES	YES
1673	tRNA nucleotidyl transferase 1	TRNT1	416103	YES	NO	YES
1674	Tropomodulin 3	TMOD3	415421	YES	YES	NO
1675	Tropomyosin 1	TPM1	396366	YES	YES	NO
1676	Tropomyosin 2	TPM2	396430	YES	YES	YES
1677	Tropomyosin 3	TPM3	770103	YES	YES	YES
1678	Tropomyosin 4	TPM4	107055375	YES	YES	NO
1679	Tryptophanyl-tRNA synthetase	WARS	423458	YES	YES	NO
1680	Tu translation elongation factor, mitochondrial	TUFM	430889	YES	YES	YES
1681	Tubulin alpha 1a	TUBA1A	429035	YES	YES	YES
1682	Tubulin alpha 4a	TUBA4A	100857247	YES	YES	YES
1683	Tubulin alpha like 3	TUBAL3	416694	YES	NO	NO
1684	Tubulin beta 1 class VI	TUBB1	396427	YES	YES	YES
1685	Tubulin beta 2B class IIb	TUBB2B	420883	YES	YES	NO
1686	Tubulin beta 3 class III	TUBB3	431043	NO	YES	NO
1687	Tubulin beta 4B class IVb	TUBB4B	417255	YES	YES	YES
1688	Tubulin beta 6 class V	TUBB6	421037	YES	NO	NO
1689	Tubulin beta class I	TUBB	396254	YES	YES	YES
1690	Tubulin folding cofactor C	TBCC	428567	YES	YES	YES
1691	Tubulin folding cofactor D	TBCD	417334	NO	YES	NO
1692	Tubulin tyrosine ligase like 12	TTLL12	418227	YES	YES	NO
1693	Tubulin, alpha 3e	TUBA3E	421169	YES	YES	YES
1694	Tubulin, alpha 8b	TUBA8B	396426	YES	NO	YES
1695	Tubulin, beta 2A class IIa	TUBB2A	768337	YES	YES	NO
1696	Tumor protein D52	TPD52	770339	YES	YES	YES
1697	Tumor protein D52 like 2	TPD52L2	419257	YES	YES	YES
1698	Tumor protein, translationally-controlled 1	TPT1	396363	YES	YES	YES
1699	Tumor suppressing subtransferable candidate 1	TSSC1	421916	YES	YES	NO
1700	Tyrosine 3-monooxygenase/tryptophan 5-monooxygenase activation protein beta	YWHAB	419190	YES	YES	YES
1701	Tyrosine 3-monooxygenase/tryptophan 5-monooxygenase activation protein epsilon	YWHAE	417554	YES	YES	YES

1702	Tyrosine 3-monooxygenase/tryptophan 5-monooxygenase activation protein eta	YWHAH	416955	YES	YES	NO
1703	Tyrosine 3-monooxygenase/tryptophan 5-monooxygenase activation protein gamma	YWHAG	427820	YES	YES	YES
1704	Tyrosine 3-monooxygenase/tryptophan 5-monooxygenase activation protein theta	YWHAQ	421932	YES	YES	YES
1705	Tyrosine 3-monooxygenase/tryptophan 5-monooxygenase activation protein zeta	YWHAZ	425619	YES	YES	YES
1706	Tyrosyl-tRNA synthetase	YARS	419666	YES	YES	YES
1707	U2 small nuclear RNA auxiliary factor 2	U2AF2	112531333	YES	NO	NO
1708	Ubiquinol-cytochrome c reductase core protein I	UQCRC1	416013	YES	YES	NO
1709	Ubiquinol-cytochrome c reductase core protein II	UQCRC2	427009	YES	YES	NO
1710	Ubiquinol-cytochrome c reductase, Rieske iron-sulfur polypeptide 1	UQCRFS1	415752	YES	YES	NO
1711	Ubiquitin conjugating enzyme E2 H	UBE2H	416678	NO	NO	YES
1712	Ubiquitin conjugating enzyme E2 I	UBE2I	374123	YES	YES	YES
1713	Ubiquitin conjugating enzyme E2 K	UBE2K	428787	YES	YES	NO
1714	Ubiquitin conjugating enzyme E2 L3	UBE2L3	416769	NO	YES	YES
1715	Ubiquitin conjugating enzyme E2 M	UBE2M	107049465	YES	YES	YES
1716	Ubiquitin conjugating enzyme E2 N	UBE2N	417898	YES	YES	YES
1717	Ubiquitin conjugating enzyme E2 O	UBE2O	417359	NO	NO	YES
1718	Ubiquitin conjugating enzyme E2 V1	UBE2V1	100538349	YES	YES	YES
1719	Ubiquitin conjugating enzyme E2 V2	UBE2V2	421109	YES	YES	YES
1720	Ubiquitin C-terminal hydrolase L1	UCHL1	770302	YES	YES	NO
1721	Ubiquitin C-terminal hydrolase L3	UCHL3	395626	YES	YES	YES
1722	Ubiquitin C-terminal hydrolase L5	UCHL5	424359	YES	YES	YES
1723	Ubiquitin fusion degradation 1 like (yeast)	UFD1L	374186	NO	NO	YES
1724	Ubiquitin like modifier activating enzyme 2	UBA2	415784	YES	YES	NO
1725	Ubiquitin like modifier activating enzyme 3	UBA3	426073	YES	YES	YES
1726	Ubiquitin specific peptidase 5	USP5	418290	YES	YES	YES
1727	Ubiquitin specific peptidase 7	USP7	395126	YES	YES	YES
1728	Ubiquitin specific peptidase 10	USP10	415817	YES	NO	NO
1729	Ubiquitin specific peptidase 14	USP14	421063	YES	YES	YES
1730	Ubiquitin specific peptidase 24	USP24	424665	NO	NO	YES
1731	Ubiquitin specific peptidase 47	USP47	423032	NO	NO	YES
1732	Ubiquitin specific peptidase 9, Y-linked	USP9Y	418569	YES	YES	YES
1733	Ubiquitin-like modifier activating enzyme 7	UBA7	395178	YES	YES	NO
1734	UDP-galactose-4-epimerase	GALE	419686	YES	YES	NO
1735	UDP-glucose 6-dehydrogenase	UGDH	422792	YES	YES	YES
1736	UDP-glucose glycoprotein glucosyltransferase 1	UGGT1	424757	YES	YES	YES
1737	UDP-glucose pyrophosphorylase 2	UGP2	373900	YES	YES	NO
1738	UDP-N-acetylglucosamine pyrophosphorylase 1 like 1	UAP1L1	417295	YES	YES	NO
1739	Ufm1-specific protease 2	UFSP2	422542	YES	NO	YES
1740	Uncharacterized LOC769729	LOC769729	769729	NO	YES	NO
1741	Uncharacterized oxidoreductase-like	LOC415661	415661	YES	NO	NO
1742	UPF1, RNA helicase and ATPase	UPF1	420119	YES	NO	YES
1743	Uracil phosphoribosyltransferase homolog	UPRT	422327	YES	YES	NO
1744	Uridine monophosphate synthetase	UMPS	424256	YES	YES	YES
1745	Uroplakin 1B	UPK1B	418345	YES	YES	NO
1746	Uroplakin 3A	UPK3A	418242	YES	NO	NO
1747	Uroplakin 3B-like	UPK3LB	417514	YES	YES	NO
1748	Uroporphyrinogen decarboxylase	UROD	424590	YES	NO	YES
1749	Uroporphyrinogen III synthase	UROS	426223	NO	YES	YES

1750	USO1 vesicle transport factor	USO1	422575	YES	YES	NO
1751	Vacuolar protein sorting 13 homolog A	VPS13A	427050	NO	YES	NO
1752	Vacuolar protein sorting 13 homolog D	VPS13D	419481	NO	NO	YES
1753	Vacuolar protein sorting 25 homolog	VPS25	420021	YES	YES	NO
1754	Vacuolar protein sorting 4 homolog B	VPS4B	420901	YES	YES	NO
1755	Valosin containing protein	VCP	427410	YES	YES	YES
1756	VAMP associated protein A	VAPA	768928	YES	YES	NO
1757	VAMP associated protein B and C	VAPB	419317	YES	YES	YES
1758	Versican	VCAN	395565	NO	YES	NO
1759	Vesicle amine transport 1	VAT1	420009	YES	YES	NO
1760	Vesicle associated membrane protein 7	VAMP7	422297	YES	YES	NO
1761	VHL binding protein 1	VBP1	422355	YES	YES	YES
1762	Villin like	VILL	420415	YES	YES	NO
1763	Vimentin	VIM	420519	YES	YES	YES
1764	Vinculin	VCL	396422	YES	YES	YES
1765	Vitamin K epoxide reductase complex, subunit 1	VKORC1	404205	YES	NO	NO
1766	Vitelline membrane outer layer 1 homolog	VMO1	418974	YES	YES	NO
1767	Vitellogenin 1	VTG1	424547	YES	NO	NO
1768	Vitellogenin 2	VTG2	424533	YES	NO	YES
1769	Vitellogenin 3	VTG3	424534	YES	NO	NO
1770	Vitronectin	VTN	395935	YES	YES	YES
1771	Voltage dependent anion channel 1	VDAC1	416320	YES	YES	NO
1772	Voltage dependent anion channel 2	VDAC2	395498	YES	YES	YES
1773	Voltage dependent anion channel 3	VDAC3	4269793	YES	YES	YES
1774	von Willebrand factor	VWF	419031	YES	YES	NO
1775	VPS26, retromer complex component A	VPS26A	423696	YES	YES	YES
1776	VPS29, retromer complex component	VPS29	416867	YES	YES	NO
1777	VPS35, retromer complex component	VPS35	415750	YES	YES	YES
1778	VRK serine/threonine kinase 1	VRK1	423443	YES	NO	YES
1779	V-type proton ATPase catalytic subunit A-like	LOC776719	776719	NO	YES	NO
1780	WAPL cohesin release factor	WAPL	423608	NO	NO	YES
1781	WAS protein family member 2	WASF2	426359	NO	YES	NO
1782	WD repeat domain 1	WDR1	422842	YES	YES	YES
1783	WD repeat domain 48	WDR48	420427	YES	NO	NO
1784	WD repeat domain 77	WDR77	419865	YES	NO	YES
1785	WD repeat domain 82	WDR82	415942	YES	YES	NO
1786	WW domain binding protein 4	WBP4	418829	NO	NO	YES
1787	X-prolyl aminopeptidase (aminopeptidase P) 1, soluble	XPNPEP1	423886	YES	YES	YES
1788	Y-box binding protein 1	YBX1	386575	YES	YES	YES
1789	Zinc finger CCCH-type containing 18	ZC3H18	415838	YES	NO	YES
1790	Zinc finger protein 185-like	ZNF185L	422301	YES	YES	NO
1791	Zinc finger protein 207	ZNF207	417413	NO	NO	YES
1792	Zinc finger protein 518B	ZNF518B	422841	NO	NO	YES
1793	Zinc finger RANBP2-type containing 2	ZRANB2	424717	NO	YES	NO
1794	Zona pellucida glycoprotein 1	ZP1	395418	YES	YES	NO
1795	Zona pellucida sperm-binding protein 3	ZP3	378906	YES	YES	NO
1796	Zyxin	ZYX	418300	YES	NO	NO
