# Supplementary material for: Proteomic Analysis of Chicken Chorioallantoic Membrane (CAM) during Embryonic Development Provides Functional Insight
Source: Biomed Res Int. 2022 Jun 19;2022:7813921. doi: 10.1155/2022/7813921 (PMC9237712; doi:10.1155/2022/7813921)
Supplement: Supplementary 5 — Protein families using Uniprot. [file 7813921.f5.pdf]

Protein families using uniprot

Entry	Cross-reference (GeneID)	Gene names	Protein names	Protein families	Organism	Cross-reference (Pfam)
R4GF89	408037;	SFN	14_3_3 domain-containing protein	14-3-3 family	Gallus gallus (Chicken)	PF00244;
Q5ZLQ6	419190;	YWHAB RCJMB04_5d11	14-3-3 protein beta/alpha	14-3-3 family	Gallus gallus (Chicken)	PF00244;
Q5ZMT0	417554;	YWHAE RCJMB04_1e8	14-3-3 protein epsilon (14-3-3E)	14-3-3 family	Gallus gallus (Chicken)	PF00244;
Q5ZKJ2	416955;	YWHAH RCJMB04_10g10	14_3_3 domain-containing protein	14-3-3 family	Gallus gallus (Chicken)	PF00244;
Q5F3W6	427820;	YWHAG RCJMB04_5e12	14-3-3 protein gamma	14-3-3 family	Gallus gallus (Chicken)	PF00244;
Q5ZMD1	421932;	YWHAQ RCJMB04_2i3	14-3-3 protein theta	14-3-3 family	Gallus gallus (Chicken)	PF00244;
Q5ZKC9	425619;	YWHAZ RCJMB04_11l21	14-3-3 protein zeta	14-3-3 family	Gallus gallus (Chicken)	PF00244;
O57389	395921;	CNP	2',3'-cyclic-nucleotide 3'-phosphodiesterase (CNP) (CNPase) (EC 3.1.4.37)	2H phosphoesterase superfamily, CNPase family	Gallus gallus (Chicken)	PF05881;
Q98UJ6	395374;		Dihydrolipoamide acetyltransferase component of pyruvate dehydrogenase complex (EC 2.3.1.-)	2-oxoacid dehydrogenase family	Gallus gallus (Chicken)	PF00198;PF00364;PF02817;
E1C6N5	419796;	DLAT	Acetyltransferase component of pyruvate dehydrogenase complex (EC 2.3.1.12)	2-oxoacid dehydrogenase family	Gallus gallus (Chicken)	PF00198;PF00364;PF02817;
F1NQH8	423357;	DLST	Dihydrolipoyllysine-residue succinyltransferase component of 2-oxoglutarate dehydrogenase complex, mitochondrial (EC 2.3.1.61) (E2K)	2-oxoacid dehydrogenase family	Gallus gallus (Chicken)	PF00198;PF00364;
Q5F326	423357;	RCJMB04_39i8	Dihydrolipoyllysine-residue succinyltransferase component of 2-oxoglutarate dehydrogenase complex, mitochondrial (EC 2.3.1.61) (E2K)	2-oxoacid dehydrogenase family	Gallus gallus (Chicken)	PF00198;PF00364;
F1P156	418953;	CRYL1	Uncharacterized protein	3-hydroxyacyl-CoA dehydrogenase family	Gallus gallus (Chicken)	PF00725;PF02737;
E1BZH9	420290;	HADH	Uncharacterized protein	3-hydroxyacyl-CoA dehydrogenase family	Gallus gallus (Chicken)	PF00725;PF02737;
Q5F3M8	427190;	RCJMB04_12o19	Succinyl-CoA:3-ketoacid-coenzyme A transferase (EC 2.8.3.5)	3-oxoacid CoA-transferase family	Gallus gallus (Chicken)	PF01144;
A0A1D5PJ03	415895;	NT5DC2	Uncharacterized protein	5'(3')-deoxyribonucleotidase family	Gallus gallus (Chicken)	PF05761;
Q5ZIZ4	423871;	NT5C2 RCJMB04_22h21	Cytosolic purine 5'-nucleotidase (EC 3.1.3.5) (Cytosolic 5'-nucleotidase II)	5'(3')-deoxyribonucleotidase family	Gallus gallus (Chicken)	PF05761;
Q5ZIZ0	419450;	PGD RCJMB04_22k15	6-phosphogluconate dehydrogenase, decarboxylating (EC 1.1.1.44)	6-phosphogluconate dehydrogenase family	Gallus gallus (Chicken)	PF00393;PF03446;
E1BQU4	419972;	NSF	Vesicle-fusing ATPase (EC 3.6.4.6)	AAA ATPase family	Gallus gallus (Chicken)	PF00004;PF17862;PF02933;PF02359;
Q90732	395804;	PSMC1	26S proteasome regulatory subunit 4 (P26s4) (26S proteasome AAA-ATPase subunit RPT2) (Proteasome 26S subunit ATPase 1)	AAA ATPase family	Gallus gallus (Chicken)	PF00004;PF17862;PF16450;
Q5ZMB8	417716;	PSMC2 RCJMB04_2j13	26S proteasome AAA-ATPase subunit RPT1 (26S proteasome regulatory subunit 7) (Proteasome 26S subunit ATPase 2)	AAA ATPase family	Gallus gallus (Chicken)	PF00004;PF17862;
Q5ZIT7	423182;	PSMC3 RCJMB04_23k2	AAA domain-containing protein	AAA ATPase family	Gallus gallus (Chicken)	PF00004;PF17862;PF16450;
F1NU79	428274;	PSMC5	AAA domain-containing protein	AAA ATPase family	Gallus gallus (Chicken)	PF00004;PF17862;PF16450;
Q5ZKX2	423586;	RCJMB04_8n6	AAA domain-containing protein	AAA ATPase family	Gallus gallus (Chicken)	PF00004;PF17862;PF16450;
Q5ZMI9	420901;	VPS4B RCJMB04_1o9	Vesicle-fusing ATPase (EC 3.6.4.6)	AAA ATPase family	Gallus gallus (Chicken)	PF00004;PF17862;PF04212;PF09336;
Q5ZMU9	427410;	VCP vcp RCJMB04_1c3	Transitional endoplasmic reticulum ATPase (EC 3.6.4.6) (Valosin-containing protein)	AAA ATPase family	Gallus gallus (Chicken)	PF00004;PF17862;PF02933;PF02359;PF09336;
E1BRI5	419685;	LYPLA2	Abhydrolase_2 domain-containing protein	AB hydrolase superfamily, AB hydrolase 2 family	Gallus gallus (Chicken)	PF02230;
P53760	396136;	LCAT	Phosphatidylcholine-sterol acyltransferase (EC 2.3.1.43) (Lecithin-cholesterol acyltransferase) (Phospholipid-cholesterol acyltransferase) (Fragment)	AB hydrolase superfamily, Lipase family	Gallus gallus (Chicken)	PF02450;
Q90ZI2	100857237;	gcn5	Histone acetyltransferase (EC 2.3.1.48)	Acetyltransferase family, GCN5 subfamily	Gallus gallus (Chicken)	PF00583;PF00439;PF06466;
E1C0V1	423588;	GNPNAT1	Glucosamine 6-phosphate N-acetyltransferase (EC 2.3.1.4)	Acetyltransferase family, GNA1 subfamily	Gallus gallus (Chicken)	PF00583;
Q5ZL43	421918;	ADI1 MTCBP1 RCJMB04_7o8	1,2-dihydroxy-3-keto-5-methylthiopentene dioxygenase (EC 1.13.11.54) (Acireductone dioxygenase (Fe(2+)-requiring)) (ARD) (Fe-ARD) (Membrane-type 1 matrix metalloproteinase cytoplasmic tail-binding protein 1) (MTCBP-1)	Acireductone dioxygenase (ARD) family	Gallus gallus (Chicken)	PF03079;

Q90875	373916;	ACO1 IREB1	Cytoplasmic aconitate hydratase (Aconitase) (EC 4.2.1.3) (Citrate hydro-lyase) (Iron-responsive element-binding protein 1) (IRE-BP 1)	Aconitase/IPM isomerase family	Gallus gallus (Chicken)	PF00330;PF00694;
Q8AYI3	374009;	ACO2	Aconitate hydratase, mitochondrial (Aconitase) (EC 4.2.1.-)	Aconitase/IPM isomerase family	Gallus gallus (Chicken)	PF00330;PF00694;
E1BR36	424975;	ACTL6A	Uncharacterized protein	Actin family	Gallus gallus (Chicken)	PF00022;
P08023	423787;	ACTA2	Actin, aortic smooth muscle (Alpha-actin) [Cleaved into: Actin, aortic smooth muscle, intermediate form]	Actin family	Gallus gallus (Chicken)	PF00022;
F1P476	423787;	ACTA2	Actin, aortic smooth muscle	Actin family	Gallus gallus (Chicken)	PF00022;
P68034	423298;	ACTC1 ACTC	Actin, alpha cardiac muscle 1 (Alpha-cardiac actin) [Cleaved into: Actin, alpha cardiac muscle 1, intermediate form]	Actin family	Gallus gallus (Chicken)	PF00022;
A0A1D5NV17	426652;	ACTBL2	Uncharacterized protein	Actin family	Gallus gallus (Chicken)	PF00022;
P53478	415296;		Actin, cytoplasmic type 5	Actin family	Gallus gallus (Chicken)	PF00022;
Q5ZM58	423862;	ACTR1A RCJMB04_3a24	Uncharacterized protein	Actin family	Gallus gallus (Chicken)	PF00022;
P53488	396147;	ACTR2 ARP2	Actin-related protein 2 (Actin-like protein 2) (Actin-like protein ACTL)	Actin family, ARP2 subfamily	Gallus gallus (Chicken)	PF00022;
Q90WD0	374197;	ACTR3 ARP3	Actin-related protein 3 (Actin-like protein 3)	Actin family, ARP3 subfamily	Gallus gallus (Chicken)	PF00022;
P21566	423320;	CFL2	Cofilin-2 (Cofilin, muscle isoform)	Actin-binding proteins ADF family	Gallus gallus (Chicken)	PF00241;
P18359	396539;	DSTN DSN RCJMB04_5f14	Destrin (Actin-depolymerizing factor) (ADF)	Actin-binding proteins ADF family	Gallus gallus (Chicken)	PF00241;
Q5ZHL1	424958;	RCJMB04_37a17	AAA domain-containing protein	Activator 1 small subunits family	Gallus gallus (Chicken)	PF00004;PF08542;
Q5ZJ68	415974;	RCJMB04_20f2	Uncharacterized protein	Acyl-CoA dehydrogenase family	Gallus gallus (Chicken)	PF00441;PF02770;PF02771;
Q5ZL56	416969;	ACADS RCJMB04_7j20	Uncharacterized protein	Acyl-CoA dehydrogenase family	Gallus gallus (Chicken)	PF00441;PF02770;PF02771;
Q5ZJ93	424005;	RCJMB04_19o8	Long-chain specific acyl-CoA dehydrogenase, mitochondrial (EC 1.3.8.8)	Acyl-CoA dehydrogenase family	Gallus gallus (Chicken)	PF00441;PF02770;PF02771;
Q5ZK70	423947;	RCJMB04_12m17	Uncharacterized protein	Acyl-CoA dehydrogenase family	Gallus gallus (Chicken)	PF00441;PF02770;PF02771;
F1NDA9	415880;	AP1G1	AP-1 complex subunit gamma	Adaptor complexes large subunit family	Gallus gallus (Chicken)	PF01602;PF02883;
Q5ZJ83	415880;	RCJMB04_20c5	AP-1 complex subunit gamma	Adaptor complexes large subunit family	Gallus gallus (Chicken)	PF01602;PF02883;
A0A1D5NV94	423102;	AP2A2	AP-2 complex subunit alpha	Adaptor complexes large subunit family	Gallus gallus (Chicken)	PF01602;PF02296;PF02883;
Q5F3T4	423102;	RCJMB04_7j8	AP-2 complex subunit alpha	Adaptor complexes large subunit family	Gallus gallus (Chicken)	PF01602;PF02296;PF02883;
Q5ZMG7	420149;	AP1M1 RCJMB04_2b13	AP-1 complex subunit mu-1	Adaptor complexes medium subunit family	Gallus gallus (Chicken)	PF00928;PF01217;
Q5ZMP6	770246;	AP2M1 RCJMB04_1h23	AP-2 complex subunit mu (AP-2 mu chain) (Clathrin assembly protein complex 2 mu medium chain) (Clathrin coat assembly protein AP50) (Clathrin coat-associated protein AP50) (HA2 50 kDa subunit) (Mu2-adaptin) (Plasma membrane adaptor AP-2 50 kDa protein)	Adaptor complexes medium subunit family	Gallus gallus (Chicken)	PF00928;PF01217;
Q5ZL57	770561;	ARCN1 COPD RCJMB04_7j3	Coatomer subunit delta (Archain) (Delta-coat protein) (Delta-COP)	Adaptor complexes medium subunit family, Delta-COP subfamily	Gallus gallus (Chicken)	PF00928;PF01217;
A0A1D5PPA8	419146;	AHCY	AdoHcyase_NAD domain-containing protein	Adenosylhomocysteinase family	Gallus gallus (Chicken)	PF05221;PF00670;
Q5F3Q3	419803;	RCJMB04_10b17	AdoHcyase_NAD domain-containing protein	Adenosylhomocysteinase family	Gallus gallus (Chicken)	PF05221;PF00670;
P05081	396002;	AK1	Adenylate kinase isoenzyme 1 (AK 1) (EC 2.7.4.3) (EC 2.7.4.6) (ATP-AMP transphosphorylase 1) (ATP:AMP phosphotransferase) (Adenylate monophosphate kinase) (Myokinase)	Adenylate kinase family, AK1 subfamily	Gallus gallus (Chicken)	
Q5ZKE7	429100;	CMPK CMPK1 RCJMB04_11f2	UMP-CMP kinase (EC 2.7.4.14) (Deoxycytidylate kinase) (CK) (dCMP kinase) (Nucleoside-diphosphate kinase) (EC 2.7.4.6) (Uridine monophosphate/cytidine monophosphate kinase) (UMP/CMP kinase) (UMP/CMPK)	Adenylate kinase family, UMP-CMP kinase subfamily	Gallus gallus (Chicken)	
F1NJ73	428227;	AK2	Adenylate kinase 2, mitochondrial (AK 2) (EC 2.7.4.3) (ATP-AMP transphosphorylase 2) (ATP:AMP phosphotransferase) (Adenylate monophosphate kinase)	Adenylate kinase family; Adenylate kinase family, AK2 subfamily	Gallus gallus (Chicken)	PF05191;
F1NVD4	776127;	ADSSL1 ADSS1	Adenylosuccinate synthetase isozyme 1 (AMPSase 1) (AdSS 1) (EC 6.3.4.4) (Adenylosuccinate synthetase, basic isozyme) (Adenylosuccinate synthetase, muscle isozyme) (M-type adenylosuccinate synthetase) (IMP--aspartate ligase 1)	Adenylosuccinate synthetase family	Gallus gallus (Chicken)	PF00709;
Q5ZJL5	428579;	ADSS2 ADSS RCJMB04_17e23	Adenylosuccinate synthetase isozyme 2 (AMPSase 2) (AdSS 2) (EC 6.3.4.4) (Adenylosuccinate synthetase, acidic isozyme) (Adenylosuccinate synthetase, liver isozyme) (L-type adenylosuccinate synthetase) (IMP--aspartate ligase 2)	Adenylosuccinate synthetase family	Gallus gallus (Chicken)	PF00709;
E1C735	423628;	MAT1A	S-adenosylmethionine synthase (EC 2.5.1.6)	AdoMet synthase family	Gallus gallus (Chicken)	PF02773;PF02772;PF00438;
A0A1D5PH07	396534;	PAICS	AIR carboxylase (EC 4.1.1.21) (EC 6.3.2.6) (Multifunctional protein ADE2) (Phosphoribosylaminoimidazole carboxylase) (Phosphoribosylaminoimidazole-succinocarboxamide synthase) (SAICAR synthetase)	AIR carboxylase family, Class II subfamily; SAICAR synthetase family	Gallus gallus (Chicken)	PF00731;PF01259;

E1BWX4	417757;	AASS	Lysine ketoglutarate reductase (EC 1.5.1.8) (EC 1.5.1.9) (Saccharopine dehydrogenase)	AlaDH/PNT family	Gallus gallus (Chicken)	PF01262;PF05222;PF16653;PF03435;
E1C6A1	427196;	NNT	Proton-translocating NAD(P)(+) transhydrogenase (EC 7.1.1.1)	AlaDH/PNT family	Gallus gallus (Chicken)	PF01262;PF05222;PF02233;PF12769;
Q9DD46	395389;	ALDH6 ALDH1A3	Aldehyde dehydrogenase	Aldehyde dehydrogenase family	Gallus gallus (Chicken)	PF00171;
Q5ZLE2	417615;	RCJMB04_6j4	Aldehyde dehydrogenase	Aldehyde dehydrogenase family	Gallus gallus (Chicken)	PF00171;
A0A1D5P553	428813;	ALDH3B2	Aldehyde dehydrogenase	Aldehyde dehydrogenase family	Gallus gallus (Chicken)	PF00171;
A0A1D5PXI1	419467;	ALDH4A1	Multifunctional fusion protein [Includes: L-glutamate gamma-semialdehyde dehydrogenase (EC 1.2.1.88) (L-glutamate gamma-semialdehyde dehydrogenase); Delta-1-pyrroline-5-carboxylate dehydrogenase (P5C dehydrogenase)]	Aldehyde dehydrogenase family	Gallus gallus (Chicken)	PF00171;
A0A1D5PFY7	420818;	ALDH5A1	Succinate-semialdehyde dehydrogenase (EC 1.2.1.24)	Aldehyde dehydrogenase family	Gallus gallus (Chicken)	PF00171;
E1C4W4	426812;	ALDH7A1	Aldehyde dehydrogenase (NAD(+)) (EC 1.2.1.3)	Aldehyde dehydrogenase family	Gallus gallus (Chicken)	PF00171;
Q5ZK84	424599;	AKR1A1 RCJMB04_12g8	Aldo-keto reductase family 1 member A1 (EC 1.1.1.2) (Alcohol dehydrogenase [NADP(+)]) (Aldehyde reductase)	Aldo/keto reductase family	Gallus gallus (Chicken)	PF00248;
Q90W83	395338;	akr AKR1B10	Aldo-keto reductase (EC 1.1.1.2)	Aldo/keto reductase family	Gallus gallus (Chicken)	PF00248;
E1BVD1	418170;	LOC418170	Aldo_ket_red domain-containing protein	Aldo/keto reductase family	Gallus gallus (Chicken)	PF00248;
Q5ZL03	424492;	BCAR3 RCJMB04_8g18	GCS light chain (Gamma-ECS regulatory subunit) (Gamma-glutamylcysteine synthetase regulatory subunit) (Glutamate--cysteine ligase modifier subunit)	Aldo/keto reductase family, Glutamate--cysteine ligase light chain subfamily	Gallus gallus (Chicken)	
F1ND55	422882;	ADD1	Aldolase_II domain-containing protein	Aldolase class II family, Adducin subfamily	Gallus gallus (Chicken)	PF00596;
P05094	373918;	ACTN1	Alpha-actinin-1 (Alpha-actinin cytoskeletal isoform) (F-actin cross-linking protein) (Non-muscle alpha-actinin-1)	Alpha-actinin family	Gallus gallus (Chicken)	PF00307;PF08726;PF00435;
A0A1D5P9P3	373918;	ACTN1	Alpha-actinin-1	Alpha-actinin family	Gallus gallus (Chicken)	PF00307;PF13833;PF08726;PF00435;
Q90734	396024;	ACTN4	Alpha-actinin-4 (Non-muscle alpha-actinin 4)	Alpha-actinin family	Gallus gallus (Chicken)	PF00307;PF08726;PF00435;
P07630	396257;	CA2	Carbonic anhydrase 2 (EC 4.2.1.1) (Carbonate dehydratase II) (Carbonic anhydrase II) (CA-II)	Alpha-carbonic anhydrase family	Gallus gallus (Chicken)	PF00194;
A0A1D5NX31	100858989;	CA13	Carbonic anhydrase (EC 4.2.1.1)	Alpha-carbonic anhydrase family	Gallus gallus (Chicken)	PF00194;
Q5ZJA7	426429;	LOC107054982 RCJMB04_19j12	Oxoglutarate dehydrogenase (succinyl-transferring) (EC 1.2.4.2)	Alpha-ketoglutarate dehydrogenase family	Gallus gallus (Chicken)	PF16078;PF00676;PF16870;PF02779;
Q6XL41	415490;	RHCG	Ammonium transporter Rh type C (Rhesus blood group family type C glycoprotein) (Rh family type C glycoprotein) (Rh type C glycoprotein)	Ammonium transporter (TC 2.A.49) family, Rh subfamily	Gallus gallus (Chicken)	PF00909;
F1NFG6	395118;	RHAG	Ammonium_transp domain-containing protein	Ammonium transporter (TC 2.A.49) family, Rh subfamily	Gallus gallus (Chicken)	PF00909;
Q7T066	395118;	RHAG	Rh blood group-associated glycoprotein	Ammonium transporter (TC 2.A.49) family, Rh subfamily	Gallus gallus (Chicken)	PF00909;
P15575	396532;	SLC4A1	Band 3 anion transport protein (Solute carrier family 4 member 1)	Anion exchanger (TC 2.A.31) family	Gallus gallus (Chicken)	PF07565;PF00955;
F1N9S7	404271;	ANXA1	Annexin	Annexin family	Gallus gallus (Chicken)	PF00191;
Q6QAZ9	404271;		Annexin	Annexin family	Gallus gallus (Chicken)	PF00191;
P17785	396297;	ANXA2 ANX2	Annexin A2 (Annexin II) (Annexin-2) (Calpactin I heavy chain) (Calpactin-1 heavy chain) (Chromobindin-8) (Lipocortin II) (Placental anticoagulant protein IV) (PAP-IV) (Protein I) (p36)	Annexin family	Gallus gallus (Chicken)	PF00191;
A0A1C9KD18	396297;	ANXA2	Annexin	Annexin family	Gallus gallus (Chicken)	PF00191;
P17153	428767;	ANXA5 ANX5	Annexin A5 (Anchorin CII) (Annexin V) (Annexin-5) (Calphobindin I) (CBP-I) (Endonexin II) (Lipocortin V) (Placental anticoagulant protein I) (PAP-I) (Thromboplastin inhibitor) (Vascular anticoagulant-alpha) (VAC-alpha)	Annexin family	Gallus gallus (Chicken)	PF00191;
P51901	395481;	ANXA6 ANX6	Annexin A6 (67 kDa calelectrin) (Annexin VI) (Annexin-6) (Calphobindin-II) (CPB-II) (Chromobindin-20) (Lipocortin VI) (P68) (P70) (Protein III)	Annexin family	Gallus gallus (Chicken)	PF00191;
E1C1D1	423747;	ANXA7	Annexin	Annexin family	Gallus gallus (Chicken)	PF00191;
E1C8K3	423774;	ANXA8L1	Annexin	Annexin family	Gallus gallus (Chicken)	PF00191;
A0A1D5PN32	423637;	ANXA11	Annexin	Annexin family	Gallus gallus (Chicken)	PF00191;
Q5ZLG6	423637;	RCJMB04_6f1	Annexin	Annexin family	Gallus gallus (Chicken)	PF00191;
Q5ZMN0	420087;	ANP32B RCJMB04_1j7	Acidic leucine-rich nuclear phosphoprotein 32 family member B	ANP32 family	Gallus gallus (Chicken)	

Q5F4A3	426109;	ANP32E RCJMB04_1l3	Acidic leucine-rich nuclear phosphoprotein 32 family member E	ANP32 family	Gallus gallus (Chicken)	
Q5ZMW3	423168;	API5 RCJMB04_1a12	Apoptosis inhibitor 5 (API-5)	API5 family	Gallus gallus (Chicken)	PF05918;
P08250	396536;	APOA1	Apolipoprotein A-I (Apo-AI) (ApoA-I) (Apolipoprotein A1) [Cleaved into: Proapolipoprotein A-I (ProapoA-I)]	Apolipoprotein A1/A4/E family	Gallus gallus (Chicken)	PF01442;
O93601	395780;	apoAIV	Apolipoprotein AIV	Apolipoprotein A1/A4/E family	Gallus gallus (Chicken)	PF01442;
A0A1D5NXD7	418599;	APOO	MICOS complex subunit	Apolipoprotein O/MICOS complex subunit Mic27 family	Gallus gallus (Chicken)	PF09769;
A0A1D5PXD8	428222;	AGO1	Uncharacterized protein	Argonaute family	Gallus gallus (Chicken)	PF08699;PF16488;PF16487;PF16486;PF02170;PF02171;
Q5ZLG4	419628;	AGO3 EIF2C3 RCJMB04_6f12	Protein argonaute-3 (Argonaute3) (EC 3.1.26.n2) (Argonaute RISC catalytic component 3) (Eukaryotic translation initiation factor 2C 3) (eIF-2C 3) (eIF2C 3)	Argonaute family, Ago subfamily	Gallus gallus (Chicken)	PF08699;PF16488;PF16487;PF16486;PF02170;PF02171;
F1P1K3	429041;	ARPC2	Arp2/3 complex 34 kDa subunit	ARPC2 family	Gallus gallus (Chicken)	PF04045;
E1C8Y3	416837;	ARPC3	Actin-related protein 2/3 complex subunit 3	ARPC3 family	Gallus gallus (Chicken)	PF04062;
F1P010	416051;	ARPC4	Actin-related protein 2/3 complex subunit 4	ARPC4 family	Gallus gallus (Chicken)	PF05856;
Q5ZMV5	429075;	ARPC5 RCJMB04_1b5	Actin-related protein 2/3 complex subunit 5	ARPC5 family	Gallus gallus (Chicken)	PF04699;
P13914	415809;		Arylamine N-acetyltransferase, pineal gland isozyme NAT-3 (Arylamine acetylase) (EC 2.3.1.5)	Arylamine N-acetyltransferase family	Gallus gallus (Chicken)	PF00797;
P05122	396248;	CKB	Creatine kinase B-type (EC 2.7.3.2) (B-CK) (Creatine kinase B chain) (Creatine phosphokinase M-type) (CPK-B)	ATP:guanido phosphotransferase family	Gallus gallus (Chicken)	PF00217;PF02807;
E1BUZ7	416511;	ATPAF2	Uncharacterized protein	ATP12 family	Gallus gallus (Chicken)	PF07542;
A0A1L1RZJ6	431564;	ATP5A1W	ATP synthase subunit alpha	ATPase alpha/beta chains family	Gallus gallus (Chicken)	PF00006;PF00306;PF02874;
Q5ZLC5	426673;	ATP5F1B ATP5B RCJMB04_6l18	ATP synthase subunit beta, mitochondrial (EC 7.1.2.2) (ATP synthase F1 subunit beta)	ATPase alpha/beta chains family	Gallus gallus (Chicken)	PF00006;PF02874;
Q90647	395821;	ATP6V1A	V-type proton ATPase catalytic subunit A (V-ATPase subunit A) (EC 7.1.2.2) (V-ATPase 69 kDa subunit) (Vacuolar proton pump subunit alpha)	ATPase alpha/beta chains family	Gallus gallus (Chicken)	PF00006;PF02874;PF16886;
F1NBW2	395821;	ATP6V1A	H(+)-transporting two-sector ATPase (EC 7.1.2.2)	ATPase alpha/beta chains family	Gallus gallus (Chicken)	PF00006;PF02874;PF16886;
A0A1D5PP57	395497;	ATP6V1B2	Vacuolar proton pump subunit B (V-ATPase subunit B) (Vacuolar proton pump subunit B)	ATPase alpha/beta chains family	Gallus gallus (Chicken)	PF00006;PF02874;
Q9I8A2	395497;	VATB	Vacuolar proton pump subunit B (V-ATPase subunit B) (Vacuolar proton pump subunit B) (Fragment)	ATPase alpha/beta chains family	Gallus gallus (Chicken)	PF00006;PF02874;
H9KYP2	776719;	LOC776719	H(+)-transporting two-sector ATPase (EC 7.1.2.2)	ATPase alpha/beta chains family	Gallus gallus (Chicken)	PF00006;PF02874;PF16886;
E1C658	422115;	ATP5H	ATP synthase subunit d, mitochondrial	ATPase d subunit family	Gallus gallus (Chicken)	PF05873;
A0A1D5P810	418508;	ATP5O	ATP synthase peripheral stalk subunit OSCP (ATP synthase subunit O, mitochondrial) (Oligomycin sensitivity conferral protein)	ATPase delta chain family	Gallus gallus (Chicken)	PF00213;
A0A1D5P0U8	419108;	ATP5C1	ATP synthase subunit gamma	ATPase gamma chain family	Gallus gallus (Chicken)	PF00231;
A0A1L1RYK0	419108;	ATP5C1	ATP synthase subunit gamma	ATPase gamma chain family	Gallus gallus (Chicken)	PF00231;
F1NLR1	416714;	ACSS1A	Acetyl-coenzyme A synthetase (EC 6.2.1.1)	ATP-dependent AMP-binding enzyme family	Gallus gallus (Chicken)	PF16177;PF00501;PF13193;
A0A1D5PNV7	420957;	TMEM245	Uncharacterized protein	Autoinducer-2 exporter (AI-2E) (TC 2.A.86) family	Gallus gallus (Chicken)	PF01594;
E1BSU4	420957;	TMEM245	Uncharacterized protein	Autoinducer-2 exporter (AI-2E) (TC 2.A.86) family	Gallus gallus (Chicken)	PF01594;
Q5ZML0	421417;	BABAM2 BRE RCJMB04_1l17	BRISC and BRCA1-A complex member 2 (BRCA1-A complex subunit BRE) (BRCA1/BRCA2-containing complex subunit 45) (Brain and reproductive organ-expressed protein)	BABAM2 family	Gallus gallus (Chicken)	PF06113;
F1NJ94	423670;	ERLIN1	PHB domain-containing protein	Band 7/mec-2 family	Gallus gallus (Chicken)	PF01145;
F1NBD0	426769;	ERLIN2	PHB domain-containing protein	Band 7/mec-2 family	Gallus gallus (Chicken)	PF01145;
E1BTV1	417118;	STOM	PHB domain-containing protein	Band 7/mec-2 family	Gallus gallus (Chicken)	PF01145;
A0A1D5PCD1	417579;	FLOT2	Flotillin	Band 7/mec-2 family, Flotillin subfamily	Gallus gallus (Chicken)	PF01145;PF15975;
A0A1L1RV80	417579;	FLOT2	Flotillin	Band 7/mec-2 family, Flotillin subfamily	Gallus gallus (Chicken)	PF01145;PF15975;

Q5ZHQ3	417579;	FLOT2 RCJMB04_34i9	Flotillin	Band 7/mec-2 family, Flotillin subfamily	Gallus gallus (Chicken)	PF01145;PF15975;
P23614	373905;	BASP1	Brain acid soluble protein 1 homolog (23 kDa cortical cytoskeleton-associated protein) (CAP-23)	BASP1 family	Gallus gallus (Chicken)	PF05466;
E1C310	417702;	BCAP29	Endoplasmic reticulum transmembrane protein	BCAP29/BCAP31 family	Gallus gallus (Chicken)	PF05529;PF18035;
Q07816	373954;	BCL2L1 BCL-X BCLX	Bcl-2-like protein 1 (Bcl2-L-1) (Apoptosis regulator Bcl-X)	Bcl-2 family	Gallus gallus (Chicken)	PF00452;PF02180;
F1NY54	419623;	THRAP3	Uncharacterized protein	BCLAF1/THRAP3 family	Gallus gallus (Chicken)	PF15440;
A0A1D5NYE8	395964;	CTNNB1	Uncharacterized protein	Beta-catenin family	Gallus gallus (Chicken)	PF00514;
A0A1D5PQN0	395964;	CTNNB1	Uncharacterized protein	Beta-catenin family	Gallus gallus (Chicken)	PF00514;
O42486	395964;	Bcat	Beta catenin	Beta-catenin family	Gallus gallus (Chicken)	PF00514;
E1C1V3	429710;	JUP	Junction plakoglobin	Beta-catenin family	Gallus gallus (Chicken)	PF00514;
A0A1D5PGF0	423621;	GHITM	Uncharacterized protein	BI1 family	Gallus gallus (Chicken)	PF01027;
E1BY06	421334;	BROX	BRO1 domain- and CAAX motif-containing protein (BRO1 domain-containing protein BROX)	BROX family	Gallus gallus (Chicken)	PF03097;
E1BV47	416492;	BUD31	Protein BUD31 homolog	BUD31 (G10) family	Gallus gallus (Chicken)	PF01125;
Q5ZLT7	424073;	BZW1 RCJMB04_4o16	Basic leucine zipper and W2 domain-containing protein 1	BZW family	Gallus gallus (Chicken)	PF02020;
Q5ZL42	420594;	BZW2 RCJMB04_7o12	Basic leucine zipper and W2 domain-containing protein 2	BZW family	Gallus gallus (Chicken)	PF02020;
F1NM03	420594;	BZW2	Basic leucine zipper and W2 domain-containing protein 2	BZW family	Gallus gallus (Chicken)	PF02020;
Q5ZM44	423211;	CHP1 CHP RCJMB04_3d7	Calcineurin B homologous protein 1 (Calcineurin B-like protein) (Calcium-binding protein CHP) (Calcium-binding protein p22) (EF-hand calcium-binding domain-containing protein p22)	Calcineurin regulatory subunit family, CHP subfamily	Gallus gallus (Chicken)	PF13499;
E1BST5	768444;	CACNA2D1	VWFA domain-containing protein	Calcium channel subunit alpha-2/delta family	Gallus gallus (Chicken)	PF08473;PF00092;PF08399;
P12957	373965;	CALD1 CAD	Caldesmon (CDM)	Caldesmon family	Gallus gallus (Chicken)	PF02029;
P05419	396523;		Neo-calmodulin (NeoCaM) (Fragment)	Calmodulin family	Gallus gallus (Chicken)	PF13499;
P26932	396522;	CNN1	Calponin-1 (Calponin, smooth muscle)	Calponin family	Gallus gallus (Chicken)	PF00402;PF00307;
Q5ZKU6	100216000;	CNN2 RCJMB04_9b22	Calponin	Calponin family	Gallus gallus (Chicken)	PF00402;PF00307;
P19966	396490;	TAGLN SM22	Transgelin (Smooth muscle protein 22-alpha) (SM22-alpha)	Calponin family	Gallus gallus (Chicken)	PF00402;PF00307;
A0A1D5PF08	416288;	CANX	Uncharacterized protein	Calreticulin family	Gallus gallus (Chicken)	PF00262;
Q5ZMF5	416288;	RCJMB04_2d15	Uncharacterized protein	Calreticulin family	Gallus gallus (Chicken)	PF00262;
Q6DRR5	419557;	FABP	Fatty acid-binding protein	Calycin superfamily, Fatty-acid binding protein (FABP) family	Gallus gallus (Chicken)	PF00061;
Q5ZIR7	420197;	RCJMB04_23p16	FABP domain-containing protein	Calycin superfamily, Fatty-acid binding protein (FABP) family	Gallus gallus (Chicken)	PF00061;
Q05423	396246;	FABP7	Fatty acid-binding protein, brain (Brain-type fatty acid-binding protein) (B-FABP) (Fatty acid-binding protein 7) (Fatty acid-binding protein, retina) (R-FABP)	Calycin superfamily, Fatty-acid binding protein (FABP) family	Gallus gallus (Chicken)	PF00061;
Q5G8Y9	424893;	ApoD APOD	Apolipoprotein D	Calycin superfamily, Lipocalin family	Gallus gallus (Chicken)	PF08212;
P21760	396393;	EXFABP	Extracellular fatty acid-binding protein (Ex-FABP) (Protein Ch21) (Quiescence-specific protein) (p20K)	Calycin superfamily, Lipocalin family	Gallus gallus (Chicken)	PF00061;
Q8QFM7	374110;	L-PGDS	Chondrogenesis associated lipocalin (Lipocalin-type prostaglandin D synthase)	Calycin superfamily, Lipocalin family	Gallus gallus (Chicken)	PF00061;
P41263	396166;	RBP4	Retinol-binding protein 4 (Plasma retinol-binding protein) (PRBP) (RBP)	Calycin superfamily, Lipocalin family	Gallus gallus (Chicken)	PF00061;
Q8JIG5	395220;	ogchi OGCHI ORM1	Alpha-1-acid glycoprotein	Calycin superfamily, Lipocalin family; Calycin superfamily, Lipocalin family	Gallus gallus (Chicken)	PF11032;
Q5ZM91	417438;	PRKAR1A RCJMB04_2n5	cAMP-dependent protein kinase type I-alpha regulatory subunit	CAMP-dependent kinase regulatory chain family	Gallus gallus (Chicken)	PF00027;PF02197;
F1NHL2	417837;	CAND1	TIP120 domain-containing protein	CAND family	Gallus gallus (Chicken)	PF08623;

Q5XNV3	426151;	CAPRIN1	Cytoplasmic activation-proliferation-associated protein 1	Caprin family	Gallus gallus (Chicken)	PF12287;PF18293;
A0A1D5NZY6	423735;	ADK	Adenosine kinase (EC 2.7.1.20)	Carbohydrate kinase PfkB family; Carbohydrate kinase pfkB family	Gallus gallus (Chicken)	PF00294;
Q5ZMK9	423735;	ADK RCJMB04_1l21	Adenosine kinase (EC 2.7.1.20)	Carbohydrate kinase PfkB family; Carbohydrate kinase pfkB family	Gallus gallus (Chicken)	PF00294;
Q6B842	423118;	CPT1A	Carnitine O-palmitoyltransferase (EC 2.3.1.21)	Carnitine/choline acetyltransferase family	Gallus gallus (Chicken)	PF00755;PF16484;
F1P1U3	424649;	CPT2	Carn_acyltransf domain-containing protein	Carnitine/choline acetyltransferase family	Gallus gallus (Chicken)	PF00755;
Q5ZK83	424649;	RCJMB04_12h19	Carn_acyltransf domain-containing protein	Carnitine/choline acetyltransferase family	Gallus gallus (Chicken)	PF00755;
Q5ZMM4	423600;	RCJMB04_1j22	Catalase (EC 1.11.1.6)	Catalase family	Gallus gallus (Chicken)	PF00199;PF06628;
Q2IAL7	420407;	CATHL2 CMAP27	Cathelicidin-2 (CATH-2) (Fowlicidin-2) (Myeloid antimicrobial peptide 27)	Cathelicidin family	Gallus gallus (Chicken)	
C4PFJ8	420407;	CATHL2	Fowlicidin-2	Cathelicidin family	Gallus gallus (Chicken)	
Q6QLQ5	414337;	CATHL1 CATH	Cathelicidin-1 (CATH-1) (Fowlicidin-1)	Cathelicidin family	Gallus gallus (Chicken)	
C4PFJ7	414337;	CATHL1 CathL1	Fowlicidin-1	Cathelicidin family	Gallus gallus (Chicken)	
Q03669	396446;	ATP2A2	Sarcoplasmic/endoplasmic reticulum calcium ATPase 2 (SERCA2) (SR Ca(2+)-ATPase 2) (EC 7.2.2.10) (Calcium pump 2) (Calcium-transporting ATPase sarcoplasmic reticulum type, slow twitch skeletal muscle isoform) (Endoplasmic reticulum class 1/2 Ca(2+) ATPase)	Cation transport ATPase (P-type) (TC 3.A.3) family, Type IIA subfamily	Gallus gallus (Chicken)	PF00689;PF00690;
A0A1L1RKB7	396446;	ATP2A2	Calcium-transporting ATPase (EC 7.2.2.10)	Cation transport ATPase (P-type) (TC 3.A.3) family, Type IIA subfamily	Gallus gallus (Chicken)	PF00689;PF00690;
A0A1D5PJX0	419934;	ATP2B4	Calcium-transporting ATPase (EC 7.2.2.10)	Cation transport ATPase (P-type) (TC 3.A.3) family, Type IIB subfamily	Gallus gallus (Chicken)	PF12424;PF00689;PF00690;
A0A1D5PU07	419934;	ATP2B4	Calcium-transporting ATPase (EC 7.2.2.10)	Cation transport ATPase (P-type) (TC 3.A.3) family, Type IIB subfamily	Gallus gallus (Chicken)	PF12424;PF00689;PF00690;
P09572	396530;	ATP1A1	Sodium/potassium-transporting ATPase subunit alpha-1 (Na(+)/K(+) ATPase alpha-1 subunit) (EC 7.2.2.13) (Sodium pump subunit alpha-1)	Cation transport ATPase (P-type) (TC 3.A.3) family, Type IIC subfamily	Gallus gallus (Chicken)	PF00689;PF00690;
P35431	373996;	CAV1	Caveolin-1	Caveolin family	Gallus gallus (Chicken)	PF01146;
Q90885	396006;		Leucine zipper protein	CAVIN family	Gallus gallus (Chicken)	PF15237;
E1BRQ5	422005;	CCDC25	Coiled-coil domain-containing protein 25	CCDC25 family	Gallus gallus (Chicken)	PF05670;
Q5ZKI4	424277;	CCDC93 RCJMB04_10i21	Coiled-coil domain-containing protein 93	CCDC93 family	Gallus gallus (Chicken)	PF09762;
O57476	395430;	CDC37	Hsp90 co-chaperone Cdc37 (Hsp90 chaperone protein kinase-targeting subunit) (p50Cdc37)	CDC37 family	Gallus gallus (Chicken)	PF08564;PF08565;PF03234;
Q5ZL72	424059;	HSPD1 HSP60 RCJMB04_7g5	60 kDa heat shock protein, mitochondrial (EC 5.6.1.7) (60 kDa chaperonin) (Chaperonin 60) (CPN60) (Heat shock protein 60) (HSP-60) (Hsp60)	Chaperonin (HSP60) family	Gallus gallus (Chicken)	PF00118;
Q5ZKI1	422178;	CLIC2 RCJMB04_10k5	Chloride intracellular channel protein	Chloride channel CLIC family	Gallus gallus (Chicken)	PF13409;
A0A1D5PD90	417293;	CLIC3	GST N-terminal domain-containing protein	Chloride channel CLIC family	Gallus gallus (Chicken)	PF13417;
F1NYZ7	419595;	CLIC4	GST N-terminal domain-containing protein	Chloride channel CLIC family	Gallus gallus (Chicken)	PF13417;
P07341	427308;	ALDOB	Fructose-bisphosphate aldolase B (EC 4.1.2.13) (Liver-type aldolase)	Class I fructose-bisphosphate aldolase family	Gallus gallus (Chicken)	PF00274;
R4GM10	395492;	ALDOC	Fructose-bisphosphate aldolase (EC 4.1.2.13)	Class I fructose-bisphosphate aldolase family	Gallus gallus (Chicken)	PF00274;
Q5ZJZ9	101750306;	RCJMB04_14b8	Protein arginine N-methyltransferase 5 (EC 2.1.1.-)	Class I-like SAM-binding methyltransferase superfamily	Gallus gallus (Chicken)	PF05185;PF17286;PF17285;
E1BYM1	421046;	RNMT	mRNA cap guanine-N7 methyltransferase (EC 2.1.1.56)	Class I-like SAM-binding methyltransferase superfamily, mRNA cap 0 methyltransferase family; DsDNA virus mRNA	Gallus gallus (Chicken)	PF03291;
F1NGW2	420830;	TPMT	Thiopurine S-methyltransferase (EC 2.1.1.67)	Class I-like SAM-binding methyltransferase superfamily, TPMT family	Gallus gallus (Chicken)	PF05724;
F1N832	418292;	EMG1	Uncharacterized protein	Class IV-like SAM-binding methyltransferase superfamily, RNA methyltransferase NEP1 family	Gallus gallus (Chicken)	PF03587;
Q5ZM11	416168;	RARS1 RARS RCJMB04_3h11	Arginine--tRNA ligase, cytoplasmic (EC 6.1.1.19) (Arginyl-tRNA synthetase) (ArgRS)	Class-I aminoacyl-tRNA synthetase family	Gallus gallus (Chicken)	PF03485;PF05746;PF00750;
Q5F408	423086;	CARS1 CARS RCJMB04_3o5	Cysteine--tRNA ligase, cytoplasmic (EC 6.1.1.16) (Cysteinyl-tRNA synthetase) (CysRS)	Class-I aminoacyl-tRNA synthetase family	Gallus gallus (Chicken)	PF01406;
Q5F3C0	416057;	RCJMB04_22h1	Glutaminyl-tRNA synthetase (EC 6.1.1.18)	Class-I aminoacyl-tRNA synthetase family	Gallus gallus (Chicken)	PF00749;PF03950;PF04558;PF04557;

A0A1D5P2D5	415957;	IARS	Isoleucyl-tRNA synthetase (EC 6.1.1.5)	Class-I aminoacyl-tRNA synthetase family	Gallus gallus (Chicken)	PF08264;PF00133;
A0A3Q2UG33	415957;	IARS	Isoleucyl-tRNA synthetase (EC 6.1.1.5)	Class-I aminoacyl-tRNA synthetase family	Gallus gallus (Chicken)	PF08264;PF00133;
Q5ZKA2	421346;	IARS2 RCJMB04_12b19	Isoleucine--tRNA ligase, mitochondrial (EC 6.1.1.5) (Isoleucyl-tRNA synthetase) (IleRS)	Class-I aminoacyl-tRNA synthetase family	Gallus gallus (Chicken)	PF08264;PF00133;
E1C2I9	416347;	LARS	Leucyl-tRNA synthetase (EC 6.1.1.4)	Class-I aminoacyl-tRNA synthetase family	Gallus gallus (Chicken)	PF08264;PF00133;
E1C2Z5	423458;	WARS	T1-TrpRS (EC 6.1.1.2) (T2-TrpRS) (Tryptophan--tRNA ligase, cytoplasmic) (Tryptophanyl-tRNA synthetase)	Class-I aminoacyl-tRNA synthetase family	Gallus gallus (Chicken)	PF00579;PF00458;
Q5ZJ08	419666;	YARS1 YARS RCJMB04_21p3	Tyrosine--tRNA ligase, cytoplasmic (EC 6.1.1.1) (Tyrosyl-tRNA synthetase) (TyrRS)	Class-I aminoacyl-tRNA synthetase family	Gallus gallus (Chicken)	PF00579;PF01588;
Q5ZM32	417699;	DLD RCJMB04_3f8	Dihydrolipoyl dehydrogenase (EC 1.8.1.4)	Class-I pyridine nucleotide-disulfide oxidoreductase family	Gallus gallus (Chicken)	PF07992;PF02852;
A0A1D5P338	771783;	GSR	Glutathione reductase (EC 1.8.1.7)	Class-I pyridine nucleotide-disulfide oxidoreductase family	Gallus gallus (Chicken)	PF07992;PF02852;
P00504	396261;	GOT1	Aspartate aminotransferase, cytoplasmic (cAspAT) (EC 2.6.1.1) (EC 2.6.1.3) (Cysteine aminotransferase, cytoplasmic) (Cysteine transaminase, cytoplasmic) (cCAT) (Glutamate oxaloacetate transaminase 1) (Transaminase A)	Class-I pyridoxal-phosphate-dependent aminotransferase family	Gallus gallus (Chicken)	PF00155;
P00508	396533;	GOT2	Aspartate aminotransferase, mitochondrial (mAspAT) (EC 2.6.1.1) (EC 2.6.1.7) (Glutamate oxaloacetate transaminase 2) (Kynurenine aminotransferase 4) (Kynurenine aminotransferase IV) (Kynurenine--oxoglutarate transaminase 4) (Kynurenine--oxoglutarate transaminase IV) (Transaminase A)	Class-I pyridoxal-phosphate-dependent aminotransferase family	Gallus gallus (Chicken)	PF00155;
Q5ZK08	426856;	RCJMB04_13p14	Asparagine--tRNA ligase (EC 6.1.1.22)	Class-II aminoacyl-tRNA synthetase family	Gallus gallus (Chicken)	PF00152;PF01336;
Q5ZHR4	428403;	GARS RCJMB04_34b10	Diadenosine tetraphosphate synthetase (EC 6.1.1.14) (Glycine--tRNA ligase)	Class-II aminoacyl-tRNA synthetase family	Gallus gallus (Chicken)	PF03129;PF00587;PF00458;
Q5ZK86	416132;	RCJMB04_12f15	Histidine--tRNA ligase (EC 6.1.1.21)	Class-II aminoacyl-tRNA synthetase family	Gallus gallus (Chicken)	PF03129;PF13393;PF00458;
Q5ZKP8	415885;	RCJMB04_9m1	Lysine--tRNA ligase (EC 6.1.1.6) (Lysyl-tRNA synthetase)	Class-II aminoacyl-tRNA synthetase family	Gallus gallus (Chicken)	PF00152;PF01336;
Q5ZLW1	427427;	RCJMB04_4k14	Threonyl-tRNA synthetase (EC 6.1.1.3)	Class-II aminoacyl-tRNA synthetase family	Gallus gallus (Chicken)	PF03129;PF02824;PF00587;PF07973;
Q5ZJQ2	100859604;	FARSA FARSLA RCJMB04_16g22	Phenylalanine--tRNA ligase alpha subunit (EC 6.1.1.20) (Phenylalanyl-tRNA synthetase alpha subunit) (PheRS)	Class-II aminoacyl-tRNA synthetase family, Phe-tRNA synthetase alpha subunit type 2 subfamily	Gallus gallus (Chicken)	PF18552;PF18554;PF18553;PF01409;
Q5ZJQ5	424296;	RCJMB04_16g5	Aspartate--tRNA ligase, cytoplasmic (EC 6.1.1.12) (Aspartyl-tRNA synthetase)	Class-II aminoacyl-tRNA synthetase family, Type 2 subfamily	Gallus gallus (Chicken)	PF00152;PF01336;
Q5ZM75	426697;	RCJMB04_2o24	Seryl-tRNA synthetase (EC 6.1.1.11)	Class-II aminoacyl-tRNA synthetase family, Type-1 seryl-tRNA synthetase subfamily	Gallus gallus (Chicken)	PF02403;PF00587;
F1P5J5	415668;	AARS	Alanine--tRNA ligase (EC 6.1.1.7)	Class-II aminoacyl-tRNA synthetase family; Class-II aminoacyl-tRNA synthetase family, Alax-L subfamily	Gallus gallus (Chicken)	PF02272;PF01411;PF07973;
Q5ZKF3	415668;	AARS RCJMB04_11d4	Alanine--tRNA ligase (EC 6.1.1.7)	Class-II aminoacyl-tRNA synthetase family; Class-II aminoacyl-tRNA synthetase family, Alax-L subfamily	Gallus gallus (Chicken)	PF01411;PF07973;
Q5ZLD1	420969;	FH RCJMB04_6k20	Fumarate hydratase, mitochondrial (EC 4.2.1.2)	Class-II fumarase/aspartase family, Fumarase subfamily	Gallus gallus (Chicken)	PF10415;PF00206;
F1NBE3	101750333;	GCAT	Aminotran_1_2 domain-containing protein	Class-II pyridoxal-phosphate-dependent aminotransferase family	Gallus gallus (Chicken)	PF00155;
E1C8M8	416642;	ABAT	(S)-3-amino-2-methylpropionate transaminase (EC 2.6.1.19) (EC 2.6.1.22) (4-aminobutyrate aminotransferase, mitochondrial) (GABA aminotransferase) (Gamma-amino-N-butyrate transaminase) (L-AIBAT)	Class-III pyridoxal-phosphate-dependent aminotransferase family	Gallus gallus (Chicken)	PF00202;
Q5ZJ29	426430;	OAT RCJMB04_21f9	Ornithine--oxo-acid aminotransferase (EC 2.6.1.13)	Class-III pyridoxal-phosphate-dependent aminotransferase family	Gallus gallus (Chicken)	PF00202;
Q5ZKP5	419133;	RCJMB04_9m12	Cysteine desulfurase, mitochondrial (EC 2.8.1.7)	Class-V pyridoxal-phosphate-dependent aminotransferase family; Class-V pyridoxal-phosphate-dependent aminotransferase	Gallus gallus (Chicken)	PF00266;
F1NW23	395272;	CLTC	Clathrin heavy chain	Clathrin heavy chain family	Gallus gallus (Chicken)	PF00637;PF09268;PF01394;
Q8UUR1	395272;	CHC	Clathrin heavy-chain (Fragment)	Clathrin heavy chain family	Gallus gallus (Chicken)	PF00637;PF09268;PF01394;
A0A1D5P650	416765;	CLTCL1	Clathrin heavy chain	Clathrin heavy chain family	Gallus gallus (Chicken)	PF00637;PF09268;PF01394;
A0A1D5PAU8	416765;	CLTCL1	Clathrin heavy chain	Clathrin heavy chain family	Gallus gallus (Chicken)	PF00637;PF09268;PF01394;
A0A1D5PDC1	416765;	CLTCL1	Clathrin heavy chain	Clathrin heavy chain family	Gallus gallus (Chicken)	PF00637;PF09268;PF01394;
A0A1D5PW75	416765;	CLTCL1	Clathrin heavy chain	Clathrin heavy chain family	Gallus gallus (Chicken)	PF00637;PF09268;PF01394;
A0A3Q2TVE4	427284;	CLTA	Clathrin light chain	Clathrin light chain family	Gallus gallus (Chicken)	PF01086;
A0A3Q2U5N2	427284;	CLTA	Clathrin light chain	Clathrin light chain family	Gallus gallus (Chicken)	PF01086;
Q5ZHR7	427284;	CLTA RCJMB04_33p3	Clathrin light chain	Clathrin light chain family	Gallus gallus (Chicken)	PF01086;

A0A1D5PJ40	416226;	CLTB	Clathrin light chain	Clathrin light chain family	Gallus gallus (Chicken)	PF01086;
A0A1D5PL88	416226;	CLTB	Clathrin light chain	Clathrin light chain family	Gallus gallus (Chicken)	PF01086;
Q5ZMG2	424910;	CLDN1 RCJMB04_2c8	Claudin	Claudin family	Gallus gallus (Chicken)	PF00822;
F1N988	417675;	CLUH	Clustered mitochondria protein homolog	CLU family	Gallus gallus (Chicken)	PF13236;PF15044;PF12807;
Q9YGP0	395722;		Clusterin	Clusterin family	Gallus gallus (Chicken)	PF01093;
Q5F475	418275;	RCJMB04_2h1	Condensin complex subunit 1	CND1 (condensin subunit 1) family	Gallus gallus (Chicken)	PF12717;PF12922;
A0A1D5PE41	769492;	NDUFS8	Complex I-23kD (NADH dehydrogenase [ubiquinone] iron-sulfur protein 8, mitochondrial) (NADH-ubiquinone oxidoreductase 23 kDa subunit)	Complex I 23 kDa subunit family	Gallus gallus (Chicken)	PF12838;
A0A1D5PI06	426488;	NDUFV2	NADH dehydrogenase [ubiquinone] flavoprotein 2, mitochondrial (NADH-ubiquinone oxidoreductase 24 kDa subunit)	Complex I 24 kDa subunit family	Gallus gallus (Chicken)	
F1ND23	423179;	NDUFS3	Complex I-30kD (NADH dehydrogenase [ubiquinone] iron-sulfur protein 3, mitochondrial) (NADH-ubiquinone oxidoreductase 30 kDa subunit)	Complex I 30 kDa subunit family	Gallus gallus (Chicken)	PF00329;
Q5ZHS2	430210;	RCJMB04_33n14	NADH dehydrogenase [ubiquinone] flavoprotein 1, mitochondrial (EC 7.1.1.2)	Complex I 51 kDa subunit family	Gallus gallus (Chicken)	PF01512;PF10589;PF10531;
F1NXN8	424102;	NDUFS1	NADH-ubiquinone oxidoreductase 75 kDa subunit, mitochondrial	Complex I 75 kDa subunit family	Gallus gallus (Chicken)	PF00384;PF10588;PF09326;
Q5ZJ57	424102;	RCJMB04_20j17	NADH-ubiquinone oxidoreductase 75 kDa subunit, mitochondrial	Complex I 75 kDa subunit family	Gallus gallus (Chicken)	PF00384;PF10588;PF09326;
A0A1D5PNM0	420337;	NDUFB9	Complex I-B22 (NADH dehydrogenase [ubiquinone] 1 beta subcomplex subunit 9) (NADH-ubiquinone oxidoreductase B22 subunit)	Complex I LYR family	Gallus gallus (Chicken)	PF05347;
Q5ZIQ5	424032;	RCJMB04_24e8	NADH dehydrogenase [ubiquinone] 1 alpha subcomplex subunit 10, mitochondrial	Complex I NDUFA10 subunit family	Gallus gallus (Chicken)	PF01712;
D5M8S5	417907;	NDUFA12	NADH dehydrogenase [ubiquinone] 1 alpha subcomplex subunit 12	Complex I NDUFA12 subunit family	Gallus gallus (Chicken)	PF05071;
E1BRT9	417753;	NDUFA5	Complex I subunit B13 (Complex I-13kD-B) (NADH dehydrogenase [ubiquinone] 1 alpha subcomplex subunit 5) (NADH-ubiquinone oxidoreductase 13 kDa-B subunit)	Complex I NDUFA5 subunit family	Gallus gallus (Chicken)	PF04716;
A0A1D5P3K4	417112;	NDUFA8	NADH dehydrogenase [ubiquinone] 1 alpha subcomplex subunit 8	Complex I NDUFA8 subunit family	Gallus gallus (Chicken)	PF06747;
E1C6C9	416543;	NDUFB10	Complex I-PDSW (NADH dehydrogenase [ubiquinone] 1 beta subcomplex subunit 10) (NADH-ubiquinone oxidoreductase PDSW subunit)	Complex I NDUFB10 subunit family	Gallus gallus (Chicken)	PF10249;
E1BT94	416391;	NDUFB6	Complex I-B17 (NADH dehydrogenase [ubiquinone] 1 beta subcomplex subunit 6) (NADH-ubiquinone oxidoreductase B17 subunit)	Complex I NDUFB6 subunit family	Gallus gallus (Chicken)	PF09782;
Q5ZJW2	423763;	NDUFB8 RCJMB04_15e3	NADH dehydrogenase [ubiquinone] 1 beta subcomplex subunit 8, mitochondrial (Complex I-ASHI) (NADH-ubiquinone oxidoreductase ASHI subunit)	Complex I NDUFB8 subunit family	Gallus gallus (Chicken)	PF05821;
A0A1L1RM23	374122;	NDUFS4	NADH dehydrogenase [ubiquinone] iron-sulfur protein 4, mitochondrial	Complex I NDUFS4 subunit family	Gallus gallus (Chicken)	PF04800;
Q5F402	416014;	RCJMB04_3p13	Coatomer subunit gamma	COPG family	Gallus gallus (Chicken)	PF01602;PF16381;PF08752;
F1NKY6	419134;	CPNE1	Uncharacterized protein	Copine family	Gallus gallus (Chicken)	PF00168;PF07002;
R4GH54	421493;	COX20	Cytochrome c oxidase assembly protein COX20, mitochondrial	COX20 family	Gallus gallus (Chicken)	PF12597;
Q5ZLU7	417382;	RCJMB04_4m4	PCI domain-containing protein	CSN1 family	Gallus gallus (Chicken)	PF01399;PF10602;
Q5ZI80	430917;	RCJMB04_29h3	COP9 signalosome complex subunit 2	CSN2 family	Gallus gallus (Chicken)	PF01399;
Q5ZJV0	422594;	COPS4 RCJMB04_15i11	COP9 signalosome complex subunit 4	CSN4 family	Gallus gallus (Chicken)	PF18420;PF01399;
Q5ZJA1	418283;	COPS7A RCJMB04_19l10	PCI domain-containing protein	CSN7/EIF3M family, CSN7 subfamily	Gallus gallus (Chicken)	PF18392;PF01399;
A0A3Q2U280	427301;	SLC44A1	Uncharacterized protein	CTL (choline transporter-like) family	Gallus gallus (Chicken)	PF04515;
Q5ZKW2	419445;	LZIC RCJMB04_8o19	Protein LZIC (Leucine zipper and CTNNBIP1 domain-containing protein) (Leucine zipper and ICAT homologous domain-containing protein)	CTNNBIP1 family	Gallus gallus (Chicken)	PF06384;
A0A1D5PSR2	419561;	CTPS1	CTP synthase (EC 6.3.4.2) (UTP--ammonia ligase)	CTP synthase family	Gallus gallus (Chicken)	PF06418;PF00117;
E1BYQ3	424804;	CUL3	CULLIN_2 domain-containing protein	Cullin family	Gallus gallus (Chicken)	PF00888;PF10557;
P80566	395938;	SOD1	Superoxide dismutase [Cu-Zn] (EC 1.15.1.1)	Cu-Zn superoxide dismutase family	Gallus gallus (Chicken)	PF00080;
D0EKR3	776282;	PPIA	Peptidyl-prolyl cis-trans isomerase (PPIase) (EC 5.2.1.8)	Cyclophilin-type PPIase family	Gallus gallus (Chicken)	PF00160;

E1BXG0	419507;	PPIH	Peptidyl-prolyl cis-trans isomerase (PPIase) (EC 5.2.1.8)	Cyclophilin-type PPIase family	Gallus gallus (Chicken)	PF00160;
A0A1D5PW44	100859332;	PPIL1	Peptidyl-prolyl cis-trans isomerase (PPIase) (EC 5.2.1.8)	Cyclophilin-type PPIase family	Gallus gallus (Chicken)	PF00160;
P24367	396447;	PPIB	Peptidyl-prolyl cis-trans isomerase B (PPIase B) (EC 5.2.1.8) (Cyclophilin B) (Rotamase B) (S-cyclophilin) (SCYLP)	Cyclophilin-type PPIase family, PPIase B subfamily	Gallus gallus (Chicken)	PF00160;
Q5ZI04	421949;	CYRIA FAM49A RCJMB04_31l4	CYFIP-related Rac1 interactor A	CYRI family	Gallus gallus (Chicken)	PF07159;
A0A1D5PP47	421949;	FAM49A	DUF1394 domain-containing protein	CYRI family	Gallus gallus (Chicken)	PF07159;
A0A1D5PZ00	420330;	FAM49B	DUF1394 domain-containing protein	CYRI family	Gallus gallus (Chicken)	PF07159;
A0A3Q2TTC4	420330;	FAM49B	DUF1394 domain-containing protein	CYRI family	Gallus gallus (Chicken)	PF07159;
F1NHH1	418267;	CSTB	Cystatin domain-containing protein	Cystatin family	Gallus gallus (Chicken)	PF00031;
P01038	396497;		Cystatin (Egg-white cystatin) (Ovocystatin)	Cystatin family	Gallus gallus (Chicken)	PF00031;
A0A3Q2UDX4	418545;	CBSL	Cystathionine beta-synthase (EC 4.2.1.22)	Cysteine synthase/cystathionine beta-synthase family	Gallus gallus (Chicken)	PF00571;PF00291;
E1BYG4	418545;	CBSL	Cystathionine beta-synthase (EC 4.2.1.22)	Cysteine synthase/cystathionine beta-synthase family	Gallus gallus (Chicken)	PF00571;PF00291;
P00174	414798;	CYB5A CYB5	Cytochrome b5	Cytochrome b5 family	Gallus gallus (Chicken)	PF00173;
Q5ZI85	415865;	RCJMB04_29f20	Cytochrome b5 heme-binding domain-containing protein	Cytochrome b5 family	Gallus gallus (Chicken)	PF00173;
Q5ZKN2	772196;	PGRMC1 RCJMB04_7g20 RCJMB04_9p4	Membrane-associated progesterone receptor component 1 (mPR)	Cytochrome b5 family, MAPR subfamily	Gallus gallus (Chicken)	PF00173;
P67881	420624;	CYC	Cytochrome c	Cytochrome c family	Gallus gallus (Chicken)	PF00034;
Q5ZJV5	415826;	COX4I1 RCJMB04_15g7	Cytochrome c oxidase subunit 4	Cytochrome c oxidase IV family	Gallus gallus (Chicken)	PF02936;
Q805F9	374050;	DDB1 RCJMB04_6h2	DNA damage-binding protein 1 (DDB p127 subunit) (Damage-specific DNA-binding protein 1) (UV-damaged DNA-binding factor)	DDB1 family	Gallus gallus (Chicken)	PF03178;PF10433;
A0A1D5NXK6	425541;	DDI2	Uncharacterized protein	DDI1 family	Gallus gallus (Chicken)	PF09668;PF00240;
F1NNS1	425542;	DDOST	Dolichyl-diphosphooligosaccharide--protein glycosyltransferase 48 kDa subunit (Oligosaccharyl transferase 48 kDa subunit)	DDOST 48 kDa subunit family	Gallus gallus (Chicken)	PF03345;
Q5F491	418567;	DDX3X RCJMB04_2a4	RNA helicase (EC 3.6.4.13)	DEAD box helicase family	Gallus gallus (Chicken)	PF00270;PF00271;
A0A1D5PD32	418024;	DDX17	RNA helicase (EC 3.6.4.13)	DEAD box helicase family	Gallus gallus (Chicken)	PF00270;PF00271;
Q90WU3	395249;	DDX1	ATP-dependent RNA helicase DDX1 (EC 3.6.4.13) (DEAD box protein 1)	DEAD box helicase family, DDX1 subfamily	Gallus gallus (Chicken)	PF00270;PF00271;PF00622;
Q5ZKB9	419783;	DDX6 RCJMB04_11n24	Probable ATP-dependent RNA helicase DDX6 (EC 3.6.4.13) (DEAD box protein 6)	DEAD box helicase family, DDX6/DHH1 subfamily	Gallus gallus (Chicken)	PF00270;PF00271;
Q8JFP1	395232;	EIF4A2 RCJMB04_14a6	Eukaryotic initiation factor 4A-II (eIF-4A-II) (eIF4A-II) (EC 3.6.4.13) (ATP-dependent RNA helicase eIF4A-2)	DEAD box helicase family, eIF4A subfamily	Gallus gallus (Chicken)	PF00270;PF00271;
Q5ZM36	416704;	EIF4A3 DDX48 RCJMB04_3e17	Eukaryotic initiation factor 4A-III (eIF-4A-III) (eIF4A-III) (EC 3.6.4.13) (ATP-dependent RNA helicase DDX48) (ATP-dependent RNA helicase eIF4A-3) (DEAD box protein 48) (Eukaryotic translation initiation factor 4A isoform 3)	DEAD box helicase family, eIF4A subfamily	Gallus gallus (Chicken)	PF00270;PF00271;
A0A3S5ZPN3	395629;	DDX5	DEAD box protein 5 (EC 3.6.4.13) (Probable ATP-dependent RNA helicase DDX5)	DEAD box helicase family; DEAD box helicase family, DDX5/DBP2 subfamily	Gallus gallus (Chicken)	PF00270;PF00271;PF08061;
Q9W744	395629;		DEAD box protein 5 (EC 3.6.4.13) (Probable ATP-dependent RNA helicase DDX5)	DEAD box helicase family; DEAD box helicase family, DDX5/DBP2 subfamily	Gallus gallus (Chicken)	PF00270;PF00271;PF08061;
E1BXS8	420928;	CMBL	Carboxymethylenebutenolidase homolog	Dienelactone hydrolase family	Gallus gallus (Chicken)	PF01738;
A3RKL3	427317;	DHFR	Dihydrofolate reductase (EC 1.5.1.3)	Dihydrofolate reductase family	Gallus gallus (Chicken)	PF00186;
Q5ZIZ6	422904;	RCJMB04_22g9	Uncharacterized protein	D-isomer specific 2-hydroxyacid dehydrogenase family	Gallus gallus (Chicken)	PF00389;PF02826;
B3Y932	100431102;	chApex1	DNA-(apurinic or apyrimidinic site) endonuclease (EC 3.1.-.-)	DNA repair enzymes AP/ExoA family	Gallus gallus (Chicken)	PF03372;
E1C0J4	420119;	UPF1	Uncharacterized protein	DNA2/NAM7 helicase family	Gallus gallus (Chicken)	PF13086;PF13087;PF18141;PF04851;PF09416;
Q5ZKD7	419872;	MOV10 RCJMB04_11i10	Putative helicase MOV-10 (EC 3.6.4.13)	DNA2/NAM7 helicase family, SDE3 subfamily	Gallus gallus (Chicken)	PF13086;PF13087;
A0A3Q2UE17	424803;	DOCK10	Uncharacterized protein	DOCK family	Gallus gallus (Chicken)	PF06920;PF14429;PF11878;PF00169;

A0A3Q2UEP9	424803;	DOCK10	Uncharacterized protein	DOCK family	Gallus gallus (Chicken)	PF06920;PF14429;PF11878;PF00169;
A0A3Q3AYC4	424803;	DOCK10	Uncharacterized protein	DOCK family	Gallus gallus (Chicken)	PF06920;PF14429;PF11878;PF00169;
E1BZN6	770021;	REEP5	Receptor expression-enhancing protein	DP1 family	Gallus gallus (Chicken)	PF03134;
A0A1D5NUH3	769000;	LOC769000	Mothers against decapentaplegic homolog (MAD homolog) (Mothers against DPP homolog) (SMAD family member)	Dwarfin/SMAD family	Gallus gallus (Chicken)	PF03165;PF03166;
P35458	426238;	DCTN1	Dynactin subunit 1 (150 kDa dynein-associated polypeptide) (DAP-150) (DP-150) (p150-glued)	Dynactin 150 kDa subunit family	Gallus gallus (Chicken)	PF01302;PF12455;
A0A1D5PEZ3	426238;	DCTN1	Dynactin subunit 1	Dynactin 150 kDa subunit family	Gallus gallus (Chicken)	PF01302;PF12455;
A0A1D5PFB8	395587;	DCTN2	Dynactin subunit 2	Dynactin subunit 2 family	Gallus gallus (Chicken)	PF04912;
F1NKL4	423461;	DYNC1H1	Uncharacterized protein	Dynein heavy chain family	Gallus gallus (Chicken)	PF12774;PF12780;PF12781;PF18198;PF08385;PF08393;PF17852;PF18199;PF03028;PF12777;
Q5ZK94	424151;	RCJMB04_12d24	WD_REPEATS_REGION domain-containing protein	Dynein intermediate chain family	Gallus gallus (Chicken)	PF11540;PF00400;
Q90828	420668;	DYNC1LI1 DNCLI1	Cytoplasmic dynein 1 light intermediate chain 1 (Dynein light chain A) (DLC-A) (Dynein light intermediate chain 1, cytosolic) (LIC57/59)	Dynein light intermediate chain family	Gallus gallus (Chicken)	PF05783;
Q5ZMQ4	415793;	DYNC1LI2 RCJMB04_1g23	Dynein light intermediate chain	Dynein light intermediate chain family	Gallus gallus (Chicken)	PF05783;
Q9YGQ1	395723;	EEF1B	Elongation factor 1-beta (EF-1-beta)	EF-1-beta/EF-1-delta family	Gallus gallus (Chicken)	PF10587;PF00736;
Q5ZIE2	418606;	EIF1AX RCJMB04_27j6	Eukaryotic translation initiation factor 4C	EIF-1A family	Gallus gallus (Chicken)	PF01176;
Q6K1L7	414740;	eif1ad	Probable RNA-binding protein EIF1AD (Eukaryotic translation initiation factor 1A domain-containing protein) (Protein Obelix)	EIF1AD family	Gallus gallus (Chicken)	PF01176;
Q5ZLX2	423279;	EIF2S1 EIF2A RCJMB04_4i21	Eukaryotic translation initiation factor 2 subunit 1 (Eukaryotic translation initiation factor 2 subunit alpha) (eIF-2-alpha) (eIF-2A) (eIF-2alpha)	EIF-2-alpha family	Gallus gallus (Chicken)	PF07541;PF00575;
Q9DEQ6	395295;	EIF2B EIF2S2	Eukaryote initiation factor 2 beta	EIF-2-beta/eIF-5 family	Gallus gallus (Chicken)	PF01873;
Q5ZIE0	423479;	EIF5 RCJMB04_27j21	Eukaryotic translation initiation factor 5	EIF-2-beta/eIF-5 family	Gallus gallus (Chicken)	PF01873;PF02020;
F1NDA1	419845;	EIF2D	Eukaryotic translation initiation factor 2D (Ligatin)	EIF2D family	Gallus gallus (Chicken)	PF17832;PF01253;
E1BSS3	423927;	EIF3A EIF3S10	Eukaryotic translation initiation factor 3 subunit A (eIF3a) (Eukaryotic translation initiation factor 3 subunit 10) (eIF-3-theta)	EIF-3 subunit A family	Gallus gallus (Chicken)	PF01399;
F1NCE1	771356;	EIF3D EIF3S7	Eukaryotic translation initiation factor 3 subunit D (eIF3d) (Eukaryotic translation initiation factor 3 subunit 7)	EIF-3 subunit D family	Gallus gallus (Chicken)	PF05091;
Q5ZLA5	420272;	EIF3E EIF3S6 RCJMB04_6o19	Eukaryotic translation initiation factor 3 subunit E (eIF3e) (Eukaryotic translation initiation factor 3 subunit 6)	EIF-3 subunit E family	Gallus gallus (Chicken)	PF09440;PF01399;
Q5ZKA4	415573;	EIF3J EIF3S1 RCJMB04_12b11	Eukaryotic translation initiation factor 3 subunit J (eIF3j) (Eukaryotic translation initiation factor 3 subunit 1) (eIF-3-alpha) (eIF3 p35)	EIF-3 subunit J family	Gallus gallus (Chicken)	PF08597;
Q5F428	418033;	EIF3L EIF3EIP EIF3S6IP RCJMB04_3l6	Eukaryotic translation initiation factor 3 subunit L (eIF3l)	EIF-3 subunit L family	Gallus gallus (Chicken)	PF10255;
Q5ZJ64	421602;	EIF3M RCJMB04_20g2	Eukaryotic translation initiation factor 3 subunit M (eIF3m)	EIF-3 subunit M family	Gallus gallus (Chicken)	PF18005;PF01399;
G3UQA9	100550619;	EIF6	Eukaryotic translation initiation factor 6 (eIF-6)	EIF-6 family	Meleagris gallopavo (Wild turkey)	PF01912;
Q5F345	424564;	ELOVL1 RCJMB04_35f19	Elongation of very long chain fatty acids protein (EC 2.3.1.199) (Very-long-chain 3-oxoacyl-CoA synthase)	ELO family	Gallus gallus (Chicken)	PF01151;
E3VVM5	424564;	ELOVL1	Elongation of very long chain fatty acids protein 1 (EC 2.3.1.199) (3-keto acyl-CoA synthase ELOVL1) (ELOVL fatty acid elongase 1) (ELOVL FA elongase 1) (Very long chain 3-ketoacyl-CoA synthase 1) (Very long chain 3-oxoacyl-CoA synthase 1)	ELO family; ELO family, ELOVL1 subfamily	Gallus gallus (Chicken)	PF01151;
Q5ZL00	419470;	EMC1 RCJMB04_8i12	ER membrane protein complex subunit 1	EMC1 family	Gallus gallus (Chicken)	PF07774;PF13360;
A0A3Q2UDL9	420273;	EMC2	ER membrane protein complex subunit 2	EMC2 family	Gallus gallus (Chicken)	
E1BQV4	415936;	EMC3	ER membrane protein complex subunit 3	EMC3 family	Gallus gallus (Chicken)	PF01956;
Q5ZMT4	424499;	TMED5 RCJMB04_1d20	GOLD domain-containing protein	EMP24/GP25L family	Gallus gallus (Chicken)	PF01105;
Q5ZLF6	769360;	RCJMB04_6g23	GOLD domain-containing protein	EMP24/GP25L family	Gallus gallus (Chicken)	PF01105;
Q5ZIR6	423362;	RCJMB04_24a4	GOLD domain-containing protein	EMP24/GP25L family	Gallus gallus (Chicken)	PF01105;
O93263	374180;		Avena	Ena/VASP family	Gallus gallus (Chicken)	PF08776;PF00568;

Q90YB5	374180;		AvEna neural variant	Ena/VASP family	Gallus gallus (Chicken)	PF08776;PF00568;
Q9DEG2	374180;	avenaII	AvenaII	Ena/VASP family	Gallus gallus (Chicken)	PF08776;PF00568;
Q8AXV0	395202;	SH3GL1 SH3P8	Endophilin-A2 (Endophilin-2) (SH3 domain-containing GRB2-like protein 2) (SH3p8)	Endophilin family	Gallus gallus (Chicken)	PF03114;PF00018;
Q5ZIR1	424522;	SH3GLB1 RCJMB04_24b23	Endophilin-B1 (SH3 domain-containing GRB2-like protein B1)	Endophilin family	Gallus gallus (Chicken)	PF03114;PF14604;
P51913	396017;	ENO1	Alpha-enolase (EC 4.2.1.11) (2-phospho-D-glycerate hydro-lyase) (Phosphopyruvate hydratase)	Enolase family	Gallus gallus (Chicken)	PF00113;PF03952;
Q5ZJ60	423979;	HIBCH RCJMB04_20j11	3-hydroxyisobutyryl-CoA hydrolase, mitochondrial (EC 3.1.2.4) (3-hydroxyisobutyryl-coenzyme A hydrolase) (HIB-CoA hydrolase) (HIBYL-CoA-H)	Enoyl-CoA hydratase/isomerase family	Gallus gallus (Chicken)	PF16113;
A0A1D5NTK1	423979;	HIBCH	3-hydroxyisobutyryl-CoA hydrolase, mitochondrial (EC 3.1.2.4) (3-hydroxyisobutyryl-coenzyme A hydrolase)	Enoyl-CoA hydratase/isomerase family	Gallus gallus (Chicken)	PF16113;
A0A3Q2U419	427269;	AUH	Uncharacterized protein	Enoyl-CoA hydratase/isomerase family	Gallus gallus (Chicken)	PF00378;
E1BTQ9	427269;	AUH	Uncharacterized protein	Enoyl-CoA hydratase/isomerase family	Gallus gallus (Chicken)	PF00378;
F1NSS6	424646;	ECHDC2	Uncharacterized protein	Enoyl-CoA hydratase/isomerase family	Gallus gallus (Chicken)	PF00378;
F1NI29	395929;	HADHA	Enoyl-CoA hydratase (EC 4.2.1.17)	Enoyl-CoA hydratase/isomerase family; Enoyl-CoA hydratase/isomerase family	Gallus gallus (Chicken)	PF00725;PF02737;PF00378;
Q5F381	421292;	EPCAM TACSTD1 RCJMB04_29h4	Epithelial cell adhesion molecule (Ep-CAM) (Tumor-associated calcium signal transducer 1)	EPCAM family	Gallus gallus (Chicken)	PF00086;
P23913	396285;	LBR	Delta(14)-sterol reductase LBR (Delta-14-SR) (EC 1.3.1.70) (3-beta-hydroxysterol Delta (14)-reductase) (C-14 sterol reductase) (C14SR) (Integral nuclear envelope inner membrane protein) (Lamin-B receptor) (Sterol C14-reductase)	ERG4/ERG24 family	Gallus gallus (Chicken)	PF01222;PF09465;
E1C2I3	416205;	ERGIC1	Uncharacterized protein	ERGIC family	Gallus gallus (Chicken)	PF07970;PF13850;
E1BXC2	418856;	ESD	S-formylglutathione hydrolase (EC 3.1.2.12)	Esterase D family	Gallus gallus (Chicken)	PF00756;
Q5ZJ46	418478;	GABPA RCJMB04_20n8	Uncharacterized protein	ETS family	Gallus gallus (Chicken)	PF00178;PF11620;PF02198;
F1NLE4	428135;	GSS	Glutathione synthetase (GSH-S) (EC 6.3.2.3)	Eukaryotic GSH synthase family	Gallus gallus (Chicken)	PF03917;PF03199;
A0A1D5PPQ7	422703;	EIF4E	Uncharacterized protein	Eukaryotic initiation factor 4E family	Gallus gallus (Chicken)	PF01652;
A0A1D5P490	424953;	EIF4G1	Uncharacterized protein	Eukaryotic initiation factor 4G family	Gallus gallus (Chicken)	PF02847;PF02854;PF02020;
A0A1D5P6I5	424953;	EIF4G1	Uncharacterized protein	Eukaryotic initiation factor 4G family	Gallus gallus (Chicken)	PF02847;PF02854;PF02020;
A0A1D5PPZ0	424953;	EIF4G1	Uncharacterized protein	Eukaryotic initiation factor 4G family	Gallus gallus (Chicken)	PF02847;PF02854;PF02020;
E1BSG5	424953;	EIF4G1	Uncharacterized protein	Eukaryotic initiation factor 4G family	Gallus gallus (Chicken)	PF02847;PF02854;PF02020;
Q5KTT9	395905;	NAT1	eIF4G-related protein NAT1	Eukaryotic initiation factor 4G family	Gallus gallus (Chicken)	PF02847;PF02854;PF02020;
E1BYN7	416320;	VDAC1	Voltage-dependent anion-selective channel protein 1	Eukaryotic mitochondrial porin family	Gallus gallus (Chicken)	PF01459;
F6T197	395498;	VDAC2	Voltage-dependent anion-selective channel protein 2	Eukaryotic mitochondrial porin family	Gallus gallus (Chicken)	PF01459;
Q9I9D1	395498;	VDAC2	Voltage-dependent anion-selective channel protein 2	Eukaryotic mitochondrial porin family	Gallus gallus (Chicken)	PF01459;
Q5ZJ50	427679;	RCJMB04_20k18	Phosphomannomutase (EC 5.4.2.8)	Eukaryotic PMM family	Gallus gallus (Chicken)	PF03332;
A0A1L1RX26	416182;	ETF1	Eukaryotic peptide chain release factor subunit 1	Eukaryotic release factor 1 family	Gallus gallus (Chicken)	PF03463;PF03464;PF03465;
P41125	395849;	RPL13 BBC1	60S ribosomal protein L13 (Breast basic conserved protein 1)	Eukaryotic ribosomal protein eL13 family	Gallus gallus (Chicken)	PF01294;
F1NQG5	428442;	RPL15	Ribosomal protein L15	Eukaryotic ribosomal protein eL15 family	Gallus gallus (Chicken)	PF00827;
Q5ZKK8	420003;	RPL19 RCJMB04_10e3	Ribosomal protein L19	Eukaryotic ribosomal protein eL19 family	Gallus gallus (Chicken)	PF01280;
R4GIQ2	418933;	RPL21	60S ribosomal protein L21	Eukaryotic ribosomal protein eL21 family	Gallus gallus (Chicken)	PF01157;
Q98TF8	373937;	RPL22	60S ribosomal protein L22	Eukaryotic ribosomal protein eL22 family	Gallus gallus (Chicken)	PF01776;
P61355	396280;	RPL27	60S ribosomal protein L27	Eukaryotic ribosomal protein eL27 family	Gallus gallus (Chicken)	PF00467;PF01777;

P67883	425416;	RPL30	60S ribosomal protein L30	Eukaryotic ribosomal protein eL30 family	Gallus gallus (Chicken)	PF01248;
F1NQ35	424924;	RPL35A	60S ribosomal protein L35a	Eukaryotic ribosomal protein eL33 family	Gallus gallus (Chicken)	PF01247;
Q98TF6	373936;	RPL36	60S ribosomal protein L36	Eukaryotic ribosomal protein eL36 family	Gallus gallus (Chicken)	PF01158;
Q8UWG7	373957;	RPL6	60S ribosomal protein L6	Eukaryotic ribosomal protein eL6 family	Gallus gallus (Chicken)	PF01159;PF03868;
P32429	417158;	RPL7A SURF-3	60S ribosomal protein L7a	Eukaryotic ribosomal protein eL8 family	Gallus gallus (Chicken)	PF01248;
F2Z4K7	422477;	RPS3A	40S ribosomal protein S3a	Eukaryotic ribosomal protein eS1 family	Gallus gallus (Chicken)	PF01015;
E1C4N0	419904;	RPS10	S10_plectin domain-containing protein	Eukaryotic ribosomal protein eS10 family	Gallus gallus (Chicken)	PF03501;
P84175	421698;	RPS12	40S ribosomal protein S12	Eukaryotic ribosomal protein eS12 family	Gallus gallus (Chicken)	PF01248;
A0A1I7Q419	421698;	RPS12	40S ribosomal protein S12	Eukaryotic ribosomal protein eS12 family	Gallus gallus (Chicken)	PF01248;
P08636	374053;	RPS17	40S ribosomal protein S17	Eukaryotic ribosomal protein eS17 family	Gallus gallus (Chicken)	PF00833;
F1NF89	423726;	RPS24	40S ribosomal protein S24	Eukaryotic ribosomal protein eS24 family	Gallus gallus (Chicken)	PF01282;
F1NU56	770722;	RPS25	40S ribosomal protein S25	Eukaryotic ribosomal protein eS25 family	Gallus gallus (Chicken)	PF03297;
Q5ZM66	100857770;	RPS26 RCJMB04_2p17	40S ribosomal protein S26	Eukaryotic ribosomal protein eS26 family	Gallus gallus (Chicken)	PF01283;
P47836	396001;	RPS4	40S ribosomal protein S4	Eukaryotic ribosomal protein eS4 family	Gallus gallus (Chicken)	PF16121;PF00467;PF00900;PF08071;PF01479;
P47838	396148;	RPS6	40S ribosomal protein S6	Eukaryotic ribosomal protein eS6 family	Gallus gallus (Chicken)	PF01092;
F1NN16	421919;	RPS7	40S ribosomal protein S7	Eukaryotic ribosomal protein eS7 family	Gallus gallus (Chicken)	PF01251;
P18660	396262;	RPLP1	60S acidic ribosomal protein P1	Eukaryotic ribosomal protein P1/P2 family	Gallus gallus (Chicken)	
A0A1D5PMT8	426492;	RPLP2	60S acidic ribosomal protein P2	Eukaryotic ribosomal protein P1/P2 family	Gallus gallus (Chicken)	
A0A1D5PCT2	424954;	POLR2H	Uncharacterized protein	Eukaryotic RPB8 RNA polymerase subunit family	Gallus gallus (Chicken)	PF03870;
A0A1D5PED8	424954;	POLR2H	Uncharacterized protein	Eukaryotic RPB8 RNA polymerase subunit family	Gallus gallus (Chicken)	PF03870;
E1BSD8	424954;	POLR2H	DNA-directed RNA polymerases I, II, and III subunit RPABC3	Eukaryotic RPB8 RNA polymerase subunit family	Gallus gallus (Chicken)	PF03870;
A0A1D5P8H7	421192;	XPO1	Importin N-terminal domain-containing protein	Exportin family	Gallus gallus (Chicken)	PF08767;PF18777;PF18784;PF18787;PF03810;PF08389;
Q5ZLT0	426925;	XPO7 RCJMB04_4p4	Exportin-7	Exportin family	Gallus gallus (Chicken)	PF03810;
A0A1D5PWR9	426925;	XPO7	Exportin-7	Exportin family	Gallus gallus (Chicken)	PF03810;
P13127	396521;	CAPZA1	F-actin-capping protein subunit alpha-1 (Beta-actinin subunit I) (CapZ 36/32)	F-actin-capping protein alpha subunit family	Gallus gallus (Chicken)	PF01267;
P28497	417771;	CAPZA2	F-actin-capping protein subunit alpha-2 (Beta-actinin subunit I) (CapZ 36/32)	F-actin-capping protein alpha subunit family	Gallus gallus (Chicken)	PF01267;
A0M8U0	417771;	CAPZA2	F-actin-capping protein subunit alpha	F-actin-capping protein alpha subunit family	Gallus gallus (Chicken)	PF01267;
P14315	396418;	CAPZB	F-actin-capping protein subunit beta isoforms 1 and 2 (Beta-actinin subunit II) (CapZ 36/32) (CapZ B1 and B2)	F-actin-capping protein beta subunit family	Gallus gallus (Chicken)	PF01115;
Q9YHT1	395758;	SDHA	Succinate dehydrogenase [ubiquinone] flavoprotein subunit, mitochondrial (EC 1.3.5.1) (Flavoprotein subunit of complex II) (Fp)	FAD-dependent oxidoreductase 2 family, FRD/SDH subfamily	Gallus gallus (Chicken)	PF00890;PF02910;
F1NPJ4	395758;	SDHA	Succinate dehydrogenase [ubiquinone] flavoprotein subunit, mitochondrial (EC 1.3.5.1)	FAD-dependent oxidoreductase 2 family, FRD/SDH subfamily	Gallus gallus (Chicken)	PF00890;PF02910;
Q5ZLF0	418003;	ST13 FAM10A1 RCJMB04_6h13	Hsc70-interacting protein (Hip) (Protein FAM10A1) (Protein ST13 homolog)	FAM10 family	Gallus gallus (Chicken)	PF18253;PF17830;PF13181;
D5LPR1	416485;	FSCN1	Fascin	Fascin family	Gallus gallus (Chicken)	PF06268;
P08267	395970;	FTH	Ferritin heavy chain (Ferritin H subunit) (EC 1.16.3.1)	Ferritin family	Gallus gallus (Chicken)	PF00210;
O42479	374020;	FECH	Ferrochelatase, mitochondrial (EC 4.99.1.1) (Heme synthase) (Protoheme ferro-lyase)	Ferrochelatase family	Gallus gallus (Chicken)	PF00762;

O73775	373979;	FBLN1	Fibulin-1 (FIBL-1)	Fibulin family	Gallus gallus (Chicken)	PF01821;PF12662;PF07645;
A0A1L1RU28	373979;	FBLN1	Fibulin-1	Fibulin family	Gallus gallus (Chicken)	PF01821;PF12662;PF07645;
F1NWN4	427583;	FBLN2	Uncharacterized protein	Fibulin family	Gallus gallus (Chicken)	PF12662;PF07645;
Q90WF1	395261;		Filamin	Filamin family	Gallus gallus (Chicken)	PF00307;PF00630;
A0A1D5NYG3	378913;	FLNB	Uncharacterized protein	Filamin family	Gallus gallus (Chicken)	PF00307;PF00630;
A0A1D5PXM1	378913;	FLNB	Uncharacterized protein	Filamin family	Gallus gallus (Chicken)	PF00307;PF00630;
Q90WF0	395260;		CgABP260	Filamin family	Gallus gallus (Chicken)	PF00307;PF00630;
A0A1D5P6W8	419571;	KDM1A	Lysine-specific histone demethylase (EC 1.-.-.-)	Flavin monoamine oxidase family	Gallus gallus (Chicken)	PF01593;PF04433;
F1NIY7	418563;	MAOA	Amine oxidase (EC 1.4.3.-)	Flavin monoamine oxidase family	Gallus gallus (Chicken)	PF01593;
Q5F4B5	418563;	RCJMB04_1d13	Amine oxidase (EC 1.4.3.-)	Flavin monoamine oxidase family	Gallus gallus (Chicken)	PF01593;
F1NZY9	418220;	CYB5R1	NADH-cytochrome b5 reductase (EC 1.6.2.2)	Flavoprotein pyridine nucleotide cytochrome reductase family	Gallus gallus (Chicken)	PF00970;PF00175;
Q8QH01	395267;	FMO3	Dimethylaniline monooxygenase [N-oxide-forming] (EC 1.14.13.8)	FMO family	Gallus gallus (Chicken)	PF00743;
P02752	396449;		Riboflavin-binding protein (RBP) [Cleaved into: Riboflavin-binding protein, plasma form; Riboflavin-binding protein, yolk major form; Riboflavin-binding protein, yolk minor form]	Folate receptor family	Gallus gallus (Chicken)	PF03024;
A0A140T8H8	396449;	RBP	Folate_rec domain-containing protein	Folate receptor family	Gallus gallus (Chicken)	PF03024;
F1NMC3	423508;	MTHFD1	C-1-tetrahydrofolate synthase, cytoplasmic (EC 1.5.1.5) (EC 3.5.4.9) (EC 6.3.4.3) (Formyltetrahydrofolate synthetase) (Methenyltetrahydrofolate cyclohydrolase) (Methylenetetrahydrofolate dehydrogenase)	Formate--tetrahydrofolate ligase family; Tetrahydrofolate dehydrogenase/cyclohydrolase family	Gallus gallus (Chicken)	PF01268;PF00763;PF02882;
Q5ZI76	423508;	RCJMB04_29j22	C-1-tetrahydrofolate synthase, cytoplasmic (EC 1.5.1.5) (EC 3.5.4.9) (EC 6.3.4.3) (Formyltetrahydrofolate synthetase) (Methenyltetrahydrofolate cyclohydrolase) (Methylenetetrahydrofolate dehydrogenase)	Formate--tetrahydrofolate ligase family; Tetrahydrofolate dehydrogenase/cyclohydrolase family	Gallus gallus (Chicken)	PF01268;PF00763;PF02882;
R4GMB6	425061;	FDPS	Farnesyl pyrophosphate synthase	FPP/GGPP synthase family	Gallus gallus (Chicken)	PF00348;
A0A1D5NWX3	427092;	GGPS1	Uncharacterized protein	FPP/GGPP synthase family	Gallus gallus (Chicken)	PF00348;
P50147	396367;	GNAI2	Guanine nucleotide-binding protein G(i) subunit alpha-2 (Adenylate cyclase-inhibiting G alpha protein)	G-alpha family, G(i/o/t/z) subfamily	Gallus gallus (Chicken)	PF00503;
Q71RI7	374077;	GNA11	Guanine nucleotide-binding protein G11 alpha-subunit	G-alpha family, G(q) subfamily	Gallus gallus (Chicken)	PF00503;
E1BS74	474379;	GNAL	Uncharacterized protein	G-alpha family, G(s) subfamily	Gallus gallus (Chicken)	PF00503;
Q5F4B3	422296;	TMLHE RCJMB04_1d18	Trimethyllysine dioxygenase, mitochondrial (EC 1.14.11.8) (Epsilon-trimethyllysine 2-oxoglutarate dioxygenase) (TML hydroxylase) (TML-alpha-ketoglutarate dioxygenase) (TML dioxygenase) (TMLD)	Gamma-BBH/TMLD family	Gallus gallus (Chicken)	PF06155;PF02668;
P21872	395315;	GART	Trifunctional purine biosynthetic protein adenosine-3 [Includes: Phosphoribosylamine--glycine ligase (EC 6.3.4.13) (Glycinamide ribonucleotide synthetase) (GARS) (Phosphoribosylglycinamide synthetase); Phosphoribosylformylglycinamidine cyclo-ligase (EC 6.3.3.1) (AIR synthase) (AIRS) (Phosphoribosyl-aminoimidazole synthetase); Phosphoribosylglycinamide formyltransferase (EC 2.1.2.2) (5'-phosphoribosylglycinamide [illegible]	GARS family; AIR synthase family; GART family	Gallus gallus (Chicken)	PF00586;PF02769;PF00551;PF01071;PF02843;PF02844;
A0A547	395315;	GART	Trifunctional purine biosynthetic protein adenosine-3 [Includes: Phosphoribosylamine--glycine ligase (EC 6.3.4.13) (Glycinamide ribonucleotide synthetase) (GARS) (Phosphoribosylglycinamide synthetase); Phosphoribosylformylglycinamidine cyclo-ligase (EC 6.3.3.1) (AIR synthase) (AIRS) (Phosphoribosyl-aminoimidazole synthetase); Phosphoribosylglycinamide formyltransferase (EC 2.1.2.2) (5'-phosphoribosylglycinamide [illegible]	GART family; GARS family; AIR synthase family	Gallus gallus (Chicken)	PF00586;PF02769;PF00551;PF01071;PF02843;PF02844;
F1N9W3	422480;	GATB PET112 PET112L	Glutamyl-tRNA(Gln) amidotransferase subunit B, mitochondrial (Glu-AdT subunit B) (EC 6.3.5.-) (Cytochrome oxidase assembly factor PET112 homolog) (PET112-like)	GatB/GatE family, GatB subfamily	Gallus gallus (Chicken)	PF02934;PF02637;
F1NAK4	416982;	GCN1	TOG domain-containing protein	GCN1 family	Gallus gallus (Chicken)	
A0A1D5PBT2	423826;	ENTPD1	Uncharacterized protein	GDA1/CD39 NTPase family	Gallus gallus (Chicken)	PF01150;
F1N851	423826;	ENTPD1	Uncharacterized protein	GDA1/CD39 NTPase family	Gallus gallus (Chicken)	PF01150;
Q5ZMN7	415596;	RCJMB04_1i18	Uncharacterized protein	GHMP kinase family, GalK subfamily	Gallus gallus (Chicken)	PF10509;PF08544;PF00288;
A0A1D5NUY9	395956;	GLI2	Zinc finger protein GLI2	GLI C2H2-type zinc-finger protein family	Gallus gallus (Chicken)	PF00096;
A0A1D5P8Y5	395956;	GLI2	Zinc finger protein GLI2	GLI C2H2-type zinc-finger protein family	Gallus gallus (Chicken)	PF00096;
F1P5L2	395956;	GLI2	Zinc finger protein GLI2	GLI C2H2-type zinc-finger protein family	Gallus gallus (Chicken)	PF00096;
Q9PSZ4	395956;	gli2/gli4	Zn finger transcription factor (Fragment)	GLI C2H2-type zinc-finger protein family	Gallus gallus (Chicken)	PF00096;
Q5QRU7	427802;	CYGB	Cytoglobin	Globin family	Gallus gallus (Chicken)	PF00042;

P02112	396485;	HBB	Hemoglobin subunit beta (Beta-globin) (Hemoglobin beta chain)	Globin family	Gallus gallus (Chicken)	PF00042;
P02127	419079;		Hemoglobin subunit rho (Hemoglobin rho chain) (Rho-globin)	Globin family	Gallus gallus (Chicken)	PF00042;
P01994	416652;	HBAA	Hemoglobin subunit alpha-A (Alpha-A-globin) (Hemoglobin alpha-A chain)	Globin family	Gallus gallus (Chicken)	PF00042;
P02001	416651;	HBAD	Hemoglobin subunit alpha-D (Alpha-D-globin) (Hemoglobin alpha-D chain)	Globin family	Gallus gallus (Chicken)	PF00042;
P02128	107049060;	HBE	Hemoglobin subunit epsilon (Epsilon-globin) (Hemoglobin epsilon chain)	Globin family	Gallus gallus (Chicken)	PF00042;
Q90864	428114;	HBE1	Beta-H globin	Globin family	Gallus gallus (Chicken)	PF00042;
P02007	416650;		Hemoglobin subunit pi (Hemoglobin pi chain) (Hemoglobin pi' chain) (Pi-globin)	Globin family	Gallus gallus (Chicken)	PF00042;
A0A1D5NT61	423612;	GLUD2	Glutamate dehydrogenase (NAD(P)(+)) (EC 1.4.1.3)	Glu/Leu/Phe/Val dehydrogenases family	Gallus gallus (Chicken)	PF00208;PF02812;
Q5ZLA0	416341;	GNPDA1 RCJMB04_7a23	Glucosamine-6-phosphate isomerase (EC 3.5.99.6) (Glucosamine-6-phosphate deaminase)	Glucosamine/galactosamine-6-phosphate isomerase family	Gallus gallus (Chicken)	PF01182;
E1C878	422772;	GNPDA2	Glucosamine-6-phosphate isomerase (EC 3.5.99.6) (Glucosamine-6-phosphate deaminase)	Glucosamine/galactosamine-6-phosphate isomerase family	Gallus gallus (Chicken)	PF01182;
Q5ZJY1	430442;	PGLS RCJMB04_14h20	6-phosphogluconolactonase (6PGL) (EC 3.1.1.31)	Glucosamine/galactosamine-6-phosphate isomerase family, 6-phosphogluconolactonase subfamily	Gallus gallus (Chicken)	PF01182;
F1NQZ9	421894;	GCLC	Glutamate--cysteine ligase (EC 6.3.2.2) (Gamma-ECS) (Gamma-glutamylcysteine synthetase)	Glutamate--cysteine ligase type 3 family	Gallus gallus (Chicken)	PF03074;
F1P0D2	417253;	GSL	Glutamine synthetase (EC 6.3.1.2)	Glutamine synthetase family	Gallus gallus (Chicken)	PF00120;PF03951;
P79764	396069;	GLRX GRX	Glutaredoxin-1 (Thioltransferase-1) (TTase-1)	Glutaredoxin family	Gallus gallus (Chicken)	PF00462;
Q8QG67	374056;	GPX4	Glutathione peroxidase	Glutathione peroxidase family	Gallus gallus (Chicken)	PF00255;
E1C697	424643;	GPX7	Glutathione peroxidase	Glutathione peroxidase family	Gallus gallus (Chicken)	PF00255;
P00356	374193;	GAPDH GAPD	Glyceraldehyde-3-phosphate dehydrogenase (GAPDH) (EC 1.2.1.12) (Peptidyl-cysteine S-nitrosylase GAPDH) (EC 2.6.99.-)	Glyceraldehyde-3-phosphate dehydrogenase family	Gallus gallus (Chicken)	PF02800;PF00044;
E1BYP8	421311;	GPCPD1	Uncharacterized protein	Glycerophosphoryl diester phosphodiesterase family	Gallus gallus (Chicken)	PF00686;PF03009;
S5ZB27	421311;		Glycerophosphocholine phosphodiesterase GDE1-like protein	Glycerophosphoryl diester phosphodiesterase family	Gallus gallus (Chicken)	PF00686;PF03009;
A0A1D5P5T7	422217;	GDPD2	GP-PDE domain-containing protein	Glycerophosphoryl diester phosphodiesterase family	Gallus gallus (Chicken)	PF03009;
Q7ZZK3	378909;		Alpha-1,4 glucan phosphorylase (EC 2.4.1.1)	Glycogen phosphorylase family	Gallus gallus (Chicken)	PF00343;
Q5ZME4	421248;	RCJMB04_2f16	Alpha-1,4 glucan phosphorylase (EC 2.4.1.1)	Glycogen phosphorylase family	Gallus gallus (Chicken)	PF00343;
A0A1D5P6M3	427964;	GBE1	1,4-alpha-glucan branching enzyme (EC 2.4.1.18)	Glycosyl hydrolase 13 family, GlgB subfamily	Gallus gallus (Chicken)	PF00128;PF02806;PF02922;
A0A1D5PHJ9	427964;	GBE1	1,4-alpha-glucan branching enzyme (EC 2.4.1.18)	Glycosyl hydrolase 13 family, GlgB subfamily	Gallus gallus (Chicken)	PF00128;PF02806;PF02922;
A0A3Q2UNP2	427964;	GBE1	1,4-alpha-glucan branching enzyme (EC 2.4.1.18)	Glycosyl hydrolase 13 family, GlgB subfamily	Gallus gallus (Chicken)	PF00128;PF02806;PF02922;
F1NTQ2	427204;	HEXB	Beta-hexosaminidase (EC 3.2.1.52)	Glycosyl hydrolase 20 family	Gallus gallus (Chicken)	PF00728;PF14845;
P00698	396218;	LYZ	Lysozyme C (EC 3.2.1.17) (1,4-beta-N-acetylmuramidase C) (Allergen Gal d IV) (allergen Gal d 4)	Glycosyl hydrolase 22 family	Gallus gallus (Chicken)	PF00062;
P27042	395708;		Lysozyme g (EC 3.2.1.17) (1,4-beta-N-acetylmuramidase) (Goose-type lysozyme)	Glycosyl hydrolase 23 family	Gallus gallus (Chicken)	
F1P5H6	395708;	LYG2	Lysozyme g (EC 3.2.1.17)	Glycosyl hydrolase 23 family	Gallus gallus (Chicken)	
F1NJF8	396547;	NAGA	Alpha-galactosidase (EC 3.2.1.-)	Glycosyl hydrolase 27 family	Gallus gallus (Chicken)	PF16499;PF17450;
E1BT44	422188;	GLA	Alpha-galactosidase (EC 3.2.1.-)	Glycosyl hydrolase 27 family	Gallus gallus (Chicken)	PF16499;PF17450;
E1BS94	421668;	FUCA2	Alpha-L-fucosidase (EC 3.2.1.51)	Glycosyl hydrolase 29 family	Gallus gallus (Chicken)	PF01120;PF16757;
A0A1D5PLC0	107049056;	NEU2	Exo-alpha-sialidase (EC 3.2.1.18)	Glycosyl hydrolase 33 family	Gallus gallus (Chicken)	PF13088;
Q5ZLM4	420720;	GLB1 RCJMB04_5i4	Glyco_hydro_35 domain-containing protein	Glycosyl hydrolase 35 family	Gallus gallus (Chicken)	PF01301;

A0A3Q2U9V6	422561;	GALNT7	Polypeptide N-acetylgalactosaminyltransferase (EC 2.4.1.-) (Protein-UDP acetylgalactosaminyltransferase)	Glycosyltransferase 2 family, GalNAc-T subfamily	Gallus gallus (Chicken)	PF00535;PF00652;
F1P077	417733;	ALG12	Mannosyltransferase (EC 2.4.1.-)	Glycosyltransferase 22 family	Gallus gallus (Chicken)	PF03901;
Q8AWB4	395070;	POFUT1 RCJMB04_15p12	GDP-fucose protein O-fucosyltransferase 1 (EC 2.4.1.221) (Peptide-O-fucosyltransferase 1)	Glycosyltransferase 65 family	Gallus gallus (Chicken)	PF10250;
A0A1L1RZK1	421428;	GLO1	Lactoylglutathione lyase (EC 4.4.1.5) (Glyoxalase I)	Glyoxalase I family	Gallus gallus (Chicken)	PF00903;
A0A1D5PKI8	422234;	GPC4	Uncharacterized protein	Glypican family	Gallus gallus (Chicken)	PF01153;
E1C3N9	427462;	GOLM1	Uncharacterized protein	GOLM family	Gallus gallus (Chicken)	
Q5ZMU3	415783;	RCJMB04_1c14	Glucose-6-phosphate isomerase (EC 5.3.1.9)	GPI family	Gallus gallus (Chicken)	PF00342;
Q07883	386572;	GRB2	Growth factor receptor-bound protein 2 (Adapter protein GRB2) (SH2/SH3 adapter GRB2)	GRB2/sem-5/DRK family	Gallus gallus (Chicken)	PF00017;PF00018;
O42283	395948;	HSPE1	10 kDa heat shock protein, mitochondrial (Chaperonin 10)	GroES chaperonin family	Gallus gallus (Chicken)	PF00166;
Q5ZHV6	422865;	RCJMB04_32n8	GrpE protein homolog	GrpE family	Gallus gallus (Chicken)	PF01025;
Q08393	414895;		Glutathione S-transferase (EC 2.5.1.18) (Class-alpha)	GST superfamily, Alpha family	Gallus gallus (Chicken)	PF14497;PF02798;
Q08392	414896;		Glutathione S-transferase (EC 2.5.1.18) (GST class-alpha)	GST superfamily, Alpha family	Gallus gallus (Chicken)	PF14497;PF02798;
Q9W6J2	395612;	GSTA4	Glutathione transferase (EC 2.5.1.18)	GST superfamily, Alpha family	Gallus gallus (Chicken)	PF14497;PF02798;
A0A1D5NT70	395611;	LOC395611	Glutathione transferase (EC 2.5.1.18)	GST superfamily, Alpha family	Gallus gallus (Chicken)	PF14497;PF02798;
P26697	396380;		Glutathione S-transferase 3 (EC 2.5.1.18) (GST class-alpha) (GST-CL3)	GST superfamily, Alpha family	Gallus gallus (Chicken)	PF14497;PF02798;
F1NQS2	396380;	LOC396380	Glutathione transferase (EC 2.5.1.18)	GST superfamily, Alpha family	Gallus gallus (Chicken)	PF14497;PF02798;
F1N9G6	418302;	GSTK1	Glutathione S-transferase kappa (EC 2.5.1.18)	GST superfamily, Kappa family	Gallus gallus (Chicken)	PF01323;
P20136	395976;	GSTM2	Glutathione S-transferase 2 (EC 2.5.1.18) (GST class-mu) (GST-CL2) (GSTM1-1)	GST superfamily, Mu family	Gallus gallus (Chicken)	PF00043;PF02798;
O73888	395863;	HPGDS GSTS PGDS PTGDS2	Hematopoietic prostaglandin D synthase (H-PGDS) (EC 5.3.99.2) (GST class-sigma) (Glutathione S-transferase) (EC 2.5.1.18) (Glutathione-dependent PGD synthase) (Glutathione-requiring prostaglandin D synthase) (Prostaglandin-H2 D-isomerase)	GST superfamily, Sigma family	Gallus gallus (Chicken)	PF14497;PF02798;
A0A1D5PFS2	422311;	RRAGA	Ras-related GTP-binding protein	GTR/RAG GTP-binding protein family	Gallus gallus (Chicken)	PF04670;
A0A1D5P856	416385;	HDHD2	STAS domain-containing protein	HAD-like hydrolase superfamily	Gallus gallus (Chicken)	PF13344;
F1NC58	771207;	PDXP	Uncharacterized protein	HAD-like hydrolase superfamily	Gallus gallus (Chicken)	PF13344;
Q5F4B1	416559;	PGP RCJMB04_1e2	Glycerol-3-phosphate phosphatase (G3PP) (EC 3.1.3.21) (Aspartate-based ubiquitous Mg(2+)-dependent phosphatase) (AUM) (EC 3.1.3.48) (Phosphoglycolate phosphatase) (PGP)	HAD-like hydrolase superfamily, CbbY/CbbZ/Gph/YieH family	Gallus gallus (Chicken)	PF13344;
F1NLH9	424390;	ITPA	Inosine triphosphate pyrophosphatase (ITPase) (Inosine triphosphatase) (EC 3.6.1.9) (Non-canonical purine NTP pyrophosphatase) (Non-standard purine NTP pyrophosphatase) (Nucleoside-triphosphate diphosphatase) (Nucleoside-triphosphate pyrophosphatase) (NTPase)	HAM1 NTPase family	Gallus gallus (Chicken)	PF01725;
Q5XXA9	431605;	PSIP1 LEDGF	Lens epithelium-derived growth factor	HDGF family	Gallus gallus (Chicken)	PF11467;PF00855;
F1NXS9	431605;	PSIP1	Lens epithelium-derived growth factor	HDGF family	Gallus gallus (Chicken)	PF11467;PF00855;
P08106	423504;		Heat shock 70 kDa protein (HSP70)	Heat shock protein 70 family	Gallus gallus (Chicken)	PF00012;
A0A346RQZ3	423504;	HSP70	Heat shock protein 70	Heat shock protein 70 family	Gallus gallus (Chicken)	PF00012;
Q90593	396487;	HSPA5 GRP78	Endoplasmic reticulum chaperone BiP (EC 3.6.4.10) (78 kDa glucose-regulated protein) (GRP-78) (Binding-immunoglobulin protein) (BiP) (Heat shock protein 70 family protein 5) (HSP70 family protein 5) (Heat shock protein family A member 5) (Immunoglobulin heavy chain-binding protein)	Heat shock protein 70 family	Gallus gallus (Chicken)	PF00012;
O73885	395853;	HSPA8 HSC70	Heat shock cognate 71 kDa protein (Heat shock 70 kDa protein 8)	Heat shock protein 70 family	Gallus gallus (Chicken)	PF00012;
A0A1D5PPF8	416339;	HSPA4	Heat shock 70 kDa protein 4	Heat shock protein 70 family	Gallus gallus (Chicken)	PF00012;
Q5F3J8	422496;	RCJMB04_15d24	Uncharacterized protein	Heat shock protein 70 family	Gallus gallus (Chicken)	PF00012;
Q5ZM98	416183;	HSPA9 RCJMB04_2m8	Stress-70 protein, mitochondrial (75 kDa glucose-regulated protein) (GRP-75) (Heat shock 70 kDa protein 9)	Heat shock protein 70 family	Gallus gallus (Chicken)	PF00012;
Q5ZLK7	428251;	HYOU1 RCJMB04_5l9	Hypoxia up-regulated protein 1	Heat shock protein 70 family	Gallus gallus (Chicken)	PF00012;

P11501	423463;	HSP90AA1 HSPCA	Heat shock protein HSP 90-alpha	Heat shock protein 90 family	Gallus gallus (Chicken)	PF02518;PF00183;
Q04619	396188;	HSP90AB1 HSPCB	Heat shock cognate protein HSP 90-beta	Heat shock protein 90 family	Gallus gallus (Chicken)	PF02518;PF00183;
F1NC33	396188;	HSP90AB1	Heat shock cognate protein HSP 90-beta	Heat shock protein 90 family	Gallus gallus (Chicken)	PF02518;PF00183;
P08110	374163;	HSP90B1 TRA1	Endoplasmin (Heat shock 108 kDa protein) (HSP 108) (HSP108) (Heat shock protein 90 kDa beta member 1) (Transferrin-binding protein)	Heat shock protein 90 family	Gallus gallus (Chicken)	PF02518;PF00183;
Q5ZMB2	417961;	HEBP1 RCJMB04_2k3	Heme-binding protein 1	HEBP family	Gallus gallus (Chicken)	PF04832;
E1C7J0	416663;	HMOX2	Heme oxygenase (EC 1.14.14.18)	Heme oxygenase family	Gallus gallus (Chicken)	PF01126;
Q8AYP8	373889;	HK1	Hexokinase (EC 2.7.1.1)	Hexokinase family	Gallus gallus (Chicken)	PF00349;PF03727;
Q8AYP7	374044;	HK2	Hexokinase (EC 2.7.1.1)	Hexokinase family	Gallus gallus (Chicken)	PF00349;PF03727;
Q5ZLI9	420632;	HIBADH RCJMB04_5p10	3-hydroxyisobutyrate dehydrogenase (HIBADH) (EC 1.1.1.31)	HIBADH-related family; HIBADH-related family, 3-hydroxyisobutyrate dehydrogenase subfamily	Gallus gallus (Chicken)	PF14833;PF03446;
P56517	373961;	HDAC1 HDAC1A	Histone deacetylase 1 (HD1) (EC 3.5.1.98)	Histone deacetylase family, HD type 1 subfamily	Gallus gallus (Chicken)	PF00850;
P02259	693250;		Histone H5	Histone H1/H5 family	Gallus gallus (Chicken)	PF00538;
P08285	100858354;		Histone H1.03	Histone H1/H5 family	Gallus gallus (Chicken)	PF00538;
P08287	427892;		Histone H1.11L	Histone H1/H5 family	Gallus gallus (Chicken)	PF00538;
P08288	427896;		Histone H1.11R	Histone H1/H5 family	Gallus gallus (Chicken)	PF00538;
P02272	426617;	H2AZ2 H2AF	Histone H2A.V (H2A.F/Z)	Histone H2A family	Gallus gallus (Chicken)	PF00125;PF16211;
P02263	100858459;101749238;404299;417955;427881;427891;427895;		Histone H2A-IV	Histone H2A family	Gallus gallus (Chicken)	PF00125;PF16211;
Q92069	100858459;101749238;404299;417955;427881;427891;427895;	H2A-VII H2A-VI H2A-VIII	Histone H2A	Histone H2A family	Gallus gallus (Chicken)	PF00125;PF16211;
P0C1H3	100858607;417956;769973;770188;770267;	H2B-I; H2B-II; H2B-III; H2B-IV; H2B-VI	Histone H2B 1/2/3/4/6 (H2B I) (H2B II) (H2B III) (H2B IV) (H2B VI)	Histone H2B family	Gallus gallus (Chicken)	PF00125;
Q9PSW9	427886;	H2B-VIII	Histone H2B 8 (H2B VIII)	Histone H2B family	Gallus gallus (Chicken)	PF00125;
P84229	100857439;100858681;417953;768333;769809;769852;770022;	H3-I; H3-II; H3-III; H3-IV; H3-V; H3-VI; H3-VII; H3-VIII	Histone H3.2 (Histone H3 class I)	Histone H3 family	Gallus gallus (Chicken)	PF00125;
P62801	100858049;100858319;417946;417950;427884;770005;770142;	H4-I; H4-II; H4-III; H4-IV; H4-V; H4-VI; H4-VII	Histone H4	Histone H4 family	Gallus gallus (Chicken)	PF15511;
A0A1D5NZ54	419701;	HMBS	Hydroxymethylbilane synthase (EC 2.5.1.61)	HMBS family	Gallus gallus (Chicken)	PF01379;PF03900;
Q9YH06	395724;	HMGB1 HMG1 RCJMB04_15a21	High mobility group protein B1 (High mobility group protein 1) (HMG-1)	HMGB family	Gallus gallus (Chicken)	PF00505;PF09011;
P26584	396482;	HMGB2 HMG2	High mobility group protein B2 (High mobility group protein 2) (HMG-2)	HMGB family	Gallus gallus (Chicken)	PF00505;PF09011;
P40618	396232;	HMGB3 HMG2A HMG4	High mobility group protein B3 (High mobility group protein 2a) (HMG-2a) (High mobility group protein 4) (HMG-4)	HMGB family	Gallus gallus (Chicken)	PF00505;PF09011;
E1C5K0	420115;	HOMER3	WH1 domain-containing protein	Homer family	Gallus gallus (Chicken)	PF00568;
Q5F4A4	416058;	IMPDH2 IMPDH RCJMB04_1j11	Inosine-5'-monophosphate dehydrogenase (IMP dehydrogenase) (IMPD) (IMPDH) (EC 1.1.1.205)	IMPDH/GMPR family	Gallus gallus (Chicken)	PF00571;PF00478;
Q5ZJA6	420842;	GMPR RCJMB04_19j20	GMP reductase (GMPR) (EC 1.7.1.7) (Guanosine 5'-monophosphate oxidoreductase) (Guanosine monophosphate reductase)	IMPDH/GMPR family, GuaC type 1 subfamily	Gallus gallus (Chicken)	PF00478;
C6KIB2	418870;	KPNA3	Importin alpha 4 (Fragment)	Importin alpha family	Gallus gallus (Chicken)	PF00514;PF16186;PF01749;
F1NV68	418870;	KPNA3	Importin subunit alpha	Importin alpha family	Gallus gallus (Chicken)	PF00514;PF16186;PF01749;
Q5ZLF7	425012;	KPNA4 RCJMB04_6g17	Importin subunit alpha	Importin alpha family	Gallus gallus (Chicken)	PF00514;PF16186;PF01749;
A0A1D5P1W7	426499;	KPNB1	Importin N-terminal domain-containing protein	Importin beta family, Importin beta-1 subfamily	Gallus gallus (Chicken)	PF03810;
A0A1D5PHE2	420199;	IMPA1	Inositol-1-monophosphatase (EC 3.1.3.25)	Inositol monophosphatase superfamily	Gallus gallus (Chicken)	PF00459;
A0A1D5P2S9	418212;	ITPR2	Uncharacterized protein	InsP3 receptor family	Gallus gallus (Chicken)	PF08709;PF00520;PF02815;PF08454;PF01365;

A0A3Q3ATV6	418212;	ITPR2	Uncharacterized protein	InsP3 receptor family	Gallus gallus (Chicken)	PF08709;PF00520;PF02815;PF08454;PF01365;
F1P1X4	418212;	ITPR2	Uncharacterized protein	InsP3 receptor family	Gallus gallus (Chicken)	PF08709;PF00520;PF02815;PF08454;PF01365;
O42094	395951;		Alpha1 integrin	Integrin alpha chain family	Gallus gallus (Chicken)	PF01839;PF08441;PF00092;
Q98TT7	373946;		Integrin alpha 3A subunit cytoplasmic domain variant	Integrin alpha chain family	Gallus gallus (Chicken)	PF01839;PF08441;
P26007	396226;	ITGA6	Integrin alpha-6 (VLA-6) [Cleaved into: Integrin alpha-6 heavy chain; Integrin alpha-6 light chain]	Integrin alpha chain family	Gallus gallus (Chicken)	PF00357;PF08441;PF13517;
P26009	396225;	ITGA8	Integrin alpha-8 [Cleaved into: Integrin alpha-8 heavy chain; Integrin alpha-8 light chain]	Integrin alpha chain family	Gallus gallus (Chicken)	PF01839;PF00357;PF08441;
A0A1D5PM20	396225;	ITGA8	Integrin alpha-8	Integrin alpha chain family	Gallus gallus (Chicken)	PF01839;PF00357;PF08441;
Q5ZM22	420757;	RCJMB04_3g3	Integrin_alpha2 domain-containing protein	Integrin alpha chain family	Gallus gallus (Chicken)	PF01839;PF08441;
P26008	396420;	ITGAV	Integrin alpha-V (Vitronectin receptor subunit alpha) [Cleaved into: Integrin alpha-V heavy chain; Integrin alpha-V light chain]	Integrin alpha chain family	Gallus gallus (Chicken)	PF01839;PF00357;PF08441;
F1N8N7	374058;	ITGB1	Integrin beta	Integrin beta chain family	Gallus gallus (Chicken)	PF07974;PF18372;PF08725;PF07965;PF00362;PF17205;
Q92071	374209;	ITGB3	Integrin beta	Integrin beta chain family	Gallus gallus (Chicken)	PF07974;PF18372;PF08725;PF07965;PF00362;PF17205;
Q6PVZ5	407779;	KRT5	Type II alpha-keratin IIA	Intermediate filament family	Gallus gallus (Chicken)	PF00038;PF16208;
A0A1D5PXP0	408041;	KRT6A	IF rod domain-containing protein	Intermediate filament family	Gallus gallus (Chicken)	PF00038;PF16208;
O93532	395772;		Keratin, type II cytoskeletal cochleal (Cytokeratin otokeratin)	Intermediate filament family	Gallus gallus (Chicken)	PF00038;PF16208;
Q6PVZ1	408039;	KRT14	Keratin, type I cytoskeletal 14 (Cytokeratin-14) (CK-14) (Keratin-14) (K14)	Intermediate filament family	Gallus gallus (Chicken)	PF00038;
A0A1D5PZ89	408039;	KRT14	Keratin, type I cytoskeletal 14	Intermediate filament family	Gallus gallus (Chicken)	PF00038;
O93256	395861;	KRT19	Keratin, type I cytoskeletal 19 (Cytokeratin-19) (CK-19) (GK-19) (Keratin-19) (K19)	Intermediate filament family	Gallus gallus (Chicken)	PF00038;
A0A1L1RWG9	395861;	KRT24	IF rod domain-containing protein	Intermediate filament family	Gallus gallus (Chicken)	PF00038;
A0A1D5PQ92	112529929;	LOC112529929	IF rod domain-containing protein	Intermediate filament family	Gallus gallus (Chicken)	PF00038;PF16208;
P13648	396224;	LMNA	Lamin-A	Intermediate filament family	Gallus gallus (Chicken)	PF00038;PF00932;
P14731	396223;	LMNB1	Lamin-B1	Intermediate filament family	Gallus gallus (Chicken)	PF00038;PF00932;
P14732	396222;	LMNB2	Lamin-B2	Intermediate filament family	Gallus gallus (Chicken)	PF00038;PF00932;
O57613	395890;		Paranemin	Intermediate filament family	Gallus gallus (Chicken)	PF00038;
F1NJM8	395599;	SYNM	IF rod domain-containing protein	Intermediate filament family	Gallus gallus (Chicken)	PF00038;
Q90662	395599;		Synemin	Intermediate filament family	Gallus gallus (Chicken)	PF00038;
F1NJ08	420519;	VIM	Vimentin	Intermediate filament family	Gallus gallus (Chicken)	PF00038;PF04732;
A0A1D5NX91	429422;	C5H14orf4	IRF-2BP1_2 domain-containing protein	IRF2BP family	Gallus gallus (Chicken)	PF11261;
Q9DDJ1	374042;	MNSOD	Superoxide dismutase (EC 1.15.1.1)	Iron/manganese superoxide dismutase family	Gallus gallus (Chicken)	PF02777;PF00081;
F1NS88	415601;	ISOC1	Isochorismatase domain-containing protein	Isochorismatase family	Gallus gallus (Chicken)	PF00857;
F1NPG2	424112;	IDH1	Isocitrate dehydrogenase [NADP] (EC 1.1.1.42)	Isocitrate and isopropylmalate dehydrogenases family	Gallus gallus (Chicken)	PF00180;
Q5ZL82	431056;	RCJMB04_7e11	Isocitrate dehydrogenase [NADP] (EC 1.1.1.42)	Isocitrate and isopropylmalate dehydrogenases family	Gallus gallus (Chicken)	PF00180;
A0A1L1RX65	415362;	IDH3A	Isocitrate dehydrogenase [NAD] subunit, mitochondrial	Isocitrate and isopropylmalate dehydrogenases family	Gallus gallus (Chicken)	PF00180;
Q5ZI29	415362;	RCJMB04_31a13	Isocitrate dehydrogenase [NAD] subunit, mitochondrial	Isocitrate and isopropylmalate dehydrogenases family	Gallus gallus (Chicken)	PF00180;
B3VE14	419110;	ITIH2	Inter-alpha inhibitor heavy chain 2	ITIH family	Gallus gallus (Chicken)	PF06668;PF08487;PF00092;

A0A1D5PET9	422095;	SPAG9	Uncharacterized protein	JIP scaffold family	Gallus gallus (Chicken)	PF16471;PF09744;
A0A3S5ZP73	416763;		HN1-like protein	JUPITER family	Gallus gallus (Chicken)	PF17054;
E1BSM9	418766;	KDELC1	CAP10 domain-containing protein	KDELC family	Gallus gallus (Chicken)	PF00630;PF05686;
Q8UVD9	374140;	KHSRP FUBP2 ZPB2	Far upstream element-binding protein 2 (FUSE-binding protein 2) (KH type-splicing regulatory protein) (KSRP) (Zipcode-binding protein 2)	KHSRP family	Gallus gallus (Chicken)	PF09005;PF00013;
A0A1D5PU09	423589;	FERMT2	PH domain-containing protein	Kindlin family	Gallus gallus (Chicken)	PF00373;PF18124;PF00169;
Q90631	396335;	KTN1	Kinectin	Kinectin family	Gallus gallus (Chicken)	PF05104;
A0A1D5PNZ4	423484;	KLC1	Kinesin light chain	Kinesin light chain family	Gallus gallus (Chicken)	PF13176;
A0A1L1RR51	423484;	KLC1	Kinesin light chain	Kinesin light chain family	Gallus gallus (Chicken)	PF13176;
A0A3Q3AI12	769965;	KLC2	Kinesin light chain	Kinesin light chain family	Gallus gallus (Chicken)	
A0A1D5PYA4	424006;	LANCL1	Uncharacterized protein	LanC-like protein family	Gallus gallus (Chicken)	PF05147;
P00340	396221;	LDHA	L-lactate dehydrogenase A chain (LDH-A) (EC 1.1.1.27)	LDH/MDH superfamily, LDH family	Gallus gallus (Chicken)	PF02866;PF00056;
P00337	373997;	LDHB	L-lactate dehydrogenase B chain (LDH-B) (EC 1.1.1.27)	LDH/MDH superfamily, LDH family	Gallus gallus (Chicken)	PF02866;PF00056;
Q5ZME2	421281;	MDH1 RCJMB04_2g5	Malate dehydrogenase, cytoplasmic (EC 1.1.1.37) (Cytosolic malate dehydrogenase)	LDH/MDH superfamily, MDH type 2 family	Gallus gallus (Chicken)	PF02866;PF00056;
E1BTT8	396221;	LDHA	L-lactate dehydrogenase (EC 1.1.1.27)	LDH/MDH superfamily; LDH/MDH superfamily, LDH family	Gallus gallus (Chicken)	PF02866;PF00056;
A0A1L1RX46	417923;	TMPO	Uncharacterized protein	LEM family	Gallus gallus (Chicken)	PF03020;PF08198;
A0A3Q2UJ35	417923;	TMPO	Uncharacterized protein	LEM family	Gallus gallus (Chicken)	PF03020;PF08198;
Q5ZMN6	417923;	RCJMB04_1i19	Uncharacterized protein	LEM family	Gallus gallus (Chicken)	PF03020;PF08198;
Q90830	395992;	CRTAP CASP	Cartilage-associated protein (Dualin)	Leprecan family	Gallus gallus (Chicken)	
Q6JHU8	414142;	P3H1 LEPRE1	Prolyl 3-hydroxylase 1 (EC 1.14.11.7) (Leucine- and proline-enriched proteoglycan 1 homolog) (Leprecan-1 homolog)	Leprecan family	Gallus gallus (Chicken)	PF13640;
F1NGG8	418289;	P3H3	Procollagen-proline 3-dioxygenase (EC 1.14.11.7)	Leprecan family	Gallus gallus (Chicken)	PF13640;
Q5F425	421608;	LIN7C RCJMB04_3l24	Protein lin-7 homolog C (Lin-7C)	Lin-7 family	Gallus gallus (Chicken)	PF02828;PF00595;
Q5ZKZ9	429380;	LMF2 RCJMB04_8i13	Lipase maturation factor 2	Lipase maturation factor family	Gallus gallus (Chicken)	PF06762;
A0A1D5P8M7	429380;	LMF2	Lipase maturation factor	Lipase maturation factor family	Gallus gallus (Chicken)	PF06762;
A0A3Q2U4S7	418219;	PPFIBP1	Uncharacterized protein	Liprin family, Liprin-beta subfamily	Gallus gallus (Chicken)	PF00536;PF07647;
Q5F3X7	418219;	RCJMB04_4k2	Uncharacterized protein	Liprin family, Liprin-beta subfamily	Gallus gallus (Chicken)	PF00536;PF07647;
Q5ZKG5	421909;	ACP1 RCJMB04_11a4	Low molecular weight phosphotyrosine protein phosphatase (LMW-PTP) (LMW-PTPase) (EC 3.1.3.48) (Low molecular weight cytosolic acid phosphatase) (EC 3.1.3.2)	Low molecular weight phosphotyrosine protein phosphatase family	Gallus gallus (Chicken)	PF01451;
A0A1D5P9Z1	421909;	ACP1	Low molecular weight cytosolic acid phosphatase (EC 3.1.3.2) (EC 3.1.3.48) (Low molecular weight phosphotyrosine protein phosphatase)	Low molecular weight phosphotyrosine protein phosphatase family	Gallus gallus (Chicken)	PF01451;
P21265	396540;	ADSL	Adenylosuccinate lyase (ADSL) (ASL) (EC 4.3.2.2) (Adenylosuccinase) (ASase)	Lyase 1 family, Adenylosuccinate lyase subfamily	Gallus gallus (Chicken)	PF10397;PF00206;
P05083	417545;	ASL2	Argininosuccinate lyase (ASAL) (EC 4.3.2.1) (Arginosuccinase) (Delta crystallin II) (Delta-2 crystallin)	Lyase 1 family, Argininosuccinate lyase subfamily	Gallus gallus (Chicken)	PF14698;PF00206;
A0A1L1RT49	374226;	MAGOH	Protein mago nashi homolog	Mago nashi family	Gallus gallus (Chicken)	PF02792;
A0A1D5PB73	418565;	CASK	Uncharacterized protein	MAGUK family	Gallus gallus (Chicken)	PF00625;PF02828;PF00595;PF00069;PF00018;
A0A3Q2ULY1	418565;	CASK	Uncharacterized protein	MAGUK family	Gallus gallus (Chicken)	PF00625;PF02828;PF00595;PF00069;PF00018;
Q5ZLZ6	419875;	SLC16A1 RCJMB04_4e8	Monocarboxylate transporter 1 (Solute carrier family 16 member 1)	Major facilitator superfamily, Monocarboxylate porter (TC 2.A.1.13) family	Gallus gallus (Chicken)	PF07690;
F1NVU4	417815;	SLC16A7	Monocarboxylate transporter 2 (Solute carrier family 16 member 7)	Major facilitator superfamily, Monocarboxylate porter (TC 2.A.1.13) family	Gallus gallus (Chicken)	PF07690;

A0A1D5Q008	769765;	MPI	Mannose-6-phosphate isomerase (EC 5.3.1.8)	Mannose-6-phosphate isomerase type 1 family	Gallus gallus (Chicken)	PF01238;
B4X9P5	418178;	MGST1	Glutathione transferase (EC 2.5.1.18)	MAPEG family	Gallus gallus (Chicken)	PF01124;
F1NT14	418178;	MGST1	Glutathione transferase (EC 2.5.1.18)	MAPEG family	Gallus gallus (Chicken)	PF01124;
Q5ZLC7	419288;	MAPRE1 RCJMB04_6l6	Microtubule-associated protein RP/EB family member 1	MAPRE family	Gallus gallus (Chicken)	PF00307;PF03271;
Q5ZKS0	770764;	MARCKSL1 RCJMB04_9h20	Uncharacterized protein	MARCKS family	Gallus gallus (Chicken)	PF02063;
P16527	396473;	MARCKS	Myristoylated alanine-rich C-kinase substrate (MARCKS)	MARCKS family	Gallus gallus (Chicken)	PF02063;
A0A1D5PDE6	396473;	MARCKS	Myristoylated alanine-rich C-kinase substrate	MARCKS family	Gallus gallus (Chicken)	PF02063;
E1C2U4	426764;	MCM4	DNA helicase (EC 3.6.4.12)	MCM family	Gallus gallus (Chicken)	PF00493;PF17855;PF14551;PF17207;
Q5ZKR8	424295;	MCM6 RCJMB04_9i8	DNA helicase (EC 3.6.4.12)	MCM family	Gallus gallus (Chicken)	PF00493;PF18263;PF17855;PF14551;PF17207;
F1NEL0	422698;	MCUB	Calcium uniporter regulatory subunit MCUb	MCU (TC 1.A.77) family	Gallus gallus (Chicken)	PF04678;
R4GI40	415475;	MESDC2	LDLR chaperone MESD (LRP chaperone MESD) (Mesoderm development candidate 2) (Mesoderm development protein)	MESD family	Gallus gallus (Chicken)	PF10185;
F1NS60	423842;	MMS19	MMS19 nucleotide excision repair protein	MET18/MMS19 family	Gallus gallus (Chicken)	PF12460;PF14500;
Q5ZI23	416537;	HAGH RCJMB04_31d24	Hydroxyacylglutathione hydrolase, mitochondrial (EC 3.1.2.6) (Glyoxalase II) (Glx II)	Metallo-beta-lactamase superfamily, Glyoxalase II family	Gallus gallus (Chicken)	PF16123;PF00753;
A0A3Q2U554	416537;	HAGH	Hydroxyacylglutathione hydrolase, mitochondrial	Metallo-beta-lactamase superfamily, Glyoxalase II family	Gallus gallus (Chicken)	PF16123;PF00753;
Q5ZKP6	419194;	ADA RCJMB04_9m8	Adenosine deaminase (EC 3.5.4.4) (Adenosine aminohydrolase)	Metallo-dependent hydrolases superfamily, Adenosine and AMP deaminases family	Gallus gallus (Chicken)	PF00962;
F1NG97	423041;	AMPD3	AMP deaminase (EC 3.5.4.6)	Metallo-dependent hydrolases superfamily, Adenosine and AMP deaminases family	Gallus gallus (Chicken)	PF00962;
Q90635	395155;	DPYSL2	Dihydropyrimidinase-related protein 2 (DRP-2) (Collapsin response mediator protein CRMP-62)	Metallo-dependent hydrolases superfamily, Hydantoinase/dihydropyrimidinase family	Gallus gallus (Chicken)	PF01979;
Q71SG2	395155;	CRMP2A DPYSL2	Dihydropyrimidinase-related protein 2	Metallo-dependent hydrolases superfamily, Hydantoinase/dihydropyrimidinase family	Gallus gallus (Chicken)	PF01979;
A0A1D5PZ51	395154;	DPYSL3	Amidohydro-rel domain-containing protein	Metallo-dependent hydrolases superfamily, Hydantoinase/dihydropyrimidinase family	Gallus gallus (Chicken)	PF01979;
Q71SF8	395154;	CRMP4B	Collapsin response mediator protein-4B	Metallo-dependent hydrolases superfamily, Hydantoinase/dihydropyrimidinase family	Gallus gallus (Chicken)	PF01979;
E1BWG7	420525;	PTER	Parathion hydrolase-related protein (Phosphotriesterase-related protein)	Metallo-dependent hydrolases superfamily, Phosphotriesterase family	Gallus gallus (Chicken)	PF02126;
E1BY22	422049;	MUT	Methylmalonyl-CoA isomerase (EC 5.4.99.2) (Methylmalonyl-CoA mutase, mitochondrial)	Methylmalonyl-CoA mutase family	Gallus gallus (Chicken)	PF02310;PF01642;
Q5F3N1	428607;	PCMT1 RCJMB04_11o11	Protein-L-isoaspartate(D-aspartate) O-methyltransferase (PIMT) (EC 2.1.1.77) (L-isoaspartyl protein carboxyl methyltransferase) (Protein L-isoaspartyl/D-aspartyl methyltransferase) (Protein-beta-aspartate methyltransferase)	Methyltransferase superfamily, L-isoaspartyl/D-aspartyl protein methyltransferase family	Gallus gallus (Chicken)	
A0A3Q3AHD7	422912;	IMMT	MICOS complex subunit MIC60 (Mitofilin)	MICOS complex subunit Mic60 family	Gallus gallus (Chicken)	PF09731;
Q5ZMI2	422912;	RCJMB04_1p19	MICOS complex subunit MIC60 (Mitofilin)	MICOS complex subunit Mic60 family	Gallus gallus (Chicken)	PF09731;
Q02960	100857237;	MIF	Macrophage migration inhibitory factor (MIF) (EC 5.3.2.1) (L-dopachrome isomerase) (L-dopachrome tautomerase) (EC 5.3.3.12) (Phenylpyruvate tautomerase)	MIF family	Gallus gallus (Chicken)	PF01187;
A0A1L1RPT9	420526;	FAM188A	Ubiquitin carboxyl-terminal hydrolase MINDY (EC 3.4.19.12)	MINDY deubiquitinase family, FAM188 subfamily	Gallus gallus (Chicken)	PF13898;
F1NWH5	426894;	AQP3	Aquaglyceroporin-3 (Aquaporin-3)	MIP/aquaporin (TC 1.A.8) family	Gallus gallus (Chicken)	PF00230;
Q5ZJP2	415571;	RCJMB04_16m9	FeS_assembly_P domain-containing protein	MIP18 family	Gallus gallus (Chicken)	PF01883;
Q9PVL6	395597;	Mtch2	Mitochondrial carrier homolog 2	Mitochondrial carrier (TC 2.A.29) family	Gallus gallus (Chicken)	PF00153;
A0A1D5PX68	416764;	SLC25A1	Uncharacterized protein	Mitochondrial carrier (TC 2.A.29) family	Gallus gallus (Chicken)	PF00153;
A0A3S5ZPB7	417924;	SLC25A3	Phosphate carrier protein, mitochondrial (Phosphate transport protein) (Solute carrier family 25 member 3)	Mitochondrial carrier (TC 2.A.29) family	Gallus gallus (Chicken)	PF00153;
Q5ZLZ0	417924;	RCJMB04_4e21	Phosphate carrier protein, mitochondrial (Phosphate transport protein) (Solute carrier family 25 member 3)	Mitochondrial carrier (TC 2.A.29) family	Gallus gallus (Chicken)	PF00153;
Q5ZMJ6	422546;	SLC25A4 RCJMB04_1n4	ADP/ATP translocase (ADP,ATP carrier protein)	Mitochondrial carrier (TC 2.A.29) family	Gallus gallus (Chicken)	PF00153;

A0A1D5PCU1	772225;	SLC25A5	ADP/ATP translocase (ADP,ATP carrier protein)	Mitochondrial carrier (TC 2.A.29) family	Gallus gallus (Chicken)	PF00153;
Q5ZLG7	374072;	SLC25A6 RCJMB04_6e4	ADP/ATP translocase (ADP,ATP carrier protein)	Mitochondrial carrier (TC 2.A.29) family	Gallus gallus (Chicken)	PF00153;
Q5ZIF4	428427;	SLC25A13 RCJMB04_27c17	Uncharacterized protein	Mitochondrial carrier (TC 2.A.29) family	Gallus gallus (Chicken)	PF00153;
R4GLG2	416062;	SLC25A20	Uncharacterized protein	Mitochondrial carrier (TC 2.A.29) family	Gallus gallus (Chicken)	PF00153;
A0A3Q2TTG3	424337;	SLC25A24	Uncharacterized protein	Mitochondrial carrier (TC 2.A.29) family	Gallus gallus (Chicken)	PF13499;PF00153;
R4GI86	424830;	CAB39	Uncharacterized protein	Mo25 family	Gallus gallus (Chicken)	PF08569;
F1NWD1	419686;	GALE	UDP-glucose 4-epimerase (EC 5.1.3.2)	NAD(P)-dependent epimerase/dehydratase family	Gallus gallus (Chicken)	PF16363;
A0A1D5PUF7	420292;	TSTA3	GDP-4-keto-6-deoxy-D-mannose-3,5-epimerase-4-reductase (EC 1.1.1.271)	NAD(P)-dependent epimerase/dehydratase family, Fucose synthase subfamily	Gallus gallus (Chicken)	PF01370;
R4GLI9	420886;	NQO2	Flavodoxin_2 domain-containing protein	NAD(P)H dehydrogenase (quinone) family	Gallus gallus (Chicken)	PF02525;
F1P0W8	420664;	GPD1L	Glycerol-3-phosphate dehydrogenase [NAD(+)] (EC 1.1.1.8)	NAD-dependent glycerol-3-phosphate dehydrogenase family	Gallus gallus (Chicken)	PF07479;PF01210;
Q5ZII4	427337;	RCJMB04_25o1	15-oxoprostaglandin 13-reductase (EC 1.3.1.48) (EC 1.3.1.74) (Dithiolethione-inducible gene 1 protein) (Leukotriene B4 12-hydroxydehydrogenase) (NAD(P)H-dependent alkenal/one oxidoreductase) (Prostaglandin reductase 1)	NADP-dependent oxidoreductase L4BD family	Gallus gallus (Chicken)	PF16884;PF00107;
F1P2T2	417520;	POR	NADPH--cytochrome P450 reductase (CPR) (P450R) (EC 1.6.2.4)	NADPH--cytochrome P450 reductase family; Flavoprotein pyridine nucleotide cytochrome reductase family; Flavodoxin family	Gallus gallus (Chicken)	PF00667;PF00258;PF00175;
Q58I02	417707;	PBEF1 NAMPTP1	Nicotinamide phosphoribosyltransferase (NAmPRTase) (EC 2.4.2.12)	NAPRTase family	Gallus gallus (Chicken)	PF18127;PF04095;
O57535	395916;		Nucleoside diphosphate kinase (NDK) (NDP kinase) (EC 2.7.4.6)	NDK family	Gallus gallus (Chicken)	PF00334;
P79795	395560;	NRP1 NRP	Neuropilin-1 (A5 protein)	Neuropilin family	Gallus gallus (Chicken)	PF00431;PF11980;PF00754;PF00629;
F1NZE1	771307;	FAM129B	PH domain-containing protein	Niban family	Gallus gallus (Chicken)	
H9L023	426048;	NCLN	Nicalin	Nicastrin family	Gallus gallus (Chicken)	PF04389;
Q5ZJ12	417539;	RCJMB04_21m21	NIPSNAP domain-containing protein	NipSnap family	Gallus gallus (Chicken)	PF07978;
Q5ZID0	416672;	NMRAL1 RCJMB04_27o15	NmrA-like family domain-containing protein 1	NmrA-type oxidoreductase family	Gallus gallus (Chicken)	PF05368;
A0A1L1RKT0	419966;	NMT1	Glycylpeptide N-tetradecanoyltransferase (EC 2.3.1.97)	NMT family	Gallus gallus (Chicken)	PF01233;PF02799;
A0A3Q2TUU0	418755;	NAXD	ATP-dependent (S)-NAD(P)H-hydrate dehydratase (EC 4.2.1.93) (ATP-dependent NAD(P)HX dehydratase)	NnrD/CARKD family	Gallus gallus (Chicken)	PF01256;
F1P2P4	418755;	NAXD	ATP-dependent (S)-NAD(P)H-hydrate dehydratase (EC 4.2.1.93) (ATP-dependent NAD(P)HX dehydratase)	NnrD/CARKD family	Gallus gallus (Chicken)	PF01256;
Q5ZLM5	422249;	TM9SF2L RCJMB04_5h24	Transmembrane 9 superfamily member	Nonaspanin (TM9SF) (TC 9.A.2) family	Gallus gallus (Chicken)	PF02990;
F1NRG5	395460;	TM9SF3	Transmembrane 9 superfamily member	Nonaspanin (TM9SF) (TC 9.A.2) family	Gallus gallus (Chicken)	PF02990;
F1NPS0	427443;	NUP155	Uncharacterized protein	Non-repetitive/WGA-negative nucleoporin family	Gallus gallus (Chicken)	PF03177;PF08801;
Q5ZMD0	426574;	RCJMB04_2i9	Nop domain-containing protein	NOP5/NOP56 family	Gallus gallus (Chicken)	PF01798;PF08156;
E1BUS2	424087;	NOP58	Nop domain-containing protein	NOP5/NOP56 family	Gallus gallus (Chicken)	PF01798;PF08156;
A0A1L1RME9	417456;	NPLOC4	Uncharacterized protein	NPL4 family	Gallus gallus (Chicken)	PF05021;PF11543;PF05020;
Q5ZK10	419268;	NSFL1C RCJMB04_13o20	NSFL1 cofactor p47 (p97 cofactor p47)	NSFL1C family	Gallus gallus (Chicken)	PF08059;PF00789;
A0A1D5P2B5	417143;	RXRA	Uncharacterized protein	Nuclear hormone receptor family	Gallus gallus (Chicken)	PF00104;PF11825;PF00105;
Q5ZHR1	423071;	RCJMB04_34c21	Uncharacterized protein	Nucleobindin family	Gallus gallus (Chicken)	PF13499;
P16039	396203;	NPM1	Nucleophosmin (NPM) (Nucleolar phosphoprotein B23) (Nucleolar protein NO38) (Numatrin)	Nucleoplasmin family	Gallus gallus (Chicken)	PF16276;PF03066;
A0A1D5PLH4	770430;	NPM3	Nucleoplasmin domain-containing protein	Nucleoplasmin family	Gallus gallus (Chicken)	PF03066;
A0A1D5PFL9	415693;	NUP93	Nuclear pore complex protein Nup93	Nucleoporin interacting component (NIC) family	Gallus gallus (Chicken)	PF04097;

Q5F386	415693;	RCJMB04_28g18	Nuclear pore complex protein Nup93	Nucleoporin interacting component (NIC) family	Gallus gallus (Chicken)	PF04097;
Q5ZIU6	422120;	RCJMB04_23h18	Nuclear pore complex protein Nup85	Nucleoporin Nup85 family	Gallus gallus (Chicken)	PF07575;
E1BZS2	417864;	NAP1L1	Uncharacterized protein	Nucleosome assembly protein (NAP) family	Gallus gallus (Chicken)	PF00956;
Q5ZI86	423087;	RCJMB04_29e19	Uncharacterized protein	Nucleosome assembly protein (NAP) family	Gallus gallus (Chicken)	PF00956;
F2Z4L4	417210;	SET	Uncharacterized protein	Nucleosome assembly protein (NAP) family	Gallus gallus (Chicken)	PF00956;
Q5ZIN1	419578;	NUDC RCJMB04_24m10	Nuclear migration protein nudC (Nuclear distribution protein C homolog)	NudC family	Gallus gallus (Chicken)	PF04969;PF16273;PF14050;
F7BGT1	416467;	NUDT1	Nudix hydrolase domain-containing protein	Nudix hydrolase family	Gallus gallus (Chicken)	PF00293;
Q9IAY5	395557;	NUDT16L1 SDOS TIRR	Tudor-interacting repair regulator protein (NUDT16-like protein 1) (Protein syndesmos)	Nudix hydrolase family, TIRR subfamily	Gallus gallus (Chicken)	
E1BV83	417935;	NUP205	Uncharacterized protein	NUP186/NUP192/NUP205 family	Gallus gallus (Chicken)	PF11894;
Q5ZMR2	424256;	RCJMB04_1g10	OMPdecase (EC 2.4.2.10) (EC 4.1.1.23) (Orotate phosphoribosyltransferase) (Orotidine 5'-phosphate decarboxylase) (Uridine 5'-monophosphate synthase)	OMP decarboxylase family; Purine/pyrimidine phosphoribosyltransferase family	Gallus gallus (Chicken)	PF00215;PF00156;
Q5ZK09	427034;	HIKESHI RCJMB04_13p7	Protein Hikeshi	OPI10 family	Gallus gallus (Chicken)	PF05603;
A0A1D5PGC8	417866;	OSBPL8	Oxysterol-binding protein	OSBP family	Gallus gallus (Chicken)	PF01237;PF00169;
A0A3Q3AQG0	417866;	OSBPL8	Oxysterol-binding protein	OSBP family	Gallus gallus (Chicken)	PF01237;PF00169;
Q90955	100859133;	PTGES3 TEBP	Prostaglandin E synthase 3 (EC 5.3.99.3) (Cytosolic prostaglandin E2 synthase) (cPGES) (Hsp90 co-chaperone) (Progesterone receptor complex p23) (Telomerase-binding protein p23) (Fragment)	P23/wos2 family	Gallus gallus (Chicken)	PF04969;
A0A1D5P4R2	423704;	P4HA1	Procollagen-proline 4-dioxygenase (EC 1.14.11.2)	P4HA family	Gallus gallus (Chicken)	PF13640;PF08336;
E1BY52	423704;	P4HA1	Procollagen-proline 4-dioxygenase (EC 1.14.11.2)	P4HA family	Gallus gallus (Chicken)	PF13640;PF08336;
O13154	395975;	PACSIN2	Protein kinase C and casein kinase substrate in neurons protein 2 (Focal adhesion protein of 52 kDa) (FAP52)	PACSIN family	Gallus gallus (Chicken)	PF00611;PF14604;
Q8JHZ8	373898;	PALD1 PALD RCJMB04_11g7	Paladin	Paladin family	Gallus gallus (Chicken)	
Q5ZJT3	419681;	RCJMB04_15o4	Palmitoyl-protein hydrolase 1 (EC 3.1.2.22) (Palmitoyl-protein thioesterase 1)	Palmitoyl-protein thioesterase family	Gallus gallus (Chicken)	
P30374	423668;		Ribonuclease homolog (EC 3.1.27.-) (RSFR)	Pancreatic ribonuclease family	Gallus gallus (Chicken)	PF00074;
Q27J90	423668;		Leukocyte ribonuclease A-2	Pancreatic ribonuclease family	Gallus gallus (Chicken)	PF00074;
F1NCU9	395830;	PON1	Paraoxonase (EC 3.1.1.2)	Paraoxonase family	Gallus gallus (Chicken)	PF01731;
Q9DEA3	373984;	PCNA	Proliferating cell nuclear antigen (PCNA)	PCNA family	Gallus gallus (Chicken)	PF02747;PF00705;
Q98TX3	374191;	PDCD4	Programmed cell death protein 4 (Protein I11/6)	PDCD4 family	Gallus gallus (Chicken)	PF02847;
F1NIY3	374191;	PDCD4	Programmed cell death protein 4	PDCD4 family	Gallus gallus (Chicken)	PF02847;
A0A1D5P2A3	424932;	PDE6D	Retinal rod rhodopsin-sensitive cGMP 3',5'-cyclic phosphodiesterase subunit delta	PDE6D/unc-119 family	Gallus gallus (Chicken)	PF05351;
Q05744	396090;	CTSD	Cathepsin D (EC 3.4.23.5) [Cleaved into: Cathepsin D light chain; Cathepsin D heavy chain]	Peptidase A1 family	Gallus gallus (Chicken)	PF00026;
E1C897	417848;	CTSEAL	CathepsinE-A-like protein	Peptidase A1 family	Gallus gallus (Chicken)	PF07966;PF00026;
P87362	395996;	BLMH	Bleomycin hydrolase (BH) (BLM hydrolase) (BMH) (EC 3.4.22.40) (Aminopeptidase H)	Peptidase C1 family	Gallus gallus (Chicken)	PF03051;
E1C4M3	419311;	CTSZ	Cathepsin X (EC 3.4.18.1)	Peptidase C1 family	Gallus gallus (Chicken)	PF00112;
A1IMF0	770302;	UCH-L1 UCHL1	Ubiquitin carboxyl-terminal hydrolase (EC 3.4.19.12)	Peptidase C12 family	Gallus gallus (Chicken)	PF01088;
Q9PW67	395626;	UCH-6	Ubiquitin carboxyl-terminal hydrolase (EC 3.4.19.12)	Peptidase C12 family	Gallus gallus (Chicken)	PF01088;
Q5ZKF1	424359;	RCJMB04_11d7	Ubiquitin carboxyl-terminal hydrolase (EC 3.4.19.12)	Peptidase C12 family	Gallus gallus (Chicken)	PF01088;PF18031;
E1C958	423418;	LGMN	Asparaginyl endopeptidase (EC 3.4.22.34) (Legumain) (Protease, cysteine 1)	Peptidase C13 family	Gallus gallus (Chicken)	PF01650;

O93417	395476;	CASP3	Caspase-3	Peptidase C14A family	Gallus gallus (Chicken)	
E1C8W4	418290;	USP5	Ubiquitin carboxyl-terminal hydrolase (EC 3.4.19.12)	Peptidase C19 family	Gallus gallus (Chicken)	PF00627;PF00443;PF02148;PF17807;
Q6U7I1	395126;	USP7	Ubiquitin carboxyl-terminal hydrolase 7 (EC 3.4.19.12) (Deubiquitinating enzyme 7) (Ubiquitin thioesterase 7) (Ubiquitin-specific-processing protease 7)	Peptidase C19 family	Gallus gallus (Chicken)	PF00917;PF00443;PF14533;PF12436;
A0A1D5PPD3	395126;	USP7	Ubiquitin carboxyl-terminal hydrolase 7 (EC 3.4.19.12) (Ubiquitin thioesterase 7) (Ubiquitin-specific-processing protease 7)	Peptidase C19 family	Gallus gallus (Chicken)	PF00917;PF00443;PF14533;PF12436;
E1BWJ5	418569;	USP9Y	Ubiquitinyl hydrolase 1 (EC 3.4.19.12)	Peptidase C19 family	Gallus gallus (Chicken)	PF12030;PF00443;
Q5ZJN4	415817;	USP10 RCJMB04_16o18	Ubiquitin carboxyl-terminal hydrolase 10 (EC 3.4.19.12) (Deubiquitinating enzyme 10) (Ubiquitin thioesterase 10) (Ubiquitin-specific-processing protease 10)	Peptidase C19 family, USP10 subfamily	Gallus gallus (Chicken)	PF07145;PF00443;
O42133	693249;		Calcium-activated neutral proteinase 1 (EC 3.4.22.52) (Calpain mu-type) (Calpain-1 catalytic subunit) (Calpain-1 large subunit) (Micromolar-calpain)	Peptidase C2 family	Gallus gallus (Chicken)	PF01067;PF13833;PF00648;
P00789	396240;		Calpain-1 catalytic subunit (EC 3.4.22.52) (Calcium-activated neutral proteinase) (CANP) (Calpain-1 large subunit) (Mu/M-type)	Peptidase C2 family	Gallus gallus (Chicken)	PF01067;PF00648;
Q8UW59	395277;	PARK7	Protein/nucleic acid deglycase DJ-1 (EC 3.1.2.-) (EC 3.5.1.-) (EC 3.5.1.124) (Maillard deglycase) (Parkinson disease protein 7 homolog) (Parkinsonism-associated deglycase) (Protein DJ-1) (DJ-1)	Peptidase C56 family	Gallus gallus (Chicken)	PF01965;
D5M8S2	395277;	DJ-1 PARK7	Maillard deglycase (EC 3.5.1.124) (Parkinsonism-associated deglycase) (Protein/nucleic acid deglycase DJ-1)	Peptidase C56 family	Gallus gallus (Chicken)	PF01965;
A0A1D5NTA0	777320;	OTUB1	Ubiquitin thioesterase (EC 3.4.19.12)	Peptidase C65 family	Gallus gallus (Chicken)	PF10275;
Q5ZIF3	422542;	UFSP2 RCJMB04_27c22	Ufm1-specific protease 2 (UfSP2) (EC 3.4.22.-)	Peptidase C78 family	Gallus gallus (Chicken)	PF07910;
O57579	395667;	ANPEP APDE	Aminopeptidase Ey (EC 3.4.11.20) (Aminopeptidase N)	Peptidase M1 family	Gallus gallus (Chicken)	PF11838;PF01433;
A0A1L1RVF4	427122;	ERAP1	Aminopeptidase (EC 3.4.11.-)	Peptidase M1 family	Gallus gallus (Chicken)	PF11838;PF01433;
Q5ZJJ6	417918;	RCJMB04_17k12	Leukotriene A(4) hydrolase (LTA-4 hydrolase) (EC 3.3.2.6)	Peptidase M1 family	Gallus gallus (Chicken)	PF09127;PF01433;
Q90611	386583;	MMP2	72 kDa type IV collagenase (EC 3.4.24.24) (72 kDa gelatinase) (Gelatinase A) (Matrix metalloproteinase-2) (MMP-2)	Peptidase M10A family	Gallus gallus (Chicken)	PF00040;PF00045;PF00413;PF01471;
E1BYS4	417590;	CPD	Carboxypeptidase D (EC 3.4.17.22) (Metallocarboxypeptidase D)	Peptidase M14 family	Gallus gallus (Chicken)	PF00246;
E1C041	417843;	CPM	Peptidase_M14 domain-containing protein	Peptidase M14 family	Gallus gallus (Chicken)	PF00246;
Q5ZJ49	417134;	PMPCA RCJMB04_20l2	Alpha-MPP (Inactive zinc metalloprotease alpha) (Mitochondrial-processing peptidase subunit alpha)	Peptidase M16 family	Gallus gallus (Chicken)	PF00675;PF05193;
E1C9F5	420462;	PITRM1	Pitrilysin metalloproteinase 1 (Presequence protease, mitochondrial)	Peptidase M16 family, PreP subfamily	Gallus gallus (Chicken)	PF08367;PF05193;
F1NSZ4	419314;	NPEPL1	CYTOSOL_AP domain-containing protein	Peptidase M17 family	Gallus gallus (Chicken)	PF18295;PF00883;
Q5ZJU9	425306;	RCJMB04_15i13	Cysteinylglycine-S-conjugate dipeptidase (EC 3.4.11.1) (EC 3.4.11.5) (EC 3.4.13.23) (Cytosol aminopeptidase) (Leucine aminopeptidase 3) (Leucyl aminopeptidase) (Proline aminopeptidase) (Prolyl aminopeptidase)	Peptidase M17 family	Gallus gallus (Chicken)	PF00883;PF02789;
Q5ZJ47	424200;	RCJMB04_20m7	Aspartyl aminopeptidase (EC 3.4.11.21)	Peptidase M18 family	Gallus gallus (Chicken)	PF02127;
A0A1D5PAU7	100858836;	ACY1	N-acyl-L-amino-acid amidohydrolase (EC 3.5.1.14)	Peptidase M20A family	Gallus gallus (Chicken)	PF07687;PF01546;
Q5ZLV5	421013;	RCJMB04_4l9	M20_dimer domain-containing protein	Peptidase M20A family	Gallus gallus (Chicken)	PF07687;PF01546;
Q5ZL18	419838;	PM20D1 RCJMB04_8d17	N-fatty-acyl-amino acid synthase/hydrolase PM20D1 (EC 3.5.1.114) (EC 3.5.1.14) (Peptidase M20 domain-containing protein 1)	Peptidase M20A family	Gallus gallus (Chicken)	PF07687;PF01546;
A0A1D5P7C6	425279;	PA2G4	Peptidase_M24 domain-containing protein	Peptidase M24 family	Gallus gallus (Chicken)	PF00557;
Q5ZIL5	417912;	METAP2 RCJMB04_25d21	Methionine aminopeptidase 2 (MAP 2) (MetAP 2) (EC 3.4.11.18) (Initiation factor 2-associated 67 kDa glycoprotein) (Peptidase M) (p67) (p67eIF2)	Peptidase M24 family; Peptidase M24A family; Peptidase M24A family, Methionine aminopeptidase eukaryotic type 2 subfamily	Gallus gallus (Chicken)	PF00557;
Q5ZKL3	415776;	RCJMB04_10d1	AMP_N domain-containing protein	Peptidase M24B family	Gallus gallus (Chicken)	PF05195;PF00557;
F1P0A1	423886;	XPNPEP1	Uncharacterized protein	Peptidase M24B family	Gallus gallus (Chicken)	PF01321;PF00557;PF16188;
Q90997	396191;	TFRC	Transferrin receptor protein 1 (TR) (TfR) (TfR1) (Trfr)	Peptidase M28 family, M28B subfamily	Gallus gallus (Chicken)	PF02225;PF04389;PF04253;
E1C0D8	427167;	NLN	Peptidase_M3 domain-containing protein	Peptidase M3 family	Gallus gallus (Chicken)	PF01432;
A0A1D5PIZ1	426398;	THOP1	Peptidase_M3 domain-containing protein	Peptidase M3 family	Gallus gallus (Chicken)	PF01432;
F1NR71	415869;	PSMD7	MPN domain-containing protein	Peptidase M67A family	Gallus gallus (Chicken)	PF01398;PF13012;

E1C8A6	422201;	BRCC3	Lys-63-specific deubiquitinase (EC 3.4.19.-)	Peptidase M67A family, BRCC36 subfamily	Gallus gallus (Chicken)	PF18110;PF01398;
Q5ZLC3	426579;	RCJMB04_6l21	MPN domain-containing protein	Peptidase M67A family, CSN5 subfamily	Gallus gallus (Chicken)	PF18323;PF01398;
Q90865	396135;	HGF1 MSP	Hepatocyte growth factor-like/macrophage stimulating protein	Peptidase S1 family, Plasminogen subfamily	Gallus gallus (Chicken)	PF00051;PF00024;PF00089;
F1NWX6	421580;	PLG	Plasminogen (EC 3.4.21.7)	Peptidase S1 family, Plasminogen subfamily	Gallus gallus (Chicken)	PF00051;PF00024;PF00089;
A0A1L1RKJ5	428163;	CTSA	Carboxypeptidase (EC 3.4.16.-)	Peptidase S10 family	Gallus gallus (Chicken)	PF00450;
Q5ZIJ5	428163;	RCJMB04_25l7	Carboxypeptidase (EC 3.4.16.-)	Peptidase S10 family	Gallus gallus (Chicken)	PF00450;
Q5F3W4	417403;	SCPEP1 RCJMB04_5g1	Carboxypeptidase (EC 3.4.16.-)	Peptidase S10 family	Gallus gallus (Chicken)	PF00450;
A0A1D5NYJ2	768942;	LONP1	Lon protease homolog, mitochondrial (EC 3.4.21.53)	Peptidase S16 family	Gallus gallus (Chicken)	PF00004;PF05362;PF02190;
A0A1D5PRA3	426850;	SEC11C	Signal peptidase complex catalytic subunit SEC11 (EC 3.4.21.89)	Peptidase S26B family	Gallus gallus (Chicken)	PF00717;
A0A1D5PVG8	428096;	PRCP	Uncharacterized protein	Peptidase S28 family	Gallus gallus (Chicken)	PF05577;
E1C7F4	421447;	EPHX1L	Epoxide hydratase (EC 3.3.2.9) (Epoxide hydrolase 1) (Microsomal epoxide hydrolase)	Peptidase S33 family	Gallus gallus (Chicken)	PF06441;
Q5ZMI7	421785;	RCJMB04_1o16	Prolyl endopeptidase (EC 3.4.21.-)	Peptidase S9A family	Gallus gallus (Chicken)	PF00326;PF02897;
A0A1D5PJA5	424187;	DPP4	Dipeptidyl peptidase 4 (EC 3.4.14.5) (Dipeptidyl peptidase 4 membrane form) (Dipeptidyl peptidase 4 soluble form) (Dipeptidyl peptidase IV) (Dipeptidyl peptidase IV membrane form) (Dipeptidyl peptidase IV soluble form) (T-cell activation antigen CD26)	Peptidase S9B family, DPPIV subfamily	Gallus gallus (Chicken)	PF00930;PF18811;PF00326;
O42265	395874;	PSMA1	Proteasome subunit alpha type-1 (Macropain subunit C2) (Multicatalytic endopeptidase complex subunit C2) (Proteasome component C2)	Peptidase T1A family	Gallus gallus (Chicken)	PF00227;PF10584;
A0A1D5PHL0	420772;	PSMA2	Proteasome subunit alpha type	Peptidase T1A family	Gallus gallus (Chicken)	PF00227;PF10584;
Q5ZLI2	423542;	PSMA3 RCJMB04_6b22	Proteasome subunit alpha type	Peptidase T1A family	Gallus gallus (Chicken)	PF00227;PF10584;
F1NC02	415357;	PSMA4	Proteasome subunit alpha type	Peptidase T1A family	Gallus gallus (Chicken)	PF00227;PF10584;
Q5ZJX9	426937;	PSMA5 RCJMB04_14i9	Proteasome subunit alpha type	Peptidase T1A family	Gallus gallus (Chicken)	PF00227;PF10584;
F1NEQ6	423326;	PSMA6	Proteasome subunit alpha type	Peptidase T1A family	Gallus gallus (Chicken)	PF00227;PF10584;
O13268	395318;	PSMA7	Proteasome subunit alpha type-7 (GPRO-28)	Peptidase T1A family	Gallus gallus (Chicken)	PF00227;PF10584;
Q6JLB2	421551;	PSMB1	Proteasome subunit beta	Peptidase T1B family	Gallus gallus (Chicken)	PF00227;
R4GLB3	419630;	PSMB2	Proteasome subunit beta	Peptidase T1B family	Gallus gallus (Chicken)	PF00227;
E1BYW9	419997;	PSMB3	Proteasome subunit beta	Peptidase T1B family	Gallus gallus (Chicken)	PF00227;
H9L0U6	429986;	PSMB4	Proteasome subunit beta	Peptidase T1B family	Gallus gallus (Chicken)	PF00227;
P34065	396003;	PSMB5	Proteasome subunit beta type-5 (EC 3.4.25.1) (Macropain chain 1) (Multicatalytic endopeptidase complex chain 1) (Proteasome chain 1) (Proteasome subunit C1) (Fragment)	Peptidase T1B family	Gallus gallus (Chicken)	PF00227;
Q7ZT63	378915;	cpsmb7 PSMB7	Proteasome subunit beta	Peptidase T1B family	Gallus gallus (Chicken)	PF12465;PF00227;
E1BW27	422779;	TMEM33	Uncharacterized protein	PER33/POM33 family	Gallus gallus (Chicken)	PF03661;
A0A1D5P6V5	100857433;	PLIN4	Perilipin	Perilipin family	Gallus gallus (Chicken)	PF03036;
P0CB50	424598;	PRDX1	Peroxiredoxin-1 (EC 1.11.1.24) (Thioredoxin-dependent peroxiredoxin 1)	Peroxiredoxin family, AhpC/Prx1 subfamily	Gallus gallus (Chicken)	PF10417;PF00578;
Q5ZJF4	429062;	PRDX6 RCJMB04_18k11	Peroxiredoxin-6 (EC 1.11.1.27) (1-Cys peroxiredoxin) (1-Cys PRX) (Acidic calcium-independent phospholipase A2) (aiPLA2) (EC 3.1.1.4) (Glutathione-dependent peroxiredoxin) (Lysophosphatidylcholine acyltransferase 5) (LPC acyltransferase 5) (LPCAT-5) (Lyso-PC acyltransferase 5) (EC 2.3.1.23) (Non-selenium glutathione peroxidase) (NSGPx)	Peroxiredoxin family, Prx6 subfamily	Gallus gallus (Chicken)	PF10417;PF00578;
Q5ZI34	423625;	PRXL2A FAM213A PAMM RCJMB04_30m16	Peroxiredoxin-like 2A (Peroxiredoxin-like 2 activated in M-CSF stimulated monocytes) (Protein PAMM) (Redox-regulatory protein FAM213A)	Peroxiredoxin-like PRXL2 family, PRXL2A subfamily	Gallus gallus (Chicken)	PF13911;
A0A0F6YFH3	423625;		Peroxiredoxin-like 2 activated in M-CSF stimulated monocytes (Peroxiredoxin-like 2A) (Redox-regulatory protein FAM213A)	Peroxiredoxin-like PRXL2 family, PRXL2A subfamily	Gallus gallus (Chicken)	PF13911;
Q5ZJ61	424812;	FARSB RCJMB04_20i16	Phenylalanyl-tRNA synthetase beta subunit (EC 6.1.1.20)	Phenylalanyl-tRNA synthetase beta subunit family, Type 2 subfamily	Gallus gallus (Chicken)	PF03483;PF03484;PF18262;PF17759;
F1NBJ2	419523;	PPP2R2A	Serine/threonine-protein phosphatase 2A 55 kDa regulatory subunit B	Phosphatase 2A regulatory subunit B family	Gallus gallus (Chicken)	

Q5ZLP4	419523;	PPP2R2A RCJMB04_5f2	Serine/threonine-protein phosphatase 2A 55 kDa regulatory subunit B	Phosphatase 2A regulatory subunit B family	Gallus gallus (Chicken)	
P51903	395833;	PGK	Phosphoglycerate kinase (EC 2.7.2.3)	Phosphoglycerate kinase family	Gallus gallus (Chicken)	PF00162;
Q5ZHV4	418172;	BPGM RCJMB04_32o10	Phosphoglycerate mutase (EC 5.4.2.11) (EC 5.4.2.4)	Phosphoglycerate mutase family, BPG-dependent PGAM subfamily	Gallus gallus (Chicken)	PF00300;
Q5ZLN1	428969;	PGAM1 RCJMB04_5g20	Phosphoglycerate mutase 1 (EC 5.4.2.11) (EC 5.4.2.4) (BPG-dependent PGAM 1)	Phosphoglycerate mutase family, BPG-dependent PGAM subfamily	Gallus gallus (Chicken)	PF00300;
F1NN63	424691;	PGM1	Uncharacterized protein	Phosphohexose mutase family	Gallus gallus (Chicken)	PF02878;PF02879;PF02880;PF00408;
Q2UZR2	424691;		Phosphoglucomutase 1	Phosphohexose mutase family	Gallus gallus (Chicken)	PF02878;PF02879;PF02880;PF00408;
Q5ZHU2	426435;	RCJMB04_33e1	Uncharacterized protein	Phosphohexose mutase family	Gallus gallus (Chicken)	PF02878;PF02879;PF02880;PF00408;
F1NAA5	419052;	PGM2L1	Uncharacterized protein	Phosphohexose mutase family	Gallus gallus (Chicken)	PF02878;PF02879;PF02880;PF00408;
E1BQU2	421841;	PGM3	Phosphoacetylglucosamine mutase (PAGM) (EC 5.4.2.3) (Acetylglucosamine phosphomutase) (N-acetylglucosamine-phosphate mutase)	Phosphohexose mutase family	Gallus gallus (Chicken)	PF02878;PF00408;
F1NM36	427215;	PGM5	Uncharacterized protein	Phosphohexose mutase family	Gallus gallus (Chicken)	PF02878;PF02879;PF02880;
A0A1D5P8X9	428635;	SNAP91	ENTH domain-containing protein	PICALM/SNAP91 family	Gallus gallus (Chicken)	PF07651;
Q5ZLX6	428635;	RCJMB04_4i4	ENTH domain-containing protein	PICALM/SNAP91 family	Gallus gallus (Chicken)	PF07651;
F1N804	416030;	PLXNA1	Sema domain-containing protein	Plexin family	Gallus gallus (Chicken)	PF08337;PF01437;PF01403;PF01833;PF18020;PF17960;
F1NCV7	431261;	MTAP	S-methyl-5'-thioadenosine phosphorylase (EC 2.4.2.28) (5'-methylthioadenosine phosphorylase) (MTA phosphorylase) (MTAP) (MTAPase)	PNP/MTAP phosphorylase family; PNP/MTAP phosphorylase family, MTAP subfamily	Gallus gallus (Chicken)	PF01048;
Q5ZHQ7	431261;	MTAP RCJMB04_34g8	S-methyl-5'-thioadenosine phosphorylase (EC 2.4.2.28) (5'-methylthioadenosine phosphorylase) (MTA phosphorylase) (MTAP) (MTAPase)	PNP/MTAP phosphorylase family; PNP/MTAP phosphorylase family, MTAP subfamily	Gallus gallus (Chicken)	PF01048;
O57604	395755;	PODXL MEP21 PCLP PCLP1	Podocalyxin (Podocalyxin-like protein 1) (PC) (PCLP-1) (Thrombomucin)	Podocalyxin family	Gallus gallus (Chicken)	PF06365;
A0A3Q2UBE7	395755;	PODXL	Podocalyxin (Podocalyxin-like protein 1)	Podocalyxin family	Gallus gallus (Chicken)	PF06365;
F1NXC1	395755;	PODXL	Podocalyxin (Podocalyxin-like protein 1)	Podocalyxin family	Gallus gallus (Chicken)	PF06365;
Q5ZL53	430997;	RCJMB04_7l20	Polyadenylate-binding protein (PABP)	Polyadenylate-binding protein type-1 family	Gallus gallus (Chicken)	PF00658;PF00076;
A0A1D5PF07	421404;	PPM1B RCJMB04_26p17	Protein-serine/threonine phosphatase (EC 3.1.3.16)	PP2C family	Gallus gallus (Chicken)	PF00481;PF07830;
F1NCD7	422134;	PIN4	Peptidyl-prolyl cis-trans isomerase (EC 5.2.1.8)	PpiC/parvulin rotamase family, PIN4 subfamily	Gallus gallus (Chicken)	
Q5ZIV0	772354;	RCJMB04_23h2	Serine/threonine-protein phosphatase (EC 3.1.3.16)	PPP phosphatase family	Gallus gallus (Chicken)	PF00149;
P62207	396019;	PPP1CB	Serine/threonine-protein phosphatase PP1-beta catalytic subunit (PP-1B) (EC 3.1.3.16) (EC 3.1.3.53)	PPP phosphatase family, PP-1 subfamily	Gallus gallus (Chicken)	PF00149;PF16891;
P48463	396021;	PPP2CA	Serine/threonine-protein phosphatase 2A catalytic subunit alpha isoform (PP2A-alpha) (EC 3.1.3.16)	PPP phosphatase family, PP-1 subfamily	Gallus gallus (Chicken)	PF00149;
A0A3Q2U159	395113;	PPP3CA	Serine/threonine-protein phosphatase (EC 3.1.3.16)	PPP phosphatase family; PPP phosphatase family, PP-2B subfamily	Gallus gallus (Chicken)	PF00149;
F1NP43	419337;	PFDN4	Prefoldin subunit 4	Prefoldin subunit beta family	Gallus gallus (Chicken)	PF01920;
F1NYT7	426297;	PCYOX1	Prenylcys_lyase domain-containing protein	Prenylcysteine oxidase family	Gallus gallus (Chicken)	PF07156;
Q90YH9	395332;	TES	Testin	Prickle / espinas / testin family	Gallus gallus (Chicken)	PF00412;PF06297;
Q5ZL50	771904;	PFN2 RCJMB04_7m18	Profilin	Profilin family	Gallus gallus (Chicken)	PF00235;
D5M8S3	419980;	PHB	Prohibitin	Prohibitin family	Gallus gallus (Chicken)	PF01145;
Q5ZMN3	771124;	PHB2 RCJMB04_1i23	Prohibitin-2	Prohibitin family	Gallus gallus (Chicken)	PF01145;
A0A1D5PM03	427752;	PTGS1	Cyclooxygenase-1 (EC 1.14.99.1) (Prostaglandin G/H synthase 1) (Prostaglandin H2 synthase 1) (Prostaglandin-endoperoxide synthase 1)	Prostaglandin G/H synthase family	Gallus gallus (Chicken)	PF03098;PF00008;
B6V3I0	427121;	CAST	Calpain inhibitor (Calpastatin)	Protease inhibitor I27 (calpastatin) family	Gallus gallus (Chicken)	PF00748;
P26652	396483;	TIMP3 IMP-3	Metalloproteinase inhibitor 3 (21 kDa protein of extracellular matrix) (Tissue inhibitor of metalloproteinases 3) (TIMP-3)	Protease inhibitor I35 (TIMP) family	Gallus gallus (Chicken)	PF00965;

F1NK40	100858010;	A2ML4	Uncharacterized protein	Protease inhibitor I39 (alpha-2-macroglobulin) family	Gallus gallus (Chicken)	PF00207;PF07703;PF07677;PF01835;PF17791;PF17789;PF07678;
F1NX21	421862;	CD109	Uncharacterized protein	Protease inhibitor I39 (alpha-2-macroglobulin) family	Gallus gallus (Chicken)	PF00207;PF07703;PF07677;PF01835;PF17791;PF07678;
Q5ZJL3	430115;	PSMF1 RCJMB04_17f8	Proteasome inhibitor PI31 subunit	Proteasome inhibitor PI31 family	Gallus gallus (Chicken)	PF08577;PF11566;
Q5ZJS1	416853;	RCJMB04_16d3	PDZ domain-containing protein	Proteasome subunit p27 family	Gallus gallus (Chicken)	PF18265;PF13180;
Q5ZI37	417425;	RCJMB04_30k11	PCI domain-containing protein	Proteasome subunit p55 family	Gallus gallus (Chicken)	PF01399;PF18098;
Q5F418	424926;	PSMD1 RCJMB04_3m24	26S proteasome non-ATPase regulatory subunit 1 (26S proteasome regulatory subunit RPN2) (26S proteasome regulatory subunit S1)	Proteasome subunit S1 family	Gallus gallus (Chicken)	PF01851;PF18004;
A0A1D5PI39	424926;	PSMD1	26S proteasome non-ATPase regulatory subunit 1	Proteasome subunit S1 family	Gallus gallus (Chicken)	PF01851;PF18004;
F1N903	416079;	PSMD6	26S proteasome non-ATPase regulatory subunit 6 (26S proteasome regulatory subunit RPN7)	Proteasome subunit S10 family	Gallus gallus (Chicken)	PF01399;PF10602;
Q5ZLU4	425294;	RCJMB04_4n15	26S proteasome non-ATPase regulatory subunit 2	Proteasome subunit S2 family	Gallus gallus (Chicken)	PF01851;PF18051;PF17781;
Q5ZMD9	426133;	RCJMB04_2h4	PCI domain-containing protein	Proteasome subunit S3 family	Gallus gallus (Chicken)	PF01399;PF08375;
Q5ZI14	100216364;	RCJMB04_31h10	26S proteasome non-ATPase regulatory subunit 4	Proteasome subunit S5A family	Gallus gallus (Chicken)	PF02809;PF13519;
R4GLK3	100216364;	PSMD4	26S proteasome non-ATPase regulatory subunit 4	Proteasome subunit S5A family	Gallus gallus (Chicken)	PF02809;PF13519;
F1NPA2	430878;	PSMD11	PCI domain-containing protein	Proteasome subunit S9 family	Gallus gallus (Chicken)	PF01399;PF18503;PF18055;
A0A1D5PV06	374091;	P4HB	Protein disulfide-isomerase (EC 5.3.4.1)	Protein disulfide isomerase family	Gallus gallus (Chicken)	PF00085;
Q8JG64	373899;	PDIA3 ERP57 GRP58	Protein disulfide-isomerase A3 (EC 5.3.4.1) (Endoplasmic reticulum resident protein 57) (ER protein 57) (ERp57) (Glucose-regulated thiol oxidoreductase 58 kDa protein)	Protein disulfide isomerase family	Gallus gallus (Chicken)	PF00085;
Q5ZK20	420785;	RCJMB04_13l7	Protein disulfide-isomerase A4 (EC 5.3.4.1)	Protein disulfide isomerase family	Gallus gallus (Chicken)	PF00085;
F1NK96	421940;	PDIA6	Protein disulfide-isomerase A6 (EC 5.3.4.1)	Protein disulfide isomerase family	Gallus gallus (Chicken)	PF00085;
Q5ZIM7	420867;	RCJMB04_24o2	Uncharacterized protein	Protein disulfide isomerase family	Gallus gallus (Chicken)	PF00085;
F1NBD7	396252;	CDK1	Cyclin-dependent kinase 1	Protein kinase superfamily	Gallus gallus (Chicken)	PF00069;
B6E1W1	100190948;	Cdk5	Cyclin-dependent kinase 5	Protein kinase superfamily	Gallus gallus (Chicken)	PF00069;
F1NGM2	416745;	COQ8A	Atypical kinase COQ8A, mitochondrial (Chaperone activity of bc1 complex-like) (Coenzyme Q protein 8A) (aarF domain-containing protein kinase 3)	Protein kinase superfamily, ADCK protein kinase family	Gallus gallus (Chicken)	PF03109;
A0A1D5NWB8	374182;	ROCK2	Rho-associated protein kinase 2 (EC 2.7.11.1) (Rho-associated, coiled-coil-containing protein kinase 2) (Rho-associated, coiled-coil-containing protein kinase II) (p164 ROCK-2)	Protein kinase superfamily, AGC Ser/Thr protein kinase family	Gallus gallus (Chicken)	PF00069;PF08912;
A0A3Q2TTH6	374182;	ROCK2	Rho-associated protein kinase 2 (EC 2.7.11.1) (Rho-associated, coiled-coil-containing protein kinase 2) (Rho-associated, coiled-coil-containing protein kinase II) (p164 ROCK-2)	Protein kinase superfamily, AGC Ser/Thr protein kinase family	Gallus gallus (Chicken)	PF00069;PF08912;
F1NK88	374182;	ROCK2	Rho-associated protein kinase 2 (EC 2.7.11.1) (Rho-associated, coiled-coil-containing protein kinase 2) (Rho-associated, coiled-coil-containing protein kinase II) (p164 ROCK-2)	Protein kinase superfamily, AGC Ser/Thr protein kinase family	Gallus gallus (Chicken)	PF00069;PF08912;
A0A1D5PK41	373970;	ROCK1	Rho-associated protein kinase (EC 2.7.11.1)	Protein kinase superfamily, AGC Ser/Thr protein kinase family	Gallus gallus (Chicken)	PF00069;PF08912;
F1NRH6	373970;	ROCK1	Rho-associated protein kinase (EC 2.7.11.1)	Protein kinase superfamily, AGC Ser/Thr protein kinase family	Gallus gallus (Chicken)	PF00069;PF08912;
Q5F3U4	416588;	PDPK1 RCJMB04_6p10	3-phosphoinositide-dependent protein kinase 1 (EC 2.7.11.1)	Protein kinase superfamily, AGC Ser/Thr protein kinase family, PDPK1 subfamily	Gallus gallus (Chicken)	PF14593;PF00069;
A0A1D5PXN4	415905;	PRKCD	Protein kinase C delta type (EC 2.7.11.13) (nPKC-delta)	Protein kinase superfamily, AGC Ser/Thr protein kinase family, PKC subfamily	Gallus gallus (Chicken)	PF00130;PF00069;PF00433;
Q5ZKE2	415905;	RCJMB04_11g12	Protein kinase C delta type (EC 2.7.11.13) (nPKC-delta)	Protein kinase superfamily, AGC Ser/Thr protein kinase family, PKC subfamily	Gallus gallus (Chicken)	PF00130;PF00069;PF00433;
A0A1L1RRF3	396445;	MYLK	Myosin light chain kinase, smooth muscle (EC 2.7.11.18) (Myosin light chain kinase, smooth muscle, deglutamylated form) (Telokin)	Protein kinase superfamily, CAMK Ser/Thr protein kinase family	Gallus gallus (Chicken)	PF00041;PF07679;PF00069;
E1C906	396445;	MYLK	Myosin light chain kinase, smooth muscle (EC 2.7.11.18) (Myosin light chain kinase, smooth muscle, deglutamylated form) (Telokin)	Protein kinase superfamily, CAMK Ser/Thr protein kinase family	Gallus gallus (Chicken)	PF00041;PF07679;PF00069;
A2NBE2	396356;		Myosin light chain kinase 2, skeletal/cardiac muscle (EC 2.7.11.18)	Protein kinase superfamily, CAMK Ser/Thr protein kinase family	Gallus gallus (Chicken)	PF00069;
Q5ZKI0	422688;	CAMK2D RCJMB04_10k21	Calcium/calmodulin-dependent protein kinase type II delta chain (CaM kinase II subunit delta) (CaM-kinase II delta chain) (CaMK-II subunit delta) (EC 2.7.11.17)	Protein kinase superfamily, CAMK Ser/Thr protein kinase family, CaMK subfamily	Gallus gallus (Chicken)	PF08332;PF00069;

P13863	396252;	CDK1 CDC2	Cyclin-dependent kinase 1 (CDK1) (EC 2.7.11.22) (EC 2.7.11.23) (Cell division control protein 2 homolog) (Cell division protein kinase 1) (p34 protein kinase)	Protein kinase superfamily, CMGC Ser/Thr protein kinase family, CDC2/CDKX subfamily	Gallus gallus (Chicken)	PF00069;
A0A1D5PIQ5	421183;	MAPK14	Mitogen-activated protein kinase (EC 2.7.11.24)	Protein kinase superfamily, CMGC Ser/Thr protein kinase family, MAP kinase subfamily	Gallus gallus (Chicken)	PF00069;
A0A3Q2U670	421183;	MAPK14	Mitogen-activated protein kinase (EC 2.7.11.24)	Protein kinase superfamily, CMGC Ser/Thr protein kinase family, MAP kinase subfamily	Gallus gallus (Chicken)	PF00069;
P21868	432370;	CSNK2A1	Casein kinase II subunit alpha (CK II) (EC 2.7.11.1)	Protein kinase superfamily, Ser/Thr protein kinase family, CK2 subfamily	Gallus gallus (Chicken)	PF00069;
Q90891	396349;	MAP2K2 MEK2 MKK2 PRKMK2	Dual specificity mitogen-activated protein kinase kinase 2 (MAP kinase kinase 2) (MAPKK 2) (EC 2.7.12.2) (ERK activator kinase 2) (MAPK/ERK kinase 2) (MEK2)	Protein kinase superfamily, STE Ser/Thr protein kinase family, MAP kinase kinase subfamily	Gallus gallus (Chicken)	PF00069;
Q9DF58	374018;	ILK ILK1 ILK2	Integrin-linked protein kinase (EC 2.7.11.1) (59 kDa serine/threonine-protein kinase) (Beta-integrin-linked kinase) (ILK-1) (ILK-2) (p59ILK)	Protein kinase superfamily, TKL Ser/Thr protein kinase family	Gallus gallus (Chicken)	PF12796;PF07714;
A0A1D5PXS9	396396;	CSK	Tyrosine-protein kinase (EC 2.7.10.2)	Protein kinase superfamily, Tyr protein kinase family	Gallus gallus (Chicken)	PF07714;PF00017;PF00018;
P41239	396396;	CSK	Tyrosine-protein kinase CSK (EC 2.7.10.2) (C-Src kinase)	Protein kinase superfamily, Tyr protein kinase family, CSK subfamily	Gallus gallus (Chicken)	PF07714;PF00017;PF00018;
P00523	396442;	SRC	Proto-oncogene tyrosine-protein kinase Src (EC 2.7.10.2) (Proto-oncogene c-Src) (pp60c-src) (p60-Src)	Protein kinase superfamily, Tyr protein kinase family, SRC subfamily	Gallus gallus (Chicken)	PF07714;PF00017;PF00018;
A0A1D5NW04	424542;	PRKACB	cAMP-dependent protein kinase (EC 2.7.11.11)	Protein kinase superfamily; Protein kinase superfamily, AGC Ser/Thr protein kinase family, cAMP subfamily	Gallus gallus (Chicken)	PF00069;
A0A1D5P0J5	424542;	PRKACB	cAMP-dependent protein kinase (EC 2.7.11.11)	Protein kinase superfamily; Protein kinase superfamily, AGC Ser/Thr protein kinase family, cAMP subfamily	Gallus gallus (Chicken)	PF00069;
A0A1D5PLV3	424542;	PRKACB	cAMP-dependent protein kinase (EC 2.7.11.11)	Protein kinase superfamily; Protein kinase superfamily, AGC Ser/Thr protein kinase family, cAMP subfamily	Gallus gallus (Chicken)	PF00069;
O57513	395928;	akt1	RAC-PK-alpha (EC 2.7.11.1) (RAC-alpha serine/threonine-protein kinase)	Protein kinase superfamily; Protein kinase superfamily, AGC Ser/Thr protein kinase family, RAC subfamily	Gallus gallus (Chicken)	PF00169;PF00069;PF00433;
A0A3Q2TVV5	418335;	GSK3A	Glycogen synthase kinase-3 beta (EC 2.7.11.26)	Protein kinase superfamily; Protein kinase superfamily, CMGC Ser/Thr protein kinase family, GSK-3 subfamily	Gallus gallus (Chicken)	PF00069;
Q8UWG6	373953;		Mitogen-activated protein kinase (EC 2.7.11.24)	Protein kinase superfamily; Protein kinase superfamily, CMGC Ser/Thr protein kinase family, MAP kinase subfamily; Protein kinase	Gallus gallus (Chicken)	PF00069;
Q90687	395815;	PTPN11	Tyrosine-protein phosphatase non-receptor type 11 (EC 3.1.3.48) (SH-PTP2) (cSH-PTP2)	Protein-tyrosine phosphatase family, Non-receptor class 2 subfamily	Gallus gallus (Chicken)	PF00017;PF00102;
A0A1D5PFX7	431602;	PTPRD	Protein-tyrosine-phosphatase (EC 3.1.3.48)	Protein-tyrosine phosphatase family, Receptor class 2A subfamily	Gallus gallus (Chicken)	PF00041;PF07679;PF00102;
Q5F3I0	427127;	PRRC1 RCJMB04_16h2	Protein PRRC1 (Proline-rich and coiled-coil-containing protein 1)	PRRC1 family	Gallus gallus (Chicken)	PF01931;
Q5ZJH9	422196;	DKC1 RCJMB04_17p9	H/ACA ribonucleoprotein complex subunit DKC1 (EC 5.4.99.-) (Dyskerin)	Pseudouridine synthase TruB family	Gallus gallus (Chicken)	PF08068;PF01472;PF16198;PF01509;
Q5F3H4	418520;	PSMG1 RCJMB04_16p8	Proteasome assembly chaperone 1	PSMG1 family	Gallus gallus (Chicken)	PF16094;
E1BZ56	421040;	PSMG2	Proteasome assembly chaperone 2	PSMG2 family	Gallus gallus (Chicken)	PF09754;
Q5ZK88	418955;	PSPC1 RCJMB04_12f9	Paraspeckle component 1	PSPC family	Gallus gallus (Chicken)	PF08075;PF00076;
Q04205	396439;	TNS	Tensin	PTEN phosphatase protein family	Gallus gallus (Chicken)	PF08416;PF10409;PF00017;
Q5ZM56	426329;	PTPA RCJMB04_3b8	Serine/threonine-protein phosphatase 2A activator (EC 5.2.1.8) (Phosphotyrosyl phosphatase activator)	PTPA-type PPIase family	Gallus gallus (Chicken)	PF03095;
P31335	396091;	ATIC PURH	Bifunctional purine biosynthesis protein ATIC (AICAR transformylase/inosine monophosphate cyclohydrolase) (ATIC) [Includes: Phosphoribosylaminoimidazolecarboxamide formyltransferase (EC 2.1.2.3) (5-aminoimidazole-4-carboxamide ribonucleotide formyltransferase) (AICAR formyltransferase) (AICAR transformylase); IMP cyclohydrolase (EC 3.5.4.10) (IMP synthase) (Inosinicase)]	PurH family	Gallus gallus (Chicken)	PF01808;PF02142;
A0A1D5NUK5	100857155;	APRT	Adenine phosphoribosyltransferase (EC 2.4.2.7)	Purine/pyrimidine phosphoribosyltransferase family	Gallus gallus (Chicken)	PF00156;
Q9W719	395653;	HPRT1 HPRT	Hypoxanthine-guanine phosphoribosyltransferase (HGPRT) (HGPRTase) (EC 2.4.2.8)	Purine/pyrimidine phosphoribosyltransferase family	Gallus gallus (Chicken)	PF00156;
A0A1D5PKK0	769203;	PYCR1	Pyrroline-5-carboxylate reductase (EC 1.5.1.2)	Pyrroline-5-carboxylate reductase family	Gallus gallus (Chicken)	PF03807;PF14748;
A0A1D5NV93	420293;	PYCRL	Pyrroline-5-carboxylate reductase (EC 1.5.1.2)	Pyrroline-5-carboxylate reductase family	Gallus gallus (Chicken)	PF03807;PF14748;
P00548	396456;	PKM	Pyruvate kinase PKM (EC 2.7.1.40)	Pyruvate kinase family	Gallus gallus (Chicken)	PF00224;PF02887;
A0A1D5P9V0	396456;	PKLR	Pyruvate kinase (EC 2.7.1.40)	Pyruvate kinase family	Gallus gallus (Chicken)	PF00224;PF02887;
F1NW43	396456;	PKLR	Pyruvate kinase (EC 2.7.1.40)	Pyruvate kinase family	Gallus gallus (Chicken)	PF00224;PF02887;
O93382	395854;		Rab GDP dissociation inhibitor	Rab GDI family	Gallus gallus (Chicken)	PF00996;
Q7SZI5	395109;	RFTN1	Raftlin (Raft-linking protein)	Raftlin family	Gallus gallus (Chicken)	PF15250;

Q5ZJJ2	417563;	RPA1 RCJMB04_17l6	Replication protein A 70 kDa DNA-binding subunit (RP-A p70) (Replication factor A protein 1) (RF-A protein 1)	Replication factor A protein 1 family	Gallus gallus (Chicken)	PF04057;PF08646;PF16900;PF01336;
Q5ZHM5	419397;	RER1 RCJMB04_35f13	Protein RER1	RER1 family	Gallus gallus (Chicken)	PF03248;
F1NLT8	417941;	ARHGDIB	Uncharacterized protein	Rho GDI family	Gallus gallus (Chicken)	PF02115;
F1NIP5	422185;	PRPS1L1	Ribose-phosphate diphosphokinase (EC 2.7.6.1)	Ribose-phosphate pyrophosphokinase family	Gallus gallus (Chicken)	PF14572;PF13793;
Q5ZL26	416521;	PRPSAP2 RCJMB04_8b17	Phosphoribosyl pyrophosphate synthase-associated protein 2 (PRPP synthase-associated protein 2)	Ribose-phosphate pyrophosphokinase family	Gallus gallus (Chicken)	PF14572;PF13793;
F1NZC6	416521;	PRPSAP2	Phosphoribosyl pyrophosphate synthase-associated protein 2	Ribose-phosphate pyrophosphokinase family	Gallus gallus (Chicken)	PF14572;PF13793;
Q5ZLR5	415752;	UQCRFS1 RCJMB04_5b19	Cytochrome b-c1 complex subunit Rieske, mitochondrial (EC 7.1.1.8) (Complex III subunit 5) (Cytochrome b-c1 complex subunit 5) (Rieske iron-sulfur protein) (RISP) (Rieske protein UQCRFS1) (Ubiquinol-cytochrome c reductase iron-sulfur subunit) [Cleaved into: Cytochrome b-c1 complex subunit 9 (Su9) (Subunit 9) (Complex III subunit IX) (UQCRFS1 mitochondrial targeting sequence) (UQCRFS1 MTS)]	Rieske iron-sulfur protein family	Gallus gallus (Chicken)	PF00355;PF09165;PF02921;
E1C1Y0	424468;	RTCA	RNA 3'-terminal phosphate cyclase (EC 6.5.1.4) (RNA terminal phosphate cyclase domain-containing protein 1)	RNA 3'-terminal cyclase family, Type 1 subfamily	Gallus gallus (Chicken)	PF01137;PF05189;
Q5ZMN9	422621;	RCJMB04_1i14	DNA-directed RNA polymerase subunit beta (EC 2.7.7.6)	RNA polymerase beta chain family	Gallus gallus (Chicken)	PF04563;PF04561;PF04565;PF04566;PF04567;PF00562;PF04560;
Q5ZL34	417844;	CPSF6 RCJMB04_7p13	Cleavage and polyadenylation specificity factor subunit 6	RRM CPSF6/7 family	Gallus gallus (Chicken)	PF00076;
A0A1D5P9A5	417844;	CPSF6	Cleavage and polyadenylation specificity factor subunit 6	RRM CPSF6/7 family	Gallus gallus (Chicken)	PF00076;
F1NGU9	417844;	CPSF6	Cleavage and polyadenylation specificity factor subunit 6	RRM CPSF6/7 family	Gallus gallus (Chicken)	PF00076;
Q9PW24	395637;	HuA	ELAV-like protein	RRM elav family	Gallus gallus (Chicken)	PF00076;
A0A1D5PSH2	419148;	RALY	RRM domain-containing protein	RRM HNRPC family, RALY subfamily	Gallus gallus (Chicken)	PF00076;
A0A3Q2U2Y0	419148;	RALY	RRM domain-containing protein	RRM HNRPC family, RALY subfamily	Gallus gallus (Chicken)	PF00076;
O42254	395953;	IGF2BP1 VICKZ1 ZBP1	Insulin-like growth factor 2 mRNA-binding protein 1 (IGF2 mRNA-binding protein 1) (IMP-1) (IGF-II mRNA-binding protein 1) (VICKZ family member 1) (Zip-code binding polypeptide) (Zipcode-binding protein 1) (ZBP-1)	RRM IMP/VICKZ family	Gallus gallus (Chicken)	PF00013;PF00076;
Q5ZLP8	420617;	IGF2BP3 VICKZ3 RCJMB04_5e15	Insulin-like growth factor 2 mRNA-binding protein 3 (IGF2 mRNA-binding protein 3) (IMP-3) (IGF-II mRNA-binding protein 3) (VICKZ family member 3)	RRM IMP/VICKZ family	Gallus gallus (Chicken)	PF00013;PF00076;
Q6J4Y8	414144;		FUS/TLS	RRM TET family	Gallus gallus (Chicken)	PF00076;PF00641;
Q5ZKD5	423845;	RRP12 RCJMB04_11j1	RRP12-like protein	RRP12 family	Gallus gallus (Chicken)	PF08161;
F1NYI3	418064;	RTCB	RNA-splicing ligase RtcB homolog (EC 6.5.1.8) (3'-phosphate/5'-hydroxy nucleic acid ligase)	RtcB family	Gallus gallus (Chicken)	PF01139;
Q90706	396326;	CLE7	RNA transcription, translation and transport factor protein	RTRAF family	Gallus gallus (Chicken)	PF10036;
Q5ZIC4	416022;	RCJMB04_28a17	RuvB-like helicase (EC 3.6.4.12)	RuvB family	Gallus gallus (Chicken)	PF06068;PF17856;
P24479	396075;	S100A11	Protein S100-A11 (Calgizzarin) (S100 calcium-binding protein A11)	S-100 family	Gallus gallus (Chicken)	PF00036;PF01023;
P28318	426356;		Protein MRP-126	S-100 family	Gallus gallus (Chicken)	PF01023;
P38024	396534;	AIRC	Multifunctional protein ADE2 [Includes: Phosphoribosylaminoimidazole-succinocarboxamide synthase (EC 6.3.2.6) (SAICAR synthetase); Phosphoribosylaminoimidazole carboxylase (EC 4.1.1.21) (AIR carboxylase) (AIRC)]	SAICAR synthetase family; AIR carboxylase family, Class II subfamily	Gallus gallus (Chicken)	PF00731;PF01259;
E1C8A2	418234;	SAMM50	POTRA domain-containing protein	SAM50/omp85 family	Gallus gallus (Chicken)	PF01103;
Q90ZH5	374206;	chSAP18	Histone deacetylase complex subunit SAP18	SAP18 family	Gallus gallus (Chicken)	PF06487;
F1NV32	769853;	SCAMP2	Secretory carrier-associated membrane protein (Secretory carrier membrane protein)	SCAMP family	Gallus gallus (Chicken)	PF04144;
H9L0D7	426359;	WASF2	Wiskott-Aldrich syndrome protein family member (WASP family protein member)	SCAR/WAVE family	Gallus gallus (Chicken)	PF02205;
A0A1D5PY29	422360;	STAG2	SCD domain-containing protein	SCC3 family	Gallus gallus (Chicken)	PF08514;
E1BSU3	422360;	STAG2	SCD domain-containing protein	SCC3 family	Gallus gallus (Chicken)	PF08514;
A0A3Q2U072	429671;	SCO2	Uncharacterized protein	SCO1/2 family	Gallus gallus (Chicken)	PF02630;
A0A1D5P0E2	423335;	SEC23A	Protein transport protein SEC23	SEC23/SEC24 family, SEC23 subfamily	Gallus gallus (Chicken)	PF00626;PF08033;PF04815;PF04811;PF04810;
A0A3Q2UGR5	423742;	SEC24C	Uncharacterized protein	SEC23/SEC24 family, SEC24 subfamily	Gallus gallus (Chicken)	PF00626;PF08033;PF04815;PF04811;PF04810;

E1BUD8	423742;	SEC24C	Uncharacterized protein	SEC23/SEC24 family, SEC24 subfamily	Gallus gallus (Chicken)	PF00626;PF08033;PF04815;PF04811;PF04810;
F1NBN1	416023;	SEC61A1	Plug_translocon domain-containing protein	SecY/SEC61-alpha family	Gallus gallus (Chicken)	PF10559;PF00344;
H9KYX6	425664;	SELENBP1	Methanethiol oxidase (EC 1.8.3.4)	Selenium-binding protein family	Gallus gallus (Chicken)	PF05694;
F1NBU4	420115;	HOMER3	Sema domain-containing protein	Semaphorin family	Gallus gallus (Chicken)	PF01437;PF01403;
E1C4L3	425255;	SPR	Sepiapterin reductase (EC 1.1.1.153)	Sepiapterin reductase family	Gallus gallus (Chicken)	PF00106;
F1NDH2	421543;	AGT	Angiotensin 1-10 (Angiotensin 1-4) (Angiotensin 1-5) (Angiotensin 1-7) (Angiotensin 1-8) (Angiotensin 1-9) (Angiotensin 2-8) (Angiotensin 3-8) (Angiotensin I) (Angiotensin II) (Angiotensin III) (Angiotensin IV) (Angiotensin-1) (Angiotensin-2) (Angiotensin-3) (Angiotensin-4) (Angiotensinogen) (Des-Asp[1]-angiotensin II) (Serpin A8)	Serpin family	Gallus gallus (Chicken)	PF00079;
A0A411G5W6	396058;		Ovalbumin	Serpin family	Gallus gallus (Chicken)	PF00079;
A0A1D5PI58	420898;	OVALX	SERPIN domain-containing protein	Serpin family	Gallus gallus (Chicken)	PF00079;
E1BTF4	420897;	OVALY	SERPIN domain-containing protein	Serpin family	Gallus gallus (Chicken)	PF00079;
I0J178	420897;	oval-Y	Ovalbumin-related Y	Serpin family	Gallus gallus (Chicken)	PF00079;
E1BTH3	420896;	SERPINB2	SERPIN domain-containing protein	Serpin family	Gallus gallus (Chicken)	PF00079;
P13731	396228;	SERPINH1 HSP47	Serpin H1 (47 kDa heat shock protein) (Collagen-binding protein) (Colligin)	Serpin family	Gallus gallus (Chicken)	PF00079;
E1C7T1	423434;	SPIA1	SERPIN domain-containing protein	Serpin family	Gallus gallus (Chicken)	PF00079;
E1BS56	423433;	SPIA4	SERPIN domain-containing protein	Serpin family	Gallus gallus (Chicken)	PF00079;
A0A1D5PLZ2	395877;	SERPIND1	SERPIN domain-containing protein	Serpin family	Gallus gallus (Chicken)	PF00079;
P01012	396058;	SERPINB14	Ovalbumin (Allergen Gal d II) (Egg albumin) (Plakalbumin) (allergen Gal d 2)	Serpin family, Ov-serpin subfamily	Gallus gallus (Chicken)	PF00079;
P01014	420897;	SERPINB14B Y	Ovalbumin-related protein Y (Gene Y protein)	Serpin family, Ov-serpin subfamily	Gallus gallus (Chicken)	PF00079;
O73790	395715;	SERPINB10 MENT	Heterochromatin-associated protein MENT (Myeloid and erythroid nuclear termination stage-specific protein) (Serpin B10)	Serpin family, Ov-serpin subfamily	Gallus gallus (Chicken)	PF00079;
A0A3Q2TTT6	420894;	SERPINB1	SERPIN domain-containing protein	Serpin family; Serpin family, Ov-serpin subfamily	Gallus gallus (Chicken)	PF00079;
E1BTE2	420900;	SERPINB5	SERPIN domain-containing protein	Serpin family; Serpin family, Ov-serpin subfamily	Gallus gallus (Chicken)	PF00079;
F1P1L8	420895;	SERPINB6	SERPIN domain-containing protein	Serpin family; Serpin family, Ov-serpin subfamily	Gallus gallus (Chicken)	PF00079;
Q5ZLB6	420895;	RCJMB04_6n9	SERPIN domain-containing protein	Serpin family; Serpin family, Ov-serpin subfamily	Gallus gallus (Chicken)	PF00079;
Q5F387	419608;	SF3A3 RCJMB04_28g11	Matrin-type domain-containing protein	SF3A3 family	Gallus gallus (Chicken)	PF11931;PF16837;PF12108;
E1C2C3	424056;	SF3B1	SF3b1 domain-containing protein	SF3B1 family	Gallus gallus (Chicken)	PF08920;
A0A1D5PBJ5	426396;	SGTA	TPR_REGION domain-containing protein	SGT family	Gallus gallus (Chicken)	PF16546;PF00515;PF13181;
Q5ZHW6	426396;	RCJMB04_32j5	TPR_REGION domain-containing protein	SGT family	Gallus gallus (Chicken)	PF16546;PF00515;PF13181;
Q5F3C9	422277;	SH3BGRL RCJMB04_21c16	SH3 domain-binding glutamic acid-rich-like protein	SH3BGR family	Gallus gallus (Chicken)	PF04908;
A0A3Q3AY90	423919;	SHTN1	Shootin-1	Shootin family	Gallus gallus (Chicken)	
F1NHH9	424891;	BDH1A	Uncharacterized protein	Short-chain dehydrogenases/reductases (SDR) family	Gallus gallus (Chicken)	PF00106;
F1N8Y3	418512;	CBR3	Uncharacterized protein	Short-chain dehydrogenases/reductases (SDR) family	Gallus gallus (Chicken)	PF00106;
Q4JK63	418512;		20-hydroxysteroid dehydrogenase	Short-chain dehydrogenases/reductases (SDR) family	Gallus gallus (Chicken)	PF00106;
A0A1D5PSE2	415662;	LOC107049164	Uncharacterized protein	Short-chain dehydrogenases/reductases (SDR) family	Gallus gallus (Chicken)	PF00106;
E1BV75	423527;	DHRS7	Uncharacterized protein	Short-chain dehydrogenases/reductases (SDR) family	Gallus gallus (Chicken)	PF00106;
Q8JIS3	374066;	DER	D-erythrulose reductase (EC 1.1.1.162) (Probable L-xylulose reductase) (XR) (EC 1.1.1.10)	Short-chain dehydrogenases/reductases (SDR) family	Gallus gallus (Chicken)	

E1C688	422567;	HPGD	Uncharacterized protein	Short-chain dehydrogenases/reductases (SDR) family	Gallus gallus (Chicken)	PF00106;
A0A1D5PM72	769787;	HSD17B12	Uncharacterized protein	Short-chain dehydrogenases/reductases (SDR) family	Gallus gallus (Chicken)	PF00106;
A0A1D5P648	776432;	RDH16	Uncharacterized protein	Short-chain dehydrogenases/reductases (SDR) family	Gallus gallus (Chicken)	PF00106;
F1N9C1	415661;	LOC415661 NADB-LER3	NADB-LER3	Short-chain dehydrogenases/reductases (SDR) family	Gallus gallus (Chicken)	PF00106;
F1NDD1	423866;	SFXN2	Sidoreflexin	Sideroflexin family	Gallus gallus (Chicken)	PF03820;
A0A3Q2U0F7	428972;	SFXN3	Sidoreflexin	Sideroflexin family	Gallus gallus (Chicken)	PF03820;
E1BRE2	420834;	SIRT5	NAD-dependent protein deacylase sirtuin-5, mitochondrial (EC 2.3.1.-) (Regulatory protein SIR2 homolog 5)	Sirtuin family, Class III subfamily	Gallus gallus (Chicken)	PF02146;
R9PXP3	420834;	SIRT5	NAD-dependent protein deacylase sirtuin-5, mitochondrial (EC 2.3.1.-) (Regulatory protein SIR2 homolog 5) (SIR2-like protein 5)	Sirtuin family, Class III subfamily	Gallus gallus (Chicken)	PF02146;
Q5ZKF5	416319;	SKP1 SKP1A RCJMB04_11c19	S-phase kinase-associated protein 1 (Cyclin-A/CDK2-associated protein p19) (S-phase kinase-associated protein 1A) (p19A) (p19skp1)	SKP1 family	Gallus gallus (Chicken)	PF01466;PF03931;
Q5ZKR9	769725;	ARF4 RCJMB04_9i5	Uncharacterized protein	Small GTPase superfamily, Arf family	Gallus gallus (Chicken)	PF00025;
E1BVB0	417934;	ARL1	Uncharacterized protein	Small GTPase superfamily, Arf family	Gallus gallus (Chicken)	PF00025;
F1P0Z7	416109;	ARL8B	Uncharacterized protein	Small GTPase superfamily, Arf family	Gallus gallus (Chicken)	PF00025;
P26990	428927;	ARF6 CPS1	ADP-ribosylation factor 6	Small GTPase superfamily, Arf family	Gallus gallus (Chicken)	PF00025;
Q90965	396153;	RAB2A RAB2	Ras-related protein Rab-2A	Small GTPase superfamily, Rab family	Gallus gallus (Chicken)	PF00071;
A0A3Q2UFY8	424636;	RAB3B	Ras-related protein Rab-3	Small GTPase superfamily, Rab family	Gallus gallus (Chicken)	PF00071;
E1C8J9	424636;	RAB3B	Ras-related protein Rab-3	Small GTPase superfamily, Rab family	Gallus gallus (Chicken)	PF00071;
A0A1D5NWR3	421532;	RAB4A	Ras-related protein Rab-4	Small GTPase superfamily, Rab family	Gallus gallus (Chicken)	PF00071;
A0A3Q2TYP7	421532;	RAB4A	Ras-related protein Rab-4	Small GTPase superfamily, Rab family	Gallus gallus (Chicken)	PF00071;
Q5ZHW4	100529061;	RAB5B RCJMB04_32j11	Ras-related protein Rab-5B (EC 3.6.5.2)	Small GTPase superfamily, Rab family	Gallus gallus (Chicken)	PF00071;
Q98932	395197;	RAB5C	Ras-related protein Rab-5C (EC 3.6.5.2) (Rab5C-like protein)	Small GTPase superfamily, Rab family	Gallus gallus (Chicken)	PF00071;
Q1KME6	419063;	RAB6A	Ras-related protein Rab-6A	Small GTPase superfamily, Rab family	Gallus gallus (Chicken)	PF00071;
Q5F470	428352;	RAB8A RCJMB04_2k8	Ras-related protein Rab-8A	Small GTPase superfamily, Rab family	Gallus gallus (Chicken)	PF00071;
Q5ZMI5	418635;	RAB9A RCJMB04_1p1	Uncharacterized protein	Small GTPase superfamily, Rab family	Gallus gallus (Chicken)	PF00071;
Q5ZIT5	421994;	RAB10 RCJMB04_23k10	Ras-related protein Rab-10 (EC 3.6.5.2)	Small GTPase superfamily, Rab family	Gallus gallus (Chicken)	PF00071;
Q5ZKU5	417119;	RAB14 RCJMB04_9b24	Ras-related protein Rab-14	Small GTPase superfamily, Rab family	Gallus gallus (Chicken)	PF00071;
Q5ZLG1	420483;	RAB18 RCJMB04_6g4	Ras-related protein Rab-18	Small GTPase superfamily, Rab family	Gallus gallus (Chicken)	PF00071;
D2D3P4	415410;	Rab27a RAB27A	Small monomeric GTPase (EC 3.6.5.2)	Small GTPase superfamily, Rab family	Gallus gallus (Chicken)	PF00071;
F1NBL3	421616;	RAB32	Ras-related protein Rab	Small GTPase superfamily, Rab family	Gallus gallus (Chicken)	PF00071;
Q5ZHV1	422441;	RAB33B RCJMB04_32p6	Ras-related protein Rab-33B	Small GTPase superfamily, Rab family	Gallus gallus (Chicken)	PF00071;
P42558	396193;	RAN	GTP-binding nuclear protein Ran (GTPase Ran) (Ras-like protein TC4) (Ras-related nuclear protein)	Small GTPase superfamily, Ran family	Gallus gallus (Chicken)	PF00071;
P08642	396229;	HRAS HRAS1	GTPase HRas (EC 3.6.5.2) (H-Ras-1) (Transforming protein p21) (c-H-ras) (p21ras) [Cleaved into: GTPase HRas, N-terminally processed]	Small GTPase superfamily, Ras family	Gallus gallus (Chicken)	PF00071;
F1NSA8	419867;	RAP1A	Uncharacterized protein	Small GTPase superfamily, Ras family	Gallus gallus (Chicken)	PF00071;
Q5ZHX1	417840;	RAP1B RCJMB04_32g9 RCJMB04_4h13	Ras-related protein Rap-1b (EC 3.6.5.2)	Small GTPase superfamily, Ras family	Gallus gallus (Chicken)	PF00071;
E1BQI2	769797;	RAP2A	Uncharacterized protein	Small GTPase superfamily, Ras family	Gallus gallus (Chicken)	PF00071;

A0A1D5P1T0	420765;	RALA	Small monomeric GTPase (EC 3.6.5.2)	Small GTPase superfamily, Ras family	Gallus gallus (Chicken)	PF00071;
Q90694	395917;	CDC42	Cell division control protein 42 homolog (EC 3.6.5.2) (G25K GTP-binding protein)	Small GTPase superfamily, Rho family, CDC42 subfamily	Gallus gallus (Chicken)	PF00071;
A0A1D5PD33	395917;	CDC42	Cell division control protein 42 homolog (EC 3.6.5.2)	Small GTPase superfamily, Rho family, CDC42 subfamily	Gallus gallus (Chicken)	PF00071;
F1NT40	423711;	SAR1A	Uncharacterized protein	Small GTPase superfamily, SAR1 family	Gallus gallus (Chicken)	PF00025;
Q00649	396227;	HSPB1	Heat shock protein beta-1 (HspB1) (25 kDa IAP) (Actin polymerization inhibitor) (Heat shock 25 kDa protein) (HSP 25) (Heat shock 27 kDa protein) (HSP 27)	Small heat shock protein (HSP20) family	Gallus gallus (Chicken)	PF00011;
P28675	417892;	DCN	Decorin (Bone proteoglycan II) (PG-S2)	Small leucine-rich proteoglycan (SLRP) family, SLRP class I subfamily	Gallus gallus (Chicken)	PF13855;PF01462;
P51890	417891;	LUM LDC	Lumican (Keratan sulfate proteoglycan lumican) (KSPG lumican)	Small leucine-rich proteoglycan (SLRP) family, SLRP class II subfamily	Gallus gallus (Chicken)	PF13516;PF13855;PF01462;
Q9W6H0	374039;	OGN	Mimecan (Osteoglycin)	Small leucine-rich proteoglycan (SLRP) family, SLRP class III subfamily	Gallus gallus (Chicken)	PF13306;PF13855;
Q90988	396156;	SMC2 SCII SMC2L1	Structural maintenance of chromosomes protein 2 (SMC protein 2) (SMC-2) (Chromosome scaffold protein ScII)	SMC family, SMC2 subfamily	Gallus gallus (Chicken)	PF06470;PF02463;
Q8AWB7	395187;	SMC1	Structural maintenance of chromosomes protein	SMC family; SMC family, SMC1 subfamily	Gallus gallus (Chicken)	PF06470;PF02463;
Q8AWB8	395188;	SMC3	Structural maintenance of chromosomes protein	SMC family; SMC family, SMC3 subfamily	Gallus gallus (Chicken)	PF06470;PF02463;
F1NDN4	395189;	SMC4	Structural maintenance of chromosomes protein	SMC family; SMC family, SMC4 subfamily	Gallus gallus (Chicken)	PF06470;PF02463;
E1BWS2	421051;	NAPG	Uncharacterized protein	SNAP family	Gallus gallus (Chicken)	
E1BRL4	423235;	SNAP23	Synaptosomal-associated protein	SNAP-25 family	Gallus gallus (Chicken)	PF00835;
A0A3S5ZP89	422457;	SMARCA5	Uncharacterized protein	SNF2/RAD54 helicase family, ISWI subfamily	Gallus gallus (Chicken)	PF13892;PF09110;PF00271;PF09111;PF00176;
Q5ZL55	428673;	CHMP6 RCJMB04_7k13	Charged multivesicular body protein 6 (Chromatin-modifying protein 6)	SNF7 family	Gallus gallus (Chicken)	PF03357;
Q5ZL58	416947;	SNRPD3 RCJMB04_7j2	Small nuclear ribonucleoprotein Sm D3 (Sm-D3) (snRNP core protein D3)	SnRNP core protein family	Gallus gallus (Chicken)	PF01423;
P62303	396180;	SNRPE	Small nuclear ribonucleoprotein E (snRNP-E) (Sm protein E) (Sm-E) (SmE)	SnRNP Sm proteins family	Gallus gallus (Chicken)	PF01423;
E1C8T5	422466;	LSM6	Sm domain-containing protein	SnRNP Sm proteins family, SmF/LSm6 subfamily	Gallus gallus (Chicken)	PF01423;
Q9PV94	395298;	SNRPB	Small nuclear ribonucleoprotein-associated protein B' (snRNP-B') (snRPB') (Sm protein B') (Sm-B') (SmB')	SnRNP SmB/SmN family	Gallus gallus (Chicken)	PF01423;
Q5F406	426797;	RCJMB04_3o14	Sorting nexin-2	Sorting nexin family	Gallus gallus (Chicken)	PF00787;PF03700;PF09325;
Q5ZK22	771038;	SNX32 RCJMB04_13l2	Sorting nexin	Sorting nexin family	Gallus gallus (Chicken)	PF00787;PF09325;
P36377	386571;	SPARC	SPARC (Basement-membrane protein 40) (BM-40) (Osteonectin) (ON) (Secreted protein acidic and rich in cysteine)	SPARC family	Gallus gallus (Chicken)	PF09289;PF00050;PF10591;
P28687	396234;	SPC22	Signal peptidase complex subunit 3 (EC 3.4.-.-) (Microsomal signal peptidase 22/23 kDa subunit) (SPC22/23) (SPase 22/23 kDa subunit) (gp23)	SPCS3 family	Gallus gallus (Chicken)	PF04573;
P07751	374234;	SPTAN1 SPTA2	Spectrin alpha chain, non-erythrocytic 1 (Alpha-II spectrin) (Fodrin alpha chain)	Spectrin family	Gallus gallus (Chicken)	PF13499;PF08726;PF00018;PF00435;
A0A1D5P797	421216;	SPTBN1	Spectrin beta chain	Spectrin family	Gallus gallus (Chicken)	PF00307;PF15410;PF00435;
A0A1D5PJY1	421216;	SPTBN1	Spectrin beta chain	Spectrin family	Gallus gallus (Chicken)	PF00307;PF15410;PF00435;
A0A1D5NY06	771447;	SRM	PABS domain-containing protein	Spermidine/spermine synthase family	Gallus gallus (Chicken)	PF17284;
E1BTS4	419884;	BCAS2	Pre-mRNA-splicing factor SPF27	SPF27 family	Gallus gallus (Chicken)	PF05700;
Q5ZML3	772264;	SRSF1 SFRS1 RCJMB04_1l5	Serine/arginine-rich splicing factor 1 (Splicing factor, arginine/serine-rich 1)	Splicing factor SR family	Gallus gallus (Chicken)	PF00076;
F1NQW8	772264;	SRSF1	Splicing factor, arginine/serine-rich 1	Splicing factor SR family	Gallus gallus (Chicken)	PF00076;
P30352	396195;	SRSF2 SFRS2	Serine/arginine-rich splicing factor 2 (Protein PR264) (Splicing component, 35 kDa) (Splicing factor SC35) (SC-35) (Splicing factor, arginine/serine-rich 2)	Splicing factor SR family	Gallus gallus (Chicken)	PF00076;
Q5ZI08	426493;	SUPT5H RCJMB04_31j17	Transcription elongation factor SPT5 (DRB sensitivity-inducing factor large subunit) (DSIF large subunit)	SPT5 family	Gallus gallus (Chicken)	PF00467;PF03439;PF11942;
P31395	396057;	STMN1 LAP18	Stathmin	Stathmin family	Gallus gallus (Chicken)	PF00836;

E1BUG3	772063;	STEAP4	Uncharacterized protein	STEAP family	Gallus gallus (Chicken)	PF03807;PF01794;
Q5ZLA7	100857165;	STT3A RCJMB04_6o16	Dolichyl-diphosphooligosaccharide--protein glycotransferase (EC 2.4.99.18)	STT3 family	Gallus gallus (Chicken)	PF02516;
F1NH65	423307;	SCFD1	Uncharacterized protein	STXBP/unc-18/SEC1 family	Gallus gallus (Chicken)	PF00995;
A0A1D5PHU2	404293;	STXBP1	Syntaxin-binding protein 1	STXBP/unc-18/SEC1 family	Gallus gallus (Chicken)	PF00995;
A0A1D5PK28	404293;	STXBP1	Syntaxin-binding protein 1	STXBP/unc-18/SEC1 family	Gallus gallus (Chicken)	PF00995;
A0A1D5PIF0	100859720;	SDHB	Succinate dehydrogenase [ubiquinone] iron-sulfur subunit, mitochondrial (EC 1.3.5.1)	Succinate dehydrogenase/fumarate reductase iron-sulfur protein family	Gallus gallus (Chicken)	PF13085;
A0A1D5PSE5	395373;	ACLY	ATP-citrate synthase (EC 2.3.3.8) (ATP-citrate (pro-S-)-lyase) (Citrate cleavage enzyme)	Succinate/malate CoA ligase alpha subunit family; Succinate/malate CoA ligase beta subunit family	Gallus gallus (Chicken)	PF16114;PF00285;PF02629;PF00549;
Q5F3V2	395373;	RCJMB04_6f14	ATP-citrate synthase (EC 2.3.3.8) (ATP-citrate (pro-S-)-lyase) (Citrate cleavage enzyme)	Succinate/malate CoA ligase alpha subunit family; Succinate/malate CoA ligase beta subunit family	Gallus gallus (Chicken)	PF16114;PF00285;PF02629;PF00549;
Q5F3B9	418857;	SUCLA2 RCJMB04_22i13	Succinate--CoA ligase [ADP-forming] subunit beta, mitochondrial (EC 6.2.1.5) (ATP-specific succinyl-CoA synthetase subunit beta) (A-SCS) (Succinyl-CoA synthetase beta-A chain) (SCS-betaA)	Succinate/malate CoA ligase beta subunit family; Succinate/malate CoA ligase beta subunit family, ATP-specific subunit beta	Gallus gallus (Chicken)	PF08442;PF00549;
Q5ZL37	416087;	SUCLG2 RCJMB04_7p8	Succinate--CoA ligase [GDP-forming] subunit beta, mitochondrial (EC 6.2.1.4) (GTP-specific succinyl-CoA synthetase subunit beta) (G-SCS) (GTPSCS) (Succinyl-CoA synthetase beta-G chain) (SCS-betaG)	Succinate/malate CoA ligase beta subunit family; Succinate/malate CoA ligase beta subunit family, GTP-specific subunit beta	Gallus gallus (Chicken)	PF08442;PF00549;
Q90WR6	395300;	SULT1C SULT1C3	Sulfotransferase (EC 2.8.2.-)	Sulfotransferase 1 family	Gallus gallus (Chicken)	PF00685;
Q8JG30	395227;	SULT1B1 SULT1B	Sulfotransferase family cytosolic 1B member 1 (ST1B1) (Sulfotransferase 1B1) (EC 2.8.2.-)	Sulfotransferase 1 family	Gallus gallus (Chicken)	PF00685;
E1BYE0	415852;	LOC415852	Sulfotransferase (EC 2.8.2.-)	Sulfotransferase 1 family	Gallus gallus (Chicken)	PF00685;
Q800K9	374273;	SURF4 SURF-4	Surfeit locus protein 4	SURF4 family	Gallus gallus (Chicken)	PF02077;
Q5ZM80	419177;	RCJMB04_2o15	Dolichyl-diphosphooligosaccharide--protein glycosyltransferase subunit 2 (Ribophorin-2)	SWP1 family	Gallus gallus (Chicken)	PF05817;
Q5ZJW4	424377;	SEC22B RCJMB04_15c3	Vesicle-trafficking protein SEC22b (SEC22 vesicle-trafficking protein homolog B)	Synaptobrevin family	Gallus gallus (Chicken)	PF13774;PF00957;
Q5ZL74	422297;	VAMP7 SYBL1 RCJMB04_7f19	Vesicle-associated membrane protein 7 (Synaptobrevin-like protein 1)	Synaptobrevin family	Gallus gallus (Chicken)	PF13774;PF00957;
Q5ZM48	100859110;	SYNGR2 RCJMB04_3c18	Synaptogyrin	Synaptogyrin family	Gallus gallus (Chicken)	PF01284;
Q5ZMP2	426930;	STX7 RCJMB04_1i11	t-SNARE coiled-coil homology domain-containing protein	Syntaxin family	Gallus gallus (Chicken)	PF05739;PF14523;
Q5ZI87	417334;	TBCD RCJMB04_29e8	Tubulin-specific chaperone D (Beta-tubulin cofactor D) (Tubulin-folding cofactor D)	TBCD family	Gallus gallus (Chicken)	PF12612;
A0A1D5NTW7	417334;	TBCD	Tubulin-specific chaperone D	TBCD family	Gallus gallus (Chicken)	PF12612;
E1BU18	417334;	TBCD	Tubulin-specific chaperone D	TBCD family	Gallus gallus (Chicken)	PF12612;
Q5F424	417846;	CCT2 RCJMB04_3m3	CCT-beta (T-complex protein 1 subunit beta)	TCP-1 chaperonin family	Gallus gallus (Chicken)	PF00118;
Q9I8D6	395414;	tcp-1 delta CCT4	T-complex protein 1 subunit delta	TCP-1 chaperonin family	Gallus gallus (Chicken)	PF00118;
Q5F411	420930;	CCT5 RCJMB04_3n20	CCT-epsilon (T-complex protein 1 subunit epsilon)	TCP-1 chaperonin family	Gallus gallus (Chicken)	PF00118;
Q5ZJ54	417541;	CCT6 RCJMB04_20k5	T-complex protein 1 subunit zeta (TCP-1-zeta) (CCT-zeta)	TCP-1 chaperonin family	Gallus gallus (Chicken)	PF00118;
F1NK38	428806;	CCT7	T-complex protein 1 subunit eta (TCP-1-eta) (CCT-eta)	TCP-1 chaperonin family	Gallus gallus (Chicken)	PF00118;
Q6EE31	418486;	CCT8	T-complex protein 1 subunit theta (TCP-1-theta) (CCT-theta)	TCP-1 chaperonin family	Gallus gallus (Chicken)	PF00118;
A0A1D5PZJ4	418486;	CCT8	CCT-theta (T-complex protein 1 subunit theta)	TCP-1 chaperonin family	Gallus gallus (Chicken)	PF00118;
Q5ZMG9	421586;	TCP1 RCJMB04_2b5	CCT-alpha (T-complex protein 1 subunit alpha)	TCP-1 chaperonin family	Gallus gallus (Chicken)	PF00118;
P43347	396363;	TPT1	Translationally-controlled tumor protein homolog (TCTP) (p23) (pCHK23)	TCTP family	Gallus gallus (Chicken)	PF00838;
A0A1D5PDR0	424037;	LSS	Terpene cyclase/mutase family member (EC 5.4.99.-)	Terpene cyclase/mutase family	Gallus gallus (Chicken)	PF13243;PF13249;
Q9IBC9	395527;	CD9	Tetraspanin	Tetraspanin (TM4SF) family	Gallus gallus (Chicken)	PF00335;
Q5F3G3	374256;	RCJMB04_17m6	Tetraspanin	Tetraspanin (TM4SF) family	Gallus gallus (Chicken)	PF00335;

Q5ZII8	423105;	CD151 RCJMB04_25m21	Tetraspanin	Tetraspanin (TM4SF) family	Gallus gallus (Chicken)	PF00335;
E1C3Y3	417854;	TSPAN8	Tetraspanin	Tetraspanin (TM4SF) family	Gallus gallus (Chicken)	PF00335;
A0A3Q2UIT0	418345;	UPK1B	Tetraspanin	Tetraspanin (TM4SF) family	Gallus gallus (Chicken)	PF00335;
Q5ZHQ8	421119;	TCEA1 RCJMB04_34f23	Uncharacterized protein	TFS-II family	Gallus gallus (Chicken)	PF08711;PF01096;PF07500;
A0A1L1RWX6	421006;	ACOT13	4HBT domain-containing protein	Thioesterase PaaI family	Gallus gallus (Chicken)	PF03061;
E1C0Q5	418968;	ACAT1	Uncharacterized protein	Thiolase-like superfamily, Thiolase family	Gallus gallus (Chicken)	PF02803;PF00108;
F1NT20	421587;	ACAT2	Uncharacterized protein	Thiolase-like superfamily, Thiolase family	Gallus gallus (Chicken)	PF02803;PF00108;
F1NB64	770094;	ACAA1	Uncharacterized protein	Thiolase-like superfamily, Thiolase family	Gallus gallus (Chicken)	PF02803;PF00108;
Q5ZLW8	426847;	RCJMB04_4j7	Uncharacterized protein	Thiolase-like superfamily, Thiolase family	Gallus gallus (Chicken)	PF02803;PF00108;
Q07598	396550;	SCP2	Sterol carrier protein 2 (SCP-2) (Acetyl-CoA C-myristoyltransferase) (EC 2.3.1.155) (Non-specific lipid-transfer protein) (NSL-TP) (Propanoyl-CoA C-acyltransferase) (EC 2.3.1.176) (SCP-2/3-oxoacyl-CoA thiolase) (SCP-2/thiolase) (EC 2.3.1.16) (SCP-chi) (SCPX) (Sterol carrier protein X) (SCP-X) (Fragment)	Thiolase-like superfamily, Thiolase family	Gallus gallus (Chicken)	PF02036;PF02803;PF00108;
P08629	396437;	TXN	Thioredoxin (Trx)	Thioredoxin family	Gallus gallus (Chicken)	PF00085;
F1N915	416077;	THOC7	THO complex subunit 7 homolog	THOC7 family	Gallus gallus (Chicken)	PF05615;
A0A1L1RIN1	427100;	TMCO1	Calcium load-activated calcium channel (CLAC channel)	TMCO1 family	Gallus gallus (Chicken)	PF01956;
Q5ZLD4	427663;	TMEM11 RCJMB04_6k9	Transmembrane protein 11, mitochondrial	TMEM11 family	Gallus gallus (Chicken)	PF14972;
Q5ZHP4	770339;	TPD52 RCJMB04_34n23	Uncharacterized protein	TPD52 family	Gallus gallus (Chicken)	PF04201;
Q5ZI93	419257;	TPD52L2 RCJMB04_29b18	Uncharacterized protein	TPD52 family	Gallus gallus (Chicken)	PF04201;
A0A3Q2U7G4	417217;	DNM1	Dynamin GTPase (EC 3.6.5.5)	TRAFAC class dynamin-like GTPase superfamily, Dynamin/Fzo/YdjA family	Gallus gallus (Chicken)	PF01031;PF00350;PF02212;PF00169;
E1BXY4	417217;	DNM1	Dynamin GTPase (EC 3.6.5.5)	TRAFAC class dynamin-like GTPase superfamily, Dynamin/Fzo/YdjA family	Gallus gallus (Chicken)	PF01031;PF00350;PF02212;PF00169;
A0A3Q2UHM4	418132;	DNM1L	Dynamin-1-like protein (EC 3.6.5.5)	TRAFAC class dynamin-like GTPase superfamily, Dynamin/Fzo/YdjA family	Gallus gallus (Chicken)	PF01031;PF00350;PF02212;
A0A3Q3ACH0	418132;	DNM1L	Dynamin-1-like protein (EC 3.6.5.5)	TRAFAC class dynamin-like GTPase superfamily, Dynamin/Fzo/YdjA family	Gallus gallus (Chicken)	PF01031;PF00350;PF02212;
Q5F469	418132;	RCJMB04_2k14	Dynamin-1-like protein (EC 3.6.5.5)	TRAFAC class dynamin-like GTPase superfamily, Dynamin/Fzo/YdjA family	Gallus gallus (Chicken)	PF01031;PF00350;PF02212;
A0A1L1RNI5	423577;	ATL1	GB1/RHD3-type G domain-containing protein	TRAFAC class dynamin-like GTPase superfamily, GB1/RHD3 GTPase family	Gallus gallus (Chicken)	PF02263;
F1NAM6	423577;	ATL1	GB1/RHD3-type G domain-containing protein	TRAFAC class dynamin-like GTPase superfamily, GB1/RHD3 GTPase family	Gallus gallus (Chicken)	PF02263;
E1C3A1	420472;	KIF5B	Kinesin-like protein	TRAFAC class myosin-kinesin ATPase superfamily, Kinesin family	Gallus gallus (Chicken)	PF00225;
Q5ZLA6	417555;	MYO1C RCJMB04_6o17	Unconventional myosin-Ic (Myosin I beta) (MMI-beta) (MMIb)	TRAFAC class myosin-kinesin ATPase superfamily, Myosin family	Gallus gallus (Chicken)	PF00063;PF06017;
A0A1D5NWY3	417555;	MYO1C	Unconventional myosin-Ic	TRAFAC class myosin-kinesin ATPase superfamily, Myosin family	Gallus gallus (Chicken)	PF00063;PF06017;
F1NG39	417555;	MYO1C	Unconventional myosin-Ic	TRAFAC class myosin-kinesin ATPase superfamily, Myosin family	Gallus gallus (Chicken)	PF00063;PF06017;
A0A3Q2TZ02	419963;	MYO1D	Uncharacterized protein	TRAFAC class myosin-kinesin ATPase superfamily, Myosin family	Gallus gallus (Chicken)	PF00063;PF06017;
A0A3Q2TZW8	419963;	MYO1D	Uncharacterized protein	TRAFAC class myosin-kinesin ATPase superfamily, Myosin family	Gallus gallus (Chicken)	PF00063;PF06017;
E1C459	419963;	MYO1D	Uncharacterized protein	TRAFAC class myosin-kinesin ATPase superfamily, Myosin family	Gallus gallus (Chicken)	PF00063;PF06017;
Q9I8D1	395487;	MYO6 CMY6	Unconventional myosin-VI (Unconventional myosin-6)	TRAFAC class myosin-kinesin ATPase superfamily, Myosin family	Gallus gallus (Chicken)	PF16521;PF00063;
A0A3Q2UDG5	417581;	MYO18A	Uncharacterized protein	TRAFAC class myosin-kinesin ATPase superfamily, Myosin family	Gallus gallus (Chicken)	PF00612;PF00063;PF01576;PF00595;
P14105	396469;	MYH9	Myosin-9 (Cellular myosin heavy chain, type A) (Myosin heavy chain 9) (Myosin heavy chain, non-muscle IIa) (Non-muscle myosin heavy chain A) (NMMHC-A) (Non-muscle myosin heavy chain IIa) (NMMHC II-a) (NMMHC-IIA)	TRAFAC class myosin-kinesin ATPase superfamily, Myosin family	Gallus gallus (Chicken)	PF00063;PF02736;PF01576;
Q02015	396465;		Nonmuscle myosin heavy chain	TRAFAC class myosin-kinesin ATPase superfamily, Myosin family	Gallus gallus (Chicken)	PF00612;PF00063;PF02736;PF01576;

Q789A4	396465;	MYH10	Nonmuscle myosin heavy chain	TRAFAC class myosin-kinesin ATPase superfamily, Myosin family	Gallus gallus (Chicken)	PF00612;PF00063;PF02736;PF01576;
Q789A5	396465;		Nonmuscle myosin heavy chain	TRAFAC class myosin-kinesin ATPase superfamily, Myosin family	Gallus gallus (Chicken)	PF00612;PF00063;PF02736;PF01576;
Q789A6	396465;	MYH10	Nonmuscle myosin heavy chain	TRAFAC class myosin-kinesin ATPase superfamily, Myosin family	Gallus gallus (Chicken)	PF00612;PF00063;PF02736;PF01576;
R4GIG1	417306;	MYH1D	Uncharacterized protein	TRAFAC class myosin-kinesin ATPase superfamily, Myosin family	Gallus gallus (Chicken)	PF00063;PF02736;PF01576;
A0A1D5P603	768566;	MYH1F	Uncharacterized protein	TRAFAC class myosin-kinesin ATPase superfamily, Myosin family	Gallus gallus (Chicken)	PF00063;PF02736;PF01576;
Q9PTY2	768566;		Skeletal myosin heavy chain	TRAFAC class myosin-kinesin ATPase superfamily, Myosin family	Gallus gallus (Chicken)	PF00063;PF02736;PF01576;
Q5ZM25	424144;	OLA1 RCJMB04_3f20	Obg-like ATPase 1	TRAFAC class OBG-HflX-like GTPase superfamily, OBG GTPase family, YchF/OLA1 subfamily	Gallus gallus (Chicken)	PF01926;PF06071;
Q5F3X4	428281;	EFTUD2 SNRP116 RCJMB04_4m11	116 kDa U5 small nuclear ribonucleoprotein component (Elongation factor Tu GTP-binding domain protein 2) (U5 snRNP-specific protein, 116 kDa) (U5-116 kDa)	TRAFAC class translation factor GTPase superfamily, Classic translation factor GTPase family, EF-G/EF-2 subfamily	Gallus gallus (Chicken)	PF00679;PF14492;PF03764;PF16004;PF00009;PF03144;
Q90705	396325;	EEF2	Elongation factor 2 (EF-2)	TRAFAC class translation factor GTPase superfamily, Classic translation factor GTPase family, EF-G/EF-2 subfamily	Gallus gallus (Chicken)	PF00679;PF14492;PF03764;PF00009;PF03144;
Q90835	373963;	EEF1A	Elongation factor 1-alpha 1 (EF-1-alpha-1) (Elongation factor Tu) (EF-Tu)	TRAFAC class translation factor GTPase superfamily, Classic translation factor GTPase family, EF-Tu/EF-1A subfamily	Gallus gallus (Chicken)	PF00009;PF03144;PF03143;
Q5ZMS3	418597;	EIF2S3 RCJMB04_1f8	Eukaryotic translation initiation factor 2 subunit 3 (EC 3.6.5.3) (Eukaryotic translation initiation factor 2 subunit gamma) (eIF-2-gamma)	TRAFAC class translation factor GTPase superfamily, Classic translation factor GTPase family, EIF2G subfamily	Gallus gallus (Chicken)	PF09173;PF00009;PF03144;
E1C3D2	424843;	SEPT2	Septin	TRAFAC class TrmE-Era-EngA-EngB-Septin-like GTPase superfamily, Septin GTPase family	Gallus gallus (Chicken)	PF00735;
Q5ZMH1	416777;	SEPTIN2 SEPT2 RCJMB04_2a21	Septin-2	TRAFAC class TrmE-Era-EngA-EngB-Septin-like GTPase superfamily, Septin GTPase family	Gallus gallus (Chicken)	PF00735;
Q5ZM42	422369;	RCJMB04_3d14	Septin	TRAFAC class TrmE-Era-EngA-EngB-Septin-like GTPase superfamily, Septin GTPase family	Gallus gallus (Chicken)	PF00735;
Q5F3T3	420741;	RCJMB04_7k16	Septin	TRAFAC class TrmE-Era-EngA-EngB-Septin-like GTPase superfamily, Septin GTPase family	Gallus gallus (Chicken)	PF00735;
A0A3Q2U387	416333;	SEPTIN8	Septin	TRAFAC class TrmE-Era-EngA-EngB-Septin-like GTPase superfamily, Septin GTPase family	Gallus gallus (Chicken)	PF00735;
Q5F3T2	417347;	SEPTIN9 RCJMB04_7k22	Septin-type G domain-containing protein	TRAFAC class TrmE-Era-EngA-EngB-Septin-like GTPase superfamily, Septin GTPase family	Gallus gallus (Chicken)	PF00735;
A0A1D5PJI5	422635;	SEPTIN11	Septin	TRAFAC class TrmE-Era-EngA-EngB-Septin-like GTPase superfamily, Septin GTPase family	Gallus gallus (Chicken)	PF00735;
F1P0X3	422635;	SEPTIN11	Septin	TRAFAC class TrmE-Era-EngA-EngB-Septin-like GTPase superfamily, Septin GTPase family	Gallus gallus (Chicken)	PF00735;
Q8AXQ0	378918;	TRAM	Translocating chain-associated membrane protein	TRAM family	Gallus gallus (Chicken)	PF08390;PF03798;
Q5ZKN8	423019;	RCJMB04_9n21	Transaldolase (EC 2.2.1.2)	Transaldolase family, Type 1 subfamily	Gallus gallus (Chicken)	PF00923;
Q6DV79	420027;	STAT3 RCJMB04_38l20	Signal transducer and activator of transcription 3	Transcription factor STAT family	Gallus gallus (Chicken)	PF00017;PF01017;PF02864;PF02865;
A0A1D5P521	107055444;	LOC107055444	Activated RNA polymerase II transcriptional coactivator p15 (SUB1 homolog)	Transcriptional coactivator PC4 family	Gallus gallus (Chicken)	PF02229;
F1NYN9	769292;	GMPPA	NTP_transferase domain-containing protein	Transferase hexapeptide repeat family	Gallus gallus (Chicken)	PF00132;PF00483;
F1P574	415924;	GMPPB	NTP_transferase domain-containing protein	Transferase hexapeptide repeat family	Gallus gallus (Chicken)	PF00132;PF00483;
P02789	396241;		Ovotransferrin (Allergen Gal d III) (Conalbumin) (Serum transferrin) (allergen Gal d 3)	Transferrin family	Gallus gallus (Chicken)	PF00405;
Q9DER6	395420;		Coagulation factor XIIIA	Transglutaminase superfamily, Transglutaminase family	Gallus gallus (Chicken)	PF00927;PF01841;PF00868;
E1BQZ4	419216;	EPB42	TGc domain-containing protein	Transglutaminase superfamily, Transglutaminase family	Gallus gallus (Chicken)	PF00927;PF01841;PF00868;
Q01841	396432;	TGM2	Protein-glutamine gamma-glutamyltransferase 2 (EC 2.3.2.13) (Erythrocyte transglutaminase) (Isopeptidase TGM2) (EC 3.4.-.-) (Protein-glutamine deamidase TGM2) (EC 3.5.1.44) (Protein-glutamine dopaminyltransferase TGM2) (EC 2.3.1.-) (Protein-glutamine histaminyltransferase TGM2) (EC 2.3.1.-) (Protein-glutamine noradrenalinyltransferase TGM2) (EC 2.3.1.-) (Protein-glutamine serotonyltransferase TGM2) (EC 2.3.1.-) (Tissue	Transglutaminase superfamily, Transglutaminase family	Gallus gallus (Chicken)	PF00927;PF01841;PF00868;
Q5ZK99	420706;	RCJMB04_12c19	TGc domain-containing protein	Transglutaminase superfamily, Transglutaminase family	Gallus gallus (Chicken)	PF00927;PF01841;PF00868;
P79769	395955;	TSN	Translin (EC 3.1.-.-) (Component 3 of promoter of RISC) (C3PO)	Translin family	Gallus gallus (Chicken)	PF01997;
E1BYF1	424716;	CTH	Cystathionine gamma-lyase (EC 4.4.1.1) (Cysteine-protein sulfhydrase) (Gamma-cystathionase)	Trans-sulfuration enzymes family	Gallus gallus (Chicken)	PF01053;
P27731	396277;	TTR	Transthyretin (Prealbumin) (TBPA)	Transthyretin family	Gallus gallus (Chicken)	PF00576;
A0A1I7Q422	396277;	TTR	Transthyretin	Transthyretin family	Gallus gallus (Chicken)	PF00576;

Q5ZM63	420871;	RCJMB04_2p21	Signal sequence receptor subunit alpha (Translocon-associated protein subunit alpha)	TRAP-alpha family	Gallus gallus (Chicken)	PF03896;
Q5ZHS6	419788;	RCJMB04_33k2	Trafficking protein particle complex subunit	TRAPP small subunits family	Gallus gallus (Chicken)	PF04099;
Q5ZI57	419625;	TRAPPC3 RCJMB04_30c23	Trafficking protein particle complex subunit 3	TRAPP small subunits family, BET3 subfamily	Gallus gallus (Chicken)	PF04051;
Q5F359	100858483;	TRAPPC5 RCJMB04_33f11	Trafficking protein particle complex subunit 5	TRAPP small subunits family, BET3 subfamily	Gallus gallus (Chicken)	PF04051;
Q5ZI89	428748;	TRAPPC11 RCJMB04_29e4	Trafficking protein particle complex subunit 11	TRAPPC11 family	Gallus gallus (Chicken)	PF11817;PF12742;
P00940	396435;	TPI1	Triosephosphate isomerase (TIM) (EC 5.3.1.1) (Methylglyoxal synthase) (EC 4.2.3.3) (Triose-phosphate isomerase)	Triosephosphate isomerase family	Gallus gallus (Chicken)	PF00121;
A0A1D5P668	416103;	TRNT1	Uncharacterized protein	TRNA nucleotidyltransferase/poly(A) polymerase family	Gallus gallus (Chicken)	PF01743;PF12627;
P04268	396366;	TPM1	Tropomyosin alpha-1 chain (Alpha-tropomyosin) (Tropomyosin-1)	Tropomyosin family	Gallus gallus (Chicken)	PF00261;
A0A1D5P342	396366;	TPM1	Tropomyosin alpha-1 chain	Tropomyosin family	Gallus gallus (Chicken)	PF00261;
A0A1L1RU52	396366;	TPM1	Tropomyosin alpha-1 chain	Tropomyosin family	Gallus gallus (Chicken)	PF00261;
A0A452J839	396366;	TPM1	Tropomyosin alpha-1 chain	Tropomyosin family	Gallus gallus (Chicken)	PF00261;
Q8AWI4	396366;		Alpha-tropomyosin 2	Tropomyosin family	Gallus gallus (Chicken)	PF00261;
Q91005	396366;	alpha-fTM	Alpha tropomyosin of brain	Tropomyosin family	Gallus gallus (Chicken)	PF00261;
P19352	396430;	TPM2	Tropomyosin beta chain (Beta-tropomyosin) (Tropomyosin-2)	Tropomyosin family	Gallus gallus (Chicken)	PF00261;
Q05705	396430;	BRT-2	Beta-tropomyosin	Tropomyosin family	Gallus gallus (Chicken)	PF00261;
Q05706	396430;	BRT-1 TPM2	Beta-tropomyosin (Tropomyosin beta chain)	Tropomyosin family	Gallus gallus (Chicken)	PF00261;
H9L074	770103;	TPM3	Uncharacterized protein	Tropomyosin family	Gallus gallus (Chicken)	PF00261;
H9L3K0	770103;	TPM3	Tropomyosin 3 alpha isoform	Tropomyosin family	Gallus gallus (Chicken)	PF00261;
Q5ZLJ7	770103;	TPM3 RCJMB04_5n23	Uncharacterized protein	Tropomyosin family	Gallus gallus (Chicken)	PF00261;
F1NK75	107055375;	TPM4	Uncharacterized protein	Tropomyosin family	Gallus gallus (Chicken)	PF00261;
F1NZA2	418226;	TSPO	Uncharacterized protein	TspO/BZRP family	Gallus gallus (Chicken)	PF03073;
F1NNL8	419914;	TSPO2	Uncharacterized protein	TspO/BZRP family	Gallus gallus (Chicken)	PF03073;
F1P5B0	416694;	TUBAL3	Tubulin alpha chain	Tubulin family	Gallus gallus (Chicken)	PF00091;PF03953;
P09207	396427;		Tubulin beta-6 chain (Beta-tubulin class-VI)	Tubulin family	Gallus gallus (Chicken)	PF00091;PF03953;
P32882	420883;		Tubulin beta-2 chain (Beta-tubulin class-II)	Tubulin family	Gallus gallus (Chicken)	PF00091;PF03953;
P09652	431043;		Tubulin beta-4 chain (Beta-tubulin class-III)	Tubulin family	Gallus gallus (Chicken)	PF00091;PF03953;
F1NYB1	417255;	TUBB4B	Tubulin beta chain	Tubulin family	Gallus gallus (Chicken)	PF00091;PF03953;
P09653	421037;		Tubulin beta-5 chain (Beta-tubulin class-V)	Tubulin family	Gallus gallus (Chicken)	PF00091;PF03953;
P09244	396254;		Tubulin beta-7 chain (Tubulin beta 4')	Tubulin family	Gallus gallus (Chicken)	PF00091;PF03953;
A0A1D5PAR5	421169;	TUBA3E	Tubulin alpha chain	Tubulin family	Gallus gallus (Chicken)	PF00091;PF03953;
P08070	396426;		Tubulin alpha-2 chain (Testis-specific)	Tubulin family	Gallus gallus (Chicken)	PF00091;PF03953;
P79994	396004;		DNA topoisomerase I (EC 5.6.2.1) (DNA topoisomerase 1)	Type IB topoisomerase family	Gallus gallus (Chicken)	PF14370;PF01028;PF02919;
O42131	395966;	TOP2B	DNA topoisomerase 2-beta (EC 5.6.2.2) (DNA topoisomerase II, beta isozyme)	Type II topoisomerase family	Gallus gallus (Chicken)	PF00204;PF00521;PF08070;PF02518;PF01751;PF16898;
A0A3Q2U225	395966;	TOP2B	DNA topoisomerase 2 (EC 5.6.2.2)	Type II topoisomerase family	Gallus gallus (Chicken)	PF00204;PF00521;PF08070;PF02518;PF01751;PF16898;

E1BZ19	395966;	TOP2B	DNA topoisomerase 2 (EC 5.6.2.2)	Type II topoisomerase family	Gallus gallus (Chicken)	PF00204;PF00521;PF08070;PF02518;PF01751;PF16898;
P20785	396000;	COL6A1	Collagen alpha-1(VI) chain	Type VI collagen family	Gallus gallus (Chicken)	PF01391;PF00092;
P15988	396292;	COL6A2	Collagen alpha-2(VI) chain	Type VI collagen family	Gallus gallus (Chicken)	PF01391;PF00092;
Q5ZKP1	415523;	RCJMB04_9n8	LRRcap domain-containing protein	U2 small nuclear ribonucleoprotein A family	Gallus gallus (Chicken)	
Q8QGH2	373930;	SUMO1 RCJMB04_2j18	Small ubiquitin-related modifier 1 (SUMO-1)	Ubiquitin family, SUMO subfamily	Gallus gallus (Chicken)	PF11976;
P79781	395796;	RPS27A UBA80	Ubiquitin-40S ribosomal protein S27a (Ubiquitin carboxyl extension protein 80) [Cleaved into: Ubiquitin; 40S ribosomal protein S27a]	Ubiquitin family; Eukaryotic ribosomal protein eS31 family	Gallus gallus (Chicken)	PF01599;PF00240;
A0A3Q2U7Y4	101750090;	SAE1	ThiF domain-containing protein	Ubiquitin-activating E1 family	Gallus gallus (Chicken)	PF00899;
F1P226	415784;	UBA2	SUMO-activating enzyme subunit 2 (EC 2.3.2.-)	Ubiquitin-activating E1 family	Gallus gallus (Chicken)	PF00899;PF14732;PF16195;
Q5ZKH7	415784;	RCJMB04_10l24	ThiF domain-containing protein	Ubiquitin-activating E1 family	Gallus gallus (Chicken)	PF00899;
E1BT61	426073;	UBA3	NEDD8-activating enzyme E1 catalytic subunit (EC 6.2.1.64)	Ubiquitin-activating E1 family, UBA3 subfamily	Gallus gallus (Chicken)	PF08825;PF00899;
Q5ZLI7	416678;	UBE2H RCJMB04_5p24	UBC core domain-containing protein	Ubiquitin-conjugating enzyme family	Gallus gallus (Chicken)	PF00179;
P63283	374123;	UBE2I UBC9 UBCE9	SUMO-conjugating enzyme UBC9 (EC 2.3.2.-) (RING-type E3 SUMO transferase UBC9) (SUMO-protein ligase) (Ubiquitin carrier protein 9) (Ubiquitin carrier protein I) (Ubiquitin-conjugating enzyme E2 I) (Ubiquitin-protein ligase I)	Ubiquitin-conjugating enzyme family	Gallus gallus (Chicken)	PF00179;
Q5ZIL3	428787;	UBE2K RCJMB04_25e17	E2 ubiquitin-conjugating enzyme (EC 2.3.2.23)	Ubiquitin-conjugating enzyme family	Gallus gallus (Chicken)	PF00627;PF00179;
Q5ZKN7	416769;	RCJMB04_9n22	UBC core domain-containing protein	Ubiquitin-conjugating enzyme family	Gallus gallus (Chicken)	PF00179;
Q5F405	417898;	RCJMB04_3o20	UBC core domain-containing protein	Ubiquitin-conjugating enzyme family	Gallus gallus (Chicken)	PF00179;
Q90879	100538349;	UBE2V1	Ubiquitin-conjugating enzyme E2 variant 1 (UEV-1) (CROC-1B)	Ubiquitin-conjugating enzyme family	Gallus gallus (Chicken)	PF00179;
Q5F3Z3	421109;	UBE2V2 RCJMB04_4b12	Ubiquitin-conjugating enzyme E2 variant 2	Ubiquitin-conjugating enzyme family	Gallus gallus (Chicken)	PF00179;
Q5F3T9	422792;	UGDH RCJMB04_7d7	UDP-glucose 6-dehydrogenase (UDP-Glc dehydrogenase) (UDP-GlcDH) (UDPGDH) (EC 1.1.1.22)	UDP-glucose/GDP-mannose dehydrogenase family	Gallus gallus (Chicken)	PF00984;PF03720;PF03721;
A0A1D5P0A1	373900;	UGP2	UTP--glucose-1-phosphate uridylyltransferase (EC 2.7.7.9)	UDPGP type 1 family	Gallus gallus (Chicken)	PF01704;
A0A1L1RP39	373900;	UGP2	UTP--glucose-1-phosphate uridylyltransferase (EC 2.7.7.9)	UDPGP type 1 family	Gallus gallus (Chicken)	PF01704;
Q5ZKW4	373900;	RCJMB04_8o6	UTP--glucose-1-phosphate uridylyltransferase (EC 2.7.7.9)	UDPGP type 1 family	Gallus gallus (Chicken)	PF01704;
Q98UC3	374186;	Ufd1l UFD1L	Ubiquitin fusion-degradation 1-like protein	UFD1 family	Gallus gallus (Chicken)	PF03152;
F6SU35	419895;	RPL10A	Ribosomal protein	Universal ribosomal protein uL1 family	Gallus gallus (Chicken)	PF00687;
P47826	395835;	RPLP0	60S acidic ribosomal protein P0 (60S ribosomal protein L10E)	Universal ribosomal protein uL10 family	Gallus gallus (Chicken)	PF00466;PF17777;
E1BTG1	417264;	RPL12	60S ribosomal protein L12	Universal ribosomal protein uL11 family	Gallus gallus (Chicken)	PF00298;PF03946;
Q7ZUR9	560828;	rpl13a	60S ribosomal protein L13a	Universal ribosomal protein uL13 family	Danio rerio (Zebrafish) (Brachydanio rerio)	PF00572;
E1BY89	420001;	RPL23	60S ribosomal protein L23	Universal ribosomal protein uL14 family	Gallus gallus (Chicken)	PF00238;
F1NBX4	770018;	RPL27A	60S ribosomal protein L27a	Universal ribosomal protein uL15 family	Gallus gallus (Chicken)	PF00828;
A0A1D5PR40	107049046;426845;	LOC107049046 RPL17	60S ribosomal protein L17	Universal ribosomal protein uL22 family	Gallus gallus (Chicken)	PF00237;
E1BS06	417574;	RPL23A	Ribosomal_L23eN domain-containing protein	Universal ribosomal protein uL23 family	Gallus gallus (Chicken)	PF00276;PF03939;
F2Z4K6	396400;	RPL26L1	KOW domain-containing protein	Universal ribosomal protein uL24 family	Gallus gallus (Chicken)	PF00467;PF16906;
Q5ZJZ2	418016;	RPL3 RCJMB04_14e22	Uncharacterized protein	Universal ribosomal protein uL3 family	Gallus gallus (Chicken)	PF00297;
Q5ZJ56	420182;	RPL7 RCJMB04_20k1	60S ribosomal protein L7	Universal ribosomal protein uL30 family	Gallus gallus (Chicken)	PF00327;PF08079;
Q5ZII1	415551;	RCJMB04_25p23	Ribos_L4_asso_C domain-containing protein	Universal ribosomal protein uL4 family	Gallus gallus (Chicken)	PF14374;PF00573;

A0A1D5P3B1	419682;	RPL11	60S ribosomal protein L11	Universal ribosomal protein uL5 family	Gallus gallus (Chicken)	PF00281;PF00673;
A0A1D5NVI1	425468;	RPL9	60S ribosomal protein L9	Universal ribosomal protein uL6 family	Gallus gallus (Chicken)	PF00347;
F1NH93	430990;	RPS20	40S ribosomal protein S20	Universal ribosomal protein uS10 family	Gallus gallus (Chicken)	PF00338;
Q5ZHW8	416275;	RPS14 RCJMB04_32i2	Uncharacterized protein	Universal ribosomal protein uS11 family	Gallus gallus (Chicken)	PF00411;
F1NDC2	427323;	RPS23	40S ribosomal protein S23	Universal ribosomal protein uS12 family	Gallus gallus (Chicken)	PF00164;
Q6ITC7	414782;	RPS13	40S ribosomal protein S13	Universal ribosomal protein uS15 family	Gallus gallus (Chicken)	PF08069;PF00312;
Q98TH5	419049;	cRPS11 RPS11	40S ribosomal protein S11	Universal ribosomal protein uS17 family	Gallus gallus (Chicken)	PF00366;PF16205;
P50890	395181;	RPSA LAMR1	40S ribosomal protein SA (37 kDa laminin receptor precursor) (37LRP) (37/67 kDa laminin receptor) (LRP/LR) (67 kDa laminin receptor) (67LR) (Laminin receptor 1) (LamR) (Laminin-binding protein precursor p40) (LBP/p40)	Universal ribosomal protein uS2 family	Gallus gallus (Chicken)	PF16122;PF00318;
Q5ZJC1	419069;	RCJMB04_19g1	DNA-(apurinic or apyrimidinic site) lyase (EC 4.2.99.18)	Universal ribosomal protein uS3 family	Gallus gallus (Chicken)	PF07650;PF00189;
E1C4M0	416544;	RPS2	40S ribosomal protein S2	Universal ribosomal protein uS5 family	Gallus gallus (Chicken)	PF00333;PF03719;
A0A3Q3A7P6	427675;	RPS15A	40S ribosomal protein S15a	Universal ribosomal protein uS8 family	Gallus gallus (Chicken)	PF00410;
R4GGJ0	417871;	RPS16	Uncharacterized protein	Universal ribosomal protein uS9 family	Gallus gallus (Chicken)	PF00380;
Q5ZIJ8	422327;	UPRT RCJMB04_25k2	Uracil phosphoribosyltransferase homolog	UPRTase family	Gallus gallus (Chicken)	
E1BUH1	426223;	UROS	Hydroxymethylbilane hydrolyase [cyclizing] (EC 4.2.1.75) (Uroporphyrinogen-III cosynthase)	Uroporphyrinogen-III synthase family	Gallus gallus (Chicken)	PF02602;
A0A1D5PCU0	418104;	ATP6V0A4	V-type proton ATPase subunit a	V-ATPase 116 kDa subunit family	Gallus gallus (Chicken)	PF01496;
A0A1D5PAJ8	421939;	ATP6V1C2	V-type proton ATPase subunit C	V-ATPase C subunit family	Gallus gallus (Chicken)	PF03223;
A0A1L1S0D5	423280;	ATP6V1D	V-type proton ATPase subunit D (V-type proton ATPase subunit d) (Vacuolar proton pump subunit D)	V-ATPase D subunit family	Gallus gallus (Chicken)	PF01813;
Q5ZKJ9	418162;	ATP6V1E1 RCJMB04_10e23	Uncharacterized protein	V-ATPase E subunit family	Gallus gallus (Chicken)	PF01991;
E1C3C6	417249;	ATP6V1G1	V-type proton ATPase subunit G	V-ATPase G subunit family	Gallus gallus (Chicken)	PF03179;
F1NW50	426199;	ATP6V1H	V-type proton ATPase subunit H	V-ATPase H subunit family	Gallus gallus (Chicken)	PF11698;PF03224;
E1BVF8	415674;	ATP6V0D1	V-type proton ATPase subunit	V-ATPase V0D/AC39 subunit family	Gallus gallus (Chicken)	PF01992;
A0A1D5PRZ7	422575;	USO1	General vesicular transport factor p115	VDP/USO1/EDE1 family	Gallus gallus (Chicken)	PF18770;PF04871;PF04869;
Q5ZMS9	422575;	RCJMB04_1e12	General vesicular transport factor p115	VDP/USO1/EDE1 family	Gallus gallus (Chicken)	PF18770;PF04871;PF04869;
Q5ZM57	415539;	HACD3 PTPLAD1 RCJMB04_3b6	Very-long-chain (3R)-3-hydroxyacyl-CoA dehydratase (EC 4.2.1.134) (3-hydroxyacyl-CoA dehydratase) (HACD) (Protein-tyrosine phosphatase-like A domain-containing protein 1)	Very long-chain fatty acids dehydratase HACD family	Gallus gallus (Chicken)	PF04387;
O93510	395774;	GSN	Gelsolin (Actin-depolymerizing factor) (ADF) (Brevin) (Homogenin)	Villin/gelsolin family	Gallus gallus (Chicken)	PF00626;
A0A1D5PH32	395774;	GSN	Actin-depolymerizing factor (Brevin) (Gelsolin)	Villin/gelsolin family	Gallus gallus (Chicken)	PF00626;
A0A1L1RYW9	395774;	GSN	Actin-depolymerizing factor (Brevin) (Gelsolin)	Villin/gelsolin family	Gallus gallus (Chicken)	PF00626;
Q5ZIV9	420588;	SCIN RCJMB04_23d8	Adseverin (Scinderin)	Villin/gelsolin family	Gallus gallus (Chicken)	PF00626;
A0A3Q2U6D7	420475;	SVIL	HP domain-containing protein	Villin/gelsolin family	Gallus gallus (Chicken)	PF00626;PF02209;
A0A3Q3AIZ0	420475;	SVIL	HP domain-containing protein	Villin/gelsolin family	Gallus gallus (Chicken)	PF00626;PF02209;
E1C5U6	420415;	VILL	Villin-1	Villin/gelsolin family	Gallus gallus (Chicken)	PF00626;PF02209;
E1C7M7	416184;	CTNNA1	Uncharacterized protein	Vinculin/alpha-catenin family	Gallus gallus (Chicken)	PF01044;
P12003	396422;	VCL VINC1	Vinculin (Metavinculin)	Vinculin/alpha-catenin family	Gallus gallus (Chicken)	PF01044;
P41366	418974;	VMO1	Vitelline membrane outer layer protein 1 (VMO-1) (VMO-I) (VMOI)	VMO1 family	Gallus gallus (Chicken)	PF03762;

A0A1D5PJI9	427050;	VPS13A	Uncharacterized protein	VPS13 family	Gallus gallus (Chicken)	PF09333;PF12624;PF06650;PF16908;PF16909;PF16910;
A0A1D5NW43	420021;	VPS25	ESCRT-II complex subunit VPS25 (Vacuolar protein-sorting-associated protein 25)	VPS25 family	Gallus gallus (Chicken)	PF05871;
A0A1L1RM52	423696;	VPS26A	Uncharacterized protein	VPS26 family	Gallus gallus (Chicken)	PF03643;
Q5ZIL2	416867;	VPS29 RCJMB04_25e21	Vacuolar protein sorting-associated protein 29 (Vesicle protein sorting 29)	VPS29 family	Gallus gallus (Chicken)	PF12850;
A0A1L1RIQ6	416867;	VPS29	Vacuolar protein sorting-associated protein 29 (Vesicle protein sorting 29)	VPS29 family	Gallus gallus (Chicken)	PF12850;
Q5ZL51	415750;	RCJMB04_7m14	Vacuolar protein sorting-associated protein 35	VPS35 family	Gallus gallus (Chicken)	
F1NF28	423608;	WAPL	WAPL domain-containing protein	WAPL family	Gallus gallus (Chicken)	PF07814;
O93277	422842;	WDR1	WD repeat-containing protein 1 (Actin-interacting protein 1) (AIP1)	WD repeat AIP1 family	Gallus gallus (Chicken)	PF12894;PF00400;
Q5ZJI7	770269;	ARPC1A RCJMB04_17n4	Actin-related protein 2/3 complex subunit	WD repeat ARPC1 family	Gallus gallus (Chicken)	PF00400;
Q5ZI99	416490;	ARPC1B RCJMB04_28o6	Actin-related protein 2/3 complex subunit	WD repeat ARPC1 family	Gallus gallus (Chicken)	PF00400;
A0A1L1RUT1	424823;	COPB2	Coatomer subunit beta'	WD repeat COPB2 family	Gallus gallus (Chicken)	PF04053;PF00400;
Q5ZI60	416893;	CORO1C RCJMB04_30b2	Coronin	WD repeat coronin family	Gallus gallus (Chicken)	PF08953;PF00400;
Q5ZIB3	421916;	RCJMB04_28i4	WD_REPEATS_REGION domain-containing protein	WD repeat EIPR1 family	Gallus gallus (Chicken)	PF00400;
A0A1D5NU87	771345;	EML4	Uncharacterized protein	WD repeat EMAP family	Gallus gallus (Chicken)	PF03451;PF00400;
F1NH66	771345;	EML4	Uncharacterized protein	WD repeat EMAP family	Gallus gallus (Chicken)	PF03451;PF00400;
F1NLV4	419402;	GNB1	WD_REPEATS_REGION domain-containing protein	WD repeat G protein beta family	Gallus gallus (Chicken)	PF00400;
Q5ZLB5	419402;	RCJMB04_6n13	WD_REPEATS_REGION domain-containing protein	WD repeat G protein beta family	Gallus gallus (Chicken)	PF00400;
E1BW98	424974;	GNB4	WD_REPEATS_REGION domain-containing protein	WD repeat G protein beta family	Gallus gallus (Chicken)	PF00400;
A0A1D5P7Z5	418289;	P3H3	WD_REPEATS_REGION domain-containing protein	WD repeat G protein beta family	Gallus gallus (Chicken)	PF00400;
P63247	417044;	RACK1 GNB2L1	Receptor of activated protein C kinase 1 (Guanine nucleotide-binding protein subunit beta-like protein 12.3) (Receptor for activated C kinase) (Receptor of activated protein kinase C 1) (RACK1)	WD repeat G protein beta family, Ribosomal protein RACK1 subfamily	Gallus gallus (Chicken)	PF00400;
Q9PTR5	374224;	PAFAH1B1 LIS1	Lissencephaly-1 homolog	WD repeat LIS1/nudF family	Gallus gallus (Chicken)	PF08513;PF00400;
Q5ZMA2	430767;	PRPF19 RCJMB04_2m2	Pre-mRNA-processing factor 19 (EC 2.3.2.27) (PRP19/PSO4 homolog) (RING-type E3 ubiquitin transferase PRP19)	WD repeat PRP19 family	Gallus gallus (Chicken)	PF08606;PF04564;PF00400;
Q9W7I5	395658;	RBBP4 RBAP48	Histone-binding protein RBBP4 (Chromatin assembly factor 1 subunit C) (CAF-1 subunit C) (Chromatin assembly factor I p48 subunit) (CAF-I 48 kDa subunit) (CAF-I p48) (chCAF-1 p48) (Retinoblastoma-binding protein 4) (RBBP-4) (Retinoblastoma-binding protein p48)	WD repeat RBAP46/RBAP48/MSI1 family	Gallus gallus (Chicken)	PF12265;PF00400;
Q9I8G9	395390;	RBBP7 RBAP46	Histone-binding protein RBBP7 (Retinoblastoma-binding protein 7) (RBBP-7) (Retinoblastoma-binding protein p46)	WD repeat RBAP46/RBAP48/MSI1 family	Gallus gallus (Chicken)	PF12265;PF00400;
A0A1D5NZU0	100859886;	SEH1L	Uncharacterized protein	WD repeat SEC13 family	Gallus gallus (Chicken)	PF00400;
A0A1L1RKI2	100859886;	SEH1L	Uncharacterized protein	WD repeat SEC13 family	Gallus gallus (Chicken)	PF00400;
A0A3Q2TX69	423764;	SEC31B	WD_REPEATS_REGION domain-containing protein	WD repeat SEC31 family	Gallus gallus (Chicken)	
A0A3Q2TZ13	423764;	SEC31B	WD_REPEATS_REGION domain-containing protein	WD repeat SEC31 family	Gallus gallus (Chicken)	
Q5ZME8	427377;	SMU1 RCJMB04_2e22	WD40 repeat-containing protein SMU1 (Smu-1 suppressor of mec-8 and unc-52 protein homolog)	WD repeat SMU1 family	Gallus gallus (Chicken)	PF00400;
Q5ZL33	418175;	STRAP RCJMB04_7p19	Serine-threonine kinase receptor-associated protein	WD repeat STRAP family	Gallus gallus (Chicken)	PF00400;
Q5ZMV7	415942;	WDR82 RCJMB04_1b3	WD repeat-containing protein 82	WD repeat SWD2 family	Gallus gallus (Chicken)	PF12894;PF00400;
Q5F3K4	420427;	WDR48 UAF1 RCJMB04_14o15	WD repeat-containing protein 48 (USP1-associated factor 1)	WD repeat WDR48 family	Gallus gallus (Chicken)	PF11816;PF00400;
P08251	396529;	ATP1B1	Sodium/potassium-transporting ATPase subunit beta-1 (Sodium/potassium-dependent ATPase subunit beta-1)	X(+)/potassium ATPases subunit beta family	Gallus gallus (Chicken)	PF00287;
P33879	396549;	ATP1B3	Sodium/potassium-transporting ATPase subunit beta-3 (Sodium/potassium-dependent ATPase subunit beta-3)	X(+)/potassium ATPases subunit beta family	Gallus gallus (Chicken)	PF00287;

Q5ZLN4	769677;	FEN1 RCJMB04_5g12	Flap endonuclease 1 (FEN-1) (EC 3.1.-.-) (Flap structure-specific endonuclease 1)	XPG/RAD2 endonuclease family, FEN1 subfamily	Gallus gallus (Chicken)	PF00867;PF00752;
E1BV44	419212;	CSE1L	Chromosome segregation 1-like protein (Exportin-2) (Importin-alpha re-exporter)	XPO2/CSE1 family	Gallus gallus (Chicken)	PF03378;PF08506;PF03810;
P23991	771920;	ADH1	Alcohol dehydrogenase 1 (ADH-1) (EC 1.1.1.1) (Alcohol dehydrogenase I)	Zinc-containing alcohol dehydrogenase family, Class-I subfamily	Gallus gallus (Chicken)	PF08240;PF00107;
Q5ZK81	422705;	RCJMB04_12i19	S-(hydroxymethyl)glutathione dehydrogenase (EC 1.1.1.284)	Zinc-containing alcohol dehydrogenase family, Class-III subfamily	Gallus gallus (Chicken)	PF08240;PF00107;
F1P4I0	772289;	CRYZ	PKS_ER domain-containing protein	Zinc-containing alcohol dehydrogenase family, Quinone oxidoreductase subfamily	Gallus gallus (Chicken)	PF08240;PF00107;
Q5ZHT3	772289;	RCJMB04_33h20	PKS_ER domain-containing protein	Zinc-containing alcohol dehydrogenase family, Quinone oxidoreductase subfamily	Gallus gallus (Chicken)	PF08240;PF00107;
P79762	378906;	ZP3 ZPC	Zona pellucida sperm-binding protein 3 (Sperm receptor) (Zona pellucida C protein) (Zona pellucida glycoprotein 3) (Zp-3) [Cleaved into: Processed zona pellucida sperm-binding protein 3]	ZP domain family, ZPC subfamily	Gallus gallus (Chicken)	PF00100;
Q5ZLX5	424717;	ZRANB2 RCJMB04_4i6	Zinc finger Ran-binding domain-containing protein 2	ZRANB2 family	Gallus gallus (Chicken)	PF00641;
Q5F464	429148;	LPP RCJMB04_2l20	Lipoma-preferred partner homolog	Zyxin/ajuba family	Gallus gallus (Chicken)	PF00412;
Q04584	418300;	ZYX	Zyxin	Zyxin/ajuba family	Gallus gallus (Chicken)	PF00412;
A0A1D5PD86	420218;	DECR1	Uncharacterized protein		Gallus gallus (Chicken)	
F1NG68	100858408;	DECR2	Uncharacterized protein		Gallus gallus (Chicken)	
F1NAM7	422715;	BDH2	Uncharacterized protein		Gallus gallus (Chicken)	
F1NV37	421954;	NT5C1B	Uncharacterized protein		Gallus gallus (Chicken)	PF06189;
Q5ZHQ4	421587;	RCJMB04_34i5	Thiolase_N domain-containing protein		Gallus gallus (Chicken)	PF00108;
A0A1D5P3M1	415562;	ANP32A	LRRcap domain-containing protein		Gallus gallus (Chicken)	
P42292	396092;	ALCAM	CD166 antigen (Activated leukocyte cell adhesion molecule) (BEN glycoprotein) (Protein DM-GRASP) (Protein JC7) (SC1 glycoprotein) (CD antigen CD166)		Gallus gallus (Chicken)	PF08205;PF13895;
Q5ZJB6	415926;	RCJMB04_19g24	Acyl-peptide hydrolase (Acylamino-acid-releasing enzyme) (Acylaminoacyl-peptidase)		Gallus gallus (Chicken)	PF00326;
Q5ZJ14	421317;	RCJMB04_21m6	Uncharacterized protein		Gallus gallus (Chicken)	PF00887;PF13897;
Q5F420	422547;	ACSL1 RCJMB04_3m22	AMP-binding domain-containing protein		Gallus gallus (Chicken)	PF00501;
F1P451	424810;	ACSL3	AMP-binding domain-containing protein		Gallus gallus (Chicken)	PF00501;
A0A3Q2UMG1	418600;	ACOT9	Uncharacterized protein		Gallus gallus (Chicken)	
F1NNU7	418600;	ACOT9	Uncharacterized protein		Gallus gallus (Chicken)	
Q5F3B4	418600;	RCJMB04_23c19	Uncharacterized protein		Gallus gallus (Chicken)	
Q5F434	415391;	RCJMB04_3k8	A_deaminase domain-containing protein		Gallus gallus (Chicken)	PF00962;
F1NRD4	404536;	ADIPOQ	C1q domain-containing protein		Gallus gallus (Chicken)	PF00386;PF01391;
Q6QWE7	404536;	ADIPOQ	Adiponectin		Gallus gallus (Chicken)	PF00386;PF01391;
F1NGR5	415317;	ADPGK	Uncharacterized protein		Gallus gallus (Chicken)	PF04587;
A0A1D5PV58	418224;	ARFGAP3	Arf-GAP domain-containing protein		Gallus gallus (Chicken)	PF01412;
F1NZX4	418224;	ARFGAP3	Uncharacterized protein		Gallus gallus (Chicken)	
Q5F391	418224;	RCJMB04_27o21	Uncharacterized protein		Gallus gallus (Chicken)	
A0A1D5PHC7	396538;	AGRN	Agrin		Gallus gallus (Chicken)	PF00008;PF00050;PF07648;PF00053;PF00054;PF03146;PF01390;
A0A1L1RLW7	396538;	AGRN	Agrin		Gallus gallus (Chicken)	PF00008;PF00050;PF07648;PF00053;PF00054;PF03146;PF01390;
A0A1D5PLE4	421634;	AKAP12	Uncharacterized protein		Gallus gallus (Chicken)	PF03832;

F1P331	419471;	AKR7A2	Aldo_ket_red domain-containing protein		Gallus gallus (Chicken)	PF00248;
E1BZE1	424956;	AHSG	Uncharacterized protein		Gallus gallus (Chicken)	PF00031;
E1C2L5	769169;	ALYREF	RRM domain-containing protein		Gallus gallus (Chicken)	PF13865;PF00076;
A0A1D5P0D3	422533;	AIMP1	tRNA-binding domain-containing protein		Gallus gallus (Chicken)	PF01588;
F1NLE7	422533;	AIMP1	tRNA-binding domain-containing protein		Gallus gallus (Chicken)	PF01588;
A0A1D5PF87	424474;	AGL	4-alpha-glucanotransferase (EC 2.4.1.25) (EC 3.2.1.33) (Amylo-alpha-1,6-glucosidase) (Dextrin 6-alpha-D-glucosidase) (Glycogen debrancher) (Glycogen debranching enzyme) (Oligo-1,4-1,4-glucantransferase)		Gallus gallus (Chicken)	PF06202;PF14701;PF14702;PF14699;
F1NX83	424474;	AGL	4-alpha-glucanotransferase (EC 2.4.1.25) (EC 3.2.1.33) (Amylo-alpha-1,6-glucosidase) (Dextrin 6-alpha-D-glucosidase) (Glycogen debrancher) (Glycogen debranching enzyme) (Oligo-1,4-1,4-glucantransferase)		Gallus gallus (Chicken)	PF06202;PF14701;PF14702;PF14699;
A0A3Q2U1V0	396311;	ANK1	Uncharacterized protein		Gallus gallus (Chicken)	PF00023;PF12796;PF00531;PF17809;PF00791;
A0A3Q2UAG0	396311;	ANK1	Uncharacterized protein		Gallus gallus (Chicken)	PF12796;PF00531;PF17809;PF00791;
A0A3Q2UDZ7	396311;	ANK1	Uncharacterized protein		Gallus gallus (Chicken)	PF00023;PF12796;PF00531;PF17809;PF00791;
A0A3Q3AKQ1	396311;	ANK1	Uncharacterized protein		Gallus gallus (Chicken)	PF00023;PF12796;PF00531;PF17809;PF00791;
E1C937	420596;	AGR2	Uncharacterized protein		Gallus gallus (Chicken)	
Q197X2	396535;	APOB	Apolipoprotein B		Gallus gallus (Chicken)	PF12491;PF06448;PF09172;PF01347;
F1P1P3	417926;	APAF1	Apoptotic protease-activating factor 1 (APAF-1)		Gallus gallus (Chicken)	PF17908;PF00619;PF00931;PF00400;
Q5ZK62	424895;	ACAP2 CENTB2 RCJMB04_12p24	Arf-GAP with coiled-coil, ANK repeat and PH domain-containing protein 2 (Centaurin-beta-2) (Cnt-b2)		Gallus gallus (Chicken)	PF12796;PF01412;PF00169;
A0A3Q2U396	424895;	ACAP2	Arf-GAP with coiled-coil, ANK repeat and PH domain-containing protein 2		Gallus gallus (Chicken)	PF12796;PF01412;PF00169;
A0A3Q2UML5	424895;	ACAP2	Arf-GAP with coiled-coil, ANK repeat and PH domain-containing protein 2		Gallus gallus (Chicken)	PF12796;PF01412;PF00169;
Q5ZJU3	420574;	ASNS RCJMB04_15l3	Asparagine synthetase [glutamine-hydrolyzing] (EC 6.3.5.4) (Glutamine-dependent asparagine synthetase)		Gallus gallus (Chicken)	PF00733;PF13537;
F1NWF6	420574;	ASNS	Asparagine synthetase [glutamine-hydrolyzing] (EC 6.3.5.4)		Gallus gallus (Chicken)	PF00733;PF13537;
P0DJJ2	423176;	ASTL	Astacin-like metalloendopeptidase (EC 3.4.-.-)		Gallus gallus (Chicken)	PF01400;PF00431;
Q5ZJX6	422462;	ABCE1 RCJMB04_14j19	Uncharacterized protein		Gallus gallus (Chicken)	PF00005;PF00037;PF04068;
Q5ZM38	427269;	AUH RCJMB04_3e14	Uncharacterized protein		Gallus gallus (Chicken)	PF00378;
E1C502	418369;	ATG3	Autophagy_act_C domain-containing protein		Gallus gallus (Chicken)	PF03987;
P17790	770363;	BSG	Basigin (5A11 antigen) (Blood-brain barrier HT7 antigen) (Neurothelin)		Gallus gallus (Chicken)	
R4GL30	421880;	BAG2	BAG domain-containing protein		Gallus gallus (Chicken)	
F1NC47	416207;	BNIP1	Uncharacterized protein		Gallus gallus (Chicken)	PF03908;
A0A3Q2TYT7	772088;	BCL2L15	Uncharacterized protein		Gallus gallus (Chicken)	
Q8JGM8	395236;	BID RCJMB04_7d15	BH3-interacting domain death agonist		Gallus gallus (Chicken)	PF06393;
A0A140T8G9	395236;	BID	BH3-interacting domain death agonist		Gallus gallus (Chicken)	PF06393;
A0A3Q2U873	395236;	BID	BH3-interacting domain death agonist		Gallus gallus (Chicken)	PF06393;
O42273	395882;	TENP	Protein TENP		Gallus gallus (Chicken)	PF02886;
Q5ZHW3	423949;	BUB3 RCJMB04_32k7	WD_REPEATS_REGION domain-containing protein		Gallus gallus (Chicken)	PF00400;
P08641	415860;	CDH1	Cadherin-1 (Epithelial cadherin) (E-cadherin) (Liver cell adhesion molecule) (L-CAM)		Gallus gallus (Chicken)	PF00028;PF01049;PF08758;
E1C6M9	415860;	CDH1	Cadherin-1 (Epithelial cadherin)		Gallus gallus (Chicken)	PF00028;PF01049;PF08758;

P33150	414849;	CDH13	Cadherin-13 (Truncated cadherin) (T-cad) (T-cadherin)		Gallus gallus (Chicken)	PF00028;PF08758;
A0A3Q2UIH0	416640;	CARHSP1	CSD domain-containing protein		Gallus gallus (Chicken)	PF00313;
Q90940	396352;	P22	Calcium-binding protein		Gallus gallus (Chicken)	PF13460;
F1P596	416692;	CALML3	Uncharacterized protein		Gallus gallus (Chicken)	PF13499;
A5AA28	395956;	GLI2	GLI-Kruppel family member (Fragment)		Gallus gallus (Chicken)	
O42184	395784;	CLIP1 RSN	CAP-Gly domain-containing linker protein 1 (Cytoplasmic linker protein 170) (CLIP-170) (Restin)		Gallus gallus (Chicken)	PF01302;PF16641;
D3KCC4	100359387;	CARNS1 ATPGD1	Carnosine synthase 1 (EC 6.3.2.11) (ATP-grasp domain-containing protein 1)		Gallus gallus (Chicken)	PF18130;
A0A1D5P2L6	395992;	CRTAP	Cartilage-associated protein		Gallus gallus (Chicken)	
O42395	395731;	CNBP ZNF9	Cellular nucleic acid-binding protein (CNBP) (Zinc finger protein 9)		Gallus gallus (Chicken)	PF00098;
A0A1D6UPT3	395731;	CNBP	Cellular nucleic acid-binding protein		Gallus gallus (Chicken)	PF00098;
O57348	395731;		Cellular nucleic acid binding protein		Gallus gallus (Chicken)	PF00098;
A0A3Q2ULT8	419852;	CD55	Uncharacterized protein		Gallus gallus (Chicken)	PF00084;
A0A1L1RR39	415315;	CD276	Uncharacterized protein		Gallus gallus (Chicken)	PF08205;PF07686;
A0A1D5P2L5	422763;	CWH43	Uncharacterized protein		Gallus gallus (Chicken)	
A0A3Q2TXL1	422763;	CWH43	Uncharacterized protein		Gallus gallus (Chicken)	
A0A1D5PIH8	419138;	CEP250	Uncharacterized protein		Gallus gallus (Chicken)	
A0A1D5PEX8	395355;	CBX3	Uncharacterized protein		Gallus gallus (Chicken)	PF00385;PF01393;
O93481	395355;	CBX3	Chromobox protein (CHCB2)		Gallus gallus (Chicken)	PF00385;PF01393;
F1NY09	419001;	C1H11ORF54	DUF1907 domain-containing protein		Gallus gallus (Chicken)	PF08925;
A0A1D5NV95	416531;	CHTF18	AAA domain-containing protein		Gallus gallus (Chicken)	PF00004;
Q5ZHX0	416531;	RCJMB04_32h3	AAA domain-containing protein (Fragment)		Gallus gallus (Chicken)	PF00004;
Q5ZM53	416240;	RCJMB04_3c5	ENTH domain-containing protein		Gallus gallus (Chicken)	PF01417;
Q5F4A0	421595;	CSTF3 RCJMB04_1m7	Suf domain-containing protein		Gallus gallus (Chicken)	PF05843;
Q5F354	426906;	RCJMB04_34d13	Coatomer subunit alpha		Gallus gallus (Chicken)	PF12894;PF04053;PF06957;PF00400;
Q5ZIA5	423063;	COPB1 COPB RCJMB04_28l17	Coatomer subunit beta (Beta-coat protein) (Beta-COP)		Gallus gallus (Chicken)	PF01602;PF07718;PF14806;
A0A1L1RNV8	418435;	CHCHD3	Uncharacterized protein		Gallus gallus (Chicken)	
E1BUJ8	416037;	CHCHD4	CHCH domain-containing protein		Gallus gallus (Chicken)	PF06747;
Q5F3I2	419886;	RCJMB04_16d21	Uncharacterized protein		Gallus gallus (Chicken)	PF00313;PF12901;
A0A5H1ZRJ7	396243;	COL1A2	Collagen alpha-2(I) chain		Gallus gallus (Chicken)	PF01410;PF01391;
A0A1D5PE57	396340;	COL3A1	Collagen alpha-1(III) chain		Gallus gallus (Chicken)	PF01410;PF01391;PF00093;
A0A1D5P8P3	395530;	COL4A1	Collagen IV NC1 domain-containing protein		Gallus gallus (Chicken)	PF01413;PF01391;
F1P2Q3	418752;	COL4A2	Collagen IV NC1 domain-containing protein		Gallus gallus (Chicken)	PF01413;PF01391;
A0A3Q2UBM2	423986;	COL5A2	Uncharacterized protein		Gallus gallus (Chicken)	PF01410;PF01391;PF00093;
A0A3Q2UD12	396548;	COL6A3	Collagen alpha-3(VI) chain		Gallus gallus (Chicken)	PF01391;PF00014;PF00092;

A0A3Q2UMJ2	396548;	COL6A3	Collagen alpha-3(VI) chain		Gallus gallus (Chicken)	PF01391;PF00014;PF00092;
A0A3Q3AR07	396548;	COL6A3	Collagen alpha-3(VI) chain		Gallus gallus (Chicken)	PF01391;PF00014;PF00092;
F1P2F0	396548;	COL6A3	Collagen alpha-3(VI) chain		Gallus gallus (Chicken)	PF01391;PF00014;PF00092;
A0A1D5P8I8	395875;	COL12A1	Collagen alpha-1(XII) chain		Gallus gallus (Chicken)	PF01391;PF00041;PF00092;
A0A1L1RPW4	395875;	COL12A1	Collagen alpha-1(XII) chain		Gallus gallus (Chicken)	PF01391;PF00041;PF00092;
F1NX22	395875;	COL12A1	Collagen alpha-1(XII) chain		Gallus gallus (Chicken)	PF01391;PF00041;PF00092;
O93419	373978;		Collagen XVIII		Gallus gallus (Chicken)	PF01391;PF06482;
Q2LK54	421061;	COLEC12 CL3	Collectin-12 (Collectin-3) (CL-3) (cCL-3)		Gallus gallus (Chicken)	PF01391;PF00059;
A0A1D5NWU4	770048;	COMMD4	COMM domain-containing protein		Gallus gallus (Chicken)	PF07258;
F1NNT4	770048;	COMMD4	COMM domain-containing protein		Gallus gallus (Chicken)	PF07258;
Q5ZM89	422376;	COMMD5 RCJMB04_2n13	COMM domain-containing protein 5		Gallus gallus (Chicken)	PF07258;
F1NY50	769778;	COMMD6	COMM domain-containing protein		Gallus gallus (Chicken)	PF07258;
E1BU89	419286;	COMMD7	COMM domain-containing protein		Gallus gallus (Chicken)	PF07258;
Q90633	396370;		Complement C3		Gallus gallus (Chicken)	PF00207;PF07703;PF07677;PF01821;PF17790;PF01835;PF17791;PF17789;PF01759;PF07678;
Q9DEG0	395384;	Cremp	Complement regulatory membrane protein		Gallus gallus (Chicken)	PF00084;
A0A3Q2TRY3	429057;	CFH	Uncharacterized protein		Gallus gallus (Chicken)	PF00084;
A0A1D5NW58	769006;	COMMD1	COMM domain-containing protein 1		Gallus gallus (Chicken)	PF07258;PF17221;
A0A1D5PHX9	769006;	COMMD1	COMM domain-containing protein 1		Gallus gallus (Chicken)	PF07258;PF17221;
Q8QGE8	395257;		Core-binding factor beta		Gallus gallus (Chicken)	PF02312;
Q01406	396455;	CTTN1 EMS1 P85.25	Src substrate protein p85 (Cortactin) (p80)		Gallus gallus (Chicken)	PF02218;PF14604;
A0A3Q2UHE4	396455;	CTTN	SH3 domain-containing protein		Gallus gallus (Chicken)	PF02218;PF14604;
F1NU55	396455;	CTTN	SH3 domain-containing protein		Gallus gallus (Chicken)	PF02218;PF14604;
A0A1D5PW77	776376;	LOC776376	Ig-like domain-containing protein		Gallus gallus (Chicken)	PF13895;PF00354;
E1BYI2	416716;	CST7	Cystatin domain-containing protein		Gallus gallus (Chicken)	PF00031;
P67966	396176;	CSRP1 CSRP	Cysteine and glycine-rich protein 1 (Cysteine-rich protein 1) (CRP) (CRP1)		Gallus gallus (Chicken)	PF00412;
P50460	396128;	CSRP2	Cysteine and glycine-rich protein 2 (Beta-cysteine-rich protein) (Beta-CRP) (Cysteine-rich protein 2) (CRP2)		Gallus gallus (Chicken)	PF00412;
C4P6P8	396128;	CSRP2	Cysteine and glycine-rich protein 2		Gallus gallus (Chicken)	PF00412;
A0A3Q2UF90	423086;	CARS	Cysteinyl-tRNA synthetase (EC 6.1.1.16)		Gallus gallus (Chicken)	PF01406;
F1P360	418073;	CKAP4	Uncharacterized protein		Gallus gallus (Chicken)	
Q5ZM92	427266;	DAZAP1 RCJMB04_2n4	Uncharacterized protein		Gallus gallus (Chicken)	PF00076;
Q5F3A9	422813;	RCJMB04_24b10	RNA helicase (EC 3.6.4.13)		Gallus gallus (Chicken)	PF00270;PF04408;PF00271;PF07717;
Q5ZMC1	426504;	RP11-529K1.3 RCJMB04_2i24	RNA helicase (EC 3.6.4.13)		Gallus gallus (Chicken)	PF00270;PF00271;
A0A1D5PE45	426247;	DHRS4	Uncharacterized protein		Gallus gallus (Chicken)	
Q5F422	420828;	DEK RCJMB04_3m16	SAP domain-containing protein		Gallus gallus (Chicken)	PF08766;

A0A1D5P2N1	419967;	DCAKD	Uncharacterized protein		Gallus gallus (Chicken)	PF01121;
F1NJD5	428529;	DSG2	Uncharacterized protein		Gallus gallus (Chicken)	PF00028;
E1BWI0	420869;	DSP	SH3 domain-containing protein		Gallus gallus (Chicken)	PF00681;PF17902;PF18373;
F1NX13	416962;	DRG1	Uncharacterized protein		Gallus gallus (Chicken)	PF01926;PF16897;PF02824;
F1NT58	416860;	DIABLO	Diablo homolog, mitochondrial		Gallus gallus (Chicken)	PF09057;
F1NKV0	424550;	DNAJB4	J domain-containing protein		Gallus gallus (Chicken)	PF00226;PF01556;
Q5ZI13	418787;	DNAJC3 RCJMB04_31h14	DnaJ homolog subfamily C member 3		Gallus gallus (Chicken)	PF00226;PF13181;
F1P053	418787;	DNAJC3	DnaJ homolog subfamily C member 3		Gallus gallus (Chicken)	PF00226;PF00515;PF13181;
E1C8S9	423640;	DNAJC9	J domain-containing protein		Gallus gallus (Chicken)	PF00226;
A0A1D5NZ71	420688;	DNAJC13	J domain-containing protein		Gallus gallus (Chicken)	PF00226;PF14237;
Q5ZK19	427404;	DCTN3 RCJMB04_13l14	Uncharacterized protein		Gallus gallus (Chicken)	PF07426;
A0A1D5PBA6	416269;	DCTN4	Uncharacterized protein		Gallus gallus (Chicken)	PF05502;
E1BQQ5	416269;	DCTN4	Uncharacterized protein		Gallus gallus (Chicken)	PF05502;
A0A1D5PHV9	417896;	EEA1	Uncharacterized protein		Gallus gallus (Chicken)	PF01363;
E1BRE5	417896;	EEA1	FYVE-type domain-containing protein		Gallus gallus (Chicken)	PF01363;
E1BQW4	427445;	EGFLAM	Uncharacterized protein		Gallus gallus (Chicken)	PF00008;PF00041;PF00054;PF02210;
Q5ZM17	421306;	RCJMB04_3g13	Uncharacterized protein		Gallus gallus (Chicken)	PF18150;PF00350;PF12763;PF16880;
A0A3Q2UHT9	100859860;	EHD4	Uncharacterized protein		Gallus gallus (Chicken)	PF18150;PF12763;
Q5F3D8	428724;	RCJMB04_20k15	Electron transfer flavoprotein-ubiquinone oxidoreductase (ETF-QO) (EC 1.5.5.1)		Gallus gallus (Chicken)	PF07992;
Q5F430	415467;	RCJMB04_3k21	EFG_C domain-containing protein		Gallus gallus (Chicken)	PF00679;PF14492;PF03144;
Q6B0K7	771557;		Endoglin		Gallus gallus (Chicken)	PF00100;
E1C8W3	428090;	ENDOD1	Uncharacterized protein		Gallus gallus (Chicken)	PF01223;
P81628	416882;	ERP29 RCJMB04_24f23	Endoplasmic reticulum resident protein 29 (ERp29) (Fragment)		Gallus gallus (Chicken)	PF07749;PF07912;
F1NRM8	416882;	ERP29	Endoplasmic reticulum resident protein 29		Gallus gallus (Chicken)	PF07749;PF07912;
E1BSL7	420994;	ERP44	Thioredoxin domain-containing protein		Gallus gallus (Chicken)	PF00085;
Q45QT1	421999;		Soluble epoxide hydrolase		Gallus gallus (Chicken)	PF00561;PF13419;
Q6EE30	100526660;		Eukaryotic translation elongation factor 1		Gallus gallus (Chicken)	PF00647;PF00043;PF02798;
Q5ZMF0	419845;	RCJMB04_2e17	PUA domain-containing protein		Gallus gallus (Chicken)	PF17832;
A0A1L1S0V6	417490;	EIF4H	Eukaryotic translation initiation factor 4H		Gallus gallus (Chicken)	PF00076;
F1NYA2	417490;	EIF4H	Eukaryotic translation initiation factor 4H		Gallus gallus (Chicken)	PF00076;
Q5ZMR0	417490;	RCJMB04_1g12	Eukaryotic translation initiation factor 4H		Gallus gallus (Chicken)	PF00076;
E1BVP1	395514;	EIF5B	Eukaryotic translation initiation factor 5B (Translation initiation factor IF-2)		Gallus gallus (Chicken)	PF00009;PF03144;PF11987;
Q9W6Q4	395514;	cIF2	Initiation of translation factor 2 (Osteoblast translation factor 3F) (Fragment)		Gallus gallus (Chicken)	
Q5ZIV8	421192;	RCJMB04_23d16	Importin N-terminal domain-containing protein		Gallus gallus (Chicken)	PF03810;PF08389;

Q9YGW6	395701;	EZR	Ezrin		Gallus gallus (Chicken)	PF00769;PF09380;PF00373;PF09379;
Q5F351	423954;	FAM175B RCJMB04_34f21	MPN domain-containing protein		Gallus gallus (Chicken)	
Q5ZHW0	424551;	RCJMB04_32l4	Uncharacterized protein		Gallus gallus (Chicken)	PF09005;PF00013;
A0A3Q2TU83	417184;	FUBP3	Uncharacterized protein		Gallus gallus (Chicken)	PF09005;PF00013;
A0A3Q3AB26	417184;	FUBP3	Uncharacterized protein		Gallus gallus (Chicken)	PF09005;PF00013;
Q5ZM76	417184;	FUBP3 RCJMB04_2o21	Uncharacterized protein		Gallus gallus (Chicken)	PF00013;
F1NUY5	416225;	FAF2	UBX domain-containing protein		Gallus gallus (Chicken)	PF00789;
F1NKQ7	423146;	FADD	Uncharacterized protein		Gallus gallus (Chicken)	PF00531;PF01335;
A0A1D5P6Q5	396061;	FASN	3-hydroxyacyl-[acyl-carrier-protein] dehydratase (EC 1.1.1.100) (EC 1.3.1.39) (EC 2.3.1.38) (EC 2.3.1.39) (EC 2.3.1.41) (EC 2.3.1.85) (EC 3.1.2.14) (EC 4.2.1.59) (3-oxoacyl-[acyl-carrier-protein] reductase) (3-oxoacyl-[acyl-carrier-protein] synthase) (Acyl-[acyl-carrier-protein] hydrolase) (Enoyl-[acyl-carrier-protein] reductase) (Fatty acid synthase) ([Acyl-carrier-protein] S-acetyltransferase) ([Acyl-carrier-protein] S-malonyltransferase)		Gallus gallus (Chicken)	PF00698;PF00107;PF16197;PF00109;PF02801;PF08659;PF00550;PF14765;PF00975;
F1NJI6	419494;	FBXO44	Uncharacterized protein		Gallus gallus (Chicken)	PF12937;PF04300;
R4GFM0	418781;	FARP1	FERM, ARHGEF and pleckstrin domain-containing protein 1		Gallus gallus (Chicken)	PF08736;PF09380;PF00373;PF09379;PF00169;PF00621;
R9PXQ2	418781;	FARP1	FERM, ARHGEF and pleckstrin domain-containing protein 1		Gallus gallus (Chicken)	PF08736;PF09380;PF00373;PF09379;PF00169;PF00621;
F1NHT5	395404;	FETUB	Uncharacterized protein		Gallus gallus (Chicken)	PF00031;
Q9DE41	395404;		Apo AI promoter B-region binding protein (Fragment)		Gallus gallus (Chicken)	
F1P4V1	396307;	FGA	Fibrinogen alpha chain (Fibrinopeptide A)		Gallus gallus (Chicken)	PF08702;PF12160;PF00147;
Q02020	373926;	FGB	Fibrinogen beta chain [Cleaved into: Fibrinopeptide B; Fibrinogen beta chain] (Fragment)		Gallus gallus (Chicken)	PF08702;PF00147;
E1BV78	395837;	FGG	Fibrinogen C-terminal domain-containing protein		Gallus gallus (Chicken)	PF08702;PF00147;
P11722	396133;	FN1	Fibronectin (FN)		Gallus gallus (Chicken)	PF00039;PF00040;PF00041;
Q90ZK7	395353;	FKBP25	Peptidylprolyl isomerase (EC 5.2.1.8)		Gallus gallus (Chicken)	PF18410;PF00254;
Q5ZJT4	418261;	RCJMB04_15n8	Peptidylprolyl isomerase (EC 5.2.1.8)		Gallus gallus (Chicken)	PF00254;PF00515;PF13174;
Q646T7	421186;	FKBP51 FKBP5	Peptidylprolyl isomerase (EC 5.2.1.8)		Gallus gallus (Chicken)	PF00254;PF00515;
A0A1D5PYB1	395652;	FKBP9	Peptidylprolyl isomerase (EC 5.2.1.8)		Gallus gallus (Chicken)	PF00254;
Q9YIC3	395652;	cFKBP/SMAP	Peptidylprolyl isomerase (EC 5.2.1.8)		Gallus gallus (Chicken)	PF00254;
R4GH26	427013;	FKBP10	Peptidylprolyl isomerase (EC 5.2.1.8)		Gallus gallus (Chicken)	PF13202;PF00254;
E1C9A8	427233;	FOCAD	DUF3730 domain-containing protein		Gallus gallus (Chicken)	PF12530;PF11229;
Q90846	374097;	Galpa i3-o	G protein		Gallus gallus (Chicken)	PF00503;
Q5ZMN1	416265;	G3BP1 RCJMB04_1j5	Uncharacterized protein		Gallus gallus (Chicken)	PF02136;PF00076;
P07583	396491;	CG-1B	Beta-galactoside-binding lectin (14 kDa lectin) (C-14) (Galectin CG-1B)		Gallus gallus (Chicken)	PF00337;
Q5ZHQ2	421278;	LGALSL GRP RCJMB04_34j3	Galectin-related protein (Lectin galactoside-binding-like protein)		Gallus gallus (Chicken)	PF00337;
Q9W6F5	395696;	VTDB	Gc-globulin (Group-specific component) (Vitamin D-binding protein)		Gallus gallus (Chicken)	PF00273;PF09164;
Q02391	396492;	GLG1 CFR	Golgi apparatus protein 1 (Cysteine-rich fibroblast growth factor receptor)		Gallus gallus (Chicken)	PF00839;
R4GGF3	420999;	GLIPR2	SCP domain-containing protein		Gallus gallus (Chicken)	PF00188;
Q5ZM94	418552;	RCJMB04_2m19	Uncharacterized protein		Gallus gallus (Chicken)	
A0A1D5P980	419514;	GFPT1	Glutamine--fructose-6-phosphate transaminase (isomerizing) (EC 2.6.1.16)		Gallus gallus (Chicken)	PF01380;

A0A1D5PXH4	419514;	GFPT1	Glutamine--fructose-6-phosphate transaminase (isomerizing) (EC 2.6.1.16)		Gallus gallus (Chicken)	PF01380;
Q5ZIG5	419514;	GFPT1 RCJMB04_26h2	Glutamine--fructose-6-phosphate transaminase (isomerizing) (EC 2.6.1.16)		Gallus gallus (Chicken)	PF01380;
A0A1D5PH72	426962;	GFPT2	Glutamine--fructose-6-phosphate transaminase (isomerizing) (EC 2.6.1.16)		Gallus gallus (Chicken)	PF01380;
A0A3Q2TSP8	421348;	EPRS	Glutamyl-tRNA synthetase (EC 6.1.1.15) (EC 6.1.1.17) (Prolyl-tRNA synthetase)		Gallus gallus (Chicken)	PF03129;PF09180;PF00749;PF03950;PF00587;PF00458;
A0A3Q2UKS1	421348;	EPRS	Glutamyl-tRNA synthetase (EC 6.1.1.15) (EC 6.1.1.17) (Prolyl-tRNA synthetase)		Gallus gallus (Chicken)	PF03129;PF09180;PF00749;PF03950;PF00587;PF00458;
Q5ZJ86	421348;	RCJMB04_20b9	Glutamyl-tRNA synthetase (EC 6.1.1.15) (EC 6.1.1.17) (Prolyl-tRNA synthetase)		Gallus gallus (Chicken)	PF03129;PF09180;PF00749;PF03950;PF00587;PF00458;
F1NNP6	423968;	GLRX3	Uncharacterized protein		Gallus gallus (Chicken)	PF00462;PF00085;
Q5ZJQ0	425050;	RCJMB04_16h16	Uncharacterized protein		Gallus gallus (Chicken)	PF01501;
A0A1L1RYU0	420302;	GPIHBP1	UPAR/Ly6 domain-containing protein		Gallus gallus (Chicken)	
A0A3Q2UGP4	426868;	GOLGB1	Uncharacterized protein		Gallus gallus (Chicken)	
F1P4P9	428014;	GCC2	GRIP domain-containing protein		Gallus gallus (Chicken)	PF01465;PF16704;
A3R0S3	386572;	GRB2	Growth factor receptor-bound protein 2		Gallus gallus (Chicken)	PF00017;PF00018;
Q5ZI61	425028;	RCJMB04_30a22	Glutamine amidotransferase (EC 6.3.5.2)		Gallus gallus (Chicken)	PF00117;PF00958;PF02540;
A0A1D5NYH2	423988;	GULP1	PID domain-containing protein		Gallus gallus (Chicken)	PF00640;
A0A3Q2U954	423988;	GULP1	PID domain-containing protein		Gallus gallus (Chicken)	PF00640;
O93327	395858;	MACROH2A1	Core histone macro-H2A.1 (Histone macroH2A1) (mH2A1) (H2A.y) (H2A/y)		Gallus gallus (Chicken)	PF00125;PF16211;PF01661;
A0A1D5PAE4	776936;	HDGF	PWWP domain-containing protein		Gallus gallus (Chicken)	PF00855;
Q5ZM33	425543;	HP1BP3 RCJMB04_3f4	Heterochromatin protein 1-binding protein 3		Gallus gallus (Chicken)	PF00538;
Q5ZLB3	424136;	HNRNPA1 RCJMB04_6o1	Helix-destabilizing protein (Heterogeneous nuclear ribonucleoprotein A1) (Heterogeneous nuclear ribonucleoprotein A1, N-terminally processed) (hnRNP core protein A1)		Gallus gallus (Chicken)	PF11627;PF00076;
Q90602	396268;		Single stranded D box binding factor		Gallus gallus (Chicken)	PF08143;PF00076;
Q5ZME1	420627;	HNRNPA2B1 RCJMB04_2g17	Uncharacterized protein		Gallus gallus (Chicken)	PF00076;
A0A1D5PXH1	100859627;	HNRNPA3	Uncharacterized protein		Gallus gallus (Chicken)	PF00076;
E1BZE6	100859627;	HNRNPA3	Uncharacterized protein		Gallus gallus (Chicken)	PF00076;
A0A3Q2UHQ9	422602;	HNRNPD	Uncharacterized protein		Gallus gallus (Chicken)	PF08143;PF00076;
Q5ZIH1	422602;	HNRNPD RCJMB04_26e18	Uncharacterized protein		Gallus gallus (Chicken)	PF00076;
Q5ZI72	422601;	HNRNPDL HNRPDL RCJMB04_29l7	Heterogeneous nuclear ribonucleoprotein D-like (hnRNP D-like) (hnRNP DL)		Gallus gallus (Chicken)	PF00076;
Q6WNG8	395157;	HNRNPH2	Heterogeneous nuclear ribonucleoprotein H1-like protein		Gallus gallus (Chicken)	PF00076;PF08080;
Q5F3D2	423686;	HNRNPH3 RCJMB04_21b18	Uncharacterized protein		Gallus gallus (Chicken)	PF00076;
A0A1D5PXG0	427458;	HNRNPK	Heterogeneous nuclear ribonucleoprotein K		Gallus gallus (Chicken)	PF00013;PF08067;
E1C453	427458;	HNRNPK	Heterogeneous nuclear ribonucleoprotein K		Gallus gallus (Chicken)	PF00013;PF08067;
Q5ZIQ3	426516;	HNRNPK HNRPK RCJMB04_24e23	Heterogeneous nuclear ribonucleoprotein K (hnRNP K)		Gallus gallus (Chicken)	PF00013;PF08067;
A0A1D5PSI3	426516;	HNRNPKL	Heterogeneous nuclear ribonucleoprotein K		Gallus gallus (Chicken)	PF00013;PF08067;
Q5ZL80	420054;	HNRNPM RCJMB04_7e17	Uncharacterized protein		Gallus gallus (Chicken)	PF11532;PF00076;
E1C3A9	395984;	HDLBP	Vigilin		Gallus gallus (Chicken)	PF00013;

A0A1D5P692	424298;	HNMT	Uncharacterized protein		Gallus gallus (Chicken)	
E1C378	424298;	HNMT	Uncharacterized protein		Gallus gallus (Chicken)	
A0A3Q2UBJ7	100859112;	HINT1	HIT domain-containing protein		Gallus gallus (Chicken)	PF01230;
Q9I882	395424;	chPKCI HINT2	Protein kinase C inhibitor		Gallus gallus (Chicken)	PF01230;
F5ANT1	396229;	H-RAS	V-Ha-ras Harvey rat sarcoma viral oncogene-like protein		Gallus gallus (Chicken)	PF00071;
A0A1D5P628	100858988;	HSPBP1	Uncharacterized protein		Gallus gallus (Chicken)	PF00514;
A0A1D5NWL9	395785;	HSD17B4	Uncharacterized protein		Gallus gallus (Chicken)	PF00106;PF01575;PF02036;
O42484	395785;	HSD17B4	17-beta-hydroxysteroid dehydrogenase type IV		Gallus gallus (Chicken)	PF00106;PF01575;PF02036;
E1C7S2	100858057;	HSDL2	SCP2 domain-containing protein		Gallus gallus (Chicken)	PF00106;PF02036;
F1NBA8	423046;	IPO7	Importin N-terminal domain-containing protein		Gallus gallus (Chicken)	PF08506;PF03810;
Q90681	395817;		Cation-independent mannose-6-phosphate receptor		Gallus gallus (Chicken)	PF00878;PF00040;
A0A1D5PCF5	422620;	IGFBP7	Uncharacterized protein		Gallus gallus (Chicken)	PF07679;PF00219;PF07648;
I3VQH4	100858655;	ILF3	Interleukin enhancer binding factor 3-like protein		Gallus gallus (Chicken)	PF00035;PF07528;
A0A1D5P2U7	427211;	IQGAP2	Uncharacterized protein		Gallus gallus (Chicken)	PF00307;PF00612;PF00616;PF03836;
A0A1D5PPF6	427211;	IQGAP2	Uncharacterized protein		Gallus gallus (Chicken)	PF00307;PF00612;PF00616;PF03836;
E1C2F9	427211;	IQGAP2	Uncharacterized protein		Gallus gallus (Chicken)	PF00307;PF00612;PF00616;PF03836;
A0A1D5PIZ8	415591;	IQGAP3	Uncharacterized protein		Gallus gallus (Chicken)	PF00307;PF00612;PF00616;PF03836;
F1NKW4	421930;	IAH1	SGNH_hydro domain-containing protein		Gallus gallus (Chicken)	PF13472;
Q5ZJ26	768701;	RCJMB04_21h11	Uncharacterized protein KIAA1671 homolog		Gallus gallus (Chicken)	PF15327;
A0A1D5P9E9	396335;	KTN1	Kinectin		Gallus gallus (Chicken)	PF05104;
A0A3Q2U3D6	396335;	KTN1	Kinectin		Gallus gallus (Chicken)	PF05104;
A0A3Q2UJC9	428148;	LAMA5	Uncharacterized protein		Gallus gallus (Chicken)	PF00052;PF00053;PF00054;PF02210;PF06008;PF06009;PF00055;
F1NZZ2	428148;	LAMA5	Uncharacterized protein		Gallus gallus (Chicken)	PF00052;PF00053;PF00054;PF02210;PF06008;PF06009;PF00055;
F1NJ23	396478;	LAMB1	Laminin subunit beta-1		Gallus gallus (Chicken)	PF00053;PF00055;
F1NI05	424442;	LAMC1	Uncharacterized protein		Gallus gallus (Chicken)	PF00052;PF00053;PF00055;
A4GTP0	373917;		Galectin-3		Gallus gallus (Chicken)	PF00337;
A0A1L1RN08	426849;	LMAN1L	L-type lectin-like domain-containing protein		Gallus gallus (Chicken)	PF03388;
Q5F3E6	426849;	RCJMB04_19g16	L-type lectin-like domain-containing protein		Gallus gallus (Chicken)	PF03388;
A0A1D5PW25	100859676;	LMAN2	L-type lectin-like domain-containing protein		Gallus gallus (Chicken)	PF03388;
F1NGU3	421403;	LRPPRC	Uncharacterized protein		Gallus gallus (Chicken)	PF01535;PF13812;
Q5ZLN0	424713;	LRRC40 RCJMB04_5h5	Leucine-rich repeat-containing protein 40		Gallus gallus (Chicken)	PF13855;
F1NWD5	424713;	LRRC40	Leucine-rich repeat-containing protein 40		Gallus gallus (Chicken)	PF13855;
A0A1L1RZ25	423236;	LRRC57	Uncharacterized protein		Gallus gallus (Chicken)	PF13855;
F1NEF7	396471;	LECT2	Uncharacterized protein		Gallus gallus (Chicken)	

D2Z1L9	420002;	LASP1	LIM and SH3 domain protein 1		Gallus gallus (Chicken)	PF00412;PF00880;PF14604;
A0A1D5PTF4	426878;	LIMA1	LIM zinc-binding domain-containing protein		Gallus gallus (Chicken)	PF00412;
A0A3Q3B025	426878;	LIMA1	LIM zinc-binding domain-containing protein		Gallus gallus (Chicken)	PF00412;
Q6IYF7	414880;	PINCH1	LIM domain-containing protein		Gallus gallus (Chicken)	PF00412;
Q90890	420872;	LY86 MD1	Lymphocyte antigen 86 (Ly-86) (Protein MD-1)		Gallus gallus (Chicken)	PF02221;
Q5ZLW0	418852;	LCP1 RCJMB04_4k19	Uncharacterized protein		Gallus gallus (Chicken)	PF00307;PF13499;
A0A3Q2UDX1	374254;	LSP1P1	Uncharacterized protein		Gallus gallus (Chicken)	PF02029;
Q5F3Y0	374254;	LSP1P1 RCJMB04_4h16	Uncharacterized protein		Gallus gallus (Chicken)	PF02029;
Q5ZMI4	420049;	MVP RCJMB04_1p8	Major vault protein (MVP)		Gallus gallus (Chicken)	PF11978;PF01505;PF17794;PF17795;PF17796;
M1X8W0	420516;	MRC1L-A	MRC1L-A		Gallus gallus (Chicken)	PF00040;PF00059;PF00652;
Q8UWC5	373948;	MATR3	Nuclear protein matrin 3		Gallus gallus (Chicken)	
Q90880	448832;		L-gicerin		Gallus gallus (Chicken)	PF08205;PF13895;
A0A1D5PKN8	418048;	MPST	Uncharacterized protein		Gallus gallus (Chicken)	PF00581;
E1C0L3	421395;	MTA3	Uncharacterized protein		Gallus gallus (Chicken)	PF01426;PF01448;PF00320;PF17226;PF00249;
F1P531	427395;	MCCC2	Uncharacterized protein		Gallus gallus (Chicken)	PF01039;
Q31600	417035;	B-G NTBGa-1 NTBGa-3	B-G protein (BG protein)		Gallus gallus (Chicken)	PF07686;
Q31608	417035;	B-G NTBGa-4	B-G protein (BG protein)		Gallus gallus (Chicken)	PF07686;
A0A1D6UPR2	426737;	MAPT	Microtubule-associated protein		Gallus gallus (Chicken)	PF00418;
A0A3Q2U2W1	426737;	MAPT	Microtubule-associated protein		Gallus gallus (Chicken)	PF00418;
A0A3Q2UDR8	426737;	MAPT	Microtubule-associated protein		Gallus gallus (Chicken)	PF00418;
A0A3Q2UE88	426737;	MAPT	Microtubule-associated protein		Gallus gallus (Chicken)	PF00418;
B0LVF9	426737;	MAPT	Microtubule-associated protein		Gallus gallus (Chicken)	PF00418;
B0LVG0	426737;	MAPT	Microtubule-associated protein		Gallus gallus (Chicken)	PF00418;
R4GJG3	100858848;	MIF4GD	MIF4G domain-containing protein		Gallus gallus (Chicken)	PF02854;
A0A1L1S057	770869;	MGARP	AIF-MLS domain-containing protein		Gallus gallus (Chicken)	PF14962;
E1BVT7	421585;	MRPL18	Uncharacterized protein		Gallus gallus (Chicken)	
A0A1D5PD29	418475;	MRPL39	TGS domain-containing protein		Gallus gallus (Chicken)	
F1NND9	422114;	MRPL58	RF_PROK_I domain-containing protein		Gallus gallus (Chicken)	PF00472;
A0A3Q3AY70	420097;	MISP	AKAP2_C domain-containing protein		Gallus gallus (Chicken)	PF15304;
A0A1D5P6C3	423097;	MOB2	Uncharacterized protein		Gallus gallus (Chicken)	PF03637;
A0A3Q2U5J6	423097;	MOB2	Uncharacterized protein		Gallus gallus (Chicken)	PF03637;
A0A3Q3AP67	423097;	MOB2	Uncharacterized protein		Gallus gallus (Chicken)	PF03637;
A0A1D5NTN8	416024;	MGLL	Hydrolase_4 domain-containing protein		Gallus gallus (Chicken)	PF12146;
A0A1D5PK92	416024;	MGLL	Hydrolase_4 domain-containing protein		Gallus gallus (Chicken)	PF12146;

A0A3Q2U9E5	416024;	MGLL	Hydrolase_4 domain-containing protein		Gallus gallus (Chicken)	PF12146;
A3F957	418784;	MBNL2	Muscleblind-like 2 isoform 2		Gallus gallus (Chicken)	PF00642;
A3F958	418784;	MBNL2	Muscleblind-like 2 isoform 3		Gallus gallus (Chicken)	PF00642;
A3F959	418784;	MBNL2	Muscleblind-like 2 isoform 5		Gallus gallus (Chicken)	PF00642;
A3F960	418784;	MBNL2	Muscleblind-like 2 isoform 7		Gallus gallus (Chicken)	
A3F961	418784;	MBNL2	Muscleblind-like 2 isoform 6		Gallus gallus (Chicken)	PF00642;
A3F962	418784;	MBNL2	Muscleblind-like 2 isoform 1		Gallus gallus (Chicken)	PF00642;
A3F963	418784;	MBNL2	Muscleblind-like 2 isoform 4		Gallus gallus (Chicken)	PF00642;
A0A1L1RUU9	770011;	MYL12B	Uncharacterized protein		Gallus gallus (Chicken)	PF13405;PF13833;
Q6W5H0	654833;		Myosin light chain smooth muscle isoform		Meleagris gallopavo (Wild turkey)	
P09540	396472;		Myosin light chain, embryonic (L23)		Gallus gallus (Chicken)	
P02612	396215;		Myosin regulatory light chain 2, smooth muscle major isoform (MLC-2) (DTNB) (G1) (Isoform L20-A)		Gallus gallus (Chicken)	PF13405;PF13833;
F1NVR6	422440;	NAA15	Uncharacterized protein		Gallus gallus (Chicken)	PF12569;PF13181;
Q5ZMS8	418319;	RCJMB04_1e13	N-acetyltransferase domain-containing protein		Gallus gallus (Chicken)	PF00583;
Q5ZMH8	427283;	NANS RCJMB04_2a5	AFP-like domain-containing protein		Gallus gallus (Chicken)	PF03102;PF08666;
A0A1D5PKH6	776932;	NAT8B	N-acetyltransferase domain-containing protein		Gallus gallus (Chicken)	PF00583;
Q5ZKW7	419556;	RCJMB04_8n22	WD_REPEATS_REGION domain-containing protein		Gallus gallus (Chicken)	PF00400;
Q5ZI00	419039;	NDUFA9 RCJMB04_31o2	Epimerase domain-containing protein		Gallus gallus (Chicken)	PF01370;
Q5ZK48	418727;	RCJMB04_13d16	Cytoplasmic protein		Gallus gallus (Chicken)	PF00017;PF00018;PF14604;
A0A3Q2UHS2	424000;	NCKAP1	Uncharacterized protein		Gallus gallus (Chicken)	PF09735;
E1C6G9	424000;	NCKAP1	Uncharacterized protein		Gallus gallus (Chicken)	PF09735;
A0A1D5P310	396085;	NF1	Neurofibromin		Gallus gallus (Chicken)	PF13716;PF00616;
A0A1D5PAD7	396085;	NF1	Neurofibromin		Gallus gallus (Chicken)	PF13716;PF00616;
A0A1D5PWG6	396085;	NF1	Neurofibromin		Gallus gallus (Chicken)	PF13716;PF00616;
A0A1D5PNU7	415316;	NPTN	Uncharacterized protein		Gallus gallus (Chicken)	
Q5ZIZ5	428701;	NONO RCJMB04_22g22	Uncharacterized protein		Gallus gallus (Chicken)	PF08075;PF00076;
Q76MF7	404775;	JC7 RCJMB04_14f20	JC7		Gallus gallus (Chicken)	
D8MIU8	768717;	NUMA	Nuclear mitotic apparatus protein		Gallus gallus (Chicken)	
R4GLW9	768717;	NUMA1	Uncharacterized protein		Gallus gallus (Chicken)	
P15771	396201;	NCL	Nucleolin (Protein C23)		Gallus gallus (Chicken)	PF00076;
Q5ZKC6	416467;	RCJMB04_11m15	Uncharacterized protein		Gallus gallus (Chicken)	
R4GJX0	427399;	NUDT2	Bis(5'-nucleosyl)-tetraphosphatase [asymmetrical] (EC 3.6.1.17) (Diadenosine 5',5'''-P1,P4-tetraphosphate asymmetrical hydrolase) (Nucleoside diphosphate-linked moiety X motif 2)		Gallus gallus (Chicken)	PF00293;
E1C4K9	419905;	NUDT3	Nudix hydrolase domain-containing protein		Gallus gallus (Chicken)	PF00293;
A0A452J855	395557;	NUDT16L1	Tudor-interacting repair regulator protein		Gallus gallus (Chicken)	

Q98UI9	395381;	MUC5B	Mucin-5B (Ovomucin, alpha-subunit)		Gallus gallus (Chicken)	PF08742;PF01826;PF00094;
F1NZY2	395381;	LOC395381	Uncharacterized protein		Gallus gallus (Chicken)	PF08742;PF01826;PF00094;
A0A1D5P2Y6	100857958;	PBXIP1	Uncharacterized protein		Gallus gallus (Chicken)	
Q5F3U9	418910;	PDS5B APRIN RCJMB04_6g19	Sister chromatid cohesion protein PDS5 homolog B (Androgen-induced proliferation inhibitor)		Gallus gallus (Chicken)	
A0A1D5PEL0	418910;	PDS5B	Sister chromatid cohesion protein PDS5 homolog B		Gallus gallus (Chicken)	
A0A1D5NU14	428948;	PDLIM1	Uncharacterized protein		Gallus gallus (Chicken)	PF15936;PF00412;PF00595;
E1C852	428948;	PDLIM1	Uncharacterized protein		Gallus gallus (Chicken)	PF15936;PF00412;PF00595;
Q9PU47	414873;	PDLIM3 ALP	PDZ and LIM domain protein 3 (Alpha-actinin-associated LIM protein)		Gallus gallus (Chicken)	PF15936;PF00412;PF00595;
F1NHA9	414873;	PDLIM3	PDZ and LIM domain protein 3		Gallus gallus (Chicken)	PF15936;PF00412;PF00595;
A0A1D5P8X3	422699;	PDLIM5	PDZ domain-containing protein		Gallus gallus (Chicken)	PF15936;PF00595;
A0A3Q2U6G6	422699;	PDLIM5	PDZ domain-containing protein		Gallus gallus (Chicken)	PF15936;PF00595;
Q5F3G1	422699;	RCJMB04_18a15	Uncharacterized protein		Gallus gallus (Chicken)	PF15936;PF00412;PF00595;
A0A1D5P4Z5	419644;	PEF1	Uncharacterized protein		Gallus gallus (Chicken)	PF13202;PF13405;
E1BXG9	428725;	PPID	Uncharacterized protein		Gallus gallus (Chicken)	PF00160;
F1NV96	408044;	PPL	SH3_10 domain-containing protein		Gallus gallus (Chicken)	PF17902;
A0A1D5PLH7	418601;	PRDX4	Thioredoxin domain-containing protein		Gallus gallus (Chicken)	PF10417;PF00578;
F1NNS8	418601;	PRDX4	Thioredoxin domain-containing protein		Gallus gallus (Chicken)	PF10417;PF00578;
Q5ZIG4	424224;	RCJMB04_26i1	Uncharacterized protein		Gallus gallus (Chicken)	
Q5ZLV1	418398;	RCJMB04_4l18	PEST proteolytic signal-containing nuclear protein		Gallus gallus (Chicken)	PF15473;
A0A1D5PGJ3	373898;	PALD1	Paladin		Gallus gallus (Chicken)	
A0A3Q3AGL8	424915;	PCYT1A	CTP_transf_like domain-containing protein		Gallus gallus (Chicken)	PF01467;
A0A3Q2UAW3	395777;	PHEX	Uncharacterized protein		Gallus gallus (Chicken)	PF01431;PF05649;
Q5ZIY2	416912;	PITPNB RCJMB04_22n3	Uncharacterized protein		Gallus gallus (Chicken)	PF02121;
Q5ZHU4	428411;	PFKP RCJMB04_33d2	Uncharacterized protein		Gallus gallus (Chicken)	PF00365;
A0A3Q2TWU3	420416;	PLCD1	Phosphoinositide phospholipase C (EC 3.1.4.11)		Gallus gallus (Chicken)	PF00168;PF09279;PF16457;PF00388;PF00387;
E1C3D8	420416;	PLCD1	Phosphoinositide phospholipase C (EC 3.1.4.11)		Gallus gallus (Chicken)	PF00168;PF09279;PF16457;PF00388;PF00387;
Q98TD1	395364;	PIT 54	PIT 54		Gallus gallus (Chicken)	PF00530;
Q5ZI39	422222;	PLS3 RCJMB04_30j1	Uncharacterized protein		Gallus gallus (Chicken)	PF00307;PF13499;
A0A3Q2UC12	419765;	PAFAH1B2	Platelet-activating factor acetylhydrolase IB subunit alpha2		Gallus gallus (Chicken)	PF13472;
Q9W6Q0	395669;		Pleckstrin		Gallus gallus (Chicken)	PF00610;PF00169;
E1C7S5	421632;	PLEKHG1	Uncharacterized protein		Gallus gallus (Chicken)	PF00621;
A0A1D5PJL7	416602;	PM5	SpaA domain-containing protein		Gallus gallus (Chicken)	PF17802;
A0A1D5P893	426023;	LOC426023	Uncharacterized protein		Gallus gallus (Chicken)	PF00013;
Q5F3W7	426402;	RCJMB04_5e8	Uncharacterized protein		Gallus gallus (Chicken)	PF00076;

A0A1L1S0D8	420098;	PTBP1	Polypyrimidine tract-binding protein 1		Gallus gallus (Chicken)	PF00076;PF11835;
Q5F456	420098;	RCJMB04_3b17	Polypyrimidine tract-binding protein 1		Gallus gallus (Chicken)	PF00076;PF11835;
A0A1L1RYG0	425504;	KCTD12	BTB domain-containing protein		Gallus gallus (Chicken)	PF02214;
E1C3V1	427644;	KCTD16	BTB domain-containing protein		Gallus gallus (Chicken)	PF02214;
A0A1D5NXK7	100859376;	PFDN5	Uncharacterized protein		Gallus gallus (Chicken)	PF02996;
F1NNQ9	417270;	PRPF4	WD_REPEATS_REGION domain-containing protein		Gallus gallus (Chicken)	PF08799;PF00400;
F1N9U0	419254;	PRPF6	PRP6 homolog (Pre-mRNA-processing factor 6) (U5 snRNP-associated 102 kDa protein)		Gallus gallus (Chicken)	PF06424;
E1BZT5	417559;	PRPF8	MPN domain-containing protein		Gallus gallus (Chicken)	PF01398;PF08082;PF08083;PF08084;PF12134;PF10598;PF10597;PF10596;
Q5ZI64	107051321;	RCJMB04_29p4	Uncharacterized protein		Gallus gallus (Chicken)	
P24802	419485;	PLOD1 PLOD	Procollagen-lysine,2-oxoglutarate 5-dioxygenase 1 (EC 1.14.11.4) (Lysyl hydroxylase 1) (LH1)		Gallus gallus (Chicken)	PF03171;
Q5ZLX0	422501;	PGRMC2 RCJMB04_4j4	Cytochrome b5 heme-binding domain-containing protein		Gallus gallus (Chicken)	PF00173;
F1NHD8	420988;	PDCD6	Uncharacterized protein		Gallus gallus (Chicken)	PF13499;
A0A1D5PLK6	420725;	PDCD6IP	BRO1 domain-containing protein		Gallus gallus (Chicken)	PF13949;PF03097;
Q5ZJ70	420725;	RCJMB04_20e18	BRO1 domain-containing protein		Gallus gallus (Chicken)	PF13949;PF03097;
F1P0M2	418774;	PCCA	Propanoyl-CoA:carbon dioxide ligase subunit alpha (EC 6.4.1.3) (Propionyl-CoA carboxylase alpha chain, mitochondrial)		Gallus gallus (Chicken)	PF02785;PF00289;PF00364;PF02786;PF18140;
A0A1D5P592	768706;	PCCB	Uncharacterized protein		Gallus gallus (Chicken)	PF01039;
Q5F3P0	417125;	RCJMB04_11e7	26S proteasome non-ATPase regulatory subunit 5		Gallus gallus (Chicken)	PF10508;
F1P1R3	422351;	PSMD10	ANK_REP_REGION domain-containing protein		Gallus gallus (Chicken)	PF12796;PF13606;
E1C6N0	424189;	PSMD14	26S proteasome regulatory subunit RPN11		Gallus gallus (Chicken)	PF01398;PF13012;
A0A1D5P8C5	424844;	PPP1R7	LRRcap domain-containing protein		Gallus gallus (Chicken)	
A0A1L1RLA6	424844;	PPP1R7	LRRcap domain-containing protein		Gallus gallus (Chicken)	
Q5ZL12	421287;	PPP1R21 CCDC128 KLRAQ1 RCJMB04_8e10	Protein phosphatase 1 regulatory subunit 21 (Coiled-coil domain-containing protein 128) (KLRAQ motif-containing protein 1)		Gallus gallus (Chicken)	PF10205;PF10212;
E1C2F4	422305;	GATD3AL2	Uncharacterized protein		Gallus gallus (Chicken)	
A0A1D5P5L5	107048987;424014;	LOC107048987 LOC112532770 LOC424014	Methyltransf_11 domain-containing protein		Gallus gallus (Chicken)	PF08241;
Q8JHF6	374263;	PYC	Pyruvate carboxylase (EC 6.4.1.1)		Gallus gallus (Chicken)	PF02785;PF00289;PF00364;PF02786;PF00682;PF02436;
A0A1D5PEH3	418610;	PDHA2	Pyruvate dehydrogenase E1 component subunit alpha (EC 1.2.4.1)		Gallus gallus (Chicken)	PF00676;
Q5F426	418610;	PDHA2 RCJMB04_3l20	Pyruvate dehydrogenase E1 component subunit alpha (EC 1.2.4.1)		Gallus gallus (Chicken)	PF00676;
A0A1D5P1U2	416066;	PDHB	Pyruvate dehydrogenase E1 component subunit beta (EC 1.2.4.1)		Gallus gallus (Chicken)	PF02779;PF02780;
Q9YH18	374204;	QKI	Protein quaking		Gallus gallus (Chicken)	PF00013;PF16551;PF16544;
F1NWV0	374204;	QKI	Protein quaking		Gallus gallus (Chicken)	PF00013;PF16551;PF16544;
Q5ZKQ2	426335;	QDPR RCJMB04_9l5	Uncharacterized protein		Gallus gallus (Chicken)	
A0A1L1RP46	421273;	RAB1B	Uncharacterized protein		Gallus gallus (Chicken)	PF00071;
Q5ZIP7	420649;	RAB5A RCJMB04_24h4	Uncharacterized protein		Gallus gallus (Chicken)	PF00071;
E1C0F3	416016;	RAB7A	Uncharacterized protein		Gallus gallus (Chicken)	PF00071;

Q5F3R8	420063;	RAB11B RCJMB04_8i9	Uncharacterized protein		Gallus gallus (Chicken)	PF00071;
A0A1D5P8F7	421048;	RAB12	Uncharacterized protein		Gallus gallus (Chicken)	PF00071;
Q5ZLB8	419318;	RAB22A RCJMB04_6n2	Uncharacterized protein		Gallus gallus (Chicken)	PF00071;
A0A1L1RUW1	100858849;	RAB34	Uncharacterized protein		Gallus gallus (Chicken)	PF00071;
Q9PU45	395511;	RDX	Radixin		Gallus gallus (Chicken)	PF00769;PF09380;PF00373;PF09379;
Q5ZJ38	416787;	RANBP1 RCJMB04_20p3	RanBD1 domain-containing protein		Gallus gallus (Chicken)	PF00638;
Q5ZL86	422701;	RCJMB04_7d16	Uncharacterized protein		Gallus gallus (Chicken)	PF00514;
A0A3Q3AUL4	420434;	RHEB	Uncharacterized protein		Gallus gallus (Chicken)	PF00071;
O93467	395442;	cRhoA RHOA rhoA	GTP-binding protein (GTPase cRhoA) (RhoA GTPase)		Gallus gallus (Chicken)	PF00071;
A0A1D5PEW1	420524;	RSU1	Uncharacterized protein		Gallus gallus (Chicken)	PF13855;
Q6LC82	395871;	cRac1A RAC1	GTPase cRac1A		Gallus gallus (Chicken)	PF00071;
E6N1V8	417044;	GNB2L1	Guanine nucleotide-binding protein beta subunit2-like 1		Gallus gallus (Chicken)	PF00400;
Q5F4B4	419295;	CREPT RPRD1B RCJMB04_1d17	Cell-cycle related and expression-elevated protein in tumor		Gallus gallus (Chicken)	PF04818;PF16566;
A0A1D5P4W0	429810;	RCC1	Uncharacterized protein		Gallus gallus (Chicken)	PF00415;
A0A1D5NZM5	419361;	RCC2	Uncharacterized protein		Gallus gallus (Chicken)	PF00415;
A0A1D5PF81	420213;	RMDN1	Uncharacterized protein		Gallus gallus (Chicken)	
Q5ZIW8	423062;	RRAS RCJMB04_23b19	Uncharacterized protein		Gallus gallus (Chicken)	PF00071;
E1BXF4	428602;	RCN1	Uncharacterized protein		Gallus gallus (Chicken)	PF13202;PF13499;
A0A1D5P5K2	378790;	RTN4	Reticulon		Gallus gallus (Chicken)	PF02453;
A0A1D5PD62	378790;	RTN4	Reticulon		Gallus gallus (Chicken)	PF02453;
A0A1D5PPG9	378790;	RTN4	Reticulon		Gallus gallus (Chicken)	PF02453;
A0A3Q2U086	378790;	RTN4	Reticulon		Gallus gallus (Chicken)	PF02453;
A0A1D5PN07	428865;	ARHGAP1	Uncharacterized protein		Gallus gallus (Chicken)	PF13716;PF00620;
A0A1D5PS30	428865;	ARHGAP1	Uncharacterized protein		Gallus gallus (Chicken)	PF13716;PF00620;
Q5ZMG8	422207;	RHOGL RCJMB04_2b11	Uncharacterized protein		Gallus gallus (Chicken)	PF00071;
Q5ZIY8	423111;	RNH1 RCJMB04_22k18	Ribonuclease inhibitor (Ribonuclease/angiogenin inhibitor 1)		Gallus gallus (Chicken)	PF13516;PF18779;
F1NIX0	418568;	RPL8	60S ribosomal protein L8		Gallus gallus (Chicken)	PF00181;PF03947;
F1P0K1	396414;	RRBP1	Rib_recp_KP_reg domain-containing protein		Gallus gallus (Chicken)	PF05104;
A0A3Q3AZJ6	425789;	RBM3	RRM domain-containing protein		Gallus gallus (Chicken)	PF00076;
Q45KQ2	425789;		Aggrecan promoter binding protein		Gallus gallus (Chicken)	PF00076;
Q5ZLU8	425789;	RBM3 RCJMB04_4m1	Cold-inducible RNA-binding protein		Gallus gallus (Chicken)	PF00076;
A0A1D5PFX6	404531;	RBM12	Uncharacterized protein		Gallus gallus (Chicken)	PF00076;
Q5ZMA3	420846;	RBM24 RCJMB04_2l21	RNA-binding protein 24 (RNA-binding motif protein 24)		Gallus gallus (Chicken)	PF00076;
E1BZ17	420846;	RBM24	RNA-binding protein 24		Gallus gallus (Chicken)	PF00076;

Q5ZKQ9	422248;	RBMX RCJMB04_9j22	RNA binding motif protein, X-linked		Gallus gallus (Chicken)	PF08081;PF00076;
A0A1D5NXB3	100859276;	LOC100859276	Uncharacterized protein		Gallus gallus (Chicken)	PF00076;PF00098;
Q5ZMK3	421485;	SCCPDH RCJMB04_1m12	Sacchrp_dh_NADP domain-containing protein		Gallus gallus (Chicken)	PF03435;
Q5ZLP5	425058;	SARNP RCJMB04_3i4 RCJMB04_5e24	SAP domain-containing protein		Gallus gallus (Chicken)	PF02037;
E1BZJ9	424739;	SCLY	Aminotran_5 domain-containing protein		Gallus gallus (Chicken)	PF00266;
F1N876	426612;	SEPHS2L	Selenide, water dikinase (EC 2.7.9.3)		Gallus gallus (Chicken)	PF00586;PF02769;
E1C8Y9	419815;	SRSF3	RRM domain-containing protein		Gallus gallus (Chicken)	PF00076;
Q5ZJ59	419116;	RCJMB04_20j14	Uncharacterized protein		Gallus gallus (Chicken)	PF00076;
P01005	416236;		Ovomucoid (Allergen Gal d I) (allergen Gal d 1)		Gallus gallus (Chicken)	PF00050;
Q6V9V7	378801;		Serine/threonine kinase 25		Gallus gallus (Chicken)	PF00069;
Q5ZMA7	422231;	STK26 RCJMB04_2k24	Protein kinase domain-containing protein		Gallus gallus (Chicken)	PF00069;
F1NQI6	421498;	SDCCAG8	Uncharacterized protein		Gallus gallus (Chicken)	PF15964;
A0A1D5NZM0	424706;	SERBP1	HABP4_PAI-RBP1 domain-containing protein		Gallus gallus (Chicken)	PF04774;PF16174;
Q5F3L2	424706;	RCJMB04_14f6	HABP4_PAI-RBP1 domain-containing protein		Gallus gallus (Chicken)	PF04774;PF16174;
A0A1D5P3G5	424522;	SH3GLB1	Endophilin-B1 (SH3 domain-containing GRB2-like protein B1)		Gallus gallus (Chicken)	PF03114;PF14604;
A0A1D5NWN5	548628;	SIRT2	Deacetylase sirtuin-type domain-containing protein		Gallus gallus (Chicken)	PF02146;
Q5ZID4	548628;	RCJMB04_27m5	Deacetylase sirtuin-type domain-containing protein		Gallus gallus (Chicken)	PF02146;
F6R1X6	395268;	SSB	Uncharacterized protein		Gallus gallus (Chicken)	PF05383;PF00076;PF08777;
Q5ZM14	422108;	SLC9A3R1 NHERF NHERF1 RCJMB04_3g21	Na(+)/H(+) exchange regulatory cofactor NHE-RF1 (NHERF-1) (Ezrin-radixin-moesin-binding phosphoprotein 50) (EBP50) (Regulatory cofactor of Na(+)/H(+) exchanger) (Sodium-hydrogen exchanger regulatory factor 1) (Solute carrier family 9 isoform A3 regulatory factor 1)		Gallus gallus (Chicken)	PF09007;PF00595;
F1NQR2	416550;	SLC9A3R2	Na(+)/H(+) exchange regulatory cofactor NHE-RF		Gallus gallus (Chicken)	PF09007;PF00595;
Q5F463	503574;	RCJMB04_2m11	Helicase_PWI domain-containing protein		Gallus gallus (Chicken)	PF18149;
Q5ZM28	420538;	RCJMB04_3f15	Uncharacterized protein		Gallus gallus (Chicken)	PF13202;PF13833;
Q5ZIA3	421772;	SNX3 RCJMB04_28m8	PX domain-containing protein		Gallus gallus (Chicken)	PF00787;
Q5ZHT0	428704;	RCJMB04_33j6	PX domain-containing protein		Gallus gallus (Chicken)	PF00787;
F1NU16	427717;	SF3A1	Uncharacterized protein		Gallus gallus (Chicken)	PF12230;PF01805;PF00240;
A0A1D6UPS9	415680;	SF3B3	Uncharacterized protein		Gallus gallus (Chicken)	PF03178;PF10433;
A0A1D5P8I3	395803;	SFPQ	Uncharacterized protein		Gallus gallus (Chicken)	PF08075;PF00076;
Q5ZHV7	418871;	SPRYD7 CLLD6 RCJMB04_32l12	SPRY domain-containing protein 7 (Chronic lymphocytic leukemia deletion region gene 6 protein homolog) (CLL deletion region gene 6 protein homolog)		Gallus gallus (Chicken)	PF00622;
E1BQV7	423878;	SLK	Non-specific serine/threonine protein kinase (EC 2.7.11.1)		Gallus gallus (Chicken)	PF00069;PF12474;
E1BQK7	423889;	SMNDC1	Tudor domain-containing protein		Gallus gallus (Chicken)	PF06003;
Q5ZLP0	420047;	RCJMB04_5f21	HMG box domain-containing protein		Gallus gallus (Chicken)	PF00505;
Q5F4B2	423044;	SWAP70 RCJMB04_1e1 RCJMB04_8b3	Switch-associated protein 70 (SWAP-70)		Gallus gallus (Chicken)	PF00169;
A0A1D5PJG4	421831;	SYNCRIP	Uncharacterized protein		Gallus gallus (Chicken)	PF18360;PF00076;
Q5ZLH7	421831;	RCJMB04_6c17	Uncharacterized protein		Gallus gallus (Chicken)	PF18360;PF00076;

P54939	395194;	TLN1 TLN	Talin-1		Gallus gallus (Chicken)	PF16511;PF00373;PF01608;PF09141;PF08913;
A0A1L1RYE4	423134;	PGR2/3	C-type lectin domain-containing protein		Gallus gallus (Chicken)	PF00059;
A0A1D5P3H6	427816;	LOC427816	Fibrinogen C-terminal domain-containing protein		Gallus gallus (Chicken)	PF00147;
E1C576	427114;	TTC37	Uncharacterized protein		Gallus gallus (Chicken)	PF13181;
F1NLC7	772208;	TXNDC12	Thioredoxin domain-containing protein 12 (EC 1.8.4.2)		Gallus gallus (Chicken)	
R4GMD9	417680;	TXNDC17	Thioredoxin domain-containing protein 17		Gallus gallus (Chicken)	PF06110;
A0A1D5NUH1	426854;	TXNL1	PITH domain-containing protein		Gallus gallus (Chicken)	PF06201;PF00085;
F1P212	421027;	TMX3	Thioredoxin domain-containing protein		Gallus gallus (Chicken)	PF00085;
E1C6J9	378897;	THY1	Thy-1 antigen (Thy-1 membrane glycoprotein)		Gallus gallus (Chicken)	PF00047;
Q90679	374161;	THYN1 THY28	Thymocyte nuclear protein 1 (Thymocyte protein Thy28) (cThy28)		Gallus gallus (Chicken)	PF01878;
A0A1D5PEL7	374161;	THYN1	Thymocyte nuclear protein 1		Gallus gallus (Chicken)	PF01878;
Q8AYT2	373888;	tfam RCJMB04_9a18	Mitochondrial transcription factor A		Gallus gallus (Chicken)	PF00505;
Q5ZLY6	420618;	RCJMB04_4g4	RRM domain-containing protein		Gallus gallus (Chicken)	PF00076;
Q9DDU8	395403;	TRA2B	Transformer-2 beta		Gallus gallus (Chicken)	PF00076;
O42390	395897;		Transforming growth factor-beta-induced protein ig-h3		Gallus gallus (Chicken)	PF02469;
F1P1A5	415991;	TKTL1	TRANSKETOLASE_1 domain-containing protein		Gallus gallus (Chicken)	PF02779;PF02780;PF00456;
A0A3Q2UB54	428387;	TMEM65	Uncharacterized protein		Gallus gallus (Chicken)	PF10507;
Q5ZKY6	428387;	TMEM65 RCJMB04_8k18	Uncharacterized protein		Gallus gallus (Chicken)	PF10507;
A0A3Q3AHG4	427218;	TNPO1	Importin N-terminal domain-containing protein		Gallus gallus (Chicken)	PF03810;
Q5ZLY3	415421;	TMOD3 RCJMB04_4g21	Uncharacterized protein		Gallus gallus (Chicken)	PF03250;
Q5F3V1	418227;	RCJMB04_6f15	Uncharacterized protein		Gallus gallus (Chicken)	PF03133;
F1P582	427009;	UQCRC2	Uncharacterized protein		Gallus gallus (Chicken)	PF00675;PF05193;
R4GJH4	423032;	USP47	Ubiquitin carboxyl-terminal hydrolase 47		Gallus gallus (Chicken)	PF14560;PF00443;
R9PXM3	423032;	USP47	Ubiquitin carboxyl-terminal hydrolase 47		Gallus gallus (Chicken)	PF14560;PF00443;
A0A1D5P7D1	417295;	UAP1L1	Uncharacterized protein		Gallus gallus (Chicken)	PF01704;
E1C856	418242;	UPK3A	Uncharacterized protein		Gallus gallus (Chicken)	
A0A1L1RPC6	768928;	VAPA	MSP domain-containing protein		Gallus gallus (Chicken)	PF00635;
Q5F419	768928;	RCJMB04_3m23	MSP domain-containing protein		Gallus gallus (Chicken)	PF00635;
Q5ZJJ3	419317;	RCJMB04_17k23	MSP domain-containing protein		Gallus gallus (Chicken)	PF00635;
Q90953	395565;	VCAN CSPG2	Versican core protein (Chondroitin sulfate proteoglycan core protein 2) (Chondroitin sulfate proteoglycan 2) (Large fibroblast proteoglycan) (PG-M)		Gallus gallus (Chicken)	PF00008;PF00059;PF00084;PF07686;PF00193;
P87498	424547;	VTG1 VTGI	Vitellogenin-1 (Minor vitellogenin) (Vitellogenin I) [Cleaved into: Lipovitellin-1 (Lipovitellin I) (LVI); Phosvitin (PV); Lipovitellin-2 (Lipovitellin II) (LVII); YGP42 (allergen Gal d 6)]		Gallus gallus (Chicken)	PF09172;PF09175;PF01347;PF00094;
P02845	424533;	VTG2 VTGII	Vitellogenin-2 (Major vitellogenin) (Vitellogenin II) [Cleaved into: Lipovitellin-1 (Lipovitellin I) (LVI); Phosvitin (PV); Lipovitellin-2 (Lipovitellin II) (LVII); YGP40]		Gallus gallus (Chicken)	PF09172;PF09175;PF01347;PF00094;
O12945	395935;		Vitronectin		Gallus gallus (Chicken)	PF00045;PF01033;
F5XVB5	419031;	VWF	von Willebrand factor		Gallus gallus (Chicken)	PF08742;PF01826;PF00092;PF00093;PF00094;

Q5ZMB6	423443;	RCJMB04_2j15	Protein kinase domain-containing protein		Gallus gallus (Chicken)	PF00069;
Q5ZLD5	419865;	RCJMB04_6k8	WD_REPEATS_REGION domain-containing protein		Gallus gallus (Chicken)	PF00400;
Q5F457	418829;	WBP4 RCJMB04_3a20	WW domain-binding protein 4 (WBP-4)		Gallus gallus (Chicken)	PF00397;PF06220;
A0A1D5PN25	386575;	YBX1	Y-box-binding protein 1		Gallus gallus (Chicken)	PF00313;
Q91956	386575;		CSD domain-containing protein		Gallus gallus (Chicken)	PF00313;
A0A3Q3A9V6	422301;	ZNF185L	LIM zinc-binding domain-containing protein		Gallus gallus (Chicken)	
A0A1D5PMM8	417413;	ZNF207	Uncharacterized protein		Gallus gallus (Chicken)	
A0A1D5PN34	417413;	ZNF207	Uncharacterized protein		Gallus gallus (Chicken)	
A0A1L1RNP9	417413;	ZNF207	Uncharacterized protein		Gallus gallus (Chicken)	
F1NRI7	422841;	ZNF518B	Uncharacterized protein		Gallus gallus (Chicken)	
