# Supplementary material for: Proteomic Analysis of Chicken Chorioallantoic Membrane (CAM) during Embryonic Development Provides Functional Insight
Source: Biomed Res Int. 2022 Jun 19;2022:7813921. doi: 10.1155/2022/7813921 (PMC9237712; doi:10.1155/2022/7813921)
Supplement: Supplementary 6 — Functional annotation analysis of the identified CAM protein constituents common to ED12 and ED19 and absent from EBS. [file 7813921.f6.pdf]

Table S6. Functional annotation analysis of the identified CAM protein constituents common to ED12 and ED19, and absent from EBS.

Cluster No.	GO term	No. of proteins	Official gene symbol
1	GO:0,000,166 Nucleotide binding	102	*ACAD9, ACADL, ACTA2, AK1, AKT1, ARL8B, ASNS, ARF6, ATL1, ATP2A2, CAMK2D, CARS, CDC42, CDK5, CHTF18, CSK, CSNK2A1, DES, DLD, DNM1L, DYNC1H1, DYNC1LI1, EFTUD2, EHD3, ETFA, FMO3, GALK2, GARS, GLUD2, GMPS, GNA11, GNAI2, GNAI3, GNAS, GRPEL1, GSK3A, GSR, HARS, HIBADH, HK1, HRAS, HSP90AB1, HSP90B1, HYOU1, IARS2, IDH3A, IGF2BP1, IGF2BP3, ILF2, ILK, LONP1, MAPK3, MAPK14, MATR3, MCM6, MYLK, MYH11, MYO1B, MYO1C, MYO6, NARS, NDUFA10, NDUFV1, NDUFV2, NT5C1B, NT5C2, PC, POLQ, POR, PSMC5, RRAGA, PRKAR1A, PYGL, RAB5A, RAB6A, RAB10, RAB18, RAB22A, RAB32, RBM25, RHOA, RHOGL, RRAS, SAR1A, SDHA, SEPHS1, SEPT2L, SEPT6, SEPT7, SEPTIN8, SEPTIN9, SEPTIN11, SIRT2, SIRT5, SRC, SUCLA2, SYNCRIP, TUBB2A, TUBB2B, UBA2, UBA7, VPS4B*
2	GO:0,005,198 Structural molecule activity	41	*ANXA1, ASL2, CLDN1, CLTB, COL1A1, COL1A2, COL3A1, COL4A1, COL4A2, COL11A1, COL12A1, COL18A1, COPG1, DES, KRT7, KRT14, LAMA1, LAMC1, MYH11, RPL3, RPL4, RPL6, RPL13, RPL14, RPL15, RPL17, RPL18A, RPL21, RPL26L1, RPL31, RPL36, RPS6, RPS11, RPS12, RPS14, RPS15A, RPS17, TUBB2A, TUBB2B, VAPA, VMO1*
3	GO:0,008,104 Protein localization	32	*AKAP12, AKT1, AP1M1, AP2A2, AP2M1, ARF6, CAV1, CDC42, CHMP6, CLTB, COPG1, CTNNB1, GRPEL1, HSPG2, KPNA4, MYO6, PCNA, RAB5A, RAB6A, RAB18, RAB22A, RAB32, RRBP1, SAR1A, SEC61A1, STXBP1, VAMP7, VPS29, USO1, XPO1, YWHAH*
4	GO:0,007,242 Intracellular signaling cascade	29	*AKT1, ARF6, ARL8B, CAV1, CDC42, COL1A2, CSK, GNA11, GNAI2, GNAS, GNB1, HACD3, HRAS, MAPK3, MAPK14, MYO6, RRAS, RAB5A, RAB6A, RAB10, RAB18, RAB22A, RAB32, RPS6, RHOA, RHOGL, STMN1, STAT3, YWHAH*
5	GO:0,016,192 Vesicle-mediated transport	26	*AP1M1, AP2A2, AP2M1, ARF6, CLTB, COLEC12, COPG1, CTNNB1, GARS, HRAS, ITGAV, MYH11, MYO6, RAB5A, RAB6A, RAB22A, RER1, SAR1A, SCFD1, SH3GL1, STXBP1, TMED10, TRAPPC3, USO1, VAMP7, VPS4B*
6	GO:0,006,412 Translation	24	*CARS, EIF1AD, GARS, HARS, IARS2, NARS, RPL3, RPL4, RPL6, RPL13, RPL14, RPL15, RPL17, RPL18A, RPL21, RPL26L1, RPL31, RPL36, RPS6, RPS11, RPS12, RPS15A, RPS17, RPS14*
7	GO:0,006,091 Generation of precursor metabolites and energy	24	*ACO2, AKT1, ATP5C1, ATP5PB, ATP5O, ATP6V1E1, CYCS, DLD, DLST, ETFDH, FH, GNAS, GNPDA1, HK1, IDH3A, NDUFA7, NDUFB8, NDUFS1, NDUFS4, PGAM1, PYGL, SDHA, SLC25A13, UQCRFS1*
8	GO:0,046,907 Intracellular transport	22	*AKAP12, AKT1, AP1M1, AP2A2, AP2M1, CLTB, COPG1, GRPEL1, KPNA4, MYO6, PCNA, RER1, SAR1A, SLC25A13, TMED10, TRAPPC3, UCHL1, USO1, VAMP7, VPS4B, XPO1, YWHAH*
9	GO:0,003,735 Structural constituent of ribosome	18	*RPL3, RPL4, RPL6, RPL13, RPL14, RPL15, RPL17, RPL18A, RPL21, RPL26L1, RPL31, RPL36, RPS6, RPS11, RPS12, RPS14, RPS15A, RPS17*
10	GO:0,042,802 Identical protein binding	18	*AKT1, ALDH1A3, CLDN1, COL1A2, GRPEL1, HDAC1, HINT2, HPRT1, HSPB1, ITGB3, LCP1, MCM6, MYO6, NRP1, PRRC1, OSTF1, S100A11, SNX32*
11	GO:0,007,010 Cytoskeleton organization	15	*ARF6, CAPZA2, CDC42, CNN1, CNN2, CTNNB1, EZR, HRAS, ITGB1, KRT14, LCP1, MYH11, PFN2, RHOA, THY1*

12	GO:0,032,989 Cellular component morphogenesis	15	*CDC42, CNP, CTNNB1, EZR, HPRT1, ILK, ITGB1, LAMA1, LAMC1, MAPK14, MYH11, NRP1, RHOA, STMN1, UCHL1*
13	GO:0,004,857 Enzyme inhibitor activity	14	*A2ML1, ANXA1, ANXA2, COL6A3, CST3, CSTB, OVALX, OVALY, OVST, RNH1, SERPINH1, SERPINB6, SPINK5, SPINK7*
14	GO:0,030,029 Actin filament-based process	13	*ARF6, CDC42, CNN1, CNN2, CAPZA2, EZR, HRAS, ITGB1, LCP1, MYH11, MYO6, PFN2, RHOA*
15	GO:0,005,996 Monosaccharide metabolic process	13	*AKT1, FUCA2, GALE, GALK2, GNPDA1, HIBADH, HK1, MAPK14, PC, PGAM1, PGM1, PMM2, PYGL*
16	GO:0,001,944 Vasculature development	13	*AKT1, ANXA2, CAV1, COL1A2, CTNNB1, ITGAV, MAPK14, MMP2, NRP1, POFUT1, QKI, RTN4, THY1*
17	GO:0,033,043 Regulation of organelle organization	11	*ARF6, ARPC1B, ARPC2, CAPZA2, CDC42, CTNNB1, HSPB1, MAP4, RHOA, STMN1, XPO1*
18	GO:0,030,030 Cell projection organization	11	*ARF6, CDC42, CNP, HPRT1, ITGA6, ITGA8, MYH11, MYO6, NRP1, STMN1, UCHL1*
19	GO:0,046,034 ATP metabolic process	10	*AK1, ATP1B1, ATP1B3, ATP2A2, ATP5C1, ATP5O, ATP5PB, ATP6V1E1, NDUFS1, VPS4B*
20	GO:0,016,044 Membrane organization	10	*COLEC12, ITGAV, HRAS, MYH11, MYO6, RAB5A, RAB22A, SH3GL1, USO1, VAMP7*
21	GO:0,051,186 Cofactor metabolic process	10	*ACO2, BAAT, COQ4, DBT, DLST, FH, HIBADH, HMOX2, IDH3A, SDHA*
22	GO:0,051,129 Negative regulation of cellular component organization	10	*CAPZA2, CDC42, CLU, HSPB1, MAP4, NRP1, RTN4, STMN1, THY1, YWHAH*
23	GO:0,045,596 Negative regulation of cell differentiation	10	*CAV1, CTNNB1, ITGAV, ITGB1, ITGB3, MAPK3, NRP1, RTN4, THY1, YWHAH*
24	GO:0,016,651 Oxidoreductase activity, acting on NADH or NADPH	9	*DLD, NDUFA7, NDUFA12, NDUFB8, NDUFS1, NDUFS3, NDUFS4, NDUFS8, NDUFV1*
25	GO:0,044,087 Regulation of cellular component biogenesis	9	*ARF6, ARPC1B, ARPC2, CAPZA2, CDC42, CLU, HSPB1, RHOA, STMN1*
26	GO:0,031,589 Cell-substrate adhesion	8	*CTNNB1, ITGA6, ITGB1, ITGB3, LAMC1, NID1, RHOA, THY1*
27	GO:0,006,631 Fatty acid metabolic process	7	*ETFDH, FASN, HADH, HADHA, MAPK14, QKI, RNPEP*
28	GO:0,008,238 Exopeptidase activity	7	*CTSA, ERAP1, LAP3, METAP2, PEPD, RNPEP, SCPEP1*
29	GO:0,051,539 4 iron, 4 sulfur cluster binding	5	*ACO2, ETFDH, NDUFS1, NDUFS8, NDUFV1*
30	GO:0,042,773 ATP synthesis coupled electron transport	4	*DLD, NDUFA7, NDUFB8, NDUFS1*
31	GO:0,034,330 Cell junction organization	4	*CDC42, ITGB3, LAMC1, THY1*

32	GO:0,016,620 Oxidoreductase activity, acting on the aldehyde or oxo group of donors, NAD or NADP as acceptor	4	*ALDH1A3, ALDH3A2, ALDH3B2, ALDH5A1*
33	GO:0,032,369 Negative regulation of lipid transport	3	*AKT1, ITGB3, ITGAV*
