# Supplementary material for: Proteomic Analysis of Chicken Chorioallantoic Membrane (CAM) during Embryonic Development Provides Functional Insight
Source: Biomed Res Int. 2022 Jun 19;2022:7813921. doi: 10.1155/2022/7813921 (PMC9237712; doi:10.1155/2022/7813921)
Supplement: Supplementary 7 — Functional annotation analysis of the identified CAM protein constituents specific to either ED12 or ED19 and absent from EBS. [file 7813921.f7.pdf]

Table S7. Functional annotation analysis of the identified CAM protein constituents specific to either ED12 or ED19, and absent from EBS.

CAM (days)	Cluster No.	GO term	No. of proteins	Official gene symbol
CAM (ED12)	1	GO:0,055,114 Oxidation reduction	11	*ACADSB, BDH1A, DHFR, KDM1A, LOC415661, P3H1, P3H3, P4HA1, PLOD2, PRCP, PYCR1*
	2	GO:0,034,621 Cellular macromolecular complex subunit organization	7	*ENAH, H2AFY, HIST1H4B, MRPL58, SNAP91, TUBAL3, TUBB6*
	3	GO:0,031,406 Carboxylic acid binding	5	*P3H1, P3H3, P4HA1, PLOD2, SH3GLB1*
	4	GO:0,019,842 Vitamin binding	5	*P3H1, P3H3, P4HA1, PLOD2, SCLY*
	5	GO:0,007,015 Actin filament organization	3	*ENAH, NCK1, PDLIM3*
CAM (ED19)	1	GO:0,016,310 Phosphorylation	12	*ATP6V0A4, ATP6V1C2, CASK, LOC776719, MAP2K2, PDPK1, PRKCD, ROCK1, ROCK2, RPS6KA3, SLK, STK25*
	2	GO:0,007,264 Small GTPase mediated signal transduction	5	*CDH13, RAP2A, RAB4A, RAB8A, RHEB*
	3	GO:0,004,857 Enzyme inhibitor activity	5	*CD109, CST7, SERPINB10B, SERPINB1, TIMP3*
