# Supplementary material for: Proteomic Analysis of Chicken Chorioallantoic Membrane (CAM) during Embryonic Development Provides Functional Insight
Source: Biomed Res Int. 2022 Jun 19;2022:7813921. doi: 10.1155/2022/7813921 (PMC9237712; doi:10.1155/2022/7813921)
Supplement: Supplementary 8 — Functional annotation analysis of the identified EBS protein constituents. [file 7813921.f8.pdf]

Table S8. Functional annotation analysis of the identified EBS protein constituents.

Cluster No.	GO term	No. of proteins	Official gene symbol
1	GO:0,000,166 Nucleotide binding	157	*AARS1, ABCB10, ABCE1, ACSS1A, ACTBL2, ACTC1, ACTG1, ACTL6A, ACTR1A, ACTR2, ACTR3, ACLY, ADSS1, AK2, ALYREF, APAF1, ATP1A1, ATP5B, ATP6V1A, CAT, CCT2, CCT3, CCT4, CCT5, CCT6A, CCT7, CCT8, CKB, CMPK1, CPSF6, CTBP1, DARS, DAZAP1, DDX1, DDX3X, DDX6, DDX17, DDX19, DHX15, EEF1A1, EEF2, EFL1, EIF2S3, EIF4A2, EIF4A3, EIF4H, EIF5B, ELAVL1, EPRS, FARSB, FKBP4, FUS, G3BP1, GAPDH, GART, GNAL, GNA14, GPD1L, GSS, HK2, HAO2, HNRNPA1, HNRNPA2B1, HNRNPAB, HNRNPD, HNRNPDL, HNRNPH1, HNRNPH3, HNRNPM, HNRNPUL2, HSP90AA1, HSPA2, HSPA4L, HSPA5, HSPA8, HSPA9, HSPD1, HSPE1, IDH1, IDH2, KARS, LOC420368, MAST2, MOV10, MTHFD1, MYH1F, MYH9, MYH10, NACA, NCL, NME2, NONO, OLA1, PABPC1, PAICS, PGD, PKLR, PGK2, PFKP, PSMC1, PSMC2, PSMC3, PSMC6, PTBP1, PUF60, QARS, RAB1B, RAP1B, RAB8B, RAB9A, RAB11B, RAB14, RAB2A, RAB5C, RAB21, RAC1, RALY, RAN, RBM3, RARS, RAP1A, RBM12, RMB24, RBMXL1, RUVBL1, SARS1, SFPQ, SMC1A, SMC2, SMC3, SMC4, SNRNP200, SRSF1, SRSF2, SRSF3, SRSF6, SSB, STK26, TARS, TCP1, TOP2B, TUFM, TRA2A, TRA2B, TTN, TUBA1A, TUBA3E, TUBA8B, TUBB, TUBB1, TUBB4B, UBA3, UBE2I, UGDH, VCP, VRK1, YARS*
2	GO:0,006,508 Proteolysis	45	*APEH, BLMH, C3, CAPN1, CASP3, CNDP2, DDB1, DDI2, DNPEP, LTA4H, MYH9, NPEPL1, NPEPPS, PCNP, PLG, PM20D1, PREP, PSMA1, PSMA2, PSMA3, PSMA4, PSMA5, PSMA6, PSMA7, PSMB1, PSMB2, PSMB4, PSMB5, PSMB7, PSMC2, RAD23B, TPP2, UBE2H, UBE2I, UBE2L3, UBE2N, UBE2V2, UCHL3, UCHL5, UFD1L, UFSP2, USP7, USP9Y, USP47, XPNPEP1*
3	GO:0,009,057 Macromolecule catabolic process	39	*APOB, CASP3, DDB1, EIF3E, EIF4A3, FEN1, LYG2, MYH9, PCNP, PSMA1, PSMA2, PSMA3, PSMA4, PSMA5, PSMA6, PSMA7, PSMB1, PSMB2, PSMB4, PSMB5, PSMB7, PSMC1, PSMC2, PSMC3, PSMC6, RAD23B, SOD1, UBE2H, UBE2I, UBE2L3, UBE2N, UBE2V2, UCHL3, UCHL5, UFD1L, UFSP2, USP7, USP9Y, USP47*
4	GO:0,008,104 Protein localization	33	*ACTN4, APOB, ARCN1, BID, CLTC, COPA, COPB1, ERP29, GDI1, HSPA9, LIN7C, MYH9, NPM1, PLEK, RAB2A, RAB5C, RAB8B, RAB9A, RAB11B, RAB14, RAB21, RAN, RDX, SCAMP2, SNX2, SEC22B, TLN1, XPO7, YWHAB, YWHAE, YWHAG, YWHAQ, YWHAZ*
5	GO:0,065,003 Macromolecular complex assembly	31	*APOA4, ALDOB, ARPC4, CAT, FGA, FGB, FGG, FKBP4, GSN, H1F0, H2AZ2, HIST1H111L, HIST1H111R, HIST1H2A4, HIST1H2B7, HIST1H2B8, HIST2H4B, HP1BP3, LOC769852, MYH10, NAP1L4, NPM1, SET, SOD2, TUBA1A, TUBA3E, TUBA8B, TUBB, TUBB1, TUBB4B, XPO7*
6	GO:0,006,091 Generation of precursor metabolites and energy	30	*ACO1, ATP5B, ATP5A1W, ATP6V1A, ATP6V1B2, ATP6V1H, ALDOB, ALDOC, BPGM, CAT, CYB5A, ENO1, GAPDH, GLRX, GOT1, GPI, HK2, LDHA, LDHB, MDH1, MDH2, OGDH, PDHA2, PFKP, PGK2, PKLR, PPP1CB, SOD2, TPI1, TXN*
7	GO:0,070,011 Peptidase activity, acting on L-amino acid peptides	30	*APEH, BLMH, CAPN1, CASP3, CNDP2, COPS5, DDI2, DNPEP, LTA4H, NPEPL1, NPEPPS, PLG, PM20D1, PREP, PSMA1, PSMA2, PSMA3, PSMA4, PSMA5, PSMA6, PSMA7, PSMB1, PSMB2, PSMB4, PSMB5, PSMB7, TPP2, UFSP2, USP7, XPNPEP1*
8	GO:0,005,525 GTP binding	30	*ADSS1, EEF1A1, EEF2, EFL1, EIF5B, EIF2S3, FKBP4, GNA14, GNAL, LOC420368, OLA1, RAB11B, RAB1B, RAB2A, RAB5C, RAB8B, RAB9A, RAB14, RAB21, RAC1, RAN, RAP1A, RAP1B, TUBA1A, TUBA3E, TUBA8B, TUBB, TUBB1, TUBB4B, TUFM*
9	GO:0,042,592 Homeostatic process	30	*ACO1, ADD1, APOA4, APOB, BCL2L1, CASP3, GCLM, GLRX, GLRX3, GOT1, FTH1, HBZ, NPM1, P4HB, PARK7, PDIA3, PDIA4, PDIA6, PLG, PRDX1, PRDX6, RHAG, RPA1, RBP4A, SOD1, SOD2,*

			TCEA1, TF, TMX4, TXN
10	GO:0,044,271 Nitrogen compound biosynthetic process	28	*ADA, ADAL, ADK, ADSL, ADSS1, AMPD3, ATIC, ATP5B, ATP1A1, ATP5A1W, ATP6V1A, ATP6V1B2, ATP6V1H, CBSL, CMPK1, CPOX, FECH, GART, GOT1, GOT2, HMBS, NME2, PAICS, PRPS1L1, PRPSAP2, TMLHE, UMPS, UROD*
11	GO:0,051,276 Chromosome organization	25	*BRE, BUB3, H1F0, H2AZ2, HIST1H111L, HIST1H111R, HIST1H2A4, HIST1H2B7, HIST1H2B8, HIST2H4B, HP1BP3, LOC769852, NCAPD2, NAP1L4, NPM1, PDS5B, RPA1, RBBP4, RUVBL1, SET, SMC1A, SMC2, SMC3, SMC4, UBE2N*
12	GO:0,016,052 Carbohydrate catabolic process	23	*AKR1A1, ALDOB, ALDOC, BPGM, ENO1, GAPDH, GPD1L, GPI, HK2, LDHA, LDHB, LYG2, MDH1, MDH2, OGDH, PDHA2, PFKP, PGD, PGK2, PGLS, PKLR, TALDO1, TPI1*
13	GO:0,005,996 Monosaccharide metabolic process	23	*AKR1A1, BPGM, ALDOB, ALDOC, ENO1, GAPDH, GPI, HK2, LDHA, LDHB, MDH1, MDH2, OGDH, PDHA2, PFKP, PGD, PGLS, PGK2, PKLR, RBP4A, TPI1, PPP1CB, TALDO1*
14	GO:0,046,907 Intracellular transport	23	*ACTBL2, ACTC1, ARCN1, BCL2L1, BID, CLTC, COPA, COPB1, HSPA9, MYH9, NPM1, NUP133, NUP155, BAFAH1B1, RAN, RANBP1, SNX2, XPO7, YWHAB, YWHAE, YWHAG, YWHAQ, YWHAZ*
15	GO:0,042,981 Regulation of apoptosis	23	*AARS1, ADA, ALB, API5, APAF1, BCL2L1, BID, CAT, CIAPIN1, EEF1A1, FADD, HSPA5, CASP3, GAPDH, GCLM, HSPD1, NME2, PDIA3, PGP, PLG, RPS3, SOD1, SOD2*
16	GO:0,016,887 ATPase activity	23	*ACTBL2, ABCB10, ABCE1, ACTC1, ATP1A1, ATP5A1W, ATP5B, ATP6V1A, ATP6V1B2, ATP6V1H, DDX1, DDX3X, DDX6, DDX17, DDX19, DHX15, EIF4A2, EIF4A3, HSPA8, MYH9, PSMC2, RBBP4, SNRNP200*
17	GO:0,042,802 Identical protein binding	21	*ACTG1, ADH4, ALDOB, APOA4, ARHGDIA, BCL2L1, CAT, ELAVL1, FBP1, GPD1L, HSP90AA1, MYH9, NFS1, NONO, NPM1, PAFAH1B1, PLEK, PSMA7, TPD52, TRA2A, VAPB*
18	GO:0,006,396 RNA processing	20	*AARS1, CPSF6, CSTF3, EIFA3, HNRNPKL, MAGOH, MOV10, PRPF19, PTBP1, RPL10A, SF3A1, SNRNP200, SNRPE, SRSF1, SRSF2, SSB, STRAP, TRA2A, WBP4, YBX1*
19	GO:0,007,010 Cytoskeleton organization	20	*ACTBL2, ACTC1, ACTG1, ARPC4, CAPZA1, CAPZB, EPB41, GSN, KRT24, MYH9, MYH10, NPM1, PLEK, RAC1, BAFAH1B1, RANBP1, SMC3, SOD1, TLN1, TUBB1*
20	GO:0,033,554 Cellular response to stress	20	*AARS1, ALB, APOA4, BRE, CASP3, CAT, EIF2S1, DDB1, FEN1, HMGB2, HSPA5, PRPF19, RAD23B, RPA1, RPS3, SOD1, SOD2, UBE2N, UBE2V2, VAPB*
21	GO:0,016,071 mRNA metabolic process	18	*CPSF6, CSTF3, EIF3E, EIFA3, ELAVL1, HNRNPD, HNRNPKL, MAGOH, MOV10, PRPF19, PTBP1, SNRPE, SRSF1, SRSF2, STRAP, TRA2A, WBP4, YBX1*
22	GO:0,042,623 ATPase activity, coupled	18	*ABCB10, ATP1A1, ATP5A1W, ATP5B, ATP6V1A, ATP6V1B2, ATP6V1H, DDX1, DDX3X, DDX6, DDX17, DDX19, DHX15, EIF4A2, EIF4A3, HSPA8, RBBP4, SNRNP200*
23	GO:0,009,259 Ribonucleotide metabolic process	18	*ADA, ADAL, ADSL, AMPD3, ATIC, ATP5B, ATP1A1, ATP5A1W, ATP6V1A, ATP6V1B2, ATP6V1H, ENTPD1, GART, NME2, OLA1, PAICS, PRPS1L1, UMPS*
24	GO:0,006,163 Purine nucleotide metabolic process	18	*ADA, ADAL, ADK, ADSL, ADSS1, AMPD3, ATIC, ATP5B, ATP1A1, ATP5A1W, ATP6V1A, ATP6V1B2, ATP6V1H, ENTPD1, GART, NME2, OLA1, PAICS*
25	GO:0,008,135 Translation factor activity, nucleic acid binding	16	*EEF1A1, EEF1B2, EEF2, EIF1AX, EIF2S1, EIF2S2, EIF2S3, EIF3D, EIF3E, EIF3J, EIF3M, EIF4A2, EIF4E, EIF5, TCEA1, TUFM*

26	GO:0,003,924 GTPase activity	16	*EEF1A1, EEF2, EFL1, EIF5B, EIF2S3, RAC1, RAN, RAP1A, RAP1B, TUBA1A, TUBA3E, TUBA8B, TUBB, TUBB1, TUBB4B, TUFM*
27	GO:0,009,260 Ribonucleotide biosynthetic process	16	*ADA, ADAL, ADSL, AMPD3, ATIC, ATP1A1, ATP5A1W, ATP5B, ATP6V1A, ATP6V1B2, ATP6V1H, GART, NME2, PAICS, PRPS1L1, UMPS*
28	GO:0,016,879 Ligase activity, forming carbon-nitrogen bonds	16	*ADSS1, GART, GCLC, GCLM, GSS, MTHFD1, PAICS, PRPF19, UBA3, UBE2H, UBE2I, UBE2L3, UBE2N, UBE2V2, UBE2O, UBR4*
29	GO:0,033,043 Regulation of organelle organization	14	*ACTR3, ARPC1A, ARPC5, CAPZA1, CAPZB, DSTN, GSN, MAPRE1, NPM1, PLEK, RDX, RANBP1, SPTAN1, UBE2N*
30	GO:0,022,402 Cell cycle process	14	*BUB3, MARRE1, MYH10, NCAPD2, NPM1, PAFAH1B1, PDS5B, RANBP1, RPA1, SMC2, SMC3, SMC4, TUBB1, UBE2I*
31	GO:0,004,857 Enzyme inhibitor activity	14	*A2ML2, AGT, AMBP, ANP32E, C3, CASP3, CAST, FETUB, HSPA5, ITIH2, KNG1, OVAL, SERPINC1, SERPIND1*
32	GO:0,006,732 Coenzyme metabolic process	13	*ACO1, ALDOB, GCLC, GCLM, GSS, MDH1, MTHFD1, PGD, PGLS, SOD1, SOD2, TALDO1, TPI1*
33	GO:0,009,611 Response to wounding	13	*C3, CASP3, ENTPD1, F13A1, FGA, FGB, FGG, FN1, PLEK, PLG, MYH10, SOD1, SOD2*
34	GO:0,032,535 Regulation of cellular component size	12	*ACTR3, ARPC1A, ARPC5, CAPZA1, CAPZB, DSTN, ENO1, GSN, NPM1, PLEK, RDX, SPTAN1*
35	GO:0,001,701 In utero embryonic development	12	*ADA, ADD1, APOB, BCL2L1, GABPA, MYH10, PITPNB, PRPF19, PSMC3, RPA1, SRSF1, YBX1*
36	GO:0,051,258 Protein polymerization	11	*ARPC4, FGA, FGB, FGG, GSN, TUBA1A, TUBA5, TUBA8B, TUBB, TUBB1, TUBB4B*
37	GO:0,051,301 Cell division	11	*DSTN, MARRE1, MYH9, MYH10, PAFAH1B1, PDS5B, PPP1CB, RBP4A, SMC2, TOP1, UBE2I*
38	GO:0,006,418 tRNA aminoacylation for protein translation	10	*AARS1, DARS, EPRS, FARSB, KARS, PARS, QARS, SARS1, TARS, YARS*
39	GO:0,006,979 Response to oxidative stress	9	*APOA4, CAT, GCLM, GPX3, GPX4, PARK7, PRDX6, SOD1, SOD2*
40	GO:0,070,647 Protein modification by small protein conjugation or removal	9	*COPS5, PCNP, PRPF19, RPS27A, UBA3, UBE2H, UBE2N, UBI, USP7*
41	GO:0,016,209 Antioxidant activity	8	*APOA4, CAT, GPX3, GPX4, PRDX1, PRDX6, SOD1, SOD2*
42	GO:0,051,188 Cofactor biosynthetic process	8	*CPOX, FECH, GCLC, GCLM, GSS, MTHFD1, NFS1, UROD*
43	GO:0,015,669 Gas transport	8	*CA2, HBA1, HBAD, HBBA, HBBR, HBE1, HBZ, RHAG*
44	GO:0,016,860 Intramolecular oxidoreductase activity	7	*GPI, HPGDS, NACA, P4HB, PDIA3, PDIA4, TPI1*

45	GO:0,010,035 Response to inorganic substance	7	*APOA4, CAT, FGA, FGB, PARK7, SOD1, SOD2*
46	GO:0,016,051 Carbohydrate biosynthetic process	7	*GAPDH, GPI, GOT1, NANS, PGD, RBP4A, TPI1*
47	GO:0,019,748 Secondary metabolic process	7	*ADH4, ALDOB, PGD, PGLS, RBP4A, TALDO1, TPI1*
48	GO:0,006,790 Sulfur metabolic process	7	*CBSL, GCLC, GCLM, GSS, NFS1, SOD1, SOD2*
49	GO:0,019,825 Oxygen binding	7	*ALB, HBA1, HBAD, HBBA, HBBR, HBE1, HBZ*
50	GO:0,050,657 Nucleic acid transport	6	*EIF4A3, HNRNPA2B1, KHSRP, MAGOH, NPM1, NUP133*
51	GO:0,018,130 Heterocycle biosynthetic process	6	*ADA, ADK, CPOX, FECH, HMBS, UROD*
52	GO:0,010,927 Cellular component assembly involved in morphogenesis	6	*ACTBL2, ACTC1, ACTG1, KRT24, MYH10, PAFAH1B1*
53	GO:0,016,125 Sterol metabolic process	6	*APOA1, APOA4, APOB, CAT, FDPS, LCAT*
54	GO:0,033,013 Tetrapyrrole metabolic process	5	*BLVRA, CPOX, FECH, HMBS, UROD*
55	GO:0,046,961 Proton-transporting ATPase activity, rotational mechanism	5	*ATP5B, ATP5A1W, ATP6V1A, ATPV1B2, ATP6V1H*
56	GO:0,043,498 Cell surface binding	4	*APOA4, FGA, FGB, HSPD1*
57	GO:0,030,261 Chromosome condensation	4	*H1F0, NCAPD2, SMC2, SMC4*
58	GO:0,046,890 Regulation of lipid biosynthetic process	4	*APOA4, APOB, ATP1A1, SOD1*
59	GO:0,008,637 Apoptotic mitochondrial changes	4	*BCL2L1, BID, CASP3, SOD2*
