# Supplementary material for: Proteomic Analysis of Chicken Chorioallantoic Membrane (CAM) during Embryonic Development Provides Functional Insight
Source: Biomed Res Int. 2022 Jun 19;2022:7813921. doi: 10.1155/2022/7813921 (PMC9237712; doi:10.1155/2022/7813921)
Supplement: Supplementary 9 — Various CAM functions and the associated proteins identified in this study. [file 7813921.f9.pdf]

Table S9. Various CAM functions and the associated proteins identified in this study.

Functions		Involved proteins	Involved cell/tissue
Ca^{2+} transport	Calcium mobilization from ES (Acidification)	▪ Carbonic anhydrase (*CA2*, *CA13*)	CE, AE
		▪ Vacuolar H^+-ATPase (*ATP6V0A4*, *ATP6V1C2*, ATP6VOD1, *ATP6V1E1*, *ATP6V1G1*, *LOC776719*)	CE, AE
		▪ Cell-cell junction proteins (*CDC42*, *ITGB3*, *LAMC1*, *THY1*)	CE
	Intracellular Ca^{2+} transport (${Ca}^{2+}$ binding proteins, CaBPs)	▪ Annexin (*ANXA 1, ANXA2, ANXA5)*<br>▪ Calmodulin (*CALML3*)<br>▪ S100 calcium binding protein (*S100A11*, *S100A11*, *S100A16*)<br>▪ Calcineurin like EF-hand protein 1 (*CHP1*)<br>▪ Calcium binding protein 39 (*CAB39*)	CE
	Intracellular Ca^{2+} transport (ATPases)	▪ Ca^{2+}-ATPase (*ATP2A2)* and Na^+/K^+-ATPase (*ATP1B1*, *ATP1B3*)	CE
Protection against pathogen invasion	Antimicrobial activities	▪ Cystatins (B, C, F)<br>▪ SERPINs (*SERPINB1, SERPINB2, SERPINB5, SERBINB6, SERPINB10B, SERPINB14C, SERPINH1)*<br>▪ Histones (H2AFY, *HIST1H2BO*, HIST1H4B, HISTH2A4L2)<br>▪ SPINKs (Ovomucoid, ovoinhibitor)<br>▪ BPI fold containing family B member 2 (TENP)	CE, M, AE
Vascular system	Diffusion	▪ From external air to the blood capillaries	ES, ESM, CE
	Gaseous exchange	▪ Annexins (A1, A2, A5, A7, A8 like)<br>▪ Catenin subunits (α1, β1)<br>▪ Collagen subunits (1α1, 1α2, 3α1, 4α1, 4α2, 5α2, 6α1, 6α2, 6α3, 7α1, 11α1, 12α1, 18α1)<br>▪ Decorin<br>▪ Fibulin (1,2)<br>▪ Glypican 4<br>▪ Integrin subunits (α1, α3, α5, α6, α8, α9, αv, β1, β3, β4)<br>▪ Laminin subunits (α1, α5, β1, β2, γ1)<br>▪ Nidogen (1,2)<br>▪ Tubulin subunits (β2A, β2B, β3 class III, β6 class V, α like 3)<br>▪ Tenascin XB	M
	Lymphatics	▪ Collagen IV subunits (α1, α2)<br>▪ Laminin subunits (α1, α5, β1, β2, γ1)<br>▪ Neuropilin-1 (NRP1)<br>▪ Mitogen-activated protein kinase (MAPK3, Erk)<br>▪ Nidogen (1,2)<br>▪ Mannose receptor (*MRC1*, *MRC2*)<br>▪ Desmoplakin (DSP)<br>▪ PDZ and LIM domain 3 (*PDLIM3*)<br>▪ TIMP metallopeptidase inhibitor 3 (TIMP3)<br>▪ Proteosome assembly chaperone 1 (*PSMG1*)<br>▪ Junction plakoglobin (*JUP*)<br>▪ Integrin subunit α6 (*ITGA6*)<br>▪ Myoferlin (*MYOF*)<br>▪ Apolipoprotein D (*APOD*)<br>▪ Collectin subfamily member 12 (*COLEC12*)	M

		▪ Galectin-1 (*LGALS1*)	
	Blood serum	▪ Hemoglobins alpha subunits (π, αA, αD) and β subunits (βA, βH, ε, ρ)	B
Defense against luminal toxic contents	Glycoconjugates rich in α-fucose	▪ Protein O-fucosyltransferase (POFUT1 and POFUT1)<br>▪ α-L-fucosidase 2 (FUCA2)	AE
	Cell-cell junction	▪ Cell division cycle 42 (CDC42)<br>▪ integrin subunit β3<br>▪ Laminin subunit γ1<br>▪ Thy-1 cell surface antigen (THY1)	AE

AE: allantoic epithelium, B: blood, CE: chorionic epithelium, ES: eggshell, ESM: eggshell membrane, M: mesodermal layer
