# Supplementary figures and images for: Proteomic Analysis of Chicken Chorioallantoic Membrane (CAM) during Embryonic Development Provides Functional Insight
Source: Biomed Res Int. 2022 Jun 19;2022:7813921. doi: 10.1155/2022/7813921 (PMC9237712; doi:10.1155/2022/7813921)

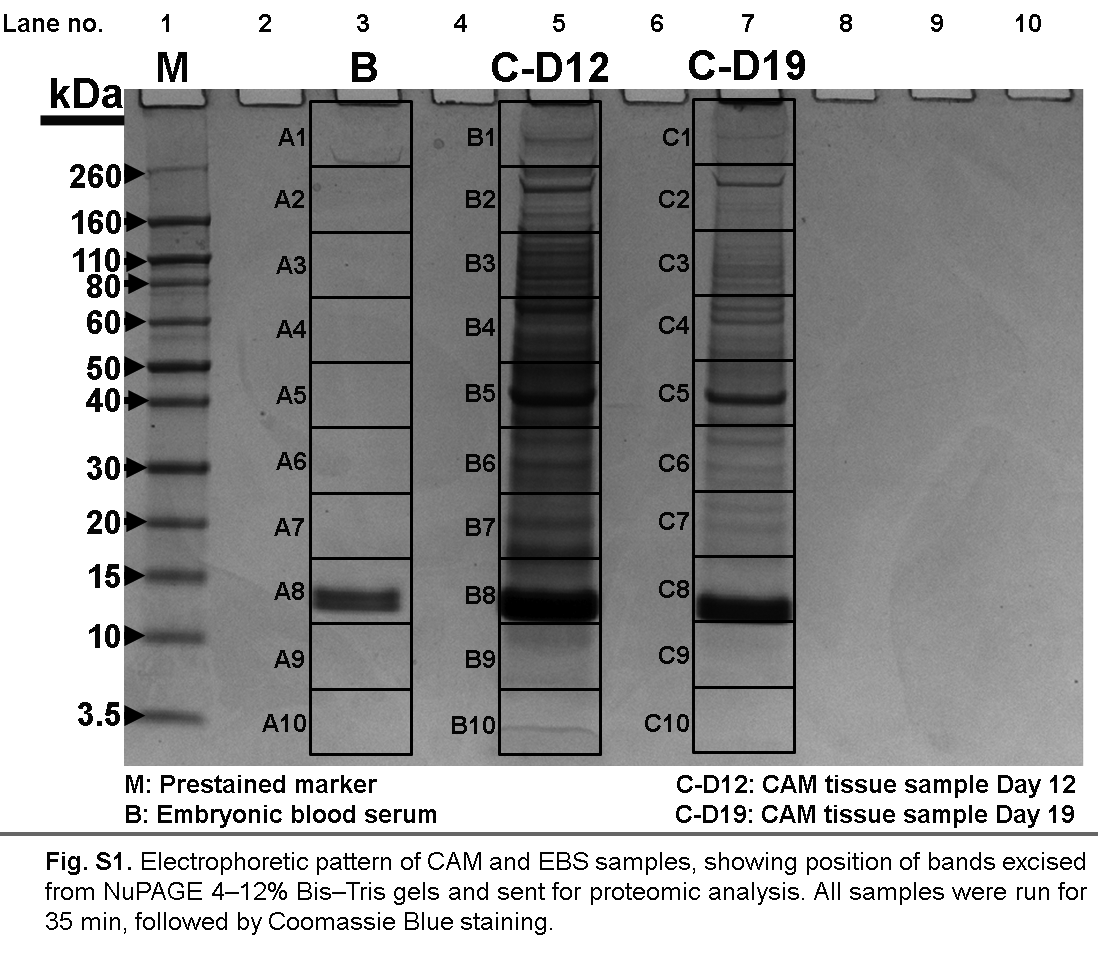

Supplement: Supplementary 10 — Electrophoretic pattern of CAM and EBS samples, showing position of bands excised from NuPAGE 4–12% Bis-Tris gels and sent for proteomic analysis. All samples were run for 35 min, followed by Coomassie blue staining. [file 7813921.f10.tiff]

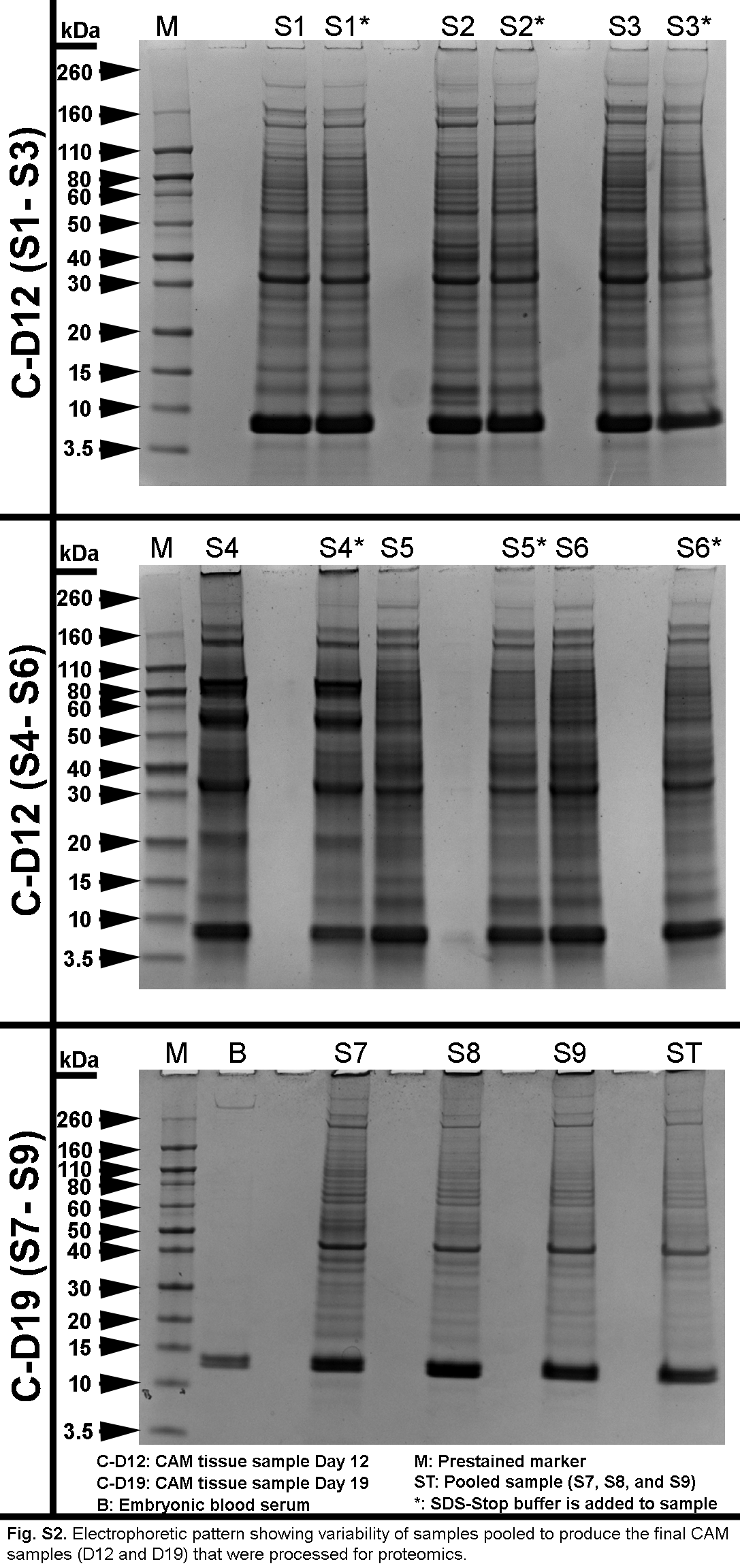

Supplement: Supplementary 11 — Electrophoretic pattern showing variability of samples pooled to produce the final CAM samples (D12 and D19) that were processed for proteomics. [file 7813921.f11.tiff]
