# Supplementary material for: Proteomic Analysis of Chicken Chorioallantoic Membrane (CAM) during Embryonic Development Provides Functional Insight
Source: Biomed Res Int. 2022 Jun 19;2022:7813921. doi: 10.1155/2022/7813921 (PMC9237712; doi:10.1155/2022/7813921)
Supplement: Supplementary 2 — Sample description and sample protein/peptide reports. [file 7813921.f2.pdf]

Sample description	
A	Embryonic blood serum ED19
B	Chorioallantoic membrane ED12
C	Chorioallantoic membrane ED19

Sample protein report

20196-20225 HinckeM --TAXchicken+archives-- 20141217, Samples report created on 05/12/2021				
Experiment: 20196-20225 HinckeM --TAXchicken+archives-- 20141217				
	Peak List Generator: unknown			
		Version: unknown		
		Charge States Calculated: unknown		
		Deisotoped: unknown		
		Textual Annotation: unknown		
	Database Set: 4 Databases			
		Database Name: a subset of the TAX_GallusGallus_9031_20141114 database		
			Version: unknown	
			Taxonomy: All Entries	
			Number of Proteins: 7028	
		Database Name: a subset of the TAX_GallusGallus_9031_20141114 database		
			Version: unknown	
			Taxonomy: All Entries	
			Number of Proteins: 7952	
		Database Name: a subset of the TAX_GallusGallus_9031_20141114 database		
			Version: unknown	
			Taxonomy: All Entries	
			Number of Proteins: 8298	
		Database Name: the TAX_GallusGallus_9031_20141114 database		
			Version: unknown	
			Taxonomy: All Entries	
			Number of Proteins: 222250	
		Does database contain common contaminants?: unknown		
	Search Engine Set: 2 Search Engines			
		Search Engine: Mascot		
			Version: 2.4.1	
			Samples: All Samples	
			Fragment Tolerance: 0.100 Da (Monoisotopic)	
			Parent Tolerance: 0.100 Da (Monoisotopic)	
			Fixed Modifications: +57 on C (Carbamidomethyl)	
			Variable Modifications: -18 on Peptide N-Terminal (Glu->pyro-Glu), -17 on Peptide N-Terminal (Gln->pyro-Glu), +1 on NQ (Deamidated), +16 on M (Oxidation)	
			Database: the TAX_GallusGallus_9031_20141114 database (unknown version, 222250 entries)	
			Digestion Enzyme: Trypsin	
			Max Missed Cleavages: 2	
			Probability Model:	
				Mudpit_Ingel20196_MGFPeaklist (F058870): LFDR Model, Classifier data: Bayes, Good (50%) m:68.8/s:15.5 m:43.8/s:1.46 m:NA m:48.3/s:21.0, Bad (50%) m:20.2/s:7.76 m:43.4/s:2.53 m:NA m:5.33/s:5.03 [all charge states]
				Mudpit_Ingel20206_MGFPeaklist (F058880): LFDR Model, Classifier data: Bayes, Good (62%) m:77.2/s:17.6 m:43.8/s:1.44 m:NA m:58.2/s:24.3, Bad (38%) m:11.3/s:7.70 m:43.5/s:1.70 m:NA m:3.42/s:3.81 [all charge states]
				Mudpit_Ingel20221_MGFPeaklist (F058864): LFDR Model, Classifier data: Bayes, Good (59%) m:73.9/s:17.1 m:43.5/s:1.80 m:NA m:54.9/s:23.4, Bad (41%) m:12.2/s:7.60 m:43.4/s:1.92 m:NA m:3.50/s:3.81 [all charge states]
		Search Engine: X! Tandem		
			Version: CYCLONE (2010.12.01.1)	
			Samples: All Samples	
			Fragment Tolerance: 0.100 Da (Monoisotopic)	
			Parent Tolerance: 0.100 Da (Monoisotopic)	
			Fixed Modifications: +57 on C (Carbamidomethyl)	
			Variable Modifications: -18 on Peptide N-Terminal (Dehydrated), -18 on Peptide N-Terminal (Glu->pyro-Glu), -17 on Peptide N-Terminal (Ammonia-loss), -17 on Peptide N-Terminal (Gln->pyro-Glu), +1 on NQ (Deamidated), +16 on M (Oxidation)	
			Database: a subset of the TAX_GallusGallus_9031_20141114 database (unknown version, 7028 entries) (Mudpit_Ingel20196_MGFPeaklist (F058870)), a subset of the TAX_GallusGallus_9031_20141114 database (unknown version, 7952 entries) (Mudpit_Ingel20206_MGFPeaklist (F058880)), a subset of the TAX_GallusGallus_9031_20141114 database (unknown version, 8298 entries) (Mudpit_Ingel20221_MGFPeaklist (F058864))	
			Digestion Enzyme: Trypsin (Mudpit_Ingel20196_MGFPeaklist (F058870)), Trypsin (Mudpit_Ingel20206_MGFPeaklist (F058880)), Trypsin (Mudpit_Ingel20221_MGFPeaklist (F058864))	
			Max Missed Cleavages: 2 (Mudpit_Ingel20196_MGFPeaklist (F058870)), 2 (Mudpit_Ingel20206_MGFPeaklist (F058880)), 2 (Mudpit_Ingel20221_MGFPeaklist (F058864))	
			Probability Model:	
				Mudpit_Ingel20196_MGFPeaklist (F058870): LFDR Model, Classifier data: Bayes, Good (82%) m:41.4/s:7.99 m:4.47/s:1.86 m:0.376/s:0.114 m:0.444/s:0.0991, Bad (18%) m:21.8/s:3.48 m:-0.721/s:0.638 m:0.0471/s:0.0489 m:0.216/s:0.113 [all charge states]
				Mudpit_Ingel20206_MGFPeaklist (F058880): LFDR Model, Classifier data: Bayes, Good (92%) m:46.7/s:7.22 m:5.12/s:1.85 m:0.405/s:0.119 m:0.487/s:0.0908, Bad (8.1%) m:25.2/s:3.81 m:-0.581/s:0.768 m:0.0489/s:0.0509 m:0.280/s:0.125 [all charge states]
				Mudpit_Ingel20221_MGFPeaklist (F058864): LFDR Model, Classifier data: Bayes, Good (89%) m:45.4/s:7.14 m:4.84/s:1.83 m:0.393/s:0.116 m:0.490/s:0.0938, Bad (11%) m:24.5/s:3.85 m:-0.647/s:0.668 m:0.0466/s:0.0478 m:0.270/s:0.120 [all charge states]
	Scaffold: Version: Scaffold_4.8.7			
		Modification Metadata Set: 1541 modifications		
			Source: C:\Program Files\Scaffold 4\parameters\unimod.xml	
			Comment:	
		Protein Grouping Strategy: MS-sample specific grouping with binary peptide-protein weights		
		Peptide Thresholds: 5.0% minimum		
		Protein Thresholds: 98.0% minimum and 2 peptides minimum		
		Peptide FDR: 0.2% (Decoy)		
		Protein FDR: 0.9% (Decoy)		
		GO Annotation Source(s):		
		Alternate ID Source(s):		

									Exclusive Unique Peptide Count	Exclusive Unique Peptide Count	Exclusive Unique Peptide Count	Quantitative Value (Normalized emPAI)	Quantitative Value (Normalized emPAI)	Quantitative Value (Normalized emPAI)
20196-20225 HinckeM --TAXchicken+archives-- 20141217									Uncategorized Sample	Uncategorized Sample	Uncategorized Sample	Uncategorized Sample	Uncategorized Sample	Uncategorized Sample
#	Visible?	Starred?	Identified Proteins (2082)	Accession Number	Alternate ID	Molecular Weight	Protein Grouping Ambiguity	Taxonomy	20196-20205_A	20206-20215_B	20216-20225_C	20196-20205_A	20206-20215_B	20216-20225_C
1	TRUE	Empty	Hemoglobin subunit beta OS=Gallus gallus GN=HBB PE=1 SV=2	HBB_CHICK		16 kDa	TRUE	unknown	28	31	34	1,134.60	905.1	987.74
2	TRUE	Empty	Hemoglobin subunit alpha-A OS=Gallus gallus GN=HBAA PE=1 SV=2	HBA_CHICK		15 kDa	TRUE	unknown	29	27	32	1,134.80	904.55	987.55

3	TRUE	Empty	Myosin-9 OS=Gallus gallus GN=MYH9 PE=4 SV=2	F1NCD4_CHICK (+4)		227 kDa	TRUE	unknown	49	77	116	1.5183	3.3779	17.259
4	TRUE	Empty	Hemoglobin subunit alpha-D OS=Gallus gallus GN=HBAD PE=1 SV=1	HBAD_CHICK		16 kDa	TRUE	unknown	22	21	20	1,133.70	904.07	986.45
5	TRUE	Empty	Apolipoprotein A-I OS=Gallus gallus GN=APOA1 PE=1 SV=2	APOA1_CHICK		31 kDa		unknown	54	25	32	1,135.00	35.994	256.08
6	TRUE	Empty	Ovalbumin OS=Gallus gallus GN=SERPINB14 PE=1 SV=2	OVAL_CHICK (+1)		43 kDa	TRUE	unknown	10	39	26	2.2233	472.68	114
7	TRUE	Empty	Filamin OS=Gallus gallus PE=2 SV=1	Q90WF1_CHICK		273 kDa	TRUE	unknown	34	75	76	0.64996	2.096	2.7531
8	TRUE	Empty	Actin, cytoplasmic type 5 OS=Gallus gallus PE=3 SV=1	ACTS_CHICK (+2)		42 kDa	TRUE	unknown	22	32	28	12.377	69.671	49.241
9	TRUE	Empty	Keratin, type II cytoskeletal cochleal OS=Gallus gallus PE=2 SV=1	K2CO_CHICK		54 kDa	TRUE	unknown	0	41	30	0	60.022	13.306
10	TRUE	Empty	Spectrin alpha chain, non-erythrocytic 1 OS=Gallus gallus GN=SPTAN1 PE=4 SV=2	F1NHT3_CHICK		285 kDa	TRUE	unknown	163	0	61	12.306	0	1.3016
11	TRUE	Empty	Vitellogenin-2 OS=Gallus gallus GN=VTG2 PE=4 SV=1	F1NFL6_CHICK		205 kDa	TRUE	unknown	3	71	0	0.061882	5.1103	0
12	TRUE	Empty	Uncharacterized protein (Fragment) OS=Gallus gallus GN=SPTB PE=4 SV=2	F1NSS0_CHICK (+6)		266 kDa	TRUE	unknown	144	11	11	12.947	0.14702	0.16039
13	TRUE	Empty	Ovotransferrin OS=Gallus gallus GN=TF PE=3 SV=2	E1BQC2_CHICK (+1)		78 kDa	TRUE	unknown	27	37	43	3.865	7.8625	14.217
14	TRUE	Empty	Tubulin beta-7 chain OS=Gallus gallus PE=2 SV=1	TBB7_CHICK		50 kDa	TRUE	unknown	9	29	23	3.679	32.648	10.531
15	TRUE	Empty	Vimentin OS=Gallus gallus GN=VIM PE=3 SV=1	F1NJ08_CHICK (+2)		53 kDa		unknown	33	24	24	12.656	10.861	7.5738
16	TRUE	Empty	Uncharacterized protein OS=Gallus gallus GN=FLNB PE=4 SV=2	F1N8D4_CHICK		277 kDa	TRUE	unknown	19	67	73	0.34037	1.3918	2.1856
17	TRUE	Empty	Nonmuscle myosin heavy chain OS=Gallus gallus PE=2 SV=2	Q02015_CHICK (+13)		231 kDa	TRUE	unknown	8	51	80	0.34699	2.2221	8.041
18	TRUE	Empty	Talin-1 OS=Gallus gallus GN=TLN1 PE=4 SV=2	E1C2S1_CHICK (+1)		272 kDa	TRUE	unknown	59	43	43	1.8364	0.74658	0.93901
19	TRUE	Empty	Fibronectin OS=Gallus gallus GN=FN1 PE=4 SV=1	F1NJT3_CHICK		273 kDa	TRUE	unknown	39	49	26	0.7972	1.0013	0.46476
20	TRUE	Empty	Serum albumin OS=Gallus gallus GN=ALB PE=4 SV=2	F2Z4L6_CHICK		64 kDa	TRUE	unknown	40	16	39	36.041	2.7901	24.837
21	TRUE	Empty	Keratin, type I cytoskeletal 19 OS=Gallus gallus GN=KRT19 PE=3 SV=1	F1NDN9_CHICK (+3)		46 kDa		unknown	4	23	11	0.41377	17.856	3.0127
22	TRUE	Empty	Elongation factor 1-alpha 1 OS=Gallus gallus GN=EEF1A PE=2 SV=1	EF1A_CHICK (+3)		50 kDa		unknown	11	13	8	2.6894	2.8742	1.3401
23	TRUE	Empty	Lamin-A OS=Gallus gallus GN=LMNA PE=2 SV=1	LMNA_CHICK (+1)		73 kDa	TRUE	unknown	28	21	25	4.6322	3.0623	4.2835
24	TRUE	Empty	Tubulin alpha-1 chain (Fragment) OS=Gallus gallus PE=1 SV=1	TBA1_CHICK		46 kDa	TRUE	unknown	15	21	15	5.7047	13.021	4.5162
25	TRUE	Empty	Vitellogenin-1 OS=Gallus gallus GN=VTG1 PE=1 SV=1	VIT1_CHICK		211 kDa	TRUE	unknown	0	56	0	0	2.4514	0
26	TRUE	Empty	Heat shock protein HSP 90-alpha OS=Gallus gallus GN=HSP90AA1 PE=3 SV=3	HS90A_CHICK (+2)		84 kDa	TRUE	unknown	17	32	35	1.5439	3.9264	6.1321
27	TRUE	Empty	Isoform Brain I-cad of Caldesmon OS=Gallus gallus GN=CALD1	sp|P12957-2|CALD1_CHICK (+10)		59 kDa		unknown	0	17	19	0	3.0269	4.1667
28	TRUE	Empty	Apolipoprotein B OS=Gallus gallus GN=APOB PE=4 SV=2	F1NV02_CHICK (+2)		523 kDa		unknown	57	21	55	0.58762	0.14962	0.50086
29	TRUE	Empty	Heat shock cognate 71 kDa protein OS=Gallus gallus GN=HSPA8 PE=3 SV=1	F1NWP3_CHICK		71 kDa	TRUE	unknown	13	28	26	3.8261	7.16	9.2617
30	TRUE	Empty	Isoform 1 of Vinculin OS=Gallus gallus GN=VCL	sp|P12003-1|VINC_CHICK (+15)		117 kDa		unknown	7	34	48	0.27455	2.6286	5.146
31	TRUE	Empty	status=active	UPI000240C70A		51 kDa	TRUE	unknown	0	14	17	0	2.8278	4.0494
32	TRUE	Empty	Peroxiredoxin-1 OS=Gallus gallus GN=PRDX1 PE=1 SV=1	PRDX1_CHICK		22 kDa		unknown	18	7	13	149.08	4.2175	18.73
33	TRUE	Empty	Uncharacterized protein OS=Gallus gallus GN=Gga.55808 PE=4 SV=2	F1NJR5_CHICK (+5)		213 kDa	TRUE	unknown	59	5	7	2.6173	0.080694	0.1254
34	TRUE	Empty	Alpha-actinin-4 OS=Gallus gallus GN=ACTN4 PE=1 SV=1	ACTN4_CHICK		104 kDa		unknown	3	36	44	0.12457	3.4129	5.9145
35	TRUE	Empty	Elongation factor 2 (Fragment) OS=Gallus gallus GN=EEF2 PE=4 SV=2	F1NFS0_CHICK (+5)		95 kDa	TRUE	unknown	15	29	26	0.87106	2.784	2.0976
36	TRUE	Empty	Carbonic anhydrase 2 OS=Gallus gallus GN=CA2 PE=2 SV=3	CAH2_CHICK (+5)		29 kDa	TRUE	unknown	21	5	15	91.735	0.98051	13.582
37	TRUE	Empty	78 kDa glucose-regulated protein OS=Gallus gallus GN=HSPA5 PE=1 SV=1	GRP78_CHICK (+1)		72 kDa	TRUE	unknown	10	24	31	0.83396	3.3709	9.4102
38	TRUE	Empty	status=active	UPI0000F0887D (+5)		192 kDa	TRUE	unknown	11	44	29	0.26294	1.3851	1.041
39	TRUE	Empty	Alpha-enolase OS=Gallus gallus GN=ENO1 PE=3 SV=2	F1NZ78_CHICK (+2)		47 kDa	TRUE	unknown	18	18	19	4.4347	6.6438	7.2478
40	TRUE	Empty	Myosin-11 OS=Gallus gallus GN=MYH11 PE=1 SV=4	MYH11_CHICK		229 kDa	TRUE	unknown	0	43	48	0	1.6351	2.8805
41	TRUE	Empty	Protein disulfide-isomerase A3 OS=Gallus gallus GN=PDIA3 PE=2 SV=1	PDIA3_CHICK (+2)		56 kDa	TRUE	unknown	21	25	23	4.1308	6.0997	5.3202
42	TRUE	Empty	Uncharacterized protein (Fragment) OS=Gallus gallus GN=KRT8 PE=3 SV=2	H9KZP2_CHICK (+3)		42 kDa		unknown	1	18	13	0.10062	8.0706	3.9741
43	TRUE	Empty	Annexin OS=Gallus gallus GN=ANXA6 PE=3 SV=2	E1BWX1_CHICK (+2)		87 kDa		unknown	2	34	34	0.098276	4.7228	4.4328
44	TRUE	Empty	Beta-H globin OS=Gallus gallus GN=HBE1 PE=3 SV=1	Q90864_CHICK		16 kDa	TRUE	unknown	7	7	8	437.51	348.92	721.54
45	TRUE	Empty	Peptidyl-prolyl cis-trans isomerase OS=Gallus gallus PE=2 SV=1	D0EKR3_CHICK (+1)		18 kDa		unknown	10	12	18	16.563	54.843	239.22
46	TRUE	Empty	Apolipoprotein AIV OS=Gallus gallus GN=apoAIV PE=2 SV=1	O93601_CHICK (+1)		41 kDa		unknown	24	6	26	14.515	0.62446	12.628
47	TRUE	Empty	14-3-3 protein epsilon OS=Gallus gallus GN=YWHAE PE=1 SV=1	1433E_CHICK		29 kDa	TRUE	unknown	14	17	22	7.8089	15.786	36.749
48	TRUE	Empty	status=inactive	UPI00024089BF (+2)		359 kDa		unknown	0	1	34	0	0.056794	0.50923
49	TRUE	Empty	Uncharacterized protein OS=Gallus gallus GN=LOC395381 PE=4 SV=1	F1NZY2_CHICK		234 kDa		unknown	0	21	18	0	0.40949	0.33943
50	TRUE	Empty	Hemoglobin subunit epsilon OS=Gallus gallus GN=HBE PE=1 SV=2	HBE_CHICK		17 kDa	TRUE	unknown	8	10	9	114.12	492.48	80.291
51	TRUE	Empty	Protein disulfide-isomerase OS=Gallus gallus GN=P4HB PE=3 SV=2	F1N9H3_CHICK (+2)		56 kDa		unknown	6	20	19	0.53569	3.9301	3.36
52	TRUE	Empty	Uncharacterized protein OS=Gallus gallus GN=RCJMB04_5n23 PE=2 SV=1	Q5ZLJ7_CHICK (+1)		29 kDa	TRUE	unknown	13	11	24	5.2386	24.337	93.487
53	TRUE	Empty	Destrin (Fragment) OS=Gallus gallus GN=DSTN PE=4 SV=1	Z4YJ88_CHICK		18 kDa	TRUE	unknown	8	14	15	6.408	57.512	92.096
54	TRUE	Empty	status=inactive	UPI0000ECA63D (+1)		282 kDa	TRUE	unknown	2	56	1	12.377	1.1088	1.267
55	TRUE	Empty	Annexin A2 OS=Gallus gallus GN=ANXA2 PE=1 SV=2	ANXA2_CHICK		39 kDa		unknown	0	25	27	0	13.563	26.489
56	TRUE	Empty	Pyruvate kinase OS=Gallus gallus GN=PKM2 PE=3 SV=2	F1NW43_CHICK (+2)		58 kDa	TRUE	unknown	10	24	21	0.97739	5.3061	3.3985
57	TRUE	Empty	Uncharacterized protein OS=Gallus gallus GN=Gga.3145 PE=4 SV=1	E1BV34_CHICK (+3)		69 kDa	TRUE	unknown	0	14	28	0	1.1939	4.6101
58	TRUE	Empty	Tubulin beta-6 chain OS=Gallus gallus PE=1 SV=1	TBB6_CHICK		50 kDa		unknown	21	11	3	13.735	5.7896	1.6978
59	TRUE	Empty	Lamin-B2 OS=Gallus gallus GN=LMNB2 PE=3 SV=1	F1NPS1_CHICK (+7)		68 kDa	TRUE	unknown	28	21	12	4.7333	2.3242	0.87758
60	TRUE	Empty	status=inactive	UPI0000447264		82 kDa	TRUE	unknown	4	12	5	0.96395	1.0903	0.29766
61	TRUE	Empty	Nucleoside diphosphate kinase OS=Gallus gallus PE=2 SV=1	NDK_CHICK		17 kDa		unknown	10	16	18	7.3358	49.533	99.586
62	TRUE	Empty	Uncharacterized protein OS=Gallus gallus GN=SPTBN1 PE=4 SV=1	R4GJH8_CHICK (+5)		274 kDa	TRUE	unknown	5	25	34	0.16089	0.37253	0.60401
63	TRUE	Empty	Band 3 anion transport protein OS=Gallus gallus GN=SLC4A1 PE=2 SV=1	B3AT_CHICK (+2)		102 kDa	TRUE	unknown	16	7	7	1.3375	0.29621	0.37036
64	TRUE	Empty	Heat shock protein 70 OS=Gallus gallus GN=HSP70 PE=3 SV=1	Q7SX63_CHICK		70 kDa	TRUE	unknown	26	13	17	4.7786	4.3236	10.168
65	TRUE	Empty	Calcium-binding protein OS=Gallus gallus GN=P22 PE=2 SV=1	Q90940_CHICK (+1)		22 kDa	TRUE	unknown	14	6	6	13.339	1.8514	1.5774
66	TRUE	Empty	ATP synthase subunit beta (Fragment) OS=Gallus gallus GN=ATP5B PE=3 SV=2	H9L340_CHICK (+4)		53 kDa		unknown	9	22	19	0.9536	5.5559	4.3871
67	TRUE	Empty	ATP synthase subunit alpha OS=Gallus gallus GN=ATP5A1 PE=3 SV=1	F1NI22_CHICK (+3)		60 kDa	TRUE	unknown	11	21	17	1.1988	3.1596	1.7545
68	TRUE	Empty	Histone H1.11L OS=Gallus gallus PE=1 SV=2	H11L_CHICK		23 kDa	TRUE	unknown	14	9	0	17.708	8.5031	0
69	TRUE	Empty	Annexin OS=Gallus gallus GN=ANXA8 PE=3 SV=1	E1C8K3_CHICK (+1)		37 kDa		unknown	0	20	21	0	8.3852	17.155
70	TRUE	Empty	status=active	UPI0000111E1A (+21)		14 kDa	TRUE	unknown	9	7	0	24.848	25.461	0
71	TRUE	Empty	Annexin OS=Gallus gallus GN=ANXA1 PE=3 SV=1	F1N957_CHICK (+1)		39 kDa		unknown	0	14	20	0	3.4668	12.229
72	TRUE	Empty	Uncharacterized protein OS=Gallus gallus GN=GDI2 PE=4 SV=2	F1NCZ2_CHICK (+3)		51 kDa		unknown	14	23	19	2.9162	7.195	5.2177
73	TRUE	Empty	60 kDa heat shock protein, mitochondrial OS=Gallus gallus GN=HSPD1 PE=1 SV=1	CH60_CHICK (+5)		61 kDa		unknown	6	22	16	0.48199	3.2918	1.5504
74	TRUE	Empty	status=active	UPI00003AA4FE (+9)		190 kDa		unknown	4	19	30	0.090041	0.42075	0.79697
75	TRUE	Empty	CgABP260 OS=Gallus gallus PE=2 SV=1	Q90WF0_CHICK		280 kDa	TRUE	unknown	0	22	30	0	0.37919	0.587
76	TRUE	Empty	Uncharacterized protein OS=Gallus gallus GN=PLS3 PE=2 SV=1	Q5ZI39_CHICK (+3)		71 kDa	TRUE	unknown	9	19	26	0.65878	1.7286	4.0404
77	TRUE	Empty	status=inactive	UPI0000ECB186 (+7)		75 kDa		unknown	9	14	11	0.61039	1.2355	0.76531
78	TRUE	Empty	Triosephosphate isomerase OS=Gallus gallus GN=TPI1 PE=1 SV=2	TPIS_CHICK (+5)		27 kDa		unknown	10	17	18	4.4455	11.966	15.047
79	TRUE	Empty	Sodium/potassium-transporting ATPase subunit alpha-1 (Fragment) OS=Gallus gallus GN=ATP1A1 PE=3 SV=2	F1NSY1_CHICK (+7)		112 kDa		unknown	5	19	22	0.19885	0.93671	1.299
80	TRUE	Empty	Fibrinogen alpha chain OS=Gallus gallus GN=FGA PE=4 SV=1	F1P4V1_CHICK (+10)		82 kDa		unknown	22	0	4	3.8975	0	0.24173
81	TRUE	Empty	status=active	UPI0000549337 (+2)		25 kDa	TRUE	unknown	5	4	1	8.5814	12.834	1.3432
82	TRUE	Empty	Fructose-bisphosphate aldolase OS=Gallus gallus GN=ALDOC PE=3 SV=1	R4GM10_CHICK		39 kDa	TRUE	unknown	14	16	18	3.7204	5.774	7.751
83	TRUE	Empty	Malate dehydrogenase OS=Gallus gallus GN=MDH2 PE=3 SV=1	E1BVT3_CHICK (+2)		36 kDa		unknown	8	17	15	1.3903	7.2604	6.3058
84	TRUE	Empty	Eukaryotic initiation factor 4A-II OS=Gallus gallus GN=EIF4A2 PE=3 SV=1	R9PXN1_CHICK (+6)		44 kDa	TRUE	unknown	14	15	15	2.7594	3.0694	2.6911

85	TRUE	Empty	status=active	UPI000240CC5B		22 kDa		unknown	2	7	14	0.42274	3.504	36.703
86	TRUE	Empty	L-lactate dehydrogenase B chain OS=Gallus gallus GN=LDHB PE=1 SV=3	LDHB_CHICK (+3)		36 kDa		unknown	8	11	9	1.6111	2.3384	2.2201
87	TRUE	Empty	status=inactive	UPI0000ECB686 (+3)		30 kDa		unknown	7	13	11	2.1208	4.3375	4.0997
88	TRUE	Empty	Decorin OS=Gallus gallus GN=DCN PE=4 SV=2	E1BRE9_CHICK (+4)		40 kDa		unknown	0	12	13	0	2.5914	4.4822
89	TRUE	Empty	Heat shock protein 108 OS=Gallus gallus PE=2 SV=1	B5AAV6_CHICK (+2)		91 kDa		unknown	0	21	15	0	1.4375	1.2818
90	TRUE	Empty	Uncharacterized protein OS=Gallus gallus GN=CAND1 PE=4 SV=2	F1NHL2_CHICK (+1)		134 kDa		unknown	26	21	15	1.2901	0.73845	0.49558
91	TRUE	Empty	Serpin H1 OS=Gallus gallus GN=SERPINH1 PE=1 SV=2	SERPH_CHICK		46 kDa	TRUE	unknown	0	18	14	0	6.6256	2.7606
92	TRUE	Empty	Uncharacterized protein (Fragment) OS=Gallus gallus GN=ALDH2 PE=3 SV=2	E1BT93_CHICK (+6)		57 kDa		unknown	6	18	23	0.51731	2.465	3.7353
93	TRUE	Empty	Glutathione S-transferase OS=Gallus gallus GN=GSTA3 PE=2 SV=1	Q9W6J2_CHICK (+1)		25 kDa	TRUE	unknown	14	3	8	9.3414	0.67241	2.4593
94	TRUE	Empty	status=inactive	UPI0000ECD355		165 kDa	TRUE	unknown	31	0	16	1.2599	0	0.47875
95	TRUE	Empty	14-3-3 protein theta OS=Gallus gallus GN=YWHAQ PE=1 SV=1	1433T_CHICK (+3)		28 kDa	TRUE	unknown	3	10	9	1.0126	5.2289	8.8249
96	TRUE	Empty	Isocitrate dehydrogenase [NADP] OS=Gallus gallus GN=IDH2 PE=3 SV=2	H9L366_CHICK (+3)		50 kDa	TRUE	unknown	7	18	16	0.86776	3.4107	2.8174
97	TRUE	Empty	Coatomer subunit alpha OS=Gallus gallus GN=LOC100859596 PE=4 SV=2	H9L3L2_CHICK (+6)		139 kDa	TRUE	unknown	18	18	13	0.68269	0.54446	0.40003
98	TRUE	Empty	Uncharacterized protein OS=Gallus gallus GN=HDGF PE=4 SV=2	H9L369_CHICK (+3)		26 kDa	TRUE	unknown	21	3	6	31.966	0.45147	1.5512
99	TRUE	Empty	Bifunctional purine biosynthesis protein PURH OS=Gallus gallus GN=ATIC PE=1 SV=1	PUR9_CHICK (+8)		64 kDa		unknown	19	15	14	2.1468	1.4353	1.2958
100	TRUE	Empty	Adenylyl cyclase-associated protein OS=Gallus gallus GN=CAP1 PE=3 SV=2	F1P2G4_CHICK (+2)		58 kDa		unknown	8	12	13	0.85438	1.8759	2.0465
101	TRUE	Empty	status=inactive	UPI0000ECAF3B (+3)		24 kDa		unknown	5	9	11	1.5745	5.991	10.638
102	TRUE	Empty	Uncharacterized protein OS=Gallus gallus GN=RCJMB04_15n19 PE=2 SV=1	Q5F3I8_CHICK (+3)		54 kDa	TRUE	unknown	12	12	7	1.7242	1.5307	0.6867
103	TRUE	Empty	Serotransferrin OS=Homo sapiens GN=TF PE=1 SV=3	P02787 (+2)		?		unknown	0	0	33	0	0	-1
104	TRUE	Empty	Collagen alpha-1(XII) chain OS=Gallus gallus GN=COL12A1 PE=4 SV=2	F1NX22_CHICK (+8)		340 kDa		unknown	0	6	24	0	0.060026	0.3168
105	TRUE	Empty	Uncharacterized protein OS=Gallus gallus GN=FGG PE=4 SV=2	E1BV78_CHICK (+8)		50 kDa		unknown	20	0	7	4.7795	0	0.6444
106	TRUE	Empty	Peroxiredoxin-6 OS=Gallus gallus GN=PRDX6 PE=4 SV=1	F1NBV0_CHICK (+6)		25 kDa		unknown	14	11	12	9.6452	6.5637	7.1604
107	TRUE	Empty	Uncharacterized protein OS=Gallus gallus GN=OVALY PE=3 SV=2	E1BTF4_CHICK (+3)		44 kDa	TRUE	unknown	0	12	8	0	3.0402	1.8718
108	TRUE	Empty	status=inactive	UPI00004484F9 (+1)		354 kDa		unknown	0	9	8	0	0.087706	0.084607
109	TRUE	Empty	status=active	UPI00003AFC27 (+1)		85 kDa		unknown	0	11	10	0	0.60107	0.58744
110	TRUE	Empty	Annexin A6 OS=Homo sapiens GN=ANXA6 PE=1 SV=3	P08133		?	TRUE	unknown	0	0	35	0	0	-1
111	TRUE	Empty	Protein disulfide-isomerase A4 OS=Gallus gallus GN=PDIA4 PE=3 SV=1	F1NDY9_CHICK (+4)		71 kDa	TRUE	unknown	0	21	21	0	2.3103	2.5203
112	TRUE	Empty	Glyceraldehyde-3-phosphate dehydrogenase (Fragment) OS=Gallus gallus GN=GAPDH PE=3 SV=1	F1NH87_CHICK (+1)		35 kDa	TRUE	unknown	0	12	11	0	4.2345	4.6195
113	TRUE	Empty	status=active	UPI0002C61449 (+6)		27 kDa		unknown	17	10	9	10.668	3.4071	2.639
114	TRUE	Empty	Uncharacterized protein (Fragment) OS=Gallus gallus GN=IDH1 PE=3 SV=1	F1NPG2_CHICK (+7)		47 kDa		unknown	8	14	14	0.95416	2.162	2.6238
115	TRUE	Empty	Uncharacterized protein (Fragment) OS=Gallus gallus GN=LOC100857226 PE=4 SV=1	H9KYX6_CHICK (+6)		52 kDa	TRUE	unknown	5	21	22	0.4622	2.8963	4.1034
116	TRUE	Empty	40S ribosomal protein S3a OS=Gallus gallus GN=RPS3A PE=3 SV=1	F2Z4K7_CHICK (+5)		30 kDa		unknown	5	13	11	0.92047	7.7444	4.2219
117	TRUE	Empty	Myristoylated alanine-rich C-kinase substrate OS=Gallus gallus GN=MARCKS PE=1 SV=2	MARCS_CHICK (+2)		28 kDa		unknown	0	8	9	0	2.3361	4.1972
118	TRUE	Empty	Catalase OS=Gallus gallus GN=RCJMB04_1j22 PE=2 SV=1	Q5ZMM4_CHICK (+2)		60 kDa		unknown	16	15	9	2.0108	1.6036	1.0403
119	TRUE	Empty	Alpha-fetoprotein OS=Gallus gallus GN=AFP PE=4 SV=1	E1BV96_CHICK		71 kDa	TRUE	unknown	6	28	8	2.1512	4.987	1.3475
120	TRUE	Empty	status=inactive	UPI000240C540		28 kDa	TRUE	unknown	0	4	14	0	0.59655	25.556
121	TRUE	Empty	Guanine nucleotide-binding protein subunit beta-2-like 1 OS=Gallus gallus GN=GNB2L1 PE=2 SV=1	GBLP_CHICK (+3)		35 kDa		unknown	7	19	12	1.1749	6.7273	2.7081
122	TRUE	Empty	Proteasome subunit beta type-5 (Fragment) OS=Gallus gallus GN=PSMB5 PE=2 SV=2	PSB5_CHICK (+3)		27 kDa		unknown	7	10	10	1.7069	5.5662	6.0722
123	TRUE	Empty	14-3-3 protein zeta OS=Gallus gallus GN=YWHAZ PE=2 SV=1	1433Z_CHICK		28 kDa	TRUE	unknown	6	7	7	2.0139	6.0637	7.6495
124	TRUE	Empty	Histone H2A-III OS=Gallus gallus PE=1 SV=2	H2A3_CHICK (+3)		14 kDa	TRUE	unknown	8	0	4	57.234	0	1.6564
125	TRUE	Empty	40S ribosomal protein SA OS=Gallus gallus GN=RPSA PE=3 SV=1	RSSA_CHICK (+2)		33 kDa		unknown	6	8	3	1.2797	1.4842	0.3772
126	TRUE	Empty	Spliceosome RNA helicase DDX39B OS=Gallus gallus GN=DDX39B PE=2 SV=1	DX39B_CHICK (+1)		49 kDa		unknown	13	8	10	2.0281	0.84413	1.3896
127	TRUE	Empty	Adenosylhomocysteinase OS=Gallus gallus GN=Gga.51956 PE=3 SV=2	F1P3F1_CHICK (+5)		74 kDa		unknown	19	6	11	1.7149	0.30538	0.77873
128	TRUE	Empty	Uncharacterized protein OS=Gallus gallus GN=RPS3 PE=3 SV=2	F1NPA9_CHICK (+1)		27 kDa		unknown	7	13	10	1.728	4.8553	2.7158
129	TRUE	Empty	Actin, gamma-enteric smooth muscle OS=Gallus gallus GN=ACTG2 PE=3 SV=2	F1P476_CHICK (+1)		42 kDa	TRUE	unknown	0	12	1	0	26.429	8.0583
130	TRUE	Empty	Collagen alpha-1(I) chain OS=Gallus gallus GN=COL1A1 PE=1 SV=3	CO1A1_CHICK		138 kDa		unknown	0	13	8	0	0.51055	0.33142
131	TRUE	Empty	GTP-binding nuclear protein Ran OS=Gallus gallus GN=RAN PE=2 SV=1	RAN_CHICK (+2)		24 kDa		unknown	8	6	6	3.0278	1.2475	1.7257
132	TRUE	Empty	Transgelin OS=Gallus gallus GN=TAGLN PE=1 SV=3	TAGL_CHICK (+3)		22 kDa	TRUE	unknown	0	9	11	0	6.1156	11.301
133	TRUE	Empty	status=inactive	UPI000240CC19 (+1)		15 kDa		unknown	0	3	3	0	2.6383	2.0916
134	TRUE	Empty	Ovalbumin-related protein X OS=Gallus gallus gallus GN=OVALX PE=2 SV=1	R9TNA6_CHICK		45 kDa	TRUE	unknown	0	10	11	0	1.8243	2.4987
135	TRUE	Empty	Uncharacterized protein OS=Gallus gallus GN=TKT PE=4 SV=2	F1P1A5_CHICK (+5)		68 kDa		unknown	7	18	16	0.50578	1.8298	1.6979
136	TRUE	Empty	Actin, alpha cardiac muscle 1 OS=Gallus gallus GN=ACTC1 PE=3 SV=1	ACTC_CHICK (+1)		42 kDa	TRUE	unknown	4	0	6	6.9171	0	14.095
137	TRUE	Empty	Calpain small subunit (Fragment) OS=Gallus gallus PE=2 SV=1	O42134_CHICK (+1)		24 kDa	TRUE	unknown	3	7	9	0.63239	2.0439	3.3351
138	TRUE	Empty	Hemoglobin subunit pi OS=Gallus gallus PE=1 SV=3	HBPI_CHICK		16 kDa	TRUE	unknown	5	18	7	11.818	228.24	8.0441
139	TRUE	Empty	Uncharacterized protein OS=Gallus gallus GN=LOC770011 PE=4 SV=2	E1C6R9_CHICK (+6)		20 kDa	TRUE	unknown	4	7	6	1.6036	5.3813	3.8336
140	TRUE	Empty	status=inactive	UPI000060E944		38 kDa	TRUE	unknown	0	22	12	0	14.643	15.975
141	TRUE	Empty	Uncharacterized protein (Fragment) OS=Gallus gallus GN=LOC100858989 PE=4 SV=1	F1P1Q1_CHICK (+5)		29 kDa		unknown	14	7	6	13.403	1.2124	1.3227
142	TRUE	Empty	Uncharacterized protein OS=Gallus gallus GN=RCJMB04_20b9 PE=2 SV=1	Q5ZJ86_CHICK (+4)		180 kDa		unknown	14	11	17	0.37023	0.24781	0.49056
143	TRUE	Empty	status=inactive	UPI0000ECA7E7 (+2)		37 kDa		unknown	12	6	5	3.6472	0.70383	0.60733
144	TRUE	Empty	status=active	UPI00003AE93D (+5)		56 kDa		unknown	8	14	15	0.76166	1.6227	2.1481
145	TRUE	Empty	Uncharacterized protein OS=Gallus gallus GN=TCP1 PE=2 SV=1	Q5ZMG9_CHICK		60 kDa		unknown	15	17	10	1.637	1.7379	0.91318
146	TRUE	Empty	Annexin A5 OS=Gallus gallus GN=ANXA5 PE=1 SV=2	ANXA5_CHICK (+1)		36 kDa		unknown	0	15	15	0	3.923	4.2796
147	TRUE	Empty	Gelsolin OS=Gallus gallus GN=GSN PE=2 SV=1	GELS_CHICK (+3)		86 kDa		unknown	7	6	18	0.45395	0.2598	1.401
148	TRUE	Empty	Uncharacterized protein OS=Gallus gallus GN=LOC426023 PE=4 SV=2	H9L011_CHICK (+22)		48 kDa		unknown	7	8	4	0.7889	1.0186	0.34754
149	TRUE	Empty	Uncharacterized protein OS=Gallus gallus GN=RCJMB04_4l9 PE=2 SV=1	Q5ZLV5_CHICK (+1)		53 kDa		unknown	13	13	10	1.7903	1.7657	1.0902
150	TRUE	Empty	Uncharacterized protein OS=Gallus gallus GN=RCJMB04_6e4 PE=2 SV=1	Q5ZLG7_CHICK (+1)		33 kDa	TRUE	unknown	0	10	12	0	1.7754	2.6462
151	TRUE	Empty	Uncharacterized protein OS=Gallus gallus GN=VCP PE=1 SV=1	Q5ZMU9_CHICK (+8)		89 kDa		unknown	3	22	17	0.14642	1.5834	1.0578
152	TRUE	Empty	Uncharacterized protein OS=Gallus gallus GN=SLC25A3 PE=3 SV=2	F1NZ24_CHICK (+5)		40 kDa	TRUE	unknown	0	10	8	0	3.2298	1.8979
153	TRUE	Empty	Histone H2B 1/2/3/4/6 OS=Gallus gallus GN=H2B-I PE=1 SV=2	H2B1_CHICK (+2)		14 kDa	TRUE	unknown	11	0	0	58.999	0	0
154	TRUE	Empty	Uncharacterized protein OS=Gallus gallus GN=CKAP4 PE=4 SV=2	F1P360_CHICK (+7)		57 kDa		unknown	2	22	11	0.1529	4.4075	1.1206
155	TRUE	Empty	Uncharacterized protein (Fragment) OS=Gallus gallus GN=DDX17 PE=3 SV=2	E1C2R8_CHICK (+5)		75 kDa	TRUE	unknown	15	14	2	1.1953	0.86626	0.099394
156	TRUE	Empty	Uncharacterized protein (Fragment) OS=Gallus gallus GN=SEPT7 PE=3 SV=2	E1BVH4_CHICK (+1)		48 kDa		unknown	0	13	11	0	2.2816	1.7965
157	TRUE	Empty	Uncharacterized protein OS=Gallus gallus GN=PEBP1 PE=4 SV=1	E1C6C1_CHICK (+1)		21 kDa		unknown	10	10	10	7.3063	3.9131	7.6935
158	TRUE	Empty	Nucleophosmin (Fragment) OS=Gallus gallus GN=NPM1 PE=4 SV=2	F1NVA4_CHICK (+7)		30 kDa		unknown	6	5	3	1.1563	1.1492	0.41554
159	TRUE	Empty	Uncharacterized protein OS=Gallus gallus GN=EPB42 PE=4 SV=2	E1BQZ4_CHICK		79 kDa	TRUE	unknown	17	5	6	1.4423	0.28455	0.43424
160	TRUE	Empty	Collagen alpha-1(VI) chain OS=Gallus gallus GN=COL6A1 PE=2 SV=1	CO6A1_CHICK (+1)		108 kDa		unknown	0	10	18	0	0.36033	0.8791
161	TRUE	Empty	status=active	UPI0000173EC7 (+2)		17 kDa		unknown	0	6	9	0	6.2002	90.931
162	TRUE	Empty	status=inactive	UPI000044ACE9 (+1)		27 kDa	TRUE	unknown	0	6	2	0	1.6366	0.66718
163	TRUE	Empty	status=active	UPI00003AD82D		65 kDa	TRUE	unknown	14	5	9	2.7378	3.1773	2.0262
164	TRUE	Empty	Glutathione peroxidase (Fragment) OS=Gallus gallus GN=GPX1 PE=3 SV=1	R4GH86_CHICK (+3)		18 kDa		unknown	9	6	8	6.8447	2.0115	3.7125
165	TRUE	Empty	GTPase cRac1A OS=Gallus gallus GN=cRac1A PE=2 SV=1	Q6LC82_CHICK (+10)		21 kDa	TRUE	unknown	4	8	9	1.0509	3.0504	4.0962
166	TRUE	Empty	Uncharacterized protein OS=Gallus gallus GN=HNRNPA2B1 PE=2 SV=1	Q5ZME1_CHICK (+4)		37 kDa		unknown	11	12	6	2.1439	2.2662	0.77376

167	TRUE	Empty	Protein disulfide-isomerase A4 OS=Homo sapiens GN=PDIA4 PE=1 SV=2	P13667		?		unknown	0	0	25	0	0	-1
168	TRUE	Empty	Transthyretin OS=Gallus gallus GN=TTR PE=1 SV=1	TTHY_CHICK (+5)		16 kDa		unknown	3	8	5	1.5226	4.0584	1.8728
169	TRUE	Empty	Uncharacterized protein OS=Gallus gallus GN=RPS16 PE=3 SV=1	R4GGJ0_CHICK (+4)		16 kDa		unknown	3	8	9	1.0045	10.53	14.424
170	TRUE	Empty	T-complex protein 1 subunit theta OS=Gallus gallus GN=CCT8 PE=1 SV=3	TCPQ_CHICK (+10)		59 kDa		unknown	0	18	11	0	2.514	1.0511
171	TRUE	Empty	Uncharacterized protein OS=Gallus gallus GN=ATP5F1 PE=4 SV=2	F1NSC1_CHICK		29 kDa		unknown	0	12	14	0	3.6156	7.1027
172	TRUE	Empty	Prohibitin-2 OS=Gallus gallus GN=PHB2 PE=2 SV=1	PHB2_CHICK (+1)		33 kDa		unknown	5	12	9	0.80094	2.7234	1.5946
173	TRUE	Empty	Uncharacterized protein OS=Gallus gallus GN=SEPT11 PE=3 SV=2	F1P0X3_CHICK (+4)		51 kDa		unknown	0	17	11	0	2.3346	1.323
174	TRUE	Empty	Ezrin OS=Gallus gallus GN=EZR PE=2 SV=1	Q9YGW6_CHICK (+1)		69 kDa	TRUE	unknown	0	9	15	0	1.3988	3.2366
175	TRUE	Empty	Erythroid protein 4.1 OS=Gallus gallus GN=EPB41 PE=4 SV=2	F1NLW8_CHICK (+7)		101 kDa	TRUE	unknown	19	1	4	1.3607	0.033003	0.15209
176	TRUE	Empty	Integrin beta OS=Gallus gallus GN=ITGB1 PE=3 SV=1	F1N8N7_CHICK (+2)		89 kDa		unknown	3	12	14	0.14758	0.6323	1.0689
177	TRUE	Empty	Uncharacterized protein OS=Gallus gallus GN=PA2G4 PE=4 SV=2	H9KZC8_CHICK (+5)		38 kDa		unknown	11	9	6	2.0461	1.1982	0.91506
178	TRUE	Empty	Proteasome subunit alpha type-7 OS=Gallus gallus GN=PSMA7 PE=4 SV=1	R4GJX7_CHICK (+2)		24 kDa		unknown	6	7	8	1.9835	2.4147	3.1969
179	TRUE	Empty	60S acidic ribosomal protein P0 OS=Gallus gallus GN=RPLP0 PE=2 SV=1	RLA0_CHICK (+2)		34 kDa		unknown	4	9	6	0.58382	2.239	1.054
180	TRUE	Empty	Ubiquitin carboxyl-terminal hydrolase OS=Gallus gallus GN=USP9X PE=3 SV=1	E1BWJ5_CHICK (+10)		290 kDa		unknown	28	10	3	0.47692	0.13374	0.037818
181	TRUE	Empty	Actin-related protein 3 OS=Gallus gallus GN=ACTR3 PE=2 SV=1	ARP3_CHICK (+2)		47 kDa	TRUE	unknown	0	13	15	0	1.7022	3.1632
182	TRUE	Empty	Lysine--tRNA ligase OS=Gallus gallus GN=KARS PE=3 SV=1	F1NE72_CHICK (+6)		68 kDa		unknown	6	11	11	0.42561	0.71773	0.8795
183	TRUE	Empty	Far upstream element-binding protein 2 OS=Gallus gallus GN=KHSRP PE=1 SV=1	FUBP2_CHICK (+1)		81 kDa		unknown	10	6	6	0.64062	0.33299	0.30414
184	TRUE	Empty	Uncharacterized protein (Fragment) OS=Gallus gallus GN=ESD PE=4 SV=2	E1BXC2_CHICK		37 kDa		unknown	10	6	7	2.4883	0.71203	0.95595
185	TRUE	Empty	status=inactive	UPI000240B7FF (+1)		243 kDa		unknown	17	13	6	0.32821	0.19405	0.10887
186	TRUE	Empty	Heterogeneous nuclear ribonucleoprotein K OS=Gallus gallus GN=HNRPK PE=4 SV=2	H9L0I0_CHICK (+7)		47 kDa		unknown	11	0	7	1.4761	0	0.69045
187	TRUE	Empty	High mobility group protein B1 OS=Gallus gallus GN=HMGB1 PE=1 SV=1	HMGB1_CHICK (+6)		25 kDa	TRUE	unknown	13	10	9	7.1406	4.0638	3.716
188	TRUE	Empty	status=active	UPI00019643CE		14 kDa	TRUE	unknown	0	3	9	0	15.381	16.78
189	TRUE	Empty	60S ribosomal protein L7a (Fragment) OS=Gallus gallus GN=RPL7A PE=4 SV=1	F2Z4L5_CHICK (+1)		30 kDa		unknown	0	12	6	0	4.4725	1.2804
190	TRUE	Empty	Uncharacterized protein OS=Gallus gallus GN=RAB7A PE=3 SV=1	E1C0F3_CHICK (+3)		24 kDa		unknown	6	7	9	2.1007	1.6753	2.8101
191	TRUE	Empty	Uncharacterized protein OS=Gallus gallus GN=BPGM PE=2 SV=1	Q5ZHV4_CHICK (+2)		30 kDa		unknown	9	2	5	2.6039	0.24369	0.8047
192	TRUE	Empty	Proteasome subunit alpha type (Fragment) OS=Gallus gallus GN=PSMA1 PE=3 SV=2	F1N954_CHICK (+2)		29 kDa		unknown	10	7	8	2.6702	1.7837	2.749
193	TRUE	Empty	Uncharacterized protein OS=Gallus gallus GN=CCT2 PE=2 SV=1	Q5F424_CHICK (+3)		57 kDa		unknown	12	15	7	1.2705	1.7184	0.54293
194	TRUE	Empty	Calreticulin (Fragment) OS=Gallus gallus PE=2 SV=1	Q6EE32_CHICK (+1)		47 kDa		unknown	4	15	9	0.40594	2.1707	1.4839
195	TRUE	Empty	Serine/arginine-rich splicing factor 1 OS=Gallus gallus GN=SRSF1 PE=1 SV=3	SRSF1_CHICK (+3)		28 kDa		unknown	6	7	8	1.2834	1.2827	2.084
196	TRUE	Empty	Vacuolar protein sorting-associated protein 35 OS=Gallus gallus GN=VPS35 PE=3 SV=1	F1NVF0_CHICK (+5)		92 kDa		unknown	3	9	16	0.14232	0.38503	0.94133
197	TRUE	Empty	Uncharacterized protein OS=Gallus gallus GN=AP2B1 PE=4 SV=1	E1BY12_CHICK		106 kDa		unknown	0	10	13	0	0.3692	0.55303
198	TRUE	Empty	Alpha-actinin-1 (Fragment) OS=Gallus gallus GN=ACTN1 PE=4 SV=1	R9PXN4_CHICK (+8)		102 kDa	TRUE	unknown	0	17	11	0	1.8161	2.0886
199	TRUE	Empty	Glucose-6-phosphate isomerase OS=Gallus gallus GN=GPI PE=3 SV=1	F1NIJ6_CHICK (+4)		62 kDa		unknown	14	17	0	1.7338	2.1519	0
200	TRUE	Empty	Uncharacterized protein OS=Gallus gallus GN=GC PE=4 SV=1	F1NVF3_CHICK (+3)		54 kDa		unknown	16	7	8	2.9036	0.53959	0.81393
201	TRUE	Empty	Malate dehydrogenase, cytoplasmic OS=Gallus gallus GN=MDH1 PE=2 SV=1	MDHC_CHICK (+3)		37 kDa		unknown	9	7	9	2.1855	1.2734	2.1977
202	TRUE	Empty	Nuclease-sensitive element-binding protein 1 (Fragment) OS=Gallus gallus GN=YBX1 PE=4 SV=2	F1NFC0_CHICK (+10)		31 kDa		unknown	1	7	5	0.13733	1.6255	0.97218
203	TRUE	Empty	Isoform 2 of Basigin OS=Gallus gallus GN=BSG	sp|P17790-2|BASI_CHICK		29 kDa		unknown	7	7	6	1.8631	1.4859	1.3227
204	TRUE	Empty	status=inactive	UPI0000ECA011 (+2)		97 kDa		unknown	7	10	5	0.33753	0.56283	0.29366
205	TRUE	Empty	Uncharacterized protein OS=Gallus gallus GN=Gga.50010 PE=4 SV=2	E1C866_CHICK (+9)		109 kDa	TRUE	unknown	0	14	15	0	0.58434	0.69234
206	TRUE	Empty	Uncharacterized protein OS=Gallus gallus GN=PDIA6 PE=3 SV=1	F1NK96_CHICK (+3)		49 kDa		unknown	6	12	9	0.6294	1.4494	1.0726
207	TRUE	Empty	Uncharacterized protein OS=Gallus gallus GN=RCJMB04_16g5 PE=2 SV=1	Q5ZJQ5_CHICK (+4)		57 kDa		unknown	6	14	14	0.51968	1.2764	1.7112
208	TRUE	Empty	Eukaryotic translation elongation factor 1 OS=Gallus gallus PE=2 SV=1	Q6EE30_CHICK (+1)		50 kDa		unknown	9	9	6	1.0333	0.953	0.6462
209	TRUE	Empty	Uncharacterized protein OS=Gallus gallus GN=KRT12 PE=3 SV=2	F1NDN6_CHICK (+8)		54 kDa	TRUE	unknown	5	0	2	0.92303	0	0.48085
210	TRUE	Empty	Uncharacterized protein OS=Gallus gallus GN=LOC101749333 PE=3 SV=1	R4GKA1_CHICK (+1)		19 kDa	TRUE	unknown	0	3	4	0	1.8262	1.0745
211	TRUE	Empty	OvoglobulinG2 type AA OS=Gallus gallus GN=G2 PE=2 SV=1	I0J172_CHICK (+2)		47 kDa		unknown	0	9	6	0	1.5124	0.56642
212	TRUE	Empty	60S ribosomal protein L7 (Fragment) OS=Gallus gallus GN=RPL7 PE=3 SV=1	F1NCI6_CHICK (+6)		29 kDa		unknown	3	12	9	0.50996	6.5108	2.415
213	TRUE	Empty	Uncharacterized protein (Fragment) OS=Gallus gallus GN=AK2 PE=3 SV=2	F1NJ73_CHICK (+5)		27 kDa		unknown	8	6	4	3.0119	1.3368	0.89993
214	TRUE	Empty	60S ribosomal protein L6 OS=Gallus gallus GN=RPL6 PE=2 SV=1	Q8UWG7_CHICK (+1)		34 kDa	TRUE	unknown	0	6	6	0	0.98369	0.86755
215	TRUE	Empty	status=inactive	UPI0000E7F7E4 (+3)		234 kDa		unknown	0	14	16	0	0.21946	0.27805
216	TRUE	Empty	status=active	UPI0003504D25		14 kDa		unknown	0	0	7	0	0	11.275
217	TRUE	Empty	6-phosphogluconate dehydrogenase, decarboxylating OS=Gallus gallus GN=PGD PE=2 SV=1	Q5ZIZ0_CHICK		53 kDa		unknown	7	9	10	0.68355	1.4188	1.5477
218	TRUE	Empty	Uncharacterized protein OS=Gallus gallus GN=COPB2 PE=4 SV=1	E1C016_CHICK (+6)		103 kDa		unknown	3	13	10	0.12583	0.52245	0.4145
219	TRUE	Empty	status=active	UPI00003AFA4C (+7)		94 kDa	TRUE	unknown	8	23	0	0.40886	1.3732	0
220	TRUE	Empty	Myosin light chain kinase, smooth muscle OS=Gallus gallus GN=Mylk PE=1 SV=2	sp|P11799|MYLK_CHICK (+2)		210 kDa		unknown	0	14	9	0	0.28771	0.20633
221	TRUE	Empty	Fibrinogen beta chain OS=Gallus gallus GN=FGB PE=4 SV=2	F1NUL9_CHICK (+10)		55 kDa		unknown	21	1	8	3.3909	0.061614	0.68492
222	TRUE	Empty	status=inactive	UPI000240C0E5 (+1)		99 kDa		unknown	0	4	15	0	0.1419	0.71751
223	TRUE	Empty	Uncharacterized protein OS=Gallus gallus GN=HNRNPAB PE=4 SV=2	F1NIB4_CHICK (+6)		25 kDa		unknown	8	6	3	3.0041	1.2393	0.5324
224	TRUE	Empty	Uncharacterized protein (Fragment) OS=Gallus gallus GN=RPS4X PE=3 SV=2	F1NFC6_CHICK (+7)		29 kDa		unknown	1	14	12	0.14536	3.9882	3.7445
225	TRUE	Empty	cDNA, FLJ95666, highly similar to Homo sapiens albumin (ALB), mRNA OS=Homo sapiens PE=2 SV=1	B2RBS8 (+3)		?		unknown	0	0	20	0	0	-1
226	TRUE	Empty	Uncharacterized protein OS=Gallus gallus GN=ALDH7A1 PE=3 SV=1	E1C4W4_CHICK (+6)		58 kDa		unknown	2	14	11	0.14953	1.687	1.2202
227	TRUE	Empty	status=active	UPI0002C87A56 (+4)		196 kDa	TRUE	unknown	0	19	0	0	0.38165	0
228	TRUE	Empty	60S ribosomal protein L5 (Fragment) OS=Gallus gallus GN=RPL5 PE=3 SV=1	F2Z4K4_CHICK (+4)		34 kDa		unknown	2	12	6	0.2645	4.4676	1.2953
229	TRUE	Empty	Uncharacterized protein (Fragment) OS=Gallus gallus GN=LOC100858328 PE=3 SV=1	R4GF85_CHICK		18 kDa	TRUE	unknown	0	5	6	0	1.4281	2.0888
230	TRUE	Empty	status=active	UPI00000FBFC1 (+1)		18 kDa		unknown	2	6	6	0.54525	2.6687	3.7566
231	TRUE	Empty	40S ribosomal protein S13 OS=Gallus gallus GN=RPS13 PE=2 SV=3	RS13_CHICK (+3)		17 kDa		unknown	5	11	7	1.9846	7.468	3.9916
232	TRUE	Empty	Lumican OS=Gallus gallus GN=LUM PE=1 SV=1	LUM_CHICK (+2)		39 kDa		unknown	4	7	8	0.50727	1.1734	1.4996
233	TRUE	Empty	status=active	UPI0000ECB3E2 (+5)		66 kDa		unknown	0	7	9	0	0.4905	0.70919
234	TRUE	Empty	Eukaryotic translation initiation factor 2 subunit 3 OS=Gallus gallus GN=EIF2S3 PE=4 SV=1	F1NPF0_CHICK (+3)		51 kDa		unknown	11	10	8	1.5013	1.3503	0.74441
235	TRUE	Empty	Adenosine deaminase OS=Gallus gallus GN=ADA PE=2 SV=1	ADA_CHICK (+6)		41 kDa		unknown	15	2	2	4.8976	0.17399	0.1898
236	TRUE	Empty	Uncharacterized protein OS=Gallus gallus GN=RCJMB04_2h4 PE=2 SV=1	Q5ZMD9_CHICK (+1)		59 kDa	TRUE	unknown	10	14	10	1.0735	1.3386	0.82111
237	TRUE	Empty	Mitochondrial ubiquinol-cytochrome-c reductase complex core protein 2 OS=Gallus gallus PE=1 SV=1	D0VX29_CHICK (+4)		47 kDa		unknown	0	9	9	0	1.046	0.98368
238	TRUE	Empty	Dihydropyrimidinase-related protein 2 OS=Gallus gallus GN=DPYSL2 PE=2 SV=1	DPYL2_CHICK (+8)		62 kDa	TRUE	unknown	0	12	10	0	0.90486	0.87631
239	TRUE	Empty	Uncharacterized protein (Fragment) OS=Gallus gallus GN=PRDX4 PE=4 SV=1	F1NNS8_CHICK (+5)		32 kDa	TRUE	unknown	0	10	11	0	2.4994	2.7267
240	TRUE	Empty	status=inactive	UPI000044994D (+2)		119 kDa		unknown	0	8	13	0	0.24978	0.62019
241	TRUE	Empty	status=inactive	UPI000060E30A (+2)		165 kDa	TRUE	unknown	0	19	5	0	0.46793	0.11444
242	TRUE	Empty	Coronin OS=Gallus gallus GN=CORO1C PE=2 SV=1	Q5ZI60_CHICK (+5)		53 kDa		unknown	7	6	10	0.68531	0.45181	1.0871
243	TRUE	Empty	Proteasome subunit alpha type OS=Gallus gallus GN=PSMA3 PE=2 SV=1	Q5ZLI2_CHICK (+2)		28 kDa		unknown	4	5	7	0.73358	1.5484	2.4471
244	TRUE	Empty	status=active	UPI00000012C8 (+8)		21 kDa	TRUE	unknown	0	6	9	0	3.3008	11.791
245	TRUE	Empty	Uncharacterized protein (Fragment) OS=Gallus gallus GN=LOC770025 PE=3 SV=2	F1NG53_CHICK (+1)		22 kDa		unknown	0	7	5	0	3.7171	2.1041
246	TRUE	Empty	Uncharacterized protein OS=Gallus gallus GN=HNRNPM PE=2 SV=1	Q5ZL80_CHICK (+3)		76 kDa		unknown	7	6	4	0.44736	0.29827	0.20648
247	TRUE	Empty	Uncharacterized protein OS=Gallus gallus GN=ACLY PE=4 SV=2	F1P269_CHICK (+6)		120 kDa		unknown	4	9	15	0.14534	0.3942	0.61158
248	TRUE	Empty	Phosphoglycerate kinase OS=Gallus gallus GN=PGK1 PE=3 SV=1	F1NU17_CHICK (+1)		45 kDa		unknown	10	9	8	1.6069	0.95748	0.88795

249	TRUE	Empty	Uncharacterized protein OS=Gallus gallus GN=ACO2 PE=2 SV=1	Q5ZMW1_CHICK (+3)		86 kDa		unknown	0	7	19	0	0.31076	1.4064
250	TRUE	Empty	Uncharacterized protein OS=Gallus gallus GN=HNRNPR PE=4 SV=1	F6SG96_CHICK (+5)		73 kDa	TRUE	unknown	8	10	5	0.5491	0.57719	0.27619
251	TRUE	Empty	Uncharacterized protein (Fragment) OS=Gallus gallus GN=LOC418811 PE=4 SV=2	E1BQD1_CHICK (+4)		26 kDa	TRUE	unknown	6	7	8	1.7606	1.7347	2.3048
252	TRUE	Empty	Uncharacterized protein OS=Gallus gallus GN=RCJMB04_7I20 PE=2 SV=1	Q5ZL53_CHICK (+1)		71 kDa		unknown	8	12	4	0.65751	0.84635	0.22237
253	TRUE	Empty	UPI000198D1F8-DECOY	UPI000198D1F8-DECOY (+1)		?		unknown	2	1	1	-1	-1	-1
254	TRUE	Empty	Uncharacterized protein OS=Gallus gallus GN=ANP32A PE=4 SV=2	E1C2A1_CHICK (+10)		32 kDa		unknown	9	5	6	2.2882	0.85022	1.4007
255	TRUE	Empty	Uncharacterized protein (Fragment) OS=Gallus gallus GN=CCT6A PE=3 SV=2	F1NWH9_CHICK (+6)		58 kDa	TRUE	unknown	12	9	6	1.4141	0.67969	0.447
256	TRUE	Empty	Eukaryotic translation initiation factor 5A-1 OS=Gallus gallus GN=EIF5A1 PE=1 SV=3	IF5A1_CHICK (+3)		16 kDa		unknown	2	6	6	1.0502	11.537	15.9
257	TRUE	Empty	status=active	UPI000060E931 (+3)		29 kDa		unknown	6	7	8	1.1979	1.1928	1.9226
258	TRUE	Empty	Uncharacterized protein OS=Gallus gallus GN=LCN8 PE=3 SV=2	E1C0K1_CHICK (+8)		20 kDa		unknown	0	5	7	0	1.2486	3.7139
259	TRUE	Empty	Uncharacterized protein OS=Gallus gallus GN=NPEPPS PE=4 SV=2	F1P455_CHICK		97 kDa	TRUE	unknown	0	0	26	0	0	1.7084
260	TRUE	Empty	Leukotriene A(4) hydrolase OS=Gallus gallus GN=LTA4H PE=3 SV=1	F1P284_CHICK (+5)		69 kDa		unknown	6	10	10	0.4972	0.69688	0.67184
261	TRUE	Empty	Uncharacterized protein OS=Gallus gallus GN=LOC101748085 PE=4 SV=1	R4GJZ8_CHICK (+1)		34 kDa		unknown	7	5	7	1.2224	0.78844	1.2893
262	TRUE	Empty	Glutathione S-transferase 2 OS=Gallus gallus GN=GSTM2 PE=1 SV=4	GSTM2_CHICK (+2)		26 kDa		unknown	2	8	11	0.35646	2.1893	5.6962
263	TRUE	Empty	Uncharacterized protein OS=Gallus gallus GN=EEF1D PE=3 SV=2	E1BZG4_CHICK (+14)		76 kDa		unknown	10	5	2	0.68831	0.24208	0.15089
264	TRUE	Empty	CFR-associated protein p70 OS=Gallus gallus PE=2 SV=1	O57660_CHICK (+6)		83 kDa		unknown	1	14	11	0.050328	0.83026	0.67464
265	TRUE	Empty	Uncharacterized protein OS=Gallus gallus GN=RPS2 PE=3 SV=1	E1C4M0_CHICK (+1)		31 kDa		unknown	0	10	6	0	1.9779	0.99124
266	TRUE	Empty	Keratin 1 OS=Homo sapiens GN=KRT1 PE=3 SV=1	H6VRF8 (+5)		?	TRUE	unknown	0	0	20	0	0	-1
267	TRUE	Empty	status=inactive	UPI0000ECA60C		52 kDa	TRUE	unknown	4	0	0	0.36275	0	0
268	TRUE	Empty	T-complex protein 1 subunit delta OS=Gallus gallus GN=tcp-1 delta PE=2 SV=1	Q9I8D6_CHICK		58 kDa		unknown	11	8	4	1.4141	0.88979	0.36044
269	TRUE	Empty	status=active	UPI0000115DA8 (+8)		54 kDa	TRUE	unknown	9	1	7	1.0816	0.062653	0.59014
270	TRUE	Empty	Uncharacterized protein OS=Gallus gallus GN=PDCD6IP PE=2 SV=1	Q5ZJ70_CHICK (+4)		97 kDa		unknown	0	10	16	0	0.45695	0.86959
271	TRUE	Empty	60S ribosomal protein L13 OS=Gallus gallus GN=RPL13 PE=2 SV=2	RL13_CHICK (+2)		24 kDa		unknown	0	7	5	0	2.4147	1.0451
272	TRUE	Empty	Rab27a OS=Gallus gallus GN=Rab27a PE=2 SV=1	D2D3P4_CHICK		25 kDa		unknown	0	4	7	0	1.8842	4.3547
273	TRUE	Empty	Fructose-bisphosphate aldolase B OS=Gallus gallus GN=ALDOB PE=3 SV=3	ALDOB_CHICK (+4)		39 kDa	TRUE	unknown	13	6	1	5.2418	0.80433	0.093884
274	TRUE	Empty	T-complex protein 1 subunit eta OS=Gallus gallus GN=CCT7 PE=3 SV=2	F1NK38_CHICK (+6)		60 kDa	TRUE	unknown	11	9	0	1.0574	1.0657	0
275	TRUE	Empty	Uncharacterized protein OS=Gallus gallus GN=ARHGDIA PE=4 SV=1	F1P3P3_CHICK (+4)		23 kDa		unknown	3	7	7	0.65424	3.8993	3.5158
276	TRUE	Empty	cDNA FLJ58187, highly similar to Protein-glutamine gamma-glutamyltransferase 2(EC 2.3.2.13) OS=Homo sapiens PE=2 SV=1	B4DIT7 (+2)		?		unknown	0	0	25	0	0	-1
277	TRUE	Empty	Uncharacterized protein (Fragment) OS=Gallus gallus GN=TGM2 PE=4 SV=2	E1BY44_CHICK (+11)		78 kDa		unknown	11	6	6	0.84561	0.29054	0.31696
278	TRUE	Empty	Uncharacterized protein OS=Gallus gallus GN=RPL4 PE=2 SV=1	Q5ZII1_CHICK		47 kDa	TRUE	unknown	0	10	5	0	1.1976	0.46097
279	TRUE	Empty	Dimethylaniline monooxygenase [N-oxide-forming] OS=Gallus gallus GN=FMO3 PE=2 SV=1	Q8QH01_CHICK (+6)		60 kDa		unknown	0	10	14	0	0.83708	1.7292
280	TRUE	Empty	Glyceraldehyde-3-phosphate dehydrogenase OS=Gallus gallus GN=GAPDH PE=2 SV=3	G3P_CHICK (+5)		36 kDa	TRUE	unknown	11	0	0	4.4844	0	0
281	TRUE	Empty	Protein syndesmos OS=Gallus gallus GN=SDOS PE=1 SV=3	SDOS_CHICK (+3)		33 kDa		unknown	6	10	5	1.262	1.7285	0.69683
282	TRUE	Empty	Uncharacterized protein OS=Gallus gallus GN=RPL12 PE=3 SV=2	E1BTG1_CHICK (+3)		18 kDa		unknown	2	4	5	0.54525	1.5087	4.785
283	TRUE	Empty	Ras-related protein Rap-1b OS=Gallus gallus GN=RAP1B PE=2 SV=1	RAP1B_CHICK (+1)		21 kDa	TRUE	unknown	4	7	8	1.0956	2.5912	3.5289
284	TRUE	Empty	status=active	UPI00003AC2BF		30 kDa		unknown	9	6	2	3.0976	1.1864	0.8086
285	TRUE	Empty	40S ribosomal protein S8 OS=Gallus gallus GN=RPS8 PE=3 SV=2	F1NB02_CHICK (+7)		24 kDa		unknown	1	10	7	0.17796	3.5894	2.1795
286	TRUE	Empty	Uncharacterized protein (Fragment) OS=Gallus gallus GN=ACAD9 PE=3 SV=2	F1NEF6_CHICK (+4)		68 kDa		unknown	0	8	16	0	0.47977	1.3243
287	TRUE	Empty	Isoform 2 of F-actin-capping protein subunit beta isoforms 1 and 2 OS=Gallus gallus GN=CAPZB	sp|P14315-2|CAPZB_CHICK (+4)		31 kDa		unknown	0	8	10	0	2.3321	4.0122
288	TRUE	Empty	Moesin OS=Homo sapiens GN=MSN PE=1 SV=3	P26038 (+1)		?	TRUE	unknown	0	0	15	0	0	-1
289	TRUE	Empty	status=inactive	UPI000044AA03		194 kDa	TRUE	unknown	7	6	7	0.15897	0.10764	0.13831
290	TRUE	Empty	Uncharacterized protein OS=Gallus gallus GN=NUMA1 PE=4 SV=2	E1BQQ4_CHICK (+3)		238 kDa		unknown	22	3	5	0.47699	0.042382	0.078256
291	TRUE	Empty	Uncharacterized protein OS=Gallus gallus GN=RAB1A PE=3 SV=1	F6UGI5_CHICK (+4)		23 kDa	TRUE	unknown	6	4	6	1.7472	1.0627	3.6805
292	TRUE	Empty	Uncharacterized protein OS=Gallus gallus GN=RCJMB04_2p17 PE=2 SV=1	Q5ZM66_CHICK (+3)		13 kDa		unknown	2	4	4	0.78951	2.4841	2.71
293	TRUE	Empty	Cofilin-2 OS=Gallus gallus GN=CFL2 PE=1 SV=2	COF2_CHICK (+3)		19 kDa		unknown	2	7	9	0.51543	3.1429	6.7575
294	TRUE	Empty	Vigilin OS=Gallus gallus GN=HDLBP PE=4 SV=1	E1C3A9_CHICK (+4)		142 kDa	TRUE	unknown	0	10	8	0	0.26398	0.25575
295	TRUE	Empty	status=inactive	UPI0000ECAD38		267 kDa	TRUE	unknown	3	6	6	0.35455	1.4073	2.023
296	TRUE	Empty	Succinyl-CoA:3-ketoacid-coenzyme A transferase OS=Gallus gallus GN=OXCT1 PE=3 SV=2	F1N9Z7_CHICK (+6)		56 kDa		unknown	2	8	7	0.1548	0.81035	0.88403
297	TRUE	Empty	Stress-70 protein, mitochondrial OS=Gallus gallus GN=HSPA9 PE=3 SV=1	F1NZ86_CHICK (+6)		73 kDa		unknown	4	10	7	0.24713	0.72945	0.5508
298	TRUE	Empty	Uncharacterized protein OS=Gallus gallus GN=TPP2 PE=4 SV=2	F1P257_CHICK (+9)		144 kDa		unknown	18	4	7	0.64893	0.095678	0.18984
299	TRUE	Empty	Brain acid soluble protein 1 homolog OS=Gallus gallus GN=BASP1 PE=2 SV=4	BASP1_CHICK (+2)		25 kDa		unknown	0	5	4	0	1.8446	1.2846
300	TRUE	Empty	Uncharacterized protein OS=Gallus gallus GN=DSP PE=4 SV=2	E1BWI0_CHICK (+7)		321 kDa	TRUE	unknown	0	13	9	0	0.14353	0.10592
301	TRUE	Empty	status=active	UPI000203494F (+7)		36 kDa	TRUE	unknown	2	7	8	0.73592	1.5546	2.6345
302	TRUE	Empty	Eukaryotic translation initiation factor 2 subunit 1 OS=Gallus gallus GN=EIF2S1 PE=2 SV=2	IF2A_CHICK (+2)		36 kDa		unknown	6	6	8	0.91411	0.72902	1.1834
303	TRUE	Empty	Ubiquitin carboxyl-terminal hydrolase OS=Gallus gallus GN=UCHL3 PE=3 SV=1	F1NY51_CHICK (+4)		26 kDa		unknown	2	8	6	0.56328	3.0626	2.321
304	TRUE	Empty	Uncharacterized protein OS=Gallus gallus GN=PRDX3 PE=4 SV=1	E1BR10_CHICK		28 kDa	TRUE	unknown	5	8	8	1.0021	1.9227	2.9864
305	TRUE	Empty	Ribosomal protein L15 OS=Gallus gallus GN=RPL15 PE=3 SV=2	F1NQG5_CHICK (+2)		24 kDa	TRUE	unknown	0	9	8	0	2.4729	2.6977
306	TRUE	Empty	Lipoma-preferred partner homolog OS=Gallus gallus GN=LPP PE=2 SV=1	LPP_CHICK (+2)		65 kDa		unknown	0	6	5	0	0.35624	0.31458
307	TRUE	Empty	Aspartate aminotransferase OS=Gallus gallus GN=GOT2 PE=3 SV=2	F1P180_CHICK (+2)		47 kDa		unknown	5	13	8	0.5225	1.7086	0.82248
308	TRUE	Empty	Uncharacterized protein OS=Gallus gallus GN=CBR1 PE=3 SV=1	F1N8Y3_CHICK (+6)		30 kDa		unknown	9	5	8	2.1208	0.72026	1.8451
309	TRUE	Empty	Uncharacterized protein OS=Gallus gallus GN=FBP1 PE=3 SV=1	F6QGI8_CHICK (+3)		45 kDa		unknown	12	8	7	1.8358	0.81395	0.74344
310	TRUE	Empty	Uncharacterized protein OS=Gallus gallus GN=LOC424014 PE=4 SV=1	R4GL55_CHICK (+2)		30 kDa		unknown	5	7	8	0.90313	1.1492	1.8451
311	TRUE	Empty	Uncharacterized protein (Fragment) OS=Gallus gallus GN=APRT PE=3 SV=1	R4GHI5_CHICK (+3)		23 kDa		unknown	11	4	4	4.7979	0.74637	1.1065
312	TRUE	Empty	Ras-related protein Rab-14 OS=Gallus gallus GN=RAB14 PE=2 SV=3	RAB14_CHICK (+5)		24 kDa	TRUE	unknown	1	5	10	0.63239	0.98853	4.8202
313	TRUE	Empty	L-lactate dehydrogenase OS=Gallus gallus GN=LDHA PE=3 SV=2	E1BTT8_CHICK (+9)		37 kDa		unknown	4	10	7	0.54191	1.743	0.96773
314	TRUE	Empty	Sulfurtransferase OS=Gallus gallus GN=MPST PE=4 SV=2	E1C8D8_CHICK (+4)		33 kDa		unknown	7	6	7	1.2678	1.0111	1.103
315	TRUE	Empty	Uncharacterized protein OS=Gallus gallus GN=RCJMB04_14h20 PE=2 SV=1	Q5ZJY1_CHICK (+3)		26 kDa		unknown	5	6	6	1.0929	1.4219	1.2308
316	TRUE	Empty	Proteasome subunit beta type OS=Gallus gallus GN=PSMB1 PE=3 SV=1	Q6JLB2_CHICK (+2)		26 kDa		unknown	6	5	6	1.4319	1.1419	1.5712
317	TRUE	Empty	Ras-related protein Rab-2A OS=Gallus gallus GN=RAB2A PE=2 SV=1	RAB2A_CHICK (+4)		24 kDa		unknown	5	6	8	1.2636	1.3166	2.2822
318	TRUE	Empty	Bleomycin hydrolase OS=Gallus gallus GN=BLMH PE=1 SV=1	BLMH_CHICK (+3)		53 kDa		unknown	8	6	4	0.96156	0.45753	0.30961
319	TRUE	Empty	DNA damage-binding protein 1 OS=Gallus gallus GN=DDB1 PE=2 SV=1	DDB1_CHICK (+6)		127 kDa	TRUE	unknown	0	9	15	0	0.26496	0.52738
320	TRUE	Empty	Uncharacterized protein OS=Gallus gallus GN=CCT5 PE=2 SV=1	Q5F411_CHICK (+4)		60 kDa		unknown	16	0	8	1.8381	0	0.61067
321	TRUE	Empty	Uncharacterized protein OS=Gallus gallus GN=SFN PE=3 SV=1	R4GF89_CHICK (+1)		28 kDa	TRUE	unknown	0	3	11	0	1.0406	10.16
322	TRUE	Empty	Isoform 2 of Tropomyosin beta chain OS=Gallus gallus GN=TPM2	sp|P19352-2|TPM2_CHICK		33 kDa	TRUE	unknown	0	9	14	0	6.1697	15.477
323	TRUE	Empty	Cathepsin D OS=Gallus gallus GN=CTSD PE=1 SV=1	CATD_CHICK (+1)		43 kDa		unknown	1	8	8	0.097722	0.99853	1.0893
324	TRUE	Empty	WD repeat-containing protein 1 OS=Gallus gallus GN=WDR1 PE=4 SV=2	F1NRI3_CHICK (+7)		67 kDa		unknown	0	9	8	0	0.56813	0.70919
325	TRUE	Empty	Heterogeneous nuclear ribonucleoprotein K OS=Gallus gallus GN=HNRNPK PE=4 SV=1	E1C453_CHICK (+3)		47 kDa	TRUE	unknown	0	12	0	0	1.3506	0
326	TRUE	Empty	Collagen alpha-2(I) chain OS=Gallus gallus GN=COL1A2 PE=4 SV=1	F1P0H9_CHICK		129 kDa	TRUE	unknown	0	12	10	0	0.36282	0.32023
327	TRUE	Empty	Uncharacterized protein (Fragment) OS=Gallus gallus GN=UBE2N PE=3 SV=1	F1NFT6_CHICK (+7)		16 kDa		unknown	5	4	6	3.0362	1.2504	3.5208
328	TRUE	Empty	Uncharacterized protein OS=Gallus gallus GN=LOC100859311 PE=3 SV=1	F1NBP2_CHICK (+10)		68 kDa	TRUE	unknown	8	4	7	0.59566	0.27308	0.44089
329	TRUE	Empty	status=inactive	UPI0000ECC6B6 (+6)		68 kDa		unknown	3	11	11	0.19521	0.71468	0.77965
330	TRUE	Empty	Lamin-B1 OS=Gallus gallus GN=LMNB1 PE=3 SV=2	F1NAM2_CHICK (+5)		74 kDa	TRUE	unknown	7	9	4	0.80983	0.80543	0.27387

331	TRUE	Empty	Superoxide dismutase [Cu-Zn] OS=Gallus gallus GN=SOD1 PE=3 SV=2	F1N8Q1_CHICK (+3)		16 kDa		unknown	6	4	6	4.2085	1.2885	2.7384
332	TRUE	Empty	Dolichyl-diphosphooligosaccharide--protein glycosyltransferase subunit 1 (Fragment) OS=Gallus gallus GN=RPN1 PE=3 SV=2	E1C0F1_CHICK (+10)		61 kDa		unknown	1	10	9	0.069348	0.73286	0.69657
333	TRUE	Empty	Eukaryotic translation initiation factor 3 subunit A OS=Gallus gallus GN=EIF3A PE=3 SV=1	E1BSS3_CHICK (+4)		163 kDa		unknown	8	9	7	0.22056	0.22498	0.166
334	TRUE	Empty	Desmin OS=Gallus gallus GN=DES PE=1 SV=1	DESM_CHICK (+3)		53 kDa	TRUE	unknown	0	9	1	0	1.5807	0.49165
335	TRUE	Empty	Uncharacterized protein OS=Gallus gallus GN=TGM4 PE=4 SV=2	F1NJH2_CHICK (+3)		79 kDa		unknown	0	0	14	0	0	1.1596
336	TRUE	Empty	Proteasome subunit alpha type (Fragment) OS=Gallus gallus GN=PSMA6 PE=3 SV=1	F1NEQ6_CHICK (+4)		28 kDa		unknown	5	5	5	1.0126	0.80759	1.7522
337	TRUE	Empty	60S ribosomal protein L27 OS=Gallus gallus GN=RPL27 PE=2 SV=2	RL27_CHICK (+3)		16 kDa		unknown	1	7	7	0.27901	4.3495	4.745
338	TRUE	Empty	Ubiquitin-conjugating enzyme E2 variant 2 OS=Gallus gallus GN=UBE2V2 PE=2 SV=1	UB2V2_CHICK (+3)		16 kDa	TRUE	unknown	1	3	8	1.0134	2.3383	7.2992
339	TRUE	Empty	ATP synthase subunit gamma OS=Gallus gallus GN=ATP5C1 PE=3 SV=2	H9KZF8_CHICK (+9)		33 kDa		unknown	2	8	8	0.27521	1.7754	1.636
340	TRUE	Empty	Uncharacterized protein OS=Gallus gallus GN=NUCB2 PE=4 SV=1	F1NGB1_CHICK (+3)		54 kDa		unknown	2	9	5	0.16186	1.1093	0.3906
341	TRUE	Empty	status=active	UPI0003506C6A (+8)		41 kDa		unknown	8	0	4	1.8124	0	0.53621
342	TRUE	Empty	Collagen alpha-3(VI) chain OS=Gallus gallus GN=COL6A3 PE=2 SV=2	CO6A3_CHICK (+1)		340 kDa		unknown	0	0	14	0	0	0.17201
343	TRUE	Empty	Uncharacterized protein OS=Gallus gallus GN=KRT6A PE=3 SV=1	H9KZ27_CHICK (+4)		57 kDa	TRUE	unknown	5	0	3	0.62997	0	1.5561
344	TRUE	Empty	Ig mu chain C region OS=Homo sapiens GN=IGHM PE=4 SV=1	A0A087X2C0		?	TRUE	unknown	0	0	12	0	0	-1
345	TRUE	Empty	26S proteasome non-ATPase regulatory subunit 13 OS=Gallus gallus GN=PSMD13 PE=1 SV=1	PSD13_CHICK (+5)		43 kDa		unknown	7	8	7	0.90349	1.0167	0.78605
346	TRUE	Empty	Glutathione peroxidase OS=Gallus gallus GN=GPX4 PE=2 SV=3	Q8QG67_CHICK (+3)		19 kDa		unknown	3	6	8	0.81278	1.7608	4.0043
347	TRUE	Empty	Uncharacterized protein OS=Gallus gallus GN=PSMD2 PE=4 SV=2	E1BX02_CHICK (+5)		100 kDa		unknown	8	7	7	0.38085	0.26079	0.2845
348	TRUE	Empty	Transaldolase OS=Gallus gallus GN=TALDO1 PE=3 SV=2	H9L1C2_CHICK (+5)		38 kDa		unknown	9	6	6	1.8011	0.69581	0.75906
349	TRUE	Empty	Uncharacterized protein (Fragment) OS=Gallus gallus GN=ATP5O PE=3 SV=2	F1P304_CHICK (+4)		25 kDa		unknown	0	5	8	0	1.5709	2.6137
350	TRUE	Empty	status=active	UPI0000447266 (+12)		21 kDa	TRUE	unknown	0	3	2	0	5.8269	0.93984
351	TRUE	Empty	status=active	UPI00035068EE (+3)		35 kDa	TRUE	unknown	0	6	9	0	0.75916	1.7377
352	TRUE	Empty	Heat shock protein beta-1 OS=Gallus gallus GN=HSPB1 PE=3 SV=2	F1P593_CHICK (+3)		22 kDa		unknown	0	6	6	0	1.468	2.5826
353	TRUE	Empty	Uncharacterized protein OS=Gallus gallus GN=PREP PE=4 SV=1	F1NUS2_CHICK (+7)		81 kDa		unknown	1	13	13	0.051798	0.71183	0.77655
354	TRUE	Empty	Uncharacterized protein OS=Gallus gallus GN=TUBB3 PE=3 SV=2	F1NYB1_CHICK		50 kDa	TRUE	unknown	3	3	2	3.0076	23.861	9.6164
355	TRUE	Empty	Annexin OS=Gallus gallus GN=LOC772393 PE=3 SV=2	H9KZI4_CHICK (+2)		36 kDa		unknown	5	7	8	0.72747	1.7863	2.5921
356	TRUE	Empty	Dihydrolipoyl dehydrogenase OS=Gallus gallus GN=DLD PE=2 SV=1	Q5ZM32_CHICK (+3)		54 kDa		unknown	2	7	8	0.16151	0.74208	0.80955
357	TRUE	Empty	Capping protein (Actin filament) muscle Z-line, alpha 2 OS=Gallus gallus GN=CAPZA2 PE=4 SV=1	A0M8U0_CHICK (+4)		33 kDa	TRUE	unknown	0	5	7	0	1.246	1.9264
358	TRUE	Empty	Uncharacterized protein (Fragment) OS=Gallus gallus GN=GSTT1L PE=3 SV=2	E1BUB6_CHICK (+3)		30 kDa		unknown	0	5	7	0	0.7341	1.5665
359	TRUE	Empty	Uncharacterized protein OS=Gallus gallus GN=NNT PE=4 SV=2	E1C6A1_CHICK (+6)		114 kDa		unknown	0	9	13	0	0.33912	0.50593
360	TRUE	Empty	status=inactive	UPI0000E7FF03 (+7)		17 kDa		unknown	2	3	3	0.57868	2.9239	2.4119
361	TRUE	Empty	Ubiquitin carboxyl-terminal hydrolase OS=Gallus gallus GN=USP5 PE=3 SV=2	E1C8W4_CHICK (+5)		96 kDa	TRUE	unknown	0	12	11	0	0.5187	0.5083
362	TRUE	Empty	Uncharacterized protein OS=Gallus gallus PE=4 SV=1	E1C270_CHICK (+3)		40 kDa		unknown	7	3	4	1.1669	0.50299	0.54872
363	TRUE	Empty	Ubiquitin-conjugating enzyme E2 variant 1 OS=Gallus gallus GN=UBE2V1 PE=2 SV=2	UB2V1_CHICK (+6)		16 kDa	TRUE	unknown	4	8	4	1.5226	4.0584	7.2992
364	TRUE	Empty	Aspartate aminotransferase, cytoplasmic OS=Gallus gallus GN=GOT1 PE=1 SV=3	AATC_CHICK (+6)		46 kDa		unknown	9	4	7	1.1531	0.43104	0.71598
365	TRUE	Empty	Uncharacterized protein OS=Gallus gallus GN=TPR PE=4 SV=2	E1BYQ6_CHICK (+11)		244 kDa		unknown	21	3	0	0.44077	0.041417	0
366	TRUE	Empty	Uncharacterized protein OS=Gallus gallus GN=ETFA PE=4 SV=2	F1N9U8_CHICK		37 kDa		unknown	0	7	6	0	1.2734	0.96773
367	TRUE	Empty	Cathepsin B OS=Gallus gallus GN=CTSB PE=3 SV=2	F1N9D8_CHICK (+10)		38 kDa		unknown	0	7	4	0	1.4364	0.45671
368	TRUE	Empty	status=active	UPI00003AAA77 (+1)		104 kDa		unknown	0	8	9	0	0.28975	0.36215
369	TRUE	Empty	Hsc70-interacting protein OS=Gallus gallus GN=ST13 PE=2 SV=1	F10A1_CHICK (+6)		40 kDa		unknown	4	5	4	0.48443	0.50637	0.42146
370	TRUE	Empty	Dolichyl-diphosphooligosaccharide--protein glycosyltransferase 48 kDa subunit OS=Gallus gallus GN=DDOST PE=3 SV=2	F1NNS1_CHICK (+6)		49 kDa		unknown	3	6	7	0.28012	0.60934	0.79081
371	TRUE	Empty	Uncharacterized protein (Fragment) OS=Gallus gallus GN=LOC100859068 PE=3 SV=2	E1C0H5_CHICK (+5)		101 kDa		unknown	6	10	4	0.27306	0.48796	0.19433
372	TRUE	Empty	status=active	UPI00001D94B7		22 kDa		unknown	0	5	4	0	1.0774	0.86142
373	TRUE	Empty	status=active	UPI00003AEA01 (+2)		37 kDa	TRUE	unknown	0	6	4	0	0.71761	0.46979
374	TRUE	Empty	status=inactive	UPI0000E7F972 (+10)		52 kDa	TRUE	unknown	8	2	4	0.82782	0.13283	0.31109
375	TRUE	Empty	Uncharacterized protein OS=Gallus gallus GN=CSPG4 PE=4 SV=2	F1NG70_CHICK (+6)		258 kDa		unknown	0	6	10	0	0.093769	0.16544
376	TRUE	Empty	Uncharacterized protein OS=Gallus gallus GN=EEF1B2 PE=3 SV=1	F1NYA9_CHICK (+4)		25 kDa	TRUE	unknown	4	3	4	1.5339	0.69541	0.75864
377	TRUE	Empty	Collagen alpha-2(VI) chain OS=Gallus gallus GN=COL6A2 PE=4 SV=1	R4GKA6_CHICK (+3)		102 kDa		unknown	0	5	9	0	0.25351	0.46812
378	TRUE	Empty	Annexin OS=Gallus gallus GN=RCJMB04_6f1 PE=2 SV=1	Q5ZLG6_CHICK (+4)		39 kDa		unknown	11	5	6	2.0003	0.67031	1.08
379	TRUE	Empty	status=inactive	UPI000060FB2C		60 kDa	TRUE	unknown	0	17	0	0	1.7587	0
380	TRUE	Empty	Proteasome subunit beta type OS=Gallus gallus GN=PSMB2 PE=3 SV=1	R4GLB3_CHICK (+4)		23 kDa		unknown	4	4	7	1.3325	0.7796	2.4338
381	TRUE	Empty	Uncharacterized protein OS=Gallus gallus GN=ACAA2 PE=3 SV=1	F1N8J0_CHICK (+5)		42 kDa		unknown	2	7	5	0.21257	1.0549	0.66528
382	TRUE	Empty	status=inactive	UPI0000ECBD9F (+3)		49 kDa		unknown	5	5	6	0.50163	0.49919	0.54458
383	TRUE	Empty	Uncharacterized protein OS=Gallus gallus GN=RPS14 PE=2 SV=1	Q5ZHW8_CHICK (+2)		16 kDa		unknown	1	6	3	0.26897	3.1072	0.88165
384	TRUE	Empty	Actin-related protein 2 OS=Gallus gallus GN=ACTR2 PE=2 SV=1	ARP2_CHICK (+9)		45 kDa		unknown	2	7	7	0.19739	1.113	0.88795
385	TRUE	Empty	Glutathione S-transferase OS=Gallus gallus PE=2 SV=1	GSTA1_CHICK (+1)		25 kDa	TRUE	unknown	2	5	4	0.58966	0.91284	0.99583
386	TRUE	Empty	Nuclear calmodulin-binding protein (Fragment) OS=Gallus gallus GN=URP PE=2 SV=1	Q9YHD2_CHICK (+1)		84 kDa		unknown	8	5	2	0.53454	0.21644	0.13555
387	TRUE	Empty	Dolichyl-diphosphooligosaccharide--protein glycosyltransferase subunit 2 OS=Gallus gallus GN=RPN2 PE=4 SV=2	E1C8Z0_CHICK (+9)		69 kDa		unknown	0	8	6	0	0.47042	0.3641
388	TRUE	Empty	status=inactive	UPI0000448513 (+2)		48 kDa		unknown	5	7	6	0.51676	0.62547	0.5616
389	TRUE	Empty	Phosphoglycerate mutase 1 OS=Gallus gallus GN=PGAM1 PE=1 SV=3	PGAM1_CHICK		29 kDa	TRUE	unknown	0	5	10	0	0.9857	3.3222
390	TRUE	Empty	Uncharacterized protein OS=Gallus gallus GN=RCJMB04_24o2 PE=2 SV=1	Q5ZIM7_CHICK		46 kDa		unknown	0	13	0	0	1.9752	0
391	TRUE	Empty	Eukaryotic initiation factor 4A-III OS=Gallus gallus GN=EIF4A3 PE=2 SV=1	IF4A3_CHICK (+3)		47 kDa	TRUE	unknown	6	6	4	1.3071	0.64246	0.46097
392	TRUE	Empty	Uncharacterized protein OS=Gallus gallus GN=OTUB1 PE=4 SV=1	R4GIN3_CHICK (+2)		38 kDa		unknown	5	9	4	0.66854	1.1783	0.44283
393	TRUE	Empty	Uncharacterized protein OS=Gallus gallus GN=RPL8 PE=3 SV=2	F1NIX0_CHICK (+4)		28 kDa		unknown	1	4	3	0.15312	1.0292	0.65079
394	TRUE	Empty	Uncharacterized protein OS=Gallus gallus GN=HMBS PE=3 SV=2	F1NKN8_CHICK (+7)		39 kDa	TRUE	unknown	13	1	1	2.5988	0.18196	0.094706
395	TRUE	Empty	Uncharacterized protein OS=Gallus gallus GN=LOC100859465 PE=4 SV=1	F1NPQ0_CHICK (+7)		53 kDa		unknown	3	9	6	0.25583	0.76051	0.49536
396	TRUE	Empty	Ribosomal protein OS=Gallus gallus GN=RPL10A PE=3 SV=1	F6SU35_CHICK (+10)		25 kDa		unknown	1	7	6	0.17462	2.3773	1.7019
397	TRUE	Empty	Heme-binding protein 1 OS=Gallus gallus GN=HEBP1 PE=2 SV=1	HEBP1_CHICK (+5)		21 kDa		unknown	9	1	4	5.9401	0.16379	0.93332
398	TRUE	Empty	Heat shock cognate protein HSP 90-beta OS=Gallus gallus GN=HSP90AB1 PE=3 SV=1	F1NC33_CHICK (+2)		83 kDa		unknown	0	2	9	0	1.0655	1.7989
399	TRUE	Empty	Uncharacterized protein OS=Gallus gallus GN=SNRPA1 PE=4 SV=1	F1NAC8_CHICK (+2)		28 kDa		unknown	4	6	5	0.73358	1.2608	0.85405
400	TRUE	Empty	Keratin, type I cytoskeletal 10 OS=Homo sapiens GN=KRT10 PE=1 SV=6	P13645 (+1)		?		unknown	0	0	9	0	0	-1
401	TRUE	Empty	status=active	UPI00035037A9		23 kDa	TRUE	unknown	0	4	0	0	1.7131	0
402	TRUE	Empty	status=inactive	UPI0000ECD78D (+8)		534 kDa		unknown	0	5	15	0	0.031399	0.10637
403	TRUE	Empty	Uncharacterized protein OS=Gallus gallus GN=MTHFD1 PE=3 SV=1	F1NMC3_CHICK (+4)		100 kDa	TRUE	unknown	6	7	6	0.27439	0.26014	0.23873
404	TRUE	Empty	Uncharacterized protein OS=Gallus gallus GN=DNPEP PE=3 SV=2	F1NWA8_CHICK (+3)		52 kDa		unknown	6	6	5	0.58254	0.56251	0.50682
405	TRUE	Empty	Prohibitin OS=Gallus gallus GN=PHB PE=1 SV=1	PHB_CHICK (+8)		30 kDa		unknown	4	8	7	0.69026	1.4359	1.2804
406	TRUE	Empty	Uncharacterized protein OS=Gallus gallus GN=RCJMB04_2o24 PE=2 SV=1	Q5ZM75_CHICK (+3)		58 kDa		unknown	4	7	4	0.31741	0.57711	0.35651
407	TRUE	Empty	Serum albumin OS=Gallus gallus GN=ALB PE=1 SV=2	ALBU_CHICK		70 kDa	TRUE	unknown	2	1	2	26.664	2.2163	20.827
408	TRUE	Empty	14-3-3 protein gamma OS=Gallus gallus GN=YWHAG PE=1 SV=1	1433G_CHICK (+1)		28 kDa	TRUE	unknown	5	3	3	1.5992	1.2754	1.7097
409	TRUE	Empty	Superoxide dismutase OS=Gallus gallus GN=SOD2 PE=3 SV=1	F1NT19_CHICK (+6)		25 kDa		unknown	2	4	5	0.37239	1.2155	2.0852
410	TRUE	Empty	Uncharacterized protein OS=Gallus gallus GN=SRSF3 PE=4 SV=1	E1C8Y9_CHICK (+6)		19 kDa	TRUE	unknown	1	5	4	0.81859	1.3326	1.9383
411	TRUE	Empty	Uncharacterized protein OS=Gallus gallus GN=PSMC2 PE=2 SV=1	Q5ZMB8_CHICK		49 kDa	TRUE	unknown	8	7	3	0.91166	0.61109	0.24432
412	TRUE	Empty	Uncharacterized protein (Fragment) OS=Gallus gallus GN=ARPC4 PE=4 SV=2	F1P010_CHICK (+2)		20 kDa		unknown	4	4	4	1.1789	0.94016	1.4181

413	TRUE	Empty	Calpain OS=Gallus gallus PE=2 SV=1	A4ZZ65_CHICK (+5)		80 kDa	TRUE	unknown	0	7	9	0	0.33405	0.49137
414	TRUE	Empty	Citrate synthase OS=Gallus gallus PE=3 SV=1	R4GLP7_CHICK (+2)		43 kDa	TRUE	unknown	0	5	8	0	0.59392	1.3018
415	TRUE	Empty	Uncharacterized protein OS=Gallus gallus GN=TMED10 PE=3 SV=1	F1NA76_CHICK (+1)		25 kDa		unknown	0	5	9	0	0.94058	3.1189
416	TRUE	Empty	Ribosomal protein S11 OS=Gallus gallus GN=cRPS11 PE=3 SV=1	Q98TH5_CHICK (+3)		18 kDa		unknown	0	6	6	0	2.5291	2.1091
417	TRUE	Empty	Uncharacterized protein OS=Gallus gallus GN=VWF PE=4 SV=2	E1C4Z6_CHICK (+10)		313 kDa	TRUE	unknown	0	9	11	0	0.099888	0.13477
418	TRUE	Empty	Basement membrane-specific heparan sulfate proteoglycan core protein OS=Gallus gallus GN=HSPG PE=2 SV=1	Q6KDZ1_CHICK (+1)		433 kDa		unknown	0	5	11	0	0.038848	0.095645
419	TRUE	Empty	High mobility group protein B3 OS=Gallus gallus GN=HMGB3 PE=2 SV=3	HMGB3_CHICK (+8)		23 kDa		unknown	11	0	4	7.1009	0	0.82938
420	TRUE	Empty	Uncharacterized protein OS=Gallus gallus GN=LECT2 PE=4 SV=2	F1NEF7_CHICK (+7)		35 kDa		unknown	6	3	4	1.1698	0.32105	0.82477
421	TRUE	Empty	Uncharacterized protein OS=Gallus gallus GN=LYG2 PE=4 SV=1	F1P5H6_CHICK (+3)		23 kDa		unknown	2	3	5	0.40021	0.75094	2.8575
422	TRUE	Empty	Ras-related protein Rab-5B OS=Gallus gallus GN=RAB5B PE=2 SV=1	RAB5B_CHICK (+1)		24 kDa	TRUE	unknown	6	5	2	2.6027	2.0757	1.4264
423	TRUE	Empty	Uncharacterized protein OS=Gallus gallus GN=RCJMB04_22h1 PE=2 SV=1	Q5F3C0_CHICK (+3)		88 kDa		unknown	3	8	6	0.14896	0.40523	0.27621
424	TRUE	Empty	Uncharacterized protein (Fragment) OS=Gallus gallus GN=PSMA5 PE=4 SV=2	H9KYP7_CHICK (+3)		25 kDa		unknown	5	6	4	1.1446	1.4979	0.99583
425	TRUE	Empty	status=inactive	UPI000240BF73 (+4)		88 kDa	TRUE	unknown	0	6	6	0	0.35142	0.3283
426	TRUE	Empty	Cytochrome b-c1 complex subunit Rieske, mitochondrial OS=Gallus gallus GN=UQCRFS1 PE=1 SV=1	UCRI_CHICK		29 kDa		unknown	0	7	7	0	1.1993	1.6024
427	TRUE	Empty	Uncharacterized protein (Fragment) OS=Gallus gallus GN=RPL18A PE=3 SV=2	F1NPD3_CHICK (+7)		21 kDa		unknown	0	6	8	0	1.5383	2.7242
428	TRUE	Empty	Uncharacterized protein OS=Gallus gallus GN=HADH PE=4 SV=1	E1BZH9_CHICK (+1)		34 kDa		unknown	0	7	7	0	1.3911	1.2713
429	TRUE	Empty	Heart fatty acid binding protein OS=Gallus gallus GN=FABP3 PE=2 SV=2	F1NUQ3_CHICK (+1)		15 kDa		unknown	0	5	6	0	2.7424	4.0324
430	TRUE	Empty	Uncharacterized protein OS=Gallus gallus PE=3 SV=2	E1BQH2_CHICK		130 kDa	TRUE	unknown	12	0	8	0.49517	0	0.24644
431	TRUE	Empty	Uncharacterized protein OS=Gallus gallus GN=HK1 PE=3 SV=2	F1NZJ2_CHICK (+1)		103 kDa		unknown	0	8	10	0	0.29439	0.41661
432	TRUE	Empty	Uncharacterized protein OS=Gallus gallus GN=RPL26L1 PE=3 SV=1	F2Z4K6_CHICK (+2)		17 kDa		unknown	0	6	7	0	2.1405	3.9916
433	TRUE	Empty	Glucose-6-phosphate isomerase OS=Gallus gallus GN=GPI PE=3 SV=1	F1NIJ6_CHICK (+2)		62 kDa	TRUE	unknown	0	0	13	0	0	1.3679
434	TRUE	Empty	Uncharacterized protein OS=Gallus gallus GN=LOC100859627 PE=4 SV=2	E1BZE6_CHICK		41 kDa	TRUE	unknown	0	4	0	0	0.76425	0
435	TRUE	Empty	Uncharacterized protein OS=Gallus gallus GN=QDPR PE=2 SV=1	Q5ZKQ2_CHICK (+3)		25 kDa		unknown	4	4	4	1.524	0.69147	0.75434
436	TRUE	Empty	Alpha-1-acid glycoprotein OS=Gallus gallus GN=OGCHI PE=2 SV=1	A7UEB0_CHICK (+4)		22 kDa		unknown	3	3	3	0.68579	0.54693	0.59666
437	TRUE	Empty	Hemoglobin subunit rho OS=Gallus gallus PE=1 SV=2	HBRH_CHICK		17 kDa	TRUE	unknown	1	8	5	16.27	323.24	52.412
438	TRUE	Empty	Uncharacterized protein OS=Gallus gallus GN=RPL9 PE=4 SV=2	F1NS91_CHICK (+7)		22 kDa		unknown	1	5	4	0.19776	2.9378	1.2172
439	TRUE	Empty	status=active	UPI0000E8096A (+3)		34 kDa		unknown	3	10	3	0.58157	1.6413	0.35923
440	TRUE	Empty	Uncharacterized protein OS=Gallus gallus GN=KIF5B PE=3 SV=1	E1C3A1_CHICK (+5)		110 kDa		unknown	0	7	9	0	0.27196	0.38386
441	TRUE	Empty	Uncharacterized protein OS=Gallus gallus GN=G3BP1 PE=2 SV=1	Q5ZMN1_CHICK (+1)		52 kDa		unknown	4	6	0	0.35756	0.5566	0
442	TRUE	Empty	Uncharacterized protein OS=Gallus gallus GN=ARL8B PE=3 SV=2	F1P0Z7_CHICK (+4)		22 kDa		unknown	0	4	4	0	0.83241	1.2438
443	TRUE	Empty	450 kDa protein (Fragment) OS=Gallus gallus PE=2 SV=1	Q90576_CHICK		47 kDa	TRUE	unknown	0	2	2	0	2.1277	2.861
444	TRUE	Empty	status=inactive	UPI0000ECCA73 (+1)		49 kDa		unknown	0	7	3	0	0.71852	0.2419
445	TRUE	Empty	status=active	UPI0003504549 (+5)		71 kDa	TRUE	unknown	5	1	3	0.40495	0.047219	0.16274
446	TRUE	Empty	status=inactive	UPI000060E7DF (+4)		45 kDa		unknown	1	9	4	0.094261	1.1052	0.37124
447	TRUE	Empty	cDNA, FLJ92973, highly similar to Homo sapiens villin 2 (ezrin) (VIL2), mRNA OS=Homo sapiens PE=2 SV=1	B2R6J2 (+3)		?	TRUE	unknown	0	0	9	0	0	-1
448	TRUE	Empty	Uncharacterized protein OS=Gallus gallus GN=ADAL PE=4 SV=1	F1NUA8_CHICK (+1)		32 kDa		unknown	9	0	0	3.0964	0	0
449	TRUE	Empty	Peptidyl-prolyl cis-trans isomerase OS=Gallus gallus GN=FKBP25 PE=2 SV=1	Q90ZK7_CHICK (+1)		25 kDa		unknown	7	5	3	2.3795	0.92929	0.52115
450	TRUE	Empty	Lissencephaly-1 homolog OS=Gallus gallus GN=PAFAH1B1 PE=2 SV=3	LIS1_CHICK (+2)		47 kDa		unknown	8	4	5	0.96313	0.3255	0.46227
451	TRUE	Empty	Uncharacterized protein OS=Gallus gallus GN=HADHB PE=3 SV=1	E1BTT4_CHICK (+4)		51 kDa		unknown	4	8	3	0.36991	0.68622	0.23267
452	TRUE	Empty	Coatomer subunit delta OS=Gallus gallus GN=ARCN1 PE=2 SV=1	COPD_CHICK (+5)		57 kDa		unknown	4	9	3	0.32428	0.68822	0.20482
453	TRUE	Empty	Uncharacterized protein OS=Gallus gallus GN=SFPQ PE=4 SV=2	F1P555_CHICK (+4)		70 kDa		unknown	2	6	5	0.12393	0.33008	0.36009
454	TRUE	Empty	60S ribosomal protein L22 (Fragment) OS=Gallus gallus GN=RPL22 PE=4 SV=1	F1N9J4_CHICK (+10)		15 kDa		unknown	1	2	2	0.3015	0.54481	0.59434
455	TRUE	Empty	Uncharacterized protein OS=Gallus gallus GN=PSMD6 PE=4 SV=2	F1N903_CHICK (+1)		46 kDa		unknown	8	5	8	0.99426	0.54577	1.0166
456	TRUE	Empty	17-beta-hydroxysteroid dehydrogenase type IV OS=Gallus gallus GN=HSD17B4 PE=2 SV=1	O42484_CHICK (+4)		80 kDa		unknown	0	9	4	0	0.51438	0.19472
457	TRUE	Empty	status=active	UPI00003AC88B (+15)		138 kDa	TRUE	unknown	0	8	9	0	0.21186	0.26358
458	TRUE	Empty	Inosine-5'-monophosphate dehydrogenase OS=Gallus gallus GN=IMPDH PE=2 SV=1	Q5F4A4_CHICK		56 kDa	TRUE	unknown	0	10	5	0	0.82338	0.46828
459	TRUE	Empty	Zona pellucida sperm-binding protein 3 OS=Gallus gallus GN=ZP3 PE=1 SV=4	ZP3_CHICK		47 kDa		unknown	0	6	8	0	0.5283	0.83533
460	TRUE	Empty	Fibulin-1 OS=Gallus gallus GN=FBLN1 PE=3 SV=1	F1NX60_CHICK (+6)		78 kDa		unknown	0	8	6	0	0.40408	0.31492
461	TRUE	Empty	Uncharacterized protein OS=Gallus gallus GN=FKBP10 PE=4 SV=1	R4GH26_CHICK (+1)		62 kDa		unknown	0	9	6	0	0.61692	0.49188
462	TRUE	Empty	Ubiquitin-40S ribosomal protein S27a OS=Gallus gallus GN=RPS27A PE=2 SV=3	RS27A_CHICK (+5)		18 kDa	TRUE	unknown	0	2	3	0	0.71399	0.7789
463	TRUE	Empty	Uncharacterized protein OS=Gallus gallus GN=PPL PE=4 SV=2	F1NV96_CHICK (+7)		180 kDa		unknown	0	2	17	0	0.037134	0.40233
464	TRUE	Empty	Kinectin OS=Gallus gallus GN=KTN1 PE=4 SV=2	E1C2P9_CHICK		156 kDa		unknown	0	9	10	0	0.2102	0.25788
465	TRUE	Empty	T-complex protein 1 subunit theta OS=Gallus gallus GN=CCT8 PE=3 SV=2	F1NEF2_CHICK (+1)		60 kDa	TRUE	unknown	14	0	0	1.5091	0	0
466	TRUE	Empty	status=inactive	UPI000060F68B		274 kDa	TRUE	unknown	20	0	0	0.34524	0	0
467	TRUE	Empty	Uncharacterized protein OS=Gallus gallus GN=SERPINA4 PE=3 SV=1	E1BS56_CHICK (+5)		48 kDa		unknown	5	5	5	0.51676	0.41213	0.5616
468	TRUE	Empty	Uncharacterized protein OS=Gallus gallus GN=ADH5 PE=4 SV=2	F1NI89_CHICK (+11)		40 kDa		unknown	5	5	5	0.64576	0.515	0.56182
469	TRUE	Empty	Uncharacterized protein OS=Gallus gallus GN=FARSB PE=2 SV=1	Q5ZJ61_CHICK (+1)		66 kDa		unknown	8	5	4	0.72139	0.28452	0.24134
470	TRUE	Empty	APEX nuclease OS=Gallus gallus GN=chApex1 PE=2 SV=1	B3Y932_CHICK (+1)		33 kDa		unknown	6	6	2	1.2562	1.0018	0.23426
471	TRUE	Empty	Uncharacterized protein OS=Gallus gallus GN=NSF PE=3 SV=2	E1BQU4_CHICK (+6)		82 kDa		unknown	3	2	7	0.15981	0.083088	0.41564
472	TRUE	Empty	status=active	UPI0003504131 (+21)		39 kDa	TRUE	unknown	3	3	2	0.36237	0.5313	0.20034
473	TRUE	Empty	status=active	UPI0003504C10 (+2)		20 kDa	TRUE	unknown	2	7	4	0.80139	2.2434	1.0256
474	TRUE	Empty	Uncharacterized protein OS=Gallus gallus GN=SNX2 PE=4 SV=2	F1NZX7_CHICK (+5)		67 kDa		unknown	2	4	9	0.12975	0.28131	0.61978
475	TRUE	Empty	Histone-binding protein RBBP4 OS=Gallus gallus GN=RBBP4 PE=4 SV=1	F2Z4M0_CHICK (+6)		48 kDa	TRUE	unknown	4	7	4	0.3984	0.74704	0.34661
476	TRUE	Empty	Uncharacterized protein OS=Gallus gallus GN=HSPA4L PE=3 SV=1	F1NC26_CHICK (+4)		95 kDa		unknown	5	6	7	0.23848	0.323	0.30222
477	TRUE	Empty	Uncharacterized protein OS=Gallus gallus GN=XPNPEP1 PE=3 SV=2	F1P0A1_CHICK (+7)		70 kDa		unknown	8	6	5	0.57856	0.32767	0.28996
478	TRUE	Empty	Carnitine palmitoyltransferase I OS=Gallus gallus GN=CPT1A PE=2 SV=1	Q6B842_CHICK (+3)		88 kDa		unknown	0	7	7	0	0.3018	0.32924
479	TRUE	Empty	Uncharacterized protein OS=Gallus gallus GN=AGR2 PE=4 SV=1	E1C937_CHICK (+2)		20 kDa		unknown	0	3	6	0	0.9261	1.8542
480	TRUE	Empty	status=active	UPI00035056AA		81 kDa		unknown	0	4	4	0	0.44378	0.42031
481	TRUE	Empty	Guanine nucleotide-binding protein G(i) subunit alpha-2 OS=Gallus gallus GN=GNAI2 PE=2 SV=2	GNAI2_CHICK (+4)		41 kDa		unknown	0	7	7	0	0.77251	1.0114
482	TRUE	Empty	Keratin, type II cytoskeletal 2 epidermal OS=Homo sapiens GN=KRT2 PE=1 SV=2	P35908 (+1)		?	TRUE	unknown	0	0	12	0	0	-1
483	TRUE	Empty	Heterochromatin protein 1-binding protein 3 OS=Homo sapiens GN=HP1BP3 PE=1 SV=1	Q5SSJ5		?		unknown	0	0	13	0	0	-1
484	TRUE	Empty	RNA binding motif protein, X-linked OS=Gallus gallus GN=RBMX PE=2 SV=1	Q5ZKQ9_CHICK (+4)		41 kDa		unknown	6	4	5	0.94151	0.37313	0.53274
485	TRUE	Empty	Serine/threonine-protein phosphatase 2A activator OS=Gallus gallus GN=PPP2R4 PE=2 SV=1	Q5ZM56_CHICK (+1)		37 kDa		unknown	5	4	6	0.70848	0.4291	0.7798
486	TRUE	Empty	status=active	UPI000240CCAB (+4)		38 kDa		unknown	3	4	5	0.51071	0.53506	0.73665
487	TRUE	Empty	Uncharacterized protein (Fragment) OS=Gallus gallus GN=RPS7 PE=4 SV=2	F1NN16_CHICK (+7)		22 kDa		unknown	2	6	5	0.42037	1.8221	1.554
488	TRUE	Empty	Uncharacterized protein OS=Gallus gallus GN=COX4I1 PE=2 SV=1	Q5ZJV5_CHICK (+2)		20 kDa		unknown	1	5	5	0.2212	1.2999	1.887
489	TRUE	Empty	Uncharacterized protein OS=Gallus gallus GN=UGP2 PE=4 SV=1	F1NNR1_CHICK (+3)		57 kDa		unknown	0	9	8	0	0.69343	0.87025
490	TRUE	Empty	Uncharacterized protein (Fragment) OS=Gallus gallus GN=EVPL PE=4 SV=2	F1P070_CHICK (+4)		235 kDa		unknown	0	5	7	0	0.088034	0.14751
491	TRUE	Empty	status=inactive	UPI00004485F0 (+12)		93 kDa		unknown	1	6	8	0.045089	0.23836	0.36113
492	TRUE	Empty	Calmodulin OS=Gallus gallus GN=CALM PE=1 SV=2	CALM_CHICK (+2)		17 kDa	TRUE	unknown	0	7	5	0	7.8263	3.1898
493	TRUE	Empty	Uncharacterized protein (Fragment) OS=Gallus gallus GN=KRT4 PE=3 SV=2	H9L107_CHICK (+4)		59 kDa	TRUE	unknown	0	0	8	0	0	0.94832
494	TRUE	Empty	status=active	UPI000060373E (+3)		76 kDa	TRUE	unknown	1	2	0	3.5907	7.9654	0

495	TRUE	Empty	Uncharacterized protein OS=Gallus gallus GN=HSD17B10 PE=3 SV=2	H9L135_CHICK (+2)		24 kDa	TRUE	unknown	0	7	5	0	2.9551	1.0516
496	TRUE	Empty	Uncharacterized protein OS=Gallus gallus GN=RUVBL1 PE=4 SV=1	F1N8Z4_CHICK (+4)		50 kDa	TRUE	unknown	3	7	6	0.27131	0.69799	0.64084
497	TRUE	Empty	Uncharacterized protein OS=Gallus gallus GN=CLIC2 PE=2 SV=1	Q5ZKI1_CHICK (+4)		28 kDa	TRUE	unknown	7	5	3	1.6084	1.0235	0.6476
498	TRUE	Empty	Translationally-controlled tumor protein homolog OS=Gallus gallus GN=TPT1 PE=2 SV=1	TCTP_CHICK (+2)		20 kDa		unknown	2	4	4	0.48868	1.7452	2.4708
499	TRUE	Empty	High mobility group protein B2 OS=Gallus gallus GN=HMGB2 PE=4 SV=1	F1NPU8_CHICK (+3)		24 kDa	TRUE	unknown	5	3	2	2.5826	0.99485	0.34092
500	TRUE	Empty	Alcohol dehydrogenase [NADP(+)] OS=Gallus gallus GN=AKR1A1 PE=2 SV=1	AK1A1_CHICK (+6)		37 kDa		unknown	2	6	7	0.24065	0.70654	0.94825
501	TRUE	Empty	Uncharacterized protein OS=Gallus gallus GN=RCJMB04_6o1 PE=2 SV=1	Q5ZLB3_CHICK (+4)		38 kDa		unknown	1	4	3	0.3671	1.1932	0.44736
502	TRUE	Empty	status=inactive	UPI00004483B2 (+7)		54 kDa		unknown	2	4	5	0.16292	0.27852	0.39342
503	TRUE	Empty	Uncharacterized protein OS=Gallus gallus GN=PPID PE=4 SV=2	E1BXG9_CHICK (+4)		40 kDa		unknown	5	5	3	0.79387	0.50299	0.29981
504	TRUE	Empty	Mitochondrial ubiquinol-cytochrome-c reductase complex core protein i OS=Gallus gallus PE=1 SV=1	D0VX31_CHICK (+6)		49 kDa		unknown	0	7	7	0	0.71224	0.77699
505	TRUE	Empty	status=active	UPI000002ACAC (+16)		42 kDa		unknown	0	5	8	0	0.47601	0.95416
506	TRUE	Empty	Platelet-activating factor acetylhydrolase IB subunit beta OS=Gallus gallus GN=PAFAH1B2 PE=2 SV=1	PA1B2_CHICK (+4)		26 kDa		unknown	0	4	4	0	1.4782	1.2767
507	TRUE	Empty	Protein pelota homolog OS=Gallus gallus GN=PELO PE=3 SV=2	E1C0V6_CHICK (+9)		130 kDa		unknown	0	3	8	0	0.079153	0.31836
508	TRUE	Empty	Major vault protein OS=Gallus gallus GN=MVP PE=4 SV=2	F1NKR8_CHICK (+6)		94 kDa		unknown	0	6	12	0	0.23554	0.5808
509	TRUE	Empty	Uncharacterized protein OS=Gallus gallus GN=PLG PE=3 SV=2	F1NWX6_CHICK (+6)		91 kDa	TRUE	unknown	10	1	5	0.54618	0.03676	0.21748
510	TRUE	Empty	Serine/threonine-protein phosphatase PP1-beta catalytic subunit OS=Gallus gallus GN=PPP1CB PE=1 SV=3	PP1B_CHICK (+5)		37 kDa		unknown	1	6	5	0.11419	1.044	0.60733
511	TRUE	Empty	Uncharacterized protein (Fragment) OS=Gallus gallus GN=PPCS PE=4 SV=2	F1NF80_CHICK		35 kDa	TRUE	unknown	0	9	5	0	1.3454	0.65034
512	TRUE	Empty	Cell division control protein 42 homolog OS=Gallus gallus GN=CDC42 PE=2 SV=1	CDC42_CHICK (+4)		21 kDa		unknown	1	6	5	0.20314	1.5261	1.6649
513	TRUE	Empty	Ras-related protein Rab-6A OS=Gallus gallus GN=RAB6A PE=2 SV=3	RAB6A_CHICK (+15)		23 kDa		unknown	2	3	5	0.39808	1.0143	2.3001
514	TRUE	Empty	Unconventional myosin-Ic OS=Gallus gallus GN=MYO1C PE=4 SV=1	F1NG39_CHICK		119 kDa	TRUE	unknown	0	0	16	0	0	0.62019
515	TRUE	Empty	DJ-1 OS=Gallus gallus GN=DJ-1 PE=2 SV=1	D5M8S2_CHICK (+3)		20 kDa		unknown	4	3	7	1.1612	0.6303	2.4017
516	TRUE	Empty	Translin OS=Gallus gallus GN=TSN PE=4 SV=1	F1NT44_CHICK (+1)		26 kDa	TRUE	unknown	5	3	5	1.1054	0.65436	1.2457
517	TRUE	Empty	Farnesyl pyrophosphate synthase OS=Gallus gallus GN=FDPS PE=1 SV=2	FPPS_CHICK (+4)		42 kDa		unknown	5	7	5	0.59875	0.73288	0.65432
518	TRUE	Empty	Uncharacterized protein OS=Gallus gallus GN=FKBP4 PE=4 SV=1	F1NIM4_CHICK (+5)		50 kDa		unknown	3	9	2	0.3736	0.93857	0.15097
519	TRUE	Empty	26S proteasome non-ATPase regulatory subunit 1 OS=Gallus gallus GN=PSMD1 PE=2 SV=1	PSMD1_CHICK (+6)		106 kDa		unknown	5	5	4	0.21099	0.16827	0.14431
520	TRUE	Empty	Uncharacterized protein (Fragment) OS=Gallus gallus GN=ETHE1 PE=4 SV=1	H9L167_CHICK (+5)		12 kDa		unknown	2	4	4	0.90124	2.9978	3.2703
521	TRUE	Empty	Uncharacterized protein OS=Gallus gallus GN=CPOX PE=4 SV=2	F1NR99_CHICK (+6)		45 kDa		unknown	7	6	2	0.83581	0.54742	0.16862
522	TRUE	Empty	Hydroxyacylglutathione hydrolase, mitochondrial OS=Gallus gallus GN=HAGH PE=3 SV=2	G1K303_CHICK (+7)		34 kDa		unknown	4	2	3	0.58608	0.20946	0.5099
523	TRUE	Empty	Uncharacterized protein OS=Gallus gallus GN=LBR PE=4 SV=1	F1NXM2_CHICK (+5)		73 kDa		unknown	8	3	2	0.72098	0.14389	0.10205
524	TRUE	Empty	Uncharacterized protein (Fragment) OS=Gallus gallus GN=PDHB PE=4 SV=1	F1N823_CHICK (+3)		39 kDa		unknown	2	5	8	0.22815	0.52576	1.0673
525	TRUE	Empty	Uncharacterized protein OS=Gallus gallus GN=OGDH PE=2 SV=1	Q5ZJA7_CHICK (+1)		115 kDa		unknown	3	4	6	0.1122	0.154	0.20488
526	TRUE	Empty	Fascin OS=Gallus gallus GN=FSCN1 PE=2 SV=1	D5LPR1_CHICK (+1)		54 kDa		unknown	0	8	5	0	0.63274	0.38782
527	TRUE	Empty	Uncharacterized protein OS=Gallus gallus GN=PRPF8 PE=4 SV=1	E1BZT5_CHICK (+9)		274 kDa		unknown	0	7	10	0	0.088167	0.15529
528	TRUE	Empty	Uncharacterized protein OS=Gallus gallus GN=PLEKHC1 PE=4 SV=2	E1C0T6_CHICK (+12)		80 kDa		unknown	0	6	8	0	0.28187	0.49548
529	TRUE	Empty	Uncharacterized protein (Fragment) OS=Gallus gallus GN=ATP5H PE=4 SV=2	E1C658_CHICK (+2)		18 kDa		unknown	0	3	5	0	0.6924	1.5579
530	TRUE	Empty	Uncharacterized protein OS=Gallus gallus GN=ECHS1 PE=3 SV=2	F1NR44_CHICK (+1)		32 kDa		unknown	0	8	8	0	1.3241	1.4445
531	TRUE	Empty	Uncharacterized protein OS=Gallus gallus GN=MYO1B PE=4 SV=2	F1NTJ5_CHICK (+19)		132 kDa		unknown	0	6	10	0	0.16193	0.4231
532	TRUE	Empty	Uncharacterized protein OS=Gallus gallus GN=RPL14 PE=4 SV=1	F6R347_CHICK (+7)		17 kDa		unknown	0	3	3	0	0.74896	0.81705
533	TRUE	Empty	UDP-glucose 6-dehydrogenase OS=Gallus gallus GN=UGDH PE=2 SV=1	UGDH_CHICK (+4)		55 kDa		unknown	1	11	6	0.076464	0.94937	0.47277
534	TRUE	Empty	Uncharacterized protein OS=Gallus gallus GN=SLC25A13 PE=3 SV=2	F1NWI3_CHICK (+9)		70 kDa	TRUE	unknown	0	7	7	0	0.3899	0.42535
535	TRUE	Empty	Uncharacterized protein OS=Gallus gallus GN=NDUFS1 PE=4 SV=1	F1NXN8_CHICK (+5)		80 kDa		unknown	0	7	10	0	0.39543	0.56597
536	TRUE	Empty	Uncharacterized protein OS=Gallus gallus GN=RRBP1 PE=4 SV=2	F1P0K1_CHICK (+4)		112 kDa		unknown	0	11	2	0	0.47272	0.065937
537	TRUE	Empty	Uncharacterized protein OS=Gallus gallus GN=GARS PE=2 SV=1	Q5ZHR4_CHICK (+4)		78 kDa		unknown	0	7	9	0	0.34848	0.51355
538	TRUE	Empty	Cysteine and glycine-rich protein 1 OS=Gallus gallus GN=CSRP1 PE=1 SV=2	CSRP1_CHICK (+4)		20 kDa		unknown	0	3	9	0	0.89268	4.5037
539	TRUE	Empty	status=inactive	UPI000240B35B (+4)		131 kDa		unknown	4	2	2	0.1682	0.051442	0.056119
540	TRUE	Empty	Acidic leucine-rich nuclear phosphoprotein 32 family member E OS=Gallus gallus GN=ANP32E PE=2 SV=1	AN32E_CHICK (+2)		29 kDa		unknown	6	5	2	1.5457	0.9857	0.43971
541	TRUE	Empty	Uncharacterized protein OS=Gallus gallus GN=DYNC1I2 PE=4 SV=1	F1P3C8_CHICK (+1)		68 kDa	TRUE	unknown	0	0	5	0	0	0.29845
542	TRUE	Empty	status=active	UPI00003AE0F2 (+3)		45 kDa	TRUE	unknown	8	6	4	1.1724	0.54742	0.36604
543	TRUE	Empty	GTP-binding protein OS=Gallus gallus GN=cRhoA PE=2 SV=1	O93467_CHICK		22 kDa	TRUE	unknown	0	0	5	0	0	1.6138
544	TRUE	Empty	Uncharacterized protein OS=Gallus gallus GN=CTH PE=3 SV=1	E1BYF1_CHICK (+4)		44 kDa		unknown	8	3	4	1.0448	0.25096	0.38097
545	TRUE	Empty	Uncharacterized protein OS=Gallus gallus GN=PTGR1 PE=4 SV=1	F1NCM5_CHICK (+6)		36 kDa		unknown	2	7	7	0.24866	1.0942	1.1937
546	TRUE	Empty	Hematopoietic prostaglandin D synthase OS=Gallus gallus GN=HPGDS PE=1 SV=3	HPGDS_CHICK (+6)		23 kDa		unknown	2	9	5	0.41117	2.734	1.1515
547	TRUE	Empty	Eukaryotic translation initiation factor 3 subunit E OS=Gallus gallus GN=EIF3E PE=2 SV=1	EIF3E_CHICK (+12)		52 kDa		unknown	4	8	3	0.36014	0.66561	0.22672
548	TRUE	Empty	Uncharacterized protein OS=Gallus gallus GN=C4BPA PE=4 SV=2	F1P3M9_CHICK (+4)		49 kDa		unknown	7	1	3	1.2328	0.14339	0.33765
549	TRUE	Empty	14-3-3 protein beta/alpha OS=Gallus gallus GN=YWHAB PE=2 SV=1	1433B_CHICK (+2)		28 kDa	TRUE	unknown	1	2	6	0.75171	1.0349	3.5493
550	TRUE	Empty	Uncharacterized protein OS=Gallus gallus GN=CPM PE=4 SV=1	E1C041_CHICK (+3)		50 kDa		unknown	0	9	7	0	0.82897	0.64982
551	TRUE	Empty	Uncharacterized protein OS=Gallus gallus GN=GSTK1 PE=4 SV=2	F1N9G6_CHICK (+6)		25 kDa		unknown	0	5	6	0	1.185	1.2927
552	TRUE	Empty	Uncharacterized protein OS=Gallus gallus GN=ACADL PE=3 SV=1	F1NC38_CHICK (+6)		48 kDa		unknown	0	7	6	0	0.87063	0.56001
553	TRUE	Empty	Aldehyde dehydrogenase OS=Gallus gallus GN=ALDH3B1 PE=3 SV=1	R4GM83_CHICK (+3)		55 kDa	TRUE	unknown	0	7	6	0	0.52227	0.56976
554	TRUE	Empty	Tubulin beta-2 chain OS=Gallus gallus PE=2 SV=1	TBB2_CHICK (+1)		50 kDa	TRUE	unknown	0	5	1	0	27.479	7.5176
555	TRUE	Empty	status=active	UPI00003AB8C5 (+5)		85 kDa		unknown	2	6	8	0.09999	0.26094	0.39674
556	TRUE	Empty	Uncharacterized protein OS=Gallus gallus GN=COL4A2 PE=4 SV=2	F1P2Q3_CHICK		169 kDa		unknown	0	4	8	0	0.10258	0.23651
557	TRUE	Empty	status=inactive	UPI0000449A9A (+3)		69 kDa	TRUE	unknown	5	5	1	1.1105	1.967	0.77145
558	TRUE	Empty	status=active	UPI00035048DF		75 kDa	TRUE	unknown	14	0	0	1.0797	0	0
559	TRUE	Empty	Mu-calpain large subunit OS=Gallus gallus PE=2 SV=1	O42133_CHICK (+1)		81 kDa		unknown	8	3	4	0.4831	0.12874	0.19156
560	TRUE	Empty	Retinol-binding protein 4 OS=Gallus gallus GN=RBP4 PE=1 SV=1	RET4_CHICK (+4)		23 kDa		unknown	5	4	5	1.3417	0.78459	1.5313
561	TRUE	Empty	Proteasome subunit beta type OS=Gallus gallus GN=PSMB4 PE=3 SV=1	H9L0U6_CHICK (+6)		28 kDa		unknown	4	2	4	0.74435	0.25958	0.6476
562	TRUE	Empty	Uncharacterized protein OS=Gallus gallus GN=RCJMB04_1l21 PE=2 SV=1	Q5ZMK9_CHICK (+4)		40 kDa		unknown	6	4	6	0.80223	0.38759	0.69796
563	TRUE	Empty	Uncharacterized protein OS=Gallus gallus GN=GPD1L PE=3 SV=2	F1P0W8_CHICK (+4)		39 kDa		unknown	5	3	7	0.66854	0.40592	1.0843
564	TRUE	Empty	Uncharacterized protein (Fragment) OS=Gallus gallus GN=VDAC3 PE=4 SV=2	E1C2E3_CHICK (+7)		31 kDa		unknown	2	6	4	0.29706	1.1373	0.58459
565	TRUE	Empty	Peptidyl-prolyl cis-trans isomerase OS=Gallus gallus GN=LOC100859332 PE=3 SV=1	R4GLM6_CHICK (+2)		16 kDa		unknown	2	4	5	0.61145	1.7536	2.6107
566	TRUE	Empty	Histone H5 OS=Gallus gallus PE=1 SV=2	H5_CHICK (+4)		21 kDa		unknown	2	3	1	0.74888	0.59724	0.18168
567	TRUE	Empty	Uncharacterized protein OS=Gallus gallus GN=RPL27A PE=3 SV=2	F1NBX4_CHICK (+2)		17 kDa		unknown	1	4	3	0.26145	1.1692	0.85175
568	TRUE	Empty	Uncharacterized protein (Fragment) OS=Gallus gallus GN=SCAMP2 PE=4 SV=2	F1NV32_CHICK (+6)		37 kDa		unknown	1	4	2	0.11561	0.4291	0.33354
569	TRUE	Empty	Uncharacterized protein OS=Gallus gallus GN=PROSC PE=3 SV=1	E1C516_CHICK (+2)		30 kDa		unknown	2	4	7	0.30186	0.54553	1.2669
570	TRUE	Empty	status=active	UPI0000000BD7 (+5)		107 kDa		unknown	4	6	4	0.16513	0.20457	0.14367
571	TRUE	Empty	Uncharacterized protein (Fragment) OS=Gallus gallus GN=RCJMB04_23b17 PE=2 SV=1	Q5F3B6_CHICK (+1)		41 kDa		unknown	1	6	5	0.10313	1.0751	0.53796
572	TRUE	Empty	Structural maintenance of chromosomes protein OS=Gallus gallus GN=SMC1 PE=2 SV=2	Q8AWB7_CHICK		143 kDa	TRUE	unknown	10	3	4	0.32724	0.071474	0.10531
573	TRUE	Empty	Transcriptional coactivator p100 (Fragment) OS=Gallus gallus PE=2 SV=1	Q90XD9_CHICK (+1)		80 kDa		unknown	2	9	4	0.10687	0.51438	0.19472
574	TRUE	Empty	5-aminoimidazole ribonucleotide/carboxylase-5-aminoimidazole-4-N-succinocarboxamide ribonucleotide synthetase OS=Gallus gallus GN=AS PE=2 SV=1	A7UEA7_CHICK (+6)		47 kDa		unknown	5	7	2	0.52395	0.6348	0.16189
575	TRUE	Empty	status=active	UPI00001D99B0 (+7)		32 kDa		unknown	4	4	2	0.64068	0.51095	0.39386
576	TRUE	Empty	Uncharacterized protein OS=Gallus gallus GN=RCJMB04_9i5 PE=2 SV=1	Q5ZKR9_CHICK (+2)		21 kDa	TRUE	unknown	2	4	5	0.46169	2.0843	6.6697

577	TRUE	Empty	Succinate dehydrogenase [ubiquinone] iron-sulfur subunit, mitochondrial OS=Gallus gallus GN=SDHB PE=4 SV=1	F1NNF7_CHICK (+7)		33 kDa		unknown	0	4	6	0	0.6581	1.3797
578	TRUE	Empty	Vacuolar protein sorting-associated protein 29 (Fragment) OS=Gallus gallus GN=VPS29 PE=4 SV=1	F1NNQ3_CHICK (+5)		21 kDa	TRUE	unknown	0	3	5	0	0.60119	1.3204
579	TRUE	Empty	Uncharacterized protein OS=Gallus gallus GN=RNH1 PE=1 SV=1	Q5ZIY8_CHICK (+2)		50 kDa		unknown	0	6	4	0	0.48842	0.32914
580	TRUE	Empty	status=active	UPI00000534B2 (+10)		24 kDa		unknown	1	4	5	0.1823	0.73737	1.0923
581	TRUE	Empty	status=active	UPI000350727F (+3)		32 kDa		unknown	4	6	4	0.82877	0.84247	0.54361
582	TRUE	Empty	Lectin-associated matrix protein (Fragment) OS=Gallus gallus GN=hLAMP-1 PE=2 SV=2	A3RL83_CHICK (+1)		33 kDa		unknown	0	7	3	0	1.2584	0.3816
583	TRUE	Empty	Uncharacterized protein OS=Gallus gallus GN=NAP1L1 PE=3 SV=1	E1BZS2_CHICK (+7)		45 kDa	TRUE	unknown	0	9	2	0	1.0898	0.16905
584	TRUE	Empty	Epithelial cell adhesion molecule OS=Gallus gallus GN=EPCAM PE=2 SV=1	EPCAM_CHICK (+5)		34 kDa		unknown	0	4	6	0	0.78175	1.054
585	TRUE	Empty	status=active	UPI000240BF1F (+11)		40 kDa		unknown	11	3	0	1.8818	0.27652	0
586	TRUE	Empty	Reticulon OS=Gallus gallus GN=RTN4 PE=4 SV=1	F1P5B1_CHICK		36 kDa		unknown	0	4	3	0	0.43689	0.47661
587	TRUE	Empty	Uncharacterized protein OS=Gallus gallus GN=MCAM PE=4 SV=2	F1NXW9_CHICK (+1)		69 kDa		unknown	0	4	6	0	0.33314	0.5926
588	TRUE	Empty	Uncharacterized protein OS=Gallus gallus GN=RCJMB04_6g23 PE=2 SV=1	Q5ZLF6_CHICK (+1)		25 kDa		unknown	0	7	7	0	1.9254	2.1004
589	TRUE	Empty	Uncharacterized protein OS=Gallus gallus GN=PHGDH PE=3 SV=2	E1C7Y3_CHICK (+4)		55 kDa		unknown	0	10	6	0	0.83454	0.47391
590	TRUE	Empty	CAST OS=Gallus gallus GN=CAST PE=2 SV=1	B6V3I0_CHICK (+1)		81 kDa	TRUE	unknown	0	0	7	0	0	0.42031
591	TRUE	Empty	Uncharacterized protein OS=Gallus gallus GN=SF3B1 PE=4 SV=1	E1C2C3_CHICK (+5)		146 kDa		unknown	13	3	0	0.43329	0.070125	0
592	TRUE	Empty	Uncharacterized protein OS=Gallus gallus GN=RAB11B PE=2 SV=1	Q5F3R8_CHICK		25 kDa	TRUE	unknown	8	1	1	2.4498	0.95214	1.352
593	TRUE	Empty	Arginine--tRNA ligase, cytoplasmic OS=Gallus gallus GN=RARS PE=3 SV=2	F1NFP5_CHICK (+1)		75 kDa	TRUE	unknown	0	10	6	0	0.6265	0.32811
594	TRUE	Empty	Glutamate dehydrogenase OS=Gallus gallus GN=GLUD1 PE=3 SV=2	F1P3F9_CHICK (+4)		48 kDa		unknown	0	2	8	0	1.0153	0.95578
595	TRUE	Empty	Uncharacterized protein OS=Gallus gallus GN=C1H11ORF54 PE=4 SV=2	F1NY09_CHICK (+4)		35 kDa		unknown	5	3	6	0.76247	0.3269	1.0402
596	TRUE	Empty	Uncharacterized protein OS=Gallus gallus GN=ARPC1A PE=2 SV=1	Q5ZJI7_CHICK (+1)		42 kDa		unknown	4	4	5	0.46497	0.4852	0.52931
597	TRUE	Empty	Protein arginine N-methyltransferase 5 OS=Gallus gallus GN=RCJMB04_14b8 PE=2 SV=1	Q5ZJZ9_CHICK (+1)		65 kDa		unknown	8	6	2	0.63703	0.35909	0.11633
598	TRUE	Empty	Uncharacterized protein (Fragment) OS=Gallus gallus GN=ARPC3 PE=4 SV=2	E1C8Y3_CHICK (+3)		21 kDa		unknown	2	3	5	0.44266	0.84382	1.6649
599	TRUE	Empty	Uncharacterized protein OS=Gallus gallus GN=HNRNPU PE=4 SV=2	F1NSP8_CHICK (+10)		75 kDa		unknown	2	1	5	0.11459	0.044596	0.26845
600	TRUE	Empty	Uncharacterized protein OS=Gallus gallus GN=SLC16A1 PE=2 SV=1	Q5ZLZ6_CHICK (+3)		54 kDa		unknown	7	1	1	0.79133	0.061871	0.067495
601	TRUE	Empty	Uncharacterized protein OS=Gallus gallus GN=RCJMB04_33j6 PE=2 SV=1	Q5ZHT0_CHICK (+3)		19 kDa	TRUE	unknown	4	2	4	1.2449	0.9928	1.5041
602	TRUE	Empty	Carboxypeptidase OS=Gallus gallus GN=CTSA PE=3 SV=1	F1NIN2_CHICK (+8)		53 kDa		unknown	0	6	7	0	0.45181	0.59624
603	TRUE	Empty	Uncharacterized protein (Fragment) OS=Gallus gallus GN=AKR7A2 PE=4 SV=1	F1P331_CHICK (+2)		41 kDa		unknown	0	7	6	0	0.76154	0.67891
604	TRUE	Empty	Uncharacterized protein OS=Gallus gallus GN=RCJMB04_33n14 PE=2 SV=1	Q5ZHS2_CHICK (+5)		50 kDa		unknown	0	6	7	0	0.4858	0.64261
605	TRUE	Empty	Uncharacterized protein OS=Gallus gallus GN=SNX3 PE=2 SV=1	Q5ZIA3_CHICK (+3)		19 kDa	TRUE	unknown	0	5	3	0	1.3908	1.0918
606	TRUE	Empty	Aldo-keto reductase OS=Gallus gallus GN=akr PE=2 SV=1	Q90W83_CHICK		36 kDa	TRUE	unknown	0	6	7	0	0.89073	1.3954
607	TRUE	Empty	Mitochondrial cytochrome c1, heme protein OS=Gallus gallus PE=1 SV=1	D0VX26_CHICK (+1)		27 kDa		unknown	0	3	4	0	0.43621	0.91464
608	TRUE	Empty	Uncharacterized protein OS=Gallus gallus GN=PDLIM1 PE=4 SV=1	E1C852_CHICK (+7)		36 kDa		unknown	3	3	5	0.39689	0.31653	0.64027
609	TRUE	Empty	116 kDa U5 small nuclear ribonucleoprotein component OS=Gallus gallus GN=EFTUD2 PE=2 SV=1	U5S1_CHICK (+3)		109 kDa	TRUE	unknown	0	6	6	0	0.19844	0.21648
610	TRUE	Empty	status=inactive	UPI000240B601 (+3)		134 kDa	TRUE	unknown	0	4	6	0	0.10324	0.20545
611	TRUE	Empty	Uncharacterized protein OS=Gallus gallus GN=ABCE1 PE=2 SV=1	Q5ZJX6_CHICK (+8)		67 kDa		unknown	5	7	1	0.34814	0.41126	0.054272
612	TRUE	Empty	status=inactive	UPI000044955A (+4)		47 kDa		unknown	4	4	4	0.40267	0.32114	0.35033
613	TRUE	Empty	status=active	UPI0003506A92 (+3)		64 kDa		unknown	1	4	5	0.065449	0.29289	0.31951
614	TRUE	Empty	Type I alpha-keratin 15 OS=Gallus gallus PE=2 SV=1	Q6PVZ2_CHICK (+1)		48 kDa	TRUE	unknown	0	0	9	0	0	1.8162
615	TRUE	Empty	Uncharacterized protein OS=Gallus gallus GN=COL4A1 PE=4 SV=1	F1P2R3_CHICK (+8)		161 kDa		unknown	0	2	2	0	0.041783	0.045582
616	TRUE	Empty	Radixin OS=Gallus gallus GN=RDX PE=2 SV=1	RADI_CHICK (+2)		69 kDa	TRUE	unknown	10	0	0	0.88857	0	0
617	TRUE	Empty	status=active	UPI00003AA5C1		135 kDa	TRUE	unknown	0	0	12	0	0	0.41204
618	TRUE	Empty	status=inactive	UPI0000ECA092		162 kDa		unknown	0	6	7	0	0.13008	0.16747
619	TRUE	Empty	Uncharacterized protein (Fragment) OS=Gallus gallus GN=NUDT21 PE=4 SV=2	E1C538_CHICK (+4)		26 kDa		unknown	3	5	4	0.56049	1.1149	0.69882
620	TRUE	Empty	Uncharacterized protein OS=Gallus gallus GN=TPD52L2 PE=2 SV=1	Q5ZI93_CHICK (+2)		22 kDa		unknown	5	4	3	1.3698	0.79992	0.60027
621	TRUE	Empty	Uncharacterized protein OS=Gallus gallus GN=EIF5 PE=2 SV=1	Q5ZIE0_CHICK (+3)		49 kDa		unknown	5	5	2	0.50029	0.39899	0.15531
622	TRUE	Empty	Uncharacterized protein OS=Gallus gallus GN=CSE1L PE=4 SV=2	E1BV44_CHICK (+6)		110 kDa		unknown	5	7	2	0.20208	0.2334	0.066889
623	TRUE	Empty	Endoplasmic reticulum resident protein 29 (Fragment) OS=Gallus gallus GN=ERP29 PE=4 SV=2	F1NRM8_CHICK (+6)		29 kDa		unknown	2	5	4	0.31323	0.97538	0.62028
624	TRUE	Empty	Uncharacterized protein OS=Gallus gallus PE=4 SV=2	G1K316_CHICK (+7)		14 kDa		unknown	2	3	3	0.75061	1.5933	1.7382
625	TRUE	Empty	Uncharacterized protein OS=Gallus gallus GN=HK2 PE=3 SV=2	H9KZN4_CHICK (+3)		103 kDa		unknown	8	2	3	0.36868	0.21259	0.23192
626	TRUE	Empty	Uncharacterized protein OS=Gallus gallus GN=Gga.22975 PE=4 SV=1	R4GF59_CHICK (+1)		164 kDa		unknown	2	5	5	0.28061	0.10572	0.13997
627	TRUE	Empty	Alcohol dehydrogenase 1 OS=Gallus gallus GN=ADH1 PE=1 SV=2	ADH1_CHICK (+10)		40 kDa		unknown	1	4	8	0.10667	0.39138	1.4375
628	TRUE	Empty	Calcium/calmodulin-dependent protein kinase type II delta chain OS=Gallus gallus GN=CAMK2D PE=2 SV=1	KCC2D_CHICK (+16)		54 kDa		unknown	0	6	4	0	0.53414	0.29965
629	TRUE	Empty	Uncharacterized protein (Fragment) OS=Gallus gallus GN=UPF1 PE=4 SV=2	E1C0J4_CHICK (+10)		123 kDa		unknown	8	5	0	0.30091	0.1746	0
630	TRUE	Empty	Protein transport protein Sec23A OS=Gallus gallus GN=SEC23A PE=4 SV=1	E1C3Y5_CHICK (+2)		86 kDa	TRUE	unknown	0	7	7	0	0.30847	0.33652
631	TRUE	Empty	Uncharacterized protein OS=Gallus gallus GN=LRPPRC PE=4 SV=2	F1NGU3_CHICK (+6)		156 kDa		unknown	0	10	4	0	0.23751	0.096459
632	TRUE	Empty	Uncharacterized protein OS=Gallus gallus GN=YWHAH PE=2 SV=1	Q5ZKJ2_CHICK (+3)		28 kDa	TRUE	unknown	0	4	7	0	1.2681	4.0359
633	TRUE	Empty	Uncharacterized protein OS=Gallus gallus GN=FH PE=2 SV=1	Q5ZLD1_CHICK (+1)		54 kDa		unknown	0	6	6	0	0.44078	0.58123
634	TRUE	Empty	Cohesin complex subunit OS=Gallus gallus GN=SMC3 PE=2 SV=1	Q8AWB8_CHICK (+3)		142 kDa		unknown	11	4	0	0.36901	0.12355	0
635	TRUE	Empty	status=active	UPI000240C6D8		53 kDa	TRUE	unknown	8	7	0	0.8081	0.54374	0
636	TRUE	Empty	Sarcoplasmic/endoplasmic reticulum calcium ATPase 2 OS=Gallus gallus GN=ATP2A2 PE=2 SV=2	sp|Q03669|AT2A2_CHICK (+8)		115 kDa	TRUE	unknown	0	6	8	0	0.1886	0.28345
637	TRUE	Empty	Ras-related protein Rab-5C OS=Gallus gallus GN=RAB5C PE=1 SV=1	RAB5C_CHICK (+2)		24 kDa	TRUE	unknown	2	0	6	1.6509	0	1.8276
638	TRUE	Empty	Integrin alpha-6 (Fragment) OS=Gallus gallus GN=ITGA6 PE=3 SV=2	F1P0B0_CHICK (+8)		115 kDa		unknown	0	1	11	0	0.028922	0.40821
639	TRUE	Empty	Uncharacterized protein (Fragment) OS=Gallus gallus PE=4 SV=2	E1C037_CHICK		453 kDa		unknown	0	7	4	0	0.052391	0.35278
640	TRUE	Empty	Uncharacterized protein (Fragment) OS=Gallus gallus GN=LOC776719 PE=3 SV=2	H9KYP2_CHICK (+3)		68 kDa	TRUE	unknown	0	0	5	0	0	0.52235
641	TRUE	Empty	Keratin, type I cytoskeletal 9 OS=Homo sapiens GN=KRT9 PE=1 SV=3	P35527 (+1)		?		unknown	0	0	8	0	0	-1
642	TRUE	Empty	status=active	UPI00003ADA7A (+8)		87 kDa		unknown	7	1	0	0.38396	0.038474	0
643	TRUE	Empty	cDNA FLJ76654, highly similar to Homo sapiens CD44 antigen (homing function and Indian blood group system) (CD44), transcript variant 4, mRNA OS=Homo sapiens PE=2 SV=1	A8K309 (+5)		?		unknown	0	0	7	0	0	-1
644	TRUE	Empty	Uncharacterized protein OS=Gallus gallus GN=CANX PE=3 SV=2	E1C8M9_CHICK (+8)		68 kDa		unknown	2	7	6	0.12602	0.47412	0.36682
645	TRUE	Empty	Angiotensinogen OS=Gallus gallus GN=AGT PE=3 SV=2	F1NDH2_CHICK (+5)		51 kDa		unknown	7	1	2	0.71673	0.065556	0.14821
646	TRUE	Empty	Fowlicidin-2 OS=Gallus gallus GN=CATHL2 PE=2 SV=1	C4PFJ8_CHICK (+7)		17 kDa		unknown	1	3	5	0.57428	0.76773	2.3858
647	TRUE	Empty	Uncharacterized protein (Fragment) OS=Gallus gallus GN=GLOD4 PE=4 SV=2	E1BQI4_CHICK (+6)		51 kDa		unknown	5	3	3	0.47504	0.21126	0.31869
648	TRUE	Empty	Insulin-like growth factor 2 mRNA-binding protein 3 OS=Gallus gallus GN=IGF2BP3 PE=2 SV=1	IF2B3_CHICK (+5)		64 kDa	TRUE	unknown	0	10	3	0	0.67756	0.1802
649	TRUE	Empty	status=inactive	UPI000240BA37 (+8)		45 kDa		unknown	4	4	4	0.42072	0.33553	0.36604
650	TRUE	Empty	Uncharacterized protein OS=Gallus gallus GN=GSR PE=4 SV=1	R4GL59_CHICK (+2)		45 kDa	TRUE	unknown	0	5	7	0	0.43986	0.87349
651	TRUE	Empty	Beta-galactoside-binding lectin OS=Gallus gallus PE=1 SV=2	LEG4_CHICK (+3)		15 kDa		unknown	0	1	5	0	0.23294	2.9151
652	TRUE	Empty	Integrin beta OS=Gallus gallus GN=Gga.55879 PE=3 SV=2	E1C9G7_CHICK (+8)		194 kDa		unknown	0	2	5	0	0.034423	0.117
653	TRUE	Empty	Heterogeneous nuclear ribonucleoprotein M OS=Homo sapiens GN=HNRNPM PE=4 SV=1	A0A087X0X3 (+2)		?		unknown	0	0	11	0	0	-1
654	TRUE	Empty	status=active	UPI00003A9D2A (+7)		57 kDa		unknown	8	3	0	0.73946	0.18735	0
655	TRUE	Empty	status=active	UPI00003AEC74 (+1)		173 kDa	TRUE	unknown	0	4	7	0	0.079228	0.18043
656	TRUE	Empty	Hypoxia up-regulated protein 1 (Fragment) OS=Gallus gallus GN=HYOU1 PE=3 SV=2	F1NY05_CHICK (+6)		108 kDa		unknown	0	6	6	0	0.20067	0.21891
657	TRUE	Empty	Uncharacterized protein OS=Gallus gallus GN=NT5DC2 PE=4 SV=2	F1NNU4_CHICK (+4)		66 kDa		unknown	3	8	2	0.20263	0.57654	0.17629
658	TRUE	Empty	Uncharacterized protein OS=Gallus gallus GN=SNRPD3 PE=2 SV=1	Q5ZL58_CHICK (+2)		14 kDa		unknown	2	3	3	0.72903	2.224	1.6761

659	TRUE	Empty	Elongation factor Tu, mitochondrial (Fragment) OS=Gallus gallus GN=TUFM PE=1 SV=1	EFTU_CHICK (+1)		38 kDa		unknown	1	6	5	0.11113	0.68029	0.58786
660	TRUE	Empty	Proteasome subunit beta type (Fragment) OS=Gallus gallus GN=PSMB7 PE=3 SV=1	F6RX73_CHICK (+1)		28 kDa		unknown	2	4	3	0.52173	0.59364	0.45392
661	TRUE	Empty	Uncharacterized protein OS=Gallus gallus GN=GNB1 PE=4 SV=1	F1NLV4_CHICK (+10)		44 kDa		unknown	1	4	4	0.094982	0.34322	0.37443
662	TRUE	Empty	Uncharacterized protein OS=Gallus gallus GN=CPNE3 PE=4 SV=1	E1BQA5_CHICK (+5)		60 kDa		unknown	4	5	3	0.48719	0.4679	0.34231
663	TRUE	Empty	Uncharacterized protein (Fragment) OS=Gallus gallus GN=STOM PE=4 SV=2	E1BTV1_CHICK (+7)		31 kDa		unknown	4	3	3	0.64891	0.36544	0.39866
664	TRUE	Empty	Cytochrome c oxidase subunit 2 OS=Gallus gallus GN=MT-CO2 PE=3 SV=1	COX2_CHICK (+8)		26 kDa		unknown	0	4	4	0	0.90219	0.98421
665	TRUE	Empty	Uncharacterized protein OS=Gallus gallus GN=TXNDC5 PE=3 SV=1	F1P4H4_CHICK (+2)		46 kDa		unknown	0	1	8	0	1.7612	0.84323
666	TRUE	Empty	Chromobox protein (CHCB2) OS=Gallus gallus GN=CBX3 PE=2 SV=1	O93481_CHICK (+2)		20 kDa	TRUE	unknown	0	5	4	0	1.2893	1.4065
667	TRUE	Empty	Uncharacterized protein (Fragment) OS=Gallus gallus GN=PFDN2 PE=4 SV=1	R4GME1_CHICK (+3)		18 kDa		unknown	0	4	5	0	1.0976	2.2627
668	TRUE	Empty	Uncharacterized protein (Fragment) OS=Gallus gallus GN=CTSC PE=3 SV=1	F1NWG2_CHICK (+5)		52 kDa	TRUE	unknown	0	2	3	0	0.13373	0.40611
669	TRUE	Empty	Uncharacterized protein OS=Gallus gallus GN=RPL23A PE=3 SV=2	E1BS06_CHICK (+8)		18 kDa		unknown	0	5	1	0	2.0529	0.21541
670	TRUE	Empty	status=inactive	UPI0000447837 (+3)		163 kDa		unknown	0	3	1	0	0.062547	0.092011
671	TRUE	Empty	status=inactive	UPI0000ECA837 (+10)		61 kDa	TRUE	unknown	0	7	6	0	0.46175	0.41845
672	TRUE	Empty	status=inactive	UPI0000E82530		51 kDa	TRUE	unknown	0	1	2	0	3.2975	2.2466
673	TRUE	Empty	status=active	UPI0001DED484		46 kDa		unknown	0	6	4	0	0.53295	0.35704
674	TRUE	Empty	status=active	UPI00035061DA		30 kDa		unknown	0	4	6	0	0.72026	1.006
675	TRUE	Empty	Uncharacterized protein OS=Gallus gallus GN=ECI1 PE=3 SV=2	E1C3T6_CHICK		33 kDa	TRUE	unknown	0	3	9	0	0.3471	1.6275
676	TRUE	Empty	Septin-2 OS=Gallus gallus GN=SEPT2 PE=3 SV=1	E1C3D2_CHICK (+4)		42 kDa	TRUE	unknown	2	8	4	0.21257	0.89363	1.1508
677	TRUE	Empty	Uncharacterized protein OS=Gallus gallus GN=PLXNB2 PE=4 SV=2	E1BST4_CHICK		184 kDa	TRUE	unknown	0	0	9	0	0	0.21579
678	TRUE	Empty	Uncharacterized protein OS=Gallus gallus GN=SYNM PE=3 SV=2	F1NJM8_CHICK (+1)		182 kDa		unknown	14	0	0	0.36458	0	0
679	TRUE	Empty	status=active	UPI0000DC3678 (+1)		22 kDa		unknown	1	3	5	0.19266	0.54366	1.9721
680	TRUE	Empty	Uncharacterized protein (Fragment) OS=Gallus gallus GN=C3 PE=4 SV=2	H9KZT6_CHICK (+5)		151 kDa		unknown	9	0	5	0.27387	0	0.12599
681	TRUE	Empty	status=active	UPI00003AEA87		69 kDa	TRUE	unknown	0	8	0	0	0.69688	0
682	TRUE	Empty	status=inactive	UPI0000449039		138 kDa	TRUE	unknown	0	4	7	0	0.12693	0.19915
683	TRUE	Empty	Eukaryotic translation initiation factor 3 subunit F OS=Gallus gallus GN=EIF3F PE=3 SV=1	E1C050_CHICK (+3)		35 kDa		unknown	5	4	3	0.74749	0.59614	0.35024
684	TRUE	Empty	status=inactive	UPI000240CB6C (+1)		23 kDa		unknown	2	3	3	0.41572	0.54042	0.58955
685	TRUE	Empty	Uncharacterized protein (Fragment) OS=Gallus gallus GN=PRPS1 PE=3 SV=2	F1NIP5_CHICK (+23)		35 kDa		unknown	4	2	2	0.56209	0.20166	0.22
686	TRUE	Empty	Uncharacterized protein OS=Gallus gallus GN=UMPS PE=3 SV=1	F1NPH9_CHICK (+5)		52 kDa		unknown	6	6	1	0.58404	0.46578	0.070736
687	TRUE	Empty	ATP-dependent RNA helicase DDX1 OS=Gallus gallus GN=DDX1 PE=1 SV=1	DDX1_CHICK (+6)		82 kDa		unknown	2	3	5	0.10375	0.17305	0.29631
688	TRUE	Empty	Protein lin-7 homolog C OS=Gallus gallus GN=LIN7C PE=1 SV=1	LIN7C_CHICK (+3)		22 kDa		unknown	2	3	5	0.43	0.56043	1.2172
689	TRUE	Empty	Actin-related protein 2/3 complex subunit 5 OS=Gallus gallus GN=RCJMB04_1b5 PE=2 SV=1	Q5ZMV5_CHICK (+5)		16 kDa		unknown	1	2	3	0.26705	0.80115	1.3121
690	TRUE	Empty	Uncharacterized protein OS=Gallus gallus GN=ELAVL1 PE=4 SV=1	F1N9I5_CHICK (+3)		36 kDa		unknown	2	2	2	0.24783	0.19765	0.21562
691	TRUE	Empty	Uncharacterized protein OS=Gallus gallus GN=RCJMB04_25e17 PE=2 SV=1	Q5ZIL3_CHICK (+7)		22 kDa		unknown	1	3	4	0.19266	0.54366	0.86142
692	TRUE	Empty	Uncharacterized protein OS=Gallus gallus GN=LAP3 PE=3 SV=2	F1NHL4_CHICK (+7)		56 kDa		unknown	0	5	6	0	0.34016	0.46061
693	TRUE	Empty	LIM and SH3 protein 1 OS=Gallus gallus GN=LASP1 PE=2 SV=1	D2Z1L9_CHICK (+7)		30 kDa		unknown	0	6	3	0	1.4522	0.42636
694	TRUE	Empty	status=active	UPI00003AE9A1 (+4)		162 kDa		unknown	0	6	6	0	0.1298	0.1416
695	TRUE	Empty	Cystatin OS=Gallus gallus PE=1 SV=2	CYT_CHICK (+7)		15 kDa		unknown	0	3	4	0	1.8953	3.8126
696	TRUE	Empty	Uncharacterized protein OS=Gallus gallus GN=RSU1 PE=4 SV=2	E1BWI3_CHICK (+3)		32 kDa		unknown	0	5	5	0	0.68472	0.74697
697	TRUE	Empty	Radixin (Fragment) OS=Gallus gallus GN=RDX PE=4 SV=2	F1NQD9_CHICK (+5)		68 kDa	TRUE	unknown	0	3	7	0	0.54758	2.1517
698	TRUE	Empty	Uncharacterized protein OS=Gallus gallus GN=CYB5R3 PE=4 SV=1	F1NZY9_CHICK (+6)		34 kDa		unknown	0	5	4	0	0.78844	0.51189
699	TRUE	Empty	Uncharacterized protein OS=Gallus gallus GN=PIT54 PE=4 SV=2	H9KZK6_CHICK (+6)		55 kDa		unknown	6	0	3	0.65976	0	0.21427
700	TRUE	Empty	Transferrin receptor protein 1 OS=Gallus gallus GN=TFRC PE=2 SV=2	TFR1_CHICK (+7)		86 kDa		unknown	8	4	0	0.45532	0.16628	0
701	TRUE	Empty	status=active	UPI00003A9DBD (+2)		42 kDa		unknown	2	6	4	0.21137	0.60578	0.40205
702	TRUE	Empty	status=active	UPI00003ACAAA		29 kDa	TRUE	unknown	0	4	4	0	4.2111	5.3291
703	TRUE	Empty	Uncharacterized protein OS=Gallus gallus GN=RCC1 PE=4 SV=1	R4GJK0_CHICK (+1)		44 kDa		unknown	6	4	0	0.70336	0.34322	0
704	TRUE	Empty	Uncharacterized protein OS=Gallus gallus GN=LOC426398 PE=3 SV=1	R4GJQ2_CHICK		78 kDa	TRUE	unknown	0	10	3	0	0.52971	0.14635
705	TRUE	Empty	status=active	UPI00001A165B		24 kDa		unknown	0	4	1	0	0.73737	0.15861
706	TRUE	Empty	status=active	UPI0000546791 (+2)		244 kDa	TRUE	unknown	10	0	1	0.18218	0	0.060585
707	TRUE	Empty	Uncharacterized protein OS=Gallus gallus GN=CYFIP1 PE=4 SV=1	E1BW56_CHICK (+5)		146 kDa		unknown	0	3	8	0	0.070235	0.21777
708	TRUE	Empty	Integrin-linked kinase OS=Gallus gallus PE=2 SV=1	Q9DF58_CHICK (+4)		52 kDa		unknown	0	6	5	0	0.56854	0.41122
709	TRUE	Empty	Uncharacterized protein OS=Gallus gallus GN=LOC100859485 PE=3 SV=1	R4GKF5_CHICK (+3)		23 kDa		unknown	0	3	3	0	0.52784	0.57583
710	TRUE	Empty	Uncharacterized protein OS=Gallus gallus GN=RPL23 PE=3 SV=2	E1BY89_CHICK (+8)		15 kDa		unknown	1	4	3	0.29672	1.9863	0.99467
711	TRUE	Empty	Uncharacterized protein OS=Gallus gallus GN=APOH PE=4 SV=2	F1NYG4_CHICK (+5)		41 kDa		unknown	7	0	3	0.9515	0	0.29439
712	TRUE	Empty	Uncharacterized protein OS=Gallus gallus GN=LOC421447 PE=4 SV=1	E1C7F4_CHICK (+1)		56 kDa		unknown	0	4	8	0	0.26671	0.66768
713	TRUE	Empty	Uncharacterized protein OS=Gallus gallus GN=MYO18A PE=4 SV=2	E1BY27_CHICK (+37)		232 kDa		unknown	2	4	4	0.036094	0.058487	0.063805
714	TRUE	Empty	Uncharacterized protein OS=Gallus gallus GN=CD109 PE=4 SV=2	F1NX21_CHICK		162 kDa		unknown	0	0	10	0	0	0.27626
715	TRUE	Empty	status=inactive	UPI00006116DA		344 kDa		unknown	0	10	0	0	0.10099	0
716	TRUE	Empty	status=inactive	UPI0000ECACA6 (+2)		207 kDa		unknown	9	2	2	0.19426	0.032343	0.035284
717	TRUE	Empty	status=inactive	UPI000240BAF5 (+1)		119 kDa	TRUE	unknown	0	5	6	0	0.14817	0.23348
718	TRUE	Empty	Uncharacterized protein OS=Gallus gallus GN=HIST1H111R PE=3 SV=1	F1NME1_CHICK (+4)		22 kDa	TRUE	unknown	1	1	2	19.329	9.1729	0.37411
719	TRUE	Empty	Uncharacterized protein OS=Gallus gallus GN=DDX3X PE=3 SV=2	F1NIX2_CHICK (+6)		73 kDa		unknown	3	7	0	0.18218	0.50963	0
720	TRUE	Empty	Radixin OS=Homo sapiens GN=RDX PE=2 SV=1	B0YJ88 (+2)		?	TRUE	unknown	0	0	8	0	0	-1
721	TRUE	Empty	Mitochondrial carrier homolog 2 OS=Gallus gallus GN=Mtch2 PE=2 SV=1	Q9PVL6_CHICK (+1)		33 kDa		unknown	0	5	5	0	0.82002	0.70274
722	TRUE	Empty	Uncharacterized protein OS=Gallus gallus GN=NPEPL1 PE=4 SV=2	F1NSZ4_CHICK (+7)		56 kDa		unknown	5	5	3	0.42847	0.34172	0.20921
723	TRUE	Empty	Uncharacterized protein OS=Gallus gallus GN=TARS PE=2 SV=1	Q5ZLW1_CHICK (+6)		82 kDa	TRUE	unknown	5	5	2	0.27992	0.22324	0.091023
724	TRUE	Empty	Uncharacterized protein OS=Gallus gallus GN=FAM49B PE=4 SV=2	E1BWS3_CHICK (+11)		37 kDa	TRUE	unknown	4	5	2	0.53613	0.71203	0.21073
725	TRUE	Empty	Uncharacterized protein OS=Gallus gallus GN=RPS25 PE=4 SV=2	F1NU56_CHICK (+7)		14 kDa		unknown	2	2	2	0.74328	0.59278	0.64667
726	TRUE	Empty	Uncharacterized protein OS=Gallus gallus GN=PSMD14 PE=4 SV=2	E1C6N0_CHICK (+5)		35 kDa		unknown	2	3	3	0.2599	0.32809	0.35792
727	TRUE	Empty	Ferritin heavy chain OS=Gallus gallus GN=FTH PE=2 SV=2	FRIH_CHICK (+4)		21 kDa		unknown	2	2	4	0.44794	0.35724	2.7492
728	TRUE	Empty	Glutathione synthetase OS=Gallus gallus GN=GSS PE=3 SV=2	F1NLE4_CHICK (+10)		52 kDa		unknown	1	3	7	0.0806	0.20686	0.72218
729	TRUE	Empty	tRNA-splicing ligase RtcB homolog OS=Gallus gallus GN=C22ORF28 PE=3 SV=2	F1NYI3_CHICK (+6)		55 kDa		unknown	1	6	3	0.076308	0.52227	0.29357
730	TRUE	Empty	Serum paraoxonase/arylesterase 2 OS=Gallus gallus GN=PON2 PE=4 SV=2	F1NCU9_CHICK (+4)		39 kDa		unknown	7	2	1	1.0085	0.18031	0.093884
731	TRUE	Empty	status=active	UPI0000437D6C		118 kDa		unknown	2	3	4	0.10913	0.11787	0.16326
732	TRUE	Empty	UMP-CMP kinase OS=Gallus gallus GN=CMPK PE=2 SV=1	KCY_CHICK (+4)		22 kDa		unknown	2	2	5	0.42274	0.33714	1.1918
733	TRUE	Empty	Succinate dehydrogenase [ubiquinone] flavoprotein subunit, mitochondrial OS=Gallus gallus GN=SDHA PE=4 SV=2	F1NPJ4_CHICK (+10)		73 kDa		unknown	0	6	6	0	0.31228	0.40794
734	TRUE	Empty	status=active	UPI00003AE8CD (+6)		38 kDa		unknown	4	4	5	0.52494	0.41865	0.6007
735	TRUE	Empty	Serine/threonine-protein phosphatase 2A 55 kDa regulatory subunit B OS=Gallus gallus GN=RCJMB04_5f2 PE=2 SV=1	Q5ZLP4_CHICK (+8)		52 kDa		unknown	3	5	4	0.26242	0.37506	0.3156
736	TRUE	Empty	Uncharacterized protein OS=Gallus gallus GN=CLIC4 PE=4 SV=2	F1NYZ7_CHICK		28 kDa	TRUE	unknown	0	7	5	0	1.3131	1.1416
737	TRUE	Empty	Uncharacterized protein (Fragment) OS=Gallus gallus GN=ILF2 PE=4 SV=1	H9KZT7_CHICK (+8)		43 kDa		unknown	0	5	5	0	0.46715	0.50963
738	TRUE	Empty	Adenylate kinase isoenzyme 1 OS=Gallus gallus GN=AK1 PE=1 SV=1	KAD1_CHICK (+1)		22 kDa		unknown	0	4	4	0	0.82682	0.90199
739	TRUE	Empty	status=inactive	UPI000044A120 (+1)		35 kDa		unknown	0	3	4	0	0.31877	0.48903
740	TRUE	Empty	Phosphomannomutase (Fragment) OS=Gallus gallus GN=PMM2 PE=3 SV=2	E1C4B8_CHICK (+5)		28 kDa	TRUE	unknown	0	4	6	0	0.5995	1.129

741	TRUE	Empty	60S ribosomal protein L18 (Fragment) OS=Gallus gallus PE=2 SV=1	Q6EE60_CHICK (+1)		19 kDa		unknown	0	3	3	0	0.67205	1.0831
742	TRUE	Empty	Uncharacterized protein OS=Gallus gallus GN=SEPT9 PE=2 SV=1	Q5F3T2_CHICK		66 kDa		unknown	0	3	3	0	0.22247	0.24269
743	TRUE	Empty	Tetraspanin OS=Gallus gallus GN=UPK1B PE=3 SV=2	E1C992_CHICK (+5)		29 kDa		unknown	0	2	2	0	0.24981	0.43581
744	TRUE	Empty	Ferrochelatase OS=Gallus gallus GN=FECH PE=3 SV=1	F1NBT4_CHICK (+5)		45 kDa		unknown	11	2	0	1.5665	0.15456	0
745	TRUE	Empty	NADPH--cytochrome P450 reductase OS=Gallus gallus GN=POR PE=3 SV=2	F1P2T2_CHICK (+5)		77 kDa		unknown	0	6	5	0	0.29387	0.26071
746	TRUE	Empty	Uncharacterized protein OS=Gallus gallus GN=RCJMB04_13p14 PE=2 SV=1	Q5ZK08_CHICK (+6)		64 kDa		unknown	0	6	4	0	0.43596	0.24872
747	TRUE	Empty	Uncharacterized protein (Fragment) OS=Gallus gallus GN=RPLP2 PE=3 SV=2	E1BUZ5_CHICK (+4)		14 kDa		unknown	0	4	3	0	2.1391	1.0569
748	TRUE	Empty	Uncharacterized protein OS=Gallus gallus GN=UAP1L1 PE=4 SV=1	E1BZG2_CHICK (+5)		57 kDa	TRUE	unknown	0	8	4	0	0.59407	0.3661
749	TRUE	Empty	Osteoclast-stimulating factor 1 OS=Gallus gallus GN=OSTF1 PE=4 SV=2	F1NHK3_CHICK (+5)		24 kDa		unknown	1	3	4	0.1823	0.73737	1.4264
750	TRUE	Empty	status=inactive	UPI000240CD9D (+3)		322 kDa		unknown	2	6	2	0.025957	0.07455	0.022583
751	TRUE	Empty	Annexin OS=Gallus gallus GN=ANXA7 PE=3 SV=2	E1C1D1_CHICK (+7)		49 kDa		unknown	1	7	4	0.0856	0.60072	0.33334
752	TRUE	Empty	Uncharacterized protein OS=Gallus gallus GN=LOC771930 PE=4 SV=1	R4GJF2_CHICK (+2)		32 kDa		unknown	0	5	4	0	0.85022	0.72736
753	TRUE	Empty	Galectin OS=Gallus gallus PE=2 SV=1	A4GTP0_CHICK (+8)		26 kDa		unknown	0	0	7	0	0	1.9844
754	TRUE	Empty	cDNA FLJ57154, highly similar to Alpha-fetoprotein OS=Homo sapiens PE=2 SV=1	B4DMX4 (+2)		?		unknown	0	0	9	0	0	-1
755	TRUE	Empty	A-kinase anchor protein 12 OS=Homo sapiens GN=AKAP12 PE=1 SV=4	Q02952 (+2)		?		unknown	0	0	11	0	0	-1
756	TRUE	Empty	Dopamine receptor interacting protein 4 OS=Homo sapiens GN=DRIP4 PE=2 SV=1	Q4W4Y1 (+2)		?		unknown	0	0	13	0	0	-1
757	TRUE	Empty	Uncharacterized protein OS=Gallus gallus GN=LOC100857165 PE=2 SV=1	Q5ZLA7_CHICK (+5)		81 kDa	TRUE	unknown	0	10	0	0	0.5109	0
758	TRUE	Empty	Uncharacterized protein OS=Gallus gallus GN=EPB49 PE=4 SV=1	R4GJU4_CHICK (+2)		37 kDa		unknown	6	0	0	1.3313	0	0
759	TRUE	Empty	status=active	UPI0000175F57 (+1)		35 kDa	TRUE	unknown	0	0	7	0	0	1.0311
760	TRUE	Empty	status=active	UPI00003ACF48 (+5)		14 kDa		unknown	0	5	4	0	2.195	1.6564
761	TRUE	Empty	Myristoylated alanine-rich C-kinase substrate OS=Homo sapiens GN=MARCKS PE=4 SV=1	A0A087WZH7 (+2)		?		unknown	0	0	6	0	0	-1
762	TRUE	Empty	H2-calponin OS=Gallus gallus GN=RCJMB04_9b22 PE=2 SV=1	Q5ZKU6_CHICK (+1)		33 kDa	TRUE	unknown	0	4	2	0	0.63609	0.37148
763	TRUE	Empty	status=inactive	UPI0000ECAD50		137 kDa	TRUE	unknown	0	10	0	0	0.30635	0
764	TRUE	Empty	Serine-threonine kinase receptor-associated protein OS=Gallus gallus GN=STRAP PE=2 SV=2	STRAP_CHICK (+3)		38 kDa		unknown	3	4	3	0.36831	0.41148	0.32044
765	TRUE	Empty	Uncharacterized protein OS=Gallus gallus GN=LOC100858988 PE=4 SV=1	R4GHG6_CHICK (+1)		26 kDa		unknown	2	3	3	0.35477	0.65436	0.49749
766	TRUE	Empty	Protein mago nashi homolog OS=Gallus gallus GN=MAGOH PE=2 SV=2	MGN_CHICK (+4)		17 kDa		unknown	2	3	2	0.56995	0.76137	0.49587
767	TRUE	Empty	NSFL1 cofactor p47 OS=Gallus gallus GN=NSFL1C PE=2 SV=1	NSF1C_CHICK (+5)		41 kDa		unknown	7	2	3	0.96516	0.17399	0.29798
768	TRUE	Empty	Trimethyllysine dioxygenase, mitochondrial OS=Gallus gallus GN=TMLHE PE=2 SV=1	TMLH_CHICK (+2)		49 kDa		unknown	2	6	3	0.18023	0.50335	0.24432
769	TRUE	Empty	Uncharacterized protein (Fragment) OS=Gallus gallus GN=ERP44 PE=4 SV=2	E1BSL7_CHICK (+7)		47 kDa		unknown	2	4	4	0.18748	0.32374	0.35318
770	TRUE	Empty	Uncharacterized protein (Fragment) OS=Gallus gallus GN=GLRX3 PE=4 SV=1	F1NNP6_CHICK (+3)		37 kDa		unknown	4	4	4	0.52862	0.42158	0.60511
771	TRUE	Empty	Uncharacterized protein OS=Gallus gallus GN=VTN PE=4 SV=2	E1C7A7_CHICK (+7)		52 kDa		unknown	4	1	2	0.36363	0.065125	0.22885
772	TRUE	Empty	Uncharacterized protein (Fragment) OS=Gallus gallus GN=RAB21 PE=3 SV=1	F1NC27_CHICK		25 kDa	TRUE	unknown	5	3	2	1.1794	0.48282	0.52672
773	TRUE	Empty	Uncharacterized protein OS=Gallus gallus GN=GLIPR2 PE=4 SV=1	R4GGF3_CHICK (+3)		17 kDa		unknown	1	3	3	0.25963	0.77419	0.84458
774	TRUE	Empty	Adapter molecule crk OS=Gallus gallus GN=CRK PE=1 SV=1	CRK_CHICK (+1)		34 kDa		unknown	2	1	5	0.26638	0.10063	0.68532
775	TRUE	Empty	Arylamine N-acetyltransferase, pineal gland isozyme NAT-3 OS=Gallus gallus PE=2 SV=1	ARY2_CHICK (+2)		34 kDa		unknown	0	5	4	0	0.63081	0.68816
776	TRUE	Empty	Calponin-1 OS=Gallus gallus GN=CNN1 PE=1 SV=2	sp|P26932|CNN1_CHICK (+5)		32 kDa	TRUE	unknown	0	3	3	0	0.66384	0.72419
777	TRUE	Empty	status=inactive	UPI0000ECCAC9 (+4)		22 kDa		unknown	0	3	4	0	0.56743	1.2348
778	TRUE	Empty	Uncharacterized protein OS=Gallus gallus GN=ATP6V1E1 PE=2 SV=1	Q5ZKJ9_CHICK (+1)		26 kDa	TRUE	unknown	0	4	4	0	0.87658	0.71003
779	TRUE	Empty	Uncharacterized protein OS=Gallus gallus GN=DECR1 PE=4 SV=1	E1BXS5_CHICK (+2)		36 kDa		unknown	0	6	3	0	0.9133	0.34409
780	TRUE	Empty	40S ribosomal protein S6 OS=Gallus gallus GN=RPS6 PE=4 SV=1	F7BYQ2_CHICK (+6)		34 kDa		unknown	0	4	3	0	0.47291	0.36594
781	TRUE	Empty	Putative helicase MOV-10 OS=Gallus gallus GN=MOV10 PE=2 SV=1	MOV10_CHICK (+1)		109 kDa	TRUE	unknown	8	2	0	0.39484	0.062084	0
782	TRUE	Empty	Ubiquitin carboxyl-terminal hydrolase OS=Gallus gallus GN=UCH-L1 PE=2 SV=1	A1IMF0_CHICK (+2)		25 kDa		unknown	0	5	1	0	0.92374	0.14914
783	TRUE	Empty	status=inactive	UPI0000449904 (+1)		33 kDa		unknown	7	0	1	1.2857	0	0.11283
784	TRUE	Empty	Cytoglobin OS=Gallus gallus GN=CYGB PE=2 SV=1	Q5QRU7_CHICK (+3)		21 kDa	TRUE	unknown	0	4	4	0	0.88627	0.96685
785	TRUE	Empty	Integrin alpha 3A subunit cytoplasmic domain variant OS=Gallus gallus PE=2 SV=1	Q98TT7_CHICK (+1)		114 kDa		unknown	0	3	7	0	0.090661	0.28625
786	TRUE	Empty	status=inactive	UPI0000447437 (+8)		35 kDa		unknown	1	4	1	0.12285	0.46027	0.10688
787	TRUE	Empty	Microtubule-associated protein (Fragment) OS=Gallus gallus GN=MAP4 PE=2 SV=1	Q98906_CHICK		96 kDa	TRUE	unknown	0	1	5	0	0.10775	0.20358
788	TRUE	Empty	Uncharacterized protein (Fragment) OS=Gallus gallus GN=FKBP9 PE=4 SV=2	F1NEL2_CHICK (+5)		64 kDa		unknown	0	6	4	0	0.36418	0.24968
789	TRUE	Empty	ATP-dependent RNA helicase DDX1 OS=Homo sapiens GN=DDX1 PE=4 SV=1	A0A087X2G1 (+4)		?		unknown	0	0	9	0	0	-1
790	TRUE	Empty	cDNA FLJ78504, highly similar to Homo sapiens keratin 6A (KRT6A), mRNA OS=Homo sapiens PE=2 SV=1	A8K2I0 (+7)		?		unknown	0	0	10	0	0	-1
791	TRUE	Empty	Uncharacterized protein OS=Gallus gallus GN=HSDL2 PE=4 SV=2	E1C7S2_CHICK (+8)		44 kDa		unknown	0	6	4	0	0.56619	0.37767
792	TRUE	Empty	78 kDa glucose-regulated protein OS=Homo sapiens GN=HSPA5 PE=1 SV=2	P11021 (+1)		?		unknown	0	0	7	0	0	-1
793	TRUE	Empty	status=active	UPI0003505EE3		147 kDa	TRUE	unknown	0	12	0	0	0.31264	0
794	TRUE	Empty	Keratin, type I cytoskeletal 14 OS=Gallus gallus GN=KRT14 PE=2 SV=1	K1C14_CHICK (+1)		51 kDa	TRUE	unknown	0	2	2	0	2.5425	4.3604
795	TRUE	Empty	Epididymis secretory protein Li 103 OS=Homo sapiens GN=HEL-S-103 PE=2 SV=1	A8K5I0 (+3)		?		unknown	0	0	9	0	0	-1
796	TRUE	Empty	Scavenger receptor class B, member 2, isoform CRA_a OS=Homo sapiens GN=SCARB2 PE=4 SV=1	A0A024RDG6 (+1)		?		unknown	0	0	7	0	0	-1
797	TRUE	Empty	BG12 OS=Gallus gallus GN=BG12 PE=4 SV=1	A0A023PSR8_CHICK (+2)		45 kDa	TRUE	unknown	6	0	0	0.68848	0	0
798	TRUE	Empty	status=active	UPI0000248024		26 kDa		unknown	0	3	3	0	0.45374	0.49499
799	TRUE	Empty	Tubulin beta-5 chain OS=Gallus gallus PE=3 SV=1	TBB5_CHICK		50 kDa	TRUE	unknown	0	8	0	0	5.8484	0
800	TRUE	Empty	Obg-like ATPase 1 OS=Gallus gallus GN=OLA1 PE=2 SV=1	OLA1_CHICK (+1)		45 kDa		unknown	4	5	2	0.42791	0.44506	0.17128
801	TRUE	Empty	Low molecular weight phosphotyrosine protein phosphatase OS=Gallus gallus GN=ACP1 PE=2 SV=3	PPAC_CHICK (+4)		18 kDa	TRUE	unknown	3	1	3	0.88152	0.42262	0.76694
802	TRUE	Empty	Eukaryotic translation initiation factor 3 subunit M OS=Gallus gallus GN=EIF3M PE=2 SV=1	EIF3M_CHICK (+2)		43 kDa		unknown	2	6	4	0.20784	0.72543	0.39477
803	TRUE	Empty	Uncharacterized protein (Fragment) OS=Gallus gallus GN=VBP1 PE=4 SV=2	E1BXR1_CHICK (+4)		23 kDa		unknown	2	5	4	0.40452	1.0345	0.82938
804	TRUE	Empty	Uncharacterized protein OS=Gallus gallus GN=RAD23B PE=4 SV=2	F1N9B7_CHICK (+1)		43 kDa	TRUE	unknown	4	2	4	0.447	0.16348	0.50805
805	TRUE	Empty	Uncharacterized protein OS=Gallus gallus GN=Gga.22231 PE=3 SV=2	F1NDW0_CHICK (+3)		62 kDa		unknown	2	2	1	0.13868	0.1106	0.058589
806	TRUE	Empty	Eukaryotic translation initiation factor 6 OS=Gallus gallus GN=EIF6 PE=3 SV=2	F1NGL6_CHICK (+7)		27 kDa		unknown	1	3	3	0.16175	0.85708	0.48524
807	TRUE	Empty	status=active	UPI00003AFAD3 (+3)		22 kDa	TRUE	unknown	3	5	1	0.68579	1.4245	0.59666
808	TRUE	Empty	Uncharacterized protein OS=Gallus gallus GN=DDX19B PE=2 SV=1	Q5ZMC1_CHICK (+4)		54 kDa		unknown	5	2	2	0.44895	0.12908	0.14082
809	TRUE	Empty	status=active	UPI00003AB5D4 (+8)		27 kDa		unknown	2	1	1	0.3342	0.26653	0.13601
810	TRUE	Empty	Ubiquitin carboxyl-terminal hydrolase (Fragment) OS=Gallus gallus GN=USP14 PE=3 SV=2	E1C4M6_CHICK (+8)		57 kDa		unknown	4	4	1	0.32783	0.26145	0.064668
811	TRUE	Empty	Uncharacterized protein (Fragment) OS=Gallus gallus GN=Gga.10533 PE=4 SV=1	R4GLP6_CHICK		3400 kDa	TRUE	unknown	6	6	5	0.0073219	0.0058393	0.0053056
812	TRUE	Empty	Aggrecan promoter binding protein OS=Gallus gallus PE=2 SV=1	Q45KQ2_CHICK (+10)		19 kDa		unknown	3	2	3	0.85524	0.41106	0.74408
813	TRUE	Empty	Procollagen-lysine,2-oxoglutarate 5-dioxygenase 1 OS=Gallus gallus GN=PLOD1 PE=4 SV=1	F1NMF6_CHICK (+4)		84 kDa		unknown	0	4	4	0	0.21612	0.18449
814	TRUE	Empty	Uncharacterized protein OS=Gallus gallus GN=RRAS2 PE=2 SV=1	Q5ZIW8_CHICK (+4)		23 kDa		unknown	0	7	5	0	1.7131	1.1211
815	TRUE	Empty	Uncharacterized protein OS=Gallus gallus GN=RPL3 PE=2 SV=1	Q5ZJZ2_CHICK (+7)		46 kDa	TRUE	unknown	0	5	3	0	0.53769	0.25924
816	TRUE	Empty	Uncharacterized protein OS=Gallus gallus GN=SCCPDH PE=2 SV=1	Q5ZMK3_CHICK (+3)		47 kDa		unknown	0	4	5	0	0.32374	0.45968
817	TRUE	Empty	S-phase kinase-associated protein 1 OS=Gallus gallus GN=SKP1 PE=2 SV=1	SKP1_CHICK (+2)		19 kDa		unknown	0	4	4	0	1.403	1.1006
818	TRUE	Empty	Septin-2 OS=Gallus gallus GN=SEPT2 PE=2 SV=1	SEPT2_CHICK		40 kDa	TRUE	unknown	0	4	8	0	0.78094	1.0229
819	TRUE	Empty	status=active	UPI00004370D7		14 kDa		unknown	2	0	5	0.75061	0	3.5413
820	TRUE	Empty	Uncharacterized protein OS=Gallus gallus GN=VAT1 PE=4 SV=2	F1NBS9_CHICK (+2)		42 kDa		unknown	0	3	5	0	0.37082	0.52931
821	TRUE	Empty	Uncharacterized protein OS=Gallus gallus GN=RCJMB04_24e8 PE=2 SV=1	Q5ZIQ5_CHICK (+1)		41 kDa	TRUE	unknown	0	5	5	0	0.48834	0.53274
822	TRUE	Empty	Uncharacterized protein OS=Gallus gallus GN=VRK1 PE=4 SV=1	F1NDK1_CHICK (+6)		47 kDa		unknown	5	1	0	0.52688	0.071728	0

823	TRUE	Empty	Uncharacterized protein OS=Gallus gallus GN=MRC2 PE=4 SV=2	F1NP60_CHICK (+9)		158 kDa		unknown	0	3	6	0	0.064587	0.17229
824	TRUE	Empty	Uncharacterized protein OS=Gallus gallus GN=ALDH6A1 PE=3 SV=1	E1C155_CHICK (+1)		58 kDa		unknown	0	5	5	0	0.40514	0.35651
825	TRUE	Empty	Uncharacterized protein OS=Gallus gallus GN=ATP6V1G1 PE=4 SV=1	E1C3C6_CHICK (+4)		14 kDa		unknown	0	2	5	0	1.0332	3.5413
826	TRUE	Empty	Uncharacterized protein OS=Gallus gallus GN=AKAP12 PE=4 SV=2	F1NJW0_CHICK (+10)		207 kDa		unknown	0	4	1	0	0.082807	0.01744
827	TRUE	Empty	Protein kinase C substrate 80K-H, isoform CRA_a OS=Homo sapiens GN=PRKCSH PE=4 SV=1	A0A024R7F1 (+4)		?		unknown	0	0	6	0	0	-1
828	TRUE	Empty	Uncharacterized protein OS=Gallus gallus GN=DYNC1I2 PE=4 SV=1	F1P3C8_CHICK (+6)		68 kDa	TRUE	unknown	0	4	0	0	0.21294	0
829	TRUE	Empty	status=active	UPI00003A99A5 (+2)		35 kDa	TRUE	unknown	6	0	0	1.1648	0	0
830	TRUE	Empty	status=inactive	UPI000061067C (+5)		149 kDa		unknown	0	7	2	0	0.16826	0.049291
831	TRUE	Empty	status=inactive	UPI0000ECAE1B (+6)		72 kDa	TRUE	unknown	0	3	3	0	0.25754	0.28095
832	TRUE	Empty	Microtubule-associated protein OS=Gallus gallus GN=MAPT PE=2 SV=1	B0LVF9_CHICK		36 kDa	TRUE	unknown	6	0	0	1.1169	0	0
833	TRUE	Empty	status=inactive	UPI00004483D8 (+6)		44 kDa		unknown	0	2	3	0	0.15826	0.26999
834	TRUE	Empty	status=inactive	UPI000044A1A4 (+2)		21 kDa	TRUE	unknown	5	3	1	1.4723	1.5506	0.63896
835	TRUE	Empty	Ubiquitin carboxyl-terminal hydrolase OS=Gallus gallus GN=USP47 PE=3 SV=1	R4GJH4_CHICK (+5)		157 kDa	TRUE	unknown	11	0	0	0.32764	0	0
836	TRUE	Empty	Fatty acid synthase OS=Gallus gallus GN=FASN PE=4 SV=2	F1N8A8_CHICK (+3)		320 kDa		unknown	0	8	4	0	0.086062	0.045877
837	TRUE	Empty	status=inactive	UPI00004475C3		142 kDa	TRUE	unknown	2	9	0	0.0596	0.26398	0
838	TRUE	Empty	Uncharacterized protein OS=Gallus gallus GN=PSMF1 PE=2 SV=1	Q5ZJL3_CHICK (+2)		29 kDa		unknown	4	2	3	0.71294	0.24981	0.43581
839	TRUE	Empty	Tyrosine--tRNA ligase, cytoplasmic OS=Gallus gallus GN=YARS PE=4 SV=2	F1NJU7_CHICK (+9)		59 kDa		unknown	5	4	1	0.40187	0.2484	0.061713
840	TRUE	Empty	Uncharacterized protein OS=Gallus gallus GN=RCJMB04_32I4 PE=2 SV=1	Q5ZHW0_CHICK (+9)		66 kDa		unknown	3	1	5	0.20299	0.74972	0.31158
841	TRUE	Empty	Anamorsin OS=Gallus gallus GN=CIAPIN1 PE=3 SV=2	F1P0W1_CHICK (+4)		33 kDa		unknown	5	3	3	0.81462	0.3471	0.37866
842	TRUE	Empty	D-erythrulose reductase OS=Gallus gallus GN=DER PE=1 SV=1	DER_CHICK (+1)		26 kDa		unknown	1	5	4	0.16388	0.87162	0.70625
843	TRUE	Empty	Uncharacterized protein OS=Gallus gallus GN=BCAP29 PE=4 SV=1	E1C310_CHICK (+3)		28 kDa		unknown	0	4	3	0	1.0406	0.46029
844	TRUE	Empty	Glutathione peroxidase (Fragment) OS=Gallus gallus GN=GPX3 PE=3 SV=1	F1NPJ8_CHICK (+6)		25 kDa		unknown	5	1	4	1.1866	0.13927	0.76298
845	TRUE	Empty	Uncharacterized protein OS=Gallus gallus GN=XPO1 PE=4 SV=2	F1NVE5_CHICK (+10)		123 kDa		unknown	0	7	1	0	0.20722	0.029511
846	TRUE	Empty	Cytochrome b5 OS=Gallus gallus GN=CYB5A PE=1 SV=4	CYB5_CHICK (+5)		16 kDa		unknown	0	3	3	0	0.86093	1.4201
847	TRUE	Empty	Uncharacterized protein OS=Gallus gallus GN=CTNNB1 PE=4 SV=1	F1NHT7_CHICK (+8)		85 kDa	TRUE	unknown	0	5	3	0	0.26094	0.13332
848	TRUE	Empty	Myosin regulatory light chain 2, smooth muscle major isoform OS=Gallus gallus PE=1 SV=2	MLRM_CHICK (+4)		20 kDa	TRUE	unknown	0	2	2	0	5.3813	4.7625
849	TRUE	Empty	Uncharacterized protein OS=Gallus gallus GN=ACSL1 PE=2 SV=1	Q5F420_CHICK (+3)		79 kDa	TRUE	unknown	0	7	4	0	0.34335	0.1992
850	TRUE	Empty	Sulfotransferase OS=Gallus gallus GN=SULT1C PE=2 SV=1	Q90WR6_CHICK (+2)		36 kDa		unknown	0	6	4	0	0.72902	0.47661
851	TRUE	Empty	Alpha tropomyosin of brain OS=Gallus gallus GN=alpha-fTM PE=3 SV=1	Q91005_CHICK (+3)		28 kDa	TRUE	unknown	0	3	5	0	15.017	27.614
852	TRUE	Empty	Uncharacterized protein OS=Gallus gallus GN=TXNDC17 PE=4 SV=1	R4GMD9_CHICK (+2)		14 kDa		unknown	0	5	6	0	2.1391	4.4078
853	TRUE	Empty	status=active	UPI000002B5B4 (+1)		29 kDa	TRUE	unknown	0	4	4	0	4.9258	12.415
854	TRUE	Empty	Uncharacterized protein OS=Gallus gallus GN=EHD3 PE=4 SV=1	F1NGM0_CHICK (+5)		61 kDa	TRUE	unknown	0	6	3	0	0.38522	0.33946
855	TRUE	Empty	Peptidyl-prolyl cis-trans isomerase OS=Gallus gallus GN=PPIH PE=3 SV=1	E1BXG0_CHICK (+3)		19 kDa		unknown	0	3	4	0	0.65754	1.0577
856	TRUE	Empty	Ovoinhibitor OS=Gallus gallus GN=OIH PE=1 SV=2	IOV7_CHICK (+2)		52 kDa		unknown	0	4	4	0	0.2886	0.40814
857	TRUE	Empty	Uncharacterized protein OS=Gallus gallus GN=SLC25A4 PE=2 SV=1	Q5ZMJ6_CHICK (+1)		33 kDa		unknown	0	4	4	0	1.7564	2.2468
858	TRUE	Empty	status=active	UPI00027F1E03 (+2)		56 kDa		unknown	2	5	3	0.15545	0.3425	0.20966
859	TRUE	Empty	Alkaline phosphatase OS=Homo sapiens GN=ALPL PE=3 SV=1	A0A024RAG0 (+2)		?		unknown	0	0	6	0	0	-1
860	TRUE	Empty	Endoplasmin OS=Homo sapiens GN=HSP90B1 PE=1 SV=1	P14625 (+2)		?		unknown	0	0	8	0	0	-1
861	TRUE	Empty	Tubulin beta-4 chain OS=Gallus gallus PE=1 SV=1	TBB4_CHICK		50 kDa		unknown	0	0	3	0	0	5.2923
862	TRUE	Empty	status=active	UPI00003ABB51 (+3)		72 kDa	TRUE	unknown	0	0	6	0	0	0.34671
863	TRUE	Empty	TRYP_PIG Trypsin OS=Sus scrofa PE=1 SV=1	P00761-CONT		?		unknown	0	0	2	0	0	-1
864	TRUE	Empty	status=active	UPI000005F936 (+7)		22 kDa		unknown	3	4	0	0.70272	1.1158	0
865	TRUE	Empty	Skeletal myosin heavy chain OS=Gallus gallus PE=2 SV=1	Q9PTY2_CHICK (+1)		223 kDa	TRUE	unknown	10	0	0	0.22275	0	0
866	TRUE	Empty	Uncharacterized protein OS=Gallus gallus GN=NANS PE=2 SV=1	Q5ZMH8_CHICK (+4)		40 kDa		unknown	5	2	4	0.63706	0.17656	0.42282
867	TRUE	Empty	Uncharacterized protein (Fragment) OS=Gallus gallus GN=RCJMB04_7b3 PE=2 SV=1	Q5ZL99_CHICK (+10)		37 kDa		unknown	3	4	2	0.38207	0.42758	0.21073
868	TRUE	Empty	Flap endonuclease 1 OS=Gallus gallus GN=FEN1 PE=2 SV=1	FEN1_CHICK (+2)		43 kDa		unknown	4	2	1	0.44834	0.16393	0.085695
869	TRUE	Empty	Vesicle-trafficking protein SEC22b OS=Gallus gallus GN=SEC22B PE=2 SV=1	SC22B_CHICK (+2)		25 kDa		unknown	3	1	4	0.6054	0.13862	1.0261
870	TRUE	Empty	Uncharacterized protein (Fragment) OS=Gallus gallus GN=SLC25A5 PE=3 SV=2	F1NJ10_CHICK (+16)		35 kDa		unknown	2	2	3	0.25633	1.8694	2.7232
871	TRUE	Empty	status=active	UPI00003AC634 (+2)		20 kDa	TRUE	unknown	2	2	1	0.47631	0.37986	1.0028
872	TRUE	Empty	Uncharacterized protein (Fragment) OS=Gallus gallus GN=EIF1AY PE=3 SV=2	F1NKZ6_CHICK (+9)		16 kDa		unknown	3	2	4	1.0502	0.49565	1.3777
873	TRUE	Empty	Insulin-like growth factor 2 mRNA-binding protein 1 OS=Gallus gallus GN=IGF2BP1 PE=1 SV=1	IF2B1_CHICK (+7)		63 kDa		unknown	0	4	3	0	0.29755	0.1836
874	TRUE	Empty	Uncharacterized protein OS=Gallus gallus GN=LANCL1 PE=4 SV=2	F1N9V4_CHICK (+5)		45 kDa		unknown	5	1	1	0.55153	0.074609	0.081392
875	TRUE	Empty	Uncharacterized protein (Fragment) OS=Gallus gallus GN=PFN2 PE=4 SV=2	F1NUH4_CHICK (+5)		10 kDa		unknown	0	3	4	0	1.5107	2.6684
876	TRUE	Empty	Cysteine and glycine-rich protein 2 OS=Gallus gallus GN=CSRP2 PE=2 SV=1	C4P6P8_CHICK (+5)		21 kDa		unknown	0	2	5	0	0.59335	1.7191
877	TRUE	Empty	Na(+)/H(+) exchange regulatory cofactor NHE-RF1 OS=Gallus gallus GN=SLC9A3R1 PE=2 SV=1	NHRF1_CHICK (+6)		36 kDa		unknown	0	2	3	0	0.19964	0.34409
878	TRUE	Empty	Claudin OS=Gallus gallus GN=CLDN1 PE=2 SV=1	Q5ZMG2_CHICK (+2)		23 kDa		unknown	0	2	3	0	0.32972	0.58606
879	TRUE	Empty	Trafficking protein particle complex subunit 3 OS=Gallus gallus GN=TRAPPC3 PE=2 SV=1	TPPC3_CHICK (+1)		20 kDa		unknown	0	3	4	0	0.61333	0.98092
880	TRUE	Empty	status=active	UPI0002C8788D (+9)		61 kDa		unknown	0	7	3	0	0.46686	0.26492
881	TRUE	Empty	Uncharacterized protein OS=Gallus gallus GN=MYL4 PE=4 SV=2	F1NJ37_CHICK (+6)		21 kDa		unknown	0	4	1	0	0.87995	0.39923
882	TRUE	Empty	Elongation of very long chain fatty acids protein OS=Gallus gallus GN=ELOVL1 PE=2 SV=1	E3VVM5_CHICK (+5)		34 kDa		unknown	0	2	1	0	0.47291	0.36594
883	TRUE	Empty	Plakoglobin OS=Gallus gallus GN=JUP PE=2 SV=2	E1C1V3_CHICK (+1)		82 kDa	TRUE	unknown	0	7	2	0	0.32625	0.090769
884	TRUE	Empty	Antithrombin-III OS=Gallus gallus GN=SERPINC1 PE=3 SV=2	F1NLP7_CHICK (+3)		52 kDa		unknown	7	0	1	0.70909	0	0.070891
885	TRUE	Empty	Dynactin subunit 1 OS=Gallus gallus GN=DCTN1 PE=2 SV=2	DCTN1_CHICK (+4)		136 kDa		unknown	5	0	2	0.1622	0	0.054195
886	TRUE	Empty	cDNA FLJ51907, highly similar to Stress-70 protein, mitochondrial OS=Homo sapiens PE=2 SV=1	B7Z4V2 (+2)		?		unknown	0	0	8	0	0	-1
887	TRUE	Empty	Uncharacterized protein OS=Gallus gallus GN=LARS PE=3 SV=1	E1C2I9_CHICK		134 kDa	TRUE	unknown	10	0	0	0.3515	0	0
888	TRUE	Empty	Epididymis tissue sperm binding protein Li 14m OS=Homo sapiens PE=2 SV=1	E9KL44 (+1)		?		unknown	0	0	7	0	0	-1
889	TRUE	Empty	Eukaryotic translation initiation factor 3 subunit B OS=Gallus gallus GN=EIF3B PE=3 SV=2	F1NZ25_CHICK		86 kDa		unknown	0	9	0	0	0.47473	0
890	TRUE	Empty	Calnexin OS=Homo sapiens GN=CANX PE=1 SV=2	P27824 (+2)		?		unknown	0	0	5	0	0	-1
891	TRUE	Empty	status=active	UPI000037814F (+7)		45 kDa		unknown	0	6	4	0	0.54908	0.47845
892	TRUE	Empty	status=active	UPI0003506BE0 (+7)		178 kDa	TRUE	unknown	0	4	3	0	0.076673	0.062091
893	TRUE	Empty	status=inactive	UPI000044A79A (+3)		36 kDa		unknown	0	3	3	0	0.44662	0.81471
894	TRUE	Empty	Uncharacterized protein OS=Gallus gallus GN=PGM2L1 PE=4 SV=2	F1NAA5_CHICK (+1)		70 kDa	TRUE	unknown	8	0	0	0.57525	0	0
895	TRUE	Empty	status=inactive	UPI0000E8082A (+6)		38 kDa	TRUE	unknown	0	3	5	0	0.29862	0.75906
896	TRUE	Empty	Uncharacterized protein OS=Gallus gallus GN=TMPO PE=4 SV=1	F1NZS9_CHICK (+3)		50 kDa	TRUE	unknown	8	0	0	0.8752	0	0
897	TRUE	Empty	status=inactive	UPI000060E308 (+11)		165 kDa		unknown	7	0	1	0.18887	0	0.021953
898	TRUE	Empty	Exportin-7 OS=Gallus gallus GN=XPO7 PE=4 SV=2	F1NFR4_CHICK		124 kDa	TRUE	unknown	10	0	0	0.38548	0	0
899	TRUE	Empty	Ovotransferrin OS=Gallus gallus PE=1 SV=2	TRFE_CHICK		78 kDa	TRUE	unknown	0	0	2	0	0	11.115
900	TRUE	Empty	status=inactive	UPI000060E33F		95 kDa	TRUE	unknown	8	0	0	0.40175	0	0
901	TRUE	Empty	status=active	UPI0000004977		51 kDa		unknown	3	2	2	0.26553	0.13617	0.14855
902	TRUE	Empty	status=inactive	UPI000240CB5F		14 kDa		unknown	2	1	2	0.69565	0.24439	0.60523
903	TRUE	Empty	Uncharacterized protein OS=Gallus gallus GN=RALA PE=4 SV=1	E1C524_CHICK (+2)		24 kDa	TRUE	unknown	3	3	3	0.64313	0.51291	0.55954
904	TRUE	Empty	F-actin-capping protein subunit alpha-1 OS=Gallus gallus GN=CAPZA1 PE=1 SV=1	CAZA1_CHICK (+14)		33 kDa		unknown	1	2	2	0.12924	0.34577	0.3772

905	TRUE	Empty	Coatomer subunit beta OS=Gallus gallus GN=COPB1 PE=2 SV=1	COPB_CHICK (+3)		107 kDa		unknown	1	6	3	0.038999	0.20341	0.10533
906	TRUE	Empty	Uncharacterized protein OS=Gallus gallus GN=RCJMB04_17k23 PE=2 SV=1	Q5ZLI3_CHICK (+5)		27 kDa		unknown	1	4	3	0.15699	0.61467	0.46906
907	TRUE	Empty	Uncharacterized protein OS=Gallus gallus GN=EIF2S2 PE=4 SV=1	E1BTB2_CHICK (+4)		38 kDa	TRUE	unknown	4	1	1	0.51951	0.089426	0.097556
908	TRUE	Empty	Uncharacterized protein (Fragment) OS=Gallus gallus GN=RPL31 PE=4 SV=2	F1P4V6_CHICK (+5)		15 kDa		unknown	1	1	4	0.3015	0.24045	2.2199
909	TRUE	Empty	status=inactive	UPI0000449985 (+4)		38 kDa		unknown	3	3	3	0.37196	0.29665	0.45355
910	TRUE	Empty	Uncharacterized protein OS=Gallus gallus GN=AHSG PE=4 SV=1	E1BZE1_CHICK (+2)		37 kDa		unknown	4	0	4	0.53048	0	0.46153
911	TRUE	Empty	Uncharacterized protein OS=Gallus gallus GN=ENDOD1 PE=4 SV=2	E1C8W3_CHICK (+5)		53 kDa		unknown	0	3	5	0	0.20591	0.40212
912	TRUE	Empty	Uncharacterized protein OS=Gallus gallus GN=RCJMB04_19g16 PE=2 SV=1	Q5F3E6_CHICK (+6)		57 kDa		unknown	0	6	4	0	0.59994	0.36941
913	TRUE	Empty	Glutathione S-transferase OS=Gallus gallus GN=LOC396380 PE=3 SV=1	F1NQS2_CHICK (+4)		26 kDa	TRUE	unknown	0	2	4	0	0.64397	1.2235
914	TRUE	Empty	Acetyltransferase component of pyruvate dehydrogenase complex OS=Gallus gallus GN=DLAT PE=3 SV=1	E1C6N5_CHICK (+4)		67 kDa		unknown	0	4	4	0	0.2188	0.23869
915	TRUE	Empty	Uncharacterized protein OS=Gallus gallus GN=C5H14ORF166 PE=4 SV=1	F1NA45_CHICK (+4)		27 kDa		unknown	0	4	3	0	0.82053	0.89513
916	TRUE	Empty	Uncharacterized protein OS=Gallus gallus GN=BLVRA PE=4 SV=2	F1NAF5_CHICK (+7)		33 kDa		unknown	6	2	0	1.0191	0.21552	0
917	TRUE	Empty	Coatomer subunit gamma OS=Gallus gallus GN=COPG PE=3 SV=1	F1NBS2_CHICK (+6)		98 kDa		unknown	0	5	2	0	0.18408	0.075894
918	TRUE	Empty	Uncharacterized protein (Fragment) OS=Gallus gallus GN=C1H21ORF33 PE=4 SV=2	F1NT93_CHICK (+1)		28 kDa		unknown	0	3	4	0	0.59075	0.86286
919	TRUE	Empty	Uncharacterized protein OS=Gallus gallus GN=PALLD PE=4 SV=2	F1P0G8_CHICK (+8)		150 kDa		unknown	0	4	2	0	0.09212	0.04903
920	TRUE	Empty	Uncharacterized protein OS=Gallus gallus GN=ACBD3 PE=4 SV=1	F1PSI8_CHICK (+8)		60 kDa		unknown	0	2	3	0	0.17981	0.19616
921	TRUE	Empty	status=active	UPI00035051A2 (+3)		27 kDa		unknown	0	4	2	0	0.82493	0.29206
922	TRUE	Empty	Integrin alpha-V OS=Gallus gallus GN=ITGAV PE=3 SV=1	F1NGX1_CHICK (+4)		114 kDa		unknown	0	2	6	0	0.059119	0.24477
923	TRUE	Empty	Uncharacterized protein (Fragment) OS=Gallus gallus GN=UBE2L3 PE=3 SV=2	F1NIZ6_CHICK (+9)		17 kDa		unknown	4	0	2	1.4134	0	0.49216
924	TRUE	Empty	Uncharacterized protein OS=Gallus gallus GN=GALNT7 PE=4 SV=2	F1NPU9_CHICK (+3)		73 kDa		unknown	0	4	4	0	0.19742	0.27619
925	TRUE	Empty	Uncharacterized protein OS=Gallus gallus GN=MCM6 PE=3 SV=1	F1NAG0_CHICK (+6)		103 kDa	TRUE	unknown	0	4	1	0	0.17296	0.035144
926	TRUE	Empty	status=inactive	UPI000240C7F1 (+5)		146 kDa		unknown	8	0	1	0.24884	0	0.024773
927	TRUE	Empty	Uncharacterized protein OS=Gallus gallus GN=ARPC1B PE=2 SV=1	Q5ZI99_CHICK (+1)		41 kDa		unknown	0	3	4	0	0.26986	0.6766
928	TRUE	Empty	status=inactive	UPI000044841E (+8)		53 kDa		unknown	2	6	1	0.16471	0.45408	0.069225
929	TRUE	Empty	Uncharacterized protein OS=Gallus gallus GN=DEK PE=2 SV=1	Q5F422_CHICK (+3)		42 kDa		unknown	7	0	0	0.91896	0	0
930	TRUE	Empty	Uncharacterized protein OS=Gallus gallus GN=EEA1 PE=4 SV=2	E1BRE5_CHICK (+7)		162 kDa		unknown	0	0	8	0	0	0.19404
931	TRUE	Empty	Nucleolin, isoform CRA_b OS=Homo sapiens GN=NCL PE=4 SV=1	A0A024R4A0 (+4)		?		unknown	0	0	8	0	0	-1
932	TRUE	Empty	cDNA, FLJ92849, highly similar to Homo sapiens lanosterol synthase (2,3-oxidosqualene-lanosterol cyclase) (LSS), mRNA OS=Homo sapiens PE=2 SV=1	B2R694 (+3)		?		unknown	0	0	7	0	0	-1
933	TRUE	Empty	Intercellular adhesion molecule 1 OS=Homo sapiens GN=ICAM1 PE=2 SV=1	Q5NKV8		?		unknown	0	0	5	0	0	-1
934	TRUE	Empty	cDNA FLJ56561 OS=Homo sapiens PE=2 SV=1	B4E2S3 (+1)		?		unknown	0	0	6	0	0	-1
935	TRUE	Empty	tr|E1BTS3|E1BTS3_CHICK-DECOY Uncharacterized protein OS=Gallus gallus GN=Gga.51341...	tr|E1BTS3|E1BTS3_CHICK-DECOY		?		unknown	0	2	0	0	-1	0
936	TRUE	Empty	Phosphoinositide phospholipase C OS=Gallus gallus GN=PLCG1 PE=4 SV=2	F1NN68_CHICK (+6)		147 kDa	TRUE	unknown	8	0	0	0.2474	0	0
937	TRUE	Empty	Glutamate dehydrogenase 1, mitochondrial OS=Gallus gallus GN=GLUD1 PE=1 SV=1	DHE3_CHICK (+1)		56 kDa	TRUE	unknown	0	9	0	0	0.71138	0
938	TRUE	Empty	status=inactive	UPI000240C5CD		74 kDa	TRUE	unknown	0	2	5	0	0.14297	0.27341
939	TRUE	Empty	status=active	UPI00005FF0BE (+2)		20 kDa	TRUE	unknown	0	3	4	0	0.61749	0.98811
940	TRUE	Empty	Uncharacterized protein OS=Gallus gallus GN=LOC100859771 PE=4 SV=1	R4GLE1_CHICK (+4)		26 kDa		unknown	3	2	2	0.5661	0.28028	0.30576
941	TRUE	Empty	Uncharacterized protein OS=Gallus gallus GN=ACAT2 PE=3 SV=2	F1NT20_CHICK		41 kDa		unknown	3	4	2	0.34042	0.37902	0.1887
942	TRUE	Empty	Uncharacterized protein (Fragment) OS=Gallus gallus GN=PDCD6 PE=4 SV=1	F1NHD8_CHICK (+7)		22 kDa		unknown	2	2	5	0.43498	0.3469	1.2348
943	TRUE	Empty	Small nuclear ribonucleoprotein-associated protein B' OS=Gallus gallus GN=SNRPB PE=4 SV=1	R9PXM6_CHICK (+5)		47 kDa		unknown	3	2	1	0.29026	0.1484	0.077873
944	TRUE	Empty	status=inactive	UPI0000448EE9 (+3)		29 kDa		unknown	3	2	1	0.49651	0.24773	0.12696
945	TRUE	Empty	Uncharacterized protein (Fragment) OS=Gallus gallus GN=PEPD PE=3 SV=2	F1NIQ9_CHICK (+17)		55 kDa		unknown	0	3	4	0	0.1947	0.29291
946	TRUE	Empty	Uncharacterized protein (Fragment) OS=Gallus gallus GN=NT5C1B PE=4 SV=1	F1NV37_CHICK (+4)		40 kDa		unknown	0	4	5	0	0.3851	0.55056
947	TRUE	Empty	Pre-mRNA-processing factor 19 OS=Gallus gallus GN=PRPF19 PE=1 SV=1	PRP19_CHICK (+1)		55 kDa	TRUE	unknown	1	2	3	0.2452	0.52357	0.29424
948	TRUE	Empty	40S ribosomal protein S17 OS=Gallus gallus GN=RPS17 PE=2 SV=3	RS17_CHICK (+4)		16 kDa		unknown	0	4	3	0	1.8527	0.9392
949	TRUE	Empty	Uncharacterized protein OS=Gallus gallus GN=RNPEP PE=4 SV=2	F1NS13_CHICK (+7)		67 kDa		unknown	0	4	4	0	0.21839	0.23825
950	TRUE	Empty	Uncharacterized protein (Fragment) OS=Gallus gallus GN=RPS15A PE=3 SV=2	F1NXW3_CHICK (+6)		17 kDa		unknown	0	3	4	0	0.74896	1.2188
951	TRUE	Empty	AP-2 complex subunit mu OS=Gallus gallus GN=AP2M1 PE=2 SV=1	AP2M1_CHICK (+9)		49 kDa		unknown	0	4	4	0	0.30478	0.33249
952	TRUE	Empty	Uncharacterized protein OS=Gallus gallus GN=NDUFB6 PE=4 SV=1	E1BT94_CHICK (+1)		15 kDa	TRUE	unknown	0	2	5	0	0.53517	2.1669
953	TRUE	Empty	Uncharacterized protein (Fragment) OS=Gallus gallus GN=NDUFA7 PE=4 SV=2	E1C645_CHICK (+2)		13 kDa		unknown	0	2	4	0	0.62965	1.8518
954	TRUE	Empty	Beta-arrestin 1 (Fragment) OS=Gallus gallus PE=2 SV=1	I7AR58_CHICK (+3)		18 kDa		unknown	0	2	3	0	1.0976	0.80393
955	TRUE	Empty	Mimecan OS=Gallus gallus GN=OGN PE=2 SV=1	MIME_CHICK (+2)		33 kDa		unknown	0	4	2	0	0.4843	0.23596
956	TRUE	Empty	Uncharacterized protein OS=Gallus gallus GN=HSD17B12 PE=3 SV=1	R4GHS0_CHICK (+2)		30 kDa		unknown	0	4	2	0	0.73764	0.26584
957	TRUE	Empty	status=active	UPI00003ABD2E		28 kDa		unknown	0	3	4	0	0.41418	0.64445
958	TRUE	Empty	Uncharacterized protein OS=Gallus gallus GN=GOLM1 PE=4 SV=2	E1C3N9_CHICK (+5)		43 kDa		unknown	0	2	2	0	0.16348	0.17834
959	TRUE	Empty	Uncharacterized protein OS=Gallus gallus GN=VILL PE=4 SV=2	E1C5U6_CHICK (+10)		97 kDa		unknown	0	2	5	0	0.069897	0.20181
960	TRUE	Empty	Uncharacterized protein OS=Gallus gallus GN=IMMT PE=4 SV=1	F1P3U1_CHICK (+5)		79 kDa		unknown	0	5	3	0	0.23156	0.14461
961	TRUE	Empty	Uncharacterized protein OS=Gallus gallus GN=SARNP PE=2 SV=1	Q5ZLP5_CHICK (+1)		23 kDa		unknown	5	1	0	1.2718	0.14683	0
962	TRUE	Empty	Uncharacterized protein (Fragment) OS=Gallus gallus GN=NDUFB9 PE=4 SV=2	F1NPF3_CHICK (+5)		22 kDa		unknown	0	2	3	0	0.34293	0.89002
963	TRUE	Empty	Ras-related protein Rab-18 OS=Gallus gallus GN=RAB18 PE=2 SV=1	RAB18_CHICK		23 kDa	TRUE	unknown	0	2	5	0	0.52784	1.1361
964	TRUE	Empty	Uncharacterized protein OS=Gallus gallus GN=FBLN2 PE=4 SV=2	F1NWN4_CHICK (+8)		132 kDa		unknown	0	1	2	0	0.050957	0.055589
965	TRUE	Empty	Uncharacterized protein OS=Gallus gallus GN=COPS4 PE=2 SV=1	Q5ZIV0_CHICK (+3)		47 kDa		unknown	7	0	1	0.80557	0	0.078628
966	TRUE	Empty	status=inactive	UPI0000EC8961 (+6)		49 kDa	TRUE	unknown	5	3	0	0.62766	0.30871	0
967	TRUE	Empty	Uncharacterized protein OS=Gallus gallus GN=SCLY PE=4 SV=1	E1BZJ9_CHICK (+2)		46 kDa		unknown	1	2	1	0.092852	0.15416	0.080784
968	TRUE	Empty	Uncharacterized protein OS=Gallus gallus GN=TMEM33 PE=4 SV=1	E1BW27_CHICK (+3)		28 kDa		unknown	1	3	3	0.15126	0.41228	0.44977
969	TRUE	Empty	Basal cell adhesion molecule (Lutheran blood group) OS=Homo sapiens GN=BCAM PE=4 SV=1	A0A068W6H0 (+5)		?		unknown	0	0	6	0	0	-1
970	TRUE	Empty	NADH-ubiquinone oxidoreductase 75 kDa subunit, mitochondrial OS=Homo sapiens GN=NDUFS1 PE=1 SV=1	B4DJ81 (+6)		?		unknown	0	0	8	0	0	-1
971	TRUE	Empty	Uncharacterized protein OS=Gallus gallus GN=ROCK2 PE=4 SV=2	F1NK88_CHICK (+7)		162 kDa	TRUE	unknown	0	0	9	0	0	0.24843
972	TRUE	Empty	Uncharacterized protein (Fragment) OS=Gallus gallus GN=ANKFY1 PE=4 SV=2	F1NSB6_CHICK (+8)		129 kDa	TRUE	unknown	8	0	0	0.28651	0	0
973	TRUE	Empty	Alpha-1B-glycoprotein OS=Homo sapiens GN=A1BG PE=1 SV=4	P04217 (+1)		?		unknown	0	0	6	0	0	-1
974	TRUE	Empty	Uncharacterized protein OS=Gallus gallus GN=RHOG PE=2 SV=1	Q5ZMG8_CHICK (+1)		21 kDa		unknown	0	3	4	0	0.57826	1.2621
975	TRUE	Empty	Plexin-B2 OS=Homo sapiens GN=PLXNB2 PE=1 SV=3	O15031 (+1)		?		unknown	0	0	6	0	0	-1
976	TRUE	Empty	Uncharacterized protein OS=Gallus gallus GN=ITGA9 PE=3 SV=1	F1NI94_CHICK (+1)		115 kDa	TRUE	unknown	0	0	3	0	0	0.16817
977	TRUE	Empty	Proto-oncogene tyrosine-protein kinase Src OS=Gallus gallus GN=SRC PE=1 SV=4	SRC_CHICK (+9)		60 kDa		unknown	0	3	4	0	0.17838	0.26764
978	TRUE	Empty	status=inactive	UPI0000449021 (+4)		101 kDa		unknown	0	2	4	0	0.10278	0.23699
979	TRUE	Empty	status=inactive	UPI000044954A (+6)		110 kDa		unknown	5	0	2	0.20363	0	0.067376
980	TRUE	Empty	status=active	UPI00003A8B51 (+3)		72 kDa	TRUE	unknown	0	6	0	0	0.31781	0
981	TRUE	Empty	status=inactive	UPI000056AE97 (+4)		66 kDa		unknown	0	6	2	0	0.35275	0.11448
982	TRUE	Empty	Lymphocyte antigen 86 OS=Gallus gallus GN=LY86 PE=4 SV=1	F1P4F3_CHICK (+1)		18 kDa		unknown	0	0	5	0	0	1.6155
983	TRUE	Empty	Proliferating cell nuclear antigen OS=Gallus gallus GN=PCNA PE=1 SV=1	PCNA_CHICK (+6)		29 kDa		unknown	0	5	1	0	0.9857	0.12898
984	TRUE	Empty	Alcohol dehydrogenase IB (Class I), beta polypeptide, isoform CRA_a OS=Homo sapiens GN=ADH1B PE=4 SV=1	A0A024RDI5 (+4)		?		unknown	0	0	7	0	0	-1
985	TRUE	Empty	Uncharacterized protein OS=Gallus gallus GN=CCAR1 PE=4 SV=2	F1P4X5_CHICK		131 kDa		unknown	9	0	0	0.31993	0	0
986	TRUE	Empty	status=inactive	UPI0000610087		63 kDa	TRUE	unknown	7	0	0	0.66209	0	0

987	TRUE	Empty	status=inactive	UPI00006115EA (+1)		142 kDa	TRUE	unknown	0	0	8	0	0	0.22415
988	TRUE	Empty	status=inactive	UPI0000ECA74F		196 kDa	TRUE	unknown	6	0	0	0.1336	0	0
989	TRUE	Empty	Uncharacterized protein OS=Gallus gallus GN=PSMD9 PE=4 SV=1	F1NVH4_CHICK (+5)		23 kDa		unknown	3	2	3	0.66965	0.32791	0.58261
990	TRUE	Empty	Acetyl-coenzyme A synthetase OS=Gallus gallus GN=ACSS1 PE=3 SV=2	F1NLR1_CHICK (+6)		67 kDa		unknown	3	4	2	0.19832	0.21681	0.11191
991	TRUE	Empty	status=active	UPI00003AFA01 (+6)		34 kDa		unknown	3	2	2	0.42537	0.21397	0.23342
992	TRUE	Empty	Uncharacterized protein OS=Gallus gallus GN=ERLIN2 PE=4 SV=2	F1NBD0_CHICK (+5)		38 kDa	TRUE	unknown	2	4	2	0.23314	0.41008	0.20284
993	TRUE	Empty	Uncharacterized protein OS=Gallus gallus GN=ATP6V1B2 PE=3 SV=2	F1NID6_CHICK (+12)		56 kDa		unknown	3	4	1	0.2415	0.26552	0.065583
994	TRUE	Empty	status=active	UPI00001A2332 (+1)		21 kDa		unknown	2	2	2	0.45063	0.35938	0.39206
995	TRUE	Empty	status=active	UPI000024C519 (+2)		47 kDa		unknown	3	5	1	0.29026	0.41786	0.077873
996	TRUE	Empty	Asparagine synthetase [glutamine-hydrolyzing] OS=Gallus gallus GN=ASNS PE=2 SV=3	ASNS_CHICK (+6)		64 kDa		unknown	1	6	2	0.065564	0.43596	0.11738
997	TRUE	Empty	Uncharacterized protein OS=Gallus gallus GN=TRA2A PE=4 SV=2	F1NPM7_CHICK (+7)		32 kDa		unknown	3	2	2	0.44205	0.35255	0.3846
998	TRUE	Empty	Uncharacterized protein OS=Gallus gallus PE=3 SV=2	F1NWH0_CHICK (+3)		244 kDa		unknown	3	0	1	0.051788	0	0.014796
999	TRUE	Empty	Fatty acid-binding protein, brain OS=Gallus gallus GN=FABP7 PE=2 SV=2	FABP7_CHICK (+2)		15 kDa		unknown	0	4	4	0	1.3869	1.513
1000	TRUE	Empty	Uncharacterized protein OS=Gallus gallus GN=LOC768337 PE=3 SV=1	G1K338_CHICK (+3)		50 kDa	TRUE	unknown	0	1	3	0	25.709	7.5579
1001	TRUE	Empty	Interleukin enhancer binding factor 3-like protein OS=Gallus gallus GN=ILF3 PE=2 SV=1	I3VQH4_CHICK		95 kDa		unknown	0	4	5	0	0.18971	0.20696
1002	TRUE	Empty	Uncharacterized protein OS=Gallus gallus GN=NDUFV2 PE=4 SV=2	E1BUZ0_CHICK (+7)		27 kDa		unknown	0	3	4	0	0.43411	0.67741
1003	TRUE	Empty	Uncharacterized protein OS=Gallus gallus GN=PGM3 PE=3 SV=2	E1BQU2_CHICK (+5)		60 kDa		unknown	0	2	5	0	0.11554	0.42849
1004	TRUE	Empty	Uncharacterized protein (Fragment) OS=Gallus gallus GN=RAB32 PE=3 SV=2	F1NBL3_CHICK (+4)		25 kDa		unknown	0	3	2	0	0.47772	0.32239
1005	TRUE	Empty	Uncharacterized protein OS=Gallus gallus GN=PGRMC2 PE=2 SV=1	Q5ZLX0_CHICK (+4)		22 kDa		unknown	0	1	5	0	0.3469	1.2348
1006	TRUE	Empty	Calcineurin B homologous protein 1 OS=Gallus gallus GN=CHP1 PE=1 SV=3	CHP1_CHICK (+4)		22 kDa		unknown	0	2	2	0	0.54366	0.86142
1007	TRUE	Empty	Uncharacterized protein OS=Gallus gallus GN=GBAS PE=4 SV=1	F1NZ68_CHICK (+3)		33 kDa		unknown	0	2	4	0	0.2163	0.52833
1008	TRUE	Empty	status=active	UPI000240CAB4		11 kDa		unknown	3	0	2	2.7231	0	0.83302
1009	TRUE	Empty	Uncharacterized protein OS=Gallus gallus GN=ZP1 PE=4 SV=2	H9L0C5_CHICK (+5)		100 kDa		unknown	0	1	4	0	0.033411	0.15407
1010	TRUE	Empty	Uncharacterized protein OS=Gallus gallus GN=PGM5 PE=4 SV=2	F1NM36_CHICK (+3)		63 kDa		unknown	0	3	5	0	0.1699	0.32786
1011	TRUE	Empty	V-Ha-ras Harvey rat sarcoma viral oncogene-like protein OS=Gallus gallus GN=H-RAS PE=2 SV=1	F5ANT1_CHICK (+5)		21 kDa		unknown	0	1	5	0	0.84382	2.1397
1012	TRUE	Empty	status=active	UPI000060329B		60 kDa		unknown	0	6	1	0	0.47209	0.061016
1013	TRUE	Empty	Tyrosine-protein kinase CSK OS=Gallus gallus GN=CSK PE=4 SV=2	F1NMH8_CHICK (+8)		59 kDa		unknown	0	4	3	0	0.2484	0.19696
1014	TRUE	Empty	Mitogen-activated protein kinase OS=Gallus gallus PE=2 SV=1	Q8UWG6_CHICK (+4)		42 kDa	TRUE	unknown	0	4	6	0	0.36741	0.65866
1015	TRUE	Empty	Uncharacterized protein OS=Gallus gallus GN=GMPS PE=4 SV=2	F1NAQ8_CHICK (+6)		84 kDa		unknown	0	6	2	0	0.26796	0.089146
1016	TRUE	Empty	Serpin peptidase inhibitor, clade A (Alpha-1 antiproteinase, antitrypsin), member 1, isoform CRA_a OS=Homo sapiens GN=SERPINA1 PE=3 SV=1	A0A024R6I7 (+2)		?		unknown	0	0	7	0	0	-1
1017	TRUE	Empty	D-beta-hydroxybutyrate dehydrogenase, mitochondrial OS=Gallus gallus GN=BDH1 PE=2 SV=1	BDH_CHICK (+5)		38 kDa	TRUE	unknown	0	6	0	0	0.83555	0
1018	TRUE	Empty	Uncharacterized protein (Fragment) OS=Gallus gallus GN=HEXB PE=4 SV=2	F1NTQ2_CHICK (+2)		63 kDa	TRUE	unknown	0	5	0	0	0.29696	0
1019	TRUE	Empty	Glutamine--fructose-6-phosphate aminotransferase [isomerizing] 1 OS=Homo sapiens GN=GFPT1 PE=1 SV=3	Q06210 (+1)		?		unknown	0	0	6	0	0	-1
1020	TRUE	Empty	status=inactive	UPI00004493FE (+4)		190 kDa	TRUE	unknown	5	0	0	0.11351	0	0
1021	TRUE	Empty	status=inactive	UPI0000ECB0BB		105 kDa	TRUE	unknown	0	5	2	0	3.796	4.6974
1022	TRUE	Empty	Structural maintenance of chromosomes protein OS=Gallus gallus GN=SMC2 PE=3 SV=2	E1C612_CHICK (+3)		135 kDa		unknown	7	0	0	0.23465	0	0
1023	TRUE	Empty	Uncharacterized protein OS=Gallus gallus GN=CFH PE=4 SV=2	E1C7P4_CHICK (+2)		148 kDa	TRUE	unknown	7	0	0	0.21252	0	0
1024	TRUE	Empty	Uncharacterized protein OS=Gallus gallus GN=RCJMB04_14f6 PE=2 SV=1	Q5F3L2_CHICK (+7)		46 kDa		unknown	0	4	1	0	0.43603	0.16818
1025	TRUE	Empty	status=inactive	UPI0000ECC710		57 kDa	TRUE	unknown	0	0	6	0	0	0.54293
1026	TRUE	Empty	Uncharacterized protein (Fragment) OS=Gallus gallus GN=IPO5 PE=4 SV=2	F1P064_CHICK (+2)		125 kDa	TRUE	unknown	0	0	8	0	0	0.29368
1027	TRUE	Empty	Heat shock cognate 71 kDa protein OS=Homo sapiens GN=HSPA8 PE=1 SV=1	E9PKE3 (+5)		?		unknown	0	0	4	0	0	-1
1028	TRUE	Empty	Uncharacterized protein OS=Gallus gallus GN=VPS26A PE=4 SV=1	E1BU33_CHICK (+4)		38 kDa		unknown	3	2	3	0.36952	0.1871	0.32149
1029	TRUE	Empty	Uncharacterized protein OS=Gallus gallus GN=BROX PE=4 SV=1	E1BY06_CHICK (+2)		47 kDa		unknown	3	2	1	0.29484	0.23514	0.079012
1030	TRUE	Empty	Protein-L-isoaspartate(D-aspartate) O-methyltransferase OS=Gallus gallus GN=PCMT1 PE=2 SV=3	PIMT_CHICK (+7)		25 kDa		unknown	2	3	1	0.37613	0.48541	0.15193
1031	TRUE	Empty	Uncharacterized protein OS=Gallus gallus GN=TMX4 PE=4 SV=1	R4GFY2_CHICK (+5)		35 kDa		unknown	2	2	1	0.25286	0.20166	0.10447
1032	TRUE	Empty	Uncharacterized protein OS=Gallus gallus GN=AIMP1 PE=4 SV=2	F1NLE7_CHICK (+9)		36 kDa		unknown	2	2	3	0.24866	0.19831	0.34169
1033	TRUE	Empty	Uncharacterized protein OS=Gallus gallus GN=ETF1 PE=4 SV=1	E1BTM1_CHICK (+6)		49 kDa		unknown	2	4	1	0.17894	0.30792	0.074991
1034	TRUE	Empty	Uncharacterized protein OS=Gallus gallus GN=HNRNPH3 PE=2 SV=1	Q5F3D2_CHICK (+4)		37 kDa	TRUE	unknown	2	2	4	0.38468	0.30679	0.46979
1035	TRUE	Empty	Uncharacterized protein OS=Gallus gallus GN=NHP2 PE=4 SV=2	F1NNP9_CHICK (+4)		17 kDa		unknown	1	3	3	0.26329	0.78744	0.85903
1036	TRUE	Empty	Mannose-6-phosphate isomerase (Fragment) OS=Gallus gallus GN=MPI PE=3 SV=2	F1NIE1_CHICK (+5)		46 kDa		unknown	4	1	1	0.54203	0.073502	0.080184
1037	TRUE	Empty	Uncharacterized protein OS=Gallus gallus GN=TSSC1 PE=4 SV=1	F1NN26_CHICK (+6)		44 kDa		unknown	2	2	1	0.20166	0.25166	0.084138
1038	TRUE	Empty	Uncharacterized protein OS=Gallus gallus GN=COPS7A PE=2 SV=1	Q5ZJA1_CHICK (+4)		30 kDa		unknown	6	2	1	1.1506	0.23786	0.12218
1039	TRUE	Empty	Uncharacterized protein OS=Gallus gallus GN=ACTR1A PE=2 SV=1	Q5ZM58_CHICK (+4)		43 kDa		unknown	2	3	3	0.20726	0.25894	0.28248
1040	TRUE	Empty	Sodium/potassium-transporting ATPase subunit beta-1 OS=Gallus gallus GN=ATP1B1 PE=1 SV=1	AT1B1_CHICK (+1)		35 kDa	TRUE	unknown	0	6	1	0	0.76231	1.0266
1041	TRUE	Empty	Sodium/potassium-transporting ATPase subunit beta-3 OS=Gallus gallus GN=ATP1B3 PE=2 SV=1	AT1B3_CHICK (+2)		32 kDa		unknown	0	3	4	0	0.35959	0.55506
1042	TRUE	Empty	Uncharacterized protein (Fragment) OS=Gallus gallus GN=C1QBP PE=4 SV=2	F1NJF0_CHICK (+5)		24 kDa		unknown	0	3	3	0	0.51291	0.55954
1043	TRUE	Empty	Uncharacterized protein OS=Gallus gallus GN=PMPCA PE=2 SV=1	Q5ZJ49_CHICK (+3)		58 kDa	TRUE	unknown	0	3	4	0	0.18657	0.28031
1044	TRUE	Empty	Uncharacterized protein OS=Gallus gallus GN=LCP1 PE=2 SV=1	Q5ZLW0_CHICK		70 kDa	TRUE	unknown	0	4	2	0	0.53573	0.43014
1045	TRUE	Empty	status=inactive	UPI000240CCDE (+1)		40 kDa		unknown	0	4	3	0	0.39394	0.30735
1046	TRUE	Empty	Reticulon (Fragment) OS=Gallus gallus GN=RTN3 PE=2 SV=1	Q6IFY8_CHICK		22 kDa		unknown	0	3	3	0	0.557	0.60764
1047	TRUE	Empty	Uncharacterized protein OS=Gallus gallus GN=BDH2 PE=4 SV=2	F1NAM7_CHICK (+3)		27 kDa		unknown	0	4	2	0	0.62736	0.29739
1048	TRUE	Empty	Uncharacterized protein OS=Gallus gallus GN=CYB5B PE=3 SV=1	F1NSI3_CHICK (+4)		16 kDa		unknown	0	3	4	0	0.81532	1.8927
1049	TRUE	Empty	Uncharacterized protein OS=Gallus gallus GN=NDUFB8 PE=2 SV=1	Q5ZJW2_CHICK (+4)		22 kDa		unknown	0	2	3	0	0.557	0.60764
1050	TRUE	Empty	Uncharacterized protein OS=Gallus gallus GN=COMMD7 PE=4 SV=1	E1BU89_CHICK (+2)		22 kDa		unknown	0	2	3	0	0.33714	0.60027
1051	TRUE	Empty	status=inactive	UPI0000E81CCD		8 kDa		unknown	3	2	0	2.9903	1.2345	0
1052	TRUE	Empty	Uncharacterized protein OS=Gallus gallus GN=DCTN3 PE=2 SV=1	Q5ZK19_CHICK (+1)		21 kDa		unknown	0	2	3	0	0.35724	0.63896
1053	TRUE	Empty	Uncharacterized protein OS=Gallus gallus GN=APEH PE=4 SV=2	F1N8I0_CHICK (+1)		81 kDa		unknown	4	3	0	0.22051	0.12892	0
1054	TRUE	Empty	mRNA cap guanine-N7 methyltransferase OS=Gallus gallus GN=RNMT PE=3 SV=1	E1BYM1_CHICK (+1)		48 kDa		unknown	0	3	2	0	0.31857	0.16069
1055	TRUE	Empty	Heterochromatin protein 1-binding protein 3 OS=Gallus gallus GN=HP1BP3 PE=3 SV=1	F1N8H6_CHICK (+4)		62 kDa		unknown	2	3	0	0.13998	0.17252	0
1056	TRUE	Empty	CAST OS=Gallus gallus GN=CAST PE=2 SV=1	B6V3I0_CHICK (+1)		81 kDa	TRUE	unknown	5	3	0	0.28161	0.12874	0
1057	TRUE	Empty	Uncharacterized protein OS=Gallus gallus GN=UROS PE=4 SV=1	E1BUH1_CHICK (+4)		29 kDa		unknown	5	0	1	1.2103	0	0.12696
1058	TRUE	Empty	Uncharacterized protein OS=Gallus gallus GN=PRPSAP1 PE=4 SV=1	E1BYN9_CHICK (+8)		42 kDa	TRUE	unknown	6	0	1	0.74715	0	0.086843
1059	TRUE	Empty	Uncharacterized protein OS=Gallus gallus GN=SSR1 PE=4 SV=1	F1P4F4_CHICK (+6)		32 kDa		unknown	0	3	1	0	0.35533	0.11523
1060	TRUE	Empty	Uncharacterized protein OS=Gallus gallus GN=RPL21 PE=4 SV=1	R4GIQ2_CHICK (+3)		19 kDa		unknown	0	3	1	0	1.8964	0.20456
1061	TRUE	Empty	Alpha-1,4 glucan phosphorylase OS=Gallus gallus GN=PYGB PE=3 SV=2	E1BSN7_CHICK (+9)		97 kDa		unknown	0	1	6	0	0.070227	0.24814
1062	TRUE	Empty	Uncharacterized protein OS=Gallus gallus GN=HNMT PE=4 SV=1	E1C378_CHICK (+3)		33 kDa		unknown	0	1	6	0	0.10343	0.90259
1063	TRUE	Empty	Uncharacterized protein OS=Gallus gallus PE=3 SV=2	F1NQH8_CHICK (+1)		50 kDa	TRUE	unknown	0	4	4	0	0.3948	0.33165
1064	TRUE	Empty	Uncharacterized protein OS=Gallus gallus GN=RCJMB04_11e7 PE=2 SV=1	Q5F3P0_CHICK (+1)		56 kDa	TRUE	unknown	5	1	0	0.43143	0.06024	0
1065	TRUE	Empty	Uncharacterized protein (Fragment) OS=Gallus gallus GN=TM9SF3 PE=4 SV=2	F1NRG5_CHICK (+6)		68 kDa		unknown	0	3	2	0	0.15541	0.11002
1066	TRUE	Empty	status=active	UPI0002C87A20 (+2)		77 kDa		unknown	0	1	2	0	0.043678	0.097599
1067	TRUE	Empty	Aldehyde dehydrogenase OS=Gallus gallus GN=ALDH3A2 PE=3 SV=1	F1NH33_CHICK (+1)		54 kDa	TRUE	unknown	0	4	5	0	0.35634	0.58417
1068	TRUE	Empty	Unconventional myosin-VI OS=Gallus gallus GN=MYO6 PE=4 SV=1	F1NWJ9_CHICK (+8)		148 kDa	TRUE	unknown	2	0	5	0.057132	0	0.129

1069	TRUE	Empty	status=inactive	UPI0000E7F8DD (+11)		80 kDa		unknown	3	3	1	0.16403	0.13081	0.045444
1070	TRUE	Empty	cDNA FLJ78440, highly similar to Human lactoferrin OS=Homo sapiens PE=2 SV=1	A8K494 (+11)		?		unknown	0	0	6	0	0	-1
1071	TRUE	Empty	DNA damage-binding protein 1 OS=Gallus gallus GN=DDB1 PE=2 SV=1	DDB1_CHICK (+6)		127 kDa	TRUE	unknown	6	0	0	0.21188	0	0
1072	TRUE	Empty	Golgi apparatus protein 1 (Fragment) OS=Gallus gallus GN=GLG1 PE=4 SV=1	F1N8B2_CHICK (+1)		119 kDa	TRUE	unknown	0	0	7	0	0	0.2342
1073	TRUE	Empty	Pyrroline-5-carboxylate reductase OS=Gallus gallus GN=PYCR2 PE=3 SV=2	F1N914_CHICK		33 kDa		unknown	0	6	0	0	1.0111	0
1074	TRUE	Empty	4F2 cell-surface antigen heavy chain OS=Homo sapiens GN=SLC3A2 PE=1 SV=1	F5GZS6 (+5)		?		unknown	0	0	7	0	0	-1
1075	TRUE	Empty	EMILIN-1 OS=Homo sapiens GN=EMILIN1 PE=1 SV=2	Q9Y6C2		?		unknown	0	0	4	0	0	-1
1076	TRUE	Empty	Heterogeneous nuclear ribonucleoprotein D-like OS=Gallus gallus GN=HNRNPDL PE=2 SV=1	HNRDL_CHICK (+7)		33 kDa		unknown	5	0	0	0.79759	0	0
1077	TRUE	Empty	Uncharacterized protein OS=Gallus gallus GN=LOC100858849 PE=4 SV=1	R4GKJ2_CHICK		27 kDa		unknown	0	2	2	0	0.85234	0.48286
1078	TRUE	Empty	Riboflavin-binding protein OS=Gallus gallus PE=1 SV=2	RBP_CHICK		27 kDa	TRUE	unknown	0	3	0	0	0.43203	0
1079	TRUE	Empty	status=inactive	UPI0000ECA060 (+4)		50 kDa		unknown	0	0	4	0	0	0.42181
1080	TRUE	Empty	X-ray repair complementing defective repair in Chinese hamster cells 6 (Ku autoantigen, 70kDa), isoform CRA_a OS=Homo sapiens GN=XRCC6 PE=4 SV=1	A0A024R1N4 (+4)		?		unknown	0	0	6	0	0	-1
1081	TRUE	Empty	Uncharacterized protein (Fragment) OS=Gallus gallus GN=CHTF18 PE=4 SV=2	F1P1U8_CHICK		109 kDa	TRUE	unknown	0	0	2	0	0	0.10376
1082	TRUE	Empty	Caspase-3 OS=Gallus gallus GN=CASP3 PE=2 SV=1	O93417_CHICK (+1)		32 kDa		unknown	3	3	0	0.45453	0.36249	0
1083	TRUE	Empty	Uncharacterized protein OS=Gallus gallus GN=LOC422305 PE=4 SV=1	E1C2F4_CHICK		24 kDa	TRUE	unknown	0	3	1	0	0.71579	0.33394
1084	TRUE	Empty	Uncharacterized protein OS=Gallus gallus GN=Gga.849 PE=4 SV=1	R9PXM5_CHICK		24 kDa	TRUE	unknown	3	0	0	0.61861	0	0
1085	TRUE	Empty	Adenylsuccinate lyase OS=Gallus gallus GN=ADSL PE=2 SV=1	Q5U7A3_CHICK (+1)		55 kDa		unknown	4	3	0	0.3405	0.19685	0
1086	TRUE	Empty	status=active	UPI00003AFDE5 (+2)		187 kDa	TRUE	unknown	0	0	7	0	0	0.23477
1087	TRUE	Empty	status=inactive	UPI00004478D0		80 kDa	TRUE	unknown	0	0	5	0	0	0.30846
1088	TRUE	Empty	Tropomyosin alpha-1 chain OS=Gallus gallus GN=TPM1 PE=1 SV=2	sp|P04268|TPM1_CHICK (+3)		33 kDa	TRUE	unknown	0	3	3	0	16.076	27.616
1089	TRUE	Empty	Uncharacterized protein OS=Gallus gallus GN=FAM188A PE=4 SV=1	E1BU55_CHICK (+1)		50 kDa		unknown	3	1	2	0.27131	0.067035	0.15168
1090	TRUE	Empty	Uncharacterized protein OS=Gallus gallus GN=F13A1 PE=4 SV=2	E1BWG1_CHICK (+3)		84 kDa		unknown	4	2	1	0.21357	0.081493	0.043493
1091	TRUE	Empty	Uncharacterized protein OS=Gallus gallus GN=GCLM PE=2 SV=1	Q5ZL03_CHICK (+2)		30 kDa		unknown	1	2	3	0.1415	0.23977	0.4173
1092	TRUE	Empty	Uncharacterized protein OS=Gallus gallus GN=PEF1 PE=4 SV=2	H9KZ63_CHICK (+6)		24 kDa		unknown	1	2	2	0.17711	0.30455	0.33224
1093	TRUE	Empty	Proteasome subunit alpha type OS=Gallus gallus GN=PSMA2 PE=3 SV=1	E1C006_CHICK (+3)		26 kDa		unknown	2	2	1	0.35646	0.28428	0.14449
1094	TRUE	Empty	Uncharacterized protein OS=Gallus gallus GN=RPS23 PE=3 SV=2	F1NDC2_CHICK (+8)		16 kDa		unknown	1	1	2	0.27901	0.22251	0.54519
1095	TRUE	Empty	Uncharacterized protein OS=Gallus gallus GN=PPM1B PE=2 SV=1	Q5F397_CHICK (+3)		43 kDa	TRUE	unknown	3	1	2	0.32375	0.078971	0.17982
1096	TRUE	Empty	Uncharacterized protein OS=Gallus gallus GN=SPR PE=4 SV=1	E1C4L3_CHICK (+2)		29 kDa		unknown	0	4	4	0	0.57397	0.62615
1097	TRUE	Empty	Uncharacterized protein OS=Gallus gallus GN=IDH3A PE=3 SV=2	F1NFD9_CHICK (+9)		40 kDa		unknown	0	4	4	0	0.3901	0.42557
1098	TRUE	Empty	status=active	UPI0000112EB9 (+4)		18 kDa		unknown	0	3	3	0	1.0604	0.7789
1099	TRUE	Empty	Uncharacterized protein OS=Gallus gallus GN=TFAM PE=4 SV=1	F1NG46_CHICK (+7)		30 kDa		unknown	0	5	2	0	0.93612	0.26369
1100	TRUE	Empty	LDLR chaperone MESD OS=Gallus gallus GN=MESDC2 PE=4 SV=1	F1P222_CHICK (+8)		25 kDa		unknown	0	5	2	0	0.9349	0.32399
1101	TRUE	Empty	Putative uncharacterized protein OS=Gallus gallus GN=RCJMB04_16d21 PE=2 SV=1	Q5F3I2_CHICK (+5)		89 kDa		unknown	0	3	2	0	0.1177	0.083844
1102	TRUE	Empty	Uncharacterized protein OS=Gallus gallus GN=ECH1 PE=4 SV=1	R4GLH7_CHICK (+2)		17 kDa		unknown	0	2	3	0	0.45114	0.82377
1103	TRUE	Empty	Uncharacterized protein (Fragment) OS=Gallus gallus GN=GCN1L1 PE=4 SV=2	F1NAK4_CHICK (+7)		294 kDa		unknown	0	4	4	0	0.045921	0.050096
1104	TRUE	Empty	Uncharacterized protein OS=Gallus gallus GN=LYPLA2 PE=4 SV=1	E1BRI5_CHICK (+3)		25 kDa	TRUE	unknown	0	2	2	0	0.29407	0.51841
1105	TRUE	Empty	Uncharacterized protein (Fragment) OS=Gallus gallus GN=ALYREF PE=4 SV=2	E1C2L5_CHICK (+7)		28 kDa		unknown	2	2	0	0.52659	0.26186	0
1106	TRUE	Empty	Uncharacterized protein OS=Gallus gallus GN=RAB5A PE=2 SV=1	Q5ZIP7_CHICK (+1)		24 kDa	TRUE	unknown	0	2	1	0	2.092	1.4363
1107	TRUE	Empty	status=inactive	UPI0003506AF0 (+5)		68 kDa		unknown	0	2	2	0	0.10051	0.10964
1108	TRUE	Empty	Glutathione S-transferase OS=Gallus gallus PE=2 SV=2	GSTA2_CHICK (+4)		25 kDa	TRUE	unknown	0	3	3	0	0.46784	0.51037
1109	TRUE	Empty	status=inactive	UPI000061279B (+7)		46 kDa		unknown	0	3	2	0	0.23576	0.16477
1110	TRUE	Empty	status=active	UPI000017122E		16 kDa	TRUE	unknown	3	2	0	177.21	23.586	0
1111	TRUE	Empty	Glutathione S-transferase (Fragment) OS=Gallus gallus PE=2 SV=1	Q9W6J3_CHICK (+2)		21 kDa	TRUE	unknown	0	1	2	0	0.84382	1.2621
1112	TRUE	Empty	Uncharacterized protein OS=Gallus gallus GN=LRRC15 PE=4 SV=2	F1N822_CHICK (+5)		62 kDa		unknown	0	3	3	0	0.17153	0.18712
1113	TRUE	Empty	Leucine zipper protein OS=Gallus gallus GN=PTRF PE=2 SV=1	Q90838_CHICK (+5)		41 kDa		unknown	0	1	4	0	0.081796	0.40832
1114	TRUE	Empty	Cytochrome c OS=Gallus gallus GN=CYC PE=1 SV=2	CYC_CHICK (+5)		12 kDa		unknown	0	1	4	0	0.30722	2.1911
1115	TRUE	Empty	Uncharacterized protein OS=Gallus gallus GN=ERGIC1 PE=4 SV=2	E1C2I3_CHICK (+7)		33 kDa		unknown	0	3	4	0	0.35117	0.54137
1116	TRUE	Empty	Uncharacterized protein (Fragment) OS=Gallus gallus GN=GMPPA PE=4 SV=2	F1NYN9_CHICK (+3)		39 kDa	TRUE	unknown	0	2	2	0	0.28715	0.19911
1117	TRUE	Empty	Synaptogyrin OS=Gallus gallus GN=RCJMB04_3c18 PE=2 SV=1	Q5ZM48_CHICK (+1)		25 kDa		unknown	0	1	2	0	0.13671	0.32081
1118	TRUE	Empty	Protein S100-A11 OS=Gallus gallus GN=S100A11 PE=1 SV=2	S10AB_CHICK (+2)		11 kDa		unknown	0	2	2	0	1.3049	2.2592
1119	TRUE	Empty	Histone H2A.V OS=Gallus gallus GN=H2AFV PE=1 SV=2	H2AV_CHICK (+11)		14 kDa	TRUE	unknown	2	3	0	2.9033	2.3154	0
1120	TRUE	Empty	Uncharacterized protein OS=Gallus gallus GN=CTBP1 PE=2 SV=1	Q5ZIZ6_CHICK (+6)		47 kDa	TRUE	unknown	6	1	0	0.66242	0.072076	0
1121	TRUE	Empty	status=active	UPI00003B0054 (+10)		151 kDa		unknown	4	3	0	0.11401	0.067369	0
1122	TRUE	Empty	Uncharacterized protein OS=Gallus gallus GN=RAB8B PE=3 SV=2	E1C080_CHICK (+8)		24 kDa	TRUE	unknown	2	3	1	1.2636	1.0077	1.4363
1123	TRUE	Empty	Uncharacterized protein OS=Gallus gallus GN=GPS1 PE=4 SV=1	F1NB10_CHICK (+8)		55 kDa		unknown	5	2	0	0.43644	0.12581	0
1124	TRUE	Empty	Protein argonaute-2 OS=Gallus gallus GN=LOC100858102 PE=3 SV=2	F1NL39_CHICK (+11)		98 kDa	TRUE	unknown	4	1	0	0.18073	0.034062	0
1125	TRUE	Empty	Uncharacterized protein OS=Gallus gallus GN=RPS10 PE=4 SV=2	E1C4N0_CHICK (+4)		19 kDa	TRUE	unknown	0	4	3	0	0.9928	0.73315
1126	TRUE	Empty	Uncharacterized protein OS=Gallus gallus GN=POLQ PE=4 SV=2	F1NMU1_CHICK (+10)		290 kDa		unknown	0	3	1	0	0.034693	0.012458
1127	TRUE	Empty	Uncharacterized protein OS=Gallus gallus GN=KCTD16 PE=4 SV=1	E1C3V1_CHICK (+3)		49 kDa		unknown	0	3	3	0	0.2234	0.24371
1128	TRUE	Empty	S-methyl-5'-thioadenosine phosphorylase OS=Gallus gallus GN=MTAP PE=3 SV=2	F1NCV7_CHICK (+5)		31 kDa		unknown	0	3	4	0	0.37301	0.57692
1129	TRUE	Empty	Uncharacterized protein OS=Gallus gallus GN=NUP205 PE=4 SV=2	E1BV83_CHICK (+5)		228 kDa		unknown	7	1	0	0.13366	0.01451	0
1130	TRUE	Empty	Uncharacterized protein OS=Gallus gallus GN=TMED5 PE=2 SV=1	Q5ZMT4_CHICK (+2)		26 kDa		unknown	0	2	3	0	0.28701	0.50514
1131	TRUE	Empty	Eukaryotic translation initiation factor 3 subunit L OS=Gallus gallus GN=EIF3L PE=2 SV=1	EIF3L_CHICK (+4)		67 kDa		unknown	1	6	0	0.063013	0.34663	0
1132	TRUE	Empty	status=active	UPI0003504D4A (+6)		45 kDa		unknown	4	0	2	0.55641	0	0.17083
1133	TRUE	Empty	Poly (ADP-ribose) polymerase family, member 1, isoform CRA_a OS=Homo sapiens GN=PARP1 PE=4 SV=1	A0A024R3T8 (+2)		?		unknown	0	0	6	0	0	-1
1134	TRUE	Empty	cDNA FLJ51525, highly similar to Homo sapiens aldehyde dehydrogenase 16 family, member A1 (ALDH16A1), mRNA OS=Homo sapiens PE=2 SV=1	B4DQ15 (+3)		?		unknown	0	0	4	0	0	-1
1135	TRUE	Empty	Uncharacterized protein OS=Gallus gallus GN=SERPINB5 PE=3 SV=2	E1BTE2_CHICK (+3)		43 kDa		unknown	0	0	6	0	0	0.78871
1136	TRUE	Empty	Uncharacterized protein OS=Gallus gallus GN=NAP1L1 PE=3 SV=1	E1BZS2_CHICK (+6)		45 kDa	TRUE	unknown	6	0	0	0.68848	0	0
1137	TRUE	Empty	Golgi apparatus protein 1 OS=Gallus gallus GN=GLG1 PE=1 SV=1	GSLG1_CHICK (+2)		130 kDa	TRUE	unknown	0	7	0	0	0.19538	0
1138	TRUE	Empty	Clathrin interactor 1 OS=Homo sapiens GN=CLINT1 PE=1 SV=1	Q14677 (+2)		?		unknown	0	0	3	0	0	-1
1139	TRUE	Empty	Myoferlin OS=Homo sapiens GN=MYOF PE=1 SV=1	Q9NZM1 (+2)		?		unknown	0	0	6	0	0	-1
1140	TRUE	Empty	status=inactive	UPI0000448544 (+1)		146 kDa	TRUE	unknown	0	6	0	0	0.17201	0
1141	TRUE	Empty	status=inactive	UPI000061289A (+6)		44 kDa		unknown	3	1	0	0.31205	0.076334	0
1142	TRUE	Empty	WW domain-binding protein 4 OS=Gallus gallus GN=WBP4 PE=2 SV=1	WBP4_CHICK (+2)		45 kDa		unknown	5	0	0	0.54833	0	0
1143	TRUE	Empty	status=active	UPI00003AD621 (+1)		67 kDa		unknown	0	5	2	0	0.27713	0.11134
1144	TRUE	Empty	status=active	UPI0000E80AAD (+8)		46 kDa		unknown	4	2	0	0.41377	0.15219	0
1145	TRUE	Empty	Uncharacterized protein OS=Gallus gallus GN=LOC100857247 PE=3 SV=2	F1NXR6_CHICK		51 kDa	TRUE	unknown	0	3	0	0	7.6631	0
1146	TRUE	Empty	Uncharacterized protein OS=Gallus gallus GN=VPS4B PE=2 SV=1	Q5ZMI9_CHICK (+7)		49 kDa	TRUE	unknown	0	3	2	0	0.22396	0.15681
1147	TRUE	Empty	status=active	UPI00003AB57E (+2)		34 kDa		unknown	0	0	4	0	0	0.67417
1148	TRUE	Empty	Cellular nucleic acid-binding protein OS=Gallus gallus GN=CNBP PE=2 SV=1	CNBP_CHICK		19 kDa	TRUE	unknown	0	0	5	0	0	1.9923
1149	TRUE	Empty	Uncharacterized protein OS=Gallus gallus GN=A2ML4 PE=4 SV=2	F1NK40_CHICK (+3)		163 kDa		unknown	6	0	0	0.16169	0	0
1150	TRUE	Empty	status=active	UPI00003AC141 (+4)		93 kDa		unknown	1	2	2	0.044871	0.072987	0.079622

1151	TRUE	Empty	status=inactive	UPI0000447E4F		150 kDa		unknown	7	0	1	0.20862	0	0.024092
1152	TRUE	Empty	status=active	UPI00003AEC75		169 kDa	TRUE	unknown	7	0	0	0.18381	0	0
1153	TRUE	Empty	Uncharacterized protein (Fragment) OS=Gallus gallus GN=RCJMB04_32h3 PE=2 SV=1	Q5ZHX0_CHICK		65 kDa	TRUE	unknown	0	3	0	0	0.16427	0
1154	TRUE	Empty	Uncharacterized protein OS=Gallus gallus GN=AKR1B1L PE=4 SV=2	F1NT56_CHICK (+4)		36 kDa	TRUE	unknown	0	1	2	0	0.73193	1.9391
1155	TRUE	Empty	Uncharacterized protein OS=Gallus gallus GN=SERPINB6 PE=3 SV=1	F1P1L8_CHICK (+1)		43 kDa	TRUE	unknown	0	2	3	0	0.35649	0.77819
1156	TRUE	Empty	status=inactive	UPI00004490F3 (+3)		105 kDa		unknown	0	0	6	0	0	0.22706
1157	TRUE	Empty	P60=60 kDa stress-related protein (Fragments) OS=Gallus gallus PE=1 SV=1	Q9PSQ5_CHICK (+1)		14 kDa		unknown	2	1	1	1.8599	0.96881	1.0569
1158	TRUE	Empty	Uncharacterized protein OS=Gallus gallus GN=Gga.7815 PE=4 SV=1	E1BVD1_CHICK (+4)		36 kDa	TRUE	unknown	1	2	2	0.93263	0.58691	0.64027
1159	TRUE	Empty	Uncharacterized protein OS=Gallus gallus GN=BUD31 PE=4 SV=1	E1BV47_CHICK (+5)		17 kDa		unknown	2	1	2	0.57428	0.20563	0.49964
1160	TRUE	Empty	Uncharacterized protein OS=Gallus gallus GN=NAP1L4 PE=3 SV=1	F1P1B7_CHICK (+8)		43 kDa	TRUE	unknown	2	3	1	0.58758	0.71813	0.085922
1161	TRUE	Empty	Uncharacterized protein OS=Gallus gallus GN=NONO PE=2 SV=1	Q5ZIZ5_CHICK (+3)		54 kDa		unknown	2	1	1	0.16116	0.062129	0.067778
1162	TRUE	Empty	Fowlicidin-1 OS=Gallus gallus GN=CATHL1 PE=2 SV=1	C4PFJ7_CHICK (+8)		16 kDa		unknown	1	1	4	0.2729	0.21764	1.913
1163	TRUE	Empty	Uncharacterized protein (Fragment) OS=Gallus gallus GN=PSMD10 PE=4 SV=2	F1P1R3_CHICK (+7)		24 kDa		unknown	3	2	1	0.89752	0.30611	0.15483
1164	TRUE	Empty	Lactoylglutathione lyase (Fragment) OS=Gallus gallus GN=GLO1 PE=3 SV=2	F1N9Q2_CHICK (+4)		21 kDa		unknown	2	2	1	0.44794	0.35724	0.17868
1165	TRUE	Empty	SAP18 OS=Gallus gallus GN=chSAP18 PE=1 SV=1	Q90ZH5_CHICK (+2)		18 kDa		unknown	1	1	3	0.24759	0.19746	0.79753
1166	TRUE	Empty	Uncharacterized protein OS=Gallus gallus GN=NAA15 PE=4 SV=2	F1NVR6_CHICK (+10)		95 kDa		unknown	1	2	4	0.043867	0.071323	0.16175
1167	TRUE	Empty	Uncharacterized protein OS=Gallus gallus GN=ENG PE=4 SV=2	E1BT59_CHICK (+7)		71 kDa		unknown	0	2	3	0	0.096902	0.16274
1168	TRUE	Empty	Microsomal glutathione S-transferase 1 OS=Gallus gallus GN=MGST1 PE=2 SV=1	B4X9P4_CHICK (+6)		17 kDa		unknown	0	3	3	0	0.75512	0.82377
1169	TRUE	Empty	status=active	UPI00003ACDCD (+8)		61 kDa		unknown	4	3	0	0.30143	0.17489	0
1170	TRUE	Empty	Uncharacterized protein OS=Gallus gallus GN=FAM82B PE=4 SV=2	F1NBF8_CHICK (+3)		34 kDa		unknown	0	3	4	0	0.32929	0.50598
1171	TRUE	Empty	Integrin alpha-8 OS=Gallus gallus GN=ITGA8 PE=3 SV=2	F1NSA4_CHICK (+5)		116 kDa		unknown	0	4	3	0	0.12013	0.09674
1172	TRUE	Empty	Uncharacterized protein OS=Gallus gallus GN=AASS PE=4 SV=2	E1BWX4_CHICK (+5)		103 kDa		unknown	0	3	3	0	0.10058	0.10972
1173	TRUE	Empty	Uncharacterized protein OS=Gallus gallus GN=ALDH4A1 PE=3 SV=1	E1C8Z8_CHICK (+4)		61 kDa		unknown	0	2	3	0	0.17627	0.19229
1174	TRUE	Empty	Uncharacterized protein (Fragment) OS=Gallus gallus GN=SLC25A1 PE=3 SV=1	F1P0C8_CHICK (+4)		35 kDa		unknown	0	3	2	0	0.3269	0.22533
1175	TRUE	Empty	Thymocyte nuclear protein 1 OS=Gallus gallus GN=THYN1 PE=1 SV=1	THYN1_CHICK (+2)		28 kDa		unknown	0	2	3	0	0.26071	0.45602
1176	TRUE	Empty	SH3 domain-binding glutamic acid-rich-like protein OS=Gallus gallus GN=RCJMB04_21c16 PE=2 SV=1	Q5F3C9_CHICK (+2)		13 kDa	TRUE	unknown	0	3	2	0	1.1195	0.70142
1177	TRUE	Empty	BH3-interacting domain death agonist OS=Gallus gallus GN=BID PE=2 SV=2	BID_CHICK (+4)		22 kDa		unknown	2	2	0	0.7115	0.3469	0
1178	TRUE	Empty	Uncharacterized protein OS=Gallus gallus GN=MARCKSL1 PE=2 SV=1	Q5ZKS0_CHICK (+1)		21 kDa		unknown	0	2	2	0	0.58196	0.38741
1179	TRUE	Empty	3-hydroxyisobutyrate dehydrogenase OS=Gallus gallus GN=HIBADH PE=2 SV=1	Q5ZLI9_CHICK (+3)		35 kDa		unknown	0	2	2	0	0.20235	0.22074
1180	TRUE	Empty	status=inactive	UPI000240C845		8 kDa		unknown	0	2	2	0	2.2781	1.296
1181	TRUE	Empty	Uncharacterized protein OS=Gallus gallus GN=TMOD3 PE=2 SV=1	Q5ZLY3_CHICK (+1)		39 kDa		unknown	0	3	3	0	0.28352	0.4326
1182	TRUE	Empty	Uncharacterized protein OS=Gallus gallus GN=EML4 PE=4 SV=2	F1NH66_CHICK (+13)		109 kDa		unknown	0	4	1	0	0.12801	0.0332
1183	TRUE	Empty	Trafficking protein particle complex subunit 5 OS=Gallus gallus GN=TRAPPC5 PE=2 SV=1	TPPC5_CHICK (+2)		21 kDa		unknown	0	1	4	0	0.1647	0.93984
1184	TRUE	Empty	Uncharacterized protein (Fragment) OS=Gallus gallus GN=PSMC5 PE=3 SV=1	F1NU79_CHICK (+5)		46 kDa	TRUE	unknown	0	2	1	0	0.23701	0.16561
1185	TRUE	Empty	GrpE protein homolog OS=Gallus gallus GN=GRPEL1 PE=2 SV=1	Q5ZHV6_CHICK (+2)		25 kDa		unknown	0	3	1	0	0.48026	0.15052
1186	TRUE	Empty	status=inactive	UPI0000ECC7D9 (+6)		113 kDa		unknown	0	6	1	0	0.19163	0.032114
1187	TRUE	Empty	Uncharacterized protein OS=Gallus gallus GN=FETUB PE=4 SV=2	F1NHT5_CHICK (+5)		44 kDa		unknown	4	0	1	0.43535	0	0.083488
1188	TRUE	Empty	status=active	UPI0002C872C6 (+7)		27 kDa		unknown	0	2	1	0	0.27261	0.29739
1189	TRUE	Empty	Uncharacterized protein OS=Gallus gallus GN=REEP5 PE=4 SV=1	E1BZN6_CHICK (+4)		21 kDa		unknown	0	1	3	0	0.16113	0.91427
1190	TRUE	Empty	Uncharacterized protein OS=Gallus gallus GN=RCJMB04_6k8 PE=2 SV=1	Q5ZLD5_CHICK (+2)		39 kDa		unknown	3	3	0	0.36354	0.28993	0
1191	TRUE	Empty	Uncharacterized protein OS=Gallus gallus GN=GHITM PE=4 SV=2	F1NJJ9_CHICK (+1)		37 kDa		unknown	0	2	2	0	0.19254	0.21005
1192	TRUE	Empty	Surfeit locus protein 4 OS=Gallus gallus GN=SURF4 PE=2 SV=1	SURF4_CHICK (+3)		31 kDa		unknown	0	1	2	0	0.11116	0.41033
1193	TRUE	Empty	Serine/threonine-protein phosphatase OS=Gallus gallus GN=PPP3CA PE=3 SV=2	F1NBZ7_CHICK (+12)		61 kDa	TRUE	unknown	0	2	4	0	0.17627	0.26438
1194	TRUE	Empty	Uncharacterized protein (Fragment) OS=Gallus gallus GN=Gga.55651 PE=4 SV=2	E1BW84_CHICK (+14)		123 kDa		unknown	0	4	1	0	0.11348	0.029592
1195	TRUE	Empty	Uncharacterized protein (Fragment) OS=Gallus gallus GN=ARHGDIB PE=4 SV=1	F1NLT8_CHICK (+3)		23 kDa		unknown	1	0	5	0.18594	0	1.1211
1196	TRUE	Empty	Serine/arginine-rich splicing factor 2 OS=Gallus gallus GN=SRSF2 PE=2 SV=1	SRSF2_CHICK (+6)		26 kDa		unknown	2	1	1	0.36162	0.13424	0.14644
1197	TRUE	Empty	Thy-1 membrane glycoprotein OS=Gallus gallus GN=THY1 PE=4 SV=2	E1C6J9_CHICK (+1)		18 kDa		unknown	0	2	2	0	0.42262	0.46105
1198	TRUE	Empty	Sulfurtransferase OS=Gallus gallus GN=TST PE=4 SV=2	F1NDP5_CHICK (+7)		33 kDa		unknown	0	4	1	0	0.49021	0.11283
1199	TRUE	Empty	Adenosylhomocysteinase OS=Gallus gallus GN=AHCYL1 PE=3 SV=2	F1NUT5_CHICK (+6)		54 kDa		unknown	0	3	3	0	0.20128	0.30313
1200	TRUE	Empty	Uncharacterized protein OS=Gallus gallus GN=AQP3 PE=3 SV=2	F1NWH5_CHICK (+5)		31 kDa		unknown	0	1	3	0	0.10872	0.567
1201	TRUE	Empty	status=inactive	UPI0000447986 (+6)		87 kDa		unknown	1	4	1	0.04818	0.16377	0.041917
1202	TRUE	Empty	Uncharacterized protein (Fragment) OS=Gallus gallus GN=TOP1 PE=4 SV=1	F1NPT1_CHICK (+5)		89 kDa		unknown	1	2	0	0.047203	0.076857	0
1203	TRUE	Empty	status=active	UPI00003AEC0C (+7)		65 kDa		unknown	4	1	1	0.28158	0.051563	0.05625
1204	TRUE	Empty	Uncharacterized protein OS=Gallus gallus GN=RCJMB04_25g18 PE=2 SV=1	Q5ZIK3_CHICK (+5)		22 kDa		unknown	1	3	0	0.19467	0.55025	0
1205	TRUE	Empty	Transcription elongation factor SPT5 OS=Gallus gallus GN=SUPT5H PE=3 SV=2	H9L0H1_CHICK (+3)		120 kDa	TRUE	unknown	6	0	0	0.22531	0	0
1206	TRUE	Empty	CD166 antigen OS=Gallus gallus GN=ALCAM PE=1 SV=1	CD166_CHICK (+4)		66 kDa		unknown	0	6	0	0	0.42283	0
1207	TRUE	Empty	Uncharacterized protein OS=Gallus gallus GN=KDELC1 PE=4 SV=2	E1BSM9_CHICK (+4)		51 kDa		unknown	0	6	0	0	0.46941	0
1208	TRUE	Empty	DBH-like monooxygenase protein 1 OS=Homo sapiens GN=MOXD1 PE=2 SV=1	Q6UVY6 (+1)		?		unknown	0	0	5	0	0	-1
1209	TRUE	Empty	Specifically androgen-regulated gene protein OS=Homo sapiens GN=SARG PE=1 SV=2	Q9BW04		?		unknown	0	0	5	0	0	-1
1210	TRUE	Empty	status=inactive	UPI0000E81507		124 kDa	TRUE	unknown	6	0	0	0.21786	0	0
1211	TRUE	Empty	G protein OS=Gallus gallus GN=Galpa i3-o PE=2 SV=1	Q90846_CHICK (+3)		40 kDa		unknown	0	3	2	0	0.63313	0.69069
1212	TRUE	Empty	status=active	UPI0003504F2A		20 kDa		unknown	0	0	2	0	0	0.69723
1213	TRUE	Empty	status=active	UPI0001E3061D (+3)		46 kDa	TRUE	unknown	3	0	0	0.53435	0	0
1214	TRUE	Empty	Ras-related protein Rab-8A OS=Gallus gallus GN=RAB8A PE=2 SV=1	RAB8A_CHICK (+1)		24 kDa	TRUE	unknown	0	0	4	0	0	1.8276
1215	TRUE	Empty	Histone H3 OS=Gallus gallus PE=3 SV=1	Q92068_CHICK (+2)		15 kDa	TRUE	unknown	3	0	2	12.257	0	0.94802
1216	TRUE	Empty	Uncharacterized protein OS=Gallus gallus GN=LAMA1 PE=4 SV=2	F1NM47_CHICK (+1)		340 kDa		unknown	0	4	2	0	0.039601	0.021369
1217	TRUE	Empty	status=active	UPI00003AD850		54 kDa		unknown	0	5	2	0	0.35634	0.14021
1218	TRUE	Empty	status=inactive	UPI000044889D (+1)		74 kDa	TRUE	unknown	1	0	2	1.7424	0	1.516
1219	TRUE	Empty	Uncharacterized protein OS=Gallus gallus GN=CTSZ PE=3 SV=1	E1C4M3_CHICK		34 kDa	TRUE	unknown	0	0	4	0	0	0.5159
1220	TRUE	Empty	Apoptosis inhibitor 5 OS=Gallus gallus GN=API5 PE=2 SV=1	API5_CHICK (+1)		59 kDa	TRUE	unknown	0	5	0	0	0.40243	0
1221	TRUE	Empty	status=active	UPI0001567B5E (+4)		523 kDa		unknown	0	2	4	0	0.012694	0.027891
1222	TRUE	Empty	status=active	UPI00003AB1A2		86 kDa	TRUE	unknown	0	4	0	0	0.21092	0
1223	TRUE	Empty	status=inactive	UPI0000449B36		54 kDa	TRUE	unknown	0	0	4	0	0	0.30384
1224	TRUE	Empty	Uncharacterized protein (Fragment) OS=Gallus gallus GN=SLC25A24 PE=3 SV=2	E1BW83_CHICK (+7)		52 kDa		unknown	2	2	2	0.16805	0.13403	0.14621
1225	TRUE	Empty	Uncharacterized protein (Fragment) OS=Gallus gallus GN=PTBP1 PE=4 SV=2	F1P5K0_CHICK (+9)		60 kDa		unknown	2	2	1	0.22367	0.11532	0.061016
1226	TRUE	Empty	Uncharacterized protein OS=Gallus gallus GN=PCNP PE=4 SV=1	F1NMQ3_CHICK (+4)		19 kDa		unknown	4	1	1	1.2253	0.18179	0.19832
1227	TRUE	Empty	Cytoplasmic aconitate hydratase OS=Gallus gallus GN=ACO1 PE=2 SV=1	ACOC_CHICK (+7)		98 kDa	TRUE	unknown	2	1	4	0.086826	0.033984	0.15686
1228	TRUE	Empty	Uncharacterized protein OS=Gallus gallus GN=NUDT1 PE=3 SV=1	F7BGT1_CHICK (+3)		18 kDa		unknown	1	1	2	0.2412	0.19236	0.46431
1229	TRUE	Empty	Hypoxanthine-guanine phosphoribosyltransferase OS=Gallus gallus GN=HPRT1 PE=2 SV=1	HPRT_CHICK (+7)		25 kDa		unknown	0	1	3	0	0.13927	0.52954
1230	TRUE	Empty	CNP2 OS=Gallus gallus GN=CNP PE=2 SV=1	O57389_CHICK (+3)		47 kDa		unknown	0	2	1	0	0.1484	0.077873
1231	TRUE	Empty	Uncharacterized protein OS=Gallus gallus GN=RCJMB04_17f18 PE=2 SV=1	Q5ZJL0_CHICK (+10)		21 kDa		unknown	0	2	2	0	0.35303	0.38513
1232	TRUE	Empty	Uncharacterized protein OS=Gallus gallus GN=BUB3 PE=4 SV=2	E1C9E5_CHICK (+9)		44 kDa		unknown	3	4	0	0.3138	0.34821	0

1233	TRUE	Empty	status=active	UPI00003AD067 (+2)		63 kDa	TRUE	unknown	0	4	1	0	0.44424	0.25308
1234	TRUE	Empty	Uncharacterized protein OS=Gallus gallus GN=ALDH5A1 PE=3 SV=2	E1BRI3_CHICK (+4)		58 kDa		unknown	0	4	2	0	0.2553	0.13061
1235	TRUE	Empty	Uncharacterized protein OS=Gallus gallus GN=PTGFRN PE=4 SV=2	F1NTD7_CHICK (+6)		100 kDa		unknown	0	2	3	0	0.068056	0.11343
1236	TRUE	Empty	Serine/threonine-protein phosphatase 2A catalytic subunit alpha isoform OS=Gallus gallus GN=PPP2CA PE=2 SV=1	PP2AA_CHICK (+8)		36 kDa		unknown	0	3	2	0	0.44662	0.21926
1237	TRUE	Empty	Uncharacterized protein OS=Gallus gallus GN=HMOX2 PE=4 SV=2	E1C7J0_CHICK (+4)		35 kDa		unknown	0	2	2	0	0.20235	0.22074
1238	TRUE	Empty	Uncharacterized protein OS=Gallus gallus GN=DIABLO PE=4 SV=2	F1NT58_CHICK (+8)		26 kDa		unknown	0	2	2	0	0.27897	0.30434
1239	TRUE	Empty	status=active	UPI0002C87503 (+9)		95 kDa		unknown	0	2	2	0	0.071237	0.11884
1240	TRUE	Empty	Raftlin OS=Gallus gallus GN=RFTN1 PE=4 SV=1	F1P375_CHICK (+6)		66 kDa		unknown	0	2	3	0	0.10494	0.24224
1241	TRUE	Empty	Alpha-actinin-4 (Fragment) OS=Gallus gallus GN=ACTN4 PE=4 SV=2	H9L0H3_CHICK (+1)		72 kDa	TRUE	unknown	0	2	2	0	3.6239	6.7839
1242	TRUE	Empty	Uncharacterized protein OS=Gallus gallus GN=DNM1L PE=3 SV=1	F1NPT0_CHICK (+11)		78 kDa		unknown	0	4	2	0	0.18486	0.096152
1243	TRUE	Empty	Uncharacterized protein OS=Gallus gallus GN=SUCLA2 PE=2 SV=1	Q5F3B9_CHICK (+1)		50 kDa	TRUE	unknown	0	4	3	0	0.30095	0.23719
1244	TRUE	Empty	Uncharacterized protein (Fragment) OS=Gallus gallus GN=ERI3 PE=4 SV=2	F1NBT2_CHICK (+7)		30 kDa		unknown	0	2	2	0	0.24369	0.26584
1245	TRUE	Empty	tr|F1NEG1|F1NEG1_CHICK-DECOY Uncharacterized protein OS=Gallus gallus GN=RAD50 PE=4...	tr|F1NEG1|F1NEG1_CHICK-DECOY (+1)		?		unknown	2	0	3	-1	0	-1
1246	TRUE	Empty	Uncharacterized protein OS=Gallus gallus GN=WARS PE=3 SV=1	E1C2Z5_CHICK (+1)		54 kDa		unknown	0	5	1	0	0.35976	0.068349
1247	TRUE	Empty	Uncharacterized protein OS=Gallus gallus GN=SAMM50 PE=4 SV=1	E1C8A2_CHICK (+6)		52 kDa		unknown	0	5	1	0	0.46221	0.070276
1248	TRUE	Empty	Uncharacterized protein OS=Gallus gallus GN=OVST PE=4 SV=2	F1NU63_CHICK (+9)		166 kDa		unknown	0	4	1	0	0.082528	0.021776
1249	TRUE	Empty	Membrane-associated progesterone receptor component 1 OS=Gallus gallus GN=PGRMC1 PE=2 SV=3	PGRC1_CHICK (+6)		21 kDa	TRUE	unknown	0	3	1	0	0.57826	0.38513
1250	TRUE	Empty	Basic leucine zipper and W2 domain-containing protein 1 OS=Gallus gallus GN=BZW1 PE=2 SV=1	BZW1_CHICK (+5)		48 kDa	TRUE	unknown	0	4	1	0	0.31522	0.076585
1251	TRUE	Empty	status=active	UPI00003A9FA1		22 kDa		unknown	0	3	2	0	0.56043	0.37411
1252	TRUE	Empty	Uncharacterized protein OS=Gallus gallus GN=UBA3 PE=4 SV=1	E1BT61_CHICK (+9)		52 kDa	TRUE	unknown	3	2	0	0.26304	0.13494	0
1253	TRUE	Empty	Ubiquitin carboxyl-terminal hydrolase (Fragment) OS=Gallus gallus GN=USP7 PE=3 SV=1	F1P4U4_CHICK (+7)		125 kDa		unknown	0	1	4	0	0.02652	0.12091
1254	TRUE	Empty	Uncharacterized protein OS=Gallus gallus GN=NUP133 PE=4 SV=2	F1NDV3_CHICK (+4)		132 kDa	TRUE	unknown	3	1	0	0.097773	0.025279	0
1255	TRUE	Empty	ADP-ribosylation factor 6 OS=Gallus gallus GN=ARF6 PE=2 SV=3	ARF6_CHICK (+6)		20 kDa		unknown	0	1	3	0	0.17233	1.373
1256	TRUE	Empty	Uncharacterized protein OS=Gallus gallus GN=OAT PE=3 SV=2	E1BRW0_CHICK (+5)		48 kDa		unknown	0	3	2	0	0.22565	0.15795
1257	TRUE	Empty	Ras-related protein Rab-10 OS=Gallus gallus GN=RAB10 PE=2 SV=1	RAB10_CHICK (+1)		23 kDa	TRUE	unknown	0	1	4	0	0.54042	1.9567
1258	TRUE	Empty	status=inactive	UPI000240CCEE (+1)		15 kDa		unknown	0	1	2	0	0.52586	0.97549
1259	TRUE	Empty	Uncharacterized protein OS=Gallus gallus GN=COL18A1 PE=4 SV=2	F1NW38_CHICK (+9)		137 kDa		unknown	0	3	1	0	0.074521	0.026387
1260	TRUE	Empty	Uncharacterized protein OS=Gallus gallus GN=RCJMB04_34p20 PE=2 SV=1	Q5F346_CHICK (+1)		16 kDa		unknown	0	1	2	0	0.22087	0.54071
1261	TRUE	Empty	status=inactive	UPI00004484D8 (+11)		126 kDa		unknown	1	2	2	0.033135	0.053624	0.058499
1262	TRUE	Empty	Cadherin-1 OS=Gallus gallus GN=CDH1 PE=1 SV=2	CADH1_CHICK (+8)		98 kDa		unknown	0	1	4	0	0.034101	0.15743
1263	TRUE	Empty	Argininosuccinate lyase (Fragment) OS=Gallus gallus GN=ASL2 PE=3 SV=1	F1NI02_CHICK (+8)		51 kDa		unknown	0	1	5	0	0.065993	0.41645
1264	TRUE	Empty	Signal peptidase complex subunit 3 OS=Gallus gallus GN=SPC22 PE=1 SV=1	SPCS3_CHICK (+1)		20 kDa		unknown	0	1	2	0	0.17133	0.40922
1265	TRUE	Empty	status=inactive	UPI0000ECC801 (+1)		37 kDa	TRUE	unknown	3	1	1	0.38077	0.091631	0.099961
1266	TRUE	Empty	Uncharacterized protein OS=Gallus gallus PE=4 SV=2	F1NBA6_CHICK (+8)		294 kDa		unknown	3	5	0	0.042851	0.057672	0
1267	TRUE	Empty	Uncharacterized protein OS=Gallus gallus GN=NDUFA9 PE=2 SV=1	Q5ZI00_CHICK		43 kDa		unknown	0	2	4	0	0.16348	0.3889
1268	TRUE	Empty	Uncharacterized protein OS=Gallus gallus GN=TLN2 PE=4 SV=2	E1BR45_CHICK		272 kDa	TRUE	unknown	7	0	1	0.30883	0	0.082478
1269	TRUE	Empty	Prolyl 4-hydroxylase subunit alpha-1 OS=Gallus gallus GN=P4HA1 PE=4 SV=2	E1BY52_CHICK (+13)		62 kDa		unknown	1	3	1	0.06833	0.17353	0.059449
1270	TRUE	Empty	Small ubiquitin-related modifier 1 OS=Gallus gallus GN=SUMO1 PE=3 SV=1	SUMO1_CHICK (+4)		12 kDa		unknown	1	0	3	0.38921	0	2.2247
1271	TRUE	Empty	status=active	UPI00003BEE4D		143 kDa	TRUE	unknown	2	0	3	0.22004	0	0.10522
1272	TRUE	Empty	status=active	UPI0000E8254D (+1)		23 kDa		unknown	0	0	4	0	0	0.8398
1273	TRUE	Empty	status=active	UPI00035039AD (+7)		185 kDa		unknown	1	3	1	0.022504	0.054917	0.019579
1274	TRUE	Empty	Myosin, heavy polypeptide 9, non-muscle, isoform CRA_a OS=Homo sapiens GN=MYH9 PE=4 SV=1	A0A024R1N1 (+1)		?		unknown	0	0	5	0	0	-1
1275	TRUE	Empty	Astacin-like metalloendopeptidase OS=Gallus gallus GN=ASTL PE=2 SV=1	ASTL_CHICK (+5)		46 kDa		unknown	0	3	0	0	0.23576	0
1276	TRUE	Empty	cDNA FLJ54349, highly similar to Vesicle-fusing ATPase (EC 3.6.4.6) OS=Homo sapiens PE=2 SV=1	B4DGR3 (+3)		?		unknown	0	0	5	0	0	-1
1277	TRUE	Empty	Uncharacterized protein (Fragment) OS=Gallus gallus GN=NEU2 PE=4 SV=2	E1BRY5_CHICK (+6)		43 kDa		unknown	0	0	3	0	0	0.28086
1278	TRUE	Empty	Uncharacterized protein OS=Gallus gallus GN=MPP6 PE=4 SV=2	E1BS31_CHICK (+5)		57 kDa	TRUE	unknown	5	0	0	0.4246	0	0
1279	TRUE	Empty	Uncharacterized protein OS=Gallus gallus GN=NCKAP1 PE=4 SV=2	E1C6G9_CHICK (+14)		129 kDa		unknown	0	0	4	0	0	0.11688
1280	TRUE	Empty	Uncharacterized protein OS=Gallus gallus GN=UPK3A PE=4 SV=2	E1C856_CHICK (+1)		29 kDa		unknown	0	4	0	0	0.76721	0
1281	TRUE	Empty	status=inactive	UPI0000E8128B (+1)		141 kDa		unknown	5	0	0	0.15557	0	0
1282	TRUE	Empty	status=active	UPI0003505B21		241 kDa	TRUE	unknown	2	0	0	2.081	0	0
1283	TRUE	Empty	status=active	UPI00035047B8 (+23)		78 kDa		unknown	0	1	4	0	0.042613	0.1995
1284	TRUE	Empty	cDNA FLJ53290, highly similar to Cytoplasmic dynein 1 intermediate chain 2 OS=Homo sapiens PE=2 SV=1	B4DPZ3 (+7)		?		unknown	0	0	3	0	0	-1
1285	TRUE	Empty	GMP reductase OS=Gallus gallus GN=GMPR PE=3 SV=2	F1NWY8_CHICK (+10)		34 kDa		unknown	2	0	0	0.26264	0	0
1286	TRUE	Empty	Structural maintenance of chromosomes protein OS=Gallus gallus GN=SMC4 PE=2 SV=1	Q8AWB9_CHICK (+1)		146 kDa	TRUE	unknown	6	0	0	0.18252	0	0
1287	TRUE	Empty	status=active	UPI0003503B87 (+5)		16 kDa		unknown	0	0	2	0	0	0.54071
1288	TRUE	Empty	status=active	UPI00003ADD26 (+3)		56 kDa		unknown	4	0	2	0.33517	0	0.13638
1289	TRUE	Empty	cDNA FLJ58517, highly similar to Calpain-2 catalytic subunit (EC 3.4.22.53) OS=Homo sapiens PE=2 SV=1	B4DN77 (+3)		?		unknown	0	0	4	0	0	-1
1290	TRUE	Empty	Leukocyte ribonuclease A-2 OS=Gallus gallus PE=2 SV=1	Q27J90_CHICK (+1)		16 kDa		unknown	0	0	4	0	0	1.364
1291	TRUE	Empty	status=inactive	UPI000060FA39		73 kDa	TRUE	unknown	4	1	0	0.24632	2.1847	0
1292	TRUE	Empty	status=active	UPI0003506F25 (+2)		420 kDa	TRUE	unknown	0	0	5	0	0	0.04373
1293	TRUE	Empty	Cellular nucleic acid binding protein OS=Gallus gallus GN=CNBP PE=2 SV=1	O57348_CHICK (+1)		19 kDa	TRUE	unknown	0	4	0	0	1.367	0
1294	TRUE	Empty	status=inactive	UPI00006113DE		107 kDa	TRUE	unknown	0	2	3	0	0.063612	0.18274
1295	TRUE	Empty	status=inactive	UPI0000ECC47F		103 kDa	TRUE	unknown	0	4	0	0	0.17316	0
1296	TRUE	Empty	Uncharacterized protein OS=Gallus gallus GN=RCJMB04_3f15 PE=2 SV=1	Q5ZM28_CHICK (+5)		22 kDa		unknown	1	2	1	0.19776	0.34293	1.2172
1297	TRUE	Empty	Uncharacterized protein OS=Gallus gallus GN=YKT6 PE=4 SV=1	E1BSV7_CHICK (+2)		22 kDa		unknown	1	1	2	0.19266	0.15365	0.59308
1298	TRUE	Empty	SUMO-conjugating enzyme UBC9 OS=Gallus gallus GN=UBE2I PE=1 SV=1	UBC9_CHICK (+4)		18 kDa		unknown	1	2	2	0.24276	0.42865	0.46762
1299	TRUE	Empty	Uncharacterized protein (Fragment) OS=Gallus gallus GN=PM5 PE=4 SV=1	F1NZW1_CHICK (+6)		133 kDa		unknown	2	1	2	0.063351	0.024918	0.055116
1300	TRUE	Empty	status=active	UPI0000439B94 (+3)		58 kDa		unknown	1	2	1	0.072449	0.11925	0.063033
1301	TRUE	Empty	status=inactive	UPI000060E777 (+9)		165 kDa	TRUE	unknown	3	1	2	0.077129	0.020056	0.044244
1302	TRUE	Empty	status=active	UPI000060F1BC (+6)		191 kDa	TRUE	unknown	0	2	2	0	0.41812	0.4838
1303	TRUE	Empty	status=inactive	UPI0000E81FC7 (+1)		28 kDa		unknown	0	2	3	0	0.25734	0.44977
1304	TRUE	Empty	Pyrroline-5-carboxylate reductase OS=Gallus gallus GN=TIGD5 PE=3 SV=2	E1BZG5_CHICK (+4)		30 kDa		unknown	0	2	2	0	0.2427	0.26476
1305	TRUE	Empty	Uncharacterized protein OS=Gallus gallus GN=NDUFB10 PE=4 SV=1	E1C6C9_CHICK (+2)		20 kDa		unknown	0	2	2	0	0.36821	0.40168
1306	TRUE	Empty	Uncharacterized protein (Fragment) OS=Gallus gallus GN=COQ4 PE=3 SV=2	F1NEN7_CHICK (+6)		31 kDa		unknown	0	2	2	0	0.23505	0.25642
1307	TRUE	Empty	Uncharacterized protein OS=Gallus gallus GN=PDAP1 PE=4 SV=2	F1NIH5_CHICK (+3)		21 kDa		unknown	3	0	2	0.75883	0	0.40168
1308	TRUE	Empty	Pyruvate carboxylase OS=Gallus gallus GN=PYC PE=2 SV=1	Q8JHF6_CHICK (+1)		127 kDa		unknown	0	2	2	0	0.053002	0.057821
1309	TRUE	Empty	Histone-binding protein RBBP7 OS=Gallus gallus GN=RBBP7 PE=1 SV=1	RBBP7_CHICK (+1)		48 kDa	TRUE	unknown	0	3	2	0	0.51626	0.34661
1310	TRUE	Empty	Protein RER1 OS=Gallus gallus GN=RER1 PE=2 SV=1	RER1_CHICK (+1)		23 kDa		unknown	0	2	2	0	0.52784	0.35385
1311	TRUE	Empty	Uncharacterized protein (Fragment) OS=Gallus gallus GN=NASP PE=4 SV=2	E1C9A0_CHICK (+6)		80 kDa		unknown	0	2	2	0	0.085232	0.092981
1312	TRUE	Empty	status=active	UPI0003505689 (+4)		31 kDa		unknown	0	2	2	0	0.23505	0.25642
1313	TRUE	Empty	status=active	UPI00001A3DBC		60 kDa		unknown	0	1	3	0	0.17732	0.19344
1314	TRUE	Empty	Uncharacterized protein OS=Gallus gallus GN=DPP7 PE=4 SV=1	E1BZ81_CHICK (+5)		55 kDa		unknown	0	3	2	0	0.19772	0.13901

1315	TRUE	Empty	status=active	UPI000011760E		62 kDa		unknown	0	4	1	0	0.23847	0.059449
1316	TRUE	Empty	Uncharacterized protein OS=Gallus gallus GN=COL11A1 PE=4 SV=2	F1P4K9_CHICK (+14)		166 kDa		unknown	0	3	1	0	0.061384	0.044153
1317	TRUE	Empty	Perilipin OS=Gallus gallus GN=LOC100857433 PE=3 SV=2	H9L1F4_CHICK (+3)		45 kDa		unknown	0	3	2	0	0.24279	0.1695
1318	TRUE	Empty	Uncharacterized protein OS=Gallus gallus GN=GFPT1 PE=2 SV=1	Q5ZIG5_CHICK (+11)		79 kDa	TRUE	unknown	0	1	2	0	0.086851	0.14548
1319	TRUE	Empty	Uncharacterized protein OS=Gallus gallus GN=RANBP1 PE=2 SV=1	Q5ZJ38_CHICK (+7)		24 kDa		unknown	4	1	0	0.89752	0.14192	0
1320	TRUE	Empty	Uncharacterized protein OS=Gallus gallus GN=CSTB PE=4 SV=2	F1NHH1_CHICK (+4)		11 kDa		unknown	0	1	3	0	0.32384	1.4845
1321	TRUE	Empty	status=inactive	UPI0000449355 (+5)		18 kDa		unknown	1	1	2	0.24924	0.19877	0.48133
1322	TRUE	Empty	status=inactive	UPI000240C759 (+4)		27 kDa		unknown	0	1	3	0	0.12789	0.48051
1323	TRUE	Empty	Uncharacterized protein (Fragment) OS=Gallus gallus GN=MEMO1 PE=3 SV=2	E1C6C0_CHICK (+3)		33 kDa		unknown	2	3	0	0.27023	0.34182	0
1324	TRUE	Empty	Uncharacterized protein OS=Gallus gallus GN=AKT1 PE=4 SV=1	F1NMS0_CHICK (+4)		56 kDa	TRUE	unknown	0	3	1	0	0.26611	0.065716
1325	TRUE	Empty	cAMP-dependent protein kinase type I-alpha regulatory subunit OS=Gallus gallus GN=PRKAR1A PE=2 SV=1	KAP0_CHICK (+6)		43 kDa		unknown	0	3	3	0	0.25452	0.27766
1326	TRUE	Empty	Protein Hikeshi OS=Gallus gallus GN=RCJMB04_13p7 PE=2 SV=2	HIKES_CHICK (+2)		22 kDa		unknown	0	2	2	0	0.34491	0.37626
1327	TRUE	Empty	Cytosolic purine 5'-nucleotidase OS=Gallus gallus GN=NT5C2 PE=2 SV=1	5NTC_CHICK (+4)		66 kDa		unknown	0	2	3	0	0.10457	0.24134
1328	TRUE	Empty	Uncharacterized protein OS=Gallus gallus PE=4 SV=1	R4GHI6_CHICK (+1)		23 kDa		unknown	0	2	1	0	0.52784	0.1634
1329	TRUE	Empty	Uncharacterized protein OS=Gallus gallus GN=LOC426301 PE=4 SV=2	F1P1V2_CHICK (+34)		50 kDa		unknown	1	2	1	0.083865	0.13871	0.15132
1330	TRUE	Empty	status=inactive	UPI00004491B2		236 kDa		unknown	0	2	3	0	0.028292	0.046657
1331	TRUE	Empty	Importin alpha 4 (Fragment) OS=Gallus gallus GN=KPNA3 PE=2 SV=1	C6KIB2_CHICK (+10)		55 kDa	TRUE	unknown	0	4	1	0	0.26911	0.066389
1332	TRUE	Empty	Tetraspanin OS=Gallus gallus GN=CD151 PE=2 SV=1	Q5ZII8_CHICK (+2)		29 kDa		unknown	0	1	2	0	0.11918	0.27715
1333	TRUE	Empty	Collectin-12 OS=Gallus gallus GN=COLEC12 PE=2 SV=1	COL12_CHICK (+9)		82 kDa		unknown	0	3	2	0	0.128	0.091023
1334	TRUE	Empty	status=active	UPI00003AB65B (+6)		119 kDa		unknown	0	4	0	0	0.14921	0
1335	TRUE	Empty	cDNA FLJ53289, highly similar to Ribosomal protein S6 kinase alpha-1 (EC 2.7.11.1) OS=Homo sapiens PE=2 SV=1	B7Z2K7 (+5)		?		unknown	0	0	3	0	0	-1
1336	TRUE	Empty	Uncharacterized protein (Fragment) OS=Gallus gallus GN=C4H4ORF27 PE=4 SV=2	F1NZ67_CHICK (+6)		40 kDa		unknown	4	0	0	0.49074	0	0
1337	TRUE	Empty	status=active	UPI000240BFFF (+3)		94 kDa	TRUE	unknown	0	5	0	0	0.19265	0
1338	TRUE	Empty	Liver histone H1e OS=Homo sapiens PE=2 SV=1	A3R0T7 (+6)		?		unknown	0	0	3	0	0	-1
1339	TRUE	Empty	Uncharacterized protein OS=Gallus gallus GN=IMPA1 PE=4 SV=1	R4GF79_CHICK (+6)		37 kDa		unknown	3	0	3	0.38337	0	0.33354
1340	TRUE	Empty	Cyclin-dependent kinase 1 OS=Gallus gallus GN=CDK1 PE=1 SV=1	CDK1_CHICK (+4)		35 kDa	TRUE	unknown	0	4	0	0	0.46027	0
1341	TRUE	Empty	Uncharacterized protein OS=Gallus gallus GN=LOC415661 PE=3 SV=2	F1N9C1_CHICK (+3)		28 kDa	TRUE	unknown	0	3	0	0	0.41996	0
1342	TRUE	Empty	Uncharacterized protein OS=Gallus gallus GN=ADIPOQ PE=4 SV=1	F1NRD4_CHICK (+6)		26 kDa		unknown	0	0	2	0	0	0.30292
1343	TRUE	Empty	Uncharacterized protein OS=Gallus gallus GN=RCJMB04_2i9 PE=2 SV=1	Q5ZMD0_CHICK (+1)		60 kDa	TRUE	unknown	0	4	0	0	0.24584	0
1344	TRUE	Empty	Uncharacterized protein OS=Gallus gallus GN=VPS13D PE=4 SV=2	E1BYT6_CHICK		492 kDa	TRUE	unknown	4	0	0	0.034043	0	0
1345	TRUE	Empty	status=active	UPI0000448F45		20 kDa		unknown	2	0	0	0.47934	0	0
1346	TRUE	Empty	Gamma-globin chain (Fragment) OS=Homo sapiens GN=HBG2 PE=3 SV=1	B7UCU6 (+10)		?	TRUE	unknown	0	0	3	0	0	-1
1347	TRUE	Empty	status=inactive	UPI000240CCC0 (+1)		48 kDa		unknown	0	0	2	0	0	0.15757
1348	TRUE	Empty	status=active	UPI00035042AC (+5)		73 kDa		unknown	0	2	2	0	0.094141	0.1027
1349	TRUE	Empty	Exocyst complex component 3, isoform CRA_a OS=Homo sapiens GN=EXOC3 PE=4 SV=1	A0A024QYZ6 (+2)		?		unknown	0	0	4	0	0	-1
1350	TRUE	Empty	status=active	UPI00003AC474		33 kDa	TRUE	unknown	5	0	0	1.0282	0	0
1351	TRUE	Empty	status=active	UPI00003AFEBB (+1)		21 kDa	TRUE	unknown	0	3	0	0	0.57461	0
1352	TRUE	Empty	status=active	UPI000000B965		81 kDa	TRUE	unknown	0	0	4	0	0	0.24799
1353	TRUE	Empty	status=inactive	UPI000012CA28 (+2)		23 kDa	TRUE	unknown	0	3	0	0	0.76501	0
1354	TRUE	Empty	status=inactive	UPI000044991D		181 kDa	TRUE	unknown	0	0	3	0	0	0.082578
1355	TRUE	Empty	status=inactive	UPI000044A0B1		102 kDa		unknown	4	0	0	0.17298	0	0
1356	TRUE	Empty	status=inactive	UPI0000ECC664		66 kDa	TRUE	unknown	4	0	0	0.27843	0	0
1357	TRUE	Empty	Uncharacterized protein OS=Gallus gallus GN=ENTPD1 PE=3 SV=2	F1N851_CHICK (+8)		57 kDa		unknown	2	1	2	0.15165	0.058577	0.13194
1358	TRUE	Empty	Uncharacterized protein OS=Gallus gallus GN=PTER PE=4 SV=2	E1BWG7_CHICK (+4)		40 kDa		unknown	1	2	2	0.10607	0.17709	0.19319
1359	TRUE	Empty	Phosphoglycolate phosphatase OS=Gallus gallus GN=PGP PE=2 SV=1	PGP_CHICK (+1)		33 kDa		unknown	1	1	2	0.12924	0.10307	0.23769
1360	TRUE	Empty	Uncharacterized protein (Fragment) OS=Gallus gallus GN=TPMT PE=3 SV=1	F1NGW2_CHICK (+6)		28 kDa		unknown	2	2	1	0.33271	0.26535	0.13544
1361	TRUE	Empty	Dynactin subunit 2 OS=Gallus gallus GN=DCTN2 PE=2 SV=1	DCTN2_CHICK (+1)		45 kDa		unknown	2	1	2	0.19533	0.074797	0.16994
1362	TRUE	Empty	Uncharacterized protein OS=Gallus gallus GN=SLC25A20 PE=3 SV=1	R4GLG2_CHICK (+1)		33 kDa		unknown	0	2	2	0	0.21868	0.23856
1363	TRUE	Empty	Uncharacterized protein OS=Gallus gallus GN=GALK2 PE=3 SV=2	E1C902_CHICK (+5)		50 kDa		unknown	0	2	2	0	0.13904	0.15168
1364	TRUE	Empty	DnaJ homolog subfamily C member 3 OS=Gallus gallus GN=DNAJC3 PE=2 SV=1	DNJC3_CHICK (+4)		57 kDa		unknown	0	2	2	0	0.12095	0.13194
1365	TRUE	Empty	Proteasome assembly chaperone 2 OS=Gallus gallus GN=PSMG2 PE=3 SV=2	E1BZ56_CHICK (+7)		30 kDa		unknown	0	2	2	0	0.23881	0.26052
1366	TRUE	Empty	status=active	UPI00003AE9DA (+2)		39 kDa		unknown	1	2	2	0.10822	0.18085	0.19729
1367	TRUE	Empty	Citrate synthase, mitochondrial OS=Gallus gallus GN=CS PE=1 SV=1	CISY_CHICK		47 kDa	TRUE	unknown	0	2	1	0	0.31942	0.45333
1368	TRUE	Empty	Uncharacterized protein OS=Gallus gallus GN=LOC420368 PE=4 SV=2	F1NKV1_CHICK (+1)		33 kDa	TRUE	unknown	2	0	2	0.2722	0	0.23682
1369	TRUE	Empty	Uncharacterized protein OS=Gallus gallus GN=CHCHD4 PE=4 SV=1	E1BUJ8_CHICK		15 kDa		unknown	0	2	2	0	0.86902	0.55907
1370	TRUE	Empty	Uncharacterized protein (Fragment) OS=Gallus gallus GN=SFXN2 PE=4 SV=2	F1NDD1_CHICK (+10)		36 kDa		unknown	0	2	2	0	0.19831	0.21634
1371	TRUE	Empty	Adseverin OS=Gallus gallus GN=SCIN PE=2 SV=2	ADSV_CHICK (+8)		81 kDa		unknown	0	2	3	0	0.084867	0.14208
1372	TRUE	Empty	Uncharacterized protein OS=Gallus gallus GN=AP1M1 PE=2 SV=1	Q5ZMG7_CHICK (+4)		49 kDa		unknown	0	3	2	0	0.22452	0.15719
1373	TRUE	Empty	Uncharacterized protein OS=Gallus gallus GN=ATPAF2 PE=4 SV=1	E1BUZ7_CHICK (+3)		32 kDa		unknown	0	2	1	0	0.22787	0.24858
1374	TRUE	Empty	Glutathione peroxidase OS=Gallus gallus GN=GPX7 PE=3 SV=1	E1C697_CHICK (+3)		24 kDa		unknown	0	2	1	0	0.3158	0.15939
1375	TRUE	Empty	Alpha-N-acetylgalactosaminidase OS=Gallus gallus GN=NAGA PE=3 SV=2	F1NJF8_CHICK (+6)		47 kDa		unknown	0	2	2	0	0.14766	0.16109
1376	TRUE	Empty	Lon protease homolog (Fragment) OS=Gallus gallus GN=LONP1 PE=3 SV=2	F1NNI2_CHICK (+6)		95 kDa		unknown	0	2	1	0	0.071408	0.038211
1377	TRUE	Empty	status=active	UPI000240B288 (+2)		165 kDa	TRUE	unknown	2	0	1	1.2136	0	0.4487
1378	TRUE	Empty	Uncharacterized protein OS=Gallus gallus GN=MCCC2 PE=4 SV=2	F1P531_CHICK (+10)		56 kDa		unknown	0	1	3	0	0.06024	0.21057
1379	TRUE	Empty	Redox-regulatory protein FAM213A OS=Gallus gallus GN=FAM213A PE=2 SV=2	F213A_CHICK (+8)		25 kDa		unknown	0	1	3	0	0.13734	0.52115
1380	TRUE	Empty	Uncharacterized protein OS=Gallus gallus GN=MUT PE=4 SV=1	E1BY22_CHICK (+5)		83 kDa		unknown	0	2	2	0	0.082511	0.090013
1381	TRUE	Empty	Uncharacterized protein OS=Gallus gallus GN=TTLL12 PE=4 SV=1	F1NZA1_CHICK (+7)		73 kDa		unknown	0	1	2	0	0.045906	0.15799
1382	TRUE	Empty	FERM, RhoGEF and pleckstrin domain-containing protein 1 OS=Gallus gallus GN=FARP1 PE=1 SV=2	FARP1_CHICK (+7)		120 kDa		unknown	0	2	2	0	0.056424	0.061554
1383	TRUE	Empty	Uncharacterized protein OS=Gallus gallus GN=CFD PE=3 SV=1	R4GKG6_CHICK (+3)		29 kDa		unknown	0	1	2	0	0.11776	0.27366
1384	TRUE	Empty	status=inactive	UPI0000E817BB (+2)		157 kDa		unknown	0	2	2	0	0.042714	0.046598
1385	TRUE	Empty	Uncharacterized protein OS=Gallus gallus GN=ECHDC2 PE=3 SV=2	F1NSS6_CHICK (+6)		31 kDa		unknown	0	1	2	0	0.11033	0.25541
1386	TRUE	Empty	Sulfotransferase (Fragment) OS=Gallus gallus GN=LOC415852 PE=3 SV=2	E1BYE0_CHICK (+10)		34 kDa		unknown	0	1	3	0	0.098951	0.36055
1387	TRUE	Empty	Uncharacterized protein OS=Gallus gallus GN=COMMD5 PE=2 SV=1	Q5ZM89_CHICK (+2)		25 kDa		unknown	0	2	3	0	0.29699	0.52392
1388	TRUE	Empty	Uncharacterized protein OS=Gallus gallus GN=ACTBL2 PE=3 SV=2	F1N8P3_CHICK		42 kDa	TRUE	unknown	1	2	0	5.101	18.534	0
1389	TRUE	Empty	Uncharacterized protein OS=Gallus gallus GN=DHX15 PE=4 SV=2	F1NHI3_CHICK (+9)		88 kDa		unknown	2	2	0	0.097504	0.077762	0
1390	TRUE	Empty	Uncharacterized protein OS=Gallus gallus GN=NLN PE=3 SV=2	E1C0D8_CHICK		80 kDa	TRUE	unknown	0	2	2	0	0.13043	0.14229
1391	TRUE	Empty	Uncharacterized protein OS=Gallus gallus GN=SEPHS1 PE=4 SV=2	F1N876_CHICK (+8)		43 kDa	TRUE	unknown	0	2	1	0	0.16438	0.085922
1392	TRUE	Empty	Uncharacterized protein OS=Gallus gallus GN=SCFD1 PE=4 SV=2	F1NH65_CHICK (+7)		71 kDa		unknown	0	1	2	0	0.046847	0.10486
1393	TRUE	Empty	Uncharacterized protein OS=Gallus gallus GN=CSTF3 PE=2 SV=1	Q5F4A0_CHICK (+6)		83 kDa		unknown	3	2	0	0.158	0.082169	0
1394	TRUE	Empty	status=inactive	UPI0000ECBF5D (+4)		62 kDa	TRUE	unknown	0	3	1	0	1.0227	0.88472
1395	TRUE	Empty	status=inactive	UPI0000E811C7 (+1)		38 kDa	TRUE	unknown	0	1	2	0	0.0881	0.20158
1396	TRUE	Empty	Uncharacterized protein OS=Gallus gallus GN=TSPO PE=4 SV=2	F1NZA2_CHICK (+4)		19 kDa		unknown	0	1	2	0	0.18752	0.45152

1397	TRUE	Empty	status=active	UPI00003AB50D (+4)		49 kDa		unknown	0	1	3	0	0.06906	0.24371
1398	TRUE	Empty	Uncharacterized protein OS=Gallus gallus GN=TSTA3 PE=3 SV=2	F1NYB5_CHICK (+8)		36 kDa		unknown	0	2	1	0	0.1957	0.10153
1399	TRUE	Empty	Uncharacterized protein (Fragment) OS=Gallus gallus GN=EIF5B PE=4 SV=2	E1BVP1_CHICK		136 kDa		unknown	4	0	2	0.12713	0	0.053835
1400	TRUE	Empty	Signal transducer and activator of transcription 3 OS=Gallus gallus GN=STAT3 PE=1 SV=2	STAT3_CHICK (+3)		88 kDa		unknown	0	3	1	0	0.11832	0.041277
1401	TRUE	Empty	Uncharacterized protein OS=Gallus gallus GN=NUDCD1 PE=4 SV=2	E1BQJ3_CHICK (+7)		78 kDa		unknown	1	3	0	0.053741	0.13476	0
1402	TRUE	Empty	Uncharacterized protein OS=Gallus gallus GN=GNPNAT1 PE=4 SV=1	E1C0V1_CHICK (+5)		21 kDa		unknown	0	1	2	0	0.16201	0.38513
1403	TRUE	Empty	Uncharacterized protein (Fragment) OS=Gallus gallus GN=GSK3B PE=1 SV=2	F1NPL8_CHICK (+7)		46 kDa		unknown	0	2	2	0	0.15142	0.16518
1404	TRUE	Empty	DNA-directed RNA polymerase OS=Gallus gallus GN=POLR2B PE=3 SV=1	F1NCB0_CHICK (+6)		134 kDa		unknown	3	0	1	0.096009	0	0.05493
1405	TRUE	Empty	Nuclear migration protein nudC OS=Gallus gallus GN=NUDC PE=4 SV=1	F1NE97_CHICK (+5)		39 kDa		unknown	0	2	0	0	0.28442	0
1406	TRUE	Empty	status=inactive	UPI000060EA86 (+4)		28 kDa		unknown	2	2	0	0.32129	0.25624	0
1407	TRUE	Empty	Uncharacterized protein OS=Gallus gallus GN=SNX6 PE=4 SV=2	F1NFW0_CHICK (+6)		46 kDa		unknown	0	3	2	0	0.23576	0.16477
1408	TRUE	Empty	cDNA FLJ36374 fis, clone THYMU2008185, highly similar to Xaa-Pro aminopeptidase 1 (EC 3.4.11.9) OS=Homo sapiens PE=2 SV=1	B3KSI7 (+6)		?		unknown	0	0	4	0	0	-1
1409	TRUE	Empty	cDNA FLJ51884, highly similar to Mitochondrial inner membrane protein OS=Homo sapiens PE=2 SV=1	B4DKR1 (+9)		?		unknown	0	0	3	0	0	-1
1410	TRUE	Empty	Phosphoinositide phospholipase C OS=Gallus gallus GN=PLCD1 PE=4 SV=2	E1C3D8_CHICK (+9)		87 kDa		unknown	0	0	5	0	0	0.22689
1411	TRUE	Empty	Lipase maturation factor 2 OS=Gallus gallus GN=LMF2 PE=2 SV=2	LMF2_CHICK		82 kDa		unknown	0	5	0	0	0.22425	0
1412	TRUE	Empty	Complement C3 OS=Homo sapiens GN=C3 PE=1 SV=2	P01024 (+1)		?		unknown	0	0	5	0	0	-1
1413	TRUE	Empty	Uncharacterized protein OS=Gallus gallus GN=SF3A3 PE=2 SV=1	Q5F387_CHICK (+2)		59 kDa		unknown	0	4	0	0	0.25048	0
1414	TRUE	Empty	Deoxynucleoside triphosphate triphosphohydrolase SAMHD1 OS=Homo sapiens GN=SAMHD1 PE=1 SV=2	Q9Y3Z3 (+2)		?		unknown	0	0	4	0	0	-1
1415	TRUE	Empty	Uncharacterized protein OS=Gallus gallus PE=4 SV=1	R4GJT7_CHICK (+1)		59 kDa	TRUE	unknown	0	0	2	0	0	0.61781
1416	TRUE	Empty	status=active	UPI00003AA59B (+2)		82 kDa	TRUE	unknown	0	5	0	0	0.22191	0
1417	TRUE	Empty	status=inactive	UPI00004488D0 (+5)		79 kDa		unknown	0	3	1	0	0.13256	0.046025
1418	TRUE	Empty	status=inactive	UPI0000611098 (+6)		70 kDa		unknown	0	4	0	0	0.20666	0
1419	TRUE	Empty	status=active	UPI0002C87547 (+6)		109 kDa		unknown	0	0	3	0	0	0.10321
1420	TRUE	Empty	Vitronectin OS=Homo sapiens GN=VTN PE=4 SV=1	D9ZGG2 (+1)		?	TRUE	unknown	0	0	4	0	0	-1
1421	TRUE	Empty	Uncharacterized protein (Fragment) OS=Gallus gallus GN=LOC101749658 PE=3 SV=2	F1NGP9_CHICK (+7)		12 kDa		unknown	3	0	0	1.5115	0	0
1422	TRUE	Empty	Calpain 1, (Mu/I) large subunit, isoform CRA_a OS=Homo sapiens GN=CAPN1 PE=4 SV=1	A0A024R5B0 (+3)		?		unknown	0	0	4	0	0	-1
1423	TRUE	Empty	Uncharacterized protein OS=Gallus gallus GN=FAF2 PE=4 SV=2	F1NUY5_CHICK (+6)		53 kDa		unknown	0	3	1	0	0.20544	0.069672
1424	TRUE	Empty	Pyruvate kinase OS=Homo sapiens GN=PKM2 PE=3 SV=1	A0A024R5Z9 (+3)		?		unknown	0	0	4	0	0	-1
1425	TRUE	Empty	status=inactive	UPI0000ECB786		245 kDa	TRUE	unknown	3	0	0	6.4592	0	0
1426	TRUE	Empty	Uncharacterized protein OS=Gallus gallus GN=PLEK PE=4 SV=1	F1NSA0_CHICK (+3)		40 kDa		unknown	4	0	0	0.48287	0	0
1427	TRUE	Empty	Uncharacterized protein OS=Gallus gallus GN=LGMN PE=4 SV=1	E1C958_CHICK (+2)		49 kDa		unknown	0	3	0	0	0.22064	0
1428	TRUE	Empty	cDNA FLJ53327, highly similar to Gelsolin OS=Homo sapiens PE=2 SV=1	B7Z2X4 (+7)		?		unknown	0	0	3	0	0	-1
1429	TRUE	Empty	FUS/TLS OS=Gallus gallus PE=1 SV=1	Q6J4Y8_CHICK (+1)		52 kDa	TRUE	unknown	3	0	0	0.25939	0	0
1430	TRUE	Empty	status=active	UPI00003AB8D1 (+11)		102 kDa		unknown	0	2	2	0	0.066756	0.072825
1431	TRUE	Empty	status=active	UPI00003AE4BA (+3)		72 kDa	TRUE	unknown	0	0	2	0	0	0.10452
1432	TRUE	Empty	status=active	UPI0001508092 (+1)		185 kDa		unknown	3	0	0	0.068903	0	0
1433	TRUE	Empty	CD36 antigen (Collagen type I receptor, thrombospondin receptor) OS=Homo sapiens GN=CD36 PE=2 SV=1	A4D1B1 (+10)		?		unknown	0	0	2	0	0	-1
1434	TRUE	Empty	Phospholipid transfer protein, isoform CRA_c OS=Homo sapiens GN=PLTP PE=2 SV=1	B3KUE5 (+3)		?		unknown	0	0	2	0	0	-1
1435	TRUE	Empty	status=inactive	UPI000060EBC8 (+6)		61 kDa		unknown	3	0	2	0.22058	0	0.12412
1436	TRUE	Empty	status=active	UPI00003AE4B8 (+3)		112 kDa	TRUE	unknown	0	2	0	0	0.060442	0
1437	TRUE	Empty	Agrin OS=Gallus gallus GN=AGRN PE=4 SV=2	F1NWQ6_CHICK (+22)		223 kDa	TRUE	unknown	0	0	4	0	0	0.06642
1438	TRUE	Empty	status=inactive	UPI000240C74C (+4)		83 kDa		unknown	0	2	2	0	0.12619	0.089763
1439	TRUE	Empty	Type II alpha-keratin IIA OS=Gallus gallus GN=KRT5 PE=2 SV=1	Q6PVZ5_CHICK		62 kDa	TRUE	unknown	2	0	0	0.29432	0	0
1440	TRUE	Empty	Engulfment and cell motility 1 (Ced-12 homolog, C. elegans) OS=Homo sapiens GN=ELMO1 PE=2 SV=1	A4D1X5 (+1)		?		unknown	0	0	3	0	0	-1
1441	TRUE	Empty	RGD-CAP OS=Gallus gallus PE=2 SV=1	O42390_CHICK (+1)		74 kDa		unknown	0	4	0	0	0.19357	0
1442	TRUE	Empty	Thioredoxin OS=Gallus gallus GN=TXN PE=3 SV=2	THIO_CHICK (+2)		12 kDa		unknown	3	0	0	2.5185	0	0
1443	TRUE	Empty	RNA-binding protein 24 OS=Gallus gallus GN=RBM24 PE=4 SV=1	E1BZ17_CHICK (+3)		27 kDa		unknown	5	0	0	1.0747	0	0
1444	TRUE	Empty	Uncharacterized protein (Fragment) OS=Gallus gallus GN=RXRA PE=3 SV=2	F1NH64_CHICK		51 kDa		unknown	0	3	0	0	0.21176	0
1445	TRUE	Empty	Ras-related protein Rab-33B OS=Gallus gallus GN=RAB33B PE=2 SV=1	RB33B_CHICK		26 kDa		unknown	0	2	0	0	0.28564	0
1446	TRUE	Empty	status=active	UPI0003506E6C		70 kDa	TRUE	unknown	4	0	0	0.25913	0	0
1447	TRUE	Empty	status=inactive	UPI0000447D5A		242 kDa		unknown	0	2	2	0	0.02757	0.030077
1448	TRUE	Empty	status=active	UPI0003503EA4		32 kDa		unknown	4	0	0	0.64068	0	0
1449	TRUE	Empty	Uncharacterized protein OS=Gallus gallus GN=LOC101750034 PE=4 SV=2	E1C245_CHICK		614 kDa	TRUE	unknown	0	0	4	0	0	0.023703
1450	TRUE	Empty	Uncharacterized protein OS=Gallus gallus GN=RPRD1B PE=4 SV=1	F6QXI2_CHICK (+2)		44 kDa	TRUE	unknown	3	0	0	0.43409	0	0
1451	TRUE	Empty	Uncharacterized protein OS=Gallus gallus GN=LOC430067 PE=3 SV=1	R4GMC7_CHICK (+3)		65 kDa	TRUE	unknown	0	4	0	0	0.22541	0
1452	TRUE	Empty	status=active	UPI000000B8C2 (+1)		24 kDa	TRUE	unknown	0	2	0	0	0.31415	0
1453	TRUE	Empty	status=inactive	UPI0000447B23 (+3)		224 kDa		unknown	0	0	5	0	0	0.083281
1454	TRUE	Empty	status=inactive	UPI00004488E7		166 kDa	TRUE	unknown	0	0	4	0	0	0.090408
1455	TRUE	Empty	status=inactive	UPI0000E80AEB (+3)		309 kDa	TRUE	unknown	6	0	0	0.083132	0	0
1456	TRUE	Empty	status=inactive	UPI0000E80F73		125 kDa	TRUE	unknown	5	0	0	0.17664	0	0
1457	TRUE	Empty	status=inactive	UPI0000EC9E86		22 kDa	TRUE	unknown	0	2	1	0	4.2605	6.7476
1458	TRUE	Empty	status=inactive	UPI0000ECA1DE		73 kDa	TRUE	unknown	4	0	0	0.24919	0	0
1459	TRUE	Empty	status=active	UPI0002036DE8		33 kDa	TRUE	unknown	2	0	1	0.27319	0	0.3772
1460	TRUE	Empty	status=active	UPI000000B60E (+3)		44 kDa		unknown	1	2	1	0.097212	0.1617	0.084577
1461	TRUE	Empty	Uncharacterized protein (Fragment) OS=Gallus gallus GN=CAMK2G PE=4 SV=2	E1BZ86_CHICK (+9)		65 kDa		unknown	1	1	2	0.064766	0.16397	0.17888
1462	TRUE	Empty	Uncharacterized protein OS=Gallus gallus GN=NPM3 PE=4 SV=2	E1C540_CHICK (+3)		16 kDa		unknown	2	1	1	0.60172	0.21451	0.23401
1463	TRUE	Empty	status=active	UPI00000FCA59 (+9)		55 kDa		unknown	0	2	2	0	0.12661	0.13812
1464	TRUE	Empty	Uncharacterized protein OS=Gallus gallus GN=ACTL6A PE=3 SV=2	E1BR36_CHICK (+9)		47 kDa		unknown	1	2	1	0.088866	0.1473	0.077316
1465	TRUE	Empty	Replication protein A 70 kDa DNA-binding subunit OS=Gallus gallus GN=RPA1 PE=2 SV=1	RFA1_CHICK (+7)		68 kDa		unknown	1	2	1	0.06176	0.10119	0.053732
1466	TRUE	Empty	Uncharacterized protein (Fragment) OS=Gallus gallus GN=GALE PE=3 SV=2	F1NWE5_CHICK (+11)		39 kDa		unknown	0	2	2	0	0.1814	0.1979
1467	TRUE	Empty	status=active	UPI00003A95AF (+2)		55 kDa		unknown	0	3	2	0	0.1947	0.13696
1468	TRUE	Empty	status=active	UPI00035067E0 (+7)		81 kDa		unknown	0	2	2	0	0.084385	0.092057
1469	TRUE	Empty	status=active	UPI0000443489 (+6)		124 kDa		unknown	0	2	2	0	0.054509	0.059465
1470	TRUE	Empty	Uncharacterized protein OS=Gallus gallus GN=SLC27A4 PE=4 SV=2	E1BT18_CHICK (+9)		73 kDa		unknown	0	3	1	0	0.14529	0.050234
1471	TRUE	Empty	Uncharacterized protein (Fragment) OS=Gallus gallus GN=NIT2 PE=4 SV=2	F1NP29_CHICK (+10)		32 kDa		unknown	0	3	1	0	0.35959	0.11648
1472	TRUE	Empty	Basic leucine zipper and W2 domain-containing protein 2 OS=Gallus gallus GN=BZW2 PE=2 SV=1	BZW2_CHICK (+5)		48 kDa	TRUE	unknown	0	2	1	0	0.22795	0.076766
1473	TRUE	Empty	H/ACA ribonucleoprotein complex subunit 4 OS=Gallus gallus GN=DKC1 PE=2 SV=1	DKC1_CHICK (+7)		58 kDa		unknown	0	4	1	0	0.25368	0.06291
1474	TRUE	Empty	Uncharacterized protein OS=Gallus gallus GN=EMC2 PE=4 SV=1	E1BRW7_CHICK (+1)		35 kDa		unknown	0	2	2	0	0.20513	0.22378
1475	TRUE	Empty	Uncharacterized protein (Fragment) OS=Gallus gallus GN=FUCA2 PE=4 SV=2	E1BS94_CHICK (+6)		54 kDa		unknown	0	2	1	0	0.12936	0.068206
1476	TRUE	Empty	Uncharacterized protein OS=Gallus gallus PE=4 SV=2	E1BST9_CHICK (+5)		52 kDa		unknown	0	2	1	0	0.13313	0.070124
1477	TRUE	Empty	MOB-like protein phocein OS=Gallus gallus GN=MOB4 PE=4 SV=1	F1NCP5_CHICK (+9)		26 kDa		unknown	0	2	1	0	0.2816	0.14321
1478	TRUE	Empty	72 kDa type IV collagenase OS=Gallus gallus GN=MMP2 PE=1 SV=1	MMP2_CHICK (+3)		75 kDa		unknown	0	2	1	0	0.091391	0.048651

1479	TRUE	Empty	Nuclear protein matrin 3 OS=Gallus gallus GN=MATR3 PE=2 SV=1	Q8UWC5_CHICK (+1)		101 kDa		unknown	0	2	1	0	0.067362	0.036083
1480	TRUE	Empty	Endophilin-A2 OS=Gallus gallus GN=SH3GL1 PE=1 SV=1	SH3G1_CHICK (+3)		42 kDa		unknown	0	3	1	0	0.26507	0.088261
1481	TRUE	Empty	status=active	UPI00004399BA (+1)		35 kDa		unknown	0	2	1	0	0.20373	0.10549
1482	TRUE	Empty	Uncharacterized protein (Fragment) OS=Gallus gallus GN=GNA11 PE=4 SV=1	H9KZ66_CHICK (+3)		37 kDa		unknown	0	2	3	0	0.19379	0.33354
1483	TRUE	Empty	Uncharacterized protein OS=Gallus gallus GN=POLR2H PE=4 SV=1	E1BSD8_CHICK (+3)		17 kDa		unknown	0	1	2	0	0.20422	0.49587
1484	TRUE	Empty	Uncharacterized protein OS=Gallus gallus GN=MGLL PE=4 SV=2	E1C6V2_CHICK (+7)		34 kDa		unknown	0	1	2	0	0.099282	0.2285
1485	TRUE	Empty	Transmembrane protein 11, mitochondrial OS=Gallus gallus GN=TMEM11 PE=4 SV=2	F1NHB3_CHICK (+4)		22 kDa		unknown	0	1	2	0	0.16027	0.38064
1486	TRUE	Empty	status=active	UPI00003AFC46 (+2)		17 kDa		unknown	0	1	3	0	0.21147	0.86644
1487	TRUE	Empty	Charged multivesicular body protein 6 OS=Gallus gallus GN=CHMP6 PE=2 SV=3	CHMP6_CHICK (+7)		23 kDa		unknown	0	1	2	0	0.14829	0.35006
1488	TRUE	Empty	Uncharacterized protein OS=Gallus gallus GN=CUL4A PE=3 SV=2	F1P2U1_CHICK (+5)		88 kDa		unknown	0	1	3	0	0.037789	0.12891
1489	TRUE	Empty	status=active	UPI000240CABE (+2)		17 kDa		unknown	0	1	2	0	0.20283	0.49216
1490	TRUE	Empty	Uncharacterized protein (Fragment) OS=Gallus gallus GN=RAP1GDS1 PE=4 SV=2	F1NIB1_CHICK (+7)		66 kDa		unknown	0	2	1	0	0.10421	0.055291
1491	TRUE	Empty	Uncharacterized protein OS=Gallus gallus GN=MGST3 PE=4 SV=2	E1C297_CHICK (+4)		17 kDa		unknown	0	3	1	0	0.75512	0.22127
1492	TRUE	Empty	Uncharacterized protein (Fragment) OS=Gallus gallus GN=Gga.30479 PE=4 SV=2	E1BWU1_CHICK (+9)		61 kDa		unknown	3	0	1	0.21758	0	0.059449
1493	TRUE	Empty	status=active	UPI000240CCAD (+4)		14 kDa		unknown	0	1	2	0	0.24641	0.61083
1494	TRUE	Empty	Synaptosomal-associated protein OS=Gallus gallus GN=SNAP23 PE=3 SV=1	E1BRL4_CHICK (+6)		24 kDa		unknown	1	0	2	0.1823	0	0.34271
1495	TRUE	Empty	Uncharacterized protein (Fragment) OS=Gallus gallus GN=TMX3 PE=4 SV=2	F1P212_CHICK (+11)		52 kDa		unknown	0	3	1	0	0.20929	0.070891
1496	TRUE	Empty	status=active	UPI0003505F78 (+1)		17 kDa		unknown	0	2	1	0	0.458	0.22433
1497	TRUE	Empty	Uncharacterized protein OS=Gallus gallus GN=KRT15 PE=3 SV=2	F1NDN5_CHICK (+7)		48 kDa	TRUE	unknown	1	3	1	0.2851	0.51189	1.6206
1498	TRUE	Empty	status=active	UPI00001D94CE (+1)		16 kDa		unknown	0	1	2	0	0.21924	0.53631
1499	TRUE	Empty	status=active	UPI0002C87AA9 (+2)		45 kDa		unknown	2	2	0	0.19381	0.15456	0
1500	TRUE	Empty	status=active	UPI00003AECFF (+8)		70 kDa	TRUE	unknown	0	3	1	0	0.15146	0.052259
1501	TRUE	Empty	Uncharacterized protein OS=Gallus gallus GN=RAB9A PE=2 SV=1	Q5ZMI5_CHICK (+1)		23 kDa	TRUE	unknown	1	1	2	0.41117	0.32791	0.58261
1502	TRUE	Empty	Adenylosuccinate synthetase (Fragment) OS=Gallus gallus GN=ADSSL1 PE=3 SV=2	F1NVD4_CHICK (+3)		49 kDa		unknown	2	3	0	0.17937	0.22284	0
1503	TRUE	Empty	Neuropilin-1 OS=Gallus gallus GN=NRP1 PE=4 SV=1	F1N8P1_CHICK (+3)		103 kDa		unknown	0	2	2	0	0.066162	0.072177
1504	TRUE	Empty	Uncharacterized protein OS=Gallus gallus GN=ERAP1 PE=4 SV=2	F1NB92_CHICK (+4)		102 kDa		unknown	0	2	2	0	0.066235	0.072257
1505	TRUE	Empty	Uncharacterized protein OS=Gallus gallus GN=AUH PE=3 SV=2	E1BTQ9_CHICK (+4)		34 kDa		unknown	0	2	1	0	0.20873	0.10795
1506	TRUE	Empty	Uncharacterized protein OS=Gallus gallus GN=FUBP3 PE=2 SV=1	Q5ZM76_CHICK		58 kDa		unknown	0	2	2	0	0.18503	0.13035
1507	TRUE	Empty	Uncharacterized protein OS=Gallus gallus GN=IGFBP7 PE=4 SV=2	F1NVP4_CHICK (+6)		32 kDa		unknown	0	2	2	0	0.22444	0.24484
1508	TRUE	Empty	Uncharacterized protein (Fragment) OS=Gallus gallus GN=PDXK PE=4 SV=2	F1NKE8_CHICK (+7)		36 kDa		unknown	0	0	3	0	0	0.3453
1509	TRUE	Empty	status=inactive	UPI0000E7FD97 (+6)		103 kDa		unknown	0	3	0	0	0.10092	0
1510	TRUE	Empty	Integrin alpha 5 OS=Gallus gallus GN=Itga5 PE=2 SV=1	X2C5I8_CHICK		113 kDa		unknown	0	0	3	0	0	0.099817
1511	TRUE	Empty	Uncharacterized protein OS=Gallus gallus GN=DNAJB4 PE=4 SV=2	F1NKV0_CHICK (+3)		38 kDa		unknown	0	0	2	0	0	0.20221
1512	TRUE	Empty	status=inactive	UPI0000448DF7		136 kDa		unknown	3	2	0	0.094628	0.049637	0
1513	TRUE	Empty	status=inactive	UPI0000ECC31B (+6)		7 kDa		unknown	2	0	0	1.8325	0	0
1514	TRUE	Empty	Uncharacterized protein OS=Gallus gallus GN=PIN4 PE=4 SV=2	F1NCD7_CHICK (+6)		13 kDa		unknown	0	0	2	0	0	1.1657
1515	TRUE	Empty	status=active	UPI00003AE2AB (+12)		130 kDa		unknown	4	0	0	0.1341	0	0
1516	TRUE	Empty	status=inactive	UPI0000447FA6 (+5)		144 kDa		unknown	0	2	2	0	0.04671	0.050957
1517	TRUE	Empty	Coiled-coil domain containing 22, isoform CRA_a OS=Homo sapiens GN=CCDC22 PE=4 SV=1	A0A024QZ03 (+3)		?		unknown	0	0	3	0	0	-1
1518	TRUE	Empty	Growth factor receptor-bound protein 2 OS=Gallus gallus GN=GRB2 PE=2 SV=1	A3R0S3_CHICK (+7)		25 kDa		unknown	0	3	0	0	0.47273	0
1519	TRUE	Empty	Carnosine synthase 1 OS=Gallus gallus GN=CARNS1 PE=1 SV=1	CRNS1_CHICK (+1)		100 kDa		unknown	4	0	0	0.17646	0	0
1520	TRUE	Empty	Uncharacterized protein OS=Gallus gallus GN=RAB4A PE=3 SV=2	E1C8H0_CHICK (+7)		24 kDa	TRUE	unknown	0	0	4	0	0	1.3699
1521	TRUE	Empty	Uncharacterized protein OS=Gallus gallus GN=DPP4 PE=4 SV=2	F1NDK7_CHICK (+5)		87 kDa		unknown	0	0	3	0	0	0.13152
1522	TRUE	Empty	Ubiquitin carboxyl-terminal hydrolase (Fragment) OS=Gallus gallus GN=USP10 PE=3 SV=2	F1NG89_CHICK (+7)		86 kDa		unknown	0	3	0	0	0.12188	0
1523	TRUE	Empty	Uncharacterized protein OS=Gallus gallus GN=CRYL1 PE=4 SV=2	F1NPU2_CHICK (+8)		35 kDa		unknown	0	0	4	0	0	0.49642
1524	TRUE	Empty	Histone H4 type VIII OS=Gallus gallus GN=H4-VIII PE=3 SV=3	H48_CHICK (+1)		11 kDa	TRUE	unknown	0	4	0	0	141.68	0
1525	TRUE	Empty	Keratin, type I cytoskeletal 14 OS=Homo sapiens GN=KRT14 PE=1 SV=4	P02533 (+1)		?	TRUE	unknown	0	0	3	0	0	-1
1526	TRUE	Empty	Keratin, type II cytoskeletal 5 OS=Homo sapiens GN=KRT5 PE=1 SV=3	P13647 (+1)		?	TRUE	unknown	0	0	3	0	0	-1
1527	TRUE	Empty	Lumican OS=Homo sapiens GN=LUM PE=1 SV=2	P51884 (+1)		?		unknown	0	0	3	0	0	-1
1528	TRUE	Empty	Isoform LCRMP-4 of Dihydropyrimidinase-related protein 3 OS=Homo sapiens GN=DPYSL3	Q14195-2 (+1)		?		unknown	0	0	3	0	0	-1
1529	TRUE	Empty	Uncharacterized protein OS=Gallus gallus PE=4 SV=1	R4GMA5_CHICK (+3)		27 kDa		unknown	0	0	3	0	0	0.48286
1530	TRUE	Empty	Neuroplastin OS=Homo sapiens GN=NPTN PE=1 SV=2	Q9Y639 (+1)		?		unknown	0	0	3	0	0	-1
1531	TRUE	Empty	Signal transducer and activator of transcription OS=Homo sapiens GN=STAT1 PE=1 SV=1	J3KPM9 (+2)		?		unknown	0	0	3	0	0	-1
1532	TRUE	Empty	Single-stranded DNA-binding protein OS=Gallus gallus GN=SSBP1 PE=4 SV=2	E1BTE0_CHICK		18 kDa		unknown	0	0	3	0	0	0.80393
1533	TRUE	Empty	Cartilage-associated protein OS=Gallus gallus GN=CRTAP PE=1 SV=1	CRTAP_CHICK (+10)		31 kDa		unknown	0	3	0	0	0.36396	0
1534	TRUE	Empty	status=active	UPI0000E80D5D		27 kDa	TRUE	unknown	0	0	2	0	0	0.66718
1535	TRUE	Empty	Uncharacterized protein OS=Gallus gallus GN=LOC100859909 PE=4 SV=1	E1C9A8_CHICK		200 kDa		unknown	0	0	4	0	0	0.074256
1536	TRUE	Empty	EH-domain containing 2, isoform CRA_a OS=Homo sapiens GN=EHD2 PE=4 SV=1	A0A024R0S6 (+4)		?		unknown	0	0	3	0	0	-1
1537	TRUE	Empty	Uncharacterized protein OS=Gallus gallus GN=SEC24C PE=4 SV=1	E1BUD8_CHICK (+6)		122 kDa		unknown	0	3	0	0	0.084477	0
1538	TRUE	Empty	Uncharacterized protein OS=Gallus gallus GN=MYOF PE=4 SV=2	E1BW21_CHICK		234 kDa		unknown	0	0	3	0	0	0.04704
1539	TRUE	Empty	Uncharacterized protein OS=Gallus gallus GN=RHAG PE=4 SV=2	F1NFG6_CHICK (+5)		44 kDa		unknown	2	0	0	0.20112	0	0
1540	TRUE	Empty	Uncharacterized protein OS=Gallus gallus GN=SEPT8 PE=3 SV=2	F1NIL0_CHICK (+10)		63 kDa	TRUE	unknown	0	2	1	0	0.23426	0.25556
1541	TRUE	Empty	Uncharacterized protein (Fragment) OS=Gallus gallus GN=TSPO2 PE=4 SV=2	F1NNL8_CHICK (+7)		19 kDa		unknown	0	0	2	0	0	0.43942
1542	TRUE	Empty	Brain acid soluble protein 1 OS=Homo sapiens GN=BASP1 PE=1 SV=2	P80723		?		unknown	0	0	2	0	0	-1
1543	TRUE	Empty	Uncharacterized protein OS=Gallus gallus GN=COPS5 PE=2 SV=1	Q5ZLC3_CHICK (+2)		38 kDa		unknown	3	0	0	0.37073	0	0
1544	TRUE	Empty	Uncharacterized protein (Fragment) OS=Gallus gallus PE=4 SV=1	R4GGS3_CHICK (+3)		17 kDa		unknown	0	0	2	0	0	0.49587
1545	TRUE	Empty	status=inactive	UPI0000449922 (+6)		231 kDa	TRUE	unknown	0	3	1	0	0.043755	0.015661
1546	TRUE	Empty	Translocating chain-associated membrane protein OS=Gallus gallus GN=TRAM1 PE=3 SV=2	F1NCN4_CHICK (+5)		43 kDa	TRUE	unknown	0	3	0	0	0.2538	0
1547	TRUE	Empty	status=active	UPI00003ACC98 (+8)		67 kDa	TRUE	unknown	0	4	0	0	0.2188	0
1548	TRUE	Empty	Uncharacterized protein OS=Gallus gallus GN=SLK PE=4 SV=2	E1BQV7_CHICK (+3)		144 kDa		unknown	0	0	3	0	0	0.077475
1549	TRUE	Empty	Uncharacterized protein OS=Gallus gallus GN=MAST2 PE=4 SV=2	F1NAH4_CHICK		202 kDa		unknown	6	0	0	0.12942	0	0
1550	TRUE	Empty	Prolow-density lipoprotein receptor-related protein 1 OS=Homo sapiens GN=LRP1 PE=1 SV=2	Q07954 (+1)		?		unknown	0	0	4	0	0	-1
1551	TRUE	Empty	Ammonium transporter Rh type C OS=Gallus gallus GN=RHCG PE=4 SV=1	F1P2I1_CHICK (+1)		52 kDa		unknown	0	0	3	0	0	0.22885
1552	TRUE	Empty	Hemopexin OS=Homo sapiens GN=HPX PE=1 SV=2	P02790		?		unknown	0	0	4	0	0	-1
1553	TRUE	Empty	Uncharacterized protein OS=Gallus gallus GN=DNM3 PE=3 SV=2	F1NQQ1_CHICK		96 kDa	TRUE	unknown	0	0	4	0	0	0.16075
1554	TRUE	Empty	status=inactive	UPI0000447184		258 kDa		unknown	0	0	4	0	0	0.05714
1555	TRUE	Empty	status=inactive	UPI0000ECC98C (+4)		16 kDa		unknown	0	0	2	0	0	0.54519
1556	TRUE	Empty	Uncharacterized protein OS=Gallus gallus GN=KNG1 PE=4 SV=2	E1BX43_CHICK (+5)		65 kDa	TRUE	unknown	2	0	0	0.13252	0	0
1557	TRUE	Empty	Uncharacterized protein (Fragment) OS=Gallus gallus PE=3 SV=2	E1BQ63_CHICK (+1)		43 kDa	TRUE	unknown	0	0	4	0	0	0.38775
1558	TRUE	Empty	Uncharacterized protein OS=Gallus gallus GN=CTTN PE=4 SV=2	F1NU55_CHICK (+8)		63 kDa		unknown	0	2	0	0	0.1683	0
1559	TRUE	Empty	Uncharacterized protein (Fragment) OS=Gallus gallus GN=RPL11 PE=3 SV=2	E1C3K3_CHICK (+2)		19 kDa		unknown	0	0	4	0	0	1.4912
1560	TRUE	Empty	Salt-tolerant protein OS=Homo sapiens GN=STP PE=2 SV=1	A7VJC9 (+6)		?		unknown	0	0	3	0	0	-1

1561	TRUE	Empty	Uncharacterized protein OS=Gallus gallus GN=NDUFS8 PE=3 SV=2	F1NAF3_CHICK (+3)		24 kDa		unknown	0	1	2	0	0.14468	0.34092
1562	TRUE	Empty	status=active	UPI00003AB84A (+7)		34 kDa		unknown	0	4	1	0	0.47854	0.11052
1563	TRUE	Empty	Uncharacterized protein OS=Gallus gallus GN=ITPR3 PE=4 SV=2	F1N9Y1_CHICK (+6)		305 kDa		unknown	0	1	4	0	0.010856	0.048232
1564	TRUE	Empty	Putative uncharacterized protein DKFZp686C11235 OS=Homo sapiens GN=DKFZp686C11235 PE=1 SV=1	Q6MZV7		?	TRUE	unknown	0	0	4	0	0	-1
1565	TRUE	Empty	status=inactive	UPI0000F1F53E (+1)		71 kDa		unknown	2	1	1	0.12111	0.047069	0.051349
1566	TRUE	Empty	Uncharacterized protein (Fragment) OS=Gallus gallus GN=STK25 PE=4 SV=2	F1N8L1_CHICK (+1)		49 kDa	TRUE	unknown	0	0	4	0	0	0.33765
1567	TRUE	Empty	Complement decay-accelerating factor OS=Homo sapiens GN=CD55 PE=1 SV=1	B1AP13 (+10)		?		unknown	0	0	2	0	0	-1
1568	TRUE	Empty	WD40 repeat-containing protein SMU1 OS=Gallus gallus GN=SMU1 PE=2 SV=1	SMU1_CHICK		57 kDa		unknown	0	4	0	0	0.25695	0
1569	TRUE	Empty	status=active	UPI00003AE91D		19 kDa		unknown	0	0	4	0	0	1.0577
1570	TRUE	Empty	Uncharacterized protein (Fragment) OS=Gallus gallus GN=DDX5 PE=3 SV=1	F1NM08_CHICK (+1)		67 kDa		unknown	0	4	0	0	0.82422	0
1571	TRUE	Empty	Uncharacterized protein OS=Gallus gallus GN=TUBA8 PE=3 SV=1	F1NMP3_CHICK		50 kDa	TRUE	unknown	3	1	0	2.4616	3.5156	0
1572	TRUE	Empty	Uncharacterized protein OS=Gallus gallus GN=RAB12 PE=3 SV=1	R4GIH4_CHICK (+1)		28 kDa	TRUE	unknown	2	0	0	0.52905	0	0
1573	TRUE	Empty	status=inactive	UPI0000448041		188 kDa	TRUE	unknown	4	0	0	0.091031	0	0
1574	TRUE	Empty	status=inactive	UPI000044973E		136 kDa		unknown	0	2	2	0	0.049554	0.054059
1575	TRUE	Empty	status=active	UPI00035061D2 (+7)		189 kDa		unknown	0	3	0	0	0.053823	0
1576	TRUE	Empty	Protein phosphatase 1 regulatory subunit 21 OS=Gallus gallus GN=PPP1R21 PE=4 SV=2	E1BX04_CHICK (+6)		87 kDa		unknown	1	2	1	0.04818	0.07848	0.041917
1577	TRUE	Empty	Coiled-coil domain-containing protein 93 OS=Gallus gallus GN=CCDC93 PE=2 SV=1	CCD93_CHICK (+6)		72 kDa		unknown	1	1	2	0.058464	0.046626	0.10435
1578	TRUE	Empty	Uncharacterized protein OS=Gallus gallus GN=SEPT6 PE=3 SV=1	F1NLX2_CHICK (+6)		49 kDa		unknown	0	2	1	0	0.85191	0.33853
1579	TRUE	Empty	Uncharacterized protein OS=Gallus gallus GN=GNB4 PE=4 SV=2	E1BW98_CHICK (+6)		38 kDa		unknown	0	2	1	0	0.41865	0.32577
1580	TRUE	Empty	Uncharacterized protein OS=Gallus gallus GN=NDUFA5 PE=4 SV=1	E1BRT9_CHICK (+1)		13 kDa		unknown	0	2	2	0	0.60459	0.65955
1581	TRUE	Empty	Integrin beta OS=Gallus gallus GN=ITGB3 PE=2 SV=1	Q92071_CHICK (+5)		86 kDa		unknown	0	2	2	0	0.079212	0.086414
1582	TRUE	Empty	Uncharacterized protein OS=Gallus gallus GN=RCJMB04_27m5 PE=2 SV=1	Q5ZID4_CHICK (+5)		45 kDa		unknown	0	2	2	0	0.15701	0.17128
1583	TRUE	Empty	Testin OS=Gallus gallus GN=TES PE=4 SV=1	R9PXN3_CHICK (+7)		47 kDa		unknown	0	2	2	0	0.14877	0.16229
1584	TRUE	Empty	Uncharacterized protein OS=Gallus gallus GN=GMPPB PE=4 SV=2	F1P574_CHICK (+4)		39 kDa	TRUE	unknown	0	3	1	0	0.28262	0.093612
1585	TRUE	Empty	status=active	UPI0002C86CFC (+7)		55 kDa	TRUE	unknown	0	2	1	0	0.26911	0.066389
1586	TRUE	Empty	Uncharacterized protein OS=Gallus gallus GN=TMCO1 PE=4 SV=1	E1C8A8_CHICK (+1)		21 kDa		unknown	0	2	1	0	0.35303	0.17674
1587	TRUE	Empty	Uncharacterized protein (Fragment) OS=Gallus gallus PE=4 SV=2	F1NS59_CHICK (+10)		41 kDa		unknown	0	2	1	0	0.17348	0.090478
1588	TRUE	Empty	Casein kinase I isoform epsilon OS=Gallus gallus GN=DBT PE=3 SV=1	F1P1X9_CHICK (+4)		54 kDa		unknown	0	2	1	0	0.12853	0.067778
1589	TRUE	Empty	Uncharacterized protein OS=Gallus gallus GN=HDHD2 PE=4 SV=1	R4GI96_CHICK (+5)		29 kDa		unknown	0	2	1	0	0.25406	0.13001
1590	TRUE	Empty	Probable ATP-dependent RNA helicase DDX6 OS=Gallus gallus GN=DDX6 PE=2 SV=1	DDX6_CHICK (+5)		54 kDa		unknown	2	0	1	0.16012	0	0.067355
1591	TRUE	Empty	Uncharacterized protein OS=Gallus gallus GN=DOCK1 PE=4 SV=2	F1NVR7_CHICK (+6)		215 kDa		unknown	0	1	4	0	0.015382	0.068853
1592	TRUE	Empty	Uncharacterized protein OS=Gallus gallus GN=LOC101750333 PE=3 SV=2	F1NBE3_CHICK (+5)		45 kDa		unknown	0	1	3	0	0.074609	0.26487
1593	TRUE	Empty	Cyclin-dependent kinase 5 OS=Gallus gallus GN=Cdk5 PE=2 SV=1	B6E1W1_CHICK (+5)		33 kDa	TRUE	unknown	0	1	2	0	0.21552	0.23511
1594	TRUE	Empty	Uncharacterized protein OS=Gallus gallus GN=CPD PE=4 SV=1	E1BYS4_CHICK (+4)		151 kDa		unknown	0	1	2	0	0.022035	0.048662
1595	TRUE	Empty	Probable RNA-binding protein EIF1AD OS=Gallus gallus GN=eif1ad PE=2 SV=1	EIF1A_CHICK (+1)		21 kDa		unknown	0	1	2	0	0.1629	0.38741
1596	TRUE	Empty	Uncharacterized protein (Fragment) OS=Gallus gallus GN=MAPK14 PE=4 SV=2	F1NDG1_CHICK (+6)		42 kDa		unknown	0	1	2	0	0.16905	0.28917
1597	TRUE	Empty	Uncharacterized protein OS=Gallus gallus GN=LOC776432 PE=3 SV=1	R4GKF2_CHICK (+3)		35 kDa		unknown	0	1	3	0	0.097651	0.35532
1598	TRUE	Empty	Uncharacterized protein (Fragment) OS=Gallus gallus GN=BAAT PE=4 SV=2	F1NBC1_CHICK (+8)		46 kDa		unknown	0	2	1	0	0.15377	0.080583
1599	TRUE	Empty	status=active	UPI000037819D		34 kDa		unknown	0	1	2	0	0.10029	0.23093
1600	TRUE	Empty	Uncharacterized protein OS=Gallus gallus GN=PSMG1 PE=2 SV=1	Q5F3H4_CHICK (+3)		31 kDa		unknown	0	1	2	0	0.10833	0.2505
1601	TRUE	Empty	Uncharacterized protein OS=Gallus gallus GN=CRYZ PE=4 SV=1	F1P4I0_CHICK (+5)		35 kDa		unknown	2	0	1	0.25372	0	0.10481
1602	TRUE	Empty	Serine/threonine-protein phosphatase OS=Gallus gallus GN=PPP6C PE=2 SV=1	Q5ZIV0_CHICK (+7)		35 kDa		unknown	2	1	0	0.25459	0.096385	0
1603	TRUE	Empty	tr|Q90681|Q90681_CHICK-DECOY Cation-independent mannose-6-phosphate receptor OS=Gallus...	tr|Q90681|Q90681_CHICK-DECOY		?		unknown	0	1	2	0	-1	-1
1604	TRUE	Empty	Uncharacterized protein OS=Gallus gallus GN=PLA2R1 PE=4 SV=1	E1C5Y7_CHICK (+4)		169 kDa		unknown	0	1	3	0	0.019684	0.065832
1605	TRUE	Empty	Uncharacterized protein OS=Gallus gallus GN=MST1 PE=3 SV=2	E1C8A1_CHICK (+5)		79 kDa	TRUE	unknown	0	1	2	0	0.04207	0.093924
1606	TRUE	Empty	Uncharacterized protein OS=Gallus gallus GN=DNAJC9 PE=4 SV=1	E1C8S9_CHICK (+2)		30 kDa	TRUE	unknown	1	2	0	0.14313	0.2427	0
1607	TRUE	Empty	Uncharacterized protein (Fragment) OS=Gallus gallus GN=PITPNB PE=4 SV=1	F1NGH1_CHICK (+19)		31 kDa		unknown	1	2	0	0.13835	0.23413	0
1608	TRUE	Empty	Caveolin OS=Gallus gallus GN=CAV1 PE=3 SV=1	A0M8T8_CHICK (+10)		20 kDa		unknown	0	1	2	0	0.16938	0.40416
1609	TRUE	Empty	Uncharacterized protein (Fragment) OS=Gallus gallus GN=ATL1 PE=4 SV=2	F1NAM6_CHICK (+27)		64 kDa		unknown	0	2	1	0	0.10799	0.057244
1610	TRUE	Empty	Protein LZIC OS=Gallus gallus GN=LZIC PE=2 SV=1	LZIC_CHICK (+1)		21 kDa		unknown	0	1	2	0	0.16113	0.38287
1611	TRUE	Empty	Uncharacterized protein OS=Gallus gallus GN=RAB22A PE=2 SV=1	Q5ZLB8_CHICK (+6)		22 kDa		unknown	0	1	3	0	0.15772	0.61139
1612	TRUE	Empty	F1N9V4-DECOY	F1N9V4-DECOY (+5)		?		unknown	0	2	2	0	-1	-1
1613	TRUE	Empty	status=active	UPI0001E6B52C (+9)		55 kDa		unknown	1	0	3	0.075996	0	0.21194
1614	TRUE	Empty	Uncharacterized protein (Fragment) OS=Gallus gallus GN=RAP1A PE=1 SV=1	F1NSA8_CHICK (+2)		21 kDa	TRUE	unknown	1	0	2	1.0581	0	2.1397
1615	TRUE	Empty	Uncharacterized protein OS=Gallus gallus GN=AS3MT PE=4 SV=2	F1NCJ8_CHICK (+3)		42 kDa		unknown	0	1	2	0	0.080686	0.18389
1616	TRUE	Empty	Uncharacterized protein OS=Gallus gallus GN=GCC2 PE=4 SV=2	F1P4P9_CHICK (+7)		196 kDa		unknown	1	2	2	0.021198	0.034127	0.037229
1617	TRUE	Empty	status=active	UPI00003AE31E (+10)		113 kDa		unknown	1	2	0	0.036802	0.059653	0
1618	TRUE	Empty	Quaking protein OS=Gallus gallus PE=2 SV=1	B0M1D7_CHICK (+22)		38 kDa		unknown	0	2	1	0	0.18888	0.098146
1619	TRUE	Empty	Tyrosine-protein phosphatase non-receptor type 11 OS=Gallus gallus GN=PTPN11 PE=2 SV=1	PTN11_CHICK (+7)		68 kDa		unknown	3	1	0	0.19555	0.049254	0
1620	TRUE	Empty	UPI0000F212E6-DECOY	UPI0000F212E6-DECOY		?	TRUE	unknown	2	0	2	-1	0	-1
1621	TRUE	Empty	3-hydroxyisobutyryl-CoA hydrolase, mitochondrial OS=Gallus gallus GN=HIBCH PE=4 SV=1	F1P188_CHICK (+7)		43 kDa		unknown	0	1	3	0	0.078762	0.28086
1622	TRUE	Empty	Uncharacterized protein OS=Gallus gallus GN=APOO PE=4 SV=2	F1NNV7_CHICK (+4)		24 kDa		unknown	0	2	1	0	0.30768	0.15557
1623	TRUE	Empty	status=active	UPI0000225C29 (+8)		23 kDa		unknown	0	2	1	0	0.31748	0.16017
1624	TRUE	Empty	Uncharacterized protein (Fragment) OS=Gallus gallus PE=4 SV=2	E1C8A6_CHICK (+8)		36 kDa		unknown	0	2	1	0	0.19964	0.10347
1625	TRUE	Empty	status=active	UPI00003AB87B (+8)		20 kDa		unknown	1	0	2	0.21239	0	0.66462
1626	TRUE	Empty	Uncharacterized protein (Fragment) OS=Gallus gallus GN=LOC768552 PE=4 SV=2	F1P4B2_CHICK (+1)		561 kDa		unknown	0	0	4	0	0	0.025979
1627	TRUE	Empty	status=active	UPI000000B893 (+7)		70 kDa		unknown	2	1	0	0.12372	0.048059	0
1628	TRUE	Empty	status=inactive	UPI0000448C09 (+8)		112 kDa		unknown	0	3	0	0	0.12492	0
1629	TRUE	Empty	Uncharacterized protein OS=Gallus gallus GN=SVIL PE=4 SV=2	E1C8N4_CHICK (+22)		244 kDa		unknown	0	3	0	0	0.041302	0
1630	TRUE	Empty	Uncharacterized protein OS=Gallus gallus GN=NAA50 PE=4 SV=1	F1NBN9_CHICK (+8)		20 kDa		unknown	0	1	2	0	0.17746	0.42516
1631	TRUE	Empty	Uncharacterized protein OS=Gallus gallus GN=COMMD1 PE=4 SV=2	F1NKT9_CHICK (+6)		21 kDa		unknown	0	0	3	0	0	0.62685
1632	TRUE	Empty	status=active	UPI00003AFBB4		20 kDa		unknown	0	1	2	0	0.17333	0.4144
1633	TRUE	Empty	status=inactive	UPI0000610AD7 (+6)		77 kDa		unknown	0	2	0	0	0.088797	0
1634	TRUE	Empty	Hsp90 co-chaperone Cdc37 OS=Gallus gallus GN=CDC37 PE=2 SV=1	CDC37_CHICK (+1)		46 kDa		unknown	0	3	0	0	0.24019	0
1635	TRUE	Empty	Uncharacterized protein OS=Gallus gallus GN=NDUFA8 PE=4 SV=1	E1BTV0_CHICK		20 kDa		unknown	0	0	3	0	0	0.9954
1636	TRUE	Empty	Uncharacterized protein OS=Gallus gallus GN=C4H4ORF41 PE=4 SV=2	E1C4H1_CHICK (+1)		129 kDa	TRUE	unknown	0	0	3	0	0	0.087122
1637	TRUE	Empty	Programmed cell death protein 4 OS=Gallus gallus GN=PDCD4 PE=4 SV=1	F1NIY3_CHICK (+4)		52 kDa		unknown	0	3	0	0	0.20929	0
1638	TRUE	Empty	Uncharacterized protein OS=Gallus gallus GN=CLINT1 PE=4 SV=2	F1NPP8_CHICK (+2)		71 kDa	TRUE	unknown	0	0	3	0	0	0.16301
1639	TRUE	Empty	Tetraspanin OS=Gallus gallus GN=CD9 PE=2 SV=1	Q9IBC9_CHICK (+1)		25 kDa		unknown	0	0	3	0	0	0.52115
1640	TRUE	Empty	status=active	UPI00003ABEB5		112 kDa	TRUE	unknown	0	3	0	0	0.091876	0
1641	TRUE	Empty	cDNA FLJ54588, highly similar to Major vault protein OS=Homo sapiens PE=2 SV=1	B4DDR2 (+4)		?		unknown	0	0	3	0	0	-1
1642	TRUE	Empty	CAP-Gly domain-containing linker protein 1 OS=Gallus gallus GN=CLIP1 PE=4 SV=1	R9PXL8_CHICK (+4)		161 kDa	TRUE	unknown	0	0	4	0	0	0.093134

1643	TRUE	Empty	status=active	UPI00003A9B7D (+5)		27 kDa		unknown	3	0	0	0.53914	0	0
1644	TRUE	Empty	Vacuolar protein sorting 35 variant (Fragment) OS=Homo sapiens PE=2 SV=1	Q53FR4 (+1)		?		unknown	0	0	3	0	0	-1
1645	TRUE	Empty	Uncharacterized protein OS=Gallus gallus GN=PSMD5 PE=4 SV=2	F1NKW9_CHICK (+2)		56 kDa		unknown	0	0	3	0	0	0.21057
1646	TRUE	Empty	Tetraspanin OS=Gallus gallus GN=TSPAN8 PE=3 SV=2	E1C3Y3_CHICK (+2)		27 kDa		unknown	0	0	2	0	0	0.29739
1647	TRUE	Empty	Uncharacterized protein (Fragment) OS=Gallus gallus GN=RPS6KA3 PE=4 SV=2	F1NLJ3_CHICK		81 kDa	TRUE	unknown	0	0	2	0	0	0.092451
1648	TRUE	Empty	Uncharacterized protein (Fragment) OS=Gallus gallus GN=LOC420516 PE=4 SV=1	F1NLS1_CHICK (+11)		167 kDa	TRUE	unknown	0	0	2	0	0	0.043944
1649	TRUE	Empty	Procollagen-lysine,2-oxoglutarate 5-dioxygenase 3 OS=Homo sapiens GN=PLOD3 PE=1 SV=1	O60568		?		unknown	0	0	2	0	0	-1
1650	TRUE	Empty	Uncharacterized protein OS=Gallus gallus GN=LSP1 PE=2 SV=1	Q5F3Y0_CHICK (+1)		42 kDa		unknown	0	2	0	0	0.26665	0
1651	TRUE	Empty	status=inactive	UPI00004477E3 (+4)		44 kDa		unknown	0	0	2	0	0	0.17451
1652	TRUE	Empty	status=active	UPI0003507522 (+6)		69 kDa	TRUE	unknown	0	0	2	0	0	0.1675
1653	TRUE	Empty	cDNA FLJ52934, highly similar to Tyrosine-protein phosphatase non-receptortype substrate 1 OS=Homo sapiens PE=2 SV=1	B4DP97 (+5)		?		unknown	0	0	2	0	0	-1
1654	TRUE	Empty	Exocyst complex component 8 OS=Homo sapiens GN=EXOC8 PE=1 SV=2	Q8IYI6		?		unknown	0	0	3	0	0	-1
1655	TRUE	Empty	status=inactive	UPI0000449762 (+6)		180 kDa		unknown	0	1	2	0	0.01846	0.040688
1656	TRUE	Empty	Coronin OS=Gallus gallus GN=LOC776194 PE=3 SV=1	R4GLV0_CHICK (+3)		43 kDa		unknown	0	0	2	0	0	0.3924
1657	TRUE	Empty	Uncharacterized protein OS=Gallus gallus GN=DNAJC13 PE=4 SV=1	E1BUS3_CHICK		255 kDa	TRUE	unknown	0	0	5	0	0	0.072905
1658	TRUE	Empty	tr|F1NI79|F1NI79_CHICK-DECOY Uncharacterized protein (Fragment) OS=Gallus gallus...	tr|F1NI79|F1NI79_CHICK-DECOY		?	TRUE	unknown	0	4	0	0	-1	0
1659	TRUE	Empty	Uncharacterized protein OS=Gallus gallus GN=LOC100858210 PE=3 SV=2	F1NNU3_CHICK (+6)		49 kDa		unknown	2	2	0	0.17851	0.14236	0
1660	TRUE	Empty	Uncharacterized protein (Fragment) OS=Gallus gallus GN=UFD1L PE=4 SV=1	F1NVD8_CHICK (+3)		35 kDa		unknown	2	0	0	0.2599	0	0
1661	TRUE	Empty	Phosphomannomutase OS=Gallus gallus GN=PMM1 PE=3 SV=2	F1NIG6_CHICK (+8)		25 kDa	TRUE	unknown	0	0	2	0	0	0.52954
1662	TRUE	Empty	Inter-alpha inhibitor heavy chain 2 OS=Gallus gallus GN=ITIH2 PE=2 SV=1	B3VE14_CHICK (+5)		107 kDa	TRUE	unknown	2	0	0	0.079592	0	0
1663	TRUE	Empty	status=active	UPI0001E91994 (+3)		12 kDa		unknown	0	2	2	0	0.7104	0.77498
1664	TRUE	Empty	Integrin alpha-4 OS=Homo sapiens GN=ITGA4 PE=4 SV=1	A0A087WX37 (+1)		?		unknown	0	0	2	0	0	-1
1665	TRUE	Empty	Uncharacterized protein OS=Gallus gallus GN=NCK2 PE=4 SV=1	F1P4Q4_CHICK (+2)		43 kDa		unknown	0	2	0	0	0.16393	0
1666	TRUE	Empty	status=active	UPI00003AB36E (+16)		96 kDa	TRUE	unknown	3	1	0	0.1351	0.14644	0
1667	TRUE	Empty	Coiled-coil domain-containing protein 93 OS=Homo sapiens GN=CCDC93 PE=1 SV=1	F8W9X7 (+1)		?		unknown	0	0	3	0	0	-1
1668	TRUE	Empty	Fibrinogen alpha chain OS=Homo sapiens GN=FGA PE=1 SV=2	P02671 (+1)		?		unknown	0	0	3	0	0	-1
1669	TRUE	Empty	Uncharacterized protein OS=Gallus gallus GN=TCERG1 PE=4 SV=2	F1NT10_CHICK (+9)		110 kDa	TRUE	unknown	0	1	2	0	0.030278	0.067166
1670	TRUE	Empty	Uncharacterized protein OS=Gallus gallus GN=LOC100857286 PE=4 SV=2	E1BRI2_CHICK (+4)		78 kDa	TRUE	unknown	0	0	2	0	0	0.14746
1671	TRUE	Empty	Uncharacterized protein OS=Gallus gallus GN=AMPD3 PE=4 SV=2	F1NG97_CHICK (+3)		89 kDa	TRUE	unknown	5	0	0	0.25544	0	0
1672	TRUE	Empty	Uncharacterized protein OS=Gallus gallus GN=SGTA PE=4 SV=2	H9L0E7_CHICK (+5)		34 kDa		unknown	0	2	0	0	0.21244	0
1673	TRUE	Empty	status=active	UPI00003AA7E3 (+3)		68 kDa		unknown	0	1	2	0	0.049254	0.11039
1674	TRUE	Empty	status=active	UPI000350734C (+5)		119 kDa		unknown	0	1	2	0	0.027916	0.061848
1675	TRUE	Empty	Uncharacterized protein OS=Gallus gallus GN=TTC37 PE=4 SV=2	E1C576_CHICK (+6)		177 kDa		unknown	1	0	2	0.023468	0	0.041258
1676	TRUE	Empty	status=active	UPI000000B7E8 (+1)		52 kDa	TRUE	unknown	3	0	0	0.26242	0	0
1677	TRUE	Empty	status=active	UPI000002B0E9 (+3)		289 kDa		unknown	0	0	4	0	0	0.050963
1678	TRUE	Empty	Uncharacterized protein OS=Gallus gallus GN=GAR1 PE=4 SV=2	E1C818_CHICK (+5)		19 kDa		unknown	0	3	0	0	0.65284	0
1679	TRUE	Empty	status=active	UPI00003AEDCD (+3)		74 kDa		unknown	4	0	0	0.24471	0	0
1680	TRUE	Empty	status=active	UPI000060EE92 (+7)		28 kDa	TRUE	unknown	0	1	2	0	0.25958	0.28318
1681	TRUE	Empty	cDNA FLJ53282, highly similar to Dipeptidyl-peptidase 3 (EC 3.4.14.4) OS=Homo sapiens PE=2 SV=1	B4E357 (+6)		?		unknown	0	0	3	0	0	-1
1682	TRUE	Empty	Uncharacterized protein OS=Gallus gallus GN=PLXNA1 PE=4 SV=2	F1N804_CHICK		213 kDa		unknown	0	0	4	0	0	0.06963
1683	TRUE	Empty	Uncharacterized protein OS=Gallus gallus GN=SF3A1 PE=4 SV=1	F1NU16_CHICK (+1)		88 kDa	TRUE	unknown	4	0	0	0.20147	0	0
1684	TRUE	Empty	Uncharacterized protein (Fragment) OS=Gallus gallus GN=Gga.14530 PE=4 SV=2	F1P2G5_CHICK		568 kDa	TRUE	unknown	5	0	0	0.036964	0	0
1685	TRUE	Empty	Uncharacterized protein (Fragment) OS=Gallus gallus GN=RPL35A PE=4 SV=2	F1NQ35_CHICK (+4)		16 kDa		unknown	0	1	2	0	0.22251	0.54519
1686	TRUE	Empty	status=active	UPI00003AC5D4		84 kDa	TRUE	unknown	0	1	2	0	0.039497	0.088057
1687	TRUE	Empty	Uncharacterized protein OS=Gallus gallus GN=RCJMB04_31h10 PE=2 SV=1	Q5ZI14_CHICK (+2)		40 kDa	TRUE	unknown	0	4	0	0	0.38387	0
1688	TRUE	Empty	Uncharacterized protein OS=Gallus gallus GN=LOC776067 PE=4 SV=2	F1NA05_CHICK		334 kDa	TRUE	unknown	0	2	0	0	0.019897	0
1689	TRUE	Empty	Uncharacterized protein OS=Gallus gallus GN=USO1 PE=4 SV=2	F1NJ69_CHICK (+1)		106 kDa	TRUE	unknown	0	0	3	0	0	0.10625
1690	TRUE	Empty	Muscleblind-like 2 isoform 2 OS=Gallus gallus GN=MBNL2 PE=2 SV=1	A3F957_CHICK (+4)		40 kDa	TRUE	unknown	3	0	0	0.34673	0	0
1691	TRUE	Empty	Uncharacterized protein (Fragment) OS=Gallus gallus GN=PSMD11 PE=4 SV=2	F1NPA2_CHICK		48 kDa	TRUE	unknown	0	0	3	0	0	0.24868
1692	TRUE	Empty	Lens epithelium-derived growth factor OS=Gallus gallus GN=PSIP1 PE=4 SV=2	F1NKN6_CHICK (+8)		63 kDa	TRUE	unknown	0	4	1	0	0.2338	0.058378
1693	TRUE	Empty	Uncharacterized protein OS=Gallus gallus GN=HNRNPD PE=2 SV=1	Q5ZIH1_CHICK		29 kDa		unknown	3	0	0	0.9431	0	0
1694	TRUE	Empty	status=inactive	UPI0000ECAD6F		44 kDa	TRUE	unknown	0	0	3	0	0	0.27532
1695	TRUE	Empty	Cullin-5 OS=Homo sapiens GN=CUL5 PE=1 SV=4	Q93034		?		unknown	0	0	3	0	0	-1
1696	TRUE	Empty	Uncharacterized protein (Fragment) OS=Gallus gallus GN=NPTN PE=4 SV=2	F1NI46_CHICK (+6)		42 kDa		unknown	0	2	0	0	0.16809	0
1697	TRUE	Empty	status=inactive	UPI000060F06E		41 kDa	TRUE	unknown	3	0	0	0.47079	0	0
1698	TRUE	Empty	status=active	UPI0001A595C0		210 kDa	TRUE	unknown	0	3	0	0	0.048205	0
1699	TRUE	Empty	status=active	UPI0003503D84 (+1)		64 kDa		unknown	0	3	0	0	0.16767	0
1700	TRUE	Empty	status=inactive	UPI0000E7FEA5 (+4)		325 kDa		unknown	0	1	2	0	0.01019	0.022357
1701	TRUE	Empty	status=active	UPI000019D5EC		46 kDa	TRUE	unknown	0	2	0	0	0.23638	0
1702	TRUE	Empty	status=inactive	UPI0000ECA65B		218 kDa	TRUE	unknown	2	0	0	0.038521	0	0
1703	TRUE	Empty	COL6A3 protein OS=Homo sapiens GN=COL6A3 PE=2 SV=1	B7ZW00 (+5)		?		unknown	0	0	3	0	0	-1
1704	TRUE	Empty	status=active	UPI00003A9996 (+1)		25 kDa	TRUE	unknown	0	0	4	0	0	0.74588
1705	TRUE	Empty	status=active	UPI00001D6E26 (+2)		40 kDa	TRUE	unknown	2	0	0	0.22271	0	0
1706	TRUE	Empty	Histone H1.03 OS=Gallus gallus PE=3 SV=2	H103_CHICK		23 kDa	TRUE	unknown	2	0	0	6.2506	0	0
1707	TRUE	Empty	Epididymis luminal secretory protein 52 OS=Homo sapiens GN=EL52 PE=2 SV=1	K9JA46 (+4)		?	TRUE	unknown	0	0	2	0	0	-1
1708	TRUE	Empty	status=active	UPI00000FB989 (+2)		47 kDa	TRUE	unknown	3	0	0	0.28877	0	0
1709	TRUE	Empty	status=active	UPI0000171389		193 kDa	TRUE	unknown	0	0	4	0	0	0.077218
1710	TRUE	Empty	status=active	UPI00003AB431 (+1)		224 kDa	TRUE	unknown	0	3	0	0	0.06076	0
1711	TRUE	Empty	status=active	UPI00003AC304 (+4)		46 kDa	TRUE	unknown	0	1	2	0	0.15142	0.25787
1712	TRUE	Empty	status=inactive	UPI00004489EA		44 kDa	TRUE	unknown	0	3	0	0	0.34421	0
1713	TRUE	Empty	status=inactive	UPI000044941B		183 kDa	TRUE	unknown	2	0	0	0.046016	0	0
1714	TRUE	Empty	status=inactive	UPI0000ECBC2A		34 kDa	TRUE	unknown	3	0	0	0.42218	0	0
1715	TRUE	Empty	status=inactive	UPI0000ECC377		189 kDa	TRUE	unknown	0	3	0	0	0.072334	0
1716	TRUE	Empty	BRCA1-A complex subunit BRE OS=Gallus gallus GN=BRE PE=2 SV=1	BRE_CHICK (+4)		43 kDa		unknown	1	2	1	0.097467	0.16214	0.084798
1717	TRUE	Empty	Aldehyde dehydrogenase-6 OS=Gallus gallus GN=ALDH6 PE=2 SV=1	Q9DD46_CHICK (+5)		56 kDa		unknown	0	2	2	0	0.1232	0.1344
1718	TRUE	Empty	Transcription factor GA binding protein, alpha subunit OS=Gallus gallus PE=2 SV=1	Q5DWF6_CHICK (+6)		51 kDa		unknown	2	2	0	0.16997	0.13555	0
1719	TRUE	Empty	Casein kinase II subunit alpha OS=Gallus gallus GN=CSNK2A1 PE=2 SV=1	CSK21_CHICK (+10)		45 kDa		unknown	0	2	1	0	0.15537	0.081392
1720	TRUE	Empty	Uncharacterized protein OS=Gallus gallus GN=EMC3 PE=4 SV=1	E1BQV4_CHICK (+3)		30 kDa		unknown	0	2	1	0	0.24074	0.12358
1721	TRUE	Empty	Uncharacterized protein OS=Gallus gallus GN=EIF2D PE=4 SV=2	F1NDA1_CHICK (+3)		66 kDa		unknown	0	2	1	0	0.10366	0.055009
1722	TRUE	Empty	Uncharacterized protein OS=Gallus gallus GN=FABP5 PE=2 SV=1	Q5ZIR7_CHICK (+3)		15 kDa		unknown	0	2	1	0	0.89419	0.25412
1723	TRUE	Empty	status=active	UPI00003A9D4A (+8)		59 kDa		unknown	0	2	1	0	0.11691	0.06183
1724	TRUE	Empty	Calmodulin, striated muscle OS=Gallus gallus GN=CCM1 PE=3 SV=2	CALMS_CHICK (+1)		17 kDa	TRUE	unknown	0	1	2	0	1.6306	1.2638

1725	TRUE	Empty	Uncharacterized protein OS=Gallus gallus GN=SNX4 PE=4 SV=1	E1BQF6_CHICK (+5)		47 kDa		unknown	0	1	2	0	0.072076	0.16352
1726	TRUE	Empty	Uncharacterized protein OS=Gallus gallus GN=ARL1 PE=3 SV=1	E1BVB0_CHICK (+4)		20 kDa		unknown	0	1	2	0	0.17035	0.40668
1727	TRUE	Empty	Uncharacterized protein (Fragment) OS=Gallus gallus GN=PTP4A2 PE=4 SV=1	F1NLG3_CHICK (+6)		20 kDa		unknown	0	1	2	0	0.17537	0.41971
1728	TRUE	Empty	Uncharacterized protein OS=Gallus gallus GN=POFUT1 PE=4 SV=1	F1NV29_CHICK (+6)		32 kDa		unknown	0	1	2	0	0.10601	0.24484
1729	TRUE	Empty	60S ribosomal protein L36 OS=Gallus gallus GN=RPL36 PE=3 SV=1	RL36_CHICK (+6)		12 kDa		unknown	0	1	2	0	0.67132	0.73235
1730	TRUE	Empty	status=active	UPI0002C88225 (+4)		31 kDa		unknown	0	1	2	0	0.11033	0.25541
1731	TRUE	Empty	status=active	UPI00003AD529 (+6)		20 kDa		unknown	0	1	2	0	0.17537	0.41971
1732	TRUE	Empty	Uncharacterized protein OS=Gallus gallus GN=PTGDS PE=3 SV=2	E1BTX1_CHICK (+3)		21 kDa		unknown	0	1	2	0	0.16654	0.39681
1733	TRUE	Empty	Uncharacterized protein OS=Gallus gallus GN=COMMD6 PE=4 SV=2	F1NY50_CHICK (+2)		23 kDa		unknown	0	1	2	0	0.15208	0.35969
1734	TRUE	Empty	Uncharacterized protein OS=Gallus gallus GN=PECR PE=4 SV=1	F1NZH6_CHICK (+5)		32 kDa		unknown	0	2	1	0	0.22614	0.11648
1735	TRUE	Empty	status=active	UPI00004438B1 (+1)		30 kDa		unknown	0	1	2	0	0.11328	0.26262
1736	TRUE	Empty	Uncharacterized protein OS=Gallus gallus GN=NDUFA12 PE=4 SV=2	F1NW95_CHICK (+3)		17 kDa		unknown	0	1	2	0	0.20563	0.49964
1737	TRUE	Empty	Uncharacterized protein OS=Gallus gallus GN=SEC61A1 PE=3 SV=2	F1NBN1_CHICK (+16)		52 kDa	TRUE	unknown	0	3	1	0	0.20686	0.070124
1738	TRUE	Empty	Methionine aminopeptidase 2 OS=Gallus gallus GN=METAP2 PE=2 SV=1	Q5ZIL5_CHICK (+7)		53 kDa		unknown	0	2	1	0	0.13136	0.069225
1739	TRUE	Empty	Protein kinase C inhibitor OS=Gallus gallus GN=chPKCI PE=2 SV=1	Q9I882_CHICK (+1)		14 kDa		unknown	0	2	1	0	0.58704	0.28038
1740	TRUE	Empty	SPRY domain-containing protein 7 OS=Gallus gallus GN=SPRYD7 PE=2 SV=1	SPRY7_CHICK (+4)		22 kDa		unknown	0	1	2	0	0.15856	0.37626
1741	TRUE	Empty	status=active	UPI0002C62355		31 kDa	TRUE	unknown	0	1	2	0	0.11075	0.25642
1742	TRUE	Empty	Uncharacterized protein OS=Gallus gallus GN=TRNT1 PE=3 SV=2	F1NMW8_CHICK (+3)		42 kDa		unknown	2	1	0	0.21318	0.17001	0
1743	TRUE	Empty	Uncharacterized protein OS=Gallus gallus GN=ISOC1 PE=4 SV=2	F1NS88_CHICK (+5)		32 kDa		unknown	1	1	2	0.13436	0.10715	0.24764
1744	TRUE	Empty	Uncharacterized protein OS=Gallus gallus GN=NUDT2 PE=3 SV=1	R4GJX0_CHICK (+3)		17 kDa		unknown	0	1	3	0	0.20563	0.83753
1745	TRUE	Empty	Stathmin (Fragment) OS=Gallus gallus PE=2 SV=1	Q9I895_CHICK (+6)		13 kDa		unknown	0	2	1	0	0.63624	0.30111
1746	TRUE	Empty	status=active	UPI00003AEB63 (+8)		73 kDa		unknown	0	2	1	0	0.09429	0.050157
1747	TRUE	Empty	Uncharacterized protein OS=Gallus gallus GN=LRRC57 PE=4 SV=1	E1C6Q6_CHICK (+2)		27 kDa		unknown	0	2	1	0	0.27512	0.14012
1748	TRUE	Empty	Uncharacterized protein OS=Gallus gallus GN=ACADS PE=2 SV=1	Q5ZL56_CHICK (+5)		45 kDa		unknown	0	1	2	0	0.075558	0.17174
1749	TRUE	Empty	Uncharacterized protein (Fragment) OS=Gallus gallus GN=UBA2 PE=3 SV=2	F1P226_CHICK (+6)		45 kDa		unknown	0	2	1	0	0.15497	0.081188
1750	TRUE	Empty	status=active	UPI0000181FCC (+9)		15 kDa		unknown	1	2	0	0.29208	0.52586	0
1751	TRUE	Empty	status=inactive	UPI000060F199 (+4)		73 kDa		unknown	0	2	0	0	0.093548	0
1752	TRUE	Empty	Uncharacterized protein (Fragment) OS=Gallus gallus GN=DYNC1LI2 PE=4 SV=2	F1P2G9_CHICK (+7)		51 kDa		unknown	0	2	2	0	0.13807	0.15062
1753	TRUE	Empty	Paraspeckle component 1 OS=Gallus gallus GN=PSPC1 PE=2 SV=1	PSPC1_CHICK (+5)		58 kDa		unknown	0	2	0	0	0.11854	0
1754	TRUE	Empty	DNA topoisomerase 2 OS=Gallus gallus GN=TOP2B PE=3 SV=2	E1BZ19_CHICK (+5)		183 kDa	TRUE	unknown	0	3	0	0	0.055361	0
1755	TRUE	Empty	Uncharacterized protein OS=Gallus gallus GN=MCM4 PE=3 SV=1	E1C2U4_CHICK (+2)		97 kDa		unknown	0	3	0	0	0.10723	0
1756	TRUE	Empty	Uncharacterized protein OS=Gallus gallus GN=AP1G1 PE=4 SV=2	F1NDA9_CHICK (+1)		91 kDa		unknown	0	0	3	0	0	0.1243
1757	TRUE	Empty	Histone H2A OS=Gallus gallus GN=H2AFY PE=2 SV=1	O93325_CHICK (+10)		24 kDa	TRUE	unknown	0	3	0	0	0.50434	0
1758	TRUE	Empty	Uncharacterized protein OS=Gallus gallus GN=CAB39 PE=4 SV=1	R4GI86_CHICK (+1)		40 kDa	TRUE	unknown	0	0	3	0	0	0.30448
1759	TRUE	Empty	status=active	UPI0000185E3C (+1)		46 kDa	TRUE	unknown	0	2	0	0	1.3949	0
1760	TRUE	Empty	Uncharacterized protein OS=Gallus gallus GN=Gga.21231 PE=4 SV=2	F1NGG8_CHICK (+10)		81 kDa		unknown	0	3	0	0	0.12911	0
1761	TRUE	Empty	Prolyl 3-hydroxylase 1 OS=Gallus gallus GN=LEPRE1 PE=1 SV=1	P3H1_CHICK		82 kDa		unknown	0	3	0	0	0.12837	0
1762	TRUE	Empty	status=active	UPI0002C86650 (+4)		62 kDa		unknown	0	3	0	0	0.17319	0
1763	TRUE	Empty	Uncharacterized protein (Fragment) OS=Gallus gallus GN=SEC11C PE=4 SV=2	F1NAZ9_CHICK		22 kDa	TRUE	unknown	0	0	3	0	0	0.60393
1764	TRUE	Empty	cDNA FLJ11308 fis, clone PLACE1010074, highly similar to Sorting nexin-2 OS=Homo sapiens PE=2 SV=1	B3KML1 (+3)		?		unknown	0	0	3	0	0	-1
1765	TRUE	Empty	Uncharacterized protein OS=Gallus gallus PE=4 SV=2	F1NX83_CHICK (+7)		174 kDa		unknown	0	0	3	0	0	0.063593
1766	TRUE	Empty	Annexin OS=Homo sapiens GN=ANXA2 PE=3 SV=1	A0A024R5Z7 (+12)		?		unknown	0	0	2	0	0	-1
1767	TRUE	Empty	HSPA12B protein OS=Homo sapiens GN=HSPA12B PE=2 SV=1	B7ZLP2 (+1)		?		unknown	0	0	2	0	0	-1
1768	TRUE	Empty	Uncharacterized protein OS=Gallus gallus GN=SF3B14 PE=4 SV=1	E1BW94_CHICK (+1)		15 kDa		unknown	0	0	2	0	0	1.0249
1769	TRUE	Empty	Uncharacterized protein OS=Gallus gallus GN=GDPD2 PE=4 SV=1	E1C392_CHICK (+8)		61 kDa		unknown	0	0	2	0	0	0.12294
1770	TRUE	Empty	Uncharacterized protein OS=Gallus gallus GN=HPGD PE=3 SV=2	E1C688_CHICK (+4)		29 kDa		unknown	0	0	2	0	0	0.27366
1771	TRUE	Empty	Uncharacterized protein OS=Gallus gallus GN=SERPINA1 PE=3 SV=1	E1C7T1_CHICK (+3)		49 kDa		unknown	2	0	0	0.1798	0	0
1772	TRUE	Empty	Uncharacterized protein (Fragment) OS=Gallus gallus GN=FBXO6 PE=4 SV=2	F1NJI6_CHICK (+4)		32 kDa		unknown	0	0	2	0	0	0.24211
1773	TRUE	Empty	Uncharacterized protein OS=Gallus gallus GN=SLC44A1 PE=4 SV=2	F1NU04_CHICK (+5)		72 kDa		unknown	0	2	0	0	0.09459	0
1774	TRUE	Empty	Uncharacterized protein OS=Gallus gallus GN=TRAPPC4 PE=4 SV=2	F1NU38_CHICK (+1)		24 kDa		unknown	0	0	2	0	0	0.33394
1775	TRUE	Empty	Uncharacterized protein (Fragment) OS=Gallus gallus GN=BCL2L15 PE=4 SV=2	F1NYS5_CHICK (+1)		19 kDa	TRUE	unknown	0	2	0	0	0.4028	0
1776	TRUE	Empty	Uncharacterized protein OS=Gallus gallus GN=PCCA PE=4 SV=2	F1P0M2_CHICK (+10)		79 kDa	TRUE	unknown	0	0	2	0	0	0.095025
1777	TRUE	Empty	Uncharacterized protein OS=Gallus gallus GN=APAF1 PE=4 SV=2	F1P1P3_CHICK (+9)		142 kDa	TRUE	unknown	2	0	0	0.059316	0	0
1778	TRUE	Empty	Cadherin-13 OS=Gallus gallus GN=CDH13 PE=4 SV=2	F1P5T8_CHICK (+7)		79 kDa		unknown	0	0	2	0	0	0.094747
1779	TRUE	Empty	Acyl-CoA synthetase short-chain family member 2, isoform CRA_c OS=Homo sapiens GN=ACSS2 PE=4 SV=1	H0UIA1 (+6)		?		unknown	0	0	2	0	0	-1
1780	TRUE	Empty	Mothers against decapentaplegic homolog OS=Gallus gallus GN=LOC769000 PE=3 SV=2	H9L0G3_CHICK (+26)		52 kDa	TRUE	unknown	0	2	0	0	0.13313	0
1781	TRUE	Empty	Galectin-related protein OS=Gallus gallus GN=LGALSL PE=2 SV=1	LEGL_CHICK (+5)		19 kDa		unknown	0	0	2	0	0	0.43942
1782	TRUE	Empty	Ectonucleoside triphosphate diphosphohydrolase 1 OS=Homo sapiens GN=ENTPD1 PE=1 SV=1	P49961 (+4)		?		unknown	0	0	2	0	0	-1
1783	TRUE	Empty	Soluble epoxide hydrolase OS=Gallus gallus GN=EPHX2 PE=2 SV=1	Q45QT1_CHICK (+5)		63 kDa		unknown	0	0	2	0	0	0.11911
1784	TRUE	Empty	Cytoplasmic activation-proliferation-associated protein 1 OS=Gallus gallus GN=CAPRIN1 PE=2 SV=1	Q5XNV3_CHICK (+5)		78 kDa		unknown	0	2	0	0	0.087749	0
1785	TRUE	Empty	Uncharacterized protein OS=Gallus gallus GN=C8H1ORF123 PE=4 SV=1	R4GKJ0_CHICK (+2)		18 kDa		unknown	0	0	2	0	0	0.76694
1786	TRUE	Empty	Uncharacterized protein OS=Gallus gallus GN=NQO2 PE=4 SV=1	R4GLI9_CHICK (+3)		27 kDa		unknown	0	0	2	0	0	0.29876
1787	TRUE	Empty	status=inactive	UPI0000611C9A (+10)		85 kDa		unknown	0	2	0	0	0.079851	0
1788	TRUE	Empty	status=active	UPI000350382F (+7)		27 kDa		unknown	0	2	0	0	0.27015	0
1789	TRUE	Empty	cDNA FLJ54176, highly similar to Signal recognition particle 68 kDa protein OS=Homo sapiens PE=2 SV=1	B4DVI7 (+3)		?		unknown	0	0	2	0	0	-1
1790	TRUE	Empty	Rho GTPase activating protein OS=Homo sapiens PE=2 SV=1	A9UK01 (+2)		?		unknown	0	0	4	0	0	-1
1791	TRUE	Empty	Ribosome-binding protein 1 OS=Homo sapiens GN=RRBP1 PE=4 SV=1	A0A087WVV2 (+7)		?		unknown	0	0	3	0	0	-1
1792	TRUE	Empty	Protein S100 OS=Gallus gallus GN=S100A16 PE=3 SV=1	R4GJQ6_CHICK (+1)		12 kDa		unknown	0	0	2	0	0	0.7405
1793	TRUE	Empty	Macrophage scavenger receptor types I and II OS=Homo sapiens GN=MSR1 PE=2 SV=1	B4DDJ5 (+2)		?		unknown	0	0	2	0	0	-1
1794	TRUE	Empty	status=inactive	UPI000044A8A6		466 kDa		unknown	0	3	0	0	0.021475	0
1795	TRUE	Empty	Proteinase 3 OS=Homo sapiens GN=PRTN3 PE=2 SV=1	D6CHE9 (+2)		?		unknown	0	0	2	0	0	-1
1796	TRUE	Empty	Sorting nexin-1 OS=Homo sapiens GN=SNX1 PE=1 SV=1	H0YK42 (+5)		?		unknown	0	0	3	0	0	-1
1797	TRUE	Empty	Uncharacterized protein OS=Gallus gallus GN=ABAT PE=3 SV=2	E1C8M8_CHICK (+6)		61 kDa		unknown	0	0	3	0	0	0.19116
1798	TRUE	Empty	tr|F1P153|F1P153_CHICK-DECOY Uncharacterized protein OS=Gallus gallus GN=Gga.56106...	tr|F1P153|F1P153_CHICK-DECOY		?	TRUE	unknown	0	3	0	0	-1	0
1799	TRUE	Empty	Uncharacterized protein (Fragment) OS=Gallus gallus GN=TMEM65 PE=4 SV=2	F1NA04_CHICK (+1)		17 kDa		unknown	0	0	3	0	0	0.81705
1800	TRUE	Empty	Procollagen-lysine 1, 2-oxoglutarate 5-dioxygenase 1, isoform CRA_a OS=Homo sapiens GN=PLOD1 PE=4 SV=1	A0A024R4H0 (+5)		?		unknown	0	0	2	0	0	-1
1801	TRUE	Empty	Lysine--tRNA ligase OS=Homo sapiens GN=KARS PE=1 SV=3	Q15046 (+1)		?		unknown	0	0	2	0	0	-1
1802	TRUE	Empty	Nicalin OS=Gallus gallus GN=NCLN PE=3 SV=2	H9L023_CHICK (+5)		63 kDa		unknown	0	0	2	0	0	0.11932
1803	TRUE	Empty	Eukaryotic translation initiation factor 4 gamma 2 (Fragment) OS=Gallus gallus GN=EIF4G2 PE=4 SV=1	F1P4A7_CHICK (+4)		102 kDa		unknown	0	3	0	0	0.10173	0
1804	TRUE	Empty	Uncharacterized protein OS=Gallus gallus GN=RCJMB04_29h3 PE=2 SV=1	Q5ZI80_CHICK (+4)		52 kDa	TRUE	unknown	3	0	0	0.26304	0	0
1805	TRUE	Empty	status=active	UPI0000443929 (+1)		83 kDa		unknown	0	2	0	0	0.082741	0
1806	TRUE	Empty	Cleavage and polyadenylation specificity factor subunit 6 OS=Gallus gallus GN=CPSF6 PE=2 SV=1	CPSF6_CHICK (+1)		59 kDa		unknown	3	0	0	0.22638	0	0

1807	TRUE	Empty	Uncharacterized protein OS=Gallus gallus GN=BUB3 PE=4 SV=2	E1C9E5_CHICK (+8)		44 kDa	TRUE	unknown	0	0	2	0	0	0.17451
1808	TRUE	Empty	Protein FAM49A OS=Gallus gallus GN=FAM49A PE=2 SV=1	FA49A_CHICK		37 kDa	TRUE	unknown	0	3	0	0	0.55468	0
1809	TRUE	Empty	Attachment region binding protein (Fragment) OS=Gallus gallus GN=ARBP PE=1 SV=1	O42403_CHICK		34 kDa		unknown	0	3	0	0	0.33796	0
1810	TRUE	Empty	Uncharacterized protein OS=Gallus gallus GN=PHEX PE=4 SV=2	E1BR88_CHICK		87 kDa		unknown	0	3	0	0	0.12056	0
1811	TRUE	Empty	Epidermal growth factor receptor kinase substrate 8-like protein 2 OS=Homo sapiens GN=EPS8L2 PE=1 SV=2	Q9H6S3 (+1)		?		unknown	0	0	3	0	0	-1
1812	TRUE	Empty	cDNA FLJ61233, highly similar to Kinesin light chain 1 OS=Homo sapiens PE=2 SV=1	B4DGB3 (+26)		?		unknown	0	0	2	0	0	-1
1813	TRUE	Empty	Small nuclear ribonucleoprotein E OS=Gallus gallus GN=SNRPE PE=3 SV=1	RUXE_CHICK (+2)		11 kDa		unknown	3	0	0	1.7823	0	0
1814	TRUE	Empty	status=active	UPI0002C6147C (+17)		127 kDa		unknown	3	0	0	0.10169	0	0
1815	TRUE	Empty	Uncharacterized protein OS=Gallus gallus GN=SMARCE1 PE=4 SV=1	F1NCC9_CHICK (+4)		47 kDa		unknown	0	2	0	0	0.14989	0
1816	TRUE	Empty	Uncharacterized protein OS=Gallus gallus GN=UBE2H PE=2 SV=1	Q5ZLI7_CHICK (+3)		21 kDa		unknown	2	0	0	0.45888	0	0
1817	TRUE	Empty	status=inactive	UPI0000447967 (+2)		87 kDa		unknown	0	1	2	0	0.038524	0.085842
1818	TRUE	Empty	status=inactive	UPI00004480A3		196 kDa	TRUE	unknown	0	0	2	0	0	0.056302
1819	TRUE	Empty	Splicing factor 3a, subunit 1, 120kDa, isoform CRA_a OS=Homo sapiens GN=SF3A1 PE=4 SV=1	A0A024R1K8 (+2)		?		unknown	0	0	2	0	0	-1
1820	TRUE	Empty	Uncharacterized protein OS=Gallus gallus GN=DOCK10 PE=4 SV=2	F1NMY8_CHICK		250 kDa		unknown	0	0	3	0	0	0.043976
1821	TRUE	Empty	cDNA FLJ50285, highly similar to Arginyl-tRNA synthetase (EC 6.1.1.19) OS=Homo sapiens PE=2 SV=1	B4DXW6 (+2)		?		unknown	0	0	2	0	0	-1
1822	TRUE	Empty	UPI000350377F-DECOY	UPI000350377F-DECOY (+7)		?	TRUE	unknown	0	3	0	0	-1	0
1823	TRUE	Empty	status=active	UPI000024A53B (+1)		34 kDa	TRUE	unknown	3	0	0	0.42218	0	0
1824	TRUE	Empty	status=active	UPI0002C881D0 (+6)		285 kDa		unknown	0	0	2	0	0	0.025477
1825	TRUE	Empty	Interleukin enhancer binding factor 3, 90kDa, isoform CRA_d OS=Homo sapiens GN=ILF3 PE=4 SV=1	A0A024R7C7 (+12)		?		unknown	0	0	2	0	0	-1
1826	TRUE	Empty	SWAP-70 protein, isoform CRA_b OS=Homo sapiens GN=SWAP70 PE=2 SV=1	B3KUB9 (+3)		?		unknown	0	0	2	0	0	-1
1827	TRUE	Empty	Integrin beta OS=Homo sapiens PE=2 SV=1	B4E0R1 (+3)		?		unknown	0	0	3	0	0	-1
1828	TRUE	Empty	Uncharacterized protein OS=Gallus gallus GN=LOC769339 PE=4 SV=2	E1BYN1_CHICK (+7)		62 kDa		unknown	0	0	2	0	0	0.12156
1829	TRUE	Empty	Uncharacterized protein (Fragment) OS=Gallus gallus GN=PFDN4 PE=4 SV=2	F1NP43_CHICK (+3)		16 kDa	TRUE	unknown	0	0	2	0	0	0.55437
1830	TRUE	Empty	Keratin, type I cytoskeletal 17 OS=Homo sapiens GN=KRT17 PE=1 SV=2	Q04695 (+1)		?		unknown	0	0	2	0	0	-1
1831	TRUE	Empty	Glucosamine-6-phosphate isomerase OS=Gallus gallus GN=RCJMB04_7a23 PE=2 SV=1	Q5ZLA0_CHICK (+3)		32 kDa		unknown	0	2	1	0	0.2236	0.11523
1832	TRUE	Empty	status=active	UPI0003506511 (+9)		38 kDa		unknown	0	0	3	0	0	0.32255
1833	TRUE	Empty	Uncharacterized protein OS=Gallus gallus GN=KLC4 PE=4 SV=2	E1C0I2_CHICK (+10)		74 kDa	TRUE	unknown	0	2	0	0	0.093108	0
1834	TRUE	Empty	Uncharacterized protein OS=Gallus gallus GN=PRPF6 PE=4 SV=2	F1N9U0_CHICK (+2)		107 kDa		unknown	0	2	0	0	0.09676	0
1835	TRUE	Empty	Uncharacterized protein OS=Gallus gallus GN=GFPT2 PE=4 SV=2	F1P1F9_CHICK (+5)		77 kDa	TRUE	unknown	0	2	0	0	0.088797	0
1836	TRUE	Empty	Uncharacterized protein OS=Gallus gallus GN=LOC422249 PE=2 SV=1	Q5ZLM5_CHICK		74 kDa	TRUE	unknown	0	3	0	0	0.14228	0
1837	TRUE	Empty	status=active	UPI000000B50D		40 kDa	TRUE	unknown	0	3	0	0	0.28173	0
1838	TRUE	Empty	status=inactive	UPI0000447A22 (+11)		49 kDa		unknown	0	1	2	0	0.068424	0.15494
1839	TRUE	Empty	status=inactive	UPI0000566FC4		180 kDa	TRUE	unknown	0	0	4	0	0	0.082948
1840	TRUE	Empty	Uncharacterized protein (Fragment) OS=Gallus gallus GN=UBE2M PE=3 SV=1	R4GFA5_CHICK		20 kDa		unknown	2	0	0	0.48553	0	0
1841	TRUE	Empty	Probable cytosolic iron-sulfur protein assembly protein CIAO1 OS=Gallus gallus GN=CIAO1 PE=3 SV=1	R4GK07_CHICK (+6)		40 kDa		unknown	2	0	0	0.2188	0	0
1842	TRUE	Empty	Uncharacterized protein OS=Gallus gallus GN=ATP6V1C2 PE=4 SV=2	F1NK97_CHICK (+3)		44 kDa		unknown	0	0	3	0	0	0.27225
1843	TRUE	Empty	status=active	UPI00003AA93F (+1)		23 kDa		unknown	0	2	0	0	0.31917	0
1844	TRUE	Empty	status=active	UPI00003ADDFB (+6)		65 kDa		unknown	0	2	1	0	0.10531	0.055862
1845	TRUE	Empty	Uncharacterized protein OS=Gallus gallus GN=CUL3 PE=3 SV=2	E1BYQ3_CHICK		87 kDa	TRUE	unknown	0	0	4	0	0	0.17744
1846	TRUE	Empty	Core-binding factor beta OS=Gallus gallus PE=2 SV=2	Q8QGE8_CHICK (+2)		22 kDa		unknown	0	0	4	0	0	0.90809
1847	TRUE	Empty	Uncharacterized protein OS=Gallus gallus GN=SPAG9 PE=4 SV=2	F1NBV9_CHICK		149 kDa		unknown	3	0	0	0.086166	0	0
1848	TRUE	Empty	Uncharacterized protein (Fragment) OS=Gallus gallus GN=POFUT2 PE=4 SV=1	F6UZB4_CHICK		51 kDa		unknown	0	0	2	0	0	0.14993
1849	TRUE	Empty	Uncharacterized protein OS=Gallus gallus GN=PCYT1A PE=4 SV=1	E1C4K8_CHICK		42 kDa	TRUE	unknown	2	0	0	0.21018	0	0
1850	TRUE	Empty	Laminin beta 2-like chain OS=Gallus gallus PE=2 SV=1	O57484_CHICK		196 kDa	TRUE	unknown	0	0	3	0	0	0.056498
1851	TRUE	Empty	Uncharacterized protein OS=Gallus gallus GN=Gga.9931 PE=4 SV=2	E1BX07_CHICK		284 kDa	TRUE	unknown	0	0	3	0	0	0.038691
1852	TRUE	Empty	Heat shock protein 90kDa alpha (Cytosolic), class B member 1, isoform CRA_a OS=Homo sapiens GN=HSP90AB1 PE=4 SV=1	A0A024RD80 (+3)		?		unknown	0	0	3	0	0	-1
1853	TRUE	Empty	Uncharacterized protein OS=Gallus gallus GN=ATP6V1D PE=4 SV=1	E1BT00_CHICK (+2)		28 kDa		unknown	0	0	2	0	0	0.28195
1854	TRUE	Empty	Uncharacterized protein OS=Gallus gallus GN=CASK PE=4 SV=2	E1BWL4_CHICK		105 kDa	TRUE	unknown	0	0	2	0	0	0.070146
1855	TRUE	Empty	Uncharacterized protein OS=Gallus gallus GN=LOC423919 PE=4 SV=2	F1P5G2_CHICK (+1)		80 kDa		unknown	0	2	1	0	0.085477	0.045572
1856	TRUE	Empty	HN1-like protein OS=Gallus gallus PE=2 SV=1	I7L9D4_CHICK (+2)		20 kDa		unknown	0	0	2	0	0	0.40922
1857	TRUE	Empty	status=inactive	UPI0000611CAD (+8)		92 kDa		unknown	0	2	0	0	0.074357	0
1858	TRUE	Empty	Uncharacterized protein OS=Gallus gallus GN=LSS PE=4 SV=2	F1NED1_CHICK (+1)		77 kDa		unknown	0	3	0	0	0.13662	0
1859	TRUE	Empty	status=active	UPI00003AE770 (+1)		51 kDa	TRUE	unknown	0	2	0	0	0.13555	0
1860	TRUE	Empty	Uncharacterized protein OS=Gallus gallus GN=CLINT1 PE=4 SV=2	F1NPP8_CHICK (+6)		71 kDa	TRUE	unknown	0	2	0	0	0.097061	0
1861	TRUE	Empty	status=active	UPI0003506CA0		25 kDa		unknown	0	0	3	0	0	0.51302
1862	TRUE	Empty	Fibulin-1 OS=Homo sapiens GN=FBLN1 PE=3 SV=1	B1AHL2		?		unknown	0	0	3	0	0	-1
1863	TRUE	Empty	Uncharacterized protein (Fragment) OS=Gallus gallus GN=PTGS1 PE=4 SV=2	F1P3I2_CHICK (+3)		68 kDa		unknown	0	0	2	0	0	0.11115
1864	TRUE	Empty	Beta-tropomyosin OS=Gallus gallus GN=BRT-2 PE=2 SV=1	Q05705_CHICK (+1)		29 kDa	TRUE	unknown	2	0	0	2.3207	0	0
1865	TRUE	Empty	status=active	UPI00001A0DAD (+2)		54 kDa		unknown	0	3	0	0	0.20082	0
1866	TRUE	Empty	status=inactive	UPI0000448E4B		95 kDa	TRUE	unknown	3	0	0	0.13709	0	0
1867	TRUE	Empty	status=inactive	UPI0000448F4A		62 kDa	TRUE	unknown	3	0	0	0.21674	0	0
1868	TRUE	Empty	status=active	UPI00004547CB		87 kDa	TRUE	unknown	0	2	0	0	0.078068	0
1869	TRUE	Empty	status=inactive	UPI00006117E4		48 kDa		unknown	3	0	0	0.28366	0	0
1870	TRUE	Empty	status=active	UPI0000D71D8C		182 kDa	TRUE	unknown	3	0	0	0.069763	0	0
1871	TRUE	Empty	status=inactive	UPI00015A799C (+1)		127 kDa	TRUE	unknown	2	0	0	0.066459	0	0
1872	TRUE	Empty	status=inactive	UPI000240B643		38 kDa		unknown	0	2	0	0	0.18478	0
1873	TRUE	Empty	status=active	UPI00035045FE (+1)		62 kDa		unknown	0	0	2	0	0	0.12179
1874	TRUE	Empty	ATP synthase subunit alpha OS=Gallus gallus GN=ATP5A1W PE=3 SV=2	F1NGA2_CHICK (+1)		60 kDa	TRUE	unknown	0	1	2	0	2.6902	1.44
1875	TRUE	Empty	Uncharacterized protein OS=Gallus gallus GN=IAH1 PE=4 SV=2	F1NKW4_CHICK (+6)		28 kDa		unknown	0	2	1	0	0.26417	0.13488
1876	TRUE	Empty	Very-long-chain (3R)-3-hydroxyacyl-CoA dehydratase OS=Gallus gallus GN=PTPLAD1 PE=4 SV=2	F1NTP4_CHICK (+8)		43 kDa		unknown	0	1	2	0	0.078346	0.17834
1877	TRUE	Empty	Uncharacterized protein OS=Gallus gallus GN=CARHSP1 PE=4 SV=1	R4GJC0_CHICK (+4)		16 kDa		unknown	0	1	2	0	0.21298	0.51938
1878	TRUE	Empty	Ufm1-specific protease 2 OS=Gallus gallus GN=UFSP2 PE=4 SV=1	F1NUY9_CHICK (+7)		52 kDa		unknown	1	2	0	0.08095	0.13373	0
1879	TRUE	Empty	Uncharacterized protein (Fragment) OS=Gallus gallus GN=ZNF207 PE=4 SV=2	E1C275_CHICK (+15)		53 kDa		unknown	2	1	0	0.16399	0.063186	0
1880	TRUE	Empty	Uncharacterized protein (Fragment) OS=Gallus gallus GN=PCYOX1 PE=4 SV=2	F1NYT7_CHICK (+5)		56 kDa		unknown	0	1	2	0	0.059516	0.13412
1881	TRUE	Empty	Uncharacterized protein OS=Gallus gallus GN=SLC9A3R2 PE=4 SV=2	F1NQR2_CHICK (+7)		39 kDa		unknown	1	0	2	0.10854	0	0.1979
1882	TRUE	Empty	Uncharacterized protein OS=Gallus gallus PE=4 SV=1	R4GJU7_CHICK (+4)		22 kDa		unknown	0	1	2	0	0.15607	0.36988
1883	TRUE	Empty	Probable carboxypeptidase PM20D1 OS=Gallus gallus GN=PM20D1 PE=4 SV=1	F1N9I7_CHICK (+4)		57 kDa		unknown	2	1	0	0.15227	0.058809	0
1884	TRUE	Empty	status=inactive	UPI0000447E4D (+10)		86 kDa		unknown	0	2	0	0	0.079637	0
1885	TRUE	Empty	Uncharacterized protein OS=Gallus gallus GN=SERPIND1 PE=3 SV=2	F1NJ16_CHICK (+4)		56 kDa		unknown	2	0	0	0.2415	0	0
1886	TRUE	Empty	Uncharacterized protein OS=Gallus gallus GN=KRAS PE=4 SV=1	E1BTS2_CHICK (+1)		21 kDa	TRUE	unknown	0	0	3	0	0	2.1218
1887	TRUE	Empty	Uncharacterized protein OS=Gallus gallus GN=MRPL18 PE=4 SV=1	E1BVT7_CHICK (+2)		21 kDa		unknown	0	0	3	0	0	0.64729
1888	TRUE	Empty	Chromosome 9 open reading frame 88, isoform CRA_a OS=Homo sapiens GN=C9orf88 PE=4 SV=1	A0A024R872 (+2)		?		unknown	0	0	2	0	0	-1

1889	TRUE	Empty	Glucosamine-6-phosphate isomerase OS=Gallus gallus GN=GNPDA2 PE=2 SV=1	E1C878_CHICK (+7)		31 kDa		unknown	0	0	2	0	0	0.25342
1890	TRUE	Empty	Uncharacterized protein OS=Gallus gallus GN=LOC417848 PE=3 SV=2	E1C897_CHICK (+6)		46 kDa		unknown	0	2	0	0	0.15298	0
1891	TRUE	Empty	Glutamyl-tRNA(Gln) amidotransferase subunit B, mitochondrial OS=Gallus gallus GN=PET112 PE=3 SV=2	F1N9W3_CHICK (+4)		56 kDa		unknown	0	2	0	0	0.12346	0
1892	TRUE	Empty	Uncharacterized protein OS=Gallus gallus GN=BNIP1 PE=4 SV=2	F1NC47_CHICK		26 kDa		unknown	0	2	0	0	0.2816	0
1893	TRUE	Empty	Glycylpeptide N-tetradecanoyltransferase OS=Gallus gallus GN=NMT1 PE=3 SV=2	F1NDA0_CHICK (+4)		57 kDa		unknown	0	2	0	0	0.12194	0
1894	TRUE	Empty	Uncharacterized protein OS=Gallus gallus GN=GGPS1 PE=3 SV=2	F1NHT9_CHICK (+7)		34 kDa		unknown	0	2	0	0	0.20873	0
1895	TRUE	Empty	Uncharacterized protein OS=Gallus gallus GN=NHP2L1 PE=4 SV=2	F1NII6_CHICK (+5)		14 kDa		unknown	0	2	0	0	0.57047	0
1896	TRUE	Empty	Uncharacterized protein OS=Gallus gallus PE=3 SV=2	F1NK75_CHICK		33 kDa	TRUE	unknown	0	2	0	0	2.4256	0
1897	TRUE	Empty	Uncharacterized protein (Fragment) OS=Gallus gallus GN=PRPF4 PE=4 SV=2	F1NNQ9_CHICK (+6)		60 kDa		unknown	0	2	0	0	0.11532	0
1898	TRUE	Empty	Uncharacterized protein (Fragment) OS=Gallus gallus GN=DNAJC7 PE=4 SV=1	F1NPY6_CHICK (+9)		55 kDa		unknown	0	2	0	0	0.12528	0
1899	TRUE	Empty	Tetraspanin OS=Gallus gallus GN=CD81 PE=3 SV=2	F1NW06_CHICK (+8)		26 kDa		unknown	0	0	2	0	0	0.30576
1900	TRUE	Empty	Uncharacterized protein OS=Gallus gallus GN=DRG1 PE=4 SV=1	F1NX13_CHICK (+2)		40 kDa		unknown	0	2	0	0	0.17501	0
1901	TRUE	Empty	Uncharacterized protein OS=Gallus gallus GN=PPT1 PE=4 SV=1	F1NYK1_CHICK (+6)		34 kDa		unknown	0	2	0	0	0.21169	0
1902	TRUE	Empty	Uncharacterized protein OS=Gallus gallus GN=STX7 PE=2 SV=1	Q5ZMP2_CHICK (+5)		29 kDa		unknown	0	2	0	0	0.24773	0
1903	TRUE	Empty	60S acidic ribosomal protein P1 OS=Gallus gallus GN=RPLP1 PE=3 SV=1	RLA1_CHICK		11 kDa		unknown	0	2	0	0	0.73606	0
1904	TRUE	Empty	RRP12-like protein OS=Gallus gallus GN=RRP12 PE=2 SV=1	RRP12_CHICK (+1)		144 kDa		unknown	0	2	0	0	0.04671	0
1905	TRUE	Empty	status=active	UPI00000424F1 (+2)		59 kDa	TRUE	unknown	0	2	0	0	0.1183	0
1906	TRUE	Empty	status=active	UPI000035199D		17 kDa		unknown	0	2	0	0	0.458	0
1907	TRUE	Empty	status=inactive	UPI000044A69D (+2)		10 kDa		unknown	0	0	2	0	0	0.925
1908	TRUE	Empty	status=inactive	UPI0000611D78		147 kDa	TRUE	unknown	0	2	0	0	0.045739	0
1909	TRUE	Empty	status=active	UPI000240CCBC		40 kDa	TRUE	unknown	0	2	0	0	0.17656	0
1910	TRUE	Empty	cDNA FLJ53160, highly similar to Zyxin OS=Homo sapiens PE=2 SV=1	B4DQR8 (+8)		?		unknown	0	0	2	0	0	-1
1911	TRUE	Empty	Protein S100 OS=Homo sapiens PE=2 SV=1	B2R4M6 (+1)		?		unknown	0	0	2	0	0	-1
1912	TRUE	Empty	Pre-mRNA-processing factor 6 OS=Homo sapiens GN=PRPF6 PE=1 SV=1	O94906 (+1)		?		unknown	0	0	2	0	0	-1
1913	TRUE	Empty	Methylenetetrahydrofolate dehydrogenase (NADP+ dependent) 1, methenyltetrahydrofolate cyclohydrolase, formyltetrahydrofolate synthetase, isoform CRA_a OS=Homo sapiens GN=MTHFD1 PE=4 SV=1	A0A024R652 (+3)		?	TRUE	unknown	0	0	2	0	0	-1
1914	TRUE	Empty	X-prolyl aminopeptidase (Aminopeptidase P) 2, membrane-bound OS=Homo sapiens GN=XPNPEP2 PE=2 SV=1	B7ZLM6 (+1)		?		unknown	0	0	2	0	0	-1
1915	TRUE	Empty	tr|E1C3A9|E1C3A9_CHICK-DECOY Vigilin OS=Gallus gallus GN=HDLBP PE=4 SV=1	tr|E1C3A9|E1C3A9_CHICK-DECOY		?		unknown	0	0	3	0	0	-1
1916	TRUE	Empty	Uncharacterized protein (Fragment) OS=Gallus gallus GN=EMG1 PE=4 SV=2	F1N832_CHICK (+3)		26 kDa		unknown	0	2	0	0	0.28028	0
1917	TRUE	Empty	Uncharacterized protein OS=Gallus gallus GN=SARDH PE=3 SV=2	F1NBJ7_CHICK (+14)		101 kDa		unknown	0	2	0	0	0.067133	0
1918	TRUE	Empty	Uncharacterized protein (Fragment) OS=Gallus gallus GN=GOLGA2 PE=4 SV=2	F1NF84_CHICK (+14)		117 kDa	TRUE	unknown	0	2	0	0	0.05774	0
1919	TRUE	Empty	Polymeric immunoglobulin receptor OS=Homo sapiens GN=PIGR PE=1 SV=4	P01833		?		unknown	0	0	2	0	0	-1
1920	TRUE	Empty	60S ribosomal protein L10 (Fragment) OS=Gallus gallus GN=RPL10 PE=2 SV=1	RL10_CHICK		24 kDa	TRUE	unknown	0	2	0	0	0.30928	0
1921	TRUE	Empty	Uncharacterized protein OS=Gallus gallus GN=SLC16A7 PE=4 SV=2	F1NVU4_CHICK (+2)		53 kDa		unknown	0	0	2	0	0	0.14458
1922	TRUE	Empty	Macrophage migration inhibitory factor OS=Gallus gallus GN=MIF PE=3 SV=3	MIF_CHICK (+2)		12 kDa		unknown	0	0	2	0	0	0.72437
1923	TRUE	Empty	Uncharacterized protein OS=Gallus gallus PE=4 SV=2	E1BZD8_CHICK (+1)		56 kDa	TRUE	unknown	0	2	0	0	0.12449	0
1924	TRUE	Empty	Dynamin-2 OS=Homo sapiens GN=DNM2 PE=1 SV=2	P50570 (+5)		?		unknown	0	0	2	0	0	-1
1925	TRUE	Empty	Uncharacterized protein OS=Gallus gallus GN=DDX23 PE=3 SV=1	R4GKV2_CHICK		45 kDa		unknown	0	2	0	0	0.15497	0
1926	TRUE	Empty	Annexin OS=Homo sapiens GN=ANXA6 PE=1 SV=1	E5RK69		?	TRUE	unknown	0	0	2	0	0	-1
1927	TRUE	Empty	Leukocyte immunoglobulin-like receptor subfamily A member 6 OS=Homo sapiens GN=LILRA6 PE=4 SV=3	D3YTC4 (+15)		?		unknown	0	0	2	0	0	-1
1928	TRUE	Empty	tr|F1P172|F1P172_CHICK-DECOY Uncharacterized protein OS=Gallus gallus GN=SACS PE=4...	tr|F1P172|F1P172_CHICK-DECOY		?		unknown	0	0	2	0	0	-1
1929	TRUE	Empty	status=active	UPI0003507326 (+13)		110 kDa		unknown	0	0	2	0	0	0.066889
1930	TRUE	Empty	Uncharacterized protein OS=Gallus gallus GN=RCJMB04_10n19 PE=2 SV=1	Q5F3P7_CHICK		15 kDa		unknown	2	0	0	0.64259	0	0
1931	TRUE	Empty	Uncharacterized protein OS=Gallus gallus GN=IPO9 PE=4 SV=2	F1NRZ6_CHICK (+4)		99 kDa		unknown	0	0	2	0	0	0.074499
1932	TRUE	Empty	status=active	UPI000019FFC4		24 kDa	TRUE	unknown	0	2	0	0	1.2726	0
1933	TRUE	Empty	Transmembrane protein 119 OS=Homo sapiens GN=TMEM119 PE=2 SV=1	Q4V9L6		?		unknown	0	0	2	0	0	-1
1934	TRUE	Empty	Uncharacterized protein OS=Gallus gallus GN=RCN1 PE=4 SV=2	E1BXF4_CHICK		52 kDa	TRUE	unknown	0	0	2	0	0	0.14621
1935	TRUE	Empty	Creatine kinase B-type OS=Gallus gallus GN=CKB PE=3 SV=2	F1NZ30_CHICK (+4)		40 kDa	TRUE	unknown	2	0	0	0.22074	0	0
1936	TRUE	Empty	Uncharacterized protein OS=Gallus gallus PE=4 SV=2	E1BSU4_CHICK		97 kDa	TRUE	unknown	0	0	2	0	0	0.076072
1937	TRUE	Empty	Cathepsin G OS=Homo sapiens GN=CTSG PE=1 SV=2	P08311		?		unknown	0	0	2	0	0	-1
1938	TRUE	Empty	Synaptogyrin OS=Gallus gallus GN=SYNGR1 PE=3 SV=2	E1BV06_CHICK		26 kDa		unknown	0	0	2	0	0	0.31013
1939	TRUE	Empty	Uncharacterized protein (Fragment) OS=Gallus gallus GN=MIF4GD PE=4 SV=1	R4GJG3_CHICK		26 kDa		unknown	0	2	0	0	0.28294	0
1940	TRUE	Empty	Gallus domesticus skeletal muscle mRNA OS=Gallus gallus PE=2 SV=1	A2NBE2_CHICK (+2)		87 kDa		unknown	2	0	0	0.098017	0	0
1941	TRUE	Empty	status=active	UPI0000E7F92C (+1)		97 kDa		unknown	0	0	2	0	0	0.076702
1942	TRUE	Empty	Phosphatidylcholine-sterol acyltransferase (Fragment) OS=Gallus gallus GN=LCAT PE=2 SV=1	LCAT_CHICK (+7)		48 kDa		unknown	2	0	0	0.18378	0	0
1943	TRUE	Empty	Uncharacterized protein OS=Gallus gallus GN=GPCPD1 PE=4 SV=2	E1BYP8_CHICK (+9)		77 kDa	TRUE	unknown	2	0	0	0.11151	0	0
1944	TRUE	Empty	Uncharacterized protein (Fragment) OS=Gallus gallus GN=LSM6 PE=4 SV=2	E1C8T5_CHICK (+6)		10 kDa		unknown	2	0	0	1.1089	0	0
1945	TRUE	Empty	Uncharacterized protein OS=Gallus gallus GN=SLC29A1 PE=4 SV=1	E1C1Q3_CHICK (+5)		51 kDa		unknown	2	0	0	0.17153	0	0
1946	TRUE	Empty	status=active	UPI000002A4C3 (+5)		21 kDa		unknown	2	0	0	0.44007	0	0
1947	TRUE	Empty	status=inactive	UPI00004481FF (+14)		101 kDa		unknown	0	0	2	0	0	0.073319
1948	TRUE	Empty	Topoisomerase (DNA) I OS=Homo sapiens GN=TOP1 PE=2 SV=1	B9EG90 (+2)		?		unknown	0	0	2	0	0	-1
1949	TRUE	Empty	Uncharacterized protein (Fragment) OS=Gallus gallus GN=MRPL39 PE=4 SV=1	F1NDS2_CHICK		40 kDa		unknown	0	2	0	0	0.17501	0
1950	TRUE	Empty	Uncharacterized protein OS=Gallus gallus GN=CWF19L1 PE=4 SV=2	E1C959_CHICK		60 kDa		unknown	0	2	0	0	0.11532	0
1951	TRUE	Empty	Paladin OS=Gallus gallus GN=PALD1 PE=4 SV=2	E1BUS7_CHICK (+10)		100 kDa		unknown	2	0	0	0.085334	0	0
1952	TRUE	Empty	Uncharacterized protein OS=Gallus gallus GN=VPS13A PE=4 SV=2	E1C549_CHICK		361 kDa	TRUE	unknown	0	0	3	0	0	0.030327
1953	TRUE	Empty	Apolipoprotein D OS=Gallus gallus GN=ApoD PE=2 SV=1	Q5G8Y9_CHICK (+2)		22 kDa		unknown	0	0	2	0	0	0.37844
1954	TRUE	Empty	status=active	UPI0002C86858 (+8)		133 kDa		unknown	2	0	0	0.063351	0	0
1955	TRUE	Empty	JC7 OS=Gallus gallus GN=JC7 PE=2 SV=1	Q76MF7_CHICK (+3)		33 kDa		unknown	2	0	0	0.27725	0	0
1956	TRUE	Empty	Uncharacterized protein (Fragment) OS=Gallus gallus GN=MOB2 PE=4 SV=2	E1C1X6_CHICK (+10)		27 kDa		unknown	0	0	2	0	0	0.29876
1957	TRUE	Empty	Cytochrome b5 OS=Gallus gallus GN=CYB5A PE=1 SV=4	CYB5_CHICK (+3)		16 kDa	TRUE	unknown	2	0	0	0.63719	0	0
1958	TRUE	Empty	Protein kinase C and casein kinase substrate in neurons protein 2 OS=Gallus gallus GN=PACSIN2 PE=4 SV=1	E1C007_CHICK (+8)		56 kDa		unknown	0	0	2	0	0	0.13496
1959	TRUE	Empty	tr|F1NLA9|F1NLA9_CHICK-DECOY Uncharacterized protein OS=Gallus gallus GN=DNAH7 PE=4...	tr|F1NLA9|F1NLA9_CHICK-DECOY		?		unknown	3	0	0	-1	0	0
1960	TRUE	Empty	Uncharacterized protein (Fragment) OS=Gallus gallus PE=4 SV=2	F1NZE6_CHICK		77 kDa	TRUE	unknown	3	0	0	0.1721	0	0
1961	TRUE	Empty	tr|F1NVV0|F1NVV0_CHICK-DECOY Uncharacterized protein OS=Gallus gallus GN=PCF11 PE=4...	tr|F1NVV0|F1NVV0_CHICK-DECOY		?		unknown	0	0	3	0	0	-1
1962	TRUE	Empty	cDNA FLJ59374, highly similar to Homo sapiens caldesmon 1 (CALD1), transcript variant 2, mRNA OS=Homo sapiens PE=2 SV=1	B4DPW5 (+11)		?		unknown	0	0	2	0	0	-1
1963	TRUE	Empty	Uncharacterized protein OS=Gallus gallus GN=Gga.55096 PE=4 SV=2	E1C6Z5_CHICK		370 kDa	TRUE	unknown	0	0	2	0	0	0.019622
1964	TRUE	Empty	ATP-dependent (S)-NAD(P)H-hydrate dehydratase OS=Gallus gallus GN=CARKD PE=3 SV=2	F1P2P4_CHICK (+8)		32 kDa		unknown	0	0	2	0	0	0.24392
1965	TRUE	Empty	status=active	UPI000240B1D3		28 kDa		unknown	0	0	2	0	0	0.28318
1966	TRUE	Empty	PDZ and LIM domain protein 3 OS=Gallus gallus GN=PDLIM3 PE=4 SV=1	F1NHA9_CHICK (+8)		34 kDa		unknown	0	2	0	0	0.208	0
1967	TRUE	Empty	Uncharacterized protein OS=Gallus gallus GN=PPP2R5D PE=4 SV=2	F1P090_CHICK (+3)		71 kDa	TRUE	unknown	0	2	0	0	0.097061	0
1968	TRUE	Empty	status=active	UPI00003AAB32 (+2)		52 kDa	TRUE	unknown	0	2	0	0	0.13283	0
1969	TRUE	Empty	Uncharacterized protein OS=Gallus gallus GN=SDCCAG8 PE=4 SV=2	F1NQI6_CHICK		85 kDa	TRUE	unknown	0	3	0	0	0.12306	0
1970	TRUE	Empty	Uncharacterized protein OS=Gallus gallus GN=GYG1 PE=4 SV=1	F1N9J1_CHICK (+4)		37 kDa		unknown	2	0	0	0.23988	0	0

1971	TRUE	Empty	Sulfite oxidase OS=Gallus gallus GN=SUOX PE=1 SV=3	SUOX_CHICK (+11)		50 kDa		unknown	2	0	0	0.17434	0	0
1972	TRUE	Empty	Uncharacterized protein OS=Gallus gallus GN=Gga.56198 PE=4 SV=2	E1C484_CHICK (+9)		136 kDa		unknown	2	0	0	0.062135	0	0
1973	TRUE	Empty	Transferrin receptor protein 1 OS=Gallus gallus GN=TFRC PE=4 SV=2	F1NTM6_CHICK (+1)		99 kDa	TRUE	unknown	0	0	2	0	0	0.074671
1974	TRUE	Empty	Uncharacterized protein OS=Gallus gallus GN=COMMD4 PE=4 SV=2	F1NNT4_CHICK (+4)		22 kDa		unknown	0	0	2	0	0	0.36988
1975	TRUE	Empty	Neurofibromin OS=Gallus gallus GN=NF1 PE=4 SV=1	E1C9F6_CHICK		320 kDa	TRUE	unknown	0	0	2	0	0	0.02271
1976	TRUE	Empty	Podocalyxin OS=Gallus gallus GN=PODXL PE=4 SV=2	F1NXC1_CHICK (+1)		61 kDa		unknown	0	0	2	0	0	0.12341
1977	TRUE	Empty	F1NV96-DECOY	F1NV96-DECOY (+7)		?	TRUE	unknown	0	2	1	0	-1	-1
1978	TRUE	Empty	Uncharacterized protein OS=Gallus gallus GN=SMARCA5 PE=4 SV=2	E1C0M8_CHICK		117 kDa	TRUE	unknown	0	2	0	0	0.057909	0
1979	TRUE	Empty	Uncharacterized protein (Fragment) OS=Gallus gallus GN=LOC771207 PE=4 SV=2	F1NC58_CHICK (+5)		33 kDa		unknown	0	0	2	0	0	0.3831
1980	TRUE	Empty	Uncharacterized protein (Fragment) OS=Gallus gallus GN=LOC100858408 PE=4 SV=2	F1NG68_CHICK		33 kDa	TRUE	unknown	0	2	0	0	0.21948	0
1981	TRUE	Empty	Uncharacterized protein OS=Gallus gallus GN=SUCLG2 PE=2 SV=1	Q5ZL37_CHICK (+3)		47 kDa	TRUE	unknown	0	0	2	0	0	0.25518
1982	TRUE	Empty	Transformer-2 beta OS=Gallus gallus GN=TRA2B PE=2 SV=1	Q9DDU8_CHICK		34 kDa		unknown	2	0	0	0.26638	0	0
1983	TRUE	Empty	Uncharacterized protein OS=Gallus gallus GN=CCDC109B PE=4 SV=2	F1NEL0_CHICK		46 kDa	TRUE	unknown	0	2	0	0	0.15337	0
1984	TRUE	Empty	Uncharacterized protein OS=Gallus gallus GN=ACOT7 PE=4 SV=2	F1NSJ2_CHICK		44 kDa		unknown	0	2	0	0	0.16126	0
1985	TRUE	Empty	Uncharacterized protein (Fragment) OS=Gallus gallus GN=DCAKD PE=3 SV=2	F1ND04_CHICK		26 kDa	TRUE	unknown	0	0	2	0	0	0.30866
1986	TRUE	Empty	Alanine--tRNA ligase, cytoplasmic OS=Gallus gallus GN=AARS PE=3 SV=2	F1P5J5_CHICK		101 kDa	TRUE	unknown	3	0	0	0.12829	0	0
1987	TRUE	Empty	status=active	UPI000004445DC (+1)		15 kDa	TRUE	unknown	0	0	2	0	0	0.56385
1988	TRUE	Empty	Uncharacterized protein OS=Gallus gallus GN=PDPK1 PE=2 SV=1	Q5F3U4_CHICK (+4)		63 kDa		unknown	0	0	2	0	0	0.11932
1989	TRUE	Empty	status=active	UPI0000183E9F (+2)		37 kDa		unknown	0	1	2	0	0.090515	0.20737
1990	TRUE	Empty	status=inactive	UPI000176034C		95 kDa	TRUE	unknown	0	0	2	0	0	0.078466
1991	TRUE	Empty	status=inactive	UPI0000612112		69 kDa	TRUE	unknown	2	0	0	0.26324	0	0
1992	TRUE	Empty	status=active	UPI00003AE485		109 kDa	TRUE	unknown	0	0	2	0	0	0.067728
1993	TRUE	Empty	status=inactive	UPI0000ECD04E		25 kDa		unknown	0	0	2	0	0	0.32239
1994	TRUE	Empty	status=inactive	UPI0000ECCE51		12 kDa		unknown	0	0	2	0	0	0.75736
1995	TRUE	Empty	status=active	UPI0000455FFF (+1)		44 kDa	TRUE	unknown	2	0	0	0.1995	0	0
1996	TRUE	Empty	status=inactive	UPI00004495B4		190 kDa	TRUE	unknown	0	0	2	0	0	0.038444
1997	TRUE	Empty	status=active	UPI00003AD337		12 kDa	TRUE	unknown	0	2	0	0	0.70223	0
1998	TRUE	Empty	Aminomethyltransferase, mitochondrial OS=Gallus gallus GN=AMT PE=2 SV=2	GCST_CHICK (+1)		42 kDa	TRUE	unknown	0	0	2	0	0	0.18286
1999	TRUE	Empty	Septin 9, isoform CRA_a OS=Homo sapiens GN=SEPT9 PE=3 SV=1	A0A024R8V0 (+6)		?		unknown	0	0	2	0	0	-1
2000	TRUE	Empty	Uncharacterized protein (Fragment) OS=Gallus gallus GN=MST4 PE=4 SV=1	F6SQB5_CHICK (+5)		47 kDa	TRUE	unknown	2	0	0	0.18701	0	0
2001	TRUE	Empty	Myeloperoxidase OS=Homo sapiens GN=MPO PE=1 SV=1	P05164 (+2)		?		unknown	0	0	2	0	0	-1
2002	TRUE	Empty	status=active	UPI00003ACD73		43 kDa		unknown	0	2	0	0	0.16438	0
2003	TRUE	Empty	status=active	UPI00004ED2A3		40 kDa		unknown	0	2	0	0	0.17656	0
2004	TRUE	Empty	status=inactive	UPI000056BF6D		55 kDa		unknown	0	2	0	0	0.12688	0
2005	TRUE	Empty	status=active	UPI0002C86756 (+1)		158 kDa		unknown	2	0	0	0.053405	0	0
2006	TRUE	Empty	Uncharacterized protein OS=Gallus gallus GN=SERPINB2 PE=3 SV=2	E1BTH3_CHICK		47 kDa		unknown	0	0	2	0	0	0.25584
2007	TRUE	Empty	Uncharacterized protein OS=Gallus gallus GN=ATP6V0D1 PE=4 SV=2	E1BVF8_CHICK (+11)		43 kDa		unknown	0	2	0	0	0.16393	0
2008	TRUE	Empty	Uncharacterized protein OS=Gallus gallus GN=CD276 PE=4 SV=2	F1NIS6_CHICK (+5)		34 kDa		unknown	0	2	0	0	0.21244	0
2009	TRUE	Empty	Uncharacterized protein OS=Gallus gallus GN=RFC4 PE=4 SV=2	F1NIY0_CHICK (+4)		40 kDa		unknown	0	2	0	0	0.17815	0
2010	TRUE	Empty	SPARC OS=Gallus gallus GN=SPARC PE=4 SV=1	F1P291_CHICK (+4)		34 kDa		unknown	0	2	0	0	0.2102	0
2011	TRUE	Empty	Uncharacterized protein OS=Gallus gallus GN=FAHD2A PE=4 SV=2	H9KYV1_CHICK (+3)		27 kDa		unknown	0	2	0	0	0.26653	0
2012	TRUE	Empty	Uncharacterized protein OS=Gallus gallus GN=PRKACB PE=4 SV=1	R4GIN8_CHICK (+5)		39 kDa	TRUE	unknown	0	2	0	0	0.18085	0
2013	TRUE	Empty	status=active	UPI00003AE498 (+8)		80 kDa		unknown	0	2	0	0	0.085848	0
2014	TRUE	Empty	status=active	UPI000350696A		13 kDa		unknown	0	2	0	0	0.6232	0
2015	TRUE	Empty	Uncharacterized protein OS=Gallus gallus GN=ACADSB PE=3 SV=2	F1NZB1_CHICK (+6)		48 kDa		unknown	0	2	0	0	0.14693	0
2016	TRUE	Empty	AP-3 complex subunit beta OS=Homo sapiens PE=2 SV=1	A8K586 (+4)		?		unknown	0	0	2	0	0	-1
2017	TRUE	Empty	Uncharacterized protein OS=Gallus gallus GN=KCTD12 PE=4 SV=1	E1C165_CHICK		33 kDa		unknown	0	0	2	0	0	0.24032
2018	TRUE	Empty	Ectonucleoside triphosphate diphosphohydrolase 5 OS=Gallus gallus GN=ENTPD5 PE=3 SV=1	ENTP5_CHICK		48 kDa		unknown	2	0	0	0.18288	0	0
2019	TRUE	Empty	status=active	UPI000240C67A		35 kDa		unknown	2	0	0	0.25633	0	0
2020	TRUE	Empty	Zinc finger Ran-binding domain-containing protein 2 OS=Gallus gallus GN=ZRANB2 PE=2 SV=1	ZRAB2_CHICK		38 kDa		unknown	0	0	2	0	0	0.20475
2021	TRUE	Empty	tr|F1NMK5|F1NMK5_CHICK-DECOY Uncharacterized protein OS=Gallus gallus GN=KIAA0100...	tr|F1NMK5|F1NMK5_CHICK-DECOY		?		unknown	0	0	2	0	0	-1
2022	TRUE	Empty	WD repeat-containing protein 48 OS=Gallus gallus GN=WDR48 PE=2 SV=1	WDR48_CHICK		76 kDa	TRUE	unknown	0	2	0	0	0.089735	0
2023	TRUE	Empty	tr|R4GLV4|R4GLV4_CHICK-DECOY Uncharacterized protein OS=Gallus gallus GN=Gga.51531...	tr|R4GLV4|R4GLV4_CHICK-DECOY		?		unknown	2	0	0	-1	0	0
2024	TRUE	Empty	Dihydrofolate reductase OS=Gallus gallus GN=DHFR PE=2 SV=1	A3RKL3_CHICK (+8)		22 kDa		unknown	0	2	0	0	0.34491	0
2025	TRUE	Empty	Uncharacterized protein OS=Gallus gallus GN=GULP1 PE=4 SV=2	E1C2M8_CHICK		34 kDa		unknown	0	0	2	0	0	0.2277
2026	TRUE	Empty	Uncharacterized protein OS=Gallus gallus GN=FBXO3 PE=4 SV=2	F1P439_CHICK		54 kDa	TRUE	unknown	0	2	0	0	0.1277	0
2027	TRUE	Empty	Oxysterol-binding protein OS=Gallus gallus GN=OSBPL8 PE=3 SV=2	F1N9F3_CHICK (+11)		101 kDa		unknown	0	2	0	0	0.067133	0
2028	TRUE	Empty	Protein kinase C delta type OS=Gallus gallus GN=PRKCD PE=3 SV=2	E1C5S5_CHICK (+1)		78 kDa		unknown	0	0	2	0	0	0.095025
2029	TRUE	Empty	Uncharacterized protein OS=Gallus gallus GN=PPFIBP1 PE=4 SV=2	E1C1C3_CHICK (+2)		116 kDa		unknown	0	2	0	0	0.058307	0
2030	TRUE	Empty	Uncharacterized protein OS=Gallus gallus PE=3 SV=2	H9L1C1_CHICK (+5)		188 kDa		unknown	0	2	0	0	0.035599	0
2031	TRUE	Empty	Uncharacterized protein OS=Gallus gallus GN=C14ORF4 PE=4 SV=2	F1NB26_CHICK (+1)		68 kDa		unknown	0	2	0	0	0.10154	0
2032	TRUE	Empty	status=active	UPI0002C8768E (+6)		37 kDa		unknown	0	2	0	0	0.19192	0
2033	TRUE	Empty	Tubulin alpha-3 chain (Fragment) OS=Gallus gallus PE=3 SV=1	TBA3_CHICK		36 kDa	TRUE	unknown	0	2	0	0	7.047	0
2034	TRUE	Empty	Uncharacterized protein OS=Gallus gallus GN=SNRNP40 PE=4 SV=2	F1NUK8_CHICK (+5)		41 kDa		unknown	0	2	0	0	0.17099	0
2035	TRUE	Empty	ATPase, Na+/K+ transporting, beta 1 polypeptide, isoform CRA_a OS=Homo sapiens GN=ATP1B1 PE=2 SV=1	A3KLL5 (+6)		?		unknown	0	0	2	0	0	-1
2036	TRUE	Empty	Scavenger receptor cysteine-rich type 1 protein M130 OS=Homo sapiens GN=CD163 PE=4 SV=1	C9JHR8 (+7)		?		unknown	0	0	2	0	0	-1
2037	TRUE	Empty	Uncharacterized protein OS=Gallus gallus GN=RPRD1B PE=4 SV=1	F6QXI2_CHICK (+1)		44 kDa	TRUE	unknown	0	2	0	0	0.15911	0
2038	TRUE	Empty	status=inactive	UPI00004495F4 (+14)		312 kDa		unknown	0	2	0	0	0.021364	0
2039	TRUE	Empty	Uncharacterized protein OS=Gallus gallus GN=EGFLAM PE=4 SV=2	E1BQW4_CHICK (+8)		114 kDa		unknown	0	0	2	0	0	0.064946
2040	TRUE	Empty	tr|F1NZI3|F1NZI3_CHICK-DECOY Uncharacterized protein OS=Gallus gallus GN=SHROOM3...	tr|F1NZI3|F1NZI3_CHICK-DECOY		?		unknown	0	2	0	0	-1	0
2041	TRUE	Empty	Uncharacterized protein OS=Gallus gallus GN=ZNF518B PE=4 SV=2	F1NRI7_CHICK (+4)		122 kDa		unknown	2	0	0	0.069496	0	0
2042	TRUE	Empty	status=active	UPI00003ACD6D (+2)		12 kDa		unknown	0	2	0	0	0.7104	0
2043	TRUE	Empty	tr|E1C4H7|E1C4H7_CHICK-DECOY Uncharacterized protein OS=Gallus gallus GN=HERC1 PE=4...	tr|E1C4H7|E1C4H7_CHICK-DECOY		?		unknown	2	0	0	-1	0	0
2044	TRUE	Empty	Uncharacterized protein OS=Gallus gallus GN=WASF2 PE=4 SV=2	H9L0D7_CHICK		54 kDa	TRUE	unknown	0	0	2	0	0	0.13901
2045	TRUE	Empty	Uncharacterized protein (Fragment) OS=Gallus gallus GN=LOC100857133 PE=4 SV=1	R4GFR4_CHICK (+5)		20 kDa		unknown	0	0	2	0	0	0.4144
2046	TRUE	Empty	Uncharacterized protein OS=Gallus gallus GN=COPS2 PE=4 SV=2	E1C3U6_CHICK (+4)		52 kDa	TRUE	unknown	0	2	0	0	0.13524	0
2047	TRUE	Empty	Uncharacterized protein OS=Gallus gallus PE=4 SV=2	F1NLQ1_CHICK (+4)		94 kDa	TRUE	unknown	0	2	0	0	0.072365	0
2048	TRUE	Empty	Endophilin-B1 OS=Gallus gallus GN=SH3GLB1 PE=2 SV=1	SHLB1_CHICK (+5)		41 kDa	TRUE	unknown	0	2	0	0	0.17348	0
2049	TRUE	Empty	Band 4.1-like protein 2 OS=Homo sapiens GN=EPB41L2 PE=1 SV=1	E9PHY5 (+10)		?		unknown	0	0	2	0	0	-1
2050	TRUE	Empty	Uncharacterized protein OS=Gallus gallus GN=TCEA1 PE=4 SV=2	F1NP36_CHICK (+7)		32 kDa		unknown	2	0	0	0.27828	0	0
2051	TRUE	Empty	status=inactive	UPI000044943F		401 kDa	TRUE	unknown	0	0	2	0	0	0.036478
2052	TRUE	Empty	CTP synthase OS=Gallus gallus GN=CTPS PE=3 SV=2	E1C458_CHICK (+10)		70 kDa	TRUE	unknown	2	0	0	0.12352	0	0

2053	TRUE	Empty	Uncharacterized protein OS=Gallus gallus GN=PDLIM5 PE=4 SV=1	F1NTC8_CHICK (+4)		64 kDa	TRUE	unknown	0	2	0	0	0.1076	0
2054	TRUE	Empty	UPI0003504F46-DECOY	UPI0003504F46-DECOY		?		unknown	2	0	0	-1	0	0
2055	TRUE	Empty	Uncharacterized protein OS=Gallus gallus GN=PRCP PE=4 SV=2	F1NWF2_CHICK		54 kDa		unknown	0	2	0	0	0.12825	0
2056	TRUE	Empty	Uncharacterized protein OS=Gallus gallus GN=ARFGAP3 PE=4 SV=1	F1NZX4_CHICK (+1)		49 kDa		unknown	0	2	0	0	0.14409	0
2057	TRUE	Empty	MHC class I antigen (Fragment) OS=Homo sapiens GN=HLA-C PE=3 SV=1	M9PAE6		?		unknown	0	0	2	0	0	-1
2058	TRUE	Empty	Uncharacterized protein (Fragment) OS=Gallus gallus GN=HAO2 PE=4 SV=2	E1C0E1_CHICK		41 kDa		unknown	2	0	0	0.21502	0	0
2059	TRUE	Empty	Uncharacterized protein OS=Gallus gallus GN=PDE6D PE=4 SV=2	E1BRH7_CHICK		17 kDa		unknown	0	0	2	0	0	0.4885
2060	TRUE	Empty	status=inactive	UPI000044A20F		61 kDa	TRUE	unknown	0	0	2	0	0	0.12341
2061	TRUE	Empty	status=active	UPI0001E90712		160 kDa	TRUE	unknown	2	0	0	0.05265	0	0
2062	TRUE	Empty	status=active	UPI00035043A4 (+2)		142 kDa		unknown	2	0	0	0.0596	0	0
2063	TRUE	Empty	Uncharacterized protein OS=Gallus gallus GN=FAM96A PE=4 SV=2	E1C7B4_CHICK (+1)		14 kDa		unknown	2	0	0	0.72903	0	0
2064	TRUE	Empty	Uncharacterized protein OS=Gallus gallus PE=4 SV=2	F1NQ49_CHICK		220 kDa	TRUE	unknown	0	2	0	0	0.030406	0
2065	TRUE	Empty	status=active	UPI00003AA540 (+3)		43 kDa		unknown	0	0	2	0	0	0.17785
2066	TRUE	Empty	Uncharacterized protein OS=Gallus gallus GN=MYO1D PE=4 SV=2	E1C459_CHICK (+15)		119 kDa		unknown	0	0	2	0	0	0.062025
2067	TRUE	Empty	status=active	UPI0000436C84		76 kDa		unknown	0	2	0	0	0.090144	0
2068	TRUE	Empty	Uncharacterized protein (Fragment) OS=Gallus gallus GN=SMNDC1 PE=4 SV=2	E1BQK7_CHICK (+4)		27 kDa	TRUE	unknown	2	0	0	0.33721	0	0
2069	TRUE	Empty	status=active	UPI000024B6F2 (+1)		90 kDa	TRUE	unknown	0	2	0	0	0.075875	0
2070	TRUE	Empty	status=active	UPI000024848F (+1)		44 kDa		unknown	0	0	2	0	0	0.17404
2071	TRUE	Empty	Uncharacterized protein OS=Gallus gallus GN=LIMS1 PE=4 SV=1	F1NZW4_CHICK		37 kDa	TRUE	unknown	0	0	2	0	0	0.2087
2072	TRUE	Empty	Uncharacterized protein OS=Gallus gallus GN=PSMC3 PE=2 SV=1	Q5ZIT7_CHICK (+4)		47 kDa	TRUE	unknown	2	0	0	0.18607	0	0
2073	TRUE	Empty	status=active	UPI00003A9A17		296 kDa	TRUE	unknown	0	0	2	0	0	0.024541
2074	TRUE	Empty	status=active	UPI00003AB475		137 kDa		unknown	0	0	2	0	0	0.053524
2075	TRUE	Empty	status=active	UPI00003ABCD9		42 kDa		unknown	0	0	2	0	0	0.29003
2076	TRUE	Empty	status=active	UPI00003ABCDA		43 kDa	TRUE	unknown	0	0	2	0	0	0.64164
2077	TRUE	Empty	status=active	UPI00003AC88B (+7)		138 kDa	TRUE	unknown	2	0	0	0.061267	0	0
2078	TRUE	Empty	status=active	UPI00003AFE79 (+1)		41 kDa	TRUE	unknown	2	0	0	0.21379	0	0
2079	TRUE	Empty	status=active	UPI0000403E86 (+2)		145 kDa	TRUE	unknown	0	2	0	0	0.046418	0
2080	TRUE	Empty	status=inactive	UPI0000545E10		110 kDa		unknown	0	2	0	0	0.094106	0
2081	TRUE	Empty	status=active	UPI00035075E3		101 kDa		unknown	2	0	0	0.084464	0	0
2082	TRUE	Empty	Vitellogenin-2 OS=Gallus gallus GN=VTG2 PE=1 SV=1	VIT2_CHICK		205 kDa	TRUE	unknown	0	2	0	0	4.7157	0
END OF FILE														

Peptides report

20196-20225 HinckeM --TAXchicken, Peptide report created on 05/13/2021

Experiment: 20196-20225 HinckeM --TAXchicken

Peak List Generator: unknown

Version: unknown

Charge States Calculated: unknown

Deisotoped: unknown

Textual Annotation: unknown

Database Set: 4 Databases

Database Name: a subset of the TAX_GallusGallus_9031 _20141114 database

Version: unknown

Taxonomy: All Entries

Number of Proteins: 7028

Database Name: a subset of the TAX_GallusGallus_9031

Version: unknown

Taxonomy: All Entries

Number of Proteins: 7952

Database Name: a subset of the

Version: unknown

Taxonomy: All Entries

Number of Proteins: 8298

Database Name: the TAX_GallusGallus_9031 _20141114 database

Version: unknown

Taxonomy: All Entries

Number of Proteins: 222250

Does database contain common contaminants?:

Search Engine Set: 2 Search Engines

Search Engine: Mascot

Version: 2.4.1

Samples: All Samples

Fragment Tolerance: 0.100 Da (Monoisotopic)

Parent Tolerance: 0.100 Da (Monoisotopic)

Fixed Modifications: +57 on C (Carbamidomethyl)

Variable Modifications: -18 on Peptide N-Terminal (Glu->pyro-Glu), -17 on Peptide N-

Database: the TAX_GallusGallus_9031_20141114

Digestion Enzyme: Trypsin

Max Missed Cleavages: 2

Probability Model:

Mudpit_Ingel20196_MGFPeaklist (F058870): LFDR Model, Classifier data: Bayes, Good (50%) m:68.8/s:15.5 m:43.8/s:1.46 m:NA m:48.3/s:21.0, Bad (50%) m:20.2/s:7.76 m:43.4/s:2.53 m:NA m:5.33/s:5.03 [all charge states]

Mudpit_Ingel20206_MGFPeaklist (F058880): LFDR Model, Classifier data: Bayes, Good (62%) m:77.2/s:17.6 m:43.8/s:1.44 m:NA m:58.2/s:24.3, Bad (38%) m:11.3/s:7.70 m:43.5/s:1.70 m:NA m:3.42/s:3.81 [all charge states]

Mudpit_Ingel20221_MGFPeaklist (F058864): LFDR Model, Classifier data: Bayes, Good (59%) m:73.9/s:17.1 m:43.5/s:1.80 m:NA m:54.9/s:23.4, Bad (41%) m:12.2/s:7.60 m:43.4/s:1.92 m:NA m:3.50/s:3.81 [all charge states]

Search Engine: X! Tandem

Version: CYCLONE (2010.12.01.1)

Samples: All Samples

Fragment Tolerance: 0.100 Da (Monoisotopic)

Parent Tolerance: 0.100 Da (Monoisotopic)

Fixed Modifications: +57 on C (Carbamidomethyl)

Variable Modifications: -18 on Peptide N-Terminal (Dehydrated), -18 on Peptide N-Terminal (Glu->pyro-Glu), -17 on Peptide N-Terminal (Ammonia-loss), -17 on Peptide N-Terminal (Gln->pyro-Glu), +1 on NQ (Deamidated), +16 on M (Oxidation)

Database: a subset of the TAX_GallusGallus_9031_20141114 database (unknown version, 7028 entries) (Mudpit_Ingel20196_MGFPeaklist (F058870)), a subset of the TAX_GallusGallus_9031_20141114

Digestion Enzyme: Trypsin (Mudpit_Ingel20196_MGFPeaklist (F058870)), Trypsin (Mudpit_Ingel20206_MGFPeaklist (F058880)), Trypsin (Mudpit_Ingel20221_MGFPeaklist

Max Missed Cleavages: 2 (Mudpit_Ingel20196_MGFPeaklist (F058870)), 2 (Mudpit_Ingel20206_MGFPeaklist (F058880)), 2

Probability Model:

Mudpit_Ingel20196_MGFPeaklist (F058870): LFDR Model, Classifier data: Bayes, Good (82%) m:41.4/s:7.99 m:4.47/s:1.86 m:0.376/s:0.114 m:0.444/s:0.0991, Bad (18%) m:21.8/s:3.48 m:-0.721/s:0.638 m:0.0471/s:0.0489 m:0.216/s:0.113 [all charge states]

Mudpit_Ingel20206_MGFPeaklist (F058880): LFDR Model, Classifier data: Bayes, Good (92%) m:46.7/s:7.22 m:5.12/s:1.85 m:0.405/s:0.119 m:0.487/s:0.0908, Bad (8.1%) m:25.2/s:3.81 m:-0.581/s:0.768 m:0.0489/s:0.0509 m:0.280/s:0.125 [all charge states]

Mudpit_Ingel20221_MGFPeaklist (F058864): LFDR Model, Classifier data: Bayes, Good (89%) m:45.4/s:7.14 m:4.84/s:1.83 m:0.393/s:0.116 m:0.490/s:0.0938, Bad (11%) m:24.5/s:3.85 m:-0.647/s:0.668 m:0.0466/s:0.0478 m:0.270/s:0.120 [all charge states]

Scaffold: Version: Scaffold_4.8.7

Modification Metadata Set: 1541 modifications

Source: C:\Program Files\Scaffold 4\parameters\unimod.xml

Comment:

Protein Grouping Strategy: MS-sample

Peptide Thresholds: 5.0% minimum

Protein Thresholds: 98.0% minimum and 2

Peptide FDR: 0.2% (Decoy)

Protein FDR: 0.9% (Decoy)

GO Annotation Source(s):

Alternate ID Source(s):

Experiment name	Biological sample category	Biological sample name	MS/MS sample name	Protein name	Protein accession numbers	Database sources	Protein molecular weight (Da)	Protein identification probability	Exclusive unique peptide count	Exclusive unique spectrum count	Exclusive spectrum count	Percentage of total spectra	Percentage sequence coverage	Peptide sequence	Previous amino acid	Next amino acid	Best Peptide identification probability	Best Mascot Ion Score	Best Mascot Identity Score	Best Mascot Homology Score	Best Mascot Delta Ion Score	Best X! Tandem Hyper Score	Best X! Tandem -Log Expect Value	Best X! Tandem DeltaCN	Best X! Tandem Ladder Score	Number of identified +1H spectra	Number of identified +2H spectra	Number of identified +3H spectra	Number of identified +4H spectra	Number of enzymatic termini	Calculated +1H Peptide Mass (AMU)	Median Retention Time	Total Precursor Intensity	Total TIC	Peptide start index	Peptide stop index	Star Category	Assigned	Other Proteins
20196-20225 HinckeM --TAXchicken	Uncategorized Sample	20196-20205_A	Mudpit_Ingel20196_MGFPeaklist (F058870)	Uncharacterized protein OS=Gallus gallus GN=NUMA1 PE=4 SV=2	E1BQQ4_CHICK,R4GLW9_CHICK,UPI0002C8711B,UPI0002C8843B	TAX_GallusGallus_9031_20141114.fasta,UPARC14_2_GallusG_9031_20140417.fasta	238,106.40	100.00%	22	22	36	0.07%	11.80%	AAESLEGNLAR	R	I	99.70%	45	45.1	40.9	30	32	2.47	0.353	0.409	0	2	0	0	2	1,130.58	1,819.50		801.14	728	738	Empty	TRUE	
20196-20225 HinckeM --TAXchicken	Uncategorized Sample	20196-20205_A	Mudpit_Ingel20196_MGFPeaklist (F058870)	Uncharacterized protein OS=Gallus gallus GN=NUMA1 PE=4 SV=2	E1BQQ4_CHICK,R4GLW9_CHICK,UPI0002C8711B,UPI0002C8843B	TAX_GallusGallus_9031_20141114.fasta,UPARC14_2_GallusG_9031_20140417.fasta	238,106.40	100.00%	22	22	36	0.07%	11.80%	AAFICGFLQK	R	L	99.70%	45.7	43.9	21.6	38	25.8	1.7	0.213	0.35	0	1	0	0	2	1,154.60	2,575.00		208.37	62	71	Empty	TRUE	
20196-20225 HinckeM --TAXchicken	Uncategorized Sample	20196-20205_A	Mudpit_Ingel20196_MGFPeaklist (F058870)	Uncharacterized protein OS=Gallus gallus GN=NUMA1 PE=4 SV=2	E1BQQ4_CHICK,R4GLW9_CHICK,UPI0002C8711B,UPI0002C8843B	TAX_GallusGallus_9031_20141114.fasta,UPARC14_2_GallusG_9031_20140417.fasta	238,106.40	100.00%	22	22	36	0.07%	11.80%	ATTALQAELAR	K	A	99.70%	55.8	44.7	33.1	33.6	37.9	3.41	0.346	0.455	0	2	0	0	2	1,144.63	2,013.50		899.26	1376	1386	Empty	TRUE	
20196-20225 HinckeM --TAXchicken	Uncategorized Sample	20196-20205_A	Mudpit_Ingel20196_MGFPeaklist (F058870)	Uncharacterized protein OS=Gallus gallus GN=NUMA1 PE=4 SV=2	E1BQQ4_CHICK,R4GLW9_CHICK,UPI0002C8711B,UPI0002C8843B	TAX_GallusGallus_9031_20141114.fasta,UPARC14_2_GallusG_9031_20140417.fasta	238,106.40	100.00%	22	22	36	0.07%	11.80%	AVAEGSQQQQAELEASQR	K	E	99.70%	51.4	44.2	21.2	37.9	42	6.64	0.486	0.389	0	0	2	0	2	1,929.93	1,633.00		729.27	1102	1119	Empty	TRUE	
20196-20225 HinckeM --TAXchicken	Uncategorized Sample	20196-20205_A	Mudpit_Ingel20196_MGFPeaklist (F058870)	Uncharacterized protein OS=Gallus gallus GN=NUMA1 PE=4 SV=2	E1BQQ4_CHICK,R4GLW9_CHICK,UPI0002C8711B,UPI0002C8843B	TAX_GallusGallus_9031_20141114.fasta,UPARC14_2_GallusG_9031_20140417.fasta	238,106.40	100.00%	22	22	36	0.07%	11.80%	ELQQSVLQAK	K	E	71.70%	23.2	44.6	24.3	11.3					0	2	0	0	2	1,143.64	1,833.50		652.34	1640	1649	Empty	TRUE	
20196-20225 HinckeM --TAXchicken	Uncategorized Sample	20196-20205_A	Mudpit_Ingel20196_MGFPeaklist (F058870)	Uncharacterized protein OS=Gallus gallus GN=NUMA1 PE=4 SV=2	E1BQQ4_CHICK,R4GLW9_CHICK,UPI0002C8711B,UPI0002C8843B	TAX_GallusGallus_9031_20141114.fasta,UPARC14_2_GallusG_9031_20140417.fasta	238,106.40	100.00%	22	22	36	0.07%	11.80%	EQAKQVEELNK	K	K	24.40%	13.3	44.2	22.6	1.51					0	2	0	0	2	1,298.66	1,632.50		810.43	1531	1541	Empty	TRUE	
20196-20225 HinckeM --TAXchicken	Uncategorized Sample	20196-20205_A	Mudpit_Ingel20196_MGFPeaklist (F058870)	Uncharacterized protein OS=Gallus gallus GN=NUMA1 PE=4 SV=2	E1BQQ4_CHICK,R4GLW9_CHICK,UPI0002C8711B,UPI0002C8843B	TAX_GallusGallus_9031_20141114.fasta,UPARC14_2_GallusG_9031_20140417.fasta	238,106.40	100.00%	22	22	36	0.07%	11.80%	GIELQASVLR	K	L	99.70%	52.8	43.4	29.2	27.7	31.7	0.42	0.3	0.4	0	2	0	0	2	1,085.63	2,290.50		801.93	429	438	Empty	TRUE	
20196-20225 HinckeM --TAXchicken	Uncategorized Sample	20196-20205_A	Mudpit_Ingel20196_MGFPeaklist (F058870)	Uncharacterized protein OS=Gallus gallus GN=NUMA1 PE=4 SV=2	E1BQQ4_CHICK,R4GLW9_CHICK,UPI0002C8711B,UPI0002C8843B	TAX_GallusGallus_9031_20141114.fasta,UPARC14_2_GallusG_9031_20140417.fasta	238,106.40	100.00%	22	22	36	0.07%	11.80%	ISLLEDQLVK	K	L	81.20%	33.5	43.6	39.4	4.56					0	2	0	0	2	1,157.68	2,466.50		504.14	387	396	Empty	TRUE	
20196-20225 HinckeM --TAXchicken	Uncategorized Sample	20196-20205_A	Mudpit_Ingel20196_MGFPeaklist (F058870)	Uncharacterized protein OS=Gallus gallus GN=NUMA1 PE=4 SV=2	E1BQQ4_CHICK,R4GLW9_CHICK,UPI0002C8711B,UPI0002C8843B	TAX_GallusGallus_9031_20141114.fasta,UPARC14_2_GallusG_9031_20140417.fasta	238,106.40	100.00%	22	22	36	0.07%	11.80%	LALLNEK	R	Q	74.20%	35.5	44.1	44.7	10.1					0	1	0	0	2	800.4877	2,048.00		268.19	261	267	Empty	TRUE	
20196-20225 HinckeM --TAXchicken	Uncategorized Sample	20196-20205_A	Mudpit_Ingel20196_MGFPeaklist (F058870)	Uncharacterized protein OS=Gallus gallus GN=NUMA1 PE=4 SV=2	E1BQQ4_CHICK,R4GLW9_CHICK,UPI0002C8711B,UPI0002C8843B	TAX_GallusGallus_9031_20141114.fasta,UPARC14_2_GallusG_9031_20140417.fasta	238,106.40	100.00%	22	22	36	0.07%	11.80%	LKGELASVEAVKER	K	A	83.40%	25.5	42.7	19.4	19.9					0	0	1	0	2	1,528.87	1,842.00		172.69	1027	1040	Empty	TRUE	
20196-20225 HinckeM --TAXchicken	Uncategorized Sample	20196-20205_A	Mudpit_Ingel20196_MGFPeaklist (F058870)	Uncharacterized protein OS=Gallus gallus GN=NUMA1 PE=4 SV=2	E1BQQ4_CHICK,R4GLW9_CHICK,UPI0002C8711B,UPI0002C8843B	TAX_GallusGallus_9031_20141114.fasta,UPARC14_2_GallusG_9031_20140417.fasta	238,106.40	100.00%	22	22	36	0.07%	11.80%	LLEGEELELAK	K	V	22.80%	27.1	44.6	25.1	10.1					0	1	0	0	2	1,243.68	2,272.00		393.22	88	98	Empty	TRUE	
20196-20225 HinckeM --TAXchicken	Uncategorized Sample	20196-20205_A	Mudpit_Ingel20196_MGFPeaklist (F058870)	Uncharacterized protein OS=Gallus gallus GN=NUMA1 PE=4 SV=2	E1BQQ4_CHICK,R4GLW9_CHICK,UPI0002C8711B,UPI0002C8843B	TAX_GallusGallus_9031_20141114.fasta,UPARC14_2_GallusG_9031_20140417.fasta	238,106.40	100.00%	22	22	36	0.07%	11.80%	LLFLEESVR	R	N	99.70%	57.8	44.2	31.7	35.1	36.4	1.34	0.288	0.556	0	2	0	0	2	1,105.63	2,593.50		1,068.68	707	715	Empty	TRUE	
20196-20225 HinckeM --TAXchicken	Uncategorized Sample	20196-20205_A	Mudpit_Ingel20196_MGFPeaklist (F058870)	Uncharacterized protein OS=Gallus gallus GN=NUMA1 PE=4 SV=2	E1BQQ4_CHICK,R4GLW9_CHICK,UPI0002C8711B,UPI0002C8843B	TAX_GallusGallus_9031_20141114.fasta,UPARC14_2_GallusG_9031_20140417.fasta	238,106.40	100.00%	22	22	36	0.07%	11.80%	LQQVAEER	R	R	27.70%	23.5	45.2	38.2	0					0	1	0	0	2	973.495	1,822.00		318.07	774	781	Empty	TRUE	
20196-20225 HinckeM --TAXchicken	Uncategorized Sample	20196-20205_A	Mudpit_Ingel20196_MGFPeaklist (F058870)	Uncharacterized protein OS=Gallus gallus GN=NUMA1 PE=4 SV=2	E1BQQ4_CHICK,R4GLW9_CHICK,UPI0002C8711B,UPI0002C8843B	TAX_GallusGallus_9031_20141114.fasta,UPARC14_2_GallusG_9031_20140417.fasta	238,106.40	100.00%	22	22	36	0.07%	11.80%	LQQVAEERREETAALQR	R	Q	24.70%	0.34	43.7	13.2	-1.07					0	0	0	2	2	2,027.06	1,700.00		476.38	774	790	Empty	TRUE	
20196-20225 HinckeM --TAXchicken	Uncategorized Sample	20196-20205_A	Mudpit_Ingel20196_MGFPeaklist (F058870)	Uncharacterized protein OS=Gallus gallus GN=NUMA1 PE=4 SV=2	E1BQQ4_CHICK,R4GLW9_CHICK,UPI0002C8711B,UPI0002C8843B	TAX_GallusGallus_9031_20141114.fasta,UPARC14_2_GallusG_9031_20140417.fasta	238,106.40	100.00%	22	22	36	0.07%	11.80%	LTAQADALTAEVGK	R	L	24.80%	10.3	44.6	22.9	1.06					0	1	0	0	2	1,387.74	2,084.00		222.21	496	509	Empty	TRUE	
20196-20225 HinckeM --TAXchicken	Uncategorized Sample	20196-20205_A	Mudpit_Ingel20196_MGFPeaklist (F058870)	Uncharacterized protein OS=Gallus gallus GN=NUMA1 PE=4 SV=2	E1BQQ4_CHICK,R4GLW9_CHICK,UPI0002C8711B,UPI0002C8843B	TAX_GallusGallus_9031_20141114.fasta,UPARC14_2_GallusG_9031_20140417.fasta	238,106.40	100.00%	22	22	36	0.07%	11.80%	QAQLEGELR	K	A	60.40%	25.8	44.7	16.4	12.5					0	1	0	0	2	1,043.55	1,794.00		193.64	359	367	Empty	TRUE	
20196-20225 HinckeM --TAXchicken	Uncategorized Sample	20196-20205_A	Mudpit_Ingel20196_MGFPeaklist (F058870)	Uncharacterized protein OS=Gallus gallus GN=NUMA1 PE=4 SV=2	E1BQQ4_CHICK,R4GLW9_CHICK,UPI0002C8711B,UPI0002C8843B	TAX_GallusGallus_9031_20141114.fasta,UPARC14_2_GallusG_9031_20140417.fasta	238,106.40	100.00%	22	22	36	0.07%	11.80%	SAQALQDGTVESLRK	R	E	99.70%	45.6	44.5	26	37.1	43.5	4.8	0.43	0.5	0	0	2	0	2	1,602.84	1,836.00		1,356.14	1140	1154	Empty	TRUE	
20196-20225 HinckeM --TAXchicken	Uncategorized Sample	20196-20205_A	Mudpit_Ingel20196_MGFPeaklist (F058870)	Uncharacterized protein OS=Gallus gallus GN=NUMA1 PE=4 SV=2	E1BQQ4_CHICK,R4GLW9_CHICK,UPI0002C8711B,UPI0002C8843B	TAX_GallusGallus_9031_20141114.fasta,UPARC14_2_GallusG_9031_20140417.fasta	238,106.40	100.00%	22	22	36	0.07%	11.80%	SLASFPSQDSLSK	R	L	52.80%	20.3	44.4	22.3	9.63					0	2	0	0	2	1,366.68	2,117.50		646.66	1841	1853	Empty	TRUE	
20196-20225 HinckeM --TAXchicken	Uncategorized Sample	20196-20205_A	Mudpit_Ingel20196_MGFPeaklist (F058870)	Uncharacterized protein OS=Gallus gallus GN=NUMA1 PE=4 SV=2	E1BQQ4_CHICK,R4GLW9_CHICK,UPI0002C8711B,UPI0002C8843B	TAX_GallusGallus_9031_20141114.fasta,UPARC14_2_GallusG_9031_20140417.fasta	238,106.40	100.00%	22	22	36	0.07%	11.80%	TAVAAAEKEVASLR	K	A	99.70%	65.4	43.8	32.2	42.4	36.1	3.66	0.38	0.393	0	0	2	0	2	1,415.79	2,260.50		857.42	1168	1181	Empty	TRUE	
20196-20225 HinckeM --TAXchicken	Uncategorized Sample	20196-20205_A	Mudpit_Ingel20196_MGFPeaklist (F058870)	Uncharacterized protein OS=Gallus gallus GN=NUMA1 PE=4 SV=2	E1BQQ4_CHICK,R4GLW9_CHICK,UPI0002C8711B,UPI0002C8843B	TAX_GallusGallus_9031_20141114.fasta,UPARC14_2_GallusG_9031_20140417.fasta	238,106.40	100.00%	22	22	36	0.07%	11.80%	VKVLEGELQSEATR	R	Q	16.90%	23.9	44	26.1	8.58					0	0	1	0	2	1,558.84	1,945.00		269.3	1557	1570	Empty	TRUE	
20196-20225 HinckeM --TAXchicken	Uncategorized Sample	20196-20205_A	Mudpit_Ingel20196_MGFPeaklist (F058870)	Uncharacterized protein OS=Gallus gallus GN=NUMA1 PE=4 SV=2	E1BQQ4_CHICK,R4GLW9_CHICK,UPI0002C8711B,UPI0002C8843B	TAX_GallusGallus_9031_20141114.fasta,UPARC14_2_GallusG_9031_20140417.fasta	238,106.40	100.00%	22	22	36	0.07%	11.80%	VLEGELQSEATR	K	Q	99.70%	58	45.1	26.3	42.2	28.3	0.638	0.29	0.25	0	2	0	0	2	1,331.68	1,854.00		329.18	1559	1570	Empty	TRUE	
20196-20225 HinckeM --TAXchicken	Uncategorized Sample	20196-20205_A	Mudpit_Ingel20196_MGFPeaklist (F058870)	Uncharacterized protein OS=Gallus gallus GN=NUMA1 PE=4 SV=2	E1BQQ4_CHICK,R4GLW9_CHICK,UPI0002C8711B,UPI0002C8843B	TAX_GallusGallus_9031_20141114.fasta,UPARC14_2_GallusG_9031_20140417.fasta	238,106.40	100.00%	22	22	36	0.07%	11.80%	VLELSAQCQQSSAQAGSAETLR	R	A	99.70%	52.5	44.3	21.9	38.9	38	5.64	0.445	0.273	0	0	1	0	2	2,334.14	2,062.00		591.14	652	673	Empty	TRUE	
20196-20225 HinckeM --TAXchicken	Uncategorized Sample	20196-20205_A	Mudpit_Ingel20196_MGFPeaklist (F058870)	Uncharacterized protein OS=Gallus gallus GN=NUMA1 PE=4 SV=2	E1BQQ4_CHICK,R4GLW9_CHICK,UPI0002C8711B,UPI0002C8843B	TAX_GallusGallus_9031_20141114.fasta,UPARC14_2_GallusG_9031_20140417.fasta	238,106.40	100.00%	22	22	36	0.07%	11.80%	AAESLEGNLAR	R	I	99.70%	45	45.1	40.9	30	32	2.47	0.353	0.409	0	2	0	0	2	1,130.58	1,819.50		801.14	728	738	Empty	TRUE	
20196-20225 HinckeM --TAXchicken	Uncategorized Sample	20196-20205_A	Mudpit_Ingel20196_MGFPeaklist (F058870)	Uncharacterized protein OS=Gallus gallus GN=NUMA1 PE=4 SV=2	E1BQQ4_CHICK,R4GLW9_CHICK,UPI0002C8711B,UPI0002C8843B	TAX_GallusGallus_9031_20141114.fasta,UPARC14_2_GallusG_9031_20140417.fasta	238,106.40	100.00%	22	22	36	0.07%	11.80%	AAFICGFLQK	R	L	99.70%	45.7	43.9	21.6	38	25.8	1.7	0.213	0.35	0	1	0	0	2	1,154.60	2,575.00		208.37	62	71	Empty	TRUE	
20196-20225 HinckeM --TAXchicken	Uncategorized Sample	20196-20205_A	Mudpit_Ingel20196_MGFPeaklist (F058870)	Uncharacterized protein OS=Gallus gallus GN=NUMA1 PE=4 SV=2	E1BQQ4_CHICK,R4GLW9_CHICK,UPI0002C8711B,UPI0002C8843B	TAX_GallusGallus_9031_20141114.fasta,UPARC14_2_GallusG_9031_20140417.fasta	238,106.40	100.00%	22	22	36	0.07%	11.80%	ATTALQAELAR	K	A	99.70%	55.8	44.7	33.1	33.6	37.9	3.41	0.346	0.455	0	2	0	0	2	1,144.63	2,013.50		899.26	1376	1386	Empty	TRUE	
20196-20225 HinckeM --TAXchicken	Uncategorized Sample	20196-20205_A	Mudpit_Ingel20196_MGFPeaklist (F058870)	Uncharacterized protein OS=Gallus gallus GN=NUMA1 PE=4 SV=2	E1BQQ4_CHICK,R4GLW9_CHICK,UPI0002C8711B,UPI0002C8843B	TAX_GallusGallus_9031_20141114.fasta,UPARC14_2_GallusG_9031_20140417.fasta	238,106.40	100.00%	22	22	36	0.07%	11.80%	AVAEGSQQQQAELEASQR	K	E	99.70%	51.4	44.2	21.2	37.9	42	6.64	0.486	0.389	0	0	2	0	2	1,929.93	1,633.00		729.27	1102	1119	Empty	TRUE	
20196-20225 HinckeM --TAXchicken	Uncategorized Sample	20196-20205_A	Mudpit_Ingel20196_MGFPeaklist (F058870)	Uncharacterized protein OS=Gallus gallus GN=NUMA1 PE=4 SV=2	E1BQQ4_CHICK,R4GLW9_CHICK,UPI0002C8711B,UPI0002C8843B	TAX_GallusGallus_9031_20141114.fasta,UPARC14_2_GallusG_9031_20140417.fasta	238,106.40	100.00%	22	22	36	0.07%	11.80%	ELQQSVLQAK	K	E	71.70%	23.2	44.6	24.3	11.3					0	2	0	0	2	1,143.64	1,833.50		652.34	1640	1649	Empty	TRUE	
20196-20225 HinckeM --TAXchicken	Uncategorized Sample	20196-20205_A	Mudpit_Ingel20196_MGFPeaklist (F058870)	Uncharacterized protein OS=Gallus gallus GN=NUMA1 PE=4 SV=2	E1BQQ4_CHICK,R4GLW9_CHICK,UPI0002C8711B,UPI0002C8843B	TAX_GallusGallus_9031_20141114.fasta,UPARC14_2_GallusG_9031_20140417.fasta	238,106.40	100.00%	22	22	36	0.07%	11.80%	EQAKQVEELNK	K	K	24.40%	13.3	44.2	22.6	1.51					0	2	0	0	2	1,298.66	1,632.50		810.43	1531	1541	Empty	TRUE	
20196-20225 HinckeM --TAXchicken	Uncategorized Sample	20196-20205_A	Mudpit_Ingel20196_MGFPeaklist (F058870)	Uncharacterized protein OS=Gallus gallus GN=NUMA1 PE=4 SV=2	E1BQQ4_CHICK,R4GLW9_CHICK,UPI0002C8711B,UPI0002C8843B	TAX_GallusGallus_9031_20141114.fasta,UPARC14_2_GallusG_9031_20140417.fasta	238,106.40	100.00%	22	22	36	0.07%	11.80%	GIELQASVLR	K	L	99.70%	52.8	43.4	29.2	27.7	31.7	0.42	0.3	0.4	0	2	0	0	2	1,085.63	2,290.50		801.93	429	438	Empty	TRUE	
20196-20225 HinckeM --TAXchicken	Uncategorized Sample	20196-20205_A	Mudpit_Ingel20196_MGFPeaklist (F058870)	Uncharacterized protein OS=Gallus gallus GN=NUMA1 PE=4 SV=2	E1BQQ4_CHICK,R4GLW9_CHICK,UPI0002C8711B,UPI0002C8843B	TAX_GallusGallus_9031_20141114.fasta,UPARC14_2_GallusG_9031_20140417.fasta	238,106.40	100.00%	22	22	36	0.07%	11.80%	ISLLEDQLVK	K	L	81.20%	33.5	43.6	39.4	4.56					0	2	0	0	2	1,157.68	2,466.50		504.14	387	396	Empty	TRUE	
20196-20225 HinckeM --TAXchicken	Uncategorized Sample	20196-20205_A	Mudpit_Ingel20196_MGFPeaklist (F058870)	Uncharacterized protein OS=Gallus gallus GN=NUMA1 PE=4 SV=2	E1BQQ4_CHICK,R4GLW9_CHICK,UPI0002C8711B,UPI0002C8843B	TAX_GallusGallus_9031_20141114.fasta,UPARC14_2_GallusG_9031_20140417.fasta	238,106.40	100.00%	22	22	36	0.07%	11.80%	LALLNEK	R	Q	74.20%	35.5	44.1	44.7	10.1					0	1	0	0	2	800.4877	2,048.00		268.19	261	267	Empty	TRUE	
20196-20225 HinckeM --TAXchicken	Uncategorized Sample	20196-20205_A	Mudpit_Ingel20196_MGFPeaklist (F058870)	Uncharacterized protein OS=Gallus gallus GN=NUMA1 PE=4 SV=2	E1BQQ4_CHICK,R4GLW9_CHICK,UPI0002C8711B,UPI0002C8843B	TAX_GallusGallus_9031_20141114.fasta,UPARC14_2_GallusG_9031_20140417.fasta	238,106.40	100.00%	22	22	36	0.07%	11.80%	LKGELASVEAVKER	K	A	83.40%	25.5	42.7	19.4	19.9					0	0	1	0	2	1,528.87	1,842.00		172.69	1027	1040	Empty	TRUE	
20196-20225 HinckeM --TAXchicken	Uncategorized Sample	20196-20205_A	Mudpit_Ingel20196_MGFPeaklist (F058870)	Uncharacterized protein OS=Gallus gallus GN=NUMA1 PE=4 SV=2	E1BQQ4_CHICK,R4GLW9_CHICK,UPI0002C8711B,UPI0002C8843B	TAX_GallusGallus_9031_20141114.fasta,UPARC14_2_GallusG_9031_20140417.fasta	238,106.40	100.00%	22	22	36	0.07%	11.80%	LLEGEELELAK	K	V	22.80%	27.1	44.6	25.1	10.1					0	1	0	0	2	1,243.68	2,272.00		393.22	88	98	Empty	TRUE	
20196-20225 HinckeM --TAXchicken	Uncategorized Sample	20196-20205_A	Mudpit_Ingel20196_MGFPeaklist (F058870)	Uncharacterized protein OS=Gallus gallus GN=NUMA1 PE=4 SV=2	E1BQQ4_CHICK,R4GLW9_CHICK,UPI0002C8711B,UPI0002C8843B	TAX_GallusGallus_9031_20141114.fasta,UPARC14_2_GallusG_9031_20140417.fasta	238,106.40	100.00%	22	22	36	0.07%	11.80%	LLFLEESVR	R	N	99.70%	57.8	44.2	31.7	35.1	36.4	1.34	0.288	0.556	0	2	0	0	2	1,105.63	2,593.50		1,068.68	707	715	Empty	TRUE	
20196-20225 HinckeM --TAXchicken	Uncategorized Sample	20196-20205_A	Mudpit_Ingel20196_MGFPeaklist (F058870)	Uncharacterized protein OS=Gallus gallus GN=NUMA1 PE=4 SV=2	E1BQQ4_CHICK,R4GLW9_CHICK,UPI0002C8711B,UPI0002C8843B	TAX_GallusGallus_9031_20141114.fasta,UPARC14_2_GallusG_9031_20140417.fasta	238,106.40	100.00%	22	22	36	0.07%	11.80%	LQQVAEER	R	R	27.70%	23.5	45.2	38.2	0					0	1	0	0	2	973.495	1,822.00		318.07	774	781	Empty	TRUE	
20196-20225 HinckeM --TAXchicken	Uncategorized Sample	20196-20205_A	Mudpit_Ingel20196_MGFPeaklist (F058870)	Uncharacterized protein OS=Gallus gallus GN=NUMA1 PE=4 SV=2	E1BQQ4_CHICK,R4GLW9_CHICK,UPI0002C8711B,UPI0002C8843B	TAX_GallusGallus_9031_20141114.fasta,UPARC14_2_GallusG_9031_20140417.fasta	238,106.40	100.00%	22	22	36	0.07%	11.80%	LQQVAEERREETAALQR	R	Q	24.70%	0.34	43.7	13.2	-1.07					0	0	0	2	2	2,027.06	1,700.00		476.38	774	790	Empty	TRUE	
20196-20225 HinckeM --TAXchicken	Uncategorized Sample	20196-20205_A	Mudpit_Ingel20196_MGFPeaklist (F058870)	Uncharacterized protein OS=Gallus gallus GN=NUMA1 PE=4 SV=2	E1BQQ4_CHICK,R4GLW9_CHICK,UPI0002C8711B,UPI0002C8843B	TAX_GallusGallus_9031_20141114.fasta,UPARC14_2_GallusG_9031_20140417.fasta	238,106.40	100.00%	22	22	36	0.07%	11.80%	LTAQADALTAEVGK	R	L	24.80%	10.3	44.6	22.9	1.06					0	1	0	0	2	1,387.74	2,084.00		222.21	496	509	Empty	TRUE	
20196-20225 HinckeM --TAXchicken	Uncategorized Sample	20196-20205_A	Mudpit_Ingel20196_MGFPeaklist (F058870)	Uncharacterized protein OS=Gallus gallus GN=NUMA1 PE=4 SV=2	E1BQQ4_CHICK,R4GLW9_CHICK,UPI0002C8711B,UPI0002C8843B	TAX_GallusGallus_9031_20141114.fasta,UPARC14_2_GallusG_9031_20140417.fasta	238,106.40	100.00%	22	22	36	0.07%	11.80%	QAQLEGELR	K	A	60.40%	25.8	44.7	16.4	12.5					0	1	0	0	2	1,043.55	1,794.00		193.64	359	367	Empty	TRUE	
20196-20225 HinckeM --TAXchicken	Uncategorized Sample	20196-20205_A	Mudpit_Ingel20196_MGFPeaklist (F058870)	Uncharacterized protein OS=Gallus gallus GN=NUMA1 PE=4 SV=2	E1BQQ4_CHICK,R4GLW9_CHICK,UPI0002C8711B,UPI0002C8843B	TAX_GallusGallus_9031_20141114.fasta,UPARC14_2_GallusG_9031_20140417.fasta	238,106.40	100.00%	22	22	36	0.07%	11.80%	SAQALQDGTVESLRK	R	E	99.70%	45.6	44.5	26	37.1	43.5	4.8	0.43	0.5	0	0	2	0	2	1,602.84	1,836.00		1,356.14	1140	1154	Empty	TRUE	
20196-20225 HinckeM --TAXchicken	Uncategorized Sample	20196-20205_A	Mudpit_Ingel20196_MGFPeaklist (F058870)	Uncharacterized protein OS=Gallus gallus GN=NUMA1 PE=4 SV=2	E1BQQ4_CHICK,R4GLW9_CHICK,UPI0002C8711B,UPI0002C8843B	TAX_GallusGallus_9031_20141114.fasta,UPARC14_2_GallusG_9031_20140417.fasta	238,106.40	100.00%	22	22	36	0.07%	11.80%	SLASFPSQDSLSK	R	L	52.80%	20.3	44.4	22.3	9.63					0	2	0	0	2	1,366.68	2,117.50		646.66	1841	1853	Empty	TRUE	
20196-20225 HinckeM --TAXchicken	Uncategorized Sample	20196-20205_A	Mudpit_Ingel20196_MGFPeaklist (F058870)	Uncharacterized protein OS=Gallus gallus GN=NUMA1 PE=4 SV=2	E1BQQ4_CHICK,R4GLW9_CHICK,UPI0002C8711B,UPI0002C8843B	TAX_GallusGallus_9031_20141114.fasta,UPARC14_2_GallusG_9031_20140417.fasta	238,106.40	100.00%	22	22	36	0.07%	11.80%	TAVAAAEKEVASLR	K	A	99.70%	65.4	43.8	32.2	42.4	36.1	3.66	0.38	0.393	0	0	2	0	2	1,415.79	2,260.50		857.42	1168	1181	Empty	TRUE	
20196-20225 HinckeM --TAXchicken	Uncategorized Sample	20196-20205_A	Mudpit_Ingel20196_MGFPeaklist (F058870)	Uncharacterized protein OS=Gallus gallus GN=NUMA1 PE=4 SV=2	E1BQQ4_CHICK,R4GLW9_CHICK,UPI0002C8711B,UPI0002C8843B	TAX_GallusGallus_9031_20141114.fasta,UPARC14_2_GallusG_9031_20140417.fasta	238,106.40	100.00%	22	22	36	0.07%	11.80%	VKVLEGELQSEATR	R	Q	16.90%	23.9	44	26.1	8.58					0	0	1	0	2	1,558.84	1,945.00		269.3	1557	1570	Empty	TRUE	
20196-20225 HinckeM --TAXchicken	Uncategorized Sample	20196-20205_A	Mudpit_Ingel20196_MGFPeaklist (F058870)	Uncharacterized protein OS=Gallus gallus GN=NUMA1 PE=4 SV=2	E1BQQ4_CHICK,R4GLW9_CHICK,UPI0002C8711B,UPI0002C8843B	TAX_GallusGallus_9031_20141114.fasta,UPARC14_2_GallusG_9031_20140417.fasta	238,106.40	100.00%	22	22	36	0.07%	11.80%	VLEGELQSEATR	K	Q	99.70%	58	45.1	26.3	42.2	28.3	0.638	0.29	0.25	0	2	0	0	2	1,331.68	1,854.00		329.18	1559	1570	Empty	TRUE	
20196-20225 HinckeM --TAXchicken	Uncategorized Sample	20196-20205_A	Mudpit_Ingel20196_MGFPeaklist (F058870)	Uncharacterized protein OS=Gallus gallus GN=NUMA1 PE=4 SV=2	E1BQQ4_CHICK,R4GLW9_CHICK,UPI0002C8711B,UPI0002C8843B	TAX_GallusGallus_9031_20141114.fasta,UPARC14_2_GallusG_9031_20140417.fasta	238,106.40	100.00%	22	22	36	0.07%	11.80%	VLELSAQCQQSSAQAGSAETLR	R	A	99.70%	52.5	44.3	21.9	38.9	38	5.64	0.445	0.273	0	0	1	0	2	2,334.14	2,062.00		591.14	652	673	Empty	TRUE	
20196-20225 HinckeM --TAXchicken	Uncategorized Sample	20196-20205_A	Mudpit_Ingel20196_MGFPeaklist (F058870)	Uncharacterized protein OS=Gallus gallus GN=NUMA1 PE=4 SV=2	E1BQQ4_CHICK,R4GLW9_CHICK,UPI0002C8711B,UPI0002C8843B	TAX_GallusGallus_9031_20141114.fasta,UPARC14_2_GallusG_9031_20140417.fasta	238,106.40	100.00%	22	22	36	0.07%	11.80%	AAESLEGNLAR	R	I	99.70%	45	45.1	40.9	30	32	2.47	0.353	0.409	0	2	0	0	2	1,130.58	1,819.50		801.14	728	738	Empty	TRUE	

20196-20225 HinckeM –TAXchicken	Uncategorized Sample	20196-20205_A	Mudpit_Ingel20196_MGFPeaklist (F058870)	Uncharacterized protein OS=Gallus gallus GN=NUMA1 PE=4 SV=2	E1BQQ4_CHICK,R4GLW9_CHICK,UPI0002C8711B,UPI0002C8843B	TAX_GallusGallus_9031_20141114.fasta,UPARC14_2_GallusG_9031_20140417.fasta	238,106.40	100.00%	22	22	36	0.07%	11.80%	AAFICGFLQK	R	L	99.70%	45.7	43.9	21.6	38	25.8	1.7	0.213	0.35	0	1	0	0	2	1,154.60	2,575.00		208.37	62	71	Empty	TRUE	
20196-20225 HinckeM –TAXchicken	Uncategorized Sample	20196-20205_A	Mudpit_Ingel20196_MGFPeaklist (F058870)	Uncharacterized protein OS=Gallus gallus GN=NUMA1 PE=4 SV=2	E1BQQ4_CHICK,R4GLW9_CHICK,UPI0002C8711B,UPI0002C8843B	TAX_GallusGallus_9031_20141114.fasta,UPARC14_2_GallusG_9031_20140417.fasta	238,106.40	100.00%	22	22	36	0.07%	11.80%	ATTALQAELAR	K	A	99.70%	55.8	44.7	33.1	33.6	37.9	3.41	0.346	0.455	0	2	0	0	2	1,144.63	2,013.50		899.26	1376	1386	Empty	TRUE	
20196-20225 HinckeM –TAXchicken	Uncategorized Sample	20196-20205_A	Mudpit_Ingel20196_MGFPeaklist (F058870)	Uncharacterized protein OS=Gallus gallus GN=NUMA1 PE=4 SV=2	E1BQQ4_CHICK,R4GLW9_CHICK,UPI0002C8711B,UPI0002C8843B	TAX_GallusGallus_9031_20141114.fasta,UPARC14_2_GallusG_9031_20140417.fasta	238,106.40	100.00%	22	22	36	0.07%	11.80%	AVAEGSQQQQAELEASQR	K	E	99.70%	51.4	44.2	21.2	37.9	42	6.64	0.486	0.389	0	0	2	0	2	1,929.93	1,633.00		729.27	1102	1119	Empty	TRUE	
20196-20225 HinckeM –TAXchicken	Uncategorized Sample	20196-20205_A	Mudpit_Ingel20196_MGFPeaklist (F058870)	Uncharacterized protein OS=Gallus gallus GN=NUMA1 PE=4 SV=2	E1BQQ4_CHICK,R4GLW9_CHICK,UPI0002C8711B,UPI0002C8843B	TAX_GallusGallus_9031_20141114.fasta,UPARC14_2_GallusG_9031_20140417.fasta	238,106.40	100.00%	22	22	36	0.07%	11.80%	ELQQSVLQAK	K	E	71.70%	23.2	44.6	24.3	11.3					0	2	0	0	2	1,143.64	1,833.50		652.34	1640	1649	Empty	TRUE	
20196-20225 HinckeM –TAXchicken	Uncategorized Sample	20196-20205_A	Mudpit_Ingel20196_MGFPeaklist (F058870)	Uncharacterized protein OS=Gallus gallus GN=NUMA1 PE=4 SV=2	E1BQQ4_CHICK,R4GLW9_CHICK,UPI0002C8711B,UPI0002C8843B	TAX_GallusGallus_9031_20141114.fasta,UPARC14_2_GallusG_9031_20140417.fasta	238,106.40	100.00%	22	22	36	0.07%	11.80%	EQAKQVEELNK	K	K	24.40%	13.3	44.2	22.6	1.51					0	2	0	0	2	1,298.66	1,632.50		810.43	1531	1541	Empty	TRUE	
20196-20225 HinckeM –TAXchicken	Uncategorized Sample	20196-20205_A	Mudpit_Ingel20196_MGFPeaklist (F058870)	Uncharacterized protein OS=Gallus gallus GN=NUMA1 PE=4 SV=2	E1BQQ4_CHICK,R4GLW9_CHICK,UPI0002C8711B,UPI0002C8843B	TAX_GallusGallus_9031_20141114.fasta,UPARC14_2_GallusG_9031_20140417.fasta	238,106.40	100.00%	22	22	36	0.07%	11.80%	GIELQASVLR	K	L	99.70%	52.8	43.4	29.2	27.7	31.7	0.42	0.3	0.4	0	2	0	0	2	1,085.63	2,290.50		801.93	429	438	Empty	TRUE	
20196-20225 HinckeM –TAXchicken	Uncategorized Sample	20196-20205_A	Mudpit_Ingel20196_MGFPeaklist (F058870)	Uncharacterized protein OS=Gallus gallus GN=NUMA1 PE=4 SV=2	E1BQQ4_CHICK,R4GLW9_CHICK,UPI0002C8711B,UPI0002C8843B	TAX_GallusGallus_9031_20141114.fasta,UPARC14_2_GallusG_9031_20140417.fasta	238,106.40	100.00%	22	22	36	0.07%	11.80%	ISLLEDQLVK	K	L	81.20%	33.5	43.6	39.4	4.56					0	2	0	0	2	1,157.68	2,466.50		504.14	387	396	Empty	TRUE	
20196-20225 HinckeM –TAXchicken	Uncategorized Sample	20196-20205_A	Mudpit_Ingel20196_MGFPeaklist (F058870)	Uncharacterized protein OS=Gallus gallus GN=NUMA1 PE=4 SV=2	E1BQQ4_CHICK,R4GLW9_CHICK,UPI0002C8711B,UPI0002C8843B	TAX_GallusGallus_9031_20141114.fasta,UPARC14_2_GallusG_9031_20140417.fasta	238,106.40	100.00%	22	22	36	0.07%	11.80%	LALLNEK	R	Q	74.20%	35.5	44.1	44.7	10.1					0	1	0	0	2	800.4877	2,048.00		268.19	261	267	Empty	TRUE	
20196-20225 HinckeM –TAXchicken	Uncategorized Sample	20196-20205_A	Mudpit_Ingel20196_MGFPeaklist (F058870)	Uncharacterized protein OS=Gallus gallus GN=NUMA1 PE=4 SV=2	E1BQQ4_CHICK,R4GLW9_CHICK,UPI0002C8711B,UPI0002C8843B	TAX_GallusGallus_9031_20141114.fasta,UPARC14_2_GallusG_9031_20140417.fasta	238,106.40	100.00%	22	22	36	0.07%	11.80%	LKGELASVEAVKER	K	A	83.40%	25.5	42.7	19.4	19.9					0	0	1	0	2	1,528.87	1,842.00		172.69	1027	1040	Empty	TRUE	
20196-20225 HinckeM –TAXchicken	Uncategorized Sample	20196-20205_A	Mudpit_Ingel20196_MGFPeaklist (F058870)	Uncharacterized protein OS=Gallus gallus GN=NUMA1 PE=4 SV=2	E1BQQ4_CHICK,R4GLW9_CHICK,UPI0002C8711B,UPI0002C8843B	TAX_GallusGallus_9031_20141114.fasta,UPARC14_2_GallusG_9031_20140417.fasta	238,106.40	100.00%	22	22	36	0.07%	11.80%	LLEGEELELAK	K	V	22.80%	27.1	44.6	25.1	10.1					0	1	0	0	2	1,243.68	2,272.00		393.22	88	98	Empty	TRUE	
20196-20225 HinckeM –TAXchicken	Uncategorized Sample	20196-20205_A	Mudpit_Ingel20196_MGFPeaklist (F058870)	Uncharacterized protein OS=Gallus gallus GN=NUMA1 PE=4 SV=2	E1BQQ4_CHICK,R4GLW9_CHICK,UPI0002C8711B,UPI0002C8843B	TAX_GallusGallus_9031_20141114.fasta,UPARC14_2_GallusG_9031_20140417.fasta	238,106.40	100.00%	22	22	36	0.07%	11.80%	LLFLEESVR	R	N	99.70%	57.8	44.2	31.7	35.1	36.4	1.34	0.288	0.556	0	2	0	0	2	1,105.63	2,593.50		1,068.68	707	715	Empty	TRUE	
20196-20225 HinckeM –TAXchicken	Uncategorized Sample	20196-20205_A	Mudpit_Ingel20196_MGFPeaklist (F058870)	Uncharacterized protein OS=Gallus gallus GN=NUMA1 PE=4 SV=2	E1BQQ4_CHICK,R4GLW9_CHICK,UPI0002C8711B,UPI0002C8843B	TAX_GallusGallus_9031_20141114.fasta,UPARC14_2_GallusG_9031_20140417.fasta	238,106.40	100.00%	22	22	36	0.07%	11.80%	LQQVAEER	R	R	27.70%	23.5	45.2	38.2	0					0	1	0	0	2	973.495	1,822.00		318.07	774	781	Empty	TRUE	
20196-20225 HinckeM –TAXchicken	Uncategorized Sample	20196-20205_A	Mudpit_Ingel20196_MGFPeaklist (F058870)	Uncharacterized protein OS=Gallus gallus GN=NUMA1 PE=4 SV=2	E1BQQ4_CHICK,R4GLW9_CHICK,UPI0002C8711B,UPI0002C8843B	TAX_GallusGallus_9031_20141114.fasta,UPARC14_2_GallusG_9031_20140417.fasta	238,106.40	100.00%	22	22	36	0.07%	11.80%	LQQVAEERREETAALQR	R	Q	24.70%	0.34	43.7	13.2	-1.07					0	0	0	2	2	2,027.06	1,700.00		476.38	774	790	Empty	TRUE	
20196-20225 HinckeM –TAXchicken	Uncategorized Sample	20196-20205_A	Mudpit_Ingel20196_MGFPeaklist (F058870)	Uncharacterized protein OS=Gallus gallus GN=NUMA1 PE=4 SV=2	E1BQQ4_CHICK,R4GLW9_CHICK,UPI0002C8711B,UPI0002C8843B	TAX_GallusGallus_9031_20141114.fasta,UPARC14_2_GallusG_9031_20140417.fasta	238,106.40	100.00%	22	22	36	0.07%	11.80%	LTAQADALTAEVGK	R	L	24.80%	10.3	44.6	22.9	1.06					0	1	0	0	2	1,387.74	2,084.00		222.21	496	509	Empty	TRUE	
20196-20225 HinckeM –TAXchicken	Uncategorized Sample	20196-20205_A	Mudpit_Ingel20196_MGFPeaklist (F058870)	Uncharacterized protein OS=Gallus gallus GN=NUMA1 PE=4 SV=2	E1BQQ4_CHICK,R4GLW9_CHICK,UPI0002C8711B,UPI0002C8843B	TAX_GallusGallus_9031_20141114.fasta,UPARC14_2_GallusG_9031_20140417.fasta	238,106.40	100.00%	22	22	36	0.07%	11.80%	QAQLEGELR	K	A	60.40%	25.8	44.7	16.4	12.5					0	1	0	0	2	1,043.55	1,794.00		193.64	359	367	Empty	TRUE	
20196-20225 HinckeM –TAXchicken	Uncategorized Sample	20196-20205_A	Mudpit_Ingel20196_MGFPeaklist (F058870)	Uncharacterized protein OS=Gallus gallus GN=NUMA1 PE=4 SV=2	E1BQQ4_CHICK,R4GLW9_CHICK,UPI0002C8711B,UPI0002C8843B	TAX_GallusGallus_9031_20141114.fasta,UPARC14_2_GallusG_9031_20140417.fasta	238,106.40	100.00%	22	22	36	0.07%	11.80%	SAQALQDGTVESLRK	R	E	99.70%	45.6	44.5	26	37.1	43.5	4.8	0.43	0.5	0	0	2	0	2	1,602.84	1,836.00		1,356.14	1140	1154	Empty	TRUE	
20196-20225 HinckeM –TAXchicken	Uncategorized Sample	20196-20205_A	Mudpit_Ingel20196_MGFPeaklist (F058870)	Uncharacterized protein OS=Gallus gallus GN=NUMA1 PE=4 SV=2	E1BQQ4_CHICK,R4GLW9_CHICK,UPI0002C8711B,UPI0002C8843B	TAX_GallusGallus_9031_20141114.fasta,UPARC14_2_GallusG_9031_20140417.fasta	238,106.40	100.00%	22	22	36	0.07%	11.80%	SLASFPSQDSLSK	R	L	52.80%	20.3	44.4	22.3	9.63					0	2	0	0	2	1,366.68	2,117.50		646.66	1841	1853	Empty	TRUE	
20196-20225 HinckeM –TAXchicken	Uncategorized Sample	20196-20205_A	Mudpit_Ingel20196_MGFPeaklist (F058870)	Uncharacterized protein OS=Gallus gallus GN=NUMA1 PE=4 SV=2	E1BQQ4_CHICK,R4GLW9_CHICK,UPI0002C8711B,UPI0002C8843B	TAX_GallusGallus_9031_20141114.fasta,UPARC14_2_GallusG_9031_20140417.fasta	238,106.40	100.00%	22	22	36	0.07%	11.80%	TAVAAAEKEVASLR	K	A	99.70%	65.4	43.8	32.2	42.4	36.1	3.66	0.38	0.393	0	0	2	0	2	1,415.79	2,260.50		857.42	1168	1181	Empty	TRUE	
20196-20225 HinckeM –TAXchicken	Uncategorized Sample	20196-20205_A	Mudpit_Ingel20196_MGFPeaklist (F058870)	Uncharacterized protein OS=Gallus gallus GN=NUMA1 PE=4 SV=2	E1BQQ4_CHICK,R4GLW9_CHICK,UPI0002C8711B,UPI0002C8843B	TAX_GallusGallus_9031_20141114.fasta,UPARC14_2_GallusG_9031_20140417.fasta	238,106.40	100.00%	22	22	36	0.07%	11.80%	VKVLEGELQSEATR	R	Q	16.90%	23.9	44	26.1	8.58					0	0	1	0	2	1,558.84	1,945.00		269.3	1557	1570	Empty	TRUE	
20196-20225 HinckeM –TAXchicken	Uncategorized Sample	20196-20205_A	Mudpit_Ingel20196_MGFPeaklist (F058870)	Uncharacterized protein OS=Gallus gallus GN=NUMA1 PE=4 SV=2	E1BQQ4_CHICK,R4GLW9_CHICK,UPI0002C8711B,UPI0002C8843B	TAX_GallusGallus_9031_20141114.fasta,UPARC14_2_GallusG_9031_20140417.fasta	238,106.40	100.00%	22	22	36	0.07%	11.80%	VLEGELQSEATR	K	Q	99.70%	58	45.1	26.3	42.2	28.3	0.638	0.29	0.25	0	2	0	0	2	1,331.68	1,854.00		329.18	1559	1570	Empty	TRUE	
20196-20225 HinckeM –TAXchicken	Uncategorized Sample	20196-20205_A	Mudpit_Ingel20196_MGFPeaklist (F058870)	Uncharacterized protein OS=Gallus gallus GN=NUMA1 PE=4 SV=2	E1BQQ4_CHICK,R4GLW9_CHICK,UPI0002C8711B,UPI0002C8843B	TAX_GallusGallus_9031_20141114.fasta,UPARC14_2_GallusG_9031_20140417.fasta	238,106.40	100.00%	22	22	36	0.07%	11.80%	VLELSAQCQQSSAQAGSAETLR	R	A	99.70%	52.5	44.3	21.9	38.9	38	5.64	0.445	0.273	0	0	1	0	2	2,334.14	2,062.00		591.14	652	673	Empty	TRUE	
20196-20225 HinckeM –TAXchicken	Uncategorized Sample	20196-20205_A	Mudpit_Ingel20196_MGFPeaklist (F058870)	Uncharacterized protein OS=Gallus gallus GN=NUMA1 PE=4 SV=2	E1BQQ4_CHICK,R4GLW9_CHICK,UPI0002C8711B,UPI0002C8843B	TAX_GallusGallus_9031_20141114.fasta,UPARC14_2_GallusG_9031_20140417.fasta	238,106.40	100.00%	22	22	36	0.07%	11.80%	AAESLEGNLAR	R	I	99.70%	45	45.1	40.9	30	32	2.47	0.353	0.409	0	2	0	0	2	1,130.58	1,819.50		801.14	728	738	Empty	TRUE	
20196-20225 HinckeM –TAXchicken	Uncategorized Sample	20196-20205_A	Mudpit_Ingel20196_MGFPeaklist (F058870)	Uncharacterized protein OS=Gallus gallus GN=NUMA1 PE=4 SV=2	E1BQQ4_CHICK,R4GLW9_CHICK,UPI0002C8711B,UPI0002C8843B	TAX_GallusGallus_9031_20141114.fasta,UPARC14_2_GallusG_9031_20140417.fasta	238,106.40	100.00%	22	22	36	0.07%	11.80%	AAFICGFLQK	R	L	99.70%	45.7	43.9	21.6	38	25.8	1.7	0.213	0.35	0	1	0	0	2	1,154.60	2,575.00		208.37	62	71	Empty	TRUE	
20196-20225 HinckeM –TAXchicken	Uncategorized Sample	20196-20205_A	Mudpit_Ingel20196_MGFPeaklist (F058870)	Uncharacterized protein OS=Gallus gallus GN=NUMA1 PE=4 SV=2	E1BQQ4_CHICK,R4GLW9_CHICK,UPI0002C8711B,UPI0002C8843B	TAX_GallusGallus_9031_20141114.fasta,UPARC14_2_GallusG_9031_20140417.fasta	238,106.40	100.00%	22	22	36	0.07%	11.80%	ATTALQAELAR	K	A	99.70%	55.8	44.7	33.1	33.6	37.9	3.41	0.346	0.455	0	2	0	0	2	1,144.63	2,013.50		899.26	1376	1386	Empty	TRUE	
20196-20225 HinckeM –TAXchicken	Uncategorized Sample	20196-20205_A	Mudpit_Ingel20196_MGFPeaklist (F058870)	Uncharacterized protein OS=Gallus gallus GN=NUMA1 PE=4 SV=2	E1BQQ4_CHICK,R4GLW9_CHICK,UPI0002C8711B,UPI0002C8843B	TAX_GallusGallus_9031_20141114.fasta,UPARC14_2_GallusG_9031_20140417.fasta	238,106.40	100.00%	22	22	36	0.07%	11.80%	AVAEGSQQQQAELEASQR	K	E	99.70%	51.4	44.2	21.2	37.9	42	6.64	0.486	0.389	0	0	2	0	2	1,929.93	1,633.00		729.27	1102	1119	Empty	TRUE	
20196-20225 HinckeM –TAXchicken	Uncategorized Sample	20196-20205_A	Mudpit_Ingel20196_MGFPeaklist (F058870)	Uncharacterized protein OS=Gallus gallus GN=NUMA1 PE=4 SV=2	E1BQQ4_CHICK,R4GLW9_CHICK,UPI0002C8711B,UPI0002C8843B	TAX_GallusGallus_9031_20141114.fasta,UPARC14_2_GallusG_9031_20140417.fasta	238,106.40	100.00%	22	22	36	0.07%	11.80%	ELQQSVLQAK	K	E	71.70%	23.2	44.6	24.3	11.3					0	2	0	0	2	1,143.64	1,833.50		652.34	1640	1649	Empty	TRUE	
20196-20225 HinckeM –TAXchicken	Uncategorized Sample	20196-20205_A	Mudpit_Ingel20196_MGFPeaklist (F058870)	Uncharacterized protein OS=Gallus gallus GN=NUMA1 PE=4 SV=2	E1BQQ4_CHICK,R4GLW9_CHICK,UPI0002C8711B,UPI0002C8843B	TAX_GallusGallus_9031_20141114.fasta,UPARC14_2_GallusG_9031_20140417.fasta	238,106.40	100.00%	22	22	36	0.07%	11.80%	EQAKQVEELNK	K	K	24.40%	13.3	44.2	22.6	1.51					0	2	0	0	2	1,298.66	1,632.50		810.43	1531	1541	Empty	TRUE	
20196-20225 HinckeM –TAXchicken	Uncategorized Sample	20196-20205_A	Mudpit_Ingel20196_MGFPeaklist (F058870)	Uncharacterized protein OS=Gallus gallus GN=NUMA1 PE=4 SV=2	E1BQQ4_CHICK,R4GLW9_CHICK,UPI0002C8711B,UPI0002C8843B	TAX_GallusGallus_9031_20141114.fasta,UPARC14_2_GallusG_9031_20140417.fasta	238,106.40	100.00%	22	22	36	0.07%	11.80%	GIELQASVLR	K	L	99.70%	52.8	43.4	29.2	27.7	31.7	0.42	0.3	0.4	0	2	0	0	2	1,085.63	2,290.50		801.93	429	438	Empty	TRUE	
20196-20225 HinckeM –TAXchicken	Uncategorized Sample	20196-20205_A	Mudpit_Ingel20196_MGFPeaklist (F058870)	Uncharacterized protein OS=Gallus gallus GN=NUMA1 PE=4 SV=2	E1BQQ4_CHICK,R4GLW9_CHICK,UPI0002C8711B,UPI0002C8843B	TAX_GallusGallus_9031_20141114.fasta,UPARC14_2_GallusG_9031_20140417.fasta	238,106.40	100.00%	22	22	36	0.07%	11.80%	ISLLEDQLVK	K	L	81.20%	33.5	43.6	39.4	4.56					0	2	0	0	2	1,157.68	2,466.50		504.14	387	396	Empty	TRUE	
20196-20225 HinckeM –TAXchicken	Uncategorized Sample	20196-20205_A	Mudpit_Ingel20196_MGFPeaklist (F058870)	Uncharacterized protein OS=Gallus gallus GN=NUMA1 PE=4 SV=2	E1BQQ4_CHICK,R4GLW9_CHICK,UPI0002C8711B,UPI0002C8843B	TAX_GallusGallus_9031_20141114.fasta,UPARC14_2_GallusG_9031_20140417.fasta	238,106.40	100.00%	22	22	36	0.07%	11.80%	LALLNEK	R	Q	74.20%	35.5	44.1	44.7	10.1					0	1	0	0	2	800.4877	2,048.00		268.19	261	267	Empty	TRUE	
20196-20225 HinckeM –TAXchicken	Uncategorized Sample	20196-20205_A	Mudpit_Ingel20196_MGFPeaklist (F058870)	Uncharacterized protein OS=Gallus gallus GN=NUMA1 PE=4 SV=2	E1BQQ4_CHICK,R4GLW9_CHICK,UPI0002C8711B,UPI0002C8843B	TAX_GallusGallus_9031_20141114.fasta,UPARC14_2_GallusG_9031_20140417.fasta	238,106.40	100.00%	22	22	36	0.07%	11.80%	LKGELASVEAVKER	K	A	83.40%	25.5	42.7	19.4	19.9					0	0	1	0	2	1,528.87	1,842.00		172.69	1027	1040	Empty	TRUE	
20196-20225 HinckeM –TAXchicken	Uncategorized Sample	20196-20205_A	Mudpit_Ingel20196_MGFPeaklist (F058870)	Uncharacterized protein OS=Gallus gallus GN=NUMA1 PE=4 SV=2	E1BQQ4_CHICK,R4GLW9_CHICK,UPI0002C8711B,UPI0002C8843B	TAX_GallusGallus_9031_20141114.fasta,UPARC14_2_GallusG_9031_20140417.fasta	238,106.40	100.00%	22	22	36	0.07%	11.80%	LLEGEELELAK	K	V	22.80%	27.1	44.6	25.1	10.1					0	1	0	0	2	1,243.68	2,272.00		393.22	88	98	Empty	TRUE	
20196-20225 HinckeM –TAXchicken	Uncategorized Sample	20196-20205_A	Mudpit_Ingel20196_MGFPeaklist (F058870)	Uncharacterized protein OS=Gallus gallus GN=NUMA1 PE=4 SV=2	E1BQQ4_CHICK,R4GLW9_CHICK,UPI0002C8711B,UPI0002C8843B	TAX_GallusGallus_9031_20141114.fasta,UPARC14_2_GallusG_9031_20140417.fasta	238,106.40	100.00%	22	22	36	0.07%	11.80%	LLFLEESVR	R	N	99.70%	57.8	44.2	31.7	35.1	36.4	1.34	0.288	0.556	0	2	0	0	2	1,105.63	2,593.50		1,068.68	707	715	Empty	TRUE	
20196-20225 HinckeM –TAXchicken	Uncategorized Sample	20196-20205_A	Mudpit_Ingel20196_MGFPeaklist (F058870)	Uncharacterized protein OS=Gallus gallus GN=NUMA1 PE=4 SV=2	E1BQQ4_CHICK,R4GLW9_CHICK,UPI0002C8711B,UPI0002C8843B	TAX_GallusGallus_9031_20141114.fasta,UPARC14_2_GallusG_9031_20140417.fasta	238,106.40	100.00%	22	22	36	0.07%	11.80%	LQQVAEER	R	R	27.70%	23.5	45.2	38.2	0					0	1	0	0	2	973.495	1,822.00		318.07	774	781	Empty	TRUE	
20196-20225 HinckeM –TAXchicken	Uncategorized Sample	20196-20205_A	Mudpit_Ingel20196_MGFPeaklist (F058870)	Uncharacterized protein OS=Gallus gallus GN=NUMA1 PE=4 SV=2	E1BQQ4_CHICK,R4GLW9_CHICK,UPI0002C8711B,UPI0002C8843B	TAX_GallusGallus_9031_20141114.fasta,UPARC14_2_GallusG_9031_20140417.fasta	238,106.40	100.00%	22	22	36	0.07%	11.80%	LQQVAEERREETAALQR	R	Q	24.70%	0.34	43.7	13.2	-1.07					0	0	0	2	2	2,027.06	1,700.00		476.38	774	790	Empty	TRUE	
20196-20225 HinckeM –TAXchicken	Uncategorized Sample	20196-20205_A	Mudpit_Ingel20196_MGFPeaklist (F058870)	Uncharacterized protein OS=Gallus gallus GN=NUMA1 PE=4 SV=2	E1BQQ4_CHICK,R4GLW9_CHICK,UPI0002C8711B,UPI0002C8843B	TAX_GallusGallus_9031_20141114.fasta,UPARC14_2_GallusG_9031_20140417.fasta	238,106.40	100.00%	22	22	36	0.07%	11.80%	LTAQADALTAEVGK	R	L	24.80%	10.3	44.6	22.9	1.06					0	1	0	0	2	1,387.74	2,084.00		222.21	496	509	Empty	TRUE	
20196-20225 HinckeM –TAXchicken	Uncategorized Sample	20196-20205_A	Mudpit_Ingel20196_MGFPeaklist (F058870)	Uncharacterized protein OS=Gallus gallus GN=NUMA1 PE=4 SV=2	E1BQQ4_CHICK,R4GLW9_CHICK,UPI0002C8711B,UPI0002C8843B	TAX_GallusGallus_9031_20141114.fasta,UPARC14_2_GallusG_9031_20140417.fasta	238,106.40	100.00%	22	22	36	0.07%	11.80%	QAQLEGELR	K	A	60.40%	25.8	44.7	16.4	12.5					0	1	0	0	2	1,043.55	1,794.00		193.64	359	367	Empty	TRUE	
20196-20225 HinckeM –TAXchicken	Uncategorized Sample	20196-20205_A	Mudpit_Ingel20196_MGFPeaklist (F058870)	Uncharacterized protein OS=Gallus gallus GN=NUMA1 PE=4 SV=2	E1BQQ4_CHICK,R4GLW9_CHICK,UPI0002C8711B,UPI0002C8843B	TAX_GallusGallus_9031_20141114.fasta,UPARC14_2_GallusG_9031_20140417.fasta	238,106.40	100.00%	22	22	36	0.07%	11.80%	SAQALQDGTVESLRK	R	E	99.70%	45.6	44.5	26	37.1	43.5	4.8	0.43	0.5	0	0	2	0	2	1,602.84	1,836.00		1,356.14	1140	1154	Empty	TRUE	
20196-20225 HinckeM –TAXchicken	Uncategorized Sample	20196-20205_A	Mudpit_Ingel20196_MGFPeaklist (F058870)	Uncharacterized protein OS=Gallus gallus GN=NUMA1 PE=4 SV=2	E1BQQ4_CHICK,R4GLW9_CHICK,UPI0002C8711B,UPI0002C8843B	TAX_GallusGallus_9031_20141114.fasta,UPARC14_2_GallusG_9031_20140417.fasta	238,106.40	100.00%	22	22	36	0.07%	11.80%	SLASFPSQDSLSK	R	L	52.80%	20.3	44.4	22.3	9.63					0	2	0	0	2	1,366.68	2,117.50		646.66	1841	1853	Empty	TRUE	
20196-20225 HinckeM –TAXchicken	Uncategorized Sample	20196-20205_A	Mudpit_Ingel20196_MGFPeaklist (F058870)	Uncharacterized protein OS=Gallus gallus GN=NUMA1 PE=4 SV=2	E1BQQ4_CHICK,R4GLW9_CHICK,UPI0002C8711B,UPI0002C8843B	TAX_GallusGallus_9031_20141114.fasta,UPARC14_2_GallusG_9031_20140417.fasta	238,106.40	100.00%	22	22	36	0.07%	11.80%	TAVAAAEKEVASLR	K	A	99.70%	65.4	43.8	32.2	42.4	36.1	3.66	0.38	0.393	0	0	2	0	2	1,415.79	2,260.50		857.42	1168	1181	Empty	TRUE	
20196-20225 HinckeM –TAXchicken	Uncategorized Sample	20196-20205_A	Mudpit_Ingel20196_MGFPeaklist (F058870)	Uncharacterized protein OS=Gallus gallus GN=NUMA1 PE=4 SV=2	E1BQQ4_CHICK,R4GLW9_CHICK,UPI0002C8711B,UPI0002C8843B	TAX_GallusGallus_9031_20141114.fasta,UPARC14_2_GallusG_9031_20140417.fasta	238,106.40	100.00%	22	22	36	0.07%	11.80%	VKVLEGELQSEATR	R	Q	16.90%	23.9	44	26.1	8.58					0	0	1	0	2	1,558.84	1,945.00		269.3	1557	1570	Empty	TRUE	
20196-20225 HinckeM –TAXchicken	Uncategorized Sample	20196-20205_A	Mudpit_Ingel20196_MGFPeaklist (F058870)	Uncharacterized protein OS=Gallus gallus GN=NUMA1 PE=4 SV=2	E1BQQ4_CHICK,R4GLW9_CHICK,UPI0002C8711B,UPI0002C8843B	TAX_GallusGallus_9031_20141114.fasta,UPARC14_2_GallusG_9031_20140417.fasta	238,106.40	100.00%	22	22	36	0.07%	11.80%	VLEGELQSEATR	K	Q	99.70%	58	45.1	26.3	42.2	28.3	0.638	0.29	0.25	0	2	0	0	2	1,331.68	1,854.00		329.18	1559	1570	Empty	TRUE	
20196-20225 HinckeM –TAXchicken	Uncategorized Sample	20196-20205_A	Mudpit_Ingel20196_MGFPeaklist (F058870)	Uncharacterized protein OS=Gallus gallus GN=NUMA1 PE=4 SV=2	E1BQQ4_CHICK,R4GLW9_CHICK,UPI0002C8711B,UPI0002C8843B	TAX_GallusGallus_9031_20141114.fasta,UPARC14_2_GallusG_9031_20140417.fasta	238,106.40	100.00%	22	22	36	0.07%	11.80%	VLELSAQCQQSSAQAGSAETLR	R	A	99.70%	52.5	44.3	21.9	38.9	38	5.64	0.445	0.273	0	0	1	0	2	2,334.14	2,062.00		591.14	652	673	Empty	TRUE	
20196-20225 HinckeM –TAXchicken	Uncategorized Sample	20196-20205_A	Mudpit_Ingel20196_MGFPeaklist (F058870)	Uncharacterized protein (Fragment) OS=Gallus gallus GN=SPTB PE=4 SV=2	F1NSS0_CHICK	TAX_GallusGallus_9031_20141114.fasta	265,530.20	100.00%	144	184	507	1.02%	54.00%	AASEELGHDEVHTQALLR	R	E	99.70%	60.4	44.5	13.9	57	43.1	5.92	0.439	0.472	0	0	0	4	2	1,975.98	2,003.50		5,895.69	763	780	Empty	TRUE	UPI000240B81D,UPI00035064DB,U[illegible]
20196-20225 HinckeM –TAXchicken	Uncategorized Sample	20196-20205_A	Mudpit_Ingel20196_MGFPeaklist (F058870)	Uncharacterized protein (Fragment) OS=Gallus gallus GN=SPTB PE=4 SV=2	F1NSS0_CHICK	TAX_GallusGallus_9031_20141114.fasta	265,530.20	100.00%	144	184	507	1.02%	54.00%	AEGMELASCKPQYGAVVGQR	K	L	99.70%	60.2	43.8	16.3	51	48.9	7.28	0.554	0.4	0	0	4	0	2	2,167.03	1,977.00		4,524.34	1332	1351	Empty	TRUE	UPI000240B81D,UPI00035064DB,U[illegible]
20196-20225 HinckeM –TAXchicken	Uncategorized Sample	20196-20205_A	Mudpit_Ingel20196_MGFPeaklist (F058870)	Uncharacterized protein (Fragment) OS=Gallus gallus GN=SPTB PE=4 SV=2	F1NSS0_CHICK	TAX_GallusGallus_9031_20141114.fasta	265,530.20	100.00%	144	184	507	1.02%	54.00%	AKDLTSANLLLK	R	K	99.70%	62.5	42.2	28.3	43	43.3	3.46	0.312	0.542	0	1	1	0	2	1,286.77	2,200.50		4,092.46	1406	1417	Empty	TRUE	UPI000240B81D,UPI00035064DB,U[illegible]
20196-20225 HinckeM –TAXchicken	Uncategorized Sample	20196-20205_A	Mudpit_Ingel20196_MGFPeaklist (F058870)	Uncharacterized protein (Fragment) OS=Gallus gallus GN=SPTB PE=4 SV=2	F1NSS0_CHICK	TAX_GallusGallus_9031_20141114.fasta	265,530.20	100.00%	144	184	507	1.02%	54.00%	AKDLTSANLLLKK	R	L	99.70%	62.3	40	36.6	43.3	43.9	4.21	0.378	0.577	0	0	2	0	2	1,414.86	2,019.50		2,700.79	1406	1418	Empty	TRUE	UPI000240B81D,UPI00035064DB,U[illegible]
20196-20225 HinckeM –TAXchicken	Uncategorized Sample	20196-20205_A	Mudpit_Ingel20196_MGFPeaklist (F058870)	Uncharacterized protein (Fragment) OS=Gallus gallus GN=SPTB PE=4 SV=2	F1NSS0_CHICK	TAX_GallusGallus_9031_20141114.fasta	265,530.20	100.00%	144	184	507	1.02%	54.00%	ALGVPCQGEEPLGLR	R	N	99.70%	67.8	44.4	23.1	58.8	45.6	5.06	0.401	0.433	0	2	0	0	2	1,595.82	2,314.00		3,245.87	2225	2239	Empty	TRUE	UPI000240B81D,UPI00035064DB,U[illegible]
20196-20225 HinckeM –TAXchicken	Uncategorized Sample	20196-20205_A	Mudpit_Ingel20196_MGFPeaklist (F058870)	Uncharacterized protein (Fragment) OS=Gallus gallus GN=SPTB PE=4 SV=2	F1NSS0_CHICK	TAX_GallusGallus_9031_20141114.fasta	265,530.20	100.00%	144	184	507	1.02%	54.00%	ALQALLEACSGR	R	R	99.70%	66.6	45	29.2	49.4	39.1	4.55	0.46	0.542	0	3	7	0	2	1,288.67	2,310.00		12,763.70	1879	1890	Empty	TRUE	UPI000240B81D,UPI00035064DB,U[illegible]
20196-20225 HinckeM –TAXchicken	Uncategorized Sample	20196-20205_A	Mudpit_Ingel20196_MGFPeaklist (F058870)	Uncharacterized protein (Fragment) OS=Gallus gallus GN=SPTB PE=4 SV=2	F1NSS0_CHICK	TAX_GallusGallus_9031_20141114.fasta	265,530.20	100.00%	144	184	507	1.02%	54.00%	ALQALLEACSGRR	R	A	99.70%	26	44.5	20.7	15.7	32.4	1.03	0.0833	0.462	0	0	4	0	2	1,444.77	2,199.50		3,335.56	1879	1891	Empty	TRUE	UPI000240B81D,UPI00035064DB,U[illegible]
20196-20225 HinckeM –TAXchicken	Uncategorized Sample	20196-20205_A	Mudpit_Ingel20196_MGFPeaklist (F058870)	Uncharacterized protein (Fragment) OS=Gallus gallus GN=SPTB PE=4 SV=2	F1NSS0_CHICK	TAX_GallusGallus_9031_20141114.fasta	265,530.20	100.00%	144	184	507	1.02%	54.00%	ALQFLKEK	K	Q	92.40%	42.8	43.7	46.9	13.7					0	1	0	0	2	976.5827	2,011.00		169.17	116	123	Empty	TRUE	UPI000240B81D,UPI00035064DB,U[illegible]
20196-20225 HinckeM –TAXchicken	Uncategorized Sample	20196-20205_A	Mudpit_Ingel20196_MGFPeaklist (F058870)	Uncharacterized protein (Fragment) OS=Gallus gallus GN=SPTB PE=4 SV=2	F1NSS0_CHICK	TAX_GallusGallus_9031_20141114.fasta	265,530.20	100.00%	144	184	507	1.02%	54.00%	ALVMAEQLAAAGR	K	E	99.70%	64.6	44.8	23.9	49.5	43.8	3.8	0.4	0.462	0	6	0	0	2	1,316.70	2,067.50		3,899.46	694	706	Empty	TRUE	UPI000240B81D,UPI00035064DB,U[illegible]
20196-20225 HinckeM –TAXchicken	Uncategorized Sample	20196-20205_A	Mudpit_Ingel20196_MGFPeaklist (F058870)	Uncharacterized protein (Fragment) OS=Gallus gallus GN=SPTB PE=4 SV=2	F1NSS0_CHICK	TAX_GallusGallus_9031_20141114.fasta	265,530.20	100.00%	144	184	507	1.02%	54.00%	AMYQLGR	K	D	98.80%	68.8	42.9	38.8	39.4	26.7	2.3	0.27	0.429	0	2	0	0	2	838.424	1,840.00		1,232.00	1477	1483	Empty	TRUE	UPI000240B81D,UPI00035064DB,U[illegible]
20196-20225 HinckeM –TAXchicken	Uncategorized Sample	20196-20205_A	Mudpit_Ingel20196_MGFPeaklist (F058870)	Uncharacterized protein (Fragment) OS=Gallus gallus GN=SPTB PE=4 SV=2	F1NSS0_CHICK	TAX_GallusGallus_9031_20141114.fasta	265,530.20	100.00%	144	184	507	1.02%	54.00%	APLAGVAPEPDAQEEK	K	L	99.70%	64.5	44.6	16.2	54.7	56	7.41	0.564	0.5	0	3	0	0	2	1,621.81	1,868.00		7,127.31	1438	1453	Empty	TRUE	UPI000240B81D,UPI00035064DB,U[illegible]
20196-20225 HinckeM –TAXchicken	Uncategorized Sample	20196-20205_A	Mudpit_Ingel20196_MGFPeaklist (F058870)	Uncharacterized protein (Fragment) OS=Gallus gallus GN=SPTB PE=4 SV=2	F1NSS0_CHICK	TAX_GallusGallus_9031_20141114.fasta	265,530.20	100.00%	144	184	507	1.02%	54.00%	APLAGVAPEPDAQEEKLQQR	K	F	99.70%	102	43.8	15.5	96.9	67	9.12	0.59	0.525	0	0	2	0	2	2,147.11	1,966.50		15,907.70	1438	1457	Empty	TRUE	UPI000240B81D,UPI00035064DB,U[illegible]
20196-20225 HinckeM –TAXchicken	Uncategorized Sample	20196-20205_A	Mudpit_Ingel20196_MGFPeaklist (F058870)	Uncharacterized protein (Fragment) OS=Gallus gallus GN=SPTB PE=4 SV=2	F1NSS0_CHICK	TAX_GallusGallus_9031_20141114.fasta	265,530.20	100.00%	144	184	507	1.02%	54.00%	AQEAAGLLQDNHR	K	L	99.70%	63.9	44.6	24.3	50.9	45.4	3.8	0.396	0.577	0	0	8	0	2	1,422.71	1,766.50		23,805.00	1261	1273	Empty	TRUE	UPI000240B81D,UPI00035064DB,U[illegible]
20196-20225 HinckeM –TAXchicken	Uncategorized Sample	20196-20205_A	Mudpit_Ingel20196_MGFPeaklist (F058870)	Uncharacterized protein (Fragment) OS=Gallus gallus GN=SPTB PE=4 SV=2	F1NSS0_CHICK	TAX_GallusGallus_9031_20141114.fasta	265,530.20	100.00%	144	184	507	1.02%	54.00%	AQERPELAEDTAR	L	R	99.70%					40.4	4.72	0.453	0.5	0	0	2	0	1	1,485.73	1,571.50		464.03	1019	1031	Empty	TRUE	
20196-20225 HinckeM –TAXchicken	Uncategorized Sample	20196-20205_A	Mudpit_Ingel20196_MGFPeaklist (F058870)	Uncharacterized protein (Fragment) OS=Gallus gallus GN=SPTB PE=4 SV=2	F1NSS0_CHICK	TAX_GallusGallus_9031_20141114.fasta	265,530.20	100.00%	144	184	507	1.02%	54.00%	AQLVDTADKFR	R	F	99.70%	67.6	44.6	23.8	58.4	48.7	4.4	0.405	0.591	0	3	0	0	2	1,263.67	1,922.00		3,577.37	1892	1902	Empty	TRUE	UPI000240B81D,UPI00035064DB,U[illegible]
20196-20225 HinckeM –TAXchicken	Uncategorized Sample	20196-20205_A	Mudpit_Ingel20196_MGFPeaklist (F058870)	Uncharacterized protein (Fragment) OS=Gallus gallus GN=SPTB PE=4 SV=2	F1NSS0_CHICK	TAX_GallusGallus_9031_20141114.fasta	265,530.20	100.00%	144	184	507	1.02%	54.00%	AQQLLSAGHPEGEQIIR	R	L	99.70%	68.8	44	19.3	57.9	44.4	5.54	0.502	0.412	0	0	5	0	2	1,846.98	2,096.00		12,767.90	1642	1658	Empty	TRUE	UPI000240B81D,UPI00035064DB,U[illegible]
20196-20225 HinckeM –TAXchicken	Uncategorized Sample	20196-20205_A	Mudpit_Ingel20196_MGFPeaklist (F058870)	Uncharacterized protein (Fragment) OS=Gallus gallus GN=SPTB PE=4 SV=2	F1NSS0_CHICK	TAX_GallusGallus_9031_20141114.fasta	265,530.20	100.00%	144	184	507	1.02%	54.00%	ASMESSAEKVPSVVAAGSK	R	L	99.70%	66.8	44.6	19.4	58	44.9	6.48	0.448	0.342	0	0	5	0	2	1,850.92	1,787.00		8,353.31	1213	1231	Empty	TRUE	UPI000240B81D,UPI00035064DB,U[illegible]
20196-20225 HinckeM –TAXchicken	Uncategorized Sample	20196-20205_A	Mudpit_Ingel20196_MGFPeaklist (F058870)	Uncharacterized protein (Fragment) OS=Gallus gallus GN=SPTB PE=4 SV=2	F1NSS0_CHICK	TAX_GallusGallus_9031_20141114.fasta	265,530.20	100.00%	144	184	507	1.02%	54.00%	AVAGTEEVPTSVGEAEELLR	K	K	99.70%	86.7	44.4	20.2	77	53.6	6.08	0.526	0.4	0	0	2	0	2	2,057.04	2,662.00		2,268.26	1080	1099	Empty	TRUE	UPI000240B81D,UPI00035064DB,U[illegible]
20196-20225 HinckeM –TAXchicken	Uncategorized Sample	20196-20205_A	Mudpit_Ingel20196_MGFPeaklist (F058870)	Uncharacterized protein (Fragment) OS=Gallus gallus GN=SPTB PE=4 SV=2	F1NSS0_CHICK	TAX_GallusGallus_9031_20141114.fasta	265,530.20	100.00%	144	184	507	1.02%	54.00%	AVAGTEEVPTSVGEAEELLRK	K	H	99.70%	74.3	43.7	16	68.9	49.6	7.29	0.482	0.405	0	0	2	0	2	2,185.13	2,556.50		5,122.07	1080	1100	Empty	TRUE	UPI000240B81D,UPI00035064DB,U[illegible]
20196-20225 HinckeM –TAXchicken	Uncategorized Sample	20196-20205_A	Mudpit_Ingel20196_MGFPeaklist (F058870)	Uncharacterized protein (Fragment) OS=Gallus gallus GN=SPTB PE=4 SV=2	F1NSS0_CHICK	TAX_GallusGallus_9031_20141114.fasta	265,530.20	100.00%	144	184	507	1.02%	54.00%	AVSIQQQEQEVAR	K	A	99.70%	69.4	44.4	20.3	51.3	48	3.7	0.394	0.5	0	4	0	0	2	1,485.77	1,816.50		19,328.20	1866	1878	Empty	TRUE	UPI000240B81D,UPI00035064DB,U[illegible]
20196-20225 HinckeM –TAXchicken	Uncategorized Sample	20196-20205_A	Mudpit_Ingel20196_MGFPeaklist (F058870)	Uncharacterized protein (Fragment) OS=Gallus gallus GN=SPTB PE=4 SV=2	F1NSS0_CHICK	TAX_GallusGallus_9031_20141114.fasta	265,530.20	100.00%	144	184	507	1.02%	54.00%	DGLAFNALIHK	K	H	99.70%	39.1	43.7	19.6	34.3	26.1	1.29	0.264	0.273	0	0	5	0	2	1,198.66	2,449.00		4,115.12	201	211	Empty	TRUE	UPI000240B81D,UPI00035064DB,U[illegible]
20196-20225 HinckeM –TAXchicken	Uncategorized Sample	20196-20205_A	Mudpit_Ingel20196_MGFPeaklist (F058870)	Uncharacterized protein (Fragment) OS=Gallus gallus GN=SPTB PE=4 SV=2	F1NSS0_CHICK	TAX_GallusGallus_9031_20141114.fasta	265,530.20	100.00%	144	184	507	1.02%	54.00%	DGLLESGHPR	A	S	99.10%					31	1.41	0.219	0.4	0	4	0	0	1	1,080.54	1,746.00		2,011.17	909	918	Empty	TRUE	
20196-20225 HinckeM –TAXchicken	Uncategorized Sample	20196-20205_A	Mudpit_Ingel20196_MGFPeaklist (F058870)	Uncharacterized protein (Fragment) OS=Gallus gallus GN=SPTB PE=4 SV=2	F1NSS0_CHICK	TAX_GallusGallus_9031_20141114.fasta	265,530.20	100.00%	144	184	507	1.02%	54.00%	DKSFDACIELGK	R	K	85.00%	21.7	44.1	15.4	16.9					0	0	1	0	2	1,382.66	2,112.00		461.48	1951	1962	Empty	TRUE	UPI000240B81D,UPI00035064DB,U[illegible]
20196-20225 HinckeM –TAXchicken	Uncategorized Sample	20196-20205_A	Mudpit_Ingel20196_MGFPeaklist (F058870)	Uncharacterized protein (Fragment) OS=Gallus gallus GN=SPTB PE=4 SV=2	F1NSS0_CHICK	TAX_GallusGallus_9031_20141114.fasta	265,530.20	100.00%	144	184	507	1.02%	54.00%	DKSFDACIELGKK	R	L	99.70%	57.2	44.5	22.3	42.8	36.7	3.68	0.387	0.462	0	0	2	0	2	1,510.76	1,973.00		2,453.69	1951	1963	Empty	TRUE	UPI000240B81D,UPI00035064DB,U[illegible]
20196-20225 HinckeM –TAXchicken	Uncategorized Sample	20196-20205_A	Mudpit_Ingel20196_MGFPeaklist (F058870)	Uncharacterized protein (Fragment) OS=Gallus gallus GN=SPTB PE=4 SV=2	F1NSS0_CHICK	TAX_GallusGallus_9031_20141114.fasta	265,530.20	100.00%	144	184	507	1.02%	54.00%	DLAGVLATQR	R	K	99.70%	64.3	44.4	32.9	41.8	34.7	2.55	0.282	0.5	0	8	0	0	2	1,043.58	2,177.00		26,298.10	983	992	Empty	TRUE	UPI000240B81D,UPI00035064DB,U[illegible]
20196-20225 HinckeM –TAXchicken	Uncategorized Sample	20196-20205_A	Mudpit_Ingel20196_MGFPeaklist (F058870)	Uncharacterized protein (Fragment) OS=Gallus gallus GN=SPTB PE=4 SV=2	F1NSS0_CHICK	TAX_GallusGallus_9031_20141114.fasta	265,530.20	100.00%	144	184	507	1.02%	54.00%	DLAGVLLLR	K	R	99.70%	64.1	40.9	36	32	39.8	1.8	0.296	0.667	0	4	0	0	2	969.6093	2,792.00		3,089.15	666	674	Empty	TRUE	UPI000240B81D,UPI00035064DB,U[illegible]
20196-20225 HinckeM –TAXchicken	Uncategorized Sample	20196-20205_A	Mudpit_Ingel20196_MGFPeaklist (F058870)	Uncharacterized protein (Fragment) OS=Gallus gallus GN=SPTB PE=4 SV=2	F1NSS0_CHICK	TAX_GallusGallus_9031_20141114.fasta	265,530.20	100.00%	144	184	507	1.02%	54.00%	DLEDEMLWVQER	R	M	99.70%	73.4	43.5	21.9	60.3	35.8	3.4	0.36	0.417	0	2	0	0	2	1,578.71	2,364.00		1,944.37	1484	1495	Empty	TRUE	UPI000240B81D,UPI00035064DB,U[illegible]
20196-20225 HinckeM –TAXchicken	Uncategorized Sample	20196-20205_A	Mudpit_Ingel20196_MGFPeaklist (F058870)	Uncharacterized protein (Fragment) OS=Gallus gallus GN=SPTB PE=4 SV=2	F1NSS0_CHICK	TAX_GallusGallus_9031_20141114.fasta	265,530.20	100.00%	144	184	507	1.02%	54.00%	DLLSWMESTVR	R	Q	99.70%	71.7	44.2	23.6	54	39.9	2.77	0.393	0.5	0	4	0	0	2	1,352.65	2,482.00		2,787.55	1909	1919	Empty	TRUE	UPI000240B81D,UPI00035064DB,UPI0003505F8D,UPI0002C86DF1,UPI00035042E9,UPI0000ECB786,UPI00035069AB
20196-20225 HinckeM –TAXchicken	Uncategorized Sample	20196-20205_A	Mudpit_Ingel20196_MGFPeaklist (F058870)	Uncharacterized protein (Fragment) OS=Gallus gallus GN=SPTB PE=4 SV=2	F1NSS0_CHICK	TAX_GallusGallus_9031_20141114.fasta	265,530.20	100.00%	144	184	507	1.02%	54.00%	DLQLLEGQVQR	R	F	99.70%	66	43.8	31.8	40.3	40.8	1.72	0.336	0.545	0	4	0	0	2	1,298.71	2,290.00		21,976.70	1839	1849	Empty	TRUE	UPI000240B81D,UPI00035064DB,UPI0003505F8D,UPI0002C86DF1,UPI00035042E9,UPI0000ECB786,UPI00035069AB
20196-20225 HinckeM –TAXchicken	Uncategorized Sample	20196-20205_A	Mudpit_Ingel20196_MGFPeaklist (F058870)	Uncharacterized protein (Fragment) OS=Gallus gallus GN=SPTB PE=4 SV=2	F1NSS0_CHICK	TAX_GallusGallus_9031_20141114.fasta	265,530.20	100.00%	144	184	507	1.02%	54.00%	DLTSANLLLK	K	K	99.70%	44.6	44.3	22.1	0	31.5	0.432	0.149	0.45	0	2	0	0	2	1,087.64	2,436.00		8,573.91	1408	1417	Empty	TRUE	UPI000240B81D,UPI00035064DB,UPI0003505F8D,UPI0002C86DF1,UPI00035042E9,UPI0000ECB786,UPI00035069AB
20196-20225 HinckeM –TAXchicken	Uncategorized Sample	20196-20205_A	Mudpit_Ingel20196_MGFPeaklist (F058870)	Uncharacterized protein (Fragment) OS=Gallus gallus GN=SPTB PE=4 SV=2	F1NSS0_CHICK	TAX_GallusGallus_9031_20141114.fasta	265,530.20	100.00%	144	184	507	1.02%	54.00%	DLTSANLLLKK	K	L	99.70%	39.4	42.7	25.6	0	30.9	1.64	0.269	0.409	0	0	3	0	2	1,215.73	2,234.00		2,722.44	1408	1418	Empty	TRUE	UPI000240B81D,UPI00035064DB,UPI0003505F8D,UPI0002C86DF1,UPI00035042E9,UPI0000ECB786,UPI00035069AB
20196-20225 HinckeM –TAXchicken	Uncategorized Sample	20196-20205_A	Mudpit_Ingel20196_MGFPeaklist (F058870)	Uncharacterized protein (Fragment) OS=Gallus gallus GN=SPTB PE=4 SV=2	F1NSS0_CHICK	TAX_GallusGallus_9031_20141114.fasta	265,530.20	100.00%	144	184	507	1.02%	54.00%	DVAASSQELGQDLDHVTILR	R	D	99.70%	95	44	18.9	85.3	57.9	9.74	0.537	0.4	0	0	2	0	2	2,167.10	2,629.50		4,769.00	1705	1724	Empty	TRUE	UPI000240B81D,UPI00035064DB,UPI0003505F8D,UPI0002C86DF1,UPI00035042E9,UPI0000ECB786,UPI00035069AB
20196-20225 HinckeM –TAXchicken	Uncategorized Sample	20196-20205_A	Mudpit_Ingel20196_MGFPeaklist (F058870)	Uncharacterized protein (Fragment) OS=Gallus gallus GN=SPTB PE=4 SV=2	F1NSS0_CHICK	TAX_GallusGallus_9031_20141114.fasta	265,530.20	100.00%	144	184	507	1.02%	54.00%	DVSSVELLMK	R	Y	99.70%	48.9	44.3	26.9	35.1	33.1	1.89	0.341	0.5	0	7	0	0	2	1,136.59	2,128.00		9,614.66	1929	1938	Empty	TRUE	UPI000240B81D,UPI00035064DB,UPI0003505F8D,UPI0002C86DF1,UPI00035042E9,UPI0000ECB786,UPI00035069AB
20196-20225 HinckeM –TAXchicken	Uncategorized Sample	20196-20205_A	Mudpit_Ingel20196_MGFPeaklist (F058870)	Uncharacterized protein (Fragment) OS=Gallus gallus GN=SPTB PE=4 SV=2	F1NSS0_CHICK	TAX_GallusGallus_9031_20141114.fasta	265,530.20	100.00%	144	184	507	1.02%	54.00%	EAAGLLQDNHR	Q	L	99.70%					34.5	3.77	0.406	0.364	0	2	0	0	1	1,223.61	1,754.00		580.29	1263	1273	Empty	TRUE	
20196-20225 HinckeM –TAXchicken	Uncategorized Sample	20196-20205_A	Mudpit_Ingel20196_MGFPeaklist (F058870)	Uncharacterized protein (Fragment) OS=Gallus gallus GN=SPTB PE=4 SV=2	F1NSS0_CHICK	TAX_GallusGallus_9031_20141114.fasta	265,530.20	100.00%	144	184	507	1.02%	54.00%	EALLLWCQMK	R	T	99.70%	39.2	44.7	22.5	31.3	33.3	2.02	0.309	0.5	0	5	0	0	2	1,291.65	2,532.00		3,399.51	174	183	Empty	TRUE	UPI000240B81D,UPI00035064DB,UPI0003505F8D,UPI0002C86DF1,UPI00035042E9,UPI0000ECB786,UPI00035069AB
20196-20225 HinckeM –TAXchicken	Uncategorized Sample	20196-20205_A	Mudpit_Ingel20196_MGFPeaklist (F058870)	Uncharacterized protein (Fragment) OS=Gallus gallus GN=SPTB PE=4 SV=2	F1NSS0_CHICK	TAX_GallusGallus_9031_20141114.fasta	265,530.20	100.00%	144	184	507	1.02%	54.00%	EASEAQQYYLDADEAK	R	A	99.70%	81.4	41.8	19.4	74	46.3	4.92	0.482	0.406	0	2	0	0	2	1,830.80	1,979.00		8,734.17	1577	1592	Empty	TRUE	UPI000240B81D,UPI00035064DB,UPI0003505F8D,UPI0002C86DF1,UPI00035042E9,UPI0000ECB786,UPI00035069AB
20196-20225 HinckeM –TAXchicken	Uncategorized Sample	20196-20205_A	Mudpit_Ingel20196_MGFPeaklist (F058870)	Uncharacterized protein (Fragment) OS=Gallus gallus GN=SPTB PE=4 SV=2	F1NSS0_CHICK	TAX_GallusGallus_9031_20141114.fasta	265,530.20	100.00%	144	184	507	1.02%	54.00%	EAWLSENQR	R	L	99.70%	61.2	44.8	36.2	43	36.2	2	0.276	0.611	0	6	0	0	2	1,132.54	1,888.50		21,500.50	432	440	Empty	TRUE	UPI000240B81D,UPI00035064DB,UPI0003505F8D,UPI0002C86DF1,UPI00035042E9,UPI00035069AB
20196-20225 HinckeM –TAXchicken	Uncategorized Sample	20196-20205_A	Mudpit_Ingel20196_MGFPeaklist (F058870)	Uncharacterized protein (Fragment) OS=Gallus gallus GN=SPTB PE=4 SV=2	F1NSS0_CHICK	TAX_GallusGallus_9031_20141114.fasta	265,530.20	100.00%	144	184	507	1.02%	54.00%	EHQELLEELAAAQQLLER	R	L	99.70%	94.9	43.8	16.6	85.4	58.2	10.2	0.579	0.472	0	0	1	0	2	2,120.10	2,879.00		904.16	781	798	Empty	TRUE	UPI000240B81D,UPI00035064DB,UPI0003505F8D,UPI0002C86DF1,UPI00035042E9,UPI0000ECB786,UPI00035069AB
20196-20225 HinckeM –TAXchicken	Uncategorized Sample	20196-20205_A	Mudpit_Ingel20196_MGFPeaklist (F058870)	Uncharacterized protein (Fragment) OS=Gallus gallus GN=SPTB PE=4 SV=2	F1NSS0_CHICK	TAX_GallusGallus_9031_20141114.fasta	265,530.20	100.00%	144	184	507	1.02%	54.00%	EKGQQLFEANRTELYSQSYSDLER	K	W	82.00%	12.2	43.4	15.3	7.04	30.5	0.658	0.138	0.167	0	0	0	1	2	2,891.38	2,298.00		1,047.66	1368	1391	Empty	TRUE	UPI000240B81D,UPI00035064DB,UPI0003505F8D,UPI0002C86DF1,UPI00035042E9,UPI00035069AB
20196-20225 HinckeM –TAXchicken	Uncategorized Sample	20196-20205_A	Mudpit_Ingel20196_MGFPeaklist (F058870)	Uncharacterized protein (Fragment) OS=Gallus gallus GN=SPTB PE=4 SV=2	F1NSS0_CHICK	TAX_GallusGallus_9031_20141114.fasta	265,530.20	100.00%	144	184	507	1.02%	54.00%	ELAAWVDEK	R	M	99.70%	38.2	45.4	37	13.9	34.5	0.921	0.287	0.556	0	4	0	0	2	1,060.53	2,133.00		11,054.30	1283	1291	Empty	TRUE	UPI000240B81D,UPI00035064DB,UPI0003505F8D,UPI0002C86DF1,UPI00035042E9,UPI0000ECB786,UPI00035069AB
20196-20225 HinckeM –TAXchicken	Uncategorized Sample	20196-20205_A	Mudpit_Ingel20196_MGFPeaklist (F058870)	Uncharacterized protein (Fragment) OS=Gallus gallus GN=SPTB PE=4 SV=2	F1NSS0_CHICK	TAX_GallusGallus_9031_20141114.fasta	265,530.20	100.00%	144	184	507	1.02%	54.00%	ELALRNELIR	R	Q	94.70%	36.6	42.1	35	16					0	0	3	0	2	1,226.72	2,201.00		3,768.55	404	413	Empty	TRUE	R4GJH8_CHICK,UPI0000ECC737,UPI00003ACAFC,UPI00035042E9,UPI00035069AB,UPI0000447B9B,F1ND51_CHICK,UPI000240B81D,UPI00035064DB,UPI0003505F8D,UPI0002C88352,UPI0002C86DF1
20196-20225 HinckeM –TAXchicken	Uncategorized Sample	20196-20205_A	Mudpit_Ingel20196_MGFPeaklist (F058870)	Uncharacterized protein (Fragment) OS=Gallus gallus GN=SPTB PE=4 SV=2	F1NSS0_CHICK	TAX_GallusGallus_9031_20141114.fasta	265,530.20	100.00%	144	184	507	1.02%	54.00%	ELALRNELIRQEK	R	L	17.30%	8.92	41.6	15.4	3.31					0	0	1	0	2	1,611.92	2,053.00		373.05	404	416	Empty	TRUE	R4GJH8_CHICK,UPI0000ECC737,UPI00003ACAFC,UPI00035042E9,UPI00035069AB,UPI0000447B9B,F1ND51_CHICK,UPI000240B81D,UPI00035064DB,UPI0003505F8D,UPI0002C88352,UPI0002C86DF1
20196-20225 HinckeM –TAXchicken	Uncategorized Sample	20196-20205_A	Mudpit_Ingel20196_MGFPeaklist (F058870)	Uncharacterized protein (Fragment) OS=Gallus gallus GN=SPTB PE=4 SV=2	F1NSS0_CHICK	TAX_GallusGallus_9031_20141114.fasta	265,530.20	100.00%	144	184	507	1.02%	54.00%	ELEREGYHDIKR	K	I	99.70%	59.9	44.7	28.8	46.6	46	5.8	0.42	0.625	0	0	1	0	2	1,544.78	1,457.00		288.78	485	496	Empty	TRUE	UPI000240B81D,UPI00035064DB,UPI0003505F8D,UPI0002C86DF1,UPI00035042E9,UPI00035069AB
20196-20225 HinckeM –TAXchicken	Uncategorized Sample	20196-20205_A	Mudpit_Ingel20196_MGFPeaklist (F058870)	Uncharacterized protein (Fragment) OS=Gallus gallus GN=SPTB PE=4 SV=2	F1NSS0_CHICK	TAX_GallusGallus_9031_20141114.fasta	265,530.20	100.00%	144	184	507	1.02%	54.00%	ELGRDLAGVLATQR	K	K	99.70%	44.3	43	35.8	8.66	36.2	2.8	0.249	0.429	0	0	2	0	2	1,498.83	2,311.00		1,872.44	979	992	Empty	TRUE	UPI000240B81D,UPI00035064DB,UPI0003505F8D,UPI0002C86DF1,UPI00035042E9,UPI00035069AB
20196-20225 HinckeM –TAXchicken	Uncategorized Sample	20196-20205_A	Mudpit_Ingel20196_MGFPeaklist (F058870)	Uncharacterized protein (Fragment) OS=Gallus gallus GN=SPTB PE=4 SV=2	F1NSS0_CHICK	TAX_GallusGallus_9031_20141114.fasta	265,530.20	100.00%	144	184	507	1.02%	54.00%	ELQALAAR	R	R	31.10%	35.7	43.8	41.8	4.72					0	2	0	0	2	871.4996	1,808.50		332.16	619	626	Empty	TRUE	UPI000240B81D,UPI00035064DB,UPI0003505F8D,UPI0002C86DF1,UPI00035042E9,UPI0000ECB786,UPI00035069AB
20196-20225 HinckeM –TAXchicken	Uncategorized Sample	20196-20205_A	Mudpit_Ingel20196_MGFPeaklist (F058870)	Uncharacterized protein (Fragment) OS=Gallus gallus GN=SPTB PE=4 SV=2	F1NSS0_CHICK	TAX_GallusGallus_9031_20141114.fasta	265,530.20	100.00%	144	184	507	1.02%	54.00%	ELQAQWEALREEVVAR	R	Q	99.70%	36	43.8	19.1	16.5	32.3	1.48	0.291	0.312	0	0	2	0	2	1,927.00	2,571.50		964.07	1556	1571	Empty	TRUE	UPI000240B81D,UPI00035064DB,UPI0003505F8D,UPI0002C86DF1,UPI00035042E9,UPI0000ECB786,UPI00035069AB
20196-20225 HinckeM –TAXchicken	Uncategorized Sample	20196-20205_A	Mudpit_Ingel20196_MGFPeaklist (F058870)	Uncharacterized protein (Fragment) OS=Gallus gallus GN=SPTB PE=4 SV=2	F1NSS0_CHICK	TAX_GallusGallus_9031_20141114.fasta	265,530.20	100.00%	144	184	507	1.02%	54.00%	ELVSQWR	R	S	74.20%	53	45.8	44.6	16.9					0	4	0	0	2	917.4839	1,967.00		9,914.38	938	944	Empty	TRUE	UPI000240B81D,UPI00035064DB,UPI0003505F8D,UPI0002C86DF1,UPI00035042E9,UPI00035069AB
20196-20225 HinckeM –TAXchicken	Uncategorized Sample	20196-20205_A	Mudpit_Ingel20196_MGFPeaklist (F058870)	Uncharacterized protein (Fragment) OS=Gallus gallus GN=SPTB PE=4 SV=2	F1NSS0_CHICK	TAX_GallusGallus_9031_20141114.fasta	265,530.20	100.00%	144	184	507	1.02%	54.00%	EQEQIYSALDYGK	K	D	99.60%	24.5	43.9	24.3	13.6	25.7	0.569	0.249	0.269	0	2	0	0	2	1,543.73	2,202.00		680.03	653	665	Empty	TRUE	UPI000240B81D,UPI00035064DB,UPI0003505F8D,UPI0002C86DF1,UPI00035042E9,UPI0000ECB786,UPI00035069AB
20196-20225 HinckeM –TAXchicken	Uncategorized Sample	20196-20205_A	Mudpit_Ingel20196_MGFPeaklist (F058870)	Uncharacterized protein (Fragment) OS=Gallus gallus GN=SPTB PE=4 SV=2	F1NSS0_CHICK	TAX_GallusGallus_9031_20141114.fasta	265,530.20	100.00%	144	184	507	1.02%	54.00%	EVEDLEQWIAER	R	D	99.70%	70.2	44.6	29.8	19.5	44.9	4	0.347	0.5	0	2	0	0	2	1,516.73	2,693.50		2,151.14	1693	1704	Empty	TRUE	UPI000240B81D,UPI00035064DB,UPI0003505F8D,UPI0002C86DF1,UPI00035042E9,UPI0000ECB786,UPI00035069AB
20196-20225 HinckeM –TAXchicken	Uncategorized Sample	20196-20205_A	Mudpit_Ingel20196_MGFPeaklist (F058870)	Uncharacterized protein (Fragment) OS=Gallus gallus GN=SPTB PE=4 SV=2	F1NSS0_CHICK	TAX_GallusGallus_9031_20141114.fasta	265,530.20	100.00%	144	184	507	1.02%	54.00%	FLAQCLGFQEFLR	R	D	99.70%	69.7	44.8	23.5	59.1	43.3	3.52	0.42	0.423	0	1	0	0	2	1,628.83	2,876.00		1,190.25	1161	1173	Empty	TRUE	UPI000240B81D,UPI00035064DB,UPI0003505F8D,UPI0002C86DF1,UPI00035042E9,UPI00035069AB
20196-20225 HinckeM –TAXchicken	Uncategorized Sample	20196-20205_A	Mudpit_Ingel20196_MGFPeaklist (F058870)	Uncharacterized protein (Fragment) OS=Gallus gallus GN=SPTB PE=4 SV=2	F1NSS0_CHICK	TAX_GallusGallus_9031_20141114.fasta	265,530.20	100.00%	144	184	507	1.02%	54.00%	FLDLLEPLR	R	K	99.70%	57.5	44.2	32.7	21.6	42.8	2.8	0.332	0.667	0	2	0	0	2	1,115.65	2,874.00		33,097.70	1458	1466	Empty	TRUE	UPI000240B81D,UPI00035064DB,UPI0003505F8D,UPI0002C86DF1,UPI00035042E9,UPI0000ECB786,UPI00035069AB
20196-20225 HinckeM –TAXchicken	Uncategorized Sample	20196-20205_A	Mudpit_Ingel20196_MGFPeaklist (F058870)	Uncharacterized protein (Fragment) OS=Gallus gallus GN=SPTB PE=4 SV=2	F1NSS0_CHICK	TAX_GallusGallus_9031_20141114.fasta	265,530.20	100.00%	144	184	507	1.02%	54.00%	FLDLLEPLRK	R	K	99.70%	44.4	42.8	34.5	24.8	37.1	1.28	0.197	0.65	0	0	4	0	2	1,243.74	2,756.50		7,044.27	1458	1467	Empty	TRUE	UPI000240B81D,UPI00035064DB,U[illegible]
20196-20225 HinckeM –TAXchicken	Uncategorized Sample	20196-20205_A	Mudpit_Ingel20196_MGFPeaklist (F058870)	Uncharacterized protein (Fragment) OS=Gallus gallus GN=SPTB PE=4 SV=2	F1NSS0_CHICK	TAX_GallusGallus_9031_20141114.fasta	265,530.20	100.00%	144	184	507	1.02%	54.00%	FLDLLEPLRKK	R	R	99.70%	22.6	40.5	26.4	7.78	26.7	0.658	0.225	0.364	0	0	2	0	2	1,371.84	2,628.00		862.6	1458	1468	Empty	TRUE	UPI000240B81D,UPI00035064DB,U[illegible]
20196-20225 HinckeM –TAXchicken	Uncategorized Sample	20196-20205_A	Mudpit_Ingel20196_MGFPeaklist (F058870)	Uncharacterized protein (Fragment) OS=Gallus gallus GN=SPTB PE=4 SV=2	F1NSS0_CHICK	TAX_GallusGallus_9031_20141114.fasta	265,530.20	100.00%	144	184	507	1.02%	54.00%	FQVQGVIEDMEEDTETR	R	S	99.70%	52	41.9	29.1	41	32.9	2.96	0.353	0.294	0	0	2	0	2	2,041.90	2,221.50		1,385.11	154	170	Empty	TRUE	UPI000240B81D,UPI00035064DB,U[illegible]
20196-20225 HinckeM –TAXchicken	Uncategorized Sample	20196-20205_A	Mudpit_Ingel20196_MGFPeaklist (F058870)	Uncharacterized protein (Fragment) OS=Gallus gallus GN=SPTB PE=4 SV=2	F1NSS0_CHICK	TAX_GallusGallus_9031_20141114.fasta	265,530.20	100.00%	144	184	507	1.02%	54.00%	FQVQGVIEDMEEDTETRSPR	R	E	98.70%	29.6	42.9	16.4	28.2					0	0	2	0	2	2,382.09	2,177.00		1,892.35	154	173	Empty	TRUE	UPI000240B81D,UPI00035064DB,U[illegible]
20196-20225 HinckeM –TAXchicken	Uncategorized Sample	20196-20205_A	Mudpit_Ingel20196_MGFPeaklist (F058870)	Uncharacterized protein (Fragment) OS=Gallus gallus GN=SPTB PE=4 SV=2	F1NSS0_CHICK	TAX_GallusGallus_9031_20141114.fasta	265,530.20	100.00%	144	184	507	1.02%	54.00%	FRELVSQWR	R	S	99.70%	40.7	44.6	32.4	2.66	30.2	1.1	0.215	0.5	0	0	2	0	2	1,220.65	2,123.50		2,528.98	936	944	Empty	TRUE	UPI000240B81D,UPI00035064DB,U[illegible]
20196-20225 HinckeM –TAXchicken	Uncategorized Sample	20196-20205_A	Mudpit_Ingel20196_MGFPeaklist (F058870)	Uncharacterized protein (Fragment) OS=Gallus gallus GN=SPTB PE=4 SV=2	F1NSS0_CHICK	TAX_GallusGallus_9031_20141114.fasta	265,530.20	100.00%	144	184	507	1.02%	54.00%	GEAAAGGPCPELAER	R	A	99.70%	77.8	43.3	27.7	60.2	54.3	7.96	0.595	0.5	0	4	0	0	2	1,484.68	1,756.50		12,341.20	1539	1553	Empty	TRUE	UPI000240B81D,UPI00035064DB,U[illegible]
20196-20225 HinckeM –TAXchicken	Uncategorized Sample	20196-20205_A	Mudpit_Ingel20196_MGFPeaklist (F058870)	Uncharacterized protein (Fragment) OS=Gallus gallus GN=SPTB PE=4 SV=2	F1NSS0_CHICK	TAX_GallusGallus_9031_20141114.fasta	265,530.20	100.00%	144	184	507	1.02%	54.00%	GNLEVLLFTIQSR	K	M	99.70%	74.3	43.1	22.9	58.9	44.8	5.46	0.402	0.5	0	2	0	0	2	1,489.84	2,880.50		4,055.85	357	369	Empty	TRUE	UPI000240B81D,UPI00035064DB,U[illegible]
20196-20225 HinckeM –TAXchicken	Uncategorized Sample	20196-20205_A	Mudpit_Ingel20196_MGFPeaklist (F058870)	Uncharacterized protein (Fragment) OS=Gallus gallus GN=SPTB PE=4 SV=2	F1NSS0_CHICK	TAX_GallusGallus_9031_20141114.fasta	265,530.20	100.00%	144	184	507	1.02%	54.00%	GQQLFEANR	K	T	99.70%	44.2	45.3	25.3	20.5	27.3	1.55	0.245	0.389	0	4	0	0	2	1,062.53	1,833.50		1,428.80	1370	1378	Empty	TRUE	UPI000240B81D,UPI00035064DB,U[illegible]
20196-20225 HinckeM –TAXchicken	Uncategorized Sample	20196-20205_A	Mudpit_Ingel20196_MGFPeaklist (F058870)	Uncharacterized protein (Fragment) OS=Gallus gallus GN=SPTB PE=4 SV=2	F1NSS0_CHICK	TAX_GallusGallus_9031_20141114.fasta	265,530.20	100.00%	144	184	507	1.02%	54.00%	GQQLFEANRTELYSQSYSDLER	K	W	99.70%	58	43.6	13.3	55.9	56.8	8.44	0.533	0.409	0	0	2	0	2	2,634.24	2,369.00		2,884.32	1370	1391	Empty	TRUE	UPI000240B81D,UPI00035064DB,U[illegible]
20196-20225 HinckeM –TAXchicken	Uncategorized Sample	20196-20205_A	Mudpit_Ingel20196_MGFPeaklist (F058870)	Uncharacterized protein (Fragment) OS=Gallus gallus GN=SPTB PE=4 SV=2	F1NSS0_CHICK	TAX_GallusGallus_9031_20141114.fasta	265,530.20	100.00%	144	184	507	1.02%	54.00%	GVEAGWGALHK	R	M	99.70%	45.7	43.8	22.6	28.9	32.2	1.24	0.146	0.409	0	2	0	0	2	1,124.58	1,947.00		2,370.78	1143	1153	Empty	TRUE	UPI000240B81D,UPI00035064DB,U[illegible]
20196-20225 HinckeM –TAXchicken	Uncategorized Sample	20196-20205_A	Mudpit_Ingel20196_MGFPeaklist (F058870)	Uncharacterized protein (Fragment) OS=Gallus gallus GN=SPTB PE=4 SV=2	F1NSS0_CHICK	TAX_GallusGallus_9031_20141114.fasta	265,530.20	100.00%	144	184	507	1.02%	54.00%	GYPHVNVTNFTSSWK	K	D	99.70%	40.8	44.5	21.8	33.8	32.1	3.17	0.321	0.333	0	0	2	0	2	1,736.84	2,446.00		1,094.65	186	200	Empty	TRUE	UPI000240B81D,UPI00035064DB,U[illegible]
20196-20225 HinckeM –TAXchicken	Uncategorized Sample	20196-20205_A	Mudpit_Ingel20196_MGFPeaklist (F058870)	Uncharacterized protein (Fragment) OS=Gallus gallus GN=SPTB PE=4 SV=2	F1NSS0_CHICK	TAX_GallusGallus_9031_20141114.fasta	265,530.20	100.00%	144	184	507	1.02%	54.00%	HLGITPLLDPEDVFTENPDEK	R	S	99.70%	54	44.1	15.5	50.8	55.3	9.24	0.552	0.429	0	0	2	0	2	2,379.17	2,862.00		6,812.03	241	261	Empty	TRUE	UPI000240B81D,UPI00035064DB,U[illegible]
20196-20225 HinckeM –TAXchicken	Uncategorized Sample	20196-20205_A	Mudpit_Ingel20196_MGFPeaklist (F058870)	Uncharacterized protein (Fragment) OS=Gallus gallus GN=SPTB PE=4 SV=2	F1NSS0_CHICK	TAX_GallusGallus_9031_20141114.fasta	265,530.20	100.00%	144	184	507	1.02%	54.00%	HLLEVEELLETHR	K	L	99.70%	66.5	44.3	22	53	43.6	5.55	0.489	0.5	0	0	4	3	2	1,617.86	2,601.00		4,605.51	557	569	Empty	TRUE	UPI000240B81D,UPI00035064DB,U[illegible]
20196-20225 HinckeM –TAXchicken	Uncategorized Sample	20196-20205_A	Mudpit_Ingel20196_MGFPeaklist (F058870)	Uncharacterized protein (Fragment) OS=Gallus gallus GN=SPTB PE=4 SV=2	F1NSS0_CHICK	TAX_GallusGallus_9031_20141114.fasta	265,530.20	100.00%	144	184	507	1.02%	54.00%	HNLEHAFSMAER	R	H	99.70%	46.3	42.9	24.1	38.5	34.7	4.51	0.435	0.5	0	0	6	0	2	1,457.66	1,675.50		4,419.23	229	240	Empty	TRUE	UPI000240B81D,UPI00035064DB,U[illegible]
20196-20225 HinckeM –TAXchicken	Uncategorized Sample	20196-20205_A	Mudpit_Ingel20196_MGFPeaklist (F058870)	Uncharacterized protein (Fragment) OS=Gallus gallus GN=SPTB PE=4 SV=2	F1NSS0_CHICK	TAX_GallusGallus_9031_20141114.fasta	265,530.20	100.00%	144	184	507	1.02%	54.00%	HQELPQDLGDDSGSVEAFHR	R	M	99.70%	58.1	43.1	15.5	54.1	45.5	6.96	0.47	0.35	0	0	1	4	2	2,237.02	2,080.00		27,532.20	1812	1831	Empty	TRUE	UPI000240B81D,UPI00035064DB,U[illegible]
20196-20225 HinckeM –TAXchicken	Uncategorized Sample	20196-20205_A	Mudpit_Ingel20196_MGFPeaklist (F058870)	Uncharacterized protein (Fragment) OS=Gallus gallus GN=SPTB PE=4 SV=2	F1NSS0_CHICK	TAX_GallusGallus_9031_20141114.fasta	265,530.20	100.00%	144	184	507	1.02%	54.00%	HRPELFDFK	K	T	99.70%	25	45	27.3	5.91	32.3	3.51	0.353	0.5	0	2	0	0	2	1,188.62	2,206.00		1,405.56	212	220	Empty	TRUE	UPI000240B81D,UPI00035064DB,U[illegible]
20196-20225 HinckeM –TAXchicken	Uncategorized Sample	20196-20205_A	Mudpit_Ingel20196_MGFPeaklist (F058870)	Uncharacterized protein (Fragment) OS=Gallus gallus GN=SPTB PE=4 SV=2	F1NSS0_CHICK	TAX_GallusGallus_9031_20141114.fasta	265,530.20	100.00%	144	184	507	1.02%	54.00%	HRPELFDFKTLTK	K	S	99.70%	24.2	43.7	15.8	19.9	27.7	2.77	0.264	0.385	0	0	0	2	2	1,631.89	2,335.00		1,220.78	212	224	Empty	TRUE	UPI000240B81D,UPI00035064DB,U[illegible]
20196-20225 HinckeM –TAXchicken	Uncategorized Sample	20196-20205_A	Mudpit_Ingel20196_MGFPeaklist (F058870)	Uncharacterized protein (Fragment) OS=Gallus gallus GN=SPTB PE=4 SV=2	F1NSS0_CHICK	TAX_GallusGallus_9031_20141114.fasta	265,530.20	100.00%	144	184	507	1.02%	54.00%	HTALEAELEAR	R	G	99.70%	63.5	44	41.7	39.1	40	3.74	0.38	0.545	0	2	2	0	2	1,239.63	1,964.00		7,979.34	677	687	Empty	TRUE	UPI000240B81D,UPI00035064DB,U[illegible]
20196-20225 HinckeM –TAXchicken	Uncategorized Sample	20196-20205_A	Mudpit_Ingel20196_MGFPeaklist (F058870)	Uncharacterized protein (Fragment) OS=Gallus gallus GN=SPTB PE=4 SV=2	F1NSS0_CHICK	TAX_GallusGallus_9031_20141114.fasta	265,530.20	100.00%	144	184	507	1.02%	54.00%	IKAEGMELASCKPQYGAVVGQR	K	L	99.70%	60.7	43.9	19.1	48.6	46.6	4.62	0.479	0.364	0	0	0	4	2	2,408.21	1,984.00		10,110.40	1330	1351	Empty	TRUE	UPI000240B81D,UPI00035064DB,U[illegible]
20196-20225 HinckeM –TAXchicken	Uncategorized Sample	20196-20205_A	Mudpit_Ingel20196_MGFPeaklist (F058870)	Uncharacterized protein (Fragment) OS=Gallus gallus GN=SPTB PE=4 SV=2	F1NSS0_CHICK	TAX_GallusGallus_9031_20141114.fasta	265,530.20	100.00%	144	184	507	1.02%	54.00%	IKALADEREAVQK	R	K	35.40%	17.4	43.1	26.8	6.95					0	0	1	0	2	1,470.83	1,598.00		310.22	42	54	Empty	TRUE	UPI000240B81D,UPI00035064DB,R[illegible]
20196-20225 HinckeM –TAXchicken	Uncategorized Sample	20196-20205_A	Mudpit_Ingel20196_MGFPeaklist (F058870)	Uncharacterized protein (Fragment) OS=Gallus gallus GN=SPTB PE=4 SV=2	F1NSS0_CHICK	TAX_GallusGallus_9031_20141114.fasta	265,530.20	100.00%	144	184	507	1.02%	54.00%	ISDLYLDLR	R	D	99.70%	67.1	44.1	37.8	37	40.3	2.7	0.352	0.667	0	4	0	0	2	1,107.60	2,473.50		8,313.08	72	80	Empty	TRUE	UPI000240B81D,UPI00035064DB,U[illegible]
20196-20225 HinckeM –TAXchicken	Uncategorized Sample	20196-20205_A	Mudpit_Ingel20196_MGFPeaklist (F058870)	Uncharacterized protein (Fragment) OS=Gallus gallus GN=SPTB PE=4 SV=2	F1NSS0_CHICK	TAX_GallusGallus_9031_20141114.fasta	265,530.20	100.00%	144	184	507	1.02%	54.00%	ISDLYLDLRDGR	R	M	99.70%	24.5	44.5	19.1	16.3	35.3	3.55	0.323	0.542	0	2	3	0	2	1,435.75	2,332.00		4,657.60	72	83	Empty	TRUE	UPI000240B81D,UPI00035064DB,U[illegible]
20196-20225 HinckeM –TAXchicken	Uncategorized Sample	20196-20205_A	Mudpit_Ingel20196_MGFPeaklist (F058870)	Uncharacterized protein (Fragment) OS=Gallus gallus GN=SPTB PE=4 SV=2	F1NSS0_CHICK	TAX_GallusGallus_9031_20141114.fasta	265,530.20	100.00%	144	184	507	1.02%	54.00%	KHEAIETDTAAYMER	K	V	99.70%	65.3	43.9	27.2	49.9	48.7	6.24	0.472	0.533	0	0	4	0	2	1,764.82	1,713.50		3,615.07	461	475	Empty	TRUE	UPI000240B81D,UPI00035064DB,U[illegible]

20196-20225 HinckeM –TAXchicken	Uncategorized Sample	20196-20205_A	Mudpit_Ingel20196_MGFPeaklist (F058870)	Uncharacterized protein (Fragment) OS=Gallus gallus GN=SPTB PE=4 SV=2	F1NSS0_CHICK	TAX_GallusGallus_9031_20141114.fasta	265,530.20	100.00%	144	184	507	1.02%	54.00%	LEVPNKLEDLDVVK	K	H	99.70%	34	42.6	19.9	23.2	37	2.08	0.17	0.393	0	0	2	0	2	1,610.90	2,443.00		1,587.36	871	884	Empty	TRUE	UPI000240B81D,UPI00035064DB,UPI0003505F8D,UPI0002C86DF1,UPI00035042E9,UPI00035069AB
20196-20225 HinckeM –TAXchicken	Uncategorized Sample	20196-20205_A	Mudpit_Ingel20196_MGFPeaklist (F058870)	Uncharacterized protein (Fragment) OS=Gallus gallus GN=SPTB PE=4 SV=2	F1NSS0_CHICK	TAX_GallusGallus_9031_20141114.fasta	265,530.20	100.00%	144	184	507	1.02%	54.00%	LGALRPQAALLAQERPELAEDTAR	R	R	99.70%	34.9	39.9	0	34.8	52.2	6.17	0.529	0.438	0	0	0	2	2	2,589.41	2,336.00		7,732.76	1008	1031	Empty	TRUE	UPI000240B81D,UPI00035064DB,UPI0003505F8D,UPI0002C86DF1,UPI00035042E9,UPI00035069AB
20196-20225 HinckeM –TAXchicken	Uncategorized Sample	20196-20205_A	Mudpit_Ingel20196_MGFPeaklist (F058870)	Uncharacterized protein (Fragment) OS=Gallus gallus GN=SPTB PE=4 SV=2	F1NSS0_CHICK	TAX_GallusGallus_9031_20141114.fasta	265,530.20	100.00%	144	184	507	1.02%	54.00%	LGHQAEGFPPELR	R	A	99.70%	47.9	44.5	25	35.6	39.5	3.92	0.435	0.5	0	0	25	0	2	1,450.74	2,152.00		26,445.80	799	811	Empty	TRUE	UPI000240B81D,UPI00035064DB,UPI0003505F8D,UPI0002C86DF1,UPI00035042E9,UPI0000ECB786,UPI00035069AB
20196-20225 HinckeM –TAXchicken	Uncategorized Sample	20196-20205_A	Mudpit_Ingel20196_MGFPeaklist (F058870)	Uncharacterized protein (Fragment) OS=Gallus gallus GN=SPTB PE=4 SV=2	F1NSS0_CHICK	TAX_GallusGallus_9031_20141114.fasta	265,530.20	100.00%	144	184	507	1.02%	54.00%	LLESDMALQAEK	R	V	99.70%	63.6	44.9	24	50.8	38.9	3.68	0.401	0.417	0	7	0	0	2	1,363.68	1,880.00		4,204.19	570	581	Empty	TRUE	UPI000240B81D,UPI00035064DB,UPI0003505F8D,UPI0002C86DF1,UPI00035042E9,UPI0000ECB786,UPI00035069AB
20196-20225 HinckeM –TAXchicken	Uncategorized Sample	20196-20205_A	Mudpit_Ingel20196_MGFPeaklist (F058870)	Uncharacterized protein (Fragment) OS=Gallus gallus GN=SPTB PE=4 SV=2	F1NSS0_CHICK	TAX_GallusGallus_9031_20141114.fasta	265,530.20	100.00%	144	184	507	1.02%	54.00%	LLESDMALQAEKVR	R	A	99.70%	72.7	44.3	24.6	62.7	44.6	5.96	0.305	0.464	0	0	2	0	2	1,602.85	2,163.50		871.14	570	583	Empty	TRUE	UPI000240B81D,UPI00035064DB,UPI0003505F8D,UPI0002C86DF1,UPI00035042E9,UPI0000ECB786,UPI00035069AB
20196-20225 HinckeM –TAXchicken	Uncategorized Sample	20196-20205_A	Mudpit_Ingel20196_MGFPeaklist (F058870)	Uncharacterized protein (Fragment) OS=Gallus gallus GN=SPTB PE=4 SV=2	F1NSS0_CHICK	TAX_GallusGallus_9031_20141114.fasta	265,530.20	100.00%	144	184	507	1.02%	54.00%	LLEVLSK	K	E	74.20%	42.2	44	41.6	0					0	1	0	0	2	801.5081	2,181.00		242.39	88	94	Empty	TRUE	UPI000240B81D,UPI00035064DB,UPI0003505F8D,UPI0002C86DF1,UPI00035042E9,UPI0000ECB786,UPI00035069AB
20196-20225 HinckeM –TAXchicken	Uncategorized Sample	20196-20205_A	Mudpit_Ingel20196_MGFPeaklist (F058870)	Uncharacterized protein (Fragment) OS=Gallus gallus GN=SPTB PE=4 SV=2	F1NSS0_CHICK	TAX_GallusGallus_9031_20141114.fasta	265,530.20	100.00%	144	184	507	1.02%	54.00%	LLEVLSKEMLPKPTK	K	G	16.20%	11.8	40.1	20.5	8.53					0	0	2	1	2	1,742.01	2,237.00		1,325.69	88	102	Empty	TRUE	UPI000240B81D,UPI00035064DB,UPI0003505F8D,UPI0002C86DF1,UPI00035042E9,UPI0000ECB786,UPI00035069AB
20196-20225 HinckeM –TAXchicken	Uncategorized Sample	20196-20205_A	Mudpit_Ingel20196_MGFPeaklist (F058870)	Uncharacterized protein (Fragment) OS=Gallus gallus GN=SPTB PE=4 SV=2	F1NSS0_CHICK	TAX_GallusGallus_9031_20141114.fasta	265,530.20	100.00%	144	184	507	1.02%	54.00%	LLLEVCQFSR	R	D	99.70%	72.7	44.4	36.1	50.9	39.4	2.33	0.365	0.5	0	6	1	0	2	1,264.67	2,513.00		6,894.41	2002	2011	Empty	TRUE	UPI000240B81D,UPI00035064DB,UPI0003505F8D,UPI0002C86DF1,UPI00035042E9,UPI0000ECB786,UPI00035069AB
20196-20225 HinckeM –TAXchicken	Uncategorized Sample	20196-20205_A	Mudpit_Ingel20196_MGFPeaklist (F058870)	Uncharacterized protein (Fragment) OS=Gallus gallus GN=SPTB PE=4 SV=2	F1NSS0_CHICK	TAX_GallusGallus_9031_20141114.fasta	265,530.20	100.00%	144	184	507	1.02%	54.00%	LQGQVDKYYAGLK	R	E	99.70%	62.4	43.5	20.9	49.7	39.6	4.41	0.394	0.5	0	0	2	0	2	1,482.80	1,961.00		3,513.43	1659	1671	Empty	TRUE	UPI000240B81D,UPI00035064DB,UPI0003505F8D,UPI0002C86DF1,UPI00035042E9,UPI0000ECB786,UPI00035069AB
20196-20225 HinckeM –TAXchicken	Uncategorized Sample	20196-20205_A	Mudpit_Ingel20196_MGFPeaklist (F058870)	Uncharacterized protein (Fragment) OS=Gallus gallus GN=SPTB PE=4 SV=2	F1NSS0_CHICK	TAX_GallusGallus_9031_20141114.fasta	265,530.20	100.00%	144	184	507	1.02%	54.00%	LQGQVDKYYAGLKEAAEER	R	R	99.70%	64.8	43.9	18.9	50.4	47.1	6.17	0.463	0.368	0	0	0	2	2	2,168.10	2,191.00		2,839.74	1659	1677	Empty	TRUE	UPI000240B81D,UPI00035064DB,UPI0003505F8D,UPI0002C86DF1,UPI00035042E9,UPI0000ECB786,UPI00035069AB
20196-20225 HinckeM –TAXchicken	Uncategorized Sample	20196-20205_A	Mudpit_Ingel20196_MGFPeaklist (F058870)	Uncharacterized protein (Fragment) OS=Gallus gallus GN=SPTB PE=4 SV=2	F1NSS0_CHICK	TAX_GallusGallus_9031_20141114.fasta	265,530.20	100.00%	144	184	507	1.02%	54.00%	LQSFLQSCR	R	E	99.70%	64.3	44.1	30	47.3	35.6	1.85	0.323	0.556	0	6	0	0	2	1,138.57	2,064.50		14,343.50	1274	1282	Empty	TRUE	UPI000240B81D,UPI00035064DB,UPI0003505F8D,UPI0002C86DF1,UPI00035042E9,UPI0000ECB786,UPI00035069AB
20196-20225 HinckeM –TAXchicken	Uncategorized Sample	20196-20205_A	Mudpit_Ingel20196_MGFPeaklist (F058870)	Uncharacterized protein (Fragment) OS=Gallus gallus GN=SPTB PE=4 SV=2	F1NSS0_CHICK	TAX_GallusGallus_9031_20141114.fasta	265,530.20	100.00%	144	184	507	1.02%	54.00%	LREASEAQQYYLDADEAK	R	A	99.70%	73.1	43.8	20.4	59.4	39.4	4.06	0.391	0.333	0	0	3	0	2	2,099.99	2,002.00		12,402.80	1575	1592	Empty	TRUE	UPI000240B81D,UPI00035064DB,UPI0003505F8D,UPI0002C86DF1,UPI00035042E9,UPI0000ECB786,UPI00035069AB
20196-20225 HinckeM –TAXchicken	Uncategorized Sample	20196-20205_A	Mudpit_Ingel20196_MGFPeaklist (F058870)	Uncharacterized protein (Fragment) OS=Gallus gallus GN=SPTB PE=4 SV=2	F1NSS0_CHICK	TAX_GallusGallus_9031_20141114.fasta	265,530.20	100.00%	144	184	507	1.02%	54.00%	LRGVEAGWGALHK	R	M	99.70%	38	43.6	24.2	14.5	35.1	2.47	0.339	0.423	0	0	3	0	2	1,393.77	2,009.00		2,080.12	1141	1153	Empty	TRUE	UPI000240B81D,UPI00035064DB,UPI0003505F8D,UPI0002C86DF1,UPI00035042E9,UPI00035069AB
20196-20225 HinckeM –TAXchicken	Uncategorized Sample	20196-20205_A	Mudpit_Ingel20196_MGFPeaklist (F058870)	Uncharacterized protein (Fragment) OS=Gallus gallus GN=SPTB PE=4 SV=2	F1NSS0_CHICK	TAX_GallusGallus_9031_20141114.fasta	265,530.20	100.00%	144	184	507	1.02%	54.00%	LTRLEEQVAAR	K	Q	99.70%	52.4	43.5	43.4	25.9	36.1	2.38	0.247	0.5	0	0	4	0	2	1,285.72	1,755.00		4,317.68	1419	1429	Empty	TRUE	UPI000240B81D,UPI00035064DB,UPI0003505F8D,UPI0002C86DF1,UPI00035042E9,UPI0000ECB786,UPI00035069AB
20196-20225 HinckeM –TAXchicken	Uncategorized Sample	20196-20205_A	Mudpit_Ingel20196_MGFPeaklist (F058870)	Uncharacterized protein (Fragment) OS=Gallus gallus GN=SPTB PE=4 SV=2	F1NSS0_CHICK	TAX_GallusGallus_9031_20141114.fasta	265,530.20	100.00%	144	184	507	1.02%	54.00%	LTTLELMGGR	K	T	99.70%	59	45	38.5	32	33.6	3	0.369	0.45	0	5	0	0	2	1,106.59	2,337.00		4,869.93	2066	2075	Empty	TRUE	UPI000240B81D,UPI00035064DB,UPI0003505F8D,UPI0002C86DF1,UPI00035042E9,UPI00035069AB
20196-20225 HinckeM –TAXchicken	Uncategorized Sample	20196-20205_A	Mudpit_Ingel20196_MGFPeaklist (F058870)	Uncharacterized protein (Fragment) OS=Gallus gallus GN=SPTB PE=4 SV=2	F1NSS0_CHICK	TAX_GallusGallus_9031_20141114.fasta	265,530.20	100.00%	144	184	507	1.02%	54.00%	LVAEGNIFSEK	K	I	99.70%	69	44.8	24.8	55.1	43.3	3.57	0.423	0.5	0	5	0	0	2	1,206.64	2,210.00		15,620.50	1232	1242	Empty	TRUE	UPI000240B81D,UPI00035064DB,UPI0003505F8D,UPI0002C86DF1,UPI00035042E9,UPI0000ECB786,UPI00035069AB
20196-20225 HinckeM –TAXchicken	Uncategorized Sample	20196-20205_A	Mudpit_Ingel20196_MGFPeaklist (F058870)	Uncharacterized protein (Fragment) OS=Gallus gallus GN=SPTB PE=4 SV=2	F1NSS0_CHICK	TAX_GallusGallus_9031_20141114.fasta	265,530.20	100.00%	144	184	507	1.02%	54.00%	LVAEGNIFSEKISEK	K	N	99.70%	39.4	43.9	22.2	32	31.8	2.72	0.33	0.3	0	0	2	0	2	1,663.89	2,264.00		1,851.71	1232	1246	Empty	TRUE	UPI000240B81D,UPI00035064DB,UPI0003505F8D,UPI0002C86DF1,UPI00035042E9,UPI0000ECB786,UPI00035069AB
20196-20225 HinckeM –TAXchicken	Uncategorized Sample	20196-20205_A	Mudpit_Ingel20196_MGFPeaklist (F058870)	Uncharacterized protein (Fragment) OS=Gallus gallus GN=SPTB PE=4 SV=2	F1NSS0_CHICK	TAX_GallusGallus_9031_20141114.fasta	265,530.20	100.00%	144	184	507	1.02%	54.00%	LVAEGNIFSEKISEKNQALQER	K	H	99.70%	28.8	42.7	18.5	16.5	29	1.38	0.217	0.227	0	0	0	2	2	2,503.32	2,249.50		892.25	1232	1253	Empty	TRUE	UPI000240B81D,UPI00035064DB,UPI0003505F8D,UPI0002C86DF1,UPI00035042E9,UPI0000ECB786,UPI00035069AB
20196-20225 HinckeM –TAXchicken	Uncategorized Sample	20196-20205_A	Mudpit_Ingel20196_MGFPeaklist (F058870)	Uncharacterized protein (Fragment) OS=Gallus gallus GN=SPTB PE=4 SV=2	F1NSS0_CHICK	TAX_GallusGallus_9031_20141114.fasta	265,530.20	100.00%	144	184	507	1.02%	54.00%	LVAQQDNFGHDLTAVEAAK	R	K	99.70%	24.4	44.4	14.2	14.8	35.9	3.4	0.262	0.263	0	0	2	0	2	2,027.02	2,239.50		1,916.08	441	459	Empty	TRUE	UPI00035064DB,UPI0002C86DF1,UPI00035042E9
20196-20225 HinckeM –TAXchicken	Uncategorized Sample	20196-20205_A	Mudpit_Ingel20196_MGFPeaklist (F058870)	Uncharacterized protein (Fragment) OS=Gallus gallus GN=SPTB PE=4 SV=2	F1NSS0_CHICK	TAX_GallusGallus_9031_20141114.fasta	265,530.20	100.00%	144	184	507	1.02%	54.00%	LVAQQDNFGHDLTAVEAAKK	R	K	99.70%	15.1	43.8	13.1	14	33.7	2.15	0.234	0.25	0	0	0	1	2	2,155.11	2,133.00		622.76	441	460	Empty	TRUE	UPI00035064DB,UPI0002C86DF1,UPI00035042E9
20196-20225 HinckeM –TAXchicken	Uncategorized Sample	20196-20205_A	Mudpit_Ingel20196_MGFPeaklist (F058870)	Uncharacterized protein (Fragment) OS=Gallus gallus GN=SPTB PE=4 SV=2	F1NSS0_CHICK	TAX_GallusGallus_9031_20141114.fasta	265,530.20	100.00%	144	184	507	1.02%	54.00%	MLTVQDASYGGAR	K	G	99.70%	79.4	43.9	25.3	63.1	44.3	4.66	0.377	0.462	0	7	1	0	2	1,384.65	1,884.50		12,454.10	1292	1304	Empty	TRUE	UPI000240B81D,UPI00035064DB,UPI0003505F8D,UPI0002C86DF1,UPI00035042E9,UPI0000ECB786,UPI00035069AB
20196-20225 HinckeM –TAXchicken	Uncategorized Sample	20196-20205_A	Mudpit_Ingel20196_MGFPeaklist (F058870)	Uncharacterized protein (Fragment) OS=Gallus gallus GN=SPTB PE=4 SV=2	F1NSS0_CHICK	TAX_GallusGallus_9031_20141114.fasta	265,530.20	100.00%	144	184	507	1.02%	54.00%	MQLLAASYDLR	R	K	99.70%	67.1	44.2	28.4	48.9	36.4	2	0.341	0.455	0	8	0	0	2	1,297.65	2,479.00		4,941.83	1785	1795	Empty	TRUE	UPI000240B81D,UPI00035064DB,UPI0003505F8D,UPI0002C86DF1,UPI00035042E9,UPI0000ECB786,UPI00035069AB
20196-20225 HinckeM –TAXchicken	Uncategorized Sample	20196-20205_A	Mudpit_Ingel20196_MGFPeaklist (F058870)	Uncharacterized protein (Fragment) OS=Gallus gallus GN=SPTB PE=4 SV=2	F1NSS0_CHICK	TAX_GallusGallus_9031_20141114.fasta	265,530.20	100.00%	144	184	507	1.02%	54.00%	MQLLAASYDLRK	R	Y	99.70%	39.9	43.9	24.9	26.7	33.3	2.74	0.366	0.417	0	0	4	0	2	1,406.75	2,105.00		2,722.04	1785	1796	Empty	TRUE	UPI000240B81D,UPI00035064DB,UPI0003505F8D,UPI0002C86DF1,UPI00035042E9,UPI0000ECB786,UPI00035069AB
20196-20225 HinckeM –TAXchicken	Uncategorized Sample	20196-20205_A	Mudpit_Ingel20196_MGFPeaklist (F058870)	Uncharacterized protein (Fragment) OS=Gallus gallus GN=SPTB PE=4 SV=2	F1NSS0_CHICK	TAX_GallusGallus_9031_20141114.fasta	265,530.20	100.00%	144	184	507	1.02%	54.00%	NETLQKELQGHAPR	R	L	99.70%	63.5	44.4	26.2	44.8	39.3	4.82	0.42	0.393	0	0	0	3	2	1,620.85	1,829.00		2,586.13	1518	1531	Empty	TRUE	UPI000240B81D,UPI0002C86DF1,UPI00035042E9,UPI0000ECB786,UPI00035069AB
20196-20225 HinckeM –TAXchicken	Uncategorized Sample	20196-20205_A	Mudpit_Ingel20196_MGFPeaklist (F058870)	Uncharacterized protein (Fragment) OS=Gallus gallus GN=SPTB PE=4 SV=2	F1NSS0_CHICK	TAX_GallusGallus_9031_20141114.fasta	265,530.20	100.00%	144	184	507	1.02%	54.00%	QCQQQLNERWDR	R	F	99.70%	24.7	43.9	20.7	16.2	28.9	1.68	0.221	0.375	0	0	2	0	2	1,660.76	1,695.50		1,011.62	924	935	Empty	TRUE	UPI000240B81D,UPI00035064DB,UPI0003505F8D,UPI0002C86DF1,UPI00035042E9,UPI00035069AB
20196-20225 HinckeM –TAXchicken	Uncategorized Sample	20196-20205_A	Mudpit_Ingel20196_MGFPeaklist (F058870)	Uncharacterized protein (Fragment) OS=Gallus gallus GN=SPTB PE=4 SV=2	F1NSS0_CHICK	TAX_GallusGallus_9031_20141114.fasta	265,530.20	100.00%	144	184	507	1.02%	54.00%	QEKLEQLAR	R	R	99.70%	58.9	43.5	37.4	17.2	31	1.01	0.0194	0.444	0	4	0	0	2	1,114.62	1,815.50		3,443.02	414	422	Empty	TRUE	R4GJH8_CHICK,UPI0000ECC737,UPI00003ACAFC,UPI00035042E9,UPI00035069AB,UPI0000447B9B,F1ND51_CHICK,UPI000240B81D,UPI00035064DB,UPI0003505F8D,UPI0002C88352,UPI0002C86DF1
20196-20225 HinckeM –TAXchicken	Uncategorized Sample	20196-20205_A	Mudpit_Ingel20196_MGFPeaklist (F058870)	Uncharacterized protein (Fragment) OS=Gallus gallus GN=SPTB PE=4 SV=2	F1NSS0_CHICK	TAX_GallusGallus_9031_20141114.fasta	265,530.20	100.00%	144	184	507	1.02%	54.00%	QIQTQEKPRDVSSVELLMK	R	Y	23.30%	2.68	43.3	14.3	0					0	0	0	2	2	2,245.19	2,114.50		1,611.13	1920	1938	Empty	TRUE	UPI000240B81D,UPI00035064DB,UPI0003505F8D,UPI0002C86DF1,UPI00035042E9,UPI0000ECB786,UPI00035069AB
20196-20225 HinckeM –TAXchicken	Uncategorized Sample	20196-20205_A	Mudpit_Ingel20196_MGFPeaklist (F058870)	Uncharacterized protein (Fragment) OS=Gallus gallus GN=SPTB PE=4 SV=2	F1NSS0_CHICK	TAX_GallusGallus_9031_20141114.fasta	265,530.20	100.00%	144	184	507	1.02%	54.00%	QKELMELKAPLAGVAPEPDAQEEK	R	L	99.70%	22	43.2	18.3	20	29.9	2.55	0.221	0.229	0	0	0	2	2	2,637.34	2,282.50		1,274.24	1430	1453	Empty	TRUE	UPI000240B81D,UPI00035064DB,UPI0003505F8D,UPI0002C86DF1,UPI00035042E9,UPI0000ECB786,UPI00035069AB
20196-20225 HinckeM –TAXchicken	Uncategorized Sample	20196-20205_A	Mudpit_Ingel20196_MGFPeaklist (F058870)	Uncharacterized protein (Fragment) OS=Gallus gallus GN=SPTB PE=4 SV=2	F1NSS0_CHICK	TAX_GallusGallus_9031_20141114.fasta	265,530.20	100.00%	144	184	507	1.02%	54.00%	QLDLDAELEAGSEEEEEKRK	R	G	99.70%	21.6	43.9	14.9	12.8	27.9	1.6	0.172	0.275	0	0	0	1	2	2,318.10	2,225.00		418.36	2091	2110	Empty	TRUE	UPI000240B81D,UPI00035064DB,UPI0003505F8D,UPI0002C86DF1,UPI00035042E9,UPI00035069AB
20196-20225 HinckeM –TAXchicken	Uncategorized Sample	20196-20205_A	Mudpit_Ingel20196_MGFPeaklist (F058870)	Uncharacterized protein (Fragment) OS=Gallus gallus GN=SPTB PE=4 SV=2	F1NSS0_CHICK	TAX_GallusGallus_9031_20141114.fasta	265,530.20	100.00%	144	184	507	1.02%	54.00%	QLLSAGHPEGEQIIR	Q	L	99.70%					30.8	3	0.289	0.267	0	2	0	0	1	1,647.88	2,090.50		1,146.81	1644	1658	Empty	TRUE	
20196-20225 HinckeM –TAXchicken	Uncategorized Sample	20196-20205_A	Mudpit_Ingel20196_MGFPeaklist (F058870)	Uncharacterized protein (Fragment) OS=Gallus gallus GN=SPTB PE=4 SV=2	F1NSS0_CHICK	TAX_GallusGallus_9031_20141114.fasta	265,530.20	100.00%	144	184	507	1.02%	54.00%	QVHLENMGSHDIVDGNHR	K	L	99.70%	47.9	43.2	16.2	38	41.2	5.06	0.357	0.389	0	0	0	2	2	2,073.95	1,723.50		5,040.36	124	141	Empty	TRUE	UPI000240B81D,UPI00035064DB,UPI0003505F8D,UPI0002C86DF1,UPI00035042E9,UPI0000ECB786,UPI00035069AB
20196-20225 HinckeM –TAXchicken	Uncategorized Sample	20196-20205_A	Mudpit_Ingel20196_MGFPeaklist (F058870)	Uncharacterized protein (Fragment) OS=Gallus gallus GN=SPTB PE=4 SV=2	F1NSS0_CHICK	TAX_GallusGallus_9031_20141114.fasta	265,530.20	100.00%	144	184	507	1.02%	54.00%	RAQLVDTADKFR	R	F	99.70%	53.8	44.4	24.4	30.7	39.9	4.15	0.396	0.542	0	0	2	0	2	1,419.77	1,840.00		4,070.37	1891	1902	Empty	TRUE	UPI000240B81D,UPI00035064DB,UPI0003505F8D,UPI0002C86DF1,UPI00035042E9,UPI0000ECB786,UPI00035069AB
20196-20225 HinckeM –TAXchicken	Uncategorized Sample	20196-20205_A	Mudpit_Ingel20196_MGFPeaklist (F058870)	Uncharacterized protein (Fragment) OS=Gallus gallus GN=SPTB PE=4 SV=2	F1NSS0_CHICK	TAX_GallusGallus_9031_20141114.fasta	265,530.20	100.00%	144	184	507	1.02%	54.00%	RHTALEAELEAR	R	G	99.70%	44.9	44.2	33.6	26	34.8	2.55	0.261	0.417	0	0	4	0	2	1,395.73	1,850.00		2,275.43	676	687	Empty	TRUE	UPI000240B81D,UPI00035064DB,UPI0003505F8D,UPI0002C86DF1,UPI00035042E9,UPI0000ECB786,UPI00035069AB
20196-20225 HinckeM –TAXchicken	Uncategorized Sample	20196-20205_A	Mudpit_Ingel20196_MGFPeaklist (F058870)	Uncharacterized protein (Fragment) OS=Gallus gallus GN=SPTB PE=4 SV=2	F1NSS0_CHICK	TAX_GallusGallus_9031_20141114.fasta	265,530.20	100.00%	144	184	507	1.02%	54.00%	RLAGAQDAMDGLQGALR	R	D	99.70%	64.8	44.8	26.2	49.5	49.9	4.49	0.437	0.441	0	0	4	0	2	1,742.90	2,190.50		7,181.33	1032	1048	Empty	TRUE	UPI000240B81D,UPI00035064DB,UPI0003505F8D,UPI0002C86DF1,UPI00035042E9,UPI00035069AB
20196-20225 HinckeM –TAXchicken	Uncategorized Sample	20196-20205_A	Mudpit_Ingel20196_MGFPeaklist (F058870)	Uncharacterized protein (Fragment) OS=Gallus gallus GN=SPTB PE=4 SV=2	F1NSS0_CHICK	TAX_GallusGallus_9031_20141114.fasta	265,530.20	100.00%	144	184	507	1.02%	54.00%	RLAGAQDAMDGLQGALRDR	R	A	99.30%	31	44.4	22.8	21.5	24.6	0.398	0.159	0.211	0	0	0	2	2	2,030.02	2,030.00		1,455.22	1032	1050	Empty	TRUE	UPI000240B81D,UPI00035064DB,UPI0003505F8D,UPI0002C86DF1,UPI00035042E9,UPI00035069AB
20196-20225 HinckeM –TAXchicken	Uncategorized Sample	20196-20205_A	Mudpit_Ingel20196_MGFPeaklist (F058870)	Uncharacterized protein (Fragment) OS=Gallus gallus GN=SPTB PE=4 SV=2	F1NSS0_CHICK	TAX_GallusGallus_9031_20141114.fasta	265,530.20	100.00%	144	184	507	1.02%	54.00%	RLENMCHLFQLK	R	R	99.50%	23.7	44.8	19.3	17	24.3	0.658	0.193	0.333	0	0	2	0	2	1,588.81	2,255.00		1,048.59	1680	1691	Empty	TRUE	UPI000240B81D,UPI00035064DB,UPI0003505F8D,UPI0002C86DF1,UPI00035042E9,UPI0000ECB786,UPI00035069AB
20196-20225 HinckeM –TAXchicken	Uncategorized Sample	20196-20205_A	Mudpit_Ingel20196_MGFPeaklist (F058870)	Uncharacterized protein (Fragment) OS=Gallus gallus GN=SPTB PE=4 SV=2	F1NSS0_CHICK	TAX_GallusGallus_9031_20141114.fasta	265,530.20	100.00%	144	184	507	1.02%	54.00%	RLENMCHLFQLKR	R	E	99.70%	25	44.7	19.6	15.6	30.4	3.05	0.237	0.346	0	0	0	2	2	1,760.90	1,957.50		1,477.70	1680	1692	Empty	TRUE	UPI000240B81D,UPI00035064DB,UPI0003505F8D,UPI0002C86DF1,UPI00035042E9,UPI0000ECB786,UPI00035069AB
20196-20225 HinckeM –TAXchicken	Uncategorized Sample	20196-20205_A	Mudpit_Ingel20196_MGFPeaklist (F058870)	Uncharacterized protein (Fragment) OS=Gallus gallus GN=SPTB PE=4 SV=2	F1NSS0_CHICK	TAX_GallusGallus_9031_20141114.fasta	265,530.20	100.00%	144	184	507	1.02%	54.00%	RNETLQKELQGHAPR	K	L	99.70%	59.5	43.9	19	44	42.6	5.51	0.434	0.467	0	0	0	4	2	1,776.95	1,717.50		1,822.17	1517	1531	Empty	TRUE	UPI000240B81D,UPI0002C86DF1,UPI00035042E9,UPI0000ECB786,UPI00035069AB
20196-20225 HinckeM –TAXchicken	Uncategorized Sample	20196-20205_A	Mudpit_Ingel20196_MGFPeaklist (F058870)	Uncharacterized protein (Fragment) OS=Gallus gallus GN=SPTB PE=4 SV=2	F1NSS0_CHICK	TAX_GallusGallus_9031_20141114.fasta	265,530.20	100.00%	144	184	507	1.02%	54.00%	RRHTALEAELEAR	R	G	99.70%	102	43.8	27.4	79.1	51.5	5.82	0.476	0.615	0	0	4	0	2	1,551.84	1,788.50		2,815.00	675	687	Empty	TRUE	UPI000240B81D,UPI00035064DB,UPI0003505F8D,UPI0002C86DF1,UPI00035042E9,UPI0000ECB786,UPI00035069AB
20196-20225 HinckeM –TAXchicken	Uncategorized Sample	20196-20205_A	Mudpit_Ingel20196_MGFPeaklist (F058870)	Uncharacterized protein (Fragment) OS=Gallus gallus GN=SPTB PE=4 SV=2	F1NSS0_CHICK	TAX_GallusGallus_9031_20141114.fasta	265,530.20	100.00%	144	184	507	1.02%	54.00%	SADGLLESGHPR	R	S	99.70%	64.4	44.2	25.8	47.8	38.7	3.89	0.408	0.542	0	0	11	0	2	1,238.61	1,763.00		38,088.80	907	918	Empty	TRUE	UPI000240B81D,UPI00035064DB,UPI0003505F8D,UPI0002C86DF1,UPI00035042E9,UPI00035069AB
20196-20225 HinckeM –TAXchicken	Uncategorized Sample	20196-20205_A	Mudpit_Ingel20196_MGFPeaklist (F058870)	Uncharacterized protein (Fragment) OS=Gallus gallus GN=SPTB PE=4 SV=2	F1NSS0_CHICK	TAX_GallusGallus_9031_20141114.fasta	265,530.20	100.00%	144	184	507	1.02%	54.00%	SAVGSALNLLSFQLECEETR	R	A	99.70%					34.4	3.08	0.311	0.2	0	0	1	0	2	2,206.08	2,874.00		672.67	945	964	Empty	TRUE	
20196-20225 HinckeM –TAXchicken	Uncategorized Sample	20196-20205_A	Mudpit_Ingel20196_MGFPeaklist (F058870)	Uncharacterized protein (Fragment) OS=Gallus gallus GN=SPTB PE=4 SV=2	F1NSS0_CHICK	TAX_GallusGallus_9031_20141114.fasta	265,530.20	100.00%	144	184	507	1.02%	54.00%	SFDACIELGK	K	K	99.70%	35.6	43.5	21.2	29.2	27.2	2.42	0.294	0.35	0	1	0	0	2	1,139.54	2,215.00		384.16	1953	1962	Empty	TRUE	UPI000240B81D,UPI00035064DB,UPI0003505F8D,UPI0002C86DF1,UPI00035042E9,UPI0000ECB786,UPI00035069AB
20196-20225 HinckeM –TAXchicken	Uncategorized Sample	20196-20205_A	Mudpit_Ingel20196_MGFPeaklist (F058870)	Uncharacterized protein (Fragment) OS=Gallus gallus GN=SPTB PE=4 SV=2	F1NSS0_CHICK	TAX_GallusGallus_9031_20141114.fasta	265,530.20	100.00%	144	184	507	1.02%	54.00%	SFDACIELGKK	K	L	31.20%	14.4	44.1	24.8	0					0	0	2	0	2	1,267.64	2,059.00		563.95	1953	1963	Empty	TRUE	UPI000240B81D,UPI00035064DB,UPI0003505F8D,UPI0002C86DF1,UPI00035042E9,UPI0000ECB786,UPI00035069AB
20196-20225 HinckeM –TAXchicken	Uncategorized Sample	20196-20205_A	Mudpit_Ingel20196_MGFPeaklist (F058870)	Uncharacterized protein (Fragment) OS=Gallus gallus GN=SPTB PE=4 SV=2	F1NSS0_CHICK	TAX_GallusGallus_9031_20141114.fasta	265,530.20	100.00%	144	184	507	1.02%	54.00%	SGKHLLEVEELLETHR	K	L	99.70%	27.9	43.7	17.9	21.9	27.9	2.11	0.269	0.312	0	0	0	2	2	1,890.01	2,442.50		779.48	554	569	Empty	TRUE	UPI000240B81D,UPI00035064DB,UPI0003505F8D,UPI0002C86DF1,UPI00035042E9,UPI0000ECB786,UPI00035069AB
20196-20225 HinckeM –TAXchicken	Uncategorized Sample	20196-20205_A	Mudpit_Ingel20196_MGFPeaklist (F058870)	Uncharacterized protein (Fragment) OS=Gallus gallus GN=SPTB PE=4 SV=2	F1NSS0_CHICK	TAX_GallusGallus_9031_20141114.fasta	265,530.20	100.00%	144	184	507	1.02%	54.00%	SIEDYGQTIK	R	E	99.70%	40.7	44	24.9	31	37.3	3.14	0.394	0.5	0	7	0	0	2	1,153.57	1,863.00		19,181.20	1627	1636	Empty	TRUE	UPI000240B81D,UPI00035064DB,UPI0003505F8D,UPI0002C86DF1,UPI00035042E9,UPI0000ECB786,UPI00035069AB
20196-20225 HinckeM –TAXchicken	Uncategorized Sample	20196-20205_A	Mudpit_Ingel20196_MGFPeaklist (F058870)	Uncharacterized protein (Fragment) OS=Gallus gallus GN=SPTB PE=4 SV=2	F1NSS0_CHICK	TAX_GallusGallus_9031_20141114.fasta	265,530.20	100.00%	144	184	507	1.02%	54.00%	SIEDYGQTIKELAGR	R	A	99.70%	62.1	44.5	20.3	51.9	49.7	6	0.507	0.5	0	0	2	0	2	1,679.86	2,443.00		5,509.56	1627	1641	Empty	TRUE	UPI000240B81D,UPI00035064DB,UPI0003505F8D,UPI0002C86DF1,UPI00035042E9,UPI0000ECB786,UPI00035069AB
20196-20225 HinckeM –TAXchicken	Uncategorized Sample	20196-20205_A	Mudpit_Ingel20196_MGFPeaklist (F058870)	Uncharacterized protein (Fragment) OS=Gallus gallus GN=SPTB PE=4 SV=2	F1NSS0_CHICK	TAX_GallusGallus_9031_20141114.fasta	265,530.20	100.00%	144	184	507	1.02%	54.00%	SLPPPHAPPEPR	R	R	99.70%	48.8	44.3	21.2	38.6	39.4	4.7	0.327	0.542	0	0	4	0	2	1,294.69	1,660.00		1,630.23	2299	2310	Empty	TRUE	UPI000240B81D,UPI0002C86DF1
20196-20225 HinckeM –TAXchicken	Uncategorized Sample	20196-20205_A	Mudpit_Ingel20196_MGFPeaklist (F058870)	Uncharacterized protein (Fragment) OS=Gallus gallus GN=SPTB PE=4 SV=2	F1NSS0_CHICK	TAX_GallusGallus_9031_20141114.fasta	265,530.20	100.00%	144	184	507	1.02%	54.00%	STENGTNLQTVQR	R	L	99.70%	77.5	44.6	28	42.7	47.4	4.92	0.184	0.462	0	7	3	0	2	1,448.70	1,591.00		11,066.10	1501	1513	Empty	TRUE	UPI000240B81D,UPI00035064DB,UPI0003505F8D,UPI0002C86DF1,UPI00035042E9,UPI00035069AB
20196-20225 HinckeM –TAXchicken	Uncategorized Sample	20196-20205_A	Mudpit_Ingel20196_MGFPeaklist (F058870)	Uncharacterized protein (Fragment) OS=Gallus gallus GN=SPTB PE=4 SV=2	F1NSS0_CHICK	TAX_GallusGallus_9031_20141114.fasta	265,530.20	100.00%	144	184	507	1.02%	54.00%	TELYSQSYSDLER	R	W	99.70%	71.3	43.6	22.8	56.5	46.3	5.96	0.413	0.5	0	0	2	0	2	1,590.73	2,058.00		3,743.80	1379	1391	Empty	TRUE	UPI000240B81D,UPI00035064DB,UPI0003505F8D,UPI0002C86DF1,UPI00035042E9,UPI00035069AB
20196-20225 HinckeM –TAXchicken	Uncategorized Sample	20196-20205_A	Mudpit_Ingel20196_MGFPeaklist (F058870)	Uncharacterized protein (Fragment) OS=Gallus gallus GN=SPTB PE=4 SV=2	F1NSS0_CHICK	TAX_GallusGallus_9031_20141114.fasta	265,530.20	100.00%	144	184	507	1.02%	54.00%	TLREEDVVHDAAPER	R	Q	99.70%	48.9	44.7	23.4	30.2	48	5.11	0.442	0.6	0	0	4	3	2	1,736.86	1,766.00		56,177.40	2076	2090	Empty	TRUE	UPI000240B81D,UPI00035064DB,UPI0003505F8D,UPI0002C86DF1,UPI00035042E9,UPI00035069AB
20196-20225 HinckeM –TAXchicken	Uncategorized Sample	20196-20205_A	Mudpit_Ingel20196_MGFPeaklist (F058870)	Uncharacterized protein (Fragment) OS=Gallus gallus GN=SPTB PE=4 SV=2	F1NSS0_CHICK	TAX_GallusGallus_9031_20141114.fasta	265,530.20	100.00%	144	184	507	1.02%	54.00%	TVEKPPKFQEK	R	G	99.70%	43.2	44.3	32.6	25.4	43	3.96	0.342	0.636	0	0	4	0	2	1,330.74	1,509.50		1,312.54	346	356	Empty	TRUE	UPI000240B81D,UPI00035064DB,UPI0003505F8D,UPI0002C86DF1,UPI00035042E9,UPI0000ECB786,UPI00035069AB
20196-20225 HinckeM –TAXchicken	Uncategorized Sample	20196-20205_A	Mudpit_Ingel20196_MGFPeaklist (F058870)	Uncharacterized protein (Fragment) OS=Gallus gallus GN=SPTB PE=4 SV=2	F1NSS0_CHICK	TAX_GallusGallus_9031_20141114.fasta	265,530.20	100.00%	144	184	507	1.02%	54.00%	VAQQDNFGHDLTAVEAAK	L	K	99.70%					50.3	6.89	0.421	0.361	0	0	2	0	1	1,896.91	2,274.00		9,178.10	442	459	Empty	TRUE	
20196-20225 HinckeM –TAXchicken	Uncategorized Sample	20196-20205_A	Mudpit_Ingel20196_MGFPeaklist (F058870)	Uncharacterized protein (Fragment) OS=Gallus gallus GN=SPTB PE=4 SV=2	F1NSS0_CHICK	TAX_GallusGallus_9031_20141114.fasta	265,530.20	100.00%	144	184	507	1.02%	54.00%	VAQQDNFGHDLTAVEAAKK	L	K	99.70%					45	4.36	0.427	0.342	0	0	0	3	1	2,025.00	2,164.00		6,122.75	442	460	Empty	TRUE	
20196-20225 HinckeM –TAXchicken	Uncategorized Sample	20196-20205_A	Mudpit_Ingel20196_MGFPeaklist (F058870)	Uncharacterized protein (Fragment) OS=Gallus gallus GN=SPTB PE=4 SV=2	F1NSS0_CHICK	TAX_GallusGallus_9031_20141114.fasta	265,530.20	100.00%	144	184	507	1.02%	54.00%	VQAIDAVAKELER	R	E	99.70%	50.7	43.4	18.3	35.2	32.7	2.72	0.346	0.346	0	0	3	0	2	1,441.80	2,399.00		1,565.19	476	488	Empty	TRUE	UPI000240B81D,UPI00035064DB,UPI0003505F8D,UPI0002C86DF1,UPI00035042E9,UPI00035069AB
20196-20225 HinckeM –TAXchicken	Uncategorized Sample	20196-20205_A	Mudpit_Ingel20196_MGFPeaklist (F058870)	Uncharacterized protein (Fragment) OS=Gallus gallus GN=SPTB PE=4 SV=2	F1NSS0_CHICK	TAX_GallusGallus_9031_20141114.fasta	265,530.20	100.00%	144	184	507	1.02%	54.00%	VTSNQEDPEYEQLR	R	E	99.70%	91.9	43.8	26.6	72.3	39.8	4.51	0.437	0.393	0	1	3	0	2	1,707.78	1,798.50		4,754.39	1125	1138	Empty	TRUE	UPI000240B81D,UPI00035064DB,UPI0003505F8D,UPI0002C86DF1,UPI00035042E9,UPI00035069AB
20196-20225 HinckeM –TAXchicken	Uncategorized Sample	20196-20205_A	Mudpit_Ingel20196_MGFPeaklist (F058870)	Uncharacterized protein (Fragment) OS=Gallus gallus GN=SPTB PE=4 SV=2	F1NSS0_CHICK	TAX_GallusGallus_9031_20141114.fasta	265,530.20	100.00%	144	184	507	1.02%	54.00%	VTSNQEDPEYEQLRER	R	L	99.70%	46.6	43.7	20.1	41.2	44.5	6.03	0.404	0.438	0	0	4	0	2	1,992.93	1,749.00		8,182.76	1125	1140	Empty	TRUE	UPI000240B81D,UPI00035064DB,UPI0003505F8D,UPI0002C86DF1,UPI00035042E9,UPI00035069AB
20196-20225 HinckeM –TAXchicken	Uncategorized Sample	20196-20205_A	Mudpit_Ingel20196_MGFPeaklist (F058870)	Uncharacterized protein (Fragment) OS=Gallus gallus GN=SPTB PE=4 SV=2	F1NSS0_CHICK	TAX_GallusGallus_9031_20141114.fasta	265,530.20	100.00%	144	184	507	1.02%	54.00%	WLGQVEGELHTTER	R	A	99.70%	64.8	44.3	17.1	51.2	38.6	2.44	0.399	0.393	0	0	4	0	2	1,654.82	2,225.00		5,236.70	1392	1405	Empty	TRUE	UPI000240B81D,UPI00035064DB,UPI0003505F8D,UPI0002C86DF1,UPI00035042E9,UPI0000ECB786,UPI00035069AB
20196-20225 HinckeM –TAXchicken	Uncategorized Sample	20196-20205_A	Mudpit_Ingel20196_MGFPeaklist (F058870)	Uncharacterized protein (Fragment) OS=Gallus gallus GN=SPTB PE=4 SV=2	F1NSS0_CHICK	TAX_GallusGallus_9031_20141114.fasta	265,530.20	100.00%	144	184	507	1.02%	54.00%	WVNLHLACVTCR	K	I	99.70%	48.7	44.4	27.3	38	40.6	6.14	0.466	0.5	0	2	0	0	2	1,528.75	2,376.50		1,131.42	60	71	Empty	TRUE	UPI000240B81D,UPI00035064DB,UPI0003505F8D,UPI0002C86DF1,UPI00035042E9,UPI0000ECB786,UPI00035069AB
20196-20225 HinckeM –TAXchicken	Uncategorized Sample	20196-20205_A	Mudpit_Ingel20196_MGFPeaklist (F058870)	Uncharacterized protein (Fragment) OS=Gallus gallus GN=SPTB PE=4 SV=2	F1NSS0_CHICK	TAX_GallusGallus_9031_20141114.fasta	265,530.20	100.00%	144	184	507	1.02%	54.00%	YHQGIHAEIDAR	K	D	99.70%	77	44.3	24.7	68.3	43.8	5.36	0.463	0.667	0	0	7	0	2	1,409.69	1,620.00		7,647.29	1939	1950	Empty	TRUE	UPI000240B81D,UPI00035064DB,UPI0003505F8D,UPI0002C86DF1,UPI00035042E9,UPI00035069AB
20196-20225 HinckeM –TAXchicken	Uncategorized Sample	20196-20205_A	Mudpit_Ingel20196_MGFPeaklist (F058870)	Uncharacterized protein (Fragment) OS=Gallus gallus GN=SPTB PE=4 SV=2	F1NSS0_CHICK	TAX_GallusGallus_9031_20141114.fasta	265,530.20	100.00%	144	184	507	1.02%	54.00%	YYAGLKEAAEER	K	R	99.70%	44	44.4	27.8	31.9	32.7	4.05	0.391	0.417	0	0	4	0	2	1,399.69	1,769.00		1,660.34	1666	1677	Empty	TRUE	UPI000240B81D,UPI00035064DB,UPI0003505F8D,UPI0002C86DF1,UPI00035042E9,UPI0000ECB786,UPI00035069AB
20196-20225 HinckeM –TAXchicken	Uncategorized Sample	20196-20205_A	Mudpit_Ingel20196_MGFPeaklist (F058870)	status=inactive	UPI0000ECB786	UPARC14_2_GallusG_9031_20140417.fasta	244,653.90	99.90%	3	4	7	0.01%	42.00%	AASEELGHDEVHTQALLR	R	E	99.70%	60.4	44.5	13.9	57	43.1	5.92	0.439	0.472	0	0	0	4	2	1,975.98	2,003.50		5,895.69	767	784	Empty	FALSE	UPI000240B81D,UPI00035064DB,UPI0003505F8D,UPI0002C86DF1,UPI00035042E9,UPI00035069AB,F1NSS0_CHICK
20196-20225 HinckeM –TAXchicken	Uncategorized Sample	20196-20205_A	Mudpit_Ingel20196_MGFPeaklist (F058870)	status=inactive	UPI0000ECB786	UPARC14_2_GallusG_9031_20140417.fasta	244,653.90	99.90%	3	4	7	0.01%	42.00%	AEGMELASCKPQYGAVVGQR	K	L	99.70%	60.2	43.8	16.3	51	48.9	7.28	0.554	0.4	0	0	4	0	2	2,167.03	1,977.00		4,524.34	1189	1208	Empty	FALSE	UPI000240B81D,UPI00035064DB,UPI0003505F8D,UPI0002C86DF1,UPI00035042E9,UPI00035069AB,F1NSS0_CHICK
20196-20225 HinckeM –TAXchicken	Uncategorized Sample	20196-20205_A	Mudpit_Ingel20196_MGFPeaklist (F058870)	status=inactive	UPI0000ECB786	UPARC14_2_GallusG_9031_20140417.fasta	244,653.90	99.90%	3	4	7	0.01%	42.00%	AKDLTSANLLLK	R	K	99.70%	62.5	42.2	28.3	43	43.3	3.46	0.312	0.542	0	1	1	0	2	1,286.77	2,200.50		4,092.46	1263	1274	Empty	FALSE	UPI000240B81D,UPI00035064DB,UPI0003505F8D,UPI0002C86DF1,UPI00035042E9,UPI00035069AB,F1NSS0_CHICK
20196-20225 HinckeM –TAXchicken	Uncategorized Sample	20196-20205_A	Mudpit_Ingel20196_MGFPeaklist (F058870)	status=inactive	UPI0000ECB786	UPARC14_2_GallusG_9031_20140417.fasta	244,653.90	99.90%	3	4	7	0.01%	42.00%	AKDLTSANLLLKK	R	L	99.70%	62.3	40	36.6	43.3	43.9	4.21	0.378	0.577	0	0	2	0	2	1,414.86	2,019.50		2,700.79	1263	1275	Empty	FALSE	UPI000240B81D,UPI00035064DB,UPI0003505F8D,UPI0002C86DF1,UPI00035042E9,UPI00035069AB,F1NSS0_CHICK
20196-20225 HinckeM –TAXchicken	Uncategorized Sample	20196-20205_A	Mudpit_Ingel20196_MGFPeaklist (F058870)	status=inactive	UPI0000ECB786	UPARC14_2_GallusG_9031_20140417.fasta	244,653.90	99.90%	3	4	7	0.01%	42.00%	ALGVPCQGEEPLGLR	R	N	99.70%	67.8	44.4	23.1	58.8	45.6	5.06	0.401	0.433	0	2	0	0	2	1,595.82	2,314.00		3,245.87	2101	2115	Empty	FALSE	UPI000240B81D,UPI00035064DB,UPI0003505F8D,UPI0002C86DF1,UPI00035042E9,UPI00035069AB,F1NSS0_CHICK
20196-20225 HinckeM –TAXchicken	Uncategorized Sample	20196-20205_A	Mudpit_Ingel20196_MGFPeaklist (F058870)	status=inactive	UPI0000ECB786	UPARC14_2_GallusG_9031_20140417.fasta	244,653.90	99.90%	3	4	7	0.01%	42.00%	ALQALLEACSGR	R	R	99.70%	66.6	45	29.2	49.4	39.1	4.55	0.46	0.542	0	3	7	0	2	1,288.67	2,215.00		12,763.70	1735	1746	Empty	FALSE	UPI000240B81D,UPI00035064DB,UPI0003505F8D,UPI0002C86DF1,UPI00035042E9,UPI00035069AB,F1NSS0_CHICK
20196-20225 HinckeM –TAXchicken	Uncategorized Sample	20196-20205_A	Mudpit_Ingel20196_MGFPeaklist (F058870)	status=inactive	UPI0000ECB786	UPARC14_2_GallusG_9031_20140417.fasta	244,653.90	99.90%	3	4	7	0.01%	42.00%	ALQALLEACSGRR	R	A	99.70%	26	44.5	20.7	15.7	32.4	1.03	0.0833	0.462	0	0	4	0	2	1,444.77	2,199.50		3,335.56	1735	1747	Empty	FALSE	UPI000240B81D,UPI00035064DB,UPI0003505F8D,UPI0002C86DF1,UPI00035042E9,UPI00035069AB,F1NSS0_CHICK
20196-20225 HinckeM –TAXchicken	Uncategorized Sample	20196-20205_A	Mudpit_Ingel20196_MGFPeaklist (F058870)	status=inactive	UPI0000ECB786	UPARC14_2_GallusG_9031_20140417.fasta	244,653.90	99.90%	3	4	7	0.01%	42.00%	ALQFLKEK	K	Q	92.40%	42.8	43.7	46.9	13.7					0	1	0	0	2	976.5827	2,011.00		169.17	118	125	Empty	FALSE	UPI000240B81D,UPI00035064DB,UPI0003505F8D,UPI0002C86DF1,UPI00035042E9,UPI00035069AB,F1NSS0_CHICK
20196-20225 HinckeM –TAXchicken	Uncategorized Sample	20196-20205_A	Mudpit_Ingel20196_MGFPeaklist (F058870)	status=inactive	UPI0000ECB786	UPARC14_2_GallusG_9031_20140417.fasta	244,653.90	99.90%	3	4	7	0.01%	42.00%	ALVMAEQLAAAGHEPGR	K	L	99.70%	79.5	44.6	19.3	71.3					0	0	4	0	2	1,736.88	2,128.00		12,895.70	698	714	Empty	TRUE	
20196-20225 HinckeM –TAXchicken	Uncategorized Sample	20196-20205_A	Mudpit_Ingel20196_MGFPeaklist (F058870)	status=inactive	UPI0000ECB786	UPARC14_2_GallusG_9031_20140417.fasta	244,653.90	99.90%	3	4	7	0.01%	42.00%	ALVMAEQLAAAGHEPGRLR	K	D	58.50%	15.6	43.5	16.6	13.5					0	0	0	2	2	2,006.06	2,076.00		900.84	698	716	Empty	TRUE	
20196-20225 HinckeM –TAXchicken	Uncategorized Sample	20196-20205_A	Mudpit_Ingel20196_MGFPeaklist (F058870)	status=inactive	UPI0000ECB786	UPARC14_2_GallusG_9031_20140417.fasta	244,653.90	99.90%	3	4	7	0.01%	42.00%	AMYQLGR	K	D	98.80%	68.8	42.9	38.8	39.4	26.7	2.3	0.27	0.429	0	2	0	0	2	838.424	1,840.00		1,232.00	1334	1340	Empty	FALSE	UPI000240B81D,UPI00035064DB,UPI0003505F8D,UPI0002C86DF1,UPI00035042E9,UPI00035069AB,F1NSS0_CHICK
20196-20225 HinckeM –TAXchicken	Uncategorized Sample	20196-20205_A	Mudpit_Ingel20196_MGFPeaklist (F058870)	status=inactive	UPI0000ECB786	UPARC14_2_GallusG_9031_20140417.fasta	244,653.90	99.90%	3	4	7	0.01%	42.00%	APLAGVAPEPDAQEEK	K	L	99.70%	64.5	44.6	16.2	54.7	56	7.41	0.564	0.5	0	3	0	0	2	1,621.81	1,868.00		7,127.31	1295	1310	Empty	FALSE	UPI000240B81D,UPI00035064DB,UPI0003505F8D,UPI0002C86DF1,UPI00035042E9,UPI00035069AB,F1NSS0_CHICK
20196-20225 HinckeM –TAXchicken	Uncategorized Sample	20196-20205_A	Mudpit_Ingel20196_MGFPeaklist (F058870)	status=inactive	UPI0000ECB786	UPARC14_2_GallusG_9031_20140417.fasta	244,653.90	99.90%	3	4	7	0.01%	42.00%	APLAGVAPEPDAQEEKLQQR	K	F	99.70%	102	43.8	15.5	96.9	67	9.12	0.59	0.525	0	0	2	0	2	2,147.11	1,966.50		15,907.70	1295	1314	Empty	FALSE	UPI000240B81D,UPI00035064DB,UPI0003505F8D,UPI0002C86DF1,UPI00035042E9,UPI00035069AB,F1NSS0_CHICK
20196-20225 HinckeM –TAXchicken	Uncategorized Sample	20196-20205_A	Mudpit_Ingel20196_MGFPeaklist (F058870)	status=inactive	UPI0000ECB786	UPARC14_2_GallusG_9031_20140417.fasta	244,653.90	99.90%	3	4	7	0.01%	42.00%	AQEAAGLLQDNHR	K	L	99.70%	63.9	44.6	24.3	50.9	45.4	3.8	0.396	0.577	0	0	8	0	2	1,422.71	1,766.50		23,805.00	1118	1130	Empty	FALSE	UPI000240B81D,UPI00035064DB,UPI0003505F8D,UPI0002C86DF1,UPI00035042E9,UPI00035069AB,F1NSS0_CHICK
20196-20225 HinckeM –TAXchicken	Uncategorized Sample	20196-20205_A	Mudpit_Ingel20196_MGFPeaklist (F058870)	status=inactive	UPI0000ECB786	UPARC14_2_GallusG_9031_20140417.fasta	244,653.90	99.90%	3	4	7	0.01%	42.00%	AQLVDTADKFR	R	F	99.70%	67.6	44.6	23.8	58.4	48.7	4.4	0.405	0.591	0	3	0	0	2	1,263.67	1,922.00		3,577.37	1748	1758	Empty	FALSE	UPI000240B81D,UPI00035064DB,UPI0003505F8D,UPI0002C86DF1,UPI00035042E9,UPI00035069AB,F1NSS0_CHICK
20196-20225 HinckeM –TAXchicken	Uncategorized Sample	20196-20205_A	Mudpit_Ingel20196_MGFPeaklist (F058870)	status=inactive	UPI0000ECB786	UPARC14_2_GallusG_9031_20140417.fasta	244,653.90	99.90%	3	4	7	0.01%	42.00%	AQQLLSAGHPEGEQIIR	R	L	99.70%	68.8	44	19.3	57.9	44.4	5.54	0.502	0.412	0	0	5	0	2	1,846.98	2,096.00		12,767.90	1498	1514	Empty	FALSE	UPI000240B81D,UPI00035064DB,UPI0003505F8D,UPI0002C86DF1,UPI00035042E9,UPI00035069AB,F1NSS0_CHICK
20196-20225 HinckeM –TAXchicken	Uncategorized Sample	20196-20205_A	Mudpit_Ingel20196_MGFPeaklist (F058870)	status=inactive	UPI0000ECB786	UPARC14_2_GallusG_9031_20140417.fasta	244,653.90	99.90%	3	4	7	0.01%	42.00%	ASMESSAEKVPSVVAAGSK	R	L	99.70%	66.8	44.6	19.4	58	44.9	6.48	0.448	0.342	0	0	5	0	2	1,850.92	1,787.00		8,353.31	1070	1088	Empty	FALSE	UPI000240B81D,UPI00035064DB,UPI0003505F8D,UPI0002C86DF1,UPI00035042E9,UPI00035069AB,F1NSS0_CHICK
20196-20225 HinckeM –TAXchicken	Uncategorized Sample	20196-20205_A	Mudpit_Ingel20196_MGFPeaklist (F058870)	status=inactive	UPI0000ECB786	UPARC14_2_GallusG_9031_20140417.fasta	244,653.90	99.90%	3	4	7	0.01%	42.00%	AVSIQQQEQEVAR	K	A	99.70%	69.4	44.4	20.3	51.3	48	3.7	0.394	0.5	0	4	0	0	2	1,485.77	1,816.50		19,328.20	1722	1734	Empty	FALSE	UPI000240B81D,UPI00035064DB,UPI0003505F8D,UPI0002C86DF1,UPI00035042E9,UPI00035069AB,F1NSS0_CHICK
20196-20225 HinckeM –TAXchicken	Uncategorized Sample	20196-20205_A	Mudpit_Ingel20196_MGFPeaklist (F058870)	status=inactive	UPI0000ECB786	UPARC14_2_GallusG_9031_20140417.fasta	244,653.90	99.90%	3	4	7	0.01%	42.00%	DGLAFNALIHK	K	H	99.70%	39.1	43.7	19.6	34.3	26.1	1.29	0.264	0.273	0	0	5	0	2	1,198.66	2,449.00		4,115.12	203	213	Empty	FALSE	UPI000240B81D,UPI00035064DB,UPI0003505F8D,UPI0002C86DF1,UPI00035042E9,UPI00035069AB,F1NSS0_CHICK
20196-20225 HinckeM –TAXchicken	Uncategorized Sample	20196-20205_A	Mudpit_Ingel20196_MGFPeaklist (F058870)	status=inactive	UPI0000ECB786	UPARC14_2_GallusG_9031_20140417.fasta	244,653.90	99.90%	3	4	7	0.01%	42.00%	DKSFDACIELGK	R	K	85.00%	21.7	44.1	15.4	16.9					0	0	1	0	2	1,382.66	2,112.00		461.48	1807	1818	Empty	FALSE	UPI000240B81D,UPI00035064DB,UPI0003505F8D,UPI0002C86DF1,UPI00035042E9,UPI00035069AB,F1NSS0_CHICK
20196-20225 HinckeM –TAXchicken	Uncategorized Sample	20196-20205_A	Mudpit_Ingel20196_MGFPeaklist (F058870)	status=inactive	UPI0000ECB786	UPARC14_2_GallusG_9031_20140417.fasta	244,653.90	99.90%	3	4	7	0.01%	42.00%	DKSFDACIELGKK	R	L	99.70%	57.2	44.5	22.3	42.8	36.7	3.68	0.387	0.462	0	0	2	0	2	1,510.76	1,973.00		2,453.69	1807	1819	Empty	FALSE	UPI000240B81D,UPI00035064DB,UPI0003505F8D,UPI0002C86DF1,UPI00035042E9,UPI00035069AB,F1NSS0_CHICK
20196-20225 HinckeM –TAXchicken	Uncategorized Sample	20196-20205_A	Mudpit_Ingel20196_MGFPeaklist (F058870)	status=inactive	UPI0000ECB786	UPARC14_2_GallusG_9031_20140417.fasta	244,653.90	99.90%	3	4	7	0.01%	42.00%	DLAGVLLLR	K	R	99.70%	64.1	40.9	36	32	39.8	1.8	0.296	0.667	0	4	0	0	2	969.6093	2,792.00		3,089.15	670	678	Empty	FALSE	UPI000240B81D,UPI00035064DB,UPI0003505F8D,UPI0002C86DF1,UPI00035042E9,UPI00035069AB,F1NSS0_CHICK
20196-20225 HinckeM –TAXchicken	Uncategorized Sample	20196-20205_A	Mudpit_Ingel20196_MGFPeaklist (F058870)	status=inactive	UPI0000ECB786	UPARC14_2_GallusG_9031_20140417.fasta	244,653.90	99.90%	3	4	7	0.01%	42.00%	DLEDEMLWVQER	R	M	99.70%	73.4	43.5	21.9	60.3	35.8	3.4	0.36	0.417	0	2	0	0	2	1,578.71	2,364.00		1,944.37	1341	1352	Empty	FALSE	UPI000240B81D,UPI00035064DB,UPI0003505F8D,UPI0002C86DF1,UPI00035042E9,UPI00035069AB,F1NSS0_CHICK
20196-20225 HinckeM –TAXchicken	Uncategorized Sample	20196-20205_A	Mudpit_Ingel20196_MGFPeaklist (F058870)	status=inactive	UPI0000ECB786	UPARC14_2_GallusG_9031_20140417.fasta	244,653.90	99.90%	3	4	7	0.01%	42.00%	DLLSWMESTVR	R	Q	99.70%	71.7	44.2	23.6	54	39.9	2.77	0.393	0.5	0	4	0	0	2	1,352.65	2,482.00		2,787.55	1765	1775	Empty	FALSE	UPI000240B81D,UPI00035064DB,UPI0003505F8D,UPI0002C86DF1,UPI00035042E9,UPI00035069AB,F1NSS0_CHICK
20196-20225 HinckeM –TAXchicken	Uncategorized Sample	20196-20205_A	Mudpit_Ingel20196_MGFPeaklist (F058870)	status=inactive	UPI0000ECB786	UPARC14_2_GallusG_9031_20140417.fasta	244,653.90	99.90%	3	4	7	0.01%	42.00%	DLQLLEGQVQR	R	F	99.70%	66	43.8	31.8	40.3	40.8	1.72	0.336	0.545	0	4	0	0	2	1,298.71	2,290.00		21,976.70	1695	1705	Empty	FALSE	UPI000240B81D,UPI00035064DB,UPI0003505F8D,UPI0002C86DF1,UPI00035042E9,UPI00035069AB,F1NSS0_CHICK
20196-20225 HinckeM –TAXchicken	Uncategorized Sample	20196-20205_A	Mudpit_Ingel20196_MGFPeaklist (F058870)	status=inactive	UPI0000ECB786	UPARC14_2_GallusG_9031_20140417.fasta	244,653.90	99.90%	3	4	7	0.01%	42.00%	DLTSANLLLK	K	K	99.70%	44.6	44.3	22.1	0	31.5	0.432	0.149	0.45	0	2	0	0	2	1,087.64	2,436.00		8,573.91	1265	1274	Empty	FALSE	UPI000240B81D,UPI00035064DB,UPI0003505F8D,UPI0002C86DF1,UPI00035042E9,UPI00035069AB,F1NSS0_CHICK
20196-20225 HinckeM –TAXchicken	Uncategorized Sample	20196-20205_A	Mudpit_Ingel20196_MGFPeaklist (F058870)	status=inactive	UPI0000ECB786	UPARC14_2_GallusG_9031_20140417.fasta	244,653.90	99.90%	3	4	7	0.01%	42.00%	DLTSANLLLKK	K	L	99.70%	39.4	42.7	25.6	0	30.9	1.64	0.269	0.409	0	0	3	0	2	1,215.73	2,234.00		2,722.44	1265	1275	Empty	FALSE	UPI000240B81D,UPI00035064DB,UPI0003505F8D,UPI0002C86DF1,UPI00035042E9,UPI00035069AB,F1NSS0_CHICK
20196-20225 HinckeM –TAXchicken	Uncategorized Sample	20196-20205_A	Mudpit_Ingel20196_MGFPeaklist (F058870)	status=inactive	UPI0000ECB786	UPARC14_2_GallusG_9031_20140417.fasta	244,653.90	99.90%	3	4	7	0.01%	42.00%	DVAASSQELGQDLDHVTILR	R	D	99.70%	95	44	18.9	85.3	57.9	9.74	0.537	0.4	0	0	2	0	2	2,167.10	2,629.50		4,769.00	1561	1580	Empty	FALSE	UPI000240B81D,UPI00035064DB,UPI0003505F8D,UPI0002C86DF1,UPI00035042E9,UPI00035069AB,F1NSS0_CHICK
20196-20225 HinckeM –TAXchicken	Uncategorized Sample	20196-20205_A	Mudpit_Ingel20196_MGFPeaklist (F058870)	status=inactive	UPI0000ECB786	UPARC14_2_GallusG_9031_20140417.fasta	244,653.90	99.90%	3	4	7	0.01%	42.00%	DVSSVELLMK	R	Y	99.70%	48.9	44.3	26.9	35.1	33.1	1.89	0.341	0.5	0	7	0	0	2	1,136.59	2,128.00		9,614.66	1785	1794	Empty	FALSE	UPI000240B81D,UPI00035064DB,UPI0003505F8D,UPI0002C86DF1,UPI00035042E9,UPI00035069AB,F1NSS0_CHICK
20196-20225 HinckeM –TAXchicken	Uncategorized Sample	20196-20205_A	Mudpit_Ingel20196_MGFPeaklist (F058870)	status=inactive	UPI0000ECB786	UPARC14_2_GallusG_9031_20140417.fasta	244,653.90	99.90%	3	4	7	0.01%	42.00%	EALLWGQMK	R	T	99.70%	39.2	44.7	22.5	31.3	33.3	2.02	0.309	0.5	0	5	0	0	2	1,291.65	2,532.00		3,399.51	176	185	Empty	FALSE	UPI000240B81D,UPI00035064DB,UPI0003505F8D,UPI0002C86DF1,UPI00035042E9,UPI00035069AB,F1NSS0_CHICK
20196-20225 HinckeM –TAXchicken	Uncategorized Sample	20196-20205_A	Mudpit_Ingel20196_MGFPeaklist (F058870)	status=inactive	UPI0000ECB786	UPARC14_2_GallusG_9031_20140417.fasta	244,653.90	99.90%	3	4	7	0.01%	42.00%	EASEAQQYYLDADEAK	R	A	99.70%	81.4	41.8	19.4	74	46.3	4.92	0.482	0.406	0	2	0	0	2	1,830.80	1,979.00		8,734.17	1433	1448	Empty	FALSE	UPI000240B81D,UPI00035064DB,UPI0003505F8D,UPI0002C86DF1,UPI00035042E9,UPI00035069AB,F1NSS0_CHICK
20196-20225 HinckeM –TAXchicken	Uncategorized Sample	20196-20205_A	Mudpit_Ingel20196_MGFPeaklist (F058870)	status=inactive	UPI0000ECB786	UPARC14_2_GallusG_9031_20140417.fasta	244,653.90	99.90%	3	4	7	0.01%	42.00%	EHQELLEELAAAQQLLER	R	L	99.70%	94.9	43.8	16.6	85.4	58.2	10.2	0.579	0.472	0	0	1	0	2	2,120.10	2,879.00		904.16	785	802	Empty	FALSE	UPI000240B81D,UPI00035064DB,UPI0003505F8D,UPI0002C86DF1,UPI00035042E9,UPI00035069AB,F1NSS0_CHICK
20196-20225 HinckeM –TAXchicken	Uncategorized Sample	20196-20205_A	Mudpit_Ingel20196_MGFPeaklist (F058870)	status=inactive	UPI0000ECB786	UPARC14_2_GallusG_9031_20140417.fasta	244,653.90	99.90%	3	4	7	0.01%	42.00%	ELAAWVDEK	R	M	99.70%	38.2	45.4	37	13.9	34.5	0.921	0.287	0.556	0	4	0	0	2	1,060.53	2,133.00		11,054.30	1140	1148	Empty	FALSE	UPI000240B81D,UPI00035064DB,UPI0003505F8D,UPI0002C86DF1,UPI00035042E9,UPI00035069AB,F1NSS0_CHICK
20196-20225 HinckeM –TAXchicken	Uncategorized Sample	20196-20205_A	Mudpit_Ingel20196_MGFPeaklist (F058870)	status=inactive	UPI0000ECB786	UPARC14_2_GallusG_9031_20140417.fasta	244,653.90	99.90%	3	4	7	0.01%	42.00%	ELQALAAR	R	R	31.10%	35.7	43.8	41.8	4.72					0	2	0	0	2	871.4996	1,808.50		332.16	623	630	Empty	FALSE	UPI000240B81D,UPI00035064DB,UPI0003505F8D,UPI0002C86DF1,UPI00035042E9,UPI00035069AB,F1NSS0_CHICK
20196-20225 HinckeM –TAXchicken	Uncategorized Sample	20196-20205_A	Mudpit_Ingel20196_MGFPeaklist (F058870)	status=inactive	UPI0000ECB786	UPARC14_2_GallusG_9031_20140417.fasta	244,653.90	99.90%	3	4	7	0.01%	42.00%	ELQAQWEALREEVVAR	R	Q	99.70%	36	43.8	19.1	16.5	32.3	1.48	0.291	0.312	0	0	2	0	2	1,927.00	2,571.50		964.07	1412	1427	Empty	FALSE	UPI000240B81D,UPI00035064DB,UPI0003505F8D,UPI0002C86DF1,UPI00035042E9,UPI00035069AB,F1NSS0_CHICK
20196-20225 HinckeM –TAXchicken	Uncategorized Sample	20196-20205_A	Mudpit_Ingel20196_MGFPeaklist (F058870)	status=inactive	UPI0000ECB786	UPARC14_2_GallusG_9031_20140417.fasta	244,653.90	99.90%	3	4	7	0.01%	42.00%	EQEQIYSALDYGK	K	D	99.60%	24.5	43.9	24.3	13.6	25.7	0.569	0.249	0.269	0	2	0	0	2	1,543.73	2,202.00		680.03	657	669	Empty	FALSE	UPI000240B81D,UPI00035064DB,UPI0003505F8D,UPI0002C86DF1,UPI00035042E9,UPI00035069AB,F1NSS0_CHICK
20196-20225 HinckeM –TAXchicken	Uncategorized Sample	20196-20205_A	Mudpit_Ingel20196_MGFPeaklist (F058870)	status=inactive	UPI0000ECB786	UPARC14_2_GallusG_9031_20140417.fasta	244,653.90	99.90%	3	4	7	0.01%	42.00%	EVEDLEQWIAER	R	D	99.70%	70.2	44.6	29.8	19.5	44.9	4	0.347	0.5	0	2	0	0	2	1,516.73	2,693.50		2,151.14	1549	1560	Empty	FALSE	UPI000240B81D,UPI00035064DB,UPI0003505F8D,UPI0002C86DF1,UPI00035042E9,UPI00035069AB,F1NSS0_CHICK
20196-20225 HinckeM –TAXchicken	Uncategorized Sample	20196-20205_A	Mudpit_Ingel20196_MGFPeaklist (F058870)	status=inactive	UPI0000ECB786	UPARC14_2_GallusG_9031_20140417.fasta	244,653.90	99.90%	3	4	7	0.01%	42.00%	FLDLLEPLR	R	K	99.70%	57.5	44.2	32.7	21.6	42.8	2.8	0.332	0.667	0	2	0	0	2	1,115.65	2,874.00		33,097.70	1315	1323	Empty	FALSE	UPI000240B81D,UPI00035064DB,UPI0003505F8D,UPI0002C86DF1,UPI00035042E9,UPI00035069AB,F1NSS0_CHICK
20196-20225 HinckeM –TAXchicken	Uncategorized Sample	20196-20205_A	Mudpit_Ingel20196_MGFPeaklist (F058870)	status=inactive	UPI0000ECB786	UPARC14_2_GallusG_9031_20140417.fasta	244,653.90	99.90%	3	4	7	0.01%	42.00%	FLDLLEPLRK	R	K	99.70%	44.4	42.8	34.5	24.8	37.1	1.28	0.197	0.65	0	0	4	0	2	1,243.74	2,756.50		7,044.27	1315	1324	Empty	FALSE	UPI000240B81D,UPI00035064DB,UPI0003505F8D,UPI0002C86DF1,UPI00035042E9,UPI00035069AB,F1NSS0_CHICK
20196-20225 HinckeM –TAXchicken	Uncategorized Sample	20196-20205_A	Mudpit_Ingel20196_MGFPeaklist (F058870)	status=inactive	UPI0000ECB786	UPARC14_2_GallusG_9031_20140417.fasta	244,653.90	99.90%	3	4	7	0.01%	42.00%	FLDLLEPLRKK	R	R	99.70%	22.6	40.5	26.4	7.78	26.7	0.658	0.225	0.364	0	0	2	0	2	1,371.84	2,628.00		862.6	1315	1325	Empty	FALSE	UPI000240B81D,UPI00035064DB,UPI0003505F8D,UPI0002C86DF1,UPI00035042E9,UPI00035069AB,F1NSS0_CHICK
20196-20225 HinckeM –TAXchicken	Uncategorized Sample	20196-20205_A	Mudpit_Ingel20196_MGFPeaklist (F058870)	status=inactive	UPI0000ECB786	UPARC14_2_GallusG_9031_20140417.fasta	244,653.90	99.90%	3	4	7	0.01%	42.00%	FQVQGVIEDMEEDTETR	R	S	99.70%	52	41.9	29.1	41	32.9	2.96	0.353	0.294	0	0	2	0	2	2,041.90	2,221.50		1,385.11	156	172	Empty	FALSE	UPI000240B81D,UPI00035064DB,UPI0003505F8D,UPI0002C86DF1,UPI00035042E9,UPI00035069AB,F1NSS0_CHICK
20196-20225 HinckeM –TAXchicken	Uncategorized Sample	20196-20205_A	Mudpit_Ingel20196_MGFPeaklist (F058870)	status=inactive	UPI0000ECB786	UPARC14_2_GallusG_9031_20140417.fasta	244,653.90	99.90%	3	4	7	0.01%	42.00%	FQVQGVIEDMEEDTETRSPR	R	E	98.70%	29.6	42.9	16.4	28.2					0	0	2	0	2	2,382.09	2,177.00		1,892.35	156	175	Empty	FALSE	UPI000240B81D,UPI00035064DB,UPI0003505F8D,UPI0002C86DF1,UPI00035042E9,UPI00035069AB,F1NSS0_CHICK
20196-20225 HinckeM –TAXchicken	Uncategorized Sample	20196-20205_A	Mudpit_Ingel20196_MGFPeaklist (F058870)	status=inactive	UPI0000ECB786	UPARC14_2_GallusG_9031_20140417.fasta	244,653.90	99.90%	3	4	7	0.01%	42.00%	GEAAAGGPCPELAER	R	A	99.70%	77.8	43.3	27.7	60.2	54.3	7.96	0.595	0.5	0	4	0	0	2	1,484.68	1,756.50		12,341.20	1395	1409	Empty	FALSE	UPI000240B81D,UPI00035064DB,UPI0003505F8D,UPI0002C86DF1,UPI00035042E9,UPI00035069AB,F1NSS0_CHICK
20196-20225 HinckeM –TAXchicken	Uncategorized Sample	20196-20205_A	Mudpit_Ingel20196_MGFPeaklist (F058870)	status=inactive	UPI0000ECB786	UPARC14_2_GallusG_9031_20140417.fasta	244,653.90	99.90%	3	4	7	0.01%	42.00%	GNLEVLLFTIQSR	K	M	99.70%	74.3	43.1	22.9	58.9	44.8	5.46	0.402	0.5	0	2	0	0	2	1,489.84	2,880.50		4,055.85	359	371	Empty	FALSE	UPI000240B81D,UPI00035064DB,UPI0003505F8D,UPI0002C86DF1,UPI00035042E9,UPI00035069AB,F1NSS0_CHICK
20196-20225 HinckeM –TAXchicken	Uncategorized Sample	20196-20205_A	Mudpit_Ingel20196_MGFPeaklist (F058870)	status=inactive	UPI0000ECB786	UPARC14_2_GallusG_9031_20140417.fasta	244,653.90	99.90%	3	4	7	0.01%	42.00%	GQQLFEANR	K	T	99.70%	44.2	45.3	25.3	20.5	27.3	1.55	0.245	0.389	0	4	0	0	2	1,062.53	1,833.50		1,428.80	1227	1235	Empty	FALSE	UPI000240B81D,UPI00035064DB,UPI0003505F8D,UPI0002C86DF1,UPI00035042E9,UPI00035069AB,F1NSS0_CHICK
20196-20225 HinckeM –TAXchicken	Uncategorized Sample	20196-20205_A	Mudpit_Ingel20196_MGFPeaklist (F058870)	status=inactive	UPI0000ECB786	UPARC14_2_GallusG_9031_20140417.fasta	244,653.90	99.90%	3	4	7	0.01%	42.00%	GYPHVNVTNFTSSWK	K	D	99.70%	40.8	44.5	21.8	33.8	32.1	3.17	0.321	0.333	0	0	2	0	2	1,736.84	2,446.00		1,094.65	188	202	Empty	FALSE	UPI000240B81D,UPI00035064DB,UPI0003505F8D,UPI0002C86DF1,UPI00035042E9,UPI00035069AB,F1NSS0_CHICK
20196-20225 HinckeM –TAXchicken	Uncategorized Sample	20196-20205_A	Mudpit_Ingel20196_MGFPeaklist (F058870)	status=inactive	UPI0000ECB786	UPARC14_2_GallusG_9031_20140417.fasta	244,653.90	99.90%	3	4	7	0.01%	42.00%	HLGITPLLDPEDVFTENPDEK	R	S	99.70%	54	44.1	15.5	50.8	55.3	9.24	0.552	0.429	0	0	2	0	2	2,379.17	2,862.00		6,812.03	243	263	Empty	FALSE	UPI000240B81D,UPI00035064DB,UPI0003505F8D,UPI0002C86DF1,UPI00035042E9,UPI00035069AB,F1NSS0_CHICK
20196-20225 HinckeM –TAXchicken	Uncategorized Sample	20196-20205_A	Mudpit_Ingel20196_MGFPeaklist (F058870)	status=inactive	UPI0000ECB786	UPARC14_2_GallusG_9031_20140417.fasta	244,653.90	99.90%	3	4	7	0.01%	42.00%	HLLEVEELLETHR	K	L	99.70%	66.5	44.3	22	53	43.6	5.55	0.489	0.5	0	0	4	3	2	1,617.86	2,601.00		4,605.51	561	573	Empty	FALSE	UPI000240B81D,UPI00035064DB,UPI0003505F8D,UPI0002C86DF1,UPI00035042E9,UPI00035069AB,F1NSS0_CHICK
20196-20225 HinckeM –TAXchicken	Uncategorized Sample	20196-20205_A	Mudpit_Ingel20196_MGFPeaklist (F058870)	status=inactive	UPI0000ECB786	UPARC14_2_GallusG_9031_20140417.fasta	244,653.90	99.90%	3	4	7	0.01%	42.00%	HNLEHAFSMAER	R	H	99.70%	46.3	42.9	24.1	38.5	34.7	4.51	0.435	0.5	0	0	6	0	2	1,457.66	1,675.50		4,419.23	231	242	Empty	FALSE	UPI000240B81D,UPI00035064DB,UPI0003505F8D,UPI0002C86DF1,UPI00035042E9,UPI00035069AB,F1NSS0_CHICK
20196-20225 HinckeM –TAXchicken	Uncategorized Sample	20196-20205_A	Mudpit_Ingel20196_MGFPeaklist (F058870)	status=inactive	UPI0000ECB786	UPARC14_2_GallusG_9031_20140417.fasta	244,653.90	99.90%	3	4	7	0.01%	42.00%	HQELPQDLGDDSGSVEAFHR	R	M	99.70%	58.1	43.1	15.5	54.1	45.5	6.96	0.47	0.35	0	0	1	4	2	2,237.02	2,080.00		27,532.20	1668	1687	Empty	FALSE	UPI000240B81D,UPI00035064DB,UPI0003505F8D,UPI0002C86DF1,UPI00035042E9,UPI00035069AB,F1NSS0_CHICK
20196-20225 HinckeM –TAXchicken	Uncategorized Sample	20196-20205_A	Mudpit_Ingel20196_MGFPeaklist (F058870)	status=inactive	UPI0000ECB786	UPARC14_2_GallusG_9031_20140417.fasta	244,653.90	99.90%	3	4	7	0.01%	42.00%	HRPELFDFK	K	T	99.70%	25	45	27.3	5.91	32.3	3.51	0.353	0.5	0	2	0	0	2	1,188.62	2,206.00		1,405.56	214	222	Empty	FALSE	UPI000240B81D,UPI00035064DB,UPI0003505F8D,UPI0002C86DF1,UPI00035042E9,UPI00035069AB,F1NSS0_CHICK
20196-20225 HinckeM –TAXchicken	Uncategorized Sample	20196-20205_A	Mudpit_Ingel20196_MGFPeaklist (F058870)	status=inactive	UPI0000ECB786	UPARC14_2_GallusG_9031_20140417.fasta	244,653.90	99.90%	3	4	7	0.01%	42.00%	HRPELFDFKTLTK	K	S	99.70%	24.2	43.7	15.8	19.9	27.7	2.77	0.264	0.385	0	0	0	2	2	1,631.89	2,335.00		1,220.78	214	226	Empty	FALSE	UPI000240B81D,UPI00035064DB,UPI0003505F8D,UPI0002C86DF1,UPI00035042E9,UPI00035069AB,F1NSS0_CHICK
20196-20225 HinckeM –TAXchicken	Uncategorized Sample	20196-20205_A	Mudpit_Ingel20196_MGFPeaklist (F058870)	status=inactive	UPI0000ECB786	UPARC14_2_GallusG_9031_20140417.fasta	244,653.90	99.90%	3	4	7	0.01%	42.00%	HTALEAELEAR	R	G	99.70%	63.5	44	41.7	39.1	40	3.74	0.38	0.545	0	2	2	0	2	1,239.63	1,964.00		7,979.34	681	691	Empty	FALSE	UPI000240B81D,UPI00035064DB,UPI0003505F8D,UPI0002C86DF1,UPI00035042E9,UPI00035069AB,F1NSS0_CHICK
20196-20225 HinckeM –TAXchicken	Uncategorized Sample	20196-20205_A	Mudpit_Ingel20196_MGFPeaklist (F058870)	status=inactive	UPI0000ECB786	UPARC14_2_GallusG_9031_20140417.fasta	244,653.90	99.90%	3	4	7	0.01%	42.00%	IKAEGMELASCKPQYGAVVGQR	K	L	99.70%	60.7	43.9	19.1	48.6	46.6	4.62	0.479	0.364	0	0	0	4	2	2,408.21	1,984.00		10,110.40	1187	1208	Empty	FALSE	UPI000240B81D,UPI00035064DB,UPI0003505F8D,UPI0002C86DF1,UPI00035042E9,UPI00035069AB,F1NSS0_CHICK
20196-20225 HinckeM –TAXchicken	Uncategorized Sample	20196-20205_A	Mudpit_Ingel20196_MGFPeaklist (F058870)	status=inactive	UPI0000ECB786	UPARC14_2_GallusG_9031_20140417.fasta	244,653.90	99.90%	3	4	7	0.01%	42.00%	IKALADEREAVQK	R	K	35.40%	17.4	43.1	26.8	6.95					0	0	1	0	2	1,470.83	1,598.00		310.22	44	56	Empty	FALSE	UPI000240B81D,UPI00035064DB,R4GJH8_CHICK,UPI0000ECC737,UPI0003505F8D,UPI00003ACAFC,UPI0002C86DF1,UPI00035042E9,UPI00035069AB,F1NSS0_CHICK,UPI0000447B9B
20196-20225 HinckeM –TAXchicken	Uncategorized Sample	20196-20205_A	Mudpit_Ingel20196_MGFPeaklist (F058870)	status=inactive	UPI0000ECB786	UPARC14_2_GallusG_9031_20140417.fasta	244,653.90	99.90%	3	4	7	0.01%	42.00%	ISDLYLDLR	R	D	99.70%	67.1	44.1	37.8	37	40.3	2.7	0.352	0.667	0	4	0	0	2	1,107.60	2,473.50		8,313.08	74	82	Empty	FALSE	UPI000240B81D,UPI00035064DB,UPI0003505F8D,UPI0002C86DF1,UPI00035042E9,UPI00035069AB,F1NSS0_CHICK
20196-20225 HinckeM –TAXchicken	Uncategorized Sample	20196-20205_A	Mudpit_Ingel20196_MGFPeaklist (F058870)	status=inactive	UPI0000ECB786	UPARC14_2_GallusG_9031_20140417.fasta	244,653.90	99.90%	3	4	7	0.01%	42.00%	ISDLYLDLRDGR	R	M	99.70%	24.5	44.5	19.1	16.3	35.3	3.55	0.323	0.542	0	2	3	0	2	1,435.75	2,332.00		4,657.60	74	85	Empty	FALSE	UPI000240B81D,UPI00035064DB,UPI0003505F8D,UPI0002C86DF1,UPI00035042E9,UPI00035069AB,F1NSS0_CHICK
20196-20225 HinckeM –TAXchicken	Uncategorized Sample	20196-20205_A	Mudpit_Ingel20196_MGFPeaklist (F058870)	status=inactive	UPI0000ECB786	UPARC14_2_GallusG_9031_20140417.fasta	244,653.90	99.90%	3	4	7	0.01%	42.00%	KLTRLEEQVAAR	K	Q	99.70%	40.5	42.2	31.1	22.5	34.4	2.4	0.308	0.5	0	0	3	0	2	1,413.82	1,706.00		1,182.23	1275	1286	Empty	FALSE	UPI000240B81D,UPI00035064DB,UPI0003505F8D,UPI0002C86DF1,UPI00035042E9,UPI00035069AB,F1NSS0_CHICK
20196-20225 HinckeM –TAXchicken	Uncategorized Sample	20196-20205_A	Mudpit_Ingel20196_MGFPeaklist (F058870)	status=inactive	UPI0000ECB786	UPARC14_2_GallusG_9031_20140417.fasta	244,653.90	99.90%	3	4	7	0.01%	42.00%	LAEVLER	R	G	98.80%	61.6	44.7	42.9	18.4	27.8	0.638	0.119	0.429	0	5	0	0	2	829.4778	1,844.00		7,658.57	1388	1394	Empty	FALSE	UPI000240B81D,UPI00035064DB,UPI0003505F8D,UPI0002C86DF1,UPI00035042E9,UPI00035069AB,F1NSS0_CHICK
20196-20225 HinckeM –TAXchicken	Uncategorized Sample	20196-20205_A	Mudpit_Ingel20196_MGFPeaklist (F058870)	status=inactive	UPI0000ECB786	UPARC14_2_GallusG_9031_20140417.fasta	244,653.90	99.90%	3	4	7	0.01%	42.00%	LAEVLERGEAAAGGPCPELAER	R	A	99.70%	56.5	44.2	20.4	43.2	61.4	7.52	0.568	0.432	0	0	2	0	2	2,295.14	2,199.00		15,766.60	1388	1409	Empty	FALSE	UPI000240B81D,UPI00035064DB,UPI0003505F8D,UPI0002C86DF1,UPI00035042E9,UPI00035069AB,F1NSS0_CHICK
20196-20225 HinckeM –TAXchicken	Uncategorized Sample	20196-20205_A	Mudpit_Ingel20196_MGFPeaklist (F058870)	status=inactive	UPI0000ECB786	UPARC14_2_GallusG_9031_20140417.fasta	244,653.90	99.90%	3	4	7	0.01%	42.00%	LDELQALWNGLHDAAK	R	E	99.70%	41.7	44.7	18.1	36.9	42.4	4.89	0.337	0.406	0	0	3	0	2	1,794.90	2,648.00		1,124.77	1209	1224	Empty	FALSE	UPI000240B81D,UPI00035064DB,UPI0003505F8D,UPI0002C86DF1,UPI00035042E9,UPI00035069AB,F1NSS0_CHICK
20196-20225 HinckeM –TAXchicken	Uncategorized Sample	20196-20205_A	Mudpit_Ingel20196_MGFPeaklist (F058870)	status=inactive	UPI0000ECB786	UPARC14_2_GallusG_9031_20140417.fasta	244,653.90	99.90%	3	4	7	0.01%	42.00%	LDKALVMAEQLAAAGHEPGR	R	L	24.20%	15	43.9	17.2	9.35					0	0	0	1	2	2,093.08	2,210.00		274.62	695	714	Empty	TRUE	
20196-20225 HinckeM –TAXchicken	Uncategorized Sample	20196-20205_A	Mudpit_Ingel20196_MGFPeaklist (F058870)	status=inactive	UPI0000ECB786	UPARC14_2_GallusG_9031_20140417.fasta	244,653.90	99.90%	3	4	7	0.01%	42.00%	LENMCHLFQLKR	R	E	67.10%	25.7	44.8	19.4	8.4					0	0	0	1	2	1,604.80	2,033.00		228.25	1537	1548	Empty	FALSE	UPI000240B81D,UPI00035064DB,UPI0003505F8D,UPI0002C86DF1,UPI00035042E9,UPI00035069AB,F1NSS0_CHICK
20196-20225 HinckeM –TAXchicken	Uncategorized Sample	20196-20205_A	Mudpit_Ingel20196_MGFPeaklist (F058870)	status=inactive	UPI0000ECB786	UPARC14_2_GallusG_9031_20140417.fasta	244,653.90	99.90%	3	4	7	0.01%	42.00%	LGHQAEGFPPELR	R	A	99.70%	47.9	44.5	25	35.6	39.5	3.92	0.435	0.5	0	0	25	0	2	1,450.74	2,152.00		26,445.80	803	815	Empty	FALSE	UPI000240B81D,UPI00035064DB,UPI0003505F8D,UPI0002C86DF1,UPI00035042E9,UPI00035069AB,F1NSS0_CHICK
20196-20225 HinckeM –TAXchicken	Uncategorized Sample	20196-20205_A	Mudpit_Ingel20196_MGFPeaklist (F058870)	status=inactive	UPI0000ECB786	UPARC14_2_GallusG_9031_20140417.fasta	244,653.90	99.90%	3	4	7	0.01%	42.00%	LLESDMALQAEK	R	V	99.70%	63.6	44.9	24	50.8	38.9	3.68	0.401	0.417	0	7	0	0	2	1,363.68	1,880.00		4,204.19	574	585	Empty	FALSE	UPI000240B81D,UPI00035064DB,UPI0003505F8D,UPI0002C86DF1,UPI00035042E9,UPI00035069AB,F1NSS0_CHICK
20196-20225 HinckeM –TAXchicken	Uncategorized Sample	20196-20205_A	Mudpit_Ingel20196_MGFPeaklist (F058870)	status=inactive	UPI0000ECB786	UPARC14_2_GallusG_9031_20140417.fasta	244,653.90	99.90%	3	4	7	0.01%	42.00%	LLESDMALQAEKVR	R	A	99.70%	72.7	44.3	24.6	62.7	44.6	5.96	0.305	0.464	0	0	2	0	2	1,602.85	2,163.50		871.14	574	587	Empty	FALSE	UPI000240B81D,UPI00035064DB,UPI0003505F8D,UPI0002C86DF1,UPI00035042E9,UPI00035069AB,F1NSS0_CHICK
20196-20225 HinckeM –TAXchicken	Uncategorized Sample	20196-20205_A	Mudpit_Ingel20196_MGFPeaklist (F058870)	status=inactive	UPI0000ECB786	UPARC14_2_GallusG_9031_20140417.fasta	244,653.90	99.90%	3	4	7	0.01%	42.00%	LLEVLSK	K	E	74.20%	42.2	44	41.6	0					0	1	0	0	2	801.5081	2,181.00		242.39	90	96	Empty	FALSE	UPI000240B81D,UPI00035064DB,UPI0003505F8D,UPI0002C86DF1,UPI00035042E9,UPI00035069AB,F1NSS0_CHICK

20196-20225 HinckeM –TAXchicken	Uncategorized Sample	20196-20205_A	Mudpit_Ingel20196_MGFPeaklist (F058870)	status=inactive	UPI0000ECB786	UPARC14_2_GallusG_9031_20140417.fasta	244,653.90	99.90%	3	4	7	0.01%	42.00%	LLEVLSKEMLPKPTK	K	G	16.20%	11.8	40.1	20.5	8.53					0	0	2	1	2	1,742.01	2,237.00	1,325.69	90	104	Empty	FALSE	UPI000240B81D,UPI00035064DB,UPI0003505F8D,UPI0002C86DF1,UPI00035042E9,UPI00035069AB,F1NSS0_CHICK
20196-20225 HinckeM –TAXchicken	Uncategorized Sample	20196-20205_A	Mudpit_Ingel20196_MGFPeaklist (F058870)	status=inactive	UPI0000ECB786	UPARC14_2_GallusG_9031_20140417.fasta	244,653.90	99.90%	3	4	7	0.01%	42.00%	LLLEVCQFSR	R	D	99.70%	72.7	44.4	36.1	50.9	39.4	2.33	0.365	0.5	0	6	1	0	2	1,264.67	2,513.00	6,894.41	1858	1867	Empty	FALSE	UPI000240B81D,UPI00035064DB,UPI0003505F8D,UPI0002C86DF1,UPI00035042E9,UPI00035069AB,F1NSS0_CHICK
20196-20225 HinckeM –TAXchicken	Uncategorized Sample	20196-20205_A	Mudpit_Ingel20196_MGFPeaklist (F058870)	status=inactive	UPI0000ECB786	UPARC14_2_GallusG_9031_20140417.fasta	244,653.90	99.90%	3	4	7	0.01%	42.00%	LQGQVDKYYAGLK	R	E	99.70%	62.4	43.5	20.9	49.7	39.6	4.41	0.394	0.5	0	0	2	0	2	1,482.80	1,961.00	3,513.43	1515	1527	Empty	FALSE	UPI000240B81D,UPI00035064DB,UPI0003505F8D,UPI0002C86DF1,UPI00035042E9,UPI00035069AB,F1NSS0_CHICK
20196-20225 HinckeM –TAXchicken	Uncategorized Sample	20196-20205_A	Mudpit_Ingel20196_MGFPeaklist (F058870)	status=inactive	UPI0000ECB786	UPARC14_2_GallusG_9031_20140417.fasta	244,653.90	99.90%	3	4	7	0.01%	42.00%	LQGQVDKYYAGLKEAAEER	R	R	99.70%	64.8	43.9	18.9	50.4	47.1	6.17	0.463	0.368	0	0	0	2	2	2,168.10	2,191.00	2,839.74	1515	1533	Empty	FALSE	UPI000240B81D,UPI00035064DB,UPI0003505F8D,UPI0002C86DF1,UPI00035042E9,UPI00035069AB,F1NSS0_CHICK
20196-20225 HinckeM –TAXchicken	Uncategorized Sample	20196-20205_A	Mudpit_Ingel20196_MGFPeaklist (F058870)	status=inactive	UPI0000ECB786	UPARC14_2_GallusG_9031_20140417.fasta	244,653.90	99.90%	3	4	7	0.01%	42.00%	LQSFLQSCR	R	E	99.70%	64.3	44.1	30	47.3	35.6	1.85	0.323	0.556	0	6	0	0	2	1,138.57	2,064.50	14,343.50	1131	1139	Empty	FALSE	UPI000240B81D,UPI00035064DB,UPI0003505F8D,UPI0002C86DF1,UPI00035042E9,UPI00035069AB,F1NSS0_CHICK
20196-20225 HinckeM –TAXchicken	Uncategorized Sample	20196-20205_A	Mudpit_Ingel20196_MGFPeaklist (F058870)	status=inactive	UPI0000ECB786	UPARC14_2_GallusG_9031_20140417.fasta	244,653.90	99.90%	3	4	7	0.01%	42.00%	LREASEAQQYYLDADEAK	R	A	99.70%	73.1	43.8	20.4	59.4	39.4	4.06	0.391	0.333	0	0	3	0	2	2,099.99	2,002.00	12,402.80	1431	1448	Empty	FALSE	UPI000240B81D,UPI00035064DB,UPI0003505F8D,UPI0002C86DF1,UPI00035042E9,UPI00035069AB,F1NSS0_CHICK
20196-20225 HinckeM –TAXchicken	Uncategorized Sample	20196-20205_A	Mudpit_Ingel20196_MGFPeaklist (F058870)	status=inactive	UPI0000ECB786	UPARC14_2_GallusG_9031_20140417.fasta	244,653.90	99.90%	3	4	7	0.01%	42.00%	LTRLEEQVAAR	K	Q	99.70%	52.4	43.5	43.4	25.9	36.1	2.38	0.247	0.5	0	0	4	0	2	1,285.72	1,755.00	4,317.68	1276	1286	Empty	FALSE	UPI000240B81D,UPI00035064DB,UPI0003505F8D,UPI0002C86DF1,UPI00035042E9,UPI00035069AB,F1NSS0_CHICK
20196-20225 HinckeM –TAXchicken	Uncategorized Sample	20196-20205_A	Mudpit_Ingel20196_MGFPeaklist (F058870)	status=inactive	UPI0000ECB786	UPARC14_2_GallusG_9031_20140417.fasta	244,653.90	99.90%	3	4	7	0.01%	42.00%	LVAEGNIFSEK	K	I	99.70%	69	44.8	24.8	55.1	43.3	3.57	0.423	0.5	0	5	0	0	2	1,206.64	2,210.00	15,620.50	1089	1099	Empty	FALSE	UPI000240B81D,UPI00035064DB,UPI0003505F8D,UPI0002C86DF1,UPI00035042E9,UPI00035069AB,F1NSS0_CHICK
20196-20225 HinckeM –TAXchicken	Uncategorized Sample	20196-20205_A	Mudpit_Ingel20196_MGFPeaklist (F058870)	status=inactive	UPI0000ECB786	UPARC14_2_GallusG_9031_20140417.fasta	244,653.90	99.90%	3	4	7	0.01%	42.00%	LVAEGNIFSEKISEK	K	N	99.70%	39.4	43.9	22.2	32	31.8	2.72	0.33	0.3	0	0	2	0	2	1,663.89	2,264.00	1,851.71	1089	1103	Empty	FALSE	UPI000240B81D,UPI00035064DB,UPI0003505F8D,UPI0002C86DF1,UPI00035042E9,UPI00035069AB,F1NSS0_CHICK
20196-20225 HinckeM –TAXchicken	Uncategorized Sample	20196-20205_A	Mudpit_Ingel20196_MGFPeaklist (F058870)	status=inactive	UPI0000ECB786	UPARC14_2_GallusG_9031_20140417.fasta	244,653.90	99.90%	3	4	7	0.01%	42.00%	LVAEGNIFSEKISEKNQALQER	K	H	99.70%	28.8	42.7	18.5	16.5	29	1.38	0.217	0.227	0	0	0	2	2	2,503.32	2,249.50	892.25	1089	1110	Empty	FALSE	UPI000240B81D,UPI00035064DB,UPI0003505F8D,UPI0002C86DF1,UPI00035042E9,UPI00035069AB,F1NSS0_CHICK
20196-20225 HinckeM –TAXchicken	Uncategorized Sample	20196-20205_A	Mudpit_Ingel20196_MGFPeaklist (F058870)	status=inactive	UPI0000ECB786	UPARC14_2_GallusG_9031_20140417.fasta	244,653.90	99.90%	3	4	7	0.01%	42.00%	MLTVQDASYGGAR	K	G	99.70%	79.4	43.9	25.3	63.1	44.3	4.66	0.377	0.462	0	7	1	0	2	1,384.65	1,884.50	12,454.10	1149	1161	Empty	FALSE	UPI000240B81D,UPI00035064DB,UPI0003505F8D,UPI0002C86DF1,UPI00035042E9,UPI00035069AB,F1NSS0_CHICK
20196-20225 HinckeM –TAXchicken	Uncategorized Sample	20196-20205_A	Mudpit_Ingel20196_MGFPeaklist (F058870)	status=inactive	UPI0000ECB786	UPARC14_2_GallusG_9031_20140417.fasta	244,653.90	99.90%	3	4	7	0.01%	42.00%	MQLLAASYDLR	R	K	99.70%	67.1	44.2	28.4	48.9	36.4	2	0.341	0.455	0	7	0	0	2	1,296.66	2,480.00	4,623.64	1641	1651	Empty	FALSE	UPI000240B81D,UPI00035064DB,UPI0003505F8D,UPI0002C86DF1,UPI00035042E9,UPI00035069AB,F1NSS0_CHICK
20196-20225 HinckeM –TAXchicken	Uncategorized Sample	20196-20205_A	Mudpit_Ingel20196_MGFPeaklist (F058870)	status=inactive	UPI0000ECB786	UPARC14_2_GallusG_9031_20140417.fasta	244,653.90	99.90%	3	4	7	0.01%	42.00%	MQLLAASYDLRK	R	Y	99.70%	39.9	43.9	24.9	26.7	33.3	2.74	0.366	0.417	0	0	3	0	2	1,424.76	2,085.00	2,385.82	1641	1652	Empty	FALSE	UPI000240B81D,UPI00035064DB,UPI0003505F8D,UPI0002C86DF1,UPI00035042E9,UPI00035069AB,F1NSS0_CHICK
20196-20225 HinckeM –TAXchicken	Uncategorized Sample	20196-20205_A	Mudpit_Ingel20196_MGFPeaklist (F058870)	status=inactive	UPI0000ECB786	UPARC14_2_GallusG_9031_20140417.fasta	244,653.90	99.90%	3	4	7	0.01%	42.00%	NETLQKELQGHAPR	R	L	99.70%	63.5	44.4	26.2	44.8	39.3	4.82	0.42	0.393	0	0	0	3	2	1,620.85	1,829.00	2,586.13	1374	1387	Empty	FALSE	UPI000240B81D,UPI0002C86DF1,UPI00035042E9,UPI00035069AB,F1NSS0_CHICK
20196-20225 HinckeM –TAXchicken	Uncategorized Sample	20196-20205_A	Mudpit_Ingel20196_MGFPeaklist (F058870)	status=inactive	UPI0000ECB786	UPARC14_2_GallusG_9031_20140417.fasta	244,653.90	99.90%	3	4	7	0.01%	42.00%	QIQTQEKPRDVSSVELLMK	R	Y	23.30%	2.68	43.3	14.3	0					0	0	0	2	2	2,245.19	2,114.50	1,611.13	1776	1794	Empty	FALSE	UPI000240B81D,UPI00035064DB,UPI0003505F8D,UPI0002C86DF1,UPI00035042E9,UPI00035069AB,F1NSS0_CHICK
20196-20225 HinckeM –TAXchicken	Uncategorized Sample	20196-20205_A	Mudpit_Ingel20196_MGFPeaklist (F058870)	status=inactive	UPI0000ECB786	UPARC14_2_GallusG_9031_20140417.fasta	244,653.90	99.90%	3	4	7	0.01%	42.00%	QKELMELKAPLAGVAPEPDAQEEK	R	L	99.70%	22	43.2	18.3	20	29.9	2.55	0.221	0.229	0	0	0	2	2	2,637.34	2,282.50	1,274.24	1287	1310	Empty	FALSE	UPI000240B81D,UPI00035064DB,UPI0003505F8D,UPI0002C86DF1,UPI00035042E9,UPI00035069AB,F1NSS0_CHICK
20196-20225 HinckeM –TAXchicken	Uncategorized Sample	20196-20205_A	Mudpit_Ingel20196_MGFPeaklist (F058870)	status=inactive	UPI0000ECB786	UPARC14_2_GallusG_9031_20140417.fasta	244,653.90	99.90%	3	4	7	0.01%	42.00%	QVHLENMGSHDIVDGNHR	K	L	99.70%	47.9	43.2	16.2	38	41.2	5.06	0.357	0.389	0	0	0	2	2	2,073.95	1,723.50	5,040.36	126	143	Empty	FALSE	UPI000240B81D,UPI00035064DB,UPI0003505F8D,UPI0002C86DF1,UPI00035042E9,UPI00035069AB,F1NSS0_CHICK
20196-20225 HinckeM –TAXchicken	Uncategorized Sample	20196-20205_A	Mudpit_Ingel20196_MGFPeaklist (F058870)	status=inactive	UPI0000ECB786	UPARC14_2_GallusG_9031_20140417.fasta	244,653.90	99.90%	3	4	7	0.01%	42.00%	RAQLVDTADKFR	R	F	99.70%	53.8	44.4	24.4	30.7	39.9	4.15	0.396	0.542	0	0	2	0	2	1,419.77	1,840.00	4,070.37	1747	1758	Empty	FALSE	UPI000240B81D,UPI00035064DB,UPI0003505F8D,UPI0002C86DF1,UPI00035042E9,UPI00035069AB,F1NSS0_CHICK
20196-20225 HinckeM –TAXchicken	Uncategorized Sample	20196-20205_A	Mudpit_Ingel20196_MGFPeaklist (F058870)	status=inactive	UPI0000ECB786	UPARC14_2_GallusG_9031_20140417.fasta	244,653.90	99.90%	3	4	7	0.01%	42.00%	RHTALEAELEAR	R	G	99.70%	44.9	44.2	33.6	26	34.8	2.55	0.261	0.417	0	0	4	0	2	1,395.73	1,850.00	2,275.43	680	691	Empty	FALSE	UPI000240B81D,UPI00035064DB,UPI0003505F8D,UPI0002C86DF1,UPI00035042E9,UPI00035069AB,F1NSS0_CHICK
20196-20225 HinckeM –TAXchicken	Uncategorized Sample	20196-20205_A	Mudpit_Ingel20196_MGFPeaklist (F058870)	status=inactive	UPI0000ECB786	UPARC14_2_GallusG_9031_20140417.fasta	244,653.90	99.90%	3	4	7	0.01%	42.00%	RLENMCHLFQLK	R	R	99.50%	23.7	44.8	19.3	17	24.3	0.658	0.193	0.333	0	0	2	0	2	1,588.81	2,255.00	1,048.59	1536	1547	Empty	FALSE	UPI000240B81D,UPI00035064DB,UPI0003505F8D,UPI0002C86DF1,UPI00035042E9,UPI00035069AB,F1NSS0_CHICK
20196-20225 HinckeM –TAXchicken	Uncategorized Sample	20196-20205_A	Mudpit_Ingel20196_MGFPeaklist (F058870)	status=inactive	UPI0000ECB786	UPARC14_2_GallusG_9031_20140417.fasta	244,653.90	99.90%	3	4	7	0.01%	42.00%	RLENMCHLFQLKR	R	E	99.70%	25	44.7	19.6	15.6	30.4	3.05	0.237	0.346	0	0	0	2	2	1,760.90	1,957.50	1,477.70	1536	1548	Empty	FALSE	UPI000240B81D,UPI00035064DB,UPI0003505F8D,UPI0002C86DF1,UPI00035042E9,UPI00035069AB,F1NSS0_CHICK
20196-20225 HinckeM –TAXchicken	Uncategorized Sample	20196-20205_A	Mudpit_Ingel20196_MGFPeaklist (F058870)	status=inactive	UPI0000ECB786	UPARC14_2_GallusG_9031_20140417.fasta	244,653.90	99.90%	3	4	7	0.01%	42.00%	RNETLQKELQGHAPR	K	L	99.70%	59.5	43.9	19	44	42.6	5.51	0.434	0.467	0	0	0	4	2	1,776.95	1,717.50	1,822.17	1373	1387	Empty	FALSE	UPI000240B81D,UPI0002C86DF1,UPI00035042E9,UPI00035069AB,F1NSS0_CHICK
20196-20225 HinckeM –TAXchicken	Uncategorized Sample	20196-20205_A	Mudpit_Ingel20196_MGFPeaklist (F058870)	status=inactive	UPI0000ECB786	UPARC14_2_GallusG_9031_20140417.fasta	244,653.90	99.90%	3	4	7	0.01%	42.00%	RRHTALEAELEAR	R	G	99.70%	102	43.8	27.4	79.1	51.5	5.82	0.476	0.615	0	0	4	0	2	1,551.84	1,788.50	2,815.00	679	691	Empty	FALSE	UPI000240B81D,UPI00035064DB,UPI0003505F8D,UPI0002C86DF1,UPI00035042E9,UPI00035069AB,F1NSS0_CHICK
20196-20225 HinckeM –TAXchicken	Uncategorized Sample	20196-20205_A	Mudpit_Ingel20196_MGFPeaklist (F058870)	status=inactive	UPI0000ECB786	UPARC14_2_GallusG_9031_20140417.fasta	244,653.90	99.90%	3	4	7	0.01%	42.00%	SFDACIELGK	K	K	99.70%	35.6	43.5	21.2	29.2	27.2	2.42	0.294	0.35	0	1	0	0	2	1,139.54	2,215.00	384.16	1809	1818	Empty	FALSE	UPI000240B81D,UPI00035064DB,UPI0003505F8D,UPI0002C86DF1,UPI00035042E9,UPI00035069AB,F1NSS0_CHICK
20196-20225 HinckeM –TAXchicken	Uncategorized Sample	20196-20205_A	Mudpit_Ingel20196_MGFPeaklist (F058870)	status=inactive	UPI0000ECB786	UPARC14_2_GallusG_9031_20140417.fasta	244,653.90	99.90%	3	4	7	0.01%	42.00%	SFDACIELGKK	K	L	31.20%	14.4	44.1	24.8	0					0	0	2	0	2	1,267.64	2,059.00	563.95	1809	1819	Empty	FALSE	UPI000240B81D,UPI00035064DB,UPI0003505F8D,UPI0002C86DF1,UPI00035042E9,UPI00035069AB,F1NSS0_CHICK
20196-20225 HinckeM –TAXchicken	Uncategorized Sample	20196-20205_A	Mudpit_Ingel20196_MGFPeaklist (F058870)	status=inactive	UPI0000ECB786	UPARC14_2_GallusG_9031_20140417.fasta	244,653.90	99.90%	3	4	7	0.01%	42.00%	SGKHLLEVEELLETHR	K	L	99.70%	27.9	43.7	17.9	21.9	27.9	2.11	0.269	0.312	0	0	0	2	2	1,890.01	2,442.50	779.48	558	573	Empty	FALSE	UPI000240B81D,UPI00035064DB,UPI0003505F8D,UPI0002C86DF1,UPI00035042E9,UPI00035069AB,F1NSS0_CHICK
20196-20225 HinckeM –TAXchicken	Uncategorized Sample	20196-20205_A	Mudpit_Ingel20196_MGFPeaklist (F058870)	status=inactive	UPI0000ECB786	UPARC14_2_GallusG_9031_20140417.fasta	244,653.90	99.90%	3	4	7	0.01%	42.00%	SIEDYGQTIK	R	E	99.70%	40.7	44	24.9	31	37.3	3.14	0.394	0.5	0	7	0	0	2	1,153.57	1,863.00	19,181.20	1483	1492	Empty	FALSE	UPI000240B81D,UPI00035064DB,UPI0003505F8D,UPI0002C86DF1,UPI00035042E9,UPI00035069AB,F1NSS0_CHICK
20196-20225 HinckeM –TAXchicken	Uncategorized Sample	20196-20205_A	Mudpit_Ingel20196_MGFPeaklist (F058870)	status=inactive	UPI0000ECB786	UPARC14_2_GallusG_9031_20140417.fasta	244,653.90	99.90%	3	4	7	0.01%	42.00%	SIEDYGQTIKELAGR	R	A	99.70%	62.1	44.5	20.3	51.9	49.7	6	0.507	0.5	0	0	2	0	2	1,679.86	2,443.00	5,509.56	1483	1497	Empty	FALSE	UPI000240B81D,UPI00035064DB,UPI0003505F8D,UPI0002C86DF1,UPI00035042E9,UPI00035069AB,F1NSS0_CHICK
20196-20225 HinckeM –TAXchicken	Uncategorized Sample	20196-20205_A	Mudpit_Ingel20196_MGFPeaklist (F058870)	status=inactive	UPI0000ECB786	UPARC14_2_GallusG_9031_20140417.fasta	244,653.90	99.90%	3	4	7	0.01%	42.00%	TVEKPPKFQEK	R	G	99.70%	43.2	44.3	32.6	25.4	43	3.96	0.342	0.636	0	0	4	0	2	1,330.74	1,509.50	1,312.54	348	358	Empty	FALSE	UPI000240B81D,UPI00035064DB,UPI0003505F8D,UPI0002C86DF1,UPI00035042E9,UPI00035069AB,F1NSS0_CHICK
20196-20225 HinckeM –TAXchicken	Uncategorized Sample	20196-20205_A	Mudpit_Ingel20196_MGFPeaklist (F058870)	status=inactive	UPI0000ECB786	UPARC14_2_GallusG_9031_20140417.fasta	244,653.90	99.90%	3	4	7	0.01%	42.00%	WLGQVEGELHTTER	R	A	99.70%	64.8	44.3	17.1	51.2	38.6	2.44	0.399	0.393	0	0	4	0	2	1,654.82	2,225.00	5,236.70	1249	1262	Empty	FALSE	UPI000240B81D,UPI00035064DB,UPI0003505F8D,UPI0002C86DF1,UPI00035042E9,UPI00035069AB,F1NSS0_CHICK
20196-20225 HinckeM –TAXchicken	Uncategorized Sample	20196-20205_A	Mudpit_Ingel20196_MGFPeaklist (F058870)	status=inactive	UPI0000ECB786	UPARC14_2_GallusG_9031_20140417.fasta	244,653.90	99.90%	3	4	7	0.01%	42.00%	WVNLHLACVTCR	K	I	99.70%	48.7	44.4	27.3	38	40.6	6.14	0.466	0.5	0	2	0	0	2	1,528.75	2,376.50	1,131.42	62	73	Empty	FALSE	UPI000240B81D,UPI00035064DB,UPI0003505F8D,UPI0002C86DF1,UPI00035042E9,UPI00035069AB,F1NSS0_CHICK
20196-20225 HinckeM –TAXchicken	Uncategorized Sample	20196-20205_A	Mudpit_Ingel20196_MGFPeaklist (F058870)	status=inactive	UPI0000ECB786	UPARC14_2_GallusG_9031_20140417.fasta	244,653.90	99.90%	3	4	7	0.01%	42.00%	YHQGIHAEIDAR	K	D	99.70%	77	44.3	24.7	68.3	43.8	5.36	0.463	0.667	0	0	7	0	2	1,409.69	1,620.00	7,647.29	1795	1806	Empty	FALSE	UPI000240B81D,UPI00035064DB,UPI0003505F8D,UPI0002C86DF1,UPI00035042E9,UPI00035069AB,F1NSS0_CHICK
20196-20225 HinckeM –TAXchicken	Uncategorized Sample	20196-20205_A	Mudpit_Ingel20196_MGFPeaklist (F058870)	status=inactive	UPI0000ECB786	UPARC14_2_GallusG_9031_20140417.fasta	244,653.90	99.90%	3	4	7	0.01%	42.00%	YYAGLKEAAEER	K	R	99.70%	44	44.4	27.8	31.9	32.7	4.05	0.391	0.417	0	0	4	0	2	1,399.69	1,769.00	1,660.34	1522	1533	Empty	FALSE	UPI000240B81D,UPI00035064DB,UPI0003505F8D,UPI0002C86DF1,UPI00035042E9,UPI00035069AB,F1NSS0_CHICK
20196-20225 HinckeM –TAXchicken	Uncategorized Sample	20196-20205_A	Mudpit_Ingel20196_MGFPeaklist (F058870)	Spectrin alpha chain, non-erythrocytic 1 OS=Gallus gallus GN=SPTAN1 PE=4 SV=2	F1NHT3_CHICK	TAX_GallusGallus_9031_20141114.fasta	285,330.20	100.00%	163	194	497	1.00%	56.40%	ADVVESWIGEK	K	E	99.70%	52.8	45.2	26.2	33.4	35.3	3.8	0.286	0.455	0	3	0	0	2	1,232.62	2,355.00	830.65	1990	2000	Empty	TRUE	UPI0000ECA63D,UPI0000ECA63C
20196-20225 HinckeM –TAXchicken	Uncategorized Sample	20196-20205_A	Mudpit_Ingel20196_MGFPeaklist (F058870)	Spectrin alpha chain, non-erythrocytic 1 OS=Gallus gallus GN=SPTAN1 PE=4 SV=2	F1NHT3_CHICK	TAX_GallusGallus_9031_20141114.fasta	285,330.20	100.00%	163	194	497	1.00%	56.40%	ADVVESWIGEKENSLK	K	T	99.70%	37.6	44.6	19.6	27.5	34.3	4	0.297	0.344	0	0	2	0	2	1,803.91	2,426.00	1,319.73	1990	2005	Empty	TRUE	UPI0000ECA63D,UPI0000ECA63C
20196-20225 HinckeM –TAXchicken	Uncategorized Sample	20196-20205_A	Mudpit_Ingel20196_MGFPeaklist (F058870)	Spectrin alpha chain, non-erythrocytic 1 OS=Gallus gallus GN=SPTAN1 PE=4 SV=2	F1NHT3_CHICK	TAX_GallusGallus_9031_20141114.fasta	285,330.20	100.00%	163	194	497	1.00%	56.40%	AGTFQAFEQFGQQLLAR	R	G	99.70%	27.4	44.2	0	19.1	30.4	1.72	0.322	0.265	0	0	1	0	2	1,911.97	2,884.00	143.7	1392	1408	Empty	TRUE	UPI0000ECA63D,UPI0000ECA63C
20196-20225 HinckeM –TAXchicken	Uncategorized Sample	20196-20205_A	Mudpit_Ingel20196_MGFPeaklist (F058870)	Spectrin alpha chain, non-erythrocytic 1 OS=Gallus gallus GN=SPTAN1 PE=4 SV=2	F1NHT3_CHICK	TAX_GallusGallus_9031_20141114.fasta	285,330.20	100.00%	163	194	497	1.00%	56.40%	ALCAEADRLQQSHPINASQIQVK	K	R	99.70%	9.04	43.1	0	9.04	36.6	2.38	0.303	0.239	0	0	0	2	2	2,577.32	2,102.50	8,838.04	313	335	Empty	TRUE	UPI0000ECA63D,UPI0000ECA63C
20196-20225 HinckeM –TAXchicken	Uncategorized Sample	20196-20205_A	Mudpit_Ingel20196_MGFPeaklist (F058870)	Spectrin alpha chain, non-erythrocytic 1 OS=Gallus gallus GN=SPTAN1 PE=4 SV=2	F1NHT3_CHICK	TAX_GallusGallus_9031_20141114.fasta	285,330.20	100.00%	163	194	497	1.00%	56.40%	ALSSEGKPYVTK	R	E	99.70%	66.2	44	23.9	53.1	48.7	5.33	0.398	0.625	0	0	2	0	2	1,279.69	1,555.50	618.77	2420	2431	Empty	TRUE	UPI0000ECA63D,UPI0000ECA63C
20196-20225 HinckeM –TAXchicken	Uncategorized Sample	20196-20205_A	Mudpit_Ingel20196_MGFPeaklist (F058870)	Spectrin alpha chain, non-erythrocytic 1 OS=Gallus gallus GN=SPTAN1 PE=4 SV=2	F1NHT3_CHICK	TAX_GallusGallus_9031_20141114.fasta	285,330.20	100.00%	163	194	497	1.00%	56.40%	ALSSEGKPYVTKEELYQNLTR	R	E	99.70%	88.8	43.1	14.9	78.8	40	3.85	0.405	0.333	0	0	0	2	2	2,426.26	2,196.50	8,205.40	2420	2440	Empty	TRUE	UPI0000ECA63D,UPI0000ECA63C
20196-20225 HinckeM –TAXchicken	Uncategorized Sample	20196-20205_A	Mudpit_Ingel20196_MGFPeaklist (F058870)	Spectrin alpha chain, non-erythrocytic 1 OS=Gallus gallus GN=SPTAN1 PE=4 SV=2	F1NHT3_CHICK	TAX_GallusGallus_9031_20141114.fasta	285,330.20	100.00%	163	194	497	1.00%	56.40%	AQLADSFHLQQFFR	R	D	99.70%	72.7	44.6	16.7	67.9	41.6	4.59	0.418	0.464	0	0	2	0	2	1,707.86	2,785.50	2,140.49	567	580	Empty	TRUE	UPI0000ECA63D,UPI0000ECA63C
20196-20225 HinckeM –TAXchicken	Uncategorized Sample	20196-20205_A	Mudpit_Ingel20196_MGFPeaklist (F058870)	Spectrin alpha chain, non-erythrocytic 1 OS=Gallus gallus GN=SPTAN1 PE=4 SV=2	F1NHT3_CHICK	TAX_GallusGallus_9031_20141114.fasta	285,330.20	100.00%	163	194	497	1.00%	56.40%	CNSLEEIKALR	R	E	49.50%	23	45	31.9	5.69					0	0	1	0	2	1,332.69	2,102.00	330.76	2125	2135	Empty	TRUE	UPI0000ECA63D,UPI0000ECA63C
20196-20225 HinckeM –TAXchicken	Uncategorized Sample	20196-20205_A	Mudpit_Ingel20196_MGFPeaklist (F058870)	Spectrin alpha chain, non-erythrocytic 1 OS=Gallus gallus GN=SPTAN1 PE=4 SV=2	F1NHT3_CHICK	TAX_GallusGallus_9031_20141114.fasta	285,330.20	100.00%	163	194	497	1.00%	56.40%	CTELNQAWSSLGK	K	R	99.70%	69.3	44.3	26.2	47.6	47.4	4.82	0.414	0.538	0	2	0	0	2	1,493.71	2,349.00	2,238.28	1314	1326	Empty	TRUE	UPI0000ECA63D,UPI0000ECA63C
20196-20225 HinckeM –TAXchicken	Uncategorized Sample	20196-20205_A	Mudpit_Ingel20196_MGFPeaklist (F058870)	Spectrin alpha chain, non-erythrocytic 1 OS=Gallus gallus GN=SPTAN1 PE=4 SV=2	F1NHT3_CHICK	TAX_GallusGallus_9031_20141114.fasta	285,330.20	100.00%	163	194	497	1.00%	56.40%	CTELNQAWSSLGKR	K	A	99.70%	63	45	27.1	43.6	46	3.82	0.393	0.536	0	0	3	0	2	1,649.81	2,252.00	3,580.82	1314	1327	Empty	TRUE	UPI0000ECA63D,UPI0000ECA63C
20196-20225 HinckeM –TAXchicken	Uncategorized Sample	20196-20205_A	Mudpit_Ingel20196_MGFPeaklist (F058870)	Spectrin alpha chain, non-erythrocytic 1 OS=Gallus gallus GN=SPTAN1 PE=4 SV=2	F1NHT3_CHICK	TAX_GallusGallus_9031_20141114.fasta	285,330.20	100.00%	163	194	497	1.00%	56.40%	DADELEKWIQEK	R	L	99.70%	50.5	44.9	31.3	28.9	39.3	3.21	0.351	0.542	0	0	2	0	2	1,503.73	2,487.50	902.74	52	63	Empty	TRUE	UPI0000ECA63D,UPI0000ECA63C
20196-20225 HinckeM –TAXchicken	Uncategorized Sample	20196-20205_A	Mudpit_Ingel20196_MGFPeaklist (F058870)	Spectrin alpha chain, non-erythrocytic 1 OS=Gallus gallus GN=SPTAN1 PE=4 SV=2	F1NHT3_CHICK	TAX_GallusGallus_9031_20141114.fasta	285,330.20	100.00%	163	194	497	1.00%	56.40%	DADETKEWIEEK	R	N	99.70%	50.1	44	24.6	34.5	38.4	2.15	0.258	0.583	0	0	2	0	2	1,492.68	1,917.50	2,873.01	1241	1252	Empty	TRUE	UPI0000ECA63D,UPI0000ECA63C
20196-20225 HinckeM –TAXchicken	Uncategorized Sample	20196-20205_A	Mudpit_Ingel20196_MGFPeaklist (F058870)	Spectrin alpha chain, non-erythrocytic 1 OS=Gallus gallus GN=SPTAN1 PE=4 SV=2	F1NHT3_CHICK	TAX_GallusGallus_9031_20141114.fasta	285,330.20	100.00%	163	194	497	1.00%	56.40%	DIEDEETWIR	R	E	99.70%	45.2	44.2	25.3	27.6	34.1	2.72	0.331	0.4	0	4	0	0	2	1,305.60	2,285.00	3,917.74	792	801	Empty	TRUE	UPI0000ECA63D,UPI0000ECA63C
20196-20225 HinckeM –TAXchicken	Uncategorized Sample	20196-20205_A	Mudpit_Ingel20196_MGFPeaklist (F058870)	Spectrin alpha chain, non-erythrocytic 1 OS=Gallus gallus GN=SPTAN1 PE=4 SV=2	F1NHT3_CHICK	TAX_GallusGallus_9031_20141114.fasta	285,330.20	100.00%	163	194	497	1.00%	56.40%	DIEDEETWIREKEPIAASTNR	R	G	99.70%	49.9	44	20.4	35.8	30.4	3.54	0.273	0.19	0	0	0	2	2	2,502.21	2,313.50	1,027.85	792	812	Empty	TRUE	UPI0000ECA63D,UPI0000ECA63C
20196-20225 HinckeM –TAXchicken	Uncategorized Sample	20196-20205_A	Mudpit_Ingel20196_MGFPeaklist (F058870)	Spectrin alpha chain, non-erythrocytic 1 OS=Gallus gallus GN=SPTAN1 PE=4 SV=2	F1NHT3_CHICK	TAX_GallusGallus_9031_20141114.fasta	285,330.20	100.00%	163	194	497	1.00%	56.40%	DLAALEDKVK	R	A	99.70%	47	44.7	29.6	31.6	28.2	1.07	0.234	0.35	0	4	0	0	2	1,101.62	2,105.50	3,056.35	303	312	Empty	TRUE	UPI0000ECA63D,UPI0000ECA63C
20196-20225 HinckeM –TAXchicken	Uncategorized Sample	20196-20205_A	Mudpit_Ingel20196_MGFPeaklist (F058870)	Spectrin alpha chain, non-erythrocytic 1 OS=Gallus gallus GN=SPTAN1 PE=4 SV=2	F1NHT3_CHICK	TAX_GallusGallus_9031_20141114.fasta	285,330.20	100.00%	163	194	497	1.00%	56.40%	DLAALGDKVNSLGETAQR	R	L	99.70%	65.1	44.2	15.5	49	41.2	5.2	0.335	0.389	0	0	4	1	2	1,857.97	2,289.00	10,407.20	1281	1298	Empty	TRUE	UPI0000ECA63D,UPI0000ECA63C
20196-20225 HinckeM –TAXchicken	Uncategorized Sample	20196-20205_A	Mudpit_Ingel20196_MGFPeaklist (F058870)	Spectrin alpha chain, non-erythrocytic 1 OS=Gallus gallus GN=SPTAN1 PE=4 SV=2	F1NHT3_CHICK	TAX_GallusGallus_9031_20141114.fasta	285,330.20	100.00%	163	194	497	1.00%	56.40%	DLASVQALLR	R	K	99.70%	59.5	43.5	34.7	42.1	38.6	1.51	0.293	0.55	0	3	0	0	2	1,085.63	2,622.00	2,066.49	286	295	Empty	TRUE	UPI0000ECA63D,UPI0000ECA63C
20196-20225 HinckeM –TAXchicken	Uncategorized Sample	20196-20205_A	Mudpit_Ingel20196_MGFPeaklist (F058870)	Spectrin alpha chain, non-erythrocytic 1 OS=Gallus gallus GN=SPTAN1 PE=4 SV=2	F1NHT3_CHICK	TAX_GallusGallus_9031_20141114.fasta	285,330.20	100.00%	163	194	497	1.00%	56.40%	DLIGVQNLLK	K	K	99.70%	37.7	41.9	25.3	19	28	0.377	0.104	0.4	0	2	0	0	2	1,112.67	2,701.00	549.42	815	824	Empty	TRUE	UPI0000ECA63D,UPI0000ECA63C
20196-20225 HinckeM –TAXchicken	Uncategorized Sample	20196-20205_A	Mudpit_Ingel20196_MGFPeaklist (F058870)	Spectrin alpha chain, non-erythrocytic 1 OS=Gallus gallus GN=SPTAN1 PE=4 SV=2	F1NHT3_CHICK	TAX_GallusGallus_9031_20141114.fasta	285,330.20	100.00%	163	194	497	1.00%	56.40%	DLIGVQNLLKK	K	H	36.00%	26.5	40.3	32.1	6.71					0	0	2	0	2	1,240.76	2,513.00	302.19	815	825	Empty	TRUE	UPI0000ECA63D,UPI0000ECA63C
20196-20225 HinckeM –TAXchicken	Uncategorized Sample	20196-20205_A	Mudpit_Ingel20196_MGFPeaklist (F058870)	Spectrin alpha chain, non-erythrocytic 1 OS=Gallus gallus GN=SPTAN1 PE=4 SV=2	F1NHT3_CHICK	TAX_GallusGallus_9031_20141114.fasta	285,330.20	100.00%	163	194	497	1.00%	56.40%	DLNSQADSLMTSSAFDTSQVK	K	D	99.70%	38.3	42.9	16	32.4	32.7	2.74	0.349	0.262	0	0	2	0	2	2,261.02	2,204.00	1,428.41	1721	1741	Empty	TRUE	UPI0000ECA63D,UPI0000ECA63C
20196-20225 HinckeM –TAXchicken	Uncategorized Sample	20196-20205_A	Mudpit_Ingel20196_MGFPeaklist (F058870)	Spectrin alpha chain, non-erythrocytic 1 OS=Gallus gallus GN=SPTAN1 PE=4 SV=2	F1NHT3_CHICK	TAX_GallusGallus_9031_20141114.fasta	285,330.20	100.00%	163	194	497	1.00%	56.40%	DLSSVQTLLTK	R	Q	99.70%	58.2	44.2	22.1	37.2	39.6	2.39	0.263	0.5	0	3	0	0	2	1,204.68	2,498.00	4,868.60	2012	2022	Empty	TRUE	UPI0000ECA63D,UPI0000ECA63C
20196-20225 HinckeM –TAXchicken	Uncategorized Sample	20196-20205_A	Mudpit_Ingel20196_MGFPeaklist (F058870)	Spectrin alpha chain, non-erythrocytic 1 OS=Gallus gallus GN=SPTAN1 PE=4 SV=2	F1NHT3_CHICK	TAX_GallusGallus_9031_20141114.fasta	285,330.20	100.00%	163	194	497	1.00%	56.40%	DLTGVQNLR	R	K	99.70%	56	45	38.4	30.3	36.1	0.456	0.222	0.556	0	5	0	0	2	1,015.55	1,931.00	7,457.21	1800	1808	Empty	TRUE	UPI0000ECA63D,UPI0000ECA63C
20196-20225 HinckeM –TAXchicken	Uncategorized Sample	20196-20205_A	Mudpit_Ingel20196_MGFPeaklist (F058870)	Spectrin alpha chain, non-erythrocytic 1 OS=Gallus gallus GN=SPTAN1 PE=4 SV=2	F1NHT3_CHICK	TAX_GallusGallus_9031_20141114.fasta	285,330.20	100.00%	163	194	497	1.00%	56.40%	DLTSVQNLQK	K	K	99.70%	38.1	45.1	28	11.8	28.4	1.77	0.151	0.35	0	2	0	0	2	1,145.62	1,873.50	1,020.59	709	718	Empty	TRUE	UPI0000ECA63D,UPI0000ECA63C
20196-20225 HinckeM –TAXchicken	Uncategorized Sample	20196-20205_A	Mudpit_Ingel20196_MGFPeaklist (F058870)	Spectrin alpha chain, non-erythrocytic 1 OS=Gallus gallus GN=SPTAN1 PE=4 SV=2	F1NHT3_CHICK	TAX_GallusGallus_9031_20141114.fasta	285,330.20	100.00%	163	194	497	1.00%	56.40%	DLTSWVTEMK	R	A	99.50%	40.2	43.5	28.7	32.1					0	2	0	0	2	1,225.58	2,378.50	899.24	372	381	Empty	TRUE	UPI0000ECA63D,UPI0000ECA63C
20196-20225 HinckeM –TAXchicken	Uncategorized Sample	20196-20205_A	Mudpit_Ingel20196_MGFPeaklist (F058870)	Spectrin alpha chain, non-erythrocytic 1 OS=Gallus gallus GN=SPTAN1 PE=4 SV=2	F1NHT3_CHICK	TAX_GallusGallus_9031_20141114.fasta	285,330.20	100.00%	163	194	497	1.00%	56.40%	DMDDEESWIKEK	R	K	21.00%	11.7	41.6	19.6	0					0	0	3	0	2	1,524.65	1,885.00	1,111.19	1777	1788	Empty	TRUE	UPI0000ECA63D,UPI0000ECA63C
20196-20225 HinckeM –TAXchicken	Uncategorized Sample	20196-20205_A	Mudpit_Ingel20196_MGFPeaklist (F058870)	Spectrin alpha chain, non-erythrocytic 1 OS=Gallus gallus GN=SPTAN1 PE=4 SV=2	F1NHT3_CHICK	TAX_GallusGallus_9031_20141114.fasta	285,330.20	100.00%	163	194	497	1.00%	56.40%	DSDELKSWVNEK	R	M	99.70%	54.9	44.4	26.9	35.9	43.5	3.59	0.354	0.542	0	2	0	0	2	1,449.69	2,068.00	1,798.64	581	592	Empty	TRUE	UPI0000ECA63D,UPI0000ECA63C
20196-20225 HinckeM –TAXchicken	Uncategorized Sample	20196-20205_A	Mudpit_Ingel20196_MGFPeaklist (F058870)	Spectrin alpha chain, non-erythrocytic 1 OS=Gallus gallus GN=SPTAN1 PE=4 SV=2	F1NHT3_CHICK	TAX_GallusGallus_9031_20141114.fasta	285,330.20	100.00%	163	194	497	1.00%	56.40%	DVDEIEAWISEK	R	L	99.70%	71.5	44.5	21.9	46.3	41.7	3.85	0.369	0.5	0	2	0	0	2	1,433.68	2,739.00	1,182.10	1559	1570	Empty	TRUE	UPI0000ECA63D,UPI0000ECA63C
20196-20225 HinckeM –TAXchicken	Uncategorized Sample	20196-20205_A	Mudpit_Ingel20196_MGFPeaklist (F058870)	Spectrin alpha chain, non-erythrocytic 1 OS=Gallus gallus GN=SPTAN1 PE=4 SV=2	F1NHT3_CHICK	TAX_GallusGallus_9031_20141114.fasta	285,330.20	100.00%	163	194	497	1.00%	56.40%	DVDETISWIK	R	E	99.70%	44.3	45	25.4	31.4	35.5	3.68	0.33	0.5	0	2	0	0	2	1,205.61	2,458.50	957.68	263	272	Empty	TRUE	UPI0000ECA63D,UPI0000ECA63C
20196-20225 HinckeM –TAXchicken	Uncategorized Sample	20196-20205_A	Mudpit_Ingel20196_MGFPeaklist (F058870)	Spectrin alpha chain, non-erythrocytic 1 OS=Gallus gallus GN=SPTAN1 PE=4 SV=2	F1NHT3_CHICK	TAX_GallusGallus_9031_20141114.fasta	285,330.20	100.00%	163	194	497	1.00%	56.40%	DVDETISWIKEK	R	G	90.00%	28.4	44.8	21.8	17					0	0	2	0	2	1,462.74	2,355.00	545.19	263	274	Empty	TRUE	UPI0000ECA63D,UPI0000ECA63C
20196-20225 HinckeM –TAXchicken	Uncategorized Sample	20196-20205_A	Mudpit_Ingel20196_MGFPeaklist (F058870)	Spectrin alpha chain, non-erythrocytic 1 OS=Gallus gallus GN=SPTAN1 PE=4 SV=2	F1NHT3_CHICK	TAX_GallusGallus_9031_20141114.fasta	285,330.20	100.00%	163	194	497	1.00%	56.40%	DVTGAEALLER	K	H	99.70%	67.8	45.2	33.8	51.6	40.6	3.1	0.411	0.5	0	4	0	0	2	1,173.61	2,270.00	3,192.11	1370	1380	Empty	TRUE	UPI0000ECA63D,UPI0000ECA63C
20196-20225 HinckeM –TAXchicken	Uncategorized Sample	20196-20205_A	Mudpit_Ingel20196_MGFPeaklist (F058870)	Spectrin alpha chain, non-erythrocytic 1 OS=Gallus gallus GN=SPTAN1 PE=4 SV=2	F1NHT3_CHICK	TAX_GallusGallus_9031_20141114.fasta	285,330.20	100.00%	163	194	497	1.00%	56.40%	EAALTNEEVGADLEQVEVLQK	K	K	99.70%	61.3	44.1	21.7	44.8	45.5	3.24	0.367	0.357	0	0	2	0	2	2,285.15	2,586.50	3,455.38	1111	1131	Empty	TRUE	UPI0000ECA63D,UPI0000ECA63C
20196-20225 HinckeM –TAXchicken	Uncategorized Sample	20196-20205_A	Mudpit_Ingel20196_MGFPeaklist (F058870)	Spectrin alpha chain, non-erythrocytic 1 OS=Gallus gallus GN=SPTAN1 PE=4 SV=2	F1NHT3_CHICK	TAX_GallusGallus_9031_20141114.fasta	285,330.20	100.00%	163	194	497	1.00%	56.40%	EAALTNEEVGADLEQVEVLQKK	K	F	99.70%	66.3	43.3	15.6	54.8	46.4	5.77	0.459	0.318	0	0	1	0	2	2,413.25	2,516.00	644.68	1111	1132	Empty	TRUE	UPI0000ECA63D,UPI0000ECA63C
20196-20225 HinckeM –TAXchicken	Uncategorized Sample	20196-20205_A	Mudpit_Ingel20196_MGFPeaklist (F058870)	Spectrin alpha chain, non-erythrocytic 1 OS=Gallus gallus GN=SPTAN1 PE=4 SV=2	F1NHT3_CHICK	TAX_GallusGallus_9031_20141114.fasta	285,330.20	100.00%	163	194	497	1.00%	56.40%	EAFLNTEDKGDSLDSVEALIK	R	K	99.70%	69.5	44.2	17.1	60.3	42.9	3.42	0.329	0.31	0	0	2	0	2	2,294.14	2,728.50	3,342.88	1465	1485	Empty	TRUE	UPI0000ECA63D,UPI0000ECA63C
20196-20225 HinckeM –TAXchicken	Uncategorized Sample	20196-20205_A	Mudpit_Ingel20196_MGFPeaklist (F058870)	Spectrin alpha chain, non-erythrocytic 1 OS=Gallus gallus GN=SPTAN1 PE=4 SV=2	F1NHT3_CHICK	TAX_GallusGallus_9031_20141114.fasta	285,330.20	100.00%	163	194	497	1.00%	56.40%	EANELQQWINEK	R	E	99.70%	58.1	44.5	28.1	41.3	39.7	2.96	0.199	0.458	0	4	0	0	2	1,501.73	2,446.50	7,052.73	1099	1110	Empty	TRUE	UPI0000ECA63D,UPI0000ECA63C
20196-20225 HinckeM –TAXchicken	Uncategorized Sample	20196-20205_A	Mudpit_Ingel20196_MGFPeaklist (F058870)	Spectrin alpha chain, non-erythrocytic 1 OS=Gallus gallus GN=SPTAN1 PE=4 SV=2	F1NHT3_CHICK	TAX_GallusGallus_9031_20141114.fasta	285,330.20	100.00%	163	194	497	1.00%	56.40%	EELIANWEQIR	R	T	99.70%	66.7	44.8	26.4	53.1	36.2	3.04	0.312	0.455	0	2	0	0	2	1,400.72	2,516.50	1,200.15	337	347	Empty	TRUE	UPI0000ECA63D,UPI0000ECA63C
20196-20225 HinckeM –TAXchicken	Uncategorized Sample	20196-20205_A	Mudpit_Ingel20196_MGFPeaklist (F058870)	Spectrin alpha chain, non-erythrocytic 1 OS=Gallus gallus GN=SPTAN1 PE=4 SV=2	F1NHT3_CHICK	TAX_GallusGallus_9031_20141114.fasta	285,330.20	100.00%	163	194	497	1.00%	56.40%	EELYQNLTR	K	E	99.70%	34.6	44.5	28.6	22.4	25.1	0.602	0.207	0.333	0	2	0	0	2	1,165.58	1,995.00	1,252.27	2432	2440	Empty	TRUE	UPI0000ECA63D,UPI0000ECA63C
20196-20225 HinckeM –TAXchicken	Uncategorized Sample	20196-20205_A	Mudpit_Ingel20196_MGFPeaklist (F058870)	Spectrin alpha chain, non-erythrocytic 1 OS=Gallus gallus GN=SPTAN1 PE=4 SV=2	F1NHT3_CHICK	TAX_GallusGallus_9031_20141114.fasta	285,330.20	100.00%	163	194	497	1.00%	56.40%	EKEPIVGSTDYGKDEDSAEALLK	R	K	99.70%	65.7	43.9	14.1	65.7	62.1	7.82	0.539	0.413	0	0	0	1	2	2,494.22	2,255.00	1,241.24	908	930	Empty	TRUE	UPI0000ECA63D,UPI0000ECA63C
20196-20225 HinckeM –TAXchicken	Uncategorized Sample	20196-20205_A	Mudpit_Ingel20196_MGFPeaklist (F058870)	Spectrin alpha chain, non-erythrocytic 1 OS=Gallus gallus GN=SPTAN1 PE=4 SV=2	F1NHT3_CHICK	TAX_GallusGallus_9031_20141114.fasta	285,330.20	100.00%	163	194	497	1.00%	56.40%	EKLDILDQER	K	T	83.40%	33.7	44.9	27.2	0					0	0	4	0	2	1,258.66	2,055.00	1,988.67	1418	1427	Empty	TRUE	UPI0000ECA63D,UPI0000ECA63C
20196-20225 HinckeM –TAXchicken	Uncategorized Sample	20196-20205_A	Mudpit_Ingel20196_MGFPeaklist (F058870)	Spectrin alpha chain, non-erythrocytic 1 OS=Gallus gallus GN=SPTAN1 PE=4 SV=2	F1NHT3_CHICK	TAX_GallusGallus_9031_20141114.fasta	285,330.20	100.00%	163	194	497	1.00%	56.40%	EKLDILDQERTDLEK	K	A	99.70%	19.6	44.4	26.8	1.26	33	0.854	0.212	0.433	0	0	1	1	2	1,844.96	2,203.00	3,854.79	1418	1432	Empty	TRUE	UPI0000ECA63D,UPI0000ECA63C
20196-20225 HinckeM –TAXchicken	Uncategorized Sample	20196-20205_A	Mudpit_Ingel20196_MGFPeaklist (F058870)	Spectrin alpha chain, non-erythrocytic 1 OS=Gallus gallus GN=SPTAN1 PE=4 SV=2	F1NHT3_CHICK	TAX_GallusGallus_9031_20141114.fasta	285,330.20	100.00%	163	194	497	1.00%	56.40%	ELPSAYDYIEFTR	R	S	99.70%	64.8	44.4	18.8	59.2	52.8	6.02	0.468	0.5	0	2	0	0	2	1,603.76	2,609.00	5,535.00	2460	2472	Empty	TRUE	UPI0000ECA63D,UPI0000ECA63C
20196-20225 HinckeM –TAXchicken	Uncategorized Sample	20196-20205_A	Mudpit_Ingel20196_MGFPeaklist (F058870)	Spectrin alpha chain, non-erythrocytic 1 OS=Gallus gallus GN=SPTAN1 PE=4 SV=2	F1NHT3_CHICK	TAX_GallusGallus_9031_20141114.fasta	285,330.20	100.00%	163	194	497	1.00%	56.40%	ENLLEEQGSIALR	R	Q	99.70%	68.9	44.4	29.3	49	46.7	3.66	0.345	0.5	0	2	0	0	2	1,471.78	2,391.50	1,931.07	1033	1045	Empty	TRUE	UPI0000ECA63D,UPI0000ECA63C
20196-20225 HinckeM –TAXchicken	Uncategorized Sample	20196-20205_A	Mudpit_Ingel20196_MGFPeaklist (F058870)	Spectrin alpha chain, non-erythrocytic 1 OS=Gallus gallus GN=SPTAN1 PE=4 SV=2	F1NHT3_CHICK	TAX_GallusGallus_9031_20141114.fasta	285,330.20	100.00%	163	194	497	1.00%	56.40%	ETENVKSSEEIESAFR	R	A	99.70%	92.7	43.9	24	78.8	46.2	6.72	0.437	0.406	0	0	3	0	2	1,854.87	2,071.00	5,899.01	2404	2419	Empty	TRUE	UPI0000ECA63D,UPI0000ECA63C
20196-20225 HinckeM –TAXchicken	Uncategorized Sample	20196-20205_A	Mudpit_Ingel20196_MGFPeaklist (F058870)	Spectrin alpha chain, non-erythrocytic 1 OS=Gallus gallus GN=SPTAN1 PE=4 SV=2	F1NHT3_CHICK	TAX_GallusGallus_9031_20141114.fasta	285,330.20	100.00%	163	194	497	1.00%	56.40%	FEEFQTDLAAHEER	K	V	99.70%	76.2	43.6	22.9	57.2	47.8	7.85	0.556	0.571	0	0	3	0	2	1,721.78	2,103.00	2,941.94	191	204	Empty	TRUE	UPI0000ECA63D,UPI0000ECA63C
20196-20225 HinckeM –TAXchicken	Uncategorized Sample	20196-20205_A	Mudpit_Ingel20196_MGFPeaklist (F058870)	Spectrin alpha chain, non-erythrocytic 1 OS=Gallus gallus GN=SPTAN1 PE=4 SV=2	F1NHT3_CHICK	TAX_GallusGallus_9031_20141114.fasta	285,330.20	100.00%	163	194	497	1.00%	56.40%	FHRDADETKEWIEEK	R	N	31.80%	15.8	44	21.1	3.91					0	0	0	2	2	1,932.91	1,813.00	1,544.53	1238	1252	Empty	TRUE	UPI0000ECA63D,UPI0000ECA63C
20196-20225 HinckeM –TAXchicken	Uncategorized Sample	20196-20205_A	Mudpit_Ingel20196_MGFPeaklist (F058870)	Spectrin alpha chain, non-erythrocytic 1 OS=Gallus gallus GN=SPTAN1 PE=4 SV=2	F1NHT3_CHICK	TAX_GallusGallus_9031_20141114.fasta	285,330.20	100.00%	163	194	497	1.00%	56.40%	FKELSSLR	R	R	92.50%	39.2	43.5	35.2	7.63					0	1	0	0	2	979.5571	1,859.00	429.66	29	36	Empty	TRUE	UPI0000ECA63D,UPI0000ECA63C
20196-20225 HinckeM –TAXchicken	Uncategorized Sample	20196-20205_A	Mudpit_Ingel20196_MGFPeaklist (F058870)	Spectrin alpha chain, non-erythrocytic 1 OS=Gallus gallus GN=SPTAN1 PE=4 SV=2	F1NHT3_CHICK	TAX_GallusGallus_9031_20141114.fasta	285,330.20	100.00%	163	194	497	1.00%	56.40%	FKELSSLRR	R	Q	99.70%	18.7	42.9	32	-1.14	30.7	0.523	0.111	0.5	0	1	0	0	2	1,135.66	1,717.00	480.35	29	37	Empty	TRUE	UPI0000ECA63D,UPI0000ECA63C
20196-20225 HinckeM –TAXchicken	Uncategorized Sample	20196-20205_A	Mudpit_Ingel20196_MGFPeaklist (F058870)	Spectrin alpha chain, non-erythrocytic 1 OS=Gallus gallus GN=SPTAN1 PE=4 SV=2	F1NHT3_CHICK	TAX_GallusGallus_9031_20141114.fasta	285,330.20	100.00%	163	194	497	1.00%	56.40%	FNRDVDETISWIK	R	E	99.70%	60.8	44.6	29.7	43	47.3	5.21	0.438	0.538	0	0	2	0	2	1,622.82	2,445.50	2,421.98	260	272	Empty	TRUE	UPI0000ECA63D,UPI0000ECA63C
20196-20225 HinckeM –TAXchicken	Uncategorized Sample	20196-20205_A	Mudpit_Ingel20196_MGFPeaklist (F058870)	Spectrin alpha chain, non-erythrocytic 1 OS=Gallus gallus GN=SPTAN1 PE=4 SV=2	F1NHT3_CHICK	TAX_GallusGallus_9031_20141114.fasta	285,330.20	100.00%	163	194	497	1.00%	56.40%	FQFFQR	R	D	90.00%	32.9	44.6	35.5	0	22.5	0.62	0.151	0.5	0	1	0	0	2	872.4413	2,321.00	2,158.44	46	51	Empty	TRUE	UPI0000ECA63D,UPI0000ECA63C
20196-20225 HinckeM –TAXchicken	Uncategorized Sample	20196-20205_A	Mudpit_Ingel20196_MGFPeaklist (F058870)	Spectrin alpha chain, non-erythrocytic 1 OS=Gallus gallus GN=SPTAN1 PE=4 SV=2	F1NHT3_CHICK	TAX_GallusGallus_9031_20141114.fasta	285,330.20	100.00%	163	194	497	1.00%	56.40%	GEIDAHEDSFR	K	S	99.70%	54.2	42.8	19.4	43.8	36.9	4.27	0.363	0.5	0	0	3	0	2	1,275.56	1,681.00	1,341.73	408	418	Empty	TRUE	UPI0000ECA63D,UPI0000ECA63C
20196-20225 HinckeM –TAXchicken	Uncategorized Sample	20196-20205_A	Mudpit_Ingel20196_MGFPeaklist (F058870)	Spectrin alpha chain, non-erythrocytic 1 OS=Gallus gallus GN=SPTAN1 PE=4 SV=2	F1NHT3_CHICK	TAX_GallusGallus_9031_20141114.fasta	285,330.20	100.00%	163	194	497	1.00%	56.40%	GKDLIGVQNLLK	R	K	99.70%	34.5	40.2	30.1	20	30.3	0.569	0.0726	0.375	0	0	3	0	2	1,297.78	2,464.00	1,473.99	813	824	Empty	TRUE	UPI0000ECA63D,UPI0000ECA63C
20196-20225 HinckeM –TAXchicken	Uncategorized Sample	20196-20205_A	Mudpit_Ingel20196_MGFPeaklist (F058870)	Spectrin alpha chain, non-erythrocytic 1 OS=Gallus gallus GN=SPTAN1 PE=4 SV=2	F1NHT3_CHICK	TAX_GallusGallus_9031_20141114.fasta	285,330.20	100.00%	163	194	497	1.00%	56.40%	GKDLIGVQNLLKK	R	H	99.70%	45.5	37.7	27.1	31.6	34.3	1.85	0.324	0.462	0	0	1	0	2	1,425.88	2,274.00	607.93	813	825	Empty	TRUE	UPI0000ECA63D,UPI0000ECA63C
20196-20225 HinckeM –TAXchicken	Uncategorized Sample	20196-20205_A	Mudpit_Ingel20196_MGFPeaklist (F058870)	Spectrin alpha chain, non-erythrocytic 1 OS=Gallus gallus GN=SPTAN1 PE=4 SV=2	F1NHT3_CHICK	TAX_GallusGallus_9031_20141114.fasta	285,330.20	100.00%	163	194	497	1.00%	56.40%	GLVSSDELAK	R	D	99.70%	33.3	45.5	21.5	20.8	32.7	0.77	0.269	0.45	0	4	0	0	2	1,018.54	1,876.00	3,728.83	1360	1369	Empty	TRUE	UPI0000ECA63D,UPI0000ECA63C
20196-20225 HinckeM –TAXchicken	Uncategorized Sample	20196-20205_A	Mudpit_Ingel20196_MGFPeaklist (F058870)	Spectrin alpha chain, non-erythrocytic 1 OS=Gallus gallus GN=SPTAN1 PE=4 SV=2	F1NHT3_CHICK	TAX_GallusGallus_9031_20141114.fasta	285,330.20	100.00%	163	194	497	1.00%	56.40%	GLVSSDELAKDVTGAEALLER	R	H	99.70%	16.7	43.5	0	16.7	30.6	2.43	0.307	0.19	0	0	1	0	2	2,173.13	2,866.00	118.97	1360	1380	Empty	TRUE	UPI0000ECA63D,UPI0000ECA63C
20196-20225 HinckeM –TAXchicken	Uncategorized Sample	20196-20205_A	Mudpit_Ingel20196_MGFPeaklist (F058870)	Spectrin alpha chain, non-erythrocytic 1 OS=Gallus gallus GN=SPTAN1 PE=4 SV=2	F1NHT3_CHICK	TAX_GallusGallus_9031_20141114.fasta	285,330.20	100.00%	163	194	497	1.00%	56.40%	GNAMVEEGHFAAEDVK	K	I	99.70%	32.3	43.4	19.2	25.2	39.8	5	0.093	0.438	0	0	4	0	2	1,703.77	1,883.00	2,892.17	847	862	Empty	TRUE	UPI0000ECA63D,UPI0000ECA63C
20196-20225 HinckeM –TAXchicken	Uncategorized Sample	20196-20205_A	Mudpit_Ingel20196_MGFPeaklist (F058870)	Spectrin alpha chain, non-erythrocytic 1 OS=Gallus gallus GN=SPTAN1 PE=4 SV=2	F1NHT3_CHICK	TAX_GallusGallus_9031_20141114.fasta	285,330.20	100.00%	163	194	497	1.00%	56.40%	GQLMASDDFGR	K	D	17.90%	16.1	43	17	4.02					0	1	0	0	2	1,196.54	1,997.00	232.87	275	285	Empty	TRUE	UPI0000ECA63D,UPI0000ECA63C
20196-20225 HinckeM –TAXchicken	Uncategorized Sample	20196-20205_A	Mudpit_Ingel20196_MGFPeaklist (F058870)	Spectrin alpha chain, non-erythrocytic 1 OS=Gallus gallus GN=SPTAN1 PE=4 SV=2	F1NHT3_CHICK	TAX_GallusGallus_9031_20141114.fasta	285,330.20	100.00%	163	194	497	1.00%	56.40%	GRELPSAYDYIEFTR	K	S	99.70%	45.8	44.6	20	38.3	39.9	5.82	0.451	0.433	0	0	2	0	2	1,816.89	2,471.00	1,677.70	2458	2472	Empty	TRUE	UPI0000ECA63D,UPI0000ECA63C
20196-20225 HinckeM –TAXchicken	Uncategorized Sample	20196-20205_A	Mudpit_Ingel20196_MGFPeaklist (F058870)	Spectrin alpha chain, non-erythrocytic 1 OS=Gallus gallus GN=SPTAN1 PE=4 SV=2	F1NHT3_CHICK	TAX_GallusGallus_9031_20141114.fasta	285,330.20	100.00%	163	194	497	1.00%	56.40%	GVIEMGNSLIER	R	G	99.70%	66.6	45	38.5	47.6	40.3	4.08	0.362	0.5	0	12	0	0	2	1,333.68	2,204.00	7,825.97	1613	1624	Empty	TRUE	UPI0000ECA63D,UPI0000ECA63C
20196-20225 HinckeM –TAXchicken	Uncategorized Sample	20196-20205_A	Mudpit_Ingel20196_MGFPeaklist (F058870)	Spectrin alpha chain, non-erythrocytic 1 OS=Gallus gallus GN=SPTAN1 PE=4 SV=2	F1NHT3_CHICK	TAX_GallusGallus_9031_20141114.fasta	285,330.20	100.00%	163	194	497	1.00%	56.40%	HALLEADVAAHQDR	K	I	99.70%	64.1	44.8	20.6	54.9	48.4	7.38	0.525	0.536	0	0	2	0	2	1,545.78	1,778.00	998.16	720	733	Empty	TRUE	UPI0000ECA63D,UPI0000ECA63C
20196-20225 HinckeM –TAXchicken	Uncategorized Sample	20196-20205_A	Mudpit_Ingel20196_MGFPeaklist (F058870)	Spectrin alpha chain, non-erythrocytic 1 OS=Gallus gallus GN=SPTAN1 PE=4 SV=2	F1NHT3_CHICK	TAX_GallusGallus_9031_20141114.fasta	285,330.20	100.00%	163	194	497	1.00%	56.40%	HEAFETDFTVHKDR	K	V	99.70%	53.8	43.8	16.3	42.9	41.6	7.57	0.459	0.571	0	0	0	2	2	1,731.81	1,862.50	2,458.96	1917	1930	Empty	TRUE	UPI0000ECA63D,UPI0000ECA63C
20196-20225 HinckeM –TAXchicken	Uncategorized Sample	20196-20205_A	Mudpit_Ingel20196_MGFPeaklist (F058870)	Spectrin alpha chain, non-erythrocytic 1 OS=Gallus gallus GN=SPTAN1 PE=4 SV=2	F1NHT3_CHICK	TAX_GallusGallus_9031_20141114.fasta	285,330.20	100.00%	163	194	497	1.00%	56.40%	HEDFDKAINVQEEK	K	I	99.70%	49.5	44.1	25.4	41.5	39.8	3.92	0.314	0.464	0	0	2	0	2	1,701.81	1,806.00	2,218.76	1487	1500	Empty	TRUE	UPI0000ECA63D,UPI0000ECA63C
20196-20225 HinckeM –TAXchicken	Uncategorized Sample	20196-20205_A	Mudpit_Ingel20196_MGFPeaklist (F058870)	Spectrin alpha chain, non-erythrocytic 1 OS=Gallus gallus GN=SPTAN1 PE=4 SV=2	F1NHT3_CHICK	TAX_GallusGallus_9031_20141114.fasta	285,330.20	100.00%	163	194	497	1.00%	56.40%	HQAFEAELHANADR	K	I	99.70%	62.2	44.4	19.1	47.2	39.9	4.89	0.429	0.536	0	0	4	7	2	1,608.75	1,756.00	40,595.10	1597	1610	Empty	TRUE	UPI0000ECA63D,UPI0000ECA63C
20196-20225 HinckeM –TAXchicken	Uncategorized Sample	20196-20205_A	Mudpit_Ingel20196_MGFPeaklist (F058870)	Spectrin alpha chain, non-erythrocytic 1 OS=Gallus gallus GN=SPTAN1 PE=4 SV=2	F1NHT3_CHICK	TAX_GallusGallus_9031_20141114.fasta	285,330.20	100.00%	163	194	497	1.00%	56.40%	HQAFEAELHANADRIR	K	G	99.70%	47.5	44.4	18.6	36.5	57	10.1	0.549	0.625	0	0	0	2	2	1,877.94	1,925.00	2,524.63	1597	1612	Empty	TRUE	UPI0000ECA63D,UPI0000ECA63C
20196-20225 HinckeM –TAXchicken	Uncategorized Sample	20196-20205_A	Mudpit_Ingel20196_MGFPeaklist (F058870)	Spectrin alpha chain, non-erythrocytic 1 OS=Gallus gallus GN=SPTAN1 PE=4 SV=2	F1NHT3_CHICK	TAX_GallusGallus_9031_20141114.fasta	285,330.20	100.00%	163	194	497	1.00%	56.40%	HQAFEAELSANQSR	K	I	99.70%	66.5	44.3	30	45.2	45.9	5.14	0.497	0.5	0	0	5	0	2	1,587.75	1,830.00	11,856.60	614	627	Empty	TRUE	UPI0000ECA63D,UPI0000ECA63C
20196-20225 HinckeM –TAXchicken	Uncategorized Sample	20196-20205_A	Mudpit_Ingel20196_MGFPeaklist (F058870)	Spectrin alpha chain, non-erythrocytic 1 OS=Gallus gallus GN=SPTAN1 PE=4 SV=2	F1NHT3_CHICK	TAX_GallusGallus_9031_20141114.fasta	285,330.20	100.00%	163	194	497	1.00%	56.40%	HQAFEAEVQANSGAIVK	K	L	99.70%	61.9	44.5	17.6	50.4	46	6.64	0.47	0.441	0	0	2	0	2	1,798.91	2,037.50	2,840.66	85	101	Empty	TRUE	UPI0000ECA63D,UPI0000ECA63C
20196-20225 HinckeM –TAXchicken	Uncategorized Sample	20196-20205_A	Mudpit_Ingel20196_MGFPeaklist (F058870)	Spectrin alpha chain, non-erythrocytic 1 OS=Gallus gallus GN=SPTAN1 PE=4 SV=2	F1NHT3_CHICK	TAX_GallusGallus_9031_20141114.fasta	285,330.20	100.00%	163	194	497	1.00%	56.40%	HQALQAEIAGHEPR	K	I	99.70%	74.2	44.5	26	57.2	54.2	6.68	0.491	0.607	0	0	4	0	2	1,556.79	1,758.00	8,943.66	826	839	Empty	TRUE	UPI0000ECA63D,UPI0000ECA63C
20196-20225 HinckeM –TAXchicken	Uncategorized Sample	20196-20205_A	Mudpit_Ingel20196_MGFPeaklist (F058870)	Spectrin alpha chain, non-erythrocytic 1 OS=Gallus gallus GN=SPTAN1 PE=4 SV=2	F1NHT3_CHICK	TAX_GallusGallus_9031_20141114.fasta	285,330.20	100.00%	163	194	497	1.00%	56.40%	HQEHKGEIDAHEDSFR	R	S	99.70%	72.9	42.9	21.1	65.1	39.8	4.54	0.412	0.469	0	0	0	4	2	1,934.87	1,482.50	1,869.44	403	418	Empty	TRUE	UPI0000ECA63D,UPI0000ECA63C
20196-20225 HinckeM –TAXchicken	Uncategorized Sample	20196-20205_A	Mudpit_Ingel20196_MGFPeaklist (F058870)	Spectrin alpha chain, non-erythrocytic 1 OS=Gallus gallus GN=SPTAN1 PE=4 SV=2	F1NHT3_CHICK	TAX_GallusGallus_9031_20141114.fasta	285,330.20	100.00%	163	194	497	1.00%	56.40%	HQKHQAFEAELHANADR	K	I	99.70%	47	44.2	15.5	38.2	44.4	6.32	0.432	0.5	0	0	0	1	2	2,001.96	1,605.00	3,536.86	1594	1610	Empty	TRUE	UPI0000ECA63D,UPI0000ECA63C
20196-20225 HinckeM –TAXchicken	Uncategorized Sample	20196-20205_A	Mudpit_Ingel20196_MGFPeaklist (F058870)	Spectrin alpha chain, non-erythrocytic 1 OS=Gallus gallus GN=SPTAN1 PE=4 SV=2	F1NHT3_CHICK	TAX_GallusGallus_9031_20141114.fasta	285,330.20	100.00%	163	194	497	1.00%	56.40%	HQLLEADISAHEDR	K	L	99.70%	66.6	44.7	20.9	52	43.3	6.59	0.434	0.571	0	0	2	1	2	1,633.79	1,877.00	4,314.61	1705	1718	Empty	TRUE	UPI0000ECA63D,UPI0000ECA63C
20196-20225 HinckeM –TAXchicken	Uncategorized Sample	20196-20205_A	Mudpit_Ingel20196_MGFPeaklist (F058870)	Spectrin alpha chain, non-erythrocytic 1 OS=Gallus gallus GN=SPTAN1 PE=4 SV=2	F1NHT3_CHICK	TAX_GallusGallus_9031_20141114.fasta	285,330.20	100.00%	163	194	497	1.00%	56.40%	IAVLQSFADQLIAADHYAK	K	G	99.70%	64.2	43.4	19.6	53.3	36.7	3.12	0.256	0.289	0	0	1	0	2	2,074.10	2,878.00	2,845.93	1501	1519	Empty	TRUE	UPI0000ECA63D,UPI0000ECA63C
20196-20225 HinckeM –TAXchicken	Uncategorized Sample	20196-20205_A	Mudpit_Ingel20196_MGFPeaklist (F058870)	Spectrin alpha chain, non-erythrocytic 1 OS=Gallus gallus GN=SPTAN1 PE=4 SV=2	F1NHT3_CHICK	TAX_GallusGallus_9031_20141114.fasta	285,330.20	100.00%	163	194	497	1.00%	56.40%	IKLNELNQKWDSLK	K	A	20.70%	9.82	42.7	24.8	[illegible]	[illegible]	[illegible]	[illegible]	[illegible]	[illegible]	[illegible]	[illegible]	[illegible]	[illegible]	[illegible]	[illegible]	[illegible]	[illegible]	[illegible]	[illegible]	[illegible]	[illegible]	[illegible]

20196-20225 HinckeM –TAXchicken	Uncategorized Sample	20196-20205_A	Mudpit_Ingel20196_MGFPeaklist (F058870)	Spectrin alpha chain, non-erythrocytic 1 OS=Gallus gallus GN=SPTAN1 PE=4 SV=2	F1NHT3_CHICK	TAX_GallusGallus_9031_20141114.fasta	285,330.20	100.00%	163	194	497	1.00%	56.40%	LDETGNQMINEGHFASETIR	K	T	99.70%	67.9	43.1	15.8	56.8	46	5.8	0.385	0.35	0	0	2	0	2	2,278.04	2,003.50		4,213.23	102	121	Empty	TRUE	UPI0000ECA63D,UPI0000ECA63C
20196-20225 HinckeM –TAXchicken	Uncategorized Sample	20196-20205_A	Mudpit_Ingel20196_MGFPeaklist (F058870)	Spectrin alpha chain, non-erythrocytic 1 OS=Gallus gallus GN=SPTAN1 PE=4 SV=2	F1NHT3_CHICK	TAX_GallusGallus_9031_20141114.fasta	285,330.20	100.00%	163	194	497	1.00%	56.40%	LDILDQER	K	T	99.70%	60.4	45.2	46.3	15.6	32.3	0.721	0.201	0.5	0	2	0	0	2	1,001.53	2,098.00		952.38	1420	1427	Empty	TRUE	UPI0000ECA63D,UPI0000ECA63C
20196-20225 HinckeM –TAXchicken	Uncategorized Sample	20196-20205_A	Mudpit_Ingel20196_MGFPeaklist (F058870)	Spectrin alpha chain, non-erythrocytic 1 OS=Gallus gallus GN=SPTAN1 PE=4 SV=2	F1NHT3_CHICK	TAX_GallusGallus_9031_20141114.fasta	285,330.20	100.00%	163	194	497	1.00%	56.40%	LDILDQERTDLEK	K	A	99.70%	41.5	44.7	29.8	16.1	41.1	1.12	0.185	0.462	0	0	1	0	2	1,587.82	2,269.00		3,310.69	1420	1432	Empty	TRUE	UPI0000ECA63D,UPI0000ECA63C
20196-20225 HinckeM –TAXchicken	Uncategorized Sample	20196-20205_A	Mudpit_Ingel20196_MGFPeaklist (F058870)	Spectrin alpha chain, non-erythrocytic 1 OS=Gallus gallus GN=SPTAN1 PE=4 SV=2	F1NHT3_CHICK	TAX_GallusGallus_9031_20141114.fasta	285,330.20	100.00%	163	194	497	1.00%	56.40%	LEAELAAHEPAIQGVLDTGKK	R	L	99.70%	30.8	42.4	19.8	22.5	37.4	4.1	0.34	0.31	0	0	0	1	2	2,190.18	2,340.00		1,117.91	1814	1834	Empty	TRUE	UPI0000ECA63D,UPI0000ECA63C
20196-20225 HinckeM –TAXchicken	Uncategorized Sample	20196-20205_A	Mudpit_Ingel20196_MGFPeaklist (F058870)	Spectrin alpha chain, non-erythrocytic 1 OS=Gallus gallus GN=SPTAN1 PE=4 SV=2	F1NHT3_CHICK	TAX_GallusGallus_9031_20141114.fasta	285,330.20	100.00%	163	194	497	1.00%	56.40%	LFGAAEVQR	K	F	99.70%	67.3	45.1	36.8	48.6	34.7	1.77	0.337	0.556	0	4	0	0	2	990.5367	1,929.00		10,580.30	251	259	Empty	TRUE	UPI0000ECA63D,UPI0000ECA63C
20196-20225 HinckeM –TAXchicken	Uncategorized Sample	20196-20205_A	Mudpit_Ingel20196_MGFPeaklist (F058870)	Spectrin alpha chain, non-erythrocytic 1 OS=Gallus gallus GN=SPTAN1 PE=4 SV=2	F1NHT3_CHICK	TAX_GallusGallus_9031_20141114.fasta	285,330.20	100.00%	163	194	497	1.00%	56.40%	LGESQTLQQFSR	K	D	99.70%	60.8	44.7	24.7	36.5	49.5	5.12	0.469	0.583	0	7	1	0	2	1,393.71	2,032.00		36,229.30	1547	1558	Empty	TRUE	UPI0000ECA63D,UPI0000ECA63C
20196-20225 HinckeM –TAXchicken	Uncategorized Sample	20196-20205_A	Mudpit_Ingel20196_MGFPeaklist (F058870)	Spectrin alpha chain, non-erythrocytic 1 OS=Gallus gallus GN=SPTAN1 PE=4 SV=2	F1NHT3_CHICK	TAX_GallusGallus_9031_20141114.fasta	285,330.20	100.00%	163	194	497	1.00%	56.40%	LIDVNHYASDEVAAR	K	M	99.70%	62.4	44.7	25.1	51.9	48	5.25	0.463	0.533	0	0	9	0	2	1,673.81	2,017.00		15,639.90	638	652	Empty	TRUE	UPI0000ECA63D,UPI0000ECA63C
20196-20225 HinckeM –TAXchicken	Uncategorized Sample	20196-20205_A	Mudpit_Ingel20196_MGFPeaklist (F058870)	Spectrin alpha chain, non-erythrocytic 1 OS=Gallus gallus GN=SPTAN1 PE=4 SV=2	F1NHT3_CHICK	TAX_GallusGallus_9031_20141114.fasta	285,330.20	100.00%	163	194	497	1.00%	56.40%	LIQEQHPEEELIK	K	S	99.70%	39.4	44.4	25.6	29.3	37.4	1.68	0.193	0.462	0	0	3	0	2	1,605.85	1,970.00		11,713.00	215	227	Empty	TRUE	UPI0000ECA63D,UPI0000ECA63C
20196-20225 HinckeM –TAXchicken	Uncategorized Sample	20196-20205_A	Mudpit_Ingel20196_MGFPeaklist (F058870)	Spectrin alpha chain, non-erythrocytic 1 OS=Gallus gallus GN=SPTAN1 PE=4 SV=2	F1NHT3_CHICK	TAX_GallusGallus_9031_20141114.fasta	285,330.20	100.00%	163	194	497	1.00%	56.40%	LIQEQHPEEELIKSK	K	Q	27.20%	12.6	43.6	15.4	5.54					0	0	0	2	2	1,820.98	1,860.00		920.77	215	229	Empty	TRUE	UPI0000ECA63D,UPI0000ECA63C
20196-20225 HinckeM –TAXchicken	Uncategorized Sample	20196-20205_A	Mudpit_Ingel20196_MGFPeaklist (F058870)	Spectrin alpha chain, non-erythrocytic 1 OS=Gallus gallus GN=SPTAN1 PE=4 SV=2	F1NHT3_CHICK	TAX_GallusGallus_9031_20141114.fasta	285,330.20	100.00%	163	194	497	1.00%	56.40%	LIQNNHYAMDDVATR	K	R	99.70%	68.1	44.4	13	59.9	38.8	4.4	0.371	0.367	0	0	4	0	2	1,776.83	1,754.50		3,023.16	533	547	Empty	TRUE	UPI0000ECA63D,UPI0000ECA63C
20196-20225 HinckeM –TAXchicken	Uncategorized Sample	20196-20205_A	Mudpit_Ingel20196_MGFPeaklist (F058870)	Spectrin alpha chain, non-erythrocytic 1 OS=Gallus gallus GN=SPTAN1 PE=4 SV=2	F1NHT3_CHICK	TAX_GallusGallus_9031_20141114.fasta	285,330.20	100.00%	163	194	497	1.00%	56.40%	LIQNNHYAMDDVATRR	K	D	99.70%	34.4	44.5	13.1	30.5	40.7	3.49	0.364	0.406	0	0	0	6	2	1,916.94	1,626.50		7,812.11	533	548	Empty	TRUE	UPI0000ECA63D,UPI0000ECA63C
20196-20225 HinckeM –TAXchicken	Uncategorized Sample	20196-20205_A	Mudpit_Ingel20196_MGFPeaklist (F058870)	Spectrin alpha chain, non-erythrocytic 1 OS=Gallus gallus GN=SPTAN1 PE=4 SV=2	F1NHT3_CHICK	TAX_GallusGallus_9031_20141114.fasta	285,330.20	100.00%	163	194	497	1.00%	56.40%	LIQSHPESAEDLQEK	R	C	99.70%	71.4	44.6	22.4	57.1	45.9	4.37	0.401	0.5	0	0	5	0	2	1,723.85	1,669.00		16,131.80	1299	1313	Empty	TRUE	UPI0000ECA63D,UPI0000ECA63C
20196-20225 HinckeM –TAXchicken	Uncategorized Sample	20196-20205_A	Mudpit_Ingel20196_MGFPeaklist (F058870)	Spectrin alpha chain, non-erythrocytic 1 OS=Gallus gallus GN=SPTAN1 PE=4 SV=2	F1NHT3_CHICK	TAX_GallusGallus_9031_20141114.fasta	285,330.20	100.00%	163	194	497	1.00%	56.40%	LKDLNSQADSLMTSSAFDTSQVK	R	D	99.70%	67.5	44	22.2	61.8	41.7	5.11	0.446	0.261	0	0	2	0	2	2,502.20	2,157.00		2,188.60	1719	1741	Empty	TRUE	UPI0000ECA63D,UPI0000ECA63C
20196-20225 HinckeM –TAXchicken	Uncategorized Sample	20196-20205_A	Mudpit_Ingel20196_MGFPeaklist (F058870)	Spectrin alpha chain, non-erythrocytic 1 OS=Gallus gallus GN=SPTAN1 PE=4 SV=2	F1NHT3_CHICK	TAX_GallusGallus_9031_20141114.fasta	285,330.20	100.00%	163	194	497	1.00%	56.40%	LLEAQEHFR	K	K	99.70%	61.3	44.5	29.9	38.5	38.4	2.21	0.279	0.611	0	4	0	0	2	1,142.60	1,810.50		2,783.16	2084	2092	Empty	TRUE	UPI0000ECA63D,UPI0000ECA63C
20196-20225 HinckeM –TAXchicken	Uncategorized Sample	20196-20205_A	Mudpit_Ingel20196_MGFPeaklist (F058870)	Spectrin alpha chain, non-erythrocytic 1 OS=Gallus gallus GN=SPTAN1 PE=4 SV=2	F1NHT3_CHICK	TAX_GallusGallus_9031_20141114.fasta	285,330.20	100.00%	163	194	497	1.00%	56.40%	LLEATELK	K	G	74.40%	30.9	44.9	31.7	3.73					0	2	0	0	2	916.5351	1,999.50		1,326.52	663	670	Empty	TRUE	UPI0000ECA63D,UPI0000ECA63C
20196-20225 HinckeM –TAXchicken	Uncategorized Sample	20196-20205_A	Mudpit_Ingel20196_MGFPeaklist (F058870)	Spectrin alpha chain, non-erythrocytic 1 OS=Gallus gallus GN=SPTAN1 PE=4 SV=2	F1NHT3_CHICK	TAX_GallusGallus_9031_20141114.fasta	285,330.20	100.00%	163	194	497	1.00%	56.40%	LLEATELKGIK	K	L	99.70%	57	42.1	23.7	45.5	35	2.89	0.297	0.455	0	0	2	0	2	1,214.74	2,118.50		1,772.63	663	673	Empty	TRUE	UPI0000ECA63D,UPI0000ECA63C
20196-20225 HinckeM –TAXchicken	Uncategorized Sample	20196-20205_A	Mudpit_Ingel20196_MGFPeaklist (F058870)	Spectrin alpha chain, non-erythrocytic 1 OS=Gallus gallus GN=SPTAN1 PE=4 SV=2	F1NHT3_CHICK	TAX_GallusGallus_9031_20141114.fasta	285,330.20	100.00%	163	194	497	1.00%	56.40%	LLGSAHEVQR	Q	F	99.70%					44.4	1.77	0.412	0.6	0	2	0	0	1	1,109.61	1,686.00		645.63	1228	1237	Empty	TRUE	
20196-20225 HinckeM –TAXchicken	Uncategorized Sample	20196-20205_A	Mudpit_Ingel20196_MGFPeaklist (F058870)	Spectrin alpha chain, non-erythrocytic 1 OS=Gallus gallus GN=SPTAN1 PE=4 SV=2	F1NHT3_CHICK	TAX_GallusGallus_9031_20141114.fasta	285,330.20	100.00%	163	194	497	1.00%	56.40%	LLVSSEDYGR	K	D	99.70%	72.3	44.1	30.2	45	39.3	3.3	0.435	0.5	0	4	0	0	2	1,138.57	1,881.00		5,696.86	1790	1799	Empty	TRUE	UPI0000ECA63D,UPI0000ECA63C
20196-20225 HinckeM –TAXchicken	Uncategorized Sample	20196-20205_A	Mudpit_Ingel20196_MGFPeaklist (F058870)	Spectrin alpha chain, non-erythrocytic 1 OS=Gallus gallus GN=SPTAN1 PE=4 SV=2	F1NHT3_CHICK	TAX_GallusGallus_9031_20141114.fasta	285,330.20	100.00%	163	194	497	1.00%	56.40%	LLVSSEDYGRDLTGVQNLR	K	K	99.70%	57.2	43.9	21.7	46.9	49.3	7.52	0.46	0.447	0	0	2	0	2	2,135.11	2,381.50		3,116.02	1790	1808	Empty	TRUE	UPI0000ECA63D,UPI0000ECA63C
20196-20225 HinckeM –TAXchicken	Uncategorized Sample	20196-20205_A	Mudpit_Ingel20196_MGFPeaklist (F058870)	Spectrin alpha chain, non-erythrocytic 1 OS=Gallus gallus GN=SPTAN1 PE=4 SV=2	F1NHT3_CHICK	TAX_GallusGallus_9031_20141114.fasta	285,330.20	100.00%	163	194	497	1.00%	56.40%	LLVSSEDYGRDLTGVQNLRK	K	K	99.70%	23.5	42.8	15.5	21.9	31	0.678	0.187	0.3	0	0	0	2	2	2,263.20	2,268.50		1,286.29	1790	1809	Empty	TRUE	UPI0000ECA63D,UPI0000ECA63C
20196-20225 HinckeM –TAXchicken	Uncategorized Sample	20196-20205_A	Mudpit_Ingel20196_MGFPeaklist (F058870)	Spectrin alpha chain, non-erythrocytic 1 OS=Gallus gallus GN=SPTAN1 PE=4 SV=2	F1NHT3_CHICK	TAX_GallusGallus_9031_20141114.fasta	285,330.20	100.00%	163	194	497	1.00%	56.40%	LNELNQKWDSLK	K	A	99.70%	32.2	44.5	21.2	24.7	30.4	1.18	0.23	0.417	0	0	2	0	2	1,487.79	2,156.00		655.62	865	876	Empty	TRUE	UPI0000ECA63D,UPI0000ECA63C
20196-20225 HinckeM –TAXchicken	Uncategorized Sample	20196-20205_A	Mudpit_Ingel20196_MGFPeaklist (F058870)	Spectrin alpha chain, non-erythrocytic 1 OS=Gallus gallus GN=SPTAN1 PE=4 SV=2	F1NHT3_CHICK	TAX_GallusGallus_9031_20141114.fasta	285,330.20	100.00%	163	194	497	1.00%	56.40%	LQIASDENYKDPSNLQGK	K	L	99.70%	66.1	44.6	19.5	57.2	43.3	4.48	0.425	0.389	0	0	2	0	2	2,020.00	1,904.00		6,653.79	64	81	Empty	TRUE	UPI0000ECA63D,UPI0000ECA63C
20196-20225 HinckeM –TAXchicken	Uncategorized Sample	20196-20205_A	Mudpit_Ingel20196_MGFPeaklist (F058870)	Spectrin alpha chain, non-erythrocytic 1 OS=Gallus gallus GN=SPTAN1 PE=4 SV=2	F1NHT3_CHICK	TAX_GallusGallus_9031_20141114.fasta	285,330.20	100.00%	163	194	497	1.00%	56.40%	LQQLFR	R	D	24.20%	46	45	50.6	0					0	1	0	0	2	804.4727	2,087.00		1,798.04	786	791	Empty	TRUE	UPI0000ECA63D,UPI0000ECA63C
20196-20225 HinckeM –TAXchicken	Uncategorized Sample	20196-20205_A	Mudpit_Ingel20196_MGFPeaklist (F058870)	Spectrin alpha chain, non-erythrocytic 1 OS=Gallus gallus GN=SPTAN1 PE=4 SV=2	F1NHT3_CHICK	TAX_GallusGallus_9031_20141114.fasta	285,330.20	100.00%	163	194	497	1.00%	56.40%	LQQSHPINASQIQVK	R	R	58.10%	16.8	43.4	15.8	12.3					0	0	1	0	2	1,690.92	1,835.00		2,269.03	321	335	Empty	TRUE	UPI0000ECA63D,UPI0000ECA63C
20196-20225 HinckeM –TAXchicken	Uncategorized Sample	20196-20205_A	Mudpit_Ingel20196_MGFPeaklist (F058870)	Spectrin alpha chain, non-erythrocytic 1 OS=Gallus gallus GN=SPTAN1 PE=4 SV=2	F1NHT3_CHICK	TAX_GallusGallus_9031_20141114.fasta	285,330.20	100.00%	163	194	497	1.00%	56.40%	LSDDNTIGKEEIQQR	K	L	99.70%	86.1	44.8	34.6	0	52.4	4.66	0.41	0.5	0	0	6	0	2	1,745.87	1,719.50		20,857.20	1835	1849	Empty	TRUE	UPI0000ECA63D,UPI0000ECA63C
20196-20225 HinckeM –TAXchicken	Uncategorized Sample	20196-20205_A	Mudpit_Ingel20196_MGFPeaklist (F058870)	Spectrin alpha chain, non-erythrocytic 1 OS=Gallus gallus GN=SPTAN1 PE=4 SV=2	F1NHT3_CHICK	TAX_GallusGallus_9031_20141114.fasta	285,330.20	100.00%	163	194	497	1.00%	56.40%	LTILSDER	K	S	99.70%	82.2	45.3	46.6	21.2	39.7	2.17	0.224	0.688	0	8	0	0	2	946.5205	2,023.00		14,285.40	442	449	Empty	TRUE	UPI0000ECA63D,UPI0000ECA63C
20196-20225 HinckeM –TAXchicken	Uncategorized Sample	20196-20205_A	Mudpit_Ingel20196_MGFPeaklist (F058870)	Spectrin alpha chain, non-erythrocytic 1 OS=Gallus gallus GN=SPTAN1 PE=4 SV=2	F1NHT3_CHICK	TAX_GallusGallus_9031_20141114.fasta	285,330.20	100.00%	163	194	497	1.00%	56.40%	LWELLLEK	R	M	99.70%	44.7	43.8	40.6	14.8	36.7	1.32	0.18	0.688	0	2	0	0	2	1,043.61	2,822.00		1,411.94	130	137	Empty	TRUE	UPI0000ECA63D,UPI0000ECA63C
20196-20225 HinckeM –TAXchicken	Uncategorized Sample	20196-20205_A	Mudpit_Ingel20196_MGFPeaklist (F058870)	Spectrin alpha chain, non-erythrocytic 1 OS=Gallus gallus GN=SPTAN1 PE=4 SV=2	F1NHT3_CHICK	TAX_GallusGallus_9031_20141114.fasta	285,330.20	100.00%	163	194	497	1.00%	56.40%	MKQVEELYHSLLELGEK	R	R	99.70%					41.8	3.82	0.416	0.412	0	0	1	0	2	2,044.04	2,415.00		1,918.78	1066	1082	Empty	TRUE	
20196-20225 HinckeM –TAXchicken	Uncategorized Sample	20196-20205_A	Mudpit_Ingel20196_MGFPeaklist (F058870)	Spectrin alpha chain, non-erythrocytic 1 OS=Gallus gallus GN=SPTAN1 PE=4 SV=2	F1NHT3_CHICK	TAX_GallusGallus_9031_20141114.fasta	285,330.20	100.00%	163	194	497	1.00%	56.40%	MKTATDEAYKDPSNLQGK	K	V	99.70%	46.5	44	17.7	35.1	39.6	4.8	0.351	0.417	0	0	0	4	2	2,012.96	1,579.00		2,578.80	593	610	Empty	TRUE	UPI0000ECA63D,UPI0000ECA63C
20196-20225 HinckeM –TAXchicken	Uncategorized Sample	20196-20205_A	Mudpit_Ingel20196_MGFPeaklist (F058870)	Spectrin alpha chain, non-erythrocytic 1 OS=Gallus gallus GN=SPTAN1 PE=4 SV=2	F1NHT3_CHICK	TAX_GallusGallus_9031_20141114.fasta	285,330.20	100.00%	163	194	497	1.00%	56.40%	MMVHTVATFNSIK	R	E	99.70%	34.1	44.4	16.1	25.6	34.1	4.15	0.361	0.385	0	0	3	0	2	1,510.74	1,940.00		1,575.17	1197	1209	Empty	TRUE	UPI0000ECA63D,UPI0000ECA63C
20196-20225 HinckeM –TAXchicken	Uncategorized Sample	20196-20205_A	Mudpit_Ingel20196_MGFPeaklist (F058870)	Spectrin alpha chain, non-erythrocytic 1 OS=Gallus gallus GN=SPTAN1 PE=4 SV=2	F1NHT3_CHICK	TAX_GallusGallus_9031_20141114.fasta	285,330.20	100.00%	163	194	497	1.00%	56.40%	MMVHTVATFNSIKELNER	R	W	99.70%	53.3	44.2	25.9	40.6	38.9	4.11	0.262	0.361	0	0	0	2	2	2,152.05	2,142.00		1,346.02	1197	1214	Empty	TRUE	UPI0000ECA63D,UPI0000ECA63C
20196-20225 HinckeM –TAXchicken	Uncategorized Sample	20196-20205_A	Mudpit_Ingel20196_MGFPeaklist (F058870)	Spectrin alpha chain, non-erythrocytic 1 OS=Gallus gallus GN=SPTAN1 PE=4 SV=2	F1NHT3_CHICK	TAX_GallusGallus_9031_20141114.fasta	285,330.20	100.00%	163	194	497	1.00%	56.40%	MNEVISLWK	R	K	99.70%	70.2	45	33.2	39	37.9	1.82	0.106	0.611	0	6	0	0	2	1,119.59	2,522.50		3,261.81	653	661	Empty	TRUE	UPI0000ECA63D,UPI0000ECA63C
20196-20225 HinckeM –TAXchicken	Uncategorized Sample	20196-20205_A	Mudpit_Ingel20196_MGFPeaklist (F058870)	Spectrin alpha chain, non-erythrocytic 1 OS=Gallus gallus GN=SPTAN1 PE=4 SV=2	F1NHT3_CHICK	TAX_GallusGallus_9031_20141114.fasta	285,330.20	100.00%	163	194	497	1.00%	56.40%	MNEVISLWKK	R	L	47.00%	22.5	44.5	30.2	3.6					0	0	2	0	2	1,263.68	2,242.50		623.89	653	662	Empty	TRUE	UPI0000ECA63D,UPI0000ECA63C
20196-20225 HinckeM –TAXchicken	Uncategorized Sample	20196-20205_A	Mudpit_Ingel20196_MGFPeaklist (F058870)	Spectrin alpha chain, non-erythrocytic 1 OS=Gallus gallus GN=SPTAN1 PE=4 SV=2	F1NHT3_CHICK	TAX_GallusGallus_9031_20141114.fasta	285,330.20	100.00%	163	194	497	1.00%	56.40%	MQHNLEQQIQAR	R	N	99.70%	52.6	44.3	29.8	36.6	43.1	2.82	0.394	0.583	0	0	8	0	2	1,511.74	1,728.50		7,950.78	2309	2320	Empty	TRUE	UPI0000ECA63D,UPI0000ECA63C
20196-20225 HinckeM –TAXchicken	Uncategorized Sample	20196-20205_A	Mudpit_Ingel20196_MGFPeaklist (F058870)	Spectrin alpha chain, non-erythrocytic 1 OS=Gallus gallus GN=SPTAN1 PE=4 SV=2	F1NHT3_CHICK	TAX_GallusGallus_9031_20141114.fasta	285,330.20	100.00%	163	194	497	1.00%	56.40%	NQALNTDNYGHDLASVQALQR	K	K	99.70%	77.3	44.1	21.8	74.6	52.8	8.52	0.568	0.333	0	0	2	0	2	2,328.13	2,251.50		10,715.10	1253	1273	Empty	TRUE	UPI0000ECA63D,UPI0000ECA63C
20196-20225 HinckeM –TAXchicken	Uncategorized Sample	20196-20205_A	Mudpit_Ingel20196_MGFPeaklist (F058870)	Spectrin alpha chain, non-erythrocytic 1 OS=Gallus gallus GN=SPTAN1 PE=4 SV=2	F1NHT3_CHICK	TAX_GallusGallus_9031_20141114.fasta	285,330.20	100.00%	163	194	497	1.00%	56.40%	NTTGVTEEALKEFSMMFK	R	H	99.60%	41.4	43.6	13	40.4					0	0	2	0	2	2,094.97	2,776.50		1,235.05	2321	2338	Empty	TRUE	UPI0000ECA63D,UPI0000ECA63C
20196-20225 HinckeM –TAXchicken	Uncategorized Sample	20196-20205_A	Mudpit_Ingel20196_MGFPeaklist (F058870)	Spectrin alpha chain, non-erythrocytic 1 OS=Gallus gallus GN=SPTAN1 PE=4 SV=2	F1NHT3_CHICK	TAX_GallusGallus_9031_20141114.fasta	285,330.20	100.00%	163	194	497	1.00%	56.40%	QDEVNASWQR	K	L	99.70%	71.8	44.3	32.8	57.5	41.1	2.96	0.355	0.55	0	5	0	0	2	1,232.57	1,742.00		2,096.38	230	239	Empty	TRUE	UPI0000ECA63D,UPI0000ECA63C
20196-20225 HinckeM –TAXchicken	Uncategorized Sample	20196-20205_A	Mudpit_Ingel20196_MGFPeaklist (F058870)	Spectrin alpha chain, non-erythrocytic 1 OS=Gallus gallus GN=SPTAN1 PE=4 SV=2	F1NHT3_CHICK	TAX_GallusGallus_9031_20141114.fasta	285,330.20	100.00%	163	194	497	1.00%	56.40%	QEFAQHANAFHQWIQETR	R	T	99.70%	53	43.7	16.1	0	33.9	3.02	0.354	0.25	0	0	0	2	2	2,241.06	2,283.50		1,438.84	2213	2230	Empty	TRUE	UPI0000ECA63D,UPI0000ECA63C
20196-20225 HinckeM –TAXchicken	Uncategorized Sample	20196-20205_A	Mudpit_Ingel20196_MGFPeaklist (F058870)	Spectrin alpha chain, non-erythrocytic 1 OS=Gallus gallus GN=SPTAN1 PE=4 SV=2	F1NHT3_CHICK	TAX_GallusGallus_9031_20141114.fasta	285,330.20	100.00%	163	194	497	1.00%	56.40%	QEQIDNQTLITK	R	E	33.00%	27.9	44.2	0	15.2					0	0	1	0	2	1,413.72	1,650.00		60.03	1046	1057	Empty	TRUE	UPI0000ECA63C
20196-20225 HinckeM –TAXchicken	Uncategorized Sample	20196-20205_A	Mudpit_Ingel20196_MGFPeaklist (F058870)	Spectrin alpha chain, non-erythrocytic 1 OS=Gallus gallus GN=SPTAN1 PE=4 SV=2	F1NHT3_CHICK	TAX_GallusGallus_9031_20141114.fasta	285,330.20	100.00%	163	194	497	1.00%	56.40%	QFQDAGHFDADNIK	R	K	99.70%	64	42.5	21.5	55.9	54.7	5.25	0.00183	0.571	0	2	2	0	2	1,588.70	2,092.50		10,466.30	743	756	Empty	TRUE	UPI0000ECA63D,UPI0000ECA63C
20196-20225 HinckeM –TAXchicken	Uncategorized Sample	20196-20205_A	Mudpit_Ingel20196_MGFPeaklist (F058870)	Spectrin alpha chain, non-erythrocytic 1 OS=Gallus gallus GN=SPTAN1 PE=4 SV=2	F1NHT3_CHICK	TAX_GallusGallus_9031_20141114.fasta	285,330.20	100.00%	163	194	497	1.00%	56.40%	QFQDAGHFDADNIKK	R	K	99.70%	16	43.7	19.7	9.85	30.8	1.22	0.00325	0.433	0	0	2	0	2	1,716.80	2,087.00		3,109.36	743	757	Empty	TRUE	UPI0000ECA63D,UPI0000ECA63C
20196-20225 HinckeM –TAXchicken	Uncategorized Sample	20196-20205_A	Mudpit_Ingel20196_MGFPeaklist (F058870)	Spectrin alpha chain, non-erythrocytic 1 OS=Gallus gallus GN=SPTAN1 PE=4 SV=2	F1NHT3_CHICK	TAX_GallusGallus_9031_20141114.fasta	285,330.20	100.00%	163	194	497	1.00%	56.40%	QGFVPAAYVK	R	K	99.70%	41.1	44.1	26.3	21.9	38.4	3.28	0.237	0.5	0	8	0	0	2	1,079.59	2,175.50		9,621.08	1013	1022	Empty	TRUE	UPI0000ECA63D,UPI0000ECA63C
20196-20225 HinckeM –TAXchicken	Uncategorized Sample	20196-20205_A	Mudpit_Ingel20196_MGFPeaklist (F058870)	Spectrin alpha chain, non-erythrocytic 1 OS=Gallus gallus GN=SPTAN1 PE=4 SV=2	F1NHT3_CHICK	TAX_GallusGallus_9031_20141114.fasta	285,330.20	100.00%	163	194	497	1.00%	56.40%	QGKLFGAAEVQR	R	F	99.70%	53.8	44.4	25.1	33.7	30.3	1.24	0.218	0.333	0	0	2	0	2	1,303.71	1,865.00		655.81	248	259	Empty	TRUE	UPI0000ECA63D,UPI0000ECA63C
20196-20225 HinckeM –TAXchicken	Uncategorized Sample	20196-20205_A	Mudpit_Ingel20196_MGFPeaklist (F058870)	Spectrin alpha chain, non-erythrocytic 1 OS=Gallus gallus GN=SPTAN1 PE=4 SV=2	F1NHT3_CHICK	TAX_GallusGallus_9031_20141114.fasta	285,330.20	100.00%	163	194	497	1.00%	56.40%	QKLADSLR	K	L	96.10%	37.9	43.6	39.3	16.5					0	4	0	0	2	913.5103	1,860.50		1,083.35	778	785	Empty	TRUE	UPI0000ECA63D,UPI0000ECA63C
20196-20225 HinckeM –TAXchicken	Uncategorized Sample	20196-20205_A	Mudpit_Ingel20196_MGFPeaklist (F058870)	Spectrin alpha chain, non-erythrocytic 1 OS=Gallus gallus GN=SPTAN1 PE=4 SV=2	F1NHT3_CHICK	TAX_GallusGallus_9031_20141114.fasta	285,330.20	100.00%	163	194	497	1.00%	56.40%	QKLEDSYR	R	F	99.70%	43.5	44.8	35.7	18.9	37.3	2.14	0.00536	0.688	0	4	0	0	2	1,021.50	1,670.50		1,559.49	38	45	Empty	TRUE	UPI0000ECA63D,UPI0000ECA63C
20196-20225 HinckeM –TAXchicken	Uncategorized Sample	20196-20205_A	Mudpit_Ingel20196_MGFPeaklist (F058870)	Spectrin alpha chain, non-erythrocytic 1 OS=Gallus gallus GN=SPTAN1 PE=4 SV=2	F1NHT3_CHICK	TAX_GallusGallus_9031_20141114.fasta	285,330.20	100.00%	163	194	497	1.00%	56.40%	QQVAPTDDETGKELVLALYDYQEK	R	S	99.70%	67.1	43.7	14.3	65.9	46.1	5.89	0.482	0.333	0	0	1	0	2	2,753.35	2,733.00		626.58	958	981	Empty	TRUE	UPI0000ECA63D,UPI0000ECA63C
20196-20225 HinckeM –TAXchicken	Uncategorized Sample	20196-20205_A	Mudpit_Ingel20196_MGFPeaklist (F058870)	Spectrin alpha chain, non-erythrocytic 1 OS=Gallus gallus GN=SPTAN1 PE=4 SV=2	F1NHT3_CHICK	TAX_GallusGallus_9031_20141114.fasta	285,330.20	100.00%	163	194	497	1.00%	56.40%	REELIANWEQIR	K	T	99.70%	43.2	44.3	27.9	22.1	33.1	1.77	0.251	0.458	0	0	4	0	2	1,556.82	2,390.00		4,629.97	336	347	Empty	TRUE	UPI0000ECA63D,UPI0000ECA63C
20196-20225 HinckeM –TAXchicken	Uncategorized Sample	20196-20205_A	Mudpit_Ingel20196_MGFPeaklist (F058870)	Spectrin alpha chain, non-erythrocytic 1 OS=Gallus gallus GN=SPTAN1 PE=4 SV=2	F1NHT3_CHICK	TAX_GallusGallus_9031_20141114.fasta	285,330.20	100.00%	163	194	497	1.00%	56.40%	RLEAELAAHEPAIQGVLDTGKK	K	L	99.70%	51.6	41.1	0	50	50.5	7.68	0.554	0.386	0	0	0	3	2	2,346.28	2,305.00		3,180.64	1813	1834	Empty	TRUE	UPI0000ECA63D,UPI0000ECA63C
20196-20225 HinckeM –TAXchicken	Uncategorized Sample	20196-20205_A	Mudpit_Ingel20196_MGFPeaklist (F058870)	Spectrin alpha chain, non-erythrocytic 1 OS=Gallus gallus GN=SPTAN1 PE=4 SV=2	F1NHT3_CHICK	TAX_GallusGallus_9031_20141114.fasta	285,330.20	100.00%	163	194	497	1.00%	56.40%	RWNQLLANSAAR	K	K	99.70%	52.7	44.2	29.3	30.5	30.1	3.28	0.266	0.375	0	0	2	0	2	1,399.76	2,051.50		749.55	2069	2080	Empty	TRUE	UPI0000ECA63D,UPI0000ECA63C
20196-20225 HinckeM –TAXchicken	Uncategorized Sample	20196-20205_A	Mudpit_Ingel20196_MGFPeaklist (F058870)	Spectrin alpha chain, non-erythrocytic 1 OS=Gallus gallus GN=SPTAN1 PE=4 SV=2	F1NHT3_CHICK	TAX_GallusGallus_9031_20141114.fasta	285,330.20	100.00%	163	194	497	1.00%	56.40%	SADESGQALLAAGHYASDEVK	R	E	99.70%	54.4	44	23.2	49.2	43.5	5.24	0.441	0.357	0	0	2	0	2	2,118.99	2,227.00		1,690.92	419	439	Empty	TRUE	UPI0000ECA63D,UPI0000ECA63C
20196-20225 HinckeM –TAXchicken	Uncategorized Sample	20196-20205_A	Mudpit_Ingel20196_MGFPeaklist (F058870)	Spectrin alpha chain, non-erythrocytic 1 OS=Gallus gallus GN=SPTAN1 PE=4 SV=2	F1NHT3_CHICK	TAX_GallusGallus_9031_20141114.fasta	285,330.20	100.00%	163	194	497	1.00%	56.40%	SADESGQALLAAGHYASDEVKEK	R	L	99.70%	87.1	43.9	14.7	84.1	58	8.54	0.559	0.413	0	0	0	2	2	2,376.13	2,184.00		15,661.80	419	441	Empty	TRUE	UPI0000ECA63D,UPI0000ECA63C
20196-20225 HinckeM –TAXchicken	Uncategorized Sample	20196-20205_A	Mudpit_Ingel20196_MGFPeaklist (F058870)	Spectrin alpha chain, non-erythrocytic 1 OS=Gallus gallus GN=SPTAN1 PE=4 SV=2	F1NHT3_CHICK	TAX_GallusGallus_9031_20141114.fasta	285,330.20	100.00%	163	194	497	1.00%	56.40%	SALLELWELRR	R	Q	99.70%	30.1	43.2	29.6	14.2	41	0.796	0.249	0.636	0	0	2	0	2	1,385.79	2,758.00		1,656.26	450	460	Empty	TRUE	UPI0000ECA63D,UPI0000ECA63C
20196-20225 HinckeM –TAXchicken	Uncategorized Sample	20196-20205_A	Mudpit_Ingel20196_MGFPeaklist (F058870)	Spectrin alpha chain, non-erythrocytic 1 OS=Gallus gallus GN=SPTAN1 PE=4 SV=2	F1NHT3_CHICK	TAX_GallusGallus_9031_20141114.fasta	285,330.20	100.00%	163	194	497	1.00%	56.40%	SKLGESQTLQQFSR	R	D	99.70%	86.8	44.4	23.3	75.8	45.4	4.77	0.414	0.464	0	1	1	0	2	1,608.83	1,945.00		8,398.43	1545	1558	Empty	TRUE	UPI0000ECA63D,UPI0000ECA63C
20196-20225 HinckeM –TAXchicken	Uncategorized Sample	20196-20205_A	Mudpit_Ingel20196_MGFPeaklist (F058870)	Spectrin alpha chain, non-erythrocytic 1 OS=Gallus gallus GN=SPTAN1 PE=4 SV=2	F1NHT3_CHICK	TAX_GallusGallus_9031_20141114.fasta	285,330.20	100.00%	163	194	497	1.00%	56.40%	SKQDEVNASWQR	K	L	99.70%	61.5	44.7	31.5	35.1	42.3	1.82	0.296	0.625	0	0	7	0	2	1,447.69	1,646.00		5,075.36	228	239	Empty	TRUE	UPI0000ECA63D,UPI0000ECA63C
20196-20225 HinckeM –TAXchicken	Uncategorized Sample	20196-20205_A	Mudpit_Ingel20196_MGFPeaklist (F058870)	Spectrin alpha chain, non-erythrocytic 1 OS=Gallus gallus GN=SPTAN1 PE=4 SV=2	F1NHT3_CHICK	TAX_GallusGallus_9031_20141114.fasta	285,330.20	100.00%	163	194	497	1.00%	56.40%	SLQQLAEER	R	S	99.70%	72.3	45.3	40.8	44.3	34.8	1.96	0.316	0.556	0	6	0	0	2	1,073.56	1,925.50		25,749.10	1217	1225	Empty	TRUE	UPI0000ECA63D,UPI0000ECA63C
20196-20225 HinckeM –TAXchicken	Uncategorized Sample	20196-20205_A	Mudpit_Ingel20196_MGFPeaklist (F058870)	Spectrin alpha chain, non-erythrocytic 1 OS=Gallus gallus GN=SPTAN1 PE=4 SV=2	F1NHT3_CHICK	TAX_GallusGallus_9031_20141114.fasta	285,330.20	100.00%	163	194	497	1.00%	56.40%	SLSAQEEKITALDEFATK	K	L	99.70%	46.9	44.1	22.5	39.1	39.3	4.77	0.445	0.361	0	0	2	0	2	1,981.01	2,426.50		1,838.20	515	532	Empty	TRUE	UPI0000ECA63D,UPI0000ECA63C
20196-20225 HinckeM –TAXchicken	Uncategorized Sample	20196-20205_A	Mudpit_Ingel20196_MGFPeaklist (F058870)	Spectrin alpha chain, non-erythrocytic 1 OS=Gallus gallus GN=SPTAN1 PE=4 SV=2	F1NHT3_CHICK	TAX_GallusGallus_9031_20141114.fasta	285,330.20	100.00%	163	194	497	1.00%	56.40%	SQLLGSAHEVQR	R	F	99.70%	67.1	43.9	23.2	54.5	48.6	5.38	0.438	0.708	0	0	13	0	2	1,324.70	1,714.00		26,876.90	1226	1237	Empty	TRUE	UPI0000ECA63D,UPI0000ECA63C
20196-20225 HinckeM –TAXchicken	Uncategorized Sample	20196-20205_A	Mudpit_Ingel20196_MGFPeaklist (F058870)	Spectrin alpha chain, non-erythrocytic 1 OS=Gallus gallus GN=SPTAN1 PE=4 SV=2	F1NHT3_CHICK	TAX_GallusGallus_9031_20141114.fasta	285,330.20	100.00%	163	194	497	1.00%	56.40%	SSEEIESAFR	K	A	99.70%	66.5	43.3	38.2	29.9	43.9	3.7	0.362	0.65	0	4	0	0	2	1,154.53	2,006.50		9,866.21	2410	2419	Empty	TRUE	UPI0000ECA63D,UPI0000ECA63C
20196-20225 HinckeM –TAXchicken	Uncategorized Sample	20196-20205_A	Mudpit_Ingel20196_MGFPeaklist (F058870)	Spectrin alpha chain, non-erythrocytic 1 OS=Gallus gallus GN=SPTAN1 PE=4 SV=2	F1NHT3_CHICK	TAX_GallusGallus_9031_20141114.fasta	285,330.20	100.00%	163	194	497	1.00%	56.40%	SSLSSAQADFNQLAELDR	R	Q	99.70%	80.4	44.3	25.6	65.7	40.6	3.31	0.337	0.333	0	0	2	0	2	1,951.94	2,631.50		1,349.63	2143	2160	Empty	TRUE	UPI0000ECA63D,UPI0000ECA63C
20196-20225 HinckeM –TAXchicken	Uncategorized Sample	20196-20205_A	Mudpit_Ingel20196_MGFPeaklist (F058870)	Spectrin alpha chain, non-erythrocytic 1 OS=Gallus gallus GN=SPTAN1 PE=4 SV=2	F1NHT3_CHICK	TAX_GallusGallus_9031_20141114.fasta	285,330.20	100.00%	163	194	497	1.00%	56.40%	TATDEAYKDPSNLQGK	K	V	99.70%	75.7	44.4	20.1	64.8	41.7	5.12	0.405	0.406	0	0	5	0	2	1,737.83	1,614.00		14,904.40	595	610	Empty	TRUE	UPI0000ECA63D,UPI0000ECA63C
20196-20225 HinckeM –TAXchicken	Uncategorized Sample	20196-20205_A	Mudpit_Ingel20196_MGFPeaklist (F058870)	Spectrin alpha chain, non-erythrocytic 1 OS=Gallus gallus GN=SPTAN1 PE=4 SV=2	F1NHT3_CHICK	TAX_GallusGallus_9031_20141114.fasta	285,330.20	100.00%	163	194	497	1.00%	56.40%	VEDLFLTFAK	K	K	99.70%	68.1	43.6	24.1	50.7	42.8	2.89	0.318	0.6	0	2	0	0	2	1,182.64	2,814.50		721.68	2094	2103	Empty	TRUE	UPI0000ECA63D,UPI0000ECA63C
20196-20225 HinckeM –TAXchicken	Uncategorized Sample	20196-20205_A	Mudpit_Ingel20196_MGFPeaklist (F058870)	Spectrin alpha chain, non-erythrocytic 1 OS=Gallus gallus GN=SPTAN1 PE=4 SV=2	F1NHT3_CHICK	TAX_GallusGallus_9031_20141114.fasta	285,330.20	100.00%	163	194	497	1.00%	56.40%	VLETAEDIQER	K	R	99.70%	67.4	44.9	31.8	41.6	46.8	3.13	0.442	0.591	0	8	0	0	2	1,302.65	1,794.00		16,668.30	8	18	Empty	TRUE	UPI0000ECA63D,UPI0000ECA63C
20196-20225 HinckeM –TAXchicken	Uncategorized Sample	20196-20205_A	Mudpit_Ingel20196_MGFPeaklist (F058870)	Spectrin alpha chain, non-erythrocytic 1 OS=Gallus gallus GN=SPTAN1 PE=4 SV=2	F1NHT3_CHICK	TAX_GallusGallus_9031_20141114.fasta	285,330.20	100.00%	163	194	497	1.00%	56.40%	VLETAEDIQERR	K	Q	99.70%	45.4	44.7	25.2	32	43.4	3.8	0.369	0.625	0	0	5	0	2	1,458.75	1,732.00		6,237.60	8	19	Empty	TRUE	UPI0000ECA63D,UPI0000ECA63C
20196-20225 HinckeM –TAXchicken	Uncategorized Sample	20196-20205_A	Mudpit_Ingel20196_MGFPeaklist (F058870)	Spectrin alpha chain, non-erythrocytic 1 OS=Gallus gallus GN=SPTAN1 PE=4 SV=2	F1NHT3_CHICK	TAX_GallusGallus_9031_20141114.fasta	285,330.20	100.00%	163	194	497	1.00%	56.40%	VNDVCANGEDLIK	R	K	99.70%	64.1	44.3	22.9	46.6	44	4.52	0.423	0.462	0	2	1	0	2	1,447.67	1,987.00		2,175.97	1931	1943	Empty	TRUE	UPI0000ECA63D,UPI0000ECA63C
20196-20225 HinckeM –TAXchicken	Uncategorized Sample	20196-20205_A	Mudpit_Ingel20196_MGFPeaklist (F058870)	Spectrin alpha chain, non-erythrocytic 1 OS=Gallus gallus GN=SPTAN1 PE=4 SV=2	F1NHT3_CHICK	TAX_GallusGallus_9031_20141114.fasta	285,330.20	100.00%	163	194	497	1.00%	56.40%	VNDVCANGEDLIKK	R	N	99.70%	69.8	44.9	20.1	60.2	43.9	5.19	0.424	0.5	0	0	6	0	2	1,574.78	1,858.00		9,724.62	1931	1944	Empty	TRUE	UPI0000ECA63D,UPI0000ECA63C
20196-20225 HinckeM –TAXchicken	Uncategorized Sample	20196-20205_A	Mudpit_Ingel20196_MGFPeaklist (F058870)	Spectrin alpha chain, non-erythrocytic 1 OS=Gallus gallus GN=SPTAN1 PE=4 SV=2	F1NHT3_CHICK	TAX_GallusGallus_9031_20141114.fasta	285,330.20	100.00%	163	194	497	1.00%	56.40%	VNEVNQFAGK	R	L	99.70%	60.7	45.1	24.8	44.4	38.5	3.89	0.374	0.55	0	6	0	0	2	1,105.56	1,728.00		3,154.14	205	214	Empty	TRUE	UPI0000ECA63D,UPI0000ECA63C
20196-20225 HinckeM –TAXchicken	Uncategorized Sample	20196-20205_A	Mudpit_Ingel20196_MGFPeaklist (F058870)	Spectrin alpha chain, non-erythrocytic 1 OS=Gallus gallus GN=SPTAN1 PE=4 SV=2	F1NHT3_CHICK	TAX_GallusGallus_9031_20141114.fasta	285,330.20	100.00%	163	194	497	1.00%	56.40%	VQKHQAFEAELSANQSR	K	I	99.70%	33.5	44.2	19.1	25.6	33.7	1.89	0.285	0.412	0	0	0	2	2	1,942.97	1,752.50		2,844.57	611	627	Empty	TRUE	UPI0000ECA63D,UPI0000ECA63C
20196-20225 HinckeM –TAXchicken	Uncategorized Sample	20196-20205_A	Mudpit_Ingel20196_MGFPeaklist (F058870)	Spectrin alpha chain, non-erythrocytic 1 OS=Gallus gallus GN=SPTAN1 PE=4 SV=2	F1NHT3_CHICK	TAX_GallusGallus_9031_20141114.fasta	285,330.20	100.00%	163	194	497	1.00%	56.40%	WNQLLANSAAR	R	K	99.70%	61.5	44.7	24.6	24.8	41.9	3.89	0.403	0.545	0	5	0	0	2	1,243.65	2,066.00		7,855.65	2070	2080	Empty	TRUE	UPI0000ECA63D,UPI0000ECA63C
20196-20225 HinckeM –TAXchicken	Uncategorized Sample	20196-20205_A	Mudpit_Ingel20196_MGFPeaklist (F058870)	Spectrin alpha chain, non-erythrocytic 1 OS=Gallus gallus GN=SPTAN1 PE=4 SV=2	F1NHT3_CHICK	TAX_GallusGallus_9031_20141114.fasta	285,330.20	100.00%	163	194	497	1.00%	56.40%	YEALKDPMVAR	R	K	99.70%	56.9	44.8	28.4	45.5	37.4	4.08	0.404	0.5	0	0	6	0	2	1,292.67	1,732.00		6,967.50	766	776	Empty	TRUE	UPI0000ECA63D,UPI0000ECA63C
20196-20225 HinckeM –TAXchicken	Uncategorized Sample	20196-20205_A	Mudpit_Ingel20196_MGFPeaklist (F058870)	status=inactive	UPI0000ECA63D	UPARC14_2_GallusG_9031_20140417.fasta	281,818.00	100.00%	2	2	5	0.01%	57.40%	ADVVESWIGEK	K	E	99.70%	52.8	45.2	26.2	33.4	35.3	3.8	0.286	0.455	0	3	0	0	2	1,232.62	2,355.00		830.65	1958	1968	Empty	FALSE	UPI0000ECA63C,F1NHT3_CHICK
20196-20225 HinckeM –TAXchicken	Uncategorized Sample	20196-20205_A	Mudpit_Ingel20196_MGFPeaklist (F058870)	status=inactive	UPI0000ECA63D	UPARC14_2_GallusG_9031_20140417.fasta	281,818.00	100.00%	2	2	5	0.01%	57.40%	ADVVESWIGEKENSLK	K	T	99.70%	37.6	44.6	19.6	27.5	34.3	4	0.297	0.344	0	0	2	0	2	1,803.91	2,426.00		1,319.73	1958	1973	Empty	FALSE	UPI0000ECA63C,F1NHT3_CHICK
20196-20225 HinckeM –TAXchicken	Uncategorized Sample	20196-20205_A	Mudpit_Ingel20196_MGFPeaklist (F058870)	status=inactive	UPI0000ECA63D	UPARC14_2_GallusG_9031_20140417.fasta	281,818.00	100.00%	2	2	5	0.01%	57.40%	AGTFQAFEQFGQQLLAR	R	G	99.70%	27.4	44.2	0	19.1	30.4	1.72	0.322	0.265	0	0	1	0	2	1,911.97	2,884.00		143.7	1365	1381	Empty	FALSE	UPI0000ECA63C,F1NHT3_CHICK
20196-20225 HinckeM –TAXchicken	Uncategorized Sample	20196-20205_A	Mudpit_Ingel20196_MGFPeaklist (F058870)	status=inactive	UPI0000ECA63D	UPARC14_2_GallusG_9031_20140417.fasta	281,818.00	100.00%	2	2	5	0.01%	57.40%	ALCAEADRLQQSHPINASQIQVK	K	R	99.70%	9.04	43.1	0	9.04	36.6	2.38	0.303	0.239	0	0	0	2	2	2,577.32	2,102.50		8,838.04	306	328	Empty	FALSE	UPI0000ECA63C,F1NHT3_CHICK
20196-20225 HinckeM –TAXchicken	Uncategorized Sample	20196-20205_A	Mudpit_Ingel20196_MGFPeaklist (F058870)	status=inactive	UPI0000ECA63D	UPARC14_2_GallusG_9031_20140417.fasta	281,818.00	100.00%	2	2	5	0.01%	57.40%	ALSSEGKPYVTK	R	E	99.70%	66.2	44	23.9	53.1	48.7	5.33	0.398	0.625	0	0	2	0	2	1,279.69	1,555.50		618.77	2388	2399	Empty	FALSE	UPI0000ECA63C,F1NHT3_CHICK
20196-20225 HinckeM –TAXchicken	Uncategorized Sample	20196-20205_A	Mudpit_Ingel20196_MGFPeaklist (F058870)	status=inactive	UPI0000ECA63D	UPARC14_2_GallusG_9031_20140417.fasta	281,818.00	100.00%	2	2	5	0.01%	57.40%	ALSSEGKPYVTKEELYQNLTR	R	E	99.70%	88.8	43.1	14.9	78.8	40	3.85	0.405	0.333	0	0	0	2	2	2,426.26	2,196.50		8,205.40	2388	2408	Empty	FALSE	UPI0000ECA63C,F1NHT3_CHICK
20196-20225 HinckeM –TAXchicken	Uncategorized Sample	20196-20205_A	Mudpit_Ingel20196_MGFPeaklist (F058870)	status=inactive	UPI0000ECA63D	UPARC14_2_GallusG_9031_20140417.fasta	281,818.00	100.00%	2	2	5	0.01%	57.40%	AQLADSFHLQQFFR	R	D	99.70%	72.7	44.6	16.7	67.9	41.6	4.59	0.418	0.464	0	0	2	0	2	1,707.86	2,785.50		2,140.49	560	573	Empty	FALSE	UPI0000ECA63C,F1NHT3_CHICK
20196-20225 HinckeM –TAXchicken	Uncategorized Sample	20196-20205_A	Mudpit_Ingel20196_MGFPeaklist (F058870)	status=inactive	UPI0000ECA63D	UPARC14_2_GallusG_9031_20140417.fasta	281,818.00	100.00%	2	2	5	0.01%	57.40%	CNSLEEIKALR	R	E	49.50%	23	45	31.9	5.69					0	0	1	0	2	1,332.69	2,102.00		330.76	2093	2103	Empty	FALSE	UPI0000ECA63C,F1NHT3_CHICK
20196-20225 HinckeM –TAXchicken	Uncategorized Sample	20196-20205_A	Mudpit_Ingel20196_MGFPeaklist (F058870)	status=inactive	UPI0000ECA63D	UPARC14_2_GallusG_9031_20140417.fasta	281,818.00	100.00%	2	2	5	0.01%	57.40%	CTELNQAWSSLGK	K	R	99.70%	69.3	44.3	26.2	47.6	47.4	4.82	0.414	0.538	0	2	0	0	2	1,493.71	2,349.00		2,238.28	1287	1299	Empty	FALSE	UPI0000ECA63C,F1NHT3_CHICK
20196-20225 HinckeM –TAXchicken	Uncategorized Sample	20196-20205_A	Mudpit_Ingel20196_MGFPeaklist (F058870)	status=inactive	UPI0000ECA63D	UPARC14_2_GallusG_9031_20140417.fasta	281,818.00	100.00%	2	2	5	0.01%	57.40%	CTELNQAWSSLGKR	K	A	99.70%	63	45	27.1	43.6	46	3.82	0.393	0.536	0	0	3	0	2	1,649.81	2,252.00		3,580.82	1287	1300	Empty	FALSE	UPI0000ECA63C,F1NHT3_CHICK
20196-20225 HinckeM –TAXchicken	Uncategorized Sample	20196-20205_A	Mudpit_Ingel20196_MGFPeaklist (F058870)	status=inactive	UPI0000ECA63D	UPARC14_2_GallusG_9031_20140417.fasta	281,818.00	100.00%	2	2	5	0.01%	57.40%	DADELEKWIQEK	R	L	99.70%	50.5	44.9	31.3	28.9	39.3	3.21	0.351	0.542	0	0	2	0	2	1,503.73	2,487.50		902.74	45	56	Empty	FALSE	UPI0000ECA63C,F1NHT3_CHICK
20196-20225 HinckeM –TAXchicken	Uncategorized Sample	20196-20205_A	Mudpit_Ingel20196_MGFPeaklist (F058870)	status=inactive	UPI0000ECA63D	UPARC14_2_GallusG_9031_20140417.fasta	281,818.00	100.00%	2	2	5	0.01%	57.40%	DADETKEWIEEK	R	N	99.70%	50.1	44	24.6	34.5	38.4	2.15	0.258	0.583	0	0	2	0	2	1,492.68	1,917.50		2,873.01	1214	1225	Empty	FALSE	UPI0000ECA63C,F1NHT3_CHICK
20196-20225 HinckeM –TAXchicken	Uncategorized Sample	20196-20205_A	Mudpit_Ingel20196_MGFPeaklist (F058870)	status=inactive	UPI0000ECA63D	UPARC14_2_GallusG_9031_20140417.fasta	281,818.00	100.00%	2	2	5	0.01%	57.40%	DIEDEETWIR	R	E	99.70%	45.2	44.2	25.3	27.6	34.1	2.72	0.331	0.4	0	4	0	0	2	1,305.60	2,285.00		3,917.74	785	794	Empty	FALSE	UPI0000ECA63C,F1NHT3_CHICK
20196-20225 HinckeM –TAXchicken	Uncategorized Sample	20196-20205_A	Mudpit_Ingel20196_MGFPeaklist (F058870)	status=inactive	UPI0000ECA63D	UPARC14_2_GallusG_9031_20140417.fasta	281,818.00	100.00%	2	2	5	0.01%	57.40%	DIEDEETWIREKEPIAASTNR	R	G	99.70%	49.9	44	20.4	35.8	30.4	3.54	0.273	0.19	0	0	0	2	2	2,502.21	2,313.50		1,027.85	785	805	Empty	FALSE	UPI0000ECA63C,F1NHT3_CHICK
20196-20225 HinckeM –TAXchicken	Uncategorized Sample	20196-20205_A	Mudpit_Ingel20196_MGFPeaklist (F058870)	status=inactive	UPI0000ECA63D	UPARC14_2_GallusG_9031_20140417.fasta	281,818.00	100.00%	2	2	5	0.01%	57.40%	DLAALEDKVK	R	A	99.70%	47	44.7	29.6	31.6	28.2	1.07	0.234	0.35	0	4	0	0	2	1,101.62	2,105.50		3,056.35	296	305	Empty	FALSE	UPI0000ECA63C,F1NHT3_CHICK
20196-20225 HinckeM –TAXchicken	Uncategorized Sample	20196-20205_A	Mudpit_Ingel20196_MGFPeaklist (F058870)	status=inactive	UPI0000ECA63D	UPARC14_2_GallusG_9031_20140417.fasta	281,818.00	100.00%	2	2	5	0.01%	57.40%	DLAALGDKVNSLGETAQR	R	L	99.70%	65.1	44.2	15.5	49	41.2	5.2	0.335	0.389	0	0	4	1	2	1,857.97	2,289.00		10,407.20	1254	1271	Empty	FALSE	UPI0000ECA63C,F1NHT3_CHICK
20196-20225 HinckeM –TAXchicken	Uncategorized Sample	20196-20205_A	Mudpit_Ingel20196_MGFPeaklist (F058870)	status=inactive	UPI0000ECA63D	UPARC14_2_GallusG_9031_20140417.fasta	281,818.00	100.00%	2	2	5	0.01%	57.40%	DLASVQALLR	R	K	99.70%	59.5	43.5	34.7	42.1	38.6	1.51	0.293	0.55	0	3	0	0	2	1,085.63	2,622.00		2,066.49	279	288	Empty	FALSE	UPI0000ECA63C,F1NHT3_CHICK
20196-20225 HinckeM –TAXchicken	Uncategorized Sample	20196-20205_A	Mudpit_Ingel20196_MGFPeaklist (F058870)	status=inactive	UPI0000ECA63D	UPARC14_2_GallusG_9031_20140417.fasta	281,818.00	100.00%	2	2	5	0.01%	57.40%	DLIGVQNLLK	K	K	99.70%	37.7	41.9	25.3	19	28	0.377	0.104	0.4	0	2	0	0	2	1,112.67	2,701.00		549.42	808	817	Empty	FALSE	UPI0000ECA63C,F1NHT3_CHICK
20196-20225 HinckeM –TAXchicken	Uncategorized Sample	20196-20205_A	Mudpit_Ingel20196_MGFPeaklist (F058870)	status=inactive	UPI0000ECA63D	UPARC14_2_GallusG_9031_20140417.fasta	281,818.00	100.00%	2	2	5	0.01%	57.40%	DLIGVQNLLKK	K	H	36.00%	26.5	40.3	32.1	6.71					0	0	2	0	2	1,240.76	2,513.00		302.19	808	818	Empty	FALSE	UPI0000ECA63C,F1NHT3_CHICK
20196-20225 HinckeM –TAXchicken	Uncategorized Sample	20196-20205_A	Mudpit_Ingel20196_MGFPeaklist (F058870)	status=inactive	UPI0000ECA63D	UPARC14_2_GallusG_9031_20140417.fasta	281,818.00	100.00%	2	2	5	0.01%	57.40%	DLNSQADSLMTSSAFDTSQVK	K	D	99.70%	38.3	42.9	16	32.4	32.7	2.74	0.349	0.262	0	0	2	0	2	2,261.02	2,204.00		1,428.41	1689	1709	Empty	FALSE	UPI0000ECA63C,F1NHT3_CHICK
20196-20225 HinckeM –TAXchicken	Uncategorized Sample	20196-20205_A	Mudpit_Ingel20196_MGFPeaklist (F058870)	status=inactive	UPI0000ECA63D	UPARC14_2_GallusG_9031_20140417.fasta	281,818.00	100.00%	2	2	5	0.01%	57.40%	DLSSVQTLLTK	R	Q	99.70%	58.2	44.2	22.1	37.2	39.6	2.39	0.263	0.5	0	3	0	0	2	1,204.68	2,498.00		4,868.60	1980	1990	Empty	FALSE	UPI0000ECA63C,F1NHT3_CHICK
20196-20225 HinckeM –TAXchicken	Uncategorized Sample	20196-20205_A	Mudpit_Ingel20196_MGFPeaklist (F058870)	status=inactive	UPI0000ECA63D	UPARC14_2_GallusG_9031_20140417.fasta	281,818.00	100.00%	2	2	5	0.01%	57.40%	DLTGVQNLR	R	K	99.70%	56	45	38.4	30.3	36.1	0.456	0.222	0.556	0	5	0	0	2	1,015.55	1,931.00		7,457.21	1768	1776	Empty	FALSE	UPI0000ECA63C,F1NHT3_CHICK
20196-20225 HinckeM –TAXchicken	Uncategorized Sample	20196-20205_A	Mudpit_Ingel20196_MGFPeaklist (F058870)	status=inactive	UPI0000ECA63D	UPARC14_2_GallusG_9031_20140417.fasta	281,818.00	100.00%	2	2	5	0.01%	57.40%	DLTSVQNLQK	K	K	99.70%	38.1	45.1	28	11.8	28.4	1.77	0.151	0.35	0	2	0	0	2	1,145.62	1,873.50		1,020.59	702	711	Empty	FALSE	UPI0000ECA63C,F1NHT3_CHICK
20196-20225 HinckeM –TAXchicken	Uncategorized Sample	20196-20205_A	Mudpit_Ingel20196_MGFPeaklist (F058870)	status=inactive	UPI0000ECA63D	UPARC14_2_GallusG_9031_20140417.fasta	281,818.00	100.00%	2	2	5	0.01%	57.40%	DLTSWVTEMK	R	A	99.50%	40.2	43.5	28.7	32.1					0	2	0	0	2	1,225.58	2,378.50		899.24	365	374	Empty	FALSE	UPI0000ECA63C,F1NHT3_CHICK
20196-20225 HinckeM –TAXchicken	Uncategorized Sample	20196-20205_A	Mudpit_Ingel20196_MGFPeaklist (F058870)	status=inactive	UPI0000ECA63D	UPARC14_2_GallusG_9031_20140417.fasta	281,818.00	100.00%	2	2	5	0.01%	57.40%	DMDDEESWIKEK	R	K	21.00%	11.7	41.6	19.6	0					0	0	3	0	2	1,524.65	1,885.00		1,111.19	1745	1756	Empty	FALSE	UPI0000ECA63C,F1NHT3_CHICK
20196-20225 HinckeM –TAXchicken	Uncategorized Sample	20196-20205_A	Mudpit_Ingel20196_MGFPeaklist (F058870)	status=inactive	UPI0000ECA63D	UPARC14_2_GallusG_9031_20140417.fasta	281,818.00	100.00%	2	2	5	0.01%	57.40%	DSDELKSWVNEK	R	M	99.70%	54.9	44.4	26.9	35.9	43.5	3.59	0.354	0.542	0	2	0	0	2	1,449.69	2,068.00		1,798.64	574	585	Empty	FALSE	UPI0000ECA63C,F1NHT3_CHICK
20196-20225 HinckeM –TAXchicken	Uncategorized Sample	20196-20205_A	Mudpit_Ingel20196_MGFPeaklist (F058870)	status=inactive	UPI0000ECA63D	UPARC14_2_GallusG_9031_20140417.fasta	281,818.00	100.00%	2	2	5	0.01%	57.40%	DVDEIEAWISEK	R	L	99.70%	71.5	44.5	21.9	46.3	41.7	3.85	0.369	0.5	0	2	0	0	2	1,433.68	2,739.00		1,182.10	1532	1543	Empty	FALSE	UPI0000ECA63C,F1NHT3_CHICK
20196-20225 HinckeM –TAXchicken	Uncategorized Sample	20196-20205_A	Mudpit_Ingel20196_MGFPeaklist (F058870)	status=inactive	UPI0000ECA63D	UPARC14_2_GallusG_9031_20140417.fasta	281,818.00	100.00%	2	2	5	0.01%	57.40%	DVDETISWIK	R	E	99.70%	44.3	45	25.4	31.4	35.5	3.68	0.33	0.5	0	2	0	0	2	1,205.61	2,458.50		957.68	256	265	Empty	FALSE	UPI0000ECA63C,F1NHT3_CHICK
20196-20225 HinckeM –TAXchicken	Uncategorized Sample	20196-20205_A	Mudpit_Ingel20196_MGFPeaklist (F058870)	status=inactive	UPI0000ECA63D	UPARC14_2_GallusG_9031_20140417.fasta	281,818.00	100.00%	2	2	5	0.01%	57.40%	DVDETISWIKEK	R	G	90.00%	28.4	44.8	21.8	17					0	0	2	0	2	1,462.74	2,355.00		545.19	256	267	Empty	FALSE	UPI0000ECA63C,F1NHT3_CHICK
20196-20225 HinckeM –TAXchicken	Uncategorized Sample	20196-20205_A	Mudpit_Ingel20196_MGFPeaklist (F058870)	status=inactive	UPI0000ECA63D	UPARC14_2_GallusG_9031_20140417.fasta	281,818.00	100.00%	2	2	5	0.01%	57.40%	DVTGAEALLER	K	H	99.70%	67.8	45.2	33.8	51.6	40.6	3.1	0.411	0.5	0	4	0	0	2	1,173.61	2,270.00		3,192.11	1343	1353	Empty	FALSE	UPI0000ECA63C,F1NHT3_CHICK
20196-20225 HinckeM –TAXchicken	Uncategorized Sample	20196-20205_A	Mudpit_Ingel20196_MGFPeaklist (F058870)	status=inactive	UPI0000ECA63D	UPARC14_2_GallusG_9031_20140417.fasta	281,818.00	100.00%	2	2	5	0.01%	57.40%	EAALTNEEVGADLEQVEVLQK	K	K	99.70%	61.3	44.1	21.7	44.8	45.5	3.24	0.367	0.357	0	0	2	0	2	2,285.15	2,586.50		3,455.38	1084	1104	Empty	FALSE	UPI0000ECA63C,F1NHT3_CHICK
20196-20225 HinckeM –TAXchicken	Uncategorized Sample	20196-20205_A	Mudpit_Ingel20196_MGFPeaklist (F058870)	status=inactive	UPI0000ECA63D	UPARC14_2_GallusG_9031_20140417.fasta	281,818.00	100.00%	2	2	5	0.01%	57.40%	EAALTNEEVGADLEQVEVLQKK	K	F	99.70%	66.3	43.3	15.6	54.8	46.4	5.77	0.459	0.318	0	0	1	0	2	2,413.25	2,516.00		644.68	1084	1105	Empty	FALSE	UPI0000ECA63C,F1NHT3_CHICK
20196-20225 HinckeM –TAXchicken	Uncategorized Sample	20196-20205_A	Mudpit_Ingel20196_MGFPeaklist (F058870)	status=inactive	UPI0000ECA63D	UPARC14_2_GallusG_9031_20140417.fasta	281,818.00	100.00%	2	2	5	0.01%	57.40%	EAFLNTEDKGDSLDSVEALIK	R	K	99.70%	69.5	44.2	17.1	60.3	42.9	3.42	0.329	0.31	0	0	2	0	2	2,294.14	2,728.50		3,342.88	1438	1458	Empty	FALSE	UPI0000ECA63C,F1NHT3_CHICK
20196-20225 HinckeM –TAXchicken	Uncategorized Sample	20196-20205_A	Mudpit_Ingel20196_MGFPeaklist (F058870)	status=inactive	UPI0000ECA63D	UPARC14_2_GallusG_9031_20140417.fasta	281,818.00	100.00%	2	2	5	0.01%	57.40%	EANELQQWINEK	R	E	99.70%	58.1	44.5	28.1	41.3	39.7	2.96	0.199	0.458	0	4	0	0	2	1,501.73	2,446.50		7,052.73	1072	1083	Empty	FALSE	UPI0000ECA63C,F1NHT3_CHICK
20196-20225 HinckeM –TAXchicken	Uncategorized Sample	20196-20205_A	Mudpit_Ingel20196_MGFPeaklist (F058870)	status=inactive	UPI0000ECA63D	UPARC14_2_GallusG_9031_20140417.fasta	281,818.00	100.00%	2	2	5	0.01%	57.40%	EELIANWEQIR	R	T	99.70%	66.7	44.8	26.4	53.1	36.2	3.04	0.312	0.455	0	2	0	0	2	1,400.72	2,516.50		1,200.15	330	340	Empty	FALSE	UPI0000ECA63C,F1NHT3_CHICK
20196-20225 HinckeM –TAXchicken	Uncategorized Sample	20196-20205_A	Mudpit_Ingel20196_MGFPeaklist (F058870)	status=inactive	UPI0000ECA63D	UPARC14_2_GallusG_9031_20140417.fasta	281,818.00	100.00%	2	2	5	0.01%	57.40%	EELYQNLTR	K	E	99.70%	34.6	44.5	28.6	22.4	25.1	0.602	0.207	0.333	0	2	0	0	2	1,165.58	1,995.00		1,252.27	2400	2408	Empty	FALSE	UPI0000ECA63C,F1NHT3_CHICK
20196-20225 HinckeM –TAXchicken	Uncategorized Sample	20196-20205_A	Mudpit_Ingel20196_MGFPeaklist (F058870)	status=inactive	UPI0000ECA63D	UPARC14_2_GallusG_9031_20140417.fasta	281,818.00	100.00%	2	2	5	0.01%	57.40%	EKEPIVGSTDYGKDEDSAEALLK	R	K	99.70%	65.7	43.9	14.1	65.7	62.1	7.82	0.539	0.413	0	0	0	1	2	2,494.22	2,255.00		1,241.24	901	923	Empty	FALSE	UPI0000ECA63C,F1NHT3_CHICK
20196-20225 HinckeM –TAXchicken	Uncategorized Sample	20196-20205_A	Mudpit_Ingel20196_MGFPeaklist (F058870)	status=inactive	UPI0000ECA63D	UPARC14_2_GallusG_9031_20140417.fasta	281,818.00	100.00%	2	2	5	0.01%	57.40%	EKLDILDQER	K	T	83.40%	33.7	44.9	27.2	0					0	0	4	0	2	1,258.66	2,055.00		1,988.67	1391	1400	Empty	FALSE	UPI0000ECA63C,F1NHT3_CHICK
20196-20225 HinckeM –TAXchicken	Uncategorized Sample	20196-20205_A	Mudpit_Ingel20196_MGFPeaklist (F058870)	status=inactive	UPI0000ECA63D	UPARC14_2_GallusG_9031_20140417.fasta	281,818.00	100.00%	2	2	5	0.01%	57.40%	EKLDILDQERTDLEK	K	A	99.70%	19.6	44.4	26.8	1.26	33	0.854	0.212	0.433	0	0	1	1	2	1,844.96	2,203.00		3,854.79	1391	1405	Empty	FALSE	UPI0000ECA63C,F1NHT3_CHICK
20196-20225 HinckeM –TAXchicken	Uncategorized Sample	20196-20205_A	Mudpit_Ingel20196_MGFPeaklist (F058870)	status=inactive	UPI0000ECA63D	UPARC14_2_GallusG_9031_20140417.fasta	281,818.00	100.00%	2	2	5	0.01%	57.40%	ELPSAYDYIEFTR	R	S	99.70%	64.8	44.4	18.8	59.2	52.8	6.02	0.468	0.5	0	2	0	0	2	1,603.76	2,609.00		5,535.00	2428	2440	Empty	FALSE	UPI0000ECA63C,F1NHT3_CHICK
20196-20225 HinckeM –TAXchicken	Uncategorized Sample	20196-20205_A	Mudpit_Ingel20196_MGFPeaklist (F058870)	status=inactive	UPI0000ECA63D	UPARC14_2_GallusG_9031_20140417.fasta	281,818.00	100.00%	2	2	5	0.01%	57.40%	ENLLEEQGSIALR	R	Q	99.70%	68.9	44.4	29.3	49	46.7	3.66	0.345	0.5	0	2	0	0	2	1,471.78	2,391.50		1,931.07	1026	1038	Empty	FALSE	UPI0000ECA63C,F1NHT3_CHICK
20196-20225 HinckeM –TAXchicken	Uncategorized Sample	20196-20205_A	Mudpit_Ingel20196_MGFPeaklist (F058870)	status=inactive	UPI0000ECA63D	UPARC14_2_GallusG_9031_20140417.fasta	281,818.00	100.00%	2	2	5	0.01%	57.40%	ETENVKSSEEIESAFR	R	A	99.70%	92.7	43.9	24	78.8	46.2	6.72	0.437	0.406	0	0	3	0	2	1,854.87	2,071.00		5,899.01	2372	2387	Empty	FALSE	UPI0000ECA63C,F1NHT3_CHICK
20196-20225 HinckeM –TAXchicken	Uncategorized Sample	20196-20205_A	Mudpit_Ingel20196_MGFPeaklist (F058870)	status=inactive	UPI0000ECA63D	UPARC14_2_GallusG_9031_20140417.fasta	281,818.00	100.00%	2	2	5	0.01%	57.40%	FEEFQTDLAAHEER	K	V	99.70%	76.2	43.6	22.9	57.2	47.8	7.85	0.556	0.571	0	0	3	0	2	1,721.78	2,103.00		2,941.94	184	197	Empty	FALSE	UPI0000ECA63C,F1NHT3_CHICK
20196-20225 HinckeM –TAXchicken	Uncategorized Sample	20196-20205_A	Mudpit_Ingel20196_MGFPeaklist (F058870)	status=inactive	UPI0000ECA63D	UPARC14_2_GallusG_9031_20140417.fasta	281,818.00	100.00%	2	2	5	0.01%	57.40%	FHRDADETKEWIEEK	R	N	31.80%	15.8	44	21.1	3.91					0	0	0	2	2	1,932.91	1,813.00		1,544.53	1211	1225	Empty	FALSE	UPI0000ECA63C,F1NHT3_CHICK
20196-20225 HinckeM –TAXchicken	Uncategorized Sample	20196-20205_A	Mudpit_Ingel20196_MGFPeaklist (F058870)	status=inactive	UPI0000ECA63D	UPARC14_2_GallusG_9031_20140417.fasta	281,818.00	100.00%	2	2	5	0.01%	57.40%	FKELSSLR	R	R	92.50%	39.2	43.5	35.2	7.63					0	1	0	0	2	979.5571	1,859.00		429.66	22	29	Empty	FALSE	UPI0000ECA63C,F1NHT3_CHICK
20196-20225 HinckeM –TAXchicken	Uncategorized Sample	20196-20205_A	Mudpit_Ingel20196_MGFPeaklist (F058870)	status=inactive	UPI0000ECA63D	UPARC14_2_GallusG_9031_20140417.fasta	281,818.00	100.00%	2	2	5	0.01%	57.40%	FKELSSLRR	R	Q	99.70%	18.7	42.9	32	-1.14	30.7	0.523	0.111	0.5	0	1	0	0	2	1,135.66	1,717.00		480.35	22	30	Empty	FALSE	UPI0000ECA63C,F1NHT3_CHICK
20196-20225 HinckeM –TAXchicken	Uncategorized Sample	20196-20205_A	Mudpit_Ingel20196_MGFPeaklist (F058870)	status=inactive	UPI0000ECA63D	UPARC14_2_GallusG_9031_20140417.fasta	281,818.00	100.00%	2	2	5	0.01%	57.40%	FNRDVDETISWIK	R	E	99.70%	60.8	44.6	29.7	43	47.3	5.21	0.438	0.538	0	0	2	0	2	1,622.82	2,445.50		2,421.98	253	265	Empty	FALSE	UPI0000ECA63C,F1NHT3_CHICK
20196-20225 HinckeM –TAXchicken	Uncategorized Sample	20196-20205_A	Mudpit_Ingel20196_MGFPeaklist (F058870)	status=inactive	UPI0000ECA63D	UPARC14_2_GallusG_9031_20140417.fasta	281,818.00	100.00%	2	2	5	0.01%	57.40%	FQFFQR	R	D	90.00%	32.9	44.6	35.5	0	22.5	0.62	0.151	0.5	0	1	0	0	2	872.4413	2,321.00		2,158.44	39	44	Empty	FALSE	UPI0000ECA63C,F1NHT3_CHICK
20196-20225 HinckeM –TAXchicken	Uncategorized Sample	20196-20205_A	Mudpit_Ingel20196_MGFPeaklist (F058870)	status=inactive	UPI0000ECA63D	UPARC14_2_GallusG_9031_20140417.fasta	281,818.00	100.00%	2	2	5	0.01%	57.40%	GEIDAHEDSFR	K	S	99.70%	54.2	42.8	19.4	43.8	36.9	4.27	0.363	0.5	0	0	3	0	2	1,275.56	1,681.00		1,341.73	401	411	Empty	FALSE	UPI0000ECA63C,F1NHT3_CHICK
20196-20225 HinckeM –TAXchicken	Uncategorized Sample	20196-20205_A	Mudpit_Ingel20196_MGFPeaklist (F058870)	status=inactive	UPI0000ECA63D	UPARC14_2_GallusG_9031_20140417.fasta	281,818.00	100.00%	2	2	5	0.01%	57.40%	GKDLIGVQNLLK	R	K	99.70%	34.5	40.2	30.1	20	30.3	0.569	0.0726	0.375	0	0	3	0	2	1,297.78	2,464.00		1,473.99	806	817	Empty	FALSE	UPI0000ECA63C,F1NHT3_CHICK

20196-20225 HinckeM –TAXchicken	Uncategorized Sample	20196-20205_A	Mudpit_Ingel20196_MGFPeaklist (F058870)	status=inactive	UPI0000ECA63D	UPARC14_2_GallusG_9031_20140417.fasta	281,818.00	100.00%	2	2	5	0.01%	57.40%	HEAFETDFTVHKDR	K	V	99.70%	53.8	43.8	16.3	42.9	41.6	7.57	0.459	0.571	0	0	0	2	2	1,731.81	1,862.50		2,458.96	1885	1898	Empty	FALSE	UPI0000ECA63C,F1NHT3_CHICK
20196-20225 HinckeM –TAXchicken	Uncategorized Sample	20196-20205_A	Mudpit_Ingel20196_MGFPeaklist (F058870)	status=inactive	UPI0000ECA63D	UPARC14_2_GallusG_9031_20140417.fasta	281,818.00	100.00%	2	2	5	0.01%	57.40%	HEDFDKAINVQEEK	K	I	99.70%	49.5	44.1	25.4	41.5	39.8	3.92	0.314	0.464	0	0	2	0	2	1,701.81	1,806.00		2,218.76	1460	1473	Empty	FALSE	UPI0000ECA63C,F1NHT3_CHICK
20196-20225 HinckeM –TAXchicken	Uncategorized Sample	20196-20205_A	Mudpit_Ingel20196_MGFPeaklist (F058870)	status=inactive	UPI0000ECA63D	UPARC14_2_GallusG_9031_20140417.fasta	281,818.00	100.00%	2	2	5	0.01%	57.40%	HQAFEAELHANADR	K	I	99.70%	62.2	44.4	19.1	47.2	39.9	4.89	0.429	0.536	0	0	4	7	2	1,608.75	1,756.00		40,595.10	1565	1578	Empty	FALSE	UPI0000ECA63C,F1NHT3_CHICK
20196-20225 HinckeM –TAXchicken	Uncategorized Sample	20196-20205_A	Mudpit_Ingel20196_MGFPeaklist (F058870)	status=inactive	UPI0000ECA63D	UPARC14_2_GallusG_9031_20140417.fasta	281,818.00	100.00%	2	2	5	0.01%	57.40%	HQAFEAELHANADRIR	K	G	99.70%	47.5	44.4	18.6	36.5	57	10.1	0.549	0.625	0	0	0	2	2	1,877.94	1,925.00		2,524.63	1565	1580	Empty	FALSE	UPI0000ECA63C,F1NHT3_CHICK
20196-20225 HinckeM –TAXchicken	Uncategorized Sample	20196-20205_A	Mudpit_Ingel20196_MGFPeaklist (F058870)	status=inactive	UPI0000ECA63D	UPARC14_2_GallusG_9031_20140417.fasta	281,818.00	100.00%	2	2	5	0.01%	57.40%	HQAFEAELSANQSR	K	I	99.70%	66.5	44.3	30	45.2	45.9	5.14	0.497	0.5	0	0	5	0	2	1,587.75	1,830.00		11,856.60	607	620	Empty	FALSE	UPI0000ECA63C,F1NHT3_CHICK
20196-20225 HinckeM –TAXchicken	Uncategorized Sample	20196-20205_A	Mudpit_Ingel20196_MGFPeaklist (F058870)	status=inactive	UPI0000ECA63D	UPARC14_2_GallusG_9031_20140417.fasta	281,818.00	100.00%	2	2	5	0.01%	57.40%	HQAFEAEVQANSGAIVK	K	L	99.70%	61.9	44.5	17.6	50.4	46	6.64	0.47	0.441	0	0	2	0	2	1,798.91	2,037.50		2,840.66	78	94	Empty	FALSE	UPI0000ECA63C,F1NHT3_CHICK
20196-20225 HinckeM –TAXchicken	Uncategorized Sample	20196-20205_A	Mudpit_Ingel20196_MGFPeaklist (F058870)	status=inactive	UPI0000ECA63D	UPARC14_2_GallusG_9031_20140417.fasta	281,818.00	100.00%	2	2	5	0.01%	57.40%	HQALQAEIAGHEPR	K	I	99.70%	74.2	44.5	26	57.2	54.2	6.68	0.491	0.607	0	0	4	0	2	1,556.79	1,758.00		8,943.66	819	832	Empty	FALSE	UPI0000ECA63C,F1NHT3_CHICK
20196-20225 HinckeM –TAXchicken	Uncategorized Sample	20196-20205_A	Mudpit_Ingel20196_MGFPeaklist (F058870)	status=inactive	UPI0000ECA63D	UPARC14_2_GallusG_9031_20140417.fasta	281,818.00	100.00%	2	2	5	0.01%	57.40%	HQEHKGEIDAHEDSFR	R	S	99.70%	72.9	42.9	21.1	65.1	39.8	4.54	0.412	0.469	0	0	0	4	2	1,934.87	1,482.50		1,869.44	396	411	Empty	FALSE	UPI0000ECA63C,F1NHT3_CHICK
20196-20225 HinckeM –TAXchicken	Uncategorized Sample	20196-20205_A	Mudpit_Ingel20196_MGFPeaklist (F058870)	status=inactive	UPI0000ECA63D	UPARC14_2_GallusG_9031_20140417.fasta	281,818.00	100.00%	2	2	5	0.01%	57.40%	HQKHQAFEAELHANADR	K	I	99.70%	47	44.2	15.5	38.2	44.4	6.32	0.432	0.5	0	0	0	1	2	2,001.96	1,605.00		3,536.86	1562	1578	Empty	FALSE	UPI0000ECA63C,F1NHT3_CHICK
20196-20225 HinckeM –TAXchicken	Uncategorized Sample	20196-20205_A	Mudpit_Ingel20196_MGFPeaklist (F058870)	status=inactive	UPI0000ECA63D	UPARC14_2_GallusG_9031_20140417.fasta	281,818.00	100.00%	2	2	5	0.01%	57.40%	HQLLEADISAHEDR	K	L	99.70%	66.6	44.7	20.9	52	43.3	6.59	0.434	0.571	0	0	2	1	2	1,633.79	1,877.00		4,314.61	1673	1686	Empty	FALSE	UPI0000ECA63C,F1NHT3_CHICK
20196-20225 HinckeM –TAXchicken	Uncategorized Sample	20196-20205_A	Mudpit_Ingel20196_MGFPeaklist (F058870)	status=inactive	UPI0000ECA63D	UPARC14_2_GallusG_9031_20140417.fasta	281,818.00	100.00%	2	2	5	0.01%	57.40%	IAVLQSFADQLIAADHYAK	K	G	99.70%	64.2	43.4	19.6	53.3	36.7	3.12	0.256	0.289	0	0	1	0	2	2,074.10	2,878.00		2,845.93	1474	1492	Empty	FALSE	UPI0000ECA63C,F1NHT3_CHICK
20196-20225 HinckeM –TAXchicken	Uncategorized Sample	20196-20205_A	Mudpit_Ingel20196_MGFPeaklist (F058870)	status=inactive	UPI0000ECA63D	UPARC14_2_GallusG_9031_20140417.fasta	281,818.00	100.00%	2	2	5	0.01%	57.40%	IKLNELNQKWDSLK	K	A	20.70%	9.82	42.7	24.8	-0.63					0	0	0	1	2	1,728.96	2,309.00		137.77	856	869	Empty	FALSE	UPI0000ECA63C,F1NHT3_CHICK
20196-20225 HinckeM –TAXchicken	Uncategorized Sample	20196-20205_A	Mudpit_Ingel20196_MGFPeaklist (F058870)	status=inactive	UPI0000ECA63D	UPARC14_2_GallusG_9031_20140417.fasta	281,818.00	100.00%	2	2	5	0.01%	57.40%	IRGVIEMGNSLIER	R	G	48.10%	20.1	44.3	22.1	17.6					0	0	2	0	2	1,602.86	2,094.00		854.89	1579	1592	Empty	FALSE	UPI0000ECA63C,F1NHT3_CHICK
20196-20225 HinckeM –TAXchicken	Uncategorized Sample	20196-20205_A	Mudpit_Ingel20196_MGFPeaklist (F058870)	status=inactive	UPI0000ECA63D	UPARC14_2_GallusG_9031_20140417.fasta	281,818.00	100.00%	2	2	5	0.01%	57.40%	ITALDEFATK	K	L	99.70%	64.9	44.2	25	48.9	38.6	2.77	0.345	0.5	0	6	0	0	2	1,108.59	2,244.00		14,048.90	516	525	Empty	FALSE	UPI0000ECA63C,F1NHT3_CHICK
20196-20225 HinckeM –TAXchicken	Uncategorized Sample	20196-20205_A	Mudpit_Ingel20196_MGFPeaklist (F058870)	status=inactive	UPI0000ECA63D	UPARC14_2_GallusG_9031_20140417.fasta	281,818.00	100.00%	2	2	5	0.01%	57.40%	KFDDFQKDLK	K	A	99.70%	32.9	44	27.9	7.56	29.2	1.36	0.161	0.5	0	0	1	0	2	1,283.66	1,889.00		845.31	1105	1114	Empty	FALSE	UPI0000ECA63C,F1NHT3_CHICK
20196-20225 HinckeM –TAXchicken	Uncategorized Sample	20196-20205_A	Mudpit_Ingel20196_MGFPeaklist (F058870)	status=inactive	UPI0000ECA63D	UPARC14_2_GallusG_9031_20140417.fasta	281,818.00	100.00%	2	2	5	0.01%	57.40%	KFEEFQTDLAAHEER	K	V	99.70%	42.4	44.1	22.9	28.1	32.9	3.05	0.347	0.433	0	0	0	2	2	1,849.87	2,032.00		1,295.27	183	197	Empty	FALSE	UPI0000ECA63C,F1NHT3_CHICK
20196-20225 HinckeM –TAXchicken	Uncategorized Sample	20196-20205_A	Mudpit_Ingel20196_MGFPeaklist (F058870)	status=inactive	UPI0000ECA63D	UPARC14_2_GallusG_9031_20140417.fasta	281,818.00	100.00%	2	2	5	0.01%	57.40%	KFEEFQTDLAAHEERVNEVNQFAGK	K	L	23.20%	2.23	43.3	13.1	0.9					0	0	0	1	2	2,936.42	2,265.00		663.01	183	207	Empty	FALSE	UPI0000ECA63C,F1NHT3_CHICK
20196-20225 HinckeM –TAXchicken	Uncategorized Sample	20196-20205_A	Mudpit_Ingel20196_MGFPeaklist (F058870)	status=inactive	UPI0000ECA63D	UPARC14_2_GallusG_9031_20140417.fasta	281,818.00	100.00%	2	2	5	0.01%	57.40%	KHALLEADVAAHQDR	K	I	99.70%	45.7	44.5	13.2	45.2	45.9	6.48	0.431	0.567	0	0	0	2	2	1,673.87	1,665.00		1,022.33	712	726	Empty	FALSE	UPI0000ECA63C,F1NHT3_CHICK
20196-20225 HinckeM –TAXchicken	Uncategorized Sample	20196-20205_A	Mudpit_Ingel20196_MGFPeaklist (F058870)	status=inactive	UPI0000ECA63D	UPARC14_2_GallusG_9031_20140417.fasta	281,818.00	100.00%	2	2	5	0.01%	57.40%	KHEAFETDFTVHK	K	D	20.10%	5.08	44.7	15.8	0.55					0	0	1	0	2	1,588.78	1,858.00		611.04	1884	1896	Empty	FALSE	UPI0000ECA63C,F1NHT3_CHICK
20196-20225 HinckeM –TAXchicken	Uncategorized Sample	20196-20205_A	Mudpit_Ingel20196_MGFPeaklist (F058870)	status=inactive	UPI0000ECA63D	UPARC14_2_GallusG_9031_20140417.fasta	281,818.00	100.00%	2	2	5	0.01%	57.40%	KHEAFETDFTVHKDR	K	V	99.70%	44.1	44.6	18.5	32.1	31.4	2.82	0.338	0.4	0	0	0	4	2	1,859.90	1,766.00		3,425.77	1884	1898	Empty	FALSE	UPI0000ECA63C,F1NHT3_CHICK
20196-20225 HinckeM –TAXchicken	Uncategorized Sample	20196-20205_A	Mudpit_Ingel20196_MGFPeaklist (F058870)	status=inactive	UPI0000ECA63D	UPARC14_2_GallusG_9031_20140417.fasta	281,818.00	100.00%	2	2	5	0.01%	57.40%	KHEALMSDLSAYGSSIQALR	K	E	99.70%	28	44.2	20.6	18.2	33.7	2.89	0.347	0.3	0	0	0	1	2	2,193.10	2,340.00		707.19	924	943	Empty	FALSE	UPI0000ECA63C,F1NHT3_CHICK
20196-20225 HinckeM –TAXchicken	Uncategorized Sample	20196-20205_A	Mudpit_Ingel20196_MGFPeaklist (F058870)	status=inactive	UPI0000ECA63D	UPARC14_2_GallusG_9031_20140417.fasta	281,818.00	100.00%	2	2	5	0.01%	57.40%	KHEDFDKAINVQEEK	K	I	99.70%	35.1	44.6	24.1	15.5	39	2.34	0.313	0.467	0	0	0	3	2	1,829.90	1,698.00		2,665.58	1459	1473	Empty	FALSE	UPI0000ECA63C,F1NHT3_CHICK
20196-20225 HinckeM –TAXchicken	Uncategorized Sample	20196-20205_A	Mudpit_Ingel20196_MGFPeaklist (F058870)	status=inactive	UPI0000ECA63D	UPARC14_2_GallusG_9031_20140417.fasta	281,818.00	100.00%	2	2	5	0.01%	57.40%	KHQALQAEIAGHEPR	K	I	99.70%	70.8	44	19.2	62	47.2	6.12	0.453	0.567	0	0	0	4	2	1,684.89	1,660.00		11,168.80	818	832	Empty	FALSE	UPI0000ECA63C,F1NHT3_CHICK
20196-20225 HinckeM –TAXchicken	Uncategorized Sample	20196-20205_A	Mudpit_Ingel20196_MGFPeaklist (F058870)	status=inactive	UPI0000ECA63D	UPARC14_2_GallusG_9031_20140417.fasta	281,818.00	100.00%	2	2	5	0.01%	57.40%	KHQLLEADISAHEDR	K	L	99.70%	84.3	44.8	23.8	64.5	43.6	6.66	0.472	0.5	0	0	0	2	2	1,761.89	1,767.50		1,441.77	1672	1686	Empty	FALSE	UPI0000ECA63C,F1NHT3_CHICK
20196-20225 HinckeM –TAXchicken	Uncategorized Sample	20196-20205_A	Mudpit_Ingel20196_MGFPeaklist (F058870)	status=inactive	UPI0000ECA63D	UPARC14_2_GallusG_9031_20140417.fasta	281,818.00	100.00%	2	2	5	0.01%	57.40%	KHQLLEADISAHEDRLK	K	D	35.80%	14.6	43.3	16.1	9.17					0	0	0	0	2	2,003.07	1,876.00		597.04	1672	1688	Empty	FALSE	UPI0000ECA63C,F1NHT3_CHICK
20196-20225 HinckeM –TAXchicken	Uncategorized Sample	20196-20205_A	Mudpit_Ingel20196_MGFPeaklist (F058870)	status=inactive	UPI0000ECA63D	UPARC14_2_GallusG_9031_20140417.fasta	281,818.00	100.00%	2	2	5	0.01%	57.40%	KLLEAQEHFR	K	K	99.70%	43.4	44.1	26.4	28.4	27.4	1.74	0.274	0.35	0	0	4	0	2	1,270.69	1,739.50		1,212.70	2051	2060	Empty	FALSE	UPI0000ECA63C,F1NHT3_CHICK
20196-20225 HinckeM –TAXchicken	Uncategorized Sample	20196-20205_A	Mudpit_Ingel20196_MGFPeaklist (F058870)	status=inactive	UPI0000ECA63D	UPARC14_2_GallusG_9031_20140417.fasta	281,818.00	100.00%	2	2	5	0.01%	57.40%	KLLEATELK	K	G	99.70%	70.3	43.8	51.3	32.8	41	1.54	0.171	0.667	0	3	0	0	2	1,044.63	1,885.00		1,104.69	655	663	Empty	FALSE	UPI0000ECA63C,F1NHT3_CHICK
20196-20225 HinckeM –TAXchicken	Uncategorized Sample	20196-20205_A	Mudpit_Ingel20196_MGFPeaklist (F058870)	status=inactive	UPI0000ECA63D	UPARC14_2_GallusG_9031_20140417.fasta	281,818.00	100.00%	2	2	5	0.01%	57.40%	KLLEATELKGIK	K	L	23.10%	23	40	30.5	6.19					0	0	2	0	2	1,342.83	2,024.50		420.94	655	666	Empty	FALSE	UPI0000ECA63C,F1NHT3_CHICK
20196-20225 HinckeM –TAXchicken	Uncategorized Sample	20196-20205_A	Mudpit_Ingel20196_MGFPeaklist (F058870)	status=inactive	UPI0000ECA63D	UPARC14_2_GallusG_9031_20140417.fasta	281,818.00	100.00%	2	2	5	0.01%	57.40%	KLLVSSEDYGR	K	D	99.70%	58.7	44.1	26.3	47.3	42.6	7.62	0.516	0.591	0	0	4	0	2	1,266.67	1,802.00		2,674.78	1757	1767	Empty	FALSE	UPI0000ECA63C,F1NHT3_CHICK
20196-20225 HinckeM –TAXchicken	Uncategorized Sample	20196-20205_A	Mudpit_Ingel20196_MGFPeaklist (F058870)	status=inactive	UPI0000ECA63D	UPARC14_2_GallusG_9031_20140417.fasta	281,818.00	100.00%	2	2	5	0.01%	57.40%	KLLVSSEDYGRDLTGVQNLR	K	K	99.70%	56.4	42.8	24.6	46.2	47.2	4.89	0.453	0.4	0	0	0	2	2	2,263.20	2,300.00		2,778.94	1757	1776	Empty	FALSE	UPI0000ECA63C,F1NHT3_CHICK
20196-20225 HinckeM –TAXchicken	Uncategorized Sample	20196-20205_A	Mudpit_Ingel20196_MGFPeaklist (F058870)	status=inactive	UPI0000ECA63D	UPARC14_2_GallusG_9031_20140417.fasta	281,818.00	100.00%	2	2	5	0.01%	57.40%	KLSDDNTIGKEEIQQR	K	L	99.70%	68.2	44.4	33.5	0	56.2	5.8	0.493	0.75	0	0	0	2	2	1,873.96	1,670.50		2,456.98	1802	1817	Empty	FALSE	UPI0000ECA63C,F1NHT3_CHICK
20196-20225 HinckeM –TAXchicken	Uncategorized Sample	20196-20205_A	Mudpit_Ingel20196_MGFPeaklist (F058870)	status=inactive	UPI0000ECA63D	UPARC14_2_GallusG_9031_20140417.fasta	281,818.00	100.00%	2	2	5	0.01%	57.40%	KVEDLFLTFAK	R	K	99.70%	51.7	42.9	25	38.8	37.8	3.77	0.275	0.636	0	0	4	0	2	1,310.74	2,707.50		3,971.32	2061	2071	Empty	FALSE	UPI0000ECA63C,F1NHT3_CHICK
20196-20225 HinckeM –TAXchicken	Uncategorized Sample	20196-20205_A	Mudpit_Ingel20196_MGFPeaklist (F058870)	status=inactive	UPI0000ECA63D	UPARC14_2_GallusG_9031_20140417.fasta	281,818.00	100.00%	2	2	5	0.01%	57.40%	KVEDLFLTFAKK	R	A	99.70%	58.9	41.7	23.1	47.7	38.7	4.96	0.328	0.5	0	0	2	0	2	1,438.83	2,540.50		1,192.55	2061	2072	Empty	FALSE	UPI0000ECA63C,F1NHT3_CHICK
20196-20225 HinckeM –TAXchicken	Uncategorized Sample	20196-20205_A	Mudpit_Ingel20196_MGFPeaklist (F058870)	status=inactive	UPI0000ECA63D	UPARC14_2_GallusG_9031_20140417.fasta	281,818.00	100.00%	2	2	5	0.01%	57.40%	LAALADQWEFLVQK	R	S	99.70%	82	44.3	20.6	74.4	39.2	3.24	0.357	0.393	0	2	0	0	2	1,631.88	2,862.50		3,039.42	1606	1619	Empty	FALSE	UPI0000ECA63C,F1NHT3_CHICK
20196-20225 HinckeM –TAXchicken	Uncategorized Sample	20196-20205_A	Mudpit_Ingel20196_MGFPeaklist (F058870)	status=inactive	UPI0000ECA63D	UPARC14_2_GallusG_9031_20140417.fasta	281,818.00	100.00%	2	2	5	0.01%	57.40%	LAQFVDHWK	R	E	99.70%	46.3	44.9	25.1	36.1	30.5	3.32	0.252	0.389	0	4	0	0	2	1,143.59	2,122.00		1,768.97	1818	1826	Empty	FALSE	UPI0000ECA63C,F1NHT3_CHICK
20196-20225 HinckeM –TAXchicken	Uncategorized Sample	20196-20205_A	Mudpit_Ingel20196_MGFPeaklist (F058870)	status=inactive	UPI0000ECA63D	UPARC14_2_GallusG_9031_20140417.fasta	281,818.00	100.00%	2	2	5	0.01%	57.40%	LAQFVDHWKELK	R	Q	99.70%	51.6	44	16.9	45.5	40.1	4.09	0.404	0.5	0	0	2	0	2	1,513.82	2,235.50		919.47	1818	1829	Empty	FALSE	UPI0000ECA63C,F1NHT3_CHICK
20196-20225 HinckeM –TAXchicken	Uncategorized Sample	20196-20205_A	Mudpit_Ingel20196_MGFPeaklist (F058870)	status=inactive	UPI0000ECA63D	UPARC14_2_GallusG_9031_20140417.fasta	281,818.00	100.00%	2	2	5	0.01%	57.40%	LDETGNQMINEGHFASETIR	K	T	99.70%	67.9	43.1	15.8	56.8	46	5.8	0.385	0.35	0	0	2	0	2	2,278.04	2,003.50		4,213.23	95	114	Empty	FALSE	UPI0000ECA63C,F1NHT3_CHICK
20196-20225 HinckeM –TAXchicken	Uncategorized Sample	20196-20205_A	Mudpit_Ingel20196_MGFPeaklist (F058870)	status=inactive	UPI0000ECA63D	UPARC14_2_GallusG_9031_20140417.fasta	281,818.00	100.00%	2	2	5	0.01%	57.40%	LDILDQER	K	T	99.70%	60.4	45.2	46.3	15.6	32.3	0.721	0.201	0.5	0	2	0	0	2	1,001.53	2,098.00		952.38	1393	1400	Empty	FALSE	UPI0000ECA63C,F1NHT3_CHICK
20196-20225 HinckeM –TAXchicken	Uncategorized Sample	20196-20205_A	Mudpit_Ingel20196_MGFPeaklist (F058870)	status=inactive	UPI0000ECA63D	UPARC14_2_GallusG_9031_20140417.fasta	281,818.00	100.00%	2	2	5	0.01%	57.40%	LDILDQERTDLEK	K	A	99.70%	41.5	44.7	29.8	16.1	41.1	1.12	0.185	0.462	0	0	1	0	2	1,587.82	2,269.00		3,310.69	1393	1405	Empty	FALSE	UPI0000ECA63C,F1NHT3_CHICK
20196-20225 HinckeM –TAXchicken	Uncategorized Sample	20196-20205_A	Mudpit_Ingel20196_MGFPeaklist (F058870)	status=inactive	UPI0000ECA63D	UPARC14_2_GallusG_9031_20140417.fasta	281,818.00	100.00%	2	2	5	0.01%	57.40%	LEAELAAHEPAIQGVLDTGKK	R	L	99.70%	30.8	42.4	19.8	22.5	37.4	4.1	0.34	0.31	0	0	0	1	2	2,190.18	2,340.00		1,117.91	1782	1802	Empty	FALSE	UPI0000ECA63C,F1NHT3_CHICK
20196-20225 HinckeM –TAXchicken	Uncategorized Sample	20196-20205_A	Mudpit_Ingel20196_MGFPeaklist (F058870)	status=inactive	UPI0000ECA63D	UPARC14_2_GallusG_9031_20140417.fasta	281,818.00	100.00%	2	2	5	0.01%	57.40%	LFGAAEVQR	K	F	99.70%	67.3	45.1	36.8	48.6	34.7	1.77	0.337	0.556	0	4	0	0	2	990.5367	1,929.00		10,580.30	244	252	Empty	FALSE	UPI0000ECA63C,F1NHT3_CHICK
20196-20225 HinckeM –TAXchicken	Uncategorized Sample	20196-20205_A	Mudpit_Ingel20196_MGFPeaklist (F058870)	status=inactive	UPI0000ECA63D	UPARC14_2_GallusG_9031_20140417.fasta	281,818.00	100.00%	2	2	5	0.01%	57.40%	LGESQTLQQFSR	K	D	99.70%	60.8	44.7	24.7	36.5	49.5	5.12	0.469	0.583	0	7	1	0	2	1,393.71	2,032.00		36,229.30	1520	1531	Empty	FALSE	UPI0000ECA63C,F1NHT3_CHICK
20196-20225 HinckeM –TAXchicken	Uncategorized Sample	20196-20205_A	Mudpit_Ingel20196_MGFPeaklist (F058870)	status=inactive	UPI0000ECA63D	UPARC14_2_GallusG_9031_20140417.fasta	281,818.00	100.00%	2	2	5	0.01%	57.40%	LIDVNHYASDEVAAR	K	M	99.70%	62.4	44.7	25.1	51.9	48	5.25	0.463	0.533	0	0	9	0	2	1,673.81	2,017.00		15,639.90	631	645	Empty	FALSE	UPI0000ECA63C,F1NHT3_CHICK
20196-20225 HinckeM –TAXchicken	Uncategorized Sample	20196-20205_A	Mudpit_Ingel20196_MGFPeaklist (F058870)	status=inactive	UPI0000ECA63D	UPARC14_2_GallusG_9031_20140417.fasta	281,818.00	100.00%	2	2	5	0.01%	57.40%	LIQEQHPEEELIK	K	S	99.70%	39.4	44.4	25.6	29.3	37.4	1.68	0.193	0.462	0	0	3	0	2	1,605.85	1,970.00		11,713.00	208	220	Empty	FALSE	UPI0000ECA63C,F1NHT3_CHICK
20196-20225 HinckeM –TAXchicken	Uncategorized Sample	20196-20205_A	Mudpit_Ingel20196_MGFPeaklist (F058870)	status=inactive	UPI0000ECA63D	UPARC14_2_GallusG_9031_20140417.fasta	281,818.00	100.00%	2	2	5	0.01%	57.40%	LIQEQHPEEELIKSK	K	Q	27.20%	12.6	43.6	15.4	5.54					0	0	0	2	2	1,820.98	1,860.00		920.77	208	222	Empty	FALSE	UPI0000ECA63C,F1NHT3_CHICK
20196-20225 HinckeM –TAXchicken	Uncategorized Sample	20196-20205_A	Mudpit_Ingel20196_MGFPeaklist (F058870)	status=inactive	UPI0000ECA63D	UPARC14_2_GallusG_9031_20140417.fasta	281,818.00	100.00%	2	2	5	0.01%	57.40%	LIQNNHYAMDDVATR	K	R	99.70%	68.1	44.4	13	59.9	38.8	4.4	0.371	0.367	0	0	4	0	2	1,776.83	1,754.50		3,023.16	526	540	Empty	FALSE	UPI0000ECA63C,F1NHT3_CHICK
20196-20225 HinckeM –TAXchicken	Uncategorized Sample	20196-20205_A	Mudpit_Ingel20196_MGFPeaklist (F058870)	status=inactive	UPI0000ECA63D	UPARC14_2_GallusG_9031_20140417.fasta	281,818.00	100.00%	2	2	5	0.01%	57.40%	LIQNNHYAMDDVATRR	K	D	99.70%	34.4	44.5	13.1	30.5	40.7	3.49	0.364	0.406	0	0	0	6	2	1,916.94	1,626.50		7,812.11	526	541	Empty	FALSE	UPI0000ECA63C,F1NHT3_CHICK
20196-20225 HinckeM –TAXchicken	Uncategorized Sample	20196-20205_A	Mudpit_Ingel20196_MGFPeaklist (F058870)	status=inactive	UPI0000ECA63D	UPARC14_2_GallusG_9031_20140417.fasta	281,818.00	100.00%	2	2	5	0.01%	57.40%	LIQSHPESAEDLQEK	R	C	99.70%	71.4	44.6	22.4	57.1	45.9	4.37	0.401	0.5	0	0	5	0	2	1,723.85	1,669.00		16,131.80	1272	1286	Empty	FALSE	UPI0000ECA63C,F1NHT3_CHICK
20196-20225 HinckeM –TAXchicken	Uncategorized Sample	20196-20205_A	Mudpit_Ingel20196_MGFPeaklist (F058870)	status=inactive	UPI0000ECA63D	UPARC14_2_GallusG_9031_20140417.fasta	281,818.00	100.00%	2	2	5	0.01%	57.40%	LKDLNSQADSLMTSSAFDTSQVK	R	D	99.70%	67.5	44	22.2	61.8	41.7	5.11	0.446	0.261	0	0	2	0	2	2,502.20	2,157.00		2,188.60	1687	1709	Empty	FALSE	UPI0000ECA63C,F1NHT3_CHICK
20196-20225 HinckeM –TAXchicken	Uncategorized Sample	20196-20205_A	Mudpit_Ingel20196_MGFPeaklist (F058870)	status=inactive	UPI0000ECA63D	UPARC14_2_GallusG_9031_20140417.fasta	281,818.00	100.00%	2	2	5	0.01%	57.40%	LLEAQEHFR	K	K	99.70%	61.3	44.5	29.9	38.5	38.4	2.21	0.279	0.611	0	4	0	0	2	1,142.60	1,810.50		2,783.16	2052	2060	Empty	FALSE	UPI0000ECA63C,F1NHT3_CHICK
20196-20225 HinckeM –TAXchicken	Uncategorized Sample	20196-20205_A	Mudpit_Ingel20196_MGFPeaklist (F058870)	status=inactive	UPI0000ECA63D	UPARC14_2_GallusG_9031_20140417.fasta	281,818.00	100.00%	2	2	5	0.01%	57.40%	LLEATELK	K	G	74.40%	30.9	44.9	31.7	3.73					0	2	0	0	2	916.5351	1,999.50		1,326.52	656	663	Empty	FALSE	UPI0000ECA63C,F1NHT3_CHICK
20196-20225 HinckeM –TAXchicken	Uncategorized Sample	20196-20205_A	Mudpit_Ingel20196_MGFPeaklist (F058870)	status=inactive	UPI0000ECA63D	UPARC14_2_GallusG_9031_20140417.fasta	281,818.00	100.00%	2	2	5	0.01%	57.40%	LLEATELKGIK	K	L	99.70%	57	42.1	23.7	45.5	35	2.89	0.297	0.455	0	0	2	0	2	1,214.74	2,118.50		1,772.63	656	666	Empty	FALSE	UPI0000ECA63C,F1NHT3_CHICK
20196-20225 HinckeM –TAXchicken	Uncategorized Sample	20196-20205_A	Mudpit_Ingel20196_MGFPeaklist (F058870)	status=inactive	UPI0000ECA63D	UPARC14_2_GallusG_9031_20140417.fasta	281,818.00	100.00%	2	2	5	0.01%	57.40%	LLVSSEDYGR	K	D	99.70%	72.3	44.1	30.2	45	39.3	3.3	0.435	0.5	0	4	0	0	2	1,138.57	1,881.00		5,696.86	1758	1767	Empty	FALSE	UPI0000ECA63C,F1NHT3_CHICK
20196-20225 HinckeM –TAXchicken	Uncategorized Sample	20196-20205_A	Mudpit_Ingel20196_MGFPeaklist (F058870)	status=inactive	UPI0000ECA63D	UPARC14_2_GallusG_9031_20140417.fasta	281,818.00	100.00%	2	2	5	0.01%	57.40%	LLVSSEDYGRDLTGVQNLR	K	K	99.70%	57.2	43.9	21.7	46.9	49.3	7.52	0.46	0.447	0	0	2	0	2	2,135.11	2,381.50		3,116.02	1758	1776	Empty	FALSE	UPI0000ECA63C,F1NHT3_CHICK
20196-20225 HinckeM –TAXchicken	Uncategorized Sample	20196-20205_A	Mudpit_Ingel20196_MGFPeaklist (F058870)	status=inactive	UPI0000ECA63D	UPARC14_2_GallusG_9031_20140417.fasta	281,818.00	100.00%	2	2	5	0.01%	57.40%	LLVSSEDYGRDLTGVQNLRK	K	K	99.70%	23.5	42.8	15.5	21.9	31	0.678	0.187	0.3	0	0	0	2	2	2,263.20	2,268.50		1,286.29	1758	1777	Empty	FALSE	UPI0000ECA63C,F1NHT3_CHICK
20196-20225 HinckeM –TAXchicken	Uncategorized Sample	20196-20205_A	Mudpit_Ingel20196_MGFPeaklist (F058870)	status=inactive	UPI0000ECA63D	UPARC14_2_GallusG_9031_20140417.fasta	281,818.00	100.00%	2	2	5	0.01%	57.40%	LNELNQKWDSLK	K	A	99.70%	32.2	44.5	21.2	24.7	30.4	1.18	0.23	0.417	0	0	2	0	2	1,487.79	2,156.00		655.62	858	869	Empty	FALSE	UPI0000ECA63C,F1NHT3_CHICK
20196-20225 HinckeM –TAXchicken	Uncategorized Sample	20196-20205_A	Mudpit_Ingel20196_MGFPeaklist (F058870)	status=inactive	UPI0000ECA63D	UPARC14_2_GallusG_9031_20140417.fasta	281,818.00	100.00%	2	2	5	0.01%	57.40%	LQIASDENYKDPSNLQGK	K	L	99.70%	66.1	44.6	19.5	57.2	43.3	4.48	0.425	0.389	0	0	2	0	2	2,020.00	1,904.00		6,653.79	57	74	Empty	FALSE	UPI0000ECA63C,F1NHT3_CHICK
20196-20225 HinckeM –TAXchicken	Uncategorized Sample	20196-20205_A	Mudpit_Ingel20196_MGFPeaklist (F058870)	status=inactive	UPI0000ECA63D	UPARC14_2_GallusG_9031_20140417.fasta	281,818.00	100.00%	2	2	5	0.01%	57.40%	LQQLFR	R	D	24.20%	46	45	50.6	0					0	1	0	0	2	804.4727	2,087.00		1,798.04	779	784	Empty	FALSE	UPI0000ECA63C,F1NHT3_CHICK
20196-20225 HinckeM –TAXchicken	Uncategorized Sample	20196-20205_A	Mudpit_Ingel20196_MGFPeaklist (F058870)	status=inactive	UPI0000ECA63D	UPARC14_2_GallusG_9031_20140417.fasta	281,818.00	100.00%	2	2	5	0.01%	57.40%	LQQSHPINASQIQVK	R	R	58.10%	16.8	43.4	15.8	12.3					0	0	1	0	2	1,690.92	1,835.00		2,269.03	314	328	Empty	FALSE	UPI0000ECA63C,F1NHT3_CHICK
20196-20225 HinckeM –TAXchicken	Uncategorized Sample	20196-20205_A	Mudpit_Ingel20196_MGFPeaklist (F058870)	status=inactive	UPI0000ECA63D	UPARC14_2_GallusG_9031_20140417.fasta	281,818.00	100.00%	2	2	5	0.01%	57.40%	LQTASDESYKDPTNIQSK	K	H	99.70%	57.9	44.4	13.2	54.7					0	0	4	0	2	2,024.98	1,691.00		5,214.14	1544	1561	Empty	TRUE	UPI0000ECA63C
20196-20225 HinckeM –TAXchicken	Uncategorized Sample	20196-20205_A	Mudpit_Ingel20196_MGFPeaklist (F058870)	status=inactive	UPI0000ECA63D	UPARC14_2_GallusG_9031_20140417.fasta	281,818.00	100.00%	2	2	5	0.01%	57.40%	LSDDNTIGKEEIQQR	K	L	99.70%	86.1	44.8	34.6	0	52.4	4.66	0.41	0.5	0	0	6	0	2	1,745.87	1,719.50		20,857.20	1803	1817	Empty	FALSE	UPI0000ECA63C,F1NHT3_CHICK
20196-20225 HinckeM –TAXchicken	Uncategorized Sample	20196-20205_A	Mudpit_Ingel20196_MGFPeaklist (F058870)	status=inactive	UPI0000ECA63D	UPARC14_2_GallusG_9031_20140417.fasta	281,818.00	100.00%	2	2	5	0.01%	57.40%	LTILSDER	K	S	99.70%	82.2	45.3	46.6	21.2	39.7	2.17	0.224	0.688	0	8	0	0	2	946.5205	2,023.00		14,285.40	435	442	Empty	FALSE	UPI0000ECA63C,F1NHT3_CHICK
20196-20225 HinckeM –TAXchicken	Uncategorized Sample	20196-20205_A	Mudpit_Ingel20196_MGFPeaklist (F058870)	status=inactive	UPI0000ECA63D	UPARC14_2_GallusG_9031_20140417.fasta	281,818.00	100.00%	2	2	5	0.01%	57.40%	LWELLLEK	R	M	99.70%	44.7	43.8	40.6	14.8	36.7	1.32	0.18	0.688	0	2	0	0	2	1,043.61	2,822.00		1,411.94	123	130	Empty	FALSE	UPI0000ECA63C,F1NHT3_CHICK
20196-20225 HinckeM –TAXchicken	Uncategorized Sample	20196-20205_A	Mudpit_Ingel20196_MGFPeaklist (F058870)	status=inactive	UPI0000ECA63D	UPARC14_2_GallusG_9031_20140417.fasta	281,818.00	100.00%	2	2	5	0.01%	57.40%	MKTATDEAYKDPSNLQGK	K	V	99.70%	46.5	44	17.7	35.1	39.6	4.8	0.351	0.417	0	0	0	4	2	2,012.96	1,579.00		2,578.80	586	603	Empty	FALSE	UPI0000ECA63C,F1NHT3_CHICK
20196-20225 HinckeM –TAXchicken	Uncategorized Sample	20196-20205_A	Mudpit_Ingel20196_MGFPeaklist (F058870)	status=inactive	UPI0000ECA63D	UPARC14_2_GallusG_9031_20140417.fasta	281,818.00	100.00%	2	2	5	0.01%	57.40%	MMVHTVATFNSIK	R	E	99.70%	34.1	44.4	16.1	25.6	34.1	4.15	0.361	0.385	0	0	3	0	2	1,510.74	1,940.00		1,575.17	1170	1182	Empty	FALSE	UPI0000ECA63C,F1NHT3_CHICK
20196-20225 HinckeM –TAXchicken	Uncategorized Sample	20196-20205_A	Mudpit_Ingel20196_MGFPeaklist (F058870)	status=inactive	UPI0000ECA63D	UPARC14_2_GallusG_9031_20140417.fasta	281,818.00	100.00%	2	2	5	0.01%	57.40%	MMVHTVATFNSIKELNER	R	W	99.70%	53.3	44.2	25.9	40.6	38.9	4.11	0.262	0.361	0	0	0	2	2	2,152.05	2,142.00		1,346.02	1170	1187	Empty	FALSE	UPI0000ECA63C,F1NHT3_CHICK
20196-20225 HinckeM –TAXchicken	Uncategorized Sample	20196-20205_A	Mudpit_Ingel20196_MGFPeaklist (F058870)	status=inactive	UPI0000ECA63D	UPARC14_2_GallusG_9031_20140417.fasta	281,818.00	100.00%	2	2	5	0.01%	57.40%	MNEVISLWK	R	K	99.70%	70.2	45	33.2	39	36.8	0.854	0.149	0.556	0	4	0	0	2	1,119.59	2,570.50		1,925.99	646	654	Empty	FALSE	UPI0000ECA63C,F1NHT3_CHICK
20196-20225 HinckeM –TAXchicken	Uncategorized Sample	20196-20205_A	Mudpit_Ingel20196_MGFPeaklist (F058870)	status=inactive	UPI0000ECA63D	UPARC14_2_GallusG_9031_20140417.fasta	281,818.00	100.00%	2	2	5	0.01%	57.40%	MNEVISLWKK	R	L	47.00%	22.5	44.5	30.2	3.6					0	0	2	0	2	1,263.68	2,242.50		623.89	646	655	Empty	FALSE	UPI0000ECA63C,F1NHT3_CHICK
20196-20225 HinckeM –TAXchicken	Uncategorized Sample	20196-20205_A	Mudpit_Ingel20196_MGFPeaklist (F058870)	status=inactive	UPI0000ECA63D	UPARC14_2_GallusG_9031_20140417.fasta	281,818.00	100.00%	2	2	5	0.01%	57.40%	MQHNLEQQIQAR	R	N	99.70%	52.6	44.3	29.8	36.6	43.1	2.82	0.394	0.583	0	0	8	0	2	1,511.74	1,728.50		7,950.78	2277	2288	Empty	FALSE	UPI0000ECA63C,F1NHT3_CHICK
20196-20225 HinckeM –TAXchicken	Uncategorized Sample	20196-20205_A	Mudpit_Ingel20196_MGFPeaklist (F058870)	status=inactive	UPI0000ECA63D	UPARC14_2_GallusG_9031_20140417.fasta	281,818.00	100.00%	2	2	5	0.01%	57.40%	NQALNTDNYGHDLASVQALQR	K	K	99.70%	77.3	44.1	21.8	74.6	52.8	8.52	0.568	0.333	0	0	2	0	2	2,328.13	2,251.50		10,715.10	1226	1246	Empty	FALSE	UPI0000ECA63C,F1NHT3_CHICK
20196-20225 HinckeM –TAXchicken	Uncategorized Sample	20196-20205_A	Mudpit_Ingel20196_MGFPeaklist (F058870)	status=inactive	UPI0000ECA63D	UPARC14_2_GallusG_9031_20140417.fasta	281,818.00	100.00%	2	2	5	0.01%	57.40%	NTTGVTEEALKEFSMMFK	R	H	99.60%	41.4	43.6	13	40.4					0	0	2	0	2	2,094.97	2,776.50		1,235.05	2289	2306	Empty	FALSE	UPI0000ECA63C,F1NHT3_CHICK
20196-20225 HinckeM –TAXchicken	Uncategorized Sample	20196-20205_A	Mudpit_Ingel20196_MGFPeaklist (F058870)	status=inactive	UPI0000ECA63D	UPARC14_2_GallusG_9031_20140417.fasta	281,818.00	100.00%	2	2	5	0.01%	57.40%	QDEVNASWQR	K	L	99.70%	71.8	44.3	32.8	57.5	41.1	2.96	0.355	0.55	0	5	0	0	2	1,232.57	1,742.00		2,096.38	223	232	Empty	FALSE	UPI0000ECA63C,F1NHT3_CHICK
20196-20225 HinckeM –TAXchicken	Uncategorized Sample	20196-20205_A	Mudpit_Ingel20196_MGFPeaklist (F058870)	status=inactive	UPI0000ECA63D	UPARC14_2_GallusG_9031_20140417.fasta	281,818.00	100.00%	2	2	5	0.01%	57.40%	QEFAQHANAFHQWIQETR	R	T	99.70%	53	43.7	16.1	0	33.9	3.02	0.354	0.25	0	0	0	2	2	2,241.06	2,283.50		1,438.84	2181	2198	Empty	FALSE	UPI0000ECA63C,F1NHT3_CHICK
20196-20225 HinckeM –TAXchicken	Uncategorized Sample	20196-20205_A	Mudpit_Ingel20196_MGFPeaklist (F058870)	status=inactive	UPI0000ECA63D	UPARC14_2_GallusG_9031_20140417.fasta	281,818.00	100.00%	2	2	5	0.01%	57.40%	QEQIDNQYHSLLELGEK	R	R	98.90%	30.5	44.3	15	29					0	0	1	0	2	2,044.00	2,413.00		1,265.04	1039	1055	Empty	TRUE	
20196-20225 HinckeM –TAXchicken	Uncategorized Sample	20196-20205_A	Mudpit_Ingel20196_MGFPeaklist (F058870)	status=inactive	UPI0000ECA63D	UPARC14_2_GallusG_9031_20140417.fasta	281,818.00	100.00%	2	2	5	0.01%	57.40%	QFQDAGHFDADNIK	R	K	99.70%	64	42.5	21.5	55.9	54.7	5.25	0.00183	0.571	0	2	2	0	2	1,588.70	2,092.50		10,466.30	736	749	Empty	FALSE	UPI0000ECA63C,F1NHT3_CHICK
20196-20225 HinckeM –TAXchicken	Uncategorized Sample	20196-20205_A	Mudpit_Ingel20196_MGFPeaklist (F058870)	status=inactive	UPI0000ECA63D	UPARC14_2_GallusG_9031_20140417.fasta	281,818.00	100.00%	2	2	5	0.01%	57.40%	QFQDAGHFDADNIKK	R	K	99.70%	16	43.7	19.7	9.85	30.8	1.22	0.00325	0.433	0	0	2	0	2	1,716.80	2,087.00		3,109.36	736	750	Empty	FALSE	UPI0000ECA63C,F1NHT3_CHICK
20196-20225 HinckeM –TAXchicken	Uncategorized Sample	20196-20205_A	Mudpit_Ingel20196_MGFPeaklist (F058870)	status=inactive	UPI0000ECA63D	UPARC14_2_GallusG_9031_20140417.fasta	281,818.00	100.00%	2	2	5	0.01%	57.40%	QGFVPAAYVK	R	K	99.70%	41.1	44.1	26.3	21.9	38.4	3.28	0.237	0.5	0	8	0	0	2	1,079.59	2,175.50		9,621.08	1006	1015	Empty	FALSE	UPI0000ECA63C,F1NHT3_CHICK
20196-20225 HinckeM –TAXchicken	Uncategorized Sample	20196-20205_A	Mudpit_Ingel20196_MGFPeaklist (F058870)	status=inactive	UPI0000ECA63D	UPARC14_2_GallusG_9031_20140417.fasta	281,818.00	100.00%	2	2	5	0.01%	57.40%	QGKLFGAAEVQR	R	F	99.70%	53.8	44.4	25.1	33.7	30.3	1.24	0.218	0.333	0	0	2	0	2	1,303.71	1,865.00		655.81	241	252	Empty	FALSE	UPI0000ECA63C,F1NHT3_CHICK
20196-20225 HinckeM –TAXchicken	Uncategorized Sample	20196-20205_A	Mudpit_Ingel20196_MGFPeaklist (F058870)	status=inactive	UPI0000ECA63D	UPARC14_2_GallusG_9031_20140417.fasta	281,818.00	100.00%	2	2	5	0.01%	57.40%	QKLADSLR	K	L	96.10%	37.9	43.6	39.3	16.5					0	4	0	0	2	913.5103	1,860.50		1,083.35	771	778	Empty	FALSE	UPI0000ECA63C,F1NHT3_CHICK
20196-20225 HinckeM –TAXchicken	Uncategorized Sample	20196-20205_A	Mudpit_Ingel20196_MGFPeaklist (F058870)	status=inactive	UPI0000ECA63D	UPARC14_2_GallusG_9031_20140417.fasta	281,818.00	100.00%	2	2	5	0.01%	57.40%	QKLEDSYR	R	F	99.70%	43.5	44.8	35.7	18.9	37.3	2.14	0.00536	0.688	0	4	0	0	2	1,021.50	1,670.50		1,559.49	31	38	Empty	FALSE	UPI0000ECA63C,F1NHT3_CHICK
20196-20225 HinckeM –TAXchicken	Uncategorized Sample	20196-20205_A	Mudpit_Ingel20196_MGFPeaklist (F058870)	status=inactive	UPI0000ECA63D	UPARC14_2_GallusG_9031_20140417.fasta	281,818.00	100.00%	2	2	5	0.01%	57.40%	QQVAPTDDETGKELVLALYDYQEK	R	S	99.70%	67.1	43.7	14.3	65.9	46.1	5.89	0.482	0.333	0	0	1	0	2	2,753.35	2,733.00		626.58	951	974	Empty	FALSE	UPI0000ECA63C,F1NHT3_CHICK
20196-20225 HinckeM –TAXchicken	Uncategorized Sample	20196-20205_A	Mudpit_Ingel20196_MGFPeaklist (F058870)	status=inactive	UPI0000ECA63D	UPARC14_2_GallusG_9031_20140417.fasta	281,818.00	100.00%	2	2	5	0.01%	57.40%	REELIANWEQIR	K	T	99.70%	43.2	44.3	27.9	22.1	33.1	1.77	0.251	0.458	0	0	4	0	2	1,556.82	2,390.00		4,629.97	329	340	Empty	FALSE	UPI0000ECA63C,F1NHT3_CHICK
20196-20225 HinckeM –TAXchicken	Uncategorized Sample	20196-20205_A	Mudpit_Ingel20196_MGFPeaklist (F058870)	status=inactive	UPI0000ECA63D	UPARC14_2_GallusG_9031_20140417.fasta	281,818.00	100.00%	2	2	5	0.01%	57.40%	RLEAELAAHEPAIQGVLDTGKK	K	L	99.70%	51.6	41.1	0	50	50.5	7.68	0.554	0.386	0	0	0	3	2	2,346.28	2,305.00		3,180.64	1781	1802	Empty	FALSE	UPI0000ECA63C,F1NHT3_CHICK
20196-20225 HinckeM –TAXchicken	Uncategorized Sample	20196-20205_A	Mudpit_Ingel20196_MGFPeaklist (F058870)	status=inactive	UPI0000ECA63D	UPARC14_2_GallusG_9031_20140417.fasta	281,818.00	100.00%	2	2	5	0.01%	57.40%	RWNQLLANSAAR	K	K	99.70%	52.7	44.2	29.3	30.5	30.1	3.28	0.266	0.375	0	0	2	0	2	1,399.76	2,051.50		749.55	2037	2048	Empty	FALSE	UPI0000ECA63C,F1NHT3_CHICK
20196-20225 HinckeM –TAXchicken	Uncategorized Sample	20196-20205_A	Mudpit_Ingel20196_MGFPeaklist (F058870)	status=inactive	UPI0000ECA63D	UPARC14_2_GallusG_9031_20140417.fasta	281,818.00	100.00%	2	2	5	0.01%	57.40%	SADESGQALLAAGHYASDEVK	R	E	99.70%	54.4	44	23.2	49.2	43.5	5.24	0.441	0.357	0	0	2	0	2	2,118.99	2,227.00		1,690.92	412	432	Empty	FALSE	UPI0000ECA63C,F1NHT3_CHICK
20196-20225 HinckeM –TAXchicken	Uncategorized Sample	20196-20205_A	Mudpit_Ingel20196_MGFPeaklist (F058870)	status=inactive	UPI0000ECA63D	UPARC14_2_GallusG_9031_20140417.fasta	281,818.00	100.00%	2	2	5	0.01%	57.40%	SADESGQALLAAGHYASDEVKEK	R	L	99.70%	87.1	43.9	14.7	84.1	58	8.54	0.559	0.413	0	0	0	2	2	2,376.13	2,184.00		15,661.80	412	434	Empty	FALSE	UPI0000ECA63C,F1NHT3_CHICK
20196-20225 HinckeM –TAXchicken	Uncategorized Sample	20196-20205_A	Mudpit_Ingel20196_MGFPeaklist (F058870)	status=inactive	UPI0000ECA63D	UPARC14_2_GallusG_9031_20140417.fasta	281,818.00	100.00%	2	2	5	0.01%	57.40%	SALLELWELRR	R	Q	99.70%	30.1	43.2	29.6	14.2	41	0.796	0.249	0.636	0	0	2	0	2	1,385.79	2,758.00		1,656.26	443	453	Empty	FALSE	UPI0000ECA63C,F1NHT3_CHICK
20196-20225 HinckeM –TAXchicken	Uncategorized Sample	20196-20205_A	Mudpit_Ingel20196_MGFPeaklist (F058870)	status=inactive	UPI0000ECA63D	UPARC14_2_GallusG_9031_20140417.fasta	281,818.00	100.00%	2	2	5	0.01%	57.40%	SKLGESQTLQQFSR	R	D	99.70%	86.8	44.4	23.3	75.8	45.4	4.77	0.414	0.464	0	1	1	0	2	1,608.83	1,945.00		8,398.43	1518	1531	Empty	FALSE	UPI0000ECA63C,F1NHT3_CHICK
20196-20225 HinckeM –TAXchicken	Uncategorized Sample	20196-20205_A	Mudpit_Ingel20196_MGFPeaklist (F058870)	status=inactive	UPI0000ECA63D	UPARC14_2_GallusG_9031_20140417.fasta	281,818.00	100.00%	2	2	5	0.01%	57.40%	SKQDEVNASWQR	K	L	99.70%	61.5	44.7	31.5	35.1	42.3	1.82	0.296	0.625	0	0	7	0	2	1,447.69	1,646.00		5,075.36	221	232	Empty	FALSE	UPI0000ECA63C,F1NHT3_CHICK
20196-20225 HinckeM –TAXchicken	Uncategorized Sample	20196-20205_A	Mudpit_Ingel20196_MGFPeaklist (F058870)	status=inactive	UPI0000ECA63D	UPARC14_2_GallusG_9031_20140417.fasta	281,818.00	100.00%	2	2	5	0.01%	57.40%	SLQQLAEER	R	S	99.70%	72.3	45.3	40.8	44.3	34.8	1.96	0.316	0.556	0	6	0	0	2	1,073.56	1,925.50		25,749.10	1190	1198	Empty	FALSE	UPI0000ECA63C,F1NHT3_CHICK
20196-20225 HinckeM –TAXchicken	Uncategorized Sample	20196-20205_A	Mudpit_Ingel20196_MGFPeaklist (F058870)	status=inactive	UPI0000ECA63D	UPARC14_2_GallusG_9031_20140417.fasta	281,818.00	100.00%	2	2	5	0.01%	57.40%	SLSAQEEKITALDEFATK	K	L	99.70%	46.9	44.1	22.5	39.1	39.3	4.77	0.445	0.361	0	0	2	0	2	1,981.01	2,426.50		1,838.20	508	525	Empty	FALSE	UPI0000ECA63C,F1NHT3_CHICK
20196-20225 HinckeM –TAXchicken	Uncategorized Sample	20196-20205_A	Mudpit_Ingel20196_MGFPeaklist (F058870)	status=inactive	UPI0000ECA63D	UPARC14_2_GallusG_9031_20140417.fasta	281,818.00	100.00%	2	2	5	0.01%	57.40%	SQLLGSAHEVQR	R	F	99.70%	67.1	43.9	23.2	54.5	48.6	5.38	0.438	0.708	0	0	13	0	2	1,324.70	1,714.00		26,876.90	1199	1210	Empty	FALSE	UPI0000ECA63C,F1NHT3_CHICK
20196-20225 HinckeM –TAXchicken	Uncategorized Sample	20196-20205_A	Mudpit_Ingel20196_MGFPeaklist (F058870)	status=inactive	UPI0000ECA63D	UPARC14_2_GallusG_9031_20140417.fasta	281,818.00	100.00%	2	2	5	0.01%	57.40%	SSEEIESAFR	K	A	99.70%	66.5	43.3	38.2	29.9	43.9	3.7	0.362	0.65	0	4	0	0	2	1,154.53	2,006.50		9,866.21	2378	2387	Empty	FALSE	UPI0000ECA63C,F1NHT3_CHICK
20196-20225 HinckeM –TAXchicken	Uncategorized Sample	20196-20205_A	Mudpit_Ingel20196_MGFPeaklist (F058870)	status=inactive	UPI0000ECA63D	UPARC14_2_GallusG_9031_20140417.fasta	281,818.00	100.00%	2	2	5	0.01%	57.40%	SSLSSAQADFNQLAELDR	R	Q	99.70%	80.4	44.3	25.6	65.7	40.6	3.31	0.337	0.333	0	0	2	0	2	1,951.94	2,631.50		1,349.63	2111	2128	Empty	FALSE	UPI0000ECA63C,F1NHT3_CHICK
20196-20225 HinckeM –TAXchicken	Uncategorized Sample	20196-20205_A	Mudpit_Ingel20196_MGFPeaklist (F058870)	status=inactive	UPI0000ECA63D	UPARC14_2_GallusG_9031_20140417.fasta	281,818.00	100.00%	2	2	5	0.01%	57.40%	TATDEAYKDPSNLQGK	K	V	99.70%	75.7	44.4	20.1	64.8	41.7	5.12	0.405	0.406	0	0	5	0	2	1,737.83	1,614.00		14,904.40	588	603	Empty	FALSE	UPI0000ECA63C,F1NHT3_CHICK
20196-20225 HinckeM –TAXchicken	Uncategorized Sample	20196-20205_A	Mudpit_Ingel20196_MGFPeaklist (F058870)	status=inactive	UPI0000ECA63D	UPARC14_2_GallusG_9031_20140417.fasta	281,818.00	100.00%	2	2	5	0.01%	57.40%	VEDLFLTFAK	K	K	99.70%	68.1	43.6	24.1	50.7	42.8	2.89	0.318	0.6	0	2	0	0	2	1,182.64	2,814.50		721.68	2062	2071	Empty	FALSE	UPI0000ECA63C,F1NHT3_CHICK
20196-20225 HinckeM –TAXchicken	Uncategorized Sample	20196-20205_A	Mudpit_Ingel20196_MGFPeaklist (F058870)	status=inactive	UPI0000ECA63D	UPARC14_2_GallusG_9031_20140417.fasta	281,818.00	100.00%	2	2	5	0.01%	57.40%	VLETAEDIQER	-	R	99.70%	67.4	44.9	31.8	41.6	46.8	3.13	0.442	0.591	0	8	0	0	2	1,302.65	1,794.00		16,668.30	1	11	Empty	FALSE	UPI0000ECA63C,F1NHT3_CHICK
20196-20225 HinckeM –TAXchicken	Uncategorized Sample	20196-20205_A	Mudpit_Ingel20196_MGFPeaklist (F058870)	status=inactive	UPI0000ECA63D	UPARC14_2_GallusG_9031_20140417.fasta	281,818.00	100.00%	2	2	5	0.01%	57.40%	VLETAEDIQERR	-	Q	99.70%	45.4	44.7	25.2	32	43.4	3.8	0.369	0.625	0	0	5	0	2	1,458.75	1,732.00		6,237.60	1	12	Empty	FALSE	UPI0000ECA63C,F1NHT3_CHICK
20196-20225 HinckeM –TAXchicken	Uncategorized Sample	20196-20205_A	Mudpit_Ingel20196_MGFPeaklist (F058870)	status=inactive	UPI0000ECA63D	UPARC14_2_GallusG_9031_20140417.fasta	281,818.00	100.00%	2	2	5	0.01%	57.40%	VNDVCANGEDLIK	R	K	99.70%	64.1	44.3	22.9	46.6	44	4.52	0.423	0.462	0	2	1	0	2	1,447.67	1,987.00		2,175.97	1899	1911	Empty	FALSE	UPI0000ECA63C,F1NHT3_CHICK
20196-20225 HinckeM –TAXchicken	Uncategorized Sample	20196-20205_A	Mudpit_Ingel20196_MGFPeaklist (F058870)	status=inactive	UPI0000ECA63D	UPARC14_2_GallusG_9031_20140417.fasta	281,818.00	100.00%	2	2	5	0.01%	57.40%	VNDVCANGEDLIKK	R	N	99.70%	69.8	44.9	20.1	60.2	43.9	5.19	0.424	0.5	0	0	6	0	2	1,574.78	1,858.00		9,724.62	1899	1912	Empty	FALSE	UPI0000ECA63C,F1NHT3_CHICK
20196-20225 HinckeM –TAXchicken	Uncategorized Sample	20196-20205_A	Mudpit_Ingel20196_MGFPeaklist (F058870)	status=inactive	UPI0000ECA63D	UPARC14_2_GallusG_9031_20140417.fasta	281,818.00	100.00%	2	2	5	0.01%	57.40%	VNEVNQFAGK	R	L	99.70%	60.7	45.1	24.8	44.4	38.5	3.89	0.374	0.55	0	6	0	0	2	1,105.56	1,728.00		3,154.14	198	207	Empty	FALSE	UPI0000ECA63C,F1NHT3_CHICK
20196-20225 HinckeM –TAXchicken	Uncategorized Sample	20196-20205_A	Mudpit_Ingel20196_MGFPeaklist (F058870)	status=inactive	UPI0000ECA63D	UPARC14_2_GallusG_9031_20140417.fasta	281,818.00	100.00%	2	2	5	0.01%	57.40%	VQKHQAFEAELSANQSR	K	I	99.70%	33.5	44.2	19.1	25.6	33.7	1.89	0.285	0.412	0	0	0	2	2	1,942.97	1,752.50		2,844.57	604	620	Empty	FALSE	UPI0000ECA63C,F1NHT3_CHICK
20196-20225 HinckeM –TAXchicken	Uncategorized Sample	20196-20205_A	Mudpit_Ingel20196_MGFPeaklist (F058870)	status=inactive	UPI0000ECA63D	UPARC14_2_GallusG_9031_20140417.fasta	281,818.00	100.00%	2	2	5	0.01%	57.40%	WNQLLANSAAR	R	K	99.70%	61.5	44.7	24.6	24.8	41.9	3.89	0.403	0.545	0	5	0	0	2	1,243.65	2,066.00		7,855.65	2038	2048	Empty	FALSE	UPI0000ECA63C,F1NHT3_CHICK
20196-20225 HinckeM –TAXchicken	Uncategorized Sample	20196-20205_A	Mudpit_Ingel20196_MGFPeaklist (F058870)	status=inactive	UPI0000ECA63D	UPARC14_2_GallusG_9031_20140417.fasta	281,818.00	100.00%	2	2	5	0.01%	57.40%	YEALKDPMVAR	R	K	99.70%	56.9	44.8	28.4	45.5	37.4	4.08	0.404	0.5	0	0	6	0	2	1,292.67	1,732.00		6,967.50	759	769	Empty	FALSE	UPI0000ECA63C,F1NHT3_CHICK
20196-20225 HinckeM –TAXchicken	Uncategorized Sample	20196-20205_A	Mudpit_Ingel20196_MGFPeaklist (F058870)	status=inactive	UPI000060F68B	UPARC14_2_GallusG_9031_20140417.fasta	273,727.60	100.00%	20	20	32	0.06%	8.43%	ADWWTNTAHYNR	K	E	99.30%	29.5	43.3	28.2	2.46	24.9	1.1	0.133	0.292	0	0	1	0	2	1,534.68	2,138.00		152.29	747	758	Empty	TRUE	UPI00003AB669,UPI00003AB668,UPI0000EB6CF0,UPI00003AB667,UPI0000449548,E1BZT5_CHICK,UPI00003AB66A,UPI0001A2C44C,UPI0003503F58
20196-20225 HinckeM –TAXchicken	Uncategorized Sample	20196-20205_A	Mudpit_Ingel20196_MGFPeaklist (F058870)	status=inactive	UPI000060F68B	UPARC14_2_GallusG_9031_20140417.fasta	273,727.60	100.00%	20	20	32	0.06%	8.43%	ALDIPLVK	R	N	16.80%	8.97	40.5	23.3	-0.78					0	1	0	0	2	868.5504	2,426.00		282.87	421	428	Empty	TRUE	UPI00003AB669,UPI00003AB668,UPI00003AB667,UPI0000449548,E1BZT5_CHICK,UPI00003AB66A,UPI0003503F58
20196-20225 HinckeM –TAXchicken	Uncategorized Sample	20196-20205_A	Mudpit_Ingel20196_MGFPeaklist (F058870)	status=inactive	UPI000060F68B	UPARC14_2_GallusG_9031_20140417.fasta	273,727.60	100.00%	20	20	32	0.06%	8.43%	ANPALYVLR	K	E	99.70%	43.5	44.7	20.2	32.6	33	2.48	0.285	0.389	0	2	0	0	2	1,016.59	2,313.00		1,166.65	1737	1745	Empty	TRUE	UPI00003AB669,UPI00003AB668,UPI0000EB6CF0,UPI00003AB667,UPI0000449548,E1BZT5_CHICK,UPI00003AB66A,UPI0001A2C44C,UPI0003503F58
20196-20225 HinckeM –TAXchicken	Uncategorized Sample	20196-20205_A	Mudpit_Ingel20196_MGFPeaklist (F058870)	status=inactive	UPI000060F68B	UPARC14_2_GallusG_9031_20140417.fasta	273,727.60	100.00%	20	20	32	0.06%	8.43%	AVFWDIK	R	N	53.60%	27.4	44.4	30	8.78					0	2	0	0	2	878.4771	2,497.50		536.32	1152	1158	Empty	TRUE	UPI00003AB669,UPI00003AB668,UPI0000EB6CF0,UPI00003AB667,UPI0000449548,E1BZT5_CHICK,UPI00003AB66A,UPI0001A2C44C,UPI0003503F58
20196-20225 HinckeM –TAXchicken	Uncategorized Sample	20196-20205_A	Mudpit_Ingel20196_MGFPeaklist (F058870)	status=inactive	UPI000060F68B	UPARC14_2_GallusG_9031_20140417.fasta	273,727.60	100.00%	20	20	32	0.06%	8.43%	ETGYTYILPK	K	N	44.20%	20.6	44.1	24.9	0					0	1	0	0	2	1,184.62	2,213.00		372.06	2100	2109	Empty	TRUE	UPI00003AB669,UPI00003AB668,UPI0000EB6CF0,UPI00003AB667,UPI0000449548,E1BZT5_CHICK,UPI00003AB66A,UPI0001A2C44C,UPI0003503F58
20196-20225 HinckeM –TAXchicken	Uncategorized Sample	20196-20205_A	Mudpit_Ingel20196_MGFPeaklist (F058870)	status=inactive	UPI000060F68B	UPARC14_2_GallusG_9031_20140417.fasta	273,727.60	100.00%	20	20	32	0.06%	8.43%	FGDLILK	K	A	74.20%	22.6	44.1	22.8	13.9					0	2	0	0	2	805.482	2,332.50		1,076.56	1903	1909	Empty	TRUE	UPI00003AB669,UPI00003AB668,UPI0000EB6CF0,UPI00003AB667,UPI0000449548,E1BZT5_CHICK,UPI00003AB66A,UPI0001A2C44C,UPI0003503F58
20196-20225 HinckeM –TAXchicken	Uncategorized Sample	20196-20205_A	Mudpit_Ingel20196_MGFPeaklist (F058870)	status=inactive	UPI000060F68B	UPARC14_2_GallusG_9031_20140417.fasta	273,727.60	100.00%	20	20	32	0.06%	8.43%	FICISDLR	K	A	99.70%	43	43.9	30.4	28.3	25.2	0.538	0.218	0.312	0	2	0	0	2	1,023.53	2,400.00		654.76	2115	2122	Empty	TRUE	UPI00003AB669,UPI00003AB668,UPI0000EB6CF0,UPI00003AB667,UPI0000449548,E1BZT5_CHICK,UPI00003AB66A,UPI0001A2C44C,UPI0003503F58
20196-20225 HinckeM –TAXchicken	Uncategorized Sample	20196-20205_A	Mudpit_Ingel20196_MGFPeaklist (F058870)	status=inactive	UPI000060F68B	UPARC14_2_GallusG_9031_20140417.fasta	273,727.60	100.00%	20	20	32	0.06%	8.43%	IDLTLLNR	K	L	99.70%	46.5	43.7	38.1	3.23	30.2	0.357	0.159	0.5	0	1	0	0	2	957.5729	2,439.00		350.61	988	995	Empty	TRUE	UPI00003AB669,UPI00003AB668,UPI0000EB6CF0,UPI00003AB667,UPI0000449548,E1BZT5_CHICK,UPI00003AB66A,UPI0001A2C44C,UPI0003503F58
20196-20225 HinckeM –TAXchicken	Uncategorized Sample	20196-20205_A	Mudpit_Ingel20196_MGFPeaklist (F058870)	status=inactive	UPI000060F68B	UPARC14_2_GallusG_9031_20140417.fasta	273,727.60	100.00%	20	20	32	0.06%	8.43%	IIHTSVWAGQK	K	R	18.00%	13.1	43.2	16.9	7.39					0	0	2	0	2	1,239.68	1,852.50		476.63	1822	1832	Empty	TRUE	UPI00003AB669,UPI00003AB668,UPI0000EB6CF0,UPI00003AB667,UPI0000449548,E1BZT5_CHICK,UPI00003AB66A,UPI0001A2C44C,UPI0003503F58
20196-20225 HinckeM –TAXchicken	Uncategorized Sample	20196-20205_A	Mudpit_Ingel20196_MGFPeaklist (F058870)	status=inactive	UPI000060F68B	UPARC14_2_GallusG_9031_20140417.fasta	273,727.60	100.00%	20	20	32	0.06%	8.43%	ISLIQIFR	K	A	99.70%	59.6	42.5	37.4	33.6	29	1.89	0.262	0.5	0	2	0	0	2	989.6143	2,871.50		1,107.17	1572	1579	Empty	TRUE	UPI00003AB669,UPI00003AB668,UPI0000EB6CF0,UPI00003AB667,UPI0000449548,E1BZT5_CHICK,UPI00003AB66A,UPI0001A2C44C,UPI0003503F58
20196-20225 HinckeM –TAXchicken	Uncategorized Sample	20196-20205_A	Mudpit_Ingel20196_MGFPeaklist (F058870)	status=inactive	UPI000060F68B	UPARC14_2_GallusG_9031_20140417.fasta	273,727.60	100.00%	20	20	32	0.06%	8.43%	LLILALER	K	L	99.70%	57.6	38.7	57.1	22.5	34.9	0.745	0.244	0.625	0	2	0	0	2	940.6191	2,674.00		754.2	838	845	Empty	TRUE	UPI00003AB669,UPI00003AB668,UPI0000EB6CF0,UPI00003AB667,UPI0000449548,E1BZT5_CHICK,UPI00003AB66A,UPI0001A2C44C,UPI0003503F58
20196-20225 HinckeM –TAXchicken	Uncategorized Sample	20196-20205_A	Mudpit_Ingel20196_MGFPeaklist (F058870)	status=inactive	UPI000060F68B	UPARC14_2_GallusG_9031_20140417.fasta	273,727.60	100.00%	20	20	32	0.06%	8.43%	LVVDSHVQYR	K	L	99.70%	55.3	44.9	22.4	48	32.9	3.3	0.371	0.45	0	0	2	0	2	1,215.65	1,767.00		896.62	556	565	Empty	TRUE	UPI00003AB669,UPI00003AB668,UPI0000EB6CF0,UPI00003AB667,UPI0000449548,E1BZT5_CHICK,UPI00003AB66A,UPI0001A2C44C,UPI0003503F58
20196-20225 HinckeM –TAXchicken	Uncategorized Sample	20196-20205_A	Mudpit_Ingel20196_MGFPeaklist (F058870)	status=inactive	UPI000060F68B	UPARC14_2_GallusG_9031_20140417.fasta	273,727.60	100.00%	20	20	32	0.06%	8.43%	QNPFWWTHQR	K	H	99.70%	30.5	44	16.2	19	25.2	2.36	0.278	0.3	0	0	2	0	2	1,399.67	2,382.50		363.54	1451	1460	Empty	TRUE	UPI00003AB669,UPI00003AB668,UPI0000EB6CF0,UPI00003AB667,UPI0000449548,E1BZT5_CHICK,UPI00003AB66A,UPI0001A2C44C,UPI0003503F58
20196-20225 HinckeM –TAXchicken	Uncategorized Sample	20196-20205_A	Mudpit_Ingel20196_MGFPeaklist (F058870)	status=inactive	UPI000060F68B	UPARC14_2_GallusG_9031_20140417.fasta	273,727.60	100.00%	20	20	32	0.06%	8.43%	QTDVGITHFR	K	S	27.20%	27.3	44.2	15.5	0					0	1	0	0	2	1,156.57	2,055.00		261.61	1345	1354	Empty	TRUE	UPI00003AB669,UPI00003AB668,UPI0000EB6CF0,UPI00003AB667,UPI0000449548,E1BZT5_CHICK,UPI00003AB66A,UPI0001A2C44C,UPI0003503F58
20196-20225 HinckeM –TAXchicken	Uncategorized Sample	20196-20205_A	Mudpit_Ingel20196_MGFPeaklist (F058870)	status=inactive	UPI000060F68B	UPARC14_2_GallusG_9031_20140417.fasta	273,727.60	100.00%	20	20	32	0.06%	8.43%	TAEEVAALIR	K	S	99.70%	74.7	44.8	29.8	52.9	34.8	2.89	0.365	0.45	0	2	0	0	2	1,072.60	2,240.50		1,009.33	1842	1851	Empty	TRUE	UPI00003AB669,UPI00003AB668,UPI0000EB6CF0,UPI00003AB667,UPI0000449548,E1BZT5_CHICK,UPI00003AB66A,UPI0001A2C44C,UPI0003503F58
20196-20225 HinckeM –TAXchicken	Uncategorized Sample	20196-20205_A	Mudpit_Ingel20196_MGFPeaklist (F058870)	status=inactive	UPI000060F68B	UPARC14_2_GallusG_9031_20140417.fasta	273,727.60	100.00%	20	20	32	0.06%	8.43%	TISSYTAFSR	K	L	99.70%	33.5	45.1	23.5	21.1	27.4	1.28	0.277	0.35	0	2	0	0	2	1,132.56	1,985.00		996.03	1927	1936	Empty	TRUE	UPI00003AB669,UPI00003AB668,UPI0000EB6CF0,UPI00003AB667,UPI0000449548,E1BZT5_CHICK,UPI00003AB66A,UPI0001A2C44C,UPI0003503F58
20196-20225 HinckeM –TAXchicken	Uncategorized Sample	20196-20205_A	Mudpit_Ingel20196_MGFPeaklist (F058870)	status=inactive	UPI000060F68B	UPARC14_2_GallusG_9031_20140417.fasta	273,727.60	100.00%	20	20	32	0.06%	8.43%	TNHIYVSSDDIK	R	E	6.80%	8.11	45	14.6	3.39					0	0	1	0	2	1,391.68	1,784.00		273.05	2088	2099	Empty	TRUE	UPI00003AB669,UPI00003AB668,UPI0000EB6CF0,UPI00003AB667,UPI0000449548,E1BZT5_CHICK,UPI00003AB66A,UPI0001A2C44C,UPI0003503F58
20196-20225 HinckeM –TAXchicken	Uncategorized Sample	20196-20205_A	Mudpit_Ingel20196_MGFPeaklist (F058870)	status=inactive	UPI000060F68B	UPARC14_2_GallusG_9031_20140417.fasta	273,727.60	100.00%	20	20	32	0.06%	8.43%	WGDYDSHDIER	R	Y	99.70%	25.8	41.4	26.2	22.8	24.5	2.01	0.265	0.227	0	0	2	0	2	1,392.58	1,812.50		429.29	1669	1679	Empty	TRUE	UPI00003AB669,UPI00003AB668,UPI0000EB6CF0,UPI00003AB667,UPI0000449548,E1BZT5_CHICK,UPI00003AB66A,UPI0001A2C44C,UPI0003503F58
20196-20225 HinckeM –TAXchicken	Uncategorized Sample	20196-20205_A	Mudpit_Ingel20196_MGFPeaklist (F058870)	status=inactive	UPI000060F68B	UPARC14_2_GallusG_9031_20140417.fasta	273,727.60	100.00%	20	20	32	0.06%	8.43%	YLTEHPDPNNENIVGYNNKK	R	C	51.50%	12.9	44	14.6	12.9					0	0	0	1	2	2,359.13	1,860.00		885.88	1113	1132	Empty	TRUE	UPI00003AB669,UPI00003AB668,UPI0000EB6CF0,UPI00003AB667,UPI0000449548,E1BZT5_CHICK,UPI00003AB66A,UPI0001A2C44C,UPI0003503F58
20196-20225 HinckeM –TAXchicken	Uncategorized Sample	20196-20205_A	Mudpit_Ingel20196_MGFPeaklist (F058870)	status=inactive	UPI000060F68B	UPARC14_2_GallusG_9031_20140417.fasta	273,727.60	100.00%	20	20	32	0.06%	8.43%	YYVLNALK	K	H	99.50%	28.3	42.8	22.2	14.1	22.6	0.495	0.15	0.312	0	1	0	0	2	983.556	2,339.00		335.5	453	460	Empty	TRUE	UPI00003AB669,UPI00003AB668,UPI0000EB6CF0,UPI00003AB667,UPI0000449548,E1BZT5_CHICK,UPI00003AB66A,UPI0001A2C44C,UPI0003503F58
20196-20225 HinckeM –TAXchicken	Uncategorized Sample	20196-20205_A	Mudpit_Ingel20196_MGFPeaklist (F058870)	Uncharacterized protein OS=Gallus gallus GN=NSF PE=3 SV=2	E1BQU4_CHICK	TAX_GallusGallus_9031_20141114.fasta	82,179.50	100.00%	3	3	4	0.01%	3.24%	DYVPIGPR	L	F	89.80%					33.7	0.62	0.122	0.625	0	1	0	0	1	899.4623	1,921.00		2,071.73	610	617	Empty	TRUE	
20196-20225 HinckeM –TAXchicken	Uncategorized Sample	20196-20205_A	Mudpit_Ingel20196_MGFPeaklist (F058870)	Uncharacterized protein OS=Gallus gallus GN=NSF PE=3 SV=2	E1BQU4_CHICK	TAX_GallusGallus_9031_20141114.fasta	82,179.50	100.00%	3	3	4	0.01%	3.24%	YIFTLR	K	T	24.20%	33.3	42.9	32.3	4.28					0	1	0	0	2	812.4665	2,112.00		263.69	45	50	Empty	TRUE	UPI0000E81765,UPI0000610346,UPI0000449985,UPI0000EC9FF7,UPI0000EC9FF8,UPI00003AC8F3
20196-20225 HinckeM –TAXchicken	Uncategorized Sample	20196-20205_A	Mudpit_Ingel20196_MGFPeaklist (F058870)	Uncharacterized protein OS=Gallus gallus GN=NSF PE=3 SV=2	E1BQU4_CHICK	TAX_GallusGallus_9031_20141114.fasta	82,179.50	100.00%	3	3	4	0.01%	3.24%	YVGESEANIR	K	K	99.70%	26.3	44.1	18.6	15.3	24	1.07	0.221	0.25	0	2	0	0	2	1,137.55	1,663.00		725.91	294	303	Empty	TRUE	UPI0000E81765,UPI0000610346,UPI0000449985,UPI0000EC9FF7,UPI0000EC9FF8,UPI00003AC8F3

20196-20225 HinckeM –TAXchicken	Uncategorized Sample	20196-20205_A	Mudpit_Ingel20196_MGFPeaklist (F058870)	Nonmuscle myosin heavy chain OS=Gallus gallus PE=2 SV=2	Q02015_CHICK,Q789A4_CHICK,Q789A5_CHICK,Q789A6_CHICK,UPI00000FC6BE,UPI0000171380,UPI0000171381,UPI0000171382,UPI00003AB452,UPI00003AB453,UPI00003AB454,UPI000060F510,UPI000060F511,UPI0000ECA50D	TAX_GallusGallus_9031_20141114.fasta,UPARC14_2_GallusG_9031_20140417.fasta	215,888.80	100.00%	8	8	18	0.04%	8.01%	ADFCIIHYAGK	K	V	45.90%	18.6	44.4	14.7	11.1					0	0	2	0	2	1,294.63	2,223.00		989.84	458	468	Empty	FALSE	UPI000060E1E4,UPI000012FB7F,MYH9_CHICK,F1NCD4_CHICK,UPI0002C868F4
20196-20225 HinckeM –TAXchicken	Uncategorized Sample	20196-20205_A	Mudpit_Ingel20196_MGFPeaklist (F058870)	Nonmuscle myosin heavy chain OS=Gallus gallus PE=2 SV=2	Q02015_CHICK,Q789A4_CHICK,Q789A5_CHICK,Q789A6_CHICK,UPI00000FC6BE,UPI0000171380,UPI0000171381,UPI0000171382,UPI00003AB452,UPI00003AB453,UPI00003AB454,UPI000060F510,UPI000060F511,UPI0000ECA50D	TAX_GallusGallus_9031_20141114.fasta,UPARC14_2_GallusG_9031_20140417.fasta	215,888.80	100.00%	8	8	18	0.04%	8.01%	AGKLDPHLVLDQLR	R	C	99.70%	52.2	42.3	31.5	35.1	37.5	3.54	0.344	0.5	0	0	2	0	2	1,574.90	2,445.50		970.79	572	585	Empty	FALSE	UPI000060E1E4,UPI000012FB7F,MYH9_CHICK,F1NCD4_CHICK,UPI0002C868F4
20196-20225 HinckeM –TAXchicken	Uncategorized Sample	20196-20205_A	Mudpit_Ingel20196_MGFPeaklist (F058870)	Nonmuscle myosin heavy chain OS=Gallus gallus PE=2 SV=2	Q02015_CHICK,Q789A4_CHICK,Q789A5_CHICK,Q789A6_CHICK,UPI00000FC6BE,UPI0000171380,UPI0000171381,UPI0000171382,UPI00003AB452,UPI00003AB453,UPI00003AB454,UPI000060F510,UPI000060F511,UPI0000ECA50D	TAX_GallusGallus_9031_20141114.fasta,UPARC14_2_GallusG_9031_20140417.fasta	215,888.80	100.00%	8	8	18	0.04%	8.01%	AGVLAHLEEER	R	D	99.70%	71.3	44.3	36.2	36.2	34.7	3.4	0.317	0.409	0	0	2	0	2	1,223.64	2,031.50		1,163.70	657	667	Empty	FALSE	UPI000060E1E4,UPI000012FB7F,MYH9_CHICK,F1NCD4_CHICK,UPI0002C868F4
20196-20225 HinckeM –TAXchicken	Uncategorized Sample	20196-20205_A	Mudpit_Ingel20196_MGFPeaklist (F058870)	Nonmuscle myosin heavy chain OS=Gallus gallus PE=2 SV=2	Q02015_CHICK,Q789A4_CHICK,Q789A5_CHICK,Q789A6_CHICK,UPI00000FC6BE,UPI0000171380,UPI0000171381,UPI0000171382,UPI00003AB452,UPI00003AB453,UPI00003AB454,UPI000060F510,UPI000060F511,UPI0000ECA50D	TAX_GallusGallus_9031_20141114.fasta,UPARC14_2_GallusG_9031_20140417.fasta	215,888.80	100.00%	8	8	18	0.04%	8.01%	AGVLAHLEEERDLK	R	I	99.70%	43.7	43.9	28.2	36.5	38.3	3.7	0.371	0.464	0	0	2	0	2	1,579.84	2,107.00		1,024.73	657	670	Empty	FALSE	UPI000060E1E4,UPI000012FB7F,MYH9_CHICK,F1NCD4_CHICK,UPI0002C868F4
20196-20225 HinckeM –TAXchicken	Uncategorized Sample	20196-20205_A	Mudpit_Ingel20196_MGFPeaklist (F058870)	Nonmuscle myosin heavy chain OS=Gallus gallus PE=2 SV=2	Q02015_CHICK,Q789A4_CHICK,Q789A5_CHICK,Q789A6_CHICK,UPI00000FC6BE,UPI0000171380,UPI0000171381,UPI0000171382,UPI00003AB452,UPI00003AB453,UPI00003AB454,UPI000060F510,UPI000060F511,UPI0000ECA50D	TAX_GallusGallus_9031_20141114.fasta,UPARC14_2_GallusG_9031_20140417.fasta	215,888.80	100.00%	8	8	18	0.04%	8.01%	AIEEETKNHEAQIQEIR	K	Q	99.70%	29	44.4	22.1	12.1	28.6	1.7	0.192	0.265	0	0	0	2	2	2,038.02	1,772.50		811.37	1067	1083	Empty	TRUE	
20196-20225 HinckeM –TAXchicken	Uncategorized Sample	20196-20205_A	Mudpit_Ingel20196_MGFPeaklist (F058870)	Nonmuscle myosin heavy chain OS=Gallus gallus PE=2 SV=2	Q02015_CHICK,Q789A4_CHICK,Q789A5_CHICK,Q789A6_CHICK,UPI00000FC6BE,UPI0000171380,UPI0000171381,UPI0000171382,UPI00003AB452,UPI00003AB453,UPI00003AB454,UPI000060F510,UPI000060F511,UPI0000ECA50D	TAX_GallusGallus_9031_20141114.fasta,UPARC14_2_GallusG_9031_20140417.fasta	215,888.80	100.00%	8	8	18	0.04%	8.01%	ALEEALEAKEEFER	R	Q	99.70%	49	44.8	23.2	35.1	34.9	3.29	0.39	0.286	0	0	2	0	2	1,663.82	2,175.50		1,480.83	1376	1389	Empty	TRUE	
20196-20225 HinckeM –TAXchicken	Uncategorized Sample	20196-20205_A	Mudpit_Ingel20196_MGFPeaklist (F058870)	Nonmuscle myosin heavy chain OS=Gallus gallus PE=2 SV=2	Q02015_CHICK,Q789A4_CHICK,Q789A5_CHICK,Q789A6_CHICK,UPI00000FC6BE,UPI0000171380,UPI0000171381,UPI0000171382,UPI00003AB452,UPI00003AB453,UPI00003AB454,UPI000060F510,UPI000060F511,UPI0000ECA50D	TAX_GallusGallus_9031_20141114.fasta,UPARC14_2_GallusG_9031_20140417.fasta	215,888.80	100.00%	8	8	18	0.04%	8.01%	ALELDPNLYR	R	I	99.70%	42.1	45.2	34.3	22.2	32.7	2.42	0.343	0.4	0	5	0	0	2	1,203.64	2,376.00		1,867.67	638	647	Empty	TRUE	MYH11_CHICK
20196-20225 HinckeM –TAXchicken	Uncategorized Sample	20196-20205_A	Mudpit_Ingel20196_MGFPeaklist (F058870)	Nonmuscle myosin heavy chain OS=Gallus gallus PE=2 SV=2	Q02015_CHICK,Q789A4_CHICK,Q789A5_CHICK,Q789A6_CHICK,UPI00000FC6BE,UPI0000171380,UPI0000171381,UPI0000171382,UPI00003AB452,UPI00003AB453,UPI00003AB454,UPI000060F510,UPI000060F511,UPI0000ECA50D	TAX_GallusGallus_9031_20141114.fasta,UPARC14_2_GallusG_9031_20140417.fasta	215,888.80	100.00%	8	8	18	0.04%	8.01%	CNGVLEGIR	R	I	28.90%	28.9	45.5	29.6	12.6					0	1	0	0	2	1,018.50	2,046.00		210.55	586	594	Empty	FALSE	UPI000060E1E4,MYH11_CHICK,Q9PTY2_CHICK,F1NCD4_CHICK,UPI0002C868F4,UPI000012FB7F,MYH9_CHICK,UPI000044943F,UPI00003AB434,UPI00000FB7E2,UPI00003AB431
20196-20225 HinckeM –TAXchicken	Uncategorized Sample	20196-20205_A	Mudpit_Ingel20196_MGFPeaklist (F058870)	Nonmuscle myosin heavy chain OS=Gallus gallus PE=2 SV=2	Q02015_CHICK,Q789A4_CHICK,Q789A5_CHICK,Q789A6_CHICK,UPI00000FC6BE,UPI0000171380,UPI0000171381,UPI0000171382,UPI00003AB452,UPI00003AB453,UPI00003AB454,UPI000060F510,UPI000060F511,UPI0000ECA50D	TAX_GallusGallus_9031_20141114.fasta,UPARC14_2_GallusG_9031_20140417.fasta	215,888.80	100.00%	8	8	18	0.04%	8.01%	ELDDATEANEGLSR	R	E	99.70%	68.7	43.6	23	0	39.4	3.44	0.327	0.357	0	2	0	0	2	1,519.69	1,739.00		940.74	1791	1804	Empty	TRUE	
20196-20225 HinckeM –TAXchicken	Uncategorized Sample	20196-20205_A	Mudpit_Ingel20196_MGFPeaklist (F058870)	Nonmuscle myosin heavy chain OS=Gallus gallus PE=2 SV=2	Q02015_CHICK,Q789A4_CHICK,Q789A5_CHICK,Q789A6_CHICK,UPI00000FC6BE,UPI0000171380,UPI0000171381,UPI0000171382,UPI00003AB452,UPI00003AB453,UPI00003AB454,UPI000060F510,UPI000060F511,UPI0000ECA50D	TAX_GallusGallus_9031_20141114.fasta,UPARC14_2_GallusG_9031_20140417.fasta	215,888.80	100.00%	8	8	18	0.04%	8.01%	GGPITFSSSR	R	S	95.00%	30.3	44	27.7	19.6					0	1	0	0	2	1,008.51	1,869.00		125.86	1816	1825	Empty	TRUE	
20196-20225 HinckeM –TAXchicken	Uncategorized Sample	20196-20205_A	Mudpit_Ingel20196_MGFPeaklist (F058870)	Nonmuscle myosin heavy chain OS=Gallus gallus PE=2 SV=2	Q02015_CHICK,Q789A4_CHICK,Q789A5_CHICK,Q789A6_CHICK,UPI00000FC6BE,UPI0000171380,UPI0000171381,UPI0000171382,UPI00003AB452,UPI00003AB453,UPI00003AB454,UPI000060F510,UPI000060F511,UPI0000ECA50D	TAX_GallusGallus_9031_20141114.fasta,UPARC14_2_GallusG_9031_20140417.fasta	215,888.80	100.00%	8	8	18	0.04%	8.01%	IAQLEEQLEQEAKER	K	A	99.70%	59.3	44.3	25.4	45.9	46.4	4.08	0.369	0.4	0	0	2	0	2	1,813.93	2,231.00		777.93	1708	1722	Empty	TRUE	
20196-20225 HinckeM –TAXchicken	Uncategorized Sample	20196-20205_A	Mudpit_Ingel20196_MGFPeaklist (F058870)	Nonmuscle myosin heavy chain OS=Gallus gallus PE=2 SV=2	Q02015_CHICK,Q789A4_CHICK,Q789A5_CHICK,Q789A6_CHICK,UPI00000FC6BE,UPI0000171380,UPI0000171381,UPI0000171382,UPI00003AB452,UPI00003AB453,UPI00003AB454,UPI000060F510,UPI000060F511,UPI0000ECA50D	TAX_GallusGallus_9031_20141114.fasta,UPARC14_2_GallusG_9031_20140417.fasta	215,888.80	100.00%	8	8	18	0.04%	8.01%	IVFQEFR	R	Q	98.80%	47	43.1	42.2	19.8	27.9	0.585	0.222	0.5	0	3	0	0	2	938.5094	2,257.00		900.74	604	610	Empty	TRUE	MYH11_CHICK
20196-20225 HinckeM –TAXchicken	Uncategorized Sample	20196-20205_A	Mudpit_Ingel20196_MGFPeaklist (F058870)	Nonmuscle myosin heavy chain OS=Gallus gallus PE=2 SV=2	Q02015_CHICK,Q789A4_CHICK,Q789A5_CHICK,Q789A6_CHICK,UPI00000FC6BE,UPI0000171380,UPI0000171381,UPI0000171382,UPI00003AB452,UPI00003AB453,UPI00003AB454,UPI000060F510,UPI000060F511,UPI0000ECA50D	TAX_GallusGallus_9031_20141114.fasta,UPARC14_2_GallusG_9031_20140417.fasta	215,888.80	100.00%	8	8	18	0.04%	8.01%	KLDAQVQELTAK	K	V	13.90%	14	44	25.3	0.53					0	0	1	0	2	1,343.75	1,897.00		288.44	1141	1152	Empty	TRUE	
20196-20225 HinckeM –TAXchicken	Uncategorized Sample	20196-20205_A	Mudpit_Ingel20196_MGFPeaklist (F058870)	Nonmuscle myosin heavy chain OS=Gallus gallus PE=2 SV=2	Q02015_CHICK,Q789A4_CHICK,Q789A5_CHICK,Q789A6_CHICK,UPI00000FC6BE,UPI0000171380,UPI0000171381,UPI0000171382,UPI00003AB452,UPI00003AB453,UPI00003AB454,UPI000060F510,UPI000060F511,UPI0000ECA50D	TAX_GallusGallus_9031_20141114.fasta,UPARC14_2_GallusG_9031_20140417.fasta	215,888.80	100.00%	8	8	18	0.04%	8.01%	LDPHLVLDQLR	K	C	99.70%	34.6	43.8	18.6	22.1	32.5	2.47	0.366	0.455	0	0	1	0	2	1,318.75	2,534.00		1,121.62	575	585	Empty	FALSE	UPI000060E1E4,UPI000012FB7F,MYH9_CHICK,F1NCD4_CHICK,UPI0002C868F4
20196-20225 HinckeM –TAXchicken	Uncategorized Sample	20196-20205_A	Mudpit_Ingel20196_MGFPeaklist (F058870)	Nonmuscle myosin heavy chain OS=Gallus gallus PE=2 SV=2	Q02015_CHICK,Q789A4_CHICK,Q789A5_CHICK,Q789A6_CHICK,UPI00000FC6BE,UPI0000171380,UPI0000171381,UPI0000171382,UPI00003AB452,UPI00003AB453,UPI00003AB454,UPI000060F510,UPI000060F511,UPI0000ECA50D	TAX_GallusGallus_9031_20141114.fasta,UPARC14_2_GallusG_9031_20140417.fasta	215,888.80	100.00%	8	8	18	0.04%	8.01%	QRYEILTPNAIPK	R	G	99.70%	44.8	43.5	22.5	30.7	33.5	1.96	0.358	0.423	0	2	4	0	2	1,542.86	2,240.50		4,800.05	611	623	Empty	FALSE	UPI000060E1E4,UPI000012FB7F,MYH9_CHICK,F1NCD4_CHICK,UPI0002C868F4
20196-20225 HinckeM –TAXchicken	Uncategorized Sample	20196-20205_A	Mudpit_Ingel20196_MGFPeaklist (F058870)	Nonmuscle myosin heavy chain OS=Gallus gallus PE=2 SV=2	Q02015_CHICK,Q789A4_CHICK,Q789A5_CHICK,Q789A6_CHICK,UPI00000FC6BE,UPI0000171380,UPI0000171381,UPI0000171382,UPI00003AB452,UPI00003AB453,UPI00003AB454,UPI000060F510,UPI000060F511,UPI0000ECA50D	TAX_GallusGallus_9031_20141114.fasta,UPARC14_2_GallusG_9031_20140417.fasta	215,888.80	100.00%	8	8	18	0.04%	8.01%	YEILTPNAIPK	R	G	99.70%	20.6	44.4	14.6	7.9	28.5	1.57	0.277	0.318	0	3	0	0	2	1,258.70	2,337.00		1,618.82	613	623	Empty	FALSE	UPI000060E1E4,UPI000012FB7F,MYH9_CHICK,F1NCD4_CHICK,UPI0002C868F4
20196-20225 HinckeM –TAXchicken	Uncategorized Sample	20196-20205_A	Mudpit_Ingel20196_MGFPeaklist (F058870)	Nonmuscle myosin heavy chain OS=Gallus gallus PE=2 SV=2	Q02015_CHICK,Q789A4_CHICK,Q789A5_CHICK,Q789A6_CHICK,UPI00000FC6BE,UPI0000171380,UPI0000171381,UPI0000171382,UPI00003AB452,UPI00003AB453,UPI00003AB454,UPI000060F510,UPI000060F511,UPI0000ECA50D	TAX_GallusGallus_9031_20141114.fasta,UPARC14_2_GallusG_9031_20140417.fasta	215,888.80	100.00%	8	8	18	0.04%	8.01%	ADFCIIHYAGK	K	V	45.90%	18.6	44.4	14.7	11.1					0	0	2	0	2	1,294.63	2,223.00		989.84	458	468	Empty	FALSE	UPI000060E1E4,UPI000012FB7F,MYH9_CHICK,F1NCD4_CHICK,UPI0002C868F4
20196-20225 HinckeM –TAXchicken	Uncategorized Sample	20196-20205_A	Mudpit_Ingel20196_MGFPeaklist (F058870)	Nonmuscle myosin heavy chain OS=Gallus gallus PE=2 SV=2	Q02015_CHICK,Q789A4_CHICK,Q789A5_CHICK,Q789A6_CHICK,UPI00000FC6BE,UPI0000171380,UPI0000171381,UPI0000171382,UPI00003AB452,UPI00003AB453,UPI00003AB454,UPI000060F510,UPI000060F511,UPI0000ECA50D	TAX_GallusGallus_9031_20141114.fasta,UPARC14_2_GallusG_9031_20140417.fasta	215,888.80	100.00%	8	8	18	0.04%	8.01%	AGKLDPHLVLDQLR	R	C	99.70%	52.2	42.3	31.5	35.1	37.5	3.54	0.344	0.5	0	0	2	0	2	1,574.90	2,445.50		970.79	572	585	Empty	FALSE	UPI000060E1E4,UPI000012FB7F,MYH9_CHICK,F1NCD4_CHICK,UPI0002C868F4
20196-20225 HinckeM –TAXchicken	Uncategorized Sample	20196-20205_A	Mudpit_Ingel20196_MGFPeaklist (F058870)	Nonmuscle myosin heavy chain OS=Gallus gallus PE=2 SV=2	Q02015_CHICK,Q789A4_CHICK,Q789A5_CHICK,Q789A6_CHICK,UPI00000FC6BE,UPI0000171380,UPI0000171381,UPI0000171382,UPI00003AB452,UPI00003AB453,UPI00003AB454,UPI000060F510,UPI000060F511,UPI0000ECA50D	TAX_GallusGallus_9031_20141114.fasta,UPARC14_2_GallusG_9031_20140417.fasta	215,888.80	100.00%	8	8	18	0.04%	8.01%	AGVLAHLEEER	R	D	99.70%	71.3	44.3	36.2	36.2	34.7	3.4	0.317	0.409	0	0	2	0	2	1,223.64	2,031.50		1,163.70	657	667	Empty	FALSE	UPI000060E1E4,UPI000012FB7F,MYH9_CHICK,F1NCD4_CHICK,UPI0002C868F4
20196-20225 HinckeM –TAXchicken	Uncategorized Sample	20196-20205_A	Mudpit_Ingel20196_MGFPeaklist (F058870)	Nonmuscle myosin heavy chain OS=Gallus gallus PE=2 SV=2	Q02015_CHICK,Q789A4_CHICK,Q789A5_CHICK,Q789A6_CHICK,UPI00000FC6BE,UPI0000171380,UPI0000171381,UPI0000171382,UPI00003AB452,UPI00003AB453,UPI00003AB454,UPI000060F510,UPI000060F511,UPI0000ECA50D	TAX_GallusGallus_9031_20141114.fasta,UPARC14_2_GallusG_9031_20140417.fasta	215,888.80	100.00%	8	8	18	0.04%	8.01%	AGVLAHLEEERDLK	R	I	99.70%	43.7	43.9	28.2	36.5	38.3	3.7	0.371	0.464	0	0	2	0	2	1,579.84	2,107.00		1,024.73	657	670	Empty	FALSE	UPI000060E1E4,UPI000012FB7F,MYH9_CHICK,F1NCD4_CHICK,UPI0002C868F4
20196-20225 HinckeM –TAXchicken	Uncategorized Sample	20196-20205_A	Mudpit_Ingel20196_MGFPeaklist (F058870)	Nonmuscle myosin heavy chain OS=Gallus gallus PE=2 SV=2	Q02015_CHICK,Q789A4_CHICK,Q789A5_CHICK,Q789A6_CHICK,UPI00000FC6BE,UPI0000171380,UPI0000171381,UPI0000171382,UPI00003AB452,UPI00003AB453,UPI00003AB454,UPI000060F510,UPI000060F511,UPI0000ECA50D	TAX_GallusGallus_9031_20141114.fasta,UPARC14_2_GallusG_9031_20140417.fasta	215,888.80	100.00%	8	8	18	0.04%	8.01%	AIEEETKNHEAQIQEIR	K	Q	99.70%	29	44.4	22.1	12.1	28.6	1.7	0.192	0.265	0	0	0	2	2	2,038.02	1,772.50		811.37	1067	1083	Empty	TRUE	
20196-20225 HinckeM –TAXchicken	Uncategorized Sample	20196-20205_A	Mudpit_Ingel20196_MGFPeaklist (F058870)	Nonmuscle myosin heavy chain OS=Gallus gallus PE=2 SV=2	Q02015_CHICK,Q789A4_CHICK,Q789A5_CHICK,Q789A6_CHICK,UPI00000FC6BE,UPI0000171380,UPI0000171381,UPI0000171382,UPI00003AB452,UPI00003AB453,UPI00003AB454,UPI000060F510,UPI000060F511,UPI0000ECA50D	TAX_GallusGallus_9031_20141114.fasta,UPARC14_2_GallusG_9031_20140417.fasta	215,888.80	100.00%	8	8	18	0.04%	8.01%	ALEEALEAKEEFER	R	Q	99.70%	49	44.8	23.2	35.1	34.9	3.29	0.39	0.286	0	0	2	0	2	1,663.82	2,175.50		1,480.83	1376	1389	Empty	TRUE	
20196-20225 HinckeM –TAXchicken	Uncategorized Sample	20196-20205_A	Mudpit_Ingel20196_MGFPeaklist (F058870)	Nonmuscle myosin heavy chain OS=Gallus gallus PE=2 SV=2	Q02015_CHICK,Q789A4_CHICK,Q789A5_CHICK,Q789A6_CHICK,UPI00000FC6BE,UPI0000171380,UPI0000171381,UPI0000171382,UPI00003AB452,UPI00003AB453,UPI00003AB454,UPI000060F510,UPI000060F511,UPI0000ECA50D	TAX_GallusGallus_9031_20141114.fasta,UPARC14_2_GallusG_9031_20140417.fasta	215,888.80	100.00%	8	8	18	0.04%	8.01%	ALELDPNLYR	R	I	99.70%	42.1	45.2	34.3	22.2	32.7	2.42	0.343	0.4	0	5	0	0	2	1,203.64	2,376.00		1,867.67	638	647	Empty	TRUE	MYH11_CHICK
20196-20225 HinckeM –TAXchicken	Uncategorized Sample	20196-20205_A	Mudpit_Ingel20196_MGFPeaklist (F058870)	Nonmuscle myosin heavy chain OS=Gallus gallus PE=2 SV=2	Q02015_CHICK,Q789A4_CHICK,Q789A5_CHICK,Q789A6_CHICK,UPI00000FC6BE,UPI0000171380,UPI0000171381,UPI0000171382,UPI00003AB452,UPI00003AB453,UPI00003AB454,UPI000060F510,UPI000060F511,UPI0000ECA50D	TAX_GallusGallus_9031_20141114.fasta,UPARC14_2_GallusG_9031_20140417.fasta	215,888.80	100.00%	8	8	18	0.04%	8.01%	CNGVLEGIR	R	I	28.90%	28.9	45.5	29.6	12.6					0	1	0	0	2	1,018.50	2,046.00		210.55	586	594	Empty	FALSE	UPI000060E1E4,MYH11_CHICK,Q9PTY2_CHICK,F1NCD4_CHICK,UPI0002C868F4,UPI000012FB7F,MYH9_CHICK,UPI000044943F,UPI00003AB434,UPI00000FB7E2,UPI00003AB431
20196-20225 HinckeM –TAXchicken	Uncategorized Sample	20196-20205_A	Mudpit_Ingel20196_MGFPeaklist (F058870)	Nonmuscle myosin heavy chain OS=Gallus gallus PE=2 SV=2	Q02015_CHICK,Q789A4_CHICK,Q789A5_CHICK,Q789A6_CHICK,UPI00000FC6BE,UPI0000171380,UPI0000171381,UPI0000171382,UPI00003AB452,UPI00003AB453,UPI00003AB454,UPI000060F510,UPI000060F511,UPI0000ECA50D	TAX_GallusGallus_9031_20141114.fasta,UPARC14_2_GallusG_9031_20140417.fasta	215,888.80	100.00%	8	8	18	0.04%	8.01%	ELDDATEANEGLSR	R	E	99.70%	68.7	43.6	23	0	39.4	3.44	0.327	0.357	0	2	0	0	2	1,519.69	1,739.00		940.74	1791	1804	Empty	TRUE	
20196-20225 HinckeM –TAXchicken	Uncategorized Sample	20196-20205_A	Mudpit_Ingel20196_MGFPeaklist (F058870)	Nonmuscle myosin heavy chain OS=Gallus gallus PE=2 SV=2	Q02015_CHICK,Q789A4_CHICK,Q789A5_CHICK,Q789A6_CHICK,UPI00000FC6BE,UPI0000171380,UPI0000171381,UPI0000171382,UPI00003AB452,UPI00003AB453,UPI00003AB454,UPI000060F510,UPI000060F511,UPI0000ECA50D	TAX_GallusGallus_9031_20141114.fasta,UPARC14_2_GallusG_9031_20140417.fasta	215,888.80	100.00%	8	8	18	0.04%	8.01%	GGPITFSSSR	R	S	95.00%	30.3	44	27.7	19.6					0	1	0	0	2	1,008.51	1,869.00		125.86	1816	1825	Empty	TRUE	
20196-20225 HinckeM –TAXchicken	Uncategorized Sample	20196-20205_A	Mudpit_Ingel20196_MGFPeaklist (F058870)	Nonmuscle myosin heavy chain OS=Gallus gallus PE=2 SV=2	Q02015_CHICK,Q789A4_CHICK,Q789A5_CHICK,Q789A6_CHICK,UPI00000FC6BE,UPI0000171380,UPI0000171381,UPI0000171382,UPI00003AB452,UPI00003AB453,UPI00003AB454,UPI000060F510,UPI000060F511,UPI0000ECA50D	TAX_GallusGallus_9031_20141114.fasta,UPARC14_2_GallusG_9031_20140417.fasta	215,888.80	100.00%	8	8	18	0.04%	8.01%	IAQLEEQLEQEAKER	K	A	99.70%	59.3	44.3	25.4	45.9	46.4	4.08	0.369	0.4	0	0	2	0	2	1,813.93	2,231.00		777.93	1708	1722	Empty	TRUE	
20196-20225 HinckeM –TAXchicken	Uncategorized Sample	20196-20205_A	Mudpit_Ingel20196_MGFPeaklist (F058870)	Nonmuscle myosin heavy chain OS=Gallus gallus PE=2 SV=2	Q02015_CHICK,Q789A4_CHICK,Q789A5_CHICK,Q789A6_CHICK,UPI00000FC6BE,UPI0000171380,UPI0000171381,UPI0000171382,UPI00003AB452,UPI00003AB453,UPI00003AB454,UPI000060F510,UPI000060F511,UPI0000ECA50D	TAX_GallusGallus_9031_20141114.fasta,UPARC14_2_GallusG_9031_20140417.fasta	215,888.80	100.00%	8	8	18	0.04%	8.01%	IVFQEFR	R	Q	98.80%	47	43.1	42.2	19.8	27.9	0.585	0.222	0.5	0	3	0	0	2	938.5094	2,257.00		900.74	604	610	Empty	TRUE	MYH11_CHICK
20196-20225 HinckeM –TAXchicken	Uncategorized Sample	20196-20205_A	Mudpit_Ingel20196_MGFPeaklist (F058870)	Nonmuscle myosin heavy chain OS=Gallus gallus PE=2 SV=2	Q02015_CHICK,Q789A4_CHICK,Q789A5_CHICK,Q789A6_CHICK,UPI00000FC6BE,UPI0000171380,UPI0000171381,UPI0000171382,UPI00003AB452,UPI00003AB453,UPI00003AB454,UPI000060F510,UPI000060F511,UPI0000ECA50D	TAX_GallusGallus_9031_20141114.fasta,UPARC14_2_GallusG_9031_20140417.fasta	215,888.80	100.00%	8	8	18	0.04%	8.01%	KLDAQVQELTAK	K	V	13.90%	14	44	25.3	0.53					0	0	1	0	2	1,343.75	1,897.00		288.44	1141	1152	Empty	TRUE	
20196-20225 HinckeM –TAXchicken	Uncategorized Sample	20196-20205_A	Mudpit_Ingel20196_MGFPeaklist (F058870)	Nonmuscle myosin heavy chain OS=Gallus gallus PE=2 SV=2	Q02015_CHICK,Q789A4_CHICK,Q789A5_CHICK,Q789A6_CHICK,UPI00000FC6BE,UPI0000171380,UPI0000171381,UPI0000171382,UPI00003AB452,UPI00003AB453,UPI00003AB454,UPI000060F510,UPI000060F511,UPI0000ECA50D	TAX_GallusGallus_9031_20141114.fasta,UPARC14_2_GallusG_9031_20140417.fasta	215,888.80	100.00%	8	8	18	0.04%	8.01%	LDPHLVLDQLR	K	C	99.70%	34.6	43.8	18.6	22.1	32.5	2.47	0.366	0.455	0	0	1	0	2	1,318.75	2,534.00		1,121.62	575	585	Empty	FALSE	UPI000060E1E4,UPI000012FB7F,MYH9_CHICK,F1NCD4_CHICK,UPI0002C868F4
20196-20225 HinckeM –TAXchicken	Uncategorized Sample	20196-20205_A	Mudpit_Ingel20196_MGFPeaklist (F058870)	Nonmuscle myosin heavy chain OS=Gallus gallus PE=2 SV=2	Q02015_CHICK,Q789A4_CHICK,Q789A5_CHICK,Q789A6_CHICK,UPI00000FC6BE,UPI0000171380,UPI0000171381,UPI0000171382,UPI00003AB452,UPI00003AB453,UPI00003AB454,UPI000060F510,UPI000060F511,UPI0000ECA50D	TAX_GallusGallus_9031_20141114.fasta,UPARC14_2_GallusG_9031_20140417.fasta	215,888.80	100.00%	8	8	18	0.04%	8.01%	QRYEILTPNAIPK	R	G	99.70%	44.8	43.5	22.5	30.7	33.5	1.96	0.358	0.423	0	2	4	0	2	1,542.86	2,240.50		4,800.05	611	623	Empty	FALSE	UPI000060E1E4,UPI000012FB7F,MYH9_CHICK,F1NCD4_CHICK,UPI0002C868F4
20196-20225 HinckeM –TAXchicken	Uncategorized Sample	20196-20205_A	Mudpit_Ingel20196_MGFPeaklist (F058870)	Nonmuscle myosin heavy chain OS=Gallus gallus PE=2 SV=2	Q02015_CHICK,Q789A4_CHICK,Q789A5_CHICK,Q789A6_CHICK,UPI00000FC6BE,UPI0000171380,UPI0000171381,UPI0000171382,UPI00003AB452,UPI00003AB453,UPI00003AB454,UPI000060F510,UPI000060F511,UPI0000ECA50D	TAX_GallusGallus_9031_20141114.fasta,UPARC14_2_GallusG_9031_20140417.fasta	215,888.80	100.00%	8	8	18	0.04%	8.01%	YEILTPNAIPK	R	G	99.70%	20.6	44.4	14.6	7.9	28.5	1.57	0.277	0.318	0	3	0	0	2	1,258.70	2,337.00		1,618.82	613	623	Empty	FALSE	UPI000060E1E4,UPI000012FB7F,MYH9_CHICK,F1NCD4_CHICK,UPI0002C868F4
20196-20225 HinckeM –TAXchicken	Uncategorized Sample	20196-20205_A	Mudpit_Ingel20196_MGFPeaklist (F058870)	Nonmuscle myosin heavy chain OS=Gallus gallus PE=2 SV=2	Q02015_CHICK,Q789A4_CHICK,Q789A5_CHICK,Q789A6_CHICK,UPI00000FC6BE,UPI0000171380,UPI0000171381,UPI0000171382,UPI00003AB452,UPI00003AB453,UPI00003AB454,UPI000060F510,UPI000060F511,UPI0000ECA50D	TAX_GallusGallus_9031_20141114.fasta,UPARC14_2_GallusG_9031_20140417.fasta	215,888.80	100.00%	8	8	18	0.04%	8.01%	ADFCIIHYAGK	K	V	45.90%	18.6	44.4	14.7	11.1					0	0	2	0	2	1,294.63	2,223.00		989.84	458	468	Empty	FALSE	UPI000060E1E4,UPI000012FB7F,MYH9_CHICK,F1NCD4_CHICK,UPI0002C868F4
20196-20225 HinckeM –TAXchicken	Uncategorized Sample	20196-20205_A	Mudpit_Ingel20196_MGFPeaklist (F058870)	Nonmuscle myosin heavy chain OS=Gallus gallus PE=2 SV=2	Q02015_CHICK,Q789A4_CHICK,Q789A5_CHICK,Q789A6_CHICK,UPI00000FC6BE,UPI0000171380,UPI0000171381,UPI0000171382,UPI00003AB452,UPI00003AB453,UPI00003AB454,UPI000060F510,UPI000060F511,UPI0000ECA50D	TAX_GallusGallus_9031_20141114.fasta,UPARC14_2_GallusG_9031_20140417.fasta	215,888.80	100.00%	8	8	18	0.04%	8.01%	AGKLDPHLVLDQLR	R	C	99.70%	52.2	42.3	31.5	35.1	37.5	3.54	0.344	0.5	0	0	2	0	2	1,574.90	2,445.50		970.79	572	585	Empty	FALSE	UPI000060E1E4,UPI000012FB7F,MYH9_CHICK,F1NCD4_CHICK,UPI0002C868F4
20196-20225 HinckeM –TAXchicken	Uncategorized Sample	20196-20205_A	Mudpit_Ingel20196_MGFPeaklist (F058870)	Nonmuscle myosin heavy chain OS=Gallus gallus PE=2 SV=2	Q02015_CHICK,Q789A4_CHICK,Q789A5_CHICK,Q789A6_CHICK,UPI00000FC6BE,UPI0000171380,UPI0000171381,UPI0000171382,UPI00003AB452,UPI00003AB453,UPI00003AB454,UPI000060F510,UPI000060F511,UPI0000ECA50D	TAX_GallusGallus_9031_20141114.fasta,UPARC14_2_GallusG_9031_20140417.fasta	215,888.80	100.00%	8	8	18	0.04%	8.01%	AGVLAHLEEER	R	D	99.70%	71.3	44.3	36.2	36.2	34.7	3.4	0.317	0.409	0	0	2	0	2	1,223.64	2,031.50		1,163.70	657	667	Empty	FALSE	UPI000060E1E4,UPI000012FB7F,MYH9_CHICK,F1NCD4_CHICK,UPI0002C868F4
20196-20225 HinckeM –TAXchicken	Uncategorized Sample	20196-20205_A	Mudpit_Ingel20196_MGFPeaklist (F058870)	Nonmuscle myosin heavy chain OS=Gallus gallus PE=2 SV=2	Q02015_CHICK,Q789A4_CHICK,Q789A5_CHICK,Q789A6_CHICK,UPI00000FC6BE,UPI0000171380,UPI0000171381,UPI0000171382,UPI00003AB452,UPI00003AB453,UPI00003AB454,UPI000060F510,UPI000060F511,UPI0000ECA50D	TAX_GallusGallus_9031_20141114.fasta,UPARC14_2_GallusG_9031_20140417.fasta	215,888.80	100.00%	8	8	18	0.04%	8.01%	AGVLAHLEEERDLK	R	I	99.70%	43.7	43.9	28.2	36.5	38.3	3.7	0.371	0.464	0	0	2	0	2	1,579.84	2,107.00		1,024.73	657	670	Empty	FALSE	UPI000060E1E4,UPI000012FB7F,MYH9_CHICK,F1NCD4_CHICK,UPI0002C868F4
20196-20225 HinckeM –TAXchicken	Uncategorized Sample	20196-20205_A	Mudpit_Ingel20196_MGFPeaklist (F058870)	Nonmuscle myosin heavy chain OS=Gallus gallus PE=2 SV=2	Q02015_CHICK,Q789A4_CHICK,Q789A5_CHICK,Q789A6_CHICK,UPI00000FC6BE,UPI0000171380,UPI0000171381,UPI0000171382,UPI00003AB452,UPI00003AB453,UPI00003AB454,UPI000060F510,UPI000060F511,UPI0000ECA50D	TAX_GallusGallus_9031_20141114.fasta,UPARC14_2_GallusG_9031_20140417.fasta	215,888.80	100.00%	8	8	18	0.04%	8.01%	AIEEETKNHEAQIQEIR	K	Q	99.70%	29	44.4	22.1	12.1	28.6	1.7	0.192	0.265	0	0	0	2	2	2,038.02	1,772.50		811.37	1067	1083	Empty	TRUE	
20196-20225 HinckeM –TAXchicken	Uncategorized Sample	20196-20205_A	Mudpit_Ingel20196_MGFPeaklist (F058870)	Nonmuscle myosin heavy chain OS=Gallus gallus PE=2 SV=2	Q02015_CHICK,Q789A4_CHICK,Q789A5_CHICK,Q789A6_CHICK,UPI00000FC6BE,UPI0000171380,UPI0000171381,UPI0000171382,UPI00003AB452,UPI00003AB453,UPI00003AB454,UPI000060F510,UPI000060F511,UPI0000ECA50D	TAX_GallusGallus_9031_20141114.fasta,UPARC14_2_GallusG_9031_20140417.fasta	215,888.80	100.00%	8	8	18	0.04%	8.01%	ALEEALEAKEEFER	R	Q	99.70%	49	44.8	23.2	35.1	34.9	3.29	0.39	0.286	0	0	2	0	2	1,663.82	2,175.50		1,480.83	1376	1389	Empty	TRUE	
20196-20225 HinckeM –TAXchicken	Uncategorized Sample	20196-20205_A	Mudpit_Ingel20196_MGFPeaklist (F058870)	Nonmuscle myosin heavy chain OS=Gallus gallus PE=2 SV=2	Q02015_CHICK,Q789A4_CHICK,Q789A5_CHICK,Q789A6_CHICK,UPI00000FC6BE,UPI0000171380,UPI0000171381,UPI0000171382,UPI00003AB452,UPI00003AB453,UPI00003AB454,UPI000060F510,UPI000060F511,UPI0000ECA50D	TAX_GallusGallus_9031_20141114.fasta,UPARC14_2_GallusG_9031_20140417.fasta	215,888.80	100.00%	8	8	18	0.04%	8.01%	ALELDPNLYR	R	I	99.70%	42.1	45.2	34.3	22.2	32.7	2.42	0.343	0.4	0	5	0	0	2	1,203.64	2,376.00		1,867.67	638	647	Empty	TRUE	MYH11_CHICK
20196-20225 HinckeM –TAXchicken	Uncategorized Sample	20196-20205_A	Mudpit_Ingel20196_MGFPeaklist (F058870)	Nonmuscle myosin heavy chain OS=Gallus gallus PE=2 SV=2	Q02015_CHICK,Q789A4_CHICK,Q789A5_CHICK,Q789A6_CHICK,UPI00000FC6BE,UPI0000171380,UPI0000171381,UPI0000171382,UPI00003AB452,UPI00003AB453,UPI00003AB454,UPI000060F510,UPI000060F511,UPI0000ECA50D	TAX_GallusGallus_9031_20141114.fasta,UPARC14_2_GallusG_9031_20140417.fasta	215,888.80	100.00%	8	8	18	0.04%	8.01%	CNGVLEGIR	R	I	28.90%	28.9	45.5	29.6	12.6					0	1	0	0	2	1,018.50	2,046.00		210.55	586	594	Empty	FALSE	UPI000060E1E4,MYH11_CHICK,Q9PTY2_CHICK,F1NCD4_CHICK,UPI0002C868F4,UPI000012FB7F,MYH9_CHICK,UPI000044943F,UPI00003AB434,UPI00000FB7E2,UPI00003AB431
20196-20225 HinckeM –TAXchicken	Uncategorized Sample	20196-20205_A	Mudpit_Ingel20196_MGFPeaklist (F058870)	Nonmuscle myosin heavy chain OS=Gallus gallus PE=2 SV=2	Q02015_CHICK,Q789A4_CHICK,Q789A5_CHICK,Q789A6_CHICK,UPI00000FC6BE,UPI0000171380,UPI0000171381,UPI0000171382,UPI00003AB452,UPI00003AB453,UPI00003AB454,UPI000060F510,UPI000060F511,UPI0000ECA50D	TAX_GallusGallus_9031_20141114.fasta,UPARC14_2_GallusG_9031_20140417.fasta	215,888.80	100.00%	8	8	18	0.04%	8.01%	ELDDATEANEGLSR	R	E	99.70%	68.7	43.6	23	0	39.4	3.44	0.327	0.357	0	2	0	0	2	1,519.69	1,739.00		940.74	1791	1804	Empty	TRUE	
20196-20225 HinckeM –TAXchicken	Uncategorized Sample	20196-20205_A	Mudpit_Ingel20196_MGFPeaklist (F058870)	Nonmuscle myosin heavy chain OS=Gallus gallus PE=2 SV=2	Q02015_CHICK,Q789A4_CHICK,Q789A5_CHICK,Q789A6_CHICK,UPI00000FC6BE,UPI0000171380,UPI0000171381,UPI0000171382,UPI00003AB452,UPI00003AB453,UPI00003AB454,UPI000060F510,UPI000060F511,UPI0000ECA50D	TAX_GallusGallus_9031_20141114.fasta,UPARC14_2_GallusG_9031_20140417.fasta	215,888.80	100.00%	8	8	18	0.04%	8.01%	GGPITFSSSR	R	S	95.00%	30.3	44	27.7	19.6					0	1	0	0	2	1,008.51	1,869.00		125.86	1816	1825	Empty	TRUE	
20196-20225 HinckeM –TAXchicken	Uncategorized Sample	20196-20205_A	Mudpit_Ingel20196_MGFPeaklist (F058870)	Nonmuscle myosin heavy chain OS=Gallus gallus PE=2 SV=2	Q02015_CHICK,Q789A4_CHICK,Q789A5_CHICK,Q789A6_CHICK,UPI00000FC6BE,UPI0000171380,UPI0000171381,UPI0000171382,UPI00003AB452,UPI00003AB453,UPI00003AB454,UPI000060F510,UPI000060F511,UPI0000ECA50D	TAX_GallusGallus_9031_20141114.fasta,UPARC14_2_GallusG_9031_20140417.fasta	215,888.80	100.00%	8	8	18	0.04%	8.01%	IAQLEEQLEQEAKER	K	A	99.70%	59.3	44.3	25.4	45.9	46.4	4.08	0.369	0.4	0	0	2	0	2	1,813.93	2,231.00		777.93	1708	1722	Empty	TRUE	
20196-20225 HinckeM –TAXchicken	Uncategorized Sample	20196-20205_A	Mudpit_Ingel20196_MGFPeaklist (F058870)	Nonmuscle myosin heavy chain OS=Gallus gallus PE=2 SV=2	Q02015_CHICK,Q789A4_CHICK,Q789A5_CHICK,Q789A6_CHICK,UPI00000FC6BE,UPI0000171380,UPI0000171381,UPI0000171382,UPI00003AB452,UPI00003AB453,UPI00003AB454,UPI000060F510,UPI000060F511,UPI0000ECA50D	TAX_GallusGallus_9031_20141114.fasta,UPARC14_2_GallusG_9031_20140417.fasta	215,888.80	100.00%	8	8	18	0.04%	8.01%	IVFQEFR	R	Q	98.80%	47	43.1	42.2	19.8	27.9	0.585	0.222	0.5	0	3	0	0	2	938.5094	2,257.00		900.74	604	610	Empty	TRUE	MYH11_CHICK
20196-20225 HinckeM –TAXchicken	Uncategorized Sample	20196-20205_A	Mudpit_Ingel20196_MGFPeaklist (F058870)	Nonmuscle myosin heavy chain OS=Gallus gallus PE=2 SV=2	Q02015_CHICK,Q789A4_CHICK,Q789A5_CHICK,Q789A6_CHICK,UPI00000FC6BE,UPI0000171380,UPI0000171381,UPI0000171382,UPI00003AB452,UPI00003AB453,UPI00003AB454,UPI000060F510,UPI000060F511,UPI0000ECA50D	TAX_GallusGallus_9031_20141114.fasta,UPARC14_2_GallusG_9031_20140417.fasta	215,888.80	100.00%	8	8	18	0.04%	8.01%	KLDAQVQELTAK	K	V	13.90%	14	44	25.3	0.53					0	0	1	0	2	1,343.75	1,897.00		288.44	1141	1152	Empty	TRUE	
20196-20225 HinckeM –TAXchicken	Uncategorized Sample	20196-20205_A	Mudpit_Ingel20196_MGFPeaklist (F058870)	Nonmuscle myosin heavy chain OS=Gallus gallus PE=2 SV=2	Q02015_CHICK,Q789A4_CHICK,Q789A5_CHICK,Q789A6_CHICK,UPI00000FC6BE,UPI0000171380,UPI0000171381,UPI0000171382,UPI00003AB452,UPI00003AB453,UPI00003AB454,UPI000060F510,UPI000060F511,UPI0000ECA50D	TAX_GallusGallus_9031_20141114.fasta,UPARC14_2_GallusG_9031_20140417.fasta	215,888.80	100.00%	8	8	18	0.04%	8.01%	LDPHLVLDQLR	K	C	99.70%	34.6	43.8	18.6	22.1	32.5	2.47	0.366	0.455	0	0	1	0	2	1,318.75	2,534.00		1,121.62	575	585	Empty	FALSE	UPI000060E1E4,UPI000012FB7F,MYH9_CHICK,F1NCD4_CHICK,UPI0002C868F4
20196-20225 HinckeM –TAXchicken	Uncategorized Sample	20196-20205_A	Mudpit_Ingel20196_MGFPeaklist (F058870)	Nonmuscle myosin heavy chain OS=Gallus gallus PE=2 SV=2	Q02015_CHICK,Q789A4_CHICK,Q789A5_CHICK,Q789A6_CHICK,UPI00000FC6BE,UPI0000171380,UPI0000171381,UPI0000171382,UPI00003AB452,UPI00003AB453,UPI00003AB454,UPI000060F510,UPI000060F511,UPI0000ECA50D	TAX_GallusGallus_9031_20141114.fasta,UPARC14_2_GallusG_9031_20140417.fasta	215,888.80	100.00%	8	8	18	0.04%	8.01%	QRYEILTPNAIPK	R	G	99.70%	44.8	43.5	22.5	30.7	33.5	1.96	0.358	0.423	0	2	4	0	2	1,542.86	2,240.50		4,800.05	611	623	Empty	FALSE	UPI000060E1E4,UPI000012FB7F,MYH9_CHICK,F1NCD4_CHICK,UPI0002C868F4
20196-20225 HinckeM –TAXchicken	Uncategorized Sample	20196-20205_A	Mudpit_Ingel20196_MGFPeaklist (F058870)	Nonmuscle myosin heavy chain OS=Gallus gallus PE=2 SV=2	Q02015_CHICK,Q789A4_CHICK,Q789A5_CHICK,Q789A6_CHICK,UPI00000FC6BE,UPI0000171380,UPI0000171381,UPI0000171382,UPI00003AB452,UPI00003AB453,UPI00003AB454,UPI000060F510,UPI000060F511,UPI0000ECA50D	TAX_GallusGallus_9031_20141114.fasta,UPARC14_2_GallusG_9031_20140417.fasta	215,888.80	100.00%	8	8	18	0.04%	8.01%	YEILTPNAIPK	R	G	99.70%	20.6	44.4	14.6	7.9	28.5	1.57	0.277	0.318	0	3	0	0	2	1,258.70	2,337.00		1,618.82	613	623	Empty	FALSE	UPI000060E1E4,UPI000012FB7F,MYH9_CHICK,F1NCD4_CHICK,UPI0002C868F4
20196-20225 HinckeM –TAXchicken	Uncategorized Sample	20196-20205_A	Mudpit_Ingel20196_MGFPeaklist (F058870)	Nonmuscle myosin heavy chain OS=Gallus gallus PE=2 SV=2	Q02015_CHICK,Q789A4_CHICK,Q789A5_CHICK,Q789A6_CHICK,UPI00000FC6BE,UPI0000171380,UPI0000171381,UPI0000171382,UPI00003AB452,UPI00003AB453,UPI00003AB454,UPI000060F510,UPI000060F511,UPI0000ECA50D	TAX_GallusGallus_9031_20141114.fasta,UPARC14_2_GallusG_9031_20140417.fasta	215,888.80	100.00%	8	8	18	0.04%	8.01%	ADFCIIHYAGK	K	V	45.90%	18.6	44.4	14.7	11.1					0	0	2	0	2	1,294.63	2,223.00		989.84	458	468	Empty	FALSE	UPI000060E1E4,UPI000012FB7F,MYH9_CHICK,F1NCD4_CHICK,UPI0002C868F4
20196-20225 HinckeM –TAXchicken	Uncategorized Sample	20196-20205_A	Mudpit_Ingel20196_MGFPeaklist (F058870)	Nonmuscle myosin heavy chain OS=Gallus gallus PE=2 SV=2	Q02015_CHICK,Q789A4_CHICK,Q789A5_CHICK,Q789A6_CHICK,UPI00000FC6BE,UPI0000171380,UPI0000171381,UPI0000171382,UPI00003AB452,UPI00003AB453,UPI00003AB454,UPI000060F510,UPI000060F511,UPI0000ECA50D	TAX_GallusGallus_9031_20141114.fasta,UPARC14_2_GallusG_9031_20140417.fasta	215,888.80	100.00%	8	8	18	0.04%	8.01%	AGKLDPHLVLDQLR	R	C	99.70%	52.2	42.3	31.5	35.1	37.5	3.54	0.344	0.5	0	0	2	0	2	1,574.90	2,445.50		970.79	572	585	Empty	FALSE	UPI000060E1E4,UPI000012FB7F,MYH9_CHICK,F1NCD4_CHICK,UPI0002C868F4
20196-20225 HinckeM –TAXchicken	Uncategorized Sample	20196-20205_A	Mudpit_Ingel20196_MGFPeaklist (F058870)	Nonmuscle myosin heavy chain OS=Gallus gallus PE=2 SV=2	Q02015_CHICK,Q789A4_CHICK,Q789A5_CHICK,Q789A6_CHICK,UPI00000FC6BE,UPI0000171380,UPI0000171381,UPI0000171382,UPI00003AB452,UPI00003AB453,UPI00003AB454,UPI000060F510,UPI000060F511,UPI0000ECA50D	TAX_GallusGallus_9031_20141114.fasta,UPARC14_2_GallusG_9031_20140417.fasta	215,888.80	100.00%	8	8	18	0.04%	8.01%	AGVLAHLEEER	R	D	99.70%	71.3	44.3	36.2	36.2	34.7	3.4	0.317	0.409	0	0	2	0	2	1,223.64	2,031.50		1,163.70	657	667	Empty	FALSE	UPI000060E1E4,UPI000012FB7F,MYH9_CHICK,F1NCD4_CHICK,UPI0002C868F4
20196-20225 HinckeM –TAXchicken	Uncategorized Sample	20196-20205_A	Mudpit_Ingel20196_MGFPeaklist (F058870)	Nonmuscle myosin heavy chain OS=Gallus gallus PE=2 SV=2	Q02015_CHICK,Q789A4_CHICK,Q789A5_CHICK,Q789A6_CHICK,UPI00000FC6BE,UPI0000171380,UPI0000171381,UPI0000171382,UPI00003AB452,UPI00003AB453,UPI00003AB454,UPI000060F510,UPI000060F511,UPI0000ECA50D	TAX_GallusGallus_9031_20141114.fasta,UPARC14_2_GallusG_9031_20140417.fasta	215,888.80	100.00%	8	8	18	0.04%	8.01%	AGVLAHLEEERDLK	R	I	99.70%	43.7	43.9	28.2	36.5	38.3	3.7	0.371	0.464	0	0	2	0	2	1,579.84	2,107.00		1,024.73	657	670	Empty	FALSE	UPI000060E1E4,UPI000012FB7F,MYH9_CHICK,F1NCD4_CHICK,UPI0002C868F4
20196-20225 HinckeM –TAXchicken	Uncategorized Sample	20196-20205_A	Mudpit_Ingel20196_MGFPeaklist (F058870)	Nonmuscle myosin heavy chain OS=Gallus gallus PE=2 SV=2	Q02015_CHICK,Q789A4_CHICK,Q789A5_CHICK,Q789A6_CHICK,UPI00000FC6BE,UPI0000171380,UPI0000171381,UPI0000171382,UPI00003AB452,UPI00003AB453,UPI00003AB454,UPI000060F510,UPI000060F511,UPI0000ECA50D	TAX_GallusGallus_9031_20141114.fasta,UPARC14_2_GallusG_9031_20140417.fasta	215,888.80	100.00%	8	8	18	0.04%	8.01%	AIEEETKNHEAQIQEIR	K	Q	99.70%	29	44.4	22.1	12.1	28.6	1.7	0.192	0.265	0	0	0	2	2	2,038.02	1,772.50		811.37	1067	1083	Empty	TRUE	
20196-20225 HinckeM –TAXchicken	Uncategorized Sample	20196-20205_A	Mudpit_Ingel20196_MGFPeaklist (F058870)	Nonmuscle myosin heavy chain OS=Gallus gallus PE=2 SV=2	Q02015_CHICK,Q789A4_CHICK,Q789A5_CHICK,Q789A6_CHICK,UPI00000FC6BE,UPI0000171380,UPI0000171381,UPI0000171382,UPI00003AB452,UPI00003AB453,UPI00003AB454,UPI000060F510,UPI000060F511,UPI0000ECA50D	TAX_GallusGallus_9031_20141114.fasta,UPARC14_2_GallusG_9031_20140417.fasta	215,888.80	100.00%	8	8	18	0.04%	8.01%	ALEEALEAKEEFER	R	Q	99.70%	49	44.8	23.2	35.1	34.9	3.29	0.39	0.286	0	0	2	0	2	1,663.82	2,175.50		1,480.83	1376	1389	Empty	TRUE	
20196-20225 HinckeM –TAXchicken	Uncategorized Sample	20196-20205_A	Mudpit_Ingel20196_MGFPeaklist (F058870)	Nonmuscle myosin heavy chain OS=Gallus gallus PE=2 SV=2	Q02015_CHICK,Q789A4_CHICK,Q789A5_CHICK,Q789A6_CHICK,UPI00000FC6BE,UPI0000171380,UPI0000171381,UPI0000171382,UPI00003AB452,UPI00003AB453,UPI00003AB454,UPI000060F510,UPI000060F511,UPI0000ECA50D	TAX_GallusGallus_9031_20141114.fasta,UPARC14_2_GallusG_9031_20140417.fasta	215,888.80	100.00%	8	8	18	0.04%	8.01%	ALELDPNLYR	R	I	99.70%	42.1	45.2	34.3	22.2	32.7	2.42	0.343	0.4	0	5	0	0	2	1,203.64	2,376.00		1,867.67	638	647	Empty	TRUE	MYH11_CHICK
20196-20225 HinckeM –TAXchicken	Uncategorized Sample	20196-20205_A	Mudpit_Ingel20196_MGFPeaklist (F058870)	Nonmuscle myosin heavy chain OS=Gallus gallus PE=2 SV=2	Q02015_CHICK,Q789A4_CHICK,Q789A5_CHICK,Q789A6_CHICK,UPI00000FC6BE,UPI0000171380,UPI0000171381,UPI0000171382,UPI00003AB452,UPI00003AB453,UPI00003AB454,UPI000060F510,UPI000060F511,UPI0000ECA50D	TAX_GallusGallus_9031_20141114.fasta,UPARC14_2_GallusG_9031_20140417.fasta	215,888.80	100.00%	8	8	18	0.04%	8.01%	CNGVLEGIR	R	I	28.90%	28.9	45.5	29.6	12.6					0	1	0	0	2	1,018.50	2,046.00		210.55	586	594	Empty	FALSE	UPI000060E1E4,MYH11_CHICK,Q9PTY2_CHICK,F1NCD4_CHICK,UPI0002C868F4,UPI000012FB7F,MYH9_CHICK,UPI000044943F,UPI00003AB434,UPI00000FB7E2,UPI00003AB431
20196-20225 HinckeM –TAXchicken	Uncategorized Sample	20196-20205_A	Mudpit_Ingel20196_MGFPeaklist (F058870)	Nonmuscle myosin heavy chain OS=Gallus gallus PE=2 SV=2	Q02015_CHICK,Q789A4_CHICK,Q789A5_CHICK,Q789A6_CHICK,UPI00000FC6BE,UPI0000171380,UPI0000171381,UPI0000171382,UPI00003AB452,UPI00003AB453,UPI00003AB454,UPI000060F510,UPI000060F511,UPI0000ECA50D	TAX_GallusGallus_9031_20141114.fasta,UPARC14_2_GallusG_9031_20140417.fasta	215,888.80	100.00%	8	8	18	0.04%	8.01%	ELDDATEANEGLSR	R	E	99.70%	68.7	43.6	23	0	39.4	3.44	0.327	0.357	0	2	0	0	2	1,519.69	1,739.00		940.74	1791	1804	Empty	TRUE	
20196-20225 HinckeM –TAXchicken	Uncategorized Sample	20196-20205_A	Mudpit_Ingel20196_MGFPeaklist (F058870)	Nonmuscle myosin heavy chain OS=Gallus gallus PE=2 SV=2	Q02015_CHICK,Q789A4_CHICK,Q789A5_CHICK,Q789A6_CHICK,UPI00000FC6BE,UPI0000171380,UPI0000171381,UPI0000171382,UPI00003AB452,UPI00003AB453,UPI00003AB454,UPI000060F510,UPI000060F511,UPI0000ECA50D	TAX_GallusGallus_9031_20141114.fasta,UPARC14_2_GallusG_9031_20140417.fasta	215,888.80	100.00%	8	8	18	0.04%	8.01%	GGPITFSSSR	R	S	95.00%	30.3	44	27.7	19.6					0	1	0	0	2	1,008.51	1,869.00		125.86	1816	1825	Empty	TRUE	
20196-20225 HinckeM –TAXchicken	Uncategorized Sample	20196-20205_A	Mudpit_Ingel20196_MGFPeaklist (F058870)	Nonmuscle myosin heavy chain OS=Gallus gallus PE=2 SV=2	Q02015_CHICK,Q789A4_CHICK,Q789A5_CHICK,Q789A6_CHICK,UPI00000FC6BE,UPI0000171380,UPI0000171381,UPI0000171382,UPI00003AB452,UPI00003AB453,UPI00003AB454,UPI000060F510,UPI000060F511,UPI0000ECA50D	TAX_GallusGallus_9031_20141114.fasta,UPARC14_2_GallusG_9031_20140417.fasta	215,888.80	100.00%	8	8	18	0.04%	8.01%	IAQLEEQLEQEAKER	K	A	99.70%	59.3	44.3	25.4	45.9	46.4	4.08	0.369	0.4	0	0	2	0	2	1,813.93	2,231.00		777.93	1708	1722	Empty	TRUE	
20196-20225 HinckeM –TAXchicken	Uncategorized Sample	20196-20205_A	Mudpit_Ingel20196_MGFPeaklist (F058870)	Nonmuscle myosin heavy chain OS=Gallus gallus PE=2 SV=2	Q02015_CHICK,Q789A4_CHICK,Q789A5_CHICK,Q789A6_CHICK,UPI00000FC6BE,UPI0000171380,UPI0000171381,UPI0000171382,UPI00003AB452,UPI00003AB453,UPI00003AB454,UPI000060F510,UPI000060F511,UPI0000ECA50D	TAX_GallusGallus_9031_20141114.fasta,UPARC14_2_GallusG_9031_20140417.fasta	215,888.80	100.00%	8	8	18	0.04%	8.01%	IVFQEFR	R	Q	98.80%	47	43.1	42.2	19.8	27.9	0.585	0.222	0.5	0	3	0	0	2	938.5094	2,257.00		900.74	604	610	Empty	TRUE	MYH11_CHICK
20196-20225 HinckeM –TAXchicken	Uncategorized Sample	20196-20205_A	Mudpit_Ingel20196_MGFPeaklist (F058870)	Nonmuscle myosin heavy chain OS=Gallus gallus PE=2 SV=2	Q02015_CHICK,Q789A4_CHICK,Q789A5_CHICK,Q789A6_CHICK,UPI00000FC6BE,UPI0000171380,UPI0000171381,UPI0000171382,UPI00003AB452,UPI00003AB453,UPI00003AB454,UPI000060F510,UPI000060F511,UPI0000ECA50D	TAX_GallusGallus_9031_20141114.fasta,UPARC14_2_GallusG_9031_20140417.fasta	215,888.80	100.00%	8	8	18	0.04%	8.01%	KLDAQVQELTAK	K	V	13.90%	14	44	25.3	0.53					0	0	1	0	2	1,343.75	1,897.00		288.44	1141	1152	Empty	TRUE	
20196-20225 HinckeM –TAXchicken	Uncategorized Sample	20196-20205_A	Mudpit_Ingel20196_MGFPeaklist (F058870)	Nonmuscle myosin heavy chain OS=Gallus gallus PE=2 SV=2	Q02015_CHICK,Q789A4_CHICK,Q789A5_CHICK,Q789A6_CHICK,UPI00000FC6BE,UPI0000171380,UPI0000171381,UPI0000171382,UPI00003AB452,UPI00003AB453,UPI00003AB454,UPI000060F510,UPI000060F511,UPI0000ECA50D	TAX_GallusGallus_9031_20141114.fasta,UPARC14_2_GallusG_9031_20140417.fasta	215,888.80	100.00%	8	8	18	0.04%	8.01%	LDPHLVLDQLR	K	C	99.70%	34.6	43.8	18.6	22.1	32.5	2.47	0.366	0.455	0	0	1	0	2	1,318.75	2,534.00		1,121.62	575	585	Empty	FALSE	UPI000060E1E4,UPI000012FB7F,MYH9_CHICK,F1NCD4_CHICK,UPI0002C868F4
20196-20225 HinckeM –TAXchicken	Uncategorized Sample	20196-20205_A	Mudpit_Ingel20196_MGFPeaklist (F058870)	Nonmuscle myosin heavy chain OS=Gallus gallus PE=2 SV=2	Q02015_CHICK,Q789A4_CHICK,Q789A5_CHICK,Q789A6_CHICK,UPI00000FC6BE,UPI0000171380,UPI0000171381,UPI0000171382,UPI00003AB452,UPI00003AB453,UPI00003AB454,UPI000060F510,UPI000060F511,UPI0000ECA50D	TAX_GallusGallus_9031_20141114.fasta,UPARC14_2_GallusG_9031_20140417.fasta	215,888.80	100.00%	8	8	18	0.04%	8.01%	QRYEILTPNAIPK	R	G	99.70%	44.8	43.5	22.5	30.7	33.5	1.96	0.358	0.423	0	2	4	0	2	1,542.86	2,240.50		4,800.05	611	623	Empty	FALSE	UPI000060E1E4,UPI000012FB7F,MYH9_CHICK,F1NCD4_CHICK,UPI0002C868F4
20196-20225 HinckeM –TAXchicken	Uncategorized Sample	20196-20205_A	Mudpit_Ingel20196_MGFPeaklist (F058870)	Nonmuscle myosin heavy chain OS=Gallus gallus PE=2 SV=2	Q02015_CHICK,Q789A4_CHICK,Q789A5_CHICK,Q789A6_CHICK,UPI00000FC6BE,UPI0000171380,UPI0000171381,UPI0000171382,UPI00003AB452,UPI00003AB453,UPI00003AB454,UPI000060F510,UPI000060F511,UPI0000ECA50D	TAX_GallusGallus_9031_20141114.fasta,UPARC14_2_GallusG_9031_20140417.fasta	215,888.80	100.00%	8	8	18	0.04%	8.01%	YEILTPNAIPK	R	G	99.70%	20.6	44.4	14.6	7.9	28.5	1.57	0.277	0.318	0	3	0	0	2	1,258.70	2,337.00		1,618.82	613	623	Empty	FALSE	UPI000060E1E4,UPI000012FB7F,MYH9_CHICK,F1NCD4_CHICK,UPI0002C868F4
20196-20225 HinckeM –TAXchicken	Uncategorized Sample	20196-20205_A	Mudpit_Ingel20196_MGFPeaklist (F058870)	Nonmuscle myosin heavy chain OS=Gallus gallus PE=2 SV=2	Q02015_CHICK,Q789A4_CHICK,Q789A5_CHICK,Q789A6_CHICK,UPI00000FC6BE,UPI0000171380,UPI0000171381,UPI0000171382,UPI00003AB452,UPI00003AB453,UPI00003AB454,UPI000060F510,UPI000060F511,UPI0000ECA50D	TAX_GallusGallus_9031_20141114.fasta,UPARC14_2_GallusG_9031_20140417.fasta	215,888.80	100.00%	8	8	18	0.04%	8.01%	ADFCIIHYAGK	K	V	45.90%	18.6	44.4	14.7	11.1					0	0	2	0	2	1,294.63	2,223.00		989.84	458	468	Empty	FALSE	UPI000060E1E4,UPI000012FB7F,MYH9_CHICK,F1NCD4_CHICK,UPI0002C868F4
20196-20225 HinckeM –TAXchicken	Uncategorized Sample	20196-20205_A	Mudpit_Ingel20196_MGFPeaklist (F058870)	Nonmuscle myosin heavy chain OS=Gallus gallus PE=2 SV=2	Q02015_CHICK,Q789A4_CHICK,Q789A5_CHICK,Q789A6_CHICK,UPI00000FC6BE,UPI0000171380,UPI0000171381,UPI0000171382,UPI00003AB452,UPI00003AB453,UPI00003AB454,UPI000060F510,UPI000060F511,UPI0000ECA50D	TAX_GallusGallus_9031_20141114.fasta,UPARC14_2_GallusG_9031_20140417.fasta	215,888.80	100.00%	8	8	18	0.04%	8.01%	AGKLDPHLVLDQLR	R	C	99.70%	52.2	42.3	31.5	35.1	37.5	3.54	0.344	0.5	0	0	2	0	2	1,574.90	2,445.50		970.79	572	585	Empty	FALSE	UPI000060E1E4,UPI000012FB7F,MYH9_CHICK,F1NCD4_CHICK,UPI0002C868F4
20196-20225 HinckeM –TAXchicken	Uncategorized Sample	20196-20205_A	Mudpit_Ingel20196_MGFPeaklist (F058870)	Nonmuscle myosin heavy chain OS=Gallus gallus PE=2 SV=2	Q02015_CHICK,Q789A4_CHICK,Q789A5_CHICK,Q789A6_CHICK,UPI00000FC6BE,UPI0000171380,UPI0000171381,UPI0000171382,UPI00003AB452,UPI00003AB453,UPI00003AB454,UPI000060F510,UPI000060F511,UPI0000ECA50D	TAX_GallusGallus_9031_20141114.fasta,UPARC14_2_GallusG_9031_20140417.fasta	215,888.80	100.00%	8	8	18	0.04%	8.01%	AGVLAHLEEER	R	D	99.70%	71.3	44.3	36.2	36.2	34.7	3.4	0.317	0.409	0	0	2	0	2	1,223.64	2,031.50		1,163.70	657	667	Empty	FALSE	UPI000060E1E4,UPI000012FB7F,MYH9_CHICK,F1NCD4_CHICK,UPI0002C868F4
20196-20225 HinckeM –TAXchicken	Uncategorized Sample	20196-20205_A	Mudpit_Ingel20196_MGFPeaklist (F058870)	Nonmuscle myosin heavy chain OS=Gallus gallus PE=2 SV=2	Q02015_CHICK,Q789A4_CHICK,Q789A5_CHICK,Q789A6_CHICK,UPI00000FC6BE,UPI0000171380,UPI0000171381,UPI0000171382,UPI00003AB452,UPI00003AB453,UPI00003AB454,UPI000060F510,UPI000060F511,UPI0000ECA50D	TAX_GallusGallus_9031_20141114.fasta,UPARC14_2_GallusG_9031_20140417.fasta	215,888.80	100.00%	8	8	18	0.04%	8.01%	AGVLAHLEEERDLK	R	I	99.70%	43.7	43.9	28.2	36.5	38.3	3.7	0.371	0.464	0	0	2	0	2	1,579.84	2,107.00		1,024.73	657	670	Empty	FALSE	UPI000060E1E4,UPI000012FB7F,MYH9_CHICK,F1NCD4_CHICK,UPI0002C868F4
20196-20225 HinckeM –TAXchicken	Uncategorized Sample	20196-20205_A	Mudpit_Ingel20196_MGFPeaklist (F058870)	Nonmuscle myosin heavy chain OS=Gallus gallus PE=2 SV=2	Q02015_CHICK,Q789A4_CHICK,Q789A5_CHICK,Q789A6_CHICK,UPI00000FC6BE,UPI0000171380,UPI0000171381,UPI0000171382,UPI00003AB452,UPI00003AB453,UPI00003AB454,UPI000060F510,UPI000060F511,UPI0000ECA50D	TAX_GallusGallus_9031_20141114.fasta,UPARC14_2_GallusG_9031_20140417.fasta	215,888.80	100.00%	8	8	18	0.04%	8.01%	AIEEETKNHEAQIQEIR	K	Q	99.70%	29	44.4	22.1	12.1	28.6	1.7	0.192	0.265	0	0	0	2	2	2,038.02	1,772.50		811.37	1067	1083	Empty	TRUE	
20196-20225 HinckeM –TAXchicken	Uncategorized Sample	20196-20205_A	Mudpit_Ingel20196_MGFPeaklist (F058870)	Nonmuscle myosin heavy chain OS=Gallus gallus PE=2 SV=2	Q02015_CHICK,Q789A4_CHICK,Q789A5_CHICK,Q789A6_CHICK,UPI00000FC6BE,UPI0000171380,UPI0000171381,UPI0000171382,UPI00003AB452,UPI00003AB453,UPI00003AB454,UPI000060F510,UPI000060F511,UPI0000ECA50D	TAX_GallusGallus_9031_20141114.fasta,UPARC14_2_GallusG_9031_20140417.fasta	215,888.80	100.00%	8	8	18	0.04%	8.01%	ALEEALEAKEEFER	R	Q	99.70%	49	44.8	23.2	35.1	34.9	3.29	0.39	0.286	0	0	2	0	2	1,663.82	2,175.50		1,480.83	1376	1389	Empty	TRUE	
20196-20225 HinckeM –TAXchicken	Uncategorized Sample	20196-20205_A	Mudpit_Ingel20196_MGFPeaklist (F058870)	Nonmuscle myosin heavy chain OS=Gallus gallus PE=2 SV=2	Q02015_CHICK,Q789A4_CHICK,Q789A5_CHICK,Q789A6_CHICK,UPI00000FC6BE,UPI0000171380,UPI0000171381,UPI0000171382,UPI00003AB452,UPI00003AB453,UPI00003AB454,UPI000060F510,UPI000060F511,UPI0000ECA50D	TAX_GallusGallus_9031_20141114.fasta,UPARC14_2_GallusG_9031_20140417.fasta	215,888.80	100.00%	8	8	18	0.04%	8.01%	ALELDPNLYR	R	I	99.70%	42.1	45.2	34.3	22.2	32.7	2.42	0.343	0.4	0	5	0	0	2	1,203.64	2,376.00		1,867.67	638	647	Empty	TRUE	MYH11_CHICK
20196-20225 HinckeM –TAXchicken	Uncategorized Sample	20196-20205_A	Mudpit_Ingel20196_MGFPeaklist (F058870)	Nonmuscle myosin heavy chain OS=Gallus gallus PE=2 SV=2	Q02015_CHICK,Q789A4_CHICK,Q789A5_CHICK,Q789A6_CHICK,UPI00000FC6BE,UPI0000171380,UPI0000171381,UPI0000171382,UPI00003AB452,UPI00003AB453,UPI00003AB454,UPI000060F510,UPI000060F511,UPI0000ECA50D	TAX_GallusGallus_9031_20141114.fasta,UPARC14_2_GallusG_9031_20140417.fasta	215,888.80	100.00%	8	8	18	0.04%	8.01%	CNGVLEGIR	R	I	28.90%	28.9	45.5	29.6	12.6					0	1	0	0	2	1,018.50	2,046.00		210.55	586	594	Empty	FALSE	UPI000060E1E4,MYH11_CHICK,Q9PTY2_CHICK,F1NCD4_CHICK,UPI0002C868F4,UPI000012FB7F,MYH9_CHICK,UPI000044943F,UPI00003AB434,UPI00000FB7E2,UPI00003AB431
20196-20225 HinckeM –TAXchicken	Uncategorized Sample	20196-20205_A	Mudpit_Ingel20196_MGFPeaklist (F058870)	Nonmuscle myosin heavy chain OS=Gallus gallus PE=2 SV=2	Q02015_CHICK,Q789A4_CHICK,Q789A5_CHICK,Q789A6_CHICK,UPI00000FC6BE,UPI0000171380,UPI0000171381,UPI0000171382,UPI00003AB452,UPI00003AB453,UPI00003AB454,UPI000060F510,UPI000060F511,UPI0000ECA50D	TAX_GallusGallus_9031_20141114.fasta,UPARC14_2_GallusG_9031_20140417.fasta	215,888.80	100.00%	8	8	18	0.04%	8.01%	ELDDATEANEGLSR	R	E	99.70%	68.7	43.6	23	0	39.4	3.44	0.327	0.357	0	2	0	0	2	1,519.69	1,739.00		940.74	1791	1804	Empty	TRUE	
20196-20225 HinckeM –TAXchicken	Uncategorized Sample	20196-20205_A	Mudpit_Ingel20196_MGFPeaklist (F058870)	Nonmuscle myosin heavy chain OS=Gallus gallus PE=2 SV=2	Q02015_CHICK,Q789A4_CHICK,Q789A5_CHICK,Q789A6_CHICK,UPI00000FC6BE,UPI0000171380,UPI0000171381,UPI0000171382,UPI00003AB452,UPI00003AB453,UPI00003AB454,UPI000060F510,UPI000060F511,UPI0000ECA50D	TAX_GallusGallus_9031_20141114.fasta,UPARC14_2_GallusG_9031_20140417.fasta	215,888.80	100.00%	8	8	18	0.04%	8.01%	GGPITFSSSR	R	S	95.00%	30.3	44	27.7	19.6					0	1	0	0	2	1,008.51	1,869.00		125.86	1816	1825	Empty	TRUE	
20196-20225 HinckeM –TAXchicken	Uncategorized Sample	20196-20205_A	Mudpit_Ingel20196_MGFPeaklist (F058870)	Nonmuscle myosin heavy chain OS=Gallus gallus PE=2 SV=2	Q02015_CHICK,Q789A4_CHICK,Q789A5_CHICK,Q789A6_CHICK,UPI00000FC6BE,UPI0000171380,UPI0000171381,UPI0000171382,UPI00003AB452,UPI00003AB453,UPI00003AB454,UPI000060F510,UPI000060F511,UPI0000ECA50D	TAX_GallusGallus_9031_20141114.fasta,UPARC14_2_GallusG_9031_20140417.fasta	215,888.80	100.00%	8	8	18	0.04%	8.01%	IAQLEEQLEQEAKER	K	A	99.70%	59.3	44.3	25.4	45.9	46.4	4.08	0.369	0.4	0	0	2	0	2	1,813.93	2,231.00		777.93	1708	1722	Empty	TRUE	
20196-20225 HinckeM –TAXchicken	Uncategorized Sample	20196-20205_A	Mudpit_Ingel20196_MGFPeaklist (F058870)	Nonmuscle myosin heavy chain OS=Gallus gallus PE=2 SV=2	Q02015_CHICK,Q789A4_CHICK,Q789A5_CHICK,Q789A6_CHICK,UPI00000FC6BE,UPI0000171380,UPI0000171381,UPI0000171382,UPI00003AB452,UPI00003AB453,UPI00003AB454,UPI000060F510,UPI000060F511,UPI0000ECA50D	TAX_GallusGallus_9031_20141114.fasta,UPARC14_2_GallusG_9031_20140417.fasta	215,888.80	100.00%	8	8	18	0.04%	8.01%	IVFQEFR	R	Q	98.80%	47	43.1	42.2	19.8	27.9	0.585	0.222	0.5	0	3	0	0	2	938.5094	2,257.00		900.74	604	610	Empty	TRUE	MYH11_CHICK
20196-20225 HinckeM –TAXchicken	Uncategorized Sample	20196-20205_A	Mudpit_Ingel20196_MGFPeaklist (F058870)	Nonmuscle myosin heavy chain OS=Gallus gallus PE=2 SV=2	Q02015_CHICK,Q789A4_CHICK,Q789A5_CHICK,Q789A6_CHICK,UPI00000FC6BE,UPI0000171380,UPI0000171381,UPI0000171382,UPI00003AB452,UPI00003AB453,UPI00003AB454,UPI000060F510,UPI000060F511,UPI0000ECA50D	TAX_GallusGallus_9031_20141114.fasta,UPARC14_2_GallusG_9031_20140417.fasta	215,888.80	100.00%	8	8	18	0.04%	8.01%	KLDAQVQELTAK	K	V	13.90%	14	44	25.3	0.53					0	0	1	0	2	1,343.75	1,897.00		288.44	1141	1152	Empty	TRUE	
20196-20225 HinckeM –TAXchicken	Uncategorized Sample	20196-20205_A	Mudpit_Ingel20196_MGFPeaklist (F058870)	Nonmuscle myosin heavy chain OS=Gallus gallus PE=2 SV=2	Q02015_CHICK,Q789A4_CHICK,Q789A5_CHICK,Q789A6_CHICK,UPI00000FC6BE,UPI0000171380,UPI0000171381,UPI0000171382,UPI00003AB452,UPI00003AB453,UPI00003AB454,UPI000060F510,UPI000060F511,UPI0000ECA50D	TAX_GallusGallus_9031_20141114.fasta,UPARC14_2_GallusG_9031_20140417.fasta	215,888.80	100.00%	8	8	18	0.04%	8.01%	LDPHLVLDQLR	K	C	99.70%	34.6	43.8	18.6	22.1	32.5	2.47	0.366	0.455	0	0	1	0	2	1,318.75	2,534.00		1,121.62	575	585	Empty	FALSE	UPI000060E1E4,UPI000012FB7F,MYH9_CHICK,F1NCD4_CHICK,UPI0002C868F4
20196-20225 HinckeM –TAXchicken	Uncategorized Sample	20196-20205_A	Mudpit_Ingel20196_MGFPeaklist (F058870)	Nonmuscle myosin heavy chain OS=Gallus gallus PE=2 SV=2	Q02015_CHICK,Q789A4_CHICK,Q789A5_CHICK,Q789A6_CHICK,UPI00000FC6BE,UPI0000171380,UPI0000171381,UPI0000171382,UPI00003AB452,UPI00003AB453,UPI00003AB454,UPI000060F510,UPI000060F511,UPI0000ECA50D	TAX_GallusGallus_9031_20141114.fasta,UPARC14_2_GallusG_9031_20140417.fasta	215,888.80	100.00%	8	8	18	0.04%	8.01%	QRYEILTPNAIPK	R	G	99.70%	44.8	43.5	22.5	30.7	33.5	1.96	0.358	0.423	0	2	4	0	2	1,542.86	2,240.50		4,800.05	611	623	Empty	FALSE	UPI000060E1E4,UPI000012FB7F,MYH9_CHICK,F1NCD4_CHICK,UPI0002C868F4
20196-20225 HinckeM –TAXchicken	Uncategorized Sample	20196-20205_A	Mudpit_Ingel20196_MGFPeaklist (F058870)	Nonmuscle myosin heavy chain OS=Gallus gallus PE=2 SV=2	Q02015_CHICK,Q789A4_CHICK,Q789A5_CHICK,Q789A6_CHICK,UPI00000FC6BE,UPI0000171380,UPI0000171381,UPI0000171382,UPI00003AB452,UPI00003AB453,UPI00003AB454,UPI000060F510,UPI000060F511,UPI0000ECA50D	TAX_GallusGallus_9031_20141114.fasta,UPARC14_2_GallusG_9031_20140417.fasta	215,888.80	100.00%	8	8	18	0.04%	8.01%	YEILTPNAIPK	R	G	99.70%	20.6	44.4	14.6	7.9	28.5	1.57	0.277	0.318	0	3	0	0	2	1,258.70	2,337.00		1,618.82	613	623	Empty	FALSE	UPI000060E1E4,UPI000012FB7F,MYH9_CHICK,F1NCD4_CHICK,UPI0002C868F4
20196-20225 HinckeM –TAXchicken	Uncategorized Sample	20196-20205_A	Mudpit_Ingel20196_MGFPeaklist (F058870)	Nonmuscle myosin heavy chain OS=Gallus gallus PE=2 SV=2	Q02015_CHICK,Q789A4_CHICK,Q789A5_CHICK,Q789A6_CHICK,UPI00000FC6BE,UPI0000171380,UPI0000171381,UPI0000171382,UPI00003AB452,UPI00003AB453,UPI00003AB454,UPI000060F510,UPI000060F511,UPI0000ECA50D	TAX_GallusGallus_9031_20141114.fasta,UPARC14_2_GallusG_9031_20140417.fasta	215,888.80	100.00%	8	8	18	0.04%	8.01%	ADFCIIHYAGK	K	V	45.90%	18.6	44.4	14.7	11.1					0	0	2	0	2	1,294.63	2,223.00		989.84	458	468	Empty	FALSE	UPI000060E1E4,UPI000012FB7F,MYH9_CHICK,F1NCD4_CHICK,UPI0002C868F4
20196-20225 HinckeM –TAXchicken	Uncategorized Sample	20196-20205_A	Mudpit_Ingel20196_MGFPeaklist (F058870)	Nonmuscle myosin heavy chain OS=Gallus gallus PE=2 SV=2	Q02015_CHICK,Q789A4_CHICK,Q789A5_CHICK,Q789A6_CHICK,UPI00000FC6BE,UPI0000171380,UPI0000171381,UPI0000171382,UPI00003AB452,UPI00003AB453,UPI00003AB454,UPI000060F510,UPI000060F511,UPI0000ECA50D	TAX_GallusGallus_9031_20141114.fasta,UPARC14_2_GallusG_9031_20140417.fasta	215,888.80	100.00%	8	8	18	0.04%	8.01%	AGKLDPHLVLDQLR	R	C	99.70%	52.2	42.3	31.5	35.1	37.5	3.54	0.344	0.5	0	0	2	0	2	1,574.90	2,445.50		970.79	572	585	Empty	FALSE	UPI000060E1E4,UPI000012FB7F,MYH9_CHICK,F1NCD4_CHICK,UPI0002C868F4
20196-20225 HinckeM –TAXchicken	Uncategorized Sample	20196-20205_A	Mudpit_Ingel20196_MGFPeaklist (F058870)	Nonmuscle myosin heavy chain OS=Gallus gallus PE=2 SV=2	Q02015_CHICK,Q789A4_CHICK,Q789A5_CHICK,Q789A6_CHICK,UPI00000FC6BE,UPI0000171380,UPI0000171381,UPI0000171382,UPI00003AB452,UPI00003AB453,UPI00003AB454,UPI000060F510,UPI000060F511,UPI0000ECA50D	TAX_GallusGallus_9031_20141114.fasta,UPARC14_2_GallusG_9031_20140417.fasta	215,888.80	100.00%	8	8	18	0.04%	8.01%	AGVLAHLEEER	R	D	99.70%	71.3	44.3	36.2	36.2	34.7	3.4	0.317	0.409	0	0	2	0	2	1,223.64	2,031.50		1,163.70	657	667	Empty	FALSE	UPI000060E1E4,UPI000012FB7F,MYH9_CHICK,F1NCD4_CHICK,UPI0002C868F4
20196-20225 HinckeM –TAXchicken	Uncategorized Sample	20196-20205_A	Mudpit_Ingel20196_MGFPeaklist (F058870)	Nonmuscle myosin heavy chain OS=Gallus gallus PE=2 SV=2	Q02015_CHICK,Q789A4_CHICK,Q789A5_CHICK,Q789A6_CHICK,UPI00000FC6BE,UPI0000171380,UPI0000171381,UPI0000171382,UPI00003AB452,UPI00003AB453,UPI00003AB454,UPI000060F510,UPI000060F511,UPI0000ECA50D	TAX_GallusGallus_9031_20141114.fasta,UPARC14_2_GallusG_9031_20140417.fasta	215,888.80	100.00%	8	8	18	0.04%	8.01%	AGVLAHLEEERDLK	R	I	99.70%	43.7	43.9	28.2	36.5	38.3	3.7	0.371	0.464	0	0	2	0	2	1,579.84	2,107.00		1,024.73	657	670	Empty	FALSE	UPI000060E1E4,UPI000012FB7F,MYH9_CHICK,F1NCD4_CHICK,UPI0002C868F4
20196-20225 HinckeM –TAXchicken	Uncategorized Sample	20196-20205_A	Mudpit_Ingel20196_MGFPeaklist (F058870)	Nonmuscle myosin heavy chain OS=Gallus gallus PE=2 SV=2	Q02015_CHICK,Q789A4_CHICK,Q789A5_CHICK,Q789A6_CHICK,UPI00000FC6BE,UPI0000171380,UPI0000171381,UPI0000171382,UPI00003AB452,UPI00003AB453,UPI00003AB454,UPI000060F510,UPI000060F511,UPI0000ECA50D	TAX_GallusGallus_9031_20141114.fasta,UPARC14_2_GallusG_9031_20140417.fasta	215,888.80	100.00%	8	8	18	0.04%	8.01%	AIEEETKNHEAQIQEIR	K	Q	99.70%	29	44.4	22.1	12.1	28.6	1.7	0.192	0.265	0	0	0	2	2	2,038.02	1,772.50		811.37	1067	1083	Empty	TRUE	
20196-20225 HinckeM –TAXchicken	Uncategorized Sample	20196-20205_A	Mudpit_Ingel20196_MGFPeaklist (F058870)	Nonmuscle myosin heavy chain OS=Gallus gallus PE=2 SV=2	Q02015_CHICK,Q789A4_CHICK,Q789A5_CHICK,Q789A6_CHICK,UPI00000FC6BE,UPI0000171380,UPI0000171381,UPI0000171382,UPI00003AB452,UPI00003AB453,UPI00003AB454,UPI000060F510,UPI000060F511,UPI0000ECA50D	TAX_GallusGallus_9031_20141114.fasta,UPARC14_2_GallusG_9031_20140417.fasta	215,888.80	100.00%	8	8	18	0.04%	8.01%	ALEEALEAKEEFER	R	Q	99.70%	49	44.8	23.2	35.1	34.9	3.29	0.39	0.286	0	0	2	0	2	1,663.82	2,175.50		1,480.83	1376	1389	Empty	TRUE	
20196-20225 HinckeM –TAXchicken	Uncategorized Sample	20196-20205_A	Mudpit_Ingel20196_MGFPeaklist (F058870)	Nonmuscle myosin heavy chain OS=Gallus gallus PE=2 SV=2	Q02015_CHICK,Q789A4_CHICK,Q789A5_CHICK,Q789A6_CHICK,UPI00000FC6BE,UPI0000171380,UPI0000171381,UPI0000171382,UPI00003AB452,UPI00003AB453,UPI00003AB454,UPI000060F510,UPI000060F511,UPI0000ECA50D	TAX_GallusGallus_9031_20141114.fasta,UPARC14_2_GallusG_9031_20140417.fasta	215,888.80	100.00%	8	8	18	0.04%	8.01%	ALELDPNLYR	R	I	99.70%	42.1	45.2	34.3	22.2	32.7	2.42	0.343	0.4	0	5	0	0	2	1,203.64	2,376.00		1,867.67	638	647	Empty	TRUE	MYH11_CHICK
20196-20225 HinckeM –TAXchicken	Uncategorized Sample	20196-20205_A	Mudpit_Ingel20196_MGFPeaklist (F058870)	Nonmuscle myosin heavy chain OS=Gallus gallus PE=2 SV=2	Q02015_CHICK,Q789A4_CHICK,Q789A5_CHICK,Q789A6_CHICK,UPI00000FC6BE,UPI0000171380,UPI0000171381,UPI0000171382,UPI00003AB452,UPI00003AB453,UPI00003AB454,UPI000060F510,UPI000060F511,UPI0000ECA50D	TAX_GallusGallus_9031_20141114.fasta,UPARC14_2_GallusG_9031_20140417.fasta	215,888.80	100.00%	8	8	18	0.04%	8.01%	CNGVLEGIR	R	I	28.90%	28.9	45.5	29.6	12.6					0	1	0	0	2	1,018.50	2,046.00		210.55	586	594	Empty	FALSE	UPI000060E1E4,MYH11_CHICK,Q9PTY2_CHICK,F1NCD4_CHICK,UPI0002C868F4,UPI000012FB7F,MYH9_CHICK,UPI000044943F,UPI00003AB434,UPI00000FB7E2,UPI00003AB431
20196-20225 HinckeM –TAXchicken	Uncategorized Sample	20196-20205_A	Mudpit_Ingel20196_MGFPeaklist (F058870)	Nonmuscle myosin heavy chain OS=Gallus gallus PE=2 SV=2	Q02015_CHICK,Q789A4_CHICK,Q789A5_CHICK,Q789A6_CHICK,UPI00000FC6BE,UPI0000171380,UPI0000171381,UPI0000171382,UPI00003AB452,UPI00003AB453,UPI00003AB454,UPI000060F510,UPI000060F511,UPI0000ECA50D	TAX_GallusGallus_9031_20141114.fasta,UPARC14_2_GallusG_9031_20140417.fasta	215,888.80	100.00%	8	8	18	0.04%	8.01%	ELDDATEANEGLSR	R	E	99.70%	68.7	43.6	23	0	39.4	3.44	0.327	0.357	0	2	0	0	2	1,519.69	1,739.00		940.74	1791	1804	Empty	TRUE	
20196-20225 HinckeM –TAXchicken	Uncategorized Sample	20196-20205_A	Mudpit_Ingel20196_MGFPeaklist (F058870)	Nonmuscle myosin heavy chain OS=Gallus gallus PE=2 SV=2	Q02015_CHICK,Q789A4_CHICK,Q789A5_CHICK,Q789A6_CHICK,UPI00000FC6BE,UPI0000171380,UPI0000171381,UPI0000171382,UPI00003AB452,UPI00003AB453,UPI00003AB454,UPI000060F510,UPI000060F511,UPI0000ECA50D	TAX_GallusGallus_9031_20141114.fasta,UPARC14_2_GallusG_9031_20140417.fasta	215,888.80	100.00%	8	8	18	0.04%	8.01%	GGPITFSSSR	R	S	95.00%	30.3	44	27.7	19.6					0	1	0	0	2	1,008.51	1,869.00		125.86	1816	1825	Empty	TRUE	
20196-20225 HinckeM –TAXchicken	Uncategorized Sample	20196-20205_A	Mudpit_Ingel20196_MGFPeaklist (F058870)	Nonmuscle myosin heavy chain OS=Gallus gallus PE=2 SV=2	Q02015_CHICK,Q789A4_CHICK,Q789A5_CHICK,Q789A6_CHICK,UPI00000FC6BE,UPI0000171380,UPI0000171381,UPI0000171382,UPI00003AB452,UPI00003AB453,UPI00003AB454,UPI000060F510,UPI000060F511,UPI0000ECA50D	TAX_GallusGallus_9031_20141114.fasta,UPARC14_2_GallusG_9031_20140417.fasta	215,888.80	100.00%	8	8	18	0.04%	8.01%	IAQLEEQLEQEAKER	K	A	99.70%	59.3	44.3	25.4	45.9	46.4	4.08	0.369	0.4	0	0	2	0	2	1,813.93	2,231.00		777.93	1708	1722	Empty	TRUE	
20196-20225 HinckeM –TAXchicken	Uncategorized Sample	20196-20205_A	Mudpit_Ingel20196_MGFPeaklist (F058870)	Nonmuscle myosin heavy chain OS=Gallus gallus PE=2 SV=2	Q02015_CHICK,Q789A4_CHICK,Q789A5_CHICK,Q789A6_CHICK,UPI00000FC6BE,UPI0000171380,UPI0000171381,UPI0000171382,UPI00003AB452,UPI00003AB453,UPI00003AB454,UPI000060F510,UPI000060F511,UPI0000ECA50D	TAX_GallusGallus_9031_20141114.fasta,UPARC14_2_GallusG_9031_20140417.fasta	215,888.80	100.00%	8	8	18	0.04%	8.01%	IVFQEFR	R	Q	98.80%	47	43.1	42.2	19.8	27.9	0.585	0.222	0.5	0	3	0	0	2	938.5094	2,257.00		900.74	604	610	Empty	TRUE	MYH11_CHICK
20196-20225 HinckeM –TAXchicken	Uncategorized Sample	20196-20205_A	Mudpit_Ingel20196_MGFPeaklist (F058870)	Nonmuscle myosin heavy chain OS=Gallus gallus PE=2 SV=2	Q02015_CHICK,Q789A4_CHICK,Q789A5_CHICK,Q789A6_CHICK,UPI00000FC6BE,UPI0000171380,UPI0000171381,UPI0000171382,UPI00003AB452,UPI00003AB453,UPI00003AB454,UPI000060F510,UPI000060F511,UPI0000ECA50D	TAX_GallusGallus_9031_20141114.fasta,UPARC14_2_GallusG_9031_20140417.fasta	215,888.80	100.00%	8	8	18	0.04%	8.01%	KLDAQVQELTAK	K	V	13.90%	14	44	25.3	0.53					0	0	1	0	2	1,343.75	1,897.00		288.44	1141	1152	Empty	TRUE	
20196-20225 HinckeM –TAXchicken	Uncategorized Sample	20196-20205_A	Mudpit_Ingel20196_MGFPeaklist (F058870)	Nonmuscle myosin heavy chain OS=Gallus gallus PE=2 SV=2	Q02015_CHICK,Q789A4_CHICK,Q789A5_CHICK,Q789A6_CHICK,UPI00000FC6BE,UPI0000171380,UPI0000171381,UPI0000171382,UPI00003AB452,UPI00003AB453,UPI00003AB454,UPI000060F510,UPI000060F511,UPI0000ECA50D	TAX_GallusGallus_9031_20141114.fasta,UPARC14_2_GallusG_9031_20140417.fasta	215,888.80	100.00%	8	8	18	0.04%	8.01%	LDPHLVLDQLR	K	C	99.70%	34.6	43.8	18.6	22.1	32.5	2.47	0.366	0.455	0	0	1	0	2	1,318.75	2,534.00		1,121.62	575	585	Empty	FALSE	UPI000060E1E4,UPI000012FB7F,MYH9_CHICK,F1NCD4_CHICK,UPI0002C868F4
20196-20225 HinckeM –TAXchicken	Uncategorized Sample	20196-20205_A	Mudpit_Ingel20196_MGFPeaklist (F058870)	Nonmuscle myosin heavy chain OS=Gallus gallus PE=2 SV=2	Q02015_CHICK,Q789A4_CHICK,Q789A5_CHICK,Q789A6_CHICK,UPI00000FC6BE,UPI0000171380,UPI0000171381,UPI0000171382,UPI00003AB452,UPI00003AB453,UPI00003AB454,UPI000060F510,UPI000060F511,UPI0000ECA50D	TAX_GallusGallus_9031_20141114.fasta,UPARC14_2_GallusG_9031_20140417.fasta	215,888.80	100.00%	8	8	18	0.04%	8.01%	QRYEILTPNAIPK	R	G	99.70%	44.8	43.5	22.5	30.7	33.5	1.96	0.358	0.423	0	2	4	0	2	1,542.86	2,240.50		4,800.05	611	623	Empty	FALSE	UPI000060E1E4,UPI000012FB7F,MYH9_CHICK,F1NCD4_CHICK,UPI0002C868F4
20196-20225 HinckeM –TAXchicken	Uncategorized Sample	20196-20205_A	Mudpit_Ingel20196_MGFPeaklist (F058870)	Nonmuscle myosin heavy chain OS=Gallus gallus PE=2 SV=2	Q02015_CHICK,Q789A4_CHICK,Q789A5_CHICK,Q789A6_CHICK,UPI00000FC6BE,UPI0000171380,UPI0000171381,UPI0000171382,UPI00003AB452,UPI00003AB453,UPI00003AB454,UPI000060F510,UPI000060F511,UPI0000ECA50D	TAX_GallusGallus_9031_20141114.fasta,UPARC14_2_GallusG_9031_20140417.fasta	215,888.80	100.00%	8	8	18	0.04%	8.01%	YEILTPNAIPK	R	G	99.70%	20.6	44.4	14.6	7.9	28.5	1.57	0.277	0.318	0	3	0	0	2	1,258.70	2,337.00		1,618.82	613	623	Empty	FALSE	UPI000060E1E4,UPI000012FB7F,MYH9_CHICK,F1NCD4_CHICK,UPI0002C868F4
20196-20225 HinckeM –TAXchicken	Uncategorized Sample	20196-20205_A	Mudpit_Ingel20196_MGFPeaklist (F058870)	Nonmuscle myosin heavy chain OS=Gallus gallus PE=2 SV=2	Q02015_CHICK,Q789A4_CHICK,Q789A5_CHICK,Q789A6_CHICK,UPI00000FC6BE,UPI0000171380,UPI0000171381,UPI0000171382,UPI00003AB452,UPI00003AB453,UPI00003AB454,UPI000060F510,UPI000060F511,UPI0000ECA50D	TAX_GallusGallus_9031_20141114.fasta,UPARC14_2_GallusG_9031_20140417.fasta	215,888.80	100.00%	8	8	18	0.04%	8.01%	ADFCIIHYAGK	K	V	45.90%	18.6	44.4	14.7	11.1					0	0	2	0	2	1,294.63	2,223.00		989.84	458	468	Empty	FALSE	UPI000060E1E4,UPI000012FB7F,MYH9_CHICK,F1NCD4_CHICK,UPI0002C868F4
20196-20225 HinckeM –TAXchicken	Uncategorized Sample	20196-20205_A	Mudpit_Ingel20196_MGFPeaklist (F058870)	Nonmuscle myosin heavy chain OS=Gallus gallus PE=2 SV=2	Q02015_CHICK,Q789A4_CHICK,Q789A5_CHICK,Q789A6_CHICK,UPI00000FC6BE,UPI0000171380,UPI0000171381,UPI0000171382,UPI00003AB452,UPI00003AB453,UPI00003AB454,UPI000060F510,UPI000060F511,UPI0000ECA50D	TAX_GallusGallus_9031_20141114.fasta,UPARC14_2_GallusG_9031_20140417.fasta	215,888.80	100.00%	8	8	18	0.04%	8.01%	AGKLDPHLVLDQLR	R	C	99.70%	52.2	42.3	31.5	35.1	37.5	3.54	0.344	0.5	0	0	2	0	2	1,574.90	2,445.50		970.79	572	585	Empty	FALSE	UPI000060E1E4,UPI000012FB7F,MYH9_CHICK,F1NCD4_CHICK,UPI0002C868F4
20196-20225 HinckeM –TAXchicken	Uncategorized Sample	20196-20205_A	Mudpit_Ingel20196_MGFPeaklist (F058870)	Nonmuscle myosin heavy chain OS=Gallus gallus PE=2 SV=2	Q02015_CHICK,Q789A4_CHICK,Q789A5_CHICK,Q789A6_CHICK,UPI00000FC6BE,UPI0000171380,UPI0000171381,UPI0000171382,UPI00003AB452,UPI00003AB453,UPI00003AB454,UPI000060F510,UPI000060F511,UPI0000ECA50D	TAX_GallusGallus_9031_20141114.fasta,UPARC14_2_GallusG_9031_20140417.fasta	215,888.80	100.00%	8	8	18	0.04%	8.01%	AGVLAHLEEER	R	D	99.70%	71.3	44.3	36.2	36.2	34.7	3.4	0.317	0.409	0	0	2	0	2	1,223.64	2,031.50		1,163.70	657	667	Empty	FALSE	UPI000060E1E4,UPI000012FB7F,MYH9_CHICK,F1NCD4_CHICK,UPI0002C868F4
20196-20225 HinckeM –TAXchicken	Uncategorized Sample	20196-20205_A	Mudpit_Ingel20196_MGFPeaklist (F058870)	Nonmuscle myosin heavy chain OS=Gallus gallus PE=2 SV=2	Q02015_CHICK,Q789A4_CHICK,Q789A5_CHICK,Q789A6_CHICK,UPI00000FC6BE,UPI0000171380,UPI0000171381,UPI0000171382,UPI00003AB452,UPI00003AB453,UPI00003AB454,UPI000060F510,UPI000060F511,UPI0000ECA50D	TAX_GallusGallus_9031_20141114.fasta,UPARC14_2_GallusG_9031_20140417.fasta	215,888.80	100.00%	8	8	18	0.04%	8.01%	AGVLAHLEEERDLK	R	I	99.70%	43.7	43.9	28.2	36.5	38.3	3.7	0.371	0.464	0	0	2	0	2	1,579.84	2,107.00		1,024.73	657	670	Empty	FALSE	UPI000060E1E4,UPI000012FB7F,MYH9_CHICK,F1NCD4_CHICK,UPI0002C868F4
20196-20225 HinckeM –TAXchicken	Uncategorized Sample	20196-20205_A	Mudpit_Ingel20196_MGFPeaklist (F058870)	Nonmuscle myosin heavy chain OS=Gallus gallus PE=2 SV=2	Q02015_CHICK,Q789A4_CHICK,Q789A5_CHICK,Q789A6_CHICK,UPI00000FC6BE,UPI0000171380,UPI0000171381,UPI0000171382,UPI00003AB452,UPI00003AB453,UPI00003AB454,UPI000060F510,UPI000060F511,UPI0000ECA50D	TAX_GallusGallus_9031_20141114.fasta,UPARC14_2_GallusG_9031_20140417.fasta	215,888.80	100.00%	8	8	18	0.04%	8.01%	AIEEETKNHEAQIQEIR	K	Q	99.70%	29	44.4	22.1	12.1	28.6	1.7	0.192	0.265	0	0	0	2	2	2,038.02	1,772.50		811.37	1067	1083	Empty	TRUE	
20196-20225 HinckeM –TAXchicken	Uncategorized Sample	20196-20205_A	Mudpit_Ingel20196_MGFPeaklist (F058870)	Nonmuscle myosin heavy chain OS=Gallus gallus PE=2 SV=2	Q02015_CHICK,Q789A4_CHICK,Q789A5_CHICK,Q789A6_CHICK,UPI00000FC6BE,UPI0000171380,UPI0000171381,UPI0000171382,UPI00003AB452,UPI00003AB453,UPI00003AB454,UPI000060F510,UPI000060F511,UPI0000ECA50D	TAX_GallusGallus_9031_20141114.fasta,UPARC14_2_GallusG_9031_20140417.fasta	215,888.80	100.00%	8	8	18	0.04%	8.01%	ALEEALEAKEEFER	R	Q	99.70%	49	44.8	23.2	35.1	34.9	3.29	0.39	0.286	0	0	2	0	2	1,663.82	2,175.50		1,480.83	1376	1389	Empty	TRUE	
20196-20225 HinckeM –TAXchicken	Uncategorized Sample	20196-20205_A	Mudpit_Ingel20196_MGFPeaklist (F058870)	Nonmuscle myosin heavy chain OS=Gallus gallus PE=2 SV=2	Q02015_CHICK,Q789A4_CHICK,Q789A5_CHICK,Q789A6_CHICK,UPI00000FC6BE,UPI0000171380,UPI0000171381,UPI0000171382,UPI00003AB452,UPI00003AB453,UPI00003AB454,UPI000060F510,UPI000060F511,UPI0000ECA50D	TAX_GallusGallus_9031_20141114.fasta,UPARC14_2_GallusG_9031_20140417.fasta	215,888.80	100.00%	8	8	18	0.04%	8.01%	ALELDPNLYR	R	I	99.70%	42.1	45.2	34.3	22.2	32.7	2.42	0.343	0.4	0	5	0	0	2	1,203.64	2,376.00		1,867.67	638	647	Empty	TRUE	MYH11_CHICK
20196-20225 HinckeM –TAXchicken	Uncategorized Sample	20196-20205_A	Mudpit_Ingel20196_MGFPeaklist (F058870)	Nonmuscle myosin heavy chain OS=Gallus gallus PE=2 SV=2	Q02015_CHICK,Q789A4_CHICK,Q789A5_CHICK,Q789A6_CHICK,UPI00000FC6BE,UPI0000171380,UPI0000171381,UPI0000171382,UPI00003AB452,UPI00003AB453,UPI00003AB454,UPI000060F510,UPI000060F511,UPI0000ECA50D	TAX_GallusGallus_9031_20141114.fasta,UPARC14_2_GallusG_9031_20140417.fasta	215,888.80	100.00%	8	8	18	0.04%	8.01%	CNGVLEGIR	R	I	28.90%	28.9	45.5	29.6	12.6					0	1	0	0	2	1,018.50	2,046.00		210.55	586	594	Empty	FALSE	UPI000060E1E4,MYH11_CHICK,Q9PTY2_CHICK,F1NCD4_CHICK,UPI0002C868F4,UPI000012FB7F,MYH9_CHICK,UPI000044943F,UPI00003AB434,UPI00000FB7E2,UPI00003AB431
20196-20225 HinckeM –TAXchicken	Uncategorized Sample	20196-20205_A	Mudpit_Ingel20196_MGFPeaklist (F058870)	Nonmuscle myosin heavy chain OS=Gallus gallus PE=2 SV=2	Q02015_CHICK,Q789A4_CHICK,Q789A5_CHICK,Q789A6_CHICK,UPI00000FC6BE,UPI0000171380,UPI0000171381,UPI0000171382,UPI00003AB452,UPI00003AB453,UPI00003AB454,UPI000060F510,UPI000060F511,UPI0000ECA50D	TAX_GallusGallus_9031_20141114.fasta,UPARC14_2_GallusG_9031_20140417.fasta	215,888.80	100.00%	8	8	18	0.04%	8.01%	ELDDATEANEGLSR	R	E	99.70%	68.7	43.6	23	0	39.4	3.44	0.327	0.357	0	2	0	0	2	1,519.69	1,739.00		940.74	1791	1804	Empty	TRUE	
20196-20225 HinckeM –TAXchicken	Uncategorized Sample	20196-20205_A	Mudpit_Ingel20196_MGFPeaklist (F058870)	Nonmuscle myosin heavy chain OS=Gallus gallus PE=2 SV=2	Q02015_CHICK,Q789A4_CHICK,Q789A5_CHICK,Q789A6_CHICK,UPI00000FC6BE,UPI0000171380,UPI0000171381,UPI0000171382,UPI00003AB452,UPI00003AB453,UPI00003AB454,UPI000060F510,UPI000060F511,UPI0000ECA50D	TAX_GallusGallus_9031_20141114.fasta,UPARC14_2_GallusG_9031_20140417.fasta	215,888.80	100.00%	8	8	18	0.04%	8.01%	GGPITFSSSR	R	S	95.00%	30.3	44	27.7	19.6					0	1	0	0	2	1,008.51	1,869.00		125.86	1816	1825	Empty	TRUE	
20196-20225 HinckeM –TAXchicken	Uncategorized Sample	20196-20205_A	Mudpit_Ingel20196_MGFPeaklist (F058870)	Nonmuscle myosin heavy chain OS=Gallus gallus PE=2 SV=2	Q02015_CHICK,Q789A4_CHICK,Q789A5_CHICK,Q789A6_CHICK,UPI00000FC6BE,UPI0000171380,UPI0000171381,UPI0000171382,UPI00003AB452,UPI00003AB453,UPI00003AB454,UPI000060F510,UPI000060F511,UPI0000ECA50D	TAX_GallusGallus_9031_20141114.fasta,UPARC14_2_GallusG_9031_20140417.fasta	215,888.80	100.00%	8	8	18	0.04%	8.01%	IAQLEEQLEQEAKER	K	A	99.70%	59.3	44.3	25.4	45.9	46.4	4.08	0.369	0.4	0	0	2	0	2	1,813.93	2,231.00		777.93	1708	1722	Empty	TRUE	
20196-20225 HinckeM –TAXchicken	Uncategorized Sample	20196-20205_A	Mudpit_Ingel20196_MGFPeaklist (F058870)	Nonmuscle myosin heavy chain OS=Gallus gallus PE=2 SV=2	Q02015_CHICK,Q789A4_CHICK,Q789A5_CHICK,Q789A6_CHICK,UPI00000FC6BE,UPI0000171380,UPI0000171381,UPI0000171382,UPI00003AB452,UPI00003AB453,UPI00003AB454,UPI000060F510,UPI000060F511,UPI0000ECA50D	TAX_GallusGallus_9031_20141114.fasta,UPARC14_2_GallusG_9031_20140417.fasta	215,888.80	100.00%	8	8	18	0.04%	8.01%	IVFQEFR	R	Q	98.80%	47	43.1	42.2	19.8	27.9	0.585	0.222	0.5	0	3	0	0	2	938.5094	2,257.00		900.74	604	610	Empty	TRUE	MYH11_CHICK
20196-20225 HinckeM –TAXchicken	Uncategorized Sample	20196-20205_A	Mudpit_Ingel20196_MGFPeaklist (F058870)	Nonmuscle myosin heavy chain OS=Gallus gallus PE=2 SV=2	Q02015_CHICK,Q789A4_CHICK,Q789A5_CHICK,Q789A6_CHICK,UPI00000FC6BE,UPI0000171380,UPI0000171381,UPI0000171382,UPI00003AB452,UPI00003AB453,UPI00003AB454,UPI000060F510,UPI000060F511,UPI0000ECA50D	TAX_GallusGallus_9031_20141114.fasta,UPARC14_2_GallusG_9031_20140417.fasta	215,888.80	100.00%	8	8	18	0.04%	8.01%	KLDAQVQELTAK	K	V	13.90%	14	44	25.3	0.53					0	0	1	0	2	1,343.75	1,897.00		288.44	1141	1152	Empty	TRUE	
20196-20225 HinckeM –TAXchicken	Uncategorized Sample	20196-20205_A	Mudpit_Ingel20196_MGFPeaklist (F058870)	Nonmuscle myosin heavy chain OS=Gallus gallus PE=2 SV=2	Q02015_CHICK,Q789A4_CHICK,Q789A5_CHICK,Q789A6_CHICK,UPI00000FC6BE,UPI0000171380,UPI0000171381,UPI0000171382,UPI00003AB452,UPI00003AB453,UPI00003AB454,UPI000060F510,UPI000060F511,UPI0000ECA50D	TAX_GallusGallus_9031_20141114.fasta,UPARC14_2_GallusG_9031_20140417.fasta	215,888.80	100.00%	8	8	18	0.04%	8.01%	LDPHLVLDQLR	K	C	99.70%	34.6	43.8	18.6	22.1	32.5	2.47	0.366	0.455	0	0	1	0	2	1,318.75	2,534.00		1,121.62	575	585	Empty	FALSE	UPI000060E1E4,UPI000012FB7F,MYH9_CHICK,F1NCD4_CHICK,UPI0002C868F4
20196-20225 HinckeM –TAXchicken	Uncategorized Sample	20196-20205_A	Mudpit_Ingel20196_MGFPeaklist (F058870)	Nonmuscle myosin heavy chain OS=Gallus gallus PE=2 SV=2	Q02015_CHICK,Q789A4_CHICK,Q789A5_CHICK,Q789A6_CHICK,UPI00000FC6BE,UPI0000171380,UPI0000171381,UPI0000171382,UPI00003AB452,UPI00003AB453,UPI00003AB454,UPI000060F510,UPI000060F511,UPI0000ECA50D	TAX_GallusGallus_9031_20141114.fasta,UPARC14_2_GallusG_9031_20140417.fasta	215,888.80	100.00%	8	8	18	0.04%	8.01%	QRYEILTPNAIPK	R	G	99.70%	44.8	43.5	22.5	30.7	33.5	1.96	0.358	0.423	0	2	4	0	2	1,542.86	2,240.50		4,800.05	611	623	Empty	FALSE	UPI000060E1E4,UPI000012FB7F,MYH9_CHICK,F1NCD4_CHICK,UPI0002C868F4

20196-20225 HinckeM –TAXchicken	Uncategorized Sample	20196-20205_A	Mudpit_Ingel20196_MGFPeaklist (F058870)	Nonmuscle myosin heavy chain OS=Gallus gallus PE=2 SV=2	Q02015_CHICK,Q789A4_CHICK,Q789A5_CHICK,Q789A6_CHICK,UPI00000FC6BE,UPI0000171380,UPI0000171381,UPI0000171382,UPI00003AB452,UPI00003AB453,UPI00003AB454,UPI000060F510,UPI000060F511,UPI0000ECA50D	TAX_GallusGallus_9031_20141114.fasta,UPARC14_2_GallusG_9031_20140417.fasta	215,888.80	100.00%	8	8	18	0.04%	8.01%	YEILTPNAIPK	R	G	99.70%	20.6	44.4	14.6	7.9	28.5	1.57	0.277	0.318	0	3	0	0	2	1,258.70	2,337.00		1,618.82	613	623	Empty	FALSE	UPI000060E1E4,UPI000012FB7F,MYH9_CHICK,F1NCD4_CHICK,UPI0002C868F4
20196-20225 HinckeM –TAXchicken	Uncategorized Sample	20196-20205_A	Mudpit_Ingel20196_MGFPeaklist (F058870)	Hemoglobin subunit alpha-D OS=Gallus gallus GN=HBAD PE=1 SV=1	HBAD_CHICK	TAX_GallusGallus_9031_20141114.fasta	15,694.90	100.00%	22	32	376	0.76%	95.00%	AASHQEEFGAEALTR	K	M	99.70%	69.4	44.1	31.5	0	49.9	5.28	0.417	0.567	0	27	68	0	2	1,598.76	1,961.00		2.73E+07	17	31	Empty	TRUE	UPI000017122E
20196-20225 HinckeM –TAXchicken	Uncategorized Sample	20196-20205_A	Mudpit_Ingel20196_MGFPeaklist (F058870)	Hemoglobin subunit alpha-D OS=Gallus gallus GN=HBAD PE=1 SV=1	HBAD_CHICK	TAX_GallusGallus_9031_20141114.fasta	15,694.90	100.00%	22	32	376	0.76%	95.00%	DYTPEVHAAFDK	K	F	99.70%	56.8	44.1	16.6	53.7	38.6	4.51	0.402	0.542	0	4	3	0	2	1,392.64	1,958.00		5336610	116	127	Empty	TRUE	UPI000017122E
20196-20225 HinckeM –TAXchicken	Uncategorized Sample	20196-20205_A	Mudpit_Ingel20196_MGFPeaklist (F058870)	Hemoglobin subunit alpha-D OS=Gallus gallus GN=HBAD PE=1 SV=1	HBAD_CHICK	TAX_GallusGallus_9031_20141114.fasta	15,694.90	100.00%	22	32	376	0.76%	95.00%	DYTPEVHAAFDKFLSAVSAVLAEK	K	Y	99.70%	66.6	43.1	13.4	61.4	41.4	4.14	0.384	0.25	0	0	1	0	2	2,608.33	2,919.00		1143300	116	139	Empty	TRUE	UPI000017122E
20196-20225 HinckeM –TAXchicken	Uncategorized Sample	20196-20205_A	Mudpit_Ingel20196_MGFPeaklist (F058870)	Hemoglobin subunit alpha-D OS=Gallus gallus GN=HBAD PE=1 SV=1	HBAD_CHICK	TAX_GallusGallus_9031_20141114.fasta	15,694.90	100.00%	22	32	376	0.76%	95.00%	EFGAEALTR	E	M	97.80%					27	1.07	0.226	0.333	0	2	0	0	1	993.5	1,981.50		848.25	23	31	Empty	TRUE	
20196-20225 HinckeM –TAXchicken	Uncategorized Sample	20196-20205_A	Mudpit_Ingel20196_MGFPeaklist (F058870)	Hemoglobin subunit alpha-D OS=Gallus gallus GN=HBAD PE=1 SV=1	HBAD_CHICK	TAX_GallusGallus_9031_20141114.fasta	15,694.90	100.00%	22	32	376	0.76%	95.00%	FLSAVSAVLAEK	K	Y	99.70%	69.5	43.6	20.9	53.2	43.1	3.21	0.392	0.5	0	15	0	0	2	1,234.70	2,620.00		6919080	128	139	Empty	TRUE	UPI000017122E
20196-20225 HinckeM –TAXchicken	Uncategorized Sample	20196-20205_A	Mudpit_Ingel20196_MGFPeaklist (F058870)	Hemoglobin subunit alpha-D OS=Gallus gallus GN=HBAD PE=1 SV=1	HBAD_CHICK	TAX_GallusGallus_9031_20141114.fasta	15,694.90	100.00%	22	32	376	0.76%	95.00%	FLSAVSAVLAEKYR	K	-	99.70%	71.3	42.6	20.7	46.3	44.8	4.66	0.433	0.536	0	0	3	0	2	1,553.87	2,592.00		70,633.30	128	141	Empty	TRUE	UPI000017122E
20196-20225 HinckeM –TAXchicken	Uncategorized Sample	20196-20205_A	Mudpit_Ingel20196_MGFPeaklist (F058870)	Hemoglobin subunit alpha-D OS=Gallus gallus GN=HBAD PE=1 SV=1	HBAD_CHICK	TAX_GallusGallus_9031_20141114.fasta	15,694.90	100.00%	22	32	376	0.76%	95.00%	KLIQQAWEK	K	A	99.70%	48.3	44.3	46.8	27.9	40.2	1.7	0.234	0.667	0	4	0	0	2	1,143.65	1,866.50		9,047.07	8	16	Empty	TRUE	UPI000017122E
20196-20225 HinckeM –TAXchicken	Uncategorized Sample	20196-20205_A	Mudpit_Ingel20196_MGFPeaklist (F058870)	Hemoglobin subunit alpha-D OS=Gallus gallus GN=HBAD PE=1 SV=1	HBAD_CHICK	TAX_GallusGallus_9031_20141114.fasta	15,694.90	100.00%	22	32	376	0.76%	95.00%	KVLGALGNAVK	K	N	99.70%	65.8	41.6	37.6	40.9	49.8	4.21	0.458	0.682	0	20	0	0	2	1,070.66	1,904.00		63,983.80	61	71	Empty	TRUE	UPI000017122E
20196-20225 HinckeM –TAXchicken	Uncategorized Sample	20196-20205_A	Mudpit_Ingel20196_MGFPeaklist (F058870)	Hemoglobin subunit alpha-D OS=Gallus gallus GN=HBAD PE=1 SV=1	HBAD_CHICK	TAX_GallusGallus_9031_20141114.fasta	15,694.90	100.00%	22	32	376	0.76%	95.00%	LIQQAWEK	K	A	99.70%	52.1	45	34.6	13.2	30.3	0.357	0.116	0.438	0	43	0	0	2	1,015.56	1,910.00		500091	9	16	Empty	TRUE	UPI000017122E
20196-20225 HinckeM –TAXchicken	Uncategorized Sample	20196-20205_A	Mudpit_Ingel20196_MGFPeaklist (F058870)	Hemoglobin subunit alpha-D OS=Gallus gallus GN=HBAD PE=1 SV=1	HBAD_CHICK	TAX_GallusGallus_9031_20141114.fasta	15,694.90	100.00%	22	32	376	0.76%	95.00%	LLSQCIQVVLAVHMGK	K	D	99.70%	69.4	42.7	13	60.3	46	5.48	0.424	0.406	0	0	9	0	2	1,811.99	2,685.00		1793970	100	115	Empty	TRUE	
20196-20225 HinckeM –TAXchicken	Uncategorized Sample	20196-20205_A	Mudpit_Ingel20196_MGFPeaklist (F058870)	Hemoglobin subunit alpha-D OS=Gallus gallus GN=HBAD PE=1 SV=1	HBAD_CHICK	TAX_GallusGallus_9031_20141114.fasta	15,694.90	100.00%	22	32	376	0.76%	95.00%	LLSQCIQVVLAVHMGKDYTPEVHAAFDKFLSAVSAVLAEK	K	Y	74.70%	27.1	36.6	0	27.1					0	0	0	0	2	4,385.30	2,911.00		314730	100	139	Empty	TRUE	
20196-20225 HinckeM –TAXchicken	Uncategorized Sample	20196-20205_A	Mudpit_Ingel20196_MGFPeaklist (F058870)	Hemoglobin subunit alpha-D OS=Gallus gallus GN=HBAD PE=1 SV=1	HBAD_CHICK	TAX_GallusGallus_9031_20141114.fasta	15,694.90	100.00%	22	32	376	0.76%	95.00%	LRVDPVNFK	N	L	99.70%	54.6	44.5	27.1	37.1	44.3	3.82	0.424	0.722	0	34	0	0	1	1,087.63	2,070.50		3435130	91	99	Empty	FALSE	HBA_CHICK,HBPI_CHICK
20196-20225 HinckeM –TAXchicken	Uncategorized Sample	20196-20205_A	Mudpit_Ingel20196_MGFPeaklist (F058870)	Hemoglobin subunit alpha-D OS=Gallus gallus GN=HBAD PE=1 SV=1	HBAD_CHICK	TAX_GallusGallus_9031_20141114.fasta	15,694.90	100.00%	22	32	376	0.76%	95.00%	LSAVSAVLAEK	F	Y	98.40%					26.7	1.4	0.221	0.318	0	1	0	0	1	1,087.64	2,244.00		391	129	139	Empty	TRUE	
20196-20225 HinckeM –TAXchicken	Uncategorized Sample	20196-20205_A	Mudpit_Ingel20196_MGFPeaklist (F058870)	Hemoglobin subunit alpha-D OS=Gallus gallus GN=HBAD PE=1 SV=1	HBAD_CHICK	TAX_GallusGallus_9031_20141114.fasta	15,694.90	100.00%	22	32	376	0.76%	95.00%	MFTTYPQTK	R	T	99.70%	51.6	44.7	25.2	41	38.8	2.8	0.345	0.611	0	57	0	0	2	1,132.53	1,741.00		191880	32	40	Empty	TRUE	
20196-20225 HinckeM –TAXchicken	Uncategorized Sample	20196-20205_A	Mudpit_Ingel20196_MGFPeaklist (F058870)	Hemoglobin subunit alpha-D OS=Gallus gallus GN=HBAD PE=1 SV=1	HBAD_CHICK	TAX_GallusGallus_9031_20141114.fasta	15,694.90	100.00%	22	32	376	0.76%	95.00%	NVDNLSQAMAELSNLHAYNLR	K	V	99.70%	75.3	44.4	16.1	0	50.2	6.42	0.498	0.333	0	0	16	0	2	2,389.16	2,716.00		1.10E+07	72	92	Empty	TRUE	
20196-20225 HinckeM –TAXchicken	Uncategorized Sample	20196-20205_A	Mudpit_Ingel20196_MGFPeaklist (F058870)	Hemoglobin subunit alpha-D OS=Gallus gallus GN=HBAD PE=1 SV=1	HBAD_CHICK	TAX_GallusGallus_9031_20141114.fasta	15,694.90	100.00%	22	32	376	0.76%	95.00%	PEVHAAFDK	T	F	99.70%					50.5	4.1	0.337	0.833	0	2	0	0	1	1,013.51	1,930.00		59,102.00	119	127	Empty	TRUE	
20196-20225 HinckeM –TAXchicken	Uncategorized Sample	20196-20205_A	Mudpit_Ingel20196_MGFPeaklist (F058870)	Hemoglobin subunit alpha-D OS=Gallus gallus GN=HBAD PE=1 SV=1	HBAD_CHICK	TAX_GallusGallus_9031_20141114.fasta	15,694.90	100.00%	22	32	376	0.76%	95.00%	RMFTTYPQTK	T	T	97.00%					29	1.7	0.279	0.35	0	3	0	0	1	1,255.61	1,900.00		34,867.50	31	40	Empty	TRUE	
20196-20225 HinckeM –TAXchicken	Uncategorized Sample	20196-20205_A	Mudpit_Ingel20196_MGFPeaklist (F058870)	Hemoglobin subunit alpha-D OS=Gallus gallus GN=HBAD PE=1 SV=1	HBAD_CHICK	TAX_GallusGallus_9031_20141114.fasta	15,694.90	100.00%	22	32	376	0.76%	95.00%	SAVLAEKYR	V	-	95.30%					36.6	1.16	0.0902	0.611	0	1	0	0	1	1,036.58	2,495.00		38,122.20	133	141	Empty	TRUE	
20196-20225 HinckeM –TAXchicken	Uncategorized Sample	20196-20205_A	Mudpit_Ingel20196_MGFPeaklist (F058870)	Hemoglobin subunit alpha-D OS=Gallus gallus GN=HBAD PE=1 SV=1	HBAD_CHICK	TAX_GallusGallus_9031_20141114.fasta	15,694.90	100.00%	22	32	376	0.76%	95.00%	SHQEEFGAEALTR	A	M	99.70%					53.4	5.1	0.404	0.615	0	1	2	0	1	1,474.69	1,933.00		18,360.20	19	31	Empty	TRUE	
20196-20225 HinckeM –TAXchicken	Uncategorized Sample	20196-20205_A	Mudpit_Ingel20196_MGFPeaklist (F058870)	Hemoglobin subunit alpha-D OS=Gallus gallus GN=HBAD PE=1 SV=1	HBAD_CHICK	TAX_GallusGallus_9031_20141114.fasta	15,694.90	100.00%	22	32	376	0.76%	95.00%	TYFPHFDLSPGSDQVR	K	G	99.70%	79.3	44.3	18.9	15.8	49.2	5.32	0.463	0.5	0	0	29	0	2	1,865.88	2,509.00		1.75E+07	41	56	Empty	TRUE	UPI000017122E
20196-20225 HinckeM –TAXchicken	Uncategorized Sample	20196-20205_A	Mudpit_Ingel20196_MGFPeaklist (F058870)	Hemoglobin subunit alpha-D OS=Gallus gallus GN=HBAD PE=1 SV=1	HBAD_CHICK	TAX_GallusGallus_9031_20141114.fasta	15,694.90	100.00%	22	32	376	0.76%	95.00%	TYFPHFDLSPGSDQVRGHGK	K	K	99.70%	42.4	44	15.8	9.16	42.5	4.77	0.409	0.35	0	0	0	2	2	2,245.08	2,329.50		3,671.14	41	60	Empty	TRUE	UPI000017122E
20196-20225 HinckeM –TAXchicken	Uncategorized Sample	20196-20205_A	Mudpit_Ingel20196_MGFPeaklist (F058870)	Hemoglobin subunit alpha-D OS=Gallus gallus GN=HBAD PE=1 SV=1	HBAD_CHICK	TAX_GallusGallus_9031_20141114.fasta	15,694.90	100.00%	22	32	376	0.76%	95.00%	VDPVNFK	R	L	98.80%	66.1	45.6	44.3	36.6	34.6	2.42	0.266	0.5	0	71	0	0	2	818.4407	1,898.00		430355	93	99	Empty	FALSE	HBA_CHICK,UPI000017122E,HBPI_CHICK
20196-20225 HinckeM –TAXchicken	Uncategorized Sample	20196-20205_A	Mudpit_Ingel20196_MGFPeaklist (F058870)	Hemoglobin subunit alpha-D OS=Gallus gallus GN=HBAD PE=1 SV=1	HBAD_CHICK	TAX_GallusGallus_9031_20141114.fasta	15,694.90	100.00%	22	32	376	0.76%	95.00%	VDPVNFKLLSQ	R	C	97.30%					30.1	0.495	0.193	0.364	0	1	0	0	1	1,259.70	2,539.00		571.4	93	103	Empty	TRUE	
20196-20225 HinckeM –TAXchicken	Uncategorized Sample	20196-20205_A	Mudpit_Ingel20196_MGFPeaklist (F058870)	Hemoglobin subunit alpha-D OS=Gallus gallus GN=HBAD PE=1 SV=1	HBAD_CHICK	TAX_GallusGallus_9031_20141114.fasta	15,694.90	100.00%	22	32	376	0.76%	95.00%	VLGALGNAVK	K	N	99.70%	62.8	42.6	38.8	26.5	48.5	2.44	0.355	0.7	0	61	0	0	2	942.562	2,055.00		1255410	62	71	Empty	TRUE	UPI000017122E
20196-20225 HinckeM –TAXchicken	Uncategorized Sample	20196-20205_A	Mudpit_Ingel20196_MGFPeaklist (F058870)	Hemoglobin subunit alpha-A OS=Gallus gallus GN=HBAA PE=1 SV=2	HBA_CHICK	TAX_GallusGallus_9031_20141114.fasta	15,429.00	100.00%	29	43	679	1.37%	92.30%	AANHIDDIAGTLSK	E	L	99.70%					46.5	4.77	0.381	0.5	0	1	0	0	1	1,425.73	1,945.00		8,757.37	70	83	Empty	TRUE	
20196-20225 HinckeM –TAXchicken	Uncategorized Sample	20196-20205_A	Mudpit_Ingel20196_MGFPeaklist (F058870)	Hemoglobin subunit alpha-A OS=Gallus gallus GN=HBAA PE=1 SV=2	HBA_CHICK	TAX_GallusGallus_9031_20141114.fasta	15,429.00	100.00%	29	43	679	1.37%	92.30%	AEEYGAETLER	H	M	99.70%					41	3.06	0.307	0.5	0	2	0	0	1	1,267.58	1,807.00		6,879.73	22	32	Empty	TRUE	
20196-20225 HinckeM –TAXchicken	Uncategorized Sample	20196-20205_A	Mudpit_Ingel20196_MGFPeaklist (F058870)	Hemoglobin subunit alpha-A OS=Gallus gallus GN=HBAA PE=1 SV=2	HBA_CHICK	TAX_GallusGallus_9031_20141114.fasta	15,429.00	100.00%	29	43	679	1.37%	92.30%	EAANHIDDIAGTLSK	I	L	99.70%					53.7	4.89	0.501	0.533	0	2	0	0	1	1,554.78	2,835.50		75,163.40	69	83	Empty	TRUE	
20196-20225 HinckeM –TAXchicken	Uncategorized Sample	20196-20205_A	Mudpit_Ingel20196_MGFPeaklist (F058870)	Hemoglobin subunit alpha-A OS=Gallus gallus GN=HBAA PE=1 SV=2	HBA_CHICK	TAX_GallusGallus_9031_20141114.fasta	15,429.00	100.00%	29	43	679	1.37%	92.30%	FLCAVGTVLTAK	K	Y	99.70%	64.9	43.6	17	54.8	45.5	4.74	0.429	0.542	0	33	0	0	2	1,279.71	2,476.00		9530240	129	140	Empty	TRUE	
20196-20225 HinckeM –TAXchicken	Uncategorized Sample	20196-20205_A	Mudpit_Ingel20196_MGFPeaklist (F058870)	Hemoglobin subunit alpha-A OS=Gallus gallus GN=HBAA PE=1 SV=2	HBA_CHICK	TAX_GallusGallus_9031_20141114.fasta	15,429.00	100.00%	29	43	679	1.37%	92.30%	FPHFDLSHGSAQIK	Y	G	99.70%					33.6	2.43	0.336	0.429	0	0	1	0	1	1,583.80	2,400.00		1,512.20	44	57	Empty	TRUE	
20196-20225 HinckeM –TAXchicken	Uncategorized Sample	20196-20205_A	Mudpit_Ingel20196_MGFPeaklist (F058870)	Hemoglobin subunit alpha-A OS=Gallus gallus GN=HBAA PE=1 SV=2	HBA_CHICK	TAX_GallusGallus_9031_20141114.fasta	15,429.00	100.00%	29	43	679	1.37%	92.30%	GHAEEYGAETLER	A	M	99.70%					55.3	9.15	0.465	0.615	0	1	0	0	1	1,461.66	1,845.00		3,014.07	20	32	Empty	TRUE	
20196-20225 HinckeM –TAXchicken	Uncategorized Sample	20196-20205_A	Mudpit_Ingel20196_MGFPeaklist (F058870)	Hemoglobin subunit alpha-A OS=Gallus gallus GN=HBAA PE=1 SV=2	HBA_CHICK	TAX_GallusGallus_9031_20141114.fasta	15,429.00	100.00%	29	43	679	1.37%	92.30%	GHGKKVVAALIEAANHIDDIAGTLSK	K	L	99.70%	79.2	39.2	13.9	79.2	62	9.89	0.577	0.404	0	0	0	2	2	2,628.45	2,716.50		354105	58	83	Empty	TRUE	
20196-20225 HinckeM –TAXchicken	Uncategorized Sample	20196-20205_A	Mudpit_Ingel20196_MGFPeaklist (F058870)	Hemoglobin subunit alpha-A OS=Gallus gallus GN=HBAA PE=1 SV=2	HBA_CHICK	TAX_GallusGallus_9031_20141114.fasta	15,429.00	100.00%	29	43	679	1.37%	92.30%	GIFTKIAGHAEEYGAETLER	K	M	99.70%	103	44.2	15.5	97.6	68.6	11.5	0.633	0.5	0	0	2	0	2	2,192.10	2,397.00		292059	13	32	Empty	TRUE	
20196-20225 HinckeM –TAXchicken	Uncategorized Sample	20196-20205_A	Mudpit_Ingel20196_MGFPeaklist (F058870)	Hemoglobin subunit alpha-A OS=Gallus gallus GN=HBAA PE=1 SV=2	HBA_CHICK	TAX_GallusGallus_9031_20141114.fasta	15,429.00	100.00%	29	43	679	1.37%	92.30%	HAEEYGAETLER	G	M	99.70%					34.3	4.07	0.3	0.458	0	0	2	0	1	1,404.64	1,782.00		1,197.51	21	32	Empty	TRUE	
20196-20225 HinckeM –TAXchicken	Uncategorized Sample	20196-20205_A	Mudpit_Ingel20196_MGFPeaklist (F058870)	Hemoglobin subunit alpha-A OS=Gallus gallus GN=HBAA PE=1 SV=2	HBA_CHICK	TAX_GallusGallus_9031_20141114.fasta	15,429.00	100.00%	29	43	679	1.37%	92.30%	IAGHAEEYGAETLER	K	M	99.70%	68.8	44.5	28.6	51.5	49.8	4.96	0.42	0.567	0	36	215	3	2	1,645.78	2,133.50		7.56E+07	18	32	Empty	TRUE	
20196-20225 HinckeM –TAXchicken	Uncategorized Sample	20196-20205_A	Mudpit_Ingel20196_MGFPeaklist (F058870)	Hemoglobin subunit alpha-A OS=Gallus gallus GN=HBAA PE=1 SV=2	HBA_CHICK	TAX_GallusGallus_9031_20141114.fasta	15,429.00	100.00%	29	43	679	1.37%	92.30%	IDDIAGTLSK	H	L	95.60%					28.4	1.55	0.215	0.35	0	1	0	0	1	1,032.56	1,992.00		616.14	74	83	Empty	TRUE	
20196-20225 HinckeM –TAXchicken	Uncategorized Sample	20196-20205_A	Mudpit_Ingel20196_MGFPeaklist (F058870)	Hemoglobin subunit alpha-A OS=Gallus gallus GN=HBAA PE=1 SV=2	HBA_CHICK	TAX_GallusGallus_9031_20141114.fasta	15,429.00	100.00%	29	43	679	1.37%	92.30%	IEAANHIDDIAGTLSK	L	L	99.70%					38.9	3.59	0.301	0.344	0	0	1	0	1	1,667.86	2,219.00		1,311.82	68	83	Empty	TRUE	
20196-20225 HinckeM –TAXchicken	Uncategorized Sample	20196-20205_A	Mudpit_Ingel20196_MGFPeaklist (F058870)	Hemoglobin subunit alpha-A OS=Gallus gallus GN=HBAA PE=1 SV=2	HBA_CHICK	TAX_GallusGallus_9031_20141114.fasta	15,429.00	100.00%	29	43	679	1.37%	92.30%	KVVAALIEAAN	K	H	99.30%					28.8	1.85	0.26	0.364	0	1	0	0	1	1,098.65	2,144.00		542.68	62	72	Empty	TRUE	
20196-20225 HinckeM –TAXchicken	Uncategorized Sample	20196-20205_A	Mudpit_Ingel20196_MGFPeaklist (F058870)	Hemoglobin subunit alpha-A OS=Gallus gallus GN=HBAA PE=1 SV=2	HBA_CHICK	TAX_GallusGallus_9031_20141114.fasta	15,429.00	100.00%	29	43	679	1.37%	92.30%	KVVAALIEAANHIDDIAGTLSK	K	L	99.70%	77.4	40.2	13.2	77.4	55.8	7.72	0.548	0.409	0	0	11	2	2	2,249.25	2,812.00		1.10E+07	62	83	Empty	TRUE	
20196-20225 HinckeM –TAXchicken	Uncategorized Sample	20196-20205_A	Mudpit_Ingel20196_MGFPeaklist (F058870)	Hemoglobin subunit alpha-A OS=Gallus gallus GN=HBAA PE=1 SV=2	HBA_CHICK	TAX_GallusGallus_9031_20141114.fasta	15,429.00	100.00%	29	43	679	1.37%	92.30%	LLGQCFLVVVAIHHPAALTPEVHASLDK	K	F	99.70%	82.5	38.4	0	82.5	39.4	4.43	0.322	0.196	0	0	0	3	2	3,035.65	2,726.00		4580310	101	128	Empty	TRUE	
20196-20225 HinckeM –TAXchicken	Uncategorized Sample	20196-20205_A	Mudpit_Ingel20196_MGFPeaklist (F058870)	Hemoglobin subunit alpha-A OS=Gallus gallus GN=HBAA PE=1 SV=2	HBA_CHICK	TAX_GallusGallus_9031_20141114.fasta	15,429.00	100.00%	29	43	679	1.37%	92.30%	LLGQCFLVVVAIHHPAALTPEVHASLDKFLCAVGTVLTAK	K	Y	99.70%	63.9	32.3	13.1	63.9					0	0	0	0	2	4,296.34	2,907.50		3320500	101	140	Empty	TRUE	
20196-20225 HinckeM –TAXchicken	Uncategorized Sample	20196-20205_A	Mudpit_Ingel20196_MGFPeaklist (F058870)	Hemoglobin subunit alpha-A OS=Gallus gallus GN=HBAA PE=1 SV=2	HBA_CHICK	TAX_GallusGallus_9031_20141114.fasta	15,429.00	100.00%	29	43	679	1.37%	92.30%	LRVDPVNFK	K	L	99.70%	54.6	44.5	27.1	37.1	44.3	3.82	0.424	0.722	0	41	0	0	2	1,087.63	2,071.00		3451980	92	100	Empty	TRUE	HBAD_CHICK,HBPI_CHICK
20196-20225 HinckeM –TAXchicken	Uncategorized Sample	20196-20205_A	Mudpit_Ingel20196_MGFPeaklist (F058870)	Hemoglobin subunit alpha-A OS=Gallus gallus GN=HBAA PE=1 SV=2	HBA_CHICK	TAX_GallusGallus_9031_20141114.fasta	15,429.00	100.00%	29	43	679	1.37%	92.30%	MFTTYPPTK	R	T	99.70%	67.7	44.6	25.8	49.6	37.2	3.72	0.384	0.611	0	188	0	0	2	1,083.52	1,822.00		2067460	33	41	Empty	TRUE	
20196-20225 HinckeM –TAXchicken	Uncategorized Sample	20196-20205_A	Mudpit_Ingel20196_MGFPeaklist (F058870)	Hemoglobin subunit alpha-A OS=Gallus gallus GN=HBAA PE=1 SV=2	HBA_CHICK	TAX_GallusGallus_9031_20141114.fasta	15,429.00	100.00%	29	43	679	1.37%	92.30%	NHIDDIAGTLSK	A	L	99.70%					44.7	4.62	0.447	0.583	0	1	0	0	1	1,283.66	1,939.00		41,707.30	72	83	Empty	TRUE	
20196-20225 HinckeM –TAXchicken	Uncategorized Sample	20196-20205_A	Mudpit_Ingel20196_MGFPeaklist (F058870)	Hemoglobin subunit alpha-A OS=Gallus gallus GN=HBAA PE=1 SV=2	HBA_CHICK	TAX_GallusGallus_9031_20141114.fasta	15,429.00	100.00%	29	43	679	1.37%	92.30%	RMFTTYPPTK	E	T	83.10%					30.2	0.658	0.169	0.35	0	1	0	0	1	1,224.61	1,930.00		17,890.70	32	41	Empty	TRUE	
20196-20225 HinckeM –TAXchicken	Uncategorized Sample	20196-20205_A	Mudpit_Ingel20196_MGFPeaklist (F058870)	Hemoglobin subunit alpha-A OS=Gallus gallus GN=HBAA PE=1 SV=2	HBA_CHICK	TAX_GallusGallus_9031_20141114.fasta	15,429.00	100.00%	29	43	679	1.37%	92.30%	TYFPHFDLSHGSAQIK	K	G	99.70%	43.5	44.7	13	39	41.1	5.28	0.433	0.5	0	0	7	14	2	1,847.91	2,389.00		1.63E+07	42	57	Empty	TRUE	
20196-20225 HinckeM –TAXchicken	Uncategorized Sample	20196-20205_A	Mudpit_Ingel20196_MGFPeaklist (F058870)	Hemoglobin subunit alpha-A OS=Gallus gallus GN=HBAA PE=1 SV=2	HBA_CHICK	TAX_GallusGallus_9031_20141114.fasta	15,429.00	100.00%	29	43	679	1.37%	92.30%	TYFPHFDLSHGSAQIKGHGK	K	K	99.70%	55.1	44.2	0	55.1	41.1	5.24	0.392	0.375	0	0	0	2	2	2,227.10	2,135.00		339388	42	61	Empty	TRUE	
20196-20225 HinckeM –TAXchicken	Uncategorized Sample	20196-20205_A	Mudpit_Ingel20196_MGFPeaklist (F058870)	Hemoglobin subunit alpha-A OS=Gallus gallus GN=HBAA PE=1 SV=2	HBA_CHICK	TAX_GallusGallus_9031_20141114.fasta	15,429.00	100.00%	29	43	679	1.37%	92.30%	TYFPHFDLSHGSAQIKGHGKK	K	V	99.40%	36.1	43.9	0	36.1					0	0	0	0	2	2,355.20	1,940.00		118234	42	62	Empty	TRUE	
20196-20225 HinckeM –TAXchicken	Uncategorized Sample	20196-20205_A	Mudpit_Ingel20196_MGFPeaklist (F058870)	Hemoglobin subunit alpha-A OS=Gallus gallus GN=HBAA PE=1 SV=2	HBA_CHICK	TAX_GallusGallus_9031_20141114.fasta	15,429.00	100.00%	29	43	679	1.37%	92.30%	VDPVNFK	R	L	98.80%	66.1	45.6	44.3	36.6	34.6	2.42	0.266	0.5	0	71	0	0	2	818.4407	1,898.00		430355	94	100	Empty	TRUE	HBAD_CHICK,UPI000017122E,HBPI_CHICK
20196-20225 HinckeM –TAXchicken	Uncategorized Sample	20196-20205_A	Mudpit_Ingel20196_MGFPeaklist (F058870)	Hemoglobin subunit alpha-A OS=Gallus gallus GN=HBAA PE=1 SV=2	HBA_CHICK	TAX_GallusGallus_9031_20141114.fasta	15,429.00	100.00%	29	43	679	1.37%	92.30%	VDPVNFKLLGQCFLVVVAIHHPAALTPEVHASLDKFLCAVGTVLTAK	R	Y	98.80%	42.3	30.1	0	42.3					0	0	0	0	2	5,095.76	2,912.00		317631	94	140	Empty	TRUE	
20196-20225 HinckeM –TAXchicken	Uncategorized Sample	20196-20205_A	Mudpit_Ingel20196_MGFPeaklist (F058870)	Hemoglobin subunit alpha-A OS=Gallus gallus GN=HBAA PE=1 SV=2	HBA_CHICK	TAX_GallusGallus_9031_20141114.fasta	15,429.00	100.00%	29	43	679	1.37%	92.30%	VLSAADKNNVK	M	G	99.70%	64.2	44.5	24.2	0	46.7	3.52	0.385	0.591	0	6	0	0	1	1,158.65	1,425.50		2,087.33	2	12	Empty	TRUE	
20196-20225 HinckeM –TAXchicken	Uncategorized Sample	20196-20205_A	Mudpit_Ingel20196_MGFPeaklist (F058870)	Hemoglobin subunit alpha-A OS=Gallus gallus GN=HBAA PE=1 SV=2	HBA_CHICK	TAX_GallusGallus_9031_20141114.fasta	15,429.00	100.00%	29	43	679	1.37%	92.30%	VLSAADKNNVKGIFTK	M	I	99.70%	88.1	42.2	14	86.7	51.3	5.42	0.53	0.5	0	0	4	0	1	1,704.96	1,964.00		121913	2	17	Empty	TRUE	
20196-20225 HinckeM –TAXchicken	Uncategorized Sample	20196-20205_A	Mudpit_Ingel20196_MGFPeaklist (F058870)	Hemoglobin subunit alpha-A OS=Gallus gallus GN=HBAA PE=1 SV=2	HBA_CHICK	TAX_GallusGallus_9031_20141114.fasta	15,429.00	100.00%	29	43	679	1.37%	92.30%	VVAALIEAANHID	K	D	99.70%					37	2.08	0.292	0.423	0	1	0	0	1	1,335.73	2,481.00		26,610.60	63	75	Empty	TRUE	
20196-20225 HinckeM –TAXchicken	Uncategorized Sample	20196-20205_A	Mudpit_Ingel20196_MGFPeaklist (F058870)	Hemoglobin subunit alpha-A OS=Gallus gallus GN=HBAA PE=1 SV=2	HBA_CHICK	TAX_GallusGallus_9031_20141114.fasta	15,429.00	100.00%	29	43	679	1.37%	92.30%	VVAALIEAANHIDDIAGTLSK	K	L	99.70%	64	42.3	13	60.8	43.2	4.68	0.435	0.333	0	2	15	0	2	2,121.16	2,877.00		5061400	63	83	Empty	TRUE	
20196-20225 HinckeM –TAXchicken	Uncategorized Sample	20196-20205_A	Mudpit_Ingel20196_MGFPeaklist (F058870)	Hemoglobin subunit pi OS=Gallus gallus PE=1 SV=3	HBPI_CHICK	TAX_GallusGallus_9031_20141114.fasta	15,709.40	100.00%	5	8	10	0.02%	51.40%	FLSSISSVLTEK	K	Y	99.70%	68.8	43.4	31	55.6	43.4	3.96	0.449	0.5	0	1	0	0	2	1,310.72	2,523.00		32,544.70	129	140	Empty	TRUE	
20196-20225 HinckeM –TAXchicken	Uncategorized Sample	20196-20205_A	Mudpit_Ingel20196_MGFPeaklist (F058870)	Hemoglobin subunit pi OS=Gallus gallus PE=1 SV=3	HBPI_CHICK	TAX_GallusGallus_9031_20141114.fasta	15,709.40	100.00%	5	8	10	0.02%	51.40%	LLSHCILCSVAAR	K	Y	99.70%	70	44.2	20.3	54	43	4.04	0.426	0.577	0	1	1	0	2	1,499.78	2,170.00		81,851.30	101	113	Empty	TRUE	
20196-20225 HinckeM –TAXchicken	Uncategorized Sample	20196-20205_A	Mudpit_Ingel20196_MGFPeaklist (F058870)	Hemoglobin subunit pi OS=Gallus gallus PE=1 SV=3	HBPI_CHICK	TAX_GallusGallus_9031_20141114.fasta	15,709.40	100.00%	5	8	10	0.02%	51.40%	LRVDPVNFK	I	L	99.70%	54.6	44.5	27.1	37.1	44.3	3.82	0.424	0.722	0	34	0	0	1	1,087.63	2,070.50		3435130	92	100	Empty	FALSE	HBAD_CHICK,HBA_CHICK
20196-20225 HinckeM –TAXchicken	Uncategorized Sample	20196-20205_A	Mudpit_Ingel20196_MGFPeaklist (F058870)	Hemoglobin subunit pi OS=Gallus gallus PE=1 SV=3	HBPI_CHICK	TAX_GallusGallus_9031_20141114.fasta	15,709.40	100.00%	5	8	10	0.02%	51.40%	LSELHAYILR	K	V	99.70%	37	43.8	26.6	26.3	51.4	4.77	0.286	0.75	0	1	0	0	2	1,214.69	2,176.00		39,693.50	84	93	Empty	TRUE	
20196-20225 HinckeM –TAXchicken	Uncategorized Sample	20196-20205_A	Mudpit_Ingel20196_MGFPeaklist (F058870)	Hemoglobin subunit pi OS=Gallus gallus PE=1 SV=3	HBPI_CHICK	TAX_GallusGallus_9031_20141114.fasta	15,709.40	100.00%	5	8	10	0.02%	51.40%	TYFPHFDVSQGSVQLR	K	G	99.70%	101	44.4	15.8	97.7	52.7	5.21	0.526	0.5	0	1	2	0	2	1,880.93	2,373.00		254560	42	57	Empty	TRUE	
20196-20225 HinckeM –TAXchicken	Uncategorized Sample	20196-20205_A	Mudpit_Ingel20196_MGFPeaklist (F058870)	Hemoglobin subunit pi OS=Gallus gallus PE=1 SV=3	HBPI_CHICK	TAX_GallusGallus_9031_20141114.fasta	15,709.40	100.00%	5	8	10	0.02%	51.40%	VATQIESIGLESLER	K	L	99.70%	65.6	44.4	28.8	44.2	48.1	6.51	0.441	0.433	0	3	0	0	2	1,644.88	2,506.00		196047	18	32	Empty	TRUE	
20196-20225 HinckeM –TAXchicken	Uncategorized Sample	20196-20205_A	Mudpit_Ingel20196_MGFPeaklist (F058870)	Hemoglobin subunit pi OS=Gallus gallus PE=1 SV=3	HBPI_CHICK	TAX_GallusGallus_9031_20141114.fasta	15,709.40	100.00%	5	8	10	0.02%	51.40%	VDPVNFK	R	L	98.80%	66.1	45.6	44.3	36.6	34.6	2.42	0.266	0.5	0	71	0	0	2	818.4407	1,898.00		430355	94	100	Empty	FALSE	HBAD_CHICK,HBA_CHICK,UPI000017122E
20196-20225 HinckeM –TAXchicken	Uncategorized Sample	20196-20205_A	Mudpit_Ingel20196_MGFPeaklist (F058870)	status=active	UPI000017122E	UPARC14_2_GallusG_9031_20140417.fasta	15,747.00	99.60%	3	5	9	0.02%	77.30%	AASHQEEFGAEALTR	K	M	99.70%	69.4	44.1	31.5	0	49.9	5.28	0.417	0.567	0	27	67	0	2	1,616.77	1,968.00		2.73E+07	17	31	Empty	FALSE	HBAD_CHICK
20196-20225 HinckeM –TAXchicken	Uncategorized Sample	20196-20205_A	Mudpit_Ingel20196_MGFPeaklist (F058870)	status=active	UPI000017122E	UPARC14_2_GallusG_9031_20140417.fasta	15,747.00	99.60%	3	5	9	0.02%	77.30%	DYTPEVHAAFDK	K	F	99.70%	56.8	44.1	16.6	53.7	38.6	4.51	0.402	0.542	0	4	3	0	2	1,392.64	1,958.00		5336610	116	127	Empty	FALSE	HBAD_CHICK
20196-20225 HinckeM –TAXchicken	Uncategorized Sample	20196-20205_A	Mudpit_Ingel20196_MGFPeaklist (F058870)	status=active	UPI000017122E	UPARC14_2_GallusG_9031_20140417.fasta	15,747.00	99.60%	3	5	9	0.02%	77.30%	DYTPEVHAAFDKFLSAVSAVLAEK	K	Y	99.70%	66.6	43.1	13.4	61.4	41.4	4.14	0.384	0.25	0	0	1	0	2	2,608.33	2,919.00		1143300	116	139	Empty	FALSE	HBAD_CHICK
20196-20225 HinckeM –TAXchicken	Uncategorized Sample	20196-20205_A	Mudpit_Ingel20196_MGFPeaklist (F058870)	status=active	UPI000017122E	UPARC14_2_GallusG_9031_20140417.fasta	15,747.00	99.60%	3	5	9	0.02%	77.30%	EVDNLSQAMAELSNLHAYNLR	K	V	7.90%	20.8	43.9	15.8	0					0	0	1	0	2	2,405.14	2,336.00		548.17	72	92	Empty	TRUE	
20196-20225 HinckeM –TAXchicken	Uncategorized Sample	20196-20205_A	Mudpit_Ingel20196_MGFPeaklist (F058870)	status=active	UPI000017122E	UPARC14_2_GallusG_9031_20140417.fasta	15,747.00	99.60%	3	5	9	0.02%	77.30%	FLSAVSAVLAEK	K	Y	99.70%	69.5	43.6	20.9	53.2	43.1	3.21	0.392	0.5	0	15	0	0	2	1,234.70	2,620.00		6919080	128	139	Empty	FALSE	HBAD_CHICK
20196-20225 HinckeM –TAXchicken	Uncategorized Sample	20196-20205_A	Mudpit_Ingel20196_MGFPeaklist (F058870)	status=active	UPI000017122E	UPARC14_2_GallusG_9031_20140417.fasta	15,747.00	99.60%	3	5	9	0.02%	77.30%	FLSAVSAVLAEKYR	K	-	99.70%	71.3	42.6	20.7	46.3	44.8	4.66	0.433	0.536	0	0	3	0	2	1,553.87	2,592.00		70,633.30	128	141	Empty	FALSE	HBAD_CHICK
20196-20225 HinckeM –TAXchicken	Uncategorized Sample	20196-20205_A	Mudpit_Ingel20196_MGFPeaklist (F058870)	status=active	UPI000017122E	UPARC14_2_GallusG_9031_20140417.fasta	15,747.00	99.60%	3	5	9	0.02%	77.30%	KLIQQAWEK	K	A	99.70%	48.3	44.3	46.8	27.9	40.2	1.7	0.234	0.667	0	4	0	0	2	1,143.65	1,866.50		9,047.07	8	16	Empty	FALSE	HBAD_CHICK
20196-20225 HinckeM –TAXchicken	Uncategorized Sample	20196-20205_A	Mudpit_Ingel20196_MGFPeaklist (F058870)	status=active	UPI000017122E	UPARC14_2_GallusG_9031_20140417.fasta	15,747.00	99.60%	3	5	9	0.02%	77.30%	KVLGALGNAVK	K	X	99.70%	65.8	41.6	37.6	40.9	49.8	4.21	0.458	0.682	0	20	0	0	2	1,070.66	1,904.00		63,983.80	61	71	Empty	FALSE	HBAD_CHICK
20196-20225 HinckeM –TAXchicken	Uncategorized Sample	20196-20205_A	Mudpit_Ingel20196_MGFPeaklist (F058870)	status=active	UPI000017122E	UPARC14_2_GallusG_9031_20140417.fasta	15,747.00	99.60%	3	5	9	0.02%	77.30%	LIQQAWEK	K	A	99.70%	52.1	45	34.6	13.2	30.3	0.357	0.116	0.438	0	43	0	0	2	1,015.56	1,910.00		500091	9	16	Empty	FALSE	HBAD_CHICK
20196-20225 HinckeM –TAXchicken	Uncategorized Sample	20196-20205_A	Mudpit_Ingel20196_MGFPeaklist (F058870)	status=active	UPI000017122E	UPARC14_2_GallusG_9031_20140417.fasta	15,747.00	99.60%	3	5	9	0.02%	77.30%	RVDNLSQAMAELSNLHAYNLR	K	V	99.30%	54.1	44	16.7	50.5					0	0	2	2	2	2,431.21	2,509.50		668432	72	92	Empty	TRUE	
20196-20225 HinckeM –TAXchicken	Uncategorized Sample	20196-20205_A	Mudpit_Ingel20196_MGFPeaklist (F058870)	status=active	UPI000017122E	UPARC14_2_GallusG_9031_20140417.fasta	15,747.00	99.60%	3	5	9	0.02%	77.30%	TYFPHFDLSPGSDQVR	K	G	99.70%	79.3	44.3	18.9	15.8	49.2	5.32	0.463	0.5	0	0	29	0	2	1,865.88	2,509.00		1.75E+07	41	56	Empty	FALSE	HBAD_CHICK
20196-20225 HinckeM –TAXchicken	Uncategorized Sample	20196-20205_A	Mudpit_Ingel20196_MGFPeaklist (F058870)	status=active	UPI000017122E	UPARC14_2_GallusG_9031_20140417.fasta	15,747.00	99.60%	3	5	9	0.02%	77.30%	TYFPHFDLSPGSDQVRGHGK	K	K	99.70%	42.4	44	15.8	9.16	42.5	4.77	0.409	0.35	0	0	0	2	2	2,245.08	2,329.50		3,671.14	41	60	Empty	FALSE	HBAD_CHICK
20196-20225 HinckeM –TAXchicken	Uncategorized Sample	20196-20205_A	Mudpit_Ingel20196_MGFPeaklist (F058870)	status=active	UPI000017122E	UPARC14_2_GallusG_9031_20140417.fasta	15,747.00	99.60%	3	5	9	0.02%	77.30%	VDPVNFK	R	L	98.80%	66.1	45.6	44.3	36.6	34.6	2.42	0.266	0.5	0	71	0	0	2	818.4407	1,898.00		430355	93	99	Empty	FALSE	HBAD_CHICK,HBA_CHICK,HBPI_CHICK
20196-20225 HinckeM –TAXchicken	Uncategorized Sample	20196-20205_A	Mudpit_Ingel20196_MGFPeaklist (F058870)	status=active	UPI000017122E	UPARC14_2_GallusG_9031_20140417.fasta	15,747.00	99.60%	3	5	9	0.02%	77.30%	VLGALGNAVK	K	X	99.70%	62.8	42.6	38.8	26.5	48.5	2.44	0.355	0.7	0	61	0	0	2	942.562	2,055.00		1255410	62	71	Empty	FALSE	HBAD_CHICK
20196-20225 HinckeM –TAXchicken	Uncategorized Sample	20196-20205_A	Mudpit_Ingel20196_MGFPeaklist (F058870)	status=active	UPI000017122E	UPARC14_2_GallusG_9031_20140417.fasta	15,747.00	99.60%	3	5	9	0.02%	77.30%	WVDNLSQAMAELSNLHAYNLR	K	V	35.50%	26.5	43.9	14.6	0					0	0	3	1	2	2,446.18	2,510.00		368045	72	92	Empty	TRUE	
20196-20225 HinckeM –TAXchicken	Uncategorized Sample	20196-20205_A	Mudpit_Ingel20196_MGFPeaklist (F058870)	Filamin OS=Gallus gallus PE=2 SV=1	Q90WF1_CHICK	TAX_GallusGallus_9031_20141114.fasta	272,939.20	100.00%	34	34	62	0.13%	15.60%	AALDVEVSGPGKGDAVR	R	D	99.70%	83.4	44.1	20.4	67.7	57.1	7.48	0.529	0.559	0	0	1	0	2	1,640.86	1,941.00		1,700.18	889	905	Empty	TRUE	
20196-20225 HinckeM –TAXchicken	Uncategorized Sample	20196-20205_A	Mudpit_Ingel20196_MGFPeaklist (F058870)	Filamin OS=Gallus gallus PE=2 SV=1	Q90WF1_CHICK	TAX_GallusGallus_9031_20141114.fasta	272,939.20	100.00%	34	34	62	0.13%	15.60%	AGSNMLLVGIQGPR	K	A	98.10%	35	43.6	22.9	18.6	26.6	0.678	0.177	0.25	0	0	3	0	2	1,429.75	1,648.00		1,552.41	2548	2561	Empty	TRUE	
20196-20225 HinckeM –TAXchicken	Uncategorized Sample	20196-20205_A	Mudpit_Ingel20196_MGFPeaklist (F058870)	Filamin OS=Gallus gallus PE=2 SV=1	Q90WF1_CHICK	TAX_GallusGallus_9031_20141114.fasta	272,939.20	100.00%	34	34	62	0.13%	15.60%	ALGAAGGAAVTAR	R	I	99.70%	78.1	44.1	35.5	51.3	40.1	2.21	0.379	0.423	0	2	0	0	2	1,085.61	1,648.00		582.58	1268	1280	Empty	TRUE	Q90576_CHICK
20196-20225 HinckeM –TAXchicken	Uncategorized Sample	20196-20205_A	Mudpit_Ingel20196_MGFPeaklist (F058870)	Filamin OS=Gallus gallus PE=2 SV=1	Q90WF1_CHICK	TAX_GallusGallus_9031_20141114.fasta	272,939.20	100.00%	34	34	62	0.13%	15.60%	APCEELQVK	R	H	88.50%					27.1	0.538	0.0148	0.333	0	1	0	0	2	1,073.53	1,742.00		380.01	2562	2570	Empty	TRUE	
20196-20225 HinckeM –TAXchicken	Uncategorized Sample	20196-20205_A	Mudpit_Ingel20196_MGFPeaklist (F058870)	Filamin OS=Gallus gallus PE=2 SV=1	Q90WF1_CHICK	TAX_GallusGallus_9031_20141114.fasta	272,939.20	100.00%	34	34	62	0.13%	15.60%	APVAAPFDPTK	R	V	99.70%	33.3	44.2	22.8	20.8	25.1	2.03	0.255	0.227	0	2	0	0	2	1,113.59	1,991.50		354.67	1136	1146	Empty	TRUE	
20196-20225 HinckeM –TAXchicken	Uncategorized Sample	20196-20205_A	Mudpit_Ingel20196_MGFPeaklist (F058870)	Filamin OS=Gallus gallus PE=2 SV=1	Q90WF1_CHICK	TAX_GallusGallus_9031_20141114.fasta	272,939.20	100.00%	34	34	62	0.13%	15.60%	AVPCRVEAGPGAESSVVR	K	W	99.70%	53.6	44.3	15.9	42.2	45.6	6.07	0.478	0.361	0	0	2	0	2	1,840.93	1,781.50		1,515.21	999	1016	Empty	TRUE	
20196-20225 HinckeM –TAXchicken	Uncategorized Sample	20196-20205_A	Mudpit_Ingel20196_MGFPeaklist (F058870)	Filamin OS=Gallus gallus PE=2 SV=1	Q90WF1_CHICK	TAX_GallusGallus_9031_20141114.fasta	272,939.20	100.00%	34	34	62	0.13%	15.60%	AYGPGIEPTGNVVK	R	Q	99.70%	28.4	44.8	20.6	21.7	35.7	3.57	0.294	0.321	0	2	0	0	2	1,401.74	1,962.00		2,666.30	272	285	Empty	TRUE	
20196-20225 HinckeM –TAXchicken	Uncategorized Sample	20196-20205_A	Mudpit_Ingel20196_MGFPeaklist (F058870)	Filamin OS=Gallus gallus PE=2 SV=1	Q90WF1_CHICK	TAX_GallusGallus_9031_20141114.fasta	272,939.20	100.00%	34	34	62	0.13%	15.60%	DAGEGLLAVQITDPEGKPK	K	K	99.70%	43.4	43.8	30.3	29.5	34.3	3.6	0.341	0.263	0	0	1	0	2	1,938.02	2,418.00		197.55	1564	1582	Empty	TRUE	Q90576_CHICK
20196-20225 HinckeM –TAXchicken	Uncategorized Sample	20196-20205_A	Mudpit_Ingel20196_MGFPeaklist (F058870)	Filamin OS=Gallus gallus PE=2 SV=1	Q90WF1_CHICK	TAX_GallusGallus_9031_20141114.fasta	272,939.20	100.00%	34	34	62	0.13%	15.60%	EGPYSISVR	R	Y	99.70%	49.4	44.5	34	26.8	30.2	0.959	0.278	0.389	0	1	0	0	2	1,007.52	1,912.00		496.4	1506	1514	Empty	TRUE	Q90576_CHICK
20196-20225 HinckeM –TAXchicken	Uncategorized Sample	20196-20205_A	Mudpit_Ingel20196_MGFPeaklist (F058870)	Filamin OS=Gallus gallus PE=2 SV=1	Q90WF1_CHICK	TAX_GallusGallus_9031_20141114.fasta	272,939.20	100.00%	34	34	62	0.13%	15.60%	FDGAHIPGSPFK	K	V	26.00%	12.5	44.8	20.2	2.52					0	0	1	0	2	1,272.64	2,196.00		413.33	2355	2366	Empty	TRUE	
20196-20225 HinckeM –TAXchicken	Uncategorized Sample	20196-20205_A	Mudpit_Ingel20196_MGFPeaklist (F058870)	Filamin OS=Gallus gallus PE=2 SV=1	Q90WF1_CHICK	TAX_GallusGallus_9031_20141114.fasta	272,939.20	100.00%	34	34	62	0.13%	15.60%	FVPEESGVHSVSVKDR	R	G	99.70%	39.8	44.6	19.9	31	31.5	2.8	0.381	0.281	0	0	0	2	2	1,771.90	1,760.00		712.38	2151	2166	Empty	TRUE	
20196-20225 HinckeM –TAXchicken	Uncategorized Sample	20196-20205_A	Mudpit_Ingel20196_MGFPeaklist (F058870)	Filamin OS=Gallus gallus PE=2 SV=1	Q90WF1_CHICK	TAX_GallusGallus_9031_20141114.fasta	272,939.20	100.00%	34	34	62	0.13%	15.60%	GALTHFEVDAR	R	A	97.30%	33.5	44.9	24.6	22.1					0	0	2	0	2	1,215.61	1,994.00		821.67	1257	1267	Empty	TRUE	Q90576_CHICK
20196-20225 HinckeM –TAXchicken	Uncategorized Sample	20196-20205_A	Mudpit_Ingel20196_MGFPeaklist (F058870)	Filamin OS=Gallus gallus PE=2 SV=1	Q90WF1_CHICK	TAX_GallusGallus_9031_20141114.fasta	272,939.20	100.00%	34	34	62	0.13%	15.60%	GVVEPVEVTDNGDGTR	K	S	99.70%	62.6	44.5	14.6	59.6	43.3	4.1	0.467	0.375	0	2	0	0	2	1,643.79	1,889.00		2,570.65	1482	1497	Empty	TRUE	Q90576_CHICK
20196-20225 HinckeM –TAXchicken	Uncategorized Sample	20196-20205_A	Mudpit_Ingel20196_MGFPeaklist (F058870)	Filamin OS=Gallus gallus PE=2 SV=1	Q90WF1_CHICK	TAX_GallusGallus_9031_20141114.fasta	272,939.20	100.00%	34	34	62	0.13%	15.60%	ISQAELGDAAR	S	V	86.60%					34.9	0.469	0.115	0.5	0	1	0	0	1	1,113.55	2,183.00		27,723.80	1988	1998	Empty	TRUE	
20196-20225 HinckeM –TAXchicken	Uncategorized Sample	20196-20205_A	Mudpit_Ingel20196_MGFPeaklist (F058870)	Filamin OS=Gallus gallus PE=2 SV=1	Q90WF1_CHICK	TAX_GallusGallus_9031_20141114.fasta	272,939.20	100.00%	34	34	62	0.13%	15.60%	ISQAELGDAARVR	S	V	94.70%					27.1	0.886	0.284	0.346	0	1	0	0	1	1,385.75	2,136.00		1,895.63	1988	2000	Empty	TRUE	
20196-20225 HinckeM –TAXchicken	Uncategorized Sample	20196-20205_A	Mudpit_Ingel20196_MGFPeaklist (F058870)	Filamin OS=Gallus gallus PE=2 SV=1	Q90WF1_CHICK	TAX_GallusGallus_9031_20141114.fasta	272,939.20	100.00%	34	34	62	0.13%	15.60%	LIALLEVLSQK	R	K	99.70%	67.6	39.5	29.4	28.6	38.4	2.66	0.182	0.5	0	1	0	0	2	1,226.77	2,894.00		134.32	63	73	Empty	TRUE	Q90WF0_CHICK,F1N8D4_CHICK
20196-20225 HinckeM –TAXchicken	Uncategorized Sample	20196-20205_A	Mudpit_Ingel20196_MGFPeaklist (F058870)	Filamin OS=Gallus gallus PE=2 SV=1	Q90WF1_CHICK	TAX_GallusGallus_9031_20141114.fasta	272,939.20	100.00%	34	34	62	0.13%	15.60%	LPQLPVTNFSR	R	D	99.70%	50.9	44.1	34.7	33.7	33.2	0.377	0.256	0.409	0	2	0	0	2	1,271.71	2,453.50		1,298.77	166	176	Empty	TRUE	
20196-20225 HinckeM –TAXchicken	Uncategorized Sample	20196-20205_A	Mudpit_Ingel20196_MGFPeaklist (F058870)	Filamin OS=Gallus gallus PE=2 SV=1	Q90WF1_CHICK	TAX_GallusGallus_9031_20141114.fasta	272,939.20	100.00%	34	34	62	0.13%	15.60%	QLYSVAYVVR	R	E	99.70%	52.1	43.5	30.2	33.4	30.7	1.89	0.3	0.4	0	2	0	0	2	1,197.66	2,293.50		1,220.45	2575	2584	Empty	TRUE	
20196-20225 HinckeM –TAXchicken	Uncategorized Sample	20196-20205_A	Mudpit_Ingel20196_MGFPeaklist (F058870)	Filamin OS=Gallus gallus PE=2 SV=1	Q90WF1_CHICK	TAX_GallusGallus_9031_20141114.fasta	272,939.20	100.00%	34	34	62	0.13%	15.60%	QPNCFTVETR	R	G	99.70%	46	43.8	27	27.9	31.3	1.82	0.198	0.35	0	3	0	0	2	1,251.58	1,792.00		663.54	1356	1365	Empty	TRUE	Q90576_CHICK
20196-20225 HinckeM –TAXchicken	Uncategorized Sample	20196-20205_A	Mudpit_Ingel20196_MGFPeaklist (F058870)	Filamin OS=Gallus gallus PE=2 SV=1	Q90WF1_CHICK	TAX_GallusGallus_9031_20141114.fasta	272,939.20	100.00%	34	34	62	0.13%	15.60%	RVEALLEGR	R	G	99.70%	23.5	43.7	29.9	11.9	25	0.409	0.136	0.389	0	2	0	0	2	1,042.60	1,863.50		488.62	413	421	Empty	TRUE	
20196-20225 HinckeM –TAXchicken	Uncategorized Sample	20196-20205_A	Mudpit_Ingel20196_MGFPeaklist (F058870)	Filamin OS=Gallus gallus PE=2 SV=1	Q90WF1_CHICK	TAX_GallusGallus_9031_20141114.fasta	272,939.20	100.00%	34	34	62	0.13%	15.60%	RVEALLEGRGDGAFR	R	C	77.70%	24.8	44.5	20.2	13.4					0	0	1	0	2	1,645.88	2,065.00		326.97	413	427	Empty	TRUE	
20196-20225 HinckeM –TAXchicken	Uncategorized Sample	20196-20205_A	Mudpit_Ingel20196_MGFPeaklist (F058870)	Filamin OS=Gallus gallus PE=2 SV=1	Q90WF1_CHICK	TAX_GallusGallus_9031_20141114.fasta	272,939.20	100.00%	34	34	62	0.13%	15.60%	SPFAVGVGQACNPSQCR	R	A	99.70%	41.2	43.3	22.3	31.9	39.6	4.34	0.452	0.353	0	0	2	0	2	1,834.83	2,040.50		1,410.66	452	468	Empty	TRUE	
20196-20225 HinckeM –TAXchicken	Uncategorized Sample	20196-20205_A	Mudpit_Ingel20196_MGFPeaklist (F058870)	Filamin OS=Gallus gallus PE=2 SV=1	Q90WF1_CHICK	TAX_GallusGallus_9031_20141114.fasta	272,939.20	100.00%	34	34	62	0.13%	15.60%	SPFEVAVGR	K	A	99.70%	30.5	45.2	28.5	13.4	29.6	1.24	0.203	0.444	0	6	0	0	2	961.5101	2,081.50		2,270.85	354	362	Empty	TRUE	
20196-20225 HinckeM –TAXchicken	Uncategorized Sample	20196-20205_A	Mudpit_Ingel20196_MGFPeaklist (F058870)	Filamin OS=Gallus gallus PE=2 SV=1	Q90WF1_CHICK	TAX_GallusGallus_9031_20141114.fasta	272,939.20	100.00%	34	34	62	0.13%	15.60%	SPFEVQVAPAGGAPK	R	V	99.70%	59.7	44.1	22.6	43.5	52	5.33	0.438	0.5	0	2	0	0	2	1,454.76	2,127.50		2,590.26	548	562	Empty	TRUE	
20196-20225 HinckeM –TAXchicken	Uncategorized Sample	20196-20205_A	Mudpit_Ingel20196_MGFPeaklist (F058870)	Filamin OS=Gallus gallus PE=2 SV=1	Q90WF1_CHICK	TAX_GallusGallus_9031_20141114.fasta	272,939.20	100.00%	34	34	62	0.13%	15.60%	SPFGVTVSPAVELGR	K	I	99.70%	54.6	44.2	22.8	45.3	43.8	5.4	0.461	0.4	0	2	0	0	2	1,515.82	2,519.00		2,735.96	943	957	Empty	TRUE	
20196-20225 HinckeM –TAXchicken	Uncategorized Sample	20196-20205_A	Mudpit_Ingel20196_MGFPeaklist (F058870)	Filamin OS=Gallus gallus PE=2 SV=1	Q90WF1_CHICK	TAX_GallusGallus_9031_20141114.fasta	272,939.20	100.00%	34	34	62	0.13%	15.60%	SPITVSISQAELGDAAR	R	V	99.70%	56.3	44.4	24.4	39.4	39.5	5.46	0.441	0.353	0	0	2	0	2	1,714.90	2,380.50		975.95	1982	1998	Empty	TRUE	
20196-20225 HinckeM –TAXchicken	Uncategorized Sample	20196-20205_A	Mudpit_Ingel20196_MGFPeaklist (F058870)	Filamin OS=Gallus gallus PE=2 SV=1	Q90WF1_CHICK	TAX_GallusGallus_9031_20141114.fasta	272,939.20	100.00%	34	34	62	0.13%	15.60%	TFSVSYVPK	R	V	24.90%	11.1	44	14.4	3.83					0	1	0	0	2	1,027.55	2,098.00		403.15	327	335	Empty	TRUE	
20196-20225 HinckeM –TAXchicken	Uncategorized Sample	20196-20205_A	Mudpit_Ingel20196_MGFPeaklist (F058870)	Filamin OS=Gallus gallus PE=2 SV=1	Q90WF1_CHICK	TAX_GallusGallus_9031_20141114.fasta	272,939.20	100.00%	34	34	62	0.13%	15.60%	TGLELGKPTQFTVLSK	R	G	99.70%	57.2	42.1	16.5	49.3	42	5.68	0.457	0.375	0	0	2	0	2	1,718.97	2,441.00		1,371.18	869	884	Empty	TRUE	
20196-20225 HinckeM –TAXchicken	Uncategorized Sample	20196-20205_A	Mudpit_Ingel20196_MGFPeaklist (F058870)	Filamin OS=Gallus gallus PE=2 SV=1	Q90WF1_CHICK	TAX_GallusGallus_9031_20141114.fasta	272,939.20	100.00%	34	34	62	0.13%	15.60%	VCVEPAGDAAAVK	K	V	99.70%	29	44.5	28.6	14.3	37.1	2.82	0.218	0.385	0	2	0	0	2	1,286.64	1,738.00		742.33	1231	1243	Empty	TRUE	Q90576_CHICK
20196-20225 HinckeM –TAXchicken	Uncategorized Sample	20196-20205_A	Mudpit_Ingel20196_MGFPeaklist (F058870)	Filamin OS=Gallus gallus PE=2 SV=1	Q90WF1_CHICK	TAX_GallusGallus_9031_20141114.fasta	272,939.20	100.00%	34	34	62	0.13%	15.60%	VEAGPGAESSVVR	R	W	99.70%	61.6	44.7	30.5	41.2	35.8	3.2	0.349	0.346	0	2	0	0	2	1,257.64	1,646.00		387.88	1004	1016	Empty	TRUE	
20196-20225 HinckeM –TAXchicken	Uncategorized Sample	20196-20205_A	Mudpit_Ingel20196_MGFPeaklist (F058870)	Filamin OS=Gallus gallus PE=2 SV=1	Q90WF1_CHICK	TAX_GallusGallus_9031_20141114.fasta	272,939.20	100.00%	34	34	62	0.13%	15.60%	VGNLQTDLGDGLR	R	L	99.70%	67.5	44.7	26.5	47.1	36.3	4.47	0.388	0.385	0	2	0	0	2	1,357.71	2,204.00		1,429.77	50	62	Empty	TRUE	
20196-20225 HinckeM –TAXchicken	Uncategorized Sample	20196-20205_A	Mudpit_Ingel20196_MGFPeaklist (F058870)	Filamin OS=Gallus gallus PE=2 SV=1	Q90WF1_CHICK	TAX_GallusGallus_9031_20141114.fasta	272,939.20	100.00%	34	34	62	0.13%	15.60%	VHQPASFAVSLNGAK	K	G	5.30%	9.8	44.2	0	0					0	0	1	0	2	1,525.81	2,095.00		102.97	2296	2310	Empty	TRUE	
20196-20225 HinckeM –TAXchicken	Uncategorized Sample	20196-20205_A	Mudpit_Ingel20196_MGFPeaklist (F058870)	Filamin OS=Gallus gallus PE=2 SV=1	Q90WF1_CHICK	TAX_GallusGallus_9031_20141114.fasta	272,939.20	100.00%	34	34	62	0.13%	15.60%	VTAQGPGIEPTGNVANR	R	T	99.70%	55.2	44.4	18.8	40.6	38.7	4.28	0.395	0.324	0	0	2	0	2	1,680.87	1,768.50		3,174.98	370	386	Empty	TRUE	
20196-20225 HinckeM –TAXchicken	Uncategorized Sample	20196-20205_A	Mudpit_Ingel20196_MGFPeaklist (F058870)	Filamin OS=Gallus gallus PE=2 SV=1	Q90WF1_CHICK	TAX_GallusGallus_9031_20141114.fasta	272,939.20	100.00%	34	34	62	0.13%	15.60%	YGGDEIPYSPYR	K	I	99.70%	33	43.6	23.4	24.6	29.8	2.92	0.389	0.333	0	1	0	0	2	1,416.64	2,104.00		281.9	1612	1623	Empty	TRUE	
20196-20225 HinckeM –TAXchicken	Uncategorized Sample	20196-20205_A	Mudpit_Ingel20196_MGFPeaklist (F058870)	Apolipoprotein B OS=Gallus gallus GN=APOB PE=4 SV=2	F1NV02_CHICK	TAX_GallusGallus_9031_20141114.fasta	523,366.00	100.00%	57	60	132	0.27%	12.80%	AEPLAFTFSHDYR	K	G	99.70%	82.6	44.1	18	74.7	44.5	7.96	0.548	0.462	0	0	2	0	2	1,553.74	2,413.00		797.78	1927	1939	Empty	TRUE	UPI0000612A36,UPI0002C8726F
20196-20225 HinckeM –TAXchicken	Uncategorized Sample	20196-20205_A	Mudpit_Ingel20196_MGFPeaklist (F058870)	Apolipoprotein B OS=Gallus gallus GN=APOB PE=4 SV=2	F1NV02_CHICK	TAX_GallusGallus_9031_20141114.fasta	523,366.00	100.00%	57	60	132	0.27%	12.80%	ALADLSVVDTAIR	R	L	99.70%	61.3	43.9	24.1	42.5	32.7	2.42	0.398	0.346	0	2	0	0	2	1,343.75	2,539.00		420.22	2005	2017	Empty	TRUE	UPI0002C8726F
20196-20225 HinckeM –TAXchicken	Uncategorized Sample	20196-20205_A	Mudpit_Ingel20196_MGFPeaklist (F058870)	Apolipoprotein B OS=Gallus gallus GN=APOB PE=4 SV=2	F1NV02_CHICK	TAX_GallusGallus_9031_20141114.fasta	523,366.00	100.00%	57	60	132	0.27%	12.80%	ALIQQGVEQGTR	R	K	99.70%	63.7	44.3	29.1	35.2	34.5	2.47	0.31	0.375	0	2	0	0	2	1,299.70	1,766.00		525.84	4587	4598	Empty	TRUE	UPI0002C8726F
20196-20225 HinckeM –TAXchicken	Uncategorized Sample	20196-20205_A	Mudpit_Ingel20196_MGFPeaklist (F058870)	Apolipoprotein B OS=Gallus gallus GN=APOB PE=4 SV=2	F1NV02_CHICK	TAX_GallusGallus_9031_20141114.fasta	523,366.00	100.00%	57	60	132	0.27%	12.80%	ALYWVDGK	K	V	29.50%	14.5	44.1	25.9	0					0	1	0	0	2	951.4935	2,197.00		71.34	710	717	Empty	TRUE	UPI0000612A36,UPI0002C8726F
20196-20225 HinckeM –TAXchicken	Uncategorized Sample	20196-20205_A	Mudpit_Ingel20196_MGFPeaklist (F058870)	Apolipoprotein B OS=Gallus gallus GN=APOB PE=4 SV=2	F1NV02_CHICK	TAX_GallusGallus_9031_20141114.fasta	523,366.00	100.00%	57	60	132	0.27%	12.80%	ALYWVDGKVPEQVSK	K	A	99.70%	42	44.1	18.2	30.4	33.6	3.22	0.315	0.333	0	0	2	0	2	1,718.91	2,264.00		2,148.78	710	724	Empty	TRUE	UPI0000612A36,UPI0002C8726F
20196-20225 HinckeM –TAXchicken	Uncategorized Sample	20196-20205_A	Mudpit_Ingel20196_MGFPeaklist (F058870)	Apolipoprotein B OS=Gallus gallus GN=APOB PE=4 SV=2	F1NV02_CHICK	TAX_GallusGallus_9031_20141114.fasta	523,366.00	100.00%	57	60	132	0.27%	12.80%	APSPVLSTPEFR	K	V	99.70%	75.2	44.7	24.8	64.2	32.2	3.42	0.339	0.333	0	3	0	0	2	1,300.69	2,243.00		520.24	3735	3746	Empty	TRUE	UPI0002C8726F
20196-20225 HinckeM –TAXchicken	Uncategorized Sample	20196-20205_A	Mudpit_Ingel20196_MGFPeaklist (F058870)	Apolipoprotein B OS=Gallus gallus GN=APOB PE=4 SV=2	F1NV02_CHICK	TAX_GallusGallus_9031_20141114.fasta	523,366.00	100.00%	57	60	132	0.27%	12.80%	ASFYGLSHAVTK	R	F	71.70%	15.7	44.3	15.4	14.9					0	0	3	0	2	1,280.66	2,053.00		1,101.90	432	443	Empty	TRUE	UPI0000612A36,UPI0002C8726F
20196-20225 HinckeM –TAXchicken	Uncategorized Sample	20196-20205_A	Mudpit_Ingel20196_MGFPeaklist (F058870)	Apolipoprotein B OS=Gallus gallus GN=APOB PE=4 SV=2	F1NV02_CHICK	TAX_GallusGallus_9031_20141114.fasta	523,366.00	100.00%	57	60	132	0.27%	12.80%	ASKDVPYAQTLQAK	K	L	99.70%	30.4	44.3	26.5	15.3	29.4	1.57	0.207	0.286	0	0	2	0	2	1,519.81	1,803.00		767.11	1233	1246	Empty	TRUE	UPI0002C8726F
20196-20225 HinckeM –TAXchicken	Uncategorized Sample	20196-20205_A	Mudpit_Ingel20196_MGFPeaklist (F058870)	Apolipoprotein B OS=Gallus gallus GN=APOB PE=4 SV=2	F1NV02_CHICK	TAX_GallusGallus_9031_20141114.fasta	523,366.00	100.00%	57	60	132	0.27%	12.80%	AVILNLEK	K	L	99.70%	40.3	42.7	37.1	25	24.2	0.699	0.12	0.375	0	2	0	0	2	899.5561	2,175.50		637.98	745	752	Empty	TRUE	UPI0000612A36,UPI0002C8726F
20196-20225 HinckeM –TAXchicken	Uncategorized Sample	20196-20205_A	Mudpit_Ingel20196_MGFPeaklist (F058870)	Apolipoprotein B OS=Gallus gallus GN=APOB PE=4 SV=2	F1NV02_CHICK	TAX_GallusGallus_9031_20141114.fasta	523,366.00	100.00%	57	60	132	0.27%	12.80%	DSVSEPQEFSISGSVK	R	Y	99.70%	34.5	44.3	20.6	24.9	28.5	1.64	0.253	0.219	0	1	1	0	2	1,695.81	2,173.50		854	2035	2050	Empty	TRUE	UPI0002C8726F
20196-20225 HinckeM –TAXchicken	Uncategorized Sample	20196-20205_A	Mudpit_Ingel20196_MGFPeaklist (F058870)	Apolipoprotein B OS=Gallus gallus GN=APOB PE=4 SV=2	F1NV02_CHICK	TAX_GallusGallus_9031_20141114.fasta	523,366.00	100.00%	57	60	132	0.27%	12.80%	DVPYAQTLQAK	K	L	99.70%	60.4	44.9	23.4	45.6	39.3	4.29	0.333	0.409	0	4	0	0	2	1,233.65	1,957.50		1,384.83	1236	1246	Empty	TRUE	UPI0002C8726F
20196-20225 HinckeM –TAXchicken	Uncategorized Sample	20196-20205_A	Mudpit_Ingel20196_MGFPeaklist (F058870)	Apolipoprotein B OS=Gallus gallus GN=APOB PE=4 SV=2	F1NV02_CHICK	TAX_GallusGallus_9031_20141114.fasta	523,366.00	100.00%	57	60	132	0.27%	12.80%	DVPYAQTLQAKL	K	S	68.30%					29.8	0.745	0.0369	0.292	0	1	0	0	1	1,346.73	1,949.00		1,039.51	1236	1247	Empty	TRUE	
20196-20225 HinckeM –TAXchicken	Uncategorized Sample	20196-20205_A	Mudpit_Ingel20196_MGFPeaklist (F058870)	Apolipoprotein B OS=Gallus gallus GN=APOB PE=4 SV=2	F1NV02_CHICK	TAX_GallusGallus_9031_20141114.fasta	523,366.00	100.00%	57	60	132	0.27%	12.80%	EFEWYLQK	K	I	13.50%	5.77	44	24.5	0					0	1	0	0	2	1,142.55	2,260.00		30,367.20	2522	2529	Empty	TRUE	UPI0000612A36,UPI0002C8726F
20196-20225 HinckeM –TAXchicken	Uncategorized Sample	20196-20205_A	Mudpit_Ingel20196_MGFPeaklist (F058870)	Apolipoprotein B OS=Gallus gallus GN=APOB PE=4 SV=2	F1NV02_CHICK	TAX_GallusGallus_9031_20141114.fasta	523,366.00	100.00%	57	60	132	0.27%	12.80%	EGTDLVDTLK	R	F	99.70%	58.5	45.2	26.8	38.1	30.8	3	0.24	0.35	0	4	0	0	2	1,090.56	2,128.00		1,712.02	1017	1026	Empty	TRUE	UPI0000612A36,UPI0002C8726F
20196-20225 HinckeM –TAXchicken	Uncategorized Sample	20196-20205_A	Mudpit_Ingel20196_MGFPeaklist (F058870)	Apolipoprotein B OS=Gallus gallus GN=APOB PE=4 SV=2	F1NV02_CHICK	TAX_GallusGallus_9031_20141114.fasta	523,366.00	100.00%	57	60	132	0.27%	12.80%	FALSGIVTPGAK	K	V	99.70%	70.6	44.1	25.1	52.8	35.2	2.54	0.366	0.417	0	4	0	0	2	1,160.67	2,427.50		2,638.08	840	851	Empty	TRUE	UPI0000612A36,UPI0002C8726F
20196-20225 HinckeM –TAXchicken	Uncategorized Sample	20196-20205_A	Mudpit_Ingel20196_MGFPeaklist (F058870)	Apolipoprotein B OS=Gallus gallus GN=APOB PE=4 SV=2	F1NV02_CHICK	TAX_GallusGallus_9031_20141114.fasta	523,366.00	100.00%	57	60	132	0.27%	12.80%	FSNNLQGSLK	K	E	99.70%	52	44.5	27.4	30.1	28.7	1.62	0.303	0.35	0	3	0	0	2	1,107.58	1,885.00		1,593.66	1730	1739	Empty	TRUE	UPI0000612A36,UPI0002C8726F
20196-20225 HinckeM –TAXchicken	Uncategorized Sample	20196-20205_A	Mudpit_Ingel20196_MGFPeaklist (F058870)	Apolipoprotein B OS=Gallus gallus GN=APOB PE=4 SV=2	F1NV02_CHICK	TAX_GallusGallus_9031_20141114.fasta	523,366.00	100.00%	57	60	132	0.27%	12.80%	GEGQIQSLSLR	K	H	39.30%	21.8	44.9	31.3	4.76					0	1	0	0	2	1,187.64	2,068.00		164.74	3629	3639	Empty	TRUE	UPI0000612A36,UPI0002C8726F

20196-20225 HinckeM –TAXchicken	Uncategorized Sample	20196-20205_A	Mudpit_Ingel20196_MGFPeaklist (F058870)	Apolipoprotein B OS=Gallus gallus GN=APOB PE=4 SV=2	F1NV02_CHICK	TAX_GallusGallus_9031_20141114.fasta	523,366.00	[illegible]
20196-20225 HinckeM –TAXchicken	Uncategorized Sample	20196-20205_A	Mudpit_Ingel20196_MGFPeaklist (F058870)	Apolipoprotein B OS=Gallus gallus GN=APOB PE=4 SV=2	F1NV02_CHICK	TAX_GallusGallus_9031_20141114.fasta	523,366.00	[illegible]
20196-20225 HinckeM –TAXchicken	Uncategorized Sample	20196-20205_A	Mudpit_Ingel20196_MGFPeaklist (F058870)	Apolipoprotein B OS=Gallus gallus GN=APOB PE=4 SV=2	F1NV02_CHICK	TAX_GallusGallus_9031_20141114.fasta	523,366.00	[illegible]
20196-20225 HinckeM –TAXchicken	Uncategorized Sample	20196-20205_A	Mudpit_Ingel20196_MGFPeaklist (F058870)	Apolipoprotein B OS=Gallus gallus GN=APOB PE=4 SV=2	F1NV02_CHICK	TAX_GallusGallus_9031_20141114.fasta	523,366.00	[illegible]
20196-20225 HinckeM –TAXchicken	Uncategorized Sample	20196-20205_A	Mudpit_Ingel20196_MGFPeaklist (F058870)	Apolipoprotein B OS=Gallus gallus GN=APOB PE=4 SV=2	F1NV02_CHICK	TAX_GallusGallus_9031_20141114.fasta	523,366.00	[illegible]
20196-20225 HinckeM –TAXchicken	Uncategorized Sample	20196-20205_A	Mudpit_Ingel20196_MGFPeaklist (F058870)	Apolipoprotein B OS=Gallus gallus GN=APOB PE=4 SV=2	F1NV02_CHICK	TAX_GallusGallus_9031_20141114.fasta	523,366.00	[illegible]
20196-20225 HinckeM –TAXchicken	Uncategorized Sample	20196-20205_A	Mudpit_Ingel20196_MGFPeaklist (F058870)	Apolipoprotein B OS=Gallus gallus GN=APOB PE=4 SV=2	F1NV02_CHICK	TAX_GallusGallus_9031_20141114.fasta	523,366.00	[illegible]
20196-20225 HinckeM –TAXchicken	Uncategorized Sample	20196-20205_A	Mudpit_Ingel20196_MGFPeaklist (F058870)	Apolipoprotein B OS=Gallus gallus GN=APOB PE=4 SV=2	F1NV02_CHICK	TAX_GallusGallus_9031_20141114.fasta	523,366.00	[illegible]
20196-20225 HinckeM –TAXchicken	Uncategorized Sample	20196-20205_A	Mudpit_Ingel20196_MGFPeaklist (F058870)	Apolipoprotein B OS=Gallus gallus GN=APOB PE=4 SV=2	F1NV02_CHICK	TAX_GallusGallus_9031_20141114.fasta	523,366.00	[illegible]
20196-20225 HinckeM –TAXchicken	Uncategorized Sample	20196-20205_A	Mudpit_Ingel20196_MGFPeaklist (F058870)	Apolipoprotein B OS=Gallus gallus GN=APOB PE=4 SV=2	F1NV02_CHICK	TAX_GallusGallus_9031_20141114.fasta	523,366.00	[illegible]
20196-20225 HinckeM –TAXchicken	Uncategorized Sample	20196-20205_A	Mudpit_Ingel20196_MGFPeaklist (F058870)	Apolipoprotein B OS=Gallus gallus GN=APOB PE=4 SV=2	F1NV02_CHICK	TAX_GallusGallus_9031_20141114.fasta	523,366.00	[illegible]
20196-20225 HinckeM –TAXchicken	Uncategorized Sample	20196-20205_A	Mudpit_Ingel20196_MGFPeaklist (F058870)	Apolipoprotein B OS=Gallus gallus GN=APOB PE=4 SV=2	F1NV02_CHICK	TAX_GallusGallus_9031_20141114.fasta	523,366.00	[illegible]
20196-20225 HinckeM –TAXchicken	Uncategorized Sample	20196-20205_A	Mudpit_Ingel20196_MGFPeaklist (F058870)	Apolipoprotein B OS=Gallus gallus GN=APOB PE=4 SV=2	F1NV02_CHICK	TAX_GallusGallus_9031_20141114.fasta	523,366.00	[illegible]
20196-20225 HinckeM –TAXchicken	Uncategorized Sample	20196-20205_A	Mudpit_Ingel20196_MGFPeaklist (F058870)	Apolipoprotein B OS=Gallus gallus GN=APOB PE=4 SV=2	F1NV02_CHICK	TAX_GallusGallus_9031_20141114.fasta	523,366.00	[illegible]
20196-20225 HinckeM –TAXchicken	Uncategorized Sample	20196-20205_A	Mudpit_Ingel20196_MGFPeaklist (F058870)	Apolipoprotein B OS=Gallus gallus GN=APOB PE=4 SV=2	F1NV02_CHICK	TAX_GallusGallus_9031_20141114.fasta	523,366.00	[illegible]
20196-20225 HinckeM –TAXchicken	Uncategorized Sample	20196-20205_A	Mudpit_Ingel20196_MGFPeaklist (F058870)	Apolipoprotein B OS=Gallus gallus GN=APOB PE=4 SV=2	F1NV02_CHICK	TAX_GallusGallus_9031_20141114.fasta	523,366.00	[illegible]
20196-20225 HinckeM –TAXchicken	Uncategorized Sample	20196-20205_A	Mudpit_Ingel20196_MGFPeaklist (F058870)	Apolipoprotein B OS=Gallus gallus GN=APOB PE=4 SV=2	F1NV02_CHICK	TAX_GallusGallus_9031_20141114.fasta	523,366.00	[illegible]
20196-20225 HinckeM –TAXchicken	Uncategorized Sample	20196-20205_A	Mudpit_Ingel20196_MGFPeaklist (F058870)	Apolipoprotein B OS=Gallus gallus GN=APOB PE=4 SV=2	F1NV02_CHICK	TAX_GallusGallus_9031_20141114.fasta	523,366.00	[illegible]
20196-20225 HinckeM –TAXchicken	Uncategorized Sample	20196-20205_A	Mudpit_Ingel20196_MGFPeaklist (F058870)	Apolipoprotein B OS=Gallus gallus GN=APOB PE=4 SV=2	F1NV02_CHICK	TAX_GallusGallus_9031_20141114.fasta	523,366.00	[illegible]
20196-20225 HinckeM –TAXchicken	Uncategorized Sample	20196-20205_A	Mudpit_Ingel20196_MGFPeaklist (F058870)	Apolipoprotein B OS=Gallus gallus GN=APOB PE=4 SV=2	F1NV02_CHICK	TAX_GallusGallus_9031_20141114.fasta	523,366.00	[illegible]
20196-20225 HinckeM –TAXchicken	Uncategorized Sample	20196-20205_A	Mudpit_Ingel20196_MGFPeaklist (F058870)	Apolipoprotein B OS=Gallus gallus GN=APOB PE=4 SV=2	F1NV02_CHICK	TAX_GallusGallus_9031_20141114.fasta	523,366.00	[illegible]
20196-20225 HinckeM –TAXchicken	Uncategorized Sample	20196-20205_A	Mudpit_Ingel20196_MGFPeaklist (F058870)	Apolipoprotein B OS=Gallus gallus GN=APOB PE=4 SV=2	F1NV02_CHICK	TAX_GallusGallus_9031_20141114.fasta	523,366.00	[illegible]
20196-20225 HinckeM –TAXchicken	Uncategorized Sample	20196-20205_A	Mudpit_Ingel20196_MGFPeaklist (F058870)	Apolipoprotein B OS=Gallus gallus GN=APOB PE=4 SV=2	F1NV02_CHICK	TAX_GallusGallus_9031_20141114.fasta	523,366.00	[illegible]
20196-20225 HinckeM –TAXchicken	Uncategorized Sample	20196-20205_A	Mudpit_Ingel20196_MGFPeaklist (F058870)	Apolipoprotein B OS=Gallus gallus GN=APOB PE=4 SV=2	F1NV02_CHICK	TAX_GallusGallus_9031_20141114.fasta	523,366.00	[illegible]
20196-20225 HinckeM –TAXchicken	Uncategorized Sample	20196-20205_A	Mudpit_Ingel20196_MGFPeaklist (F058870)	Apolipoprotein B OS=Gallus gallus GN=APOB PE=4 SV=2	F1NV02_CHICK	TAX_GallusGallus_9031_20141114.fasta	523,366.00	[illegible]
20196-20225 HinckeM –TAXchicken	Uncategorized Sample	20196-20205_A	Mudpit_Ingel20196_MGFPeaklist (F058870)	Apolipoprotein B OS=Gallus gallus GN=APOB PE=4 SV=2	F1NV02_CHICK	TAX_GallusGallus_9031_20141114.fasta	523,366.00	[illegible]
20196-20225 HinckeM –TAXchicken	Uncategorized Sample	20196-20205_A	Mudpit_Ingel20196_MGFPeaklist (F058870)	Apolipoprotein B OS=Gallus gallus GN=APOB PE=4 SV=2	F1NV02_CHICK	TAX_GallusGallus_9031_20141114.fasta	523,366.00	[illegible]
20196-20225 HinckeM –TAXchicken	Uncategorized Sample	20196-20205_A	Mudpit_Ingel20196_MGFPeaklist (F058870)	Apolipoprotein B OS=Gallus gallus GN=APOB PE=4 SV=2	F1NV02_CHICK	TAX_GallusGallus_9031_20141114.fasta	523,366.00	[illegible]
20196-20225 HinckeM –TAXchicken	Uncategorized Sample	20196-20205_A	Mudpit_Ingel20196_MGFPeaklist (F058870)	Apolipoprotein B OS=Gallus gallus GN=APOB PE=4 SV=2	F1NV02_CHICK	TAX_GallusGallus_9031_20141114.fasta	523,366.00	[illegible]
20196-20225 HinckeM –TAXchicken	Uncategorized Sample	20196-20205_A	Mudpit_Ingel20196_MGFPeaklist (F058870)	Apolipoprotein B OS=Gallus gallus GN=APOB PE=4 SV=2	F1NV02_CHICK	TAX_GallusGallus_9031_20141114.fasta	523,366.00	[illegible]
20196-20225 HinckeM –TAXchicken	Uncategorized Sample	20196-20205_A	Mudpit_Ingel20196_MGFPeaklist (F058870)	Apolipoprotein B OS=Gallus gallus GN=APOB PE=4 SV=2	F1NV02_CHICK	TAX_GallusGallus_9031_20141114.fasta	523,366.00	[illegible]
20196-20225 HinckeM –TAXchicken	Uncategorized Sample	20196-20205_A	Mudpit_Ingel20196_MGFPeaklist (F058870)	Apolipoprotein B OS=Gallus gallus GN=APOB PE=4 SV=2	F1NV02_CHICK	TAX_GallusGallus_9031_20141114.fasta	523,366.00	[illegible]
20196-20225 HinckeM –TAXchicken	Uncategorized Sample	20196-20205_A	Mudpit_Ingel20196_MGFPeaklist (F058870)	Apolipoprotein B OS=Gallus gallus GN=APOB PE=4 SV=2	F1NV02_CHICK	TAX_GallusGallus_9031_20141114.fasta	523,366.00	[illegible]
20196-20225 HinckeM –TAXchicken	Uncategorized Sample	20196-20205_A	Mudpit_Ingel20196_MGFPeaklist (F058870)	Apolipoprotein B OS=Gallus gallus GN=APOB PE=4 SV=2	F1NV02_CHICK	TAX_GallusGallus_9031_20141114.fasta	523,366.00	[illegible]
20196-20225 HinckeM –TAXchicken	Uncategorized Sample	20196-20205_A	Mudpit_Ingel20196_MGFPeaklist (F058870)	Apolipoprotein B OS=Gallus gallus GN=APOB PE=4 SV=2	F1NV02_CHICK	TAX_GallusGallus_9031_20141114.fasta	523,366.00	[illegible]
20196-20225 HinckeM –TAXchicken	Uncategorized Sample	20196-20205_A	Mudpit_Ingel20196_MGFPeaklist (F058870)	Apolipoprotein B OS=Gallus gallus GN=APOB PE=4 SV=2	F1NV02_CHICK	TAX_GallusGallus_9031_20141114.fasta	523,366.00	[illegible]
20196-20225 HinckeM –TAXchicken	Uncategorized Sample	20196-20205_A	Mudpit_Ingel20196_MGFPeaklist (F058870)	Apolipoprotein B OS=Gallus gallus GN=APOB PE=4 SV=2	F1NV02_CHICK	TAX_GallusGallus_9031_20141114.fasta	523,366.00	[illegible]
20196-20225 HinckeM –TAXchicken	Uncategorized Sample	20196-20205_A	Mudpit_Ingel20196_MGFPeaklist (F058870)	Apolipoprotein B OS=Gallus gallus GN=APOB PE=4 SV=2	F1NV02_CHICK	TAX_GallusGallus_9031_20141114.fasta	523,366.00	[illegible]
20196-20225 HinckeM –TAXchicken	Uncategorized Sample	20196-20205_A	Mudpit_Ingel20196_MGFPeaklist (F058870)	Apolipoprotein B OS=Gallus gallus GN=APOB PE=4 SV=2	F1NV02_CHICK	TAX_GallusGallus_9031_20141114.fasta	523,366.00	[illegible]
20196-20225 HinckeM –TAXchicken	Uncategorized Sample	20196-20205_A	Mudpit_Ingel20196_MGFPeaklist (F058870)	Apolipoprotein B OS=Gallus gallus GN=APOB PE=4 SV=2	F1NV02_CHICK	TAX_GallusGallus_9031_20141114.fasta	523,366.00	[illegible]
20196-20225 HinckeM –TAXchicken	Uncategorized Sample	20196-20205_A	Mudpit_Ingel20196_MGFPeaklist (F058870)	Radixin OS=Gallus gallus GN=RDX PE=2 SV=1	RADI_CHICK,UPI00000FD97F,UPI0000ECD5FA	TAX_GallusGallus_9031_20141114.fasta,UPARC14_2_GallusG_9031_20140417.fasta	68,614.50	[illegible]
20196-20225 HinckeM –TAXchicken	Uncategorized Sample	20196-20205_A	Mudpit_Ingel20196_MGFPeaklist (F058870)	Radixin OS=Gallus gallus GN=RDX PE=2 SV=1	RADI_CHICK,UPI00000FD97F,UPI0000ECD5FA	TAX_GallusGallus_9031_20141114.fasta,UPARC14_2_GallusG_9031_20140417.fasta	68,614.50	[illegible]
20196-20225 HinckeM –TAXchicken	Uncategorized Sample	20196-20205_A	Mudpit_Ingel20196_MGFPeaklist (F058870)	Radixin OS=Gallus gallus GN=RDX PE=2 SV=1	RADI_CHICK,UPI00000FD97F,UPI0000ECD5FA	TAX_GallusGallus_9031_20141114.fasta,UPARC14_2_GallusG_9031_20140417.fasta	68,614.50	[illegible]
20196-20225 HinckeM –TAXchicken	Uncategorized Sample	20196-20205_A	Mudpit_Ingel20196_MGFPeaklist (F058870)	Radixin OS=Gallus gallus GN=RDX PE=2 SV=1	RADI_CHICK,UPI00000FD97F,UPI0000ECD5FA	TAX_GallusGallus_9031_20141114.fasta,UPARC14_2_GallusG_9031_20140417.fasta	68,614.50	[illegible]
20196-20225 HinckeM –TAXchicken	Uncategorized Sample	20196-20205_A	Mudpit_Ingel20196_MGFPeaklist (F058870)	Radixin OS=Gallus gallus GN=RDX PE=2 SV=1	RADI_CHICK,UPI00000FD97F,UPI0000ECD5FA	TAX_GallusGallus_9031_20141114.fasta,UPARC14_2_GallusG_9031_20140417.fasta	68,614.50	[illegible]
20196-20225 HinckeM –TAXchicken	Uncategorized Sample	20196-20205_A	Mudpit_Ingel20196_MGFPeaklist (F058870)	Radixin OS=Gallus gallus GN=RDX PE=2 SV=1	RADI_CHICK,UPI00000FD97F,UPI0000ECD5FA	TAX_GallusGallus_9031_20141114.fasta,UPARC14_2_GallusG_9031_20140417.fasta	68,614.50	[illegible]
20196-20225 HinckeM –TAXchicken	Uncategorized Sample	20196-20205_A	Mudpit_Ingel20196_MGFPeaklist (F058870)	Radixin OS=Gallus gallus GN=RDX PE=2 SV=1	RADI_CHICK,UPI00000FD97F,UPI0000ECD5FA	TAX_GallusGallus_9031_20141114.fasta,UPARC14_2_GallusG_9031_20140417.fasta	68,614.50	[illegible]
20196-20225 HinckeM –TAXchicken	Uncategorized Sample	20196-20205_A	Mudpit_Ingel20196_MGFPeaklist (F058870)	Radixin OS=Gallus gallus GN=RDX PE=2 SV=1	RADI_CHICK,UPI00000FD97F,UPI0000ECD5FA	TAX_GallusGallus_9031_20141114.fasta,UPARC14_2_GallusG_9031_20140417.fasta	68,614.50	[illegible]
20196-20225 HinckeM –TAXchicken	Uncategorized Sample	20196-20205_A	Mudpit_Ingel20196_MGFPeaklist (F058870)	Radixin OS=Gallus gallus GN=RDX PE=2 SV=1	RADI_CHICK,UPI00000FD97F,UPI0000ECD5FA	TAX_GallusGallus_9031_20141114.fasta,UPARC14_2_GallusG_9031_20140417.fasta	68,614.50	[illegible]
20196-20225 HinckeM –TAXchicken	Uncategorized Sample	20196-20205_A	Mudpit_Ingel20196_MGFPeaklist (F058870)	Radixin OS=Gallus gallus GN=RDX PE=2 SV=1	RADI_CHICK,UPI00000FD97F,UPI0000ECD5FA	TAX_GallusGallus_9031_20141114.fasta,UPARC14_2_GallusG_9031_20140417.fasta	68,614.50	[illegible]
20196-20225 HinckeM –TAXchicken	Uncategorized Sample	20196-20205_A	Mudpit_Ingel20196_MGFPeaklist (F058870)	Radixin OS=Gallus gallus GN=RDX PE=2 SV=1	RADI_CHICK,UPI00000FD97F,UPI0000ECD5FA	TAX_GallusGallus_9031_20141114.fasta,UPARC14_2_GallusG_9031_20140417.fasta	68,614.50	[illegible]
20196-20225 HinckeM –TAXchicken	Uncategorized Sample	20196-20205_A	Mudpit_Ingel20196_MGFPeaklist (F058870)	Radixin OS=Gallus gallus GN=RDX PE=2 SV=1	RADI_CHICK,UPI00000FD97F,UPI0000ECD5FA	TAX_GallusGallus_9031_20141114.fasta,UPARC14_2_GallusG_9031_20140417.fasta	68,614.50	[illegible]
20196-20225 HinckeM –TAXchicken	Uncategorized Sample	20196-20205_A	Mudpit_Ingel20196_MGFPeaklist (F058870)	Radixin OS=Gallus gallus GN=RDX PE=2 SV=1	RADI_CHICK,UPI00000FD97F,UPI0000ECD5FA	TAX_GallusGallus_9031_20141114.fasta,UPARC14_2_GallusG_9031_20140417.fasta	68,614.50	[illegible]
20196-20225 HinckeM –TAXchicken	Uncategorized Sample	20196-20205_A	Mudpit_Ingel20196_MGFPeaklist (F058870)	Radixin OS=Gallus gallus GN=RDX PE=2 SV=1	RADI_CHICK,UPI00000FD97F,UPI0000ECD5FA	TAX_GallusGallus_9031_20141114.fasta,UPARC14_2_GallusG_9031_20140417.fasta	68,614.50	[illegible]
20196-20225 HinckeM –TAXchicken	Uncategorized Sample	20196-20205_A	Mudpit_Ingel20196_MGFPeaklist (F058870)	Radixin OS=Gallus gallus GN=RDX PE=2 SV=1	RADI_CHICK,UPI00000FD97F,UPI0000ECD5FA	TAX_GallusGallus_9031_20141114.fasta,UPARC14_2_GallusG_9031_20140417.fasta	68,614.50	[illegible]
20196-20225 HinckeM –TAXchicken	Uncategorized Sample	20196-20205_A	Mudpit_Ingel20196_MGFPeaklist (F058870)	Radixin OS=Gallus gallus GN=RDX PE=2 SV=1	RADI_CHICK,UPI00000FD97F,UPI0000ECD5FA	TAX_GallusGallus_9031_20141114.fasta,UPARC14_2_GallusG_9031_20140417.fasta	68,614.50	[illegible]
20196-20225 HinckeM –TAXchicken	Uncategorized Sample	20196-20205_A	Mudpit_Ingel20196_MGFPeaklist (F058870)	Radixin OS=Gallus gallus GN=RDX PE=2 SV=1	RADI_CHICK,UPI00000FD97F,UPI0000ECD5FA	TAX_GallusGallus_9031_20141114.fasta,UPARC14_2_GallusG_9031_20140417.fasta	68,614.50	[illegible]
20196-20225 HinckeM –TAXchicken	Uncategorized Sample	20196-20205_A	Mudpit_Ingel20196_MGFPeaklist (F058870)	Radixin OS=Gallus gallus GN=RDX PE=2 SV=1	RADI_CHICK,UPI00000FD97F,UPI0000ECD5FA	TAX_GallusGallus_9031_20141114.fasta,UPARC14_2_GallusG_9031_20140417.fasta	68,614.50	[illegible]
20196-20225 HinckeM –TAXchicken	Uncategorized Sample	20196-20205_A	Mudpit_Ingel20196_MGFPeaklist (F058870)	Radixin OS=Gallus gallus GN=RDX PE=2 SV=1	RADI_CHICK,UPI00000FD97F,UPI0000ECD5FA	TAX_GallusGallus_9031_20141114.fasta,UPARC14_2_GallusG_9031_20140417.fasta	68,614.50	[illegible]
20196-20225 HinckeM –TAXchicken	Uncategorized Sample	20196-20205_A	Mudpit_Ingel20196_MGFPeaklist (F058870)	Radixin OS=Gallus gallus GN=RDX PE=2 SV=1	RADI_CHICK,UPI00000FD97F,UPI0000ECD5FA	TAX_GallusGallus_9031_20141114.fasta,UPARC14_2_GallusG_9031_20140417.fasta	68,614.50	[illegible]
20196-20225 HinckeM –TAXchicken	Uncategorized Sample	20196-20205_A	Mudpit_Ingel20196_MGFPeaklist (F058870)	Radixin OS=Gallus gallus GN=RDX PE=2 SV=1	RADI_CHICK,UPI00000FD97F,UPI0000ECD5FA	TAX_GallusGallus_9031_20141114.fasta,UPARC14_2_GallusG_9031_20140417.fasta	68,614.50	[illegible]
20196-20225 HinckeM –TAXchicken	Uncategorized Sample	20196-20205_A	Mudpit_Ingel20196_MGFPeaklist (F058870)	Radixin OS=Gallus gallus GN=RDX PE=2 SV=1	RADI_CHICK,UPI00000FD97F,UPI0000ECD5FA	TAX_GallusGallus_9031_20141114.fasta,UPARC14_2_GallusG_9031_20140417.fasta	68,614.50	[illegible]
20196-20225 HinckeM –TAXchicken	Uncategorized Sample	20196-20205_A	Mudpit_Ingel20196_MGFPeaklist (F058870)	Radixin OS=Gallus gallus GN=RDX PE=2 SV=1	RADI_CHICK,UPI00000FD97F,UPI0000ECD5FA	TAX_GallusGallus_9031_20141114.fasta,UPARC14_2_GallusG_9031_20140417.fasta	68,614.50	[illegible]
20196-20225 HinckeM –TAXchicken	Uncategorized Sample	20196-20205_A	Mudpit_Ingel20196_MGFPeaklist (F058870)	Radixin OS=Gallus gallus GN=RDX PE=2 SV=1	RADI_CHICK,UPI00000FD97F,UPI0000ECD5FA	TAX_GallusGallus_9031_20141114.fasta,UPARC14_2_GallusG_9031_20140417.fasta	68,614.50	[illegible]
20196-20225 HinckeM –TAXchicken	Uncategorized Sample	20196-20205_A	Mudpit_Ingel20196_MGFPeaklist (F058870)	Radixin OS=Gallus gallus GN=RDX PE=2 SV=1	RADI_CHICK,UPI00000FD97F,UPI0000ECD5FA	TAX_GallusGallus_9031_20141114.fasta,UPARC14_2_GallusG_9031_20140417.fasta	68,614.50	[illegible]
20196-20225 HinckeM –TAXchicken	Uncategorized Sample	20196-20205_A	Mudpit_Ingel20196_MGFPeaklist (F058870)	Radixin OS=Gallus gallus GN=RDX PE=2 SV=1	RADI_CHICK,UPI00000FD97F,UPI0000ECD5FA	TAX_GallusGallus_9031_20141114.fasta,UPARC14_2_GallusG_9031_20140417.fasta	68,614.50	[illegible]
20196-20225 HinckeM –TAXchicken	Uncategorized Sample	20196-20205_A	Mudpit_Ingel20196_MGFPeaklist (F058870)	Radixin OS=Gallus gallus GN=RDX PE=2 SV=1	RADI_CHICK,UPI00000FD97F,UPI0000ECD5FA	TAX_GallusGallus_9031_20141114.fasta,UPARC14_2_GallusG_9031_20140417.fasta	68,614.50	[illegible]
20196-20225 HinckeM –TAXchicken	Uncategorized Sample	20196-20205_A	Mudpit_Ingel20196_MGFPeaklist (F058870)	Radixin OS=Gallus gallus GN=RDX PE=2 SV=1	RADI_CHICK,UPI00000FD97F,UPI0000ECD5FA	TAX_GallusGallus_9031_20141114.fasta,UPARC14_2_GallusG_9031_20140417.fasta	68,614.50	[illegible]
20196-20225 HinckeM –TAXchicken	Uncategorized Sample	20196-20205_A	Mudpit_Ingel20196_MGFPeaklist (F058870)	Radixin OS=Gallus gallus GN=RDX PE=2 SV=1	RADI_CHICK,UPI00000FD97F,UPI0000ECD5FA	TAX_GallusGallus_9031_20141114.fasta,UPARC14_2_GallusG_9031_20140417.fasta	68,614.50	[illegible]
20196-20225 HinckeM –TAXchicken	Uncategorized Sample	20196-20205_A	Mudpit_Ingel20196_MGFPeaklist (F058870)	Radixin OS=Gallus gallus GN=RDX PE=2 SV=1	RADI_CHICK,UPI00000FD97F,UPI0000ECD5FA	TAX_GallusGallus_9031_20141114.fasta,UPARC14_2_GallusG_9031_20140417.fasta	68,614.50	[illegible]
20196-20225 HinckeM –TAXchicken	Uncategorized Sample	20196-20205_A	Mudpit_Ingel20196_MGFPeaklist (F058870)	Radixin OS=Gallus gallus GN=RDX PE=2 SV=1	RADI_CHICK,UPI00000FD97F,UPI0000ECD5FA	TAX_GallusGallus_9031_20141114.fasta,UPARC14_2_GallusG_9031_20140417.fasta	68,614.50	[illegible]
20196-20225 HinckeM –TAXchicken	Uncategorized Sample	20196-20205_A	Mudpit_Ingel20196_MGFPeaklist (F058870)	Radixin OS=Gallus gallus GN=RDX PE=2 SV=1	RADI_CHICK,UPI00000FD97F,UPI0000ECD5FA	TAX_GallusGallus_9031_20141114.fasta,UPARC14_2_GallusG_9031_20140417.fasta	68,614.50	[illegible]
20196-20225 HinckeM –TAXchicken	Uncategorized Sample	20196-20205_A	Mudpit_Ingel20196_MGFPeaklist (F058870)	Ras-related protein Rap-1b OS=Gallus gallus GN=RAP1B PE=2 SV=1	RAP1B_CHICK,UPI0000001221	TAX_GallusGallus_9031_20141114.fasta,UPARC14_2_GallusG_9031_20140417.fasta	20,824.90	[illegible]
20196-20225 HinckeM –TAXchicken	Uncategorized Sample	20196-20205_A	Mudpit_Ingel20196_MGFPeaklist (F058870)	Ras-related protein Rap-1b OS=Gallus gallus GN=RAP1B PE=2 SV=1	RAP1B_CHICK,UPI0000001221	TAX_GallusGallus_9031_20141114.fasta,UPARC14_2_GallusG_9031_20140417.fasta	20,824.90	[illegible]
20196-20225 HinckeM –TAXchicken	Uncategorized Sample	20196-20205_A	Mudpit_Ingel20196_MGFPeaklist (F058870)	Ras-related protein Rap-1b OS=Gallus gallus GN=RAP1B PE=2 SV=1	RAP1B_CHICK,UPI0000001221	TAX_GallusGallus_9031_20141114.fasta,UPARC14_2_GallusG_9031_20140417.fasta	20,824.90	[illegible]
20196-20225 HinckeM –TAXchicken	Uncategorized Sample	20196-20205_A	Mudpit_Ingel20196_MGFPeaklist (F058870)	Ras-related protein Rap-1b OS=Gallus gallus GN=RAP1B PE=2 SV=1	RAP1B_CHICK,UPI0000001221	TAX_GallusGallus_9031_20141114.fasta,UPARC14_2_GallusG_9031_20140417.fasta	20,824.90	[illegible]
20196-20225 HinckeM –TAXchicken	Uncategorized Sample	20196-20205_A	Mudpit_Ingel20196_MGFPeaklist (F058870)	Ras-related protein Rap-1b OS=Gallus gallus GN=RAP1B PE=2 SV=1	RAP1B_CHICK,UPI0000001221	TAX_GallusGallus_9031_20141114.fasta,UPARC14_2_GallusG_9031_20140417.fasta	20,824.90	[illegible]
20196-20225 HinckeM –TAXchicken	Uncategorized Sample	20196-20205_A	Mudpit_Ingel20196_MGFPeaklist (F058870)	Ras-related protein Rap-1b OS=Gallus gallus GN=RAP1B PE=2 SV=1	RAP1B_CHICK,UPI0000001221	TAX_GallusGallus_9031_20141114.fasta,UPARC14_2_GallusG_9031_20140417.fasta	20,824.90	[illegible]
20196-20225 HinckeM –TAXchicken	Uncategorized Sample	20196-20205_A	Mudpit_Ingel20196_MGFPeaklist (F058870)	Ras-related protein Rap-1b OS=Gallus gallus GN=RAP1B PE=2 SV=1	RAP1B_CHICK,UPI0000001221	TAX_GallusGallus_9031_20141114.fasta,UPARC14_2_GallusG_9031_20140417.fasta	20,824.90	[illegible]
20196-20225 HinckeM –TAXchicken	Uncategorized Sample	20196-20205_A	Mudpit_Ingel20196_MGFPeaklist (F058870)	Ras-related protein Rap-1b OS=Gallus gallus GN=RAP1B PE=2 SV=1	RAP1B_CHICK,UPI0000001221	TAX_GallusGallus_9031_20141114.fasta,UPARC14_2_GallusG_9031_20140417.fasta	20,824.90	[illegible]
20196-20225 HinckeM –TAXchicken	Uncategorized Sample	20196-20205_A	Mudpit_Ingel20196_MGFPeaklist (F058870)	status=inactive	UPI0000E80AEB,UPI0000ECB34C,UPI000240C082,UPI000350741B	UPARC14_2_GallusG_9031_20140417.fasta	299,043.50	[illegible]
20196-20225 HinckeM –TAXchicken	Uncategorized Sample	20196-20205_A	Mudpit_Ingel20196_MGFPeaklist (F058870)	status=inactive	UPI0000E80AEB,UPI0000ECB34C,UPI000240C082,UPI000350741B	UPARC14_2_GallusG_9031_20140417.fasta	299,043.50	[illegible]
20196-20225 HinckeM –TAXchicken	Uncategorized Sample	20196-20205_A	Mudpit_Ingel20196_MGFPeaklist (F058870)	status=inactive	UPI0000E80AEB,UPI0000ECB34C,UPI000240C082,UPI000350741B	UPARC14_2_GallusG_9031_20140417.fasta	299,043.50	[illegible]
20196-20225 HinckeM –TAXchicken	Uncategorized Sample	20196-20205_A	Mudpit_Ingel20196_MGFPeaklist (F058870)	status=inactive	UPI0000E80AEB,UPI0000ECB34C,UPI000240C082,UPI000350741B	UPARC14_2_GallusG_9031_20140417.fasta	299,043.50	[illegible]
20196-20225 HinckeM –TAXchicken	Uncategorized Sample	20196-20205_A	Mudpit_Ingel20196_MGFPeaklist (F058870)	status=inactive	UPI0000E80AEB,UPI0000ECB34C,UPI000240C082,UPI000350741B	UPARC14_2_GallusG_9031_20140417.fasta	299,043.50	[illegible]
20196-20225 HinckeM –TAXchicken	Uncategorized Sample	20196-20205_A	Mudpit_Ingel20196_MGFPeaklist (F058870)	status=inactive	UPI0000E80AEB,UPI0000ECB34C,UPI000240C082,UPI000350741B	UPARC14_2_GallusG_9031_20140417.fasta	299,043.50	[illegible]
20196-20225 HinckeM –TAXchicken	Uncategorized Sample	20196-20205_A	Mudpit_Ingel20196_MGFPeaklist (F058870)	status=inactive	UPI0000E80AEB,UPI0000ECB34C,UPI000240C082,UPI000350741B	UPARC14_2_GallusG_9031_20140417.fasta	299,043.50	[illegible]
20196-20225 HinckeM –TAXchicken	Uncategorized Sample	20196-20205_A	Mudpit_Ingel20196_MGFPeaklist (F058870)	status=inactive	UPI0000E80AEB,UPI0000ECB34C,UPI000240C082,UPI000350741B	UPARC14_2_GallusG_9031_20140417.fasta	299,043.50	[illegible]
20196-20225 HinckeM –TAXchicken	Uncategorized Sample	20196-20205_A	Mudpit_Ingel20196_MGFPeaklist (F058870)	status=inactive	UPI0000E80AEB,UPI0000ECB34C,UPI000240C082,UPI000350741B	UPARC14_2_GallusG_9031_20140417.fasta	299,043.50	[illegible]
20196-20225 HinckeM –TAXchicken	Uncategorized Sample	20196-20205_A	Mudpit_Ingel20196_MGFPeaklist (F058870)	status=inactive	UPI0000E80AEB,UPI0000ECB34C,UPI000240C082,UPI000350741B	UPARC14_2_GallusG_9031_20140417.fasta	299,043.50	[illegible]
20196-20225 HinckeM –TAXchicken	Uncategorized Sample	20196-20205_A	Mudpit_Ingel20196_MGFPeaklist (F058870)	status=inactive	UPI0000E80AEB,UPI0000ECB34C,UPI000240C082,UPI000350741B	UPARC14_2_GallusG_9031_20140417.fasta	299,043.50	[illegible]
20196-20225 HinckeM –TAXchicken	Uncategorized Sample	20196-20205_A	Mudpit_Ingel20196_MGFPeaklist (F058870)	status=inactive	UPI0000E80AEB,UPI0000ECB34C,UPI000240C082,UPI000350741B	UPARC14_2_GallusG_9031_20140417.fasta	299,043.50	[illegible]
20196-20225 HinckeM –TAXchicken	Uncategorized Sample	20196-20205_A	Mudpit_Ingel20196_MGFPeaklist (F058870)	status=inactive	UPI0000E80AEB,UPI0000ECB34C,UPI000240C082,UPI000350741B	UPARC14_2_GallusG_9031_20140417.fasta	299,043.50	[illegible]
20196-20225 HinckeM –TAXchicken	Uncategorized Sample	20196-20205_A	Mudpit_Ingel20196_MGFPeaklist (F058870)	status=inactive	UPI0000E80AEB,UPI0000ECB34C,UPI000240C082,UPI000350741B	UPARC14_2_GallusG_9031_20140417.fasta	299,043.50	[illegible]
20196-20225 HinckeM –TAXchicken	Uncategorized Sample	20196-20205_A	Mudpit_Ingel20196_MGFPeaklist (F058870)	status=inactive	UPI0000E80AEB,UPI0000ECB34C,UPI000240C082,UPI000350741B	UPARC14_2_GallusG_9031_20140417.fasta	299,043.50	[illegible]
20196-20225 HinckeM –TAXchicken	Uncategorized Sample	20196-20205_A	Mudpit_Ingel20196_MGFPeaklist (F058870)	status=inactive	UPI0000E80AEB,UPI0000ECB34C,UPI000240C082,UPI000350741B	UPARC14_2_GallusG_9031_20140417.fasta	299,043.50	[illegible]
20196-20225 HinckeM –TAXchicken	Uncategorized Sample	20196-20205_A	Mudpit_Ingel20196_MGFPeaklist (F058870)	status=inactive	UPI0000E80AEB,UPI0000ECB34C,UPI000240C082,UPI000350741B	UPARC14_2_GallusG_9031_20140417.fasta	299,043.50	[illegible]
20196-20225 HinckeM –TAXchicken	Uncategorized Sample	20196-20205_A	Mudpit_Ingel20196_MGFPeaklist (F058870)	status=inactive	UPI0000E80AEB,UPI0000ECB34C,UPI000240C082,UPI000350741B	UPARC14_2_GallusG_9031_20140417.fasta	299,043.50	[illegible]
20196-20225 HinckeM –TAXchicken	Uncategorized Sample	20196-20205_A	Mudpit_Ingel20196_MGFPeaklist (F058870)	status=inactive	UPI0000E80AEB,UPI0000ECB34C,UPI000240C082,UPI000350741B	UPARC14_2_GallusG_9031_20140417.fasta	299,043.50	[illegible]
20196-20225 HinckeM –TAXchicken	Uncategorized Sample	20196-20205_A	Mudpit_Ingel20196_MGFPeaklist (F058870)	status=inactive	UPI0000E80AEB,UPI0000ECB34C,UPI000240C082,UPI000350741B	UPARC14_2_GallusG_9031_20140417.fasta	299,043.50	[illegible]
20196-20225 HinckeM –TAXchicken	Uncategorized Sample	20196-20205_A	Mudpit_Ingel20196_MGFPeaklist (F058870)	status=inactive	UPI0000E80AEB,UPI0000ECB34C,UPI000240C082,UPI000350741B	UPARC14_2_GallusG_9031_20140417.fasta	299,043.50	[illegible]
20196-20225 HinckeM –TAXchicken	Uncategorized Sample	20196-20205_A	Mudpit_Ingel20196_MGFPeaklist (F058870)	status=inactive	UPI0000E80AEB,UPI0000ECB34C,UPI000240C082,UPI000350741B	UPARC14_2_GallusG_9031_20140417.fasta	299,043.50	[illegible]
20196-20225 HinckeM –TAXchicken	Uncategorized Sample	20196-20205_A	Mudpit_Ingel20196_MGFPeaklist (F058870)	status=inactive	UPI0000E80AEB,UPI0000ECB34C,UPI000240C082,UPI000350741B	UPARC14_2_GallusG_9031_20140417.fasta	299,043.50	[illegible]
20196-20225 HinckeM –TAXchicken	Uncategorized Sample	20196-20205_A	Mudpit_Ingel20196_MGFPeaklist (F058870)	status=inactive	UPI0000E80AEB,UPI0000ECB34C,UPI000240C082,UPI000350741B	UPARC14_2_GallusG_9031_20140417.fasta	299,043.50	[illegible]
20196-20225 HinckeM –TAXchicken	Uncategorized Sample	20196-20205_A	Mudpit_Ingel20196_MGFPeaklist (F058870)	Talin-1 OS=Gallus gallus GN=TLN1 PE=4 SV=2	E1C2S1_CHICK,TLN1_CHICK	TAX_GallusGallus_9031_20141114.fasta	271,840.20	[illegible]
20196-20225 HinckeM –TAXchicken	Uncategorized Sample	20196-20205_A	Mudpit_Ingel20196_MGFPeaklist (F058870)	Talin-1 OS=Gallus gallus GN=TLN1 PE=4 SV=2	E1C2S1_CHICK,TLN1_CHICK	TAX_GallusGallus_9031_20141114.fasta	271,840.20	[illegible]
20196-20225 HinckeM –TAXchicken	Uncategorized Sample	20196-20205_A	Mudpit_Ingel20196_MGFPeaklist (F058870)	Talin-1 OS=Gallus gallus GN=TLN1 PE=4 SV=2	E1C2S1_CHICK,TLN1_CHICK	TAX_GallusGallus_9031_20141114.fasta	271,840.20	[illegible]
20196-20225 HinckeM –TAXchicken	Uncategorized Sample	20196-20205_A	Mudpit_Ingel20196_MGFPeaklist (F058870)	Talin-1 OS=Gallus gallus GN=TLN1 PE=4 SV=2	E1C2S1_CHICK,TLN1_CHICK	TAX_GallusGallus_9031_20141114.fasta	271,840.20	[illegible]
20196-20225 HinckeM –TAXchicken	Uncategorized Sample	20196-20205_A	Mudpit_Ingel20196_MGFPeaklist (F058870)	Talin-1 OS=Gallus gallus GN=TLN1 PE=4 SV=2	E1C2S1_CHICK,TLN1_CHICK	TAX_GallusGallus_9031_20141114.fasta	271,840.20	[illegible]
20196-20225 HinckeM –TAXchicken	Uncategorized Sample	20196-20205_A	Mudpit_Ingel20196_MGFPeaklist (F058870)	Talin-1 OS=Gallus gallus GN=TLN1 PE=4 SV=2	E1C2S1_CHICK,TLN1_CHICK	TAX_GallusGallus_9031_20141114.fasta	271,840.20	[illegible]
20196-20225 HinckeM –TAXchicken	Uncategorized Sample	20196-20205_A	Mudpit_Ingel20196_MGFPeaklist (F058870)	Talin-1 OS=Gallus gallus GN=TLN1 PE=4 SV=2	E1C2S1_CHICK,TLN1_CHICK	TAX_GallusGallus_9031_20141114.fasta	271,840.20	[illegible]
20196-20225 HinckeM –TAXchicken	Uncategorized Sample	20196-20205_A	Mudpit_Ingel20196_MGFPeaklist (F058870)	Talin-1 OS=Gallus gallus GN=TLN1 PE=4 SV=2	E1C2S1_CHICK,TLN1_CHICK	TAX_GallusGallus_9031_20141114.fasta	271,840.20	[illegible]
20196-20225 HinckeM –TAXchicken	Uncategorized Sample	20196-20205_A	Mudpit_Ingel20196_MGFPeaklist (F058870)	Talin-1 OS=Gallus gallus GN=TLN1 PE=4 SV=2	E1C2S1_CHICK,TLN1_CHICK	TAX_GallusGallus_9031_20141114.fasta	271,840.20	[illegible]
20196-20225 HinckeM –TAXchicken	Uncategorized Sample	20196-20205_A	Mudpit_Ingel20196_MGFPeaklist (F058870)	Talin-1 OS=Gallus gallus GN=TLN1 PE=4 SV=2	E1C2S1_CHICK,TLN1_CHICK	TAX_GallusGallus_9031_20141114.fasta	271,840.20	[illegible]
20196-20225 HinckeM –TAXchicken	Uncategorized Sample	20196-20205_A	Mudpit_Ingel20196_MGFPeaklist (F058870)	Talin-1 OS=Gallus gallus GN=TLN1 PE=4 SV=2	E1C2S1_CHICK,TLN1_CHICK	TAX_GallusGallus_9031_20141114.fasta	271,840.20	[illegible]
20196-20225 HinckeM –TAXchicken	Uncategorized Sample	20196-20205_A	Mudpit_Ingel20196_MGFPeaklist (F058870)	Talin-1 OS=Gallus gallus GN=TLN1 PE=4 SV=2	E1C2S1_CHICK,TLN1_CHICK	TAX_GallusGallus_9031_20141114.fasta	271,840.20	[illegible]
20196-20225 HinckeM –TAXchicken	Uncategorized Sample	20196-20205_A	Mudpit_Ingel20196_MGFPeaklist (F058870)	Talin-1 OS=Gallus gallus GN=TLN1 PE=4 SV=2	E1C2S1_CHICK,TLN1_CHICK	TAX_GallusGallus_9031_20141114.fasta	271,840.20	[illegible]
20196-20225 HinckeM –TAXchicken	Uncategorized Sample	20196-20205_A	Mudpit_Ingel20196_MGFPeaklist (F058870)	Talin-1 OS=Gallus gallus GN=TLN1 PE=4 SV=2	E1C2S1_CHICK,TLN1_CHICK	TAX_GallusGallus_9031_20141114.fasta	271,840.20	[illegible]
20196-20225 HinckeM –TAXchicken	Uncategorized Sample	20196-20205_A	Mudpit_Ingel20196_MGFPeaklist (F058870)	Talin-1 OS=Gallus gallus GN=TLN1 PE=4 SV=2	E1C2S1_CHICK,TLN1_CHICK	TAX_GallusGallus_9031_20141114.fasta	271,840.20	[illegible]

20196-20225 HinckeM –TAXchicken	Uncategorized Sample	20196-20205_A	Mudpit_Ingel20196_MGFPeaklist (F058870)	Talin-1 OS=Gallus gallus GN=TLN1 PE=4 SV=2	E1C2S1_CHICK,TLN1_CHICK	TAX_GallusGallus_9031_20141114.fasta	271,840.20	100.00%	59	72	143	0.29%	25.90%	ISIGNVVK	K	T	83.50%	34.3	43.9	30.6	14.3					0	2	0	0	2	829.5142	2,051.00		3,254.37	8	15	Empty	TRUE	
20196-20225 HinckeM –TAXchicken	Uncategorized Sample	20196-20205_A	Mudpit_Ingel20196_MGFPeaklist (F058870)	Talin-1 OS=Gallus gallus GN=TLN1 PE=4 SV=2	E1C2S1_CHICK,TLN1_CHICK	TAX_GallusGallus_9031_20141114.fasta	271,840.20	100.00%	59	72	143	0.29%	25.90%	KSTVLQQQFNR	K	V	62.60%	25.1	44.3	33.6	6.33					0	0	1	0	2	1,348.73	1,762.00		346.62	428	438	Empty	TRUE	
20196-20225 HinckeM –TAXchicken	Uncategorized Sample	20196-20205_A	Mudpit_Ingel20196_MGFPeaklist (F058870)	Talin-1 OS=Gallus gallus GN=TLN1 PE=4 SV=2	E1C2S1_CHICK,TLN1_CHICK	TAX_GallusGallus_9031_20141114.fasta	271,840.20	100.00%	59	72	143	0.29%	25.90%	LAQAAQSSVSTITR	K	L	99.70%	91	44.5	28.5	23.3	52	7.89	0.513	0.5	0	3	0	0	2	1,432.78	1,788.00		3,740.29	2043	2056	Empty	TRUE	
20196-20225 HinckeM –TAXchicken	Uncategorized Sample	20196-20205_A	Mudpit_Ingel20196_MGFPeaklist (F058870)	Talin-1 OS=Gallus gallus GN=TLN1 PE=4 SV=2	E1C2S1_CHICK,TLN1_CHICK	TAX_GallusGallus_9031_20141114.fasta	271,840.20	100.00%	59	72	143	0.29%	25.90%	LHTDDELNWLDHGR	K	T	99.70%	50.3	44.2	23	39.3	42.9	6.89	0.524	0.464	0	0	1	1	2	1,720.80	2,278.50		1,166.09	165	178	Empty	TRUE	
20196-20225 HinckeM –TAXchicken	Uncategorized Sample	20196-20205_A	Mudpit_Ingel20196_MGFPeaklist (F058870)	Talin-1 OS=Gallus gallus GN=TLN1 PE=4 SV=2	E1C2S1_CHICK,TLN1_CHICK	TAX_GallusGallus_9031_20141114.fasta	271,840.20	100.00%	59	72	143	0.29%	25.90%	LLAALMEDEGGNGR	K	Q	99.70%	73.7	44.7	31.6	60	42.6	5.55	0.465	0.429	0	6	0	0	2	1,462.68	2,062.50		3,549.60	593	606	Empty	TRUE	
20196-20225 HinckeM –TAXchicken	Uncategorized Sample	20196-20205_A	Mudpit_Ingel20196_MGFPeaklist (F058870)	Talin-1 OS=Gallus gallus GN=TLN1 PE=4 SV=2	E1C2S1_CHICK,TLN1_CHICK	TAX_GallusGallus_9031_20141114.fasta	271,840.20	100.00%	59	72	143	0.29%	25.90%	LLSDSFPPSNK	R	T	74.60%	27.1	45.1	22.3	9.36					0	1	0	0	2	1,204.62	2,136.00		154.75	1222	1232	Empty	TRUE	
20196-20225 HinckeM –TAXchicken	Uncategorized Sample	20196-20205_A	Mudpit_Ingel20196_MGFPeaklist (F058870)	Talin-1 OS=Gallus gallus GN=TLN1 PE=4 SV=2	E1C2S1_CHICK,TLN1_CHICK	TAX_GallusGallus_9031_20141114.fasta	271,840.20	100.00%	59	72	143	0.29%	25.90%	LPEFGESISAASK	K	A	99.70%	37.6	44.8	22.7	27.7	30.4	1.72	0.319	0.308	0	2	0	0	2	1,335.68	2,221.50		805.77	1418	1430	Empty	TRUE	
20196-20225 HinckeM –TAXchicken	Uncategorized Sample	20196-20205_A	Mudpit_Ingel20196_MGFPeaklist (F058870)	Talin-1 OS=Gallus gallus GN=TLN1 PE=4 SV=2	E1C2S1_CHICK,TLN1_CHICK	TAX_GallusGallus_9031_20141114.fasta	271,840.20	100.00%	59	72	143	0.29%	25.90%	NLAAALAELR	K	T	99.70%	78.6	42.9	34.7	51	38.3	2.17	0.316	0.55	0	3	0	0	2	1,041.61	2,641.00		1,588.89	1025	1034	Empty	TRUE	
20196-20225 HinckeM –TAXchicken	Uncategorized Sample	20196-20205_A	Mudpit_Ingel20196_MGFPeaklist (F058870)	Talin-1 OS=Gallus gallus GN=TLN1 PE=4 SV=2	E1C2S1_CHICK,TLN1_CHICK	TAX_GallusGallus_9031_20141114.fasta	271,840.20	100.00%	59	72	143	0.29%	25.90%	NLASAVSDLLK	K	T	99.70%	64	44.6	19.9	55.2	44.1	2.64	0.395	0.545	0	2	0	0	2	1,130.64	2,852.50		4,184.46	614	624	Empty	TRUE	
20196-20225 HinckeM –TAXchicken	Uncategorized Sample	20196-20205_A	Mudpit_Ingel20196_MGFPeaklist (F058870)	Talin-1 OS=Gallus gallus GN=TLN1 PE=4 SV=2	E1C2S1_CHICK,TLN1_CHICK	TAX_GallusGallus_9031_20141114.fasta	271,840.20	100.00%	59	72	143	0.29%	25.90%	NSKLPEFGESISAASK	K	A	99.70%	72.8	44.5	29.2	65.9	42.3	5.13	0.428	0.406	0	0	2	0	2	1,664.85	2,142.00		3,563.25	1415	1430	Empty	TRUE	
20196-20225 HinckeM –TAXchicken	Uncategorized Sample	20196-20205_A	Mudpit_Ingel20196_MGFPeaklist (F058870)	Talin-1 OS=Gallus gallus GN=TLN1 PE=4 SV=2	E1C2S1_CHICK,TLN1_CHICK	TAX_GallusGallus_9031_20141114.fasta	271,840.20	100.00%	59	72	143	0.29%	25.90%	PAALTAENEEIGSHIK	K	R	99.70%					45.7	5.17	0.481	0.438	0	0	2	0	1	1,679.86	1,990.50		3,158.20	1901	1916	Empty	TRUE	
20196-20225 HinckeM –TAXchicken	Uncategorized Sample	20196-20205_A	Mudpit_Ingel20196_MGFPeaklist (F058870)	Talin-1 OS=Gallus gallus GN=TLN1 PE=4 SV=2	E1C2S1_CHICK,TLN1_CHICK	TAX_GallusGallus_9031_20141114.fasta	271,840.20	100.00%	59	72	143	0.29%	25.90%	QEDVIATANLSR	R	R	99.70%	55.5	45	31.3	30.3	32.4	2.89	0.34	0.375	0	3	0	0	2	1,316.68	2,208.00		786.49	2197	2208	Empty	TRUE	E1BR45_CHICK
20196-20225 HinckeM –TAXchicken	Uncategorized Sample	20196-20205_A	Mudpit_Ingel20196_MGFPeaklist (F058870)	Talin-1 OS=Gallus gallus GN=TLN1 PE=4 SV=2	E1C2S1_CHICK,TLN1_CHICK	TAX_GallusGallus_9031_20141114.fasta	271,840.20	100.00%	59	72	143	0.29%	25.90%	QQQYKFLPTEL	R	R	52.00%					25.3	0.921	0.0791	0.227	0	1	0	0	1	1,394.73	2,160.00		882.16	2525	2535	Empty	TRUE	E1BR45_CHICK
20196-20225 HinckeM –TAXchicken	Uncategorized Sample	20196-20205_A	Mudpit_Ingel20196_MGFPeaklist (F058870)	Talin-1 OS=Gallus gallus GN=TLN1 PE=4 SV=2	E1C2S1_CHICK,TLN1_CHICK	TAX_GallusGallus_9031_20141114.fasta	271,840.20	100.00%	59	72	143	0.29%	25.90%	QVAASTAQLLVACK	K	V	99.70%	62	44.5	21.5	57.6	44.2	7.42	0.493	0.429	0	4	0	0	2	1,459.79	2,312.00		3,135.98	2429	2442	Empty	TRUE	E1BR45_CHICK
20196-20225 HinckeM –TAXchicken	Uncategorized Sample	20196-20205_A	Mudpit_Ingel20196_MGFPeaklist (F058870)	Talin-1 OS=Gallus gallus GN=TLN1 PE=4 SV=2	E1C2S1_CHICK,TLN1_CHICK	TAX_GallusGallus_9031_20141114.fasta	271,840.20	100.00%	59	72	143	0.29%	25.90%	RVQELGHGCAALVTK	R	A	99.70%	24.4	43.9	18.1	16.2	26.7	1.13	0.288	0.3	0	0	2	0	2	1,638.87	1,733.50		1,243.05	1918	1932	Empty	TRUE	
20196-20225 HinckeM –TAXchicken	Uncategorized Sample	20196-20205_A	Mudpit_Ingel20196_MGFPeaklist (F058870)	Talin-1 OS=Gallus gallus GN=TLN1 PE=4 SV=2	E1C2S1_CHICK,TLN1_CHICK	TAX_GallusGallus_9031_20141114.fasta	271,840.20	100.00%	59	72	143	0.29%	25.90%	SIAAATSALVK	K	A	99.70%	38.2	44.2	26.7	14.2	34.1	0.921	0.311	0.455	0	3	0	0	2	1,031.61	2,081.00		2,086.72	2350	2360	Empty	TRUE	E1BR45_CHICK
20196-20225 HinckeM –TAXchicken	Uncategorized Sample	20196-20205_A	Mudpit_Ingel20196_MGFPeaklist (F058870)	Talin-1 OS=Gallus gallus GN=TLN1 PE=4 SV=2	E1C2S1_CHICK,TLN1_CHICK	TAX_GallusGallus_9031_20141114.fasta	271,840.20	100.00%	59	72	143	0.29%	25.90%	SLAVNPKDPPQWSVLAGHSR	R	T	99.70%	49.3	43.4	15.6	0	41.7	6.51	0.417	0.35	0	0	0	2	2	2,159.14	2,315.50		2,558.67	1618	1637	Empty	TRUE	
20196-20225 HinckeM –TAXchicken	Uncategorized Sample	20196-20205_A	Mudpit_Ingel20196_MGFPeaklist (F058870)	Talin-1 OS=Gallus gallus GN=TLN1 PE=4 SV=2	E1C2S1_CHICK,TLN1_CHICK	TAX_GallusGallus_9031_20141114.fasta	271,840.20	100.00%	59	72	143	0.29%	25.90%	STVLQQQFNR	K	V	99.70%	72	44.7	30.8	55.8	34.2	2.15	0.342	0.5	0	2	0	0	2	1,220.64	1,929.50		1,592.61	429	438	Empty	TRUE	
20196-20225 HinckeM –TAXchicken	Uncategorized Sample	20196-20205_A	Mudpit_Ingel20196_MGFPeaklist (F058870)	Talin-1 OS=Gallus gallus GN=TLN1 PE=4 SV=2	E1C2S1_CHICK,TLN1_CHICK	TAX_GallusGallus_9031_20141114.fasta	271,840.20	100.00%	59	72	143	0.29%	25.90%	TFQEAQSQLNR	K	A	99.70%	62.6	44.7	22.1	36.3	35.7	4.31	0.403	0.455	0	3	0	0	2	1,321.65	1,695.00		1,334.70	1233	1243	Empty	TRUE	
20196-20225 HinckeM –TAXchicken	Uncategorized Sample	20196-20205_A	Mudpit_Ingel20196_MGFPeaklist (F058870)	Talin-1 OS=Gallus gallus GN=TLN1 PE=4 SV=2	E1C2S1_CHICK,TLN1_CHICK	TAX_GallusGallus_9031_20141114.fasta	271,840.20	100.00%	59	72	143	0.29%	25.90%	TKEVIQEWSLTNIKR	K	W	98.50%	34.8	42.4	21.1	26.2					0	0	0	2	2	1,845.02	2,302.50		792.84	344	358	Empty	TRUE	
20196-20225 HinckeM –TAXchicken	Uncategorized Sample	20196-20205_A	Mudpit_Ingel20196_MGFPeaklist (F058870)	Talin-1 OS=Gallus gallus GN=TLN1 PE=4 SV=2	E1C2S1_CHICK,TLN1_CHICK	TAX_GallusGallus_9031_20141114.fasta	271,840.20	100.00%	59	72	143	0.29%	25.90%	TLAESALQMLYTAK	K	E	99.70%	40.9	44.4	16.9	31.1	28.1	1.3	0.249	0.286	0	1	1	0	2	1,555.80	2,593.50		662.51	1766	1779	Empty	TRUE	
20196-20225 HinckeM –TAXchicken	Uncategorized Sample	20196-20205_A	Mudpit_Ingel20196_MGFPeaklist (F058870)	Talin-1 OS=Gallus gallus GN=TLN1 PE=4 SV=2	E1C2S1_CHICK,TLN1_CHICK	TAX_GallusGallus_9031_20141114.fasta	271,840.20	100.00%	59	72	143	0.29%	25.90%	TLREQGIDDNETLLLR	R	R	99.70%	60.9	43.9	25.7	49.3	46.5	4.74	0.376	0.438	0	0	2	0	2	1,886.00	2,306.50		1,561.49	179	194	Empty	TRUE	
20196-20225 HinckeM –TAXchicken	Uncategorized Sample	20196-20205_A	Mudpit_Ingel20196_MGFPeaklist (F058870)	Talin-1 OS=Gallus gallus GN=TLN1 PE=4 SV=2	E1C2S1_CHICK,TLN1_CHICK	TAX_GallusGallus_9031_20141114.fasta	271,840.20	100.00%	59	72	143	0.29%	25.90%	TLREQGIDDNETLLLRR	R	K	26.60%	17.5	42.7	16.9	11.1					0	0	1	3	2	2,042.10	2,178.00		1,555.46	179	195	Empty	TRUE	
20196-20225 HinckeM –TAXchicken	Uncategorized Sample	20196-20205_A	Mudpit_Ingel20196_MGFPeaklist (F058870)	Talin-1 OS=Gallus gallus GN=TLN1 PE=4 SV=2	E1C2S1_CHICK,TLN1_CHICK	TAX_GallusGallus_9031_20141114.fasta	271,840.20	100.00%	59	72	143	0.29%	25.90%	TMLESSAGLIQTAR	K	S	99.70%	74.1	44.7	24.7	57.9	50.1	6.28	0.475	0.5	0	4	0	0	2	1,493.76	2,152.50		3,334.01	1604	1617	Empty	TRUE	
20196-20225 HinckeM –TAXchicken	Uncategorized Sample	20196-20205_A	Mudpit_Ingel20196_MGFPeaklist (F058870)	Talin-1 OS=Gallus gallus GN=TLN1 PE=4 SV=2	E1C2S1_CHICK,TLN1_CHICK	TAX_GallusGallus_9031_20141114.fasta	271,840.20	100.00%	59	72	143	0.29%	25.90%	TPNHQQQMNLLDQTK	K	T	99.60%	24.9	44.1	22.5	12.6	27	0.538	0.156	0.3	0	0	2	0	2	1,811.87	1,693.50		528.1	1751	1765	Empty	TRUE	
20196-20225 HinckeM –TAXchicken	Uncategorized Sample	20196-20205_A	Mudpit_Ingel20196_MGFPeaklist (F058870)	Talin-1 OS=Gallus gallus GN=TLN1 PE=4 SV=2	E1C2S1_CHICK,TLN1_CHICK	TAX_GallusGallus_9031_20141114.fasta	271,840.20	100.00%	59	72	143	0.29%	25.90%	TYGVSFFLVK	K	E	99.70%	33.4	44.9	19.4	27.8	33.4	1.8	0.338	0.5	0	2	0	0	2	1,160.64	2,786.50		1,278.77	307	316	Empty	TRUE	E1BR45_CHICK
20196-20225 HinckeM –TAXchicken	Uncategorized Sample	20196-20205_A	Mudpit_Ingel20196_MGFPeaklist (F058870)	Talin-1 OS=Gallus gallus GN=TLN1 PE=4 SV=2	E1C2S1_CHICK,TLN1_CHICK	TAX_GallusGallus_9031_20141114.fasta	271,840.20	100.00%	59	72	143	0.29%	25.90%	VDQASLAAISQQLAPR	E	E	79.70%					25.1	0.367	0.0438	0.219	0	0	0	1	1	1,650.88	1,875.00		1,016.88	1674	1689	Empty	TRUE	
20196-20225 HinckeM –TAXchicken	Uncategorized Sample	20196-20205_A	Mudpit_Ingel20196_MGFPeaklist (F058870)	Talin-1 OS=Gallus gallus GN=TLN1 PE=4 SV=2	E1C2S1_CHICK,TLN1_CHICK	TAX_GallusGallus_9031_20141114.fasta	271,840.20	100.00%	59	72	143	0.29%	25.90%	VGKAELGSVALPAIMR	R	T	92.40%	24.2	42.2	15.9	20.1					0	0	2	0	2	1,627.92	2,320.50		1,034.67	439	454	Empty	TRUE	
20196-20225 HinckeM –TAXchicken	Uncategorized Sample	20196-20205_A	Mudpit_Ingel20196_MGFPeaklist (F058870)	Talin-1 OS=Gallus gallus GN=TLN1 PE=4 SV=2	E1C2S1_CHICK,TLN1_CHICK	TAX_GallusGallus_9031_20141114.fasta	271,840.20	100.00%	59	72	143	0.29%	25.90%	VLGESMAGISQNAK	K	N	99.70%	67.2	44.8	22.7	58.7	45.9	4.92	0.46	0.464	0	4	0	0	2	1,420.71	1,833.50		1,448.49	1401	1414	Empty	TRUE	
20196-20225 HinckeM –TAXchicken	Uncategorized Sample	20196-20205_A	Mudpit_Ingel20196_MGFPeaklist (F058870)	Talin-1 OS=Gallus gallus GN=TLN1 PE=4 SV=2	E1C2S1_CHICK,TLN1_CHICK	TAX_GallusGallus_9031_20141114.fasta	271,840.20	100.00%	59	72	143	0.29%	25.90%	VMVTNVTSLLK	K	T	99.70%	55.6	43.6	25.1	36.5	41.1	5.66	0.392	0.545	0	4	0	0	2	1,204.70	2,523.50		1,708.14	2119	2129	Empty	TRUE	E1BR45_CHICK
20196-20225 HinckeM –TAXchicken	Uncategorized Sample	20196-20205_A	Mudpit_Ingel20196_MGFPeaklist (F058870)	Talin-1 OS=Gallus gallus GN=TLN1 PE=4 SV=2	E1C2S1_CHICK,TLN1_CHICK	TAX_GallusGallus_9031_20141114.fasta	271,840.20	100.00%	59	72	143	0.29%	25.90%	VQELGHGCAALVTK	R	A	13.70%	7.12	44	20.5	0					0	1	0	0	2	1,482.77	1,817.00		133.58	1919	1932	Empty	TRUE	
20196-20225 HinckeM –TAXchicken	Uncategorized Sample	20196-20205_A	Mudpit_Ingel20196_MGFPeaklist (F058870)	Talin-1 OS=Gallus gallus GN=TLN1 PE=4 SV=2	E1C2S1_CHICK,TLN1_CHICK	TAX_GallusGallus_9031_20141114.fasta	271,840.20	100.00%	59	72	143	0.29%	25.90%	VSHVLAALQAGNR	K	G	99.70%	32.2	43.3	22	23.7	26.7	1.11	0.281	0.346	0	0	2	0	2	1,335.75	1,989.50		1,887.53	1960	1972	Empty	TRUE	
20196-20225 HinckeM –TAXchicken	Uncategorized Sample	20196-20205_A	Mudpit_Ingel20196_MGFPeaklist (F058870)	Talin-1 OS=Gallus gallus GN=TLN1 PE=4 SV=2	E1C2S1_CHICK,TLN1_CHICK	TAX_GallusGallus_9031_20141114.fasta	271,840.20	100.00%	59	72	143	0.29%	25.90%	VVADQIPMLVQGVR	K	G	82.30%	25.2	43.1	15.5	13.8					0	1	2	0	2	1,540.85	2,452.00		1,196.64	957	970	Empty	TRUE	
20196-20225 HinckeM –TAXchicken	Uncategorized Sample	20196-20205_A	Mudpit_Ingel20196_MGFPeaklist (F058870)	Talin-1 OS=Gallus gallus GN=TLN1 PE=4 SV=2	E1C2S1_CHICK,TLN1_CHICK	TAX_GallusGallus_9031_20141114.fasta	271,840.20	100.00%	59	72	143	0.29%	25.90%	VVAPTISSPVCQEQLIEAGK	K	L	99.70%	64.1	43.4	15.6	54.3	55	7.55	0.193	0.4	0	0	2	0	2	2,126.12	2,379.00		2,707.90	722	741	Empty	TRUE	
20196-20225 HinckeM –TAXchicken	Uncategorized Sample	20196-20205_A	Mudpit_Ingel20196_MGFPeaklist (F058870)	Talin-1 OS=Gallus gallus GN=TLN1 PE=4 SV=2	E1C2S1_CHICK,TLN1_CHICK	TAX_GallusGallus_9031_20141114.fasta	271,840.20	100.00%	59	72	143	0.29%	25.90%	AAAFQDHDETVVVKEK	K	M	96.20%					30.1	1.37	0.183	0.312	0	0	0	1	2	1,786.90	1,726.00		1,193.44	2476	2491	Empty	TRUE	
20196-20225 HinckeM –TAXchicken	Uncategorized Sample	20196-20205_A	Mudpit_Ingel20196_MGFPeaklist (F058870)	Talin-1 OS=Gallus gallus GN=TLN1 PE=4 SV=2	E1C2S1_CHICK,TLN1_CHICK	TAX_GallusGallus_9031_20141114.fasta	271,840.20	100.00%	59	72	143	0.29%	25.90%	AAAGLNQSANELVQASR	R	G	99.70%	56.1	44.5	21.8	48	49.8	7.96	0.528	0.471	0	0	2	0	2	1,699.87	2,099.50		4,622.52	1244	1260	Empty	TRUE	
20196-20225 HinckeM –TAXchicken	Uncategorized Sample	20196-20205_A	Mudpit_Ingel20196_MGFPeaklist (F058870)	Talin-1 OS=Gallus gallus GN=TLN1 PE=4 SV=2	E1C2S1_CHICK,TLN1_CHICK	TAX_GallusGallus_9031_20141114.fasta	271,840.20	100.00%	59	72	143	0.29%	25.90%	AELGSVALPAIMR	K	T	97.50%	33.9	44.3	24.5	22					0	2	0	0	2	1,343.74	2,406.00		813.56	442	454	Empty	TRUE	
20196-20225 HinckeM –TAXchicken	Uncategorized Sample	20196-20205_A	Mudpit_Ingel20196_MGFPeaklist (F058870)	Talin-1 OS=Gallus gallus GN=TLN1 PE=4 SV=2	E1C2S1_CHICK,TLN1_CHICK	TAX_GallusGallus_9031_20141114.fasta	271,840.20	100.00%	59	72	143	0.29%	25.90%	AGALQCSPSDAYTKK	K	E	99.70%	74.4	44.3	23.9	46.2	47.3	6.34	0.516	0.5	0	0	4	0	2	1,596.77	1,601.50		1,410.57	1933	1947	Empty	TRUE	
20196-20225 HinckeM –TAXchicken	Uncategorized Sample	20196-20205_A	Mudpit_Ingel20196_MGFPeaklist (F058870)	Talin-1 OS=Gallus gallus GN=TLN1 PE=4 SV=2	E1C2S1_CHICK,TLN1_CHICK	TAX_GallusGallus_9031_20141114.fasta	271,840.20	100.00%	59	72	143	0.29%	25.90%	AGDDPAVYQLK	K	N	99.70%	54.7	45.2	31.6	37.9	53.6	5.04	0.437	0.682	0	3	0	0	2	1,176.59	1,908.00		3,351.51	2104	2114	Empty	TRUE	
20196-20225 HinckeM –TAXchicken	Uncategorized Sample	20196-20205_A	Mudpit_Ingel20196_MGFPeaklist (F058870)	Talin-1 OS=Gallus gallus GN=TLN1 PE=4 SV=2	E1C2S1_CHICK,TLN1_CHICK	TAX_GallusGallus_9031_20141114.fasta	271,840.20	100.00%	59	72	143	0.29%	25.90%	AIAVTVQEMVTK	K	S	99.70%	59.7	44.6	21.8	52.3	35.9	3.06	0.318	0.417	0	4	0	0	2	1,305.71	2,135.00		2,285.47	1862	1873	Empty	TRUE	
20196-20225 HinckeM –TAXchicken	Uncategorized Sample	20196-20205_A	Mudpit_Ingel20196_MGFPeaklist (F058870)	Talin-1 OS=Gallus gallus GN=TLN1 PE=4 SV=2	E1C2S1_CHICK,TLN1_CHICK	TAX_GallusGallus_9031_20141114.fasta	271,840.20	100.00%	59	72	143	0.29%	25.90%	ALDGAFNEENRER	K	C	99.30%	20.9	44.3	17.5	7.16	25.4	0.523	0.181	0.308	0	0	2	0	2	1,520.71	1,759.50		1,514.04	1544	1556	Empty	TRUE	
20196-20225 HinckeM –TAXchicken	Uncategorized Sample	20196-20205_A	Mudpit_Ingel20196_MGFPeaklist (F058870)	Talin-1 OS=Gallus gallus GN=TLN1 PE=4 SV=2	E1C2S1_CHICK,TLN1_CHICK	TAX_GallusGallus_9031_20141114.fasta	271,840.20	100.00%	59	72	143	0.29%	25.90%	ALDYYMLR	K	N	99.70%	31.9	45	24.7	21	24.9	0.495	0.205	0.312	0	1	0	0	2	1,044.52	2,354.00		430.54	67	74	Empty	TRUE	
20196-20225 HinckeM –TAXchicken	Uncategorized Sample	20196-20205_A	Mudpit_Ingel20196_MGFPeaklist (F058870)	Talin-1 OS=Gallus gallus GN=TLN1 PE=4 SV=2	E1C2S1_CHICK,TLN1_CHICK	TAX_GallusGallus_9031_20141114.fasta	271,840.20	100.00%	59	72	143	0.29%	25.90%	ALGDLIGATK	K	A	99.70%	59.1	44.8	45.9	31.3	31.6	1.92	0.177	0.4	0	3	0	0	2	958.557	2,214.00		2,372.76	2089	2098	Empty	TRUE	
20196-20225 HinckeM –TAXchicken	Uncategorized Sample	20196-20205_A	Mudpit_Ingel20196_MGFPeaklist (F058870)	Talin-1 OS=Gallus gallus GN=TLN1 PE=4 SV=2	E1C2S1_CHICK,TLN1_CHICK	TAX_GallusGallus_9031_20141114.fasta	271,840.20	100.00%	59	72	143	0.29%	25.90%	ALSADPTSPNLK	K	S	99.70%	51.8	44.3	26.7	33	34.1	1.89	0.279	0.375	0	4	0	0	2	1,213.64	1,793.50		2,398.45	1320	1331	Empty	TRUE	
20196-20225 HinckeM –TAXchicken	Uncategorized Sample	20196-20205_A	Mudpit_Ingel20196_MGFPeaklist (F058870)	Talin-1 OS=Gallus gallus GN=TLN1 PE=4 SV=2	E1C2S1_CHICK,TLN1_CHICK	TAX_GallusGallus_9031_20141114.fasta	271,840.20	100.00%	59	72	143	0.29%	25.90%	ALVEDTKVLVQNATASQEK	K	L	99.70%	30	43.2	14	20.9	28.3	1.77	0.244	0.211	0	0	2	0	2	2,044.09	2,251.50		855.25	2024	2042	Empty	TRUE	
20196-20225 HinckeM –TAXchicken	Uncategorized Sample	20196-20205_A	Mudpit_Ingel20196_MGFPeaklist (F058870)	Talin-1 OS=Gallus gallus GN=TLN1 PE=4 SV=2	E1C2S1_CHICK,TLN1_CHICK	TAX_GallusGallus_9031_20141114.fasta	271,840.20	100.00%	59	72	143	0.29%	25.90%	ATVPTITDQASAMQLSQCAK	K	N	99.70%	37	44.2	17.7	32.5	29.7	2.8	0.323	0.175	0	0	2	0	2	2,137.03	2,075.50		1,122.77	1005	1024	Empty	TRUE	
20196-20225 HinckeM –TAXchicken	Uncategorized Sample	20196-20205_A	Mudpit_Ingel20196_MGFPeaklist (F058870)	Talin-1 OS=Gallus gallus GN=TLN1 PE=4 SV=2	E1C2S1_CHICK,TLN1_CHICK	TAX_GallusGallus_9031_20141114.fasta	271,840.20	100.00%	59	72	143	0.29%	25.90%	AVAAGNSCRQEDVIATANLSR	K	R	99.70%	38.5	44.4	14.6	34.5	41.6	4.23	0.37	0.333	0	0	2	0	2	2,203.09	1,979.00		1,839.93	2188	2208	Empty	TRUE	E1BR45_CHICK
20196-20225 HinckeM –TAXchicken	Uncategorized Sample	20196-20205_A	Mudpit_Ingel20196_MGFPeaklist (F058870)	Talin-1 OS=Gallus gallus GN=TLN1 PE=4 SV=2	E1C2S1_CHICK,TLN1_CHICK	TAX_GallusGallus_9031_20141114.fasta	271,840.20	100.00%	59	72	143	0.29%	25.90%	AVASAAAALVLK	K	A	99.70%	36.2	41.2	18.8	18.8	34.4	2.55	0.302	0.375	0	2	0	0	2	1,084.67	2,344.00		2,770.26	674	685	Empty	TRUE	
20196-20225 HinckeM –TAXchicken	Uncategorized Sample	20196-20205_A	Mudpit_Ingel20196_MGFPeaklist (F058870)	Talin-1 OS=Gallus gallus GN=TLN1 PE=4 SV=2	E1C2S1_CHICK,TLN1_CHICK	TAX_GallusGallus_9031_20141114.fasta	271,840.20	100.00%	59	72	143	0.29%	25.90%	DDILNGSHPVSFDK	R	A	25.50%	11.5	43.9	16.6	4.11					0	0	2	0	2	1,544.72	2,120.50		529.93	221	234	Empty	TRUE	
20196-20225 HinckeM –TAXchicken	Uncategorized Sample	20196-20205_A	Mudpit_Ingel20196_MGFPeaklist (F058870)	Talin-1 OS=Gallus gallus GN=TLN1 PE=4 SV=2	E1C2S1_CHICK,TLN1_CHICK	TAX_GallusGallus_9031_20141114.fasta	271,840.20	100.00%	59	72	143	0.29%	25.90%	EAAYHPEVSADVR	K	Q	99.70%	61.5	44.1	28	46.8	35.3	3.06	0.371	0.346	0	0	4	0	2	1,443.69	1,725.00		1,833.45	2220	2232	Empty	TRUE	
20196-20225 HinckeM –TAXchicken	Uncategorized Sample	20196-20205_A	Mudpit_Ingel20196_MGFPeaklist (F058870)	Talin-1 OS=Gallus gallus GN=TLN1 PE=4 SV=2	E1C2S1_CHICK,TLN1_CHICK	TAX_GallusGallus_9031_20141114.fasta	271,840.20	100.00%	59	72	143	0.29%	25.90%	ECDEAIDVLNR	R	C	99.70%	56.4	43.9	27.5	35.9	35.3	3.42	0.394	0.409	0	4	0	0	2	1,333.61	2,120.00		2,063.70	1659	1669	Empty	TRUE	
20196-20225 HinckeM –TAXchicken	Uncategorized Sample	20196-20205_A	Mudpit_Ingel20196_MGFPeaklist (F058870)	Talin-1 OS=Gallus gallus GN=TLN1 PE=4 SV=2	E1C2S1_CHICK,TLN1_CHICK	TAX_GallusGallus_9031_20141114.fasta	271,840.20	100.00%	59	72	143	0.29%	25.90%	EQGIDDNETLLLR	R	R	99.70%	93.7	44.7	26.1	35.2	48.3	5.34	0.182	0.5	0	2	0	0	2	1,515.77	2,324.00		1,807.04	182	194	Empty	TRUE	
20196-20225 HinckeM –TAXchicken	Uncategorized Sample	20196-20205_A	Mudpit_Ingel20196_MGFPeaklist (F058870)	Talin-1 OS=Gallus gallus GN=TLN1 PE=4 SV=2	E1C2S1_CHICK,TLN1_CHICK	TAX_GallusGallus_9031_20141114.fasta	271,840.20	100.00%	59	72	143	0.29%	25.90%	EQGIDDNETLLLRR	R	K	99.70%	27.8	44.4	17.4	7.22	28	1.89	0.0893	0.286	0	0	1	0	2	1,671.87	2,165.00		694.61	182	195	Empty	TRUE	
20196-20225 HinckeM –TAXchicken	Uncategorized Sample	20196-20205_A	Mudpit_Ingel20196_MGFPeaklist (F058870)	Talin-1 OS=Gallus gallus GN=TLN1 PE=4 SV=2	E1C2S1_CHICK,TLN1_CHICK	TAX_GallusGallus_9031_20141114.fasta	271,840.20	100.00%	59	72	143	0.29%	25.90%	EVANSTANLVK	K	T	99.70%	47.5	45.1	22.3	31.4	30.5	1.14	0.256	0.364	0	2	0	0	2	1,145.62	1,720.50		713.07	1530	1540	Empty	TRUE	E1BR45_CHICK
20196-20225 HinckeM –TAXchicken	Uncategorized Sample	20196-20205_A	Mudpit_Ingel20196_MGFPeaklist (F058870)	Talin-1 OS=Gallus gallus GN=TLN1 PE=4 SV=2	E1C2S1_CHICK,TLN1_CHICK	TAX_GallusGallus_9031_20141114.fasta	271,840.20	100.00%	59	72	143	0.29%	25.90%	EVDQASLAAISQQLAPR	R	E	99.70%	66.2	43.9	26	57.1	40.2	4.62	0.398	0.412	0	0	2	0	2	1,796.95	2,572.00		1,044.72	1673	1689	Empty	TRUE	
20196-20225 HinckeM –TAXchicken	Uncategorized Sample	20196-20205_A	Mudpit_Ingel20196_MGFPeaklist (F058870)	Talin-1 OS=Gallus gallus GN=TLN1 PE=4 SV=2	E1C2S1_CHICK,TLN1_CHICK	TAX_GallusGallus_9031_20141114.fasta	271,840.20	100.00%	59	72	143	0.29%	25.90%	EVIQEWSLTNIKR	K	W	99.70%	56	43.6	31.1	39.7	32.5	2.85	0.345	0.385	0	0	2	0	2	1,615.88	2,388.00		617.43	346	358	Empty	TRUE	
20196-20225 HinckeM –TAXchicken	Uncategorized Sample	20196-20205_A	Mudpit_Ingel20196_MGFPeaklist (F058870)	Talin-1 OS=Gallus gallus GN=TLN1 PE=4 SV=2	E1C2S1_CHICK,TLN1_CHICK	TAX_GallusGallus_9031_20141114.fasta	271,840.20	100.00%	59	72	143	0.29%	25.90%	FLPTELRDEEQN	K	-	25.80%	10.7	44.6	21.9	1.6					0	1	0	0	2	1,490.71	2,111.00		356.12	2530	2541	Empty	TRUE	
20196-20225 HinckeM –TAXchicken	Uncategorized Sample	20196-20205_A	Mudpit_Ingel20196_MGFPeaklist (F058870)	Talin-1 OS=Gallus gallus GN=TLN1 PE=4 SV=2	E1C2S1_CHICK,TLN1_CHICK	TAX_GallusGallus_9031_20141114.fasta	271,840.20	100.00%	59	72	143	0.29%	25.90%	GIWLEAGK	K	A	99.70%	28.3	45.7	30.6	12.4	24.7	0.523	0.13	0.312	0	4	0	0	2	873.483	2,210.50		1,043.61	59	66	Empty	TRUE	
20196-20225 HinckeM –TAXchicken	Uncategorized Sample	20196-20205_A	Mudpit_Ingel20196_MGFPeaklist (F058870)	Talin-1 OS=Gallus gallus GN=TLN1 PE=4 SV=2	E1C2S1_CHICK,TLN1_CHICK	TAX_GallusGallus_9031_20141114.fasta	271,840.20	100.00%	59	72	143	0.29%	25.90%	IGITNYDEYSLVR	R	E	99.70%	86.2	44.5	27.5	74	54.1	8.92	0.499	0.577	0	2	0	0	2	1,542.78	2,431.50		2,544.59	119	131	Empty	TRUE	
20196-20225 HinckeM –TAXchicken	Uncategorized Sample	20196-20205_A	Mudpit_Ingel20196_MGFPeaklist (F058870)	Talin-1 OS=Gallus gallus GN=TLN1 PE=4 SV=2	E1C2S1_CHICK,TLN1_CHICK	TAX_GallusGallus_9031_20141114.fasta	271,840.20	100.00%	59	72	143	0.29%	25.90%	ISIGNVVK	K	T	83.50%	34.3	43.9	30.6	14.3					0	2	0	0	2	829.5142	2,051.00		3,254.37	8	15	Empty	TRUE	
20196-20225 HinckeM –TAXchicken	Uncategorized Sample	20196-20205_A	Mudpit_Ingel20196_MGFPeaklist (F058870)	Talin-1 OS=Gallus gallus GN=TLN1 PE=4 SV=2	E1C2S1_CHICK,TLN1_CHICK	TAX_GallusGallus_9031_20141114.fasta	271,840.20	100.00%	59	72	143	0.29%	25.90%	KSTVLQQQFNR	K	V	62.60%	25.1	44.3	33.6	6.33					0	0	1	0	2	1,348.73	1,762.00		346.62	428	438	Empty	TRUE	
20196-20225 HinckeM –TAXchicken	Uncategorized Sample	20196-20205_A	Mudpit_Ingel20196_MGFPeaklist (F058870)	Talin-1 OS=Gallus gallus GN=TLN1 PE=4 SV=2	E1C2S1_CHICK,TLN1_CHICK	TAX_GallusGallus_9031_20141114.fasta	271,840.20	100.00%	59	72	143	0.29%	25.90%	LAQAAQSSVSTITR	K	L	99.70%	91	44.5	28.5	23.3	52	7.89	0.513	0.5	0	3	0	0	2	1,432.78	1,788.00		3,740.29	2043	2056	Empty	TRUE	
20196-20225 HinckeM –TAXchicken	Uncategorized Sample	20196-20205_A	Mudpit_Ingel20196_MGFPeaklist (F058870)	Talin-1 OS=Gallus gallus GN=TLN1 PE=4 SV=2	E1C2S1_CHICK,TLN1_CHICK	TAX_GallusGallus_9031_20141114.fasta	271,840.20	100.00%	59	72	143	0.29%	25.90%	LHTDDELNWLDHGR	K	T	99.70%	50.3	44.2	23	39.3	42.9	6.89	0.524	0.464	0	0	1	1	2	1,720.80	2,278.50		1,166.09	165	178	Empty	TRUE	
20196-20225 HinckeM –TAXchicken	Uncategorized Sample	20196-20205_A	Mudpit_Ingel20196_MGFPeaklist (F058870)	Talin-1 OS=Gallus gallus GN=TLN1 PE=4 SV=2	E1C2S1_CHICK,TLN1_CHICK	TAX_GallusGallus_9031_20141114.fasta	271,840.20	100.00%	59	72	143	0.29%	25.90%	LLAALMEDEGGNGR	K	Q	99.70%	73.7	44.7	31.6	60	42.6	5.55	0.465	0.429	0	6	0	0	2	1,462.68	2,062.50		3,549.60	593	606	Empty	TRUE	
20196-20225 HinckeM –TAXchicken	Uncategorized Sample	20196-20205_A	Mudpit_Ingel20196_MGFPeaklist (F058870)	Talin-1 OS=Gallus gallus GN=TLN1 PE=4 SV=2	E1C2S1_CHICK,TLN1_CHICK	TAX_GallusGallus_9031_20141114.fasta	271,840.20	100.00%	59	72	143	0.29%	25.90%	LLSDSFPPSNK	R	T	74.60%	27.1	45.1	22.3	9.36					0	1	0	0	2	1,204.62	2,136.00		154.75	1222	1232	Empty	TRUE	
20196-20225 HinckeM –TAXchicken	Uncategorized Sample	20196-20205_A	Mudpit_Ingel20196_MGFPeaklist (F058870)	Talin-1 OS=Gallus gallus GN=TLN1 PE=4 SV=2	E1C2S1_CHICK,TLN1_CHICK	TAX_GallusGallus_9031_20141114.fasta	271,840.20	100.00%	59	72	143	0.29%	25.90%	LPEFGESISAASK	K	A	99.70%	37.6	44.8	22.7	27.7	30.4	1.72	0.319	0.308	0	2	0	0	2	1,335.68	2,221.50		805.77	1418	1430	Empty	TRUE	
20196-20225 HinckeM –TAXchicken	Uncategorized Sample	20196-20205_A	Mudpit_Ingel20196_MGFPeaklist (F058870)	Talin-1 OS=Gallus gallus GN=TLN1 PE=4 SV=2	E1C2S1_CHICK,TLN1_CHICK	TAX_GallusGallus_9031_20141114.fasta	271,840.20	100.00%	59	72	143	0.29%	25.90%	NLAAALAELR	K	T	99.70%	78.6	42.9	34.7	51	38.3	2.17	0.316	0.55	0	3	0	0	2	1,041.61	2,641.00		1,588.89	1025	1034	Empty	TRUE	
20196-20225 HinckeM –TAXchicken	Uncategorized Sample	20196-20205_A	Mudpit_Ingel20196_MGFPeaklist (F058870)	Talin-1 OS=Gallus gallus GN=TLN1 PE=4 SV=2	E1C2S1_CHICK,TLN1_CHICK	TAX_GallusGallus_9031_20141114.fasta	271,840.20	100.00%	59	72	143	0.29%	25.90%	NLASAVSDLLK	K	T	99.70%	64	44.6	19.9	55.2	44.1	2.64	0.395	0.545	0	2	0	0	2	1,130.64	2,852.50		4,184.46	614	624	Empty	TRUE	
20196-20225 HinckeM –TAXchicken	Uncategorized Sample	20196-20205_A	Mudpit_Ingel20196_MGFPeaklist (F058870)	Talin-1 OS=Gallus gallus GN=TLN1 PE=4 SV=2	E1C2S1_CHICK,TLN1_CHICK	TAX_GallusGallus_9031_20141114.fasta	271,840.20	100.00%	59	72	143	0.29%	25.90%	NSKLPEFGESISAASK	K	A	99.70%	72.8	44.5	29.2	65.9	42.3	5.13	0.428	0.406	0	0	2	0	2	1,664.85	2,142.00		3,563.25	1415	1430	Empty	TRUE	
20196-20225 HinckeM –TAXchicken	Uncategorized Sample	20196-20205_A	Mudpit_Ingel20196_MGFPeaklist (F058870)	Talin-1 OS=Gallus gallus GN=TLN1 PE=4 SV=2	E1C2S1_CHICK,TLN1_CHICK	TAX_GallusGallus_9031_20141114.fasta	271,840.20	100.00%	59	72	143	0.29%	25.90%	PAALTAENEEIGSHIK	K	R	99.70%					45.7	5.17	0.481	0.438	0	0	2	0	1	1,679.86	1,990.50		3,158.20	1901	1916	Empty	TRUE	
20196-20225 HinckeM –TAXchicken	Uncategorized Sample	20196-20205_A	Mudpit_Ingel20196_MGFPeaklist (F058870)	Talin-1 OS=Gallus gallus GN=TLN1 PE=4 SV=2	E1C2S1_CHICK,TLN1_CHICK	TAX_GallusGallus_9031_20141114.fasta	271,840.20	100.00%	59	72	143	0.29%	25.90%	QEDVIATANLSR	R	R	99.70%	55.5	45	31.3	30.3	32.4	2.89	0.34	0.375	0	3	0	0	2	1,316.68	2,208.00		786.49	2197	2208	Empty	TRUE	E1BR45_CHICK
20196-20225 HinckeM –TAXchicken	Uncategorized Sample	20196-20205_A	Mudpit_Ingel20196_MGFPeaklist (F058870)	Talin-1 OS=Gallus gallus GN=TLN1 PE=4 SV=2	E1C2S1_CHICK,TLN1_CHICK	TAX_GallusGallus_9031_20141114.fasta	271,840.20	100.00%	59	72	143	0.29%	25.90%	QQQYKFLPTEL	R	R	52.00%					25.3	0.921	0.0791	0.227	0	1	0	0	1	1,394.73	2,160.00		882.16	2525	2535	Empty	TRUE	E1BR45_CHICK
20196-20225 HinckeM –TAXchicken	Uncategorized Sample	20196-20205_A	Mudpit_Ingel20196_MGFPeaklist (F058870)	Talin-1 OS=Gallus gallus GN=TLN1 PE=4 SV=2	E1C2S1_CHICK,TLN1_CHICK	TAX_GallusGallus_9031_20141114.fasta	271,840.20	100.00%	59	72	143	0.29%	25.90%	QVAASTAQLLVACK	K	V	99.70%	62	44.5	21.5	57.6	44.2	7.42	0.493	0.429	0	4	0	0	2	1,459.79	2,312.00		3,135.98	2429	2442	Empty	TRUE	E1BR45_CHICK
20196-20225 HinckeM –TAXchicken	Uncategorized Sample	20196-20205_A	Mudpit_Ingel20196_MGFPeaklist (F058870)	Talin-1 OS=Gallus gallus GN=TLN1 PE=4 SV=2	E1C2S1_CHICK,TLN1_CHICK	TAX_GallusGallus_9031_20141114.fasta	271,840.20	100.00%	59	72	143	0.29%	25.90%	RVQELGHGCAALVTK	R	A	99.70%	24.4	43.9	18.1	16.2	26.7	1.13	0.288	0.3	0	0	2	0	2	1,638.87	1,733.50		1,243.05	1918	1932	Empty	TRUE	
20196-20225 HinckeM –TAXchicken	Uncategorized Sample	20196-20205_A	Mudpit_Ingel20196_MGFPeaklist (F058870)	Talin-1 OS=Gallus gallus GN=TLN1 PE=4 SV=2	E1C2S1_CHICK,TLN1_CHICK	TAX_GallusGallus_9031_20141114.fasta	271,840.20	100.00%	59	72	143	0.29%	25.90%	SIAAATSALVK	K	A	99.70%	38.2	44.2	26.7	14.2	34.1	0.921	0.311	0.455	0	3	0	0	2	1,031.61	2,081.00		2,086.72	2350	2360	Empty	TRUE	E1BR45_CHICK
20196-20225 HinckeM –TAXchicken	Uncategorized Sample	20196-20205_A	Mudpit_Ingel20196_MGFPeaklist (F058870)	Talin-1 OS=Gallus gallus GN=TLN1 PE=4 SV=2	E1C2S1_CHICK,TLN1_CHICK	TAX_GallusGallus_9031_20141114.fasta	271,840.20	100.00%	59	72	143	0.29%	25.90%	SLAVNPKDPPQWSVLAGHSR	R	T	99.70%	49.3	43.4	15.6	0	41.7	6.51	0.417	0.35	0	0	0	2	2	2,159.14	2,315.50		2,558.67	1618	1637	Empty	TRUE	
20196-20225 HinckeM –TAXchicken	Uncategorized Sample	20196-20205_A	Mudpit_Ingel20196_MGFPeaklist (F058870)	Talin-1 OS=Gallus gallus GN=TLN1 PE=4 SV=2	E1C2S1_CHICK,TLN1_CHICK	TAX_GallusGallus_9031_20141114.fasta	271,840.20	100.00%	59	72	143	0.29%	25.90%	STVLQQQFNR	K	V	99.70%	72	44.7	30.8	55.8	34.2	2.15	0.342	0.5	0	2	0	0	2	1,220.64	1,929.50		1,592.61	429	438	Empty	TRUE	
20196-20225 HinckeM –TAXchicken	Uncategorized Sample	20196-20205_A	Mudpit_Ingel20196_MGFPeaklist (F058870)	Talin-1 OS=Gallus gallus GN=TLN1 PE=4 SV=2	E1C2S1_CHICK,TLN1_CHICK	TAX_GallusGallus_9031_20141114.fasta	271,840.20	100.00%	59	72	143	0.29%	25.90%	TFQEAQSQLNR	K	A	99.70%	62.6	44.7	22.1	36.3	35.7	4.31	0.403	0.455	0	3	0	0	2	1,321.65	1,695.00		1,334.70	1233	1243	Empty	TRUE	
20196-20225 HinckeM –TAXchicken	Uncategorized Sample	20196-20205_A	Mudpit_Ingel20196_MGFPeaklist (F058870)	Talin-1 OS=Gallus gallus GN=TLN1 PE=4 SV=2	E1C2S1_CHICK,TLN1_CHICK	TAX_GallusGallus_9031_20141114.fasta	271,840.20	100.00%	59	72	143	0.29%	25.90%	TKEVIQEWSLTNIKR	K	W	98.50%	34.8	42.4	21.1	26.2					0	0	0	2	2	1,845.02	2,302.50		792.84	344	358	Empty	TRUE	
20196-20225 HinckeM –TAXchicken	Uncategorized Sample	20196-20205_A	Mudpit_Ingel20196_MGFPeaklist (F058870)	Talin-1 OS=Gallus gallus GN=TLN1 PE=4 SV=2	E1C2S1_CHICK,TLN1_CHICK	TAX_GallusGallus_9031_20141114.fasta	271,840.20	100.00%	59	72	143	0.29%	25.90%	TLAESALQMLYTAK	K	E	99.70%	40.9	44.4	16.9	31.1	28.1	1.3	0.249	0.286	0	1	1	0	2	1,555.80	2,593.50		662.51	1766	1779	Empty	TRUE	
20196-20225 HinckeM –TAXchicken	Uncategorized Sample	20196-20205_A	Mudpit_Ingel20196_MGFPeaklist (F058870)	Talin-1 OS=Gallus gallus GN=TLN1 PE=4 SV=2	E1C2S1_CHICK,TLN1_CHICK	TAX_GallusGallus_9031_20141114.fasta	271,840.20	100.00%	59	72	143	0.29%	25.90%	TLREQGIDDNETLLLR	R	R	99.70%	60.9	43.9	25.7	49.3	46.5	4.74	0.376	0.438	0	0	2	0	2	1,886.00	2,306.50		1,561.49	179	194	Empty	TRUE	
20196-20225 HinckeM –TAXchicken	Uncategorized Sample	20196-20205_A	Mudpit_Ingel20196_MGFPeaklist (F058870)	Talin-1 OS=Gallus gallus GN=TLN1 PE=4 SV=2	E1C2S1_CHICK,TLN1_CHICK	TAX_GallusGallus_9031_20141114.fasta	271,840.20	100.00%	59	72	143	0.29%	25.90%	TLREQGIDDNETLLLRR	R	K	26.60%	17.5	42.7	16.9	11.1					0	0	1	3	2	2,042.10	2,178.00		1,555.46	179	195	Empty	TRUE	
20196-20225 HinckeM –TAXchicken	Uncategorized Sample	20196-20205_A	Mudpit_Ingel20196_MGFPeaklist (F058870)	Talin-1 OS=Gallus gallus GN=TLN1 PE=4 SV=2	E1C2S1_CHICK,TLN1_CHICK	TAX_GallusGallus_9031_20141114.fasta	271,840.20	100.00%	59	72	143	0.29%	25.90%	TMLESSAGLIQTAR	K	S	99.70%	74.1	44.7	24.7	57.9	50.1	6.28	0.475	0.5	0	4	0	0	2	1,493.76	2,152.50		3,334.01	1604	1617	Empty	TRUE	
20196-20225 HinckeM –TAXchicken	Uncategorized Sample	20196-20205_A	Mudpit_Ingel20196_MGFPeaklist (F058870)	Talin-1 OS=Gallus gallus GN=TLN1 PE=4 SV=2	E1C2S1_CHICK,TLN1_CHICK	TAX_GallusGallus_9031_20141114.fasta	271,840.20	100.00%	59	72	143	0.29%	25.90%	TPNHQQQMNLLDQTK	K	T	99.60%	24.9	44.1	22.5	12.6	27	0.538	0.156	0.3	0	0	2	0	2	1,811.87	1,693.50		528.1	1751	1765	Empty	TRUE	
20196-20225 HinckeM –TAXchicken	Uncategorized Sample	20196-20205_A	Mudpit_Ingel20196_MGFPeaklist (F058870)	Talin-1 OS=Gallus gallus GN=TLN1 PE=4 SV=2	E1C2S1_CHICK,TLN1_CHICK	TAX_GallusGallus_9031_20141114.fasta	271,840.20	100.00%	59	72	143	0.29%	25.90%	TYGVSFFLVK	K	E	99.70%	33.4	44.9	19.4	27.8	33.4	1.8	0.338	0.5	0	2	0	0	2	1,160.64	2,786.50		1,278.77	307	316	Empty	TRUE	E1BR45_CHICK
20196-20225 HinckeM –TAXchicken	Uncategorized Sample	20196-20205_A	Mudpit_Ingel20196_MGFPeaklist (F058870)	Talin-1 OS=Gallus gallus GN=TLN1 PE=4 SV=2	E1C2S1_CHICK,TLN1_CHICK	TAX_GallusGallus_9031_20141114.fasta	271,840.20	100.00%	59	72	143	0.29%	25.90%	VDQASLAAISQQLAPR	E	E	79.70%					25.1	0.367	0.0438	0.219	0	0	0	1	1	1,650.88	1,875.00		1,016.88	1674	1689	Empty	TRUE	
20196-20225 HinckeM –TAXchicken	Uncategorized Sample	20196-20205_A	Mudpit_Ingel20196_MGFPeaklist (F058870)	Talin-1 OS=Gallus gallus GN=TLN1 PE=4 SV=2	E1C2S1_CHICK,TLN1_CHICK	TAX_GallusGallus_9031_20141114.fasta	271,840.20	100.00%	59	72	143	0.29%	25.90%	VGKAELGSVALPAIMR	R	T	92.40%	24.2	42.2	15.9	20.1					0	0	2	0	2	1,627.92	2,320.50		1,034.67	439	454	Empty	TRUE	
20196-20225 HinckeM –TAXchicken	Uncategorized Sample	20196-20205_A	Mudpit_Ingel20196_MGFPeaklist (F058870)	Talin-1 OS=Gallus gallus GN=TLN1 PE=4 SV=2	E1C2S1_CHICK,TLN1_CHICK	TAX_GallusGallus_9031_20141114.fasta	271,840.20	100.00%	59	72	143	0.29%	25.90%	VLGESMAGISQNAK	K	N	99.70%	67.2	44.8	22.7	58.7	45.9	4.92	0.46	0.464	0	4	0	0	2	1,420.71	1,833.50		1,448.49	1401	1414	Empty	TRUE	
20196-20225 HinckeM –TAXchicken	Uncategorized Sample	20196-20205_A	Mudpit_Ingel20196_MGFPeaklist (F058870)	Talin-1 OS=Gallus gallus GN=TLN1 PE=4 SV=2	E1C2S1_CHICK,TLN1_CHICK	TAX_GallusGallus_9031_20141114.fasta	271,840.20	100.00%	59	72	143	0.29%	25.90%	VMVTNVTSLLK	K	T	99.70%	55.6	43.6	25.1	36.5	41.1	5.66	0.392	0.545	0	4	0	0	2	1,204.70	2,523.50		1,708.14	2119	2129	Empty	TRUE	E1BR45_CHICK
20196-20225 HinckeM –TAXchicken	Uncategorized Sample	20196-20205_A	Mudpit_Ingel20196_MGFPeaklist (F058870)	Talin-1 OS=Gallus gallus GN=TLN1 PE=4 SV=2	E1C2S1_CHICK,TLN1_CHICK	TAX_GallusGallus_9031_20141114.fasta	271,840.20	100.00%	59	72	143	0.29%	25.90%	VQELGHGCAALVTK	R	A	13.70%	7.12	44	20.5	0					0	1	0	0	2	1,482.77	1,817.00		133.58	1919	1932	Empty	TRUE	
20196-20225 HinckeM –TAXchicken	Uncategorized Sample	20196-20205_A	Mudpit_Ingel20196_MGFPeaklist (F058870)	Talin-1 OS=Gallus gallus GN=TLN1 PE=4 SV=2	E1C2S1_CHICK,TLN1_CHICK	TAX_GallusGallus_9031_20141114.fasta	271,840.20	100.00%	59	72	143	0.29%	25.90%	VSHVLAALQAGNR	K	G	99.70%	32.2	43.3	22	23.7	26.7	1.11	0.281	0.346	0	0	2	0	2	1,335.75	1,989.50		1,887.53	1960	1972	Empty	TRUE	
20196-20225 HinckeM –TAXchicken	Uncategorized Sample	20196-20205_A	Mudpit_Ingel20196_MGFPeaklist (F058870)	Talin-1 OS=Gallus gallus GN=TLN1 PE=4 SV=2	E1C2S1_CHICK,TLN1_CHICK	TAX_GallusGallus_9031_20141114.fasta	271,840.20	100.00%	59	72	143	0.29%	25.90%	VVADQIPMLVQGVR	K	G	82.30%	25.2	43.1	15.5	13.8					0	1	2	0	2	1,540.85	2,452.00		1,196.64	957	970	Empty	TRUE	
20196-20225 HinckeM –TAXchicken	Uncategorized Sample	20196-20205_A	Mudpit_Ingel20196_MGFPeaklist (F058870)	Talin-1 OS=Gallus gallus GN=TLN1 PE=4 SV=2	E1C2S1_CHICK,TLN1_CHICK	TAX_GallusGallus_9031_20141114.fasta	271,840.20	100.00%	59	72	143	0.29%	25.90%	VVAPTISSPVCQEQLIEAGK	K	L	99.70%	64.1	43.4	15.6	54.3	55	7.55	0.193	0.4	0	0	2	0	2	2,126.12	2,379.00		2,707.90	722	741	Empty	TRUE	
20196-20225 HinckeM –TAXchicken	Uncategorized Sample	20196-20205_A	Mudpit_Ingel20196_MGFPeaklist (F058870)	Fibronectin OS=Gallus gallus GN=FN1 PE=4 SV=1	F1NJT3_CHICK	TAX_GallusGallus_9031_20141114.fasta	273,242.20	100.00%	39	40	119	0.24%	20.20%	AQTASVPQATPAQGK	Q	Q	99.70%					46.7	5.42	0.458	0.433	0	2	0	0	1	1,454.76	1,511.00		1,261.52	37	51	Empty	TRUE	
20196-20225 HinckeM –TAXchicken	Uncategorized Sample	20196-20205_A	Mudpit_Ingel20196_MGFPeaklist (F058870)	Fibronectin OS=Gallus gallus GN=FN1 PE=4 SV=1	F1NJT3_CHICK	TAX_GallusGallus_9031_20141114.fasta	273,242.20	100.00%	39	40	119	0.24%	20.20%	DLQFVEVSDVK	K	V	99.70%	53.1	44.8	28.6	32.1	41.1	3.54	0.384	0.545	0	4	0	0	2	1,278.66	2,367.50		1,916.97	916	926	Empty	TRUE	
20196-20225 HinckeM –TAXchicken	Uncategorized Sample	20196-20205_A	Mudpit_Ingel20196_MGFPeaklist (F058870)	Fibronectin OS=Gallus gallus GN=FN1 PE=4 SV=1	F1NJT3_CHICK	TAX_GallusGallus_9031_20141114.fasta	273,242.20	100.00%	39	40	119	0.24%	20.20%	ESKPLTGEQTTKLDAPTNLR	R	F	99.70%	36.8	42.8	23.1	27.6	34.6	2.15	0.309	0.225	0	0	0	1	2	2,199.16	1,927.00		1,173.90	989	1008	Empty	TRUE	
20196-20225 HinckeM –TAXchicken	Uncategorized Sample	20196-20205_A	Mudpit_Ingel20196_MGFPeaklist (F058870)	Fibronectin OS=Gallus gallus GN=FN1 PE=4 SV=1	F1NJT3_CHICK	TAX_GallusGallus_9031_20141114.fasta	273,242.20	100.00%	39	40	119	0.24%	20.20%	FTNVGPDTMR	R	V	99.70%	51.4	43.8	23.3	38.5	33.7	2.96	0.356	0.4	0	2	0	0	2	1,137.54	1,816.00		1,113.82	1370	1379	Empty	TRUE	
20196-20225 HinckeM –TAXchicken	Uncategorized Sample	20196-20205_A	Mudpit_Ingel20196_MGFPeaklist (F058870)	Fibronectin OS=Gallus gallus GN=FN1 PE=4 SV=1	F1NJT3_CHICK	TAX_GallusGallus_9031_20141114.fasta	273,242.20	100.00%	39	40	119	0.24%	20.20%	GATYNIIVEALK	R	D	99.70%	41.1	43.8	22.8	26.6	30.6	2.77	0.297	0.333	0	1	0	0	2	1,291.73	2,739.00		150.75	2261	2272	Empty	TRUE	
20196-20225 HinckeM –TAXchicken	Uncategorized Sample	20196-20205_A	Mudpit_Ingel20196_MGFPeaklist (F058870)	Fibronectin OS=Gallus gallus GN=FN1 PE=4 SV=1	F1NJT3_CHICK	TAX_GallusGallus_9031_20141114.fasta	273,242.20	100.00%	39	40	119	0.24%	20.20%	GDSPASSKPVTVTYK	R	T	99.70%	56.2	44.5	16.7	48.2	39	3.24	0.382	0.4	0	0	3	0	2	1,536.79	1,631.00		963.94	1620	1634	Empty	TRUE	
20196-20225 HinckeM –TAXchicken	Uncategorized Sample	20196-20205_A	Mudpit_Ingel20196_MGFPeaklist (F058870)	Fibronectin OS=Gallus gallus GN=FN1 PE=4 SV=1	F1NJT3_CHICK	TAX_GallusGallus_9031_20141114.fasta	273,242.20	100.00%	39	40	119	0.24%	20.20%	GEWTCIAYSQLR	R	D	99.70%	50.1	43.8	30.1	30	34	4.05	0.332	0.375	0	2	0	0	2	1,483.70	2,399.00		1,167.70	509	520	Empty	TRUE	
20196-20225 HinckeM –TAXchicken	Uncategorized Sample	20196-20205_A	Mudpit_Ingel20196_MGFPeaklist (F058870)	Fibronectin OS=Gallus gallus GN=FN1 PE=4 SV=1	F1NJT3_CHICK	TAX_GallusGallus_9031_20141114.fasta	273,242.20	100.00%	39	40	119	0.24%	20.20%	GEWTCKPLAER	K	C	99.70%	34.6	45	23.1	22.6	24	1.21	0.242	0.227	0	0	1	0	2	1,346.65	1,809.00		316.76	177	187	Empty	TRUE	
20196-20225 HinckeM –TAXchicken	Uncategorized Sample	20196-20205_A	Mudpit_Ingel20196_MGFPeaklist (F058870)	Fibronectin OS=Gallus gallus GN=FN1 PE=4 SV=1	F1NJT3_CHICK	TAX_GallusGallus_9031_20141114.fasta	273,242.20	100.00%	39	40	119	0.24%	20.20%	GFNCESKPEPEETCFDK	R	Y	99.70%	44.2	38.6	16.1	15.3	28	3.96	0.364	0.206	0	0	1	0	2	2,073.85	1,833.00		310.45	86	102	Empty	TRUE	
20196-20225 HinckeM –TAXchicken	Uncategorized Sample	20196-20205_A	Mudpit_Ingel20196_MGFPeaklist (F058870)	Fibronectin OS=Gallus gallus GN=FN1 PE=4 SV=1	F1NJT3_CHICK	TAX_GallusGallus_9031_20141114.fasta	273,242.20	100.00%	39	40	119	0.24%	20.20%	GFNCESKPEPEETCFDKYTGSTYR	R	V	99.70%	51.7	38.6	15.2	49.3	39.7	5.68	0.499	0.229	0	0	0	2	2	2,902.23	2,053.00		2,053.11	86	109	Empty	TRUE	
20196-20225 HinckeM –TAXchicken	Uncategorized Sample	20196-20205_A	Mudpit_Ingel20196_MGFPeaklist (F058870)	Fibronectin OS=Gallus gallus GN=FN1 PE=4 SV=1	F1NJT3_CHICK	TAX_GallusGallus_9031_20141114.fasta	273,242.20	100.00%	39	40	119	0.24%	20.20%	GLTFTEVDVDSIK	K	I	99.70%	73	44.4	18.7	58	40.4	4.82	0.327	0.423	0	2	0	0	2	1,423.73	2,507.00		1,003.68	1732	1744	Empty	TRUE	
20196-20225 HinckeM –TAXchicken	Uncategorized Sample	20196-20205_A	Mudpit_Ingel20196_MGFPeaklist (F058870)	Fibronectin OS=Gallus gallus GN=FN1 PE=4 SV=1	F1NJT3_CHICK	TAX_GallusGallus_9031_20141114.fasta	273,242.20	100.00%	39	40	119	0.24%	20.20%	GNLLQCICTGNGR	R	G	99.70%	65.8	44	22.7	54.7	43.9	6.89	0.478	0.462	0	6	0	0	2	1,463.67	2,304.00		4,015.33	255	267	Empty	TRUE	
20196-20225 HinckeM –TAXchicken	Uncategorized Sample	20196-20205_A	Mudpit_Ingel20196_MGFPeaklist (F058870)	Fibronectin OS=Gallus gallus GN=FN1 PE=4 SV=1	F1NJT3_CHICK	TAX_GallusGallus_9031_20141114.fasta	273,242.20	100.00%	39	40	119	0.24%	20.20%	IAWESPQGQVTR	K	Y	99.70%	55.4	44.8	31.4	38.3	30.6	2.46	0.346	0.292	0	3	0	0	2	1,371.70	1,987.00		981.89	1745	1756	Empty	TRUE	
20196-20225 HinckeM –TAXchicken	Uncategorized Sample	20196-20205_A	Mudpit_Ingel20196_MGFPeaklist (F058870)	Fibronectin OS=Gallus gallus GN=FN1 PE=4 SV=1	F1NJT3_CHICK	TAX_GallusGallus_9031_20141114.fasta	273,242.20	100.00%	39	40	119	0.24%	20.20%	ITGYIIR	K	Y	74.20%	31.1	42.7	39	8.08					0	4	0	0	2	835.5037	2,015.00		2,563.39	2026	2032	Empty	TRUE	
20196-20225 HinckeM –TAXchicken	Uncategorized Sample	20196-20205_A	Mudpit_Ingel20196_MGFPeaklist (F058870)	Fibronectin OS=Gallus gallus GN=FN1 PE=4 SV=1	F1NJT3_CHICK	TAX_GallusGallus_9031_20141114.fasta	273,242.20	100.00%	39	40	119	0.24%	20.20%	ITGYIIRYDKPGSPAK	K	E	99.70%	28.9	42.4	18	19.2	32.7	1.17	0.229	0.375	0	0	0	2	2	1,778.98	1,964.50		2,295.64	2026	2041	Empty	TRUE	
20196-20225 HinckeM –TAXchicken	Uncategorized Sample	20196-20205_A	Mudpit_Ingel20196_MGFPeaklist (F058870)	Fibronectin OS=Gallus gallus GN=FN1 PE=4 SV=1	F1NJT3_CHICK	TAX_GallusGallus_9031_20141114.fasta	273,242.20	100.00%	39	40	119	0.24%	20.20%	ITYGETGGSSPVQEFTVPGTMSR	R	A	99.60%	28.2	43.7	15.6	21.3	27.6	1.4	0.12	0.196	0	0	2	0	2	2,417.13	2,359.00		1,964.12	1575	1597	Empty	TRUE	
20196-20225 HinckeM –TAXchicken	Uncategorized Sample	20196-20205_A	Mudpit_Ingel20196_MGFPeaklist (F058870)	Fibronectin OS=Gallus gallus GN=FN1 PE=4 SV=1	F1NJT3_CHICK	TAX_GallusGallus_9031_20141114.fasta	273,242.20	100.00%	39	40	119	0.24%	20.20%	IYLGNTLVCTCYGGSR	R	G	99.70%	44.5	44.2	15.2	42.3	33.8	2.85	0.402	0.281	0	0	2	0	2	1,833.86	2,378.00		1,167.60	70	85	Empty	TRUE	
20196-20225 HinckeM –TAXchicken	Uncategorized Sample	20196-20205_A	Mudpit_Ingel20196_MGFPeaklist (F058870)	Fibronectin OS=Gallus gallus GN=FN1 PE=4 SV=1	F1NJT3_CHICK	TAX_GallusGallus_9031_20141114.fasta	273,242.20	100.00%	39	40	119	0.24%	20.20%	IYLYTLNENAR	K	S	99.70%	68.9	44.3	27.4	51.2	39.1	3.14	0.419	0.5	0	6	0	0	2	1,369.71	2,312.50		3,592.48	1977	1987	Empty	TRUE	
20196-20225 HinckeM –TAXchicken	Uncategorized Sample	20196-20205_A	Mudpit_Ingel20196_MGFPeaklist (F058870)	Fibronectin OS=Gallus gallus GN=FN1 PE=4 SV=1	F1NJT3_CHICK	TAX_GallusGallus_9031_20141114.fasta	273,242.20	100.00%	39	40	119	0.24%	20.20%	LDAPTNLR	K	F	99.70%	45.8	43.8	47.7	17.1	29.6	1.19	0.25	0.438	0	6	0	0	2	899.4946	1,796.50		5,727.33	1001	1008	Empty	TRUE	
20196-20225 HinckeM –TAXchicken	Uncategorized Sample	20196-20205_A	Mudpit_Ingel20196_MGFPeaklist (F058870)	Fibronectin OS=Gallus gallus GN=FN1 PE=4 SV=1	F1NJT3_CHICK	TAX_GallusGallus_9031_20141114.fasta	273,242.20	100.00%	39	40	119	0.24%	20.20%	LGVRPSQGGEAPR	K	E	99.70%	49.1	43.7	20.4	31.1	44.2	4.89	0.328	0.615	0	0	2	0	2	1,323.71	1,488.50		841.61	1121	1133	Empty	TRUE	
20196-20225 HinckeM –TAXchicken	Uncategorized Sample	20196-20205_A	Mudpit_Ingel20196_MGFPeaklist (F058870)	Fibronectin OS=Gallus gallus GN=FN1 PE=4 SV=1	F1NJT3_CHICK	TAX_GallusGallus_9031_20141114.fasta	273,242.20	100.00%	39	40	119	0.24%	20.20%	QAQTASVPQATPAQGK	R	Q	99.70%	49.8	44.5	30.5	41.5	42.1	5.4	0	0.375	0	2	0	0	2	1,565.79	1,646.00		1,426.09	36	51	Empty	TRUE	
20196-20225 HinckeM –TAXchicken	Uncategorized Sample	20196-20205_A	Mudpit_Ingel20196_MGFPeaklist (F058870)	Fibronectin OS=Gallus gallus GN=FN1 PE=4 SV=1	F1NJT3_CHICK	TAX_GallusGallus_9031_20141114.fasta	273,242.20	100.00%	39	40	119	0.24%	20.20%	RPSVEVAPEGSAGHTYPQFTQR	R	Y	99.70%	74.6	44.1	15.7	70.2	55.3	8.66	0.562	0.386	0	0	0	3	2	2,414.18	1,963.00		17,807.90	2431	2452	Empty	TRUE	
20196-20225 HinckeM –TAXchicken	Uncategorized Sample	20196-20205_A	Mudpit_Ingel20196_MGFPeaklist (F058870)	Fibronectin OS=Gallus gallus GN=FN1 PE=4 SV=1	F1NJT3_CHICK	TAX_GallusGallus_9031_20141114.fasta	273,242.20	100.00%	39	40	119	0.24%	20.20%	SSPVVIDASTAIDAPSNLR	R	F	99.70%	43.8	43.8	26.5	31.6	38.3	4	0.324	0.316	0	0	2	0	2	1,913.00	2,413.50		1,784.82	1988	2006	Empty	TRUE	
20196-20225 HinckeM –TAXchicken	Uncategorized Sample	20196-20205_A	Mudpit_Ingel20196_MGFPeaklist (F058870)	Fibronectin OS=Gallus gallus GN=FN1 PE=4 SV=1	F1NJT3_CHICK	TAX_GallusGallus_9031_20141114.fasta	273,242.20	100.00%	39	40	119	0.24%	20.20%	STTPGISGYR	R	V	99.70%	55	43.8	22.8	34.1	34.2	2.3	0.371	0.4	0	5	0	0	2	1,038.52	1,709.00		3,681.27	1202	1211	Empty	TRUE	

20196-20225 HinckeM –TAXchicken	Uncategorized Sample	20196-20205_A	Mudpit_Ingel20196_MGFPeaklist (F058870)	Fibronectin OS=Gallus gallus GN=FN1 PE=4 SV=1	F1NJT3_CHICK	TAX_GallusGallus_9031_20141114.fasta	273,242.20	100.00%	39	40	119	0.24%	20.20%	WKCDPVDQCQDSETR	R	T	99.70%	67.2	39.1	16.1	54	43.9	4.89	0.508	0.433	0	0	2	0	2	1,923.80	1,653.50		1,268.06	558	572	Empty	TRUE	
20196-20225 HinckeM –TAXchicken	Uncategorized Sample	20196-20205_A	Mudpit_Ingel20196_MGFPeaklist (F058870)	Fibronectin OS=Gallus gallus GN=FN1 PE=4 SV=1	F1NJT3_CHICK	TAX_GallusGallus_9031_20141114.fasta	273,242.20	100.00%	39	40	119	0.24%	20.20%	WLPSSSPVTGYR	R	V	99.70%	60.8	44.8	21.6	52.2	49.2	4.28	0.52	0.5	0	8	0	0	2	1,349.68	2,196.50		4,128.56	1657	1668	Empty	TRUE	
20196-20225 HinckeM –TAXchicken	Uncategorized Sample	20196-20205_A	Mudpit_Ingel20196_MGFPeaklist (F058870)	Fibronectin OS=Gallus gallus GN=FN1 PE=4 SV=1	F1NJT3_CHICK	TAX_GallusGallus_9031_20141114.fasta	273,242.20	100.00%	39	40	119	0.24%	20.20%	YEVSVYALK	K	D	99.70%	25.8	44.7	18.3	20	23	0.824	0.235	0.333	0	2	0	0	2	1,071.57	2,226.00		921.75	1883	1891	Empty	TRUE	
20196-20225 HinckeM –TAXchicken	Uncategorized Sample	20196-20205_A	Mudpit_Ingel20196_MGFPeaklist (F058870)	Fibronectin OS=Gallus gallus GN=FN1 PE=4 SV=1	F1NJT3_CHICK	TAX_GallusGallus_9031_20141114.fasta	273,242.20	100.00%	39	40	119	0.24%	20.20%	YQCYCYGR	R	G	99.70%	37.5	38.1	17.7	31.5	30.9	2.62	0.44	0.562	0	4	0	0	2	1,169.45	1,619.50		1,598.56	590	597	Empty	TRUE	
20196-20225 HinckeM –TAXchicken	Uncategorized Sample	20196-20205_A	Mudpit_Ingel20196_MGFPeaklist (F058870)	Fibronectin OS=Gallus gallus GN=FN1 PE=4 SV=1	F1NJT3_CHICK	TAX_GallusGallus_9031_20141114.fasta	273,242.20	100.00%	39	40	119	0.24%	20.20%	YYQINQQWER	R	I	99.70%	54.2	43.8	22.2	42.5	37.2	2	0.32	0.55	0	3	0	0	2	1,427.67	2,084.00		3,710.12	60	69	Empty	TRUE	
20196-20225 HinckeM –TAXchicken	Uncategorized Sample	20196-20205_A	Mudpit_Ingel20196_MGFPeaklist (F058870)	Uncharacterized protein (Fragment) OS=Gallus gallus GN=Gga.14530 PE=4 SV=2	F1P2G5_CHICK	TAX_GallusGallus_9031_20141114.fasta	567,768.90	100.00%	5	5	5	0.01%	1.43%	AKAKIQDMVAIR	P	H	96.60%					34	1.64	0.232	0.417	0	0	1	0	1	1,343.78	1,945.00		4,217.47	2146	2157	Empty	TRUE	
20196-20225 HinckeM –TAXchicken	Uncategorized Sample	20196-20205_A	Mudpit_Ingel20196_MGFPeaklist (F058870)	Uncharacterized protein (Fragment) OS=Gallus gallus GN=Gga.14530 PE=4 SV=2	F1P2G5_CHICK	TAX_GallusGallus_9031_20141114.fasta	567,768.90	100.00%	5	5	5	0.01%	1.43%	GALDNLLSQLIAELCMEK	K	K	15.90%	12.3	44.6	17.4	5.59					0	0	0	1	2	2,020.00	1,678.00		405.84	2955	2972	Empty	TRUE	
20196-20225 HinckeM –TAXchicken	Uncategorized Sample	20196-20205_A	Mudpit_Ingel20196_MGFPeaklist (F058870)	Uncharacterized protein (Fragment) OS=Gallus gallus GN=Gga.14530 PE=4 SV=2	F1P2G5_CHICK	TAX_GallusGallus_9031_20141114.fasta	567,768.90	100.00%	5	5	5	0.01%	1.43%	ILGPAESDEFLAR	R	V	99.70%	41.5	44.9	21.6	29	28.4	2.29	0.285	0.269	0	1	0	0	2	1,417.73	2,355.00		207.77	1277	1289	Empty	TRUE	
20196-20225 HinckeM –TAXchicken	Uncategorized Sample	20196-20205_A	Mudpit_Ingel20196_MGFPeaklist (F058870)	Uncharacterized protein (Fragment) OS=Gallus gallus GN=Gga.14530 PE=4 SV=2	F1P2G5_CHICK	TAX_GallusGallus_9031_20141114.fasta	567,768.90	100.00%	5	5	5	0.01%	1.43%	KNERSPVNEVHLVVMR	K	L	5.60%	10.9	43.8	17.8	0					0	0	0	1	2	1,908.01	1,502.00		96.54	2978	2993	Empty	TRUE	
20196-20225 HinckeM –TAXchicken	Uncategorized Sample	20196-20205_A	Mudpit_Ingel20196_MGFPeaklist (F058870)	Uncharacterized protein (Fragment) OS=Gallus gallus GN=Gga.14530 PE=4 SV=2	F1P2G5_CHICK	TAX_GallusGallus_9031_20141114.fasta	567,768.90	100.00%	5	5	5	0.01%	1.43%	QKALGTLGMTTNEK	R	G	7.10%	5.15	44.7	0	0					0	0	1	0	2	1,508.76	1,709.00		115.38	4738	4751	Empty	TRUE	
20196-20225 HinckeM –TAXchicken	Uncategorized Sample	20196-20205_A	Mudpit_Ingel20196_MGFPeaklist (F058870)	Uncharacterized protein OS=Gallus gallus GN=Gga.55808 PE=4 SV=2	F1NJR5_CHICK	TAX_GallusGallus_9031_20141114.fasta	212,630.20	100.00%	59	69	237	0.48%	33.90%	ADAATSFLR	G	A	99.70%	63.7	44.1	37.5	33.7	40.6	4.28	0.313	0.667	0	7	0	0	1	951.4894	2,075.00		6,949.04	48	56	Empty	TRUE	UPI0001849F10,UPI0000EC9F07
20196-20225 HinckeM –TAXchicken	Uncategorized Sample	20196-20205_A	Mudpit_Ingel20196_MGFPeaklist (F058870)	Uncharacterized protein OS=Gallus gallus GN=Gga.55808 PE=4 SV=2	F1NJR5_CHICK	TAX_GallusGallus_9031_20141114.fasta	212,630.20	100.00%	59	69	237	0.48%	33.90%	AEILLDHGAPIQAK	I	T	99.70%					33.9	4	0.372	0.357	0	1	0	0	1	1,475.82	2,321.00		264.12	326	339	Empty	TRUE	
20196-20225 HinckeM –TAXchicken	Uncategorized Sample	20196-20205_A	Mudpit_Ingel20196_MGFPeaklist (F058870)	Uncharacterized protein OS=Gallus gallus GN=Gga.55808 PE=4 SV=2	F1NJR5_CHICK	TAX_GallusGallus_9031_20141114.fasta	212,630.20	100.00%	59	69	237	0.48%	33.90%	ALNGFTPLHIACK	R	K	99.70%	47.1	44.6	20.6	42	29	3.23	0.0552	0.346	0	0	4	0	2	1,442.75	2,350.50		1,073.19	406	418	Empty	TRUE	UPI0001849F10,UPI0000EC9F07,UPI0003505B21,UPI0002C86B7B,UPI000240C443
20196-20225 HinckeM –TAXchicken	Uncategorized Sample	20196-20205_A	Mudpit_Ingel20196_MGFPeaklist (F058870)	Uncharacterized protein OS=Gallus gallus GN=Gga.55808 PE=4 SV=2	F1NJR5_CHICK	TAX_GallusGallus_9031_20141114.fasta	212,630.20	100.00%	59	69	237	0.48%	33.90%	ALNGFTPLHIACKK	R	N	35.90%	16.1	43.8	15.3	7.87					0	0	2	0	2	1,570.84	2,209.00		995.15	406	419	Empty	TRUE	UPI0001849F10,UPI0000EC9F07,UPI0003505B21,UPI0002C86B7B,UPI000240C443
20196-20225 HinckeM –TAXchicken	Uncategorized Sample	20196-20205_A	Mudpit_Ingel20196_MGFPeaklist (F058870)	Uncharacterized protein OS=Gallus gallus GN=Gga.55808 PE=4 SV=2	F1NJR5_CHICK	TAX_GallusGallus_9031_20141114.fasta	212,630.20	100.00%	59	69	237	0.48%	33.90%	EASGSLSFLR	R	K	99.70%	78.9	44.1	28.6	58	43.6	4.62	0.433	0.65	0	7	0	0	2	1,066.55	2,283.00		8,871.14	1375	1384	Empty	TRUE	UPI0001849F10,UPI0000EC9F07,UPI0003505B21,UPI0002C86B7B,UPI000240C443
20196-20225 HinckeM –TAXchicken	Uncategorized Sample	20196-20205_A	Mudpit_Ingel20196_MGFPeaklist (F058870)	Uncharacterized protein OS=Gallus gallus GN=Gga.55808 PE=4 SV=2	F1NJR5_CHICK	TAX_GallusGallus_9031_20141114.fasta	212,630.20	100.00%	59	69	237	0.48%	33.90%	EASGSLSFLRK	R	A	99.70%	44.1	43.7	23	25.4	35.2	3.57	0.341	0.455	0	2	0	0	2	1,194.65	2,069.00		909.82	1375	1385	Empty	TRUE	UPI0001849F10,UPI0000EC9F07,UPI0003505B21,UPI0002C86B7B,UPI000240C443
20196-20225 HinckeM –TAXchicken	Uncategorized Sample	20196-20205_A	Mudpit_Ingel20196_MGFPeaklist (F058870)	Uncharacterized protein OS=Gallus gallus GN=Gga.55808 PE=4 SV=2	F1NJR5_CHICK	TAX_GallusGallus_9031_20141114.fasta	212,630.20	100.00%	59	69	237	0.48%	33.90%	ECANFTTNVSAR	K	F	99.70%	72.7	42.9	29.8	57.9	38.1	4.28	0.462	0.417	0	2	0	0	2	1,369.62	1,766.50		1,175.46	1239	1250	Empty	TRUE	UPI0001849F10,UPI0000EC9F07,UPI0003505B21,UPI0002C86B7B,UPI000240C443
20196-20225 HinckeM –TAXchicken	Uncategorized Sample	20196-20205_A	Mudpit_Ingel20196_MGFPeaklist (F058870)	Uncharacterized protein OS=Gallus gallus GN=Gga.55808 PE=4 SV=2	F1NJR5_CHICK	TAX_GallusGallus_9031_20141114.fasta	212,630.20	100.00%	59	69	237	0.48%	33.90%	EGHVDTALALLEK	R	G	99.70%	65.7	44	31.1	32.8	39.3	3.08	0.316	0.462	0	0	6	0	2	1,395.75	2,417.00		3,110.98	551	563	Empty	TRUE	UPI0003505B21,UPI0002C86B7B,UPI000240C443
20196-20225 HinckeM –TAXchicken	Uncategorized Sample	20196-20205_A	Mudpit_Ingel20196_MGFPeaklist (F058870)	Uncharacterized protein OS=Gallus gallus GN=Gga.55808 PE=4 SV=2	F1NJR5_CHICK	TAX_GallusGallus_9031_20141114.fasta	212,630.20	100.00%	59	69	237	0.48%	33.90%	EIVLETTTK	K	K	99.70%	60.5	44.7	33.5	39.9	35.2	2.23	0.352	0.556	0	4	0	0	2	1,033.58	1,895.00		2,729.35	106	114	Empty	TRUE	UPI0001849F10,UPI0000EC9F07,UPI0003505B21,UPI0002C86B7B,UPI000240C443
20196-20225 HinckeM –TAXchicken	Uncategorized Sample	20196-20205_A	Mudpit_Ingel20196_MGFPeaklist (F058870)	Uncharacterized protein OS=Gallus gallus GN=Gga.55808 PE=4 SV=2	F1NJR5_CHICK	TAX_GallusGallus_9031_20141114.fasta	212,630.20	100.00%	59	69	237	0.48%	33.90%	ELQFGVDDINR	R	I	99.70%	61.2	45	28.1	38.2	39.3	2.12	0.341	0.455	0	4	0	0	2	1,305.64	2,292.50		4,691.50	1464	1474	Empty	TRUE	UPI0001849F10,UPI0000EC9F07,UPI0003505B21,UPI0002C86B7B,UPI000240C443
20196-20225 HinckeM –TAXchicken	Uncategorized Sample	20196-20205_A	Mudpit_Ingel20196_MGFPeaklist (F058870)	Uncharacterized protein OS=Gallus gallus GN=Gga.55808 PE=4 SV=2	F1NJR5_CHICK	TAX_GallusGallus_9031_20141114.fasta	212,630.20	100.00%	59	69	237	0.48%	33.90%	ELTAVPYMAK	K	F	99.70%	33.8	44.1	24	18.5	34.9	2.38	0.338	0.5	0	4	0	0	2	1,138.58	1,888.00		2,823.91	1272	1281	Empty	TRUE	UPI0001849F10,UPI0000EC9F07,UPI0003505B21,UPI0002C86B7B,UPI000240C443
20196-20225 HinckeM –TAXchicken	Uncategorized Sample	20196-20205_A	Mudpit_Ingel20196_MGFPeaklist (F058870)	Uncharacterized protein OS=Gallus gallus GN=Gga.55808 PE=4 SV=2	F1NJR5_CHICK	TAX_GallusGallus_9031_20141114.fasta	212,630.20	100.00%	59	69	237	0.48%	33.90%	ELVNYGANVNAQSQK	R	G	99.70%	67.4	44.7	17.9	58.1	47.7	6	0.499	0.433	0	3	2	0	2	1,634.81	1,820.00		4,181.44	134	148	Empty	TRUE	UPI0001849F10,UPI0000EC9F07,UPI0003505B21,UPI0002C86B7B,UPI000240C443
20196-20225 HinckeM –TAXchicken	Uncategorized Sample	20196-20205_A	Mudpit_Ingel20196_MGFPeaklist (F058870)	Uncharacterized protein OS=Gallus gallus GN=Gga.55808 PE=4 SV=2	F1NJR5_CHICK	TAX_GallusGallus_9031_20141114.fasta	212,630.20	100.00%	59	69	237	0.48%	33.90%	FLLQHQADVNAK	K	T	99.70%	40	44	17.8	37.5	37.7	2.32	0.202	0.458	0	0	6	0	2	1,383.74	1,887.00		4,995.51	757	768	Empty	TRUE	UPI0001849F10,UPI0000EC9F07,UPI0003505B21,UPI0002C86B7B,UPI000240C443
20196-20225 HinckeM –TAXchicken	Uncategorized Sample	20196-20205_A	Mudpit_Ingel20196_MGFPeaklist (F058870)	Uncharacterized protein OS=Gallus gallus GN=Gga.55808 PE=4 SV=2	F1NJR5_CHICK	TAX_GallusGallus_9031_20141114.fasta	212,630.20	100.00%	59	69	237	0.48%	33.90%	FWLADCPR	R	T	99.70%	64.9	44.4	40.5	27.4	32.1	1.54	0.209	0.562	0	7	0	0	2	1,064.50	2,319.00		6,976.92	1251	1258	Empty	TRUE	UPI0001849F10,UPI0000EC9F07,UPI0003505B21,UPI0002C86B7B,UPI000240C443
20196-20225 HinckeM –TAXchicken	Uncategorized Sample	20196-20205_A	Mudpit_Ingel20196_MGFPeaklist (F058870)	Uncharacterized protein OS=Gallus gallus GN=Gga.55808 PE=4 SV=2	F1NJR5_CHICK	TAX_GallusGallus_9031_20141114.fasta	212,630.20	100.00%	59	69	237	0.48%	33.90%	GASPNVSNVKVETPLHMAAR	R	A	99.70%	59.7	43.9	0	54.2	42.8	4.51	0.414	0.375	0	0	0	3	2	2,094.08	1,933.00		9,717.63	465	484	Empty	TRUE	UPI0001849F10,UPI0000EC9F07,UPI0003505B21,UPI0002C86B7B,UPI000240C443
20196-20225 HinckeM –TAXchicken	Uncategorized Sample	20196-20205_A	Mudpit_Ingel20196_MGFPeaklist (F058870)	Uncharacterized protein OS=Gallus gallus GN=Gga.55808 PE=4 SV=2	F1NJR5_CHICK	TAX_GallusGallus_9031_20141114.fasta	212,630.20	100.00%	59	69	237	0.48%	33.90%	GASVNFTPQNGITPLHIASR	R	R	99.70%	50.6	43.5	16	40.6	35.9	4	0.407	0.275	0	0	4	0	2	2,080.09	2,432.00		3,108.59	267	286	Empty	TRUE	UPI0001849F10,UPI0000EC9F07,UPI0003505B21,UPI0002C86B7B,UPI000240C443
20196-20225 HinckeM –TAXchicken	Uncategorized Sample	20196-20205_A	Mudpit_Ingel20196_MGFPeaklist (F058870)	Uncharacterized protein OS=Gallus gallus GN=Gga.55808 PE=4 SV=2	F1NJR5_CHICK	TAX_GallusGallus_9031_20141114.fasta	212,630.20	100.00%	59	69	237	0.48%	33.90%	GDRELVVLR	R	S	99.70%	42	43.3	37.9	18.3	36.4	2.4	0.341	0.611	0	2	0	0	2	1,056.62	1,987.00		990.51	1042	1050	Empty	TRUE	UPI0001849F10,UPI0000EC9F07,UPI0003505B21,UPI0002C86B7B,UPI000240C443
20196-20225 HinckeM –TAXchicken	Uncategorized Sample	20196-20205_A	Mudpit_Ingel20196_MGFPeaklist (F058870)	Uncharacterized protein OS=Gallus gallus GN=Gga.55808 PE=4 SV=2	F1NJR5_CHICK	TAX_GallusGallus_9031_20141114.fasta	212,630.20	100.00%	59	69	237	0.48%	33.90%	GFTPLYMAAQENHLEVVK	K	F	99.70%	30.9	44.7	13	30.9	36.6	3.7	0.383	0.25	0	0	2	0	2	2,063.03	2,436.00		1,557.72	149	166	Empty	TRUE	UPI0001849F10,UPI0000EC9F07,UPI0003505B21,UPI0002C86B7B,UPI000240C443
20196-20225 HinckeM –TAXchicken	Uncategorized Sample	20196-20205_A	Mudpit_Ingel20196_MGFPeaklist (F058870)	Uncharacterized protein OS=Gallus gallus GN=Gga.55808 PE=4 SV=2	F1NJR5_CHICK	TAX_GallusGallus_9031_20141114.fasta	212,630.20	100.00%	59	69	237	0.48%	33.90%	GNTALHIAALAGQQDVVR	K	E	99.70%	66.1	43.6	27.3	56.8	47.6	4.85	0.447	0.417	0	0	2	0	2	1,833.99	2,315.00		1,588.92	116	133	Empty	TRUE	UPI0001849F10,UPI0000EC9F07,UPI0003505B21,UPI0002C86B7B,UPI000240C443
20196-20225 HinckeM –TAXchicken	Uncategorized Sample	20196-20205_A	Mudpit_Ingel20196_MGFPeaklist (F058870)	Uncharacterized protein OS=Gallus gallus GN=Gga.55808 PE=4 SV=2	F1NJR5_CHICK	TAX_GallusGallus_9031_20141114.fasta	212,630.20	100.00%	59	69	237	0.48%	33.90%	GSSPHSSAWNGYTPLHIAAK	K	Q	99.70%	27.1	44.3	14.1	18.8	34.9	3.24	0.218	0.375	0	0	1	5	2	2,081.02	2,216.50		5,273.57	630	649	Empty	TRUE	UPI0001849F10,UPI0000EC9F07,UPI0003505B21,UPI0002C86B7B,UPI000240C443
20196-20225 HinckeM –TAXchicken	Uncategorized Sample	20196-20205_A	Mudpit_Ingel20196_MGFPeaklist (F058870)	Uncharacterized protein OS=Gallus gallus GN=Gga.55808 PE=4 SV=2	F1NJR5_CHICK	TAX_GallusGallus_9031_20141114.fasta	212,630.20	100.00%	59	69	237	0.48%	33.90%	GVKIENLYTALR	K	N	99.70%	45	42.8	25.7	31.1	28.4	2.72	0.271	0.333	0	0	2	0	2	1,376.79	2,432.00		715.69	1501	1512	Empty	TRUE	UPI0001849F10,UPI0000EC9F07,UPI0003505B21,UPI0002C86B7B,UPI000240C443
20196-20225 HinckeM –TAXchicken	Uncategorized Sample	20196-20205_A	Mudpit_Ingel20196_MGFPeaklist (F058870)	Uncharacterized protein OS=Gallus gallus GN=Gga.55808 PE=4 SV=2	F1NJR5_CHICK	TAX_GallusGallus_9031_20141114.fasta	212,630.20	100.00%	59	69	237	0.48%	33.90%	HGASPNEISTNGTTPLAIAK	K	R	99.70%	62.7	44.2	14.2	41.1	46.4	7.29	0.487	0.4	0	0	8	0	2	1,979.02	2,105.00		13,283.10	794	813	Empty	TRUE	UPI0001849F10,UPI0000EC9F07,UPI0003505B21,UPI0002C86B7B,UPI000240C443
20196-20225 HinckeM –TAXchicken	Uncategorized Sample	20196-20205_A	Mudpit_Ingel20196_MGFPeaklist (F058870)	Uncharacterized protein OS=Gallus gallus GN=Gga.55808 PE=4 SV=2	F1NJR5_CHICK	TAX_GallusGallus_9031_20141114.fasta	212,630.20	100.00%	59	69	237	0.48%	33.90%	HGASPNEISTNGTTPLAIAKR	K	L	99.70%	59.4	44	13.3	23.9	30.5	3.7	0.308	0.238	0	0	0	2	2	2,135.12	2,014.50		835.46	794	814	Empty	TRUE	UPI0001849F10,UPI0000EC9F07,UPI0003505B21,UPI0002C86B7B,UPI000240C443
20196-20225 HinckeM –TAXchicken	Uncategorized Sample	20196-20205_A	Mudpit_Ingel20196_MGFPeaklist (F058870)	Uncharacterized protein OS=Gallus gallus GN=Gga.55808 PE=4 SV=2	F1NJR5_CHICK	TAX_GallusGallus_9031_20141114.fasta	212,630.20	100.00%	59	69	237	0.48%	33.90%	IAEILLDHGAPIQAK	R	T	99.70%	75.9	42.3	27.4	62.8	46.8	4.96	0.44	0.533	0	0	6	0	2	1,588.91	2,308.00		9,481.71	325	339	Empty	TRUE	UPI0001849F10,UPI0000EC9F07,UPI0003505B21,UPI0002C86B7B,UPI000240C443
20196-20225 HinckeM –TAXchicken	Uncategorized Sample	20196-20205_A	Mudpit_Ingel20196_MGFPeaklist (F058870)	Uncharacterized protein OS=Gallus gallus GN=Gga.55808 PE=4 SV=2	F1NJR5_CHICK	TAX_GallusGallus_9031_20141114.fasta	212,630.20	100.00%	59	69	237	0.48%	33.90%	ICQDCDMIGPEGGCLK	R	S	99.70%	33.6	38.9	21.4	33.6	40.6	5.8	0.475	0.406	0	0	2	0	2	1,868.77	1,875.00		1,721.33	1109	1124	Empty	TRUE	UPI0001849F10,UPI0000EC9F07,UPI0003505B21,UPI0002C86B7B,UPI000240C443
20196-20225 HinckeM –TAXchicken	Uncategorized Sample	20196-20205_A	Mudpit_Ingel20196_MGFPeaklist (F058870)	Uncharacterized protein OS=Gallus gallus GN=Gga.55808 PE=4 SV=2	F1NJR5_CHICK	TAX_GallusGallus_9031_20141114.fasta	212,630.20	100.00%	59	69	237	0.48%	33.90%	IENLYTALR	K	N	99.70%	63.2	44.3	35.4	43	38.4	2.3	0.221	0.611	0	6	0	0	2	1,092.60	2,315.00		6,139.52	1504	1512	Empty	TRUE	UPI0001849F10,UPI0000EC9F07,UPI0003505B21,UPI0002C86B7B,UPI000240C443
20196-20225 HinckeM –TAXchicken	Uncategorized Sample	20196-20205_A	Mudpit_Ingel20196_MGFPeaklist (F058870)	Uncharacterized protein OS=Gallus gallus GN=Gga.55808 PE=4 SV=2	F1NJR5_CHICK	TAX_GallusGallus_9031_20141114.fasta	212,630.20	100.00%	59	69	237	0.48%	33.90%	IPLPPSWK	R	D	99.70%	36.5	42.3	23.9	21.9	25.7	0.854	0.21	0.312	0	6	0	0	2	937.5507	2,341.00		1,160.21	1189	1196	Empty	TRUE	UPI0001849F10,UPI0000EC9F07,UPI0003505B21,UPI0002C86B7B,UPI000240C443
20196-20225 HinckeM –TAXchicken	Uncategorized Sample	20196-20205_A	Mudpit_Ingel20196_MGFPeaklist (F058870)	Uncharacterized protein OS=Gallus gallus GN=Gga.55808 PE=4 SV=2	F1NJR5_CHICK	TAX_GallusGallus_9031_20141114.fasta	212,630.20	100.00%	59	69	237	0.48%	33.90%	IPLPPSWKDNPR	R	D	99.70%	64.9	44.2	30.4	44.7	42.4	3.66	0.392	0.625	0	1	9	0	2	1,419.77	2,167.00		4,524.49	1189	1200	Empty	TRUE	UPI0001849F10,UPI0000EC9F07,UPI0003505B21,UPI0002C86B7B,UPI000240C443
20196-20225 HinckeM –TAXchicken	Uncategorized Sample	20196-20205_A	Mudpit_Ingel20196_MGFPeaklist (F058870)	Uncharacterized protein OS=Gallus gallus GN=Gga.55808 PE=4 SV=2	F1NJR5_CHICK	TAX_GallusGallus_9031_20141114.fasta	212,630.20	100.00%	59	69	237	0.48%	33.90%	IVTEETTIPPFSDK	K	Y	99.70%	51	44.9	20.4	37.7	36.6	4.3	0.448	0.357	0	2	0	0	2	1,576.81	2,257.50		1,123.95	826	839	Empty	TRUE	UPI0001849F10,UPI0000EC9F07,UPI0003505B21,UPI0002C86B7B,UPI000240C443
20196-20225 HinckeM –TAXchicken	Uncategorized Sample	20196-20205_A	Mudpit_Ingel20196_MGFPeaklist (F058870)	Uncharacterized protein OS=Gallus gallus GN=Gga.55808 PE=4 SV=2	F1NJR5_CHICK	TAX_GallusGallus_9031_20141114.fasta	212,630.20	100.00%	59	69	237	0.48%	33.90%	IVTEETTIPPFSDKYR	K	M	99.70%	62	44.1	15.6	53.4	42.6	5	0.369	0.406	0	0	8	0	2	1,895.98	2,258.00		6,912.73	826	841	Empty	TRUE	UPI0001849F10,UPI0000EC9F07,UPI0003505B21,UPI0002C86B7B,UPI000240C443
20196-20225 HinckeM –TAXchicken	Uncategorized Sample	20196-20205_A	Mudpit_Ingel20196_MGFPeaklist (F058870)	Uncharacterized protein OS=Gallus gallus GN=Gga.55808 PE=4 SV=2	F1NJR5_CHICK	TAX_GallusGallus_9031_20141114.fasta	212,630.20	100.00%	59	69	237	0.48%	33.90%	KGNTALHIAALAGQQDVVR	K	E	99.70%	46.5	41.8	19	34	38.5	5.21	0.364	0.368	0	0	0	3	2	1,962.09	2,195.00		1,635.70	115	133	Empty	TRUE	UPI0001849F10,UPI0000EC9F07,UPI0003505B21,UPI0002C86B7B,UPI000240C443
20196-20225 HinckeM –TAXchicken	Uncategorized Sample	20196-20205_A	Mudpit_Ingel20196_MGFPeaklist (F058870)	Uncharacterized protein OS=Gallus gallus GN=Gga.55808 PE=4 SV=2	F1NJR5_CHICK	TAX_GallusGallus_9031_20141114.fasta	212,630.20	100.00%	59	69	237	0.48%	33.90%	LAIPIKVR	R	D	99.70%	56.7	35.2	29	33.2	32.2	1.54	0.193	0.5	0	2	0	0	2	909.6245	2,185.50		683.48	1363	1370	Empty	TRUE	UPI0001849F10,UPI0000EC9F07,UPI0003505B21,UPI0002C86B7B,UPI000240C443
20196-20225 HinckeM –TAXchicken	Uncategorized Sample	20196-20205_A	Mudpit_Ingel20196_MGFPeaklist (F058870)	Uncharacterized protein OS=Gallus gallus GN=Gga.55808 PE=4 SV=2	F1NJR5_CHICK	TAX_GallusGallus_9031_20141114.fasta	212,630.20	100.00%	59	69	237	0.48%	33.90%	LALQAQPVPDELVTK	R	L	99.70%	59.2	41.8	24.4	54.7	44.9	4.12	0.47	0.433	0	6	0	0	2	1,621.92	2,355.50		17,846.80	1146	1160	Empty	TRUE	UPI0001849F10,UPI0000EC9F07,UPI0003505B21,UPI0002C86B7B,UPI000240C443
20196-20225 HinckeM –TAXchicken	Uncategorized Sample	20196-20205_A	Mudpit_Ingel20196_MGFPeaklist (F058870)	Uncharacterized protein OS=Gallus gallus GN=Gga.55808 PE=4 SV=2	F1NJR5_CHICK	TAX_GallusGallus_9031_20141114.fasta	212,630.20	100.00%	59	69	237	0.48%	33.90%	LGYISVTDVLK	R	I	99.70%	63.7	43.1	26.8	26	42.4	3.7	0.193	0.545	0	5	0	0	2	1,207.69	2,658.00		2,351.51	815	825	Empty	TRUE	UPI0001849F10,UPI0000EC9F07,UPI0003505B21,UPI0002C86B7B,UPI000240C443
20196-20225 HinckeM –TAXchicken	Uncategorized Sample	20196-20205_A	Mudpit_Ingel20196_MGFPeaklist (F058870)	Uncharacterized protein OS=Gallus gallus GN=Gga.55808 PE=4 SV=2	F1NJR5_CHICK	TAX_GallusGallus_9031_20141114.fasta	212,630.20	100.00%	59	69	237	0.48%	33.90%	LLGNQATFSPIVTVEPR	K	R	99.70%	66.8	42.6	25.3	0	49.1	4.85	0.424	0.441	0	0	4	0	2	1,842.01	2,587.00		3,716.59	1161	1177	Empty	TRUE	UPI0001849F10,UPI0000EC9F07,UPI0003505B21,UPI0002C86B7B,UPI000240C443
20196-20225 HinckeM –TAXchicken	Uncategorized Sample	20196-20205_A	Mudpit_Ingel20196_MGFPeaklist (F058870)	Uncharacterized protein OS=Gallus gallus GN=Gga.55808 PE=4 SV=2	F1NJR5_CHICK	TAX_GallusGallus_9031_20141114.fasta	212,630.20	100.00%	59	69	237	0.48%	33.90%	LPALHIAAR	R	N	99.70%	48.2	42.9	30.1	31.9	32.3	2.54	0.359	0.389	0	3	0	0	2	961.5942	2,080.00		641.69	212	220	Empty	TRUE	UPI0001849F10,UPI0000EC9F07,UPI0003505B21,UPI0002C86B7B,UPI000240C443
20196-20225 HinckeM –TAXchicken	Uncategorized Sample	20196-20205_A	Mudpit_Ingel20196_MGFPeaklist (F058870)	Uncharacterized protein OS=Gallus gallus GN=Gga.55808 PE=4 SV=2	F1NJR5_CHICK	TAX_GallusGallus_9031_20141114.fasta	212,630.20	100.00%	59	69	237	0.48%	33.90%	LPAPPPLAEEEGLASR	K	I	99.70%	51.5	44.5	27	39.1	47.1	7.51	0.507	0.438	0	1	1	0	2	1,646.88	2,370.50		2,729.64	999	1014	Empty	TRUE	UPI0001849F10,UPI0000EC9F07,UPI0003505B21,UPI0002C86B7B,UPI000240C443
20196-20225 HinckeM –TAXchicken	Uncategorized Sample	20196-20205_A	Mudpit_Ingel20196_MGFPeaklist (F058870)	Uncharacterized protein OS=Gallus gallus GN=Gga.55808 PE=4 SV=2	F1NJR5_CHICK	TAX_GallusGallus_9031_20141114.fasta	212,630.20	100.00%	59	69	237	0.48%	33.90%	LVKPQKLPAPPPLAEEEGLASR	R	I	99.70%	48.4	38	13.1	47.2	44.6	4.8	0.446	0.318	0	0	0	4	2	2,340.33	2,298.50		2,553.23	993	1014	Empty	TRUE	UPI0001849F10,UPI0000EC9F07,UPI0003505B21,UPI0002C86B7B,UPI000240C443
20196-20225 HinckeM –TAXchicken	Uncategorized Sample	20196-20205_A	Mudpit_Ingel20196_MGFPeaklist (F058870)	Uncharacterized protein OS=Gallus gallus GN=Gga.55808 PE=4 SV=2	F1NJR5_CHICK	TAX_GallusGallus_9031_20141114.fasta	212,630.20	100.00%	59	69	237	0.48%	33.90%	MGYTSLHIASHYGNIK	R	L	24.90%	11.9	44.6	18.2	2.47					0	0	0	1	2	1,807.88	2,012.00		387.07	738	753	Empty	TRUE	UPI0001849F10,UPI0000EC9F07,UPI0003505B21,UPI0002C86B7B,UPI000240C443
20196-20225 HinckeM –TAXchicken	Uncategorized Sample	20196-20205_A	Mudpit_Ingel20196_MGFPeaklist (F058870)	Uncharacterized protein OS=Gallus gallus GN=Gga.55808 PE=4 SV=2	F1NJR5_CHICK	TAX_GallusGallus_9031_20141114.fasta	212,630.20	100.00%	59	69	237	0.48%	33.90%	MPASDHQLGTGEQNPESEVSFVSVQQK	K	V	99.70%	64.5	42.6	0	64.5	58.9	11.2	0.594	0.315	0	0	4	0	2	2,945.36	2,189.50		5,582.27	1690	1716	Empty	TRUE	UPI0001849F10,UPI0000EC9F07,UPI0003505B21,UPI0002C86B7B,UPI000240C443
20196-20225 HinckeM –TAXchicken	Uncategorized Sample	20196-20205_A	Mudpit_Ingel20196_MGFPeaklist (F058870)	Uncharacterized protein OS=Gallus gallus GN=Gga.55808 PE=4 SV=2	F1NJR5_CHICK	TAX_GallusGallus_9031_20141114.fasta	212,630.20	100.00%	59	69	237	0.48%	33.90%	MVVELLHK	K	E	99.40%	26.2	43.3	14.3	16	21.6	0.638	0.162	0.25	0	1	0	0	2	984.5548	1,901.00		107.1	98	105	Empty	TRUE	UPI0001849F10,UPI0000EC9F07,UPI0003505B21,UPI0002C86B7B,UPI000240C443
20196-20225 HinckeM –TAXchicken	Uncategorized Sample	20196-20205_A	Mudpit_Ingel20196_MGFPeaklist (F058870)	Uncharacterized protein OS=Gallus gallus GN=Gga.55808 PE=4 SV=2	F1NJR5_CHICK	TAX_GallusGallus_9031_20141114.fasta	212,630.20	100.00%	59	69	237	0.48%	33.90%	NGLSPIHMAAQGDHLDCVR	K	L	99.70%	54.6	43.4	17	41.4	35.7	5.64	0	0.316	0	0	0	3	2	2,107.97	2,040.00		1,322.63	342	360	Empty	TRUE	UPI0001849F10,UPI0000EC9F07,UPI0003505B21,UPI0002C86B7B,UPI000240C443
20196-20225 HinckeM –TAXchicken	Uncategorized Sample	20196-20205_A	Mudpit_Ingel20196_MGFPeaklist (F058870)	Uncharacterized protein OS=Gallus gallus GN=Gga.55808 PE=4 SV=2	F1NJR5_CHICK	TAX_GallusGallus_9031_20141114.fasta	212,630.20	100.00%	59	69	237	0.48%	33.90%	NGVDINTCNQNGLNALHLASK	R	E	99.70%	67.1	43.8	13.6	9.84	40.3	5.14	0.00248	0.262	0	0	4	0	2	2,254.09	2,361.50		2,034.30	72	92	Empty	TRUE	UPI0001849F10,UPI0000EC9F07,UPI0003505B21,UPI0002C86B7B,UPI000240C443
20196-20225 HinckeM –TAXchicken	Uncategorized Sample	20196-20205_A	Mudpit_Ingel20196_MGFPeaklist (F058870)	Uncharacterized protein OS=Gallus gallus GN=Gga.55808 PE=4 SV=2	F1NJR5_CHICK	TAX_GallusGallus_9031_20141114.fasta	212,630.20	100.00%	59	69	237	0.48%	33.90%	NIDRSEIISTLEGSGR	R	Q	99.70%	67.7	44.7	23.7	53.9	49.3	4.72	0.424	0.5	0	0	4	0	2	1,746.90	2,393.50		7,921.80	1513	1528	Empty	TRUE	UPI0001849F10,UPI0000EC9F07,UPI0003505B21,UPI0002C86B7B,UPI000240C443
20196-20225 HinckeM –TAXchicken	Uncategorized Sample	20196-20205_A	Mudpit_Ingel20196_MGFPeaklist (F058870)	Uncharacterized protein OS=Gallus gallus GN=Gga.55808 PE=4 SV=2	F1NJR5_CHICK	TAX_GallusGallus_9031_20141114.fasta	212,630.20	100.00%	59	69	237	0.48%	33.90%	QEQEQVLAPDVELMR	G	A	65.10%					28.2	0.699	0.0319	0.3	0	0	0	1	1	1,784.88	1,724.00		775.63	1780	1794	Empty	TRUE	
20196-20225 HinckeM –TAXchicken	Uncategorized Sample	20196-20205_A	Mudpit_Ingel20196_MGFPeaklist (F058870)	Uncharacterized protein OS=Gallus gallus GN=Gga.55808 PE=4 SV=2	F1NJR5_CHICK	TAX_GallusGallus_9031_20141114.fasta	212,630.20	100.00%	59	69	237	0.48%	33.90%	RLGYISVTDVLK	K	I	99.70%	33	41.9	21.8	13.1	34.9	1.14	0.175	0.458	0	0	2	0	2	1,363.79	2,535.50		847.45	814	825	Empty	TRUE	UPI0001849F10,UPI0000EC9F07,UPI0003505B21,UPI0002C86B7B,UPI000240C443
20196-20225 HinckeM –TAXchicken	Uncategorized Sample	20196-20205_A	Mudpit_Ingel20196_MGFPeaklist (F058870)	Uncharacterized protein OS=Gallus gallus GN=Gga.55808 PE=4 SV=2	F1NJR5_CHICK	TAX_GallusGallus_9031_20141114.fasta	212,630.20	100.00%	59	69	237	0.48%	33.90%	SEIISTLEGSGR	R	Q	99.70%	63.1	45	26.5	44.3	32.8	2.8	0.354	0.375	0	6	0	0	2	1,248.64	2,178.00		2,185.44	1517	1528	Empty	TRUE	UPI0001849F10,UPI0000EC9F07,UPI0003505B21,UPI0002C86B7B,UPI000240C443
20196-20225 HinckeM –TAXchicken	Uncategorized Sample	20196-20205_A	Mudpit_Ingel20196_MGFPeaklist (F058870)	Uncharacterized protein OS=Gallus gallus GN=Gga.55808 PE=4 SV=2	F1NJR5_CHICK	TAX_GallusGallus_9031_20141114.fasta	212,630.20	100.00%	59	69	237	0.48%	33.90%	STLVPMVQATFPDTAVTK	K	K	99.70%	51.7	44.2	20.3	49	44.8	3.8	0.437	0.417	0	0	2	0	2	1,921.99	2,479.00		1,377.22	1125	1142	Empty	TRUE	UPI0001849F10,UPI0000EC9F07,UPI0003505B21,UPI0002C86B7B,UPI000240C443
20196-20225 HinckeM –TAXchicken	Uncategorized Sample	20196-20205_A	Mudpit_Ingel20196_MGFPeaklist (F058870)	Uncharacterized protein OS=Gallus gallus GN=Gga.55808 PE=4 SV=2	F1NJR5_CHICK	TAX_GallusGallus_9031_20141114.fasta	212,630.20	100.00%	59	69	237	0.48%	33.90%	STLVPMVQATFPDTAVTKK	K	V	99.70%	48.4	43.2	13.2	39.7	49.9	5.62	0.529	0.421	0	0	3	0	2	2,050.09	2,373.00		1,250.18	1125	1143	Empty	TRUE	UPI0001849F10,UPI0000EC9F07,UPI0003505B21,UPI0002C86B7B,UPI000240C443
20196-20225 HinckeM –TAXchicken	Uncategorized Sample	20196-20205_A	Mudpit_Ingel20196_MGFPeaklist (F058870)	Uncharacterized protein OS=Gallus gallus GN=Gga.55808 PE=4 SV=2	F1NJR5_CHICK	TAX_GallusGallus_9031_20141114.fasta	212,630.20	100.00%	59	69	237	0.48%	33.90%	TAAVLLQNDPNADVLSK	R	T	99.70%	61.2	43.6	18.3	52.6	48.6	4.24	0.409	0.441	0	0	6	0	2	1,768.94	2,298.00		9,936.45	226	242	Empty	TRUE	UPI0001849F10,UPI0000EC9F07,UPI0003505B21,UPI0002C86B7B,UPI000240C443
20196-20225 HinckeM –TAXchicken	Uncategorized Sample	20196-20205_A	Mudpit_Ingel20196_MGFPeaklist (F058870)	Uncharacterized protein OS=Gallus gallus GN=Gga.55808 PE=4 SV=2	F1NJR5_CHICK	TAX_GallusGallus_9031_20141114.fasta	212,630.20	100.00%	59	69	237	0.48%	33.90%	TAEAVHFATVLYK	R	E	99.70%	28.4	44.2	18.5	19.5	36.1	2.27	0.244	0.462	0	0	3	0	2	1,449.77	2,318.00		1,677.86	1259	1271	Empty	TRUE	UPI0001849F10,UPI0000EC9F07,UPI0003505B21,UPI0002C86B7B,UPI000240C443
20196-20225 HinckeM –TAXchicken	Uncategorized Sample	20196-20205_A	Mudpit_Ingel20196_MGFPeaklist (F058870)	Uncharacterized protein OS=Gallus gallus GN=Gga.55808 PE=4 SV=2	F1NJR5_CHICK	TAX_GallusGallus_9031_20141114.fasta	212,630.20	100.00%	59	69	237	0.48%	33.90%	TFFFQSFR	R	E	99.70%	46.9	44.4	22.5	34.3	30.6	2.12	0.16	0.562	0	6	0	0	2	1,079.53	2,703.50		5,930.91	1352	1359	Empty	TRUE	UPI0001849F10,UPI0000EC9F07,UPI0003505B21,UPI0002C86B7B,UPI000240C443
20196-20225 HinckeM –TAXchicken	Uncategorized Sample	20196-20205_A	Mudpit_Ingel20196_MGFPeaklist (F058870)	Uncharacterized protein OS=Gallus gallus GN=Gga.55808 PE=4 SV=2	F1NJR5_CHICK	TAX_GallusGallus_9031_20141114.fasta	212,630.20	100.00%	59	69	237	0.48%	33.90%	TKDELTPLHCAAR	R	N	99.70%	64.4	44.4	21.3	49.3	42.6	4.59	0.256	0.538	0	0	4	0	2	1,511.76	1,699.00		3,120.21	307	319	Empty	TRUE	UPI0001849F10,UPI0000EC9F07,UPI0003505B21,UPI0002C86B7B,UPI000240C443
20196-20225 HinckeM –TAXchicken	Uncategorized Sample	20196-20205_A	Mudpit_Ingel20196_MGFPeaklist (F058870)	Uncharacterized protein OS=Gallus gallus GN=Gga.55808 PE=4 SV=2	F1NJR5_CHICK	TAX_GallusGallus_9031_20141114.fasta	212,630.20	100.00%	59	69	237	0.48%	33.90%	TLEHHENFTEVAR	K	S	99.70%	71.1	44.2	19.3	59.6	49.4	6.33	0.478	0.654	0	0	6	0	2	1,582.76	1,690.00		4,086.56	1309	1321	Empty	TRUE	UPI0001849F10,UPI0000EC9F07,UPI0003505B21,UPI0002C86B7B,UPI000240C443
20196-20225 HinckeM –TAXchicken	Uncategorized Sample	20196-20205_A	Mudpit_Ingel20196_MGFPeaklist (F058870)	Uncharacterized protein OS=Gallus gallus GN=Gga.55808 PE=4 SV=2	F1NJR5_CHICK	TAX_GallusGallus_9031_20141114.fasta	212,630.20	100.00%	59	69	237	0.48%	33.90%	VDVAELLLVHDAHPNAAGK	K	N	99.70%	41.9	43.2	14.3	37.3	36.5	5.03	0.323	0.342	0	0	0	2	2	1,969.05	2,558.00		397.43	587	605	Empty	TRUE	UPI0001849F10,UPI0000EC9F07,UPI0003505B21,UPI0002C86B7B,UPI000240C443
20196-20225 HinckeM –TAXchicken	Uncategorized Sample	20196-20205_A	Mudpit_Ingel20196_MGFPeaklist (F058870)	Uncharacterized protein OS=Gallus gallus GN=Gga.55808 PE=4 SV=2	F1NJR5_CHICK	TAX_GallusGallus_9031_20141114.fasta	212,630.20	100.00%	59	69	237	0.48%	33.90%	VMELLLK	R	T	41.10%	20.7	44.9	30.9	0					0	1	0	0	2	845.5166	2,429.00		184.13	424	430	Empty	TRUE	UPI0001849F10,UPI0000EC9F07,UPI0003505B21,UPI0002C86B7B,UPI000240C443
20196-20225 HinckeM –TAXchicken	Uncategorized Sample	20196-20205_A	Mudpit_Ingel20196_MGFPeaklist (F058870)	Uncharacterized protein OS=Gallus gallus GN=Gga.55808 PE=4 SV=2	F1NJR5_CHICK	TAX_GallusGallus_9031_20141114.fasta	212,630.20	100.00%	59	69	237	0.48%	33.90%	VQATIVPSPTVSHTVER	K	S	99.70%	68.5	43.2	22.5	62	40.1	5.09	0.406	0.382	0	0	10	0	2	1,820.99	2,001.00		19,921.60	1717	1733	Empty	TRUE	UPI0001849F10,UPI0000EC9F07,UPI0003505B21,UPI0002C86B7B,UPI000240C443
20196-20225 HinckeM –TAXchicken	Uncategorized Sample	20196-20205_A	Mudpit_Ingel20196_MGFPeaklist (F058870)	Uncharacterized protein OS=Gallus gallus GN=Gga.55808 PE=4 SV=2	F1NJR5_CHICK	TAX_GallusGallus_9031_20141114.fasta	212,630.20	100.00%	59	69	237	0.48%	33.90%	VRLPALHIAAR	K	N	99.70%	38.6	40.6	21.2	27.3	29.8	2.89	0.326	0.409	0	0	5	0	2	1,216.76	2,186.00		3,104.58	210	220	Empty	TRUE	UPI0001849F10,UPI0000EC9F07,UPI0003505B21,UPI0002C86B7B,UPI000240C443
20196-20225 HinckeM –TAXchicken	Uncategorized Sample	20196-20205_A	Mudpit_Ingel20196_MGFPeaklist (F058870)	Uncharacterized protein OS=Gallus gallus GN=Gga.55808 PE=4 SV=2	F1NJR5_CHICK	TAX_GallusGallus_9031_20141114.fasta	212,630.20	100.00%	59	69	237	0.48%	33.90%	YGKVDVAELLLVHDAHPNAAGK	K	N	99.70%	25.9	42.6	13.3	24.8	36.8	3.7	0.38	0.273	0	0	0	2	2	2,317.23	2,490.50		1,064.95	584	605	Empty	TRUE	UPI0001849F10,UPI0000EC9F07,UPI0003505B21,UPI0002C86B7B,UPI000240C443
20196-20225 HinckeM –TAXchicken	Uncategorized Sample	20196-20205_A	Mudpit_Ingel20196_MGFPeaklist (F058870)	status=active	UPI0003505B21	UPARC14_2_GallusG_9031_20140417.fasta	240,659.00	99.00%	2	2	9	0.02%	30.00%	ALNGFTPLHIACK	R	K	99.70%	47.1	44.6	20.6	42	29	3.23	0.0552	0.346	0	0	4	0	2	1,442.75	2,350.50		1,073.19	432	444	Empty	FALSE	UPI0001849F10,UPI0000EC9F07,UPI0002C86B7B,UPI000240C443,F1NJR5_CHICK
20196-20225 HinckeM –TAXchicken	Uncategorized Sample	20196-20205_A	Mudpit_Ingel20196_MGFPeaklist (F058870)	status=active	UPI0003505B21	UPARC14_2_GallusG_9031_20140417.fasta	240,659.00	99.00%	2	2	9	0.02%	30.00%	ALNGFTPLHIACKK	R	N	35.90%	16.1	43.8	15.3	7.87					0	0	2	0	2	1,570.84	2,209.00		995.15	432	445	Empty	FALSE	UPI0001849F10,UPI0000EC9F07,UPI0002C86B7B,UPI000240C443,F1NJR5_CHICK
20196-20225 HinckeM –TAXchicken	Uncategorized Sample	20196-20205_A	Mudpit_Ingel20196_MGFPeaklist (F058870)	status=active	UPI0003505B21	UPARC14_2_GallusG_9031_20140417.fasta	240,659.00	99.00%	2	2	9	0.02%	30.00%	EASGSLSFLR	R	K	99.70%	78.9	44.1	28.6	58	43.6	4.62	0.433	0.65	0	7	0	0	2	1,066.55	2,283.00		8,871.14	1493	1502	Empty	FALSE	UPI0001849F10,UPI0000EC9F07,UPI0002C86B7B,UPI000240C443,F1NJR5_CHICK
20196-20225 HinckeM –TAXchicken	Uncategorized Sample	20196-20205_A	Mudpit_Ingel20196_MGFPeaklist (F058870)	status=active	UPI0003505B21	UPARC14_2_GallusG_9031_20140417.fasta	240,659.00	99.00%	2	2	9	0.02%	30.00%	EASGSLSFLRK	R	A	99.70%	44.1	43.7	23	25.4	35.2	3.57	0.341	0.455	0	2	0	0	2	1,194.65	2,069.00		909.82	1493	1503	Empty	FALSE	UPI0001849F10,UPI0000EC9F07,UPI0002C86B7B,UPI000240C443,F1NJR5_CHICK
20196-20225 HinckeM –TAXchicken	Uncategorized Sample	20196-20205_A	Mudpit_Ingel20196_MGFPeaklist (F058870)	status=active	UPI0003505B21	UPARC14_2_GallusG_9031_20140417.fasta	240,659.00	99.00%	2	2	9	0.02%	30.00%	ECANFTTNVSAR	K	F	99.70%	72.7	42.9	29.8	57.9	38.1	4.28	0.462	0.417	0	2	0	0	2	1,369.62	1,766.50		1,175.46	1357	1368	Empty	FALSE	UPI0001849F10,UPI0000EC9F07,UPI0002C86B7B,UPI000240C443,F1NJR5_CHICK
20196-20225 HinckeM –TAXchicken	Uncategorized Sample	20196-20205_A	Mudpit_Ingel20196_MGFPeaklist (F058870)	status=active	UPI0003505B21	UPARC14_2_GallusG_9031_20140417.fasta	240,659.00	99.00%	2	2	9	0.02%	30.00%	EGHVDTALALLEK	R	G	99.70%	65.7	44	31.1	32.8	39.3	3.08	0.316	0.462	0	0	6	0	2	1,395.75	2,417.00		3,110.98	577	589	Empty	FALSE	UPI0002C86B7B,UPI000240C443,F1NJR5_CHICK
20196-20225 HinckeM –TAXchicken	Uncategorized Sample	20196-20205_A	Mudpit_Ingel20196_MGFPeaklist (F058870)	status=active	UPI0003505B21	UPARC14_2_GallusG_9031_20140417.fasta	240,659.00	99.00%	2	2	9	0.02%	30.00%	EIVLETTTK	K	K	99.70%	60.5	44.7	33.5	39.9	35.2	2.23	0.352	0.556	0	4	0	0	2	1,033.58	1,895.00		2,729.35	132	140	Empty	FALSE	UPI0001849F10,UPI0000EC9F07,UPI0002C86B7B,UPI000240C443,F1NJR5_CHICK
20196-20225 HinckeM –TAXchicken	Uncategorized Sample	20196-20205_A	Mudpit_Ingel20196_MGFPeaklist (F058870)	status=active	UPI0003505B21	UPARC14_2_GallusG_9031_20140417.fasta	240,659.00	99.00%	2	2	9	0.02%	30.00%	ELQFGVDDINR	R	I	99.70%	61.2	45	28.1	38.2	39.3	2.12	0.341	0.455	0	4	0	0	2	1,305.64	2,292.50		4,691.50	1584	1594	Empty	FALSE	UPI0001849F10,UPI0000EC9F07,UPI0002C86B7B,UPI000240C443,F1NJR5_CHICK
20196-20225 HinckeM –TAXchicken	Uncategorized Sample	20196-20205_A	Mudpit_Ingel20196_MGFPeaklist (F058870)	status=active	UPI0003505B21	UPARC14_2_GallusG_9031_20140417.fasta	240,659.00	99.00%	2	2	9	0.02%	30.00%	ELTAVPYMAK	K	F	99.70%	33.8	44.1	24	18.5	34.9	2.38	0.338	0.5	0	4	0	0	2	1,138.58	1,888.00		2,823.91	1390	1399	Empty	FALSE	UPI0001849F10,UPI0000EC9F07,UPI0002C86B7B,UPI000240C443,F1NJR5_CHICK
20196-20225 HinckeM –TAXchicken	Uncategorized Sample	20196-20205_A	Mudpit_Ingel20196_MGFPeaklist (F058870)	status=active	UPI0003505B21	UPARC14_2_GallusG_9031_20140417.fasta	240,659.00	99.00%	2	2	9	0.02%	30.00%	ELVNYGANVNAQSQK	R	G	99.70%	67.4	44.7	17.9	58.1	47.7	6	0.499	0.433	0	3	2	0	2	1,634.81	1,820.00		4,181.44	160	174	Empty	FALSE	UPI0001849F10,UPI0000EC9F07,UPI0002C86B7B,UPI000240C443,F1NJR5_CHICK
20196-20225 HinckeM –TAXchicken	Uncategorized Sample	20196-20205_A	Mudpit_Ingel20196_MGFPeaklist (F058870)	status=active	UPI0003505B21	UPARC14_2_GallusG_9031_20140417.fasta	240,659.00	99.00%	2	2	9	0.02%	30.00%	FLLQHQADVNAK	K	T	99.70%	40	44	17.8	37.5	37.7	2.32	0.202	0.458	0	0	6	0	2	1,383.74	1,887.00		4,995.51	783	794	Empty	FALSE	UPI0001849F10,UPI0000EC9F07,UPI0002C86B7B,UPI000240C443,F1NJR5_CHICK
20196-20225 HinckeM –TAXchicken	Uncategorized Sample	20196-20205_A	Mudpit_Ingel20196_MGFPeaklist (F058870)	status=active	UPI0003505B21	UPARC14_2_GallusG_9031_20140417.fasta	240,659.00	99.00%	2	2	9	0.02%	30.00%	FWLADCPR	R	T	99.70%	64.9	44.4	40.5	27.4	32.1	1.54	0.209	0.562	0	7	0	0	2	1,064.50	2,319.00		6,976.92	1369	1376	Empty	FALSE	UPI0001849F10,UPI0000EC9F07,UPI0002C86B7B,UPI000240C443,F1NJR5_CHICK
20196-20225 HinckeM –TAXchicken	Uncategorized Sample	20196-20205_A	Mudpit_Ingel20196_MGFPeaklist (F058870)	status=active	UPI0003505B21	UPARC14_2_GallusG_9031_20140417.fasta	240,659.00	99.00%	2	2	9	0.02%	30.00%	GASPNVSNVKVETPLHMAAR	R	A	99.70%	59.7	43.9	0	54.2	42.8	4.51	0.414	0.375	0	0	0	3	2	2,094.08	1,933.00		9,717.63	491	510	Empty	FALSE	UPI0001849F10,UPI0000EC9F07,UPI0002C86B7B,UPI000240C443,F1NJR5_CHICK
20196-20225 HinckeM –TAXchicken	Uncategorized Sample	20196-20205_A	Mudpit_Ingel20196_MGFPeaklist (F058870)	status=active	UPI0003505B21	UPARC14_2_GallusG_9031_20140417.fasta	240,659.00	99.00%	2	2	9	0.02%	30.00%	GASVNFTPQNGITPLHIASR	R	R	99.70%	50.6	43.5	16	40.6	35.9	4	0.407	0.275	0	0	4	0	2	2,080.09	2,432.00		3,108.59	293	312	Empty	FALSE	UPI0001849F10,UPI0000EC9F07,UPI0002C86B7B,UPI000240C443,F1NJR5_CHICK
20196-20225 HinckeM –TAXchicken	Uncategorized Sample	20196-20205_A	Mudpit_Ingel20196_MGFPeaklist (F058870)	status=active	UPI0003505B21	UPARC14_2_GallusG_9031_20140417.fasta	240,659.00	99.00%	2	2	9	0.02%	30.00%	GDRELVVLR	R	S	99.70%	42	43.3	37.9	18.3	36.4	2.4	0.341	0.611	0	2	0	0	2	1,056.62	1,987.00		990.51	1160	1168	Empty	FALSE	UPI0001849F10,UPI0000EC9F07,UPI0002C86B7B,UPI000240C443,F1NJR5_CHICK
20196-20225 HinckeM –TAXchicken	Uncategorized Sample	20196-20205_A	Mudpit_Ingel20196_MGFPeaklist (F058870)	status=active	UPI0003505B21	UPARC14_2_GallusG_9031_20140417.fasta	240,659.00	99.00%	2	2	9	0.02%	30.00%	GFTPLYMAAQENHLEVVK	K	F	99.70%	30.9	44.7	13	30.9	36.6	3.7	0.383	0.25	0	0	2	0	2	2,063.03	2,436.00		1,557.72	175	192	Empty	FALSE	UPI0001849F10,UPI0000EC9F07,UPI0002C86B7B,UPI000240C443,F1NJR5_CHICK
20196-20225 HinckeM –TAXchicken	Uncategorized Sample	20196-20205_A	Mudpit_Ingel20196_MGFPeaklist (F058870)	status=active	UPI0003505B21	UPARC14_2_GallusG_9031_20140417.fasta	240,659.00	99.00%	2	2	9	0.02%	30.00%	GNTALHIAALAGQQDVVR	K	E	99.70%	66.1	43.6	27.3	56.8	47.6	4.85	0.447	0.417	0	0	2	0	2	1,833.99	2,315.00		1,588.92	142	159	Empty	FALSE	UPI0001849F10,UPI0000EC9F07,UPI0002C86B7B,UPI000240C443,F1NJR5_CHICK
20196-20225 HinckeM –TAXchicken	Uncategorized Sample	20196-20205_A	Mudpit_Ingel20196_MGFPeaklist (F058870)	status=active	UPI0003505B21	UPARC14_2_GallusG_9031_20140417.fasta	240,659.00	99.00%	2	2	9	0.02%	30.00%	GSSPHSSAWNGYTPLHIAAK	K	Q	99.70%	27.1	44.3	14.1	18.8	34.9	3.24	0.218	0.375	0	0	1	4	2	2,081.02	2,221.00		4,303.70	656	675	Empty	FALSE	UPI0001849F10,UPI0000EC9F07,UPI0002C86B7B,UPI000240C443,F1NJR5_CHICK
20196-20225 HinckeM –TAXchicken	Uncategorized Sample	20196-20205_A	Mudpit_Ingel20196_MGFPeaklist (F058870)	status=active	UPI0003505B21	UPARC14_2_GallusG_9031_20140417.fasta	240,659.00	99.00%	2	2	9	0.02%	30.00%	GVKIENLYTALR	K	N	99.70%	45	42.8	25.7	31.1	28.4	2.72	0.271	0.333	0	0	2	0	2	1,376.79	2,432.00		715.69	1621	1632	Empty	FALSE	UPI0001849F10,UPI0000EC9F07,UPI0002C86B7B,UPI000240C443,F1NJR5_CHICK
20196-20225 HinckeM –TAXchicken	Uncategorized Sample	20196-20205_A	Mudpit_Ingel20196_MGFPeaklist (F058870)	status=active	UPI0003505B21	UPARC14_2_GallusG_9031_20140417.fasta	240,659.00	99.00%	2	2	9	0.02%	30.00%	HGASPNEISTNGTTPLAIAK	K	R	99.70%	62.7	44.2	14.2	41.1	46.4	7.29	0.487	0.4	0	0	8	0	2	1,979.02	2,105.00		13,283.10	820	839	Empty	FALSE	UPI0001849F10,UPI0000EC9F07,UPI0002C86B7B,UPI000240C443,F1NJR5_CHICK
20196-20225 HinckeM –TAXchicken	Uncategorized Sample	20196-20205_A	Mudpit_Ingel20196_MGFPeaklist (F058870)	status=active	UPI0003505B21	UPARC14_2_GallusG_9031_20140417.fasta	240,659.00	99.00%	2	2	9	0.02%	30.00%	HGASPNEISTNGTTPLAIAKR	K	L	99.70%	59.4	44	13.3	23.9	30.5	3.7	0.308	0.238	0	0	0	2	2	2,135.12	2,014.50		835.46	820	840	Empty	FALSE	UPI0001849F10,UPI0000EC9F07,UPI0002C86B7B,UPI000240C443,F1NJR5_CHICK
20196-20225 HinckeM –TAXchicken	Uncategorized Sample	20196-20205_A	Mudpit_Ingel20196_MGFPeaklist (F058870)	status=active	UPI0003505B21	UPARC14_2_GallusG_9031_20140417.fasta	240,659.00	99.00%	2	2	9	0.02%	30.00%	IAEILLDHGAPIQAK	R	T	99.70%	75.9	42.3	27.4	62.8	46.8	4.96	0.44	0.533	0	0	6	0	2	1,588.91	2,308.00		9,481.71	351	365	Empty	FALSE	UPI0001849F10,UPI0000EC9F07,UPI0002C86B7B,UPI000240C443,F1NJR5_CHICK
20196-20225 HinckeM –TAXchicken	Uncategorized Sample	20196-20205_A	Mudpit_Ingel20196_MGFPeaklist (F058870)	status=active	UPI0003505B21	UPARC14_2_GallusG_9031_20140417.fasta	240,659.00	99.00%	2	2	9	0.02%	30.00%	ICQDCDMIGPEGGCLK	R	S	99.70%	33.6	38.9	21.4	33.6	40.6	5.8	0.475	0.406	0	0	2	0	2	1,868.77	1,875.00		1,721.33	1227	1242	Empty	FALSE	UPI0001849F10,UPI0000EC9F07,UPI0002C86B7B,UPI000240C443,F1NJR5_CHICK
20196-20225 HinckeM –TAXchicken	Uncategorized Sample	20196-20205_A	Mudpit_Ingel20196_MGFPeaklist (F058870)	status=active	UPI0003505B21	UPARC14_2_GallusG_9031_20140417.fasta	240,659.00	99.00%	2	2	9	0.02%	30.00%	IENLYTALR	K	N	99.70%	63.2	44.3	35.4	43	38.4	2.3	0.221	0.611	0	6	0	0	2	1,092.60	2,315.00		6,139.52	1624	1632	Empty	FALSE	UPI0001849F10,UPI0000EC9F07,UPI0002C86B7B,UPI000240C443,F1NJR5_CHICK
20196-20225 HinckeM –TAXchicken	Uncategorized Sample	20196-20205_A	Mudpit_Ingel20196_MGFPeaklist (F058870)	status=active	UPI0003505B21	UPARC14_2_GallusG_9031_20140417.fasta	240,659.00	99.00%	2	2	9	0.02%	30.00%	IPLPPSWK	R	D	99.70%	36.5	42.3	23.9	21.9	25.7	0.854	0.21	0.312	0	6	0	0	2	937.5507	2,341.00		1,160.21	1307	1314	Empty	FALSE	UPI0001849F10,UPI0000EC9F07,UPI0002C86B7B,UPI000240C443,F1NJR5_CHICK
20196-20225 HinckeM –TAXchicken	Uncategorized Sample	20196-20205_A	Mudpit_Ingel20196_MGFPeaklist (F058870)	status=active	UPI0003505B21	UPARC14_2_GallusG_9031_20140417.fasta	240,659.00	99.00%	2	2	9	0.02%	30.00%	IPLPPSWKDNPR	R	D	99.70%	64.9	44.2	30.4	44.7	42.4	3.66	0.392	0.625	0	1	9	0	2	1,419.77	2,167.00		4,524.49	1307	1318	Empty	FALSE	UPI0001849F10,UPI0000EC9F07,UPI0002C86B7B,UPI000240C443,F1NJR5_CHICK
20196-20225 HinckeM –TAXchicken	Uncategorized Sample	20196-20205_A	Mudpit_Ingel20196_MGFPeaklist (F058870)	status=active	UPI0003505B21	UPARC14_2_GallusG_9031_20140417.fasta	240,659.00	99.00%	2	2	9	0.02%	30.00%	IVTEETTIPPFSDK	K	Y	99.70%	51	44.9	20.4	37.7	36.6	4.3	0.448	0.357	0	2	0	0	2	1,576.81	2,257.50		1,123.95	852	865	Empty	FALSE	UPI0001849F10,UPI0000EC9F07,UPI0002C86B7B,UPI000240C443,F1NJR5_CHICK
20196-20225 HinckeM –TAXchicken	Uncategorized Sample	20196-20205_A	Mudpit_Ingel20196_MGFPeaklist (F058870)	status=active	UPI0003505B21	UPARC14_2_GallusG_9031_20140417.fasta	240,659.00	99.00%	2	2	9	0.02%	30.00%	IVTEETTIPPFSDKYR	K	M	99.70%	62	44.1	15.6	53.4	42.6	5	0.369	0.406	0	0	8	0	2	1,895.98	2,258.00		6,912.73	852	867	Empty	FALSE	UPI0001849F10,UPI0000EC9F07,UPI0002C86B7B,UPI000240C443,F1NJR5_CHICK
20196-20225 HinckeM –TAXchicken	Uncategorized Sample	20196-20205_A	Mudpit_Ingel20196_MGFPeaklist (F058870)	status=active	UPI0003505B21	UPARC14_2_GallusG_9031_20140417.fasta	240,659.00	99.00%	2	2	9	0.02%	30.00%	KGNTALHIAALAGQQDVVR	K	E	99.70%	46.5	41.8	19	34	38.5	5.21	0.364	0.368	0	0	0	3	2	1,962.09	2,195.00		1,635.70	141	159	Empty	FALSE	UPI0001849F10,UPI0000EC9F07,UPI0002C86B7B,UPI000240C443,F1NJR5_CHICK
20196-20225 HinckeM –TAXchicken	Uncategorized Sample	20196-20205_A	Mudpit_Ingel20196_MGFPeaklist (F058870)	status=active	UPI0003505B21	UPARC14_2_GallusG_9031_20140417.fasta	240,659.00	99.00%	2	2	9	0.02%	30.00%	LAIPIKVR	R	D	99.70%	56.7	35.2	29	33.2	32.2	1.54	0.193	0.5	0	2	0	0	2	909.6245	2,185.50		683.48	1481	1488	Empty	FALSE	UPI0001849F10,UPI0000EC9F07,UPI0002C86B7B,UPI000240C443,F1NJR5_CHICK
20196-20225 HinckeM –TAXchicken	Uncategorized Sample	20196-20205_A	Mudpit_Ingel20196_MGFPeaklist (F058870)	status=active	UPI0003505B21	UPARC14_2_GallusG_9031_20140417.fasta	240,659.00	99.00%	2	2	9	0.02%	30.00%	LALQAQPVPDELVTK	R	L	99.70%	59.2	41.8	24.4	54.7	44.9	4.12	0.47	0.433	0	6	0	0	2	1,621.92	2,355.50		17,846.80	1264	1278	Empty	FALSE	UPI0001849F10,UPI0000EC9F07,UPI0002C86B7B,UPI000240C443,F1NJR5_CHICK
20196-20225 HinckeM –TAXchicken	Uncategorized Sample	20196-20205_A	Mudpit_Ingel20196_MGFPeaklist (F058870)	status=active	UPI0003505B21	UPARC14_2_GallusG_9031_20140417.fasta	240,659.00	99.00%	2	2	9	0.02%	30.00%	LGYISVTDVLK	R	I	99.70%	63.7	43.1	26.8	26	42.4	3.7	0.193	0.545	0	5	0	0	2	1,207.69	2,658.00		2,351.51	841	851	Empty	FALSE	UPI0001849F10,UPI0000EC9F07,UPI0002C86B7B,UPI000240C443,F1NJR5_CHICK
20196-20225 HinckeM –TAXchicken	Uncategorized Sample	20196-20205_A	Mudpit_Ingel20196_MGFPeaklist (F058870)	status=active	UPI0003505B21	UPARC14_2_GallusG_9031_20140417.fasta	240,659.00	99.00%	2	2	9	0.02%	30.00%	LLGNQATFSPIVTVEPR	K	R	99.70%	66.8	42.6	25.3	0	49.1	4.85	0.424	0.441	0	0	4	0	2	1,842.01	2,587.00		3,716.59	1279	1295	Empty	FALSE	UPI0001849F10,UPI0000EC9F07,UPI0002C86B7B,UPI000240C443,F1NJR5_CHICK
20196-20225 HinckeM –TAXchicken	Uncategorized Sample	20196-20205_A	Mudpit_Ingel20196_MGFPeaklist (F058870)	status=active	UPI0003505B21	UPARC14_2_GallusG_9031_20140417.fasta	240,659.00	99.00%	2	2	9	0.02%	30.00%	LPALHIAAR	R	N	99.70%	48.2	42.9	30.1	31.9	32.3	2.54	0.359	0.389	0	3	0	0	2	961.5942	2,080.00		641.69	238	246	Empty	FALSE	UPI0001849F10,UPI0000EC9F07,UPI0002C86B7B,UPI000240C443,F1NJR5_CHICK
20196-20225 HinckeM –TAXchicken	Uncategorized Sample	20196-20205_A	Mudpit_Ingel20196_MGFPeaklist (F058870)	status=active	UPI0003505B21	UPARC14_2_GallusG_9031_20140417.fasta	240,659.00	99.00%	2	2	9	0.02%	30.00%	LPAPPPLAEEEGLASR	K	I	99.70%	51.5	44.5	27	39.1	47.1	7.51	0.507	0.438	0	1	1	0	2	1,646.88	2,370.50		2,729.64	1117	1132	Empty	FALSE	UPI0001849F10,UPI0000EC9F07,UPI0002C86B7B,UPI000240C443,F1NJR5_CHICK
20196-20225 HinckeM –TAXchicken	Uncategorized Sample	20196-20205_A	Mudpit_Ingel20196_MGFPeaklist (F058870)	status=active	UPI0003505B21	UPARC14_2_GallusG_9031_20140417.fasta	240,659.00	99.00%	2	2	9	0.02%	30.00%	LVKPQKLPAPPPLAEEEGLASR	R	I	99.70%	48.4	38	13.1	47.2	44.6	4.8	0.446	0.318	0	0	0	4	2	2,340.33	2,298.50		2,553.23	1111	1132	Empty	FALSE	UPI0001849F10,UPI0000EC9F07,UPI0002C86B7B,UPI000240C443,F1NJR5_CHICK
20196-20225 HinckeM –TAXchicken	Uncategorized Sample	20196-20205_A	Mudpit_Ingel20196_MGFPeaklist (F058870)	status=active	UPI0003505B21	UPARC14_2_GallusG_9031_20140417.fasta	240,659.00	99.00%	2	2	9	0.02%	30.00%	MGYTSLHIASHYGNIK	R	L	24.90%	11.9	44.6	18.2	2.47					0	0	0	1	2	1,807.88	2,012.00		387.07	764	779	Empty	FALSE	UPI0001849F10,UPI0000EC9F07,UPI0002C86B7B,UPI000240C443,F1NJR5_CHICK
20196-20225 HinckeM –TAXchicken	Uncategorized Sample	20196-20205_A	Mudpit_Ingel20196_MGFPeaklist (F058870)	status=active	UPI0003505B21	UPARC14_2_GallusG_9031_20140417.fasta	240,659.00	99.00%	2	2	9	0.02%	30.00%	MPASDHQLGTGEQNPESEVSFVSVQQK	K	V	99.70%	64.5	42.6	0	64.5	58.9	11.2	0.594	0.315	0	0	4	0	2	2,945.36	2,189.50		5,582.27	1810	1836	Empty	FALSE	UPI0001849F10,UPI0000EC9F07,UPI0002C86B7B,UPI000240C443,F1NJR5_CHICK
20196-20225 HinckeM –TAXchicken	Uncategorized Sample	20196-20205_A	Mudpit_Ingel20196_MGFPeaklist (F058870)	status=active	UPI0003505B21	UPARC14_2_GallusG_9031_20140417.fasta	240,659.00	99.00%	2	2	9	0.02%	30.00%	MVVELLHK	K	E	99.40%	26.2	43.3	14.3	16	21.6	0.638	0.162	0.25	0	1	0	0	2	984.5548	1,901.00		107.1	124	131	Empty	FALSE	UPI0001849F10,UPI0000EC9F07,UPI0002C86B7B,UPI000240C443,F1NJR5_CHICK
20196-20225 HinckeM –TAXchicken	Uncategorized Sample	20196-20205_A	Mudpit_Ingel20196_MGFPeaklist (F058870)	status=active	UPI0003505B21	UPARC14_2_GallusG_9031_20140417.fasta	240,659.00	99.00%	2	2	9	0.02%	30.00%	NGLSPIHMAAQGDHLDCVR	K	L	99.70%	54.6	43.4	17	41.4	35.7	5.64	0	0.316	0	0	0	3	2	2,107.97	2,040.00		1,322.63	368	386	Empty	FALSE	UPI0001849F10,UPI0000EC9F07,UPI0002C86B7B,UPI000240C443,F1NJR5_CHICK
20196-20225 HinckeM –TAXchicken	Uncategorized Sample	20196-20205_A	Mudpit_Ingel20196_MGFPeaklist (F058870)	status=active	UPI0003505B21	UPARC14_2_GallusG_9031_20140417.fasta	240,659.00	99.00%	2	2	9	0.02%	30.00%	NGVDINTCNQNGLNALHLASK	R	E	99.70%	67.1	43.8	13.6	9.84	40.3	5.14	0.00248	0.262	0	0	4	0	2	2,254.09	2,361.50		2,034.30	98	118	Empty	FALSE	UPI0001849F10,UPI0000EC9F07,UPI0002C86B7B,UPI000240C443,F1NJR5_CHICK
20196-20225 HinckeM –TAXchicken	Uncategorized Sample	20196-20205_A	Mudpit_Ingel20196_MGFPeaklist (F058870)	status=active	UPI0003505B21	UPARC14_2_GallusG_9031_20140417.fasta	240,659.00	99.00%	2	2	9	0.02%	30.00%	NIDRSEIISTLEGSGR	R	Q	99.70%	67.7	44.7	23.7	53.9	49.3	4.72	0.424	0.5	0	0	4	0	2	1,746.90	2,393.50		7,921.80	1633	1648	Empty	FALSE	UPI0001849F10,UPI0000EC9F07,UPI0002C86B7B,UPI000240C443,F1NJR5_CHICK
20196-20225 HinckeM –TAXchicken	Uncategorized Sample	20196-20205_A	Mudpit_Ingel20196_MGFPeaklist (F058870)	status=active	UPI0003505B21	UPARC14_2_GallusG_9031_20140417.fasta	240,659.00	99.00%	2	2	9	0.02%	30.00%	QEVLAELLPWPLGGLLAQNR	R	M	68.50%	28.8	43.4	22.7	22.9					0	0	0	1	2	2,217.24	2,565.00		1,647.67	1960	1979	Empty	TRUE	
20196-20225 HinckeM –TAXchicken	Uncategorized Sample	20196-20205_A	Mudpit_Ingel20196_MGFPeaklist (F058870)	status=active	UPI0003505B21	UPARC14_2_GallusG_9031_20140417.fasta	240,659.00	99.00%	2	2	9	0.02%	30.00%	RLGYISVTDVLK	K	I	99.70%	33	41.9	21.8	13.1	34.9	1.14	0.175	0.458	0	0	2	0	2	1,363.79	2,535.50		847.45	840	851	Empty	FALSE	UPI0001849F10,UPI0000EC9F07,UPI0002C86B7B,UPI000240C443,F1NJR5_CHICK
20196-20225 HinckeM –TAXchicken	Uncategorized Sample	20196-20205_A	Mudpit_Ingel20196_MGFPeaklist (F058870)	status=active	UPI0003505B21	UPARC14_2_GallusG_9031_20140417.fasta	240,659.00	99.00%	2	2	9	0.02%	30.00%	SEIISTLEGSGR	R	Q	99.70%	63.1	45	26.5	44.3	32.8	2.8	0.354	0.375	0	6	0	0	2	1,248.64	2,178.00		2,185.44	1637	1648	Empty	FALSE	UPI0001849F10,UPI0000EC9F07,UPI0002C86B7B,UPI000240C443,F1NJR5_CHICK
20196-20225 HinckeM –TAXchicken	Uncategorized Sample	20196-20205_A	Mudpit_Ingel20196_MGFPeaklist (F058870)	status=active	UPI0003505B21	UPARC14_2_GallusG_9031_20140417.fasta	240,659.00	99.00%	2	2	9	0.02%	30.00%	STLVPMVQATFPDTAVTK	K	K	99.70%	51.7	44.2	20.3	49	44.8	3.8	0.437	0.417	0	0	2	0	2	1,921.99	2,479.00		1,377.22	1243	1260	Empty	FALSE	UPI0001849F10,UPI0000EC9F07,UPI0002C86B7B,UPI000240C443,F1NJR5_CHICK
20196-20225 HinckeM –TAXchicken	Uncategorized Sample	20196-20205_A	Mudpit_Ingel20196_MGFPeaklist (F058870)	status=active	UPI0003505B21	UPARC14_2_GallusG_9031_20140417.fasta	240,659.00	99.00%	2	2	9	0.02%	30.00%	STLVPMVQATFPDTAVTKK	K	V	99.70%	48.4	43.2	13.2	39.7	49.9	5.62	0.529	0.421	0	0	3	0	2	2,050.09	2,373.00		1,250.18	1243	1261	Empty	FALSE	UPI0001849F10,UPI0000EC9F07,UPI0002C86B7B,UPI000240C443,F1NJR5_CHICK
20196-20225 HinckeM –TAXchicken	Uncategorized Sample	20196-20205_A	Mudpit_Ingel20196_MGFPeaklist (F058870)	status=active	UPI0003505B21	UPARC14_2_GallusG_9031_20140417.fasta	240,659.00	99.00%	2	2	9	0.02%	30.00%	TAAVLLQNDPNADVLSK	R	T	99.70%	61.2	43.6	18.3	52.6	48.6	4.24	0.409	0.441	0	0	6	0	2	1,768.94	2,298.00		9,936.45	252	268	Empty	FALSE	UPI0001849F10,UPI0000EC9F07,UPI0002C86B7B,UPI000240C443,F1NJR5_CHICK
20196-20225 HinckeM –TAXchicken	Uncategorized Sample	20196-20205_A	Mudpit_Ingel20196_MGFPeaklist (F058870)	status=active	UPI0003505B21	UPARC14_2_GallusG_9031_20140417.fasta	240,659.00	99.00%	2	2	9	0.02%	30.00%	TAEAVHFATVLYK	R	E	99.70%	28.4	44.2	18.5	19.5	36.1	2.27	0.244	0.462	0	0	3	0	2	1,449.77	2,318.00		1,677.86	1377	1389	Empty	FALSE	UPI0001849F10,UPI0000EC9F07,UPI0002C86B7B,UPI000240C443,F1NJR5_CHICK
20196-20225 HinckeM –TAXchicken	Uncategorized Sample	20196-20205_A	Mudpit_Ingel20196_MGFPeaklist (F058870)	status=active	UPI0003505B21	UPARC14_2_GallusG_9031_20140417.fasta	240,659.00	99.00%	2	2	9	0.02%	30.00%	TFFFQSFR	R	E	99.70%	46.9	44.4	22.5	34.3	30.6	2.12	0.16	0.562	0	6	0	0	2	1,079.53	2,703.50		5,930.91	1470	1477	Empty	FALSE	UPI0001849F10,UPI0000EC9F07,UPI0002C86B7B,UPI000240C443,F1NJR5_CHICK
20196-20225 HinckeM –TAXchicken	Uncategorized Sample	20196-20205_A	Mudpit_Ingel20196_MGFPeaklist (F058870)	status=active	UPI0003505B21	UPARC14_2_GallusG_9031_20140417.fasta	240,659.00	99.00%	2	2	9	0.02%	30.00%	TKDELTPLHCAAR	R	N	99.70%	64.4	44.4	21.3	49.3	42.6	4.59	0.256	0.538	0	0	4	0	2	1,511.76	1,699.00		3,120.21	333	345	Empty	FALSE	UPI0001849F10,UPI0000EC9F07,UPI0002C86B7B,UPI000240C443,F1NJR5_CHICK
20196-20225 HinckeM –TAXchicken	Uncategorized Sample	20196-20205_A	Mudpit_Ingel20196_MGFPeaklist (F058870)	status=active	UPI0003505B21	UPARC14_2_GallusG_9031_20140417.fasta	240,659.00	99.00%	2	2	9	0.02%	30.00%	TLEHHENFTEVAR	K	S	99.70%	71.1	44.2	19.3	59.6	49.4	6.33	0.478	0.654	0	0	6	0	2	1,582.76	1,690.00		4,086.56	1427	1439	Empty	FALSE	UPI0001849F10,UPI0000EC9F07,UPI0002C86B7B,UPI000240C443,F1NJR5_CHICK
20196-20225 HinckeM –TAXchicken	Uncategorized Sample	20196-20205_A	Mudpit_Ingel20196_MGFPeaklist (F058870)	status=active	UPI0003505B21	UPARC14_2_GallusG_9031_20140417.fasta	240,659.00	99.00%	2	2	9	0.02%	30.00%	TLTPLSLR	R	E	96.90%	56.9	43.5	48.8	34.4					0	8	0	0	2	900.5514	2,248.50		13,467.90	1536	1543	Empty	TRUE	UPI000240C443
20196-20225 HinckeM –TAXchicken	Uncategorized Sample	20196-20205_A	Mudpit_Ingel20196_MGFPeaklist (F058870)	status=active	UPI0003505B21	UPARC14_2_GallusG_9031_20140417.fasta	240,659.00	99.00%	2	2	9	0.02%	30.00%	VDVAELLLVHDAHPNAAGK	K	N	99.70%	41.9	43.2	14.3	37.3	36.5	5.03	0.323	0.342	0	0	0	2	2	1,969.05	2,558.00		397.43	613	631	Empty	FALSE	UPI0001849F10,UPI0000EC9F07,UPI0002C86B7B,UPI000240C443,F1NJR5_CHICK
20196-20225 HinckeM –TAXchicken	Uncategorized Sample	20196-20205_A	Mudpit_Ingel20196_MGFPeaklist (F058870)	status=active	UPI0003505B21	UPARC14_2_GallusG_9031_20140417.fasta	240,659.00	99.00%	2	2	9	0.02%	30.00%	VMELLLK	R	T	41.10%	20.7	44.9	30.9	0					0	1	0	0	2	845.5166	2,429.00		184.13	450	456	Empty	FALSE	UPI0001849F10,UPI0000EC9F07,UPI0002C86B7B,UPI000240C443,F1NJR5_CHICK
20196-20225 HinckeM –TAXchicken	Uncategorized Sample	20196-20205_A	Mudpit_Ingel20196_MGFPeaklist (F058870)	status=active	UPI0003505B21	UPARC14_2_GallusG_9031_20140417.fasta	240,659.00	99.00%	2	2	9	0.02%	30.00%	VQATIVPSPTVSHTVER	K	S	99.70%	68.5	43.2	22.5	62	40.1	5.09	0.406	0.382	0	0	10	0	2	1,820.99	2,001.00		19,921.60	1837	1853	Empty	FALSE	UPI0001849F10,UPI0000EC9F07,UPI0002C86B7B,UPI000240C443,F1NJR5_CHICK
20196-20225 HinckeM –TAXchicken	Uncategorized Sample	20196-20205_A	Mudpit_Ingel20196_MGFPeaklist (F058870)	status=active	UPI0003505B21	UPARC14_2_GallusG_9031_20140417.fasta	240,659.00	99.00%	2	2	9	0.02%	30.00%	VRLPALHIAAR	K	N	99.70%	38.6	40.6	21.2	27.3	29.8	2.89	0.326	0.409	0	0	5	0	2	1,216.76	2,186.00		3,104.58	236	246	Empty	FALSE	UPI0001849F10,UPI0000EC9F07,UPI0002C86B7B,UPI000240C443,F1NJR5_CHICK
[illegible]	[illegible]	[illegible]	[illegible]	[illegible]	[illegible]	[illegible]	[illegible]	[illegible]	[illegible]	[illegible]	[illegible]	[illegible]	[illegible]	[illegible]	[illegible]	[illegible]	[illegible]	[illegible]	[illegible]	[illegible]	[illegible]	[illegible]	[illegible]	[illegible]	[illegible]	[illegible]	[illegible]	[illegible]	[illegible]	[illegible]	[illegible]	[illegible]		[illegible]	[illegible]	[illegible]	[illegible]	[illegible]	[illegible]

20196-20225 HinckeM –TAXchicken	Uncategorized Sample	20196-20205_A	Mudpit_Ingel20196_MGFPeaklist (F058870)	Gelsolin OS=Gallus gallus GN=GSN PE=2 SV=1	F1NKF3_CHICK,GELS_CHICK,UPI00000FE618,UPI00003AB298	TAX_GallusGallus_9031_20141114.fasta,UPARC14_2_GallusG_9031_20140417.fasta	85,876.20	100.00%	7	8	15	0.03%	9.25%	AGKEPGLQIWR	K	I	99.70%	33	43.4	20.3	17	27.8	2.11	0.248	0.409	0	0	2	0	2	1,254.70	2,216.00		1,871.22	58	68	Empty	TRUE	
20196-20225 HinckeM –TAXchicken	Uncategorized Sample	20196-20205_A	Mudpit_Ingel20196_MGFPeaklist (F058870)	Gelsolin OS=Gallus gallus GN=GSN PE=2 SV=1	F1NKF3_CHICK,GELS_CHICK,UPI00000FE618,UPI00003AB298	TAX_GallusGallus_9031_20141114.fasta,UPARC14_2_GallusG_9031_20140417.fasta	85,876.20	100.00%	7	8	15	0.03%	9.25%	HVVPNEVTVQR	R	L	99.70%	45	44.4	25	24.8	36.7	5.01	0.403	0.5	0	0	2	0	2	1,277.70	1,649.00		1,661.20	174	184	Empty	TRUE	
20196-20225 HinckeM –TAXchicken	Uncategorized Sample	20196-20205_A	Mudpit_Ingel20196_MGFPeaklist (F058870)	Gelsolin OS=Gallus gallus GN=GSN PE=2 SV=1	F1NKF3_CHICK,GELS_CHICK,UPI00000FE618,UPI00003AB298	TAX_GallusGallus_9031_20141114.fasta,UPARC14_2_GallusG_9031_20140417.fasta	85,876.20	100.00%	7	8	15	0.03%	9.25%	IEKFDLVPVPK	R	N	44.60%	24.4	42	28.9	0.58					0	0	2	0	2	1,284.76	2,389.50		4,668.27	69	79	Empty	TRUE	
20196-20225 HinckeM –TAXchicken	Uncategorized Sample	20196-20205_A	Mudpit_Ingel20196_MGFPeaklist (F058870)	Gelsolin OS=Gallus gallus GN=GSN PE=2 SV=1	F1NKF3_CHICK,GELS_CHICK,UPI00000FE618,UPI00003AB298	TAX_GallusGallus_9031_20141114.fasta,UPARC14_2_GallusG_9031_20140417.fasta	85,876.20	100.00%	7	8	15	0.03%	9.25%	IEKVPFDAATLHTSR	K	A	99.70%	47.1	43.6	21.3	32.9	39	5.59	0.431	0.4	0	0	1	2	2	1,684.90	2,084.00		3,393.43	413	427	Empty	TRUE	
20196-20225 HinckeM –TAXchicken	Uncategorized Sample	20196-20205_A	Mudpit_Ingel20196_MGFPeaklist (F058870)	Gelsolin OS=Gallus gallus GN=GSN PE=2 SV=1	F1NKF3_CHICK,GELS_CHICK,UPI00000FE618,UPI00003AB298	TAX_GallusGallus_9031_20141114.fasta,UPARC14_2_GallusG_9031_20140417.fasta	85,876.20	100.00%	7	8	15	0.03%	9.25%	TPSAAYLWVGR	K	G	99.70%	63.6	44.7	27.7	53.8	48.7	6.18	0.341	0.591	0	2	0	0	2	1,220.64	2,428.50		2,917.42	594	604	Empty	TRUE	
20196-20225 HinckeM –TAXchicken	Uncategorized Sample	20196-20205_A	Mudpit_Ingel20196_MGFPeaklist (F058870)	Gelsolin OS=Gallus gallus GN=GSN PE=2 SV=1	F1NKF3_CHICK,GELS_CHICK,UPI00000FE618,UPI00003AB298	TAX_GallusGallus_9031_20141114.fasta,UPARC14_2_GallusG_9031_20140417.fasta	85,876.20	100.00%	7	8	15	0.03%	9.25%	VLGARPVQVSEGR	K	E	86.50%	30.2	42.5	23	14					0	0	2	0	2	1,367.78	1,711.00		551.98	620	632	Empty	TRUE	
20196-20225 HinckeM –TAXchicken	Uncategorized Sample	20196-20205_A	Mudpit_Ingel20196_MGFPeaklist (F058870)	Gelsolin OS=Gallus gallus GN=GSN PE=2 SV=1	F1NKF3_CHICK,GELS_CHICK,UPI00000FE618,UPI00003AB298	TAX_GallusGallus_9031_20141114.fasta,UPARC14_2_GallusG_9031_20140417.fasta	85,876.20	100.00%	7	8	15	0.03%	9.25%	VPFDAATLHTSR	K	A	99.70%	49.2	44.6	21.8	37.1	33.7	4.36	0.386	0.333	0	0	2	0	2	1,314.68	1,960.00		1,383.10	416	427	Empty	TRUE	
20196-20225 HinckeM –TAXchicken	Uncategorized Sample	20196-20205_A	Mudpit_Ingel20196_MGFPeaklist (F058870)	Gelsolin OS=Gallus gallus GN=GSN PE=2 SV=1	F1NKF3_CHICK,GELS_CHICK,UPI00000FE618,UPI00003AB298	TAX_GallusGallus_9031_20141114.fasta,UPARC14_2_GallusG_9031_20140417.fasta	85,876.20	100.00%	7	8	15	0.03%	9.25%	AGKEPGLQIWR	K	I	99.70%	33	43.4	20.3	17	27.8	2.11	0.248	0.409	0	0	2	0	2	1,254.70	2,216.00		1,871.22	58	68	Empty	TRUE	
20196-20225 HinckeM –TAXchicken	Uncategorized Sample	20196-20205_A	Mudpit_Ingel20196_MGFPeaklist (F058870)	Gelsolin OS=Gallus gallus GN=GSN PE=2 SV=1	F1NKF3_CHICK,GELS_CHICK,UPI00000FE618,UPI00003AB298	TAX_GallusGallus_9031_20141114.fasta,UPARC14_2_GallusG_9031_20140417.fasta	85,876.20	100.00%	7	8	15	0.03%	9.25%	HVVPNEVTVQR	R	L	99.70%	45	44.4	25	24.8	36.7	5.01	0.403	0.5	0	0	2	0	2	1,277.70	1,649.00		1,661.20	174	184	Empty	TRUE	
20196-20225 HinckeM –TAXchicken	Uncategorized Sample	20196-20205_A	Mudpit_Ingel20196_MGFPeaklist (F058870)	Gelsolin OS=Gallus gallus GN=GSN PE=2 SV=1	F1NKF3_CHICK,GELS_CHICK,UPI00000FE618,UPI00003AB298	TAX_GallusGallus_9031_20141114.fasta,UPARC14_2_GallusG_9031_20140417.fasta	85,876.20	100.00%	7	8	15	0.03%	9.25%	IEKFDLVPVPK	R	N	44.60%	24.4	42	28.9	0.58					0	0	2	0	2	1,284.76	2,389.50		4,668.27	69	79	Empty	TRUE	
20196-20225 HinckeM –TAXchicken	Uncategorized Sample	20196-20205_A	Mudpit_Ingel20196_MGFPeaklist (F058870)	Gelsolin OS=Gallus gallus GN=GSN PE=2 SV=1	F1NKF3_CHICK,GELS_CHICK,UPI00000FE618,UPI00003AB298	TAX_GallusGallus_9031_20141114.fasta,UPARC14_2_GallusG_9031_20140417.fasta	85,876.20	100.00%	7	8	15	0.03%	9.25%	IEKVPFDAATLHTSR	K	A	99.70%	47.1	43.6	21.3	32.9	39	5.59	0.431	0.4	0	0	1	2	2	1,684.90	2,084.00		3,393.43	413	427	Empty	TRUE	
20196-20225 HinckeM –TAXchicken	Uncategorized Sample	20196-20205_A	Mudpit_Ingel20196_MGFPeaklist (F058870)	Gelsolin OS=Gallus gallus GN=GSN PE=2 SV=1	F1NKF3_CHICK,GELS_CHICK,UPI00000FE618,UPI00003AB298	TAX_GallusGallus_9031_20141114.fasta,UPARC14_2_GallusG_9031_20140417.fasta	85,876.20	100.00%	7	8	15	0.03%	9.25%	TPSAAYLWVGR	K	G	99.70%	63.6	44.7	27.7	53.8	48.7	6.18	0.341	0.591	0	2	0	0	2	1,220.64	2,428.50		2,917.42	594	604	Empty	TRUE	
20196-20225 HinckeM –TAXchicken	Uncategorized Sample	20196-20205_A	Mudpit_Ingel20196_MGFPeaklist (F058870)	Gelsolin OS=Gallus gallus GN=GSN PE=2 SV=1	F1NKF3_CHICK,GELS_CHICK,UPI00000FE618,UPI00003AB298	TAX_GallusGallus_9031_20141114.fasta,UPARC14_2_GallusG_9031_20140417.fasta	85,876.20	100.00%	7	8	15	0.03%	9.25%	VLGARPVQVSEGR	K	E	86.50%	30.2	42.5	23	14					0	0	2	0	2	1,367.78	1,711.00		551.98	620	632	Empty	TRUE	
20196-20225 HinckeM –TAXchicken	Uncategorized Sample	20196-20205_A	Mudpit_Ingel20196_MGFPeaklist (F058870)	Gelsolin OS=Gallus gallus GN=GSN PE=2 SV=1	F1NKF3_CHICK,GELS_CHICK,UPI00000FE618,UPI00003AB298	TAX_GallusGallus_9031_20141114.fasta,UPARC14_2_GallusG_9031_20140417.fasta	85,876.20	100.00%	7	8	15	0.03%	9.25%	VPFDAATLHTSR	K	A	99.70%	49.2	44.6	21.8	37.1	33.7	4.36	0.386	0.333	0	0	2	0	2	1,314.68	1,960.00		1,383.10	416	427	Empty	TRUE	
20196-20225 HinckeM –TAXchicken	Uncategorized Sample	20196-20205_A	Mudpit_Ingel20196_MGFPeaklist (F058870)	Gelsolin OS=Gallus gallus GN=GSN PE=2 SV=1	F1NKF3_CHICK,GELS_CHICK,UPI00000FE618,UPI00003AB298	TAX_GallusGallus_9031_20141114.fasta,UPARC14_2_GallusG_9031_20140417.fasta	85,876.20	100.00%	7	8	15	0.03%	9.25%	AGKEPGLQIWR	K	I	99.70%	33	43.4	20.3	17	27.8	2.11	0.248	0.409	0	0	2	0	2	1,254.70	2,216.00		1,871.22	58	68	Empty	TRUE	
20196-20225 HinckeM –TAXchicken	Uncategorized Sample	20196-20205_A	Mudpit_Ingel20196_MGFPeaklist (F058870)	Gelsolin OS=Gallus gallus GN=GSN PE=2 SV=1	F1NKF3_CHICK,GELS_CHICK,UPI00000FE618,UPI00003AB298	TAX_GallusGallus_9031_20141114.fasta,UPARC14_2_GallusG_9031_20140417.fasta	85,876.20	100.00%	7	8	15	0.03%	9.25%	HVVPNEVTVQR	R	L	99.70%	45	44.4	25	24.8	36.7	5.01	0.403	0.5	0	0	2	0	2	1,277.70	1,649.00		1,661.20	174	184	Empty	TRUE	
20196-20225 HinckeM –TAXchicken	Uncategorized Sample	20196-20205_A	Mudpit_Ingel20196_MGFPeaklist (F058870)	Gelsolin OS=Gallus gallus GN=GSN PE=2 SV=1	F1NKF3_CHICK,GELS_CHICK,UPI00000FE618,UPI00003AB298	TAX_GallusGallus_9031_20141114.fasta,UPARC14_2_GallusG_9031_20140417.fasta	85,876.20	100.00%	7	8	15	0.03%	9.25%	IEKFDLVPVPK	R	N	44.60%	24.4	42	28.9	0.58					0	0	2	0	2	1,284.76	2,389.50		4,668.27	69	79	Empty	TRUE	
20196-20225 HinckeM –TAXchicken	Uncategorized Sample	20196-20205_A	Mudpit_Ingel20196_MGFPeaklist (F058870)	Gelsolin OS=Gallus gallus GN=GSN PE=2 SV=1	F1NKF3_CHICK,GELS_CHICK,UPI00000FE618,UPI00003AB298	TAX_GallusGallus_9031_20141114.fasta,UPARC14_2_GallusG_9031_20140417.fasta	85,876.20	100.00%	7	8	15	0.03%	9.25%	IEKVPFDAATLHTSR	K	A	99.70%	47.1	43.6	21.3	32.9	39	5.59	0.431	0.4	0	0	1	2	2	1,684.90	2,084.00		3,393.43	413	427	Empty	TRUE	
20196-20225 HinckeM –TAXchicken	Uncategorized Sample	20196-20205_A	Mudpit_Ingel20196_MGFPeaklist (F058870)	Gelsolin OS=Gallus gallus GN=GSN PE=2 SV=1	F1NKF3_CHICK,GELS_CHICK,UPI00000FE618,UPI00003AB298	TAX_GallusGallus_9031_20141114.fasta,UPARC14_2_GallusG_9031_20140417.fasta	85,876.20	100.00%	7	8	15	0.03%	9.25%	TPSAAYLWVGR	K	G	99.70%	63.6	44.7	27.7	53.8	48.7	6.18	0.341	0.591	0	2	0	0	2	1,220.64	2,428.50		2,917.42	594	604	Empty	TRUE	
20196-20225 HinckeM –TAXchicken	Uncategorized Sample	20196-20205_A	Mudpit_Ingel20196_MGFPeaklist (F058870)	Gelsolin OS=Gallus gallus GN=GSN PE=2 SV=1	F1NKF3_CHICK,GELS_CHICK,UPI00000FE618,UPI00003AB298	TAX_GallusGallus_9031_20141114.fasta,UPARC14_2_GallusG_9031_20140417.fasta	85,876.20	100.00%	7	8	15	0.03%	9.25%	VLGARPVQVSEGR	K	E	86.50%	30.2	42.5	23	14					0	0	2	0	2	1,367.78	1,711.00		551.98	620	632	Empty	TRUE	
20196-20225 HinckeM –TAXchicken	Uncategorized Sample	20196-20205_A	Mudpit_Ingel20196_MGFPeaklist (F058870)	Gelsolin OS=Gallus gallus GN=GSN PE=2 SV=1	F1NKF3_CHICK,GELS_CHICK,UPI00000FE618,UPI00003AB298	TAX_GallusGallus_9031_20141114.fasta,UPARC14_2_GallusG_9031_20140417.fasta	85,876.20	100.00%	7	8	15	0.03%	9.25%	VPFDAATLHTSR	K	A	99.70%	49.2	44.6	21.8	37.1	33.7	4.36	0.386	0.333	0	0	2	0	2	1,314.68	1,960.00		1,383.10	416	427	Empty	TRUE	
20196-20225 HinckeM –TAXchicken	Uncategorized Sample	20196-20205_A	Mudpit_Ingel20196_MGFPeaklist (F058870)	Gelsolin OS=Gallus gallus GN=GSN PE=2 SV=1	F1NKF3_CHICK,GELS_CHICK,UPI00000FE618,UPI00003AB298	TAX_GallusGallus_9031_20141114.fasta,UPARC14_2_GallusG_9031_20140417.fasta	85,876.20	100.00%	7	8	15	0.03%	9.25%	AGKEPGLQIWR	K	I	99.70%	33	43.4	20.3	17	27.8	2.11	0.248	0.409	0	0	2	0	2	1,254.70	2,216.00		1,871.22	58	68	Empty	TRUE	
20196-20225 HinckeM –TAXchicken	Uncategorized Sample	20196-20205_A	Mudpit_Ingel20196_MGFPeaklist (F058870)	Gelsolin OS=Gallus gallus GN=GSN PE=2 SV=1	F1NKF3_CHICK,GELS_CHICK,UPI00000FE618,UPI00003AB298	TAX_GallusGallus_9031_20141114.fasta,UPARC14_2_GallusG_9031_20140417.fasta	85,876.20	100.00%	7	8	15	0.03%	9.25%	HVVPNEVTVQR	R	L	99.70%	45	44.4	25	24.8	36.7	5.01	0.403	0.5	0	0	2	0	2	1,277.70	1,649.00		1,661.20	174	184	Empty	TRUE	
20196-20225 HinckeM –TAXchicken	Uncategorized Sample	20196-20205_A	Mudpit_Ingel20196_MGFPeaklist (F058870)	Gelsolin OS=Gallus gallus GN=GSN PE=2 SV=1	F1NKF3_CHICK,GELS_CHICK,UPI00000FE618,UPI00003AB298	TAX_GallusGallus_9031_20141114.fasta,UPARC14_2_GallusG_9031_20140417.fasta	85,876.20	100.00%	7	8	15	0.03%	9.25%	IEKFDLVPVPK	R	N	44.60%	24.4	42	28.9	0.58					0	0	2	0	2	1,284.76	2,389.50		4,668.27	69	79	Empty	TRUE	
20196-20225 HinckeM –TAXchicken	Uncategorized Sample	20196-20205_A	Mudpit_Ingel20196_MGFPeaklist (F058870)	Gelsolin OS=Gallus gallus GN=GSN PE=2 SV=1	F1NKF3_CHICK,GELS_CHICK,UPI00000FE618,UPI00003AB298	TAX_GallusGallus_9031_20141114.fasta,UPARC14_2_GallusG_9031_20140417.fasta	85,876.20	100.00%	7	8	15	0.03%	9.25%	IEKVPFDAATLHTSR	K	A	99.70%	47.1	43.6	21.3	32.9	39	5.59	0.431	0.4	0	0	1	2	2	1,684.90	2,084.00		3,393.43	413	427	Empty	TRUE	
20196-20225 HinckeM –TAXchicken	Uncategorized Sample	20196-20205_A	Mudpit_Ingel20196_MGFPeaklist (F058870)	Gelsolin OS=Gallus gallus GN=GSN PE=2 SV=1	F1NKF3_CHICK,GELS_CHICK,UPI00000FE618,UPI00003AB298	TAX_GallusGallus_9031_20141114.fasta,UPARC14_2_GallusG_9031_20140417.fasta	85,876.20	100.00%	7	8	15	0.03%	9.25%	TPSAAYLWVGR	K	G	99.70%	63.6	44.7	27.7	53.8	48.7	6.18	0.341	0.591	0	2	0	0	2	1,220.64	2,428.50		2,917.42	594	604	Empty	TRUE	
20196-20225 HinckeM –TAXchicken	Uncategorized Sample	20196-20205_A	Mudpit_Ingel20196_MGFPeaklist (F058870)	Gelsolin OS=Gallus gallus GN=GSN PE=2 SV=1	F1NKF3_CHICK,GELS_CHICK,UPI00000FE618,UPI00003AB298	TAX_GallusGallus_9031_20141114.fasta,UPARC14_2_GallusG_9031_20140417.fasta	85,876.20	100.00%	7	8	15	0.03%	9.25%	VLGARPVQVSEGR	K	E	86.50%	30.2	42.5	23	14					0	0	2	0	2	1,367.78	1,711.00		551.98	620	632	Empty	TRUE	
20196-20225 HinckeM –TAXchicken	Uncategorized Sample	20196-20205_A	Mudpit_Ingel20196_MGFPeaklist (F058870)	Gelsolin OS=Gallus gallus GN=GSN PE=2 SV=1	F1NKF3_CHICK,GELS_CHICK,UPI00000FE618,UPI00003AB298	TAX_GallusGallus_9031_20141114.fasta,UPARC14_2_GallusG_9031_20140417.fasta	85,876.20	100.00%	7	8	15	0.03%	9.25%	VPFDAATLHTSR	K	A	99.70%	49.2	44.6	21.8	37.1	33.7	4.36	0.386	0.333	0	0	2	0	2	1,314.68	1,960.00		1,383.10	416	427	Empty	TRUE	
20196-20225 HinckeM –TAXchicken	Uncategorized Sample	20196-20205_A	Mudpit_Ingel20196_MGFPeaklist (F058870)	Ubiquitin carboxyl-terminal hydrolase OS=Gallus gallus GN=USP9X PE=3 SV=1	E1BWJ5_CHICK,V9GVG9_CHICK	TAX_GallusGallus_9031_20141114.fasta	290,113.20	100.00%	28	28	53	0.11%	12.90%	DDVFGYPHQYEDKPALSK	R	T	21.30%	8.84	44	15.6	2.04					0	0	0	2	2	2,108.99	2,094.00		1,019.79	1615	1632	Empty	TRUE	UPI000060E52A,UPI0003503F85,UPI0000E7FA8E,UPI0000E7FA8D,UPI0000ECD4F9,UPI0000ECD4F8,UPI00003A9D5C,UPI00003A9D5B,UPI0003DDF37A
20196-20225 HinckeM –TAXchicken	Uncategorized Sample	20196-20205_A	Mudpit_Ingel20196_MGFPeaklist (F058870)	Ubiquitin carboxyl-terminal hydrolase OS=Gallus gallus GN=USP9X PE=3 SV=1	E1BWJ5_CHICK,V9GVG9_CHICK	TAX_GallusGallus_9031_20141114.fasta	290,113.20	100.00%	28	28	53	0.11%	12.90%	DGLTISFTK	R	I	66.40%	22.9	43.6	23.3	11.1					0	2	0	0	2	981.5253	2,308.50		220.56	122	130	Empty	TRUE	UPI000060E52A,UPI0003503F85,UPI0000E7FA8E,UPI0000E7FA8D,UPI0000ECD4F9,UPI0000ECD4F8,UPI00003A9D5C,UPI00003A9D5B,UPI0003DDF37A
20196-20225 HinckeM –TAXchicken	Uncategorized Sample	20196-20205_A	Mudpit_Ingel20196_MGFPeaklist (F058870)	Ubiquitin carboxyl-terminal hydrolase OS=Gallus gallus GN=USP9X PE=3 SV=1	E1BWJ5_CHICK,V9GVG9_CHICK	TAX_GallusGallus_9031_20141114.fasta	290,113.20	100.00%	28	28	53	0.11%	12.90%	EHGEYDPQTVRPGSR	K	Y	99.70%	20	43.8	13.3	18.1	28.3	2.02	0.265	0.3	0	0	0	2	2	1,727.81	1,508.50		849.99	633	647	Empty	TRUE	UPI000060E52A,UPI0003503F85,UPI0000E7FA8E,UPI0000E7FA8D,UPI0000ECD4F9,UPI0000ECD4F8,UPI00003A9D5C,UPI00003A9D5B,UPI0003DDF37A
20196-20225 HinckeM –TAXchicken	Uncategorized Sample	20196-20205_A	Mudpit_Ingel20196_MGFPeaklist (F058870)	Ubiquitin carboxyl-terminal hydrolase OS=Gallus gallus GN=USP9X PE=3 SV=1	E1BWJ5_CHICK,V9GVG9_CHICK	TAX_GallusGallus_9031_20141114.fasta	290,113.20	100.00%	28	28	53	0.11%	12.90%	ELLAFQTPEKK	K	Y	26.30%	10.3	44.1	16.2	0					0	0	1	0	2	1,303.73	2,150.00		322.95	1436	1446	Empty	TRUE	UPI000060E52A,UPI0003503F85,UPI0000E7FA8E,UPI0000E7FA8D,UPI0000ECD4F9,UPI0000ECD4F8,UPI00003A9D5C,UPI00003A9D5B,UPI0003DDF37A
20196-20225 HinckeM –TAXchicken	Uncategorized Sample	20196-20205_A	Mudpit_Ingel20196_MGFPeaklist (F058870)	Ubiquitin carboxyl-terminal hydrolase OS=Gallus gallus GN=USP9X PE=3 SV=1	E1BWJ5_CHICK,V9GVG9_CHICK	TAX_GallusGallus_9031_20141114.fasta	290,113.20	100.00%	28	28	53	0.11%	12.90%	EYINECDSDYHEER	R	T	99.70%	62.5	35.2	21.9	52.9	44.2	6.37	0.466	0.464	0	0	2	0	2	1,858.72	1,628.00		684.22	859	872	Empty	TRUE	UPI000060E52A,UPI0003503F85,UPI0000E7FA8E,UPI0000E7FA8D,UPI0000ECD4F9,UPI0000ECD4F8,UPI00003A9D5C,UPI00003A9D5B,UPI0003DDF37A
20196-20225 HinckeM –TAXchicken	Uncategorized Sample	20196-20205_A	Mudpit_Ingel20196_MGFPeaklist (F058870)	Ubiquitin carboxyl-terminal hydrolase OS=Gallus gallus GN=USP9X PE=3 SV=1	E1BWJ5_CHICK,V9GVG9_CHICK	TAX_GallusGallus_9031_20141114.fasta	290,113.20	100.00%	28	28	53	0.11%	12.90%	EYNIGVLR	K	H	28.50%	13.3	44.6	14.6	0					0	1	0	0	2	963.5258	2,154.00		183.05	1638	1645	Empty	TRUE	UPI000060E52A,UPI0003503F85,UPI0000E7FA8E,UPI0000E7FA8D,UPI0000ECD4F9,UPI0000ECD4F8,UPI00003A9D5C,UPI00003A9D5B,UPI0003DDF37A
20196-20225 HinckeM –TAXchicken	Uncategorized Sample	20196-20205_A	Mudpit_Ingel20196_MGFPeaklist (F058870)	Ubiquitin carboxyl-terminal hydrolase OS=Gallus gallus GN=USP9X PE=3 SV=1	E1BWJ5_CHICK,V9GVG9_CHICK	TAX_GallusGallus_9031_20141114.fasta	290,113.20	100.00%	28	28	53	0.11%	12.90%	FLFTTGFHTK	R	K	38.90%	13.9	44	19.2	9.75					0	0	2	0	2	1,198.63	2,395.00		1,000.16	2050	2059	Empty	TRUE	UPI000060E52A,UPI0003503F85,UPI0000E7FA8E,UPI0000E7FA8D,UPI0000ECD4F9,UPI0000ECD4F8,UPI00003A9D5C,UPI00003A9D5B,UPI0003DDF37A
20196-20225 HinckeM –TAXchicken	Uncategorized Sample	20196-20205_A	Mudpit_Ingel20196_MGFPeaklist (F058870)	Ubiquitin carboxyl-terminal hydrolase OS=Gallus gallus GN=USP9X PE=3 SV=1	E1BWJ5_CHICK,V9GVG9_CHICK	TAX_GallusGallus_9031_20141114.fasta	290,113.20	100.00%	28	28	53	0.11%	12.90%	GDLLEGANAYHCEK	K	C	99.40%	21.1	43.4	15.5	17.5	23.4	0.921	0.209	0.214	0	0	3	0	2	1,576.71	1,902.00		1,065.95	1760	1773	Empty	TRUE	UPI000060E52A,UPI0003503F85,UPI0000E7FA8E,UPI0000E7FA8D,UPI0000ECD4F9,UPI0000ECD4F8,UPI00003A9D5C,UPI00003A9D5B,UPI0003DDF37A
20196-20225 HinckeM –TAXchicken	Uncategorized Sample	20196-20205_A	Mudpit_Ingel20196_MGFPeaklist (F058870)	Ubiquitin carboxyl-terminal hydrolase OS=Gallus gallus GN=USP9X PE=3 SV=1	E1BWJ5_CHICK,V9GVG9_CHICK	TAX_GallusGallus_9031_20141114.fasta	290,113.20	100.00%	28	28	53	0.11%	12.90%	GIPDDRDGLFDTIQR	K	S	99.70%	35.4	44.6	17.2	33.7	36.9	5.1	0.371	0.367	0	0	2	0	2	1,717.85	2,338.50		1,623.56	2360	2374	Empty	TRUE	UPI000060E52A,UPI0003503F85,UPI0000E7FA8E,UPI0000E7FA8D,UPI0000ECD4F9,UPI0000ECD4F8,UPI00003A9D5C,UPI00003A9D5B,UPI0003DDF37A
20196-20225 HinckeM –TAXchicken	Uncategorized Sample	20196-20205_A	Mudpit_Ingel20196_MGFPeaklist (F058870)	Ubiquitin carboxyl-terminal hydrolase OS=Gallus gallus GN=USP9X PE=3 SV=1	E1BWJ5_CHICK,V9GVG9_CHICK	TAX_GallusGallus_9031_20141114.fasta	290,113.20	100.00%	28	28	53	0.11%	12.90%	HGNPEEEEWLTAER	R	M	99.70%	38.3	42.9	16.7	26.2	36.1	4.92	0.374	0.429	0	0	2	0	2	1,696.76	2,074.50		1,479.11	372	385	Empty	TRUE	UPI000060E52A,UPI0003503F85,UPI0000E7FA8E,UPI0000E7FA8D,UPI0000ECD4F9,UPI0000ECD4F8,UPI00003A9D5C,UPI00003A9D5B,UPI0003DDF37A
20196-20225 HinckeM –TAXchicken	Uncategorized Sample	20196-20205_A	Mudpit_Ingel20196_MGFPeaklist (F058870)	Ubiquitin carboxyl-terminal hydrolase OS=Gallus gallus GN=USP9X PE=3 SV=1	E1BWJ5_CHICK,V9GVG9_CHICK	TAX_GallusGallus_9031_20141114.fasta	290,113.20	100.00%	28	28	53	0.11%	12.90%	IIEDCSNSEETIK	K	L	99.70%	19.2	43.9	13	12.4	28.4	1.82	0.306	0.269	0	1	0	0	2	1,537.71	1,682.00		287.64	2288	2300	Empty	TRUE	UPI000060E52A,UPI0003503F85,UPI0000E7FA8E,UPI0000E7FA8D,UPI0000ECD4F9,UPI0000ECD4F8,UPI00003A9D5C,UPI00003A9D5B,UPI0003DDF37A
20196-20225 HinckeM –TAXchicken	Uncategorized Sample	20196-20205_A	Mudpit_Ingel20196_MGFPeaklist (F058870)	Ubiquitin carboxyl-terminal hydrolase OS=Gallus gallus GN=USP9X PE=3 SV=1	E1BWJ5_CHICK,V9GVG9_CHICK	TAX_GallusGallus_9031_20141114.fasta	290,113.20	100.00%	28	28	53	0.11%	12.90%	ILTDEAVSGWK	K	F	99.70%	50.9	45.2	23.6	32.5	28	0.854	0.225	0.273	0	2	0	0	2	1,218.64	2,194.50		598.06	131	141	Empty	TRUE	UPI000060E52A,UPI0003503F85,UPI0000E7FA8E,UPI0000E7FA8D,UPI0000ECD4F9,UPI0000ECD4F8,UPI00003A9D5C,UPI00003A9D5B,UPI0003DDF37A
20196-20225 HinckeM –TAXchicken	Uncategorized Sample	20196-20205_A	Mudpit_Ingel20196_MGFPeaklist (F058870)	Ubiquitin carboxyl-terminal hydrolase OS=Gallus gallus GN=USP9X PE=3 SV=1	E1BWJ5_CHICK,V9GVG9_CHICK	TAX_GallusGallus_9031_20141114.fasta	290,113.20	100.00%	28	28	53	0.11%	12.90%	LIGQLNLK	K	D	20.30%	13.9	41.1	26.5	0					0	1	0	0	2	898.5722	2,189.00		146.15	949	956	Empty	TRUE	UPI000060E52A,UPI0003503F85,UPI0000E7FA8E,UPI0000E7FA8D,UPI0000ECD4F9,UPI0000ECD4F8,UPI00003A9D5C,UPI00003A9D5B,UPI0003DDF37A
20196-20225 HinckeM –TAXchicken	Uncategorized Sample	20196-20205_A	Mudpit_Ingel20196_MGFPeaklist (F058870)	Ubiquitin carboxyl-terminal hydrolase OS=Gallus gallus GN=USP9X PE=3 SV=1	E1BWJ5_CHICK,V9GVG9_CHICK	TAX_GallusGallus_9031_20141114.fasta	290,113.20	100.00%	28	28	53	0.11%	12.90%	LLLTAIGYGHVR	K	A	99.70%	45	41.7	20.6	37.7	30.8	4.49	0.38	0.333	0	0	2	0	2	1,312.77	2,422.50		795.67	1157	1168	Empty	TRUE	UPI000060E52A,UPI0003503F85,UPI0000E7FA8E,UPI0000E7FA8D,UPI0000ECD4F9,UPI0000ECD4F8,UPI00003A9D5C,UPI00003A9D5B,UPI0003DDF37A
20196-20225 HinckeM –TAXchicken	Uncategorized Sample	20196-20205_A	Mudpit_Ingel20196_MGFPeaklist (F058870)	Ubiquitin carboxyl-terminal hydrolase OS=Gallus gallus GN=USP9X PE=3 SV=1	E1BWJ5_CHICK,V9GVG9_CHICK	TAX_GallusGallus_9031_20141114.fasta	290,113.20	100.00%	28	28	53	0.11%	12.90%	LLQISSFNGK	R	M	99.70%	74.6	44.1	28.3	43.7	34.4	2.55	0.314	0.45	0	4	0	0	2	1,107.60	2,299.00		1,168.02	342	351	Empty	TRUE	UPI000060E52A,UPI0003503F85,UPI0000E7FA8E,UPI0000E7FA8D,UPI0000ECD4F9,UPI0000ECD4F8,UPI00003A9D5C,UPI00003A9D5B,UPI0003DDF37A
20196-20225 HinckeM –TAXchicken	Uncategorized Sample	20196-20205_A	Mudpit_Ingel20196_MGFPeaklist (F058870)	Ubiquitin carboxyl-terminal hydrolase OS=Gallus gallus GN=USP9X PE=3 SV=1	E1BWJ5_CHICK,V9GVG9_CHICK	TAX_GallusGallus_9031_20141114.fasta	290,113.20	100.00%	28	28	53	0.11%	12.90%	LPPVLAIQLK	K	R	99.70%	38.4	36.4	20.5	26.9	38.8	2.8	0.369	0.5	0	1	0	0	2	1,091.72	2,676.00		190.47	1789	1798	Empty	TRUE	UPI000060E52A,UPI0003503F85,UPI0000E7FA8E,UPI0000E7FA8D,UPI0000ECD4F9,UPI0000ECD4F8,UPI00003A9D5C,UPI00003A9D5B,UPI0003DDF37A
20196-20225 HinckeM –TAXchicken	Uncategorized Sample	20196-20205_A	Mudpit_Ingel20196_MGFPeaklist (F058870)	Ubiquitin carboxyl-terminal hydrolase OS=Gallus gallus GN=USP9X PE=3 SV=1	E1BWJ5_CHICK,V9GVG9_CHICK	TAX_GallusGallus_9031_20141114.fasta	290,113.20	100.00%	28	28	53	0.11%	12.90%	LQYYVPR	R	G	74.20%	41.1	43.1	24.8	14.3					0	2	0	0	2	938.5094	1,955.50		1,062.37	1659	1665	Empty	TRUE	UPI000060E52A,UPI0003503F85,UPI0000E7FA8E,UPI0000E7FA8D,UPI0000ECD4F9,UPI0000ECD4F8,UPI00003A9D5C,UPI00003A9D5B,UPI0003DDF37A
20196-20225 HinckeM –TAXchicken	Uncategorized Sample	20196-20205_A	Mudpit_Ingel20196_MGFPeaklist (F058870)	Ubiquitin carboxyl-terminal hydrolase OS=Gallus gallus GN=USP9X PE=3 SV=1	E1BWJ5_CHICK,V9GVG9_CHICK	TAX_GallusGallus_9031_20141114.fasta	290,113.20	100.00%	28	28	53	0.11%	12.90%	LYTVVSQLIR	K	C	99.70%	68.3	42.1	33.1	55.5	39.4	4.85	0.409	0.55	0	2	0	0	2	1,191.71	2,613.50		585.45	2227	2236	Empty	TRUE	UPI000060E52A,UPI0003503F85,UPI0000E7FA8E,UPI0000E7FA8D,UPI0000ECD4F9,UPI0000ECD4F8,UPI00003A9D5C,UPI00003A9D5B,UPI0003DDF37A
20196-20225 HinckeM –TAXchicken	Uncategorized Sample	20196-20205_A	Mudpit_Ingel20196_MGFPeaklist (F058870)	Ubiquitin carboxyl-terminal hydrolase OS=Gallus gallus GN=USP9X PE=3 SV=1	E1BWJ5_CHICK,V9GVG9_CHICK	TAX_GallusGallus_9031_20141114.fasta	290,113.20	100.00%	28	28	53	0.11%	12.90%	SFNGKMNALNEVNK	S	V	77.50%					26.1	1	0.165	0.214	0	1	0	0	1	1,565.77	2,091.00		629.86	347	360	Empty	TRUE	
20196-20225 HinckeM –TAXchicken	Uncategorized Sample	20196-20205_A	Mudpit_Ingel20196_MGFPeaklist (F058870)	Ubiquitin carboxyl-terminal hydrolase OS=Gallus gallus GN=USP9X PE=3 SV=1	E1BWJ5_CHICK,V9GVG9_CHICK	TAX_GallusGallus_9031_20141114.fasta	290,113.20	100.00%	28	28	53	0.11%	12.90%	SGGLPLVLSMLTR	K	N	99.70%	59.9	44	27	44.1	35.2	2.47	0.293	0.385	0	2	0	0	2	1,359.77	2,859.00		888.17	1120	1132	Empty	TRUE	UPI000060E52A,UPI0003503F85,UPI0000E7FA8E,UPI0000E7FA8D,UPI0000ECD4F9,UPI0000ECD4F8,UPI00003A9D5C,UPI00003A9D5B,UPI0003DDF37A
20196-20225 HinckeM –TAXchicken	Uncategorized Sample	20196-20205_A	Mudpit_Ingel20196_MGFPeaklist (F058870)	Ubiquitin carboxyl-terminal hydrolase OS=Gallus gallus GN=USP9X PE=3 SV=1	E1BWJ5_CHICK,V9GVG9_CHICK	TAX_GallusGallus_9031_20141114.fasta	290,113.20	100.00%	28	28	53	0.11%	12.90%	TGETGVEETILEGHLGVTK	R	E	99.70%	45	44.2	26.2	35.2	35.6	3.6	0.306	0.289	0	0	2	0	2	1,970.01	2,454.00		832.83	1417	1435	Empty	TRUE	UPI000060E52A,UPI0003503F85,UPI0000E7FA8E,UPI0000E7FA8D,UPI0000ECD4F9,UPI0000ECD4F8,UPI00003A9D5C,UPI00003A9D5B,UPI0003DDF37A
20196-20225 HinckeM –TAXchicken	Uncategorized Sample	20196-20205_A	Mudpit_Ingel20196_MGFPeaklist (F058870)	Ubiquitin carboxyl-terminal hydrolase OS=Gallus gallus GN=USP9X PE=3 SV=1	E1BWJ5_CHICK,V9GVG9_CHICK	TAX_GallusGallus_9031_20141114.fasta	290,113.20	100.00%	28	28	53	0.11%	12.90%	VISSVSYYTHR	K	H	99.70%	36.6	44.1	26.7	23.5	22.7	0.854	0.233	0.227	0	0	2	0	2	1,311.67	1,825.00		655.77	361	371	Empty	TRUE	UPI000060E52A,UPI0003503F85,UPI0000E7FA8E,UPI0000E7FA8D,UPI0000ECD4F9,UPI0000ECD4F8,UPI00003A9D5C,UPI00003A9D5B,UPI0003DDF37A
20196-20225 HinckeM –TAXchicken	Uncategorized Sample	20196-20205_A	Mudpit_Ingel20196_MGFPeaklist (F058870)	Ubiquitin carboxyl-terminal hydrolase OS=Gallus gallus GN=USP9X PE=3 SV=1	E1BWJ5_CHICK,V9GVG9_CHICK	TAX_GallusGallus_9031_20141114.fasta	290,113.20	100.00%	28	28	53	0.11%	12.90%	VLGGSFADQK	K	I	96.70%	35.8	44.8	24.3	20.5					0	2	0	0	2	1,021.53	1,813.00		444.47	1713	1722	Empty	TRUE	UPI000060E52A,UPI0003503F85,UPI0000E7FA8E,UPI0000E7FA8D,UPI0000ECD4F9,UPI0000ECD4F8,UPI00003A9D5C,UPI00003A9D5B,UPI0003DDF37A
20196-20225 HinckeM –TAXchicken	Uncategorized Sample	20196-20205_A	Mudpit_Ingel20196_MGFPeaklist (F058870)	Ubiquitin carboxyl-terminal hydrolase OS=Gallus gallus GN=USP9X PE=3 SV=1	E1BWJ5_CHICK,V9GVG9_CHICK	TAX_GallusGallus_9031_20141114.fasta	290,113.20	100.00%	28	28	53	0.11%	12.90%	VPGQEETVKNLEIFR	R	L	99.70%	46.3	44.2	20.1	23.7	34.8	3.55	0.342	0.333	0	0	2	0	2	1,758.94	2,403.00		1,061.15	321	335	Empty	TRUE	UPI000060E52A,UPI0003503F85,UPI0000E7FA8E,UPI0000E7FA8D,UPI0000ECD4F9,UPI0000ECD4F8,UPI00003A9D5C,UPI00003A9D5B,UPI0003DDF37A
20196-20225 HinckeM –TAXchicken	Uncategorized Sample	20196-20205_A	Mudpit_Ingel20196_MGFPeaklist (F058870)	Ubiquitin carboxyl-terminal hydrolase OS=Gallus gallus GN=USP9X PE=3 SV=1	E1BWJ5_CHICK,V9GVG9_CHICK	TAX_GallusGallus_9031_20141114.fasta	290,113.20	100.00%	28	28	53	0.11%	12.90%	VVIQGNDDIANR	R	A	99.70%	65	44.4	30.1	49.1	35.3	3.06	0.394	0.375	0	3	0	0	2	1,313.68	1,747.00		1,241.15	777	788	Empty	TRUE	UPI000060E52A,UPI0003503F85,UPI0000E7FA8E,UPI0000E7FA8D,UPI0000ECD4F9,UPI0000ECD4F8,UPI00003A9D5C,UPI00003A9D5B,UPI0003DDF37A
20196-20225 HinckeM –TAXchicken	Uncategorized Sample	20196-20205_A	Mudpit_Ingel20196_MGFPeaklist (F058870)	Ubiquitin carboxyl-terminal hydrolase OS=Gallus gallus GN=USP9X PE=3 SV=1	E1BWJ5_CHICK,V9GVG9_CHICK	TAX_GallusGallus_9031_20141114.fasta	290,113.20	100.00%	28	28	53	0.11%	12.90%	WVVPVLPK	R	G	99.70%	25.3	40.4	20.2	14	27.6	1.92	0.239	0.438	0	2	0	0	2	937.587	2,575.50		361.59	84	91	Empty	TRUE	UPI000060E52A,UPI0003503F85,UPI0000E7FA8E,UPI0000E7FA8D,UPI0000ECD4F9,UPI0000ECD4F8,UPI00003A9D5C,UPI00003A9D5B,UPI0003DDF37A
20196-20225 HinckeM –TAXchicken	Uncategorized Sample	20196-20205_A	Mudpit_Ingel20196_MGFPeaklist (F058870)	Ubiquitin carboxyl-terminal hydrolase OS=Gallus gallus GN=USP9X PE=3 SV=1	E1BWJ5_CHICK,V9GVG9_CHICK	TAX_GallusGallus_9031_20141114.fasta	290,113.20	100.00%	28	28	53	0.11%	12.90%	YIPDICVIR	K	A	63.60%	16.4	44.7	22.4	13.2	24.5	0.553	0.106	0.222	0	2	0	0	2	1,148.61	2,441.00		551.34	1232	1240	Empty	TRUE	UPI000060E52A,UPI0003503F85,UPI0000E7FA8E,UPI0000E7FA8D,UPI0000ECD4F9,UPI0000ECD4F8,UPI00003A9D5C,UPI00003A9D5B,UPI0003DDF37A
20196-20225 HinckeM –TAXchicken	Uncategorized Sample	20196-20205_A	Mudpit_Ingel20196_MGFPeaklist (F058870)	Ubiquitin carboxyl-terminal hydrolase OS=Gallus gallus GN=USP9X PE=3 SV=1	E1BWJ5_CHICK,V9GVG9_CHICK	TAX_GallusGallus_9031_20141114.fasta	290,113.20	100.00%	28	28	53	0.11%	12.90%	YISELAITTKPHQIK	K	M	99.70%	25.1	41.8	13.3	19.9	27.3	1.32	0.289	0.2	0	0	1	0	2	1,741.98	2,061.00		399.57	1967	1981	Empty	TRUE	UPI000060E52A,UPI0003503F85,UPI0000E7FA8E,UPI0000E7FA8D,UPI0000ECD4F9,UPI0000ECD4F8,UPI00003A9D5C,UPI00003A9D5B,UPI0003DDF37A
20196-20225 HinckeM –TAXchicken	Uncategorized Sample	20196-20205_A	Mudpit_Ingel20196_MGFPeaklist (F058870)	Ubiquitin carboxyl-terminal hydrolase OS=Gallus gallus GN=USP9X PE=3 SV=1	E1BWJ5_CHICK,V9GVG9_CHICK	TAX_GallusGallus_9031_20141114.fasta	290,113.20	100.00%	28	28	53	0.11%	12.90%	DDVFGYPHQYEDKPALSK	R	T	21.30%	8.84	44	15.6	2.04					0	0	0	2	2	2,108.99	2,094.00		1,019.79	1615	1632	Empty	TRUE	UPI000060E52A,UPI0003503F85,UPI0000E7FA8E,UPI0000E7FA8D,UPI0000ECD4F9,UPI0000ECD4F8,UPI00003A9D5C,UPI00003A9D5B,UPI0003DDF37A
20196-20225 HinckeM –TAXchicken	Uncategorized Sample	20196-20205_A	Mudpit_Ingel20196_MGFPeaklist (F058870)	Ubiquitin carboxyl-terminal hydrolase OS=Gallus gallus GN=USP9X PE=3 SV=1	E1BWJ5_CHICK,V9GVG9_CHICK	TAX_GallusGallus_9031_20141114.fasta	290,113.20	100.00%	28	28	53	0.11%	12.90%	DGLTISFTK	R	I	66.40%	22.9	43.6	23.3	11.1					0	2	0	0	2	981.5253	2,308.50		220.56	122	130	Empty	TRUE	UPI000060E52A,UPI0003503F85,UPI0000E7FA8E,UPI0000E7FA8D,UPI0000ECD4F9,UPI0000ECD4F8,UPI00003A9D5C,UPI00003A9D5B,UPI0003DDF37A
20196-20225 HinckeM –TAXchicken	Uncategorized Sample	20196-20205_A	Mudpit_Ingel20196_MGFPeaklist (F058870)	Ubiquitin carboxyl-terminal hydrolase OS=Gallus gallus GN=USP9X PE=3 SV=1	E1BWJ5_CHICK,V9GVG9_CHICK	TAX_GallusGallus_9031_20141114.fasta	290,113.20	100.00%	28	28	53	0.11%	12.90%	EHGEYDPQTVRPGSR	K	Y	99.70%	20	43.8	13.3	18.1	28.3	2.02	0.265	0.3	0	0	0	2	2	1,727.81	1,508.50		849.99	633	647	Empty	TRUE	UPI000060E52A,UPI0003503F85,UPI0000E7FA8E,UPI0000E7FA8D,UPI0000ECD4F9,UPI0000ECD4F8,UPI00003A9D5C,UPI00003A9D5B,UPI0003DDF37A
20196-20225 HinckeM –TAXchicken	Uncategorized Sample	20196-20205_A	Mudpit_Ingel20196_MGFPeaklist (F058870)	Ubiquitin carboxyl-terminal hydrolase OS=Gallus gallus GN=USP9X PE=3 SV=1	E1BWJ5_CHICK,V9GVG9_CHICK	TAX_GallusGallus_9031_20141114.fasta	290,113.20	100.00%	28	28	53	0.11%	12.90%	ELLAFQTPEKK	K	Y	26.30%	10.3	44.1	16.2	0					0	0	1	0	2	1,303.73	2,150.00		322.95	1436	1446	Empty	TRUE	UPI000060E52A,UPI0003503F85,UPI0000E7FA8E,UPI0000E7FA8D,UPI0000ECD4F9,UPI0000ECD4F8,UPI00003A9D5C,UPI00003A9D5B,UPI0003DDF37A
20196-20225 HinckeM –TAXchicken	Uncategorized Sample	20196-20205_A	Mudpit_Ingel20196_MGFPeaklist (F058870)	Ubiquitin carboxyl-terminal hydrolase OS=Gallus gallus GN=USP9X PE=3 SV=1	E1BWJ5_CHICK,V9GVG9_CHICK	TAX_GallusGallus_9031_20141114.fasta	290,113.20	100.00%	28	28	53	0.11%	12.90%	EYINECDSDYHEER	R	T	99.70%	62.5	35.2	21.9	52.9	44.2	6.37	0.466	0.464	0	0	2	0	2	1,858.72	1,628.00		684.22	859	872	Empty	TRUE	UPI000060E52A,UPI0003503F85,UPI0000E7FA8E,UPI0000E7FA8D,UPI0000ECD4F9,UPI0000ECD4F8,UPI00003A9D5C,UPI00003A9D5B,UPI0003DDF37A
20196-20225 HinckeM –TAXchicken	Uncategorized Sample	20196-20205_A	Mudpit_Ingel20196_MGFPeaklist (F058870)	Ubiquitin carboxyl-terminal hydrolase OS=Gallus gallus GN=USP9X PE=3 SV=1	E1BWJ5_CHICK,V9GVG9_CHICK	TAX_GallusGallus_9031_20141114.fasta	290,113.20	100.00%	28	28	53	0.11%	12.90%	EYNIGVLR	K	H	28.50%	13.3	44.6	14.6	0					0	1	0	0	2	963.5258	2,154.00		183.05	1638	1645	Empty	TRUE	UPI000060E52A,UPI0003503F85,UPI0000E7FA8E,UPI0000E7FA8D,UPI0000ECD4F9,UPI0000ECD4F8,UPI00003A9D5C,UPI00003A9D5B,UPI0003DDF37A
20196-20225 HinckeM –TAXchicken	Uncategorized Sample	20196-20205_A	Mudpit_Ingel20196_MGFPeaklist (F058870)	Ubiquitin carboxyl-terminal hydrolase OS=Gallus gallus GN=USP9X PE=3 SV=1	E1BWJ5_CHICK,V9GVG9_CHICK	TAX_GallusGallus_9031_20141114.fasta	290,113.20	100.00%	28	28	53	0.11%	12.90%	FLFTTGFHTK	R	K	38.90%	13.9	44	19.2	9.75					0	0	2	0	2	1,198.63	2,395.00		1,000.16	2050	2059	Empty	TRUE	UPI000060E52A,UPI0003503F85,UPI0000E7FA8E,UPI0000E7FA8D,UPI0000ECD4F9,UPI0000ECD4F8,UPI00003A9D5C,UPI00003A9D5B,UPI0003DDF37A
20196-20225 HinckeM –TAXchicken	Uncategorized Sample	20196-20205_A	Mudpit_Ingel20196_MGFPeaklist (F058870)	Ubiquitin carboxyl-terminal hydrolase OS=Gallus gallus GN=USP9X PE=3 SV=1	E1BWJ5_CHICK,V9GVG9_CHICK	TAX_GallusGallus_9031_20141114.fasta	290,113.20	100.00%	28	28	53	0.11%	12.90%	GDLLEGANAYHCEK	K	C	99.40%	21.1	43.4	15.5	17.5	23.4	0.921	0.209	0.214	0	0	3	0	2	1,576.71	1,902.00		1,065.95	1760	1773	Empty	TRUE	UPI000060E52A,UPI0003503F85,UPI0000E7FA8E,UPI0000E7FA8D,UPI0000ECD4F9,UPI0000ECD4F8,UPI00003A9D5C,UPI00003A9D5B,UPI0003DDF37A
20196-20225 HinckeM –TAXchicken	Uncategorized Sample	20196-20205_A	Mudpit_Ingel20196_MGFPeaklist (F058870)	Ubiquitin carboxyl-terminal hydrolase OS=Gallus gallus GN=USP9X PE=3 SV=1	E1BWJ5_CHICK,V9GVG9_CHICK	TAX_GallusGallus_9031_20141114.fasta	290,113.20	100.00%	28	28	53	0.11%	12.90%	GIPDDRDGLFDTIQR	K	S	99.70%	35.4	44.6	17.2	33.7	36.9	5.1	0.371	0.367	0	0	2	0	2	1,717.85	2,338.50		1,623.56	2360	2374	Empty	TRUE	UPI000060E52A,UPI0003503F85,UPI0000E7FA8E,UPI0000E7FA8D,UPI0000ECD4F9,UPI0000ECD4F8,UPI00003A9D5C,UPI00003A9D5B,UPI0003DDF37A
20196-20225 HinckeM –TAXchicken	Uncategorized Sample	20196-20205_A	Mudpit_Ingel20196_MGFPeaklist (F058870)	Ubiquitin carboxyl-terminal hydrolase OS=Gallus gallus GN=USP9X PE=3 SV=1	E1BWJ5_CHICK,V9GVG9_CHICK	TAX_GallusGallus_9031_20141114.fasta	290,113.20	100.00%	28	28	53	0.11%	12.90%	HGNPEEEEWLTAER	R	M	99.70%	38.3	42.9	16.7	26.2	36.1	4.92	0.374	0.429	0	0	2	0	2	1,696.76	2,074.50		1,479.11	372	385	Empty	TRUE	UPI000060E52A,UPI0003503F85,UPI0000E7FA8E,UPI0000E7FA8D,UPI0000ECD4F9,UPI0000ECD4F8,UPI00003A9D5C,UPI00003A9D5B,UPI0003DDF37A
20196-20225 HinckeM –TAXchicken	Uncategorized Sample	20196-20205_A	Mudpit_Ingel20196_MGFPeaklist (F058870)	Ubiquitin carboxyl-terminal hydrolase OS=Gallus gallus GN=USP9X PE=3 SV=1	E1BWJ5_CHICK,V9GVG9_CHICK	TAX_GallusGallus_9031_20141114.fasta	290,113.20	100.00%	28	28	53	0.11%	12.90%	IIEDCSNSEETIK	K	L	99.70%	19.2	43.9	13	12.4	28.4	1.82	0.306	0.269	0	1	0	0	2	1,537.71	1,682.00		287.64	2288	2300	Empty	TRUE	UPI000060E52A,UPI0003503F85,UPI0000E7FA8E,UPI0000E7FA8D,UPI0000ECD4F9,UPI0000ECD4F8,UPI00003A9D5C,UPI00003A9D5B,UPI0003DDF37A
20196-20225 HinckeM –TAXchicken	Uncategorized Sample	20196-20205_A	Mudpit_Ingel20196_MGFPeaklist (F058870)	Ubiquitin carboxyl-terminal hydrolase OS=Gallus gallus GN=USP9X PE=3 SV=1	E1BWJ5_CHICK,V9GVG9_CHICK	TAX_GallusGallus_9031_20141114.fasta	290,113.20	100.00%	28	28	53	0.11%	12.90%	ILTDEAVSGWK	K	F	99.70%	50.9	45.2	23.6	32.5	28	0.854	0.225	0.273	0	2	0	0	2	1,218.64	2,194.50		598.06	131	141	Empty	TRUE	UPI000060E52A,UPI0003503F85,UPI0000E7FA8E,UPI0000E7FA8D,UPI0000ECD4F9,UPI0000ECD4F8,UPI00003A9D5C,UPI00003A9D5B,UPI0003DDF37A
20196-20225 HinckeM –TAXchicken	Uncategorized Sample	20196-20205_A	Mudpit_Ingel20196_MGFPeaklist (F058870)	Ubiquitin carboxyl-terminal hydrolase OS=Gallus gallus GN=USP9X PE=3 SV=1	E1BWJ5_CHICK,V9GVG9_CHICK	TAX_GallusGallus_9031_20141114.fasta	290,113.20	100.00%	28	28	53	0.11%	12.90%	LIGQLNLK	K	D	20.30%	13.9	41.1	26.5	0					0	1	0	0	2	898.5722	2,189.00		146.15	949	956	Empty	TRUE	UPI000060E52A,UPI0003503F85,UPI0000E7FA8E,UPI0000E7FA8D,UPI0000ECD4F9,UPI0000ECD4F8,UPI00003A9D5C,UPI00003A9D5B,UPI0003DDF37A
20196-20225 HinckeM –TAXchicken	Uncategorized Sample	20196-20205_A	Mudpit_Ingel20196_MGFPeaklist (F058870)	Ubiquitin carboxyl-terminal hydrolase OS=Gallus gallus GN=USP9X PE=3 SV=1	E1BWJ5_CHICK,V9GVG9_CHICK	TAX_GallusGallus_9031_20141114.fasta	290,113.20	100.00%	28	28	53	0.11%	12.90%	LLLTAIGYGHVR	K	A	99.70%	45	41.7	20.6	37.7	30.8	4.49	0.38	0.333	0	0	2	0	2	1,312.77	2,422.50		795.67	1157	1168	Empty	TRUE	UPI000060E52A,UPI0003503F85,UPI0000E7FA8E,UPI0000E7FA8D,UPI0000ECD4F9,UPI0000ECD4F8,UPI00003A9D5C,UPI00003A9D5B,UPI0003DDF37A
20196-20225 HinckeM –TAXchicken	Uncategorized Sample	20196-20205_A	Mudpit_Ingel20196_MGFPeaklist (F058870)	Ubiquitin carboxyl-terminal hydrolase OS=Gallus gallus GN=USP9X PE=3 SV=1	E1BWJ5_CHICK,V9GVG9_CHICK	TAX_GallusGallus_9031_20141114.fasta	290,113.20	100.00%	28	28	53	0.11%	12.90%	LLQISSFNGK	R	M	99.70%	74.6	44.1	28.3	43.7	34.4	2.55	0.314	0.45	0	4	0	0	2	1,107.60	2,299.00		1,168.02	342	351	Empty	TRUE	UPI000060E52A,UPI0003503F85,UPI0000E7FA8E,UPI0000E7FA8D,UPI0000ECD4F9,UPI0000ECD4F8,UPI00003A9D5C,UPI00003A9D5B,UPI0003DDF37A
20196-20225 HinckeM –TAXchicken	Uncategorized Sample	20196-20205_A	Mudpit_Ingel20196_MGFPeaklist (F058870)	Ubiquitin carboxyl-terminal hydrolase OS=Gallus gallus GN=USP9X PE=3 SV=1	E1BWJ5_CHICK,V9GVG9_CHICK	TAX_GallusGallus_9031_20141114.fasta	290,113.20	100.00%	28	28	53	0.11%	12.90%	LPPVLAIQLK	K	R	99.70%	38.4	36.4	20.5	26.9	38.8	2.8	0.369	0.5	0	1	0	0	2	1,091.72	2,676.00		190.47	1789	1798	Empty	TRUE	UPI000060E52A,UPI0003503F85,UPI0000E7FA8E,UPI0000E7FA8D,UPI0000ECD4F9,UPI0000ECD4F8,UPI00003A9D5C,UPI00003A9D5B,UPI0003DDF37A
20196-20225 HinckeM –TAXchicken	Uncategorized Sample	20196-20205_A	Mudpit_Ingel20196_MGFPeaklist (F058870)	Ubiquitin carboxyl-terminal hydrolase OS=Gallus gallus GN=USP9X PE=3 SV=1	E1BWJ5_CHICK,V9GVG9_CHICK	TAX_GallusGallus_9031_20141114.fasta	290,113.20	100.00%	28	28	53	0.11%	12.90%	LQYYVPR	R	G	74.20%	41.1	43.1	24.8	14.3					0	2	0	0	2	938.5094	1,955.50		1,062.37	1659	1665	Empty	TRUE	UPI000060E52A,UPI0003503F85,UPI0000E7FA8E,UPI0000E7FA8D,UPI0000ECD4F9,UPI0000ECD4F8,UPI00003A9D5C,UPI00003A9D5B,UPI0003DDF37A
20196-20225 HinckeM –TAXchicken	Uncategorized Sample	20196-20205_A	Mudpit_Ingel20196_MGFPeaklist (F058870)	Ubiquitin carboxyl-terminal hydrolase OS=Gallus gallus GN=USP9X PE=3 SV=1	E1BWJ5_CHICK,V9GVG9_CHICK	TAX_GallusGallus_9031_20141114.fasta	290,113.20	100.00%	28	28	53	0.11%	12.90%	LYTVVSQLIR	K	C	99.70%	68.3	42.1	33.1	55.5	39.4	4.85	0.409	0.55	0	2	0	0	2	1,191.71	2,613.50		585.45	2227	2236	Empty	TRUE	UPI000060E52A,UPI0003503F85,UPI0000E7FA8E,UPI0000E7FA8D,UPI0000ECD4F9,UPI0000ECD4F8,UPI00003A9D5C,UPI00003A9D5B,UPI0003DDF37A
20196-20225 HinckeM –TAXchicken	Uncategorized Sample	20196-20205_A	Mudpit_Ingel20196_MGFPeaklist (F058870)	Ubiquitin carboxyl-terminal hydrolase OS=Gallus gallus GN=USP9X PE=3 SV=1	E1BWJ5_CHICK,V9GVG9_CHICK	TAX_GallusGallus_9031_20141114.fasta	290,113.20	100.00%	28	28	53	0.11%	12.90%	SFNGKMNALNEVNK	S	V	77.50%					26.1	1	0.165	0.214	0	1	0	0	1	1,565.77	2,091.00		629.86	347	360	Empty	TRUE	
20196-20225 HinckeM –TAXchicken	Uncategorized Sample	20196-20205_A	Mudpit_Ingel20196_MGFPeaklist (F058870)	Ubiquitin carboxyl-terminal hydrolase OS=Gallus gallus GN=USP9X PE=3 SV=1	E1BWJ5_CHICK,V9GVG9_CHICK	TAX_GallusGallus_9031_20141114.fasta	290,113.20	100.00%	28	28	53	0.11%	12.90%	SGGLPLVLSMLTR	K	N	99.70%	59.9	44	27	44.1	35.2	2.47	0.293	0.385	0	2	0	0	2	1,359.77	2,859.00		888.17	1120	1132	Empty	TRUE	UPI000060E52A,UPI0003503F85,UPI0000E7FA8E,UPI0000E7FA8D,UPI0000ECD4F9,UPI0000ECD4F8,UPI00003A9D5C,UPI00003A9D5B,UPI0003DDF37A
20196-20225 HinckeM –TAXchicken	Uncategorized Sample	20196-20205_A	Mudpit_Ingel20196_MGFPeaklist (F058870)	Ubiquitin carboxyl-terminal hydrolase OS=Gallus gallus GN=USP9X PE=3 SV=1	E1BWJ5_CHICK,V9GVG9_CHICK	TAX_GallusGallus_9031_20141114.fasta	290,113.20	100.00%	28	28	53	0.11%	12.90%	TGETGVEETILEGHLGVTK	R	E	99.70%	45	44.2	26.2	35.2	35.6	3.6	0.306	0.289	0	0	2	0	2	1,970.01	2,454.00		832.83	1417	1435	Empty	TRUE	UPI000060E52A,UPI0003503F85,UPI0000E7FA8E,UPI0000E7FA8D,UPI0000ECD4F9,UPI0000ECD4F8,UPI00003A9D5C,UPI00003A9D5B,UPI0003DDF37A
20196-20225 HinckeM –TAXchicken	Uncategorized Sample	20196-20205_A	Mudpit_Ingel20196_MGFPeaklist (F058870)	Ubiquitin carboxyl-terminal hydrolase OS=Gallus gallus GN=USP9X PE=3 SV=1	E1BWJ5_CHICK,V9GVG9_CHICK	TAX_GallusGallus_9031_20141114.fasta	290,113.20	100.00%	28	28	53	0.11%	12.90%	VISSVSYYTHR	K	H	99.70%	36.6	44.1	26.7	23.5	22.7	0.854	0.233	0.227	0	0	2	0	2	1,311.67	1,825.00		655.77	361	371	Empty	TRUE	UPI000060E52A,UPI0003503F85,UPI0000E7FA8E,UPI0000E7FA8D,UPI0000ECD4F9,UPI0000ECD4F8,UPI00003A9D5C,UPI00003A9D5B,UPI0003DDF37A
20196-20225 HinckeM –TAXchicken	Uncategorized Sample	20196-20205_A	Mudpit_Ingel20196_MGFPeaklist (F058870)	Ubiquitin carboxyl-terminal hydrolase OS=Gallus gallus GN=USP9X PE=3 SV=1	E1BWJ5_CHICK,V9GVG9_CHICK	TAX_GallusGallus_9031_20141114.fasta	290,113.20	100.00%	28	28	53	0.11%	12.90%	VLGGSFADQK	K	I	96.70%	35.8	44.8	24.3	20.5					0	2	0	0	2	1,021.53	1,813.00		444.47	1713	1722	Empty	TRUE	UPI000060E52A,UPI0003503F85,UPI0000E7FA8E,UPI0000E7FA8D,UPI0000ECD4F9,UPI0000ECD4F8,UPI00003A9D5C,UPI00003A9D5B,UPI0003DDF37A
20196-20225 HinckeM –TAXchicken	Uncategorized Sample	20196-20205_A	Mudpit_Ingel20196_MGFPeaklist (F058870)	Ubiquitin carboxyl-terminal hydrolase OS=Gallus gallus GN=USP9X PE=3 SV=1	E1BWJ5_CHICK,V9GVG9_CHICK	TAX_GallusGallus_9031_20141114.fasta	290,113.20	100.00%	28	28	53	0.11%	12.90%	VPGQEETVKNLEIFR	R	L	99.70%	46.3	44.2	20.1	23.7	34.8	3.55	0.342	0.333	0	0	2	0	2	1,758.94	2,403.00		1,061.15	321	335	Empty	TRUE	UPI000060E52A,UPI0003503F85,UPI0000E7FA8E,UPI0000E7FA8D,UPI0000ECD4F9,UPI0000ECD4F8,UPI00003A9D5C,UPI00003A9D5B,UPI0003DDF37A
20196-20225 HinckeM –TAXchicken	Uncategorized Sample	20196-20205_A	Mudpit_Ingel20196_MGFPeaklist (F058870)	Ubiquitin carboxyl-terminal hydrolase OS=Gallus gallus GN=USP9X PE=3 SV=1	E1BWJ5_CHICK,V9GVG9_CHICK	TAX_GallusGallus_9031_20141114.fasta	290,113.20	100.00%	28	28	53	0.11%	12.90%	VVIQGNDDIANR	R	A	99.70%	65	44.4	30.1	49.1	35.3	3.06	0.394	0.375	0	3	0	0	2	1,313.68	1,747.00		1,241.15	777	788	Empty	TRUE	UPI000060E52A,UPI0003503F85,UPI0000E7FA8E,UPI0000E7FA8D,UPI0000ECD4F9,UPI0000ECD4F8,UPI00003A9D5C,UPI00003A9D5B,UPI0003DDF37A
20196-20225 HinckeM –TAXchicken	Uncategorized Sample	20196-20205_A	Mudpit_Ingel20196_MGFPeaklist (F058870)	Ubiquitin carboxyl-terminal hydrolase OS=Gallus gallus GN=USP9X PE=3 SV=1	E1BWJ5_CHICK,V9GVG9_CHICK	TAX_GallusGallus_9031_20141114.fasta	290,113.20	100.00%	28	28	53	0.11%	12.90%	WVVPVLPK	R	G	99.70%	25.3	40.4	20.2	14	27.6	1.92	0.239	0.438	0	2	0	0	2	937.587	2,575.50		361.59	84	91	Empty	TRUE	UPI000060E52A,UPI0003503F85,UPI0000E7FA8E,UPI0000E7FA8D,UPI0000ECD4F9,UPI0000ECD4F8,UPI00003A9D5C,UPI00003A9D5B,UPI0003DDF37A
20196-20225 HinckeM –TAXchicken	Uncategorized Sample	20196-20205_A	Mudpit_Ingel20196_MGFPeaklist (F058870)	Ubiquitin carboxyl-terminal hydrolase OS=Gallus gallus GN=USP9X PE=3 SV=1	E1BWJ5_CHICK,V9GVG9_CHICK	TAX_GallusGallus_9031_20141114.fasta	290,113.20	100.00%	28	28	53	0.11%	12.90%	YIPDICVIR	K	A	63.60%	16.4	44.7	22.4	13.2	24.5	0.553	0.106	0.222	0	2	0	0	2	1,148.61	2,441.00		551.34	1232	1240	Empty	TRUE	UPI000060E52A,UPI0003503F85,UPI0000E7FA8E,UPI0000E7FA8D,UPI0000ECD4F9,UPI0000ECD4F8,UPI00003A9D5C,UPI00003A9D5B,UPI0003DDF37A
20196-20225 HinckeM –TAXchicken	Uncategorized Sample	20196-20205_A	Mudpit_Ingel20196_MGFPeaklist (F058870)	Ubiquitin carboxyl-terminal hydrolase OS=Gallus gallus GN=USP9X PE=3 SV=1	E1BWJ5_CHICK,V9GVG9_CHICK	TAX_GallusGallus_9031_20141114.fasta	290,113.20	100.00%	28	28	53	0.11%	12.90%	YISELAITTKPHQIK	K	M	99.70%	25.1	41.8	13.3	19.9	27.3	1.32	0.289	0.2	0	0	1	0	2	1,741.98	2,061.00		399.57	1967	1981	Empty	TRUE	UPI000060E52A,UPI0003503F85,UPI0000E7FA8E,UPI0000E7FA8D,UPI0000ECD4F9,UPI0000ECD4F8,UPI00003A9D5C,UPI00003A9D5B,UPI0003DDF37A
20196-20225 HinckeM –TAXchicken	Uncategorized Sample	20196-20205_A	Mudpit_Ingel20196_MGFPeaklist (F058870)	Uncharacterized protein OS=Gallus gallus GN=ARPC1A PE=2 SV=1	Q5ZJI7_CHICK,UPI00003AAD40	TAX_GallusGallus_9031_20141114.fasta,UPARC14_2_GallusG_9031_20140417.fasta	41,689.80	100.00%	4	4	5	0.01%	13.00%	EVDEKPASTPWGSK	K	M	99.70%	25.4	44.6	19	15.3	24.2	0.585	0.202	0.214	0	0	1	0	2	1,530.74	1,766.00		370.63	175	188	Empty	TRUE	
20196-20225 HinckeM –TAXchicken	Uncategorized Sample	20196-20205_A	Mudpit_Ingel20196_MGFPeaklist (F058870)	Uncharacterized protein OS=Gallus gallus GN=ARPC1A PE=2 SV=1	Q5ZJI7_CHICK,UPI00003AAD40	TAX_GallusGallus_9031_20141114.fasta,UPARC14_2_GallusG_9031_20140417.fasta	41,689.80	100.00%	4	4	5	0.01%	13.00%	LAWVSHDSTVSVADASK	R	N	79.80%	24.5	44.7	19.5	14.3					0	0	1	0	2	1,772.88	2,095.00		997.24	219	235	Empty	TRUE	
20196-20225 HinckeM –TAXchicken	Uncategorized Sample	20196-20205_A	Mudpit_Ingel20196_MGFPeaklist (F058870)	Uncharacterized protein OS=Gallus gallus GN=ARPC1A PE=2 SV=1	Q5ZJI7_CHICK,UPI00003AAD40	TAX_GallusGallus_9031_20141114.fasta,UPARC14_2_GallusG_9031_20140417.fasta	41,689.80	100.00%	4	4	5	0.01%	13.00%	TLESSIQGLR	K	I	93.00%	33.5	45.2	36.2	20					0	2	0	0	2	1,103.61	2,183.50		1,679.56	359	368	Empty	TRUE	
20196-20225 HinckeM –TAXchicken	Uncategorized Sample	20196-20205_A	Mudpit_Ingel20196_MGFPeaklist (F058870)	Uncharacterized protein OS=Gallus gallus GN=ARPC1A PE=2 SV=1	Q5ZJI7_CHICK,UPI00003AAD40	TAX_GallusGallus_9031_20141114.fasta,UPARC14_2_GallusG_9031_20140417.fasta	41,689.80	100.00%	4	4	5	0.01%	13.00%	VFSAYIK	R	E	33.20%	16.4	43.1	22.2	0					0	1	0	0	2	827.4661	2,041.00		243.05	168	174	Empty	TRUE	
20196-20225 HinckeM –TAXchicken	Uncategorized Sample	20196-20205_A	Mudpit_Ingel20196_MGFPeaklist (F058870)	Uncharacterized protein OS=Gallus gallus GN=ARPC1A PE=2 SV=1	Q5ZJI7_CHICK,UPI00003AAD40	TAX_GallusGallus_9031_20141114.fasta,UPARC14_2_GallusG_9031_20140417.fasta	41,689.80	100.00%	4	4	5	0.01%	13.00%	EVDEKPASTPWGSK	K	M	99.70%	25.4	44.6	19	15.3	24.2	0.585	0.202	0.214	0	0	1	0	2	1,530.74	1,766.00		370.63	175	188	Empty	TRUE	
20196-20225 HinckeM –TAXchicken	Uncategorized Sample	20196-20205_A	Mudpit_Ingel20196_MGFPeaklist (F058870)	Uncharacterized protein OS=Gallus gallus GN=ARPC1A PE=2 SV=1	Q5ZJI7_CHICK,UPI00003AAD40	TAX_GallusGallus_9031_20141114.fasta,UPARC14_2_GallusG_9031_20140417.fasta	41,689.80	100.00%	4	4	5	0.01%	13.00%	LAWVSHDSTVSVADASK	R	N	79.80%	24.5	44.7	19.5	14.3					0	0	1	0	2	1,772.88	2,095.00		997.24	219	235	Empty	TRUE	
20196-20225 HinckeM –TAXchicken	Uncategorized Sample	20196-20205_A	Mudpit_Ingel20196_MGFPeaklist (F058870)	Uncharacterized protein OS=Gallus gallus GN=ARPC1A PE=2 SV=1	Q5ZJI7_CHICK,UPI00003AAD40	TAX_GallusGallus_9031_20141114.fasta,UPARC14_2_GallusG_9031_20140417.fasta	41,689.80	100.00%	4	4	5	0.01%	13.00%	TLESSIQGLR	K	I	93.00%	33.5	45.2	36.2	20					0	2	0	0	2	1,103.61	2,183.50		1,679.56	359	368	Empty	TRUE	
20196-20225 HinckeM –TAXchicken	Uncategorized Sample	20196-20205_A	Mudpit_Ingel20196_MGFPeaklist (F058870)	Uncharacterized protein OS=Gallus gallus GN=ARPC1A PE=2 SV=1	Q5ZJI7_CHICK,UPI00003AAD40	TAX_GallusGallus_9031_20141114.fasta,UPARC14_2_GallusG_9031_20140417.fasta	41,689.80	100.00%	4	4	5	0.01%	13.00%	VFSAYIK	R	E	33.20%	16.4	43.1	22.2	0					0	1	0	0	2	827.4661	2,041.00		243.05	168	174	Empty	TRUE	
20196-20225 HinckeM –TAXchicken	Uncategorized Sample	20196-20205_A	Mudpit_Ingel20196_MGFPeaklist (F058870)	status=inactive	F1P3B8_CHICK,PDS5B_CHICK,UPI00003AA07B,UPI00004CF187,UPI000060E777,UPI000060E778,UPI0000ECD657,UPI0000ECD658,UPI000150AF44,UPI0001C48FBF	TAX_GallusGallus_9031_20141114.fasta,UPARC14_2_GallusG_9031_20140417.fasta	161,031.60	100.00%	3	3	4	0.01%	3.72%	AANVLGAVNKPLSSAGK	K	Q	99.70%	41.8	41.9	20.5	34.5	30.6	3.85	0.304	0.265	0	0	2	0	2	1,596.91	2,077.00		488.35	1127	1143	Empty	TRUE	
20196-20225 HinckeM –TAXchicken	Uncategorized Sample	20196-20205_A	Mudpit_Ingel20196_MGFPeaklist (F058870)	status=inactive	F1P3B8_CHICK,PDS5B_CHICK,UPI00003AA07B,UPI00004CF187,UPI000060E777,UPI000060E778,UPI0000ECD657,UPI0000ECD658,UPI000150AF44,UPI0001C48FBF	TAX_GallusGallus_9031_20141114.fasta,UPARC14_2_GallusG_9031_20140417.fasta	161,031.60	100.00%	3	3	4	0.01%	3.72%	METVSNASSSSNPSSPGRIK	R	G	22.80%	4.5	43.5	13.7	0					0	0	1	0	2	2,052.95	2,226.00		1,816.59	1152	1171	Empty	TRUE	
20196-20225 HinckeM –TAXchicken	Uncategorized Sample	20196-20205_A	Mudpit_Ingel20196_MGFPeaklist (F058870)	status=inactive	F1P3B8_CHICK,PDS5B_CHICK,UPI00003AA07B,UPI00004CF187,UPI000060E777,UPI000060E778,UPI0000ECD657,UPI0000ECD658,UPI000150AF44,UPI0001C48FBF	TAX_GallusGallus_9031_20141114.fasta,UPARC14_2_GallusG_9031_20140417.fasta	161,031.60	100.00%	3	3	4	0.01%	3.72%	STTYSLESPKDPVLPAR	K	Y	99.50%	19.7	44.2	24.7	7.04	23.4	1.3	0.226	0.147	0	0	1	0	2	1,860.97	2,136.00		236.71	1080	1096	Empty	TRUE	
20196-20225 HinckeM –TAXchicken	Uncategorized Sample	20196-20205_A	Mudpit_Ingel20196_MGFPeaklist (F058870)	status=inactive	F1P3B8_CHICK,PDS5B_CHICK,UPI00003AA07B,UPI00004CF187,UPI000060E777,UPI000060E778,UPI0000ECD657,UPI0000ECD658,UPI000150AF44,UPI0001C48FBF	TAX_GallusGallus_9031_20141114.fasta,UPARC14_2_GallusG_9031_20140417.fasta	161,031.60	100.00%	3	3	4	0.01%	3.72%	AANVLGAVNKPLSSAGK	K	Q	99.70%	41.8	41.9	20.5	34.5	30.6	3.85	0.304	0.265	0	0	2	0	2	1,596.91	2,077.00		488.35	1127	1143	Empty	TRUE	
20196-20225 HinckeM –TAXchicken	Uncategorized Sample	20196-20205_A	Mudpit_Ingel20196_MGFPeaklist (F058870)	status=inactive	F1P3B8_CHICK,PDS5B_CHICK,UPI00003AA07B,UPI00004CF187,UPI000060E777,UPI000060E778,UPI0000ECD657,UPI0000ECD658,UPI000150AF44,UPI0001C48FBF	TAX_GallusGallus_9031_20141114.fasta,UPARC14_2_GallusG_9031_20140417.fasta	161,031.60	100.00%	3	3	4	0.01%	3.72%	METVSNASSSSNPSSPGRIK	R	G	22.80%	4.5	43.5	13.7	0					0	0	1	0	2	2,052.95	2,226.00		1,816.59	1152	1171	Empty	TRUE	
20196-20225 HinckeM –TAXchicken	Uncategorized Sample	20196-20205_A	Mudpit_Ingel20196_MGFPeaklist (F058870)	status=inactive	F1P3B8_CHICK,PDS5B_CHICK,UPI00003AA07B,UPI00004CF187,UPI000060E777,UPI000060E778,UPI0000ECD657,UPI0000ECD658,UPI000150AF44,UPI0001C48FBF	TAX_GallusGallus_9031_20141114.fasta,UPARC14_2_GallusG_9031_20140417.fasta	161,031.60	100.00%	3	3	4	0.01%	3.72%	STTYSLESPKDPVLPAR	K	Y	99.50%	19.7	44.2	24.7	7.04	23.4	1.3	0.226	0.147	0	0	1	0	2	1,860.97	2,136.00		236.71	1080	1096	Empty	TRUE	
20196-20225 HinckeM –TAXchicken	Uncategorized Sample	20196-20205_A	Mudpit_Ingel20196_MGFPeaklist (F058870)	status=inactive	F1P3B8_CHICK,PDS5B_CHICK,UPI00003AA07B,UPI00004CF187,UPI000060E777,UPI000060E778,UPI0000ECD657,UPI0000ECD658,UPI000150AF44,UPI0001C48FBF	TAX_GallusGallus_9031_20141114.fasta,UPARC14_2_GallusG_9031_20140417.fasta	161,031.60	100.00%	3	3	4	0.01%	3.72%	AANVLGAVNKPLSSAGK	K	Q	99.70%	41.8	41.9	20.5	34.5	30.6	3.85	0.304	0.265	0	0	2	0	2	1,596.91	2,077.00		488.35	1127	1143	Empty	TRUE	
20196-20225 HinckeM –TAXchicken	Uncategorized Sample	20196-20205_A	Mudpit_Ingel20196_MGFPeaklist (F058870)	status=inactive	F1P3B8_CHICK,PDS5B_CHICK,UPI00003AA07B,UPI00004CF187,UPI000060E777,UPI000060E778,UPI0000ECD657,UPI0000ECD658,UPI000150AF44,UPI0001C48FBF	TAX_GallusGallus_9031_20141114.fasta,UPARC14_2_GallusG_9031_20140417.fasta	161,031.60	100.00%	3	3	4	0.01%	3.72%	METVSNASSSSNPSSPGRIK	R	G	22.80%	4.5	43.5	13.7	0					0	0	1	0	2	2,052.95	2,226.00		1,816.59	1152	1171	Empty	TRUE	
20196-20225 HinckeM –TAXchicken	Uncategorized Sample	20196-20205_A	Mudpit_Ingel20196_MGFPeaklist (F058870)	status=inactive	F1P3B8_CHICK,PDS5B_CHICK,UPI00003AA07B,UPI00004CF187,UPI000060E777,UPI000060E778,UPI0000ECD657,UPI0000ECD658,UPI000150AF44,UPI0001C48FBF	TAX_GallusGallus_9031_20141114.fasta,UPARC14_2_GallusG_9031_20140417.fasta	161,031.60	100.00%	3	3	4	0.01%	3.72%	STTYSLESPKDPVLPAR	K	Y	99.50%	19.7	44.2	24.7	7.04	23.4	1.3	0.226	0.147	0	0	1	0	2	1,860.97	2,136.00		236.71	1080	1096	Empty	TRUE	
20196-20225 HinckeM –TAXchicken	Uncategorized Sample	20196-20205_A	Mudpit_Ingel20196_MGFPeaklist (F058870)	status=inactive	F1P3B8_CHICK,PDS5B_CHICK,UPI00003AA07B,UPI00004CF187,UPI000060E777,UPI000060E778,UPI0000ECD657,UPI0000ECD658,UPI000150AF44,UPI0001C48FBF	TAX_GallusGallus_9031_20141114.fasta,UPARC14_2_GallusG_9031_20140417.fasta	161,031.60	100.00%	3	3	4	0.01%	3.72%	AANVLGAVNKPLSSAGK	K	Q	99.70%	41.8	41.9	20.5	34.5	30.6	3.85	0.304	0.265	0	0	2	0	2	1,596.91	2,077.00		488.35	1127	1143	Empty	TRUE	
20196-20225 HinckeM –TAXchicken	Uncategorized Sample	20196-20205_A	Mudpit_Ingel20196_MGFPeaklist (F058870)	status=inactive	F1P3B8_CHICK,PDS5B_CHICK,UPI00003AA07B,UPI00004CF187,UPI000060E777,UPI000060E778,UPI0000ECD657,UPI0000ECD658,UPI000150AF44,UPI0001C48FBF	TAX_GallusGallus_9031_20141114.fasta,UPARC14_2_GallusG_9031_20140417.fasta	161,031.60	100.00%	3	3	4	0.01%	3.72%	METVSNASSSSNPSSPGRIK	R	G	22.80%	4.5	43.5	13.7	0					0	0	1	0	2	2,052.95	2,226.00		1,816.59	1152	1171	Empty	TRUE	
20196-20225 HinckeM –TAXchicken	Uncategorized Sample	20196-20205_A	Mudpit_Ingel20196_MGFPeaklist (F058870)	status=inactive	F1P3B8_CHICK,PDS5B_CHICK,UPI00003AA07B,UPI00004CF187,UPI000060E777,UPI000060E778,UPI0000ECD657,UPI0000ECD658,UPI000150AF44,UPI0001C48FBF	TAX_GallusGallus_9031_20141114.fasta,UPARC14_2_GallusG_9031_20140417.fasta	161,031.60	100.00%	3	3	4	0.01%	3.72%	STTYSLESPKDPVLPAR	K	Y	99.50%	19.7	44.2	24.7	7.04	23.4	1.3	0.226	0.147	0	0	1	0	2	1,860.97	2,136.00		236.71	1080	1096	Empty	TRUE	
20196-20225 HinckeM –TAXchicken	Uncategorized Sample	20196-20205_A	Mudpit_Ingel20196_MGFPeaklist (F058870)	status=inactive	F1P3B8_CHICK,PDS5B_CHICK,UPI00003AA07B,UPI00004CF187,UPI000060E777,UPI000060E778,UPI0000ECD657,UPI0000ECD658,UPI000150AF44,UPI0001C48FBF	TAX_GallusGallus_9031_20141114.fasta,UPARC14_2_GallusG_9031_20140417.fasta	161,031.60	100.00%	3	3	4	0.01%	3.72%	AANVLGAVNKPLSSAGK	K	Q	99.70%	41.8	41.9	20.5	34.5	30.6	3.85	0.304	0.265	0	0	2	0	2	1,596.91	2,077.00		488.35	1127	1143	Empty	TRUE	
20196-20225 HinckeM –TAXchicken	Uncategorized Sample	20196-20205_A	Mudpit_Ingel20196_MGFPeaklist (F058870)	status=inactive	F1P3B8_CHICK,PDS5B_CHICK,UPI00003AA07B,UPI00004CF187,UPI000060E777,UPI000060E778,UPI0000ECD657,UPI0000ECD658,UPI000150AF44,UPI0001C48FBF	TAX_GallusGallus_9031_20141114.fasta,UPARC14_2_GallusG_9031_20140417.fasta	161,031.60	100.00%	3	3	4	0.01%	3.72%	METVSNASSSSNPSSPGRIK	R	G	22.80%	4.5	43.5	13.7	0					0	0	1	0	2	2,052.95	2,226.00		1,816.59	1152	1171	Empty	TRUE	
20196-20225 HinckeM –TAXchicken	Uncategorized Sample	20196-20205_A	Mudpit_Ingel20196_MGFPeaklist (F058870)	status=inactive	F1P3B8_CHICK,PDS5B_CHICK,UPI00003AA07B,UPI00004CF187,UPI000060E777,UPI000060E778,UPI0000ECD657,UPI0000ECD658,UPI000150AF44,UPI0001C48FBF	TAX_GallusGallus_9031_20141114.fasta,UPARC14_2_GallusG_9031_20140417.fasta	161,031.60	100.00%	3	3	4	0.01%	3.72%	STTYSLESPKDPVLPAR	K	Y	99.50%	19.7	44.2	24.7	7.04	23.4	1.3	0.226	0.147	0	0	1	0	2	1,860.97	2,136.00		236.71	1080	1096	Empty	TRUE	
20196-20225 HinckeM –TAXchicken	Uncategorized Sample	20196-20205_A	Mudpit_Ingel20196_MGFPeaklist (F058870)	status=inactive	F1P3B8_CHICK,PDS5B_CHICK,UPI00003AA07B,UPI00004CF187,UPI000060E777,UPI000060E778,UPI0000ECD657,UPI0000ECD658,UPI000150AF44,UPI0001C48FBF	TAX_GallusGallus_9031_20141114.fasta,UPARC14_2_GallusG_9031_20140417.fasta	161,031.60	100.00%	3	3	4	0.01%	3.72%	AANVLGAVNKPLSSAGK	K	Q	99.70%	41.8	41.9	20.5	34.5	30.6	3.85	0.304	0.265	0	0	2	0	2	1,596.91	2,077.00		488.35	1127	1143	Empty	TRUE	
20196-20225 HinckeM –TAXchicken	Uncategorized Sample	20196-20205_A	Mudpit_Ingel20196_MGFPeaklist (F058870)	status=inactive	F1P3B8_CHICK,PDS5B_CHICK,UPI00003AA07B,UPI00004CF187,UPI000060E777,UPI000060E778,UPI0000ECD657,UPI0000ECD658,UPI000150AF44,UPI0001C48FBF	TAX_GallusGallus_9031_20141114.fasta,UPARC14_2_GallusG_9031_20140417.fasta	161,031.60	100.00%	3	3	4	0.01%	3.72%	METVSNASSSSNPSSPGRIK	R	G	22.80%	4.5	43.5	13.7	0					0	0	1	0	2	2,052.95	2,226.00		1,816.59	1152	1171	Empty	TRUE	
20196-20225 HinckeM –TAXchicken	Uncategorized Sample	20196-20205_A	Mudpit_Ingel20196_MGFPeaklist (F058870)	status=inactive	F1P3B8_CHICK,PDS5B_CHICK,UPI00003AA07B,UPI00004CF187,UPI000060E777,UPI000060E778,UPI0000ECD657,UPI0000ECD658,UPI000150AF44,UPI0001C48FBF	TAX_GallusGallus_9031_20141114.fasta,UPARC14_2_GallusG_9031_20140417.fasta	161,031.60	100.00%	3	3	4	0.01%	3.72%	STTYSLESPKDPVLPAR	K	Y	99.50%	19.7	44.2	24.7	7.04	23.4	1.3	0.226	0.147	0	0	1	0	2	1,860.97	2,136.00		236.71	1080	1096	Empty	TRUE	
20196-20225 HinckeM –TAXchicken	Uncategorized Sample	20196-20205_A	Mudpit_Ingel20196_MGFPeaklist (F058870)	status=inactive	F1P3B8_CHICK,PDS5B_CHICK,UPI00003AA07B,UPI00004CF187,UPI000060E777,UPI000060E778,UPI0000ECD657,UPI0000ECD658,UPI000150AF44,UPI0001C48FBF	TAX_GallusGallus_9031_20141114.fasta,UPARC14_2_GallusG_9031_20140417.fasta	161,031.60	100.00%	3	3	4	0.01%	3.72%	AANVLGAVNKPLSSAGK	K	Q	99.70%	41.8	41.9	20.5	34.5	30.6	3.85	0.304	0.265	0	0	2	0	2	1,596.91	2,077.00		488.35	1127	1143	Empty	TRUE	
20196-20225 HinckeM –TAXchicken	Uncategorized Sample	20196-20205_A	Mudpit_Ingel20196_MGFPeaklist (F058870)	status=inactive	F1P3B8_CHICK,PDS5B_CHICK,UPI00003AA07B,UPI00004CF187,UPI000060E777,UPI000060E778,UPI0000ECD657,UPI0000ECD658,UPI000150AF44,UPI0001C48FBF	TAX_GallusGallus_9031_20141114.fasta,UPARC14_2_GallusG_9031_20140417.fasta	161,031.60	100.00%	3	3	4	0.01%	3.72%	METVSNASSSSNPSSPGRIK	R	G	22.80%	4.5	43.5	13.7	0					0	0	1	0	2	2,052.95	2,226.00		1,816.59	1152	1171	Empty	TRUE	
20196-20225 HinckeM –TAXchicken	Uncategorized Sample	20196-20205_A	Mudpit_Ingel20196_MGFPeaklist (F058870)	status=inactive	F1P3B8_CHICK,PDS5B_CHICK,UPI00003AA07B,UPI00004CF187,UPI000060E777,UPI000060E778,UPI0000ECD657,UPI0000ECD658,UPI000150AF44,UPI0001C48FBF	TAX_GallusGallus_9031_20141114.fasta,UPARC14_2_GallusG_9031_20140417.fasta	161,031.60	100.00%	3	3	4	0.01%	3.72%	STTYSLESPKDPVLPAR	K	Y	99.50%	19.7	44.2	24.7	7.04	23.4	1.3	0.226	0.147	0	0	1	0	2	1,860.97	2,136.00		236.71	1080	1096	Empty	TRUE	
20196-20225 HinckeM –TAXchicken	Uncategorized Sample	20196-20205_A	Mudpit_Ingel20196_MGFPeaklist (F058870)	status=inactive	F1P3B8_CHICK,PDS5B_CHICK,UPI00003AA07B,UPI00004CF187,UPI000060E777,UPI000060E778,UPI0000ECD657,UPI0000ECD658,UPI000150AF44,UPI0001C48FBF	TAX_GallusGallus_9031_20141114.fasta,UPARC14_2_GallusG_9031_20140417.fasta	161,031.60	100.00%	3	3	4	0.01%	3.72%	AANVLGAVNKPLSSAGK	K	Q	99.70%	41.8	41.9	20.5	34.5	30.6	3.85	0.304	0.265	0	0	2	0	2	1,596.91	2,077.00		488.35	1127	1143	Empty	TRUE	
20196-20225 HinckeM –TAXchicken	Uncategorized Sample	20196-20205_A	Mudpit_Ingel20196_MGFPeaklist (F058870)	status=inactive	F1P3B8_CHICK,PDS5B_CHICK,UPI00003AA07B,UPI00004CF187,UPI000060E777,UPI000060E778,UPI0000ECD657,UPI0000ECD658,UPI000150AF44,UPI0001C48FBF	TAX_GallusGallus_9031_20141114.fasta,UPARC14_2_GallusG_9031_20140417.fasta	161,031.60	100.00%	3	3	4	0.01%	3.72%	METVSNASSSSNPSSPGRIK	R	G	22.80%	4.5	43.5	13.7	0					0	0	1	0	2	2,052.95	2,226.00		1,816.59	1152	1171	Empty	TRUE	
20196-20225 HinckeM –TAXchicken	Uncategorized Sample	20196-20205_A	Mudpit_Ingel20196_MGFPeaklist (F058870)	status=inactive	F1P3B8_CHICK,PDS5B_CHICK,UPI00003AA07B,UPI00004CF187,UPI000060E777,UPI000060E778,UPI0000ECD657,UPI0000ECD658,UPI000150AF44,UPI0001C48FBF	TAX_GallusGallus_9031_20141114.fasta,UPARC14_2_GallusG_9031_20140417.fasta	161,031.60	100.00%	3	3	4	0.01%	3.72%	STTYSLESPKDPVLPAR	K	Y	99.50%	19.7	44.2	24.7	7.04	23.4	1.3	0.226	0.147	0	0	1	0	2	1,860.97	2,136.00		236.71	1080	1096	Empty	TRUE	
20196-20225 HinckeM –TAXchicken	Uncategorized Sample	20196-20205_A	Mudpit_Ingel20196_MGFPeaklist (F058870)	status=inactive	F1P3B8_CHICK,PDS5B_CHICK,UPI00003AA07B,UPI00004CF187,UPI000060E777,UPI000060E778,UPI0000ECD657,UPI0000ECD658,UPI000150AF44,UPI0001C48FBF	TAX_GallusGallus_9031_20141114.fasta,UPARC14_2_GallusG_9031_20140417.fasta	161,031.60	100.00%	3	3	4	0.01%	3.72%	AANVLGAVNKPLSSAGK	K	Q	99.70%	41.8	41.9	20.5	34.5	30.6	3.85	0.304	0.265	0	0	2	0	2	1,596.91	2,077.00		488.35	1127	1143	Empty	TRUE	
20196-20225 HinckeM –TAXchicken	Uncategorized Sample	20196-20205_A	Mudpit_Ingel20196_MGFPeaklist (F058870)	status=inactive	F1P3B8_CHICK,PDS5B_CHICK,UPI00003AA07B,UPI00004CF187,UPI000060E777,UPI000060E778,UPI0000ECD657,UPI0000ECD658,UPI000150AF44,UPI0001C48FBF	TAX_GallusGallus_9031_20141114.fasta,UPARC14_2_GallusG_9031_20140417.fasta	161,031.60	100.00%	3	3	4	0.01%	3.72%	METVSNASSSSNPSSPGRIK	R	G	22.80%	4.5	43.5	13.7	0					0	0	1	0	2	2,052.95	2,226.00		1,816.59	1152	1171	Empty	TRUE	
20196-20225 HinckeM –TAXchicken	Uncategorized Sample	20196-20205_A	Mudpit_Ingel20196_MGFPeaklist (F058870)	status=inactive	F1P3B8_CHICK,PDS5B_CHICK,UPI00003AA07B,UPI00004CF187,UPI000060E777,UPI000060E778,UPI0000ECD657,UPI0000ECD658,UPI000150AF44,UPI0001C48FBF	TAX_GallusGallus_9031_20141114.fasta,UPARC14_2_GallusG_9031_20140417.fasta	161,031.60	100.00%	3	3	4	0.01%	3.72%	STTYSLESPKDPVLPAR	K	Y	99.50%	19.7	44.2	24.7	7.04	23.4	1.3	0.226	0.147	0	0	1	0	2	1,860.97	2,136.00		236.71	1080	1096	Empty	TRUE	
20196-20225 HinckeM –TAXchicken	Uncategorized Sample	20196-20205_A	Mudpit_Ingel20196_MGFPeaklist (F058870)	status=inactive	F1P3B8_CHICK,PDS5B_CHICK,UPI00003AA07B,UPI00004CF187,UPI000060E777,UPI000060E778,UPI0000ECD657,UPI0000ECD658,UPI000150AF44,UPI0001C48FBF	TAX_GallusGallus_9031_20141114.fasta,UPARC14_2_GallusG_9031_20140417.fasta	161,031.60	100.00%	3	3	4	0.01%	3.72%	AANVLGAVNKPLSSAGK	K	Q	99.70%	41.8	41.9	20.5	34.5	30.6	3.85	0.304	0.265	0	0	2	0	2	1,596.91	2,077.00		488.35	1127	1143	Empty	TRUE	
20196-20225 HinckeM –TAXchicken	Uncategorized Sample	20196-20205_A	Mudpit_Ingel20196_MGFPeaklist (F058870)	status=inactive	F1P3B8_CHICK,PDS5B_CHICK,UPI00003AA07B,UPI00004CF187,UPI000060E777,UPI000060E778,UPI0000ECD657,UPI0000ECD658,UPI000150AF44,UPI0001C48FBF	TAX_GallusGallus_9031_20141114.fasta,UPARC14_2_GallusG_9031_20140417.fasta	161,031.60	100.00%	3	3	4	0.01%	3.72%	METVSNASSSSNPSSPGRIK	R	G	22.80%	4.5	43.5	13.7	0					0	0	1	0	2	2,052.95	2,226.00		1,816.59	1152	1171	Empty	TRUE	
20196-20225 HinckeM –TAXchicken	Uncategorized Sample	20196-20205_A	Mudpit_Ingel20196_MGFPeaklist (F058870)	status=inactive	F1P3B8_CHICK,PDS5B_CHICK,UPI00003AA07B,UPI00004CF187,UPI000060E777,UPI000060E778,UPI0000ECD657,UPI0000ECD658,UPI000150AF44,UPI0001C48FBF	TAX_GallusGallus_9031_20141114.fasta,UPARC14_2_GallusG_9031_20140417.fasta	161,031.60	100.00%	3	3	4	0.01%	3.72%	STTYSLESPKDPVLPAR	K	Y	99.50%	19.7	44.2	24.7	7.04	23.4	1.3	0.226	0.147	0	0	1	0	2	1,860.97	2,136.00		236.71	1080	1096	Empty	TRUE	
20196-20225 HinckeM –TAXchicken	Uncategorized Sample	20196-20205_A	Mudpit_Ingel20196_MGFPeaklist (F058870)	UDP-glucose 6-dehydrogenase OS=Gallus gallus GN=UGDH PE=2 SV=1	UGDH_CHICK,UPI00003AD9C2,UPI0000610F1A,UPI0000E80467,UPI0000ECC684	TAX_GallusGallus_9031_20141114.fasta,UPARC14_2_GallusG_9031_20140417.fasta	46,620.30	97.60%	1	1	1	0.00%	2.43%	IQVTVVDVNEAR	K	I	99.70%	49	44.1	23.4	31.9	28.1	1.82	0.267	0.25	0	1	0	0	2	1,342.73	2,137.00		649.07	30	41	Empty	TRUE	
20196-20225 HinckeM –TAXchicken	Uncategorized Sample	20196-20205_A	Mudpit_Ingel20196_MGFPeaklist (F058870)	UDP-glucose 6-dehydrogenase OS=Gallus gallus GN=UGDH PE=2 SV=1	UGDH_CHICK,UPI00003AD9C2,UPI0000610F1A,UPI0000E80467,UPI0000ECC684	TAX_GallusGallus_9031_20141114.fasta,UPARC14_2_GallusG_9031_20140417.fasta	46,620.30	97.60%	1	1	1	0.00%	2.43%	IQVTVVDVNEAR	K	I	99.70%	49	44.1	23.4	31.9	28.1	1.82	0.267	0.25	0	1	0	0	2	1,342.73	2,137.00		649.07	30	41	Empty	TRUE	
20196-20225 HinckeM –TAXchicken	Uncategorized Sample	20196-20205_A	Mudpit_Ingel20196_MGFPeaklist (F058870)	UDP-glucose 6-dehydrogenase OS=Gallus gallus GN=UGDH PE=2 SV=1	UGDH_CHICK,UPI00003AD9C2,UPI0000610F1A,UPI0000E80467,UPI0000ECC684	TAX_GallusGallus_9031_20141114.fasta,UPARC14_2_GallusG_9031_20140417.fasta	46,620.30	97.60%	1	1	1	0.00%	2.43%	IQVTVVDVNEAR	K	I	99.70%	49	44.1	23.4	31.9	28.1	1.82	0.267	0.25	0	1	0	0	2	1,342.73	2,137.00		649.07	30	41	Empty	TRUE	
20196-20225 HinckeM –TAXchicken	Uncategorized Sample	20196-20205_A	Mudpit_Ingel20196_MGFPeaklist (F058870)	UDP-glucose 6-dehydrogenase OS=Gallus gallus GN=UGDH PE=2 SV=1	UGDH_CHICK,UPI00003AD9C2,UPI0000610F1A,UPI0000E80467,UPI0000ECC684	TAX_GallusGallus_9031_20141114.fasta,UPARC14_2_GallusG_9031_20140417.fasta	46,620.30	97.60%	1	1	1	0.00%	2.43%	IQVTVVDVNEAR	K	I	99.70%	49	44.1	23.4	31.9	28.1	1.82	0.267	0.25	0	1	0	0	2	1,342.73	2,137.00		649.07	30	41	Empty	TRUE	
20196-20225 HinckeM –TAXchicken	Uncategorized Sample	20196-20205_A	Mudpit_Ingel20196_MGFPeaklist (F058870)	UDP-glucose 6-dehydrogenase OS=Gallus gallus GN=UGDH PE=2 SV=1	UGDH_CHICK,UPI00003AD9C2,UPI0000610F1A,UPI0000E80467,UPI0000ECC684	TAX_GallusGallus_9031_20141114.fasta,UPARC14_2_GallusG_9031_20140417.fasta	46,620.30	97.60%	1	1	1	0.00%	2.43%	IQVTVVDVNEAR	K	I	99.70%	49	44.1	23.4	31.9	28.1	1.82	0.267	0.25	0	1	0	0	2	1,342.73	2,137.00		649.07	30	41	Empty	TRUE	

20196-20225 HinckeM –TAXchicken	Uncategorized Sample	20196-20205_A	Mudpit_Ingel20196_MGFPeaklist (F058870)	Alanine--tRNA ligase, cytoplasmic OS=Gallus gallus GN=AARS PE=3 SV=2	F1P5J5_CHICK	TAX_GallusGallus_9031_20141114.fasta	101,349.40	97.90%	3	3	3	0.01%	2.85%	EAVKALKK	R	V	5.50%	13.2	42.6	23.2	0					0	1	0	0	2	886.5721	2,021.00		81.59	817	824	Empty	TRUE	
20196-20225 HinckeM –TAXchicken	Uncategorized Sample	20196-20205_A	Mudpit_Ingel20196_MGFPeaklist (F058870)	Alanine--tRNA ligase, cytoplasmic OS=Gallus gallus GN=AARS PE=3 SV=2	F1P5J5_CHICK	TAX_GallusGallus_9031_20141114.fasta	101,349.40	97.90%	3	3	3	0.01%	2.85%	ITCLCQVPQR	R	R	43.50%					22.4	0.602	0.0759	0.25	0	0	1	0	2	1,274.63	1,752.00		425.92	899	908	Empty	TRUE	
20196-20225 HinckeM –TAXchicken	Uncategorized Sample	20196-20205_A	Mudpit_Ingel20196_MGFPeaklist (F058870)	Alanine--tRNA ligase, cytoplasmic OS=Gallus gallus GN=AARS PE=3 SV=2	F1P5J5_CHICK	TAX_GallusGallus_9031_20141114.fasta	101,349.40	97.90%	3	3	3	0.01%	2.85%	LALDLLTK	K	E	96.10%	36.7	42.8	37.2	27					0	1	0	0	2	886.561	2,541.00		1,458.75	117	124	Empty	TRUE	UPI00003AA59C,UPI00003AA59B,UPI00003AA59E
20196-20225 HinckeM –TAXchicken	Uncategorized Sample	20196-20205_A	Mudpit_Ingel20196_MGFPeaklist (F058870)	Uncharacterized protein OS=Gallus gallus GN=MAST2 PE=4 SV=2	F1NAH4_CHICK	TAX_GallusGallus_9031_20141114.fasta	201,717.10	100.00%	6	6	6	0.01%	5.09%	ASQSMAAGTVTSTLLVPCAIER	K	A	17.80%	0.72	44.2	13.5	0					0	0	0	1	2	2,280.12	2,117.00		1,325.85	1645	1666	Empty	TRUE	
20196-20225 HinckeM –TAXchicken	Uncategorized Sample	20196-20205_A	Mudpit_Ingel20196_MGFPeaklist (F058870)	Uncharacterized protein OS=Gallus gallus GN=MAST2 PE=4 SV=2	F1NAH4_CHICK	TAX_GallusGallus_9031_20141114.fasta	201,717.10	100.00%	6	6	6	0.01%	5.09%	EAISCSTK	K	A	6.70%	4.9	42.8	15.5	0					0	1	0	0	2	895.419	1,906.00		174.8	1766	1773	Empty	TRUE	
20196-20225 HinckeM –TAXchicken	Uncategorized Sample	20196-20205_A	Mudpit_Ingel20196_MGFPeaklist (F058870)	Uncharacterized protein OS=Gallus gallus GN=MAST2 PE=4 SV=2	F1NAH4_CHICK	TAX_GallusGallus_9031_20141114.fasta	201,717.10	100.00%	6	6	6	0.01%	5.09%	SLSSGESVPGSPTHNLSPR	R	S	99.70%	46.4	44.5	19	38.4	35.6	4.51	0.413	0.211	0	0	1	0	2	1,908.94	1,825.00		546	1125	1143	Empty	TRUE	
20196-20225 HinckeM –TAXchicken	Uncategorized Sample	20196-20205_A	Mudpit_Ingel20196_MGFPeaklist (F058870)	Uncharacterized protein OS=Gallus gallus GN=MAST2 PE=4 SV=2	F1NAH4_CHICK	TAX_GallusGallus_9031_20141114.fasta	201,717.10	100.00%	6	6	6	0.01%	5.09%	SPRRKSAGNIPLSP	R	L	84.80%					24.3	0.77	0.259	0.25	0	1	0	0	1	1,479.84	1,897.00		654.67	1201	1214	Empty	TRUE	
20196-20225 HinckeM –TAXchicken	Uncategorized Sample	20196-20205_A	Mudpit_Ingel20196_MGFPeaklist (F058870)	Uncharacterized protein OS=Gallus gallus GN=MAST2 PE=4 SV=2	F1NAH4_CHICK	TAX_GallusGallus_9031_20141114.fasta	201,717.10	100.00%	6	6	6	0.01%	5.09%	SSTTTLTTGSTADFSDPR	V	A	93.60%					30	1.21	0.263	0.25	0	0	0	1	1	1,844.85	1,418.00		130.08	855	872	Empty	TRUE	
20196-20225 HinckeM –TAXchicken	Uncategorized Sample	20196-20205_A	Mudpit_Ingel20196_MGFPeaklist (F058870)	Uncharacterized protein OS=Gallus gallus GN=MAST2 PE=4 SV=2	F1NAH4_CHICK	TAX_GallusGallus_9031_20141114.fasta	201,717.10	100.00%	6	6	6	0.01%	5.09%	TQDFPLSHDGAVK	K	K	22.60%	9.38	44.4	22.8	0					0	0	1	0	2	1,414.70	1,892.00		472.64	1831	1843	Empty	TRUE	
20196-20225 HinckeM –TAXchicken	Uncategorized Sample	20196-20205_A	Mudpit_Ingel20196_MGFPeaklist (F058870)	status=inactive	UPI000240B7FF	UPARC14_2_GallusG_9031_20140417.fasta	243,079.30	100.00%	17	17	30	0.06%	8.20%	AQWTPFEGR	K	N	97.50%	35.7	44.9	25.5	20.9					0	2	0	0	2	1,091.53	2,203.50		501.15	1775	1783	Empty	TRUE	UPI0003507688
20196-20225 HinckeM –TAXchicken	Uncategorized Sample	20196-20205_A	Mudpit_Ingel20196_MGFPeaklist (F058870)	status=inactive	UPI000240B7FF	UPARC14_2_GallusG_9031_20140417.fasta	243,079.30	100.00%	17	17	30	0.06%	8.20%	ATGYPLAYVAAK	K	L	76.30%	19.3	43.7	19.8	14.9					0	2	0	0	2	1,224.66	2,233.50		750.71	697	708	Empty	TRUE	UPI0003507688
20196-20225 HinckeM –TAXchicken	Uncategorized Sample	20196-20205_A	Mudpit_Ingel20196_MGFPeaklist (F058870)	status=inactive	UPI000240B7FF	UPARC14_2_GallusG_9031_20140417.fasta	243,079.30	100.00%	17	17	30	0.06%	8.20%	DLGILPVVK	R	Q	99.70%	45.6	39.7	31.3	29.4	33.3	2.89	0.354	0.5	0	2	0	0	2	953.6032	2,542.50		511.03	896	904	Empty	TRUE	UPI0003507688
20196-20225 HinckeM –TAXchicken	Uncategorized Sample	20196-20205_A	Mudpit_Ingel20196_MGFPeaklist (F058870)	status=inactive	UPI000240B7FF	UPARC14_2_GallusG_9031_20140417.fasta	243,079.30	100.00%	17	17	30	0.06%	8.20%	EHNVPGLEGVDTR	K	A	44.50%	21	44.6	21.3	10.7					0	0	2	0	2	1,422.70	1,866.00		403.45	111	123	Empty	TRUE	UPI0003507688
20196-20225 HinckeM –TAXchicken	Uncategorized Sample	20196-20205_A	Mudpit_Ingel20196_MGFPeaklist (F058870)	status=inactive	UPI000240B7FF	UPARC14_2_GallusG_9031_20140417.fasta	243,079.30	100.00%	17	17	30	0.06%	8.20%	GRLVLQDGSVLR	M	G	99.70%	34.6	41.8	25.1	23.5	27.8	0.886	0.201	0.333	0	0	1	0	1	1,312.77	2,110.00		575.84	2	13	Empty	TRUE	
20196-20225 HinckeM –TAXchicken	Uncategorized Sample	20196-20205_A	Mudpit_Ingel20196_MGFPeaklist (F058870)	status=inactive	UPI000240B7FF	UPARC14_2_GallusG_9031_20140417.fasta	243,079.30	100.00%	17	17	30	0.06%	8.20%	HLVQEVSLKEPR	R	V	99.70%	43.8	43.2	24.1	31	29.3	1.49	0.345	0.417	0	0	2	0	2	1,434.81	1,828.00		833.71	158	169	Empty	TRUE	UPI0003507688
20196-20225 HinckeM –TAXchicken	Uncategorized Sample	20196-20205_A	Mudpit_Ingel20196_MGFPeaklist (F058870)	status=inactive	UPI000240B7FF	UPARC14_2_GallusG_9031_20140417.fasta	243,079.30	100.00%	17	17	30	0.06%	8.20%	HPQPGAVELAATHCR	R	K	99.70%	53.8	44.7	24.5	42.1					0	0	2	0	2	1,643.81	1,692.50		1,385.08	2020	2034	Empty	TRUE	UPI0003507688
20196-20225 HinckeM –TAXchicken	Uncategorized Sample	20196-20205_A	Mudpit_Ingel20196_MGFPeaklist (F058870)	status=inactive	UPI000240B7FF	UPARC14_2_GallusG_9031_20140417.fasta	243,079.30	100.00%	17	17	30	0.06%	8.20%	IFVLAAALR	R	A	99.70%	56.9	43	30.6	30.8	28.7	1.68	0.223	0.444	0	2	0	0	2	973.6193	2,720.50		639.53	811	819	Empty	TRUE	UPI0003507688
20196-20225 HinckeM –TAXchicken	Uncategorized Sample	20196-20205_A	Mudpit_Ingel20196_MGFPeaklist (F058870)	status=inactive	UPI000240B7FF	UPARC14_2_GallusG_9031_20140417.fasta	243,079.30	100.00%	17	17	30	0.06%	8.20%	ILGTSPEAIDSAENR	R	F	99.70%	68.1	44.7	21.3	58.5	47.1	4.66	0.406	0.433	0	2	0	0	2	1,572.79	1,996.00		2,083.70	1037	1051	Empty	TRUE	UPI0003507688
20196-20225 HinckeM –TAXchicken	Uncategorized Sample	20196-20205_A	Mudpit_Ingel20196_MGFPeaklist (F058870)	status=inactive	UPI000240B7FF	UPARC14_2_GallusG_9031_20140417.fasta	243,079.30	100.00%	17	17	30	0.06%	8.20%	KNILLTIGSYK	K	N	62.30%	21.8	41.1	17.7	17.8					0	0	2	0	2	1,249.75	2,404.50		446.1	1316	1326	Empty	TRUE	UPI0003507688
20196-20225 HinckeM –TAXchicken	Uncategorized Sample	20196-20205_A	Mudpit_Ingel20196_MGFPeaklist (F058870)	status=inactive	UPI000240B7FF	UPARC14_2_GallusG_9031_20140417.fasta	243,079.30	100.00%	17	17	30	0.06%	8.20%	LALGIPLPVLR	K	N	99.70%	56.7	34.4	23.7	44.5	42.5	4.27	0.536	0.545	0	1	0	0	2	1,161.77	2,887.00		743.99	709	719	Empty	TRUE	UPI0003507688
20196-20225 HinckeM –TAXchicken	Uncategorized Sample	20196-20205_A	Mudpit_Ingel20196_MGFPeaklist (F058870)	status=inactive	UPI000240B7FF	UPARC14_2_GallusG_9031_20140417.fasta	243,079.30	100.00%	17	17	30	0.06%	8.20%	LCQQTVANLR	R	A	99.70%	48.7	45.2	36.2	29.1	31.8	2.28	0.189	0.45	0	2	0	0	2	1,202.63	1,798.50		658.57	229	238	Empty	TRUE	UPI0003507688
20196-20225 HinckeM –TAXchicken	Uncategorized Sample	20196-20205_A	Mudpit_Ingel20196_MGFPeaklist (F058870)	status=inactive	UPI000240B7FF	UPARC14_2_GallusG_9031_20140417.fasta	243,079.30	100.00%	17	17	30	0.06%	8.20%	LGYPVLVR	R	S	99.70%	52.4	44.3	39.6	35.7	30.9	4.25	0.275	0.438	0	2	0	0	2	916.5615	2,275.50		823.84	548	555	Empty	TRUE	UPI0003507688
20196-20225 HinckeM –TAXchicken	Uncategorized Sample	20196-20205_A	Mudpit_Ingel20196_MGFPeaklist (F058870)	status=inactive	UPI000240B7FF	UPARC14_2_GallusG_9031_20140417.fasta	243,079.30	100.00%	17	17	30	0.06%	8.20%	LLPDEAPEESIPFQDPNER	K	H	18.30%	1.55	43.4	15.6	-0.92					0	0	0	0	2	2,198.01	1,383.00		480.34	139	157	Empty	TRUE	UPI0003507688
20196-20225 HinckeM –TAXchicken	Uncategorized Sample	20196-20205_A	Mudpit_Ingel20196_MGFPeaklist (F058870)	status=inactive	UPI000240B7FF	UPARC14_2_GallusG_9031_20140417.fasta	243,079.30	100.00%	17	17	30	0.06%	8.20%	LTVEDIILR	R	L	99.70%	72	43.4	52.6	45.8	38.1	1.92	0.202	0.611	0	2	0	0	2	1,071.64	2,690.50		545.75	1729	1737	Empty	TRUE	UPI0003507688
20196-20225 HinckeM –TAXchicken	Uncategorized Sample	20196-20205_A	Mudpit_Ingel20196_MGFPeaklist (F058870)	status=inactive	UPI000240B7FF	UPARC14_2_GallusG_9031_20140417.fasta	243,079.30	100.00%	17	17	30	0.06%	8.20%	LVLQDGSVLR	R	G	99.70%	50.2	43	37.2	22.3	31.5	0.585	0.187	0.4	0	2	0	0	2	1,099.65	2,181.50		817.69	4	13	Empty	TRUE	UPI0003507688
20196-20225 HinckeM –TAXchicken	Uncategorized Sample	20196-20205_A	Mudpit_Ingel20196_MGFPeaklist (F058870)	status=inactive	UPI000240B7FF	UPARC14_2_GallusG_9031_20140417.fasta	243,079.30	100.00%	17	17	30	0.06%	8.20%	SELLPTVR	K	T	38.60%	18.2	44.1	0	0					0	1	0	0	2	914.5306	2,095.00		124.92	1329	1336	Empty	TRUE	UPI0003507688
20196-20225 HinckeM –TAXchicken	Uncategorized Sample	20196-20205_A	Mudpit_Ingel20196_MGFPeaklist (F058870)	Putative helicase MOV-10 OS=Gallus gallus GN=MOV10 PE=2 SV=1	MOV10_CHICK	TAX_GallusGallus_9031_20141114.fasta	109,052.50	100.00%	8	9	15	0.03%	9.72%	ALPDIGQLK	R	V	99.70%	18.2	42.1	19.2	15.9	32.1	1.36	0.296	0.444	0	2	0	0	2	954.5621	2,267.00		2,049.36	850	858	Empty	TRUE	UPI0000443677
20196-20225 HinckeM –TAXchicken	Uncategorized Sample	20196-20205_A	Mudpit_Ingel20196_MGFPeaklist (F058870)	Putative helicase MOV-10 OS=Gallus gallus GN=MOV10 PE=2 SV=1	MOV10_CHICK	TAX_GallusGallus_9031_20141114.fasta	109,052.50	100.00%	8	9	15	0.03%	9.72%	GDHLFASLSS	R	E	89.90%					25.1	1.85	0.231	0.25	0	1	0	0	1	1,016.47	1,844.00		421.3	386	395	Empty	TRUE	
20196-20225 HinckeM –TAXchicken	Uncategorized Sample	20196-20205_A	Mudpit_Ingel20196_MGFPeaklist (F058870)	Putative helicase MOV-10 OS=Gallus gallus GN=MOV10 PE=2 SV=1	MOV10_CHICK	TAX_GallusGallus_9031_20141114.fasta	109,052.50	100.00%	8	9	15	0.03%	9.72%	LLTNIAPR	R	Y	62.90%	28.9	42.7	40.8	0.41					0	4	0	0	2	898.5358	2,448.00		1,462.82	559	566	Empty	TRUE	UPI0000443677
20196-20225 HinckeM –TAXchicken	Uncategorized Sample	20196-20205_A	Mudpit_Ingel20196_MGFPeaklist (F058870)	Putative helicase MOV-10 OS=Gallus gallus GN=MOV10 PE=2 SV=1	MOV10_CHICK	TAX_GallusGallus_9031_20141114.fasta	109,052.50	100.00%	8	9	15	0.03%	9.72%	RSLFTGIFQPR	Q	W	83.20%					30.6	1.15	0.108	0.409	0	1	0	0	1	1,304.71	2,465.00		832.16	473	483	Empty	TRUE	
20196-20225 HinckeM –TAXchicken	Uncategorized Sample	20196-20205_A	Mudpit_Ingel20196_MGFPeaklist (F058870)	Putative helicase MOV-10 OS=Gallus gallus GN=MOV10 PE=2 SV=1	MOV10_CHICK	TAX_GallusGallus_9031_20141114.fasta	109,052.50	100.00%	8	9	15	0.03%	9.72%	SIAAIAQSPLAR	R	E	99.70%	36.5	42.5	17.4	28.4	48.9	5.15	0.423	0.625	0	2	0	0	2	1,197.70	2,242.50		3,988.15	238	249	Empty	TRUE	UPI0000443677
20196-20225 HinckeM –TAXchicken	Uncategorized Sample	20196-20205_A	Mudpit_Ingel20196_MGFPeaklist (F058870)	Putative helicase MOV-10 OS=Gallus gallus GN=MOV10 PE=2 SV=1	MOV10_CHICK	TAX_GallusGallus_9031_20141114.fasta	109,052.50	100.00%	8	9	15	0.03%	9.72%	TDTEVSAPAPGQQPSSGPPAPQSR	R	A	99.70%	57.6	43.9	20.4	52.3	42	4.8	0.476	0.271	0	0	2	0	2	2,362.13	1,699.50		1,875.60	93	116	Empty	TRUE	UPI0000443677
20196-20225 HinckeM –TAXchicken	Uncategorized Sample	20196-20205_A	Mudpit_Ingel20196_MGFPeaklist (F058870)	Putative helicase MOV-10 OS=Gallus gallus GN=MOV10 PE=2 SV=1	MOV10_CHICK	TAX_GallusGallus_9031_20141114.fasta	109,052.50	100.00%	8	9	15	0.03%	9.72%	VGSVEEFQGQER	K	R	99.70%	43.6	44.4	25.6	26.1	34.6	2.18	0.315	0.333	0	2	0	0	2	1,364.64	1,742.50		747.35	859	870	Empty	TRUE	UPI0000443677
20196-20225 HinckeM –TAXchicken	Uncategorized Sample	20196-20205_A	Mudpit_Ingel20196_MGFPeaklist (F058870)	Putative helicase MOV-10 OS=Gallus gallus GN=MOV10 PE=2 SV=1	MOV10_CHICK	TAX_GallusGallus_9031_20141114.fasta	109,052.50	100.00%	8	9	15	0.03%	9.72%	VQLGFSPK	R	L	72.00%	41.8	45.4	30.6	17					0	1	0	0	2	875.4986	2,022.00		219.53	417	424	Empty	TRUE	UPI0000443677
20196-20225 HinckeM –TAXchicken	Uncategorized Sample	20196-20205_A	Mudpit_Ingel20196_MGFPeaklist (F058870)	status=active	UPI0000448F45	UPARC14_2_GallusG_9031_20140417.fasta	19,848.30	98.30%	2	2	8	0.02%	9.04%	RNVSLPGR	M	G	45.00%	36.2	43.3	0	11					0	6	0	0	1	899.5058	1,950.00		40,092.30	2	9	Empty	TRUE	
20196-20225 HinckeM –TAXchicken	Uncategorized Sample	20196-20205_A	Mudpit_Ingel20196_MGFPeaklist (F058870)	status=active	UPI0000448F45	UPARC14_2_GallusG_9031_20140417.fasta	19,848.30	98.30%	2	2	8	0.02%	9.04%	VALSVCGR	R	E	96.90%	33.6	45.4	25	25.3					0	2	0	0	2	861.4611	1,793.50		342.34	72	79	Empty	TRUE	
20196-20225 HinckeM –TAXchicken	Uncategorized Sample	20196-20205_A	Mudpit_Ingel20196_MGFPeaklist (F058870)	Uncharacterized protein OS=Gallus gallus GN=SPTBN1 PE=4 SV=1	R4GJH8_CHICK	TAX_GallusGallus_9031_20141114.fasta	274,115.50	98.40%	5	5	5	0.01%	4.11%	EKWLNNMQIPEKLEDLEVIQHR	K	F	7.10%	1.23	43.4	13.1	0.2					0	0	0	0	2	2,781.38	2,107.00		3,563.37	869	890	Empty	TRUE	UPI0000ECC737,UPI00003ACAFC,UPI0002C88352,UPI0000447B9B,F1ND51_CHICK
20196-20225 HinckeM –TAXchicken	Uncategorized Sample	20196-20205_A	Mudpit_Ingel20196_MGFPeaklist (F058870)	Uncharacterized protein OS=Gallus gallus GN=SPTBN1 PE=4 SV=1	R4GJH8_CHICK	TAX_GallusGallus_9031_20141114.fasta	274,115.50	98.40%	5	5	5	0.01%	4.11%	ELALRNELIR	R	Q	94.70%	36.6	42.1	35	16					0	0	3	0	2	1,226.72	2,201.00		3,768.55	407	416	Empty	FALSE	UPI0000ECC737,UPI00003ACAFC,UPI00035042E9,UPI00035069AB,UPI0000447B9B,F1ND51_CHICK,UPI000240B81D,UPI00035064DB,UPI0003505F8D,UPI0002C88352,UPI0002C86DF1,F1NSS0_CHICK
20196-20225 HinckeM –TAXchicken	Uncategorized Sample	20196-20205_A	Mudpit_Ingel20196_MGFPeaklist (F058870)	Uncharacterized protein OS=Gallus gallus GN=SPTBN1 PE=4 SV=1	R4GJH8_CHICK	TAX_GallusGallus_9031_20141114.fasta	274,115.50	98.40%	5	5	5	0.01%	4.11%	ELALRNELIRQEK	R	L	17.30%	8.92	41.6	15.4	3.31					0	0	1	0	2	1,611.92	2,053.00		373.05	407	419	Empty	FALSE	UPI0000ECC737,UPI00003ACAFC,UPI00035042E9,UPI00035069AB,UPI0000447B9B,F1ND51_CHICK,UPI000240B81D,UPI00035064DB,UPI0003505F8D,UPI0002C88352,UPI0002C86DF1,F1NSS0_CHICK
20196-20225 HinckeM –TAXchicken	Uncategorized Sample	20196-20205_A	Mudpit_Ingel20196_MGFPeaklist (F058870)	Uncharacterized protein OS=Gallus gallus GN=SPTBN1 PE=4 SV=1	R4GJH8_CHICK	TAX_GallusGallus_9031_20141114.fasta	274,115.50	98.40%	5	5	5	0.01%	4.11%	IDDIFER	R	S	55.60%	24.5	44.8	29.6	0					0	1	0	0	2	907.4521	2,162.00		213.62	1541	1547	Empty	TRUE	UPI0000ECC737,UPI00003ACAFC,UPI0002C88352,UPI0000447B9B,F1ND51_CHICK
20196-20225 HinckeM –TAXchicken	Uncategorized Sample	20196-20205_A	Mudpit_Ingel20196_MGFPeaklist (F058870)	Uncharacterized protein OS=Gallus gallus GN=SPTBN1 PE=4 SV=1	R4GJH8_CHICK	TAX_GallusGallus_9031_20141114.fasta	274,115.50	98.40%	5	5	5	0.01%	4.11%	IKALADEREAVQK	R	K	35.40%	17.4	43.1	26.8	6.95					0	0	1	0	2	1,470.83	1,598.00		310.22	45	57	Empty	FALSE	UPI000240B81D,UPI00035064DB,UPI0000ECC737,UPI0003505F8D,UPI00003ACAFC,UPI0002C86DF1,UPI00035042E9,UPI0000ECB786,UPI00035069AB,F1NSS0_CHICK,UPI0000447B9B
20196-20225 HinckeM –TAXchicken	Uncategorized Sample	20196-20205_A	Mudpit_Ingel20196_MGFPeaklist (F058870)	Uncharacterized protein OS=Gallus gallus GN=SPTBN1 PE=4 SV=1	R4GJH8_CHICK	TAX_GallusGallus_9031_20141114.fasta	274,115.50	98.40%	5	5	5	0.01%	4.11%	LILEVHQFSR	R	D	95.30%					27.6	1.49	0.21	0.4	0	0	1	0	2	1,241.70	2,244.00		264.96	2014	2023	Empty	TRUE	F1ND51_CHICK
20196-20225 HinckeM –TAXchicken	Uncategorized Sample	20196-20205_A	Mudpit_Ingel20196_MGFPeaklist (F058870)	Uncharacterized protein OS=Gallus gallus GN=SPTBN1 PE=4 SV=1	R4GJH8_CHICK	TAX_GallusGallus_9031_20141114.fasta	274,115.50	98.40%	5	5	5	0.01%	4.11%	LLEVLSGERLPKPTKGR	K	M	7.20%	20.1	42.3	24.1	10.4					0	0	1	0	2	1,893.13	2,467.00		1,174.27	91	107	Empty	TRUE	UPI0000ECC737,UPI00003ACAFC,UPI0002C88352,UPI0000447B9B,F1ND51_CHICK
20196-20225 HinckeM –TAXchicken	Uncategorized Sample	20196-20205_A	Mudpit_Ingel20196_MGFPeaklist (F058870)	Uncharacterized protein OS=Gallus gallus GN=SPTBN1 PE=4 SV=1	R4GJH8_CHICK	TAX_GallusGallus_9031_20141114.fasta	274,115.50	98.40%	5	5	5	0.01%	4.11%	QEKLEQLAR	R	R	99.70%	58.9	43.5	37.4	17.2	31	1.01	0.0194	0.444	0	4	0	0	2	1,114.62	1,815.50		3,443.02	417	425	Empty	FALSE	UPI0000ECC737,UPI00003ACAFC,UPI00035042E9,UPI00035069AB,UPI0000447B9B,F1ND51_CHICK,UPI000240B81D,UPI00035064DB,UPI0003505F8D,UPI0002C88352,UPI0002C86DF1,F1NSS0_CHICK
20196-20225 HinckeM –TAXchicken	Uncategorized Sample	20196-20205_A	Mudpit_Ingel20196_MGFPeaklist (F058870)	Uncharacterized protein OS=Gallus gallus GN=SPTBN1 PE=4 SV=1	R4GJH8_CHICK	TAX_GallusGallus_9031_20141114.fasta	274,115.50	98.40%	5	5	5	0.01%	4.11%	VAVVNQIAR	R	Q	9.90%	11.1	42.2	22.8	3.96					0	1	0	0	2	969.5839	1,832.00		574.33	905	913	Empty	TRUE	UPI0000ECC737,UPI00003ACAFC,UPI0002C88352,UPI0000447B9B,F1ND51_CHICK
20196-20225 HinckeM –TAXchicken	Uncategorized Sample	20196-20205_A	Mudpit_Ingel20196_MGFPeaklist (F058870)	status=active	H9L2J4_CHICK,UPI000240CCAA,UPI0002C87AA9	TAX_GallusGallus_9031_20141114.fasta,UPARC14_2_GallusG_9031_20140417.fasta	45,476.40	100.00%	2	2	4	0.01%	8.21%	AQEAASLIAQRPGNPR	K	D	99.70%	36.9	43.8	15.4	35.2	29.2	1.48	0.253	0.281	0	0	2	0	2	1,678.90	1,845.00		4,160.22	223	238	Empty	TRUE	
20196-20225 HinckeM –TAXchicken	Uncategorized Sample	20196-20205_A	Mudpit_Ingel20196_MGFPeaklist (F058870)	status=active	H9L2J4_CHICK,UPI000240CCAA,UPI0002C87AA9	TAX_GallusGallus_9031_20141114.fasta,UPARC14_2_GallusG_9031_20140417.fasta	45,476.40	100.00%	2	2	4	0.01%	8.21%	EQDEVPTPTPTTPDPSR	R	V	99.70%	17.4	43.6	17.5	10.4	29.3	2.12	0.324	0.206	0	0	2	0	2	1,866.87	1,833.00		2,043.24	269	285	Empty	TRUE	
20196-20225 HinckeM –TAXchicken	Uncategorized Sample	20196-20205_A	Mudpit_Ingel20196_MGFPeaklist (F058870)	status=active	H9L2J4_CHICK,UPI000240CCAA,UPI0002C87AA9	TAX_GallusGallus_9031_20141114.fasta,UPARC14_2_GallusG_9031_20140417.fasta	45,476.40	100.00%	2	2	4	0.01%	8.21%	AQEAASLIAQRPGNPR	K	D	99.70%	36.9	43.8	15.4	35.2	29.2	1.48	0.253	0.281	0	0	2	0	2	1,678.90	1,845.00		4,160.22	223	238	Empty	TRUE	
20196-20225 HinckeM –TAXchicken	Uncategorized Sample	20196-20205_A	Mudpit_Ingel20196_MGFPeaklist (F058870)	status=active	H9L2J4_CHICK,UPI000240CCAA,UPI0002C87AA9	TAX_GallusGallus_9031_20141114.fasta,UPARC14_2_GallusG_9031_20140417.fasta	45,476.40	100.00%	2	2	4	0.01%	8.21%	EQDEVPTPTPTTPDPSR	R	V	99.70%	17.4	43.6	17.5	10.4	29.3	2.12	0.324	0.206	0	0	2	0	2	1,866.87	1,833.00		2,043.24	269	285	Empty	TRUE	
20196-20225 HinckeM –TAXchicken	Uncategorized Sample	20196-20205_A	Mudpit_Ingel20196_MGFPeaklist (F058870)	status=active	H9L2J4_CHICK,UPI000240CCAA,UPI0002C87AA9	TAX_GallusGallus_9031_20141114.fasta,UPARC14_2_GallusG_9031_20140417.fasta	45,476.40	100.00%	2	2	4	0.01%	8.21%	AQEAASLIAQRPGNPR	K	D	99.70%	36.9	43.8	15.4	35.2	29.2	1.48	0.253	0.281	0	0	2	0	2	1,678.90	1,845.00		4,160.22	223	238	Empty	TRUE	
20196-20225 HinckeM –TAXchicken	Uncategorized Sample	20196-20205_A	Mudpit_Ingel20196_MGFPeaklist (F058870)	status=active	H9L2J4_CHICK,UPI000240CCAA,UPI0002C87AA9	TAX_GallusGallus_9031_20141114.fasta,UPARC14_2_GallusG_9031_20140417.fasta	45,476.40	100.00%	2	2	4	0.01%	8.21%	EQDEVPTPTPTTPDPSR	R	V	99.70%	17.4	43.6	17.5	10.4	29.3	2.12	0.324	0.206	0	0	2	0	2	1,866.87	1,833.00		2,043.24	269	285	Empty	TRUE	
20196-20225 HinckeM –TAXchicken	Uncategorized Sample	20196-20205_A	Mudpit_Ingel20196_MGFPeaklist (F058870)	status=active	UPI000240CC5B	UPARC14_2_GallusG_9031_20140417.fasta	22,243.60	99.30%	2	2	5	0.01%	10.10%	LINSLYPR	R	G	96.90%	42.5	44.8	31	15.5					0	3	0	0	2	975.5622	2,082.00		1,445.97	65	72	Empty	TRUE	
20196-20225 HinckeM –TAXchicken	Uncategorized Sample	20196-20205_A	Mudpit_Ingel20196_MGFPeaklist (F058870)	status=active	UPI000240CC5B	UPARC14_2_GallusG_9031_20140417.fasta	22,243.60	99.30%	2	2	5	0.01%	10.10%	TLMNLGSLAVAK	R	G	77.00%	27.5	44.2	22.6	12.7					0	2	0	0	2	1,233.69	2,249.00		939.32	128	139	Empty	TRUE	
20196-20225 HinckeM –TAXchicken	Uncategorized Sample	20196-20205_A	Mudpit_Ingel20196_MGFPeaklist (F058870)	Myosin-9 OS=Gallus gallus GN=MYH9 PE=4 SV=2	F1NCD4_CHICK,MYH9_CHICK,UPI000012FB7F,UPI000060E1E4,UPI0002C868F4	TAX_GallusGallus_9031_20141114.fasta,UPARC14_2_GallusG_9031_20140417.fasta	226,643.40	100.00%	49	53	115	0.23%	24.40%	ADFCIIHYAGK	K	V	45.90%	18.6	44.4	14.7	11.1					0	0	2	0	2	1,294.63	2,223.00		989.84	566	576	Empty	TRUE	UPI00000FC6BE,Q789A5_CHICK,Q789A4_CHICK,UPI0000171382,UPI000060F510,UPI0000ECA50D,UPI000060F511,UPI00003AB452,Q789A6_CHICK,UPI0000171380,Q02015_CHICK,UPI0000171381,UPI00003AB454,UPI00003AB453
20196-20225 HinckeM –TAXchicken	Uncategorized Sample	20196-20205_A	Mudpit_Ingel20196_MGFPeaklist (F058870)	Myosin-9 OS=Gallus gallus GN=MYH9 PE=4 SV=2	F1NCD4_CHICK,MYH9_CHICK,UPI000012FB7F,UPI000060E1E4,UPI0002C868F4	TAX_GallusGallus_9031_20141114.fasta,UPARC14_2_GallusG_9031_20140417.fasta	226,643.40	100.00%	49	53	115	0.23%	24.40%	AGKLDPHLVLDQLR	K	C	99.70%	52.2	42.3	31.5	35.1	37.5	3.54	0.344	0.5	0	0	2	0	2	1,574.90	2,445.50		970.79	680	693	Empty	TRUE	UPI00000FC6BE,Q789A5_CHICK,Q789A4_CHICK,UPI0000171382,UPI000060F510,UPI0000ECA50D,UPI000060F511,UPI00003AB452,Q789A6_CHICK,UPI0000171380,Q02015_CHICK,UPI0000171381,UPI00003AB454,UPI00003AB453
20196-20225 HinckeM –TAXchicken	Uncategorized Sample	20196-20205_A	Mudpit_Ingel20196_MGFPeaklist (F058870)	Myosin-9 OS=Gallus gallus GN=MYH9 PE=4 SV=2	F1NCD4_CHICK,MYH9_CHICK,UPI000012FB7F,UPI000060E1E4,UPI0002C868F4	TAX_GallusGallus_9031_20141114.fasta,UPARC14_2_GallusG_9031_20140417.fasta	226,643.40	100.00%	49	53	115	0.23%	24.40%	AGVLAHLEEER	R	D	99.70%	71.3	44.3	36.2	36.2	34.7	3.4	0.317	0.409	0	0	2	0	2	1,223.64	2,031.50		1,163.70	765	775	Empty	TRUE	UPI00000FC6BE,Q789A5_CHICK,Q789A4_CHICK,UPI0000171382,UPI000060F510,UPI0000ECA50D,UPI000060F511,UPI00003AB452,Q789A6_CHICK,UPI0000171380,Q02015_CHICK,UPI0000171381,UPI00003AB454,UPI00003AB453
20196-20225 HinckeM –TAXchicken	Uncategorized Sample	20196-20205_A	Mudpit_Ingel20196_MGFPeaklist (F058870)	Myosin-9 OS=Gallus gallus GN=MYH9 PE=4 SV=2	F1NCD4_CHICK,MYH9_CHICK,UPI000012FB7F,UPI000060E1E4,UPI0002C868F4	TAX_GallusGallus_9031_20141114.fasta,UPARC14_2_GallusG_9031_20140417.fasta	226,643.40	100.00%	49	53	115	0.23%	24.40%	AGVLAHLEEERDLK	R	I	99.70%	43.7	43.9	28.2	36.5	38.3	3.7	0.371	0.464	0	0	2	0	2	1,579.84	2,107.00		1,024.73	765	778	Empty	TRUE	UPI00000FC6BE,Q789A5_CHICK,Q789A4_CHICK,UPI0000171382,UPI000060F510,UPI0000ECA50D,UPI000060F511,UPI00003AB452,Q789A6_CHICK,UPI0000171380,Q02015_CHICK,UPI0000171381,UPI00003AB454,UPI00003AB453
20196-20225 HinckeM –TAXchicken	Uncategorized Sample	20196-20205_A	Mudpit_Ingel20196_MGFPeaklist (F058870)	Myosin-9 OS=Gallus gallus GN=MYH9 PE=4 SV=2	F1NCD4_CHICK,MYH9_CHICK,UPI000012FB7F,UPI000060E1E4,UPI0002C868F4	TAX_GallusGallus_9031_20141114.fasta,UPARC14_2_GallusG_9031_20140417.fasta	226,643.40	100.00%	49	53	115	0.23%	24.40%	ALEEAIEQK	R	A	84.60%	33.1	45.3	36.6	8.49					0	3	0	0	2	1,030.54	1,744.00		686.34	1484	1492	Empty	TRUE	
20196-20225 HinckeM –TAXchicken	Uncategorized Sample	20196-20205_A	Mudpit_Ingel20196_MGFPeaklist (F058870)	Myosin-9 OS=Gallus gallus GN=MYH9 PE=4 SV=2	F1NCD4_CHICK,MYH9_CHICK,UPI000012FB7F,UPI000060E1E4,UPI0002C868F4	TAX_GallusGallus_9031_20141114.fasta,UPARC14_2_GallusG_9031_20140417.fasta	226,643.40	100.00%	49	53	115	0.23%	24.40%	ALEEAIEQKAELER	R	V	99.70%	72.1	44.6	34.9	46.5	50.4	4.02	0.423	0.607	0	1	4	0	2	1,628.85	2,104.00		4,055.28	1484	1497	Empty	TRUE	
20196-20225 HinckeM –TAXchicken	Uncategorized Sample	20196-20205_A	Mudpit_Ingel20196_MGFPeaklist (F058870)	Myosin-9 OS=Gallus gallus GN=MYH9 PE=4 SV=2	F1NCD4_CHICK,MYH9_CHICK,UPI000012FB7F,UPI000060E1E4,UPI0002C868F4	TAX_GallusGallus_9031_20141114.fasta,UPARC14_2_GallusG_9031_20140417.fasta	226,643.40	100.00%	49	53	115	0.23%	24.40%	ALELDSNLYR	K	I	99.70%	58.5	44.7	27.2	33.6	40.4	3.82	0.351	0.55	0	5	0	0	2	1,193.62	2,244.00		3,596.17	746	755	Empty	TRUE	
20196-20225 HinckeM –TAXchicken	Uncategorized Sample	20196-20205_A	Mudpit_Ingel20196_MGFPeaklist (F058870)	Myosin-9 OS=Gallus gallus GN=MYH9 PE=4 SV=2	F1NCD4_CHICK,MYH9_CHICK,UPI000012FB7F,UPI000060E1E4,UPI0002C868F4	TAX_GallusGallus_9031_20141114.fasta,UPARC14_2_GallusG_9031_20140417.fasta	226,643.40	100.00%	49	53	115	0.23%	24.40%	ANLQIDQMNADLNAER	K	S	99.70%	61.6	44.3	23.7	51.8	27.8	1.57	0.237	0.219	0	0	1	0	2	1,831.86	1,969.00		215.45	1755	1770	Empty	TRUE	
20196-20225 HinckeM –TAXchicken	Uncategorized Sample	20196-20205_A	Mudpit_Ingel20196_MGFPeaklist (F058870)	Myosin-9 OS=Gallus gallus GN=MYH9 PE=4 SV=2	F1NCD4_CHICK,MYH9_CHICK,UPI000012FB7F,UPI000060E1E4,UPI0002C868F4	TAX_GallusGallus_9031_20141114.fasta,UPARC14_2_GallusG_9031_20140417.fasta	226,643.40	100.00%	49	53	115	0.23%	24.40%	ATITALEAK	K	I	14.20%	16.5	45.4	22.1	0.69					0	2	0	0	2	917.5303	1,880.00		993.4	1807	1815	Empty	TRUE	
20196-20225 HinckeM –TAXchicken	Uncategorized Sample	20196-20205_A	Mudpit_Ingel20196_MGFPeaklist (F058870)	Myosin-9 OS=Gallus gallus GN=MYH9 PE=4 SV=2	F1NCD4_CHICK,MYH9_CHICK,UPI000012FB7F,UPI000060E1E4,UPI0002C868F4	TAX_GallusGallus_9031_20141114.fasta,UPARC14_2_GallusG_9031_20140417.fasta	226,643.40	100.00%	49	53	115	0.23%	24.40%	CNGVLEGIR	R	I	28.90%	28.9	45.5	29.6	12.6					0	1	0	0	2	1,018.50	2,046.00		210.55	694	702	Empty	TRUE	MYH11_CHICK,Q789A4_CHICK,Q9PTY2_CHICK,UPI0000ECA50D,Q789A6_CHICK,UPI00000FC6BE,Q789A5_CHICK,UPI0000171382,UPI000044943F,UPI000060F510,UPI000060F511,UPI00003AB452,UPI0000171380,Q02015_CHICK,UPI0000171381,UPI00003AB434,UPI00000FB7E2,UPI00003AB454,UPI00003AB431,UPI00003AB453
20196-20225 HinckeM –TAXchicken	Uncategorized Sample	20196-20205_A	Mudpit_Ingel20196_MGFPeaklist (F058870)	Myosin-9 OS=Gallus gallus GN=MYH9 PE=4 SV=2	F1NCD4_CHICK,MYH9_CHICK,UPI000012FB7F,UPI000060E1E4,UPI0002C868F4	TAX_GallusGallus_9031_20141114.fasta,UPARC14_2_GallusG_9031_20140417.fasta	226,643.40	100.00%	49	53	115	0.23%	24.40%	DADKYLYVDK	R	N	47.90%	22.8	44.7	32.5	0					0	0	1	0	2	1,229.61	1,861.00		140	5	14	Empty	TRUE	
20196-20225 HinckeM –TAXchicken	Uncategorized Sample	20196-20205_A	Mudpit_Ingel20196_MGFPeaklist (F058870)	Myosin-9 OS=Gallus gallus GN=MYH9 PE=4 SV=2	F1NCD4_CHICK,MYH9_CHICK,UPI000012FB7F,UPI000060E1E4,UPI0002C868F4	TAX_GallusGallus_9031_20141114.fasta,UPARC14_2_GallusG_9031_20140417.fasta	226,643.40	100.00%	49	53	115	0.23%	24.40%	DLESLTQRYEEK	K	I	56.20%	18.3	44.4	21.2	11.9					0	0	3	0	2	1,510.74	2,123.00		788.9	1393	1404	Empty	TRUE	
20196-20225 HinckeM –TAXchicken	Uncategorized Sample	20196-20205_A	Mudpit_Ingel20196_MGFPeaklist (F058870)	Myosin-9 OS=Gallus gallus GN=MYH9 PE=4 SV=2	F1NCD4_CHICK,MYH9_CHICK,UPI000012FB7F,UPI000060E1E4,UPI0002C868F4	TAX_GallusGallus_9031_20141114.fasta,UPARC14_2_GallusG_9031_20140417.fasta	226,643.40	100.00%	49	53	115	0.23%	24.40%	EQADFAIEALAK	K	A	99.70%	57.8	45	27.9	43.9	37.5	3.8	0.331	0.375	0	4	0	0	2	1,305.67	2,461.50		1,501.85	408	419	Empty	TRUE	MYH11_CHICK
20196-20225 HinckeM –TAXchicken	Uncategorized Sample	20196-20205_A	Mudpit_Ingel20196_MGFPeaklist (F058870)	Myosin-9 OS=Gallus gallus GN=MYH9 PE=4 SV=2	F1NCD4_CHICK,MYH9_CHICK,UPI000012FB7F,UPI000060E1E4,UPI0002C868F4	TAX_GallusGallus_9031_20141114.fasta,UPARC14_2_GallusG_9031_20140417.fasta	226,643.40	100.00%	49	53	115	0.23%	24.40%	FDQLLAEEK	K	N	62.50%	25.1	44.8	29.4	6.88					0	2	0	0	2	1,092.56	2,017.50		508.72	1446	1454	Empty	TRUE	
20196-20225 HinckeM –TAXchicken	Uncategorized Sample	20196-20205_A	Mudpit_Ingel20196_MGFPeaklist (F058870)	Myosin-9 OS=Gallus gallus GN=MYH9 PE=4 SV=2	F1NCD4_CHICK,MYH9_CHICK,UPI000012FB7F,UPI000060E1E4,UPI0002C868F4	TAX_GallusGallus_9031_20141114.fasta,UPARC14_2_GallusG_9031_20140417.fasta	226,643.40	100.00%	49	53	115	0.23%	24.40%	HSQAIEELAEQLEQTKR	K	V	83.90%	21.7	43.9	18	16.7					0	0	0	2	2	2,010.03	2,465.50		456.01	1194	1210	Empty	TRUE	
20196-20225 HinckeM –TAXchicken	Uncategorized Sample	20196-20205_A	Mudpit_Ingel20196_MGFPeaklist (F058870)	Myosin-9 OS=Gallus gallus GN=MYH9 PE=4 SV=2	F1NCD4_CHICK,MYH9_CHICK,UPI000012FB7F,UPI000060E1E4,UPI0002C868F4	TAX_GallusGallus_9031_20141114.fasta,UPARC14_2_GallusG_9031_20140417.fasta	226,643.40	100.00%	49	53	115	0.23%	24.40%	IMGIPDEEQIGLLK	R	V	99.70%	33.4	44.2	14.4	29.2	36.2	1.96	0.318	0.357	0	2	0	0	2	1,571.84	2,519.50		2,423.65	328	341	Empty	TRUE	
20196-20225 HinckeM –TAXchicken	Uncategorized Sample	20196-20205_A	Mudpit_Ingel20196_MGFPeaklist (F058870)	Myosin-9 OS=Gallus gallus GN=MYH9 PE=4 SV=2	F1NCD4_CHICK,MYH9_CHICK,UPI000012FB7F,UPI000060E1E4,UPI0002C868F4	TAX_GallusGallus_9031_20141114.fasta,UPARC14_2_GallusG_9031_20140417.fasta	226,643.40	100.00%	49	53	115	0.23%	24.40%	IVQLEEQLDMETKER	K	Q	98.40%	37.5	44.6	22.6	23.5					0	0	2	0	2	1,876.93	2,189.50		318.2	1816	1830	Empty	TRUE	
20196-20225 HinckeM –TAXchicken	Uncategorized Sample	20196-20205_A	Mudpit_Ingel20196_MGFPeaklist (F058870)	Myosin-9 OS=Gallus gallus GN=MYH9 PE=4 SV=2	F1NCD4_CHICK,MYH9_CHICK,UPI000012FB7F,UPI000060E1E4,UPI0002C868F4	TAX_GallusGallus_9031_20141114.fasta,UPARC14_2_GallusG_9031_20140417.fasta	226,643.40	100.00%	49	53	115	0.23%	24.40%	KFDQLLAEEK	K	N	93.10%	39.1	44.6	40.3	9.11					0	0	4	0	2	1,220.65	2,002.50		2,060.83	1445	1454	Empty	TRUE	
20196-20225 HinckeM –TAXchicken	Uncategorized Sample	20196-20205_A	Mudpit_Ingel20196_MGFPeaklist (F058870)	Myosin-9 OS=Gallus gallus GN=MYH9 PE=4 SV=2	F1NCD4_CHICK,MYH9_CHICK,UPI000012FB7F,UPI000060E1E4,UPI0002C868F4	TAX_GallusGallus_9031_20141114.fasta,UPARC14_2_GallusG_9031_20140417.fasta	226,643.40	100.00%	49	53	115	0.23%	24.40%	KFDQLLAEEKNISAK	K	Y	99.70%	26	43.5	21.7	15.4	29.3	1.7	0.147	0.367	0	0	1	0	2	1,733.94	2,153.00		416.04	1445	1459	Empty	TRUE	
20196-20225 HinckeM –TAXchicken	Uncategorized Sample	20196-20205_A	Mudpit_Ingel20196_MGFPeaklist (F058870)	Myosin-9 OS=Gallus gallus GN=MYH9 PE=4 SV=2	F1NCD4_CHICK,MYH9_CHICK,UPI000012FB7F,UPI000060E1E4,UPI0002C868F4	TAX_GallusGallus_9031_20141114.fasta,UPARC14_2_GallusG_9031_20140417.fasta	226,643.40	100.00%	49	53	115	0.23%	24.40%	KKVDAQLQELQVK	R	F	67.80%	28.2	40.7	28.4	11.1					0	0	1	0	2	1,526.89	1,881.00		329.09	1248	1260	Empty	TRUE	
20196-20225 HinckeM –TAXchicken	Uncategorized Sample	20196-20205_A	Mudpit_Ingel20196_MGFPeaklist (F058870)	Myosin-9 OS=Gallus gallus GN=MYH9 PE=4 SV=2	F1NCD4_CHICK,MYH9_CHICK,UPI000012FB7F,UPI000060E1E4,UPI0002C868F4	TAX_GallusGallus_9031_20141114.fasta,UPARC14_2_GallusG_9031_20140417.fasta	226,643.40	100.00%	49	53	115	0.23%	24.40%	KLEEDVIVLEDQNLK	K	L	99.70%	41	43.3	24.6	26.1	36.6	4	0.363	0.367	0	0	2	0	2	1,784.96	2,380.50		1,109.02	975	989	Empty	TRUE	
20196-20225 HinckeM –TAXchicken	Uncategorized Sample	20196-20205_A	Mudpit_Ingel20196_MGFPeaklist (F058870)	Myosin-9 OS=Gallus gallus GN=MYH9 PE=4 SV=2	F1NCD4_CHICK,MYH9_CHICK,UPI000012FB7F,UPI000060E1E4,UPI0002C868F4	TAX_GallusGallus_9031_20141114.fasta,UPARC14_2_GallusG_9031_20140417.fasta	226,643.40	100.00%	49	53	115	0.23%	24.40%	KLVWVPSEK	K	S	29.70%	13.5	43.2	17.5	0					0	1	0	0	2	1,085.64	2,060.00		390.18	30	38	Empty	TRUE	MYH11_CHICK
20196-20225 HinckeM –TAXchicken	Uncategorized Sample	20196-20205_A	Mudpit_Ingel20196_MGFPeaklist (F058870)	Myosin-9 OS=Gallus gallus GN=MYH9 PE=4 SV=2	F1NCD4_CHICK,MYH9_CHICK,UPI000012FB7F,UPI000060E1E4,UPI0002C868F4	TAX_GallusGallus_9031_20141114.fasta,UPARC14_2_GallusG_9031_20140417.fasta	226,643.40	100.00%	49	53	115	0.23%	24.40%	KQELEEICHDLEAR	K	V	99.70%	48.3	44.3	23.2	40.8	35.2	3.44	0.347	0.357	0	0	2	1	2	1,769.85	2,175.00		1,534.37	910	923	Empty	TRUE	
20196-20225 HinckeM –TAXchicken	Uncategorized Sample	20196-20205_A	Mudpit_Ingel20196_MGFPeaklist (F058870)	Myosin-9 OS=Gallus gallus GN=MYH9 PE=4 SV=2	F1NCD4_CHICK,MYH9_CHICK,UPI000012FB7F,UPI000060E1E4,UPI0002C868F4	TAX_GallusGallus_9031_20141114.fasta,UPARC14_2_GallusG_9031_20140417.fasta	226,643.40	100.00%	49	53	115	0.23%	24.40%	KVDAQLQELQVK	K	F	99.70%	33.1	42.7	21.6	9.14	27.3	1.21	0.22	0.333	0	0	2	0	2	1,398.80	2,003.00		643.7	1249	1260	Empty	TRUE	
20196-20225 HinckeM –TAXchicken	Uncategorized Sample	20196-20205_A	Mudpit_Ingel20196_MGFPeaklist (F058870)	Myosin-9 OS=Gallus gallus GN=MYH9 PE=4 SV=2	F1NCD4_CHICK,MYH9_CHICK,UPI000012FB7F,UPI000060E1E4,UPI0002C868F4	TAX_GallusGallus_9031_20141114.fasta,UPARC14_2_GallusG_9031_20140417.fasta	226,643.40	100.00%	49	53	115	0.23%	24.40%	LDPHLVLDQLR	K	C	99.70%	34.6	43.8	18.6	22.1	32.5	2.47	0.366	0.455	0	0	1	0	2	1,318.75	2,534.00		1,121.62	683	693	Empty	TRUE	UPI00000FC6BE,Q789A5_CHICK,Q789A4_CHICK,UPI0000171382,UPI000060F510,UPI0000ECA50D,UPI000060F511,UPI00003AB452,Q789A6_CHICK,UPI0000171380,Q02015_CHICK,UPI0000171381,UPI00003AB454,UPI00003AB453
20196-20225 HinckeM –TAXchicken	Uncategorized Sample	20196-20205_A	Mudpit_Ingel20196_MGFPeaklist (F058870)	Myosin-9 OS=Gallus gallus GN=MYH9 PE=4 SV=2	F1NCD4_CHICK,MYH9_CHICK,UPI000012FB7F,UPI000060E1E4,UPI0002C868F4	TAX_GallusGallus_9031_20141114.fasta,UPARC14_2_GallusG_9031_20140417.fasta	226,643.40	100.00%	49	53	115	0.23%	24.40%	LKDILLQVDDERR	K	N	27.70%	15.6	42.7	21.5	4.84					0	0	0	1	2	1,612.90	2,214.00		458.07	1844	1856	Empty	TRUE	
20196-20225 HinckeM –TAXchicken	Uncategorized Sample	20196-20205_A	Mudpit_Ingel20196_MGFPeaklist (F058870)	Myosin-9 OS=Gallus gallus GN=MYH9 PE=4 SV=2	F1NCD4_CHICK,MYH9_CHICK,UPI000012FB7F,UPI000060E1E4,UPI0002C868F4	TAX_GallusGallus_9031_20141114.fasta,UPARC14_2_GallusG_9031_20140417.fasta	226,643.40	100.00%	49	53	115	0.23%	24.40%	LQQELDDIAVDLDHQR	R	Q	99.70%	68.1	44.4	19.5	56.3	44.1	5.77	0.515	0.438	0	0	2	0	2	1,907.95	2,474.00		1,640.23	1418	1433	Empty	TRUE	
20196-20225 HinckeM –TAXchicken	Uncategorized Sample	20196-20205_A	Mudpit_Ingel20196_MGFPeaklist (F058870)	Myosin-9 OS=Gallus gallus GN=MYH9 PE=4 SV=2	F1NCD4_CHICK,MYH9_CHICK,UPI000012FB7F,UPI000060E1E4,UPI0002C868F4	TAX_GallusGallus_9031_20141114.fasta,UPARC14_2_GallusG_9031_20140417.fasta	226,643.40	100.00%	49	53	115	0.23%	24.40%	LRLEVNQQAMK	K	A	73.00%	17.5	44.8	15.5	15.2					0	0	1	0	2	1,345.73	1,759.00		157.86	1556	1566	Empty	TRUE	
20196-20225 HinckeM –TAXchicken	Uncategorized Sample	20196-20205_A	Mudpit_Ingel20196_MGFPeaklist (F058870)	Myosin-9 OS=Gallus gallus GN=MYH9 PE=4 SV=2	F1NCD4_CHICK,MYH9_CHICK,UPI000012FB7F,UPI000060E1E4,UPI0002C868F4	TAX_GallusGallus_9031_20141114.fasta,UPARC14_2_GallusG_9031_20140417.fasta	226,643.40	100.00%	49	53	115	0.23%	24.40%	LVWVPSEK	K	S	99.70%	26.7	44.4	23.8	14.6	22.7	0.638	0.123	0.312	0	2	0	0	2	957.5404	2,164.50		372.91	31	38	Empty	TRUE	MYH11_CHICK
20196-20225 HinckeM –TAXchicken	Uncategorized Sample	20196-20205_A	Mudpit_Ingel20196_MGFPeaklist (F058870)	Myosin-9 OS=Gallus gallus GN=MYH9 PE=4 SV=2	F1NCD4_CHICK,MYH9_CHICK,UPI000012FB7F,UPI000060E1E4,UPI0002C868F4	TAX_GallusGallus_9031_20141114.fasta,UPARC14_2_GallusG_9031_20140417.fasta	226,643.40	100.00%	49	53	115	0.23%	24.40%	MSEFTTNLTEEEEKSK	R	S	99.70%	32.2	43	16.9	29.3	30.3	2.14	0.0066	0.25	0	0	2	0	2	1,902.86	2,035.00		827.04	1001	1016	Empty	TRUE	
20196-20225 HinckeM –TAXchicken	Uncategorized Sample	20196-20205_A	Mudpit_Ingel20196_MGFPeaklist (F058870)	Myosin-9 OS=Gallus gallus GN=MYH9 PE=4 SV=2	F1NCD4_CHICK,MYH9_CHICK,UPI000012FB7F,UPI000060E1E4,UPI0002C868F4	TAX_GallusGallus_9031_20141114.fasta,UPARC14_2_GallusG_9031_20140417.fasta	226,643.40	100.00%	49	53	115	0.23%	24.40%	NWQWWR	R	L	24.20%	12.7	45.1	22.7	3.78					0	1	0	0	2	975.4583	2,584.00		138.57	824	829	Empty	TRUE	MYH11_CHICK
20196-20225 HinckeM –TAXchicken	Uncategorized Sample	20196-20205_A	Mudpit_Ingel20196_MGFPeaklist (F058870)	Myosin-9 OS=Gallus gallus GN=MYH9 PE=4 SV=2	F1NCD4_CHICK,MYH9_CHICK,UPI000012FB7F,UPI000060E1E4,UPI0002C868F4	TAX_GallusGallus_9031_20141114.fasta,UPARC14_2_GallusG_9031_20140417.fasta	226,643.40	100.00%	49	53	115	0.23%	24.40%	QACVLMIK	K	A	17.50%	25.6	44	27.9	9.47					0	1	0	0	2	961.4847	2,343.00		270	738	745	Empty	TRUE	
20196-20225 HinckeM –TAXchicken	Uncategorized Sample	20196-20205_A	Mudpit_Ingel20196_MGFPeaklist (F058870)	Myosin-9 OS=Gallus gallus GN=MYH9 PE=4 SV=2	F1NCD4_CHICK,MYH9_CHICK,UPI000012FB7F,UPI000060E1E4,UPI0002C868F4	TAX_GallusGallus_9031_20141114.fasta,UPARC14_2_GallusG_9031_20140417.fasta	226,643.40	100.00%	49	53	115	0.23%	24.40%	QAQQERDELADEIANSSGK	R	G	99.70%	56.6	44.1	18.7	50.3	48	6.68	0.515	0.395	0	0	4	0	2	2,088.98	1,982.00		4,035.47	1698	1716	Empty	TRUE	
20196-20225 HinckeM –TAXchicken	Uncategorized Sample	20196-20205_A	Mudpit_Ingel20196_MGFPeaklist (F058870)	Myosin-9 OS=Gallus gallus GN=MYH9 PE=4 SV=2	F1NCD4_CHICK,MYH9_CHICK,UPI000012FB7F,UPI000060E1E4,UPI0002C868F4	TAX_GallusGallus_9031_20141114.fasta,UPARC14_2_GallusG_9031_20140417.fasta	226,643.40	100.00%	49	53	115	0.23%	24.40%	QISVLQQQAVEAR	K	K	99.70%	71.5	43.7	29.1	53.9	45.3	3.96	0.422	0.5	0	6	0	0	2	1,469.81	2,037.50		6,070.84	1358	1370	Empty	TRUE	
20196-20225 HinckeM –TAXchicken	Uncategorized Sample	20196-20205_A	Mudpit_Ingel20196_MGFPeaklist (F058870)	Myosin-9 OS=Gallus gallus GN=MYH9 PE=4 SV=2	F1NCD4_CHICK,MYH9_CHICK,UPI000012FB7F,UPI000060E1E4,UPI0002C868F4	TAX_GallusGallus_9031_20141114.fasta,UPARC14_2_GallusG_9031_20140417.fasta	226,643.40	100.00%	49	53	115	0.23%	24.40%	QKHSQAIEELAEQLEQTKR	R	V	99.70%	38.5	43.4	23.6	26	30.7	2.18	0.192	0.184	0	0	0	2	2	2,266.18	2,363.00		795.44	1192	1210	Empty	TRUE	
20196-20225 HinckeM –TAXchicken	Uncategorized Sample	20196-20205_A	Mudpit_Ingel20196_MGFPeaklist (F058870)	Myosin-9 OS=Gallus gallus GN=MYH9 PE=4 SV=2	F1NCD4_CHICK,MYH9_CHICK,UPI000012FB7F,UPI000060E1E4,UPI0002C868F4	TAX_GallusGallus_9031_20141114.fasta,UPARC14_2_GallusG_9031_20140417.fasta	226,643.40	100.00%	49	53	115	0.23%	24.40%	QLLQANPILEAFGNAK	R	T	33.80%	11.9	42.9	17.8	9.6					0	1	0	0	2	1,726.95	2,877.00		107.82	210	225	Empty	TRUE	MYH11_CHICK
20196-20225 HinckeM –TAXchicken	Uncategorized Sample	20196-20205_A	Mudpit_Ingel20196_MGFPeaklist (F058870)	Myosin-9 OS=Gallus gallus GN=MYH9 PE=4 SV=2	F1NCD4_CHICK,MYH9_CHICK,UPI000012FB7F,UPI000060E1E4,UPI0002C868F4	TAX_GallusGallus_9031_20141114.fasta,UPARC14_2_GallusG_9031_20140417.fasta	226,643.40	100.00%	49	53	115	0.23%	24.40%	QRYEILTPNAIPK	R	G	99.70%	44.8	43.5	22.5	30.7	33.5	1.96	0.358	0.423	0	2	4	0	2	1,542.86	2,240.50		4,800.05	719	731	Empty	TRUE	UPI00000FC6BE,Q789A5_CHICK,Q789A4_CHICK,UPI0000171382,UPI000060F510,UPI0000ECA50D,UPI000060F511,UPI00003AB452,Q789A6_CHICK,UPI0000171380,Q02015_CHICK,UPI0000171381,UPI00003AB454,UPI00003AB453
20196-20225 HinckeM –TAXchicken	Uncategorized Sample	20196-20205_A	Mudpit_Ingel20196_MGFPeaklist (F058870)	Myosin-9 OS=Gallus gallus GN=MYH9 PE=4 SV=2	F1NCD4_CHICK,MYH9_CHICK,UPI000012FB7F,UPI000060E1E4,UPI0002C868F4	TAX_GallusGallus_9031_20141114.fasta,UPARC14_2_GallusG_9031_20140417.fasta	226,643.40	100.00%	49	53	115	0.23%	24.40%	RGDLPFVVTR	R	R	99.70%	45.9	44.6	39	28	34.9	1.66	0.301	0.5	0	2	0	0	2	1,159.66	2,210.00		561.14	1923	1932	Empty	TRUE	
20196-20225 HinckeM –TAXchicken	Uncategorized Sample	20196-20205_A	Mudpit_Ingel20196_MGFPeaklist (F058870)	Myosin-9 OS=Gallus gallus GN=MYH9 PE=4 SV=2	F1NCD4_CHICK,MYH9_CHICK,UPI000012FB7F,UPI000060E1E4,UPI0002C868F4	TAX_GallusGallus_9031_20141114.fasta,UPARC14_2_GallusG_9031_20140417.fasta	226,643.40	100.00%	49	53	115	0.23%	24.40%	TDLLLEPYNK	K	Y	99.70%	34.2	44.8	29.3	12.7	27.4	1.57	0.234	0.35	0	2	0	0	2	1,205.64	2,293.50		465.92	290	299	Empty	TRUE	
20196-20225 HinckeM –TAXchicken	Uncategorized Sample	20196-20205_A	Mudpit_Ingel20196_MGFPeaklist (F058870)	Myosin-9 OS=Gallus gallus GN=MYH9 PE=4 SV=2	F1NCD4_CHICK,MYH9_CHICK,UPI000012FB7F,UPI000060E1E4,UPI0002C868F4	TAX_GallusGallus_9031_20141114.fasta,UPARC14_2_GallusG_9031_20140417.fasta	226,643.40	100.00%	49	53	115	0.23%	24.40%	TDLLLEPYNKYR	K	F	99.70%	24.5	44.3	23.3	10.2	26.9	2.44	0.208	0.333	0	0	2	0	2	1,524.81	2,290.00		819.24	290	301	Empty	TRUE	
20196-20225 HinckeM –TAXchicken	Uncategorized Sample	20196-20205_A	Mudpit_Ingel20196_MGFPeaklist (F058870)	Myosin-9 OS=Gallus gallus GN=MYH9 PE=4 SV=2	F1NCD4_CHICK,MYH9_CHICK,UPI000012FB7F,UPI000060E1E4,UPI0002C868F4	TAX_GallusGallus_9031_20141114.fasta,UPARC14_2_GallusG_9031_20140417.fasta	226,643.40	100.00%	49	53	115	0.23%	24.40%	TLEDEAKTHEAQIQEMR	K	Q	98.60%	43.9	44	22.9	33.3					0	0	0	2	2	2,044.96	1,846.50		641.82	1175	1191	Empty	TRUE	
20196-20225 HinckeM –TAXchicken	Uncategorized Sample	20196-20205_A	Mudpit_Ingel20196_MGFPeaklist (F058870)	Myosin-9 OS=Gallus gallus GN=MYH9 PE=4 SV=2	F1NCD4_CHICK,MYH9_CHICK,UPI000012FB7F,UPI000060E1E4,UPI0002C868F4	TAX_GallusGallus_9031_20141114.fasta,UPARC14_2_GallusG_9031_20140417.fasta	226,643.40	100.00%	49	53	115	0.23%	24.40%	TSREEILAQAK	R	E	92.20%	37	44.7	34.3	9.74					0	0	3	0	2	1,245.68	1,668.00		1,425.65	1659	1669	Empty	TRUE	
20196-20225 HinckeM –TAXchicken	Uncategorized Sample	20196-20205_A	Mudpit_Ingel20196_MGFPeaklist (F058870)	Myosin-9 OS=Gallus gallus GN=MYH9 PE=4 SV=2	F1NCD4_CHICK,MYH9_CHICK,UPI000012FB7F,UPI000060E1E4,UPI0002C868F4	TAX_GallusGallus_9031_20141114.fasta,UPARC14_2_GallusG_9031_20140417.fasta	226,643.40	100.00%	49	53	115	0.23%	24.40%	TSREEILAQAKENEK	R	K	99.70%	49.7	44.6	29.6	30.6	42.7	3.42	0.335	0.5	0	0	0	2	2	1,745.90	1,637.00		624	1659	1673	Empty	TRUE	
20196-20225 HinckeM –TAXchicken	Uncategorized Sample	20196-20205_A	Mudpit_Ingel20196_MGFPeaklist (F058870)	Myosin-9 OS=Gallus gallus GN=MYH9 PE=4 SV=2	F1NCD4_CHICK,MYH9_CHICK,UPI000012FB7F,UPI000060E1E4,UPI0002C868F4	TAX_GallusGallus_9031_20141114.fasta,UPARC14_2_GallusG_9031_20140417.fasta	226,643.40	100.00%	49	53	115	0.23%	24.40%	TVGQLYKEQLAK	R	L	99.70%	37	43.6	25.2	25.9	29.3	1.14	0.266	0.417	0	0	4	0	2	1,377.77	1,866.50		2,477.43	645	656	Empty	TRUE	
20196-20225 HinckeM –TAXchicken	Uncategorized Sample	20196-20205_A	Mudpit_Ingel20196_MGFPeaklist (F058870)	Myosin-9 OS=Gallus gallus GN=MYH9 PE=4 SV=2	F1NCD4_CHICK,MYH9_CHICK,UPI000012FB7F,UPI000060E1E4,UPI0002C868F4	TAX_GallusGallus_9031_20141114.fasta,UPARC14_2_GallusG_9031_20140417.fasta	226,643.40	100.00%	49	53	115	0.23%	24.40%	VDAQLQELQVK	K	F	50.50%	23.2	43.9	38.2	1.53					0	1	0	0	2	1,270.70	2,098.00		128.78	1250	1260	Empty	TRUE	
20196-20225 HinckeM –TAXchicken	Uncategorized Sample	20196-20205_A	Mudpit_Ingel20196_MGFPeaklist (F058870)	Myosin-9 OS=Gallus gallus GN=MYH9 PE=4 SV=2	F1NCD4_CHICK,MYH9_CHICK,UPI000012FB7F,UPI000060E1E4,UPI0002C868F4	TAX_GallusGallus_9031_20141114.fasta,UPARC14_2_GallusG_9031_20140417.fasta	226,643.40	100.00%	49	53	115	0.23%	24.40%	VISGVLQLGNIVFKK	K	E	99.70%	64.9	36.9	20.4	56.6	45.8	3.11	0.162	0.5	0	0	2	0	2	1,614.99	2,854.50		1,206.55	342	356	Empty	TRUE	
20196-20225 HinckeM –TAXchicken	Uncategorized Sample	20196-20205_A	Mudpit_Ingel20196_MGFPeaklist (F058870)	Myosin-9 OS=Gallus gallus GN=MYH9 PE=4 SV=2	F1NCD4_CHICK,MYH9_CHICK,UPI000012FB7F,UPI000060E1E4,UPI0002C868F4	TAX_GallusGallus_9031_20141114.fasta,UPARC14_2_GallusG_9031_20140417.fasta	226,643.40	100.00%	49	53	115	0.23%	24.40%	VSHLLGINVTDFTR	K	G	99.70%	50.9	43.8	29.7	35.6	39.2	4.7	0.487	0.429	0	0	3	0	2	1,571.85	2,599.00		1,284.73	374	387	Empty	TRUE	
20196-20225 HinckeM –TAXchicken	Uncategorized Sample	20196-20205_A	Mudpit_Ingel20196_MGFPeaklist (F058870)	Myosin-9 OS=Gallus gallus GN=MYH9 PE=4 SV=2	F1NCD4_CHICK,MYH9_CHICK,UPI000012FB7F,UPI000060E1E4,UPI0002C868F4	TAX_GallusGallus_9031_20141114.fasta,UPARC14_2_GallusG_9031_20140417.fasta	226,643.40	100.00%	49	53	115	0.23%	24.40%	VVFQEFR	R	Q	74.20%	30.9	43.3	26.1	16.6					0	4	0	0	2	924.4937	2,156.00		1,480.25	712	718	Empty	TRUE	
20196-20225 HinckeM –TAXchicken	Uncategorized Sample	20196-20205_A	Mudpit_Ingel20196_MGFPeaklist (F058870)	Myosin-9 OS=Gallus gallus GN=MYH9 PE=4 SV=2	F1NCD4_CHICK,MYH9_CHICK,UPI000012FB7F,UPI000060E1E4,UPI0002C868F4	TAX_GallusGallus_9031_20141114.fasta,UPARC14_2_GallusG_9031_20140417.fasta	226,643.40	100.00%	49	53	115	0.23%	24.40%	YEILTPNAIPK	R	G	99.70%	20.6	44.4	14.6	7.9	28.5	1.57	0.277	0.318	0	3	0	0	2	1,258.70	2,337.00		1,618.82	721	731	Empty	TRUE	UPI00000FC6BE,Q789A5_CHICK,Q789A4_CHICK,UPI0000171382,UPI000060F510,UPI0000ECA50D,UPI000060F511,UPI00003AB452,Q789A6_CHICK,UPI0000171380,Q02015_CHICK,UPI0000171381,UPI00003AB454,UPI00003AB453
20196-20225 HinckeM –TAXchicken	Uncategorized Sample	20196-20205_A	Mudpit_Ingel20196_MGFPeaklist (F058870)	Myosin-9 OS=Gallus gallus GN=MYH9 PE=4 SV=2	F1NCD4_CHICK,MYH9_CHICK,UPI000012FB7F,UPI000060E1E4,UPI0002C868F4	TAX_GallusGallus_9031_20141114.fasta,UPARC14_2_GallusG_9031_20140417.fasta	226,643.40	100.00%	49	53	115	0.23%	24.40%	ADFCIIHYAGK	K	V	45.90%	18.6	44.4	14.7	11.1					0	0	2	0	2	1,294.63	2,223.00		989.84	566	576	Empty	TRUE	UPI00000FC6BE,Q789A5_CHICK,Q789A4_CHICK,UPI0000171382,UPI000060F510,UPI0000ECA50D,UPI000060F511,UPI00003AB452,Q789A6_CHICK,UPI0000171380,Q02015_CHICK,UPI0000171381,UPI00003AB454,UPI00003AB453
20196-20225 HinckeM –TAXchicken	Uncategorized Sample	20196-20205_A	Mudpit_Ingel20196_MGFPeaklist (F058870)	Myosin-9 OS=Gallus gallus GN=MYH9 PE=4 SV=2	F1NCD4_CHICK,MYH9_CHICK,UPI000012FB7F,UPI000060E1E4,UPI0002C868F4	TAX_GallusGallus_9031_20141114.fasta,UPARC14_2_GallusG_9031_20140417.fasta	226,643.40	100.00%	49	53	115	0.23%	24.40%	AGKLDPHLVLDQLR	K	C	99.70%	52.2	42.3	31.5	35.1	37.5	3.54	0.344	0.5	0	0	2	0	2	1,574.90	2,445.50		970.79	680	693	Empty	TRUE	UPI00000FC6BE,Q789A5_CHICK,Q789A4_CHICK,UPI0000171382,UPI000060F510,UPI0000ECA50D,UPI000060F511,UPI00003AB452,Q789A6_CHICK,UPI0000171380,Q02015_CHICK,UPI0000171381,UPI00003AB454,UPI00003AB453
20196-20225 HinckeM –TAXchicken	Uncategorized Sample	20196-20205_A	Mudpit_Ingel20196_MGFPeaklist (F058870)	Myosin-9 OS=Gallus gallus GN=MYH9 PE=4 SV=2	F1NCD4_CHICK,MYH9_CHICK,UPI000012FB7F,UPI000060E1E4,UPI0002C868F4	TAX_GallusGallus_9031_20141114.fasta,UPARC14_2_GallusG_9031_20140417.fasta	226,643.40	100.00%	49	53	115	0.23%	24.40%	AGVLAHLEEER	R	D	99.70%	71.3	44.3	36.2	36.2	34.7	3.4	0.317	0.409	0	0	2	0	2	1,223.64	2,031.50		1,163.70	765	775	Empty	TRUE	UPI00000FC6BE,Q789A5_CHICK,Q789A4_CHICK,UPI0000171382,UPI000060F510,UPI0000ECA50D,UPI000060F511,UPI00003AB452,Q789A6_CHICK,UPI0000171380,Q02015_CHICK,UPI0000171381,UPI00003AB454,UPI00003AB453
20196-20225 HinckeM –TAXchicken	Uncategorized Sample	20196-20205_A	Mudpit_Ingel20196_MGFPeaklist (F058870)	Myosin-9 OS=Gallus gallus GN=MYH9 PE=4 SV=2	F1NCD4_CHICK,MYH9_CHICK,UPI000012FB7F,UPI000060E1E4,UPI0002C868F4	TAX_GallusGallus_9031_20141114.fasta,UPARC14_2_GallusG_9031_20140417.fasta	226,643.40	100.00%	49	53	115	0.23%	24.40%	AGVLAHLEEERDLK	R	I	99.70%	43.7	43.9	28.2	36.5	38.3	3.7	0.371	0.464	0	0	2	0	2	1,579.84	2,107.00		1,024.73	765	778	Empty	TRUE	UPI00000FC6BE,Q789A5_CHICK,Q789A4_CHICK,UPI0000171382,UPI000060F510,UPI0000ECA50D,UPI000060F511,UPI00003AB452,Q789A6_CHICK,UPI0000171380,Q02015_CHICK,UPI0000171381,UPI00003AB454,UPI00003AB453
20196-20225 HinckeM –TAXchicken	Uncategorized Sample	20196-20205_A	Mudpit_Ingel20196_MGFPeaklist (F058870)	Myosin-9 OS=Gallus gallus GN=MYH9 PE=4 SV=2	F1NCD4_CHICK,MYH9_CHICK,UPI000012FB7F,UPI000060E1E4,UPI0002C868F4	TAX_GallusGallus_9031_20141114.fasta,UPARC14_2_GallusG_9031_20140417.fasta	226,643.40	100.00%	49	53	115	0.23%	24.40%	ALEEAIEQK	R	A	84.60%	33.1	45.3	36.6	8.49					0	3	0	0	2	1,030.54	1,744.00		686.34	1484	1492	Empty	TRUE	
20196-20225 HinckeM –TAXchicken	Uncategorized Sample	20196-20205_A	Mudpit_Ingel20196_MGFPeaklist (F058870)	Myosin-9 OS=Gallus gallus GN=MYH9 PE=4 SV=2	F1NCD4_CHICK,MYH9_CHICK,UPI000012FB7F,UPI000060E1E4,UPI0002C868F4	TAX_GallusGallus_9031_20141114.fasta,UPARC14_2_GallusG_9031_20140417.fasta	226,643.40	100.00%	49	53	115	0.23%	24.40%	ALEEAIEQKAELER	R	V	99.70%	72.1	44.6	34.9	46.5	50.4	4.02	0.423	0.607	0	1	4	0	2	1,628.85	2,104.00		4,055.28	1484	1497	Empty	TRUE	
20196-20225 HinckeM –TAXchicken	Uncategorized Sample	20196-20205_A	Mudpit_Ingel20196_MGFPeaklist (F058870)	Myosin-9 OS=Gallus gallus GN=MYH9 PE=4 SV=2	F1NCD4_CHICK,MYH9_CHICK,UPI000012FB7F,UPI000060E1E4,UPI0002C868F4	TAX_GallusGallus_9031_20141114.fasta,UPARC14_2_GallusG_9031_20140417.fasta	226,643.40	100.00%	49	53	115	0.23%	24.40%	ALELDSNLYR	K	I	99.70%	58.5	44.7	27.2	33.6	40.4	3.82	0.351	0.55	0	5	0	0	2	1,193.62	2,244.00		3,596.17	746	755	Empty	TRUE	
20196-20225 HinckeM –TAXchicken	Uncategorized Sample	20196-20205_A	Mudpit_Ingel20196_MGFPeaklist (F058870)	Myosin-9 OS=Gallus gallus GN=MYH9 PE=4 SV=2	F1NCD4_CHICK,MYH9_CHICK,UPI000012FB7F,UPI000060E1E4,UPI0002C868F4	TAX_GallusGallus_9031_20141114.fasta,UPARC14_2_GallusG_9031_20140417.fasta	226,643.40	100.00%	49	53	115	0.23%	24.40%	ANLQIDQMNADLNAER	K	S	99.70%	61.6	44.3	23.7	51.8	27.8	1.57	0.237	0.219	0	0	1	0	2	1,831.86	1,969.00		215.45	1755	1770	Empty	TRUE	
20196-20225 HinckeM –TAXchicken	Uncategorized Sample	20196-20205_A	Mudpit_Ingel20196_MGFPeaklist (F058870)	Myosin-9 OS=Gallus gallus GN=MYH9 PE=4 SV=2	F1NCD4_CHICK,MYH9_CHICK,UPI000012FB7F,UPI000060E1E4,UPI0002C868F4	TAX_GallusGallus_9031_20141114.fasta,UPARC14_2_GallusG_9031_20140417.fasta	226,643.40	100.00%	49	53	115	0.23%	24.40%	ATITALEAK	K	I	14.20%	16.5	45.4	22.1	0.69					0	2	0	0	2	917.5303	1,880.00		993.4	1807	1815	Empty	TRUE	
20196-20225 HinckeM –TAXchicken	Uncategorized Sample	20196-20205_A	Mudpit_Ingel20196_MGFPeaklist (F058870)	Myosin-9 OS=Gallus gallus GN=MYH9 PE=4 SV=2	F1NCD4_CHICK,MYH9_CHICK,UPI000012FB7F,UPI000060E1E4,UPI0002C868F4	TAX_GallusGallus_9031_20141114.fasta,UPARC14_2_GallusG_9031_20140417.fasta	226,643.40	100.00%	49	53	115	0.23%	24.40%	CNGVLEGIR	R	I	28.90%	28.9	45.5	29.6	12.6					0	1	0	0	2	1,018.50	2,046.00		210.55	694	702	Empty	TRUE	MYH11_CHICK,Q789A4_CHICK,Q9PTY2_CHICK,UPI0000ECA50D,Q789A6_CHICK,UPI00000FC6BE,Q789A5_CHICK,UPI0000171382,UPI000044943F,UPI000060F510,UPI000060F511,UPI00003AB452,UPI0000171380,Q02015_CHICK,UPI0000171381,UPI00003AB434,UPI00000FB7E2,UPI00003AB454,UPI00003AB431,UPI00003AB453
20196-20225 HinckeM –TAXchicken	Uncategorized Sample	20196-20205_A	Mudpit_Ingel20196_MGFPeaklist (F058870)	Myosin-9 OS=Gallus gallus GN=MYH9 PE=4 SV=2	F1NCD4_CHICK,MYH9_CHICK,UPI000012FB7F,UPI000060E1E4,UPI0002C868F4	TAX_GallusGallus_9031_20141114.fasta,UPARC14_2_GallusG_9031_20140417.fasta	226,643.40	100.00%	49	53	115	0.23%	24.40%	DADKYLYVDK	R	N	47.90%	22.8	44.7	32.5	0					0	0	1	0	2	1,229.61	1,861.00		140	5	14	Empty	TRUE	
20196-20225 HinckeM –TAXchicken	Uncategorized Sample	20196-20205_A	Mudpit_Ingel20196_MGFPeaklist (F058870)	Myosin-9 OS=Gallus gallus GN=MYH9 PE=4 SV=2	F1NCD4_CHICK,MYH9_CHICK,UPI000012FB7F,UPI000060E1E4,UPI0002C868F4	TAX_GallusGallus_9031_20141114.fasta,UPARC14_2_GallusG_9031_20140417.fasta	226,643.40	100.00%	49	53	115	0.23%	24.40%	DLESLTQRYEEK	K	I	56.20%	18.3	44.4	21.2	11.9					0	0	3	0	2	1,510.74	2,123.00		788.9	1393	1404	Empty	TRUE	
20196-20225 HinckeM –TAXchicken	Uncategorized Sample	20196-20205_A	Mudpit_Ingel20196_MGFPeaklist (F058870)	Myosin-9 OS=Gallus gallus GN=MYH9 PE=4 SV=2	F1NCD4_CHICK,MYH9_CHICK,UPI000012FB7F,UPI000060E1E4,UPI0002C868F4	TAX_GallusGallus_9031_20141114.fasta,UPARC14_2_GallusG_9031_20140417.fasta	226,643.40	100.00%	49	53	115	0.23%	24.40%	EQADFAIEALAK	K	A	99.70%	57.8	45	27.9	43.9	37.5	3.8	0.331	0.375	0	4	0	0	2	1,305.67	2,461.50		1,501.85	408	419	Empty	TRUE	MYH11_CHICK
20196-20225 HinckeM –TAXchicken	Uncategorized Sample	20196-20205_A	Mudpit_Ingel20196_MGFPeaklist (F058870)	Myosin-9 OS=Gallus gallus GN=MYH9 PE=4 SV=2	F1NCD4_CHICK,MYH9_CHICK,UPI000012FB7F,UPI000060E1E4,UPI0002C868F4	TAX_GallusGallus_9031_20141114.fasta,UPARC14_2_GallusG_9031_20140417.fasta	226,643.40	100.00%	49	53	115	0.23%	24.40%	FDQLLAEEK	K	N	62.50%	25.1	44.8	29.4	6.88					0	2	0	0	2	1,092.56	2,017.50		508.72	1446	1454	Empty	TRUE	
20196-20225 HinckeM –TAXchicken	Uncategorized Sample	20196-20205_A	Mudpit_Ingel20196_MGFPeaklist (F058870)	Myosin-9 OS=Gallus gallus GN=MYH9 PE=4 SV=2	F1NCD4_CHICK,MYH9_CHICK,UPI000012FB7F,UPI000060E1E4,UPI0002C868F4	TAX_GallusGallus_9031_20141114.fasta,UPARC14_2_GallusG_9031_20140417.fasta	226,643.40	100.00%	49	53	115	0.23%	24.40%	HSQAIEELAEQLEQTKR	K	V	83.90%	21.7	43.9	18	16.7					0	0	0	2	2	2,010.03	2,465.50		456.01	1194	1210	Empty	TRUE	
20196-20225 HinckeM –TAXchicken	Uncategorized Sample	20196-20205_A	Mudpit_Ingel20196_MGFPeaklist (F058870)	Myosin-9 OS=Gallus gallus GN=MYH9 PE=4 SV=2	F1NCD4_CHICK,MYH9_CHICK,UPI000012FB7F,UPI000060E1E4,UPI0002C868F4	TAX_GallusGallus_9031_20141114.fasta,UPARC14_2_GallusG_9031_20140417.fasta	226,643.40	100.00%	49	53	115	0.23%	24.40%	IMGIPDEEQIGLLK	R	V	99.70%	33.4	44.2	14.4	29.2	36.2	1.96	0.318	0.357	0	2	0	0	2	1,571.84	2,519.50		2,423.65	328	341	Empty	TRUE	
20196-20225 HinckeM –TAXchicken	Uncategorized Sample	20196-20205_A	Mudpit_Ingel20196_MGFPeaklist (F058870)	Myosin-9 OS=Gallus gallus GN=MYH9 PE=4 SV=2	F1NCD4_CHICK,MYH9_CHICK,UPI000012FB7F,UPI000060E1E4,UPI0002C868F4	TAX_GallusGallus_9031_20141114.fasta,UPARC14_2_GallusG_9031_20140417.fasta	226,643.40	100.00%	49	53	115	0.23%	24.40%	IVQLEEQLDMETKER	K	Q	98.40%	37.5	44.6	22.6	23.5					0	0	2	0	2	1,876.93	2,189.50		318.2	1816	1830	Empty	TRUE	
20196-20225 HinckeM –TAXchicken	Uncategorized Sample	20196-20205_A	Mudpit_Ingel20196_MGFPeaklist (F058870)	Myosin-9 OS=Gallus gallus GN=MYH9 PE=4 SV=2	F1NCD4_CHICK,MYH9_CHICK,UPI000012FB7F,UPI000060E1E4,UPI0002C868F4	TAX_GallusGallus_9031_20141114.fasta,UPARC14_2_GallusG_9031_20140417.fasta	226,643.40	100.00%	49	53	115	0.23%	24.40%	KFDQLLAEEK	K	N	93.10%	39.1	44.6	40.3	9.11					0	0	4	0	2	1,220.65	2,002.50		2,060.83	1445	1454	Empty	TRUE	
20196-20225 HinckeM –TAXchicken	Uncategorized Sample	20196-20205_A	Mudpit_Ingel20196_MGFPeaklist (F058870)	Myosin-9 OS=Gallus gallus GN=MYH9 PE=4 SV=2	F1NCD4_CHICK,MYH9_CHICK,UPI000012FB7F,UPI000060E1E4,UPI0002C868F4	TAX_GallusGallus_9031_20141114.fasta,UPARC14_2_GallusG_9031_20140417.fasta	226,643.40	100.00%	49	53	115	0.23%	24.40%	KFDQLLAEEKNISAK	K	Y	99.70%	26	43.5	21.7	15.4	29.3	1.7	0.147	0.367	0	0	1	0	2	1,733.94	2,153.00		416.04	1445	1459	Empty	TRUE	
20196-20225 HinckeM –TAXchicken	Uncategorized Sample	20196-20205_A	Mudpit_Ingel20196_MGFPeaklist (F058870)	Myosin-9 OS=Gallus gallus GN=MYH9 PE=4 SV=2	F1NCD4_CHICK,MYH9_CHICK,UPI000012FB7F,UPI000060E1E4,UPI0002C868F4	TAX_GallusGallus_9031_20141114.fasta,UPARC14_2_GallusG_9031_20140417.fasta	226,643.40	100.00%	49	53	115	0.23%	24.40%	KKVDAQLQELQVK	R	F	67.80%	28.2	40.7	28.4	11.1					0	0	1	0	2	1,526.89	1,881.00		329.09	1248	1260	Empty	TRUE	
20196-20225 HinckeM –TAXchicken	Uncategorized Sample	20196-20205_A	Mudpit_Ingel20196_MGFPeaklist (F058870)	Myosin-9 OS=Gallus gallus GN=MYH9 PE=4 SV=2	F1NCD4_CHICK,MYH9_CHICK,UPI000012FB7F,UPI000060E1E4,UPI0002C868F4	TAX_GallusGallus_9031_20141114.fasta,UPARC14_2_GallusG_9031_20140417.fasta	226,643.40	100.00%	49	53	115	0.23%	24.40%	KLEEDVIVLEDQNLK	K	L	99.70%	41	43.3	24.6	26.1	36.6	4	0.363	0.367	0	0	2	0	2	1,784.96	2,380.50		1,109.02	975	989	Empty	TRUE	
20196-20225 HinckeM –TAXchicken	Uncategorized Sample	20196-20205_A	Mudpit_Ingel20196_MGFPeaklist (F058870)	Myosin-9 OS=Gallus gallus GN=MYH9 PE=4 SV=2	F1NCD4_CHICK,MYH9_CHICK,UPI000012FB7F,UPI000060E1E4,UPI0002C868F4	TAX_GallusGallus_9031_20141114.fasta,UPARC14_2_GallusG_9031_20140417.fasta	226,643.40	100.00%	49	53	115	0.23%	24.40%	KLVWVPSEK	K	S	29.70%	13.5	43.2	17.5	0					0	1	0	0	2	1,085.64	2,060.00		390.18	30	38	Empty	TRUE	MYH11_CHICK
20196-20225 HinckeM –TAXchicken	Uncategorized Sample	20196-20205_A	Mudpit_Ingel20196_MGFPeaklist (F058870)	Myosin-9 OS=Gallus gallus GN=MYH9 PE=4 SV=2	F1NCD4_CHICK,MYH9_CHICK,UPI000012FB7F,UPI000060E1E4,UPI0002C868F4	TAX_GallusGallus_9031_20141114.fasta,UPARC14_2_GallusG_9031_20140417.fasta	226,643.40	100.00%	49	53	115	0.23%	24.40%	KQELEEICHDLEAR	K	V	99.70%	48.3	44.3	23.2	40.8	35.2	3.44	0.347	0.357	0	0	2	1	2	1,769.85	2,175.00		1,534.37	910	923	Empty	TRUE	
20196-20225 HinckeM –TAXchicken	Uncategorized Sample	20196-20205_A	Mudpit_Ingel20196_MGFPeaklist (F058870)	Myosin-9 OS=Gallus gallus GN=MYH9 PE=4 SV=2	F1NCD4_CHICK,MYH9_CHICK,UPI000012FB7F,UPI000060E1E4,UPI0002C868F4	TAX_GallusGallus_9031_20141114.fasta,UPARC14_2_GallusG_9031_20140417.fasta	226,643.40	100.00%	49	53	115	0.23%	24.40%	KVDAQLQELQVK	K	F	99.70%	33.1	42.7	21.6	9.14	27.3	1.21	0.22	0.333	0	0	2	0	2	1,398.80	2,003.00		643.7	1249	1260	Empty	TRUE	
20196-20225 HinckeM –TAXchicken	Uncategorized Sample	20196-20205_A	Mudpit_Ingel20196_MGFPeaklist (F058870)	Myosin-9 OS=Gallus gallus GN=MYH9 PE=4 SV=2	F1NCD4_CHICK,MYH9_CHICK,UPI000012FB7F,UPI000060E1E4,UPI0002C868F4	TAX_GallusGallus_9031_20141114.fasta,UPARC14_2_GallusG_9031_20140417.fasta	226,643.40	100.00%	49	53	115	0.23%	24.40%	LDPHLVLDQLR	K	C	99.70%	34.6	43.8	18.6	22.1	32.5	2.47	0.366	0.455	0	0	1	0	2	1,318.75	2,534.00		1,121.62	683	693	Empty	TRUE	UPI00000FC6BE,Q789A5_CHICK,Q789A4_CHICK,UPI0000171382,UPI000060F510,UPI0000ECA50D,UPI000060F511,UPI00003AB452,Q789A6_CHICK,UPI0000171380,Q02015_CHICK,UPI0000171381,UPI00003AB454,UPI00003AB453

20196-20225 HinckeM –TAXchicken	Uncategorized Sample	20196-20205_A	Mudpit_Ingel20196_MGFPeaklist (F058870)	Myosin-9 OS=Gallus gallus GN=MYH9 PE=4 SV=2	F1NCD4_CHICK,MYH9_CHICK,UPI000012FB7F,UPI000060E1E4,UPI0002C868F4	TAX_GallusGallus_9031_20141114.fasta,UPARC14_2_GallusG_9031_20140417.fasta	226,643.40	100.00%	49	53	115	0.23%	24.40%	LKDILLQVDDERR	K	N	27.70%	15.6	42.7	21.5	4.84					0	0	0	1	2	1,612.90	2,214.00		458.07	1844	1856	Empty	TRUE	
20196-20225 HinckeM –TAXchicken	Uncategorized Sample	20196-20205_A	Mudpit_Ingel20196_MGFPeaklist (F058870)	Myosin-9 OS=Gallus gallus GN=MYH9 PE=4 SV=2	F1NCD4_CHICK,MYH9_CHICK,UPI000012FB7F,UPI000060E1E4,UPI0002C868F4	TAX_GallusGallus_9031_20141114.fasta,UPARC14_2_GallusG_9031_20140417.fasta	226,643.40	100.00%	49	53	115	0.23%	24.40%	LQQELDDIAVDLDHQR	R	Q	99.70%	68.1	44.4	19.5	56.3	44.1	5.77	0.515	0.438	0	0	2	0	2	1,907.95	2,474.00		1,640.23	1418	1433	Empty	TRUE	
20196-20225 HinckeM –TAXchicken	Uncategorized Sample	20196-20205_A	Mudpit_Ingel20196_MGFPeaklist (F058870)	Myosin-9 OS=Gallus gallus GN=MYH9 PE=4 SV=2	F1NCD4_CHICK,MYH9_CHICK,UPI000012FB7F,UPI000060E1E4,UPI0002C868F4	TAX_GallusGallus_9031_20141114.fasta,UPARC14_2_GallusG_9031_20140417.fasta	226,643.40	100.00%	49	53	115	0.23%	24.40%	LRLEVNQQAMK	K	A	73.00%	17.5	44.8	15.5	15.2					0	0	1	0	2	1,345.73	1,759.00		157.86	1556	1566	Empty	TRUE	
20196-20225 HinckeM –TAXchicken	Uncategorized Sample	20196-20205_A	Mudpit_Ingel20196_MGFPeaklist (F058870)	Myosin-9 OS=Gallus gallus GN=MYH9 PE=4 SV=2	F1NCD4_CHICK,MYH9_CHICK,UPI000012FB7F,UPI000060E1E4,UPI0002C868F4	TAX_GallusGallus_9031_20141114.fasta,UPARC14_2_GallusG_9031_20140417.fasta	226,643.40	100.00%	49	53	115	0.23%	24.40%	LVWVPSEK	K	S	99.70%	26.7	44.4	23.8	14.6	22.7	0.638	0.123	0.312	0	2	0	0	2	957.5404	2,164.50		372.91	31	38	Empty	TRUE	MYH11_CHICK
20196-20225 HinckeM –TAXchicken	Uncategorized Sample	20196-20205_A	Mudpit_Ingel20196_MGFPeaklist (F058870)	Myosin-9 OS=Gallus gallus GN=MYH9 PE=4 SV=2	F1NCD4_CHICK,MYH9_CHICK,UPI000012FB7F,UPI000060E1E4,UPI0002C868F4	TAX_GallusGallus_9031_20141114.fasta,UPARC14_2_GallusG_9031_20140417.fasta	226,643.40	100.00%	49	53	115	0.23%	24.40%	MSEFTTNLTEEEEKSK	R	S	99.70%	32.2	43	16.9	29.3	30.3	2.14	0.0066	0.25	0	0	2	0	2	1,902.86	2,035.00		827.04	1001	1016	Empty	TRUE	
20196-20225 HinckeM –TAXchicken	Uncategorized Sample	20196-20205_A	Mudpit_Ingel20196_MGFPeaklist (F058870)	Myosin-9 OS=Gallus gallus GN=MYH9 PE=4 SV=2	F1NCD4_CHICK,MYH9_CHICK,UPI000012FB7F,UPI000060E1E4,UPI0002C868F4	TAX_GallusGallus_9031_20141114.fasta,UPARC14_2_GallusG_9031_20140417.fasta	226,643.40	100.00%	49	53	115	0.23%	24.40%	NWQWWR	R	L	24.20%	12.7	45.1	22.7	3.78					0	1	0	0	2	975.4583	2,584.00		138.57	824	829	Empty	TRUE	MYH11_CHICK
20196-20225 HinckeM –TAXchicken	Uncategorized Sample	20196-20205_A	Mudpit_Ingel20196_MGFPeaklist (F058870)	Myosin-9 OS=Gallus gallus GN=MYH9 PE=4 SV=2	F1NCD4_CHICK,MYH9_CHICK,UPI000012FB7F,UPI000060E1E4,UPI0002C868F4	TAX_GallusGallus_9031_20141114.fasta,UPARC14_2_GallusG_9031_20140417.fasta	226,643.40	100.00%	49	53	115	0.23%	24.40%	QACVLMIK	K	A	17.50%	25.6	44	27.9	9.47					0	1	0	0	2	961.4847	2,343.00		270	738	745	Empty	TRUE	
20196-20225 HinckeM –TAXchicken	Uncategorized Sample	20196-20205_A	Mudpit_Ingel20196_MGFPeaklist (F058870)	Myosin-9 OS=Gallus gallus GN=MYH9 PE=4 SV=2	F1NCD4_CHICK,MYH9_CHICK,UPI000012FB7F,UPI000060E1E4,UPI0002C868F4	TAX_GallusGallus_9031_20141114.fasta,UPARC14_2_GallusG_9031_20140417.fasta	226,643.40	100.00%	49	53	115	0.23%	24.40%	QAQQERDELADEIANSSGK	R	G	99.70%	56.6	44.1	18.7	50.3	48	6.68	0.515	0.395	0	0	4	0	2	2,088.98	1,982.00		4,035.47	1698	1716	Empty	TRUE	
20196-20225 HinckeM –TAXchicken	Uncategorized Sample	20196-20205_A	Mudpit_Ingel20196_MGFPeaklist (F058870)	Myosin-9 OS=Gallus gallus GN=MYH9 PE=4 SV=2	F1NCD4_CHICK,MYH9_CHICK,UPI000012FB7F,UPI000060E1E4,UPI0002C868F4	TAX_GallusGallus_9031_20141114.fasta,UPARC14_2_GallusG_9031_20140417.fasta	226,643.40	100.00%	49	53	115	0.23%	24.40%	QISVLQQQAVEAR	K	K	99.70%	71.5	43.7	29.1	53.9	45.3	3.96	0.422	0.5	0	6	0	0	2	1,469.81	2,037.50		6,070.84	1358	1370	Empty	TRUE	
20196-20225 HinckeM –TAXchicken	Uncategorized Sample	20196-20205_A	Mudpit_Ingel20196_MGFPeaklist (F058870)	Myosin-9 OS=Gallus gallus GN=MYH9 PE=4 SV=2	F1NCD4_CHICK,MYH9_CHICK,UPI000012FB7F,UPI000060E1E4,UPI0002C868F4	TAX_GallusGallus_9031_20141114.fasta,UPARC14_2_GallusG_9031_20140417.fasta	226,643.40	100.00%	49	53	115	0.23%	24.40%	QKHSQAIEELAEQLEQTKR	R	V	99.70%	38.5	43.4	23.6	26	30.7	2.18	0.192	0.184	0	0	0	2	2	2,266.18	2,363.00		795.44	1192	1210	Empty	TRUE	
20196-20225 HinckeM –TAXchicken	Uncategorized Sample	20196-20205_A	Mudpit_Ingel20196_MGFPeaklist (F058870)	Myosin-9 OS=Gallus gallus GN=MYH9 PE=4 SV=2	F1NCD4_CHICK,MYH9_CHICK,UPI000012FB7F,UPI000060E1E4,UPI0002C868F4	TAX_GallusGallus_9031_20141114.fasta,UPARC14_2_GallusG_9031_20140417.fasta	226,643.40	100.00%	49	53	115	0.23%	24.40%	QLLQANPILEAFGNAK	R	T	33.80%	11.9	42.9	17.8	9.6					0	1	0	0	2	1,726.95	2,877.00		107.82	210	225	Empty	TRUE	MYH11_CHICK
20196-20225 HinckeM –TAXchicken	Uncategorized Sample	20196-20205_A	Mudpit_Ingel20196_MGFPeaklist (F058870)	Myosin-9 OS=Gallus gallus GN=MYH9 PE=4 SV=2	F1NCD4_CHICK,MYH9_CHICK,UPI000012FB7F,UPI000060E1E4,UPI0002C868F4	TAX_GallusGallus_9031_20141114.fasta,UPARC14_2_GallusG_9031_20140417.fasta	226,643.40	100.00%	49	53	115	0.23%	24.40%	QRYEILTPNAIPK	R	G	99.70%	44.8	43.5	22.5	30.7	33.5	1.96	0.358	0.423	0	2	4	0	2	1,542.86	2,240.50		4,800.05	719	731	Empty	TRUE	UPI00000FC6BE,Q789A5_CHICK,Q789A4_CHICK,UPI0000171382,UPI000060F510,UPI0000ECA50D,UPI000060F511,UPI00003AB452,Q789A6_CHICK,UPI0000171380,Q02015_CHICK,UPI0000171381,UPI00003AB454,UPI00003AB453
20196-20225 HinckeM –TAXchicken	Uncategorized Sample	20196-20205_A	Mudpit_Ingel20196_MGFPeaklist (F058870)	Myosin-9 OS=Gallus gallus GN=MYH9 PE=4 SV=2	F1NCD4_CHICK,MYH9_CHICK,UPI000012FB7F,UPI000060E1E4,UPI0002C868F4	TAX_GallusGallus_9031_20141114.fasta,UPARC14_2_GallusG_9031_20140417.fasta	226,643.40	100.00%	49	53	115	0.23%	24.40%	RGDLPFVVTR	R	R	99.70%	45.9	44.6	39	28	34.9	1.66	0.301	0.5	0	2	0	0	2	1,159.66	2,210.00		561.14	1923	1932	Empty	TRUE	
20196-20225 HinckeM –TAXchicken	Uncategorized Sample	20196-20205_A	Mudpit_Ingel20196_MGFPeaklist (F058870)	Myosin-9 OS=Gallus gallus GN=MYH9 PE=4 SV=2	F1NCD4_CHICK,MYH9_CHICK,UPI000012FB7F,UPI000060E1E4,UPI0002C868F4	TAX_GallusGallus_9031_20141114.fasta,UPARC14_2_GallusG_9031_20140417.fasta	226,643.40	100.00%	49	53	115	0.23%	24.40%	TDLLLEPYNK	K	Y	99.70%	34.2	44.8	29.3	12.7	27.4	1.57	0.234	0.35	0	2	0	0	2	1,205.64	2,293.50		465.92	290	299	Empty	TRUE	
20196-20225 HinckeM –TAXchicken	Uncategorized Sample	20196-20205_A	Mudpit_Ingel20196_MGFPeaklist (F058870)	Myosin-9 OS=Gallus gallus GN=MYH9 PE=4 SV=2	F1NCD4_CHICK,MYH9_CHICK,UPI000012FB7F,UPI000060E1E4,UPI0002C868F4	TAX_GallusGallus_9031_20141114.fasta,UPARC14_2_GallusG_9031_20140417.fasta	226,643.40	100.00%	49	53	115	0.23%	24.40%	TDLLLEPYNKYR	K	F	99.70%	24.5	44.3	23.3	10.2	26.9	2.44	0.208	0.333	0	0	2	0	2	1,524.81	2,290.00		819.24	290	301	Empty	TRUE	
20196-20225 HinckeM –TAXchicken	Uncategorized Sample	20196-20205_A	Mudpit_Ingel20196_MGFPeaklist (F058870)	Myosin-9 OS=Gallus gallus GN=MYH9 PE=4 SV=2	F1NCD4_CHICK,MYH9_CHICK,UPI000012FB7F,UPI000060E1E4,UPI0002C868F4	TAX_GallusGallus_9031_20141114.fasta,UPARC14_2_GallusG_9031_20140417.fasta	226,643.40	100.00%	49	53	115	0.23%	24.40%	TLEDEAKTHEAQIQEMR	K	Q	98.60%	43.9	44	22.9	33.3					0	0	0	2	2	2,044.96	1,846.50		641.82	1175	1191	Empty	TRUE	
20196-20225 HinckeM –TAXchicken	Uncategorized Sample	20196-20205_A	Mudpit_Ingel20196_MGFPeaklist (F058870)	Myosin-9 OS=Gallus gallus GN=MYH9 PE=4 SV=2	F1NCD4_CHICK,MYH9_CHICK,UPI000012FB7F,UPI000060E1E4,UPI0002C868F4	TAX_GallusGallus_9031_20141114.fasta,UPARC14_2_GallusG_9031_20140417.fasta	226,643.40	100.00%	49	53	115	0.23%	24.40%	TSREEILAQAK	R	E	92.20%	37	44.7	34.3	9.74					0	0	3	0	2	1,245.68	1,668.00		1,425.65	1659	1669	Empty	TRUE	
20196-20225 HinckeM –TAXchicken	Uncategorized Sample	20196-20205_A	Mudpit_Ingel20196_MGFPeaklist (F058870)	Myosin-9 OS=Gallus gallus GN=MYH9 PE=4 SV=2	F1NCD4_CHICK,MYH9_CHICK,UPI000012FB7F,UPI000060E1E4,UPI0002C868F4	TAX_GallusGallus_9031_20141114.fasta,UPARC14_2_GallusG_9031_20140417.fasta	226,643.40	100.00%	49	53	115	0.23%	24.40%	TSREEILAQAKENEK	R	K	99.70%	49.7	44.6	29.6	30.6	42.7	3.42	0.335	0.5	0	0	0	2	2	1,745.90	1,637.00		624	1659	1673	Empty	TRUE	
20196-20225 HinckeM –TAXchicken	Uncategorized Sample	20196-20205_A	Mudpit_Ingel20196_MGFPeaklist (F058870)	Myosin-9 OS=Gallus gallus GN=MYH9 PE=4 SV=2	F1NCD4_CHICK,MYH9_CHICK,UPI000012FB7F,UPI000060E1E4,UPI0002C868F4	TAX_GallusGallus_9031_20141114.fasta,UPARC14_2_GallusG_9031_20140417.fasta	226,643.40	100.00%	49	53	115	0.23%	24.40%	TVGQLYKEQLAK	R	L	99.70%	37	43.6	25.2	25.9	29.3	1.14	0.266	0.417	0	0	4	0	2	1,377.77	1,866.50		2,477.43	645	656	Empty	TRUE	
20196-20225 HinckeM –TAXchicken	Uncategorized Sample	20196-20205_A	Mudpit_Ingel20196_MGFPeaklist (F058870)	Myosin-9 OS=Gallus gallus GN=MYH9 PE=4 SV=2	F1NCD4_CHICK,MYH9_CHICK,UPI000012FB7F,UPI000060E1E4,UPI0002C868F4	TAX_GallusGallus_9031_20141114.fasta,UPARC14_2_GallusG_9031_20140417.fasta	226,643.40	100.00%	49	53	115	0.23%	24.40%	VDAQLQELQVK	K	F	50.50%	23.2	43.9	38.2	1.53					0	1	0	0	2	1,270.70	2,098.00		128.78	1250	1260	Empty	TRUE	
20196-20225 HinckeM –TAXchicken	Uncategorized Sample	20196-20205_A	Mudpit_Ingel20196_MGFPeaklist (F058870)	Myosin-9 OS=Gallus gallus GN=MYH9 PE=4 SV=2	F1NCD4_CHICK,MYH9_CHICK,UPI000012FB7F,UPI000060E1E4,UPI0002C868F4	TAX_GallusGallus_9031_20141114.fasta,UPARC14_2_GallusG_9031_20140417.fasta	226,643.40	100.00%	49	53	115	0.23%	24.40%	VISGVLQLGNIVFKK	K	E	99.70%	64.9	36.9	20.4	56.6	45.8	3.11	0.162	0.5	0	0	2	0	2	1,614.99	2,854.50		1,206.55	342	356	Empty	TRUE	
20196-20225 HinckeM –TAXchicken	Uncategorized Sample	20196-20205_A	Mudpit_Ingel20196_MGFPeaklist (F058870)	Myosin-9 OS=Gallus gallus GN=MYH9 PE=4 SV=2	F1NCD4_CHICK,MYH9_CHICK,UPI000012FB7F,UPI000060E1E4,UPI0002C868F4	TAX_GallusGallus_9031_20141114.fasta,UPARC14_2_GallusG_9031_20140417.fasta	226,643.40	100.00%	49	53	115	0.23%	24.40%	VSHLLGINVTDFTR	K	G	99.70%	50.9	43.8	29.7	35.6	39.2	4.7	0.487	0.429	0	0	3	0	2	1,571.85	2,599.00		1,284.73	374	387	Empty	TRUE	
20196-20225 HinckeM –TAXchicken	Uncategorized Sample	20196-20205_A	Mudpit_Ingel20196_MGFPeaklist (F058870)	Myosin-9 OS=Gallus gallus GN=MYH9 PE=4 SV=2	F1NCD4_CHICK,MYH9_CHICK,UPI000012FB7F,UPI000060E1E4,UPI0002C868F4	TAX_GallusGallus_9031_20141114.fasta,UPARC14_2_GallusG_9031_20140417.fasta	226,643.40	100.00%	49	53	115	0.23%	24.40%	VVFQEFR	R	Q	74.20%	30.9	43.3	26.1	16.6					0	4	0	0	2	924.4937	2,156.00		1,480.25	712	718	Empty	TRUE	
20196-20225 HinckeM –TAXchicken	Uncategorized Sample	20196-20205_A	Mudpit_Ingel20196_MGFPeaklist (F058870)	Myosin-9 OS=Gallus gallus GN=MYH9 PE=4 SV=2	F1NCD4_CHICK,MYH9_CHICK,UPI000012FB7F,UPI000060E1E4,UPI0002C868F4	TAX_GallusGallus_9031_20141114.fasta,UPARC14_2_GallusG_9031_20140417.fasta	226,643.40	100.00%	49	53	115	0.23%	24.40%	YEILTPNAIPK	R	G	99.70%	20.6	44.4	14.6	7.9	28.5	1.57	0.277	0.318	0	3	0	0	2	1,258.70	2,337.00		1,618.82	721	731	Empty	TRUE	UPI00000FC6BE,Q789A5_CHICK,Q789A4_CHICK,UPI0000171382,UPI000060F510,UPI0000ECA50D,UPI000060F511,UPI00003AB452,Q789A6_CHICK,UPI0000171380,Q02015_CHICK,UPI0000171381,UPI00003AB454,UPI00003AB453
20196-20225 HinckeM –TAXchicken	Uncategorized Sample	20196-20205_A	Mudpit_Ingel20196_MGFPeaklist (F058870)	Myosin-9 OS=Gallus gallus GN=MYH9 PE=4 SV=2	F1NCD4_CHICK,MYH9_CHICK,UPI000012FB7F,UPI000060E1E4,UPI0002C868F4	TAX_GallusGallus_9031_20141114.fasta,UPARC14_2_GallusG_9031_20140417.fasta	226,643.40	100.00%	49	53	115	0.23%	24.40%	ADFCIIHYAGK	K	V	45.90%	18.6	44.4	14.7	11.1					0	0	2	0	2	1,294.63	2,223.00		989.84	566	576	Empty	TRUE	UPI00000FC6BE,Q789A5_CHICK,Q789A4_CHICK,UPI0000171382,UPI000060F510,UPI0000ECA50D,UPI000060F511,UPI00003AB452,Q789A6_CHICK,UPI0000171380,Q02015_CHICK,UPI0000171381,UPI00003AB454,UPI00003AB453
20196-20225 HinckeM –TAXchicken	Uncategorized Sample	20196-20205_A	Mudpit_Ingel20196_MGFPeaklist (F058870)	Myosin-9 OS=Gallus gallus GN=MYH9 PE=4 SV=2	F1NCD4_CHICK,MYH9_CHICK,UPI000012FB7F,UPI000060E1E4,UPI0002C868F4	TAX_GallusGallus_9031_20141114.fasta,UPARC14_2_GallusG_9031_20140417.fasta	226,643.40	100.00%	49	53	115	0.23%	24.40%	AGKLDPHLVLDQLR	K	C	99.70%	52.2	42.3	31.5	35.1	37.5	3.54	0.344	0.5	0	0	2	0	2	1,574.90	2,445.50		970.79	680	693	Empty	TRUE	UPI00000FC6BE,Q789A5_CHICK,Q789A4_CHICK,UPI0000171382,UPI000060F510,UPI0000ECA50D,UPI000060F511,UPI00003AB452,Q789A6_CHICK,UPI0000171380,Q02015_CHICK,UPI0000171381,UPI00003AB454,UPI00003AB453
20196-20225 HinckeM –TAXchicken	Uncategorized Sample	20196-20205_A	Mudpit_Ingel20196_MGFPeaklist (F058870)	Myosin-9 OS=Gallus gallus GN=MYH9 PE=4 SV=2	F1NCD4_CHICK,MYH9_CHICK,UPI000012FB7F,UPI000060E1E4,UPI0002C868F4	TAX_GallusGallus_9031_20141114.fasta,UPARC14_2_GallusG_9031_20140417.fasta	226,643.40	100.00%	49	53	115	0.23%	24.40%	AGVLAHLEEER	R	D	99.70%	71.3	44.3	36.2	36.2	34.7	3.4	0.317	0.409	0	0	2	0	2	1,223.64	2,031.50		1,163.70	765	775	Empty	TRUE	UPI00000FC6BE,Q789A5_CHICK,Q789A4_CHICK,UPI0000171382,UPI000060F510,UPI0000ECA50D,UPI000060F511,UPI00003AB452,Q789A6_CHICK,UPI0000171380,Q02015_CHICK,UPI0000171381,UPI00003AB454,UPI00003AB453
20196-20225 HinckeM –TAXchicken	Uncategorized Sample	20196-20205_A	Mudpit_Ingel20196_MGFPeaklist (F058870)	Myosin-9 OS=Gallus gallus GN=MYH9 PE=4 SV=2	F1NCD4_CHICK,MYH9_CHICK,UPI000012FB7F,UPI000060E1E4,UPI0002C868F4	TAX_GallusGallus_9031_20141114.fasta,UPARC14_2_GallusG_9031_20140417.fasta	226,643.40	100.00%	49	53	115	0.23%	24.40%	AGVLAHLEEERDLK	R	I	99.70%	43.7	43.9	28.2	36.5	38.3	3.7	0.371	0.464	0	0	2	0	2	1,579.84	2,107.00		1,024.73	765	778	Empty	TRUE	UPI00000FC6BE,Q789A5_CHICK,Q789A4_CHICK,UPI0000171382,UPI000060F510,UPI0000ECA50D,UPI000060F511,UPI00003AB452,Q789A6_CHICK,UPI0000171380,Q02015_CHICK,UPI0000171381,UPI00003AB454,UPI00003AB453
20196-20225 HinckeM –TAXchicken	Uncategorized Sample	20196-20205_A	Mudpit_Ingel20196_MGFPeaklist (F058870)	Myosin-9 OS=Gallus gallus GN=MYH9 PE=4 SV=2	F1NCD4_CHICK,MYH9_CHICK,UPI000012FB7F,UPI000060E1E4,UPI0002C868F4	TAX_GallusGallus_9031_20141114.fasta,UPARC14_2_GallusG_9031_20140417.fasta	226,643.40	100.00%	49	53	115	0.23%	24.40%	ALEEAIEQK	R	A	84.60%	33.1	45.3	36.6	8.49					0	3	0	0	2	1,030.54	1,744.00		686.34	1484	1492	Empty	TRUE	
20196-20225 HinckeM –TAXchicken	Uncategorized Sample	20196-20205_A	Mudpit_Ingel20196_MGFPeaklist (F058870)	Myosin-9 OS=Gallus gallus GN=MYH9 PE=4 SV=2	F1NCD4_CHICK,MYH9_CHICK,UPI000012FB7F,UPI000060E1E4,UPI0002C868F4	TAX_GallusGallus_9031_20141114.fasta,UPARC14_2_GallusG_9031_20140417.fasta	226,643.40	100.00%	49	53	115	0.23%	24.40%	ALEEAIEQKAELER	R	V	99.70%	72.1	44.6	34.9	46.5	50.4	4.02	0.423	0.607	0	1	4	0	2	1,628.85	2,104.00		4,055.28	1484	1497	Empty	TRUE	
20196-20225 HinckeM –TAXchicken	Uncategorized Sample	20196-20205_A	Mudpit_Ingel20196_MGFPeaklist (F058870)	Myosin-9 OS=Gallus gallus GN=MYH9 PE=4 SV=2	F1NCD4_CHICK,MYH9_CHICK,UPI000012FB7F,UPI000060E1E4,UPI0002C868F4	TAX_GallusGallus_9031_20141114.fasta,UPARC14_2_GallusG_9031_20140417.fasta	226,643.40	100.00%	49	53	115	0.23%	24.40%	ALELDSNLYR	K	I	99.70%	58.5	44.7	27.2	33.6	40.4	3.82	0.351	0.55	0	5	0	0	2	1,193.62	2,244.00		3,596.17	746	755	Empty	TRUE	
20196-20225 HinckeM –TAXchicken	Uncategorized Sample	20196-20205_A	Mudpit_Ingel20196_MGFPeaklist (F058870)	Myosin-9 OS=Gallus gallus GN=MYH9 PE=4 SV=2	F1NCD4_CHICK,MYH9_CHICK,UPI000012FB7F,UPI000060E1E4,UPI0002C868F4	TAX_GallusGallus_9031_20141114.fasta,UPARC14_2_GallusG_9031_20140417.fasta	226,643.40	100.00%	49	53	115	0.23%	24.40%	ANLQIDQMNADLNAER	K	S	99.70%	61.6	44.3	23.7	51.8	27.8	1.57	0.237	0.219	0	0	1	0	2	1,831.86	1,969.00		215.45	1755	1770	Empty	TRUE	
20196-20225 HinckeM –TAXchicken	Uncategorized Sample	20196-20205_A	Mudpit_Ingel20196_MGFPeaklist (F058870)	Myosin-9 OS=Gallus gallus GN=MYH9 PE=4 SV=2	F1NCD4_CHICK,MYH9_CHICK,UPI000012FB7F,UPI000060E1E4,UPI0002C868F4	TAX_GallusGallus_9031_20141114.fasta,UPARC14_2_GallusG_9031_20140417.fasta	226,643.40	100.00%	49	53	115	0.23%	24.40%	ATITALEAK	K	I	14.20%	16.5	45.4	22.1	0.69					0	2	0	0	2	917.5303	1,880.00		993.4	1807	1815	Empty	TRUE	
20196-20225 HinckeM –TAXchicken	Uncategorized Sample	20196-20205_A	Mudpit_Ingel20196_MGFPeaklist (F058870)	Myosin-9 OS=Gallus gallus GN=MYH9 PE=4 SV=2	F1NCD4_CHICK,MYH9_CHICK,UPI000012FB7F,UPI000060E1E4,UPI0002C868F4	TAX_GallusGallus_9031_20141114.fasta,UPARC14_2_GallusG_9031_20140417.fasta	226,643.40	100.00%	49	53	115	0.23%	24.40%	CNGVLEGIR	R	I	28.90%	28.9	45.5	29.6	12.6					0	1	0	0	2	1,018.50	2,046.00		210.55	694	702	Empty	TRUE	MYH11_CHICK,Q789A4_CHICK,Q9PTY2_CHICK,UPI0000ECA50D,Q789A6_CHICK,UPI00000FC6BE,Q789A5_CHICK,UPI0000171382,UPI000044943F,UPI000060F510,UPI000060F511,UPI00003AB452,UPI0000171380,Q02015_CHICK,UPI0000171381,UPI00003AB434,UPI00000FB7E2,UPI00003AB454,UPI00003AB431,UPI00003AB453
20196-20225 HinckeM –TAXchicken	Uncategorized Sample	20196-20205_A	Mudpit_Ingel20196_MGFPeaklist (F058870)	Myosin-9 OS=Gallus gallus GN=MYH9 PE=4 SV=2	F1NCD4_CHICK,MYH9_CHICK,UPI000012FB7F,UPI000060E1E4,UPI0002C868F4	TAX_GallusGallus_9031_20141114.fasta,UPARC14_2_GallusG_9031_20140417.fasta	226,643.40	100.00%	49	53	115	0.23%	24.40%	DADKYLYVDK	R	N	47.90%	22.8	44.7	32.5	0					0	0	1	0	2	1,229.61	1,861.00		140	5	14	Empty	TRUE	
20196-20225 HinckeM –TAXchicken	Uncategorized Sample	20196-20205_A	Mudpit_Ingel20196_MGFPeaklist (F058870)	Myosin-9 OS=Gallus gallus GN=MYH9 PE=4 SV=2	F1NCD4_CHICK,MYH9_CHICK,UPI000012FB7F,UPI000060E1E4,UPI0002C868F4	TAX_GallusGallus_9031_20141114.fasta,UPARC14_2_GallusG_9031_20140417.fasta	226,643.40	100.00%	49	53	115	0.23%	24.40%	DLESLTQRYEEK	K	I	56.20%	18.3	44.4	21.2	11.9					0	0	3	0	2	1,510.74	2,123.00		788.9	1393	1404	Empty	TRUE	
20196-20225 HinckeM –TAXchicken	Uncategorized Sample	20196-20205_A	Mudpit_Ingel20196_MGFPeaklist (F058870)	Myosin-9 OS=Gallus gallus GN=MYH9 PE=4 SV=2	F1NCD4_CHICK,MYH9_CHICK,UPI000012FB7F,UPI000060E1E4,UPI0002C868F4	TAX_GallusGallus_9031_20141114.fasta,UPARC14_2_GallusG_9031_20140417.fasta	226,643.40	100.00%	49	53	115	0.23%	24.40%	EQADFAIEALAK	K	A	99.70%	57.8	45	27.9	43.9	37.5	3.8	0.331	0.375	0	4	0	0	2	1,305.67	2,461.50		1,501.85	408	419	Empty	TRUE	MYH11_CHICK
20196-20225 HinckeM –TAXchicken	Uncategorized Sample	20196-20205_A	Mudpit_Ingel20196_MGFPeaklist (F058870)	Myosin-9 OS=Gallus gallus GN=MYH9 PE=4 SV=2	F1NCD4_CHICK,MYH9_CHICK,UPI000012FB7F,UPI000060E1E4,UPI0002C868F4	TAX_GallusGallus_9031_20141114.fasta,UPARC14_2_GallusG_9031_20140417.fasta	226,643.40	100.00%	49	53	115	0.23%	24.40%	FDQLLAEEK	K	N	62.50%	25.1	44.8	29.4	6.88					0	2	0	0	2	1,092.56	2,017.50		508.72	1446	1454	Empty	TRUE	
20196-20225 HinckeM –TAXchicken	Uncategorized Sample	20196-20205_A	Mudpit_Ingel20196_MGFPeaklist (F058870)	Myosin-9 OS=Gallus gallus GN=MYH9 PE=4 SV=2	F1NCD4_CHICK,MYH9_CHICK,UPI000012FB7F,UPI000060E1E4,UPI0002C868F4	TAX_GallusGallus_9031_20141114.fasta,UPARC14_2_GallusG_9031_20140417.fasta	226,643.40	100.00%	49	53	115	0.23%	24.40%	HSQAIEELAEQLEQTKR	K	V	83.90%	21.7	43.9	18	16.7					0	0	0	2	2	2,010.03	2,465.50		456.01	1194	1210	Empty	TRUE	
20196-20225 HinckeM –TAXchicken	Uncategorized Sample	20196-20205_A	Mudpit_Ingel20196_MGFPeaklist (F058870)	Myosin-9 OS=Gallus gallus GN=MYH9 PE=4 SV=2	F1NCD4_CHICK,MYH9_CHICK,UPI000012FB7F,UPI000060E1E4,UPI0002C868F4	TAX_GallusGallus_9031_20141114.fasta,UPARC14_2_GallusG_9031_20140417.fasta	226,643.40	100.00%	49	53	115	0.23%	24.40%	IMGIPDEEQIGLLK	R	V	99.70%	33.4	44.2	14.4	29.2	36.2	1.96	0.318	0.357	0	2	0	0	2	1,571.84	2,519.50		2,423.65	328	341	Empty	TRUE	
20196-20225 HinckeM –TAXchicken	Uncategorized Sample	20196-20205_A	Mudpit_Ingel20196_MGFPeaklist (F058870)	Myosin-9 OS=Gallus gallus GN=MYH9 PE=4 SV=2	F1NCD4_CHICK,MYH9_CHICK,UPI000012FB7F,UPI000060E1E4,UPI0002C868F4	TAX_GallusGallus_9031_20141114.fasta,UPARC14_2_GallusG_9031_20140417.fasta	226,643.40	100.00%	49	53	115	0.23%	24.40%	IVQLEEQLDMETKER	K	Q	98.40%	37.5	44.6	22.6	23.5					0	0	2	0	2	1,876.93	2,189.50		318.2	1816	1830	Empty	TRUE	
20196-20225 HinckeM –TAXchicken	Uncategorized Sample	20196-20205_A	Mudpit_Ingel20196_MGFPeaklist (F058870)	Myosin-9 OS=Gallus gallus GN=MYH9 PE=4 SV=2	F1NCD4_CHICK,MYH9_CHICK,UPI000012FB7F,UPI000060E1E4,UPI0002C868F4	TAX_GallusGallus_9031_20141114.fasta,UPARC14_2_GallusG_9031_20140417.fasta	226,643.40	100.00%	49	53	115	0.23%	24.40%	KFDQLLAEEK	K	N	93.10%	39.1	44.6	40.3	9.11					0	0	4	0	2	1,220.65	2,002.50		2,060.83	1445	1454	Empty	TRUE	
20196-20225 HinckeM –TAXchicken	Uncategorized Sample	20196-20205_A	Mudpit_Ingel20196_MGFPeaklist (F058870)	Myosin-9 OS=Gallus gallus GN=MYH9 PE=4 SV=2	F1NCD4_CHICK,MYH9_CHICK,UPI000012FB7F,UPI000060E1E4,UPI0002C868F4	TAX_GallusGallus_9031_20141114.fasta,UPARC14_2_GallusG_9031_20140417.fasta	226,643.40	100.00%	49	53	115	0.23%	24.40%	KFDQLLAEEKNISAK	K	Y	99.70%	26	43.5	21.7	15.4	29.3	1.7	0.147	0.367	0	0	1	0	2	1,733.94	2,153.00		416.04	1445	1459	Empty	TRUE	
20196-20225 HinckeM –TAXchicken	Uncategorized Sample	20196-20205_A	Mudpit_Ingel20196_MGFPeaklist (F058870)	Myosin-9 OS=Gallus gallus GN=MYH9 PE=4 SV=2	F1NCD4_CHICK,MYH9_CHICK,UPI000012FB7F,UPI000060E1E4,UPI0002C868F4	TAX_GallusGallus_9031_20141114.fasta,UPARC14_2_GallusG_9031_20140417.fasta	226,643.40	100.00%	49	53	115	0.23%	24.40%	KKVDAQLQELQVK	R	F	67.80%	28.2	40.7	28.4	11.1					0	0	1	0	2	1,526.89	1,881.00		329.09	1248	1260	Empty	TRUE	
20196-20225 HinckeM –TAXchicken	Uncategorized Sample	20196-20205_A	Mudpit_Ingel20196_MGFPeaklist (F058870)	Myosin-9 OS=Gallus gallus GN=MYH9 PE=4 SV=2	F1NCD4_CHICK,MYH9_CHICK,UPI000012FB7F,UPI000060E1E4,UPI0002C868F4	TAX_GallusGallus_9031_20141114.fasta,UPARC14_2_GallusG_9031_20140417.fasta	226,643.40	100.00%	49	53	115	0.23%	24.40%	KLEEDVIVLEDQNLK	K	L	99.70%	41	43.3	24.6	26.1	36.6	4	0.363	0.367	0	0	2	0	2	1,784.96	2,380.50		1,109.02	975	989	Empty	TRUE	
20196-20225 HinckeM –TAXchicken	Uncategorized Sample	20196-20205_A	Mudpit_Ingel20196_MGFPeaklist (F058870)	Myosin-9 OS=Gallus gallus GN=MYH9 PE=4 SV=2	F1NCD4_CHICK,MYH9_CHICK,UPI000012FB7F,UPI000060E1E4,UPI0002C868F4	TAX_GallusGallus_9031_20141114.fasta,UPARC14_2_GallusG_9031_20140417.fasta	226,643.40	100.00%	49	53	115	0.23%	24.40%	KLVWVPSEK	K	S	29.70%	13.5	43.2	17.5	0					0	1	0	0	2	1,085.64	2,060.00		390.18	30	38	Empty	TRUE	MYH11_CHICK
20196-20225 HinckeM –TAXchicken	Uncategorized Sample	20196-20205_A	Mudpit_Ingel20196_MGFPeaklist (F058870)	Myosin-9 OS=Gallus gallus GN=MYH9 PE=4 SV=2	F1NCD4_CHICK,MYH9_CHICK,UPI000012FB7F,UPI000060E1E4,UPI0002C868F4	TAX_GallusGallus_9031_20141114.fasta,UPARC14_2_GallusG_9031_20140417.fasta	226,643.40	100.00%	49	53	115	0.23%	24.40%	KQELEEICHDLEAR	K	V	99.70%	48.3	44.3	23.2	40.8	35.2	3.44	0.347	0.357	0	0	2	1	2	1,769.85	2,175.00		1,534.37	910	923	Empty	TRUE	
20196-20225 HinckeM –TAXchicken	Uncategorized Sample	20196-20205_A	Mudpit_Ingel20196_MGFPeaklist (F058870)	Myosin-9 OS=Gallus gallus GN=MYH9 PE=4 SV=2	F1NCD4_CHICK,MYH9_CHICK,UPI000012FB7F,UPI000060E1E4,UPI0002C868F4	TAX_GallusGallus_9031_20141114.fasta,UPARC14_2_GallusG_9031_20140417.fasta	226,643.40	100.00%	49	53	115	0.23%	24.40%	KVDAQLQELQVK	K	F	99.70%	33.1	42.7	21.6	9.14	27.3	1.21	0.22	0.333	0	0	2	0	2	1,398.80	2,003.00		643.7	1249	1260	Empty	TRUE	
20196-20225 HinckeM –TAXchicken	Uncategorized Sample	20196-20205_A	Mudpit_Ingel20196_MGFPeaklist (F058870)	Myosin-9 OS=Gallus gallus GN=MYH9 PE=4 SV=2	F1NCD4_CHICK,MYH9_CHICK,UPI000012FB7F,UPI000060E1E4,UPI0002C868F4	TAX_GallusGallus_9031_20141114.fasta,UPARC14_2_GallusG_9031_20140417.fasta	226,643.40	100.00%	49	53	115	0.23%	24.40%	LDPHLVLDQLR	K	C	99.70%	34.6	43.8	18.6	22.1	32.5	2.47	0.366	0.455	0	0	1	0	2	1,318.75	2,534.00		1,121.62	683	693	Empty	TRUE	UPI00000FC6BE,Q789A5_CHICK,Q789A4_CHICK,UPI0000171382,UPI000060F510,UPI0000ECA50D,UPI000060F511,UPI00003AB452,Q789A6_CHICK,UPI0000171380,Q02015_CHICK,UPI0000171381,UPI00003AB454,UPI00003AB453
20196-20225 HinckeM –TAXchicken	Uncategorized Sample	20196-20205_A	Mudpit_Ingel20196_MGFPeaklist (F058870)	Myosin-9 OS=Gallus gallus GN=MYH9 PE=4 SV=2	F1NCD4_CHICK,MYH9_CHICK,UPI000012FB7F,UPI000060E1E4,UPI0002C868F4	TAX_GallusGallus_9031_20141114.fasta,UPARC14_2_GallusG_9031_20140417.fasta	226,643.40	100.00%	49	53	115	0.23%	24.40%	LKDILLQVDDERR	K	N	27.70%	15.6	42.7	21.5	4.84					0	0	0	1	2	1,612.90	2,214.00		458.07	1844	1856	Empty	TRUE	
20196-20225 HinckeM –TAXchicken	Uncategorized Sample	20196-20205_A	Mudpit_Ingel20196_MGFPeaklist (F058870)	Myosin-9 OS=Gallus gallus GN=MYH9 PE=4 SV=2	F1NCD4_CHICK,MYH9_CHICK,UPI000012FB7F,UPI000060E1E4,UPI0002C868F4	TAX_GallusGallus_9031_20141114.fasta,UPARC14_2_GallusG_9031_20140417.fasta	226,643.40	100.00%	49	53	115	0.23%	24.40%	LQQELDDIAVDLDHQR	R	Q	99.70%	68.1	44.4	19.5	56.3	44.1	5.77	0.515	0.438	0	0	2	0	2	1,907.95	2,474.00		1,640.23	1418	1433	Empty	TRUE	
20196-20225 HinckeM –TAXchicken	Uncategorized Sample	20196-20205_A	Mudpit_Ingel20196_MGFPeaklist (F058870)	Myosin-9 OS=Gallus gallus GN=MYH9 PE=4 SV=2	F1NCD4_CHICK,MYH9_CHICK,UPI000012FB7F,UPI000060E1E4,UPI0002C868F4	TAX_GallusGallus_9031_20141114.fasta,UPARC14_2_GallusG_9031_20140417.fasta	226,643.40	100.00%	49	53	115	0.23%	24.40%	LRLEVNQQAMK	K	A	73.00%	17.5	44.8	15.5	15.2					0	0	1	0	2	1,345.73	1,759.00		157.86	1556	1566	Empty	TRUE	
20196-20225 HinckeM –TAXchicken	Uncategorized Sample	20196-20205_A	Mudpit_Ingel20196_MGFPeaklist (F058870)	Myosin-9 OS=Gallus gallus GN=MYH9 PE=4 SV=2	F1NCD4_CHICK,MYH9_CHICK,UPI000012FB7F,UPI000060E1E4,UPI0002C868F4	TAX_GallusGallus_9031_20141114.fasta,UPARC14_2_GallusG_9031_20140417.fasta	226,643.40	100.00%	49	53	115	0.23%	24.40%	LVWVPSEK	K	S	99.70%	26.7	44.4	23.8	14.6	22.7	0.638	0.123	0.312	0	2	0	0	2	957.5404	2,164.50		372.91	31	38	Empty	TRUE	MYH11_CHICK
20196-20225 HinckeM –TAXchicken	Uncategorized Sample	20196-20205_A	Mudpit_Ingel20196_MGFPeaklist (F058870)	Myosin-9 OS=Gallus gallus GN=MYH9 PE=4 SV=2	F1NCD4_CHICK,MYH9_CHICK,UPI000012FB7F,UPI000060E1E4,UPI0002C868F4	TAX_GallusGallus_9031_20141114.fasta,UPARC14_2_GallusG_9031_20140417.fasta	226,643.40	100.00%	49	53	115	0.23%	24.40%	MSEFTTNLTEEEEKSK	R	S	99.70%	32.2	43	16.9	29.3	30.3	2.14	0.0066	0.25	0	0	2	0	2	1,902.86	2,035.00		827.04	1001	1016	Empty	TRUE	
20196-20225 HinckeM –TAXchicken	Uncategorized Sample	20196-20205_A	Mudpit_Ingel20196_MGFPeaklist (F058870)	Myosin-9 OS=Gallus gallus GN=MYH9 PE=4 SV=2	F1NCD4_CHICK,MYH9_CHICK,UPI000012FB7F,UPI000060E1E4,UPI0002C868F4	TAX_GallusGallus_9031_20141114.fasta,UPARC14_2_GallusG_9031_20140417.fasta	226,643.40	100.00%	49	53	115	0.23%	24.40%	NWQWWR	R	L	24.20%	12.7	45.1	22.7	3.78					0	1	0	0	2	975.4583	2,584.00		138.57	824	829	Empty	TRUE	MYH11_CHICK
20196-20225 HinckeM –TAXchicken	Uncategorized Sample	20196-20205_A	Mudpit_Ingel20196_MGFPeaklist (F058870)	Myosin-9 OS=Gallus gallus GN=MYH9 PE=4 SV=2	F1NCD4_CHICK,MYH9_CHICK,UPI000012FB7F,UPI000060E1E4,UPI0002C868F4	TAX_GallusGallus_9031_20141114.fasta,UPARC14_2_GallusG_9031_20140417.fasta	226,643.40	100.00%	49	53	115	0.23%	24.40%	QACVLMIK	K	A	17.50%	25.6	44	27.9	9.47					0	1	0	0	2	961.4847	2,343.00		270	738	745	Empty	TRUE	
20196-20225 HinckeM –TAXchicken	Uncategorized Sample	20196-20205_A	Mudpit_Ingel20196_MGFPeaklist (F058870)	Myosin-9 OS=Gallus gallus GN=MYH9 PE=4 SV=2	F1NCD4_CHICK,MYH9_CHICK,UPI000012FB7F,UPI000060E1E4,UPI0002C868F4	TAX_GallusGallus_9031_20141114.fasta,UPARC14_2_GallusG_9031_20140417.fasta	226,643.40	100.00%	49	53	115	0.23%	24.40%	QAQQERDELADEIANSSGK	R	G	99.70%	56.6	44.1	18.7	50.3	48	6.68	0.515	0.395	0	0	4	0	2	2,088.98	1,982.00		4,035.47	1698	1716	Empty	TRUE	
20196-20225 HinckeM –TAXchicken	Uncategorized Sample	20196-20205_A	Mudpit_Ingel20196_MGFPeaklist (F058870)	Myosin-9 OS=Gallus gallus GN=MYH9 PE=4 SV=2	F1NCD4_CHICK,MYH9_CHICK,UPI000012FB7F,UPI000060E1E4,UPI0002C868F4	TAX_GallusGallus_9031_20141114.fasta,UPARC14_2_GallusG_9031_20140417.fasta	226,643.40	100.00%	49	53	115	0.23%	24.40%	QISVLQQQAVEAR	K	K	99.70%	71.5	43.7	29.1	53.9	45.3	3.96	0.422	0.5	0	6	0	0	2	1,469.81	2,037.50		6,070.84	1358	1370	Empty	TRUE	
20196-20225 HinckeM –TAXchicken	Uncategorized Sample	20196-20205_A	Mudpit_Ingel20196_MGFPeaklist (F058870)	Myosin-9 OS=Gallus gallus GN=MYH9 PE=4 SV=2	F1NCD4_CHICK,MYH9_CHICK,UPI000012FB7F,UPI000060E1E4,UPI0002C868F4	TAX_GallusGallus_9031_20141114.fasta,UPARC14_2_GallusG_9031_20140417.fasta	226,643.40	100.00%	49	53	115	0.23%	24.40%	QKHSQAIEELAEQLEQTKR	R	V	99.70%	38.5	43.4	23.6	26	30.7	2.18	0.192	0.184	0	0	0	2	2	2,266.18	2,363.00		795.44	1192	1210	Empty	TRUE	
20196-20225 HinckeM –TAXchicken	Uncategorized Sample	20196-20205_A	Mudpit_Ingel20196_MGFPeaklist (F058870)	Myosin-9 OS=Gallus gallus GN=MYH9 PE=4 SV=2	F1NCD4_CHICK,MYH9_CHICK,UPI000012FB7F,UPI000060E1E4,UPI0002C868F4	TAX_GallusGallus_9031_20141114.fasta,UPARC14_2_GallusG_9031_20140417.fasta	226,643.40	100.00%	49	53	115	0.23%	24.40%	QLLQANPILEAFGNAK	R	T	33.80%	11.9	42.9	17.8	9.6					0	1	0	0	2	1,726.95	2,877.00		107.82	210	225	Empty	TRUE	MYH11_CHICK
20196-20225 HinckeM –TAXchicken	Uncategorized Sample	20196-20205_A	Mudpit_Ingel20196_MGFPeaklist (F058870)	Myosin-9 OS=Gallus gallus GN=MYH9 PE=4 SV=2	F1NCD4_CHICK,MYH9_CHICK,UPI000012FB7F,UPI000060E1E4,UPI0002C868F4	TAX_GallusGallus_9031_20141114.fasta,UPARC14_2_GallusG_9031_20140417.fasta	226,643.40	100.00%	49	53	115	0.23%	24.40%	QRYEILTPNAIPK	R	G	99.70%	44.8	43.5	22.5	30.7	33.5	1.96	0.358	0.423	0	2	4	0	2	1,542.86	2,240.50		4,800.05	719	731	Empty	TRUE	UPI00000FC6BE,Q789A5_CHICK,Q789A4_CHICK,UPI0000171382,UPI000060F510,UPI0000ECA50D,UPI000060F511,UPI00003AB452,Q789A6_CHICK,UPI0000171380,Q02015_CHICK,UPI0000171381,UPI00003AB454,UPI00003AB453
20196-20225 HinckeM –TAXchicken	Uncategorized Sample	20196-20205_A	Mudpit_Ingel20196_MGFPeaklist (F058870)	Myosin-9 OS=Gallus gallus GN=MYH9 PE=4 SV=2	F1NCD4_CHICK,MYH9_CHICK,UPI000012FB7F,UPI000060E1E4,UPI0002C868F4	TAX_GallusGallus_9031_20141114.fasta,UPARC14_2_GallusG_9031_20140417.fasta	226,643.40	100.00%	49	53	115	0.23%	24.40%	RGDLPFVVTR	R	R	99.70%	45.9	44.6	39	28	34.9	1.66	0.301	0.5	0	2	0	0	2	1,159.66	2,210.00		561.14	1923	1932	Empty	TRUE	
20196-20225 HinckeM –TAXchicken	Uncategorized Sample	20196-20205_A	Mudpit_Ingel20196_MGFPeaklist (F058870)	Myosin-9 OS=Gallus gallus GN=MYH9 PE=4 SV=2	F1NCD4_CHICK,MYH9_CHICK,UPI000012FB7F,UPI000060E1E4,UPI0002C868F4	TAX_GallusGallus_9031_20141114.fasta,UPARC14_2_GallusG_9031_20140417.fasta	226,643.40	100.00%	49	53	115	0.23%	24.40%	TDLLLEPYNK	K	Y	99.70%	34.2	44.8	29.3	12.7	27.4	1.57	0.234	0.35	0	2	0	0	2	1,205.64	2,293.50		465.92	290	299	Empty	TRUE	
20196-20225 HinckeM –TAXchicken	Uncategorized Sample	20196-20205_A	Mudpit_Ingel20196_MGFPeaklist (F058870)	Myosin-9 OS=Gallus gallus GN=MYH9 PE=4 SV=2	F1NCD4_CHICK,MYH9_CHICK,UPI000012FB7F,UPI000060E1E4,UPI0002C868F4	TAX_GallusGallus_9031_20141114.fasta,UPARC14_2_GallusG_9031_20140417.fasta	226,643.40	100.00%	49	53	115	0.23%	24.40%	TDLLLEPYNKYR	K	F	99.70%	24.5	44.3	23.3	10.2	26.9	2.44	0.208	0.333	0	0	2	0	2	1,524.81	2,290.00		819.24	290	301	Empty	TRUE	
20196-20225 HinckeM –TAXchicken	Uncategorized Sample	20196-20205_A	Mudpit_Ingel20196_MGFPeaklist (F058870)	Myosin-9 OS=Gallus gallus GN=MYH9 PE=4 SV=2	F1NCD4_CHICK,MYH9_CHICK,UPI000012FB7F,UPI000060E1E4,UPI0002C868F4	TAX_GallusGallus_9031_20141114.fasta,UPARC14_2_GallusG_9031_20140417.fasta	226,643.40	100.00%	49	53	115	0.23%	24.40%	TLEDEAKTHEAQIQEMR	K	Q	98.60%	43.9	44	22.9	33.3					0	0	0	2	2	2,044.96	1,846.50		641.82	1175	1191	Empty	TRUE	
20196-20225 HinckeM –TAXchicken	Uncategorized Sample	20196-20205_A	Mudpit_Ingel20196_MGFPeaklist (F058870)	Myosin-9 OS=Gallus gallus GN=MYH9 PE=4 SV=2	F1NCD4_CHICK,MYH9_CHICK,UPI000012FB7F,UPI000060E1E4,UPI0002C868F4	TAX_GallusGallus_9031_20141114.fasta,UPARC14_2_GallusG_9031_20140417.fasta	226,643.40	100.00%	49	53	115	0.23%	24.40%	TSREEILAQAK	R	E	92.20%	37	44.7	34.3	9.74					0	0	3	0	2	1,245.68	1,668.00		1,425.65	1659	1669	Empty	TRUE	
20196-20225 HinckeM –TAXchicken	Uncategorized Sample	20196-20205_A	Mudpit_Ingel20196_MGFPeaklist (F058870)	Myosin-9 OS=Gallus gallus GN=MYH9 PE=4 SV=2	F1NCD4_CHICK,MYH9_CHICK,UPI000012FB7F,UPI000060E1E4,UPI0002C868F4	TAX_GallusGallus_9031_20141114.fasta,UPARC14_2_GallusG_9031_20140417.fasta	226,643.40	100.00%	49	53	115	0.23%	24.40%	TSREEILAQAKENEK	R	K	99.70%	49.7	44.6	29.6	30.6	42.7	3.42	0.335	0.5	0	0	0	2	2	1,745.90	1,637.00		624	1659	1673	Empty	TRUE	
20196-20225 HinckeM –TAXchicken	Uncategorized Sample	20196-20205_A	Mudpit_Ingel20196_MGFPeaklist (F058870)	Myosin-9 OS=Gallus gallus GN=MYH9 PE=4 SV=2	F1NCD4_CHICK,MYH9_CHICK,UPI000012FB7F,UPI000060E1E4,UPI0002C868F4	TAX_GallusGallus_9031_20141114.fasta,UPARC14_2_GallusG_9031_20140417.fasta	226,643.40	100.00%	49	53	115	0.23%	24.40%	TVGQLYKEQLAK	R	L	99.70%	37	43.6	25.2	25.9	29.3	1.14	0.266	0.417	0	0	4	0	2	1,377.77	1,866.50		2,477.43	645	656	Empty	TRUE	
20196-20225 HinckeM –TAXchicken	Uncategorized Sample	20196-20205_A	Mudpit_Ingel20196_MGFPeaklist (F058870)	Myosin-9 OS=Gallus gallus GN=MYH9 PE=4 SV=2	F1NCD4_CHICK,MYH9_CHICK,UPI000012FB7F,UPI000060E1E4,UPI0002C868F4	TAX_GallusGallus_9031_20141114.fasta,UPARC14_2_GallusG_9031_20140417.fasta	226,643.40	100.00%	49	53	115	0.23%	24.40%	VDAQLQELQVK	K	F	50.50%	23.2	43.9	38.2	1.53					0	1	0	0	2	1,270.70	2,098.00		128.78	1250	1260	Empty	TRUE	
20196-20225 HinckeM –TAXchicken	Uncategorized Sample	20196-20205_A	Mudpit_Ingel20196_MGFPeaklist (F058870)	Myosin-9 OS=Gallus gallus GN=MYH9 PE=4 SV=2	F1NCD4_CHICK,MYH9_CHICK,UPI000012FB7F,UPI000060E1E4,UPI0002C868F4	TAX_GallusGallus_9031_20141114.fasta,UPARC14_2_GallusG_9031_20140417.fasta	226,643.40	100.00%	49	53	115	0.23%	24.40%	VISGVLQLGNIVFKK	K	E	99.70%	64.9	36.9	20.4	56.6	45.8	3.11	0.162	0.5	0	0	2	0	2	1,614.99	2,854.50		1,206.55	342	356	Empty	TRUE	
20196-20225 HinckeM –TAXchicken	Uncategorized Sample	20196-20205_A	Mudpit_Ingel20196_MGFPeaklist (F058870)	Myosin-9 OS=Gallus gallus GN=MYH9 PE=4 SV=2	F1NCD4_CHICK,MYH9_CHICK,UPI000012FB7F,UPI000060E1E4,UPI0002C868F4	TAX_GallusGallus_9031_20141114.fasta,UPARC14_2_GallusG_9031_20140417.fasta	226,643.40	100.00%	49	53	115	0.23%	24.40%	VSHLLGINVTDFTR	K	G	99.70%	50.9	43.8	29.7	35.6	39.2	4.7	0.487	0.429	0	0	3	0	2	1,571.85	2,599.00		1,284.73	374	387	Empty	TRUE	
20196-20225 HinckeM –TAXchicken	Uncategorized Sample	20196-20205_A	Mudpit_Ingel20196_MGFPeaklist (F058870)	Myosin-9 OS=Gallus gallus GN=MYH9 PE=4 SV=2	F1NCD4_CHICK,MYH9_CHICK,UPI000012FB7F,UPI000060E1E4,UPI0002C868F4	TAX_GallusGallus_9031_20141114.fasta,UPARC14_2_GallusG_9031_20140417.fasta	226,643.40	100.00%	49	53	115	0.23%	24.40%	VVFQEFR	R	Q	74.20%	30.9	43.3	26.1	16.6					0	4	0	0	2	924.4937	2,156.00		1,480.25	712	718	Empty	TRUE	
20196-20225 HinckeM –TAXchicken	Uncategorized Sample	20196-20205_A	Mudpit_Ingel20196_MGFPeaklist (F058870)	Myosin-9 OS=Gallus gallus GN=MYH9 PE=4 SV=2	F1NCD4_CHICK,MYH9_CHICK,UPI000012FB7F,UPI000060E1E4,UPI0002C868F4	TAX_GallusGallus_9031_20141114.fasta,UPARC14_2_GallusG_9031_20140417.fasta	226,643.40	100.00%	49	53	115	0.23%	24.40%	YEILTPNAIPK	R	G	99.70%	20.6	44.4	14.6	7.9	28.5	1.57	0.277	0.318	0	3	0	0	2	1,258.70	2,337.00		1,618.82	721	731	Empty	TRUE	UPI00000FC6BE,Q789A5_CHICK,Q789A4_CHICK,UPI0000171382,UPI000060F510,UPI0000ECA50D,UPI000060F511,UPI00003AB452,Q789A6_CHICK,UPI0000171380,Q02015_CHICK,UPI0000171381,UPI00003AB454,UPI00003AB453
20196-20225 HinckeM –TAXchicken	Uncategorized Sample	20196-20205_A	Mudpit_Ingel20196_MGFPeaklist (F058870)	Myosin-9 OS=Gallus gallus GN=MYH9 PE=4 SV=2	F1NCD4_CHICK,MYH9_CHICK,UPI000012FB7F,UPI000060E1E4,UPI0002C868F4	TAX_GallusGallus_9031_20141114.fasta,UPARC14_2_GallusG_9031_20140417.fasta	226,643.40	100.00%	49	53	115	0.23%	24.40%	ADFCIIHYAGK	K	V	45.90%	18.6	44.4	14.7	11.1					0	0	2	0	2	1,294.63	2,223.00		989.84	566	576	Empty	TRUE	UPI00000FC6BE,Q789A5_CHICK,Q789A4_CHICK,UPI0000171382,UPI000060F510,UPI0000ECA50D,UPI000060F511,UPI00003AB452,Q789A6_CHICK,UPI0000171380,Q02015_CHICK,UPI0000171381,UPI00003AB454,UPI00003AB453
20196-20225 HinckeM –TAXchicken	Uncategorized Sample	20196-20205_A	Mudpit_Ingel20196_MGFPeaklist (F058870)	Myosin-9 OS=Gallus gallus GN=MYH9 PE=4 SV=2	F1NCD4_CHICK,MYH9_CHICK,UPI000012FB7F,UPI000060E1E4,UPI0002C868F4	TAX_GallusGallus_9031_20141114.fasta,UPARC14_2_GallusG_9031_20140417.fasta	226,643.40	100.00%	49	53	115	0.23%	24.40%	AGKLDPHLVLDQLR	K	C	99.70%	52.2	42.3	31.5	35.1	37.5	3.54	0.344	0.5	0	0	2	0	2	1,574.90	2,445.50		970.79	680	693	Empty	TRUE	UPI00000FC6BE,Q789A5_CHICK,Q789A4_CHICK,UPI0000171382,UPI000060F510,UPI0000ECA50D,UPI000060F511,UPI00003AB452,Q789A6_CHICK,UPI0000171380,Q02015_CHICK,UPI0000171381,UPI00003AB454,UPI00003AB453
20196-20225 HinckeM –TAXchicken	Uncategorized Sample	20196-20205_A	Mudpit_Ingel20196_MGFPeaklist (F058870)	Myosin-9 OS=Gallus gallus GN=MYH9 PE=4 SV=2	F1NCD4_CHICK,MYH9_CHICK,UPI000012FB7F,UPI000060E1E4,UPI0002C868F4	TAX_GallusGallus_9031_20141114.fasta,UPARC14_2_GallusG_9031_20140417.fasta	226,643.40	100.00%	49	53	115	0.23%	24.40%	AGVLAHLEEER	R	D	99.70%	71.3	44.3	36.2	36.2	34.7	3.4	0.317	0.409	0	0	2	0	2	1,223.64	2,031.50		1,163.70	765	775	Empty	TRUE	UPI00000FC6BE,Q789A5_CHICK,Q789A4_CHICK,UPI0000171382,UPI000060F510,UPI0000ECA50D,UPI000060F511,UPI00003AB452,Q789A6_CHICK,UPI0000171380,Q02015_CHICK,UPI0000171381,UPI00003AB454,UPI00003AB453
20196-20225 HinckeM –TAXchicken	Uncategorized Sample	20196-20205_A	Mudpit_Ingel20196_MGFPeaklist (F058870)	Myosin-9 OS=Gallus gallus GN=MYH9 PE=4 SV=2	F1NCD4_CHICK,MYH9_CHICK,UPI000012FB7F,UPI000060E1E4,UPI0002C868F4	TAX_GallusGallus_9031_20141114.fasta,UPARC14_2_GallusG_9031_20140417.fasta	226,643.40	100.00%	49	53	115	0.23%	24.40%	AGVLAHLEEERDLK	R	I	99.70%	43.7	43.9	28.2	36.5	38.3	3.7	0.371	0.464	0	0	2	0	2	1,579.84	2,107.00		1,024.73	765	778	Empty	TRUE	UPI00000FC6BE,Q789A5_CHICK,Q789A4_CHICK,UPI0000171382,UPI000060F510,UPI0000ECA50D,UPI000060F511,UPI00003AB452,Q789A6_CHICK,UPI0000171380,Q02015_CHICK,UPI0000171381,UPI00003AB454,UPI00003AB453
20196-20225 HinckeM –TAXchicken	Uncategorized Sample	20196-20205_A	Mudpit_Ingel20196_MGFPeaklist (F058870)	Myosin-9 OS=Gallus gallus GN=MYH9 PE=4 SV=2	F1NCD4_CHICK,MYH9_CHICK,UPI000012FB7F,UPI000060E1E4,UPI0002C868F4	TAX_GallusGallus_9031_20141114.fasta,UPARC14_2_GallusG_9031_20140417.fasta	226,643.40	100.00%	49	53	115	0.23%	24.40%	ALEEAIEQK	R	A	84.60%	33.1	45.3	36.6	8.49					0	3	0	0	2	1,030.54	1,744.00		686.34	1484	1492	Empty	TRUE	
20196-20225 HinckeM –TAXchicken	Uncategorized Sample	20196-20205_A	Mudpit_Ingel20196_MGFPeaklist (F058870)	Myosin-9 OS=Gallus gallus GN=MYH9 PE=4 SV=2	F1NCD4_CHICK,MYH9_CHICK,UPI000012FB7F,UPI000060E1E4,UPI0002C868F4	TAX_GallusGallus_9031_20141114.fasta,UPARC14_2_GallusG_9031_20140417.fasta	226,643.40	100.00%	49	53	115	0.23%	24.40%	ALEEAIEQKAELER	R	V	99.70%	72.1	44.6	34.9	46.5	50.4	4.02	0.423	0.607	0	1	4	0	2	1,628.85	2,104.00		4,055.28	1484	1497	Empty	TRUE	
20196-20225 HinckeM –TAXchicken	Uncategorized Sample	20196-20205_A	Mudpit_Ingel20196_MGFPeaklist (F058870)	Myosin-9 OS=Gallus gallus GN=MYH9 PE=4 SV=2	F1NCD4_CHICK,MYH9_CHICK,UPI000012FB7F,UPI000060E1E4,UPI0002C868F4	TAX_GallusGallus_9031_20141114.fasta,UPARC14_2_GallusG_9031_20140417.fasta	226,643.40	100.00%	49	53	115	0.23%	24.40%	ALELDSNLYR	K	I	99.70%	58.5	44.7	27.2	33.6	40.4	3.82	0.351	0.55	0	5	0	0	2	1,193.62	2,244.00		3,596.17	746	755	Empty	TRUE	
20196-20225 HinckeM –TAXchicken	Uncategorized Sample	20196-20205_A	Mudpit_Ingel20196_MGFPeaklist (F058870)	Myosin-9 OS=Gallus gallus GN=MYH9 PE=4 SV=2	F1NCD4_CHICK,MYH9_CHICK,UPI000012FB7F,UPI000060E1E4,UPI0002C868F4	TAX_GallusGallus_9031_20141114.fasta,UPARC14_2_GallusG_9031_20140417.fasta	226,643.40	100.00%	49	53	115	0.23%	24.40%	ANLQIDQMNADLNAER	K	S	99.70%	61.6	44.3	23.7	51.8	27.8	1.57	0.237	0.219	0	0	1	0	2	1,831.86	1,969.00		215.45	1755	1770	Empty	TRUE	
20196-20225 HinckeM –TAXchicken	Uncategorized Sample	20196-20205_A	Mudpit_Ingel20196_MGFPeaklist (F058870)	Myosin-9 OS=Gallus gallus GN=MYH9 PE=4 SV=2	F1NCD4_CHICK,MYH9_CHICK,UPI000012FB7F,UPI000060E1E4,UPI0002C868F4	TAX_GallusGallus_9031_20141114.fasta,UPARC14_2_GallusG_9031_20140417.fasta	226,643.40	100.00%	49	53	115	0.23%	24.40%	ATITALEAK	K	I	14.20%	16.5	45.4	22.1	0.69					0	2	0	0	2	917.5303	1,880.00		993.4	1807	1815	Empty	TRUE	
20196-20225 HinckeM –TAXchicken	Uncategorized Sample	20196-20205_A	Mudpit_Ingel20196_MGFPeaklist (F058870)	Myosin-9 OS=Gallus gallus GN=MYH9 PE=4 SV=2	F1NCD4_CHICK,MYH9_CHICK,UPI000012FB7F,UPI000060E1E4,UPI0002C868F4	TAX_GallusGallus_9031_20141114.fasta,UPARC14_2_GallusG_9031_20140417.fasta	226,643.40	100.00%	49	53	115	0.23%	24.40%	CNGVLEGIR	R	I	28.90%	28.9	45.5	29.6	12.6					0	1	0	0	2	1,018.50	2,046.00		210.55	694	702	Empty	TRUE	MYH11_CHICK,Q789A4_CHICK,Q9PTY2_CHICK,UPI0000ECA50D,Q789A6_CHICK,UPI00000FC6BE,Q789A5_CHICK,UPI0000171382,UPI000044943F,UPI000060F510,UPI000060F511,UPI00003AB452,UPI0000171380,Q02015_CHICK,UPI0000171381,UPI00003AB434,UPI00000FB7E2,UPI00003AB454,UPI00003AB431,UPI00003AB453
20196-20225 HinckeM –TAXchicken	Uncategorized Sample	20196-20205_A	Mudpit_Ingel20196_MGFPeaklist (F058870)	Myosin-9 OS=Gallus gallus GN=MYH9 PE=4 SV=2	F1NCD4_CHICK,MYH9_CHICK,UPI000012FB7F,UPI000060E1E4,UPI0002C868F4	TAX_GallusGallus_9031_20141114.fasta,UPARC14_2_GallusG_9031_20140417.fasta	226,643.40	100.00%	49	53	115	0.23%	24.40%	DADKYLYVDK	R	N	47.90%	22.8	44.7	32.5	0					0	0	1	0	2	1,229.61	1,861.00		140	5	14	Empty	TRUE	
20196-20225 HinckeM –TAXchicken	Uncategorized Sample	20196-20205_A	Mudpit_Ingel20196_MGFPeaklist (F058870)	Myosin-9 OS=Gallus gallus GN=MYH9 PE=4 SV=2	F1NCD4_CHICK,MYH9_CHICK,UPI000012FB7F,UPI000060E1E4,UPI0002C868F4	TAX_GallusGallus_9031_20141114.fasta,UPARC14_2_GallusG_9031_20140417.fasta	226,643.40	100.00%	49	53	115	0.23%	24.40%	DLESLTQRYEEK	K	I	56.20%	18.3	44.4	21.2	11.9					0	0	3	0	2	1,510.74	2,123.00		788.9	1393	1404	Empty	TRUE	
20196-20225 HinckeM –TAXchicken	Uncategorized Sample	20196-20205_A	Mudpit_Ingel20196_MGFPeaklist (F058870)	Myosin-9 OS=Gallus gallus GN=MYH9 PE=4 SV=2	F1NCD4_CHICK,MYH9_CHICK,UPI000012FB7F,UPI000060E1E4,UPI0002C868F4	TAX_GallusGallus_9031_20141114.fasta,UPARC14_2_GallusG_9031_20140417.fasta	226,643.40	100.00%	49	53	115	0.23%	24.40%	EQADFAIEALAK	K	A	99.70%	57.8	45	27.9	43.9	37.5	3.8	0.331	0.375	0	4	0	0	2	1,305.67	2,461.50		1,501.85	408	419	Empty	TRUE	MYH11_CHICK
20196-20225 HinckeM –TAXchicken	Uncategorized Sample	20196-20205_A	Mudpit_Ingel20196_MGFPeaklist (F058870)	Myosin-9 OS=Gallus gallus GN=MYH9 PE=4 SV=2	F1NCD4_CHICK,MYH9_CHICK,UPI000012FB7F,UPI000060E1E4,UPI0002C868F4	TAX_GallusGallus_9031_20141114.fasta,UPARC14_2_GallusG_9031_20140417.fasta	226,643.40	100.00%	49	53	115	0.23%	24.40%	FDQLLAEEK	K	N	62.50%	25.1	44.8	29.4	6.88					0	2	0	0	2	1,092.56	2,017.50		508.72	1446	1454	Empty	TRUE	
20196-20225 HinckeM –TAXchicken	Uncategorized Sample	20196-20205_A	Mudpit_Ingel20196_MGFPeaklist (F058870)	Myosin-9 OS=Gallus gallus GN=MYH9 PE=4 SV=2	F1NCD4_CHICK,MYH9_CHICK,UPI000012FB7F,UPI000060E1E4,UPI0002C868F4	TAX_GallusGallus_9031_20141114.fasta,UPARC14_2_GallusG_9031_20140417.fasta	226,643.40	100.00%	49	53	115	0.23%	24.40%	HSQAIEELAEQLEQTKR	K	V	83.90%	21.7	43.9	18	16.7					0	0	0	2	2	2,010.03	2,465.50		456.01	1194	1210	Empty	TRUE	
20196-20225 HinckeM –TAXchicken	Uncategorized Sample	20196-20205_A	Mudpit_Ingel20196_MGFPeaklist (F058870)	Myosin-9 OS=Gallus gallus GN=MYH9 PE=4 SV=2	F1NCD4_CHICK,MYH9_CHICK,UPI000012FB7F,UPI000060E1E4,UPI0002C868F4	TAX_GallusGallus_9031_20141114.fasta,UPARC14_2_GallusG_9031_20140417.fasta	226,643.40	100.00%	49	53	115	0.23%	24.40%	IMGIPDEEQIGLLK	R	V	99.70%	33.4	44.2	14.4	29.2	36.2	1.96	0.318	0.357	0	2	0	0	2	1,571.84	2,519.50		2,423.65	328	341	Empty	TRUE	
20196-20225 HinckeM –TAXchicken	Uncategorized Sample	20196-20205_A	Mudpit_Ingel20196_MGFPeaklist (F058870)	Myosin-9 OS=Gallus gallus GN=MYH9 PE=4 SV=2	F1NCD4_CHICK,MYH9_CHICK,UPI000012FB7F,UPI000060E1E4,UPI0002C868F4	TAX_GallusGallus_9031_20141114.fasta,UPARC14_2_GallusG_9031_20140417.fasta	226,643.40	100.00%	49	53	115	0.23%	24.40%	IVQLEEQLDMETKER	K	Q	98.40%	37.5	44.6	22.6	23.5					0	0	2	0	2	1,876.93	2,189.50		318.2	1816	1830	Empty	TRUE	
20196-20225 HinckeM –TAXchicken	Uncategorized Sample	20196-20205_A	Mudpit_Ingel20196_MGFPeaklist (F058870)	Myosin-9 OS=Gallus gallus GN=MYH9 PE=4 SV=2	F1NCD4_CHICK,MYH9_CHICK,UPI000012FB7F,UPI000060E1E4,UPI0002C868F4	TAX_GallusGallus_9031_20141114.fasta,UPARC14_2_GallusG_9031_20140417.fasta	226,643.40	100.00%	49	53	115	0.23%	24.40%	KFDQLLAEEK	K	N	93.10%	39.1	44.6	40.3	9.11					0	0	4	0	2	1,220.65	2,002.50		2,060.83	1445	1454	Empty	TRUE	
20196-20225 HinckeM –TAXchicken	Uncategorized Sample	20196-20205_A	Mudpit_Ingel20196_MGFPeaklist (F058870)	Myosin-9 OS=Gallus gallus GN=MYH9 PE=4 SV=2	F1NCD4_CHICK,MYH9_CHICK,UPI000012FB7F,UPI000060E1E4,UPI0002C868F4	TAX_GallusGallus_9031_20141114.fasta,UPARC14_2_GallusG_9031_20140417.fasta	226,643.40	100.00%	49	53	115	0.23%	24.40%	KFDQLLAEEKNISAK	K	Y	99.70%	26	43.5	21.7	15.4	29.3	1.7	0.147	0.367	0	0	1	0	2	1,733.94	2,153.00		416.04	1445	1459	Empty	TRUE	
20196-20225 HinckeM –TAXchicken	Uncategorized Sample	20196-20205_A	Mudpit_Ingel20196_MGFPeaklist (F058870)	Myosin-9 OS=Gallus gallus GN=MYH9 PE=4 SV=2	F1NCD4_CHICK,MYH9_CHICK,UPI000012FB7F,UPI000060E1E4,UPI0002C868F4	TAX_GallusGallus_9031_20141114.fasta,UPARC14_2_GallusG_9031_20140417.fasta	226,643.40	100.00%	49	53	115	0.23%	24.40%	KKVDAQLQELQVK	R	F	67.80%	28.2	40.7	28.4	11.1					0	0	1	0	2	1,526.89	1,881.00		329.09	1248	1260	Empty	TRUE	
20196-20225 HinckeM –TAXchicken	Uncategorized Sample	20196-20205_A	Mudpit_Ingel20196_MGFPeaklist (F058870)	Myosin-9 OS=Gallus gallus GN=MYH9 PE=4 SV=2	F1NCD4_CHICK,MYH9_CHICK,UPI000012FB7F,UPI000060E1E4,UPI0002C868F4	TAX_GallusGallus_9031_20141114.fasta,UPARC14_2_GallusG_9031_20140417.fasta	226,643.40	100.00%	49	53	115	0.23%	24.40%	KLEEDVIVLEDQNLK	K	L	99.70%	41	43.3	24.6	26.1	36.6	4	0.363	0.367	0	0	2	0	2	1,784.96	2,380.50		1,109.02	975	989	Empty	TRUE	
20196-20225 HinckeM –TAXchicken	Uncategorized Sample	20196-20205_A	Mudpit_Ingel20196_MGFPeaklist (F058870)	Myosin-9 OS=Gallus gallus GN=MYH9 PE=4 SV=2	F1NCD4_CHICK,MYH9_CHICK,UPI000012FB7F,UPI000060E1E4,UPI0002C868F4	TAX_GallusGallus_9031_20141114.fasta,UPARC14_2_GallusG_9031_20140417.fasta	226,643.40	100.00%	49	53	115	0.23%	24.40%	KLVWVPSEK	K	S	29.70%	13.5	43.2	17.5	0					0	1	0	0	2	1,085.64	2,060.00		390.18	30	38	Empty	TRUE	MYH11_CHICK
20196-20225 HinckeM –TAXchicken	Uncategorized Sample	20196-20205_A	Mudpit_Ingel20196_MGFPeaklist (F058870)	Myosin-9 OS=Gallus gallus GN=MYH9 PE=4 SV=2	F1NCD4_CHICK,MYH9_CHICK,UPI000012FB7F,UPI000060E1E4,UPI0002C868F4	TAX_GallusGallus_9031_20141114.fasta,UPARC14_2_GallusG_9031_20140417.fasta	226,643.40	100.00%	49	53	115	0.23%	24.40%	KQELEEICHDLEAR	K	V	99.70%	48.3	44.3	23.2	40.8	35.2	3.44	0.347	0.357	0	0	2	1	2	1,769.85	2,175.00		1,534.37	910	923	Empty	TRUE	
20196-20225 HinckeM –TAXchicken	Uncategorized Sample	20196-20205_A	Mudpit_Ingel20196_MGFPeaklist (F058870)	Myosin-9 OS=Gallus gallus GN=MYH9 PE=4 SV=2	F1NCD4_CHICK,MYH9_CHICK,UPI000012FB7F,UPI000060E1E4,UPI0002C868F4	TAX_GallusGallus_9031_20141114.fasta,UPARC14_2_GallusG_9031_20140417.fasta	226,643.40	100.00%	49	53	115	0.23%	24.40%	KVDAQLQELQVK	K	F	99.70%	33.1	42.7	21.6	9.14	27.3	1.21	0.22	0.333	0	0	2	0	2	1,398.80	2,003.00		643.7	1249	1260	Empty	TRUE	
20196-20225 HinckeM –TAXchicken	Uncategorized Sample	20196-20205_A	Mudpit_Ingel20196_MGFPeaklist (F058870)	Myosin-9 OS=Gallus gallus GN=MYH9 PE=4 SV=2	F1NCD4_CHICK,MYH9_CHICK,UPI000012FB7F,UPI000060E1E4,UPI0002C868F4	TAX_GallusGallus_9031_20141114.fasta,UPARC14_2_GallusG_9031_20140417.fasta	226,643.40	100.00%	49	53	115	0.23%	24.40%	LDPHLVLDQLR	K	C	99.70%	34.6	43.8	18.6	22.1	32.5	2.47	0.366	0.455	0	0	1	0	2	1,318.75	2,534.00		1,121.62	683	693	Empty	TRUE	UPI00000FC6BE,Q789A5_CHICK,Q789A4_CHICK,UPI0000171382,UPI000060F510,UPI0000ECA50D,UPI000060F511,UPI00003AB452,Q789A6_CHICK,UPI0000171380,Q02015_CHICK,UPI0000171381,UPI00003AB454,UPI00003AB453
20196-20225 HinckeM –TAXchicken	Uncategorized Sample	20196-20205_A	Mudpit_Ingel20196_MGFPeaklist (F058870)	Myosin-9 OS=Gallus gallus GN=MYH9 PE=4 SV=2	F1NCD4_CHICK,MYH9_CHICK,UPI000012FB7F,UPI000060E1E4,UPI0002C868F4	TAX_GallusGallus_9031_20141114.fasta,UPARC14_2_GallusG_9031_20140417.fasta	226,643.40	100.00%	49	53	115	0.23%	24.40%	LKDILLQVDDERR	K	N	27.70%	15.6	42.7	21.5	4.84					0	0	0	1	2	1,612.90	2,214.00		458.07	1844	1856	Empty	TRUE	
20196-20225 HinckeM –TAXchicken	Uncategorized Sample	20196-20205_A	Mudpit_Ingel20196_MGFPeaklist (F058870)	Myosin-9 OS=Gallus gallus GN=MYH9 PE=4 SV=2	F1NCD4_CHICK,MYH9_CHICK,UPI000012FB7F,UPI000060E1E4,UPI0002C868F4	TAX_GallusGallus_9031_20141114.fasta,UPARC14_2_GallusG_9031_20140417.fasta	226,643.40	100.00%	49	53	115	0.23%	24.40%	LQQELDDIAVDLDHQR	R	Q	99.70%	68.1	44.4	19.5	56.3	44.1	5.77	0.515	0.438	0	0	2	0	2	1,907.95	2,474.00		1,640.23	1418	1433	Empty	TRUE	
20196-20225 HinckeM –TAXchicken	Uncategorized Sample	20196-20205_A	Mudpit_Ingel20196_MGFPeaklist (F058870)	Myosin-9 OS=Gallus gallus GN=MYH9 PE=4 SV=2	F1NCD4_CHICK,MYH9_CHICK,UPI000012FB7F,UPI000060E1E4,UPI0002C868F4	TAX_GallusGallus_9031_20141114.fasta,UPARC14_2_GallusG_9031_20140417.fasta	226,643.40	100.00%	49	53	115	0.23%	24.40%	LRLEVNQQAMK	K	A	73.00%	17.5	44.8	15.5	15.2					0	0	1	0	2	1,345.73	1,759.00		157.86	1556	1566	Empty	TRUE	
20196-20225 HinckeM –TAXchicken	Uncategorized Sample	20196-20205_A	Mudpit_Ingel20196_MGFPeaklist (F058870)	Myosin-9 OS=Gallus gallus GN=MYH9 PE=4 SV=2	F1NCD4_CHICK,MYH9_CHICK,UPI000012FB7F,UPI000060E1E4,UPI0002C868F4	TAX_GallusGallus_9031_20141114.fasta,UPARC14_2_GallusG_9031_20140417.fasta	226,643.40	100.00%	49	53	115	0.23%	24.40%	LVWVPSEK	K	S	99.70%	26.7	44.4	23.8	14.6	22.7	0.638	0.123	0.312	0	2	0	0	2	957.5404	2,164.50		372.91	31	38	Empty	TRUE	MYH11_CHICK
20196-20225 HinckeM –TAXchicken	Uncategorized Sample	20196-20205_A	Mudpit_Ingel20196_MGFPeaklist (F058870)	Myosin-9 OS=Gallus gallus GN=MYH9 PE=4 SV=2	F1NCD4_CHICK,MYH9_CHICK,UPI000012FB7F,UPI000060E1E4,UPI0002C868F4	TAX_GallusGallus_9031_20141114.fasta,UPARC14_2_GallusG_9031_20140417.fasta	226,643.40	100.00%	49	53	115	0.23%	24.40%	MSEFTTNLTEEEEKSK	R	S	99.70%	32.2	43	16.9	29.3	30.3	2.14	0.0066	0.25	0	0	2	0	2	1,902.86	2,035.00		827.04	1001	1016	Empty	TRUE	
20196-20225 HinckeM –TAXchicken	Uncategorized Sample	20196-20205_A	Mudpit_Ingel20196_MGFPeaklist (F058870)	Myosin-9 OS=Gallus gallus GN=MYH9 PE=4 SV=2	F1NCD4_CHICK,MYH9_CHICK,UPI000012FB7F,UPI000060E1E4,UPI0002C868F4	TAX_GallusGallus_9031_20141114.fasta,UPARC14_2_GallusG_9031_20140417.fasta	226,643.40	100.00%	49	53	115	0.23%	24.40%	NWQWWR	R	L	24.20%	12.7	45.1	22.7	3.78					0	1	0	0	2	975.4583	2,584.00		138.57	824	829	Empty	TRUE	MYH11_CHICK
20196-20225 HinckeM –TAXchicken	Uncategorized Sample	20196-20205_A	Mudpit_Ingel20196_MGFPeaklist (F058870)	Myosin-9 OS=Gallus gallus GN=MYH9 PE=4 SV=2	F1NCD4_CHICK,MYH9_CHICK,UPI000012FB7F,UPI000060E1E4,UPI0002C868F4	TAX_GallusGallus_9031_20141114.fasta,UPARC14_2_GallusG_9031_20140417.fasta	226,643.40	100.00%	49	53	115	0.23%	24.40%	QACVLMIK	K	A	17.50%	25.6	44	27.9	9.47					0	1	0	0	2	961.4847	2,343.00		270	738	745	Empty	TRUE	
20196-20225 HinckeM –TAXchicken	Uncategorized Sample	20196-20205_A	Mudpit_Ingel20196_MGFPeaklist (F058870)	Myosin-9 OS=Gallus gallus GN=MYH9 PE=4 SV=2	F1NCD4_CHICK,MYH9_CHICK,UPI000012FB7F,UPI000060E1E4,UPI0002C868F4	TAX_GallusGallus_9031_20141114.fasta,UPARC14_2_GallusG_9031_20140417.fasta	226,643.40	100.00%	49	53	115	0.23%	24.40%	QAQQERDELADEIANSSGK	R	G	99.70%	56.6	44.1	18.7	50.3	48	6.68	0.515	0.395	0	0	4	0	2	2,088.98	1,982.00		4,035.47	1698	1716	Empty	TRUE	
20196-20225 HinckeM –TAXchicken	Uncategorized Sample	20196-20205_A	Mudpit_Ingel20196_MGFPeaklist (F058870)	Myosin-9 OS=Gallus gallus GN=MYH9 PE=4 SV=2	F1NCD4_CHICK,MYH9_CHICK,UPI000012FB7F,UPI000060E1E4,UPI0002C868F4	TAX_GallusGallus_9031_20141114.fasta,UPARC14_2_GallusG_9031_20140417.fasta	226,643.40	100.00%	49	53	115	0.23%	24.40%	QISVLQQQAVEAR	K	K	99.70%	71.5	43.7	29.1	53.9	45.3	3.96	0.422	0.5	0	6	0	0	2	1,469.81	2,037.50		6,070.84	1358	1370	Empty	TRUE	
20196-20225 HinckeM –TAXchicken	Uncategorized Sample	20196-20205_A	Mudpit_Ingel20196_MGFPeaklist (F058870)	Myosin-9 OS=Gallus gallus GN=MYH9 PE=4 SV=2	F1NCD4_CHICK,MYH9_CHICK,UPI000012FB7F,UPI000060E1E4,UPI0002C868F4	TAX_GallusGallus_9031_20141114.fasta,UPARC14_2_GallusG_9031_20140417.fasta	226,643.40	100.00%	49	53	115	0.23%	24.40%	QKHSQAIEELAEQLEQTKR	R	V	99.70%	38.5	43.4	23.6	26	30.7	2.18	0.192	0.184	0	0	0	2	2	2,266.18	2,363.00		795.44	1192	1210	Empty	TRUE	
20196-20225 HinckeM –TAXchicken	Uncategorized Sample	20196-20205_A	Mudpit_Ingel20196_MGFPeaklist (F058870)	Myosin-9 OS=Gallus gallus GN=MYH9 PE=4 SV=2	F1NCD4_CHICK,MYH9_CHICK,UPI000012FB7F,UPI000060E1E4,UPI0002C868F4	TAX_GallusGallus_9031_20141114.fasta,UPARC14_2_GallusG_9031_20140417.fasta	226,643.40	100.00%	49	53	115	0.23%	24.40%	QLLQANPILEAFGNAK	R	T	33.80%	11.9	42.9	17.8	9.6					0	1	0	0	2	1,726.95	2,877.00		107.82	210	225	Empty	TRUE	MYH11_CHICK
20196-20225 HinckeM –TAXchicken	Uncategorized Sample	20196-20205_A	Mudpit_Ingel20196_MGFPeaklist (F058870)	Myosin-9 OS=Gallus gallus GN=MYH9 PE=4 SV=2	F1NCD4_CHICK,MYH9_CHICK,UPI000012FB7F,UPI000060E1E4,UPI0002C868F4	TAX_GallusGallus_9031_20141114.fasta,UPARC14_2_GallusG_9031_20140417.fasta	226,643.40	100.00%	49	53	115	0.23%	24.40%	QRYEILTPNAIPK	R	G	99.70%	44.8	43.5	22.5	30.7	33.5	1.96	0.358	0.423	0	2	4	0	2	1,542.86	2,240.50		4,800.05	719	731	Empty	TRUE	UPI00000FC6BE,Q789A5_CHICK,Q789A4_CHICK,UPI0000171382,UPI000060F510,UPI0000ECA50D,UPI000060F511,UPI00003AB452,Q789A6_CHICK,UPI0000171380,Q02015_CHICK,UPI0000171381,UPI00003AB454,UPI00003AB453
20196-20225 HinckeM –TAXchicken	Uncategorized Sample	20196-20205_A	Mudpit_Ingel20196_MGFPeaklist (F058870)	Myosin-9 OS=Gallus gallus GN=MYH9 PE=4 SV=2	F1NCD4_CHICK,MYH9_CHICK,UPI000012FB7F,UPI000060E1E4,UPI0002C868F4	TAX_GallusGallus_9031_20141114.fasta,UPARC14_2_GallusG_9031_20140417.fasta	226,643.40	100.00%	49	53	115	0.23%	24.40%	RGDLPFVVTR	R	R	99.70%	45.9	44.6	39	28	34.9	1.66	0.301	0.5	0	2	0	0	2	1,159.66	2,210.00		561.14	1923	1932	Empty	TRUE	
20196-20225 HinckeM –TAXchicken	Uncategorized Sample	20196-20205_A	Mudpit_Ingel20196_MGFPeaklist (F058870)	Myosin-9 OS=Gallus gallus GN=MYH9 PE=4 SV=2	F1NCD4_CHICK,MYH9_CHICK,UPI000012FB7F,UPI000060E1E4,UPI0002C868F4	TAX_GallusGallus_9031_20141114.fasta,UPARC14_2_GallusG_9031_20140417.fasta	226,643.40	100.00%	49	53	115	0.23%	24.40%	TDLLLEPYNK	K	Y	99.70%	34.2	44.8	29.3	12.7	27.4	1.57	0.234	0.35	0	2	0	0	2	1,205.64	2,293.50		465.92	290	299	Empty	TRUE	
20196-20225 HinckeM –TAXchicken	Uncategorized Sample	20196-20205_A	Mudpit_Ingel20196_MGFPeaklist (F058870)	Myosin-9 OS=Gallus gallus GN=MYH9 PE=4 SV=2	F1NCD4_CHICK,MYH9_CHICK,UPI000012FB7F,UPI000060E1E4,UPI0002C868F4	TAX_GallusGallus_9031_20141114.fasta,UPARC14_2_GallusG_9031_20140417.fasta	226,643.40	100.00%	49	53	115	0.23%	24.40%	TDLLLEPYNKYR	K	F	99.70%	24.5	44.3	23.3	10.2	26.9	2.44	0.208	0.333	0	0	2	0	2	1,524.81	2,290.00		819.24	290	301	Empty	TRUE	
20196-20225 HinckeM –TAXchicken	Uncategorized Sample	20196-20205_A	Mudpit_Ingel20196_MGFPeaklist (F058870)	Myosin-9 OS=Gallus gallus GN=MYH9 PE=4 SV=2	F1NCD4_CHICK,MYH9_CHICK,UPI000012FB7F,UPI000060E1E4,UPI0002C868F4	TAX_GallusGallus_9031_20141114.fasta,UPARC14_2_GallusG_9031_20140417.fasta	226,643.40	100.00%	49	53	115	0.23%	24.40%	TLEDEAKTHEAQIQEMR	K	Q	98.60%	43.9	44	22.9	33.3					0	0	0	2	2	2,044.96	1,846.50		641.82	1175	1191	Empty	TRUE	
20196-20225 HinckeM –TAXchicken	Uncategorized Sample	20196-20205_A	Mudpit_Ingel20196_MGFPeaklist (F058870)	Myosin-9 OS=Gallus gallus GN=MYH9 PE=4 SV=2	F1NCD4_CHICK,MYH9_CHICK,UPI000012FB7F,UPI000060E1E4,UPI0002C868F4	TAX_GallusGallus_9031_20141114.fasta,UPARC14_2_GallusG_9031_20140417.fasta	226,643.40	100.00%	49	53	115	0.23%	24.40%	TSREEILAQAK	R	E	92.20%	37	44.7	34.3	9.74					0	0	3	0	2	1,245.68	1,668.00		1,425.65	1659	1669	Empty	TRUE	
20196-20225 HinckeM –TAXchicken	Uncategorized Sample	20196-20205_A	Mudpit_Ingel20196_MGFPeaklist (F058870)	Myosin-9 OS=Gallus gallus GN=MYH9 PE=4 SV=2	F1NCD4_CHICK,MYH9_CHICK,UPI000012FB7F,UPI000060E1E4,UPI0002C868F4	TAX_GallusGallus_9031_20141114.fasta,UPARC14_2_GallusG_9031_20140417.fasta	226,643.40	100.00%	49	53	115	0.23%	24.40%	TSREEILAQAKENEK	R	K	99.70%	49.7	44.6	29.6	30.6	42.7	3.42	0.335	0.5	0	0	0	2	2	1,745.90	1,637.00		624	1659	1673	Empty	TRUE	
20196-20225 HinckeM –TAXchicken	Uncategorized Sample	20196-20205_A	Mudpit_Ingel20196_MGFPeaklist (F058870)	Myosin-9 OS=Gallus gallus GN=MYH9 PE=4 SV=2	F1NCD4_CHICK,MYH9_CHICK,UPI000012FB7F,UPI000060E1E4,UPI0002C868F4	TAX_GallusGallus_9031_20141114.fasta,UPARC14_2_GallusG_9031_20140417.fasta	226,643.40	100.00%	49	53	115	0.23%	24.40%	TVGQLYKEQLAK	R	L	99.70%	37	43.6	25.2	25.9	29.3	1.14	0.266	0.417	0	0	4	0	2	1,377.77	1,866.50		2,477.43	645	656	Empty	TRUE	
20196-20225 HinckeM –TAXchicken	Uncategorized Sample	20196-20205_A	Mudpit_Ingel20196_MGFPeaklist (F058870)	Myosin-9 OS=Gallus gallus GN=MYH9 PE=4 SV=2	F1NCD4_CHICK,MYH9_CHICK,UPI000012FB7F,UPI000060E1E4,UPI0002C868F4	TAX_GallusGallus_9031_20141114.fasta,UPARC14_2_GallusG_9031_20140417.fasta	226,643.40	100.00%	49	53	115	0.23%	24.40%	VDAQLQELQVK	K	F	50.50%	23.2	43.9	38.2	1.53					0	1	0	0	2	1,270.70	2,098.00		128.78	1250	1260	Empty	TRUE	
20196-20225 HinckeM –TAXchicken	Uncategorized Sample	20196-20205_A	Mudpit_Ingel20196_MGFPeaklist (F058870)	Myosin-9 OS=Gallus gallus GN=MYH9 PE=4 SV=2	F1NCD4_CHICK,MYH9_CHICK,UPI000012FB7F,UPI000060E1E4,UPI0002C868F4	TAX_GallusGallus_9031_20141114.fasta,UPARC14_2_GallusG_9031_20140417.fasta	226,643.40	100.00%	49	53	115	0.23%	24.40%	VISGVLQLGNIVFKK	K	E	99.70%	64.9	36.9	20.4	56.6	45.8	3.11	0.162	0.5	0	0	2	0	2	1,614.99	2,854.50		1,206.55	342	356	Empty	TRUE	
20196-20225 HinckeM –TAXchicken	Uncategorized Sample	20196-20205_A	Mudpit_Ingel20196_MGFPeaklist (F058870)	Myosin-9 OS=Gallus gallus GN=MYH9 PE=4 SV=2	F1NCD4_CHICK,MYH9_CHICK,UPI000012FB7F,UPI000060E1E4,UPI0002C868F4	TAX_GallusGallus_9031_20141114.fasta,UPARC14_2_GallusG_9031_20140417.fasta	226,643.40	100.00%	49	53	115	0.23%	24.40%	VSHLLGINVTDFTR	K	G	99.70%	50.9	43.8	29.7	35.6	39.2	4.7	0.487	0.429	0	0	3	0	2	1,571.85	2,599.00		1,284.73	374	387	Empty	TRUE	
20196-20225 HinckeM –TAXchicken	Uncategorized Sample	20196-20205_A	Mudpit_Ingel20196_MGFPeaklist (F058870)	Myosin-9 OS=Gallus gallus GN=MYH9 PE=4 SV=2	F1NCD4_CHICK,MYH9_CHICK,UPI000012FB7F,UPI000060E1E4,UPI0002C868F4	TAX_GallusGallus_9031_20141114.fasta,UPARC14_2_GallusG_9031_20140417.fasta	226,643.40	100.00%	49	53	115	0.23%	24.40%	VVFQEFR	R	Q	74.20%	30.9	43.3	26.1	16.6					0	4	0	0	2	924.4937	2,156.00		1,480.25	712	718	Empty	TRUE	
20196-20225 HinckeM –TAXchicken	Uncategorized Sample	20196-20205_A	Mudpit_Ingel20196_MGFPeaklist (F058870)	Myosin-9 OS=Gallus gallus GN=MYH9 PE=4 SV=2	F1NCD4_CHICK,MYH9_CHICK,UPI000012FB7F,UPI000060E1E4,UPI0002C868F4	TAX_GallusGallus_9031_20141114.fasta,UPARC14_2_GallusG_9031_20140417.fasta	226,643.40	100.00%	49	53	115	0.23%	24.40%	YEILTPNAIPK	R	G	99.70%	20.6	44.4	14.6	7.9	28.5	1.57	0.277	0.318	0	3	0	0	2	1,258.70	2,337.00		1,618.82	721	731	Empty	TRUE	UPI00000FC6BE,Q789A5_CHICK,Q789A4_CHICK,UPI0000171382,UPI000060F510,UPI0000ECA50D,UPI000060F511,UPI00003AB452,Q789A6_CHICK,UPI0000171380,Q02015_CHICK,UPI0000171381,UPI00003AB454,UPI00003AB453
20196-20225 HinckeM –TAXchicken	Uncategorized Sample	20196-20205_A	Mudpit_Ingel20196_MGFPeaklist (F058870)	Myosin-9 OS=Gallus gallus GN=MYH9 PE=4 SV=2	F1NCD4_CHICK,MYH9_CHICK,UPI000012FB7F,UPI000060E1E4,UPI0002C868F4	TAX_GallusGallus_9031_20141114.fasta,UPARC14_2_GallusG_9031_20140417.fasta	226,643.40	100.00%	49	53	115	0.23%	24.40%	ADFCIIHYAGK	K	V	45.90%	18.6	44.4	14.7	11.1					0	0	2	0	2	1,294.63	2,223.00		989.84	566	576	Empty	TRUE	UPI00000FC6BE,Q789A5_CHICK,Q789A4_CHICK,UPI0000171382,UPI000060F510,UPI0000ECA50D,UPI000060F511,UPI00003AB452,Q789A6_CHICK,UPI0000171380,Q02015_CHICK,UPI0000171381,UPI00003AB454,UPI00003AB453
20196-20225 HinckeM –TAXchicken	Uncategorized Sample	20196-20205_A	Mudpit_Ingel20196_MGFPeaklist (F058870)	Myosin-9 OS=Gallus gallus GN=MYH9 PE=4 SV=2	F1NCD4_CHICK,MYH9_CHICK,UPI000012FB7F,UPI000060E1E4,UPI0002C868F4	TAX_GallusGallus_9031_20141114.fasta,UPARC14_2_GallusG_9031_20140417.fasta	226,643.40	100.00%	49	53	115	0.23%	24.40%	AGKLDPHLVLDQLR	K	C	99.70%	52.2	42.3	31.5	35.1	37.5	3.54	0.344	0.5	0	0	2	0	2	1,574.90	2,445.50		970.79	680	693	Empty	TRUE	UPI00000FC6BE,Q789A5_CHICK,Q789A4_CHICK,UPI0000171382,UPI000060F510,UPI0000ECA50D,UPI000060F511,UPI00003AB452,Q789A6_CHICK,UPI0000171380,Q02015_CHICK,UPI0000171381,UPI00003AB454,UPI00003AB453
20196-20225 HinckeM –TAXchicken	Uncategorized Sample	20196-20205_A	Mudpit_Ingel20196_MGFPeaklist (F058870)	Myosin-9 OS=Gallus gallus GN=MYH9 PE=4 SV=2	F1NCD4_CHICK,MYH9_CHICK,UPI000012FB7F,UPI000060E1E4,UPI0002C868F4	TAX_GallusGallus_9031_20141114.fasta,UPARC14_2_GallusG_9031_20140417.fasta	226,643.40	100.00%	49	53	115	0.23%	24.40%	AGVLAHLEEER	R	D	99.70%	71.3	44.3	36.2	36.2	34.7	3.4	0.317	0.409	0	0	2	0	2	1,223.64	2,031.50		1,163.70	765	775	Empty	TRUE	UPI00000FC6BE,Q789A5_CHICK,Q789A4_CHICK,UPI0000171382,UPI000060F510,UPI0000ECA50D,UPI000060F511,UPI00003AB452,Q789A6_CHICK,UPI0000171380,Q02015_CHICK,UPI0000171381,UPI00003AB454,UPI00003AB453
20196-20225 HinckeM –TAXchicken	Uncategorized Sample	20196-20205_A	Mudpit_Ingel20196_MGFPeaklist (F058870)	Myosin-9 OS=Gallus gallus GN=MYH9 PE=4 SV=2	F1NCD4_CHICK,MYH9_CHICK,UPI000012FB7F,UPI000060E1E4,UPI0002C868F4	TAX_GallusGallus_9031_20141114.fasta,UPARC14_2_GallusG_9031_20140417.fasta	226,643.40	100.00%	49	53	115	0.23%	24.40%	AGVLAHLEEERDLK	R	I	99.70%	43.7	43.9	28.2	36.5	38.3	3.7	0.371	0.464	0	0	2	0	2	1,579.84	2,107.00		1,024.73	765	778	Empty	TRUE	UPI00000FC6BE,Q789A5_CHICK,Q789A4_CHICK,UPI0000171382,UPI000060F510,UPI0000ECA50D,UPI000060F511,UPI00003AB452,Q789A6_CHICK,UPI0000171380,Q02015_CHICK,UPI0000171381,UPI00003AB454,UPI00003AB453
20196-20225 HinckeM –TAXchicken	Uncategorized Sample	20196-20205_A	Mudpit_Ingel20196_MGFPeaklist (F058870)	Myosin-9 OS=Gallus gallus GN=MYH9 PE=4 SV=2	F1NCD4_CHICK,MYH9_CHICK,UPI000012FB7F,UPI000060E1E4,UPI0002C868F4	TAX_GallusGallus_9031_20141114.fasta,UPARC14_2_GallusG_9031_20140417.fasta	226,643.40	100.00%	49	53	115	0.23%	24.40%	ALEEAIEQK	R	A	84.60%	33.1	45.3	36.6	8.49					0	3	0	0	2	1,030.54	1,744.00		686.34	1484	1492	Empty	TRUE	

20196-20225 HinckeM –TAXchicken	Uncategorized Sample	20196-20205_A	Mudpit_Ingel20196_MGFPeaklist (F058870)	Myosin-9 OS=Gallus gallus GN=MYH9 PE=4 SV=2	F1NCD4_CHICK,MYH9_CHICK,UPI000012FB7F,UPI000060E1E4,UPI0002C868F4	TAX_GallusGallus_9031_20141114.fasta,UPARC14_2_GallusG_9031_20140417.fasta	226,643.40	100.00%	49	53	115	0.23%	24.40%	ALEEAIEQKAELER	R	V	99.70%	72.1	44.6	34.9	46.5	50.4	4.02	0.423	0.607	0	1	4	0	2	1,628.85	2,104.00		4,055.28	1484	1497	Empty	TRUE	
20196-20225 HinckeM –TAXchicken	Uncategorized Sample	20196-20205_A	Mudpit_Ingel20196_MGFPeaklist (F058870)	Myosin-9 OS=Gallus gallus GN=MYH9 PE=4 SV=2	F1NCD4_CHICK,MYH9_CHICK,UPI000012FB7F,UPI000060E1E4,UPI0002C868F4	TAX_GallusGallus_9031_20141114.fasta,UPARC14_2_GallusG_9031_20140417.fasta	226,643.40	100.00%	49	53	115	0.23%	24.40%	ALELDSNLYR	K	I	99.70%	58.5	44.7	27.2	33.6	40.4	3.82	0.351	0.55	0	5	0	0	2	1,193.62	2,244.00		3,596.17	746	755	Empty	TRUE	
20196-20225 HinckeM –TAXchicken	Uncategorized Sample	20196-20205_A	Mudpit_Ingel20196_MGFPeaklist (F058870)	Myosin-9 OS=Gallus gallus GN=MYH9 PE=4 SV=2	F1NCD4_CHICK,MYH9_CHICK,UPI000012FB7F,UPI000060E1E4,UPI0002C868F4	TAX_GallusGallus_9031_20141114.fasta,UPARC14_2_GallusG_9031_20140417.fasta	226,643.40	100.00%	49	53	115	0.23%	24.40%	ANLQIDQMNADLNAER	K	S	99.70%	61.6	44.3	23.7	51.8	27.8	1.57	0.237	0.219	0	0	1	0	2	1,831.86	1,969.00		215.45	1755	1770	Empty	TRUE	
20196-20225 HinckeM –TAXchicken	Uncategorized Sample	20196-20205_A	Mudpit_Ingel20196_MGFPeaklist (F058870)	Myosin-9 OS=Gallus gallus GN=MYH9 PE=4 SV=2	F1NCD4_CHICK,MYH9_CHICK,UPI000012FB7F,UPI000060E1E4,UPI0002C868F4	TAX_GallusGallus_9031_20141114.fasta,UPARC14_2_GallusG_9031_20140417.fasta	226,643.40	100.00%	49	53	115	0.23%	24.40%	ATITALEAK	K	I	14.20%	16.5	45.4	22.1	0.69					0	2	0	0	2	917.5303	1,880.00		993.4	1807	1815	Empty	TRUE	
20196-20225 HinckeM –TAXchicken	Uncategorized Sample	20196-20205_A	Mudpit_Ingel20196_MGFPeaklist (F058870)	Myosin-9 OS=Gallus gallus GN=MYH9 PE=4 SV=2	F1NCD4_CHICK,MYH9_CHICK,UPI000012FB7F,UPI000060E1E4,UPI0002C868F4	TAX_GallusGallus_9031_20141114.fasta,UPARC14_2_GallusG_9031_20140417.fasta	226,643.40	100.00%	49	53	115	0.23%	24.40%	CNGVLEGIR	R	I	28.90%	28.9	45.5	29.6	12.6					0	1	0	0	2	1,018.50	2,046.00		210.55	694	702	Empty	TRUE	MYH11_CHICK,Q789A4_CHICK,Q9PTY2_CHICK,UPI0000ECA50D,Q789A6_CHICK,UPI00000FC6BE,Q789A5_CHICK,UPI0000171382,UPI000044943F,UPI000060F510,UPI000060F511,UPI00003AB452,UPI0000171380,Q02015_CHICK,UPI0000171381,UPI00003AB434,UPI00000FB7E2,UPI00003AB454,UPI00003AB431,UPI00003AB453
20196-20225 HinckeM –TAXchicken	Uncategorized Sample	20196-20205_A	Mudpit_Ingel20196_MGFPeaklist (F058870)	Myosin-9 OS=Gallus gallus GN=MYH9 PE=4 SV=2	F1NCD4_CHICK,MYH9_CHICK,UPI000012FB7F,UPI000060E1E4,UPI0002C868F4	TAX_GallusGallus_9031_20141114.fasta,UPARC14_2_GallusG_9031_20140417.fasta	226,643.40	100.00%	49	53	115	0.23%	24.40%	DADKYLYVDK	R	N	47.90%	22.8	44.7	32.5	0					0	0	1	0	2	1,229.61	1,861.00		140	5	14	Empty	TRUE	
20196-20225 HinckeM –TAXchicken	Uncategorized Sample	20196-20205_A	Mudpit_Ingel20196_MGFPeaklist (F058870)	Myosin-9 OS=Gallus gallus GN=MYH9 PE=4 SV=2	F1NCD4_CHICK,MYH9_CHICK,UPI000012FB7F,UPI000060E1E4,UPI0002C868F4	TAX_GallusGallus_9031_20141114.fasta,UPARC14_2_GallusG_9031_20140417.fasta	226,643.40	100.00%	49	53	115	0.23%	24.40%	DLESLTQRYEEK	K	I	56.20%	18.3	44.4	21.2	11.9					0	0	3	0	2	1,510.74	2,123.00		788.9	1393	1404	Empty	TRUE	
20196-20225 HinckeM –TAXchicken	Uncategorized Sample	20196-20205_A	Mudpit_Ingel20196_MGFPeaklist (F058870)	Myosin-9 OS=Gallus gallus GN=MYH9 PE=4 SV=2	F1NCD4_CHICK,MYH9_CHICK,UPI000012FB7F,UPI000060E1E4,UPI0002C868F4	TAX_GallusGallus_9031_20141114.fasta,UPARC14_2_GallusG_9031_20140417.fasta	226,643.40	100.00%	49	53	115	0.23%	24.40%	EQADFAIEALAK	K	A	99.70%	57.8	45	27.9	43.9	37.5	3.8	0.331	0.375	0	4	0	0	2	1,305.67	2,461.50		1,501.85	408	419	Empty	TRUE	MYH11_CHICK
20196-20225 HinckeM –TAXchicken	Uncategorized Sample	20196-20205_A	Mudpit_Ingel20196_MGFPeaklist (F058870)	Myosin-9 OS=Gallus gallus GN=MYH9 PE=4 SV=2	F1NCD4_CHICK,MYH9_CHICK,UPI000012FB7F,UPI000060E1E4,UPI0002C868F4	TAX_GallusGallus_9031_20141114.fasta,UPARC14_2_GallusG_9031_20140417.fasta	226,643.40	100.00%	49	53	115	0.23%	24.40%	FDQLLAEEK	K	N	62.50%	25.1	44.8	29.4	6.88					0	2	0	0	2	1,092.56	2,017.50		508.72	1446	1454	Empty	TRUE	
20196-20225 HinckeM –TAXchicken	Uncategorized Sample	20196-20205_A	Mudpit_Ingel20196_MGFPeaklist (F058870)	Myosin-9 OS=Gallus gallus GN=MYH9 PE=4 SV=2	F1NCD4_CHICK,MYH9_CHICK,UPI000012FB7F,UPI000060E1E4,UPI0002C868F4	TAX_GallusGallus_9031_20141114.fasta,UPARC14_2_GallusG_9031_20140417.fasta	226,643.40	100.00%	49	53	115	0.23%	24.40%	HSQAIEELAEQLEQTKR	K	V	83.90%	21.7	43.9	18	16.7					0	0	0	2	2	2,010.03	2,465.50		456.01	1194	1210	Empty	TRUE	
20196-20225 HinckeM –TAXchicken	Uncategorized Sample	20196-20205_A	Mudpit_Ingel20196_MGFPeaklist (F058870)	Myosin-9 OS=Gallus gallus GN=MYH9 PE=4 SV=2	F1NCD4_CHICK,MYH9_CHICK,UPI000012FB7F,UPI000060E1E4,UPI0002C868F4	TAX_GallusGallus_9031_20141114.fasta,UPARC14_2_GallusG_9031_20140417.fasta	226,643.40	100.00%	49	53	115	0.23%	24.40%	IMGIPDEEQIGLLK	R	V	99.70%	33.4	44.2	14.4	29.2	36.2	1.96	0.318	0.357	0	2	0	0	2	1,571.84	2,519.50		2,423.65	328	341	Empty	TRUE	
20196-20225 HinckeM –TAXchicken	Uncategorized Sample	20196-20205_A	Mudpit_Ingel20196_MGFPeaklist (F058870)	Myosin-9 OS=Gallus gallus GN=MYH9 PE=4 SV=2	F1NCD4_CHICK,MYH9_CHICK,UPI000012FB7F,UPI000060E1E4,UPI0002C868F4	TAX_GallusGallus_9031_20141114.fasta,UPARC14_2_GallusG_9031_20140417.fasta	226,643.40	100.00%	49	53	115	0.23%	24.40%	IVQLEEQLDMETKER	K	Q	98.40%	37.5	44.6	22.6	23.5					0	0	2	0	2	1,876.93	2,189.50		318.2	1816	1830	Empty	TRUE	
20196-20225 HinckeM –TAXchicken	Uncategorized Sample	20196-20205_A	Mudpit_Ingel20196_MGFPeaklist (F058870)	Myosin-9 OS=Gallus gallus GN=MYH9 PE=4 SV=2	F1NCD4_CHICK,MYH9_CHICK,UPI000012FB7F,UPI000060E1E4,UPI0002C868F4	TAX_GallusGallus_9031_20141114.fasta,UPARC14_2_GallusG_9031_20140417.fasta	226,643.40	100.00%	49	53	115	0.23%	24.40%	KFDQLLAEEK	K	N	93.10%	39.1	44.6	40.3	9.11					0	0	4	0	2	1,220.65	2,002.50		2,060.83	1445	1454	Empty	TRUE	
20196-20225 HinckeM –TAXchicken	Uncategorized Sample	20196-20205_A	Mudpit_Ingel20196_MGFPeaklist (F058870)	Myosin-9 OS=Gallus gallus GN=MYH9 PE=4 SV=2	F1NCD4_CHICK,MYH9_CHICK,UPI000012FB7F,UPI000060E1E4,UPI0002C868F4	TAX_GallusGallus_9031_20141114.fasta,UPARC14_2_GallusG_9031_20140417.fasta	226,643.40	100.00%	49	53	115	0.23%	24.40%	KFDQLLAEEKNISAK	K	Y	99.70%	26	43.5	21.7	15.4	29.3	1.7	0.147	0.367	0	0	1	0	2	1,733.94	2,153.00		416.04	1445	1459	Empty	TRUE	
20196-20225 HinckeM –TAXchicken	Uncategorized Sample	20196-20205_A	Mudpit_Ingel20196_MGFPeaklist (F058870)	Myosin-9 OS=Gallus gallus GN=MYH9 PE=4 SV=2	F1NCD4_CHICK,MYH9_CHICK,UPI000012FB7F,UPI000060E1E4,UPI0002C868F4	TAX_GallusGallus_9031_20141114.fasta,UPARC14_2_GallusG_9031_20140417.fasta	226,643.40	100.00%	49	53	115	0.23%	24.40%	KKVDAQLQELQVK	R	F	67.80%	28.2	40.7	28.4	11.1					0	0	1	0	2	1,526.89	1,881.00		329.09	1248	1260	Empty	TRUE	
20196-20225 HinckeM –TAXchicken	Uncategorized Sample	20196-20205_A	Mudpit_Ingel20196_MGFPeaklist (F058870)	Myosin-9 OS=Gallus gallus GN=MYH9 PE=4 SV=2	F1NCD4_CHICK,MYH9_CHICK,UPI000012FB7F,UPI000060E1E4,UPI0002C868F4	TAX_GallusGallus_9031_20141114.fasta,UPARC14_2_GallusG_9031_20140417.fasta	226,643.40	100.00%	49	53	115	0.23%	24.40%	KLEEDVIVLEDQNLK	K	L	99.70%	41	43.3	24.6	26.1	36.6	4	0.363	0.367	0	0	2	0	2	1,784.96	2,380.50		1,109.02	975	989	Empty	TRUE	
20196-20225 HinckeM –TAXchicken	Uncategorized Sample	20196-20205_A	Mudpit_Ingel20196_MGFPeaklist (F058870)	Myosin-9 OS=Gallus gallus GN=MYH9 PE=4 SV=2	F1NCD4_CHICK,MYH9_CHICK,UPI000012FB7F,UPI000060E1E4,UPI0002C868F4	TAX_GallusGallus_9031_20141114.fasta,UPARC14_2_GallusG_9031_20140417.fasta	226,643.40	100.00%	49	53	115	0.23%	24.40%	KLVWVPSEK	K	S	29.70%	13.5	43.2	17.5	0					0	1	0	0	2	1,085.64	2,060.00		390.18	30	38	Empty	TRUE	MYH11_CHICK
20196-20225 HinckeM –TAXchicken	Uncategorized Sample	20196-20205_A	Mudpit_Ingel20196_MGFPeaklist (F058870)	Myosin-9 OS=Gallus gallus GN=MYH9 PE=4 SV=2	F1NCD4_CHICK,MYH9_CHICK,UPI000012FB7F,UPI000060E1E4,UPI0002C868F4	TAX_GallusGallus_9031_20141114.fasta,UPARC14_2_GallusG_9031_20140417.fasta	226,643.40	100.00%	49	53	115	0.23%	24.40%	KQELEEICHDLEAR	K	V	99.70%	48.3	44.3	23.2	40.8	35.2	3.44	0.347	0.357	0	0	2	1	2	1,769.85	2,175.00		1,534.37	910	923	Empty	TRUE	
20196-20225 HinckeM –TAXchicken	Uncategorized Sample	20196-20205_A	Mudpit_Ingel20196_MGFPeaklist (F058870)	Myosin-9 OS=Gallus gallus GN=MYH9 PE=4 SV=2	F1NCD4_CHICK,MYH9_CHICK,UPI000012FB7F,UPI000060E1E4,UPI0002C868F4	TAX_GallusGallus_9031_20141114.fasta,UPARC14_2_GallusG_9031_20140417.fasta	226,643.40	100.00%	49	53	115	0.23%	24.40%	KVDAQLQELQVK	K	F	99.70%	33.1	42.7	21.6	9.14	27.3	1.21	0.22	0.333	0	0	2	0	2	1,398.80	2,003.00		643.7	1249	1260	Empty	TRUE	
20196-20225 HinckeM –TAXchicken	Uncategorized Sample	20196-20205_A	Mudpit_Ingel20196_MGFPeaklist (F058870)	Myosin-9 OS=Gallus gallus GN=MYH9 PE=4 SV=2	F1NCD4_CHICK,MYH9_CHICK,UPI000012FB7F,UPI000060E1E4,UPI0002C868F4	TAX_GallusGallus_9031_20141114.fasta,UPARC14_2_GallusG_9031_20140417.fasta	226,643.40	100.00%	49	53	115	0.23%	24.40%	LDPHLVLDQLR	K	C	99.70%	34.6	43.8	18.6	22.1	32.5	2.47	0.366	0.455	0	0	1	0	2	1,318.75	2,534.00		1,121.62	683	693	Empty	TRUE	UPI00000FC6BE,Q789A5_CHICK,Q789A4_CHICK,UPI0000171382,UPI000060F510,UPI0000ECA50D,UPI000060F511,UPI00003AB452,Q789A6_CHICK,UPI0000171380,Q02015_CHICK,UPI0000171381,UPI00003AB454,UPI00003AB453
20196-20225 HinckeM –TAXchicken	Uncategorized Sample	20196-20205_A	Mudpit_Ingel20196_MGFPeaklist (F058870)	Myosin-9 OS=Gallus gallus GN=MYH9 PE=4 SV=2	F1NCD4_CHICK,MYH9_CHICK,UPI000012FB7F,UPI000060E1E4,UPI0002C868F4	TAX_GallusGallus_9031_20141114.fasta,UPARC14_2_GallusG_9031_20140417.fasta	226,643.40	100.00%	49	53	115	0.23%	24.40%	LKDILLQVDDERR	K	N	27.70%	15.6	42.7	21.5	4.84					0	0	0	1	2	1,612.90	2,214.00		458.07	1844	1856	Empty	TRUE	
20196-20225 HinckeM –TAXchicken	Uncategorized Sample	20196-20205_A	Mudpit_Ingel20196_MGFPeaklist (F058870)	Myosin-9 OS=Gallus gallus GN=MYH9 PE=4 SV=2	F1NCD4_CHICK,MYH9_CHICK,UPI000012FB7F,UPI000060E1E4,UPI0002C868F4	TAX_GallusGallus_9031_20141114.fasta,UPARC14_2_GallusG_9031_20140417.fasta	226,643.40	100.00%	49	53	115	0.23%	24.40%	LQQELDDIAVDLDHQR	R	Q	99.70%	68.1	44.4	19.5	56.3	44.1	5.77	0.515	0.438	0	0	2	0	2	1,907.95	2,474.00		1,640.23	1418	1433	Empty	TRUE	
20196-20225 HinckeM –TAXchicken	Uncategorized Sample	20196-20205_A	Mudpit_Ingel20196_MGFPeaklist (F058870)	Myosin-9 OS=Gallus gallus GN=MYH9 PE=4 SV=2	F1NCD4_CHICK,MYH9_CHICK,UPI000012FB7F,UPI000060E1E4,UPI0002C868F4	TAX_GallusGallus_9031_20141114.fasta,UPARC14_2_GallusG_9031_20140417.fasta	226,643.40	100.00%	49	53	115	0.23%	24.40%	LRLEVNQQAMK	K	A	73.00%	17.5	44.8	15.5	15.2					0	0	1	0	2	1,345.73	1,759.00		157.86	1556	1566	Empty	TRUE	
20196-20225 HinckeM –TAXchicken	Uncategorized Sample	20196-20205_A	Mudpit_Ingel20196_MGFPeaklist (F058870)	Myosin-9 OS=Gallus gallus GN=MYH9 PE=4 SV=2	F1NCD4_CHICK,MYH9_CHICK,UPI000012FB7F,UPI000060E1E4,UPI0002C868F4	TAX_GallusGallus_9031_20141114.fasta,UPARC14_2_GallusG_9031_20140417.fasta	226,643.40	100.00%	49	53	115	0.23%	24.40%	LVWVPSEK	K	S	99.70%	26.7	44.4	23.8	14.6	22.7	0.638	0.123	0.312	0	2	0	0	2	957.5404	2,164.50		372.91	31	38	Empty	TRUE	MYH11_CHICK
20196-20225 HinckeM –TAXchicken	Uncategorized Sample	20196-20205_A	Mudpit_Ingel20196_MGFPeaklist (F058870)	Myosin-9 OS=Gallus gallus GN=MYH9 PE=4 SV=2	F1NCD4_CHICK,MYH9_CHICK,UPI000012FB7F,UPI000060E1E4,UPI0002C868F4	TAX_GallusGallus_9031_20141114.fasta,UPARC14_2_GallusG_9031_20140417.fasta	226,643.40	100.00%	49	53	115	0.23%	24.40%	MSEFTTNLTEEEEKSK	R	S	99.70%	32.2	43	16.9	29.3	30.3	2.14	0.0066	0.25	0	0	2	0	2	1,902.86	2,035.00		827.04	1001	1016	Empty	TRUE	
20196-20225 HinckeM –TAXchicken	Uncategorized Sample	20196-20205_A	Mudpit_Ingel20196_MGFPeaklist (F058870)	Myosin-9 OS=Gallus gallus GN=MYH9 PE=4 SV=2	F1NCD4_CHICK,MYH9_CHICK,UPI000012FB7F,UPI000060E1E4,UPI0002C868F4	TAX_GallusGallus_9031_20141114.fasta,UPARC14_2_GallusG_9031_20140417.fasta	226,643.40	100.00%	49	53	115	0.23%	24.40%	NWQWWR	R	L	24.20%	12.7	45.1	22.7	3.78					0	1	0	0	2	975.4583	2,584.00		138.57	824	829	Empty	TRUE	MYH11_CHICK
20196-20225 HinckeM –TAXchicken	Uncategorized Sample	20196-20205_A	Mudpit_Ingel20196_MGFPeaklist (F058870)	Myosin-9 OS=Gallus gallus GN=MYH9 PE=4 SV=2	F1NCD4_CHICK,MYH9_CHICK,UPI000012FB7F,UPI000060E1E4,UPI0002C868F4	TAX_GallusGallus_9031_20141114.fasta,UPARC14_2_GallusG_9031_20140417.fasta	226,643.40	100.00%	49	53	115	0.23%	24.40%	QACVLMIK	K	A	17.50%	25.6	44	27.9	9.47					0	1	0	0	2	961.4847	2,343.00		270	738	745	Empty	TRUE	
20196-20225 HinckeM –TAXchicken	Uncategorized Sample	20196-20205_A	Mudpit_Ingel20196_MGFPeaklist (F058870)	Myosin-9 OS=Gallus gallus GN=MYH9 PE=4 SV=2	F1NCD4_CHICK,MYH9_CHICK,UPI000012FB7F,UPI000060E1E4,UPI0002C868F4	TAX_GallusGallus_9031_20141114.fasta,UPARC14_2_GallusG_9031_20140417.fasta	226,643.40	100.00%	49	53	115	0.23%	24.40%	QAQQERDELADEIANSSGK	R	G	99.70%	56.6	44.1	18.7	50.3	48	6.68	0.515	0.395	0	0	4	0	2	2,088.98	1,982.00		4,035.47	1698	1716	Empty	TRUE	
20196-20225 HinckeM –TAXchicken	Uncategorized Sample	20196-20205_A	Mudpit_Ingel20196_MGFPeaklist (F058870)	Myosin-9 OS=Gallus gallus GN=MYH9 PE=4 SV=2	F1NCD4_CHICK,MYH9_CHICK,UPI000012FB7F,UPI000060E1E4,UPI0002C868F4	TAX_GallusGallus_9031_20141114.fasta,UPARC14_2_GallusG_9031_20140417.fasta	226,643.40	100.00%	49	53	115	0.23%	24.40%	QISVLQQQAVEAR	K	K	99.70%	71.5	43.7	29.1	53.9	45.3	3.96	0.422	0.5	0	6	0	0	2	1,469.81	2,037.50		6,070.84	1358	1370	Empty	TRUE	
20196-20225 HinckeM –TAXchicken	Uncategorized Sample	20196-20205_A	Mudpit_Ingel20196_MGFPeaklist (F058870)	Myosin-9 OS=Gallus gallus GN=MYH9 PE=4 SV=2	F1NCD4_CHICK,MYH9_CHICK,UPI000012FB7F,UPI000060E1E4,UPI0002C868F4	TAX_GallusGallus_9031_20141114.fasta,UPARC14_2_GallusG_9031_20140417.fasta	226,643.40	100.00%	49	53	115	0.23%	24.40%	QKHSQAIEELAEQLEQTKR	R	V	99.70%	38.5	43.4	23.6	26	30.7	2.18	0.192	0.184	0	0	0	2	2	2,266.18	2,363.00		795.44	1192	1210	Empty	TRUE	
20196-20225 HinckeM –TAXchicken	Uncategorized Sample	20196-20205_A	Mudpit_Ingel20196_MGFPeaklist (F058870)	Myosin-9 OS=Gallus gallus GN=MYH9 PE=4 SV=2	F1NCD4_CHICK,MYH9_CHICK,UPI000012FB7F,UPI000060E1E4,UPI0002C868F4	TAX_GallusGallus_9031_20141114.fasta,UPARC14_2_GallusG_9031_20140417.fasta	226,643.40	100.00%	49	53	115	0.23%	24.40%	QLLQANPILEAFGNAK	R	T	33.80%	11.9	42.9	17.8	9.6					0	1	0	0	2	1,726.95	2,877.00		107.82	210	225	Empty	TRUE	MYH11_CHICK
20196-20225 HinckeM –TAXchicken	Uncategorized Sample	20196-20205_A	Mudpit_Ingel20196_MGFPeaklist (F058870)	Myosin-9 OS=Gallus gallus GN=MYH9 PE=4 SV=2	F1NCD4_CHICK,MYH9_CHICK,UPI000012FB7F,UPI000060E1E4,UPI0002C868F4	TAX_GallusGallus_9031_20141114.fasta,UPARC14_2_GallusG_9031_20140417.fasta	226,643.40	100.00%	49	53	115	0.23%	24.40%	QRYEILTPNAIPK	R	G	99.70%	44.8	43.5	22.5	30.7	33.5	1.96	0.358	0.423	0	2	4	0	2	1,542.86	2,240.50		4,800.05	719	731	Empty	TRUE	UPI00000FC6BE,Q789A5_CHICK,Q789A4_CHICK,UPI0000171382,UPI000060F510,UPI0000ECA50D,UPI000060F511,UPI00003AB452,Q789A6_CHICK,UPI0000171380,Q02015_CHICK,UPI0000171381,UPI00003AB454,UPI00003AB453
20196-20225 HinckeM –TAXchicken	Uncategorized Sample	20196-20205_A	Mudpit_Ingel20196_MGFPeaklist (F058870)	Myosin-9 OS=Gallus gallus GN=MYH9 PE=4 SV=2	F1NCD4_CHICK,MYH9_CHICK,UPI000012FB7F,UPI000060E1E4,UPI0002C868F4	TAX_GallusGallus_9031_20141114.fasta,UPARC14_2_GallusG_9031_20140417.fasta	226,643.40	100.00%	49	53	115	0.23%	24.40%	RGDLPFVVTR	R	R	99.70%	45.9	44.6	39	28	34.9	1.66	0.301	0.5	0	2	0	0	2	1,159.66	2,210.00		561.14	1923	1932	Empty	TRUE	
20196-20225 HinckeM –TAXchicken	Uncategorized Sample	20196-20205_A	Mudpit_Ingel20196_MGFPeaklist (F058870)	Myosin-9 OS=Gallus gallus GN=MYH9 PE=4 SV=2	F1NCD4_CHICK,MYH9_CHICK,UPI000012FB7F,UPI000060E1E4,UPI0002C868F4	TAX_GallusGallus_9031_20141114.fasta,UPARC14_2_GallusG_9031_20140417.fasta	226,643.40	100.00%	49	53	115	0.23%	24.40%	TDLLLEPYNK	K	Y	99.70%	34.2	44.8	29.3	12.7	27.4	1.57	0.234	0.35	0	2	0	0	2	1,205.64	2,293.50		465.92	290	299	Empty	TRUE	
20196-20225 HinckeM –TAXchicken	Uncategorized Sample	20196-20205_A	Mudpit_Ingel20196_MGFPeaklist (F058870)	Myosin-9 OS=Gallus gallus GN=MYH9 PE=4 SV=2	F1NCD4_CHICK,MYH9_CHICK,UPI000012FB7F,UPI000060E1E4,UPI0002C868F4	TAX_GallusGallus_9031_20141114.fasta,UPARC14_2_GallusG_9031_20140417.fasta	226,643.40	100.00%	49	53	115	0.23%	24.40%	TDLLLEPYNKYR	K	F	99.70%	24.5	44.3	23.3	10.2	26.9	2.44	0.208	0.333	0	0	2	0	2	1,524.81	2,290.00		819.24	290	301	Empty	TRUE	
20196-20225 HinckeM –TAXchicken	Uncategorized Sample	20196-20205_A	Mudpit_Ingel20196_MGFPeaklist (F058870)	Myosin-9 OS=Gallus gallus GN=MYH9 PE=4 SV=2	F1NCD4_CHICK,MYH9_CHICK,UPI000012FB7F,UPI000060E1E4,UPI0002C868F4	TAX_GallusGallus_9031_20141114.fasta,UPARC14_2_GallusG_9031_20140417.fasta	226,643.40	100.00%	49	53	115	0.23%	24.40%	TLEDEAKTHEAQIQEMR	K	Q	98.60%	43.9	44	22.9	33.3					0	0	0	2	2	2,044.96	1,846.50		641.82	1175	1191	Empty	TRUE	
20196-20225 HinckeM –TAXchicken	Uncategorized Sample	20196-20205_A	Mudpit_Ingel20196_MGFPeaklist (F058870)	Myosin-9 OS=Gallus gallus GN=MYH9 PE=4 SV=2	F1NCD4_CHICK,MYH9_CHICK,UPI000012FB7F,UPI000060E1E4,UPI0002C868F4	TAX_GallusGallus_9031_20141114.fasta,UPARC14_2_GallusG_9031_20140417.fasta	226,643.40	100.00%	49	53	115	0.23%	24.40%	TSREEILAQAK	R	E	92.20%	37	44.7	34.3	9.74					0	0	3	0	2	1,245.68	1,668.00		1,425.65	1659	1669	Empty	TRUE	
20196-20225 HinckeM –TAXchicken	Uncategorized Sample	20196-20205_A	Mudpit_Ingel20196_MGFPeaklist (F058870)	Myosin-9 OS=Gallus gallus GN=MYH9 PE=4 SV=2	F1NCD4_CHICK,MYH9_CHICK,UPI000012FB7F,UPI000060E1E4,UPI0002C868F4	TAX_GallusGallus_9031_20141114.fasta,UPARC14_2_GallusG_9031_20140417.fasta	226,643.40	100.00%	49	53	115	0.23%	24.40%	TSREEILAQAKENEK	R	K	99.70%	49.7	44.6	29.6	30.6	42.7	3.42	0.335	0.5	0	0	0	2	2	1,745.90	1,637.00		624	1659	1673	Empty	TRUE	
20196-20225 HinckeM –TAXchicken	Uncategorized Sample	20196-20205_A	Mudpit_Ingel20196_MGFPeaklist (F058870)	Myosin-9 OS=Gallus gallus GN=MYH9 PE=4 SV=2	F1NCD4_CHICK,MYH9_CHICK,UPI000012FB7F,UPI000060E1E4,UPI0002C868F4	TAX_GallusGallus_9031_20141114.fasta,UPARC14_2_GallusG_9031_20140417.fasta	226,643.40	100.00%	49	53	115	0.23%	24.40%	TVQQLYKEQLAK	R	L	99.70%	37	43.6	25.2	25.9	29.3	1.14	0.266	0.417	0	0	4	0	2	1,377.77	1,866.50		2,477.43	645	656	Empty	TRUE	
20196-20225 HinckeM –TAXchicken	Uncategorized Sample	20196-20205_A	Mudpit_Ingel20196_MGFPeaklist (F058870)	Myosin-9 OS=Gallus gallus GN=MYH9 PE=4 SV=2	F1NCD4_CHICK,MYH9_CHICK,UPI000012FB7F,UPI000060E1E4,UPI0002C868F4	TAX_GallusGallus_9031_20141114.fasta,UPARC14_2_GallusG_9031_20140417.fasta	226,643.40	100.00%	49	53	115	0.23%	24.40%	VDAQLQELQVK	K	F	50.50%	23.2	43.9	38.2	1.53					0	1	0	0	2	1,270.70	2,098.00		128.78	1250	1260	Empty	TRUE	
20196-20225 HinckeM –TAXchicken	Uncategorized Sample	20196-20205_A	Mudpit_Ingel20196_MGFPeaklist (F058870)	Myosin-9 OS=Gallus gallus GN=MYH9 PE=4 SV=2	F1NCD4_CHICK,MYH9_CHICK,UPI000012FB7F,UPI000060E1E4,UPI0002C868F4	TAX_GallusGallus_9031_20141114.fasta,UPARC14_2_GallusG_9031_20140417.fasta	226,643.40	100.00%	49	53	115	0.23%	24.40%	VISGVLQLGNIVFKK	K	E	99.70%	64.9	36.9	20.4	56.6	45.8	3.11	0.162	0.5	0	0	2	0	2	1,614.99	2,854.50		1,206.55	342	356	Empty	TRUE	
20196-20225 HinckeM –TAXchicken	Uncategorized Sample	20196-20205_A	Mudpit_Ingel20196_MGFPeaklist (F058870)	Myosin-9 OS=Gallus gallus GN=MYH9 PE=4 SV=2	F1NCD4_CHICK,MYH9_CHICK,UPI000012FB7F,UPI000060E1E4,UPI0002C868F4	TAX_GallusGallus_9031_20141114.fasta,UPARC14_2_GallusG_9031_20140417.fasta	226,643.40	100.00%	49	53	115	0.23%	24.40%	VSHLLGINVTDFTR	K	G	99.70%	50.9	43.8	29.7	35.6	39.2	4.7	0.487	0.429	0	0	3	0	2	1,571.85	2,599.00		1,284.73	374	387	Empty	TRUE	
20196-20225 HinckeM –TAXchicken	Uncategorized Sample	20196-20205_A	Mudpit_Ingel20196_MGFPeaklist (F058870)	Myosin-9 OS=Gallus gallus GN=MYH9 PE=4 SV=2	F1NCD4_CHICK,MYH9_CHICK,UPI000012FB7F,UPI000060E1E4,UPI0002C868F4	TAX_GallusGallus_9031_20141114.fasta,UPARC14_2_GallusG_9031_20140417.fasta	226,643.40	100.00%	49	53	115	0.23%	24.40%	VVFQEFR	R	Q	74.20%	30.9	43.3	26.1	16.6					0	4	0	0	2	924.4937	2,156.00		1,480.25	712	718	Empty	TRUE	
20196-20225 HinckeM –TAXchicken	Uncategorized Sample	20196-20205_A	Mudpit_Ingel20196_MGFPeaklist (F058870)	Myosin-9 OS=Gallus gallus GN=MYH9 PE=4 SV=2	F1NCD4_CHICK,MYH9_CHICK,UPI000012FB7F,UPI000060E1E4,UPI0002C868F4	TAX_GallusGallus_9031_20141114.fasta,UPARC14_2_GallusG_9031_20140417.fasta	226,643.40	100.00%	49	53	115	0.23%	24.40%	YEILTPNAIPK	R	G	99.70%	20.6	44.4	14.6	7.9	28.5	1.57	0.277	0.318	0	3	0	0	2	1,258.70	2,337.00		1,618.82	721	731	Empty	TRUE	UPI00000FC6BE,Q789A5_CHICK,Q789A4_CHICK,UPI0000171382,UPI000060F510,UPI0000ECA50D,UPI000060F511,UPI00003AB452,Q789A6_CHICK,UPI0000171380,Q02015_CHICK,UPI0000171381,UPI00003AB454,UPI00003AB453
20196-20225 HinckeM –TAXchicken	Uncategorized Sample	20196-20205_A	Mudpit_Ingel20196_MGFPeaklist (F058870)	Nonmuscle myosin heavy chain OS=Gallus gallus PE=2 SV=2	Q02015_CHICK,Q789A4_CHICK,Q789A5_CHICK,Q789A6_CHICK,UPI00000FC6BE,UPI0000171380,UPI0000171381,UPI0000171382,UPI00003AB452,UPI00003AB453,UPI00003AB454,UPI000060F510,UPI000060F511,UPI0000ECA50D	TAX_GallusGallus_9031_20141114.fasta,UPARC14_2_GallusG_9031_20140417.fasta	215,888.80	100.00%	8	8	18	0.04%	8.01%	ADFCIIHYAGK	K	V	45.90%	18.6	44.4	14.7	11.1					0	0	2	0	2	1,294.63	2,223.00		989.84	458	468	Empty	FALSE	UPI000060E1E4,UPI000012FB7F,MYH9_CHICK,F1NCD4_CHICK,UPI0002C868F4
20196-20225 HinckeM –TAXchicken	Uncategorized Sample	20196-20205_A	Mudpit_Ingel20196_MGFPeaklist (F058870)	Nonmuscle myosin heavy chain OS=Gallus gallus PE=2 SV=2	Q02015_CHICK,Q789A4_CHICK,Q789A5_CHICK,Q789A6_CHICK,UPI00000FC6BE,UPI0000171380,UPI0000171381,UPI0000171382,UPI00003AB452,UPI00003AB453,UPI00003AB454,UPI000060F510,UPI000060F511,UPI0000ECA50D	TAX_GallusGallus_9031_20141114.fasta,UPARC14_2_GallusG_9031_20140417.fasta	215,888.80	100.00%	8	8	18	0.04%	8.01%	AGKLDPHLVLDQLR	R	C	99.70%	52.2	42.3	31.5	35.1	37.5	3.54	0.344	0.5	0	0	2	0	2	1,574.90	2,445.50		970.79	572	585	Empty	FALSE	UPI000060E1E4,UPI000012FB7F,MYH9_CHICK,F1NCD4_CHICK,UPI0002C868F4
20196-20225 HinckeM –TAXchicken	Uncategorized Sample	20196-20205_A	Mudpit_Ingel20196_MGFPeaklist (F058870)	Nonmuscle myosin heavy chain OS=Gallus gallus PE=2 SV=2	Q02015_CHICK,Q789A4_CHICK,Q789A5_CHICK,Q789A6_CHICK,UPI00000FC6BE,UPI0000171380,UPI0000171381,UPI0000171382,UPI00003AB452,UPI00003AB453,UPI00003AB454,UPI000060F510,UPI000060F511,UPI0000ECA50D	TAX_GallusGallus_9031_20141114.fasta,UPARC14_2_GallusG_9031_20140417.fasta	215,888.80	100.00%	8	8	18	0.04%	8.01%	AGVLAHLEEER	R	D	99.70%	71.3	44.3	36.2	36.2	34.7	3.4	0.317	0.409	0	0	2	0	2	1,223.64	2,031.50		1,163.70	657	667	Empty	FALSE	UPI000060E1E4,UPI000012FB7F,MYH9_CHICK,F1NCD4_CHICK,UPI0002C868F4
20196-20225 HinckeM –TAXchicken	Uncategorized Sample	20196-20205_A	Mudpit_Ingel20196_MGFPeaklist (F058870)	Nonmuscle myosin heavy chain OS=Gallus gallus PE=2 SV=2	Q02015_CHICK,Q789A4_CHICK,Q789A5_CHICK,Q789A6_CHICK,UPI00000FC6BE,UPI0000171380,UPI0000171381,UPI0000171382,UPI00003AB452,UPI00003AB453,UPI00003AB454,UPI000060F510,UPI000060F511,UPI0000ECA50D	TAX_GallusGallus_9031_20141114.fasta,UPARC14_2_GallusG_9031_20140417.fasta	215,888.80	100.00%	8	8	18	0.04%	8.01%	AGVLAHLEEERDLK	R	I	99.70%	43.7	43.9	28.2	36.5	38.3	3.7	0.371	0.464	0	0	2	0	2	1,579.84	2,107.00		1,024.73	657	670	Empty	FALSE	UPI000060E1E4,UPI000012FB7F,MYH9_CHICK,F1NCD4_CHICK,UPI0002C868F4
20196-20225 HinckeM –TAXchicken	Uncategorized Sample	20196-20205_A	Mudpit_Ingel20196_MGFPeaklist (F058870)	Nonmuscle myosin heavy chain OS=Gallus gallus PE=2 SV=2	Q02015_CHICK,Q789A4_CHICK,Q789A5_CHICK,Q789A6_CHICK,UPI00000FC6BE,UPI0000171380,UPI0000171381,UPI0000171382,UPI00003AB452,UPI00003AB453,UPI00003AB454,UPI000060F510,UPI000060F511,UPI0000ECA50D	TAX_GallusGallus_9031_20141114.fasta,UPARC14_2_GallusG_9031_20140417.fasta	215,888.80	100.00%	8	8	18	0.04%	8.01%	AIEEETKNHEAQIQEIR	K	Q	99.70%	29	44.4	22.1	12.1	28.6	1.7	0.192	0.265	0	0	0	2	2	2,038.02	1,772.50		811.37	1067	1083	Empty	TRUE	
20196-20225 HinckeM –TAXchicken	Uncategorized Sample	20196-20205_A	Mudpit_Ingel20196_MGFPeaklist (F058870)	Nonmuscle myosin heavy chain OS=Gallus gallus PE=2 SV=2	Q02015_CHICK,Q789A4_CHICK,Q789A5_CHICK,Q789A6_CHICK,UPI00000FC6BE,UPI0000171380,UPI0000171381,UPI0000171382,UPI00003AB452,UPI00003AB453,UPI00003AB454,UPI000060F510,UPI000060F511,UPI0000ECA50D	TAX_GallusGallus_9031_20141114.fasta,UPARC14_2_GallusG_9031_20140417.fasta	215,888.80	100.00%	8	8	18	0.04%	8.01%	ALEEALEAKEEFER	R	Q	99.70%	49	44.8	23.2	35.1	34.9	3.29	0.39	0.286	0	0	2	0	2	1,663.82	2,175.50		1,480.83	1376	1389	Empty	TRUE	
20196-20225 HinckeM –TAXchicken	Uncategorized Sample	20196-20205_A	Mudpit_Ingel20196_MGFPeaklist (F058870)	Nonmuscle myosin heavy chain OS=Gallus gallus PE=2 SV=2	Q02015_CHICK,Q789A4_CHICK,Q789A5_CHICK,Q789A6_CHICK,UPI00000FC6BE,UPI0000171380,UPI0000171381,UPI0000171382,UPI00003AB452,UPI00003AB453,UPI00003AB454,UPI000060F510,UPI000060F511,UPI0000ECA50D	TAX_GallusGallus_9031_20141114.fasta,UPARC14_2_GallusG_9031_20140417.fasta	215,888.80	100.00%	8	8	18	0.04%	8.01%	ALELDPNLYR	R	I	99.70%	42.1	45.2	34.3	22.2	32.7	2.42	0.343	0.4	0	5	0	0	2	1,203.64	2,376.00		1,867.67	638	647	Empty	TRUE	MYH11_CHICK
20196-20225 HinckeM –TAXchicken	Uncategorized Sample	20196-20205_A	Mudpit_Ingel20196_MGFPeaklist (F058870)	Nonmuscle myosin heavy chain OS=Gallus gallus PE=2 SV=2	Q02015_CHICK,Q789A4_CHICK,Q789A5_CHICK,Q789A6_CHICK,UPI00000FC6BE,UPI0000171380,UPI0000171381,UPI0000171382,UPI00003AB452,UPI00003AB453,UPI00003AB454,UPI000060F510,UPI000060F511,UPI0000ECA50D	TAX_GallusGallus_9031_20141114.fasta,UPARC14_2_GallusG_9031_20140417.fasta	215,888.80	100.00%	8	8	18	0.04%	8.01%	CNGVLEGIR	R	I	28.90%	28.9	45.5	29.6	12.6					0	1	0	0	2	1,018.50	2,046.00		210.55	586	594	Empty	FALSE	UPI000060E1E4,MYH11_CHICK,Q9PTY2_CHICK,F1NCD4_CHICK,UPI0002C868F4,UPI000012FB7F,MYH9_CHICK,UPI000044943F,UPI00003AB434,UPI00000FB7E2,UPI00003AB431
20196-20225 HinckeM –TAXchicken	Uncategorized Sample	20196-20205_A	Mudpit_Ingel20196_MGFPeaklist (F058870)	Nonmuscle myosin heavy chain OS=Gallus gallus PE=2 SV=2	Q02015_CHICK,Q789A4_CHICK,Q789A5_CHICK,Q789A6_CHICK,UPI00000FC6BE,UPI0000171380,UPI0000171381,UPI0000171382,UPI00003AB452,UPI00003AB453,UPI00003AB454,UPI000060F510,UPI000060F511,UPI0000ECA50D	TAX_GallusGallus_9031_20141114.fasta,UPARC14_2_GallusG_9031_20140417.fasta	215,888.80	100.00%	8	8	18	0.04%	8.01%	ELDDATEANEGLSR	R	E	99.70%	68.7	43.6	23	0	39.4	3.44	0.327	0.357	0	2	0	0	2	1,519.69	1,739.00		940.74	1791	1804	Empty	TRUE	
20196-20225 HinckeM –TAXchicken	Uncategorized Sample	20196-20205_A	Mudpit_Ingel20196_MGFPeaklist (F058870)	Nonmuscle myosin heavy chain OS=Gallus gallus PE=2 SV=2	Q02015_CHICK,Q789A4_CHICK,Q789A5_CHICK,Q789A6_CHICK,UPI00000FC6BE,UPI0000171380,UPI0000171381,UPI0000171382,UPI00003AB452,UPI00003AB453,UPI00003AB454,UPI000060F510,UPI000060F511,UPI0000ECA50D	TAX_GallusGallus_9031_20141114.fasta,UPARC14_2_GallusG_9031_20140417.fasta	215,888.80	100.00%	8	8	18	0.04%	8.01%	GGPITFSSSR	R	S	95.00%	30.3	44	27.7	19.6					0	1	0	0	2	1,008.51	1,869.00		125.86	1816	1825	Empty	TRUE	
20196-20225 HinckeM –TAXchicken	Uncategorized Sample	20196-20205_A	Mudpit_Ingel20196_MGFPeaklist (F058870)	Nonmuscle myosin heavy chain OS=Gallus gallus PE=2 SV=2	Q02015_CHICK,Q789A4_CHICK,Q789A5_CHICK,Q789A6_CHICK,UPI00000FC6BE,UPI0000171380,UPI0000171381,UPI0000171382,UPI00003AB452,UPI00003AB453,UPI00003AB454,UPI000060F510,UPI000060F511,UPI0000ECA50D	TAX_GallusGallus_9031_20141114.fasta,UPARC14_2_GallusG_9031_20140417.fasta	215,888.80	100.00%	8	8	18	0.04%	8.01%	IAQLEEQLEQEAKER	K	A	99.70%	59.3	44.3	25.4	45.9	46.4	4.08	0.369	0.4	0	0	2	0	2	1,813.93	2,231.00		777.93	1708	1722	Empty	TRUE	
20196-20225 HinckeM –TAXchicken	Uncategorized Sample	20196-20205_A	Mudpit_Ingel20196_MGFPeaklist (F058870)	Nonmuscle myosin heavy chain OS=Gallus gallus PE=2 SV=2	Q02015_CHICK,Q789A4_CHICK,Q789A5_CHICK,Q789A6_CHICK,UPI00000FC6BE,UPI0000171380,UPI0000171381,UPI0000171382,UPI00003AB452,UPI00003AB453,UPI00003AB454,UPI000060F510,UPI000060F511,UPI0000ECA50D	TAX_GallusGallus_9031_20141114.fasta,UPARC14_2_GallusG_9031_20140417.fasta	215,888.80	100.00%	8	8	18	0.04%	8.01%	IVFQEFR	R	Q	98.80%	47	43.1	42.2	19.8	27.9	0.585	0.222	0.5	0	3	0	0	2	938.5094	2,257.00		900.74	604	610	Empty	TRUE	MYH11_CHICK
20196-20225 HinckeM –TAXchicken	Uncategorized Sample	20196-20205_A	Mudpit_Ingel20196_MGFPeaklist (F058870)	Nonmuscle myosin heavy chain OS=Gallus gallus PE=2 SV=2	Q02015_CHICK,Q789A4_CHICK,Q789A5_CHICK,Q789A6_CHICK,UPI00000FC6BE,UPI0000171380,UPI0000171381,UPI0000171382,UPI00003AB452,UPI00003AB453,UPI00003AB454,UPI000060F510,UPI000060F511,UPI0000ECA50D	TAX_GallusGallus_9031_20141114.fasta,UPARC14_2_GallusG_9031_20140417.fasta	215,888.80	100.00%	8	8	18	0.04%	8.01%	KLDAQVQELTAK	K	V	13.90%	14	44	25.3	0.53					0	0	1	0	2	1,343.75	1,897.00		288.44	1141	1152	Empty	TRUE	
20196-20225 HinckeM –TAXchicken	Uncategorized Sample	20196-20205_A	Mudpit_Ingel20196_MGFPeaklist (F058870)	Nonmuscle myosin heavy chain OS=Gallus gallus PE=2 SV=2	Q02015_CHICK,Q789A4_CHICK,Q789A5_CHICK,Q789A6_CHICK,UPI00000FC6BE,UPI0000171380,UPI0000171381,UPI0000171382,UPI00003AB452,UPI00003AB453,UPI00003AB454,UPI000060F510,UPI000060F511,UPI0000ECA50D	TAX_GallusGallus_9031_20141114.fasta,UPARC14_2_GallusG_9031_20140417.fasta	215,888.80	100.00%	8	8	18	0.04%	8.01%	LDPHLVLDQLR	K	C	99.70%	34.6	43.8	18.6	22.1	32.5	2.47	0.366	0.455	0	0	1	0	2	1,318.75	2,534.00		1,121.62	575	585	Empty	FALSE	UPI000060E1E4,UPI000012FB7F,MYH9_CHICK,F1NCD4_CHICK,UPI0002C868F4
20196-20225 HinckeM –TAXchicken	Uncategorized Sample	20196-20205_A	Mudpit_Ingel20196_MGFPeaklist (F058870)	Nonmuscle myosin heavy chain OS=Gallus gallus PE=2 SV=2	Q02015_CHICK,Q789A4_CHICK,Q789A5_CHICK,Q789A6_CHICK,UPI00000FC6BE,UPI0000171380,UPI0000171381,UPI0000171382,UPI00003AB452,UPI00003AB453,UPI00003AB454,UPI000060F510,UPI000060F511,UPI0000ECA50D	TAX_GallusGallus_9031_20141114.fasta,UPARC14_2_GallusG_9031_20140417.fasta	215,888.80	100.00%	8	8	18	0.04%	8.01%	QRYEILTPNAIPK	R	G	99.70%	44.8	43.5	22.5	30.7	33.5	1.96	0.358	0.423	0	2	4	0	2	1,542.86	2,240.50		4,800.05	611	623	Empty	FALSE	UPI000060E1E4,UPI000012FB7F,MYH9_CHICK,F1NCD4_CHICK,UPI0002C868F4
20196-20225 HinckeM –TAXchicken	Uncategorized Sample	20196-20205_A	Mudpit_Ingel20196_MGFPeaklist (F058870)	Nonmuscle myosin heavy chain OS=Gallus gallus PE=2 SV=2	Q02015_CHICK,Q789A4_CHICK,Q789A5_CHICK,Q789A6_CHICK,UPI00000FC6BE,UPI0000171380,UPI0000171381,UPI0000171382,UPI00003AB452,UPI00003AB453,UPI00003AB454,UPI000060F510,UPI000060F511,UPI0000ECA50D	TAX_GallusGallus_9031_20141114.fasta,UPARC14_2_GallusG_9031_20140417.fasta	215,888.80	100.00%	8	8	18	0.04%	8.01%	YEILTPNAIPK	R	G	99.70%	20.6	44.4	14.6	7.9	28.5	1.57	0.277	0.318	0	3	0	0	2	1,258.70	2,337.00		1,618.82	613	623	Empty	FALSE	UPI000060E1E4,UPI000012FB7F,MYH9_CHICK,F1NCD4_CHICK,UPI0002C868F4
20196-20225 HinckeM –TAXchicken	Uncategorized Sample	20196-20205_A	Mudpit_Ingel20196_MGFPeaklist (F058870)	Nonmuscle myosin heavy chain OS=Gallus gallus PE=2 SV=2	Q02015_CHICK,Q789A4_CHICK,Q789A5_CHICK,Q789A6_CHICK,UPI00000FC6BE,UPI0000171380,UPI0000171381,UPI0000171382,UPI00003AB452,UPI00003AB453,UPI00003AB454,UPI000060F510,UPI000060F511,UPI0000ECA50D	TAX_GallusGallus_9031_20141114.fasta,UPARC14_2_GallusG_9031_20140417.fasta	215,888.80	100.00%	8	8	18	0.04%	8.01%	ADFCIIHYAGK	K	V	45.90%	18.6	44.4	14.7	11.1					0	0	2	0	2	1,294.63	2,223.00		989.84	458	468	Empty	FALSE	UPI000060E1E4,UPI000012FB7F,MYH9_CHICK,F1NCD4_CHICK,UPI0002C868F4
20196-20225 HinckeM –TAXchicken	Uncategorized Sample	20196-20205_A	Mudpit_Ingel20196_MGFPeaklist (F058870)	Nonmuscle myosin heavy chain OS=Gallus gallus PE=2 SV=2	Q02015_CHICK,Q789A4_CHICK,Q789A5_CHICK,Q789A6_CHICK,UPI00000FC6BE,UPI0000171380,UPI0000171381,UPI0000171382,UPI00003AB452,UPI00003AB453,UPI00003AB454,UPI000060F510,UPI000060F511,UPI0000ECA50D	TAX_GallusGallus_9031_20141114.fasta,UPARC14_2_GallusG_9031_20140417.fasta	215,888.80	100.00%	8	8	18	0.04%	8.01%	AGKLDPHLVLDQLR	R	C	99.70%	52.2	42.3	31.5	35.1	37.5	3.54	0.344	0.5	0	0	2	0	2	1,574.90	2,445.50		970.79	572	585	Empty	FALSE	UPI000060E1E4,UPI000012FB7F,MYH9_CHICK,F1NCD4_CHICK,UPI0002C868F4
20196-20225 HinckeM –TAXchicken	Uncategorized Sample	20196-20205_A	Mudpit_Ingel20196_MGFPeaklist (F058870)	Nonmuscle myosin heavy chain OS=Gallus gallus PE=2 SV=2	Q02015_CHICK,Q789A4_CHICK,Q789A5_CHICK,Q789A6_CHICK,UPI00000FC6BE,UPI0000171380,UPI0000171381,UPI0000171382,UPI00003AB452,UPI00003AB453,UPI00003AB454,UPI000060F510,UPI000060F511,UPI0000ECA50D	TAX_GallusGallus_9031_20141114.fasta,UPARC14_2_GallusG_9031_20140417.fasta	215,888.80	100.00%	8	8	18	0.04%	8.01%	AGVLAHLEEER	R	D	99.70%	71.3	44.3	36.2	36.2	34.7	3.4	0.317	0.409	0	0	2	0	2	1,223.64	2,031.50		1,163.70	657	667	Empty	FALSE	UPI000060E1E4,UPI000012FB7F,MYH9_CHICK,F1NCD4_CHICK,UPI0002C868F4
20196-20225 HinckeM –TAXchicken	Uncategorized Sample	20196-20205_A	Mudpit_Ingel20196_MGFPeaklist (F058870)	Nonmuscle myosin heavy chain OS=Gallus gallus PE=2 SV=2	Q02015_CHICK,Q789A4_CHICK,Q789A5_CHICK,Q789A6_CHICK,UPI00000FC6BE,UPI0000171380,UPI0000171381,UPI0000171382,UPI00003AB452,UPI00003AB453,UPI00003AB454,UPI000060F510,UPI000060F511,UPI0000ECA50D	TAX_GallusGallus_9031_20141114.fasta,UPARC14_2_GallusG_9031_20140417.fasta	215,888.80	100.00%	8	8	18	0.04%	8.01%	AGVLAHLEEERDLK	R	I	99.70%	43.7	43.9	28.2	36.5	38.3	3.7	0.371	0.464	0	0	2	0	2	1,579.84	2,107.00		1,024.73	657	670	Empty	FALSE	UPI000060E1E4,UPI000012FB7F,MYH9_CHICK,F1NCD4_CHICK,UPI0002C868F4
20196-20225 HinckeM –TAXchicken	Uncategorized Sample	20196-20205_A	Mudpit_Ingel20196_MGFPeaklist (F058870)	Nonmuscle myosin heavy chain OS=Gallus gallus PE=2 SV=2	Q02015_CHICK,Q789A4_CHICK,Q789A5_CHICK,Q789A6_CHICK,UPI00000FC6BE,UPI0000171380,UPI0000171381,UPI0000171382,UPI00003AB452,UPI00003AB453,UPI00003AB454,UPI000060F510,UPI000060F511,UPI0000ECA50D	TAX_GallusGallus_9031_20141114.fasta,UPARC14_2_GallusG_9031_20140417.fasta	215,888.80	100.00%	8	8	18	0.04%	8.01%	AIEEETKNHEAQIQEIR	K	Q	99.70%	29	44.4	22.1	12.1	28.6	1.7	0.192	0.265	0	0	0	2	2	2,038.02	1,772.50		811.37	1067	1083	Empty	TRUE	
20196-20225 HinckeM –TAXchicken	Uncategorized Sample	20196-20205_A	Mudpit_Ingel20196_MGFPeaklist (F058870)	Nonmuscle myosin heavy chain OS=Gallus gallus PE=2 SV=2	Q02015_CHICK,Q789A4_CHICK,Q789A5_CHICK,Q789A6_CHICK,UPI00000FC6BE,UPI0000171380,UPI0000171381,UPI0000171382,UPI00003AB452,UPI00003AB453,UPI00003AB454,UPI000060F510,UPI000060F511,UPI0000ECA50D	TAX_GallusGallus_9031_20141114.fasta,UPARC14_2_GallusG_9031_20140417.fasta	215,888.80	100.00%	8	8	18	0.04%	8.01%	ALEEALEAKEEFER	R	Q	99.70%	49	44.8	23.2	35.1	34.9	3.29	0.39	0.286	0	0	2	0	2	1,663.82	2,175.50		1,480.83	1376	1389	Empty	TRUE	
20196-20225 HinckeM –TAXchicken	Uncategorized Sample	20196-20205_A	Mudpit_Ingel20196_MGFPeaklist (F058870)	Nonmuscle myosin heavy chain OS=Gallus gallus PE=2 SV=2	Q02015_CHICK,Q789A4_CHICK,Q789A5_CHICK,Q789A6_CHICK,UPI00000FC6BE,UPI0000171380,UPI0000171381,UPI0000171382,UPI00003AB452,UPI00003AB453,UPI00003AB454,UPI000060F510,UPI000060F511,UPI0000ECA50D	TAX_GallusGallus_9031_20141114.fasta,UPARC14_2_GallusG_9031_20140417.fasta	215,888.80	100.00%	8	8	18	0.04%	8.01%	ALELDPNLYR	R	I	99.70%	42.1	45.2	34.3	22.2	32.7	2.42	0.343	0.4	0	5	0	0	2	1,203.64	2,376.00		1,867.67	638	647	Empty	TRUE	MYH11_CHICK
20196-20225 HinckeM –TAXchicken	Uncategorized Sample	20196-20205_A	Mudpit_Ingel20196_MGFPeaklist (F058870)	Nonmuscle myosin heavy chain OS=Gallus gallus PE=2 SV=2	Q02015_CHICK,Q789A4_CHICK,Q789A5_CHICK,Q789A6_CHICK,UPI00000FC6BE,UPI0000171380,UPI0000171381,UPI0000171382,UPI00003AB452,UPI00003AB453,UPI00003AB454,UPI000060F510,UPI000060F511,UPI0000ECA50D	TAX_GallusGallus_9031_20141114.fasta,UPARC14_2_GallusG_9031_20140417.fasta	215,888.80	100.00%	8	8	18	0.04%	8.01%	CNGVLEGIR	R	I	28.90%	28.9	45.5	29.6	12.6					0	1	0	0	2	1,018.50	2,046.00		210.55	586	594	Empty	FALSE	UPI000060E1E4,MYH11_CHICK,Q9PTY2_CHICK,F1NCD4_CHICK,UPI0002C868F4,UPI000012FB7F,MYH9_CHICK,UPI000044943F,UPI00003AB434,UPI00000FB7E2,UPI00003AB431
20196-20225 HinckeM –TAXchicken	Uncategorized Sample	20196-20205_A	Mudpit_Ingel20196_MGFPeaklist (F058870)	Nonmuscle myosin heavy chain OS=Gallus gallus PE=2 SV=2	Q02015_CHICK,Q789A4_CHICK,Q789A5_CHICK,Q789A6_CHICK,UPI00000FC6BE,UPI0000171380,UPI0000171381,UPI0000171382,UPI00003AB452,UPI00003AB453,UPI00003AB454,UPI000060F510,UPI000060F511,UPI0000ECA50D	TAX_GallusGallus_9031_20141114.fasta,UPARC14_2_GallusG_9031_20140417.fasta	215,888.80	100.00%	8	8	18	0.04%	8.01%	ELDDATEANEGLSR	R	E	99.70%	68.7	43.6	23	0	39.4	3.44	0.327	0.357	0	2	0	0	2	1,519.69	1,739.00		940.74	1791	1804	Empty	TRUE	
20196-20225 HinckeM –TAXchicken	Uncategorized Sample	20196-20205_A	Mudpit_Ingel20196_MGFPeaklist (F058870)	Nonmuscle myosin heavy chain OS=Gallus gallus PE=2 SV=2	Q02015_CHICK,Q789A4_CHICK,Q789A5_CHICK,Q789A6_CHICK,UPI00000FC6BE,UPI0000171380,UPI0000171381,UPI0000171382,UPI00003AB452,UPI00003AB453,UPI00003AB454,UPI000060F510,UPI000060F511,UPI0000ECA50D	TAX_GallusGallus_9031_20141114.fasta,UPARC14_2_GallusG_9031_20140417.fasta	215,888.80	100.00%	8	8	18	0.04%	8.01%	GGPITFSSSR	R	S	95.00%	30.3	44	27.7	19.6					0	1	0	0	2	1,008.51	1,869.00		125.86	1816	1825	Empty	TRUE	
20196-20225 HinckeM –TAXchicken	Uncategorized Sample	20196-20205_A	Mudpit_Ingel20196_MGFPeaklist (F058870)	Nonmuscle myosin heavy chain OS=Gallus gallus PE=2 SV=2	Q02015_CHICK,Q789A4_CHICK,Q789A5_CHICK,Q789A6_CHICK,UPI00000FC6BE,UPI0000171380,UPI0000171381,UPI0000171382,UPI00003AB452,UPI00003AB453,UPI00003AB454,UPI000060F510,UPI000060F511,UPI0000ECA50D	TAX_GallusGallus_9031_20141114.fasta,UPARC14_2_GallusG_9031_20140417.fasta	215,888.80	100.00%	8	8	18	0.04%	8.01%	IAQLEEQLEQEAKER	K	A	99.70%	59.3	44.3	25.4	45.9	46.4	4.08	0.369	0.4	0	0	2	0	2	1,813.93	2,231.00		777.93	1708	1722	Empty	TRUE	
20196-20225 HinckeM –TAXchicken	Uncategorized Sample	20196-20205_A	Mudpit_Ingel20196_MGFPeaklist (F058870)	Nonmuscle myosin heavy chain OS=Gallus gallus PE=2 SV=2	Q02015_CHICK,Q789A4_CHICK,Q789A5_CHICK,Q789A6_CHICK,UPI00000FC6BE,UPI0000171380,UPI0000171381,UPI0000171382,UPI00003AB452,UPI00003AB453,UPI00003AB454,UPI000060F510,UPI000060F511,UPI0000ECA50D	TAX_GallusGallus_9031_20141114.fasta,UPARC14_2_GallusG_9031_20140417.fasta	215,888.80	100.00%	8	8	18	0.04%	8.01%	IVFQEFR	R	Q	98.80%	47	43.1	42.2	19.8	27.9	0.585	0.222	0.5	0	3	0	0	2	938.5094	2,257.00		900.74	604	610	Empty	TRUE	MYH11_CHICK
20196-20225 HinckeM –TAXchicken	Uncategorized Sample	20196-20205_A	Mudpit_Ingel20196_MGFPeaklist (F058870)	Nonmuscle myosin heavy chain OS=Gallus gallus PE=2 SV=2	Q02015_CHICK,Q789A4_CHICK,Q789A5_CHICK,Q789A6_CHICK,UPI00000FC6BE,UPI0000171380,UPI0000171381,UPI0000171382,UPI00003AB452,UPI00003AB453,UPI00003AB454,UPI000060F510,UPI000060F511,UPI0000ECA50D	TAX_GallusGallus_9031_20141114.fasta,UPARC14_2_GallusG_9031_20140417.fasta	215,888.80	100.00%	8	8	18	0.04%	8.01%	KLDAQVQELTAK	K	V	13.90%	14	44	25.3	0.53					0	0	1	0	2	1,343.75	1,897.00		288.44	1141	1152	Empty	TRUE	
20196-20225 HinckeM –TAXchicken	Uncategorized Sample	20196-20205_A	Mudpit_Ingel20196_MGFPeaklist (F058870)	Nonmuscle myosin heavy chain OS=Gallus gallus PE=2 SV=2	Q02015_CHICK,Q789A4_CHICK,Q789A5_CHICK,Q789A6_CHICK,UPI00000FC6BE,UPI0000171380,UPI0000171381,UPI0000171382,UPI00003AB452,UPI00003AB453,UPI00003AB454,UPI000060F510,UPI000060F511,UPI0000ECA50D	TAX_GallusGallus_9031_20141114.fasta,UPARC14_2_GallusG_9031_20140417.fasta	215,888.80	100.00%	8	8	18	0.04%	8.01%	LDPHLVLDQLR	K	C	99.70%	34.6	43.8	18.6	22.1	32.5	2.47	0.366	0.455	0	0	1	0	2	1,318.75	2,534.00		1,121.62	575	585	Empty	FALSE	UPI000060E1E4,UPI000012FB7F,MYH9_CHICK,F1NCD4_CHICK,UPI0002C868F4
20196-20225 HinckeM –TAXchicken	Uncategorized Sample	20196-20205_A	Mudpit_Ingel20196_MGFPeaklist (F058870)	Nonmuscle myosin heavy chain OS=Gallus gallus PE=2 SV=2	Q02015_CHICK,Q789A4_CHICK,Q789A5_CHICK,Q789A6_CHICK,UPI00000FC6BE,UPI0000171380,UPI0000171381,UPI0000171382,UPI00003AB452,UPI00003AB453,UPI00003AB454,UPI000060F510,UPI000060F511,UPI0000ECA50D	TAX_GallusGallus_9031_20141114.fasta,UPARC14_2_GallusG_9031_20140417.fasta	215,888.80	100.00%	8	8	18	0.04%	8.01%	QRYEILTPNAIPK	R	G	99.70%	44.8	43.5	22.5	30.7	33.5	1.96	0.358	0.423	0	2	4	0	2	1,542.86	2,240.50		4,800.05	611	623	Empty	FALSE	UPI000060E1E4,UPI000012FB7F,MYH9_CHICK,F1NCD4_CHICK,UPI0002C868F4
20196-20225 HinckeM –TAXchicken	Uncategorized Sample	20196-20205_A	Mudpit_Ingel20196_MGFPeaklist (F058870)	Nonmuscle myosin heavy chain OS=Gallus gallus PE=2 SV=2	Q02015_CHICK,Q789A4_CHICK,Q789A5_CHICK,Q789A6_CHICK,UPI00000FC6BE,UPI0000171380,UPI0000171381,UPI0000171382,UPI00003AB452,UPI00003AB453,UPI00003AB454,UPI000060F510,UPI000060F511,UPI0000ECA50D	TAX_GallusGallus_9031_20141114.fasta,UPARC14_2_GallusG_9031_20140417.fasta	215,888.80	100.00%	8	8	18	0.04%	8.01%	YEILTPNAIPK	R	G	99.70%	20.6	44.4	14.6	7.9	28.5	1.57	0.277	0.318	0	3	0	0	2	1,258.70	2,337.00		1,618.82	613	623	Empty	FALSE	UPI000060E1E4,UPI000012FB7F,MYH9_CHICK,F1NCD4_CHICK,UPI0002C868F4
20196-20225 HinckeM –TAXchicken	Uncategorized Sample	20196-20205_A	Mudpit_Ingel20196_MGFPeaklist (F058870)	Nonmuscle myosin heavy chain OS=Gallus gallus PE=2 SV=2	Q02015_CHICK,Q789A4_CHICK,Q789A5_CHICK,Q789A6_CHICK,UPI00000FC6BE,UPI0000171380,UPI0000171381,UPI0000171382,UPI00003AB452,UPI00003AB453,UPI00003AB454,UPI000060F510,UPI000060F511,UPI0000ECA50D	TAX_GallusGallus_9031_20141114.fasta,UPARC14_2_GallusG_9031_20140417.fasta	215,888.80	100.00%	8	8	18	0.04%	8.01%	ADFCIIHYAGK	K	V	45.90%	18.6	44.4	14.7	11.1					0	0	2	0	2	1,294.63	2,223.00		989.84	458	468	Empty	FALSE	UPI000060E1E4,UPI000012FB7F,MYH9_CHICK,F1NCD4_CHICK,UPI0002C868F4
20196-20225 HinckeM –TAXchicken	Uncategorized Sample	20196-20205_A	Mudpit_Ingel20196_MGFPeaklist (F058870)	Nonmuscle myosin heavy chain OS=Gallus gallus PE=2 SV=2	Q02015_CHICK,Q789A4_CHICK,Q789A5_CHICK,Q789A6_CHICK,UPI00000FC6BE,UPI0000171380,UPI0000171381,UPI0000171382,UPI00003AB452,UPI00003AB453,UPI00003AB454,UPI000060F510,UPI000060F511,UPI0000ECA50D	TAX_GallusGallus_9031_20141114.fasta,UPARC14_2_GallusG_9031_20140417.fasta	215,888.80	100.00%	8	8	18	0.04%	8.01%	AGKLDPHLVLDQLR	R	C	99.70%	52.2	42.3	31.5	35.1	37.5	3.54	0.344	0.5	0	0	2	0	2	1,574.90	2,445.50		970.79	572	585	Empty	FALSE	UPI000060E1E4,UPI000012FB7F,MYH9_CHICK,F1NCD4_CHICK,UPI0002C868F4
20196-20225 HinckeM –TAXchicken	Uncategorized Sample	20196-20205_A	Mudpit_Ingel20196_MGFPeaklist (F058870)	Nonmuscle myosin heavy chain OS=Gallus gallus PE=2 SV=2	Q02015_CHICK,Q789A4_CHICK,Q789A5_CHICK,Q789A6_CHICK,UPI00000FC6BE,UPI0000171380,UPI0000171381,UPI0000171382,UPI00003AB452,UPI00003AB453,UPI00003AB454,UPI000060F510,UPI000060F511,UPI0000ECA50D	TAX_GallusGallus_9031_20141114.fasta,UPARC14_2_GallusG_9031_20140417.fasta	215,888.80	100.00%	8	8	18	0.04%	8.01%	AGVLAHLEEER	R	D	99.70%	71.3	44.3	36.2	36.2	34.7	3.4	0.317	0.409	0	0	2	0	2	1,223.64	2,031.50		1,163.70	657	667	Empty	FALSE	UPI000060E1E4,UPI000012FB7F,MYH9_CHICK,F1NCD4_CHICK,UPI0002C868F4
20196-20225 HinckeM –TAXchicken	Uncategorized Sample	20196-20205_A	Mudpit_Ingel20196_MGFPeaklist (F058870)	Nonmuscle myosin heavy chain OS=Gallus gallus PE=2 SV=2	Q02015_CHICK,Q789A4_CHICK,Q789A5_CHICK,Q789A6_CHICK,UPI00000FC6BE,UPI0000171380,UPI0000171381,UPI0000171382,UPI00003AB452,UPI00003AB453,UPI00003AB454,UPI000060F510,UPI000060F511,UPI0000ECA50D	TAX_GallusGallus_9031_20141114.fasta,UPARC14_2_GallusG_9031_20140417.fasta	215,888.80	100.00%	8	8	18	0.04%	8.01%	AGVLAHLEEERDLK	R	I	99.70%	43.7	43.9	28.2	36.5	38.3	3.7	0.371	0.464	0	0	2	0	2	1,579.84	2,107.00		1,024.73	657	670	Empty	FALSE	UPI000060E1E4,UPI000012FB7F,MYH9_CHICK,F1NCD4_CHICK,UPI0002C868F4
20196-20225 HinckeM –TAXchicken	Uncategorized Sample	20196-20205_A	Mudpit_Ingel20196_MGFPeaklist (F058870)	Nonmuscle myosin heavy chain OS=Gallus gallus PE=2 SV=2	Q02015_CHICK,Q789A4_CHICK,Q789A5_CHICK,Q789A6_CHICK,UPI00000FC6BE,UPI0000171380,UPI0000171381,UPI0000171382,UPI00003AB452,UPI00003AB453,UPI00003AB454,UPI000060F510,UPI000060F511,UPI0000ECA50D	TAX_GallusGallus_9031_20141114.fasta,UPARC14_2_GallusG_9031_20140417.fasta	215,888.80	100.00%	8	8	18	0.04%	8.01%	AIEEETKNHEAQIQEIR	K	Q	99.70%	29	44.4	22.1	12.1	28.6	1.7	0.192	0.265	0	0	0	2	2	2,038.02	1,772.50		811.37	1067	1083	Empty	TRUE	
20196-20225 HinckeM –TAXchicken	Uncategorized Sample	20196-20205_A	Mudpit_Ingel20196_MGFPeaklist (F058870)	Nonmuscle myosin heavy chain OS=Gallus gallus PE=2 SV=2	Q02015_CHICK,Q789A4_CHICK,Q789A5_CHICK,Q789A6_CHICK,UPI00000FC6BE,UPI0000171380,UPI0000171381,UPI0000171382,UPI00003AB452,UPI00003AB453,UPI00003AB454,UPI000060F510,UPI000060F511,UPI0000ECA50D	TAX_GallusGallus_9031_20141114.fasta,UPARC14_2_GallusG_9031_20140417.fasta	215,888.80	100.00%	8	8	18	0.04%	8.01%	ALEEALEAKEEFER	R	Q	99.70%	49	44.8	23.2	35.1	34.9	3.29	0.39	0.286	0	0	2	0	2	1,663.82	2,175.50		1,480.83	1376	1389	Empty	TRUE	
20196-20225 HinckeM –TAXchicken	Uncategorized Sample	20196-20205_A	Mudpit_Ingel20196_MGFPeaklist (F058870)	Nonmuscle myosin heavy chain OS=Gallus gallus PE=2 SV=2	Q02015_CHICK,Q789A4_CHICK,Q789A5_CHICK,Q789A6_CHICK,UPI00000FC6BE,UPI0000171380,UPI0000171381,UPI0000171382,UPI00003AB452,UPI00003AB453,UPI00003AB454,UPI000060F510,UPI000060F511,UPI0000ECA50D	TAX_GallusGallus_9031_20141114.fasta,UPARC14_2_GallusG_9031_20140417.fasta	215,888.80	100.00%	8	8	18	0.04%	8.01%	ALELDPNLYR	R	I	99.70%	42.1	45.2	34.3	22.2	32.7	2.42	0.343	0.4	0	5	0	0	2	1,203.64	2,376.00		1,867.67	638	647	Empty	TRUE	MYH11_CHICK
20196-20225 HinckeM –TAXchicken	Uncategorized Sample	20196-20205_A	Mudpit_Ingel20196_MGFPeaklist (F058870)	Nonmuscle myosin heavy chain OS=Gallus gallus PE=2 SV=2	Q02015_CHICK,Q789A4_CHICK,Q789A5_CHICK,Q789A6_CHICK,UPI00000FC6BE,UPI0000171380,UPI0000171381,UPI0000171382,UPI00003AB452,UPI00003AB453,UPI00003AB454,UPI000060F510,UPI000060F511,UPI0000ECA50D	TAX_GallusGallus_9031_20141114.fasta,UPARC14_2_GallusG_9031_20140417.fasta	215,888.80	100.00%	8	8	18	0.04%	8.01%	CNGVLEGIR	R	I	28.90%	28.9	45.5	29.6	12.6					0	1	0	0	2	1,018.50	2,046.00		210.55	586	594	Empty	FALSE	UPI000060E1E4,MYH11_CHICK,Q9PTY2_CHICK,F1NCD4_CHICK,UPI0002C868F4,UPI000012FB7F,MYH9_CHICK,UPI000044943F,UPI00003AB434,UPI00000FB7E2,UPI00003AB431
20196-20225 HinckeM –TAXchicken	Uncategorized Sample	20196-20205_A	Mudpit_Ingel20196_MGFPeaklist (F058870)	Nonmuscle myosin heavy chain OS=Gallus gallus PE=2 SV=2	Q02015_CHICK,Q789A4_CHICK,Q789A5_CHICK,Q789A6_CHICK,UPI00000FC6BE,UPI0000171380,UPI0000171381,UPI0000171382,UPI00003AB452,UPI00003AB453,UPI00003AB454,UPI000060F510,UPI000060F511,UPI0000ECA50D	TAX_GallusGallus_9031_20141114.fasta,UPARC14_2_GallusG_9031_20140417.fasta	215,888.80	100.00%	8	8	18	0.04%	8.01%	ELDDATEANEGLSR	R	E	99.70%	68.7	43.6	23	0	39.4	3.44	0.327	0.357	0	2	0	0	2	1,519.69	1,739.00		940.74	1791	1804	Empty	TRUE	
20196-20225 HinckeM –TAXchicken	Uncategorized Sample	20196-20205_A	Mudpit_Ingel20196_MGFPeaklist (F058870)	Nonmuscle myosin heavy chain OS=Gallus gallus PE=2 SV=2	Q02015_CHICK,Q789A4_CHICK,Q789A5_CHICK,Q789A6_CHICK,UPI00000FC6BE,UPI0000171380,UPI0000171381,UPI0000171382,UPI00003AB452,UPI00003AB453,UPI00003AB454,UPI000060F510,UPI000060F511,UPI0000ECA50D	TAX_GallusGallus_9031_20141114.fasta,UPARC14_2_GallusG_9031_20140417.fasta	215,888.80	100.00%	8	8	18	0.04%	8.01%	GGPITFSSSR	R	S	95.00%	30.3	44	27.7	19.6					0	1	0	0	2	1,008.51	1,869.00		125.86	1816	1825	Empty	TRUE	
20196-20225 HinckeM –TAXchicken	Uncategorized Sample	20196-20205_A	Mudpit_Ingel20196_MGFPeaklist (F058870)	Nonmuscle myosin heavy chain OS=Gallus gallus PE=2 SV=2	Q02015_CHICK,Q789A4_CHICK,Q789A5_CHICK,Q789A6_CHICK,UPI00000FC6BE,UPI0000171380,UPI0000171381,UPI0000171382,UPI00003AB452,UPI00003AB453,UPI00003AB454,UPI000060F510,UPI000060F511,UPI0000ECA50D	TAX_GallusGallus_9031_20141114.fasta,UPARC14_2_GallusG_9031_20140417.fasta	215,888.80	100.00%	8	8	18	0.04%	8.01%	IAQLEEQLEQEAKER	K	A	99.70%	59.3	44.3	25.4	45.9	46.4	4.08	0.369	0.4	0	0	2	0	2	1,813.93	2,231.00		777.93	1708	1722	Empty	TRUE	
20196-20225 HinckeM –TAXchicken	Uncategorized Sample	20196-20205_A	Mudpit_Ingel20196_MGFPeaklist (F058870)	Nonmuscle myosin heavy chain OS=Gallus gallus PE=2 SV=2	Q02015_CHICK,Q789A4_CHICK,Q789A5_CHICK,Q789A6_CHICK,UPI00000FC6BE,UPI0000171380,UPI0000171381,UPI0000171382,UPI00003AB452,UPI00003AB453,UPI00003AB454,UPI000060F510,UPI000060F511,UPI0000ECA50D	TAX_GallusGallus_9031_20141114.fasta,UPARC14_2_GallusG_9031_20140417.fasta	215,888.80	100.00%	8	8	18	0.04%	8.01%	IVFQEFR	R	Q	98.80%	47	43.1	42.2	19.8	27.9	0.585	0.222	0.5	0	3	0	0	2	938.5094	2,257.00		900.74	604	610	Empty	TRUE	MYH11_CHICK
20196-20225 HinckeM –TAXchicken	Uncategorized Sample	20196-20205_A	Mudpit_Ingel20196_MGFPeaklist (F058870)	Nonmuscle myosin heavy chain OS=Gallus gallus PE=2 SV=2	Q02015_CHICK,Q789A4_CHICK,Q789A5_CHICK,Q789A6_CHICK,UPI00000FC6BE,UPI0000171380,UPI0000171381,UPI0000171382,UPI00003AB452,UPI00003AB453,UPI00003AB454,UPI000060F510,UPI000060F511,UPI0000ECA50D	TAX_GallusGallus_9031_20141114.fasta,UPARC14_2_GallusG_9031_20140417.fasta	215,888.80	100.00%	8	8	18	0.04%	8.01%	KLDAQVQELTAK	K	V	13.90%	14	44	25.3	0.53					0	0	1	0	2	1,343.75	1,897.00		288.44	1141	1152	Empty	TRUE	
20196-20225 HinckeM –TAXchicken	Uncategorized Sample	20196-20205_A	Mudpit_Ingel20196_MGFPeaklist (F058870)	Nonmuscle myosin heavy chain OS=Gallus gallus PE=2 SV=2	Q02015_CHICK,Q789A4_CHICK,Q789A5_CHICK,Q789A6_CHICK,UPI00000FC6BE,UPI0000171380,UPI0000171381,UPI0000171382,UPI00003AB452,UPI00003AB453,UPI00003AB454,UPI000060F510,UPI000060F511,UPI0000ECA50D	TAX_GallusGallus_9031_20141114.fasta,UPARC14_2_GallusG_9031_20140417.fasta	215,888.80	100.00%	8	8	18	0.04%	8.01%	LDPHLVLDQLR	K	C	99.70%	34.6	43.8	18.6	22.1	32.5	2.47	0.366	0.455	0	0	1	0	2	1,318.75	2,534.00		1,121.62	575	585	Empty	FALSE	UPI000060E1E4,UPI000012FB7F,MYH9_CHICK,F1NCD4_CHICK,UPI0002C868F4
20196-20225 HinckeM –TAXchicken	Uncategorized Sample	20196-20205_A	Mudpit_Ingel20196_MGFPeaklist (F058870)	Nonmuscle myosin heavy chain OS=Gallus gallus PE=2 SV=2	Q02015_CHICK,Q789A4_CHICK,Q789A5_CHICK,Q789A6_CHICK,UPI00000FC6BE,UPI0000171380,UPI0000171381,UPI0000171382,UPI00003AB452,UPI00003AB453,UPI00003AB454,UPI000060F510,UPI000060F511,UPI0000ECA50D	TAX_GallusGallus_9031_20141114.fasta,UPARC14_2_GallusG_9031_20140417.fasta	215,888.80	100.00%	8	8	18	0.04%	8.01%	QRYEILTPNAIPK	R	G	99.70%	44.8	43.5	22.5	30.7	33.5	1.96	0.358	0.423	0	2	4	0	2	1,542.86	2,240.50		4,800.05	611	623	Empty	FALSE	UPI000060E1E4,UPI000012FB7F,MYH9_CHICK,F1NCD4_CHICK,UPI0002C868F4
20196-20225 HinckeM –TAXchicken	Uncategorized Sample	20196-20205_A	Mudpit_Ingel20196_MGFPeaklist (F058870)	Nonmuscle myosin heavy chain OS=Gallus gallus PE=2 SV=2	Q02015_CHICK,Q789A4_CHICK,Q789A5_CHICK,Q789A6_CHICK,UPI00000FC6BE,UPI0000171380,UPI0000171381,UPI0000171382,UPI00003AB452,UPI00003AB453,UPI00003AB454,UPI000060F510,UPI000060F511,UPI0000ECA50D	TAX_GallusGallus_9031_20141114.fasta,UPARC14_2_GallusG_9031_20140417.fasta	215,888.80	100.00%	8	8	18	0.04%	8.01%	YEILTPNAIPK	R	G	99.70%	20.6	44.4	14.6	7.9	28.5	1.57	0.277	0.318	0	3	0	0	2	1,258.70	2,337.00		1,618.82	613	623	Empty	FALSE	UPI000060E1E4,UPI000012FB7F,MYH9_CHICK,F1NCD4_CHICK,UPI0002C868F4
20196-20225 HinckeM –TAXchicken	Uncategorized Sample	20196-20205_A	Mudpit_Ingel20196_MGFPeaklist (F058870)	Nonmuscle myosin heavy chain OS=Gallus gallus PE=2 SV=2	Q02015_CHICK,Q789A4_CHICK,Q789A5_CHICK,Q789A6_CHICK,UPI00000FC6BE,UPI0000171380,UPI0000171381,UPI0000171382,UPI00003AB452,UPI00003AB453,UPI00003AB454,UPI000060F510,UPI000060F511,UPI0000ECA50D	TAX_GallusGallus_9031_20141114.fasta,UPARC14_2_GallusG_9031_20140417.fasta	215,888.80	100.00%	8	8	18	0.04%	8.01%	ADFCIIHYAGK	K	V	45.90%	18.6	44.4	14.7	11.1					0	0	2	0	2	1,294.63	2,223.00		989.84	458	468	Empty	FALSE	UPI000060E1E4,UPI000012FB7F,MYH9_CHICK,F1NCD4_CHICK,UPI0002C868F4
20196-20225 HinckeM –TAXchicken	Uncategorized Sample	20196-20205_A	Mudpit_Ingel20196_MGFPeaklist (F058870)	Nonmuscle myosin heavy chain OS=Gallus gallus PE=2 SV=2	Q02015_CHICK,Q789A4_CHICK,Q789A5_CHICK,Q789A6_CHICK,UPI00000FC6BE,UPI0000171380,UPI0000171381,UPI0000171382,UPI00003AB452,UPI00003AB453,UPI00003AB454,UPI000060F510,UPI000060F511,UPI0000ECA50D	TAX_GallusGallus_9031_20141114.fasta,UPARC14_2_GallusG_9031_20140417.fasta	215,888.80	100.00%	8	8	18	0.04%	8.01%	AGKLDPHLVLDQLR	R	C	99.70%	52.2	42.3	31.5	35.1	37.5	3.54	0.344	0.5	0	0	2	0	2	1,574.90	2,445.50		970.79	572	585	Empty	FALSE	UPI000060E1E4,UPI000012FB7F,MYH9_CHICK,F1NCD4_CHICK,UPI0002C868F4
20196-20225 HinckeM –TAXchicken	Uncategorized Sample	20196-20205_A	Mudpit_Ingel20196_MGFPeaklist (F058870)	Nonmuscle myosin heavy chain OS=Gallus gallus PE=2 SV=2	Q02015_CHICK,Q789A4_CHICK,Q789A5_CHICK,Q789A6_CHICK,UPI00000FC6BE,UPI0000171380,UPI0000171381,UPI0000171382,UPI00003AB452,UPI00003AB453,UPI00003AB454,UPI000060F510,UPI000060F511,UPI0000ECA50D	TAX_GallusGallus_9031_20141114.fasta,UPARC14_2_GallusG_9031_20140417.fasta	215,888.80	100.00%	8	8	18	0.04%	8.01%	AGVLAHLEEER	R	D	99.70%	71.3	44.3	36.2	36.2	34.7	3.4	0.317	0.409	0	0	2	0	2	1,223.64	2,031.50		1,163.70	657	667	Empty	FALSE	UPI000060E1E4,UPI000012FB7F,MYH9_CHICK,F1NCD4_CHICK,UPI0002C868F4
20196-20225 HinckeM –TAXchicken	Uncategorized Sample	20196-20205_A	Mudpit_Ingel20196_MGFPeaklist (F058870)	Nonmuscle myosin heavy chain OS=Gallus gallus PE=2 SV=2	Q02015_CHICK,Q789A4_CHICK,Q789A5_CHICK,Q789A6_CHICK,UPI00000FC6BE,UPI0000171380,UPI0000171381,UPI0000171382,UPI00003AB452,UPI00003AB453,UPI00003AB454,UPI000060F510,UPI000060F511,UPI0000ECA50D	TAX_GallusGallus_9031_20141114.fasta,UPARC14_2_GallusG_9031_20140417.fasta	215,888.80	100.00%	8	8	18	0.04%	8.01%	AGVLAHLEEERDLK	R	I	99.70%	43.7	43.9	28.2	36.5	38.3	3.7	0.371	0.464	0	0	2	0	2	1,579.84	2,107.00		1,024.73	657	670	Empty	FALSE	UPI000060E1E4,UPI000012FB7F,MYH9_CHICK,F1NCD4_CHICK,UPI0002C868F4
20196-20225 HinckeM –TAXchicken	Uncategorized Sample	20196-20205_A	Mudpit_Ingel20196_MGFPeaklist (F058870)	Nonmuscle myosin heavy chain OS=Gallus gallus PE=2 SV=2	Q02015_CHICK,Q789A4_CHICK,Q789A5_CHICK,Q789A6_CHICK,UPI00000FC6BE,UPI0000171380,UPI0000171381,UPI0000171382,UPI00003AB452,UPI00003AB453,UPI00003AB454,UPI000060F510,UPI000060F511,UPI0000ECA50D	TAX_GallusGallus_9031_20141114.fasta,UPARC14_2_GallusG_9031_20140417.fasta	215,888.80	100.00%	8	8	18	0.04%	8.01%	AIEEETKNHEAQIQEIR	K	Q	99.70%	29	44.4	22.1	12.1	28.6	1.7	0.192	0.265	0	0	0	2	2	2,038.02	1,772.50		811.37	1067	1083	Empty	TRUE	
20196-20225 HinckeM –TAXchicken	Uncategorized Sample	20196-20205_A	Mudpit_Ingel20196_MGFPeaklist (F058870)	Nonmuscle myosin heavy chain OS=Gallus gallus PE=2 SV=2	Q02015_CHICK,Q789A4_CHICK,Q789A5_CHICK,Q789A6_CHICK,UPI00000FC6BE,UPI0000171380,UPI0000171381,UPI0000171382,UPI00003AB452,UPI00003AB453,UPI00003AB454,UPI000060F510,UPI000060F511,UPI0000ECA50D	TAX_GallusGallus_9031_20141114.fasta,UPARC14_2_GallusG_9031_20140417.fasta	215,888.80	100.00%	8	8	18	0.04%	8.01%	ALEEALEAKEEFER	R	Q	99.70%	49	44.8	23.2	35.1	34.9	3.29	0.39	0.286	0	0	2	0	2	1,663.82	2,175.50		1,480.83	1376	1389	Empty	TRUE	
20196-20225 HinckeM –TAXchicken	Uncategorized Sample	20196-20205_A	Mudpit_Ingel20196_MGFPeaklist (F058870)	Nonmuscle myosin heavy chain OS=Gallus gallus PE=2 SV=2	Q02015_CHICK,Q789A4_CHICK,Q789A5_CHICK,Q789A6_CHICK,UPI00000FC6BE,UPI0000171380,UPI0000171381,UPI0000171382,UPI00003AB452,UPI00003AB453,UPI00003AB454,UPI000060F510,UPI000060F511,UPI0000ECA50D	TAX_GallusGallus_9031_20141114.fasta,UPARC14_2_GallusG_9031_20140417.fasta	215,888.80	100.00%	8	8	18	0.04%	8.01%	ALELDPNLYR	R	I	99.70%	42.1	45.2	34.3	22.2	32.7	2.42	0.343	0.4	0	5	0	0	2	1,203.64	2,376.00		1,867.67	638	647	Empty	TRUE	MYH11_CHICK
20196-20225 HinckeM –TAXchicken	Uncategorized Sample	20196-20205_A	Mudpit_Ingel20196_MGFPeaklist (F058870)	Nonmuscle myosin heavy chain OS=Gallus gallus PE=2 SV=2	Q02015_CHICK,Q789A4_CHICK,Q789A5_CHICK,Q789A6_CHICK,UPI00000FC6BE,UPI0000171380,UPI0000171381,UPI0000171382,UPI00003AB452,UPI00003AB453,UPI00003AB454,UPI000060F510,UPI000060F511,UPI0000ECA50D	TAX_GallusGallus_9031_20141114.fasta,UPARC14_2_GallusG_9031_20140417.fasta	215,888.80	100.00%	8	8	18	0.04%	8.01%	CNGVLEGIR	R	I	28.90%	28.9	45.5	29.6	12.6					0	1	0	0	2	1,018.50	2,046.00		210.55	586	594	Empty	FALSE	UPI000060E1E4,MYH11_CHICK,Q9PTY2_CHICK,F1NCD4_CHICK,UPI0002C868F4,UPI000012FB7F,MYH9_CHICK,UPI000044943F,UPI00003AB434,UPI00000FB7E2,UPI00003AB431
20196-20225 HinckeM –TAXchicken	Uncategorized Sample	20196-20205_A	Mudpit_Ingel20196_MGFPeaklist (F058870)	Nonmuscle myosin heavy chain OS=Gallus gallus PE=2 SV=2	Q02015_CHICK,Q789A4_CHICK,Q789A5_CHICK,Q789A6_CHICK,UPI00000FC6BE,UPI0000171380,UPI0000171381,UPI0000171382,UPI00003AB452,UPI00003AB453,UPI00003AB454,UPI000060F510,UPI000060F511,UPI0000ECA50D	TAX_GallusGallus_9031_20141114.fasta,UPARC14_2_GallusG_9031_20140417.fasta	215,888.80	100.00%	8	8	18	0.04%	8.01%	ELDDATEANEGLSR	R	E	99.70%	68.7	43.6	23	0	39.4	3.44	0.327	0.357	0	2	0	0	2	1,519.69	1,739.00		940.74	1791	1804	Empty	TRUE	
20196-20225 HinckeM –TAXchicken	Uncategorized Sample	20196-20205_A	Mudpit_Ingel20196_MGFPeaklist (F058870)	Nonmuscle myosin heavy chain OS=Gallus gallus PE=2 SV=2	Q02015_CHICK,Q789A4_CHICK,Q789A5_CHICK,Q789A6_CHICK,UPI00000FC6BE,UPI0000171380,UPI0000171381,UPI0000171382,UPI00003AB452,UPI00003AB453,UPI00003AB454,UPI000060F510,UPI000060F511,UPI0000ECA50D	TAX_GallusGallus_9031_20141114.fasta,UPARC14_2_GallusG_9031_20140417.fasta	215,888.80	100.00%	8	8	18	0.04%	8.01%	GGPITFSSSR	R	S	95.00%	30.3	44	27.7	19.6					0	1	0	0	2	1,008.51	1,869.00		125.86	1816	1825	Empty	TRUE	
20196-20225 HinckeM –TAXchicken	Uncategorized Sample	20196-20205_A	Mudpit_Ingel20196_MGFPeaklist (F058870)	Nonmuscle myosin heavy chain OS=Gallus gallus PE=2 SV=2	Q02015_CHICK,Q789A4_CHICK,Q789A5_CHICK,Q789A6_CHICK,UPI00000FC6BE,UPI0000171380,UPI0000171381,UPI0000171382,UPI00003AB452,UPI00003AB453,UPI00003AB454,UPI000060F510,UPI000060F511,UPI0000ECA50D	TAX_GallusGallus_9031_20141114.fasta,UPARC14_2_GallusG_9031_20140417.fasta	215,888.80	100.00%	8	8	18	0.04%	8.01%	IAQLEEQLEQEAKER	K	A	99.70%	59.3	44.3	25.4	45.9	46.4	4.08	0.369	0.4	0	0	2	0	2	1,813.93	2,231.00		777.93	1708	1722	Empty	TRUE	
20196-20225 HinckeM –TAXchicken	Uncategorized Sample	20196-20205_A	Mudpit_Ingel20196_MGFPeaklist (F058870)	Nonmuscle myosin heavy chain OS=Gallus gallus PE=2 SV=2	Q02015_CHICK,Q789A4_CHICK,Q789A5_CHICK,Q789A6_CHICK,UPI00000FC6BE,UPI0000171380,UPI0000171381,UPI0000171382,UPI00003AB452,UPI00003AB453,UPI00003AB454,UPI000060F510,UPI000060F511,UPI0000ECA50D	TAX_GallusGallus_9031_20141114.fasta,UPARC14_2_GallusG_9031_20140417.fasta	215,888.80	100.00%	8	8	18	0.04%	8.01%	IVFQEFR	R	Q	98.80%	47	43.1	42.2	19.8	27.9	0.585	0.222	0.5	0	3	0	0	2	938.5094	2,257.00		900.74	604	610	Empty	TRUE	MYH11_CHICK

20196-20225 HinckeM --TAXchicken	Uncategorized Sample	20196-20205_A	Mudpit_Ingel20196_MGFPeaklist (F058870)	Nonmuscle myosin heavy chain OS=Gallus gallus PE=2 SV=2	Q02015_CHICK,Q789A4_CHICK,Q789A5_CHICK,Q789A6_CHICK,UPI00000FC6BE,UPI0000171380,UPI0000171381,UPI0000171382,UPI00003AB452,UPI00003AB453,UPI00003AB454,UPI000060F510,UPI000060F511,UPI0000ECA50D	TAX_GallusGallus_9031_20141114.fasta,UPARC14_2_GallusG_9031_20140417.fasta	215,888.80	100.00%	8	8	18	0.04%	8.01%	AGVLAHLEEERDLK	R	I	99.70%	43.7	43.9	28.2	36.5	38.3	3.7	0.371	0.464	0	0	2	0	2	1,579.84	2,107.00		1,024.73	657	670	Empty	FALSE	UPI000060E1E4,UPI000012FB7F,MYH9_CHICK,F1NCD4_CHICK,UPI0002C868F4
20196-20225 HinckeM --TAXchicken	Uncategorized Sample	20196-20205_A	Mudpit_Ingel20196_MGFPeaklist (F058870)	Nonmuscle myosin heavy chain OS=Gallus gallus PE=2 SV=2	Q02015_CHICK,Q789A4_CHICK,Q789A5_CHICK,Q789A6_CHICK,UPI00000FC6BE,UPI0000171380,UPI0000171381,UPI0000171382,UPI00003AB452,UPI00003AB453,UPI00003AB454,UPI000060F510,UPI000060F511,UPI0000ECA50D	TAX_GallusGallus_9031_20141114.fasta,UPARC14_2_GallusG_9031_20140417.fasta	215,888.80	100.00%	8	8	18	0.04%	8.01%	AIEEETKNHEAQIQEIR	K	Q	99.70%	29	44.4	22.1	12.1	28.6	1.7	0.192	0.265	0	0	0	2	2	2,038.02	1,772.50		811.37	1067	1083	Empty	TRUE	
20196-20225 HinckeM --TAXchicken	Uncategorized Sample	20196-20205_A	Mudpit_Ingel20196_MGFPeaklist (F058870)	Nonmuscle myosin heavy chain OS=Gallus gallus PE=2 SV=2	Q02015_CHICK,Q789A4_CHICK,Q789A5_CHICK,Q789A6_CHICK,UPI00000FC6BE,UPI0000171380,UPI0000171381,UPI0000171382,UPI00003AB452,UPI00003AB453,UPI00003AB454,UPI000060F510,UPI000060F511,UPI0000ECA50D	TAX_GallusGallus_9031_20141114.fasta,UPARC14_2_GallusG_9031_20140417.fasta	215,888.80	100.00%	8	8	18	0.04%	8.01%	ALEEALEAKEEFER	R	Q	99.70%	49	44.8	23.2	35.1	34.9	3.29	0.39	0.286	0	0	2	0	2	1,663.82	2,175.50		1,480.83	1376	1389	Empty	TRUE	
20196-20225 HinckeM --TAXchicken	Uncategorized Sample	20196-20205_A	Mudpit_Ingel20196_MGFPeaklist (F058870)	Nonmuscle myosin heavy chain OS=Gallus gallus PE=2 SV=2	Q02015_CHICK,Q789A4_CHICK,Q789A5_CHICK,Q789A6_CHICK,UPI00000FC6BE,UPI0000171380,UPI0000171381,UPI0000171382,UPI00003AB452,UPI00003AB453,UPI00003AB454,UPI000060F510,UPI000060F511,UPI0000ECA50D	TAX_GallusGallus_9031_20141114.fasta,UPARC14_2_GallusG_9031_20140417.fasta	215,888.80	100.00%	8	8	18	0.04%	8.01%	ALELDPNLYR	R	I	99.70%	42.1	45.2	34.3	22.2	32.7	2.42	0.343	0.4	0	5	0	0	2	1,203.64	2,376.00		1,867.67	638	647	Empty	TRUE	MYH11_CHICK
20196-20225 HinckeM --TAXchicken	Uncategorized Sample	20196-20205_A	Mudpit_Ingel20196_MGFPeaklist (F058870)	Nonmuscle myosin heavy chain OS=Gallus gallus PE=2 SV=2	Q02015_CHICK,Q789A4_CHICK,Q789A5_CHICK,Q789A6_CHICK,UPI00000FC6BE,UPI0000171380,UPI0000171381,UPI0000171382,UPI00003AB452,UPI00003AB453,UPI00003AB454,UPI000060F510,UPI000060F511,UPI0000ECA50D	TAX_GallusGallus_9031_20141114.fasta,UPARC14_2_GallusG_9031_20140417.fasta	215,888.80	100.00%	8	8	18	0.04%	8.01%	CNGVLEGIR	R	I	28.90%	28.9	45.5	29.6	12.6					0	1	0	0	2	1,018.50	2,046.00		210.55	586	594	Empty	FALSE	UPI000060E1E4,MYH11_CHICK,Q9PTY2_CHICK,F1NCD4_CHICK,UPI0002C868F4,UPI000012FB7F,MYH9_CHICK,UPI000044943F,UPI00003AB434,UPI00000FB7E2,UPI00003AB431
20196-20225 HinckeM --TAXchicken	Uncategorized Sample	20196-20205_A	Mudpit_Ingel20196_MGFPeaklist (F058870)	Nonmuscle myosin heavy chain OS=Gallus gallus PE=2 SV=2	Q02015_CHICK,Q789A4_CHICK,Q789A5_CHICK,Q789A6_CHICK,UPI00000FC6BE,UPI0000171380,UPI0000171381,UPI0000171382,UPI00003AB452,UPI00003AB453,UPI00003AB454,UPI000060F510,UPI000060F511,UPI0000ECA50D	TAX_GallusGallus_9031_20141114.fasta,UPARC14_2_GallusG_9031_20140417.fasta	215,888.80	100.00%	8	8	18	0.04%	8.01%	ELDDATEANEGLSR	R	E	99.70%	68.7	43.6	23	0	39.4	3.44	0.327	0.357	0	2	0	0	2	1,519.69	1,739.00		940.74	1791	1804	Empty	TRUE	
20196-20225 HinckeM --TAXchicken	Uncategorized Sample	20196-20205_A	Mudpit_Ingel20196_MGFPeaklist (F058870)	Nonmuscle myosin heavy chain OS=Gallus gallus PE=2 SV=2	Q02015_CHICK,Q789A4_CHICK,Q789A5_CHICK,Q789A6_CHICK,UPI00000FC6BE,UPI0000171380,UPI0000171381,UPI0000171382,UPI00003AB452,UPI00003AB453,UPI00003AB454,UPI000060F510,UPI000060F511,UPI0000ECA50D	TAX_GallusGallus_9031_20141114.fasta,UPARC14_2_GallusG_9031_20140417.fasta	215,888.80	100.00%	8	8	18	0.04%	8.01%	GGPITFSSSR	R	S	95.00%	30.3	44	27.7	19.6					0	1	0	0	2	1,008.51	1,869.00		125.86	1816	1825	Empty	TRUE	
20196-20225 HinckeM --TAXchicken	Uncategorized Sample	20196-20205_A	Mudpit_Ingel20196_MGFPeaklist (F058870)	Nonmuscle myosin heavy chain OS=Gallus gallus PE=2 SV=2	Q02015_CHICK,Q789A4_CHICK,Q789A5_CHICK,Q789A6_CHICK,UPI00000FC6BE,UPI0000171380,UPI0000171381,UPI0000171382,UPI00003AB452,UPI00003AB453,UPI00003AB454,UPI000060F510,UPI000060F511,UPI0000ECA50D	TAX_GallusGallus_9031_20141114.fasta,UPARC14_2_GallusG_9031_20140417.fasta	215,888.80	100.00%	8	8	18	0.04%	8.01%	IAQLEEQLEQEAKER	K	A	99.70%	59.3	44.3	25.4	45.9	46.4	4.08	0.369	0.4	0	0	2	0	2	1,813.93	2,231.00		777.93	1708	1722	Empty	TRUE	
20196-20225 HinckeM --TAXchicken	Uncategorized Sample	20196-20205_A	Mudpit_Ingel20196_MGFPeaklist (F058870)	Nonmuscle myosin heavy chain OS=Gallus gallus PE=2 SV=2	Q02015_CHICK,Q789A4_CHICK,Q789A5_CHICK,Q789A6_CHICK,UPI00000FC6BE,UPI0000171380,UPI0000171381,UPI0000171382,UPI00003AB452,UPI00003AB453,UPI00003AB454,UPI000060F510,UPI000060F511,UPI0000ECA50D	TAX_GallusGallus_9031_20141114.fasta,UPARC14_2_GallusG_9031_20140417.fasta	215,888.80	100.00%	8	8	18	0.04%	8.01%	IVFQEFR	R	Q	98.80%	47	43.1	42.2	19.8	27.9	0.585	0.222	0.5	0	3	0	0	2	938.5094	2,257.00		900.74	604	610	Empty	TRUE	MYH11_CHICK
20196-20225 HinckeM --TAXchicken	Uncategorized Sample	20196-20205_A	Mudpit_Ingel20196_MGFPeaklist (F058870)	Nonmuscle myosin heavy chain OS=Gallus gallus PE=2 SV=2	Q02015_CHICK,Q789A4_CHICK,Q789A5_CHICK,Q789A6_CHICK,UPI00000FC6BE,UPI0000171380,UPI0000171381,UPI0000171382,UPI00003AB452,UPI00003AB453,UPI00003AB454,UPI000060F510,UPI000060F511,UPI0000ECA50D	TAX_GallusGallus_9031_20141114.fasta,UPARC14_2_GallusG_9031_20140417.fasta	215,888.80	100.00%	8	8	18	0.04%	8.01%	KLDAQVQELTAK	K	V	13.90%	14	44	25.3	0.53					0	0	1	0	2	1,343.75	1,897.00		288.44	1141	1152	Empty	TRUE	
20196-20225 HinckeM --TAXchicken	Uncategorized Sample	20196-20205_A	Mudpit_Ingel20196_MGFPeaklist (F058870)	Nonmuscle myosin heavy chain OS=Gallus gallus PE=2 SV=2	Q02015_CHICK,Q789A4_CHICK,Q789A5_CHICK,Q789A6_CHICK,UPI00000FC6BE,UPI0000171380,UPI0000171381,UPI0000171382,UPI00003AB452,UPI00003AB453,UPI00003AB454,UPI000060F510,UPI000060F511,UPI0000ECA50D	TAX_GallusGallus_9031_20141114.fasta,UPARC14_2_GallusG_9031_20140417.fasta	215,888.80	100.00%	8	8	18	0.04%	8.01%	LDPHLVLDQLR	K	C	99.70%	34.6	43.8	18.6	22.1	32.5	2.47	0.366	0.455	0	0	1	0	2	1,318.75	2,534.00		1,121.62	575	585	Empty	FALSE	UPI000060E1E4,UPI000012FB7F,MYH9_CHICK,F1NCD4_CHICK,UPI0002C868F4
20196-20225 HinckeM --TAXchicken	Uncategorized Sample	20196-20205_A	Mudpit_Ingel20196_MGFPeaklist (F058870)	Nonmuscle myosin heavy chain OS=Gallus gallus PE=2 SV=2	Q02015_CHICK,Q789A4_CHICK,Q789A5_CHICK,Q789A6_CHICK,UPI00000FC6BE,UPI0000171380,UPI0000171381,UPI0000171382,UPI00003AB452,UPI00003AB453,UPI00003AB454,UPI000060F510,UPI000060F511,UPI0000ECA50D	TAX_GallusGallus_9031_20141114.fasta,UPARC14_2_GallusG_9031_20140417.fasta	215,888.80	100.00%	8	8	18	0.04%	8.01%	QRYEILTPNAIPK	R	G	99.70%	44.8	43.5	22.5	30.7	33.5	1.96	0.358	0.423	0	2	4	0	2	1,542.86	2,240.50		4,800.05	611	623	Empty	FALSE	UPI000060E1E4,UPI000012FB7F,MYH9_CHICK,F1NCD4_CHICK,UPI0002C868F4
20196-20225 HinckeM --TAXchicken	Uncategorized Sample	20196-20205_A	Mudpit_Ingel20196_MGFPeaklist (F058870)	Nonmuscle myosin heavy chain OS=Gallus gallus PE=2 SV=2	Q02015_CHICK,Q789A4_CHICK,Q789A5_CHICK,Q789A6_CHICK,UPI00000FC6BE,UPI0000171380,UPI0000171381,UPI0000171382,UPI00003AB452,UPI00003AB453,UPI00003AB454,UPI000060F510,UPI000060F511,UPI0000ECA50D	TAX_GallusGallus_9031_20141114.fasta,UPARC14_2_GallusG_9031_20140417.fasta	215,888.80	100.00%	8	8	18	0.04%	8.01%	YEILTPNAIPK	R	G	99.70%	20.6	44.4	14.6	7.9	28.5	1.57	0.277	0.318	0	3	0	0	2	1,258.70	2,337.00		1,618.82	613	623	Empty	FALSE	UPI000060E1E4,UPI000012FB7F,MYH9_CHICK,F1NCD4_CHICK,UPI0002C868F4
20196-20225 HinckeM --TAXchicken	Uncategorized Sample	20196-20205_A	Mudpit_Ingel20196_MGFPeaklist (F058870)	Histone H2A-III OS=Gallus gallus PE=1 SV=2	H2A3_CHICK,H2A4_CHICK,H2AJ_CHICK,Q92069_CHICK	TAX_GallusGallus_9031_20141114.fasta	13,941.00	100.00%	8	16	83	0.17%	36.40%	AGLQFPVGR	R	V	99.70%	63.3	45	39	28	37.3	1.85	0.322	0.611	0	43	0	0	2	944.5313	2,236.00		1565700	22	30	Empty	TRUE	UPI0000185E44,H2AZ_CHICK,UPI00000001D4,UPI000012BF68,UPI00004433FD,UPI0000248BE4,UPI0000DAC2AB,UPI00035037A9,H2AV_CHICK,UPI00005EE2B9,UPI000061263D,UPI00003AFB0F,UPI0000600042
20196-20225 HinckeM --TAXchicken	Uncategorized Sample	20196-20205_A	Mudpit_Ingel20196_MGFPeaklist (F058870)	Histone H2A-III OS=Gallus gallus PE=1 SV=2	H2A3_CHICK,H2A4_CHICK,H2AJ_CHICK,Q92069_CHICK	TAX_GallusGallus_9031_20141114.fasta	13,941.00	100.00%	8	16	83	0.17%	36.40%	HLQLAIR	R	N	98.80%	36	41.9	38.1	9.46	35.9	3.21	0.251	0.786	0	10	0	0	2	850.5258	1,979.00		130250	83	89	Empty	TRUE	UPI0000185E44,H2AZ_CHICK,UPI00000001D4,UPI000012BF68,UPI00004433FD,UPI0000248BE4,UPI0000DAC2AB,UPI00035037A9,H2AV_CHICK,UPI00005EE2B9,UPI000061263D,UPI00003AFB0F,UPI0000600042
20196-20225 HinckeM --TAXchicken	Uncategorized Sample	20196-20205_A	Mudpit_Ingel20196_MGFPeaklist (F058870)	Histone H2A-III OS=Gallus gallus PE=1 SV=2	H2A3_CHICK,H2A4_CHICK,H2AJ_CHICK,Q92069_CHICK	TAX_GallusGallus_9031_20141114.fasta	13,941.00	100.00%	8	16	83	0.17%	36.40%	HLQLAIRNDEELNK	R	L	99.70%	18.5	44.1	19.7	16.3	40.1	2.89	0.262	0.464	0	0	4	3	2	1,692.90	1,973.00		84,567.40	83	96	Empty	TRUE	UPI00035037A9
20196-20225 HinckeM --TAXchicken	Uncategorized Sample	20196-20205_A	Mudpit_Ingel20196_MGFPeaklist (F058870)	Histone H2A-III OS=Gallus gallus PE=1 SV=2	H2A3_CHICK,H2A4_CHICK,H2AJ_CHICK,Q92069_CHICK	TAX_GallusGallus_9031_20141114.fasta	13,941.00	100.00%	8	16	83	0.17%	36.40%	HLQLAIRNDEELNKLLGK	R	V	99.70%	16.8	39.8	13.1	16.3	36.5	3.6	0.296	0.389	0	0	0	2	2	2,104.19	2,509.00		10,209.70	83	100	Empty	TRUE	UPI00035037A9
20196-20225 HinckeM --TAXchicken	Uncategorized Sample	20196-20205_A	Mudpit_Ingel20196_MGFPeaklist (F058870)	Histone H2A-III OS=Gallus gallus PE=1 SV=2	H2A3_CHICK,H2A4_CHICK,H2AJ_CHICK,Q92069_CHICK	TAX_GallusGallus_9031_20141114.fasta	13,941.00	100.00%	8	16	83	0.17%	36.40%	LPNIQAVLLPK	V	K	99.70%					42	6.19	0.438	0.545	0	1	0	0	1	1,205.76	2,743.00		593.02	109	119	Empty	TRUE	
[illegible]	[illegible]	[illegible]	[illegible]	[illegible]	[illegible]	[illegible]	[illegible]	[illegible]	[illegible]	[illegible]	[illegible]	[illegible]	[illegible]	[illegible]	[illegible]	[illegible]	[illegible]	[illegible]	[illegible]	[illegible]	[illegible]	[illegible]	[illegible]	[illegible]	[illegible]	[illegible]	[illegible]	[illegible]	[illegible]	[illegible]	[illegible]	[illegible]	[illegible]	[illegible]	[illegible]	[illegible]	[illegible]	[illegible]	[illegible]

20196-20225 HinckeM –TAXchicken	Uncategorized Sample	20196-20205_A	Mudpit_Ingel20196_MGFPeaklist (F058870)	status=active	UPI0000549337,UPI0001A2BC0F,UPI0001A2BE55	UPARC14_2_GallusG_9031_20140417.fasta	24,621.60	100.00%	5	6	72	0.15%	44.40%	DNIQGITKPAIR	R	R	99.70%	59.9	42.6	20.3	45	43.2	3.37	0.419	0.625	0	0	20	0	2	1,325.75	1,883.50		630057	25	36	Empty	TRUE	UPI0000447264,H48_CHICK,H4_CHICK
20196-20225 HinckeM –TAXchicken	Uncategorized Sample	20196-20205_A	Mudpit_Ingel20196_MGFPeaklist (F058870)	status=active	UPI0000549337,UPI0001A2BC0F,UPI0001A2BE55	UPARC14_2_GallusG_9031_20140417.fasta	24,621.60	100.00%	5	6	72	0.15%	44.40%	HAVSEGTKAVTKYTSSK	K	-	99.70%	64.9	44.2	15.8	56.6	43.3	5.8	0.46	0.441	0	0	0	3	2	1,793.94	1,514.00		1,578.84	207	223	Empty	FALSE	UPI0000111B89,UPI0000ECD1D2,H2B5_CHICK,UPI0000447267,UPI0000447266,UPI0000171242,UPI00003AF50B,UPI0000ECD1F7,UPI00001113BC,UPI000060E152,H2B8_CHICK,UPI0000DA5A49,H2B1_CHICK,UPI0000449F22
20196-20225 HinckeM –TAXchicken	Uncategorized Sample	20196-20205_A	Mudpit_Ingel20196_MGFPeaklist (F058870)	status=active	UPI0000549337,UPI0001A2BC0F,UPI0001A2BE55	UPARC14_2_GallusG_9031_20140417.fasta	24,621.60	100.00%	5	6	72	0.15%	44.40%	ISGLIYEETR	R	G	99.70%	70.1	44	39.6	43.2	45.9	3	0.32	0.65	0	24	0	0	2	1,180.62	2,104.50		255163	47	56	Empty	TRUE	UPI0000447264,H48_CHICK,H4_CHICK
20196-20225 HinckeM –TAXchicken	Uncategorized Sample	20196-20205_A	Mudpit_Ingel20196_MGFPeaklist (F058870)	status=active	UPI0000549337,UPI0001A2BC0F,UPI0001A2BE55	UPARC14_2_GallusG_9031_20140417.fasta	24,621.60	100.00%	5	6	72	0.15%	44.40%	LLLPGELAK	R	H	99.70%	55.3	39.6	36.7	25.2	39.8	1.48	0.274	0.556	0	38	0	0	2	953.6032	2,393.00		1601450	198	206	Empty	FALSE	UPI0000111B89,UPI0000ECD1D2,H2B5_CHICK,UPI0000447267,UPI0000447266,UPI0000171242,UPI00003AF50B,UPI0000ECD1F7,UPI00001113BC,UPI000060E152,H2B8_CHICK,UPI0000DA5A49,H2B1_CHICK,UPI0000449F22
20196-20225 HinckeM –TAXchicken	Uncategorized Sample	20196-20205_A	Mudpit_Ingel20196_MGFPeaklist (F058870)	status=active	UPI0000549337,UPI0001A2BC0F,UPI0001A2BE55	UPARC14_2_GallusG_9031_20140417.fasta	24,621.60	100.00%	5	6	72	0.15%	44.40%	LLLPGELAKHAVSEGTK	R	A	99.70%	50.6	40.7	14.6	44.3	36.8	3.57	0.37	0.471	0	0	0	3	2	1,763.01	2,318.00		4,885.14	198	214	Empty	FALSE	UPI0000111B89,UPI0000ECD1D2,H2B5_CHICK,UPI0000447267,UPI0000447266,UPI0000171242,UPI00003AF50B,UPI0000ECD1F7,UPI00001113BC,UPI000060E152,H2B8_CHICK,UPI0000DA5A49,H2B1_CHICK,UPI0000449F22
20196-20225 HinckeM –TAXchicken	Uncategorized Sample	20196-20205_A	Mudpit_Ingel20196_MGFPeaklist (F058870)	status=active	UPI0000549337,UPI0001A2BC0F,UPI0001A2BE55	UPARC14_2_GallusG_9031_20140417.fasta	24,621.60	100.00%	5	6	72	0.15%	44.40%	RISGLIYEETR	K	G	99.70%	49	44.2	21.5	27.3	50.1	4.44	0.377	0.682	0	2	0	0	2	1,336.72	1,938.00		54,590.90	46	56	Empty	TRUE	UPI0000447264,H48_CHICK,H4_CHICK
20196-20225 HinckeM –TAXchicken	Uncategorized Sample	20196-20205_A	Mudpit_Ingel20196_MGFPeaklist (F058870)	status=active	UPI0000549337,UPI0001A2BC0F,UPI0001A2BE55	UPARC14_2_GallusG_9031_20140417.fasta	24,621.60	100.00%	5	6	72	0.15%	44.40%	TVTAMDVVYALKR	K	Q	99.70%	71.7	43.2	19.3	62.8	43.7	5.89	0.432	0.538	0	0	9	0	2	1,482.80	2,366.00		420466	81	93	Empty	TRUE	UPI0000447264,H48_CHICK,H4_CHICK
20196-20225 HinckeM –TAXchicken	Uncategorized Sample	20196-20205_A	Mudpit_Ingel20196_MGFPeaklist (F058870)	status=active	UPI0000549337,UPI0001A2BC0F,UPI0001A2BE55	UPARC14_2_GallusG_9031_20140417.fasta	24,621.60	100.00%	5	6	72	0.15%	44.40%	VFLENVIR	K	D	99.70%	46	44.5	32.2	29.4	31.7	2.35	0.123	0.562	0	17	0	0	2	989.5778	2,441.00		238111	61	68	Empty	TRUE	UPI0000447264,H48_CHICK,H4_CHICK
20196-20225 HinckeM –TAXchicken	Uncategorized Sample	20196-20205_A	Mudpit_Ingel20196_MGFPeaklist (F058870)	status=active	UPI0000549337,UPI0001A2BC0F,UPI0001A2BE55	UPARC14_2_GallusG_9031_20140417.fasta	24,621.60	100.00%	5	6	72	0.15%	44.40%	VLKQVHPDTGISSK	K	A	99.70%	52.2	42.9	26	35.8	41.7	5.92	0.408	0.464	0	0	4	0	2	1,508.84	1,583.50		1,094.40	142	155	Empty	FALSE	UPI0000111B89,UPI0000ECD1D2,H2B5_CHICK,UPI0000447267,UPI0000447266,UPI0000171242,UPI00003AF50B,UPI0000ECD1F7,UPI00001113BC,UPI000060E152,H2B8_CHICK,UPI0000DA5A49,H2B1_CHICK,UPI0000449F22
20196-20225 HinckeM –TAXchicken	Uncategorized Sample	20196-20205_A	Mudpit_Ingel20196_MGFPeaklist (F058870)	status=active	UPI0000549337,UPI0001A2BC0F,UPI0001A2BE55	UPARC14_2_GallusG_9031_20140417.fasta	24,621.60	100.00%	5	6	72	0.15%	44.40%	AMGIMNSFVNDIFER	K	I	99.70%	75.5	44	22.2	66.3	54.2	7.14	0.196	0.5	0	14	1	0	2	1,776.79	2,772.00		147735	156	170	Empty	FALSE	UPI0000111B89,UPI0000ECD1D2,H2B5_CHICK,UPI0000447267,UPI0000447266,UPI0000171242,UPI00003AF50B,UPI0000ECD1F7,UPI00001113BC,UPI000060E152,H2B8_CHICK,UPI0000DA5A49,H2B1_CHICK,UPI0000449F22
20196-20225 HinckeM –TAXchicken	Uncategorized Sample	20196-20205_A	Mudpit_Ingel20196_MGFPeaklist (F058870)	status=active	UPI0000549337,UPI0001A2BC0F,UPI0001A2BE55	UPARC14_2_GallusG_9031_20140417.fasta	24,621.60	100.00%	5	6	72	0.15%	44.40%	DNIQGITKPAIR	R	R	99.70%	59.9	42.6	20.3	45	43.2	3.37	0.419	0.625	0	0	20	0	2	1,325.75	1,883.50		630057	25	36	Empty	TRUE	UPI0000447264,H48_CHICK,H4_CHICK
20196-20225 HinckeM –TAXchicken	Uncategorized Sample	20196-20205_A	Mudpit_Ingel20196_MGFPeaklist (F058870)	status=active	UPI0000549337,UPI0001A2BC0F,UPI0001A2BE55	UPARC14_2_GallusG_9031_20140417.fasta	24,621.60	100.00%	5	6	72	0.15%	44.40%	HAVSEGTKAVTKYTSSK	K	-	99.70%	64.9	44.2	15.8	56.6	43.3	5.8	0.46	0.441	0	0	0	3	2	1,793.94	1,514.00		1,578.84	207	223	Empty	FALSE	UPI0000111B89,UPI0000ECD1D2,H2B5_CHICK,UPI0000447267,UPI0000447266,UPI0000171242,UPI00003AF50B,UPI0000ECD1F7,UPI00001113BC,UPI000060E152,H2B8_CHICK,UPI0000DA5A49,H2B1_CHICK,UPI0000449F22
20196-20225 HinckeM –TAXchicken	Uncategorized Sample	20196-20205_A	Mudpit_Ingel20196_MGFPeaklist (F058870)	status=active	UPI0000549337,UPI0001A2BC0F,UPI0001A2BE55	UPARC14_2_GallusG_9031_20140417.fasta	24,621.60	100.00%	5	6	72	0.15%	44.40%	ISGLIYEETR	R	G	99.70%	70.1	44	39.6	43.2	45.9	3	0.32	0.65	0	24	0	0	2	1,180.62	2,104.50		255163	47	56	Empty	TRUE	UPI0000447264,H48_CHICK,H4_CHICK
20196-20225 HinckeM –TAXchicken	Uncategorized Sample	20196-20205_A	Mudpit_Ingel20196_MGFPeaklist (F058870)	status=active	UPI0000549337,UPI0001A2BC0F,UPI0001A2BE55	UPARC14_2_GallusG_9031_20140417.fasta	24,621.60	100.00%	5	6	72	0.15%	44.40%	LLLPGELAK	R	H	99.70%	55.3	39.6	36.7	25.2	39.8	1.48	0.274	0.556	0	38	0	0	2	953.6032	2,393.00		1601450	198	206	Empty	FALSE	UPI0000111B89,UPI0000ECD1D2,H2B5_CHICK,UPI0000447267,UPI0000447266,UPI0000171242,UPI00003AF50B,UPI0000ECD1F7,UPI00001113BC,UPI000060E152,H2B8_CHICK,UPI0000DA5A49,H2B1_CHICK,UPI0000449F22
20196-20225 HinckeM –TAXchicken	Uncategorized Sample	20196-20205_A	Mudpit_Ingel20196_MGFPeaklist (F058870)	status=active	UPI0000549337,UPI0001A2BC0F,UPI0001A2BE55	UPARC14_2_GallusG_9031_20140417.fasta	24,621.60	100.00%	5	6	72	0.15%	44.40%	LLLPGELAKHAVSEGTK	R	A	99.70%	50.6	40.7	14.6	44.3	36.8	3.57	0.37	0.471	0	0	0	3	2	1,763.01	2,318.00		4,885.14	198	214	Empty	FALSE	UPI0000111B89,UPI0000ECD1D2,H2B5_CHICK,UPI0000447267,UPI0000447266,UPI0000171242,UPI00003AF50B,UPI0000ECD1F7,UPI00001113BC,UPI000060E152,H2B8_CHICK,UPI0000DA5A49,H2B1_CHICK,UPI0000449F22
20196-20225 HinckeM –TAXchicken	Uncategorized Sample	20196-20205_A	Mudpit_Ingel20196_MGFPeaklist (F058870)	status=active	UPI0000549337,UPI0001A2BC0F,UPI0001A2BE55	UPARC14_2_GallusG_9031_20140417.fasta	24,621.60	100.00%	5	6	72	0.15%	44.40%	RISGLIYEETR	K	G	99.70%	49	44.2	21.5	27.3	50.1	4.44	0.377	0.682	0	2	0	0	2	1,336.72	1,938.00		54,590.90	46	56	Empty	TRUE	UPI0000447264,H48_CHICK,H4_CHICK
20196-20225 HinckeM –TAXchicken	Uncategorized Sample	20196-20205_A	Mudpit_Ingel20196_MGFPeaklist (F058870)	status=active	UPI0000549337,UPI0001A2BC0F,UPI0001A2BE55	UPARC14_2_GallusG_9031_20140417.fasta	24,621.60	100.00%	5	6	72	0.15%	44.40%	TVTAMDVVYALKR	K	Q	99.70%	71.7	43.2	19.3	62.8	43.7	5.89	0.432	0.538	0	0	9	0	2	1,482.80	2,366.00		420466	81	93	Empty	TRUE	UPI0000447264,H48_CHICK,H4_CHICK
20196-20225 HinckeM –TAXchicken	Uncategorized Sample	20196-20205_A	Mudpit_Ingel20196_MGFPeaklist (F058870)	status=active	UPI0000549337,UPI0001A2BC0F,UPI0001A2BE55	UPARC14_2_GallusG_9031_20140417.fasta	24,621.60	100.00%	5	6	72	0.15%	44.40%	VFLENVIR	K	D	99.70%	46	44.5	32.2	29.4	31.7	2.35	0.123	0.562	0	17	0	0	2	989.5778	2,441.00		238111	61	68	Empty	TRUE	UPI0000447264,H48_CHICK,H4_CHICK
20196-20225 HinckeM –TAXchicken	Uncategorized Sample	20196-20205_A	Mudpit_Ingel20196_MGFPeaklist (F058870)	status=active	UPI0000549337,UPI0001A2BC0F,UPI0001A2BE55	UPARC14_2_GallusG_9031_20140417.fasta	24,621.60	100.00%	5	6	72	0.15%	44.40%	VLKQVHPDTGISSK	K	A	99.70%	52.2	42.9	26	35.8	41.7	5.92	0.408	0.464	0	0	4	0	2	1,508.84	1,583.50		1,094.40	142	155	Empty	FALSE	UPI0000111B89,UPI0000ECD1D2,H2B5_CHICK,UPI0000447267,UPI0000447266,UPI0000171242,UPI00003AF50B,UPI0000ECD1F7,UPI00001113BC,UPI000060E152,H2B8_CHICK,UPI0000DA5A49,H2B1_CHICK,UPI0000449F22
20196-20225 HinckeM –TAXchicken	Uncategorized Sample	20196-20205_A	Mudpit_Ingel20196_MGFPeaklist (F058870)	status=active	UPI0000549337,UPI0001A2BC0F,UPI0001A2BE55	UPARC14_2_GallusG_9031_20140417.fasta	24,621.60	100.00%	5	6	72	0.15%	44.40%	AMGIMNSFVNDIFER	K	I	99.70%	75.5	44	22.2	66.3	54.2	7.14	0.196	0.5	0	14	1	0	2	1,776.79	2,772.00		147735	156	170	Empty	FALSE	UPI0000111B89,UPI0000ECD1D2,H2B5_CHICK,UPI0000447267,UPI0000447266,UPI0000171242,UPI00003AF50B,UPI0000ECD1F7,UPI00001113BC,UPI000060E152,H2B8_CHICK,UPI0000DA5A49,H2B1_CHICK,UPI0000449F22
20196-20225 HinckeM –TAXchicken	Uncategorized Sample	20196-20205_A	Mudpit_Ingel20196_MGFPeaklist (F058870)	status=active	UPI0000549337,UPI0001A2BC0F,UPI0001A2BE55	UPARC14_2_GallusG_9031_20140417.fasta	24,621.60	100.00%	5	6	72	0.15%	44.40%	DNIQGITKPAIR	R	R	99.70%	59.9	42.6	20.3	45	43.2	3.37	0.419	0.625	0	0	20	0	2	1,325.75	1,883.50		630057	25	36	Empty	TRUE	UPI0000447264,H48_CHICK,H4_CHICK
20196-20225 HinckeM –TAXchicken	Uncategorized Sample	20196-20205_A	Mudpit_Ingel20196_MGFPeaklist (F058870)	status=active	UPI0000549337,UPI0001A2BC0F,UPI0001A2BE55	UPARC14_2_GallusG_9031_20140417.fasta	24,621.60	100.00%	5	6	72	0.15%	44.40%	HAVSEGTKAVTKYTSSK	K	-	99.70%	64.9	44.2	15.8	56.6	43.3	5.8	0.46	0.441	0	0	0	3	2	1,793.94	1,514.00		1,578.84	207	223	Empty	FALSE	UPI0000111B89,UPI0000ECD1D2,H2B5_CHICK,UPI0000447267,UPI0000447266,UPI0000171242,UPI00003AF50B,UPI0000ECD1F7,UPI00001113BC,UPI000060E152,H2B8_CHICK,UPI0000DA5A49,H2B1_CHICK,UPI0000449F22
20196-20225 HinckeM –TAXchicken	Uncategorized Sample	20196-20205_A	Mudpit_Ingel20196_MGFPeaklist (F058870)	status=active	UPI0000549337,UPI0001A2BC0F,UPI0001A2BE55	UPARC14_2_GallusG_9031_20140417.fasta	24,621.60	100.00%	5	6	72	0.15%	44.40%	ISGLIYEETR	R	G	99.70%	70.1	44	39.6	43.2	45.9	3	0.32	0.65	0	24	0	0	2	1,180.62	2,104.50		255163	47	56	Empty	TRUE	UPI0000447264,H48_CHICK,H4_CHICK
20196-20225 HinckeM –TAXchicken	Uncategorized Sample	20196-20205_A	Mudpit_Ingel20196_MGFPeaklist (F058870)	status=active	UPI0000549337,UPI0001A2BC0F,UPI0001A2BE55	UPARC14_2_GallusG_9031_20140417.fasta	24,621.60	100.00%	5	6	72	0.15%	44.40%	LLLPGELAK	R	H	99.70%	55.3	39.6	36.7	25.2	39.8	1.48	0.274	0.556	0	38	0	0	2	953.6032	2,393.00		1601450	198	206	Empty	FALSE	UPI0000111B89,UPI0000ECD1D2,H2B5_CHICK,UPI0000447267,UPI0000447266,UPI0000171242,UPI00003AF50B,UPI0000ECD1F7,UPI00001113BC,UPI000060E152,H2B8_CHICK,UPI0000DA5A49,H2B1_CHICK,UPI0000449F22
20196-20225 HinckeM –TAXchicken	Uncategorized Sample	20196-20205_A	Mudpit_Ingel20196_MGFPeaklist (F058870)	status=active	UPI0000549337,UPI0001A2BC0F,UPI0001A2BE55	UPARC14_2_GallusG_9031_20140417.fasta	24,621.60	100.00%	5	6	72	0.15%	44.40%	LLLPGELAKHAVSEGTK	R	A	99.70%	50.6	40.7	14.6	44.3	36.8	3.57	0.37	0.471	0	0	0	3	2	1,763.01	2,318.00		4,885.14	198	214	Empty	FALSE	UPI0000111B89,UPI0000ECD1D2,H2B5_CHICK,UPI0000447267,UPI0000447266,UPI0000171242,UPI00003AF50B,UPI0000ECD1F7,UPI00001113BC,UPI000060E152,H2B8_CHICK,UPI0000DA5A49,H2B1_CHICK,UPI0000449F22
20196-20225 HinckeM –TAXchicken	Uncategorized Sample	20196-20205_A	Mudpit_Ingel20196_MGFPeaklist (F058870)	status=active	UPI0000549337,UPI0001A2BC0F,UPI0001A2BE55	UPARC14_2_GallusG_9031_20140417.fasta	24,621.60	100.00%	5	6	72	0.15%	44.40%	RISGLIYEETR	K	G	99.70%	49	44.2	21.5	27.3	50.1	4.44	0.377	0.682	0	2	0	0	2	1,336.72	1,938.00		54,590.90	46	56	Empty	TRUE	UPI0000447264,H48_CHICK,H4_CHICK
20196-20225 HinckeM –TAXchicken	Uncategorized Sample	20196-20205_A	Mudpit_Ingel20196_MGFPeaklist (F058870)	status=active	UPI0000549337,UPI0001A2BC0F,UPI0001A2BE55	UPARC14_2_GallusG_9031_20140417.fasta	24,621.60	100.00%	5	6	72	0.15%	44.40%	TVTAMDVVYALKR	K	Q	99.70%	71.7	43.2	19.3	62.8	43.7	5.89	0.432	0.538	0	0	9	0	2	1,482.80	2,366.00		420466	81	93	Empty	TRUE	UPI0000447264,H48_CHICK,H4_CHICK
20196-20225 HinckeM –TAXchicken	Uncategorized Sample	20196-20205_A	Mudpit_Ingel20196_MGFPeaklist (F058870)	status=active	UPI0000549337,UPI0001A2BC0F,UPI0001A2BE55	UPARC14_2_GallusG_9031_20140417.fasta	24,621.60	100.00%	5	6	72	0.15%	44.40%	VFLENVIR	K	D	99.70%	46	44.5	32.2	29.4	31.7	2.35	0.123	0.562	0	17	0	0	2	989.5778	2,441.00		238111	61	68	Empty	TRUE	UPI0000447264,H48_CHICK,H4_CHICK
20196-20225 HinckeM –TAXchicken	Uncategorized Sample	20196-20205_A	Mudpit_Ingel20196_MGFPeaklist (F058870)	status=active	UPI0000549337,UPI0001A2BC0F,UPI0001A2BE55	UPARC14_2_GallusG_9031_20140417.fasta	24,621.60	100.00%	5	6	72	0.15%	44.40%	VLKQVHPDTGISSK	K	A	99.70%	52.2	42.9	26	35.8	41.7	5.92	0.408	0.464	0	0	4	0	2	1,508.84	1,583.50		1,094.40	142	155	Empty	FALSE	UPI0000111B89,UPI0000ECD1D2,H2B5_CHICK,UPI0000447267,UPI0000447266,UPI0000171242,UPI00003AF50B,UPI0000ECD1F7,UPI00001113BC,UPI000060E152,H2B8_CHICK,UPI0000DA5A49,H2B1_CHICK,UPI0000449F22
20196-20225 HinckeM –TAXchicken	Uncategorized Sample	20196-20205_A	Mudpit_Ingel20196_MGFPeaklist (F058870)	Uncharacterized protein OS=Gallus gallus GN=CSE1L PE=4 SV=2	E1BV44_CHICK,UPI0000E81400,UPI0000ECA915,UPI0001C99C16,UPI0003506C88	TAX_GallusGallus_9031_20141114.fasta,UPARC14_2_GallusG_9031_20140417.fasta	106,529.80	100.00%	5	5	11	0.02%	4.74%	IIIPEIQK	K	V	96.90%	56.8	39.7	39.4	15.8					0	5	0	0	2	953.6032	2,374.00		4,096.05	789	796	Empty	TRUE	UPI00003AC206,UPI000060FE73
20196-20225 HinckeM –TAXchicken	Uncategorized Sample	20196-20205_A	Mudpit_Ingel20196_MGFPeaklist (F058870)	Uncharacterized protein OS=Gallus gallus GN=CSE1L PE=4 SV=2	E1BV44_CHICK,UPI0000E81400,UPI0000ECA915,UPI0001C99C16,UPI0003506C88	TAX_GallusGallus_9031_20141114.fasta,UPARC14_2_GallusG_9031_20140417.fasta	106,529.80	100.00%	5	5	11	0.02%	4.74%	SASVNEFPVLK	K	A	99.70%	33.9	44.9	15.2	26.7	33.7	3.4	0.282	0.409	0	2	0	0	2	1,190.64	2,310.50		3,612.52	435	445	Empty	TRUE	UPI00003AC206,UPI000060FE73
20196-20225 HinckeM –TAXchicken	Uncategorized Sample	20196-20205_A	Mudpit_Ingel20196_MGFPeaklist (F058870)	Uncharacterized protein OS=Gallus gallus GN=CSE1L PE=4 SV=2	E1BV44_CHICK,UPI0000E81400,UPI0000ECA915,UPI0001C99C16,UPI0003506C88	TAX_GallusGallus_9031_20141114.fasta,UPARC14_2_GallusG_9031_20140417.fasta	106,529.80	100.00%	5	5	11	0.02%	4.74%	TGNIPPLVR	R	L	8.90%	2.62	42	13.2	1.04					0	1	0	0	2	966.5732	2,035.00		457.5	662	670	Empty	TRUE	UPI00003AC206,UPI000060FE73
20196-20225 HinckeM –TAXchicken	Uncategorized Sample	20196-20205_A	Mudpit_Ingel20196_MGFPeaklist (F058870)	Uncharacterized protein OS=Gallus gallus GN=CSE1L PE=4 SV=2	E1BV44_CHICK,UPI0000E81400,UPI0000ECA915,UPI0001C99C16,UPI0003506C88	TAX_GallusGallus_9031_20141114.fasta,UPARC14_2_GallusG_9031_20140417.fasta	106,529.80	100.00%	5	5	11	0.02%	4.74%	TLDPDPAIR	K	R	99.70%	34.5	43.5	24.7	21.7	26.6	1.55	0.286	0.333	0	2	0	0	2	997.5315	1,868.00		1,580.69	18	26	Empty	TRUE	UPI00003AC206,UPI000060FE73
20196-20225 HinckeM –TAXchicken	Uncategorized Sample	20196-20205_A	Mudpit_Ingel20196_MGFPeaklist (F058870)	Uncharacterized protein OS=Gallus gallus GN=CSE1L PE=4 SV=2	E1BV44_CHICK,UPI0000E81400,UPI0000ECA915,UPI0001C99C16,UPI0003506C88	TAX_GallusGallus_9031_20141114.fasta,UPARC14_2_GallusG_9031_20140417.fasta	106,529.80	100.00%	5	5	11	0.02%	4.74%	VIVPNMEFR	K	A	30.10%	15.5	44.9	21.1	0					0	1	0	0	2	1,120.58	2,015.00		525.74	311	319	Empty	TRUE	
20196-20225 HinckeM –TAXchicken	Uncategorized Sample	20196-20205_A	Mudpit_Ingel20196_MGFPeaklist (F058870)	Uncharacterized protein OS=Gallus gallus GN=CSE1L PE=4 SV=2	E1BV44_CHICK,UPI0000E81400,UPI0000ECA915,UPI0001C99C16,UPI0003506C88	TAX_GallusGallus_9031_20141114.fasta,UPARC14_2_GallusG_9031_20140417.fasta	106,529.80	100.00%	5	5	11	0.02%	4.74%	IIIPEIQK	K	V	96.90%	56.8	39.7	39.4	15.8					0	5	0	0	2	953.6032	2,374.00		4,096.05	789	796	Empty	TRUE	UPI00003AC206,UPI000060FE73
20196-20225 HinckeM –TAXchicken	Uncategorized Sample	20196-20205_A	Mudpit_Ingel20196_MGFPeaklist (F058870)	Uncharacterized protein OS=Gallus gallus GN=CSE1L PE=4 SV=2	E1BV44_CHICK,UPI0000E81400,UPI0000ECA915,UPI0001C99C16,UPI0003506C88	TAX_GallusGallus_9031_20141114.fasta,UPARC14_2_GallusG_9031_20140417.fasta	106,529.80	100.00%	5	5	11	0.02%	4.74%	SASVNEFPVLK	K	A	99.70%	33.9	44.9	15.2	26.7	33.7	3.4	0.282	0.409	0	2	0	0	2	1,190.64	2,310.50		3,612.52	435	445	Empty	TRUE	UPI00003AC206,UPI000060FE73
20196-20225 HinckeM –TAXchicken	Uncategorized Sample	20196-20205_A	Mudpit_Ingel20196_MGFPeaklist (F058870)	Uncharacterized protein OS=Gallus gallus GN=CSE1L PE=4 SV=2	E1BV44_CHICK,UPI0000E81400,UPI0000ECA915,UPI0001C99C16,UPI0003506C88	TAX_GallusGallus_9031_20141114.fasta,UPARC14_2_GallusG_9031_20140417.fasta	106,529.80	100.00%	5	5	11	0.02%	4.74%	TGNIPPLVR	R	L	8.90%	2.62	42	13.2	1.04					0	1	0	0	2	966.5732	2,035.00		457.5	662	670	Empty	TRUE	UPI00003AC206,UPI000060FE73
20196-20225 HinckeM –TAXchicken	Uncategorized Sample	20196-20205_A	Mudpit_Ingel20196_MGFPeaklist (F058870)	Uncharacterized protein OS=Gallus gallus GN=CSE1L PE=4 SV=2	E1BV44_CHICK,UPI0000E81400,UPI0000ECA915,UPI0001C99C16,UPI0003506C88	TAX_GallusGallus_9031_20141114.fasta,UPARC14_2_GallusG_9031_20140417.fasta	106,529.80	100.00%	5	5	11	0.02%	4.74%	TLDPDPAIR	K	R	99.70%	34.5	43.5	24.7	21.7	26.6	1.55	0.286	0.333	0	2	0	0	2	997.5315	1,868.00		1,580.69	18	26	Empty	TRUE	UPI00003AC206,UPI000060FE73
20196-20225 HinckeM –TAXchicken	Uncategorized Sample	20196-20205_A	Mudpit_Ingel20196_MGFPeaklist (F058870)	Uncharacterized protein OS=Gallus gallus GN=CSE1L PE=4 SV=2	E1BV44_CHICK,UPI0000E81400,UPI0000ECA915,UPI0001C99C16,UPI0003506C88	TAX_GallusGallus_9031_20141114.fasta,UPARC14_2_GallusG_9031_20140417.fasta	106,529.80	100.00%	5	5	11	0.02%	4.74%	VIVPNMEFR	K	A	30.10%	15.5	44.9	21.1	0					0	1	0	0	2	1,120.58	2,015.00		525.74	311	319	Empty	TRUE	
20196-20225 HinckeM –TAXchicken	Uncategorized Sample	20196-20205_A	Mudpit_Ingel20196_MGFPeaklist (F058870)	Uncharacterized protein OS=Gallus gallus GN=CSE1L PE=4 SV=2	E1BV44_CHICK,UPI0000E81400,UPI0000ECA915,UPI0001C99C16,UPI0003506C88	TAX_GallusGallus_9031_20141114.fasta,UPARC14_2_GallusG_9031_20140417.fasta	106,529.80	100.00%	5	5	11	0.02%	4.74%	IIIPEIQK	K	V	96.90%	56.8	39.7	39.4	15.8					0	5	0	0	2	953.6032	2,374.00		4,096.05	789	796	Empty	TRUE	UPI00003AC206,UPI000060FE73
20196-20225 HinckeM –TAXchicken	Uncategorized Sample	20196-20205_A	Mudpit_Ingel20196_MGFPeaklist (F058870)	Uncharacterized protein OS=Gallus gallus GN=CSE1L PE=4 SV=2	E1BV44_CHICK,UPI0000E81400,UPI0000ECA915,UPI0001C99C16,UPI0003506C88	TAX_GallusGallus_9031_20141114.fasta,UPARC14_2_GallusG_9031_20140417.fasta	106,529.80	100.00%	5	5	11	0.02%	4.74%	SASVNEFPVLK	K	A	99.70%	33.9	44.9	15.2	26.7	33.7	3.4	0.282	0.409	0	2	0	0	2	1,190.64	2,310.50		3,612.52	435	445	Empty	TRUE	UPI00003AC206,UPI000060FE73
20196-20225 HinckeM –TAXchicken	Uncategorized Sample	20196-20205_A	Mudpit_Ingel20196_MGFPeaklist (F058870)	Uncharacterized protein OS=Gallus gallus GN=CSE1L PE=4 SV=2	E1BV44_CHICK,UPI0000E81400,UPI0000ECA915,UPI0001C99C16,UPI0003506C88	TAX_GallusGallus_9031_20141114.fasta,UPARC14_2_GallusG_9031_20140417.fasta	106,529.80	100.00%	5	5	11	0.02%	4.74%	TGNIPPLVR	R	L	8.90%	2.62	42	13.2	1.04					0	1	0	0	2	966.5732	2,035.00		457.5	662	670	Empty	TRUE	UPI00003AC206,UPI000060FE73
20196-20225 HinckeM –TAXchicken	Uncategorized Sample	20196-20205_A	Mudpit_Ingel20196_MGFPeaklist (F058870)	Uncharacterized protein OS=Gallus gallus GN=CSE1L PE=4 SV=2	E1BV44_CHICK,UPI0000E81400,UPI0000ECA915,UPI0001C99C16,UPI0003506C88	TAX_GallusGallus_9031_20141114.fasta,UPARC14_2_GallusG_9031_20140417.fasta	106,529.80	100.00%	5	5	11	0.02%	4.74%	TLDPDPAIR	K	R	99.70%	34.5	43.5	24.7	21.7	26.6	1.55	0.286	0.333	0	2	0	0	2	997.5315	1,868.00		1,580.69	18	26	Empty	TRUE	UPI00003AC206,UPI000060FE73
20196-20225 HinckeM –TAXchicken	Uncategorized Sample	20196-20205_A	Mudpit_Ingel20196_MGFPeaklist (F058870)	Uncharacterized protein OS=Gallus gallus GN=CSE1L PE=4 SV=2	E1BV44_CHICK,UPI0000E81400,UPI0000ECA915,UPI0001C99C16,UPI0003506C88	TAX_GallusGallus_9031_20141114.fasta,UPARC14_2_GallusG_9031_20140417.fasta	106,529.80	100.00%	5	5	11	0.02%	4.74%	VIVPNMEFR	K	A	30.10%	15.5	44.9	21.1	0					0	1	0	0	2	1,120.58	2,015.00		525.74	311	319	Empty	TRUE	
20196-20225 HinckeM –TAXchicken	Uncategorized Sample	20196-20205_A	Mudpit_Ingel20196_MGFPeaklist (F058870)	Uncharacterized protein OS=Gallus gallus GN=CSE1L PE=4 SV=2	E1BV44_CHICK,UPI0000E81400,UPI0000ECA915,UPI0001C99C16,UPI0003506C88	TAX_GallusGallus_9031_20141114.fasta,UPARC14_2_GallusG_9031_20140417.fasta	106,529.80	100.00%	5	5	11	0.02%	4.74%	IIIPEIQK	K	V	96.90%	56.8	39.7	39.4	15.8					0	5	0	0	2	953.6032	2,374.00		4,096.05	789	796	Empty	TRUE	UPI00003AC206,UPI000060FE73
20196-20225 HinckeM –TAXchicken	Uncategorized Sample	20196-20205_A	Mudpit_Ingel20196_MGFPeaklist (F058870)	Uncharacterized protein OS=Gallus gallus GN=CSE1L PE=4 SV=2	E1BV44_CHICK,UPI0000E81400,UPI0000ECA915,UPI0001C99C16,UPI0003506C88	TAX_GallusGallus_9031_20141114.fasta,UPARC14_2_GallusG_9031_20140417.fasta	106,529.80	100.00%	5	5	11	0.02%	4.74%	SASVNEFPVLK	K	A	99.70%	33.9	44.9	15.2	26.7	33.7	3.4	0.282	0.409	0	2	0	0	2	1,190.64	2,310.50		3,612.52	435	445	Empty	TRUE	UPI00003AC206,UPI000060FE73
20196-20225 HinckeM –TAXchicken	Uncategorized Sample	20196-20205_A	Mudpit_Ingel20196_MGFPeaklist (F058870)	Uncharacterized protein OS=Gallus gallus GN=CSE1L PE=4 SV=2	E1BV44_CHICK,UPI0000E81400,UPI0000ECA915,UPI0001C99C16,UPI0003506C88	TAX_GallusGallus_9031_20141114.fasta,UPARC14_2_GallusG_9031_20140417.fasta	106,529.80	100.00%	5	5	11	0.02%	4.74%	TGNIPPLVR	R	L	8.90%	2.62	42	13.2	1.04					0	1	0	0	2	966.5732	2,035.00		457.5	662	670	Empty	TRUE	UPI00003AC206,UPI000060FE73
20196-20225 HinckeM –TAXchicken	Uncategorized Sample	20196-20205_A	Mudpit_Ingel20196_MGFPeaklist (F058870)	Uncharacterized protein OS=Gallus gallus GN=CSE1L PE=4 SV=2	E1BV44_CHICK,UPI0000E81400,UPI0000ECA915,UPI0001C99C16,UPI0003506C88	TAX_GallusGallus_9031_20141114.fasta,UPARC14_2_GallusG_9031_20140417.fasta	106,529.80	100.00%	5	5	11	0.02%	4.74%	TLDPDPAIR	K	R	99.70%	34.5	43.5	24.7	21.7	26.6	1.55	0.286	0.333	0	2	0	0	2	997.5315	1,868.00		1,580.69	18	26	Empty	TRUE	UPI00003AC206,UPI000060FE73
20196-20225 HinckeM –TAXchicken	Uncategorized Sample	20196-20205_A	Mudpit_Ingel20196_MGFPeaklist (F058870)	Uncharacterized protein OS=Gallus gallus GN=CSE1L PE=4 SV=2	E1BV44_CHICK,UPI0000E81400,UPI0000ECA915,UPI0001C99C16,UPI0003506C88	TAX_GallusGallus_9031_20141114.fasta,UPARC14_2_GallusG_9031_20140417.fasta	106,529.80	100.00%	5	5	11	0.02%	4.74%	VIVPNMEFR	K	A	30.10%	15.5	44.9	21.1	0					0	1	0	0	2	1,120.58	2,015.00		525.74	311	319	Empty	TRUE	
20196-20225 HinckeM –TAXchicken	Uncategorized Sample	20196-20205_A	Mudpit_Ingel20196_MGFPeaklist (F058870)	Uncharacterized protein OS=Gallus gallus GN=CSE1L PE=4 SV=2	E1BV44_CHICK,UPI0000E81400,UPI0000ECA915,UPI0001C99C16,UPI0003506C88	TAX_GallusGallus_9031_20141114.fasta,UPARC14_2_GallusG_9031_20140417.fasta	106,529.80	100.00%	5	5	11	0.02%	4.74%	IIIPEIQK	K	V	96.90%	56.8	39.7	39.4	15.8					0	5	0	0	2	953.6032	2,374.00		4,096.05	789	796	Empty	TRUE	UPI00003AC206,UPI000060FE73
20196-20225 HinckeM –TAXchicken	Uncategorized Sample	20196-20205_A	Mudpit_Ingel20196_MGFPeaklist (F058870)	Uncharacterized protein OS=Gallus gallus GN=CSE1L PE=4 SV=2	E1BV44_CHICK,UPI0000E81400,UPI0000ECA915,UPI0001C99C16,UPI0003506C88	TAX_GallusGallus_9031_20141114.fasta,UPARC14_2_GallusG_9031_20140417.fasta	106,529.80	100.00%	5	5	11	0.02%	4.74%	SASVNEFPVLK	K	A	99.70%	33.9	44.9	15.2	26.7	33.7	3.4	0.282	0.409	0	2	0	0	2	1,190.64	2,310.50		3,612.52	435	445	Empty	TRUE	UPI00003AC206,UPI000060FE73
20196-20225 HinckeM –TAXchicken	Uncategorized Sample	20196-20205_A	Mudpit_Ingel20196_MGFPeaklist (F058870)	Uncharacterized protein OS=Gallus gallus GN=CSE1L PE=4 SV=2	E1BV44_CHICK,UPI0000E81400,UPI0000ECA915,UPI0001C99C16,UPI0003506C88	TAX_GallusGallus_9031_20141114.fasta,UPARC14_2_GallusG_9031_20140417.fasta	106,529.80	100.00%	5	5	11	0.02%	4.74%	TGNIPPLVR	R	L	8.90%	2.62	42	13.2	1.04					0	1	0	0	2	966.5732	2,035.00		457.5	662	670	Empty	TRUE	UPI00003AC206,UPI000060FE73
20196-20225 HinckeM –TAXchicken	Uncategorized Sample	20196-20205_A	Mudpit_Ingel20196_MGFPeaklist (F058870)	Uncharacterized protein OS=Gallus gallus GN=CSE1L PE=4 SV=2	E1BV44_CHICK,UPI0000E81400,UPI0000ECA915,UPI0001C99C16,UPI0003506C88	TAX_GallusGallus_9031_20141114.fasta,UPARC14_2_GallusG_9031_20140417.fasta	106,529.80	100.00%	5	5	11	0.02%	4.74%	TLDPDPAIR	K	R	99.70%	34.5	43.5	24.7	21.7	26.6	1.55	0.286	0.333	0	2	0	0	2	997.5315	1,868.00		1,580.69	18	26	Empty	TRUE	UPI00003AC206,UPI000060FE73
20196-20225 HinckeM –TAXchicken	Uncategorized Sample	20196-20205_A	Mudpit_Ingel20196_MGFPeaklist (F058870)	Uncharacterized protein OS=Gallus gallus GN=CSE1L PE=4 SV=2	E1BV44_CHICK,UPI0000E81400,UPI0000ECA915,UPI0001C99C16,UPI0003506C88	TAX_GallusGallus_9031_20141114.fasta,UPARC14_2_GallusG_9031_20140417.fasta	106,529.80	100.00%	5	5	11	0.02%	4.74%	VIVPNMEFR	K	A	30.10%	15.5	44.9	21.1	0					0	1	0	0	2	1,120.58	2,015.00		525.74	311	319	Empty	TRUE	
20196-20225 HinckeM –TAXchicken	Uncategorized Sample	20196-20205_A	Mudpit_Ingel20196_MGFPeaklist (F058870)	status=inactive	UPI000060F06E	UPARC14_2_GallusG_9031_20140417.fasta	41,157.90	100.00%	3	3	5	0.01%	13.40%	EKPPITVVGDVGGR	K	I	98.00%	29.2	42.8	25	17.4	23.6	0.523	0.136	0.179	0	0	3	0	2	1,423.79	2,012.00		2,687.18	255	268	Empty	FALSE	UPI000000B50D,UPI0003504520,E1BYN9_CHICK,UPI00003AB4A8,UPI000044946D,UPI00003AB4AB
20196-20225 HinckeM –TAXchicken	Uncategorized Sample	20196-20205_A	Mudpit_Ingel20196_MGFPeaklist (F058870)	status=inactive	UPI000060F06E	UPARC14_2_GallusG_9031_20140417.fasta	41,157.90	100.00%	3	3	5	0.01%	13.40%	GAIMNITK	-	G	20.80%	7.73	44.6	13.5	0					0	1	0	0	2	863.4657	1,871.00		150.18	1	8	Empty	TRUE	
20196-20225 HinckeM –TAXchicken	Uncategorized Sample	20196-20205_A	Mudpit_Ingel20196_MGFPeaklist (F058870)	status=inactive	UPI000060F06E	UPARC14_2_GallusG_9031_20140417.fasta	41,157.90	100.00%	3	3	5	0.01%	13.40%	IFVMATHGLLSSDAPR	K	L	99.70%	29.8	44.5	15.7	0	31.3	1.37	0.268	0.312	0	0	1	0	2	1,730.89	2,255.00		893.53	298	313	Empty	TRUE	
20196-20225 HinckeM –TAXchicken	Uncategorized Sample	20196-20205_A	Mudpit_Ingel20196_MGFPeaklist (F058870)	status=inactive	UPI000060F06E	UPARC14_2_GallusG_9031_20140417.fasta	41,157.90	100.00%	3	3	5	0.01%	13.40%	VQVYQEPNRETR	K	V	99.70%	42.7	44.9	19.9	26.2	35.5	2.19	0.293	0.375	0	0	3	0	2	1,518.77	1,528.00		1,639.90	40	51	Empty	TRUE	
20196-20225 HinckeM –TAXchicken	Uncategorized Sample	20196-20205_A	Mudpit_Ingel20196_MGFPeaklist (F058870)	Uncharacterized protein OS=Gallus gallus GN=TPR PE=4 SV=2	E1BYQ6_CHICK	TAX_GallusGallus_9031_20141114.fasta	243,592.20	100.00%	21	22	33	0.07%	11.90%	AMVLSLEESLNKEK	R	Q	25.60%	13.9	44.7	33.9	0					0	0	1	0	2	1,606.84	2,202.00		370.9	1056	1069	Empty	TRUE	UPI0000448941,UPI0003504067,UPI0003506653,UPI00003AE9C4,UPI00006119F3,UPI0002C87FFA,UPI0003504732,UPI00035049CE,UPI0000E80A39,UPI0003503FF9,UPI000350570C
20196-20225 HinckeM –TAXchicken	Uncategorized Sample	20196-20205_A	Mudpit_Ingel20196_MGFPeaklist (F058870)	Uncharacterized protein OS=Gallus gallus GN=TPR PE=4 SV=2	E1BYQ6_CHICK	TAX_GallusGallus_9031_20141114.fasta	243,592.20	100.00%	21	22	33	0.07%	11.90%	AVENMEQQLSELK	K	K	16.30%	11.9	44.4	29.6	-3.42					0	0	1	0	2	1,535.73	2,376.00		459.47	1114	1126	Empty	TRUE	UPI0000448941,UPI0003504067,UPI0003506653,UPI00003AE9C4,UPI00006119F3,UPI0002C87FFA,UPI0003504732,UPI00035049CE,UPI0000E80A39,UPI0003503FF9,UPI000350570C
20196-20225 HinckeM –TAXchicken	Uncategorized Sample	20196-20205_A	Mudpit_Ingel20196_MGFPeaklist (F058870)	Uncharacterized protein OS=Gallus gallus GN=TPR PE=4 SV=2	E1BYQ6_CHICK	TAX_GallusGallus_9031_20141114.fasta	243,592.20	100.00%	21	22	33	0.07%	11.90%	DEIVQEVEAK	L	A	72.10%					25.2	0.444	0.0635	0.35	0	1	0	0	1	1,159.58	2,247.00		1,153.23	510	519	Empty	TRUE	
20196-20225 HinckeM –TAXchicken	Uncategorized Sample	20196-20205_A	Mudpit_Ingel20196_MGFPeaklist (F058870)	Uncharacterized protein OS=Gallus gallus GN=TPR PE=4 SV=2	E1BYQ6_CHICK	TAX_GallusGallus_9031_20141114.fasta	243,592.20	100.00%	21	22	33	0.07%	11.90%	ENEEWKQKSNSLEEQK	K	T	23.50%	1.34	43.7	16.4	-0.47					0	0	1	0	2	2,006.93	2,388.00		478.35	1667	1682	Empty	TRUE	UPI0000448941,UPI0003504067,UPI0003506653,UPI00003AE9C4,UPI00006119F3,UPI0002C87FFA,UPI0003504732,UPI00035049CE,UPI0000E80A39,UPI0003503FF9,UPI000350570C
20196-20225 HinckeM –TAXchicken	Uncategorized Sample	20196-20205_A	Mudpit_Ingel20196_MGFPeaklist (F058870)	Uncharacterized protein OS=Gallus gallus GN=TPR PE=4 SV=2	E1BYQ6_CHICK	TAX_GallusGallus_9031_20141114.fasta	243,592.20	100.00%	21	22	33	0.07%	11.90%	ERLEQELQQMQAK	K	V	56.20%					23.2	0.638	0.129	0.192	0	1	0	0	2	1,631.76	2,147.00		729.49	1373	1385	Empty	TRUE	
20196-20225 HinckeM –TAXchicken	Uncategorized Sample	20196-20205_A	Mudpit_Ingel20196_MGFPeaklist (F058870)	Uncharacterized protein OS=Gallus gallus GN=TPR PE=4 SV=2	E1BYQ6_CHICK	TAX_GallusGallus_9031_20141114.fasta	243,592.20	100.00%	21	22	33	0.07%	11.90%	ERLRTSSSNVEQYR	R	A	86.80%	18.5	44.3	18.1	13	29.8	0.553	0.238	0.25	0	0	3	0	2	1,707.84	1,958.00		28,718.20	1042	1055	Empty	TRUE	UPI0000448941,UPI0003504067,UPI0003506653,UPI00003AE9C4,UPI00006119F3,UPI0002C87FFA,UPI0003504732,UPI00035049CE,UPI0000E80A39,UPI0003503FF9,UPI000350570C
20196-20225 HinckeM –TAXchicken	Uncategorized Sample	20196-20205_A	Mudpit_Ingel20196_MGFPeaklist (F058870)	Uncharacterized protein OS=Gallus gallus GN=TPR PE=4 SV=2	E1BYQ6_CHICK	TAX_GallusGallus_9031_20141114.fasta	243,592.20	100.00%	21	22	33	0.07%	11.90%	ESLLVEQR	R	G	99.40%	54.6	45.2	40.2	18.5	21.5	0.553	0.0651	0.25	0	3	0	0	2	973.5313	1,879.00		335.15	891	898	Empty	TRUE	UPI0000448941,UPI0003504067,UPI0003506653,UPI00003AE9C4,UPI00006119F3,UPI0002C87FFA,UPI0003504732,UPI00035049CE,UPI0000E80A39,UPI0003503FF9,UPI000350570C
20196-20225 HinckeM –TAXchicken	Uncategorized Sample	20196-20205_A	Mudpit_Ingel20196_MGFPeaklist (F058870)	Uncharacterized protein OS=Gallus gallus GN=TPR PE=4 SV=2	E1BYQ6_CHICK	TAX_GallusGallus_9031_20141114.fasta	243,592.20	100.00%	21	22	33	0.07%	11.90%	FLADQQSEIDGLR	R	A	99.70%	72.5	44.9	33.3	57.5	45	4.43	0.453	0.462	0	2	0	0	2	1,491.74	2,194.50		1,576.11	114	126	Empty	TRUE	UPI0000448941,UPI0003504067,UPI0003506653,UPI00003AE9C4,UPI00006119F3,UPI0002C87FFA,UPI0003504732,UPI00035049CE,UPI0000E80A39,UPI0003503FF9,UPI000350570C
20196-20225 HinckeM –TAXchicken	Uncategorized Sample	20196-20205_A	Mudpit_Ingel20196_MGFPeaklist (F058870)	Uncharacterized protein OS=Gallus gallus GN=TPR PE=4 SV=2	E1BYQ6_CHICK	TAX_GallusGallus_9031_20141114.fasta	243,592.20	100.00%	21	22	33	0.07%	11.90%	GQNLLLTNLR	R	T	99.70%	55.9	42.8	30.7	43.3	30.5	1.1	0.223	0.45	0	2	0	0	2	1,141.67	2,405.50		1,011.28	899	908	Empty	TRUE	UPI0000448941,UPI0003504067,UPI0003506653,UPI00003AE9C4,UPI00006119F3,UPI0002C87FFA,UPI0003504732,UPI00035049CE,UPI0000E80A39,UPI0003503FF9,UPI000350570C
20196-20225 HinckeM –TAXchicken	Uncategorized Sample	20196-20205_A	Mudpit_Ingel20196_MGFPeaklist (F058870)	Uncharacterized protein OS=Gallus gallus GN=TPR PE=4 SV=2	E1BYQ6_CHICK	TAX_GallusGallus_9031_20141114.fasta	243,592.20	100.00%	21	22	33	0.07%	11.90%	GQPGTSEDVDDLVSR	K	L	99.70%	91.4	44.1	26.5	82.2	48.9	8.89	0.505	0.367	0	1	0	0	2	1,574.73	2,007.00		214.16	1015	1029	Empty	TRUE	UPI0000448941,UPI00003AE9C4,UPI00006119F3,UPI0002C87FFA,UPI0000E80A39,UPI0003503FF9,UPI000350570C
20196-20225 HinckeM –TAXchicken	Uncategorized Sample	20196-20205_A	Mudpit_Ingel20196_MGFPeaklist (F058870)	Uncharacterized protein OS=Gallus gallus GN=TPR PE=4 SV=2	E1BYQ6_CHICK	TAX_GallusGallus_9031_20141114.fasta	243,592.20	100.00%	21	22	33	0.07%	11.90%	HHQLQLVESIVR	R	Q	99.70%	44.5	43.5	31.1	27.4	28.1	1.54	0.256	0.375	0	0	2	0	2	1,458.82	2,225.00		598.65	684	695	Empty	TRUE	UPI0000448941,UPI0003504067,UPI0003506653,UPI00003AE9C4,UPI00006119F3,UPI0002C87FFA,UPI0003504732,UPI00035049CE,UPI0000E80A39,UPI0003503FF9,UPI000350570C
20196-20225 HinckeM –TAXchicken	Uncategorized Sample	20196-20205_A	Mudpit_Ingel20196_MGFPeaklist (F058870)	Uncharacterized protein OS=Gallus gallus GN=TPR PE=4 SV=2	E1BYQ6_CHICK	TAX_GallusGallus_9031_20141114.fasta	243,592.20	100.00%	21	22	33	0.07%	11.90%	ISTQLEFASK	K	R	99.70%	41.2	43.8	20.9	19.2	27.2	1.8	0.305	0.3	0	2	0	0	2	1,123.60	2,039.50		568.07	802	811	Empty	TRUE	UPI0000448941,UPI0003504067,UPI0003506653,UPI00003AE9C4,UPI00006119F3,UPI0002C87FFA,UPI0003504732,UPI00035049CE,UPI0000E80A39,UPI0003503FF9,UPI000350570C
20196-20225 HinckeM –TAXchicken	Uncategorized Sample	20196-20205_A	Mudpit_Ingel20196_MGFPeaklist (F058870)	Uncharacterized protein OS=Gallus gallus GN=TPR PE=4 SV=2	E1BYQ6_CHICK	TAX_GallusGallus_9031_20141114.fasta	243,592.20	100.00%	21	22	33	0.07%	11.90%	KLEADILPLQESNAELSEK	R	S	99.70%	46.7	43.4	16.4	34.4	37.2	4.26	0.406	0.289	0	0	2	0	2	2,127.12	2,451.50		907.94	1388	1406	Empty	TRUE	UPI0000448941,UPI0003504067,UPI0003506653,UPI00003AE9C4,UPI00006119F3,UPI0002C87FFA,UPI0003504732,UPI00035049CE,UPI0000E80A39,UPI0003503FF9,UPI000350570C
20196-20225 HinckeM –TAXchicken	Uncategorized Sample	20196-20205_A	Mudpit_Ingel20196_MGFPeaklist (F058870)	Uncharacterized protein OS=Gallus gallus GN=TPR PE=4 SV=2	E1BYQ6_CHICK	TAX_GallusGallus_9031_20141114.fasta	243,592.20	100.00%	21	22	33	0.07%	11.90%	LDELQTSDVTVK	K	Y	99.70%	38.7	45.1	28	22.4	37.5	2.85	0.368	0.417	0	1	0	0	2	1,347.70	1,973.00		280.07	252	263	Empty	TRUE	UPI0000448941,UPI0003504067,UPI0003506653,UPI00003AE9C4,UPI00006119F3,UPI0002C87FFA,UPI0003504732,UPI00035049CE,UPI0000E80A39,UPI0003503FF9,UPI000350570C
20196-20225 HinckeM –TAXchicken	Uncategorized Sample	20196-20205_A	Mudpit_Ingel20196_MGFPeaklist (F058870)	Uncharacterized protein OS=Gallus gallus GN=TPR PE=4 SV=2	E1BYQ6_CHICK	TAX_GallusGallus_9031_20141114.fasta	243,592.20	100.00%	21	22	33	0.07%	11.90%	LLHEQLESLSDK	R	M	34.80%	13.9	44.1	21	-2.8					0	0	1	0	2	1,411.74	2,011.00		217.86	1247	1258	Empty	TRUE	UPI0000448941,UPI0003504067,UPI0003506653,UPI00003AE9C4,UPI00006119F3,UPI0002C87FFA,UPI0003504732,UPI00035049CE,UPI0000E80A39,UPI0003503FF9,UPI000350570C
20196-20225 HinckeM –TAXchicken	Uncategorized Sample	20196-20205_A	Mudpit_Ingel20196_MGFPeaklist (F058870)	Uncharacterized protein OS=Gallus gallus GN=TPR PE=4 SV=2	E1BYQ6_CHICK	TAX_GallusGallus_9031_20141114.fasta	243,592.20	100.00%	21	22	33	0.07%	11.90%	LQEQVTELR	K	S	99.70%	45.2	44.9	43.2	16.9	34.6	1.29	0.211	0.444	0	2	0	0	2	1,115.61	1,836.00		974.6	788	796	Empty	TRUE	UPI0000448941,UPI0003504067,UPI0003506653,UPI00003AE9C4,UPI00006119F3,UPI0002C87FFA,UPI0003504732,UPI00035049CE,UPI0000E80A39,UPI0003503FF9,UPI000350570C
20196-20225 HinckeM –TAXchicken	Uncategorized Sample	20196-20205_A	Mudpit_Ingel20196_MGFPeaklist (F058870)	Uncharacterized protein OS=Gallus gallus GN=TPR PE=4 SV=2	E1BYQ6_CHICK	TAX_GallusGallus_9031_20141114.fasta	243,592.20	100.00%	21	22	33	0.07%	11.90%	MSDVRLTQQR	K	E	64.00%	14.2	44.8	27.4	0	24.1	0.42	0.116	0.35	0	1	0	0	2	1,235.61	2,290.00		797.5	881	890	Empty	TRUE	UPI0000448941,UPI0003504067,UPI0003506653,UPI00003AE9C4,UPI00006119F3,UPI0002C87FFA,UPI0003504732,UPI00035049CE,UPI0000E80A39,UPI0003503FF9,UPI000350570C
20196-20225 HinckeM –TAXchicken	Uncategorized Sample	20196-20205_A	Mudpit_Ingel20196_MGFPeaklist (F058870)	Uncharacterized protein OS=Gallus gallus GN=TPR PE=4 SV=2	E1BYQ6_CHICK	TAX_GallusGallus_9031_20141114.fasta	243,592.20	100.00%	21	22	33	0.07%	11.90%	QKIAQLAGTK	K	E	19.10%	7.9	43.3	21.7	-1.57					0	1	0	0	2	1,041.59	2,194.00		760.04	1652	1661	Empty	TRUE	UPI0000448941,UPI0003504067,UPI0003506653,UPI00003AE9C4,UPI00006119F3,UPI0002C87FFA,UPI0003504732,UPI00035049CE,UPI0000E80A39,UPI0003503FF9,UPI000350570C
20196-20225 HinckeM –TAXchicken	Uncategorized Sample	20196-20205_A	Mudpit_Ingel20196_MGFPeaklist (F058870)	Uncharacterized protein OS=Gallus gallus GN=TPR PE=4 SV=2	E1BYQ6_CHICK	TAX_GallusGallus_9031_20141114.fasta	243,592.20	100.00%	21	22	33	0.07%	11.90%	SNSLEEQKTELEVR	K	M	99.70%	72.5	45	32.4	54.8	39.2	4.77	0.383	0.393	0	0	2	0	2	1,661.83	1,841.00		1,257.86	1675	1688	Empty	TRUE	UPI0000448941,UPI0003504067,UPI0003506653,UPI00003AE9C4,UPI00006119F3,UPI0002C87FFA,UPI0003504732,UPI00035049CE,UPI0000E80A39,UPI0003503FF9,UPI000350570C
20196-20225 HinckeM –TAXchicken	Uncategorized Sample	20196-20205_A	Mudpit_Ingel20196_MGFPeaklist (F058870)	Uncharacterized protein OS=Gallus gallus GN=TPR PE=4 SV=2	E1BYQ6_CHICK	TAX_GallusGallus_9031_20141114.fasta	243,592.20	100.00%	21	22	33	0.07%	11.90%	TLENQVENLQK	K	T	99.70%	27.6	44.8	23.1	14.4	33.6	2.32	0.318	0.455	0	2	0	0	2	1,315.69	1,972.00		599.21	1582	1592	Empty	TRUE	UPI0000448941,UPI0003504067,UPI0003506653,UPI00003AE9C4,UPI00006119F3,UPI0002C87FFA,UPI0003504732,UPI00035049CE,UPI0000E80A39,UPI0003503FF9,UPI000350570C
20196-20225 HinckeM –TAXchicken	Uncategorized Sample	20196-20205_A	Mudpit_Ingel20196_MGFPeaklist (F058870)	Uncharacterized protein OS=Gallus gallus GN=TPR PE=4 SV=2	E1BYQ6_CHICK	TAX_GallusGallus_9031_20141114.fasta	243,592.20	100.00%	21	22	33	0.07%	11.90%	TQHLLSQQKDTDLEEYRK	R	L	32.40%	14.5	44.1	26.7	7.37					0	0	0	1	2	2,232.13	1,780.00		380.91	1428	1445	Empty	TRUE	UPI0000448941,UPI0003504067,UPI0003506653,UPI00003AE9C4,UPI00006119F3,UPI0002C87FFA,UPI0003504732,UPI00035049CE,UPI0000E80A39,UPI0003503FF9,UPI000350570C
20196-20225 HinckeM –TAXchicken	Uncategorized Sample	20196-20205_A	Mudpit_Ingel20196_MGFPeaklist (F058870)	status=inactive	UPI0000447264	UPARC14_2_GallusG_9031_20140417.fasta	82,246.60	100.00%	4	8	45	0.09%	11.00%	DNIQGITKPAIR	R	R	99.70%	59.9	42.6	20.3	45	43.2	3.37	0.419	0.625	0	0	20	0	2	1,325.75	1,883.50		630057	136	147	Empty	FALSE	UPI0001A2BE55,UPI0000549337,UPI0001A2BC0F,H48_CHICK,H4_CHICK
20196-20225 HinckeM –TAXchicken	Uncategorized Sample	20196-20205_A	Mudpit_Ingel20196_MGFPeaklist (F058870)	status=inactive	UPI0000447264	UPARC14_2_GallusG_9031_20140417.fasta	82,246.60	100.00%	4	8	45	0.09%	11.00%	EIAQDFKTDLR	R	F	99.70%	68.7	44.7	30.3	0	41.6	3.24	0.43	0.545	0	4	11	0	2	1,317.68	2,040.00		219304	694	704	Empty	TRUE	H33_CHICK,H32_CHICK,Q92068_CHICK
20196-20225 HinckeM –TAXchicken	Uncategorized Sample	20196-20205_A	Mudpit_Ingel20196_MGFPeaklist (F058870)	status=inactive	UPI0000447264	UPARC14_2_GallusG_9031_20140417.fasta	82,246.60	100.00%	4	8	45	0.09%	11.00%	ISGLIYEETR	R	G	99.70%	70.1	44	39.6	43.2	45.9	3	0.32	0.65	0	24	0	0	2	1,180.62	2,104.50		255163	158	167	Empty	FALSE	UPI0001A2BE55,UPI0000549337,UPI0001A2BC0F,H48_CHICK,H4_CHICK
20196-20225 HinckeM –TAXchicken	Uncategorized Sample	20196-20205_A	Mudpit_Ingel20196_MGFPeaklist (F058870)	status=inactive	UPI0000447264	UPARC14_2_GallusG_9031_20140417.fasta	82,246.60	100.00%	4	8	45	0.09%	11.00%	RISGLIYEETR	K	G	99.70%	49	44.2	21.5	27.3	50.1	4.44	0.377	0.682	0	2	0	0	2	1,336.72	1,938.00		54,590.90	157	167	Empty	FALSE	UPI0001A2BE55,UPI0000549337,UPI0001A2BC0F,H48_CHICK,H4_CHICK
20196-20225 HinckeM –TAXchicken	Uncategorized Sample	20196-20205_A	Mudpit_Ingel20196_MGFPeaklist (F058870)	status=inactive	UPI0000447264	UPARC14_2_GallusG_9031_20140417.fasta	82,246.60	100.00%	4	8	45	0.09%	11.00%	STELLIR	K	K	98.80%	63.5	45.2	42.3	23.8	28.7	1.29	0.233	0.571	0	22	0	0	2	831.4935	2,138.00		394581	678	684	Empty	TRUE	H33_CHICK,H32_CHICK,Q92068_CHICK
20196-20225 HinckeM –TAXchicken	Uncategorized Sample	20196-20205_A	Mudpit_Ingel20196_MGFPeaklist (F058870)	status=inactive	UPI0000447264	UPARC14_2_GallusG_9031_20140417.fasta	82,246.60	100.00%	4	8	45	0.09%	11.00%	TVTAMDVVYALKR	K	Q	99.70%	71.7	43.2	19.3	62.8	43.7	5.89	0.432	0.538	0	0	9	0	2	1,482.80	2,366.00		420466	192	204	Empty	FALSE	UPI0001A2BE55,UPI0000549337,UPI0001A2BC0F,H48_CHICK,H4_CHICK
20196-20225 HinckeM –TAXchicken	Uncategorized Sample	20196-20205_A	Mudpit_Ingel20196_MGFPeaklist (F058870)	status=inactive	UPI0000447264	UPARC14_2_GallusG_9031_20140417.fasta	82,246.60	100.00%	4	8	45	0.09%	11.00%	VFLENVIR	K	D	99.70%	46	44.5	32.2	29.4	31.7	2.35	0.123	0.562	0	17	0	0	2	989.5778	2,441.00		238111	172	179	Empty	FALSE	UPI0001A2BE55,UPI0000549337,UPI0001A2BC0F,H48_CHICK,H4_CHICK
20196-20225 HinckeM –TAXchicken	Uncategorized Sample	20196-20205_A	Mudpit_Ingel20196_MGFPeaklist (F058870)	status=inactive	UPI0000447264	UPARC14_2_GallusG_9031_20140417.fasta	82,246.60	100.00%	4	8	45	0.09%	11.00%	VTIMPKDIQLAR	R	R	99.70%	40.3	43.3	23	23.4	29	1.96	0.266	0.333	0	0	2	0	2	1,400.79	2,095.00		1,160.31	738	749	Empty	TRUE	H33_CHICK,H32_CHICK,Q92068_CHICK
20196-20225 HinckeM –TAXchicken	Uncategorized Sample	20196-20205_A	Mudpit_Ingel20196_MGFPeaklist (F058870)	status=inactive	UPI0000447264	UPARC14_2_GallusG_9031_20140417.fasta	82,246.60	100.00%	4	8	45	0.09%	11.00%	YRPGTVALR	R	E	99.70%	38.8	44.5	31.6	17.5	44	2.64	0.375	0.722	0	6	0	0	2	1,032.59	1,726.00		79,831.00	662	670	Empty	TRUE	H33_CHICK,H32_CHICK,Q92068_CHICK
20196-20225 HinckeM –TAXchicken	Uncategorized Sample	20196-20205_A	Mudpit_Ingel20196_MGFPeaklist (F058870)	Hemoglobin subunit beta OS=Gallus gallus GN=HBB PE=1 SV=2	HBB_CHICK	TAX_GallusGallus_9031_20141114.fasta	16,466.30	100.00%	28	45	669	1.35%	92.50%	DFTPECQAAWQK	K	L	99.70%	48.4	42.5	18.8	39.4	46	4.19	0.324	0.583	0	33	0	0	2	1,480.65	2,028.00		1.01E+07	122	133	Empty	TRUE	
20196-20225 HinckeM –TAXchicken	Uncategorized Sample	20196-20205_A	Mudpit_Ingel20196_MGFPeaklist (F058870)	Hemoglobin subunit beta OS=Gallus gallus GN=HBB PE=1 SV=2	HBB_CHICK	TAX_GallusGallus_9031_20141114.fasta	16,466.30	100.00%	28	45	669	1.35%	92.50%	FFASFGNLSSPTAILGNPMVR	R	A	99.70%	70.9	44.1	14.2	52	36.9	2.57	0.317	0.286	0	5	5	0	2	2,226.14	2,873.00		1.50E+07	42	62	Empty	TRUE	
20196-20225 HinckeM –TAXchicken	Uncategorized Sample	20196-20205_A	Mudpit_Ingel20196_MGFPeaklist (F058870)	Hemoglobin subunit beta OS=Gallus gallus GN=HBB PE=1 SV=2	HBB_CHICK	TAX_GallusGallus_9031_20141114.fasta	16,466.30	100.00%	28	45	669	1.35%	92.50%	GDILIIVLAAHFSK	L	D	99.70%					43.4	5.12	0.279	0.464	0	1	0	0	1	1,496.88	2,928.00		32,565.60	108	121	Empty	TRUE	
20196-20225 HinckeM –TAXchicken	Uncategorized Sample	20196-20205_A	Mudpit_Ingel20196_MGFPeaklist (F058870)	Hemoglobin subunit beta OS=Gallus gallus GN=HBB PE=1 SV=2	HBB_CHICK	TAX_GallusGallus_9031_20141114.fasta	16,466.30	100.00%	28	45	669	1.35%	92.50%	KQLITGLWGK	E	V	99.70%					43.4	3.12	0.26	0.65	0	1	0	0	1	1,143.69	2,203.00		21,576.00	9	18	Empty	TRUE	Q90864_CHICK
20196-20225 HinckeM –TAXchicken	Uncategorized Sample	20196-20205_A	Mudpit_Ingel20196_MGFPeaklist (F058870)	Hemoglobin subunit beta OS=Gallus gallus GN=HBB PE=1 SV=2	HBB_CHICK	TAX_GallusGallus_9031_20141114.fasta	16,466.30	100.00%	28	45	669	1.35%	92.50%	KVLTSFGDAVK	K	N	99.70%	73.8	43.3	41.6	45.1	54	5.77	0.335	0.727	0	28	0	0	2	1,164.66	1,949.50		141830	67	77	Empty	TRUE	
20196-20225 HinckeM –TAXchicken	Uncategorized Sample	20196-20205_A	Mudpit_Ingel20196_MGFPeaklist (F058870)	Hemoglobin subunit beta OS=Gallus gallus GN=HBB PE=1 SV=2	HBB_CHICK	TAX_GallusGallus_9031_20141114.fasta	16,466.30	100.00%	28	45	669	1.35%	92.50%	KVNVAECGAEALAR	G	L	99.70%					49.3	5.18	0.495	0.571	0	0	2	0	1	1,487.76	1,869.00		131800	18	31	Empty	TRUE	Q90864_CHICK
20196-20225 HinckeM –TAXchicken	Uncategorized Sample	20196-20205_A	Mudpit_Ingel20196_MGFPeaklist (F058870)	Hemoglobin subunit beta OS=Gallus gallus GN=HBB PE=1 SV=2	HBB_CHICK	TAX_GallusGallus_9031_20141114.fasta	16,466.30	100.00%	28	45	669	1.35%	92.50%	LHCDKLHVDPENFR	E	L	99.70%	62.2	44.6	15.6	57	41.2	4.62	0.427	0.429	0	1	1	12	1	1,779.86	1,883.50		5643460	92	105	Empty	TRUE	HBE_CHICK,Q90864_CHICK
20196-20225 HinckeM –TAXchicken	Uncategorized Sample	20196-20205_A	Mudpit_Ingel20196_MGFPeaklist (F058870)	Hemoglobin subunit beta OS=Gallus gallus GN=HBB PE=1 SV=2	HBB_CHICK	TAX_GallusGallus_9031_20141114.fasta	16,466.30	100.00%	28	45	669	1.35%	92.50%	LHVDPENFR	K	L	99.70%	40.5	43.8	28.9	24.1	35.8	3.35	0.419	0.556	0	16	0	0	2	1,126.56	1,945.00		2591890	97	105	Empty	TRUE	HBE_CHICK,HBRH_CHICK,Q90864_CHICK
20196-20225 HinckeM –TAXchicken	Uncategorized Sample	20196-20205_A	Mudpit_Ingel20196_MGFPeaklist (F058870)	Hemoglobin subunit beta OS=Gallus gallus GN=HBB PE=1 SV=2	HBB_CHICK	TAX_GallusGallus_9031_20141114.fasta	16,466.30	100.00%	28	45	669	1.35%	92.50%	LLGDILIIVLAAHFSK	R	D	99.70%	73.4	34.6	19.4	65.7	47.4	4.59	0.443	0.469	0	0	18	0	2	1,723.05	3,212.50		1989610	106	121	Empty	TRUE	
20196-20225 HinckeM –TAXchicken	Uncategorized Sample	20196-20205_A	Mudpit_Ingel20196_MGFPeaklist (F058870)	Hemoglobin subunit beta OS=Gallus gallus GN=HBB PE=1 SV=2	HBB_CHICK	TAX_GallusGallus_9031_20141114.fasta	16,466.30	100.00%	28	45	669	1.35%	92.50%	LLGDILIIVLAAHFSKDFTPECQAAWQK	R	L	99.70%	29	39.5	13.4	28.5	34.5	2.21	0.2	0.232	0	0	0	1	2	3,184.69	2,919.00		450687	106	133	Empty	TRUE	
20196-20225 HinckeM –TAXchicken	Uncategorized Sample	20196-20205_A	Mudpit_Ingel20196_MGFPeaklist (F058870)	Hemoglobin subunit beta OS=Gallus gallus GN=HBB PE=1 SV=2	HBB_CHICK	TAX_GallusGallus_9031_20141114.fasta	16,466.30	100.00%	28	45	669	1.35%	92.50%	LLIVYPWTQR	R	F	99.70%	64.1	43.6	25.4	48.8	44.4	3.38	0.351	0.6	0	78	0	0	2	1,288.74	2,774.00		4.25E+07	32	41	Empty	TRUE	HBE_CHICK,HBRH_CHICK,Q90864_CHICK
20196-20225 HinckeM –TAXchicken	Uncategorized Sample	20196-20205_A	Mudpit_Ingel20196_MGFPeaklist (F058870)	Hemoglobin subunit beta OS=Gallus gallus GN=HBB PE=1 SV=2	HBB_CHICK	TAX_GallusGallus_9031_20141114.fasta	16,466.30	100.00%	28	45	669	1.35%	92.50%	LLIVYPWTQRFFASFGNLSSPTAILGNPMVR	R	A	99.70%	59.3	38.6	13	54	48.8	6.04	0.443	0.258	0	0	1	1	2	3,495.86	2,912.00		303825	32	62	Empty	TRUE	
20196-20225 HinckeM –TAXchicken	Uncategorized Sample	20196-20205_A	Mudpit_Ingel20196_MGFPeaklist (F058870)	Hemoglobin subunit beta OS=Gallus gallus GN=HBB PE=1 SV=2	HBB_CHICK	TAX_GallusGallus_9031_20141114.fasta	16,466.30	100.00%	28	45	669	1.35%	92.50%	LSELHCDKLHVDPENFR	Q	L	99.70%	53.1	44.3	21.5	48.7	37.3	3.03	0.0322	0.324	0	0	0	1	1	2,109.02	2,097.00		145014	89	105	Empty	TRUE	HBE_CHICK
20196-20225 HinckeM –TAXchicken	Uncategorized Sample	20196-20205_A	Mudpit_Ingel20196_MGFPeaklist (F058870)	Hemoglobin subunit beta OS=Gallus gallus GN=HBB PE=1 SV=2	HBB_CHICK	TAX_GallusGallus_9031_20141114.fasta	16,466.30	100.00%	28	45	669	1.35%	92.50%	NLDNIKNTFSQLSELHCDK	K	L	99.70%	79.8	44.2	13.4	15.4	36	3.12	0.103	0.289	0	0	0	4	2	2,276.10	2,520.50		353514	78	96	Empty	TRUE	
20196-20225 HinckeM –TAXchicken	Uncategorized Sample	20196-20205_A	Mudpit_Ingel20196_MGFPeaklist (F058870)	Hemoglobin subunit beta OS=Gallus gallus GN=HBB PE=1 SV=2	HBB_CHICK	TAX_GallusGallus_9031_20141114.fasta	16,466.30	100.00%	28	45	669	1.35%	92.50%	NTFSQLSELHCDK	K	L	99.70%	77.8	43.8	27.8	23.2	41.6	4.48	0.142	0.462	0	14	33	0	2	1,578.72	2,087.00		1.66E+07	84	96	Empty	TRUE	
20196-20225 HinckeM –TAXchicken	Uncategorized Sample	20196-20205_A	Mudpit_Ingel20196_MGFPeaklist (F058870)	Hemoglobin subunit beta OS=Gallus gallus GN=HBB PE=1 SV=2	HBB_CHICK	TAX_GallusGallus_9031_20141114.fasta	16,466.30	100.00%	28	45	669	1.35%	92.50%	NTFSQLSELHCDKLHVDPENFR	K	L	99.70%	83.8	43.3	14.8	14.1	50.5	6.41	0.0238	0.341	0	0	0	12	2	2,686.27	2,413.00		1.29E+07	84	105	Empty	TRUE	
20196-20225 HinckeM –TAXchicken	Uncategorized Sample	20196-20205_A	Mudpit_Ingel20196_MGFPeaklist (F058870)	Hemoglobin subunit beta OS=Gallus gallus GN=HBB PE=1 SV=2	HBB_CHICK	TAX_GallusGallus_9031_20141114.fasta	16,466.30	100.00%	28	45	669	1.35%	92.50%	QLITGLWGK	K	V	99.70%	62.3	44.4	26.8	39	38.3	1.74	0.324	0.611	0	95	0	0	2	1,015.59	2,525.00		2.67E+07	10	18	Empty	TRUE	Q90864_CHICK
20196-20225 HinckeM –TAXchicken	Uncategorized Sample	20196-20205_A	Mudpit_Ingel20196_MGFPeaklist (F058870)	Hemoglobin subunit beta OS=Gallus gallus GN=HBB PE=1 SV=2	HBB_CHICK	TAX_GallusGallus_9031_20141114.fasta	16,466.30	100.00%	28	45	669	1.35%	92.50%	QLITGLWGKVNVAECGAEALAR	K	L	99.70%	102	42.9	14.1	98.6	68.3	10.6	0.621	0.432	0	0	2	0	2	2,356.24	2,715.00		1028340	10	31	Empty	TRUE	Q90864_CHICK
20196-20225 HinckeM –TAXchicken	Uncategorized Sample	20196-20205_A	Mudpit_Ingel20196_MGFPeaklist (F058870)	Hemoglobin subunit beta OS=Gallus gallus GN=HBB PE=1 SV=2	HBB_CHICK	TAX_GallusGallus_9031_20141114.fasta	16,466.30	100.00%	28	45	669	1.35%	92.50%	SELHCDKLHVDPENFR	L	L	99.40%					37	3.11	0.0865	0.375	0	0	0	1	1	1,995.93	1,926.00		10,369.20	90	105	Empty	TRUE	HBE_CHICK
20196-20225 HinckeM –TAXchicken	Uncategorized Sample	20196-20205_A	Mudpit_Ingel20196_MGFPeaklist (F058870)	Hemoglobin subunit beta OS=Gallus gallus GN=HBB PE=1 SV=2	HBB_CHICK	TAX_GallusGallus_9031_20141114.fasta	16,466.30	100.00%	28	45	669	1.35%	92.50%	SSPTAILGNPMVR	L	A	99.70%					35.6	1.89	0.348	0.385	0	1	0	0	1	1,342.71	2,225.00		53,420.80	50	62	Empty	TRUE	
20196-20225 HinckeM –TAXchicken	Uncategorized Sample	20196-20205_A	Mudpit_Ingel20196_MGFPeaklist (F058870)	Hemoglobin subunit beta OS=Gallus gallus GN=HBB PE=1 SV=2	HBB_CHICK	TAX_GallusGallus_9031_20141114.fasta	16,466.30	100.00%	28	45	669	1.35%	92.50%	VAECGAEALAR	N	L	99.70%					42.7	3.11	0.328	0.5	0	3	0	0	1	1,146.56	1,772.00		6,356.38	21	31	Empty	TRUE	Q90864_CHICK
20196-20225 HinckeM –TAXchicken	Uncategorized Sample	20196-20205_A	Mudpit_Ingel20196_MGFPeaklist (F058870)	Hemoglobin subunit beta OS=Gallus gallus GN=HBB PE=1 SV=2	HBB_CHICK	TAX_GallusGallus_9031_20141114.fasta	16,466.30	100.00%	28	45	669	1.35%	92.50%	VHWTAEEK	M	Q	99.70%	41.7	43.6	27	28	37.2	1.59	0.344	0.688	0	18	0	0	1	999.4894	1,668.50		14,341.90	2	9	Empty	TRUE	Q90864_CHICK
20196-20225 HinckeM –TAXchicken	Uncategorized Sample	20196-20205_A	Mudpit_Ingel20196_MGFPeaklist (F058870)	Hemoglobin subunit beta OS=Gallus gallus GN=HBB PE=1 SV=2	HBB_CHICK	TAX_GallusGallus_9031_20141114.fasta	16,466.30	100.00%	28	45	669	1.35%	92.50%	VHWTAEEKQLITGLWGK	M	V	99.70%	71.9	42.9	19	71.9	43.5	6.03	0.497	0.382	0	0	2	0	1	1,996.07	2,560.50		675133	2	18	Empty	TRUE	Q90864_CHICK
20196-20225 HinckeM –TAXchicken	Uncategorized Sample	20196-20205_A	Mudpit_Ingel20196_MGFPeaklist (F058870)	Hemoglobin subunit beta OS=Gallus gallus GN=HBB PE=1 SV=2	HBB_CHICK	TAX_GallusGallus_9031_20141114.fasta	16,466.30	100.00%	28	45	669	1.35%	92.50%	VHWTAEEKQLITGLWGKVNVAECGAEALAR	M	L	99.70%	60	41.3	0	60	45.5	6.1	0.484	0.25	0	0	0	2	1	3,336.72	2,857.00		1250740	2	31	Empty	TRUE	Q90864_CHICK
20196-20225 HinckeM –TAXchicken	Uncategorized Sample	20196-20205_A	Mudpit_Ingel20196_MGFPeaklist (F058870)	Hemoglobin subunit beta OS=Gallus gallus GN=HBB PE=1 SV=2	HBB_CHICK	TAX_GallusGallus_9031_20141114.fasta	16,466.30	100.00%	28	45	669	1.35%	92.50%	VLTSFGDAVK	K	N	99.70%	67.1	44	27.7	47.9	40	3.33	0.353	0.55	0	126	0	0	2	1,036.57	2,116.50		2.74E+07	68	77	Empty	TRUE	
20196-20225 HinckeM –TAXchicken	Uncategorized Sample	20196-20205_A	Mudpit_Ingel20196_MGFPeaklist (F058870)	Hemoglobin subunit beta OS=Gallus gallus GN=HBB PE=1 SV=2	HBB_CHICK	TAX_GallusGallus_9031_20141114.fasta	16,466.30	100.00%	28	45	669	1.35%	92.50%	VLTSFGDAVKNLDNIK	K	N	99.70%	89.5	43.5	18.2	56	52.6	7.49	0.262	0.5	0	0	4	0	2	1,733.94	2,549.00		246261	68	83	Empty	TRUE	
20196-20225 HinckeM –TAXchicken	Uncategorized Sample	20196-20205_A	Mudpit_Ingel20196_MGFPeaklist (F058870)	Hemoglobin subunit beta OS=Gallus gallus GN=HBB PE=1 SV=2	HBB_CHICK	TAX_GallusGallus_9031_20141114.fasta	16,466.30	100.00%	28	45	669	1.35%	92.50%	VNVAECGAEALAR	K	L	99.70%	64.8	44.7	22.1	52.5	48.1	4.96	0.395	0.5	0	126	2	0	2	1,359.67	2,097.50		4.86E+07	19	31	Empty	TRUE	Q90864_CHICK
20196-20225 HinckeM –TAXchicken	Uncategorized Sample	20196-20205_A	Mudpit_Ingel20196_MGFPeaklist (F058870)	Hemoglobin subunit beta OS=Gallus gallus GN=HBB PE=1 SV=2	HBB_CHICK	TAX_GallusGallus_9031_20141114.fasta	16,466.30	100.00%	28	45	669	1.35%	92.50%	VVAHALAR	R	K	99.70%	55	42.2	29.3	43.4	46	4.06	0.411	0.812	0	17	0	0	2	836.51	1,517.00		6,330.30	137	144	Empty	TRUE	HBE_CHICK
20196-20225 HinckeM –TAXchicken	Uncategorized Sample	20196-20205_A	Mudpit_Ingel20196_MGFPeaklist (F058870)	Beta-H globin OS=Gallus gallus GN=HBE1 PE=3 SV=1	Q90864_CHICK	TAX_GallusGallus_9031_20141114.fasta	16,365.20	100.00%	7	8	90	0.18%	75.50%	DFTPASQAAWQK	K	M	99.70%	49	44.7	19.4	39.8	48.1	6.57	0.478	0.625	0	24	0	0	2	1,349.65	2,000.00		2606680	122	133	Empty	TRUE	
20196-20225 HinckeM –TAXchicken	Uncategorized Sample	20196-20205_A	Mudpit_Ingel20196_MGFPeaklist (F058870)	Beta-H globin OS=Gallus gallus GN=HBE1 PE=3 SV=1	Q90864_CHICK	TAX_GallusGallus_9031_20141114.fasta	16,365.20	100.00%	7	8	90	0.18%	75.50%	KQLITGLWGK	E	V	99.70%					43.4	3.12	0.26	0.65	0	1	0	0	1	1,143.69	2,203.00		21,576.00	9	18	Empty	FALSE	HBB_CHICK
20196-20225 HinckeM –TAXchicken	Uncategorized Sample	20196-20205_A	Mudpit_Ingel20196_MGFPeaklist (F058870)	Beta-H globin OS=Gallus gallus GN=HBE1 PE=3 SV=1	Q90864_CHICK	TAX_GallusGallus_9031_20141114.fasta	16,365.20	100.00%	7	8	90	0.18%	75.50%	KVLSSFGEAVK	K	N	99.70%	67.4	43.3	37.4	42.5	47.1	4.59	0.34	0.636	0	7	0	0	2	1,164.66	1,909.00		10,064.40	67	77	Empty	TRUE	HBE_CHICK,HBRH_CHICK
20196-20225 HinckeM –TAXchicken	Uncategorized Sample	20196-20205_A	Mudpit_Ingel20196_MGFPeaklist (F058870)	Beta-H globin OS=Gallus gallus GN=HBE1 PE=3 SV=1	Q90864_CHICK	TAX_GallusGallus_9031_20141114.fasta	16,365.20	100.00%	7	8	90	0.18%	75.50%	KVNVAECGAEALAR	G	L	99.70%					49.3	5.18	0.495	0.571	0	0	2	0	1	1,487.76	1,869.00		131800	18	31	Empty	FALSE	HBB_CHICK
20196-20225 HinckeM –TAXchicken	Uncategorized Sample	20196-20205_A	Mudpit_Ingel20196_MGFPeaklist (F058870)	Beta-H globin OS=Gallus gallus GN=HBE1 PE=3 SV=1	Q90864_CHICK	TAX_GallusGallus_9031_20141114.fasta	16,365.20	100.00%	7	8	90	0.18%	75.50%	LHCDKLHVDPENFR	K	L	99.70%	62.2	44.6	15.6	57	41.2	4.62	0.427	0.429	0	1	1	14	2	1,779.86	1,886.00		5650870	92	105	Empty	FALSE	HBE_CHICK,HBB_CHICK
20196-20225 HinckeM –TAXchicken	Uncategorized Sample	20196-20205_A	Mudpit_Ingel20196_MGFPeaklist (F058870)	Beta-H globin OS=Gallus gallus GN=HBE1 PE=3 SV=1	Q90864_CHICK	TAX_GallusGallus_9031_20141114.fasta	16,365.20	100.00%	7	8	90	0.18%	75.50%	LHVDPENFR	K	L	99.70%	40.5	43.8	28.9	24.1	35.8	3.35	0.419	0.556	0	16	0	0	2	1,126.56	1,945.00		2591890	97	105	Empty	FALSE	HBE_CHICK,HBB_CHICK,HBRH_CHICK
20196-20225 HinckeM –TAXchicken	Uncategorized Sample	20196-20205_A	Mudpit_Ingel20196_MGFPeaklist (F058870)	Beta-H globin OS=Gallus gallus GN=HBE1 PE=3 SV=1	Q90864_CHICK	TAX_GallusGallus_9031_20141114.fasta	16,365.20	100.00%	7	8	90	0.18%	75.50%	LLGDILIIVLASHFSK	R	D	99.70%	86.6	36.5	19	77.8	44.6	4.74	0.466	0.406	0	0	2	0	2	1,739.05	2,926.00		1542810	106	121	Empty	TRUE	
20196-20225 HinckeM –TAXchicken	Uncategorized Sample	20196-20205_A	Mudpit_Ingel20196_MGFPeaklist (F058870)	Beta-H globin OS=Gallus gallus GN=HBE1 PE=3 SV=1	Q90864_CHICK	TAX_GallusGallus_9031_20141114.fasta	16,365.20	100.00%	7	8	90	0.18%	75.50%	LLGDILIIVLASHFSKDFTPASQAAWQK	R	M	99.70%	59.4	37.6	13.2	56.9	50.7	6.09	0.436	0.286	0	0	0	2	2	3,069.68	2,918.00		617486	106	133	Empty	TRUE	
20196-20225 HinckeM –TAXchicken	Uncategorized Sample	20196-20205_A	Mudpit_Ingel20196_MGFPeaklist (F058870)	Beta-H globin OS=Gallus gallus GN=HBE1 PE=3 SV=1	Q90864_CHICK	TAX_GallusGallus_9031_20141114.fasta	16,365.20	100.00%	7	8	90	0.18%	75.50%	LLIVYPWTQR	R	F	99.70%	64.1	43.6	25.4	48.8	44.4	3.38	0.351	0.6	0	78	0	0	2	1,288.74	2,774.00		4.25E+07	32	41	Empty	FALSE	HBE_CHICK,HBB_CHICK,HBRH_CHICK
20196-20225 HinckeM –TAXchicken	Uncategorized Sample	20196-20205_A	Mudpit_Ingel20196_MGFPeaklist (F058870)	Beta-H globin OS=Gallus gallus GN=HBE1 PE=3 SV=1	Q90864_CHICK	TAX_GallusGallus_9031_20141114.fasta	16,365.20	100.00%	7	8	90	0.18%	75.50%	QLITGLWGK	K	V	99.70%	62.3	44.4	26.8	39	38.3	1.74	0.324	0.611	0	95	0	0	2	1,015.59	2,525.00		2.67E+07	10	18	Empty	FALSE	HBB_CHICK
20196-20225 HinckeM –TAXchicken	Uncategorized Sample	20196-20205_A	Mudpit_Ingel20196_MGFPeaklist (F058870)	Beta-H globin OS=Gallus gallus GN=HBE1 PE=3 SV=1	Q90864_CHICK	TAX_GallusGallus_9031_20141114.fasta	16,365.20	100.00%	7	8	90	0.18%	75.50%	QLITGLWGKVNVAECGAEALAR	K	L	99.70%	102	42.9	14.1	98.6	68.3	10.6	0.621	0.432	[illegible]	[illegible]	[illegible]	[illegible]	[illegible]	2,356.24	2,715.00		1028340	10	31	Empty	FALSE	HBB_CHICK

20196-20225 HinckeM –TAXchicken	Uncategorized Sample	20196-20205_A	Mudpit_Ingel20196_MGFPeaklist (F058870)	Beta-H globin OS=Gallus gallus GN=HBE1 PE=3 SV=1	Q90864_CHICK	TAX_GallusGallus_9031_20141114.fasta	16,365.20	100.00%	7	8	90	0.18%	75.50%	VAECGAEALAR	N	L	99.70%					42.7	3.11	0.328	0.5	0	3	0	0	1	1,146.56	1,772.00		6,356.38	21	31	Empty	FALSE	HBB_CHICK
20196-20225 HinckeM –TAXchicken	Uncategorized Sample	20196-20205_A	Mudpit_Ingel20196_MGFPeaklist (F058870)	Beta-H globin OS=Gallus gallus GN=HBE1 PE=3 SV=1	Q90864_CHICK	TAX_GallusGallus_9031_20141114.fasta	16,365.20	100.00%	7	8	90	0.18%	75.50%	VHWTAEEK	M	Q	99.70%	41.7	43.6	27	28	37.2	1.59	0.344	0.688	0	18	0	0	1	999.4894	1,668.50		14,341.90	2	9	Empty	FALSE	HBB_CHICK
20196-20225 HinckeM –TAXchicken	Uncategorized Sample	20196-20205_A	Mudpit_Ingel20196_MGFPeaklist (F058870)	Beta-H globin OS=Gallus gallus GN=HBE1 PE=3 SV=1	Q90864_CHICK	TAX_GallusGallus_9031_20141114.fasta	16,365.20	100.00%	7	8	90	0.18%	75.50%	VHWTAEEKQLITGLWGK	M	V	99.70%	71.9	42.9	19	71.9	43.5	6.03	0.497	0.382	0	0	2	0	1	1,996.07	2,560.50		675133	2	18	Empty	FALSE	HBB_CHICK
20196-20225 HinckeM –TAXchicken	Uncategorized Sample	20196-20205_A	Mudpit_Ingel20196_MGFPeaklist (F058870)	Beta-H globin OS=Gallus gallus GN=HBE1 PE=3 SV=1	Q90864_CHICK	TAX_GallusGallus_9031_20141114.fasta	16,365.20	100.00%	7	8	90	0.18%	75.50%	VHWTAEEKQLITGLWGKVNVAECGAEALAR	M	L	99.70%	60	41.3	0	60	45.5	6.1	0.484	0.25	0	0	0	2	1	3,336.72	2,857.00		1250740	2	31	Empty	FALSE	HBB_CHICK
20196-20225 HinckeM –TAXchicken	Uncategorized Sample	20196-20205_A	Mudpit_Ingel20196_MGFPeaklist (F058870)	Beta-H globin OS=Gallus gallus GN=HBE1 PE=3 SV=1	Q90864_CHICK	TAX_GallusGallus_9031_20141114.fasta	16,365.20	100.00%	7	8	90	0.18%	75.50%	VLSSFGEAVK	K	N	99.70%	65.8	44	31.1	39	40.2	2.55	0.373	0.55	0	32	0	0	2	1,036.57	2,022.00		1438060	68	77	Empty	TRUE	HBE_CHICK,HBRH_CHICK
20196-20225 HinckeM –TAXchicken	Uncategorized Sample	20196-20205_A	Mudpit_Ingel20196_MGFPeaklist (F058870)	Beta-H globin OS=Gallus gallus GN=HBE1 PE=3 SV=1	Q90864_CHICK	TAX_GallusGallus_9031_20141114.fasta	16,365.20	100.00%	7	8	90	0.18%	75.50%	VLSSFGEAVKNLDNIKK	K	S	99.70%	89.4	41.9	21	71.3	49.9	6.43	0.471	0.382	0	0	1	3	2	1,862.04	2,356.50		219774	68	84	Empty	TRUE	
20196-20225 HinckeM –TAXchicken	Uncategorized Sample	20196-20205_A	Mudpit_Ingel20196_MGFPeaklist (F058870)	Beta-H globin OS=Gallus gallus GN=HBE1 PE=3 SV=1	Q90864_CHICK	TAX_GallusGallus_9031_20141114.fasta	16,365.20	100.00%	7	8	90	0.18%	75.50%	VNVAECGAEALAR	K	L	99.70%	64.8	44.7	22.1	52.5	48.1	4.96	0.395	0.5	0	126	2	0	2	1,359.67	2,097.50		4.86E+07	19	31	Empty	FALSE	HBB_CHICK
20196-20225 HinckeM –TAXchicken	Uncategorized Sample	20196-20205_A	Mudpit_Ingel20196_MGFPeaklist (F058870)	Beta-H globin OS=Gallus gallus GN=HBE1 PE=3 SV=1	Q90864_CHICK	TAX_GallusGallus_9031_20141114.fasta	16,365.20	100.00%	7	8	90	0.18%	75.50%	VVAHALAHEYH	R	-	99.70%	58.7	45.2	15.4	52.7	39.8	5.26	0.467	0.591	0	0	19	0	2	1,246.63	1,639.00		17,920.70	137	147	Empty	TRUE	
20196-20225 HinckeM –TAXchicken	Uncategorized Sample	20196-20205_A	Mudpit_Ingel20196_MGFPeaklist (F058870)	Transcription elongation factor SPT5 OS=Gallus gallus GN=SUPT5H PE=3 SV=2	H9L0H1_CHICK,SPT5H_CHICK,UPI000044395E,UPI0002C86EDC	TAX_GallusGallus_9031_20141114.fasta,UPARC14_2_GallusG_9031_20140417.fasta	120,009.80	100.00%	6	6	9	0.02%	6.02%	DMLEFPAQELR	K	K	18.50%	8.38	44.2	21.7	0					0	1	0	0	2	1,365.64	1,651.00		267.3	456	466	Empty	TRUE	
20196-20225 HinckeM –TAXchicken	Uncategorized Sample	20196-20205_A	Mudpit_Ingel20196_MGFPeaklist (F058870)	Transcription elongation factor SPT5 OS=Gallus gallus GN=SUPT5H PE=3 SV=2	H9L0H1_CHICK,SPT5H_CHICK,UPI000044395E,UPI0002C86EDC	TAX_GallusGallus_9031_20141114.fasta,UPARC14_2_GallusG_9031_20140417.fasta	120,009.80	100.00%	6	6	9	0.02%	6.02%	DMLEFPAQELRK	K	Y	8.20%	11.5	44.7	23.3	0.1					0	0	1	0	2	1,493.73	1,585.00		178.38	456	467	Empty	TRUE	
20196-20225 HinckeM –TAXchicken	Uncategorized Sample	20196-20205_A	Mudpit_Ingel20196_MGFPeaklist (F058870)	Transcription elongation factor SPT5 OS=Gallus gallus GN=SUPT5H PE=3 SV=2	H9L0H1_CHICK,SPT5H_CHICK,UPI000044395E,UPI0002C86EDC	TAX_GallusGallus_9031_20141114.fasta,UPARC14_2_GallusG_9031_20140417.fasta	120,009.80	100.00%	6	6	9	0.02%	6.02%	EVTNLKPKSWVR	K	L	7.00%	2.6	43.2	17.7	0					0	0	1	0	2	1,457.81	2,097.00		315.68	266	277	Empty	TRUE	
20196-20225 HinckeM –TAXchicken	Uncategorized Sample	20196-20205_A	Mudpit_Ingel20196_MGFPeaklist (F058870)	Transcription elongation factor SPT5 OS=Gallus gallus GN=SUPT5H PE=3 SV=2	H9L0H1_CHICK,SPT5H_CHICK,UPI000044395E,UPI0002C86EDC	TAX_GallusGallus_9031_20141114.fasta,UPARC14_2_GallusG_9031_20140417.fasta	120,009.80	100.00%	6	6	9	0.02%	6.02%	FAVALDSEQNNIHVK	R	D	19.80%	16	44.2	16.8	0					0	0	2	0	2	1,684.87	2,166.50		392.56	580	594	Empty	TRUE	
20196-20225 HinckeM –TAXchicken	Uncategorized Sample	20196-20205_A	Mudpit_Ingel20196_MGFPeaklist (F058870)	Transcription elongation factor SPT5 OS=Gallus gallus GN=SUPT5H PE=3 SV=2	H9L0H1_CHICK,SPT5H_CHICK,UPI000044395E,UPI0002C86EDC	TAX_GallusGallus_9031_20141114.fasta,UPARC14_2_GallusG_9031_20140417.fasta	120,009.80	100.00%	6	6	9	0.02%	6.02%	QAIEGVGNLR	K	M	99.50%	37.3	44.2	26.2	15.6	24.2	0.62	0.0826	0.25	0	2	0	0	2	1,056.58	1,919.50		253.63	234	243	Empty	TRUE	
20196-20225 HinckeM –TAXchicken	Uncategorized Sample	20196-20205_A	Mudpit_Ingel20196_MGFPeaklist (F058870)	Transcription elongation factor SPT5 OS=Gallus gallus GN=SUPT5H PE=3 SV=2	H9L0H1_CHICK,SPT5H_CHICK,UPI000044395E,UPI0002C86EDC	TAX_GallusGallus_9031_20141114.fasta,UPARC14_2_GallusG_9031_20140417.fasta	120,009.80	100.00%	6	6	9	0.02%	6.02%	VVSISSEHLEPVTPTK	K	S	99.70%	43.8	43.5	21.8	31.9	38.1	2.74	0.315	0.375	0	0	2	0	2	1,722.93	2,064.50		1,177.08	1014	1029	Empty	TRUE	
20196-20225 HinckeM –TAXchicken	Uncategorized Sample	20196-20205_A	Mudpit_Ingel20196_MGFPeaklist (F058870)	Transcription elongation factor SPT5 OS=Gallus gallus GN=SUPT5H PE=3 SV=2	H9L0H1_CHICK,SPT5H_CHICK,UPI000044395E,UPI0002C86EDC	TAX_GallusGallus_9031_20141114.fasta,UPARC14_2_GallusG_9031_20140417.fasta	120,009.80	100.00%	6	6	9	0.02%	6.02%	DMLEFPAQELR	K	K	18.50%	8.38	44.2	21.7	0					0	1	0	0	2	1,365.64	1,651.00		267.3	456	466	Empty	TRUE	
20196-20225 HinckeM –TAXchicken	Uncategorized Sample	20196-20205_A	Mudpit_Ingel20196_MGFPeaklist (F058870)	Transcription elongation factor SPT5 OS=Gallus gallus GN=SUPT5H PE=3 SV=2	H9L0H1_CHICK,SPT5H_CHICK,UPI000044395E,UPI0002C86EDC	TAX_GallusGallus_9031_20141114.fasta,UPARC14_2_GallusG_9031_20140417.fasta	120,009.80	100.00%	6	6	9	0.02%	6.02%	DMLEFPAQELRK	K	Y	8.20%	11.5	44.7	23.3	0.1					0	0	1	0	2	1,493.73	1,585.00		178.38	456	467	Empty	TRUE	
20196-20225 HinckeM –TAXchicken	Uncategorized Sample	20196-20205_A	Mudpit_Ingel20196_MGFPeaklist (F058870)	Transcription elongation factor SPT5 OS=Gallus gallus GN=SUPT5H PE=3 SV=2	H9L0H1_CHICK,SPT5H_CHICK,UPI000044395E,UPI0002C86EDC	TAX_GallusGallus_9031_20141114.fasta,UPARC14_2_GallusG_9031_20140417.fasta	120,009.80	100.00%	6	6	9	0.02%	6.02%	EVTNLKPKSWVR	K	L	7.00%	2.6	43.2	17.7	0					0	0	1	0	2	1,457.81	2,097.00		315.68	266	277	Empty	TRUE	
20196-20225 HinckeM –TAXchicken	Uncategorized Sample	20196-20205_A	Mudpit_Ingel20196_MGFPeaklist (F058870)	Transcription elongation factor SPT5 OS=Gallus gallus GN=SUPT5H PE=3 SV=2	H9L0H1_CHICK,SPT5H_CHICK,UPI000044395E,UPI0002C86EDC	TAX_GallusGallus_9031_20141114.fasta,UPARC14_2_GallusG_9031_20140417.fasta	120,009.80	100.00%	6	6	9	0.02%	6.02%	FAVALDSEQNNIHVK	R	D	19.80%	16	44.2	16.8	0					0	0	2	0	2	1,684.87	2,166.50		392.56	580	594	Empty	TRUE	
20196-20225 HinckeM –TAXchicken	Uncategorized Sample	20196-20205_A	Mudpit_Ingel20196_MGFPeaklist (F058870)	Transcription elongation factor SPT5 OS=Gallus gallus GN=SUPT5H PE=3 SV=2	H9L0H1_CHICK,SPT5H_CHICK,UPI000044395E,UPI0002C86EDC	TAX_GallusGallus_9031_20141114.fasta,UPARC14_2_GallusG_9031_20140417.fasta	120,009.80	100.00%	6	6	9	0.02%	6.02%	QAIEGVGNLR	K	M	99.50%	37.3	44.2	26.2	15.6	24.2	0.62	0.0826	0.25	0	2	0	0	2	1,056.58	1,919.50		253.63	234	243	Empty	TRUE	
20196-20225 HinckeM –TAXchicken	Uncategorized Sample	20196-20205_A	Mudpit_Ingel20196_MGFPeaklist (F058870)	Transcription elongation factor SPT5 OS=Gallus gallus GN=SUPT5H PE=3 SV=2	H9L0H1_CHICK,SPT5H_CHICK,UPI000044395E,UPI0002C86EDC	TAX_GallusGallus_9031_20141114.fasta,UPARC14_2_GallusG_9031_20140417.fasta	120,009.80	100.00%	6	6	9	0.02%	6.02%	VVSISSEHLEPVTPTK	K	S	99.70%	43.8	43.5	21.8	31.9	38.1	2.74	0.315	0.375	0	0	2	0	2	1,722.93	2,064.50		1,177.08	1014	1029	Empty	TRUE	
20196-20225 HinckeM –TAXchicken	Uncategorized Sample	20196-20205_A	Mudpit_Ingel20196_MGFPeaklist (F058870)	Transcription elongation factor SPT5 OS=Gallus gallus GN=SUPT5H PE=3 SV=2	H9L0H1_CHICK,SPT5H_CHICK,UPI000044395E,UPI0002C86EDC	TAX_GallusGallus_9031_20141114.fasta,UPARC14_2_GallusG_9031_20140417.fasta	120,009.80	100.00%	6	6	9	0.02%	6.02%	DMLEFPAQELR	K	K	18.50%	8.38	44.2	21.7	0					0	1	0	0	2	1,365.64	1,651.00		267.3	456	466	Empty	TRUE	
20196-20225 HinckeM –TAXchicken	Uncategorized Sample	20196-20205_A	Mudpit_Ingel20196_MGFPeaklist (F058870)	Transcription elongation factor SPT5 OS=Gallus gallus GN=SUPT5H PE=3 SV=2	H9L0H1_CHICK,SPT5H_CHICK,UPI000044395E,UPI0002C86EDC	TAX_GallusGallus_9031_20141114.fasta,UPARC14_2_GallusG_9031_20140417.fasta	120,009.80	100.00%	6	6	9	0.02%	6.02%	DMLEFPAQELRK	K	Y	8.20%	11.5	44.7	23.3	0.1					0	0	1	0	2	1,493.73	1,585.00		178.38	456	467	Empty	TRUE	
20196-20225 HinckeM –TAXchicken	Uncategorized Sample	20196-20205_A	Mudpit_Ingel20196_MGFPeaklist (F058870)	Transcription elongation factor SPT5 OS=Gallus gallus GN=SUPT5H PE=3 SV=2	H9L0H1_CHICK,SPT5H_CHICK,UPI000044395E,UPI0002C86EDC	TAX_GallusGallus_9031_20141114.fasta,UPARC14_2_GallusG_9031_20140417.fasta	120,009.80	100.00%	6	6	9	0.02%	6.02%	EVTNLKPKSWVR	K	L	7.00%	2.6	43.2	17.7	0					0	0	1	0	2	1,457.81	2,097.00		315.68	266	277	Empty	TRUE	
20196-20225 HinckeM –TAXchicken	Uncategorized Sample	20196-20205_A	Mudpit_Ingel20196_MGFPeaklist (F058870)	Transcription elongation factor SPT5 OS=Gallus gallus GN=SUPT5H PE=3 SV=2	H9L0H1_CHICK,SPT5H_CHICK,UPI000044395E,UPI0002C86EDC	TAX_GallusGallus_9031_20141114.fasta,UPARC14_2_GallusG_9031_20140417.fasta	120,009.80	100.00%	6	6	9	0.02%	6.02%	FAVALDSEQNNIHVK	R	D	19.80%	16	44.2	16.8	0					0	0	2	0	2	1,684.87	2,166.50		392.56	580	594	Empty	TRUE	
20196-20225 HinckeM –TAXchicken	Uncategorized Sample	20196-20205_A	Mudpit_Ingel20196_MGFPeaklist (F058870)	Transcription elongation factor SPT5 OS=Gallus gallus GN=SUPT5H PE=3 SV=2	H9L0H1_CHICK,SPT5H_CHICK,UPI000044395E,UPI0002C86EDC	TAX_GallusGallus_9031_20141114.fasta,UPARC14_2_GallusG_9031_20140417.fasta	120,009.80	100.00%	6	6	9	0.02%	6.02%	QAIEGVGNLR	K	M	99.50%	37.3	44.2	26.2	15.6	24.2	0.62	0.0826	0.25	0	2	0	0	2	1,056.58	1,919.50		253.63	234	243	Empty	TRUE	
20196-20225 HinckeM –TAXchicken	Uncategorized Sample	20196-20205_A	Mudpit_Ingel20196_MGFPeaklist (F058870)	Transcription elongation factor SPT5 OS=Gallus gallus GN=SUPT5H PE=3 SV=2	H9L0H1_CHICK,SPT5H_CHICK,UPI000044395E,UPI0002C86EDC	TAX_GallusGallus_9031_20141114.fasta,UPARC14_2_GallusG_9031_20140417.fasta	120,009.80	100.00%	6	6	9	0.02%	6.02%	VVSISSEHLEPVTPTK	K	S	99.70%	43.8	43.5	21.8	31.9	38.1	2.74	0.315	0.375	0	0	2	0	2	1,722.93	2,064.50		1,177.08	1014	1029	Empty	TRUE	
20196-20225 HinckeM –TAXchicken	Uncategorized Sample	20196-20205_A	Mudpit_Ingel20196_MGFPeaklist (F058870)	Transcription elongation factor SPT5 OS=Gallus gallus GN=SUPT5H PE=3 SV=2	H9L0H1_CHICK,SPT5H_CHICK,UPI000044395E,UPI0002C86EDC	TAX_GallusGallus_9031_20141114.fasta,UPARC14_2_GallusG_9031_20140417.fasta	120,009.80	100.00%	6	6	9	0.02%	6.02%	DMLEFPAQELR	K	K	18.50%	8.38	44.2	21.7	0					0	1	0	0	2	1,365.64	1,651.00		267.3	456	466	Empty	TRUE	
20196-20225 HinckeM –TAXchicken	Uncategorized Sample	20196-20205_A	Mudpit_Ingel20196_MGFPeaklist (F058870)	Transcription elongation factor SPT5 OS=Gallus gallus GN=SUPT5H PE=3 SV=2	H9L0H1_CHICK,SPT5H_CHICK,UPI000044395E,UPI0002C86EDC	TAX_GallusGallus_9031_20141114.fasta,UPARC14_2_GallusG_9031_20140417.fasta	120,009.80	100.00%	6	6	9	0.02%	6.02%	DMLEFPAQELRK	K	Y	8.20%	11.5	44.7	23.3	0.1					0	0	1	0	2	1,493.73	1,585.00		178.38	456	467	Empty	TRUE	
20196-20225 HinckeM –TAXchicken	Uncategorized Sample	20196-20205_A	Mudpit_Ingel20196_MGFPeaklist (F058870)	Transcription elongation factor SPT5 OS=Gallus gallus GN=SUPT5H PE=3 SV=2	H9L0H1_CHICK,SPT5H_CHICK,UPI000044395E,UPI0002C86EDC	TAX_GallusGallus_9031_20141114.fasta,UPARC14_2_GallusG_9031_20140417.fasta	120,009.80	100.00%	6	6	9	0.02%	6.02%	EVTNLKPKSWVR	K	L	7.00%	2.6	43.2	17.7	0					0	0	1	0	2	1,457.81	2,097.00		315.68	266	277	Empty	TRUE	
20196-20225 HinckeM –TAXchicken	Uncategorized Sample	20196-20205_A	Mudpit_Ingel20196_MGFPeaklist (F058870)	Transcription elongation factor SPT5 OS=Gallus gallus GN=SUPT5H PE=3 SV=2	H9L0H1_CHICK,SPT5H_CHICK,UPI000044395E,UPI0002C86EDC	TAX_GallusGallus_9031_20141114.fasta,UPARC14_2_GallusG_9031_20140417.fasta	120,009.80	100.00%	6	6	9	0.02%	6.02%	FAVALDSEQNNIHVK	R	D	19.80%	16	44.2	16.8	0					0	0	2	0	2	1,684.87	2,166.50		392.56	580	594	Empty	TRUE	
20196-20225 HinckeM –TAXchicken	Uncategorized Sample	20196-20205_A	Mudpit_Ingel20196_MGFPeaklist (F058870)	Transcription elongation factor SPT5 OS=Gallus gallus GN=SUPT5H PE=3 SV=2	H9L0H1_CHICK,SPT5H_CHICK,UPI000044395E,UPI0002C86EDC	TAX_GallusGallus_9031_20141114.fasta,UPARC14_2_GallusG_9031_20140417.fasta	120,009.80	100.00%	6	6	9	0.02%	6.02%	QAIEGVGNLR	K	M	99.50%	37.3	44.2	26.2	15.6	24.2	0.62	0.0826	0.25	0	2	0	0	2	1,056.58	1,919.50		253.63	234	243	Empty	TRUE	
20196-20225 HinckeM –TAXchicken	Uncategorized Sample	20196-20205_A	Mudpit_Ingel20196_MGFPeaklist (F058870)	Transcription elongation factor SPT5 OS=Gallus gallus GN=SUPT5H PE=3 SV=2	H9L0H1_CHICK,SPT5H_CHICK,UPI000044395E,UPI0002C86EDC	TAX_GallusGallus_9031_20141114.fasta,UPARC14_2_GallusG_9031_20140417.fasta	120,009.80	100.00%	6	6	9	0.02%	6.02%	VVSISSEHLEPVTPTK	K	S	99.70%	43.8	43.5	21.8	31.9	38.1	2.74	0.315	0.375	0	0	2	0	2	1,722.93	2,064.50		1,177.08	1014	1029	Empty	TRUE	
20196-20225 HinckeM –TAXchicken	Uncategorized Sample	20196-20205_A	Mudpit_Ingel20196_MGFPeaklist (F058870)	Uncharacterized protein OS=Gallus gallus GN=LOC770011 PE=4 SV=2	E1C6R9_CHICK,MLRN_CHICK,UPI000012F21C,UPI000017124A,UPI00001776DD,UPI000060FC08,UPI0000ECCE36	TAX_GallusGallus_9031_20141114.fasta,UPARC14_2_GallusG_9031_20140417.fasta	20,295.80	100.00%	4	5	6	0.01%	23.80%	FTDEEVDELYR	R	E	99.70%	31.3	42.9	19.7	17.3	27.3	2.04	0.264	0.318	0	1	0	0	2	1,415.63	2,230.00		433.54	138	148	Empty	TRUE	
20196-20225 HinckeM –TAXchicken	Uncategorized Sample	20196-20205_A	Mudpit_Ingel20196_MGFPeaklist (F058870)	Uncharacterized protein OS=Gallus gallus GN=LOC770011 PE=4 SV=2	E1C6R9_CHICK,MLRN_CHICK,UPI000012F21C,UPI000017124A,UPI00001776DD,UPI000060FC08,UPI0000ECCE36	TAX_GallusGallus_9031_20141114.fasta,UPARC14_2_GallusG_9031_20140417.fasta	20,295.80	100.00%	4	5	6	0.01%	23.80%	GNFNYIEFTR	K	I	99.70%	39.7	44.7	18.4	33.2	35.9	3.01	0.362	0.5	0	2	0	0	2	1,260.60	2,406.00		4,305.46	156	165	Empty	TRUE	
20196-20225 HinckeM –TAXchicken	Uncategorized Sample	20196-20205_A	Mudpit_Ingel20196_MGFPeaklist (F058870)	Uncharacterized protein OS=Gallus gallus GN=LOC770011 PE=4 SV=2	E1C6R9_CHICK,MLRN_CHICK,UPI000012F21C,UPI000017124A,UPI00001776DD,UPI000060FC08,UPI0000ECCE36	TAX_GallusGallus_9031_20141114.fasta,UPARC14_2_GallusG_9031_20140417.fasta	20,295.80	100.00%	4	5	6	0.01%	23.80%	ILKHGAKDK	R	D	7.70%	5.39	41	22	-0.36					0	1	0	0	2	1,009.62	2,147.00		206.13	166	174	Empty	TRUE	MLRM_CHICK,UPI00005FF04B,UPI0000171367,UPI000012F21A,UPI000060FDE7
20196-20225 HinckeM –TAXchicken	Uncategorized Sample	20196-20205_A	Mudpit_Ingel20196_MGFPeaklist (F058870)	Uncharacterized protein OS=Gallus gallus GN=LOC770011 PE=4 SV=2	E1C6R9_CHICK,MLRN_CHICK,UPI000012F21C,UPI000017124A,UPI00001776DD,UPI000060FC08,UPI0000ECCE36	TAX_GallusGallus_9031_20141114.fasta,UPARC14_2_GallusG_9031_20140417.fasta	20,295.80	100.00%	4	5	6	0.01%	23.80%	LNGTDPEDVIR	K	N	99.50%	34.1	44.8	25.4	20.6	29.3	1.96	0.113	0.318	0	2	0	0	2	1,229.60	2,000.00		1,013.09	98	108	Empty	TRUE	MLRM_CHICK,UPI00005FF04B,UPI0000171367,UPI000012F21A,UPI000060FDE7
20196-20225 HinckeM –TAXchicken	Uncategorized Sample	20196-20205_A	Mudpit_Ingel20196_MGFPeaklist (F058870)	Uncharacterized protein OS=Gallus gallus GN=LOC770011 PE=4 SV=2	E1C6R9_CHICK,MLRN_CHICK,UPI000012F21C,UPI000017124A,UPI00001776DD,UPI000060FC08,UPI0000ECCE36	TAX_GallusGallus_9031_20141114.fasta,UPARC14_2_GallusG_9031_20140417.fasta	20,295.80	100.00%	4	5	6	0.01%	23.80%	FTDEEVDELYR	R	E	99.70%	31.3	42.9	19.7	17.3	27.3	2.04	0.264	0.318	0	1	0	0	2	1,415.63	2,230.00		433.54	138	148	Empty	TRUE	
20196-20225 HinckeM –TAXchicken	Uncategorized Sample	20196-20205_A	Mudpit_Ingel20196_MGFPeaklist (F058870)	Uncharacterized protein OS=Gallus gallus GN=LOC770011 PE=4 SV=2	E1C6R9_CHICK,MLRN_CHICK,UPI000012F21C,UPI000017124A,UPI00001776DD,UPI000060FC08,UPI0000ECCE36	TAX_GallusGallus_9031_20141114.fasta,UPARC14_2_GallusG_9031_20140417.fasta	20,295.80	100.00%	4	5	6	0.01%	23.80%	GNFNYIEFTR	K	I	99.70%	39.7	44.7	18.4	33.2	35.9	3.01	0.362	0.5	0	2	0	0	2	1,260.60	2,406.00		4,305.46	156	165	Empty	TRUE	
20196-20225 HinckeM –TAXchicken	Uncategorized Sample	20196-20205_A	Mudpit_Ingel20196_MGFPeaklist (F058870)	Uncharacterized protein OS=Gallus gallus GN=LOC770011 PE=4 SV=2	E1C6R9_CHICK,MLRN_CHICK,UPI000012F21C,UPI000017124A,UPI00001776DD,UPI000060FC08,UPI0000ECCE36	TAX_GallusGallus_9031_20141114.fasta,UPARC14_2_GallusG_9031_20140417.fasta	20,295.80	100.00%	4	5	6	0.01%	23.80%	ILKHGAKDK	R	D	7.70%	5.39	41	22	-0.36					0	1	0	0	2	1,009.62	2,147.00		206.13	166	174	Empty	TRUE	MLRM_CHICK,UPI00005FF04B,UPI0000171367,UPI000012F21A,UPI000060FDE7
20196-20225 HinckeM –TAXchicken	Uncategorized Sample	20196-20205_A	Mudpit_Ingel20196_MGFPeaklist (F058870)	Uncharacterized protein OS=Gallus gallus GN=LOC770011 PE=4 SV=2	E1C6R9_CHICK,MLRN_CHICK,UPI000012F21C,UPI000017124A,UPI00001776DD,UPI000060FC08,UPI0000ECCE36	TAX_GallusGallus_9031_20141114.fasta,UPARC14_2_GallusG_9031_20140417.fasta	20,295.80	100.00%	4	5	6	0.01%	23.80%	LNGTDPEDVIR	K	N	99.50%	34.1	44.8	25.4	20.6	29.3	1.96	0.113	0.318	0	2	0	0	2	1,229.60	2,000.00		1,013.09	98	108	Empty	TRUE	MLRM_CHICK,UPI00005FF04B,UPI0000171367,UPI000012F21A,UPI000060FDE7
20196-20225 HinckeM –TAXchicken	Uncategorized Sample	20196-20205_A	Mudpit_Ingel20196_MGFPeaklist (F058870)	Uncharacterized protein OS=Gallus gallus GN=LOC770011 PE=4 SV=2	E1C6R9_CHICK,MLRN_CHICK,UPI000012F21C,UPI000017124A,UPI00001776DD,UPI000060FC08,UPI0000ECCE36	TAX_GallusGallus_9031_20141114.fasta,UPARC14_2_GallusG_9031_20140417.fasta	20,295.80	100.00%	4	5	6	0.01%	23.80%	FTDEEVDELYR	R	E	99.70%	31.3	42.9	19.7	17.3	27.3	2.04	0.264	0.318	0	1	0	0	2	1,415.63	2,230.00		433.54	138	148	Empty	TRUE	
20196-20225 HinckeM –TAXchicken	Uncategorized Sample	20196-20205_A	Mudpit_Ingel20196_MGFPeaklist (F058870)	Uncharacterized protein OS=Gallus gallus GN=LOC770011 PE=4 SV=2	E1C6R9_CHICK,MLRN_CHICK,UPI000012F21C,UPI000017124A,UPI00001776DD,UPI000060FC08,UPI0000ECCE36	TAX_GallusGallus_9031_20141114.fasta,UPARC14_2_GallusG_9031_20140417.fasta	20,295.80	100.00%	4	5	6	0.01%	23.80%	GNFNYIEFTR	K	I	99.70%	39.7	44.7	18.4	33.2	35.9	3.01	0.362	0.5	0	2	0	0	2	1,260.60	2,406.00		4,305.46	156	165	Empty	TRUE	
20196-20225 HinckeM –TAXchicken	Uncategorized Sample	20196-20205_A	Mudpit_Ingel20196_MGFPeaklist (F058870)	Uncharacterized protein OS=Gallus gallus GN=LOC770011 PE=4 SV=2	E1C6R9_CHICK,MLRN_CHICK,UPI000012F21C,UPI000017124A,UPI00001776DD,UPI000060FC08,UPI0000ECCE36	TAX_GallusGallus_9031_20141114.fasta,UPARC14_2_GallusG_9031_20140417.fasta	20,295.80	100.00%	4	5	6	0.01%	23.80%	ILKHGAKDK	R	D	7.70%	5.39	41	22	-0.36					0	1	0	0	2	1,009.62	2,147.00		206.13	166	174	Empty	TRUE	MLRM_CHICK,UPI00005FF04B,UPI0000171367,UPI000012F21A,UPI000060FDE7
20196-20225 HinckeM –TAXchicken	Uncategorized Sample	20196-20205_A	Mudpit_Ingel20196_MGFPeaklist (F058870)	Uncharacterized protein OS=Gallus gallus GN=LOC770011 PE=4 SV=2	E1C6R9_CHICK,MLRN_CHICK,UPI000012F21C,UPI000017124A,UPI00001776DD,UPI000060FC08,UPI0000ECCE36	TAX_GallusGallus_9031_20141114.fasta,UPARC14_2_GallusG_9031_20140417.fasta	20,295.80	100.00%	4	5	6	0.01%	23.80%	LNGTDPEDVIR	K	N	99.50%	34.1	44.8	25.4	20.6	29.3	1.96	0.113	0.318	0	2	0	0	2	1,229.60	2,000.00		1,013.09	98	108	Empty	TRUE	MLRM_CHICK,UPI00005FF04B,UPI0000171367,UPI000012F21A,UPI000060FDE7
20196-20225 HinckeM –TAXchicken	Uncategorized Sample	20196-20205_A	Mudpit_Ingel20196_MGFPeaklist (F058870)	Uncharacterized protein OS=Gallus gallus GN=LOC770011 PE=4 SV=2	E1C6R9_CHICK,MLRN_CHICK,UPI000012F21C,UPI000017124A,UPI00001776DD,UPI000060FC08,UPI0000ECCE36	TAX_GallusGallus_9031_20141114.fasta,UPARC14_2_GallusG_9031_20140417.fasta	20,295.80	100.00%	4	5	6	0.01%	23.80%	FTDEEVDELYR	R	E	99.70%	31.3	42.9	19.7	17.3	27.3	2.04	0.264	0.318	0	1	0	0	2	1,415.63	2,230.00		433.54	138	148	Empty	TRUE	
20196-20225 HinckeM –TAXchicken	Uncategorized Sample	20196-20205_A	Mudpit_Ingel20196_MGFPeaklist (F058870)	Uncharacterized protein OS=Gallus gallus GN=LOC770011 PE=4 SV=2	E1C6R9_CHICK,MLRN_CHICK,UPI000012F21C,UPI000017124A,UPI00001776DD,UPI000060FC08,UPI0000ECCE36	TAX_GallusGallus_9031_20141114.fasta,UPARC14_2_GallusG_9031_20140417.fasta	20,295.80	100.00%	4	5	6	0.01%	23.80%	GNFNYIEFTR	K	I	99.70%	39.7	44.7	18.4	33.2	35.9	3.01	0.362	0.5	0	2	0	0	2	1,260.60	2,406.00		4,305.46	156	165	Empty	TRUE	
20196-20225 HinckeM –TAXchicken	Uncategorized Sample	20196-20205_A	Mudpit_Ingel20196_MGFPeaklist (F058870)	Uncharacterized protein OS=Gallus gallus GN=LOC770011 PE=4 SV=2	E1C6R9_CHICK,MLRN_CHICK,UPI000012F21C,UPI000017124A,UPI00001776DD,UPI000060FC08,UPI0000ECCE36	TAX_GallusGallus_9031_20141114.fasta,UPARC14_2_GallusG_9031_20140417.fasta	20,295.80	100.00%	4	5	6	0.01%	23.80%	ILKHGAKDK	R	D	7.70%	5.39	41	22	-0.36					0	1	0	0	2	1,009.62	2,147.00		206.13	166	174	Empty	TRUE	MLRM_CHICK,UPI00005FF04B,UPI0000171367,UPI000012F21A,UPI000060FDE7
20196-20225 HinckeM –TAXchicken	Uncategorized Sample	20196-20205_A	Mudpit_Ingel20196_MGFPeaklist (F058870)	Uncharacterized protein OS=Gallus gallus GN=LOC770011 PE=4 SV=2	E1C6R9_CHICK,MLRN_CHICK,UPI000012F21C,UPI000017124A,UPI00001776DD,UPI000060FC08,UPI0000ECCE36	TAX_GallusGallus_9031_20141114.fasta,UPARC14_2_GallusG_9031_20140417.fasta	20,295.80	100.00%	4	5	6	0.01%	23.80%	LNGTDPEDVIR	K	N	99.50%	34.1	44.8	25.4	20.6	29.3	1.96	0.113	0.318	0	2	0	0	2	1,229.60	2,000.00		1,013.09	98	108	Empty	TRUE	MLRM_CHICK,UPI00005FF04B,UPI0000171367,UPI000012F21A,UPI000060FDE7
20196-20225 HinckeM –TAXchicken	Uncategorized Sample	20196-20205_A	Mudpit_Ingel20196_MGFPeaklist (F058870)	Uncharacterized protein OS=Gallus gallus GN=LOC770011 PE=4 SV=2	E1C6R9_CHICK,MLRN_CHICK,UPI000012F21C,UPI000017124A,UPI00001776DD,UPI000060FC08,UPI0000ECCE36	TAX_GallusGallus_9031_20141114.fasta,UPARC14_2_GallusG_9031_20140417.fasta	20,295.80	100.00%	4	5	6	0.01%	23.80%	FTDEEVDELYR	R	E	99.70%	31.3	42.9	19.7	17.3	27.3	2.04	0.264	0.318	0	1	0	0	2	1,415.63	2,230.00		433.54	138	148	Empty	TRUE	
20196-20225 HinckeM –TAXchicken	Uncategorized Sample	20196-20205_A	Mudpit_Ingel20196_MGFPeaklist (F058870)	Uncharacterized protein OS=Gallus gallus GN=LOC770011 PE=4 SV=2	E1C6R9_CHICK,MLRN_CHICK,UPI000012F21C,UPI000017124A,UPI00001776DD,UPI000060FC08,UPI0000ECCE36	TAX_GallusGallus_9031_20141114.fasta,UPARC14_2_GallusG_9031_20140417.fasta	20,295.80	100.00%	4	5	6	0.01%	23.80%	GNFNYIEFTR	K	I	99.70%	39.7	44.7	18.4	33.2	35.9	3.01	0.362	0.5	0	2	0	0	2	1,260.60	2,406.00		4,305.46	156	165	Empty	TRUE	
20196-20225 HinckeM –TAXchicken	Uncategorized Sample	20196-20205_A	Mudpit_Ingel20196_MGFPeaklist (F058870)	Uncharacterized protein OS=Gallus gallus GN=LOC770011 PE=4 SV=2	E1C6R9_CHICK,MLRN_CHICK,UPI000012F21C,UPI000017124A,UPI00001776DD,UPI000060FC08,UPI0000ECCE36	TAX_GallusGallus_9031_20141114.fasta,UPARC14_2_GallusG_9031_20140417.fasta	20,295.80	100.00%	4	5	6	0.01%	23.80%	ILKHGAKDK	R	D	7.70%	5.39	41	22	-0.36					0	1	0	0	2	1,009.62	2,147.00		206.13	166	174	Empty	TRUE	MLRM_CHICK,UPI00005FF04B,UPI0000171367,UPI000012F21A,UPI000060FDE7
20196-20225 HinckeM –TAXchicken	Uncategorized Sample	20196-20205_A	Mudpit_Ingel20196_MGFPeaklist (F058870)	Uncharacterized protein OS=Gallus gallus GN=LOC770011 PE=4 SV=2	E1C6R9_CHICK,MLRN_CHICK,UPI000012F21C,UPI000017124A,UPI00001776DD,UPI000060FC08,UPI0000ECCE36	TAX_GallusGallus_9031_20141114.fasta,UPARC14_2_GallusG_9031_20140417.fasta	20,295.80	100.00%	4	5	6	0.01%	23.80%	LNGTDPEDVIR	K	N	99.50%	34.1	44.8	25.4	20.6	29.3	1.96	0.113	0.318	0	2	0	0	2	1,229.60	2,000.00		1,013.09	98	108	Empty	TRUE	MLRM_CHICK,UPI00005FF04B,UPI0000171367,UPI000012F21A,UPI000060FDE7
20196-20225 HinckeM –TAXchicken	Uncategorized Sample	20196-20205_A	Mudpit_Ingel20196_MGFPeaklist (F058870)	Uncharacterized protein OS=Gallus gallus GN=LOC770011 PE=4 SV=2	E1C6R9_CHICK,MLRN_CHICK,UPI000012F21C,UPI000017124A,UPI00001776DD,UPI000060FC08,UPI0000ECCE36	TAX_GallusGallus_9031_20141114.fasta,UPARC14_2_GallusG_9031_20140417.fasta	20,295.80	100.00%	4	5	6	0.01%	23.80%	FTDEEVDELYR	R	E	99.70%	31.3	42.9	19.7	17.3	27.3	2.04	0.264	0.318	0	1	0	0	2	1,415.63	2,230.00		433.54	138	148	Empty	TRUE	
20196-20225 HinckeM –TAXchicken	Uncategorized Sample	20196-20205_A	Mudpit_Ingel20196_MGFPeaklist (F058870)	Uncharacterized protein OS=Gallus gallus GN=LOC770011 PE=4 SV=2	E1C6R9_CHICK,MLRN_CHICK,UPI000012F21C,UPI000017124A,UPI00001776DD,UPI000060FC08,UPI0000ECCE36	TAX_GallusGallus_9031_20141114.fasta,UPARC14_2_GallusG_9031_20140417.fasta	20,295.80	100.00%	4	5	6	0.01%	23.80%	GNFNYIEFTR	K	I	99.70%	39.7	44.7	18.4	33.2	35.9	3.01	0.362	0.5	0	2	0	0	2	1,260.60	2,406.00		4,305.46	156	165	Empty	TRUE	
20196-20225 HinckeM –TAXchicken	Uncategorized Sample	20196-20205_A	Mudpit_Ingel20196_MGFPeaklist (F058870)	Uncharacterized protein OS=Gallus gallus GN=LOC770011 PE=4 SV=2	E1C6R9_CHICK,MLRN_CHICK,UPI000012F21C,UPI000017124A,UPI00001776DD,UPI000060FC08,UPI0000ECCE36	TAX_GallusGallus_9031_20141114.fasta,UPARC14_2_GallusG_9031_20140417.fasta	20,295.80	100.00%	4	5	6	0.01%	23.80%	ILKHGAKDK	R	D	7.70%	5.39	41	22	-0.36					0	1	0	0	2	1,009.62	2,147.00		206.13	166	174	Empty	TRUE	MLRM_CHICK,UPI00005FF04B,UPI0000171367,UPI000012F21A,UPI000060FDE7
20196-20225 HinckeM –TAXchicken	Uncategorized Sample	20196-20205_A	Mudpit_Ingel20196_MGFPeaklist (F058870)	Uncharacterized protein OS=Gallus gallus GN=LOC770011 PE=4 SV=2	E1C6R9_CHICK,MLRN_CHICK,UPI000012F21C,UPI000017124A,UPI00001776DD,UPI000060FC08,UPI0000ECCE36	TAX_GallusGallus_9031_20141114.fasta,UPARC14_2_GallusG_9031_20140417.fasta	20,295.80	100.00%	4	5	6	0.01%	23.80%	LNGTDPEDVIR	K	N	99.50%	34.1	44.8	25.4	20.6	29.3	1.96	0.113	0.318	0	2	0	0	2	1,229.60	2,000.00		1,013.09	98	108	Empty	TRUE	MLRM_CHICK,UPI00005FF04B,UPI0000171367,UPI000012F21A,UPI000060FDE7
20196-20225 HinckeM –TAXchicken	Uncategorized Sample	20196-20205_A	Mudpit_Ingel20196_MGFPeaklist (F058870)	Uncharacterized protein OS=Gallus gallus GN=LOC770011 PE=4 SV=2	E1C6R9_CHICK,MLRN_CHICK,UPI000012F21C,UPI000017124A,UPI00001776DD,UPI000060FC08,UPI0000ECCE36	TAX_GallusGallus_9031_20141114.fasta,UPARC14_2_GallusG_9031_20140417.fasta	20,295.80	100.00%	4	5	6	0.01%	23.80%	FTDEEVDELYR	R	E	99.70%	31.3	42.9	19.7	17.3	27.3	2.04	0.264	0.318	0	1	0	0	2	1,415.63	2,230.00		433.54	138	148	Empty	TRUE	
20196-20225 HinckeM –TAXchicken	Uncategorized Sample	20196-20205_A	Mudpit_Ingel20196_MGFPeaklist (F058870)	Uncharacterized protein OS=Gallus gallus GN=LOC770011 PE=4 SV=2	E1C6R9_CHICK,MLRN_CHICK,UPI000012F21C,UPI000017124A,UPI00001776DD,UPI000060FC08,UPI0000ECCE36	TAX_GallusGallus_9031_20141114.fasta,UPARC14_2_GallusG_9031_20140417.fasta	20,295.80	100.00%	4	5	6	0.01%	23.80%	GNFNYIEFTR	K	I	99.70%	39.7	44.7	18.4	33.2	35.9	3.01	0.362	0.5	0	2	0	0	2	1,260.60	2,406.00		4,305.46	156	165	Empty	TRUE	
20196-20225 HinckeM –TAXchicken	Uncategorized Sample	20196-20205_A	Mudpit_Ingel20196_MGFPeaklist (F058870)	Uncharacterized protein OS=Gallus gallus GN=LOC770011 PE=4 SV=2	E1C6R9_CHICK,MLRN_CHICK,UPI000012F21C,UPI000017124A,UPI00001776DD,UPI000060FC08,UPI0000ECCE36	TAX_GallusGallus_9031_20141114.fasta,UPARC14_2_GallusG_9031_20140417.fasta	20,295.80	100.00%	4	5	6	0.01%	23.80%	ILKHGAKDK	R	D	7.70%	5.39	41	22	-0.36					0	1	0	0	2	1,009.62	2,147.00		206.13	166	174	Empty	TRUE	MLRM_CHICK,UPI00005FF04B,UPI0000171367,UPI000012F21A,UPI000060FDE7
20196-20225 HinckeM –TAXchicken	Uncategorized Sample	20196-20205_A	Mudpit_Ingel20196_MGFPeaklist (F058870)	Uncharacterized protein OS=Gallus gallus GN=LOC770011 PE=4 SV=2	E1C6R9_CHICK,MLRN_CHICK,UPI000012F21C,UPI000017124A,UPI00001776DD,UPI000060FC08,UPI0000ECCE36	TAX_GallusGallus_9031_20141114.fasta,UPARC14_2_GallusG_9031_20140417.fasta	20,295.80	100.00%	4	5	6	0.01%	23.80%	LNGTDPEDVIR	K	N	99.50%	34.1	44.8	25.4	20.6	29.3	1.96	0.113	0.318	0	2	0	0	2	1,229.60	2,000.00		1,013.09	98	108	Empty	TRUE	MLRM_CHICK,UPI00005FF04B,UPI0000171367,UPI000012F21A,UPI000060FDE7
20196-20225 HinckeM –TAXchicken	Uncategorized Sample	20196-20205_A	Mudpit_Ingel20196_MGFPeaklist (F058870)	Uncharacterized protein OS=Gallus gallus GN=LOC770011 PE=4 SV=2	E1C6R9_CHICK,MLRN_CHICK,UPI000012F21C,UPI000017124A,UPI00001776DD,UPI000060FC08,UPI0000ECCE36	TAX_GallusGallus_9031_20141114.fasta,UPARC14_2_GallusG_9031_20140417.fasta	20,295.80	100.00%	4	5	6	0.01%	23.80%	FTDEEVDELYR	R	E	99.70%	31.3	42.9	19.7	17.3	27.3	2.04	0.264	0.318	0	1	0	0	2	1,415.63	2,230.00		433.54	138	148	Empty	TRUE	
20196-20225 HinckeM –TAXchicken	Uncategorized Sample	20196-20205_A	Mudpit_Ingel20196_MGFPeaklist (F058870)	Uncharacterized protein OS=Gallus gallus GN=LOC770011 PE=4 SV=2	E1C6R9_CHICK,MLRN_CHICK,UPI000012F21C,UPI000017124A,UPI00001776DD,UPI000060FC08,UPI0000ECCE36	TAX_GallusGallus_9031_20141114.fasta,UPARC14_2_GallusG_9031_20140417.fasta	20,295.80	100.00%	4	5	6	0.01%	23.80%	GNFNYIEFTR	K	I	99.70%	39.7	44.7	18.4	33.2	35.9	3.01	0.362	0.5	0	2	0	0	2	1,260.60	2,406.00		4,305.46	156	165	Empty	TRUE	
20196-20225 HinckeM –TAXchicken	Uncategorized Sample	20196-20205_A	Mudpit_Ingel20196_MGFPeaklist (F058870)	Uncharacterized protein OS=Gallus gallus GN=LOC770011 PE=4 SV=2	E1C6R9_CHICK,MLRN_CHICK,UPI000012F21C,UPI000017124A,UPI00001776DD,UPI000060FC08,UPI0000ECCE36	TAX_GallusGallus_9031_20141114.fasta,UPARC14_2_GallusG_9031_20140417.fasta	20,295.80	100.00%	4	5	6	0.01%	23.80%	ILKHGAKDK	R	D	7.70%	5.39	41	22	-0.36					0	1	0	0	2	1,009.62	2,147.00		206.13	166	174	Empty	TRUE	MLRM_CHICK,UPI00005FF04B,UPI0000171367,UPI000012F21A,UPI000060FDE7
20196-20225 HinckeM –TAXchicken	Uncategorized Sample	20196-20205_A	Mudpit_Ingel20196_MGFPeaklist (F058870)	Uncharacterized protein OS=Gallus gallus GN=LOC770011 PE=4 SV=2	E1C6R9_CHICK,MLRN_CHICK,UPI000012F21C,UPI000017124A,UPI00001776DD,UPI000060FC08,UPI0000ECCE36	TAX_GallusGallus_9031_20141114.fasta,UPARC14_2_GallusG_9031_20140417.fasta	20,295.80	100.00%	4	5	6	0.01%	23.80%	LNGTDPEDVIR	K	N	99.50%	34.1	44.8	25.4	20.6	29.3	1.96	0.113	0.318	0	2	0	0	2	1,229.60	2,000.00		1,013.09	98	108	Empty	TRUE	MLRM_CHICK,UPI00005FF04B,UPI0000171367,UPI000012F21A,UPI000060FDE7
20196-20225 HinckeM –TAXchicken	Uncategorized Sample	20196-20205_A	Mudpit_Ingel20196_MGFPeaklist (F058870)	Uncharacterized protein OS=Gallus gallus GN=FLNB PE=4 SV=2	F1N8D4_CHICK	TAX_GallusGallus_9031_20141114.fasta	277,135.60	100.00%	19	19	34	0.07%	10.60%	AGVAPLEVVVAGPR	K	G	99.70%	64.4	42	21.5	51.1	34.8	3.19	0.362	0.321	0	2	0	0	2	1,334.78	2,497.00		765.82	1464	1477	Empty	TRUE	UPI0000ECAD38
20196-20225 HinckeM –TAXchicken	Uncategorized Sample	20196-20205_A	Mudpit_Ingel20196_MGFPeaklist (F058870)	Uncharacterized protein OS=Gallus gallus GN=FLNB PE=4 SV=2	F1N8D4_CHICK	TAX_GallusGallus_9031_20141114.fasta	277,135.60	100.00%	19	19	34	0.07%	10.60%	AVPCLVEPVLGK	K	E	82.80%	27.5	43	28.4	13.3					0	1	0	0	2	1,281.72	2,455.00		242.77	1003	1014	Empty	TRUE	UPI0000ECAD38
20196-20225 HinckeM –TAXchicken	Uncategorized Sample	20196-20205_A	Mudpit_Ingel20196_MGFPeaklist (F058870)	Uncharacterized protein OS=Gallus gallus GN=FLNB PE=4 SV=2	F1N8D4_CHICK	TAX_GallusGallus_9031_20141114.fasta	277,135.60	100.00%	19	19	34	0.07%	10.60%	AWGPGLHEGIVGR	R	S	99.70%	27.8	44.8	19.8	17.5	26.8	1.66	0.168	0.308	0	0	2	0	2	1,348.71	2,118.50		778.14	569	581	Empty	TRUE	UPI0000ECAD38
20196-20225 HinckeM –TAXchicken	Uncategorized Sample	20196-20205_A	Mudpit_Ingel20196_MGFPeaklist (F058870)	Uncharacterized protein OS=Gallus gallus GN=FLNB PE=4 SV=2	F1N8D4_CHICK	TAX_GallusGallus_9031_20141114.fasta	277,135.60	100.00%	19	19	34	0.07%	10.60%	CLATGPGIAPTVR	K	T	99.70%	66.9	43.8	25.2	55.5	38.4	6	0.474	0.423	0	2	0	0	2	1,312.70	1,993.50		458.05	1627	1639	Empty	TRUE	UPI0000ECAD38
20196-20225 HinckeM –TAXchicken	Uncategorized Sample	20196-20205_A	Mudpit_Ingel20196_MGFPeaklist (F058870)	Uncharacterized protein OS=Gallus gallus GN=FLNB PE=4 SV=2	F1N8D4_CHICK	TAX_GallusGallus_9031_20141114.fasta	277,135.60	100.00%	19	19	34	0.07%	10.60%	DAGQGLLTVQITDQEGKPK	K	R	99.70%	61	43.5	19.8	49.5	44.6	8.82	0.448	0.342	0	0	2	0	2	1,998.05	2,266.50		445.49	1556	1574	Empty	TRUE	UPI0000ECAD38
20196-20225 HinckeM –TAXchicken	Uncategorized Sample	20196-20205_A	Mudpit_Ingel20196_MGFPeaklist (F058870)	Uncharacterized protein OS=Gallus gallus GN=FLNB PE=4 SV=2	F1N8D4_CHICK	TAX_GallusGallus_9031_20141114.fasta	277,135.60	100.00%	19	19	34	0.07%	10.60%	DMVIPFAVRK	F	G	42.30%					24.6	0.538	0.0447	0.25	0	1	0	0	1	1,158.63	2,205.00		447.33	1734	1743	Empty	TRUE	
20196-20225 HinckeM –TAXchicken	Uncategorized Sample	20196-20205_A	Mudpit_Ingel20196_MGFPeaklist (F058870)	Uncharacterized protein OS=Gallus gallus GN=FLNB PE=4 SV=2	F1N8D4_CHICK	TAX_GallusGallus_9031_20141114.fasta	277,135.60	100.00%	19	19	34	0.07%	10.60%	GAGIGGLGITVEGPSESK	R	I	99.70%	66.6	44.6	19.6	52.9	38.7	4.96	0.403	0.278	0	2	0	0	2	1,628.85	2,371.50		1,176.08	1369	1386	Empty	TRUE	UPI0000ECAD38
20196-20225 HinckeM –TAXchicken	Uncategorized Sample	20196-20205_A	Mudpit_Ingel20196_MGFPeaklist (F058870)	Uncharacterized protein OS=Gallus gallus GN=FLNB PE=4 SV=2	F1N8D4_CHICK	TAX_GallusGallus_9031_20141114.fasta	277,135.60	100.00%	19	19	34	0.07%	10.60%	GAGTGGLGLTVEGPCEAK	K	I	99.70%	55.1	44.6	17.8	42	34	4.15	0.432	0.222	0	2	0	0	2	1,673.82	2,152.00		472.77	1081	1098	Empty	TRUE	UPI0000ECAD38
20196-20225 HinckeM –TAXchicken	Uncategorized Sample	20196-20205_A	Mudpit_Ingel20196_MGFPeaklist (F058870)	Uncharacterized protein OS=Gallus gallus GN=FLNB PE=4 SV=2	F1N8D4_CHICK	TAX_GallusGallus_9031_20141114.fasta	277,135.60	100.00%	19	19	34	0.07%	10.60%	IAGPGLGTAVR	K	A	99.70%	40.9	42.4	19.6	17.9	27	2.06	0.259	0.318	0	2	0	0	2	1,011.59	1,885.50		606.91	1440	1450	Empty	TRUE	UPI0000ECAD38
20196-20225 HinckeM –TAXchicken	Uncategorized Sample	20196-20205_A	Mudpit_Ingel20196_MGFPeaklist (F058870)	Uncharacterized protein OS=Gallus gallus GN=FLNB PE=4 SV=2	F1N8D4_CHICK	TAX_GallusGallus_9031_20141114.fasta	277,135.60	100.00%	19	19	34	0.07%	10.60%	IGNLQHDLSDGLR	R	L	99.70%	79.5	44.4	34.1	61.4	39.6	6.21	0.429	0.5	0	0	2	0	2	1,437.74	2,148.50		689.58	56	68	Empty	TRUE	
20196-20225 HinckeM –TAXchicken	Uncategorized Sample	20196-20205_A	Mudpit_Ingel20196_MGFPeaklist (F058870)	Uncharacterized protein OS=Gallus gallus GN=FLNB PE=4 SV=2	F1N8D4_CHICK	TAX_GallusGallus_9031_20141114.fasta	277,135.60	100.00%	19	19	34	0.07%	10.60%	LIALLEVLSQK	R	R	99.70%	67.6	39.5	29.4	28.6	38.4	2.66	0.182	0.5	0	1	0	0	2	1,226.77	2,894.00		134.32	69	79	Empty	FALSE	Q90WF0_CHICK,Q90WF1_CHICK
20196-20225 HinckeM –TAXchicken	Uncategorized Sample	20196-20205_A	Mudpit_Ingel20196_MGFPeaklist (F058870)	Uncharacterized protein OS=Gallus gallus GN=FLNB PE=4 SV=2	F1N8D4_CHICK	TAX_GallusGallus_9031_20141114.fasta	277,135.60	100.00%	19	19	34	0.07%	10.60%	LPNNHIGISFIPR	R	E	99.70%	60.4	43	20.8	0	34.1	3.62	0.387	0.346	0	0	2	0	2	1,477.83	2,433.00		205.14	1943	1955	Empty	TRUE	UPI0000ECAD38
20196-20225 HinckeM –TAXchicken	Uncategorized Sample	20196-20205_A	Mudpit_Ingel20196_MGFPeaklist (F058870)	Uncharacterized protein OS=Gallus gallus GN=FLNB PE=4 SV=2	F1N8D4_CHICK	TAX_GallusGallus_9031_20141114.fasta	277,135.60	100.00%	19	19	34	0.07%	10.60%	SGCIVNNPAEFTVETK	R	D	99.70%	59	44.4	19.1	49.8	43.9	6.37	0.519	0.375	0	2	0	0	2	1,765.84	2,217.00		1,433.91	673	688	Empty	TRUE	UPI0000ECAD38
20196-20225 HinckeM –TAXchicken	Uncategorized Sample	20196-20205_A	Mudpit_Ingel20196_MGFPeaklist (F058870)	Uncharacterized protein OS=Gallus gallus GN=FLNB PE=4 SV=2	F1N8D4_CHICK	TAX_GallusGallus_9031_20141114.fasta	277,135.60	100.00%	19	19	34	0.07%	10.60%	SPFEVNVDKAQGDASK	K	V	99.70%	38.9	44.8	15.5	34.2	31.1	2.96	0.277	0.219	0	0	1	0	2	1,691.82	1,905.00		516	360	375	Empty	TRUE	UPI0000ECAD38
20196-20225 HinckeM –TAXchicken	Uncategorized Sample	20196-20205_A	Mudpit_Ingel20196_MGFPeaklist (F058870)	Uncharacterized protein OS=Gallus gallus GN=FLNB PE=4 SV=2	F1N8D4_CHICK	TAX_GallusGallus_9031_20141114.fasta	277,135.60	100.00%	19	19	34	0.07%	10.60%	SPFTVGVAAPLDLSK	K	V	99.70%	70	43.8	22.1	43.8	48.2	6.21	0.463	0.467	0	2	0	0	2	1,501.83	2,624.50		847.92	947	961	Empty	TRUE	UPI0000ECAD38
20196-20225 HinckeM –TAXchicken	Uncategorized Sample	20196-20205_A	Mudpit_Ingel20196_MGFPeaklist (F058870)	Uncharacterized protein OS=Gallus gallus GN=FLNB PE=4 SV=2	F1N8D4_CHICK	TAX_GallusGallus_9031_20141114.fasta	277,135.60	100.00%	19	19	34	0.07%	10.60%	TGEEVGFVVDAK	R	S	99.70%	45.1	44.7	24.4	32.4	30.8	0.824	0.315	0.375	0	2	0	0	2	1,250.63	2,115.00		678.35	1640	1651	Empty	TRUE	UPI0000ECAD38
20196-20225 HinckeM –TAXchicken	Uncategorized Sample	20196-20205_A	Mudpit_Ingel20196_MGFPeaklist (F058870)	Uncharacterized protein OS=Gallus gallus GN=FLNB PE=4 SV=2	F1N8D4_CHICK	TAX_GallusGallus_9031_20141114.fasta	277,135.60	100.00%	19	19	34	0.07%	10.60%	TPCSVLIGEACNPNACR	K	A	99.70%	48.8	42.6	26	38.1	36.6	5.2	0.407	0.294	0	0	2	0	2	1,918.86	2,145.00		469.83	459	475	Empty	TRUE	UPI0000ECAD38
20196-20225 HinckeM –TAXchicken	Uncategorized Sample	20196-20205_A	Mudpit_Ingel20196_MGFPeaklist (F058870)	Uncharacterized protein OS=Gallus gallus GN=FLNB PE=4 SV=2	F1N8D4_CHICK	TAX_GallusGallus_9031_20141114.fasta	277,135.60	100.00%	19	19	34	0.07%	10.60%	VIATGPGLER	K	G	99.70%	47.8	43	26.5	28.6	31.4	1.16	0.264	0.4	0	2	0	0	2	1,012.58	1,772.00		715.27	1150	1159	Empty	TRUE	UPI0000ECAD38
20196-20225 HinckeM –TAXchicken	Uncategorized Sample	20196-20205_A	Mudpit_Ingel20196_MGFPeaklist (F058870)	Uncharacterized protein OS=Gallus gallus GN=FLNB PE=4 SV=2	F1N8D4_CHICK	TAX_GallusGallus_9031_20141114.fasta	277,135.60	100.00%	19	19	34	0.07%	10.60%	VNQPASFAIR	K	L	82.20%	30.7	45	25.7	13.1					0	1	0	0	2	1,102.60	1,994.00		265.03	2285	2294	Empty	TRUE	UPI0000ECAD38
20196-20225 HinckeM –TAXchicken	Uncategorized Sample	20196-20205_A	Mudpit_Ingel20196_MGFPeaklist (F058870)	Uncharacterized protein OS=Gallus gallus GN=FLNB PE=4 SV=2	F1N8D4_CHICK	TAX_GallusGallus_9031_20141114.fasta	277,135.60	100.00%	19	19	34	0.07%	10.60%	YGGDDIPSSPYR	K	I	99.70%	39.2	42.9	29.5	27.6	34.3	3.24	0.42	0.417	0	2	0	0	2	1,326.60	1,874.00		1,093.78	1604	1615	Empty	TRUE	UPI0000ECAD38
20196-20225 HinckeM –TAXchicken	Uncategorized Sample	20196-20205_A	Mudpit_Ingel20196_MGFPeaklist (F058870)	status=inactive	UPI0000ECAD38	UPARC14_2_GallusG_9031_20140417.fasta	267,337.00	99.80%	3	3	3	0.01%	11.20%	AGVAPLEVVVAGPR	K	G	99.70%	64.4	42	21.5	51.1	34.8	3.19	0.362	0.321	0	2	0	0	2	1,334.78	2,497.00		765.82	1353	1366	Empty	FALSE	F1N8D4_CHICK
20196-20225 HinckeM –TAXchicken	Uncategorized Sample	20196-20205_A	Mudpit_Ingel20196_MGFPeaklist (F058870)	status=inactive	UPI0000ECAD38	UPARC14_2_GallusG_9031_20140417.fasta	267,337.00	99.80%	3	3	3	0.01%	11.20%	AVPCLVEPVLGK	K	E	82.80%	27.5	43	28.4	13.3					0	1	0	0	2	1,281.72	2,455.00		242.77	891	902	Empty	FALSE	F1N8D4_CHICK
20196-20225 HinckeM –TAXchicken	Uncategorized Sample	20196-20205_A	Mudpit_Ingel20196_MGFPeaklist (F058870)	status=inactive	UPI0000ECAD38	UPARC14_2_GallusG_9031_20140417.fasta	267,337.00	99.80%	3	3	3	0.01%	11.20%	AWGPGLHEGIVGR	R	S	99.70%	27.8	44.8	19.8	17.5	26.8	1.66	0.168	0.308	0	0	2	0	2	1,348.71	2,118.50		778.14	457	469	Empty	FALSE	F1N8D4_CHICK
20196-20225 HinckeM –TAXchicken	Uncategorized Sample	20196-20205_A	Mudpit_Ingel20196_MGFPeaklist (F058870)	status=inactive	UPI0000ECAD38	UPARC14_2_GallusG_9031_20140417.fasta	267,337.00	99.80%	3	3	3	0.01%	11.20%	CLATGPGIAPTVR	K	T	99.70%	66.9	43.8	25.2	55.5	38.4	6	0.474	0.423	0	2	0	0	2	1,312.70	1,993.50		458.05	1520	1532	Empty	FALSE	F1N8D4_CHICK
20196-20225 HinckeM –TAXchicken	Uncategorized Sample	20196-20205_A	Mudpit_Ingel20196_MGFPeaklist (F058870)	status=inactive	UPI0000ECAD38	UPARC14_2_GallusG_9031_20140417.fasta	267,337.00	99.80%	3	3	3	0.01%	11.20%	DAGQGLLTVQITDQEGKPK	K	R	99.70%	61	43.5	19.8	49.5	44.6	8.82	0.448	0.342	0	0	2	0	2	1,998.05	2,266.50		445.49	1449	1467	Empty	FALSE	F1N8D4_CHICK
20196-20225 HinckeM –TAXchicken	Uncategorized Sample	20196-20205_A	Mudpit_Ingel20196_MGFPeaklist (F058870)	status=inactive	UPI0000ECAD38	UPARC14_2_GallusG_9031_20140417.fasta	267,337.00	99.80%	3	3	3	0.01%	11.20%	FADEHIPGSPFTVK	K	I	22.50%	10.7	44.7	19.5	0					0	0	1	0	2	1,544.77	2,282.00		673.26	1979	1992	Empty	TRUE	
20196-20225 HinckeM –TAXchicken	Uncategorized Sample	20196-20205_A	Mudpit_Ingel20196_MGFPeaklist (F058870)	status=inactive	UPI0000ECAD38	UPARC14_2_GallusG_9031_20140417.fasta	267,337.00	99.80%	3	3	3	0.01%	11.20%	FNGSHVVGSPFK	K	V	11.40%	14.6	44.9	16.3	8.16					0	0	1	0	2	1,276.63	2,065.00		225.09	2265	2276	Empty	TRUE	
20196-20225 HinckeM –TAXchicken	Uncategorized Sample	20196-20205_A	Mudpit_Ingel20196_MGFPeaklist (F058870)	status=inactive	UPI0000ECAD38	UPARC14_2_GallusG_9031_20140417.fasta	267,337.00	99.80%	3	3	3	0.01%	11.20%	GAGIGGLGITVEGPSESK	R	I	99.70%	66.6	44.6	19.6	52.9	38.7	4.96	0.403	0.278	0	2	0	0	2	1,628.85	2,371.50		1,176.08	1258	1275	Empty	FALSE	F1N8D4_CHICK
20196-20225 HinckeM –TAXchicken	Uncategorized Sample	20196-20205_A	Mudpit_Ingel20196_MGFPeaklist (F058870)	status=inactive	UPI0000ECAD38	UPARC14_2_GallusG_9031_20140417.fasta	267,337.00	99.80%	3	3	3	0.01%	11.20%	GAGTGGLGLTVEGPCEAK	K	I	99.70%	55.1	44.6	17.8	42	34	4.15	0.432	0.222	0	2	0	0	2	1,673.82	2,152.00		472.77	970	987	Empty	FALSE	F1N8D4_CHICK
20196-20225 HinckeM –TAXchicken	Uncategorized Sample	20196-20205_A	Mudpit_Ingel20196_MGFPeaklist (F058870)	status=inactive	UPI0000ECAD38	UPARC14_2_GallusG_9031_20140417.fasta	267,337.00	99.80%	3	3	3	0.01%	11.20%	GGLVGKPAQFTIDTK	R	G	99.70%	52.9	43.9	27	44.7					0	0	1	0	2	1,531.85	2,133.00		375.13	955	969	Empty	TRUE	
20196-20225 HinckeM –TAXchicken	Uncategorized Sample	20196-20205_A	Mudpit_Ingel20196_MGFPeaklist (F058870)	status=inactive	UPI0000ECAD38	UPARC14_2_GallusG_9031_20140417.fasta	267,337.00	99.80%	3	3	3	0.01%	11.20%	IAGPGLGTAVR	K	A	99.70%	40.9	42.4	19.6	17.9	27	2.06	0.259	0.318	0	2	0	0	2	1,011.59	1,885.50		606.91	1329	1339	Empty	FALSE	F1N8D4_CHICK
20196-20225 HinckeM –TAXchicken	Uncategorized Sample	20196-20205_A	Mudpit_Ingel20196_MGFPeaklist (F058870)	status=inactive	UPI0000ECAD38	UPARC14_2_GallusG_9031_20140417.fasta	267,337.00	99.80%	3	3	3	0.01%	11.20%	LPNNHIGISFIPR	R	E	99.70%	60.4	43	20.8	0	34.1	3.62	0.387	0.346	0	0	2	0	2	1,477.83	2,433.00		205.14	1861	1873	Empty	FALSE	F1N8D4_CHICK
20196-20225 HinckeM –TAXchicken	Uncategorized Sample	20196-20205_A	Mudpit_Ingel20196_MGFPeaklist (F058870)	status=inactive	UPI0000ECAD38	UPARC14_2_GallusG_9031_20140417.fasta	267,337.00	99.80%	3	3	3	0.01%	11.20%	SGCIVNNPAEFTVETK	R	D	99.70%	59	44.4	19.1	49.8	43.9	6.37	0.519	0.375	0	2	0	0	2	1,765.84	2,217.00		1,433.91	561	576	Empty	FALSE	F1N8D4_CHICK
20196-20225 HinckeM –TAXchicken	Uncategorized Sample	20196-20205_A	Mudpit_Ingel20196_MGFPeaklist (F058870)	status=inactive	UPI0000ECAD38	UPARC14_2_GallusG_9031_20140417.fasta	267,337.00	99.80%	3	3	3	0.01%	11.20%	SPFEVNVDKAQGDASK	K	V	99.70%	38.9	44.8	15.5	34.2	31.1	2.96	0.277	0.219	0	0	1	0	2	1,691.82	1,905.00		516	244	259	Empty	FALSE	F1N8D4_CHICK
20196-20225 HinckeM –TAXchicken	Uncategorized Sample	20196-20205_A	Mudpit_Ingel20196_MGFPeaklist (F058870)	status=inactive	UPI0000ECAD38	UPARC14_2_GallusG_9031_20140417.fasta	267,337.00	99.80%	3	3	3	0.01%	11.20%	SPFTVGVAAPLDLSK	K	V	99.70%	70	43.8	22.1	43.8	48.2	6.21	0.463	0.467	0	2	0	0	2	1,501.83	2,624.50		847.92	835	849	Empty	FALSE	F1N8D4_CHICK
20196-20225 HinckeM –TAXchicken	Uncategorized Sample	20196-20205_A	Mudpit_Ingel20196_MGFPeaklist (F058870)	status=inactive	UPI0000ECAD38	UPARC14_2_GallusG_9031_20140417.fasta	267,337.00	99.80%	3	3	3	0.01%	11.20%	TGEEVGFVVDAK	R	S	99.70%	45.1	44.7	24.4	32.4	30.8	0.824	0.315	0.375	0	2	0	0	2	1,250.63	2,115.00		678.35	1533	1544	Empty	FALSE	F1N8D4_CHICK
20196-20225 HinckeM –TAXchicken	Uncategorized Sample	20196-20205_A	Mudpit_Ingel20196_MGFPeaklist (F058870)	status=inactive	UPI0000ECAD38	UPARC14_2_GallusG_9031_20140417.fasta	267,337.00	99.80%	3	3	3	0.01%	11.20%	TPCSVLIGEACNPNACR	K	A	99.70%	48.8	42.6	26	38.1	36.6	5.2	0.407	0.294	0	0	2	0	2	1,918.86	2,145.00		469.83	343	359	Empty	FALSE	F1N8D4_CHICK
20196-20225 HinckeM –TAXchicken	Uncategorized Sample	20196-20205_A	Mudpit_Ingel20196_MGFPeaklist (F058870)	status=inactive	UPI0000ECAD38	UPARC14_2_GallusG_9031_20140417.fasta	267,337.00	99.80%	3	3	3	0.01%	11.20%	VIATGPGLER	K	G	99.70%	47.8	43	26.5	28.6	31.4	1.16	0.264	0.4	0	2	0	0	2	1,012.58	1,772.00		715.27	1039	1048	Empty	FALSE	F1N8D4_CHICK
20196-20225 HinckeM –TAXchicken	Uncategorized Sample	20196-20205_A	Mudpit_Ingel20196_MGFPeaklist (F058870)	status=inactive	UPI0000ECAD38	UPARC14_2_GallusG_9031_20140417.fasta	267,337.00	99.80%	3	3	3	0.01%	11.20%	VNQPASFAIR	K	L	82.20%	30.7	45	25.7	13.1					0	1	0	0	2	1,102.60	1,994.00		265.03	2206	2215	Empty	FALSE	F1N8D4_CHICK
20196-20225 HinckeM –TAXchicken	Uncategorized Sample	20196-20205_A	Mudpit_Ingel20196_MGFPeaklist (F058870)	status=inactive	UPI0000ECAD38	UPARC14_2_GallusG_9031_20140417.fasta	267,337.00	99.80%	3	3	3	0.01%	11.20%	YGGDDIPSSPYR	K	I	99.70%	39.2	42.9	29.5	27.6	34.3	3.24	0.42	0.417	0	2	0	0	2	1,326.60	1,874.00		1,093.78	1497	1508	Empty	FALSE	F1N8D4_CHICK
20196-20225 HinckeM –TAXchicken	Uncategorized Sample	20196-20205_A	Mudpit_Ingel20196_MGFPeaklist (F058870)	JC7 OS=Gallus gallus GN=JC7 PE=2 SV=1	Q76MF7_CHICK,UPI00001BE925,UPI00003AC7AC,UPI0000ECA104	TAX_GallusGallus_9031_20141114.fasta,UPARC14_2_GallusG_9031_20140417.fasta	32,625.80	99.60%	2	2	3	0.01%	6.51%	LKATVTPSPVK	R	G	99.70%	45.8	40.8	34.5	26.1	34.2	1.1	0.24	0.455	0	2	0	0	2	1,140.70	1,653.50		697.5	223	233	Empty	TRUE	
20196-20225 HinckeM –TAXchicken	Uncategorized Sample	20196-20205_A	Mudpit_Ingel20196_MGFPeaklist (F058870)	JC7 OS=Gallus gallus GN=JC7 PE=2 SV=1	Q76MF7_CHICK,UPI00001BE925,UPI00003AC7AC,UPI0000ECA104	TAX_GallusGallus_9031_20141114.fasta,UPARC14_2_GallusG_9031_20140417.fasta	32,625.80	99.60%	2	2	3	0.01%	6.51%	MSRPVRNR	-	K	10.30%	2.99	45	14.6	0					0	1	0	0	2	1,015.56	2,330.00		52.05	1	8	Empty	TRUE	
20196-20225 HinckeM –TAXchicken	Uncategorized Sample	20196-20205_A	Mudpit_Ingel20196_MGFPeaklist (F058870)	JC7 OS=Gallus gallus GN=JC7 PE=2 SV=1	Q76MF7_CHICK,UPI00001BE925,UPI00003AC7AC,UPI0000ECA104	TAX_GallusGallus_9031_20141114.fasta,UPARC14_2_GallusG_9031_20140417.fasta	32,625.80	99.60%	2	2	3	0.01%	6.51%	LKATVTPSPVK	R	G	99.70%	45.8	40.8	34.5	26.1	34.2	1.1	0.24	0.455	0	2	0	0	2	1,140.70	1,653.50		697.5	223	233	Empty	TRUE	
20196-20225 HinckeM –TAXchicken	Uncategorized Sample	20196-20205_A	Mudpit_Ingel20196_MGFPeaklist (F058870)	JC7 OS=Gallus gallus GN=JC7 PE=2 SV=1	Q76MF7_CHICK,UPI00001BE925,UPI00003AC7AC,UPI0000ECA104	TAX_GallusGallus_9031_20141114.fasta,UPARC14_2_GallusG_9031_20140417.fasta	32,625.80	99.60%	2	2	3	0.01%	6.51%	MSRPVRNR	-	K	10.30%	2.99	45	14.6	0					0	1	0	0	2	1,015.56	2,330.00		52.05	1	8	Empty	TRUE	
20196-20225 HinckeM –TAXchicken	Uncategorized Sample	20196-20205_A	Mudpit_Ingel20196_MGFPeaklist (F058870)	JC7 OS=Gallus gallus GN=JC7 PE=2 SV=1	Q76MF7_CHICK,UPI00001BE925,UPI00003AC7AC,UPI0000ECA104	TAX_GallusGallus_9031_20141114.fasta,UPARC14_2_GallusG_9031_20140417.fasta	32,625.80	99.60%	2	2	3	0.01%	6.51%	LKATVTPSPVK	R	G	99.70%	45.8	40.8	34.5	26.1	34.2	1.1	0.24	0.455	0	2	0	0	2	1,140.70	1,653.50		697.5	223	233	Empty	TRUE	
20196-20225 HinckeM –TAXchicken	Uncategorized Sample	20196-20205_A	Mudpit_Ingel20196_MGFPeaklist (F058870)	JC7 OS=Gallus gallus GN=JC7 PE=2 SV=1	Q76MF7_CHICK,UPI00001BE925,UPI00003AC7AC,UPI0000ECA104	TAX_GallusGallus_9031_20141114.fasta,UPARC14_2_GallusG_9031_20140417.fasta	32,625.80	99.60%	2	2	3	0.01%	6.51%	MSRPVRNR	-	K	10.30%	2.99	45	14.6	0					0	1	0	0	2	1,015.56	2,330.00		52.05	1	8	Empty	TRUE	
20196-20225 HinckeM –TAXchicken	Uncategorized Sample	20196-20205_A	Mudpit_Ingel20196_MGFPeaklist (F058870)	JC7 OS=Gallus gallus GN=JC7 PE=2 SV=1	Q76MF7_CHICK,UPI00001BE925,UPI00003AC7AC,UPI0000ECA104	TAX_GallusGallus_9031_20141114.fasta,UPARC14_2_GallusG_9031_20140417.fasta	32,625.80	99.60%	2	2	3	0.01%	6.51%	LKATVTPSPVK	R	G	99.70%	45.8	40.8	34.5	26.1	34.2	1.1	0.24	0.455	0	2	0	0	2	1,140.70	1,653.50		697.5	223	233	Empty	TRUE	
20196-20225 HinckeM –TAXchicken	Uncategorized Sample	20196-20205_A	Mudpit_Ingel20196_MGFPeaklist (F058870)	JC7 OS=Gallus gallus GN=JC7 PE=2 SV=1	Q76MF7_CHICK,UPI00001BE925,UPI00003AC7AC,UPI0000ECA104	TAX_GallusGallus_9031_20141114.fasta,UPARC14_2_GallusG_9031_20140417.fasta	32,625.80	99.60%	2	2	3	0.01%	6.51%	MSRPVRNR	-	K	10.30%	2.99	45	14.6	0					0	1	0	0	2	1,015.56	2,330.00		52.05	1	8	Empty	TRUE	
20196-20225 HinckeM –TAXchicken	Uncategorized Sample	20196-20205_A	Mudpit_Ingel20196_MGFPeaklist (F058870)	Skeletal myosin heavy chain OS=Gallus gallus PE=2 SV=1	Q9PTY2_CHICK,UPI00000FB7E2	TAX_GallusGallus_9031_20141114.fasta,UPARC14_2_GallusG_9031_20140417.fasta	222,947.70	100.00%	10	10	17	0.03%	6.45%	CIIPNETK	R	T	99.20%	23.7	45.5	33.8	0	23.3	0.921	0.142	0.312	0	1	0	0	2	974.4977	1,785.00		252.69	676	683	Empty	TRUE	UPI00003AB434,UPI00003AB431
20196-20225 HinckeM –TAXchicken	Uncategorized Sample	20196-20205_A	Mudpit_Ingel20196_MGFPeaklist (F058870)	Skeletal myosin heavy chain OS=Gallus gallus PE=2 SV=1	Q9PTY2_CHICK,UPI00000FB7E2	TAX_GallusGallus_9031_20141114.fasta,UPARC14_2_GallusG_9031_20140417.fasta	222,947.70	100.00%	10	10	17	0.03%	6.45%	CNGVLEGIR	R	I	28.90%	28.9	45.5	29.6	12.6					0	1	0	0	2	1,018.50	2,046.00		210.55	699	707	Empty	FALSE	UPI000060E1E4,MYH11_CHICK,Q789A4_CHICK,UPI0000ECA50D,F1NCD4_CHICK,Q789A6_CHICK,UPI0002C868F4,UPI00000FC6BE,Q789A5_CHICK,UPI000012FB7F,UPI0000171382,MYH9_CHICK,UPI000044943F,UPI000060F510,UPI000060F511,UPI00003AB452,UPI0000171380,Q02015_CHICK,UPI0000171381,UPI00003AB434,UPI00003AB454,UPI00003AB431,UPI00003AB45
20196-20225 HinckeM –TAXchicken	Uncategorized Sample	20196-20205_A	Mudpit_Ingel20196_MGFPeaklist (F058870)	Skeletal myosin heavy chain OS=Gallus gallus PE=2 SV=1	Q9PTY2_CHICK,UPI00000FB7E2	TAX_GallusGallus_9031_20141114.fasta,UPARC14_2_GallusG_9031_20140417.fasta	222,947.70	100.00%	10	10	17	0.03%	6.45%	DLEEATLQHEATAAALR	R	K	99.70%	58.2	44.3	13.9	56.7	44.7	5.92	0.472	0.412	0	0	2	0	2	1,838.92	2,300.00		4,451.73	1181	1197	Empty	TRUE	UPI000044943F,UPI00003AB434,UPI00003AB431
20196-20225 HinckeM –TAXchicken	Uncategorized Sample	20196-20205_A	Mudpit_Ingel20196_MGFPeaklist (F058870)	Skeletal myosin heavy chain OS=Gallus gallus PE=2 SV=1	Q9PTY2_CHICK,UPI00000FB7E2	TAX_GallusGallus_9031_20141114.fasta,UPARC14_2_GallusG_9031_20140417.fasta	222,947.70	100.00%	10	10	17	0.03%	6.45%	ENLNKLMTNLR	R	S	39.00%	25.1	44.9	19.8	10.4					0	2	0	0	2	1,362.70	2,190.50		3,293.41	657	667	Empty	TRUE	UPI00003AB434,UPI00003AB431
20196-20225 HinckeM –TAXchicken	Uncategorized Sample	20196-20205_A	Mudpit_Ingel20196_MGFPeaklist (F058870)	Skeletal myosin heavy chain OS=Gallus gallus PE=2 SV=1	Q9PTY2_CHICK,UPI00000FB7E2	TAX_GallusGallus_9031_20141114.fasta,UPARC14_2_GallusG_9031_20140417.fasta	222,947.70	100.00%	10	10	17	0.03%	6.45%	IAEKDEEIDQLKR	K	N	99.70%	65.5	44.4	28.4	29.1	40.3	3.72	0.218	0.462	0	0	2	0	2	1,586.84	1,695.50		1,096.81	1580	1592	Empty	TRUE	UPI000044943F,UPI00003AB434,UPI00003AB431
20196-20225 HinckeM –TAXchicken	Uncategorized Sample	20196-20205_A	Mudpit_Ingel20196_MGFPeaklist (F058870)	Skeletal myosin heavy chain OS=Gallus gallus PE=2 SV=1	Q9PTY2_CHICK,UPI00000FB7E2	TAX_GallusGallus_9031_20141114.fasta,UPARC14_2_GallusG_9031_20140417.fasta	222,947.70	100.00%	10	10	17	0.03%	6.45%	ILYADFK	R	Q	98.80%	30.4	43.1	23.1	13.9	25	0.77	0.204	0.357	0	2	0	0	2	869.4768	2,157.00		1,085.21	717	723	Empty	TRUE	UPI000044943F
20196-20225 HinckeM –TAXchicken	Uncategorized Sample	20196-20205_A	Mudpit_Ingel20196_MGFPeaklist (F058870)	Skeletal myosin heavy chain OS=Gallus gallus PE=2 SV=1	Q9PTY2_CHICK,UPI00000FB7E2	TAX_GallusGallus_9031_20141114.fasta,UPARC14_2_GallusG_9031_20140417.fasta	222,947.70	100.00%	10	10	17	0.03%	6.45%	LQLELNQVK	R	S	99.70%	29.3	43	30.2	13	31.9	0.409	0.0815	0.444	0	2	0	0	2	1,084.64	2,194.50		2,256.33	1565	1573	Empty	TRUE	
20196-20225 HinckeM –TAXchicken	Uncategorized Sample	20196-20205_A	Mudpit_Ingel20196_MGFPeaklist (F058870)	Skeletal myosin heavy chain OS=Gallus gallus PE=2 SV=1	Q9PTY2_CHICK,UPI00000FB7E2	TAX_GallusGallus_9031_20141114.fasta,UPARC14_2_GallusG_9031_20140417.fasta	222,947.70	100.00%	10	10	17	0.03%	6.45%	MAAEAQKNLR	R	N	57.40%	25.3	45.2	33.6	-3.28					0	2	0	0	2	1,132.58	1,865.00		3,909.80	1639	1648	Empty	TRUE	
20196-20225 HinckeM –TAXchicken	Uncategorized Sample	20196-20205_A	Mudpit_Ingel20196_MGFPeaklist (F058870)	Skeletal myosin heavy chain OS=Gallus gallus PE=2 SV=1	Q9PTY2_CHICK,UPI00000FB7E2	TAX_GallusGallus_9031_20141114.fasta,UPARC14_2_GallusG_9031_20140417.fasta	222,947.70	100.00%	10	10	17	0.03%	6.45%	QAFTQQIEELKR	K	H	99.70%	58.4	44.4	28.9	0	30.6	2.02	0.206	0.292	0	0	1	0	2	1,490.80	2,145.00		587.69	1310	1321	Empty	TRUE	UPI000044943F,UPI00003AB434,UPI00003AB431
20196-20225 HinckeM –TAXchicken	Uncategorized Sample	20196-20205_A	Mudpit_Ingel20196_MGFPeaklist (F058870)	Skeletal myosin heavy chain OS=Gallus gallus PE=2 SV=1	Q9PTY2_CHICK,UPI00000FB7E2	TAX_GallusGallus_9031_20141114.fasta,UPARC14_2_GallusG_9031_20140417.fasta	222,947.70	100.00%	10	10	17	0.03%	6.45%	QVEEKDALISQLSR	R	G	69.20%	27.5	44.1	32	0					0	0	1	0	2	1,615.87	2,168.00		681.93	1294	1307	Empty	TRUE	UPI000044943F,UPI00003AB434,UPI00003AB431
20196-20225 HinckeM –TAXchicken	Uncategorized Sample	20196-20205_A	Mudpit_Ingel20196_MGFPeaklist (F058870)	Skeletal myosin heavy chain OS=Gallus gallus PE=2 SV=1	Q9PTY2_CHICK,UPI00000FB7E2	TAX_GallusGallus_9031_20141114.fasta,UPARC14_2_GallusG_9031_20140417.fasta	222,947.70	100.00%	10	10	17	0.03%	6.45%	VQLLHTQNTSLINTK	R	K	99.70%	21.8	42.1	0	21.8	29.7	1.8	0.306	0.267	0	0	2	0	2	1,709.95	2,152.50		4,565.09	1717	1731	Empty	TRUE	UPI000044943F,UPI00003AB434,UPI00003AB431
20196-20225 HinckeM –TAXchicken	Uncategorized Sample	20196-20205_A	Mudpit_Ingel20196_MGFPeaklist (F058870)	Skeletal myosin heavy chain OS=Gallus gallus PE=2 SV=1	Q9PTY2_CHICK,UPI00000FB7E2	TAX_GallusGallus_9031_20141114.fasta,UPARC14_2_GallusG_9031_20140417.fasta	222,947.70	100.00%	10	10	17	0.03%	6.45%	CIIPNETK	R	T	99.20%	23.7	45.5	33.8	0	23.3	0.921	0.142	0.312	0	1	0	0	2	974.4977	1,785.00		252.69	676	683	Empty	TRUE	UPI00003AB434,UPI00003AB431
20196-20225 HinckeM –TAXchicken	Uncategorized Sample	20196-20205_A	Mudpit_Ingel20196_MGFPeaklist (F058870)	Skeletal myosin heavy chain OS=Gallus gallus PE=2 SV=1	Q9PTY2_CHICK,UPI00000FB7E2	TAX_GallusGallus_9031_20141114.fasta,UPARC14_2_GallusG_9031_20140417.fasta	222,947.70	100.00%	10	10	17	0.03%	6.45%	CNGVLEGIR	R	I	28.90%	28.9	45.5	29.6	12.6					0	1	0	0	2	1,018.50	2,046.00		210.55	699	707	Empty	FALSE	UPI000060E1E4,MYH11_CHICK,Q789A4_CHICK,UPI0000ECA50D,F1NCD4_CHICK,Q789A6_CHICK,UPI0002C868F4,UPI00000FC6BE,Q789A5_CHICK,UPI000012FB7F,UPI0000171382,MYH9_CHICK,UPI000044943F,UPI000060F510,UPI000060F511,UPI00003AB452,UPI0000171380,Q02015_CHICK,UPI0000171381,UPI00003AB434,UPI00003AB454,UPI00003AB431,UPI00003AB45
20196-20225 HinckeM –TAXchicken	Uncategorized Sample	20196-20205_A	Mudpit_Ingel20196_MGFPeaklist (F058870)	Skeletal myosin heavy chain OS=Gallus gallus PE=2 SV=1	Q9PTY2_CHICK,UPI00000FB7E2	TAX_GallusGallus_9031_20141114.fasta,UPARC14_2_GallusG_9031_20140417.fasta	222,947.70	100.00%	10	10	17	0.03%	6.45%	DLEEATLQHEATAAALR	R	K	99.70%	58.2	44.3	13.9	56.7	44.7	5.92	0.472	0.412	0	0	2	0	2	1,838.92	2,300.00		4,451.73	1181	1197	Empty	TRUE	UPI000044943F,UPI00003AB434,UPI00003AB431
20196-20225 HinckeM –TAXchicken	Uncategorized Sample	20196-20205_A	Mudpit_Ingel20196_MGFPeaklist (F058870)	Skeletal myosin heavy chain OS=Gallus gallus PE=2 SV=1	Q9PTY2_CHICK,UPI00000FB7E2	TAX_GallusGallus_9031_20141114.fasta,UPARC14_2_GallusG_9031_20140417.fasta	222,947.70	100.00%	10	10	17	0.03%	6.45%	ENLNKLMTNLR	R	S	39.00%	25.1	44.9	19.8	10.4					0	2	0	0	2	1,362.70	2,190.50		3,293.41	657	667	Empty	TRUE	UPI00003AB434,UPI00003AB431
20196-20225 HinckeM –TAXchicken	Uncategorized Sample	20196-20205_A	Mudpit_Ingel20196_MGFPeaklist (F058870)	Skeletal myosin heavy chain OS=Gallus gallus PE=2 SV=1	Q9PTY2_CHICK,UPI00000FB7E2	TAX_GallusGallus_9031_20141114.fasta,UPARC14_2_GallusG_9031_20140417.fasta	222,947.70	100.00%	10	10	17	0.03%	6.45%	IAEKDEEIDQLKR	K	N	99.70%	65.5	44.4	28.4	29.1	40.3	3.72	0.218	0.462	0	0	2	0	2	1,586.84	1,695.50		1,096.81	1580	1592	Empty	TRUE	UPI000044943F,UPI00003AB434,UPI00003AB431
20196-20225 HinckeM –TAXchicken	Uncategorized Sample	20196-20205_A	Mudpit_Ingel20196_MGFPeaklist (F058870)	Skeletal myosin heavy chain OS=Gallus gallus PE=2 SV=1	Q9PTY2_CHICK,UPI00000FB7E2	TAX_GallusGallus_9031_20141114.fasta,UPARC14_2_GallusG_9031_20140417.fasta	222,947.70	100.00%	10	10	17	0.03%	6.45%	ILYADFK	R	Q	98.80%	30.4	43.1	23.1	13.9	25	0.77	0.204	0.357	0	2	0	0	2	869.4768	2,157.00		1,085.21	717	723	Empty	TRUE	UPI000044943F
20196-20225 HinckeM –TAXchicken	Uncategorized Sample	20196-20205_A	Mudpit_Ingel20196_MGFPeaklist (F058870)	Skeletal myosin heavy chain OS=Gallus gallus PE=2 SV=1	Q9PTY2_CHICK,UPI00000FB7E2	TAX_GallusGallus_9031_20141114.fasta,UPARC14_2_GallusG_9031_20140417.fasta	222,947.70	100.00%	10	10	17	0.03%	6.45%	LQLELNQVK	R	S	99.70%	29.3	43	30.2	13	31.9	0.409	0.0815	0.444	0	2	0	0	2	1,084.64	2,194.50		2,256.33	1565	1573	Empty	TRUE	
20196-20225 HinckeM –TAXchicken	Uncategorized Sample	20196-20205_A	Mudpit_Ingel20196_MGFPeaklist (F058870)	Skeletal myosin heavy chain OS=Gallus gallus PE=2 SV=1	Q9PTY2_CHICK,UPI00000FB7E2	TAX_GallusGallus_9031_20141114.fasta,UPARC14_2_GallusG_9031_20140417.fasta	222,947.70	100.00%	10	10	17	0.03%	6.45%	MAAEAQKNLR	R	N	57.40%	25.3	45.2	33.6	-3.28					0	2	0	0	2	1,132.58	1,865.00		3,909.80	1639	1648	Empty	TRUE	

20196-20225 HinckeM –TAXchicken	Uncategorized Sample	20196-20205_A	Mudpit_Ingel20196_MGFPeaklist (F058870)	Skeletal myosin heavy chain OS=Gallus gallus PE=2 SV=1	Q9PTY2_CHICK,UPI00000FB7E2	TAX_GallusGallus_9031_20141114.fasta,UPARC14_2_GallusG_9031_20140417.fasta	222,947.70	100.00%	10	10	17	0.03%	6.45%	QAFTQQIEELKR	K	H	99.70%	58.4	44.4	28.9	0	30.6	2.02	0.206	0.292	0	0	1	0	2	1,490.80	2,145.00		587.69	1310	1321	Empty	TRUE	UPI000044943F,UPI00003AB434,UPI00003AB431
20196-20225 HinckeM –TAXchicken	Uncategorized Sample	20196-20205_A	Mudpit_Ingel20196_MGFPeaklist (F058870)	Skeletal myosin heavy chain OS=Gallus gallus PE=2 SV=1	Q9PTY2_CHICK,UPI00000FB7E2	TAX_GallusGallus_9031_20141114.fasta,UPARC14_2_GallusG_9031_20140417.fasta	222,947.70	100.00%	10	10	17	0.03%	6.45%	QVEEKDALISQLSR	R	G	69.20%	27.5	44.1	32	0					0	0	1	0	2	1,615.87	2,168.00		681.93	1294	1307	Empty	TRUE	UPI000044943F,UPI00003AB434,UPI00003AB431
20196-20225 HinckeM –TAXchicken	Uncategorized Sample	20196-20205_A	Mudpit_Ingel20196_MGFPeaklist (F058870)	Skeletal myosin heavy chain OS=Gallus gallus PE=2 SV=1	Q9PTY2_CHICK,UPI00000FB7E2	TAX_GallusGallus_9031_20141114.fasta,UPARC14_2_GallusG_9031_20140417.fasta	222,947.70	100.00%	10	10	17	0.03%	6.45%	VQLLHTQNTSLINTK	R	K	99.70%	21.8	42.1	0	21.8	29.7	1.8	0.306	0.267	0	0	2	0	2	1,709.95	2,152.50		4,565.09	1717	1731	Empty	TRUE	UPI000044943F,UPI00003AB434,UPI00003AB431
20196-20225 HinckeM –TAXchicken	Uncategorized Sample	20196-20205_A	Mudpit_Ingel20196_MGFPeaklist (F058870)	Uncharacterized protein OS=Gallus gallus GN=TLN2 PE=4 SV=2	E1BR45_CHICK	TAX_GallusGallus_9031_20141114.fasta	271,758.20	100.00%	7	7	8	0.02%	7.00%	ANPVAKRHFVQSAK	T	E	68.90%					25.7	0.569	0.167	0.214	0	1	0	0	1	1,535.84	1,849.00		1,758.21	1519	1532	Empty	TRUE	
20196-20225 HinckeM –TAXchicken	Uncategorized Sample	20196-20205_A	Mudpit_Ingel20196_MGFPeaklist (F058870)	Uncharacterized protein OS=Gallus gallus GN=TLN2 PE=4 SV=2	E1BR45_CHICK	TAX_GallusGallus_9031_20141114.fasta	271,758.20	100.00%	7	7	8	0.02%	7.00%	AVAAGNSCRQEDVIATANLSR	K	K	99.70%	38.5	44.4	14.6	34.5	41.6	4.23	0.37	0.333	0	0	2	0	2	2,203.09	1,979.00		1,839.93	2190	2210	Empty	FALSE	E1C2S1_CHICK,TLN1_CHICK
20196-20225 HinckeM –TAXchicken	Uncategorized Sample	20196-20205_A	Mudpit_Ingel20196_MGFPeaklist (F058870)	Uncharacterized protein OS=Gallus gallus GN=TLN2 PE=4 SV=2	E1BR45_CHICK	TAX_GallusGallus_9031_20141114.fasta	271,758.20	100.00%	7	7	8	0.02%	7.00%	EVANSTANLVK	K	T	99.70%	47.5	45.1	22.3	31.4	30.5	1.14	0.256	0.364	0	2	0	0	2	1,145.62	1,720.50		713.07	1533	1543	Empty	FALSE	E1C2S1_CHICK,TLN1_CHICK
20196-20225 HinckeM –TAXchicken	Uncategorized Sample	20196-20205_A	Mudpit_Ingel20196_MGFPeaklist (F058870)	Uncharacterized protein OS=Gallus gallus GN=TLN2 PE=4 SV=2	E1BR45_CHICK	TAX_GallusGallus_9031_20141114.fasta	271,758.20	100.00%	7	7	8	0.02%	7.00%	FLPTELREDEG	K	-	99.70%	26.6	44.9	31.4	13.1	24.3	0.854	0.243	0.227	0	2	0	0	2	1,305.63	2,135.00		426.29	2532	2542	Empty	TRUE	
20196-20225 HinckeM –TAXchicken	Uncategorized Sample	20196-20205_A	Mudpit_Ingel20196_MGFPeaklist (F058870)	Uncharacterized protein OS=Gallus gallus GN=TLN2 PE=4 SV=2	E1BR45_CHICK	TAX_GallusGallus_9031_20141114.fasta	271,758.20	100.00%	7	7	8	0.02%	7.00%	NLATSLAELR	K	T	99.70%	31.7	44.8	21.7	22	25.1	0.469	0.108	0.3	0	1	0	0	2	1,087.61	2,494.00		187.03	1028	1037	Empty	TRUE	
20196-20225 HinckeM –TAXchicken	Uncategorized Sample	20196-20205_A	Mudpit_Ingel20196_MGFPeaklist (F058870)	Uncharacterized protein OS=Gallus gallus GN=TLN2 PE=4 SV=2	E1BR45_CHICK	TAX_GallusGallus_9031_20141114.fasta	271,758.20	100.00%	7	7	8	0.02%	7.00%	NVAQVAEDTVLQNR	K	V	7.40%	18.6	44.6	21	9.51					0	1	0	0	2	1,558.77	2,140.00		31,743.90	691	704	Empty	TRUE	
20196-20225 HinckeM –TAXchicken	Uncategorized Sample	20196-20205_A	Mudpit_Ingel20196_MGFPeaklist (F058870)	Uncharacterized protein OS=Gallus gallus GN=TLN2 PE=4 SV=2	E1BR45_CHICK	TAX_GallusGallus_9031_20141114.fasta	271,758.20	100.00%	7	7	8	0.02%	7.00%	QEDVIATANLSR	R	K	99.70%	55.5	45	31.3	30.3	32.4	2.89	0.34	0.375	0	3	0	0	2	1,316.68	2,208.00		786.49	2199	2210	Empty	FALSE	E1C2S1_CHICK,TLN1_CHICK
20196-20225 HinckeM –TAXchicken	Uncategorized Sample	20196-20205_A	Mudpit_Ingel20196_MGFPeaklist (F058870)	Uncharacterized protein OS=Gallus gallus GN=TLN2 PE=4 SV=2	E1BR45_CHICK	TAX_GallusGallus_9031_20141114.fasta	271,758.20	100.00%	7	7	8	0.02%	7.00%	QQQYKFLPTEL	R	R	52.00%					25.3	0.921	0.0791	0.227	0	1	0	0	1	1,394.73	2,160.00		882.16	2527	2537	Empty	FALSE	E1C2S1_CHICK,TLN1_CHICK
20196-20225 HinckeM –TAXchicken	Uncategorized Sample	20196-20205_A	Mudpit_Ingel20196_MGFPeaklist (F058870)	Uncharacterized protein OS=Gallus gallus GN=TLN2 PE=4 SV=2	E1BR45_CHICK	TAX_GallusGallus_9031_20141114.fasta	271,758.20	100.00%	7	7	8	0.02%	7.00%	QVAASTAQLLVACK	K	V	99.70%	62	44.5	21.5	57.6	44.2	7.42	0.493	0.429	0	4	0	0	2	1,459.79	2,312.00		3,135.98	2431	2444	Empty	FALSE	E1C2S1_CHICK,TLN1_CHICK
20196-20225 HinckeM –TAXchicken	Uncategorized Sample	20196-20205_A	Mudpit_Ingel20196_MGFPeaklist (F058870)	Uncharacterized protein OS=Gallus gallus GN=TLN2 PE=4 SV=2	E1BR45_CHICK	TAX_GallusGallus_9031_20141114.fasta	271,758.20	100.00%	7	7	8	0.02%	7.00%	RFGLEGDEESTMLEESVSPK	D	K	37.40%					24.6	0.42	0.0528	0.2	0	0	0	1	1	2,240.04	1,767.00		1,205.89	411	430	Empty	TRUE	
20196-20225 HinckeM –TAXchicken	Uncategorized Sample	20196-20205_A	Mudpit_Ingel20196_MGFPeaklist (F058870)	Uncharacterized protein OS=Gallus gallus GN=TLN2 PE=4 SV=2	E1BR45_CHICK	TAX_GallusGallus_9031_20141114.fasta	271,758.20	100.00%	7	7	8	0.02%	7.00%	SIAAATSALVK	K	S	99.70%	38.2	44.2	26.7	14.2	34.1	0.921	0.311	0.455	0	3	0	0	2	1,031.61	2,081.00		2,086.72	2352	2362	Empty	FALSE	E1C2S1_CHICK,TLN1_CHICK
20196-20225 HinckeM –TAXchicken	Uncategorized Sample	20196-20205_A	Mudpit_Ingel20196_MGFPeaklist (F058870)	Uncharacterized protein OS=Gallus gallus GN=TLN2 PE=4 SV=2	E1BR45_CHICK	TAX_GallusGallus_9031_20141114.fasta	271,758.20	100.00%	7	7	8	0.02%	7.00%	SLITSIRDKAPG	K	Q	56.80%					25	0.42	0.14	0.25	0	1	0	0	1	1,257.72	1,826.00		536.92	1648	1659	Empty	TRUE	
20196-20225 HinckeM –TAXchicken	Uncategorized Sample	20196-20205_A	Mudpit_Ingel20196_MGFPeaklist (F058870)	Uncharacterized protein OS=Gallus gallus GN=TLN2 PE=4 SV=2	E1BR45_CHICK	TAX_GallusGallus_9031_20141114.fasta	271,758.20	100.00%	7	7	8	0.02%	7.00%	TMLESSSLLIKTAR	K	S	88.20%					27.7	0.854	0.253	0.286	0	0	1	0	2	1,532.84	1,882.00		520.47	1607	1620	Empty	TRUE	
20196-20225 HinckeM –TAXchicken	Uncategorized Sample	20196-20205_A	Mudpit_Ingel20196_MGFPeaklist (F058870)	Uncharacterized protein OS=Gallus gallus GN=TLN2 PE=4 SV=2	E1BR45_CHICK	TAX_GallusGallus_9031_20141114.fasta	271,758.20	100.00%	7	7	8	0.02%	7.00%	TYGVSFFLVK	R	E	99.70%	33.4	44.9	19.4	27.8	33.4	1.8	0.338	0.5	0	2	0	0	2	1,160.64	2,786.50		1,278.77	310	319	Empty	FALSE	E1C2S1_CHICK,TLN1_CHICK
20196-20225 HinckeM –TAXchicken	Uncategorized Sample	20196-20205_A	Mudpit_Ingel20196_MGFPeaklist (F058870)	Uncharacterized protein OS=Gallus gallus GN=TLN2 PE=4 SV=2	E1BR45_CHICK	TAX_GallusGallus_9031_20141114.fasta	271,758.20	100.00%	7	7	8	0.02%	7.00%	VMVTNVTSLLK	K	T	99.70%	55.6	43.6	25.1	36.5	41.1	5.66	0.392	0.545	0	4	0	0	2	1,204.70	2,523.50		1,708.14	2121	2131	Empty	FALSE	E1C2S1_CHICK,TLN1_CHICK
20196-20225 HinckeM –TAXchicken	Uncategorized Sample	20196-20205_A	Mudpit_Ingel20196_MGFPeaklist (F058870)	status=active	UPI0000546791,UPI0000546792,UPI0001A2D229	UPARC14_2_GallusG_9031_20140417.fasta	244,148.70	100.00%	10	10	19	0.04%	5.39%	ANSNLVLQADR	K	S	99.70%	35.2	44.8	22	25.1	28.3	2.09	0.357	0.364	0	2	0	0	2	1,200.63	1,859.00		705.65	15	25	Empty	TRUE	UPI0000612112
20196-20225 HinckeM –TAXchicken	Uncategorized Sample	20196-20205_A	Mudpit_Ingel20196_MGFPeaklist (F058870)	status=active	UPI0000546791,UPI0000546792,UPI0001A2D229	UPARC14_2_GallusG_9031_20140417.fasta	244,148.70	100.00%	10	10	19	0.04%	5.39%	DIDAFWLQR	R	Q	99.70%	51.3	44.9	30.1	27.5	32.8	2.4	0.131	0.5	0	2	0	0	2	1,163.58	2,692.00		533.08	261	269	Empty	TRUE	UPI0000612112
20196-20225 HinckeM –TAXchicken	Uncategorized Sample	20196-20205_A	Mudpit_Ingel20196_MGFPeaklist (F058870)	status=active	UPI0000546791,UPI0000546792,UPI0001A2D229	UPARC14_2_GallusG_9031_20140417.fasta	244,148.70	100.00%	10	10	19	0.04%	5.39%	IVALSSSLSNAK	R	D	99.70%	62.6	44.3	27.6	43	37.1	3.23	0.313	0.417	0	2	0	0	2	1,189.68	2,035.00		728.08	1485	1496	Empty	TRUE	UPI0002C87FD9,R4GF59_CHICK
20196-20225 HinckeM –TAXchicken	Uncategorized Sample	20196-20205_A	Mudpit_Ingel20196_MGFPeaklist (F058870)	status=active	UPI0000546791,UPI0000546792,UPI0001A2D229	UPARC14_2_GallusG_9031_20140417.fasta	244,148.70	100.00%	10	10	19	0.04%	5.39%	LYDLNHNEIGELIR	R	M	99.70%	44.8	44.3	15.8	36.2	35.8	3.68	0.402	0.393	0	0	1	0	2	1,698.88	2,527.00		263.29	1152	1165	Empty	TRUE	UPI0002C87FD9,R4GF59_CHICK
20196-20225 HinckeM –TAXchicken	Uncategorized Sample	20196-20205_A	Mudpit_Ingel20196_MGFPeaklist (F058870)	status=active	UPI0000546791,UPI0000546792,UPI0001A2D229	UPARC14_2_GallusG_9031_20140417.fasta	244,148.70	100.00%	10	10	19	0.04%	5.39%	TICAEFAILR	K	M	99.70%	48.1	44.5	21	36.9	34.8	2.49	0.336	0.4	0	2	0	0	2	1,193.63	2,608.00		512.65	1355	1364	Empty	TRUE	UPI0002C87FD9,R4GF59_CHICK
20196-20225 HinckeM –TAXchicken	Uncategorized Sample	20196-20205_A	Mudpit_Ingel20196_MGFPeaklist (F058870)	status=active	UPI0000546791,UPI0000546792,UPI0001A2D229	UPARC14_2_GallusG_9031_20140417.fasta	244,148.70	100.00%	10	10	19	0.04%	5.39%	TNLLLQAHLSR	K	M	99.70%	32.1	42.1	24.2	25.2	26	2.62	0.208	0.364	0	0	2	0	2	1,265.73	2,220.50		723.4	1889	1899	Empty	TRUE	UPI0002C87FD9,R4GF59_CHICK
20196-20225 HinckeM –TAXchicken	Uncategorized Sample	20196-20205_A	Mudpit_Ingel20196_MGFPeaklist (F058870)	status=active	UPI0000546791,UPI0000546792,UPI0001A2D229	UPARC14_2_GallusG_9031_20140417.fasta	244,148.70	100.00%	10	10	19	0.04%	5.39%	VFSLSSEFR	R	N	99.70%	57.7	44.6	26.1	45.2	30	2.44	0.4	0.444	0	2	0	0	2	1,071.55	2,418.00		1,052.61	1014	1022	Empty	TRUE	UPI0002C87FD9,R4GF59_CHICK
20196-20225 HinckeM –TAXchicken	Uncategorized Sample	20196-20205_A	Mudpit_Ingel20196_MGFPeaklist (F058870)	status=active	UPI0000546791,UPI0000546792,UPI0001A2D229	UPARC14_2_GallusG_9031_20140417.fasta	244,148.70	100.00%	10	10	19	0.04%	5.39%	VPIPVKESIEEPSAK	R	I	99.70%	36.6	42.7	16.8	26.9	28.6	3.77	0.353	0.233	0	0	2	0	2	1,622.90	1,997.50		603.05	1041	1055	Empty	TRUE	UPI0002C87FD9,R4GF59_CHICK
20196-20225 HinckeM –TAXchicken	Uncategorized Sample	20196-20205_A	Mudpit_Ingel20196_MGFPeaklist (F058870)	status=active	UPI0000546791,UPI0000546792,UPI0001A2D229	UPARC14_2_GallusG_9031_20140417.fasta	244,148.70	100.00%	10	10	19	0.04%	5.39%	VVLLTGETSTDLK	K	L	99.70%	65.6	44	22.2	53	37.3	4.62	0.432	0.385	0	2	0	0	2	1,375.77	2,226.50		690.58	1403	1415	Empty	TRUE	UPI0002C87FD9,R4GF59_CHICK
20196-20225 HinckeM –TAXchicken	Uncategorized Sample	20196-20205_A	Mudpit_Ingel20196_MGFPeaklist (F058870)	status=active	UPI0000546791,UPI0000546792,UPI0001A2D229	UPARC14_2_GallusG_9031_20140417.fasta	244,148.70	100.00%	10	10	19	0.04%	5.39%	YISSQIERPIR	R	I	99.70%	22.3	44.2	21.9	14.9	22.5	0.721	0.164	0.273	0	0	2	0	2	1,361.75	1,850.50		835.53	1474	1484	Empty	TRUE	UPI0002C87FD9,R4GF59_CHICK
20196-20225 HinckeM –TAXchicken	Uncategorized Sample	20196-20205_A	Mudpit_Ingel20196_MGFPeaklist (F058870)	status=active	UPI0000546791,UPI0000546792,UPI0001A2D229	UPARC14_2_GallusG_9031_20140417.fasta	244,148.70	100.00%	10	10	19	0.04%	5.39%	ANSNLVLQADR	K	S	99.70%	35.2	44.8	22	25.1	28.3	2.09	0.357	0.364	0	2	0	0	2	1,200.63	1,859.00		705.65	15	25	Empty	TRUE	UPI0000612112
20196-20225 HinckeM –TAXchicken	Uncategorized Sample	20196-20205_A	Mudpit_Ingel20196_MGFPeaklist (F058870)	status=active	UPI0000546791,UPI0000546792,UPI0001A2D229	UPARC14_2_GallusG_9031_20140417.fasta	244,148.70	100.00%	10	10	19	0.04%	5.39%	DIDAFWLQR	R	Q	99.70%	51.3	44.9	30.1	27.5	32.8	2.4	0.131	0.5	0	2	0	0	2	1,163.58	2,692.00		533.08	261	269	Empty	TRUE	UPI0000612112
20196-20225 HinckeM –TAXchicken	Uncategorized Sample	20196-20205_A	Mudpit_Ingel20196_MGFPeaklist (F058870)	status=active	UPI0000546791,UPI0000546792,UPI0001A2D229	UPARC14_2_GallusG_9031_20140417.fasta	244,148.70	100.00%	10	10	19	0.04%	5.39%	IVALSSSLSNAK	R	D	99.70%	62.6	44.3	27.6	43	37.1	3.23	0.313	0.417	0	2	0	0	2	1,189.68	2,035.00		728.08	1485	1496	Empty	TRUE	UPI0002C87FD9,R4GF59_CHICK
20196-20225 HinckeM –TAXchicken	Uncategorized Sample	20196-20205_A	Mudpit_Ingel20196_MGFPeaklist (F058870)	status=active	UPI0000546791,UPI0000546792,UPI0001A2D229	UPARC14_2_GallusG_9031_20140417.fasta	244,148.70	100.00%	10	10	19	0.04%	5.39%	LYDLNHNEIGELIR	R	M	99.70%	44.8	44.3	15.8	36.2	35.8	3.68	0.402	0.393	0	0	1	0	2	1,698.88	2,527.00		263.29	1152	1165	Empty	TRUE	UPI0002C87FD9,R4GF59_CHICK
20196-20225 HinckeM –TAXchicken	Uncategorized Sample	20196-20205_A	Mudpit_Ingel20196_MGFPeaklist (F058870)	status=active	UPI0000546791,UPI0000546792,UPI0001A2D229	UPARC14_2_GallusG_9031_20140417.fasta	244,148.70	100.00%	10	10	19	0.04%	5.39%	TICAEFAILR	K	M	99.70%	48.1	44.5	21	36.9	34.8	2.49	0.336	0.4	0	2	0	0	2	1,193.63	2,608.00		512.65	1355	1364	Empty	TRUE	UPI0002C87FD9,R4GF59_CHICK
20196-20225 HinckeM –TAXchicken	Uncategorized Sample	20196-20205_A	Mudpit_Ingel20196_MGFPeaklist (F058870)	status=active	UPI0000546791,UPI0000546792,UPI0001A2D229	UPARC14_2_GallusG_9031_20140417.fasta	244,148.70	100.00%	10	10	19	0.04%	5.39%	TNLLLQAHLSR	K	M	99.70%	32.1	42.1	24.2	25.2	26	2.62	0.208	0.364	0	0	2	0	2	1,265.73	2,220.50		723.4	1889	1899	Empty	TRUE	UPI0002C87FD9,R4GF59_CHICK
20196-20225 HinckeM –TAXchicken	Uncategorized Sample	20196-20205_A	Mudpit_Ingel20196_MGFPeaklist (F058870)	status=active	UPI0000546791,UPI0000546792,UPI0001A2D229	UPARC14_2_GallusG_9031_20140417.fasta	244,148.70	100.00%	10	10	19	0.04%	5.39%	VFSLSSEFR	R	N	99.70%	57.7	44.6	26.1	45.2	30	2.44	0.4	0.444	0	2	0	0	2	1,071.55	2,418.00		1,052.61	1014	1022	Empty	TRUE	UPI0002C87FD9,R4GF59_CHICK
20196-20225 HinckeM –TAXchicken	Uncategorized Sample	20196-20205_A	Mudpit_Ingel20196_MGFPeaklist (F058870)	status=active	UPI0000546791,UPI0000546792,UPI0001A2D229	UPARC14_2_GallusG_9031_20140417.fasta	244,148.70	100.00%	10	10	19	0.04%	5.39%	VPIPVKESIEEPSAK	R	I	99.70%	36.6	42.7	16.8	26.9	28.6	3.77	0.353	0.233	0	0	2	0	2	1,622.90	1,997.50		603.05	1041	1055	Empty	TRUE	UPI0002C87FD9,R4GF59_CHICK
20196-20225 HinckeM –TAXchicken	Uncategorized Sample	20196-20205_A	Mudpit_Ingel20196_MGFPeaklist (F058870)	status=active	UPI0000546791,UPI0000546792,UPI0001A2D229	UPARC14_2_GallusG_9031_20140417.fasta	244,148.70	100.00%	10	10	19	0.04%	5.39%	VVLLTGETSTDLK	K	L	99.70%	65.6	44	22.2	53	37.3	4.62	0.432	0.385	0	2	0	0	2	1,375.77	2,226.50		690.58	1403	1415	Empty	TRUE	UPI0002C87FD9,R4GF59_CHICK
20196-20225 HinckeM –TAXchicken	Uncategorized Sample	20196-20205_A	Mudpit_Ingel20196_MGFPeaklist (F058870)	status=active	UPI0000546791,UPI0000546792,UPI0001A2D229	UPARC14_2_GallusG_9031_20140417.fasta	244,148.70	100.00%	10	10	19	0.04%	5.39%	YISSQIERPIR	R	I	99.70%	22.3	44.2	21.9	14.9	22.5	0.721	0.164	0.273	0	0	2	0	2	1,361.75	1,850.50		835.53	1474	1484	Empty	TRUE	UPI0002C87FD9,R4GF59_CHICK
20196-20225 HinckeM –TAXchicken	Uncategorized Sample	20196-20205_A	Mudpit_Ingel20196_MGFPeaklist (F058870)	status=inactive	UPI0000612112	UPARC14_2_GallusG_9031_20140417.fasta	69,099.10	99.80%	2	2	3	0.01%	6.07%	ANSNLVLQADR	K	S	99.70%	35.2	44.8	22	25.1	28.3	2.09	0.357	0.364	0	2	0	0	2	1,200.63	1,859.00		705.65	15	25	Empty	FALSE	UPI0000546791,UPI0000546792,UPI0001A2D229
20196-20225 HinckeM –TAXchicken	Uncategorized Sample	20196-20205_A	Mudpit_Ingel20196_MGFPeaklist (F058870)	status=inactive	UPI0000612112	UPARC14_2_GallusG_9031_20140417.fasta	69,099.10	99.80%	2	2	3	0.01%	6.07%	DIDAFWLQR	R	Q	99.70%	51.3	44.9	30.1	27.5	32.8	2.4	0.131	0.5	0	2	0	0	2	1,163.58	2,692.00		533.08	262	270	Empty	FALSE	UPI0000546791,UPI0000546792,UPI0001A2D229
20196-20225 HinckeM –TAXchicken	Uncategorized Sample	20196-20205_A	Mudpit_Ingel20196_MGFPeaklist (F058870)	status=inactive	UPI0000612112	UPARC14_2_GallusG_9031_20140417.fasta	69,099.10	99.80%	2	2	3	0.01%	6.07%	FYDDAIVSQK	R	K	99.70%	36.7	44.1	22	0	33.1	2.32	0.344	0.4	0	2	0	0	2	1,185.58	1,957.00		1,113.67	275	284	Empty	TRUE	
20196-20225 HinckeM –TAXchicken	Uncategorized Sample	20196-20205_A	Mudpit_Ingel20196_MGFPeaklist (F058870)	status=inactive	UPI0000612112	UPARC14_2_GallusG_9031_20140417.fasta	69,099.10	99.80%	2	2	3	0.01%	6.07%	MKQPKTK	K	V	23.00%	12.8	44.6	0	-1.86					0	1	0	0	2	877.4814	2,057.00		100	101	107	Empty	TRUE	
20196-20225 HinckeM –TAXchicken	Uncategorized Sample	20196-20205_A	Mudpit_Ingel20196_MGFPeaklist (F058870)	status=active	UPI0000546791,UPI0000546792,UPI0001A2D229	UPARC14_2_GallusG_9031_20140417.fasta	244,148.70	100.00%	10	10	19	0.04%	5.39%	ANSNLVLQADR	K	S	99.70%	35.2	44.8	22	25.1	28.3	2.09	0.357	0.364	0	2	0	0	2	1,200.63	1,859.00		705.65	15	25	Empty	TRUE	UPI0000612112
20196-20225 HinckeM –TAXchicken	Uncategorized Sample	20196-20205_A	Mudpit_Ingel20196_MGFPeaklist (F058870)	status=active	UPI0000546791,UPI0000546792,UPI0001A2D229	UPARC14_2_GallusG_9031_20140417.fasta	244,148.70	100.00%	10	10	19	0.04%	5.39%	DIDAFWLQR	R	Q	99.70%	51.3	44.9	30.1	27.5	32.8	2.4	0.131	0.5	0	2	0	0	2	1,163.58	2,692.00		533.08	261	269	Empty	TRUE	UPI0000612112
20196-20225 HinckeM –TAXchicken	Uncategorized Sample	20196-20205_A	Mudpit_Ingel20196_MGFPeaklist (F058870)	status=active	UPI0000546791,UPI0000546792,UPI0001A2D229	UPARC14_2_GallusG_9031_20140417.fasta	244,148.70	100.00%	10	10	19	0.04%	5.39%	IVALSSSLSNAK	R	D	99.70%	62.6	44.3	27.6	43	37.1	3.23	0.313	0.417	0	2	0	0	2	1,189.68	2,035.00		728.08	1485	1496	Empty	TRUE	UPI0002C87FD9,R4GF59_CHICK
20196-20225 HinckeM –TAXchicken	Uncategorized Sample	20196-20205_A	Mudpit_Ingel20196_MGFPeaklist (F058870)	status=active	UPI0000546791,UPI0000546792,UPI0001A2D229	UPARC14_2_GallusG_9031_20140417.fasta	244,148.70	100.00%	10	10	19	0.04%	5.39%	LYDLNHNEIGELIR	R	M	99.70%	44.8	44.3	15.8	36.2	35.8	3.68	0.402	0.393	0	0	1	0	2	1,698.88	2,527.00		263.29	1152	1165	Empty	TRUE	UPI0002C87FD9,R4GF59_CHICK
20196-20225 HinckeM –TAXchicken	Uncategorized Sample	20196-20205_A	Mudpit_Ingel20196_MGFPeaklist (F058870)	status=active	UPI0000546791,UPI0000546792,UPI0001A2D229	UPARC14_2_GallusG_9031_20140417.fasta	244,148.70	100.00%	10	10	19	0.04%	5.39%	TICAEFAILR	K	M	99.70%	48.1	44.5	21	36.9	34.8	2.49	0.336	0.4	0	2	0	0	2	1,193.63	2,608.00		512.65	1355	1364	Empty	TRUE	UPI0002C87FD9,R4GF59_CHICK
20196-20225 HinckeM –TAXchicken	Uncategorized Sample	20196-20205_A	Mudpit_Ingel20196_MGFPeaklist (F058870)	status=active	UPI0000546791,UPI0000546792,UPI0001A2D229	UPARC14_2_GallusG_9031_20140417.fasta	244,148.70	100.00%	10	10	19	0.04%	5.39%	TNLLLQAHLSR	K	M	99.70%	32.1	42.1	24.2	25.2	26	2.62	0.208	0.364	0	0	2	0	2	1,265.73	2,220.50		723.4	1889	1899	Empty	TRUE	UPI0002C87FD9,R4GF59_CHICK
20196-20225 HinckeM –TAXchicken	Uncategorized Sample	20196-20205_A	Mudpit_Ingel20196_MGFPeaklist (F058870)	status=active	UPI0000546791,UPI0000546792,UPI0001A2D229	UPARC14_2_GallusG_9031_20140417.fasta	244,148.70	100.00%	10	10	19	0.04%	5.39%	VFSLSSEFR	R	N	99.70%	57.7	44.6	26.1	45.2	30	2.44	0.4	0.444	0	2	0	0	2	1,071.55	2,418.00		1,052.61	1014	1022	Empty	TRUE	UPI0002C87FD9,R4GF59_CHICK
20196-20225 HinckeM –TAXchicken	Uncategorized Sample	20196-20205_A	Mudpit_Ingel20196_MGFPeaklist (F058870)	status=active	UPI0000546791,UPI0000546792,UPI0001A2D229	UPARC14_2_GallusG_9031_20140417.fasta	244,148.70	100.00%	10	10	19	0.04%	5.39%	VPIPVKESIEEPSAK	R	I	99.70%	36.6	42.7	16.8	26.9	28.6	3.77	0.353	0.233	0	0	2	0	2	1,622.90	1,997.50		603.05	1041	1055	Empty	TRUE	UPI0002C87FD9,R4GF59_CHICK
20196-20225 HinckeM –TAXchicken	Uncategorized Sample	20196-20205_A	Mudpit_Ingel20196_MGFPeaklist (F058870)	status=active	UPI0000546791,UPI0000546792,UPI0001A2D229	UPARC14_2_GallusG_9031_20140417.fasta	244,148.70	100.00%	10	10	19	0.04%	5.39%	VVLLTGETSTDLK	K	L	99.70%	65.6	44	22.2	53	37.3	4.62	0.432	0.385	0	2	0	0	2	1,375.77	2,226.50		690.58	1403	1415	Empty	TRUE	UPI0002C87FD9,R4GF59_CHICK
20196-20225 HinckeM –TAXchicken	Uncategorized Sample	20196-20205_A	Mudpit_Ingel20196_MGFPeaklist (F058870)	status=active	UPI0000546791,UPI0000546792,UPI0001A2D229	UPARC14_2_GallusG_9031_20140417.fasta	244,148.70	100.00%	10	10	19	0.04%	5.39%	YISSQIERPIR	R	I	99.70%	22.3	44.2	21.9	14.9	22.5	0.721	0.164	0.273	0	0	2	0	2	1,361.75	1,850.50		835.53	1474	1484	Empty	TRUE	UPI0002C87FD9,R4GF59_CHICK
20196-20225 HinckeM –TAXchicken	Uncategorized Sample	20196-20205_A	Mudpit_Ingel20196_MGFPeaklist (F058870)	Keratin, type I cytoskeletal 19 OS=Gallus gallus GN=KRT19 PE=3 SV=1	F1NDN9_CHICK,K1C19_CHICK,UPI00000FD637,UPI0000ECA065	TAX_GallusGallus_9031_20141114.fasta,UPARC14_2_GallusG_9031_20140417.fasta	46,040.00	100.00%	4	4	22	0.04%	9.46%	GALESTLADTEGR	K	Y	6.70%	3.52	44.9	13.2	2.07					0	0	1	0	2	1,319.64	1,676.00		196.61	332	344	Empty	TRUE	
20196-20225 HinckeM –TAXchicken	Uncategorized Sample	20196-20205_A	Mudpit_Ingel20196_MGFPeaklist (F058870)	Keratin, type I cytoskeletal 19 OS=Gallus gallus GN=KRT19 PE=3 SV=1	F1NDN9_CHICK,K1C19_CHICK,UPI00000FD637,UPI0000ECA065	TAX_GallusGallus_9031_20141114.fasta,UPARC14_2_GallusG_9031_20140417.fasta	46,040.00	100.00%	4	4	22	0.04%	9.46%	LASYLDKVR	R	A	99.70%	64.8	43.4	33.9	43.9	38.6	2.31	0.207	0.5	0	8	0	0	2	1,064.61	1,896.00		6,459.27	105	113	Empty	TRUE	
20196-20225 HinckeM –TAXchicken	Uncategorized Sample	20196-20205_A	Mudpit_Ingel20196_MGFPeaklist (F058870)	Keratin, type I cytoskeletal 19 OS=Gallus gallus GN=KRT19 PE=3 SV=1	F1NDN9_CHICK,K1C19_CHICK,UPI00000FD637,UPI0000ECA065	TAX_GallusGallus_9031_20141114.fasta,UPARC14_2_GallusG_9031_20140417.fasta	46,040.00	100.00%	4	4	22	0.04%	9.46%	VLDELTLAR	R	T	99.70%	56.8	44.3	36.6	38.5	33.1	1.47	0.326	0.5	0	12	0	0	2	1,029.59	2,259.50		7,117.95	203	211	Empty	TRUE	
20196-20225 HinckeM –TAXchicken	Uncategorized Sample	20196-20205_A	Mudpit_Ingel20196_MGFPeaklist (F058870)	Keratin, type I cytoskeletal 19 OS=Gallus gallus GN=KRT19 PE=3 SV=1	F1NDN9_CHICK,K1C19_CHICK,UPI00000FD637,UPI0000ECA065	TAX_GallusGallus_9031_20141114.fasta,UPARC14_2_GallusG_9031_20140417.fasta	46,040.00	100.00%	4	4	22	0.04%	9.46%	VVLQIDNAR	K	L	13.00%	22.1	44.3	26.5	0					0	1	0	0	2	1,028.57	1,770.00		170.04	165	173	Empty	TRUE	Q6PVZ2_CHICK,UPI00006103B5,UPI0002C86D9D,UPI00006103B6,UPI0003287CD7,UPI000035A84B,F1NDN5_CHICK,UPI00003AC98C
20196-20225 HinckeM –TAXchicken	Uncategorized Sample	20196-20205_A	Mudpit_Ingel20196_MGFPeaklist (F058870)	Keratin, type I cytoskeletal 19 OS=Gallus gallus GN=KRT19 PE=3 SV=1	F1NDN9_CHICK,K1C19_CHICK,UPI00000FD637,UPI0000ECA065	TAX_GallusGallus_9031_20141114.fasta,UPARC14_2_GallusG_9031_20140417.fasta	46,040.00	100.00%	4	4	22	0.04%	9.46%	GALESTLADTEGR	K	Y	6.70%	3.52	44.9	13.2	2.07					0	0	1	0	2	1,319.64	1,676.00		196.61	332	344	Empty	TRUE	
20196-20225 HinckeM –TAXchicken	Uncategorized Sample	20196-20205_A	Mudpit_Ingel20196_MGFPeaklist (F058870)	Keratin, type I cytoskeletal 19 OS=Gallus gallus GN=KRT19 PE=3 SV=1	F1NDN9_CHICK,K1C19_CHICK,UPI00000FD637,UPI0000ECA065	TAX_GallusGallus_9031_20141114.fasta,UPARC14_2_GallusG_9031_20140417.fasta	46,040.00	100.00%	4	4	22	0.04%	9.46%	LASYLDKVR	R	A	99.70%	64.8	43.4	33.9	43.9	38.6	2.31	0.207	0.5	0	8	0	0	2	1,064.61	1,896.00		6,459.27	105	113	Empty	TRUE	
20196-20225 HinckeM –TAXchicken	Uncategorized Sample	20196-20205_A	Mudpit_Ingel20196_MGFPeaklist (F058870)	Keratin, type I cytoskeletal 19 OS=Gallus gallus GN=KRT19 PE=3 SV=1	F1NDN9_CHICK,K1C19_CHICK,UPI00000FD637,UPI0000ECA065	TAX_GallusGallus_9031_20141114.fasta,UPARC14_2_GallusG_9031_20140417.fasta	46,040.00	100.00%	4	4	22	0.04%	9.46%	VLDELTLAR	R	T	99.70%	56.8	44.3	36.6	38.5	33.1	1.47	0.326	0.5	0	12	0	0	2	1,029.59	2,259.50		7,117.95	203	211	Empty	TRUE	
20196-20225 HinckeM –TAXchicken	Uncategorized Sample	20196-20205_A	Mudpit_Ingel20196_MGFPeaklist (F058870)	Keratin, type I cytoskeletal 19 OS=Gallus gallus GN=KRT19 PE=3 SV=1	F1NDN9_CHICK,K1C19_CHICK,UPI00000FD637,UPI0000ECA065	TAX_GallusGallus_9031_20141114.fasta,UPARC14_2_GallusG_9031_20140417.fasta	46,040.00	100.00%	4	4	22	0.04%	9.46%	VVLQIDNAR	K	L	13.00%	22.1	44.3	26.5	0					0	1	0	0	2	1,028.57	1,770.00		170.04	165	173	Empty	TRUE	Q6PVZ2_CHICK,UPI00006103B5,UPI0002C86D9D,UPI00006103B6,UPI0003287CD7,UPI000035A84B,F1NDN5_CHICK,UPI00003AC98C
20196-20225 HinckeM –TAXchicken	Uncategorized Sample	20196-20205_A	Mudpit_Ingel20196_MGFPeaklist (F058870)	Keratin, type I cytoskeletal 19 OS=Gallus gallus GN=KRT19 PE=3 SV=1	F1NDN9_CHICK,K1C19_CHICK,UPI00000FD637,UPI0000ECA065	TAX_GallusGallus_9031_20141114.fasta,UPARC14_2_GallusG_9031_20140417.fasta	46,040.00	100.00%	4	4	22	0.04%	9.46%	GALESTLADTEGR	K	Y	6.70%	3.52	44.9	13.2	2.07					0	0	1	0	2	1,319.64	1,676.00		196.61	332	344	Empty	TRUE	
20196-20225 HinckeM –TAXchicken	Uncategorized Sample	20196-20205_A	Mudpit_Ingel20196_MGFPeaklist (F058870)	Keratin, type I cytoskeletal 19 OS=Gallus gallus GN=KRT19 PE=3 SV=1	F1NDN9_CHICK,K1C19_CHICK,UPI00000FD637,UPI0000ECA065	TAX_GallusGallus_9031_20141114.fasta,UPARC14_2_GallusG_9031_20140417.fasta	46,040.00	100.00%	4	4	22	0.04%	9.46%	LASYLDKVR	R	A	99.70%	64.8	43.4	33.9	43.9	38.6	2.31	0.207	0.5	0	8	0	0	2	1,064.61	1,896.00		6,459.27	105	113	Empty	TRUE	
20196-20225 HinckeM –TAXchicken	Uncategorized Sample	20196-20205_A	Mudpit_Ingel20196_MGFPeaklist (F058870)	Keratin, type I cytoskeletal 19 OS=Gallus gallus GN=KRT19 PE=3 SV=1	F1NDN9_CHICK,K1C19_CHICK,UPI00000FD637,UPI0000ECA065	TAX_GallusGallus_9031_20141114.fasta,UPARC14_2_GallusG_9031_20140417.fasta	46,040.00	100.00%	4	4	22	0.04%	9.46%	VLDELTLAR	R	T	99.70%	56.8	44.3	36.6	38.5	33.1	1.47	0.326	0.5	0	12	0	0	2	1,029.59	2,259.50		7,117.95	203	211	Empty	TRUE	
20196-20225 HinckeM –TAXchicken	Uncategorized Sample	20196-20205_A	Mudpit_Ingel20196_MGFPeaklist (F058870)	Keratin, type I cytoskeletal 19 OS=Gallus gallus GN=KRT19 PE=3 SV=1	F1NDN9_CHICK,K1C19_CHICK,UPI00000FD637,UPI0000ECA065	TAX_GallusGallus_9031_20141114.fasta,UPARC14_2_GallusG_9031_20140417.fasta	46,040.00	100.00%	4	4	22	0.04%	9.46%	VVLQIDNAR	K	L	13.00%	22.1	44.3	26.5	0					0	1	0	0	2	1,028.57	1,770.00		170.04	165	173	Empty	TRUE	Q6PVZ2_CHICK,UPI00006103B5,UPI0002C86D9D,UPI00006103B6,UPI0003287CD7,UPI000035A84B,F1NDN5_CHICK,UPI00003AC98C
20196-20225 HinckeM –TAXchicken	Uncategorized Sample	20196-20205_A	Mudpit_Ingel20196_MGFPeaklist (F058870)	Keratin, type I cytoskeletal 19 OS=Gallus gallus GN=KRT19 PE=3 SV=1	F1NDN9_CHICK,K1C19_CHICK,UPI00000FD637,UPI0000ECA065	TAX_GallusGallus_9031_20141114.fasta,UPARC14_2_GallusG_9031_20140417.fasta	46,040.00	100.00%	4	4	22	0.04%	9.46%	GALESTLADTEGR	K	Y	6.70%	3.52	44.9	13.2	2.07					0	0	1	0	2	1,319.64	1,676.00		196.61	332	344	Empty	TRUE	
20196-20225 HinckeM –TAXchicken	Uncategorized Sample	20196-20205_A	Mudpit_Ingel20196_MGFPeaklist (F058870)	Keratin, type I cytoskeletal 19 OS=Gallus gallus GN=KRT19 PE=3 SV=1	F1NDN9_CHICK,K1C19_CHICK,UPI00000FD637,UPI0000ECA065	TAX_GallusGallus_9031_20141114.fasta,UPARC14_2_GallusG_9031_20140417.fasta	46,040.00	100.00%	4	4	22	0.04%	9.46%	LASYLDKVR	R	A	99.70%	64.8	43.4	33.9	43.9	38.6	2.31	0.207	0.5	0	8	0	0	2	1,064.61	1,896.00		6,459.27	105	113	Empty	TRUE	
20196-20225 HinckeM –TAXchicken	Uncategorized Sample	20196-20205_A	Mudpit_Ingel20196_MGFPeaklist (F058870)	Keratin, type I cytoskeletal 19 OS=Gallus gallus GN=KRT19 PE=3 SV=1	F1NDN9_CHICK,K1C19_CHICK,UPI00000FD637,UPI0000ECA065	TAX_GallusGallus_9031_20141114.fasta,UPARC14_2_GallusG_9031_20140417.fasta	46,040.00	100.00%	4	4	22	0.04%	9.46%	VLDELTLAR	R	T	99.70%	56.8	44.3	36.6	38.5	33.1	1.47	0.326	0.5	0	12	0	0	2	1,029.59	2,259.50		7,117.95	203	211	Empty	TRUE	
20196-20225 HinckeM –TAXchicken	Uncategorized Sample	20196-20205_A	Mudpit_Ingel20196_MGFPeaklist (F058870)	Keratin, type I cytoskeletal 19 OS=Gallus gallus GN=KRT19 PE=3 SV=1	F1NDN9_CHICK,K1C19_CHICK,UPI00000FD637,UPI0000ECA065	TAX_GallusGallus_9031_20141114.fasta,UPARC14_2_GallusG_9031_20140417.fasta	46,040.00	100.00%	4	4	22	0.04%	9.46%	VVLQIDNAR	K	L	13.00%	22.1	44.3	26.5	0					0	1	0	0	2	1,028.57	1,770.00		170.04	165	173	Empty	TRUE	Q6PVZ2_CHICK,UPI00006103B5,UPI0002C86D9D,UPI00006103B6,UPI0003287CD7,UPI000035A84B,F1NDN5_CHICK,UPI00003AC98C
20196-20225 HinckeM –TAXchicken	Uncategorized Sample	20196-20205_A	Mudpit_Ingel20196_MGFPeaklist (F058870)	Alpha-enolase OS=Gallus gallus GN=ENO1 PE=3 SV=2	F1NZ78_CHICK,UPI0000ECA2FD,UPI0002C86C58	TAX_GallusGallus_9031_20141114.fasta,UPARC14_2_GallusG_9031_20140417.fasta	47,334.10	100.00%	18	21	68	0.14%	47.00%	AAVPSGASTGIYEALELRDNDKTR	R	Y	99.70%	42.8	43.5	0	42.8	38.4	3.72	0.372	0.292	0	0	0	3	2	2,534.28	2,397.00		16,739.40	33	56	Empty	TRUE	
20196-20225 HinckeM –TAXchicken	Uncategorized Sample	20196-20205_A	Mudpit_Ingel20196_MGFPeaklist (F058870)	Alpha-enolase OS=Gallus gallus GN=ENO1 PE=3 SV=2	F1NZ78_CHICK,UPI0000ECA2FD,UPI0002C86C58	TAX_GallusGallus_9031_20141114.fasta,UPARC14_2_GallusG_9031_20140417.fasta	47,334.10	100.00%	18	21	68	0.14%	47.00%	DATNVGDEGGFAPNILENK	K	E	99.70%	66.7	43.9	15.4	57	45.6	4.23	0.408	0.421	0	0	4	0	2	1,960.92	2,402.50		8,502.20	203	221	Empty	TRUE	
20196-20225 HinckeM –TAXchicken	Uncategorized Sample	20196-20205_A	Mudpit_Ingel20196_MGFPeaklist (F058870)	Alpha-enolase OS=Gallus gallus GN=ENO1 PE=3 SV=2	F1NZ78_CHICK,UPI0000ECA2FD,UPI0002C86C58	TAX_GallusGallus_9031_20141114.fasta,UPARC14_2_GallusG_9031_20140417.fasta	47,334.10	100.00%	18	21	68	0.14%	47.00%	DATNVGDEGGFAPNILENKEALELLK	K	T	99.70%	29.9	43.2	13	29.5	33.8	1.74	0.178	0.212	0	0	1	0	2	2,757.39	2,858.00		1,959.71	203	228	Empty	TRUE	
20196-20225 HinckeM –TAXchicken	Uncategorized Sample	20196-20205_A	Mudpit_Ingel20196_MGFPeaklist (F058870)	Alpha-enolase OS=Gallus gallus GN=ENO1 PE=3 SV=2	F1NZ78_CHICK,UPI0000ECA2FD,UPI0002C86C58	TAX_GallusGallus_9031_20141114.fasta,UPARC14_2_GallusG_9031_20140417.fasta	47,334.10	100.00%	18	21	68	0.14%	47.00%	DGKYDLDFKSPDDPSR	R	Y	99.70%	52.7	43.5	19.6	38.4	41.3	5.44	0.494	0.375	0	0	3	3	2	1,854.85	1,992.50		11,237.20	254	269	Empty	TRUE	
20196-20225 HinckeM –TAXchicken	Uncategorized Sample	20196-20205_A	Mudpit_Ingel20196_MGFPeaklist (F058870)	Alpha-enolase OS=Gallus gallus GN=ENO1 PE=3 SV=2	F1NZ78_CHICK,UPI0000ECA2FD,UPI0002C86C58	TAX_GallusGallus_9031_20141114.fasta,UPARC14_2_GallusG_9031_20140417.fasta	47,334.10	100.00%	18	21	68	0.14%	47.00%	EALELLK	K	T	74.20%	54.6	44.3	56	0					0	5	0	0	2	815.4874	2,221.00		7,201.26	222	228	Empty	TRUE	
20196-20225 HinckeM –TAXchicken	Uncategorized Sample	20196-20205_A	Mudpit_Ingel20196_MGFPeaklist (F058870)	Alpha-enolase OS=Gallus gallus GN=ENO1 PE=3 SV=2	F1NZ78_CHICK,UPI0000ECA2FD,UPI0002C86C58	TAX_GallusGallus_9031_20141114.fasta,UPARC14_2_GallusG_9031_20140417.fasta	47,334.10	100.00%	18	21	68	0.14%	47.00%	GNPTVEVDLYTNK	R	G	99.70%	63.5	44.8	18.6	58	44.1	5.92	0.506	0.423	0	6	0	0	2	1,449.72	2,113.50		10,158.60	16	28	Empty	TRUE	
20196-20225 HinckeM –TAXchicken	Uncategorized Sample	20196-20205_A	Mudpit_Ingel20196_MGFPeaklist (F058870)	Alpha-enolase OS=Gallus gallus GN=ENO1 PE=3 SV=2	F1NZ78_CHICK,UPI0000ECA2FD,UPI0002C86C58	TAX_GallusGallus_9031_20141114.fasta,UPARC14_2_GallusG_9031_20140417.fasta	47,334.10	100.00%	18	21	68	0.14%	47.00%	IDKLMLEMDGTENK	K	S	29.70%	15	43.8	24.8	4.25					0	0	1	0	2	1,668.78	1,807.00		546.6	90	103	Empty	TRUE	
20196-20225 HinckeM –TAXchicken	Uncategorized Sample	20196-20205_A	Mudpit_Ingel20196_MGFPeaklist (F058870)	Alpha-enolase OS=Gallus gallus GN=ENO1 PE=3 SV=2	F1NZ78_CHICK,UPI0000ECA2FD,UPI0002C86C58	TAX_GallusGallus_9031_20141114.fasta,UPARC14_2_GallusG_9031_20140417.fasta	47,334.10	100.00%	18	21	68	0.14%	47.00%	IGAEVYHNLK	R	N	99.70%	48.7	44.9	29	26.5	38.3	2.47	0.292	0.45	0	4	0	0	2	1,143.62	1,769.50		4,096.00	184	193	Empty	TRUE	
20196-20225 HinckeM –TAXchicken	Uncategorized Sample	20196-20205_A	Mudpit_Ingel20196_MGFPeaklist (F058870)	Alpha-enolase OS=Gallus gallus GN=ENO1 PE=3 SV=2	F1NZ78_CHICK,UPI0000ECA2FD,UPI0002C86C58	TAX_GallusGallus_9031_20141114.fasta,UPARC14_2_GallusG_9031_20140417.fasta	47,334.10	100.00%	18	21	68	0.14%	47.00%	LAMQEFMILPVGADNFK	K	E	99.70%	41.8	44.3	21.8	35.9	40.3	4.62	0.444	0.382	0	0	2	0	2	1,955.96	2,738.50		2,626.63	163	179	Empty	TRUE	
20196-20225 HinckeM –TAXchicken	Uncategorized Sample	20196-20205_A	Mudpit_Ingel20196_MGFPeaklist (F058870)	Alpha-enolase OS=Gallus gallus GN=ENO1 PE=3 SV=2	F1NZ78_CHICK,UPI0000ECA2FD,UPI0002C86C58	TAX_GallusGallus_9031_20141114.fasta,UPARC14_2_GallusG_9031_20140417.fasta	47,334.10	100.00%	18	21	68	0.14%	47.00%	LAQSNGWGVMVSHR	K	S	99.40%	37.2	44.4	19.4	29.2	26.5	1.11	0.223	0.214	0	0	4	0	2	1,558.74	1,898.00		2,638.81	359	372	Empty	TRUE	
20196-20225 HinckeM –TAXchicken	Uncategorized Sample	20196-20205_A	Mudpit_Ingel20196_MGFPeaklist (F058870)	Alpha-enolase OS=Gallus gallus GN=ENO1 PE=3 SV=2	F1NZ78_CHICK,UPI0000ECA2FD,UPI0002C86C58	TAX_GallusGallus_9031_20141114.fasta,UPARC14_2_GallusG_9031_20140417.fasta	47,334.10	100.00%	18	21	68	0.14%	47.00%	NVNVVEQEKIDK	K	L	35.00%	17.7	44.3	20.6	1.23					0	0	2	0	2	1,414.75	1,699.50		1,351.85	81	92	Empty	TRUE	
20196-20225 HinckeM –TAXchicken	Uncategorized Sample	20196-20205_A	Mudpit_Ingel20196_MGFPeaklist (F058870)	Alpha-enolase OS=Gallus gallus GN=ENO1 PE=3 SV=2	F1NZ78_CHICK,UPI0000ECA2FD,UPI0002C86C58	TAX_GallusGallus_9031_20141114.fasta,UPARC14_2_GallusG_9031_20140417.fasta	47,334.10	100.00%	18	21	68	0.14%	47.00%	NVNVVEQEKIDKLMLEMDGTENK	K	S	81.90%	20.6	43.9	14.1	18.3					0	0	0	1	2	2,708.31	2,118.00		741.65	81	103	Empty	TRUE	
20196-20225 HinckeM –TAXchicken	Uncategorized Sample	20196-20205_A	Mudpit_Ingel20196_MGFPeaklist (F058870)	Alpha-enolase OS=Gallus gallus GN=ENO1 PE=3 SV=2	F1NZ78_CHICK,UPI0000ECA2FD,UPI0002C86C58	TAX_GallusGallus_9031_20141114.fasta,UPARC14_2_GallusG_9031_20140417.fasta	47,334.10	100.00%	18	21	68	0.14%	47.00%	SCNCLLLK	K	V	82.80%	29.3	44.5	27.2	16					0	5	0	0	2	1,007.50	2,039.00		4,903.86	336	343	Empty	TRUE	
20196-20225 HinckeM –TAXchicken	Uncategorized Sample	20196-20205_A	Mudpit_Ingel20196_MGFPeaklist (F058870)	Alpha-enolase OS=Gallus gallus GN=ENO1 PE=3 SV=2	F1NZ78_CHICK,UPI0000ECA2FD,UPI0002C86C58	TAX_GallusGallus_9031_20141114.fasta,UPARC14_2_GallusG_9031_20140417.fasta	47,334.10	100.00%	18	21	68	0.14%	47.00%	TIAPALISK	K	N	99.70%	45	42.2	18.3	32.2	39.6	3.22	0.343	0.611	0	9	0	0	2	913.5718	2,044.00		32,337.00	72	80	Empty	TRUE	
20196-20225 HinckeM –TAXchicken	Uncategorized Sample	20196-20205_A	Mudpit_Ingel20196_MGFPeaklist (F058870)	Alpha-enolase OS=Gallus gallus GN=ENO1 PE=3 SV=2	F1NZ78_CHICK,UPI0000ECA2FD,UPI0002C86C58	TAX_GallusGallus_9031_20141114.fasta,UPARC14_2_GallusG_9031_20140417.fasta	47,334.10	100.00%	18	21	68	0.14%	47.00%	VNQIGSVTESLQACK	K	L	99.70%	81.1	44.8	28.6	0	42.2	4.24	0.417	0.4	0	4	1	0	2	1,633.82	2,130.00		13,218.20	344	358	Empty	TRUE	
20196-20225 HinckeM –TAXchicken	Uncategorized Sample	20196-20205_A	Mudpit_Ingel20196_MGFPeaklist (F058870)	Alpha-enolase OS=Gallus gallus GN=ENO1 PE=3 SV=2	F1NZ78_CHICK,UPI0000ECA2FD,UPI0002C86C58	TAX_GallusGallus_9031_20141114.fasta,UPARC14_2_GallusG_9031_20140417.fasta	47,334.10	100.00%	18	21	68	0.14%	47.00%	VVIGMDVAASEFYR	K	D	99.70%	74	44.6	14.6	63.3	43.8	3.96	0.224	0.429	0	2	0	0	2	1,572.77	2,354.00		2,837.46	240	253	Empty	TRUE	
20196-20225 HinckeM –TAXchicken	Uncategorized Sample	20196-20205_A	Mudpit_Ingel20196_MGFPeaklist (F058870)	Alpha-enolase OS=Gallus gallus GN=ENO1 PE=3 SV=2	F1NZ78_CHICK,UPI0000ECA2FD,UPI0002C86C58	TAX_GallusGallus_9031_20141114.fasta,UPARC14_2_GallusG_9031_20140417.fasta	47,334.10	100.00%	18	21	68	0.14%	47.00%	YGKDATNVGDEGGFAPNILENK	K	E	99.70%	47.5	44.1	13.2	0	43.4	4.74	0.399	0.318	0	0	2	0	2	2,309.10	2,329.50		2,540.55	200	221	Empty	TRUE	
20196-20225 HinckeM –TAXchicken	Uncategorized Sample	20196-20205_A	Mudpit_Ingel20196_MGFPeaklist (F058870)	Alpha-enolase OS=Gallus gallus GN=ENO1 PE=3 SV=2	F1NZ78_CHICK,UPI0000ECA2FD,UPI0002C86C58	TAX_GallusGallus_9031_20141114.fasta,UPARC14_2_GallusG_9031_20140417.fasta	47,334.10	100.00%	18	21	68	0.14%	47.00%	YISPDQLADLYK	R	G	99.70%	70.2	44.3	20.5	0	47.7	3.92	0.47	0.542	0	6	0	0	2	1,425.73	2,536.50		20,864.30	270	281	Empty	TRUE	
20196-20225 HinckeM –TAXchicken	Uncategorized Sample	20196-20205_A	Mudpit_Ingel20196_MGFPeaklist (F058870)	Alpha-enolase OS=Gallus gallus GN=ENO1 PE=3 SV=2	F1NZ78_CHICK,UPI0000ECA2FD,UPI0002C86C58	TAX_GallusGallus_9031_20141114.fasta,UPARC14_2_GallusG_9031_20140417.fasta	47,334.10	100.00%	18	21	68	0.14%	47.00%	AAVPSGASTGIYEALELRDNDKTR	R	Y	99.70%	42.8	43.5	0	42.8	38.4	3.72	0.372	0.292	0	0	0	3	2	2,534.28	2,397.00		16,739.40	33	56	Empty	TRUE	
20196-20225 HinckeM –TAXchicken	Uncategorized Sample	20196-20205_A	Mudpit_Ingel20196_MGFPeaklist (F058870)	Alpha-enolase OS=Gallus gallus GN=ENO1 PE=3 SV=2	F1NZ78_CHICK,UPI0000ECA2FD,UPI0002C86C58	TAX_GallusGallus_9031_20141114.fasta,UPARC14_2_GallusG_9031_20140417.fasta	47,334.10	100.00%	18	21	68	0.14%	47.00%	DATNVGDEGGFAPNILENK	K	E	99.70%	66.7	43.9	15.4	57	45.6	4.23	0.408	0.421	0	0	4	0	2	1,960.92	2,402.50		8,502.20	203	221	Empty	TRUE	
20196-20225 HinckeM –TAXchicken	Uncategorized Sample	20196-20205_A	Mudpit_Ingel20196_MGFPeaklist (F058870)	Alpha-enolase OS=Gallus gallus GN=ENO1 PE=3 SV=2	F1NZ78_CHICK,UPI0000ECA2FD,UPI0002C86C58	TAX_GallusGallus_9031_20141114.fasta,UPARC14_2_GallusG_9031_20140417.fasta	47,334.10	100.00%	18	21	68	0.14%	47.00%	DATNVGDEGGFAPNILENKEALELLK	K	T	99.70%	29.9	43.2	13	29.5	33.8	1.74	0.178	0.212	0	0	1	0	2	2,757.39	2,858.00		1,959.71	203	228	Empty	TRUE	
20196-20225 HinckeM –TAXchicken	Uncategorized Sample	20196-20205_A	Mudpit_Ingel20196_MGFPeaklist (F058870)	Alpha-enolase OS=Gallus gallus GN=ENO1 PE=3 SV=2	F1NZ78_CHICK,UPI0000ECA2FD,UPI0002C86C58	TAX_GallusGallus_9031_20141114.fasta,UPARC14_2_GallusG_9031_20140417.fasta	47,334.10	100.00%	18	21	68	0.14%	47.00%	DGKYDLDFKSPDDPSR	R	Y	99.70%	52.7	43.5	19.6	38.4	41.3	5.44	0.494	0.375	0	0	3	3	2	1,854.85	1,992.50		11,237.20	254	269	Empty	TRUE	
20196-20225 HinckeM –TAXchicken	Uncategorized Sample	20196-20205_A	Mudpit_Ingel20196_MGFPeaklist (F058870)	Alpha-enolase OS=Gallus gallus GN=ENO1 PE=3 SV=2	F1NZ78_CHICK,UPI0000ECA2FD,UPI0002C86C58	TAX_GallusGallus_9031_20141114.fasta,UPARC14_2_GallusG_9031_20140417.fasta	47,334.10	100.00%	18	21	68	0.14%	47.00%	EALELLK	K	T	74.20%	54.6	44.3	56	0					0	5	0	0	2	815.4874	2,221.00		7,201.26	222	228	Empty	TRUE	
20196-20225 HinckeM –TAXchicken	Uncategorized Sample	20196-20205_A	Mudpit_Ingel20196_MGFPeaklist (F058870)	Alpha-enolase OS=Gallus gallus GN=ENO1 PE=3 SV=2	F1NZ78_CHICK,UPI0000ECA2FD,UPI0002C86C58	TAX_GallusGallus_9031_20141114.fasta,UPARC14_2_GallusG_9031_20140417.fasta	47,334.10	100.00%	18	21	68	0.14%	47.00%	GNPTVEVDLYTNK	R	G	99.70%	63.5	44.8	18.6	58	44.1	5.92	0.506	0.423	0	6	0	0	2	1,449.72	2,113.50		10,158.60	16	28	Empty	TRUE	
20196-20225 HinckeM –TAXchicken	Uncategorized Sample	20196-20205_A	Mudpit_Ingel20196_MGFPeaklist (F058870)	Alpha-enolase OS=Gallus gallus GN=ENO1 PE=3 SV=2	F1NZ78_CHICK,UPI0000ECA2FD,UPI0002C86C58	TAX_GallusGallus_9031_20141114.fasta,UPARC14_2_GallusG_9031_20140417.fasta	47,334.10	100.00%	18	21	68	0.14%	47.00%	IDKLMLEMDGTENK	K	S	29.70%	15	43.8	24.8	4.25					0	0	1	0	2	1,668.78	1,807.00		546.6	90	103	Empty	TRUE	
20196-20225 HinckeM –TAXchicken	Uncategorized Sample	20196-20205_A	Mudpit_Ingel20196_MGFPeaklist (F058870)	Alpha-enolase OS=Gallus gallus GN=ENO1 PE=3 SV=2	F1NZ78_CHICK,UPI0000ECA2FD,UPI0002C86C58	TAX_GallusGallus_9031_20141114.fasta,UPARC14_2_GallusG_9031_20140417.fasta	47,334.10	100.00%	18	21	68	0.14%	47.00%	IGAEVYHNLK	R	N	99.70%	48.7	44.9	29	26.5	38.3	2.47	0.292	0.45	0	4	0	0	2	1,143.62	1,769.50		4,096.00	184	193	Empty	TRUE	
20196-20225 HinckeM –TAXchicken	Uncategorized Sample	20196-20205_A	Mudpit_Ingel20196_MGFPeaklist (F058870)	Alpha-enolase OS=Gallus gallus GN=ENO1 PE=3 SV=2	F1NZ78_CHICK,UPI0000ECA2FD,UPI0002C86C58	TAX_GallusGallus_9031_20141114.fasta,UPARC14_2_GallusG_9031_20140417.fasta	47,334.10	100.00%	18	21	68	0.14%	47.00%	LAMQEFMILPVGADNFK	K	E	99.70%	41.8	44.3	21.8	35.9	40.3	4.62	0.444	0.382	0	0	2	0	2	1,955.96	2,738.50		2,626.63	163	179	Empty	TRUE	
20196-20225 HinckeM –TAXchicken	Uncategorized Sample	20196-20205_A	Mudpit_Ingel20196_MGFPeaklist (F058870)	Alpha-enolase OS=Gallus gallus GN=ENO1 PE=3 SV=2	F1NZ78_CHICK,UPI0000ECA2FD,UPI0002C86C58	TAX_GallusGallus_9031_20141114.fasta,UPARC14_2_GallusG_9031_20140417.fasta	47,334.10	100.00%	18	21	68	0.14%	47.00%	LAQSNGWGVMVSHR	K	S	99.40%	37.2	44.4	19.4	29.2	26.5	1.11	0.223	0.214	0	0	4	0	2	1,558.74	1,898.00		2,638.81	359	372	Empty	TRUE	
20196-20225 HinckeM –TAXchicken	Uncategorized Sample	20196-20205_A	Mudpit_Ingel20196_MGFPeaklist (F058870)	Alpha-enolase OS=Gallus gallus GN=ENO1 PE=3 SV=2	F1NZ78_CHICK,UPI0000ECA2FD,UPI0002C86C58	TAX_GallusGallus_9031_20141114.fasta,UPARC14_2_GallusG_9031_20140417.fasta	47,334.10	100.00%	18	21	68	0.14%	47.00%	NVNVVEQEKIDK	K	L	35.00%	17.7	44.3	20.6	1.23					0	0	2	0	2	1,414.75	1,699.50		1,351.85	81	92	Empty	TRUE	
20196-20225 HinckeM –TAXchicken	Uncategorized Sample	20196-20205_A	Mudpit_Ingel20196_MGFPeaklist (F058870)	Alpha-enolase OS=Gallus gallus GN=ENO1 PE=3 SV=2	F1NZ78_CHICK,UPI0000ECA2FD,UPI0002C86C58	TAX_GallusGallus_9031_20141114.fasta,UPARC14_2_GallusG_9031_20140417.fasta	47,334.10	100.00%	18	21	68	0.14%	47.00%	NVNVVEQEKIDKLMLEMDGTENK	K	S	81.90%	20.6	43.9	14.1	18.3					0	0	0	1	2	2,708.31	2,118.00		741.65	81	103	Empty	TRUE	
20196-20225 HinckeM –TAXchicken	Uncategorized Sample	20196-20205_A	Mudpit_Ingel20196_MGFPeaklist (F058870)	Alpha-enolase OS=Gallus gallus GN=ENO1 PE=3 SV=2	F1NZ78_CHICK,UPI0000ECA2FD,UPI0002C86C58	TAX_GallusGallus_9031_20141114.fasta,UPARC14_2_GallusG_9031_20140417.fasta	47,334.10	100.00%	18	21	68	0.14%	47.00%	SCNCLLLK	K	V	82.80%	29.3	44.5	27.2	16					0	5	0	0	2	1,007.50	2,039.00		4,903.86	336	343	Empty	TRUE	
20196-20225 HinckeM –TAXchicken	Uncategorized Sample	20196-20205_A	Mudpit_Ingel20196_MGFPeaklist (F058870)	Alpha-enolase OS=Gallus gallus GN=ENO1 PE=3 SV=2	F1NZ78_CHICK,UPI0000ECA2FD,UPI0002C86C58	TAX_GallusGallus_9031_20141114.fasta,UPARC14_2_GallusG_9031_20140417.fasta	47,334.10	100.00%	18	21	68	0.14%	47.00%	TIAPALISK	K	N	99.70%	45	42.2	18.3	32.2	39.6	3.22	0.343	0.611	0	9	0	0	2	913.5718	2,044.00		32,337.00	72	80	Empty	TRUE	
20196-20225 HinckeM –TAXchicken	Uncategorized Sample	20196-20205_A	Mudpit_Ingel20196_MGFPeaklist (F058870)	Alpha-enolase OS=Gallus gallus GN=ENO1 PE=3 SV=2	F1NZ78_CHICK,UPI0000ECA2FD,UPI0002C86C58	TAX_GallusGallus_9031_20141114.fasta,UPARC14_2_GallusG_9031_20140417.fasta	47,334.10	100.00%	18	21	68	0.14%	47.00%	VNQIGSVTESLQACK	K	L	99.70%	81.1	44.8	28.6	0	42.2	4.24	0.417	0.4	0	4	1	0	2	1,633.82	2,130.00		13,218.20	344	358	Empty	TRUE	
20196-20225 HinckeM –TAXchicken	Uncategorized Sample	20196-20205_A	Mudpit_Ingel20196_MGFPeaklist (F058870)	Alpha-enolase OS=Gallus gallus GN=ENO1 PE=3 SV=2	F1NZ78_CHICK,UPI0000ECA2FD,UPI0002C86C58	TAX_GallusGallus_9031_20141114.fasta,UPARC14_2_GallusG_9031_20140417.fasta	47,334.10	100.00%	18	21	68	0.14%	47.00%	VVIGMDVAASEFYR	K	D	99.70%	74	44.6	14.6	63.3	43.8	3.96	0.224	0.429	0	2	0	0	2	1,572.77	2,354.00		2,837.46	240	253	Empty	TRUE	
20196-20225 HinckeM –TAXchicken	Uncategorized Sample	20196-20205_A	Mudpit_Ingel20196_MGFPeaklist (F058870)	Alpha-enolase OS=Gallus gallus GN=ENO1 PE=3 SV=2	F1NZ78_CHICK,UPI0000ECA2FD,UPI0002C86C58	TAX_GallusGallus_9031_20141114.fasta,UPARC14_2_GallusG_9031_20140417.fasta	47,334.10	100.00%	18	21	68	0.14%	47.00%	YGKDATNVGDEGGFAPNILENK	K	E	99.70%	47.5	44.1	13.2	0	43.4	4.74	0.399	0.318	0	0	2	0	2	2,309.10	2,329.50		2,540.55	200	221	Empty	TRUE	
20196-20225 HinckeM –TAXchicken	Uncategorized Sample	20196-20205_A	Mudpit_Ingel20196_MGFPeaklist (F058870)	Alpha-enolase OS=Gallus gallus GN=ENO1 PE=3 SV=2	F1NZ78_CHICK,UPI0000ECA2FD,UPI0002C86C58	TAX_GallusGallus_9031_20141114.fasta,UPARC14_2_GallusG_9031_20140417.fasta	47,334.10	100.00%	18	21	68	0.14%	47.00%	YISPDQLADLYK	R	G	99.70%	70.2	44.3	20.5	0	47.7	3.92	0.47	0.542	0	6	0	0	2	1,425.73	2,536.50		20,864.30	270	281	Empty	TRUE	
20196-20225 HinckeM –TAXchicken	Uncategorized Sample	20196-20205_A	Mudpit_Ingel20196_MGFPeaklist (F058870)	Alpha-enolase OS=Gallus gallus GN=ENO1 PE=3 SV=2	F1NZ78_CHICK,UPI0000ECA2FD,UPI0002C86C58	TAX_GallusGallus_9031_20141114.fasta,UPARC14_2_GallusG_9031_20140417.fasta	47,334.10	100.00%	18	21	68	0.14%	47.00%	AAVPSGASTGIYEALELRDNDKTR	R	Y	99.70%	42.8	43.5	0	42.8	38.4	3.72	0.372	0.292	0	0	0	3	2	2,534.28	2,397.00		16,739.40	33	56	Empty	TRUE	
20196-20225 HinckeM –TAXchicken	Uncategorized Sample	20196-20205_A	Mudpit_Ingel20196_MGFPeaklist (F058870)	Alpha-enolase OS=Gallus gallus GN=ENO1 PE=3 SV=2	F1NZ78_CHICK,UPI0000ECA2FD,UPI0002C86C58	TAX_GallusGallus_9031_20141114.fasta,UPARC14_2_GallusG_9031_20140417.fasta	47,334.10	100.00%	18	21	68	0.14%	47.00%	DATNVGDEGGFAPNILENK	K	E	99.70%	66.7	43.9	15.4	57	45.6	4.23	0.408	0.421	0	0	4	0	2	1,960.92	2,402.50		8,502.20	203	221	Empty	TRUE	
20196-20225 HinckeM –TAXchicken	Uncategorized Sample	20196-20205_A	Mudpit_Ingel20196_MGFPeaklist (F058870)	Alpha-enolase OS=Gallus gallus GN=ENO1 PE=3 SV=2	F1NZ78_CHICK,UPI0000ECA2FD,UPI0002C86C58	TAX_GallusGallus_9031_20141114.fasta,UPARC14_2_GallusG_9031_20140417.fasta	47,334.10	100.00%	18	21	68	0.14%	47.00%	DATNVGDEGGFAPNILENKEALELLK	K	T	99.70%	29.9	43.2	13	29.5	33.8	1.74	0.178	0.212	0	0	1	0	2	2,757.39	2,858.00		1,959.71	203	228	Empty	TRUE	
20196-20225 HinckeM –TAXchicken	Uncategorized Sample	20196-20205_A	Mudpit_Ingel20196_MGFPeaklist (F058870)	Alpha-enolase OS=Gallus gallus GN=ENO1 PE=3 SV=2	F1NZ78_CHICK,UPI0000ECA2FD,UPI0002C86C58	TAX_GallusGallus_9031_20141114.fasta,UPARC14_2_GallusG_9031_20140417.fasta	47,334.10	100.00%	18	21	68	0.14%	47.00%	DGKYDLDFKSPDDPSR	R	Y	99.70%	52.7	43.5	19.6	38.4	41.3	5.44	0.494	0.375	0	0	3	3	2	1,854.85	1,992.50		11,237.20	254	269	Empty	TRUE	
20196-20225 HinckeM –TAXchicken	Uncategorized Sample	20196-20205_A	Mudpit_Ingel20196_MGFPeaklist (F058870)	Alpha-enolase OS=Gallus gallus GN=ENO1 PE=3 SV=2	F1NZ78_CHICK,UPI0000ECA2FD,UPI0002C86C58	TAX_GallusGallus_9031_20141114.fasta,UPARC14_2_GallusG_9031_20140417.fasta	47,334.10	100.00%	18	21	68	0.14%	47.00%	EALELLK	K	T	74.20%	54.6	44.3	56	0					0	5	0	0	2	815.4874	2,221.00		7,201.26	222	228	Empty	TRUE	
20196-20225 HinckeM –TAXchicken	Uncategorized Sample	20196-20205_A	Mudpit_Ingel20196_MGFPeaklist (F058870)	Alpha-enolase OS=Gallus gallus GN=ENO1 PE=3 SV=2	F1NZ78_CHICK,UPI0000ECA2FD,UPI0002C86C58	TAX_GallusGallus_9031_20141114.fasta,UPARC14_2_GallusG_9031_20140417.fasta	47,334.10	100.00%	18	21	68	0.14%	47.00%	GNPTVEVDLYTNK	R	G	99.70%	63.5	44.8	18.6	58	44.1	5.92	0.506	0.423	0	6	0	0	2	1,449.72	2,113.50		10,158.60	16	28	Empty	TRUE	
20196-20225 HinckeM –TAXchicken	Uncategorized Sample	20196-20205_A	Mudpit_Ingel20196_MGFPeaklist (F058870)	Alpha-enolase OS=Gallus gallus GN=ENO1 PE=3 SV=2	F1NZ78_CHICK,UPI0000ECA2FD,UPI0002C86C58	TAX_GallusGallus_9031_20141114.fasta,UPARC14_2_GallusG_9031_20140417.fasta	47,334.10	100.00%	18	21	68	0.14%	47.00%	IDKLMLEMDGTENK	K	S	29.70%	15	43.8	24.8	4.25					0	0	1	0	2	1,668.78	1,807.00		546.6	90	103	Empty	TRUE	
20196-20225 HinckeM –TAXchicken	Uncategorized Sample	20196-20205_A	Mudpit_Ingel20196_MGFPeaklist (F058870)	Alpha-enolase OS=Gallus gallus GN=ENO1 PE=3 SV=2	F1NZ78_CHICK,UPI0000ECA2FD,UPI0002C86C58	TAX_GallusGallus_9031_20141114.fasta,UPARC14_2_GallusG_9031_20140417.fasta	47,334.10	100.00%	18	21	68	0.14%	47.00%	IGAEVYHNLK	R	N	99.70%	48.7	44.9	29	26.5	38.3	2.47	0.292	0.45	0	4	0	0	2	1,143.62	1,769.50		4,096.00	184	193	Empty	TRUE	
20196-20225 HinckeM –TAXchicken	Uncategorized Sample	20196-20205_A	Mudpit_Ingel20196_MGFPeaklist (F058870)	Alpha-enolase OS=Gallus gallus GN=ENO1 PE=3 SV=2	F1NZ78_CHICK,UPI0000ECA2FD,UPI0002C86C58	TAX_GallusGallus_9031_20141114.fasta,UPARC14_2_GallusG_9031_20140417.fasta	47,334.10	100.00%	18	21	68	0.14%	47.00%	LAMQEFMILPVGADNFK	K	E	99.70%	41.8	44.3	21.8	35.9	40.3	4.62	0.444	0.382	0	0	2	0	2	1,955.96	2,738.50		2,626.63	163	179	Empty	TRUE	
20196-20225 HinckeM –TAXchicken	Uncategorized Sample	20196-20205_A	Mudpit_Ingel20196_MGFPeaklist (F058870)	Alpha-enolase OS=Gallus gallus GN=ENO1 PE=3 SV=2	F1NZ78_CHICK,UPI0000ECA2FD,UPI0002C86C58	TAX_GallusGallus_9031_20141114.fasta,UPARC14_2_GallusG_9031_20140417.fasta	47,334.10	100.00%	18	21	68	0.14%	47.00%	LAQSNGWGVMVSHR	K	S	99.40%	37.2	44.4	19.4	29.2	26.5	1.11	0.223	0.214	0	0	4	0	2	1,558.74	1,898.00		2,638.81	359	372	Empty	TRUE	
20196-20225 HinckeM –TAXchicken	Uncategorized Sample	20196-20205_A	Mudpit_Ingel20196_MGFPeaklist (F058870)	Alpha-enolase OS=Gallus gallus GN=ENO1 PE=3 SV=2	F1NZ78_CHICK,UPI0000ECA2FD,UPI0002C86C58	TAX_GallusGallus_9031_20141114.fasta,UPARC14_2_GallusG_9031_20140417.fasta	47,334.10	100.00%	18	21	68	0.14%	47.00%	NVNVVEQEKIDK	K	L	35.00%	17.7	44.3	20.6	1.23					0	0	2	0	2	1,414.75	1,699.50		1,351.85	81	92	Empty	TRUE	
20196-20225 HinckeM –TAXchicken	Uncategorized Sample	20196-20205_A	Mudpit_Ingel20196_MGFPeaklist (F058870)	Alpha-enolase OS=Gallus gallus GN=ENO1 PE=3 SV=2	F1NZ78_CHICK,UPI0000ECA2FD,UPI0002C86C58	TAX_GallusGallus_9031_20141114.fasta,UPARC14_2_GallusG_9031_20140417.fasta	47,334.10	100.00%	18	21	68	0.14%	47.00%	NVNVVEQEKIDKLMLEMDGTENK	K	S	81.90%	20.6	43.9	14.1	18.3					0	0	0	1	2	2,708.31	2,118.00		741.65	81	103	Empty	TRUE	
20196-20225 HinckeM –TAXchicken	Uncategorized Sample	20196-20205_A	Mudpit_Ingel20196_MGFPeaklist (F058870)	Alpha-enolase OS=Gallus gallus GN=ENO1 PE=3 SV=2	F1NZ78_CHICK,UPI0000ECA2FD,UPI0002C86C58	TAX_GallusGallus_9031_20141114.fasta,UPARC14_2_GallusG_9031_20140417.fasta	47,334.10	100.00%	18	21	68	0.14%	47.00%	SCNCLLLK	K	V	82.80%	29.3	44.5	27.2	16					0	5	0	0	2	1,007.50	2,039.00		4,903.86	336	343	Empty	TRUE	
20196-20225 HinckeM –TAXchicken	Uncategorized Sample	20196-20205_A	Mudpit_Ingel20196_MGFPeaklist (F058870)	Alpha-enolase OS=Gallus gallus GN=ENO1 PE=3 SV=2	F1NZ78_CHICK,UPI0000ECA2FD,UPI0002C86C58	TAX_GallusGallus_9031_20141114.fasta,UPARC14_2_GallusG_9031_20140417.fasta	47,334.10	100.00%	18	21	68	0.14%	47.00%	TIAPALISK	K	N	99.70%	45	42.2	18.3	32.2	39.6	3.22	0.343	0.611	0	9	0	0	2	913.5718	2,044.00		32,337.00	72	80	Empty	TRUE	
20196-20225 HinckeM –TAXchicken	Uncategorized Sample	20196-20205_A	Mudpit_Ingel20196_MGFPeaklist (F058870)	Alpha-enolase OS=Gallus gallus GN=ENO1 PE=3 SV=2	F1NZ78_CHICK,UPI0000ECA2FD,UPI0002C86C58	TAX_GallusGallus_9031_20141114.fasta,UPARC14_2_GallusG_9031_20140417.fasta	47,334.10	100.00%	18	21	68	0.14%	47.00%	VNQIGSVTESLQACK	K	L	99.70%	81.1	44.8	28.6	0	42.2	4.24	0.417	0.4	0	4	1	0	2	1,633.82	2,130.00		13,218.20	344	358	Empty	TRUE	
20196-20225 HinckeM –TAXchicken	Uncategorized Sample	20196-20205_A	Mudpit_Ingel20196_MGFPeaklist (F058870)	Alpha-enolase OS=Gallus gallus GN=ENO1 PE=3 SV=2	F1NZ78_CHICK,UPI0000ECA2FD,UPI0002C86C58	TAX_GallusGallus_9031_20141114.fasta,UPARC14_2_GallusG_9031_20140417.fasta	47,334.10	100.00%	18	21	68	0.14%	47.00%	VVIGMDVAASEFYR	K	D	99.70%	74	44.6	14.6	63.3	43.8	3.96	0.224	0.429	0	2	0	0	2	1,572.77	2,354.00		2,837.46	240	253	Empty	TRUE	
20196-20225 HinckeM –TAXchicken	Uncategorized Sample	20196-20205_A	Mudpit_Ingel20196_MGFPeaklist (F058870)	Alpha-enolase OS=Gallus gallus GN=ENO1 PE=3 SV=2	F1NZ78_CHICK,UPI0000ECA2FD,UPI0002C86C58	TAX_GallusGallus_9031_20141114.fasta,UPARC14_2_GallusG_9031_20140417.fasta	47,334.10	100.00%	18	21	68	0.14%	47.00%	YGKDATNVGDEGGFAPNILENK	K	E	99.70%	47.5	44.1	13.2	0	43.4	4.74	0.399	0.318	0	0	2	0	2	2,309.10	2,329.50		2,540.55	200	221	Empty	TRUE	
20196-20225 HinckeM –TAXchicken	Uncategorized Sample	20196-20205_A	Mudpit_Ingel20196_MGFPeaklist (F058870)	Alpha-enolase OS=Gallus gallus GN=ENO1 PE=3 SV=2	F1NZ78_CHICK,UPI0000ECA2FD,UPI0002C86C58	TAX_GallusGallus_9031_20141114.fasta,UPARC14_2_GallusG_9031_20140417.fasta	47,334.10	100.00%	18	21	68	0.14%	47.00%	YISPDQLADLYK	R	G	99.70%	70.2	44.3	20.5	0	47.7	3.92	0.47	0.542	0	6	0	0	2	1,425.73	2,536.50		20,864.30	270	281	Empty	TRUE	
20196-20225 HinckeM –TAXchicken	Uncategorized Sample	20196-20205_A	Mudpit_Ingel20196_MGFPeaklist (F058870)	Hemoglobin subunit epsilon OS=Gallus gallus GN=HBE PE=1 SV=2	HBE_CHICK	TAX_GallusGallus_9031_20141114.fasta	16,603.30	100.00%	8	10	30	0.06%	83.70%	DFTPACQFAWQK	R	L	99.70%	37.2	42.9	20.1	17.2	43.6	5.37	0.429	0.583	0	2	0	0	2	1,498.68	2,295.50		208153	122	133	Empty	TRUE	
20196-20225 HinckeM –TAXchicken	Uncategorized Sample	20196-20205_A	Mudpit_Ingel20196_MGFPeaklist (F058870)	Hemoglobin subunit epsilon OS=Gallus gallus GN=HBE PE=1 SV=2	HBE_CHICK	TAX_GallusGallus_9031_20141114.fasta	16,603.30	100.00%	8	10	30	0.06%	83.70%	FFASFGNLSSPTAIMGNPR	R	V	99.70%	64.9	44.2	13.8	63.2	39.1	3.77	0.414	0.316	0	1	2	0	2	2,013.99	2,567.00		209584	42	60	Empty	TRUE	
20196-20225 HinckeM –TAXchicken	Uncategorized Sample	20196-20205_A	Mudpit_Ingel20196_MGFPeaklist (F058870)	Hemoglobin subunit epsilon OS=Gallus gallus GN=HBE PE=1 SV=2	HBE_CHICK	TAX_GallusGallus_9031_20141114.fasta	16,603.30	100.00%	8	10	30	0.06%	83.70%	KVLSSFGEAVK	K	N	99.70%	67.4	43.3	37.4	42.5	47.1	4.59	0.34	0.636	0	7	0	0	2	1,164.66	1,909.00		10,064.40	67	77	Empty	FALSE	HBRH_CHICK,Q90864_CHICK
20196-20225 HinckeM –TAXchicken	Uncategorized Sample	20196-20205_A	Mudpit_Ingel20196_MGFPeaklist (F058870)	Hemoglobin subunit epsilon OS=Gallus gallus GN=HBE PE=1 SV=2	HBE_CHICK	TAX_GallusGallus_9031_20141114.fasta	16,603.30	100.00%	8	10	30	0.06%	83.70%	LHCDKLHVDPENFR	E	L	99.70%	62.2	44.6	15.6	57	41.2	4.62	0.427	0.429	0	1	1	12	1	1,779.86	1,883.50		5643460	92	105	Empty	FALSE	HBB_CHICK,Q90864_CHICK
20196-20225 HinckeM –TAXchicken	Uncategorized Sample	20196-20205_A	Mudpit_Ingel20196_MGFPeaklist (F058870)	Hemoglobin subunit epsilon OS=Gallus gallus GN=HBE PE=1 SV=2	HBE_CHICK	TAX_GallusGallus_9031_20141114.fasta	16,603.30	100.00%	8	10	30	0.06%	83.70%	LHVDPENFR	K	L	99.70%	40.5	43.8	28.9	24.1	35.8	3.35	0.419	0.556	0	16	0	0	2	1,126.56	1,945.00		2591890	97	105	Empty	FALSE	HBB_CHICK,HBRH_CHICK,Q90864_CHICK

20196-20225 HinckeM –TAXchicken	Uncategorized Sample	20196-20205_A	Mudpit_Ingel20196_MGFPeaklist (F058870)	Hemoglobin subunit epsilon OS=Gallus gallus GN=HBE PE=1 SV=2	HBE_CHICK	TAX_GallusGallus_9031_20141114.fasta	16,603.30	100.00%	8	10	30	0.06%	83.70%	LLGDILIIVLASHFAR	R	D	99.70%	64.7	36.2	28.7	37.2	53	5.6	0.443	0.5	0	0	1	0	2	1,751.06	2,935.00		20,476.40	106	121	Empty	TRUE	
20196-20225 HinckeM –TAXchicken	Uncategorized Sample	20196-20205_A	Mudpit_Ingel20196_MGFPeaklist (F058870)	Hemoglobin subunit epsilon OS=Gallus gallus GN=HBE PE=1 SV=2	HBE_CHICK	TAX_GallusGallus_9031_20141114.fasta	16,603.30	100.00%	8	10	30	0.06%	83.70%	LLIVYPWTQR	R	F	99.70%	64.1	43.6	25.4	48.8	44.4	3.38	0.351	0.6	0	78	0	0	2	1,288.74	2,774.00		4.25E+07	32	41	Empty	FALSE	HBB_CHICK,HBRH_CHICK,Q90864_CHICK
20196-20225 HinckeM –TAXchicken	Uncategorized Sample	20196-20205_A	Mudpit_Ingel20196_MGFPeaklist (F058870)	Hemoglobin subunit epsilon OS=Gallus gallus GN=HBE PE=1 SV=2	HBE_CHICK	TAX_GallusGallus_9031_20141114.fasta	16,603.30	100.00%	8	10	30	0.06%	83.70%	LSELHCDKLHVDPENFR	K	L	99.70%	64.6	44.3	15.9	58.2	37.3	3.03	0.0322	0.324	0	0	0	2	2	2,109.02	2,096.00		250383	89	105	Empty	FALSE	HBB_CHICK
20196-20225 HinckeM –TAXchicken	Uncategorized Sample	20196-20205_A	Mudpit_Ingel20196_MGFPeaklist (F058870)	Hemoglobin subunit epsilon OS=Gallus gallus GN=HBE PE=1 SV=2	HBE_CHICK	TAX_GallusGallus_9031_20141114.fasta	16,603.30	100.00%	8	10	30	0.06%	83.70%	LVNVVAHALAR	K	K	99.70%	50.5	41.2	19.2	43.6	38.3	3.07	0.423	0.5	0	2	0	0	2	1,162.71	2,140.00		91,800.40	134	144	Empty	TRUE	
20196-20225 HinckeM –TAXchicken	Uncategorized Sample	20196-20205_A	Mudpit_Ingel20196_MGFPeaklist (F058870)	Hemoglobin subunit epsilon OS=Gallus gallus GN=HBE PE=1 SV=2	HBE_CHICK	TAX_GallusGallus_9031_20141114.fasta	16,603.30	100.00%	8	10	30	0.06%	83.70%	NTYAKLSELHCDK	K	L	6.50%	16.9	43.8	15.8	11.1					0	0	1	0	2	1,578.76	2,178.00		35,777.00	84	96	Empty	TRUE	
20196-20225 HinckeM –TAXchicken	Uncategorized Sample	20196-20205_A	Mudpit_Ingel20196_MGFPeaklist (F058870)	Hemoglobin subunit epsilon OS=Gallus gallus GN=HBE PE=1 SV=2	HBE_CHICK	TAX_GallusGallus_9031_20141114.fasta	16,603.30	100.00%	8	10	30	0.06%	83.70%	NVEECGAEALAR	V	L	60.00%					27.9	0.495	0.0502	0.333	0	1	0	0	1	1,301.58	1,891.00		8,841.88	20	31	Empty	TRUE	HBRH_CHICK
20196-20225 HinckeM –TAXchicken	Uncategorized Sample	20196-20205_A	Mudpit_Ingel20196_MGFPeaklist (F058870)	Hemoglobin subunit epsilon OS=Gallus gallus GN=HBE PE=1 SV=2	HBE_CHICK	TAX_GallusGallus_9031_20141114.fasta	16,603.30	100.00%	8	10	30	0.06%	83.70%	QLITSVWSK	K	V	99.70%	60.2	44.8	28.7	31.7	38.6	2.57	0.264	0.611	0	11	0	0	2	1,061.60	2,326.00		538317	10	18	Empty	TRUE	HBRH_CHICK
20196-20225 HinckeM –TAXchicken	Uncategorized Sample	20196-20205_A	Mudpit_Ingel20196_MGFPeaklist (F058870)	Hemoglobin subunit epsilon OS=Gallus gallus GN=HBE PE=1 SV=2	HBE_CHICK	TAX_GallusGallus_9031_20141114.fasta	16,603.30	100.00%	8	10	30	0.06%	83.70%	SELHCDKLHVDPENFR	L	L	99.40%					37	3.11	0.0865	0.375	0	0	0	1	1	1,995.93	1,926.00		10,369.20	90	105	Empty	FALSE	HBB_CHICK
20196-20225 HinckeM –TAXchicken	Uncategorized Sample	20196-20205_A	Mudpit_Ingel20196_MGFPeaklist (F058870)	Hemoglobin subunit epsilon OS=Gallus gallus GN=HBE PE=1 SV=2	HBE_CHICK	TAX_GallusGallus_9031_20141114.fasta	16,603.30	100.00%	8	10	30	0.06%	83.70%	VLSSFGEAVK	K	N	99.70%	65.8	44	31.1	39	40.2	2.55	0.373	0.55	0	32	0	0	2	1,036.57	2,022.00		1438060	68	77	Empty	FALSE	HBRH_CHICK,Q90864_CHICK
20196-20225 HinckeM –TAXchicken	Uncategorized Sample	20196-20205_A	Mudpit_Ingel20196_MGFPeaklist (F058870)	Hemoglobin subunit epsilon OS=Gallus gallus GN=HBE PE=1 SV=2	HBE_CHICK	TAX_GallusGallus_9031_20141114.fasta	16,603.30	100.00%	8	10	30	0.06%	83.70%	VNVEECGAEALAR	K	L	99.70%	58.5	44.5	21.1	47.6	50.7	3.92	0.46	0.538	0	9	0	0	2	1,417.67	2,012.00		1808460	19	31	Empty	TRUE	HBRH_CHICK
20196-20225 HinckeM –TAXchicken	Uncategorized Sample	20196-20205_A	Mudpit_Ingel20196_MGFPeaklist (F058870)	Hemoglobin subunit epsilon OS=Gallus gallus GN=HBE PE=1 SV=2	HBE_CHICK	TAX_GallusGallus_9031_20141114.fasta	16,603.30	100.00%	8	10	30	0.06%	83.70%	VVAHALAR	N	K	99.70%	55	42.2	29.3	43.4	46	4.06	0.411	0.812	0	16	0	0	1	836.51	1,507.50		6,079.58	137	144	Empty	FALSE	HBB_CHICK
20196-20225 HinckeM –TAXchicken	Uncategorized Sample	20196-20205_A	Mudpit_Ingel20196_MGFPeaklist (F058870)	Hemoglobin subunit rho OS=Gallus gallus PE=1 SV=2	HBRH_CHICK	TAX_GallusGallus_9031_20141114.fasta	16,589.20	77.60%	1	1	1	0.00%	44.20%	KVLSSFGEAVK	K	N	99.70%	67.4	43.3	37.4	42.5	47.1	4.59	0.34	0.636	0	7	0	0	2	1,164.66	1,909.00		10,064.40	67	77	Empty	FALSE	HBE_CHICK,Q90864_CHICK
20196-20225 HinckeM –TAXchicken	Uncategorized Sample	20196-20205_A	Mudpit_Ingel20196_MGFPeaklist (F058870)	Hemoglobin subunit rho OS=Gallus gallus PE=1 SV=2	HBRH_CHICK	TAX_GallusGallus_9031_20141114.fasta	16,589.20	77.60%	1	1	1	0.00%	44.20%	LHVDPENFR	K	L	99.70%	40.5	43.8	28.9	24.1	35.8	3.35	0.419	0.556	0	16	0	0	2	1,126.56	1,945.00		2591890	97	105	Empty	FALSE	HBE_CHICK,HBB_CHICK,Q90864_CHICK
20196-20225 HinckeM –TAXchicken	Uncategorized Sample	20196-20205_A	Mudpit_Ingel20196_MGFPeaklist (F058870)	Hemoglobin subunit rho OS=Gallus gallus PE=1 SV=2	HBRH_CHICK	TAX_GallusGallus_9031_20141114.fasta	16,589.20	77.60%	1	1	1	0.00%	44.20%	LLIVYPWTQR	R	F	99.70%	64.1	43.6	25.4	48.8	44.4	3.38	0.351	0.6	0	78	0	0	2	1,288.74	2,774.00		4.25E+07	32	41	Empty	FALSE	HBE_CHICK,HBB_CHICK,Q90864_CHICK
20196-20225 HinckeM –TAXchicken	Uncategorized Sample	20196-20205_A	Mudpit_Ingel20196_MGFPeaklist (F058870)	Hemoglobin subunit rho OS=Gallus gallus PE=1 SV=2	HBRH_CHICK	TAX_GallusGallus_9031_20141114.fasta	16,589.20	77.60%	1	1	1	0.00%	44.20%	NTYAKLSELHCEK	K	L	99.70%	46.9	44.1	22.5	29.5	30.5	1.72	0.157	0.308	0	1	1	0	2	1,592.77	2,065.50		65,491.20	84	96	Empty	TRUE	
20196-20225 HinckeM –TAXchicken	Uncategorized Sample	20196-20205_A	Mudpit_Ingel20196_MGFPeaklist (F058870)	Hemoglobin subunit rho OS=Gallus gallus PE=1 SV=2	HBRH_CHICK	TAX_GallusGallus_9031_20141114.fasta	16,589.20	77.60%	1	1	1	0.00%	44.20%	NTYAKLSELHCEKLHVDPENFR	K	L	96.90%					43.9	4.51	0	0.273	0	0	0	1	2	2,682.31	2,281.00		379519	84	105	Empty	TRUE	
20196-20225 HinckeM –TAXchicken	Uncategorized Sample	20196-20205_A	Mudpit_Ingel20196_MGFPeaklist (F058870)	Hemoglobin subunit rho OS=Gallus gallus PE=1 SV=2	HBRH_CHICK	TAX_GallusGallus_9031_20141114.fasta	16,589.20	77.60%	1	1	1	0.00%	44.20%	NVEECGAEALAR	V	L	60.00%					27.9	0.495	0.0502	0.333	0	1	0	0	1	1,301.58	1,891.00		8,841.88	20	31	Empty	FALSE	HBE_CHICK
20196-20225 HinckeM –TAXchicken	Uncategorized Sample	20196-20205_A	Mudpit_Ingel20196_MGFPeaklist (F058870)	Hemoglobin subunit rho OS=Gallus gallus PE=1 SV=2	HBRH_CHICK	TAX_GallusGallus_9031_20141114.fasta	16,589.20	77.60%	1	1	1	0.00%	44.20%	QLITSVWSK	K	V	99.70%	60.2	44.8	28.7	31.7	38.6	2.57	0.264	0.611	0	11	0	0	2	1,061.60	2,326.00		538317	10	18	Empty	FALSE	HBE_CHICK
20196-20225 HinckeM –TAXchicken	Uncategorized Sample	20196-20205_A	Mudpit_Ingel20196_MGFPeaklist (F058870)	Hemoglobin subunit rho OS=Gallus gallus PE=1 SV=2	HBRH_CHICK	TAX_GallusGallus_9031_20141114.fasta	16,589.20	77.60%	1	1	1	0.00%	44.20%	VLSSFGEAVK	K	N	99.70%	65.8	44	31.1	39	40.2	2.55	0.373	0.55	0	32	0	0	2	1,036.57	2,022.00		1438060	68	77	Empty	FALSE	HBE_CHICK,Q90864_CHICK
20196-20225 HinckeM –TAXchicken	Uncategorized Sample	20196-20205_A	Mudpit_Ingel20196_MGFPeaklist (F058870)	Hemoglobin subunit rho OS=Gallus gallus PE=1 SV=2	HBRH_CHICK	TAX_GallusGallus_9031_20141114.fasta	16,589.20	77.60%	1	1	1	0.00%	44.20%	VNVEECGAEALAR	K	L	99.70%	58.5	44.5	21.1	47.6	50.7	3.92	0.46	0.538	0	9	0	0	2	1,417.67	2,012.00		1808460	19	31	Empty	FALSE	HBE_CHICK
20196-20225 HinckeM –TAXchicken	Uncategorized Sample	20196-20205_A	Mudpit_Ingel20196_MGFPeaklist (F058870)	Uncharacterized protein OS=Gallus gallus GN=Gga.22975 PE=4 SV=1	R4GF59_CHICK	TAX_GallusGallus_9031_20141114.fasta	164,160.80	99.90%	2	2	3	0.01%	7.40%	IVALSSSLSNAK	R	D	99.70%	62.6	44.3	27.6	43	37.1	3.23	0.313	0.417	0	2	0	0	2	1,189.68	2,035.00		728.08	784	795	Empty	FALSE	UPI0002C87FD9,UPI0000546791,UPI0000546792,UPI0001A2D229
20196-20225 HinckeM –TAXchicken	Uncategorized Sample	20196-20205_A	Mudpit_Ingel20196_MGFPeaklist (F058870)	Uncharacterized protein OS=Gallus gallus GN=Gga.22975 PE=4 SV=1	R4GF59_CHICK	TAX_GallusGallus_9031_20141114.fasta	164,160.80	99.90%	2	2	3	0.01%	7.40%	LYDLNHNEIGELIR	R	M	99.70%	44.8	44.3	15.8	36.2	35.8	3.68	0.402	0.393	0	0	1	0	2	1,698.88	2,527.00		263.29	450	463	Empty	FALSE	UPI0002C87FD9,UPI0000546791,UPI0000546792,UPI0001A2D229
20196-20225 HinckeM –TAXchicken	Uncategorized Sample	20196-20205_A	Mudpit_Ingel20196_MGFPeaklist (F058870)	Uncharacterized protein OS=Gallus gallus GN=Gga.22975 PE=4 SV=1	R4GF59_CHICK	TAX_GallusGallus_9031_20141114.fasta	164,160.80	99.90%	2	2	3	0.01%	7.40%	TGNFQVTELGR	K	I	99.70%	58.2	44.7	32.4	38.6	33.9	3.68	0.401	0.455	0	2	0	0	2	1,221.62	2,092.00		818.97	273	283	Empty	TRUE	UPI0002C87FD9
20196-20225 HinckeM –TAXchicken	Uncategorized Sample	20196-20205_A	Mudpit_Ingel20196_MGFPeaklist (F058870)	Uncharacterized protein OS=Gallus gallus GN=Gga.22975 PE=4 SV=1	R4GF59_CHICK	TAX_GallusGallus_9031_20141114.fasta	164,160.80	99.90%	2	2	3	0.01%	7.40%	TICAEFAILR	K	M	99.70%	48.1	44.5	21	36.9	34.8	2.49	0.336	0.4	0	2	0	0	2	1,193.63	2,608.00		512.65	654	663	Empty	FALSE	UPI0002C87FD9,UPI0000546791,UPI0000546792,UPI0001A2D229
20196-20225 HinckeM –TAXchicken	Uncategorized Sample	20196-20205_A	Mudpit_Ingel20196_MGFPeaklist (F058870)	Uncharacterized protein OS=Gallus gallus GN=Gga.22975 PE=4 SV=1	R4GF59_CHICK	TAX_GallusGallus_9031_20141114.fasta	164,160.80	99.90%	2	2	3	0.01%	7.40%	TNLLLQAHLSR	K	M	99.70%	32.1	42.1	24.2	25.2	26	2.62	0.208	0.364	0	0	2	0	2	1,265.73	2,220.50		723.4	1188	1198	Empty	FALSE	UPI0002C87FD9,UPI0000546791,UPI0000546792,UPI0001A2D229
20196-20225 HinckeM –TAXchicken	Uncategorized Sample	20196-20205_A	Mudpit_Ingel20196_MGFPeaklist (F058870)	Uncharacterized protein OS=Gallus gallus GN=Gga.22975 PE=4 SV=1	R4GF59_CHICK	TAX_GallusGallus_9031_20141114.fasta	164,160.80	99.90%	2	2	3	0.01%	7.40%	VFSLSSEFR	R	N	99.70%	57.7	44.6	26.1	45.2	30	2.44	0.4	0.444	0	2	0	0	2	1,071.55	2,418.00		1,052.61	314	322	Empty	FALSE	UPI0002C87FD9,UPI0000546791,UPI0000546792,UPI0001A2D229
20196-20225 HinckeM –TAXchicken	Uncategorized Sample	20196-20205_A	Mudpit_Ingel20196_MGFPeaklist (F058870)	Uncharacterized protein OS=Gallus gallus GN=Gga.22975 PE=4 SV=1	R4GF59_CHICK	TAX_GallusGallus_9031_20141114.fasta	164,160.80	99.90%	2	2	3	0.01%	7.40%	VPIPVKESIEEPSAK	R	I	99.70%	36.6	42.7	16.8	26.9	28.6	3.77	0.353	0.233	0	0	2	0	2	1,622.90	1,997.50		603.05	341	355	Empty	FALSE	UPI0002C87FD9,UPI0000546791,UPI0000546792,UPI0001A2D229
20196-20225 HinckeM –TAXchicken	Uncategorized Sample	20196-20205_A	Mudpit_Ingel20196_MGFPeaklist (F058870)	Uncharacterized protein OS=Gallus gallus GN=Gga.22975 PE=4 SV=1	R4GF59_CHICK	TAX_GallusGallus_9031_20141114.fasta	164,160.80	99.90%	2	2	3	0.01%	7.40%	VVLLTGET	K	S	71.80%					22.5	0.569	0.164	0.375	0	1	0	0	1	831.4823	2,875.00		768.76	702	709	Empty	TRUE	
20196-20225 HinckeM –TAXchicken	Uncategorized Sample	20196-20205_A	Mudpit_Ingel20196_MGFPeaklist (F058870)	Uncharacterized protein OS=Gallus gallus GN=Gga.22975 PE=4 SV=1	R4GF59_CHICK	TAX_GallusGallus_9031_20141114.fasta	164,160.80	99.90%	2	2	3	0.01%	7.40%	VVLLTGETSTDLK	K	L	99.70%	65.6	44	22.2	53	37.3	4.62	0.432	0.385	0	2	0	0	2	1,375.77	2,226.50		690.58	702	714	Empty	FALSE	UPI0002C87FD9,UPI0000546791,UPI0000546792,UPI0001A2D229
20196-20225 HinckeM –TAXchicken	Uncategorized Sample	20196-20205_A	Mudpit_Ingel20196_MGFPeaklist (F058870)	Uncharacterized protein OS=Gallus gallus GN=Gga.22975 PE=4 SV=1	R4GF59_CHICK	TAX_GallusGallus_9031_20141114.fasta	164,160.80	99.90%	2	2	3	0.01%	7.40%	YISSQIERPIR	R	I	99.70%	22.3	44.2	21.9	14.9	22.5	0.721	0.164	0.273	0	0	2	0	2	1,361.75	1,850.50		835.53	773	783	Empty	FALSE	UPI0002C87FD9,UPI0000546791,UPI0000546792,UPI0001A2D229
20196-20225 HinckeM –TAXchicken	Uncategorized Sample	20196-20205_A	Mudpit_Ingel20196_MGFPeaklist (F058870)	status=inactive	UPI0000ECA60C	UPARC14_2_GallusG_9031_20140417.fasta	51,706.80	100.00%	4	4	55	0.11%	10.20%	ESTLHLVLR	K	L	99.70%	52.2	42.3	23.1	0	34.9	2.8	0.307	0.444	0	9	0	0	2	1,067.62	2,195.00		5,669.74	65	73	Empty	TRUE	UPI00001713D2,UPI00006104B3,UPI0000185E41,RS27A_CHICK,UPI00000FC6C2,UPI0000E2690A
20196-20225 HinckeM –TAXchicken	Uncategorized Sample	20196-20205_A	Mudpit_Ingel20196_MGFPeaklist (F058870)	status=inactive	UPI0000ECA60C	UPARC14_2_GallusG_9031_20140417.fasta	51,706.80	100.00%	4	4	55	0.11%	10.20%	IQDKEGIPPDQQR	K	L	99.70%	62.1	44.4	30.2	31.7	35	4	0.303	0.423	0	0	22	0	2	1,523.78	1,504.50		8,450.47	31	43	Empty	TRUE	UPI00001713D2,UPI00006104B3,UPI0000185E41,RS27A_CHICK,UPI00000FC6C2,UPI0000E2690A
20196-20225 HinckeM –TAXchicken	Uncategorized Sample	20196-20205_A	Mudpit_Ingel20196_MGFPeaklist (F058870)	status=inactive	UPI0000ECA60C	UPARC14_2_GallusG_9031_20140417.fasta	51,706.80	100.00%	4	4	55	0.11%	10.20%	TITLEVEPSDTIENVK	K	A	99.70%	66.6	44.7	29.3	53.1	48.5	6.35	0.452	0.438	0	14	0	0	2	1,787.93	2,408.50		51,048.90	13	28	Empty	TRUE	UPI00001713D2,UPI00006104B3,UPI0000185E41,RS27A_CHICK,UPI00000FC6C2
20196-20225 HinckeM –TAXchicken	Uncategorized Sample	20196-20205_A	Mudpit_Ingel20196_MGFPeaklist (F058870)	status=inactive	UPI0000ECA60C	UPARC14_2_GallusG_9031_20140417.fasta	51,706.80	100.00%	4	4	55	0.11%	10.20%	TLSDYNIQK	R	E	99.70%	29.8	43.8	20.5	19.5	30.5	1.26	0.302	0.444	0	10	0	0	2	1,081.55	1,781.00		10,696.00	56	64	Empty	TRUE	UPI00001713D2,UPI00006104B3,UPI0000185E41,RS27A_CHICK,UPI00000FC6C2,UPI0000E2690A
20196-20225 HinckeM –TAXchicken	Uncategorized Sample	20196-20205_A	Mudpit_Ingel20196_MGFPeaklist (F058870)	Vitellogenin-2 OS=Gallus gallus GN=VTG2 PE=4 SV=1	F1NFL6_CHICK	TAX_GallusGallus_9031_20141114.fasta	205,150.60	100.00%	3	3	4	0.01%	2.32%	KQSHSSREDLRRSTGK	R	R	81.50%					28.2	1	0.181	0.281	0	0	1	0	2	1,871.98	2,511.00		859950	967	982	Empty	TRUE	VIT2_CHICK
20196-20225 HinckeM –TAXchicken	Uncategorized Sample	20196-20205_A	Mudpit_Ingel20196_MGFPeaklist (F058870)	Vitellogenin-2 OS=Gallus gallus GN=VTG2 PE=4 SV=1	F1NFL6_CHICK	TAX_GallusGallus_9031_20141114.fasta	205,150.60	100.00%	3	3	4	0.01%	2.32%	QQLTLVEVR	R	S	99.70%	64.4	43.4	38.2	16.6	33	1.23	0.261	0.5	0	1	0	0	2	1,085.63	2,185.00		199.21	260	268	Empty	TRUE	VIT2_CHICK
20196-20225 HinckeM –TAXchicken	Uncategorized Sample	20196-20205_A	Mudpit_Ingel20196_MGFPeaklist (F058870)	Vitellogenin-2 OS=Gallus gallus GN=VTG2 PE=4 SV=1	F1NFL6_CHICK	TAX_GallusGallus_9031_20141114.fasta	205,150.60	100.00%	3	3	4	0.01%	2.32%	RSSSSSSSSSSSSSSSR	K	S	99.70%					40.8	4.48	0.284	0.278	0	2	0	0	2	1,705.72	2,291.50		23,954.70	1192	1209	Empty	TRUE	
20196-20225 HinckeM –TAXchicken	Uncategorized Sample	20196-20205_A	Mudpit_Ingel20196_MGFPeaklist (F058870)	status=inactive	UPI0000E80F73	UPARC14_2_GallusG_9031_20140417.fasta	125,161.30	100.00%	5	5	7	0.01%	4.88%	GPPPPPTASEPTRR	R	T	99.70%	22.6	44.8	22	13.9	40.5	3.92	0.198	0.5	0	0	2	0	2	1,459.77	1,449.50		492.07	1090	1103	Empty	TRUE	UPI0000ECAE17,UPI0003504CCA,UPI00035076B1,UPI00035036DB,UPI0003506FE7,UPI000044903B,F1NT10_CHICK,UPI0002C87F22,UPI000240C2B2
20196-20225 HinckeM –TAXchicken	Uncategorized Sample	20196-20205_A	Mudpit_Ingel20196_MGFPeaklist (F058870)	status=inactive	UPI0000E80F73	UPARC14_2_GallusG_9031_20140417.fasta	125,161.30	100.00%	5	5	7	0.01%	4.88%	LIQESDQHLKDVEK	K	I	99.70%	26.7	44.2	17.1	16.4	35.1	1.8	0.194	0.357	0	0	0	2	2	1,681.88	1,674.00		718.56	1044	1057	Empty	TRUE	UPI0000ECAE17,UPI0003504CCA,UPI00035076B1,UPI00035036DB,UPI0003506FE7,UPI000044903B,F1NT10_CHICK,UPI0002C87F22,UPI000240C2B2
20196-20225 HinckeM –TAXchicken	Uncategorized Sample	20196-20205_A	Mudpit_Ingel20196_MGFPeaklist (F058870)	status=inactive	UPI0000E80F73	UPARC14_2_GallusG_9031_20140417.fasta	125,161.30	100.00%	5	5	7	0.01%	4.88%	QYIEKIAK	K	N	20.10%	6.17	43	17.2	0					0	1	0	0	2	992.5776	2,272.00		230	850	857	Empty	TRUE	UPI0000ECAE17,UPI0003504CCA,UPI00035076B1,UPI00035036DB,UPI0003506FE7,UPI000044903B,F1NT10_CHICK,UPI0002C87F22,UPI000240C2B2
20196-20225 HinckeM –TAXchicken	Uncategorized Sample	20196-20205_A	Mudpit_Ingel20196_MGFPeaklist (F058870)	status=inactive	UPI0000E80F73	UPARC14_2_GallusG_9031_20140417.fasta	125,161.30	100.00%	5	5	7	0.01%	4.88%	TTFSEFAAK	R	H	27.50%	12.8	44.9	25.1	-0.03					0	1	0	0	2	1,001.49	2,008.00		239.56	753	761	Empty	TRUE	UPI0000ECAE17,UPI0003504CCA,UPI00035076B1,UPI00035036DB,UPI0003506FE7,UPI000044903B,F1NT10_CHICK,UPI0002C87F22,UPI000240C2B2
20196-20225 HinckeM –TAXchicken	Uncategorized Sample	20196-20205_A	Mudpit_Ingel20196_MGFPeaklist (F058870)	status=inactive	UPI0000E80F73	UPARC14_2_GallusG_9031_20140417.fasta	125,161.30	100.00%	5	5	7	0.01%	4.88%	VFFYNPTTR	R	L	99.70%	25.9	45	20.2	9.94	27.7	1.28	0.213	0.333	0	1	0	0	2	1,144.58	2,289.00		447.95	552	560	Empty	TRUE	UPI0000ECAE17,UPI0003504CCA,UPI00035076B1,UPI00035036DB,UPI0003506FE7,UPI000044903B,F1NT10_CHICK,UPI0002C87F22,UPI000240C2B2
20196-20225 HinckeM –TAXchicken	Uncategorized Sample	20196-20205_A	Mudpit_Ingel20196_MGFPeaklist (F058870)	status=active	UPI00000FB989,UPI000035A875,UPI0000520A79	UPARC14_2_GallusG_9031_20140417.fasta	45,745.00	99.90%	3	3	5	0.01%	8.61%	FSYVCPDLVK	R	E	40.30%	15.6	44.1	13.1	9.39					0	1	0	0	2	1,227.61	2,322.00		533.63	231	240	Empty	TRUE	UPI00000FBDA3,UPI00003AE7E4,ARP3_CHICK
20196-20225 HinckeM –TAXchicken	Uncategorized Sample	20196-20205_A	Mudpit_Ingel20196_MGFPeaklist (F058870)	status=active	UPI00000FB989,UPI000035A875,UPI0000520A79	UPARC14_2_GallusG_9031_20140417.fasta	45,745.00	99.90%	3	3	5	0.01%	8.61%	LPACVVDCGTGYTK	R	L	99.70%	58.4	43.7	16.3	52.9	48.4	4.89	0.469	0.429	0	2	0	0	2	1,540.71	1,952.50		1,718.43	5	18	Empty	TRUE	UPI00000FBDA3,UPI00003AE7E4,ARP3_CHICK
20196-20225 HinckeM –TAXchicken	Uncategorized Sample	20196-20205_A	Mudpit_Ingel20196_MGFPeaklist (F058870)	status=active	UPI00000FB989,UPI000035A875,UPI0000520A79	UPARC14_2_GallusG_9031_20140417.fasta	45,745.00	99.90%	3	3	5	0.01%	8.61%	NIVLSGGSTMFR	K	D	39.10%	17.9	44	18.3	7.57					0	2	0	0	2	1,297.66	2,124.00		727.64	318	329	Empty	TRUE	UPI00000FBDA3,UPI00003AE7E4,ARP3_CHICK
20196-20225 HinckeM –TAXchicken	Uncategorized Sample	20196-20205_A	Mudpit_Ingel20196_MGFPeaklist (F058870)	status=active	UPI00000FB989,UPI000035A875,UPI0000520A79	UPARC14_2_GallusG_9031_20140417.fasta	45,745.00	99.90%	3	3	5	0.01%	8.61%	FSYVCPDLVK	R	E	40.30%	15.6	44.1	13.1	9.39					0	1	0	0	2	1,227.61	2,322.00		533.63	231	240	Empty	TRUE	UPI00000FBDA3,UPI00003AE7E4,ARP3_CHICK
20196-20225 HinckeM –TAXchicken	Uncategorized Sample	20196-20205_A	Mudpit_Ingel20196_MGFPeaklist (F058870)	status=active	UPI00000FB989,UPI000035A875,UPI0000520A79	UPARC14_2_GallusG_9031_20140417.fasta	45,745.00	99.90%	3	3	5	0.01%	8.61%	LPACVVDCGTGYTK	R	L	99.70%	58.4	43.7	16.3	52.9	48.4	4.89	0.469	0.429	0	2	0	0	2	1,540.71	1,952.50		1,718.43	5	18	Empty	TRUE	UPI00000FBDA3,UPI00003AE7E4,ARP3_CHICK
20196-20225 HinckeM –TAXchicken	Uncategorized Sample	20196-20205_A	Mudpit_Ingel20196_MGFPeaklist (F058870)	status=active	UPI00000FB989,UPI000035A875,UPI0000520A79	UPARC14_2_GallusG_9031_20140417.fasta	45,745.00	99.90%	3	3	5	0.01%	8.61%	NIVLSGGSTMFR	K	D	39.10%	17.9	44	18.3	7.57					0	2	0	0	2	1,297.66	2,124.00		727.64	318	329	Empty	TRUE	UPI00000FBDA3,UPI00003AE7E4,ARP3_CHICK
20196-20225 HinckeM –TAXchicken	Uncategorized Sample	20196-20205_A	Mudpit_Ingel20196_MGFPeaklist (F058870)	status=active	UPI00000FB989,UPI000035A875,UPI0000520A79	UPARC14_2_GallusG_9031_20140417.fasta	45,745.00	99.90%	3	3	5	0.01%	8.61%	FSYVCPDLVK	R	E	40.30%	15.6	44.1	13.1	9.39					0	1	0	0	2	1,227.61	2,322.00		533.63	231	240	Empty	TRUE	UPI00000FBDA3,UPI00003AE7E4,ARP3_CHICK
20196-20225 HinckeM –TAXchicken	Uncategorized Sample	20196-20205_A	Mudpit_Ingel20196_MGFPeaklist (F058870)	status=active	UPI00000FB989,UPI000035A875,UPI0000520A79	UPARC14_2_GallusG_9031_20140417.fasta	45,745.00	99.90%	3	3	5	0.01%	8.61%	LPACVVDCGTGYTK	R	L	99.70%	58.4	43.7	16.3	52.9	48.4	4.89	0.469	0.429	0	2	0	0	2	1,540.71	1,952.50		1,718.43	5	18	Empty	TRUE	UPI00000FBDA3,UPI00003AE7E4,ARP3_CHICK
20196-20225 HinckeM –TAXchicken	Uncategorized Sample	20196-20205_A	Mudpit_Ingel20196_MGFPeaklist (F058870)	status=active	UPI00000FB989,UPI000035A875,UPI0000520A79	UPARC14_2_GallusG_9031_20140417.fasta	45,745.00	99.90%	3	3	5	0.01%	8.61%	NIVLSGGSTMFR	K	D	39.10%	17.9	44	18.3	7.57					0	2	0	0	2	1,297.66	2,124.00		727.64	318	329	Empty	TRUE	UPI00000FBDA3,UPI00003AE7E4,ARP3_CHICK
20196-20225 HinckeM –TAXchicken	Uncategorized Sample	20196-20205_A	Mudpit_Ingel20196_MGFPeaklist (F058870)	Actin, cytoplasmic type 5 OS=Gallus gallus PE=3 SV=1	ACT5_CHICK,ACTB_CHICK,ACTG_CHICK	TAX_GallusGallus_9031_20141114.fasta	41,793.90	100.00%	22	29	121	0.24%	45.70%	AVFPSIVGR	R	P	99.70%					31.2	2.59	0.279	0.389	0	3	0	0	1	945.5516	2,317.00		6,506.17	29	37	Empty	TRUE	ACTA_CHICK,F1N8P3_CHICK,F1P476_CHICK,ACTS_CHICK,ACTC_CHICK
20196-20225 HinckeM –TAXchicken	Uncategorized Sample	20196-20205_A	Mudpit_Ingel20196_MGFPeaklist (F058870)	Actin, cytoplasmic type 5 OS=Gallus gallus PE=3 SV=1	ACT5_CHICK,ACTB_CHICK,ACTG_CHICK	TAX_GallusGallus_9031_20141114.fasta	41,793.90	100.00%	22	29	121	0.24%	45.70%	AVFPSIVGRPR	R	H	99.70%	63.7	42.3	21	52.4	41	5.02	0.429	0.591	0	1	21	0	2	1,198.71	2,202.00		267262	29	39	Empty	TRUE	ACTA_CHICK,F1N8P3_CHICK,F1P476_CHICK,ACTS_CHICK,ACTC_CHICK
20196-20225 HinckeM –TAXchicken	Uncategorized Sample	20196-20205_A	Mudpit_Ingel20196_MGFPeaklist (F058870)	Actin, cytoplasmic type 5 OS=Gallus gallus PE=3 SV=1	ACT5_CHICK,ACTB_CHICK,ACTG_CHICK	TAX_GallusGallus_9031_20141114.fasta	41,793.90	100.00%	22	29	121	0.24%	45.70%	DLTDYLMK	R	I	99.70%	37.6	43.3	20.1	28.9	30.3	1.38	0.32	0.438	0	10	0	0	2	998.4865	2,207.50		21,013.60	184	191	Empty	TRUE	ACTA_CHICK,F1N8P3_CHICK,F1P476_CHICK,ACTS_CHICK,ACTC_CHICK
20196-20225 HinckeM –TAXchicken	Uncategorized Sample	20196-20205_A	Mudpit_Ingel20196_MGFPeaklist (F058870)	Actin, cytoplasmic type 5 OS=Gallus gallus PE=3 SV=1	ACT5_CHICK,ACTB_CHICK,ACTG_CHICK	TAX_GallusGallus_9031_20141114.fasta	41,793.90	100.00%	22	29	121	0.24%	45.70%	DLYANTVLSGGTTMYPGIADR	K	M	99.70%	61.2	43.9	19.8	53.8	44	5.85	0.241	0.333	0	0	2	0	2	2,231.07	2,469.00		5,341.63	292	312	Empty	TRUE	
20196-20225 HinckeM –TAXchicken	Uncategorized Sample	20196-20205_A	Mudpit_Ingel20196_MGFPeaklist (F058870)	Actin, cytoplasmic type 5 OS=Gallus gallus PE=3 SV=1	ACT5_CHICK,ACTB_CHICK,ACTG_CHICK	TAX_GallusGallus_9031_20141114.fasta	41,793.90	100.00%	22	29	121	0.24%	45.70%	EITALAPSTMK	K	I	99.70%	62.3	45.1	28.9	43.7	39.2	3.43	0.383	0.455	0	15	0	0	2	1,161.62	1,857.00		61,507.60	316	326	Empty	TRUE	ACTA_CHICK,F1N8P3_CHICK,F1P476_CHICK,ACTS_CHICK,ACTC_CHICK
20196-20225 HinckeM –TAXchicken	Uncategorized Sample	20196-20205_A	Mudpit_Ingel20196_MGFPeaklist (F058870)	Actin, cytoplasmic type 5 OS=Gallus gallus PE=3 SV=1	ACT5_CHICK,ACTB_CHICK,ACTG_CHICK	TAX_GallusGallus_9031_20141114.fasta	41,793.90	100.00%	22	29	121	0.24%	45.70%	FPSIVGRPR	V	H	99.70%					40.8	3.55	0.397	0.611	0	1	0	0	1	1,028.60	2,189.00		5,430.63	31	39	Empty	TRUE	ACTA_CHICK,F1N8P3_CHICK,F1P476_CHICK,ACTS_CHICK,ACTC_CHICK
20196-20225 HinckeM –TAXchicken	Uncategorized Sample	20196-20205_A	Mudpit_Ingel20196_MGFPeaklist (F058870)	Actin, cytoplasmic type 5 OS=Gallus gallus PE=3 SV=1	ACT5_CHICK,ACTB_CHICK,ACTG_CHICK	TAX_GallusGallus_9031_20141114.fasta	41,793.90	100.00%	22	29	121	0.24%	45.70%	GYSFTTTAER	R	E	99.70%	55.3	44.6	31.2	37.9	36.7	2.55	0.335	0.55	0	11	0	0	2	1,132.53	1,854.00		117699	197	206	Empty	TRUE	
20196-20225 HinckeM –TAXchicken	Uncategorized Sample	20196-20205_A	Mudpit_Ingel20196_MGFPeaklist (F058870)	Actin, cytoplasmic type 5 OS=Gallus gallus PE=3 SV=1	ACT5_CHICK,ACTB_CHICK,ACTG_CHICK	TAX_GallusGallus_9031_20141114.fasta	41,793.90	100.00%	22	29	121	0.24%	45.70%	GYSFTTTAEREIVR	R	D	99.70%	55	45	17.4	47.9	34.7	2.96	0.256	0.429	0	0	2	0	2	1,629.82	2,217.50		13,050.70	197	210	Empty	TRUE	
20196-20225 HinckeM –TAXchicken	Uncategorized Sample	20196-20205_A	Mudpit_Ingel20196_MGFPeaklist (F058870)	Actin, cytoplasmic type 5 OS=Gallus gallus PE=3 SV=1	ACT5_CHICK,ACTB_CHICK,ACTG_CHICK	TAX_GallusGallus_9031_20141114.fasta	41,793.90	100.00%	22	29	121	0.24%	45.70%	HQGVMVGMGQKDSYVGDEAQSK	R	R	99.70%	63.9	42.1	14.8	58.8	46.4	5.19	0.463	0.364	0	0	0	2	2	2,383.07	1,577.00		1,673.99	40	61	Empty	TRUE	ACTA_CHICK,F1N8P3_CHICK,F1P476_CHICK,ACTS_CHICK,ACTC_CHICK
20196-20225 HinckeM –TAXchicken	Uncategorized Sample	20196-20205_A	Mudpit_Ingel20196_MGFPeaklist (F058870)	Actin, cytoplasmic type 5 OS=Gallus gallus PE=3 SV=1	ACT5_CHICK,ACTB_CHICK,ACTG_CHICK	TAX_GallusGallus_9031_20141114.fasta	41,793.90	100.00%	22	29	121	0.24%	45.70%	HQGVMVGMGQKDSYVGDEAQSKR	R	G	99.70%	72.8	43.1	14.9	69.1	57.5	7.01	0.59	0.435	0	0	0	2	2	2,539.17	1,534.00		1,171.87	40	62	Empty	TRUE	ACTA_CHICK,F1N8P3_CHICK,F1P476_CHICK,ACTS_CHICK,ACTC_CHICK
20196-20225 HinckeM –TAXchicken	Uncategorized Sample	20196-20205_A	Mudpit_Ingel20196_MGFPeaklist (F058870)	Actin, cytoplasmic type 5 OS=Gallus gallus PE=3 SV=1	ACT5_CHICK,ACTB_CHICK,ACTG_CHICK	TAX_GallusGallus_9031_20141114.fasta	41,793.90	100.00%	22	29	121	0.24%	45.70%	IKIIAPPER	K	K	55.90%	29.8	39.4	28.9	13.7					0	2	0	0	2	1,036.65	1,888.00		1,766.94	327	335	Empty	TRUE	ACTA_CHICK,F1P476_CHICK,ACTS_CHICK,ACTC_CHICK
20196-20225 HinckeM –TAXchicken	Uncategorized Sample	20196-20205_A	Mudpit_Ingel20196_MGFPeaklist (F058870)	Actin, cytoplasmic type 5 OS=Gallus gallus PE=3 SV=1	ACT5_CHICK,ACTB_CHICK,ACTG_CHICK	TAX_GallusGallus_9031_20141114.fasta	41,793.90	100.00%	22	29	121	0.24%	45.70%	IWHHTFYNELR	K	V	99.70%	71	44.6	20	58.1	37.9	3.96	0.377	0.455	0	0	4	0	2	1,515.75	2,059.00		7,272.64	85	95	Empty	TRUE	ACTS_CHICK,ACTC_CHICK
20196-20225 HinckeM –TAXchicken	Uncategorized Sample	20196-20205_A	Mudpit_Ingel20196_MGFPeaklist (F058870)	Actin, cytoplasmic type 5 OS=Gallus gallus PE=3 SV=1	ACT5_CHICK,ACTB_CHICK,ACTG_CHICK	TAX_GallusGallus_9031_20141114.fasta	41,793.90	100.00%	22	29	121	0.24%	45.70%	KDLYANTVLSGGTTMYPGIADR	R	M	99.70%	99	44.2	19.8	86.9	56.9	6.52	0.316	0.432	0	0	2	0	2	2,359.16	2,319.00		6,603.07	291	312	Empty	TRUE	
20196-20225 HinckeM –TAXchicken	Uncategorized Sample	20196-20205_A	Mudpit_Ingel20196_MGFPeaklist (F058870)	Actin, cytoplasmic type 5 OS=Gallus gallus PE=3 SV=1	ACT5_CHICK,ACTB_CHICK,ACTG_CHICK	TAX_GallusGallus_9031_20141114.fasta	41,793.90	100.00%	22	29	121	0.24%	45.70%	LDLAGRDLTDYLMK	R	I	99.70%	17.9	44.4	25.4	12.9	38.2	2.6	0.212	0.464	0	0	2	0	2	1,639.84	2,510.50		4,373.75	178	191	Empty	TRUE	ACTA_CHICK,F1N8P3_CHICK,F1P476_CHICK,ACTS_CHICK,ACTC_CHICK
20196-20225 HinckeM –TAXchicken	Uncategorized Sample	20196-20205_A	Mudpit_Ingel20196_MGFPeaklist (F058870)	Actin, cytoplasmic type 5 OS=Gallus gallus PE=3 SV=1	ACT5_CHICK,ACTB_CHICK,ACTG_CHICK	TAX_GallusGallus_9031_20141114.fasta	41,793.90	100.00%	22	29	121	0.24%	45.70%	MQKEITALAPSTMK	R	I	57.80%	22.7	44.4	24.8	8.39					0	0	2	0	2	1,580.80	1,773.00		1,387.25	313	326	Empty	TRUE	ACTA_CHICK,F1N8P3_CHICK,F1P476_CHICK,ACTS_CHICK,ACTC_CHICK
20196-20225 HinckeM –TAXchicken	Uncategorized Sample	20196-20205_A	Mudpit_Ingel20196_MGFPeaklist (F058870)	Actin, cytoplasmic type 5 OS=Gallus gallus PE=3 SV=1	ACT5_CHICK,ACTB_CHICK,ACTG_CHICK	TAX_GallusGallus_9031_20141114.fasta	41,793.90	100.00%	22	29	121	0.24%	45.70%	PSIVGRPR	F	H	96.90%					36.4	0.367	0.236	0.688	0	1	0	0	1	881.5316	2,194.00		3,706.31	32	39	Empty	TRUE	ACTA_CHICK,F1N8P3_CHICK,F1P476_CHICK,ACTS_CHICK,ACTC_CHICK
20196-20225 HinckeM –TAXchicken	Uncategorized Sample	20196-20205_A	Mudpit_Ingel20196_MGFPeaklist (F058870)	Actin, cytoplasmic type 5 OS=Gallus gallus PE=3 SV=1	ACT5_CHICK,ACTB_CHICK,ACTG_CHICK	TAX_GallusGallus_9031_20141114.fasta	41,793.90	100.00%	22	29	121	0.24%	45.70%	PVLLTEAPLNPK	H	A	99.70%					33.5	3.19	0.349	0.375	0	1	0	0	1	1,291.76	2,352.00		297.78	102	113	Empty	TRUE	F1N8P3_CHICK
20196-20225 HinckeM –TAXchicken	Uncategorized Sample	20196-20205_A	Mudpit_Ingel20196_MGFPeaklist (F058870)	Actin, cytoplasmic type 5 OS=Gallus gallus PE=3 SV=1	ACT5_CHICK,ACTB_CHICK,ACTG_CHICK	TAX_GallusGallus_9031_20141114.fasta	41,793.90	100.00%	22	29	121	0.24%	45.70%	QEYDESGPSIVHR	K	K	99.70%	77.5	44	22.5	67.1	43.8	7.51	0.466	0.5	0	4	8	0	2	1,516.70	1,731.00		36,310.00	360	372	Empty	TRUE	
20196-20225 HinckeM –TAXchicken	Uncategorized Sample	20196-20205_A	Mudpit_Ingel20196_MGFPeaklist (F058870)	Actin, cytoplasmic type 5 OS=Gallus gallus PE=3 SV=1	ACT5_CHICK,ACTB_CHICK,ACTG_CHICK	TAX_GallusGallus_9031_20141114.fasta	41,793.90	100.00%	22	29	121	0.24%	45.70%	QEYDESGPSIVHRK	K	C	99.70%	65.9	44.2	19.6	58.9	40.7	5.41	0.4	0.5	0	0	2	4	2	1,627.77	1,606.00		7,227.04	360	373	Empty	TRUE	
20196-20225 HinckeM –TAXchicken	Uncategorized Sample	20196-20205_A	Mudpit_Ingel20196_MGFPeaklist (F058870)	Actin, cytoplasmic type 5 OS=Gallus gallus PE=3 SV=1	ACT5_CHICK,ACTB_CHICK,ACTG_CHICK	TAX_GallusGallus_9031_20141114.fasta	41,793.90	100.00%	22	29	121	0.24%	45.70%	RGILTLK	K	Y	56.70%	26.4	42.2	39.8	0.95					0	2	0	0	2	800.5354	1,975.00		5,469.59	62	68	Empty	TRUE	ACTA_CHICK,F1N8P3_CHICK,F1P476_CHICK,ACTS_CHICK,ACTC_CHICK
20196-20225 HinckeM –TAXchicken	Uncategorized Sample	20196-20205_A	Mudpit_Ingel20196_MGFPeaklist (F058870)	Actin, cytoplasmic type 5 OS=Gallus gallus PE=3 SV=1	ACT5_CHICK,ACTB_CHICK,ACTG_CHICK	TAX_GallusGallus_9031_20141114.fasta	41,793.90	100.00%	22	29	121	0.24%	45.70%	SYELPDGQVITIGNER	K	F	99.70%	75.4	44.7	18.4	65.2	46.5	5.42	0.514	0.438	0	2	2	0	2	1,790.89	2,514.50		26,845.70	239	254	Empty	TRUE	ACTA_CHICK,F1N8P3_CHICK,F1P476_CHICK,ACTS_CHICK,ACTC_CHICK
20196-20225 HinckeM –TAXchicken	Uncategorized Sample	20196-20205_A	Mudpit_Ingel20196_MGFPeaklist (F058870)	Actin, cytoplasmic type 5 OS=Gallus gallus PE=3 SV=1	ACT5_CHICK,ACTB_CHICK,ACTG_CHICK	TAX_GallusGallus_9031_20141114.fasta	41,793.90	100.00%	22	29	121	0.24%	45.70%	VAPEEHPVLLTEAPLNPK	R	A	99.70%	61.8	42.3	14.9	59.6	41.3	3.28	0.339	0.389	0	0	10	3	2	1,954.06	2,333.00		220139	96	113	Empty	TRUE	
20196-20225 HinckeM –TAXchicken	Uncategorized Sample	20196-20205_A	Mudpit_Ingel20196_MGFPeaklist (F058870)	Actin, cytoplasmic type 5 OS=Gallus gallus PE=3 SV=1	ACT5_CHICK,ACTB_CHICK,ACTG_CHICK	TAX_GallusGallus_9031_20141114.fasta	41,793.90	100.00%	22	29	121	0.24%	45.70%	AVFPSIVGR	R	P	99.70%					31.2	2.59	0.279	0.389	0	3	0	0	1	945.5516	2,317.00		6,506.17	29	37	Empty	TRUE	ACTA_CHICK,F1N8P3_CHICK,F1P476_CHICK,ACTS_CHICK,ACTC_CHICK
20196-20225 HinckeM –TAXchicken	Uncategorized Sample	20196-20205_A	Mudpit_Ingel20196_MGFPeaklist (F058870)	Actin, cytoplasmic type 5 OS=Gallus gallus PE=3 SV=1	ACT5_CHICK,ACTB_CHICK,ACTG_CHICK	TAX_GallusGallus_9031_20141114.fasta	41,793.90	100.00%	22	29	121	0.24%	45.70%	AVFPSIVGRPR	R	H	99.70%	63.7	42.3	21	52.4	41	5.02	0.429	0.591	0	1	21	0	2	1,198.71	2,202.00		267262	29	39	Empty	TRUE	ACTA_CHICK,F1N8P3_CHICK,F1P476_CHICK,ACTS_CHICK,ACTC_CHICK
20196-20225 HinckeM –TAXchicken	Uncategorized Sample	20196-20205_A	Mudpit_Ingel20196_MGFPeaklist (F058870)	Actin, cytoplasmic type 5 OS=Gallus gallus PE=3 SV=1	ACT5_CHICK,ACTB_CHICK,ACTG_CHICK	TAX_GallusGallus_9031_20141114.fasta	41,793.90	100.00%	22	29	121	0.24%	45.70%	DLTDYLMK	R	I	99.70%	37.6	43.3	20.1	28.9	30.3	1.38	0.32	0.438	0	10	0	0	2	998.4865	2,207.50		21,013.60	184	191	Empty	TRUE	ACTA_CHICK,F1N8P3_CHICK,F1P476_CHICK,ACTS_CHICK,ACTC_CHICK
20196-20225 HinckeM –TAXchicken	Uncategorized Sample	20196-20205_A	Mudpit_Ingel20196_MGFPeaklist (F058870)	Actin, cytoplasmic type 5 OS=Gallus gallus PE=3 SV=1	ACT5_CHICK,ACTB_CHICK,ACTG_CHICK	TAX_GallusGallus_9031_20141114.fasta	41,793.90	100.00%	22	29	121	0.24%	45.70%	DLYANTVLSGGTTMYPGIADR	K	M	99.70%	61.2	43.9	19.8	53.8	44	5.85	0.241	0.333	0	0	2	0	2	2,231.07	2,469.00		5,341.63	292	312	Empty	TRUE	
20196-20225 HinckeM –TAXchicken	Uncategorized Sample	20196-20205_A	Mudpit_Ingel20196_MGFPeaklist (F058870)	Actin, cytoplasmic type 5 OS=Gallus gallus PE=3 SV=1	ACT5_CHICK,ACTB_CHICK,ACTG_CHICK	TAX_GallusGallus_9031_20141114.fasta	41,793.90	100.00%	22	29	121	0.24%	45.70%	EITALAPSTMK	K	I	99.70%	62.3	45.1	28.9	43.7	39.2	3.43	0.383	0.455	0	15	0	0	2	1,161.62	1,857.00		61,507.60	316	326	Empty	TRUE	ACTA_CHICK,F1N8P3_CHICK,F1P476_CHICK,ACTS_CHICK,ACTC_CHICK
20196-20225 HinckeM –TAXchicken	Uncategorized Sample	20196-20205_A	Mudpit_Ingel20196_MGFPeaklist (F058870)	Actin, cytoplasmic type 5 OS=Gallus gallus PE=3 SV=1	ACT5_CHICK,ACTB_CHICK,ACTG_CHICK	TAX_GallusGallus_9031_20141114.fasta	41,793.90	100.00%	22	29	121	0.24%	45.70%	FPSIVGRPR	V	H	99.70%					40.8	3.55	0.397	0.611	0	1	0	0	1	1,028.60	2,189.00		5,430.63	31	39	Empty	TRUE	ACTA_CHICK,F1N8P3_CHICK,F1P476_CHICK,ACTS_CHICK,ACTC_CHICK
20196-20225 HinckeM –TAXchicken	Uncategorized Sample	20196-20205_A	Mudpit_Ingel20196_MGFPeaklist (F058870)	Actin, cytoplasmic type 5 OS=Gallus gallus PE=3 SV=1	ACT5_CHICK,ACTB_CHICK,ACTG_CHICK	TAX_GallusGallus_9031_20141114.fasta	41,793.90	100.00%	22	29	121	0.24%	45.70%	GYSFTTTAER	R	E	99.70%	55.3	44.6	31.2	37.9	36.7	2.55	0.335	0.55	0	11	0	0	2	1,132.53	1,854.00		117699	197	206	Empty	TRUE	
20196-20225 HinckeM –TAXchicken	Uncategorized Sample	20196-20205_A	Mudpit_Ingel20196_MGFPeaklist (F058870)	Actin, cytoplasmic type 5 OS=Gallus gallus PE=3 SV=1	ACT5_CHICK,ACTB_CHICK,ACTG_CHICK	TAX_GallusGallus_9031_20141114.fasta	41,793.90	100.00%	22	29	121	0.24%	45.70%	GYSFTTTAEREIVR	R	D	99.70%	55	45	17.4	47.9	34.7	2.96	0.256	0.429	0	0	2	0	2	1,629.82	2,217.50		13,050.70	197	210	Empty	TRUE	
20196-20225 HinckeM –TAXchicken	Uncategorized Sample	20196-20205_A	Mudpit_Ingel20196_MGFPeaklist (F058870)	Actin, cytoplasmic type 5 OS=Gallus gallus PE=3 SV=1	ACT5_CHICK,ACTB_CHICK,ACTG_CHICK	TAX_GallusGallus_9031_20141114.fasta	41,793.90	100.00%	22	29	121	0.24%	45.70%	HQGVMVGMGQKDSYVGDEAQSK	R	R	99.70%	63.9	42.1	14.8	58.8	46.4	5.19	0.463	0.364	0	0	0	2	2	2,383.07	1,577.00		1,673.99	40	61	Empty	TRUE	ACTA_CHICK,F1N8P3_CHICK,F1P476_CHICK,ACTS_CHICK,ACTC_CHICK
20196-20225 HinckeM –TAXchicken	Uncategorized Sample	20196-20205_A	Mudpit_Ingel20196_MGFPeaklist (F058870)	Actin, cytoplasmic type 5 OS=Gallus gallus PE=3 SV=1	ACT5_CHICK,ACTB_CHICK,ACTG_CHICK	TAX_GallusGallus_9031_20141114.fasta	41,793.90	100.00%	22	29	121	0.24%	45.70%	HQGVMVGMGQKDSYVGDEAQSKR	R	G	99.70%	72.8	43.1	14.9	69.1	57.5	7.01	0.59	0.435	0	0	0	2	2	2,539.17	1,534.00		1,171.87	40	62	Empty	TRUE	ACTA_CHICK,F1N8P3_CHICK,F1P476_CHICK,ACTS_CHICK,ACTC_CHICK
20196-20225 HinckeM –TAXchicken	Uncategorized Sample	20196-20205_A	Mudpit_Ingel20196_MGFPeaklist (F058870)	Actin, cytoplasmic type 5 OS=Gallus gallus PE=3 SV=1	ACT5_CHICK,ACTB_CHICK,ACTG_CHICK	TAX_GallusGallus_9031_20141114.fasta	41,793.90	100.00%	22	29	121	0.24%	45.70%	IKIIAPPER	K	K	55.90%	29.8	39.4	28.9	13.7					0	2	0	0	2	1,036.65	1,888.00		1,766.94	327	335	Empty	TRUE	ACTA_CHICK,F1P476_CHICK,ACTS_CHICK,ACTC_CHICK
20196-20225 HinckeM –TAXchicken	Uncategorized Sample	20196-20205_A	Mudpit_Ingel20196_MGFPeaklist (F058870)	Actin, cytoplasmic type 5 OS=Gallus gallus PE=3 SV=1	ACT5_CHICK,ACTB_CHICK,ACTG_CHICK	TAX_GallusGallus_9031_20141114.fasta	41,793.90	100.00%	22	29	121	0.24%	45.70%	IWHHTFYNELR	K	V	99.70%	71	44.6	20	58.1	37.9	3.96	0.377	0.455	0	0	4	0	2	1,515.75	2,059.00		7,272.64	85	95	Empty	TRUE	ACTS_CHICK,ACTC_CHICK
20196-20225 HinckeM –TAXchicken	Uncategorized Sample	20196-20205_A	Mudpit_Ingel20196_MGFPeaklist (F058870)	Actin, cytoplasmic type 5 OS=Gallus gallus PE=3 SV=1	ACT5_CHICK,ACTB_CHICK,ACTG_CHICK	TAX_GallusGallus_9031_20141114.fasta	41,793.90	100.00%	22	29	121	0.24%	45.70%	KDLYANTVLSGGTTMYPGIADR	R	M	99.70%	99	44.2	19.8	86.9	56.9	6.52	0.316	0.432	0	0	2	0	2	2,359.16	2,319.00		6,603.07	291	312	Empty	TRUE	
20196-20225 HinckeM –TAXchicken	Uncategorized Sample	20196-20205_A	Mudpit_Ingel20196_MGFPeaklist (F058870)	Actin, cytoplasmic type 5 OS=Gallus gallus PE=3 SV=1	ACT5_CHICK,ACTB_CHICK,ACTG_CHICK	TAX_GallusGallus_9031_20141114.fasta	41,793.90	100.00%	22	29	121	0.24%	45.70%	LDLAGRDLTDYLMK	R	I	99.70%	17.9	44.4	25.4	12.9	38.2	2.6	0.212	0.464	0	0	2	0	2	1,639.84	2,510.50		4,373.75	178	191	Empty	TRUE	ACTA_CHICK,F1N8P3_CHICK,F1P476_CHICK,ACTS_CHICK,ACTC_CHICK
20196-20225 HinckeM –TAXchicken	Uncategorized Sample	20196-20205_A	Mudpit_Ingel20196_MGFPeaklist (F058870)	Actin, cytoplasmic type 5 OS=Gallus gallus PE=3 SV=1	ACT5_CHICK,ACTB_CHICK,ACTG_CHICK	TAX_GallusGallus_9031_20141114.fasta	41,793.90	100.00%	22	29	121	0.24%	45.70%	MQKEITALAPSTMK	R	I	57.80%	22.7	44.4	24.8	8.39					0	0	2	0	2	1,580.80	1,773.00		1,387.25	313	326	Empty	TRUE	ACTA_CHICK,F1N8P3_CHICK,F1P476_CHICK,ACTS_CHICK,ACTC_CHICK
20196-20225 HinckeM –TAXchicken	Uncategorized Sample	20196-20205_A	Mudpit_Ingel20196_MGFPeaklist (F058870)	Actin, cytoplasmic type 5 OS=Gallus gallus PE=3 SV=1	ACT5_CHICK,ACTB_CHICK,ACTG_CHICK	TAX_GallusGallus_9031_20141114.fasta	41,793.90	100.00%	22	29	121	0.24%	45.70%	PSIVGRPR	F	H	96.90%					36.4	0.367	0.236	0.688	0	1	0	0	1	881.5316	2,194.00		3,706.31	32	39	Empty	TRUE	ACTA_CHICK,F1N8P3_CHICK,F1P476_CHICK,ACTS_CHICK,ACTC_CHICK
20196-20225 HinckeM –TAXchicken	Uncategorized Sample	20196-20205_A	Mudpit_Ingel20196_MGFPeaklist (F058870)	Actin, cytoplasmic type 5 OS=Gallus gallus PE=3 SV=1	ACT5_CHICK,ACTB_CHICK,ACTG_CHICK	TAX_GallusGallus_9031_20141114.fasta	41,793.90	100.00%	22	29	121	0.24%	45.70%	PVLLTEAPLNPK	H	A	99.70%					33.5	3.19	0.349	0.375	0	1	0	0	1	1,291.76	2,352.00		297.78	102	113	Empty	TRUE	F1N8P3_CHICK
20196-20225 HinckeM –TAXchicken	Uncategorized Sample	20196-20205_A	Mudpit_Ingel20196_MGFPeaklist (F058870)	Actin, cytoplasmic type 5 OS=Gallus gallus PE=3 SV=1	ACT5_CHICK,ACTB_CHICK,ACTG_CHICK	TAX_GallusGallus_9031_20141114.fasta	41,793.90	100.00%	22	29	121	0.24%	45.70%	QEYDESGPSIVHR	K	K	99.70%	77.5	44	22.5	67.1	43.8	7.51	0.466	0.5	0	4	8	0	2	1,516.70	1,731.00		36,310.00	360	372	Empty	TRUE	
20196-20225 HinckeM –TAXchicken	Uncategorized Sample	20196-20205_A	Mudpit_Ingel20196_MGFPeaklist (F058870)	Actin, cytoplasmic type 5 OS=Gallus gallus PE=3 SV=1	ACT5_CHICK,ACTB_CHICK,ACTG_CHICK	TAX_GallusGallus_9031_20141114.fasta	41,793.90	100.00%	22	29	121	0.24%	45.70%	QEYDESGPSIVHRK	K	C	99.70%	65.9	44.2	19.6	58.9	40.7	5.41	0.4	0.5	0	0	2	4	2	1,627.77	1,606.00		7,227.04	360	373	Empty	TRUE	
20196-20225 HinckeM –TAXchicken	Uncategorized Sample	20196-20205_A	Mudpit_Ingel20196_MGFPeaklist (F058870)	Actin, cytoplasmic type 5 OS=Gallus gallus PE=3 SV=1	ACT5_CHICK,ACTB_CHICK,ACTG_CHICK	TAX_GallusGallus_9031_20141114.fasta	41,793.90	100.00%	22	29	121	0.24%	45.70%	RGILTLK	K	Y	56.70%	26.4	42.2	39.8	0.95					0	2	0	0	2	800.5354	1,975.00		5,469.59	62	68	Empty	TRUE	ACTA_CHICK,F1N8P3_CHICK,F1P476_CHICK,ACTS_CHICK,ACTC_CHICK
20196-20225 HinckeM –TAXchicken	Uncategorized Sample	20196-20205_A	Mudpit_Ingel20196_MGFPeaklist (F058870)	Actin, cytoplasmic type 5 OS=Gallus gallus PE=3 SV=1	ACT5_CHICK,ACTB_CHICK,ACTG_CHICK	TAX_GallusGallus_9031_20141114.fasta	41,793.90	100.00%	22	29	121	0.24%	45.70%	SYELPDGQVITIGNER	K	F	99.70%	75.4	44.7	18.4	65.2	46.5	5.42	0.514	0.438	0	2	2	0	2	1,790.89	2,514.50		26,845.70	239	254	Empty	TRUE	ACTA_CHICK,F1N8P3_CHICK,F1P476_CHICK,ACTS_CHICK,ACTC_CHICK
20196-20225 HinckeM –TAXchicken	Uncategorized Sample	20196-20205_A	Mudpit_Ingel20196_MGFPeaklist (F058870)	Actin, cytoplasmic type 5 OS=Gallus gallus PE=3 SV=1	ACT5_CHICK,ACTB_CHICK,ACTG_CHICK	TAX_GallusGallus_9031_20141114.fasta	41,793.90	100.00%	22	29	121	0.24%	45.70%	VAPEEHPVLLTEAPLNPK	R	A	99.70%	61.8	42.3	14.9	59.6	41.3	3.28	0.339	0.389	0	0	10	3	2	1,954.06	2,333.00		220139	96	113	Empty	TRUE	
20196-20225 HinckeM –TAXchicken	Uncategorized Sample	20196-20205_A	Mudpit_Ingel20196_MGFPeaklist (F058870)	Actin, cytoplasmic type 5 OS=Gallus gallus PE=3 SV=1	ACT5_CHICK,ACTB_CHICK,ACTG_CHICK	TAX_GallusGallus_9031_20141114.fasta	41,793.90	100.00%	22	29	121	0.24%	45.70%	AVFPSIVGR	R	P	99.70%					31.2	2.59	0.279	0.389	0	3	0	0	1	945.5516	2,317.00		6,506.17	29	37	Empty	TRUE	ACTA_CHICK,F1N8P3_CHICK,F1P476_CHICK,ACTS_CHICK,ACTC_CHICK
20196-20225 HinckeM –TAXchicken	Uncategorized Sample	20196-20205_A	Mudpit_Ingel20196_MGFPeaklist (F058870)	Actin, cytoplasmic type 5 OS=Gallus gallus PE=3 SV=1	ACT5_CHICK,ACTB_CHICK,ACTG_CHICK	TAX_GallusGallus_9031_20141114.fasta	41,793.90	100.00%	22	29	121	0.24%	45.70%	AVFPSIVGRPR	R	H	99.70%	63.7	42.3	21	52.4	41	5.02	0.429	0.591	0	1	21	0	2	1,198.71	2,202.00		267262	29	39	Empty	TRUE	ACTA_CHICK,F1N8P3_CHICK,F1P476_CHICK,ACTS_CHICK,ACTC_CHICK
20196-20225 HinckeM –TAXchicken	Uncategorized Sample	20196-20205_A	Mudpit_Ingel20196_MGFPeaklist (F058870)	Actin, cytoplasmic type 5 OS=Gallus gallus PE=3 SV=1	ACT5_CHICK,ACTB_CHICK,ACTG_CHICK	TAX_GallusGallus_9031_20141114.fasta	41,793.90	100.00%	22	29	121	0.24%	45.70%	DLTDYLMK	R	I	99.70%	37.6	43.3	20.1	28.9	30.3	1.38	0.32	0.438	0	10	0	0	2	998.4865	2,207.50		21,013.60	184	191	Empty	TRUE	ACTA_CHICK,F1N8P3_CHICK,F1P476_CHICK,ACTS_CHICK,ACTC_CHICK
20196-20225 HinckeM –TAXchicken	Uncategorized Sample	20196-20205_A	Mudpit_Ingel20196_MGFPeaklist (F058870)	Actin, cytoplasmic type 5 OS=Gallus gallus PE=3 SV=1	ACT5_CHICK,ACTB_CHICK,ACTG_CHICK	TAX_GallusGallus_9031_20141114.fasta	41,793.90	100.00%	22	29	121	0.24%	45.70%	DLYANTVLSGGTTMYPGIADR	K	M	99.70%	61.2	43.9	19.8	53.8	44	5.85	0.241	0.333	0	0	2	0	2	2,231.07	2,469.00		5,341.63	292	312	Empty	TRUE	
20196-20225 HinckeM –TAXchicken	Uncategorized Sample	20196-20205_A	Mudpit_Ingel20196_MGFPeaklist (F058870)	Actin, cytoplasmic type 5 OS=Gallus gallus PE=3 SV=1	ACT5_CHICK,ACTB_CHICK,ACTG_CHICK	TAX_GallusGallus_9031_20141114.fasta	41,793.90	100.00%	22	29	121	0.24%	45.70%	EITALAPSTMK	K	I	99.70%	62.3	45.1	28.9	43.7	39.2	3.43	0.383	0.455	0	15	0	0	2	1,161.62	1,857.00		61,507.60	316	326	Empty	TRUE	ACTA_CHICK,F1N8P3_CHICK,F1P476_CHICK,ACTS_CHICK,ACTC_CHICK
20196-20225 HinckeM –TAXchicken	Uncategorized Sample	20196-20205_A	Mudpit_Ingel20196_MGFPeaklist (F058870)	Actin, cytoplasmic type 5 OS=Gallus gallus PE=3 SV=1	ACT5_CHICK,ACTB_CHICK,ACTG_CHICK	TAX_GallusGallus_9031_20141114.fasta	41,793.90	100.00%	22	29	121	0.24%	45.70%	FPSIVGRPR	V	H	99.70%					40.8	3.55	0.397	0.611	0	1	0	0	1	1,028.60	2,189.00		5,430.63	31	39	Empty	TRUE	ACTA_CHICK,F1N8P3_CHICK,F1P476_CHICK,ACTS_CHICK,ACTC_CHICK
20196-20225 HinckeM –TAXchicken	Uncategorized Sample	20196-20205_A	Mudpit_Ingel20196_MGFPeaklist (F058870)	Actin, cytoplasmic type 5 OS=Gallus gallus PE=3 SV=1	ACT5_CHICK,ACTB_CHICK,ACTG_CHICK	TAX_GallusGallus_9031_20141114.fasta	41,793.90	100.00%	22	29	121	0.24%	45.70%	GYSFTTTAER	R	E	99.70%	55.3	44.6	31.2	37.9	36.7	2.55	0.335	0.55	0	11	0	0	2	1,132.53	1,854.00		117699	197	206	Empty	TRUE	
20196-20225 HinckeM –TAXchicken	Uncategorized Sample	20196-20205_A	Mudpit_Ingel20196_MGFPeaklist (F058870)	Actin, cytoplasmic type 5 OS=Gallus gallus PE=3 SV=1	ACT5_CHICK,ACTB_CHICK,ACTG_CHICK	TAX_GallusGallus_9031_20141114.fasta	41,793.90	100.00%	22	29	121	0.24%	45.70%	GYSFTTTAEREIVR	R	D	99.70%	55	45	17.4	47.9	34.7	2.96	0.256	0.429	0	0	2	0	2	1,629.82	2,217.50		13,050.70	197	210	Empty	TRUE	
20196-20225 HinckeM –TAXchicken	Uncategorized Sample	20196-20205_A	Mudpit_Ingel20196_MGFPeaklist (F058870)	Actin, cytoplasmic type 5 OS=Gallus gallus PE=3 SV=1	ACT5_CHICK,ACTB_CHICK,ACTG_CHICK	TAX_GallusGallus_9031_20141114.fasta	41,793.90	100.00%	22	29	121	0.24%	45.70%	HQGVMVGMGQKDSYVGDEAQSK	R	R	99.70%	63.9	42.1	14.8	58.8	46.4	5.19	0.463	0.364	0	0	0	2	2	2,383.07	1,577.00		1,673.99	40	61	Empty	TRUE	ACTA_CHICK,F1N8P3_CHICK,F1P476_CHICK,ACTS_CHICK,ACTC_CHICK
20196-20225 HinckeM –TAXchicken	Uncategorized Sample	20196-20205_A	Mudpit_Ingel20196_MGFPeaklist (F058870)	Actin, cytoplasmic type 5 OS=Gallus gallus PE=3 SV=1	ACT5_CHICK,ACTB_CHICK,ACTG_CHICK	TAX_GallusGallus_9031_20141114.fasta	41,793.90	100.00%	22	29	121	0.24%	45.70%	HQGVMVGMGQKDSYVGDEAQSKR	R	G	99.70%	72.8	43.1	14.9	69.1	57.5	7.01	0.59	0.435	0	0	0	2	2	2,539.17	1,534.00		1,171.87	40	62	Empty	TRUE	ACTA_CHICK,F1N8P3_CHICK,F1P476_CHICK,ACTS_CHICK,ACTC_CHICK
20196-20225 HinckeM –TAXchicken	Uncategorized Sample	20196-20205_A	Mudpit_Ingel20196_MGFPeaklist (F058870)	Actin, cytoplasmic type 5 OS=Gallus gallus PE=3 SV=1	ACT5_CHICK,ACTB_CHICK,ACTG_CHICK	TAX_GallusGallus_9031_20141114.fasta	41,793.90	100.00%	22	29	121	0.24%	45.70%	IKIIAPPER	K	K	55.90%	29.8	39.4	28.9	13.7					0	2	0	0	2	1,036.65	1,888.00		1,766.94	327	335	Empty	TRUE	ACTA_CHICK,F1P476_CHICK,ACTS_CHICK,ACTC_CHICK
20196-20225 HinckeM –TAXchicken	Uncategorized Sample	20196-20205_A	Mudpit_Ingel20196_MGFPeaklist (F058870)	Actin, cytoplasmic type 5 OS=Gallus gallus PE=3 SV=1	ACT5_CHICK,ACTB_CHICK,ACTG_CHICK	TAX_GallusGallus_9031_20141114.fasta	41,793.90	100.00%	22	29	121	0.24%	45.70%	IWHHTFYNELR	K	V	99.70%	71	44.6	20	58.1	37.9	3.96	0.377	0.455	0	0	4	0	2	1,515.75	2,059.00		7,272.64	85	95	Empty	TRUE	ACTS_CHICK,ACTC_CHICK
20196-20225 HinckeM –TAXchicken	Uncategorized Sample	20196-20205_A	Mudpit_Ingel20196_MGFPeaklist (F058870)	Actin, cytoplasmic type 5 OS=Gallus gallus PE=3 SV=1	ACT5_CHICK,ACTB_CHICK,ACTG_CHICK	TAX_GallusGallus_9031_20141114.fasta	41,793.90	100.00%	22	29	121	0.24%	45.70%	KDLYANTVLSGGTTMYPGIADR	R	M	99.70%	99	44.2	19.8	86.9	56.9	6.52	0.316	0.432	0	0	2	0	2	2,359.16	2,319.00		6,603.07	291	312	Empty	TRUE	
20196-20225 HinckeM –TAXchicken	Uncategorized Sample	20196-20205_A	Mudpit_Ingel20196_MGFPeaklist (F058870)	Actin, cytoplasmic type 5 OS=Gallus gallus PE=3 SV=1	ACT5_CHICK,ACTB_CHICK,ACTG_CHICK	TAX_GallusGallus_9031_20141114.fasta	41,793.90	100.00%	22	29	121	0.24%	45.70%	LDLAGRDLTDYLMK	R	I	99.70%	17.9	44.4	25.4	12.9	38.2	2.6	0.212	0.464	0	0	2	0	2	1,639.84	2,510.50		4,373.75	178	191	Empty	TRUE	ACTA_CHICK,F1N8P3_CHICK,F1P476_CHICK,ACTS_CHICK,ACTC_CHICK
20196-20225 HinckeM –TAXchicken	Uncategorized Sample	20196-20205_A	Mudpit_Ingel20196_MGFPeaklist (F058870)	Actin, cytoplasmic type 5 OS=Gallus gallus PE=3 SV=1	ACT5_CHICK,ACTB_CHICK,ACTG_CHICK	TAX_GallusGallus_9031_20141114.fasta	41,793.90	100.00%	22	29	121	0.24%	45.70%	MQKEITALAPSTMK	R	I	57.80%	22.7	44.4	24.8	8.39					0	0	2	0	2	1,580.80	1,773.00		1,387.25	313	326	Empty	TRUE	ACTA_CHICK,F1N8P3_CHICK,F1P476_CHICK,ACTS_CHICK,ACTC_CHICK
20196-20225 HinckeM –TAXchicken	Uncategorized Sample	20196-20205_A	Mudpit_Ingel20196_MGFPeaklist (F058870)	Actin, cytoplasmic type 5 OS=Gallus gallus PE=3 SV=1	ACT5_CHICK,ACTB_CHICK,ACTG_CHICK	TAX_GallusGallus_9031_20141114.fasta	41,793.90	100.00%	22	29	121	0.24%	45.70%	PSIVGRPR	F	H	96.90%					36.4	0.367	0.236	0.688	0	1	0	0	1	881.5316	2,194.00		3,706.31	32	39	Empty	TRUE	ACTA_CHICK,F1N8P3_CHICK,F1P476_CHICK,ACTS_CHICK,ACTC_CHICK
20196-20225 HinckeM –TAXchicken	Uncategorized Sample	20196-20205_A	Mudpit_Ingel20196_MGFPeaklist (F058870)	Actin, cytoplasmic type 5 OS=Gallus gallus PE=3 SV=1	ACT5_CHICK,ACTB_CHICK,ACTG_CHICK	TAX_GallusGallus_9031_20141114.fasta	41,793.90	100.00%	22	29	121	0.24%	45.70%	PVLLTEAPLNPK	H	A	99.70%					33.5	3.19	0.349	0.375	0	1	0	0	1	1,291.76	2,352.00		297.78	102	113	Empty	TRUE	F1N8P3_CHICK
20196-20225 HinckeM –TAXchicken	Uncategorized Sample	20196-20205_A	Mudpit_Ingel20196_MGFPeaklist (F058870)	Actin, cytoplasmic type 5 OS=Gallus gallus PE=3 SV=1	ACT5_CHICK,ACTB_CHICK,ACTG_CHICK	TAX_GallusGallus_9031_20141114.fasta	41,793.90	100.00%	22	29	121	0.24%	45.70%	QEYDESGPSIVHR	K	K	99.70%	77.5	44	22.5	67.1	43.8	7.51	0.466	0.5	0	4	8	0	2	1,516.70	1,731.00		36,310.00	360	372	Empty	TRUE	
20196-20225 HinckeM –TAXchicken	Uncategorized Sample	20196-20205_A	Mudpit_Ingel20196_MGFPeaklist (F058870)	Actin, cytoplasmic type 5 OS=Gallus gallus PE=3 SV=1	ACT5_CHICK,ACTB_CHICK,ACTG_CHICK	TAX_GallusGallus_9031_20141114.fasta	41,793.90	100.00%	22	29	121	0.24%	45.70%	QEYDESGPSIVHRK	K	C	99.70%	65.9	44.2	19.6	58.9	40.7	5.41	0.4	0.5	0	0	2	4	2	1,627.77	1,606.00		7,227.04	360	373	Empty	TRUE	
20196-20225 HinckeM –TAXchicken	Uncategorized Sample	20196-20205_A	Mudpit_Ingel20196_MGFPeaklist (F058870)	Actin, cytoplasmic type 5 OS=Gallus gallus PE=3 SV=1	ACT5_CHICK,ACTB_CHICK,ACTG_CHICK	TAX_GallusGallus_9031_20141114.fasta	41,793.90	100.00%	22	29	121	0.24%	45.70%	RGILTLK	K	Y	56.70%	26.4	42.2	39.8	0.95					0	2	0	0	2	800.5354	1,975.00		5,469.59	62	68	Empty	TRUE	ACTA_CHICK,F1N8P3_CHICK,F1P476_CHICK,ACTS_CHICK,ACTC_CHICK
20196-20225 HinckeM –TAXchicken	Uncategorized Sample	20196-20205_A	Mudpit_Ingel20196_MGFPeaklist (F058870)	Actin, cytoplasmic type 5 OS=Gallus gallus PE=3 SV=1	ACT5_CHICK,ACTB_CHICK,ACTG_CHICK	TAX_GallusGallus_9031_20141114.fasta	41,793.90	100.00%	22	29	121	0.24%	45.70%	SYELPDGQVITIGNER	K	F	99.70%	75.4	44.7	18.4	65.2	46.5	5.42	0.514	0.438	0	2	2	0	2	1,790.89	2,514.50		26,845.70	239	254	Empty	TRUE	ACTA_CHICK,F1N8P3_CHICK,F1P476_CHICK,ACTS_CHICK,ACTC_CHICK
20196-20225 HinckeM –TAXchicken	Uncategorized Sample	20196-20205_A	Mudpit_Ingel20196_MGFPeaklist (F058870)	Actin, cytoplasmic type 5 OS=Gallus gallus PE=3 SV=1	ACT5_CHICK,ACTB_CHICK,ACTG_CHICK	TAX_GallusGallus_9031_20141114.fasta	41,793.90	100.00%	22	29	121	0.24%	45.70%	VAPEEHPVLLTEAPLNPK	R	A	99.70%	61.8	42.3	14.9	59.6	41.3	3.28	0.339	0.389	0	0	10	3	2	1,954.06	2,333.00		220139	96	113	Empty	TRUE	

20196-20225 HinckeM –TAXchicken	Uncategorized Sample	20196-20205_A	Mudpit_Ingel20196_MGFPeaklist (F058870)	Uncharacterized protein OS=Gallus gallus GN=DHX15 PE=4 SV=2	F1NHI3_CHICK,Q5F3A9_CHICK,UPI00004CF227,UPI0000610F33,UPI0000ECC700,UPI0002C87744	TAX_GallusGallus_9031_20141114.fasta,UPARC14_2_GallusG_9031_20140417.fasta	87,678.60	99.70%	2	2	4	0.01%	1.97%	FTDILIR	R	H	22.20%	9.52	43.8	17.8	0					0	2	0	0	2	877.5143	2,356.50		2,737.72	113	119	Empty	TRUE	UPI0000449D13,UPI00003AD9E7,UPI00003AF458
20196-20225 HinckeM –TAXchicken	Uncategorized Sample	20196-20205_A	Mudpit_Ingel20196_MGFPeaklist (F058870)	Uncharacterized protein OS=Gallus gallus GN=DHX15 PE=4 SV=2	F1NHI3_CHICK,Q5F3A9_CHICK,UPI00004CF227,UPI0000610F33,UPI0000ECC700,UPI0002C87744	TAX_GallusGallus_9031_20141114.fasta,UPARC14_2_GallusG_9031_20140417.fasta	87,678.60	99.70%	2	2	4	0.01%	1.97%	IFEPPPPK	R	K	99.60%	35	42.7	24.7	11	22.6	0.585	0.128	0.25	0	2	0	0	2	924.5191	1,787.50		312.87	367	374	Empty	TRUE	
20196-20225 HinckeM –TAXchicken	Uncategorized Sample	20196-20205_A	Mudpit_Ingel20196_MGFPeaklist (F058870)	Actin, alpha cardiac muscle 1 OS=Gallus gallus GN=ACTC1 PE=3 SV=1	ACTC_CHICK,ACTS_CHICK	TAX_GallusGallus_9031_20141114.fasta	42,052.20	100.00%	4	5	13	0.03%	42.70%	AVFPSIVGR	R	P	99.70%					31.2	2.59	0.279	0.389	0	3	0	0	1	945.5516	2,317.00		6,506.17	31	39	Empty	FALSE	ACTB_CHICK,ACTA_CHICK,ACTG_CHICK,F1N8P3_CHICK,F1P476_CHICK,ACT5_CHICK
20196-20225 HinckeM –TAXchicken	Uncategorized Sample	20196-20205_A	Mudpit_Ingel20196_MGFPeaklist (F058870)	Actin, alpha cardiac muscle 1 OS=Gallus gallus GN=ACTC1 PE=3 SV=1	ACTC_CHICK,ACTS_CHICK	TAX_GallusGallus_9031_20141114.fasta	42,052.20	100.00%	4	5	13	0.03%	42.70%	AVFPSIVGRPR	R	H	99.70%	63.7	42.3	21	52.4	41	5.02	0.429	0.591	0	1	21	0	2	1,198.71	2,202.00		267262	31	41	Empty	FALSE	ACTB_CHICK,ACTA_CHICK,ACTG_CHICK,F1N8P3_CHICK,F1P476_CHICK,ACT5_CHICK
20196-20225 HinckeM –TAXchicken	Uncategorized Sample	20196-20205_A	Mudpit_Ingel20196_MGFPeaklist (F058870)	Actin, alpha cardiac muscle 1 OS=Gallus gallus GN=ACTC1 PE=3 SV=1	ACTC_CHICK,ACTS_CHICK	TAX_GallusGallus_9031_20141114.fasta	42,052.20	100.00%	4	5	13	0.03%	42.70%	DLTDYLMK	R	I	99.70%	37.6	43.3	20.1	28.9	30.3	1.38	0.32	0.438	0	10	0	0	2	998.4865	2,207.50		21,013.60	186	193	Empty	FALSE	ACTB_CHICK,ACTA_CHICK,ACTG_CHICK,F1N8P3_CHICK,F1P476_CHICK,ACT5_CHICK
20196-20225 HinckeM –TAXchicken	Uncategorized Sample	20196-20205_A	Mudpit_Ingel20196_MGFPeaklist (F058870)	Actin, alpha cardiac muscle 1 OS=Gallus gallus GN=ACTC1 PE=3 SV=1	ACTC_CHICK,ACTS_CHICK	TAX_GallusGallus_9031_20141114.fasta	42,052.20	100.00%	4	5	13	0.03%	42.70%	EITALAPSTMK	K	I	99.70%	62.3	45.1	28.9	43.7	39.2	3.43	0.383	0.455	0	15	0	0	2	1,161.62	1,857.00		61,507.60	318	328	Empty	FALSE	ACTB_CHICK,ACTA_CHICK,ACTG_CHICK,F1N8P3_CHICK,F1P476_CHICK,ACT5_CHICK
20196-20225 HinckeM –TAXchicken	Uncategorized Sample	20196-20205_A	Mudpit_Ingel20196_MGFPeaklist (F058870)	Actin, alpha cardiac muscle 1 OS=Gallus gallus GN=ACTC1 PE=3 SV=1	ACTC_CHICK,ACTS_CHICK	TAX_GallusGallus_9031_20141114.fasta	42,052.20	100.00%	4	5	13	0.03%	42.70%	FPSIVGRPR	V	H	99.70%					40.8	3.55	0.397	0.611	0	1	0	0	1	1,028.60	2,189.00		5,430.63	33	41	Empty	FALSE	ACTB_CHICK,ACTA_CHICK,ACTG_CHICK,F1N8P3_CHICK,F1P476_CHICK,ACT5_CHICK
20196-20225 HinckeM –TAXchicken	Uncategorized Sample	20196-20205_A	Mudpit_Ingel20196_MGFPeaklist (F058870)	Actin, alpha cardiac muscle 1 OS=Gallus gallus GN=ACTC1 PE=3 SV=1	ACTC_CHICK,ACTS_CHICK	TAX_GallusGallus_9031_20141114.fasta	42,052.20	100.00%	4	5	13	0.03%	42.70%	GYSFVTTAER	R	E	99.70%	33.9	44.8	18.6	27.7	26.4	1.17	0.265	0.3	0	4	0	0	2	1,130.55	1,993.00		3,547.80	199	208	Empty	TRUE	ACTA_CHICK,F1N8P3_CHICK,F1P476_CHICK
20196-20225 HinckeM –TAXchicken	Uncategorized Sample	20196-20205_A	Mudpit_Ingel20196_MGFPeaklist (F058870)	Actin, alpha cardiac muscle 1 OS=Gallus gallus GN=ACTC1 PE=3 SV=1	ACTC_CHICK,ACTS_CHICK	TAX_GallusGallus_9031_20141114.fasta	42,052.20	100.00%	4	5	13	0.03%	42.70%	HQGVMVGMGQKDSYVGDEAQSK	R	R	99.70%	63.9	42.1	14.8	58.8	46.4	5.19	0.463	0.364	0	0	0	2	2	2,383.07	1,577.00		1,673.99	42	63	Empty	FALSE	ACTB_CHICK,ACTA_CHICK,ACTG_CHICK,F1N8P3_CHICK,F1P476_CHICK,ACT5_CHICK
20196-20225 HinckeM –TAXchicken	Uncategorized Sample	20196-20205_A	Mudpit_Ingel20196_MGFPeaklist (F058870)	Actin, alpha cardiac muscle 1 OS=Gallus gallus GN=ACTC1 PE=3 SV=1	ACTC_CHICK,ACTS_CHICK	TAX_GallusGallus_9031_20141114.fasta	42,052.20	100.00%	4	5	13	0.03%	42.70%	HQGVMVGMGQKDSYVGDEAQSKR	R	G	99.70%	72.8	43.1	14.9	69.1	57.5	7.01	0.59	0.435	0	0	0	2	2	2,539.17	1,534.00		1,171.87	42	64	Empty	FALSE	ACTB_CHICK,ACTA_CHICK,ACTG_CHICK,F1N8P3_CHICK,F1P476_CHICK,ACT5_CHICK
20196-20225 HinckeM –TAXchicken	Uncategorized Sample	20196-20205_A	Mudpit_Ingel20196_MGFPeaklist (F058870)	Actin, alpha cardiac muscle 1 OS=Gallus gallus GN=ACTC1 PE=3 SV=1	ACTC_CHICK,ACTS_CHICK	TAX_GallusGallus_9031_20141114.fasta	42,052.20	100.00%	4	5	13	0.03%	42.70%	IKIIAPPER	K	K	55.90%	29.8	39.4	28.9	13.7					0	2	0	0	2	1,036.65	1,888.00		1,766.94	329	337	Empty	FALSE	ACTB_CHICK,ACTA_CHICK,ACTG_CHICK,F1P476_CHICK,ACT5_CHICK
20196-20225 HinckeM –TAXchicken	Uncategorized Sample	20196-20205_A	Mudpit_Ingel20196_MGFPeaklist (F058870)	Actin, alpha cardiac muscle 1 OS=Gallus gallus GN=ACTC1 PE=3 SV=1	ACTC_CHICK,ACTS_CHICK	TAX_GallusGallus_9031_20141114.fasta	42,052.20	100.00%	4	5	13	0.03%	42.70%	IWHHTFYNELR	K	V	99.70%	71	44.6	20	58.1	37.9	3.96	0.377	0.455	0	0	4	0	2	1,515.75	2,059.00		7,272.64	87	97	Empty	FALSE	ACTB_CHICK,ACTG_CHICK,ACT5_CHICK
20196-20225 HinckeM –TAXchicken	Uncategorized Sample	20196-20205_A	Mudpit_Ingel20196_MGFPeaklist (F058870)	Actin, alpha cardiac muscle 1 OS=Gallus gallus GN=ACTC1 PE=3 SV=1	ACTC_CHICK,ACTS_CHICK	TAX_GallusGallus_9031_20141114.fasta	42,052.20	100.00%	4	5	13	0.03%	42.70%	LDLAGRDLTDYLMK	R	I	99.70%	17.9	44.4	25.4	12.9	38.2	2.6	0.212	0.464	0	0	2	0	2	1,639.84	2,510.50		4,373.75	180	193	Empty	FALSE	ACTB_CHICK,ACTA_CHICK,ACTG_CHICK,F1N8P3_CHICK,F1P476_CHICK,ACT5_CHICK
20196-20225 HinckeM –TAXchicken	Uncategorized Sample	20196-20205_A	Mudpit_Ingel20196_MGFPeaklist (F058870)	Actin, alpha cardiac muscle 1 OS=Gallus gallus GN=ACTC1 PE=3 SV=1	ACTC_CHICK,ACTS_CHICK	TAX_GallusGallus_9031_20141114.fasta	42,052.20	100.00%	4	5	13	0.03%	42.70%	MQKEITALAPSTMK	R	I	57.80%	22.7	44.4	24.8	8.39					0	0	2	0	2	1,580.80	1,773.00		1,387.25	315	328	Empty	FALSE	ACTB_CHICK,ACTA_CHICK,ACTG_CHICK,F1N8P3_CHICK,F1P476_CHICK,ACT5_CHICK
20196-20225 HinckeM –TAXchicken	Uncategorized Sample	20196-20205_A	Mudpit_Ingel20196_MGFPeaklist (F058870)	Actin, alpha cardiac muscle 1 OS=Gallus gallus GN=ACTC1 PE=3 SV=1	ACTC_CHICK,ACTS_CHICK	TAX_GallusGallus_9031_20141114.fasta	42,052.20	100.00%	4	5	13	0.03%	42.70%	PSIVGRPR	F	H	96.90%					36.4	0.367	0.236	0.688	0	1	0	0	1	881.5316	2,194.00		3,706.31	34	41	Empty	FALSE	ACTB_CHICK,ACTA_CHICK,ACTG_CHICK,F1N8P3_CHICK,F1P476_CHICK,ACT5_CHICK
20196-20225 HinckeM –TAXchicken	Uncategorized Sample	20196-20205_A	Mudpit_Ingel20196_MGFPeaklist (F058870)	Actin, alpha cardiac muscle 1 OS=Gallus gallus GN=ACTC1 PE=3 SV=1	ACTC_CHICK,ACTS_CHICK	TAX_GallusGallus_9031_20141114.fasta	42,052.20	100.00%	4	5	13	0.03%	42.70%	QEYDEAGPSIVHR	K	K	99.70%	61.9	43.6	18.6	53.8	44.6	7.24	0.137	0.5	0	1	2	0	2	1,483.68	1,765.00		2,586.35	362	374	Empty	TRUE	ACTA_CHICK,F1P476_CHICK
20196-20225 HinckeM –TAXchicken	Uncategorized Sample	20196-20205_A	Mudpit_Ingel20196_MGFPeaklist (F058870)	Actin, alpha cardiac muscle 1 OS=Gallus gallus GN=ACTC1 PE=3 SV=1	ACTC_CHICK,ACTS_CHICK	TAX_GallusGallus_9031_20141114.fasta	42,052.20	100.00%	4	5	13	0.03%	42.70%	RGILTLK	K	Y	56.70%	26.4	42.2	39.8	0.95					0	2	0	0	2	800.5354	1,975.00		5,469.59	64	70	Empty	FALSE	ACTB_CHICK,ACTA_CHICK,ACTG_CHICK,F1N8P3_CHICK,F1P476_CHICK,ACT5_CHICK
20196-20225 HinckeM –TAXchicken	Uncategorized Sample	20196-20205_A	Mudpit_Ingel20196_MGFPeaklist (F058870)	Actin, alpha cardiac muscle 1 OS=Gallus gallus GN=ACTC1 PE=3 SV=1	ACTC_CHICK,ACTS_CHICK	TAX_GallusGallus_9031_20141114.fasta	42,052.20	100.00%	4	5	13	0.03%	42.70%	SYELPDGQVITIGNER	K	F	99.70%	75.4	44.7	18.4	65.2	46.5	5.42	0.514	0.438	0	2	2	0	2	1,790.89	2,514.50		26,845.70	241	256	Empty	FALSE	ACTB_CHICK,ACTA_CHICK,ACTG_CHICK,F1N8P3_CHICK,F1P476_CHICK,ACT5_CHICK
20196-20225 HinckeM –TAXchicken	Uncategorized Sample	20196-20205_A	Mudpit_Ingel20196_MGFPeaklist (F058870)	Actin, alpha cardiac muscle 1 OS=Gallus gallus GN=ACTC1 PE=3 SV=1	ACTC_CHICK,ACTS_CHICK	TAX_GallusGallus_9031_20141114.fasta	42,052.20	100.00%	4	5	13	0.03%	42.70%	VAPEEHPTLLTEAPLNPK	R	A	99.70%	50.1	43.3	14.7	42.4	40	3.7	0.102	0.333	0	0	4	0	2	1,956.04	2,310.50		14,238.60	98	115	Empty	TRUE	ACTA_CHICK,F1P476_CHICK
20196-20225 HinckeM –TAXchicken	Uncategorized Sample	20196-20205_A	Mudpit_Ingel20196_MGFPeaklist (F058870)	Actin, alpha cardiac muscle 1 OS=Gallus gallus GN=ACTC1 PE=3 SV=1	ACTC_CHICK,ACTS_CHICK	TAX_GallusGallus_9031_20141114.fasta	42,052.20	100.00%	4	5	13	0.03%	42.70%	YPIEHGIITNWDDMEK	K	I	94.90%	23.5	43.4	15.4	22.8					0	0	2	0	2	1,976.91	2,248.50		7,598.36	71	86	Empty	TRUE	ACTA_CHICK,F1N8P3_CHICK,F1P476_CHICK
20196-20225 HinckeM –TAXchicken	Uncategorized Sample	20196-20205_A	Mudpit_Ingel20196_MGFPeaklist (F058870)	Actin, alpha cardiac muscle 1 OS=Gallus gallus GN=ACTC1 PE=3 SV=1	ACTC_CHICK,ACTS_CHICK	TAX_GallusGallus_9031_20141114.fasta	42,052.20	100.00%	4	5	13	0.03%	42.70%	AVFPSIVGR	R	P	99.70%					31.2	2.59	0.279	0.389	0	3	0	0	1	945.5516	2,317.00		6,506.17	31	39	Empty	FALSE	ACTB_CHICK,ACTA_CHICK,ACTG_CHICK,F1N8P3_CHICK,F1P476_CHICK,ACT5_CHICK
20196-20225 HinckeM –TAXchicken	Uncategorized Sample	20196-20205_A	Mudpit_Ingel20196_MGFPeaklist (F058870)	Actin, alpha cardiac muscle 1 OS=Gallus gallus GN=ACTC1 PE=3 SV=1	ACTC_CHICK,ACTS_CHICK	TAX_GallusGallus_9031_20141114.fasta	42,052.20	100.00%	4	5	13	0.03%	42.70%	AVFPSIVGRPR	R	H	99.70%	63.7	42.3	21	52.4	41	5.02	0.429	0.591	0	1	21	0	2	1,198.71	2,202.00		267262	31	41	Empty	FALSE	ACTB_CHICK,ACTA_CHICK,ACTG_CHICK,F1N8P3_CHICK,F1P476_CHICK,ACT5_CHICK
20196-20225 HinckeM –TAXchicken	Uncategorized Sample	20196-20205_A	Mudpit_Ingel20196_MGFPeaklist (F058870)	Actin, alpha cardiac muscle 1 OS=Gallus gallus GN=ACTC1 PE=3 SV=1	ACTC_CHICK,ACTS_CHICK	TAX_GallusGallus_9031_20141114.fasta	42,052.20	100.00%	4	5	13	0.03%	42.70%	DLTDYLMK	R	I	99.70%	37.6	43.3	20.1	28.9	30.3	1.38	0.32	0.438	0	10	0	0	2	998.4865	2,207.50		21,013.60	186	193	Empty	FALSE	ACTB_CHICK,ACTA_CHICK,ACTG_CHICK,F1N8P3_CHICK,F1P476_CHICK,ACT5_CHICK
20196-20225 HinckeM –TAXchicken	Uncategorized Sample	20196-20205_A	Mudpit_Ingel20196_MGFPeaklist (F058870)	Actin, alpha cardiac muscle 1 OS=Gallus gallus GN=ACTC1 PE=3 SV=1	ACTC_CHICK,ACTS_CHICK	TAX_GallusGallus_9031_20141114.fasta	42,052.20	100.00%	4	5	13	0.03%	42.70%	EITALAPSTMK	K	I	99.70%	62.3	45.1	28.9	43.7	39.2	3.43	0.383	0.455	0	15	0	0	2	1,161.62	1,857.00		61,507.60	318	328	Empty	FALSE	ACTB_CHICK,ACTA_CHICK,ACTG_CHICK,F1N8P3_CHICK,F1P476_CHICK,ACT5_CHICK
20196-20225 HinckeM –TAXchicken	Uncategorized Sample	20196-20205_A	Mudpit_Ingel20196_MGFPeaklist (F058870)	Actin, alpha cardiac muscle 1 OS=Gallus gallus GN=ACTC1 PE=3 SV=1	ACTC_CHICK,ACTS_CHICK	TAX_GallusGallus_9031_20141114.fasta	42,052.20	100.00%	4	5	13	0.03%	42.70%	FPSIVGRPR	V	H	99.70%					40.8	3.55	0.397	0.611	0	1	0	0	1	1,028.60	2,189.00		5,430.63	33	41	Empty	FALSE	ACTB_CHICK,ACTA_CHICK,ACTG_CHICK,F1N8P3_CHICK,F1P476_CHICK,ACT5_CHICK
20196-20225 HinckeM –TAXchicken	Uncategorized Sample	20196-20205_A	Mudpit_Ingel20196_MGFPeaklist (F058870)	Actin, alpha cardiac muscle 1 OS=Gallus gallus GN=ACTC1 PE=3 SV=1	ACTC_CHICK,ACTS_CHICK	TAX_GallusGallus_9031_20141114.fasta	42,052.20	100.00%	4	5	13	0.03%	42.70%	GYSFVTTAER	R	E	99.70%	33.9	44.8	18.6	27.7	26.4	1.17	0.265	0.3	0	4	0	0	2	1,130.55	1,993.00		3,547.80	199	208	Empty	TRUE	ACTA_CHICK,F1N8P3_CHICK,F1P476_CHICK
20196-20225 HinckeM –TAXchicken	Uncategorized Sample	20196-20205_A	Mudpit_Ingel20196_MGFPeaklist (F058870)	Actin, alpha cardiac muscle 1 OS=Gallus gallus GN=ACTC1 PE=3 SV=1	ACTC_CHICK,ACTS_CHICK	TAX_GallusGallus_9031_20141114.fasta	42,052.20	100.00%	4	5	13	0.03%	42.70%	HQGVMVGMGQKDSYVGDEAQSK	R	R	99.70%	63.9	42.1	14.8	58.8	46.4	5.19	0.463	0.364	0	0	0	2	2	2,383.07	1,577.00		1,673.99	42	63	Empty	FALSE	ACTB_CHICK,ACTA_CHICK,ACTG_CHICK,F1N8P3_CHICK,F1P476_CHICK,ACT5_CHICK
20196-20225 HinckeM –TAXchicken	Uncategorized Sample	20196-20205_A	Mudpit_Ingel20196_MGFPeaklist (F058870)	Actin, alpha cardiac muscle 1 OS=Gallus gallus GN=ACTC1 PE=3 SV=1	ACTC_CHICK,ACTS_CHICK	TAX_GallusGallus_9031_20141114.fasta	42,052.20	100.00%	4	5	13	0.03%	42.70%	HQGVMVGMGQKDSYVGDEAQSKR	R	G	99.70%	72.8	43.1	14.9	69.1	57.5	7.01	0.59	0.435	0	0	0	2	2	2,539.17	1,534.00		1,171.87	42	64	Empty	FALSE	ACTB_CHICK,ACTA_CHICK,ACTG_CHICK,F1N8P3_CHICK,F1P476_CHICK,ACT5_CHICK
20196-20225 HinckeM –TAXchicken	Uncategorized Sample	20196-20205_A	Mudpit_Ingel20196_MGFPeaklist (F058870)	Actin, alpha cardiac muscle 1 OS=Gallus gallus GN=ACTC1 PE=3 SV=1	ACTC_CHICK,ACTS_CHICK	TAX_GallusGallus_9031_20141114.fasta	42,052.20	100.00%	4	5	13	0.03%	42.70%	IKIIAPPER	K	K	55.90%	29.8	39.4	28.9	13.7					0	2	0	0	2	1,036.65	1,888.00		1,766.94	329	337	Empty	FALSE	ACTB_CHICK,ACTA_CHICK,ACTG_CHICK,F1P476_CHICK,ACT5_CHICK
20196-20225 HinckeM –TAXchicken	Uncategorized Sample	20196-20205_A	Mudpit_Ingel20196_MGFPeaklist (F058870)	Actin, alpha cardiac muscle 1 OS=Gallus gallus GN=ACTC1 PE=3 SV=1	ACTC_CHICK,ACTS_CHICK	TAX_GallusGallus_9031_20141114.fasta	42,052.20	100.00%	4	5	13	0.03%	42.70%	IWHHTFYNELR	K	V	99.70%	71	44.6	20	58.1	37.9	3.96	0.377	0.455	0	0	4	0	2	1,515.75	2,059.00		7,272.64	87	97	Empty	FALSE	ACTB_CHICK,ACTG_CHICK,ACT5_CHICK
20196-20225 HinckeM –TAXchicken	Uncategorized Sample	20196-20205_A	Mudpit_Ingel20196_MGFPeaklist (F058870)	Actin, alpha cardiac muscle 1 OS=Gallus gallus GN=ACTC1 PE=3 SV=1	ACTC_CHICK,ACTS_CHICK	TAX_GallusGallus_9031_20141114.fasta	42,052.20	100.00%	4	5	13	0.03%	42.70%	LDLAGRDLTDYLMK	R	I	99.70%	17.9	44.4	25.4	12.9	38.2	2.6	0.212	0.464	0	0	2	0	2	1,639.84	2,510.50		4,373.75	180	193	Empty	FALSE	ACTB_CHICK,ACTA_CHICK,ACTG_CHICK,F1N8P3_CHICK,F1P476_CHICK,ACT5_CHICK
20196-20225 HinckeM –TAXchicken	Uncategorized Sample	20196-20205_A	Mudpit_Ingel20196_MGFPeaklist (F058870)	Actin, alpha cardiac muscle 1 OS=Gallus gallus GN=ACTC1 PE=3 SV=1	ACTC_CHICK,ACTS_CHICK	TAX_GallusGallus_9031_20141114.fasta	42,052.20	100.00%	4	5	13	0.03%	42.70%	MQKEITALAPSTMK	R	I	57.80%	22.7	44.4	24.8	8.39					0	0	2	0	2	1,580.80	1,773.00		1,387.25	315	328	Empty	FALSE	ACTB_CHICK,ACTA_CHICK,ACTG_CHICK,F1N8P3_CHICK,F1P476_CHICK,ACT5_CHICK
20196-20225 HinckeM –TAXchicken	Uncategorized Sample	20196-20205_A	Mudpit_Ingel20196_MGFPeaklist (F058870)	Actin, alpha cardiac muscle 1 OS=Gallus gallus GN=ACTC1 PE=3 SV=1	ACTC_CHICK,ACTS_CHICK	TAX_GallusGallus_9031_20141114.fasta	42,052.20	100.00%	4	5	13	0.03%	42.70%	PSIVGRPR	F	H	96.90%					36.4	0.367	0.236	0.688	0	1	0	0	1	881.5316	2,194.00		3,706.31	34	41	Empty	FALSE	ACTB_CHICK,ACTA_CHICK,ACTG_CHICK,F1N8P3_CHICK,F1P476_CHICK,ACT5_CHICK
20196-20225 HinckeM –TAXchicken	Uncategorized Sample	20196-20205_A	Mudpit_Ingel20196_MGFPeaklist (F058870)	Actin, alpha cardiac muscle 1 OS=Gallus gallus GN=ACTC1 PE=3 SV=1	ACTC_CHICK,ACTS_CHICK	TAX_GallusGallus_9031_20141114.fasta	42,052.20	100.00%	4	5	13	0.03%	42.70%	QEYDEAGPSIVHR	K	K	99.70%	61.9	43.6	18.6	53.8	44.6	7.24	0.137	0.5	0	1	2	0	2	1,483.68	1,765.00		2,586.35	362	374	Empty	TRUE	ACTA_CHICK,F1P476_CHICK
20196-20225 HinckeM –TAXchicken	Uncategorized Sample	20196-20205_A	Mudpit_Ingel20196_MGFPeaklist (F058870)	Actin, alpha cardiac muscle 1 OS=Gallus gallus GN=ACTC1 PE=3 SV=1	ACTC_CHICK,ACTS_CHICK	TAX_GallusGallus_9031_20141114.fasta	42,052.20	100.00%	4	5	13	0.03%	42.70%	RGILTLK	K	Y	56.70%	26.4	42.2	39.8	0.95					0	2	0	0	2	800.5354	1,975.00		5,469.59	64	70	Empty	FALSE	ACTB_CHICK,ACTA_CHICK,ACTG_CHICK,F1N8P3_CHICK,F1P476_CHICK,ACT5_CHICK
20196-20225 HinckeM –TAXchicken	Uncategorized Sample	20196-20205_A	Mudpit_Ingel20196_MGFPeaklist (F058870)	Actin, alpha cardiac muscle 1 OS=Gallus gallus GN=ACTC1 PE=3 SV=1	ACTC_CHICK,ACTS_CHICK	TAX_GallusGallus_9031_20141114.fasta	42,052.20	100.00%	4	5	13	0.03%	42.70%	SYELPDGQVITIGNER	K	F	99.70%	75.4	44.7	18.4	65.2	46.5	5.42	0.514	0.438	0	2	2	0	2	1,790.89	2,514.50		26,845.70	241	256	Empty	FALSE	ACTB_CHICK,ACTA_CHICK,ACTG_CHICK,F1N8P3_CHICK,F1P476_CHICK,ACT5_CHICK
20196-20225 HinckeM –TAXchicken	Uncategorized Sample	20196-20205_A	Mudpit_Ingel20196_MGFPeaklist (F058870)	Actin, alpha cardiac muscle 1 OS=Gallus gallus GN=ACTC1 PE=3 SV=1	ACTC_CHICK,ACTS_CHICK	TAX_GallusGallus_9031_20141114.fasta	42,052.20	100.00%	4	5	13	0.03%	42.70%	VAPEEHPTLLTEAPLNPK	R	A	99.70%	50.1	43.3	14.7	42.4	40	3.7	0.102	0.333	0	0	4	0	2	1,956.04	2,310.50		14,238.60	98	115	Empty	TRUE	ACTA_CHICK,F1P476_CHICK
20196-20225 HinckeM –TAXchicken	Uncategorized Sample	20196-20205_A	Mudpit_Ingel20196_MGFPeaklist (F058870)	Actin, alpha cardiac muscle 1 OS=Gallus gallus GN=ACTC1 PE=3 SV=1	ACTC_CHICK,ACTS_CHICK	TAX_GallusGallus_9031_20141114.fasta	42,052.20	100.00%	4	5	13	0.03%	42.70%	YPIEHGIITNWDDMEK	K	I	94.90%	23.5	43.4	15.4	22.8					0	0	2	0	2	1,976.91	2,248.50		7,598.36	71	86	Empty	TRUE	ACTA_CHICK,F1N8P3_CHICK,F1P476_CHICK
20196-20225 HinckeM –TAXchicken	Uncategorized Sample	20196-20205_A	Mudpit_Ingel20196_MGFPeaklist (F058870)	Uncharacterized protein OS=Gallus gallus GN=ACTBL2 PE=3 SV=2	F1N8P3_CHICK	TAX_GallusGallus_9031_20141114.fasta	42,015.10	97.60%	1	1	2	0.00%	34.00%	AVFPSIVGR	R	P	99.70%					31.2	2.59	0.279	0.389	0	3	0	0	1	945.5516	2,317.00		6,506.17	31	39	Empty	FALSE	ACTB_CHICK,ACTA_CHICK,ACTG_CHICK,F1P476_CHICK,ACT5_CHICK,ACTS_CHICK,ACTC_CHICK
20196-20225 HinckeM –TAXchicken	Uncategorized Sample	20196-20205_A	Mudpit_Ingel20196_MGFPeaklist (F058870)	Uncharacterized protein OS=Gallus gallus GN=ACTBL2 PE=3 SV=2	F1N8P3_CHICK	TAX_GallusGallus_9031_20141114.fasta	42,015.10	97.60%	1	1	2	0.00%	34.00%	AVFPSIVGRPR	R	H	99.70%	63.7	42.3	21	52.4	41	5.02	0.429	0.591	0	1	21	0	2	1,198.71	2,202.00		267262	31	41	Empty	FALSE	ACTB_CHICK,ACTA_CHICK,ACTG_CHICK,F1P476_CHICK,ACT5_CHICK,ACTS_CHICK,ACTC_CHICK
20196-20225 HinckeM –TAXchicken	Uncategorized Sample	20196-20205_A	Mudpit_Ingel20196_MGFPeaklist (F058870)	Uncharacterized protein OS=Gallus gallus GN=ACTBL2 PE=3 SV=2	F1N8P3_CHICK	TAX_GallusGallus_9031_20141114.fasta	42,015.10	97.60%	1	1	2	0.00%	34.00%	DLTDYLMK	R	I	99.70%	37.6	43.3	20.1	28.9	30.3	1.38	0.32	0.438	0	10	0	0	2	998.4865	2,207.50		21,013.60	186	193	Empty	FALSE	ACTB_CHICK,ACTA_CHICK,ACTG_CHICK,F1P476_CHICK,ACT5_CHICK,ACTS_CHICK,ACTC_CHICK
20196-20225 HinckeM –TAXchicken	Uncategorized Sample	20196-20205_A	Mudpit_Ingel20196_MGFPeaklist (F058870)	Uncharacterized protein OS=Gallus gallus GN=ACTBL2 PE=3 SV=2	F1N8P3_CHICK	TAX_GallusGallus_9031_20141114.fasta	42,015.10	97.60%	1	1	2	0.00%	34.00%	EITALAPSTMK	K	I	99.70%	62.3	45.1	28.9	43.7	39.2	3.43	0.383	0.455	0	15	0	0	2	1,161.62	1,857.00		61,507.60	318	328	Empty	FALSE	ACTB_CHICK,ACTA_CHICK,ACTG_CHICK,F1P476_CHICK,ACT5_CHICK,ACTS_CHICK,ACTC_CHICK
20196-20225 HinckeM –TAXchicken	Uncategorized Sample	20196-20205_A	Mudpit_Ingel20196_MGFPeaklist (F058870)	Uncharacterized protein OS=Gallus gallus GN=ACTBL2 PE=3 SV=2	F1N8P3_CHICK	TAX_GallusGallus_9031_20141114.fasta	42,015.10	97.60%	1	1	2	0.00%	34.00%	FPSIVGRPR	V	H	99.70%					40.8	3.55	0.397	0.611	0	1	0	0	1	1,028.60	2,189.00		5,430.63	33	41	Empty	FALSE	ACTB_CHICK,ACTA_CHICK,ACTG_CHICK,F1P476_CHICK,ACT5_CHICK,ACTS_CHICK,ACTC_CHICK
20196-20225 HinckeM –TAXchicken	Uncategorized Sample	20196-20205_A	Mudpit_Ingel20196_MGFPeaklist (F058870)	Uncharacterized protein OS=Gallus gallus GN=ACTBL2 PE=3 SV=2	F1N8P3_CHICK	TAX_GallusGallus_9031_20141114.fasta	42,015.10	97.60%	1	1	2	0.00%	34.00%	GYSFVTTAER	R	E	99.70%	33.9	44.8	18.6	27.7	26.4	1.17	0.265	0.3	0	4	0	0	2	1,130.55	1,993.00		3,547.80	199	208	Empty	FALSE	ACTA_CHICK,F1P476_CHICK,ACTS_CHICK,ACTC_CHICK
20196-20225 HinckeM –TAXchicken	Uncategorized Sample	20196-20205_A	Mudpit_Ingel20196_MGFPeaklist (F058870)	Uncharacterized protein OS=Gallus gallus GN=ACTBL2 PE=3 SV=2	F1N8P3_CHICK	TAX_GallusGallus_9031_20141114.fasta	42,015.10	97.60%	1	1	2	0.00%	34.00%	HQGVMVGMGQKDSYVGDEAQSK	R	R	99.70%	63.9	42.1	14.8	58.8	46.4	5.19	0.463	0.364	0	0	0	2	2	2,383.07	1,577.00		1,673.99	42	63	Empty	FALSE	ACTB_CHICK,ACTA_CHICK,ACTG_CHICK,F1P476_CHICK,ACT5_CHICK,ACTS_CHICK,ACTC_CHICK
20196-20225 HinckeM –TAXchicken	Uncategorized Sample	20196-20205_A	Mudpit_Ingel20196_MGFPeaklist (F058870)	Uncharacterized protein OS=Gallus gallus GN=ACTBL2 PE=3 SV=2	F1N8P3_CHICK	TAX_GallusGallus_9031_20141114.fasta	42,015.10	97.60%	1	1	2	0.00%	34.00%	HQGVMVGMGQKDSYVGDEAQSKR	R	G	99.70%	72.8	43.1	14.9	69.1	57.5	7.01	0.59	0.435	0	0	0	2	2	2,539.17	1,534.00		1,171.87	42	64	Empty	FALSE	ACTB_CHICK,ACTA_CHICK,ACTG_CHICK,F1P476_CHICK,ACT5_CHICK,ACTS_CHICK,ACTC_CHICK
20196-20225 HinckeM –TAXchicken	Uncategorized Sample	20196-20205_A	Mudpit_Ingel20196_MGFPeaklist (F058870)	Uncharacterized protein OS=Gallus gallus GN=ACTBL2 PE=3 SV=2	F1N8P3_CHICK	TAX_GallusGallus_9031_20141114.fasta	42,015.10	97.60%	1	1	2	0.00%	34.00%	IAPEEHPVLLTEAPLNPK	R	A	99.70%					39.3	5.19	0.397	0.25	0	0	2	0	2	1,968.08	2,316.00		2,276.18	98	115	Empty	TRUE	
20196-20225 HinckeM –TAXchicken	Uncategorized Sample	20196-20205_A	Mudpit_Ingel20196_MGFPeaklist (F058870)	Uncharacterized protein OS=Gallus gallus GN=ACTBL2 PE=3 SV=2	F1N8P3_CHICK	TAX_GallusGallus_9031_20141114.fasta	42,015.10	97.60%	1	1	2	0.00%	34.00%	LDLAGRDLTDYLMK	R	I	99.70%	17.9	44.4	25.4	12.9	38.2	2.6	0.212	0.464	0	0	2	0	2	1,639.84	2,510.50		4,373.75	180	193	Empty	FALSE	ACTB_CHICK,ACTA_CHICK,ACTG_CHICK,F1P476_CHICK,ACT5_CHICK,ACTS_CHICK,ACTC_CHICK
20196-20225 HinckeM –TAXchicken	Uncategorized Sample	20196-20205_A	Mudpit_Ingel20196_MGFPeaklist (F058870)	Uncharacterized protein OS=Gallus gallus GN=ACTBL2 PE=3 SV=2	F1N8P3_CHICK	TAX_GallusGallus_9031_20141114.fasta	42,015.10	97.60%	1	1	2	0.00%	34.00%	MQKEITALAPSTMK	R	I	57.80%	22.7	44.4	24.8	8.39					0	0	2	0	2	1,580.80	1,773.00		1,387.25	315	328	Empty	FALSE	ACTB_CHICK,ACTA_CHICK,ACTG_CHICK,F1P476_CHICK,ACT5_CHICK,ACTS_CHICK,ACTC_CHICK
20196-20225 HinckeM –TAXchicken	Uncategorized Sample	20196-20205_A	Mudpit_Ingel20196_MGFPeaklist (F058870)	Uncharacterized protein OS=Gallus gallus GN=ACTBL2 PE=3 SV=2	F1N8P3_CHICK	TAX_GallusGallus_9031_20141114.fasta	42,015.10	97.60%	1	1	2	0.00%	34.00%	PSIVGRPR	F	H	96.90%					36.4	0.367	0.236	0.688	0	1	0	0	1	881.5316	2,194.00		3,706.31	34	41	Empty	FALSE	ACTB_CHICK,ACTA_CHICK,ACTG_CHICK,F1P476_CHICK,ACT5_CHICK,ACTS_CHICK,ACTC_CHICK
20196-20225 HinckeM –TAXchicken	Uncategorized Sample	20196-20205_A	Mudpit_Ingel20196_MGFPeaklist (F058870)	Uncharacterized protein OS=Gallus gallus GN=ACTBL2 PE=3 SV=2	F1N8P3_CHICK	TAX_GallusGallus_9031_20141114.fasta	42,015.10	97.60%	1	1	2	0.00%	34.00%	PVLLTEAPLNPK	H	A	99.70%					33.5	3.19	0.349	0.375	0	1	0	0	1	1,291.76	2,352.00		297.78	104	115	Empty	FALSE	ACTB_CHICK,ACTG_CHICK,ACT5_CHICK
20196-20225 HinckeM –TAXchicken	Uncategorized Sample	20196-20205_A	Mudpit_Ingel20196_MGFPeaklist (F058870)	Uncharacterized protein OS=Gallus gallus GN=ACTBL2 PE=3 SV=2	F1N8P3_CHICK	TAX_GallusGallus_9031_20141114.fasta	42,015.10	97.60%	1	1	2	0.00%	34.00%	RGILTLK	K	Y	56.70%	26.4	42.2	39.8	0.95					0	2	0	0	2	800.5354	1,975.00		5,469.59	64	70	Empty	FALSE	ACTB_CHICK,ACTA_CHICK,ACTG_CHICK,F1P476_CHICK,ACT5_CHICK,ACTS_CHICK,ACTC_CHICK
20196-20225 HinckeM –TAXchicken	Uncategorized Sample	20196-20205_A	Mudpit_Ingel20196_MGFPeaklist (F058870)	Uncharacterized protein OS=Gallus gallus GN=ACTBL2 PE=3 SV=2	F1N8P3_CHICK	TAX_GallusGallus_9031_20141114.fasta	42,015.10	97.60%	1	1	2	0.00%	34.00%	SYELPDGQVITIGNER	K	F	99.70%	75.4	44.7	18.4	65.2	46.5	5.42	0.514	0.438	0	2	2	0	2	1,790.89	2,514.50		26,845.70	241	256	Empty	FALSE	ACTB_CHICK,ACTA_CHICK,ACTG_CHICK,F1P476_CHICK,ACT5_CHICK,ACTS_CHICK,ACTC_CHICK
20196-20225 HinckeM –TAXchicken	Uncategorized Sample	20196-20205_A	Mudpit_Ingel20196_MGFPeaklist (F058870)	Uncharacterized protein OS=Gallus gallus GN=ACTBL2 PE=3 SV=2	F1N8P3_CHICK	TAX_GallusGallus_9031_20141114.fasta	42,015.10	97.60%	1	1	2	0.00%	34.00%	YPIEHGIITNWDDMEK	K	I	94.90%	23.5	43.4	15.4	22.8					0	0	2	0	2	1,976.91	2,248.50		7,598.36	71	86	Empty	FALSE	ACTA_CHICK,F1P476_CHICK,ACTS_CHICK,ACTC_CHICK
20196-20225 HinckeM –TAXchicken	Uncategorized Sample	20196-20205_A	Mudpit_Ingel20196_MGFPeaklist (F058870)	Lamin-A OS=Gallus gallus GN=LMNA PE=2 SV=1	LMNA_CHICK,UPI000012E20B	TAX_GallusGallus_9031_20141114.fasta,UPARC14_2_GallusG_9031_20140417.fasta	73,165.80	100.00%	28	33	135	0.27%	40.00%	AAYEAELADAR	K	K	99.70%	73	44.2	29.9	51.2	39.5	4.7	0.458	0.5	0	6	0	0	2	1,179.56	1,860.50		7,279.28	78	88	Empty	TRUE	
20196-20225 HinckeM –TAXchicken	Uncategorized Sample	20196-20205_A	Mudpit_Ingel20196_MGFPeaklist (F058870)	Lamin-A OS=Gallus gallus GN=LMNA PE=2 SV=1	LMNA_CHICK,UPI000012E20B	TAX_GallusGallus_9031_20141114.fasta,UPARC14_2_GallusG_9031_20140417.fasta	73,165.80	100.00%	28	33	135	0.27%	40.00%	AAYEAELADARK	K	T	99.70%	49.5	44.7	25.9	32.1	32	2.7	0.278	0.375	0	0	4	0	2	1,307.66	1,739.50		4,574.56	78	89	Empty	TRUE	
20196-20225 HinckeM –TAXchicken	Uncategorized Sample	20196-20205_A	Mudpit_Ingel20196_MGFPeaklist (F058870)	Lamin-A OS=Gallus gallus GN=LMNA PE=2 SV=1	LMNA_CHICK,UPI000012E20B	TAX_GallusGallus_9031_20141114.fasta,UPARC14_2_GallusG_9031_20140417.fasta	73,165.80	100.00%	28	33	135	0.27%	40.00%	AQSSWGSGDSLR	K	T	99.70%	56.7	44.2	25.8	42.7	30.3	2.25	0.35	0.333	0	5	0	0	2	1,250.58	1,826.00		5,462.14	517	528	Empty	TRUE	
20196-20225 HinckeM –TAXchicken	Uncategorized Sample	20196-20205_A	Mudpit_Ingel20196_MGFPeaklist (F058870)	Lamin-A OS=Gallus gallus GN=LMNA PE=2 SV=1	LMNA_CHICK,UPI000012E20B	TAX_GallusGallus_9031_20141114.fasta,UPARC14_2_GallusG_9031_20140417.fasta	73,165.80	100.00%	28	33	135	0.27%	40.00%	EAALSTALGEK	K	R	99.70%	42.8	45.3	27.1	30.9	28.8	2.34	0.299	0.364	0	3	0	0	2	1,089.58	1,950.00		1,622.42	144	154	Empty	TRUE	
20196-20225 HinckeM –TAXchicken	Uncategorized Sample	20196-20205_A	Mudpit_Ingel20196_MGFPeaklist (F058870)	Lamin-A OS=Gallus gallus GN=LMNA PE=2 SV=1	LMNA_CHICK,UPI000012E20B	TAX_GallusGallus_9031_20141114.fasta,UPARC14_2_GallusG_9031_20140417.fasta	73,165.80	100.00%	28	33	135	0.27%	40.00%	EAALSTALGEKR	K	N	99.70%	73.7	44.7	34.8	39.7	37.4	3.39	0.374	0.542	0	0	4	0	2	1,245.68	1,829.50		4,846.77	144	155	Empty	TRUE	
20196-20225 HinckeM –TAXchicken	Uncategorized Sample	20196-20205_A	Mudpit_Ingel20196_MGFPeaklist (F058870)	Lamin-A OS=Gallus gallus GN=LMNA PE=2 SV=1	LMNA_CHICK,UPI000012E20B	TAX_GallusGallus_9031_20141114.fasta,UPARC14_2_GallusG_9031_20140417.fasta	73,165.80	100.00%	28	33	135	0.27%	40.00%	EADLLAAQAR	K	L	99.70%	45.4	44.6	37.7	16	27.5	1.3	0.276	0.3	0	5	0	0	2	1,057.56	1,899.00		3,656.01	123	132	Empty	TRUE	
20196-20225 HinckeM –TAXchicken	Uncategorized Sample	20196-20205_A	Mudpit_Ingel20196_MGFPeaklist (F058870)	Lamin-A OS=Gallus gallus GN=LMNA PE=2 SV=1	LMNA_CHICK,UPI000012E20B	TAX_GallusGallus_9031_20141114.fasta,UPARC14_2_GallusG_9031_20140417.fasta	73,165.80	100.00%	28	33	135	0.27%	40.00%	IDSLSAELSQLQK	R	Q	99.70%	67.7	44.6	30	50.4	45.4	3.72	0.355	0.5	0	4	0	0	2	1,431.77	2,526.50		20,911.30	298	310	Empty	TRUE	
20196-20225 HinckeM –TAXchicken	Uncategorized Sample	20196-20205_A	Mudpit_Ingel20196_MGFPeaklist (F058870)	Lamin-A OS=Gallus gallus GN=LMNA PE=2 SV=1	LMNA_CHICK,UPI000012E20B	TAX_GallusGallus_9031_20141114.fasta,UPARC14_2_GallusG_9031_20140417.fasta	73,165.80	100.00%	28	33	135	0.27%	40.00%	ITESEEVVSR	R	E	99.70%	69.6	44.9	30.5	51.6	38.5	3.62	0.338	0.5	0	10	0	0	2	1,148.58	1,613.00		9,195.70	62	71	Empty	TRUE	
20196-20225 HinckeM –TAXchicken	Uncategorized Sample	20196-20205_A	Mudpit_Ingel20196_MGFPeaklist (F058870)	Lamin-A OS=Gallus gallus GN=LMNA PE=2 SV=1	LMNA_CHICK,UPI000012E20B	TAX_GallusGallus_9031_20141114.fasta,UPARC14_2_GallusG_9031_20140417.fasta	73,165.80	100.00%	28	33	135	0.27%	40.00%	LAEALQDLR	K	R	99.70%	70.7	44.2	46.7	46.5	35.1	1.74	0.251	0.5	0	5	0	0	2	1,028.57	2,117.00		9,169.64	240	248	Empty	TRUE	
20196-20225 HinckeM –TAXchicken	Uncategorized Sample	20196-20205_A	Mudpit_Ingel20196_MGFPeaklist (F058870)	Lamin-A OS=Gallus gallus GN=LMNA PE=2 SV=1	LMNA_CHICK,UPI000012E20B	TAX_GallusGallus_9031_20141114.fasta,UPARC14_2_GallusG_9031_20140417.fasta	73,165.80	100.00%	28	33	135	0.27%	40.00%	LAVYIDK	R	V	98.80%	36	43.8	29.2	18.2	27.5	0.77	0.218	0.5	0	3	0	0	2	821.4768	1,956.00		2,455.14	41	47	Empty	TRUE	UPI000012E202,F1NAM2_CHICK,LMNB1_CHICK,UPI0002C87565,UPI0000ECC2CB,UPI0000ECC2CA
20196-20225 HinckeM –TAXchicken	Uncategorized Sample	20196-20205_A	Mudpit_Ingel20196_MGFPeaklist (F058870)	Lamin-A OS=Gallus gallus GN=LMNA PE=2 SV=1	LMNA_CHICK,UPI000012E20B	TAX_GallusGallus_9031_20141114.fasta,UPARC14_2_GallusG_9031_20140417.fasta	73,165.80	100.00%	28	33	135	0.27%	40.00%	LKDLEALLNSK	R	E	99.70%	81.2	43.3	42	57.8	36	1.41	0.311	0.545	0	0	2	0	2	1,243.73	2,422.50		9,184.08	133	143	Empty	TRUE	
20196-20225 HinckeM –TAXchicken	Uncategorized Sample	20196-20205_A	Mudpit_Ingel20196_MGFPeaklist (F058870)	Lamin-A OS=Gallus gallus GN=LMNA PE=2 SV=1	LMNA_CHICK,UPI000012E20B	TAX_GallusGallus_9031_20141114.fasta,UPARC14_2_GallusG_9031_20140417.fasta	73,165.80	100.00%	28	33	135	0.27%	40.00%	LLEGEEERLR	K	L	36.60%	18.3	44.7	34.8	-2.17					0	0	2	0	2	1,243.66	1,818.00		1,931.03	378	387	Empty	TRUE	
20196-20225 HinckeM –TAXchicken	Uncategorized Sample	20196-20205_A	Mudpit_Ingel20196_MGFPeaklist (F058870)	Lamin-A OS=Gallus gallus GN=LMNA PE=2 SV=1	LMNA_CHICK,UPI000012E20B	TAX_GallusGallus_9031_20141114.fasta,UPARC14_2_GallusG_9031_20140417.fasta	73,165.80	100.00%	28	33	135	0.27%	40.00%	LQEKEDLQELNDR	R	L	99.70%	67.6	44.9	39.8	43.8	47.4	3.13	0.369	0.615	0	0	6	0	2	1,629.81	1,812.00		20,646.10	28	40	Empty	TRUE	
20196-20225 HinckeM –TAXchicken	Uncategorized Sample	20196-20205_A	Mudpit_Ingel20196_MGFPeaklist (F058870)	Lamin-A OS=Gallus gallus GN=LMNA PE=2 SV=1	LMNA_CHICK,UPI000012E20B	TAX_GallusGallus_9031_20141114.fasta,UPARC14_2_GallusG_9031_20140417.fasta	73,165.80	100.00%	28	33	135	0.27%	40.00%	LQTLKEELEFQK	R	N	99.70%	65.9	43.8	28.3	0	35.9	2.11	0.298	0.417	0	0	2	0	2	1,505.82	2,194.50		8,532.50	196	207	Empty	TRUE	
20196-20225 HinckeM –TAXchicken	Uncategorized Sample	20196-20205_A	Mudpit_Ingel20196_MGFPeaklist (F058870)	Lamin-A OS=Gallus gallus GN=LMNA PE=2 SV=1	LMNA_CHICK,UPI000012E20B	TAX_GallusGallus_9031_20141114.fasta,UPARC14_2_GallusG_9031_20140417.fasta	73,165.80	100.00%	28	33	135	0.27%	40.00%	LREVEEALSR	K	E	99.70%	46	44.8	34.2	18.6	33.4	2.52	0.251	0.5	0	0	8	0	2	1,201.65	1,823.50		10,410.60	319	328	Empty	TRUE	
20196-20225 HinckeM –TAXchicken	Uncategorized Sample	20196-20205_A	Mudpit_Ingel20196_MGFPeaklist (F058870)	Lamin-A OS=Gallus gallus GN=LMNA PE=2 SV=1	LMNA_CHICK,UPI000012E20B	TAX_GallusGallus_9031_20141114.fasta,UPARC14_2_GallusG_9031_20140417.fasta	73,165.80	100.00%	28	33	135	0.27%	40.00%	LRITESEEVVSR	R	E	37.60%	18.5	44.5	0	0					0	0	2	0	2	1,417.76	1,880.50		468.49	60	71	Empty	TRUE	
20196-20225 HinckeM –TAXchicken	Uncategorized Sample	20196-20205_A	Mudpit_Ingel20196_MGFPeaklist (F058870)	Lamin-A OS=Gallus gallus GN=LMNA PE=2 SV=1	LMNA_CHICK,UPI000012E20B	TAX_GallusGallus_9031_20141114.fasta,UPARC14_2_GallusG_9031_20140417.fasta	73,165.80	100.00%	28	33	135	0.27%	40.00%	LVEIDNGRQQEFESK	R	L	99.70%	29.4	44.7	19.7	4.75	41.4	2.8	0.15	0.467	0	0	5	0	2	1,792.87	1,897.00		12,188.10	225	239	Empty	TRUE	
20196-20225 HinckeM –TAXchicken	Uncategorized Sample	20196-20205_A	Mudpit_Ingel20196_MGFPeaklist (F058870)	Lamin-A OS=Gallus gallus GN=LMNA PE=2 SV=1	LMNA_CHICK,UPI000012E20B	TAX_GallusGallus_9031_20141114.fasta,UPARC14_2_GallusG_9031_20140417.fasta	73,165.80	100.00%	28	33	135	0.27%	40.00%	NIYSEELR	K	E	99.70%	37.5	43.9	34.8	15.9	24.5	0.602	0.208	0.312	0	4	0	0	2	1,023.51	1,892.50		2,481.92	208	215	Empty	TRUE	
20196-20225 HinckeM –TAXchicken	Uncategorized Sample	20196-20205_A	Mudpit_Ingel20196_MGFPeaklist (F058870)	Lamin-A OS=Gallus gallus GN=LMNA PE=2 SV=1	LMNA_CHICK,UPI000012E20B	TAX_GallusGallus_9031_20141114.fasta,UPARC14_2_GallusG_9031_20140417.fasta	73,165.80	100.00%	28	33	135	0.27%	40.00%	NIYSEELRETK	K	R	82.40%	20	44.4	15	16.5					0	0	2	0	2	1,381.70	1,824.00		1,242.96	208	218	Empty	TRUE	
20196-20225 HinckeM –TAXchicken	Uncategorized Sample	20196-20205_A	Mudpit_Ingel20196_MGFPeaklist (F058870)	Lamin-A OS=Gallus gallus GN=LMNA PE=2 SV=1	LMNA_CHICK,UPI000012E20B	TAX_GallusGallus_9031_20141114.fasta,UPARC14_2_GallusG_9031_20140417.fasta	73,165.80	100.00%	28	33	135	0.27%	40.00%	NKSNEDQALGNWQVK	R	R	99.70%	19.6	44.6	24.6	4.03	29.6	1.85	0.206	0.333	0	0	1	0	2	1,730.85	2,017.00		1,170.90	457	471	Empty	TRUE	
20196-20225 HinckeM –TAXchicken	Uncategorized Sample	20196-20205_A	Mudpit_Ingel20196_MGFPeaklist (F058870)	Lamin-A OS=Gallus gallus GN=LMNA PE=2 SV=1	LMNA_CHICK,UPI000012E20B	TAX_GallusGallus_9031_20141114.fasta,UPARC14_2_GallusG_9031_20140417.fasta	73,165.80	100.00%	28	33	135	0.27%	40.00%	NSSMAGAAHEELQQTHIR	R	I	99.70%	56.1	43.5	19.3	47.5	37.6	3.54	0.378	0.306	0	0	0	5	2	1,995.93	1,633.00		3,012.13	280	297	Empty	TRUE	
20196-20225 HinckeM –TAXchicken	Uncategorized Sample	20196-20205_A	Mudpit_Ingel20196_MGFPeaklist (F058870)	Lamin-A OS=Gallus gallus GN=LMNA PE=2 SV=1	LMNA_CHICK,UPI000012E20B	TAX_GallusGallus_9031_20141114.fasta,UPARC14_2_GallusG_9031_20140417.fasta	73,165.80	100.00%	28	33	135	0.27%	40.00%	QNGDDPPLTYR	R	F	99.70%	27.9	44.4	19.5	22.3	25.8	1.11	0.283	0.227	0	6	0	0	2	1,275.60	1,805.50		2,294.84	473	483	Empty	TRUE	
20196-20225 HinckeM –TAXchicken	Uncategorized Sample	20196-20205_A	Mudpit_Ingel20196_MGFPeaklist (F058870)	Lamin-A OS=Gallus gallus GN=LMNA PE=2 SV=1	LMNA_CHICK,UPI000012E20B	TAX_GallusGallus_9031_20141114.fasta,UPARC14_2_GallusG_9031_20140417.fasta	73,165.80	100.00%	28	33	135	0.27%	40.00%	SLELENAGLR	R	L	99.70%	59.6	45	41.9	32.8	36.2	1.77	0.221	0.5	0	10	0	0	2	1,101.59	2,110.50		17,733.70	50	59	Empty	TRUE	
20196-20225 HinckeM –TAXchicken	Uncategorized Sample	20196-20205_A	Mudpit_Ingel20196_MGFPeaklist (F058870)	Lamin-A OS=Gallus gallus GN=LMNA PE=2 SV=1	LMNA_CHICK,UPI000012E20B	TAX_GallusGallus_9031_20141114.fasta,UPARC14_2_GallusG_9031_20140417.fasta	73,165.80	100.00%	28	33	135	0.27%	40.00%	SNEDQALGNWQVKR	K	Q	99.70%	37.1	44.9	25.6	22.4	30.7	1.96	0.156	0.321	0	0	1	0	2	1,644.81	1,977.00		1,898.16	459	472	Empty	TRUE	
20196-20225 HinckeM –TAXchicken	Uncategorized Sample	20196-20205_A	Mudpit_Ingel20196_MGFPeaklist (F058870)	Lamin-A OS=Gallus gallus GN=LMNA PE=2 SV=1	LMNA_CHICK,UPI000012E20B	TAX_GallusGallus_9031_20141114.fasta,UPARC14_2_GallusG_9031_20140417.fasta	73,165.80	100.00%	28	33	135	0.27%	40.00%	SSGGGIGEGLLGR	R	S	99.70%	72.7	45.2	40.7	46.7	47.1	4.14	0.348	0.538	0	11	0	0	2	1,159.61	2,189.00		19,604.70	623	635	Empty	TRUE	
20196-20225 HinckeM –TAXchicken	Uncategorized Sample	20196-20205_A	Mudpit_Ingel20196_MGFPeaklist (F058870)	Lamin-A OS=Gallus gallus GN=LMNA PE=2 SV=1	LMNA_CHICK,UPI000012E20B	TAX_GallusGallus_9031_20141114.fasta,UPARC14_2_GallusG_9031_20140417.fasta	73,165.80	100.00%	28	33	135	0.27%	40.00%	TALINSNGEEVAMR	R	K	98.70%	36.4	44.7	22.7	24.9					0	3	0	0	2	1,520.74	1,830.00		1,452.45	529	542	Empty	TRUE	
20196-20225 HinckeM –TAXchicken	Uncategorized Sample	20196-20205_A	Mudpit_Ingel20196_MGFPeaklist (F058870)	Lamin-A OS=Gallus gallus GN=LMNA PE=2 SV=1	LMNA_CHICK,UPI000012E20B	TAX_GallusGallus_9031_20141114.fasta,UPARC14_2_GallusG_9031_20140417.fasta	73,165.80	100.00%	28	33	135	0.27%	40.00%	TALINSNGEEVAMRK	R	L	99.70%	43.4	44.9	22.5	30.5	25.4	1.07	0.177	0.233	0	0	2	0	2	1,648.83	1,732.00		1,499.35	529	543	Empty	TRUE	
20196-20225 HinckeM –TAXchicken	Uncategorized Sample	20196-20205_A	Mudpit_Ingel20196_MGFPeaklist (F058870)	Lamin-A OS=Gallus gallus GN=LMNA PE=2 SV=1	LMNA_CHICK,UPI000012E20B	TAX_GallusGallus_9031_20141114.fasta,UPARC14_2_GallusG_9031_20140417.fasta	73,165.80	100.00%	28	33	135	0.27%	40.00%	VGVEEVDLEGR	R	F	99.70%	70	45	30.9	50.3	36.3	4.28	0.424	0.455	0	12	0	0	2	1,201.61	2,016.50		16,566.70	441	451	Empty	TRUE	
20196-20225 HinckeM –TAXchicken	Uncategorized Sample	20196-20205_A	Mudpit_Ingel20196_MGFPeaklist (F058870)	Lamin-A OS=Gallus gallus GN=LMNA PE=2 SV=1	LMNA_CHICK,UPI000012E20B	TAX_GallusGallus_9031_20141114.fasta,UPARC14_2_GallusG_9031_20140417.fasta	73,165.80	100.00%	28	33	135	0.27%	40.00%	AAYEAELADAR	K	K	99.70%	73	44.2	29.9	51.2	39.5	4.7	0.458	0.5	0	6	0	0	2	1,179.56	1,860.50		7,279.28	78	88	Empty	TRUE	
20196-20225 HinckeM –TAXchicken	Uncategorized Sample	20196-20205_A	Mudpit_Ingel20196_MGFPeaklist (F058870)	Lamin-A OS=Gallus gallus GN=LMNA PE=2 SV=1	LMNA_CHICK,UPI000012E20B	TAX_GallusGallus_9031_20141114.fasta,UPARC14_2_GallusG_9031_20140417.fasta	73,165.80	100.00%	28	33	135	0.27%	40.00%	AAYEAELADARK	K	T	99.70%	49.5	44.7	25.9	32.1	32	2.7	0.278	0.375	0	0	4	0	2	1,307.66	1,739.50		4,574.56	78	89	Empty	TRUE	
20196-20225 HinckeM –TAXchicken	Uncategorized Sample	20196-20205_A	Mudpit_Ingel20196_MGFPeaklist (F058870)	Lamin-A OS=Gallus gallus GN=LMNA PE=2 SV=1	LMNA_CHICK,UPI000012E20B	TAX_GallusGallus_9031_20141114.fasta,UPARC14_2_GallusG_9031_20140417.fasta	73,165.80	100.00%	28	33	135	0.27%	40.00%	AQSSWGSGDSLR	K	T	99.70%	56.7	44.2	25.8	42.7	30.3	2.25	0.35	0.333	0	5	0	0	2	1,250.58	1,826.00		5,462.14	517	528	Empty	TRUE	
20196-20225 HinckeM –TAXchicken	Uncategorized Sample	20196-20205_A	Mudpit_Ingel20196_MGFPeaklist (F058870)	Lamin-A OS=Gallus gallus GN=LMNA PE=2 SV=1	LMNA_CHICK,UPI000012E20B	TAX_GallusGallus_9031_20141114.fasta,UPARC14_2_GallusG_9031_20140417.fasta	73,165.80	100.00%	28	33	135	0.27%	40.00%	EAALSTALGEK	K	R	99.70%	42.8	45.3	27.1	30.9	28.8	2.34	0.299	0.364	0	3	0	0	2	1,089.58	1,950.00		1,622.42	144	154	Empty	TRUE	
20196-20225 HinckeM –TAXchicken	Uncategorized Sample	20196-20205_A	Mudpit_Ingel20196_MGFPeaklist (F058870)	Lamin-A OS=Gallus gallus GN=LMNA PE=2 SV=1	LMNA_CHICK,UPI000012E20B	TAX_GallusGallus_9031_20141114.fasta,UPARC14_2_GallusG_9031_20140417.fasta	73,165.80	100.00%	28	33	135	0.27%	40.00%	EAALSTALGEKR	K	N	99.70%	73.7	44.7	34.8	39.7	37.4	3.39	0.374	0.542	0	0	4	0	2	1,245.68	1,829.50		4,846.77	144	155	Empty	TRUE	
20196-20225 HinckeM –TAXchicken	Uncategorized Sample	20196-20205_A	Mudpit_Ingel20196_MGFPeaklist (F058870)	Lamin-A OS=Gallus gallus GN=LMNA PE=2 SV=1	LMNA_CHICK,UPI000012E20B	TAX_GallusGallus_9031_20141114.fasta,UPARC14_2_GallusG_9031_20140417.fasta	73,165.80	100.00%	28	33	135	0.27%	40.00%	EADLLAAQAR	K	L	99.70%	45.4	44.6	37.7	16	27.5	1.3	0.276	0.3	0	5	0	0	2	1,057.56	1,899.00		3,656.01	123	132	Empty	TRUE	
20196-20225 HinckeM –TAXchicken	Uncategorized Sample	20196-20205_A	Mudpit_Ingel20196_MGFPeaklist (F058870)	Lamin-A OS=Gallus gallus GN=LMNA PE=2 SV=1	LMNA_CHICK,UPI000012E20B	TAX_GallusGallus_9031_20141114.fasta,UPARC14_2_GallusG_9031_20140417.fasta	73,165.80	100.00%	28	33	135	0.27%	40.00%	IDSLSAELSQLQK	R	Q	99.70%	67.7	44.6	30	50.4	45.4	3.72	0.355	0.5	0	4	0	0	2	1,431.77	2,526.50		20,911.30	298	310	Empty	TRUE	
20196-20225 HinckeM –TAXchicken	Uncategorized Sample	20196-20205_A	Mudpit_Ingel20196_MGFPeaklist (F058870)	Lamin-A OS=Gallus gallus GN=LMNA PE=2 SV=1	LMNA_CHICK,UPI000012E20B	TAX_GallusGallus_9031_20141114.fasta,UPARC14_2_GallusG_9031_20140417.fasta	73,165.80	100.00%	28	33	135	0.27%	40.00%	ITESEEVVSR	R	E	99.70%	69.6	44.9	30.5	51.6	38.5	3.62	0.338	0.5	0	10	0	0	2	1,148.58	1,613.00		9,195.70	62	71	Empty	TRUE	
20196-20225 HinckeM –TAXchicken	Uncategorized Sample	20196-20205_A	Mudpit_Ingel20196_MGFPeaklist (F058870)	Lamin-A OS=Gallus gallus GN=LMNA PE=2 SV=1	LMNA_CHICK,UPI000012E20B	TAX_GallusGallus_9031_20141114.fasta,UPARC14_2_GallusG_9031_20140417.fasta	73,165.80	100.00%	28	33	135	0.27%	40.00%	LAEALQDLR	K	R	99.70%	70.7	44.2	46.7	46.5	35.1	1.74	0.251	0.5	0	5	0	0	2	1,028.57	2,117.00		9,169.64	240	248	Empty	TRUE	
20196-20225 HinckeM –TAXchicken	Uncategorized Sample	20196-20205_A	Mudpit_Ingel20196_MGFPeaklist (F058870)	Lamin-A OS=Gallus gallus GN=LMNA PE=2 SV=1	LMNA_CHICK,UPI000012E20B	TAX_GallusGallus_9031_20141114.fasta,UPARC14_2_GallusG_9031_20140417.fasta	73,165.80	100.00%	28	33	135	0.27%	40.00%	LAVYIDK	R	V	98.80%	36	43.8	29.2	18.2	27.5	0.77	0.218	0.5	0	3	0	0	2	821.4768	1,956.00		2,455.14	41	47	Empty	TRUE	UPI000012E202,F1NAM2_CHICK,LMNB1_CHICK,UPI0002C87565,UPI0000ECC2CB,UPI0000ECC2CA
20196-20225 HinckeM –TAXchicken	Uncategorized Sample	20196-20205_A	Mudpit_Ingel20196_MGFPeaklist (F058870)	Lamin-A OS=Gallus gallus GN=LMNA PE=2 SV=1	LMNA_CHICK,UPI000012E20B	TAX_GallusGallus_9031_20141114.fasta,UPARC14_2_GallusG_9031_20140417.fasta	73,165.80	100.00%	28	33	135	0.27%	40.00%	LKDLEALLNSK	R	E	99.70%	81.2	43.3	42	57.8	36	1.41	0.311	0.545	0	0	2	0	2	1,243.73	2,422.50		9,184.08	133	143	Empty	TRUE	
20196-20225 HinckeM –TAXchicken	Uncategorized Sample	20196-20205_A	Mudpit_Ingel20196_MGFPeaklist (F058870)	Lamin-A OS=Gallus gallus GN=LMNA PE=2 SV=1	LMNA_CHICK,UPI000012E20B	TAX_GallusGallus_9031_20141114.fasta,UPARC14_2_GallusG_9031_20140417.fasta	73,165.80	100.00%	28	33	135	0.27%	40.00%	LLEGEEERLR	K	L	36.60%	18.3	44.7	34.8	-2.17					0	0	2	0	2	1,243.66	1,818.00		1,931.03	378	387	Empty	TRUE	
20196-20225 HinckeM –TAXchicken	Uncategorized Sample	20196-20205_A	Mudpit_Ingel20196_MGFPeaklist (F058870)	Lamin-A OS=Gallus gallus GN=LMNA PE=2 SV=1	LMNA_CHICK,UPI000012E20B	TAX_GallusGallus_9031_20141114.fasta,UPARC14_2_GallusG_9031_20140417.fasta	73,165.80	100.00%	28	33	135	0.27%	40.00%	LQEKEDLQELNDR	R	L	99.70%	67.6	44.9	39.8	43.8	47.4	3.13	0.369	0.615	0	0	6	0	2	1,629.81	1,812.00		20,646.10	28	40	Empty	TRUE	
20196-20225 HinckeM –TAXchicken	Uncategorized Sample	20196-20205_A	Mudpit_Ingel20196_MGFPeaklist (F058870)	Lamin-A OS=Gallus gallus GN=LMNA PE=2 SV=1	LMNA_CHICK,UPI000012E20B	TAX_GallusGallus_9031_20141114.fasta,UPARC14_2_GallusG_9031_20140417.fasta	73,165.80	100.00%	28	33	135	0.27%	40.00%	LQTLKEELEFQK	R	N	99.70%	65.9	43.8	28.3	0	35.9	2.11	0.298	0.417	0	0	2	0	2	1,505.82	2,194.50		8,532.50	196	207	Empty	TRUE	
20196-20225 HinckeM –TAXchicken	Uncategorized Sample	20196-20205_A	Mudpit_Ingel20196_MGFPeaklist (F058870)	Lamin-A OS=Gallus gallus GN=LMNA PE=2 SV=1	LMNA_CHICK,UPI000012E20B	TAX_GallusGallus_9031_20141114.fasta,UPARC14_2_GallusG_9031_20140417.fasta	73,165.80	100.00%	28	33	135	0.27%	40.00%	LREVEEALSR	K	E	99.70%	46	44.8	34.2	18.6	33.4	2.52	0.251	0.5	0	0	8	0	2	1,201.65	1,823.50		10,410.60	319	328	Empty	TRUE	
20196-20225 HinckeM –TAXchicken	Uncategorized Sample	20196-20205_A	Mudpit_Ingel20196_MGFPeaklist (F058870)	Lamin-A OS=Gallus gallus GN=LMNA PE=2 SV=1	LMNA_CHICK,UPI000012E20B	TAX_GallusGallus_9031_20141114.fasta,UPARC14_2_GallusG_9031_20140417.fasta	73,165.80	100.00%	28	33	135	0.27%	40.00%	LRITESEEVVSR	R	E	37.60%	18.5	44.5	0	0					0	0	2	0	2	1,417.76	1,880.50		468.49	60	71	Empty	TRUE	
20196-20225 HinckeM –TAXchicken	Uncategorized Sample	20196-20205_A	Mudpit_Ingel20196_MGFPeaklist (F058870)	Lamin-A OS=Gallus gallus GN=LMNA PE=2 SV=1	LMNA_CHICK,UPI000012E20B	TAX_GallusGallus_9031_20141114.fasta,UPARC14_2_GallusG_9031_20140417.fasta	73,165.80	100.00%	28	33	135	0.27%	40.00%	LVEIDNGRQQEFESK	R	L	99.70%	29.4	44.7	19.7	4.75	41.4	2.8	0.15	0.467	0	0	5	0	2	1,792.87	1,897.00		12,188.10	225	239	Empty	TRUE	
20196-20225 HinckeM –TAXchicken	Uncategorized Sample	20196-20205_A	Mudpit_Ingel20196_MGFPeaklist (F058870)	Lamin-A OS=Gallus gallus GN=LMNA PE=2 SV=1	LMNA_CHICK,UPI000012E20B	TAX_GallusGallus_9031_20141114.fasta,UPARC14_2_GallusG_9031_20140417.fasta	73,165.80	100.00%	28	33	135	0.27%	40.00%	NIYSEELR	K	E	99.70%	37.5	43.9	34.8	15.9	24.5	0.602	0.208	0.312	0	4	0	0	2	1,023.51	1,892.50		2,481.92	208	215	Empty	TRUE	
20196-20225 HinckeM –TAXchicken	Uncategorized Sample	20196-20205_A	Mudpit_Ingel20196_MGFPeaklist (F058870)	Lamin-A OS=Gallus gallus GN=LMNA PE=2 SV=1	LMNA_CHICK,UPI000012E20B	TAX_GallusGallus_9031_20141114.fasta,UPARC14_2_GallusG_9031_20140417.fasta	73,165.80	100.00%	28	33	135	0.27%	40.00%	NIYSEELRETK	K	R	82.40%	20	44.4	15	16.5					0	0	2	0	2	1,381.70	1,824.00		1,242.96	208	218	Empty	TRUE	
20196-20225 HinckeM –TAXchicken	Uncategorized Sample	20196-20205_A	Mudpit_Ingel20196_MGFPeaklist (F058870)	Lamin-A OS=Gallus gallus GN=LMNA PE=2 SV=1	LMNA_CHICK,UPI000012E20B	TAX_GallusGallus_9031_20141114.fasta,UPARC14_2_GallusG_9031_20140417.fasta	73,165.80	100.00%	28	33	135	0.27%	40.00%	NKSNEDQALGNWQVK	R	R	99.70%	19.6	44.6	24.6	4.03	29.6	1.85	0.206	0.333	0	0	1	0	2	1,730.85	2,017.00		1,170.90	457	471	Empty	TRUE	
20196-20225 HinckeM –TAXchicken	Uncategorized Sample	20196-20205_A	Mudpit_Ingel20196_MGFPeaklist (F058870)	Lamin-A OS=Gallus gallus GN=LMNA PE=2 SV=1	LMNA_CHICK,UPI000012E20B	TAX_GallusGallus_9031_20141114.fasta,UPARC14_2_GallusG_9031_20140417.fasta	73,165.80	100.00%	28	33	135	0.27%	40.00%	NSSMAGAAHEELQQTHIR	R	I	99.70%	56.1	43.5	19.3	47.5	37.6	3.54	0.378	0.306	0	0	0	5	2	1,995.93	1,633.00		3,012.13	280	297	Empty	TRUE	
20196-20225 HinckeM –TAXchicken	Uncategorized Sample	20196-20205_A	Mudpit_Ingel20196_MGFPeaklist (F058870)	Lamin-A OS=Gallus gallus GN=LMNA PE=2 SV=1	LMNA_CHICK,UPI000012E20B	TAX_GallusGallus_9031_20141114.fasta,UPARC14_2_GallusG_9031_20140417.fasta	73,165.80	100.00%	28	33	135	0.27%	40.00%	QNGDDPPLTYR	R	F	99.70%	27.9	44.4	19.5	22.3	25.8	1.11	0.283	0.227	0	6	0	0	2	1,275.60	1,805.50		2,294.84	473	483	Empty	TRUE	
20196-20225 HinckeM –TAXchicken	Uncategorized Sample	20196-20205_A	Mudpit_Ingel20196_MGFPeaklist (F058870)	Lamin-A OS=Gallus gallus GN=LMNA PE=2 SV=1	LMNA_CHICK,UPI000012E20B	TAX_GallusGallus_9031_20141114.fasta,UPARC14_2_GallusG_9031_20140417.fasta	73,165.80	100.00%	28	33	135	0.27%	40.00%	SLELENAGLR	R	L	99.70%	59.6	45	41.9	32.8	36.2	1.77	0.221	0.5	0	10	0	0	2	1,101.59	2,110.50		17,733.70	50	59	Empty	TRUE	
20196-20225 HinckeM –TAXchicken	Uncategorized Sample	20196-20205_A	Mudpit_Ingel20196_MGFPeaklist (F058870)	Lamin-A OS=Gallus gallus GN=LMNA PE=2 SV=1	LMNA_CHICK,UPI000012E20B	TAX_GallusGallus_9031_20141114.fasta,UPARC14_2_GallusG_9031_20140417.fasta	73,165.80	100.00%	28	33	135	0.27%	40.00%	SNEDQALGNWQVKR	K	Q	99.70%	37.1	44.9	25.6	22.4	30.7	1.96	0.156	0.321	0	0	1	0	2	1,644.81	1,977.00		1,898.16	459	472	Empty	TRUE	
20196-20225 HinckeM –TAXchicken	Uncategorized Sample	20196-20205_A	Mudpit_Ingel20196_MGFPeaklist (F058870)	Lamin-A OS=Gallus gallus GN=LMNA PE=2 SV=1	LMNA_CHICK,UPI000012E20B	TAX_GallusGallus_9031_20141114.fasta,UPARC14_2_GallusG_9031_20140417.fasta	73,165.80	100.00%	28	33	135	0.27%	40.00%	SSGGGIGEGLLGR	R	S	99.70%	72.7	45.2	40.7	46.7	47.1	4.14	0.348	0.538	0	11	0	0	2	1,159.61	2,189.00		19,604.70	623	635	Empty	TRUE	
20196-20225 HinckeM –TAXchicken	Uncategorized Sample	20196-20205_A	Mudpit_Ingel20196_MGFPeaklist (F058870)	Lamin-A OS=Gallus gallus GN=LMNA PE=2 SV=1	LMNA_CHICK,UPI000012E20B	TAX_GallusGallus_9031_20141114.fasta,UPARC14_2_GallusG_9031_20140417.fasta	73,165.80	100.00%	28	33	135	0.27%	40.00%	TALINSNGEEVAMR	R	K	98.70%	36.4	44.7	22.7	24.9					0	3	0	0	2	1,520.74	1,830.00		1,452.45	529	542	Empty	TRUE	
20196-20225 HinckeM –TAXchicken	Uncategorized Sample	20196-20205_A	Mudpit_Ingel20196_MGFPeaklist (F058870)	Lamin-A OS=Gallus gallus GN=LMNA PE=2 SV=1	LMNA_CHICK,UPI000012E20B	TAX_GallusGallus_9031_20141114.fasta,UPARC14_2_GallusG_9031_20140417.fasta	73,165.80	100.00%	28	33	135	0.27%	40.00%	TALINSNGEEVAMRK	R	L	99.70%	43.4	44.9	22.5	30.5	25.4	1.07	0.177	0.233	0	0	2	0	2	1,648.83	1,732.00		1,499.35	529	543	Empty	TRUE	
20196-20225 HinckeM –TAXchicken	Uncategorized Sample	20196-20205_A	Mudpit_Ingel20196_MGFPeaklist (F058870)	Lamin-A OS=Gallus gallus GN=LMNA PE=2 SV=1	LMNA_CHICK,UPI000012E20B	TAX_GallusGallus_9031_20141114.fasta,UPARC14_2_GallusG_9031_20140417.fasta	73,165.80	100.00%	28	33	135	0.27%	40.00%	VGVEEVDLEGR	R	F	99.70%	70	45	30.9	50.3	36.3	4.28	0.424	0.455	0	12	0	0	2	1,201.61	2,016.50		16,566.70	441	451	Empty	TRUE	
20196-20225 HinckeM –TAXchicken	Uncategorized Sample	20196-20205_A	Mudpit_Ingel20196_MGFPeaklist (F058870)	Structural maintenance of chromosomes protein OS=Gallus gallus GN=SMC4 PE=2 SV=1	Q8AWB9_CHICK,UPI00000FE3F7	TAX_GallusGallus_9031_20141114.fasta,UPARC14_2_GallusG_9031_20140417.fasta	145,862.00	100.00%	6	6	9	0.02%	5.26%	AIEFLSLENK	K	M	99.70%	39	44.5	31	22.7	35.1	1.8	0.296	0.5	0	2	0	0	2	1,163.63	2,544.50		523.7	276	285	Empty	TRUE	UPI0000611D78
20196-20225 HinckeM –TAXchicken	Uncategorized Sample	20196-20205_A	Mudpit_Ingel20196_MGFPeaklist (F058870)	Structural maintenance of chromosomes protein OS=Gallus gallus GN=SMC4 PE=2 SV=1	Q8AWB9_CHICK,UPI00000FE3F7	TAX_GallusGallus_9031_20141114.fasta,UPARC14_2_GallusG_9031_20140417.fasta	145,862.00	100.00%	6	6	9	0.02%	5.26%	DTLVVKDLEDATR	R	I	99.70%	26.9	44.9	24.1	10.5	26	0.959	0.162	0.269	0	0	1	0	2	1,474.78	2,332.00		156.03	685	697	Empty	TRUE	UPI0000611D78
20196-20225 HinckeM –TAXchicken	Uncategorized Sample	20196-20205_A	Mudpit_Ingel20196_MGFPeaklist (F058870)	Structural maintenance of chromosomes protein OS=Gallus gallus GN=SMC4 PE=2 SV=1	Q8AWB9_CHICK,UPI00000FE3F7	TAX_GallusGallus_9031_20141114.fasta,UPARC14_2_GallusG_9031_20140417.fasta	145,862.00	100.00%	6	6	9	0.02%	5.26%	ELEANVTAAAPDKNK	K	Q	17.60%	9.33	44.3	15.4	3.75					0	0	1	0	2	1,570.81	1,686.00		177.12	812	826	Empty	TRUE	UPI0000611D78
20196-20225 HinckeM –TAXchicken	Uncategorized Sample	20196-20205_A	Mudpit_Ingel20196_MGFPeaklist (F058870)	Structural maintenance of chromosomes protein OS=Gallus gallus GN=SMC4 PE=2 SV=1	Q8AWB9_CHICK,UPI00000FE3F7	TAX_GallusGallus_9031_20141114.fasta,UPARC14_2_GallusG_9031_20140417.fasta	145,862.00	100.00%	6	6	9	0.02%	5.26%	IPTPENAPR	K	L	99.70%	54.8	43.8	31.1	37.2	35.2	1.37	0.349	0.444	0	2	0	0	2	994.5317	1,632.50		444.28	655	663	Empty	TRUE	UPI0000611D78
20196-20225 HinckeM –TAXchicken	Uncategorized Sample	20196-20205_A	Mudpit_Ingel20196_MGFPeaklist (F058870)	Structural maintenance of chromosomes protein OS=Gallus gallus GN=SMC4 PE=2 SV=1	Q8AWB9_CHICK,UPI00000FE3F7	TAX_GallusGallus_9031_20141114.fasta,UPARC14_2_GallusG_9031_20140417.fasta	145,862.00	100.00%	6	6	9	0.02%	5.26%	KIFNLSGGEK	K	T	26.90%	8.4	44.3	22	-1.48					0	1	0	0	2	1,093.59	2,103.00		199.2	1158	1167	Empty	TRUE	UPI0000611D78
20196-20225 HinckeM –TAXchicken	Uncategorized Sample	20196-20205_A	Mudpit_Ingel20196_MGFPeaklist (F058870)	Structural maintenance of chromosomes protein OS=Gallus gallus GN=SMC4 PE=2 SV=1	Q8AWB9_CHICK,UPI00000FE3F7	TAX_GallusGallus_9031_20141114.fasta,UPARC14_2_GallusG_9031_20140417.fasta	145,862.00	100.00%	6	6	9	0.02%	5.26%	VLEALLEQKR	K	S	99.70%	49.5	41.8	35.8	35.7	37	1.8	0.281	0.6	0	0	2	0	2	1,198.72	2,148.50		737.88	570	579	Empty	TRUE	UPI0000611D78
20196-20225 HinckeM –TAXchicken	Uncategorized Sample	20196-20205_A	Mudpit_Ingel20196_MGFPeaklist (F058870)	Structural maintenance of chromosomes protein OS=Gallus gallus GN=SMC4 PE=2 SV=1	Q8AWB9_CHICK,UPI00000FE3F7	TAX_GallusGallus_9031_20141114.fasta,UPARC14_2_GallusG_9031_20140417.fasta	145,862.00	100.00%	6	6	9	0.02%	5.26%	AIEFLSLENK	K	M	99.70%	39	44.5	31	22.7	35.1	1.8	0.296	0.5	0	2	0	0	2	1,163.63	2,544.50		523.7	276	285	Empty	TRUE	UPI0000611D78
20196-20225 HinckeM –TAXchicken	Uncategorized Sample	20196-20205_A	Mudpit_Ingel20196_MGFPeaklist (F058870)	Structural maintenance of chromosomes protein OS=Gallus gallus GN=SMC4 PE=2 SV=1	Q8AWB9_CHICK,UPI00000FE3F7	TAX_GallusGallus_9031_20141114.fasta,UPARC14_2_GallusG_9031_20140417.fasta	145,862.00	100.00%	6	6	9	0.02%	5.26%	DTLVVKDLEDATR	R	I	99.70%	26.9	44.9	24.1	10.5	26	0.959	0.162	0.269	0	0	1	0	2	1,474.78	2,332.00		156.03	685	697	Empty	TRUE	UPI0000611D78
20196-20225 HinckeM –TAXchicken	Uncategorized Sample	20196-20205_A	Mudpit_Ingel20196_MGFPeaklist (F058870)	Structural maintenance of chromosomes protein OS=Gallus gallus GN=SMC4 PE=2 SV=1	Q8AWB9_CHICK,UPI00000FE3F7	TAX_GallusGallus_9031_20141114.fasta,UPARC14_2_GallusG_9031_20140417.fasta	145,862.00	100.00%	6	6	9	0.02%	5.26%	ELEANVTAAAPDKNK	K	Q	17.60%	9.33	44.3	15.4	3.75					0	0	1	0	2	1,570.81	1,686.00		177.12	812	826	Empty	TRUE	UPI0000611D78
20196-20225 HinckeM –TAXchicken	Uncategorized Sample	20196-20205_A	Mudpit_Ingel20196_MGFPeaklist (F058870)	Structural maintenance of chromosomes protein OS=Gallus gallus GN=SMC4 PE=2 SV=1	Q8AWB9_CHICK,UPI00000FE3F7	TAX_GallusGallus_9031_20141114.fasta,UPARC14_2_GallusG_9031_20140417.fasta	145,862.00	100.00%	6	6	9	0.02%	5.26%	IPTPENAPR	K	L	99.70%	54.8	43.8	31.1	37.2	35.2	1.37	0.349	0.444	0	2	0	0	2	994.5317	1,632.50		444.28	655	663	Empty	TRUE	UPI0000611D78
20196-20225 HinckeM –TAXchicken	Uncategorized Sample	20196-20205_A	Mudpit_Ingel20196_MGFPeaklist (F058870)	Structural maintenance of chromosomes protein OS=Gallus gallus GN=SMC4 PE=2 SV=1	Q8AWB9_CHICK,UPI00000FE3F7	TAX_GallusGallus_9031_20141114.fasta,UPARC14_2_GallusG_9031_20140417.fasta	145,862.00	100.00%	6	6	9	0.02%	5.26%	KIFNLSGGEK	K	T	26.90%	8.4	44.3	22	-1.48					0	1	0	0	2	1,093.59	2,103.00		199.2	1158	1167	Empty	TRUE	UPI0000611D78
20196-20225 HinckeM –TAXchicken	Uncategorized Sample	20196-20205_A	Mudpit_Ingel20196_MGFPeaklist (F058870)	Structural maintenance of chromosomes protein OS=Gallus gallus GN=SMC4 PE=2 SV=1	Q8AWB9_CHICK,UPI00000FE3F7	TAX_GallusGallus_9031_20141114.fasta,UPARC14_2_GallusG_9031_20140417.fasta	145,862.00	100.00%	6	6	9	0.02%	5.26%	VLEALLEQKR	K	S	99.70%	49.5	41.8	35.8	35.7	37	1.8	0.281	0.6	0	0	2	0	2	1,198.72	2,148.50		737.88	570	579	Empty	TRUE	UPI0000611D78
20196-20225 HinckeM –TAXchicken	Uncategorized Sample	20196-20205_A	Mudpit_Ingel20196_MGFPeaklist (F058870)	status=active	UPI00003ACDCD	UPARC14_2_GallusG_9031_20140417.fasta	61,089.20	100.00%	4	4	6	0.01%	6.98%	GFDTVVGEK	K	G	98.80%	25.4	44.1	27.6	11.8	20.6	0.347	0.209	0.222	0	1	0	0	2	951.4783	1,871.00		320.18	443	451	Empty	TRUE	F1NDU4_CHICK,UPI0000447CE7,UPI0003506DA1,UPI00003ACDCF,UPI0000ECC84C,UPI00003ACDCE,UPI0000E800EA,UPI0002C8675F
20196-20225 HinckeM –TAXchicken	Uncategorized Sample	20196-20205_A	Mudpit_Ingel20196_MGFPeaklist (F058870)	status=active	UPI00003ACDCD	UPARC14_2_GallusG_9031_20140417.fasta	61,089.20	100.00%	4	4	6	0.01%	6.98%	LMQKQAFLQN	K	-	17.70%	16.3	42.8	0	16.3					0	1	0	0	2	1,221.63	2,539.00		95.09	550	559	Empty	TRUE	
20196-20225 HinckeM –TAXchicken	Uncategorized Sample	20196-20205_A	Mudpit_Ingel20196_MGFPeaklist (F058870)	status=active	UPI00003ACDCD	UPARC14_2_GallusG_9031_20140417.fasta	61,089.20	100.00%	4	4	6	0.01%	6.98%	SLTENLSDGLR	R	A	99.70%	51.9	45	25.7	32	28.7	1.48	0.293	0.318	0	2	0	0	2	1,204.62	2,135.50		1,906.76	108	118	Empty	TRUE	F1NDU4_CHICK,UPI0000447CE7,UPI0003506DA1,UPI00003ACDCF,UPI0000ECC84C,UPI00003ACDCE,UPI0000E800EA,UPI0002C8675F
20196-20225 HinckeM –TAXchicken	Uncategorized Sample	20196-20205_A	Mudpit_Ingel20196_MGFPeaklist (F058870)	status=active	UPI00003ACDCD	UPARC14_2_GallusG_9031_20140417.fasta	61,089.20	100.00%	4	4	6	0.01%	6.98%	STIVSLLLR	K	L	99.70%	41.2	42.6	29.4	18.8	30.7	1.77	0.215	0.5	0	2	0	0	2	1,001.64	2,862.50		2,483.61	355	363	Empty	TRUE	F1NDU4_CHICK,UPI0000447CE7,UPI0003506DA1,UPI00003ACDCF,UPI0000ECC84C,UPI00003ACDCE,UPI0000E800EA,UPI0002C8675F
20196-20225 HinckeM –TAXchicken	Uncategorized Sample	20196-20205_A	Mudpit_Ingel20196_MGFPeaklist (F058870)	Uncharacterized protein OS=Gallus gallus GN=KRT12 PE=3 SV=2	F1NDN6_CHICK,UPI00004499EE,UPI00006103B9,UPI00006103BA,UPI0000E817AA,UPI0000ECA063,UPI0002C8713A,UPI0003504109,UPI00035050B3	TAX_GallusGallus_9031_20141114.fasta,UPARC14_2_GallusG_9031_20140417.fasta	56,959.00	100.00%	5	9	57	0.12%	5.63%	LAADDFR	R	L	98.80%	55.6	44.4	44	17.5	27.5	0.721	0.236	0.5	0	3	0	0	2	807.3996	1,812.00		868.25	251	257	Empty	TRUE	Q6PVZ2_CHICK,UPI00006103B6,K1C14_CHICK,F1NDN5_CHICK,UPI00003AC98C,UPI0000ECA06C,UPI00006103B5,UPI0002C86D9D,UPI0003287CD7,UPI000035A84B

20196-20225 HinckeM --TAXchicken	Uncategorized Sample	20196-20205_A	Mudpit_Ingel20196_MGFPeaklist (F058870)	Uncharacterized protein OS=Gallus gallus GN=KRT12 PE=3 SV=2	F1NDN6_CHICK,UPI00004499EE,UPI00006103B9,UPI00006103BA,UPI0000E817AA,UPI0000ECA063,UPI0002C8713A,UPI0003504109,UPI00035050B3	TAX_GallusGallus_9031_20141114.fasta,UPARC14_2_GallusG_9031_20140417.fasta	56,959.00	100.00%	5	9	57	0.12%	5.63%	LKYENEVALR	R	Q	99.70%	70.1	44.5	37.7	42.4	35.8	3.03	0.355	0.55	0	0	15	0	2	1,234.68	1,877.00		15,532.10	258	267	Empty	TRUE	
20196-20225 HinckeM --TAXchicken	Uncategorized Sample	20196-20205_A	Mudpit_Ingel20196_MGFPeaklist (F058870)	Uncharacterized protein OS=Gallus gallus GN=KRT12 PE=3 SV=2	F1NDN6_CHICK,UPI00004499EE,UPI00006103B9,UPI00006103BA,UPI0000E817AA,UPI0000ECA063,UPI0002C8713A,UPI0003504109,UPI00035050B3	TAX_GallusGallus_9031_20141114.fasta,UPARC14_2_GallusG_9031_20140417.fasta	56,959.00	100.00%	5	9	57	0.12%	5.63%	QSVEADINGLR	R	R	99.70%	64.7	45.1	41.1	37.3	35.1	3.04	0.316	0.364	0	5	0	0	2	1,201.62	2,092.00		3,828.14	268	278	Empty	TRUE	
20196-20225 HinckeM --TAXchicken	Uncategorized Sample	20196-20205_A	Mudpit_Ingel20196_MGFPeaklist (F058870)	Uncharacterized protein OS=Gallus gallus GN=KRT12 PE=3 SV=2	F1NDN6_CHICK,UPI00004499EE,UPI00006103B9,UPI00006103BA,UPI0000E817AA,UPI0000ECA063,UPI0002C8713A,UPI0003504109,UPI00035050B3	TAX_GallusGallus_9031_20141114.fasta,UPARC14_2_GallusG_9031_20140417.fasta	56,959.00	100.00%	5	9	57	0.12%	5.63%	QSVEADINGLRR	R	V	99.70%	41.7	44	29.2	21	40.1	4.55	0.377	0.583	0	8	19	0	2	1,358.70	1,973.00		25,419.50	268	279	Empty	TRUE	
20196-20225 HinckeM --TAXchicken	Uncategorized Sample	20196-20205_A	Mudpit_Ingel20196_MGFPeaklist (F058870)	Uncharacterized protein OS=Gallus gallus GN=KRT12 PE=3 SV=2	F1NDN6_CHICK,UPI00004499EE,UPI00006103B9,UPI00006103BA,UPI0000E817AA,UPI0000ECA063,UPI0002C8713A,UPI0003504109,UPI00035050B3	TAX_GallusGallus_9031_20141114.fasta,UPARC14_2_GallusG_9031_20140417.fasta	56,959.00	100.00%	5	9	57	0.12%	5.63%	YENEVALR	K	Q	99.70%	57.6	44.3	39.2	17.4	34.9	2	0.33	0.625	0	7	0	0	2	993.4999	1,769.00		2,322.87	260	267	Empty	TRUE	
20196-20225 HinckeM --TAXchicken	Uncategorized Sample	20196-20205_A	Mudpit_Ingel20196_MGFPeaklist (F058870)	Uncharacterized protein OS=Gallus gallus GN=KRT12 PE=3 SV=2	F1NDN6_CHICK,UPI00004499EE,UPI00006103B9,UPI00006103BA,UPI0000E817AA,UPI0000ECA063,UPI0002C8713A,UPI0003504109,UPI00035050B3	TAX_GallusGallus_9031_20141114.fasta,UPARC14_2_GallusG_9031_20140417.fasta	56,959.00	100.00%	5	9	57	0.12%	5.63%	LAADDFR	R	L	98.80%	55.6	44.4	44	17.5	27.5	0.721	0.236	0.5	0	3	0	0	2	807.3996	1,812.00		868.25	251	257	Empty	TRUE	Q6PVZ2_CHICK,UPI00006103B6,K1C14_CHICK,F1NDN5_CHICK,UPI00003AC98C,UPI0000ECA06C,UPI00006103B5,UPI0002C86D9D,UPI0003287CD7,[illegible]
20196-20225 HinckeM --TAXchicken	Uncategorized Sample	20196-20205_A	Mudpit_Ingel20196_MGFPeaklist (F058870)	Uncharacterized protein OS=Gallus gallus GN=KRT12 PE=3 SV=2	F1NDN6_CHICK,UPI00004499EE,UPI00006103B9,UPI00006103BA,UPI0000E817AA,UPI0000ECA063,UPI0002C8713A,UPI0003504109,UPI00035050B3	TAX_GallusGallus_9031_20141114.fasta,UPARC14_2_GallusG_9031_20140417.fasta	56,959.00	100.00%	5	9	57	0.12%	5.63%	LKYENEVALR	R	Q	99.70%	70.1	44.5	37.7	42.4	35.8	3.03	0.355	0.55	0	0	15	0	2	1,234.68	1,877.00		15,532.10	258	267	Empty	TRUE	
20196-20225 HinckeM --TAXchicken	Uncategorized Sample	20196-20205_A	Mudpit_Ingel20196_MGFPeaklist (F058870)	Uncharacterized protein OS=Gallus gallus GN=KRT12 PE=3 SV=2	F1NDN6_CHICK,UPI00004499EE,UPI00006103B9,UPI00006103BA,UPI0000E817AA,UPI0000ECA063,UPI0002C8713A,UPI0003504109,UPI00035050B3	TAX_GallusGallus_9031_20141114.fasta,UPARC14_2_GallusG_9031_20140417.fasta	56,959.00	100.00%	5	9	57	0.12%	5.63%	QSVEADINGLR	R	R	99.70%	64.7	45.1	41.1	37.3	35.1	3.04	0.316	0.364	0	5	0	0	2	1,201.62	2,092.00		3,828.14	268	278	Empty	TRUE	
20196-20225 HinckeM --TAXchicken	Uncategorized Sample	20196-20205_A	Mudpit_Ingel20196_MGFPeaklist (F058870)	Uncharacterized protein OS=Gallus gallus GN=KRT12 PE=3 SV=2	F1NDN6_CHICK,UPI00004499EE,UPI00006103B9,UPI00006103BA,UPI0000E817AA,UPI0000ECA063,UPI0002C8713A,UPI0003504109,UPI00035050B3	TAX_GallusGallus_9031_20141114.fasta,UPARC14_2_GallusG_9031_20140417.fasta	56,959.00	100.00%	5	9	57	0.12%	5.63%	QSVEADINGLRR	R	V	99.70%	41.7	44	29.2	21	40.1	4.55	0.377	0.583	0	8	19	0	2	1,358.70	1,973.00		25,419.50	268	279	Empty	TRUE	
20196-20225 HinckeM --TAXchicken	Uncategorized Sample	20196-20205_A	Mudpit_Ingel20196_MGFPeaklist (F058870)	Uncharacterized protein OS=Gallus gallus GN=KRT12 PE=3 SV=2	F1NDN6_CHICK,UPI00004499EE,UPI00006103B9,UPI00006103BA,UPI0000E817AA,UPI0000ECA063,UPI0002C8713A,UPI0003504109,UPI00035050B3	TAX_GallusGallus_9031_20141114.fasta,UPARC14_2_GallusG_9031_20140417.fasta	56,959.00	100.00%	5	9	57	0.12%	5.63%	YENEVALR	K	Q	99.70%	57.6	44.3	39.2	17.4	34.9	2	0.33	0.625	0	7	0	0	2	993.4999	1,769.00		2,322.87	260	267	Empty	TRUE	
20196-20225 HinckeM --TAXchicken	Uncategorized Sample	20196-20205_A	Mudpit_Ingel20196_MGFPeaklist (F058870)	Uncharacterized protein OS=Gallus gallus GN=KRT12 PE=3 SV=2	F1NDN6_CHICK,UPI00004499EE,UPI00006103B9,UPI00006103BA,UPI0000E817AA,UPI0000ECA063,UPI0002C8713A,UPI0003504109,UPI00035050B3	TAX_GallusGallus_9031_20141114.fasta,UPARC14_2_GallusG_9031_20140417.fasta	56,959.00	100.00%	5	9	57	0.12%	5.63%	LAADDFR	R	L	98.80%	55.6	44.4	44	17.5	27.5	0.721	0.236	0.5	0	3	0	0	2	807.3996	1,812.00		868.25	251	257	Empty	TRUE	Q6PVZ2_CHICK,UPI00006103B6,K1C14_CHICK,F1NDN5_CHICK,UPI00003AC98C,UPI0000ECA06C,UPI00006103B5,UPI0002C86D9D,UPI0003287CD7,[illegible]
20196-20225 HinckeM --TAXchicken	Uncategorized Sample	20196-20205_A	Mudpit_Ingel20196_MGFPeaklist (F058870)	Uncharacterized protein OS=Gallus gallus GN=KRT12 PE=3 SV=2	F1NDN6_CHICK,UPI00004499EE,UPI00006103B9,UPI00006103BA,UPI0000E817AA,UPI0000ECA063,UPI0002C8713A,UPI0003504109,UPI00035050B3	TAX_GallusGallus_9031_20141114.fasta,UPARC14_2_GallusG_9031_20140417.fasta	56,959.00	100.00%	5	9	57	0.12%	5.63%	LKYENEVALR	R	Q	99.70%	70.1	44.5	37.7	42.4	35.8	3.03	0.355	0.55	0	0	15	0	2	1,234.68	1,877.00		15,532.10	258	267	Empty	TRUE	
20196-20225 HinckeM --TAXchicken	Uncategorized Sample	20196-20205_A	Mudpit_Ingel20196_MGFPeaklist (F058870)	Uncharacterized protein OS=Gallus gallus GN=KRT12 PE=3 SV=2	F1NDN6_CHICK,UPI00004499EE,UPI00006103B9,UPI00006103BA,UPI0000E817AA,UPI0000ECA063,UPI0002C8713A,UPI0003504109,UPI00035050B3	TAX_GallusGallus_9031_20141114.fasta,UPARC14_2_GallusG_9031_20140417.fasta	56,959.00	100.00%	5	9	57	0.12%	5.63%	QSVEADINGLR	R	R	99.70%	64.7	45.1	41.1	37.3	35.1	3.04	0.316	0.364	0	5	0	0	2	1,201.62	2,092.00		3,828.14	268	278	Empty	TRUE	
20196-20225 HinckeM --TAXchicken	Uncategorized Sample	20196-20205_A	Mudpit_Ingel20196_MGFPeaklist (F058870)	Uncharacterized protein OS=Gallus gallus GN=KRT12 PE=3 SV=2	F1NDN6_CHICK,UPI00004499EE,UPI00006103B9,UPI00006103BA,UPI0000E817AA,UPI0000ECA063,UPI0002C8713A,UPI0003504109,UPI00035050B3	TAX_GallusGallus_9031_20141114.fasta,UPARC14_2_GallusG_9031_20140417.fasta	56,959.00	100.00%	5	9	57	0.12%	5.63%	QSVEADINGLRR	R	V	99.70%	41.7	44	29.2	21	40.1	4.55	0.377	0.583	0	8	19	0	2	1,358.70	1,973.00		25,419.50	268	279	Empty	TRUE	
20196-20225 HinckeM --TAXchicken	Uncategorized Sample	20196-20205_A	Mudpit_Ingel20196_MGFPeaklist (F058870)	Uncharacterized protein OS=Gallus gallus GN=KRT12 PE=3 SV=2	F1NDN6_CHICK,UPI00004499EE,UPI00006103B9,UPI00006103BA,UPI0000E817AA,UPI0000ECA063,UPI0002C8713A,UPI0003504109,UPI00035050B3	TAX_GallusGallus_9031_20141114.fasta,UPARC14_2_GallusG_9031_20140417.fasta	56,959.00	100.00%	5	9	57	0.12%	5.63%	YENEVALR	K	Q	99.70%	57.6	44.3	39.2	17.4	34.9	2	0.33	0.625	0	7	0	0	2	993.4999	1,769.00		2,322.87	260	267	Empty	TRUE	
20196-20225 HinckeM --TAXchicken	Uncategorized Sample	20196-20205_A	Mudpit_Ingel20196_MGFPeaklist (F058870)	Uncharacterized protein OS=Gallus gallus GN=KRT12 PE=3 SV=2	F1NDN6_CHICK,UPI00004499EE,UPI00006103B9,UPI00006103BA,UPI0000E817AA,UPI0000ECA063,UPI0002C8713A,UPI0003504109,UPI00035050B3	TAX_GallusGallus_9031_20141114.fasta,UPARC14_2_GallusG_9031_20140417.fasta	56,959.00	100.00%	5	9	57	0.12%	5.63%	LAADDFR	R	L	98.80%	55.6	44.4	44	17.5	27.5	0.721	0.236	0.5	0	3	0	0	2	807.3996	1,812.00		868.25	251	257	Empty	TRUE	Q6PVZ2_CHICK,UPI00006103B6,K1C14_CHICK,F1NDN5_CHICK,UPI00003AC98C,UPI0000ECA06C,UPI00006103B5,UPI0002C86D9D,UPI0003287CD7,[illegible]
20196-20225 HinckeM --TAXchicken	Uncategorized Sample	20196-20205_A	Mudpit_Ingel20196_MGFPeaklist (F058870)	Uncharacterized protein OS=Gallus gallus GN=KRT12 PE=3 SV=2	F1NDN6_CHICK,UPI00004499EE,UPI00006103B9,UPI00006103BA,UPI0000E817AA,UPI0000ECA063,UPI0002C8713A,UPI0003504109,UPI00035050B3	TAX_GallusGallus_9031_20141114.fasta,UPARC14_2_GallusG_9031_20140417.fasta	56,959.00	100.00%	5	9	57	0.12%	5.63%	LKYENEVALR	R	Q	99.70%	70.1	44.5	37.7	42.4	35.8	3.03	0.355	0.55	0	0	15	0	2	1,234.68	1,877.00		15,532.10	258	267	Empty	TRUE	
20196-20225 HinckeM --TAXchicken	Uncategorized Sample	20196-20205_A	Mudpit_Ingel20196_MGFPeaklist (F058870)	Uncharacterized protein OS=Gallus gallus GN=KRT12 PE=3 SV=2	F1NDN6_CHICK,UPI00004499EE,UPI00006103B9,UPI00006103BA,UPI0000E817AA,UPI0000ECA063,UPI0002C8713A,UPI0003504109,UPI00035050B3	TAX_GallusGallus_9031_20141114.fasta,UPARC14_2_GallusG_9031_20140417.fasta	56,959.00	100.00%	5	9	57	0.12%	5.63%	QSVEADINGLR	R	R	99.70%	64.7	45.1	41.1	37.3	35.1	3.04	0.316	0.364	0	5	0	0	2	1,201.62	2,092.00		3,828.14	268	278	Empty	TRUE	
20196-20225 HinckeM --TAXchicken	Uncategorized Sample	20196-20205_A	Mudpit_Ingel20196_MGFPeaklist (F058870)	Uncharacterized protein OS=Gallus gallus GN=KRT12 PE=3 SV=2	F1NDN6_CHICK,UPI00004499EE,UPI00006103B9,UPI00006103BA,UPI0000E817AA,UPI0000ECA063,UPI0002C8713A,UPI0003504109,UPI00035050B3	TAX_GallusGallus_9031_20141114.fasta,UPARC14_2_GallusG_9031_20140417.fasta	56,959.00	100.00%	5	9	57	0.12%	5.63%	QSVEADINGLRR	R	V	99.70%	41.7	44	29.2	21	40.1	4.55	0.377	0.583	0	8	19	0	2	1,358.70	1,973.00		25,419.50	268	279	Empty	TRUE	
20196-20225 HinckeM --TAXchicken	Uncategorized Sample	20196-20205_A	Mudpit_Ingel20196_MGFPeaklist (F058870)	Uncharacterized protein OS=Gallus gallus GN=KRT12 PE=3 SV=2	F1NDN6_CHICK,UPI00004499EE,UPI00006103B9,UPI00006103BA,UPI0000E817AA,UPI0000ECA063,UPI0002C8713A,UPI0003504109,UPI00035050B3	TAX_GallusGallus_9031_20141114.fasta,UPARC14_2_GallusG_9031_20140417.fasta	56,959.00	100.00%	5	9	57	0.12%	5.63%	YENEVALR	K	Q	99.70%	57.6	44.3	39.2	17.4	34.9	2	0.33	0.625	0	7	0	0	2	993.4999	1,769.00		2,322.87	260	267	Empty	TRUE	
20196-20225 HinckeM --TAXchicken	Uncategorized Sample	20196-20205_A	Mudpit_Ingel20196_MGFPeaklist (F058870)	Uncharacterized protein OS=Gallus gallus GN=KRT12 PE=3 SV=2	F1NDN6_CHICK,UPI00004499EE,UPI00006103B9,UPI00006103BA,UPI0000E817AA,UPI0000ECA063,UPI0002C8713A,UPI0003504109,UPI00035050B3	TAX_GallusGallus_9031_20141114.fasta,UPARC14_2_GallusG_9031_20140417.fasta	56,959.00	100.00%	5	9	57	0.12%	5.63%	LAADDFR	R	L	98.80%	55.6	44.4	44	17.5	27.5	0.721	0.236	0.5	0	3	0	0	2	807.3996	1,812.00		868.25	251	257	Empty	TRUE	Q6PVZ2_CHICK,UPI00006103B6,K1C14_CHICK,F1NDN5_CHICK,UPI00003AC98C,UPI0000ECA06C,UPI00006103B5,UPI0002C86D9D,UPI0003287CD7,[illegible]
20196-20225 HinckeM --TAXchicken	Uncategorized Sample	20196-20205_A	Mudpit_Ingel20196_MGFPeaklist (F058870)	Uncharacterized protein OS=Gallus gallus GN=KRT12 PE=3 SV=2	F1NDN6_CHICK,UPI00004499EE,UPI00006103B9,UPI00006103BA,UPI0000E817AA,UPI0000ECA063,UPI0002C8713A,UPI0003504109,UPI00035050B3	TAX_GallusGallus_9031_20141114.fasta,UPARC14_2_GallusG_9031_20140417.fasta	56,959.00	100.00%	5	9	57	0.12%	5.63%	LKYENEVALR	R	Q	99.70%	70.1	44.5	37.7	42.4	35.8	3.03	0.355	0.55	0	0	15	0	2	1,234.68	1,877.00		15,532.10	258	267	Empty	TRUE	
20196-20225 HinckeM --TAXchicken	Uncategorized Sample	20196-20205_A	Mudpit_Ingel20196_MGFPeaklist (F058870)	Uncharacterized protein OS=Gallus gallus GN=KRT12 PE=3 SV=2	F1NDN6_CHICK,UPI00004499EE,UPI00006103B9,UPI00006103BA,UPI0000E817AA,UPI0000ECA063,UPI0002C8713A,UPI0003504109,UPI00035050B3	TAX_GallusGallus_9031_20141114.fasta,UPARC14_2_GallusG_9031_20140417.fasta	56,959.00	100.00%	5	9	57	0.12%	5.63%	QSVEADINGLR	R	R	99.70%	64.7	45.1	41.1	37.3	35.1	3.04	0.316	0.364	0	5	0	0	2	1,201.62	2,092.00		3,828.14	268	278	Empty	TRUE	
20196-20225 HinckeM --TAXchicken	Uncategorized Sample	20196-20205_A	Mudpit_Ingel20196_MGFPeaklist (F058870)	Uncharacterized protein OS=Gallus gallus GN=KRT12 PE=3 SV=2	F1NDN6_CHICK,UPI00004499EE,UPI00006103B9,UPI00006103BA,UPI0000E817AA,UPI0000ECA063,UPI0002C8713A,UPI0003504109,UPI00035050B3	TAX_GallusGallus_9031_20141114.fasta,UPARC14_2_GallusG_9031_20140417.fasta	56,959.00	100.00%	5	9	57	0.12%	5.63%	QSVEADINGLRR	R	V	99.70%	41.7	44	29.2	21	40.1	4.55	0.377	0.583	0	8	19	0	2	1,358.70	1,973.00		25,419.50	268	279	Empty	TRUE	
20196-20225 HinckeM --TAXchicken	Uncategorized Sample	20196-20205_A	Mudpit_Ingel20196_MGFPeaklist (F058870)	Uncharacterized protein OS=Gallus gallus GN=KRT12 PE=3 SV=2	F1NDN6_CHICK,UPI00004499EE,UPI00006103B9,UPI00006103BA,UPI0000E817AA,UPI0000ECA063,UPI0002C8713A,UPI0003504109,UPI00035050B3	TAX_GallusGallus_9031_20141114.fasta,UPARC14_2_GallusG_9031_20140417.fasta	56,959.00	100.00%	5	9	57	0.12%	5.63%	YENEVALR	K	Q	99.70%	57.6	44.3	39.2	17.4	34.9	2	0.33	0.625	0	7	0	0	2	993.4999	1,769.00		2,322.87	260	267	Empty	TRUE	
20196-20225 HinckeM --TAXchicken	Uncategorized Sample	20196-20205_A	Mudpit_Ingel20196_MGFPeaklist (F058870)	Uncharacterized protein OS=Gallus gallus GN=KRT12 PE=3 SV=2	F1NDN6_CHICK,UPI00004499EE,UPI00006103B9,UPI00006103BA,UPI0000E817AA,UPI0000ECA063,UPI0002C8713A,UPI0003504109,UPI00035050B3	TAX_GallusGallus_9031_20141114.fasta,UPARC14_2_GallusG_9031_20140417.fasta	56,959.00	100.00%	5	9	57	0.12%	5.63%	LAADDFR	R	L	98.80%	55.6	44.4	44	17.5	27.5	0.721	0.236	0.5	0	3	0	0	2	807.3996	1,812.00		868.25	251	257	Empty	TRUE	Q6PVZ2_CHICK,UPI00006103B6,K1C14_CHICK,F1NDN5_CHICK,UPI00003AC98C,UPI0000ECA06C,UPI00006103B5,UPI0002C86D9D,UPI0003287CD7,[illegible]
20196-20225 HinckeM --TAXchicken	Uncategorized Sample	20196-20205_A	Mudpit_Ingel20196_MGFPeaklist (F058870)	Uncharacterized protein OS=Gallus gallus GN=KRT12 PE=3 SV=2	F1NDN6_CHICK,UPI00004499EE,UPI00006103B9,UPI00006103BA,UPI0000E817AA,UPI0000ECA063,UPI0002C8713A,UPI0003504109,UPI00035050B3	TAX_GallusGallus_9031_20141114.fasta,UPARC14_2_GallusG_9031_20140417.fasta	56,959.00	100.00%	5	9	57	0.12%	5.63%	LKYENEVALR	R	Q	99.70%	70.1	44.5	37.7	42.4	35.8	3.03	0.355	0.55	0	0	15	0	2	1,234.68	1,877.00		15,532.10	258	267	Empty	TRUE	
20196-20225 HinckeM --TAXchicken	Uncategorized Sample	20196-20205_A	Mudpit_Ingel20196_MGFPeaklist (F058870)	Uncharacterized protein OS=Gallus gallus GN=KRT12 PE=3 SV=2	F1NDN6_CHICK,UPI00004499EE,UPI00006103B9,UPI00006103BA,UPI0000E817AA,UPI0000ECA063,UPI0002C8713A,UPI0003504109,UPI00035050B3	TAX_GallusGallus_9031_20141114.fasta,UPARC14_2_GallusG_9031_20140417.fasta	56,959.00	100.00%	5	9	57	0.12%	5.63%	QSVEADINGLR	R	R	99.70%	64.7	45.1	41.1	37.3	35.1	3.04	0.316	0.364	0	5	0	0	2	1,201.62	2,092.00		3,828.14	268	278	Empty	TRUE	
20196-20225 HinckeM --TAXchicken	Uncategorized Sample	20196-20205_A	Mudpit_Ingel20196_MGFPeaklist (F058870)	Uncharacterized protein OS=Gallus gallus GN=KRT12 PE=3 SV=2	F1NDN6_CHICK,UPI00004499EE,UPI00006103B9,UPI00006103BA,UPI0000E817AA,UPI0000ECA063,UPI0002C8713A,UPI0003504109,UPI00035050B3	TAX_GallusGallus_9031_20141114.fasta,UPARC14_2_GallusG_9031_20140417.fasta	56,959.00	100.00%	5	9	57	0.12%	5.63%	QSVEADINGLRR	R	V	99.70%	41.7	44	29.2	21	40.1	4.55	0.377	0.583	0	8	19	0	2	1,358.70	1,973.00		25,419.50	268	279	Empty	TRUE	
20196-20225 HinckeM --TAXchicken	Uncategorized Sample	20196-20205_A	Mudpit_Ingel20196_MGFPeaklist (F058870)	Uncharacterized protein OS=Gallus gallus GN=KRT12 PE=3 SV=2	F1NDN6_CHICK,UPI00004499EE,UPI00006103B9,UPI00006103BA,UPI0000E817AA,UPI0000ECA063,UPI0002C8713A,UPI0003504109,UPI00035050B3	TAX_GallusGallus_9031_20141114.fasta,UPARC14_2_GallusG_9031_20140417.fasta	56,959.00	100.00%	5	9	57	0.12%	5.63%	YENEVALR	K	Q	99.70%	57.6	44.3	39.2	17.4	34.9	2	0.33	0.625	0	7	0	0	2	993.4999	1,769.00		2,322.87	260	267	Empty	TRUE	
20196-20225 HinckeM --TAXchicken	Uncategorized Sample	20196-20205_A	Mudpit_Ingel20196_MGFPeaklist (F058870)	Uncharacterized protein OS=Gallus gallus GN=KRT12 PE=3 SV=2	F1NDN6_CHICK,UPI00004499EE,UPI00006103B9,UPI00006103BA,UPI0000E817AA,UPI0000ECA063,UPI0002C8713A,UPI0003504109,UPI00035050B3	TAX_GallusGallus_9031_20141114.fasta,UPARC14_2_GallusG_9031_20140417.fasta	56,959.00	100.00%	5	9	57	0.12%	5.63%	LAADDFR	R	L	98.80%	55.6	44.4	44	17.5	27.5	0.721	0.236	0.5	0	3	0	0	2	807.3996	1,812.00		868.25	251	257	Empty	TRUE	Q6PVZ2_CHICK,UPI00006103B6,K1C14_CHICK,F1NDN5_CHICK,UPI00003AC98C,UPI0000ECA06C,UPI00006103B5,UPI0002C86D9D,UPI0003287CD7,[illegible]
20196-20225 HinckeM --TAXchicken	Uncategorized Sample	20196-20205_A	Mudpit_Ingel20196_MGFPeaklist (F058870)	Uncharacterized protein OS=Gallus gallus GN=KRT12 PE=3 SV=2	F1NDN6_CHICK,UPI00004499EE,UPI00006103B9,UPI00006103BA,UPI0000E817AA,UPI0000ECA063,UPI0002C8713A,UPI0003504109,UPI00035050B3	TAX_GallusGallus_9031_20141114.fasta,UPARC14_2_GallusG_9031_20140417.fasta	56,959.00	100.00%	5	9	57	0.12%	5.63%	LKYENEVALR	R	Q	99.70%	70.1	44.5	37.7	42.4	35.8	3.03	0.355	0.55	0	0	15	0	2	1,234.68	1,877.00		15,532.10	258	267	Empty	TRUE	
20196-20225 HinckeM --TAXchicken	Uncategorized Sample	20196-20205_A	Mudpit_Ingel20196_MGFPeaklist (F058870)	Uncharacterized protein OS=Gallus gallus GN=KRT12 PE=3 SV=2	F1NDN6_CHICK,UPI00004499EE,UPI00006103B9,UPI00006103BA,UPI0000E817AA,UPI0000ECA063,UPI0002C8713A,UPI0003504109,UPI00035050B3	TAX_GallusGallus_9031_20141114.fasta,UPARC14_2_GallusG_9031_20140417.fasta	56,959.00	100.00%	5	9	57	0.12%	5.63%	QSVEADINGLR	R	R	99.70%	64.7	45.1	41.1	37.3	35.1	3.04	0.316	0.364	0	5	0	0	2	1,201.62	2,092.00		3,828.14	268	278	Empty	TRUE	
20196-20225 HinckeM --TAXchicken	Uncategorized Sample	20196-20205_A	Mudpit_Ingel20196_MGFPeaklist (F058870)	Uncharacterized protein OS=Gallus gallus GN=KRT12 PE=3 SV=2	F1NDN6_CHICK,UPI00004499EE,UPI00006103B9,UPI00006103BA,UPI0000E817AA,UPI0000ECA063,UPI0002C8713A,UPI0003504109,UPI00035050B3	TAX_GallusGallus_9031_20141114.fasta,UPARC14_2_GallusG_9031_20140417.fasta	56,959.00	100.00%	5	9	57	0.12%	5.63%	QSVEADINGLRR	R	V	99.70%	41.7	44	29.2	21	40.1	4.55	0.377	0.583	0	8	19	0	2	1,358.70	1,973.00		25,419.50	268	279	Empty	TRUE	
20196-20225 HinckeM --TAXchicken	Uncategorized Sample	20196-20205_A	Mudpit_Ingel20196_MGFPeaklist (F058870)	Uncharacterized protein OS=Gallus gallus GN=KRT12 PE=3 SV=2	F1NDN6_CHICK,UPI00004499EE,UPI00006103B9,UPI00006103BA,UPI0000E817AA,UPI0000ECA063,UPI0002C8713A,UPI0003504109,UPI00035050B3	TAX_GallusGallus_9031_20141114.fasta,UPARC14_2_GallusG_9031_20140417.fasta	56,959.00	100.00%	5	9	57	0.12%	5.63%	YENEVALR	K	Q	99.70%	57.6	44.3	39.2	17.4	34.9	2	0.33	0.625	0	7	0	0	2	993.4999	1,769.00		2,322.87	260	267	Empty	TRUE	
20196-20225 HinckeM --TAXchicken	Uncategorized Sample	20196-20205_A	Mudpit_Ingel20196_MGFPeaklist (F058870)	Uncharacterized protein OS=Gallus gallus GN=KRT12 PE=3 SV=2	F1NDN6_CHICK,UPI00004499EE,UPI00006103B9,UPI00006103BA,UPI0000E817AA,UPI0000ECA063,UPI0002C8713A,UPI0003504109,UPI00035050B3	TAX_GallusGallus_9031_20141114.fasta,UPARC14_2_GallusG_9031_20140417.fasta	56,959.00	100.00%	5	9	57	0.12%	5.63%	LAADDFR	R	L	98.80%	55.6	44.4	44	17.5	27.5	0.721	0.236	0.5	0	3	0	0	2	807.3996	1,812.00		868.25	251	257	Empty	TRUE	Q6PVZ2_CHICK,UPI00006103B6,K1C14_CHICK,F1NDN5_CHICK,UPI00003AC98C,UPI0000ECA06C,UPI00006103B5,UPI0002C86D9D,UPI0003287CD7,[illegible]
20196-20225 HinckeM --TAXchicken	Uncategorized Sample	20196-20205_A	Mudpit_Ingel20196_MGFPeaklist (F058870)	Uncharacterized protein OS=Gallus gallus GN=KRT12 PE=3 SV=2	F1NDN6_CHICK,UPI00004499EE,UPI00006103B9,UPI00006103BA,UPI0000E817AA,UPI0000ECA063,UPI0002C8713A,UPI0003504109,UPI00035050B3	TAX_GallusGallus_9031_20141114.fasta,UPARC14_2_GallusG_9031_20140417.fasta	56,959.00	100.00%	5	9	57	0.12%	5.63%	LKYENEVALR	R	Q	99.70%	70.1	44.5	37.7	42.4	35.8	3.03	0.355	0.55	0	0	15	0	2	1,234.68	1,877.00		15,532.10	258	267	Empty	TRUE	
20196-20225 HinckeM --TAXchicken	Uncategorized Sample	20196-20205_A	Mudpit_Ingel20196_MGFPeaklist (F058870)	Uncharacterized protein OS=Gallus gallus GN=KRT12 PE=3 SV=2	F1NDN6_CHICK,UPI00004499EE,UPI00006103B9,UPI00006103BA,UPI0000E817AA,UPI0000ECA063,UPI0002C8713A,UPI0003504109,UPI00035050B3	TAX_GallusGallus_9031_20141114.fasta,UPARC14_2_GallusG_9031_20140417.fasta	56,959.00	100.00%	5	9	57	0.12%	5.63%	QSVEADINGLR	R	R	99.70%	64.7	45.1	41.1	37.3	35.1	3.04	0.316	0.364	0	5	0	0	2	1,201.62	2,092.00		3,828.14	268	278	Empty	TRUE	
20196-20225 HinckeM --TAXchicken	Uncategorized Sample	20196-20205_A	Mudpit_Ingel20196_MGFPeaklist (F058870)	Uncharacterized protein OS=Gallus gallus GN=KRT12 PE=3 SV=2	F1NDN6_CHICK,UPI00004499EE,UPI00006103B9,UPI00006103BA,UPI0000E817AA,UPI0000ECA063,UPI0002C8713A,UPI0003504109,UPI00035050B3	TAX_GallusGallus_9031_20141114.fasta,UPARC14_2_GallusG_9031_20140417.fasta	56,959.00	100.00%	5	9	57	0.12%	5.63%	QSVEADINGLRR	R	V	99.70%	41.7	44	29.2	21	40.1	4.55	0.377	0.583	0	8	19	0	2	1,358.70	1,973.00		25,419.50	268	279	Empty	TRUE	
20196-20225 HinckeM --TAXchicken	Uncategorized Sample	20196-20205_A	Mudpit_Ingel20196_MGFPeaklist (F058870)	Uncharacterized protein OS=Gallus gallus GN=KRT12 PE=3 SV=2	F1NDN6_CHICK,UPI00004499EE,UPI00006103B9,UPI00006103BA,UPI0000E817AA,UPI0000ECA063,UPI0002C8713A,UPI0003504109,UPI00035050B3	TAX_GallusGallus_9031_20141114.fasta,UPARC14_2_GallusG_9031_20140417.fasta	56,959.00	100.00%	5	9	57	0.12%	5.63%	YENEVALR	K	Q	99.70%	57.6	44.3	39.2	17.4	34.9	2	0.33	0.625	0	7	0	0	2	993.4999	1,769.00		2,322.87	260	267	Empty	TRUE	
20196-20225 HinckeM --TAXchicken	Uncategorized Sample	20196-20205_A	Mudpit_Ingel20196_MGFPeaklist (F058870)	Uncharacterized protein OS=Gallus gallus GN=KRT12 PE=3 SV=2	F1NDN6_CHICK,UPI00004499EE,UPI00006103B9,UPI00006103BA,UPI0000E817AA,UPI0000ECA063,UPI0002C8713A,UPI0003504109,UPI00035050B3	TAX_GallusGallus_9031_20141114.fasta,UPARC14_2_GallusG_9031_20140417.fasta	56,959.00	100.00%	5	9	57	0.12%	5.63%	LAADDFR	R	L	98.80%	55.6	44.4	44	17.5	27.5	0.721	0.236	0.5	0	3	0	0	2	807.3996	1,812.00		868.25	251	257	Empty	TRUE	Q6PVZ2_CHICK,UPI00006103B6,K1C14_CHICK,F1NDN5_CHICK,UPI00003AC98C,UPI0000ECA06C,UPI00006103B5,UPI0002C86D9D,UPI0003287CD7,[illegible]
20196-20225 HinckeM --TAXchicken	Uncategorized Sample	20196-20205_A	Mudpit_Ingel20196_MGFPeaklist (F058870)	Uncharacterized protein OS=Gallus gallus GN=KRT12 PE=3 SV=2	F1NDN6_CHICK,UPI00004499EE,UPI00006103B9,UPI00006103BA,UPI0000E817AA,UPI0000ECA063,UPI0002C8713A,UPI0003504109,UPI00035050B3	TAX_GallusGallus_9031_20141114.fasta,UPARC14_2_GallusG_9031_20140417.fasta	56,959.00	100.00%	5	9	57	0.12%	5.63%	LKYENEVALR	R	Q	99.70%	70.1	44.5	37.7	42.4	35.8	3.03	0.355	0.55	0	0	15	0	2	1,234.68	1,877.00		15,532.10	258	267	Empty	TRUE	
20196-20225 HinckeM --TAXchicken	Uncategorized Sample	20196-20205_A	Mudpit_Ingel20196_MGFPeaklist (F058870)	Uncharacterized protein OS=Gallus gallus GN=KRT12 PE=3 SV=2	F1NDN6_CHICK,UPI00004499EE,UPI00006103B9,UPI00006103BA,UPI0000E817AA,UPI0000ECA063,UPI0002C8713A,UPI0003504109,UPI00035050B3	TAX_GallusGallus_9031_20141114.fasta,UPARC14_2_GallusG_9031_20140417.fasta	56,959.00	100.00%	5	9	57	0.12%	5.63%	QSVEADINGLR	R	R	99.70%	64.7	45.1	41.1	37.3	35.1	3.04	0.316	0.364	0	5	0	0	2	1,201.62	2,092.00		3,828.14	268	278	Empty	TRUE	
20196-20225 HinckeM --TAXchicken	Uncategorized Sample	20196-20205_A	Mudpit_Ingel20196_MGFPeaklist (F058870)	Uncharacterized protein OS=Gallus gallus GN=KRT12 PE=3 SV=2	F1NDN6_CHICK,UPI00004499EE,UPI00006103B9,UPI00006103BA,UPI0000E817AA,UPI0000ECA063,UPI0002C8713A,UPI0003504109,UPI00035050B3	TAX_GallusGallus_9031_20141114.fasta,UPARC14_2_GallusG_9031_20140417.fasta	56,959.00	100.00%	5	9	57	0.12%	5.63%	QSVEADINGLRR	R	V	99.70%	41.7	44	29.2	21	40.1	4.55	0.377	0.583	0	8	19	0	2	1,358.70	1,973.00		25,419.50	268	279	Empty	TRUE	
20196-20225 HinckeM --TAXchicken	Uncategorized Sample	20196-20205_A	Mudpit_Ingel20196_MGFPeaklist (F058870)	Uncharacterized protein OS=Gallus gallus GN=KRT12 PE=3 SV=2	F1NDN6_CHICK,UPI00004499EE,UPI00006103B9,UPI00006103BA,UPI0000E817AA,UPI0000ECA063,UPI0002C8713A,UPI0003504109,UPI00035050B3	TAX_GallusGallus_9031_20141114.fasta,UPARC14_2_GallusG_9031_20140417.fasta	56,959.00	100.00%	5	9	57	0.12%	5.63%	YENEVALR	K	Q	99.70%	57.6	44.3	39.2	17.4	34.9	2	0.33	0.625	0	7	0	0	2	993.4999	1,769.00		2,322.87	260	267	Empty	TRUE	
20196-20225 HinckeM --TAXchicken	Uncategorized Sample	20196-20205_A	Mudpit_Ingel20196_MGFPeaklist (F058870)	Uncharacterized protein OS=Gallus gallus GN=HNRNPU PE=4 SV=2	F1NSP8_CHICK,UPI00003ACD6C,UPI0000447CA3,UPI0000610658,UPI0000610659,UPI0000E800C8,UPI0000ECC882,UPI0000ECC883,UPI000240B660,UPI0002C8704D,UPI00035073BE	TAX_GallusGallus_9031_20141114.fasta,UPARC14_2_GallusG_9031_20140417.fasta	67,965.30	100.00%	2	2	6	0.01%	2.53%	FIEIAAR	K	K	98.80%	32.2	44.8	35	16.7	27.5	1.15	0.124	0.429	0	4	0	0	2	819.4723	2,027.50		3,887.09	343	349	Empty	TRUE	
20196-20225 HinckeM --TAXchicken	Uncategorized Sample	20196-20205_A	Mudpit_Ingel20196_MGFPeaklist (F058870)	Uncharacterized protein OS=Gallus gallus GN=HNRNPU PE=4 SV=2	F1NSP8_CHICK,UPI00003ACD6C,UPI0000447CA3,UPI0000610658,UPI0000610659,UPI0000E800C8,UPI0000ECC882,UPI0000ECC883,UPI000240B660,UPI0002C8704D,UPI00035073BE	TAX_GallusGallus_9031_20141114.fasta,UPARC14_2_GallusG_9031_20140417.fasta	67,965.30	100.00%	2	2	6	0.01%	2.53%	NGQELGVAFK	K	I	99.70%	53.8	45.1	27.1	17.7	31.1	2	0.00643	0.4	0	2	0	0	2	1,063.54	2,141.50		569.68	201	210	Empty	TRUE	
20196-20225 HinckeM --TAXchicken	Uncategorized Sample	20196-20205_A	Mudpit_Ingel20196_MGFPeaklist (F058870)	Uncharacterized protein OS=Gallus gallus GN=HNRNPU PE=4 SV=2	F1NSP8_CHICK,UPI00003ACD6C,UPI0000447CA3,UPI0000610658,UPI0000610659,UPI0000E800C8,UPI0000ECC882,UPI0000ECC883,UPI000240B660,UPI0002C8704D,UPI00035073BE	TAX_GallusGallus_9031_20141114.fasta,UPARC14_2_GallusG_9031_20140417.fasta	67,965.30	100.00%	2	2	6	0.01%	2.53%	FIEIAAR	K	K	98.80%	32.2	44.8	35	16.7	27.5	1.15	0.124	0.429	0	4	0	0	2	819.4723	2,027.50		3,887.09	343	349	Empty	TRUE	
20196-20225 HinckeM --TAXchicken	Uncategorized Sample	20196-20205_A	Mudpit_Ingel20196_MGFPeaklist (F058870)	Uncharacterized protein OS=Gallus gallus GN=HNRNPU PE=4 SV=2	F1NSP8_CHICK,UPI00003ACD6C,UPI0000447CA3,UPI0000610658,UPI0000610659,UPI0000E800C8,UPI0000ECC882,UPI0000ECC883,UPI000240B660,UPI0002C8704D,UPI00035073BE	TAX_GallusGallus_9031_20141114.fasta,UPARC14_2_GallusG_9031_20140417.fasta	67,965.30	100.00%	2	2	6	0.01%	2.53%	NGQELGVAFK	K	I	99.70%	53.8	45.1	27.1	17.7	31.1	2	0.00643	0.4	0	2	0	0	2	1,063.54	2,141.50		569.68	201	210	Empty	TRUE	
20196-20225 HinckeM --TAXchicken	Uncategorized Sample	20196-20205_A	Mudpit_Ingel20196_MGFPeaklist (F058870)	Uncharacterized protein OS=Gallus gallus GN=HNRNPU PE=4 SV=2	F1NSP8_CHICK,UPI00003ACD6C,UPI0000447CA3,UPI0000610658,UPI0000610659,UPI0000E800C8,UPI0000ECC882,UPI0000ECC883,UPI000240B660,UPI0002C8704D,UPI00035073BE	TAX_GallusGallus_9031_20141114.fasta,UPARC14_2_GallusG_9031_20140417.fasta	67,965.30	100.00%	2	2	6	0.01%	2.53%	FIEIAAR	K	K	98.80%	32.2	44.8	35	16.7	27.5	1.15	0.124	0.429	0	4	0	0	2	819.4723	2,027.50		3,887.09	343	349	Empty	TRUE	
20196-20225 HinckeM --TAXchicken	Uncategorized Sample	20196-20205_A	Mudpit_Ingel20196_MGFPeaklist (F058870)	Uncharacterized protein OS=Gallus gallus GN=HNRNPU PE=4 SV=2	F1NSP8_CHICK,UPI00003ACD6C,UPI0000447CA3,UPI0000610658,UPI0000610659,UPI0000E800C8,UPI0000ECC882,UPI0000ECC883,UPI000240B660,UPI0002C8704D,UPI00035073BE	TAX_GallusGallus_9031_20141114.fasta,UPARC14_2_GallusG_9031_20140417.fasta	67,965.30	100.00%	2	2	6	0.01%	2.53%	NGQELGVAFK	K	I	99.70%	53.8	45.1	27.1	17.7	31.1	2	0.00643	0.4	0	2	0	0	2	1,063.54	2,141.50		569.68	201	210	Empty	TRUE	
20196-20225 HinckeM --TAXchicken	Uncategorized Sample	20196-20205_A	Mudpit_Ingel20196_MGFPeaklist (F058870)	Uncharacterized protein OS=Gallus gallus GN=HNRNPU PE=4 SV=2	F1NSP8_CHICK,UPI00003ACD6C,UPI0000447CA3,UPI0000610658,UPI0000610659,UPI0000E800C8,UPI0000ECC882,UPI0000ECC883,UPI000240B660,UPI0002C8704D,UPI00035073BE	TAX_GallusGallus_9031_20141114.fasta,UPARC14_2_GallusG_9031_20140417.fasta	67,965.30	100.00%	2	2	6	0.01%	2.53%	FIEIAAR	K	K	98.80%	32.2	44.8	35	16.7	27.5	1.15	0.124	0.429	0	4	0	0	2	819.4723	2,027.50		3,887.09	343	349	Empty	TRUE	
20196-20225 HinckeM --TAXchicken	Uncategorized Sample	20196-20205_A	Mudpit_Ingel20196_MGFPeaklist (F058870)	Uncharacterized protein OS=Gallus gallus GN=HNRNPU PE=4 SV=2	F1NSP8_CHICK,UPI00003ACD6C,UPI0000447CA3,UPI0000610658,UPI0000610659,UPI0000E800C8,UPI0000ECC882,UPI0000ECC883,UPI000240B660,UPI0002C8704D,UPI00035073BE	TAX_GallusGallus_9031_20141114.fasta,UPARC14_2_GallusG_9031_20140417.fasta	67,965.30	100.00%	2	2	6	0.01%	2.53%	NGQELGVAFK	K	I	99.70%	53.8	45.1	27.1	17.7	31.1	2	0.00643	0.4	0	2	0	0	2	1,063.54	2,141.50		569.68	201	210	Empty	TRUE	
20196-20225 HinckeM --TAXchicken	Uncategorized Sample	20196-20205_A	Mudpit_Ingel20196_MGFPeaklist (F058870)	Uncharacterized protein OS=Gallus gallus GN=HNRNPU PE=4 SV=2	F1NSP8_CHICK,UPI00003ACD6C,UPI0000447CA3,UPI0000610658,UPI0000610659,UPI0000E800C8,UPI0000ECC882,UPI0000ECC883,UPI000240B660,UPI0002C8704D,UPI00035073BE	TAX_GallusGallus_9031_20141114.fasta,UPARC14_2_GallusG_9031_20140417.fasta	67,965.30	100.00%	2	2	6	0.01%	2.53%	FIEIAAR	K	K	98.80%	32.2	44.8	35	16.7	27.5	1.15	0.124	0.429	0	4	0	0	2	819.4723	2,027.50		3,887.09	343	349	Empty	TRUE	
20196-20225 HinckeM --TAXchicken	Uncategorized Sample	20196-20205_A	Mudpit_Ingel20196_MGFPeaklist (F058870)	Uncharacterized protein OS=Gallus gallus GN=HNRNPU PE=4 SV=2	F1NSP8_CHICK,UPI00003ACD6C,UPI0000447CA3,UPI0000610658,UPI0000610659,UPI0000E800C8,UPI0000ECC882,UPI0000ECC883,UPI000240B660,UPI0002C8704D,UPI00035073BE	TAX_GallusGallus_9031_20141114.fasta,UPARC14_2_GallusG_9031_20140417.fasta	67,965.30	100.00%	2	2	6	0.01%	2.53%	NGQELGVAFK	K	I	99.70%	53.8	45.1	27.1	17.7	31.1	2	0.00643	0.4	0	2	0	0	2	1,063.54	2,141.50		569.68	201	210	Empty	TRUE	
20196-20225 HinckeM --TAXchicken	Uncategorized Sample	20196-20205_A	Mudpit_Ingel20196_MGFPeaklist (F058870)	Uncharacterized protein OS=Gallus gallus GN=HNRNPU PE=4 SV=2	F1NSP8_CHICK,UPI00003ACD6C,UPI0000447CA3,UPI0000610658,UPI0000610659,UPI0000E800C8,UPI0000ECC882,UPI0000ECC883,UPI000240B660,UPI0002C8704D,UPI00035073BE	TAX_GallusGallus_9031_20141114.fasta,UPARC14_2_GallusG_9031_20140417.fasta	67,965.30	100.00%	2	2	6	0.01%	2.53%	FIEIAAR	K	K	98.80%	32.2	44.8	35	16.7	27.5	1.15	0.124	0.429	0	4	0	0	2	819.4723	2,027.50		3,887.09	343	349	Empty	TRUE	
20196-20225 HinckeM --TAXchicken	Uncategorized Sample	20196-20205_A	Mudpit_Ingel20196_MGFPeaklist (F058870)	Uncharacterized protein OS=Gallus gallus GN=HNRNPU PE=4 SV=2	F1NSP8_CHICK,UPI00003ACD6C,UPI0000447CA3,UPI0000610658,UPI0000610659,UPI0000E800C8,UPI0000ECC882,UPI0000ECC883,UPI000240B660,UPI0002C8704D,UPI00035073BE	TAX_GallusGallus_9031_20141114.fasta,UPARC14_2_GallusG_9031_20140417.fasta	67,965.30	100.00%	2	2	6	0.01%	2.53%	NGQELGVAFK	K	I	99.70%	53.8	45.1	27.1	17.7	31.1	2	0.00643	0.4	0	2	0	0	2	1,063.54	2,141.50		569.68	201	210	Empty	TRUE	
20196-20225 HinckeM --TAXchicken	Uncategorized Sample	20196-20205_A	Mudpit_Ingel20196_MGFPeaklist (F058870)	Uncharacterized protein OS=Gallus gallus GN=HNRNPU PE=4 SV=2	F1NSP8_CHICK,UPI00003ACD6C,UPI0000447CA3,UPI0000610658,UPI0000610659,UPI0000E800C8,UPI0000ECC882,UPI0000ECC883,UPI000240B660,UPI0002C8704D,UPI00035073BE	TAX_GallusGallus_9031_20141114.fasta,UPARC14_2_GallusG_9031_20140417.fasta	67,965.30	100.00%	2	2	6	0.01%	2.53%	FIEIAAR	K	K	98.80%	32.2	44.8	35	16.7	27.5	1.15	0.124	0.429	0	4	0	0	2	819.4723	2,027.50		3,887.09	343	349	Empty	TRUE	
20196-20225 HinckeM --TAXchicken	Uncategorized Sample	20196-20205_A	Mudpit_Ingel20196_MGFPeaklist (F058870)	Uncharacterized protein OS=Gallus gallus GN=HNRNPU PE=4 SV=2	F1NSP8_CHICK,UPI00003ACD6C,UPI0000447CA3,UPI0000610658,UPI0000610659,UPI0000E800C8,UPI0000ECC882,UPI0000ECC883,UPI000240B660,UPI0002C8704D,UPI00035073BE	TAX_GallusGallus_9031_20141114.fasta,UPARC14_2_GallusG_9031_20140417.fasta	67,965.30	100.00%	2	2	6	0.01%	2.53%	NGQELGVAFK	K	I	99.70%	53.8	45.1	27.1	17.7	31.1	2	0.00643	0.4	0	2	0	0	2	1,063.54	2,141.50		569.68	201	210	Empty	TRUE	
20196-20225 HinckeM --TAXchicken	Uncategorized Sample	20196-20205_A	Mudpit_Ingel20196_MGFPeaklist (F058870)	Uncharacterized protein OS=Gallus gallus GN=HNRNPU PE=4 SV=2	F1NSP8_CHICK,UPI00003ACD6C,UPI0000447CA3,UPI0000610658,UPI0000610659,UPI0000E800C8,UPI0000ECC882,UPI0000ECC883,UPI000240B660,UPI0002C8704D,UPI00035073BE	TAX_GallusGallus_9031_20141114.fasta,UPARC14_2_GallusG_9031_20140417.fasta	67,965.30	100.00%	2	2	6	0.01%	2.53%	FIEIAAR	K	K	98.80%	32.2	44.8	35	16.7	27.5	1.15	0.124	0.429	0	4	0	0	2	819.4723	2,027.50		3,887.09	343	349	Empty	TRUE	
20196-20225 HinckeM --TAXchicken	Uncategorized Sample	20196-20205_A	Mudpit_Ingel20196_MGFPeaklist (F058870)	Uncharacterized protein OS=Gallus gallus GN=HNRNPU PE=4 SV=2	F1NSP8_CHICK,UPI00003ACD6C,UPI0000447CA3,UPI0000610658,UPI0000610659,UPI0000E800C8,UPI0000ECC882,UPI0000ECC883,UPI000240B660,UPI0002C8704D,UPI00035073BE	TAX_GallusGallus_9031_20141114.fasta,UPARC14_2_GallusG_9031_20140417.fasta	67,965.30	100.00%	2	2	6	0.01%	2.53%	NGQELGVAFK	K	I	99.70%	53.8	45.1	27.1	17.7	31.1	2	0.00643	0.4	0	2	0	0	2	1,063.54	2,141.50		569.68	201	210	Empty	TRUE	
20196-20225 HinckeM --TAXchicken	Uncategorized Sample	20196-20205_A	Mudpit_Ingel20196_MGFPeaklist (F058870)	Uncharacterized protein OS=Gallus gallus GN=HNRNPU PE=4 SV=2	F1NSP8_CHICK,UPI00003ACD6C,UPI0000447CA3,UPI0000610658,UPI0000610659,UPI0000E800C8,UPI0000ECC882,UPI0000ECC883,UPI000240B660,UPI0002C8704D,UPI00035073BE	TAX_GallusGallus_9031_20141114.fasta,UPARC14_2_GallusG_9031_20140417.fasta	67,965.30	100.00%	2	2	6	0.01%	2.53%	FIEIAAR	K	K	98.80%	32.2	44.8	35	16.7	27.5	1.15	0.124	0.429	0	4	0	0	2	819.4723	2,027.50		3,887.09	343	349	Empty	TRUE	
20196-20225 HinckeM --TAXchicken	Uncategorized Sample	20196-20205_A	Mudpit_Ingel20196_MGFPeaklist (F058870)	Uncharacterized protein OS=Gallus gallus GN=HNRNPU PE=4 SV=2	F1NSP8_CHICK,UPI00003ACD6C,UPI0000447CA3,UPI0000610658,UPI0000610659,UPI0000E800C8,UPI0000ECC882,UPI0000ECC883,UPI000240B660,UPI0002C8704D,UPI00035073BE	TAX_GallusGallus_9031_20141114.fasta,UPARC14_2_GallusG_9031_20140417.fasta	67,965.30	100.00%	2	2	6	0.01%	2.53%	NGQELGVAFK	K	I	99.70%	53.8	45.1	27.1	17.7	31.1	2	0.00643	0.4	0	2	0	0	2	1,063.54	2,141.50		569.68	201	210	Empty	TRUE	
20196-20225 HinckeM --TAXchicken	Uncategorized Sample	20196-20205_A	Mudpit_Ingel20196_MGFPeaklist (F058870)	Uncharacterized protein OS=Gallus gallus GN=HNRNPU PE=4 SV=2	F1NSP8_CHICK,UPI00003ACD6C,UPI0000447CA3,UPI0000610658,UPI0000610659,UPI0000E800C8,UPI0000ECC882,UPI0000ECC883,UPI000240B660,UPI0002C8704D,UPI00035073BE	TAX_GallusGallus_9031_20141114.fasta,UPARC14_2_GallusG_9031_20140417.fasta	67,965.30	100.00%	2	2	6	0.01%	2.53%	FIEIAAR	K	K	98.80%	32.2	44.8	35	16.7	27.5	1.15	0.124	0.429	0	4	0	0	2	819.4723	2,027.50		3,887.09	343	349	Empty	TRUE	
20196-20225 HinckeM --TAXchicken	Uncategorized Sample	20196-20205_A	Mudpit_Ingel20196_MGFPeaklist (F058870)	Uncharacterized protein OS=Gallus gallus GN=HNRNPU PE=4 SV=2	F1NSP8_CHICK,UPI00003ACD6C,UPI0000447CA3,UPI0000610658,UPI0000610659,UPI0000E800C8,UPI0000ECC882,UPI0000ECC883,UPI000240B660,UPI0002C8704D,UPI00035073BE	TAX_GallusGallus_9031_20141114.fasta,UPARC14_2_GallusG_9031_20140417.fasta	67,965.30	100.00%	2	2	6	0.01%	2.53%	NGQELGVAFK	K	I	99.70%	53.8	45.1	27.1	17.7	31.1	2	0.00643	0.4	0	2	0	0	2	1,063.54	2,141.50		569.68	201	210	Empty	TRUE	
20196-20225 HinckeM --TAXchicken	Uncategorized Sample	20196-20205_A	Mudpit_Ingel20196_MGFPeaklist (F058870)	Uncharacterized protein OS=Gallus gallus GN=HNRNPU PE=4 SV=2	F1NSP8_CHICK,UPI00003ACD6C,UPI0000447CA3,UPI0000610658,UPI0000610659,UPI0000E800C8,UPI0000ECC882,UPI0000ECC883,UPI000240B660,UPI0002C8704D,UPI00035073BE	TAX_GallusGallus_9031_20141114.fasta,UPARC14_2_GallusG_9031_20140417.fasta	67,965.30	100.00%	2	2	6	0.01%	2.53%	FIEIAAR	K	K	98.80%	32.2	44.8	35	16.7	27.5	1.15	0.124	0.429	0	4	0	0	2	819.4723	2,027.50		3,887.09	343	349	Empty	TRUE	
20196-20225 HinckeM --TAXchicken	Uncategorized Sample	20196-20205_A	Mudpit_Ingel20196_MGFPeaklist (F058870)	Uncharacterized protein OS=Gallus gallus GN=HNRNPU PE=4 SV=2	F1NSP8_CHICK,UPI00003ACD6C,UPI0000447CA3,UPI0000610658,UPI0000610659,UPI0000E800C8,UPI0000ECC882,UPI0000ECC883,UPI000240B660,UPI0002C8704D,UPI00035073BE	TAX_GallusGallus_9031_20141114.fasta,UPARC14_2_GallusG_9031_20140417.fasta	67,965.30	100.00%	2	2	6	0.01%	2.53%	NGQELGVAFK	K	I	99.70%	53.8	45.1	27.1	17.7	31.1	2	0.00643	0.4	0	2	0	0	2	1,063.54	2,141.50		569.68	201	210	Empty	TRUE	
20196-20225 HinckeM --TAXchicken	Uncategorized Sample	20196-20205_A	Mudpit_Ingel20196_MGFPeaklist (F058870)	Uncharacterized protein OS=Gallus gallus GN=HNRNPU PE=4 SV=2	F1NSP8_CHICK,UPI00003ACD6C,UPI0000447CA3,UPI0000610658,UPI0000610659,UPI0000E800C8,UPI0000ECC882,UPI0000ECC883,UPI000240B660,UPI0002C8704D,UPI00035073BE	TAX_GallusGallus_9031_20141114.fasta,UPARC14_2_GallusG_9031_20140417.fasta	67,965.30	100.00%	2	2	6	0.01%	2.53%	FIEIAAR	K	K	98.80%	32.2	44.8	35	16.7	27.5	1.15	0.124	0.429	0	4	0	0	2	819.4723	2,027.50		3,887.09	343	349	Empty	TRUE	
20196-20225 HinckeM --TAXchicken	Uncategorized Sample	20196-20205_A	Mudpit_Ingel20196_MGFPeaklist (F058870)	Uncharacterized protein OS=Gallus gallus GN=HNRNPU PE=4 SV=2	F1NSP8_CHICK,UPI00003ACD6C,UPI0000447CA3,UPI0000610658,UPI0000610659,UPI0000E800C8,UPI0000ECC882,UPI0000ECC883,UPI000240B660,UPI0002C8704D,UPI00035073BE	TAX_GallusGallus_9031_20141114.fasta,UPARC14_2_GallusG_9031_20140417.fasta	67,965.30	100.00%	2	2	6	0.01%	2.53%	NGQELGVAFK	K	I	99.70%	53.8	45.1	27.1	17.7	31.1	2	0.00643	0.4	0	2	0	0	2	1,063.54	2,141.50		569.68	201	210	Empty	TRUE	
20196-20225 HinckeM --TAXchicken	Uncategorized Sample	20196-20205_A	Mudpit_Ingel20196_MGFPeaklist (F058870)	Eukaryotic initiation factor 4A-III OS=Gallus gallus GN=EIF4A3 PE=2 SV=1	IF4A3_CHICK,UPI0000443458	TAX_GallusGallus_9031_20141114.fasta,UPARC14_2_GallusG_9031_20140417.fasta	46,817.20	100.00%	6	7	14	0.03%	17.20%	EQIYDVYR	K	Y	9.90%	15.3	43.8	19.3	3.35					0	2	0	0	2	1,085.53	1,977.50		1,150.92	200	207	Empty	TRUE	UPI000000B8EA,UPI00015A536D
20196-20225 HinckeM --TAXchicken	Uncategorized Sample	20196-20205_A	Mudpit_Ingel20196_MGFPeaklist (F058870)	Eukaryotic initiation factor 4A-III OS=Gallus gallus GN=EIF4A3 PE=2 SV=1	IF4A3_CHICK,UPI0000443458	TAX_GallusGallus_9031_20141114.fasta,UPARC14_2_GallusG_9031_20140417.fasta	46,817.20	100.00%	6	7	14	0.03%	17.20%	ETQALILAPTR	R	E	99.70%	54.9	42.9	29	37.2	31.1	2.47	0.322	0.318	0	2	0	0	2	1,212.69	2,151.00		2,167.79	107	117	Empty	TRUE	UPI000000B8EA,UPI00015A536D
20196-20225 HinckeM --TAXchicken	Uncategorized Sample	20196-20205_A	Mudpit_Ingel20196_MGFPeaklist (F058870)	Eukaryotic initiation factor 4A-III OS=Gallus gallus GN=EIF4A3 PE=2 SV=1	IF4A3_CHICK,UPI0000443458	TAX_GallusGallus_9031_20141114.fasta,UPARC14_2_GallusG_9031_20140417.fasta	46,817.20	100.00%	6	7	14	0.03%	17.20%	GFKEQIYDVYR	K	Y	99.70%	33.7	44.9	32	18.1	25.5	1.6	0.224	0.318	0	0	2	0	2	1,417.71	2,158.00		1,564.09	197	207	Empty	TRUE	UPI000000B8EA
20196-20225 HinckeM --TAXchicken	Uncategorized Sample	20196-20205_A	Mudpit_Ingel20196_MGFPeaklist (F058870)	Eukaryotic initiation factor 4A-III OS=Gallus gallus GN=EIF4A3 PE=2 SV=1	IF4A3_CHICK,UPI0000443458	TAX_GallusGallus_9031_20141114.fasta,UPARC14_2_GallusG_9031_20140417.fasta	46,817.20	100.00%	6	7	14	0.03%	17.20%	GIYAYGFEKPSAIQQR	R	A	99.70%	92.5	44.2	16.2	83.9	57.8	8.68	0.564	0.562	0	0	2	0	2	1,827.94	2,186.00		42,007.20	53	68	Empty	FALSE	UPI000000B8EA,UPI0001A2D2C8,UPI0000185E3C,IF4A2_CHICK,UPI0001E92679,UPI00000FE071,UPI00015A68B0,R9PXN1_CHICK,UPI00015A536D,UPI00015CFFCA,UPI0002C87F31
20196-20225 HinckeM --TAXchicken	Uncategorized Sample	20196-20205_A	Mudpit_Ingel20196_MGFPeaklist (F058870)	Eukaryotic initiation factor 4A-III OS=Gallus gallus GN=EIF4A3 PE=2 SV=1	IF4A3_CHICK,UPI0000443458	TAX_GallusGallus_9031_20141114.fasta,UPARC14_2_GallusG_9031_20140417.fasta	46,817.20	100.00%	6	7	14	0.03%	17.20%	KLDYGQHVVAGTPGR	R	V	99.70%	26.5	44	26.1	11.7	24.1	0.602	0.199	0.267	0	0	2	2	2	1,597.84	1,697.50		3,981.13	153	167	Empty	TRUE	UPI000000B8EA,UPI00015A536D
20196-20225 HinckeM --TAXchicken	Uncategorized Sample	20196-20205_A	Mudpit_Ingel20196_MGFPeaklist (F058870)	Eukaryotic initiation factor 4A-III OS=Gallus gallus GN=EIF4A3 PE=2 SV=1	IF4A3_CHICK,UPI0000443458	TAX_GallusGallus_9031_20141114.fasta,UPARC14_2_GallusG_9031_20140417.fasta	46,817.20	100.00%	6	7	14	0.03%	17.20%	KVDWLTEK	R	M	82.10%	30.9	45.4	32.1	8.82					0	3	0	0	2	1,018.56	2,004.00		1,260.20	290	297	Empty	FALSE	UPI000000B8EA,UPI0001A2D2C8,UPI0000185E3C,IF4A2_CHICK,UPI0001E92679,UPI00000FE071,UPI00015A68B0,R9PXN1_CHICK,UPI00015A536D,UPI00015CFFCA,UPI0002C87F31
20196-20225 HinckeM --TAXchicken	Uncategorized Sample	20196-20205_A	Mudpit_Ingel20196_MGFPeaklist (F058870)	Eukaryotic initiation factor 4A-III OS=Gallus gallus GN=EIF4A3 PE=2 SV=1	IF4A3_CHICK,UPI0000443458	TAX_GallusGallus_9031_20141114.fasta,UPARC14_2_GallusG_9031_20140417.fasta	46,817.20	100.00%	6	7	14	0.03%	17.20%	LDYGQHVVAGTPGR	K	V	99.70%	49.5	44.3	23.2	39.6	29.9	4.01	0.351	0.25	0	0	2	0	2	1,469.75	1,766.50		1,269.34	154	167	Empty	TRUE	UPI000000B8EA,UPI00015A536D
20196-20225 HinckeM --TAXchicken	Uncategorized Sample	20196-20205_A	Mudpit_Ingel20196_MGFPeaklist (F058870)	Eukaryotic initiation factor 4A-III OS=Gallus gallus GN=EIF4A3 PE=2 SV=1	IF4A3_CHICK,UPI0000443458	TAX_GallusGallus_9031_20141114.fasta,UPARC14_2_GallusG_9031_20140417.fasta	46,817.20	100.00%	6	7	14	0.03%	17.20%	VDWLTEK	K	M	32.10%	16.6	45.3	30.6	-0.71					0	1	0	0	2	890.4619	2,136.00		285.12	291	297	Empty	FALSE	UPI000000B8EA,UPI0001A2D2C8,UPI0000185E3C,IF4A2_CHICK,UPI0001E92679,UPI00000FE071,UPI00015A68B0,R9PXN1_CHICK,UPI00015A536D,UPI00015CFFCA,UPI0002C87F31
20196-20225 HinckeM --TAXchicken	Uncategorized Sample	20196-20205_A	Mudpit_Ingel20196_MGFPeaklist (F058870)	Eukaryotic initiation factor 4A-III OS=Gallus gallus GN=EIF4A3 PE=2 SV=1	IF4A3_CHICK,UPI0000443458	TAX_GallusGallus_9031_20141114.fasta,UPARC14_2_GallusG_9031_20140417.fasta	46,817.20	100.00%	6	7	14	0.03%	17.20%	VLISTDVWAR	R	G	99.70%	40.5	44.8	35.8	27.9	31.9	0.886	0.266	0.4	0	2	0	0	2	1,159.65	2,438.00		1,760.65	331	340	Empty	TRUE	UPI000000B8EA,UPI00015A536D
20196-20225 HinckeM --TAXchicken	Uncategorized Sample	20196-20205_A	Mudpit_Ingel20196_MGFPeaklist (F058870)	Eukaryotic initiation factor 4A-III OS=Gallus gallus GN=EIF4A3 PE=2 SV=1	IF4A3_CHICK,UPI0000443458	TAX_GallusGallus_9031_20141114.fasta,UPARC14_2_GallusG_9031_20140417.fasta	46,817.20	100.00%	6	7	14	0.03%	17.20%	EQIYDVYR	K	Y	9.90%	15.3	43.8	19.3	3.35					0	2	0	0	2	1,085.53	1,977.50		1,150.92	200	207	Empty	TRUE	UPI000000B8EA,UPI00015A536D
20196-20225 HinckeM --TAXchicken	Uncategorized Sample	20196-20205_A	Mudpit_Ingel20196_MGFPeaklist (F058870)	Eukaryotic initiation factor 4A-III OS=Gallus gallus GN=EIF4A3 PE=2 SV=1	IF4A3_CHICK,UPI0000443458	TAX_GallusGallus_9031_20141114.fasta,UPARC14_2_GallusG_9031_20140417.fasta	46,817.20	100.00%	6	7	14	0.03%	17.20%	ETQALILAPTR	R	E	99.70%	54.9	42.9	29	37.2	31.1	2.47	0.322	0.318	0	2	0	0	2	1,212.69	2,151.00		2,167.79	107	117	Empty	TRUE	UPI000000B8EA,UPI00015A536D
20196-20225 HinckeM --TAXchicken	Uncategorized Sample	20196-20205_A	Mudpit_Ingel20196_MGFPeaklist (F058870)	Eukaryotic initiation factor 4A-III OS=Gallus gallus GN=EIF4A3 PE=2 SV=1	IF4A3_CHICK,UPI0000443458	TAX_GallusGallus_9031_20141114.fasta,UPARC14_2_GallusG_9031_20140417.fasta	46,817.20	100.00%	6	7	14	0.03%	17.20%	GFKEQIYDVYR	K	Y	99.70%	33.7	44.9	32	18.1	25.5	1.6	0.224	0.318	0	0	2	0	2	1,417.71	2,158.00		1,564.09	197	207	Empty	TRUE	UPI000000B8EA
20196-20225 HinckeM --TAXchicken	Uncategorized Sample	20196-20205_A	Mudpit_Ingel20196_MGFPeaklist (F058870)	Eukaryotic initiation factor 4A-III OS=Gallus gallus GN=EIF4A3 PE=2 SV=1	IF4A3_CHICK,UPI0000443458	TAX_GallusGallus_9031_20141114.fasta,UPARC14_2_GallusG_9031_20140417.fasta	46,817.20	100.00%	6	7	14	0.03%	17.20%	GIYAYGFEKPSAIQQR	R	A	99.70%	92.5	44.2	16.2	83.9	57.8	8.68	0.564	0.562	0	0	2	0	2	1,827.94	2,186.00		42,007.20	53	68	Empty	FALSE	UPI000000B8EA,UPI0001A2D2C8,UPI0000185E3C,IF4A2_CHICK,UPI0001E92679,UPI00000FE071,UPI00015A68B0,R9PXN1_CHICK,UPI00015A536D,UPI00015CFFCA,UPI0002C87F31
20196-20225 HinckeM --TAXchicken	Uncategorized Sample	20196-20205_A	Mudpit_Ingel20196_MGFPeaklist (F058870)	Eukaryotic initiation factor 4A-III OS=Gallus gallus GN=EIF4A3 PE=2 SV=1	IF4A3_CHICK,UPI0000443458	TAX_GallusGallus_9031_20141114.fasta,UPARC14_2_GallusG_9031_20140417.fasta	46,817.20	100.00%	6	7	14	0.03%	17.20%	KLDYGQHVVAGTPGR	R	V	99.70%	26.5	44	26.1	11.7	24.1	0.602	0.199	0.267	0	0	2	2	2	1,597.84	1,697.50		3,981.13	153	167	Empty	TRUE	UPI000000B8EA,UPI00015A536D
20196-20225 HinckeM --TAXchicken	Uncategorized Sample	20196-20205_A	Mudpit_Ingel20196_MGFPeaklist (F058870)	Eukaryotic initiation factor 4A-III OS=Gallus gallus GN=EIF4A3 PE=2 SV=1	IF4A3_CHICK,UPI0000443458	TAX_GallusGallus_9031_20141114.fasta,UPARC14_2_GallusG_9031_20140417.fasta	46,817.20	100.00%	6	7	14	0.03%	17.20%	KVDWLTEK	R	M	82.10%	30.9	45.4	32.1	8.82					0	3	0	0	2	1,018.56	2,004.00		1,260.20	290	297	Empty	FALSE	UPI000000B8EA,UPI0001A2D2C8,UPI0000185E3C,IF4A2_CHICK,UPI0001E92679,UPI00000FE071,UPI00015A68B0,R9PXN1_CHICK,UPI00015A536D,UPI00015CFFCA,UPI0002C87F31
20196-20225 HinckeM --TAXchicken	Uncategorized Sample	20196-20205_A	Mudpit_Ingel20196_MGFPeaklist (F058870)	Eukaryotic initiation factor 4A-III OS=Gallus gallus GN=EIF4A3 PE=2 SV=1	IF4A3_CHICK,UPI0000443458	TAX_GallusGallus_9031_20141114.fasta,UPARC14_2_GallusG_9031_20140417.fasta	46,817.20	100.00%	6	7	14	0.03%	17.20%	LDYGQHVVAGTPGR	K	V	99.70%	49.5	44.3	23.2	39.6	29.9	4.01	0.351	0.25	0	0	2	0	2	1,469.75	1,766.50		1,269.34	154	167	Empty	TRUE	UPI000000B8EA,UPI00015A536D
20196-20225 HinckeM --TAXchicken	Uncategorized Sample	20196-20205_A	Mudpit_Ingel20196_MGFPeaklist (F058870)	Eukaryotic initiation factor 4A-III OS=Gallus gallus GN=EIF4A3 PE=2 SV=1	IF4A3_CHICK,UPI0000443458	TAX_GallusGallus_9031_20141114.fasta,UPARC14_2_GallusG_9031_20140417.fasta	46,817.20	100.00%	6	7	14	0.03%	17.20%	VDWLTEK	K	M	32.10%	16.6	45.3	30.6	-0.71					0	1	0	0	2	890.4619	2,136.00		285.12	291	297	Empty	FALSE	UPI000000B8EA,UPI0001A2D2C8,UPI0000185E3C,IF4A2_CHICK,UPI0001E92679,UPI00000FE071,UPI00015A68B0,R9PXN1_CHICK,UPI00015A536D,UPI00015CFFCA,UPI0002C87F31
20196-20225 HinckeM --TAXchicken	Uncategorized Sample	20196-20205_A	Mudpit_Ingel20196_MGFPeaklist (F058870)	Eukaryotic initiation factor 4A-III OS=Gallus gallus GN=EIF4A3 PE=2 SV=1	IF4A3_CHICK,UPI0000443458	TAX_GallusGallus_9031_20141114.fasta,UPARC14_2_GallusG_9031_20140417.fasta	46,817.20	100.00%	6	7	14	0.03%	17.20%	VLISTDVWAR	R	G	99.70%	40.5	44.8	35.8	27.9	31.9	0.886	0.266	0.4	0	2	0	0	2	1,159.65	2,438.00		1,760.65	331	340	Empty	TRUE	UPI000000B8EA,UPI00015A536D
20196-20225 HinckeM --TAXchicken	Uncategorized Sample	20196-20205_A	Mudpit_Ingel20196_MGFPeaklist (F058870)	Vimentin OS=Gallus gallus GN=VIM PE=3 SV=1	F1NJ08_CHICK	TAX_GallusGallus_9031_20141114.fasta	53,216.80	100.00%	33	37	104	0.21%	62.80%	DGQVINETSQHHDDLE	R	-	99.70%	22.5	42.1	14.8	22.5	34	2.48	0.344	0.375	0	0	4	0	2	1,836.80	1,715.00		8,312.95	445	460	Empty	TRUE	UPI0000ECCBDF,UPI0001849FE9
20196-20225 HinckeM --TAXchicken	Uncategorized Sample	20196-20205_A	Mudpit_Ingel20196_MGFPeaklist (F058870)	Vimentin OS=Gallus gallus GN=VIM PE=3 SV=1	F1NJ08_CHICK	TAX_GallusGallus_9031_20141114.fasta	53,216.80	100.00%	33	37	104	0.21%	62.80%	DNLADDIMR	R	L	93.00%	42.2	43.9	28.6	22.7					0	1	0	0	2	1,078.48	1,874.00		564.55	170	178	Empty	TRUE	UPI0000ECCBDF,UPI0001849FE9
20196-20225 HinckeM --TAXchicken	Uncategorized Sample	20196-20205_A	Mudpit_Ingel20196_MGFPeaklist (F058870)	Vimentin OS=Gallus gallus GN=VIM PE=3 SV=1	F1NJ08_CHICK	TAX_GallusGallus_9031_20141114.fasta	53,216.80	100.00%	33	37	104	0.21%	62.80%	EEAESTLQSFR	R	Q	99.70%	45.5	43.9	21.7	38.3	31.6	2.82	0.348	0.364	0	2	0	0	2	1,296.61	1,979.00		2,809.24	191	201	Empty	TRUE	UPI0000ECCBDF,UPI0001849FE9
20196-20225 HinckeM --TAXchicken	Uncategorized Sample	20196-20205_A	Mudpit_Ingel20196_MGFPeaklist (F058870)	Vimentin OS=Gallus gallus GN=VIM PE=3 SV=1	F1NJ08_CHICK	TAX_GallusGallus_9031_20141114.fasta	53,216.80	100.00%	33	37	104	0.21%	62.80%	EEAESTLQSFRQDVDNASLAR	R	L	99.70%	63.2	43.9	14.5	58.8	45	4.96	0.404	0.333	0	0	2	0	2	2,366.12	2,479.00		14,304.20	191	211	Empty	TRUE	UPI0000ECCBDF,UPI0001849FE9
20196-20225 HinckeM --TAXchicken	Uncategorized Sample	20196-20205_A	Mudpit_Ingel20196_MGFPeaklist (F058870)	Vimentin OS=Gallus gallus GN=VIM PE=3 SV=1	F1NJ08_CHICK	TAX_GallusGallus_9031_20141114.fasta	53,216.80	100.00%	33	37	104	0.21%	62.80%	EKLQEEMLQREEAESTLQSFR	R	Q	99.70%	30.9	43.8	16	23.4	35.4	3.57	0.379	0.262	0	0	0	3	2	2,597.25	2,210.00		11,172.80	181	201	Empty	TRUE	UPI0000ECCBDF,UPI0001849FE9
20196-20225 HinckeM --TAXchicken	Uncategorized Sample	20196-20205_A	Mudpit_Ingel20196_MGFPeaklist (F058870)	Vimentin OS=Gallus gallus GN=VIM PE=3 SV=1	F1NJ08_CHICK	TAX_GallusGallus_9031_20141114.fasta	53,216.80	100.00%	33	37	104	0.21%	62.80%	EMEENFAVEAANYQDTIGR	R	L	99.70%	89.2	41.1	18.5	84.9	60.1	7.05	0.532	0.5	0	0	2	0	2	2,202.96	2,336.00		17,008.30	340	358	Empty	TRUE	UPI0000ECCBDF,UPI0001849FE9
20196-20225 HinckeM --TAXchicken	Uncategorized Sample	20196-20205_A	Mudpit_Ingel20196_MGFPeaklist (F058870)	Vimentin OS=Gallus gallus GN=VIM PE=3 SV=1	F1NJ08_CHICK	TAX_GallusGallus_9031_20141114.fasta	53,216.80	100.00%	33	37	104	0.21%	62.80%	ETNIESQPIVDTHSK	R	R	99.70%	44.2	44.4	17.1	34	37.6	3.64	0.386	0.4	0	0	2	0	2	1,697.83	1,756.00		13,236.20	419	433	Empty	TRUE	UPI0000ECCBDF,UPI0001849FE9
20196-20225 HinckeM --TAXchicken	Uncategorized Sample	20196-20205_A	Mudpit_Ingel20196_MGFPeaklist (F058870)	Vimentin OS=Gallus gallus GN=VIM PE=3 SV=1	F1NJ08_CHICK	TAX_GallusGallus_9031_20141114.fasta	53,216.80	100.00%	33	37	104	0.21%	62.80%	ETNIESQPIVDTHSKR	R	T	99.70%	49.4	44.3	18.9	43.9	47.2	6.7	0.449	0.5	0	0	0	4	2	1,853.94	1,689.00		7,718.29	419	434	Empty	TRUE	UPI0000ECCBDF,UPI0001849FE9
20196-20225 HinckeM --TAXchicken	Uncategorized Sample	20196-20205_A	Mudpit_Ingel20196_MGFPeaklist (F058870)	Vimentin OS=Gallus gallus GN=VIM PE=3 SV=1	F1NJ08_CHICK	TAX_GallusGallus_9031_20141114.fasta	53,216.80	100.00%	33	37	104	0.21%	62.80%	EYQDLLNVK	R	M	99.70%	48.8	44.7	34	28.9	30.4	1.15	0.227	0.389	0	4	0	0	2	1,121.58	2,200.00		21,570.80	376	384	Empty	TRUE	UPI0000610087,UPI00000FB2FA,UPI00001291D3,O73665_CHICK,UPI0000ECCBDF,UPI0001849FE9,DESM_CHICK
20196-20225 HinckeM --TAXchicken	Uncategorized Sample	20196-20205_A	Mudpit_Ingel20196_MGFPeaklist (F058870)	Vimentin OS=Gallus gallus GN=VIM PE=3 SV=1	F1NJ08_CHICK	TAX_GallusGallus_9031_20141114.fasta	53,216.80	100.00%	33	37	104	0.21%	62.80%	FADLSEAANR	K	N	99.70%	68.7	44.4	32.7	19.3	41.4	3.96	0.44	0.6	0	6	0	0	2	1,093.53	1,845.50		38,473.60	289	298	Empty	TRUE	UPI0000ECCBDF,UPI0001849FE9
20196-20225 HinckeM --TAXchicken	Uncategorized Sample	20196-20205_A	Mudpit_Ingel20196_MGFPeaklist (F058870)	Vimentin OS=Gallus gallus GN=VIM PE=3 SV=1	F1NJ08_CHICK	TAX_GallusGallus_9031_20141114.fasta	53,216.80	100.00%	33	37	104	0.21%	62.80%	FADLSEAANRNNDALR	K	Q	99.70%	82.9	44.8	18	75.3	53	4.77	0.447	0.531	0	0	4	0	2	1,776.86	1,974.00		18,543.50	289	304	Empty	TRUE	UPI0000ECCBDF,UPI0001849FE9
20196-20225 HinckeM --TAXchicken	Uncategorized Sample	20196-20205_A	Mudpit_Ingel20196_MGFPeaklist (F058870)	Vimentin OS=Gallus gallus GN=VIM PE=3 SV=1	F1NJ08_CHICK	TAX_GallusGallus_9031_20141114.fasta	53,216.80	100.00%	33	37	104	0.21%	62.80%	FANYIDK	R	V	98.80%	36.4	43.1	30.2	20.2	24.5	1.05	0.22	0.5	0	4	0	0	2	870.4356	1,832.00		4,114.14	108	114	Empty	TRUE	UPI0000ECCBDF,UPI0001849FE9
20196-20225 HinckeM --TAXchicken	Uncategorized Sample	20196-20205_A	Mudpit_Ingel20196_MGFPeaklist (F058870)	Vimentin OS=Gallus gallus GN=VIM PE=3 SV=1	F1NJ08_CHICK	TAX_GallusGallus_9031_20141114.fasta	53,216.80	100.00%	33	37	104	0.21%	62.80%	FANYIDKVR	R	F	99.70%	54.4	43.6	25.7	43.6	37.7	4.3	0.326	0.611	0	2	0	0	2	1,125.61	1,930.00		4,563.37	108	116	Empty	TRUE	UPI0000ECCBDF,UPI0001849FE9
20196-20225 HinckeM --TAXchicken	Uncategorized Sample	20196-20205_A	Mudpit_Ingel20196_MGFPeaklist (F058870)	Vimentin OS=Gallus gallus GN=VIM PE=3 SV=1	F1NJ08_CHICK	TAX_GallusGallus_9031_20141114.fasta	53,216.80	100.00%	33	37	104	0.21%	62.80%	HLREYQDLLNVK	R	M	99.70%	31.6	43.8	21.5	18.8	42.4	3.11	0.285	0.667	0	0	2	0	2	1,527.83	2,145.00		5,877.99	373	384	Empty	TRUE	UPI00000FB2FA,UPI00001291D3,O73665_CHICK,UPI0000ECCBDF,UPI0001849FE9,DESM_CHICK
20196-20225 HinckeM --TAXchicken	Uncategorized Sample	20196-20205_A	Mudpit_Ingel20196_MGFPeaklist (F058870)	Vimentin OS=Gallus gallus GN=VIM PE=3 SV=1	F1NJ08_CHICK	TAX_GallusGallus_9031_20141114.fasta	53,216.80	100.00%	33	37	104	0.21%	62.80%	ILLAELEQLK	K	G	99.70%	48	40.7	36.8	15.7	40.6	2.04	0.246	0.55	0	4	0	0	2	1,169.71	2,737.00		30,303.80	124	133	Empty	TRUE	UPI0000ECCBDF,UPI0001849FE9
20196-20225 HinckeM --TAXchicken	Uncategorized Sample	20196-20205_A	Mudpit_Ingel20196_MGFPeaklist (F058870)	Vimentin OS=Gallus gallus GN=VIM PE=3 SV=1	F1NJ08_CHICK	TAX_GallusGallus_9031_20141114.fasta	53,216.80	100.00%	33	37	104	0.21%	62.80%	ILLAELEQLKGK	K	G	99.70%	59.9	38.9	17.7	48.7	51.6	3.64	0.432	0.667	0	0	2	0	2	1,354.83	2,517.50		23,224.00	124	135	Empty	TRUE	UPI0000ECCBDF,UPI0001849FE9
20196-20225 HinckeM --TAXchicken	Uncategorized Sample	20196-20205_A	Mudpit_Ingel20196_MGFPeaklist (F058870)	Vimentin OS=Gallus gallus GN=VIM PE=3 SV=1	F1NJ08_CHICK	TAX_GallusGallus_9031_20141114.fasta	53,216.80	100.00%	33	37	104	0.21%	62.80%	INMPIPTFASLNLR	R	E	99.70%	64.1	44.1	29.3	48.9	45	4.12	0.451	0.464	0	3	0	0	2	1,602.87	2,711.00		37,712.60	405	418	Empty	TRUE	UPI0000ECCBDF,UPI0001849FE9
20196-20225 HinckeM --TAXchicken	Uncategorized Sample	20196-20205_A	Mudpit_Ingel20196_MGFPeaklist (F058870)	Vimentin OS=Gallus gallus GN=VIM PE=3 SV=1	F1NJ08_CHICK	TAX_GallusGallus_9031_20141114.fasta	53,216.80	100.00%	33	37	104	0.21%	62.80%	KVESLQEEIVFLK	R	K	99.70%	66.2	42.9	29.5	47.4	46.7	4.82	0.426	0.538	0	0	2	0	2	1,561.88	2,590.00		27,761.10	217	229	Empty	TRUE	UPI0000ECCBDF,UPI0001849FE9
20196-20225 HinckeM --TAXchicken	Uncategorized Sample	20196-20205_A	Mudpit_Ingel20196_MGFPeaklist (F058870)	Vimentin OS=Gallus gallus GN=VIM PE=3 SV=1	F1NJ08_CHICK	TAX_GallusGallus_9031_20141114.fasta	53,216.80	100.00%	33	37	104	0.21%	62.80%	KVESLQEEIVFLKK	R	L	99.70%	65.3	40.7	23.7	53.6	49.6	4.72	0.452	0.536	0	0	2	0	2	1,689.98	2,398.00		19,760.60	217	230	Empty	TRUE	UPI0000ECCBDF,UPI0001849FE9
20196-20225 HinckeM --TAXchicken	Uncategorized Sample	20196-20205_A	Mudpit_Ingel20196_MGFPeaklist (F058870)	Vimentin OS=Gallus gallus GN=VIM PE=3 SV=1	F1NJ08_CHICK	TAX_GallusGallus_9031_20141114.fasta	53,216.80	100.00%	33	37	104	0.21%	62.80%	LAELEQLKGK	L	G	99.70%					40.9	2.23	0.11	0.6	0	1	0	0	1	1,128.66	2,526.00		1,005.46	126	135	Empty	TRUE	
20196-20225 HinckeM --TAXchicken	Uncategorized Sample	20196-20205_A	Mudpit_Ingel20196_MGFPeaklist (F058870)	Vimentin OS=Gallus gallus GN=VIM PE=3 SV=1	F1NJ08_CHICK	TAX_GallusGallus_9031_20141114.fasta	53,216.80	100.00%	33	37	104	0.21%	62.80%	LGDLYEEEMR	R	E	99.50%	41.7	42.4	22.6	31.9					0	2	0	0	2	1,270.56	1,868.00		1,575.29	140	149	Empty	TRUE	UPI0000ECCBDF,UPI0001849FE9
20196-20225 HinckeM --TAXchicken	Uncategorized Sample	20196-20205_A	Mudpit_Ingel20196_MGFPeaklist (F058870)	Vimentin OS=Gallus gallus GN=VIM PE=3 SV=1	F1NJ08_CHICK	TAX_GallusGallus_9031_20141114.fasta	53,216.80	100.00%	33	37	104	0.21%	62.80%	LQDEIQNMKEEMAR	R	H	99.70%	52	43.5	21.4	42.4	34	2.34	0.25	0.393	0	0	6	0	2	1,766.81	1,532.50		7,832.31	359	372	Empty	TRUE	UPI0000ECCBDF,UPI0001849FE9
20196-20225 HinckeM --TAXchicken	Uncategorized Sample	20196-20205_A	Mudpit_Ingel20196_MGFPeaklist (F058870)	Vimentin OS=Gallus gallus GN=VIM PE=3 SV=1	F1NJ08_CHICK	TAX_GallusGallus_9031_20141114.fasta	53,216.80	100.00%	33	37	104	0.21%	62.80%	LQEEMLQREEAESTLQSFR	K	Q	99.70%	80.3	44.1	15.3	78.8	53	8.29	0.525	0.395	0	0	2	0	2	2,340.11	2,268.50		13,889.60	183	201	Empty	TRUE	UPI0000ECCBDF,UPI0001849FE9
20196-20225 HinckeM --TAXchicken	Uncategorized Sample	20196-20205_A	Mudpit_Ingel20196_MGFPeaklist (F058870)	Vimentin OS=Gallus gallus GN=VIM PE=3 SV=1	F1NJ08_CHICK	TAX_GallusGallus_9031_20141114.fasta	53,216.80	100.00%	33	37	104	0.21%	62.80%	MALDIEIATYR	K	K	99.70%	53.6	44.1	29.3	38.3	42.6	3.26	0.3	0.545	0	3	0	0	2	1,311.66	2,402.00		5,515.76	385	395	Empty	TRUE	UPI0000ECCBDF,UPI0001849FE9
20196-20225 HinckeM --TAXchicken	Uncategorized Sample	20196-20205_A	Mudpit_Ingel20196_MGFPeaklist (F058870)	Vimentin OS=Gallus gallus GN=VIM PE=3 SV=1	F1NJ08_CHICK	TAX_GallusGallus_9031_20141114.fasta	53,216.80	100.00%	33	37	104	0.21%	62.80%	NLQEAEEWYK	K	S	99.70%	33.6	43.8	17.9	23.8	31.6	1.12	0.199	0.4	0	5	0	0	2	1,309.61	2,165.00		17,228.70	277	286	Empty	TRUE	UPI0000ECCBDF,UPI0001849FE9
20196-20225 HinckeM --TAXchicken	Uncategorized Sample	20196-20205_A	Mudpit_Ingel20196_MGFPeaklist (F058870)	Vimentin OS=Gallus gallus GN=VIM PE=3 SV=1	F1NJ08_CHICK	TAX_GallusGallus_9031_20141114.fasta	53,216.80	100.00%	33	37	104	0.21%	62.80%	QDVDNASLAR	R	L	99.70%	51.2	43.8	27.5	30.3	31.3	1.66	0.00319	0.4	0	1	0	0	2	1,071.51	1,758.00		1,011.94	202	211	Empty	TRUE	UPI0000ECCBDF,UPI0001849FE9
20196-20225 HinckeM --TAXchicken	Uncategorized Sample	20196-20205_A	Mudpit_Ingel20196_MGFPeaklist (F058870)	Vimentin OS=Gallus gallus GN=VIM PE=3 SV=1	F1NJ08_CHICK	TAX_GallusGallus_9031_20141114.fasta	53,216.80	100.00%	33	37	104	0.21%	62.80%	QIQSLTCEVDALK	R	G	99.70%	72.9	44.9	28.3	60.8	50.4	3.85	0.317	0.538	0	2	0	0	2	1,504.77	2,378.50		13,187.60	316	328	Empty	TRUE	UPI0000ECCBDF,UPI0001849FE9
20196-20225 HinckeM --TAXchicken	Uncategorized Sample	20196-20205_A	Mudpit_Ingel20196_MGFPeaklist (F058870)	Vimentin OS=Gallus gallus GN=VIM PE=3 SV=1	F1NJ08_CHICK	TAX_GallusGallus_9031_20141114.fasta	53,216.80	100.00%	33	37	104	0.21%	62.80%	QIQSLTCEVDALKGSNESLER	R	Q	99.70%	68.9	44.1	14.7	63	51.4	6.29	0.447	0.357	0	0	2	0	2	2,377.17	2,411.50		65,017.20	316	336	Empty	TRUE	UPI0000ECCBDF,UPI0001849FE9
20196-20225 HinckeM --TAXchicken	Uncategorized Sample	20196-20205_A	Mudpit_Ingel20196_MGFPeaklist (F058870)	Vimentin OS=Gallus gallus GN=VIM PE=3 SV=1	F1NJ08_CHICK	TAX_GallusGallus_9031_20141114.fasta	53,216.80	100.00%	33	37	104	0.21%	62.80%	QVDQLTNDKAR	R	V	99.70%	52.2	44.4	28.5	34.5	34.4	2.62	0.125	0.455	0	3	0	0	2	1,270.64	1,764.00		3,914.26	154	164	Empty	TRUE	UPI0000ECCBDF,UPI0001849FE9
20196-20225 HinckeM --TAXchicken	Uncategorized Sample	20196-20205_A	Mudpit_Ingel20196_MGFPeaklist (F058870)	Vimentin OS=Gallus gallus GN=VIM PE=3 SV=1	F1NJ08_CHICK	TAX_GallusGallus_9031_20141114.fasta	53,216.80	100.00%	33	37	104	0.21%	62.80%	TNEKVELQELNDR	R	F	99.70%	70.4	44.8	31.2	45.2	46.4	3.77	0.379	0.538	0	0	5	0	2	1,587.80	1,879.00		11,290.00	95	107	Empty	TRUE	UPI00000FB2FA,UPI00001291D3,O73665_CHICK,UPI0000ECCBDF,UPI0001849FE9,DESM_CHICK
20196-20225 HinckeM --TAXchicken	Uncategorized Sample	20196-20205_A	Mudpit_Ingel20196_MGFPeaklist (F058870)	Vimentin OS=Gallus gallus GN=VIM PE=3 SV=1	F1NJ08_CHICK	TAX_GallusGallus_9031_20141114.fasta	53,216.80	100.00%	33	37	104	0.21%	62.80%	VEVERDNLADDIMR	R	L	99.70%	46.4	44.6	23.6	33.5	38.8	3.12	0.222	0.464	0	0	5	0	2	1,674.81	1,964.00		13,562.20	165	178	Empty	TRUE	UPI0000ECCBDF,UPI0001849FE9
20196-20225 HinckeM --TAXchicken	Uncategorized Sample	20196-20205_A	Mudpit_Ingel20196_MGFPeaklist (F058870)	Vimentin OS=Gallus gallus GN=VIM PE=3 SV=1	F1NJ08_CHICK	TAX_GallusGallus_9031_20141114.fasta	53,216.80	100.00%	33	37	104	0.21%	62.80%	YSLGSALRPSSAR	R	Y	99.70%	36.7	44.2	20.4	23.2	30.2	2.28	0.268	0.385	0	0	5	0	2	1,364.73	1,967.00		16,860.20	34	46	Empty	TRUE	UPI0000ECCBDF,UPI0001849FE9
20196-20225 HinckeM --TAXchicken	Uncategorized Sample	20196-20205_A	Mudpit_Ingel20196_MGFPeaklist (F058870)	Vimentin OS=Gallus gallus GN=VIM PE=3 SV=1	F1NJ08_CHICK	TAX_GallusGallus_9031_20141114.fasta	53,216.80	100.00%	33	37	104	0.21%	62.80%	YVSASPGGVYATK	R	A	99.70%	64.2	44.4	18.7	57.5	47.3	6.04	0.529	0.5	0	7	0	0	2	1,299.66	1,749.00		38,278.50	47	59	Empty	TRUE	UPI0000ECCBDF,UPI0001849FE9
20196-20225 HinckeM --TAXchicken	Uncategorized Sample	20196-20205_A	Mudpit_Ingel20196_MGFPeaklist (F058870)	Uncharacterized protein (Fragment) OS=Gallus gallus GN=KRT8 PE=3 SV=2	H9KZP2_CHICK,UPI000240CAA1,UPI0002C86B23,UPI00035046FA	TAX_GallusGallus_9031_20141114.fasta,UPARC14_2_GallusG_9031_20140417.fasta	40,649.10	97.60%	1	1	8	0.02%	3.25%	SLDLDSIIAEVK	R	A	99.70%	68.4	44.4	32.3	0	50.9	3.96	0.00196	0.625	0	8	0	0	2	1,302.72	2,868.50		176028	136	147	Empty	TRUE	
20196-20225 HinckeM --TAXchicken	Uncategorized Sample	20196-20205_A	Mudpit_Ingel20196_MGFPeaklist (F058870)	Uncharacterized protein (Fragment) OS=Gallus gallus GN=KRT8 PE=3 SV=2	H9KZP2_CHICK,UPI000240CAA1,UPI0002C86B23,UPI00035046FA	TAX_GallusGallus_9031_20141114.fasta,UPARC14_2_GallusG_9031_20140417.fasta	40,649.10	97.60%	1	1	8	0.02%	3.25%	SLDLDSIIAEVK	R	A	99.70%	68.4	44.4	32.3	0	50.9	3.96	0.00196	0.625	0	8	0	0	2	1,302.72	2,868.50		176028	136	147	Empty	TRUE	
20196-20225 HinckeM --TAXchicken	Uncategorized Sample	20196-20205_A	Mudpit_Ingel20196_MGFPeaklist (F058870)	Uncharacterized protein (Fragment) OS=Gallus gallus GN=KRT8 PE=3 SV=2	H9KZP2_CHICK,UPI000240CAA1,UPI0002C86B23,UPI00035046FA	TAX_GallusGallus_9031_20141114.fasta,UPARC14_2_GallusG_9031_20140417.fasta	40,649.10	97.60%	1	1	8	0.02%	3.25%	SLDLDSIIAEVK	R	A	99.70%	68.4	44.4	32.3	0	50.9	3.96	0.00196	0.625	0	8	0	0	2	1,302.72	2,868.50		176028	136	147	Empty	TRUE	
20196-20225 HinckeM --TAXchicken	Uncategorized Sample	20196-20205_A	Mudpit_Ingel20196_MGFPeaklist (F058870)	Uncharacterized protein (Fragment) OS=Gallus gallus GN=KRT8 PE=3 SV=2	H9KZP2_CHICK,UPI000240CAA1,UPI0002C86B23,UPI00035046FA	TAX_GallusGallus_9031_20141114.fasta,UPARC14_2_GallusG_9031_20140417.fasta	40,649.10	97.60%	1	1	8	0.02%	3.25%	SLDLDSIIAEVK	R	A	99.70%	68.4	44.4	32.3	0	50.9	3.96	0.00196	0.625	0	8	0	0	2	1,302.72	2,868.50		176028	136	147	Empty	TRUE	
20196-20225 HinckeM --TAXchicken	Uncategorized Sample	20196-20205_A	Mudpit_Ingel20196_MGFPeaklist (F058870)	status=inactive	UPI0000448DF7	UPARC14_2_GallusG_9031_20140417.fasta	135,697.00	100.00%	3	3	4	0.01%	3.68%	EQPRSQADVTVADKVEAR	K	W	14.40%	15.2	44.3	23	3.69					0	0	0	1	2	2,000.99	1,739.00		376.32	695	712	Empty	TRUE	
20196-20225 HinckeM --TAXchicken	Uncategorized Sample	20196-20205_A	Mudpit_Ingel20196_MGFPeaklist (F058870)	status=inactive	UPI0000448DF7	UPARC14_2_GallusG_9031_20140417.fasta	135,697.00	100.00%	3	3	4	0.01%	3.68%	ITAAAPEPAKPGDNR	K	E	99.70%	63.6	44.5	21.2	49.8	48.9	10.2	0.448	0.5	0	0	1	0	2	1,507.79	1,527.00		285.13	895	909	Empty	TRUE	
20196-20225 HinckeM --TAXchicken	Uncategorized Sample	20196-20205_A	Mudpit_Ingel20196_MGFPeaklist (F058870)	status=inactive	UPI0000448DF7	UPARC14_2_GallusG_9031_20140417.fasta	135,697.00	100.00%	3	3	4	0.01%	3.68%	LAALPVPQPLAK	K	I	99.60%	24.2	39.8	19.8	11.8	27.5	1.08	0.124	0.292	0	2	0	0	2	1,217.76	2,451.00		536.15	883	894	Empty	TRUE	
20196-20225 HinckeM --TAXchicken	Uncategorized Sample	20196-20205_A	Mudpit_Ingel20196_MGFPeaklist (F058870)	Ovalbumin OS=Gallus gallus GN=SERPINB14 PE=1 SV=2	OVAL_CHICK,R9PXP5_CHICK	TAX_GallusGallus_9031_20141114.fasta	45,757.20	100.00%	10	13	52	0.11%	35.00%	ADHPFLFCIK	R	H	99.70%	27.2	45.2	24.9	0	25.7	0.62	0.191	0.35	0	0	2	0	2	1,247.62	2,527.00		2,103.75	386	395	Empty	TRUE	
20196-20225 HinckeM --TAXchicken	Uncategorized Sample	20196-20205_A	Mudpit_Ingel20196_MGFPeaklist (F058870)	Ovalbumin OS=Gallus gallus GN=SERPINB14 PE=1 SV=2	OVAL_CHICK,R9PXP5_CHICK	TAX_GallusGallus_9031_20141114.fasta	45,757.20	100.00%	10	13	52	0.11%	35.00%	AFKDEDTQAMPFR	K	V	99.70%	33.7	43	21.1	23	29.7	2.18	0.152	0.346	0	0	9	0	2	1,571.72	1,783.00		8,694.84	213	225	Empty	TRUE	
20196-20225 HinckeM --TAXchicken	Uncategorized Sample	20196-20205_A	Mudpit_Ingel20196_MGFPeaklist (F058870)	Ovalbumin OS=Gallus gallus GN=SERPINB14 PE=1 SV=2	OVAL_CHICK,R9PXP5_CHICK	TAX_GallusGallus_9031_20141114.fasta	45,757.20	100.00%	10	13	52	0.11%	35.00%	GGLEPINFQTAADQAR	R	E	99.70%	65	44.7	23.6	52.3	50.3	5.89	0.513	0.469	0	4	3	0	2	1,687.84	2,357.00		19,160.20	153	168	Empty	TRUE	

20196-20225 HinckeM –TAXchicken	Uncategorized Sample	20196-20205_A	Mudpit_Ingel20196_MGFPeaklist (F058870)	Ovalbumin OS=Gallus gallus GN=SERPINB14 PE=1 SV=2	OVAL_CHICK,R9PXP5_CHICK	TAX_GallusGallus_9031_20141114.fasta	45,757.20	100.00%	10	13	52	0.11%	35.00%	HIATNAVLFFGR	K	C	99.70%	44	44.6	22.7	37.4	39.3	4.3	0.443	0.542	0	0	5	0	2	1,345.74	2,519.00		4,188.85	396	407	Empty	TRUE	
20196-20225 HinckeM –TAXchicken	Uncategorized Sample	20196-20205_A	Mudpit_Ingel20196_MGFPeaklist (F058870)	Ovalbumin OS=Gallus gallus GN=SERPINB14 PE=1 SV=2	OVAL_CHICK,R9PXP5_CHICK	TAX_GallusGallus_9031_20141114.fasta	45,757.20	100.00%	10	13	52	0.11%	35.00%	ISQAVHAAHAEINEAGR	K	E	99.70%	60	44.7	17.6	50.5	38.9	3.37	0.326	0.412	0	0	0	11	2	1,773.90	1,620.00		7,571.48	349	365	Empty	TRUE	
20196-20225 HinckeM –TAXchicken	Uncategorized Sample	20196-20205_A	Mudpit_Ingel20196_MGFPeaklist (F058870)	Ovalbumin OS=Gallus gallus GN=SERPINB14 PE=1 SV=2	OVAL_CHICK,R9PXP5_CHICK	TAX_GallusGallus_9031_20141114.fasta	45,757.20	100.00%	10	13	52	0.11%	35.00%	LTEWTSSNVMEER	K	K	99.70%	66.3	42.9	18.9	47.2	49.8	5	0.526	0.5	0	2	1	0	2	1,597.72	1,890.00		5,034.15	290	302	Empty	TRUE	
20196-20225 HinckeM –TAXchicken	Uncategorized Sample	20196-20205_A	Mudpit_Ingel20196_MGFPeaklist (F058870)	Ovalbumin OS=Gallus gallus GN=SERPINB14 PE=1 SV=2	OVAL_CHICK,R9PXP5_CHICK	TAX_GallusGallus_9031_20141114.fasta	45,757.20	100.00%	10	13	52	0.11%	35.00%	NVLQPSSVDSQTAMVLVNAIVFK	R	G	99.70%	64	41.8	0	0	47.1	5	0.272	0.283	0	0	1	0	2	2,460.32	2,900.00		872.35	185	207	Empty	TRUE	
20196-20225 HinckeM –TAXchicken	Uncategorized Sample	20196-20205_A	Mudpit_Ingel20196_MGFPeaklist (F058870)	Ovalbumin OS=Gallus gallus GN=SERPINB14 PE=1 SV=2	OVAL_CHICK,R9PXP5_CHICK	TAX_GallusGallus_9031_20141114.fasta	45,757.20	100.00%	10	13	52	0.11%	35.00%	SQAVHAAHAEINEAGR	I	E	99.70%					31.8	3.07	0.327	0.312	0	0	1	0	1	1,660.81	1,626.00		396.71	350	365	Empty	TRUE	
20196-20225 HinckeM –TAXchicken	Uncategorized Sample	20196-20205_A	Mudpit_Ingel20196_MGFPeaklist (F058870)	Ovalbumin OS=Gallus gallus GN=SERPINB14 PE=1 SV=2	OVAL_CHICK,R9PXP5_CHICK	TAX_GallusGallus_9031_20141114.fasta	45,757.20	100.00%	10	13	52	0.11%	35.00%	VTEQESKPVQMMYQIGLFR	R	V	99.70%	64.5	44.1	13	58.9	45.6	5.23	0.445	0.368	0	0	11	0	2	2,316.14	2,849.00		27,821.80	226	244	Empty	TRUE	
20196-20225 HinckeM –TAXchicken	Uncategorized Sample	20196-20205_A	Mudpit_Ingel20196_MGFPeaklist (F058870)	Ovalbumin OS=Gallus gallus GN=SERPINB14 PE=1 SV=2	OVAL_CHICK,R9PXP5_CHICK	TAX_GallusGallus_9031_20141114.fasta	45,757.20	100.00%	10	13	52	0.11%	35.00%	YPILPEYLQCVK	R	E	99.70%	65	44.3	23.7	0	48.4	4.16	0.407	0.583	0	2	0	0	2	1,522.80	2,657.50		2,186.87	137	148	Empty	TRUE	
20196-20225 HinckeM –TAXchicken	Uncategorized Sample	20196-20205_A	Mudpit_Ingel20196_MGFPeaklist (F058870)	Ovalbumin OS=Gallus gallus GN=SERPINB14 PE=1 SV=2	OVAL_CHICK,R9PXP5_CHICK	TAX_GallusGallus_9031_20141114.fasta	45,757.20	100.00%	10	13	52	0.11%	35.00%	ADHPFLFCIK	R	H	99.70%	27.2	45.2	24.9	0	25.7	0.62	0.191	0.35	0	0	2	0	2	1,247.62	2,527.00		2,103.75	386	395	Empty	TRUE	
20196-20225 HinckeM –TAXchicken	Uncategorized Sample	20196-20205_A	Mudpit_Ingel20196_MGFPeaklist (F058870)	Ovalbumin OS=Gallus gallus GN=SERPINB14 PE=1 SV=2	OVAL_CHICK,R9PXP5_CHICK	TAX_GallusGallus_9031_20141114.fasta	45,757.20	100.00%	10	13	52	0.11%	35.00%	AFKDEDTQAMPFR	K	V	99.70%	33.7	43	21.1	23	29.7	2.18	0.152	0.346	0	0	9	0	2	1,571.72	1,783.00		8,694.84	213	225	Empty	TRUE	
20196-20225 HinckeM –TAXchicken	Uncategorized Sample	20196-20205_A	Mudpit_Ingel20196_MGFPeaklist (F058870)	Ovalbumin OS=Gallus gallus GN=SERPINB14 PE=1 SV=2	OVAL_CHICK,R9PXP5_CHICK	TAX_GallusGallus_9031_20141114.fasta	45,757.20	100.00%	10	13	52	0.11%	35.00%	GGLEPINFQTAADQAR	R	E	99.70%	65	44.7	23.6	52.3	50.3	5.89	0.513	0.469	0	4	3	0	2	1,687.84	2,357.00		19,160.20	153	168	Empty	TRUE	
20196-20225 HinckeM –TAXchicken	Uncategorized Sample	20196-20205_A	Mudpit_Ingel20196_MGFPeaklist (F058870)	Ovalbumin OS=Gallus gallus GN=SERPINB14 PE=1 SV=2	OVAL_CHICK,R9PXP5_CHICK	TAX_GallusGallus_9031_20141114.fasta	45,757.20	100.00%	10	13	52	0.11%	35.00%	HIATNAVLFFGR	K	C	99.70%	44	44.6	22.7	37.4	39.3	4.3	0.443	0.542	0	0	5	0	2	1,345.74	2,519.00		4,188.85	396	407	Empty	TRUE	
20196-20225 HinckeM –TAXchicken	Uncategorized Sample	20196-20205_A	Mudpit_Ingel20196_MGFPeaklist (F058870)	Ovalbumin OS=Gallus gallus GN=SERPINB14 PE=1 SV=2	OVAL_CHICK,R9PXP5_CHICK	TAX_GallusGallus_9031_20141114.fasta	45,757.20	100.00%	10	13	52	0.11%	35.00%	ISQAVHAAHAEINEAGR	K	E	99.70%	60	44.7	17.6	50.5	38.9	3.37	0.326	0.412	0	0	0	11	2	1,773.90	1,620.00		7,571.48	349	365	Empty	TRUE	
20196-20225 HinckeM –TAXchicken	Uncategorized Sample	20196-20205_A	Mudpit_Ingel20196_MGFPeaklist (F058870)	Ovalbumin OS=Gallus gallus GN=SERPINB14 PE=1 SV=2	OVAL_CHICK,R9PXP5_CHICK	TAX_GallusGallus_9031_20141114.fasta	45,757.20	100.00%	10	13	52	0.11%	35.00%	LTEWTSSNVMEER	K	K	99.70%	66.3	42.9	18.9	47.2	49.8	5	0.526	0.5	0	2	1	0	2	1,597.72	1,890.00		5,034.15	290	302	Empty	TRUE	
20196-20225 HinckeM –TAXchicken	Uncategorized Sample	20196-20205_A	Mudpit_Ingel20196_MGFPeaklist (F058870)	Ovalbumin OS=Gallus gallus GN=SERPINB14 PE=1 SV=2	OVAL_CHICK,R9PXP5_CHICK	TAX_GallusGallus_9031_20141114.fasta	45,757.20	100.00%	10	13	52	0.11%	35.00%	NVLQPSSVDSQTAMVLVNAIVFK	R	G	99.70%	64	41.8	0	0	47.1	5	0.272	0.283	0	0	1	0	2	2,460.32	2,900.00		872.35	185	207	Empty	TRUE	
20196-20225 HinckeM –TAXchicken	Uncategorized Sample	20196-20205_A	Mudpit_Ingel20196_MGFPeaklist (F058870)	Ovalbumin OS=Gallus gallus GN=SERPINB14 PE=1 SV=2	OVAL_CHICK,R9PXP5_CHICK	TAX_GallusGallus_9031_20141114.fasta	45,757.20	100.00%	10	13	52	0.11%	35.00%	SQAVHAAHAEINEAGR	I	E	99.70%					31.8	3.07	0.327	0.312	0	0	1	0	1	1,660.81	1,626.00		396.71	350	365	Empty	TRUE	
20196-20225 HinckeM –TAXchicken	Uncategorized Sample	20196-20205_A	Mudpit_Ingel20196_MGFPeaklist (F058870)	Ovalbumin OS=Gallus gallus GN=SERPINB14 PE=1 SV=2	OVAL_CHICK,R9PXP5_CHICK	TAX_GallusGallus_9031_20141114.fasta	45,757.20	100.00%	10	13	52	0.11%	35.00%	VTEQESKPVQMMYQIGLFR	R	V	99.70%	64.5	44.1	13	58.9	45.6	5.23	0.445	0.368	0	0	11	0	2	2,316.14	2,849.00		27,821.80	226	244	Empty	TRUE	
20196-20225 HinckeM –TAXchicken	Uncategorized Sample	20196-20205_A	Mudpit_Ingel20196_MGFPeaklist (F058870)	Ovalbumin OS=Gallus gallus GN=SERPINB14 PE=1 SV=2	OVAL_CHICK,R9PXP5_CHICK	TAX_GallusGallus_9031_20141114.fasta	45,757.20	100.00%	10	13	52	0.11%	35.00%	YPILPEYLQCVK	R	E	99.70%	65	44.3	23.7	0	48.4	4.16	0.407	0.583	0	2	0	0	2	1,522.80	2,657.50		2,186.87	137	148	Empty	TRUE	
20196-20225 HinckeM –TAXchicken	Uncategorized Sample	20196-20205_A	Mudpit_Ingel20196_MGFPeaklist (F058870)	status=active	UPI00035043A4,UPI00035049A4,UPI0003506214	UPARC14_2_GallusG_9031_20140417.fasta	141,669.00	99.70%	2	2	2	0.00%	2.29%	EPTKPWETRVEEEQK	R	K	99.60%	12.1	44.5	19.5	5.12	28.3	0.854	0.148	0.233	0	0	1	0	2	1,867.92	2,170.00		2,066.23	491	505	Empty	TRUE	
20196-20225 HinckeM –TAXchicken	Uncategorized Sample	20196-20205_A	Mudpit_Ingel20196_MGFPeaklist (F058870)	status=active	UPI00035043A4,UPI00035049A4,UPI0003506214	UPARC14_2_GallusG_9031_20140417.fasta	141,669.00	99.70%	2	2	2	0.00%	2.29%	GRFPARGSAGVPGR	R	A	25.90%	20.4	43.6	0	14.3					0	0	1	0	2	1,384.76	2,261.00		112.69	43	56	Empty	TRUE	
20196-20225 HinckeM –TAXchicken	Uncategorized Sample	20196-20205_A	Mudpit_Ingel20196_MGFPeaklist (F058870)	status=active	UPI00035043A4,UPI00035049A4,UPI0003506214	UPARC14_2_GallusG_9031_20140417.fasta	141,669.00	99.70%	2	2	2	0.00%	2.29%	EPTKPWETRVEEEQK	R	K	99.60%	12.1	44.5	19.5	5.12	28.3	0.854	0.148	0.233	0	0	1	0	2	1,867.92	2,170.00		2,066.23	491	505	Empty	TRUE	
20196-20225 HinckeM –TAXchicken	Uncategorized Sample	20196-20205_A	Mudpit_Ingel20196_MGFPeaklist (F058870)	status=active	UPI00035043A4,UPI00035049A4,UPI0003506214	UPARC14_2_GallusG_9031_20140417.fasta	141,669.00	99.70%	2	2	2	0.00%	2.29%	GRFPARGSAGVPGR	R	A	25.90%	20.4	43.6	0	14.3					0	0	1	0	2	1,384.76	2,261.00		112.69	43	56	Empty	TRUE	
20196-20225 HinckeM –TAXchicken	Uncategorized Sample	20196-20205_A	Mudpit_Ingel20196_MGFPeaklist (F058870)	status=active	UPI00035043A4,UPI00035049A4,UPI0003506214	UPARC14_2_GallusG_9031_20140417.fasta	141,669.00	99.70%	2	2	2	0.00%	2.29%	EPTKPWETRVEEEQK	R	K	99.60%	12.1	44.5	19.5	5.12	28.3	0.854	0.148	0.233	0	0	1	0	2	1,867.92	2,170.00		2,066.23	491	505	Empty	TRUE	
20196-20225 HinckeM –TAXchicken	Uncategorized Sample	20196-20205_A	Mudpit_Ingel20196_MGFPeaklist (F058870)	status=active	UPI00035043A4,UPI00035049A4,UPI0003506214	UPARC14_2_GallusG_9031_20140417.fasta	141,669.00	99.70%	2	2	2	0.00%	2.29%	GRFPARGSAGVPGR	R	A	25.90%	20.4	43.6	0	14.3					0	0	1	0	2	1,384.76	2,261.00		112.69	43	56	Empty	TRUE	
20196-20225 HinckeM –TAXchicken	Uncategorized Sample	20196-20205_A	Mudpit_Ingel20196_MGFPeaklist (F058870)	status=active	E1BZJ3_CHICK,UPI000000B893,UPI00003AB57C,UPI00004494C5,UPI0000E812D7,UPI0000ECA55E,UPI000240C217,UPI0003504750	TAX_GallusGallus_9031_20141114.fasta,UPARC14_2_GallusG_9031_20140417.fasta	65,680.10	99.60%	2	2	3	0.01%	3.53%	FVALENISCK	K	I	99.70%	77.1	44.2	25.9	62.1	36.8	2.52	0.359	0.5	0	2	0	0	2	1,180.60	2,179.00		2,277.54	181	190	Empty	TRUE	
20196-20225 HinckeM –TAXchicken	Uncategorized Sample	20196-20205_A	Mudpit_Ingel20196_MGFPeaklist (F058870)	status=active	E1BZJ3_CHICK,UPI000000B893,UPI00003AB57C,UPI00004494C5,UPI0000E812D7,UPI0000ECA55E,UPI000240C217,UPI0003504750	TAX_GallusGallus_9031_20141114.fasta,UPARC14_2_GallusG_9031_20140417.fasta	65,680.10	99.60%	2	2	3	0.01%	3.53%	YTEHKDIPLGIR	R	A	10.50%	6.37	44	18.7	2.02					0	0	1	0	2	1,441.78	1,891.00		253.87	257	268	Empty	TRUE	
20196-20225 HinckeM –TAXchicken	Uncategorized Sample	20196-20205_A	Mudpit_Ingel20196_MGFPeaklist (F058870)	status=active	E1BZJ3_CHICK,UPI000000B893,UPI00003AB57C,UPI00004494C5,UPI0000E812D7,UPI0000ECA55E,UPI000240C217,UPI0003504750	TAX_GallusGallus_9031_20141114.fasta,UPARC14_2_GallusG_9031_20140417.fasta	65,680.10	99.60%	2	2	3	0.01%	3.53%	FVALENISCK	K	I	99.70%	77.1	44.2	25.9	62.1	36.8	2.52	0.359	0.5	0	2	0	0	2	1,180.60	2,179.00		2,277.54	181	190	Empty	TRUE	
20196-20225 HinckeM –TAXchicken	Uncategorized Sample	20196-20205_A	Mudpit_Ingel20196_MGFPeaklist (F058870)	status=active	E1BZJ3_CHICK,UPI000000B893,UPI00003AB57C,UPI00004494C5,UPI0000E812D7,UPI0000ECA55E,UPI000240C217,UPI0003504750	TAX_GallusGallus_9031_20141114.fasta,UPARC14_2_GallusG_9031_20140417.fasta	65,680.10	99.60%	2	2	3	0.01%	3.53%	YTEHKDIPLGIR	R	A	10.50%	6.37	44	18.7	2.02					0	0	1	0	2	1,441.78	1,891.00		253.87	257	268	Empty	TRUE	
20196-20225 HinckeM –TAXchicken	Uncategorized Sample	20196-20205_A	Mudpit_Ingel20196_MGFPeaklist (F058870)	status=active	E1BZJ3_CHICK,UPI000000B893,UPI00003AB57C,UPI00004494C5,UPI0000E812D7,UPI0000ECA55E,UPI000240C217,UPI0003504750	TAX_GallusGallus_9031_20141114.fasta,UPARC14_2_GallusG_9031_20140417.fasta	65,680.10	99.60%	2	2	3	0.01%	3.53%	FVALENISCK	K	I	99.70%	77.1	44.2	25.9	62.1	36.8	2.52	0.359	0.5	0	2	0	0	2	1,180.60	2,179.00		2,277.54	181	190	Empty	TRUE	
20196-20225 HinckeM –TAXchicken	Uncategorized Sample	20196-20205_A	Mudpit_Ingel20196_MGFPeaklist (F058870)	status=active	E1BZJ3_CHICK,UPI000000B893,UPI00003AB57C,UPI00004494C5,UPI0000E812D7,UPI0000ECA55E,UPI000240C217,UPI0003504750	TAX_GallusGallus_9031_20141114.fasta,UPARC14_2_GallusG_9031_20140417.fasta	65,680.10	99.60%	2	2	3	0.01%	3.53%	YTEHKDIPLGIR	R	A	10.50%	6.37	44	18.7	2.02					0	0	1	0	2	1,441.78	1,891.00		253.87	257	268	Empty	TRUE	
20196-20225 HinckeM –TAXchicken	Uncategorized Sample	20196-20205_A	Mudpit_Ingel20196_MGFPeaklist (F058870)	status=active	E1BZJ3_CHICK,UPI000000B893,UPI00003AB57C,UPI00004494C5,UPI0000E812D7,UPI0000ECA55E,UPI000240C217,UPI0003504750	TAX_GallusGallus_9031_20141114.fasta,UPARC14_2_GallusG_9031_20140417.fasta	65,680.10	99.60%	2	2	3	0.01%	3.53%	FVALENISCK	K	I	99.70%	77.1	44.2	25.9	62.1	36.8	2.52	0.359	0.5	0	2	0	0	2	1,180.60	2,179.00		2,277.54	181	190	Empty	TRUE	
20196-20225 HinckeM –TAXchicken	Uncategorized Sample	20196-20205_A	Mudpit_Ingel20196_MGFPeaklist (F058870)	status=active	E1BZJ3_CHICK,UPI000000B893,UPI00003AB57C,UPI00004494C5,UPI0000E812D7,UPI0000ECA55E,UPI000240C217,UPI0003504750	TAX_GallusGallus_9031_20141114.fasta,UPARC14_2_GallusG_9031_20140417.fasta	65,680.10	99.60%	2	2	3	0.01%	3.53%	YTEHKDIPLGIR	R	A	10.50%	6.37	44	18.7	2.02					0	0	1	0	2	1,441.78	1,891.00		253.87	257	268	Empty	TRUE	
20196-20225 HinckeM –TAXchicken	Uncategorized Sample	20196-20205_A	Mudpit_Ingel20196_MGFPeaklist (F058870)	status=active	E1BZJ3_CHICK,UPI000000B893,UPI00003AB57C,UPI00004494C5,UPI0000E812D7,UPI0000ECA55E,UPI000240C217,UPI0003504750	TAX_GallusGallus_9031_20141114.fasta,UPARC14_2_GallusG_9031_20140417.fasta	65,680.10	99.60%	2	2	3	0.01%	3.53%	FVALENISCK	K	I	99.70%	77.1	44.2	25.9	62.1	36.8	2.52	0.359	0.5	0	2	0	0	2	1,180.60	2,179.00		2,277.54	181	190	Empty	TRUE	
20196-20225 HinckeM –TAXchicken	Uncategorized Sample	20196-20205_A	Mudpit_Ingel20196_MGFPeaklist (F058870)	status=active	E1BZJ3_CHICK,UPI000000B893,UPI00003AB57C,UPI00004494C5,UPI0000E812D7,UPI0000ECA55E,UPI000240C217,UPI0003504750	TAX_GallusGallus_9031_20141114.fasta,UPARC14_2_GallusG_9031_20140417.fasta	65,680.10	99.60%	2	2	3	0.01%	3.53%	YTEHKDIPLGIR	R	A	10.50%	6.37	44	18.7	2.02					0	0	1	0	2	1,441.78	1,891.00		253.87	257	268	Empty	TRUE	
20196-20225 HinckeM –TAXchicken	Uncategorized Sample	20196-20205_A	Mudpit_Ingel20196_MGFPeaklist (F058870)	status=active	E1BZJ3_CHICK,UPI000000B893,UPI00003AB57C,UPI00004494C5,UPI0000E812D7,UPI0000ECA55E,UPI000240C217,UPI0003504750	TAX_GallusGallus_9031_20141114.fasta,UPARC14_2_GallusG_9031_20140417.fasta	65,680.10	99.60%	2	2	3	0.01%	3.53%	FVALENISCK	K	I	99.70%	77.1	44.2	25.9	62.1	36.8	2.52	0.359	0.5	0	2	0	0	2	1,180.60	2,179.00		2,277.54	181	190	Empty	TRUE	
20196-20225 HinckeM –TAXchicken	Uncategorized Sample	20196-20205_A	Mudpit_Ingel20196_MGFPeaklist (F058870)	status=active	E1BZJ3_CHICK,UPI000000B893,UPI00003AB57C,UPI00004494C5,UPI0000E812D7,UPI0000ECA55E,UPI000240C217,UPI0003504750	TAX_GallusGallus_9031_20141114.fasta,UPARC14_2_GallusG_9031_20140417.fasta	65,680.10	99.60%	2	2	3	0.01%	3.53%	YTEHKDIPLGIR	R	A	10.50%	6.37	44	18.7	2.02					0	0	1	0	2	1,441.78	1,891.00		253.87	257	268	Empty	TRUE	
20196-20225 HinckeM –TAXchicken	Uncategorized Sample	20196-20205_A	Mudpit_Ingel20196_MGFPeaklist (F058870)	status=active	E1BZJ3_CHICK,UPI000000B893,UPI00003AB57C,UPI00004494C5,UPI0000E812D7,UPI0000ECA55E,UPI000240C217,UPI0003504750	TAX_GallusGallus_9031_20141114.fasta,UPARC14_2_GallusG_9031_20140417.fasta	65,680.10	99.60%	2	2	3	0.01%	3.53%	FVALENISCK	K	I	99.70%	77.1	44.2	25.9	62.1	36.8	2.52	0.359	0.5	0	2	0	0	2	1,180.60	2,179.00		2,277.54	181	190	Empty	TRUE	
20196-20225 HinckeM –TAXchicken	Uncategorized Sample	20196-20205_A	Mudpit_Ingel20196_MGFPeaklist (F058870)	status=active	E1BZJ3_CHICK,UPI000000B893,UPI00003AB57C,UPI00004494C5,UPI0000E812D7,UPI0000ECA55E,UPI000240C217,UPI0003504750	TAX_GallusGallus_9031_20141114.fasta,UPARC14_2_GallusG_9031_20140417.fasta	65,680.10	99.60%	2	2	3	0.01%	3.53%	YTEHKDIPLGIR	R	A	10.50%	6.37	44	18.7	2.02					0	0	1	0	2	1,441.78	1,891.00		253.87	257	268	Empty	TRUE	
20196-20225 HinckeM –TAXchicken	Uncategorized Sample	20196-20205_A	Mudpit_Ingel20196_MGFPeaklist (F058870)	status=active	E1BZJ3_CHICK,UPI000000B893,UPI00003AB57C,UPI00004494C5,UPI0000E812D7,UPI0000ECA55E,UPI000240C217,UPI0003504750	TAX_GallusGallus_9031_20141114.fasta,UPARC14_2_GallusG_9031_20140417.fasta	65,680.10	99.60%	2	2	3	0.01%	3.53%	FVALENISCK	K	I	99.70%	77.1	44.2	25.9	62.1	36.8	2.52	0.359	0.5	0	2	0	0	2	1,180.60	2,179.00		2,277.54	181	190	Empty	TRUE	
20196-20225 HinckeM –TAXchicken	Uncategorized Sample	20196-20205_A	Mudpit_Ingel20196_MGFPeaklist (F058870)	status=active	E1BZJ3_CHICK,UPI000000B893,UPI00003AB57C,UPI00004494C5,UPI0000E812D7,UPI0000ECA55E,UPI000240C217,UPI0003504750	TAX_GallusGallus_9031_20141114.fasta,UPARC14_2_GallusG_9031_20140417.fasta	65,680.10	99.60%	2	2	3	0.01%	3.53%	YTEHKDIPLGIR	R	A	10.50%	6.37	44	18.7	2.02					0	0	1	0	2	1,441.78	1,891.00		253.87	257	268	Empty	TRUE	
20196-20225 HinckeM –TAXchicken	Uncategorized Sample	20196-20205_A	Mudpit_Ingel20196_MGFPeaklist (F058870)	Uncharacterized protein (Fragment) OS=Gallus gallus GN=C3 PE=4 SV=2	H9KZT6_CHICK,R4GJS6_CHICK,UPI0002C875F7,UPI0002C8784D	TAX_GallusGallus_9031_20141114.fasta,UPARC14_2_GallusG_9031_20140417.fasta	150,963.90	100.00%	9	9	14	0.03%	6.22%	ADNFQGLVSTQR	K	D	99.70%	33.5	44.8	23.3	23.2	30.2	2.28	0.341	0.333	0	2	0	0	2	1,335.67	2,066.00		1,611.55	131	142	Empty	TRUE	UPI00000FC766,Q90633_CHICK
20196-20225 HinckeM –TAXchicken	Uncategorized Sample	20196-20205_A	Mudpit_Ingel20196_MGFPeaklist (F058870)	Uncharacterized protein (Fragment) OS=Gallus gallus GN=C3 PE=4 SV=2	H9KZT6_CHICK,R4GJS6_CHICK,UPI0002C875F7,UPI0002C8784D	TAX_GallusGallus_9031_20141114.fasta,UPARC14_2_GallusG_9031_20140417.fasta	150,963.90	100.00%	9	9	14	0.03%	6.22%	AELTGPVVR	K	W	34.00%	17.6	43.2	22.9	0					0	1	0	0	2	941.5415	1,841.00		775.95	979	987	Empty	TRUE	UPI00000FC766,Q90633_CHICK
20196-20225 HinckeM –TAXchicken	Uncategorized Sample	20196-20205_A	Mudpit_Ingel20196_MGFPeaklist (F058870)	Uncharacterized protein (Fragment) OS=Gallus gallus GN=C3 PE=4 SV=2	H9KZT6_CHICK,R4GJS6_CHICK,UPI0002C875F7,UPI0002C8784D	TAX_GallusGallus_9031_20141114.fasta,UPARC14_2_GallusG_9031_20140417.fasta	150,963.90	100.00%	9	9	14	0.03%	6.22%	GYTQQLAYR	K	K	99.70%	26.4	44.1	22.1	10.6	23.3	2.2	0.232	0.278	0	2	0	0	2	1,099.55	1,782.00		980.47	788	796	Empty	TRUE	UPI00000FC766,Q90633_CHICK
20196-20225 HinckeM –TAXchicken	Uncategorized Sample	20196-20205_A	Mudpit_Ingel20196_MGFPeaklist (F058870)	Uncharacterized protein (Fragment) OS=Gallus gallus GN=C3 PE=4 SV=2	H9KZT6_CHICK,R4GJS6_CHICK,UPI0002C875F7,UPI0002C8784D	TAX_GallusGallus_9031_20141114.fasta,UPARC14_2_GallusG_9031_20140417.fasta	150,963.90	100.00%	9	9	14	0.03%	6.22%	IPVKADNFQGLVSTQR	R	D	38.20%	15.5	43.4	13.9	8.72					0	0	1	0	2	1,772.97	2,205.00		1,319.70	127	142	Empty	TRUE	Q90633_CHICK
20196-20225 HinckeM –TAXchicken	Uncategorized Sample	20196-20205_A	Mudpit_Ingel20196_MGFPeaklist (F058870)	Uncharacterized protein (Fragment) OS=Gallus gallus GN=C3 PE=4 SV=2	H9KZT6_CHICK,R4GJS6_CHICK,UPI0002C875F7,UPI0002C8784D	TAX_GallusGallus_9031_20141114.fasta,UPARC14_2_GallusG_9031_20140417.fasta	150,963.90	100.00%	9	9	14	0.03%	6.22%	IVTSPYTIHFTHTPK	R	Y	99.70%	25.8	43.7	16.6	24.2	32.6	1.17	0.239	0.333	0	0	0	2	2	1,741.93	2,114.00		4,142.83	88	102	Empty	TRUE	UPI00000FC766,Q90633_CHICK
20196-20225 HinckeM –TAXchicken	Uncategorized Sample	20196-20205_A	Mudpit_Ingel20196_MGFPeaklist (F058870)	Uncharacterized protein (Fragment) OS=Gallus gallus GN=C3 PE=4 SV=2	H9KZT6_CHICK,R4GJS6_CHICK,UPI0002C875F7,UPI0002C8784D	TAX_GallusGallus_9031_20141114.fasta,UPARC14_2_GallusG_9031_20140417.fasta	150,963.90	100.00%	9	9	14	0.03%	6.22%	KGYTQQLAYR	K	K	99.70%	31.9	44.2	28.3	14.1	27.1	1.29	0.229	0.45	0	0	1	0	2	1,227.65	1,679.00		246.47	787	796	Empty	TRUE	UPI00000FC766,Q90633_CHICK
20196-20225 HinckeM –TAXchicken	Uncategorized Sample	20196-20205_A	Mudpit_Ingel20196_MGFPeaklist (F058870)	Uncharacterized protein (Fragment) OS=Gallus gallus GN=C3 PE=4 SV=2	H9KZT6_CHICK,R4GJS6_CHICK,UPI0002C875F7,UPI0002C8784D	TAX_GallusGallus_9031_20141114.fasta,UPARC14_2_GallusG_9031_20140417.fasta	150,963.90	100.00%	9	9	14	0.03%	6.22%	NSVPITVR	K	T	52.00%	23.1	43.9	23.9	4.32					0	1	0	0	2	885.5152	1,805.00		461.89	157	164	Empty	TRUE	UPI00000FC766,Q90633_CHICK
20196-20225 HinckeM –TAXchicken	Uncategorized Sample	20196-20205_A	Mudpit_Ingel20196_MGFPeaklist (F058870)	Uncharacterized protein (Fragment) OS=Gallus gallus GN=C3 PE=4 SV=2	H9KZT6_CHICK,R4GJS6_CHICK,UPI0002C875F7,UPI0002C8784D	TAX_GallusGallus_9031_20141114.fasta,UPARC14_2_GallusG_9031_20140417.fasta	150,963.90	100.00%	9	9	14	0.03%	6.22%	TEAIELIK	R	K	51.00%	21.6	44.9	25.3	8.12					0	1	0	0	2	916.5351	2,187.00		325.56	779	786	Empty	TRUE	UPI00000FC766,Q90633_CHICK
20196-20225 HinckeM –TAXchicken	Uncategorized Sample	20196-20205_A	Mudpit_Ingel20196_MGFPeaklist (F058870)	Uncharacterized protein (Fragment) OS=Gallus gallus GN=C3 PE=4 SV=2	H9KZT6_CHICK,R4GJS6_CHICK,UPI0002C875F7,UPI0002C8784D	TAX_GallusGallus_9031_20141114.fasta,UPARC14_2_GallusG_9031_20140417.fasta	150,963.90	100.00%	9	9	14	0.03%	6.22%	VKVTDGDGEAVLPMAMLR	R	Q	49.20%	11.9	44.6	16.3	0	26.6	0.638	0	0.222	0	0	1	0	2	1,933.97	2,137.00		1,266.49	33	50	Empty	TRUE	
20196-20225 HinckeM –TAXchicken	Uncategorized Sample	20196-20205_A	Mudpit_Ingel20196_MGFPeaklist (F058870)	Uncharacterized protein (Fragment) OS=Gallus gallus GN=C3 PE=4 SV=2	H9KZT6_CHICK,R4GJS6_CHICK,UPI0002C875F7,UPI0002C8784D	TAX_GallusGallus_9031_20141114.fasta,UPARC14_2_GallusG_9031_20140417.fasta	150,963.90	100.00%	9	9	14	0.03%	6.22%	ADNFQGLVSTQR	K	D	99.70%	33.5	44.8	23.3	23.2	30.2	2.28	0.341	0.333	0	2	0	0	2	1,335.67	2,066.00		1,611.55	131	142	Empty	TRUE	UPI00000FC766,Q90633_CHICK
20196-20225 HinckeM –TAXchicken	Uncategorized Sample	20196-20205_A	Mudpit_Ingel20196_MGFPeaklist (F058870)	Uncharacterized protein (Fragment) OS=Gallus gallus GN=C3 PE=4 SV=2	H9KZT6_CHICK,R4GJS6_CHICK,UPI0002C875F7,UPI0002C8784D	TAX_GallusGallus_9031_20141114.fasta,UPARC14_2_GallusG_9031_20140417.fasta	150,963.90	100.00%	9	9	14	0.03%	6.22%	AELTGPVVR	K	W	34.00%	17.6	43.2	22.9	0					0	1	0	0	2	941.5415	1,841.00		775.95	979	987	Empty	TRUE	UPI00000FC766,Q90633_CHICK
20196-20225 HinckeM –TAXchicken	Uncategorized Sample	20196-20205_A	Mudpit_Ingel20196_MGFPeaklist (F058870)	Uncharacterized protein (Fragment) OS=Gallus gallus GN=C3 PE=4 SV=2	H9KZT6_CHICK,R4GJS6_CHICK,UPI0002C875F7,UPI0002C8784D	TAX_GallusGallus_9031_20141114.fasta,UPARC14_2_GallusG_9031_20140417.fasta	150,963.90	100.00%	9	9	14	0.03%	6.22%	GYTQQLAYR	K	K	99.70%	26.4	44.1	22.1	10.6	23.3	2.2	0.232	0.278	0	2	0	0	2	1,099.55	1,782.00		980.47	788	796	Empty	TRUE	UPI00000FC766,Q90633_CHICK
20196-20225 HinckeM –TAXchicken	Uncategorized Sample	20196-20205_A	Mudpit_Ingel20196_MGFPeaklist (F058870)	Uncharacterized protein (Fragment) OS=Gallus gallus GN=C3 PE=4 SV=2	H9KZT6_CHICK,R4GJS6_CHICK,UPI0002C875F7,UPI0002C8784D	TAX_GallusGallus_9031_20141114.fasta,UPARC14_2_GallusG_9031_20140417.fasta	150,963.90	100.00%	9	9	14	0.03%	6.22%	IPVKADNFQGLVSTQR	R	D	38.20%	15.5	43.4	13.9	8.72					0	0	1	0	2	1,772.97	2,205.00		1,319.70	127	142	Empty	TRUE	Q90633_CHICK
20196-20225 HinckeM –TAXchicken	Uncategorized Sample	20196-20205_A	Mudpit_Ingel20196_MGFPeaklist (F058870)	Uncharacterized protein (Fragment) OS=Gallus gallus GN=C3 PE=4 SV=2	H9KZT6_CHICK,R4GJS6_CHICK,UPI0002C875F7,UPI0002C8784D	TAX_GallusGallus_9031_20141114.fasta,UPARC14_2_GallusG_9031_20140417.fasta	150,963.90	100.00%	9	9	14	0.03%	6.22%	IVTSPYTIHFTHTPK	R	Y	99.70%	25.8	43.7	16.6	24.2	32.6	1.17	0.239	0.333	0	0	0	2	2	1,741.93	2,114.00		4,142.83	88	102	Empty	TRUE	UPI00000FC766,Q90633_CHICK
20196-20225 HinckeM –TAXchicken	Uncategorized Sample	20196-20205_A	Mudpit_Ingel20196_MGFPeaklist (F058870)	Uncharacterized protein (Fragment) OS=Gallus gallus GN=C3 PE=4 SV=2	H9KZT6_CHICK,R4GJS6_CHICK,UPI0002C875F7,UPI0002C8784D	TAX_GallusGallus_9031_20141114.fasta,UPARC14_2_GallusG_9031_20140417.fasta	150,963.90	100.00%	9	9	14	0.03%	6.22%	KGYTQQLAYR	K	K	99.70%	31.9	44.2	28.3	14.1	27.1	1.29	0.229	0.45	0	0	1	0	2	1,227.65	1,679.00		246.47	787	796	Empty	TRUE	UPI00000FC766,Q90633_CHICK
20196-20225 HinckeM –TAXchicken	Uncategorized Sample	20196-20205_A	Mudpit_Ingel20196_MGFPeaklist (F058870)	Uncharacterized protein (Fragment) OS=Gallus gallus GN=C3 PE=4 SV=2	H9KZT6_CHICK,R4GJS6_CHICK,UPI0002C875F7,UPI0002C8784D	TAX_GallusGallus_9031_20141114.fasta,UPARC14_2_GallusG_9031_20140417.fasta	150,963.90	100.00%	9	9	14	0.03%	6.22%	NSVPITVR	K	T	52.00%	23.1	43.9	23.9	4.32					0	1	0	0	2	885.5152	1,805.00		461.89	157	164	Empty	TRUE	UPI00000FC766,Q90633_CHICK
20196-20225 HinckeM –TAXchicken	Uncategorized Sample	20196-20205_A	Mudpit_Ingel20196_MGFPeaklist (F058870)	Uncharacterized protein (Fragment) OS=Gallus gallus GN=C3 PE=4 SV=2	H9KZT6_CHICK,R4GJS6_CHICK,UPI0002C875F7,UPI0002C8784D	TAX_GallusGallus_9031_20141114.fasta,UPARC14_2_GallusG_9031_20140417.fasta	150,963.90	100.00%	9	9	14	0.03%	6.22%	TEAIELIK	R	K	51.00%	21.6	44.9	25.3	8.12					0	1	0	0	2	916.5351	2,187.00		325.56	779	786	Empty	TRUE	UPI00000FC766,Q90633_CHICK
20196-20225 HinckeM –TAXchicken	Uncategorized Sample	20196-20205_A	Mudpit_Ingel20196_MGFPeaklist (F058870)	Uncharacterized protein (Fragment) OS=Gallus gallus GN=C3 PE=4 SV=2	H9KZT6_CHICK,R4GJS6_CHICK,UPI0002C875F7,UPI0002C8784D	TAX_GallusGallus_9031_20141114.fasta,UPARC14_2_GallusG_9031_20140417.fasta	150,963.90	100.00%	9	9	14	0.03%	6.22%	VKVTDGDGEAVLPMAMLR	R	Q	49.20%	11.9	44.6	16.3	0	26.6	0.638	0	0.222	0	0	1	0	2	1,933.97	2,137.00		1,266.49	33	50	Empty	TRUE	
20196-20225 HinckeM –TAXchicken	Uncategorized Sample	20196-20205_A	Mudpit_Ingel20196_MGFPeaklist (F058870)	Uncharacterized protein (Fragment) OS=Gallus gallus GN=C3 PE=4 SV=2	H9KZT6_CHICK,R4GJS6_CHICK,UPI0002C875F7,UPI0002C8784D	TAX_GallusGallus_9031_20141114.fasta,UPARC14_2_GallusG_9031_20140417.fasta	150,963.90	100.00%	9	9	14	0.03%	6.22%	ADNFQGLVSTQR	K	D	99.70%	33.5	44.8	23.3	23.2	30.2	2.28	0.341	0.333	0	2	0	0	2	1,335.67	2,066.00		1,611.55	131	142	Empty	TRUE	UPI00000FC766,Q90633_CHICK
20196-20225 HinckeM –TAXchicken	Uncategorized Sample	20196-20205_A	Mudpit_Ingel20196_MGFPeaklist (F058870)	Uncharacterized protein (Fragment) OS=Gallus gallus GN=C3 PE=4 SV=2	H9KZT6_CHICK,R4GJS6_CHICK,UPI0002C875F7,UPI0002C8784D	TAX_GallusGallus_9031_20141114.fasta,UPARC14_2_GallusG_9031_20140417.fasta	150,963.90	100.00%	9	9	14	0.03%	6.22%	AELTGPVVR	K	W	34.00%	17.6	43.2	22.9	0					0	1	0	0	2	941.5415	1,841.00		775.95	979	987	Empty	TRUE	UPI00000FC766,Q90633_CHICK
20196-20225 HinckeM –TAXchicken	Uncategorized Sample	20196-20205_A	Mudpit_Ingel20196_MGFPeaklist (F058870)	Uncharacterized protein (Fragment) OS=Gallus gallus GN=C3 PE=4 SV=2	H9KZT6_CHICK,R4GJS6_CHICK,UPI0002C875F7,UPI0002C8784D	TAX_GallusGallus_9031_20141114.fasta,UPARC14_2_GallusG_9031_20140417.fasta	150,963.90	100.00%	9	9	14	0.03%	6.22%	GYTQQLAYR	K	K	99.70%	26.4	44.1	22.1	10.6	23.3	2.2	0.232	0.278	0	2	0	0	2	1,099.55	1,782.00		980.47	788	796	Empty	TRUE	UPI00000FC766,Q90633_CHICK
20196-20225 HinckeM –TAXchicken	Uncategorized Sample	20196-20205_A	Mudpit_Ingel20196_MGFPeaklist (F058870)	Uncharacterized protein (Fragment) OS=Gallus gallus GN=C3 PE=4 SV=2	H9KZT6_CHICK,R4GJS6_CHICK,UPI0002C875F7,UPI0002C8784D	TAX_GallusGallus_9031_20141114.fasta,UPARC14_2_GallusG_9031_20140417.fasta	150,963.90	100.00%	9	9	14	0.03%	6.22%	IPVKADNFQGLVSTQR	R	D	38.20%	15.5	43.4	13.9	8.72					0	0	1	0	2	1,772.97	2,205.00		1,319.70	127	142	Empty	TRUE	Q90633_CHICK
20196-20225 HinckeM –TAXchicken	Uncategorized Sample	20196-20205_A	Mudpit_Ingel20196_MGFPeaklist (F058870)	Uncharacterized protein (Fragment) OS=Gallus gallus GN=C3 PE=4 SV=2	H9KZT6_CHICK,R4GJS6_CHICK,UPI0002C875F7,UPI0002C8784D	TAX_GallusGallus_9031_20141114.fasta,UPARC14_2_GallusG_9031_20140417.fasta	150,963.90	100.00%	9	9	14	0.03%	6.22%	IVTSPYTIHFTHTPK	R	Y	99.70%	25.8	43.7	16.6	24.2	32.6	1.17	0.239	0.333	0	0	0	2	2	1,741.93	2,114.00		4,142.83	88	102	Empty	TRUE	UPI00000FC766,Q90633_CHICK
20196-20225 HinckeM –TAXchicken	Uncategorized Sample	20196-20205_A	Mudpit_Ingel20196_MGFPeaklist (F058870)	Uncharacterized protein (Fragment) OS=Gallus gallus GN=C3 PE=4 SV=2	H9KZT6_CHICK,R4GJS6_CHICK,UPI0002C875F7,UPI0002C8784D	TAX_GallusGallus_9031_20141114.fasta,UPARC14_2_GallusG_9031_20140417.fasta	150,963.90	100.00%	9	9	14	0.03%	6.22%	KGYTQQLAYR	K	K	99.70%	31.9	44.2	28.3	14.1	27.1	1.29	0.229	0.45	0	0	1	0	2	1,227.65	1,679.00		246.47	787	796	Empty	TRUE	UPI00000FC766,Q90633_CHICK
20196-20225 HinckeM –TAXchicken	Uncategorized Sample	20196-20205_A	Mudpit_Ingel20196_MGFPeaklist (F058870)	Uncharacterized protein (Fragment) OS=Gallus gallus GN=C3 PE=4 SV=2	H9KZT6_CHICK,R4GJS6_CHICK,UPI0002C875F7,UPI0002C8784D	TAX_GallusGallus_9031_20141114.fasta,UPARC14_2_GallusG_9031_20140417.fasta	150,963.90	100.00%	9	9	14	0.03%	6.22%	NSVPITVR	K	T	52.00%	23.1	43.9	23.9	4.32					0	1	0	0	2	885.5152	1,805.00		461.89	157	164	Empty	TRUE	UPI00000FC766,Q90633_CHICK
20196-20225 HinckeM –TAXchicken	Uncategorized Sample	20196-20205_A	Mudpit_Ingel20196_MGFPeaklist (F058870)	Uncharacterized protein (Fragment) OS=Gallus gallus GN=C3 PE=4 SV=2	H9KZT6_CHICK,R4GJS6_CHICK,UPI0002C875F7,UPI0002C8784D	TAX_GallusGallus_9031_20141114.fasta,UPARC14_2_GallusG_9031_20140417.fasta	150,963.90	100.00%	9	9	14	0.03%	6.22%	TEAIELIK	R	K	51.00%	21.6	44.9	25.3	8.12					0	1	0	0	2	916.5351	2,187.00		325.56	779	786	Empty	TRUE	UPI00000FC766,Q90633_CHICK
20196-20225 HinckeM –TAXchicken	Uncategorized Sample	20196-20205_A	Mudpit_Ingel20196_MGFPeaklist (F058870)	Uncharacterized protein (Fragment) OS=Gallus gallus GN=C3 PE=4 SV=2	H9KZT6_CHICK,R4GJS6_CHICK,UPI0002C875F7,UPI0002C8784D	TAX_GallusGallus_9031_20141114.fasta,UPARC14_2_GallusG_9031_20140417.fasta	150,963.90	100.00%	9	9	14	0.03%	6.22%	VKVTDGDGEAVLPMAMLR	R	Q	49.20%	11.9	44.6	16.3	0	26.6	0.638	0	0.222	0	0	1	0	2	1,933.97	2,137.00		1,266.49	33	50	Empty	TRUE	
20196-20225 HinckeM –TAXchicken	Uncategorized Sample	20196-20205_A	Mudpit_Ingel20196_MGFPeaklist (F058870)	Uncharacterized protein (Fragment) OS=Gallus gallus GN=C3 PE=4 SV=2	H9KZT6_CHICK,R4GJS6_CHICK,UPI0002C875F7,UPI0002C8784D	TAX_GallusGallus_9031_20141114.fasta,UPARC14_2_GallusG_9031_20140417.fasta	150,963.90	100.00%	9	9	14	0.03%	6.22%	ADNFQGLVSTQR	K	D	99.70%	33.5	44.8	23.3	23.2	30.2	2.28	0.341	0.333	0	2	0	0	2	1,335.67	2,066.00		1,611.55	131	142	Empty	TRUE	UPI00000FC766,Q90633_CHICK
20196-20225 HinckeM –TAXchicken	Uncategorized Sample	20196-20205_A	Mudpit_Ingel20196_MGFPeaklist (F058870)	Uncharacterized protein (Fragment) OS=Gallus gallus GN=C3 PE=4 SV=2	H9KZT6_CHICK,R4GJS6_CHICK,UPI0002C875F7,UPI0002C8784D	TAX_GallusGallus_9031_20141114.fasta,UPARC14_2_GallusG_9031_20140417.fasta	150,963.90	100.00%	9	9	14	0.03%	6.22%	AELTGPVVR	K	W	34.00%	17.6	43.2	22.9	0					0	1	0	0	2	941.5415	1,841.00		775.95	979	987	Empty	TRUE	UPI00000FC766,Q90633_CHICK
20196-20225 HinckeM –TAXchicken	Uncategorized Sample	20196-20205_A	Mudpit_Ingel20196_MGFPeaklist (F058870)	Uncharacterized protein (Fragment) OS=Gallus gallus GN=C3 PE=4 SV=2	H9KZT6_CHICK,R4GJS6_CHICK,UPI0002C875F7,UPI0002C8784D	TAX_GallusGallus_9031_20141114.fasta,UPARC14_2_GallusG_9031_20140417.fasta	150,963.90	100.00%	9	9	14	0.03%	6.22%	GYTQQLAYR	K	K	99.70%	26.4	44.1	22.1	10.6	23.3	2.2	0.232	0.278	0	2	0	0	2	1,099.55	1,782.00		980.47	788	796	Empty	TRUE	UPI00000FC766,Q90633_CHICK
20196-20225 HinckeM –TAXchicken	Uncategorized Sample	20196-20205_A	Mudpit_Ingel20196_MGFPeaklist (F058870)	Uncharacterized protein (Fragment) OS=Gallus gallus GN=C3 PE=4 SV=2	H9KZT6_CHICK,R4GJS6_CHICK,UPI0002C875F7,UPI0002C8784D	TAX_GallusGallus_9031_20141114.fasta,UPARC14_2_GallusG_9031_20140417.fasta	150,963.90	100.00%	9	9	14	0.03%	6.22%	IPVKADNFQGLVSTQR	R	D	38.20%	15.5	43.4	13.9	8.72					0	0	1	0	2	1,772.97	2,205.00		1,319.70	127	142	Empty	TRUE	Q90633_CHICK
20196-20225 HinckeM –TAXchicken	Uncategorized Sample	20196-20205_A	Mudpit_Ingel20196_MGFPeaklist (F058870)	Uncharacterized protein (Fragment) OS=Gallus gallus GN=C3 PE=4 SV=2	H9KZT6_CHICK,R4GJS6_CHICK,UPI0002C875F7,UPI0002C8784D	TAX_GallusGallus_9031_20141114.fasta,UPARC14_2_GallusG_9031_20140417.fasta	150,963.90	100.00%	9	9	14	0.03%	6.22%	IVTSPYTIHFTHTPK	R	Y	99.70%	25.8	43.7	16.6	24.2	32.6	1.17	0.239	0.333	0	0	0	2	2	1,741.93	2,114.00		4,142.83	88	102	Empty	TRUE	UPI00000FC766,Q90633_CHICK
20196-20225 HinckeM –TAXchicken	Uncategorized Sample	20196-20205_A	Mudpit_Ingel20196_MGFPeaklist (F058870)	Uncharacterized protein (Fragment) OS=Gallus gallus GN=C3 PE=4 SV=2	H9KZT6_CHICK,R4GJS6_CHICK,UPI0002C875F7,UPI0002C8784D	TAX_GallusGallus_9031_20141114.fasta,UPARC14_2_GallusG_9031_20140417.fasta	150,963.90	100.00%	9	9	14	0.03%	6.22%	KGYTQQLAYR	K	K	99.70%	31.9	44.2	28.3	14.1	27.1	1.29	0.229	0.45	0	0	1	0	2	1,227.65	1,679.00		246.47	787	796	Empty	TRUE	UPI00000FC766,Q90633_CHICK
20196-20225 HinckeM –TAXchicken	Uncategorized Sample	20196-20205_A	Mudpit_Ingel20196_MGFPeaklist (F058870)	Uncharacterized protein (Fragment) OS=Gallus gallus GN=C3 PE=4 SV=2	H9KZT6_CHICK,R4GJS6_CHICK,UPI0002C875F7,UPI0002C8784D	TAX_GallusGallus_9031_20141114.fasta,UPARC14_2_GallusG_9031_20140417.fasta	150,963.90	100.00%	9	9	14	0.03%	6.22%	NSVPITVR	K	T	52.00%	23.1	43.9	23.9	4.32					0	1	0	0	2	885.5152	1,805.00		461.89	157	164	Empty	TRUE	UPI00000FC766,Q90633_CHICK
20196-20225 HinckeM –TAXchicken	Uncategorized Sample	20196-20205_A	Mudpit_Ingel20196_MGFPeaklist (F058870)	Uncharacterized protein (Fragment) OS=Gallus gallus GN=C3 PE=4 SV=2	H9KZT6_CHICK,R4GJS6_CHICK,UPI0002C875F7,UPI0002C8784D	TAX_GallusGallus_9031_20141114.fasta,UPARC14_2_GallusG_9031_20140417.fasta	150,963.90	100.00%	9	9	14	0.03%	6.22%	TEAIELIK	R	K	51.00%	21.6	44.9	25.3	8.12					0	1	0	0	2	916.5351	2,187.00		325.56	779	786	Empty	TRUE	UPI00000FC766,Q90633_CHICK
20196-20225 HinckeM –TAXchicken	Uncategorized Sample	20196-20205_A	Mudpit_Ingel20196_MGFPeaklist (F058870)	Uncharacterized protein (Fragment) OS=Gallus gallus GN=C3 PE=4 SV=2	H9KZT6_CHICK,R4GJS6_CHICK,UPI0002C875F7,UPI0002C8784D	TAX_GallusGallus_9031_20141114.fasta,UPARC14_2_GallusG_9031_20140417.fasta	150,963.90	100.00%	9	9	14	0.03%	6.22%	VKVTDGDGEAVLPMAMLR	R	Q	49.20%	11.9	44.6	16.3	0	26.6	0.638	0	0.222	0	0	1	0	2	1,933.97	2,137.00		1,266.49	33	50	Empty	TRUE	
20196-20225 HinckeM –TAXchicken	Uncategorized Sample	20196-20205_A	Mudpit_Ingel20196_MGFPeaklist (F058870)	status=active	F1NH88_CHICK,UPI0002C86756	TAX_GallusGallus_9031_20141114.fasta,UPARC14_2_GallusG_9031_20140417.fasta	157,727.60	99.80%	2	2	3	0.01%	1.35%	ALAEELEQK	K	L	25.30%	8.5	45.3	25.1	-2.2					0	1	0	0	2	1,030.54	1,858.00		176.95	1286	1294	Empty	TRUE	
20196-20225 HinckeM –TAXchicken	Uncategorized Sample	20196-20205_A	Mudpit_Ingel20196_MGFPeaklist (F058870)	status=active	F1NH88_CHICK,UPI0002C86756	TAX_GallusGallus_9031_20141114.fasta,UPARC14_2_GallusG_9031_20140417.fasta	157,727.60	99.80%	2	2	3	0.01%	1.35%	ELPPAVAAFR	R	A	99.70%	74.2	44	29.5	63.9	44.9	2.82	0.408	0.5	0	2	0	0	2	1,070.60	2,357.00		549.56	36	45	Empty	TRUE	
20196-20225 HinckeM –TAXchicken	Uncategorized Sample	20196-20205_A	Mudpit_Ingel20196_MGFPeaklist (F058870)	status=active	F1NH88_CHICK,UPI0002C86756	TAX_GallusGallus_9031_20141114.fasta,UPARC14_2_GallusG_9031_20140417.fasta	157,727.60	99.80%	2	2	3	0.01%	1.35%	ALAEELEQK	K	L	25.30%	8.5	45.3	25.1	-2.2					0	1	0	0	2	1,030.54	1,858.00		176.95	1286	1294	Empty	TRUE	
20196-20225 HinckeM –TAXchicken	Uncategorized Sample	20196-20205_A	Mudpit_Ingel20196_MGFPeaklist (F058870)	status=active	F1NH88_CHICK,UPI0002C86756	TAX_GallusGallus_9031_20141114.fasta,UPARC14_2_GallusG_9031_20140417.fasta	157,727.60	99.80%	2	2	3	0.01%	1.35%	ELPPAVAAFR	R	A	99.70%	74.2	44	29.5	63.9	44.9	2.82	0.408	0.5	0	2	0	0	2	1,070.60	2,357.00		549.56	36	45	Empty	TRUE	
20196-20225 HinckeM –TAXchicken	Uncategorized Sample	20196-20205_A	Mudpit_Ingel20196_MGFPeaklist (F058870)	status=inactive	UPI0000ECA65B	UPARC14_2_GallusG_9031_20140417.fasta	217,626.00	99.60%	2	2	5	0.01%	1.42%	SLLVTPEPLPAAGER	R	A	99.70%	47.7	43.1	25.6	35.1	39.6	3.85	0.338	0.367	0	2	0	0	2	1,549.86	2,406.50		1,777.33	414	428	Empty	TRUE	
20196-20225 HinckeM –TAXchicken	Uncategorized Sample	20196-20205_A	Mudpit_Ingel20196_MGFPeaklist (F058870)	status=inactive	UPI0000ECA65B	UPARC14_2_GallusG_9031_20140417.fasta	217,626.00	99.60%	2	2	5	0.01%	1.42%	TGALTPAVSTNEWK	K	K	10.70%	7.25	44.9	28.2	-1.28					0	3	0	0	2	1,475.74	2,138.00		6,161.29	1122	1135	Empty	TRUE	
20196-20225 HinckeM –TAXchicken	Uncategorized Sample	20196-20205_A	Mudpit_Ingel20196_MGFPeaklist (F058870)	Septin-2 OS=Gallus gallus GN=SEPT2 PE=3 SV=1	E1C3D2_CHICK,UPI00003AED38,UPI0000448AD4,UPI0000611C4E,UPI000350449B	TAX_GallusGallus_9031_20141114.fasta,UPARC14_2_GallusG_9031_20140417.fasta	46,090.40	99.80%	2	2	2	0.00%	6.08%	DQILLEKEAELRR	K	M	23.20%	10.7	42.7	24.5	0					0	0	0	1	2	1,612.90	2,206.00		604.45	359	371	Empty	TRUE	
20196-20225 HinckeM –TAXchicken	Uncategorized Sample	20196-20205_A	Mudpit_Ingel20196_MGFPeaklist (F058870)	Septin-2 OS=Gallus gallus GN=SEPT2 PE=3 SV=1	E1C3D2_CHICK,UPI00003AED38,UPI0000448AD4,UPI0000611C4E,UPI000350449B	TAX_GallusGallus_9031_20141114.fasta,UPARC14_2_GallusG_9031_20140417.fasta	46,090.40	99.80%	2	2	2	0.00%	6.08%	VNIVPVIAK	K	A	99.70%	46.6	38	23.7	0	28.8	1.21	0.292	0.389	0	1	0	0	2	952.619	2,269.00		361.06	215	223	Empty	TRUE	SEPT2_CHICK
20196-20225 HinckeM –TAXchicken	Uncategorized Sample	20196-20205_A	Mudpit_Ingel20196_MGFPeaklist (F058870)	Septin-2 OS=Gallus gallus GN=SEPT2 PE=3 SV=1	E1C3D2_CHICK,UPI00003AED38,UPI0000448AD4,UPI0000611C4E,UPI000350449B	TAX_GallusGallus_9031_20141114.fasta,UPARC14_2_GallusG_9031_20140417.fasta	46,090.40	99.80%	2	2	2	0.00%	6.08%	DQILLEKEAELRR	K	M	23.20%	10.7	42.7	24.5	0					0	0	0	1	2	1,612.90	2,206.00		604.45	359	371	Empty	TRUE	
20196-20225 HinckeM –TAXchicken	Uncategorized Sample	20196-20205_A	Mudpit_Ingel20196_MGFPeaklist (F058870)	Septin-2 OS=Gallus gallus GN=SEPT2 PE=3 SV=1	E1C3D2_CHICK,UPI00003AED38,UPI0000448AD4,UPI0000611C4E,UPI000350449B	TAX_GallusGallus_9031_20141114.fasta,UPARC14_2_GallusG_9031_20140417.fasta	46,090.40	99.80%	2	2	2	0.00%	6.08%	VNIVPVIAK	K	A	99.70%	46.6	38	23.7	0	28.8	1.21	0.292	0.389	0	1	0	0	2	952.619	2,269.00		361.06	215	223	Empty	TRUE	SEPT2_CHICK
20196-20225 HinckeM –TAXchicken	Uncategorized Sample	20196-20205_A	Mudpit_Ingel20196_MGFPeaklist (F058870)	Septin-2 OS=Gallus gallus GN=SEPT2 PE=3 SV=1	E1C3D2_CHICK,UPI00003AED38,UPI0000448AD4,UPI0000611C4E,UPI000350449B	TAX_GallusGallus_9031_20141114.fasta,UPARC14_2_GallusG_9031_20140417.fasta	46,090.40	99.80%	2	2	2	0.00%	6.08%	DQILLEKEAELRR	K	M	23.20%	10.7	42.7	24.5	0					0	0	0	1	2	1,612.90	2,206.00		604.45	359	371	Empty	TRUE	
20196-20225 HinckeM –TAXchicken	Uncategorized Sample	20196-20205_A	Mudpit_Ingel20196_MGFPeaklist (F058870)	Septin-2 OS=Gallus gallus GN=SEPT2 PE=3 SV=1	E1C3D2_CHICK,UPI00003AED38,UPI0000448AD4,UPI0000611C4E,UPI000350449B	TAX_GallusGallus_9031_20141114.fasta,UPARC14_2_GallusG_9031_20140417.fasta	46,090.40	99.80%	2	2	2	0.00%	6.08%	VNIVPVIAK	K	A	99.70%	46.6	38	23.7	0	28.8	1.21	0.292	0.389	0	1	0	0	2	952.619	2,269.00		361.06	215	223	Empty	TRUE	SEPT2_CHICK
20196-20225 HinckeM –TAXchicken	Uncategorized Sample	20196-20205_A	Mudpit_Ingel20196_MGFPeaklist (F058870)	Septin-2 OS=Gallus gallus GN=SEPT2 PE=3 SV=1	E1C3D2_CHICK,UPI00003AED38,UPI0000448AD4,UPI0000611C4E,UPI000350449B	TAX_GallusGallus_9031_20141114.fasta,UPARC14_2_GallusG_9031_20140417.fasta	46,090.40	99.80%	2	2	2	0.00%	6.08%	DQILLEKEAELRR	K	M	23.20%	10.7	42.7	24.5	0					0	0	0	1	2	1,612.90	2,206.00		604.45	359	371	Empty	TRUE	
20196-20225 HinckeM –TAXchicken	Uncategorized Sample	20196-20205_A	Mudpit_Ingel20196_MGFPeaklist (F058870)	Septin-2 OS=Gallus gallus GN=SEPT2 PE=3 SV=1	E1C3D2_CHICK,UPI00003AED38,UPI0000448AD4,UPI0000611C4E,UPI000350449B	TAX_GallusGallus_9031_20141114.fasta,UPARC14_2_GallusG_9031_20140417.fasta	46,090.40	99.80%	2	2	2	0.00%	6.08%	VNIVPVIAK	K	A	99.70%	46.6	38	23.7	0	28.8	1.21	0.292	0.389	0	1	0	0	2	952.619	2,269.00		361.06	215	223	Empty	TRUE	SEPT2_CHICK
20196-20225 HinckeM –TAXchicken	Uncategorized Sample	20196-20205_A	Mudpit_Ingel20196_MGFPeaklist (F058870)	Septin-2 OS=Gallus gallus GN=SEPT2 PE=3 SV=1	E1C3D2_CHICK,UPI00003AED38,UPI0000448AD4,UPI0000611C4E,UPI000350449B	TAX_GallusGallus_9031_20141114.fasta,UPARC14_2_GallusG_9031_20140417.fasta	46,090.40	99.80%	2	2	2	0.00%	6.08%	DQILLEKEAELRR	K	M	23.20%	10.7	42.7	24.5	0					0	0	0	1	2	1,612.90	2,206.00		604.45	359	371	Empty	TRUE	
20196-20225 HinckeM –TAXchicken	Uncategorized Sample	20196-20205_A	Mudpit_Ingel20196_MGFPeaklist (F058870)	Septin-2 OS=Gallus gallus GN=SEPT2 PE=3 SV=1	E1C3D2_CHICK,UPI00003AED38,UPI0000448AD4,UPI0000611C4E,UPI000350449B	TAX_GallusGallus_9031_20141114.fasta,UPARC14_2_GallusG_9031_20140417.fasta	46,090.40	99.80%	2	2	2	0.00%	6.08%	VNIVPVIAK	K	A	99.70%	46.6	38	23.7	0	28.8	1.21	0.292	0.389	0	1	0	0	2	952.619	2,269.00		361.06	215	223	Empty	TRUE	SEPT2_CHICK
20196-20225 HinckeM –TAXchicken	Uncategorized Sample	20196-20205_A	Mudpit_Ingel20196_MGFPeaklist (F058870)	Uncharacterized protein OS=Gallus gallus GN=RCJMB04_7l20 PE=2 SV=1	Q5ZL53_CHICK,UPI0000443583	TAX_GallusGallus_9031_20141114.fasta,UPARC14_2_GallusG_9031_20140417.fasta	70,940.40	100.00%	8	9	13	0.03%	13.80%	ALDTMNFDVIK	R	G	99.70%	23.6	44	20.4	17.7	27.2	0.921	0.232	0.318	0	3	0	0	2	1,282.64	2,300.00		1,589.64	68	78	Empty	TRUE	
20196-20225 HinckeM –TAXchicken	Uncategorized Sample	20196-20205_A	Mudpit_Ingel20196_MGFPeaklist (F058870)	Uncharacterized protein OS=Gallus gallus GN=RCJMB04_7l20 PE=2 SV=1	Q5ZL53_CHICK,UPI0000443583	TAX_GallusGallus_9031_20141114.fasta,UPARC14_2_GallusG_9031_20140417.fasta	70,940.40	100.00%	8	9	13	0.03%	13.80%	AVNNPTGVPSV	K	-	22.20%	4.62	43.5	16.7	2.36					0	1	0	0	2	1,054.55	1,995.00		756.78	627	637	Empty	TRUE	
20196-20225 HinckeM –TAXchicken	Uncategorized Sample	20196-20205_A	Mudpit_Ingel20196_MGFPeaklist (F058870)	Uncharacterized protein OS=Gallus gallus GN=RCJMB04_7l20 PE=2 SV=1	Q5ZL53_CHICK,UPI0000443583	TAX_GallusGallus_9031_20141114.fasta,UPARC14_2_GallusG_9031_20140417.fasta	70,940.40	100.00%	8	9	13	0.03%	13.80%	FGPALSVK	K	V	99.70%	22	45.2	24.4	8.24	26.7	1	0.124	0.375	0	2	0	0	2	818.4771	2,049.50		2,958.70	214	221	Empty	TRUE	
20196-20225 HinckeM –TAXchicken	Uncategorized Sample	20196-20205_A	Mudpit_Ingel20196_MGFPeaklist (F058870)	Uncharacterized protein OS=Gallus gallus GN=RCJMB04_7l20 PE=2 SV=1	Q5ZL53_CHICK,UPI0000443583	TAX_GallusGallus_9031_20141114.fasta,UPARC14_2_GallusG_9031_20140417.fasta	70,940.40	100.00%	8	9	13	0.03%	13.80%	FSPAGPILSIR	K	V	99.70%	59.1	43.9	30.6	51	38.9	4.3	0.411	0.455	0	2	0	0	2	1,157.67	2,500.50		9,055.19	31	41	Empty	TRUE	
20196-20225 HinckeM –TAXchicken	Uncategorized Sample	20196-20205_A	Mudpit_Ingel20196_MGFPeaklist (F058870)	Uncharacterized protein OS=Gallus gallus GN=RCJMB04_7l20 PE=2 SV=1	Q5ZL53_CHICK,UPI0000443583	TAX_GallusGallus_9031_20141114.fasta,UPARC14_2_GallusG_9031_20140417.fasta	70,940.40	100.00%	8	9	13	0.03%	13.80%	GFGFVSFER	K	H	99.70%	31.6	45.2	23.1	12.8	31	1.49	0.219	0.5	0	1	0	0	2	1,045.51	2,561.00		4,043.89	232	240	Empty	TRUE	
20196-20225 HinckeM –TAXchicken	Uncategorized Sample	20196-20205_A	Mudpit_Ingel20196_MGFPeaklist (F058870)	Uncharacterized protein OS=Gallus gallus GN=RCJMB04_7l20 PE=2 SV=1	Q5ZL53_CHICK,UPI0000443583	TAX_GallusGallus_9031_20141114.fasta,UPARC14_2_GallusG_9031_20140417.fasta	70,940.40	100.00%	8	9	13	0.03%	13.80%	GYGFVHFETQEAAER	K	A	99.70%	25.8	43.4	14.1	24.5	26.5	1.3	0.26	0.267	0	0	1	0	2	1,740.80	2,211.00		1,605.25	139	153	Empty	TRUE	
20196-20225 HinckeM –TAXchicken	Uncategorized Sample	20196-20205_A	Mudpit_Ingel20196_MGFPeaklist (F058870)	Uncharacterized protein OS=Gallus gallus GN=RCJMB04_7l20 PE=2 SV=1	Q5ZL53_CHICK,UPI0000443583	TAX_GallusGallus_9031_20141114.fasta,UPARC14_2_GallusG_9031_20140417.fasta	70,940.40	100.00%	8	9	13	0.03%	13.80%	IVATKPLYVALAQR	R	K	99.70%	51.2	39	18.1	40.5	39.4	2.89	0.0355	0.429	0	0	1	0	2	1,542.94	2,329.00		5,110.99	357	370	Empty	TRUE	
20196-20225 HinckeM –TAXchicken	Uncategorized Sample	20196-20205_A	Mudpit_Ingel20196_MGFPeaklist (F058870)	Uncharacterized protein OS=Gallus gallus GN=RCJMB04_7l20 PE=2 SV=1	Q5ZL53_CHICK,UPI0000443583	TAX_GallusGallus_9031_20141114.fasta,UPARC14_2_GallusG_9031_20140417.fasta	70,940.40	100.00%	8	9	13	0.03%	13.80%	SGVGNIFIK	K	N	85.90%	28.3	44.2	29.6	13.3					0	2	0	0	2	934.5357	2,267.50		1,630.62	96	104	Empty	TRUE	
20196-20225 HinckeM –TAXchicken	Uncategorized Sample	20196-20205_A	Mudpit_Ingel20196_MGFPeaklist (F058870)	Uncharacterized protein OS=Gallus gallus GN=RCJMB04_7l20 PE=2 SV=1	Q5ZL53_CHICK,UPI0000443583	TAX_GallusGallus_9031_20141114.fasta,UPARC14_2_GallusG_9031_20140417.fasta	70,940.40	100.00%	8	9	13	0.03%	13.80%	ALDTMNFDVIK	R	G	99.70%	23.6	44	20.4	17.7	27.2	0.921	0.232	0.318	0	3	0	0	2	1,282.64	2,300.00		1,589.64	68	78	Empty	TRUE	
20196-20225 HinckeM –TAXchicken	Uncategorized Sample	20196-20205_A	Mudpit_Ingel20196_MGFPeaklist (F058870)	Uncharacterized protein OS=Gallus gallus GN=RCJMB04_7l20 PE=2 SV=1	Q5ZL53_CHICK,UPI0000443583	TAX_GallusGallus_9031_20141114.fasta,UPARC14_2_GallusG_9031_20140417.fasta	70,940.40	100.00%	8	9	13	0.03%	13.80%	AVNNPTGVPSV	K	-	22.20%	4.62	43.5	16.7	2.36					0	1	0	0	2	1,054.55	1,995.00		756.78	627	637	Empty	TRUE	
20196-20225 HinckeM –TAXchicken	Uncategorized Sample	20196-20205_A	Mudpit_Ingel20196_MGFPeaklist (F058870)	Uncharacterized protein OS=Gallus gallus GN=RCJMB04_7l20 PE=2 SV=1	Q5ZL53_CHICK,UPI0000443583	TAX_GallusGallus_9031_20141114.fasta,UPARC14_2_GallusG_9031_20140417.fasta	70,940.40	100.00%	8	9	13	0.03%	13.80%	FGPALSVK	K	V	99.70%	22	45.2	24.4	8.24	26.7	1	0.124	0.375	0	2	0	0	2	818.4771	2,049.50		2,958.70	214	221	Empty	TRUE	
20196-20225 HinckeM –TAXchicken	Uncategorized Sample	20196-20205_A	Mudpit_Ingel20196_MGFPeaklist (F058870)	Uncharacterized protein OS=Gallus gallus GN=RCJMB04_7l20 PE=2 SV=1	Q5ZL53_CHICK,UPI0000443583	TAX_GallusGallus_9031_20141114.fasta,UPARC14_2_GallusG_9031_20140417.fasta	70,940.40	100.00%	8	9	13	0.03%	13.80%	FSPAGPILSIR	K	V	99.70%	59.1	43.9	30.6	51	38.9	4.3	0.411	0.455	0	2	0	0	2	1,157.67	2,500.50		9,055.19	31	41	Empty	TRUE	
20196-20225 HinckeM –TAXchicken	Uncategorized Sample	20196-20205_A	Mudpit_Ingel20196_MGFPeaklist (F058870)	Uncharacterized protein OS=Gallus gallus GN=RCJMB04_7l20 PE=2 SV=1	Q5ZL53_CHICK,UPI0000443583	TAX_GallusGallus_9031_20141114.fasta,UPARC14_2_GallusG_9031_20140417.fasta	70,940.40	100.00%	8	9	13	0.03%	13.80%	GFGFVSFER	K	H	99.70%	31.6	45.2	23.1	12.8	31	1.49	0.219	0.5	0	1	0	0	2	1,045.51	2,561.00		4,043.89	232	240	Empty	TRUE	
20196-20225 HinckeM –TAXchicken	Uncategorized Sample	20196-20205_A	Mudpit_Ingel20196_MGFPeaklist (F058870)	Uncharacterized protein OS=Gallus gallus GN=RCJMB04_7l20 PE=2 SV=1	Q5ZL53_CHICK,UPI0000443583	TAX_GallusGallus_9031_20141114.fasta,UPARC14_2_GallusG_9031_20140417.fasta	70,940.40	100.00%	8	9	13	0.03%	13.80%	GYGFVHFETQEAAER	K	A	99.70%	25.8	43.4	14.1	24.5	26.5	1.3	0.26	0.267	0	0	1	0	2	1,740.80	2,211.00		1,605.25	139	153	Empty	TRUE	
20196-20225 HinckeM –TAXchicken	Uncategorized Sample	20196-20205_A	Mudpit_Ingel20196_MGFPeaklist (F058870)	Uncharacterized protein OS=Gallus gallus GN=RCJMB04_7l20 PE=2 SV=1	Q5ZL53_CHICK,UPI0000443583	TAX_GallusGallus_9031_20141114.fasta,UPARC14_2_GallusG_9031_20140417.fasta	70,940.40	100.00%	8	9	13	0.03%	13.80%	IVATKPLYVALAQR	R	K	99.70%	51.2	39	18.1	40.5	39.4	2.89	0.0355	0.429	0	0	1	0	2	1,542.94	2,329.00		5,110.99	357	370	Empty	TRUE	
20196-20225 HinckeM –TAXchicken	Uncategorized Sample	20196-20205_A	Mudpit_Ingel20196_MGFPeaklist (F058870)	Uncharacterized protein OS=Gallus gallus GN=RCJMB04_7l20 PE=2 SV=1	Q5ZL53_CHICK,UPI0000443583	TAX_GallusGallus_9031_20141114.fasta,UPARC14_2_GallusG_9031_20140417.fasta	70,940.40	100.00%	8	9	13	0.03%	13.80%	SGVGNIFIK	K	N	85.90%	28.3	44.2	29.6	13.3					0	2	0	0	2	934.5357	2,267.50		1,630.62	96	104	Empty	TRUE	
20196-20225 HinckeM –TAXchicken	Uncategorized Sample	20196-20205_A	Mudpit_Ingel20196_MGFPeaklist (F058870)	Uncharacterized protein (Fragment) OS=Gallus gallus GN=EIF5B PE=4 SV=2	E1BVP1_CHICK	TAX_GallusGallus_9031_20141114.fasta	136,485.10	100.00%	4	4	5	0.01%	3.97%	AIIVEFAK	K	Q	99.70%	44.5	44	33.3	33.7	28.2	1.77	0.248	0.438	0	2	0	0	2	890.5346	2,280.00		332.43	782	789	Empty	TRUE	
20196-20225 HinckeM –TAXchicken	Uncategorized Sample	20196-20205_A	Mudpit_Ingel20196_MGFPeaklist (F058870)	Uncharacterized protein (Fragment) OS=Gallus gallus GN=EIF5B PE=4 SV=2	E1BVP1_CHICK	TAX_GallusGallus_9031_20141114.fasta	136,485.10	100.00%	4	4	5	0.01%	3.97%	ESKPVHIEV	K	K	71.10%					26.4	0.921	0.0682	0.444	0	1	0	0	1	1,037.56	1,630.00		277.93	502	510	Empty	TRUE	
20196-20225 HinckeM –TAXchicken	Uncategorized Sample	20196-20205_A	Mudpit_Ingel20196_MGFPeaklist (F058870)	Uncharacterized protein (Fragment) OS=Gallus gallus GN=EIF5B PE=4 SV=2	E1BVP1_CHICK	TAX_GallusGallus_9031_20141114.fasta	136,485.10	100.00%	4	4	5	0.01%	3.97%	HFEATDILVSKISR	R	Q	26.80%	25.8	44.1	22	13.8					0	0	1	0	2	1,615.88	2,171.00		855.36	1164	1177	Empty	TRUE	
20196-20225 HinckeM –TAXchicken	Uncategorized Sample	20196-20205_A	Mudpit_Ingel20196_MGFPeaklist (F058870)	Uncharacterized protein (Fragment) OS=Gallus gallus GN=EIF5B PE=4 SV=2	E1BVP1_CHICK	TAX_GallusGallus_9031_20141114.fasta	136,485.10	100.00%	4	4	5	0.01%	3.97%	LLQAQGVEVPSKDSVPK	K	K	33.10%	16.3	42.3	24	8.52					0	0	1	0	2	1,795.00	2,003.00		598.38	407	423	Empty	TRUE	
20196-20225 HinckeM –TAXchicken	Uncategorized Sample	20196-20205_A	Mudpit_Ingel20196_MGFPeaklist (F058870)	Fibrinogen alpha chain OS=Gallus gallus GN=FGA PE=4 SV=1	F1P4V1_CHICK,UPI000012A756,UPI00003AD607,sp|P14448|FIBA_CHICK	TAX_GallusGallus_9031_20141114.fasta,UPARC14_2_GallusG_9031_20140417.fasta	82,438.80	100.00%	22	34	99	0.20%	37.50%	ALQNSIQEQVVEMK	K	R	99.70%	46.3	44.9	17.7	38.7	33.4	2	0.296	0.286	0	2	2	0	2	1,632.83	1,992.50		5,475.94	154	167	Empty	TRUE	UPI0000610C95,UPI000002A9B0,UPI0000111307,UPI000059C9D0,UPI0002A11C16,sp|P14448-2|FIBA_CHICK,UPI0000113356
20196-20225 HinckeM –TAXchicken	Uncategorized Sample	20196-20205_A	Mudpit_Ingel20196_MGFPeaklist (F058870)	Fibrinogen alpha chain OS=Gallus gallus GN=FGA PE=4 SV=1	F1P4V1_CHICK,UPI000012A756,UPI00003AD607,sp|P14448|FIBA_CHICK	TAX_GallusGallus_9031_20141114.fasta,UPARC14_2_GallusG_9031_20140417.fasta	82,438.80	100.00%	22	34	99	0.20%	37.50%	EGSTYHFSGTGDFHK	R	L	99.70%	31.8	41.9	15.2	22.4	26.1	0.854	0.192	0.233	0	0	0	2	2	1,669.72	1,796.00		4,880.02	349	363	Empty	TRUE	UPI0000610C95,UPI000002A9B0,UPI0000111307,UPI000059C9D0,UPI0002A11C16,sp|P14448-2|FIBA_CHICK,UPI0000113356
20196-20225 HinckeM –TAXchicken	Uncategorized Sample	20196-20205_A	Mudpit_Ingel20196_MGFPeaklist (F058870)	Fibrinogen alpha chain OS=Gallus gallus GN=FGA PE=4 SV=1	F1P4V1_CHICK,UPI000012A756,UPI00003AD607,sp|P14448|FIBA_CHICK	TAX_GallusGallus_9031_20141114.fasta,UPARC14_2_GallusG_9031_20140417.fasta	82,438.80	100.00%	22	34	99	0.20%	37.50%	EGSTYHFSGTGDFHKLDR	R	L	99.70%	59.5	43.5	15	55.3	44.7	5.49	0.407	0.417	0	0	1	2	2	2,053.94	1,941.00		4,776.58	349	366	Empty	TRUE	UPI0000610C95,UPI000002A9B0,UPI0000111307,UPI000059C9D0,UPI0002A11C16,sp|P14448-2|FIBA_CHICK,UPI0000113356
20196-20225 HinckeM –TAXchicken	Uncategorized Sample	20196-20205_A	Mudpit_Ingel20196_MGFPeaklist (F058870)	Fibrinogen alpha chain OS=Gallus gallus GN=FGA PE=4 SV=1	F1P4V1_CHICK,UPI000012A756,UPI00003AD607,sp|P14448|FIBA_CHICK	TAX_GallusGallus_9031_20141114.fasta,UPARC14_2_GallusG_9031_20140417.fasta	82,438.80	100.00%	22	34	99	0.20%	37.50%	ESPSLVDKTSSASSVHR	R	C	99.70%	63.4	44.7	17.9	52.9	39.8	5.55	0.342	0.353	0	0	1	5	2	1,786.89	1,595.50		6,891.88	293	309	Empty	TRUE	UPI0000610C95,UPI000002A9B0,UPI0000111307,UPI000059C9D0,UPI0002A11C16,sp|P14448-2|FIBA_CHICK,UPI0000113356
20196-20225 HinckeM –TAXchicken	Uncategorized Sample	20196-20205_A	Mudpit_Ingel20196_MGFPeaklist (F058870)	Fibrinogen alpha chain OS=Gallus gallus GN=FGA PE=4 SV=1	F1P4V1_CHICK,UPI000012A756,UPI00003AD607,sp|P14448|FIBA_CHICK	TAX_GallusGallus_9031_20141114.fasta,UPARC14_2_GallusG_9031_20140417.fasta	82,438.80	100.00%	22	34	99	0.20%	37.50%	ETSEQLGGVQHDQSAEDTPDFK	R	A	99.70%	99.4	41.5	0	96.6	39.1	3.72	0.34	0.25	0	0	2	0	2	2,418.07	1,883.50		16,715.50	466	487	Empty	TRUE	UPI0000610C95,UPI000002A9B0,UPI0000111307,UPI000059C9D0,UPI0002A11C16,sp|P14448-2|FIBA_CHICK,UPI0000113356
20196-20225 HinckeM –TAXchicken	Uncategorized Sample	20196-20205_A	Mudpit_Ingel20196_MGFPeaklist (F058870)	Fibrinogen alpha chain OS=Gallus gallus GN=FGA PE=4 SV=1	F1P4V1_CHICK,UPI000012A756,UPI00003AD607,sp|P14448|FIBA_CHICK	TAX_GallusGallus_9031_20141114.fasta,UPARC14_2_GallusG_9031_20140417.fasta	82,438.80	100.00%	22	34	99	0.20%	37.50%	HLTQASSIDMHPDFQTTTLSTLK	K	M	99.70%	58	43.9	13.3	53.6	51	4.4	0.5	0.37	0	0	2	5	2	2,588.27	2,262.00		29,071.40	203	225	Empty	TRUE	UPI0000610C95,UPI000002A9B0,UPI0000111307,UPI000059C9D0,UPI0002A11C16,sp|P14448-2|FIBA_CHICK,UPI0000113356
20196-20225 HinckeM –TAXchicken	Uncategorized Sample	20196-20205_A	Mudpit_Ingel20196_MGFPeaklist (F058870)	Fibrinogen alpha chain OS=Gallus gallus GN=FGA PE=4 SV=1	F1P4V1_CHICK,UPI000012A756,UPI00003AD607,sp|P14448|FIBA_CHICK	TAX_GallusGallus_9031_20141114.fasta,UPARC14_2_GallusG_9031_20140417.fasta	82,438.80	100.00%	22	34	99	0.20%	37.50%	HSIGSSTSSHVTGAGSSHLGTGGK	R	D	99.70%	68	43.9	13.1	61.9	49.8	6.54	0.544	0.396	0	0	0	11	2	2,209.06	1,477.00		40,746.60	386	409	Empty	TRUE	UPI0000610C95,UPI000002A9B0,UPI0000111307,UPI000059C9D0,UPI0002A11C16,sp|P14448-2|FIBA_CHICK,UPI0000113356
20196-20225 HinckeM –TAXchicken	Uncategorized Sample	20196-20205_A	Mudpit_Ingel20196_MGFPeaklist (F058870)	Fibrinogen alpha chain OS=Gallus gallus GN=FGA PE=4 SV=1	F1P4V1_CHICK,UPI000012A756,UPI00003AD607,sp|P14448|FIBA_CHICK	TAX_GallusGallus_9031_20141114.fasta,UPARC14_2_GallusG_9031_20140417.fasta	82,438.80	100.00%	22	34	99	0.20%	37.50%	HSIGSSTSSHVTGAGSSHLGTGGKDK	R	F	99.70%	68.3	44	0	64.6					0	0	0	0	2	2,452.18	1,410.00		6,910.85	386	411	Empty	TRUE	UPI0000610C95,UPI000002A9B0,UPI0000111307,UPI000059C9D0,UPI0002A11C16,sp|P14448-2|FIBA_CHICK,UPI0000113356
20196-20225 HinckeM –TAXchicken	Uncategorized Sample	20196-20205_A	Mudpit_Ingel20196_MGFPeaklist (F058870)	Fibrinogen alpha chain OS=Gallus gallus GN=FGA PE=4 SV=1	F1P4V1_CHICK,UPI000012A756,UPI00003AD607,sp|P14448|FIBA_CHICK	TAX_GallusGallus_9031_20141114.fasta,UPARC14_2_GallusG_9031_20140417.fasta	82,438.80	100.00%	22	34	99	0.20%	37.50%	IRPLETL	K	-	15.40%	5.93	42.1	0	5.93					0	1	0	0	2	841.5143	2,202.00		316.81	735	741	Empty	TRUE	
20196-20225 HinckeM –TAXchicken	Uncategorized Sample	20196-20205_A	Mudpit_Ingel20196_MGFPeaklist (F058870)	Fibrinogen alpha chain OS=Gallus gallus GN=FGA PE=4 SV=1	F1P4V1_CHICK,UPI000012A756,UPI00003AD607,sp|P14448|FIBA_CHICK	TAX_GallusGallus_9031_20141114.fasta,UPARC14_2_GallusG_9031_20140417.fasta	82,438.80	100.00%	22	34	99	0.20%	37.50%	KVISGPDGPREEIVEK	K	M	28.90%	13.6	43.1	23.6	4.85					0	0	0	2	2	1,752.95	1,719.00		3,162.92	317	332	Empty	TRUE	UPI0000610C95,UPI000002A9B0,UPI0000111307,UPI000059C9D0,UPI0002A11C16,sp|P14448-2|FIBA_CHICK,UPI0000113356
20196-20225 HinckeM –TAXchicken	Uncategorized Sample	20196-20205_A	Mudpit_Ingel20196_MGFPeaklist (F058870)	Fibrinogen alpha chain OS=Gallus gallus GN=FGA PE=4 SV=1	F1P4V1_CHICK,UPI000012A756,UPI00003AD607,sp|P14448|FIBA_CHICK	TAX_GallusGallus_9031_20141114.fasta,UPARC14_2_GallusG_9031_20140417.fasta	82,438.80	100.00%	22	34	99	0.20%	37.50%	LKPSPEMQAMSAFNNIK	K	Q	99.70%	34.3	44.3	16.7	29.5	38.5	3.72	0.33	0.353	0	0	3	0	2	1,937.95	2,167.00		12,094.80	240	256	Empty	TRUE	UPI0000610C95,UPI000002A9B0,UPI0000111307,UPI000059C9D0,UPI0002A11C16,sp|P14448-2|FIBA_CHICK,UPI0000113356
20196-20225 HinckeM –TAXchicken	Uncategorized Sample	20196-20205_A	Mudpit_Ingel20196_MGFPeaklist (F058870)	Fibrinogen alpha chain OS=Gallus gallus GN=FGA PE=4 SV=1	F1P4V1_CHICK,UPI000012A756,UPI00003AD607,sp|P14448|FIBA_CHICK	TAX_GallusGallus_9031_20141114.fasta,UPARC14_2_GallusG_9031_20140417.fasta	82,438.80	100.00%	22	34	99	0.20%	37.50%	LLPDLESFFTHDSVSTSSR	R	H	99.70%	96.8	44.3	13.3	90.4	62.7	10.1	0.608	0.447	0	0	2	0	2	2,138.04	2,871.00		102642	367	385	Empty	TRUE	UPI0000610C95,UPI000002A9B0,UPI0000111307,UPI000059C9D0,UPI0002A11C16,sp|P14448-2|FIBA_CHICK,UPI0000113356
20196-20225 HinckeM –TAXchicken	Uncategorized Sample	20196-20205_A	Mudpit_Ingel20196_MGFPeaklist (F058870)	Fibrinogen alpha chain OS=Gallus gallus GN=FGA PE=4 SV=1	F1P4V1_CHICK,UPI000012A756,UPI00003AD607,sp|P14448|FIBA_CHICK	TAX_GallusGallus_9031_20141114.fasta,UPARC14_2_GallusG_9031_20140417.fasta	82,438.80	100.00%	22	34	99	0.20%	37.50%	MQGIIDDTDQNYSQR	R	I	99.70%	49.5	41.8	23.8	37.7	36.8	2.96	0.304	0.367	0	0	5	0	2	1,799.79	1,820.00		6,922.48	70	84	Empty	TRUE	UPI0000610C95,UPI000002A9B0,UPI0000111307,UPI000059C9D0,UPI0002A11C16,sp|P14448-2|FIBA_CHICK,UPI0000113356
20196-20225 HinckeM –TAXchicken	Uncategorized Sample	20196-20205_A	Mudpit_Ingel20196_MGFPeaklist (F058870)	Fibrinogen alpha chain OS=Gallus gallus GN=FGA PE=4 SV=1	F1P4V1_CHICK,UPI000012A756,UPI00003AD607,sp|P14448|FIBA_CHICK	TAX_GallusGallus_9031_20141114.fasta,UPARC14_2_GallusG_9031_20140417.fasta	82,438.80	100.00%	22	34	99	0.20%	37.50%	MRPLKDSNVPEHFK	K	L	99.70%	24.4	44.5	23.9	0.19	29.8	1.28	0.211	0.393	0	0	0	4	2	1,713.87	1,593.50		4,260.60	226	239	Empty	TRUE	UPI0000610C95,UPI000002A9B0,UPI0000111307,UPI000059C9D0,UPI0002A11C16,sp|P14448-2|FIBA_CHICK,UPI0000113356
20196-20225 HinckeM –TAXchicken	Uncategorized Sample	20196-20205_A	Mudpit_Ingel20196_MGFPeaklist (F058870)	Fibrinogen alpha chain OS=Gallus gallus GN=FGA PE=4 SV=1	F1P4V1_CHICK,UPI000012A756,UPI00003AD607,sp|P14448|FIBA_CHICK	TAX_GallusGallus_9031_20141114.fasta,UPARC14_2_GallusG_9031_20140417.fasta	82,438.80	100.00%	22	34	99	0.20%	37.50%	NWPICVDDDWGTK	K	C	99.70%	55.5	42.1	16.2	52.6	48.3	6.22	0.516	0.5	0	2	0	0	2	1,605.70	2,596.00		11,252.10	51	63	Empty	TRUE	UPI0000610C95,UPI000002A9B0,UPI0000111307,UPI000059C9D0,UPI0002A11C16,sp|P14448-2|FIBA_CHICK,UPI0000113356
20196-20225 HinckeM –TAXchicken	Uncategorized Sample	20196-20205_A	Mudpit_Ingel20196_MGFPeaklist (F058870)	Fibrinogen alpha chain OS=Gallus gallus GN=FGA PE=4 SV=1	F1P4V1_CHICK,UPI000012A756,UPI00003AD607,sp|P14448|FIBA_CHICK	TAX_GallusGallus_9031_20141114.fasta,UPARC14_2_GallusG_9031_20140417.fasta	82,438.80	100.00%	22	34	99	0.20%	37.50%	QMQVVLERPETDHVAEAR	K	G	99.70%	65.6	44.5	19	55	39.5	3.85	0.362	0.389	0	0	3	6	2	2,107.02	1,939.00		39,582.90	257	274	Empty	TRUE	UPI0000610C95,UPI000002A9B0,UPI0000111307,UPI000059C9D0,UPI0002A11C16,sp|P14448-2|FIBA_CHICK,UPI0000113356

20196-20225 HinckeM --TAXchicken	Uncategorized Sample	20196-20205_A	Mudpit_Ingel20196_MGFPeaklist (F058870)	Fibrinogen alpha chain OS=Gallus gallus GN=FGA PE=4 SV=1	F1P4V1_CHICK,UPI000012A756,UPI00003AD607,sp|P14448|FIBA_CHICK	TAX_GallusGallus_9031_20141114.fasta,UPARC14_2_GallusG_9031_20140417.fasta	82,438.80	100.00%	22	34	99	0.20%	37.50%	RESPSLVDKTSSASSVHR	R	C	99.70%	61.9	44	19.3	51.3	43.3	7.41	0.446	0.389	0	0	0	2	2	1,942.99	1,525.00		1,257.36	292	309	Empty	TRUE	UPI0000610C95,UPI000002A9B0,UPI0000111307,UPI000059C9D0,UPI0002A11C16,sp|P14448-2|FIBA_CHICK,UPI0000113356
20196-20225 HinckeM --TAXchicken	Uncategorized Sample	20196-20205_A	Mudpit_Ingel20196_MGFPeaklist (F058870)	Fibrinogen alpha chain OS=Gallus gallus GN=FGA PE=4 SV=1	F1P4V1_CHICK,UPI000012A756,UPI00003AD607,sp|P14448|FIBA_CHICK	TAX_GallusGallus_9031_20141114.fasta,UPARC14_2_GallusG_9031_20140417.fasta	82,438.80	100.00%	22	34	99	0.20%	37.50%	SFDYQVDKEGYDNIQK	R	H	99.70%	52.7	43.3	14.1	43	42.2	3	0.455	0.406	0	0	6	0	2	1,948.89	2,001.50		18,549.10	187	202	Empty	TRUE	UPI0000610C95,UPI000002A9B0,UPI0000111307,UPI000059C9D0,UPI0002A11C16,sp|P14448-2|FIBA_CHICK,UPI0000113356
20196-20225 HinckeM --TAXchicken	Uncategorized Sample	20196-20205_A	Mudpit_Ingel20196_MGFPeaklist (F058870)	Fibrinogen alpha chain OS=Gallus gallus GN=FGA PE=4 SV=1	F1P4V1_CHICK,UPI000012A756,UPI00003AD607,sp|P14448|FIBA_CHICK	TAX_GallusGallus_9031_20141114.fasta,UPARC14_2_GallusG_9031_20140417.fasta	82,438.80	100.00%	22	34	99	0.20%	37.50%	TRETSEQLGGVQHDQSAEDTPDFK	K	A	99.70%	66	42.5	21.9	57.1	44.3	4.52	0.397	0.292	0	0	0	2	2	2,675.22	1,830.50		6,861.05	464	487	Empty	TRUE	UPI0000610C95,UPI000002A9B0,UPI0000111307,UPI000059C9D0,UPI0002A11C16,sp|P14448-2|FIBA_CHICK,UPI0000113356
20196-20225 HinckeM --TAXchicken	Uncategorized Sample	20196-20205_A	Mudpit_Ingel20196_MGFPeaklist (F058870)	Fibrinogen alpha chain OS=Gallus gallus GN=FGA PE=4 SV=1	F1P4V1_CHICK,UPI000012A756,UPI00003AD607,sp|P14448|FIBA_CHICK	TAX_GallusGallus_9031_20141114.fasta,UPARC14_2_GallusG_9031_20140417.fasta	82,438.80	100.00%	22	34	99	0.20%	37.50%	TVLTSSSSSFNK	K	G	99.70%	61.1	44.6	30.8	44.4	45.3	4.74	0.313	0.542	0	16	0	0	2	1,257.63	1,788.50		53,737.50	441	452	Empty	TRUE	UPI0000610C95,UPI000002A9B0,UPI0000111307,UPI000059C9D0,UPI0002A11C16,sp|P14448-2|FIBA_CHICK,UPI0000113356
20196-20225 HinckeM --TAXchicken	Uncategorized Sample	20196-20205_A	Mudpit_Ingel20196_MGFPeaklist (F058870)	Fibrinogen alpha chain OS=Gallus gallus GN=FGA PE=4 SV=1	F1P4V1_CHICK,UPI000012A756,UPI00003AD607,sp|P14448|FIBA_CHICK	TAX_GallusGallus_9031_20141114.fasta,UPARC14_2_GallusG_9031_20140417.fasta	82,438.80	100.00%	22	34	99	0.20%	37.50%	TVLTSSSSSFNKGGSTFETK	K	S	99.70%	57.3	44.4	14.4	49	49.2	7.42	0.492	0.425	0	0	2	0	2	2,065.01	1,967.00		4,339.42	441	460	Empty	TRUE	UPI0000610C95,UPI000002A9B0,UPI0000111307,UPI000059C9D0,UPI0002A11C16,sp|P14448-2|FIBA_CHICK,UPI0000113356
20196-20225 HinckeM --TAXchicken	Uncategorized Sample	20196-20205_A	Mudpit_Ingel20196_MGFPeaklist (F058870)	Fibrinogen alpha chain OS=Gallus gallus GN=FGA PE=4 SV=1	F1P4V1_CHICK,UPI000012A756,UPI00003AD607,sp|P14448|FIBA_CHICK	TAX_GallusGallus_9031_20141114.fasta,UPARC14_2_GallusG_9031_20140417.fasta	82,438.80	100.00%	22	34	99	0.20%	37.50%	VISGPDGPREEIVEK	K	M	99.70%	25.2	44.1	27.2	9.22	36	0.959	0.197	0.467	0	0	3	0	2	1,624.85	1,799.00		70,528.00	318	332	Empty	TRUE	UPI0000610C95,UPI000002A9B0,UPI0000111307,UPI000059C9D0,UPI0002A11C16,sp|P14448-2|FIBA_CHICK,UPI0000113356
20196-20225 HinckeM --TAXchicken	Uncategorized Sample	20196-20205_A	Mudpit_Ingel20196_MGFPeaklist (F058870)	Fibrinogen alpha chain OS=Gallus gallus GN=FGA PE=4 SV=1	F1P4V1_CHICK,UPI000012A756,UPI00003AD607,sp|P14448|FIBA_CHICK	TAX_GallusGallus_9031_20141114.fasta,UPARC14_2_GallusG_9031_20140417.fasta	82,438.80	100.00%	22	34	99	0.20%	37.50%	ALQNSIQEQVVEMK	K	R	99.70%	46.3	44.9	17.7	38.7	33.4	2	0.296	0.286	0	2	2	0	2	1,632.83	1,992.50		5,475.94	154	167	Empty	TRUE	UPI0000610C95,UPI000002A9B0,UPI0000111307,UPI000059C9D0,UPI0002A11C16,sp|P14448-2|FIBA_CHICK,UPI0000113356
20196-20225 HinckeM --TAXchicken	Uncategorized Sample	20196-20205_A	Mudpit_Ingel20196_MGFPeaklist (F058870)	Fibrinogen alpha chain OS=Gallus gallus GN=FGA PE=4 SV=1	F1P4V1_CHICK,UPI000012A756,UPI00003AD607,sp|P14448|FIBA_CHICK	TAX_GallusGallus_9031_20141114.fasta,UPARC14_2_GallusG_9031_20140417.fasta	82,438.80	100.00%	22	34	99	0.20%	37.50%	EGSTYHFSGTGDFHK	R	L	99.70%	31.8	41.9	15.2	22.4	26.1	0.854	0.192	0.233	0	0	0	2	2	1,669.72	1,796.00		4,880.02	349	363	Empty	TRUE	UPI0000610C95,UPI000002A9B0,UPI0000111307,UPI000059C9D0,UPI0002A11C16,sp|P14448-2|FIBA_CHICK,UPI0000113356
20196-20225 HinckeM --TAXchicken	Uncategorized Sample	20196-20205_A	Mudpit_Ingel20196_MGFPeaklist (F058870)	Fibrinogen alpha chain OS=Gallus gallus GN=FGA PE=4 SV=1	F1P4V1_CHICK,UPI000012A756,UPI00003AD607,sp|P14448|FIBA_CHICK	TAX_GallusGallus_9031_20141114.fasta,UPARC14_2_GallusG_9031_20140417.fasta	82,438.80	100.00%	22	34	99	0.20%	37.50%	EGSTYHFSGTGDFHKLDR	R	L	99.70%	59.5	43.5	15	55.3	44.7	5.49	0.407	0.417	0	0	1	2	2	2,053.94	1,941.00		4,776.58	349	366	Empty	TRUE	UPI0000610C95,UPI000002A9B0,UPI0000111307,UPI000059C9D0,UPI0002A11C16,sp|P14448-2|FIBA_CHICK,UPI0000113356
20196-20225 HinckeM --TAXchicken	Uncategorized Sample	20196-20205_A	Mudpit_Ingel20196_MGFPeaklist (F058870)	Fibrinogen alpha chain OS=Gallus gallus GN=FGA PE=4 SV=1	F1P4V1_CHICK,UPI000012A756,UPI00003AD607,sp|P14448|FIBA_CHICK	TAX_GallusGallus_9031_20141114.fasta,UPARC14_2_GallusG_9031_20140417.fasta	82,438.80	100.00%	22	34	99	0.20%	37.50%	ESPSLVDKTSSASSVHR	R	C	99.70%	63.4	44.7	17.9	52.9	39.8	5.55	0.342	0.353	0	0	1	5	2	1,786.89	1,595.50		6,891.88	293	309	Empty	TRUE	UPI0000610C95,UPI000002A9B0,UPI0000111307,UPI000059C9D0,UPI0002A11C16,sp|P14448-2|FIBA_CHICK,UPI0000113356
20196-20225 HinckeM --TAXchicken	Uncategorized Sample	20196-20205_A	Mudpit_Ingel20196_MGFPeaklist (F058870)	Fibrinogen alpha chain OS=Gallus gallus GN=FGA PE=4 SV=1	F1P4V1_CHICK,UPI000012A756,UPI00003AD607,sp|P14448|FIBA_CHICK	TAX_GallusGallus_9031_20141114.fasta,UPARC14_2_GallusG_9031_20140417.fasta	82,438.80	100.00%	22	34	99	0.20%	37.50%	ETSEQLGGVQHDQSAEDTPDFK	R	A	99.70%	99.4	41.5	0	96.6	39.1	3.72	0.34	0.25	0	0	2	0	2	2,418.07	1,883.50		16,715.50	466	487	Empty	TRUE	UPI0000610C95,UPI000002A9B0,UPI0000111307,UPI000059C9D0,UPI0002A11C16,sp|P14448-2|FIBA_CHICK,UPI0000113356
20196-20225 HinckeM --TAXchicken	Uncategorized Sample	20196-20205_A	Mudpit_Ingel20196_MGFPeaklist (F058870)	Fibrinogen alpha chain OS=Gallus gallus GN=FGA PE=4 SV=1	F1P4V1_CHICK,UPI000012A756,UPI00003AD607,sp|P14448|FIBA_CHICK	TAX_GallusGallus_9031_20141114.fasta,UPARC14_2_GallusG_9031_20140417.fasta	82,438.80	100.00%	22	34	99	0.20%	37.50%	HLTQASSIDMHPDFQTTTLSTLK	K	M	99.70%	58	43.9	13.3	53.6	51	4.4	0.5	0.37	0	0	2	5	2	2,588.27	2,262.00		29,071.40	203	225	Empty	TRUE	UPI0000610C95,UPI000002A9B0,UPI0000111307,UPI000059C9D0,UPI0002A11C16,sp|P14448-2|FIBA_CHICK,UPI0000113356
20196-20225 HinckeM --TAXchicken	Uncategorized Sample	20196-20205_A	Mudpit_Ingel20196_MGFPeaklist (F058870)	Fibrinogen alpha chain OS=Gallus gallus GN=FGA PE=4 SV=1	F1P4V1_CHICK,UPI000012A756,UPI00003AD607,sp|P14448|FIBA_CHICK	TAX_GallusGallus_9031_20141114.fasta,UPARC14_2_GallusG_9031_20140417.fasta	82,438.80	100.00%	22	34	99	0.20%	37.50%	HSIGSSTSSHVTGAGSSHLGTGGK	R	D	99.70%	68	43.9	13.1	61.9	49.8	6.54	0.544	0.396	0	0	0	11	2	2,209.06	1,477.00		40,746.60	386	409	Empty	TRUE	UPI0000610C95,UPI000002A9B0,UPI0000111307,UPI000059C9D0,UPI0002A11C16,sp|P14448-2|FIBA_CHICK,UPI0000113356
20196-20225 HinckeM --TAXchicken	Uncategorized Sample	20196-20205_A	Mudpit_Ingel20196_MGFPeaklist (F058870)	Fibrinogen alpha chain OS=Gallus gallus GN=FGA PE=4 SV=1	F1P4V1_CHICK,UPI000012A756,UPI00003AD607,sp|P14448|FIBA_CHICK	TAX_GallusGallus_9031_20141114.fasta,UPARC14_2_GallusG_9031_20140417.fasta	82,438.80	100.00%	22	34	99	0.20%	37.50%	HSIGSSTSSHVTGAGSSHLGTGGKDK	R	F	99.70%	68.3	44	0	64.6					0	0	0	0	2	2,452.18	1,410.00		6,910.85	386	411	Empty	TRUE	UPI0000610C95,UPI000002A9B0,UPI0000111307,UPI000059C9D0,UPI0002A11C16,sp|P14448-2|FIBA_CHICK,UPI0000113356
20196-20225 HinckeM --TAXchicken	Uncategorized Sample	20196-20205_A	Mudpit_Ingel20196_MGFPeaklist (F058870)	Fibrinogen alpha chain OS=Gallus gallus GN=FGA PE=4 SV=1	F1P4V1_CHICK,UPI000012A756,UPI00003AD607,sp|P14448|FIBA_CHICK	TAX_GallusGallus_9031_20141114.fasta,UPARC14_2_GallusG_9031_20140417.fasta	82,438.80	100.00%	22	34	99	0.20%	37.50%	IRPLETL	K	-	15.40%	5.93	42.1	0	5.93					0	1	0	0	2	841.5143	2,202.00		316.81	735	741	Empty	TRUE	
20196-20225 HinckeM --TAXchicken	Uncategorized Sample	20196-20205_A	Mudpit_Ingel20196_MGFPeaklist (F058870)	Fibrinogen alpha chain OS=Gallus gallus GN=FGA PE=4 SV=1	F1P4V1_CHICK,UPI000012A756,UPI00003AD607,sp|P14448|FIBA_CHICK	TAX_GallusGallus_9031_20141114.fasta,UPARC14_2_GallusG_9031_20140417.fasta	82,438.80	100.00%	22	34	99	0.20%	37.50%	KVISGPDGPREEIVEK	K	M	28.90%	13.6	43.1	23.6	4.85					0	0	0	2	2	1,752.95	1,719.00		3,162.92	317	332	Empty	TRUE	UPI0000610C95,UPI000002A9B0,UPI0000111307,UPI000059C9D0,UPI0002A11C16,sp|P14448-2|FIBA_CHICK,UPI0000113356
20196-20225 HinckeM --TAXchicken	Uncategorized Sample	20196-20205_A	Mudpit_Ingel20196_MGFPeaklist (F058870)	Fibrinogen alpha chain OS=Gallus gallus GN=FGA PE=4 SV=1	F1P4V1_CHICK,UPI000012A756,UPI00003AD607,sp|P14448|FIBA_CHICK	TAX_GallusGallus_9031_20141114.fasta,UPARC14_2_GallusG_9031_20140417.fasta	82,438.80	100.00%	22	34	99	0.20%	37.50%	LKPSPEMQAMSAFNNIK	K	Q	99.70%	34.3	44.3	16.7	29.5	38.5	3.72	0.33	0.353	0	0	3	0	2	1,937.95	2,167.00		12,094.80	240	256	Empty	TRUE	UPI0000610C95,UPI000002A9B0,UPI0000111307,UPI000059C9D0,UPI0002A11C16,sp|P14448-2|FIBA_CHICK,UPI0000113356
20196-20225 HinckeM --TAXchicken	Uncategorized Sample	20196-20205_A	Mudpit_Ingel20196_MGFPeaklist (F058870)	Fibrinogen alpha chain OS=Gallus gallus GN=FGA PE=4 SV=1	F1P4V1_CHICK,UPI000012A756,UPI00003AD607,sp|P14448|FIBA_CHICK	TAX_GallusGallus_9031_20141114.fasta,UPARC14_2_GallusG_9031_20140417.fasta	82,438.80	100.00%	22	34	99	0.20%	37.50%	LLPDLESFFTHDSVSTSSR	R	H	99.70%	96.8	44.3	13.3	90.4	62.7	10.1	0.608	0.447	0	0	2	0	2	2,138.04	2,871.00		102642	367	385	Empty	TRUE	UPI0000610C95,UPI000002A9B0,UPI0000111307,UPI000059C9D0,UPI0002A11C16,sp|P14448-2|FIBA_CHICK,UPI0000113356
20196-20225 HinckeM --TAXchicken	Uncategorized Sample	20196-20205_A	Mudpit_Ingel20196_MGFPeaklist (F058870)	Fibrinogen alpha chain OS=Gallus gallus GN=FGA PE=4 SV=1	F1P4V1_CHICK,UPI000012A756,UPI00003AD607,sp|P14448|FIBA_CHICK	TAX_GallusGallus_9031_20141114.fasta,UPARC14_2_GallusG_9031_20140417.fasta	82,438.80	100.00%	22	34	99	0.20%	37.50%	MQGIIDDTDQNYSQR	R	I	99.70%	49.5	41.8	23.8	37.7	36.8	2.96	0.304	0.367	0	0	5	0	2	1,799.79	1,820.00		6,922.48	70	84	Empty	TRUE	UPI0000610C95,UPI000002A9B0,UPI0000111307,UPI000059C9D0,UPI0002A11C16,sp|P14448-2|FIBA_CHICK,UPI0000113356
20196-20225 HinckeM --TAXchicken	Uncategorized Sample	20196-20205_A	Mudpit_Ingel20196_MGFPeaklist (F058870)	Fibrinogen alpha chain OS=Gallus gallus GN=FGA PE=4 SV=1	F1P4V1_CHICK,UPI000012A756,UPI00003AD607,sp|P14448|FIBA_CHICK	TAX_GallusGallus_9031_20141114.fasta,UPARC14_2_GallusG_9031_20140417.fasta	82,438.80	100.00%	22	34	99	0.20%	37.50%	MRPLKDSNVPEHFK	K	L	99.70%	24.4	44.5	23.9	0.19	29.8	1.28	0.211	0.393	0	0	0	4	2	1,713.87	1,593.50		4,260.60	226	239	Empty	TRUE	UPI0000610C95,UPI000002A9B0,UPI0000111307,UPI000059C9D0,UPI0002A11C16,sp|P14448-2|FIBA_CHICK,UPI0000113356
20196-20225 HinckeM --TAXchicken	Uncategorized Sample	20196-20205_A	Mudpit_Ingel20196_MGFPeaklist (F058870)	Fibrinogen alpha chain OS=Gallus gallus GN=FGA PE=4 SV=1	F1P4V1_CHICK,UPI000012A756,UPI00003AD607,sp|P14448|FIBA_CHICK	TAX_GallusGallus_9031_20141114.fasta,UPARC14_2_GallusG_9031_20140417.fasta	82,438.80	100.00%	22	34	99	0.20%	37.50%	NWPICVDDDWGTK	K	C	99.70%	55.5	42.1	16.2	52.6	48.3	6.22	0.516	0.5	0	2	0	0	2	1,605.70	2,596.00		11,252.10	51	63	Empty	TRUE	UPI0000610C95,UPI000002A9B0,UPI0000111307,UPI000059C9D0,UPI0002A11C16,sp|P14448-2|FIBA_CHICK,UPI0000113356
20196-20225 HinckeM --TAXchicken	Uncategorized Sample	20196-20205_A	Mudpit_Ingel20196_MGFPeaklist (F058870)	Fibrinogen alpha chain OS=Gallus gallus GN=FGA PE=4 SV=1	F1P4V1_CHICK,UPI000012A756,UPI00003AD607,sp|P14448|FIBA_CHICK	TAX_GallusGallus_9031_20141114.fasta,UPARC14_2_GallusG_9031_20140417.fasta	82,438.80	100.00%	22	34	99	0.20%	37.50%	QMQVVLERPETDHVAEAR	K	G	99.70%	65.6	44.5	19	55	39.5	3.85	0.362	0.389	0	0	3	6	2	2,107.02	1,939.00		39,582.90	257	274	Empty	TRUE	UPI0000610C95,UPI000002A9B0,UPI0000111307,UPI000059C9D0,UPI0002A11C16,sp|P14448-2|FIBA_CHICK,UPI0000113356
20196-20225 HinckeM --TAXchicken	Uncategorized Sample	20196-20205_A	Mudpit_Ingel20196_MGFPeaklist (F058870)	Fibrinogen alpha chain OS=Gallus gallus GN=FGA PE=4 SV=1	F1P4V1_CHICK,UPI000012A756,UPI00003AD607,sp|P14448|FIBA_CHICK	TAX_GallusGallus_9031_20141114.fasta,UPARC14_2_GallusG_9031_20140417.fasta	82,438.80	100.00%	22	34	99	0.20%	37.50%	RESPSLVDKTSSASSVHR	R	C	99.70%	61.9	44	19.3	51.3	43.3	7.41	0.446	0.389	0	0	0	2	2	1,942.99	1,525.00		1,257.36	292	309	Empty	TRUE	UPI0000610C95,UPI000002A9B0,UPI0000111307,UPI000059C9D0,UPI0002A11C16,sp|P14448-2|FIBA_CHICK,UPI0000113356
20196-20225 HinckeM --TAXchicken	Uncategorized Sample	20196-20205_A	Mudpit_Ingel20196_MGFPeaklist (F058870)	Fibrinogen alpha chain OS=Gallus gallus GN=FGA PE=4 SV=1	F1P4V1_CHICK,UPI000012A756,UPI00003AD607,sp|P14448|FIBA_CHICK	TAX_GallusGallus_9031_20141114.fasta,UPARC14_2_GallusG_9031_20140417.fasta	82,438.80	100.00%	22	34	99	0.20%	37.50%	SFDYQVDKEGYDNIQK	R	H	99.70%	52.7	43.3	14.1	43	42.2	3	0.455	0.406	0	0	6	0	2	1,948.89	2,001.50		18,549.10	187	202	Empty	TRUE	UPI0000610C95,UPI000002A9B0,UPI0000111307,UPI000059C9D0,UPI0002A11C16,sp|P14448-2|FIBA_CHICK,UPI0000113356
20196-20225 HinckeM --TAXchicken	Uncategorized Sample	20196-20205_A	Mudpit_Ingel20196_MGFPeaklist (F058870)	Fibrinogen alpha chain OS=Gallus gallus GN=FGA PE=4 SV=1	F1P4V1_CHICK,UPI000012A756,UPI00003AD607,sp|P14448|FIBA_CHICK	TAX_GallusGallus_9031_20141114.fasta,UPARC14_2_GallusG_9031_20140417.fasta	82,438.80	100.00%	22	34	99	0.20%	37.50%	TRETSEQLGGVQHDQSAEDTPDFK	K	A	99.70%	66	42.5	21.9	57.1	44.3	4.52	0.397	0.292	0	0	0	2	2	2,675.22	1,830.50		6,861.05	464	487	Empty	TRUE	UPI0000610C95,UPI000002A9B0,UPI0000111307,UPI000059C9D0,UPI0002A11C16,sp|P14448-2|FIBA_CHICK,UPI0000113356
20196-20225 HinckeM --TAXchicken	Uncategorized Sample	20196-20205_A	Mudpit_Ingel20196_MGFPeaklist (F058870)	Fibrinogen alpha chain OS=Gallus gallus GN=FGA PE=4 SV=1	F1P4V1_CHICK,UPI000012A756,UPI00003AD607,sp|P14448|FIBA_CHICK	TAX_GallusGallus_9031_20141114.fasta,UPARC14_2_GallusG_9031_20140417.fasta	82,438.80	100.00%	22	34	99	0.20%	37.50%	TVLTSSSSSFNK	K	G	99.70%	61.1	44.6	30.8	44.4	45.3	4.74	0.313	0.542	0	16	0	0	2	1,257.63	1,788.50		53,737.50	441	452	Empty	TRUE	UPI0000610C95,UPI000002A9B0,UPI0000111307,UPI000059C9D0,UPI0002A11C16,sp|P14448-2|FIBA_CHICK,UPI0000113356
20196-20225 HinckeM --TAXchicken	Uncategorized Sample	20196-20205_A	Mudpit_Ingel20196_MGFPeaklist (F058870)	Fibrinogen alpha chain OS=Gallus gallus GN=FGA PE=4 SV=1	F1P4V1_CHICK,UPI000012A756,UPI00003AD607,sp|P14448|FIBA_CHICK	TAX_GallusGallus_9031_20141114.fasta,UPARC14_2_GallusG_9031_20140417.fasta	82,438.80	100.00%	22	34	99	0.20%	37.50%	TVLTSSSSSFNKGGSTFETK	K	S	99.70%	57.3	44.4	14.4	49	49.2	7.42	0.492	0.425	0	0	2	0	2	2,065.01	1,967.00		4,339.42	441	460	Empty	TRUE	UPI0000610C95,UPI000002A9B0,UPI0000111307,UPI000059C9D0,UPI0002A11C16,sp|P14448-2|FIBA_CHICK,UPI0000113356
20196-20225 HinckeM --TAXchicken	Uncategorized Sample	20196-20205_A	Mudpit_Ingel20196_MGFPeaklist (F058870)	Fibrinogen alpha chain OS=Gallus gallus GN=FGA PE=4 SV=1	F1P4V1_CHICK,UPI000012A756,UPI00003AD607,sp|P14448|FIBA_CHICK	TAX_GallusGallus_9031_20141114.fasta,UPARC14_2_GallusG_9031_20140417.fasta	82,438.80	100.00%	22	34	99	0.20%	37.50%	VISGPDGPREEIVEK	K	M	99.70%	25.2	44.1	27.2	9.22	36	0.959	0.197	0.467	0	0	3	0	2	1,624.85	1,799.00		70,528.00	318	332	Empty	TRUE	UPI0000610C95,UPI000002A9B0,UPI0000111307,UPI000059C9D0,UPI0002A11C16,sp|P14448-2|FIBA_CHICK,UPI0000113356
20196-20225 HinckeM --TAXchicken	Uncategorized Sample	20196-20205_A	Mudpit_Ingel20196_MGFPeaklist (F058870)	Fibrinogen alpha chain OS=Gallus gallus GN=FGA PE=4 SV=1	F1P4V1_CHICK,UPI000012A756,UPI00003AD607,sp|P14448|FIBA_CHICK	TAX_GallusGallus_9031_20141114.fasta,UPARC14_2_GallusG_9031_20140417.fasta	82,438.80	100.00%	22	34	99	0.20%	37.50%	ALQNSIQEQVVEMK	K	R	99.70%	46.3	44.9	17.7	38.7	33.4	2	0.296	0.286	0	2	2	0	2	1,632.83	1,992.50		5,475.94	154	167	Empty	TRUE	UPI0000610C95,UPI000002A9B0,UPI0000111307,UPI000059C9D0,UPI0002A11C16,sp|P14448-2|FIBA_CHICK,UPI0000113356
20196-20225 HinckeM --TAXchicken	Uncategorized Sample	20196-20205_A	Mudpit_Ingel20196_MGFPeaklist (F058870)	Fibrinogen alpha chain OS=Gallus gallus GN=FGA PE=4 SV=1	F1P4V1_CHICK,UPI000012A756,UPI00003AD607,sp|P14448|FIBA_CHICK	TAX_GallusGallus_9031_20141114.fasta,UPARC14_2_GallusG_9031_20140417.fasta	82,438.80	100.00%	22	34	99	0.20%	37.50%	EGSTYHFSGTGDFHK	R	L	99.70%	31.8	41.9	15.2	22.4	26.1	0.854	0.192	0.233	0	0	0	2	2	1,669.72	1,796.00		4,880.02	349	363	Empty	TRUE	UPI0000610C95,UPI000002A9B0,UPI0000111307,UPI000059C9D0,UPI0002A11C16,sp|P14448-2|FIBA_CHICK,UPI0000113356
20196-20225 HinckeM --TAXchicken	Uncategorized Sample	20196-20205_A	Mudpit_Ingel20196_MGFPeaklist (F058870)	Fibrinogen alpha chain OS=Gallus gallus GN=FGA PE=4 SV=1	F1P4V1_CHICK,UPI000012A756,UPI00003AD607,sp|P14448|FIBA_CHICK	TAX_GallusGallus_9031_20141114.fasta,UPARC14_2_GallusG_9031_20140417.fasta	82,438.80	100.00%	22	34	99	0.20%	37.50%	EGSTYHFSGTGDFHKLDR	R	L	99.70%	59.5	43.5	15	55.3	44.7	5.49	0.407	0.417	0	0	1	2	2	2,053.94	1,941.00		4,776.58	349	366	Empty	TRUE	UPI0000610C95,UPI000002A9B0,UPI0000111307,UPI000059C9D0,UPI0002A11C16,sp|P14448-2|FIBA_CHICK,UPI0000113356
20196-20225 HinckeM --TAXchicken	Uncategorized Sample	20196-20205_A	Mudpit_Ingel20196_MGFPeaklist (F058870)	Fibrinogen alpha chain OS=Gallus gallus GN=FGA PE=4 SV=1	F1P4V1_CHICK,UPI000012A756,UPI00003AD607,sp|P14448|FIBA_CHICK	TAX_GallusGallus_9031_20141114.fasta,UPARC14_2_GallusG_9031_20140417.fasta	82,438.80	100.00%	22	34	99	0.20%	37.50%	ESPSLVDKTSSASSVHR	R	C	99.70%	63.4	44.7	17.9	52.9	39.8	5.55	0.342	0.353	0	0	1	5	2	1,786.89	1,595.50		6,891.88	293	309	Empty	TRUE	UPI0000610C95,UPI000002A9B0,UPI0000111307,UPI000059C9D0,UPI0002A11C16,sp|P14448-2|FIBA_CHICK,UPI0000113356
20196-20225 HinckeM --TAXchicken	Uncategorized Sample	20196-20205_A	Mudpit_Ingel20196_MGFPeaklist (F058870)	Fibrinogen alpha chain OS=Gallus gallus GN=FGA PE=4 SV=1	F1P4V1_CHICK,UPI000012A756,UPI00003AD607,sp|P14448|FIBA_CHICK	TAX_GallusGallus_9031_20141114.fasta,UPARC14_2_GallusG_9031_20140417.fasta	82,438.80	100.00%	22	34	99	0.20%	37.50%	ETSEQLGGVQHDQSAEDTPDFK	R	A	99.70%	99.4	41.5	0	96.6	39.1	3.72	0.34	0.25	0	0	2	0	2	2,418.07	1,883.50		16,715.50	466	487	Empty	TRUE	UPI0000610C95,UPI000002A9B0,UPI0000111307,UPI000059C9D0,UPI0002A11C16,sp|P14448-2|FIBA_CHICK,UPI0000113356
20196-20225 HinckeM --TAXchicken	Uncategorized Sample	20196-20205_A	Mudpit_Ingel20196_MGFPeaklist (F058870)	Fibrinogen alpha chain OS=Gallus gallus GN=FGA PE=4 SV=1	F1P4V1_CHICK,UPI000012A756,UPI00003AD607,sp|P14448|FIBA_CHICK	TAX_GallusGallus_9031_20141114.fasta,UPARC14_2_GallusG_9031_20140417.fasta	82,438.80	100.00%	22	34	99	0.20%	37.50%	HLTQASSIDMHPDFQTTTLSTLK	K	M	99.70%	58	43.9	13.3	53.6	51	4.4	0.5	0.37	0	0	2	5	2	2,588.27	2,262.00		29,071.40	203	225	Empty	TRUE	UPI0000610C95,UPI000002A9B0,UPI0000111307,UPI000059C9D0,UPI0002A11C16,sp|P14448-2|FIBA_CHICK,UPI0000113356
20196-20225 HinckeM --TAXchicken	Uncategorized Sample	20196-20205_A	Mudpit_Ingel20196_MGFPeaklist (F058870)	Fibrinogen alpha chain OS=Gallus gallus GN=FGA PE=4 SV=1	F1P4V1_CHICK,UPI000012A756,UPI00003AD607,sp|P14448|FIBA_CHICK	TAX_GallusGallus_9031_20141114.fasta,UPARC14_2_GallusG_9031_20140417.fasta	82,438.80	100.00%	22	34	99	0.20%	37.50%	HSIGSSTSSHVTGAGSSHLGTGGK	R	D	99.70%	68	43.9	13.1	61.9	49.8	6.54	0.544	0.396	0	0	0	11	2	2,209.06	1,477.00		40,746.60	386	409	Empty	TRUE	UPI0000610C95,UPI000002A9B0,UPI0000111307,UPI000059C9D0,UPI0002A11C16,sp|P14448-2|FIBA_CHICK,UPI0000113356
20196-20225 HinckeM --TAXchicken	Uncategorized Sample	20196-20205_A	Mudpit_Ingel20196_MGFPeaklist (F058870)	Fibrinogen alpha chain OS=Gallus gallus GN=FGA PE=4 SV=1	F1P4V1_CHICK,UPI000012A756,UPI00003AD607,sp|P14448|FIBA_CHICK	TAX_GallusGallus_9031_20141114.fasta,UPARC14_2_GallusG_9031_20140417.fasta	82,438.80	100.00%	22	34	99	0.20%	37.50%	HSIGSSTSSHVTGAGSSHLGTGGKDK	R	F	99.70%	68.3	44	0	64.6					0	0	0	0	2	2,452.18	1,410.00		6,910.85	386	411	Empty	TRUE	UPI0000610C95,UPI000002A9B0,UPI0000111307,UPI000059C9D0,UPI0002A11C16,sp|P14448-2|FIBA_CHICK,UPI0000113356
20196-20225 HinckeM --TAXchicken	Uncategorized Sample	20196-20205_A	Mudpit_Ingel20196_MGFPeaklist (F058870)	Fibrinogen alpha chain OS=Gallus gallus GN=FGA PE=4 SV=1	F1P4V1_CHICK,UPI000012A756,UPI00003AD607,sp|P14448|FIBA_CHICK	TAX_GallusGallus_9031_20141114.fasta,UPARC14_2_GallusG_9031_20140417.fasta	82,438.80	100.00%	22	34	99	0.20%	37.50%	IRPLETL	K	-	15.40%	5.93	42.1	0	5.93					0	1	0	0	2	841.5143	2,202.00		316.81	735	741	Empty	TRUE	
20196-20225 HinckeM --TAXchicken	Uncategorized Sample	20196-20205_A	Mudpit_Ingel20196_MGFPeaklist (F058870)	Fibrinogen alpha chain OS=Gallus gallus GN=FGA PE=4 SV=1	F1P4V1_CHICK,UPI000012A756,UPI00003AD607,sp|P14448|FIBA_CHICK	TAX_GallusGallus_9031_20141114.fasta,UPARC14_2_GallusG_9031_20140417.fasta	82,438.80	100.00%	22	34	99	0.20%	37.50%	KVISGPDGPREEIVEK	K	M	28.90%	13.6	43.1	23.6	4.85					0	0	0	2	2	1,752.95	1,719.00		3,162.92	317	332	Empty	TRUE	UPI0000610C95,UPI000002A9B0,UPI0000111307,UPI000059C9D0,UPI0002A11C16,sp|P14448-2|FIBA_CHICK,UPI0000113356
20196-20225 HinckeM --TAXchicken	Uncategorized Sample	20196-20205_A	Mudpit_Ingel20196_MGFPeaklist (F058870)	Fibrinogen alpha chain OS=Gallus gallus GN=FGA PE=4 SV=1	F1P4V1_CHICK,UPI000012A756,UPI00003AD607,sp|P14448|FIBA_CHICK	TAX_GallusGallus_9031_20141114.fasta,UPARC14_2_GallusG_9031_20140417.fasta	82,438.80	100.00%	22	34	99	0.20%	37.50%	LKPSPEMQAMSAFNNIK	K	Q	99.70%	34.3	44.3	16.7	29.5	38.5	3.72	0.33	0.353	0	0	3	0	2	1,937.95	2,167.00		12,094.80	240	256	Empty	TRUE	UPI0000610C95,UPI000002A9B0,UPI0000111307,UPI000059C9D0,UPI0002A11C16,sp|P14448-2|FIBA_CHICK,UPI0000113356
20196-20225 HinckeM --TAXchicken	Uncategorized Sample	20196-20205_A	Mudpit_Ingel20196_MGFPeaklist (F058870)	Fibrinogen alpha chain OS=Gallus gallus GN=FGA PE=4 SV=1	F1P4V1_CHICK,UPI000012A756,UPI00003AD607,sp|P14448|FIBA_CHICK	TAX_GallusGallus_9031_20141114.fasta,UPARC14_2_GallusG_9031_20140417.fasta	82,438.80	100.00%	22	34	99	0.20%	37.50%	LLPDLESFFTHDSVSTSSR	R	H	99.70%	96.8	44.3	13.3	90.4	62.7	10.1	0.608	0.447	0	0	2	0	2	2,138.04	2,871.00		102642	367	385	Empty	TRUE	UPI0000610C95,UPI000002A9B0,UPI0000111307,UPI000059C9D0,UPI0002A11C16,sp|P14448-2|FIBA_CHICK,UPI0000113356
20196-20225 HinckeM --TAXchicken	Uncategorized Sample	20196-20205_A	Mudpit_Ingel20196_MGFPeaklist (F058870)	Fibrinogen alpha chain OS=Gallus gallus GN=FGA PE=4 SV=1	F1P4V1_CHICK,UPI000012A756,UPI00003AD607,sp|P14448|FIBA_CHICK	TAX_GallusGallus_9031_20141114.fasta,UPARC14_2_GallusG_9031_20140417.fasta	82,438.80	100.00%	22	34	99	0.20%	37.50%	MQGIIDDTDQNYSQR	R	I	99.70%	49.5	41.8	23.8	37.7	36.8	2.96	0.304	0.367	0	0	5	0	2	1,799.79	1,820.00		6,922.48	70	84	Empty	TRUE	UPI0000610C95,UPI000002A9B0,UPI0000111307,UPI000059C9D0,UPI0002A11C16,sp|P14448-2|FIBA_CHICK,UPI0000113356
20196-20225 HinckeM --TAXchicken	Uncategorized Sample	20196-20205_A	Mudpit_Ingel20196_MGFPeaklist (F058870)	Fibrinogen alpha chain OS=Gallus gallus GN=FGA PE=4 SV=1	F1P4V1_CHICK,UPI000012A756,UPI00003AD607,sp|P14448|FIBA_CHICK	TAX_GallusGallus_9031_20141114.fasta,UPARC14_2_GallusG_9031_20140417.fasta	82,438.80	100.00%	22	34	99	0.20%	37.50%	MRPLKDSNVPEHFK	K	L	99.70%	24.4	44.5	23.9	0.19	29.8	1.28	0.211	0.393	0	0	0	4	2	1,713.87	1,593.50		4,260.60	226	239	Empty	TRUE	UPI0000610C95,UPI000002A9B0,UPI0000111307,UPI000059C9D0,UPI0002A11C16,sp|P14448-2|FIBA_CHICK,UPI0000113356
20196-20225 HinckeM --TAXchicken	Uncategorized Sample	20196-20205_A	Mudpit_Ingel20196_MGFPeaklist (F058870)	Fibrinogen alpha chain OS=Gallus gallus GN=FGA PE=4 SV=1	F1P4V1_CHICK,UPI000012A756,UPI00003AD607,sp|P14448|FIBA_CHICK	TAX_GallusGallus_9031_20141114.fasta,UPARC14_2_GallusG_9031_20140417.fasta	82,438.80	100.00%	22	34	99	0.20%	37.50%	NWPICVDDDWGTK	K	C	99.70%	55.5	42.1	16.2	52.6	48.3	6.22	0.516	0.5	0	2	0	0	2	1,605.70	2,596.00		11,252.10	51	63	Empty	TRUE	UPI0000610C95,UPI000002A9B0,UPI0000111307,UPI000059C9D0,UPI0002A11C16,sp|P14448-2|FIBA_CHICK,UPI0000113356
20196-20225 HinckeM --TAXchicken	Uncategorized Sample	20196-20205_A	Mudpit_Ingel20196_MGFPeaklist (F058870)	Fibrinogen alpha chain OS=Gallus gallus GN=FGA PE=4 SV=1	F1P4V1_CHICK,UPI000012A756,UPI00003AD607,sp|P14448|FIBA_CHICK	TAX_GallusGallus_9031_20141114.fasta,UPARC14_2_GallusG_9031_20140417.fasta	82,438.80	100.00%	22	34	99	0.20%	37.50%	QMQVVLERPETDHVAEAR	K	G	99.70%	65.6	44.5	19	55	39.5	3.85	0.362	0.389	0	0	3	6	2	2,107.02	1,939.00		39,582.90	257	274	Empty	TRUE	UPI0000610C95,UPI000002A9B0,UPI0000111307,UPI000059C9D0,UPI0002A11C16,sp|P14448-2|FIBA_CHICK,UPI0000113356
20196-20225 HinckeM --TAXchicken	Uncategorized Sample	20196-20205_A	Mudpit_Ingel20196_MGFPeaklist (F058870)	Fibrinogen alpha chain OS=Gallus gallus GN=FGA PE=4 SV=1	F1P4V1_CHICK,UPI000012A756,UPI00003AD607,sp|P14448|FIBA_CHICK	TAX_GallusGallus_9031_20141114.fasta,UPARC14_2_GallusG_9031_20140417.fasta	82,438.80	100.00%	22	34	99	0.20%	37.50%	RESPSLVDKTSSASSVHR	R	C	99.70%	61.9	44	19.3	51.3	43.3	7.41	0.446	0.389	0	0	0	2	2	1,942.99	1,525.00		1,257.36	292	309	Empty	TRUE	UPI0000610C95,UPI000002A9B0,UPI0000111307,UPI000059C9D0,UPI0002A11C16,sp|P14448-2|FIBA_CHICK,UPI0000113356
20196-20225 HinckeM --TAXchicken	Uncategorized Sample	20196-20205_A	Mudpit_Ingel20196_MGFPeaklist (F058870)	Fibrinogen alpha chain OS=Gallus gallus GN=FGA PE=4 SV=1	F1P4V1_CHICK,UPI000012A756,UPI00003AD607,sp|P14448|FIBA_CHICK	TAX_GallusGallus_9031_20141114.fasta,UPARC14_2_GallusG_9031_20140417.fasta	82,438.80	100.00%	22	34	99	0.20%	37.50%	SFDYQVDKEGYDNIQK	R	H	99.70%	52.7	43.3	14.1	43	42.2	3	0.455	0.406	0	0	6	0	2	1,948.89	2,001.50		18,549.10	187	202	Empty	TRUE	UPI0000610C95,UPI000002A9B0,UPI0000111307,UPI000059C9D0,UPI0002A11C16,sp|P14448-2|FIBA_CHICK,UPI0000113356
20196-20225 HinckeM --TAXchicken	Uncategorized Sample	20196-20205_A	Mudpit_Ingel20196_MGFPeaklist (F058870)	Fibrinogen alpha chain OS=Gallus gallus GN=FGA PE=4 SV=1	F1P4V1_CHICK,UPI000012A756,UPI00003AD607,sp|P14448|FIBA_CHICK	TAX_GallusGallus_9031_20141114.fasta,UPARC14_2_GallusG_9031_20140417.fasta	82,438.80	100.00%	22	34	99	0.20%	37.50%	TRETSEQLGGVQHDQSAEDTPDFK	K	A	99.70%	66	42.5	21.9	57.1	44.3	4.52	0.397	0.292	0	0	0	2	2	2,675.22	1,830.50		6,861.05	464	487	Empty	TRUE	UPI0000610C95,UPI000002A9B0,UPI0000111307,UPI000059C9D0,UPI0002A11C16,sp|P14448-2|FIBA_CHICK,UPI0000113356
20196-20225 HinckeM --TAXchicken	Uncategorized Sample	20196-20205_A	Mudpit_Ingel20196_MGFPeaklist (F058870)	Fibrinogen alpha chain OS=Gallus gallus GN=FGA PE=4 SV=1	F1P4V1_CHICK,UPI000012A756,UPI00003AD607,sp|P14448|FIBA_CHICK	TAX_GallusGallus_9031_20141114.fasta,UPARC14_2_GallusG_9031_20140417.fasta	82,438.80	100.00%	22	34	99	0.20%	37.50%	TVLTSSSSSFNK	K	G	99.70%	61.1	44.6	30.8	44.4	45.3	4.74	0.313	0.542	0	16	0	0	2	1,257.63	1,788.50		53,737.50	441	452	Empty	TRUE	UPI0000610C95,UPI000002A9B0,UPI0000111307,UPI000059C9D0,UPI0002A11C16,sp|P14448-2|FIBA_CHICK,UPI0000113356
20196-20225 HinckeM --TAXchicken	Uncategorized Sample	20196-20205_A	Mudpit_Ingel20196_MGFPeaklist (F058870)	Fibrinogen alpha chain OS=Gallus gallus GN=FGA PE=4 SV=1	F1P4V1_CHICK,UPI000012A756,UPI00003AD607,sp|P14448|FIBA_CHICK	TAX_GallusGallus_9031_20141114.fasta,UPARC14_2_GallusG_9031_20140417.fasta	82,438.80	100.00%	22	34	99	0.20%	37.50%	TVLTSSSSSFNKGGSTFETK	K	S	99.70%	57.3	44.4	14.4	49	49.2	7.42	0.492	0.425	0	0	2	0	2	2,065.01	1,967.00		4,339.42	441	460	Empty	TRUE	UPI0000610C95,UPI000002A9B0,UPI0000111307,UPI000059C9D0,UPI0002A11C16,sp|P14448-2|FIBA_CHICK,UPI0000113356
20196-20225 HinckeM --TAXchicken	Uncategorized Sample	20196-20205_A	Mudpit_Ingel20196_MGFPeaklist (F058870)	Fibrinogen alpha chain OS=Gallus gallus GN=FGA PE=4 SV=1	F1P4V1_CHICK,UPI000012A756,UPI00003AD607,sp|P14448|FIBA_CHICK	TAX_GallusGallus_9031_20141114.fasta,UPARC14_2_GallusG_9031_20140417.fasta	82,438.80	100.00%	22	34	99	0.20%	37.50%	VISGPDGPREEIVEK	K	M	99.70%	25.2	44.1	27.2	9.22	36	0.959	0.197	0.467	0	0	3	0	2	1,624.85	1,799.00		70,528.00	318	332	Empty	TRUE	UPI0000610C95,UPI000002A9B0,UPI0000111307,UPI000059C9D0,UPI0002A11C16,sp|P14448-2|FIBA_CHICK,UPI0000113356
20196-20225 HinckeM --TAXchicken	Uncategorized Sample	20196-20205_A	Mudpit_Ingel20196_MGFPeaklist (F058870)	Fibrinogen alpha chain OS=Gallus gallus GN=FGA PE=4 SV=1	F1P4V1_CHICK,UPI000012A756,UPI00003AD607,sp|P14448|FIBA_CHICK	TAX_GallusGallus_9031_20141114.fasta,UPARC14_2_GallusG_9031_20140417.fasta	82,438.80	100.00%	22	34	99	0.20%	37.50%	ALQNSIQEQVVEMK	K	R	99.70%	46.3	44.9	17.7	38.7	33.4	2	0.296	0.286	0	2	2	0	2	1,632.83	1,992.50		5,475.94	154	167	Empty	TRUE	UPI0000610C95,UPI000002A9B0,UPI0000111307,UPI000059C9D0,UPI0002A11C16,sp|P14448-2|FIBA_CHICK,UPI0000113356
20196-20225 HinckeM --TAXchicken	Uncategorized Sample	20196-20205_A	Mudpit_Ingel20196_MGFPeaklist (F058870)	Fibrinogen alpha chain OS=Gallus gallus GN=FGA PE=4 SV=1	F1P4V1_CHICK,UPI000012A756,UPI00003AD607,sp|P14448|FIBA_CHICK	TAX_GallusGallus_9031_20141114.fasta,UPARC14_2_GallusG_9031_20140417.fasta	82,438.80	100.00%	22	34	99	0.20%	37.50%	EGSTYHFSGTGDFHK	R	L	99.70%	31.8	41.9	15.2	22.4	26.1	0.854	0.192	0.233	0	0	0	2	2	1,669.72	1,796.00		4,880.02	349	363	Empty	TRUE	UPI0000610C95,UPI000002A9B0,UPI0000111307,UPI000059C9D0,UPI0002A11C16,sp|P14448-2|FIBA_CHICK,UPI0000113356
20196-20225 HinckeM --TAXchicken	Uncategorized Sample	20196-20205_A	Mudpit_Ingel20196_MGFPeaklist (F058870)	Fibrinogen alpha chain OS=Gallus gallus GN=FGA PE=4 SV=1	F1P4V1_CHICK,UPI000012A756,UPI00003AD607,sp|P14448|FIBA_CHICK	TAX_GallusGallus_9031_20141114.fasta,UPARC14_2_GallusG_9031_20140417.fasta	82,438.80	100.00%	22	34	99	0.20%	37.50%	EGSTYHFSGTGDFHKLDR	R	L	99.70%	59.5	43.5	15	55.3	44.7	5.49	0.407	0.417	0	0	1	2	2	2,053.94	1,941.00		4,776.58	349	366	Empty	TRUE	UPI0000610C95,UPI000002A9B0,UPI0000111307,UPI000059C9D0,UPI0002A11C16,sp|P14448-2|FIBA_CHICK,UPI0000113356
20196-20225 HinckeM --TAXchicken	Uncategorized Sample	20196-20205_A	Mudpit_Ingel20196_MGFPeaklist (F058870)	Fibrinogen alpha chain OS=Gallus gallus GN=FGA PE=4 SV=1	F1P4V1_CHICK,UPI000012A756,UPI00003AD607,sp|P14448|FIBA_CHICK	TAX_GallusGallus_9031_20141114.fasta,UPARC14_2_GallusG_9031_20140417.fasta	82,438.80	100.00%	22	34	99	0.20%	37.50%	ESPSLVDKTSSASSVHR	R	C	99.70%	63.4	44.7	17.9	52.9	39.8	5.55	0.342	0.353	0	0	1	5	2	1,786.89	1,595.50		6,891.88	293	309	Empty	TRUE	UPI0000610C95,UPI000002A9B0,UPI0000111307,UPI000059C9D0,UPI0002A11C16,sp|P14448-2|FIBA_CHICK,UPI0000113356
20196-20225 HinckeM --TAXchicken	Uncategorized Sample	20196-20205_A	Mudpit_Ingel20196_MGFPeaklist (F058870)	Fibrinogen alpha chain OS=Gallus gallus GN=FGA PE=4 SV=1	F1P4V1_CHICK,UPI000012A756,UPI00003AD607,sp|P14448|FIBA_CHICK	TAX_GallusGallus_9031_20141114.fasta,UPARC14_2_GallusG_9031_20140417.fasta	82,438.80	100.00%	22	34	99	0.20%	37.50%	ETSEQLGGVQHDQSAEDTPDFK	R	A	99.70%	99.4	41.5	0	96.6	39.1	3.72	0.34	0.25	0	0	2	0	2	2,418.07	1,883.50		16,715.50	466	487	Empty	TRUE	UPI0000610C95,UPI000002A9B0,UPI0000111307,UPI000059C9D0,UPI0002A11C16,sp|P14448-2|FIBA_CHICK,UPI0000113356
20196-20225 HinckeM --TAXchicken	Uncategorized Sample	20196-20205_A	Mudpit_Ingel20196_MGFPeaklist (F058870)	Fibrinogen alpha chain OS=Gallus gallus GN=FGA PE=4 SV=1	F1P4V1_CHICK,UPI000012A756,UPI00003AD607,sp|P14448|FIBA_CHICK	TAX_GallusGallus_9031_20141114.fasta,UPARC14_2_GallusG_9031_20140417.fasta	82,438.80	100.00%	22	34	99	0.20%	37.50%	HLTQASSIDMHPDFQTTTLSTLK	K	M	99.70%	58	43.9	13.3	53.6	51	4.4	0.5	0.37	0	0	2	5	2	2,588.27	2,262.00		29,071.40	203	225	Empty	TRUE	UPI0000610C95,UPI000002A9B0,UPI0000111307,UPI000059C9D0,UPI0002A11C16,sp|P14448-2|FIBA_CHICK,UPI0000113356
20196-20225 HinckeM --TAXchicken	Uncategorized Sample	20196-20205_A	Mudpit_Ingel20196_MGFPeaklist (F058870)	Fibrinogen alpha chain OS=Gallus gallus GN=FGA PE=4 SV=1	F1P4V1_CHICK,UPI000012A756,UPI00003AD607,sp|P14448|FIBA_CHICK	TAX_GallusGallus_9031_20141114.fasta,UPARC14_2_GallusG_9031_20140417.fasta	82,438.80	100.00%	22	34	99	0.20%	37.50%	HSIGSSTSSHVTGAGSSHLGTGGK	R	D	99.70%	68	43.9	13.1	61.9	49.8	6.54	0.544	0.396	0	0	0	11	2	2,209.06	1,477.00		40,746.60	386	409	Empty	TRUE	UPI0000610C95,UPI000002A9B0,UPI0000111307,UPI000059C9D0,UPI0002A11C16,sp|P14448-2|FIBA_CHICK,UPI0000113356
20196-20225 HinckeM --TAXchicken	Uncategorized Sample	20196-20205_A	Mudpit_Ingel20196_MGFPeaklist (F058870)	Fibrinogen alpha chain OS=Gallus gallus GN=FGA PE=4 SV=1	F1P4V1_CHICK,UPI000012A756,UPI00003AD607,sp|P14448|FIBA_CHICK	TAX_GallusGallus_9031_20141114.fasta,UPARC14_2_GallusG_9031_20140417.fasta	82,438.80	100.00%	22	34	99	0.20%	37.50%	HSIGSSTSSHVTGAGSSHLGTGGKDK	R	F	99.70%	68.3	44	0	64.6					0	0	0	0	2	2,452.18	1,410.00		6,910.85	386	411	Empty	TRUE	UPI0000610C95,UPI000002A9B0,UPI0000111307,UPI000059C9D0,UPI0002A11C16,sp|P14448-2|FIBA_CHICK,UPI0000113356
20196-20225 HinckeM --TAXchicken	Uncategorized Sample	20196-20205_A	Mudpit_Ingel20196_MGFPeaklist (F058870)	Fibrinogen alpha chain OS=Gallus gallus GN=FGA PE=4 SV=1	F1P4V1_CHICK,UPI000012A756,UPI00003AD607,sp|P14448|FIBA_CHICK	TAX_GallusGallus_9031_20141114.fasta,UPARC14_2_GallusG_9031_20140417.fasta	82,438.80	100.00%	22	34	99	0.20%	37.50%	IRPLETL	K	-	15.40%	5.93	42.1	0	5.93					0	1	0	0	2	841.5143	2,202.00		316.81	735	741	Empty	TRUE	
20196-20225 HinckeM --TAXchicken	Uncategorized Sample	20196-20205_A	Mudpit_Ingel20196_MGFPeaklist (F058870)	Fibrinogen alpha chain OS=Gallus gallus GN=FGA PE=4 SV=1	F1P4V1_CHICK,UPI000012A756,UPI00003AD607,sp|P14448|FIBA_CHICK	TAX_GallusGallus_9031_20141114.fasta,UPARC14_2_GallusG_9031_20140417.fasta	82,438.80	100.00%	22	34	99	0.20%	37.50%	KVISGPDGPREEIVEK	K	M	28.90%	13.6	43.1	23.6	4.85					0	0	0	2	2	1,752.95	1,719.00		3,162.92	317	332	Empty	TRUE	UPI0000610C95,UPI000002A9B0,UPI0000111307,UPI000059C9D0,UPI0002A11C16,sp|P14448-2|FIBA_CHICK,UPI0000113356
20196-20225 HinckeM --TAXchicken	Uncategorized Sample	20196-20205_A	Mudpit_Ingel20196_MGFPeaklist (F058870)	Fibrinogen alpha chain OS=Gallus gallus GN=FGA PE=4 SV=1	F1P4V1_CHICK,UPI000012A756,UPI00003AD607,sp|P14448|FIBA_CHICK	TAX_GallusGallus_9031_20141114.fasta,UPARC14_2_GallusG_9031_20140417.fasta	82,438.80	100.00%	22	34	99	0.20%	37.50%	LKPSPEMQAMSAFNNIK	K	Q	99.70%	34.3	44.3	16.7	29.5	38.5	3.72	0.33	0.353	0	0	3	0	2	1,937.95	2,167.00		12,094.80	240	256	Empty	TRUE	UPI0000610C95,UPI000002A9B0,UPI0000111307,UPI000059C9D0,UPI0002A11C16,sp|P14448-2|FIBA_CHICK,UPI0000113356
20196-20225 HinckeM --TAXchicken	Uncategorized Sample	20196-20205_A	Mudpit_Ingel20196_MGFPeaklist (F058870)	Fibrinogen alpha chain OS=Gallus gallus GN=FGA PE=4 SV=1	F1P4V1_CHICK,UPI000012A756,UPI00003AD607,sp|P14448|FIBA_CHICK	TAX_GallusGallus_9031_20141114.fasta,UPARC14_2_GallusG_9031_20140417.fasta	82,438.80	100.00%	22	34	99	0.20%	37.50%	LLPDLESFFTHDSVSTSSR	R	H	99.70%	96.8	44.3	13.3	90.4	62.7	10.1	0.608	0.447	0	0	2	0	2	2,138.04	2,871.00		102642	367	385	Empty	TRUE	UPI0000610C95,UPI000002A9B0,UPI0000111307,UPI000059C9D0,UPI0002A11C16,sp|P14448-2|FIBA_CHICK,UPI0000113356
20196-20225 HinckeM --TAXchicken	Uncategorized Sample	20196-20205_A	Mudpit_Ingel20196_MGFPeaklist (F058870)	Fibrinogen alpha chain OS=Gallus gallus GN=FGA PE=4 SV=1	F1P4V1_CHICK,UPI000012A756,UPI00003AD607,sp|P14448|FIBA_CHICK	TAX_GallusGallus_9031_20141114.fasta,UPARC14_2_GallusG_9031_20140417.fasta	82,438.80	100.00%	22	34	99	0.20%	37.50%	MQGIIDDTDQNYSQR	R	I	99.70%	49.5	41.8	23.8	37.7	36.8	2.96	0.304	0.367	0	0	5	0	2	1,799.79	1,820.00		6,922.48	70	84	Empty	TRUE	UPI0000610C95,UPI000002A9B0,UPI0000111307,UPI000059C9D0,UPI0002A11C16,sp|P14448-2|FIBA_CHICK,UPI0000113356
20196-20225 HinckeM --TAXchicken	Uncategorized Sample	20196-20205_A	Mudpit_Ingel20196_MGFPeaklist (F058870)	Fibrinogen alpha chain OS=Gallus gallus GN=FGA PE=4 SV=1	F1P4V1_CHICK,UPI000012A756,UPI00003AD607,sp|P14448|FIBA_CHICK	TAX_GallusGallus_9031_20141114.fasta,UPARC14_2_GallusG_9031_20140417.fasta	82,438.80	100.00%	22	34	99	0.20%	37.50%	MRPLKDSNVPEHFK	K	L	99.70%	24.4	44.5	23.9	0.19	29.8	1.28	0.211	0.393	0	0	0	4	2	1,713.87	1,593.50		4,260.60	226	239	Empty	TRUE	UPI0000610C95,UPI000002A9B0,UPI0000111307,UPI000059C9D0,UPI0002A11C16,sp|P14448-2|FIBA_CHICK,UPI0000113356
20196-20225 HinckeM --TAXchicken	Uncategorized Sample	20196-20205_A	Mudpit_Ingel20196_MGFPeaklist (F058870)	Fibrinogen alpha chain OS=Gallus gallus GN=FGA PE=4 SV=1	F1P4V1_CHICK,UPI000012A756,UPI00003AD607,sp|P14448|FIBA_CHICK	TAX_GallusGallus_9031_20141114.fasta,UPARC14_2_GallusG_9031_20140417.fasta	82,438.80	100.00%	22	34	99	0.20%	37.50%	NWPICVDDDWGTK	K	C	99.70%	55.5	42.1	16.2	52.6	48.3	6.22	0.516	0.5	0	2	0	0	2	1,605.70	2,596.00		11,252.10	51	63	Empty	TRUE	UPI0000610C95,UPI000002A9B0,UPI0000111307,UPI000059C9D0,UPI0002A11C16,sp|P14448-2|FIBA_CHICK,UPI0000113356
20196-20225 HinckeM --TAXchicken	Uncategorized Sample	20196-20205_A	Mudpit_Ingel20196_MGFPeaklist (F058870)	Fibrinogen alpha chain OS=Gallus gallus GN=FGA PE=4 SV=1	F1P4V1_CHICK,UPI000012A756,UPI00003AD607,sp|P14448|FIBA_CHICK	TAX_GallusGallus_9031_20141114.fasta,UPARC14_2_GallusG_9031_20140417.fasta	82,438.80	100.00%	22	34	99	0.20%	37.50%	QMQVVLERPETDHVAEAR	K	G	99.70%	65.6	44.5	19	55	39.5	3.85	0.362	0.389	0	0	3	6	2	2,107.02	1,939.00		39,582.90	257	274	Empty	TRUE	UPI0000610C95,UPI000002A9B0,UPI0000111307,UPI000059C9D0,UPI0002A11C16,sp|P14448-2|FIBA_CHICK,UPI0000113356
20196-20225 HinckeM --TAXchicken	Uncategorized Sample	20196-20205_A	Mudpit_Ingel20196_MGFPeaklist (F058870)	Fibrinogen alpha chain OS=Gallus gallus GN=FGA PE=4 SV=1	F1P4V1_CHICK,UPI000012A756,UPI00003AD607,sp|P14448|FIBA_CHICK	TAX_GallusGallus_9031_20141114.fasta,UPARC14_2_GallusG_9031_20140417.fasta	82,438.80	100.00%	22	34	99	0.20%	37.50%	RESPSLVDKTSSASSVHR	R	C	99.70%	61.9	44	19.3	51.3	43.3	7.41	0.446	0.389	0	0	0	2	2	1,942.99	1,525.00		1,257.36	292	309	Empty	TRUE	UPI0000610C95,UPI000002A9B0,UPI0000111307,UPI000059C9D0,UPI0002A11C16,sp|P14448-2|FIBA_CHICK,UPI0000113356
20196-20225 HinckeM --TAXchicken	Uncategorized Sample	20196-20205_A	Mudpit_Ingel20196_MGFPeaklist (F058870)	Fibrinogen alpha chain OS=Gallus gallus GN=FGA PE=4 SV=1	F1P4V1_CHICK,UPI000012A756,UPI00003AD607,sp|P14448|FIBA_CHICK	TAX_GallusGallus_9031_20141114.fasta,UPARC14_2_GallusG_9031_20140417.fasta	82,438.80	100.00%	22	34	99	0.20%	37.50%	SFDYQVDKEGYDNIQK	R	H	99.70%	52.7	43.3	14.1	43	42.2	3	0.455	0.406	0	0	6	0	2	1,948.89	2,001.50		18,549.10	187	202	Empty	TRUE	UPI0000610C95,UPI000002A9B0,UPI0000111307,UPI000059C9D0,UPI0002A11C16,sp|P14448-2|FIBA_CHICK,UPI0000113356
20196-20225 HinckeM --TAXchicken	Uncategorized Sample	20196-20205_A	Mudpit_Ingel20196_MGFPeaklist (F058870)	Fibrinogen alpha chain OS=Gallus gallus GN=FGA PE=4 SV=1	F1P4V1_CHICK,UPI000012A756,UPI00003AD607,sp|P14448|FIBA_CHICK	TAX_GallusGallus_9031_20141114.fasta,UPARC14_2_GallusG_9031_20140417.fasta	82,438.80	100.00%	22	34	99	0.20%	37.50%	TRETSEQLGGVQHDQSAEDTPDFK	K	A	99.70%	66	42.5	21.9	57.1	44.3	4.52	0.397	0.292	0	0	0	2	2	2,675.22	1,830.50		6,861.05	464	487	Empty	TRUE	UPI0000610C95,UPI000002A9B0,UPI0000111307,UPI000059C9D0,UPI0002A11C16,sp|P14448-2|FIBA_CHICK,UPI0000113356
20196-20225 HinckeM --TAXchicken	Uncategorized Sample	20196-20205_A	Mudpit_Ingel20196_MGFPeaklist (F058870)	Fibrinogen alpha chain OS=Gallus gallus GN=FGA PE=4 SV=1	F1P4V1_CHICK,UPI000012A756,UPI00003AD607,sp|P14448|FIBA_CHICK	TAX_GallusGallus_9031_20141114.fasta,UPARC14_2_GallusG_9031_20140417.fasta	82,438.80	100.00%	22	34	99	0.20%	37.50%	TVLTSSSSSFNK	K	G	99.70%	61.1	44.6	30.8	44.4	45.3	4.74	0.313	0.542	0	16	0	0	2	1,257.63	1,788.50		53,737.50	441	452	Empty	TRUE	UPI0000610C95,UPI000002A9B0,UPI0000111307,UPI000059C9D0,UPI0002A11C16,sp|P14448-2|FIBA_CHICK,UPI0000113356
20196-20225 HinckeM --TAXchicken	Uncategorized Sample	20196-20205_A	Mudpit_Ingel20196_MGFPeaklist (F058870)	Fibrinogen alpha chain OS=Gallus gallus GN=FGA PE=4 SV=1	F1P4V1_CHICK,UPI000012A756,UPI00003AD607,sp|P14448|FIBA_CHICK	TAX_GallusGallus_9031_20141114.fasta,UPARC14_2_GallusG_9031_20140417.fasta	82,438.80	100.00%	22	34	99	0.20%	37.50%	TVLTSSSSSFNKGGSTFETK	K	S	99.70%	57.3	44.4	14.4	49	49.2	7.42	0.492	0.425	0	0	2	0	2	2,065.01	1,967.00		4,339.42	441	460	Empty	TRUE	UPI0000610C95,UPI000002A9B0,UPI0000111307,UPI000059C9D0,UPI0002A11C16,sp|P14448-2|FIBA_CHICK,UPI0000113356
20196-20225 HinckeM --TAXchicken	Uncategorized Sample	20196-20205_A	Mudpit_Ingel20196_MGFPeaklist (F058870)	Fibrinogen alpha chain OS=Gallus gallus GN=FGA PE=4 SV=1	F1P4V1_CHICK,UPI000012A756,UPI00003AD607,sp|P14448|FIBA_CHICK	TAX_GallusGallus_9031_20141114.fasta,UPARC14_2_GallusG_9031_20140417.fasta	82,438.80	100.00%	22	34	99	0.20%	37.50%	VISGPDGPREEIVEK	K	M	99.70%	25.2	44.1	27.2	9.22	36	0.959	0.197	0.467	0	0	3	0	2	1,624.85	1,799.00		70,528.00	318	332	Empty	TRUE	UPI0000610C95,UPI000002A9B0,UPI0000111307,UPI000059C9D0,UPI0002A11C16,sp|P14448-2|FIBA_CHICK,UPI0000113356
20196-20225 HinckeM --TAXchicken	Uncategorized Sample	20196-20205_A	Mudpit_Ingel20196_MGFPeaklist (F058870)	status=inactive	F1NU40_CHICK,F1P336_CHICK,NUCL_CHICK,UPI00001308C6,UPI00003AEE22,UPI0000611CEE,UPI0000ECB1B6,UPI0002C881E0	TAX_GallusGallus_9031_20141114.fasta,UPARC14_2_GallusG_9031_20140417.fasta	67,216.00	100.00%	9	9	12	0.02%	12.90%	AMVIDYTGEK	R	S	99.70%	55.1	43.5	20.3	44.1	28.3	1.92	0.286	0.4	0	2	0	0	2	1,142.54	1,770.00		1,248.60	437	446	Empty	TRUE	
20196-20225 HinckeM --TAXchicken	Uncategorized Sample	20196-20205_A	Mudpit_Ingel20196_MGFPeaklist (F058870)	status=inactive	F1NU40_CHICK,F1P336_CHICK,NUCL_CHICK,UPI00001308C6,UPI00003AEE22,UPI0000611CEE,UPI0000ECB1B6,UPI0002C881E0	TAX_GallusGallus_9031_20141114.fasta,UPARC14_2_GallusG_9031_20140417.fasta	67,216.00	100.00%	9	9	12	0.02%	12.90%	AMVIDYTGEKSQQESQK	R	G	7.00%	8.96	43	17.2	1.65					0	0	1	0	2	1,959.89	1,795.00		322.45	437	453	Empty	TRUE	
20196-20225 HinckeM --TAXchicken	Uncategorized Sample	20196-20205_A	Mudpit_Ingel20196_MGFPeaklist (F058870)	status=inactive	F1NU40_CHICK,F1P336_CHICK,NUCL_CHICK,UPI00001308C6,UPI00003AEE22,UPI0000611CEE,UPI0000ECB1B6,UPI0002C881E0	TAX_GallusGallus_9031_20141114.fasta,UPARC14_2_GallusG_9031_20140417.fasta	67,216.00	100.00%	9	9	12	0.02%	12.90%	EALNSCNNTEIEGR	K	A	99.70%	64.8	43	15.2	58.9	39	5.8	0.418	0.357	0	1	0	0	2	1,606.71	1,637.00		439.8	513	526	Empty	TRUE	
20196-20225 HinckeM --TAXchicken	Uncategorized Sample	20196-20205_A	Mudpit_Ingel20196_MGFPeaklist (F058870)	status=inactive	F1NU40_CHICK,F1P336_CHICK,NUCL_CHICK,UPI00001308C6,UPI00003AEE22,UPI0000611CEE,UPI0000ECB1B6,UPI0002C881E0	TAX_GallusGallus_9031_20141114.fasta,UPARC14_2_GallusG_9031_20140417.fasta	67,216.00	100.00%	9	9	12	0.02%	12.90%	GLSEDTTEETLR	R	E	99.70%	56.9	44.5	24.8	42.2	34.9	3.28	0.433	0.333	0	1	0	0	2	1,350.64	1,815.00		357.61	559	570	Empty	TRUE	
20196-20225 HinckeM --TAXchicken	Uncategorized Sample	20196-20205_A	Mudpit_Ingel20196_MGFPeaklist (F058870)	status=inactive	F1NU40_CHICK,F1P336_CHICK,NUCL_CHICK,UPI00001308C6,UPI00003AEE22,UPI0000611CEE,UPI0000ECB1B6,UPI0002C881E0	TAX_GallusGallus_9031_20141114.fasta,UPARC14_2_GallusG_9031_20140417.fasta	67,216.00	100.00%	9	9	12	0.02%	12.90%	KTETPASAFSLFVKNLTPTK	K	D	6.70%	13.2	41.8	15.3	0					0	0	0	1	2	2,181.18	1,889.00		152.45	273	292	Empty	TRUE	
20196-20225 HinckeM --TAXchicken	Uncategorized Sample	20196-20205_A	Mudpit_Ingel20196_MGFPeaklist (F058870)	status=inactive	F1NU40_CHICK,F1P336_CHICK,NUCL_CHICK,UPI00001308C6,UPI00003AEE22,UPI0000611CEE,UPI0000ECB1B6,UPI0002C881E0	TAX_GallusGallus_9031_20141114.fasta,UPARC14_2_GallusG_9031_20140417.fasta	67,216.00	100.00%	9	9	12	0.02%	12.90%	LEFSSPSWQK	R	G	99.70%	49.8	44.4	23.8	36.6	34.7	2.24	0.346	0.45	0	1	0	0	2	1,208.59	2,226.00		372.48	530	539	Empty	TRUE	
20196-20225 HinckeM --TAXchicken	Uncategorized Sample	20196-20205_A	Mudpit_Ingel20196_MGFPeaklist (F058870)	status=inactive	F1NU40_CHICK,F1P336_CHICK,NUCL_CHICK,UPI00001308C6,UPI00003AEE22,UPI0000611CEE,UPI0000ECB1B6,UPI0002C881E0	TAX_GallusGallus_9031_20141114.fasta,UPARC14_2_GallusG_9031_20140417.fasta	67,216.00	100.00%	9	9	12	0.02%	12.90%	NLTPTKDYEELR	K	T	99.70%	71.4	44.7	34.5	51.3	38.4	4.12	0.44	0.458	0	0	2	0	2	1,478.75	1,883.50		1,478.57	287	298	Empty	TRUE	
20196-20225 HinckeM --TAXchicken	Uncategorized Sample	20196-20205_A	Mudpit_Ingel20196_MGFPeaklist (F058870)	status=inactive	F1NU40_CHICK,F1P336_CHICK,NUCL_CHICK,UPI00001308C6,UPI00003AEE22,UPI0000611CEE,UPI0000ECB1B6,UPI0002C881E0	TAX_GallusGallus_9031_20141114.fasta,UPARC14_2_GallusG_9031_20140417.fasta	67,216.00	100.00%	9	9	12	0.02%	12.90%	NVFENALEVR	K	L	99.70%	34.8	45.1	25	23.6	38.8	3.66	0.405	0.5	0	2	0	0	2	1,190.62	2,445.50		4,160.63	389	398	Empty	TRUE	
20196-20225 HinckeM --TAXchicken	Uncategorized Sample	20196-20205_A	Mudpit_Ingel20196_MGFPeaklist (F058870)	status=inactive	F1NU40_CHICK,F1P336_CHICK,NUCL_CHICK,UPI00001308C6,UPI00003AEE22,UPI0000611CEE,UPI0000ECB1B6,UPI0002C881E0	TAX_GallusGallus_9031_20141114.fasta,UPARC14_2_GallusG_9031_20140417.fasta	67,216.00	100.00%	9	9	12	0.02%	12.90%	TETPASAFSLFVK	K	N	99.70%	53.3	44.2	21.5	49.8	48.7	5.4	0.489	0.5	0	1	0	0	2	1,397.73	2,845.00		1,174.33	274	286	Empty	TRUE	
20196-20225 HinckeM --TAXchicken	Uncategorized Sample	20196-20205_A	Mudpit_Ingel20196_MGFPeaklist (F058870)	status=inactive	F1NU40_CHICK,F1P336_CHICK,NUCL_CHICK,UPI00001308C6,UPI00003AEE22,UPI0000611CEE,UPI0000ECB1B6,UPI0002C881E0	TAX_GallusGallus_9031_20141114.fasta,UPARC14_2_GallusG_9031_20140417.fasta	67,216.00	100.00%	9	9	12	0.02%	12.90%	AMVIDYTGEK	R	S	99.70%	55.1	43.5	20.3	44.1	28.3	1.92	0.286	0.4	0	2	0	0	2	1,142.54	1,770.00		1,248.60	437	446	Empty	TRUE	
20196-20225 HinckeM --TAXchicken	Uncategorized Sample	20196-20205_A	Mudpit_Ingel20196_MGFPeaklist (F058870)	status=inactive	F1NU40_CHICK,F1P336_CHICK,NUCL_CHICK,UPI00001308C6,UPI00003AEE22,UPI0000611CEE,UPI0000ECB1B6,UPI0002C881E0	TAX_GallusGallus_9031_20141114.fasta,UPARC14_2_GallusG_9031_20140417.fasta	67,216.00	100.00%	9	9	12	0.02%	12.90%	AMVIDYTGEKSQQESQK	R	G	7.00%	8.96	43	17.2	1.65					0	0	1	0	2	1,959.89	1,795.00		322.45	437	453	Empty	TRUE	
20196-20225 HinckeM --TAXchicken	Uncategorized Sample	20196-20205_A	Mudpit_Ingel20196_MGFPeaklist (F058870)	status=inactive	F1NU40_CHICK,F1P336_CHICK,NUCL_CHICK,UPI00001308C6,UPI00003AEE22,UPI0000611CEE,UPI0000ECB1B6,UPI0002C881E0	TAX_GallusGallus_9031_20141114.fasta,UPARC14_2_GallusG_9031_20140417.fasta	67,216.00	100.00%	9	9	12	0.02%	12.90%	EALNSCNNTEIEGR	K	A	99.70%	64.8	43	15.2	58.9	39	5.8	0.418	0.357	0	1	0	0	2	1,606.71	1,637.00		439.8	513	526	Empty	TRUE	
20196-20225 HinckeM --TAXchicken	Uncategorized Sample	20196-20205_A	Mudpit_Ingel20196_MGFPeaklist (F058870)	status=inactive	F1NU40_CHICK,F1P336_CHICK,NUCL_CHICK,UPI00001308C6,UPI00003AEE22,UPI0000611CEE,UPI0000ECB1B6,UPI0002C881E0	TAX_GallusGallus_9031_20141114.fasta,UPARC14_2_GallusG_9031_20140417.fasta	67,216.00	100.00%	9	9	12	0.02%	12.90%	GLSEDTTEETLR	R	E	99.70%	56.9	44.5	24.8	42.2	34.9	3.28	0.433	0.333	0	1	0	0	2	1,350.64	1,815.00		357.61	559	570	Empty	TRUE	
20196-20225 HinckeM --TAXchicken	Uncategorized Sample	20196-20205_A	Mudpit_Ingel20196_MGFPeaklist (F058870)	status=inactive	F1NU40_CHICK,F1P336_CHICK,NUCL_CHICK,UPI00001308C6,UPI00003AEE22,UPI0000611CEE,UPI0000ECB1B6,UPI0002C881E0	TAX_GallusGallus_9031_20141114.fasta,UPARC14_2_GallusG_9031_20140417.fasta	67,216.00	100.00%	9	9	12	0.02%	12.90%	KTETPASAFSLFVKNLTPTK	K	D	6.70%	13.2	41.8	15.3	0					0	0	0	1	2	2,181.18	1,889.00		152.45	273	292	Empty	TRUE	
20196-20225 HinckeM --TAXchicken	Uncategorized Sample	20196-20205_A	Mudpit_Ingel20196_MGFPeaklist (F058870)	status=inactive	F1NU40_CHICK,F1P336_CHICK,NUCL_CHICK,UPI00001308C6,UPI00003AEE22,UPI0000611CEE,UPI0000ECB1B6,UPI0002C881E0	TAX_GallusGallus_9031_20141114.fasta,UPARC14_2_GallusG_9031_20140417.fasta	67,216.00	100.00%	9	9	12	0.02%	12.90%	LEFSSPSWQK	R	G	99.70%	49.8	44.4	23.8	36.6	34.7	2.24	0.346	0.45	0	1	0	0	2	1,208.59	2,226.00		372.48	530	539	Empty	TRUE	
20196-20225 HinckeM --TAXchicken	Uncategorized Sample	20196-20205_A	Mudpit_Ingel20196_MGFPeaklist (F058870)	status=inactive	F1NU40_CHICK,F1P336_CHICK,NUCL_CHICK,UPI00001308C6,UPI00003AEE22,UPI0000611CEE,UPI0000ECB1B6,UPI0002C881E0	TAX_GallusGallus_9031_20141114.fasta,UPARC14_2_GallusG_9031_20140417.fasta	67,216.00	100.00%	9	9	12	0.02%	12.90%	NLTPTKDYEELR	K	T	99.70%	71.4	44.7	34.5	51.3	38.4	4.12	0.44	0.458	0	0	2	0	2	1,478.75	1,883.50		1,478.57	287	298	Empty	TRUE	
20196-20225 HinckeM --TAXchicken	Uncategorized Sample	20196-20205_A	Mudpit_Ingel20196_MGFPeaklist (F058870)	status=inactive	F1NU40_CHICK,F1P336_CHICK,NUCL_CHICK,UPI00001308C6,UPI00003AEE22,UPI0000611CEE,UPI0000ECB1B6,UPI0002C881E0	TAX_GallusGallus_9031_20141114.fasta,UPARC14_2_GallusG_9031_20140417.fasta	67,216.00	100.00%	9	9	12	0.02%	12.90%	NVFENALEVR	K	L	99.70%	34.8	45.1	25	23.6	38.8	3.66	0.405	0.5	0	2	0	0	2	1,190.62	2,445.50		4,160.63	389	398	Empty	TRUE	
20196-20225 HinckeM --TAXchicken	Uncategorized Sample	20196-20205_A	Mudpit_Ingel20196_MGFPeaklist (F058870)	status=inactive	F1NU40_CHICK,F1P336_CHICK,NUCL_CHICK,UPI00001308C6,UPI00003AEE22,UPI0000611CEE,UPI0000ECB1B6,UPI0002C881E0	TAX_GallusGallus_9031_20141114.fasta,UPARC14_2_GallusG_9031_20140417.fasta	67,216.00	100.00%	9	9	12	0.02%	12.90%	TETPASAFSLFVK	K	N	99.70%	53.3	44.2	21.5	49.8	48.7	5.4	0.489	0.5	0	1	0	0	2	1,397.73	2,845.00		1,174.33	274	286	Empty	TRUE	
20196-20225 HinckeM --TAXchicken	Uncategorized Sample	20196-20205_A	Mudpit_Ingel20196_MGFPeaklist (F058870)	status=inactive	F1NU40_CHICK,F1P336_CHICK,NUCL_CHICK,UPI00001308C6,UPI00003AEE22,UPI0000611CEE,UPI0000ECB1B6,UPI0002C881E0	TAX_GallusGallus_9031_20141114.fasta,UPARC14_2_GallusG_9031_20140417.fasta	67,216.00	100.00%	9	9	12	0.02%	12.90%	AMVIDYTGEK	R	S	99.70%	55.1	43.5	20.3	44.1	28.3	1.92	0.286	0.4	0	2	0	0	2	1,142.54	1,770.00		1,248.60	437	446	Empty	TRUE	
20196-20225 HinckeM --TAXchicken	Uncategorized Sample	20196-20205_A	Mudpit_Ingel20196_MGFPeaklist (F058870)	status=inactive	F1NU40_CHICK,F1P336_CHICK,NUCL_CHICK,UPI00001308C6,UPI00003AEE22,UPI0000611CEE,UPI0000ECB1B6,UPI0002C881E0	TAX_GallusGallus_9031_20141114.fasta,UPARC14_2_GallusG_9031_20140417.fasta	67,216.00	100.00%	9	9	12	0.02%	12.90%	AMVIDYTGEKSQQESQK	R	G	7.00%	8.96	43	17.2	1.65					0	0	1	0	2	1,959.89	1,795.00		322.45	437	453	Empty	TRUE	
20196-20225 HinckeM --TAXchicken	Uncategorized Sample	20196-20205_A	Mudpit_Ingel20196_MGFPeaklist (F058870)	status=inactive	F1NU40_CHICK,F1P336_CHICK,NUCL_CHICK,UPI00001308C6,UPI00003AEE22,UPI0000611CEE,UPI0000ECB1B6,UPI0002C881E0	TAX_GallusGallus_9031_20141114.fasta,UPARC14_2_GallusG_9031_20140417.fasta	67,216.00	100.00%	9	9	12	0.02%	12.90%	EALNSCNNTEIEGR	K	A	99.70%	64.8	43	15.2	58.9	39	5.8	0.418	0.357	0	1	0	0	2	1,606.71	1,637.00		439.8	513	526	Empty	TRUE	
20196-20225 HinckeM --TAXchicken	Uncategorized Sample	20196-20205_A	Mudpit_Ingel20196_MGFPeaklist (F058870)	status=inactive	F1NU40_CHICK,F1P336_CHICK,NUCL_CHICK,UPI00001308C6,UPI00003AEE22,UPI0000611CEE,UPI0000ECB1B6,UPI0002C881E0	TAX_GallusGallus_9031_20141114.fasta,UPARC14_2_GallusG_9031_20140417.fasta	67,216.00	100.00%	9	9	12	0.02%	12.90%	GLSEDTTEETLR	R	E	99.70%	56.9	44.5	24.8	42.2	34.9	3.28	0.433	0.333	0	1	0	0	2	1,350.64	1,815.00		357.61	559	570	Empty	TRUE	
20196-20225 HinckeM --TAXchicken	Uncategorized Sample	20196-20205_A	Mudpit_Ingel20196_MGFPeaklist (F058870)	status=inactive	F1NU40_CHICK,F1P336_CHICK,NUCL_CHICK,UPI00001308C6,UPI00003AEE22,UPI0000611CEE,UPI0000ECB1B6,UPI0002C881E0	TAX_GallusGallus_9031_20141114.fasta,UPARC14_2_GallusG_9031_20140417.fasta	67,216.00	100.00%	9	9	12	0.02%	12.90%	KTETPASAFSLFVKNLTPTK	K	D	6.70%	13.2	41.8	15.3	0					0	0	0	1	2	2,181.18	1,889.00		152.45	273	292	Empty	TRUE	
20196-20225 HinckeM --TAXchicken	Uncategorized Sample	20196-20205_A	Mudpit_Ingel20196_MGFPeaklist (F058870)	status=inactive	F1NU40_CHICK,F1P336_CHICK,NUCL_CHICK,UPI00001308C6,UPI00003AEE22,UPI0000611CEE,UPI0000ECB1B6,UPI0002C881E0	TAX_GallusGallus_9031_20141114.fasta,UPARC14_2_GallusG_9031_20140417.fasta	67,216.00	100.00%	9	9	12	0.02%	12.90%	LEFSSPSWQK	R	G	99.70%	49.8	44.4	23.8	36.6	34.7	2.24	0.346	0.45	0	1	0	0	2	1,208.59	2,226.00		372.48	530	539	Empty	TRUE	
20196-20225 HinckeM --TAXchicken	Uncategorized Sample	20196-20205_A	Mudpit_Ingel20196_MGFPeaklist (F058870)	status=inactive	F1NU40_CHICK,F1P336_CHICK,NUCL_CHICK,UPI00001308C6,UPI00003AEE22,UPI0000611CEE,UPI0000ECB1B6,UPI0002C881E0	TAX_GallusGallus_9031_20141114.fasta,UPARC14_2_GallusG_9031_20140417.fasta	67,216.00	100.00%	9	9	12	0.02%	12.90%	NLTPTKDYEELR	K	T	99.70%	71.4	44.7	34.5	51.3	38.4	4.12	0.44	0.458	0	0	2	0	2	1,478.75	1,883.50		1,478.57	287	298	Empty	TRUE	
20196-20225 HinckeM --TAXchicken	Uncategorized Sample	20196-20205_A	Mudpit_Ingel20196_MGFPeaklist (F058870)	status=inactive	F1NU40_CHICK,F1P336_CHICK,NUCL_CHICK,UPI00001308C6,UPI00003AEE22,UPI0000611CEE,UPI0000ECB1B6,UPI0002C881E0	TAX_GallusGallus_9031_20141114.fasta,UPARC14_2_GallusG_9031_20140417.fasta	67,216.00	100.00%	9	9	12	0.02%	12.90%	NVFENALEVR	K	L	99.70%	34.8	45.1	25	23.6	38.8	3.66	0.405	0.5	0	2	0	0	2	1,190.62	2,445.50		4,160.63	389	398	Empty	TRUE	
20196-20225 HinckeM --TAXchicken	Uncategorized Sample	20196-20205_A	Mudpit_Ingel20196_MGFPeaklist (F058870)	status=inactive	F1NU40_CHICK,F1P336_CHICK,NUCL_CHICK,UPI00001308C6,UPI00003AEE22,UPI0000611CEE,UPI0000ECB1B6,UPI0002C881E0	TAX_GallusGallus_9031_20141114.fasta,UPARC14_2_GallusG_9031_20140417.fasta	67,216.00	100.00%	9	9	12	0.02%	12.90%	TETPASAFSLFVK	K	N	99.70%	53.3	44.2	21.5	49.8	48.7	5.4	0.489	0.5	0	1	0	0	2	1,397.73	2,845.00		1,174.33	274	286	Empty	TRUE	
20196-20225 HinckeM --TAXchicken	Uncategorized Sample	20196-20205_A	Mudpit_Ingel20196_MGFPeaklist (F058870)	status=inactive	F1NU40_CHICK,F1P336_CHICK,NUCL_CHICK,UPI00001308C6,UPI00003AEE22,UPI0000611CEE,UPI0000ECB1B6,UPI0002C881E0	TAX_GallusGallus_9031_20141114.fasta,UPARC14_2_GallusG_9031_20140417.fasta	67,216.00	100.00%	9	9	12	0.02%	12.90%	AMVIDYTGEK	R	S	99.70%	55.1	43.5	20.3	44.1	28.3	1.92	0.286	0.4	0	2	0	0	2	1,142.54	1,770.00		1,248.60	437	446	Empty	TRUE	
20196-20225 HinckeM --TAXchicken	Uncategorized Sample	20196-20205_A	Mudpit_Ingel20196_MGFPeaklist (F058870)	status=inactive	F1NU40_CHICK,F1P336_CHICK,NUCL_CHICK,UPI00001308C6,UPI00003AEE22,UPI0000611CEE,UPI0000ECB1B6,UPI0002C881E0	TAX_GallusGallus_9031_20141114.fasta,UPARC14_2_GallusG_9031_20140417.fasta	67,216.00	100.00%	9	9	12	0.02%	12.90%	AMVIDYTGEKSQQESQK	R	G	7.00%	8.96	43	17.2	1.65					0	0	1	0	2	1,959.89	1,795.00		322.45	437	453	Empty	TRUE	
20196-20225 HinckeM --TAXchicken	Uncategorized Sample	20196-20205_A	Mudpit_Ingel20196_MGFPeaklist (F058870)	status=inactive	F1NU40_CHICK,F1P336_CHICK,NUCL_CHICK,UPI00001308C6,UPI00003AEE22,UPI0000611CEE,UPI0000ECB1B6,UPI0002C881E0	TAX_GallusGallus_9031_20141114.fasta,UPARC14_2_GallusG_9031_20140417.fasta	67,216.00	100.00%	9	9	12	0.02%	12.90%	EALNSCNNTEIEGR	K	A	99.70%	64.8	43	15.2	58.9	39	5.8	0.418	0.357	0	1	0	0	2	1,606.71	1,637.00		439.8	513	526	Empty	TRUE	
20196-20225 HinckeM --TAXchicken	Uncategorized Sample	20196-20205_A	Mudpit_Ingel20196_MGFPeaklist (F058870)	status=inactive	F1NU40_CHICK,F1P336_CHICK,NUCL_CHICK,UPI00001308C6,UPI00003AEE22,UPI0000611CEE,UPI0000ECB1B6,UPI0002C881E0	TAX_GallusGallus_9031_20141114.fasta,UPARC14_2_GallusG_9031_20140417.fasta	67,216.00	100.00%	9	9	12	0.02%	12.90%	GLSEDTTEETLR	R	E	99.70%	56.9	44.5	24.8	42.2	34.9	3.28	0.433	0.333	0	1	0	0	2	1,350.64	1,815.00		357.61	559	570	Empty	TRUE	
20196-20225 HinckeM --TAXchicken	Uncategorized Sample	20196-20205_A	Mudpit_Ingel20196_MGFPeaklist (F058870)	status=inactive	F1NU40_CHICK,F1P336_CHICK,NUCL_CHICK,UPI00001308C6,UPI00003AEE22,UPI0000611CEE,UPI0000ECB1B6,UPI0002C881E0	TAX_GallusGallus_9031_20141114.fasta,UPARC14_2_GallusG_9031_20140417.fasta	67,216.00	100.00%	9	9	12	0.02%	12.90%	KTETPASAFSLFVKNLTPTK	K	D	6.70%	13.2	41.8	15.3	0					0	0	0	1	2	2,181.18	1,889.00		152.45	273	292	Empty	TRUE	
20196-20225 HinckeM --TAXchicken	Uncategorized Sample	20196-20205_A	Mudpit_Ingel20196_MGFPeaklist (F058870)	status=inactive	F1NU40_CHICK,F1P336_CHICK,NUCL_CHICK,UPI00001308C6,UPI00003AEE22,UPI0000611CEE,UPI0000ECB1B6,UPI0002C881E0	TAX_GallusGallus_9031_20141114.fasta,UPARC14_2_GallusG_9031_20140417.fasta	67,216.00	100.00%	9	9	12	0.02%	12.90%	LEFSSPSWQK	R	G	99.70%	49.8	44.4	23.8	36.6	34.7	2.24	0.346	0.45	0	1	0	0	2	1,208.59	2,226.00		372.48	530	539	Empty	TRUE	
20196-20225 HinckeM --TAXchicken	Uncategorized Sample	20196-20205_A	Mudpit_Ingel20196_MGFPeaklist (F058870)	status=inactive	F1NU40_CHICK,F1P336_CHICK,NUCL_CHICK,UPI00001308C6,UPI00003AEE22,UPI0000611CEE,UPI0000ECB1B6,UPI0002C881E0	TAX_GallusGallus_9031_20141114.fasta,UPARC14_2_GallusG_9031_20140417.fasta	67,216.00	100.00%	9	9	12	0.02%	12.90%	NLTPTKDYEELR	K	T	99.70%	71.4	44.7	34.5	51.3	38.4	4.12	0.44	0.458	0	0	2	0	2	1,478.75	1,883.50		1,478.57	287	298	Empty	TRUE	
20196-20225 HinckeM --TAXchicken	Uncategorized Sample	20196-20205_A	Mudpit_Ingel20196_MGFPeaklist (F058870)	status=inactive	F1NU40_CHICK,F1P336_CHICK,NUCL_CHICK,UPI00001308C6,UPI00003AEE22,UPI0000611CEE,UPI0000ECB1B6,UPI0002C881E0	TAX_GallusGallus_9031_20141114.fasta,UPARC14_2_GallusG_9031_20140417.fasta	67,216.00	100.00%	9	9	12	0.02%	12.90%	NVFENALEVR	K	L	99.70%	34.8	45.1	25	23.6	38.8	3.66	0.405	0.5	0	2	0	0	2	1,190.62	2,445.50		4,160.63	389	398	Empty	TRUE	
20196-20225 HinckeM --TAXchicken	Uncategorized Sample	20196-20205_A	Mudpit_Ingel20196_MGFPeaklist (F058870)	status=inactive	F1NU40_CHICK,F1P336_CHICK,NUCL_CHICK,UPI00001308C6,UPI00003AEE22,UPI0000611CEE,UPI0000ECB1B6,UPI0002C881E0	TAX_GallusGallus_9031_20141114.fasta,UPARC14_2_GallusG_9031_20140417.fasta	67,216.00	100.00%	9	9	12	0.02%	12.90%	TETPASAFSLFVK	K	N	99.70%	53.3	44.2	21.5	49.8	48.7	5.4	0.489	0.5	0	1	0	0	2	1,397.73	2,845.00		1,174.33	274	286	Empty	TRUE	
20196-20225 HinckeM --TAXchicken	Uncategorized Sample	20196-20205_A	Mudpit_Ingel20196_MGFPeaklist (F058870)	status=inactive	F1NU40_CHICK,F1P336_CHICK,NUCL_CHICK,UPI00001308C6,UPI00003AEE22,UPI0000611CEE,UPI0000ECB1B6,UPI0002C881E0	TAX_GallusGallus_9031_20141114.fasta,UPARC14_2_GallusG_9031_20140417.fasta	67,216.00	100.00%	9	9	12	0.02%	12.90%	AMVIDYTGEK	R	S	99.70%	55.1	43.5	20.3	44.1	28.3	1.92	0.286	0.4	0	2	0	0	2	1,142.54	1,770.00		1,248.60	437	446	Empty	TRUE	

20196-20225 HinckeM –TAXchicken	Uncategorized Sample	20196-20205_A	Mudpit_Ingel20196_MGFPeaklist (F058870)	status=inactive	F1NU40_CHICK,F1P336_CHICK,NUCL_CHICK,UPI00001308C6,UPI00003AEE22,UPI0000611CEE,UPI0000ECB1B6,UPI0002C881E0	TAX_GallusGallus_9031_20141114.fasta,UPARC14_2_GallusG_9031_20140417.fasta	67,216.00	100.00%	9	9	12	0.02%	12.90%	AMVIDYTGEKSQQESQK	R	G	7.00%	8.96	43	17.2	1.65					0	0	1	0	2	1,959.89	1,795.00		322.45	437	453	Empty	TRUE	
20196-20225 HinckeM –TAXchicken	Uncategorized Sample	20196-20205_A	Mudpit_Ingel20196_MGFPeaklist (F058870)	status=inactive	F1NU40_CHICK,F1P336_CHICK,NUCL_CHICK,UPI00001308C6,UPI00003AEE22,UPI0000611CEE,UPI0000ECB1B6,UPI0002C881E0	TAX_GallusGallus_9031_20141114.fasta,UPARC14_2_GallusG_9031_20140417.fasta	67,216.00	100.00%	9	9	12	0.02%	12.90%	EALNSCNNTEIEGR	K	A	99.70%	64.8	43	15.2	58.9	39	5.8	0.418	0.357	0	1	0	0	2	1,606.71	1,637.00		439.8	513	526	Empty	TRUE	
20196-20225 HinckeM –TAXchicken	Uncategorized Sample	20196-20205_A	Mudpit_Ingel20196_MGFPeaklist (F058870)	status=inactive	F1NU40_CHICK,F1P336_CHICK,NUCL_CHICK,UPI00001308C6,UPI00003AEE22,UPI0000611CEE,UPI0000ECB1B6,UPI0002C881E0	TAX_GallusGallus_9031_20141114.fasta,UPARC14_2_GallusG_9031_20140417.fasta	67,216.00	100.00%	9	9	12	0.02%	12.90%	GLSEDTTEETLR	R	E	99.70%	56.9	44.5	24.8	42.2	34.9	3.28	0.433	0.333	0	1	0	0	2	1,350.64	1,815.00		357.61	559	570	Empty	TRUE	
20196-20225 HinckeM –TAXchicken	Uncategorized Sample	20196-20205_A	Mudpit_Ingel20196_MGFPeaklist (F058870)	status=inactive	F1NU40_CHICK,F1P336_CHICK,NUCL_CHICK,UPI00001308C6,UPI00003AEE22,UPI0000611CEE,UPI0000ECB1B6,UPI0002C881E0	TAX_GallusGallus_9031_20141114.fasta,UPARC14_2_GallusG_9031_20140417.fasta	67,216.00	100.00%	9	9	12	0.02%	12.90%	KTETPASAFSLFVKNLTPTK	K	D	6.70%	13.2	41.8	15.3	0					0	0	0	1	2	2,181.18	1,889.00		152.45	273	292	Empty	TRUE	
20196-20225 HinckeM –TAXchicken	Uncategorized Sample	20196-20205_A	Mudpit_Ingel20196_MGFPeaklist (F058870)	status=inactive	F1NU40_CHICK,F1P336_CHICK,NUCL_CHICK,UPI00001308C6,UPI00003AEE22,UPI0000611CEE,UPI0000ECB1B6,UPI0002C881E0	TAX_GallusGallus_9031_20141114.fasta,UPARC14_2_GallusG_9031_20140417.fasta	67,216.00	100.00%	9	9	12	0.02%	12.90%	LEFSSPSWQK	R	G	99.70%	49.8	44.4	23.8	36.6	34.7	2.24	0.346	0.45	0	1	0	0	2	1,208.59	2,226.00		372.48	530	539	Empty	TRUE	
20196-20225 HinckeM –TAXchicken	Uncategorized Sample	20196-20205_A	Mudpit_Ingel20196_MGFPeaklist (F058870)	status=inactive	F1NU40_CHICK,F1P336_CHICK,NUCL_CHICK,UPI00001308C6,UPI00003AEE22,UPI0000611CEE,UPI0000ECB1B6,UPI0002C881E0	TAX_GallusGallus_9031_20141114.fasta,UPARC14_2_GallusG_9031_20140417.fasta	67,216.00	100.00%	9	9	12	0.02%	12.90%	NLTPTKDYEELR	K	T	99.70%	71.4	44.7	34.5	51.3	38.4	4.12	0.44	0.458	0	0	2	0	2	1,478.75	1,883.50		1,478.57	287	298	Empty	TRUE	
20196-20225 HinckeM –TAXchicken	Uncategorized Sample	20196-20205_A	Mudpit_Ingel20196_MGFPeaklist (F058870)	status=inactive	F1NU40_CHICK,F1P336_CHICK,NUCL_CHICK,UPI00001308C6,UPI00003AEE22,UPI0000611CEE,UPI0000ECB1B6,UPI0002C881E0	TAX_GallusGallus_9031_20141114.fasta,UPARC14_2_GallusG_9031_20140417.fasta	67,216.00	100.00%	9	9	12	0.02%	12.90%	NVFENALEVR	K	L	99.70%	34.8	45.1	25	23.6	38.8	3.66	0.405	0.5	0	2	0	0	2	1,190.62	2,445.50		4,160.63	389	398	Empty	TRUE	
20196-20225 HinckeM –TAXchicken	Uncategorized Sample	20196-20205_A	Mudpit_Ingel20196_MGFPeaklist (F058870)	status=inactive	F1NU40_CHICK,F1P336_CHICK,NUCL_CHICK,UPI00001308C6,UPI00003AEE22,UPI0000611CEE,UPI0000ECB1B6,UPI0002C881E0	TAX_GallusGallus_9031_20141114.fasta,UPARC14_2_GallusG_9031_20140417.fasta	67,216.00	100.00%	9	9	12	0.02%	12.90%	TETPASAFSLFVK	K	N	99.70%	53.3	44.2	21.5	49.8	48.7	5.4	0.489	0.5	0	1	0	0	2	1,397.73	2,845.00		1,174.33	274	286	Empty	TRUE	
20196-20225 HinckeM –TAXchicken	Uncategorized Sample	20196-20205_A	Mudpit_Ingel20196_MGFPeaklist (F058870)	status=inactive	F1NU40_CHICK,F1P336_CHICK,NUCL_CHICK,UPI00001308C6,UPI00003AEE22,UPI0000611CEE,UPI0000ECB1B6,UPI0002C881E0	TAX_GallusGallus_9031_20141114.fasta,UPARC14_2_GallusG_9031_20140417.fasta	67,216.00	100.00%	9	9	12	0.02%	12.90%	AMVIDYTGEK	R	S	99.70%	55.1	43.5	20.3	44.1	28.3	1.92	0.286	0.4	0	2	0	0	2	1,142.54	1,770.00		1,248.60	437	446	Empty	TRUE	
20196-20225 HinckeM –TAXchicken	Uncategorized Sample	20196-20205_A	Mudpit_Ingel20196_MGFPeaklist (F058870)	status=inactive	F1NU40_CHICK,F1P336_CHICK,NUCL_CHICK,UPI00001308C6,UPI00003AEE22,UPI0000611CEE,UPI0000ECB1B6,UPI0002C881E0	TAX_GallusGallus_9031_20141114.fasta,UPARC14_2_GallusG_9031_20140417.fasta	67,216.00	100.00%	9	9	12	0.02%	12.90%	AMVIDYTGEKSQQESQK	R	G	7.00%	8.96	43	17.2	1.65					0	0	1	0	2	1,959.89	1,795.00		322.45	437	453	Empty	TRUE	
20196-20225 HinckeM –TAXchicken	Uncategorized Sample	20196-20205_A	Mudpit_Ingel20196_MGFPeaklist (F058870)	status=inactive	F1NU40_CHICK,F1P336_CHICK,NUCL_CHICK,UPI00001308C6,UPI00003AEE22,UPI0000611CEE,UPI0000ECB1B6,UPI0002C881E0	TAX_GallusGallus_9031_20141114.fasta,UPARC14_2_GallusG_9031_20140417.fasta	67,216.00	100.00%	9	9	12	0.02%	12.90%	EALNSCNNTEIEGR	K	A	99.70%	64.8	43	15.2	58.9	39	5.8	0.418	0.357	0	1	0	0	2	1,606.71	1,637.00		439.8	513	526	Empty	TRUE	
20196-20225 HinckeM –TAXchicken	Uncategorized Sample	20196-20205_A	Mudpit_Ingel20196_MGFPeaklist (F058870)	status=inactive	F1NU40_CHICK,F1P336_CHICK,NUCL_CHICK,UPI00001308C6,UPI00003AEE22,UPI0000611CEE,UPI0000ECB1B6,UPI0002C881E0	TAX_GallusGallus_9031_20141114.fasta,UPARC14_2_GallusG_9031_20140417.fasta	67,216.00	100.00%	9	9	12	0.02%	12.90%	GLSEDTTEETLR	R	E	99.70%	56.9	44.5	24.8	42.2	34.9	3.28	0.433	0.333	0	1	0	0	2	1,350.64	1,815.00		357.61	559	570	Empty	TRUE	
20196-20225 HinckeM –TAXchicken	Uncategorized Sample	20196-20205_A	Mudpit_Ingel20196_MGFPeaklist (F058870)	status=inactive	F1NU40_CHICK,F1P336_CHICK,NUCL_CHICK,UPI00001308C6,UPI00003AEE22,UPI0000611CEE,UPI0000ECB1B6,UPI0002C881E0	TAX_GallusGallus_9031_20141114.fasta,UPARC14_2_GallusG_9031_20140417.fasta	67,216.00	100.00%	9	9	12	0.02%	12.90%	KTETPASAFSLFVKNLTPTK	K	D	6.70%	13.2	41.8	15.3	0					0	0	0	1	2	2,181.18	1,889.00		152.45	273	292	Empty	TRUE	
20196-20225 HinckeM –TAXchicken	Uncategorized Sample	20196-20205_A	Mudpit_Ingel20196_MGFPeaklist (F058870)	status=inactive	F1NU40_CHICK,F1P336_CHICK,NUCL_CHICK,UPI00001308C6,UPI00003AEE22,UPI0000611CEE,UPI0000ECB1B6,UPI0002C881E0	TAX_GallusGallus_9031_20141114.fasta,UPARC14_2_GallusG_9031_20140417.fasta	67,216.00	100.00%	9	9	12	0.02%	12.90%	LEFSSPSWQK	R	G	99.70%	49.8	44.4	23.8	36.6	34.7	2.24	0.346	0.45	0	1	0	0	2	1,208.59	2,226.00		372.48	530	539	Empty	TRUE	
20196-20225 HinckeM –TAXchicken	Uncategorized Sample	20196-20205_A	Mudpit_Ingel20196_MGFPeaklist (F058870)	status=inactive	F1NU40_CHICK,F1P336_CHICK,NUCL_CHICK,UPI00001308C6,UPI00003AEE22,UPI0000611CEE,UPI0000ECB1B6,UPI0002C881E0	TAX_GallusGallus_9031_20141114.fasta,UPARC14_2_GallusG_9031_20140417.fasta	67,216.00	100.00%	9	9	12	0.02%	12.90%	NLTPTKDYEELR	K	T	99.70%	71.4	44.7	34.5	51.3	38.4	4.12	0.44	0.458	0	0	2	0	2	1,478.75	1,883.50		1,478.57	287	298	Empty	TRUE	
20196-20225 HinckeM –TAXchicken	Uncategorized Sample	20196-20205_A	Mudpit_Ingel20196_MGFPeaklist (F058870)	status=inactive	F1NU40_CHICK,F1P336_CHICK,NUCL_CHICK,UPI00001308C6,UPI00003AEE22,UPI0000611CEE,UPI0000ECB1B6,UPI0002C881E0	TAX_GallusGallus_9031_20141114.fasta,UPARC14_2_GallusG_9031_20140417.fasta	67,216.00	100.00%	9	9	12	0.02%	12.90%	NVFENALEVR	K	L	99.70%	34.8	45.1	25	23.6	38.8	3.66	0.405	0.5	0	2	0	0	2	1,190.62	2,445.50		4,160.63	389	398	Empty	TRUE	
20196-20225 HinckeM –TAXchicken	Uncategorized Sample	20196-20205_A	Mudpit_Ingel20196_MGFPeaklist (F058870)	status=inactive	F1NU40_CHICK,F1P336_CHICK,NUCL_CHICK,UPI00001308C6,UPI00003AEE22,UPI0000611CEE,UPI0000ECB1B6,UPI0002C881E0	TAX_GallusGallus_9031_20141114.fasta,UPARC14_2_GallusG_9031_20140417.fasta	67,216.00	100.00%	9	9	12	0.02%	12.90%	TETPASAFSLFVK	K	N	99.70%	53.3	44.2	21.5	49.8	48.7	5.4	0.489	0.5	0	1	0	0	2	1,397.73	2,845.00		1,174.33	274	286	Empty	TRUE	
20196-20225 HinckeM –TAXchicken	Uncategorized Sample	20196-20205_A	Mudpit_Ingel20196_MGFPeaklist (F058870)	Uncharacterized protein OS=Gallus gallus GN=ALDH7A1 PE=3 SV=1	E1C4W4_CHICK,UPI00003AF00F,UPI00003AF010,UPI00003AF011,UPI0000611E3D,UPI0000E81A00,UPI000240C9F9	TAX_GallusGallus_9031_20141114.fasta,UPARC14_2_GallusG_9031_20140417.fasta	50,972.50	9.70%	2	2	2	0.00%	4.85%	MDLLSFTGSTEVGKQVALMVQER	R	F	24.50%	9.34	43.9	20.9	2.07					0	0	0	0	2	2,556.27	1,914.00		43.72	264	286	Empty	TRUE	
20196-20225 HinckeM –TAXchicken	Uncategorized Sample	20196-20205_A	Mudpit_Ingel20196_MGFPeaklist (F058870)	Uncharacterized protein OS=Gallus gallus GN=ALDH7A1 PE=3 SV=1	E1C4W4_CHICK,UPI00003AF00F,UPI00003AF010,UPI00003AF011,UPI0000611E3D,UPI0000E81A00,UPI000240C9F9	TAX_GallusGallus_9031_20141114.fasta,UPARC14_2_GallusG_9031_20140417.fasta	50,972.50	9.70%	2	2	2	0.00%	4.85%	QVALMVQERFGR	K	S	17.20%	3.37	45	21.9	-0.28					0	1	0	0	2	1,418.71	2,151.00		603.81	278	289	Empty	TRUE	
20196-20225 HinckeM –TAXchicken	Uncategorized Sample	20196-20205_A	Mudpit_Ingel20196_MGFPeaklist (F058870)	Uncharacterized protein OS=Gallus gallus GN=ALDH7A1 PE=3 SV=1	E1C4W4_CHICK,UPI00003AF00F,UPI00003AF010,UPI00003AF011,UPI0000611E3D,UPI0000E81A00,UPI000240C9F9	TAX_GallusGallus_9031_20141114.fasta,UPARC14_2_GallusG_9031_20140417.fasta	50,972.50	9.70%	2	2	2	0.00%	4.85%	MDLLSFTGSTEVGKQVALMVQER	R	F	24.50%	9.34	43.9	20.9	2.07					0	0	0	0	2	2,556.27	1,914.00		43.72	264	286	Empty	TRUE	
20196-20225 HinckeM –TAXchicken	Uncategorized Sample	20196-20205_A	Mudpit_Ingel20196_MGFPeaklist (F058870)	Uncharacterized protein OS=Gallus gallus GN=ALDH7A1 PE=3 SV=1	E1C4W4_CHICK,UPI00003AF00F,UPI00003AF010,UPI00003AF011,UPI0000611E3D,UPI0000E81A00,UPI000240C9F9	TAX_GallusGallus_9031_20141114.fasta,UPARC14_2_GallusG_9031_20140417.fasta	50,972.50	9.70%	2	2	2	0.00%	4.85%	QVALMVQERFGR	K	S	17.20%	3.37	45	21.9	-0.28					0	1	0	0	2	1,418.71	2,151.00		603.81	278	289	Empty	TRUE	
20196-20225 HinckeM –TAXchicken	Uncategorized Sample	20196-20205_A	Mudpit_Ingel20196_MGFPeaklist (F058870)	Uncharacterized protein OS=Gallus gallus GN=ALDH7A1 PE=3 SV=1	E1C4W4_CHICK,UPI00003AF00F,UPI00003AF010,UPI00003AF011,UPI0000611E3D,UPI0000E81A00,UPI000240C9F9	TAX_GallusGallus_9031_20141114.fasta,UPARC14_2_GallusG_9031_20140417.fasta	50,972.50	9.70%	2	2	2	0.00%	4.85%	MDLLSFTGSTEVGKQVALMVQER	R	F	24.50%	9.34	43.9	20.9	2.07					0	0	0	0	2	2,556.27	1,914.00		43.72	264	286	Empty	TRUE	
20196-20225 HinckeM –TAXchicken	Uncategorized Sample	20196-20205_A	Mudpit_Ingel20196_MGFPeaklist (F058870)	Uncharacterized protein OS=Gallus gallus GN=ALDH7A1 PE=3 SV=1	E1C4W4_CHICK,UPI00003AF00F,UPI00003AF010,UPI00003AF011,UPI0000611E3D,UPI0000E81A00,UPI000240C9F9	TAX_GallusGallus_9031_20141114.fasta,UPARC14_2_GallusG_9031_20140417.fasta	50,972.50	9.70%	2	2	2	0.00%	4.85%	QVALMVQERFGR	K	S	17.20%	3.37	45	21.9	-0.28					0	1	0	0	2	1,418.71	2,151.00		603.81	278	289	Empty	TRUE	
20196-20225 HinckeM –TAXchicken	Uncategorized Sample	20196-20205_A	Mudpit_Ingel20196_MGFPeaklist (F058870)	Uncharacterized protein OS=Gallus gallus GN=ALDH7A1 PE=3 SV=1	E1C4W4_CHICK,UPI00003AF00F,UPI00003AF010,UPI00003AF011,UPI0000611E3D,UPI0000E81A00,UPI000240C9F9	TAX_GallusGallus_9031_20141114.fasta,UPARC14_2_GallusG_9031_20140417.fasta	50,972.50	9.70%	2	2	2	0.00%	4.85%	MDLLSFTGSTEVGKQVALMVQER	R	F	24.50%	9.34	43.9	20.9	2.07					0	0	0	0	2	2,556.27	1,914.00		43.72	264	286	Empty	TRUE	
20196-20225 HinckeM –TAXchicken	Uncategorized Sample	20196-20205_A	Mudpit_Ingel20196_MGFPeaklist (F058870)	Uncharacterized protein OS=Gallus gallus GN=ALDH7A1 PE=3 SV=1	E1C4W4_CHICK,UPI00003AF00F,UPI00003AF010,UPI00003AF011,UPI0000611E3D,UPI0000E81A00,UPI000240C9F9	TAX_GallusGallus_9031_20141114.fasta,UPARC14_2_GallusG_9031_20140417.fasta	50,972.50	9.70%	2	2	2	0.00%	4.85%	QVALMVQERFGR	K	S	17.20%	3.37	45	21.9	-0.28					0	1	0	0	2	1,418.71	2,151.00		603.81	278	289	Empty	TRUE	
20196-20225 HinckeM –TAXchicken	Uncategorized Sample	20196-20205_A	Mudpit_Ingel20196_MGFPeaklist (F058870)	Uncharacterized protein OS=Gallus gallus GN=ALDH7A1 PE=3 SV=1	E1C4W4_CHICK,UPI00003AF00F,UPI00003AF010,UPI00003AF011,UPI0000611E3D,UPI0000E81A00,UPI000240C9F9	TAX_GallusGallus_9031_20141114.fasta,UPARC14_2_GallusG_9031_20140417.fasta	50,972.50	9.70%	2	2	2	0.00%	4.85%	MDLLSFTGSTEVGKQVALMVQER	R	F	24.50%	9.34	43.9	20.9	2.07					0	0	0	0	2	2,556.27	1,914.00		43.72	264	286	Empty	TRUE	
20196-20225 HinckeM –TAXchicken	Uncategorized Sample	20196-20205_A	Mudpit_Ingel20196_MGFPeaklist (F058870)	Uncharacterized protein OS=Gallus gallus GN=ALDH7A1 PE=3 SV=1	E1C4W4_CHICK,UPI00003AF00F,UPI00003AF010,UPI00003AF011,UPI0000611E3D,UPI0000E81A00,UPI000240C9F9	TAX_GallusGallus_9031_20141114.fasta,UPARC14_2_GallusG_9031_20140417.fasta	50,972.50	9.70%	2	2	2	0.00%	4.85%	QVALMVQERFGR	K	S	17.20%	3.37	45	21.9	-0.28					0	1	0	0	2	1,418.71	2,151.00		603.81	278	289	Empty	TRUE	
20196-20225 HinckeM –TAXchicken	Uncategorized Sample	20196-20205_A	Mudpit_Ingel20196_MGFPeaklist (F058870)	Uncharacterized protein OS=Gallus gallus GN=ALDH7A1 PE=3 SV=1	E1C4W4_CHICK,UPI00003AF00F,UPI00003AF010,UPI00003AF011,UPI0000611E3D,UPI0000E81A00,UPI000240C9F9	TAX_GallusGallus_9031_20141114.fasta,UPARC14_2_GallusG_9031_20140417.fasta	50,972.50	9.70%	2	2	2	0.00%	4.85%	MDLLSFTGSTEVGKQVALMVQER	R	F	24.50%	9.34	43.9	20.9	2.07					0	0	0	0	2	2,556.27	1,914.00		43.72	264	286	Empty	TRUE	
20196-20225 HinckeM –TAXchicken	Uncategorized Sample	20196-20205_A	Mudpit_Ingel20196_MGFPeaklist (F058870)	Uncharacterized protein OS=Gallus gallus GN=ALDH7A1 PE=3 SV=1	E1C4W4_CHICK,UPI00003AF00F,UPI00003AF010,UPI00003AF011,UPI0000611E3D,UPI0000E81A00,UPI000240C9F9	TAX_GallusGallus_9031_20141114.fasta,UPARC14_2_GallusG_9031_20140417.fasta	50,972.50	9.70%	2	2	2	0.00%	4.85%	QVALMVQERFGR	K	S	17.20%	3.37	45	21.9	-0.28					0	1	0	0	2	1,418.71	2,151.00		603.81	278	289	Empty	TRUE	
20196-20225 HinckeM –TAXchicken	Uncategorized Sample	20196-20205_A	Mudpit_Ingel20196_MGFPeaklist (F058870)	Uncharacterized protein OS=Gallus gallus GN=ALDH7A1 PE=3 SV=1	E1C4W4_CHICK,UPI00003AF00F,UPI00003AF010,UPI00003AF011,UPI0000611E3D,UPI0000E81A00,UPI000240C9F9	TAX_GallusGallus_9031_20141114.fasta,UPARC14_2_GallusG_9031_20140417.fasta	50,972.50	9.70%	2	2	2	0.00%	4.85%	MDLLSFTGSTEVGKQVALMVQER	R	F	24.50%	9.34	43.9	20.9	2.07					0	0	0	0	2	2,556.27	1,914.00		43.72	264	286	Empty	TRUE	
20196-20225 HinckeM –TAXchicken	Uncategorized Sample	20196-20205_A	Mudpit_Ingel20196_MGFPeaklist (F058870)	Uncharacterized protein OS=Gallus gallus GN=ALDH7A1 PE=3 SV=1	E1C4W4_CHICK,UPI00003AF00F,UPI00003AF010,UPI00003AF011,UPI0000611E3D,UPI0000E81A00,UPI000240C9F9	TAX_GallusGallus_9031_20141114.fasta,UPARC14_2_GallusG_9031_20140417.fasta	50,972.50	9.70%	2	2	2	0.00%	4.85%	QVALMVQERFGR	K	S	17.20%	3.37	45	21.9	-0.28					0	1	0	0	2	1,418.71	2,151.00		603.81	278	289	Empty	TRUE	
20196-20225 HinckeM –TAXchicken	Uncategorized Sample	20196-20205_A	Mudpit_Ingel20196_MGFPeaklist (F058870)	Uncharacterized protein OS=Gallus gallus GN=TTC37 PE=4 SV=2	E1C576_CHICK,UPI0000449AC4,UPI0000E81999,UPI0000ECC33B,UPI000240C876,UPI0002C870B7,UPI00035058F5	TAX_GallusGallus_9031_20141114.fasta,UPARC14_2_GallusG_9031_20140417.fasta	174,635.00	97.60%	1	1	2	0.00%	0.63%	AVADLQAALR	K	A	99.70%	42.3	43.5	27.2	26.9	26.4	0.886	0.246	0.35	0	2	0	0	2	1,027.59	2,166.00		379.25	581	590	Empty	TRUE	
20196-20225 HinckeM –TAXchicken	Uncategorized Sample	20196-20205_A	Mudpit_Ingel20196_MGFPeaklist (F058870)	Uncharacterized protein OS=Gallus gallus GN=TTC37 PE=4 SV=2	E1C576_CHICK,UPI0000449AC4,UPI0000E81999,UPI0000ECC33B,UPI000240C876,UPI0002C870B7,UPI00035058F5	TAX_GallusGallus_9031_20141114.fasta,UPARC14_2_GallusG_9031_20140417.fasta	174,635.00	97.60%	1	1	2	0.00%	0.63%	AVADLQAALR	K	A	99.70%	42.3	43.5	27.2	26.9	26.4	0.886	0.246	0.35	0	2	0	0	2	1,027.59	2,166.00		379.25	581	590	Empty	TRUE	
20196-20225 HinckeM –TAXchicken	Uncategorized Sample	20196-20205_A	Mudpit_Ingel20196_MGFPeaklist (F058870)	Uncharacterized protein OS=Gallus gallus GN=TTC37 PE=4 SV=2	E1C576_CHICK,UPI0000449AC4,UPI0000E81999,UPI0000ECC33B,UPI000240C876,UPI0002C870B7,UPI00035058F5	TAX_GallusGallus_9031_20141114.fasta,UPARC14_2_GallusG_9031_20140417.fasta	174,635.00	97.60%	1	1	2	0.00%	0.63%	AVADLQAALR	K	A	99.70%	42.3	43.5	27.2	26.9	26.4	0.886	0.246	0.35	0	2	0	0	2	1,027.59	2,166.00		379.25	581	590	Empty	TRUE	
20196-20225 HinckeM –TAXchicken	Uncategorized Sample	20196-20205_A	Mudpit_Ingel20196_MGFPeaklist (F058870)	Uncharacterized protein OS=Gallus gallus GN=TTC37 PE=4 SV=2	E1C576_CHICK,UPI0000449AC4,UPI0000E81999,UPI0000ECC33B,UPI000240C876,UPI0002C870B7,UPI00035058F5	TAX_GallusGallus_9031_20141114.fasta,UPARC14_2_GallusG_9031_20140417.fasta	174,635.00	97.60%	1	1	2	0.00%	0.63%	AVADLQAALR	K	A	99.70%	42.3	43.5	27.2	26.9	26.4	0.886	0.246	0.35	0	2	0	0	2	1,027.59	2,166.00		379.25	581	590	Empty	TRUE	
20196-20225 HinckeM –TAXchicken	Uncategorized Sample	20196-20205_A	Mudpit_Ingel20196_MGFPeaklist (F058870)	Uncharacterized protein OS=Gallus gallus GN=TTC37 PE=4 SV=2	E1C576_CHICK,UPI0000449AC4,UPI0000E81999,UPI0000ECC33B,UPI000240C876,UPI0002C870B7,UPI00035058F5	TAX_GallusGallus_9031_20141114.fasta,UPARC14_2_GallusG_9031_20140417.fasta	174,635.00	97.60%	1	1	2	0.00%	0.63%	AVADLQAALR	K	A	99.70%	42.3	43.5	27.2	26.9	26.4	0.886	0.246	0.35	0	2	0	0	2	1,027.59	2,166.00		379.25	581	590	Empty	TRUE	
20196-20225 HinckeM –TAXchicken	Uncategorized Sample	20196-20205_A	Mudpit_Ingel20196_MGFPeaklist (F058870)	Uncharacterized protein OS=Gallus gallus GN=TTC37 PE=4 SV=2	E1C576_CHICK,UPI0000449AC4,UPI0000E81999,UPI0000ECC33B,UPI000240C876,UPI0002C870B7,UPI00035058F5	TAX_GallusGallus_9031_20141114.fasta,UPARC14_2_GallusG_9031_20140417.fasta	174,635.00	97.60%	1	1	2	0.00%	0.63%	AVADLQAALR	K	A	99.70%	42.3	43.5	27.2	26.9	26.4	0.886	0.246	0.35	0	2	0	0	2	1,027.59	2,166.00		379.25	581	590	Empty	TRUE	
20196-20225 HinckeM –TAXchicken	Uncategorized Sample	20196-20205_A	Mudpit_Ingel20196_MGFPeaklist (F058870)	Uncharacterized protein OS=Gallus gallus GN=TTC37 PE=4 SV=2	E1C576_CHICK,UPI0000449AC4,UPI0000E81999,UPI0000ECC33B,UPI000240C876,UPI0002C870B7,UPI00035058F5	TAX_GallusGallus_9031_20141114.fasta,UPARC14_2_GallusG_9031_20140417.fasta	174,635.00	97.60%	1	1	2	0.00%	0.63%	AVADLQAALR	K	A	99.70%	42.3	43.5	27.2	26.9	26.4	0.886	0.246	0.35	0	2	0	0	2	1,027.59	2,166.00		379.25	581	590	Empty	TRUE	
20196-20225 HinckeM –TAXchicken	Uncategorized Sample	20196-20205_A	Mudpit_Ingel20196_MGFPeaklist (F058870)	Uncharacterized protein OS=Gallus gallus GN=CFH PE=4 SV=2	E1C7P4_CHICK,UPI0000E809E2,UPI000240C056	TAX_GallusGallus_9031_20141114.fasta,UPARC14_2_GallusG_9031_20140417.fasta	147,856.10	100.00%	7	7	13	0.03%	5.74%	AQFSHGDEVTYR	R	C	99.70%	42.1	43.7	15.3	37.7	41.3	4.96	0.453	0.542	0	0	2	0	2	1,409.64	1,641.00		742.37	710	721	Empty	TRUE	UPI00004488E7
20196-20225 HinckeM –TAXchicken	Uncategorized Sample	20196-20205_A	Mudpit_Ingel20196_MGFPeaklist (F058870)	Uncharacterized protein OS=Gallus gallus GN=CFH PE=4 SV=2	E1C7P4_CHICK,UPI0000E809E2,UPI000240C056	TAX_GallusGallus_9031_20141114.fasta,UPARC14_2_GallusG_9031_20140417.fasta	147,856.10	100.00%	7	7	13	0.03%	5.74%	ETGLYLTYK	K	C	99.70%	50.2	45.2	25	47.3	23.9	0.824	0.201	0.333	0	2	0	0	2	1,087.57	2,144.00		420.11	773	781	Empty	TRUE	UPI00004488E7
20196-20225 HinckeM –TAXchicken	Uncategorized Sample	20196-20205_A	Mudpit_Ingel20196_MGFPeaklist (F058870)	Uncharacterized protein OS=Gallus gallus GN=CFH PE=4 SV=2	E1C7P4_CHICK,UPI0000E809E2,UPI000240C056	TAX_GallusGallus_9031_20141114.fasta,UPARC14_2_GallusG_9031_20140417.fasta	147,856.10	100.00%	7	7	13	0.03%	5.74%	IGNVEDPASSPFR	K	V	99.70%	85.3	45	25.3	66.5	43.2	4.57	0.451	0.462	0	2	0	0	2	1,388.68	2,062.00		1,103.51	1211	1223	Empty	TRUE	UPI00004488E7
20196-20225 HinckeM –TAXchicken	Uncategorized Sample	20196-20205_A	Mudpit_Ingel20196_MGFPeaklist (F058870)	Uncharacterized protein OS=Gallus gallus GN=CFH PE=4 SV=2	E1C7P4_CHICK,UPI0000E809E2,UPI000240C056	TAX_GallusGallus_9031_20141114.fasta,UPARC14_2_GallusG_9031_20140417.fasta	147,856.10	100.00%	7	7	13	0.03%	5.74%	IGQTIYYR	R	C	99.70%	48.3	44	26.5	32.2	26.5	1.44	0.253	0.375	0	2	0	0	2	1,013.54	1,834.00		495.71	351	358	Empty	TRUE	UPI00004488E7
20196-20225 HinckeM –TAXchicken	Uncategorized Sample	20196-20205_A	Mudpit_Ingel20196_MGFPeaklist (F058870)	Uncharacterized protein OS=Gallus gallus GN=CFH PE=4 SV=2	E1C7P4_CHICK,UPI0000E809E2,UPI000240C056	TAX_GallusGallus_9031_20141114.fasta,UPARC14_2_GallusG_9031_20140417.fasta	147,856.10	100.00%	7	7	13	0.03%	5.74%	NKEAYEPGETIR	R	Y	99.70%	33.1	44.9	23.9	16.2	38.5	3.4	0.301	0.542	0	0	2	0	2	1,406.69	1,638.00		678.3	953	964	Empty	TRUE	UPI00004488E7
20196-20225 HinckeM –TAXchicken	Uncategorized Sample	20196-20205_A	Mudpit_Ingel20196_MGFPeaklist (F058870)	Uncharacterized protein OS=Gallus gallus GN=CFH PE=4 SV=2	E1C7P4_CHICK,UPI0000E809E2,UPI000240C056	TAX_GallusGallus_9031_20141114.fasta,UPARC14_2_GallusG_9031_20140417.fasta	147,856.10	100.00%	7	7	13	0.03%	5.74%	TVYLAGAVVK	K	F	24.80%	14	43.2	25.6	0.66					0	1	0	0	2	1,020.61	2,130.00		413.24	896	905	Empty	TRUE	UPI00004488E7
20196-20225 HinckeM –TAXchicken	Uncategorized Sample	20196-20205_A	Mudpit_Ingel20196_MGFPeaklist (F058870)	Uncharacterized protein OS=Gallus gallus GN=CFH PE=4 SV=2	E1C7P4_CHICK,UPI0000E809E2,UPI000240C056	TAX_GallusGallus_9031_20141114.fasta,UPARC14_2_GallusG_9031_20140417.fasta	147,856.10	100.00%	7	7	13	0.03%	5.74%	WLYEPVSRPEK	K	F	47.90%	18.8	45	24	10.3					0	0	2	0	2	1,403.73	2,076.00		481.98	1276	1286	Empty	TRUE	
20196-20225 HinckeM –TAXchicken	Uncategorized Sample	20196-20205_A	Mudpit_Ingel20196_MGFPeaklist (F058870)	Uncharacterized protein OS=Gallus gallus GN=CFH PE=4 SV=2	E1C7P4_CHICK,UPI0000E809E2,UPI000240C056	TAX_GallusGallus_9031_20141114.fasta,UPARC14_2_GallusG_9031_20140417.fasta	147,856.10	100.00%	7	7	13	0.03%	5.74%	AQFSHGDEVTYR	R	C	99.70%	42.1	43.7	15.3	37.7	41.3	4.96	0.453	0.542	0	0	2	0	2	1,409.64	1,641.00		742.37	710	721	Empty	TRUE	UPI00004488E7
20196-20225 HinckeM –TAXchicken	Uncategorized Sample	20196-20205_A	Mudpit_Ingel20196_MGFPeaklist (F058870)	Uncharacterized protein OS=Gallus gallus GN=CFH PE=4 SV=2	E1C7P4_CHICK,UPI0000E809E2,UPI000240C056	TAX_GallusGallus_9031_20141114.fasta,UPARC14_2_GallusG_9031_20140417.fasta	147,856.10	100.00%	7	7	13	0.03%	5.74%	ETGLYLTYK	K	C	99.70%	50.2	45.2	25	47.3	23.9	0.824	0.201	0.333	0	2	0	0	2	1,087.57	2,144.00		420.11	773	781	Empty	TRUE	UPI00004488E7
20196-20225 HinckeM –TAXchicken	Uncategorized Sample	20196-20205_A	Mudpit_Ingel20196_MGFPeaklist (F058870)	Uncharacterized protein OS=Gallus gallus GN=CFH PE=4 SV=2	E1C7P4_CHICK,UPI0000E809E2,UPI000240C056	TAX_GallusGallus_9031_20141114.fasta,UPARC14_2_GallusG_9031_20140417.fasta	147,856.10	100.00%	7	7	13	0.03%	5.74%	IGNVEDPASSPFR	K	V	99.70%	85.3	45	25.3	66.5	43.2	4.57	0.451	0.462	0	2	0	0	2	1,388.68	2,062.00		1,103.51	1211	1223	Empty	TRUE	UPI00004488E7
20196-20225 HinckeM –TAXchicken	Uncategorized Sample	20196-20205_A	Mudpit_Ingel20196_MGFPeaklist (F058870)	Uncharacterized protein OS=Gallus gallus GN=CFH PE=4 SV=2	E1C7P4_CHICK,UPI0000E809E2,UPI000240C056	TAX_GallusGallus_9031_20141114.fasta,UPARC14_2_GallusG_9031_20140417.fasta	147,856.10	100.00%	7	7	13	0.03%	5.74%	IGQTIYYR	R	C	99.70%	48.3	44	26.5	32.2	26.5	1.44	0.253	0.375	0	2	0	0	2	1,013.54	1,834.00		495.71	351	358	Empty	TRUE	UPI00004488E7
20196-20225 HinckeM –TAXchicken	Uncategorized Sample	20196-20205_A	Mudpit_Ingel20196_MGFPeaklist (F058870)	Uncharacterized protein OS=Gallus gallus GN=CFH PE=4 SV=2	E1C7P4_CHICK,UPI0000E809E2,UPI000240C056	TAX_GallusGallus_9031_20141114.fasta,UPARC14_2_GallusG_9031_20140417.fasta	147,856.10	100.00%	7	7	13	0.03%	5.74%	NKEAYEPGETIR	R	Y	99.70%	33.1	44.9	23.9	16.2	38.5	3.4	0.301	0.542	0	0	2	0	2	1,406.69	1,638.00		678.3	953	964	Empty	TRUE	UPI00004488E7
20196-20225 HinckeM –TAXchicken	Uncategorized Sample	20196-20205_A	Mudpit_Ingel20196_MGFPeaklist (F058870)	Uncharacterized protein OS=Gallus gallus GN=CFH PE=4 SV=2	E1C7P4_CHICK,UPI0000E809E2,UPI000240C056	TAX_GallusGallus_9031_20141114.fasta,UPARC14_2_GallusG_9031_20140417.fasta	147,856.10	100.00%	7	7	13	0.03%	5.74%	TVYLAGAVVK	K	F	24.80%	14	43.2	25.6	0.66					0	1	0	0	2	1,020.61	2,130.00		413.24	896	905	Empty	TRUE	UPI00004488E7
20196-20225 HinckeM –TAXchicken	Uncategorized Sample	20196-20205_A	Mudpit_Ingel20196_MGFPeaklist (F058870)	Uncharacterized protein OS=Gallus gallus GN=CFH PE=4 SV=2	E1C7P4_CHICK,UPI0000E809E2,UPI000240C056	TAX_GallusGallus_9031_20141114.fasta,UPARC14_2_GallusG_9031_20140417.fasta	147,856.10	100.00%	7	7	13	0.03%	5.74%	WLYEPVSRPEK	K	F	47.90%	18.8	45	24	10.3					0	0	2	0	2	1,403.73	2,076.00		481.98	1276	1286	Empty	TRUE	
20196-20225 HinckeM –TAXchicken	Uncategorized Sample	20196-20205_A	Mudpit_Ingel20196_MGFPeaklist (F058870)	Uncharacterized protein OS=Gallus gallus GN=CFH PE=4 SV=2	E1C7P4_CHICK,UPI0000E809E2,UPI000240C056	TAX_GallusGallus_9031_20141114.fasta,UPARC14_2_GallusG_9031_20140417.fasta	147,856.10	100.00%	7	7	13	0.03%	5.74%	AQFSHGDEVTYR	R	C	99.70%	42.1	43.7	15.3	37.7	41.3	4.96	0.453	0.542	0	0	2	0	2	1,409.64	1,641.00		742.37	710	721	Empty	TRUE	UPI00004488E7
20196-20225 HinckeM –TAXchicken	Uncategorized Sample	20196-20205_A	Mudpit_Ingel20196_MGFPeaklist (F058870)	Uncharacterized protein OS=Gallus gallus GN=CFH PE=4 SV=2	E1C7P4_CHICK,UPI0000E809E2,UPI000240C056	TAX_GallusGallus_9031_20141114.fasta,UPARC14_2_GallusG_9031_20140417.fasta	147,856.10	100.00%	7	7	13	0.03%	5.74%	ETGLYLTYK	K	C	99.70%	50.2	45.2	25	47.3	23.9	0.824	0.201	0.333	0	2	0	0	2	1,087.57	2,144.00		420.11	773	781	Empty	TRUE	UPI00004488E7
20196-20225 HinckeM –TAXchicken	Uncategorized Sample	20196-20205_A	Mudpit_Ingel20196_MGFPeaklist (F058870)	Uncharacterized protein OS=Gallus gallus GN=CFH PE=4 SV=2	E1C7P4_CHICK,UPI0000E809E2,UPI000240C056	TAX_GallusGallus_9031_20141114.fasta,UPARC14_2_GallusG_9031_20140417.fasta	147,856.10	100.00%	7	7	13	0.03%	5.74%	IGNVEDPASSPFR	K	V	99.70%	85.3	45	25.3	66.5	43.2	4.57	0.451	0.462	0	2	0	0	2	1,388.68	2,062.00		1,103.51	1211	1223	Empty	TRUE	UPI00004488E7
20196-20225 HinckeM –TAXchicken	Uncategorized Sample	20196-20205_A	Mudpit_Ingel20196_MGFPeaklist (F058870)	Uncharacterized protein OS=Gallus gallus GN=CFH PE=4 SV=2	E1C7P4_CHICK,UPI0000E809E2,UPI000240C056	TAX_GallusGallus_9031_20141114.fasta,UPARC14_2_GallusG_9031_20140417.fasta	147,856.10	100.00%	7	7	13	0.03%	5.74%	IGQTIYYR	R	C	99.70%	48.3	44	26.5	32.2	26.5	1.44	0.253	0.375	0	2	0	0	2	1,013.54	1,834.00		495.71	351	358	Empty	TRUE	UPI00004488E7
20196-20225 HinckeM –TAXchicken	Uncategorized Sample	20196-20205_A	Mudpit_Ingel20196_MGFPeaklist (F058870)	Uncharacterized protein OS=Gallus gallus GN=CFH PE=4 SV=2	E1C7P4_CHICK,UPI0000E809E2,UPI000240C056	TAX_GallusGallus_9031_20141114.fasta,UPARC14_2_GallusG_9031_20140417.fasta	147,856.10	100.00%	7	7	13	0.03%	5.74%	NKEAYEPGETIR	R	Y	99.70%	33.1	44.9	23.9	16.2	38.5	3.4	0.301	0.542	0	0	2	0	2	1,406.69	1,638.00		678.3	953	964	Empty	TRUE	UPI00004488E7
20196-20225 HinckeM –TAXchicken	Uncategorized Sample	20196-20205_A	Mudpit_Ingel20196_MGFPeaklist (F058870)	Uncharacterized protein OS=Gallus gallus GN=CFH PE=4 SV=2	E1C7P4_CHICK,UPI0000E809E2,UPI000240C056	TAX_GallusGallus_9031_20141114.fasta,UPARC14_2_GallusG_9031_20140417.fasta	147,856.10	100.00%	7	7	13	0.03%	5.74%	TVYLAGAVVK	K	F	24.80%	14	43.2	25.6	0.66					0	1	0	0	2	1,020.61	2,130.00		413.24	896	905	Empty	TRUE	UPI00004488E7
20196-20225 HinckeM –TAXchicken	Uncategorized Sample	20196-20205_A	Mudpit_Ingel20196_MGFPeaklist (F058870)	Uncharacterized protein OS=Gallus gallus GN=CFH PE=4 SV=2	E1C7P4_CHICK,UPI0000E809E2,UPI000240C056	TAX_GallusGallus_9031_20141114.fasta,UPARC14_2_GallusG_9031_20140417.fasta	147,856.10	100.00%	7	7	13	0.03%	5.74%	WLYEPVSRPEK	K	F	47.90%	18.8	45	24	10.3					0	0	2	0	2	1,403.73	2,076.00		481.98	1276	1286	Empty	TRUE	
20196-20225 HinckeM –TAXchicken	Uncategorized Sample	20196-20205_A	Mudpit_Ingel20196_MGFPeaklist (F058870)	UPI000198D1F8-DECOY	UPI000198D1F8-DECOY		0	97.90%	2	2	42	0.08%	0.00%	APIASIPR	?	?	96.90%	41.5	41.1	33.3	23.8					0	41	0	0	2	824.4989	1,932.00		127499	0	0	Empty	TRUE	UPI0001CF1DBC-DECOY
20196-20225 HinckeM –TAXchicken	Uncategorized Sample	20196-20205_A	Mudpit_Ingel20196_MGFPeaklist (F058870)	UPI000198D1F8-DECOY	UPI000198D1F8-DECOY		0	97.90%	2	2	42	0.08%	0.00%	KQGTFITSSYK	?	?	30.10%	13.9	45	26.1	-0.59					0	0	1	0	2	1,259.66	1,609.00		356.56	0	0	Empty	TRUE	
20196-20225 HinckeM –TAXchicken	Uncategorized Sample	20196-20205_A	Mudpit_Ingel20196_MGFPeaklist (F058870)	UPI0003504F46-DECOY	UPI0003504F46-DECOY		0	97.70%	2	2	2	0.00%	0.00%	QMFGRNEQAR	?	?	23.80%	7.58	43.2	20.5	0					0	1	0	0	2	1,236.54	1,797.00		671.3	0	0	Empty	TRUE	
20196-20225 HinckeM –TAXchicken	Uncategorized Sample	20196-20205_A	Mudpit_Ingel20196_MGFPeaklist (F058870)	UPI0003504F46-DECOY	UPI0003504F46-DECOY		0	97.70%	2	2	2	0.00%	0.00%	VQLIDEQR	?	?	96.90%	41.1	45.1	40	16.6					0	1	0	0	2	1,001.53	2,086.00		235.38	0	0	Empty	TRUE	
20196-20225 HinckeM –TAXchicken	Uncategorized Sample	20196-20205_A	Mudpit_Ingel20196_MGFPeaklist (F058870)	Ubiquitin carboxyl-terminal hydrolase OS=Gallus gallus GN=USP47 PE=3 SV=1	R4GJH4_CHICK,R9PXM3_CHICK,UBP47_CHICK,UPI000061108D,UPI0000E80563,UPI0002C87F52	TAX_GallusGallus_9031_20141114.fasta,UPARC14_2_GallusG_9031_20140417.fasta	157,952.40	100.00%	11	11	18	0.04%	8.50%	AYLNQTVSEFK	R	Q	99.70%	29.4	44.4	21.9	23.7	23.8	0.959	0.269	0.273	0	2	0	0	2	1,299.66	2,179.50		461.7	741	751	Empty	TRUE	
20196-20225 HinckeM –TAXchicken	Uncategorized Sample	20196-20205_A	Mudpit_Ingel20196_MGFPeaklist (F058870)	Ubiquitin carboxyl-terminal hydrolase OS=Gallus gallus GN=USP47 PE=3 SV=1	R4GJH4_CHICK,R9PXM3_CHICK,UBP47_CHICK,UPI000061108D,UPI0000E80563,UPI0002C87F52	TAX_GallusGallus_9031_20141114.fasta,UPARC14_2_GallusG_9031_20140417.fasta	157,952.40	100.00%	11	11	18	0.04%	8.50%	FIGPLPR	R	E	69.20%	29.6	43	27.6	12.8					0	2	0	0	2	799.4826	2,113.00		860.44	158	164	Empty	TRUE	
20196-20225 HinckeM –TAXchicken	Uncategorized Sample	20196-20205_A	Mudpit_Ingel20196_MGFPeaklist (F058870)	Ubiquitin carboxyl-terminal hydrolase OS=Gallus gallus GN=USP47 PE=3 SV=1	R4GJH4_CHICK,R9PXM3_CHICK,UBP47_CHICK,UPI000061108D,UPI0000E80563,UPI0002C87F52	TAX_GallusGallus_9031_20141114.fasta,UPARC14_2_GallusG_9031_20140417.fasta	157,952.40	100.00%	11	11	18	0.04%	8.50%	FLLDAVFAK	K	G	99.70%	56.7	42.8	28.5	26.3	34.8	1.72	0.247	0.5	0	1	0	0	2	1,023.59	2,853.00		262.92	1146	1154	Empty	TRUE	
20196-20225 HinckeM –TAXchicken	Uncategorized Sample	20196-20205_A	Mudpit_Ingel20196_MGFPeaklist (F058870)	Ubiquitin carboxyl-terminal hydrolase OS=Gallus gallus GN=USP47 PE=3 SV=1	R4GJH4_CHICK,R9PXM3_CHICK,UBP47_CHICK,UPI000061108D,UPI0000E80563,UPI0002C87F52	TAX_GallusGallus_9031_20141114.fasta,UPARC14_2_GallusG_9031_20140417.fasta	157,952.40	100.00%	11	11	18	0.04%	8.50%	IYLDGAPNKDLTQD	K	-	99.20%	23.5	44.8	18.2	12.3	28	0.569	0.132	0.286	0	1	0	0	2	1,562.77	2,081.00		575.69	1368	1381	Empty	TRUE	
20196-20225 HinckeM –TAXchicken	Uncategorized Sample	20196-20205_A	Mudpit_Ingel20196_MGFPeaklist (F058870)	Ubiquitin carboxyl-terminal hydrolase OS=Gallus gallus GN=USP47 PE=3 SV=1	R4GJH4_CHICK,R9PXM3_CHICK,UBP47_CHICK,UPI000061108D,UPI0000E80563,UPI0002C87F52	TAX_GallusGallus_9031_20141114.fasta,UPARC14_2_GallusG_9031_20140417.fasta	157,952.40	100.00%	11	11	18	0.04%	8.50%	LFEDVASK	K	V	29.10%	20.8	44.2	17.3	6.52					0	1	0	0	2	908.4724	1,880.00		89.86	78	85	Empty	TRUE	
20196-20225 HinckeM –TAXchicken	Uncategorized Sample	20196-20205_A	Mudpit_Ingel20196_MGFPeaklist (F058870)	Ubiquitin carboxyl-terminal hydrolase OS=Gallus gallus GN=USP47 PE=3 SV=1	R4GJH4_CHICK,R9PXM3_CHICK,UBP47_CHICK,UPI000061108D,UPI0000E80563,UPI0002C87F52	TAX_GallusGallus_9031_20141114.fasta,UPARC14_2_GallusG_9031_20140417.fasta	157,952.40	100.00%	11	11	18	0.04%	8.50%	LFVLLQTSK	R	K	99.70%	53.7	42	35	41.4	27.5	1.28	0.142	0.389	0	2	0	0	2	1,048.64	2,538.00		572.8	244	252	Empty	TRUE	
20196-20225 HinckeM –TAXchicken	Uncategorized Sample	20196-20205_A	Mudpit_Ingel20196_MGFPeaklist (F058870)	Ubiquitin carboxyl-terminal hydrolase OS=Gallus gallus GN=USP47 PE=3 SV=1	R4GJH4_CHICK,R9PXM3_CHICK,UBP47_CHICK,UPI000061108D,UPI0000E80563,UPI0002C87F52	TAX_GallusGallus_9031_20141114.fasta,UPARC14_2_GallusG_9031_20140417.fasta	157,952.40	100.00%	11	11	18	0.04%	8.50%	TESVAPPISVR	K	A	99.70%	27.2	43.6	15.3	11.6	24.7	1.38	0.166	0.227	0	1	0	0	2	1,155.64	2,006.00		261.7	730	740	Empty	TRUE	
20196-20225 HinckeM –TAXchicken	Uncategorized Sample	20196-20205_A	Mudpit_Ingel20196_MGFPeaklist (F058870)	Ubiquitin carboxyl-terminal hydrolase OS=Gallus gallus GN=USP47 PE=3 SV=1	R4GJH4_CHICK,R9PXM3_CHICK,UBP47_CHICK,UPI000061108D,UPI0000E80563,UPI0002C87F52	TAX_GallusGallus_9031_20141114.fasta,UPARC14_2_GallusG_9031_20140417.fasta	157,952.40	100.00%	11	11	18	0.04%	8.50%	VFIESSESLDR	K	H	99.70%	44.8	44.1	28.8	27	24.2	1.04	0.149	0.273	0	2	0	0	2	1,281.63	2,029.50		341.37	797	807	Empty	TRUE	
20196-20225 HinckeM –TAXchicken	Uncategorized Sample	20196-20205_A	Mudpit_Ingel20196_MGFPeaklist (F058870)	Ubiquitin carboxyl-terminal hydrolase OS=Gallus gallus GN=USP47 PE=3 SV=1	R4GJH4_CHICK,R9PXM3_CHICK,UBP47_CHICK,UPI000061108D,UPI0000E80563,UPI0002C87F52	TAX_GallusGallus_9031_20141114.fasta,UPARC14_2_GallusG_9031_20140417.fasta	157,952.40	100.00%	11	11	18	0.04%	8.50%	VLCIIQDTTNSK	R	T	99.70%	48.3	44.9	24.1	37.2	31.3	2.59	0.249	0.375	0	2	0	0	2	1,391.72	2,147.00		482.19	48	59	Empty	TRUE	
20196-20225 HinckeM –TAXchicken	Uncategorized Sample	20196-20205_A	Mudpit_Ingel20196_MGFPeaklist (F058870)	Ubiquitin carboxyl-terminal hydrolase OS=Gallus gallus GN=USP47 PE=3 SV=1	R4GJH4_CHICK,R9PXM3_CHICK,UBP47_CHICK,UPI000061108D,UPI0000E80563,UPI0002C87F52	TAX_GallusGallus_9031_20141114.fasta,UPARC14_2_GallusG_9031_20140417.fasta	157,952.40	100.00%	11	11	18	0.04%	8.50%	VTLNVPASTPLKK	R	L	99.70%	43.7	39.8	20.6	39.4	29.8	3.04	0.309	0.308	0	0	2	0	2	1,367.83	2,046.00		1,215.01	65	77	Empty	TRUE	
20196-20225 HinckeM –TAXchicken	Uncategorized Sample	20196-20205_A	Mudpit_Ingel20196_MGFPeaklist (F058870)	Ubiquitin carboxyl-terminal hydrolase OS=Gallus gallus GN=USP47 PE=3 SV=1	R4GJH4_CHICK,R9PXM3_CHICK,UBP47_CHICK,UPI000061108D,UPI0000E80563,UPI0002C87F52	TAX_GallusGallus_9031_20141114.fasta,UPARC14_2_GallusG_9031_20140417.fasta	157,952.40	100.00%	11	11	18	0.04%	8.50%	VYASNQEFESVR	R	L	99.70%	76.2	44	18.1	69.2	42.2	5.49	0.521	0.458	0	2	0	0	2	1,428.68	1,887.50		485.93	1091	1102	Empty	TRUE	
20196-20225 HinckeM –TAXchicken	Uncategorized Sample	20196-20205_A	Mudpit_Ingel20196_MGFPeaklist (F058870)	Ubiquitin carboxyl-terminal hydrolase OS=Gallus gallus GN=USP47 PE=3 SV=1	R4GJH4_CHICK,R9PXM3_CHICK,UBP47_CHICK,UPI000061108D,UPI0000E80563,UPI0002C87F52	TAX_GallusGallus_9031_20141114.fasta,UPARC14_2_GallusG_9031_20140417.fasta	157,952.40	100.00%	11	11	18	0.04%	8.50%	AYLNQTVSEFK	R	Q	99.70%	29.4	44.4	21.9	23.7	23.8	0.959	0.269	0.273	0	2	0	0	2	1,299.66	2,179.50		461.7	741	751	Empty	TRUE	
20196-20225 HinckeM –TAXchicken	Uncategorized Sample	20196-20205_A	Mudpit_Ingel20196_MGFPeaklist (F058870)	Ubiquitin carboxyl-terminal hydrolase OS=Gallus gallus GN=USP47 PE=3 SV=1	R4GJH4_CHICK,R9PXM3_CHICK,UBP47_CHICK,UPI000061108D,UPI0000E80563,UPI0002C87F52	TAX_GallusGallus_9031_20141114.fasta,UPARC14_2_GallusG_9031_20140417.fasta	157,952.40	100.00%	11	11	18	0.04%	8.50%	FIGPLPR	R	E	69.20%	29.6	43	27.6	12.8					0	2	0	0	2	799.4826	2,113.00		860.44	158	164	Empty	TRUE	
20196-20225 HinckeM –TAXchicken	Uncategorized Sample	20196-20205_A	Mudpit_Ingel20196_MGFPeaklist (F058870)	Ubiquitin carboxyl-terminal hydrolase OS=Gallus gallus GN=USP47 PE=3 SV=1	R4GJH4_CHICK,R9PXM3_CHICK,UBP47_CHICK,UPI000061108D,UPI0000E80563,UPI0002C87F52	TAX_GallusGallus_9031_20141114.fasta,UPARC14_2_GallusG_9031_20140417.fasta	157,952.40	100.00%	11	11	18	0.04%	8.50%	FLLDAVFAK	K	G	99.70%	56.7	42.8	28.5	26.3	34.8	1.72	0.247	0.5	0	1	0	0	2	1,023.59	2,853.00		262.92	1146	1154	Empty	TRUE	
20196-20225 HinckeM –TAXchicken	Uncategorized Sample	20196-20205_A	Mudpit_Ingel20196_MGFPeaklist (F058870)	Ubiquitin carboxyl-terminal hydrolase OS=Gallus gallus GN=USP47 PE=3 SV=1	R4GJH4_CHICK,R9PXM3_CHICK,UBP47_CHICK,UPI000061108D,UPI0000E80563,UPI0002C87F52	TAX_GallusGallus_9031_20141114.fasta,UPARC14_2_GallusG_9031_20140417.fasta	157,952.40	100.00%	11	11	18	0.04%	8.50%	IYLDGAPNKDLTQD	K	-	99.20%	23.5	44.8	18.2	12.3	28	0.569	0.132	0.286	0	1	0	0	2	1,562.77	2,081.00		575.69	1368	1381	Empty	TRUE	
20196-20225 HinckeM –TAXchicken	Uncategorized Sample	20196-20205_A	Mudpit_Ingel20196_MGFPeaklist (F058870)	Ubiquitin carboxyl-terminal hydrolase OS=Gallus gallus GN=USP47 PE=3 SV=1	R4GJH4_CHICK,R9PXM3_CHICK,UBP47_CHICK,UPI000061108D,UPI0000E80563,UPI0002C87F52	TAX_GallusGallus_9031_20141114.fasta,UPARC14_2_GallusG_9031_20140417.fasta	157,952.40	100.00%	11	11	18	0.04%	8.50%	LFEDVASK	K	V	29.10%	20.8	44.2	17.3	6.52					0	1	0	0	2	908.4724	1,880.00		89.86	78	85	Empty	TRUE	
20196-20225 HinckeM –TAXchicken	Uncategorized Sample	20196-20205_A	Mudpit_Ingel20196_MGFPeaklist (F058870)	Ubiquitin carboxyl-terminal hydrolase OS=Gallus gallus GN=USP47 PE=3 SV=1	R4GJH4_CHICK,R9PXM3_CHICK,UBP47_CHICK,UPI000061108D,UPI0000E80563,UPI0002C87F52	TAX_GallusGallus_9031_20141114.fasta,UPARC14_2_GallusG_9031_20140417.fasta	157,952.40	100.00%	11	11	18	0.04%	8.50%	LFVLLQTSK	R	K	99.70%	53.7	42	35	41.4	27.5	1.28	0.142	0.389	0	2	0	0	2	1,048.64	2,538.00		572.8	244	252	Empty	TRUE	
20196-20225 HinckeM –TAXchicken	Uncategorized Sample	20196-20205_A	Mudpit_Ingel20196_MGFPeaklist (F058870)	Ubiquitin carboxyl-terminal hydrolase OS=Gallus gallus GN=USP47 PE=3 SV=1	R4GJH4_CHICK,R9PXM3_CHICK,UBP47_CHICK,UPI000061108D,UPI0000E80563,UPI0002C87F52	TAX_GallusGallus_9031_20141114.fasta,UPARC14_2_GallusG_9031_20140417.fasta	157,952.40	100.00%	11	11	18	0.04%	8.50%	TESVAPPISVR	K	A	99.70%	27.2	43.6	15.3	11.6	24.7	1.38	0.166	0.227	0	1	0	0	2	1,155.64	2,006.00		261.7	730	740	Empty	TRUE	
20196-20225 HinckeM –TAXchicken	Uncategorized Sample	20196-20205_A	Mudpit_Ingel20196_MGFPeaklist (F058870)	Ubiquitin carboxyl-terminal hydrolase OS=Gallus gallus GN=USP47 PE=3 SV=1	R4GJH4_CHICK,R9PXM3_CHICK,UBP47_CHICK,UPI000061108D,UPI0000E80563,UPI0002C87F52	TAX_GallusGallus_9031_20141114.fasta,UPARC14_2_GallusG_9031_20140417.fasta	157,952.40	100.00%	11	11	18	0.04%	8.50%	VFIESSESLDR	K	H	99.70%	44.8	44.1	28.8	27	24.2	1.04	0.149	0.273	0	2	0	0	2	1,281.63	2,029.50		341.37	797	807	Empty	TRUE	
20196-20225 HinckeM –TAXchicken	Uncategorized Sample	20196-20205_A	Mudpit_Ingel20196_MGFPeaklist (F058870)	Ubiquitin carboxyl-terminal hydrolase OS=Gallus gallus GN=USP47 PE=3 SV=1	R4GJH4_CHICK,R9PXM3_CHICK,UBP47_CHICK,UPI000061108D,UPI0000E80563,UPI0002C87F52	TAX_GallusGallus_9031_20141114.fasta,UPARC14_2_GallusG_9031_20140417.fasta	157,952.40	100.00%	11	11	18	0.04%	8.50%	VLCIIQDTTNSK	R	T	99.70%	48.3	44.9	24.1	37.2	31.3	2.59	0.249	0.375	0	2	0	0	2	1,391.72	2,147.00		482.19	48	59	Empty	TRUE	
20196-20225 HinckeM –TAXchicken	Uncategorized Sample	20196-20205_A	Mudpit_Ingel20196_MGFPeaklist (F058870)	Ubiquitin carboxyl-terminal hydrolase OS=Gallus gallus GN=USP47 PE=3 SV=1	R4GJH4_CHICK,R9PXM3_CHICK,UBP47_CHICK,UPI000061108D,UPI0000E80563,UPI0002C87F52	TAX_GallusGallus_9031_20141114.fasta,UPARC14_2_GallusG_9031_20140417.fasta	157,952.40	100.00%	11	11	18	0.04%	8.50%	VTLNVPASTPLKK	R	L	99.70%	43.7	39.8	20.6	39.4	29.8	3.04	0.309	0.308	0	0	2	0	2	1,367.83	2,046.00		1,215.01	65	77	Empty	TRUE	
20196-20225 HinckeM –TAXchicken	Uncategorized Sample	20196-20205_A	Mudpit_Ingel20196_MGFPeaklist (F058870)	Ubiquitin carboxyl-terminal hydrolase OS=Gallus gallus GN=USP47 PE=3 SV=1	R4GJH4_CHICK,R9PXM3_CHICK,UBP47_CHICK,UPI000061108D,UPI0000E80563,UPI0002C87F52	TAX_GallusGallus_9031_20141114.fasta,UPARC14_2_GallusG_9031_20140417.fasta	157,952.40	100.00%	11	11	18	0.04%	8.50%	VYASNQEFESVR	R	L	99.70%	76.2	44	18.1	69.2	42.2	5.49	0.521	0.458	0	2	0	0	2	1,428.68	1,887.50		485.93	1091	1102	Empty	TRUE	
20196-20225 HinckeM –TAXchicken	Uncategorized Sample	20196-20205_A	Mudpit_Ingel20196_MGFPeaklist (F058870)	Ubiquitin carboxyl-terminal hydrolase OS=Gallus gallus GN=USP47 PE=3 SV=1	R4GJH4_CHICK,R9PXM3_CHICK,UBP47_CHICK,UPI000061108D,UPI0000E80563,UPI0002C87F52	TAX_GallusGallus_9031_20141114.fasta,UPARC14_2_GallusG_9031_20140417.fasta	157,952.40	100.00%	11	11	18	0.04%	8.50%	AYLNQTVSEFK	R	Q	99.70%	29.4	44.4	21.9	23.7	23.8	0.959	0.269	0.273	0	2	0	0	2	1,299.66	2,179.50		461.7	741	751	Empty	TRUE	
20196-20225 HinckeM –TAXchicken	Uncategorized Sample	20196-20205_A	Mudpit_Ingel20196_MGFPeaklist (F058870)	Ubiquitin carboxyl-terminal hydrolase OS=Gallus gallus GN=USP47 PE=3 SV=1	R4GJH4_CHICK,R9PXM3_CHICK,UBP47_CHICK,UPI000061108D,UPI0000E80563,UPI0002C87F52	TAX_GallusGallus_9031_20141114.fasta,UPARC14_2_GallusG_9031_20140417.fasta	157,952.40	100.00%	11	11	18	0.04%	8.50%	FIGPLPR	R	E	69.20%	29.6	43	27.6	12.8					0	2	0	0	2	799.4826	2,113.00		860.44	158	164	Empty	TRUE	
20196-20225 HinckeM –TAXchicken	Uncategorized Sample	20196-20205_A	Mudpit_Ingel20196_MGFPeaklist (F058870)	Ubiquitin carboxyl-terminal hydrolase OS=Gallus gallus GN=USP47 PE=3 SV=1	R4GJH4_CHICK,R9PXM3_CHICK,UBP47_CHICK,UPI000061108D,UPI0000E80563,UPI0002C87F52	TAX_GallusGallus_9031_20141114.fasta,UPARC14_2_GallusG_9031_20140417.fasta	157,952.40	100.00%	11	11	18	0.04%	8.50%	FLLDAVFAK	K	G	99.70%	56.7	42.8	28.5	26.3	34.8	1.72	0.247	0.5	0	1	0	0	2	1,023.59	2,853.00		262.92	1146	1154	Empty	TRUE	
20196-20225 HinckeM –TAXchicken	Uncategorized Sample	20196-20205_A	Mudpit_Ingel20196_MGFPeaklist (F058870)	Ubiquitin carboxyl-terminal hydrolase OS=Gallus gallus GN=USP47 PE=3 SV=1	R4GJH4_CHICK,R9PXM3_CHICK,UBP47_CHICK,UPI000061108D,UPI0000E80563,UPI0002C87F52	TAX_GallusGallus_9031_20141114.fasta,UPARC14_2_GallusG_9031_20140417.fasta	157,952.40	100.00%	11	11	18	0.04%	8.50%	IYLDGAPNKDLTQD	K	-	99.20%	23.5	44.8	18.2	12.3	28	0.569	0.132	0.286	0	1	0	0	2	1,562.77	2,081.00		575.69	1368	1381	Empty	TRUE	
20196-20225 HinckeM –TAXchicken	Uncategorized Sample	20196-20205_A	Mudpit_Ingel20196_MGFPeaklist (F058870)	Ubiquitin carboxyl-terminal hydrolase OS=Gallus gallus GN=USP47 PE=3 SV=1	R4GJH4_CHICK,R9PXM3_CHICK,UBP47_CHICK,UPI000061108D,UPI0000E80563,UPI0002C87F52	TAX_GallusGallus_9031_20141114.fasta,UPARC14_2_GallusG_9031_20140417.fasta	157,952.40	100.00%	11	11	18	0.04%	8.50%	LFEDVASK	K	V	29.10%	20.8	44.2	17.3	6.52					0	1	0	0	2	908.4724	1,880.00		89.86	78	85	Empty	TRUE	
20196-20225 HinckeM –TAXchicken	Uncategorized Sample	20196-20205_A	Mudpit_Ingel20196_MGFPeaklist (F058870)	Ubiquitin carboxyl-terminal hydrolase OS=Gallus gallus GN=USP47 PE=3 SV=1	R4GJH4_CHICK,R9PXM3_CHICK,UBP47_CHICK,UPI000061108D,UPI0000E80563,UPI0002C87F52	TAX_GallusGallus_9031_20141114.fasta,UPARC14_2_GallusG_9031_20140417.fasta	157,952.40	100.00%	11	11	18	0.04%	8.50%	LFVLLQTSK	R	K	99.70%	53.7	42	35	41.4	27.5	1.28	0.142	0.389	0	2	0	0	2	1,048.64	2,538.00		572.8	244	252	Empty	TRUE	
20196-20225 HinckeM –TAXchicken	Uncategorized Sample	20196-20205_A	Mudpit_Ingel20196_MGFPeaklist (F058870)	Ubiquitin carboxyl-terminal hydrolase OS=Gallus gallus GN=USP47 PE=3 SV=1	R4GJH4_CHICK,R9PXM3_CHICK,UBP47_CHICK,UPI000061108D,UPI0000E80563,UPI0002C87F52	TAX_GallusGallus_9031_20141114.fasta,UPARC14_2_GallusG_9031_20140417.fasta	157,952.40	100.00%	11	11	18	0.04%	8.50%	TESVAPPISVR	K	A	99.70%	27.2	43.6	15.3	11.6	24.7	1.38	0.166	0.227	0	1	0	0	2	1,155.64	2,006.00		261.7	730	740	Empty	TRUE	
20196-20225 HinckeM –TAXchicken	Uncategorized Sample	20196-20205_A	Mudpit_Ingel20196_MGFPeaklist (F058870)	Ubiquitin carboxyl-terminal hydrolase OS=Gallus gallus GN=USP47 PE=3 SV=1	R4GJH4_CHICK,R9PXM3_CHICK,UBP47_CHICK,UPI000061108D,UPI0000E80563,UPI0002C87F52	TAX_GallusGallus_9031_20141114.fasta,UPARC14_2_GallusG_9031_20140417.fasta	157,952.40	100.00%	11	11	18	0.04%	8.50%	VFIESSESLDR	K	H	99.70%	44.8	44.1	28.8	27	24.2	1.04	0.149	0.273	0	2	0	0	2	1,281.63	2,029.50		341.37	797	807	Empty	TRUE	
20196-20225 HinckeM –TAXchicken	Uncategorized Sample	20196-20205_A	Mudpit_Ingel20196_MGFPeaklist (F058870)	Ubiquitin carboxyl-terminal hydrolase OS=Gallus gallus GN=USP47 PE=3 SV=1	R4GJH4_CHICK,R9PXM3_CHICK,UBP47_CHICK,UPI000061108D,UPI0000E80563,UPI0002C87F52	TAX_GallusGallus_9031_20141114.fasta,UPARC14_2_GallusG_9031_20140417.fasta	157,952.40	100.00%	11	11	18	0.04%	8.50%	VLCIIQDTTNSK	R	T	99.70%	48.3	44.9	24.1	37.2	31.3	2.59	0.249	0.375	0	2	0	0	2	1,391.72	2,147.00		482.19	48	59	Empty	TRUE	
20196-20225 HinckeM –TAXchicken	Uncategorized Sample	20196-20205_A	Mudpit_Ingel20196_MGFPeaklist (F058870)	Ubiquitin carboxyl-terminal hydrolase OS=Gallus gallus GN=USP47 PE=3 SV=1	R4GJH4_CHICK,R9PXM3_CHICK,UBP47_CHICK,UPI000061108D,UPI0000E80563,UPI0002C87F52	TAX_GallusGallus_9031_20141114.fasta,UPARC14_2_GallusG_9031_20140417.fasta	157,952.40	100.00%	11	11	18	0.04%	8.50%	VTLNVPASTPLKK	R	L	99.70%	43.7	39.8	20.6	39.4	29.8	3.04	0.309	0.308	0	0	2	0	2	1,367.83	2,046.00		1,215.01	65	77	Empty	TRUE	
20196-20225 HinckeM –TAXchicken	Uncategorized Sample	20196-20205_A	Mudpit_Ingel20196_MGFPeaklist (F058870)	Ubiquitin carboxyl-terminal hydrolase OS=Gallus gallus GN=USP47 PE=3 SV=1	R4GJH4_CHICK,R9PXM3_CHICK,UBP47_CHICK,UPI000061108D,UPI0000E80563,UPI0002C87F52	TAX_GallusGallus_9031_20141114.fasta,UPARC14_2_GallusG_9031_20140417.fasta	157,952.40	100.00%	11	11	18	0.04%	8.50%	VYASNQEFESVR	R	L	99.70%	76.2	44	18.1	69.2	42.2	5.49	0.521	0.458	0	2	0	0	2	1,428.68	1,887.50		485.93	1091	1102	Empty	TRUE	
20196-20225 HinckeM –TAXchicken	Uncategorized Sample	20196-20205_A	Mudpit_Ingel20196_MGFPeaklist (F058870)	Ubiquitin carboxyl-terminal hydrolase OS=Gallus gallus GN=USP47 PE=3 SV=1	R4GJH4_CHICK,R9PXM3_CHICK,UBP47_CHICK,UPI000061108D,UPI0000E80563,UPI0002C87F52	TAX_GallusGallus_9031_20141114.fasta,UPARC14_2_GallusG_9031_20140417.fasta	157,952.40	100.00%	11	11	18	0.04%	8.50%	AYLNQTVSEFK	R	Q	99.70%	29.4	44.4	21.9	23.7	23.8	0.959	0.269	0.273	0	2	0	0	2	1,299.66	2,179.50		461.7	741	751	Empty	TRUE	
20196-20225 HinckeM –TAXchicken	Uncategorized Sample	20196-20205_A	Mudpit_Ingel20196_MGFPeaklist (F058870)	Ubiquitin carboxyl-terminal hydrolase OS=Gallus gallus GN=USP47 PE=3 SV=1	R4GJH4_CHICK,R9PXM3_CHICK,UBP47_CHICK,UPI000061108D,UPI0000E80563,UPI0002C87F52	TAX_GallusGallus_9031_20141114.fasta,UPARC14_2_GallusG_9031_20140417.fasta	157,952.40	100.00%	11	11	18	0.04%	8.50%	FIGPLPR	R	E	69.20%	29.6	43	27.6	12.8					0	2	0	0	2	799.4826	2,113.00		860.44	158	164	Empty	TRUE	
20196-20225 HinckeM –TAXchicken	Uncategorized Sample	20196-20205_A	Mudpit_Ingel20196_MGFPeaklist (F058870)	Ubiquitin carboxyl-terminal hydrolase OS=Gallus gallus GN=USP47 PE=3 SV=1	R4GJH4_CHICK,R9PXM3_CHICK,UBP47_CHICK,UPI000061108D,UPI0000E80563,UPI0002C87F52	TAX_GallusGallus_9031_20141114.fasta,UPARC14_2_GallusG_9031_20140417.fasta	157,952.40	100.00%	11	11	18	0.04%	8.50%	FLLDAVFAK	K	G	99.70%	56.7	42.8	28.5	26.3	34.8	1.72	0.247	0.5	0	1	0	0	2	1,023.59	2,853.00		262.92	1146	1154	Empty	TRUE	
20196-20225 HinckeM –TAXchicken	Uncategorized Sample	20196-20205_A	Mudpit_Ingel20196_MGFPeaklist (F058870)	Ubiquitin carboxyl-terminal hydrolase OS=Gallus gallus GN=USP47 PE=3 SV=1	R4GJH4_CHICK,R9PXM3_CHICK,UBP47_CHICK,UPI000061108D,UPI0000E80563,UPI0002C87F52	TAX_GallusGallus_9031_20141114.fasta,UPARC14_2_GallusG_9031_20140417.fasta	157,952.40	100.00%	11	11	18	0.04%	8.50%	IYLDGAPNKDLTQD	K	-	99.20%	23.5	44.8	18.2	12.3	28	0.569	0.132	0.286	0	1	0	0	2	1,562.77	2,081.00		575.69	1368	1381	Empty	TRUE	
20196-20225 HinckeM –TAXchicken	Uncategorized Sample	20196-20205_A	Mudpit_Ingel20196_MGFPeaklist (F058870)	Ubiquitin carboxyl-terminal hydrolase OS=Gallus gallus GN=USP47 PE=3 SV=1	R4GJH4_CHICK,R9PXM3_CHICK,UBP47_CHICK,UPI000061108D,UPI0000E80563,UPI0002C87F52	TAX_GallusGallus_9031_20141114.fasta,UPARC14_2_GallusG_9031_20140417.fasta	157,952.40	100.00%	11	11	18	0.04%	8.50%	LFEDVASK	K	V	29.10%	20.8	44.2	17.3	6.52					0	1	0	0	2	908.4724	1,880.00		89.86	78	85	Empty	TRUE	
20196-20225 HinckeM –TAXchicken	Uncategorized Sample	20196-20205_A	Mudpit_Ingel20196_MGFPeaklist (F058870)	Ubiquitin carboxyl-terminal hydrolase OS=Gallus gallus GN=USP47 PE=3 SV=1	R4GJH4_CHICK,R9PXM3_CHICK,UBP47_CHICK,UPI000061108D,UPI0000E80563,UPI0002C87F52	TAX_GallusGallus_9031_20141114.fasta,UPARC14_2_GallusG_9031_20140417.fasta	157,952.40	100.00%	11	11	18	0.04%	8.50%	LFVLLQTSK	R	K	99.70%	53.7	42	35	41.4	27.5	1.28	0.142	0.389	0	2	0	0	2	1,048.64	2,538.00		572.8	244	252	Empty	TRUE	
20196-20225 HinckeM –TAXchicken	Uncategorized Sample	20196-20205_A	Mudpit_Ingel20196_MGFPeaklist (F058870)	Ubiquitin carboxyl-terminal hydrolase OS=Gallus gallus GN=USP47 PE=3 SV=1	R4GJH4_CHICK,R9PXM3_CHICK,UBP47_CHICK,UPI000061108D,UPI0000E80563,UPI0002C87F52	TAX_GallusGallus_9031_20141114.fasta,UPARC14_2_GallusG_9031_20140417.fasta	157,952.40	100.00%	11	11	18	0.04%	8.50%	TESVAPPISVR	K	A	99.70%	27.2	43.6	15.3	11.6	24.7	1.38	0.166	0.227	0	1	0	0	2	1,155.64	2,006.00		261.7	730	740	Empty	TRUE	
20196-20225 HinckeM –TAXchicken	Uncategorized Sample	20196-20205_A	Mudpit_Ingel20196_MGFPeaklist (F058870)	Ubiquitin carboxyl-terminal hydrolase OS=Gallus gallus GN=USP47 PE=3 SV=1	R4GJH4_CHICK,R9PXM3_CHICK,UBP47_CHICK,UPI000061108D,UPI0000E80563,UPI0002C87F52	TAX_GallusGallus_9031_20141114.fasta,UPARC14_2_GallusG_9031_20140417.fasta	157,952.40	100.00%	11	11	18	0.04%	8.50%	VFIESSESLDR	K	H	99.70%	44.8	44.1	28.8	27	24.2	1.04	0.149	0.273	0	2	0	0	2	1,281.63	2,029.50		341.37	797	807	Empty	TRUE	
20196-20225 HinckeM –TAXchicken	Uncategorized Sample	20196-20205_A	Mudpit_Ingel20196_MGFPeaklist (F058870)	Ubiquitin carboxyl-terminal hydrolase OS=Gallus gallus GN=USP47 PE=3 SV=1	R4GJH4_CHICK,R9PXM3_CHICK,UBP47_CHICK,UPI000061108D,UPI0000E80563,UPI0002C87F52	TAX_GallusGallus_9031_20141114.fasta,UPARC14_2_GallusG_9031_20140417.fasta	157,952.40	100.00%	11	11	18	0.04%	8.50%	VLCIIQDTTNSK	R	T	99.70%	48.3	44.9	24.1	37.2	31.3	2.59	0.249	0.375	0	2	0	0	2	1,391.72	2,147.00		482.19	48	59	Empty	TRUE	
20196-20225 HinckeM –TAXchicken	Uncategorized Sample	20196-20205_A	Mudpit_Ingel20196_MGFPeaklist (F058870)	Ubiquitin carboxyl-terminal hydrolase OS=Gallus gallus GN=USP47 PE=3 SV=1	R4GJH4_CHICK,R9PXM3_CHICK,UBP47_CHICK,UPI000061108D,UPI0000E80563,UPI0002C87F52	TAX_GallusGallus_9031_20141114.fasta,UPARC14_2_GallusG_9031_20140417.fasta	157,952.40	100.00%	11	11	18	0.04%	8.50%	VTLNVPASTPLKK	R	L	99.70%	43.7	39.8	20.6	39.4	29.8	3.04	0.309	0.308	0	0	2	0	2	1,367.83	2,046.00		1,215.01	65	77	Empty	TRUE	
20196-20225 HinckeM –TAXchicken	Uncategorized Sample	20196-20205_A	Mudpit_Ingel20196_MGFPeaklist (F058870)	Ubiquitin carboxyl-terminal hydrolase OS=Gallus gallus GN=USP47 PE=3 SV=1	R4GJH4_CHICK,R9PXM3_CHICK,UBP47_CHICK,UPI000061108D,UPI0000E80563,UPI0002C87F52	TAX_GallusGallus_9031_20141114.fasta,UPARC14_2_GallusG_9031_20140417.fasta	157,952.40	100.00%	11	11	18	0.04%	8.50%	VYASNQEFESVR	R	L	99.70%	76.2	44	18.1	69.2	42.2	5.49	0.521	0.458	0	2	0	0	2	1,428.68	1,887.50		485.93	1091	1102	Empty	TRUE	
20196-20225 HinckeM –TAXchicken	Uncategorized Sample	20196-20205_A	Mudpit_Ingel20196_MGFPeaklist (F058870)	Ubiquitin carboxyl-terminal hydrolase OS=Gallus gallus GN=USP47 PE=3 SV=1	R4GJH4_CHICK,R9PXM3_CHICK,UBP47_CHICK,UPI000061108D,UPI0000E80563,UPI0002C87F52	TAX_GallusGallus_9031_20141114.fasta,UPARC14_2_GallusG_9031_20140417.fasta	157,952.40	100.00%	11	11	18	0.04%	8.50%	AYLNQTVSEFK	R	Q	99.70%	29.4	44.4	21.9	23.7	23.8	0.959	0.269	0.273	0	2	0	0	2	1,299.66	2,179.50		461.7	741	751	Empty	TRUE	
20196-20225 HinckeM –TAXchicken	Uncategorized Sample	20196-20205_A	Mudpit_Ingel20196_MGFPeaklist (F058870)	Ubiquitin carboxyl-terminal hydrolase OS=Gallus gallus GN=USP47 PE=3 SV=1	R4GJH4_CHICK,R9PXM3_CHICK,UBP47_CHICK,UPI000061108D,UPI0000E80563,UPI0002C87F52	TAX_GallusGallus_9031_20141114.fasta,UPARC14_2_GallusG_9031_20140417.fasta	157,952.40	100.00%	11	11	18	0.04%	8.50%	FIGPLPR	R	E	69.20%	29.6	43	27.6	12.8					0	2	0	0	2	799.4826	2,113.00		860.44	158	164	Empty	TRUE	
20196-20225 HinckeM –TAXchicken	Uncategorized Sample	20196-20205_A	Mudpit_Ingel20196_MGFPeaklist (F058870)	Ubiquitin carboxyl-terminal hydrolase OS=Gallus gallus GN=USP47 PE=3 SV=1	R4GJH4_CHICK,R9PXM3_CHICK,UBP47_CHICK,UPI000061108D,UPI0000E80563,UPI0002C87F52	TAX_GallusGallus_9031_20141114.fasta,UPARC14_2_GallusG_9031_20140417.fasta	157,952.40	100.00%	11	11	18	0.04%	8.50%	FLLDAVFAK	K	G	99.70%	56.7	42.8	28.5	26.3	34.8	1.72	0.247	0.5	0	1	0	0	2	1,023.59	2,853.00		262.92	1146	1154	Empty	TRUE	
20196-20225 HinckeM –TAXchicken	Uncategorized Sample	20196-20205_A	Mudpit_Ingel20196_MGFPeaklist (F058870)	Ubiquitin carboxyl-terminal hydrolase OS=Gallus gallus GN=USP47 PE=3 SV=1	R4GJH4_CHICK,R9PXM3_CHICK,UBP47_CHICK,UPI000061108D,UPI0000E80563,UPI0002C87F52	TAX_GallusGallus_9031_20141114.fasta,UPARC14_2_GallusG_9031_20140417.fasta	157,952.40	100.00%	11	11	18	0.04%	8.50%	IYLDGAPNKDLTQD	K	-	99.20%	23.5	44.8	18.2	12.3	28	0.569	0.132	0.286	0	1	0	0	2	1,562.77	2,081.00		575.69	1368	1381	Empty	TRUE	
20196-20225 HinckeM –TAXchicken	Uncategorized Sample	20196-20205_A	Mudpit_Ingel20196_MGFPeaklist (F058870)	Ubiquitin carboxyl-terminal hydrolase OS=Gallus gallus GN=USP47 PE=3 SV=1	R4GJH4_CHICK,R9PXM3_CHICK,UBP47_CHICK,UPI000061108D,UPI0000E80563,UPI0002C87F52	TAX_GallusGallus_9031_20141114.fasta,UPARC14_2_GallusG_9031_20140417.fasta	157,952.40	100.00%	11	11	18	0.04%	8.50%	LFEDVASK	K	V	29.10%	20.8	44.2	17.3	6.52					0	1	0	0	2	908.4724	1,880.00		89.86	78	85	Empty	TRUE	
20196-20225 HinckeM –TAXchicken	Uncategorized Sample	20196-20205_A	Mudpit_Ingel20196_MGFPeaklist (F058870)	Ubiquitin carboxyl-terminal hydrolase OS=Gallus gallus GN=USP47 PE=3 SV=1	R4GJH4_CHICK,R9PXM3_CHICK,UBP47_CHICK,UPI000061108D,UPI0000E80563,UPI0002C87F52	TAX_GallusGallus_9031_20141114.fasta,UPARC14_2_GallusG_9031_20140417.fasta	157,952.40	100.00%	11	11	18	0.04%	8.50%	LFVLLQTSK	R	K	99.70%	53.7	42	35	41.4	27.5	1.28	0.142	0.389	0	2	0	0	2	1,048.64	2,538.00		572.8	244	252	Empty	TRUE	
20196-20225 HinckeM –TAXchicken	Uncategorized Sample	20196-20205_A	Mudpit_Ingel20196_MGFPeaklist (F058870)	Ubiquitin carboxyl-terminal hydrolase OS=Gallus gallus GN=USP47 PE=3 SV=1	R4GJH4_CHICK,R9PXM3_CHICK,UBP47_CHICK,UPI000061108D,UPI0000E80563,UPI0002C87F52	TAX_GallusGallus_9031_20141114.fasta,UPARC14_2_GallusG_9031_20140417.fasta	157,952.40	100.00%	11	11	18	0.04%	8.50%	TESVAPPISVR	K	A	99.70%	27.2	43.6	15.3	11.6	24.7	1.38	0.166	0.227	0	1	0	0	2	1,155.64	2,006.00		261.7	730	740	Empty	TRUE	
20196-20225 HinckeM –TAXchicken	Uncategorized Sample	20196-20205_A	Mudpit_Ingel20196_MGFPeaklist (F058870)	Ubiquitin carboxyl-terminal hydrolase OS=Gallus gallus GN=USP47 PE=3 SV=1	R4GJH4_CHICK,R9PXM3_CHICK,UBP47_CHICK,UPI000061108D,UPI0000E80563,UPI0002C87F52	TAX_GallusGallus_9031_20141114.fasta,UPARC14_2_GallusG_9031_20140417.fasta	157,952.40	100.00%	11	11	18	0.04%	8.50%	VFIESSESLDR	K	H	99.70%	44.8	44.1	28.8	27	24.2	1.04	0.149	0.273	0	2	0	0	2	1,281.63	2,029.50		341.37	797	807	Empty	TRUE	
20196-20225 HinckeM –TAXchicken	Uncategorized Sample	20196-20205_A	Mudpit_Ingel20196_MGFPeaklist (F058870)	Ubiquitin carboxyl-terminal hydrolase OS=Gallus gallus GN=USP47 PE=3 SV=1	R4GJH4_CHICK,R9PXM3_CHICK,UBP47_CHICK,UPI000061108D,UPI0000E80563,UPI0002C87F52	TAX_GallusGallus_9031_20141114.fasta,UPARC14_2_GallusG_9031_20140417.fasta	157,952.40	100.00%	11	11	18	0.04%	8.50%	VLCIIQDTTNSK	R	T	99.70%	48.3	44.9	24.1	37.2	31.3	2.59	0.249	0.375	0	2	0	0	2	1,391.72	2,147.00		482.19	48	59	Empty	TRUE	
20196-20225 HinckeM –TAXchicken	Uncategorized Sample	20196-20205_A	Mudpit_Ingel20196_MGFPeaklist (F058870)	Ubiquitin carboxyl-terminal hydrolase OS=Gallus gallus GN=USP47 PE=3 SV=1	R4GJH4_CHICK,R9PXM3_CHICK,UBP47_CHICK,UPI000061108D,UPI0000E80563,UPI0002C87F52	TAX_GallusGallus_9031_20141114.fasta,UPARC14_2_GallusG_9031_20140417.fasta	157,952.40	100.00%	11	11	18	0.04%	8.50%	VTLNVPASTPLKK	R	L	99.70%	43.7	39.8	20.6	39.4	29.8	3.04	0.309	0.308	0	0	2	0	2	1,367.83	2,046.00		1,215.01	65	77	Empty	TRUE	
20196-20225 HinckeM –TAXchicken	Uncategorized Sample	20196-20205_A	Mudpit_Ingel20196_MGFPeaklist (F058870)	Ubiquitin carboxyl-terminal hydrolase OS=Gallus gallus GN=USP47 PE=3 SV=1	R4GJH4_CHICK,R9PXM3_CHICK,UBP47_CHICK,UPI000061108D,UPI0000E80563,UPI0002C87F52	TAX_GallusGallus_9031_20141114.fasta,UPARC14_2_GallusG_9031_20140417.fasta	157,952.40	100.00%	11	11	18	0.04%	8.50%	VYASNQEFESVR	R	L	99.70%	76.2	44	18.1	69.2	42.2	5.49	0.521	0.458	0	2	0	0	2	1,428.68	1,887.50		485.93	1091	1102	Empty	TRUE	
20196-20225 HinckeM –TAXchicken	Uncategorized Sample	20196-20205_A	Mudpit_Ingel20196_MGFPeaklist (F058870)	Ubiquitin carboxyl-terminal hydrolase OS=Gallus gallus GN=USP47 PE=3 SV=1	R4GJH4_CHICK,R9PXM3_CHICK,UBP47_CHICK,UPI000061108D,UPI0000E80563,UPI0002C87F52	TAX_GallusGallus_9031_20141114.fasta,UPARC14_2_GallusG_9031_20140417.fasta	157,952.40	100.00%	11	11	18	0.04%	8.50%	AYLNQTVSEFK	R	Q	99.70%	29.4	44.4	21.9	23.7	23.8	0.959	0.269	0.273	0	2	0	0	2	1,299.66	2,179.50		461.7	741	751	Empty	TRUE	
20196-20225 HinckeM –TAXchicken	Uncategorized Sample	20196-20205_A	Mudpit_Ingel20196_MGFPeaklist (F058870)	Ubiquitin carboxyl-terminal hydrolase OS=Gallus gallus GN=USP47 PE=3 SV=1	R4GJH4_CHICK,R9PXM3_CHICK,UBP47_CHICK,UPI000061108D,UPI0000E80563,UPI0002C87F52	TAX_GallusGallus_9031_20141114.fasta,UPARC14_2_GallusG_9031_20140417.fasta	157,952.40	100.00%	11	11	18	0.04%	8.50%	FIGPLPR	R	E	69.20%	29.6	43	27.6	12.8					0	2	0	0	2	799.4826	2,113.00		860.44	158	164	Empty	TRUE	
20196-20225 HinckeM –TAXchicken	Uncategorized Sample	20196-20205_A	Mudpit_Ingel20196_MGFPeaklist (F058870)	Ubiquitin carboxyl-terminal hydrolase OS=Gallus gallus GN=USP47 PE=3 SV=1	R4GJH4_CHICK,R9PXM3_CHICK,UBP47_CHICK,UPI000061108D,UPI0000E80563,UPI0002C87F52	TAX_GallusGallus_9031_20141114.fasta,UPARC14_2_GallusG_9031_20140417.fasta	157,952.40	100.00%	11	11	18	0.04%	8.50%	FLLDAVFAK	K	G	99.70%	56.7	42.8	28.5	26.3	34.8	1.72	0.247	0.5	0	1	0	0	2	1,023.59	2,853.00		262.92	1146	1154	Empty	TRUE	
20196-20225 HinckeM –TAXchicken	Uncategorized Sample	20196-20205_A	Mudpit_Ingel20196_MGFPeaklist (F058870)	Ubiquitin carboxyl-terminal hydrolase OS=Gallus gallus GN=USP47 PE=3 SV=1	R4GJH4_CHICK,R9PXM3_CHICK,UBP47_CHICK,UPI000061108D,UPI0000E80563,UPI0002C87F52	TAX_GallusGallus_9031_20141114.fasta,UPARC14_2_GallusG_9031_20140417.fasta	157,952.40	100.00%	11	11	18	0.04%	8.50%	IYLDGAPNKDLTQD	K	-	99.20%	23.5	44.8	18.2	12.3	28	0.569	0.132	0.286	0	1	0	0	2	1,562.77	2,081.00		575.69	1368	1381	Empty	TRUE	
20196-20225 HinckeM –TAXchicken	Uncategorized Sample	20196-20205_A	Mudpit_Ingel20196_MGFPeaklist (F058870)	Ubiquitin carboxyl-terminal hydrolase OS=Gallus gallus GN=USP47 PE=3 SV=1	R4GJH4_CHICK,R9PXM3_CHICK,UBP47_CHICK,UPI000061108D,UPI0000E80563,UPI0002C87F52	TAX_GallusGallus_9031_20141114.fasta,UPARC14_2_GallusG_9031_20140417.fasta	157,952.40	100.00%	11	11	18	0.04%	8.50%	LFEDVASK	K	V	29.10%	20.8	44.2	17.3	6.52					0	1	0	0	2	908.4724	1,880.00		89.86	78	85	Empty	TRUE	
20196-20225 HinckeM –TAXchicken	Uncategorized Sample	20196-20205_A	Mudpit_Ingel20196_MGFPeaklist (F058870)	Ubiquitin carboxyl-terminal hydrolase OS=Gallus gallus GN=USP47 PE=3 SV=1	R4GJH4_CHICK,R9PXM3_CHICK,UBP47_CHICK,UPI000061108D,UPI0000E80563,UPI0002C87F52	TAX_GallusGallus_9031_20141114.fasta,UPARC14_2_GallusG_9031_20140417.fasta	157,952.40	100.00%	11	11	18	0.04%	8.50%	LFVLLQTSK	R	K	99.70%	53.7	42	35	41.4	27.5	1.28	0.142	0.389	0	2	0	0	2	1,048.64	2,538.00		572.8	244	252	Empty	TRUE	
20196-20225 HinckeM –TAXchicken	Uncategorized Sample	20196-20205_A	Mudpit_Ingel20196_MGFPeaklist (F058870)	Ubiquitin carboxyl-terminal hydrolase OS=Gallus gallus GN=USP47 PE=3 SV=1	R4GJH4_CHICK,R9PXM3_CHICK,UBP47_CHICK,UPI000061108D,UPI0000E80563,UPI0002C87F52	TAX_GallusGallus_9031_20141114.fasta,UPARC14_2_GallusG_9031_20140417.fasta	157,952.40	100.00%	11	11	18	0.04%	8.50%	TESVAPPISVR	K	A	99.70%	27.2	43.6	15.3	11.6	24.7	1.38	0.166	0.227	0	1	0	0	2	1,155.64	2,006.00		261.7	730	740	Empty	TRUE	
20196-20225 HinckeM –TAXchicken	Uncategorized Sample	20196-20205_A	Mudpit_Ingel20196_MGFPeaklist (F058870)	Ubiquitin carboxyl-terminal hydrolase OS=Gallus gallus GN=USP47 PE=3 SV=1	R4GJH4_CHICK,R9PXM3_CHICK,UBP47_CHICK,UPI000061108D,UPI0000E80563,UPI0002C87F52	TAX_GallusGallus_9031_20141114.fasta,UPARC14_2_GallusG_9031_20140417.fasta	157,952.40	100.00%	11	11	18	0.04%	8.50%	VFIESSESLDR	K	H	99.70%	44.8	44.1	28.8	27	24.2	1.04	0.149	0.273	0	2	0	0	2	1,281.63	2,029.50		341.37	797	807	Empty	TRUE	
20196-20225 HinckeM –TAXchicken	Uncategorized Sample	20196-20205_A	Mudpit_Ingel20196_MGFPeaklist (F058870)	Ubiquitin carboxyl-terminal hydrolase OS=Gallus gallus GN=USP47 PE=3 SV=1	R4GJH4_CHICK,R9PXM3_CHICK,UBP47_CHICK,UPI000061108D,UPI0000E80563,UPI0002C87F52	TAX_GallusGallus_9031_20141114.fasta,UPARC14_2_GallusG_9031_20140417.fasta	157,952.40	100.00%	11	11	18	0.04%	8.50%	VLCIIQDTTNSK	R	T	99.70%	48.3	44.9	24.1	37.2	31.3	2.59	0.249	0.375	0	2	0	0	2	1,391.72	2,147.00		482.19	48	59	Empty	TRUE	
20196-20225 HinckeM –TAXchicken	Uncategorized Sample	20196-20205_A	Mudpit_Ingel20196_MGFPeaklist (F058870)	Ubiquitin carboxyl-terminal hydrolase OS=Gallus gallus GN=USP47 PE=3 SV=1	R4GJH4_CHICK,R9PXM3_CHICK,UBP47_CHICK,UPI000061108D,UPI0000E80563,UPI0002C87F52	TAX_GallusGallus_9031_20141114.fasta,UPARC14_2_GallusG_9031_20140417.fasta	157,952.40	100.00%	11	11	18	0.04%	8.50%	VTLNVPASTPLKK	R	L	99.70%	43.7	39.8	20.6	39.4	29.8	3.04	0.309	0.308	0	0	2	0	2	1,367.83	2,046.00		1,215.01	65	77	Empty	TRUE	
20196-20225 HinckeM –TAXchicken	Uncategorized Sample	20196-20205_A	Mudpit_Ingel20196_MGFPeaklist (F058870)	Ubiquitin carboxyl-terminal hydrolase OS=Gallus gallus GN=USP47 PE=3 SV=1	R4GJH4_CHICK,R9PXM3_CHICK,UBP47_CHICK,UPI000061108D,UPI0000E80563,UPI0002C87F52	TAX_GallusGallus_9031_20141114.fasta,UPARC14_2_GallusG_9031_20140417.fasta	157,952.40	100.00%	11	11	18	0.04%	8.50%	VYASNQEFESVR	R	L	99.70%	76.2	44	18.1	69.2	42.2	5.49	0.521	0.458	0	2	0	0	2	1,428.68	1,887.50		485.93	1091	1102	Empty	TRUE	
20196-20225 HinckeM –TAXchicken	Uncategorized Sample	20196-20205_A	Mudpit_Ingel20196_MGFPeaklist (F058870)	Ubiquitin carboxyl-terminal hydrolase OS=Gallus gallus GN=USP47 PE=3 SV=1	R4GJH4_CHICK,R9PXM3_CHICK,UBP47_CHICK,UPI000061108D,UPI0000E80563,UPI0002C87F52	TAX_GallusGallus_9031_20141114.fasta,UPARC14_2_GallusG_9031_20140417.fasta	157,952.40	100.00%	11	11	18	0.04%	8.50%	AYLNQTVSEFK	R	Q	99.70%	29.4	44.4	21.9	23.7	23.8	0.959	0.269	0.273	0	2	0	0	2	1,299.66	2,179.50		461.7	741	751	Empty	TRUE	
20196-20225 HinckeM –TAXchicken	Uncategorized Sample	20196-20205_A	Mudpit_Ingel20196_MGFPeaklist (F058870)	Ubiquitin carboxyl-terminal hydrolase OS=Gallus gallus GN=USP47 PE=3 SV=1	R4GJH4_CHICK,R9PXM3_CHICK,UBP47_CHICK,UPI000061108D,UPI0000E80563,UPI0002C87F52	TAX_GallusGallus_9031_20141114.fasta,UPARC14_2_GallusG_9031_20140417.fasta	157,952.40	100.00%	11	11	18	0.04%	8.50%	FIGPLPR	R	E	69.20%	29.6	43	27.6	12.8					0	2	0	0	2	799.4826	2,113.00		860.44	158	164	Empty	TRUE	
20196-20225 HinckeM –TAXchicken	Uncategorized Sample	20196-20205_A	Mudpit_Ingel20196_MGFPeaklist (F058870)	Ubiquitin carboxyl-terminal hydrolase OS=Gallus gallus GN=USP47 PE=3 SV=1	R4GJH4_CHICK,R9PXM3_CHICK,UBP47_CHICK,UPI000061108D,UPI0000E80563,UPI0002C87F52	TAX_GallusGallus_9031_20141114.fasta,UPARC14_2_GallusG_9031_20140417.fasta	157,952.40	100.00%	11	11	18	0.04%	8.50%	FLLDAVFAK	K	G	99.70%	56.7	42.8	28.5	26.3	34.8	1.72	0.247	0.5	0	1	0	0	2	1,023.59	2,853.00		262.92	1146	1154	Empty	TRUE	
20196-20225 HinckeM –TAXchicken	Uncategorized Sample	20196-20205_A	Mudpit_Ingel20196_MGFPeaklist (F058870)	Ubiquitin carboxyl-terminal hydrolase OS=Gallus gallus GN=USP47 PE=3 SV=1	R4GJH4_CHICK,R9PXM3_CHICK,UBP47_CHICK,UPI000061108D,UPI0000E80563,UPI0002C87F52	TAX_GallusGallus_9031_20141114.fasta,UPARC14_2_GallusG_9031_20140417.fasta	157,952.40	100.00%	11	11	18	0.04%	8.50%	IYLDGAPNKDLTQD	K	-	99.20%	23.5	44.8	18.2	12.3	28	0.569	0.132	0.286	0	1	0	0	2	1,562.77	2,081.00		575.69	1368	1381	Empty	TRUE	
20196-20225 HinckeM –TAXchicken	Uncategorized Sample	20196-20205_A	Mudpit_Ingel20196_MGFPeaklist (F058870)	Ubiquitin carboxyl-terminal hydrolase OS=Gallus gallus GN=USP47 PE=3 SV=1	R4GJH4_CHICK,R9PXM3_CHICK,UBP47_CHICK,UPI000061108D,UPI0000E80563,UPI0002C87F52	TAX_GallusGallus_9031_20141114.fasta,UPARC14_2_GallusG_9031_20140417.fasta	157,952.40	100.00%	11	11	18	0.04%	8.50%	LFEDVASK	K	V	29.10%	20.8	44.2	17.3	6.52					0	1	0	0	2	908.4724	1,880.00		89.86	78	85	Empty	TRUE	
20196-20225 HinckeM –TAXchicken	Uncategorized Sample	20196-20205_A	Mudpit_Ingel20196_MGFPeaklist (F058870)	Ubiquitin carboxyl-terminal hydrolase OS=Gallus gallus GN=USP47 PE=3 SV=1	R4GJH4_CHICK,R9PXM3_CHICK,UBP47_CHICK,UPI000061108D,UPI0000E80563,UPI0002C87F52	TAX_GallusGallus_9031_20141114.fasta,UPARC14_2_GallusG_9031_20140417.fasta	157,952.40	100.00%	11	11	18	0.04%	8.50%	LFVLLQTSK	R	K	99.70%	53.7	42	35	41.4	27.5	1.28	0.142	0.389	0	2	0	0	2	1,048.64	2,538.00		572.8	244	252	Empty	TRUE	
20196-20225 HinckeM –TAXchicken	Uncategorized Sample	20196-20205_A	Mudpit_Ingel20196_MGFPeaklist (F058870)	Ubiquitin carboxyl-terminal hydrolase OS=Gallus gallus GN=USP47 PE=3 SV=1	R4GJH4_CHICK,R9PXM3_CHICK,UBP47_CHICK,UPI000061108D,UPI0000E80563,UPI0002C87F52	TAX_GallusGallus_9031_20141114.fasta,UPARC14_2_GallusG_9031_20140417.fasta	157,952.40	100.00%	11	11	18	0.04%	8.50%	TESVAPPISVR	K	A	99.70%	27.2	43.6	15.3	11.6	24.7	1.38	0.166	0.227	0	1	0	0	2	1,155.64	2,006.00		261.7	730	740	Empty	TRUE	
20196-20225 HinckeM –TAXchicken	Uncategorized Sample	20196-20205_A	Mudpit_Ingel20196_MGFPeaklist (F058870)	Ubiquitin carboxyl-terminal hydrolase OS=Gallus gallus GN=USP47 PE=3 SV=1	R4GJH4_CHICK,R9PXM3_CHICK,UBP47_CHICK,UPI000061108D,UPI0000E80563,UPI0002C87F52	TAX_GallusGallus_9031_20141114.fasta,UPARC14_2_GallusG_9031_20140417.fasta	157,952.40	100.00%	11	11	18	0.04%	8.50%	VFIESSESLDR	K	H	99.70%	44.8	44.1	28.8	27	24.2	1.04	0.149	0.273	0	2	0	0	2	1,281.63	2,029.50		341.37	797	807	Empty	TRUE	
20196-20225 HinckeM –TAXchicken	Uncategorized Sample	20196-20205_A	Mudpit_Ingel20196_MGFPeaklist (F058870)	Ubiquitin carboxyl-terminal hydrolase OS=Gallus gallus GN=USP47 PE=3 SV=1	R4GJH4_CHICK,R9PXM3_CHICK,UBP47_CHICK,UPI000061108D,UPI0000E80563,UPI0002C87F52	TAX_GallusGallus_9031_20141114.fasta,UPARC14_2_GallusG_9031_20140417.fasta	157,952.40	100.00%	11	11	18	0.04%	8.50%	VLCIIQDTTNSK	R	T	99.70%	48.3	44.9	24.1	37.2	31.3	2.59	0.249	0.375	0	2	0	0	2	1,391.72	2,147.00		482.19	48	59	Empty	TRUE	
20196-20225 HinckeM –TAXchicken	Uncategorized Sample	20196-20205_A	Mudpit_Ingel20196_MGFPeaklist (F058870)	Ubiquitin carboxyl-terminal hydrolase OS=Gallus gallus GN=USP47 PE=3 SV=1	R4GJH4_CHICK,R9PXM3_CHICK,UBP47_CHICK,UPI000061108D,UPI0000E80563,UPI0002C87F52	TAX_GallusGallus_9031_20141114.fasta,UPARC14_2_GallusG_9031_20140417.fasta	157,952.40	100.00%	11	11	18	0.04%	8.50%	VTLNVPASTPLKK	R	L	99.70%	43.7	39.8	20.6	39.4	29.8	3.04	0.309	0.308	0	0	2	0	2	1,367.83	2,046.00		1,215.01	65	77	Empty	TRUE	
20196-20225 HinckeM –TAXchicken	Uncategorized Sample	20196-20205_A	Mudpit_Ingel20196_MGFPeaklist (F058870)	Ubiquitin carboxyl-terminal hydrolase OS=Gallus gallus GN=USP47 PE=3 SV=1	R4GJH4_CHICK,R9PXM3_CHICK,UBP47_CHICK,UPI000061108D,UPI0000E80563,UPI0002C87F52	TAX_GallusGallus_9031_20141114.fasta,UPARC14_2_GallusG_9031_20140417.fasta	157,952.40	100.00%	11	11	18	0.04%	8.50%	VYASNQEFESVR	R	L	99.70%	76.2	44	18.1	69.2	42.2	5.49	0.521	0.458	0	2	0	0	2	1,428.68	1,887.50		485.93	1091	1102	Empty	TRUE	
20196-20225 HinckeM –TAXchicken	Uncategorized Sample	20196-20205_A	Mudpit_Ingel20196_MGFPeaklist (F058870)	Ubiquitin carboxyl-terminal hydrolase OS=Gallus gallus GN=USP47 PE=3 SV=1	R4GJH4_CHICK,R9PXM3_CHICK,UBP47_CHICK,UPI000061108D,UPI0000E80563,UPI0002C87F52	TAX_GallusGallus_9031_20141114.fasta,UPARC14_2_GallusG_9031_20140417.fasta	157,952.40	100.00%	11	11	18	0.04%	8.50%	AYLNQTVSEFK	R	Q	99.70%	29.4	44.4	21.9	23.7	23.8	0.959	0.269	0.273	0	2	0	0	2	1,299.66	2,179.50		461.7	741	751	Empty	TRUE	
20196-20225 HinckeM –TAXchicken	Uncategorized Sample	20196-20205_A	Mudpit_Ingel20196_MGFPeaklist (F058870)	Ubiquitin carboxyl-terminal hydrolase OS=Gallus gallus GN=USP47 PE=3 SV=1	R4GJH4_CHICK,R9PXM3_CHICK,UBP47_CHICK,UPI000061108D,UPI0000E80563,UPI0002C87F52	TAX_GallusGallus_9031_20141114.fasta,UPARC14_2_GallusG_9031_20140417.fasta	157,952.40	100.00%	11	11	18	0.04%	8.50%	FIGPLPR	R	E	69.20%	29.6	43	27.6	12.8					0	2	0	0	2	799.4826	2,113.00		860.44	158	164	Empty	TRUE	
20196-20225 HinckeM –TAXchicken	Uncategorized Sample	20196-20205_A	Mudpit_Ingel20196_MGFPeaklist (F058870)	Ubiquitin carboxyl-terminal hydrolase OS=Gallus gallus GN=USP47 PE=3 SV=1	R4GJH4_CHICK,R9PXM3_CHICK,UBP47_CHICK,UPI000061108D,UPI0000E80563,UPI0002C87F52	TAX_GallusGallus_9031_20141114.fasta,UPARC14_2_GallusG_9031_20140417.fasta	157,952.40	100.00%	11	11	18	0.04%	8.50%	FLLDAVFAK	K	G	99.70%	56.7	42.8	28.5	26.3	34.8	1.72	0.247	0.5	0	1	0	0	2	1,023.59	2,853.00		262.92	1146	1154	Empty	TRUE	
20196-20225 HinckeM –TAXchicken	Uncategorized Sample	20196-20205_A	Mudpit_Ingel20196_MGFPeaklist (F058870)	Ubiquitin carboxyl-terminal hydrolase OS=Gallus gallus GN=USP47 PE=3 SV=1	R4GJH4_CHICK,R9PXM3_CHICK,UBP47_CHICK,UPI000061108D,UPI0000E80563,UPI0002C87F52	TAX_GallusGallus_9031_20141114.fasta,UPARC14_2_GallusG_9031_20140417.fasta	157,952.40	100.00%	11	11	18	0.04%	8.50%	IYLDGAPNKDLTQD	K	-	99.20%	23.5	44.8	18.2	12.3	28	0.569	0.132	0.286	0	1	0	0	2	1,562.77	2,081.00		575.69	1368	1381	Empty	TRUE	
20196-20225 HinckeM –TAXchicken	Uncategorized Sample	20196-20205_A	Mudpit_Ingel20196_MGFPeaklist (F058870)	Ubiquitin carboxyl-terminal hydrolase OS=Gallus gallus GN=USP47 PE=3 SV=1	R4GJH4_CHICK,R9PXM3_CHICK,UBP47_CHICK,UPI000061108D,UPI0000E80563,UPI0002C87F52	TAX_GallusGallus_9031_20141114.fasta,UPARC14_2_GallusG_9031_20140417.fasta	157,952.40	100.00%	11	11	18	0.04%	8.50%	LFEDVASK	K	V	29.10%	20.8	44.2	17.3	6.52					0	1	0	0	2	908.4724	1,880.00		89.86	78	85	Empty	TRUE	
20196-20225 HinckeM –TAXchicken	Uncategorized Sample	20196-20205_A	Mudpit_Ingel20196_MGFPeaklist (F058870)	Ubiquitin carboxyl-terminal hydrolase OS=Gallus gallus GN=USP47 PE=3 SV=1	R4GJH4_CHICK,R9PXM3_CHICK,UBP47_CHICK,UPI000061108D,UPI0000E80563,UPI0002C87F52	TAX_GallusGallus_9031_20141114.fasta,UPARC14_2_GallusG_9031_20140417.fasta	157,952.40	100.00%	11	11	18	0.04%	8.50%	LFVLLQTSK	R	K	99.70%	53.7	42	35	41.4	27.5	1.28	0.142	0.389	0	2	0	0	2	1,048.64	2,538.00		572.8	244	252	Empty	TRUE	
20196-20225 HinckeM –TAXchicken	Uncategorized Sample	20196-20205_A	Mudpit_Ingel20196_MGFPeaklist (F058870)	Ubiquitin carboxyl-terminal hydrolase OS=Gallus gallus GN=USP47 PE=3 SV=1	R4GJH4_CHICK,R9PXM3_CHICK,UBP47_CHICK,UPI000061108D,UPI0000E80563,UPI0002C87F52	TAX_GallusGallus_9031_20141114.fasta,UPARC14_2_GallusG_9031_20140417.fasta	157,952.40	100.00%	11	11	18	0.04%	8.50%	TESVAPPISVR	K	A	99.70%	27.2	43.6	15.3	11.6	24.7	1.38	0.166	0.227	0	1	0	0	2	1,155.64	2,006.00		261.7	730	740	Empty	TRUE	
20196-20225 HinckeM –TAXchicken	Uncategorized Sample	20196-20205_A	Mudpit_Ingel20196_MGFPeaklist (F058870)	Ubiquitin carboxyl-terminal hydrolase OS=Gallus gallus GN=USP47 PE=3 SV=1	R4GJH4_CHICK,R9PXM3_CHICK,UBP47_CHICK,UPI000061108D,UPI0000E80563,UPI0002C87F52	TAX_GallusGallus_9031_20141114.fasta,UPARC14_2_GallusG_9031_20140417.fasta	157,952.40	100.00%	11	11	18	0.04%	8.50%	VFIESSESLDR	K	H	99.70%	44.8	44.1	28.8	27	24.2	1.04	0.149	0.273	0	2	0	0	2	1,281.63	2,029.50		341.37	797	807	Empty	TRUE	
20196-20225 HinckeM –TAXchicken	Uncategorized Sample	20196-20205_A	Mudpit_Ingel20196_MGFPeaklist (F058870)	Ubiquitin carboxyl-terminal hydrolase OS=Gallus gallus GN=USP47 PE=3 SV=1	R4GJH4_CHICK,R9PXM3_CHICK,UBP47_CHICK,UPI000061108D,UPI0000E80563,UPI0002C87F52	TAX_GallusGallus_9031_20141114.fasta,UPARC14_2_GallusG_9031_20140417.fasta	157,952.40	100.00%	11	11	18	0.04%	8.50%	VLCIIQDTTNSK	R	T	99.70%	48.3	44.9	24.1	37.2	31.3	2.59	0.249	0.375	0	2	0	0	2	1,391.72	2,147.00		482.19	48	59	Empty	TRUE	

20196-20225 HinckeM –TAXchicken	Uncategorized Sample	20196-20205_A	Mudpit_Ingel20196_MGFPeaklist (F058870)	Ubiquitin carboxyl-terminal hydrolase OS=Gallus gallus GN=USP47 PE=3 SV=1	R4GJH4_CHICK,R9PXM3_CHICK,UBP47_CHICK,UPI000061108D,UPI0000E80563,UPI0002C87F52	TAX_GallusGallus_9031_20141114.fasta,UPARC14_2_GallusG_9031_20140417.fasta	157,952.40	100.00%	11	11	18	0.04%	8.50%	VTLNVPASTPLKK	R	L	99.70%	43.7	39.8	20.6	39.4	29.8	3.04	0.309	0.308	0	0	2	0	2	1,367.83	2,046.00		1,215.01	65	77	Empty	TRUE	
20196-20225 HinckeM –TAXchicken	Uncategorized Sample	20196-20205_A	Mudpit_Ingel20196_MGFPeaklist (F058870)	Ubiquitin carboxyl-terminal hydrolase OS=Gallus gallus GN=USP47 PE=3 SV=1	R4GJH4_CHICK,R9PXM3_CHICK,UBP47_CHICK,UPI000061108D,UPI0000E80563,UPI0002C87F52	TAX_GallusGallus_9031_20141114.fasta,UPARC14_2_GallusG_9031_20140417.fasta	157,952.40	100.00%	11	11	18	0.04%	8.50%	VYASNQEFESVR	R	L	99.70%	76.2	44	18.1	69.2	42.2	5.49	0.521	0.458	0	2	0	0	2	1,428.68	1,887.50		485.93	1091	1102	Empty	TRUE	
20196-20225 HinckeM –TAXchicken	Uncategorized Sample	20196-20205_A	Mudpit_Ingel20196_MGFPeaklist (F058870)	Uncharacterized protein OS=Gallus gallus GN=NUP205 PE=4 SV=2	E1BV83_CHICK	TAX_GallusGallus_9031_20141114.fasta	228,337.10	100.00%	7	7	10	0.02%	3.43%	DLPSADSVQYR	K	H	99.70%	38.3	44.5	22.1	21.9	36.8	1.74	0.337	0.409	0	2	0	0	2	1,250.60	1,893.00		424.71	572	582	Empty	TRUE	UPI0000E7F894,UPI00003A978F,UPI0000447258,UPI000060E12B,UPI0000ECD1BC
20196-20225 HinckeM –TAXchicken	Uncategorized Sample	20196-20205_A	Mudpit_Ingel20196_MGFPeaklist (F058870)	Uncharacterized protein OS=Gallus gallus GN=NUP205 PE=4 SV=2	E1BV83_CHICK	TAX_GallusGallus_9031_20141114.fasta	228,337.10	100.00%	7	7	10	0.02%	3.43%	LLPEQLIR	R	E	40.80%	28.4	40.3	29.6	0					0	1	0	0	2	981.6093	2,390.00		244.34	85	92	Empty	TRUE	UPI0000E7F894,UPI00003A978F,UPI0000447258,UPI000060E12B,UPI0000ECD1BC
20196-20225 HinckeM –TAXchicken	Uncategorized Sample	20196-20205_A	Mudpit_Ingel20196_MGFPeaklist (F058870)	Uncharacterized protein OS=Gallus gallus GN=NUP205 PE=4 SV=2	E1BV83_CHICK	TAX_GallusGallus_9031_20141114.fasta	228,337.10	100.00%	7	7	10	0.02%	3.43%	LPLLAER	R	Q	53.20%	29.2	40.9	39.4	-0.14					0	1	0	0	2	811.5037	2,140.00		249.33	298	304	Empty	TRUE	UPI0000E7F894,UPI00003A978F,UPI0000447258,UPI000060E12B,UPI0000ECD1BC
20196-20225 HinckeM –TAXchicken	Uncategorized Sample	20196-20205_A	Mudpit_Ingel20196_MGFPeaklist (F058870)	Uncharacterized protein OS=Gallus gallus GN=NUP205 PE=4 SV=2	E1BV83_CHICK	TAX_GallusGallus_9031_20141114.fasta	228,337.10	100.00%	7	7	10	0.02%	3.43%	LTAPEDVFSK	R	L	99.70%	50.6	44.8	21.2	41.5	34.8	2.49	0.351	0.45	0	2	0	0	2	1,106.57	2,168.50		601.52	1454	1463	Empty	TRUE	UPI0000E7F894,UPI00003A978F,UPI0000447258,UPI000060E12B,UPI0000ECD1BC
20196-20225 HinckeM –TAXchicken	Uncategorized Sample	20196-20205_A	Mudpit_Ingel20196_MGFPeaklist (F058870)	Uncharacterized protein OS=Gallus gallus GN=NUP205 PE=4 SV=2	E1BV83_CHICK	TAX_GallusGallus_9031_20141114.fasta	228,337.10	100.00%	7	7	10	0.02%	3.43%	SDFISLFR	R	N	99.70%	48.8	43.6	26	28.4	28.8	1.48	0.247	0.5	0	1	0	0	2	984.5149	2,767.00		177.93	48	55	Empty	TRUE	UPI0000E7F894,UPI00003A978F,UPI0000447258,UPI000060E12B,UPI0000ECD1BC
20196-20225 HinckeM –TAXchicken	Uncategorized Sample	20196-20205_A	Mudpit_Ingel20196_MGFPeaklist (F058870)	Uncharacterized protein OS=Gallus gallus GN=NUP205 PE=4 SV=2	E1BV83_CHICK	TAX_GallusGallus_9031_20141114.fasta	228,337.10	100.00%	7	7	10	0.02%	3.43%	SQQGALELLR	K	S	99.70%	52	44.4	34.1	32.3	33.3	3.55	0.318	0.5	0	2	0	0	2	1,114.62	2,299.50		383.25	1568	1577	Empty	TRUE	UPI0000E7F894,UPI00003A978F,UPI0000447258,UPI000060E12B,UPI0000ECD1BC
20196-20225 HinckeM –TAXchicken	Uncategorized Sample	20196-20205_A	Mudpit_Ingel20196_MGFPeaklist (F058870)	Uncharacterized protein OS=Gallus gallus GN=NUP205 PE=4 SV=2	E1BV83_CHICK	TAX_GallusGallus_9031_20141114.fasta	228,337.10	100.00%	7	7	10	0.02%	3.43%	VLKAELLETLTAFGK	P	S	86.20%					27.3	0.959	0.216	0.3	0	0	1	0	1	1,632.96	2,860.00		807.32	641	655	Empty	TRUE	
20196-20225 HinckeM –TAXchicken	Uncategorized Sample	20196-20205_A	Mudpit_Ingel20196_MGFPeaklist (F058870)	status=active	F1NW23_CHICK,UPI00003AB706,UPI000044958C,UPI000060F708,UPI0000E8139D,UPI0000F0887D	TAX_GallusGallus_9031_20141114.fasta,UPARC14_2_GallusG_9031_20140417.fasta	191,615.00	100.00%	11	11	18	0.04%	7.04%	AVTFFTK	R	V	30.50%	15.9	43.5	0	4.62					0	1	0	0	2	813.4505	2,156.00		113.59	1435	1441	Empty	TRUE	
20196-20225 HinckeM –TAXchicken	Uncategorized Sample	20196-20205_A	Mudpit_Ingel20196_MGFPeaklist (F058870)	status=active	F1NW23_CHICK,UPI00003AB706,UPI000044958C,UPI000060F708,UPI0000E8139D,UPI0000F0887D	TAX_GallusGallus_9031_20141114.fasta,UPARC14_2_GallusG_9031_20140417.fasta	191,615.00	100.00%	11	11	18	0.04%	7.04%	FLRENPYYDSR	R	V	30.80%	16.2	44.5	24.9	2.72					0	0	2	0	2	1,459.70	1,841.00		492.9	893	903	Empty	TRUE	UPI0003503C23,UPI000060F1BC,F1NYC4_CHICK,UPI000060F1BD,UPI0000E81090,UPI000060F1BB,UPI00003AAF6D
20196-20225 HinckeM –TAXchicken	Uncategorized Sample	20196-20205_A	Mudpit_Ingel20196_MGFPeaklist (F058870)	status=active	F1NW23_CHICK,UPI00003AB706,UPI000044958C,UPI000060F708,UPI0000E8139D,UPI0000F0887D	TAX_GallusGallus_9031_20141114.fasta,UPARC14_2_GallusG_9031_20140417.fasta	191,615.00	100.00%	11	11	18	0.04%	7.04%	IAAYLFK	R	G	98.80%	43.2	41.9	30.4	27.5	27.2	0.585	0.239	0.5	0	2	0	0	2	825.4869	2,268.50		441.22	1510	1516	Empty	TRUE	
20196-20225 HinckeM –TAXchicken	Uncategorized Sample	20196-20205_A	Mudpit_Ingel20196_MGFPeaklist (F058870)	status=active	F1NW23_CHICK,UPI00003AB706,UPI000044958C,UPI000060F708,UPI0000E8139D,UPI0000F0887D	TAX_GallusGallus_9031_20141114.fasta,UPARC14_2_GallusG_9031_20140417.fasta	191,615.00	100.00%	11	11	18	0.04%	7.04%	IYIDSNNNPER	K	F	99.70%	63.7	44.7	22.9	50.2	34.9	4.42	0.372	0.409	0	2	0	0	2	1,334.63	1,703.50		379.2	882	892	Empty	TRUE	UPI0003503C23,UPI000060F1BC,F1NYC4_CHICK,UPI000060F1BD,UPI0000E81090,UPI000060F1BB,UPI00003AAF6D
20196-20225 HinckeM –TAXchicken	Uncategorized Sample	20196-20205_A	Mudpit_Ingel20196_MGFPeaklist (F058870)	status=active	F1NW23_CHICK,UPI00003AB706,UPI000044958C,UPI000060F708,UPI0000E8139D,UPI0000F0887D	TAX_GallusGallus_9031_20141114.fasta,UPARC14_2_GallusG_9031_20140417.fasta	191,615.00	100.00%	11	11	18	0.04%	7.04%	KFNALFAQGNYSEAAK	R	V	99.70%	47.9	44.8	28.8	40	43.7	5.51	0.444	0.469	0	0	2	0	2	1,758.88	2,202.00		815.8	367	382	Empty	TRUE	
20196-20225 HinckeM –TAXchicken	Uncategorized Sample	20196-20205_A	Mudpit_Ingel20196_MGFPeaklist (F058870)	status=active	F1NW23_CHICK,UPI00003AB706,UPI000044958C,UPI000060F708,UPI0000E8139D,UPI0000F0887D	TAX_GallusGallus_9031_20141114.fasta,UPARC14_2_GallusG_9031_20140417.fasta	191,615.00	100.00%	11	11	18	0.04%	7.04%	LLYNNVSNFGR	K	L	99.70%	71.2	44.3	24.2	60.3	36.6	2.11	0.404	0.455	0	2	0	0	2	1,296.67	2,211.50		535.55	1216	1226	Empty	TRUE	
20196-20225 HinckeM –TAXchicken	Uncategorized Sample	20196-20205_A	Mudpit_Ingel20196_MGFPeaklist (F058870)	status=active	F1NW23_CHICK,UPI00003AB706,UPI000044958C,UPI000060F708,UPI0000E8139D,UPI0000F0887D	TAX_GallusGallus_9031_20141114.fasta,UPARC14_2_GallusG_9031_20140417.fasta	191,615.00	100.00%	11	11	18	0.04%	7.04%	NNLAGAEELFAR	R	K	99.70%	72	45.1	39.9	37.3	35.8	2.85	0.355	0.417	0	2	0	0	2	1,304.66	2,436.00		530.53	355	366	Empty	TRUE	
20196-20225 HinckeM –TAXchicken	Uncategorized Sample	20196-20205_A	Mudpit_Ingel20196_MGFPeaklist (F058870)	status=active	F1NW23_CHICK,UPI00003AB706,UPI000044958C,UPI000060F708,UPI0000E8139D,UPI0000F0887D	TAX_GallusGallus_9031_20141114.fasta,UPARC14_2_GallusG_9031_20140417.fasta	191,615.00	100.00%	11	11	18	0.04%	7.04%	TLQIFNIEMK	K	S	71.70%	29.6	43.9	26.3	3.61					0	1	0	0	2	1,252.66	2,538.00		107.23	87	96	Empty	TRUE	UPI0003503C23,UPI000060F1BC,F1NYC4_CHICK,UPI000060F1BD,UPI0000E81090,UPI000060F1BB,UPI00003AAF6D
20196-20225 HinckeM –TAXchicken	Uncategorized Sample	20196-20205_A	Mudpit_Ingel20196_MGFPeaklist (F058870)	status=active	F1NW23_CHICK,UPI00003AB706,UPI000044958C,UPI000060F708,UPI0000E8139D,UPI0000F0887D	TAX_GallusGallus_9031_20141114.fasta,UPARC14_2_GallusG_9031_20140417.fasta	191,615.00	100.00%	11	11	18	0.04%	7.04%	VANVELYYK	K	A	99.70%	45.5	44	30.3	28.7	29	2.49	0.314	0.444	0	2	0	0	2	1,098.58	2,049.50		500.42	1398	1406	Empty	TRUE	UPI0003503C23,UPI000060F1BC,F1NYC4_CHICK,UPI000060F1BD,UPI0000E81090,UPI000060F1BB,UPI00003AAF6D
20196-20225 HinckeM –TAXchicken	Uncategorized Sample	20196-20205_A	Mudpit_Ingel20196_MGFPeaklist (F058870)	status=active	F1NW23_CHICK,UPI00003AB706,UPI000044958C,UPI000060F708,UPI0000E8139D,UPI0000F0887D	TAX_GallusGallus_9031_20141114.fasta,UPARC14_2_GallusG_9031_20140417.fasta	191,615.00	100.00%	11	11	18	0.04%	7.04%	VSQPIEGHAASFAQFK	K	M	95.20%	14.1	44.6	17.5	6.8	26.9	0.523	0.23	0.281	0	0	1	0	2	1,716.87	2,138.00		789.19	190	205	Empty	TRUE	
20196-20225 HinckeM –TAXchicken	Uncategorized Sample	20196-20205_A	Mudpit_Ingel20196_MGFPeaklist (F058870)	status=active	F1NW23_CHICK,UPI00003AB706,UPI000044958C,UPI000060F708,UPI0000E8139D,UPI0000F0887D	TAX_GallusGallus_9031_20141114.fasta,UPARC14_2_GallusG_9031_20140417.fasta	191,615.00	100.00%	11	11	18	0.04%	7.04%	YIEIYVQK	K	V	14.10%	8.67	43.6	17.1	0					0	1	0	0	2	1,055.58	2,126.00		248.12	799	806	Empty	TRUE	UPI0003503C23,UPI000060F1BC,F1NYC4_CHICK,UPI000060F1BD,UPI0000E81090,UPI000060F1BB,UPI00003AAF6D
20196-20225 HinckeM –TAXchicken	Uncategorized Sample	20196-20205_A	Mudpit_Ingel20196_MGFPeaklist (F058870)	status=active	F1NW23_CHICK,UPI00003AB706,UPI000044958C,UPI000060F708,UPI0000E8139D,UPI0000F0887D	TAX_GallusGallus_9031_20141114.fasta,UPARC14_2_GallusG_9031_20140417.fasta	191,615.00	100.00%	11	11	18	0.04%	7.04%	AVTFFTK	R	V	30.50%	15.9	43.5	0	4.62					0	1	0	0	2	813.4505	2,156.00		113.59	1435	1441	Empty	TRUE	
20196-20225 HinckeM –TAXchicken	Uncategorized Sample	20196-20205_A	Mudpit_Ingel20196_MGFPeaklist (F058870)	status=active	F1NW23_CHICK,UPI00003AB706,UPI000044958C,UPI000060F708,UPI0000E8139D,UPI0000F0887D	TAX_GallusGallus_9031_20141114.fasta,UPARC14_2_GallusG_9031_20140417.fasta	191,615.00	100.00%	11	11	18	0.04%	7.04%	FLRENPYYDSR	R	V	30.80%	16.2	44.5	24.9	2.72					0	0	2	0	2	1,459.70	1,841.00		492.9	893	903	Empty	TRUE	UPI0003503C23,UPI000060F1BC,F1NYC4_CHICK,UPI000060F1BD,UPI0000E81090,UPI000060F1BB,UPI00003AAF6D
20196-20225 HinckeM –TAXchicken	Uncategorized Sample	20196-20205_A	Mudpit_Ingel20196_MGFPeaklist (F058870)	status=active	F1NW23_CHICK,UPI00003AB706,UPI000044958C,UPI000060F708,UPI0000E8139D,UPI0000F0887D	TAX_GallusGallus_9031_20141114.fasta,UPARC14_2_GallusG_9031_20140417.fasta	191,615.00	100.00%	11	11	18	0.04%	7.04%	IAAYLFK	R	G	98.80%	43.2	41.9	30.4	27.5	27.2	0.585	0.239	0.5	0	2	0	0	2	825.4869	2,268.50		441.22	1510	1516	Empty	TRUE	
20196-20225 HinckeM –TAXchicken	Uncategorized Sample	20196-20205_A	Mudpit_Ingel20196_MGFPeaklist (F058870)	status=active	F1NW23_CHICK,UPI00003AB706,UPI000044958C,UPI000060F708,UPI0000E8139D,UPI0000F0887D	TAX_GallusGallus_9031_20141114.fasta,UPARC14_2_GallusG_9031_20140417.fasta	191,615.00	100.00%	11	11	18	0.04%	7.04%	IYIDSNNNPER	K	F	99.70%	63.7	44.7	22.9	50.2	34.9	4.42	0.372	0.409	0	2	0	0	2	1,334.63	1,703.50		379.2	882	892	Empty	TRUE	UPI0003503C23,UPI000060F1BC,F1NYC4_CHICK,UPI000060F1BD,UPI0000E81090,UPI000060F1BB,UPI00003AAF6D
20196-20225 HinckeM –TAXchicken	Uncategorized Sample	20196-20205_A	Mudpit_Ingel20196_MGFPeaklist (F058870)	status=active	F1NW23_CHICK,UPI00003AB706,UPI000044958C,UPI000060F708,UPI0000E8139D,UPI0000F0887D	TAX_GallusGallus_9031_20141114.fasta,UPARC14_2_GallusG_9031_20140417.fasta	191,615.00	100.00%	11	11	18	0.04%	7.04%	KFNALFAQGNYSEAAK	R	V	99.70%	47.9	44.8	28.8	40	43.7	5.51	0.444	0.469	0	0	2	0	2	1,758.88	2,202.00		815.8	367	382	Empty	TRUE	
20196-20225 HinckeM –TAXchicken	Uncategorized Sample	20196-20205_A	Mudpit_Ingel20196_MGFPeaklist (F058870)	status=active	F1NW23_CHICK,UPI00003AB706,UPI000044958C,UPI000060F708,UPI0000E8139D,UPI0000F0887D	TAX_GallusGallus_9031_20141114.fasta,UPARC14_2_GallusG_9031_20140417.fasta	191,615.00	100.00%	11	11	18	0.04%	7.04%	LLYNNVSNFGR	K	L	99.70%	71.2	44.3	24.2	60.3	36.6	2.11	0.404	0.455	0	2	0	0	2	1,296.67	2,211.50		535.55	1216	1226	Empty	TRUE	
20196-20225 HinckeM –TAXchicken	Uncategorized Sample	20196-20205_A	Mudpit_Ingel20196_MGFPeaklist (F058870)	status=active	F1NW23_CHICK,UPI00003AB706,UPI000044958C,UPI000060F708,UPI0000E8139D,UPI0000F0887D	TAX_GallusGallus_9031_20141114.fasta,UPARC14_2_GallusG_9031_20140417.fasta	191,615.00	100.00%	11	11	18	0.04%	7.04%	NNLAGAEELFAR	R	K	99.70%	72	45.1	39.9	37.3	35.8	2.85	0.355	0.417	0	2	0	0	2	1,304.66	2,436.00		530.53	355	366	Empty	TRUE	
20196-20225 HinckeM –TAXchicken	Uncategorized Sample	20196-20205_A	Mudpit_Ingel20196_MGFPeaklist (F058870)	status=active	F1NW23_CHICK,UPI00003AB706,UPI000044958C,UPI000060F708,UPI0000E8139D,UPI0000F0887D	TAX_GallusGallus_9031_20141114.fasta,UPARC14_2_GallusG_9031_20140417.fasta	191,615.00	100.00%	11	11	18	0.04%	7.04%	TLQIFNIEMK	K	S	71.70%	29.6	43.9	26.3	3.61					0	1	0	0	2	1,252.66	2,538.00		107.23	87	96	Empty	TRUE	UPI0003503C23,UPI000060F1BC,F1NYC4_CHICK,UPI000060F1BD,UPI0000E81090,UPI000060F1BB,UPI00003AAF6D
20196-20225 HinckeM –TAXchicken	Uncategorized Sample	20196-20205_A	Mudpit_Ingel20196_MGFPeaklist (F058870)	status=active	F1NW23_CHICK,UPI00003AB706,UPI000044958C,UPI000060F708,UPI0000E8139D,UPI0000F0887D	TAX_GallusGallus_9031_20141114.fasta,UPARC14_2_GallusG_9031_20140417.fasta	191,615.00	100.00%	11	11	18	0.04%	7.04%	VANVELYYK	K	A	99.70%	45.5	44	30.3	28.7	29	2.49	0.314	0.444	0	2	0	0	2	1,098.58	2,049.50		500.42	1398	1406	Empty	TRUE	UPI0003503C23,UPI000060F1BC,F1NYC4_CHICK,UPI000060F1BD,UPI0000E81090,UPI000060F1BB,UPI00003AAF6D
20196-20225 HinckeM –TAXchicken	Uncategorized Sample	20196-20205_A	Mudpit_Ingel20196_MGFPeaklist (F058870)	status=active	F1NW23_CHICK,UPI00003AB706,UPI000044958C,UPI000060F708,UPI0000E8139D,UPI0000F0887D	TAX_GallusGallus_9031_20141114.fasta,UPARC14_2_GallusG_9031_20140417.fasta	191,615.00	100.00%	11	11	18	0.04%	7.04%	VSQPIEGHAASFAQFK	K	M	95.20%	14.1	44.6	17.5	6.8	26.9	0.523	0.23	0.281	0	0	1	0	2	1,716.87	2,138.00		789.19	190	205	Empty	TRUE	
20196-20225 HinckeM –TAXchicken	Uncategorized Sample	20196-20205_A	Mudpit_Ingel20196_MGFPeaklist (F058870)	status=active	F1NW23_CHICK,UPI00003AB706,UPI000044958C,UPI000060F708,UPI0000E8139D,UPI0000F0887D	TAX_GallusGallus_9031_20141114.fasta,UPARC14_2_GallusG_9031_20140417.fasta	191,615.00	100.00%	11	11	18	0.04%	7.04%	YIEIYVQK	K	V	14.10%	8.67	43.6	17.1	0					0	1	0	0	2	1,055.58	2,126.00		248.12	799	806	Empty	TRUE	UPI0003503C23,UPI000060F1BC,F1NYC4_CHICK,UPI000060F1BD,UPI0000E81090,UPI000060F1BB,UPI00003AAF6D
20196-20225 HinckeM –TAXchicken	Uncategorized Sample	20196-20205_A	Mudpit_Ingel20196_MGFPeaklist (F058870)	status=active	F1NW23_CHICK,UPI00003AB706,UPI000044958C,UPI000060F708,UPI0000E8139D,UPI0000F0887D	TAX_GallusGallus_9031_20141114.fasta,UPARC14_2_GallusG_9031_20140417.fasta	191,615.00	100.00%	11	11	18	0.04%	7.04%	AVTFFTK	R	V	30.50%	15.9	43.5	0	4.62					0	1	0	0	2	813.4505	2,156.00		113.59	1435	1441	Empty	TRUE	
20196-20225 HinckeM –TAXchicken	Uncategorized Sample	20196-20205_A	Mudpit_Ingel20196_MGFPeaklist (F058870)	status=active	F1NW23_CHICK,UPI00003AB706,UPI000044958C,UPI000060F708,UPI0000E8139D,UPI0000F0887D	TAX_GallusGallus_9031_20141114.fasta,UPARC14_2_GallusG_9031_20140417.fasta	191,615.00	100.00%	11	11	18	0.04%	7.04%	FLRENPYYDSR	R	V	30.80%	16.2	44.5	24.9	2.72					0	0	2	0	2	1,459.70	1,841.00		492.9	893	903	Empty	TRUE	UPI0003503C23,UPI000060F1BC,F1NYC4_CHICK,UPI000060F1BD,UPI0000E81090,UPI000060F1BB,UPI00003AAF6D
20196-20225 HinckeM –TAXchicken	Uncategorized Sample	20196-20205_A	Mudpit_Ingel20196_MGFPeaklist (F058870)	status=active	F1NW23_CHICK,UPI00003AB706,UPI000044958C,UPI000060F708,UPI0000E8139D,UPI0000F0887D	TAX_GallusGallus_9031_20141114.fasta,UPARC14_2_GallusG_9031_20140417.fasta	191,615.00	100.00%	11	11	18	0.04%	7.04%	IAAYLFK	R	G	98.80%	43.2	41.9	30.4	27.5	27.2	0.585	0.239	0.5	0	2	0	0	2	825.4869	2,268.50		441.22	1510	1516	Empty	TRUE	
20196-20225 HinckeM –TAXchicken	Uncategorized Sample	20196-20205_A	Mudpit_Ingel20196_MGFPeaklist (F058870)	status=active	F1NW23_CHICK,UPI00003AB706,UPI000044958C,UPI000060F708,UPI0000E8139D,UPI0000F0887D	TAX_GallusGallus_9031_20141114.fasta,UPARC14_2_GallusG_9031_20140417.fasta	191,615.00	100.00%	11	11	18	0.04%	7.04%	IYIDSNNNPER	K	F	99.70%	63.7	44.7	22.9	50.2	34.9	4.42	0.372	0.409	0	2	0	0	2	1,334.63	1,703.50		379.2	882	892	Empty	TRUE	UPI0003503C23,UPI000060F1BC,F1NYC4_CHICK,UPI000060F1BD,UPI0000E81090,UPI000060F1BB,UPI00003AAF6D
20196-20225 HinckeM –TAXchicken	Uncategorized Sample	20196-20205_A	Mudpit_Ingel20196_MGFPeaklist (F058870)	status=active	F1NW23_CHICK,UPI00003AB706,UPI000044958C,UPI000060F708,UPI0000E8139D,UPI0000F0887D	TAX_GallusGallus_9031_20141114.fasta,UPARC14_2_GallusG_9031_20140417.fasta	191,615.00	100.00%	11	11	18	0.04%	7.04%	KFNALFAQGNYSEAAK	R	V	99.70%	47.9	44.8	28.8	40	43.7	5.51	0.444	0.469	0	0	2	0	2	1,758.88	2,202.00		815.8	367	382	Empty	TRUE	
20196-20225 HinckeM –TAXchicken	Uncategorized Sample	20196-20205_A	Mudpit_Ingel20196_MGFPeaklist (F058870)	status=active	F1NW23_CHICK,UPI00003AB706,UPI000044958C,UPI000060F708,UPI0000E8139D,UPI0000F0887D	TAX_GallusGallus_9031_20141114.fasta,UPARC14_2_GallusG_9031_20140417.fasta	191,615.00	100.00%	11	11	18	0.04%	7.04%	LLYNNVSNFGR	K	L	99.70%	71.2	44.3	24.2	60.3	36.6	2.11	0.404	0.455	0	2	0	0	2	1,296.67	2,211.50		535.55	1216	1226	Empty	TRUE	
20196-20225 HinckeM –TAXchicken	Uncategorized Sample	20196-20205_A	Mudpit_Ingel20196_MGFPeaklist (F058870)	status=active	F1NW23_CHICK,UPI00003AB706,UPI000044958C,UPI000060F708,UPI0000E8139D,UPI0000F0887D	TAX_GallusGallus_9031_20141114.fasta,UPARC14_2_GallusG_9031_20140417.fasta	191,615.00	100.00%	11	11	18	0.04%	7.04%	NNLAGAEELFAR	R	K	99.70%	72	45.1	39.9	37.3	35.8	2.85	0.355	0.417	0	2	0	0	2	1,304.66	2,436.00		530.53	355	366	Empty	TRUE	
20196-20225 HinckeM –TAXchicken	Uncategorized Sample	20196-20205_A	Mudpit_Ingel20196_MGFPeaklist (F058870)	status=active	F1NW23_CHICK,UPI00003AB706,UPI000044958C,UPI000060F708,UPI0000E8139D,UPI0000F0887D	TAX_GallusGallus_9031_20141114.fasta,UPARC14_2_GallusG_9031_20140417.fasta	191,615.00	100.00%	11	11	18	0.04%	7.04%	TLQIFNIEMK	K	S	71.70%	29.6	43.9	26.3	3.61					0	1	0	0	2	1,252.66	2,538.00		107.23	87	96	Empty	TRUE	UPI0003503C23,UPI000060F1BC,F1NYC4_CHICK,UPI000060F1BD,UPI0000E81090,UPI000060F1BB,UPI00003AAF6D
20196-20225 HinckeM –TAXchicken	Uncategorized Sample	20196-20205_A	Mudpit_Ingel20196_MGFPeaklist (F058870)	status=active	F1NW23_CHICK,UPI00003AB706,UPI000044958C,UPI000060F708,UPI0000E8139D,UPI0000F0887D	TAX_GallusGallus_9031_20141114.fasta,UPARC14_2_GallusG_9031_20140417.fasta	191,615.00	100.00%	11	11	18	0.04%	7.04%	VANVELYYK	K	A	99.70%	45.5	44	30.3	28.7	29	2.49	0.314	0.444	0	2	0	0	2	1,098.58	2,049.50		500.42	1398	1406	Empty	TRUE	UPI0003503C23,UPI000060F1BC,F1NYC4_CHICK,UPI000060F1BD,UPI0000E81090,UPI000060F1BB,UPI00003AAF6D
20196-20225 HinckeM –TAXchicken	Uncategorized Sample	20196-20205_A	Mudpit_Ingel20196_MGFPeaklist (F058870)	status=active	F1NW23_CHICK,UPI00003AB706,UPI000044958C,UPI000060F708,UPI0000E8139D,UPI0000F0887D	TAX_GallusGallus_9031_20141114.fasta,UPARC14_2_GallusG_9031_20140417.fasta	191,615.00	100.00%	11	11	18	0.04%	7.04%	VSQPIEGHAASFAQFK	K	M	95.20%	14.1	44.6	17.5	6.8	26.9	0.523	0.23	0.281	0	0	1	0	2	1,716.87	2,138.00		789.19	190	205	Empty	TRUE	
20196-20225 HinckeM –TAXchicken	Uncategorized Sample	20196-20205_A	Mudpit_Ingel20196_MGFPeaklist (F058870)	status=active	F1NW23_CHICK,UPI00003AB706,UPI000044958C,UPI000060F708,UPI0000E8139D,UPI0000F0887D	TAX_GallusGallus_9031_20141114.fasta,UPARC14_2_GallusG_9031_20140417.fasta	191,615.00	100.00%	11	11	18	0.04%	7.04%	YIEIYVQK	K	V	14.10%	8.67	43.6	17.1	0					0	1	0	0	2	1,055.58	2,126.00		248.12	799	806	Empty	TRUE	UPI0003503C23,UPI000060F1BC,F1NYC4_CHICK,UPI000060F1BD,UPI0000E81090,UPI000060F1BB,UPI00003AAF6D
20196-20225 HinckeM –TAXchicken	Uncategorized Sample	20196-20205_A	Mudpit_Ingel20196_MGFPeaklist (F058870)	status=active	F1NW23_CHICK,UPI00003AB706,UPI000044958C,UPI000060F708,UPI0000E8139D,UPI0000F0887D	TAX_GallusGallus_9031_20141114.fasta,UPARC14_2_GallusG_9031_20140417.fasta	191,615.00	100.00%	11	11	18	0.04%	7.04%	AVTFFTK	R	V	30.50%	15.9	43.5	0	4.62					0	1	0	0	2	813.4505	2,156.00		113.59	1435	1441	Empty	TRUE	
20196-20225 HinckeM –TAXchicken	Uncategorized Sample	20196-20205_A	Mudpit_Ingel20196_MGFPeaklist (F058870)	status=active	F1NW23_CHICK,UPI00003AB706,UPI000044958C,UPI000060F708,UPI0000E8139D,UPI0000F0887D	TAX_GallusGallus_9031_20141114.fasta,UPARC14_2_GallusG_9031_20140417.fasta	191,615.00	100.00%	11	11	18	0.04%	7.04%	FLRENPYYDSR	R	V	30.80%	16.2	44.5	24.9	2.72					0	0	2	0	2	1,459.70	1,841.00		492.9	893	903	Empty	TRUE	UPI0003503C23,UPI000060F1BC,F1NYC4_CHICK,UPI000060F1BD,UPI0000E81090,UPI000060F1BB,UPI00003AAF6D
20196-20225 HinckeM –TAXchicken	Uncategorized Sample	20196-20205_A	Mudpit_Ingel20196_MGFPeaklist (F058870)	status=active	F1NW23_CHICK,UPI00003AB706,UPI000044958C,UPI000060F708,UPI0000E8139D,UPI0000F0887D	TAX_GallusGallus_9031_20141114.fasta,UPARC14_2_GallusG_9031_20140417.fasta	191,615.00	100.00%	11	11	18	0.04%	7.04%	IAAYLFK	R	G	98.80%	43.2	41.9	30.4	27.5	27.2	0.585	0.239	0.5	0	2	0	0	2	825.4869	2,268.50		441.22	1510	1516	Empty	TRUE	
20196-20225 HinckeM –TAXchicken	Uncategorized Sample	20196-20205_A	Mudpit_Ingel20196_MGFPeaklist (F058870)	status=active	F1NW23_CHICK,UPI00003AB706,UPI000044958C,UPI000060F708,UPI0000E8139D,UPI0000F0887D	TAX_GallusGallus_9031_20141114.fasta,UPARC14_2_GallusG_9031_20140417.fasta	191,615.00	100.00%	11	11	18	0.04%	7.04%	IYIDSNNNPER	K	F	99.70%	63.7	44.7	22.9	50.2	34.9	4.42	0.372	0.409	0	2	0	0	2	1,334.63	1,703.50		379.2	882	892	Empty	TRUE	UPI0003503C23,UPI000060F1BC,F1NYC4_CHICK,UPI000060F1BD,UPI0000E81090,UPI000060F1BB,UPI00003AAF6D
20196-20225 HinckeM –TAXchicken	Uncategorized Sample	20196-20205_A	Mudpit_Ingel20196_MGFPeaklist (F058870)	status=active	F1NW23_CHICK,UPI00003AB706,UPI000044958C,UPI000060F708,UPI0000E8139D,UPI0000F0887D	TAX_GallusGallus_9031_20141114.fasta,UPARC14_2_GallusG_9031_20140417.fasta	191,615.00	100.00%	11	11	18	0.04%	7.04%	KFNALFAQGNYSEAAK	R	V	99.70%	47.9	44.8	28.8	40	43.7	5.51	0.444	0.469	0	0	2	0	2	1,758.88	2,202.00		815.8	367	382	Empty	TRUE	
20196-20225 HinckeM –TAXchicken	Uncategorized Sample	20196-20205_A	Mudpit_Ingel20196_MGFPeaklist (F058870)	status=active	F1NW23_CHICK,UPI00003AB706,UPI000044958C,UPI000060F708,UPI0000E8139D,UPI0000F0887D	TAX_GallusGallus_9031_20141114.fasta,UPARC14_2_GallusG_9031_20140417.fasta	191,615.00	100.00%	11	11	18	0.04%	7.04%	LLYNNVSNFGR	K	L	99.70%	71.2	44.3	24.2	60.3	36.6	2.11	0.404	0.455	0	2	0	0	2	1,296.67	2,211.50		535.55	1216	1226	Empty	TRUE	
20196-20225 HinckeM –TAXchicken	Uncategorized Sample	20196-20205_A	Mudpit_Ingel20196_MGFPeaklist (F058870)	status=active	F1NW23_CHICK,UPI00003AB706,UPI000044958C,UPI000060F708,UPI0000E8139D,UPI0000F0887D	TAX_GallusGallus_9031_20141114.fasta,UPARC14_2_GallusG_9031_20140417.fasta	191,615.00	100.00%	11	11	18	0.04%	7.04%	NNLAGAEELFAR	R	K	99.70%	72	45.1	39.9	37.3	35.8	2.85	0.355	0.417	0	2	0	0	2	1,304.66	2,436.00		530.53	355	366	Empty	TRUE	
20196-20225 HinckeM –TAXchicken	Uncategorized Sample	20196-20205_A	Mudpit_Ingel20196_MGFPeaklist (F058870)	status=active	F1NW23_CHICK,UPI00003AB706,UPI000044958C,UPI000060F708,UPI0000E8139D,UPI0000F0887D	TAX_GallusGallus_9031_20141114.fasta,UPARC14_2_GallusG_9031_20140417.fasta	191,615.00	100.00%	11	11	18	0.04%	7.04%	TLQIFNIEMK	K	S	71.70%	29.6	43.9	26.3	3.61					0	1	0	0	2	1,252.66	2,538.00		107.23	87	96	Empty	TRUE	UPI0003503C23,UPI000060F1BC,F1NYC4_CHICK,UPI000060F1BD,UPI0000E81090,UPI000060F1BB,UPI00003AAF6D
20196-20225 HinckeM –TAXchicken	Uncategorized Sample	20196-20205_A	Mudpit_Ingel20196_MGFPeaklist (F058870)	status=active	F1NW23_CHICK,UPI00003AB706,UPI000044958C,UPI000060F708,UPI0000E8139D,UPI0000F0887D	TAX_GallusGallus_9031_20141114.fasta,UPARC14_2_GallusG_9031_20140417.fasta	191,615.00	100.00%	11	11	18	0.04%	7.04%	VANVELYYK	K	A	99.70%	45.5	44	30.3	28.7	29	2.49	0.314	0.444	0	2	0	0	2	1,098.58	2,049.50		500.42	1398	1406	Empty	TRUE	UPI0003503C23,UPI000060F1BC,F1NYC4_CHICK,UPI000060F1BD,UPI0000E81090,UPI000060F1BB,UPI00003AAF6D
20196-20225 HinckeM –TAXchicken	Uncategorized Sample	20196-20205_A	Mudpit_Ingel20196_MGFPeaklist (F058870)	status=active	F1NW23_CHICK,UPI00003AB706,UPI000044958C,UPI000060F708,UPI0000E8139D,UPI0000F0887D	TAX_GallusGallus_9031_20141114.fasta,UPARC14_2_GallusG_9031_20140417.fasta	191,615.00	100.00%	11	11	18	0.04%	7.04%	VSQPIEGHAASFAQFK	K	M	95.20%	14.1	44.6	17.5	6.8	26.9	0.523	0.23	0.281	0	0	1	0	2	1,716.87	2,138.00		789.19	190	205	Empty	TRUE	
20196-20225 HinckeM –TAXchicken	Uncategorized Sample	20196-20205_A	Mudpit_Ingel20196_MGFPeaklist (F058870)	status=active	F1NW23_CHICK,UPI00003AB706,UPI000044958C,UPI000060F708,UPI0000E8139D,UPI0000F0887D	TAX_GallusGallus_9031_20141114.fasta,UPARC14_2_GallusG_9031_20140417.fasta	191,615.00	100.00%	11	11	18	0.04%	7.04%	YIEIYVQK	K	V	14.10%	8.67	43.6	17.1	0					0	1	0	0	2	1,055.58	2,126.00		248.12	799	806	Empty	TRUE	UPI0003503C23,UPI000060F1BC,F1NYC4_CHICK,UPI000060F1BD,UPI0000E81090,UPI000060F1BB,UPI00003AAF6D
20196-20225 HinckeM –TAXchicken	Uncategorized Sample	20196-20205_A	Mudpit_Ingel20196_MGFPeaklist (F058870)	status=active	F1NW23_CHICK,UPI00003AB706,UPI000044958C,UPI000060F708,UPI0000E8139D,UPI0000F0887D	TAX_GallusGallus_9031_20141114.fasta,UPARC14_2_GallusG_9031_20140417.fasta	191,615.00	100.00%	11	11	18	0.04%	7.04%	AVTFFTK	R	V	30.50%	15.9	43.5	0	4.62					0	1	0	0	2	813.4505	2,156.00		113.59	1435	1441	Empty	TRUE	
20196-20225 HinckeM –TAXchicken	Uncategorized Sample	20196-20205_A	Mudpit_Ingel20196_MGFPeaklist (F058870)	status=active	F1NW23_CHICK,UPI00003AB706,UPI000044958C,UPI000060F708,UPI0000E8139D,UPI0000F0887D	TAX_GallusGallus_9031_20141114.fasta,UPARC14_2_GallusG_9031_20140417.fasta	191,615.00	100.00%	11	11	18	0.04%	7.04%	FLRENPYYDSR	R	V	30.80%	16.2	44.5	24.9	2.72					0	0	2	0	2	1,459.70	1,841.00		492.9	893	903	Empty	TRUE	UPI0003503C23,UPI000060F1BC,F1NYC4_CHICK,UPI000060F1BD,UPI0000E81090,UPI000060F1BB,UPI00003AAF6D
20196-20225 HinckeM –TAXchicken	Uncategorized Sample	20196-20205_A	Mudpit_Ingel20196_MGFPeaklist (F058870)	status=active	F1NW23_CHICK,UPI00003AB706,UPI000044958C,UPI000060F708,UPI0000E8139D,UPI0000F0887D	TAX_GallusGallus_9031_20141114.fasta,UPARC14_2_GallusG_9031_20140417.fasta	191,615.00	100.00%	11	11	18	0.04%	7.04%	IAAYLFK	R	G	98.80%	43.2	41.9	30.4	27.5	27.2	0.585	0.239	0.5	0	2	0	0	2	825.4869	2,268.50		441.22	1510	1516	Empty	TRUE	
20196-20225 HinckeM –TAXchicken	Uncategorized Sample	20196-20205_A	Mudpit_Ingel20196_MGFPeaklist (F058870)	status=active	F1NW23_CHICK,UPI00003AB706,UPI000044958C,UPI000060F708,UPI0000E8139D,UPI0000F0887D	TAX_GallusGallus_9031_20141114.fasta,UPARC14_2_GallusG_9031_20140417.fasta	191,615.00	100.00%	11	11	18	0.04%	7.04%	IYIDSNNNPER	K	F	99.70%	63.7	44.7	22.9	50.2	34.9	4.42	0.372	0.409	0	2	0	0	2	1,334.63	1,703.50		379.2	882	892	Empty	TRUE	UPI0003503C23,UPI000060F1BC,F1NYC4_CHICK,UPI000060F1BD,UPI0000E81090,UPI000060F1BB,UPI00003AAF6D
20196-20225 HinckeM –TAXchicken	Uncategorized Sample	20196-20205_A	Mudpit_Ingel20196_MGFPeaklist (F058870)	status=active	F1NW23_CHICK,UPI00003AB706,UPI000044958C,UPI000060F708,UPI0000E8139D,UPI0000F0887D	TAX_GallusGallus_9031_20141114.fasta,UPARC14_2_GallusG_9031_20140417.fasta	191,615.00	100.00%	11	11	18	0.04%	7.04%	KFNALFAQGNYSEAAK	R	V	99.70%	47.9	44.8	28.8	40	43.7	5.51	0.444	0.469	0	0	2	0	2	1,758.88	2,202.00		815.8	367	382	Empty	TRUE	
20196-20225 HinckeM –TAXchicken	Uncategorized Sample	20196-20205_A	Mudpit_Ingel20196_MGFPeaklist (F058870)	status=active	F1NW23_CHICK,UPI00003AB706,UPI000044958C,UPI000060F708,UPI0000E8139D,UPI0000F0887D	TAX_GallusGallus_9031_20141114.fasta,UPARC14_2_GallusG_9031_20140417.fasta	191,615.00	100.00%	11	11	18	0.04%	7.04%	LLYNNVSNFGR	K	L	99.70%	71.2	44.3	24.2	60.3	36.6	2.11	0.404	0.455	0	2	0	0	2	1,296.67	2,211.50		535.55	1216	1226	Empty	TRUE	
20196-20225 HinckeM –TAXchicken	Uncategorized Sample	20196-20205_A	Mudpit_Ingel20196_MGFPeaklist (F058870)	status=active	F1NW23_CHICK,UPI00003AB706,UPI000044958C,UPI000060F708,UPI0000E8139D,UPI0000F0887D	TAX_GallusGallus_9031_20141114.fasta,UPARC14_2_GallusG_9031_20140417.fasta	191,615.00	100.00%	11	11	18	0.04%	7.04%	NNLAGAEELFAR	R	K	99.70%	72	45.1	39.9	37.3	35.8	2.85	0.355	0.417	0	2	0	0	2	1,304.66	2,436.00		530.53	355	366	Empty	TRUE	
20196-20225 HinckeM –TAXchicken	Uncategorized Sample	20196-20205_A	Mudpit_Ingel20196_MGFPeaklist (F058870)	status=active	F1NW23_CHICK,UPI00003AB706,UPI000044958C,UPI000060F708,UPI0000E8139D,UPI0000F0887D	TAX_GallusGallus_9031_20141114.fasta,UPARC14_2_GallusG_9031_20140417.fasta	191,615.00	100.00%	11	11	18	0.04%	7.04%	TLQIFNIEMK	K	S	71.70%	29.6	43.9	26.3	3.61					0	1	0	0	2	1,252.66	2,538.00		107.23	87	96	Empty	TRUE	UPI0003503C23,UPI000060F1BC,F1NYC4_CHICK,UPI000060F1BD,UPI0000E81090,UPI000060F1BB,UPI00003AAF6D
20196-20225 HinckeM –TAXchicken	Uncategorized Sample	20196-20205_A	Mudpit_Ingel20196_MGFPeaklist (F058870)	status=active	F1NW23_CHICK,UPI00003AB706,UPI000044958C,UPI000060F708,UPI0000E8139D,UPI0000F0887D	TAX_GallusGallus_9031_20141114.fasta,UPARC14_2_GallusG_9031_20140417.fasta	191,615.00	100.00%	11	11	18	0.04%	7.04%	VANVELYYK	K	A	99.70%	45.5	44	30.3	28.7	29	2.49	0.314	0.444	0	2	0	0	2	1,098.58	2,049.50		500.42	1398	1406	Empty	TRUE	UPI0003503C23,UPI000060F1BC,F1NYC4_CHICK,UPI000060F1BD,UPI0000E81090,UPI000060F1BB,UPI00003AAF6D
20196-20225 HinckeM –TAXchicken	Uncategorized Sample	20196-20205_A	Mudpit_Ingel20196_MGFPeaklist (F058870)	status=active	F1NW23_CHICK,UPI00003AB706,UPI000044958C,UPI000060F708,UPI0000E8139D,UPI0000F0887D	TAX_GallusGallus_9031_20141114.fasta,UPARC14_2_GallusG_9031_20140417.fasta	191,615.00	100.00%	11	11	18	0.04%	7.04%	VSQPIEGHAASFAQFK	K	M	95.20%	14.1	44.6	17.5	6.8	26.9	0.523	0.23	0.281	0	0	1	0	2	1,716.87	2,138.00		789.19	190	205	Empty	TRUE	
20196-20225 HinckeM –TAXchicken	Uncategorized Sample	20196-20205_A	Mudpit_Ingel20196_MGFPeaklist (F058870)	status=active	F1NW23_CHICK,UPI00003AB706,UPI000044958C,UPI000060F708,UPI0000E8139D,UPI0000F0887D	TAX_GallusGallus_9031_20141114.fasta,UPARC14_2_GallusG_9031_20140417.fasta	191,615.00	100.00%	11	11	18	0.04%	7.04%	YIEIYVQK	K	V	14.10%	8.67	43.6	17.1	0					0	1	0	0	2	1,055.58	2,126.00		248.12	799	806	Empty	TRUE	UPI0003503C23,UPI000060F1BC,F1NYC4_CHICK,UPI000060F1BD,UPI0000E81090,UPI000060F1BB,UPI00003AAF6D
20196-20225 HinckeM –TAXchicken	Uncategorized Sample	20196-20205_A	Mudpit_Ingel20196_MGFPeaklist (F058870)	status=active	F1NW23_CHICK,UPI00003AB706,UPI000044958C,UPI000060F708,UPI0000E8139D,UPI0000F0887D	TAX_GallusGallus_9031_20141114.fasta,UPARC14_2_GallusG_9031_20140417.fasta	191,615.00	100.00%	11	11	18	0.04%	7.04%	AVTFFTK	R	V	30.50%	15.9	43.5	0	4.62					0	1	0	0	2	813.4505	2,156.00		113.59	1435	1441	Empty	TRUE	
20196-20225 HinckeM –TAXchicken	Uncategorized Sample	20196-20205_A	Mudpit_Ingel20196_MGFPeaklist (F058870)	status=active	F1NW23_CHICK,UPI00003AB706,UPI000044958C,UPI000060F708,UPI0000E8139D,UPI0000F0887D	TAX_GallusGallus_9031_20141114.fasta,UPARC14_2_GallusG_9031_20140417.fasta	191,615.00	100.00%	11	11	18	0.04%	7.04%	FLRENPYYDSR	R	V	30.80%	16.2	44.5	24.9	2.72					0	0	2	0	2	1,459.70	1,841.00		492.9	893	903	Empty	TRUE	UPI0003503C23,UPI000060F1BC,F1NYC4_CHICK,UPI000060F1BD,UPI0000E81090,UPI000060F1BB,UPI00003AAF6D
20196-20225 HinckeM –TAXchicken	Uncategorized Sample	20196-20205_A	Mudpit_Ingel20196_MGFPeaklist (F058870)	status=active	F1NW23_CHICK,UPI00003AB706,UPI000044958C,UPI000060F708,UPI0000E8139D,UPI0000F0887D	TAX_GallusGallus_9031_20141114.fasta,UPARC14_2_GallusG_9031_20140417.fasta	191,615.00	100.00%	11	11	18	0.04%	7.04%	IAAYLFK	R	G	98.80%	43.2	41.9	30.4	27.5	27.2	0.585	0.239	0.5	0	2	0	0	2	825.4869	2,268.50		441.22	1510	1516	Empty	TRUE	
20196-20225 HinckeM –TAXchicken	Uncategorized Sample	20196-20205_A	Mudpit_Ingel20196_MGFPeaklist (F058870)	status=active	F1NW23_CHICK,UPI00003AB706,UPI000044958C,UPI000060F708,UPI0000E8139D,UPI0000F0887D	TAX_GallusGallus_9031_20141114.fasta,UPARC14_2_GallusG_9031_20140417.fasta	191,615.00	100.00%	11	11	18	0.04%	7.04%	IYIDSNNNPER	K	F	99.70%	63.7	44.7	22.9	50.2	34.9	4.42	0.372	0.409	0	2	0	0	2	1,334.63	1,703.50		379.2	882	892	Empty	TRUE	UPI0003503C23,UPI000060F1BC,F1NYC4_CHICK,UPI000060F1BD,UPI0000E81090,UPI000060F1BB,UPI00003AAF6D
20196-20225 HinckeM –TAXchicken	Uncategorized Sample	20196-20205_A	Mudpit_Ingel20196_MGFPeaklist (F058870)	status=active	F1NW23_CHICK,UPI00003AB706,UPI000044958C,UPI000060F708,UPI0000E8139D,UPI0000F0887D	TAX_GallusGallus_9031_20141114.fasta,UPARC14_2_GallusG_9031_20140417.fasta	191,615.00	100.00%	11	11	18	0.04%	7.04%	KFNALFAQGNYSEAAK	R	V	99.70%	47.9	44.8	28.8	40	43.7	5.51	0.444	0.469	0	0	2	0	2	1,758.88	2,202.00		815.8	367	382	Empty	TRUE	
20196-20225 HinckeM –TAXchicken	Uncategorized Sample	20196-20205_A	Mudpit_Ingel20196_MGFPeaklist (F058870)	status=active	F1NW23_CHICK,UPI00003AB706,UPI000044958C,UPI000060F708,UPI0000E8139D,UPI0000F0887D	TAX_GallusGallus_9031_20141114.fasta,UPARC14_2_GallusG_9031_20140417.fasta	191,615.00	100.00%	11	11	18	0.04%	7.04%	LLYNNVSNFGR	K	L	99.70%	71.2	44.3	24.2	60.3	36.6	2.11	0.404	0.455	0	2	0	0	2	1,296.67	2,211.50		535.55	1216	1226	Empty	TRUE	
20196-20225 HinckeM –TAXchicken	Uncategorized Sample	20196-20205_A	Mudpit_Ingel20196_MGFPeaklist (F058870)	status=active	F1NW23_CHICK,UPI00003AB706,UPI000044958C,UPI000060F708,UPI0000E8139D,UPI0000F0887D	TAX_GallusGallus_9031_20141114.fasta,UPARC14_2_GallusG_9031_20140417.fasta	191,615.00	100.00%	11	11	18	0.04%	7.04%	NNLAGAEELFAR	R	K	99.70%	72	45.1	39.9	37.3	35.8	2.85	0.355	0.417	0	2	0	0	2	1,304.66	2,436.00		530.53	355	366	Empty	TRUE	
20196-20225 HinckeM –TAXchicken	Uncategorized Sample	20196-20205_A	Mudpit_Ingel20196_MGFPeaklist (F058870)	status=active	F1NW23_CHICK,UPI00003AB706,UPI000044958C,UPI000060F708,UPI0000E8139D,UPI0000F0887D	TAX_GallusGallus_9031_20141114.fasta,UPARC14_2_GallusG_9031_20140417.fasta	191,615.00	100.00%	11	11	18	0.04%	7.04%	TLQIFNIEMK	K	S	71.70%	29.6	43.9	26.3	3.61					0	1	0	0	2	1,252.66	2,538.00		107.23	87	96	Empty	TRUE	UPI0003503C23,UPI000060F1BC,F1NYC4_CHICK,UPI000060F1BD,UPI0000E81090,UPI000060F1BB,UPI00003AAF6D
20196-20225 HinckeM –TAXchicken	Uncategorized Sample	20196-20205_A	Mudpit_Ingel20196_MGFPeaklist (F058870)	status=active	F1NW23_CHICK,UPI00003AB706,UPI000044958C,UPI000060F708,UPI0000E8139D,UPI0000F0887D	TAX_GallusGallus_9031_20141114.fasta,UPARC14_2_GallusG_9031_20140417.fasta	191,615.00	100.00%	11	11	18	0.04%	7.04%	VANVELYYK	K	A	99.70%	45.5	44	30.3	28.7	29	2.49	0.314	0.444	0	2	0	0	2	1,098.58	2,049.50		500.42	1398	1406	Empty	TRUE	UPI0003503C23,UPI000060F1BC,F1NYC4_CHICK,UPI000060F1BD,UPI0000E81090,UPI000060F1BB,UPI00003AAF6D
20196-20225 HinckeM –TAXchicken	Uncategorized Sample	20196-20205_A	Mudpit_Ingel20196_MGFPeaklist (F058870)	status=active	F1NW23_CHICK,UPI00003AB706,UPI000044958C,UPI000060F708,UPI0000E8139D,UPI0000F0887D	TAX_GallusGallus_9031_20141114.fasta,UPARC14_2_GallusG_9031_20140417.fasta	191,615.00	100.00%	11	11	18	0.04%	7.04%	VSQPIEGHAASFAQFK	K	M	95.20%	14.1	44.6	17.5	6.8	26.9	0.523	0.23	0.281	0	0	1	0	2	1,716.87	2,138.00		789.19	190	205	Empty	TRUE	
20196-20225 HinckeM –TAXchicken	Uncategorized Sample	20196-20205_A	Mudpit_Ingel20196_MGFPeaklist (F058870)	status=active	F1NW23_CHICK,UPI00003AB706,UPI000044958C,UPI000060F708,UPI0000E8139D,UPI0000F0887D	TAX_GallusGallus_9031_20141114.fasta,UPARC14_2_GallusG_9031_20140417.fasta	191,615.00	100.00%	11	11	18	0.04%	7.04%	YIEIYVQK	K	V	14.10%	8.67	43.6	17.1	0					0	1	0	0	2	1,055.58	2,126.00		248.12	799	806	Empty	TRUE	UPI0003503C23,UPI000060F1BC,F1NYC4_CHICK,UPI000060F1BD,UPI0000E81090,UPI000060F1BB,UPI00003AAF6D
20196-20225 HinckeM –TAXchicken	Uncategorized Sample	20196-20205_A	Mudpit_Ingel20196_MGFPeaklist (F058870)	status=active	F1NW23_CHICK,UPI00003AB706,UPI000044958C,UPI000060F708,UPI0000E8139D,UPI0000F0887D	TAX_GallusGallus_9031_20141114.fasta,UPARC14_2_GallusG_9031_20140417.fasta	191,615.00	100.00%	11	11	18	0.04%	7.04%	AVTFFTK	R	V	30.50%	15.9	43.5	0	4.62					0	1	0	0	2	813.4505	2,156.00		113.59	1435	1441	Empty	TRUE	
20196-20225 HinckeM –TAXchicken	Uncategorized Sample	20196-20205_A	Mudpit_Ingel20196_MGFPeaklist (F058870)	status=active	F1NW23_CHICK,UPI00003AB706,UPI000044958C,UPI000060F708,UPI0000E8139D,UPI0000F0887D	TAX_GallusGallus_9031_20141114.fasta,UPARC14_2_GallusG_9031_20140417.fasta	191,615.00	100.00%	11	11	18	0.04%	7.04%	FLRENPYYDSR	R	V	30.80%	16.2	44.5	24.9	2.72					0	0	2	0	2	1,459.70	1,841.00		492.9	893	903	Empty	TRUE	UPI0003503C23,UPI000060F1BC,F1NYC4_CHICK,UPI000060F1BD,UPI0000E81090,UPI000060F1BB,UPI00003AAF6D
20196-20225 HinckeM –TAXchicken	Uncategorized Sample	20196-20205_A	Mudpit_Ingel20196_MGFPeaklist (F058870)	status=active	F1NW23_CHICK,UPI00003AB706,UPI000044958C,UPI000060F708,UPI0000E8139D,UPI0000F0887D	TAX_GallusGallus_9031_20141114.fasta,UPARC14_2_GallusG_9031_20140417.fasta	191,615.00	100.00%	11	11	18	0.04%	7.04%	IAAYLFK	R	G	98.80%	43.2	41.9	30.4	27.5	27.2	0.585	0.239	0.5	0	2	0	0	2	825.4869	2,268.50		441.22	1510	1516	Empty	TRUE	
20196-20225 HinckeM –TAXchicken	Uncategorized Sample	20196-20205_A	Mudpit_Ingel20196_MGFPeaklist (F058870)	status=active	F1NW23_CHICK,UPI00003AB706,UPI000044958C,UPI000060F708,UPI0000E8139D,UPI0000F0887D	TAX_GallusGallus_9031_20141114.fasta,UPARC14_2_GallusG_9031_20140417.fasta	191,615.00	100.00%	11	11	18	0.04%	7.04%	IYIDSNNNPER	K	F	99.70%	63.7	44.7	22.9	50.2	34.9	4.42	0.372	0.409	0	2	0	0	2	1,334.63	1,703.50		379.2	882	892	Empty	TRUE	UPI0003503C23,UPI000060F1BC,F1NYC4_CHICK,UPI000060F1BD,UPI0000E81090,UPI000060F1BB,UPI00003AAF6D
20196-20225 HinckeM –TAXchicken	Uncategorized Sample	20196-20205_A	Mudpit_Ingel20196_MGFPeaklist (F058870)	status=active	F1NW23_CHICK,UPI00003AB706,UPI000044958C,UPI000060F708,UPI0000E8139D,UPI0000F0887D	TAX_GallusGallus_9031_20141114.fasta,UPARC14_2_GallusG_9031_20140417.fasta	191,615.00	100.00%	11	11	18	0.04%	7.04%	KFNALFAQGNYSEAAK	R	V	99.70%	47.9	44.8	28.8	40	43.7	5.51	0.444	0.469	0	0	2	0	2	1,758.88	2,202.00		815.8	367	382	Empty	TRUE	
20196-20225 HinckeM –TAXchicken	Uncategorized Sample	20196-20205_A	Mudpit_Ingel20196_MGFPeaklist (F058870)	status=active	F1NW23_CHICK,UPI00003AB706,UPI000044958C,UPI000060F708,UPI0000E8139D,UPI0000F0887D	TAX_GallusGallus_9031_20141114.fasta,UPARC14_2_GallusG_9031_20140417.fasta	191,615.00	100.00%	11	11	18	0.04%	7.04%	LLYNNVSNFGR	K	L	99.70%	71.2	44.3	24.2	60.3	36.6	2.11	0.404	0.455	0	2	0	0	2	1,296.67	2,211.50		535.55	1216	1226	Empty	TRUE	
20196-20225 HinckeM –TAXchicken	Uncategorized Sample	20196-20205_A	Mudpit_Ingel20196_MGFPeaklist (F058870)	status=active	F1NW23_CHICK,UPI00003AB706,UPI000044958C,UPI000060F708,UPI0000E8139D,UPI0000F0887D	TAX_GallusGallus_9031_20141114.fasta,UPARC14_2_GallusG_9031_20140417.fasta	191,615.00	100.00%	11	11	18	0.04%	7.04%	NNLAGAEELFAR	R	K	99.70%	72	45.1	39.9	37.3	35.8	2.85	0.355	0.417	0	2	0	0	2	1,304.66	2,436.00		530.53	355	366	Empty	TRUE	
20196-20225 HinckeM –TAXchicken	Uncategorized Sample	20196-20205_A	Mudpit_Ingel20196_MGFPeaklist (F058870)	status=active	F1NW23_CHICK,UPI00003AB706,UPI000044958C,UPI000060F708,UPI0000E8139D,UPI0000F0887D	TAX_GallusGallus_9031_20141114.fasta,UPARC14_2_GallusG_9031_20140417.fasta	191,615.00	100.00%	11	11	18	0.04%	7.04%	TLQIFNIEMK	K	S	71.70%	29.6	43.9	26.3	3.61					0	1	0	0	2	1,252.66	2,538.00		107.23	87	96	Empty	TRUE	UPI0003503C23,UPI000060F1BC,F1NYC4_CHICK,UPI000060F1BD,UPI0000E81090,UPI000060F1BB,UPI00003AAF6D
20196-20225 HinckeM –TAXchicken	Uncategorized Sample	20196-20205_A	Mudpit_Ingel20196_MGFPeaklist (F058870)	status=active	F1NW23_CHICK,UPI00003AB706,UPI000044958C,UPI000060F708,UPI0000E8139D,UPI0000F0887D	TAX_GallusGallus_9031_20141114.fasta,UPARC14_2_GallusG_9031_20140417.fasta	191,615.00	100.00%	11	11	18	0.04%	7.04%	VANVELYYK	K	A	99.70%	45.5	44	30.3	28.7	29	2.49	0.314	0.444	0	2	0	0	2	1,098.58	2,049.50		500.42	1398	1406	Empty	TRUE	UPI0003503C23,UPI000060F1BC,F1NYC4_CHICK,UPI000060F1BD,UPI0000E81090,UPI000060F1BB,UPI00003AAF6D
20196-20225 HinckeM –TAXchicken	Uncategorized Sample	20196-20205_A	Mudpit_Ingel20196_MGFPeaklist (F058870)	status=active	F1NW23_CHICK,UPI00003AB706,UPI000044958C,UPI000060F708,UPI0000E8139D,UPI0000F0887D	TAX_GallusGallus_9031_20141114.fasta,UPARC14_2_GallusG_9031_20140417.fasta	191,615.00	100.00%	11	11	18	0.04%	7.04%	VSQPIEGHAASFAQFK	K	M	95.20%	14.1	44.6	17.5	6.8	26.9	0.523	0.23	0.281	0	0	1	0	2	1,716.87	2,138.00		789.19	190	205	Empty	TRUE	
20196-20225 HinckeM –TAXchicken	Uncategorized Sample	20196-20205_A	Mudpit_Ingel20196_MGFPeaklist (F058870)	status=active	F1NW23_CHICK,UPI00003AB706,UPI000044958C,UPI000060F708,UPI0000E8139D,UPI0000F0887D	TAX_GallusGallus_9031_20141114.fasta,UPARC14_2_GallusG_9031_20140417.fasta	191,615.00	100.00%	11	11	18	0.04%	7.04%	YIEIYVQK	K	V	14.10%	8.67	43.6	17.1	0					0	1	0	0	2	1,055.58	2,126.00		248.12	799	806	Empty	TRUE	UPI0003503C23,UPI000060F1BC,F1NYC4_CHICK,UPI000060F1BD,UPI0000E81090,UPI000060F1BB,UPI00003AAF6D
20196-20225 HinckeM –TAXchicken	Uncategorized Sample	20196-20205_A	Mudpit_Ingel20196_MGFPeaklist (F058870)	Uncharacterized protein OS=Gallus gallus GN=OGDH PE=2 SV=1	Q5ZJA7_CHICK,UPI00004437CA	TAX_GallusGallus_9031_20141114.fasta,UPARC14_2_GallusG_9031_20140417.fasta	115,182.30	100.00%	3	3	3	0.01%	3.25%	SSPYPTDVAR	R	V	99.70%	37	44.8	25.8	14.5	27.8	2.27	0.302	0.35	0	1	0	0	2	1,092.53	1,690.00		312.86	452	461	Empty	TRUE	
20196-20225 HinckeM –TAXchicken	Uncategorized Sample	20196-20205_A	Mudpit_Ingel20196_MGFPeaklist (F058870)	Uncharacterized protein OS=Gallus gallus GN=OGDH PE=2 SV=1	Q5ZJA7_CHICK,UPI00004437CA	TAX_GallusGallus_9031_20141114.fasta,UPARC14_2_GallusG_9031_20140417.fasta	115,182.30	100.00%	3	3	3	0.01%	3.25%	VIPDGGPAAQSPQNVR	R	R	85.70%	23.7	44.8	22.9	17.8					0	0	1	0	2	1,605.83	1,733.00		395.99	877	892	Empty	TRUE	
20196-20225 HinckeM –TAXchicken	Uncategorized Sample	20196-20205_A	Mudpit_Ingel20196_MGFPeaklist (F058870)	Uncharacterized protein OS=Gallus gallus GN=OGDH PE=2 SV=1	Q5ZJA7_CHICK,UPI00004437CA	TAX_GallusGallus_9031_20141114.fasta,UPARC14_2_GallusG_9031_20140417.fasta	115,182.30	100.00%	3	3	3	0.01%	3.25%	VYYDLTR	K	E	9.10%	24.3	44.9	0	0					0	1	0	0	2	929.4727	1,895.00		92.69	901	907	Empty	TRUE	
20196-20225 HinckeM –TAXchicken	Uncategorized Sample	20196-20205_A	Mudpit_Ingel20196_MGFPeaklist (F058870)	Uncharacterized protein OS=Gallus gallus GN=OGDH PE=2 SV=1	Q5ZJA7_CHICK,UPI00004437CA	TAX_GallusGallus_9031_20141114.fasta,UPARC14_2_GallusG_9031_20140417.fasta	115,182.30	100.00%	3	3	3	0.01%	3.25%	SSPYPTDVAR	R	V	99.70%	37	44.8	25.8	14.5	27.8	2.27	0.302	0.35	0	1	0	0	2	1,092.53	1,690.00		312.86	452	461	Empty	TRUE	
20196-20225 HinckeM –TAXchicken	Uncategorized Sample	20196-20205_A	Mudpit_Ingel20196_MGFPeaklist (F058870)	Uncharacterized protein OS=Gallus gallus GN=OGDH PE=2 SV=1	Q5ZJA7_CHICK,UPI00004437CA	TAX_GallusGallus_9031_20141114.fasta,UPARC14_2_GallusG_9031_20140417.fasta	115,182.30	100.00%	3	3	3	0.01%	3.25%	VIPDGGPAAQSPQNVR	R	R	85.70%	23.7	44.8	22.9	17.8					0	0	1	0	2	1,605.83	1,733.00		395.99	877	892	Empty	TRUE	
20196-20225 HinckeM –TAXchicken	Uncategorized Sample	20196-20205_A	Mudpit_Ingel20196_MGFPeaklist (F058870)	Uncharacterized protein OS=Gallus gallus GN=OGDH PE=2 SV=1	Q5ZJA7_CHICK,UPI00004437CA	TAX_GallusGallus_9031_20141114.fasta,UPARC14_2_GallusG_9031_20140417.fasta	115,182.30	100.00%	3	3	3	0.01%	3.25%	VYYDLTR	K	E	9.10%	24.3	44.9	0	0					0	1	0	0	2	929.4727	1,895.00		92.69	901	907	Empty	TRUE	
20196-20225 HinckeM –TAXchicken	Uncategorized Sample	20196-20205_A	Mudpit_Ingel20196_MGFPeaklist (F058870)	Serum albumin OS=Gallus gallus GN=ALB PE=1 SV=2	ALBU_CHICK	TAX_GallusGallus_9031_20141114.fasta	69,918.70	100.00%	2	4	12	0.02%	62.30%	ADPERNECFLSFK	K	V	99.70%	22.2	43.7	16	7.94	43	6.02	0.453	0.462	0	0	5	0	2	1,612.74	2,261.00		122894	121	133	Empty	FALSE	F2Z4L6_CHICK,UPI00003AD82D
20196-20225 HinckeM –TAXchicken	Uncategorized Sample	20196-20205_A	Mudpit_Ingel20196_MGFPeaklist (F058870)	Serum albumin OS=Gallus gallus GN=ALB PE=1 SV=2	ALBU_CHICK	TAX_GallusGallus_9031_20141114.fasta	69,918.70	100.00%	2	4	12	0.02%	62.30%	AVAMITFAQYLQR	K	C	99.70%	64.8	44.2	23.8	56.4	41	3.68	0.407	0.462	0	4	0	0	2	1,511.80	2,725.00		77,452.50	48	60	Empty	TRUE	
20196-20225 HinckeM –TAXchicken	Uncategorized Sample	20196-20205_A	Mudpit_Ingel20196_MGFPeaklist (F058870)	Serum albumin OS=Gallus gallus GN=ALB PE=1 SV=2	ALBU_CHICK	TAX_GallusGallus_9031_20141114.fasta	69,918.70	100.00%	2	4	12	0.02%	62.30%	CVANEDAPECSKPLPSIIDEICQVEK	K	L	99.70%	47.1	42.7	14.1	36	45.2	4.68	0.491	0.241	0	0	1	0	2	3,114.48	2,878.00		6,950.78	80	106	Empty	FALSE	F2Z4L6_CHICK,UPI00003AD82D
20196-20225 HinckeM –TAXchicken	Uncategorized Sample	20196-20205_A	Mudpit_Ingel20196_MGFPeaklist (F058870)	Serum albumin OS=Gallus gallus GN=ALB PE=1 SV=2	ALBU_CHICK	TAX_GallusGallus_9031_20141114.fasta	69,918.70	100.00%	2	4	12	0.02%	62.30%	FVHDSIGVHKECCEGDMVECMDDMAR	K	M	83.50%	21.7	31.3	0	21.7					0	0	0	0	2	3,175.24	1,698.00		2,339.16	262	287	Empty	FALSE	F2Z4L6_CHICK
20196-20225 HinckeM –TAXchicken	Uncategorized Sample	20196-20205_A	Mudpit_Ingel20196_MGFPeaklist (F058870)	Serum albumin OS=Gallus gallus GN=ALB PE=1 SV=2	ALBU_CHICK	TAX_GallusGallus_9031_20141114.fasta	69,918.70	100.00%	2	4	12	0.02%	62.30%	GYESLLEK	K	C	99.70%	41.7	43.4	26.1	27	30.2	1.28	0.328	0.562	0	4	0	0	2	938.483	2,061.00		54,763.90	380	387	Empty	FALSE	F2Z4L6_CHICK
20196-20225 HinckeM –TAXchicken	Uncategorized Sample	20196-20205_A	Mudpit_Ingel20196_MGFPeaklist (F058870)	Serum albumin OS=Gallus gallus GN=ALB PE=1 SV=2	ALBU_CHICK	TAX_GallusGallus_9031_20141114.fasta	69,918.70	100.00%	2	4	12	0.02%	62.30%	HPEFSIQLIMR	R	I	99.70%	33	44.8	22.7	21.9	33	0.854	0.261	0.455	0	0	2	0	2	1,386.72	2,417.50		10,061.40	366	376	Empty	FALSE	F2Z4L6_CHICK
20196-20225 HinckeM –TAXchicken	Uncategorized Sample	20196-20205_A	Mudpit_Ingel20196_MGFPeaklist (F058870)	Serum albumin OS=Gallus gallus GN=ALB PE=1 SV=2	ALBU_CHICK	TAX_GallusGallus_9031_20141114.fasta	69,918.70	100.00%	2	4	12	0.02%	62.30%	IKDCCEKPIVER	K	S	99.70%	72.1	44.9	23.4	48.2	43.4	6.03	0.493	0.542	0	0	8	0	2	1,546.77	1,525.00		5,379.69	303	314	Empty	FALSE	F2Z4L6_CHICK
20196-20225 HinckeM –TAXchicken	Uncategorized Sample	20196-20205_A	Mudpit_Ingel20196_MGFPeaklist (F058870)	Serum albumin OS=Gallus gallus GN=ALB PE=1 SV=2	ALBU_CHICK	TAX_GallusGallus_9031_20141114.fasta	69,918.70	100.00%	2	4	12	0.02%	62.30%	KMPQVPTDLLLETGK	K	K	99.70%	35.4	43.3	15.9	32.5	41.2	4.39	0.369	0.4	0	0	2	0	2	1,685.91	2,400.00		10,039.90	442	456	Empty	FALSE	F2Z4L6_CHICK
20196-20225 HinckeM –TAXchicken	Uncategorized Sample	20196-20205_A	Mudpit_Ingel20196_MGFPeaklist (F058870)	Serum albumin OS=Gallus gallus GN=ALB PE=1 SV=2	ALBU_CHICK	TAX_GallusGallus_9031_20141114.fasta	69,918.70	100.00%	2	4	12	0.02%	62.30%	KMPQVPTDLLLETGKK	K	M	99.70%	64.5	42.3	22.7	57	53.2	6.44	0.515	0.562	0	0	2	4	2	1,814.01	2,269.50		70,515.40	442	457	Empty	FALSE	F2Z4L6_CHICK
20196-20225 HinckeM –TAXchicken	Uncategorized Sample	20196-20205_A	Mudpit_Ingel20196_MGFPeaklist (F058870)	Serum albumin OS=Gallus gallus GN=ALB PE=1 SV=2	ALBU_CHICK	TAX_GallusGallus_9031_20141114.fasta	69,918.70	100.00%	2	4	12	0.02%	62.30%	LCSAPAEERVGQMK	K	L	99.70%	42.8	44.2	17.2	35.3	30.7	1.47	0.186	0.3	0	0	6	0	2	1,720.80	1,548.00		27,210.00	541	555	Empty	FALSE	F2Z4L6_CHICK
20196-20225 HinckeM –TAXchicken	Uncategorized Sample	20196-20205_A	Mudpit_Ingel20196_MGFPeaklist (F058870)	Serum albumin OS=Gallus gallus GN=ALB PE=1 SV=2	ALBU_CHICK	TAX_GallusGallus_9031_20141114.fasta	69,918.70	100.00%	2	4	12	0.02%	62.30%	LLINLIK	K	R	37.00%	41.1	37.2	47.5	0					0	7	0	0	2	826.5762	2,649.00		304972	556	562	Empty	FALSE	F2Z4L6_CHICK
20196-20225 HinckeM –TAXchicken	Uncategorized Sample	20196-20205_A	Mudpit_Ingel20196_MGFPeaklist (F058870)	Serum albumin OS=Gallus gallus GN=ALB PE=1 SV=2	ALBU_CHICK	TAX_GallusGallus_9031_20141114.fasta	69,918.70	100.00%	2	4	12	0.02%	62.30%	LLINLIKR	K	K	99.70%	61.2	35.1	45.9	26.8	36.3	1.06	0.218	0.625	0	2	0	0	2	982.6773	2,405.00		6,493.08	556	563	Empty	FALSE	F2Z4L6_CHICK
20196-20225 HinckeM –TAXchicken	Uncategorized Sample	20196-20205_A	Mudpit_Ingel20196_MGFPeaklist (F058870)	Serum albumin OS=Gallus gallus GN=ALB PE=1 SV=2	ALBU_CHICK	TAX_GallusGallus_9031_20141114.fasta	69,918.70	100.00%	2	4	12	0.02%	62.30%	LVKDVVDLAQK	K	C	99.70%	50.6	41.9	25.1	33.5	40.7	2.39	0.322	0.591	0	0	4	0	2	1,227.73	2,016.00		43,147.80	69	79	Empty	FALSE	F2Z4L6_CHICK,UPI00003AD82D
20196-20225 HinckeM –TAXchicken	Uncategorized Sample	20196-20205_A	Mudpit_Ingel20196_MGFPeaklist (F058870)	Serum albumin OS=Gallus gallus GN=ALB PE=1 SV=2	ALBU_CHICK	TAX_GallusGallus_9031_20141114.fasta	69,918.70	100.00%	2	4	12	0.02%	62.30%	MMSNLCSQQDVFSGK	R	I	99.70%	52	41.1	15.7	49.2	43.1	3.89	0.425	0.433	0	0	3	0	2	1,763.74	1,914.00		11,655.70	288	302	Empty	FALSE	F2Z4L6_CHICK
20196-20225 HinckeM –TAXchicken	Uncategorized Sample	20196-20205_A	Mudpit_Ingel20196_MGFPeaklist (F058870)	Serum albumin OS=Gallus gallus GN=ALB PE=1 SV=2	ALBU_CHICK	TAX_GallusGallus_9031_20141114.fasta	69,918.70	100.00%	2	4	12	0.02%	62.30%	MMSNLCSQQDVFSGKIKDCCEKPIVER	R	S	6.30%	8.62	42.1	16.8	0					0	0	0	0	2	3,275.50	1,737.00		1,855.76	288	314	Empty	FALSE	F2Z4L6_CHICK
20196-20225 HinckeM –TAXchicken	Uncategorized Sample	20196-20205_A	Mudpit_Ingel20196_MGFPeaklist (F058870)	Serum albumin OS=Gallus gallus GN=ALB PE=1 SV=2	ALBU_CHICK	TAX_GallusGallus_9031_20141114.fasta	69,918.70	100.00%	2	4	12	0.02%	62.30%	MPQVPTDLLLETGK	K	K	99.70%	71.6	44.4	22.9	65.7	49	4.43	0.492	0.5	0	2	0	0	2	1,557.82	2,501.50		7,772.04	443	456	Empty	FALSE	F2Z4L6_CHICK
20196-20225 HinckeM –TAXchicken	Uncategorized Sample	20196-20205_A	Mudpit_Ingel20196_MGFPeaklist (F058870)	Serum albumin OS=Gallus gallus GN=ALB PE=1 SV=2	ALBU_CHICK	TAX_GallusGallus_9031_20141114.fasta	69,918.70	100.00%	2	4	12	0.02%	62.30%	MPQVPTDLLLETGKK	K	M	99.70%	66.4	43.3	20.8	35.1	45.7	3.39	0.392	0.5	0	3	5	0	2	1,685.91	2,380.50		117935	443	457	Empty	FALSE	F2Z4L6_CHICK
20196-20225 HinckeM –TAXchicken	Uncategorized Sample	20196-20205_A	Mudpit_Ingel20196_MGFPeaklist (F058870)	Serum albumin OS=Gallus gallus GN=ALB PE=1 SV=2	ALBU_CHICK	TAX_GallusGallus_9031_20141114.fasta	69,918.70	100.00%	2	4	12	0.02%	62.30%	NECFLSFK	R	V	99.70%	35.4	43.9	24.5	25.7	31.5	1.08	0.21	0.5	0	6	0	0	2	1,044.48	2,342.50		20,841.10	126	133	Empty	FALSE	F2Z4L6_CHICK,UPI00003AD82D
20196-20225 HinckeM –TAXchicken	Uncategorized Sample	20196-20205_A	Mudpit_Ingel20196_MGFPeaklist (F058870)	Serum albumin OS=Gallus gallus GN=ALB PE=1 SV=2	ALBU_CHICK	TAX_GallusGallus_9031_20141114.fasta	69,918.70	100.00%	2	4	12	0.02%	62.30%	PERNECFLSFK	D	V	99.70%					39.6	4.26	0.323	0.545	0	2	0	0	1	1,426.68	2,248.50		4,669.79	123	133	Empty	FALSE	F2Z4L6_CHICK
20196-20225 HinckeM –TAXchicken	Uncategorized Sample	20196-20205_A	Mudpit_Ingel20196_MGFPeaklist (F058870)	Serum albumin OS=Gallus gallus GN=ALB PE=1 SV=2	ALBU_CHICK	TAX_GallusGallus_9031_20141114.fasta	69,918.70	100.00%	2	4	12	0.02%	62.30%	PTDLLLETGKK	V	M	99.60%					31.5	2.32	0.222	0.364	0	1	0	0	1	1,214.70	2,107.00		1,189.22	447	457	Empty	FALSE	F2Z4L6_CHICK
20196-20225 HinckeM –TAXchicken	Uncategorized Sample	20196-20205_A	Mudpit_Ingel20196_MGFPeaklist (F058870)	Serum albumin OS=Gallus gallus GN=ALB PE=1 SV=2	ALBU_CHICK	TAX_GallusGallus_9031_20141114.fasta	69,918.70	100.00%	2	4	12	0.02%	62.30%	QFGDRVFQAR	K	Q	28.00%	14.5	44.4	22	2.58					0	0	2	0	2	1,223.63	1,940.50		5,186.86	233	242	Empty	FALSE	F2Z4L6_CHICK
20196-20225 HinckeM –TAXchicken	Uncategorized Sample	20196-20205_A	Mudpit_Ingel20196_MGFPeaklist (F058870)	Serum albumin OS=Gallus gallus GN=ALB PE=1 SV=2	ALBU_CHICK	TAX_GallusGallus_9031_20141114.fasta	69,918.70	100.00%	2	4	12	0.02%	62.30%	QLIYLSQK	R	Y	99.70%	39.6	43	27.5	25.1	31.2	0.699	0.112	0.562	0	13	0	0	2	992.5776	2,111.00		135537	243	250	Empty	FALSE	F2Z4L6_CHICK
20196-20225 HinckeM –TAXchicken	Uncategorized Sample	20196-20205_A	Mudpit_Ingel20196_MGFPeaklist (F058870)	Serum albumin OS=Gallus gallus GN=ALB PE=1 SV=2	ALBU_CHICK	TAX_GallusGallus_9031_20141114.fasta	69,918.70	100.00%	2	4	12	0.02%	62.30%	QLIYLSQKYPK	R	A	99.70%	53.2	43.4	28.5	44.5	36.8	3.26	0.356	0.545	0	5	6	0	2	1,363.76	2,138.00		75,567.90	243	253	Empty	FALSE	F2Z4L6_CHICK
20196-20225 HinckeM –TAXchicken	Uncategorized Sample	20196-20205_A	Mudpit_Ingel20196_MGFPeaklist (F058870)	Serum albumin OS=Gallus gallus GN=ALB PE=1 SV=2	ALBU_CHICK	TAX_GallusGallus_9031_20141114.fasta	69,918.70	100.00%	2	4	12	0.02%	62.30%	QQYFCGILK	K	Q	99.70%	33	44.3	26.4	13.8	32.1	0.959	0.259	0.5	0	7	0	0	2	1,156.58	2,362.00		119525	224	232	Empty	FALSE	F2Z4L6_CHICK
20196-20225 HinckeM –TAXchicken	Uncategorized Sample	20196-20205_A	Mudpit_Ingel20196_MGFPeaklist (F058870)	Serum albumin OS=Gallus gallus GN=ALB PE=1 SV=2	ALBU_CHICK	TAX_GallusGallus_9031_20141114.fasta	69,918.70	100.00%	2	4	12	0.02%	62.30%	QRPASDVICQEYQDNR	Y	V	99.70%					46.4	8.66	0.442	0.469	0	0	1	0	1	1,978.90	1,814.00		4,221.45	144	159	Empty	FALSE	F2Z4L6_CHICK
20196-20225 HinckeM –TAXchicken	Uncategorized Sample	20196-20205_A	Mudpit_Ingel20196_MGFPeaklist (F058870)	Serum albumin OS=Gallus gallus GN=ALB PE=1 SV=2	ALBU_CHICK	TAX_GallusGallus_9031_20141114.fasta	69,918.70	100.00%	2	4	12	0.02%	62.30%	QSDINTCFGEEGANLIVQSR	K	A	99.70%	92.7	43.6	19.2	80.7	55.3	7.92	0.561	0.425	0	0	2	0	2	2,238.05	2,449.50		49,886.00	589	608	Empty	FALSE	F2Z4L6_CHICK
20196-20225 HinckeM –TAXchicken	Uncategorized Sample	20196-20205_A	Mudpit_Ingel20196_MGFPeaklist (F058870)	Serum albumin OS=Gallus gallus GN=ALB PE=1 SV=2	ALBU_CHICK	TAX_GallusGallus_9031_20141114.fasta	69,918.70	100.00%	2	4	12	0.02%	62.30%	RHPEFSIQLIMR	R	I	99.70%	74.5	43.4	29.6	47.2	50.3	6.12	0.441	0.708	0	0	5	0	2	1,526.83	2,322.00		51,589.10	365	376	Empty	FALSE	F2Z4L6_CHICK
20196-20225 HinckeM –TAXchicken	Uncategorized Sample	20196-20205_A	Mudpit_Ingel20196_MGFPeaklist (F058870)	Serum albumin OS=Gallus gallus GN=ALB PE=1 SV=2	ALBU_CHICK	TAX_GallusGallus_9031_20141114.fasta	69,918.70	100.00%	2	4	12	0.02%	62.30%	RPCFTAMGVDTK	R	Y	99.70%	56.5	43.7	22.1	45.7	39.5	4.17	0.43	0.542	0	0	9	0	2	1,398.65	1,743.00		35,953.20	513	524	Empty	FALSE	F2Z4L6_CHICK
20196-20225 HinckeM –TAXchicken	Uncategorized Sample	20196-20205_A	Mudpit_Ingel20196_MGFPeaklist (F058870)	Serum albumin OS=Gallus gallus GN=ALB PE=1 SV=2	ALBU_CHICK	TAX_GallusGallus_9031_20141114.fasta	69,918.70	100.00%	2	4	12	0.02%	62.30%	SQCIMEAEFDEKPADLPSLVEK	R	Y	99.70%	62.8	43.5	0	54.6	44.8	4.85	0.482	0.295	0	0	6	1	2	2,552.19	2,463.00		134522	315	336	Empty	FALSE	F2Z4L6_CHICK
20196-20225 HinckeM –TAXchicken	Uncategorized Sample	20196-20205_A	Mudpit_Ingel20196_MGFPeaklist (F058870)	Serum albumin OS=Gallus gallus GN=ALB PE=1 SV=2	ALBU_CHICK	TAX_GallusGallus_9031_20141114.fasta	69,918.70	100.00%	2	4	12	0.02%	62.30%	TDNPAECYANAQEQLNQHIK	K	E	99.70%	62.2	42.8	13	59.9	40.7	4.92	0.396	0.3	0	0	7	1	2	2,344.06	2,201.00		149818	391	410	Empty	FALSE	F2Z4L6_CHICK
20196-20225 HinckeM –TAXchicken	Uncategorized Sample	20196-20205_A	Mudpit_Ingel20196_MGFPeaklist (F058870)	Serum albumin OS=Gallus gallus GN=ALB PE=1 SV=2	ALBU_CHICK	TAX_GallusGallus_9031_20141114.fasta	69,918.70	100.00%	2	4	12	0.02%	62.30%	TDNPAECYANAQEQLNQHIKETQDVVK	K	T	99.70%	39.7	42.8	0	39.7	38.9	3.72	0.35	0.259	0	0	0	2	2	3,143.47	2,443.50		38,124.80	391	417	Empty	FALSE	F2Z4L6_CHICK
20196-20225 HinckeM –TAXchicken	Uncategorized Sample	20196-20205_A	Mudpit_Ingel20196_MGFPeaklist (F058870)	Serum albumin OS=Gallus gallus GN=ALB PE=1 SV=2	ALBU_CHICK	TAX_GallusGallus_9031_20141114.fasta	69,918.70	100.00%	2	4	12	0.02%	62.30%	TIADGFTAMVDK	K	C	99.70%	65.2	43.9	22.5	55.1	43.2	4.21	0.4	0.5	0	7	0	0	2	1,268.62	2,077.00		50,401.60	574	585	Empty	FALSE	F2Z4L6_CHICK
20196-20225 HinckeM –TAXchicken	Uncategorized Sample	20196-20205_A	Mudpit_Ingel20196_MGFPeaklist (F058870)	Serum albumin OS=Gallus gallus GN=ALB PE=1 SV=2	ALBU_CHICK	TAX_GallusGallus_9031_20141114.fasta	69,918.70	100.00%	2	4	12	0.02%	62.30%	TIADGFTAMVDKCCK	K	Q	99.70%	14.9	42.7	18.8	11.4	22.4	1.21	0.21	0.2	0	0	1	0	2	1,732.77	2,032.00		898.62	574	588	Empty	FALSE	F2Z4L6_CHICK
20196-20225 HinckeM –TAXchicken	Uncategorized Sample	20196-20205_A	Mudpit_Ingel20196_MGFPeaklist (F058870)	Serum albumin OS=Gallus gallus GN=ALB PE=1 SV=2	ALBU_CHICK	TAX_GallusGallus_9031_20141114.fasta	69,918.70	100.00%	2	4	12	0.02%	62.30%	TNCDLLHDHGEADFLK	K	S	99.70%	31.4	43.2	17.6	28.9	42.5	4.64	0.414	0.375	0	0	1	6	2	1,884.85	2,234.00		51,883.70	418	433	Empty	FALSE	F2Z4L6_CHICK
20196-20225 HinckeM –TAXchicken	Uncategorized Sample	20196-20205_A	Mudpit_Ingel20196_MGFPeaklist (F058870)	Serum albumin OS=Gallus gallus GN=ALB PE=1 SV=2	ALBU_CHICK	TAX_GallusGallus_9031_20141114.fasta	69,918.70	100.00%	2	4	12	0.02%	62.30%	VSFLGHFIYSVAR	R	R	99.70%	60.6	43.7	17.6	53	47.1	6.89	0.397	0.615	0	0	2	0	2	1,495.81	2,828.50		26,073.30	160	172	Empty	FALSE	F2Z4L6_CHICK,UPI00003AD82D
20196-20225 HinckeM –TAXchicken	Uncategorized Sample	20196-20205_A	Mudpit_Ingel20196_MGFPeaklist (F058870)	Serum albumin OS=Gallus gallus GN=ALB PE=1 SV=2	ALBU_CHICK	TAX_GallusGallus_9031_20141114.fasta	69,918.70	100.00%	2	4	12	0.02%	62.30%	VSQPDFVQPYQR	K	P	99.70%					32.1	2.08	0.283	0.333	0	1	0	0	1	1,463.73	2,037.00		5,675.80	134	145	Empty	FALSE	F2Z4L6_CHICK
20196-20225 HinckeM –TAXchicken	Uncategorized Sample	20196-20205_A	Mudpit_Ingel20196_MGFPeaklist (F058870)	Serum albumin OS=Gallus gallus GN=ALB PE=1 SV=2	ALBU_CHICK	TAX_GallusGallus_9031_20141114.fasta	69,918.70	100.00%	2	4	12	0.02%	62.30%	VSQPDFVQPYQRPASDVICQEYQDNR	K	V	99.70%	52.9	42.4	0	52.9	49	6.52	0.482	0.288	0	0	4	0	2	3,139.45	2,306.50		44,303.40	134	159	Empty	FALSE	F2Z4L6_CHICK,UPI00003AD82D
20196-20225 HinckeM –TAXchicken	Uncategorized Sample	20196-20205_A	Mudpit_Ingel20196_MGFPeaklist (F058870)	Serum albumin OS=Gallus gallus GN=ALB PE=1 SV=2	ALBU_CHICK	TAX_GallusGallus_9031_20141114.fasta	69,918.70	100.00%	2	4	12	0.02%	62.30%	YNDLKEETFK	R	A	99.70%	56.5	44.3	26.2	32.9	41.6	3.52	0.397	0.75	0	1	7	0	2	1,286.63	1,783.50		75,619.50	38	47	Empty	TRUE	
20196-20225 HinckeM –TAXchicken	Uncategorized Sample	20196-20205_A	Mudpit_Ingel20196_MGFPeaklist (F058870)	Serum albumin OS=Gallus gallus GN=ALB PE=1 SV=2	ALBU_CHICK	TAX_GallusGallus_9031_20141114.fasta	69,918.70	100.00%	2	4	12	0.02%	62.30%	YVPPPFNPDMFSFDEK	K	L	99.70%	62.8	42.7	13.3	55.8	44.1	4.85	0.467	0.375	0	0	8	0	2	1,945.87	2,721.50		112513	525	540	Empty	FALSE	F2Z4L6_CHICK
20196-20225 HinckeM –TAXchicken	Uncategorized Sample	20196-20205_A	Mudpit_Ingel20196_MGFPeaklist (F058870)	Serum albumin OS=Gallus gallus GN=ALB PE=4 SV=2	F2Z4L6_CHICK	TAX_GallusGallus_9031_20141114.fasta	64,004.60	100.00%	40	62	179	0.36%	69.20%	ADPERNECFLSFK	K	V	99.70%	22.2	43.7	16	7.94	43	6.02	0.453	0.462	0	0	5	0	2	1,612.74	2,261.00		122894	71	83	Empty	TRUE	UPI00003AD82D,ALBU_CHICK
20196-20225 HinckeM –TAXchicken	Uncategorized Sample	20196-20205_A	Mudpit_Ingel20196_MGFPeaklist (F058870)	Serum albumin OS=Gallus gallus GN=ALB PE=4 SV=2	F2Z4L6_CHICK	TAX_GallusGallus_9031_20141114.fasta	64,004.60	100.00%	40	62	179	0.36%	69.20%	CCQLPEDRR	K	M	98.20%					31.4	1.54	0.0732	0.5	0	1	0	0	2	1,216.52	1,681.00		15,439.40	415	423	Empty	TRUE	
[illegible]	[illegible]	[illegible]	[illegible]	[illegible]	[illegible]	[illegible]	[illegible]	[illegible]	[illegible]	[illegible]	[illegible]	[illegible]	[illegible]	[illegible]	[illegible]	[illegible]	[illegible]	[illegible]	[illegible]	[illegible]	[illegible]	[illegible]	[illegible]	[illegible]	[illegible]	[illegible]	[illegible]	[illegible]	[illegible]	[illegible]	[illegible]	[illegible]		[illegible]	[illegible]	[illegible]	[illegible]	[illegible]	[illegible]
[illegible]	[illegible]	[illegible]	[illegible]	[illegible]	[illegible]	[illegible]	[illegible]	[illegible]	[illegible]	[illegible]	[illegible]	[illegible]	[illegible]	[illegible]	[illegible]	[illegible]	[illegible]	[illegible]	[illegible]	[illegible]	[illegible]	[illegible]	[illegible]	[illegible]	[illegible]	[illegible]	[illegible]	[illegible]	[illegible]	[illegible]	[illegible]	[illegible]		[illegible]	[illegible]	[illegible]	[illegible]	[illegible]	[illegible]
[illegible]	[illegible]	[illegible]	[illegible]	[illegible]	[illegible]	[illegible]	[illegible]	[illegible]	[illegible]	[illegible]	[illegible]	[illegible]	[illegible]	[illegible]	[illegible]	[illegible]	[illegible]	[illegible]	[illegible]	[illegible]	[illegible]	[illegible]	[illegible]	[illegible]	[illegible]	[illegible]	[illegible]	[illegible]	[illegible]	[illegible]	[illegible]	[illegible]		[illegible]	[illegible]	[illegible]	[illegible]	[illegible]	[illegible]
[illegible]	[illegible]	[illegible]	[illegible]	[illegible]	[illegible]	[illegible]	[illegible]	[illegible]	[illegible]	[illegible]	[illegible]	[illegible]	[illegible]	[illegible]	[illegible]	[illegible]	[illegible]	[illegible]	[illegible]	[illegible]	[illegible]	[illegible]	[illegible]	[illegible]	[illegible]	[illegible]	[illegible]	[illegible]	[illegible]	[illegible]	[illegible]	[illegible]		[illegible]	[illegible]	[illegible]	[illegible]	[illegible]	[illegible]

20196-20225 HinckeM --TAXchicken	Uncategorized Sample	20196-20205_A	Mudpit_Ingel20196_MGFPeaklist (F058870)	Serum albumin OS=Gallus gallus GN=ALB PE=4 SV=2	F2Z4L6_CHICK	TAX_GallusGallus_9031_20141114.fasta	64,004.60	100.00%	40	62	179	0.36%	69.20%	KMPQVPTDLLLETGK	K	K	99.70%	35.4	43.3	[illegible]
20196-20225 HinckeM --TAXchicken	Uncategorized Sample	20196-20205_A	Mudpit_Ingel20196_MGFPeaklist (F058870)	Serum albumin OS=Gallus gallus GN=ALB PE=4 SV=2	F2Z4L6_CHICK	TAX_GallusGallus_9031_20141114.fasta	64,004.60	100.00%	40	62	179	0.36%	69.20%	KMPQVPTDLLLETGKK	K	M	99.70%	64.5	42.3	[illegible]
20196-20225 HinckeM --TAXchicken	Uncategorized Sample	20196-20205_A	Mudpit_Ingel20196_MGFPeaklist (F058870)	Serum albumin OS=Gallus gallus GN=ALB PE=4 SV=2	F2Z4L6_CHICK	TAX_GallusGallus_9031_20141114.fasta	64,004.60	100.00%	40	62	179	0.36%	69.20%	KQETTPINDNVSQCCSSSYANR	R	R	99.70%	81.8	41.4	[illegible]
20196-20225 HinckeM --TAXchicken	Uncategorized Sample	20196-20205_A	Mudpit_Ingel20196_MGFPeaklist (F058870)	Serum albumin OS=Gallus gallus GN=ALB PE=4 SV=2	F2Z4L6_CHICK	TAX_GallusGallus_9031_20141114.fasta	64,004.60	100.00%	40	62	179	0.36%	69.20%	LCSAPAEEREVGQMK	K	L	99.70%	42.8	44.2	[illegible]
20196-20225 HinckeM --TAXchicken	Uncategorized Sample	20196-20205_A	Mudpit_Ingel20196_MGFPeaklist (F058870)	Serum albumin OS=Gallus gallus GN=ALB PE=4 SV=2	F2Z4L6_CHICK	TAX_GallusGallus_9031_20141114.fasta	64,004.60	100.00%	40	62	179	0.36%	69.20%	LLINLIK	K	R	37.00%	41.1	37.2	[illegible]
20196-20225 HinckeM --TAXchicken	Uncategorized Sample	20196-20205_A	Mudpit_Ingel20196_MGFPeaklist (F058870)	Serum albumin OS=Gallus gallus GN=ALB PE=4 SV=2	F2Z4L6_CHICK	TAX_GallusGallus_9031_20141114.fasta	64,004.60	100.00%	40	62	179	0.36%	69.20%	LLINLIKR	K	K	99.70%	61.2	35.1	[illegible]
20196-20225 HinckeM --TAXchicken	Uncategorized Sample	20196-20205_A	Mudpit_Ingel20196_MGFPeaklist (F058870)	Serum albumin OS=Gallus gallus GN=ALB PE=4 SV=2	F2Z4L6_CHICK	TAX_GallusGallus_9031_20141114.fasta	64,004.60	100.00%	40	62	179	0.36%	69.20%	LVKDVVDLAQK	K	C	99.70%	50.6	41.9	[illegible]
20196-20225 HinckeM --TAXchicken	Uncategorized Sample	20196-20205_A	Mudpit_Ingel20196_MGFPeaklist (F058870)	Serum albumin OS=Gallus gallus GN=ALB PE=4 SV=2	F2Z4L6_CHICK	TAX_GallusGallus_9031_20141114.fasta	64,004.60	100.00%	40	62	179	0.36%	69.20%	MMSNLCSQQDVFSGK	R	I	99.70%	52	41.1	[illegible]
20196-20225 HinckeM --TAXchicken	Uncategorized Sample	20196-20205_A	Mudpit_Ingel20196_MGFPeaklist (F058870)	Serum albumin OS=Gallus gallus GN=ALB PE=4 SV=2	F2Z4L6_CHICK	TAX_GallusGallus_9031_20141114.fasta	64,004.60	100.00%	40	62	179	0.36%	69.20%	MMSNLCSQQDVFSGKIKDCCEKPIVER	R	S	6.30%	8.62	42.1	[illegible]
20196-20225 HinckeM --TAXchicken	Uncategorized Sample	20196-20205_A	Mudpit_Ingel20196_MGFPeaklist (F058870)	Serum albumin OS=Gallus gallus GN=ALB PE=4 SV=2	F2Z4L6_CHICK	TAX_GallusGallus_9031_20141114.fasta	64,004.60	100.00%	40	62	179	0.36%	69.20%	MPQVPTDLLLETGK	K	K	99.70%	71.6	44.4	[illegible]
20196-20225 HinckeM --TAXchicken	Uncategorized Sample	20196-20205_A	Mudpit_Ingel20196_MGFPeaklist (F058870)	Serum albumin OS=Gallus gallus GN=ALB PE=4 SV=2	F2Z4L6_CHICK	TAX_GallusGallus_9031_20141114.fasta	64,004.60	100.00%	40	62	179	0.36%	69.20%	MPQVPTDLLLETGKK	K	M	99.70%	66.4	43.3	[illegible]
20196-20225 HinckeM --TAXchicken	Uncategorized Sample	20196-20205_A	Mudpit_Ingel20196_MGFPeaklist (F058870)	Serum albumin OS=Gallus gallus GN=ALB PE=4 SV=2	F2Z4L6_CHICK	TAX_GallusGallus_9031_20141114.fasta	64,004.60	100.00%	40	62	179	0.36%	69.20%	NECFLSFK	R	V	99.70%	35.4	43.9	[illegible]
20196-20225 HinckeM --TAXchicken	Uncategorized Sample	20196-20205_A	Mudpit_Ingel20196_MGFPeaklist (F058870)	Serum albumin OS=Gallus gallus GN=ALB PE=4 SV=2	F2Z4L6_CHICK	TAX_GallusGallus_9031_20141114.fasta	64,004.60	100.00%	40	62	179	0.36%	69.20%	PERNECFLSFK	D	V	99.70%			[illegible]
20196-20225 HinckeM --TAXchicken	Uncategorized Sample	20196-20205_A	Mudpit_Ingel20196_MGFPeaklist (F058870)	Serum albumin OS=Gallus gallus GN=ALB PE=4 SV=2	F2Z4L6_CHICK	TAX_GallusGallus_9031_20141114.fasta	64,004.60	100.00%	40	62	179	0.36%	69.20%	PTDLLLETGKK	V	M	99.60%			[illegible]
20196-20225 HinckeM --TAXchicken	Uncategorized Sample	20196-20205_A	Mudpit_Ingel20196_MGFPeaklist (F058870)	Serum albumin OS=Gallus gallus GN=ALB PE=4 SV=2	F2Z4L6_CHICK	TAX_GallusGallus_9031_20141114.fasta	64,004.60	100.00%	40	62	179	0.36%	69.20%	QETTPINDNVSQCCSSSYANR	K	R	99.70%	65.6	39.3	[illegible]
20196-20225 HinckeM --TAXchicken	Uncategorized Sample	20196-20205_A	Mudpit_Ingel20196_MGFPeaklist (F058870)	Serum albumin OS=Gallus gallus GN=ALB PE=4 SV=2	F2Z4L6_CHICK	TAX_GallusGallus_9031_20141114.fasta	64,004.60	100.00%	40	62	179	0.36%	69.20%	QFGDRVFQAR	K	Q	28.00%	14.5	44.4	[illegible]
20196-20225 HinckeM --TAXchicken	Uncategorized Sample	20196-20205_A	Mudpit_Ingel20196_MGFPeaklist (F058870)	Serum albumin OS=Gallus gallus GN=ALB PE=4 SV=2	F2Z4L6_CHICK	TAX_GallusGallus_9031_20141114.fasta	64,004.60	100.00%	40	62	179	0.36%	69.20%	QLIYLSQK	R	Y	99.70%	39.6	43	[illegible]
20196-20225 HinckeM --TAXchicken	Uncategorized Sample	20196-20205_A	Mudpit_Ingel20196_MGFPeaklist (F058870)	Serum albumin OS=Gallus gallus GN=ALB PE=4 SV=2	F2Z4L6_CHICK	TAX_GallusGallus_9031_20141114.fasta	64,004.60	100.00%	40	62	179	0.36%	69.20%	QLIYLSQKYPK	R	A	99.70%	53.2	43.4	[illegible]
20196-20225 HinckeM --TAXchicken	Uncategorized Sample	20196-20205_A	Mudpit_Ingel20196_MGFPeaklist (F058870)	Serum albumin OS=Gallus gallus GN=ALB PE=4 SV=2	F2Z4L6_CHICK	TAX_GallusGallus_9031_20141114.fasta	64,004.60	100.00%	40	62	179	0.36%	69.20%	QQYFCGILK	K	Q	99.70%	33	44.3	[illegible]
20196-20225 HinckeM --TAXchicken	Uncategorized Sample	20196-20205_A	Mudpit_Ingel20196_MGFPeaklist (F058870)	Serum albumin OS=Gallus gallus GN=ALB PE=4 SV=2	F2Z4L6_CHICK	TAX_GallusGallus_9031_20141114.fasta	64,004.60	100.00%	40	62	179	0.36%	69.20%	QRPASDVICQEYQDNR	Y	V	99.70%			[illegible]
20196-20225 HinckeM --TAXchicken	Uncategorized Sample	20196-20205_A	Mudpit_Ingel20196_MGFPeaklist (F058870)	Serum albumin OS=Gallus gallus GN=ALB PE=4 SV=2	F2Z4L6_CHICK	TAX_GallusGallus_9031_20141114.fasta	64,004.60	100.00%	40	62	179	0.36%	69.20%	QSDINTCFGEEGANLIVQSR	K	A	99.70%	92.7	43.6	[illegible]
20196-20225 HinckeM --TAXchicken	Uncategorized Sample	20196-20205_A	Mudpit_Ingel20196_MGFPeaklist (F058870)	Serum albumin OS=Gallus gallus GN=ALB PE=4 SV=2	F2Z4L6_CHICK	TAX_GallusGallus_9031_20141114.fasta	64,004.60	100.00%	40	62	179	0.36%	69.20%	RHPEFSIQLIMR	R	I	99.70%	74.5	43.4	[illegible]
20196-20225 HinckeM --TAXchicken	Uncategorized Sample	20196-20205_A	Mudpit_Ingel20196_MGFPeaklist (F058870)	Serum albumin OS=Gallus gallus GN=ALB PE=4 SV=2	F2Z4L6_CHICK	TAX_GallusGallus_9031_20141114.fasta	64,004.60	100.00%	40	62	179	0.36%	69.20%	RPCFTAMGVDTK	R	Y	99.70%	56.5	43.7	[illegible]
20196-20225 HinckeM --TAXchicken	Uncategorized Sample	20196-20205_A	Mudpit_Ingel20196_MGFPeaklist (F058870)	Serum albumin OS=Gallus gallus GN=ALB PE=4 SV=2	F2Z4L6_CHICK	TAX_GallusGallus_9031_20141114.fasta	64,004.60	100.00%	40	62	179	0.36%	69.20%	SQCIMEAEFDEKPADLPSLVEK	R	Y	99.70%	62.8	43.5	[illegible]
20196-20225 HinckeM --TAXchicken	Uncategorized Sample	20196-20205_A	Mudpit_Ingel20196_MGFPeaklist (F058870)	Serum albumin OS=Gallus gallus GN=ALB PE=4 SV=2	F2Z4L6_CHICK	TAX_GallusGallus_9031_20141114.fasta	64,004.60	100.00%	40	62	179	0.36%	69.20%	TDNPAECYANAQEQLNQHIK	K	E	99.70%	62.2	42.8	[illegible]
20196-20225 HinckeM --TAXchicken	Uncategorized Sample	20196-20205_A	Mudpit_Ingel20196_MGFPeaklist (F058870)	Serum albumin OS=Gallus gallus GN=ALB PE=4 SV=2	F2Z4L6_CHICK	TAX_GallusGallus_9031_20141114.fasta	64,004.60	100.00%	40	62	179	0.36%	69.20%	TDNPAECYANAQEQLNQHIKETQDVVK	K	T	99.70%	39.7	42.8	[illegible]
20196-20225 HinckeM --TAXchicken	Uncategorized Sample	20196-20205_A	Mudpit_Ingel20196_MGFPeaklist (F058870)	Serum albumin OS=Gallus gallus GN=ALB PE=4 SV=2	F2Z4L6_CHICK	TAX_GallusGallus_9031_20141114.fasta	64,004.60	100.00%	40	62	179	0.36%	69.20%	TIADGFTAMVDK	K	C	99.70%	65.2	43.9	[illegible]
20196-20225 HinckeM --TAXchicken	Uncategorized Sample	20196-20205_A	Mudpit_Ingel20196_MGFPeaklist (F058870)	Serum albumin OS=Gallus gallus GN=ALB PE=4 SV=2	F2Z4L6_CHICK	TAX_GallusGallus_9031_20141114.fasta	64,004.60	100.00%	40	62	179	0.36%	69.20%	TIADGFTAMVDKCCK	K	Q	99.70%	14.9	42.7	[illegible]
20196-20225 HinckeM --TAXchicken	Uncategorized Sample	20196-20205_A	Mudpit_Ingel20196_MGFPeaklist (F058870)	Serum albumin OS=Gallus gallus GN=ALB PE=4 SV=2	F2Z4L6_CHICK	TAX_GallusGallus_9031_20141114.fasta	64,004.60	100.00%	40	62	179	0.36%	69.20%	TNCDLLHDHGEADFLK	K	S	99.70%	31.4	43.2	[illegible]
20196-20225 HinckeM --TAXchicken	Uncategorized Sample	20196-20205_A	Mudpit_Ingel20196_MGFPeaklist (F058870)	Serum albumin OS=Gallus gallus GN=ALB PE=4 SV=2	F2Z4L6_CHICK	TAX_GallusGallus_9031_20141114.fasta	64,004.60	100.00%	40	62	179	0.36%	69.20%	VSFLGHFIYSVAR	R	R	99.70%	60.6	43.7	[illegible]
20196-20225 HinckeM --TAXchicken	Uncategorized Sample	20196-20205_A	Mudpit_Ingel20196_MGFPeaklist (F058870)	Serum albumin OS=Gallus gallus GN=ALB PE=4 SV=2	F2Z4L6_CHICK	TAX_GallusGallus_9031_20141114.fasta	64,004.60	100.00%	40	62	179	0.36%	69.20%	VSQPDFVQPYQR	K	P	99.70%			[illegible]
20196-20225 HinckeM --TAXchicken	Uncategorized Sample	20196-20205_A	Mudpit_Ingel20196_MGFPeaklist (F058870)	Serum albumin OS=Gallus gallus GN=ALB PE=4 SV=2	F2Z4L6_CHICK	TAX_GallusGallus_9031_20141114.fasta	64,004.60	100.00%	40	62	179	0.36%	69.20%	VSQPDFVQPYQRPASDVICQEYQDNR	K	V	99.70%	52.9	42.4	[illegible]
20196-20225 HinckeM --TAXchicken	Uncategorized Sample	20196-20205_A	Mudpit_Ingel20196_MGFPeaklist (F058870)	Serum albumin OS=Gallus gallus GN=ALB PE=4 SV=2	F2Z4L6_CHICK	TAX_GallusGallus_9031_20141114.fasta	64,004.60	100.00%	40	62	179	0.36%	69.20%	YVPPPFNPDMFSFDEK	K	L	99.70%	62.8	42.7	[illegible]
20196-20225 HinckeM --TAXchicken	Uncategorized Sample	20196-20205_A	Mudpit_Ingel20196_MGFPeaklist (F058870)	status=active	UPI00003AA4FE,UPI00003AA4FF,UPI00003AA500,UPI00003AA501,UPI0000448CEC,UPI000060EAA3,UPI0000E80D04,UPI0000ECB083,UPI000240BE0E,UPI0003505DE6	UPARC14_2_GallusG_9031_20140417.fasta	189,631.40	100.00%	4	4	7	0.01%	2.22%	LGNFFSPK	K	V	48.30%	18.9	43.5	[illegible]
20196-20225 HinckeM --TAXchicken	Uncategorized Sample	20196-20205_A	Mudpit_Ingel20196_MGFPeaklist (F058870)	status=active	UPI00003AA4FE,UPI00003AA4FF,UPI00003AA500,UPI00003AA501,UPI0000448CEC,UPI000060EAA3,UPI0000E80D04,UPI0000ECB083,UPI000240BE0E,UPI0003505DE6	UPARC14_2_GallusG_9031_20140417.fasta	189,631.40	100.00%	4	4	7	0.01%	2.22%	LIVDVIR	R	F	74.20%	34	41	[illegible]
20196-20225 HinckeM --TAXchicken	Uncategorized Sample	20196-20205_A	Mudpit_Ingel20196_MGFPeaklist (F058870)	status=active	UPI00003AA4FE,UPI00003AA4FF,UPI00003AA500,UPI00003AA501,UPI0000448CEC,UPI000060EAA3,UPI0000E80D04,UPI0000ECB083,UPI000240BE0E,UPI0003505DE6	UPARC14_2_GallusG_9031_20140417.fasta	189,631.40	100.00%	4	4	7	0.01%	2.22%	TLQALQIPAAK	K	L	99.70%	28	41.1	[illegible]
20196-20225 HinckeM --TAXchicken	Uncategorized Sample	20196-20205_A	Mudpit_Ingel20196_MGFPeaklist (F058870)	status=active	UPI00003AA4FE,UPI00003AA4FF,UPI00003AA500,UPI00003AA501,UPI0000448CEC,UPI000060EAA3,UPI0000E80D04,UPI0000ECB083,UPI000240BE0E,UPI0003505DE6	UPARC14_2_GallusG_9031_20140417.fasta	189,631.40	100.00%	4	4	7	0.01%	2.22%	VAQHYQDTLLR	K	A	99.70%	31.2	44.7	[illegible]
20196-20225 HinckeM --TAXchicken	Uncategorized Sample	20196-20205_A	Mudpit_Ingel20196_MGFPeaklist (F058870)	status=active	UPI00003AA4FE,UPI00003AA4FF,UPI00003AA500,UPI00003AA501,UPI0000448CEC,UPI000060EAA3,UPI0000E80D04,UPI0000ECB083,UPI000240BE0E,UPI0003505DE6	UPARC14_2_GallusG_9031_20140417.fasta	189,631.40	100.00%	4	4	7	0.01%	2.22%	LGNFFSPK	K	V	48.30%	18.9	43.5	[illegible]
20196-20225 HinckeM --TAXchicken	Uncategorized Sample	20196-20205_A	Mudpit_Ingel20196_MGFPeaklist (F058870)	status=active	UPI00003AA4FE,UPI00003AA4FF,UPI00003AA500,UPI00003AA501,UPI0000448CEC,UPI000060EAA3,UPI0000E80D04,UPI0000ECB083,UPI000240BE0E,UPI0003505DE6	UPARC14_2_GallusG_9031_20140417.fasta	189,631.40	100.00%	4	4	7	0.01%	2.22%	LIVDVIR	R	F	74.20%	34	41	[illegible]
20196-20225 HinckeM --TAXchicken	Uncategorized Sample	20196-20205_A	Mudpit_Ingel20196_MGFPeaklist (F058870)	status=active	UPI00003AA4FE,UPI00003AA4FF,UPI00003AA500,UPI00003AA501,UPI0000448CEC,UPI000060EAA3,UPI0000E80D04,UPI0000ECB083,UPI000240BE0E,UPI0003505DE6	UPARC14_2_GallusG_9031_20140417.fasta	189,631.40	100.00%	4	4	7	0.01%	2.22%	TLQALQIPAAK	K	L	99.70%	28	41.1	[illegible]
20196-20225 HinckeM --TAXchicken	Uncategorized Sample	20196-20205_A	Mudpit_Ingel20196_MGFPeaklist (F058870)	status=active	UPI00003AA4FE,UPI00003AA4FF,UPI00003AA500,UPI00003AA501,UPI0000448CEC,UPI000060EAA3,UPI0000E80D04,UPI0000ECB083,UPI000240BE0E,UPI0003505DE6	UPARC14_2_GallusG_9031_20140417.fasta	189,631.40	100.00%	4	4	7	0.01%	2.22%	VAQHYQDTLLR	K	A	99.70%	31.2	44.7	[illegible]
20196-20225 HinckeM --TAXchicken	Uncategorized Sample	20196-20205_A	Mudpit_Ingel20196_MGFPeaklist (F058870)	status=active	UPI00003AA4FE,UPI00003AA4FF,UPI00003AA500,UPI00003AA501,UPI0000448CEC,UPI000060EAA3,UPI0000E80D04,UPI0000ECB083,UPI000240BE0E,UPI0003505DE6	UPARC14_2_GallusG_9031_20140417.fasta	189,631.40	100.00%	4	4	7	0.01%	2.22%	LGNFFSPK	K	V	48.30%	18.9	43.5	[illegible]
20196-20225 HinckeM --TAXchicken	Uncategorized Sample	20196-20205_A	Mudpit_Ingel20196_MGFPeaklist (F058870)	status=active	UPI00003AA4FE,UPI00003AA4FF,UPI00003AA500,UPI00003AA501,UPI0000448CEC,UPI000060EAA3,UPI0000E80D04,UPI0000ECB083,UPI000240BE0E,UPI0003505DE6	UPARC14_2_GallusG_9031_20140417.fasta	189,631.40	100.00%	4	4	7	0.01%	2.22%	LIVDVIR	R	F	74.20%	34	41	[illegible]
20196-20225 HinckeM --TAXchicken	Uncategorized Sample	20196-20205_A	Mudpit_Ingel20196_MGFPeaklist (F058870)	status=active	UPI00003AA4FE,UPI00003AA4FF,UPI00003AA500,UPI00003AA501,UPI0000448CEC,UPI000060EAA3,UPI0000E80D04,UPI0000ECB083,UPI000240BE0E,UPI0003505DE6	UPARC14_2_GallusG_9031_20140417.fasta	189,631.40	100.00%	4	4	7	0.01%	2.22%	TLQALQIPAAK	K	L	99.70%	28	41.1	[illegible]
20196-20225 HinckeM --TAXchicken	Uncategorized Sample	20196-20205_A	Mudpit_Ingel20196_MGFPeaklist (F058870)	status=active	UPI00003AA4FE,UPI00003AA4FF,UPI00003AA500,UPI00003AA501,UPI0000448CEC,UPI000060EAA3,UPI0000E80D04,UPI0000ECB083,UPI000240BE0E,UPI0003505DE6	UPARC14_2_GallusG_9031_20140417.fasta	189,631.40	100.00%	4	4	7	0.01%	2.22%	VAQHYQDTLLR	K	A	99.70%	31.2	44.7	[illegible]
20196-20225 HinckeM --TAXchicken	Uncategorized Sample	20196-20205_A	Mudpit_Ingel20196_MGFPeaklist (F058870)	status=active	UPI00003AA4FE,UPI00003AA4FF,UPI00003AA500,UPI00003AA501,UPI0000448CEC,UPI000060EAA3,UPI0000E80D04,UPI0000ECB083,UPI000240BE0E,UPI0003505DE6	UPARC14_2_GallusG_9031_20140417.fasta	189,631.40	100.00%	4	4	7	0.01%	2.22%	LGNFFSPK	K	V	48.30%	18.9	43.5	[illegible]
20196-20225 HinckeM --TAXchicken	Uncategorized Sample	20196-20205_A	Mudpit_Ingel20196_MGFPeaklist (F058870)	status=active	UPI00003AA4FE,UPI00003AA4FF,UPI00003AA500,UPI00003AA501,UPI0000448CEC,UPI000060EAA3,UPI0000E80D04,UPI0000ECB083,UPI000240BE0E,UPI0003505DE6	UPARC14_2_GallusG_9031_20140417.fasta	189,631.40	100.00%	4	4	7	0.01%	2.22%	LIVDVIR	R	F	74.20%	34	41	[illegible]
20196-20225 HinckeM --TAXchicken	Uncategorized Sample	20196-20205_A	Mudpit_Ingel20196_MGFPeaklist (F058870)	status=active	UPI00003AA4FE,UPI00003AA4FF,UPI00003AA500,UPI00003AA501,UPI0000448CEC,UPI000060EAA3,UPI0000E80D04,UPI0000ECB083,UPI000240BE0E,UPI0003505DE6	UPARC14_2_GallusG_9031_20140417.fasta	189,631.40	100.00%	4	4	7	0.01%	2.22%	TLQALQIPAAK	K	L	99.70%	28	41.1	[illegible]
20196-20225 HinckeM --TAXchicken	Uncategorized Sample	20196-20205_A	Mudpit_Ingel20196_MGFPeaklist (F058870)	status=active	UPI00003AA4FE,UPI00003AA4FF,UPI00003AA500,UPI00003AA501,UPI0000448CEC,UPI000060EAA3,UPI0000E80D04,UPI0000ECB083,UPI000240BE0E,UPI0003505DE6	UPARC14_2_GallusG_9031_20140417.fasta	189,631.40	100.00%	4	4	7	0.01%	2.22%	VAQHYQDTLLR	K	A	99.70%	31.2	44.7	[illegible]
20196-20225 HinckeM --TAXchicken	Uncategorized Sample	20196-20205_A	Mudpit_Ingel20196_MGFPeaklist (F058870)	status=active	UPI00003AA4FE,UPI00003AA4FF,UPI00003AA500,UPI00003AA501,UPI0000448CEC,UPI000060EAA3,UPI0000E80D04,UPI0000ECB083,UPI000240BE0E,UPI0003505DE6	UPARC14_2_GallusG_9031_20140417.fasta	189,631.40	100.00%	4	4	7	0.01%	2.22%	LGNFFSPK	K	V	48.30%	18.9	43.5	[illegible]
20196-20225 HinckeM --TAXchicken	Uncategorized Sample	20196-20205_A	Mudpit_Ingel20196_MGFPeaklist (F058870)	status=active	UPI00003AA4FE,UPI00003AA4FF,UPI00003AA500,UPI00003AA501,UPI0000448CEC,UPI000060EAA3,UPI0000E80D04,UPI0000ECB083,UPI000240BE0E,UPI0003505DE6	UPARC14_2_GallusG_9031_20140417.fasta	189,631.40	100.00%	4	4	7	0.01%	2.22%	LIVDVIR	R	F	74.20%	34	41	[illegible]
20196-20225 HinckeM --TAXchicken	Uncategorized Sample	20196-20205_A	Mudpit_Ingel20196_MGFPeaklist (F058870)	status=active	UPI00003AA4FE,UPI00003AA4FF,UPI00003AA500,UPI00003AA501,UPI0000448CEC,UPI000060EAA3,UPI0000E80D04,UPI0000ECB083,UPI000240BE0E,UPI0003505DE6	UPARC14_2_GallusG_9031_20140417.fasta	189,631.40	100.00%	4	4	7	0.01%	2.22%	TLQALQIPAAK	K	L	99.70%	28	41.1	[illegible]
20196-20225 HinckeM --TAXchicken	Uncategorized Sample	20196-20205_A	Mudpit_Ingel20196_MGFPeaklist (F058870)	status=active	UPI00003AA4FE,UPI00003AA4FF,UPI00003AA500,UPI00003AA501,UPI0000448CEC,UPI000060EAA3,UPI0000E80D04,UPI0000ECB083,UPI000240BE0E,UPI0003505DE6	UPARC14_2_GallusG_9031_20140417.fasta	189,631.40	100.00%	4	4	7	0.01%	2.22%	VAQHYQDTLLR	K	A	99.70%	31.2	44.7	[illegible]
20196-20225 HinckeM --TAXchicken	Uncategorized Sample	20196-20205_A	Mudpit_Ingel20196_MGFPeaklist (F058870)	status=active	UPI00003AA4FE,UPI00003AA4FF,UPI00003AA500,UPI00003AA501,UPI0000448CEC,UPI000060EAA3,UPI0000E80D04,UPI0000ECB083,UPI000240BE0E,UPI0003505DE6	UPARC14_2_GallusG_9031_20140417.fasta	189,631.40	100.00%	4	4	7	0.01%	2.22%	LGNFFSPK	K	V	48.30%	18.9	43.5	[illegible]
20196-20225 HinckeM --TAXchicken	Uncategorized Sample	20196-20205_A	Mudpit_Ingel20196_MGFPeaklist (F058870)	status=active	UPI00003AA4FE,UPI00003AA4FF,UPI00003AA500,UPI00003AA501,UPI0000448CEC,UPI000060EAA3,UPI0000E80D04,UPI0000ECB083,UPI000240BE0E,UPI0003505DE6	UPARC14_2_GallusG_9031_20140417.fasta	189,631.40	100.00%	4	4	7	0.01%	2.22%	LIVDVIR	R	F	74.20%	34	41	[illegible]
20196-20225 HinckeM --TAXchicken	Uncategorized Sample	20196-20205_A	Mudpit_Ingel20196_MGFPeaklist (F058870)	status=active	UPI00003AA4FE,UPI00003AA4FF,UPI00003AA500,UPI00003AA501,UPI0000448CEC,UPI000060EAA3,UPI0000E80D04,UPI0000ECB083,UPI000240BE0E,UPI0003505DE6	UPARC14_2_GallusG_9031_20140417.fasta	189,631.40	100.00%	4	4	7	0.01%	2.22%	TLQALQIPAAK	K	L	99.70%	28	41.1	[illegible]
20196-20225 HinckeM --TAXchicken	Uncategorized Sample	20196-20205_A	Mudpit_Ingel20196_MGFPeaklist (F058870)	status=active	UPI00003AA4FE,UPI00003AA4FF,UPI00003AA500,UPI00003AA501,UPI0000448CEC,UPI000060EAA3,UPI0000E80D04,UPI0000ECB083,UPI000240BE0E,UPI0003505DE6	UPARC14_2_GallusG_9031_20140417.fasta	189,631.40	100.00%	4	4	7	0.01%	2.22%	VAQHYQDTLLR	K	A	99.70%	31.2	44.7	[illegible]
20196-20225 HinckeM --TAXchicken	Uncategorized Sample	20196-20205_A	Mudpit_Ingel20196_MGFPeaklist (F058870)	status=active	UPI00003AA4FE,UPI00003AA4FF,UPI00003AA500,UPI00003AA501,UPI0000448CEC,UPI000060EAA3,UPI0000E80D04,UPI0000ECB083,UPI000240BE0E,UPI0003505DE6	UPARC14_2_GallusG_9031_20140417.fasta	189,631.40	100.00%	4	4	7	0.01%	2.22%	LGNFFSPK	K	V	48.30%	18.9	43.5	[illegible]
20196-20225 HinckeM --TAXchicken	Uncategorized Sample	20196-20205_A	Mudpit_Ingel20196_MGFPeaklist (F058870)	status=active	UPI00003AA4FE,UPI00003AA4FF,UPI00003AA500,UPI00003AA501,UPI0000448CEC,UPI000060EAA3,UPI0000E80D04,UPI0000ECB083,UPI000240BE0E,UPI0003505DE6	UPARC14_2_GallusG_9031_20140417.fasta	189,631.40	100.00%	4	4	7	0.01%	2.22%	LIVDVIR	R	F	74.20%	34	41	[illegible]
20196-20225 HinckeM --TAXchicken	Uncategorized Sample	20196-20205_A	Mudpit_Ingel20196_MGFPeaklist (F058870)	status=active	UPI00003AA4FE,UPI00003AA4FF,UPI00003AA500,UPI00003AA501,UPI0000448CEC,UPI000060EAA3,UPI0000E80D04,UPI0000ECB083,UPI000240BE0E,UPI0003505DE6	UPARC14_2_GallusG_9031_20140417.fasta	189,631.40	100.00%	4	4	7	0.01%	2.22%	TLQALQIPAAK	K	L	99.70%	28	41.1	[illegible]
20196-20225 HinckeM --TAXchicken	Uncategorized Sample	20196-20205_A	Mudpit_Ingel20196_MGFPeaklist (F058870)	status=active	UPI00003AA4FE,UPI00003AA4FF,UPI00003AA500,UPI00003AA501,UPI0000448CEC,UPI000060EAA3,UPI0000E80D04,UPI0000ECB083,UPI000240BE0E,UPI0003505DE6	UPARC14_2_GallusG_9031_20140417.fasta	189,631.40	100.00%	4	4	7	0.01%	2.22%	VAQHYQDTLLR	K	A	99.70%	31.2	44.7	[illegible]
20196-20225 HinckeM --TAXchicken	Uncategorized Sample	20196-20205_A	Mudpit_Ingel20196_MGFPeaklist (F058870)	status=active	UPI00003AA4FE,UPI00003AA4FF,UPI00003AA500,UPI00003AA501,UPI0000448CEC,UPI000060EAA3,UPI0000E80D04,UPI0000ECB083,UPI000240BE0E,UPI0003505DE6	UPARC14_2_GallusG_9031_20140417.fasta	189,631.40	100.00%	4	4	7	0.01%	2.22%	LGNFFSPK	K	V	48.30%	18.9	43.5	[illegible]
20196-20225 HinckeM --TAXchicken	Uncategorized Sample	20196-20205_A	Mudpit_Ingel20196_MGFPeaklist (F058870)	status=active	UPI00003AA4FE,UPI00003AA4FF,UPI00003AA500,UPI00003AA501,UPI0000448CEC,UPI000060EAA3,UPI0000E80D04,UPI0000ECB083,UPI000240BE0E,UPI0003505DE6	UPARC14_2_GallusG_9031_20140417.fasta	189,631.40	100.00%	4	4	7	0.01%	2.22%	LIVDVIR	R	F	74.20%	34	41	[illegible]
20196-20225 HinckeM --TAXchicken	Uncategorized Sample	20196-20205_A	Mudpit_Ingel20196_MGFPeaklist (F058870)	status=active	UPI00003AA4FE,UPI00003AA4FF,UPI00003AA500,UPI00003AA501,UPI0000448CEC,UPI000060EAA3,UPI0000E80D04,UPI0000ECB083,UPI000240BE0E,UPI0003505DE6	UPARC14_2_GallusG_9031_20140417.fasta	189,631.40	100.00%	4	4	7	0.01%	2.22%	TLQALQIPAAK	K	L	99.70%	28	41.1	[illegible]
20196-20225 HinckeM --TAXchicken	Uncategorized Sample	20196-20205_A	Mudpit_Ingel20196_MGFPeaklist (F058870)	status=active	UPI00003AA4FE,UPI00003AA4FF,UPI00003AA500,UPI00003AA501,UPI0000448CEC,UPI000060EAA3,UPI0000E80D04,UPI0000ECB083,UPI000240BE0E,UPI0003505DE6	UPARC14_2_GallusG_9031_20140417.fasta	189,631.40	100.00%	4	4	7	0.01%	2.22%	VAQHYQDTLLR	K	A	99.70%	31.2	44.7	[illegible]
20196-20225 HinckeM --TAXchicken	Uncategorized Sample	20196-20205_A	Mudpit_Ingel20196_MGFPeaklist (F058870)	status=active	UPI00003AA4FE,UPI00003AA4FF,UPI00003AA500,UPI00003AA501,UPI0000448CEC,UPI000060EAA3,UPI0000E80D04,UPI0000ECB083,UPI000240BE0E,UPI0003505DE6	UPARC14_2_GallusG_9031_20140417.fasta	189,631.40	100.00%	4	4	7	0.01%	2.22%	LGNFFSPK	K	V	48.30%	18.9	43.5	[illegible]
20196-20225 HinckeM --TAXchicken	Uncategorized Sample	20196-20205_A	Mudpit_Ingel20196_MGFPeaklist (F058870)	status=active	UPI00003AA4FE,UPI00003AA4FF,UPI00003AA500,UPI00003AA501,UPI0000448CEC,UPI000060EAA3,UPI0000E80D04,UPI0000ECB083,UPI000240BE0E,UPI0003505DE6	UPARC14_2_GallusG_9031_20140417.fasta	189,631.40	100.00%	4	4	7	0.01%	2.22%	LIVDVIR	R	F	74.20%	34	41	[illegible]
20196-20225 HinckeM --TAXchicken	Uncategorized Sample	20196-20205_A	Mudpit_Ingel20196_MGFPeaklist (F058870)	status=active	UPI00003AA4FE,UPI00003AA4FF,UPI00003AA500,UPI00003AA501,UPI0000448CEC,UPI000060EAA3,UPI0000E80D04,UPI0000ECB083,UPI000240BE0E,UPI0003505DE6	UPARC14_2_GallusG_9031_20140417.fasta	189,631.40	100.00%	4	4	7	0.01%	2.22%	TLQALQIPAAK	K	L	99.70%	28	41.1	[illegible]
20196-20225 HinckeM --TAXchicken	Uncategorized Sample	20196-20205_A	Mudpit_Ingel20196_MGFPeaklist (F058870)	status=active	UPI00003AA4FE,UPI00003AA4FF,UPI00003AA500,UPI00003AA501,UPI0000448CEC,UPI000060EAA3,UPI0000E80D04,UPI0000ECB083,UPI000240BE0E,UPI0003505DE6	UPARC14_2_GallusG_9031_20140417.fasta	189,631.40	100.00%	4	4	7	0.01%	2.22%	VAQHYQDTLLR	K	A	99.70%	31.2	44.7	[illegible]
20196-20225 HinckeM --TAXchicken	Uncategorized Sample	20196-20205_A	Mudpit_Ingel20196_MGFPeaklist (F058870)	Structural maintenance of chromosomes protein OS=Gallus gallus GN=SMC1 PE=2 SV=2	Q8AWB7_CHICK	TAX_GallusGallus_9031_20141114.fasta	143,033.70	100.00%	10	10	15	0.03%	7.78%	AAAPAEEEIKQEMNT	K	L	93.80%			[illegible]
20196-20225 HinckeM --TAXchicken	Uncategorized Sample	20196-20205_A	Mudpit_Ingel20196_MGFPeaklist (F058870)	Structural maintenance of chromosomes protein OS=Gallus gallus GN=SMC1 PE=2 SV=2	Q8AWB7_CHICK	TAX_GallusGallus_9031_20141114.fasta	143,033.70	100.00%	10	10	15	0.03%	7.78%	AQVQLQLFK	R	L	99.70%	34.4	44.1	[illegible]
20196-20225 HinckeM --TAXchicken	Uncategorized Sample	20196-20205_A	Mudpit_Ingel20196_MGFPeaklist (F058870)	Structural maintenance of chromosomes protein OS=Gallus gallus GN=SMC1 PE=2 SV=2	Q8AWB7_CHICK	TAX_GallusGallus_9031_20141114.fasta	143,033.70	100.00%	10	10	15	0.03%	7.78%	DLTLEENQVKK	R	Y	24.10%	7.58	44.7	[illegible]
20196-20225 HinckeM --TAXchicken	Uncategorized Sample	20196-20205_A	Mudpit_Ingel20196_MGFPeaklist (F058870)	Structural maintenance of chromosomes protein OS=Gallus gallus GN=SMC1 PE=2 SV=2	Q8AWB7_CHICK	TAX_GallusGallus_9031_20141114.fasta	143,033.70	100.00%	10	10	15	0.03%	7.78%	LIEIENFK	K	S	67.60%	28.5	44.5	[illegible]
20196-20225 HinckeM --TAXchicken	Uncategorized Sample	20196-20205_A	Mudpit_Ingel20196_MGFPeaklist (F058870)	Structural maintenance of chromosomes protein OS=Gallus gallus GN=SMC1 PE=2 SV=2	Q8AWB7_CHICK	TAX_GallusGallus_9031_20141114.fasta	143,033.70	100.00%	10	10	15	0.03%	7.78%	LIVGSSSEYK	R	I	7.00%	16	43.7	[illegible]
20196-20225 HinckeM --TAXchicken	Uncategorized Sample	20196-20205_A	Mudpit_Ingel20196_MGFPeaklist (F058870)	Structural maintenance of chromosomes protein OS=Gallus gallus GN=SMC1 PE=2 SV=2	Q8AWB7_CHICK	TAX_GallusGallus_9031_20141114.fasta	143,033.70	100.00%	10	10	15	0.03%	7.78%	LIVGSSSEYKINNR	R	V	99.40%	24.4	44.6	[illegible]
20196-20225 HinckeM --TAXchicken	Uncategorized Sample	20196-20205_A	Mudpit_Ingel20196_MGFPeaklist (F058870)	Structural maintenance of chromosomes protein OS=Gallus gallus GN=SMC1 PE=2 SV=2	Q8AWB7_CHICK	TAX_GallusGallus_9031_20141114.fasta	143,033.70	100.00%	10	10	15	0.03%	7.78%	LVIDVIR	K	Y	74.20%	36.4	41	[illegible]
20196-20225 HinckeM --TAXchicken	Uncategorized Sample	20196-20205_A	Mudpit_Ingel20196_MGFPeaklist (F058870)	Structural maintenance of chromosomes protein OS=Gallus gallus GN=SMC1 PE=2 SV=2	Q8AWB7_CHICK	TAX_GallusGallus_9031_20141114.fasta	143,033.70	100.00%	10	10	15	0.03%	7.78%	LYPGSVYGR	R	L	99.70%	26	43.7	[illegible]
20196-20225 HinckeM --TAXchicken	Uncategorized Sample	20196-20205_A	Mudpit_Ingel20196_MGFPeaklist (F058870)	Structural maintenance of chromosomes protein OS=Gallus gallus GN=SMC1 PE=2 SV=2	Q8AWB7_CHICK	TAX_GallusGallus_9031_20141114.fasta	143,033.70	100.00%	10	10	15	0.03%	7.78%	SGVISGGASDLK	K	A	99.70%	35.5	45.2	[illegible]
20196-20225 HinckeM --TAXchicken	Uncategorized Sample	20196-20205_A	Mudpit_Ingel20196_MGFPeaklist (F058870)	Structural maintenance of chromosomes protein OS=Gallus gallus GN=SMC1 PE=2 SV=2	Q8AWB7_CHICK	TAX_GallusGallus_9031_20141114.fasta	143,033.70	100.00%	10	10	15	0.03%	7.78%	TVALDGTLFQK	K	S	26.70%	15.1	44.1	[illegible]
20196-20225 HinckeM --TAXchicken	Uncategorized Sample	20196-20205_A	Mudpit_Ingel20196_MGFPeaklist (F058870)	status=active	UPI00003BEE4D	UPARC14_2_GallusG_9031_20140417.fasta	143,146.40	100.00%	2	2	4	0.01%	5.68%	ADQDRLDLEER	K	K	99.70%	49.6	44.6	[illegible]
20196-20225 HinckeM --TAXchicken	Uncategorized Sample	20196-20205_A	Mudpit_Ingel20196_MGFPeaklist (F058870)	status=active	UPI00003BEE4D	UPARC14_2_GallusG_9031_20140417.fasta	143,146.40	100.00%	2	2	4	0.01%	5.68%	FTAIIGPNGSGK	K	S	99.70%	47	44.9	[illegible]
20196-20225 HinckeM --TAXchicken	Uncategorized Sample	20196-20205_A	Mudpit_Ingel20196_MGFPeaklist (F058870)	status=active	UPI00003BEE4D	UPARC14_2_GallusG_9031_20140417.fasta	143,146.40	100.00%	2	2	4	0.01%	5.68%	LIEIENFK	K	S	67.60%	28.5	44.5	[illegible]
20196-20225 HinckeM --TAXchicken	Uncategorized Sample	20196-20205_A	Mudpit_Ingel20196_MGFPeaklist (F058870)	status=active	UPI00003BEE4D	UPARC14_2_GallusG_9031_20140417.fasta	143,146.40	100.00%	2	2	4	0.01%	5.68%	LVIDVIR	K	Y	74.20%	36.4	41	[illegible]
20196-20225 HinckeM --TAXchicken	Uncategorized Sample	20196-20205_A	Mudpit_Ingel20196_MGFPeaklist (F058870)	status=active	UPI00003BEE4D	UPARC14_2_GallusG_9031_20140417.fasta	143,146.40	100.00%	2	2	4	0.01%	5.68%	LYPGSVYGR	R	L	99.70%	26	43.7	[illegible]
20196-20225 HinckeM --TAXchicken	Uncategorized Sample	20196-20205_A	Mudpit_Ingel20196_MGFPeaklist (F058870)	status=active	UPI00003BEE4D	UPARC14_2_GallusG_9031_20140417.fasta	143,146.40	100.00%	2	2	4	0.01%	5.68%	SGVISGGASDLK	K	A	99.70%	35.5	45.2	[illegible]
20196-20225 HinckeM --TAXchicken	Uncategorized Sample	20196-20205_A	Mudpit_Ingel20196_MGFPeaklist (F058870)	status=active	UPI00003BEE4D	UPARC14_2_GallusG_9031_20140417.fasta	143,146.40	100.00%	2	2	4	0.01%	5.68%	TVALDGTLFQK	K	S	26.70%	15.1	44.1	[illegible]
20196-20225 HinckeM --TAXchicken	Uncategorized Sample	20196-20205_A	Mudpit_Ingel20196_MGFPeaklist (F058870)	status=active	UPI0001E90712	UPARC14_2_GallusG_9031_20140417.fasta	159,935.30	99.30%	2	2	2	0.00%	1.35%	ALFLIPR	R	N	98.80%	49.5	43.5	[illegible]
20196-20225 HinckeM --TAXchicken	Uncategorized Sample	20196-20205_A	Mudpit_Ingel20196_MGFPeaklist (F058870)	status=active	UPI0001E90712	UPARC14_2_GallusG_9031_20140417.fasta	159,935.30	99.30%	2	2	2	0.00%	1.35%	KHHSFNSPGDKR	K	V	39.30%	14.4	44.1	[illegible]
20196-20225 HinckeM --TAXchicken	Uncategorized Sample	20196-20205_A	Mudpit_Ingel20196_MGFPeaklist (F058870)	Histone H3 OS=Gallus gallus PE=3 SV=1	Q92068_CHICK	TAX_GallusGallus_9031_20141114.fasta	15,388.80	100.00%	3	3	4	0.01%	28.70%	AQDFKTDLR	I	F	99.20%			[illegible]
20196-20225 HinckeM --TAXchicken	Uncategorized Sample	20196-20205_A	Mudpit_Ingel20196_MGFPeaklist (F058870)	Histone H3 OS=Gallus gallus PE=3 SV=1	Q92068_CHICK	TAX_GallusGallus_9031_20141114.fasta	15,388.80	100.00%	3	3	4	0.01%	28.70%	EIAQDFKTDLR	R	F	99.70%	68.7	44.7	[illegible]
20196-20225 HinckeM --TAXchicken	Uncategorized Sample	20196-20205_A	Mudpit_Ingel20196_MGFPeaklist (F058870)	Histone H3 OS=Gallus gallus PE=3 SV=1	Q92068_CHICK	TAX_GallusGallus_9031_20141114.fasta	15,388.80	100.00%	3	3	4	0.01%	28.70%	QDFKTDLR	A	F	96.90%			[illegible]
20196-20225 HinckeM --TAXchicken	Uncategorized Sample	20196-20205_A	Mudpit_Ingel20196_MGFPeaklist (F058870)	Histone H3 OS=Gallus gallus PE=3 SV=1	Q92068_CHICK	TAX_GallusGallus_9031_20141114.fasta	15,388.80	100.00%	3	3	4	0.01%	28.70%	STELLIR	K	K	98.80%	63.5	45.2	[illegible]
20196-20225 HinckeM --TAXchicken	Uncategorized Sample	20196-20205_A	Mudpit_Ingel20196_MGFPeaklist (F058870)	Histone H3 OS=Gallus gallus PE=3 SV=1	Q92068_CHICK	TAX_GallusGallus_9031_20141114.fasta	15,388.80	100.00%	3	3	4	0.01%	28.70%	VTIMPKDIQLAR	R	R	99.70%	40.3	43.3	[illegible]
20196-20225 HinckeM --TAXchicken	Uncategorized Sample	20196-20205_A	Mudpit_Ingel20196_MGFPeaklist (F058870)	Histone H3 OS=Gallus gallus PE=3 SV=1	Q92068_CHICK	TAX_GallusGallus_9031_20141114.fasta	15,388.80	100.00%	3	3	4	0.01%	28.70%	YRPGTVAL	R	R	90.00%			[illegible]
20196-20225 HinckeM --TAXchicken	Uncategorized Sample	20196-20205_A	Mudpit_Ingel20196_MGFPeaklist (F058870)	Histone H3 OS=Gallus gallus PE=3 SV=1	Q92068_CHICK	TAX_GallusGallus_9031_20141114.fasta	15,388.80	100.00%	3	3	4	0.01%	28.70%	YRPGTVALR	R	E	99.70%	38.8	44.5	[illegible]
20196-20225 HinckeM --TAXchicken	Uncategorized Sample	20196-20205_A	Mudpit_Ingel20196_MGFPeaklist (F058870)	status=active	UPI00003B0054,UPI00003B0055,UPI00006129E8,UPI0000E818B9,UPI000350706F	UPARC14_2_GallusG_9031_20140417.fasta	154,147.20	100.00%	4	4	7	0.01%	4.49%	HLLVSNTGGDGEEIER	R	F	99.70%	70.7	44.3	[illegible]
20196-20225 HinckeM --TAXchicken	Uncategorized Sample	20196-20205_A	Mudpit_Ingel20196_MGFPeaklist (F058870)	status=active	UPI00003B0054,UPI00003B0055,UPI00006129E8,UPI0000E818B9,UPI000350706F	UPARC14_2_GallusG_9031_20140417.fasta	154,147.20	100.00%	4	4	7	0.01%	4.49%	LADLHSTEISLQQR	K	L	99.70%	58.1	44.1	[illegible]
20196-20225 HinckeM --TAXchicken	Uncategorized Sample	20196-20205_A	Mudpit_Ingel20196_MGFPeaklist (F058870)	status=active	UPI00003B0054,UPI00003B0055,UPI00006129E8,UPI0000E818B9,UPI000350706F	UPARC14_2_GallusG_9031_20140417.fasta	154,147.20	100.00%	4	4	7	0.01%	4.49%	NSLQGIQQLVR	K	K	99.70%	43	43.2	[illegible]
20196-20225 HinckeM --TAXchicken	Uncategorized Sample	20196-20205_A	Mudpit_Ingel20196_MGFPeaklist (F058870)	status=active	UPI00003B0054,UPI00003B0055,UPI00006129E8,UPI0000E818B9,UPI000350706F	UPARC14_2_GallusG_9031_20140417.fasta	154,147.20	100.00%	4	4	7	0.01%	4.49%	PAAMSPTVPAGHEALEIAGR	M	I	99.70%	47	44.5	[illegible]
20196-20225 HinckeM --TAXchicken	Uncategorized Sample	20196-20205_A	Mudpit_Ingel20196_MGFPeaklist (F058870)	status=active	UPI00003B0054,UPI00003B0055,UPI00006129E8,UPI0000E818B9,UPI000350706F	UPARC14_2_GallusG_9031_20140417.fasta	154,147.20	100.00%	4	4	7	0.01%	4.49%	HLLVSNTGGDGEEIER	R	F	99.70%	70.7	44.3	[illegible]
20196-20225 HinckeM --TAXchicken	Uncategorized Sample	20196-20205_A	Mudpit_Ingel20196_MGFPeaklist (F058870)	status=active	UPI00003B0054,UPI00003B0055,UPI00006129E8,UPI0000E818B9,UPI000350706F	UPARC14_2_GallusG_9031_20140417.fasta	154,147.20	100.00%	4	4	7	0.01%	4.49%	LADLHSTEISLQQR	K	L	99.70%	58.1	44.1	[illegible]
20196-20225 HinckeM --TAXchicken	Uncategorized Sample	20196-20205_A	Mudpit_Ingel20196_MGFPeaklist (F058870)	status=active	UPI00003B0054,UPI00003B0055,UPI00006129E8,UPI0000E818B9,UPI000350706F	UPARC14_2_GallusG_9031_20140417.fasta	154,147.20	100.00%	4	4	7	0.01%	4.49%	NSLQGIQQLVR	K	K	99.70%	43	43.2	[illegible]
20196-20225 HinckeM --TAXchicken	Uncategorized Sample	20196-20205_A	Mudpit_Ingel20196_MGFPeaklist (F058870)	status=active	UPI00003B0054,UPI00003B0055,UPI00006129E8,UPI0000E818B9,UPI000350706F	UPARC14_2_GallusG_9031_20140417.fasta	154,147.20	100.00%	4	4	7	0.01%	4.49%	PAAMSPTVPAGHEALEIAGR	M	I	99.70%	47	44.5	[illegible]
20196-20225 HinckeM --TAXchicken	Uncategorized Sample	20196-20205_A	Mudpit_Ingel20196_MGFPeaklist (F058870)	status=active	UPI00003B0054,UPI00003B0055,UPI00006129E8,UPI0000E818B9,UPI000350706F	UPARC14_2_GallusG_9031_20140417.fasta	154,147.20	100.00%	4	4	7	0.01%	4.49%	HLLVSNTGGDGEEIER	R	F	99.70%	70.7	44.3	[illegible]
20196-20225 HinckeM --TAXchicken	Uncategorized Sample	20196-20205_A	Mudpit_Ingel20196_MGFPeaklist (F058870)	status=active	UPI00003B0054,UPI00003B0055,UPI00006129E8,UPI0000E818B9,UPI000350706F	UPARC14_2_GallusG_9031_20140417.fasta	154,147.20	100.00%	4	4	7	0.01%	4.49%	LADLHSTEISLQQR	K	L	99.70%	58.1	44.1	[illegible]
20196-20225 HinckeM --TAXchicken	Uncategorized Sample	20196-20205_A	Mudpit_Ingel20196_MGFPeaklist (F058870)	status=active	UPI00003B0054,UPI00003B0055,UPI00006129E8,UPI0000E818B9,UPI000350706F	UPARC14_2_GallusG_9031_20140417.fasta	154,147.20	100.00%	4	4	7	0.01%	4.49%	NSLQGIQQLVR	K	K	99.70%	43	43.2	[illegible]
20196-20225 HinckeM --TAXchicken	Uncategorized Sample	20196-20205_A	Mudpit_Ingel20196_MGFPeaklist (F058870)	status=active	UPI00003B0054,UPI00003B0055,UPI00006129E8,UPI0000E818B9,UPI000350706F	UPARC14_2_GallusG_9031_20140417.fasta	154,147.20	100.00%	4	4	7	0.01%	4.49%	PAAMSPTVPAGHEALEIAGR	M	I	99.70%	47	44.5	[illegible]
20196-20225 HinckeM --TAXchicken	Uncategorized Sample	20196-20205_A	Mudpit_Ingel20196_MGFPeaklist (F058870)	status=active	UPI00003B0054,UPI00003B0055,UPI00006129E8,UPI0000E818B9,UPI000350706F	UPARC14_2_GallusG_9031_20140417.fasta	154,147.20	100.00%	4	4	7	0.01%	4.49%	HLLVSNTGGDGEEIER	R	F	99.70%	70.7	44.3	[illegible]
20196-20225 HinckeM --TAXchicken	Uncategorized Sample	20196-20205_A	Mudpit_Ingel20196_MGFPeaklist (F058870)	status=active	UPI00003B0054,UPI00003B0055,UPI00006129E8,UPI0000E818B9,UPI000350706F	UPARC14_2_GallusG_9031_20140417.fasta	154,147.20	100.00%	4	4	7	0.01%	4.49%	LADLHSTEISLQQR	K	L	99.70%	58.1	44.1	[illegible]
20196-20225 HinckeM --TAXchicken	Uncategorized Sample	20196-20205_A	Mudpit_Ingel20196_MGFPeaklist (F058870)	status=active	UPI00003B0054,UPI00003B0055,UPI00006129E8,UPI0000E818B9,UPI000350706F	UPARC14_2_GallusG_9031_20140417.fasta	154,147.20	100.00%	4	4	7	0.01%	4.49%	NSLQGIQQLVR	K	K	99.70%	43	43.2	[illegible]
20196-20225 HinckeM --TAXchicken	Uncategorized Sample	20196-20205_A	Mudpit_Ingel20196_MGFPeaklist (F058870)	status=active	UPI00003B0054,UPI00003B0055,UPI00006129E8,UPI0000E818B9,UPI000350706F	UPARC14_2_GallusG_9031_20140417.fasta	154,147.20	100.00%	4	4	7	0.01%	4.49%	PAAMSPTVPAGHEALEIAGR	M	I	99.70%	47	44.5	[illegible]
20196-20225 HinckeM --TAXchicken	Uncategorized Sample	20196-20205_A	Mudpit_Ingel20196_MGFPeaklist (F058870)	status=active	UPI00003B0054,UPI00003B0055,UPI00006129E8,UPI0000E818B9,UPI000350706F	UPARC14_2_GallusG_9031_20140417.fasta	154,147.20	100.00%	4	4	7	0.01%	4.49%	HLLVSNTGGDGEEIER	R	F	99.70%	70.7	44.3	[illegible]
20196-20225 HinckeM --TAXchicken	Uncategorized Sample	20196-20205_A	Mudpit_Ingel20196_MGFPeaklist (F058870)	status=active	UPI00003B0054,UPI00003B0055,UPI00006129E8,UPI0000E818B9,UPI000350706F	UPARC14_2_GallusG_9031_20140417.fasta	154,147.20	100.00%	4	4	7	0.01%	4.49%	LADLHSTEISLQQR	K	L	99.70%	58.1	44.1	[illegible]
20196-20225 HinckeM --TAXchicken	Uncategorized Sample	20196-20205_A	Mudpit_Ingel20196_MGFPeaklist (F058870)	status=active	UPI00003B0054,UPI00003B0055,UPI00006129E8,UPI0000E818B9,UPI000350706F	UPARC14_2_GallusG_9031_20140417.fasta	154,147.20	100.00%	4	4	7	0.01%	4.49%	NSLQGIQQLVR	K	K	99.70%	43	43.2	[illegible]
20196-20225 HinckeM --TAXchicken	Uncategorized Sample	20196-20205_A	Mudpit_Ingel20196_MGFPeaklist (F058870)	status=active	UPI00003B0054,UPI00003B0055,UPI00006129E8,UPI0000E818B9,UPI000350706F	UPARC14_2_GallusG_9031_20140417.fasta	154,147.20	100.00%	4	4	7	0.01%	4.49%	PAAMSPTVPAGHEALEIAGR	M	I	99.70%	47	44.5	[illegible]
20196-20225 HinckeM --TAXchicken	Uncategorized Sample	20196-20205_A	Mudpit_Ingel20196_MGFPeaklist (F058870)	status=inactive	UPI0000ECD355	UPARC14_2_GallusG_9031_20140417.fasta	165,116.80	100.00%	31	34	82	0.17%	20.80%	AIGYLVSGYQR	K	Q	99.70%	62.5	44	[illegible]
20196-20225 HinckeM --TAXchicken	Uncategorized Sample	20196-20205_A	Mudpit_Ingel20196_MGFPeaklist (F058870)	status=inactive	UPI0000ECD355	UPARC14_2_GallusG_9031_20140417.fasta	165,116.80	100.00%	31	34	82	0.17%	20.80%	EFLSSLEK	K	E	92.60%	39.3	43.6	[illegible]
20196-20225 HinckeM --TAXchicken	Uncategorized Sample	20196-20205_A	Mudpit_Ingel20196_MGFPeaklist (F058870)	status=inactive	UPI0000ECD355	UPARC14_2_GallusG_9031_20140417.fasta	165,116.80	100.00%	31	34	82	0.17%	20.80%	EVIADSTKFNIEK	R	C	99.70%	66.9	44.3	[illegible]
20196-20225 HinckeM --TAXchicken	Uncategorized Sample	20196-20205_A	Mudpit_Ingel20196_MGFPeaklist (F058870)	status=inactive	UPI0000ECD355	UPARC14_2_GallusG_9031_20140417.fasta	165,116.80	100.00%	31	34	82	0.17%	20.80%	FEHPATACYGEEAK	K	A	99.70%	27.2	42.2	[illegible]
20196-20225 HinckeM --TAXchicken	Uncategorized Sample	20196-20205_A	Mudpit_Ingel20196_MGFPeaklist (F058870)	status=inactive	UPI0000ECD355	UPARC14_2_GallusG_9031_20140417.fasta	165,116.80	100.00%	31	34	82	0.17%	20.80%	FSLLLPSNVVQDSGR	K	A	99.70%	95.4	44.3	[illegible]
20196-20225 HinckeM --TAXchicken	Uncategorized Sample	20196-20205_A	Mudpit_Ingel20196_MGFPeaklist (F058870)	status=inactive	UPI0000ECD355	UPARC14_2_GallusG_9031_20140417.fasta	165,116.80	100.00%	31	34	82	0.17%	20.80%	GCVYLQTSLR	K	Y	99.70%	71.2	43.9	[illegible]
20196-20225 HinckeM --TAXchicken	Uncategorized Sample	20196-20205_A	Mudpit_Ingel20196_MGFPeaklist (F058870)	status=inactive	UPI0000ECD355	UPARC14_2_GallusG_9031_20140417.fasta	165,116.80	100.00%	31	34	82	0.17%	20.80%	GETLQFR	K	N	17.20%	13	44.2	[illegible]
20196-20225 HinckeM --TAXchicken	Uncategorized Sample	20196-20205_A	Mudpit_Ingel20196_MGFPeaklist (F058870)	status=inactive	UPI0000ECD355	UPARC14_2_GallusG_9031_20140417.fasta	165,116.80	100.00%	31	34	82	0.17%	20.80%	HPDGSYSTFGPR	K	Y	99.70%	38.4	43.8	[illegible]

20196-20225 HinckeM –TAXchicken	Uncategorized Sample	20196-20205_A	Mudpit_Ingel20196_MGFPeaklist (F058870)	status=inactive	UPI0000ECD355	UPARC14_2_GallusG_9031_20140417.fasta	165,116.80	100.00%	31	34	82	0.17%	20.80%	IEPSPETLSCGSSAEVR	K	V	99.60%	103	44.3	16.2	95.9					0	2	0	0	2	1,818.85	1,956.00		3,529.38	461	477	Empty	TRUE	
20196-20225 HinckeM –TAXchicken	Uncategorized Sample	20196-20205_A	Mudpit_Ingel20196_MGFPeaklist (F058870)	status=inactive	UPI0000ECD355	UPARC14_2_GallusG_9031_20140417.fasta	165,116.80	100.00%	31	34	82	0.17%	20.80%	IHETSSTPEEVTETIR	R	K	99.70%	76.9	44.3	28.5	62.1	38.6	5.32	0.443	0.344	0	0	3	0	2	1,828.89	1,926.00		9,440.85	731	746	Empty	TRUE	UPI0003505F95,UPI0003504D4E,UPI000240B288
20196-20225 HinckeM –TAXchicken	Uncategorized Sample	20196-20205_A	Mudpit_Ingel20196_MGFPeaklist (F058870)	status=inactive	UPI0000ECD355	UPARC14_2_GallusG_9031_20140417.fasta	165,116.80	100.00%	31	34	82	0.17%	20.80%	IHETSSTPEEVTETIRK	R	Y	99.70%	32.9	44.3	18.4	21.8	43.7	3.51	0.412	0.529	0	0	0	3	2	1,956.99	1,832.00		3,523.49	731	747	Empty	TRUE	UPI0003505F95,UPI0003504D4E,UPI000240B288
20196-20225 HinckeM –TAXchicken	Uncategorized Sample	20196-20205_A	Mudpit_Ingel20196_MGFPeaklist (F058870)	status=inactive	UPI0000ECD355	UPARC14_2_GallusG_9031_20140417.fasta	165,116.80	100.00%	31	34	82	0.17%	20.80%	IVFYYLVMAK	K	G	99.70%	45.7	44.6	17.1	40.7	41	4.18	0.344	0.6	0	2	0	0	2	1,262.69	2,711.00		596.52	493	502	Empty	TRUE	UPI0003505F95,UPI0003504D4E,UPI000240B288
20196-20225 HinckeM –TAXchicken	Uncategorized Sample	20196-20205_A	Mudpit_Ingel20196_MGFPeaklist (F058870)	status=inactive	UPI0000ECD355	UPARC14_2_GallusG_9031_20140417.fasta	165,116.80	100.00%	31	34	82	0.17%	20.80%	KFEHPATACYGEEAK	R	A	99.70%	39.9	43.6	16.9	36.3	38.6	4.66	0.36	0.467	0	0	0	4	2	1,737.79	1,559.00		2,885.26	270	284	Empty	TRUE	UPI0003505F95,UPI0003504D4E,UPI000240B288
20196-20225 HinckeM –TAXchicken	Uncategorized Sample	20196-20205_A	Mudpit_Ingel20196_MGFPeaklist (F058870)	status=inactive	UPI0000ECD355	UPARC14_2_GallusG_9031_20140417.fasta	165,116.80	100.00%	31	34	82	0.17%	20.80%	KTVAWLVTPK	R	S	99.70%	49.9	42.1	33.7	26.7	36.8	1.96	0.22	0.55	0	2	0	0	2	1,142.69	2,219.00		571.21	868	877	Empty	TRUE	UPI0003505F95,UPI0003504D4E,UPI000240B288
20196-20225 HinckeM –TAXchicken	Uncategorized Sample	20196-20205_A	Mudpit_Ingel20196_MGFPeaklist (F058870)	status=inactive	UPI0000ECD355	UPARC14_2_GallusG_9031_20140417.fasta	165,116.80	100.00%	31	34	82	0.17%	20.80%	LYQWTK	R	A	24.20%	31.2	42.8	33.3	12.1					0	1	0	0	2	838.4458	1,907.00		318.01	174	179	Empty	TRUE	UPI0003505F95,UPI0003504D4E,UPI000240B288
20196-20225 HinckeM –TAXchicken	Uncategorized Sample	20196-20205_A	Mudpit_Ingel20196_MGFPeaklist (F058870)	status=inactive	UPI0000ECD355	UPARC14_2_GallusG_9031_20140417.fasta	165,116.80	100.00%	31	34	82	0.17%	20.80%	MLSGFIPVK	K	S	93.10%	34.1	44.1	29.6	15					0	4	0	0	2	991.5646	2,395.00		1,072.54	1393	1401	Empty	TRUE	UPI0003505F95,UPI0003504D4E,UPI000240B288
20196-20225 HinckeM –TAXchicken	Uncategorized Sample	20196-20205_A	Mudpit_Ingel20196_MGFPeaklist (F058870)	status=inactive	UPI0000ECD355	UPARC14_2_GallusG_9031_20140417.fasta	165,116.80	100.00%	31	34	82	0.17%	20.80%	NALFCLETAGNGKENHVYTK	R	A	99.70%	27.9	43.9	18.4	13.6	35.3	3.72	0.108	0.275	0	0	0	3	2	2,267.08	2,261.00		1,982.07	1136	1155	Empty	TRUE	UPI0003505F95,UPI0003504D4E,UPI000240B288
20196-20225 HinckeM –TAXchicken	Uncategorized Sample	20196-20205_A	Mudpit_Ingel20196_MGFPeaklist (F058870)	status=inactive	UPI0000ECD355	UPARC14_2_GallusG_9031_20140417.fasta	165,116.80	100.00%	31	34	82	0.17%	20.80%	QESAPFLLHVHTSPETCEDSK	K	A	99.70%	48.8	43.4	13.5	48.8	36.2	2.72	0.343	0.262	0	0	0	2	2	2,412.11	2,196.50		3,096.82	1344	1364	Empty	TRUE	UPI0003505F95,UPI0003504D4E,UPI000240B288
20196-20225 HinckeM –TAXchicken	Uncategorized Sample	20196-20205_A	Mudpit_Ingel20196_MGFPeaklist (F058870)	status=inactive	UPI0000ECD355	UPARC14_2_GallusG_9031_20140417.fasta	165,116.80	100.00%	31	34	82	0.17%	20.80%	QLLVEPEGVEK	R	E	99.70%	36.6	43.7	27.5	23.9	31.7	2.48	0.237	0.409	0	2	0	0	2	1,240.68	2,079.00		1,166.45	916	926	Empty	TRUE	UPI0003505F95,UPI0003504D4E,UPI000240B288
20196-20225 HinckeM –TAXchicken	Uncategorized Sample	20196-20205_A	Mudpit_Ingel20196_MGFPeaklist (F058870)	status=inactive	UPI0000ECD355	UPARC14_2_GallusG_9031_20140417.fasta	165,116.80	100.00%	31	34	82	0.17%	20.80%	QMNYKHPDGSYSTFGPR	R	Y	99.70%	36.7	41.8	16.9	33.5	32.1	4.03	0.0436	0.265	0	0	2	2	2	1,983.87	1,785.00		2,916.49	1029	1045	Empty	TRUE	UPI0003505F95,UPI0003504D4E,UPI000240B288
20196-20225 HinckeM –TAXchicken	Uncategorized Sample	20196-20205_A	Mudpit_Ingel20196_MGFPeaklist (F058870)	status=inactive	UPI0000ECD355	UPARC14_2_GallusG_9031_20140417.fasta	165,116.80	100.00%	31	34	82	0.17%	20.80%	RKEFLSSLEK	K	E	99.70%	32.9	43.3	34.7	11	42.2	3.02	0.379	0.65	0	0	2	0	2	1,236.69	1,893.50		1,694.23	1171	1180	Empty	TRUE	UPI0003505F95,UPI0003504D4E,UPI000240B288
20196-20225 HinckeM –TAXchicken	Uncategorized Sample	20196-20205_A	Mudpit_Ingel20196_MGFPeaklist (F058870)	status=inactive	UPI0000ECD355	UPARC14_2_GallusG_9031_20140417.fasta	165,116.80	100.00%	31	34	82	0.17%	20.80%	SGGDFQQDFHVDPSNR	R	L	99.70%	42.2	41.9	17	30.4	52.8	10.3	0.612	0.562	0	0	4	0	2	1,805.78	2,048.00		3,891.32	1290	1305	Empty	TRUE	UPI0003505F95,UPI0003504D4E,UPI000240B288
20196-20225 HinckeM –TAXchicken	Uncategorized Sample	20196-20205_A	Mudpit_Ingel20196_MGFPeaklist (F058870)	status=inactive	UPI0000ECD355	UPARC14_2_GallusG_9031_20140417.fasta	165,116.80	100.00%	31	34	82	0.17%	20.80%	SLIDDVVSEK	R	D	99.70%	47.8	45.3	30.1	27.7	27.6	1.37	0.254	0.4	0	2	0	0	2	1,104.58	2,304.50		854.85	71	80	Empty	TRUE	UPI0003505F95,UPI0003504D4E,UPI000240B288
20196-20225 HinckeM –TAXchicken	Uncategorized Sample	20196-20205_A	Mudpit_Ingel20196_MGFPeaklist (F058870)	status=inactive	UPI0000ECD355	UPARC14_2_GallusG_9031_20140417.fasta	165,116.80	100.00%	31	34	82	0.17%	20.80%	SVSNMVIIDVK	R	M	99.70%	59	44.6	27.7	41.1	34.9	3.43	0.312	0.364	0	4	0	0	2	1,220.66	2,248.50		1,585.02	1382	1392	Empty	TRUE	UPI0003505F95,UPI0003504D4E,UPI000240B288
20196-20225 HinckeM –TAXchicken	Uncategorized Sample	20196-20205_A	Mudpit_Ingel20196_MGFPeaklist (F058870)	status=inactive	UPI0000ECD355	UPARC14_2_GallusG_9031_20140417.fasta	165,116.80	100.00%	31	34	82	0.17%	20.80%	TEFSTNHVLVYVEK	R	L	99.70%	39.1	44.6	20.5	33.7	43.3	5.6	0.342	0.429	0	0	2	0	2	1,665.85	2,292.50		2,234.79	1416	1429	Empty	TRUE	UPI0003505F95,UPI0003504D4E,UPI000240B288
20196-20225 HinckeM –TAXchicken	Uncategorized Sample	20196-20205_A	Mudpit_Ingel20196_MGFPeaklist (F058870)	status=inactive	UPI0000ECD355	UPARC14_2_GallusG_9031_20140417.fasta	165,116.80	100.00%	31	34	82	0.17%	20.80%	TIHHPFSVEEYVLPK	K	F	39.30%	14.2	44.1	17.9	10.1					0	0	1	0	2	1,795.94	2,369.00		972.15	213	227	Empty	TRUE	UPI0003505F95,UPI0003504D4E,UPI000240B288
20196-20225 HinckeM –TAXchicken	Uncategorized Sample	20196-20205_A	Mudpit_Ingel20196_MGFPeaklist (F058870)	status=inactive	UPI0000ECD355	UPARC14_2_GallusG_9031_20140417.fasta	165,116.80	100.00%	31	34	82	0.17%	20.80%	TVAWLVTPK	K	S	99.70%	47.7	43.7	32.2	30.8	31.1	0.77	0.177	0.5	0	4	0	0	2	1,014.60	2,417.00		4,353.17	869	877	Empty	TRUE	UPI0003505F95,UPI0003504D4E,UPI000240B288
20196-20225 HinckeM –TAXchicken	Uncategorized Sample	20196-20205_A	Mudpit_Ingel20196_MGFPeaklist (F058870)	status=inactive	UPI0000ECD355	UPARC14_2_GallusG_9031_20140417.fasta	165,116.80	100.00%	31	34	82	0.17%	20.80%	VHYILTPEAVGEK	R	K	99.70%	55.2	44	23.2	42.3					0	0	2	0	2	1,455.78	2,151.50		2,357.73	478	490	Empty	TRUE	
20196-20225 HinckeM –TAXchicken	Uncategorized Sample	20196-20205_A	Mudpit_Ingel20196_MGFPeaklist (F058870)	status=inactive	UPI0000ECD355	UPARC14_2_GallusG_9031_20140417.fasta	165,116.80	100.00%	31	34	82	0.17%	20.80%	VHYILTPEAVGEKK	R	K	77.80%	27.2	43	24.7	10.5					0	0	1	0	2	1,583.88	2,023.00		210.51	478	491	Empty	TRUE	
20196-20225 HinckeM –TAXchicken	Uncategorized Sample	20196-20205_A	Mudpit_Ingel20196_MGFPeaklist (F058870)	status=inactive	UPI0000ECD355	UPARC14_2_GallusG_9031_20140417.fasta	165,116.80	100.00%	31	34	82	0.17%	20.80%	VITILDEK	K	L	99.70%	56.6	44.7	46	30.7	31.7	1.14	0.215	0.5	0	4	0	0	2	930.5508	2,185.50		1,418.52	237	244	Empty	TRUE	UPI0003505F95,UPI0003504D4E,UPI000240B288
20196-20225 HinckeM –TAXchicken	Uncategorized Sample	20196-20205_A	Mudpit_Ingel20196_MGFPeaklist (F058870)	status=inactive	UPI0000ECD355	UPARC14_2_GallusG_9031_20140417.fasta	165,116.80	100.00%	31	34	82	0.17%	20.80%	VITILDEKLK	K	V	20.30%	9.93	41.4	21.4	-1.3					0	1	0	0	2	1,171.73	2,327.00		232.95	237	246	Empty	TRUE	UPI0003505F95,UPI0003504D4E,UPI000240B288
20196-20225 HinckeM –TAXchicken	Uncategorized Sample	20196-20205_A	Mudpit_Ingel20196_MGFPeaklist (F058870)	status=active	UPI000240B288,UPI0003504D4E,UPI0003505F95	UPARC14_2_GallusG_9031_20140417.fasta	170,579.30	100.00%	2	2	4	0.01%	21.20%	AIGYLVSGYQR	K	Q	99.70%	62.5	44	29.3	52	44.6	3.54	0.457	0.591	0	8	0	0	2	1,226.65	2,172.00		28,243.70	1012	1022	Empty	FALSE	UPI0000ECD355
20196-20225 HinckeM –TAXchicken	Uncategorized Sample	20196-20205_A	Mudpit_Ingel20196_MGFPeaklist (F058870)	status=active	UPI000240B288,UPI0003504D4E,UPI0003505F95	UPARC14_2_GallusG_9031_20140417.fasta	170,579.30	100.00%	2	2	4	0.01%	21.20%	AVCDEFSGQTDNHGCISK	K	L	99.70%	71.9	39.3	15.2	68.2					0	0	2	0	2	2,024.84	1,749.00		3,701.33	285	302	Empty	TRUE	
20196-20225 HinckeM –TAXchicken	Uncategorized Sample	20196-20205_A	Mudpit_Ingel20196_MGFPeaklist (F058870)	status=active	UPI000240B288,UPI0003504D4E,UPI0003505F95	UPARC14_2_GallusG_9031_20140417.fasta	170,579.30	100.00%	2	2	4	0.01%	21.20%	EFLSSLEK	K	E	92.60%	39.3	43.6	31.6	3.06					0	4	0	0	2	952.4986	2,198.50		1,250.61	1167	1174	Empty	FALSE	UPI0000ECD355
20196-20225 HinckeM –TAXchicken	Uncategorized Sample	20196-20205_A	Mudpit_Ingel20196_MGFPeaklist (F058870)	status=active	UPI000240B288,UPI0003504D4E,UPI0003505F95	UPARC14_2_GallusG_9031_20140417.fasta	170,579.30	100.00%	2	2	4	0.01%	21.20%	EVIADSTKFNIEK	R	C	99.70%	66.9	44.3	18.6	55.4	43	5.24	0.381	0.423	0	0	2	0	2	1,493.78	2,043.50		1,434.84	547	559	Empty	FALSE	UPI0000ECD355
20196-20225 HinckeM –TAXchicken	Uncategorized Sample	20196-20205_A	Mudpit_Ingel20196_MGFPeaklist (F058870)	status=active	UPI000240B288,UPI0003504D4E,UPI0003505F95	UPARC14_2_GallusG_9031_20140417.fasta	170,579.30	100.00%	2	2	4	0.01%	21.20%	FEHPATACYGEEAK	K	A	99.70%	27.2	42.2	13.2	20.7	39.3	4.64	0.351	0.464	0	0	1	0	2	1,609.70	1,645.00		536.81	271	284	Empty	FALSE	UPI0000ECD355
20196-20225 HinckeM –TAXchicken	Uncategorized Sample	20196-20205_A	Mudpit_Ingel20196_MGFPeaklist (F058870)	status=active	UPI000240B288,UPI0003504D4E,UPI0003505F95	UPARC14_2_GallusG_9031_20140417.fasta	170,579.30	100.00%	2	2	4	0.01%	21.20%	FSLLLPSNVVQDSGR	K	A	99.70%	95.4	44.3	24.1	87.7	48.8	5.15	0.477	0.467	0	2	0	0	2	1,631.88	2,755.00		4,728.80	939	953	Empty	FALSE	UPI0000ECD355
20196-20225 HinckeM –TAXchicken	Uncategorized Sample	20196-20205_A	Mudpit_Ingel20196_MGFPeaklist (F058870)	status=active	UPI000240B288,UPI0003504D4E,UPI0003505F95	UPARC14_2_GallusG_9031_20140417.fasta	170,579.30	100.00%	2	2	4	0.01%	21.20%	GCVYLQTSLR	K	Y	99.70%	71.2	43.9	26.9	47.5	37.5	3.54	0.379	0.55	0	2	0	0	2	1,196.61	2,086.50		1,336.44	1322	1331	Empty	FALSE	UPI0000ECD355
20196-20225 HinckeM –TAXchicken	Uncategorized Sample	20196-20205_A	Mudpit_Ingel20196_MGFPeaklist (F058870)	status=active	UPI000240B288,UPI0003504D4E,UPI0003505F95	UPARC14_2_GallusG_9031_20140417.fasta	170,579.30	100.00%	2	2	4	0.01%	21.20%	GETLQFR	K	N	17.20%	13	44.2	20.9	6.14					0	1	0	0	2	850.4418	1,922.00		469.72	108	114	Empty	FALSE	UPI0000ECD355
20196-20225 HinckeM –TAXchicken	Uncategorized Sample	20196-20205_A	Mudpit_Ingel20196_MGFPeaklist (F058870)	status=active	UPI000240B288,UPI0003504D4E,UPI0003505F95	UPARC14_2_GallusG_9031_20140417.fasta	170,579.30	100.00%	2	2	4	0.01%	21.20%	HPDGSYSTFGPR	K	Y	99.70%	38.4	43.8	23.3	31.9	39.9	5.32	0.464	0.542	0	0	3	0	2	1,320.60	1,707.00		1,369.29	1028	1039	Empty	FALSE	UPI0000ECD355
20196-20225 HinckeM –TAXchicken	Uncategorized Sample	20196-20205_A	Mudpit_Ingel20196_MGFPeaklist (F058870)	status=active	UPI000240B288,UPI0003504D4E,UPI0003505F95	UPARC14_2_GallusG_9031_20140417.fasta	170,579.30	100.00%	2	2	4	0.01%	21.20%	IEPSSETLSCGSSAEVR	K	V	99.70%	121	43.7	22.7	109	66.1	6	0.551	0.471	0	2	0	0	2	1,808.83	1,822.50		2,976.62	460	476	Empty	TRUE	
20196-20225 HinckeM –TAXchicken	Uncategorized Sample	20196-20205_A	Mudpit_Ingel20196_MGFPeaklist (F058870)	status=active	UPI000240B288,UPI0003504D4E,UPI0003505F95	UPARC14_2_GallusG_9031_20140417.fasta	170,579.30	100.00%	2	2	4	0.01%	21.20%	IHETSSTPEEVTETIR	R	K	99.70%	76.9	44.3	28.5	62.1	38.6	5.32	0.443	0.344	0	0	3	0	2	1,828.89	1,926.00		9,440.85	725	740	Empty	FALSE	UPI0000ECD355
20196-20225 HinckeM –TAXchicken	Uncategorized Sample	20196-20205_A	Mudpit_Ingel20196_MGFPeaklist (F058870)	status=active	UPI000240B288,UPI0003504D4E,UPI0003505F95	UPARC14_2_GallusG_9031_20140417.fasta	170,579.30	100.00%	2	2	4	0.01%	21.20%	IHETSSTPEEVTETIRK	R	Y	99.70%	32.9	44.3	18.4	21.8	43.7	3.51	0.412	0.529	0	0	0	3	2	1,956.99	1,832.00		3,523.49	725	741	Empty	FALSE	UPI0000ECD355
20196-20225 HinckeM –TAXchicken	Uncategorized Sample	20196-20205_A	Mudpit_Ingel20196_MGFPeaklist (F058870)	status=active	UPI000240B288,UPI0003504D4E,UPI0003505F95	UPARC14_2_GallusG_9031_20140417.fasta	170,579.30	100.00%	2	2	4	0.01%	21.20%	IVFYYLVMAK	K	G	99.70%	45.7	44.6	17.1	40.7	41	4.18	0.344	0.6	0	2	0	0	2	1,262.69	2,711.00		596.52	490	499	Empty	FALSE	UPI0000ECD355
20196-20225 HinckeM –TAXchicken	Uncategorized Sample	20196-20205_A	Mudpit_Ingel20196_MGFPeaklist (F058870)	status=active	UPI000240B288,UPI0003504D4E,UPI0003505F95	UPARC14_2_GallusG_9031_20140417.fasta	170,579.30	100.00%	2	2	4	0.01%	21.20%	KFEHPATACYGEEAK	R	A	99.70%	39.9	43.6	16.9	36.3	38.6	4.66	0.36	0.467	0	0	0	4	2	1,737.79	1,559.00		2,885.26	270	284	Empty	FALSE	UPI0000ECD355
20196-20225 HinckeM –TAXchicken	Uncategorized Sample	20196-20205_A	Mudpit_Ingel20196_MGFPeaklist (F058870)	status=active	UPI000240B288,UPI0003504D4E,UPI0003505F95	UPARC14_2_GallusG_9031_20140417.fasta	170,579.30	100.00%	2	2	4	0.01%	21.20%	KTVAWLVTPK	R	S	99.70%	49.9	42.1	33.7	26.7	36.8	1.96	0.22	0.55	0	2	0	0	2	1,142.69	2,219.00		571.21	862	871	Empty	FALSE	UPI0000ECD355
20196-20225 HinckeM –TAXchicken	Uncategorized Sample	20196-20205_A	Mudpit_Ingel20196_MGFPeaklist (F058870)	status=active	UPI000240B288,UPI0003504D4E,UPI0003505F95	UPARC14_2_GallusG_9031_20140417.fasta	170,579.30	100.00%	2	2	4	0.01%	21.20%	LYQWTK	R	A	24.20%	31.2	42.8	33.3	12.1					0	1	0	0	2	838.4458	1,907.00		318.01	174	179	Empty	FALSE	UPI0000ECD355
20196-20225 HinckeM –TAXchicken	Uncategorized Sample	20196-20205_A	Mudpit_Ingel20196_MGFPeaklist (F058870)	status=active	UPI000240B288,UPI0003504D4E,UPI0003505F95	UPARC14_2_GallusG_9031_20140417.fasta	170,579.30	100.00%	2	2	4	0.01%	21.20%	MLSGFIPVK	K	S	93.10%	34.1	44.1	29.6	15					0	4	0	0	2	991.5646	2,395.00		1,072.54	1387	1395	Empty	FALSE	UPI0000ECD355
20196-20225 HinckeM –TAXchicken	Uncategorized Sample	20196-20205_A	Mudpit_Ingel20196_MGFPeaklist (F058870)	status=active	UPI000240B288,UPI0003504D4E,UPI0003505F95	UPARC14_2_GallusG_9031_20140417.fasta	170,579.30	100.00%	2	2	4	0.01%	21.20%	NALFCLETAGNGKENHVYTK	R	A	99.70%	27.9	43.9	18.4	13.6	35.3	3.72	0.108	0.275	0	0	0	3	2	2,267.08	2,261.00		1,982.07	1130	1149	Empty	FALSE	UPI0000ECD355
20196-20225 HinckeM –TAXchicken	Uncategorized Sample	20196-20205_A	Mudpit_Ingel20196_MGFPeaklist (F058870)	status=active	UPI000240B288,UPI0003504D4E,UPI0003505F95	UPARC14_2_GallusG_9031_20140417.fasta	170,579.30	100.00%	2	2	4	0.01%	21.20%	QESAPFLLHVHTSPETCEDSK	K	A	99.70%	48.8	43.4	13.5	48.8	36.2	2.72	0.343	0.262	0	0	0	2	2	2,412.11	2,196.50		3,096.82	1338	1358	Empty	FALSE	UPI0000ECD355
20196-20225 HinckeM –TAXchicken	Uncategorized Sample	20196-20205_A	Mudpit_Ingel20196_MGFPeaklist (F058870)	status=active	UPI000240B288,UPI0003504D4E,UPI0003505F95	UPARC14_2_GallusG_9031_20140417.fasta	170,579.30	100.00%	2	2	4	0.01%	21.20%	QLLVEPEGVEK	R	E	99.70%	36.6	43.7	27.5	23.9	31.7	2.48	0.237	0.409	0	2	0	0	2	1,240.68	2,079.00		1,166.45	910	920	Empty	FALSE	UPI0000ECD355
20196-20225 HinckeM –TAXchicken	Uncategorized Sample	20196-20205_A	Mudpit_Ingel20196_MGFPeaklist (F058870)	status=active	UPI000240B288,UPI0003504D4E,UPI0003505F95	UPARC14_2_GallusG_9031_20140417.fasta	170,579.30	100.00%	2	2	4	0.01%	21.20%	QMNYKHPDGSYSTFGPR	R	Y	99.70%	36.7	41.8	16.9	33.5	32.1	4.03	0.0436	0.265	0	0	2	2	2	1,983.87	1,785.00		2,916.49	1023	1039	Empty	FALSE	UPI0000ECD355
20196-20225 HinckeM –TAXchicken	Uncategorized Sample	20196-20205_A	Mudpit_Ingel20196_MGFPeaklist (F058870)	status=active	UPI000240B288,UPI0003504D4E,UPI0003505F95	UPARC14_2_GallusG_9031_20140417.fasta	170,579.30	100.00%	2	2	4	0.01%	21.20%	RKEFLSSLEK	K	E	99.70%	32.9	43.3	34.7	11	42.2	3.02	0.379	0.65	0	0	2	0	2	1,236.69	1,893.50		1,694.23	1165	1174	Empty	FALSE	UPI0000ECD355
20196-20225 HinckeM –TAXchicken	Uncategorized Sample	20196-20205_A	Mudpit_Ingel20196_MGFPeaklist (F058870)	status=active	UPI000240B288,UPI0003504D4E,UPI0003505F95	UPARC14_2_GallusG_9031_20140417.fasta	170,579.30	100.00%	2	2	4	0.01%	21.20%	SGGDFQQDFHVDPSNR	R	L	99.70%	42.2	41.9	17	30.4	52.8	10.3	0.612	0.562	0	0	4	0	2	1,805.78	2,048.00		3,891.32	1284	1299	Empty	FALSE	UPI0000ECD355
20196-20225 HinckeM –TAXchicken	Uncategorized Sample	20196-20205_A	Mudpit_Ingel20196_MGFPeaklist (F058870)	status=active	UPI000240B288,UPI0003504D4E,UPI0003505F95	UPARC14_2_GallusG_9031_20140417.fasta	170,579.30	100.00%	2	2	4	0.01%	21.20%	SLIDDVVSEK	R	D	99.70%	47.8	45.3	30.1	27.7	27.6	1.37	0.254	0.4	0	2	0	0	2	1,104.58	2,304.50		854.85	71	80	Empty	FALSE	UPI0000ECD355
20196-20225 HinckeM –TAXchicken	Uncategorized Sample	20196-20205_A	Mudpit_Ingel20196_MGFPeaklist (F058870)	status=active	UPI000240B288,UPI0003504D4E,UPI0003505F95	UPARC14_2_GallusG_9031_20140417.fasta	170,579.30	100.00%	2	2	4	0.01%	21.20%	SVSNMVIIDVK	R	M	99.70%	59	44.6	27.7	41.1	34.9	3.43	0.312	0.364	0	4	0	0	2	1,220.66	2,248.50		1,585.02	1376	1386	Empty	FALSE	UPI0000ECD355
20196-20225 HinckeM –TAXchicken	Uncategorized Sample	20196-20205_A	Mudpit_Ingel20196_MGFPeaklist (F058870)	status=active	UPI000240B288,UPI0003504D4E,UPI0003505F95	UPARC14_2_GallusG_9031_20140417.fasta	170,579.30	100.00%	2	2	4	0.01%	21.20%	TEFSTNHVLVYVEK	R	L	99.70%	39.1	44.6	20.5	33.7	43.3	5.6	0.342	0.429	0	0	2	0	2	1,665.85	2,292.50		2,234.79	1410	1423	Empty	FALSE	UPI0000ECD355
20196-20225 HinckeM –TAXchicken	Uncategorized Sample	20196-20205_A	Mudpit_Ingel20196_MGFPeaklist (F058870)	status=active	UPI000240B288,UPI0003504D4E,UPI0003505F95	UPARC14_2_GallusG_9031_20140417.fasta	170,579.30	100.00%	2	2	4	0.01%	21.20%	TIHHPFSVEEYVLPK	K	F	39.30%	14.2	44.1	17.9	10.1					0	0	1	0	2	1,795.94	2,369.00		972.15	213	227	Empty	FALSE	UPI0000ECD355
20196-20225 HinckeM –TAXchicken	Uncategorized Sample	20196-20205_A	Mudpit_Ingel20196_MGFPeaklist (F058870)	status=active	UPI000240B288,UPI0003504D4E,UPI0003505F95	UPARC14_2_GallusG_9031_20140417.fasta	170,579.30	100.00%	2	2	4	0.01%	21.20%	TVAWLVTPK	K	S	99.70%	47.7	43.7	32.2	30.8	31.1	0.77	0.177	0.5	0	4	0	0	2	1,014.60	2,417.00		4,353.17	863	871	Empty	FALSE	UPI0000ECD355
20196-20225 HinckeM –TAXchicken	Uncategorized Sample	20196-20205_A	Mudpit_Ingel20196_MGFPeaklist (F058870)	status=active	UPI000240B288,UPI0003504D4E,UPI0003505F95	UPARC14_2_GallusG_9031_20140417.fasta	170,579.30	100.00%	2	2	4	0.01%	21.20%	VITILDEK	K	L	99.70%	56.6	44.7	46	30.7	31.7	1.14	0.215	0.5	0	4	0	0	2	930.5508	2,185.50		1,418.52	237	244	Empty	FALSE	UPI0000ECD355
20196-20225 HinckeM –TAXchicken	Uncategorized Sample	20196-20205_A	Mudpit_Ingel20196_MGFPeaklist (F058870)	status=active	UPI000240B288,UPI0003504D4E,UPI0003505F95	UPARC14_2_GallusG_9031_20140417.fasta	170,579.30	100.00%	2	2	4	0.01%	21.20%	VITILDEKLK	K	V	20.30%	9.93	41.4	21.4	-1.3					0	1	0	0	2	1,171.73	2,327.00		232.95	237	246	Empty	FALSE	UPI0000ECD355
20196-20225 HinckeM –TAXchicken	Uncategorized Sample	20196-20205_A	Mudpit_Ingel20196_MGFPeaklist (F058870)	status=active	UPI000240B288,UPI0003504D4E,UPI0003505F95	UPARC14_2_GallusG_9031_20140417.fasta	170,579.30	100.00%	2	2	4	0.01%	21.20%	AIGYLVSGYQR	K	Q	99.70%	62.5	44	29.3	52	44.6	3.54	0.457	0.591	0	8	0	0	2	1,226.65	2,172.00		28,243.70	1012	1022	Empty	FALSE	UPI0000ECD355
20196-20225 HinckeM –TAXchicken	Uncategorized Sample	20196-20205_A	Mudpit_Ingel20196_MGFPeaklist (F058870)	status=active	UPI000240B288,UPI0003504D4E,UPI0003505F95	UPARC14_2_GallusG_9031_20140417.fasta	170,579.30	100.00%	2	2	4	0.01%	21.20%	AVCDEFSGQTDNHGCISK	K	L	99.70%	71.9	39.3	15.2	68.2					0	0	2	0	2	2,024.84	1,749.00		3,701.33	285	302	Empty	TRUE	
20196-20225 HinckeM –TAXchicken	Uncategorized Sample	20196-20205_A	Mudpit_Ingel20196_MGFPeaklist (F058870)	status=active	UPI000240B288,UPI0003504D4E,UPI0003505F95	UPARC14_2_GallusG_9031_20140417.fasta	170,579.30	100.00%	2	2	4	0.01%	21.20%	EFLSSLEK	K	E	92.60%	39.3	43.6	31.6	3.06					0	4	0	0	2	952.4986	2,198.50		1,250.61	1167	1174	Empty	FALSE	UPI0000ECD355
20196-20225 HinckeM –TAXchicken	Uncategorized Sample	20196-20205_A	Mudpit_Ingel20196_MGFPeaklist (F058870)	status=active	UPI000240B288,UPI0003504D4E,UPI0003505F95	UPARC14_2_GallusG_9031_20140417.fasta	170,579.30	100.00%	2	2	4	0.01%	21.20%	EVIADSTKFNIEK	R	C	99.70%	66.9	44.3	18.6	55.4	43	5.24	0.381	0.423	0	0	2	0	2	1,493.78	2,043.50		1,434.84	547	559	Empty	FALSE	UPI0000ECD355
20196-20225 HinckeM –TAXchicken	Uncategorized Sample	20196-20205_A	Mudpit_Ingel20196_MGFPeaklist (F058870)	status=active	UPI000240B288,UPI0003504D4E,UPI0003505F95	UPARC14_2_GallusG_9031_20140417.fasta	170,579.30	100.00%	2	2	4	0.01%	21.20%	FEHPATACYGEEAK	K	A	99.70%	27.2	42.2	13.2	20.7	39.3	4.64	0.351	0.464	0	0	1	0	2	1,609.70	1,645.00		536.81	271	284	Empty	FALSE	UPI0000ECD355
20196-20225 HinckeM –TAXchicken	Uncategorized Sample	20196-20205_A	Mudpit_Ingel20196_MGFPeaklist (F058870)	status=active	UPI000240B288,UPI0003504D4E,UPI0003505F95	UPARC14_2_GallusG_9031_20140417.fasta	170,579.30	100.00%	2	2	4	0.01%	21.20%	FSLLLPSNVVQDSGR	K	A	99.70%	95.4	44.3	24.1	87.7	48.8	5.15	0.477	0.467	0	2	0	0	2	1,631.88	2,755.00		4,728.80	939	953	Empty	FALSE	UPI0000ECD355
20196-20225 HinckeM –TAXchicken	Uncategorized Sample	20196-20205_A	Mudpit_Ingel20196_MGFPeaklist (F058870)	status=active	UPI000240B288,UPI0003504D4E,UPI0003505F95	UPARC14_2_GallusG_9031_20140417.fasta	170,579.30	100.00%	2	2	4	0.01%	21.20%	GCVYLQTSLR	K	Y	99.70%	71.2	43.9	26.9	47.5	37.5	3.54	0.379	0.55	0	2	0	0	2	1,196.61	2,086.50		1,336.44	1322	1331	Empty	FALSE	UPI0000ECD355
20196-20225 HinckeM –TAXchicken	Uncategorized Sample	20196-20205_A	Mudpit_Ingel20196_MGFPeaklist (F058870)	status=active	UPI000240B288,UPI0003504D4E,UPI0003505F95	UPARC14_2_GallusG_9031_20140417.fasta	170,579.30	100.00%	2	2	4	0.01%	21.20%	GETLQFR	K	N	17.20%	13	44.2	20.9	6.14					0	1	0	0	2	850.4418	1,922.00		469.72	108	114	Empty	FALSE	UPI0000ECD355
20196-20225 HinckeM –TAXchicken	Uncategorized Sample	20196-20205_A	Mudpit_Ingel20196_MGFPeaklist (F058870)	status=active	UPI000240B288,UPI0003504D4E,UPI0003505F95	UPARC14_2_GallusG_9031_20140417.fasta	170,579.30	100.00%	2	2	4	0.01%	21.20%	HPDGSYSTFGPR	K	Y	99.70%	38.4	43.8	23.3	31.9	39.9	5.32	0.464	0.542	0	0	3	0	2	1,320.60	1,707.00		1,369.29	1028	1039	Empty	FALSE	UPI0000ECD355
20196-20225 HinckeM –TAXchicken	Uncategorized Sample	20196-20205_A	Mudpit_Ingel20196_MGFPeaklist (F058870)	status=active	UPI000240B288,UPI0003504D4E,UPI0003505F95	UPARC14_2_GallusG_9031_20140417.fasta	170,579.30	100.00%	2	2	4	0.01%	21.20%	IEPSSETLSCGSSAEVR	K	V	99.70%	121	43.7	22.7	109	66.1	6	0.551	0.471	0	2	0	0	2	1,808.83	1,822.50		2,976.62	460	476	Empty	TRUE	
20196-20225 HinckeM –TAXchicken	Uncategorized Sample	20196-20205_A	Mudpit_Ingel20196_MGFPeaklist (F058870)	status=active	UPI000240B288,UPI0003504D4E,UPI0003505F95	UPARC14_2_GallusG_9031_20140417.fasta	170,579.30	100.00%	2	2	4	0.01%	21.20%	IHETSSTPEEVTETIR	R	K	99.70%	76.9	44.3	28.5	62.1	38.6	5.32	0.443	0.344	0	0	3	0	2	1,828.89	1,926.00		9,440.85	725	740	Empty	FALSE	UPI0000ECD355
20196-20225 HinckeM –TAXchicken	Uncategorized Sample	20196-20205_A	Mudpit_Ingel20196_MGFPeaklist (F058870)	status=active	UPI000240B288,UPI0003504D4E,UPI0003505F95	UPARC14_2_GallusG_9031_20140417.fasta	170,579.30	100.00%	2	2	4	0.01%	21.20%	IHETSSTPEEVTETIRK	R	Y	99.70%	32.9	44.3	18.4	21.8	43.7	3.51	0.412	0.529	0	0	0	3	2	1,956.99	1,832.00		3,523.49	725	741	Empty	FALSE	UPI0000ECD355
20196-20225 HinckeM –TAXchicken	Uncategorized Sample	20196-20205_A	Mudpit_Ingel20196_MGFPeaklist (F058870)	status=active	UPI000240B288,UPI0003504D4E,UPI0003505F95	UPARC14_2_GallusG_9031_20140417.fasta	170,579.30	100.00%	2	2	4	0.01%	21.20%	IVFYYLVMAK	K	G	99.70%	45.7	44.6	17.1	40.7	41	4.18	0.344	0.6	0	2	0	0	2	1,262.69	2,711.00		596.52	490	499	Empty	FALSE	UPI0000ECD355
20196-20225 HinckeM –TAXchicken	Uncategorized Sample	20196-20205_A	Mudpit_Ingel20196_MGFPeaklist (F058870)	status=active	UPI000240B288,UPI0003504D4E,UPI0003505F95	UPARC14_2_GallusG_9031_20140417.fasta	170,579.30	100.00%	2	2	4	0.01%	21.20%	KFEHPATACYGEEAK	R	A	99.70%	39.9	43.6	16.9	36.3	38.6	4.66	0.36	0.467	0	0	0	4	2	1,737.79	1,559.00		2,885.26	270	284	Empty	FALSE	UPI0000ECD355
20196-20225 HinckeM –TAXchicken	Uncategorized Sample	20196-20205_A	Mudpit_Ingel20196_MGFPeaklist (F058870)	status=active	UPI000240B288,UPI0003504D4E,UPI0003505F95	UPARC14_2_GallusG_9031_20140417.fasta	170,579.30	100.00%	2	2	4	0.01%	21.20%	KTVAWLVTPK	R	S	99.70%	49.9	42.1	33.7	26.7	36.8	1.96	0.22	0.55	0	2	0	0	2	1,142.69	2,219.00		571.21	862	871	Empty	FALSE	UPI0000ECD355
20196-20225 HinckeM –TAXchicken	Uncategorized Sample	20196-20205_A	Mudpit_Ingel20196_MGFPeaklist (F058870)	status=active	UPI000240B288,UPI0003504D4E,UPI0003505F95	UPARC14_2_GallusG_9031_20140417.fasta	170,579.30	100.00%	2	2	4	0.01%	21.20%	LYQWTK	R	A	24.20%	31.2	42.8	33.3	12.1					0	1	0	0	2	838.4458	1,907.00		318.01	174	179	Empty	FALSE	UPI0000ECD355
20196-20225 HinckeM –TAXchicken	Uncategorized Sample	20196-20205_A	Mudpit_Ingel20196_MGFPeaklist (F058870)	status=active	UPI000240B288,UPI0003504D4E,UPI0003505F95	UPARC14_2_GallusG_9031_20140417.fasta	170,579.30	100.00%	2	2	4	0.01%	21.20%	MLSGFIPVK	K	S	93.10%	34.1	44.1	29.6	15					0	4	0	0	2	991.5646	2,395.00		1,072.54	1387	1395	Empty	FALSE	UPI0000ECD355
[truncated: 37,827,373 more chars]
